# Analysis of the Heterogeneity of the Tumor Microenvironment and the Prognosis and Immunotherapy Response of Different Immune Subtypes in Hepatocellular Carcinoma

**DOI:** 10.1155/2022/1087399

**Published:** 2022-03-29

**Authors:** Jian Hu, Feifei Mao, Lifang Li, Xiaoqian Wang, Depei Cai, Longmei He, Qian Wu, Cong Wang, Ning Zhang, Yanfen Ma, Xia Wu, Kai Qu, Xiaoqin Wang

**Affiliations:** ^1^Department of Clinical Laboratory, The First Affiliated Hospital of Xi'an Jiaotong University, Xi'an, 710061 Shaanxi Province, China; ^2^Tongji University Cancer Center, Shanghai Tenth People's Hospital, School of Medicine, Tongji University, Shanghai 200072, China; ^3^Emergency Department, The First Affiliated Hospital of Xi'an Jiaotong University, Xi'an, 710061 Shaanxi Province, China; ^4^Department of Clinical Laboratory, Xi'an Aerospace General Hospital, Xi'an, 710061 Shaanxi Province, China; ^5^Department of Clinical Laboratory, Shaanxi Provincial Hospital of Chinese Medicine, Xi'an, 710082 Shaanxi Province, China; ^6^Department of Clinical Laboratory, Xi'an Chest Hospital, Xi'an, 710061 Shaanxi Province, China; ^7^Department of Hepatobiliary Surgery, The First Affiliated Hospital of Xi'an Jiaotong University, Xi'an, 710061 Shaanxi Province, China

## Abstract

**Purpose:**

The current clinical classification of hepatocellular carcinoma (HCC) cannot well predict the patient's possible response to the treatment plan, nor can it predict the patient's prognosis. We use the gene expression patterns of patients with hepatocellular carcinoma to reveal the heterogeneity of hepatocellular carcinoma and analyze the differences in prognosis and immunotherapy response of different immune subtypes.

**Methods:**

Firstly, using the hepatocellular carcinoma expression profile data of TCGA, combined with the single sample gene set enrichment analysis (ssGSEA) algorithm, the immune enrichment of the patient's tumor microenvironment was analyzed. Subsequently, the spectral clustering algorithm was used to extract different classifications, and the cohort of hepatocellular carcinoma was divided into 3 subtypes, and the correlation between immune subtypes and clinical characteristics and survival prognosis was established. The patient's risk index is obtained through the prognostic prediction model, suggesting the correlation between the risk index and various types of immune cells.

**Results:**

We can divide the liver cancer cohort into three subtypes: stromal cell activated immune-enriched type (A-IS), general immune-enriched type (N-IS), and non-immune-enriched type (non-IS). The 3-year survival rate of TCGA's A-IS is higher than that of N-IS and non-IS, and the three components are significantly different (*p* = 0.017). The 3-year survival rates of ICGC's A-IS and N-IS groups were higher than those of the non-IS group. The analysis of the correlation between the risk index and immune cells showed that the patient's disease risk was significantly positively correlated with cancer-associated fibroblast (CAF) stimulated cell, activated stroma cell, and anti-PD-1 resistant cell.

**Conclusion:**

The tumor gene expression characteristics of patients with hepatocellular carcinoma can be used as a basis for clinical patient classification. Different immune subtypes are closely related to survival prognosis. Different immune cell states of patients may lead to different disease risk levels. All these provide important references for the clinical identification and prognosis prediction of hepatocellular carcinoma.

## 1. Introduction

Liver cancer is still a global health challenge, which is expected to have more than 1 million cases by 2025. Hepatocellular carcinoma (HCC) is the most common form of liver cancer, accounting for 90% of cases [1, 2]. Its main risk factors include hepatitis B virus (HBV) and hepatitis C virus (HCV) infections [3–6], and metabolic syndrome and alcohol intake are becoming more common risk factors [7, 8].

The classification of HCC is based on the Barcelona-Clinical-Liver Cancer (BCLC) classification [2, 9, 10]. The system defines five subcategories of HCC and provides specific treatment recommendations for each category, including surgical resection, liver transplantation, radiofrequency ablation, chemoembolization, and multikinase inhibitor sorafenib [2]. However, the high recurrence rate of resectable liver cancer leads to a poor prognosis [11]. Recurrence seriously affects the long-term survival of HCC patients [12]. Due to the emergence of primary and secondary drug resistance, sorafenib only works in some patients with HCC, and its therapeutic effect is limited. Primary drug resistance is mainly due to genetic heterogeneity [13]. To make matters worse, almost all patients will develop secondary resistance to sorafenib within 6 months, and the recurrence rate of patients has not been significantly reduced [14]. At present, it is generally believed that the high heterogeneity of HCC, including genetic heterogeneity and immune heterogeneity, is the main reason for treatment failure [15, 16]. Among them, immune heterogeneity is one of the main reasons why current therapies are ineffective against most types of cancer, including HCC. Therefore, a comprehensive and accurate understanding of the heterogeneity of the tumor immune microenvironment of HCC is essential to improve the efficiency of personalized treatment of HCC.

In recent years, analysis and research based on HCC high-throughput data expression profile have been devoted to unraveling the molecular characteristics of HCC heterogeneity [17–20]. Although researchers have stratified clinical samples based on molecular markers, they have not yet fully clarified the correlation between the new subtypes and clinic pathological characteristics. Recently, researchers have divided HCC patients into three subgroups from the perspective of metabolism, namely, metabolic subgroup (S-Mb), microenvironment disorder subgroup (S-Me), and proliferation subgroup. Among them, the S-Me subtype enriched in proteins involved in immunity and inflammation and has a worse prognosis than S-Mb [21].

We evaluated the expression profile characteristics, immune enrichment characteristics, matrix enrichment characteristics, prognostic value, and other information of the HCC cohort, aiming to characterize the molecular characteristics of HCC by developing immune and matrix-related gene expression profiles. Comprehensive analysis was performed using the metadata set of 371 HCC human samples from The Cancer Genome Atlas (TCGA), and GSE144269 (*n* = 70), GSE14520_cohort1 (*n* = 22), GSE14520_cohort2 (*n* = 225), GSE25097_GPL10687 (*n* = 268), GSE36376_GPL10558 (*n* = 240), and ICGC_LIRI_JP (*n* = 232) data sets are used to verify the enrichment of immune-related molecules.

All samples are associated with clinical information, and the correlation between patient subtype and survival rate is verified in the ICGC data set. Three subtypes of HCC have been preliminarily identified: stromal cell-activated immune-enriched type (A-IS), general immune-enriched type (N-IS), and non-immune-enriched type (non-IS). Then, we analyzed the metadata set of immune activity characteristics, clinical characteristics, and prognostic value. Subclass A-IS shows active stromal enrichment, high immunological activity, and good prognosis. The subtype N-IS exhibits normal stromal activity, average middle immune activity, and normal survival. The subtype non-IS shows low matrix enrichment, low immune-related enrichment, and poor prognosis. In this study, a new classification of HCC was established based on the gene expression profile of immunity and matrix, thereby further revealing the diversity of human HCC.

## 2. Results

### 2.1. Classification of Gene Expression Patterns in Patients Presenting with Hepatocellular Carcinoma

**Figure 1:**
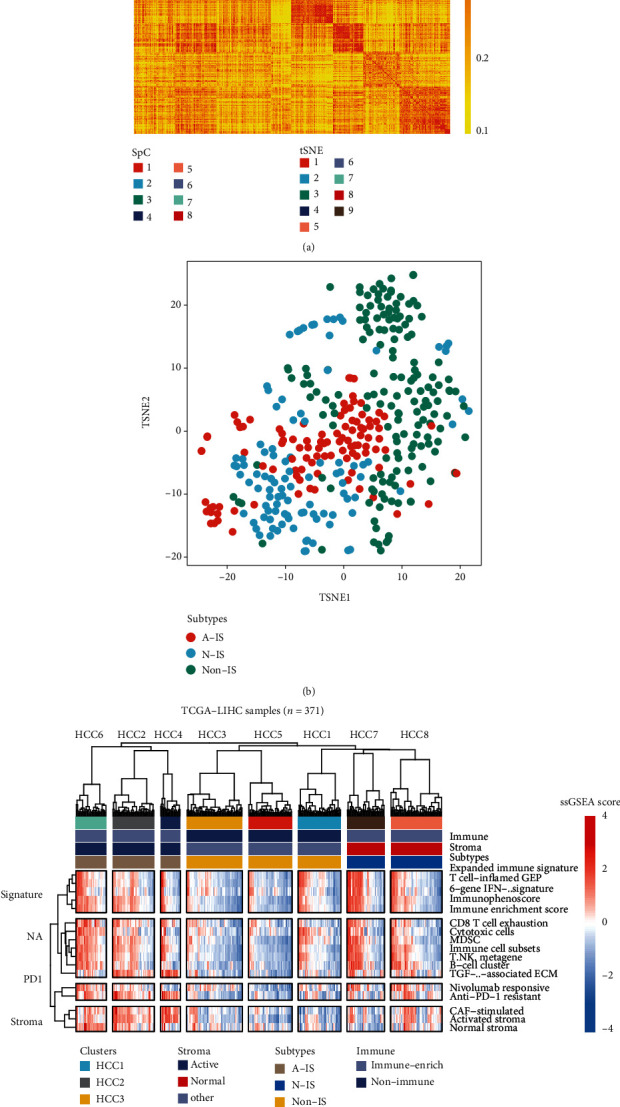
Classification of gene expression patterns in patients presenting with hepatocellular carcinoma. (a) Classification of gene expression patterns of hepatocellular carcinoma patients. The spectral clustering algorithm is used to extract expression patterns from hepatocellular carcinoma samples in TCGA cohort. According to different expression patterns, patients can be divided into 8 subgroups including HCC1, HCC2, HCC3, HCC4, HCC5, HCC6, HCC7, and HCC8. SpC stands for spectral clustering. (b) The plot shows the tSNE clustering of different subsets, and the distribution of each subtype is relatively concentrated. (c) The ssGSEA algorithm reveals the immune enrichment of the tumor microenvironment of each subtype, and divides all patients into three subgroups based on immunity and stromal-related features. The latest taxa included stromal cell-activated immune-enriched subtype (A-IS), normal immune-enriched subtype (N-IS) and non-immune-enriched subtype (non-IS).

We applied the spectral clustering algorithm to extract expression patterns from liver cancer samples in TCGA cohort, based on the expression profile data of TCGA (Figure 1(a)). At the same time, we used *t*-Distributed Stochastic Neighbor Embedding (tSNE) to show the subgroups among samples (Figure 1(b)). Based on the above classification, we further analyzed the immune enrichment situation of the tumor microenvironment of each subgroup through the single sample gene set enrichment analysis (ssGSEA) algorithm. The immune-related gene set comes from the following references (Table 1).

The analysis results showed that there was a subtype with immune-related genetic enrichment (IS) in the cohort, and the rest were non-IS types, that is, types with less immune infiltration (Figure 1(c)). We found that patients with immune-enriched subtypes were significantly enriched in the characteristics of identifying immune cells or immune responses (*p* < 0.05). In addition, even in the presence of massive immune cells, stromal cells play an essential role in tumor immune escape. Therefore, we further dissected the enrichment of stromal cells in the gene expression profile of immune enrichment subtypes. Likewise, we found that there are features of activated stromal response in the cohort by ssGSEA (Figure 1(c)). Overall, we divided hepatocellular carcinoma cohort into three subtypes: A-IS, N-IS, and non-IS.

### 2.2. Validation of Immune Subtype Classification of Cohort Patients

**Figure 2:**
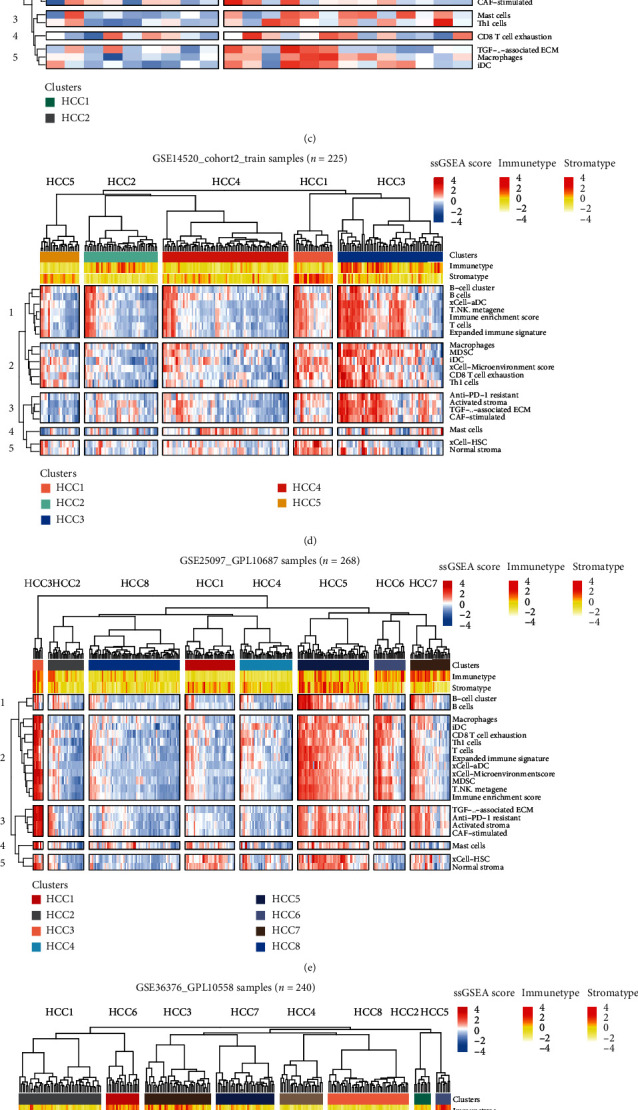
Validation of immune subtype classification of cohort patients. (a) Comparison of the striking differences in the immune microenvironment of the three subtypes. The red box represents non-immune-enriched subtype (non-IS), the blue box represents stromal cell-activated immune-enriched subtype (A-IS), and the green box represents normal immune-enriched subtype (N-IS). Anti-PD-1 resistant, B cell, immune enrichment score, macrophages, and other immune characteristics were significantly different among three subgroups (ANOVA test, *p* < 0.01). (b) The ssGSEA algorithm was performed on the GSE144269 data set (*n* = 70). Th1 cells, CD8 T cell exhaustion, MDSC, expanded immune signature, and other immune signatures were enriched in HCC2 subtypes instead of HCC1. (c) The ssGSEA algorithm was performed on the GSE14520 (cohort1_test) data set (*n* = 22). Compared with HCC1, majority of HCC2 subjects showed an enrichment of immune and stromal-related features. It specifically included B cells, immune enrichment score, MDSC, macrophages, and other signatures. (d) Molecular marker enrichment of patients in the GSE14520 (cohort2_train) data set (*n* = 225). Among existing clusters, HCC3 subclass showed strong immunity and stroma enrichment, followed by HCC1. (e) Enrichment of immune and stromal marker in subjects from the GSE25097_GPL10687 data set (*n* = 268). Among existing clusters, HCC3 subclass showed strong immunity and stroma enrichment, followed by HCC5 and HCC6. (f) Enrichment of immune and stromal marker in subjects from the GSE25097_GPL10558 data set (*n* = 240). Among existing clusters, HCC1 and HCC6 subclass showed moderate immunity and stroma enrichment. (g) Enrichment of immune and stromal marker in subjects from the ICGC_LIRI_JP data set (*n* = 232). Among existing clusters, HCC6 subclass showed strong immunity and stroma enrichment, followed by HCC4 and HCC7. (h) A stack barplot for percentage of patients in non-IS, A-IS, and N-IS subtypes among the data sets.

As shown below, the three subtypes have the following immune differences (Figure 2(a)). Compared with non-IS and N-IS (Figure 2(a), blue box), A-IS subtypes showed significant enrichment in identifying immune cells or immune response characteristics (all *p* < 0.01) including B cells, immune enrichment score, macrophages, mast cells, and Th1 cells. We further compared the differentially expressed genes of IS (including A-IS and N-IS) and non-IS subtypes, mainly using the limma algorithm and *p* < 0.05 as the criterion for significant differences (Table S1). At the same time, the genes with significant differences between A-IS and N-IS subtypes were compared (Table S2). We found that representative genes with significant differences are closely related to immune recognition and immune response. In order to verify the accuracy and consistency of the analysis method, we use the same strategy to verify it in other independent data. Our verification strategy is to select the top 50 genes that are differentially upregulated to construct a gene set and use the ssGSEA algorithm to predict the enrichment of other data. In addition, we selected cells with significant differences in immunological activity for verification. The analysis results were shown below including the GSE144269 data set (Figure 2(b), *n* = 70), the GSE14520_cohort1_test data set (Figure 2(c), *n* = 22), the GSE14520_cohort2_train samples (Figure 2(d), *n* = 225), the GSE25097_GPL10687 samples (Figure 2(e), *n* = 268), GSE36376_GPL10558 (Figure 2(f), *n* = 240) data set, and ICGC_LIRI_JP samples (Figure 2(g), *n* = 232). In the Mongolian hepatocellular carcinoma (HCC) patient cohort, we found that compared with the HCC1 patient population, immune and stromal enrichment was common in the HCC2 patient population (Figure 2(b)). Each subject in the HCC2 panel has hepatitis virus HDV and HBV infection [22]. Studies have shown that HDV RNA pattern recognition can activate immunity [23], and it has also been reported that L-HDAg, consisting of 214 amino acids, can directly induce IFN signaling [24]. Moreover, HBV-HDV coinfection shows a strong immune response [23]. xCell-aDC, B cells, immune enrichment score, myeloid-derived suppressor cells (MDSC), activated stroma, and other immune- and stromal-related features represented in most of HCC2 in GSE14520_cohort1 (Figure 2(c)). Similarly, certain subgroups of GSE14520_cohort2, mainly including HCC1 (pink box) and HCC3 (blue box), arose the enrichment of immune and stromal signatures (Figure 2(d)). HCC3, HCC5, HCC6 and HCC7 in the GSE25097_GPL10687 cohort showed biomarker enrichment on the immune and stoma response (Figure 2(e)). Certain subgroups in the ICGC_LIRI_JP cohort, mainly including HCC6, HCC4, and HCC7, have enrichment of immune and stromal features (Figure 2(f)). However, in the GSE36376_GPL10558 (Figure 2(e)) cohort, multiple subtypes have patients with both immune- and matrix-related enrichment and nonenriched. It suggests that the existing HCC classification method cannot cover all patients. Our research strategy might provide more references for the clinical classification of HCC patients.

### 2.3. Differences in Immune Subtypes Are Related to Clinical Features and Survival Prognosis

**Figure 3:**
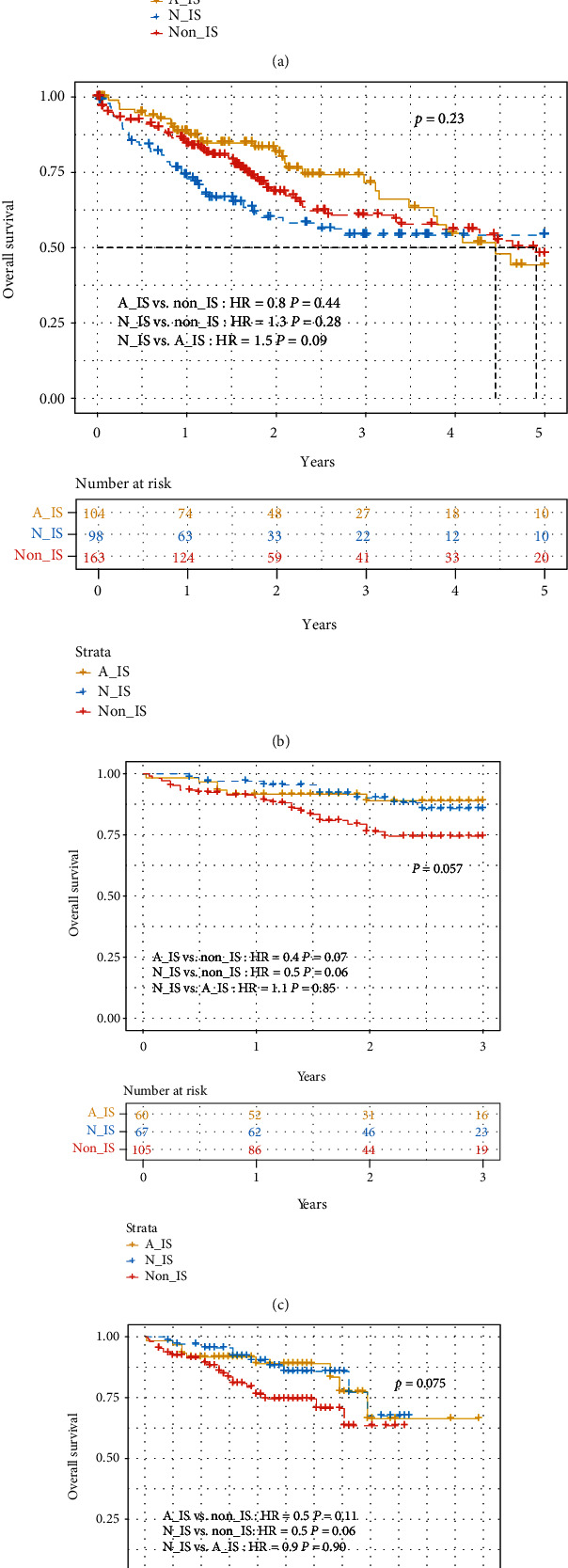
Differences in immune subtypes are related to clinical features and survival prognosis. (a) Comparison of 3-year survival among subgroups in TCGA cohort. The survival of patients was significantly different (*p* = 0.017) among the three types, and the survival of the A-IS subgroup (yellow line) was higher than that of N-IS (blue line) and non-IS (red line). (b) Comparison of 5-year survival rate of TCGA cohort. The analysis results showed that there was no significant difference among subtypes (*p* = 0.23). (c) Comparison of 3-year survival rate of the ICGC cohort. The survival was different among subgroups, and the survival of the A-IS subgroup (yellow line) was slightly higher than that of N-IS (blue line) and much higher than that of non-IS (red line). (d) Comparison of 6-year survival rates of ICGC patients. Compared with N-IS and non-IS subtypes (5 years), the overall survival of A-IS (6 years) is longer.

We have preliminarily determined that different patient subsets have differences in immune- and matrix-related signatures. So, whether or what clinical information might be associated with immune alterations? Firstly, we collected and sorted out the clinical information of all patients within three immune subtypes (Table 2). The statistical results showed that clinical indicators such as age_at_initial_pathologic_diagnosis, neoplasm_histologic_grade, and vascular_tumor_cell_type in different subgroups are strikingly different among three subpopulations (*p* < 0.01). The value of albumin_result_upper_limit of the A-IS subgroup is significantly larger than that of the N-IS and non-IS subgroups, since the age at initial pathologic diagnosis has differential survival advantages in fibrolamellar hepatocellular carcinoma (FLHCC) and hepatocellular carcinoma (HCC) [25]. And, the albumin/globulin ratio can provide guidance for the postoperative prognosis and survival prediction of HCC patients [26]. Therefore, prognostic inquiry among all subtypes matters hugely. Fortunately, we found that the three-year survival of A-IS was higher than that of N-IS and non-IS, and there is significance of intergroup variations (*p* value < 0.05) in TCGA cohort (Figure 3(a)). Nevertheless, the five-year survival of A-IS was not improving in the same cohort (Figure 3(b)). Similarly, the three-year (Figure 3(c)) and ten-year (Figure 3(d)) survival of patients in the ICGC cohort was compared in detail, which exhibited similar trends; that is, A-IS and N-IS have higher survival than non-IS.

### 2.4. Prognostic Prediction Model Based on Signatures of Tumor Microenvironment

**Figure 4:**
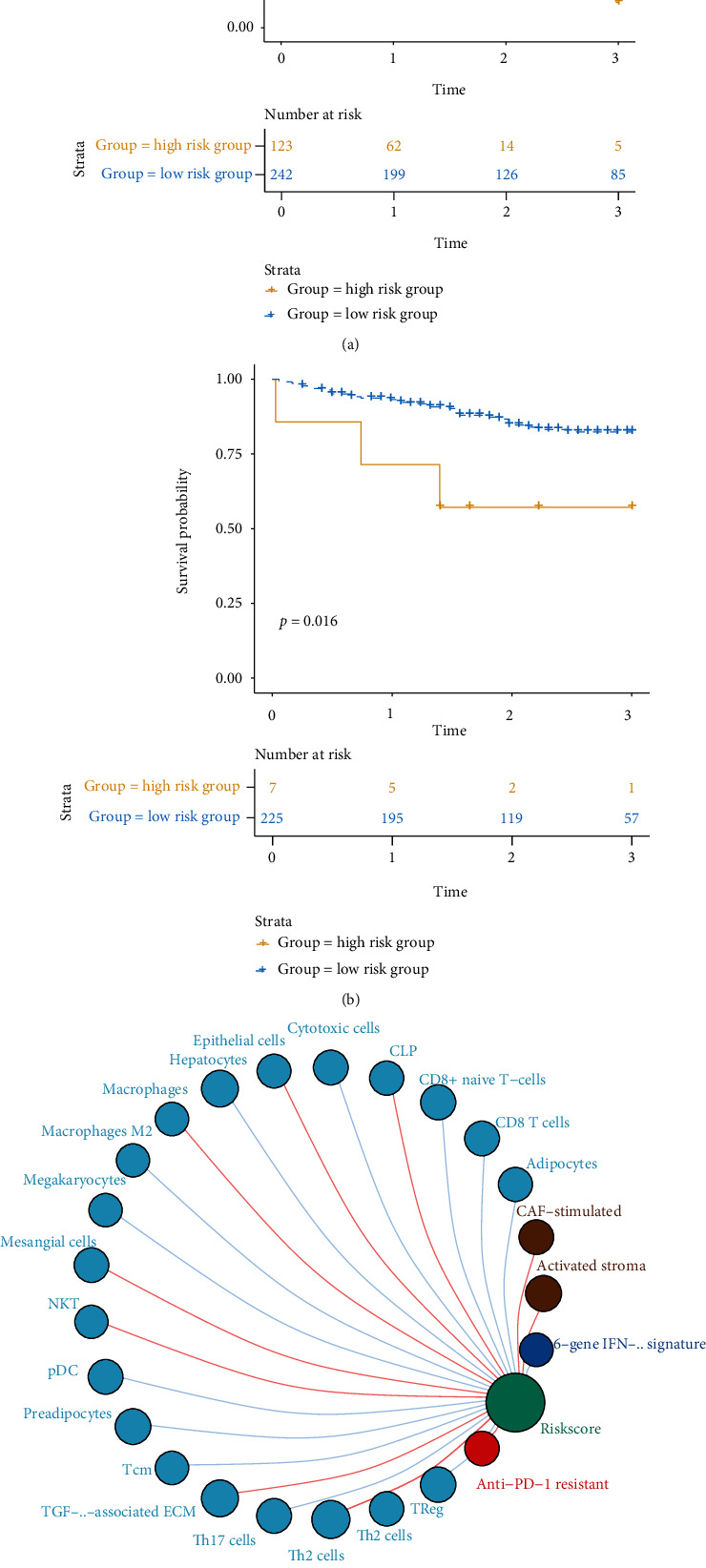
Prognostic prediction model based on signatures of tumor microenvironment. (a) Kaplan-Meier survival curve of the high- and low-risk groups in TCGA training set. The horizontal axis represents time (unit: year); the vertical axis represents survival probability. The low-risk group (blue line) presented a high three-year survival probability (35.12%); however, the high-risk group (yellow) presented a low three-year survival rate (4.07%). (b) Kaplan-Meier survival curve of the high- and low-risk groups in the ICGC testing set. The low-risk group (blue line) presented a high three-year survival probability (25.33%); however, the high-risk group (yellow) presented a low three-year survival rate (14.29%). (c). Immune cells associated with the risk index of TCGA patients. The red line indicates a positive correlation between the risk index and immune cells, and the gray line indicates a negative correlation between the risk index and immune cells. The size of the circle indicates different correlation coefficients, and the larger the area of the circle, the larger the correlation coefficient.

In order to clarify the molecular markers related to the prognosis of HCC patients, we screened the characteristic genes of immune subtypes, combined with the random forest algorithm to construct a predictive model. We took TCGA data as the training set and filter to the following signatures. At the same time, the risk coefficient (*β* value) of Cox multiple regression is introduced to predict the risk coefficient of each patient. We calculated the risk score (risk score) of each patient based on the expression of the 96-gene panel and the multiple Cox regression coefficient (Table 3). These 96 genes were enriched in the calcium signaling pathway and neuroactive ligand-receptor interaction pathway, which have been known to be involved in the HCC. The risk index is used to analyze its relationship with patient survival and to draw the K-M survival curve (Figure 4(a)). Similarly, we use the patient's risk index to verify in the test set of the ICGC database (Figure 4(b)).

Based on the patient's immune subtypes and differences in survival, we want to know which immune cells are related to the patient's disease risk. Therefore, by establishing the correlation between the patient's risk index and immune cells in TCGA cohort, and taking *p* < 0.05 as the significant correlation, the immune cells related to the patient's disease risk were screened out (Figure 4(c)).

## 3. Discussion

The HCC ecosystem, which is mainly composed of tumor cells and immune cells, is complex and dynamic. Due to drug resistance or immune escape, the heterogeneity at all levels from single cells to lesions reduces the therapeutic effect [27]. In the past decade, many efforts have been made to use multiregional and high-throughput analysis to study intratumoral heterogeneity [28–30]. Most studies focus on the genetic changes of HCC cells; this study is an attempt to use computational biology to study the immune-related heterogeneity of HCC at the genomic level. In this study, we tried to construct its correlation with survival prognosis based on the patient's immunotype and the differential genes screened. Our findings confirm that the prognostic survival of A-IS is significantly higher than that of N-IS. These findings are consistent with existing studies, namely, other microenvironmental factors (for example, angiogenesis and extracellular matrix contribute more immune heterogeneity) [27]. Therefore, intervention in the immune status of the HCC microenvironment may be a suitable strategy, because such treatments may affect all lesions of the individual and may also be applicable for a group of patients. More importantly, many new tools for immunotherapy have been developed and improved. In addition, through comprehensive analysis, we have observed that some immune cells are significantly related to patient classification and disease risk, providing a comprehensive new understanding of immunophenotyping and risk prediction, and proposed possible targets for intervention in HCC.

Enhancing host immunity may be beneficial to the cure of cancer. Researchers found infiltrating T cells in HCC and discovered the enrichment of Treg cells and the depletion of CD8+ T cells [31]. Studies have confirmed the enrichment of immunosuppressive cells in patients with HCC [32, 33]. Researchers revealed significant differences between immune cells infiltrating HCC [34]. The similarity of the immune microenvironment of some HCC patients not only facilitates classification but also facilitates the implementation of personalized treatment. In this case, according to our new classification scheme, HCC patients can be divided into three subtypes. More importantly, we use an independent cohort of HCC patients to confirm the classification results. Although there is significant heterogeneity in the immune status among patients, the three subtypes of HCC are clearly identified, indicating that this classification method can be applied to the HCC patient population. Patients with N-IS subtype generally had normal lymphocyte infiltration, but some patients have abundant expression of immune-related genes. The upregulation of features included expanded immune signature [35], T cell-inflamed gene expression profile (GEP) [36], and immune enrichment score [37]. T cell inflammation gene expression profile (GEP) contains genes related to antigen presentation, chemokine expression, cytotoxic activity, and adaptive immune resistance [35]. They can divide cancer into different subgroups and correspond to corresponding biological patterns. Capturing immune-related feature sets can provide accurate reference for reasonable construction and evaluation of treatment plans [36]. Non-IS is like a “cold” tumor, with almost no enrichment of immune- and matrix-related molecules. For such patients, combination therapy is more effective [38–40]. Enhanced T cell trafficking or suppression of inhibitory MDSC may increase the response of these HCC patients to immune checkpoint inhibitors [41, 42].

The three HCC subtypes we identified represent the clinical situation of human patients. A-IS subtype patients have relatively strong immune enrichment of stromal cell activation, although in pancreatic ductal adenocarcinoma, compared with patients with normal stromal subtype (N-IS), patients with activated stromal subtype samples (A-IS) have a worse survival [43]. But in HCC patients, the situation is different; that is, the 3-year survival performance of patients with an activated stroma subtype is better than that of patients with a normal stroma subtype (Figure 3(a)). However, the 5-year survival of different patient subgroups did not differ significantly (Figure 3(b)).

The current WHO classification of HCC highlights subtypes with stromal characteristics [44] which include lymphocyte-rich HCC. It is featured by lymphocyte infiltration into tumor and related to a better prognosis notably [45]. Studies have shown that different tumor subtypes have different types of immune microenvironments [46, 47] usually related to intratumoral heterogeneity [48]. The composition of the tumor immune microenvironment has been analyzed by methods such as gene expression analysis, single-cell RNA sequencing, and flow cytometry analysis [16, 31, 48–50]. In liver cancer, studies have shown that the number of immune cell infiltration, especially cytotoxic T cells [51–53], and the molecular classification of the immune microenvironment have clinicopathological significance [16, 48, 54]. In our study, the stroma activation of immune activity can indeed divide HCC patients into three subgroups (Figure 1(c)), and it is significantly associated with individuals' survival (Figure 3(a)).

## 4. Methods

### 4.1. Project and Sample

Data sets of 371 liver hepatocellular carcinoma donors were downloaded from TCGA database with detailed clinical information (https://xenabrowser.net/datapages/?dataset=TCGA-LIHC). The independent data sets used for verification come from the GSE144269 data set (*n* = 70) (https://www.ncbi.nlm.nih.gov/geo/query/acc.cgi?acc=GSE144269), the GSE14520_cohort1_test data set (*n* = 22), the GSE14520_cohort2_train samples (*n* = 225) (https://www.ncbi.nlm.nih.gov/geo/query/acc.cgi?acc=GSE14520), the GSE25097_GPL10687 samples (*n* = 268) (https://www.ncbi.nlm.nih.gov/geo/query/acc.cgi?acc=GSE25097), the GSE36376_GPL10558 (*n* = 240) (https://www.ncbi.nlm.nih.gov/geo/query/acc.cgi?acc=GSE36376) data set, and ICGC_LIRI_JP samples (*n* = 232) (https://dcc.icgc.org/projects/LIRI-JP).

### 4.2. Bioinformatics Analysis

1. ssGSEA algorithm: use the R package “GSVA (version 1.30.0),” and use ssGSEA to explore the HCC expression profile data of TCGA-LIHC cohort, and analyze the immune enrichment of each patient's tumor microenvironment. According to the immune enrichment status and stroma status of HCC samples, they are divided into A-IS, N-IS, and non-IS subtypes. According to the ssGSEA score obtained by each sample, the spectral clustering algorithm is used to extract different classifications. In addition, the R package “limma (version 3.41.18)” was used to analyze immunoenriched and non-immune-enriched patients, as well as the significantly different genes of stromal cell enrichment and nonmatrix enrichment, and *p* < 0.05 was taken as the significant difference
2. The unsupervised clustering of the data set was performed mainly based on tSNE which is embedded in *t*-distributed random neighborhoods [55]. In this study, we use tSNE to show the different subgroups of TCGA-LIHC cohort
3. We performed Kaplan-Meier survival analysis on the samples and plotted survival curves. Survival analysis divided the samples into high-index groups and low-index groups based on the median. Data visualization is mainly done in the R environment (version 4.1.0). Kaplan-Meier survival analysis relies on the use of the “survival (version 3.1-8)” package. The ROC curve is drawn based on the “survivalROC (version 1.0.3)” package
4. Prognosis prediction model establishment process: (a) use the training set to perform unit Cox regression on each gene to initially screen disease-related genes; (b) after obtaining all Cox significant genes in all units, perform 1000X LASSO regression to calculate the frequency of each gene and rank it; (c) according to the sorting result of the previous step, build the gene set incrementally. Use each gene set to perform multiple Cox regression to get the contribution of each gene; (d) obtain the optimal gene set according to the gene contribution degree, and perform multiple Cox regression analysis on these genes. Finally, we determined the regression coefficient of each gene; (e) calculate the death risk score of each patient through regression coefficients; (f) the death risk score model is tested in the training set (comparing the predicted situation with the actual situation); (g) the same model is tested in the independent testing set at the beginning (comparison of the predicted situation with the actual situation)
5. Construct the optimal multivariate Cox model based on the LASSO algorithm. This analysis uses the LASSO algorithm for gene screening: in the field of statistics and machine learning, LASSO algorithm (least absolute shrinkage and selection operator, also translated as minimum absolute shrinkage and selection operator, LASSO algorithm) is a regression analysis method that simultaneously performs feature selection and regularization (mathematics). It is aimed at enhancing the predictive accuracy and interpretability of statistical models. LASSO adopts the linear regression method of L1-regularization, so that the weight of some learned features is 0, so as to achieve the purpose of sparseness, selection of variables, and construction of the best model. The characteristic of LASSO regression is to perform variable selection and regularization while fitting a generalized linear model. Therefore, regardless of whether the target dependent variable (dependent/response variable) is continuous, binary, or discrete, it can be modeled by LASSO regression and then predicted
6. We use the random forest algorithm to select the best gene model based on the Cox multiple regression model and finally draw the unit Cox regression model forest diagram based on the gene panel as follows: we calculate the risk score (risk score) of each patient based on the expression of the gene panel and the multiple regression coefficient. The formula is as follows:

(1) $$\begin{array}{c} \text{Risk score}=\overset{n}{\underset{i=1}{\sum }}\beta_{i}*x_{i}. \end{array}$$

where *x*_*i*_ represents the expression level of each gene in the panel and *β*_*i*_ is the multivariate Cox regression beta value (multi_beta) corresponding to each gene

## Figures and Tables

**Table 1:** Immune-related signatures and references.

Signature name	Reference
Immune enrichment score	Yoshihara et al. Nat Commun. 2013 [37]
6-gene IFN-*γ* signature	Chow et al. J Clin Oncol. 2016 (suppl) [56]
Activated stroma	Moffitt et al. Nat Genet. 2015 [43]
Immune cell subsets	Cancer Genome Atlas Network. Cell. 2015 [57]
T cells	Bindea et al. Immunity. 2013 [58]
CD8 T cells	Bindea et al. Immunity. 2013 [58]
T. NK. metagene	Alistar et al. Genome Med. 2014 [59]
B-cell cluster	Iglesia et al. Clin Cancer Res. 2014 [60]
Macrophages	Bindea et al. Immunity. 2013 [58]
Cytotoxic cells	Bindea et al. Immunity. 2013 [58]
Immunophenoscore	Charoentong et al. Cell Rep. 2017 [61]
T cell-inflamed GEP	Cristescu et al. Science. 2018 [36]
Expanded immune signature	Ayers et al. J Clin Invest. 2017 [62]
TGF-*β*-associated ECM	Chakravarthy et al. Nat Commun. 2018 [35]
MDSC	Yaddanapudi et al. Cancer Immunol Res. 2016 [63]
CAF	Calon et al. Cancer Cell. 2012 [64]
TAM M2/M1	Beyer et al. PLoS One. 2012 [65]
CD8 T cell exhaustion	Giordano et al. EMBO J. 2015 [66]
T cell exhaustion early/late stage	Philip et al. Nature 2017 [67]
Nivolumab responsive	Riaz et al. Cell. 2017 [68]

**Table 2:** Clinical index of three subtypes.

		Stroma				
	Level	A-IS	N-IS	Non-IS	*p*	Test
*n*		16	16	38		
Adjacent_hepatic_tissue_inflammation_extent_type (%)		2 (12.5)	6 (37.5)	7 (18.4)	5.60*E*-01	Fisher_exact
	Mild	7 (43.8)	4 (25.0)	12 (31.6)		
	None	7 (43.8)	6 (37.5)	16 (42.1)		
	Severe	0 (0.0)	0 (0.0)	3 (7.9)		
Age_at_initial_pathologic_diagnosis (median [IQR])		61.50 [57.75, 67.25]	64.00 [56.00, 69.50]	64.50 [56.25, 72.00]	8.47*E*-01	Nonnorm
Albumin_result_lower_limit (median [IQR])		3.50 [3.48, 3.50]	3.50 [3.38, 3.50]	3.50 [3.50, 3.50]	3.74*E*-01	Nonnorm
Albumin_result_specified_value (median [IQR])		4.05 [3.77, 4.53]	4.00 [3.58, 4.32]	4.05 [3.50, 4.50]	7.34*E*-01	Nonnorm
Albumin_result_upper_limit (median [IQR])		5.00 [4.95, 5.00]	5.00 [5.00, 5.00]	5.00 [5.00, 5.00]	3.69*E*-01	Nonnorm
Bilirubin_lower_limit (median [IQR])		0.10 [0.10, 0.20]	0.20 [0.10, 0.20]	0.10 [0.10, 0.20]	2.08*E*-01	Nonnorm
Bilirubin_upper_limit (median [IQR])		0.70 [0.67, 0.93]	0.65 [0.38, 1.00]	0.60 [0.50, 0.90]	3.07*E*-01	Nonnorm
Cancer_first_degree_relative (median [IQR])		1.00 [1.00, 1.00]	1.00 [1.00, 2.00]	1.00 [1.00, 2.00]	2.34*E*-01	Nonnorm
Child_Pugh_classification_grade (%)		2 (12.5)	5 (31.2)	5 (13.2)	2.35*E*-01	Fisher_exact
	A	12 (75.0)	8 (50.0)	29 (76.3)		
	B	1 (6.2)	3 (18.8)	4 (10.5)		
	C	1 (6.2)	0 (0.0)	0 (0.0)		
Creatinine_lower_level (median [IQR])		0.60 [0.50, 0.80]	0.70 [0.60, 0.80]	0.70 [0.60, 0.80]	3.51*E*-01	Nonnorm
Creatinine_upper_limit (median [IQR])		1.30 [1.20, 1.40]	1.40 [1.20, 1.50]	1.25 [1.10, 1.40]	2.32*E*-01	Nonnorm
Creatinine_value_in_mg_dl (median [IQR])		0.80 [0.70, 1.00]	0.90 [0.75, 1.00]	0.95 [0.80, 1.10]	2.71*E*-01	Nonnorm
Inter_norm_ratio_lower_limit (median [IQR])		4.60 [0.88, 8.40]	8.30 [0.97, 8.33]	8.30 [0.90, 8.40]	8.18*E*-01	Nonnorm
Neoplasm_histologic_grade (%)	G1	3 (18.8)	0 (0.0)	7 (18.4)	4.14*E*-02	Fisher_exact
	G2	10 (62.5)	9 (56.2)	26 (68.4)		
	G3	2 (12.5)	7 (43.8)	3 (7.9)		
	G4	1 (6.2)	0 (0.0)	2 (5.3)		
Pathologic_M (%)	M0	7 (43.8)	10 (62.5)	29 (76.3)	7.96*E*-02	Fisher_exact
	M1	0 (0.0)	0 (0.0)	1 (2.6)		
	MX	9 (56.2)	6 (37.5)	8 (21.1)		
Pathologic_N (%)	N0	8 (50.0)	10 (62.5)	27 (71.1)	2.48*E*-01	Fisher_exact
	N1	0 (0.0)	1 (6.2)	0 (0.0)		
	NX	8 (50.0)	5 (31.2)	11 (28.9)		
Person_neoplasm_cancer_status (%)		6 (37.5)	6 (37.5)	11 (28.9)	8.52*E*-01	
	TUMOR FREE	5 (31.2)	5 (31.2)	17 (44.7)		
	WITH TUMOR	5 (31.2)	5 (31.2)	10 (26.3)		
Platelet_result_count (median [IQR])		196.00 [174.75, 232.00]	232.50 [206.25, 308.50]	206.50 [164.75, 263.25]	2.38*E*-01	Nonnorm
Prothrombin_time_result_value (median [IQR])		5.15 [1.10, 9.88]	9.40 [1.15, 10.33]	8.85 [1.02, 10.97]	8.37*E*-01	Nonnorm
Relative_family_cancer_history (%)	NO	3 (18.8)	2 (12.5)	2 (5.3)	3.28*E*-01	Fisher_exact
	YES	13 (81.2)	14 (87.5)	36 (94.7)		
Vascular_tumor_cell_type (%)		1 (6.2)	4 (25.0)	2 (5.3)	1.42*E*-02	Fisher_exact
	Macro	2 (12.5)	3 (18.8)	1 (2.6)		
	Micro	0 (0.0)	2 (12.5)	9 (23.7)		
	None	13 (81.2)	7 (43.8)	26 (68.4)		
Weight (median [IQR])		86.00 [69.00, 102.00]	72.00 [63.50, 82.50]	70.00 [61.00, 92.50]	2.09*E*-01	Nonnorm
Gender.demographic (%)	Female	3 (18.8)	11 (68.8)	15 (39.5)	1.57*E*-02	Fisher_exact
	Male	13 (81.2)	5 (31.2)	23 (60.5)		
Race.demographic (%)	Asian	2 (12.5)	1 (6.2)	10 (26.3)	3.11*E*-01	Fisher_exact
	Black or African American	0 (0.0)	2 (12.5)	2 (5.3)		
	Not reported	1 (6.2)	0 (0.0)	3 (7.9)		
	White	13 (81.2)	13 (81.2)	23 (60.5)		
Vital_status.demographic (%)	Alive	11 (68.8)	7 (43.8)	23 (60.5)	3.34*E*-01	
	Dead	5 (31.2)	9 (56.2)	15 (39.5)		
BMI.exposures (median [IQR])		29.20 [25.64, 31.92]	27.49 [24.29, 32.87]	23.74 [21.03, 30.07]	1.63*E*-01	Nonnorm

**Table 3:** Genes in the signature of survival prediction model.

Features	Multi_beta	Multi_HR	Multi_95%_CI_for_HR	Multi_*p*.value
ACRV1	0.284056	1.32851	0.974712-1.81072	0.0722009
AXDND1	0.666513	1.94743	1.493-2.54019	8.83*E*-07
B3GALT2	-1.88552	0.15175	0.0940178-0.244934	1.17*E*-14
ATP6V0D2	-0.266228	0.766265	0.628065-0.934874	0.00869992
ACPT	-0.532479	0.587148	0.465202-0.74106	7.36*E*-06
BRDT	0.890815	2.43711	1.7581-3.37837	8.98*E*-08
C10orf90	-0.321027	0.725404	0.590832-0.890626	0.00216696
BCO2	-0.606966	0.545002	0.369291-0.804318	0.00223885
ADAM32	0.740927	2.09788	1.61513-2.72492	2.81*E*-08
APOC4	-0.44861	0.638515	0.492737-0.827422	0.000692331
BSND	0.177579	1.19432	0.979831-1.45577	0.0787091
C12orf56	0.651933	1.91925	1.47251-2.50152	1.42*E*-06
C3orf36	-0.51696	0.596331	0.471639-0.753988	1.57*E*-05
CCNJL	0.807457	2.2422	1.47032-3.41929	0.000176535
DRD1	-0.731418	0.481226	0.373178-0.620558	1.72*E*-08
BAI2	1.06687	2.90626	1.99042-4.24349	3.31*E*-08
ERMN	-1.53079	0.216365	0.146571-0.319394	1.32*E*-14
ADAM12	3.4097	30.2563	12.0407-76.0287	4.08*E*-13
ADRA1A	-0.520536	0.594202	0.462577-0.763281	4.62*E*-05
GPR17	0.60065	1.8233	1.45229-2.2891	2.29*E*-07
HOXD10	0.174129	1.19021	0.989576-1.43152	0.0644995
C6orf223	-0.451106	0.636924	0.515442-0.787036	2.94*E*-05
SPAG6	-0.237856	0.788316	0.663102-0.937174	0.0070345
ACADL	-1.0514	0.349449	0.253158-0.482365	1.63*E*-10
CACNA1G	-0.410589	0.663259	0.532399-0.826285	0.000250567
CCDC36	0.37175	1.45027	1.14484-1.83719	0.00206295
CLEC2L	0.659235	1.93331	1.54772-2.41497	6.31*E*-09
CRISPLD1	-1.56237	0.209639	0.139785-0.314403	4.17*E*-14
FAM163B	0.347517	1.41555	1.09563-1.82888	0.00784528
HAVCR1	-0.281462	0.754679	0.62655-0.909011	0.0030281
MAMDC2	-1.1539	0.315405	0.204155-0.487279	2.00*E*-07
SFTPD	0.383284	1.46709	1.16916-1.84095	0.000934926
TKTL1	0.302278	1.35294	1.11067-1.64806	0.0026775
PPP2R2C	-0.546543	0.578948	0.46423-0.722013	1.23*E*-06
RTL1	0.444023	1.55897	1.22861-1.97815	0.000257703
TMC2	0.228553	1.25678	1.00585-1.57031	0.0442939
CYP19A1	0.410575	1.50768	1.23172-1.84548	6.88*E*-05
EPO	0.440563	1.55358	1.24005-1.94638	0.000127753
NKPD1	-0.297478	0.742689	0.575869-0.957833	0.0219126
SLC4A10	0.390634	1.47792	1.1312-1.9309	0.00418663
C15orf43	0.345474	1.41266	1.13902-1.75205	0.00166174
CLDN18	-0.545319	0.579657	0.462226-0.726921	2.34*E*-06
DPYSL4	1.17553	3.23985	2.29182-4.58003	2.82*E*-11
GNG4	0.383981	1.46812	1.16163-1.85547	0.0013088
GPM6A	0.777221	2.17542	1.57699-3.00094	2.19*E*-06
GPR18	-0.660017	0.516843	0.358506-0.74511	0.000405541
MYOCD	-0.409451	0.664015	0.474862-0.928514	0.0166865
NAV2.AS4	0.368257	1.44521	1.16618-1.791	0.000766567
PGA5	0.648481	1.91263	1.4807-2.47057	6.85*E*-07
SLC35F3	0.229401	1.25785	1.07694-1.46914	0.00378439
SOX8	-0.482409	0.617294	0.443993-0.858238	0.00411509
CD79A	-2.0343	0.130772	0.073325-0.233227	5.52*E*-12
HOXC6	0.250183	1.28426	1.07384-1.53591	0.0061387
MAGEA10	-0.87019	0.418872	0.317794-0.552099	6.58*E*-10
NKAIN1	-0.316253	0.728875	0.602838-0.881263	0.00109518
NKX3.2	0.497165	1.64405	1.28246-2.1076	8.74*E*-05
POU3F2	-0.604137	0.546546	0.428564-0.697007	1.12*E*-06
PSAPL1	-0.189652	0.827247	0.692495-0.988221	0.0365659
RCOR2	-0.505268	0.603344	0.455672-0.798872	0.000418987
TRAT1	1.94154	6.96945	3.92159-12.3861	3.65*E*-11
UBASH3A	-2.19459	0.111404	0.0547494-0.226683	1.41*E*-09
CDH10	0.579573	1.78528	1.39972-2.27703	3.03*E*-06
CHRND	0.165047	1.17945	1.00318-1.38669	0.0456736
CLEC17A	-0.482991	0.616935	0.468195-0.812928	0.000600521
COL25A1	-0.198214	0.820195	0.673116-0.99941	0.0493201
COLEC10	0.563006	1.75594	1.27602-2.41638	0.000547644
CRHBP	1.5943	4.92489	2.96712-8.17444	6.97*E*-10
DHH	0.719224	2.05284	1.44977-2.90677	5.06*E*-05
FAM129C	0.676126	1.96625	1.41399-2.7342	5.84*E*-05
FAM72D	0.509919	1.66516	1.16518-2.37967	0.00512327
GABRQ	0.287478	1.33306	0.99848-1.77976	0.0512178
GPR182	-0.322767	0.724142	0.533903-0.982168	0.0379242
HOXD3	0.755311	2.12827	1.62083-2.79459	5.48*E*-08
IGJ	0.333382	1.39568	0.896479-2.17286	0.139915
MAGEA6	-0.372357	0.689108	0.565764-0.839343	0.000215221
MS4A1	0.565355	1.76007	1.26789-2.44331	0.00072941
OGN	-0.228948	0.79537	0.647752-0.976629	0.0288351
OR13A1	0.299903	1.34973	1.10913-1.64252	0.00275417
SAA2	1.17003	3.22208	2.18463-4.75219	3.60*E*-09
VCX3A	0.585063	1.7951	1.36651-2.35812	2.63*E*-05
DLX2	-0.2672	0.76552	0.621962-0.942213	0.0116782
GFRA3	-0.263969	0.767997	0.644168-0.91563	0.00325573
KIF5A	0.319303	1.37617	1.09699-1.7264	0.00577724
MEP1A	0.354055	1.42483	1.18877-1.70777	0.000127605
PAGE2	-0.463863	0.62885	0.504242-0.78425	3.84*E*-05
PANX3	0.720169	2.05478	1.47722-2.85816	1.89*E*-05
PIP5K1B	-1.29461	0.274003	0.190459-0.394195	3.03*E*-12
PNCK	-0.505915	0.602954	0.485959-0.748116	4.29*E*-06
PRICKLE1	2.16419	8.70756	4.44639-17.0524	2.77*E*-10
RGS6	-0.611302	0.542644	0.41516-0.709275	7.67*E*-06
RSPO3	-0.412661	0.661887	0.493666-0.88743	0.00581211
SLC22A8	-0.259751	0.771243	0.6545-0.908811	0.00192368
SLC30A8	-0.295976	0.743806	0.565241-0.978781	0.0345933
TCF24	-0.526444	0.590702	0.448147-0.778603	0.000187066
TDRD5	0.277834	1.32027	1.11272-1.56653	0.00145269
XCR1	0.29765	1.34669	1.01721-1.78289	0.0376024

## Data Availability

The data used to support the findings of this study are included within the article.

## Supplementary Materials

Supplementary 1 Table S1: significantly differently expressed genes between immune and nonimmune subtypes.

Supplementary 2 Table S2: significantly differently expressed genes between stroma and nonstroma subtypes.
