# Supplementary material for: Analysis of the Heterogeneity of the Tumor Microenvironment and the Prognosis and Immunotherapy Response of Different Immune Subtypes in Hepatocellular Carcinoma
Source: J Oncol. 2022 Mar 29;2022:1087399. doi: 10.1155/2022/1087399 (PMC8984740; doi:10.1155/2022/1087399)
Supplement: Supplementary 1 — Table S1: significantly differently expressed genes between immune and nonimmune subtypes. [file 1087399.f1.pdf]

ID	logFC	AveExpr	t	P.Value	adj.P.Val	B
SLC6A6	0.8734976	5.3274142	14.054276	2.94E-36	1.07E-31	71.507275
ELF4	0.5961357	5.1950877	13.945791	7.99E-36	1.46E-31	70.525734
DCXR	-0.476588	7.0634485	-13.50656	4.44E-34	5.40E-30	66.575907
LRRK1	0.5808448	4.9474092	13.037092	3.10E-32	2.83E-28	62.401005
COL8A2	1.4587944	4.1075556	12.993594	4.58E-32	2.88E-28	62.016833
NBEAL2	0.2535556	5.9773846	12.989668	4.74E-32	2.88E-28	61.982182
IGDCC4	2.2199653	2.9431629	12.705202	6.03E-31	3.14E-27	59.481878
ZDHHC13	0.6536147	5.0608112	12.508904	3.44E-30	1.57E-26	57.76895
TRERF1	0.5939206	4.7750127	12.470376	4.84E-30	1.96E-26	57.433984
SPIB	2.1588437	2.8373887	12.455457	5.52E-30	2.02E-26	57.30439
SEMA3C	1.9376286	3.6124706	12.433213	6.72E-30	2.10E-26	57.111285
ABR	0.4102681	5.7597451	12.430268	6.90E-30	2.10E-26	57.08573
SEMA4D	0.4899744	5.4152249	12.31927	1.83E-29	5.15E-26	56.1243
WNK2	2.2484402	4.1369567	12.254934	3.23E-29	8.42E-26	55.568664
ALOX5	0.774389	5.0739024	12.209099	4.83E-29	1.17E-25	55.173547
KIAA1211	2.3133358	2.7836355	12.161586	7.31E-29	1.67E-25	54.764622
B4GALNT4	2.7165639	2.2178888	12.106437	1.18E-28	2.54E-25	54.290815
KIF3C	0.5891062	4.9320989	12.07343	1.58E-28	3.20E-25	54.007683
FMNL1	0.4168636	5.6489251	12.035865	2.19E-28	4.21E-25	53.685853
PTPRE	0.4806158	5.3840731	11.965272	4.05E-28	7.39E-25	53.082235
FCHO1	0.9479662	4.3844646	11.949752	4.64E-28	8.06E-25	52.949733
RASA3	0.4698675	5.5064005	11.921594	5.92E-28	9.57E-25	52.709529
PNMA1	0.3406277	5.7489378	11.919478	6.03E-28	9.57E-25	52.691489
RFNG	-0.237067	6.5394379	-11.88374	8.22E-28	1.21E-24	52.387004
DHRS4L2	-0.31876	6.1264894	-11.88232	8.32E-28	1.21E-24	52.374922
GAS7	0.5538671	5.2489873	11.872412	9.06E-28	1.27E-24	52.29057
MAP3K12	0.3559059	5.0413637	11.865006	9.66E-28	1.31E-24	52.227548
MFAP2	1.8994117	3.8639565	11.855255	1.05E-27	1.37E-24	52.144602
CTNND2	3.1352206	2.9439715	11.834859	1.25E-27	1.58E-24	51.971196
HIRIP3	-0.254055	6.0474736	-11.79854	1.72E-27	2.01E-24	51.662729
DOCK8	0.5251581	5.5226403	11.798006	1.72E-27	2.01E-24	51.658209
TOB1-AS1	-0.462839	4.734	-11.79551	1.76E-27	2.01E-24	51.636992
GLI2	2.0059735	2.9018015	11.7577	2.44E-27	2.70E-24	51.316394
NALCN	2.4631591	2.7507286	11.717294	3.46E-27	3.71E-24	50.974261
TMEM51	0.5644974	5.2931063	11.693878	4.23E-27	4.41E-24	50.776228
F3	0.9669857	4.5752839	11.689229	4.40E-27	4.47E-24	50.736935
PTPLAD2	0.5863319	4.5533608	11.684	4.61E-27	4.55E-24	50.692748
ZNF469	0.8875525	4.2716186	11.662744	5.53E-27	5.29E-24	50.513207
PHLDB1	0.4390461	5.7149805	11.660302	5.65E-27	5.29E-24	50.492591
TOR4A	0.4723015	5.3547585	11.643617	6.52E-27	5.95E-24	50.351782
AF131215.2	2.0387832	1.8043012	11.639939	6.73E-27	5.99E-24	50.320755
DZIP1L	0.9754026	4.0564915	11.633006	7.14E-27	6.10E-24	50.262278
PELI1	0.3151174	5.8693825	11.632268	7.18E-27	6.10E-24	50.256055
AFAP1	0.4689122	5.4907145	11.629235	7.37E-27	6.12E-24	50.230476
CCS	-0.25353	6.447598	-11.60195	9.32E-27	7.56E-24	50.000502
MBOAT2	0.9575267	4.3703611	11.596372	9.77E-27	7.76E-24	49.953569
MTHFD2	0.5070049	5.2282051	11.591444	1.02E-26	7.92E-24	49.912071
PCYT2	-0.2625	6.8444454	-11.58506	1.08E-26	8.19E-24	49.858361
SCARA5	2.5473483	2.1158545	11.575633	1.17E-26	8.70E-24	49.778992
ASPSCR1	-0.306701	6.5422153	-11.55368	1.41E-26	1.03E-23	49.594345

ITPR3	0.9099379	5.2017048	11.547149	1.49E-26	1.07E-23	49.539469
NAT6	-0.283228	5.651432	-11.53654	1.63E-26	1.15E-23	49.450349
ORAI2	0.3702863	5.5144904	11.520745	1.87E-26	1.29E-23	49.317682
SFXN3	0.3442517	5.7690513	11.515834	1.95E-26	1.32E-23	49.276451
PNMA2	1.6438635	3.3390871	11.508017	2.08E-26	1.38E-23	49.210849
GFPT2	1.7329039	3.8167717	11.488215	2.46E-26	1.61E-23	49.044757
SLC25A36	0.5178841	5.1470611	11.48212	2.60E-26	1.66E-23	48.993662
IGHV3-72	2.5165025	1.0256572	11.436102	3.84E-26	2.42E-23	48.608285
WDFY4	0.6544767	4.9238219	11.428776	4.09E-26	2.53E-23	48.547007
CTBP2	0.4806938	5.3959146	11.40891	4.84E-26	2.95E-23	48.380915
CCR6	2.1496464	0.6929603	11.403673	5.06E-26	3.03E-23	48.337154
PTPN13	1.2268253	3.9951402	11.384579	5.96E-26	3.51E-23	48.177686
WNT10A	1.9267052	2.3270225	11.360427	7.31E-26	4.24E-23	47.976149
EVC2	1.5729699	3.9890108	11.34734	8.17E-26	4.66E-23	47.867029
WNT9A	2.2342623	2.1018413	11.335438	9.04E-26	5.08E-23	47.767845
NCK2	0.4419136	5.870449	11.333466	9.19E-26	5.08E-23	47.751419
HINT2	-0.285874	6.4194656	-11.32101	1.02E-25	5.57E-23	47.647705
CCDC88C	0.4384165	5.4660154	11.304576	1.17E-25	6.27E-23	47.51089
SH3BP1	0.4974129	5.4013601	11.303471	1.19E-25	6.27E-23	47.501694
LOXL1-AS1	1.8119279	2.7746574	11.300408	1.22E-25	6.34E-23	47.476213
PIP4K2A	0.2074556	6.0175244	11.272889	1.54E-25	7.89E-23	47.24742
SOD1	-0.258091	7.1820241	-11.26104	1.70E-25	8.60E-23	47.148968
KIAA1377	1.3579631	3.3472414	11.258356	1.74E-25	8.68E-23	47.126699
NAP1L3	1.8361245	2.9784103	11.253226	1.81E-25	8.88E-23	47.084097
PLEKHH2	1.2862246	4.0740723	11.252525	1.82E-25	8.88E-23	47.078282
STRA13	-0.289948	6.4037345	-11.249	1.88E-25	9.02E-23	47.049042
IGHV3-15	2.698898	2.5996098	11.244091	1.96E-25	9.27E-23	47.008273
ADAM12	1.2769046	4.129941	11.242715	1.98E-25	9.27E-23	46.996855
STMN3	0.6771351	5.0434271	11.233659	2.14E-25	9.88E-23	46.921717
BASP1	0.6892885	5.1794483	11.232066	2.17E-25	9.89E-23	46.908507
ADI1	-0.293201	7.0493227	-11.22873	2.23E-25	1.00E-22	46.880858
MARVELD1	0.5193557	5.400558	11.220652	2.39E-25	1.06E-22	46.813851
ZNF532	0.3904513	5.5955199	11.203364	2.76E-25	1.21E-22	46.670579
RP11-66B24	1.6742035	2.1450872	11.202873	2.77E-25	1.21E-22	46.666513
TNRC6C-AS1	0.646279	4.6024409	11.196783	2.92E-25	1.25E-22	46.616064
FAM101A	2.1972598	2.7868883	11.182412	3.30E-25	1.40E-22	46.497079
TLR10	1.8482431	2.7067452	11.173278	3.56E-25	1.49E-22	46.421499
VANGL2	1.4765375	3.737907	11.172049	3.60E-25	1.49E-22	46.411325
PLEKHB1	1.7348947	3.940896	11.165338	3.80E-25	1.56E-22	46.355813
GALNT7	0.6883427	4.719981	11.144179	4.55E-25	1.84E-22	46.180898
EPHB3	1.4435611	4.0382429	11.138177	4.78E-25	1.92E-22	46.131311
IGLV3-10	2.7357498	1.9499848	11.129165	5.16E-25	2.05E-22	46.056881
LINC00920	1.2012295	3.0199468	11.114578	5.83E-25	2.29E-22	45.936469
TEX264	-0.190644	6.5796319	-11.10422	6.36E-25	2.47E-22	45.851015
PHC1	0.5876377	4.6243037	11.102455	6.46E-25	2.48E-22	45.836456
LRRC45	-0.269701	6.2398794	-11.09969	6.61E-25	2.51E-22	45.81367
FGD3	0.4974823	5.2071276	11.097132	6.75E-25	2.54E-22	45.792558
OLFML3	0.6713488	5.3423636	11.09202	7.05E-25	2.62E-22	45.750408
XYLT1	0.7976661	4.3826123	11.088149	7.28E-25	2.68E-22	45.7185
FHOD3	2.1525885	3.3119362	11.086109	7.41E-25	2.70E-22	45.701688
KCNH2	2.2485235	2.4752553	11.076811	8.01E-25	2.89E-22	45.625075

SEMA4A	0.6020576	4.9282559	11.060323	9.19E-25	3.29E-22	45.489292
LPAR1	1.7111518	3.612721	11.058193	9.36E-25	3.32E-22	45.471752
RASAL1	2.2670755	2.8136676	11.055846	9.54E-25	3.35E-22	45.452442
POU2AF1	1.3442474	4.0042859	11.024706	1.24E-24	4.31E-22	45.196317
MRC2	0.5827354	5.5981389	11.013174	1.36E-24	4.70E-22	45.101561
RP11-266L9	-0.619181	4.7069514	-11.00181	1.50E-24	5.12E-22	45.008235
RP11-398K2	1.7003838	2.2276832	10.996558	1.57E-24	5.30E-22	44.965122
SLC25A10	-0.285648	6.5021972	-10.98401	1.74E-24	5.83E-22	44.86216
RAB31	0.3669872	5.7315308	10.963157	2.07E-24	6.88E-22	44.691162
FBLIM1	0.4058436	6.0003642	10.956722	2.19E-24	7.19E-22	44.638432
BEX4	0.484128	5.3605241	10.955475	2.21E-24	7.20E-22	44.62821
TAF4B	1.3568616	3.3327828	10.952249	2.27E-24	7.33E-22	44.601787
CLEC17A	1.8825578	0.233312	10.937758	2.56E-24	8.20E-22	44.483116
SIGLEC10	0.7394221	4.5853476	10.936424	2.59E-24	8.22E-22	44.472198
FAM102B	0.3490057	5.5341204	10.913951	3.12E-24	9.82E-22	44.288335
DSCAML1	2.2416223	2.0734553	10.911425	3.19E-24	9.95E-22	44.267677
GEM	0.8243962	5.1752051	10.904948	3.37E-24	1.04E-21	44.214726
CHD3	0.3598246	6.0730229	10.898287	3.56E-24	1.09E-21	44.160292
ENTPD3-AS1	-0.349421	5.3389024	-10.88233	4.06E-24	1.24E-21	44.029979
DHRS4L1	-0.847635	4.076449	-10.87881	4.18E-24	1.26E-21	44.001201
CISD3	-0.235081	6.6265981	-10.86586	4.66E-24	1.39E-21	43.895485
MFSD6	0.3816804	5.6456658	10.86501	4.69E-24	1.39E-21	43.888582
IGHV3-53	2.5097664	1.4221959	10.860808	4.86E-24	1.43E-21	43.854309
LAMP5	2.1740249	2.3443314	10.855099	5.09E-24	1.49E-21	43.807744
HAS2	2.1899593	2.5863199	10.828739	6.34E-24	1.83E-21	43.592922
GPBAR1	0.8920108	4.2457913	10.828128	6.37E-24	1.83E-21	43.587941
EHD3	0.4676166	5.2594239	10.817851	6.94E-24	1.97E-21	43.504264
CTD-2256P1	-0.456996	4.8061873	-10.81739	6.96E-24	1.97E-21	43.500497
IL17RD	1.1909288	3.6824453	10.815698	7.06E-24	1.98E-21	43.48674
IGHV3-11	2.6317376	2.261632	10.797997	8.18E-24	2.28E-21	43.342716
XXyac-YX65	1.8474976	1.4794371	10.789871	8.75E-24	2.42E-21	43.276642
GRHPR	-0.271282	6.9666001	-10.78863	8.84E-24	2.43E-21	43.266558
STMN2	2.903797	2.1587921	10.77091	1.02E-23	2.79E-21	43.122568
IQGAP1	0.2356197	6.2272801	10.766301	1.06E-23	2.87E-21	43.085141
LAMA1	2.1720448	2.2623031	10.763188	1.09E-23	2.93E-21	43.059866
TGFA	0.969767	4.7237963	10.756304	1.15E-23	3.08E-21	43.00398
RP11-86H7.	1.9801186	1.8569046	10.749542	1.22E-23	3.23E-21	42.949107
CUTA	-0.209492	6.8581632	-10.74881	1.23E-23	3.23E-21	42.943151
LTK	1.152783	3.7865796	10.741488	1.30E-23	3.40E-21	42.883773
RAB9B	1.6869986	2.2403315	10.729346	1.44E-23	3.73E-21	42.785315
CBR1	-0.3048	6.8754023	-10.71938	1.57E-23	4.03E-21	42.704583
KIF26B	1.0753042	4.4723638	10.713518	1.64E-23	4.20E-21	42.657063
C6orf106	-0.162196	6.9255404	-10.7125	1.66E-23	4.20E-21	42.64885
ANO9	1.0252411	4.6339903	10.709282	1.70E-23	4.27E-21	42.622757
C7orf55	-0.319454	5.8427287	-10.70902	1.71E-23	4.27E-21	42.620601
ARHGEF3	0.4021908	5.6735667	10.699033	1.85E-23	4.60E-21	42.539783
FAM105A	0.6688113	4.7448676	10.69532	1.91E-23	4.71E-21	42.509736
IGKV1-27	2.553554	1.794614	10.690907	1.98E-23	4.84E-21	42.474023
IGHV3-74	2.4201097	2.6619809	10.689966	2.00E-23	4.84E-21	42.466414
GCSAM	1.5350591	2.763932	10.689628	2.00E-23	4.84E-21	42.46368
RRNAD1	-0.180932	6.2813681	-10.68771	2.03E-23	4.88E-21	42.448121

PRSS22	2.4911144	1.3191379	10.686929	2.05E-23	4.88E-21	42.44184
PREX1	0.2721062	6.0874515	10.67859	2.19E-23	5.20E-21	42.374402
C3orf52	2.1901227	2.3190877	10.675074	2.26E-23	5.31E-21	42.345978
FXYD2	2.2065636	3.8592872	10.661855	2.52E-23	5.89E-21	42.239139
PPAPDC1A	2.389165	1.2451292	10.660765	2.54E-23	5.90E-21	42.230329
CILP2	1.9272366	2.9346195	10.653761	2.69E-23	6.21E-21	42.173759
DHRS4	-0.279782	6.1335149	-10.6489	2.80E-23	6.43E-21	42.134498
IGKV1-16	2.5686926	1.9471489	10.646381	2.86E-23	6.52E-21	42.114168
BCL11A	1.7016344	3.3017745	10.642938	2.94E-23	6.65E-21	42.086378
KCNG1	2.1954096	1.8384445	10.642434	2.95E-23	6.65E-21	42.08231
OCEL1	-0.260817	6.2977792	-10.63952	3.02E-23	6.77E-21	42.058775
CARD11	0.6609184	4.866946	10.630977	3.24E-23	7.22E-21	41.989854
IGHV3-48	2.508893	1.3440166	10.62944	3.28E-23	7.27E-21	41.977457
FZD1	0.4761789	5.3735616	10.626974	3.35E-23	7.37E-21	41.957571
NFIA	-0.237504	6.4240187	-10.62539	3.40E-23	7.42E-21	41.944828
BEND6	2.0904116	1.244919	10.612714	3.77E-23	8.19E-21	41.8426
PLEKHG4	1.0497222	4.276377	10.610071	3.85E-23	8.32E-21	41.821296
HPSE	0.6740213	4.5711339	10.608036	3.92E-23	8.41E-21	41.804898
ZNF430	0.819404	4.5068231	10.600567	4.16E-23	8.89E-21	41.74473
PRKAR2B	1.1567426	3.9303878	10.59628	4.31E-23	9.15E-21	41.710202
MAPK13	0.7205987	5.4842095	10.584567	4.75E-23	1.00E-20	41.615905
KCND1	0.6528269	4.1824713	10.581615	4.86E-23	1.02E-20	41.592146
FZD2	1.0388362	3.8443162	10.578145	5.00E-23	1.04E-20	41.564226
RCOR2	1.8773207	2.6907735	10.572727	5.23E-23	1.08E-20	41.520635
PLAGL1	0.7323838	4.6824044	10.56808	5.43E-23	1.12E-20	41.483267
LOXL1	0.9174644	4.8642463	10.56223	5.70E-23	1.17E-20	41.436229
CXorf21	0.9357703	3.7084283	10.558804	5.86E-23	1.20E-20	41.408685
TMEM51-AS1	2.0024669	1.9947184	10.555361	6.03E-23	1.22E-20	41.381011
ACOT13	-0.223	6.4480325	-10.54029	6.82E-23	1.38E-20	41.259978
SSC5D	0.9886535	4.4274762	10.533773	7.19E-23	1.44E-20	41.207615
YEATS2	0.1817936	5.924402	10.53335	7.22E-23	1.44E-20	41.204223
SPINT1	1.1065835	5.1951776	10.532631	7.26E-23	1.44E-20	41.19845
PTPN22	0.8524327	4.1528641	10.527451	7.57E-23	1.49E-20	41.156876
DNAJA4	0.597682	4.9924632	10.525545	7.69E-23	1.51E-20	41.141576
VCAN	0.8737283	5.6433891	10.515821	8.33E-23	1.63E-20	41.06357
NXPE3	0.527468	4.9452317	10.509973	8.74E-23	1.70E-20	41.016674
TNFSF15	1.0813613	4.2543175	10.5092	8.79E-23	1.70E-20	41.010468
TACO1	-0.214774	6.418636	-10.50463	9.13E-23	1.75E-20	40.973803
B3GNT8	0.9097075	3.8931637	10.498599	9.59E-23	1.83E-20	40.925499
ECI2	-0.238132	6.847319	-10.49707	9.71E-23	1.85E-20	40.91326
HAGH	-0.288529	6.7695767	-10.48841	1.04E-22	1.97E-20	40.843901
RP11-166P1	-0.41413	4.8191195	-10.4866	1.06E-22	1.99E-20	40.829357
PAPLN	0.8505061	5.2774535	10.465323	1.26E-22	2.35E-20	40.659073
ST3GAL3	-0.236098	5.874978	-10.4587	1.33E-22	2.47E-20	40.606095
RASSF2	0.3743387	5.4786191	10.452832	1.39E-22	2.58E-20	40.559175
TAGAP	0.75054	4.5507167	10.447478	1.45E-22	2.68E-20	40.516383
OSBPL7	0.4790988	5.0402498	10.442063	1.52E-22	2.78E-20	40.473105
TSC22D1-AS	1.6588851	1.4404913	10.441951	1.52E-22	2.78E-20	40.472216
PLA2G4A	1.2931761	3.5066447	10.434163	1.62E-22	2.94E-20	40.409995
APBA2	1.2156266	4.0202446	10.431115	1.66E-22	3.00E-20	40.385652
ESRP1	2.6237381	2.0050144	10.427955	1.70E-22	3.07E-20	40.360418

SYNGR3	1.8029045	2.6437272	10.412412	1.93E-22	3.46E-20	40.23637
ACYP2	-0.251419	5.795416	-10.40647	2.03E-22	3.62E-20	40.188954
CDCP1	1.0652457	4.3574449	10.404731	2.06E-22	3.65E-20	40.175103
GPS1	-0.165666	6.749174	-10.39785	2.18E-22	3.84E-20	40.120201
AMPD3	0.5140352	4.9378011	10.389529	2.33E-22	4.07E-20	40.053919
CSF3R	0.5633495	5.1036991	10.38944	2.33E-22	4.07E-20	40.053215
ATP2A3	0.4457633	5.6227581	10.375746	2.61E-22	4.53E-20	39.944128
CPZ	2.0245244	0.2323036	10.374347	2.64E-22	4.56E-20	39.93299
RING1	-0.16763	6.4490655	-10.36746	2.79E-22	4.79E-20	39.878166
ABI3BP	0.8532494	4.7325595	10.367032	2.80E-22	4.79E-20	39.874757
IGKV1-9	2.4712372	2.2683667	10.363844	2.87E-22	4.89E-20	39.849389
KLHL6	0.5522986	4.8487927	10.361484	2.92E-22	4.97E-20	39.830612
DENND3	0.3192139	5.524155	10.35135	3.18E-22	5.35E-20	39.750001
GPANK1	-0.20832	6.1669586	-10.35114	3.18E-22	5.35E-20	39.748297
SPACA6P	0.9142808	3.9183103	10.348907	3.24E-22	5.42E-20	39.730574
KMT2E-AS1	-0.379875	5.1859949	-10.34712	3.29E-22	5.48E-20	39.716352
PCNXL2	0.7378036	4.8603743	10.33695	3.57E-22	5.92E-20	39.635525
TMCC2	0.9289682	3.7769259	10.331919	3.72E-22	6.14E-20	39.595555
RP11-81701	-0.436096	4.7859251	-10.32407	3.96E-22	6.47E-20	39.533194
FRAS1	1.5918835	4.2206474	10.323947	3.96E-22	6.47E-20	39.532235
RP11-793H1	-0.486836	4.6719372	-10.32374	3.97E-22	6.47E-20	39.530604
PMAIP1	1.3642304	3.4197239	10.319388	4.11E-22	6.67E-20	39.496032
WIPF1	0.306482	5.9013341	10.311742	4.38E-22	7.07E-20	39.435347
IGLV3-9	2.3868203	1.1624733	10.307216	4.54E-22	7.30E-20	39.399433
ZNF287	1.1830622	3.5385581	10.300222	4.80E-22	7.69E-20	39.343954
3-Mar	0.6071847	4.7586576	10.297548	4.91E-22	7.82E-20	39.322751
TMC6	0.4103786	5.842262	10.292383	5.12E-22	8.12E-20	39.281794
IGLV3-1	2.4934469	2.456126	10.290683	5.19E-22	8.17E-20	39.268317
SEL1L3	0.5328655	5.9287757	10.290609	5.19E-22	8.17E-20	39.267732
CELF2	0.4590003	5.2835661	10.28899	5.26E-22	8.24E-20	39.254898
ZNF853	0.8819956	4.2214588	10.271476	6.06E-22	9.45E-20	39.116143
DMKN	2.0882361	3.8666132	10.250968	7.15E-22	1.11E-19	38.953839
EMILIN1	0.4928184	5.931997	10.246673	7.40E-22	1.14E-19	38.91987
LOXL3	0.4296613	4.6182226	10.236956	8.00E-22	1.23E-19	38.843045
TPM4	0.2089557	6.6833918	10.236542	8.03E-22	1.23E-19	38.839774
ADAM28	1.0464532	4.2420117	10.232777	8.27E-22	1.26E-19	38.810017
C11orf71	-0.269606	5.748897	-10.23208	8.32E-22	1.27E-19	38.804492
ENO2	0.6268658	4.9428815	10.226715	8.69E-22	1.32E-19	38.76212
MAP9	1.3806043	3.3962166	10.22493	8.81E-22	1.32E-19	38.748025
FNDC1	1.7388097	4.0972743	10.22451	8.84E-22	1.32E-19	38.744706
IGLV3-25	2.2416372	3.0761272	10.2245	8.84E-22	1.32E-19	38.744622
FGFR1	0.4573463	5.6082336	10.22139	9.07E-22	1.35E-19	38.720059
IGHV5-51	2.2896181	3.1669169	10.217487	9.36E-22	1.39E-19	38.689242
CERKL	0.4490415	5.0226692	10.214589	9.58E-22	1.42E-19	38.666364
TST	-0.273116	7.0188999	-10.20124	1.07E-21	1.57E-19	38.561042
FMNL3	0.2442822	5.8447405	10.193145	1.14E-21	1.67E-19	38.49717
AKR1A1	-0.197381	6.8648475	-10.19156	1.15E-21	1.68E-19	38.48465
TSPYL5	0.6547519	4.3476512	10.190757	1.16E-21	1.69E-19	38.47834
FBXO41	0.823327	4.3462432	10.179054	1.27E-21	1.84E-19	38.386103
BANK1	1.8088261	2.6352957	10.179037	1.27E-21	1.84E-19	38.385967
RP11-253E3	1.3066487	3.2646248	10.178312	1.28E-21	1.84E-19	38.380253

BMPR1B	2.1846912	1.4846926	10.17627	1.30E-21	1.87E-19	38.364168
STK17B	0.3858836	5.5533013	10.17543	1.31E-21	1.87E-19	38.357551
CCDC136	1.3516501	2.6896787	10.174779	1.32E-21	1.87E-19	38.352422
BTD	-0.255239	6.4218142	-10.17114	1.36E-21	1.92E-19	38.323737
TSHZ3	0.5697061	4.5901052	10.170833	1.36E-21	1.92E-19	38.321346
IGHV1-18	2.4133872	2.9445364	10.16988	1.37E-21	1.93E-19	38.313837
DUS1L	-0.181193	6.7721459	-10.16845	1.39E-21	1.94E-19	38.302551
HSD17B8	-0.315122	6.2474996	-10.16761	1.40E-21	1.95E-19	38.295942
BLVRB	-0.251158	6.7872319	-10.15843	1.50E-21	2.09E-19	38.223708
RAB36	1.3383217	3.3617488	10.155762	1.54E-21	2.12E-19	38.202695
IGHV1-24	2.4783526	1.8745709	10.153937	1.56E-21	2.15E-19	38.188339
LGI2	1.0320352	4.1156894	10.152493	1.58E-21	2.16E-19	38.176978
PRUNE2	1.022742	4.0461255	10.151894	1.58E-21	2.17E-19	38.172263
EBNA1BP2	-0.184449	6.4255736	-10.14817	1.63E-21	2.22E-19	38.142986
B3GNT7	0.8251779	4.4585027	10.145614	1.67E-21	2.26E-19	38.122864
PODN	1.0907033	4.974229	10.137074	1.78E-21	2.41E-19	38.055717
NRG3	2.1676382	1.3775987	10.13413	1.83E-21	2.46E-19	38.032572
GPR161	0.5123522	4.7663024	10.131578	1.86E-21	2.50E-19	38.012521
PTGIS	1.4520783	4.5233625	10.123667	1.99E-21	2.66E-19	37.950365
ECHDC2	-0.265259	6.7772208	-10.11689	2.10E-21	2.79E-19	37.897116
STARD10	-0.2667	6.9913433	-10.11665	2.10E-21	2.79E-19	37.895276
ARMC5	-0.299601	5.9752131	-10.11627	2.11E-21	2.79E-19	37.892274
POLDIP2	-0.148806	6.9176514	-10.11457	2.14E-21	2.82E-19	37.878924
MPP2	1.5152925	3.0182552	10.101677	2.37E-21	3.11E-19	37.777737
ABHD14B	-0.234108	6.7329484	-10.10084	2.38E-21	3.11E-19	37.771133
DUSP4	0.6572656	4.9609037	10.100797	2.39E-21	3.11E-19	37.770827
SLC28A3	2.2427845	1.0091069	10.097545	2.45E-21	3.18E-19	37.745315
FUT8	0.5102643	5.053506	10.095739	2.48E-21	3.22E-19	37.731156
IGKV2-24	2.3096365	1.5754585	10.089931	2.60E-21	3.36E-19	37.685611
PLXNA1	0.2809789	5.9695694	10.085022	2.71E-21	3.48E-19	37.647129
RP11-51F16	0.8627508	3.6251565	10.084577	2.72E-21	3.48E-19	37.643637
ATOX1	-0.263609	6.7300062	-10.07405	2.95E-21	3.77E-19	37.561112
ADAP1	0.7609398	4.6386667	10.069442	3.06E-21	3.90E-19	37.525055
RECQL5	-0.15774	6.187433	-10.06156	3.26E-21	4.14E-19	37.46334
DPEP1	2.2282627	2.7713002	10.060884	3.28E-21	4.14E-19	37.45805
IGHV3-73	2.4585973	1.3130155	10.055401	3.43E-21	4.31E-19	37.415138
PYGO1	1.7821755	2.4314708	10.055015	3.44E-21	4.31E-19	37.412119
CTD-2561J2	1.7100276	1.1621455	10.054606	3.45E-21	4.31E-19	37.408916
KIRREL	0.5024366	5.5042991	10.052039	3.52E-21	4.39E-19	37.388833
RCSD1	0.3821574	5.3785428	10.051412	3.54E-21	4.39E-19	37.383931
ROR1	1.6249672	3.6576001	10.049956	3.58E-21	4.43E-19	37.372535
P2RX5	1.6443742	3.1594497	10.04905	3.61E-21	4.44E-19	37.365448
IGHV4-59	2.3426284	3.0153054	10.048864	3.61E-21	4.44E-19	37.363995
GAPT	1.517781	2.7653429	10.039114	3.90E-21	4.78E-19	37.28775
COL5A1	0.5143597	6.1230605	10.038844	3.91E-21	4.78E-19	37.28564
NTRK1	1.6654635	1.3224969	10.038022	3.94E-21	4.79E-19	37.27921
MCOLN2	1.4713244	3.2817702	10.036515	3.98E-21	4.83E-19	37.267433
LRRC75A	0.8017625	3.8062232	10.032411	4.12E-21	4.98E-19	37.235355
DOCK10	0.5021959	5.2498641	10.02983	4.20E-21	5.06E-19	37.215187
LBH	0.2986738	6.0228048	10.022432	4.46E-21	5.35E-19	37.157386
MNDA	0.6406839	4.7127339	10.020542	4.53E-21	5.42E-19	37.142625

SLC4A3	1.6714286	3.3267194	10.014219	4.76E-21	5.68E-19	37.093257
C11orf63	1.0758983	3.3406274	10.013146	4.80E-21	5.71E-19	37.084882
TNFRSF8	1.2951034	2.9699812	10.006772	5.05E-21	5.98E-19	37.035126
TNFAIP6	1.9830272	2.1213992	10.004615	5.14E-21	6.07E-19	37.018299
RP11-834C1	0.9661889	4.1471915	10.00415	5.16E-21	6.07E-19	37.014669
SLC7A7	0.3958233	5.5321094	9.9981047	5.41E-21	6.35E-19	36.967507
NOL4L	0.2289599	5.8152647	9.9876268	5.88E-21	6.88E-19	36.885805
RP11-80901	1.8352796	2.2732895	9.9804769	6.22E-21	7.26E-19	36.83008
IGHV3-30	2.3474801	2.6976289	9.9767362	6.41E-21	7.45E-19	36.800936
HDAC7	0.2208114	6.1451876	9.9759135	6.45E-21	7.46E-19	36.794527
PDPN	2.2646652	2.7303012	9.9757629	6.46E-21	7.46E-19	36.793354
OBSCN	0.8181234	5.0102345	9.9710817	6.70E-21	7.72E-19	36.756893
CLIP3	0.4409215	5.1183783	9.9703027	6.75E-21	7.74E-19	36.750826
ITGA4	0.6803749	4.793911	9.9657306	7.00E-21	8.00E-19	36.715225
EMR2	0.7075543	4.3356755	9.9654872	7.01E-21	8.00E-19	36.71333
FAAH	-0.319536	6.1674309	-9.964518	7.06E-21	8.03E-19	36.705786
CCDC152	-0.405751	5.7546963	-9.958238	7.42E-21	8.42E-19	36.656905
RP11-219B1	-1.412222	2.4082789	-9.957707	7.46E-21	8.43E-19	36.652771
GABBR1	0.6320477	4.9315775	9.9572624	7.48E-21	8.43E-19	36.649312
SURF1	-0.202911	6.4667194	-9.956276	7.54E-21	8.47E-19	36.641636
ZSCAN16-AS	-0.324652	5.8798329	-9.955828	7.57E-21	8.47E-19	36.638152
SAMD14	0.5485939	4.3036486	9.9555261	7.59E-21	8.47E-19	36.635801
EEFSEC	-0.170019	6.199649	-9.954602	7.64E-21	8.50E-19	36.628613
IGKV3-15	2.3235519	3.0472917	9.9540104	7.68E-21	8.52E-19	36.624008
IGHV2-26	2.3171145	0.878652	9.9530524	7.74E-21	8.56E-19	36.616555
CERCAM	0.6043647	5.2770812	9.9468101	8.13E-21	8.95E-19	36.568
AP006621.5	-0.402736	5.3836731	-9.946338	8.16E-21	8.95E-19	36.564327
GS1-44D20.	1.6087729	1.6419052	9.946303	8.16E-21	8.95E-19	36.564056
BOLA1	-0.213565	6.0366804	-9.942983	8.38E-21	9.16E-19	36.538236
MMP2	0.5504453	5.9344913	9.9372026	8.77E-21	9.56E-19	36.493302
RP13-650J1	-1.284325	4.4730834	-9.93565	8.88E-21	9.65E-19	36.481236
GPR68	0.7691553	4.1887635	9.926262	9.57E-21	1.04E-18	36.408292
HS6ST2	2.4243029	1.5772803	9.9233651	9.79E-21	1.06E-18	36.385791
PLA2R1	1.033564	3.9979944	9.9226761	9.84E-21	1.06E-18	36.38044
GALNT12	1.579178	3.1241021	9.921177	9.96E-21	1.07E-18	36.368798
AMPH	1.6874128	2.3149047	9.9201434	1.00E-20	1.07E-18	36.360772
COL16A1	0.8566682	5.2420388	9.9200613	1.00E-20	1.07E-18	36.360135
CCR4	1.6716245	2.87251	9.9191806	1.01E-20	1.08E-18	36.353296
MMEL1	1.7654364	1.9434375	9.9148493	1.05E-20	1.11E-18	36.319669
LRRN3	1.6656819	1.9242182	9.9056835	1.13E-20	1.19E-18	36.248537
ADAMTS3	1.7642233	2.7452265	9.9038962	1.14E-20	1.20E-18	36.234671
DDX26B	0.6991736	4.435885	9.9014264	1.16E-20	1.22E-18	36.215513
PROM1	2.4212313	2.3030364	9.8957356	1.22E-20	1.28E-18	36.171379
CEP135	0.3866187	4.9356572	9.8946946	1.23E-20	1.28E-18	36.163307
PMVK	-0.2133	6.6293286	-9.893071	1.24E-20	1.30E-18	36.150723
EPS8L1	1.1496677	4.0391793	9.8925207	1.25E-20	1.30E-18	36.146453
COL3A1	0.4101999	6.7445642	9.8898265	1.28E-20	1.32E-18	36.125567
KLHDC8A	2.0752624	1.9017457	9.8891059	1.28E-20	1.33E-18	36.119982
CD22	0.9760593	4.2061481	9.874007	1.45E-20	1.49E-18	36.003001
CBL	0.2697353	5.6827348	9.8688299	1.51E-20	1.55E-18	35.962915
CTTNBP2NL	0.2489011	5.5889112	9.8670432	1.53E-20	1.57E-18	35.949083

RP1-47M23.	1.6547702	2.1707597	9.8666169	1.53E-20	1.57E-18	35.945783
IGLV4-69	2.3363811	2.3479996	9.8607922	1.60E-20	1.64E-18	35.900703
CCDC101	-0.219057	5.9936721	-9.859226	1.62E-20	1.65E-18	35.888584
IGLV2-11	2.2433114	3.0489628	9.8589609	1.63E-20	1.65E-18	35.886533
INPP4A	0.1750267	5.7816433	9.8586174	1.63E-20	1.65E-18	35.883875
BOLA3-AS1	1.1443835	2.8998308	9.8547283	1.68E-20	1.70E-18	35.853788
BTG2	0.3089916	6.1334883	9.8523043	1.72E-20	1.73E-18	35.835038
ZDBF2	1.2596861	4.1650793	9.8495588	1.75E-20	1.76E-18	35.813805
UBASH3B	0.5761721	4.8452763	9.8430379	1.85E-20	1.85E-18	35.763389
PNMAL1	2.0932659	2.491187	9.8413789	1.87E-20	1.87E-18	35.750565
CCDC53	-0.227811	5.9970361	-9.833783	1.99E-20	1.97E-18	35.691863
ZNF691	-0.186499	5.6985216	-9.833157	2.00E-20	1.98E-18	35.687027
MED18	-0.168683	5.9033555	-9.825745	2.12E-20	2.09E-18	35.629784
GPR132	0.8471495	4.2330645	9.8229473	2.16E-20	2.13E-18	35.608178
ARL4C	0.3712475	5.8981583	9.8226012	2.17E-20	2.13E-18	35.605506
PRDM8	1.4638954	2.5776282	9.822324	2.17E-20	2.13E-18	35.603366
FENDRR	1.9261893	1.0232967	9.8190001	2.23E-20	2.18E-18	35.577705
PTK7	0.6980186	5.3727221	9.8186666	2.24E-20	2.18E-18	35.575131
HAPLN3	0.7860422	4.7481011	9.8149428	2.30E-20	2.24E-18	35.546391
HEXDC	-0.193396	6.1431577	-9.799201	2.61E-20	2.53E-18	35.42496
HSD17B10	-0.2105	6.7124556	-9.789769	2.81E-20	2.72E-18	35.35226
PEF1	-0.149404	6.4979061	-9.781837	2.99E-20	2.88E-18	35.291152
HDHD3	-0.207311	6.5200497	-9.77862	3.06E-20	2.95E-18	35.266378
SLMO1	1.1014411	3.1153407	9.7784654	3.07E-20	2.95E-18	35.265189
CADPS	2.2234782	1.2128331	9.7777079	3.09E-20	2.96E-18	35.259356
PEBP1	-0.222524	7.3995845	-9.771478	3.24E-20	3.10E-18	35.211397
PTGER4	0.5731131	4.8742033	9.7667558	3.36E-20	3.21E-18	35.175051
IGLV2-8	2.3130631	2.2054552	9.762943	3.46E-20	3.29E-18	35.145714
IDNK	-0.313438	5.818651	-9.759798	3.55E-20	3.37E-18	35.12152
RAP1GAP2	0.8365252	4.801956	9.7545547	3.70E-20	3.50E-18	35.081196
IGHV3-23	2.0803567	3.6407381	9.7523466	3.77E-20	3.55E-18	35.064218
ZEB2	0.3302569	5.6792494	9.7517087	3.78E-20	3.56E-18	35.059314
OSBPL3	0.3471306	5.5427884	9.7504672	3.82E-20	3.59E-18	35.049769
STXBP5	0.2818853	5.5711572	9.7455514	3.97E-20	3.72E-18	35.011984
KCNQ5	1.7694388	1.006111	9.7445996	4.00E-20	3.74E-18	35.004669
ADCY3	0.375973	5.3269171	9.7414164	4.10E-20	3.82E-18	34.980209
RUNX2	1.1000956	3.9551346	9.740616	4.13E-20	3.83E-18	34.974059
UQCRQ	-0.241544	6.9694929	-9.740267	4.14E-20	3.83E-18	34.971381
PEX11G	-0.394935	5.4951661	-9.738456	4.20E-20	3.88E-18	34.957464
ACBD4	-0.293889	6.3538681	-9.738159	4.21E-20	3.88E-18	34.955182
RELT	0.3901333	4.9670734	9.733625	4.36E-20	4.01E-18	34.920358
EPCAM	1.8362843	4.4392081	9.7331916	4.37E-20	4.01E-18	34.917031
COL11A1	2.3796074	2.3160439	9.7316684	4.43E-20	4.05E-18	34.905333
PSMC5	-0.161472	6.77613	-9.730898	4.45E-20	4.06E-18	34.899418
SLC16A9	1.89172	3.5927804	9.7259745	4.63E-20	4.21E-18	34.861618
MYOF	0.4077634	5.6780027	9.7221987	4.77E-20	4.33E-18	34.832637
IGHV1-58	2.050787	0.1994409	9.7170933	4.96E-20	4.49E-18	34.793461
IGKV4-1	2.0050173	4.0737316	9.7169439	4.97E-20	4.49E-18	34.792316
IGHV1-46	2.3219103	2.4686654	9.7096969	5.26E-20	4.74E-18	34.736728
SLC38A10	-0.148659	6.9553602	-9.707997	5.33E-20	4.79E-18	34.723692
PTGS2	1.3348321	3.3895854	9.706404	5.39E-20	4.84E-18	34.711479

DCAF8	-0.152139	6.6251924	-9.70599	5.41E-20	4.84E-18	34.708303
CTD-2575K1	1.8667025	0.5198833	9.7029881	5.54E-20	4.95E-18	34.685291
SVEP1	1.1541177	4.6753367	9.6926833	6.01E-20	5.35E-18	34.606324
ITGB8	1.9468894	3.018239	9.6914703	6.06E-20	5.38E-18	34.597031
CYBA	0.4051033	6.2187709	9.690219	6.12E-20	5.42E-18	34.587447
C12orf49	0.2740969	5.9993729	9.6883166	6.21E-20	5.49E-18	34.572876
TPRA1	-0.134702	6.3361254	-9.686531	6.30E-20	5.56E-18	34.559199
CR2	2.1210707	1.0301044	9.6850029	6.38E-20	5.61E-18	34.547499
CRMP1	0.8139157	4.9922895	9.6832798	6.46E-20	5.67E-18	34.534306
RLTPR	0.7286396	4.3647663	9.6829982	6.48E-20	5.67E-18	34.53215
DHRS3	-0.213424	6.948605	-9.682745	6.49E-20	5.67E-18	34.530211
CAMK4	0.7027657	4.1330522	9.6786284	6.70E-20	5.84E-18	34.498698
IGHV4-39	2.1364882	3.2568381	9.6765967	6.81E-20	5.92E-18	34.483148
P2RX1	1.0220242	3.5045316	9.6740646	6.94E-20	6.02E-18	34.463772
SFMBT2	0.4806233	4.9672998	9.6734701	6.98E-20	6.04E-18	34.459222
METTL7A	-0.245205	7.1329134	-9.669724	7.18E-20	6.20E-18	34.430561
ANK3	0.7771952	5.0698754	9.668985	7.23E-20	6.22E-18	34.424908
CALHM2	0.3629847	5.3111688	9.6671437	7.33E-20	6.30E-18	34.410823
WBP2	-0.14865	6.8379761	-9.663041	7.57E-20	6.49E-18	34.379447
COL9A2	0.798698	4.61383	9.6619344	7.63E-20	6.53E-18	34.370984
KIAA0226L	0.6825263	4.2793164	9.6614034	7.67E-20	6.54E-18	34.366925
PTGIR	0.6872335	4.6929067	9.6604667	7.72E-20	6.57E-18	34.359763
MTMR2	0.2427509	5.7074313	9.6567714	7.95E-20	6.75E-18	34.331513
MMP16	1.755505	2.7321822	9.652495	8.22E-20	6.96E-18	34.298828
IGHV3-13	2.1433034	0.8407581	9.6470492	8.57E-20	7.24E-18	34.257219
GAB3	0.4260509	4.705798	9.6464107	8.62E-20	7.26E-18	34.252341
NPFFR1	1.6640013	0.027964	9.6444402	8.75E-20	7.36E-18	34.237289
WTIP	0.5027898	4.9096731	9.6430047	8.85E-20	7.42E-18	34.226325
IGHV3-49	2.3564339	2.034155	9.6422304	8.90E-20	7.45E-18	34.220412
GPR124	0.6319175	5.5314145	9.6398454	9.07E-20	7.56E-18	34.202199
DACT1	0.9843633	4.5006051	9.6398282	9.07E-20	7.56E-18	34.202067
TENM4	1.8248716	2.3323272	9.6381616	9.19E-20	7.64E-18	34.189342
GPR55	1.5870204	2.0199208	9.6347834	9.43E-20	7.81E-18	34.163551
B3GALT2	1.6065699	2.6167122	9.6347326	9.44E-20	7.81E-18	34.163163
SV2A	0.8196115	4.189059	9.6330671	9.56E-20	7.89E-18	34.15045
DOCK2	0.4740603	5.3543296	9.6299773	9.79E-20	8.07E-18	34.126868
NUDT16	-0.176313	6.4959281	-9.629603	9.82E-20	8.07E-18	34.12401
PDE7A	0.2474839	5.6550303	9.6261964	1.01E-19	8.27E-18	34.098019
P3H3	0.710743	4.9258322	9.6246406	1.02E-19	8.35E-18	34.086149
SYK	0.4251319	5.4034019	9.6231928	1.03E-19	8.43E-18	34.075104
LINC00494	2.2741076	0.9099765	9.6209249	1.05E-19	8.56E-18	34.057805
FMOD	0.8639925	5.3373833	9.6178762	1.08E-19	8.75E-18	34.034555
RP11-499E1	-0.673929	4.0403383	-9.617362	1.08E-19	8.76E-18	34.030637
LRRC4C	1.9032031	2.0950285	9.616335	1.09E-19	8.81E-18	34.022804
FUT4	0.6490536	5.0965259	9.6155851	1.10E-19	8.85E-18	34.017085
RPA3	-0.213968	6.084143	-9.613339	1.11E-19	8.98E-18	33.999957
GNB4	0.3138063	5.4169518	9.6130395	1.12E-19	8.98E-18	33.997678
PCBP3	1.6490269	2.9421587	9.6113805	1.13E-19	9.08E-18	33.985031
SLC27A5	-0.529257	6.8606153	-9.60665	1.17E-19	9.40E-18	33.948981
IGKV3D-20	2.2376756	1.2465872	9.603143	1.21E-19	9.64E-18	33.922257
RIPK3	0.578145	4.3398147	9.5974608	1.26E-19	1.00E-17	33.878973

ADD2	1.4096746	2.9220724	9.5930704	1.30E-19	1.04E-17	33.845541
ARMCX2	0.5519041	4.8074417	9.5924859	1.31E-19	1.04E-17	33.841091
SEC14L2	-0.486659	6.5540858	-9.584202	1.40E-19	1.11E-17	33.778038
RASGEF1A	1.6281065	3.3726959	9.5833574	1.41E-19	1.11E-17	33.771609
CSRNP2	0.1703377	5.8357052	9.5795908	1.45E-19	1.14E-17	33.742951
CCBE1	1.8283522	2.6178951	9.5789397	1.46E-19	1.15E-17	33.737998
LFNG	0.4092545	5.2928544	9.5778648	1.47E-19	1.15E-17	33.729821
IGHV1-69-2	2.4012248	2.1253305	9.5759202	1.49E-19	1.17E-17	33.71503
PLEKHG2	0.2365091	5.8471676	9.5744737	1.51E-19	1.18E-17	33.704029
ARMCX6	0.6938437	4.7676658	9.5727751	1.53E-19	1.19E-17	33.691112
GAREML	0.8275937	4.4942258	9.5647216	1.63E-19	1.26E-17	33.629886
PXMP2	-0.317265	6.4196554	-9.564638	1.63E-19	1.26E-17	33.629252
FAM60A	0.3560617	5.6345473	9.5638611	1.64E-19	1.27E-17	33.623346
FHOD1	0.2735275	5.5980017	9.5635701	1.64E-19	1.27E-17	33.621135
CPXM1	0.8971949	4.5734795	9.5626474	1.65E-19	1.27E-17	33.614123
GAS1	1.1794118	3.8398685	9.5613528	1.67E-19	1.28E-17	33.604284
PLD4	1.1418536	3.963872	9.5594832	1.69E-19	1.30E-17	33.590079
RP11-326I1	-0.400184	4.9189483	-9.559431	1.69E-19	1.30E-17	33.589682
PIK3R5	0.4221091	5.082195	9.5555212	1.75E-19	1.34E-17	33.559979
CTSK	0.4177485	5.5036413	9.5537439	1.77E-19	1.35E-17	33.546479
DYNLL2	-0.132511	6.7066202	-9.553369	1.78E-19	1.35E-17	33.543632
MYOZ3	1.3409928	2.2901338	9.5527583	1.78E-19	1.35E-17	33.538994
KIAA1024	1.6988292	2.1311103	9.5527073	1.78E-19	1.35E-17	33.538607
SCD5	0.7966222	4.543374	9.5515942	1.80E-19	1.36E-17	33.530153
RP11-140K1	-0.356307	5.0936408	-9.548794	1.84E-19	1.39E-17	33.508888
ECI1	-0.213738	6.7086162	-9.5445	1.90E-19	1.43E-17	33.47629
IGHV3-66	2.2331567	0.8791939	9.5439888	1.91E-19	1.44E-17	33.47241
SPRED1	0.2976305	5.7074793	9.5408506	1.96E-19	1.47E-17	33.448592
VIM-AS1	1.175475	2.9688482	9.5380843	2.00E-19	1.50E-17	33.4276
CDR2L	0.4390224	5.1833769	9.5323949	2.09E-19	1.56E-17	33.384438
CCNJL	1.2397581	3.6165896	9.5322978	2.09E-19	1.56E-17	33.383702
MSRB3	0.4580391	5.4072113	9.5307884	2.11E-19	1.57E-17	33.372254
JAK3	0.4856378	5.3517389	9.5291596	2.14E-19	1.59E-17	33.359901
CES2	-0.370397	7.0700162	-9.527364	2.17E-19	1.61E-17	33.346287
NDUFA2	-0.213256	6.6303215	-9.525685	2.20E-19	1.63E-17	33.333555
IGF1R	0.6088453	5.0466654	9.5250636	2.21E-19	1.63E-17	33.328843
PTGDS	1.1201147	5.4587923	9.5237343	2.23E-19	1.65E-17	33.318765
FAF1	-0.128883	6.3492201	-9.522823	2.25E-19	1.65E-17	33.311856
IGKV1-5	2.0117296	3.8694167	9.5210285	2.28E-19	1.67E-17	33.298255
PHC1P1	1.7097964	1.0013944	9.5167505	2.36E-19	1.73E-17	33.265834
MXRA5	0.7734016	5.0917157	9.5165347	2.36E-19	1.73E-17	33.264199
ORAI3	-0.231421	6.1339646	-9.515519	2.38E-19	1.74E-17	33.2565
SPINT2	0.6501814	5.5696517	9.5147677	2.39E-19	1.74E-17	33.25081
PIWIL4	0.9557877	4.0414277	9.5126158	2.43E-19	1.77E-17	33.234507
RAB39B	1.3150638	2.5894385	9.5102832	2.48E-19	1.80E-17	33.216838
EGR2	0.8915035	4.5488332	9.5071339	2.54E-19	1.84E-17	33.192986
CCDC102B	0.3749335	4.9751232	9.5060595	2.56E-19	1.85E-17	33.184851
SCUBE3	0.8917232	3.3079624	9.5031639	2.62E-19	1.89E-17	33.162925
PEX16	-0.177716	6.3398898	-9.502822	2.62E-19	1.89E-17	33.160338
FZD7	0.8545182	4.7040869	9.4938922	2.81E-19	2.02E-17	33.09275
ADCY7	0.7923595	4.5038597	9.49387	2.81E-19	2.02E-17	33.092583

TANC1	0.2498443	5.7814745	9.4928424	2.83E-19	2.03E-17	33.084808
RPP25L	-0.214068	6.1672557	-9.491457	2.86E-19	2.05E-17	33.074326
IGHV4-61	2.1242644	0.6608322	9.4899069	2.90E-19	2.07E-17	33.062599
PCK2	-0.337459	6.9898332	-9.489145	2.92E-19	2.07E-17	33.056837
WDR24	-0.205134	5.9334831	-9.488936	2.92E-19	2.07E-17	33.055254
CSF2RB	0.4997122	5.0065136	9.4849505	3.01E-19	2.14E-17	33.025112
RP11-73M7.	1.8847098	0.8037807	9.4799625	3.13E-19	2.21E-17	32.987398
FMN1	0.9641863	4.1375698	9.4773072	3.20E-19	2.26E-17	32.967326
STRA6	2.0999197	2.183409	9.4753409	3.24E-19	2.29E-17	32.952464
DOK1	0.3595762	5.1573477	9.4716745	3.34E-19	2.35E-17	32.924758
TIMP2	0.3048273	6.4815423	9.4695987	3.39E-19	2.38E-17	32.909075
CYS1	1.9218457	2.967375	9.4688064	3.41E-19	2.39E-17	32.90309
LIMK1	0.2569176	5.8902354	9.4686985	3.41E-19	2.39E-17	32.902274
ANTXR1	0.5346842	5.6554742	9.4673742	3.45E-19	2.41E-17	32.892271
LBX2-AS1	-0.239095	6.1437112	-9.466534	3.47E-19	2.42E-17	32.885927
EMC3	-0.17029	6.594657	-9.463413	3.56E-19	2.47E-17	32.862353
CCDC8	1.2660491	3.6964561	9.4609611	3.62E-19	2.51E-17	32.843838
GUCY1A3	0.5670468	5.1848283	9.4608499	3.63E-19	2.51E-17	32.842998
RP11-6N17.	-0.366755	5.2513917	-9.455658	3.78E-19	2.61E-17	32.803803
ARHGEF4	1.939021	2.5565776	9.4540958	3.82E-19	2.64E-17	32.792012
ZNF280B	1.7310715	1.7174567	9.4526524	3.86E-19	2.66E-17	32.781118
GPR27	2.0955455	1.3051678	9.451369	3.90E-19	2.68E-17	32.771433
MMP23B	1.7401083	0.5560365	9.4512347	3.91E-19	2.68E-17	32.77042
IL7R	0.7879665	5.1240909	9.4505536	3.93E-19	2.69E-17	32.76528
TMEM71	0.6857059	3.7076532	9.4484578	3.99E-19	2.73E-17	32.749467
TUBB3	1.7794132	1.7660552	9.4476959	4.01E-19	2.74E-17	32.743719
GLI3	0.8488661	3.9184748	9.4469235	4.04E-19	2.75E-17	32.737892
FBLN1	0.5719222	6.0037512	9.446097	4.06E-19	2.76E-17	32.731658
ADCY5	0.6888011	5.0444245	9.4443706	4.12E-19	2.79E-17	32.718635
IGLV3-27	2.217118	0.8976803	9.4435809	4.14E-19	2.81E-17	32.712679
ALDH1L2	0.9542117	3.8885958	9.4428637	4.17E-19	2.82E-17	32.70727
FKBP10	0.42688	5.868124	9.4401599	4.25E-19	2.87E-17	32.68688
GPR65	0.5709177	4.5029186	9.4381883	4.32E-19	2.91E-17	32.672014
IL21R	1.1414985	3.6543461	9.4376514	4.34E-19	2.92E-17	32.667966
IGHV3-21	2.2353583	2.6189087	9.4302842	4.59E-19	3.08E-17	32.612435
SNAI1	0.6820662	4.6982003	9.4286665	4.65E-19	3.11E-17	32.600246
VILL	0.4507452	4.6173781	9.4255191	4.76E-19	3.18E-17	32.576532
GLIS2	0.5202476	5.5166981	9.4248989	4.78E-19	3.19E-17	32.57186
FAM129C	1.3981468	2.4910459	9.4225304	4.87E-19	3.24E-17	32.554019
IGLV1-44	2.2041801	3.2857476	9.4192623	4.99E-19	3.32E-17	32.529406
TRAV8-6	1.747904	0.7024946	9.4189681	5.01E-19	3.32E-17	32.527191
DBNDD2	0.8653598	3.9406584	9.4168385	5.09E-19	3.37E-17	32.511155
TMEM44-AS1	-0.312296	5.4314803	-9.416401	5.11E-19	3.37E-17	32.507863
IGHV3-33	2.2631403	2.1046899	9.4163917	5.11E-19	3.37E-17	32.507791
UROD	-0.174631	6.6151581	-9.415946	5.12E-19	3.38E-17	32.504438
FARS2	-0.186692	5.9897257	-9.414934	5.16E-19	3.40E-17	32.496814
PKIA	1.6845749	2.6708862	9.4147276	5.17E-19	3.40E-17	32.495263
PLTP	0.4293745	5.9796813	9.4135416	5.22E-19	3.42E-17	32.486335
DGKA	0.3706282	5.3108945	9.4080995	5.44E-19	3.56E-17	32.445376
COA4	-0.161528	6.5029786	-9.405254	5.56E-19	3.63E-17	32.423963
MRPL40	-0.196742	6.3989238	-9.403253	5.65E-19	3.68E-17	32.408911

DTX3	0.4456178	5.3141668	9.4030208	5.66E-19	3.68E-17	32.407165
IGLV3-21	2.0743294	3.4901035	9.4017598	5.71E-19	3.71E-17	32.39768
DUSP26	1.7402738	1.9559355	9.4005724	5.77E-19	3.74E-17	32.388749
RYR1	0.9176341	3.9279006	9.3995585	5.81E-19	3.76E-17	32.381123
PFKFB3	0.4248243	5.9299368	9.3988155	5.84E-19	3.78E-17	32.375536
HOXA1	1.4212172	1.8791739	9.3984561	5.86E-19	3.78E-17	32.372833
LRRN1	2.0726147	1.1148259	9.3975065	5.90E-19	3.80E-17	32.365692
LIMD2	0.412609	5.7195759	9.3939181	6.07E-19	3.90E-17	32.338711
CD37	0.369244	5.4786462	9.3924743	6.13E-19	3.94E-17	32.327857
ZNF124	0.4830213	4.7314781	9.3906622	6.22E-19	3.98E-17	32.314236
RP11-1182F	-1.797946	2.4459979	-9.390274	6.24E-19	3.99E-17	32.311316
COL1A2	0.3685309	6.7177341	9.386939	6.40E-19	4.08E-17	32.286254
CPPED1	-0.207041	6.5089714	-9.386268	6.43E-19	4.09E-17	32.281211
IGHJ3	1.8403465	-0.012201	9.3862407	6.44E-19	4.09E-17	32.281006
PCGF1	-0.147589	5.9008905	-9.383985	6.55E-19	4.16E-17	32.264056
ALDH2	-0.249441	7.2149337	-9.379513	6.78E-19	4.29E-17	32.230462
LTBP2	0.4412102	5.9518228	9.3793187	6.79E-19	4.29E-17	32.229004
SEC14L6	1.927624	2.7577904	9.3792988	6.79E-19	4.29E-17	32.228855
TMEM200A	1.1971857	3.7956929	9.3783835	6.83E-19	4.31E-17	32.22198
COL1A1	0.4238343	6.8361485	9.3770021	6.91E-19	4.35E-17	32.211606
LPAR6	0.2563079	5.7012719	9.3758698	6.97E-19	4.38E-17	32.203103
RAB3IL1	0.4299157	5.477838	9.3738281	7.08E-19	4.44E-17	32.187773
KRT19	2.2424079	3.8354466	9.3673046	7.44E-19	4.66E-17	32.138803
SYNC	1.59382	2.06335	9.3666535	7.48E-19	4.67E-17	32.133917
SELENBP1	-0.336168	6.853827	-9.36534	7.55E-19	4.71E-17	32.124056
GRIK4	1.9765582	2.4684592	9.3652398	7.56E-19	4.71E-17	32.123307
MOXD1	1.3335329	4.3747125	9.3619922	7.75E-19	4.82E-17	32.09894
EMB	0.5789484	5.0496758	9.3595301	7.90E-19	4.90E-17	32.08047
IKZF1	0.466102	5.156548	9.3583505	7.97E-19	4.94E-17	32.071622
MACROD1	-0.342147	6.0297368	-9.357125	8.04E-19	4.98E-17	32.062431
FAM225A	1.7262067	1.6934673	9.3562496	8.10E-19	5.00E-17	32.055865
BAI3	1.8805394	1.216821	9.3530303	8.30E-19	5.12E-17	32.031724
PCDHGA12	1.7152605	2.9344251	9.3523381	8.34E-19	5.14E-17	32.026534
PRTG	1.6878437	2.4287633	9.3517936	8.38E-19	5.15E-17	32.022452
TRBV20-1	1.6462378	2.3494525	9.351613	8.39E-19	5.15E-17	32.021098
PLAUR	0.4349027	5.2723815	9.3478944	8.63E-19	5.29E-17	31.993221
SRPX	0.9817275	4.3666151	9.3465888	8.72E-19	5.33E-17	31.983436
GLYCTK	-0.30336	6.7484991	-9.343954	8.90E-19	5.43E-17	31.963688
MYBPC2	1.7386867	0.6018412	9.3392114	9.22E-19	5.62E-17	31.928157
PLCB2	0.3454386	5.4735537	9.3373955	9.35E-19	5.69E-17	31.914555
RP11-131K5	-1.904489	0.088582	-9.336703	9.40E-19	5.71E-17	31.909371
C11orf45	0.9951063	3.7511166	9.3343537	9.57E-19	5.80E-17	31.891773
PKNOX2	1.4623544	3.1806887	9.3336466	9.62E-19	5.83E-17	31.886478
PDZRN3	1.5465261	3.5307227	9.3324455	9.71E-19	5.87E-17	31.877483
GPX8	0.4732032	5.0625226	9.3312219	9.80E-19	5.91E-17	31.868322
B3GNT9	0.5479128	4.8008623	9.3310248	9.82E-19	5.91E-17	31.866846
RHOH	0.7695705	4.4799235	9.3260129	1.02E-18	6.13E-17	31.829327
AXL	0.3372741	5.6410901	9.3259817	1.02E-18	6.13E-17	31.829094
GBGT1	0.4510188	4.7088885	9.3256972	1.02E-18	6.13E-17	31.826964
AC010894.3	-0.431707	4.5826644	-9.323012	1.04E-18	6.25E-17	31.806866
PLXNB3	1.3634166	3.8603147	9.3227383	1.05E-18	6.25E-17	31.804821

POLD2	-0.164013	6.6827614	-9.322595	1.05E-18	6.25E-17	31.803746
BMP5	2.1520759	1.8711179	9.3223604	1.05E-18	6.25E-17	31.801993
LINC00900	1.3645007	2.4609222	9.3210864	1.06E-18	6.30E-17	31.79246
FUT7	1.5324484	2.1307362	9.3201624	1.07E-18	6.33E-17	31.785547
ZFP92	1.2910343	3.7739294	9.3173979	1.09E-18	6.46E-17	31.764865
IGLON5	1.6925215	2.8134741	9.3167862	1.09E-18	6.48E-17	31.760289
METTL23	-0.16227	6.1393161	-9.315176	1.11E-18	6.55E-17	31.748244
HEMK1	-0.152811	6.1063492	-9.313759	1.12E-18	6.61E-17	31.73765
ZCCHC18	1.3706277	1.8225814	9.3123521	1.13E-18	6.67E-17	31.727127
QSOX1	0.450511	6.3042369	9.3119954	1.14E-18	6.67E-17	31.72446
CD226	0.7588381	4.2342806	9.3119283	1.14E-18	6.67E-17	31.723958
LCP2	0.3147882	5.6825262	9.3101165	1.15E-18	6.75E-17	31.71041
LPAR2	0.575199	4.9293385	9.3092281	1.16E-18	6.78E-17	31.703768
TMEM57	-0.163516	6.4352268	-9.30858	1.17E-18	6.81E-17	31.69892
TMEM14C	-0.141798	6.6395114	-9.308196	1.17E-18	6.82E-17	31.696053
GCDH	-0.277648	6.4268141	-9.306805	1.18E-18	6.88E-17	31.685652
C6orf226	-0.274046	5.3841021	-9.305673	1.19E-18	6.93E-17	31.677195
APEH	-0.14959	6.7161525	-9.302987	1.22E-18	7.06E-17	31.657114
RAC3	-0.405039	5.8056966	-9.301872	1.23E-18	7.11E-17	31.648782
UNC5B	0.3748834	5.7103845	9.3003915	1.24E-18	7.18E-17	31.637722
NXNL2	1.6227598	0.0736356	9.2953509	1.29E-18	7.45E-17	31.600064
F2R	0.3611553	5.9034076	9.2940519	1.30E-18	7.51E-17	31.590362
PTPRC	0.456251	5.6725584	9.2934955	1.31E-18	7.53E-17	31.586207
OSER1-AS1	-0.283425	5.694416	-9.292121	1.32E-18	7.60E-17	31.575944
PDGFRA	1.289149	4.8726659	9.2909204	1.33E-18	7.65E-17	31.566976
TRBV19	1.7037063	1.6746796	9.2899996	1.34E-18	7.69E-17	31.5601
CD19	1.7142509	2.5801423	9.2885263	1.36E-18	7.77E-17	31.5491
TMEM219	-0.194746	6.6606409	-9.287679	1.37E-18	7.81E-17	31.542777
ATP5H	-0.166307	6.8443124	-9.283122	1.41E-18	8.07E-17	31.508756
ZNF711	1.3884014	3.7495663	9.2827349	1.42E-18	8.08E-17	31.505869
RUNX3	0.4752277	5.1285031	9.2794934	1.45E-18	8.27E-17	31.481679
ZMAT2	-0.151691	6.5494356	-9.278478	1.47E-18	8.32E-17	31.474104
NTRK2	1.7685921	3.3516374	9.2770867	1.48E-18	8.40E-17	31.463723
CCR5	0.6490698	4.7949058	9.276892	1.48E-18	8.40E-17	31.46227
PLEKHA2	0.2831974	5.686934	9.2743619	1.51E-18	8.55E-17	31.443396
RP11-1334A	1.5112215	0.9443588	9.2738722	1.52E-18	8.57E-17	31.439744
SERPINE2	0.7272294	5.673057	9.271424	1.55E-18	8.71E-17	31.421484
SEPHS2	-0.189227	7.1493034	-9.268685	1.58E-18	8.88E-17	31.40106
DHPS	-0.147848	6.4007215	-9.268398	1.58E-18	8.89E-17	31.398921
FHDC1	0.9919134	3.96805	9.2670365	1.60E-18	8.97E-17	31.388769
NUDCD2	-0.15258	6.1843492	-9.266696	1.60E-18	8.98E-17	31.386227
ADAMDEC1	1.7917674	3.1817941	9.253351	1.77E-18	9.91E-17	31.286784
MFAP4	1.3552404	4.9607594	9.2532707	1.78E-18	9.91E-17	31.286186
KIAA0125	2.0573496	1.6712876	9.2503631	1.82E-18	1.01E-16	31.26453
ZNF431	0.478038	5.0194688	9.2475351	1.85E-18	1.03E-16	31.243472
EVC	1.2341762	4.8770241	9.2475342	1.85E-18	1.03E-16	31.243465
AK2	-0.140428	6.6761407	-9.245395	1.89E-18	1.05E-16	31.227538
MLF1	1.0325475	3.7473937	9.2433906	1.91E-18	1.06E-16	31.212617
CTD-2020K1	0.9962773	3.8509995	9.2376	2.00E-18	1.11E-16	31.169523
RASL11B	1.8253345	2.3234209	9.2373065	2.00E-18	1.11E-16	31.167339
MYO1G	0.4690707	5.083941	9.2370092	2.01E-18	1.11E-16	31.165127

SUSD5	1.6584745	2.6697141	9.2369199	2.01E-18	1.11E-16	31.164463
FAR1	0.329544	5.3508051	9.2363429	2.02E-18	1.11E-16	31.16017
SFXN1	-0.186419	6.5918016	-9.235813	2.03E-18	1.11E-16	31.156224
CXCL14	1.9699815	3.0533249	9.2353767	2.03E-18	1.12E-16	31.152981
PCYOX1L	0.4066866	4.9263788	9.2315675	2.09E-18	1.15E-16	31.124646
IGLV2-23	2.0353372	3.5496629	9.2308171	2.11E-18	1.15E-16	31.119064
HOXB3	0.5322378	4.7670916	9.2307985	2.11E-18	1.15E-16	31.118927
CCDC80	0.70701	5.6384947	9.2272926	2.16E-18	1.18E-16	31.092855
IKBKE	0.5548559	5.307625	9.226766	2.17E-18	1.18E-16	31.088939
FGR	0.3649008	5.2007751	9.2258669	2.19E-18	1.19E-16	31.082254
CAND2	1.0352733	3.9212137	9.2213024	2.26E-18	1.23E-16	31.048323
SCG2	1.9125151	1.6807647	9.2211888	2.27E-18	1.23E-16	31.047479
IGLV7-46	2.1604091	1.5729192	9.2207837	2.27E-18	1.23E-16	31.044468
RP5-1139B1	1.5618222	1.0859187	9.2207489	2.27E-18	1.23E-16	31.044209
IGKV3-11	1.7479436	4.2640499	9.2182504	2.32E-18	1.25E-16	31.025641
SPN	0.4959917	5.0346438	9.2174461	2.33E-18	1.25E-16	31.019664
SDC1	-0.212244	7.2748506	-9.215969	2.36E-18	1.27E-16	31.008687
COL6A3	0.3440516	6.3555172	9.2140545	2.39E-18	1.28E-16	30.994466
NQO2	-0.255947	6.5542136	-9.213398	2.40E-18	1.29E-16	30.989589
COMMD4	-0.180215	6.5756306	-9.21272	2.42E-18	1.29E-16	30.984552
BCAS3	-0.184963	6.1115555	-9.212157	2.43E-18	1.30E-16	30.98037
MYLIP	0.2889674	5.4806859	9.211194	2.44E-18	1.30E-16	30.973217
COL10A1	2.1764218	2.1988018	9.2111704	2.44E-18	1.30E-16	30.973042
ZNF439	0.726059	4.0027099	9.2106923	2.45E-18	1.31E-16	30.969491
IGLV1-47	2.1553714	3.0709218	9.2100374	2.47E-18	1.31E-16	30.964627
CACNA2D1	0.8102748	4.2936446	9.2093962	2.48E-18	1.31E-16	30.959865
IGKV3-20	1.8161202	4.250501	9.2076976	2.51E-18	1.33E-16	30.947251
CMTM3	0.294731	5.8198169	9.2067352	2.53E-18	1.34E-16	30.940104
TMEM119	1.0516566	4.5430943	9.2050014	2.56E-18	1.35E-16	30.927232
PMP22	0.3668233	5.6376613	9.2022485	2.62E-18	1.38E-16	30.906795
NAPSB	0.6357771	4.8592334	9.1975397	2.71E-18	1.43E-16	30.871847
TYRO3	0.6806224	5.2748666	9.1943714	2.78E-18	1.46E-16	30.848339
ATPIF1	-0.183721	6.67809	-9.194324	2.78E-18	1.46E-16	30.847985
PELI2	0.7670851	4.3104662	9.1936254	2.79E-18	1.46E-16	30.842804
NOTCH1	0.2152561	6.1149524	9.1931146	2.80E-18	1.47E-16	30.839014
KLF7	0.438499	5.381816	9.1913044	2.84E-18	1.49E-16	30.825586
POMGNT1	-0.139699	6.5019968	-9.191064	2.85E-18	1.49E-16	30.823803
GLP2R	2.0355634	1.4449907	9.1903917	2.86E-18	1.49E-16	30.818817
UNC13D	0.3632037	5.43273	9.1814263	3.06E-18	1.59E-16	30.752341
MGST2	-0.217469	6.6555565	-9.178612	3.13E-18	1.63E-16	30.731479
ZNF702P	1.2414042	3.3912637	9.1770486	3.17E-18	1.64E-16	30.719896
ADAMTS2	0.5192676	5.6312359	9.1721401	3.28E-18	1.70E-16	30.683528
MISP	2.3421323	2.7252011	9.1717862	3.29E-18	1.71E-16	30.680906
HIST3H2A	1.5788362	3.3625032	9.1675286	3.40E-18	1.76E-16	30.649372
SAMSN1	0.5504963	4.8197072	9.1663077	3.43E-18	1.77E-16	30.640331
ECM1	0.437763	5.3792191	9.165827	3.45E-18	1.78E-16	30.636772
PGRMC2	-0.1501	6.6636208	-9.1617	3.55E-18	1.83E-16	30.606216
NLRC3	0.3805195	4.8232714	9.1608321	3.58E-18	1.84E-16	30.599793
HOXB2	0.412597	4.6370132	9.1579176	3.66E-18	1.88E-16	30.578222
CHST3	0.4808581	5.1845358	9.1575897	3.67E-18	1.88E-16	30.575795
ARHGAP18	0.2423477	5.7075681	9.1563095	3.70E-18	1.90E-16	30.566321

CTD-3099C6	1.8357437	2.121275	9.1533952	3.78E-18	1.93E-16	30.544758
ST8SIA1	0.9889872	3.339798	9.152211	3.82E-18	1.95E-16	30.535997
GNB3	1.485134	1.8198333	9.1517133	3.83E-18	1.95E-16	30.532316
THAP7	-0.158932	6.1756134	-9.147531	3.96E-18	2.01E-16	30.501379
MRPL54	-0.224911	6.3151875	-9.146699	3.98E-18	2.02E-16	30.495231
IGHV4-34	2.0434322	2.7315484	9.1463658	3.99E-18	2.03E-16	30.492765
MMAB	-0.245928	6.5138208	-9.144878	4.04E-18	2.05E-16	30.481765
TRBV7-9	1.7153987	1.5466603	9.1429079	4.10E-18	2.07E-16	30.467197
DLG3	0.6467833	5.1228387	9.1414803	4.14E-18	2.09E-16	30.456643
BACE2	0.5650015	5.7246367	9.1347272	4.36E-18	2.20E-16	30.406734
IFRD2	-0.151757	6.4687032	-9.133298	4.40E-18	2.22E-16	30.396175
EIF2B3	-0.175562	6.083114	-9.131045	4.48E-18	2.26E-16	30.379527
TRBV6-1	1.6633381	0.6102114	9.1304667	4.50E-18	2.26E-16	30.375258
HTRA3	0.8359894	4.9993947	9.1288873	4.55E-18	2.29E-16	30.363592
CD1E	1.6073915	2.8232919	9.1276224	4.60E-18	2.31E-16	30.35425
NRSN2	0.5344284	5.5022609	9.1270306	4.62E-18	2.31E-16	30.34988
LDOC1	0.7032095	4.7908996	9.1254767	4.67E-18	2.34E-16	30.338405
CTB-133G6.	1.5274013	1.2538886	9.1230883	4.76E-18	2.37E-16	30.32077
GS1-124K5.	-0.374513	5.049065	-9.123087	4.76E-18	2.37E-16	30.320757
GAL3ST4	0.7040457	4.5863174	9.1217579	4.80E-18	2.39E-16	30.310948
LINGO1	0.7008212	5.0461702	9.1201176	4.86E-18	2.42E-16	30.29884
GEMIN6	-0.172446	5.9292574	-9.119689	4.88E-18	2.42E-16	30.295678
DCLK1	1.1155542	3.7277487	9.119187	4.90E-18	2.43E-16	30.291971
MDFI	1.0245882	4.3687046	9.1178022	4.95E-18	2.45E-16	30.28175
COL22A1	2.1235743	2.9262677	9.1175905	4.96E-18	2.45E-16	30.280187
DNAJC18	0.3574586	4.7599325	9.1175899	4.96E-18	2.45E-16	30.280183
FCRL2	1.8833886	0.7802339	9.1144363	5.08E-18	2.50E-16	30.256911
NDUFB10	-0.192992	6.6026805	-9.113625	5.11E-18	2.52E-16	30.250922
MSMO1	-0.267227	6.8894775	-9.113234	5.12E-18	2.52E-16	30.248039
BCL11B	0.8911319	4.2902382	9.1114259	5.19E-18	2.55E-16	30.234702
RAB33A	1.0981795	2.9296883	9.1101694	5.24E-18	2.57E-16	30.225433
ARHGEF2	0.2450033	6.1038081	9.1063885	5.39E-18	2.64E-16	30.197547
CASC15	1.3681351	3.3990112	9.1062496	5.40E-18	2.64E-16	30.196523
SIRT3	-0.154852	6.1589426	-9.105189	5.44E-18	2.66E-16	30.188705
C1QTNF7	1.3859051	3.0508077	9.1028512	5.54E-18	2.70E-16	30.171464
SLC1A7	1.355751	4.3241594	9.1020215	5.57E-18	2.72E-16	30.165348
IGLV1-51	1.3594272	4.0206351	9.0999921	5.66E-18	2.75E-16	30.150387
NLRP3	0.7015099	4.1934037	9.0987926	5.71E-18	2.78E-16	30.141546
AC006129.2	1.131802	3.1413482	9.0955089	5.85E-18	2.84E-16	30.117346
POLRMT	-0.157043	6.5463836	-9.09407	5.92E-18	2.87E-16	30.10674
HAAO	-0.335398	6.643642	-9.093936	5.92E-18	2.87E-16	30.105757
BICD1	0.5009997	4.9845007	9.093873	5.93E-18	2.87E-16	30.105292
WNT10B	1.6271292	2.0310617	9.0928768	5.97E-18	2.88E-16	30.097952
ARHGEF17	0.3181705	5.8198051	9.0924782	5.99E-18	2.89E-16	30.095016
SSPN	1.1039655	4.1894448	9.0922799	6.00E-18	2.89E-16	30.093554
UBXN6	-0.14167	6.7086956	-9.087376	6.22E-18	2.99E-16	30.057436
TRIM17	1.8648965	2.1121258	9.0858632	6.29E-18	3.02E-16	30.046292
PDE1A	0.952364	4.5038312	9.0821053	6.48E-18	3.11E-16	30.018622
CDK5RAP3	-0.154018	6.6938652	-9.081749	6.49E-18	3.11E-16	30.015998
DBN1	0.3630088	5.9481009	9.0813343	6.51E-18	3.12E-16	30.012946
RP11-747H7	1.6931482	2.9875576	9.0810321	6.53E-18	3.12E-16	30.010721

EMP2	-0.21765	6.5653234	-9.080145	6.57E-18	3.14E-16	30.004193
TRAF5	0.3181637	5.4401625	9.0796465	6.60E-18	3.14E-16	30.000522
TRAV1-2	1.7219571	0.499355	9.0788913	6.63E-18	3.16E-16	29.994963
ASAP1	0.2284746	6.0733109	9.0774661	6.70E-18	3.19E-16	29.984473
CTB-58E17.	-0.35312	4.9699844	-9.077152	6.72E-18	3.19E-16	29.982165
IGHV2-5	1.9953416	0.440705	9.0755252	6.80E-18	3.23E-16	29.97019
WNT7B	2.0773095	1.3616292	9.0753289	6.81E-18	3.23E-16	29.968746
GSTK1	-0.182525	6.9773045	-9.074391	6.86E-18	3.24E-16	29.961843
FAM127C	0.5787501	4.7853039	9.0722878	6.97E-18	3.29E-16	29.94637
SALL2	1.0994766	4.5717982	9.0620099	7.53E-18	3.55E-16	29.870782
BAP1	-0.151774	6.5663655	-9.060677	7.61E-18	3.58E-16	29.860981
MIR4697HG	1.588412	1.9534895	9.0585539	7.73E-18	3.64E-16	29.845378
IGKV3D-11	1.9412282	0.288136	9.0581083	7.75E-18	3.64E-16	29.842102
ZNF827	0.7883596	4.2085683	9.0562711	7.86E-18	3.69E-16	29.8286
IGKV1-17	2.2389675	2.001524	9.0514502	8.15E-18	3.82E-16	29.793179
LINC01430	-1.828216	1.5504813	-9.049883	8.25E-18	3.86E-16	29.781665
EMILIN2	0.428465	5.3325596	9.047807	8.38E-18	3.92E-16	29.766418
DOK6	1.4758347	3.2854062	9.0426517	8.71E-18	4.06E-16	29.728562
MEOX1	1.7251825	2.6155193	9.0425752	8.71E-18	4.06E-16	29.728
RASGRP1	0.7789589	4.2937832	9.0425655	8.71E-18	4.06E-16	29.727929
DOK3	0.3345491	5.1050286	9.0404779	8.85E-18	4.12E-16	29.712604
GCNT1	0.5644911	4.5742684	9.0378016	9.03E-18	4.19E-16	29.692959
ARHGEF25	0.5918887	4.8695582	9.035702	9.17E-18	4.25E-16	29.677551
C19orf26	1.0876196	3.043969	9.0329163	9.37E-18	4.33E-16	29.657111
CDH23	-0.420455	5.6922603	-9.03286	9.37E-18	4.33E-16	29.656701
INO80B	-0.253083	5.1631178	-9.025823	9.88E-18	4.56E-16	29.60508
TRBV30	1.7166873	0.3593192	9.0237187	1.00E-17	4.63E-16	29.589652
LXN	0.5311684	4.8438693	9.0151453	1.07E-17	4.93E-16	29.52681
GPX4	-0.172517	7.0830409	-9.003013	1.17E-17	5.39E-16	29.43795
SULT1C4	1.1715481	3.8119104	8.9983047	1.21E-17	5.58E-16	29.403483
HOXB4	0.6564997	4.0827306	8.997532	1.22E-17	5.60E-16	29.397828
PIK3CD	0.376113	5.3790411	8.9967597	1.23E-17	5.63E-16	29.392175
AGAP2-AS1	0.5572366	4.4671694	8.9965743	1.23E-17	5.63E-16	29.390819
EPHA3	1.0340605	4.6698141	8.9936727	1.26E-17	5.75E-16	29.369587
IGHV2-70	2.2402405	1.066506	8.993482	1.26E-17	5.75E-16	29.368192
CD207	1.6847267	2.2176528	8.9913067	1.28E-17	5.83E-16	29.352278
GPR173	1.2207378	2.8724564	8.990791	1.28E-17	5.84E-16	29.348505
HOXC4	1.5297443	2.1257297	8.9907516	1.28E-17	5.84E-16	29.348217
GLS	0.3140006	6.1685316	8.9905604	1.29E-17	5.84E-16	29.346819
CACNB1	0.5320101	4.3243372	8.990228	1.29E-17	5.85E-16	29.344387
IGHV4-28	2.1158312	1.0448944	8.9875408	1.31E-17	5.96E-16	29.324733
PRSS16	2.2742015	1.3754268	8.9869185	1.32E-17	5.98E-16	29.320182
FCHSD1	0.423659	5.1043171	8.9861131	1.33E-17	6.01E-16	29.314292
EXOC3-AS1	-0.333244	5.125612	-8.98386	1.35E-17	6.11E-16	29.297816
RP11-524D1	1.5403074	1.8429147	8.9828869	1.36E-17	6.14E-16	29.290703
PRKX	0.4601041	5.3250013	8.9825415	1.36E-17	6.15E-16	29.288178
TFEC	0.6643411	4.4717522	8.9815446	1.38E-17	6.19E-16	29.280891
HCG14	-1.601269	1.1306273	-8.981412	1.38E-17	6.19E-16	29.279924
ACSF2	-0.244997	6.4802009	-8.97941	1.40E-17	6.27E-16	29.26529
IGHV4-31	2.2648783	1.7085901	8.9790973	1.40E-17	6.28E-16	29.263002
BAI2	1.3605777	3.112116	8.9755087	1.44E-17	6.44E-16	29.236776

SCN3B	1.7350215	1.307665	8.9728881	1.47E-17	6.56E-16	29.217629
CHST11	0.444539	5.5101285	8.9686991	1.51E-17	6.76E-16	29.18703
CADM3	1.9710647	1.4536814	8.9649151	1.56E-17	6.95E-16	29.159397
TMEM87B	0.1709823	5.8702603	8.9606331	1.61E-17	7.16E-16	29.128137
HAND2-AS1	2.0679455	2.1818458	8.9584583	1.63E-17	7.27E-16	29.112263
AIM2	1.5393079	2.6528876	8.9562573	1.66E-17	7.38E-16	29.096202
TMEM205	-0.200905	6.8122654	-8.956174	1.66E-17	7.38E-16	29.095592
UQCRC1	-0.166123	6.9929053	-8.955027	1.68E-17	7.43E-16	29.087225
TUBB6	0.289564	5.7814206	8.9549424	1.68E-17	7.43E-16	29.086607
PIGV	-0.207589	6.0100043	-8.954395	1.68E-17	7.45E-16	29.082614
PLAC8	0.8774478	4.0754561	8.9521537	1.71E-17	7.57E-16	29.066262
CXCL5	2.3374491	2.1213876	8.9500508	1.74E-17	7.68E-16	29.050923
FSCN1	0.2468545	6.1267866	8.9476108	1.77E-17	7.81E-16	29.033127
ZNF358	-0.209921	6.3782666	-8.945827	1.79E-17	7.90E-16	29.020121
COL6A4P2	1.5933296	1.105564	8.9456067	1.80E-17	7.90E-16	29.018513
RP4-647J21	1.2246211	2.5846891	8.9453959	1.80E-17	7.90E-16	29.016976
OSGIN1	-0.393399	6.7617411	-8.945358	1.80E-17	7.90E-16	29.016698
SLC9A5	0.8691828	3.1263483	8.9427735	1.84E-17	8.05E-16	28.997858
ERMN	1.5213925	1.6087715	8.9412572	1.86E-17	8.13E-16	28.986804
TBCA	-0.159328	6.6419555	-8.939336	1.88E-17	8.23E-16	28.972804
CD79A	1.3762819	3.7228093	8.9376965	1.91E-17	8.32E-16	28.960853
FOXL1	1.3848114	3.2144847	8.9375962	1.91E-17	8.32E-16	28.960122
AQP9	-0.695473	6.7209853	-8.936557	1.92E-17	8.38E-16	28.952552
ZMIZ1	0.1750394	6.3397469	8.9350908	1.94E-17	8.46E-16	28.941866
MAP1A	0.6151465	4.7326301	8.934439	1.95E-17	8.49E-16	28.937117
CYP19A1	2.1587562	1.4257971	8.9336607	1.96E-17	8.53E-16	28.931446
CCR8	1.7261749	0.6009862	8.9330822	1.97E-17	8.55E-16	28.927232
ISLR	1.2569708	4.9300048	8.9317821	1.99E-17	8.63E-16	28.917761
FBXL2	0.5175271	4.1900964	8.9308777	2.01E-17	8.68E-16	28.911174
CTD-2054N2	1.7093089	1.1862381	8.9302318	2.02E-17	8.71E-16	28.906469
PTMS	-0.204521	7.1473716	-8.92897	2.03E-17	8.78E-16	28.897283
RP11-532F1	2.0557468	2.5019031	8.9228437	2.13E-17	9.18E-16	28.852673
PSME2	-0.185705	6.8452579	-8.919812	2.18E-17	9.38E-16	28.830609
SLC8A1	0.4465192	4.8731652	8.9167882	2.23E-17	9.56E-16	28.808601
HAND2	1.9658439	3.0680309	8.9167083	2.23E-17	9.56E-16	28.80802
UNC5C	0.8797317	4.1518229	8.9166623	2.23E-17	9.56E-16	28.807685
HGF	0.7376575	5.0109266	8.9161275	2.24E-17	9.59E-16	28.803794
MEG3	0.6478516	5.3978457	8.9141386	2.27E-17	9.72E-16	28.789323
IGHV7-81	1.5387383	-0.133739	8.9132058	2.29E-17	9.78E-16	28.782537
NBEA	1.0391495	4.0961227	8.9094566	2.35E-17	1.00E-15	28.755268
ALKBH2	-0.244393	6.079684	-8.907772	2.38E-17	1.02E-15	28.743018
LAMB1	0.2560771	6.449444	8.9075063	2.39E-17	1.02E-15	28.741085
SYNDIG1	1.9837404	0.8806475	8.9057573	2.42E-17	1.03E-15	28.728368
PMEPA1	0.5268313	5.6032293	8.9047295	2.44E-17	1.04E-15	28.720895
PREB	-0.168103	6.6542882	-8.903708	2.45E-17	1.04E-15	28.713468
TMEM63C	1.8114081	2.0345093	8.9029624	2.47E-17	1.05E-15	28.708049
SLC7A1	0.6401397	5.3250612	8.9022648	2.48E-17	1.05E-15	28.702978
ACOX1	-0.204188	6.8853807	-8.901905	2.49E-17	1.05E-15	28.700363
XCR1	1.6922293	2.5402137	8.8999739	2.52E-17	1.07E-15	28.686327
HDAC6	-0.184179	6.5512081	-8.898066	2.56E-17	1.08E-15	28.672464
ANO4	1.6704764	1.8301777	8.8973154	2.57E-17	1.08E-15	28.667008

FBN2	1.4242244	2.4610968	8.8967015	2.59E-17	1.09E-15	28.662548
FAIM2	1.7675226	1.8014993	8.8957966	2.60E-17	1.09E-15	28.655974
OSBPL5	0.393777	5.2481358	8.8955942	2.61E-17	1.10E-15	28.654503
TRIM46	0.9369698	3.2586662	8.8947696	2.62E-17	1.10E-15	28.648512
OMG	1.4378903	1.7148199	8.8939372	2.64E-17	1.11E-15	28.642465
ZNF714	1.0801633	3.9425448	8.8935251	2.65E-17	1.11E-15	28.639471
HDAC9	0.6090132	4.3889145	8.8899116	2.72E-17	1.14E-15	28.613226
SYT11	0.394531	5.2065715	8.8894662	2.73E-17	1.14E-15	28.609992
GJC2	1.0249168	3.4286414	8.8846001	2.83E-17	1.18E-15	28.574659
PRRX2	1.8508646	1.1326382	8.8830758	2.86E-17	1.19E-15	28.563595
ZNF860	1.7130333	0.7368853	8.8805225	2.92E-17	1.21E-15	28.545063
ICT1	-0.166858	6.2039819	-8.880422	2.92E-17	1.21E-15	28.544334
TMEM132E	1.6306492	2.7454874	8.8779483	2.97E-17	1.23E-15	28.526382
IGLV2-14	1.895323	3.6684295	8.8776779	2.98E-17	1.24E-15	28.52442
SLC35F2	0.7724314	4.1971682	8.8771312	2.99E-17	1.24E-15	28.520454
TRAV8-2	1.6151079	0.7426113	8.8759335	3.02E-17	1.25E-15	28.511764
STAP1	1.5818661	1.5394026	8.8756922	3.02E-17	1.25E-15	28.510014
QDPR	-0.243792	6.6386087	-8.874423	3.05E-17	1.26E-15	28.500808
STON1	0.4485542	4.7668993	8.8732114	3.08E-17	1.27E-15	28.492017
PLEKHG5	0.2877735	5.2576829	8.8729723	3.08E-17	1.27E-15	28.490283
AC025335.1	0.6333681	4.2479445	8.8717051	3.11E-17	1.28E-15	28.481093
NHS	0.876093	3.61197	8.8712528	3.12E-17	1.28E-15	28.477812
MIXL1	1.7454605	0.4006251	8.8707572	3.13E-17	1.29E-15	28.474218
FAM129A	0.3508073	5.4932334	8.8701933	3.15E-17	1.29E-15	28.470129
ADAMTS12	0.5472585	4.9413264	8.8661999	3.24E-17	1.33E-15	28.441173
APOC4	-1.516163	3.0312576	-8.864288	3.29E-17	1.34E-15	28.427314
CCL22	1.3986809	3.1023373	8.8642698	3.29E-17	1.34E-15	28.427181
KIAA1549L	1.8011608	1.8126588	8.8642001	3.29E-17	1.34E-15	28.426676
ST6GAL2	1.8911345	2.6870771	8.8625813	3.33E-17	1.36E-15	28.414942
DNAJC25	-0.230265	5.9766703	-8.861068	3.37E-17	1.37E-15	28.403978
RP11-758H9	-0.442604	4.5485994	-8.859821	3.40E-17	1.38E-15	28.394939
EPHX1	-0.290527	7.5244473	-8.859504	3.41E-17	1.38E-15	28.392642
VAV3	0.4935488	5.0279197	8.8574252	3.46E-17	1.40E-15	28.377578
CLNK	1.5763754	1.5267994	8.8562593	3.49E-17	1.42E-15	28.369131
RASA4	1.188033	2.5078354	8.8558095	3.50E-17	1.42E-15	28.365873
DACT3	0.8775668	4.3358631	8.8534666	3.56E-17	1.44E-15	28.348902
PRADC1	-0.221201	6.2195744	-8.852916	3.58E-17	1.45E-15	28.344915
TMEM199	-0.142649	5.9479443	-8.851304	3.62E-17	1.46E-15	28.333238
CALCOCO2	-0.114952	6.6530677	-8.847848	3.71E-17	1.50E-15	28.308214
IL20RA	2.2446206	1.0455827	8.8465957	3.75E-17	1.51E-15	28.299148
KLC4	-0.195728	6.6106849	-8.84575	3.77E-17	1.52E-15	28.293028
EVI2A	0.5138054	4.8078578	8.8447937	3.80E-17	1.53E-15	28.286104
AATK	0.6348292	4.5393705	8.844397	3.81E-17	1.53E-15	28.283232
SLIT2	0.9604702	4.5504088	8.8410803	3.90E-17	1.57E-15	28.259228
MPI	-0.174107	6.3073428	-8.839953	3.94E-17	1.58E-15	28.251068
DZIP1	0.6881539	4.6391705	8.8399188	3.94E-17	1.58E-15	28.250823
CCDC64B	2.1708239	1.5123583	8.8391757	3.96E-17	1.58E-15	28.245446
TRBV29-1	1.7019196	1.3841212	8.835691	4.06E-17	1.62E-15	28.220236
NDUFB6	-0.161576	6.3846399	-8.83522	4.08E-17	1.63E-15	28.216833
CEBPB	-0.217091	6.6737302	-8.833527	4.13E-17	1.64E-15	28.204585
TMEM26	0.8480794	3.630226	8.832781	4.15E-17	1.65E-15	28.199188

ZNHIT1	-0.205534	6.7920249	-8.83259	4.16E-17	1.65E-15	28.197807
FCRL5	1.9972149	2.0042919	8.8325821	4.16E-17	1.65E-15	28.19775
SSTR3	1.9031193	0.7266713	8.8310696	4.20E-17	1.67E-15	28.186812
SEMA3A	1.4497725	3.3481145	8.830859	4.21E-17	1.67E-15	28.185289
AKNA	0.2208771	5.9544102	8.8302963	4.23E-17	1.67E-15	28.181221
IGHV6-1	1.8126371	0.0515028	8.8285774	4.28E-17	1.69E-15	28.168792
KSR1	0.3408303	5.4437942	8.8270165	4.33E-17	1.71E-15	28.157508
HDGFRP3	0.3992839	5.1482903	8.8248273	4.40E-17	1.74E-15	28.141683
IGLV3-19	2.0269088	3.3987166	8.8242218	4.42E-17	1.74E-15	28.137307
GRAMD1B	0.8031103	5.0189855	8.824193	4.42E-17	1.74E-15	28.137099
SLC34A2	2.3961251	2.3323876	8.8191909	4.59E-17	1.80E-15	28.100953
TNXB	0.6296613	5.195138	8.8184934	4.61E-17	1.81E-15	28.095913
DAGLA	0.85127	4.9028557	8.8172224	4.66E-17	1.83E-15	28.086732
PCDH7	1.5904847	3.2148652	8.813502	4.79E-17	1.87E-15	28.05986
CRACR2A	0.6589662	4.3828805	8.8134542	4.79E-17	1.87E-15	28.059515
MIAT	0.994701	3.918331	8.812645	4.82E-17	1.88E-15	28.053671
TBX1	1.8478571	1.9032141	8.8122335	4.83E-17	1.89E-15	28.050699
MTMR4	-0.18946	6.4516448	-8.811327	4.86E-17	1.90E-15	28.044155
RP5-1071N3	1.3847481	0.3143151	8.8105526	4.89E-17	1.91E-15	28.038562
PABPC5	1.5693837	1.7614653	8.8071145	5.02E-17	1.95E-15	28.013741
MRPL57	-0.176271	6.412841	-8.805231	5.09E-17	1.98E-15	28.000144
L1CAM	1.6659215	2.1048772	8.8042815	5.12E-17	1.99E-15	27.993294
HHIP-AS1	1.7839962	1.3785507	8.8010165	5.25E-17	2.04E-15	27.969733
CDH11	0.7165219	5.0720128	8.8009043	5.25E-17	2.04E-15	27.968923
FCRL1	1.713282	0.5152584	8.7993435	5.31E-17	2.06E-15	27.957663
DNAJC19	-0.165564	6.2210685	-8.797982	5.37E-17	2.08E-15	27.947842
PDE4A	0.4225946	5.3960945	8.7972012	5.40E-17	2.09E-15	27.942209
MYH15	1.5759777	0.4220356	8.796848	5.41E-17	2.09E-15	27.939661
STAB2	1.3301226	3.2610974	8.7960142	5.44E-17	2.10E-15	27.933647
LINC00570	-1.739757	1.7747264	-8.795312	5.47E-17	2.11E-15	27.928584
NFATC2	0.3299653	5.7369804	8.7945777	5.50E-17	2.12E-15	27.923287
DSE	0.3847854	5.3424582	8.7940829	5.52E-17	2.12E-15	27.919719
HAS1	1.9952704	1.1690669	8.7912694	5.64E-17	2.16E-15	27.899431
MAML2	0.4113215	5.185363	8.7912104	5.64E-17	2.16E-15	27.899005
KCNJ8	-0.413919	6.134998	-8.790696	5.66E-17	2.17E-15	27.895297
MYADM	0.1926128	6.418254	8.7902665	5.68E-17	2.18E-15	27.8922
IGHV3-64	1.9004209	0.2708777	8.7852138	5.89E-17	2.26E-15	27.85578
PSMD4	-0.166779	6.9595915	-8.784618	5.92E-17	2.26E-15	27.851488
NUDT8	-0.293716	5.7852625	-8.781086	6.08E-17	2.32E-15	27.826039
BVES	1.1266613	3.2793024	8.7787156	6.18E-17	2.36E-15	27.80896
CHST10	0.6326795	4.7311939	8.7779061	6.22E-17	2.37E-15	27.803129
CTC-231011	1.5071771	2.5852947	8.7770988	6.26E-17	2.38E-15	27.797314
PSMG4	-0.201413	5.9124465	-8.776786	6.27E-17	2.38E-15	27.795061
TMEM110	-0.276165	5.6278776	-8.774071	6.40E-17	2.43E-15	27.775513
BAI1	1.3849046	2.8414281	8.7731448	6.44E-17	2.44E-15	27.76884
TRAV12-3	1.5995868	0.4204059	8.7713117	6.53E-17	2.47E-15	27.755642
IGSF8	-0.214298	6.6792896	-8.770964	6.55E-17	2.48E-15	27.75314
IGLV6-57	2.0795708	2.7519048	8.7707072	6.56E-17	2.48E-15	27.75129
BLCAP	-0.17107	6.5479996	-8.770248	6.58E-17	2.49E-15	27.747983
ZFP3	0.7384208	4.2172594	8.7690484	6.64E-17	2.51E-15	27.739349
RNF122	0.2736275	5.1888961	8.7678595	6.70E-17	2.53E-15	27.730792

DCHS1	0.343015	5.4646241	8.7664037	6.77E-17	2.55E-15	27.720314
KIAA1644	1.7626502	1.7129317	8.7661062	6.78E-17	2.55E-15	27.718173
LINC01215	1.6697234	0.9425631	8.7659965	6.79E-17	2.55E-15	27.717384
GLB1L2	1.7609109	2.7814002	8.7657646	6.80E-17	2.55E-15	27.715715
TRAV12-2	1.6394914	0.6670848	8.7620422	6.99E-17	2.62E-15	27.688931
ATP1A3	1.4304387	2.402322	8.7619442	6.99E-17	2.62E-15	27.688226
IGJ	1.0982788	4.9905147	8.7599405	7.10E-17	2.66E-15	27.673811
FYB	0.43713	5.365499	8.7596414	7.11E-17	2.66E-15	27.67166
SOWAHD	0.946556	3.3999058	8.7595498	7.12E-17	2.66E-15	27.671001
FAM134C	-0.142231	6.6302936	-8.75942	7.13E-17	2.66E-15	27.670065
TWIST1	1.4860389	2.2807566	8.7591877	7.14E-17	2.66E-15	27.668397
RPLP1P6	-0.527287	4.2201234	-8.756449	7.28E-17	2.71E-15	27.648701
KCTD12	0.3014121	5.943216	8.7555138	7.33E-17	2.73E-15	27.641975
KAL1	1.1018042	3.8454965	8.7548388	7.37E-17	2.74E-15	27.637121
ZNF738	0.8734202	4.0958354	8.7536653	7.43E-17	2.76E-15	27.628683
EBP	-0.238034	6.8716079	-8.75241	7.50E-17	2.78E-15	27.61966
IGKV3D-15	1.8079853	0.0361376	8.7521361	7.52E-17	2.79E-15	27.617689
FGD6	0.2873541	5.7057024	8.7510082	7.58E-17	2.81E-15	27.609581
OSBPL10	0.5428672	4.8764425	8.7499201	7.64E-17	2.83E-15	27.601759
RAB3D	0.6450618	5.0721776	8.7487266	7.71E-17	2.85E-15	27.593181
CXCL12	0.4736902	6.0665806	8.748628	7.71E-17	2.85E-15	27.592473
CYSLTR2	0.9657662	3.7869148	8.7477739	7.76E-17	2.86E-15	27.586334
LINC00265	1.1505804	3.6015252	8.7460434	7.86E-17	2.90E-15	27.573898
B3GALT5	2.0118076	1.2219905	8.7430849	8.03E-17	2.96E-15	27.552642
METTL24	1.711378	2.2523032	8.7424348	8.07E-17	2.97E-15	27.547971
IGLV1-40	1.9969885	3.4652454	8.7419333	8.10E-17	2.98E-15	27.544368
IGLV7-43	2.0955694	1.5281158	8.7415888	8.12E-17	2.98E-15	27.541894
POM121L9P	1.5094103	2.0308687	8.7411031	8.15E-17	2.99E-15	27.538405
CLEC4A	0.4343984	4.3442146	8.7394056	8.25E-17	3.02E-15	27.526212
C6orf132	1.5599023	3.5154239	8.7393501	8.26E-17	3.02E-15	27.525814
CTC-378H22	1.6410649	2.5542729	8.7329586	8.65E-17	3.16E-15	27.47992
RGL4	0.7474509	3.631618	8.731745	8.73E-17	3.19E-15	27.471208
PRKCQ-AS1	1.0594813	3.4009733	8.7309149	8.78E-17	3.20E-15	27.465249
P2RY10	1.5424065	2.5435873	8.7281219	8.97E-17	3.27E-15	27.445205
PRKCB	0.5460562	4.885421	8.7221112	9.37E-17	3.41E-15	27.402081
RTCA-AS1	-0.556013	3.9322594	-8.721516	9.41E-17	3.42E-15	27.397812
NDUFAF3	-0.160062	6.4873618	-8.72076	9.46E-17	3.44E-15	27.392392
SNPH	0.7589458	4.1102483	8.7176289	9.68E-17	3.51E-15	27.369934
DUT	-0.169724	6.2861934	-8.717177	9.71E-17	3.52E-15	27.366696
SLC24A3	1.6237854	3.0979248	8.7164553	9.77E-17	3.54E-15	27.36152
FAM19A5	1.7989987	3.6479976	8.7159341	9.80E-17	3.55E-15	27.357783
TMED3	0.5348206	5.943194	8.7149252	9.88E-17	3.57E-15	27.35055
HCG11	0.8167782	3.5840956	8.7133137	9.99E-17	3.61E-15	27.338998
RP11-126K1	-0.50036	4.2809866	-8.713193	1.00E-16	3.61E-15	27.338133
MEIS3	0.5824766	4.4147882	8.710338	1.02E-16	3.68E-15	27.317669
FDXR	-0.254211	6.1482513	-8.710156	1.02E-16	3.68E-15	27.316366
AC144831.1	1.3624618	2.3901626	8.7095331	1.03E-16	3.69E-15	27.311901
L3MBTL2	-0.115623	6.2426445	-8.708068	1.04E-16	3.73E-15	27.301401
TMEM159	0.5075827	4.7247363	8.7077595	1.04E-16	3.74E-15	27.299192
SIL1	-0.195502	6.7599392	-8.704313	1.07E-16	3.83E-15	27.2745
GNPTG	-0.164742	6.6352964	-8.703443	1.07E-16	3.85E-15	27.268266

CACNB3	0.3729448	4.9072572	8.7032768	1.08E-16	3.85E-15	27.267079
FAM159A	1.4739489	2.6896672	8.701524	1.09E-16	3.89E-15	27.254525
PGM1	-0.200234	6.7828722	-8.697884	1.12E-16	4.00E-15	27.228459
STX5	-0.134717	6.3976638	-8.691464	1.17E-16	4.18E-15	27.182506
NTRK3	1.6524701	2.191573	8.6893208	1.19E-16	4.25E-15	27.167168
TC2N	0.8539477	5.1598633	8.6888442	1.20E-16	4.26E-15	27.163758
FAP	1.1275404	4.1738763	8.6886331	1.20E-16	4.26E-15	27.162247
STK32A	1.7162029	1.3154176	8.6860504	1.22E-16	4.34E-15	27.14377
GPC4	1.06141	4.7375825	8.6829511	1.25E-16	4.43E-15	27.121603
RP5-1059L7	1.4778645	2.1674878	8.6823735	1.25E-16	4.45E-15	27.117472
WDR74	-0.14653	6.1516571	-8.67211	1.35E-16	4.79E-15	27.044099
SYDE1	0.3327215	5.4676991	8.6704404	1.37E-16	4.84E-15	27.032171
CTD-3035K2	1.4650569	0.4337638	8.6700081	1.37E-16	4.85E-15	27.029083
PTGS1	0.563116	5.0071556	8.6697415	1.37E-16	4.86E-15	27.027178
IGKV1-6	2.1729538	1.9625347	8.668981	1.38E-16	4.88E-15	27.021744
TIPARP-AS1	-0.443019	4.627167	-8.668532	1.39E-16	4.89E-15	27.018538
HPCAL4	1.4057558	-0.093798	8.6676916	1.39E-16	4.92E-15	27.012534
IGHV1-69	2.1773444	1.0168244	8.6624595	1.45E-16	5.10E-15	26.975166
KCTD17	0.5186728	5.4992889	8.6622106	1.45E-16	5.11E-15	26.973388
VN1R48P	1.732487	0.489155	8.6617284	1.46E-16	5.12E-15	26.969945
CREB3L1	0.9286526	4.6880072	8.6615292	1.46E-16	5.12E-15	26.968523
RPUSD2	-0.151983	5.7699279	-8.661212	1.46E-16	5.13E-15	26.966258
GSTA12P	-1.772874	1.1073831	-8.6588	1.49E-16	5.21E-15	26.949036
RGS19	0.2545424	5.5784559	8.6580723	1.50E-16	5.24E-15	26.943843
CDH17	1.9912582	1.242247	8.6567584	1.51E-16	5.28E-15	26.934465
IGHG2	1.1738537	5.3856476	8.6526296	1.56E-16	5.44E-15	26.905
C16orf95	-0.397672	4.592944	-8.650501	1.58E-16	5.52E-15	26.889813
CLEC11A	0.4873391	5.1600013	8.649071	1.60E-16	5.57E-15	26.879612
UBE2D4	-0.164755	6.0525261	-8.648467	1.60E-16	5.59E-15	26.875301
1-Mar	0.325548	5.0762762	8.6477632	1.61E-16	5.61E-15	26.870283
KLF8	0.9339016	3.6525088	8.6467572	1.63E-16	5.65E-15	26.863108
MEPCE	-0.137777	6.4810936	-8.644683	1.65E-16	5.73E-15	26.848316
ANK1	1.3360113	2.6560712	8.6443178	1.65E-16	5.74E-15	26.845712
AMPD1	1.5444973	-0.309698	8.6402976	1.70E-16	5.90E-15	26.817049
EPB41L3	0.4517289	5.1382548	8.6395265	1.71E-16	5.93E-15	26.811552
KLHL38	1.7098705	0.8100436	8.6350407	1.77E-16	6.12E-15	26.779582
HEG1	0.2255836	6.120612	8.634707	1.77E-16	6.13E-15	26.777205
COL14A1	0.6064659	5.6807839	8.6345538	1.78E-16	6.13E-15	26.776113
KIAA1324L	0.8009296	4.4594717	8.6335669	1.79E-16	6.17E-15	26.769082
TESPA1	0.8666158	3.8980483	8.6280581	1.86E-16	6.42E-15	26.72984
EFS	1.0593225	3.8052274	8.6278121	1.87E-16	6.42E-15	26.728088
EFNB3	1.2925318	3.1186904	8.6271675	1.87E-16	6.45E-15	26.723497
THBS2	0.8357845	5.7957128	8.6267171	1.88E-16	6.46E-15	26.72029
COL6A2	0.2738225	6.6316827	8.6265844	1.88E-16	6.46E-15	26.719345
TMEM178B	1.5072718	3.5043011	8.6250808	1.90E-16	6.53E-15	26.708638
NUBP2	-0.142161	6.462309	-8.624999	1.90E-16	6.53E-15	26.708053
RP11-44B19	-1.488429	-0.13867	-8.623934	1.92E-16	6.57E-15	26.700472
PFDN6	-0.187715	6.4457972	-8.622982	1.93E-16	6.61E-15	26.693697
CTD-307407	-0.344076	4.9226372	-8.622574	1.94E-16	6.62E-15	26.690794
HMGCL	-0.225547	6.7460034	-8.622483	1.94E-16	6.62E-15	26.69014
PDP1	0.4908826	5.2526686	8.6221498	1.94E-16	6.63E-15	26.687771

MTHFD1	-0.261479	6.8349306	-8.619605	1.98E-16	6.75E-15	26.669658
SGPP2	1.437726	3.480975	8.6181308	2.00E-16	6.82E-15	26.659165
RORB	1.7489808	0.8546754	8.6167218	2.02E-16	6.88E-15	26.649139
EMR4P	1.4943537	2.0907728	8.6159383	2.03E-16	6.91E-15	26.643564
LRFN4	0.5567667	4.5587064	8.6153719	2.04E-16	6.93E-15	26.639534
GPR20	1.7287176	0.9278155	8.6134555	2.07E-16	7.03E-15	26.6259
PIK3R6	0.5141413	4.3748846	8.6112393	2.10E-16	7.13E-15	26.610136
RP11-10K16	1.4722396	1.1879148	8.6104773	2.12E-16	7.17E-15	26.604715
MCUR1	-0.155635	6.2754287	-8.605754	2.19E-16	7.41E-15	26.571127
SULF2	0.3600478	6.2021424	8.6035337	2.23E-16	7.52E-15	26.555344
MOCS2	-0.174224	6.4412318	-8.602309	2.25E-16	7.58E-15	26.546639
POR	-0.209848	7.1729299	-8.601737	2.25E-16	7.61E-15	26.542577
BTBD3	0.1902948	5.9265824	8.6000798	2.28E-16	7.69E-15	26.530796
CDCA7	1.1367164	4.1991958	8.5975362	2.32E-16	7.83E-15	26.512721
INSL3	1.5474123	0.7920904	8.5959346	2.35E-16	7.91E-15	26.501342
RUNDC3A	1.4013327	2.5686059	8.5901219	2.45E-16	8.25E-15	26.460056
MRPS15	-0.17224	6.5821697	-8.589184	2.47E-16	8.29E-15	26.453395
PPP1R9B	0.1854939	6.1975404	8.5884043	2.48E-16	8.33E-15	26.44786
PSMC3	-0.136242	6.7736202	-8.587291	2.50E-16	8.39E-15	26.439954
KEAP1	-0.135078	6.5809088	-8.586887	2.51E-16	8.41E-15	26.437089
IMP3	-0.157324	6.3582261	-8.585666	2.53E-16	8.48E-15	26.428419
LYPD5	0.9687945	3.5108219	8.5851418	2.54E-16	8.50E-15	26.424699
RP11-259N1	1.5281422	2.0966394	8.5844804	2.56E-16	8.54E-15	26.420004
IGHV4-55	1.8672393	0.3428069	8.5840171	2.56E-16	8.56E-15	26.416715
SLC25A24	0.6334342	5.090946	8.5835009	2.57E-16	8.58E-15	26.413051
GLIPR2	0.3422658	5.4533371	8.582914	2.58E-16	8.61E-15	26.408886
CILP	1.2759154	3.4630601	8.5817207	2.61E-16	8.68E-15	26.400417
TRBV5-1	1.5944665	1.5196797	8.5813312	2.61E-16	8.69E-15	26.397653
DNAJC30	-0.191471	5.9741321	-8.581125	2.62E-16	8.70E-15	26.396189
TMC8	0.3694567	5.4195537	8.5807012	2.63E-16	8.72E-15	26.393182
TMEM222	-0.148877	6.3170039	-8.579948	2.64E-16	8.76E-15	26.38784
IGKV1-8	2.0792933	1.090808	8.5794399	2.65E-16	8.78E-15	26.384233
ODF2L	0.4091157	5.0623033	8.579249	2.65E-16	8.79E-15	26.382879
DNAH10	1.0806861	3.0171124	8.578676	2.67E-16	8.81E-15	26.378813
CHST2	0.3220005	5.1259165	8.5776833	2.68E-16	8.87E-15	26.37177
LINC01124	-0.779854	5.0504753	-8.577175	2.69E-16	8.89E-15	26.368168
RP11-635N1	-0.481565	4.7622475	-8.574995	2.74E-16	9.03E-15	26.352701
MBOAT4	1.6016355	2.5469911	8.5744144	2.75E-16	9.06E-15	26.348583
APTX	-0.126185	6.0671123	-8.567178	2.90E-16	9.54E-15	26.297276
GPR18	1.1856617	2.6768826	8.566962	2.90E-16	9.54E-15	26.295742
CD5	0.6879256	4.6770957	8.5658979	2.92E-16	9.61E-15	26.288199
FLRT2	1.1083865	4.3621273	8.5635526	2.97E-16	9.76E-15	26.271578
FUOM	-0.300398	6.5080798	-8.563438	2.98E-16	9.76E-15	26.270766
ISYNA1	0.3829109	5.8158134	8.5629681	2.99E-16	9.79E-15	26.267436
RHOF	0.7908122	4.0737922	8.5619855	3.01E-16	9.84E-15	26.260473
HACL1	-0.203425	6.2575142	-8.561963	3.01E-16	9.84E-15	26.260317
PSMD10P2	1.6473037	1.2968813	8.5605495	3.04E-16	9.93E-15	26.250299
KLF9	-0.284825	6.5515063	-8.559935	3.05E-16	9.97E-15	26.245943
ZNF365	1.6004063	0.8917218	8.5568063	3.12E-16	1.02E-14	26.223783
HADH	-0.212282	6.6571873	-8.554428	3.18E-16	1.04E-14	26.206941
LAMTOR2	-0.196847	6.5953625	-8.554044	3.19E-16	1.04E-14	26.204223

SAP30L	-0.134441	6.2577409	-8.551619	3.24E-16	1.05E-14	26.187053
INPP5D	0.2655446	5.7153029	8.5507167	3.26E-16	1.06E-14	26.180661
WNT2	2.0650863	1.0739646	8.5494882	3.29E-16	1.07E-14	26.171965
BLOC1S1	-0.204004	6.4158652	-8.548729	3.31E-16	1.07E-14	26.166588
APOBEC3C	0.3785921	5.4482707	8.5472442	3.35E-16	1.09E-14	26.156081
ATG12	-0.113274	6.2699664	-8.54573	3.38E-16	1.10E-14	26.145363
DICER1-AS1	-0.333567	4.9529277	-8.543542	3.44E-16	1.11E-14	26.129885
PPP1R3E	-0.208249	5.6333983	-8.542612	3.46E-16	1.12E-14	26.123301
SFXN5	-0.208479	6.4236745	-8.541161	3.50E-16	1.13E-14	26.113035
BAK1P1	1.3616331	1.0232084	8.5394863	3.54E-16	1.14E-14	26.101192
MS4A1	1.7228588	2.8261946	8.5383165	3.57E-16	1.15E-14	26.092917
NUDT11	1.6659466	0.7102626	8.5377061	3.59E-16	1.16E-14	26.0886
SRA1	-0.174464	6.381032	-8.534215	3.68E-16	1.18E-14	26.063911
TMPRSS13	1.4283907	2.4159599	8.5335926	3.69E-16	1.19E-14	26.059514
TRBV18	1.5575748	0.4316158	8.533532	3.70E-16	1.19E-14	26.059085
DEF6	0.3660106	5.3430208	8.5332827	3.70E-16	1.19E-14	26.057322
DECR2	-0.222185	6.6074661	-8.53178	3.74E-16	1.20E-14	26.046701
DIO3	2.1188462	1.4405143	8.5282487	3.84E-16	1.23E-14	26.02174
IGKV3-7	1.7938328	0.1568256	8.526824	3.88E-16	1.24E-14	26.011673
HINT1	-0.15374	7.0410222	-8.525926	3.90E-16	1.25E-14	26.005326
PCDHGA11	1.380142	2.5445594	8.5254104	3.92E-16	1.25E-14	26.001684
ATRNL1	1.8019186	3.3580752	8.5235715	3.97E-16	1.27E-14	25.988692
IMPDH1	0.3415369	5.6761174	8.522226	4.01E-16	1.28E-14	25.979188
NCF1	0.6760897	4.1611106	8.5198296	4.08E-16	1.30E-14	25.962262
GPR171	1.2404773	3.3638609	8.5178043	4.14E-16	1.32E-14	25.94796
SELK	-0.152206	6.3618543	-8.511048	4.35E-16	1.38E-14	25.900267
CACNA1C	0.5679752	4.8112369	8.5090871	4.41E-16	1.40E-14	25.886426
SH2D2A	0.6760319	4.6940513	8.5078285	4.45E-16	1.41E-14	25.877546
WDR17	1.5033728	0.7926743	8.5070634	4.47E-16	1.42E-14	25.872148
PTGER2	0.9018335	3.7645304	8.5063071	4.50E-16	1.43E-14	25.866812
CD96	0.5683999	4.8289105	8.5056048	4.52E-16	1.43E-14	25.861857
HYAL1	-0.293861	6.6630891	-8.505288	4.53E-16	1.43E-14	25.859624
WBSCR17	2.0340426	1.7618054	8.5050771	4.54E-16	1.44E-14	25.858134
KIF5A	1.7570704	1.3166555	8.5049831	4.54E-16	1.44E-14	25.857472
GCAT	-0.343182	6.2637572	-8.501738	4.65E-16	1.47E-14	25.83458
LINC01094	1.2409174	2.8877452	8.5010717	4.67E-16	1.47E-14	25.829885
RP11-588K2	0.5951917	5.003611	8.4990662	4.74E-16	1.49E-14	25.815743
EFEMP2	0.3371626	5.6962177	8.497728	4.79E-16	1.51E-14	25.806308
DNPH1	-0.189058	6.5240114	-8.493033	4.95E-16	1.56E-14	25.773212
CYTIP	0.5625011	4.9581678	8.4920405	4.99E-16	1.57E-14	25.766219
IRF4	1.2439217	3.5259726	8.4906725	5.03E-16	1.58E-14	25.75658
ELMSAN1	0.1931538	5.9090857	8.4904995	5.04E-16	1.58E-14	25.755361
RP11-284N8	1.205386	3.3148118	8.4896607	5.07E-16	1.59E-14	25.749451
CAMKK1	0.4060734	4.5917498	8.4885893	5.11E-16	1.60E-14	25.741903
SHISA2	1.7455509	2.0756915	8.4877686	5.14E-16	1.61E-14	25.736121
IGLV10-54	1.9585878	0.8674486	8.485457	5.23E-16	1.64E-14	25.719838
RSAD1	-0.14429	6.430423	-8.485024	5.24E-16	1.64E-14	25.716787
RAI1	0.2662015	5.8323917	8.4847214	5.26E-16	1.64E-14	25.714657
DGCR6L	-0.206481	6.4580068	-8.483141	5.32E-16	1.66E-14	25.703527
ERI3	-0.136457	6.3568785	-8.483058	5.32E-16	1.66E-14	25.702941
AKR7A2	-0.174505	6.4010707	-8.481907	5.36E-16	1.67E-14	25.694837

LINC00339	-0.183192	5.7800056	-8.481501	5.38E-16	1.67E-14	25.691982
CRLS1	-0.187676	6.6741726	-8.481225	5.39E-16	1.68E-14	25.690035
MXRA8	0.409385	5.6136873	8.4788114	5.48E-16	1.70E-14	25.673044
IGKV2D-29	1.9698962	0.88339	8.478269	5.51E-16	1.71E-14	25.669225
GPR160	0.4655024	5.0069147	8.4781825	5.51E-16	1.71E-14	25.668617
TNFSF13B	0.4232336	5.0221509	8.4761335	5.59E-16	1.73E-14	25.654194
ACSS1	0.3919649	5.7701484	8.4753452	5.62E-16	1.74E-14	25.648647
ABHD14A	-0.205952	5.9661595	-8.473879	5.68E-16	1.76E-14	25.638329
PIN4	-0.188401	6.0737661	-8.473158	5.71E-16	1.77E-14	25.633258
ZNF488	1.5915567	0.4619517	8.4714971	5.78E-16	1.79E-14	25.621569
KCNH3	1.3777909	2.8338578	8.4711219	5.80E-16	1.79E-14	25.61893
MMP11	0.5353967	5.5997021	8.4708925	5.81E-16	1.79E-14	25.617315
SAR1B	-0.193281	6.7503669	-8.468571	5.90E-16	1.82E-14	25.600982
CDK5	-0.175051	5.9884261	-8.466122	6.01E-16	1.85E-14	25.583764
NDUFA11	-0.197901	6.513356	-8.462838	6.15E-16	1.89E-14	25.560669
SIRPB2	0.9645447	3.7857192	8.4626501	6.16E-16	1.89E-14	25.559348
FAM213A	-0.196699	6.8226734	-8.46215	6.18E-16	1.90E-14	25.555835
VASH1	0.263467	5.6340674	8.4619209	6.19E-16	1.90E-14	25.554222
TRPV4	0.8023268	4.8977882	8.4617412	6.20E-16	1.90E-14	25.552958
CFL2	-0.197793	6.4787893	-8.461613	6.21E-16	1.90E-14	25.552057
FOXF2	1.4558583	2.5720075	8.4614675	6.21E-16	1.90E-14	25.551034
ZNF154	0.5464366	4.2411269	8.4603856	6.26E-16	1.91E-14	25.543429
TNFAIP1	-0.116721	6.5746812	-8.460063	6.28E-16	1.92E-14	25.541159
RP11-334A1	1.7187835	1.4974655	8.4599425	6.28E-16	1.92E-14	25.540314
IK	-0.104549	6.6212041	-8.458885	6.33E-16	1.93E-14	25.532879
NFE2L3	0.3682506	5.4011458	8.4576213	6.39E-16	1.95E-14	25.524
TBL3	-0.141863	6.3695233	-8.457451	6.39E-16	1.95E-14	25.522802
XKR6	1.3413394	1.7710758	8.4573683	6.40E-16	1.95E-14	25.522222
RGS18	0.9685812	3.7958419	8.4564895	6.44E-16	1.96E-14	25.516046
IGLV9-49	1.9956916	0.8952512	8.4564711	6.44E-16	1.96E-14	25.515917
TUT1	-0.182479	6.0783913	-8.454709	6.52E-16	1.98E-14	25.503532
SALL4	1.8014343	2.9923757	8.4525432	6.62E-16	2.01E-14	25.48832
TRG-AS1	0.7295292	3.7064624	8.4511179	6.69E-16	2.03E-14	25.478308
MX2	0.4426933	5.3727404	8.4509439	6.70E-16	2.03E-14	25.477085
BTK	0.4212907	4.8501786	8.4494925	6.77E-16	2.05E-14	25.466891
NTM	1.7846173	3.2793468	8.4494057	6.77E-16	2.05E-14	25.466282
MSC	0.794195	5.0139753	8.4493739	6.78E-16	2.05E-14	25.466058
IL1B	1.0098414	3.8115867	8.4483952	6.82E-16	2.06E-14	25.459185
ZNF610	1.0502931	3.3381796	8.4428174	7.10E-16	2.14E-14	25.420023
LUM	1.2945614	5.406014	8.4424626	7.12E-16	2.14E-14	25.417533
MEX3B	0.7738083	4.2290429	8.4422848	7.13E-16	2.15E-14	25.416285
ARMCX1	0.4703463	4.8622029	8.4380069	7.35E-16	2.21E-14	25.386263
ITGA3	0.3945079	5.6675902	8.4375486	7.38E-16	2.22E-14	25.383047
ACY1	-0.257002	5.931296	-8.436523	7.43E-16	2.23E-14	25.375854
GLT8D2	0.5778318	4.3660406	8.435921	7.46E-16	2.24E-14	25.371627
TGFB1	0.3096388	6.1043299	8.4349279	7.52E-16	2.25E-14	25.36466
CMBL	-0.38497	6.6678122	-8.432257	7.66E-16	2.29E-14	25.345927
IPCEF1	0.6646507	4.0788247	8.4315659	7.70E-16	2.30E-14	25.341079
NFIC	-0.178952	6.7944819	-8.430436	7.76E-16	2.32E-14	25.333156
RP11-24F11	1.4785643	1.4792495	8.4286728	7.86E-16	2.35E-14	25.320792
IGHV3-20	1.8494948	0.1366671	8.4285034	7.87E-16	2.35E-14	25.319604

TRBV10-3	1.4604182	0.0889713	8.4277963	7.91E-16	2.36E-14	25.314646
NEFH	0.9558801	3.2107509	8.4271543	7.95E-16	2.37E-14	25.310146
C19orf70	-0.194578	6.4038151	-8.424535	8.10E-16	2.41E-14	25.291788
TMBIM6	-0.135689	7.4381135	-8.424248	8.11E-16	2.41E-14	25.289771
RP11-368I7	-0.482021	4.1925576	-8.423476	8.16E-16	2.43E-14	25.284364
ELP6	-0.136347	6.020952	-8.422264	8.23E-16	2.44E-14	25.275871
FIS1	-0.18302	6.7378676	-8.422211	8.23E-16	2.44E-14	25.2755
ENPP5	1.856523	3.2645535	8.4217755	8.26E-16	2.45E-14	25.272445
OAF	-0.201499	6.8702169	-8.420135	8.36E-16	2.48E-14	25.260951
GRIP1	1.6285772	1.4614722	8.4187955	8.44E-16	2.50E-14	25.251564
TRAV9-2	1.4865512	0.2001462	8.4177103	8.50E-16	2.52E-14	25.243962
AURKAIP1	-0.188669	6.6569044	-8.41738	8.52E-16	2.52E-14	25.241649
SYN1	1.0575696	3.805123	8.4168304	8.56E-16	2.53E-14	25.237798
IGKV1-12	1.7037939	-0.078641	8.4164886	8.58E-16	2.53E-14	25.235404
NDN	0.5670127	5.1613611	8.415814	8.62E-16	2.54E-14	25.230679
IGLV8-61	2.1151371	1.9226577	8.4150638	8.67E-16	2.55E-14	25.225424
ZNF415	0.8585165	3.8011219	8.4123993	8.83E-16	2.60E-14	25.206764
RAC2	0.3564562	5.7664296	8.4109881	8.92E-16	2.62E-14	25.196883
LRTOMT	-0.176628	5.5456381	-8.404407	9.35E-16	2.75E-14	25.150814
PDGFRB	0.2340002	6.4309447	8.4037903	9.39E-16	2.76E-14	25.146502
MRPL2	-0.166241	6.451874	-8.402921	9.45E-16	2.77E-14	25.140418
ADRA2A	1.2659674	3.9767795	8.402297	9.50E-16	2.78E-14	25.136053
TOLLIP	-0.13801	6.7763393	-8.401783	9.53E-16	2.79E-14	25.132458
HID1	0.4149647	5.6483583	8.4013027	9.56E-16	2.80E-14	25.129096
YIF1A	-0.156785	6.6963978	-8.398924	9.73E-16	2.85E-14	25.112453
RP11-728F1	1.5883335	1.5890055	8.3986722	9.74E-16	2.85E-14	25.110694
HFM1	1.5659487	0.7064376	8.3980169	9.79E-16	2.86E-14	25.106112
HHIP	2.0392002	2.2556259	8.3974168	9.83E-16	2.87E-14	25.101914
BEX2	1.7675523	3.9965074	8.3964021	9.90E-16	2.89E-14	25.094817
TNC	0.5375223	5.6681712	8.3943314	1.01E-15	2.93E-14	25.080338
KCNN4	0.8481014	4.1419958	8.3942553	1.01E-15	2.93E-14	25.079805
TRAV8-3	1.5183683	0.244556	8.3919901	1.02E-15	2.97E-14	25.063969
GRIN2A	1.9654677	1.9455588	8.3906193	1.03E-15	3.00E-14	25.054386
MPND	-0.205067	6.3001134	-8.389866	1.04E-15	3.01E-14	25.049122
CTHRC1	0.7728985	4.9151513	8.3879719	1.05E-15	3.05E-14	25.035882
MAP4K1	0.3758767	5.0975073	8.3877139	1.05E-15	3.06E-14	25.034079
ELK3	0.2047589	5.9792569	8.3864568	1.06E-15	3.08E-14	25.025294
ZDHHC8P1	1.8749223	1.0075801	8.3852585	1.07E-15	3.10E-14	25.016921
SLC45A4	0.4764215	5.3711068	8.3851457	1.07E-15	3.10E-14	25.016133
ELOVL4	1.3696292	2.4073408	8.3846355	1.08E-15	3.11E-14	25.012569
EIF2B4	-0.130103	6.2758734	-8.384587	1.08E-15	3.11E-14	25.012232
C14orf1	-0.196563	6.5630283	-8.383906	1.08E-15	3.13E-14	25.007469
C15orf39	0.1987878	6.0183766	8.3830598	1.09E-15	3.14E-14	25.00156
ZC3HAV1L	1.3461569	2.9753076	8.3827887	1.09E-15	3.15E-14	24.999666
MSRB1	-0.196267	6.7088801	-8.381836	1.10E-15	3.16E-14	24.993011
C1orf123	-0.142973	6.1850326	-8.3813	1.10E-15	3.17E-14	24.989266
NSMCE1	-0.181294	6.4033314	-8.379075	1.12E-15	3.22E-14	24.973724
C11orf70	1.4392384	1.0494669	8.3780447	1.13E-15	3.24E-14	24.966532
SNX20	0.523479	4.7717354	8.3736593	1.17E-15	3.34E-14	24.935914
MFNG	0.240877	5.5289694	8.3716425	1.18E-15	3.39E-14	24.921838
RNF5	-0.153464	6.600217	-8.369927	1.20E-15	3.43E-14	24.909868

TCEA3	-0.254482	6.7193302	-8.368661	1.21E-15	3.46E-14	24.90103
TNFSF8	0.9122978	3.7496273	8.3666866	1.22E-15	3.50E-14	24.887255
TIMM8A	-0.228248	5.8279083	-8.366009	1.23E-15	3.52E-14	24.882526
ASB2	0.8378389	4.0261157	8.3652023	1.24E-15	3.54E-14	24.8769
LAX1	1.0506303	3.3473305	8.3646678	1.24E-15	3.54E-14	24.873172
AFMID	-0.177566	6.6426728	-8.364615	1.24E-15	3.54E-14	24.872806
LTB	0.5634015	5.3679866	8.36354	1.25E-15	3.57E-14	24.865305
TRAV2	1.4649093	0.1218438	8.3634528	1.25E-15	3.57E-14	24.864697
ANAPC11	-0.18268	6.6499409	-8.362346	1.26E-15	3.59E-14	24.85698
DPM3	-0.239993	6.2833491	-8.362048	1.27E-15	3.60E-14	24.854901
FANK1	1.3977439	1.8987163	8.3620442	1.27E-15	3.60E-14	24.854873
LCNL1	1.841002	0.95732	8.3615976	1.27E-15	3.60E-14	24.851758
CD97	0.29624	6.1375542	8.3614589	1.27E-15	3.61E-14	24.850791
SUPT4H1	-0.117731	6.4854141	-8.359614	1.29E-15	3.65E-14	24.837927
PLEKHB2	0.1514405	6.2526564	8.3559178	1.32E-15	3.74E-14	24.812158
IGLV2-18	1.8736982	0.6185511	8.3552637	1.33E-15	3.76E-14	24.807599
TMEM53	-0.204813	6.1621358	-8.350794	1.37E-15	3.88E-14	24.77645
NLGN3	0.9170007	2.9493885	8.3501975	1.38E-15	3.89E-14	24.772294
SLC9A1	0.2074588	5.6316171	8.3487621	1.39E-15	3.93E-14	24.762294
FAM174A	-0.152912	5.9120426	-8.347862	1.40E-15	3.95E-14	24.756023
ROR2	1.178366	3.9769089	8.3476312	1.40E-15	3.95E-14	24.754416
CD53	0.3317778	5.755198	8.3456875	1.42E-15	4.01E-14	24.740877
ZNF70	0.3953279	4.7218048	8.3446178	1.43E-15	4.03E-14	24.733428
RP5-1180D1	1.2147003	2.9951559	8.3442865	1.44E-15	4.04E-14	24.731121
RP11-285E2	-1.494377	1.9963309	-8.344137	1.44E-15	4.04E-14	24.73008
BNIP3	-0.192376	6.7130486	-8.34247	1.46E-15	4.09E-14	24.718475
MEX3C	0.1851749	5.9024197	8.3404267	1.48E-15	4.14E-14	24.704245
C20orf194	0.2527285	5.5810393	8.3372694	1.51E-15	4.23E-14	24.682268
PSME1	-0.145893	6.8845223	-8.336854	1.51E-15	4.24E-14	24.679378
TRBV25-1	1.4206826	1.9304975	8.3362965	1.52E-15	4.26E-14	24.675497
GPRASP1	0.4774234	4.6858862	8.3359171	1.52E-15	4.27E-14	24.672856
ZNF880	0.7230239	4.4102687	8.3346678	1.54E-15	4.30E-14	24.664163
OXLD1	-0.191678	6.0155848	-8.333824	1.55E-15	4.32E-14	24.658294
PTHLH	1.4440809	3.29722	8.3336717	1.55E-15	4.32E-14	24.657232
SIGLEC7	0.6648776	4.0785367	8.3323705	1.56E-15	4.36E-14	24.648179
SEMA4C	0.1599881	6.1450198	8.3321728	1.57E-15	4.36E-14	24.646803
IVD	-0.188261	6.7761199	-8.3319	1.57E-15	4.37E-14	24.644909
PI16	1.7373181	0.9892977	8.3307915	1.58E-15	4.40E-14	24.637195
SFRP5	2.3902966	1.8345246	8.3307717	1.58E-15	4.40E-14	24.637056
SUV39H1	-0.186235	5.9221274	-8.3302	1.59E-15	4.41E-14	24.633083
RP11-278J6	-1.07529	2.7103952	-8.327973	1.61E-15	4.48E-14	24.61759
IL4I1	0.6511114	4.7475729	8.326342	1.63E-15	4.53E-14	24.606248
ADAM17	0.3280593	5.5160012	8.3259064	1.64E-15	4.54E-14	24.603219
AP000445.1	-1.91428	1.8998327	-8.324973	1.65E-15	4.57E-14	24.596727
KAZN	0.6373709	4.4322338	8.3232028	1.67E-15	4.62E-14	24.584422
RP11-388C1	-1.0495	2.9584705	-8.320958	1.70E-15	4.69E-14	24.568819
VKORC1	-0.204355	6.4567731	-8.320726	1.70E-15	4.69E-14	24.567204
CRIP1	0.8145472	3.7272229	8.3191436	1.72E-15	4.74E-14	24.556207
NEGR1	1.6011635	1.6074479	8.3187491	1.72E-15	4.75E-14	24.553465
IGHV1-2	2.1447642	1.95793	8.3186277	1.72E-15	4.75E-14	24.552622
PLAU	0.2885418	5.515997	8.3176712	1.74E-15	4.78E-14	24.545975

NDUFA5	-0.150266	6.4341169	-8.316988	1.74E-15	4.80E-14	24.541227
SERPINB9	0.2964804	5.652682	8.3156282	1.76E-15	4.85E-14	24.53178
CLIC3	0.9692724	3.5894178	8.3145315	1.78E-15	4.88E-14	24.524161
NEDD8	-0.139608	6.4804755	-8.314262	1.78E-15	4.89E-14	24.522286
EGR3	0.8563193	3.919525	8.3127271	1.80E-15	4.94E-14	24.511627
PPP1R10	-0.126215	6.6117349	-8.311508	1.81E-15	4.97E-14	24.503156
NEURL1	0.9633472	3.8151212	8.3110256	1.82E-15	4.99E-14	24.499809
TM7SF2	-0.326501	6.7377828	-8.309561	1.84E-15	5.04E-14	24.48964
CLPP	-0.161755	6.4009237	-8.305558	1.89E-15	5.18E-14	24.461848
COL13A1	0.94911	3.7319127	8.3053524	1.89E-15	5.18E-14	24.460418
DCAF11	-0.181904	6.7433415	-8.303987	1.91E-15	5.23E-14	24.45094
IL2RA	1.0549876	3.7897345	8.2973797	2.01E-15	5.47E-14	24.405092
CRAT	-0.16715	6.7721607	-8.296775	2.01E-15	5.49E-14	24.400894
TRAV26-1	1.4241628	0.0351811	8.2967535	2.01E-15	5.49E-14	24.400747
RBP5	-0.433034	6.4523732	-8.296545	2.02E-15	5.49E-14	24.399301
FOXF1	0.7384434	4.3644629	8.2962689	2.02E-15	5.50E-14	24.397386
PPP1R18	0.1998639	6.2482771	8.2955887	2.03E-15	5.52E-14	24.392668
SPHK1	0.7068248	5.351476	8.2952727	2.04E-15	5.53E-14	24.390476
TOMM70A	-0.103178	6.6646391	-8.292429	2.08E-15	5.64E-14	24.370753
NKIRAS1	-0.210548	5.7136107	-8.287969	2.14E-15	5.82E-14	24.339835
KHK	-0.331183	6.7675305	-8.286901	2.16E-15	5.86E-14	24.332428
LMAN2	-0.145242	7.0562947	-8.286692	2.16E-15	5.86E-14	24.330982
PLCL1	0.4791625	4.4406528	8.2848453	2.19E-15	5.93E-14	24.318183
RP11-817I4	1.3267263	1.5867777	8.2823826	2.23E-15	6.03E-14	24.301118
TFAP2E	1.1233997	2.8405064	8.2823308	2.23E-15	6.03E-14	24.300759
PCBD1	-0.186675	6.7980415	-8.279905	2.27E-15	6.13E-14	24.283956
WISP1	0.9053293	4.0971557	8.2789982	2.28E-15	6.17E-14	24.277671
C3orf80	1.4934544	2.8371962	8.2786099	2.29E-15	6.18E-14	24.274982
TRAV21	1.5358654	0.2652744	8.2768616	2.32E-15	6.25E-14	24.262873
LDHD	-0.377316	6.4520557	-8.276347	2.33E-15	6.27E-14	24.259309
UBASH3A	1.0316604	3.6914057	8.2761223	2.33E-15	6.28E-14	24.257753
KCND2	1.787131	1.2054931	8.2736219	2.37E-15	6.38E-14	24.24044
FAR2	0.5023648	4.8107641	8.2734336	2.38E-15	6.39E-14	24.239137
PSMD12	-0.10188	6.4639747	-8.272294	2.40E-15	6.43E-14	24.231249
ATP6V0E2	-0.289027	6.5451333	-8.271789	2.40E-15	6.45E-14	24.227749
EHHADH	-0.356285	6.7477737	-8.271326	2.41E-15	6.47E-14	24.224546
ACSM5	-0.617334	6.2378632	-8.270087	2.43E-15	6.52E-14	24.215968
MICAL1	0.2854825	5.7928452	8.2695334	2.44E-15	6.54E-14	24.212137
PEX14	-0.179368	6.1234356	-8.269095	2.45E-15	6.56E-14	24.209103
RALY-AS1	-0.323365	4.8288041	-8.268751	2.46E-15	6.57E-14	24.206726
MRPS7	-0.133844	6.5044132	-8.268654	2.46E-15	6.57E-14	24.206049
JPH1	1.637416	3.1949271	8.2681848	2.47E-15	6.59E-14	24.202804
TRPC1	0.689904	4.3678764	8.2658024	2.51E-15	6.69E-14	24.186318
ENTPD1	0.1452202	6.0410236	8.2650411	2.52E-15	6.72E-14	24.181051
AC020571.3	1.5857781	0.9895921	8.2648142	2.53E-15	6.73E-14	24.179481
SPI1	0.2977593	5.6843098	8.2631296	2.56E-15	6.80E-14	24.167827
SYTL3	0.5573429	4.2836976	8.2630901	2.56E-15	6.80E-14	24.167553
ITGA11	0.5769733	5.0112636	8.2629671	2.56E-15	6.80E-14	24.166703
TMC7	0.5144199	4.6779495	8.2629036	2.56E-15	6.80E-14	24.166264
CD1A	1.6646963	1.1054199	8.26254	2.57E-15	6.81E-14	24.163748
LY9	0.7250501	4.111822	8.262293	2.57E-15	6.82E-14	24.162039

FAH	-0.219707	6.8073166	-8.256546	2.68E-15	7.10E-14	24.122295
SPON1	1.0112327	4.3224045	8.2553255	2.70E-15	7.15E-14	24.113858
CARD9	0.3618305	4.7581044	8.2549842	2.71E-15	7.17E-14	24.111499
PCDH18	0.7139432	4.5183749	8.2537415	2.73E-15	7.22E-14	24.102909
LINGO3	1.3644459	0.7793313	8.2525314	2.75E-15	7.28E-14	24.094545
RPL26L1	-0.163438	6.0227849	-8.252166	2.76E-15	7.30E-14	24.092016
FAM129B	0.2364965	6.3481525	8.2490874	2.82E-15	7.45E-14	24.070745
NOD1	0.1837017	5.2763868	8.2479769	2.84E-15	7.50E-14	24.063072
TSKU	-0.388669	6.7508854	-8.24713	2.86E-15	7.54E-14	24.057224
C19orf35	1.1614895	2.2917012	8.2470052	2.86E-15	7.54E-14	24.056359
SC5D	-0.25526	6.5952991	-8.246493	2.87E-15	7.57E-14	24.052821
RNF150	1.3180451	3.4816775	8.2453183	2.90E-15	7.62E-14	24.044706
IGHGP	1.8057382	3.8176578	8.2449627	2.91E-15	7.64E-14	24.04225
TPBG	1.0400642	4.1220424	8.2442054	2.92E-15	7.67E-14	24.03702
ERP27	1.11828	3.50821	8.2441729	2.92E-15	7.67E-14	24.036795
EVI2B	0.4175435	5.205747	8.2437625	2.93E-15	7.69E-14	24.033961
BGN	0.3294659	6.7289444	8.2427012	2.95E-15	7.74E-14	24.026631
OSER1	-0.127039	6.1973702	-8.240275	3.00E-15	7.87E-14	24.009874
ALDH1L1	-0.556576	6.8792857	-8.239827	3.01E-15	7.89E-14	24.006786
SMDT1	-0.175333	6.3444796	-8.239116	3.03E-15	7.92E-14	24.001875
AEBP1	0.4912141	6.1995456	8.2377446	3.06E-15	7.99E-14	23.992408
IGLV1-36	1.8527068	0.5190623	8.236862	3.08E-15	8.04E-14	23.986316
FAM43B	1.7971084	2.3052366	8.235703	3.10E-15	8.10E-14	23.978317
JAG1	0.2565873	6.1314712	8.2337322	3.15E-15	8.20E-14	23.964715
MRPL12	-0.204792	6.3264519	-8.233563	3.15E-15	8.21E-14	23.963549
RP11-1094M	1.4688925	1.2284049	8.2319284	3.19E-15	8.30E-14	23.952269
C6orf89	-0.111324	6.6765948	-8.231313	3.20E-15	8.33E-14	23.948026
B3GALNT1	0.5359522	4.7757479	8.2303614	3.22E-15	8.38E-14	23.941458
SPDYC	-1.101007	4.2133662	-8.22761	3.28E-15	8.53E-14	23.922478
AC090587.5	-0.679067	3.4979364	-8.225012	3.35E-15	8.69E-14	23.904559
TCAIM	-0.15316	6.3261499	-8.224364	3.36E-15	8.72E-14	23.900096
PADI2	1.0940702	3.8981949	8.223708	3.38E-15	8.75E-14	23.89557
CAPN6	2.0600644	2.0729214	8.2233194	3.39E-15	8.77E-14	23.892891
MCOLN3	1.8849339	2.4014285	8.2232263	3.39E-15	8.77E-14	23.892249
SRPX2	0.5682156	4.8411785	8.2231006	3.39E-15	8.77E-14	23.891383
CHPT1	-0.181155	6.6986256	-8.221671	3.43E-15	8.86E-14	23.881529
CD1C	1.0258533	3.8714676	8.2215283	3.43E-15	8.86E-14	23.880542
ZNF624	0.3517792	4.5752079	8.2204869	3.45E-15	8.92E-14	23.873364
SMARCD3	0.3947602	5.3590633	8.2195514	3.48E-15	8.97E-14	23.866915
PDCD1	1.1289772	3.894002	8.2168179	3.54E-15	9.14E-14	23.848077
PRKCQ	0.8707005	3.8256183	8.2162332	3.56E-15	9.17E-14	23.844048
ALKBH7	-0.203836	6.4667407	-8.214484	3.60E-15	9.28E-14	23.831994
TOX	1.0761804	4.0740785	8.2138708	3.62E-15	9.31E-14	23.827771
NLRC4	0.2937746	4.4830502	8.2109595	3.69E-15	9.50E-14	23.807717
GNG2	0.2758722	5.3798031	8.210858	3.70E-15	9.50E-14	23.807018
FAHD1	-0.183262	6.4841612	-8.210521	3.71E-15	9.51E-14	23.804696
NACAD	1.1147531	2.9065697	8.209756	3.73E-15	9.56E-14	23.799428
MUC12	1.536654	2.1679611	8.2093284	3.74E-15	9.58E-14	23.796483
COTL1	0.2692479	6.2073867	8.2075518	3.78E-15	9.69E-14	23.78425
PRICKLE2	0.6689385	4.8994177	8.2071742	3.79E-15	9.71E-14	23.78165
TPRG1L	-0.181653	6.5625893	-8.20649	3.81E-15	9.75E-14	23.776936

SLC4A11	1.059635	3.7487988	8.2062833	3.82E-15	9.76E-14	23.775515
RP1-28010.	-0.974194	4.1360543	-8.206109	3.82E-15	9.76E-14	23.774318
BIN2	0.3659676	5.1424619	8.2036567	3.89E-15	9.93E-14	23.757434
WNT2B	0.4748998	4.2834637	8.2034162	3.90E-15	9.94E-14	23.755779
LZTS1	0.4152222	5.2061267	8.2022718	3.93E-15	1.00E-13	23.747901
TMEM115	-0.120817	6.4370592	-8.19939	4.01E-15	1.02E-13	23.728071
MILR1	0.4679903	4.4502263	8.1974686	4.06E-15	1.03E-13	23.71485
MRPS18B	-0.156497	6.6477566	-8.1966	4.09E-15	1.04E-13	23.708876
GPR174	1.6090621	1.4066122	8.1950425	4.13E-15	1.05E-13	23.69816
ANKRD36BP2	1.776914	1.3106562	8.1941061	4.16E-15	1.06E-13	23.69172
PNOC	1.6964863	0.5404219	8.1923545	4.21E-15	1.07E-13	23.679674
STYK1	1.5579579	0.7152744	8.1911552	4.25E-15	1.08E-13	23.671427
CRY2	-0.190566	6.5178456	-8.190591	4.26E-15	1.08E-13	23.667549
C19orf73	-0.457022	4.3616317	-8.18848	4.33E-15	1.10E-13	23.653037
C11orf1	-0.262067	5.645548	-8.185396	4.42E-15	1.12E-13	23.631838
ARFGAP2	-0.109685	6.6491852	-8.184261	4.46E-15	1.13E-13	23.624035
RP11-573G6	1.4697834	1.026617	8.1818574	4.53E-15	1.15E-13	23.607519
KIAA2013	-0.15075	6.5589899	-8.181641	4.54E-15	1.15E-13	23.606033
RP11-517C1	1.2877248	-0.399362	8.1808989	4.56E-15	1.15E-13	23.600934
NFAM1	0.4066778	5.095316	8.1736458	4.80E-15	1.21E-13	23.551118
ZNF204P	1.3868129	3.470235	8.1726687	4.84E-15	1.22E-13	23.54441
APOC1	-0.289825	7.6402751	-8.172182	4.85E-15	1.22E-13	23.541071
ARHGAP15	0.3447924	5.1960601	8.1711884	4.89E-15	1.23E-13	23.534248
IGKV5-2	1.5965959	-0.177014	8.171153	4.89E-15	1.23E-13	23.534005
CYB5A	-0.237389	7.0555453	-8.170395	4.91E-15	1.24E-13	23.528805
TTC38	-0.208329	6.7873393	-8.169538	4.94E-15	1.24E-13	23.522921
RP11-750H9	1.2319366	2.7279677	8.1692132	4.95E-15	1.24E-13	23.520691
RAD54L2	-0.159695	6.0769601	-8.167498	5.01E-15	1.26E-13	23.508921
CXCR4	0.2948107	6.0237033	8.1658311	5.07E-15	1.27E-13	23.497481
RP11-336K2	1.467827	1.9041859	8.1648317	5.11E-15	1.28E-13	23.490624
AC025165.8	1.5081854	0.5797545	8.1641217	5.14E-15	1.29E-13	23.485753
RP11-136I1	-1.571383	1.6812272	-8.162527	5.19E-15	1.30E-13	23.474811
LARP4	-0.133258	6.5289036	-8.161307	5.24E-15	1.31E-13	23.466447
NTHL1	-0.25521	6.1779921	-8.159721	5.30E-15	1.32E-13	23.455567
SPPL2A	-0.132386	6.614865	-8.158241	5.35E-15	1.34E-13	23.44542
HVCN1	0.3230847	4.882729	8.1555474	5.45E-15	1.36E-13	23.42695
B3GALT4	0.4823829	4.8649645	8.1552633	5.46E-15	1.36E-13	23.425003
PEX19	-0.148943	6.7193957	-8.155045	5.47E-15	1.36E-13	23.42351
SLC25A38	-0.16015	6.2943516	-8.154697	5.49E-15	1.37E-13	23.42112
PPP2R2B	1.1469105	3.4782483	8.1544047	5.50E-15	1.37E-13	23.419117
GIPR	1.1377562	3.2520199	8.1542258	5.50E-15	1.37E-13	23.417891
PHF7	-0.251433	5.3016314	-8.154123	5.51E-15	1.37E-13	23.417185
WDR45	-0.14011	6.3779557	-8.153606	5.53E-15	1.37E-13	23.413646
ADH5	-0.151633	6.870455	-8.152225	5.58E-15	1.39E-13	23.404178
YWHAZP4	1.3003813	2.4610856	8.1516296	5.61E-15	1.39E-13	23.400097
ZNF660	0.824148	3.5985099	8.1488472	5.72E-15	1.42E-13	23.381031
PRDM1	0.3423508	5.18655	8.1479463	5.75E-15	1.42E-13	23.374858
RSPO3	1.8597125	2.0834683	8.1472627	5.78E-15	1.43E-13	23.370175
PARVG	0.3495109	5.3563307	8.1467999	5.80E-15	1.43E-13	23.367005
MED29	-0.107755	6.4904103	-8.144938	5.87E-15	1.45E-13	23.35425
KLHDC3	-0.144435	6.719186	-8.144549	5.89E-15	1.45E-13	23.35159

TMEM42	-0.181248	5.8599485	-8.14424	5.90E-15	1.46E-13	23.349473
GPR183	0.5813234	4.8791163	8.1420192	6.00E-15	1.48E-13	23.334262
NOP9	-0.110932	6.1882818	-8.141189	6.03E-15	1.49E-13	23.328575
MLXIPL	-0.323787	6.9410259	-8.141131	6.03E-15	1.49E-13	23.328181
IGSF9B	1.4837252	1.0991754	8.1408509	6.05E-15	1.49E-13	23.326262
EMC4	-0.110917	6.5105606	-8.140018	6.08E-15	1.49E-13	23.320557
PLAT	0.4125589	5.5991794	8.1400053	6.08E-15	1.49E-13	23.320473
RP11-65J21	-1.784427	0.2341371	-8.137095	6.21E-15	1.52E-13	23.300547
GPRC5C	-0.161525	6.7749194	-8.134433	6.32E-15	1.55E-13	23.282331
COX17	-0.167475	6.340695	-8.133702	6.36E-15	1.56E-13	23.277327
CCND2	0.3065076	5.5264182	8.1333967	6.37E-15	1.56E-13	23.27524
RP11-161M6	1.2014427	3.0134468	8.132943	6.39E-15	1.57E-13	23.272136
PDZD7	0.979267	3.0115542	8.132761	6.40E-15	1.57E-13	23.270891
RP11-307C1	1.6820013	1.2566734	8.1312715	6.46E-15	1.58E-13	23.2607
DNPEP	-0.119346	6.5361419	-8.1301	6.52E-15	1.59E-13	23.252689
SSBP2	0.4696676	5.0705752	8.1300024	6.52E-15	1.59E-13	23.252018
LRRC55	1.4942348	2.0266801	8.1295305	6.54E-15	1.60E-13	23.24879
FAM153A	1.7594794	1.2389556	8.1289826	6.57E-15	1.60E-13	23.245042
RAB42	0.7191084	4.1808107	8.1289396	6.57E-15	1.60E-13	23.244748
KCNAB3	0.8898166	3.1087129	8.128815	6.58E-15	1.60E-13	23.243896
XXbac-BPG5	1.3688845	0.1230414	8.1286856	6.58E-15	1.60E-13	23.243011
ZNF518B	0.513862	4.9486717	8.1284181	6.60E-15	1.61E-13	23.241181
DCN	0.8955229	5.8738565	8.1240239	6.80E-15	1.65E-13	23.211132
SEC13	-0.118589	6.6843945	-8.123835	6.81E-15	1.66E-13	23.209841
NEXN	0.5321649	4.8765888	8.1235923	6.82E-15	1.66E-13	23.208182
RCAN3	0.3663131	5.4117968	8.1231877	6.84E-15	1.66E-13	23.205415
BICC1	0.9183269	5.3303851	8.1177983	7.10E-15	1.72E-13	23.168579
MRPL46	-0.195495	6.0424892	-8.11706	7.14E-15	1.73E-13	23.163532
ELOVL7	1.4094522	4.503223	8.1146437	7.26E-15	1.76E-13	23.147025
CCL19	1.5648992	4.1773627	8.1135515	7.32E-15	1.77E-13	23.139564
FAM177A1	-0.149093	6.3524617	-8.113195	7.34E-15	1.77E-13	23.137129
FAIM3	0.4971646	4.7603574	8.109784	7.51E-15	1.82E-13	23.113832
HHLA3	-0.296896	5.5993123	-8.109076	7.55E-15	1.82E-13	23.108996
SMAD9	0.4715039	4.4473473	8.1089899	7.56E-15	1.82E-13	23.10841
ARID3A	0.4442391	5.6185714	8.1050906	7.76E-15	1.87E-13	23.081788
AP1G2	0.3253432	5.6771896	8.1050443	7.77E-15	1.87E-13	23.081472
GAMT	-0.270971	6.9556981	-8.105021	7.77E-15	1.87E-13	23.081316
MTFR1L	-0.133191	6.3776622	-8.104945	7.77E-15	1.87E-13	23.080793
FTSJ3	-0.11098	6.4432355	-8.103824	7.83E-15	1.88E-13	23.073143
LCA5	0.7090642	3.9619342	8.1001437	8.04E-15	1.93E-13	23.048028
RP11-204C1	1.2867316	2.1228797	8.1000251	8.04E-15	1.93E-13	23.047219
FAM109B	0.4617421	4.9338814	8.0993739	8.08E-15	1.94E-13	23.042776
ZNF747	-0.162171	5.9162532	-8.097884	8.16E-15	1.96E-13	23.03261
AF131215.9	0.9942852	2.9289758	8.097536	8.18E-15	1.96E-13	23.030237
PIPOX	-0.386124	6.8969779	-8.091768	8.52E-15	2.04E-13	22.9909
C8orf88	1.5004854	1.9359289	8.0906448	8.59E-15	2.06E-13	22.983243
NPRL2	-0.142501	6.0538567	-8.08833	8.73E-15	2.09E-13	22.967464
ANKRD24	-0.372596	5.2615245	-8.087747	8.76E-15	2.10E-13	22.963488
OGFRL1	0.2301088	5.6587319	8.0870687	8.80E-15	2.10E-13	22.958866
TNFRSF21	0.3374727	6.0363221	8.0866399	8.83E-15	2.11E-13	22.955944
RP11-395G2	1.1556024	2.7556963	8.0865698	8.84E-15	2.11E-13	22.955466

ITK	0.6732655	4.4757481	8.0851235	8.92E-15	2.13E-13	22.94561
MOCS1	-0.214873	6.3515153	-8.08168	9.14E-15	2.18E-13	22.92215
TRAT1	1.3267157	2.9341209	8.0810719	9.18E-15	2.19E-13	22.918006
TRAV3	1.4879906	0.3417719	8.0800178	9.25E-15	2.20E-13	22.910826
RHOV	1.8213011	2.0342393	8.077794	9.39E-15	2.24E-13	22.89568
FAM163B	2.0012171	1.7656833	8.077129	9.44E-15	2.24E-13	22.891152
TNFRSF17	1.7834188	0.9756059	8.0746342	9.60E-15	2.28E-13	22.874165
CFDP1	-0.152519	6.3457776	-8.074311	9.62E-15	2.29E-13	22.871967
UNC13A	1.5445272	2.572887	8.0738757	9.65E-15	2.29E-13	22.869001
GMPR2	-0.109761	6.3886155	-8.073838	9.66E-15	2.29E-13	22.868747
FPR3	0.3832408	5.3852088	8.073381	9.69E-15	2.30E-13	22.865633
CTB-41I6.2	1.3447379	0.6491052	8.0697084	9.94E-15	2.35E-13	22.840636
ADAM8	0.3150173	5.3380449	8.0679607	1.01E-14	2.38E-13	22.828744
RP11-1006G	-1.394192	1.208211	-8.06619	1.02E-14	2.41E-13	22.816699
ATP8B2	0.3280962	5.7336806	8.0654818	1.02E-14	2.42E-13	22.811879
TRUB2	-0.14745	6.4117856	-8.06514	1.03E-14	2.42E-13	22.809555
AP000648.5	0.4719506	4.249509	8.0638686	1.03E-14	2.44E-13	22.800905
SDC3	0.2080231	6.3184281	8.0633534	1.04E-14	2.45E-13	22.797401
PRAME	2.4568559	1.892287	8.0624885	1.04E-14	2.46E-13	22.791519
PAM	0.2805803	5.9508907	8.0617175	1.05E-14	2.48E-13	22.786275
VSIG1	1.779632	2.6917499	8.0600948	1.06E-14	2.50E-13	22.775241
TESC	0.6825019	5.4829982	8.0595602	1.07E-14	2.51E-13	22.771606
EIF1	-0.125703	7.1792529	-8.059072	1.07E-14	2.52E-13	22.768288
YPEL4	0.6576387	3.3962344	8.0589653	1.07E-14	2.52E-13	22.767561
TRAPPC3	-0.113418	6.3510285	-8.057867	1.08E-14	2.53E-13	22.760092
RP11-454H1	1.2649067	-0.37536	8.0565422	1.09E-14	2.56E-13	22.751089
RP11-729I1	-1.47667	0.7037585	-8.056296	1.09E-14	2.56E-13	22.749417
SASH3	0.366691	5.3700243	8.0536286	1.11E-14	2.61E-13	22.731286
FERMT3	0.2679672	5.7862712	8.0479828	1.16E-14	2.71E-13	22.692928
RP4-665J23	-0.347641	5.5561157	-8.041172	1.21E-14	2.84E-13	22.646678
GATA3	0.8673631	4.0174594	8.0389445	1.23E-14	2.88E-13	22.631559
SEMA6A	0.4767397	5.5383132	8.0372842	1.25E-14	2.91E-13	22.620292
ARHGAP9	0.3936295	5.1960461	8.0359759	1.26E-14	2.94E-13	22.611414
NDUFC2	-0.183038	6.5015945	-8.035711	1.26E-14	2.94E-13	22.609618
KCNIP1	1.6101472	0.5179814	8.0352111	1.26E-14	2.95E-13	22.606225
PROM2	1.4399882	2.7300017	8.0331855	1.28E-14	2.99E-13	22.592483
FAM66C	1.136835	2.3202294	8.0321928	1.29E-14	3.01E-13	22.585749
GPR63	1.4585054	1.3203402	8.0312129	1.30E-14	3.03E-13	22.579102
TSR3	-0.153813	6.3613841	-8.02783	1.33E-14	3.10E-13	22.556163
PRPSAP1	-0.149897	6.5437783	-8.026163	1.35E-14	3.13E-13	22.544861
TRAV12-1	1.4647838	0.241559	8.0258019	1.35E-14	3.14E-13	22.54241
SLC9A9	0.3691541	5.052325	8.0242637	1.36E-14	3.17E-13	22.531983
NHLRC4	0.9083105	2.941505	8.0225439	1.38E-14	3.20E-13	22.520327
SLC12A3	1.3850721	0.503921	8.02246	1.38E-14	3.20E-13	22.519758
TRBV2	1.4871603	0.6029595	8.0175374	1.43E-14	3.31E-13	22.486403
EDNRA	0.4643229	4.9957919	8.0170718	1.43E-14	3.32E-13	22.483249
NCKAP1L	0.3246999	5.6538605	8.0162954	1.44E-14	3.33E-13	22.477989
SMOX	0.387717	5.5861952	8.0162654	1.44E-14	3.33E-13	22.477786
PGM2L1	0.3329234	5.0013033	8.0155167	1.45E-14	3.35E-13	22.472715
C18orf54	0.5654511	4.3692879	8.0135719	1.47E-14	3.39E-13	22.459543
CD86	0.3986087	5.1501694	8.0112088	1.49E-14	3.45E-13	22.443542

PRICKLE1	1.0121724	3.7337518	8.0083206	1.52E-14	3.51E-13	22.42399
TSSK5P	1.3997835	2.3456962	8.0074034	1.53E-14	3.53E-13	22.417781
CLEC7A	0.6310619	4.68621	8.0064931	1.54E-14	3.55E-13	22.41162
SSTR2	0.7714706	4.7364115	8.003997	1.57E-14	3.61E-13	22.394728
CCSER1	1.4806331	1.2354991	8.0034044	1.57E-14	3.63E-13	22.390719
ICA1L	0.5751042	3.9756768	8.0032651	1.58E-14	3.63E-13	22.389776
RP11-277P1	1.4355268	0.5674772	8.0018465	1.59E-14	3.66E-13	22.380179
RP11-34A14	1.5029681	-0.026249	8.0013972	1.60E-14	3.67E-13	22.377139
C1orf147	1.2935781	0.0288227	8.0012455	1.60E-14	3.67E-13	22.376113
MIR155HG	0.9794235	3.1883577	8.0010952	1.60E-14	3.67E-13	22.375096
RP11-960L1	1.3042584	-0.02144	7.9990247	1.62E-14	3.72E-13	22.361091
CRYL1	-0.241943	6.7012173	-7.997451	1.64E-14	3.76E-13	22.350449
B4GAT1	-0.158224	6.2690562	-7.996057	1.66E-14	3.80E-13	22.341024
NDFIP1	-0.128366	6.8533388	-7.99591	1.66E-14	3.80E-13	22.340026
CTD-2231E1	1.2130914	2.2527325	7.9936208	1.69E-14	3.86E-13	22.32455
ST6GALNAC5	1.8062281	1.3491253	7.9918902	1.71E-14	3.90E-13	22.312852
REEP5	-0.136984	6.7959627	-7.990502	1.72E-14	3.94E-13	22.303468
GDF10	2.0254682	1.1565817	7.9898064	1.73E-14	3.95E-13	22.298769
PRMT2	0.1329383	6.1359429	7.9896523	1.73E-14	3.95E-13	22.297727
KCNK15	1.9729305	1.2795415	7.9884049	1.75E-14	3.99E-13	22.289298
CDH3	1.5516332	2.2503523	7.9865076	1.77E-14	4.03E-13	22.276479
FAM64A	1.0180765	3.8843314	7.986486	1.77E-14	4.03E-13	22.276333
NUDT5	-0.13196	6.6119967	-7.982951	1.81E-14	4.13E-13	22.252453
LINC01353	1.4303316	1.0840838	7.9825092	1.82E-14	4.14E-13	22.24947
GRID2IP	1.5029086	0.9034932	7.9815551	1.83E-14	4.17E-13	22.243027
AC002064.7	-1.060699	-0.774933	-7.981177	1.84E-14	4.17E-13	22.240477
C7orf31	0.6138642	4.7490745	7.9785388	1.87E-14	4.25E-13	22.22266
GSTZ1	-0.305317	6.1699812	-7.977372	1.89E-14	4.28E-13	22.214782
BEST1	0.5098819	4.5866799	7.9763041	1.90E-14	4.31E-13	22.207575
ARMCX3	0.4624334	5.7951447	7.97576	1.91E-14	4.32E-13	22.203902
IL10	1.2973989	2.1061625	7.9750103	1.92E-14	4.34E-13	22.198842
RP5-884G6.	-0.583939	3.849536	-7.97411	1.93E-14	4.37E-13	22.192767
CD48	0.4281332	5.2815807	7.9737453	1.93E-14	4.38E-13	22.190305
KRBA1	0.4707896	5.0633639	7.9732689	1.94E-14	4.39E-13	22.18709
LRRC37A4P	1.1809807	2.6720312	7.9720072	1.96E-14	4.42E-13	22.178576
HCN4	1.6894702	0.293655	7.9679519	2.01E-14	4.55E-13	22.151219
GPSM3	0.2673102	5.6720117	7.9677701	2.02E-14	4.55E-13	22.149992
TNFRSF13C	1.1500652	3.1736408	7.9656829	2.04E-14	4.61E-13	22.135915
SLITRK4	1.5625406	2.1595731	7.9640512	2.07E-14	4.66E-13	22.124913
TLR7	0.8138641	3.9604302	7.9616693	2.10E-14	4.74E-13	22.108854
SIGLEC8	1.2547049	3.1287132	7.9613124	2.11E-14	4.74E-13	22.106448
CTSF	-0.246816	6.6616039	-7.958952	2.14E-14	4.82E-13	22.090535
CPNE5	0.5344245	4.6019351	7.9571559	2.17E-14	4.88E-13	22.078434
IGKV1D-8	1.7436259	0.4400485	7.9546266	2.21E-14	4.96E-13	22.061393
RP11-247C2	1.5340473	0.2129897	7.9538147	2.22E-14	4.98E-13	22.055923
ZIK1	0.7779953	3.976974	7.9537455	2.22E-14	4.98E-13	22.055457
PDX1	2.3943967	2.3031427	7.9530689	2.23E-14	5.00E-13	22.050899
PRDM4	0.1080707	5.8882267	7.9514834	2.25E-14	5.06E-13	22.040219
RP11-404P2	1.7340664	3.22414	7.9510768	2.26E-14	5.06E-13	22.037481
KCNK3	1.5798165	2.4756432	7.9510419	2.26E-14	5.06E-13	22.037246
NPTXR	0.9119206	4.5193625	7.9494497	2.29E-14	5.12E-13	22.026524

LRRC8B	0.3773875	5.4364563	7.9492053	2.29E-14	5.12E-13	22.024878
ADRB2	-0.62969	5.1934256	-7.948132	2.31E-14	5.16E-13	22.017648
ALDH7A1	-0.219151	6.7632217	-7.947957	2.31E-14	5.16E-13	22.016473
CTD-2184C2	-0.782903	3.1911211	-7.947415	2.32E-14	5.18E-13	22.012823
WIBG	-0.118668	6.2505192	-7.947187	2.32E-14	5.18E-13	22.011286
DCAKD	-0.142633	6.1462948	-7.947115	2.32E-14	5.18E-13	22.010806
MAGEL2	1.4864746	0.5396957	7.9457206	2.35E-14	5.23E-13	22.001416
CHCHD5	-0.178147	6.0479243	-7.945526	2.35E-14	5.23E-13	22.000103
CUEDC2	-0.142047	6.3227944	-7.944603	2.36E-14	5.26E-13	21.993894
PHACTR3	1.4649483	0.5433651	7.944553	2.37E-14	5.26E-13	21.993556
MZB1	1.2979165	3.9815452	7.9429947	2.39E-14	5.31E-13	21.983068
GMIP	0.2168762	5.6332197	7.942714	2.40E-14	5.32E-13	21.981179
COL5A2	0.2587178	6.2508347	7.9423005	2.40E-14	5.33E-13	21.978397
LAMA2	1.0208331	4.9902734	7.9418082	2.41E-14	5.35E-13	21.975083
TM6SF1	1.071542	3.4414855	7.9406795	2.43E-14	5.39E-13	21.967489
TSPY26P	0.5720269	4.1070174	7.9404493	2.43E-14	5.39E-13	21.96594
CACNA2D2	0.6252307	4.0023101	7.9398076	2.44E-14	5.41E-13	21.961622
EPB41L2	0.2058896	6.1069794	7.9391215	2.46E-14	5.44E-13	21.957006
UAP1L1	0.449624	5.24607	7.9390029	2.46E-14	5.44E-13	21.956208
HCN3	-0.311878	6.2183206	-7.938776	2.46E-14	5.44E-13	21.954682
SPOP	-0.08958	6.3855076	-7.937711	2.48E-14	5.48E-13	21.947516
SLA	0.3703401	5.4365325	7.937189	2.49E-14	5.49E-13	21.944005
AFAP1L2	0.3755364	4.9007001	7.9361271	2.51E-14	5.53E-13	21.936863
HNRNPA1P21	1.3312734	2.5557694	7.9351437	2.52E-14	5.57E-13	21.930249
NCCRP1	1.5690178	1.3265417	7.9342398	2.54E-14	5.60E-13	21.92417
RP5-1172A2	1.4489692	0.0721683	7.9335658	2.55E-14	5.62E-13	21.919637
ST3GAL6	-0.348004	5.9789847	-7.933546	2.55E-14	5.62E-13	21.919504
RP11-43F13	1.8084786	1.8775129	7.9333201	2.56E-14	5.62E-13	21.917985
SPCS1	-0.142186	6.7175653	-7.932063	2.58E-14	5.67E-13	21.909536
CCR2	0.8382038	4.2278633	7.9279867	2.65E-14	5.83E-13	21.882131
GLP1R	1.9068939	0.3488784	7.9263831	2.68E-14	5.89E-13	21.871353
KCNK12	1.4595275	0.1358626	7.9263029	2.68E-14	5.89E-13	21.870815
CHCHD4	-0.134281	5.936764	-7.925365	2.70E-14	5.92E-13	21.864509
STX7	0.1245649	6.163008	7.9252509	2.70E-14	5.92E-13	21.863746
TNFRSF13B	1.6283959	1.1906683	7.9248367	2.71E-14	5.94E-13	21.860962
RIC3	1.7244785	2.0698642	7.9243672	2.72E-14	5.95E-13	21.857808
TOMM7	-0.153354	6.6810451	-7.921336	2.77E-14	6.07E-13	21.837443
C1QTNF2	1.1009993	3.2689597	7.9208916	2.78E-14	6.09E-13	21.834459
PDZD4	0.7965979	4.3997236	7.9201976	2.80E-14	6.11E-13	21.829797
ALAS1	-0.235392	6.8775824	-7.915959	2.88E-14	6.29E-13	21.801336
CD3G	0.8203819	4.1282416	7.9150402	2.90E-14	6.33E-13	21.795166
GADD45A	-0.24483	6.4033899	-7.913642	2.93E-14	6.38E-13	21.785783
IGLV5-45	1.8424702	0.9352659	7.9133968	2.93E-14	6.39E-13	21.784134
NSDHL	-0.183703	6.3482333	-7.912725	2.94E-14	6.42E-13	21.779623
PC	-0.262848	6.8549673	-7.912594	2.95E-14	6.42E-13	21.778743
FKBPL	-0.203794	5.5524301	-7.911353	2.97E-14	6.47E-13	21.770417
PITPNM3	1.0305229	3.345045	7.9110358	2.98E-14	6.48E-13	21.768288
TMEM14B	-0.130963	6.4301011	-7.910952	2.98E-14	6.48E-13	21.767728
IGF2BP2	0.9309839	5.3100194	7.9088098	3.02E-14	6.57E-13	21.753351
TMX2	-0.107139	6.5635292	-7.908481	3.03E-14	6.58E-13	21.751142
LIF	0.9131372	4.6928538	7.9071849	3.06E-14	6.64E-13	21.742449

ADAMTS6	1.1024918	3.5123708	7.9068106	3.07E-14	6.65E-13	21.739939
KLHL14	1.3825565	-0.088303	7.9065245	3.07E-14	6.66E-13	21.738019
NUMBL	0.2523156	5.4382962	7.9061723	3.08E-14	6.67E-13	21.735656
PECR	-0.238649	6.5922614	-7.906047	3.08E-14	6.67E-13	21.734817
ATPAF1	-0.135455	6.4619671	-7.904222	3.12E-14	6.75E-13	21.722572
ARNT2	1.2454706	4.2435644	7.9028923	3.15E-14	6.81E-13	21.713658
ECSIT	-0.15099	6.4197281	-7.902021	3.17E-14	6.85E-13	21.707816
CCNY	-0.101221	6.5842979	-7.901375	3.18E-14	6.88E-13	21.70348
LINC00941	1.6540187	1.4473688	7.9008664	3.19E-14	6.90E-13	21.700073
RP11-242G2	-1.181551	-0.514815	-7.900605	3.20E-14	6.90E-13	21.698323
HUNK	1.3339963	4.1517422	7.8997958	3.22E-14	6.94E-13	21.692895
LINC00086	0.9053021	3.0827993	7.8993847	3.23E-14	6.95E-13	21.690139
FBXO31	-0.247912	6.4252103	-7.899011	3.24E-14	6.97E-13	21.687635
CCDC109B	0.470242	4.9757976	7.8933579	3.36E-14	7.24E-13	21.649746
SSU72	-0.132217	6.6589885	-7.890841	3.42E-14	7.36E-13	21.632882
DDI2	-0.160818	6.4301562	-7.88823	3.48E-14	7.49E-13	21.615394
SORD	-0.292449	6.7536147	-7.886666	3.52E-14	7.57E-13	21.604921
HIBADH	-0.191383	6.6065377	-7.88631	3.53E-14	7.58E-13	21.602536
FBN1	0.3527058	5.9565628	7.8862681	3.53E-14	7.58E-13	21.602258
MFSD10	0.209958	6.0508461	7.8855193	3.55E-14	7.61E-13	21.597244
YWHAZP5	1.3128933	2.5416622	7.8853962	3.55E-14	7.61E-13	21.59642
HCK	0.3372893	5.4732267	7.8850336	3.56E-14	7.63E-13	21.593992
DGKH	0.2358322	5.3959387	7.8841319	3.58E-14	7.67E-13	21.587955
COQ7	-0.123174	5.8836291	-7.881883	3.64E-14	7.79E-13	21.572901
CD200R1	0.7074671	3.7304708	7.8806814	3.67E-14	7.84E-13	21.564859
RGS2	0.4120603	5.6757168	7.8805184	3.67E-14	7.85E-13	21.563768
CHST4	2.1273182	1.5794803	7.8797722	3.69E-14	7.88E-13	21.558774
IFI27L1	-0.247283	5.6672319	-7.879214	3.71E-14	7.91E-13	21.555037
TRPA1	1.678553	0.6809772	7.8767777	3.77E-14	8.04E-13	21.538737
SEPP1	-0.23686	7.1610652	-7.875691	3.80E-14	8.09E-13	21.531465
DAPP1	0.7622248	4.0316879	7.8717593	3.90E-14	8.31E-13	21.50517
ANK2	0.6556838	4.6037092	7.8715646	3.90E-14	8.32E-13	21.503868
ACSM2A	-0.641506	6.5870846	-7.870679	3.93E-14	8.36E-13	21.497944
CD1B	1.6056572	1.3667724	7.8701958	3.94E-14	8.38E-13	21.494715
MSI1	1.36955	4.2698425	7.8696889	3.96E-14	8.41E-13	21.491326
MRPL27	-0.174748	6.4434379	-7.869091	3.97E-14	8.44E-13	21.487327
RP5-827C21	-0.392863	4.6577898	-7.868862	3.98E-14	8.45E-13	21.485799
ST8SIA6	1.5376757	0.8569694	7.8673064	4.02E-14	8.53E-13	21.475398
TIMM17A	-0.157043	6.5547594	-7.865506	4.07E-14	8.63E-13	21.463365
TRAV13-1	1.4972597	0.6992945	7.8653223	4.08E-14	8.64E-13	21.462136
MB21D2	0.462858	4.3814742	7.8650717	4.08E-14	8.65E-13	21.460461
RHOB	-0.206633	7.094388	-7.864312	4.10E-14	8.69E-13	21.455385
RAB39A	1.4489024	1.2430254	7.8638053	4.12E-14	8.71E-13	21.451998
NCKIPSD	-0.131577	6.1936584	-7.862196	4.16E-14	8.81E-13	21.441247
RNASE4	-0.36625	5.8627443	-7.860734	4.21E-14	8.89E-13	21.431477
BMF	0.3448281	5.7336535	7.8604559	4.21E-14	8.90E-13	21.429619
GLI1	0.8269399	3.9800135	7.8548948	4.38E-14	9.24E-13	21.392477
ID4	0.8349079	4.6123715	7.8533716	4.42E-14	9.33E-13	21.382308
NMUR1	0.7789869	3.5866199	7.8531192	4.43E-14	9.34E-13	21.380622
HEXA	-0.148147	6.5819986	-7.852628	4.45E-14	9.37E-13	21.377342
PGRMC1	-0.195811	7.0385965	-7.851348	4.48E-14	9.45E-13	21.368801

PSMB7	-0.146965	6.7518112	-7.850594	4.51E-14	9.49E-13	21.363766
FAM150A	1.5568878	-0.269577	7.8482507	4.58E-14	9.64E-13	21.348128
PDE6B	0.695862	3.8790741	7.8460421	4.65E-14	9.78E-13	21.333391
TATDN2P2	1.2951186	2.0827071	7.8453916	4.67E-14	9.82E-13	21.329051
GRIN2D	0.6816842	4.0109187	7.8439271	4.72E-14	9.91E-13	21.319281
FLNA	0.2008639	6.7630461	7.8428421	4.75E-14	9.98E-13	21.312045
RP11-834C1	1.3400997	0.8092667	7.8427717	4.75E-14	9.98E-13	21.311575
GDF11	0.3404937	5.1213812	7.8422814	4.77E-14	1.00E-12	21.308305
CACNA1G	1.5609615	0.3819292	7.8398469	4.85E-14	1.02E-12	21.29207
GLO1	-0.138023	6.708191	-7.839028	4.88E-14	1.02E-12	21.28661
TMEM208	-0.178181	6.4682196	-7.838913	4.88E-14	1.02E-12	21.28584
CRABP2	0.9079282	3.659337	7.8365676	4.96E-14	1.04E-12	21.270206
RP11-522I2	-0.284812	4.9336255	-7.83652	4.96E-14	1.04E-12	21.269891
CPEB1	1.6561575	1.0433089	7.8350156	5.01E-14	1.05E-12	21.259861
ADAM19	0.2449259	5.6698169	7.8345165	5.03E-14	1.05E-12	21.256535
SIGLEC9	0.4988767	4.4446292	7.8329332	5.09E-14	1.06E-12	21.245984
C20orf62	-1.205996	-0.572526	-7.832898	5.09E-14	1.06E-12	21.245747
C22orf23	0.8577814	3.3047832	7.8320158	5.12E-14	1.07E-12	21.23987
TLDC1	0.5060683	5.0479084	7.8315811	5.13E-14	1.07E-12	21.236974
RP11-42015	-0.801419	4.7857035	-7.829533	5.20E-14	1.08E-12	21.223331
RAB34	0.4597222	5.671422	7.826338	5.32E-14	1.11E-12	21.202048
SLC1A5	0.3708789	5.8526678	7.8239871	5.41E-14	1.12E-12	21.186393
PTPRS	0.7490112	5.1456312	7.8196479	5.57E-14	1.16E-12	21.157506
MGAT3	0.7663508	3.9935481	7.8177541	5.64E-14	1.17E-12	21.144903
SUSD1	0.3393477	5.2537505	7.8168134	5.68E-14	1.18E-12	21.138643
HRH2	1.5240703	1.5565631	7.8164455	5.69E-14	1.18E-12	21.136195
GCLC	-0.189021	6.6117318	-7.815177	5.74E-14	1.19E-12	21.127757
SIDT1	0.5268009	4.1751025	7.8112848	5.89E-14	1.22E-12	21.101865
LDLRAD3	0.3615357	4.9779325	7.8101764	5.94E-14	1.23E-12	21.094493
PIK3CG	0.786038	4.0040572	7.8096355	5.96E-14	1.23E-12	21.090896
RPL36AL	-0.157175	6.7639254	-7.808976	5.99E-14	1.24E-12	21.086509
FAM3A	-0.152373	6.5000668	-7.800958	6.32E-14	1.31E-12	21.033215
IGKV2-30	1.7283272	0.372996	7.7994264	6.39E-14	1.32E-12	21.023041
HMG20B	-0.117123	6.4071867	-7.798907	6.41E-14	1.32E-12	21.01959
RP11-67C2.	1.420254	0.250757	7.7986865	6.42E-14	1.33E-12	21.018126
FIBIN	1.3770983	3.8578614	7.798518	6.43E-14	1.33E-12	21.017006
SMOC2	0.607936	5.350355	7.7981384	6.45E-14	1.33E-12	21.014485
SLC9A3R1	-0.13769	6.9519475	-7.797005	6.50E-14	1.34E-12	21.006959
ABHD10	-0.125245	6.2225611	-7.796168	6.53E-14	1.35E-12	21.0014
HMGA2	1.9421967	1.2161302	7.7944568	6.61E-14	1.36E-12	20.990033
PBX4	1.167319	2.8335606	7.7936092	6.65E-14	1.37E-12	20.984405
PAGE4	-2.431705	1.9398368	-7.792406	6.70E-14	1.38E-12	20.976413
TRAV14DV4	1.3404153	-0.075124	7.7918595	6.73E-14	1.38E-12	20.972788
RXRB	-0.118254	6.3699619	-7.786632	6.97E-14	1.43E-12	20.938095
TAPT1	-0.161651	6.2072038	-7.786563	6.97E-14	1.43E-12	20.937632
AMIGO2	0.5938855	5.0205904	7.7859483	7.00E-14	1.44E-12	20.933555
PRSS21	1.6679234	0.6237457	7.7850009	7.05E-14	1.45E-12	20.927269
MACC1	1.5163116	2.7331617	7.7843427	7.08E-14	1.45E-12	20.922902
LINC01485	-0.960888	5.8609253	-7.781551	7.22E-14	1.48E-12	20.904384
PTAFR	0.3884337	5.2383036	7.7807186	7.26E-14	1.49E-12	20.898863
TMEM14A	-0.191685	6.3626592	-7.78042	7.27E-14	1.49E-12	20.89688

HOPX	0.5016348	4.6998336	7.7798594	7.30E-14	1.49E-12	20.893165
L3MBTL3	0.361677	5.1339802	7.7795169	7.32E-14	1.50E-12	20.890894
ICAM3	0.9139452	3.5290243	7.7794005	7.32E-14	1.50E-12	20.890122
ZNF347	0.5884291	4.6768941	7.7778007	7.40E-14	1.51E-12	20.879514
ZFPM2	0.6911697	4.3453649	7.7742165	7.58E-14	1.55E-12	20.855753
ANG	-0.331918	6.9548137	-7.773218	7.64E-14	1.56E-12	20.849138
FLT3	1.267398	2.7337498	7.7726412	7.67E-14	1.56E-12	20.845314
TAF1A-AS1	-0.357668	4.7848575	-7.771778	7.71E-14	1.57E-12	20.839593
C17orf89	-0.225939	6.1221418	-7.771677	7.72E-14	1.57E-12	20.838924
CDC37L1-AS	-0.511778	3.9964551	-7.770089	7.80E-14	1.59E-12	20.8284
HIF1A	0.1836089	6.4207305	7.7691999	7.85E-14	1.60E-12	20.822511
CNTNAP1	0.4662933	4.9559771	7.7676307	7.93E-14	1.61E-12	20.812116
OPRL1	0.6234514	4.0532756	7.761181	8.29E-14	1.68E-12	20.769405
NSUN7	1.783335	2.0168561	7.7600319	8.35E-14	1.70E-12	20.761798
ROBO2	1.668831	2.6051083	7.7593507	8.39E-14	1.70E-12	20.757289
ITPRIPL1	0.6035078	3.9345944	7.7580757	8.46E-14	1.72E-12	20.748851
PKM	0.2383518	6.6476143	7.7579367	8.47E-14	1.72E-12	20.747931
PKDCC	0.3703532	5.8268295	7.7570981	8.52E-14	1.73E-12	20.742381
INTS5	-0.111341	6.15298	-7.756889	8.53E-14	1.73E-12	20.740998
SIGMAR1	-0.158041	6.8616341	-7.75682	8.53E-14	1.73E-12	20.74054
HAVCR2	0.3618496	5.3033364	7.7552098	8.63E-14	1.74E-12	20.729886
SLAMF7	0.5229947	5.096167	7.7548071	8.65E-14	1.75E-12	20.727221
ERAL1	-0.10912	6.4408915	-7.753653	8.72E-14	1.76E-12	20.719586
RAB25	1.8552775	1.7302455	7.7532864	8.74E-14	1.76E-12	20.717161
SLC35F3	1.7181495	0.8309917	7.7532208	8.75E-14	1.76E-12	20.716727
BNC2	0.9118592	3.5557561	7.7528676	8.77E-14	1.77E-12	20.714391
PEX6	-0.185282	6.4656432	-7.752517	8.79E-14	1.77E-12	20.712073
GALNT3	1.0243184	3.7014081	7.7524838	8.79E-14	1.77E-12	20.711852
SLC22A17	0.7909308	4.8125545	7.7518659	8.83E-14	1.78E-12	20.707765
POSTN	0.9994984	5.2210315	7.7473287	9.10E-14	1.83E-12	20.67776
FAU	-0.145995	7.0540625	-7.745462	9.22E-14	1.85E-12	20.665417
C2orf27A	0.8560567	3.800423	7.7452868	9.23E-14	1.85E-12	20.664261
ELF1	0.2024824	6.0843576	7.7444073	9.28E-14	1.87E-12	20.658447
ZNF239	1.2981689	3.7263995	7.743922	9.31E-14	1.87E-12	20.655239
ATP8A2	1.3443236	2.2372292	7.7424179	9.41E-14	1.89E-12	20.645299
P4HB	-0.128587	7.5539676	-7.740126	9.56E-14	1.92E-12	20.630152
LRFN1	0.6458412	4.4497753	7.7397774	9.58E-14	1.92E-12	20.627851
C16orf13	-0.177576	6.2808612	-7.739662	9.59E-14	1.92E-12	20.627085
MPST	-0.1866	7.0007954	-7.739494	9.60E-14	1.92E-12	20.625976
CTD-2026K1	-1.11579	2.1624586	-7.738787	9.64E-14	1.93E-12	20.621305
CLIC6	1.6719841	2.6534959	7.7351966	9.88E-14	1.98E-12	20.597592
SLFN11	0.321781	5.3633891	7.7340329	9.96E-14	1.99E-12	20.589907
HSPB9	-0.529288	4.6616678	-7.732788	1.00E-13	2.01E-12	20.581689
SLFN13	0.680954	4.815185	7.7326975	1.00E-13	2.01E-12	20.58109
DHRS1	-0.248549	6.4185786	-7.731465	1.01E-13	2.02E-12	20.572951
UTRN	0.169727	6.3193794	7.731441	1.01E-13	2.02E-12	20.572794
ACY3	-0.527809	5.8264741	-7.730349	1.02E-13	2.04E-12	20.565582
DPY30	-0.124531	6.2555056	-7.729924	1.02E-13	2.04E-12	20.562777
NDUFAB1	-0.159298	6.4473544	-7.729856	1.02E-13	2.04E-12	20.562327
LTBP4	0.3523278	6.0861425	7.7295468	1.03E-13	2.04E-12	20.560289
ARHGAP27	0.2277377	5.7258536	7.7291646	1.03E-13	2.05E-12	20.557767

RP4-639F20	-0.412164	5.5067825	-7.726972	1.04E-13	2.08E-12	20.543293
MAP1LC3C	1.280177	1.0057356	7.7267166	1.05E-13	2.08E-12	20.54161
GPR84	1.5796273	1.8634824	7.7260391	1.05E-13	2.09E-12	20.53714
LILRB1	0.4338057	4.9010087	7.7250329	1.06E-13	2.10E-12	20.530501
UFSP1	-0.339889	4.7628672	-7.724136	1.06E-13	2.11E-12	20.524581
MRPL24	-0.179778	6.7330134	-7.722628	1.08E-13	2.13E-12	20.514637
ITGA8	0.5517036	4.5503866	7.7225136	1.08E-13	2.13E-12	20.51388
MEA1	-0.135522	6.5161983	-7.721437	1.08E-13	2.15E-12	20.50678
RNF24	0.2312981	5.5486492	7.7204928	1.09E-13	2.16E-12	20.500551
MRPL11	-0.142853	6.3167268	-7.718048	1.11E-13	2.20E-12	20.484432
TRBV3-1	1.4447807	0.5814949	7.7152362	1.13E-13	2.24E-12	20.465892
TSPAN15	0.3104631	5.7330561	7.7151788	1.13E-13	2.24E-12	20.465514
IGLL5	1.5595683	4.0258269	7.7140044	1.14E-13	2.25E-12	20.457772
SRP68	-0.092481	6.5824229	-7.713689	1.14E-13	2.26E-12	20.455692
NBAS	-0.101207	6.2804089	-7.710327	1.17E-13	2.31E-12	20.433537
CCNG2	0.2012569	5.7423299	7.7102745	1.17E-13	2.31E-12	20.433192
GSTP1	0.2947694	6.0354673	7.7100869	1.17E-13	2.31E-12	20.431956
TMEM173	0.2478699	5.7657045	7.7075868	1.19E-13	2.35E-12	20.415486
ANAPC2	-0.116644	6.3629787	-7.70643	1.20E-13	2.36E-12	20.407869
RP11-1094M	1.0342225	3.1333051	7.7063603	1.20E-13	2.36E-12	20.407407
EBPL	-0.22339	6.3826599	-7.706227	1.20E-13	2.36E-12	20.406526
OSM	1.0217131	3.3367793	7.7059527	1.20E-13	2.37E-12	20.404722
HOMER1	0.8372426	4.778251	7.7056766	1.21E-13	2.37E-12	20.402904
RP5-1136G1	-0.257575	5.1486617	-7.703629	1.22E-13	2.40E-12	20.389417
RP11-165F2	1.4165304	0.6389448	7.7022736	1.23E-13	2.42E-12	20.380495
CORO6	0.7968482	3.5473628	7.7018816	1.24E-13	2.43E-12	20.377914
GGT6	1.8901085	1.6549372	7.7014952	1.24E-13	2.43E-12	20.37537
ALDH1A1	-0.267266	7.3058467	-7.700584	1.25E-13	2.45E-12	20.36937
NFKBIL1	-0.156402	6.1626485	-7.700234	1.25E-13	2.45E-12	20.36707
SMIM4	-0.251009	5.8557066	-7.700006	1.25E-13	2.45E-12	20.365564
ZNF391	1.295693	3.2417023	7.6998994	1.25E-13	2.45E-12	20.364865
RGS4	0.892865	4.310593	7.6996609	1.26E-13	2.46E-12	20.363295
AC010883.5	-0.705079	3.42567	-7.699366	1.26E-13	2.46E-12	20.361355
ZNF14	0.6575247	4.5073538	7.6985866	1.27E-13	2.47E-12	20.356224
NPHP4	0.581692	4.963934	7.6973761	1.28E-13	2.49E-12	20.348258
SUOX	-0.165229	6.3598587	-7.696179	1.29E-13	2.51E-12	20.340383
AC109826.1	1.2971792	1.7615996	7.6952081	1.29E-13	2.52E-12	20.333991
NMNAT2	1.230647	2.7202606	7.6949829	1.30E-13	2.53E-12	20.33251
RP11-442N2	-0.814284	3.0862505	-7.69338	1.31E-13	2.55E-12	20.321965
KIAA1244	1.0713422	4.7354796	7.690615	1.34E-13	2.60E-12	20.303777
LPAR5	0.5957774	4.2450538	7.6894552	1.35E-13	2.62E-12	20.29615
LINC00239	1.6135991	1.7084508	7.6869402	1.37E-13	2.66E-12	20.279613
SCRN2	-0.1898	6.4169276	-7.684018	1.40E-13	2.71E-12	20.260405
FGFR2	1.2111105	5.6326982	7.6834848	1.40E-13	2.72E-12	20.256899
TMEM192	-0.17157	6.2993897	-7.683254	1.40E-13	2.72E-12	20.255381
AF127936.5	1.3185046	0.3314264	7.6820767	1.41E-13	2.74E-12	20.247645
SNRNP25	-0.174962	6.1684861	-7.682014	1.41E-13	2.74E-12	20.247232
PFKP	0.4097175	5.5931489	7.681941	1.42E-13	2.74E-12	20.246753
SIRPG	0.9819919	3.8933637	7.6806519	1.43E-13	2.77E-12	20.238283
MB21D1	0.507743	4.5296134	7.6802024	1.43E-13	2.77E-12	20.235329
PDLIM4	0.9754799	3.7078763	7.6789108	1.44E-13	2.80E-12	20.226844

PLCB4	0.86229	3.8984315	7.6784827	1.45E-13	2.80E-12	20.224031
RP11-182J1	1.4739803	0.2363178	7.6766105	1.47E-13	2.84E-12	20.211734
POP5	-0.153831	6.1052127	-7.675714	1.48E-13	2.85E-12	20.205844
AC016735.1	1.7643423	1.3847794	7.6749149	1.48E-13	2.87E-12	20.200597
TIMM10	-0.174994	6.234423	-7.674818	1.49E-13	2.87E-12	20.199964
PYURF	-0.271252	4.8557129	-7.673791	1.50E-13	2.89E-12	20.193218
MEI1	0.6184563	4.0058438	7.6726319	1.51E-13	2.91E-12	20.185607
SLC25A26	-0.143121	6.0311805	-7.671795	1.52E-13	2.92E-12	20.180114
CRHR1-IT1	0.6513856	4.8251213	7.6714629	1.52E-13	2.93E-12	20.177932
LINC01140	1.1266955	2.3246617	7.6701912	1.53E-13	2.95E-12	20.169584
UCHL1	0.986831	4.1473851	7.6700807	1.53E-13	2.95E-12	20.168858
MRPL20	-0.152892	6.5102996	-7.66881	1.55E-13	2.97E-12	20.160519
DRP2	1.5492883	0.6281044	7.6677866	1.56E-13	2.99E-12	20.153801
YIPF3	-0.117247	6.8081778	-7.666336	1.57E-13	3.02E-12	20.144284
VSTM5	1.3473272	1.0002476	7.6651457	1.58E-13	3.04E-12	20.136472
FAM171A2	0.7524408	4.0444704	7.6650487	1.59E-13	3.04E-12	20.135835
GABPB1	-0.094812	6.1030778	-7.664054	1.60E-13	3.06E-12	20.129307
SLC47A1	-0.36668	6.5057895	-7.663817	1.60E-13	3.06E-12	20.127753
PM20D1	1.3241728	1.3856695	7.6638137	1.60E-13	3.06E-12	20.127733
NIT1	-0.147625	6.4433532	-7.66323	1.61E-13	3.07E-12	20.123904
TMEM203	-0.130184	6.3301916	-7.661482	1.62E-13	3.11E-12	20.11244
TRIM27	-0.116021	6.5644129	-7.659684	1.64E-13	3.15E-12	20.100645
RP5-968P14	-0.507597	4.0967736	-7.658108	1.66E-13	3.18E-12	20.090314
TUFM	-0.127455	6.9629187	-7.657839	1.66E-13	3.18E-12	20.088549
RP11-1134I	1.5200445	1.3342027	7.6576279	1.67E-13	3.18E-12	20.087165
SEPT4	-0.355263	6.0350997	-7.657495	1.67E-13	3.18E-12	20.086295
FLJ37453	-0.269279	5.0215307	-7.657473	1.67E-13	3.18E-12	20.08615
RP11-81701	-0.726932	3.3278776	-7.657188	1.67E-13	3.19E-12	20.084281
ETNK2	-0.415943	6.5645588	-7.655984	1.69E-13	3.21E-12	20.076387
TUSC2	-0.142251	6.1736546	-7.655843	1.69E-13	3.21E-12	20.075462
TNIK	0.6119249	5.1616902	7.655346	1.69E-13	3.22E-12	20.072205
LDOC1L	0.4311155	5.3515518	7.6551526	1.69E-13	3.23E-12	20.070938
IGSF23	-0.885061	5.4078184	-7.654942	1.70E-13	3.23E-12	20.069559
SCRN1	0.4033434	5.3811859	7.6546484	1.70E-13	3.23E-12	20.067633
MRPL41	-0.202662	6.5136251	-7.654254	1.71E-13	3.24E-12	20.06505
PRKG1	0.5719864	4.6532918	7.6525786	1.72E-13	3.28E-12	20.054068
RP11-1017G	-0.373477	4.483425	-7.652164	1.73E-13	3.28E-12	20.051348
FASTK	-0.132195	6.6280155	-7.651886	1.73E-13	3.29E-12	20.049527
HS3ST1	0.5458889	4.4546591	7.6467436	1.79E-13	3.40E-12	20.01584
IGKV3OR2-2	1.4455992	-0.135659	7.6467166	1.79E-13	3.40E-12	20.015663
RNF39	0.9764156	3.3563963	7.6465478	1.80E-13	3.40E-12	20.014558
ADH1B	-0.50449	7.1611574	-7.646511	1.80E-13	3.40E-12	20.014318
PTGES	1.3223225	3.9385384	7.6461518	1.80E-13	3.41E-12	20.011964
CCR7	0.752881	4.1204314	7.6461478	1.80E-13	3.41E-12	20.011938
TCEB2	-0.169904	6.7662878	-7.645748	1.81E-13	3.41E-12	20.009323
CRISPLD2	0.5154031	5.6278452	7.6449449	1.82E-13	3.43E-12	20.004061
CYP8B1	-0.94539	6.3421641	-7.644037	1.83E-13	3.45E-12	19.998114
RBP7	-0.40003	5.6123505	-7.643464	1.83E-13	3.46E-12	19.994366
SLC4A10	1.4351088	1.5652182	7.6423969	1.85E-13	3.48E-12	19.987377
ZNF710	0.2754192	5.6736684	7.642285	1.85E-13	3.48E-12	19.986644
ARSD	-0.211644	6.4178777	-7.641759	1.85E-13	3.49E-12	19.983202

RP11-278H7	-1.603444	0.2015427	-7.641276	1.86E-13	3.50E-12	19.980042
MIR181A2HG	1.4098174	1.2160052	7.6409374	1.86E-13	3.51E-12	19.977822
CMC1	-0.137919	6.0702227	-7.640424	1.87E-13	3.52E-12	19.974465
FGD2	0.3609203	5.1267878	7.6397919	1.88E-13	3.53E-12	19.970324
NDUFC1	-0.150107	6.3101508	-7.637265	1.91E-13	3.59E-12	19.953786
ARID5B	0.2160814	5.8405948	7.6347574	1.94E-13	3.65E-12	19.937379
TRAV19	1.4532488	0.476049	7.6342938	1.95E-13	3.66E-12	19.934347
FOXC2	1.1249135	3.0984899	7.6338961	1.95E-13	3.67E-12	19.931745
SHB	-0.207299	6.0783464	-7.633297	1.96E-13	3.68E-12	19.927828
TNF	1.1712817	2.5550748	7.6330327	1.97E-13	3.69E-12	19.926097
ALDH1A3	1.1206928	4.1235064	7.6312079	1.99E-13	3.73E-12	19.914162
RP11-333B6	-1.571535	1.3665464	-7.63083	2.00E-13	3.74E-12	19.911688
SAMD11	1.120929	3.8353207	7.6300109	2.01E-13	3.76E-12	19.906334
RP11-417E7	1.5500445	0.3014562	7.6290121	2.02E-13	3.78E-12	19.899803
2-Mar	-0.163683	6.351706	-7.628879	2.02E-13	3.78E-12	19.898934
MSRB2	-0.177261	6.39294	-7.627174	2.04E-13	3.82E-12	19.887786
THEMIS2	0.306296	5.5816477	7.6228325	2.11E-13	3.93E-12	19.859409
TCEAL3	0.3398545	5.2565216	7.6224456	2.11E-13	3.94E-12	19.856881
TIGD6	-0.133808	5.4733125	-7.621705	2.12E-13	3.96E-12	19.852039
CSF2RA	0.5296513	4.8398057	7.6214563	2.12E-13	3.96E-12	19.850416
QPRT	-0.231808	6.8421492	-7.620754	2.13E-13	3.98E-12	19.845826
MFI2	0.615186	5.2060324	7.6203385	2.14E-13	3.99E-12	19.843113
ZNF511	-0.173235	5.8302203	-7.619859	2.15E-13	4.00E-12	19.839978
CHMP6	-0.14051	6.1971367	-7.618691	2.16E-13	4.03E-12	19.83235
TRBV6-5	1.4386054	0.9612568	7.6180368	2.17E-13	4.04E-12	19.828077
PRKAG2-AS1	-0.564367	5.0837924	-7.616664	2.19E-13	4.08E-12	19.819109
S100B	1.1947274	3.2175975	7.6139149	2.23E-13	4.15E-12	19.801159
ITGBL1	1.3845805	4.1123108	7.6121087	2.26E-13	4.20E-12	19.789367
WIPI2	-0.108269	6.5703609	-7.611628	2.27E-13	4.21E-12	19.786231
C1orf53	-0.298241	5.4295336	-7.611465	2.27E-13	4.22E-12	19.785166
RP11-615I2	1.0846425	-0.653028	7.6112982	2.27E-13	4.22E-12	19.784076
RP11-563J2	1.4372461	1.3217112	7.6105807	2.29E-13	4.24E-12	19.779393
MMD	0.248585	5.8744881	7.6105268	2.29E-13	4.24E-12	19.779041
SIRT5	-0.166128	6.2057399	-7.608399	2.32E-13	4.29E-12	19.765154
SLC7A4	1.621302	0.5886684	7.6073384	2.34E-13	4.32E-12	19.758233
DLG4	0.2326861	5.2950088	7.6070914	2.34E-13	4.33E-12	19.756621
C20orf197	1.3422274	0.6016147	7.6055331	2.36E-13	4.37E-12	19.746454
JTB	-0.131065	6.8597929	-7.605079	2.37E-13	4.38E-12	19.743492
CEP170	0.2035554	5.6968279	7.6048808	2.37E-13	4.39E-12	19.742199
NAV1	0.2594911	5.5085008	7.6047342	2.38E-13	4.39E-12	19.741242
RP11-446N1	-0.679236	3.9387117	-7.603078	2.40E-13	4.43E-12	19.730436
UXT	-0.141186	6.3959667	-7.602952	2.40E-13	4.44E-12	19.72962
GSTA7P	-1.339911	3.9046378	-7.602522	2.41E-13	4.45E-12	19.726816
METTL9	0.1298479	6.0934659	7.6017881	2.42E-13	4.47E-12	19.722026
TGFB3	0.3366312	5.5605403	7.6006359	2.44E-13	4.50E-12	19.714513
RP11-307C1	-1.249671	1.6716569	-7.600479	2.45E-13	4.50E-12	19.713487
RP11-366L2	1.1296022	-0.625368	7.5994983	2.46E-13	4.53E-12	19.707095
COMP	1.9650152	2.8369056	7.5977763	2.49E-13	4.58E-12	19.695868
RP11-325F2	1.3583499	0.6969785	7.5972774	2.50E-13	4.59E-12	19.692616
C10orf55	1.2861443	0.8156821	7.5972157	2.50E-13	4.59E-12	19.692214
CSNK2B	-0.16047	6.5100291	-7.594647	2.54E-13	4.67E-12	19.675469

CD40LG	0.9883901	3.5423175	7.5926129	2.58E-13	4.73E-12	19.662215
ZNRF1	-0.148782	6.1614874	-7.591998	2.59E-13	4.75E-12	19.658207
NSMCE4A	-0.128455	6.1289988	-7.590311	2.62E-13	4.80E-12	19.647217
SAP30BP	-0.110193	6.3924149	-7.588806	2.64E-13	4.84E-12	19.637414
DNM3OS	1.0058857	3.3919808	7.5884352	2.65E-13	4.85E-12	19.634999
SYTL1	0.4932034	4.8310915	7.5882907	2.65E-13	4.86E-12	19.634058
AC011242.6	1.5081609	1.0049115	7.5864058	2.69E-13	4.91E-12	19.621782
PPP1R32	-0.380247	4.6872953	-7.586228	2.69E-13	4.92E-12	19.620626
PRSS35	1.2254271	3.107288	7.5858718	2.70E-13	4.93E-12	19.618305
DCTN3	-0.135439	6.2820876	-7.585715	2.70E-13	4.93E-12	19.617285
POU2F2	0.4346292	4.6831907	7.5851816	2.71E-13	4.95E-12	19.613811
TCL1A	1.6417897	1.3711506	7.5838351	2.73E-13	4.99E-12	19.605044
CCDC74B	1.4981216	1.2304571	7.5833475	2.74E-13	5.00E-12	19.60187
LAMTOR5-AS	-0.300607	4.6399769	-7.581914	2.77E-13	5.04E-12	19.592537
GLYAT	-1.305416	5.6344302	-7.581901	2.77E-13	5.04E-12	19.592456
DMBT1	1.8670871	1.4079561	7.5803865	2.80E-13	5.09E-12	19.582596
HENMT1	0.6315859	4.458896	7.5801961	2.80E-13	5.10E-12	19.581356
COQ5	-0.128464	6.3123279	-7.58	2.80E-13	5.10E-12	19.580078
TBC1D27	1.4251238	0.4115059	7.579054	2.82E-13	5.13E-12	19.573924
RP11-91G21	-0.737372	3.3900847	-7.578474	2.83E-13	5.15E-12	19.570152
TMEM108	1.1240309	3.0026553	7.5782271	2.84E-13	5.15E-12	19.568543
KCNE4	0.6254746	4.8215125	7.5762695	2.87E-13	5.22E-12	19.555806
LYPD6B	1.8736613	1.355239	7.5758187	2.88E-13	5.23E-12	19.552873
ASNA1	-0.122232	6.4225139	-7.573633	2.92E-13	5.31E-12	19.538658
CD2	0.4994262	5.2066177	7.5735718	2.93E-13	5.31E-12	19.538258
IL18BP	0.2374498	5.6948001	7.5733423	2.93E-13	5.31E-12	19.536765
ARHGAP25	0.2334112	5.4868897	7.5731439	2.93E-13	5.32E-12	19.535475
RP11-350N1	1.3144724	-0.308128	7.5716363	2.96E-13	5.37E-12	19.525671
GPR141	1.4045664	1.1811318	7.5711842	2.97E-13	5.38E-12	19.522731
CTD-2033D1	1.474372	1.0792851	7.5698462	3.00E-13	5.43E-12	19.514031
TSR2	-0.116835	6.3417078	-7.56899	3.02E-13	5.45E-12	19.508465
POLR2L	-0.163607	6.6663866	-7.568247	3.03E-13	5.48E-12	19.503635
CMC2	-0.142621	6.14983	-7.567997	3.04E-13	5.49E-12	19.502008
TTYH2	0.417373	5.0943495	7.5678773	3.04E-13	5.49E-12	19.501231
HSPA12A	0.538831	4.7908005	7.5666529	3.06E-13	5.53E-12	19.493272
RP6-65G23.	-1.250976	2.2082374	-7.564213	3.11E-13	5.62E-12	19.477416
PAPPA	1.0289595	3.275721	7.5625166	3.15E-13	5.68E-12	19.466393
RNU6-813P	-1.133512	-0.735204	-7.562435	3.15E-13	5.68E-12	19.46586
LINC00844	-1.90697	3.82733	-7.562344	3.15E-13	5.68E-12	19.465273
DES	1.5855395	2.8639405	7.5597967	3.21E-13	5.77E-12	19.448724
ADAM32	1.4253305	1.2867971	7.5566358	3.28E-13	5.89E-12	19.428196
ST6GALNAC4	0.3137287	5.4759255	7.5564406	3.28E-13	5.90E-12	19.426928
SS18L2	-0.161268	5.9341779	-7.555481	3.30E-13	5.93E-12	19.420697
SERPING1	-0.194102	7.5288365	-7.555315	3.30E-13	5.94E-12	19.419619
SIAH2	-0.180322	6.4885944	-7.554151	3.33E-13	5.98E-12	19.412066
PTPLA	1.1440142	3.1413158	7.5536236	3.34E-13	6.00E-12	19.408639
FAM26F	0.5326132	4.8660553	7.5534424	3.35E-13	6.00E-12	19.407463
DUSP2	0.5403665	4.8355638	7.5510997	3.40E-13	6.09E-12	19.392257
THEMIS	1.0225503	3.3072308	7.5507547	3.41E-13	6.10E-12	19.390018
COPRS	-0.148623	6.3618557	-7.54817	3.47E-13	6.21E-12	19.373247
CORO1A	0.295503	5.9632334	7.5463643	3.51E-13	6.28E-12	19.361531

LINC00238	-1.801666	1.5113495	-7.545185	3.54E-13	6.32E-12	19.35388
VAV1	0.3802861	5.1385558	7.5451619	3.54E-13	6.32E-12	19.353732
RP11-213H1	-1.47939	1.5192025	-7.540762	3.64E-13	6.51E-12	19.325197
DDX41	-0.111057	6.5378931	-7.540186	3.65E-13	6.53E-12	19.321464
PI4K2B	-0.179598	6.1929314	-7.539708	3.67E-13	6.55E-12	19.318367
CRLF3	0.1612089	5.5315664	7.5387442	3.69E-13	6.59E-12	19.312119
C1QTNF1	0.4427249	5.7882268	7.538692	3.69E-13	6.59E-12	19.31178
AC104654.2	1.5792098	1.0000406	7.5381483	3.70E-13	6.61E-12	19.308256
GLT8D1	-0.144358	6.4255133	-7.536821	3.74E-13	6.66E-12	19.299652
NT5DC4	1.4481986	0.7473668	7.5354378	3.77E-13	6.72E-12	19.290689
NRD1	-0.094167	6.7346907	-7.535217	3.78E-13	6.73E-12	19.289258
SNCA	1.1572649	2.8833648	7.5347714	3.79E-13	6.74E-12	19.286372
MEDAG	1.2307739	3.2577702	7.5346671	3.79E-13	6.74E-12	19.285695
C16orf86	-0.352814	4.9013936	-7.53382	3.81E-13	6.78E-12	19.280208
CSRNP3	1.2613569	3.1355847	7.5332929	3.83E-13	6.80E-12	19.276792
RP4-539M6.	-1.226972	1.8203709	-7.530527	3.90E-13	6.92E-12	19.258876
D4S234E	1.1297473	2.360539	7.5305202	3.90E-13	6.92E-12	19.25883
LINC01260	1.3715783	0.853502	7.5291682	3.93E-13	6.98E-12	19.250074
SYT13	2.0556836	2.4746697	7.5287922	3.94E-13	6.99E-12	19.247639
RWDD2B	-0.15883	5.9450425	-7.527668	3.97E-13	7.04E-12	19.240357
FKBP2	-0.177301	6.6762918	-7.527618	3.97E-13	7.04E-12	19.240034
SLC35A4	-0.094197	6.5786951	-7.527361	3.98E-13	7.05E-12	19.238371
ACOT8	-0.145274	6.1369477	-7.525844	4.02E-13	7.12E-12	19.228551
ITGA9	0.3173004	5.5927339	7.5243877	4.06E-13	7.18E-12	19.219122
LINC00884	-0.375315	5.0172265	-7.523969	4.07E-13	7.20E-12	19.216409
HSD17B4	-0.182628	6.9835604	-7.523405	4.09E-13	7.22E-12	19.21276
HLF	-0.420953	6.359866	-7.522295	4.12E-13	7.27E-12	19.205576
RALGDS	0.1280938	6.1005073	7.5219991	4.12E-13	7.28E-12	19.203662
TIMD4	1.5812653	2.4349661	7.5217542	4.13E-13	7.29E-12	19.202077
CD3E	0.4773897	5.3457879	7.5210281	4.15E-13	7.32E-12	19.197378
PRELP	0.7407545	5.4120505	7.5205304	4.16E-13	7.34E-12	19.194158
PDE10A	0.6612104	4.2349606	7.5197804	4.18E-13	7.38E-12	19.189305
OTULIN	-0.112524	6.1844816	-7.519569	4.19E-13	7.38E-12	19.187935
LINC01144	-0.463559	4.341848	-7.519365	4.20E-13	7.39E-12	19.186617
DERA	-0.155262	6.3436301	-7.519075	4.20E-13	7.40E-12	19.184743
CISD2	-0.127296	6.3570244	-7.519005	4.21E-13	7.40E-12	19.184285
HCLS1	0.2294561	6.0297494	7.5176173	4.25E-13	7.46E-12	19.175311
PLXNA4	1.6099932	2.624482	7.5175417	4.25E-13	7.47E-12	19.174821
EGLN3	0.7175577	5.0754308	7.5173987	4.25E-13	7.47E-12	19.173897
TRAC	0.4405849	5.4888456	7.5167897	4.27E-13	7.50E-12	19.169957
PDZRN4	1.6700738	0.8390254	7.515939	4.29E-13	7.53E-12	19.164455
CTD-237701	-1.432284	2.2994529	-7.514665	4.33E-13	7.59E-12	19.156218
LL22NC03-3	-1.416187	1.4021952	-7.513847	4.35E-13	7.63E-12	19.150928
LAT2	0.2840447	5.4016443	7.5135144	4.36E-13	7.65E-12	19.148775
IL18	0.4605257	5.0612021	7.5133757	4.37E-13	7.65E-12	19.147878
C17orf58	-0.169494	5.9268275	-7.513196	4.37E-13	7.65E-12	19.146713
FLJ20021	-0.271484	5.0308527	-7.512308	4.40E-13	7.70E-12	19.140976
CTD-3076M1	-1.318654	3.5303419	-7.510771	4.44E-13	7.77E-12	19.13104
CDRT4	1.2133927	1.5726714	7.5078426	4.53E-13	7.92E-12	19.112109
ADCK3	-0.186728	6.7693521	-7.507723	4.53E-13	7.92E-12	19.111335
MAPK10	0.8640978	3.906315	7.507181	4.55E-13	7.95E-12	19.107834

ALDH5A1	-0.223394	6.6626397	-7.506745	4.56E-13	7.97E-12	19.105019
ACSM2B	-0.597961	6.7279699	-7.506559	4.57E-13	7.97E-12	19.103816
MARCKS	0.1885715	6.5379125	7.5053566	4.60E-13	8.03E-12	19.096045
FAM96B	-0.155128	6.4965047	-7.505206	4.61E-13	8.04E-12	19.09507
HEYL	0.5032489	5.5523546	7.5046219	4.63E-13	8.06E-12	19.091298
ITM2C	0.2848443	6.3169622	7.5045964	4.63E-13	8.06E-12	19.091133
CNTRL	0.195398	5.6267129	7.5042244	4.64E-13	8.08E-12	19.08873
DENND2A	0.3045645	5.2253162	7.5041223	4.64E-13	8.08E-12	19.088071
TMEM200C	1.183719	3.2461511	7.503241	4.67E-13	8.12E-12	19.082377
IGHG4	1.4659438	4.6540279	7.502564	4.69E-13	8.15E-12	19.078004
CTB-39G8.3	1.3070178	1.7071295	7.5024257	4.70E-13	8.16E-12	19.077111
EAPP	-0.115923	6.1927522	-7.501793	4.71E-13	8.19E-12	19.073027
IL2RB	0.3972775	5.2952988	7.5003601	4.76E-13	8.26E-12	19.06377
GRHL2	1.7300815	1.5408351	7.500297	4.76E-13	8.26E-12	19.063362
TRAF3IP3	0.4127617	4.8026831	7.4999081	4.77E-13	8.28E-12	19.060851
EDF1	-0.157536	7.0069805	-7.499755	4.78E-13	8.28E-12	19.059861
STK16	-0.124067	6.2356021	-7.499558	4.79E-13	8.29E-12	19.058592
USP12-AS2	-1.364358	1.1263264	-7.498919	4.81E-13	8.32E-12	19.054465
KLHL35	1.2691274	1.9193514	7.498701	4.81E-13	8.33E-12	19.053056
ABHD1	-0.657317	4.5613906	-7.498504	4.82E-13	8.34E-12	19.051784
ZNF66	1.3798334	2.2948095	7.4964674	4.88E-13	8.45E-12	19.038636
DHRS12	-0.22635	5.8457324	-7.496225	4.89E-13	8.46E-12	19.03707
IGHD	1.6351785	2.8322831	7.4942324	4.96E-13	8.56E-12	19.024209
CCHCR1	-0.169325	6.1981313	-7.494179	4.96E-13	8.56E-12	19.023862
TRBV15	1.18798	-0.391908	7.4939751	4.97E-13	8.57E-12	19.022548
IKZF4	0.2197099	5.0569067	7.492713	5.01E-13	8.64E-12	19.014403
SEC61B	-0.1436	6.7306668	-7.492481	5.01E-13	8.65E-12	19.012906
SART1	-0.107912	6.5398667	-7.492418	5.02E-13	8.65E-12	19.012497
CTD-2024P1	-0.995891	2.454172	-7.490973	5.06E-13	8.72E-12	19.003178
AC012358.8	-0.445999	4.4152067	-7.490912	5.07E-13	8.72E-12	19.002779
RP11-529K1	-0.557175	3.604943	-7.490747	5.07E-13	8.73E-12	19.001716
CD80	1.1389933	2.7134341	7.489823	5.10E-13	8.78E-12	18.995756
CLEC2D	0.3700708	5.0226663	7.4884338	5.15E-13	8.86E-12	18.986795
TRBV4-1	1.3968463	0.1853912	7.4875642	5.18E-13	8.90E-12	18.981186
GPR82	1.1603468	2.2876412	7.4874239	5.19E-13	8.91E-12	18.98028
LCK	0.4544851	5.0586268	7.4867647	5.21E-13	8.94E-12	18.976029
EIF6	-0.142362	6.8246926	-7.486025	5.23E-13	8.98E-12	18.971258
CCL11	1.7095896	1.576248	7.4857558	5.24E-13	8.99E-12	18.969522
PIGP	-0.160053	5.9397281	-7.484931	5.27E-13	9.04E-12	18.964205
KLC3	1.7253324	1.8481507	7.4836543	5.32E-13	9.11E-12	18.955972
AC009303.2	-0.338799	4.7897759	-7.483391	5.33E-13	9.12E-12	18.954273
CTD-2366F1	-0.303303	5.0275765	-7.482577	5.35E-13	9.17E-12	18.949024
NDUFB1	-0.180615	6.455562	-7.481857	5.38E-13	9.21E-12	18.944385
DIAPH1	-0.131641	6.8727296	-7.481801	5.38E-13	9.21E-12	18.944024
MRPS22	-0.107167	6.2629224	-7.479322	5.47E-13	9.35E-12	18.928045
TSPAN2	0.9924696	3.3915578	7.4788216	5.49E-13	9.38E-12	18.924821
IL17RC	-0.159288	6.3862489	-7.47726	5.55E-13	9.47E-12	18.914758
LPCAT4	0.2784721	5.4180078	7.4766241	5.57E-13	9.51E-12	18.910661
TMEM130	1.5703609	1.980041	7.4751769	5.62E-13	9.60E-12	18.901337
PROSER1	0.1468189	5.816152	7.4749518	5.63E-13	9.61E-12	18.899887
CDK5RAP2	-0.11525	6.4612123	-7.4746	5.64E-13	9.62E-12	18.897618

XXbac-B135	-0.646537	3.5987432	-7.473375	5.69E-13	9.70E-12	18.889733
BTBD10	0.1062777	5.7837382	7.472977	5.70E-13	9.72E-12	18.887167
ECHS1	-0.180009	7.1444035	-7.472752	5.71E-13	9.73E-12	18.885721
CTC-277H1.	1.1475569	1.7746204	7.472408	5.73E-13	9.74E-12	18.883502
MICU1	-0.130247	6.5534878	-7.472365	5.73E-13	9.74E-12	18.883225
HLX	-0.229595	5.890791	-7.47216	5.74E-13	9.75E-12	18.881903
FTH1P22	1.3699467	1.0896399	7.4714812	5.76E-13	9.79E-12	18.877534
FER1L6	1.9950362	1.4351268	7.4707498	5.79E-13	9.83E-12	18.872824
RP11-103B5	1.4421322	0.7009224	7.4690121	5.86E-13	9.94E-12	18.861635
GUCY1B3	0.3492791	5.3844254	7.4681495	5.89E-13	1.00E-11	18.856082
PRPH2	1.2276182	2.2301284	7.4667671	5.94E-13	1.01E-11	18.847183
SLC9A7P1	1.3915163	2.375415	7.4664657	5.96E-13	1.01E-11	18.845243
RP5-1031D4	1.4121384	1.4341887	7.4658957	5.98E-13	1.01E-11	18.841574
ERCC5	-0.144329	6.1001185	-7.465469	5.99E-13	1.02E-11	18.838827
DHRS11	-0.181153	5.8580848	-7.464846	6.02E-13	1.02E-11	18.834818
ALDH6A1	-0.276582	6.7650226	-7.464778	6.02E-13	1.02E-11	18.834379
MTMR14	-0.103113	6.3674703	-7.464648	6.03E-13	1.02E-11	18.833546
CLEC12A	0.7857195	3.6226659	7.4643398	6.04E-13	1.02E-11	18.831561
AZGP1	-0.417725	7.1238351	-7.463508	6.07E-13	1.03E-11	18.826206
VN1R81P	1.4516088	0.6550727	7.4632112	6.08E-13	1.03E-11	18.824298
TRPV6	1.8724284	1.6116473	7.4622538	6.12E-13	1.03E-11	18.818139
PCDHGB7	1.2324658	3.2865615	7.4615276	6.15E-13	1.04E-11	18.813466
AC000403.4	1.2173821	1.5986924	7.4586188	6.27E-13	1.06E-11	18.794756
CTD-3157E1	1.3860114	1.5871195	7.4582863	6.29E-13	1.06E-11	18.792617
RASSF1-AS1	-0.786995	3.0886733	-7.457572	6.32E-13	1.06E-11	18.788025
CRTAC1	1.7271478	1.6559798	7.4570894	6.34E-13	1.07E-11	18.78492
TTC24	1.4411781	0.5004622	7.4570298	6.34E-13	1.07E-11	18.784537
HPR	-0.709361	6.6793638	-7.456339	6.37E-13	1.07E-11	18.780093
EFEMP1	1.0470814	5.3972524	7.4553917	6.41E-13	1.08E-11	18.774004
SLC9A3R2	-0.20501	6.8439462	-7.45468	6.44E-13	1.08E-11	18.769427
XAB2	-0.118803	6.4485333	-7.453035	6.51E-13	1.09E-11	18.758854
TMEM59	-0.108028	6.9835615	-7.452156	6.55E-13	1.10E-11	18.753202
IGHV3-7	1.5650156	0.0780035	7.4519664	6.55E-13	1.10E-11	18.751984
NDUFB7	-0.17402	6.6536653	-7.451208	6.59E-13	1.11E-11	18.747111
MRPL28	-0.160195	6.5033065	-7.450371	6.62E-13	1.11E-11	18.741731
IL27RA	0.2728569	5.4206476	7.4498066	6.65E-13	1.11E-11	18.738104
HNRNPA1P66	-1.485258	0.2609741	-7.449725	6.65E-13	1.11E-11	18.737578
KLHL3	0.6470394	4.739082	7.449508	6.66E-13	1.12E-11	18.736185
SYTL2	0.3575195	5.1948731	7.448237	6.72E-13	1.12E-11	18.728019
ZNF688	-0.183777	5.685336	-7.44814	6.72E-13	1.12E-11	18.727394
HOXB-AS1	0.7944095	3.0851908	7.4476054	6.74E-13	1.13E-11	18.723961
MEIS3P1	1.4098467	1.8495655	7.4473769	6.75E-13	1.13E-11	18.722493
OXNAD1	-0.166618	5.8694056	-7.447303	6.76E-13	1.13E-11	18.722019
RP11-1099M	0.8356786	2.7507803	7.4468281	6.78E-13	1.13E-11	18.718967
PAAF1	-0.124337	5.9515552	-7.446016	6.82E-13	1.14E-11	18.713749
NOVA1	1.1013651	3.8322599	7.4459611	6.82E-13	1.14E-11	18.713397
SMARCD2	-0.117899	6.5811165	-7.445954	6.82E-13	1.14E-11	18.713352
IPO13	-0.109127	6.2523423	-7.445531	6.84E-13	1.14E-11	18.710634
GALNT6	0.3910695	4.7832434	7.4431657	6.94E-13	1.16E-11	18.695443
ASPHD2	0.5313435	4.0760789	7.4430577	6.95E-13	1.16E-11	18.69475
RBKS	-0.204253	6.0277374	-7.443044	6.95E-13	1.16E-11	18.694664

RANBP10	-0.165844	6.1846619	-7.442854	6.96E-13	1.16E-11	18.693444
RCC2	0.1261847	6.3570869	7.4422623	6.99E-13	1.16E-11	18.689642
ESYT3	1.3616374	3.3670159	7.4413769	7.03E-13	1.17E-11	18.683958
SMAD7	0.2428591	5.6657736	7.4407039	7.06E-13	1.17E-11	18.679637
CD69	0.6813769	4.4822207	7.4406108	7.06E-13	1.17E-11	18.679039
PALD1	0.266123	5.4549432	7.4405509	7.07E-13	1.17E-11	18.678654
STXBP5-AS1	0.9176617	2.9378442	7.4400164	7.09E-13	1.18E-11	18.675223
H2AFV	-0.087063	6.67014	-7.439285	7.12E-13	1.18E-11	18.670526
TPTEP1	1.297279	1.8709048	7.4373956	7.21E-13	1.20E-11	18.6584
RP11-792A8	-0.403979	4.4942675	-7.436944	7.24E-13	1.20E-11	18.655503
TEDDM2P	-1.349924	-0.640964	-7.436583	7.25E-13	1.20E-11	18.653186
CTNNBL1	-0.15622	6.4470824	-7.435612	7.30E-13	1.21E-11	18.646954
C8orf82	-0.206539	6.4115237	-7.435242	7.32E-13	1.21E-11	18.644577
TOR1AIP2	-0.146788	6.8065549	-7.434838	7.34E-13	1.21E-11	18.641986
PRR33	1.1536913	2.4038294	7.4345565	7.35E-13	1.22E-11	18.640181
MTMR10	-0.143824	6.2429414	-7.434522	7.35E-13	1.22E-11	18.639959
EIF4EBP3	-0.297642	5.5414382	-7.43415	7.37E-13	1.22E-11	18.637574
RAVER1	0.415825	5.3670609	7.4341286	7.37E-13	1.22E-11	18.637436
AC113189.5	-0.301789	5.3105025	-7.432951	7.43E-13	1.23E-11	18.629882
MECR	-0.146137	6.0194009	-7.43261	7.44E-13	1.23E-11	18.627692
GATB	-0.163662	6.1013822	-7.432289	7.46E-13	1.23E-11	18.625631
IGHA1	0.6786425	5.9241675	7.4321096	7.47E-13	1.23E-11	18.624483
POMT1	-0.118128	6.2333472	-7.431102	7.52E-13	1.24E-11	18.618021
COPS6	-0.124217	6.6305156	-7.430511	7.55E-13	1.24E-11	18.61423
BCAS1	1.462294	3.7101787	7.4300632	7.57E-13	1.25E-11	18.611357
FFAR2	1.2151261	2.5294793	7.4296648	7.59E-13	1.25E-11	18.608802
SPSB4	1.4172396	0.2990572	7.4270633	7.72E-13	1.27E-11	18.592121
PYGM	0.6801117	3.5959552	7.4256402	7.79E-13	1.28E-11	18.582997
FAM210B	-0.162571	6.6278433	-7.425382	7.81E-13	1.28E-11	18.58134
SLC35C2	-0.105015	6.4894328	-7.423993	7.88E-13	1.29E-11	18.572436
E4F1	-0.134094	6.1254744	-7.42378	7.89E-13	1.30E-11	18.571075
RP11-798K3	-0.618393	5.7235927	-7.422913	7.93E-13	1.30E-11	18.565516
LDB1	0.1494956	5.9999453	7.4226962	7.95E-13	1.30E-11	18.564127
METRNL	0.3644175	5.5913624	7.4222383	7.97E-13	1.31E-11	18.561193
TTC36	-1.295931	4.5832385	-7.421717	8.00E-13	1.31E-11	18.557853
AC016700.5	-0.599702	4.5455714	-7.42137	8.02E-13	1.31E-11	18.555628
CRLF1	0.5398662	4.2066551	7.4206517	8.05E-13	1.32E-11	18.551025
DKK1	1.7606177	3.3867689	7.4201111	8.08E-13	1.32E-11	18.547562
IL2RG	0.3929004	5.7311782	7.4201008	8.08E-13	1.32E-11	18.547496
MORN3	0.8823544	2.7301727	7.4200197	8.09E-13	1.32E-11	18.546976
RP11-347C1	-0.330578	4.8020266	-7.419236	8.13E-13	1.33E-11	18.541953
TRBV5-4	1.3620888	0.4039188	7.4188716	8.15E-13	1.33E-11	18.539621
DPYSL4	1.4789401	2.6340635	7.417064	8.25E-13	1.35E-11	18.528042
SLC4A8	0.8237701	3.1262586	7.4164269	8.28E-13	1.35E-11	18.523961
SLFN12L	1.0376808	3.0092525	7.4151598	8.35E-13	1.36E-11	18.515846
SERP1	-0.10416	6.8421893	-7.414609	8.38E-13	1.37E-11	18.512318
TTF2	0.2355207	5.4393106	7.4139494	8.42E-13	1.37E-11	18.508096
ABCA6	-0.445689	6.3150043	-7.413681	8.43E-13	1.37E-11	18.506374
NCF2	0.3566693	5.5260723	7.4127657	8.48E-13	1.38E-11	18.500516
RP11-705C1	0.7067613	3.811108	7.4127191	8.48E-13	1.38E-11	18.500218
LINC01356	1.2843773	0.9282773	7.4124926	8.50E-13	1.38E-11	18.498768

GRINA	-0.152485	7.1144225	-7.412477	8.50E-13	1.38E-11	18.498667
LINC01057	1.2105887	3.2549618	7.4124093	8.50E-13	1.38E-11	18.498235
PPP1R16A	-0.203929	6.6073941	-7.412289	8.51E-13	1.38E-11	18.497467
PQLC1	-0.178581	6.757862	-7.411493	8.55E-13	1.39E-11	18.492366
C15orf61	-0.173111	5.5421695	-7.411175	8.57E-13	1.39E-11	18.490333
ACOX2	-0.355309	6.5294982	-7.411152	8.57E-13	1.39E-11	18.490183
AMT	-0.238603	6.320499	-7.411002	8.58E-13	1.39E-11	18.489225
HAX1	-0.127815	6.6751308	-7.410575	8.60E-13	1.40E-11	18.486493
NAIP	0.9412831	2.8998561	7.4104746	8.61E-13	1.40E-11	18.48585
LRRC4	0.6554377	3.6716536	7.4059559	8.87E-13	1.44E-11	18.456932
SLITRK6	1.4497127	-0.014734	7.4054823	8.90E-13	1.44E-11	18.453902
COX5B	-0.157874	6.7931322	-7.404001	8.98E-13	1.45E-11	18.444426
RP11-53B2.	1.2519725	1.1724342	7.4039532	8.99E-13	1.45E-11	18.44412
IGLC2	1.0202591	5.1211342	7.400734	9.18E-13	1.48E-11	18.423531
MEG8	1.4332541	-0.128475	7.3994747	9.26E-13	1.50E-11	18.415479
SMIM10	0.520185	4.3018805	7.3986791	9.30E-13	1.50E-11	18.410392
GRIP2	1.4461915	2.6530805	7.3980364	9.34E-13	1.51E-11	18.406283
RP11-407B7	-0.689778	4.5777948	-7.397256	9.39E-13	1.52E-11	18.401294
SLIT3	0.4403132	5.3642285	7.3967603	9.42E-13	1.52E-11	18.398125
CDK10	-0.135642	6.4600115	-7.395551	9.50E-13	1.53E-11	18.390394
TRMT112	-0.126801	6.6918839	-7.392618	9.68E-13	1.56E-11	18.37165
TREML2	1.4325291	0.9384159	7.3899968	9.85E-13	1.59E-11	18.354907
SUPT6H	-0.079153	6.7013808	-7.38831	9.96E-13	1.60E-11	18.344134
THAP5	-0.12736	6.0418534	-7.388228	9.96E-13	1.60E-11	18.343611
MINK1	0.1226905	6.3334427	7.3872004	1.00E-12	1.61E-11	18.337046
SOX8	1.3362891	2.086521	7.387027	1.00E-12	1.62E-11	18.335939
EFNA5	1.4447794	3.1867169	7.3862084	1.01E-12	1.62E-11	18.330712
VIPR2	1.5017148	0.8954493	7.385929	1.01E-12	1.63E-11	18.328927
ALDH9A1	-0.149474	6.7414061	-7.385715	1.01E-12	1.63E-11	18.327561
METTL20	-0.226396	5.3743758	-7.384847	1.02E-12	1.64E-11	18.322017
RP11-455F5	-0.406722	4.250615	-7.383706	1.03E-12	1.65E-11	18.314731
KCNK13	1.2253756	3.3000572	7.3833789	1.03E-12	1.65E-11	18.312646
PPP1R12A	0.1199437	6.0647656	7.3828611	1.03E-12	1.66E-11	18.309341
MRPL55	-0.184389	6.479729	-7.382749	1.03E-12	1.66E-11	18.308625
AC011899.9	0.6946047	3.8347556	7.3816416	1.04E-12	1.67E-11	18.301557
CTC-246B18	1.3532373	0.1007819	7.3803642	1.05E-12	1.68E-11	18.293405
SORCS1	1.7033125	0.2077256	7.3799602	1.05E-12	1.68E-11	18.290827
ARSI	1.1123682	3.495633	7.3799531	1.05E-12	1.68E-11	18.290781
IGLC7	1.7852693	1.1132304	7.3792765	1.06E-12	1.69E-11	18.286463
PRDX6	-0.152654	7.1734484	-7.378866	1.06E-12	1.69E-11	18.283841
CD81	-0.173364	7.1157188	-7.377672	1.07E-12	1.71E-11	18.276223
HMGB3P22	-0.894686	2.9724276	-7.377173	1.07E-12	1.71E-11	18.273046
C2orf81	0.4781652	4.1700718	7.3768143	1.07E-12	1.71E-11	18.270754
TTC1	-0.124744	6.3657443	-7.37451	1.09E-12	1.74E-11	18.256056
ACP5	0.349404	6.1256298	7.3724415	1.10E-12	1.76E-11	18.242865
TMEM154	0.8061753	4.3480126	7.3712239	1.11E-12	1.78E-11	18.235101
ADH6	-0.423106	6.7271869	-7.36946	1.13E-12	1.80E-11	18.223858
KCTD7	0.1953563	5.4724491	7.3690763	1.13E-12	1.80E-11	18.22141
RP11-930P1	1.3100015	1.376026	7.3690297	1.13E-12	1.80E-11	18.221113
IER3	0.3496123	6.0047675	7.3682798	1.14E-12	1.81E-11	18.216333
RNF212	1.2876219	1.5021638	7.36702	1.14E-12	1.82E-11	18.208303

RP11-848P1	0.8235606	3.4634703	7.3663848	1.15E-12	1.83E-11	18.204255
SHANK1	1.3563303	1.3538309	7.3623297	1.18E-12	1.88E-11	18.178418
CTD-2194A8	-1.261975	2.6792998	-7.361144	1.19E-12	1.89E-11	18.170869
SOD3	0.6224879	5.4036066	7.3600525	1.20E-12	1.90E-11	18.163914
ACPP	1.5538301	1.4406046	7.3599968	1.20E-12	1.90E-11	18.16356
DHX16	-0.098192	6.3548915	-7.359899	1.20E-12	1.90E-11	18.162938
RAP2B	0.1417809	6.0446659	7.3597737	1.20E-12	1.90E-11	18.162139
RP11-672A2	1.4092835	1.8550438	7.3592531	1.20E-12	1.91E-11	18.158823
RP11-361L1	-1.043692	-0.732793	-7.358258	1.21E-12	1.92E-11	18.152484
C8orf31	1.3513878	2.1714637	7.3580582	1.21E-12	1.92E-11	18.151215
TEF	-0.198134	6.2600643	-7.357247	1.22E-12	1.93E-11	18.146049
PCTP	-0.181168	6.4396657	-7.355839	1.23E-12	1.95E-11	18.137083
CTC-332L22	-0.711217	3.140831	-7.35495	1.24E-12	1.96E-11	18.131429
SLC7A10	1.8647928	1.2090924	7.3542493	1.24E-12	1.97E-11	18.126967
ZNF486	0.7845209	3.9354734	7.3532638	1.25E-12	1.98E-11	18.120694
IGKV2D-40	1.5866609	0.0080399	7.352823	1.26E-12	1.98E-11	18.117889
RP11-70D24	-0.590029	4.0233188	-7.352756	1.26E-12	1.98E-11	18.117461
C11orf68	-0.115072	6.2136143	-7.351756	1.26E-12	2.00E-11	18.1111
GSTM4	-0.214711	6.1734537	-7.351746	1.26E-12	2.00E-11	18.111037
PSMB4	-0.125758	7.0164997	-7.351528	1.27E-12	2.00E-11	18.109646
TPMT	-0.154906	6.4564493	-7.3509	1.27E-12	2.01E-11	18.105651
NIT2	-0.138032	6.5618452	-7.350052	1.28E-12	2.02E-11	18.100259
TRBV7-6	1.1856715	-0.26189	7.3459709	1.31E-12	2.07E-11	18.074297
SLC37A1	0.2672165	5.6674633	7.3455844	1.32E-12	2.07E-11	18.071839
BPHL	-0.201213	6.4081184	-7.34533	1.32E-12	2.08E-11	18.070219
PCLO	1.9566064	1.6306826	7.3427786	1.34E-12	2.11E-11	18.053998
ARHGAP23	0.2514844	5.6249295	7.3421739	1.35E-12	2.12E-11	18.050154
VTCN1	1.8471616	2.0625681	7.3407473	1.36E-12	2.14E-11	18.041086
SCG3	1.5262385	0.5236821	7.3407335	1.36E-12	2.14E-11	18.040998
BOK	-0.27298	6.5502065	-7.339046	1.37E-12	2.16E-11	18.030275
AC133644.2	0.914415	3.2508869	7.3386487	1.38E-12	2.16E-11	18.027748
LINC00865	1.0428539	2.6898008	7.3383261	1.38E-12	2.17E-11	18.025698
FAM192A	-0.09416	6.3514399	-7.338164	1.38E-12	2.17E-11	18.024665
STKLD1	-0.343259	4.3026475	-7.335954	1.40E-12	2.20E-11	18.010626
SIRPB1	0.869761	3.5271898	7.3356439	1.40E-12	2.20E-11	18.008656
RP11-456H1	-1.078441	3.491926	-7.334941	1.41E-12	2.21E-11	18.004189
C1orf50	-0.171409	5.5728823	-7.334928	1.41E-12	2.21E-11	18.004107
GABRP	1.636685	1.9563795	7.3349097	1.41E-12	2.21E-11	18.003991
ZNF93	0.6523139	4.1935232	7.3341331	1.42E-12	2.22E-11	17.999058
NFATC1	0.3415505	5.2947172	7.333298	1.43E-12	2.23E-11	17.993754
FAM57A	0.3280336	5.1822272	7.3327248	1.43E-12	2.24E-11	17.990113
AC006994.3	-1.424141	0.4904881	-7.329952	1.46E-12	2.28E-11	17.972506
AIG1	-0.166921	6.7092907	-7.329173	1.47E-12	2.29E-11	17.967557
RGS11	1.1252997	3.0163131	7.3291675	1.47E-12	2.29E-11	17.967525
TXN	-0.177249	6.9759279	-7.328834	1.47E-12	2.29E-11	17.965406
CAT	-0.190571	6.9982194	-7.327177	1.48E-12	2.32E-11	17.954887
CMTM7	0.2688894	5.5853965	7.3260229	1.50E-12	2.33E-11	17.947564
LGALS2	0.7211669	4.4773273	7.3259622	1.50E-12	2.33E-11	17.947178
ATP2C2	1.57125	1.4190605	7.32462	1.51E-12	2.35E-11	17.93866
RP11-5407.	1.5215235	1.4083765	7.324542	1.51E-12	2.35E-11	17.938166
CD6	0.5031108	5.0064867	7.3234825	1.52E-12	2.37E-11	17.931443

TMCO6	-0.202602	6.0699154	-7.322734	1.53E-12	2.38E-11	17.926695
THBS1	0.3048058	6.4468275	7.3225642	1.53E-12	2.38E-11	17.925616
B3GALT5-AS	1.1682346	-0.689408	7.3222562	1.53E-12	2.38E-11	17.923662
RP11-569G1	1.3281609	-0.520662	7.3216829	1.54E-12	2.39E-11	17.920025
AP003774.4	1.2470414	1.2713694	7.3200492	1.56E-12	2.42E-11	17.909662
IFT57	0.3626397	5.429682	7.3197575	1.56E-12	2.42E-11	17.907812
AR	-0.568123	6.2732099	-7.318771	1.57E-12	2.43E-11	17.901552
TPRG1-AS1	-0.92846	4.6130075	-7.318007	1.58E-12	2.44E-11	17.89671
CHAD	-0.485397	5.7632008	-7.317655	1.58E-12	2.45E-11	17.894479
IMPG1	1.2817028	0.9954862	7.3170665	1.59E-12	2.46E-11	17.890746
FPR1	0.7535232	4.3127156	7.3168188	1.59E-12	2.46E-11	17.889175
RP11-115J1	-1.345004	-0.087299	-7.315352	1.60E-12	2.48E-11	17.879878
TRAV17	1.3392312	0.2737051	7.3133362	1.62E-12	2.52E-11	17.867096
PSTPIP1	0.4804977	4.8083925	7.3128945	1.63E-12	2.52E-11	17.864297
KCNMB4	0.5524028	4.4354788	7.3128186	1.63E-12	2.52E-11	17.863815
NSA2	-0.122943	6.4975586	-7.312055	1.64E-12	2.53E-11	17.858978
RP11-497E1	1.1777646	-0.451685	7.3119176	1.64E-12	2.53E-11	17.858105
CTC-455F18	1.3109119	0.0866567	7.3116129	1.64E-12	2.54E-11	17.856174
ZBED2	1.6114652	1.5596129	7.3114506	1.64E-12	2.54E-11	17.855145
TTYH1	1.1860692	3.3836751	7.3095169	1.67E-12	2.57E-11	17.842892
BEND4	1.3912864	0.2364507	7.3087629	1.67E-12	2.58E-11	17.838115
COQ4	-0.144195	6.3786702	-7.308732	1.67E-12	2.58E-11	17.837919
PARK7	-0.151313	6.8531179	-7.306779	1.70E-12	2.61E-11	17.825547
KIF22	-0.15347	6.3147797	-7.30527	1.71E-12	2.64E-11	17.81599
KHDC1	1.4499904	1.2996138	7.3045155	1.72E-12	2.65E-11	17.811211
DEPDC7	-0.396163	5.9509551	-7.303406	1.73E-12	2.67E-11	17.804183
CTC-550B14	1.0719207	2.567139	7.3026728	1.74E-12	2.68E-11	17.799542
RP4-763G1.	-1.359142	4.3677321	-7.302434	1.74E-12	2.68E-11	17.79803
FAM117B	0.2361622	5.3890216	7.300328	1.77E-12	2.72E-11	17.784697
HSH2D	0.6540987	4.7534522	7.2998086	1.77E-12	2.73E-11	17.78141
SHFM1	-0.157576	6.7407687	-7.298765	1.79E-12	2.74E-11	17.774807
TARDBP	-0.0723	6.609868	-7.297912	1.80E-12	2.76E-11	17.769403
CNR2	1.3207085	1.3059195	7.297621	1.80E-12	2.76E-11	17.767564
NPY5R	1.5168097	0.803724	7.2968333	1.81E-12	2.78E-11	17.762579
RADIL	0.9188241	2.801522	7.296469	1.81E-12	2.78E-11	17.760274
HOMER3	0.2835117	5.6571327	7.295891	1.82E-12	2.79E-11	17.756617
RP11-21L23	-1.146289	3.2160875	-7.294917	1.83E-12	2.81E-11	17.750455
NAA60	-0.122574	6.3546068	-7.294324	1.84E-12	2.82E-11	17.7467
RP1-261D10	-0.470679	4.2915281	-7.293369	1.85E-12	2.83E-11	17.74066
CAMLG	-0.128697	6.2188922	-7.293203	1.85E-12	2.83E-11	17.739613
HK2	0.6889199	5.0138162	7.2931923	1.85E-12	2.83E-11	17.739543
LINC00539	0.9303289	2.0831811	7.2928203	1.86E-12	2.84E-11	17.737191
RP11-350G8	-0.606699	3.6703442	-7.291773	1.87E-12	2.86E-11	17.730567
DNAH2	0.9659141	3.1387427	7.2916783	1.87E-12	2.86E-11	17.729967
RP11-158I9	-0.39875	4.6090109	-7.291457	1.87E-12	2.86E-11	17.72857
ZFP28	0.6716994	4.222173	7.290525	1.88E-12	2.88E-11	17.722674
STX11	0.5731824	4.5534012	7.2888892	1.90E-12	2.91E-11	17.71233
RASA4DP	1.2904337	0.7790166	7.2878794	1.92E-12	2.92E-11	17.705946
RP11-25K19	1.5143117	2.6054135	7.2868422	1.93E-12	2.94E-11	17.699389
NPTX1	1.6107146	0.7592	7.2860098	1.94E-12	2.96E-11	17.694127
TBC1D17	-0.114008	6.4339178	-7.285238	1.95E-12	2.97E-11	17.689247

POC1A	-0.214203	5.702913	-7.284889	1.95E-12	2.98E-11	17.687046
HYAL3	-0.314364	5.3775233	-7.284687	1.96E-12	2.98E-11	17.685764
CDIP1	-0.149469	6.3784349	-7.284484	1.96E-12	2.98E-11	17.684485
PDE5A	0.3333682	5.0932544	7.283594	1.97E-12	3.00E-11	17.678859
NDRG4	0.5202809	4.371117	7.2817115	2.00E-12	3.03E-11	17.666964
CECR6	0.8506133	3.0258266	7.2809443	2.01E-12	3.05E-11	17.662117
B3GAT2	1.1168018	2.7186561	7.2802707	2.01E-12	3.06E-11	17.657862
DDR1	0.4238596	5.9551074	7.2795101	2.02E-12	3.07E-11	17.653057
DEXI	-0.207351	5.581372	-7.278619	2.04E-12	3.09E-11	17.647425
STK33	1.7502258	1.400232	7.2776028	2.05E-12	3.11E-11	17.64101
NTNG2	1.0111874	3.0302328	7.2775711	2.05E-12	3.11E-11	17.64081
CCDC74A	0.8709189	3.6510301	7.2773726	2.05E-12	3.11E-11	17.639556
CD84	0.3812691	5.1911802	7.2771114	2.06E-12	3.11E-11	17.637907
HSPB7	1.1427827	3.4938446	7.276425	2.06E-12	3.13E-11	17.633573
YBX3	0.2784891	6.044494	7.2747342	2.09E-12	3.16E-11	17.622896
CD101	0.5362344	4.0699968	7.2724889	2.12E-12	3.21E-11	17.608722
SLC6A19	1.95305	1.0345927	7.2720066	2.12E-12	3.21E-11	17.605678
IL12RB1	0.4532761	4.7129521	7.2720045	2.12E-12	3.21E-11	17.605664
ING1	-0.160987	5.9592739	-7.271725	2.13E-12	3.22E-11	17.603902
MAPK7	0.1445034	5.6363602	7.2714535	2.13E-12	3.22E-11	17.602187
MRPL14	-0.163917	6.5375489	-7.270162	2.15E-12	3.25E-11	17.594038
RP11-710C1	1.2706718	0.6911844	7.2696949	2.16E-12	3.26E-11	17.591088
KAT8	-0.105038	6.1303504	-7.268507	2.17E-12	3.28E-11	17.583593
NRROS	0.2829709	5.0918431	7.2681945	2.18E-12	3.28E-11	17.581621
AAAS	-0.084504	6.2888025	-7.267162	2.19E-12	3.31E-11	17.575106
PTPN7	0.4432876	5.0343471	7.2663956	2.20E-12	3.32E-11	17.570272
SLC7A8	0.3515352	5.2486225	7.2661909	2.21E-12	3.32E-11	17.568981
AC244250.2	1.5471616	0.0561954	7.2660611	2.21E-12	3.33E-11	17.568163
TP53BP1	0.2973384	5.6565687	7.2645617	2.23E-12	3.36E-11	17.558705
AGFG2	-0.159041	6.6463646	-7.263956	2.24E-12	3.37E-11	17.554885
SSSCA1	-0.156971	6.0384627	-7.26353	2.24E-12	3.38E-11	17.552196
RP11-266K4	1.2986699	0.932122	7.2632783	2.25E-12	3.38E-11	17.550611
LINC01121	1.604669	1.1257878	7.2631216	2.25E-12	3.38E-11	17.549623
DAD1	-0.116122	6.7270065	-7.262467	2.26E-12	3.40E-11	17.545497
SMIM12	-0.138228	6.2613457	-7.260648	2.29E-12	3.43E-11	17.534026
UBE2Q2	0.2559645	5.871202	7.2601631	2.29E-12	3.44E-11	17.530969
PCED1B	0.3829394	5.1405123	7.25972	2.30E-12	3.45E-11	17.528176
CHID1	-0.125954	6.6356711	-7.259004	2.31E-12	3.47E-11	17.523661
CD8A	0.5621053	4.9869538	7.2587552	2.31E-12	3.47E-11	17.522094
ZNF185	0.2414218	5.1055306	7.2546735	2.38E-12	3.56E-11	17.496372
C6orf195	1.1795017	-0.373118	7.2541012	2.39E-12	3.57E-11	17.492766
MARK1	0.8415468	3.7832542	7.2527999	2.41E-12	3.60E-11	17.484568
PXDN	0.2527135	5.9975715	7.2527139	2.41E-12	3.60E-11	17.484027
RP11-23J18	1.2744072	1.228226	7.2517068	2.42E-12	3.62E-11	17.477683
PFKFB1	-0.679006	5.5402996	-7.251469	2.43E-12	3.63E-11	17.476183
EPHX3	1.0785251	2.4139626	7.2512218	2.43E-12	3.63E-11	17.474628
RAB7B	0.3975452	4.9370532	7.250185	2.45E-12	3.66E-11	17.468098
TRBV6-6	1.2515366	-0.122796	7.2495624	2.46E-12	3.67E-11	17.464177
ADORA2B	0.9565316	3.6102445	7.2489635	2.47E-12	3.68E-11	17.460406
AP001046.5	0.8860274	3.1324881	7.2478124	2.48E-12	3.71E-11	17.453158
QPCT	0.5332911	4.6312297	7.2469411	2.50E-12	3.73E-11	17.447673

CCL21	1.2456188	4.9661127	7.2464315	2.51E-12	3.74E-11	17.444465
OCA2	1.791378	2.3443108	7.2458419	2.52E-12	3.75E-11	17.440753
HYKK	-0.189705	5.2994784	-7.244006	2.55E-12	3.79E-11	17.4292
TRDMT1	0.4142874	4.4543995	7.2429302	2.56E-12	3.82E-11	17.422427
ACAA2	-0.195582	6.9224954	-7.241838	2.58E-12	3.84E-11	17.415552
CD27	0.6753298	4.262776	7.2415578	2.59E-12	3.85E-11	17.413791
HS1BP3	-0.138185	6.3851311	-7.240733	2.60E-12	3.87E-11	17.4086
RP1-313I6.	-0.318794	4.6514826	-7.240072	2.61E-12	3.88E-11	17.404445
BTLA	1.2730399	2.3471871	7.239139	2.63E-12	3.91E-11	17.398574
PET100	-0.199895	6.1524413	-7.238781	2.63E-12	3.91E-11	17.396325
SLC25A4	-0.166555	6.3767489	-7.237946	2.65E-12	3.93E-11	17.391068
NRIP2	0.3249914	4.9705295	7.2377755	2.65E-12	3.94E-11	17.389998
YIPF1	-0.115381	6.3113694	-7.237615	2.65E-12	3.94E-11	17.388987
CTC-510F12	-0.706785	3.0812695	-7.237525	2.66E-12	3.94E-11	17.388419
SLA2	0.5346627	4.3254051	7.2363684	2.68E-12	3.97E-11	17.381148
NAALADL1	0.5164838	4.8244664	7.234826	2.70E-12	4.00E-11	17.371449
NMT1	-0.089511	6.6917903	-7.234754	2.70E-12	4.01E-11	17.370995
LINC01023	-0.362003	4.5005949	-7.233413	2.73E-12	4.04E-11	17.362566
SEPSECS	-0.187287	6.2038301	-7.233282	2.73E-12	4.04E-11	17.361743
SCP2	-0.195732	7.0090728	-7.232333	2.75E-12	4.06E-11	17.355776
RP11-159F2	-1.689738	0.9021947	-7.228854	2.81E-12	4.15E-11	17.33391
PTPRO	0.7408239	3.8253514	7.2287379	2.81E-12	4.16E-11	17.333181
RP11-383H1	0.8884461	4.3013609	7.2281431	2.82E-12	4.17E-11	17.329444
RP11-872J2	-0.403226	4.1272466	-7.227826	2.83E-12	4.18E-11	17.327453
PDE4DIP	-0.192458	6.4689012	-7.227551	2.83E-12	4.18E-11	17.325722
PCDHGC3	0.9419821	4.4458863	7.2263825	2.85E-12	4.21E-11	17.318382
FAM83F	1.6599574	2.1932535	7.2253331	2.87E-12	4.24E-11	17.311791
KIAA1324	0.7420672	4.1705548	7.2251701	2.88E-12	4.24E-11	17.310767
TIFAB	1.3102342	2.4089353	7.22499	2.88E-12	4.24E-11	17.309636
CCL26	1.5281185	1.565305	7.2249245	2.88E-12	4.24E-11	17.309224
LYL1	0.2855119	4.9693181	7.2248202	2.88E-12	4.25E-11	17.308569
PRDX5	-0.136776	6.9172927	-7.224324	2.89E-12	4.26E-11	17.305451
SUMO1	-0.098051	6.6626808	-7.224245	2.89E-12	4.26E-11	17.304955
SNRPC	-0.132181	6.5940068	-7.222536	2.93E-12	4.30E-11	17.294225
GHDC	-0.139777	6.2926078	-7.222403	2.93E-12	4.31E-11	17.293386
MGST1	-0.292445	7.1211538	-7.221063	2.95E-12	4.34E-11	17.284971
TRIM41	-0.120107	6.2309245	-7.221032	2.95E-12	4.34E-11	17.284777
PLOD1	-0.134818	6.9241056	-7.22099	2.95E-12	4.34E-11	17.284518
PIP5K1B	1.0248814	3.0004127	7.2204556	2.96E-12	4.35E-11	17.281161
AC016723.4	-1.556614	0.4271462	-7.220427	2.97E-12	4.35E-11	17.28098
LIMA1	0.1658711	6.0940163	7.2203435	2.97E-12	4.35E-11	17.280457
FREM1	1.6970251	1.9988277	7.2191223	2.99E-12	4.39E-11	17.272791
BRAT1	-0.109491	6.4557608	-7.219037	2.99E-12	4.39E-11	17.272254
MYO1B	-0.170298	6.8780931	-7.218756	3.00E-12	4.39E-11	17.27049
VPS37D	-0.338311	5.3097951	-7.218334	3.01E-12	4.40E-11	17.267842
RP11-563J2	1.3483124	0.6258316	7.2181632	3.01E-12	4.41E-11	17.26677
CD83	0.2590193	5.3771715	7.2180073	3.01E-12	4.41E-11	17.265791
ZNF578	1.3358236	1.2769186	7.2177598	3.02E-12	4.41E-11	17.264238
PRAM1	0.5136839	4.1284983	7.2173943	3.02E-12	4.42E-11	17.261944
RP11-96B2.	-1.642202	1.3740029	-7.217245	3.03E-12	4.42E-11	17.261007
SLC2A6	0.4466942	5.4689512	7.2171404	3.03E-12	4.42E-11	17.26035

MT-CO2	-0.148446	7.8424788	-7.21712	3.03E-12	4.42E-11	17.260223
DLX4	1.63392	1.4266457	7.2168107	3.04E-12	4.43E-11	17.258281
SMIM19	-0.212392	6.2100864	-7.216429	3.04E-12	4.44E-11	17.255886
CD300LF	0.488945	4.3635713	7.2162081	3.05E-12	4.45E-11	17.2545
GBP5	0.4782307	5.2796301	7.2157522	3.06E-12	4.46E-11	17.251638
CNPY2	-0.152966	6.3012933	-7.21201	3.13E-12	4.56E-11	17.228163
PSMC2	-0.106633	6.672804	-7.210626	3.16E-12	4.60E-11	17.219476
TMEM134	-0.137551	6.202542	-7.209868	3.17E-12	4.62E-11	17.214724
STK24	0.2165675	6.1353607	7.2090347	3.19E-12	4.65E-11	17.209499
CTD-2562J1	0.3801876	5.0686975	7.2084002	3.20E-12	4.66E-11	17.205521
MALSU1	-0.143879	6.19432	-7.208397	3.20E-12	4.66E-11	17.205499
WSB1	0.1335361	6.2792718	7.2076871	3.22E-12	4.68E-11	17.201049
CLDN11	0.7548928	4.3946851	7.2062278	3.25E-12	4.72E-11	17.1919
RP11-455F5	1.1769631	1.2244012	7.2058332	3.26E-12	4.73E-11	17.189427
ZNF85	0.647269	4.1686872	7.2057323	3.26E-12	4.73E-11	17.188794
TK2	-0.14369	6.250821	-7.204695	3.28E-12	4.76E-11	17.182295
LANCL3	1.2290544	0.9724801	7.20466	3.28E-12	4.76E-11	17.182073
PSMB3	-0.152522	6.7837298	-7.204261	3.29E-12	4.77E-11	17.179573
TSPAN33	-0.200317	6.4433377	-7.202361	3.33E-12	4.83E-11	17.167661
TCTA	-0.140815	6.4028443	-7.201574	3.35E-12	4.85E-11	17.162734
ASB13	-0.192877	6.3541895	-7.200934	3.36E-12	4.87E-11	17.15872
RP11-553L6	-0.188054	6.0813198	-7.200346	3.37E-12	4.89E-11	17.155038
YWHAZP2	1.1982933	0.8674149	7.1978532	3.43E-12	4.97E-11	17.139423
CCDC57	-0.193218	6.029781	-7.19741	3.44E-12	4.98E-11	17.136645
RAB11B-AS1	-0.304008	5.6574764	-7.19603	3.47E-12	5.02E-11	17.128001
IFI16	0.2454115	5.9787785	7.1939441	3.52E-12	5.09E-11	17.114943
CCDC112	0.5159305	4.7625707	7.193686	3.52E-12	5.09E-11	17.113327
HR	1.025839	3.5441175	7.1930609	3.54E-12	5.11E-11	17.109414
IGIP	-0.165974	5.774777	-7.192389	3.55E-12	5.13E-11	17.105205
PSMD3	-0.092743	6.7505514	-7.191897	3.56E-12	5.14E-11	17.10213
REN	-1.34593	3.5178689	-7.190916	3.59E-12	5.18E-11	17.095986
UPB1	-0.596064	6.570499	-7.190771	3.59E-12	5.18E-11	17.095082
PGAP3	-0.146187	6.4280193	-7.18977	3.61E-12	5.21E-11	17.088817
FAT3	1.1107966	2.6429592	7.1845578	3.73E-12	5.38E-11	17.056205
CTD-2527I2	1.242505	1.6770273	7.1843791	3.74E-12	5.39E-11	17.055087
ATRAID	-0.108824	6.5054702	-7.183006	3.77E-12	5.43E-11	17.046501
CXCR3	0.6641261	4.310299	7.1829433	3.77E-12	5.43E-11	17.046108
RP11-778D9	-0.81112	2.5889276	-7.18195	3.80E-12	5.47E-11	17.039897
SHMT1	-0.262466	6.842949	-7.181694	3.80E-12	5.47E-11	17.038296
COMMD1	-0.147212	6.0583175	-7.181552	3.81E-12	5.48E-11	17.037408
IGHV1OR15-	1.3430006	-0.33703	7.1808025	3.83E-12	5.50E-11	17.032721
RDH14	-0.096526	5.9487922	-7.180793	3.83E-12	5.50E-11	17.032662
MYCL	-0.346167	5.8465195	-7.180756	3.83E-12	5.50E-11	17.032429
GAA	-0.143364	6.8373763	-7.179969	3.85E-12	5.52E-11	17.027509
RBP4	-0.295155	7.6650847	-7.178997	3.87E-12	5.56E-11	17.021435
NARFL	-0.123339	6.1774023	-7.177963	3.90E-12	5.59E-11	17.01497
HCAR2	1.3159277	2.5144052	7.1778953	3.90E-12	5.59E-11	17.014547
PCED1B-AS1	0.4620061	4.7934196	7.1778597	3.90E-12	5.59E-11	17.014324
CTDSP1	-0.1024	6.793106	-7.177602	3.91E-12	5.60E-11	17.012713
TMEM177	-0.161013	5.9570334	-7.176787	3.93E-12	5.62E-11	17.007617
AP3B2	1.6003107	0.7223905	7.1753825	3.96E-12	5.67E-11	16.998843

WDR70	-0.105997	6.0296324	-7.175164	3.97E-12	5.68E-11	16.997474
CTLA4	1.2088373	3.5174053	7.1750693	3.97E-12	5.68E-11	16.996886
ABCB4	-0.486148	6.4687712	-7.174956	3.97E-12	5.68E-11	16.996176
NRXN3	1.1880851	3.9153735	7.1740409	4.00E-12	5.71E-11	16.99046
RP11-230F1	1.0918921	2.3203623	7.1721235	4.05E-12	5.78E-11	16.978481
CCL2	0.4121816	5.5516574	7.1705477	4.09E-12	5.84E-11	16.968638
TRIM59	0.4706295	4.6534421	7.1703993	4.09E-12	5.84E-11	16.967712
ZBP1	0.8327607	3.6690398	7.1681176	4.15E-12	5.93E-11	16.953463
LHFPL2	0.3060098	5.6737983	7.1669858	4.18E-12	5.97E-11	16.946397
RPL3L	-0.727476	3.5084837	-7.166509	4.19E-12	5.98E-11	16.943422
PRSS36	-0.371678	4.5975858	-7.166501	4.19E-12	5.98E-11	16.943369
IL9R	1.2206824	0.360375	7.16556	4.22E-12	6.01E-11	16.937496
SERBP1	-0.085722	6.8795994	-7.165303	4.23E-12	6.02E-11	16.935894
DBI	-0.137927	6.8683245	-7.164202	4.26E-12	6.06E-11	16.929017
ANAPC16	-0.0981	6.6056962	-7.163508	4.27E-12	6.09E-11	16.924688
ICOS	1.2869529	2.976276	7.1618182	4.32E-12	6.15E-11	16.914143
SLC16A5	0.3995663	4.5604037	7.1617296	4.32E-12	6.15E-11	16.91359
SELPLG	0.3005816	5.5584342	7.1608264	4.35E-12	6.18E-11	16.907954
TRNP1	0.6532423	5.4686181	7.1608246	4.35E-12	6.18E-11	16.907943
EIF4A1	0.3361392	4.951851	7.1605785	4.36E-12	6.19E-11	16.906407
SLC41A1	0.3137591	5.7192918	7.1603944	4.36E-12	6.19E-11	16.905259
SPATA12	1.2802539	-0.053289	7.160304	4.36E-12	6.20E-11	16.904695
RABEPK	-0.221356	6.2914639	-7.159653	4.38E-12	6.22E-11	16.900633
UQCR11	-0.166371	6.5988642	-7.159214	4.39E-12	6.23E-11	16.897898
IGHV3-43	1.6950507	1.1338071	7.159138	4.40E-12	6.23E-11	16.897421
RNF181	-0.152422	6.5966878	-7.158194	4.42E-12	6.27E-11	16.891534
ZNF708	0.3573492	4.8128296	7.1575493	4.44E-12	6.29E-11	16.887511
U52111.14	1.2280434	1.0876797	7.157505	4.44E-12	6.29E-11	16.887235
RP11-65J21	-0.640885	3.9250283	-7.156782	4.46E-12	6.32E-11	16.882729
CUL9	-0.105901	6.1857611	-7.156695	4.47E-12	6.32E-11	16.882181
AAMDC	-0.196873	6.0088645	-7.156593	4.47E-12	6.32E-11	16.881548
API5	-0.080801	6.4743748	-7.156464	4.47E-12	6.33E-11	16.88074
SLC22A15	1.1543168	4.3175174	7.1563257	4.48E-12	6.33E-11	16.87988
SWI5	-0.182856	6.0236365	-7.156006	4.49E-12	6.34E-11	16.877887
GPRIN2	1.6691155	0.4093213	7.1547783	4.52E-12	6.39E-11	16.870231
AUP1	-0.109228	6.8157839	-7.154317	4.53E-12	6.40E-11	16.867352
OXTR	0.8026116	3.8291484	7.1541649	4.54E-12	6.41E-11	16.866406
ACSS2	-0.193561	6.6121292	-7.153308	4.56E-12	6.44E-11	16.861064
HOXA2	1.1887417	1.9957429	7.1526972	4.58E-12	6.46E-11	16.857256
MRPS25	-0.139252	6.4073022	-7.151643	4.61E-12	6.50E-11	16.850682
RP11-366M4	1.3201258	0.9475123	7.1511107	4.63E-12	6.52E-11	16.847367
RP11-278H7	-1.520829	0.0183725	-7.148569	4.70E-12	6.63E-11	16.83153
NCF1C	0.5996749	3.9268871	7.1485157	4.71E-12	6.63E-11	16.831194
F2RL2	1.13239	3.6321101	7.1475027	4.74E-12	6.67E-11	16.824883
JAGN1	-0.124493	6.3905943	-7.14715	4.75E-12	6.68E-11	16.822687
CCDC94	-0.135649	6.0645431	-7.147117	4.75E-12	6.68E-11	16.82248
HHIPL1	0.5103922	4.5419678	7.1468865	4.76E-12	6.69E-11	16.821044
BSDC1	-0.113159	6.5850554	-7.146029	4.78E-12	6.72E-11	16.8157
NMRK1	-0.170727	6.048131	-7.145605	4.79E-12	6.74E-11	16.813062
FZD10	1.5301479	1.5422475	7.1448025	4.82E-12	6.77E-11	16.808061
RP11-54C4.	-0.302599	5.0957873	-7.144749	4.82E-12	6.77E-11	16.807729

MGAT5B	1.2336431	1.8270762	7.1434112	4.86E-12	6.82E-11	16.799397
FAM167A	1.4060127	2.9213024	7.1428501	4.88E-12	6.85E-11	16.795902
VCAM1	0.4181549	5.9525218	7.1411641	4.93E-12	6.92E-11	16.785404
RP11-143N1	-1.41893	1.3089448	-7.140488	4.95E-12	6.94E-11	16.781192
NCALD	0.3653581	5.7896629	7.1397504	4.98E-12	6.97E-11	16.776602
KLKB1	-0.34275	6.4516148	-7.139709	4.98E-12	6.97E-11	16.776347
FCGR1B	1.0969905	2.5336654	7.1394715	4.99E-12	6.98E-11	16.774866
GADD45G	-0.337544	6.3082031	-7.139108	5.00E-12	6.99E-11	16.772604
C1orf43	-0.101158	7.0009868	-7.139087	5.00E-12	6.99E-11	16.772472
RP1-122P22	1.3281212	1.5956634	7.1390598	5.00E-12	6.99E-11	16.772303
GNA15	0.3890335	4.8190803	7.1385093	5.02E-12	7.01E-11	16.768876
TMEM132A	0.3715647	5.4767647	7.1383441	5.02E-12	7.02E-11	16.767848
CTB-114C7.	0.9589759	-0.693061	7.1370149	5.07E-12	7.08E-11	16.759575
SGCA	1.2042483	3.6124493	7.1362219	5.09E-12	7.11E-11	16.75464
ARHGAP31-A	1.1814057	-0.346859	7.1340143	5.16E-12	7.21E-11	16.740904
PLEK	0.3442885	5.5560461	7.1339168	5.17E-12	7.21E-11	16.740297
ASPN	0.5029519	5.5754764	7.1338506	5.17E-12	7.21E-11	16.739885
RNF112	0.8743518	3.3787069	7.1338439	5.17E-12	7.21E-11	16.739843
BCL2A1	0.676497	4.1551601	7.1335435	5.18E-12	7.22E-11	16.737975
CRISPLD1	1.0169677	3.6899214	7.1335278	5.18E-12	7.22E-11	16.737877
VAC14-AS1	1.23098	3.4851446	7.1324755	5.21E-12	7.26E-11	16.731331
CRADD	-0.172998	5.9713062	-7.132374	5.22E-12	7.26E-11	16.730699
SRC	0.3042933	6.067617	7.1322346	5.22E-12	7.27E-11	16.729832
KLHDC10	-0.148637	6.4474182	-7.131798	5.24E-12	7.28E-11	16.727118
RPUSD3	-0.132508	6.1769797	-7.131754	5.24E-12	7.28E-11	16.726842
CHN2	-0.262226	6.1616146	-7.130893	5.27E-12	7.32E-11	16.721486
ZNF83	0.4517992	5.5240472	7.1305546	5.28E-12	7.33E-11	16.719383
DAK	-0.239887	6.6671575	-7.1293	5.32E-12	7.39E-11	16.711578
RP11-6F2.5	-1.519644	2.4009525	-7.127184	5.39E-12	7.49E-11	16.698426
SRD5A1	-0.243655	6.2645541	-7.12697	5.40E-12	7.50E-11	16.697095
RP11-473M2	-0.987339	3.0896165	-7.126725	5.41E-12	7.50E-11	16.695569
RNF25	-0.118409	6.0055694	-7.126682	5.41E-12	7.50E-11	16.695306
SYCP2	0.8032736	3.3132444	7.1262534	5.43E-12	7.52E-11	16.692638
ZNF273	0.3827468	4.5855685	7.126138	5.43E-12	7.52E-11	16.691921
INSR	-0.137297	6.7853758	-7.125914	5.44E-12	7.53E-11	16.690529
UQCR10	-0.151348	6.6296199	-7.125697	5.44E-12	7.54E-11	16.689182
ADAM23	1.2792022	3.5742986	7.1248524	5.47E-12	7.58E-11	16.68393
SLC26A9	1.5735185	1.3163028	7.1229295	5.54E-12	7.67E-11	16.671979
FCGR1A	0.7650025	4.1052288	7.121721	5.58E-12	7.73E-11	16.66447
RP11-1220K	1.8500435	1.4049736	7.1216559	5.59E-12	7.73E-11	16.664065
COQ10A	-0.228855	5.9795248	-7.121521	5.59E-12	7.73E-11	16.663226
RP11-48B3.	-1.52951	0.6790809	-7.121308	5.60E-12	7.74E-11	16.661901
RNF144A	0.3149742	5.3147254	7.1212549	5.60E-12	7.74E-11	16.661573
IGKV1D-16	1.5116348	0.1719536	7.1209818	5.61E-12	7.75E-11	16.659877
P2RY6	0.5943973	4.4894342	7.1208054	5.62E-12	7.75E-11	16.658781
MMACHC	-0.158586	6.0989467	-7.120666	5.62E-12	7.76E-11	16.657916
B4GALT5	0.1314378	6.3324627	7.1198752	5.65E-12	7.79E-11	16.653002
EI24	-0.124585	6.7971012	-7.11957	5.66E-12	7.81E-11	16.651106
MAPRE1	0.1116967	6.3530477	7.1193191	5.67E-12	7.81E-11	16.649547
IGHJ2	1.2564546	-0.510728	7.1191127	5.68E-12	7.82E-11	16.648265
KDM6B	0.1423418	6.068386	7.1189861	5.68E-12	7.83E-11	16.647479

RGS10	0.3283294	5.3697305	7.1182088	5.71E-12	7.86E-11	16.642651
YWHAZP6	1.1349787	0.2205202	7.1120418	5.94E-12	8.17E-11	16.604359
SLC2A4RG	-0.153858	6.7239197	-7.10916	6.05E-12	8.32E-11	16.586474
A4GNT	1.5810725	0.1074218	7.1089815	6.06E-12	8.33E-11	16.585366
AC002456.2	-0.883443	3.5824149	-7.108825	6.06E-12	8.33E-11	16.584396
ORMDL3	-0.159489	6.9234632	-7.108527	6.08E-12	8.35E-11	16.582543
RNA5SP508	-0.928743	-0.888434	-7.108332	6.08E-12	8.35E-11	16.581338
SDHB	-0.150478	6.664175	-7.107604	6.11E-12	8.39E-11	16.57682
C1orf145	1.3080568	1.7973295	7.107112	6.13E-12	8.41E-11	16.573767
LINC00910	-0.307581	4.9400807	-7.106389	6.16E-12	8.45E-11	16.569283
CLEC4F	1.3291896	2.4317199	7.1062308	6.16E-12	8.45E-11	16.568301
LARP6	0.7531526	4.6956125	7.1058407	6.18E-12	8.47E-11	16.565881
PHF21A	0.118382	5.9551455	7.1055837	6.19E-12	8.48E-11	16.564287
RTN1	0.5307726	4.6396143	7.1053254	6.20E-12	8.49E-11	16.562684
NDUFV1	-0.129381	6.8764094	-7.104932	6.22E-12	8.51E-11	16.560245
FBXO5	0.2875989	5.0970099	7.103762	6.26E-12	8.57E-11	16.552988
CXCL3	1.2421924	2.8821896	7.1022079	6.32E-12	8.65E-11	16.543351
CTAGE5	-0.161089	5.6301074	-7.101677	6.35E-12	8.68E-11	16.540061
NRP2	0.2459647	5.7974119	7.1016429	6.35E-12	8.68E-11	16.539848
ZSWIM4	0.2198877	5.3913977	7.1015311	6.35E-12	8.68E-11	16.539155
HRCT1	-0.611775	5.1316309	-7.099814	6.42E-12	8.77E-11	16.528508
RNF219	0.1739824	5.2953786	7.0993713	6.44E-12	8.79E-11	16.525765
SLC6A17	1.396157	1.2898327	7.0991189	6.45E-12	8.81E-11	16.524201
NCF1B	0.8636122	3.2033294	7.0981003	6.49E-12	8.86E-11	16.517887
TSPAN11	1.099526	3.2843718	7.0962677	6.57E-12	8.96E-11	16.50653
IQSEC2	0.3456342	4.9041401	7.0954023	6.60E-12	9.01E-11	16.501167
WBP5	0.3439036	5.9042926	7.0940708	6.66E-12	9.08E-11	16.492918
TMEM44	0.2078	5.3820329	7.0940339	6.66E-12	9.08E-11	16.492689
TNIP1	-0.128311	6.8536763	-7.092277	6.74E-12	9.18E-11	16.481803
ADAM22	0.9091464	3.9302186	7.0916291	6.77E-12	9.21E-11	16.477793
IGKV6-21	1.5565868	0.1435441	7.0914929	6.77E-12	9.22E-11	16.47695
LETM2	0.6220265	3.6595529	7.0908344	6.80E-12	9.25E-11	16.472871
CYBB	0.3436817	5.711773	7.0893494	6.86E-12	9.34E-11	16.463675
AC079767.4	1.3145195	1.8758887	7.0892909	6.87E-12	9.34E-11	16.463313
GALNT5	1.5392004	1.0358981	7.0891327	6.87E-12	9.34E-11	16.462333
GIT2	0.1321839	5.8158446	7.0874381	6.95E-12	9.44E-11	16.451842
TMEM65	0.2021869	5.9051948	7.0870394	6.97E-12	9.46E-11	16.449374
SNTA1	-0.183751	6.1601602	-7.084992	7.06E-12	9.58E-11	16.436699
SLC4A7	0.3151124	5.3621706	7.0849735	7.06E-12	9.58E-11	16.436586
SLC6A13	-0.764997	5.0853815	-7.084385	7.08E-12	9.61E-11	16.432945
NAV2-AS4	-1.426216	0.392252	-7.083956	7.10E-12	9.63E-11	16.430286
C9orf173-A	-0.747532	4.0097679	-7.083142	7.14E-12	9.68E-11	16.425254
AC073934.6	-1.049327	-0.407113	-7.082308	7.18E-12	9.73E-11	16.420092
CIB1	-0.132246	6.695553	-7.080848	7.25E-12	9.81E-11	16.411061
F10	-0.285516	6.8327531	-7.080437	7.26E-12	9.84E-11	16.408513
TRAM2-AS1	-0.227902	5.5177558	-7.080098	7.28E-12	9.85E-11	16.406419
JAKMIP1	0.9662584	3.0702674	7.079921	7.29E-12	9.86E-11	16.405323
IGKV1D-17	1.4252038	-0.350805	7.0795768	7.30E-12	9.88E-11	16.403194
KCNF1	1.6818933	2.4995781	7.0792353	7.32E-12	9.90E-11	16.401082
AC114271.2	-0.365217	4.0535943	-7.079018	7.33E-12	9.91E-11	16.39974
NDUFS7	-0.154345	6.5326935	-7.078105	7.37E-12	9.96E-11	16.394088

APRT	-0.159277	6.6293508	-7.077072	7.42E-12	1.00E-10	16.387701
IGHM	0.7978777	5.6236992	7.0763597	7.46E-12	1.01E-10	16.383298
P2RY12	1.3031201	1.8203896	7.0745185	7.54E-12	1.02E-10	16.371914
ZNF765	0.2015751	5.3003644	7.0745111	7.54E-12	1.02E-10	16.371868
LINC00667	-0.182217	6.0953562	-7.073519	7.59E-12	1.02E-10	16.365735
USP30	-0.135946	6.1026794	-7.073516	7.59E-12	1.02E-10	16.365716
MAL	1.2585933	2.5365789	7.0730885	7.61E-12	1.03E-10	16.363074
GPC5	1.703087	3.0058246	7.0713218	7.70E-12	1.04E-10	16.352155
NR1I3	-0.553771	6.1450068	-7.071037	7.71E-12	1.04E-10	16.350396
CH17-340M2	-0.265574	5.33125	-7.070996	7.71E-12	1.04E-10	16.350139
F10-AS1	-1.094415	2.0941003	-7.069939	7.77E-12	1.04E-10	16.34361
TECR	-0.13047	6.7348083	-7.069687	7.78E-12	1.05E-10	16.34205
KLRG1	0.5733169	4.0343972	7.0692398	7.80E-12	1.05E-10	16.339289
CHI3L2	1.1417018	2.9330104	7.0688305	7.82E-12	1.05E-10	16.336761
TDP2	-0.121517	6.4080974	-7.06865	7.83E-12	1.05E-10	16.335644
SOGA1	0.2112182	5.8968361	7.0683061	7.85E-12	1.05E-10	16.33352
MLC1	0.9549362	2.6174084	7.0676089	7.88E-12	1.06E-10	16.329213
TRIM52-AS1	-0.219277	5.4650737	-7.067424	7.89E-12	1.06E-10	16.328073
RP11-312J1	0.8815285	3.0849552	7.067193	7.90E-12	1.06E-10	16.326644
HNMT	-0.163376	6.6417099	-7.066578	7.93E-12	1.06E-10	16.322847
CPA4	1.4724736	1.3056662	7.0656565	7.98E-12	1.07E-10	16.317153
SMIM2-AS1	-0.608637	4.7207319	-7.06526	8.00E-12	1.07E-10	16.314705
FGGY	-0.361563	6.3861895	-7.064987	8.01E-12	1.07E-10	16.313019
ITGAX	0.2634804	5.6929233	7.0643622	8.05E-12	1.08E-10	16.309159
ITIH1	-0.359811	7.3570116	-7.061627	8.19E-12	1.10E-10	16.292269
MSX2	1.4128964	0.2033484	7.0610503	8.22E-12	1.10E-10	16.288711
PLXNA3	0.3322364	5.7511322	7.0597699	8.28E-12	1.11E-10	16.280807
C8orf48	1.1718319	1.9880699	7.0594902	8.30E-12	1.11E-10	16.279081
RP11-483F1	-0.913608	2.7836584	-7.05942	8.30E-12	1.11E-10	16.278646
CCDC159	-0.166658	5.9776207	-7.058814	8.33E-12	1.11E-10	16.27491
OXA1L	-0.102513	6.7499073	-7.05814	8.37E-12	1.12E-10	16.270744
GSTO1	-0.189663	6.9584857	-7.057798	8.39E-12	1.12E-10	16.268636
PLBD1	0.4882671	5.1861845	7.0576283	8.40E-12	1.12E-10	16.26759
FAM120A	-0.08494	6.776901	-7.05681	8.44E-12	1.13E-10	16.26254
HSPB11	-0.13633	6.0204152	-7.05577	8.50E-12	1.13E-10	16.256124
RP11-510M2	1.6036737	-0.017235	7.0553331	8.52E-12	1.14E-10	16.253427
LHPP	-0.247651	6.2900617	-7.055059	8.53E-12	1.14E-10	16.251738
MTCL1	0.6277171	4.9309032	7.054797	8.55E-12	1.14E-10	16.25012
NUDCD3	-0.092073	6.5441756	-7.051779	8.71E-12	1.16E-10	16.231503
RGS1	0.5626262	5.5984286	7.0517005	8.72E-12	1.16E-10	16.23102
ARL11	0.5703652	3.9671916	7.0496762	8.83E-12	1.18E-10	16.218538
FBXL17	-0.13593	6.0420969	-7.049533	8.84E-12	1.18E-10	16.217656
SOBP	0.5922048	5.058197	7.04891	8.87E-12	1.18E-10	16.213814
C2CD5	0.1292377	5.9896492	7.0487597	8.88E-12	1.18E-10	16.212887
WDR83OS	-0.128922	6.6102548	-7.047835	8.93E-12	1.19E-10	16.207189
FCRLA	1.4270965	2.6158053	7.0477341	8.94E-12	1.19E-10	16.206565
H3F3B	-0.094698	7.0476235	-7.047236	8.97E-12	1.19E-10	16.203494
FAM153B	1.626512	1.5147473	7.0469303	8.99E-12	1.19E-10	16.20161
NELFE	-0.146212	6.5168616	-7.045115	9.09E-12	1.21E-10	16.190419
BHLHE22	1.4786425	2.4377907	7.0426305	9.23E-12	1.22E-10	16.175112
GDF6	1.4630248	1.8248884	7.0412533	9.31E-12	1.24E-10	16.166628

CORO2B	0.7808266	4.0340352	7.0399425	9.39E-12	1.24E-10	16.158554
DOLPP1	-0.133872	6.142449	-7.038431	9.48E-12	1.26E-10	16.149243
TMEM186	-0.15214	5.7294957	-7.038306	9.49E-12	1.26E-10	16.148472
C7orf73	-0.104892	6.4337109	-7.037936	9.51E-12	1.26E-10	16.146195
SLAMF1	0.7191632	4.1752511	7.0375335	9.54E-12	1.26E-10	16.143718
SLC36A1	0.1804817	5.6206307	7.0371017	9.56E-12	1.27E-10	16.14106
INSIG1	-0.28372	7.0228343	-7.036934	9.57E-12	1.27E-10	16.140026
MXI1	-0.151814	6.2761517	-7.03597	9.63E-12	1.27E-10	16.134093
RP11-817I4	1.0562357	2.6453975	7.0358966	9.64E-12	1.27E-10	16.13364
RP11-465B2	1.6128584	0.9406071	7.0351691	9.68E-12	1.28E-10	16.129161
ZNF611	0.5760694	4.7446486	7.0350976	9.68E-12	1.28E-10	16.128721
PHKG2	-0.136086	6.1373816	-7.035064	9.69E-12	1.28E-10	16.128516
HSDL2	-0.16291	6.7142629	-7.034212	9.74E-12	1.29E-10	16.123266
NGDN	-0.098738	6.1125426	-7.033779	9.77E-12	1.29E-10	16.120604
ADAMTS9-AS	1.0932957	2.3658296	7.0323193	9.86E-12	1.30E-10	16.11162
CCDC65	0.3619739	4.1561537	7.0321051	9.87E-12	1.30E-10	16.110302
TANC2	0.4175936	5.0843668	7.0311996	9.93E-12	1.31E-10	16.10473
ZNF137P	1.2628441	2.8617229	7.0311922	9.93E-12	1.31E-10	16.104684
SELO	-0.174866	6.4809609	-7.030482	9.97E-12	1.31E-10	16.100316
RNU6-46P	-1.127768	-0.482675	-7.029426	1.00E-11	1.32E-10	16.093819
TFCP2L1	1.0226298	3.4238964	7.0288261	1.01E-11	1.33E-10	16.090126
WISP2	1.4709998	3.1594454	7.0285803	1.01E-11	1.33E-10	16.088615
ATP6V1B1	1.1606256	2.6378642	7.0274513	1.02E-11	1.34E-10	16.081669
TLR8	0.9535234	3.7376666	7.0267943	1.02E-11	1.34E-10	16.077628
RP1-137D17	1.163661	-0.255837	7.0262963	1.02E-11	1.35E-10	16.074565
DHCR24	-0.144598	7.4179984	-7.024839	1.03E-11	1.36E-10	16.065603
PSMB2	-0.113716	6.7503391	-7.023466	1.04E-11	1.37E-10	16.05716
RENBP	0.3848459	5.4666383	7.0217895	1.05E-11	1.38E-10	16.046855
TRIAP1	-0.116072	6.2589445	-7.021746	1.05E-11	1.38E-10	16.046587
SS18L1	-0.178034	6.1773997	-7.019694	1.07E-11	1.40E-10	16.033974
LAPTM4A	-0.091962	6.9599287	-7.019277	1.07E-11	1.40E-10	16.03141
GJC1	0.3626879	5.152307	7.0192388	1.07E-11	1.40E-10	16.031178
GPR78	0.9604504	-0.830311	7.018319	1.08E-11	1.41E-10	16.025525
P2RY13	0.535202	4.4127564	7.0147384	1.10E-11	1.44E-10	16.003527
EMC7	-0.116688	6.5110472	-7.011696	1.12E-11	1.47E-10	15.984842
ACOT6	-1.273999	2.2384331	-7.01163	1.12E-11	1.47E-10	15.984435
ATP1B2	0.6376087	4.7619948	7.0111654	1.13E-11	1.47E-10	15.981585
CXorf66	-1.395276	0.0414902	-7.011079	1.13E-11	1.47E-10	15.981057
SLC26A10	1.245185	1.2411379	7.0102962	1.13E-11	1.48E-10	15.976248
ME2	0.1451481	6.0297968	7.0101727	1.13E-11	1.48E-10	15.975489
WDR83	-0.161008	5.5550514	-7.009885	1.14E-11	1.48E-10	15.973726
JMJD8	-0.128443	6.2869577	-7.008116	1.15E-11	1.50E-10	15.962863
MCEE	-0.179981	5.8465841	-7.00784	1.15E-11	1.50E-10	15.961173
IGKC	0.7713822	6.1057451	7.0064112	1.16E-11	1.51E-10	15.952401
AC079922.3	-0.272702	4.6973876	-7.005021	1.17E-11	1.53E-10	15.943868
HSPG2	0.3511307	6.3904182	7.004428	1.18E-11	1.53E-10	15.940232
RP11-141M3	-1.101583	3.9862485	-7.003972	1.18E-11	1.54E-10	15.937431
RABGGTA	-0.114881	6.1224562	-7.003034	1.19E-11	1.55E-10	15.931677
SLC2A8	-0.149298	6.2129817	-7.002541	1.19E-11	1.55E-10	15.928657
FAHD2A	-0.155548	6.1886992	-7.002496	1.19E-11	1.55E-10	15.92838
PODNL1	0.7981425	3.2230018	7.0004186	1.21E-11	1.57E-10	15.915637

ARAP3	0.2266187	5.5999512	6.9999336	1.21E-11	1.57E-10	15.912663
RP11-177H2	1.1733719	0.6749722	6.9998285	1.21E-11	1.57E-10	15.912018
FAM173A	-0.186642	5.842832	-6.999661	1.21E-11	1.58E-10	15.910991
ATP13A4	1.3615025	0.857582	6.999037	1.22E-11	1.58E-10	15.907164
ATF7IP2	-0.242142	6.0038023	-6.998503	1.22E-11	1.59E-10	15.90389
MYO10	0.3310944	5.592969	6.998271	1.22E-11	1.59E-10	15.902468
RP11-35N6.	-0.831283	4.5698982	-6.996756	1.23E-11	1.60E-10	15.893183
ZNF549	0.5157347	4.5726293	6.9958501	1.24E-11	1.61E-10	15.887626
SLC5A1	1.7931514	1.5636214	6.9956219	1.24E-11	1.61E-10	15.886227
BHLHE41	0.724263	4.4814653	6.9943487	1.25E-11	1.62E-10	15.878424
SKIV2L	-0.103298	6.4984341	-6.994298	1.25E-11	1.62E-10	15.878112
IGHV1-3	1.4763288	-0.185521	6.992595	1.27E-11	1.64E-10	15.867677
MLX	-0.109139	6.4421494	-6.992458	1.27E-11	1.64E-10	15.866839
FAM78A	0.2502104	5.2412875	6.9913053	1.28E-11	1.65E-10	15.859774
DMAP1	-0.120754	6.2511408	-6.991124	1.28E-11	1.66E-10	15.858663
AMPD2	-0.132014	6.4015359	-6.991074	1.28E-11	1.66E-10	15.858359
ABCC6	-0.242243	6.6860454	-6.989742	1.29E-11	1.67E-10	15.850197
IGHV4-4	1.4864934	-0.034585	6.9895943	1.29E-11	1.67E-10	15.849292
SAMD12	1.1992106	3.9141591	6.9892331	1.29E-11	1.67E-10	15.84708
AC074212.6	-0.305818	4.5076338	-6.988936	1.30E-11	1.68E-10	15.845261
ATXN7L1	-0.22035	5.4916836	-6.988779	1.30E-11	1.68E-10	15.844297
PXDC1	-0.165002	6.6221721	-6.98831	1.30E-11	1.68E-10	15.841425
PTN	0.8941287	4.2829709	6.9872751	1.31E-11	1.69E-10	15.835088
RP13-20L14	-0.390875	4.3240968	-6.986639	1.31E-11	1.70E-10	15.831194
RGS7BP	1.5685124	2.2133054	6.9862487	1.32E-11	1.70E-10	15.828803
BCAT1	0.3101989	5.2681886	6.9855476	1.32E-11	1.71E-10	15.82451
MT-ND6	-0.202804	7.3447177	-6.985237	1.33E-11	1.71E-10	15.822609
ALDH3A2	-0.174945	6.9768037	-6.984739	1.33E-11	1.72E-10	15.819557
FAXC	1.5858863	1.0228066	6.984503	1.33E-11	1.72E-10	15.818114
CD300A	0.3194954	5.2191824	6.9834913	1.34E-11	1.73E-10	15.811921
AC009299.5	1.1912924	1.3035886	6.9825474	1.35E-11	1.74E-10	15.806142
QTRT1	-0.145543	6.3061048	-6.981414	1.36E-11	1.75E-10	15.799207
CREB3L4	-0.174208	5.9350374	-6.981098	1.36E-11	1.75E-10	15.797269
LAMA5-AS1	-1.487655	4.2143463	-6.981084	1.36E-11	1.75E-10	15.797187
POLR3GL	-0.153511	6.2532437	-6.980909	1.36E-11	1.75E-10	15.796116
RP11-669E1	-1.566587	2.7003805	-6.980545	1.37E-11	1.76E-10	15.793885
ADAMTS15	0.586749	4.2383597	6.9792168	1.38E-11	1.77E-10	15.78576
RP3-462C17	-1.275962	-0.193393	-6.979195	1.38E-11	1.77E-10	15.785625
TCEAL8	0.3325491	6.0064423	6.9783043	1.39E-11	1.78E-10	15.780177
NUB1	-0.096695	6.5076189	-6.978066	1.39E-11	1.78E-10	15.77872
CTD-2260A1	-0.345916	4.2673416	-6.977352	1.39E-11	1.79E-10	15.774349
MRPL37	-0.123163	6.6404898	-6.977266	1.39E-11	1.79E-10	15.773828
HEPH	0.4768948	5.0428811	6.9748999	1.42E-11	1.81E-10	15.759353
ANXA6	-0.184854	6.8678533	-6.974682	1.42E-11	1.82E-10	15.758021
TADA3	-0.107191	6.5131536	-6.974005	1.42E-11	1.82E-10	15.753881
MAP3K13	-0.144433	6.3137945	-6.973871	1.42E-11	1.82E-10	15.753059
C10orf91	1.553849	0.0627666	6.9735133	1.43E-11	1.83E-10	15.750873
CXCL1	1.3961692	3.9888749	6.9713072	1.45E-11	1.85E-10	15.737385
DNAJC27-AS	-0.346702	4.5057768	-6.971088	1.45E-11	1.86E-10	15.736046
CMTM1	1.0650617	2.3043317	6.970329	1.46E-11	1.86E-10	15.731405
RP11-119D9	-1.233204	4.4051648	-6.968022	1.48E-11	1.89E-10	15.717303

ST18	1.4212868	0.7006505	6.9676506	1.48E-11	1.89E-10	15.715035
AC093724.2	1.040782	2.6174422	6.9653998	1.50E-11	1.92E-10	15.701282
NUBPL	-0.21404	5.7193534	-6.9653	1.50E-11	1.92E-10	15.700672
VCL	0.1232487	6.3077328	6.9651899	1.50E-11	1.92E-10	15.7
RP11-39404	0.6167004	4.4200764	6.9649693	1.51E-11	1.92E-10	15.698652
CES4A	-0.711548	4.8031063	-6.964844	1.51E-11	1.92E-10	15.697886
GVINP1	0.4948681	4.4137047	6.9646323	1.51E-11	1.93E-10	15.696593
TRPM2	0.3634484	5.065438	6.9645539	1.51E-11	1.93E-10	15.696115
NUDT7	-0.272233	5.671402	-6.964401	1.51E-11	1.93E-10	15.695183
AK5	1.3079648	1.1690088	6.9637402	1.52E-11	1.93E-10	15.691144
TDRD5	1.587286	0.2510202	6.9632287	1.52E-11	1.94E-10	15.68802
KCNJ12	0.8717853	3.142986	6.9627358	1.53E-11	1.95E-10	15.685009
C9orf139	0.7160586	2.9627463	6.9626246	1.53E-11	1.95E-10	15.68433
CTSE	1.7698235	1.7856708	6.9623129	1.53E-11	1.95E-10	15.682427
CATIP-AS1	-0.893404	3.2401671	-6.962251	1.53E-11	1.95E-10	15.682047
ZNF816	0.7296432	4.4543209	6.9622369	1.53E-11	1.95E-10	15.681962
XXYLT1	0.1886018	5.4300459	6.9610313	1.54E-11	1.96E-10	15.6746
PLD2	0.1340421	5.8810693	6.9603528	1.55E-11	1.97E-10	15.670457
IGHA2	0.9943356	4.718891	6.9601016	1.55E-11	1.97E-10	15.668923
MON1A	-0.160556	5.8286915	-6.959572	1.56E-11	1.98E-10	15.665689
MYL5	-0.198348	5.7413066	-6.958056	1.57E-11	2.00E-10	15.656434
LAS1L	-0.106407	6.3616362	-6.957956	1.57E-11	2.00E-10	15.655825
ITIH5	0.7357352	4.8745383	6.9575441	1.58E-11	2.00E-10	15.65331
IFT52	0.1289324	5.7679532	6.9574447	1.58E-11	2.00E-10	15.652703
RPARP-AS1	-0.200549	5.5962628	-6.957056	1.58E-11	2.01E-10	15.65033
FITM1	-0.556918	4.3486101	-6.956678	1.59E-11	2.01E-10	15.648023
MFSD6L	1.3191228	0.1496023	6.9565073	1.59E-11	2.01E-10	15.646981
GFI1	0.6370233	4.146413	6.9559682	1.59E-11	2.02E-10	15.643691
ABCG2	-0.43758	5.8279211	-6.955684	1.60E-11	2.02E-10	15.641954
CWC15	-0.108513	6.3795387	-6.954564	1.61E-11	2.04E-10	15.635122
RP13-58209	-0.330952	5.1711316	-6.9542	1.61E-11	2.04E-10	15.632898
NDUFB8	-0.166239	6.3224574	-6.953839	1.62E-11	2.04E-10	15.630698
KANSL1-AS1	-0.380538	4.783562	-6.953411	1.62E-11	2.05E-10	15.628088
UBB	-0.12504	7.23349	-6.953347	1.62E-11	2.05E-10	15.627695
PASK	0.2244108	5.3605891	6.95245	1.63E-11	2.06E-10	15.622223
ACAN	1.2969706	3.4835597	6.9524428	1.63E-11	2.06E-10	15.622179
ACAA1	-0.187623	6.81333	-6.952346	1.63E-11	2.06E-10	15.621589
ST8SIA4	0.3024336	5.176245	6.9517994	1.64E-11	2.06E-10	15.618255
MCTP2	0.9755738	4.1512918	6.951736	1.64E-11	2.07E-10	15.617868
BEX1	1.8264601	1.8535943	6.9499941	1.66E-11	2.09E-10	15.607243
MRPL21	-0.157406	6.2953498	-6.949736	1.66E-11	2.09E-10	15.605669
FLI1	0.2139007	5.6062153	6.9492195	1.66E-11	2.10E-10	15.602519
FH	-0.159874	6.9018327	-6.94859	1.67E-11	2.10E-10	15.598677
BLM	0.4580671	4.8898849	6.9470847	1.69E-11	2.12E-10	15.589501
ZNF496	0.3103474	5.8014762	6.9470515	1.69E-11	2.12E-10	15.589299
TRBV7-3	1.2340521	0.0183758	6.9461397	1.70E-11	2.13E-10	15.583739
MIEN1	-0.132742	6.383993	-6.94586	1.70E-11	2.14E-10	15.582037
PKP3	1.5499821	2.55712	6.9455557	1.70E-11	2.14E-10	15.580179
GPD1L	0.3010575	5.4855151	6.9454729	1.70E-11	2.14E-10	15.579674
HELB	0.2076353	5.1142916	6.945404	1.70E-11	2.14E-10	15.579255
RP11-1191J	1.2394057	1.1366646	6.9453926	1.70E-11	2.14E-10	15.579185

SEZ6L	1.3441465	0.6340682	6.945249	1.71E-11	2.14E-10	15.57831
LSM5	-0.14575	6.1273208	-6.944839	1.71E-11	2.15E-10	15.575812
STK26	0.5734655	5.2058804	6.9446524	1.71E-11	2.15E-10	15.574673
PIR	-0.251387	6.1286394	-6.944593	1.71E-11	2.15E-10	15.57431
SAMD3	0.8412126	3.2625121	6.9438997	1.72E-11	2.16E-10	15.570085
RGMB-AS1	-0.871859	3.5086447	-6.943757	1.72E-11	2.16E-10	15.569216
RP11-770J1	-1.017407	3.1120205	-6.942829	1.73E-11	2.17E-10	15.563557
RP1-159G19	-1.632687	1.2807992	-6.941924	1.74E-11	2.18E-10	15.558045
CSDC2	1.4408426	1.8264708	6.9410077	1.75E-11	2.19E-10	15.552462
ALAD	-0.18043	6.8117823	-6.940872	1.75E-11	2.19E-10	15.551634
SERPINC1	-0.497568	7.4051136	-6.938967	1.77E-11	2.22E-10	15.540027
COA3	-0.148948	6.603913	-6.938647	1.78E-11	2.22E-10	15.538082
CD70	1.2659941	2.0915589	6.9355188	1.81E-11	2.27E-10	15.519028
CHMP2A	-0.146623	6.7642944	-6.934152	1.83E-11	2.28E-10	15.510708
AJAP1	1.4284949	1.4321839	6.9337927	1.83E-11	2.29E-10	15.508518
PPFIA4	1.2529588	2.6331037	6.9321937	1.85E-11	2.31E-10	15.498784
CDC42BPG	0.7473222	4.8001144	6.931739	1.86E-11	2.32E-10	15.496016
RARRES2	-0.252663	7.2391561	-6.93104	1.86E-11	2.33E-10	15.491761
MNX1	1.7666923	0.4005419	6.9300703	1.88E-11	2.34E-10	15.48586
TIMM8B	-0.151997	6.2578424	-6.92905	1.89E-11	2.35E-10	15.479653
RRP8	-0.105668	6.0348674	-6.929005	1.89E-11	2.35E-10	15.479381
KIF5C	0.728659	3.9033948	6.9289117	1.89E-11	2.35E-10	15.47881
C15orf59	1.1957781	3.0703403	6.9284167	1.89E-11	2.36E-10	15.475798
C12orf10	-0.110774	6.2644125	-6.928051	1.90E-11	2.37E-10	15.473573
VEPH1	1.4593424	3.0357281	6.927346	1.91E-11	2.38E-10	15.469284
MRPS33	-0.134845	6.3040205	-6.926885	1.91E-11	2.38E-10	15.466481
NSFL1C	-0.103275	6.5877884	-6.926451	1.92E-11	2.39E-10	15.46384
LAMC2	1.2183677	3.8888303	6.9254427	1.93E-11	2.40E-10	15.457706
INO80E	-0.105873	6.2295026	-6.925224	1.93E-11	2.40E-10	15.456377
SYNGAP1	0.2482138	5.2446834	6.9242696	1.94E-11	2.42E-10	15.450571
PRR16	0.8046389	3.6331938	6.9236528	1.95E-11	2.43E-10	15.44682
DPYSL3	0.3056118	5.7104204	6.9233395	1.96E-11	2.43E-10	15.444915
TARS2	-0.130989	6.4029892	-6.922922	1.96E-11	2.44E-10	15.442377
IDH1	-0.13064	6.9991373	-6.922405	1.97E-11	2.44E-10	15.439229
CHRDL1	1.7308241	2.0058902	6.9209731	1.98E-11	2.46E-10	15.430526
CPSF3	-0.090702	6.2215554	-6.920917	1.99E-11	2.46E-10	15.430185
MRPS21	-0.152413	6.5615665	-6.920836	1.99E-11	2.46E-10	15.429694
NME5	1.4248422	1.8944441	6.9200701	2.00E-11	2.47E-10	15.425037
RUNDC3B	-0.54852	5.2400168	-6.920048	2.00E-11	2.47E-10	15.424903
TNFAIP8L3	0.7534497	4.6683794	6.9192582	2.01E-11	2.49E-10	15.420102
RP11-1334A	1.1017731	1.6422278	6.9192062	2.01E-11	2.49E-10	15.419785
CIB2	0.6262837	4.5729894	6.9183286	2.02E-11	2.50E-10	15.414452
CLEC10A	0.3851524	5.0608516	6.9181996	2.02E-11	2.50E-10	15.413668
IRS1	-0.190814	6.4783372	-6.917724	2.03E-11	2.51E-10	15.410777
AC135048.1	-0.763651	3.4721954	-6.916599	2.04E-11	2.52E-10	15.403943
SCN7A	1.6052471	2.0750962	6.915189	2.06E-11	2.54E-10	15.395374
MPLKIP	-0.15555	6.3577415	-6.915122	2.06E-11	2.54E-10	15.394965
ISOC2	-0.167905	6.7297384	-6.914873	2.06E-11	2.55E-10	15.393454
CTD-319301	-1.27936	2.4520946	-6.913928	2.07E-11	2.56E-10	15.387715
ITGB2	0.2298811	6.2098714	6.910051	2.13E-11	2.62E-10	15.364166
RAB5B	-0.072427	6.7440326	-6.909816	2.13E-11	2.63E-10	15.362737

FBLN2	0.648085	5.5494961	6.9085525	2.14E-11	2.65E-10	15.355067
KIAA1755	0.9693535	4.2256221	6.9068727	2.17E-11	2.67E-10	15.34487
YPEL1	-0.376832	4.7242703	-6.906504	2.17E-11	2.68E-10	15.342634
ZNF816-ZNF	1.088581	3.0239026	6.9055952	2.18E-11	2.69E-10	15.337117
SLC16A1	-0.227903	6.5123918	-6.905572	2.19E-11	2.69E-10	15.336977
POLR2J	-0.148482	6.3431843	-6.905477	2.19E-11	2.69E-10	15.336399
TRBV12-3	1.0782847	-0.374089	6.9052922	2.19E-11	2.70E-10	15.335278
JAM3	0.1938147	5.588899	6.9047802	2.20E-11	2.70E-10	15.33217
AC005540.3	1.0900997	-0.137452	6.9047357	2.20E-11	2.70E-10	15.3319
LRRC15	1.5087994	2.2783622	6.9043348	2.20E-11	2.71E-10	15.329468
IGLV4-60	1.605217	0.4883463	6.9014052	2.24E-11	2.76E-10	15.311693
SLC46A2	1.3928661	0.4806575	6.9013388	2.24E-11	2.76E-10	15.31129
AC104820.2	1.2506025	0.7224303	6.9009186	2.25E-11	2.77E-10	15.308742
CHCHD1	-0.133741	6.2494447	-6.899821	2.27E-11	2.78E-10	15.302082
RP11-731F5	1.1237164	-0.554461	6.8996524	2.27E-11	2.79E-10	15.301061
OVOL2	1.6759144	0.8566146	6.8987867	2.28E-11	2.80E-10	15.295811
PRPF19	-0.086913	6.691594	-6.897776	2.29E-11	2.82E-10	15.289685
CNGB1	1.4097516	0.7059237	6.8972032	2.30E-11	2.83E-10	15.286209
COA5	-0.103567	6.0984736	-6.896664	2.31E-11	2.83E-10	15.282939
TRAPPC6A	-0.18272	6.1871934	-6.896502	2.31E-11	2.84E-10	15.281954
AMACR	-0.342675	6.0498258	-6.896045	2.32E-11	2.84E-10	15.279186
GPX1	-0.153358	7.0080742	-6.89575	2.32E-11	2.85E-10	15.277395
COX7C	-0.132749	6.9722781	-6.895525	2.33E-11	2.85E-10	15.276032
SEMA3D	1.0659653	3.4163911	6.8944322	2.34E-11	2.87E-10	15.26941
TRBV5-6	1.2072097	-0.001056	6.8936561	2.35E-11	2.88E-10	15.264706
RP11-1007C	-0.310849	4.1417904	-6.893403	2.36E-11	2.89E-10	15.26317
AC129492.6	1.3592557	0.1264393	6.8920984	2.38E-11	2.91E-10	15.255265
HLA-DOA	0.3758432	5.4917292	6.8919781	2.38E-11	2.91E-10	15.254536
LRRC17	0.4440959	4.5299786	6.8914499	2.39E-11	2.92E-10	15.251335
FAM200B	-0.121322	5.9274813	-6.890213	2.40E-11	2.94E-10	15.24384
LSM3	-0.119232	6.3294515	-6.889222	2.42E-11	2.96E-10	15.237837
NCEH1	0.2942285	5.4868711	6.8891954	2.42E-11	2.96E-10	15.237676
TALDO1	-0.149287	6.8850453	-6.888817	2.43E-11	2.96E-10	15.235383
ELMOD2	-0.104414	6.0969807	-6.888641	2.43E-11	2.96E-10	15.234317
CYP2S1	0.6456201	4.4449829	6.8883353	2.43E-11	2.97E-10	15.232466
FA2H	1.647556	1.9019223	6.8883049	2.43E-11	2.97E-10	15.232281
GPR56	0.2601009	5.9375582	6.8876708	2.44E-11	2.98E-10	15.228441
ADAP2	0.2271062	5.47165	6.8855895	2.48E-11	3.02E-10	15.215836
APOBEC3G	0.3385805	5.2200761	6.8852327	2.48E-11	3.02E-10	15.213675
LANCL2	-0.111693	6.0681735	-6.885225	2.48E-11	3.02E-10	15.21363
HS3ST3A1	1.2876587	2.1789175	6.8852056	2.48E-11	3.02E-10	15.213511
GLUL	-0.260213	7.3622216	-6.88494	2.49E-11	3.03E-10	15.2119
GLRB	1.7109524	3.0673464	6.8832658	2.51E-11	3.06E-10	15.201766
SLU7	-0.091921	6.2661826	-6.883071	2.51E-11	3.06E-10	15.200588
ZNF630	0.9482718	3.4130217	6.8822615	2.53E-11	3.07E-10	15.195687
LINC01410	0.993711	2.313225	6.8822554	2.53E-11	3.07E-10	15.19565
IL17REL	1.1214877	-0.401651	6.8814589	2.54E-11	3.09E-10	15.190829
SATB1-AS1	1.3467892	1.849781	6.881326	2.54E-11	3.09E-10	15.190024
DNMT1	0.152936	6.2576775	6.8778582	2.60E-11	3.15E-10	15.169039
RP11-403I1	0.6760461	3.9122436	6.8776493	2.60E-11	3.16E-10	15.167775
KCNA3	1.2880864	1.8406793	6.8761614	2.62E-11	3.19E-10	15.158774

C15orf40	-0.112442	5.9106192	-6.874145	2.66E-11	3.22E-10	15.146578
TEAD4	0.3718591	5.1913622	6.872687	2.68E-11	3.25E-10	15.13776
FMNL2	0.3335444	5.5966727	6.8709339	2.71E-11	3.29E-10	15.127161
NDUFA4	-0.13307	6.9201246	-6.869038	2.74E-11	3.33E-10	15.115702
GPRIN3	0.337894	5.4915087	6.8684622	2.75E-11	3.34E-10	15.11222
TTLL4	0.2230735	5.9699912	6.8679849	2.76E-11	3.34E-10	15.109335
LSAMP	1.3749537	2.5984832	6.8677328	2.77E-11	3.35E-10	15.107812
ASMTL	-0.164667	6.3164947	-6.867063	2.78E-11	3.36E-10	15.103761
TMOD2	0.2797602	5.2750616	6.8664796	2.79E-11	3.37E-10	15.100239
RP11-539G1	-0.496641	3.4748055	-6.865613	2.80E-11	3.39E-10	15.095
MRPS34	-0.14093	6.5046934	-6.865112	2.81E-11	3.40E-10	15.091974
RXRA	-0.145098	6.8744666	-6.86439	2.82E-11	3.41E-10	15.087614
THOP1	-0.177511	6.4229845	-6.864304	2.83E-11	3.41E-10	15.087094
PRIMA1	1.4516777	0.0226658	6.8634219	2.84E-11	3.43E-10	15.081766
C6orf99	1.4778724	0.2751134	6.863421	2.84E-11	3.43E-10	15.08176
AC006369.3	-0.496281	-1.330155	-6.86251	2.86E-11	3.45E-10	15.076259
DYRK2	0.1879304	5.9049105	6.8624509	2.86E-11	3.45E-10	15.075901
TNS4	1.2827751	1.5689461	6.8616786	2.87E-11	3.46E-10	15.071236
PRDM5	0.9544883	3.1407828	6.8613783	2.88E-11	3.47E-10	15.069422
DNAJA2	-0.123512	6.4701682	-6.860568	2.89E-11	3.49E-10	15.06453
RP11-395N3	-0.639773	3.6062192	-6.859407	2.91E-11	3.51E-10	15.057519
VDR	0.6047666	4.9257208	6.8593545	2.91E-11	3.51E-10	15.057202
OXSM	-0.126836	5.8879647	-6.857762	2.94E-11	3.54E-10	15.047589
CLSTN1	0.1868174	6.3623584	6.8567067	2.96E-11	3.57E-10	15.041218
CCNJ	0.55149	4.8497286	6.8566707	2.96E-11	3.57E-10	15.041
C16orf54	0.6060688	4.2055245	6.8526005	3.04E-11	3.66E-10	15.016439
FMO4	-0.325899	5.9592264	-6.851858	3.05E-11	3.67E-10	15.011959
TM6SF2	-0.505495	5.607857	-6.851727	3.06E-11	3.67E-10	15.011168
CTD-2353F2	1.1887747	0.2777254	6.8513235	3.06E-11	3.68E-10	15.008735
TXN2	-0.123523	6.6472012	-6.850733	3.07E-11	3.69E-10	15.005174
RP11-59C5.	0.8514994	2.9561405	6.8502357	3.08E-11	3.70E-10	15.002173
AQP11	-0.284328	5.7575031	-6.850165	3.08E-11	3.70E-10	15.001749
CFAP20	-0.118263	6.0893247	-6.849067	3.11E-11	3.73E-10	14.995122
GPATCH3	-0.133112	5.9402917	-6.848063	3.13E-11	3.75E-10	14.989069
COL12A1	0.4383453	5.5877261	6.8479855	3.13E-11	3.75E-10	14.988603
FBXO7	-0.122902	6.6958937	-6.847837	3.13E-11	3.75E-10	14.987708
CYP1A1	-1.431019	4.5873568	-6.846805	3.15E-11	3.78E-10	14.981487
CNTN1	1.7991013	1.0494226	6.8465887	3.15E-11	3.78E-10	14.980181
COX6A1	-0.132699	6.7973766	-6.846263	3.16E-11	3.79E-10	14.978215
IGKV6D-21	1.2527743	-0.479927	6.8460436	3.16E-11	3.79E-10	14.976895
SARDH	-0.298138	6.550164	-6.843815	3.21E-11	3.84E-10	14.963462
HIBCH	-0.156077	6.3729824	-6.842112	3.24E-11	3.88E-10	14.953199
SH2D1A	0.789201	3.9166831	6.8411202	3.26E-11	3.90E-10	14.947221
RET	1.2865024	3.0037604	6.8409371	3.27E-11	3.91E-10	14.946118
EDA	0.6105381	4.8286874	6.839849	3.29E-11	3.93E-10	14.939562
FTCDNL1	-0.28355	5.187376	-6.839584	3.29E-11	3.94E-10	14.937963
TPH1	1.1562013	2.2259148	6.8387906	3.31E-11	3.96E-10	14.933187
FAM171B	0.5302734	4.3993724	6.8380265	3.33E-11	3.97E-10	14.928584
RP5-1091N2	1.0292259	2.3079874	6.8377322	3.33E-11	3.98E-10	14.926812
PCDHGA9	1.2138174	2.5376195	6.8360317	3.37E-11	4.02E-10	14.91657
NGFR	0.6321559	5.2168932	6.8355235	3.38E-11	4.03E-10	14.91351

RP11-486B1	1.1160459	-0.268352	6.8350648	3.39E-11	4.04E-10	14.910748
NDUFA7	-0.216328	5.2018208	-6.834325	3.40E-11	4.06E-10	14.906296
PDE9A	0.5033874	5.2999485	6.8340765	3.41E-11	4.06E-10	14.904797
C1orf106	1.261074	4.3324385	6.8324699	3.44E-11	4.10E-10	14.895126
LSM10	-0.135136	6.1089606	-6.831864	3.46E-11	4.12E-10	14.89148
LINC00565	1.1720106	-0.065354	6.8312175	3.47E-11	4.13E-10	14.887588
GFOD2	-0.14985	6.1629287	-6.831178	3.47E-11	4.13E-10	14.88735
MAN2A1	-0.118484	6.6130655	-6.830828	3.48E-11	4.14E-10	14.885242
SIT1	0.7929062	3.973882	6.8303579	3.49E-11	4.15E-10	14.882415
SLC6A11	2.0770549	2.9532456	6.8298235	3.50E-11	4.16E-10	14.879199
CR1	1.0399926	3.3070179	6.8284012	3.53E-11	4.20E-10	14.87064
ACSM5P1	-1.264399	3.2092138	-6.827984	3.54E-11	4.21E-10	14.868131
RP11-191G2	-0.977756	2.6366457	-6.827909	3.54E-11	4.21E-10	14.867681
ZBED5-AS1	-0.188447	5.6913747	-6.826794	3.57E-11	4.24E-10	14.860974
EIF3EP1	0.9335938	2.4341423	6.8266098	3.57E-11	4.24E-10	14.859863
ATP5D	-0.175799	6.6594576	-6.82546	3.60E-11	4.27E-10	14.852949
NICN1	-0.135637	5.8504733	-6.824501	3.62E-11	4.29E-10	14.847181
TMEM55A	0.4122231	5.234971	6.8223682	3.66E-11	4.35E-10	14.834354
RP11-659E9	-1.355518	2.1723532	-6.820911	3.70E-11	4.39E-10	14.825591
PROC	-0.332001	6.9289869	-6.820708	3.70E-11	4.39E-10	14.824371
GATA5	1.7946174	1.2810459	6.8200304	3.72E-11	4.41E-10	14.8203
AC007386.4	1.155765	-0.233716	6.8196792	3.73E-11	4.41E-10	14.818189
GYPE	1.1864221	0.4593485	6.8191282	3.74E-11	4.43E-10	14.814877
CTD-2213F2	-0.836206	-1.096978	-6.818713	3.75E-11	4.44E-10	14.812384
ZG16B	1.2983836	2.8997889	6.8170578	3.79E-11	4.48E-10	14.802435
CHD5	1.2659327	0.3908513	6.8155372	3.82E-11	4.52E-10	14.793298
KCNK6	0.2793502	5.2782948	6.8151955	3.83E-11	4.53E-10	14.791246
LAMP3	0.6770293	4.6287792	6.814668	3.84E-11	4.54E-10	14.788077
ITPR2	-0.21042	6.6054185	-6.814093	3.86E-11	4.56E-10	14.784625
FAM98B	0.0968972	5.9286394	6.8135758	3.87E-11	4.57E-10	14.781516
ZNF215	1.3079008	2.5892196	6.8131988	3.88E-11	4.58E-10	14.779252
HCAR3	1.4858084	1.2019177	6.8129174	3.88E-11	4.59E-10	14.777561
PSMA2	-0.149324	6.0705057	-6.812405	3.90E-11	4.60E-10	14.774487
MRPS11	-0.113406	6.1419275	-6.812066	3.91E-11	4.61E-10	14.772445
IGHG1	0.745117	6.3785086	6.8118284	3.91E-11	4.61E-10	14.771021
CD99L2	-0.159085	6.5843887	-6.811399	3.92E-11	4.63E-10	14.768445
RP11-902B1	1.1320731	1.574737	6.8102911	3.95E-11	4.66E-10	14.76179
SCAND1	-0.153202	6.4662497	-6.808545	3.99E-11	4.70E-10	14.751309
MAB21L1	1.1974009	-0.000841	6.8075255	4.02E-11	4.73E-10	14.745187
RAB13	-0.116567	6.6322471	-6.80744	4.02E-11	4.73E-10	14.744675
LAMTOR1	-0.104888	6.5575843	-6.806956	4.03E-11	4.75E-10	14.741768
SF3B6	-0.097449	6.4114249	-6.806815	4.03E-11	4.75E-10	14.740923
RP4-800J21	1.103968	0.4591868	6.806789	4.03E-11	4.75E-10	14.740767
TLR5	0.4123254	4.9945703	6.8066755	4.04E-11	4.75E-10	14.740085
FPR2	1.3681021	1.4447906	6.8059714	4.06E-11	4.77E-10	14.73586
PRDX1	-0.131123	7.0420842	-6.805956	4.06E-11	4.77E-10	14.735768
RP4-798A17	-0.824365	3.6298374	-6.805883	4.06E-11	4.77E-10	14.735327
SCARF2	0.3478248	5.0276449	6.8057365	4.06E-11	4.77E-10	14.73445
CD14	-0.243204	6.9456075	-6.804753	4.09E-11	4.80E-10	14.728546
AP000892.6	0.6788443	3.9658665	6.8045429	4.09E-11	4.80E-10	14.727287
EID3	0.6355527	3.5164504	6.8026621	4.14E-11	4.86E-10	14.716003

SLC18A2	1.1620645	2.0800584	6.8025556	4.14E-11	4.86E-10	14.715364
ETHE1	-0.171746	6.097511	-6.802273	4.15E-11	4.87E-10	14.713669
SLC52A1	1.2157007	1.267211	6.8021914	4.15E-11	4.87E-10	14.713179
FRG1	-0.137535	5.9575312	-6.801841	4.16E-11	4.88E-10	14.71108
SMIM14	-0.167933	6.8045013	-6.801501	4.17E-11	4.89E-10	14.709037
CHGB	1.5417688	0.6145073	6.7974669	4.27E-11	5.01E-10	14.684846
SCIMP	0.398959	4.6740948	6.7965653	4.30E-11	5.03E-10	14.67944
CDKN2AIPNL	-0.154473	6.0672498	-6.792308	4.41E-11	5.17E-10	14.653923
LINC00526	-0.409252	5.3881957	-6.791977	4.42E-11	5.17E-10	14.651939
AP3M2	0.1681564	5.5543779	6.7906793	4.46E-11	5.21E-10	14.644166
RP11-863P1	1.2204938	0.0050685	6.7905203	4.46E-11	5.22E-10	14.643213
CST2	1.6512772	1.4698457	6.7898203	4.48E-11	5.24E-10	14.63902
KCNQ1	0.3258651	5.2677609	6.7887844	4.51E-11	5.27E-10	14.632815
ARHGAP28	0.9891848	3.8019405	6.788597	4.51E-11	5.27E-10	14.631693
GALNT13	1.4179446	1.5044698	6.7878075	4.54E-11	5.30E-10	14.626964
UBL5	-0.134387	6.7077747	-6.787616	4.54E-11	5.30E-10	14.625818
VWDE	1.464439	0.2096923	6.7873842	4.55E-11	5.31E-10	14.62443
RP11-61J19	-0.304613	4.627713	-6.786609	4.57E-11	5.33E-10	14.61979
HMBS	-0.126149	6.1437175	-6.786168	4.58E-11	5.35E-10	14.617145
BNC1	1.2792652	0.3891711	6.7850946	4.61E-11	5.38E-10	14.61072
RPL39P40	-1.06698	3.4064956	-6.784402	4.63E-11	5.40E-10	14.606571
GJA3	1.3665372	0.6554098	6.7826608	4.68E-11	5.46E-10	14.59615
MET	-0.162573	6.6845722	-6.780163	4.75E-11	5.54E-10	14.5812
GPR137	-0.122037	6.2354569	-6.779945	4.76E-11	5.55E-10	14.5799
RP11-363E6	1.0777814	-0.296335	6.7796799	4.77E-11	5.55E-10	14.578311
FOPNL	-0.096491	6.1494154	-6.779671	4.77E-11	5.55E-10	14.57826
ST13	-0.092899	6.9336587	-6.779176	4.78E-11	5.57E-10	14.575296
IZUMO4	-0.422478	4.7145711	-6.778561	4.80E-11	5.59E-10	14.571617
TRAV13-2	1.2287268	0.3495097	6.7778426	4.82E-11	5.61E-10	14.56732
EPHX2	-0.253703	6.5893664	-6.775529	4.89E-11	5.69E-10	14.553481
EPHB6	0.5425668	4.9083991	6.7754575	4.89E-11	5.69E-10	14.553053
FAM201A	1.8141408	2.2389637	6.7754506	4.89E-11	5.69E-10	14.553012
TIGIT	0.7076851	4.3755551	6.7712171	5.02E-11	5.83E-10	14.5277
MST1	-0.285792	6.8313651	-6.769936	5.06E-11	5.88E-10	14.520045
TMEM136	0.4173169	4.8805589	6.7694325	5.08E-11	5.90E-10	14.517034
RAB6B	0.2651999	5.1107689	6.7681987	5.12E-11	5.94E-10	14.50966
AC147651.5	1.4447129	-0.264322	6.7669617	5.16E-11	5.98E-10	14.502269
ENSA	-0.110662	6.8327042	-6.766356	5.18E-11	6.00E-10	14.498652
CD52	0.3555688	5.4861247	6.7657679	5.19E-11	6.02E-10	14.495138
C20orf166-	1.1465209	-0.265855	6.7654555	5.20E-11	6.03E-10	14.493271
ITPRIPL2	0.2352447	5.862811	6.765362	5.21E-11	6.03E-10	14.492713
GLIS1	1.2160835	-0.171752	6.7648217	5.23E-11	6.05E-10	14.489485
MRPL16	-0.10816	6.3816509	-6.763143	5.28E-11	6.11E-10	14.479462
COMMD3	-0.119601	6.1140257	-6.762181	5.31E-11	6.15E-10	14.473712
GGT5	0.3956566	5.8415291	6.7607889	5.36E-11	6.20E-10	14.465403
MYOM3	1.1120861	2.7312027	6.7591249	5.41E-11	6.26E-10	14.455469
TNFSF13	0.4244863	4.8600518	6.7562847	5.51E-11	6.37E-10	14.438518
LRRC20	-0.188094	6.3431978	-6.755323	5.54E-11	6.40E-10	14.432778
IFI30	0.3871741	4.1993659	6.7536814	5.60E-11	6.47E-10	14.422986
SPG7	-0.105119	6.4551891	-6.753103	5.62E-11	6.49E-10	14.419532
ABI2	0.1226712	6.0034068	6.7529925	5.62E-11	6.49E-10	14.418876

LINC00493	-0.135733	6.2669852	-6.752188	5.65E-11	6.52E-10	14.414076
AC005083.1	1.3804261	1.607913	6.7513169	5.68E-11	6.55E-10	14.408882
GXYLT2	1.2262886	3.1416524	6.7508294	5.69E-11	6.57E-10	14.405975
CNOT6	0.1269908	5.9409396	6.7505698	5.70E-11	6.58E-10	14.404427
FEZ1	0.3350707	5.066547	6.7498691	5.73E-11	6.60E-10	14.400248
COL4A3	1.002343	3.5667936	6.7498081	5.73E-11	6.60E-10	14.399884
OTUD5	-0.084884	6.4591268	-6.748937	5.76E-11	6.64E-10	14.394689
DDHD1	0.2035909	5.5388241	6.7478151	5.80E-11	6.68E-10	14.388002
SLC17A8	1.3363244	0.1935237	6.7475969	5.81E-11	6.69E-10	14.386701
ARHGAP22	0.4386159	4.5081902	6.7463732	5.85E-11	6.74E-10	14.379407
RP11-345P4	-0.240938	5.1191132	-6.746064	5.86E-11	6.75E-10	14.377566
SLAMF8	0.3791438	5.2697947	6.7435906	5.95E-11	6.85E-10	14.362824
ZFP82	0.5993785	4.41878	6.7434254	5.96E-11	6.85E-10	14.36184
TCP11L2	0.349689	4.7836634	6.7426345	5.99E-11	6.88E-10	14.357127
SOX9	0.6496199	5.8533963	6.742072	6.01E-11	6.91E-10	14.353776
SEPT5	0.3984711	5.3544264	6.7417137	6.02E-11	6.92E-10	14.351641
NFATC4	0.3346767	5.2922003	6.7416667	6.02E-11	6.92E-10	14.351361
PHLPP1	-0.169926	6.1970317	-6.741413	6.03E-11	6.93E-10	14.349851
RP11-22C11	1.3239351	-0.565168	6.7412198	6.04E-11	6.93E-10	14.348699
MN1	0.6207181	4.5486781	6.740744	6.06E-11	6.95E-10	14.345865
ARHGAP31	0.2294527	5.5590199	6.7403074	6.07E-11	6.97E-10	14.343264
ZNF587	0.1683341	5.5610602	6.7395078	6.10E-11	7.00E-10	14.338502
RP11-494O1	1.1053503	0.3836209	6.73902	6.12E-11	7.02E-10	14.335597
CD200	0.3618326	4.9567407	6.7388732	6.13E-11	7.02E-10	14.334722
CCNG1	-0.123558	6.6623137	-6.73874	6.13E-11	7.03E-10	14.333929
PCDH19	1.1746765	1.6694547	6.7385387	6.14E-11	7.03E-10	14.332731
ADAMTS7	0.2829382	5.1358488	6.7371859	6.19E-11	7.09E-10	14.324675
ZC3HC1	-0.112505	6.0158448	-6.737182	6.19E-11	7.09E-10	14.324651
IL16	0.2350143	5.4683335	6.7370143	6.20E-11	7.09E-10	14.323654
SLC25A44	-0.127661	6.5159656	-6.736939	6.20E-11	7.09E-10	14.323203
CTU2	-0.141996	5.8471986	-6.736626	6.21E-11	7.10E-10	14.321341
SDC2	-0.153326	7.0921516	-6.736399	6.22E-11	7.11E-10	14.319988
PGR	1.3703505	2.6034223	6.7358616	6.24E-11	7.13E-10	14.31679
CHST1	0.4771492	4.9263855	6.7341499	6.31E-11	7.21E-10	14.306601
TRAV4	1.3155836	0.8500737	6.7332437	6.34E-11	7.24E-10	14.301208
FAM90A1	1.2959918	1.742223	6.7327666	6.36E-11	7.26E-10	14.298368
ZNF771	-0.210057	5.2955051	-6.732295	6.38E-11	7.28E-10	14.295561
TMEM256-PL	0.5898431	2.8240963	6.7309485	6.43E-11	7.34E-10	14.28755
TRBV6-4	0.8755812	-0.863207	6.7309067	6.43E-11	7.34E-10	14.287301
CHRM3-AS2	1.256107	1.4731778	6.7306148	6.45E-11	7.35E-10	14.285565
LINC00883	0.72389	3.5844456	6.7304689	6.45E-11	7.35E-10	14.284697
LINC00467	-0.183631	5.8307807	-6.730097	6.47E-11	7.37E-10	14.282487
SLIT1	0.8965764	2.7320214	6.729933	6.47E-11	7.37E-10	14.281508
GORAB	0.2547823	5.6559066	6.7296766	6.48E-11	7.38E-10	14.279983
KCTD19	1.0500062	-0.551777	6.7296036	6.49E-11	7.38E-10	14.279549
PRRT2	0.6907904	3.6082581	6.7288201	6.52E-11	7.42E-10	14.274888
PPARA	-0.177027	6.5528235	-6.728676	6.52E-11	7.42E-10	14.27403
RP11-757F1	1.4411475	0.1810351	6.7285468	6.53E-11	7.43E-10	14.273262
DBP	-0.205281	6.0078568	-6.726997	6.59E-11	7.49E-10	14.264042
GRM4	1.3696335	0.2418626	6.7258086	6.64E-11	7.55E-10	14.256978
PSMB5	-0.098383	6.6880246	-6.725655	6.64E-11	7.55E-10	14.256064

GJB3	1.465209	2.145915	6.7255645	6.65E-11	7.55E-10	14.255526
PRR12	0.1314792	6.0218628	6.7240251	6.71E-11	7.62E-10	14.246374
RP13-122B2	0.9154065	2.6887942	6.7233257	6.74E-11	7.65E-10	14.242216
GOLGA7B	1.0473971	3.9569573	6.7232949	6.74E-11	7.65E-10	14.242033
BRWD1-AS2	-0.541906	3.6671655	-6.723237	6.74E-11	7.65E-10	14.241686
PAOX	-0.224796	5.8805252	-6.722738	6.76E-11	7.67E-10	14.23872
NAPRT	-0.193997	6.7103186	-6.722672	6.77E-11	7.67E-10	14.238332
PLXDC2	0.3384347	5.6086006	6.7222307	6.78E-11	7.69E-10	14.235707
PTGR2	-0.151314	5.9669232	-6.722227	6.78E-11	7.69E-10	14.235682
C4orf3	-0.127067	6.5795431	-6.721716	6.81E-11	7.71E-10	14.232645
COX14	-0.141439	6.3155935	-6.721574	6.81E-11	7.72E-10	14.231801
SPRED3	0.806047	3.3293756	6.7204953	6.86E-11	7.76E-10	14.225393
PBX1	0.4587124	5.405561	6.7202272	6.87E-11	7.77E-10	14.2238
SPEG	0.7128814	3.7600647	6.7181756	6.96E-11	7.87E-10	14.211609
ASNS	0.4908351	5.3097537	6.7172834	6.99E-11	7.91E-10	14.206309
TRAV5	1.0817077	-0.281021	6.7161947	7.04E-11	7.96E-10	14.199842
LYRM1	-0.14487	6.2780272	-6.714635	7.11E-11	8.03E-10	14.190582
ZAP70	0.3830153	5.0317237	6.7142561	7.12E-11	8.05E-10	14.188329
TBCD	-0.09821	6.557004	-6.714062	7.13E-11	8.06E-10	14.187174
BAAT	-0.441887	7.081397	-6.713623	7.15E-11	8.08E-10	14.184569
SNF8	-0.117023	6.5092146	-6.712475	7.20E-11	8.13E-10	14.177753
TRAV8-1	1.047715	-0.504832	6.7121622	7.22E-11	8.14E-10	14.175896
RP11-73M18	0.7236946	3.265473	6.7112744	7.25E-11	8.19E-10	14.170626
C4orf27	-0.115923	5.8584507	-6.711167	7.26E-11	8.19E-10	14.169988
SRP19	-0.118414	6.0610049	-6.710488	7.29E-11	8.22E-10	14.165957
MRPL34	-0.137875	6.4647524	-6.709487	7.33E-11	8.27E-10	14.160014
YWHAZP3	1.1556357	1.154352	6.7092509	7.35E-11	8.28E-10	14.158615
GLRX5	-0.139364	6.5305352	-6.709108	7.35E-11	8.28E-10	14.157767
CD33	0.3896751	4.655089	6.7089102	7.36E-11	8.29E-10	14.156593
ZNF575	-0.179568	4.8019654	-6.708738	7.37E-11	8.30E-10	14.155572
TRAV8-4	1.2165147	0.2146304	6.7083455	7.39E-11	8.31E-10	14.153242
PMF1	-0.156182	6.2974248	-6.708308	7.39E-11	8.31E-10	14.153018
DUOX2	1.506235	3.5777408	6.708102	7.40E-11	8.32E-10	14.151797
ZNF783	0.2453544	5.2522669	6.7077891	7.41E-11	8.33E-10	14.14994
CCDC89	1.1161782	0.1891301	6.7073702	7.43E-11	8.35E-10	14.147454
CTD-2270L9	-0.252904	4.7731257	-6.707184	7.44E-11	8.36E-10	14.14635
NDRG2	-0.166688	6.7924853	-6.706329	7.48E-11	8.40E-10	14.141276
LBX2	-0.403821	5.1299169	-6.705712	7.51E-11	8.43E-10	14.137619
PI4KAP2	0.248219	5.0534414	6.7056881	7.51E-11	8.43E-10	14.137475
TDRD9	1.3553211	1.7846076	6.7056379	7.51E-11	8.43E-10	14.137177
RP11-415J8	0.6526204	3.916064	6.7056006	7.51E-11	8.43E-10	14.136955
CYP4F12	-0.493569	6.0420719	-6.705324	7.52E-11	8.44E-10	14.135317
RP4-539M6.	-0.909718	3.079051	-6.705193	7.53E-11	8.44E-10	14.134539
ATP5J	-0.13628	6.7231153	-6.704467	7.56E-11	8.48E-10	14.130234
CD180	0.4610504	4.581044	6.7035144	7.61E-11	8.53E-10	14.124581
SLC35B4	-0.144072	6.1257559	-6.702265	7.67E-11	8.59E-10	14.11717
AIRE	1.2284683	0.0373612	6.700778	7.74E-11	8.66E-10	14.108354
FAM124A	0.552489	4.1521563	6.7005892	7.74E-11	8.67E-10	14.107235
FAM81A	0.7261503	3.8183762	6.6993744	7.80E-11	8.73E-10	14.100033
NT5C2	0.340637	5.6373238	6.6982653	7.85E-11	8.79E-10	14.093459
GPT	-0.354743	6.4990014	-6.698215	7.86E-11	8.79E-10	14.09316

RP5-887A1C	1.1633891	-0.343393	6.6980003	7.87E-11	8.80E-10	14.091888
NAGLU	-0.140262	6.5382166	-6.697488	7.89E-11	8.82E-10	14.088854
CACNA1D	0.5720237	5.0618592	6.6968389	7.92E-11	8.86E-10	14.085005
NDUFS4	-0.146967	6.3776728	-6.696229	7.95E-11	8.89E-10	14.081393
HIC1	0.2743986	5.2462079	6.6958438	7.97E-11	8.90E-10	14.079108
PAX5	1.3498274	2.615533	6.694305	8.05E-11	8.98E-10	14.06999
C1S	-0.184127	7.4662036	-6.694276	8.05E-11	8.98E-10	14.069816
PYCR1	0.7526268	5.3683359	6.694169	8.05E-11	8.99E-10	14.069185
LINC00996	0.9755373	2.6167802	6.6919878	8.16E-11	9.11E-10	14.056264
HTR3A	1.4819132	0.4552684	6.6917124	8.17E-11	9.12E-10	14.054632
CAMK2N1	-0.165978	6.5843521	-6.6907	8.23E-11	9.17E-10	14.048635
HIC2	0.2615359	5.4081232	6.6904786	8.24E-11	9.18E-10	14.047326
TMEM158	0.8673593	3.5796515	6.6895169	8.29E-11	9.23E-10	14.041631
B3GAT1	1.1904893	3.7047816	6.6892984	8.30E-11	9.24E-10	14.040337
FAM162A	-0.16008	6.5684541	-6.689233	8.30E-11	9.24E-10	14.039948
NDUFS8	-0.143306	6.6196564	-6.689168	8.30E-11	9.24E-10	14.039566
CTD-252902	-1.457763	1.7623135	-6.688616	8.33E-11	9.27E-10	14.036294
EMP3	0.2575709	5.6630886	6.6882206	8.35E-11	9.29E-10	14.033956
RP11-387H1	-1.032858	2.6106398	-6.688213	8.35E-11	9.29E-10	14.033911
MOGS	-0.0802	6.555062	-6.687597	8.38E-11	9.32E-10	14.030264
ANKS6	0.6351924	5.4425298	6.6867521	8.43E-11	9.37E-10	14.025263
SH3D19	-0.156196	6.4468976	-6.686071	8.46E-11	9.40E-10	14.021232
ZNF354C	0.5955513	4.2260997	6.6840251	8.57E-11	9.52E-10	14.009123
BDH1	-0.328217	6.6948824	-6.682888	8.63E-11	9.58E-10	14.002394
FRMPD3	1.1918643	0.3674775	6.6822304	8.66E-11	9.62E-10	13.998504
EXOC4	-0.094456	6.371653	-6.680436	8.76E-11	9.72E-10	13.987889
KCTD21-AS1	-0.319184	4.7152806	-6.679692	8.80E-11	9.76E-10	13.983489
KCTD21	-0.147012	6.04042	-6.678487	8.86E-11	9.83E-10	13.97636
TMC5	1.2905489	4.0141326	6.6766019	8.96E-11	9.94E-10	13.965216
NDUFS5	-0.142386	6.6812277	-6.676579	8.96E-11	9.94E-10	13.965083
PHB	-0.106507	6.7133432	-6.675329	9.03E-11	1.00E-09	13.957688
DIRAS1	1.4452743	2.5694467	6.6750806	9.05E-11	1.00E-09	13.956223
RP11-5407.	1.4754251	2.087312	6.6746836	9.07E-11	1.00E-09	13.953876
TMEM184B	0.1098876	6.2778775	6.6731255	9.15E-11	1.01E-09	13.944666
AOAH	0.3673256	5.1712982	6.6724322	9.19E-11	1.02E-09	13.94057
C19orf24	-0.167318	6.4425635	-6.67218	9.21E-11	1.02E-09	13.939077
TMEM126A	-0.129835	5.9853804	-6.672031	9.22E-11	1.02E-09	13.938199
DPP10	1.5857309	-0.023543	6.6719461	9.22E-11	1.02E-09	13.937697
HLA-DQB2	0.752704	4.9249283	6.6712098	9.26E-11	1.02E-09	13.933346
FOXO6	1.4977068	2.6644505	6.6711983	9.26E-11	1.02E-09	13.933278
GANAB	-0.075727	7.0983392	-6.670766	9.29E-11	1.03E-09	13.930723
LINC01554	-1.148361	4.8326728	-6.670304	9.31E-11	1.03E-09	13.927995
UBC	-0.094663	7.149681	-6.670184	9.32E-11	1.03E-09	13.927283
LINC00839	0.9234815	2.8156385	6.6685339	9.41E-11	1.04E-09	13.917538
ILF3-AS1	-0.180027	5.7708652	-6.66851	9.42E-11	1.04E-09	13.917395
YTHDF3-AS1	-0.371358	4.4706063	-6.668137	9.44E-11	1.04E-09	13.915192
BORA	0.356489	4.56899	6.6675715	9.47E-11	1.04E-09	13.911854
TMEM18	-0.104704	6.0005641	-6.665944	9.56E-11	1.05E-09	13.902241
CDH6	0.4613515	5.2341882	6.6658387	9.57E-11	1.05E-09	13.901621
MDS2	1.1012354	0.9215006	6.6652335	9.60E-11	1.06E-09	13.898047
LHFP	0.2775794	5.7128921	6.6638541	9.69E-11	1.07E-09	13.889904

ASPDH	-0.734567	5.8892625	-6.663467	9.71E-11	1.07E-09	13.887617
LONP2	-0.145502	6.6988736	-6.663214	9.72E-11	1.07E-09	13.886126
TMA7	-0.143227	6.5236679	-6.663107	9.73E-11	1.07E-09	13.885496
TRAPPC13P1	-1.649469	1.338762	-6.663107	9.73E-11	1.07E-09	13.885496
MRPS36	-0.136195	6.0900075	-6.662701	9.75E-11	1.07E-09	13.883095
ACSL5	-0.287493	6.5807278	-6.661909	9.80E-11	1.08E-09	13.878425
SEPT2	0.0813027	6.75259	6.6617249	9.81E-11	1.08E-09	13.877336
ZNF292	0.1810216	5.8265515	6.6613074	9.84E-11	1.08E-09	13.874872
ACOT4	-0.257428	5.7592424	-6.660136	9.91E-11	1.09E-09	13.867958
HRSP12	-0.243038	6.8726986	-6.659143	9.97E-11	1.09E-09	13.8621
DOCK11	0.3657977	5.1652144	6.6588152	9.99E-11	1.10E-09	13.860166
TMX2P1	0.7924299	3.8302543	6.6578902	1.00E-10	1.10E-09	13.854709
RP11-203F1	-1.160083	-0.372182	-6.657852	1.00E-10	1.10E-09	13.854485
SLC10A3	0.1566644	5.8056502	6.6534206	1.03E-10	1.13E-09	13.828348
MIA3	-0.140302	6.7350535	-6.653397	1.03E-10	1.13E-09	13.828207
TRBJ2-3	0.9343659	-0.594971	6.6526084	1.04E-10	1.14E-09	13.823559
TLR2	0.4493488	5.1499794	6.6522212	1.04E-10	1.14E-09	13.821277
CYP27A1	-0.253632	7.0971849	-6.651612	1.04E-10	1.14E-09	13.817683
FLNC	0.8078685	4.9814228	6.6511445	1.05E-10	1.15E-09	13.814929
RNF130	-0.119016	6.6973279	-6.650946	1.05E-10	1.15E-09	13.813757
BEND3	0.2662883	5.0997191	6.6506974	1.05E-10	1.15E-09	13.812294
CRYM	-0.432015	5.6215787	-6.650176	1.05E-10	1.15E-09	13.809218
AC017002.1	1.1485723	0.9286494	6.6494935	1.06E-10	1.16E-09	13.805198
ABCC1	0.3125177	5.7008576	6.6489051	1.06E-10	1.16E-09	13.801731
RP11-728F1	1.5464763	0.2254718	6.6485365	1.06E-10	1.16E-09	13.799559
LOXL4	0.8012581	5.5346949	6.647714	1.07E-10	1.17E-09	13.794712
ABALON	0.9902381	2.8430167	6.6469433	1.07E-10	1.17E-09	13.790171
H2AFJ	-0.185903	6.4667039	-6.645606	1.08E-10	1.18E-09	13.782292
TMEM19	-0.131916	6.1263389	-6.644714	1.09E-10	1.19E-09	13.77704
ZNF737	0.8801994	3.8876796	6.6443712	1.09E-10	1.19E-09	13.775019
PROCR	0.318507	5.1829289	6.6442084	1.09E-10	1.19E-09	13.774061
RP1-59D14.	1.0205811	2.6858937	6.6438081	1.09E-10	1.19E-09	13.771703
SOX4	0.3401482	6.0148431	6.6430296	1.10E-10	1.20E-09	13.767118
CPAMD8	0.5389123	4.2957554	6.6429744	1.10E-10	1.20E-09	13.766793
PLCXD3	1.4587102	3.09235	6.642763	1.10E-10	1.20E-09	13.765548
H2AFY2	0.489227	5.5626455	6.6417788	1.11E-10	1.21E-09	13.759753
COX4I1	-0.145995	7.0288353	-6.641775	1.11E-10	1.21E-09	13.759731
CERS1	1.5557131	1.715755	6.6417633	1.11E-10	1.21E-09	13.759662
TIAM2	0.2804351	5.1636241	6.6417197	1.11E-10	1.21E-09	13.759404
MTTP	-0.533279	6.5854713	-6.640412	1.12E-10	1.22E-09	13.751707
MGP	0.3656582	6.0441954	6.6403191	1.12E-10	1.22E-09	13.751159
NOTCH3	0.2353773	6.24962	6.6388784	1.13E-10	1.23E-09	13.742678
FITM2	-0.153211	5.9285918	-6.637976	1.13E-10	1.23E-09	13.737365
RFTN2	0.3803671	4.7235407	6.6377414	1.13E-10	1.23E-09	13.735986
CXCR6	0.6346758	4.5897338	6.6368115	1.14E-10	1.24E-09	13.730513
LINC00654	0.823466	3.6955871	6.6355464	1.15E-10	1.25E-09	13.723069
CKM	1.189381	0.7642364	6.6351794	1.15E-10	1.25E-09	13.72091
TOLLIP-AS1	-0.341738	4.2725677	-6.635028	1.15E-10	1.25E-09	13.720019
KIF26A	0.6016927	4.4831907	6.6348587	1.15E-10	1.25E-09	13.719023
CTC-251I16	0.7325956	3.587881	6.6345021	1.16E-10	1.26E-09	13.716925
SULF1	0.6408281	5.4105105	6.6344278	1.16E-10	1.26E-09	13.716488

CES1	-0.356502	7.3758703	-6.634095	1.16E-10	1.26E-09	13.714531
RPN1	-0.088567	7.0933091	-6.633752	1.16E-10	1.26E-09	13.712512
ADAM33	0.915913	3.4730726	6.6333606	1.17E-10	1.26E-09	13.71021
RP11-796E2	0.9139995	2.7729027	6.6329324	1.17E-10	1.27E-09	13.707691
DDT	-0.19988	6.6621516	-6.632201	1.17E-10	1.27E-09	13.703392
TBXAS1	0.2515362	5.4190296	6.6319284	1.18E-10	1.27E-09	13.701787
MOSPD3	-0.15087	6.1323781	-6.631835	1.18E-10	1.27E-09	13.701239
C11orf31	-0.12115	6.5010927	-6.631197	1.18E-10	1.28E-09	13.697487
CAMTA1	-0.128324	6.1396596	-6.630634	1.18E-10	1.28E-09	13.694176
PYHIN1	0.678443	3.9374109	6.6276738	1.21E-10	1.30E-09	13.67677
FAM66B	1.2247696	0.3701355	6.626754	1.21E-10	1.31E-09	13.671364
OMD	1.5662862	1.8458762	6.625331	1.22E-10	1.32E-09	13.663001
RP11-268J1	0.6142426	3.7089753	6.6247372	1.23E-10	1.33E-09	13.659511
ITGB2-AS1	0.7425045	3.9084182	6.6247098	1.23E-10	1.33E-09	13.65935
NARF	-0.13225	6.3336119	-6.624487	1.23E-10	1.33E-09	13.658039
TRBV11-2	1.2336347	0.1631585	6.6244289	1.23E-10	1.33E-09	13.6577
OGFOD3	-0.125022	6.2591161	-6.624078	1.23E-10	1.33E-09	13.655638
ABHD6	-0.229001	6.165955	-6.623152	1.24E-10	1.34E-09	13.650196
LMNTD2	-0.217894	5.355437	-6.622121	1.25E-10	1.35E-09	13.644142
RP5-968J1.	0.9780946	-0.589384	6.6213137	1.25E-10	1.35E-09	13.639398
GLRX	-0.223796	6.3402297	-6.620668	1.26E-10	1.36E-09	13.635608
RP11-488L1	0.3091264	5.4018302	6.618941	1.27E-10	1.37E-09	13.625463
ALOX5AP	0.5392062	4.7812033	6.6187455	1.27E-10	1.37E-09	13.624315
RCAN2	0.3961129	5.2819896	6.6182173	1.28E-10	1.38E-09	13.621214
CFAP57	-1.003758	3.727091	-6.617955	1.28E-10	1.38E-09	13.619676
IL12B	1.1262629	-0.033059	6.6170877	1.29E-10	1.38E-09	13.614582
TEAD2	0.280101	5.8948496	6.6170792	1.29E-10	1.38E-09	13.614532
DDR2	0.2979571	5.4350921	6.6164151	1.29E-10	1.39E-09	13.610633
C10orf107	1.2258294	0.8116763	6.6157523	1.30E-10	1.39E-09	13.606742
SRD5A2	-0.888393	5.1864931	-6.614824	1.30E-10	1.40E-09	13.601291
SLCO3A1	0.2698987	5.6760068	6.6115117	1.33E-10	1.43E-09	13.581856
RNF175	1.0411955	2.6430908	6.6098298	1.34E-10	1.44E-09	13.571989
FAM83E	1.3727416	0.384913	6.6092297	1.35E-10	1.45E-09	13.56847
ARMC3	1.1855465	-0.308979	6.6091273	1.35E-10	1.45E-09	13.567869
C14orf132	0.8867126	4.7101742	6.609123	1.35E-10	1.45E-09	13.567844
RP11-96H19	-0.368357	4.5434904	-6.608895	1.35E-10	1.45E-09	13.566507
CAAP1	-0.14338	5.9644568	-6.607466	1.36E-10	1.46E-09	13.558124
ILDR1	1.5540213	3.0576841	6.6072087	1.37E-10	1.46E-09	13.556617
CLEC5A	1.3130036	2.5153025	6.6068284	1.37E-10	1.47E-09	13.554387
IFT74-AS1	-1.030768	-0.353448	-6.606408	1.37E-10	1.47E-09	13.551919
KCNQ4	0.7565955	3.4715506	6.6059295	1.38E-10	1.47E-09	13.549116
RP4-555D20	1.157189	-0.493973	6.60579	1.38E-10	1.48E-09	13.548298
DCTN4	-0.087418	6.4233634	-6.605041	1.38E-10	1.48E-09	13.543909
GABARAPL2	-0.102162	6.4234323	-6.604266	1.39E-10	1.49E-09	13.539362
METTL2A	-0.106756	5.8615691	-6.603772	1.39E-10	1.49E-09	13.536467
RP11-379H1	-0.172742	5.6902009	-6.603554	1.40E-10	1.49E-09	13.535189
LTA	0.9637089	2.8659829	6.6033658	1.40E-10	1.49E-09	13.534087
MAMLD1	0.4213778	5.2674609	6.6026251	1.40E-10	1.50E-09	13.529746
TRGV3	1.0574219	-0.105977	6.6005235	1.42E-10	1.52E-09	13.517431
CLIC2	0.2650589	5.3479335	6.6001915	1.42E-10	1.52E-09	13.515485
TXNDC15	-0.094959	6.334343	-6.599735	1.43E-10	1.53E-09	13.512813

MEN1	-0.089908	6.3086589	-6.59973	1.43E-10	1.53E-09	13.512779
SSTR5	1.7960072	0.5549179	6.5996411	1.43E-10	1.53E-09	13.51226
OLFM1	0.6988818	4.333356	6.599036	1.43E-10	1.53E-09	13.508716
COMMD9	-0.108739	6.2832058	-6.598574	1.44E-10	1.54E-09	13.50601
CEACAMP10	-1.284174	-0.284187	-6.598379	1.44E-10	1.54E-09	13.504865
HIPK1-AS1	-0.773001	2.7431451	-6.597532	1.45E-10	1.54E-09	13.499908
CYSTM1	-0.143836	6.5447504	-6.597479	1.45E-10	1.54E-09	13.499596
UPK3A	1.7803056	2.8859674	6.5973428	1.45E-10	1.54E-09	13.498797
ULBP1	1.3625769	1.7702277	6.5971421	1.45E-10	1.55E-09	13.497622
RBM10	-0.079897	6.46416	-6.593913	1.48E-10	1.58E-09	13.478714
OTOGL	1.2275601	0.584433	6.5933676	1.48E-10	1.58E-09	13.47552
PTPRZ1	1.4105738	1.1995642	6.592677	1.49E-10	1.59E-09	13.471477
RPL29	-0.13492	7.1097142	-6.592022	1.50E-10	1.59E-09	13.467641
CTC-338M12	-0.573837	3.900156	-6.59158	1.50E-10	1.60E-09	13.465054
ADAMTS14	0.8043832	3.8679336	6.5914512	1.50E-10	1.60E-09	13.464301
ZFAND2B	-0.13918	6.3389594	-6.590934	1.51E-10	1.60E-09	13.461277
SLC37A2	0.2850952	5.2761821	6.5901597	1.51E-10	1.61E-09	13.456743
CTA-126B4.	0.9879899	-0.600111	6.590079	1.51E-10	1.61E-09	13.456271
PAQR8	0.3594625	5.1902918	6.5889397	1.52E-10	1.62E-09	13.449604
MS4A4E	0.9750194	2.2204874	6.5882349	1.53E-10	1.63E-09	13.44548
CEP290	0.1797038	5.5467207	6.5867028	1.54E-10	1.64E-09	13.436517
FAM155B	1.5540318	3.2226847	6.5842663	1.57E-10	1.66E-09	13.422266
AP001626.1	1.4648895	2.9118558	6.5840534	1.57E-10	1.67E-09	13.421021
CCBL1	-0.174824	5.8868903	-6.583987	1.57E-10	1.67E-09	13.420633
MIR600HG	0.3748614	4.7154413	6.5833596	1.58E-10	1.67E-09	13.416964
COX8A	-0.121477	6.7599093	-6.582401	1.59E-10	1.68E-09	13.41136
SDK2	1.1952974	3.3618182	6.5816567	1.59E-10	1.69E-09	13.407008
GPC2	0.9135549	2.8176275	6.5811721	1.60E-10	1.69E-09	13.404175
KIF20B	0.2412056	5.3416246	6.5810003	1.60E-10	1.69E-09	13.403171
NAGS	-0.481147	5.9780318	-6.580526	1.60E-10	1.70E-09	13.4004
SLAMF6	0.5447843	4.4260286	6.5800959	1.61E-10	1.70E-09	13.397884
PKIG	-0.119526	6.1981512	-6.579609	1.61E-10	1.71E-09	13.395038
PCBP2	-0.07752	6.9599555	-6.579243	1.62E-10	1.71E-09	13.392899
KIT	0.6443602	4.3889586	6.578604	1.62E-10	1.72E-09	13.389165
SPOCK2	0.245924	5.8109586	6.5785445	1.62E-10	1.72E-09	13.388817
AGAP3	-0.109698	6.4985854	-6.578471	1.62E-10	1.72E-09	13.388388
NADK2	-0.182745	6.812592	-6.578445	1.62E-10	1.72E-09	13.388235
RP4-583P15	-0.964368	3.1056106	-6.578187	1.63E-10	1.72E-09	13.386726
CLDN18	1.4950183	1.0351612	6.5765152	1.64E-10	1.74E-09	13.37696
PLEKHO2	0.1626863	6.0085455	6.5753764	1.65E-10	1.75E-09	13.370306
RER1	-0.111782	6.686204	-6.57472	1.66E-10	1.75E-09	13.366474
SNAP25	1.1761334	4.1251666	6.5745165	1.66E-10	1.76E-09	13.365283
VAMP5	-0.183495	6.4192708	-6.574098	1.67E-10	1.76E-09	13.362836
SAMD9L	0.3038697	5.4435686	6.5737738	1.67E-10	1.76E-09	13.360946
LINC01547	-0.236102	4.9704161	-6.5735	1.67E-10	1.76E-09	13.359348
FAM195A	-0.170613	6.4731683	-6.573027	1.68E-10	1.77E-09	13.356585
ATP6V0E1	-0.124367	6.7977052	-6.572271	1.69E-10	1.78E-09	13.352171
CTD-2619J1	-0.614902	4.1396197	-6.572158	1.69E-10	1.78E-09	13.351507
LA16c-358E	0.5909171	4.0360407	6.571202	1.70E-10	1.79E-09	13.345927
BCL9L	0.2812734	5.9165728	6.5710909	1.70E-10	1.79E-09	13.345278
NDUFA1	-0.146662	6.7336576	-6.570926	1.70E-10	1.79E-09	13.344318

HNRNPA1L2	0.2723803	4.6317933	6.5701935	1.71E-10	1.80E-09	13.340039
NDUFB11	-0.142457	6.6919512	-6.569817	1.71E-10	1.80E-09	13.337842
MORN2	-0.172564	5.5918041	-6.568908	1.72E-10	1.81E-09	13.332532
MROH5	0.9876879	-0.546226	6.5677206	1.73E-10	1.82E-09	13.325604
PLXND1	0.1280843	6.5490204	6.5669828	1.74E-10	1.83E-09	13.321298
AKT3	0.2824494	5.4684041	6.5663837	1.75E-10	1.84E-09	13.317802
TAT	-0.571908	6.8139948	-6.566007	1.75E-10	1.84E-09	13.315605
GPRC5A	1.1390145	3.1928226	6.5651463	1.76E-10	1.85E-09	13.310582
LUZP1	0.1299778	5.7917809	6.5649025	1.76E-10	1.85E-09	13.309159
ZNF677	0.4721146	4.2997444	6.5646935	1.76E-10	1.85E-09	13.30794
ZNF879	0.7021473	3.9249821	6.5640957	1.77E-10	1.86E-09	13.304452
MRPL51	-0.127659	6.5994258	-6.564081	1.77E-10	1.86E-09	13.304368
LINC01480	1.2564097	2.4790096	6.5639029	1.77E-10	1.86E-09	13.303327
EIF2B5	-0.076938	6.3668412	-6.563615	1.78E-10	1.86E-09	13.301647
IFFO2	0.2003319	5.7495134	6.5621592	1.79E-10	1.88E-09	13.293157
TBC1D12	0.1612962	5.530874	6.5617411	1.80E-10	1.88E-09	13.290718
PIGQ	-0.127173	6.2976881	-6.560519	1.81E-10	1.89E-09	13.283591
CASP5	1.1726158	1.0052629	6.5596359	1.82E-10	1.90E-09	13.278442
CAPG	0.3265295	6.003976	6.55957	1.82E-10	1.90E-09	13.278057
HSD17B11	-0.155438	6.7959853	-6.558955	1.83E-10	1.91E-09	13.274474
BARX2	1.3520797	0.3486017	6.5589382	1.83E-10	1.91E-09	13.274374
AC019206.1	0.8656566	-0.458289	6.5584359	1.83E-10	1.92E-09	13.271445
PLA2G7	0.3647918	5.247745	6.5582882	1.83E-10	1.92E-09	13.270584
ARRDC1-AS1	-0.136543	5.634929	-6.557936	1.84E-10	1.92E-09	13.268529
IFT27	-0.153868	6.0765134	-6.557428	1.84E-10	1.93E-09	13.26557
GALK1	-0.197809	6.6557936	-6.556288	1.86E-10	1.94E-09	13.258926
ASCC2	-0.097757	6.5645133	-6.556242	1.86E-10	1.94E-09	13.258656
GZMK	0.7168855	4.2137018	6.5557406	1.86E-10	1.94E-09	13.255735
M1AP	1.0647181	1.8680515	6.5554684	1.86E-10	1.95E-09	13.254148
RP11-395B7	1.2477777	0.4329612	6.5553193	1.87E-10	1.95E-09	13.253279
IGFBP6	0.3960832	4.8720926	6.5545485	1.87E-10	1.96E-09	13.248788
NR1H3	-0.128125	6.4868862	-6.55402	1.88E-10	1.96E-09	13.245708
TRGV10	1.1797001	1.1908745	6.5537012	1.88E-10	1.97E-09	13.243851
IRAK3	0.4051208	4.7818438	6.5532934	1.89E-10	1.97E-09	13.241474
CTD-2334D1	1.0916019	-0.071254	6.5531229	1.89E-10	1.97E-09	13.240481
MIR8071-2	1.1359077	-0.450657	6.5494181	1.93E-10	2.01E-09	13.218901
HSD17B7	-0.200597	5.9964211	-6.549107	1.94E-10	2.02E-09	13.217087
ARSA	-0.161924	6.4988639	-6.548667	1.94E-10	2.02E-09	13.214529
RP11-169D4	1.115734	0.8836098	6.5484506	1.94E-10	2.02E-09	13.213268
PTPN2	0.1146568	5.9985349	6.546985	1.96E-10	2.04E-09	13.204734
SYVN1	-0.114896	6.8020515	-6.546833	1.96E-10	2.04E-09	13.20385
SHMT2	-0.121253	6.8739865	-6.545959	1.97E-10	2.05E-09	13.198762
MRVI1	0.351655	5.1829316	6.5454478	1.98E-10	2.06E-09	13.195786
RMND5B	-0.100584	6.2620696	-6.545172	1.98E-10	2.06E-09	13.194178
SYNJ2	0.1819481	5.9996222	6.5447883	1.99E-10	2.07E-09	13.191947
CTC-523E23	1.0767061	1.5485477	6.5444525	1.99E-10	2.07E-09	13.189993
CERS4	-0.204038	6.501711	-6.543601	2.00E-10	2.08E-09	13.185038
OXCT1	0.5053146	4.7837294	6.5435986	2.00E-10	2.08E-09	13.185023
CELF1	-0.066409	6.6489092	-6.543444	2.00E-10	2.08E-09	13.184121
GTPBP6	-0.115553	6.4197779	-6.543243	2.01E-10	2.08E-09	13.182952
AP006216.5	-1.349048	2.5431588	-6.540973	2.03E-10	2.11E-09	13.169748

C1QTNF3	-0.473182	5.2028676	-6.540286	2.04E-10	2.12E-09	13.165748
IRS2	-0.217592	6.5276551	-6.540239	2.04E-10	2.12E-09	13.165473
IGFBP4	-0.153133	7.2844491	-6.539292	2.05E-10	2.13E-09	13.159966
C1R	-0.184224	7.3126638	-6.537347	2.08E-10	2.15E-09	13.148657
ZNF813	0.670462	4.7221353	6.5373444	2.08E-10	2.15E-09	13.148641
BHLHA15	1.094409	3.6801261	6.5371218	2.08E-10	2.16E-09	13.147347
HRASLS5	1.3198814	0.31066	6.536926	2.08E-10	2.16E-09	13.146208
RP11-418J1	-1.340472	2.5858594	-6.536823	2.08E-10	2.16E-09	13.145612
MTIF3	-0.124258	6.2600123	-6.536478	2.09E-10	2.16E-09	13.143602
EIF3G	-0.116037	6.745958	-6.536032	2.09E-10	2.17E-09	13.141012
DQX1	1.7259144	1.6897142	6.5348599	2.11E-10	2.18E-09	13.134196
TAF10	-0.127031	6.4842805	-6.534007	2.12E-10	2.19E-09	13.129239
ZNF665	0.9252185	3.254397	6.5337749	2.12E-10	2.20E-09	13.127889
OS9	-0.094481	7.057923	-6.532601	2.14E-10	2.21E-09	13.121065
CPT2	-0.173347	6.4185214	-6.532303	2.14E-10	2.21E-09	13.119336
TRBV27	1.0837968	-0.446216	6.5305326	2.16E-10	2.24E-09	13.109047
PLB1	0.4158272	4.2798924	6.5292185	2.18E-10	2.25E-09	13.101412
C1orf116	1.0501527	4.0030896	6.5290817	2.18E-10	2.25E-09	13.100617
CTD-3099C6	1.0470013	2.5751276	6.5284238	2.19E-10	2.26E-09	13.096796
PITRM1-AS1	0.9912506	2.1719018	6.5282495	2.19E-10	2.26E-09	13.095783
RP11-666F1	-1.057885	2.3086981	-6.527985	2.20E-10	2.27E-09	13.094244
ULBP3	1.2947844	0.8266126	6.5279118	2.20E-10	2.27E-09	13.093822
RP3-523E19	1.1277736	0.1382281	6.5257696	2.23E-10	2.30E-09	13.081381
POLR2E	-0.107218	6.8289044	-6.525641	2.23E-10	2.30E-09	13.080634
SPIRE1	0.6016649	4.8048977	6.5254904	2.23E-10	2.30E-09	13.079759
RCL1	-0.225615	6.1197355	-6.52533	2.23E-10	2.30E-09	13.078827
AC098614.2	0.899	2.9151159	6.5216318	2.28E-10	2.35E-09	13.057359
CTXN1	0.9820211	3.0806814	6.5210275	2.29E-10	2.36E-09	13.053852
PRMT7	-0.113129	6.1062896	-6.519007	2.32E-10	2.39E-09	13.04213
LILRB4	0.3560041	5.2370509	6.5185963	2.33E-10	2.39E-09	13.039744
ATP5G1	-0.150233	6.6742933	-6.516268	2.36E-10	2.43E-09	13.026239
SP140	0.5011128	4.5283998	6.5159578	2.36E-10	2.43E-09	13.024439
MIS18BP1	0.207115	5.4811065	6.5156513	2.37E-10	2.43E-09	13.022661
ALDH1L1-AS	-1.083544	4.051088	-6.51493	2.38E-10	2.44E-09	13.018476
RP11-408A1	0.907322	2.2167938	6.5139947	2.39E-10	2.46E-09	13.013054
LINC00641	0.3092556	4.7628382	6.5131862	2.40E-10	2.47E-09	13.008366
KRT6B	1.5556567	0.3786782	6.5129627	2.40E-10	2.47E-09	13.00707
CFB	-0.24158	7.0186044	-6.512364	2.41E-10	2.48E-09	13.003601
HIVEP3	0.297628	5.1432482	6.5113026	2.43E-10	2.49E-09	12.997446
MT-ND2	-0.136781	7.7410679	-6.510092	2.45E-10	2.51E-09	12.990429
LGALS9	0.2192828	6.078047	6.5100528	2.45E-10	2.51E-09	12.990202
GMPPA	-0.110289	6.4922654	-6.50843	2.47E-10	2.53E-09	12.980799
RUFY4	1.1129325	2.5399192	6.5077911	2.48E-10	2.54E-09	12.977095
DUSP23	-0.173859	6.5814898	-6.507784	2.48E-10	2.54E-09	12.977053
DPT	1.3582867	3.8921641	6.5071182	2.49E-10	2.55E-09	12.973197
EOMES	0.7247634	3.7736044	6.5068717	2.49E-10	2.55E-09	12.971768
MCEMP1	1.2985214	0.5290297	6.5066151	2.50E-10	2.56E-09	12.970282
THBD	0.3022844	5.5541891	6.5055741	2.51E-10	2.57E-09	12.964252
DDX56	-0.102405	6.5422525	-6.505443	2.52E-10	2.57E-09	12.96349
SEPSECS-AS	-0.255297	4.9799378	-6.504964	2.52E-10	2.58E-09	12.960719
LILRA1	0.7116219	3.520651	6.5047438	2.53E-10	2.58E-09	12.959442

TNFRSF18	0.6997778	4.1815167	6.5038874	2.54E-10	2.60E-09	12.954482
ZNF593	-0.246334	5.2950199	-6.503427	2.55E-10	2.60E-09	12.951814
RARG	0.2233341	5.3796651	6.5023188	2.56E-10	2.62E-09	12.945399
PCDH9	1.0360057	3.4776911	6.5020715	2.57E-10	2.62E-09	12.943967
HTR7	1.1002198	2.0111281	6.4988571	2.62E-10	2.67E-09	12.925359
PHACTR1	0.3138199	4.7035409	6.4983614	2.62E-10	2.68E-09	12.922489
LINC01588	-0.35721	4.8419709	-6.498119	2.63E-10	2.68E-09	12.921089
ZBTB34	0.1591954	5.4957172	6.4976212	2.64E-10	2.69E-09	12.918205
DAO	-0.651322	6.0607899	-6.496641	2.65E-10	2.70E-09	12.912531
PLIN5	-0.331456	6.2810294	-6.496446	2.65E-10	2.71E-09	12.911402
PDLIM7	0.1715376	6.0057757	6.4964352	2.65E-10	2.71E-09	12.911343
USP30-AS1	-0.511058	4.8843514	-6.495704	2.67E-10	2.72E-09	12.907109
GOLGA6D	-0.773627	-1.055304	-6.493329	2.70E-10	2.76E-09	12.893371
COQ9	-0.144353	6.4510178	-6.493225	2.71E-10	2.76E-09	12.892773
SMG6	0.2970151	5.5003669	6.4929764	2.71E-10	2.76E-09	12.891333
SLC25A1	-0.134473	6.8801273	-6.491319	2.74E-10	2.79E-09	12.88175
BTF3	-0.105512	6.9735935	-6.490897	2.74E-10	2.79E-09	12.879306
RP11-524C2	0.8783271	-0.782144	6.490376	2.75E-10	2.80E-09	12.876296
PROS1	-0.184526	6.79215	-6.489205	2.77E-10	2.82E-09	12.869523
FBXO33	-0.119502	5.6952819	-6.488925	2.78E-10	2.82E-09	12.867907
AC106786.1	1.209617	0.1627664	6.4889075	2.78E-10	2.82E-09	12.867806
IL6R	-0.200596	6.6494628	-6.488395	2.78E-10	2.83E-09	12.864841
NR3C1	-0.117882	6.4570839	-6.487412	2.80E-10	2.85E-09	12.859161
ITIH3	-0.326828	7.3727933	-6.487234	2.80E-10	2.85E-09	12.858135
PRDM16	1.137494	3.6703191	6.4863246	2.82E-10	2.86E-09	12.852877
SLC10A1	-0.847527	6.0806754	-6.485428	2.83E-10	2.88E-09	12.847694
QARS	-0.10068	6.7943638	-6.483757	2.86E-10	2.91E-09	12.838038
RP11-355F1	1.1237236	1.2463049	6.4828976	2.88E-10	2.92E-09	12.833077
ZNF114	0.9398211	1.762477	6.4827692	2.88E-10	2.92E-09	12.832335
NBPF19	0.3077162	5.1843267	6.482425	2.89E-10	2.93E-09	12.830346
RP11-848P1	0.5898732	3.2897904	6.4820188	2.89E-10	2.93E-09	12.828
SMOC1	-0.252624	6.5235543	-6.480816	2.91E-10	2.95E-09	12.821054
STAC	1.2875246	2.4862175	6.480175	2.92E-10	2.96E-09	12.817351
NDP	1.0134881	-0.650017	6.4798292	2.93E-10	2.97E-09	12.815355
MIR589	0.9870675	-0.225755	6.4791131	2.94E-10	2.98E-09	12.81122
CCNI2	1.241559	1.9263912	6.4787653	2.95E-10	2.99E-09	12.809212
CTB-113I20	-0.414078	4.058314	-6.478481	2.95E-10	2.99E-09	12.807571
SCYL1	-0.093377	6.6462905	-6.478056	2.96E-10	3.00E-09	12.805115
SLCO4C1	1.5993975	3.2638784	6.477614	2.97E-10	3.00E-09	12.802565
SUPT20H	0.1138171	5.8912507	6.4763305	2.99E-10	3.03E-09	12.795156
ZCCHC6	-0.167645	6.2208546	-6.476119	3.00E-10	3.03E-09	12.793934
RP5-1021I2	-0.362175	4.4884658	-6.475666	3.00E-10	3.04E-09	12.791319
BMP8A	1.0671142	2.1267435	6.4747094	3.02E-10	3.05E-09	12.7858
TMEM179	1.6000362	0.3929664	6.4736427	3.04E-10	3.07E-09	12.779645
GPRC5D	0.9186587	2.4988211	6.4727061	3.06E-10	3.09E-09	12.774241
LINC01550	1.3256768	1.4994019	6.472139	3.07E-10	3.10E-09	12.770969
AC046143.3	0.6412388	2.8528314	6.4719862	3.07E-10	3.10E-09	12.770087
CYP4V2	-0.19633	6.5303985	-6.471952	3.07E-10	3.10E-09	12.769893
CTSO	-0.193891	6.3864387	-6.47077	3.09E-10	3.12E-09	12.763074
IGKV1D-13	1.4370626	0.0020672	6.4701001	3.10E-10	3.13E-09	12.759208
RECK	0.3409171	5.2636039	6.4696897	3.11E-10	3.14E-09	12.756841

CTD-3064M3	1.091393	2.9309626	6.4694673	3.12E-10	3.14E-09	12.755559
APOE	-0.183071	7.8010627	-6.468956	3.13E-10	3.15E-09	12.75261
GPR155	-0.208028	5.8069615	-6.468454	3.14E-10	3.16E-09	12.749713
SUGP1	-0.086411	6.1670399	-6.465581	3.19E-10	3.21E-09	12.733149
RGS17	0.8147045	2.9735101	6.4627571	3.24E-10	3.27E-09	12.716876
CD300C	0.7218059	3.6918286	6.4624263	3.25E-10	3.27E-09	12.71497
CHADL	-0.451239	5.0651144	-6.462422	3.25E-10	3.27E-09	12.714945
MRPL43	-0.108244	6.4597558	-6.461106	3.28E-10	3.30E-09	12.70736
HAS2-AS1	1.1817353	-0.13202	6.4609636	3.28E-10	3.30E-09	12.706542
EPB41L4A	0.5438021	4.4450768	6.4606613	3.28E-10	3.30E-09	12.704801
CRTAM	0.8803912	3.0676225	6.460483	3.29E-10	3.30E-09	12.703774
NAV2-AS5	-0.893047	-0.71171	-6.459777	3.30E-10	3.32E-09	12.699705
CYP2T1P	0.8636584	2.8446547	6.4594998	3.31E-10	3.32E-09	12.69811
PACS1	0.1195942	6.3165544	6.4578026	3.34E-10	3.36E-09	12.688335
SFXN2	-0.206221	6.0014336	-6.453342	3.43E-10	3.44E-09	12.662654
MRPS16	-0.099578	6.5833471	-6.452065	3.46E-10	3.47E-09	12.655303
AP000344.3	1.183961	-0.409083	6.4519625	3.46E-10	3.47E-09	12.654715
ASCL2	0.9742911	3.0869123	6.4517772	3.46E-10	3.47E-09	12.653648
RP11-345J1	1.2338798	0.9192969	6.4515706	3.47E-10	3.48E-09	12.65246
OSCAR	0.4149832	4.3799881	6.4508788	3.48E-10	3.49E-09	12.648479
ANKDD1B	0.9567376	2.4942449	6.4499346	3.50E-10	3.51E-09	12.643047
FBXL7	0.3200713	5.2655462	6.4498726	3.50E-10	3.51E-09	12.64269
AP001331.1	-1.399283	-0.160597	-6.449582	3.51E-10	3.51E-09	12.64102
OR7E29P	-1.350326	0.6132989	-6.449404	3.51E-10	3.52E-09	12.639994
AC068580.5	1.0130222	2.2300533	6.4492396	3.51E-10	3.52E-09	12.639048
LINC00506	1.2240344	0.4659964	6.4488115	3.52E-10	3.53E-09	12.636586
LSP1	0.2475504	5.9625219	6.4485966	3.53E-10	3.53E-09	12.63535
RP11-16E12	-0.356684	4.1548821	-6.448144	3.54E-10	3.54E-09	12.632746
RP11-403I1	-1.509605	3.817177	-6.447996	3.54E-10	3.54E-09	12.631895
PEAK1	0.1352142	5.9331246	6.4479675	3.54E-10	3.54E-09	12.631731
KLF15	-0.31007	6.5131601	-6.447837	3.54E-10	3.54E-09	12.630978
HOXD1	1.3882479	0.5393978	6.4466769	3.57E-10	3.57E-09	12.624308
ZCRB1	-0.085408	6.2750462	-6.446196	3.58E-10	3.58E-09	12.62154
CNDP2	-0.115432	6.8746031	-6.446065	3.58E-10	3.58E-09	12.620787
UQCC2	-0.151778	6.3278822	-6.445937	3.58E-10	3.58E-09	12.620051
NME8	1.1953681	1.227301	6.4458085	3.59E-10	3.58E-09	12.619314
FAM95C	1.6110758	1.2437338	6.4457131	3.59E-10	3.58E-09	12.618765
IL27	-0.594467	5.0789711	-6.444844	3.61E-10	3.60E-09	12.613767
RP11-449D8	0.8908562	-0.627694	6.4439876	3.63E-10	3.62E-09	12.608845
MS4A6A	0.253285	5.8916273	6.4438863	3.63E-10	3.62E-09	12.608262
DMBX1	1.4565924	0.2774095	6.4438447	3.63E-10	3.62E-09	12.608023
C15orf56	1.1144547	-0.336658	6.4432184	3.64E-10	3.63E-09	12.604423
ARRDC5	1.0788013	1.2050094	6.4426898	3.65E-10	3.64E-09	12.601384
PIFO	1.3512085	1.8631811	6.4423913	3.66E-10	3.65E-09	12.599669
HPD	-0.593126	6.9366495	-6.442379	3.66E-10	3.65E-09	12.599598
ATG10	-0.144111	5.5592811	-6.442311	3.66E-10	3.65E-09	12.599209
WDR66	0.444984	3.9206017	6.44136	3.68E-10	3.67E-09	12.593741
PINLYP	0.5770031	3.4862571	6.4413004	3.68E-10	3.67E-09	12.593398
DHX30	-0.073006	6.5350822	-6.441042	3.69E-10	3.67E-09	12.591911
KRTAP10-6	-0.726348	-1.139263	-6.440536	3.70E-10	3.68E-09	12.589003
RP11-467L1	-0.289355	4.8301717	-6.439849	3.72E-10	3.69E-09	12.585058

ANKRD20A5F	1.1463406	-0.061477	6.4398202	3.72E-10	3.69E-09	12.584892
TCN1	1.4617802	0.1966507	6.4393536	3.73E-10	3.70E-09	12.582211
MRPL22	-0.125068	6.1766308	-6.439304	3.73E-10	3.70E-09	12.581929
ZC2HC1A	0.4724466	4.7047803	6.4389565	3.74E-10	3.71E-09	12.57993
FCGR2C	0.9388188	3.6363524	6.4388404	3.74E-10	3.71E-09	12.579262
RPS14	-0.130939	7.1974336	-6.438764	3.74E-10	3.71E-09	12.578822
RP11-686D2	0.9912952	2.6030062	6.4385778	3.74E-10	3.72E-09	12.577754
ATP10A	0.6352963	4.5731993	6.4384307	3.75E-10	3.72E-09	12.576909
MT-CYB	-0.137004	7.8211409	-6.437806	3.76E-10	3.73E-09	12.573321
RP11-686D2	1.1761754	0.3151414	6.4375878	3.77E-10	3.73E-09	12.572067
AP000997.1	-1.20505	0.0625346	-6.437545	3.77E-10	3.73E-09	12.571821
FOXRED1	-0.126346	6.3821117	-6.437543	3.77E-10	3.73E-09	12.571809
MTCH2	-0.101042	6.7879288	-6.435642	3.81E-10	3.77E-09	12.560888
EIF2AK1	-0.083592	6.8571572	-6.434805	3.83E-10	3.79E-09	12.556082
PBXIP1	-0.1105	6.7307144	-6.434733	3.83E-10	3.79E-09	12.555671
CTD-2014N1	-0.507292	-1.310077	-6.434326	3.84E-10	3.80E-09	12.553331
CCDC47	-0.095951	6.7370349	-6.43426	3.84E-10	3.80E-09	12.552952
CNTFR	1.5437489	3.7076733	6.4331995	3.86E-10	3.82E-09	12.546865
LRP1	-0.130075	7.1688627	-6.43293	3.87E-10	3.83E-09	12.545316
TRPT1	-0.138662	6.1148459	-6.431507	3.90E-10	3.86E-09	12.537149
CTD-2012J1	1.0225227	2.171836	6.4315049	3.90E-10	3.86E-09	12.537136
CTD-2130F2	-0.71403	-1.126854	-6.431366	3.91E-10	3.86E-09	12.536338
FMO1	1.0747589	3.9673683	6.4310372	3.91E-10	3.87E-09	12.534452
SDF2	-0.096539	6.2924628	-6.430801	3.92E-10	3.87E-09	12.533096
TXNDC11	-0.106596	6.5748108	-6.430308	3.93E-10	3.88E-09	12.530266
GTF2IRD2B	-0.214624	5.1466993	-6.430194	3.93E-10	3.88E-09	12.52961
RP5-1092L1	-0.676134	-1.101329	-6.430171	3.93E-10	3.88E-09	12.529483
RP11-94C24	-0.637395	3.9566004	-6.429637	3.95E-10	3.89E-09	12.526418
RP11-38902	-0.994414	2.6124156	-6.429608	3.95E-10	3.89E-09	12.52625
GTF3C5	-0.107996	6.3612116	-6.429568	3.95E-10	3.89E-09	12.526021
TRBV12-4	1.1656895	0.2216636	6.4293165	3.95E-10	3.90E-09	12.524576
FLJ27354	0.8681168	2.2066486	6.4289456	3.96E-10	3.91E-09	12.522448
SLC37A4	-0.164025	6.7478444	-6.428898	3.96E-10	3.91E-09	12.522173
MFGE8	0.2092834	6.0535617	6.4285928	3.97E-10	3.91E-09	12.520423
ST6GALNAC1	1.2381389	1.6729653	6.4280984	3.98E-10	3.92E-09	12.517587
LRRC8C	0.2055067	5.3932508	6.428072	3.98E-10	3.92E-09	12.517435
IGKV1OR2-6	1.0170387	-0.738432	6.4266657	4.02E-10	3.95E-09	12.509367
SFRP1	1.1900606	3.1602352	6.4254007	4.05E-10	3.98E-09	12.502111
CHCHD2	-0.101594	6.8482966	-6.424799	4.06E-10	4.00E-09	12.498662
ABCC4	0.5100479	5.4577947	6.4245366	4.07E-10	4.00E-09	12.497154
MLST8	-0.10873	6.3113979	-6.424425	4.07E-10	4.00E-09	12.496517
NDUFA8	-0.130469	6.3523167	-6.424387	4.07E-10	4.00E-09	12.496295
CENPB	-0.096695	6.6123512	-6.424102	4.08E-10	4.01E-09	12.494663
RGN	-0.311782	6.6485758	-6.422674	4.11E-10	4.04E-09	12.486474
RP11-78J21	1.0625388	1.3870608	6.4224394	4.12E-10	4.05E-09	12.485128
ZNF701	0.5658389	4.4980181	6.4219305	4.13E-10	4.06E-09	12.48221
CLDN12	-0.12598	6.4258107	-6.420246	4.17E-10	4.10E-09	12.472556
TMEM179B	-0.100681	6.3189384	-6.420185	4.17E-10	4.10E-09	12.472202
GGN	1.010469	2.0800063	6.4188418	4.21E-10	4.13E-09	12.464506
ANXA1	0.1949936	6.0382027	6.4187962	4.21E-10	4.13E-09	12.464244
DIS3L2	-0.080534	6.0598648	-6.418548	4.21E-10	4.13E-09	12.46282

TPPP3	0.3593794	5.2095983	6.4178226	4.23E-10	4.15E-09	12.458665
NRARP	0.3308814	4.9762736	6.4173585	4.24E-10	4.16E-09	12.456005
SNX17	-0.078464	6.6909194	-6.417338	4.24E-10	4.16E-09	12.455886
PYCRL	-0.172781	6.159494	-6.415623	4.29E-10	4.20E-09	12.446061
CAPN13	1.743824	1.4286338	6.4146806	4.31E-10	4.22E-09	12.440663
TMEM256	-0.186905	6.208518	-6.414452	4.32E-10	4.23E-09	12.439351
CALB2	1.3276417	0.4943698	6.4137409	4.34E-10	4.24E-09	12.435281
POLR2I	-0.154418	6.10344	-6.413354	4.35E-10	4.25E-09	12.433065
DLGAP1	1.1262255	2.0691899	6.4127845	4.36E-10	4.27E-09	12.429803
SLC25A42	-0.215247	6.4780185	-6.412752	4.36E-10	4.27E-09	12.429618
ZBTB48	-0.120676	5.992819	-6.412438	4.37E-10	4.27E-09	12.427817
RAVER2	0.4265522	5.2007029	6.4121841	4.38E-10	4.28E-09	12.426365
ETFA	-0.12613	6.8470575	-6.411997	4.38E-10	4.28E-09	12.425295
IGHV3OR15-	0.9889758	-0.761469	6.411751	4.39E-10	4.29E-09	12.423885
IGFN1	1.4848447	1.1320125	6.4103236	4.42E-10	4.32E-09	12.415712
MRPL52	-0.148224	6.3169959	-6.409979	4.43E-10	4.33E-09	12.413739
DMGDH	-0.387244	6.3935978	-6.409456	4.45E-10	4.34E-09	12.410743
ETFB	-0.179014	7.0153721	-6.408752	4.47E-10	4.36E-09	12.406718
APOBEC3D	0.5047272	4.4836948	6.4085165	4.47E-10	4.36E-09	12.405368
PPP2R2C	1.7976348	2.234406	6.4076778	4.49E-10	4.38E-09	12.400567
LRRC49	0.5782543	3.6453552	6.4069671	4.51E-10	4.40E-09	12.3965
TPI1	-0.104652	7.0783224	-6.406633	4.52E-10	4.41E-09	12.39459
CCRL2	0.3104773	4.6678139	6.4058959	4.54E-10	4.43E-09	12.39037
ZNHIT2	-0.151831	5.8221025	-6.404091	4.59E-10	4.47E-09	12.380042
PDGFRL	0.4827675	4.3842777	6.4040526	4.59E-10	4.47E-09	12.379824
PFDN5	-0.117629	6.8531947	-6.403038	4.62E-10	4.50E-09	12.374021
TMEM156	0.7015082	4.4884181	6.4025117	4.63E-10	4.51E-09	12.37101
TEP1	0.1180008	5.9159634	6.400825	4.68E-10	4.55E-09	12.361364
CTGF	0.2324112	6.4000489	6.4000054	4.70E-10	4.58E-09	12.356678
LINC00092	1.120114	1.9864381	6.3997804	4.71E-10	4.58E-09	12.355391
GLYATL1P3	-1.066183	-0.559538	-6.39836	4.75E-10	4.62E-09	12.347271
ROCK1P1	0.9160483	2.818493	6.3974181	4.77E-10	4.64E-09	12.341886
PANX1	-0.174785	5.933073	-6.396862	4.79E-10	4.66E-09	12.338707
RASSF3	0.2129236	6.0484237	6.3961853	4.81E-10	4.67E-09	12.334841
TRAPPC2L	-0.142335	6.2383322	-6.395447	4.83E-10	4.69E-09	12.330619
NFIB	-0.161919	6.333904	-6.395113	4.84E-10	4.70E-09	12.328711
TM2D3	-0.115733	6.280313	-6.395005	4.84E-10	4.70E-09	12.328094
CRTAP	-0.121095	6.7007801	-6.394482	4.86E-10	4.72E-09	12.325106
NBPF26	0.8598199	3.7182677	6.3938756	4.87E-10	4.73E-09	12.321643
SLC6A14	1.4885463	0.1019411	6.3937634	4.88E-10	4.73E-09	12.321002
C19orf84	1.1267995	0.2001505	6.3933218	4.89E-10	4.74E-09	12.318479
LY86	0.3519063	4.9086295	6.3922189	4.92E-10	4.77E-09	12.312179
RAB30-AS1	-0.169874	5.4477385	-6.390796	4.96E-10	4.81E-09	12.304049
PPOX	-0.12838	6.0157262	-6.390781	4.96E-10	4.81E-09	12.303967
BCRP3	0.9170403	3.2120182	6.3899783	4.99E-10	4.83E-09	12.299381
ICK	-0.190779	6.1150032	-6.389231	5.01E-10	4.85E-09	12.295117
RP4-758J18	-0.134824	6.0678655	-6.387068	5.07E-10	4.91E-09	12.282763
B4GALT1-AS	-0.398701	4.3330798	-6.38691	5.08E-10	4.92E-09	12.281866
AC093162.5	0.9878513	3.25645	6.3867036	5.09E-10	4.92E-09	12.280685
ZMPSTE24	-0.08832	6.4803277	-6.386582	5.09E-10	4.92E-09	12.27999
C20orf195	1.1118776	0.0896722	6.3863465	5.10E-10	4.93E-09	12.278646

ZNF410	0.4475009	4.180628	6.3858891	5.11E-10	4.94E-09	12.276036
MAGOH	-0.100753	6.1437265	-6.384661	5.15E-10	4.98E-09	12.269025
PPCS	-0.09445	6.3562219	-6.38282	5.20E-10	5.03E-09	12.258524
PRKCSH	-0.089053	7.0913389	-6.382514	5.21E-10	5.04E-09	12.256775
IGHG3	0.9183073	5.3903109	6.3816855	5.24E-10	5.06E-09	12.25205
SAP18	-0.102718	6.6476155	-6.38143	5.25E-10	5.07E-09	12.250594
RNF145	0.1429149	6.0418731	6.3808426	5.26E-10	5.08E-09	12.247241
DNAJC17	-0.133601	5.9283386	-6.380734	5.27E-10	5.08E-09	12.246622
NENF	-0.163053	6.5272559	-6.380709	5.27E-10	5.08E-09	12.24648
RP11-49I11	-0.323964	4.7400468	-6.379376	5.31E-10	5.12E-09	12.238879
PDE3A	0.4218014	5.1436531	6.3784482	5.34E-10	5.15E-09	12.233585
TET3	0.1630691	5.8255779	6.3782455	5.34E-10	5.15E-09	12.23243
CYP4F11	-0.46823	6.4807718	-6.377336	5.37E-10	5.18E-09	12.227242
GIMAP5	0.5093663	4.2545018	6.3770819	5.38E-10	5.19E-09	12.225795
LRRFIP2	-0.092135	6.4175504	-6.376549	5.40E-10	5.20E-09	12.222759
PCDHB18P	1.1050624	1.5355639	6.3754525	5.43E-10	5.23E-09	12.216507
ADAL	-0.193357	5.5309959	-6.375372	5.44E-10	5.24E-09	12.21605
NTF3	1.0922474	2.8576062	6.3750348	5.45E-10	5.24E-09	12.214126
PSMD10P1	0.8418392	-0.583878	6.3749388	5.45E-10	5.25E-09	12.213578
GADD45GIP1	-0.167106	6.4244294	-6.374679	5.46E-10	5.25E-09	12.212095
MRPL36	-0.131994	6.1074284	-6.374637	5.46E-10	5.25E-09	12.211859
ABCF3	-0.076019	6.4490913	-6.374296	5.47E-10	5.26E-09	12.209917
SSBP3	-0.132134	6.4677209	-6.373938	5.48E-10	5.27E-09	12.207874
CRTC3	0.100811	6.0677518	6.3727173	5.52E-10	5.31E-09	12.200918
RBMS3	0.3346904	4.9807682	6.3717513	5.55E-10	5.34E-09	12.195414
ATP5I	-0.159292	6.6860093	-6.371127	5.57E-10	5.36E-09	12.191859
ZNF423	0.5727866	4.4735496	6.3710319	5.58E-10	5.36E-09	12.191315
SNAI3	0.4505971	3.9637046	6.3709267	5.58E-10	5.36E-09	12.190716
DOPEY1	0.2094581	5.5563039	6.3704564	5.60E-10	5.37E-09	12.188037
ZYG11B	-0.136266	6.2220392	-6.369598	5.62E-10	5.40E-09	12.183146
RP11-394B2	0.9869977	3.2750539	6.3683851	5.66E-10	5.43E-09	12.176239
MNX1-AS1	1.5878457	-0.001845	6.3681076	5.67E-10	5.44E-09	12.174658
ARL14	1.5461097	2.1659017	6.367704	5.69E-10	5.45E-09	12.17236
SLC44A2	0.1630724	6.226622	6.3674032	5.70E-10	5.46E-09	12.170647
CD248	0.2333612	5.7120336	6.3673965	5.70E-10	5.46E-09	12.170609
SHISA5	-0.097553	6.8343378	-6.366864	5.71E-10	5.48E-09	12.167576
GAP43	1.3801348	1.2384893	6.3655412	5.76E-10	5.52E-09	12.160046
FCGR1C	1.2276888	0.7665003	6.3655116	5.76E-10	5.52E-09	12.159877
RP11-505K9	-0.494638	4.307462	-6.364808	5.78E-10	5.54E-09	12.15587
SPTB	0.6511513	3.9872588	6.3645973	5.79E-10	5.54E-09	12.154672
SNHG24	1.166195	-0.605942	6.3643731	5.80E-10	5.55E-09	12.153396
LDLRAP1	-0.132457	6.2360396	-6.364287	5.80E-10	5.55E-09	12.152906
TRBV24-1	1.1101362	-0.097815	6.3641058	5.81E-10	5.56E-09	12.151874
D2HGDH	-0.138365	6.206136	-6.36356	5.83E-10	5.57E-09	12.148768
TMEM163	1.2502174	3.1599205	6.3631701	5.84E-10	5.58E-09	12.146549
CEACAM21	0.5889077	3.8135975	6.3630645	5.84E-10	5.59E-09	12.145948
RP11-115C1	-1.760215	3.0113365	-6.362226	5.87E-10	5.61E-09	12.141178
USE1	-0.153418	6.1622724	-6.361473	5.90E-10	5.63E-09	12.13689
FAM186B	-0.425097	3.8370357	-6.361196	5.91E-10	5.64E-09	12.135314
EMD	-0.11108	6.4250417	-6.360789	5.92E-10	5.65E-09	12.132999
TRAV16	1.0591051	-0.196286	6.3605543	5.93E-10	5.66E-09	12.131664

RP11-883G1	-1.237811	1.4455022	-6.360488	5.93E-10	5.66E-09	12.131284
RP11-320G2	1.1245987	0.1561799	6.360347	5.94E-10	5.66E-09	12.130484
RP11-44N21	1.3525682	2.7787623	6.3603002	5.94E-10	5.66E-09	12.130218
NHP2	-0.123491	6.3464292	-6.359188	5.98E-10	5.70E-09	12.123891
RP11-367G6	1.1543192	0.9439249	6.3590597	5.98E-10	5.70E-09	12.123161
RPP14	-0.098133	6.0435254	-6.358749	5.99E-10	5.71E-09	12.121393
ANKRD18EP	0.8643273	4.2245359	6.3586937	6.00E-10	5.71E-09	12.121079
NID2	0.7153209	5.0126143	6.357827	6.03E-10	5.74E-09	12.11615
MPG	-0.130092	6.0851174	-6.357753	6.03E-10	5.74E-09	12.115726
RPL38	-0.145223	7.0289718	-6.357715	6.03E-10	5.74E-09	12.11551
HADHA	-0.096623	7.0144004	-6.356819	6.06E-10	5.77E-09	12.110419
FPGS	-0.128895	6.4681201	-6.356786	6.06E-10	5.77E-09	12.110231
RP11-21L23	-0.63459	5.2758971	-6.355261	6.12E-10	5.82E-09	12.101558
RP11-295P9	1.0391048	1.2568396	6.3549426	6.13E-10	5.83E-09	12.099747
TRGC1	1.0078774	2.590022	6.3543442	6.15E-10	5.85E-09	12.096345
HDGF	-0.089986	7.090243	-6.35432	6.15E-10	5.85E-09	12.096209
AP1S1	-0.116886	6.4164111	-6.354297	6.15E-10	5.85E-09	12.096077
ACAD9	-0.082062	6.3971532	-6.353841	6.17E-10	5.86E-09	12.093484
APCDD1	0.7321382	4.5777789	6.3536898	6.17E-10	5.86E-09	12.092625
TRBV9	1.276926	0.7294041	6.3531351	6.19E-10	5.88E-09	12.089472
FAM134A	-0.086788	6.7429192	-6.351812	6.24E-10	5.93E-09	12.081954
PCYT1B	1.3299469	0.9544835	6.3517793	6.24E-10	5.93E-09	12.081766
KDELR2	-0.093054	7.0600445	-6.350652	6.28E-10	5.96E-09	12.075361
CTH	-0.410839	6.1513112	-6.35008	6.31E-10	5.98E-09	12.072111
PCSK1N	1.7429723	2.1303297	6.3495545	6.33E-10	6.00E-09	12.069124
PLA2G2D	1.3384493	3.1329229	6.3487842	6.35E-10	6.02E-09	12.064747
UBTF	-0.063938	6.6458188	-6.348201	6.38E-10	6.04E-09	12.061432
KCNS1	1.4054604	0.566776	6.3475446	6.40E-10	6.07E-09	12.057706
PTCH2	0.3669014	4.1079409	6.347207	6.41E-10	6.08E-09	12.055788
H6PD	-0.158804	6.8641184	-6.345216	6.49E-10	6.15E-09	12.044482
RP11-473M2	1.2479183	0.2186341	6.3449975	6.50E-10	6.15E-09	12.04324
ART5	1.4100084	0.880638	6.3447097	6.51E-10	6.16E-09	12.041605
HRH1	0.3435777	4.5152108	6.3445989	6.51E-10	6.16E-09	12.040976
LPAR3	1.4752185	0.4350764	6.3443178	6.52E-10	6.17E-09	12.039381
ISOC1	-0.146451	6.3618689	-6.343305	6.56E-10	6.21E-09	12.033632
AC006548.1	-0.769154	-1.111995	-6.343208	6.56E-10	6.21E-09	12.033077
STK38L	0.1511532	5.8042952	6.3420349	6.61E-10	6.25E-09	12.02642
ADCY2	1.1656549	-0.0938	6.3405589	6.67E-10	6.30E-09	12.018043
CTD-2536I1	1.1501073	0.9838058	6.3403589	6.68E-10	6.31E-09	12.016908
AKR1C8P	-1.389432	3.4661548	-6.33939	6.71E-10	6.34E-09	12.011408
CLECL1	1.0568404	2.4449616	6.3392838	6.72E-10	6.34E-09	12.010807
F12	-0.405779	6.9786786	-6.337836	6.77E-10	6.40E-09	12.002594
MAD2L2	-0.153039	6.2228641	-6.337546	6.79E-10	6.41E-09	12.00095
MUC6	1.4513732	2.7667529	6.3364134	6.83E-10	6.45E-09	11.994523
SLC39A8	-0.20929	6.1361676	-6.336224	6.84E-10	6.45E-09	11.993451
DSTNP1	0.8818066	1.8309222	6.3360598	6.85E-10	6.46E-09	11.992517
AC002480.3	1.1437767	0.5294352	6.3357914	6.86E-10	6.47E-09	11.990995
RASAL3	0.2435571	5.3971956	6.3357581	6.86E-10	6.47E-09	11.990806
KIF7	0.3829066	4.8520688	6.3343975	6.91E-10	6.52E-09	11.983089
PSMD11	-0.075743	6.6085538	-6.333028	6.97E-10	6.57E-09	11.975325
CTD-2377D2	1.1210599	-0.295029	6.3322327	7.00E-10	6.59E-09	11.970815

LRRC52	-1.371704	-0.069044	-6.332223	7.00E-10	6.59E-09	11.97076
TNFAIP8	0.2484929	5.4987424	6.3278467	7.18E-10	6.76E-09	11.945957
AC005076.5	-0.483782	3.6608445	-6.327002	7.22E-10	6.79E-09	11.941173
SLC37A3	0.1105746	5.8577293	6.3265495	7.24E-10	6.81E-09	11.938608
TAF9	-0.100486	7.1612887	-6.325843	7.27E-10	6.84E-09	11.934607
CTC-336P14	-0.889619	2.2798848	-6.325546	7.28E-10	6.85E-09	11.932921
VPS25	-0.104278	6.4366457	-6.323938	7.35E-10	6.91E-09	11.923815
ACACB	-0.199129	6.5111314	-6.32276	7.40E-10	6.96E-09	11.917146
TMEM248	-0.066391	6.6983563	-6.322112	7.43E-10	6.98E-09	11.913478
F5	-0.247155	7.0863231	-6.321743	7.44E-10	6.99E-09	11.911387
BAIAP2	-0.196162	6.4864254	-6.321544	7.45E-10	7.00E-09	11.910261
ZNF521	0.5505837	4.4667966	6.3210574	7.47E-10	7.02E-09	11.907505
VGLL3	0.7323966	4.1731691	6.3197543	7.53E-10	7.07E-09	11.900129
PEAR1	0.316146	5.1757662	6.3187191	7.58E-10	7.11E-09	11.89427
ALDH3B1	0.2164228	5.7871992	6.3178037	7.62E-10	7.15E-09	11.88909
NBPF10	0.6097557	4.3135917	6.3170754	7.65E-10	7.18E-09	11.884968
RP11-778D9	-0.941071	1.525617	-6.316613	7.67E-10	7.19E-09	11.882352
KCNS2	1.0792961	-0.282727	6.3162237	7.69E-10	7.21E-09	11.88015
TWIST2	1.3760244	2.0469952	6.3156913	7.71E-10	7.23E-09	11.877137
RMDN3	-0.100017	6.4095942	-6.315355	7.73E-10	7.24E-09	11.875236
MT-ND3	-0.151833	7.4917542	-6.314816	7.75E-10	7.26E-09	11.872186
NOP10	-0.121265	6.4705348	-6.313973	7.79E-10	7.30E-09	11.867415
CTC-523E23	1.1082936	1.7669517	6.313153	7.83E-10	7.33E-09	11.86278
PTPN12	0.1000442	6.2953033	6.3128835	7.84E-10	7.34E-09	11.861256
TMEM41B	-0.096359	6.38068	-6.312719	7.85E-10	7.34E-09	11.860323
MEG9	1.3870642	0.8127675	6.3122616	7.87E-10	7.36E-09	11.857739
RAB19	1.2503947	0.249483	6.3111117	7.92E-10	7.41E-09	11.851237
CTD-2587H1	0.9979782	-0.712129	6.3110877	7.92E-10	7.41E-09	11.851101
RP5-966M1.	-0.3297	6.096573	-6.310592	7.94E-10	7.43E-09	11.848296
ZNF107	0.3343213	5.0126961	6.3101042	7.97E-10	7.45E-09	11.845541
VEZT	0.0955732	6.2084388	6.3098222	7.98E-10	7.46E-09	11.843947
ENPP7	-0.751479	5.5828003	-6.309796	7.98E-10	7.46E-09	11.8438
HSD11B1	-0.615805	6.3863155	-6.309646	7.99E-10	7.46E-09	11.842952
PKIB	0.5898597	4.9273322	6.3096083	7.99E-10	7.46E-09	11.842737
CTD-3157E1	0.6225404	3.3512745	6.3087464	8.03E-10	7.50E-09	11.837865
GPNMB	0.2457227	6.2197126	6.3081434	8.06E-10	7.52E-09	11.834457
KIAA0141	-0.098725	6.4771082	-6.307935	8.07E-10	7.53E-09	11.833279
CDIPT	-0.116937	6.6820598	-6.307824	8.07E-10	7.53E-09	11.832655
CDK2AP1	0.1473632	6.0900405	6.3077097	8.08E-10	7.53E-09	11.832006
BAD	-0.130163	6.2712322	-6.30603	8.16E-10	7.60E-09	11.822516
CCR1	0.3370527	5.0942711	6.306022	8.16E-10	7.60E-09	11.822469
ARHGEF10	0.3086834	5.1465465	6.3056651	8.18E-10	7.62E-09	11.820452
UNC119	0.1793646	5.9839338	6.3045449	8.23E-10	7.67E-09	11.814124
AP3S1	-0.110289	6.3704993	-6.304114	8.25E-10	7.68E-09	11.811689
CCNH	-0.112615	6.0067581	-6.30386	8.26E-10	7.69E-09	11.810252
FGFRL1	-0.139867	6.4452095	-6.30348	8.28E-10	7.71E-09	11.808108
ATG9B	0.4385693	3.9471378	6.3028388	8.31E-10	7.74E-09	11.804487
NDUFS6	-0.147307	6.6137518	-6.302208	8.34E-10	7.76E-09	11.800922
BABAM1	-0.11481	6.3267307	-6.301798	8.36E-10	7.78E-09	11.798607
ECM2	-0.318393	5.934077	-6.301777	8.36E-10	7.78E-09	11.798489
CNN2P6	-0.812162	-1.079854	-6.300608	8.42E-10	7.83E-09	11.791887

RP11-172H2	1.2946784	1.096615	6.3003412	8.43E-10	7.84E-09	11.790382
CXorf65	1.1754023	1.6262053	6.2974577	8.58E-10	7.97E-09	11.774104
FGL2	0.2600081	5.7721234	6.2973364	8.58E-10	7.97E-09	11.773419
IGKV2D-24	1.0608478	-0.588612	6.2968691	8.61E-10	7.99E-09	11.770782
HLA-DQA1	0.2770914	6.1704079	6.2961688	8.64E-10	8.02E-09	11.76683
AGMO	-0.355669	6.3202615	-6.295932	8.65E-10	8.03E-09	11.765492
SYF2	-0.093686	6.2993831	-6.295903	8.65E-10	8.03E-09	11.76533
SMPD1	-0.131313	6.5352634	-6.295841	8.66E-10	8.03E-09	11.764981
SERINC3	-0.079621	6.7724342	-6.2957	8.66E-10	8.04E-09	11.764187
CTB-41I6.1	0.9423383	-0.244258	6.2954089	8.68E-10	8.05E-09	11.762542
TMEM101	-0.124595	6.2824986	-6.293839	8.76E-10	8.12E-09	11.753686
UBALD1	-0.13765	6.1433366	-6.291735	8.87E-10	8.22E-09	11.741816
RTP3	-1.002298	5.4993888	-6.290558	8.93E-10	8.27E-09	11.735181
ADPRH	0.1890402	5.3787446	6.2903535	8.94E-10	8.28E-09	11.734025
TRIM50	1.4564603	3.0658132	6.2901815	8.95E-10	8.29E-09	11.733055
ARHGAP6	0.4591878	4.5039247	6.2900324	8.95E-10	8.29E-09	11.732214
SH3PXD2A-A	1.0312715	0.7889957	6.2897505	8.97E-10	8.30E-09	11.730625
HAVCR1	1.5434326	1.6628232	6.2893025	8.99E-10	8.32E-09	11.728099
TDG	0.1131393	5.7543996	6.2874144	9.09E-10	8.41E-09	11.717455
RPLP1	-0.124814	7.3606418	-6.287376	9.09E-10	8.41E-09	11.717236
AC005523.3	-1.102194	1.5788162	-6.287132	9.11E-10	8.42E-09	11.715863
ELL2	-0.168409	6.6655461	-6.285998	9.17E-10	8.48E-09	11.709471
AP001626.2	1.3618354	0.2789894	6.2855986	9.19E-10	8.49E-09	11.707221
RP11-130L8	-0.635423	4.7490657	-6.284988	9.22E-10	8.52E-09	11.703781
STUB1	-0.116133	6.2632463	-6.284914	9.23E-10	8.52E-09	11.703361
CYP2J2	-0.344027	6.3992392	-6.284814	9.23E-10	8.53E-09	11.702801
VDAC1P8	0.3667715	4.4894213	6.2845508	9.24E-10	8.54E-09	11.701316
INPP5J	0.7948476	3.7488996	6.2843673	9.25E-10	8.54E-09	11.700282
TMEM187	-0.18432	5.7782649	-6.281954	9.39E-10	8.66E-09	11.686688
RP5-834N19	-1.274745	2.6666344	-6.281213	9.43E-10	8.70E-09	11.682511
RP11-295M1	-1.107562	2.1473339	-6.280528	9.46E-10	8.73E-09	11.678652
CTD-2373N4	0.8290292	3.2236691	6.2804393	9.47E-10	8.73E-09	11.678154
PACSIN1	1.0594865	3.7991796	6.2800395	9.49E-10	8.75E-09	11.675903
SLC35B1	-0.100371	6.5604635	-6.278995	9.55E-10	8.80E-09	11.670018
AC115522.3	1.0980778	0.2384168	6.2787914	9.56E-10	8.81E-09	11.668875
FBXL19	0.1365206	5.6325523	6.2781435	9.60E-10	8.84E-09	11.665227
RBMS2	0.1803071	5.6272686	6.2777037	9.62E-10	8.86E-09	11.66275
SCAP	-0.099626	6.7329652	-6.277619	9.63E-10	8.86E-09	11.662273
KIAA0753	0.1582776	5.3884504	6.2762134	9.70E-10	8.93E-09	11.654361
RP11-4B16.	0.8028914	2.5448792	6.2761273	9.71E-10	8.94E-09	11.653876
CNBP	-0.081317	6.9344638	-6.275462	9.75E-10	8.97E-09	11.650134
OLFML2A	0.3179356	5.6093773	6.2751265	9.77E-10	8.98E-09	11.648244
ADARB2	1.4417593	1.1027789	6.2741324	9.82E-10	9.03E-09	11.642649
NME3	-0.143924	6.2159118	-6.273439	9.86E-10	9.07E-09	11.638746
CPXM2	0.5955012	4.7797366	6.2733478	9.87E-10	9.07E-09	11.638234
GPBP1	-0.069269	6.4956645	-6.27325	9.87E-10	9.07E-09	11.637685
DUSP5	0.2776306	5.795519	6.2723574	9.92E-10	9.11E-09	11.632662
LIN7C	-0.095335	6.231152	-6.27235	9.92E-10	9.11E-09	11.632619
C9orf156	-0.104803	5.6784853	-6.272347	9.92E-10	9.11E-09	11.632605
DCUN1D4	-0.101422	6.2709879	-6.272166	9.93E-10	9.12E-09	11.631585
ZNF333	0.1525494	5.3490104	6.2720805	9.94E-10	9.12E-09	11.631104

IGKV1D-42	0.9818702	-0.744028	6.2719069	9.95E-10	9.13E-09	11.630127
ZFAND5	-0.16864	6.8288207	-6.269839	1.01E-09	9.24E-09	11.618498
REM1	0.7836775	3.5196034	6.2694583	1.01E-09	9.26E-09	11.616354
C9orf66	1.2788641	2.1384858	6.2690812	1.01E-09	9.27E-09	11.614233
OPCML	1.2342045	0.9953044	6.2688883	1.01E-09	9.28E-09	11.613149
SCML4	0.7266958	4.2708323	6.2672903	1.02E-09	9.37E-09	11.604163
KCTD10	0.0786508	6.153418	6.2667006	1.03E-09	9.40E-09	11.600848
RUNDC3A-AS	1.1336202	0.4453677	6.2662029	1.03E-09	9.42E-09	11.59805
NPAS3	1.1498633	1.9418117	6.2659931	1.03E-09	9.43E-09	11.596871
RP11-3M1.1	-1.22572	1.38709	-6.265246	1.03E-09	9.47E-09	11.592672
CTD-2616J1	0.9009651	-0.485925	6.2650672	1.04E-09	9.48E-09	11.591666
RP11-693J1	1.1620307	0.5454418	6.2645848	1.04E-09	9.50E-09	11.588955
FAM32A	-0.087687	6.5443225	-6.264458	1.04E-09	9.51E-09	11.588242
UCA1	1.5607655	1.5774596	6.2641841	1.04E-09	9.52E-09	11.586703
APH1A	-0.084179	6.9312201	-6.263339	1.05E-09	9.56E-09	11.581954
ZDHHC1	0.3780136	5.0296042	6.2631862	1.05E-09	9.57E-09	11.581095
CBX7	-0.153922	6.1094783	-6.261639	1.06E-09	9.65E-09	11.572403
OCIAD2	-0.335203	6.3167493	-6.261416	1.06E-09	9.66E-09	11.571147
HIGD1A	-0.15435	6.5077624	-6.261073	1.06E-09	9.68E-09	11.569221
ADORA2A-AS	-0.472833	5.9436017	-6.261	1.06E-09	9.68E-09	11.568812
HSPA1A	-0.182287	6.7025763	-6.260852	1.06E-09	9.69E-09	11.567978
ZNF135	0.8428503	3.8734832	6.2607283	1.06E-09	9.69E-09	11.567285
GMNC	-1.306554	3.7741378	-6.259397	1.07E-09	9.76E-09	11.559807
ZNF519	0.6067992	4.1489942	6.2588349	1.07E-09	9.79E-09	11.55665
TNFRSF11A	0.5077685	4.7440638	6.2570598	1.08E-09	9.89E-09	11.546681
CD300LB	0.8615418	3.3382989	6.2565003	1.09E-09	9.92E-09	11.54354
RP11-147L1	0.3702027	4.2064082	6.2564426	1.09E-09	9.92E-09	11.543216
UGT2B7	-0.48804	6.6620258	-6.256148	1.09E-09	9.94E-09	11.541563
CYTH4	0.2340321	5.5494952	6.2559407	1.09E-09	9.95E-09	11.540398
SCARB1	-0.144967	7.0472376	-6.255765	1.09E-09	9.95E-09	11.539412
RP11-348P1	0.6465934	3.2564275	6.2553355	1.10E-09	9.98E-09	11.537001
PROB1	0.2601699	4.5634587	6.254638	1.10E-09	1.00E-08	11.533085
AC111186.1	-0.748102	4.2279866	-6.253498	1.11E-09	1.01E-08	11.526685
MYO1F	0.1952408	5.7510834	6.2534588	1.11E-09	1.01E-08	11.526467
RP11-488C1	1.0809185	1.0227706	6.2530517	1.11E-09	1.01E-08	11.524182
LINC01564	-0.754607	4.3805027	-6.252797	1.11E-09	1.01E-08	11.522751
FILIP1L	0.2624203	5.6807804	6.2526251	1.11E-09	1.01E-08	11.521788
LRRFIP1P1	1.0615469	2.8348643	6.2523965	1.11E-09	1.01E-08	11.520505
CD8B	0.8439539	3.8034151	6.2508986	1.12E-09	1.02E-08	11.5121
CEP55	0.4250794	4.9229487	6.2494718	1.13E-09	1.03E-08	11.504096
WDR46	-0.112306	6.4057138	-6.248478	1.14E-09	1.04E-08	11.498524
ZNF761	0.2858411	5.4939961	6.2483533	1.14E-09	1.04E-08	11.497822
SRGAP3-AS4	-0.902501	-0.947002	-6.247664	1.15E-09	1.04E-08	11.493956
SLC12A2	0.3068637	5.8457916	6.247638	1.15E-09	1.04E-08	11.493811
HOTAIRM1	0.4239315	4.0470454	6.2440256	1.17E-09	1.06E-08	11.473556
EPHB1	0.7759975	3.5592453	6.2437661	1.17E-09	1.06E-08	11.472102
IGKV1D-12	1.0658837	-0.743273	6.2433041	1.17E-09	1.07E-08	11.469512
SLC25A28	-0.127932	6.2308335	-6.243189	1.18E-09	1.07E-08	11.468865
SPTSSB	1.3387101	2.4001672	6.2419801	1.18E-09	1.07E-08	11.462092
MAGEE1	0.6779443	4.1739729	6.2414265	1.19E-09	1.08E-08	11.45899
TTC31	-0.113364	6.244444	-6.241044	1.19E-09	1.08E-08	11.456846

RP11-597D1	0.7860589	2.8657599	6.2409532	1.19E-09	1.08E-08	11.456337
RPL19	-0.110343	7.3210669	-6.240305	1.19E-09	1.08E-08	11.452707
ALDH4A1	-0.185903	6.9323495	-6.239085	1.20E-09	1.09E-08	11.445871
TMEM176B	-0.203079	7.3336842	-6.238918	1.20E-09	1.09E-08	11.444933
PLAGL2	0.155733	5.6926746	6.2387249	1.21E-09	1.09E-08	11.443854
CCDC170	-0.533753	5.0716203	-6.237282	1.22E-09	1.10E-08	11.435769
RNU6-564P	-1.012006	-0.240475	-6.236531	1.22E-09	1.10E-08	11.431565
CHMP3	0.0899051	6.1819665	6.2360923	1.22E-09	1.11E-08	11.429109
TRBV4-2	1.2075456	0.4129633	6.2358637	1.23E-09	1.11E-08	11.427829
MAPKAP1	-0.078662	6.498052	-6.235193	1.23E-09	1.11E-08	11.424071
TPRG1	-0.42211	4.9054137	-6.234929	1.23E-09	1.11E-08	11.422593
USH2A	-0.604979	5.0747587	-6.234866	1.23E-09	1.11E-08	11.422244
IGHV3OR16-	1.0240825	-0.652059	6.2344932	1.24E-09	1.12E-08	11.420156
YBEY	-0.193652	5.7928217	-6.234443	1.24E-09	1.12E-08	11.419873
PARP16	-0.114514	5.8891165	-6.234291	1.24E-09	1.12E-08	11.419024
PROSER3	0.2395477	5.0935064	6.2342675	1.24E-09	1.12E-08	11.418892
DLEU7	1.1240756	1.9892819	6.2335415	1.24E-09	1.12E-08	11.414827
TMEM120A	-0.135866	6.5912204	-6.233117	1.25E-09	1.12E-08	11.412453
SIVA1	-0.142296	6.4171341	-6.232233	1.25E-09	1.13E-08	11.407502
RP11-982M1	1.0214305	-0.40362	6.2318931	1.25E-09	1.13E-08	11.405601
RNU6-526P	-0.477491	-1.300844	-6.231853	1.25E-09	1.13E-08	11.405376
CTD-2015H6	-0.207884	5.0647364	-6.231502	1.26E-09	1.13E-08	11.403411
RIMKLB	0.2999848	5.0117609	6.2312161	1.26E-09	1.13E-08	11.401812
CTC-510F12	1.2214412	0.8457695	6.231013	1.26E-09	1.14E-08	11.400675
BCAP29	-0.08749	6.3057857	-6.230091	1.27E-09	1.14E-08	11.395518
AGL	-0.171027	6.1766859	-6.229956	1.27E-09	1.14E-08	11.394761
TUBA1A	0.1888588	6.0579139	6.2297134	1.27E-09	1.14E-08	11.393403
TMEM59L	1.2193133	1.6560052	6.2295044	1.27E-09	1.14E-08	11.392234
WNT4	1.1140145	4.2685591	6.2290585	1.28E-09	1.15E-08	11.389739
APOC3	-0.384337	7.5063615	-6.227359	1.29E-09	1.16E-08	11.38023
CACNA1H	0.6167011	5.4642745	6.2270211	1.29E-09	1.16E-08	11.378342
LINC01151	-1.363167	3.3239975	-6.225852	1.30E-09	1.17E-08	11.371805
LOXL2	0.1803448	5.8390419	6.225829	1.30E-09	1.17E-08	11.371675
CEMIP	0.6223572	4.7455807	6.2252271	1.30E-09	1.17E-08	11.368309
C19orf60	-0.160753	6.2418032	-6.22517	1.30E-09	1.17E-08	11.36799
PSMD8	-0.091855	6.7395614	-6.224845	1.31E-09	1.17E-08	11.36617
C2orf70	1.2148765	-0.318494	6.2242895	1.31E-09	1.18E-08	11.363066
SHF	-0.305314	5.8941959	-6.223324	1.32E-09	1.18E-08	11.357668
USP19	-0.076084	6.4300236	-6.22277	1.32E-09	1.19E-08	11.354572
OARD1	-0.109423	6.1950761	-6.222567	1.32E-09	1.19E-08	11.353437
UGGT1	-0.107765	6.6396421	-6.222398	1.33E-09	1.19E-08	11.352491
CLMP	0.5467886	3.860496	6.2221687	1.33E-09	1.19E-08	11.35121
CNKSR1	1.1048706	3.2982233	6.2216942	1.33E-09	1.19E-08	11.348558
PSMA4	-0.091926	6.6754986	-6.221327	1.33E-09	1.20E-08	11.346505
LRRC8E	1.1636723	2.8948635	6.2210083	1.34E-09	1.20E-08	11.344725
TUBBP5	1.7501877	1.8155039	6.2209741	1.34E-09	1.20E-08	11.344533
CDC37L1	-0.15124	6.1213179	-6.220927	1.34E-09	1.20E-08	11.34427
SOCS3	0.2918449	6.0089811	6.2205582	1.34E-09	1.20E-08	11.342209
NSUN5	-0.114996	6.1548137	-6.22011	1.34E-09	1.20E-08	11.339703
ZNF213-AS1	-0.171986	5.2317583	-6.219927	1.34E-09	1.20E-08	11.338683
ZNF337	0.6330744	4.1956833	6.2189538	1.35E-09	1.21E-08	11.333244

FLJ22763	-1.586066	2.7906115	-6.218707	1.35E-09	1.21E-08	11.331864
HEXB	-0.112766	6.8026605	-6.218654	1.35E-09	1.21E-08	11.331568
ZDHHC20	0.1292168	6.0945218	6.218346	1.36E-09	1.21E-08	11.329848
GPR15	0.9926171	-0.485039	6.2181468	1.36E-09	1.21E-08	11.328735
BRD1	0.1158038	6.0242355	6.2177948	1.36E-09	1.22E-08	11.326769
PSMF1	-0.095418	6.8001603	-6.21659	1.37E-09	1.22E-08	11.32004
CCDC144NL-	0.9198039	3.598051	6.2164579	1.37E-09	1.23E-08	11.319301
RP11-245P1	1.1718483	0.2039555	6.2163467	1.37E-09	1.23E-08	11.31868
MLYCD	-0.180094	6.0998297	-6.216191	1.37E-09	1.23E-08	11.317809
RGSL1	-1.687518	1.7140185	-6.216067	1.37E-09	1.23E-08	11.317115
RORC	-0.352932	6.5810142	-6.21543	1.38E-09	1.23E-08	11.313558
QPCTL	-0.148605	5.9813954	-6.215294	1.38E-09	1.23E-08	11.312801
NTS	1.6470877	2.5460438	6.2146457	1.39E-09	1.24E-08	11.30918
SRPRB	-0.111237	6.6434543	-6.213502	1.39E-09	1.24E-08	11.302796
SLC39A2	0.9351401	-0.534451	6.2127688	1.40E-09	1.25E-08	11.2987
SPOCD1	1.0184426	2.8822219	6.2121553	1.41E-09	1.25E-08	11.295276
PGPEP1	-0.135689	6.4634523	-6.210982	1.42E-09	1.26E-08	11.288724
CTD-2510F5	1.0274003	3.0934751	6.2104782	1.42E-09	1.26E-08	11.285914
PHB2	-0.100328	6.8685959	-6.210391	1.42E-09	1.27E-08	11.285428
NBR2	-0.184996	5.3707009	-6.209923	1.42E-09	1.27E-08	11.282813
TRBV23-1	0.8453359	-0.685348	6.209635	1.43E-09	1.27E-08	11.281208
UBXN1	-0.107972	6.5711799	-6.209582	1.43E-09	1.27E-08	11.280913
RNF14	-0.094823	6.383019	-6.209211	1.43E-09	1.27E-08	11.278844
ADRM1	-0.099262	6.726985	-6.208577	1.44E-09	1.28E-08	11.275307
HDGFRP2	-0.105152	6.4550798	-6.207971	1.44E-09	1.28E-08	11.271924
SERF1B	0.838476	2.1954157	6.2077454	1.44E-09	1.28E-08	11.270665
CTD-3252C9	0.3607396	4.8808829	6.2074214	1.44E-09	1.28E-08	11.268857
AC138430.4	-1.263337	1.313992	-6.207038	1.45E-09	1.29E-08	11.266719
SH3D21	0.3262095	4.5851199	6.2068891	1.45E-09	1.29E-08	11.265887
MGMT	-0.194205	6.5523066	-6.206581	1.45E-09	1.29E-08	11.26417
BRK1	-0.090453	6.6579032	-6.206513	1.45E-09	1.29E-08	11.26379
VGLL4	0.2448759	5.7276802	6.2064649	1.45E-09	1.29E-08	11.263521
RP1-30M3.5	-0.230506	4.6530509	-6.206285	1.45E-09	1.29E-08	11.262517
RP6-159A1.	0.8524479	2.8943548	6.2059738	1.46E-09	1.29E-08	11.260782
RP11-752L2	0.8567932	2.618334	6.205103	1.46E-09	1.30E-08	11.255925
RP11-848G1	1.0688184	1.9202681	6.2050603	1.46E-09	1.30E-08	11.255686
RP1-223H12	-0.465471	-1.350777	-6.205041	1.46E-09	1.30E-08	11.255578
RP11-1212A	1.0685762	3.2825228	6.2050079	1.46E-09	1.30E-08	11.255394
NDUFA6	-0.11994	6.5794447	-6.204897	1.47E-09	1.30E-08	11.254776
CTD-2553L1	-0.316415	4.2181617	-6.204617	1.47E-09	1.30E-08	11.253215
CTD-3184A7	-0.243436	5.0851086	-6.204544	1.47E-09	1.30E-08	11.252807
MYL3	1.0982298	2.2296133	6.2041444	1.47E-09	1.30E-08	11.250579
MRPL33	-0.120772	6.1518904	-6.203689	1.48E-09	1.31E-08	11.248041
RP11-21C4.	1.1002931	-0.063719	6.2022033	1.49E-09	1.32E-08	11.239755
TMPRSS3	1.1398505	4.3754638	6.2009462	1.50E-09	1.33E-08	11.232747
NUDT2	-0.151881	5.9831484	-6.200624	1.50E-09	1.33E-08	11.230952
MST1P2	-0.379668	5.8767432	-6.199299	1.51E-09	1.34E-08	11.223569
ATP5F1P5	0.9545806	-0.489223	6.198761	1.52E-09	1.34E-08	11.220569
RP11-344B5	0.9095689	2.6884054	6.1985264	1.52E-09	1.34E-08	11.219261
SRPR	-0.092092	6.8821353	-6.198475	1.52E-09	1.34E-08	11.218976
RERG	0.4757829	4.6516955	6.1969993	1.53E-09	1.36E-08	11.210752

APH1B	0.2008244	5.4407031	6.1968566	1.54E-09	1.36E-08	11.209957
ALG8	-0.104148	6.3986603	-6.196418	1.54E-09	1.36E-08	11.207513
APOBR	0.2119794	5.3855887	6.1958143	1.54E-09	1.36E-08	11.204151
GALE	-0.133639	6.5332036	-6.195469	1.55E-09	1.37E-08	11.202227
RGMB	-0.274703	5.3139208	-6.195397	1.55E-09	1.37E-08	11.201825
ADHFE1	-0.32425	6.0677145	-6.194452	1.56E-09	1.37E-08	11.196563
MMP12	1.5268382	2.4573416	6.1940093	1.56E-09	1.38E-08	11.194097
COG4	-0.097603	6.3891398	-6.193811	1.56E-09	1.38E-08	11.192994
CCL16	-0.844877	6.1139152	-6.19338	1.57E-09	1.38E-08	11.190595
WDR25	-0.138989	5.6220807	-6.191843	1.58E-09	1.39E-08	11.182034
GRAMD1A	0.183536	6.2922059	6.1917176	1.58E-09	1.39E-08	11.181336
ISCA1P4	-1.172605	0.932385	-6.191337	1.58E-09	1.40E-08	11.179216
SLC45A3	0.2958333	5.6665773	6.1911023	1.59E-09	1.40E-08	11.177911
AC114730.2	0.9167592	-0.323573	6.1910046	1.59E-09	1.40E-08	11.177367
CRELD1	-0.106978	6.3441564	-6.190819	1.59E-09	1.40E-08	11.176332
IGHV3-79	0.8942448	-0.697367	6.1893135	1.60E-09	1.41E-08	11.167953
AC147651.4	0.5825438	3.4969799	6.1881671	1.61E-09	1.42E-08	11.161573
LIFR-AS1	0.9924392	2.396533	6.1873587	1.62E-09	1.43E-08	11.157075
RP11-458J1	-0.159433	5.1217619	-6.187031	1.62E-09	1.43E-08	11.155253
RP11-307C1	-0.397568	4.4551525	-6.186472	1.63E-09	1.43E-08	11.152143
TXLNB	0.8504076	3.2928669	6.1858889	1.64E-09	1.44E-08	11.148897
MMP9	0.5524217	5.3785374	6.1848349	1.65E-09	1.45E-08	11.143034
SPECC1L	-0.106487	6.2507971	-6.184818	1.65E-09	1.45E-08	11.14294
RUVBL2	-0.11385	6.5714583	-6.18433	1.65E-09	1.45E-08	11.140224
RP11-705C1	0.350484	4.5499084	6.1841662	1.65E-09	1.45E-08	11.139315
EIF2B1	-0.071986	6.3522975	-6.183954	1.65E-09	1.45E-08	11.138133
NPLOC4	-0.083669	6.7350259	-6.183869	1.65E-09	1.45E-08	11.137664
RP11-700H6	-0.987864	2.7931119	-6.183711	1.66E-09	1.45E-08	11.136781
CD177	1.3484337	1.595264	6.1833523	1.66E-09	1.46E-08	11.134788
ANAPC13	-0.081162	6.3516126	-6.182906	1.66E-09	1.46E-08	11.132304
AC068580.6	0.5013269	4.5654816	6.1824662	1.67E-09	1.46E-08	11.12986
MRPS18C	-0.115156	5.7876125	-6.182363	1.67E-09	1.46E-08	11.129285
VAX2	1.1485695	2.2057369	6.1813554	1.68E-09	1.47E-08	11.123684
TAF11	-0.091322	6.1598377	-6.181208	1.68E-09	1.47E-08	11.122865
RP11-624M8	-1.27358	2.1269033	-6.180723	1.68E-09	1.48E-08	11.120167
BNIP3P11	1.059143	2.1629369	6.1799165	1.69E-09	1.48E-08	11.115684
MAT1A	-0.333473	7.1725976	-6.179779	1.69E-09	1.48E-08	11.114918
PRSS23	0.2281254	6.0330708	6.1796685	1.69E-09	1.48E-08	11.114306
FBXO44	-0.158527	6.1023431	-6.179624	1.70E-09	1.48E-08	11.114059
BAG6	-0.079553	6.9724704	-6.179275	1.70E-09	1.49E-08	11.112118
RP11-60E8.	-1.074112	-0.146133	-6.178343	1.71E-09	1.49E-08	11.106939
C12orf45	-0.155446	5.7057542	-6.177944	1.71E-09	1.50E-08	11.10472
CFTR	1.706749	2.3160871	6.1771773	1.72E-09	1.50E-08	11.10046
LINC01160	0.8744041	1.7953285	6.177105	1.72E-09	1.50E-08	11.100059
KEL	1.1077854	2.8763706	6.1769473	1.72E-09	1.50E-08	11.099182
RP11-108M9	-0.373986	5.0995024	-6.17693	1.72E-09	1.50E-08	11.099086
ASAH2B	-0.165749	5.2845586	-6.176676	1.72E-09	1.51E-08	11.097676
SAC3D1	-0.163162	5.9045771	-6.176158	1.73E-09	1.51E-08	11.094794
PRKAR2A-AS	-0.363511	4.4297113	-6.175343	1.74E-09	1.52E-08	11.090267
CCDC106	-0.199842	5.8735454	-6.174494	1.75E-09	1.52E-08	11.085554
GABPB1-AS1	-0.193496	5.5993044	-6.173476	1.76E-09	1.53E-08	11.079898

KIAA1191	-0.100598	6.573139	-6.171928	1.77E-09	1.55E-08	11.071302
PLD1	0.2403941	6.0607743	6.1716525	1.77E-09	1.55E-08	11.06977
AC010226.4	-0.321683	3.9133754	-6.171411	1.78E-09	1.55E-08	11.068428
SH3PXD2B	0.1513113	5.8162224	6.1679135	1.81E-09	1.58E-08	11.049013
MIR3685	0.860667	2.1766918	6.1677186	1.82E-09	1.58E-08	11.047932
ENDOV	-0.151729	5.7795575	-6.167621	1.82E-09	1.58E-08	11.047387
AC009403.2	-0.188907	5.4536813	-6.167256	1.82E-09	1.59E-08	11.045366
TOX4	-0.065843	6.4129408	-6.166764	1.82E-09	1.59E-08	11.042634
CDH19	1.5353601	2.5157714	6.1659112	1.83E-09	1.60E-08	11.037902
DOC2B	0.4671874	4.8016603	6.1655234	1.84E-09	1.60E-08	11.03575
COASY	-0.099134	6.6249902	-6.165515	1.84E-09	1.60E-08	11.035704
RAMP1	-0.373594	6.4344425	-6.165302	1.84E-09	1.60E-08	11.034523
AP3S2	-0.113923	6.0748667	-6.164616	1.85E-09	1.61E-08	11.030716
GPR1	1.2592235	0.7198712	6.1645755	1.85E-09	1.61E-08	11.030491
FN3K	-0.1605	6.5456445	-6.164143	1.85E-09	1.61E-08	11.028092
ADAMTSL2	0.5514162	5.6280114	6.1640765	1.85E-09	1.61E-08	11.027723
FXYD5	0.2024354	6.098017	6.1631097	1.86E-09	1.62E-08	11.02236
TARID	1.1121528	0.4466856	6.1628263	1.87E-09	1.62E-08	11.020788
CTD-3035K2	0.9648645	-0.395097	6.1626395	1.87E-09	1.62E-08	11.019752
DFNA5	0.3985961	5.3908412	6.1623615	1.87E-09	1.63E-08	11.01821
ALKBH4	-0.107049	5.7411114	-6.162063	1.87E-09	1.63E-08	11.016554
MCU	0.1151714	5.9149701	6.1619428	1.88E-09	1.63E-08	11.015888
ARMC2	0.2462566	4.4240211	6.1608737	1.89E-09	1.64E-08	11.00996
NSMAF	0.1697954	5.7446755	6.1597079	1.90E-09	1.65E-08	11.003496
AC019129.1	-1.13349	-0.252844	-6.158969	1.91E-09	1.66E-08	10.999397
RNU2-27P	0.9834658	1.3769096	6.1588419	1.91E-09	1.66E-08	10.998695
SERPINH1	0.1291436	6.6009267	6.1586303	1.91E-09	1.66E-08	10.997522
CYB5D2	-0.177402	6.0234485	-6.158416	1.91E-09	1.66E-08	10.996336
TINF2	-0.081712	6.2281457	-6.156294	1.94E-09	1.68E-08	10.984571
NLRP2	1.2004069	2.7745853	6.1561685	1.94E-09	1.68E-08	10.983878
BSPRY	0.9438608	4.2667207	6.1557437	1.94E-09	1.68E-08	10.981523
GPHN	-0.200642	6.0688534	-6.155128	1.95E-09	1.69E-08	10.978112
CYP26B1	1.0454335	3.6995332	6.1548426	1.95E-09	1.69E-08	10.97653
TIMP1	0.2957359	6.7767815	6.154305	1.96E-09	1.70E-08	10.973552
RP11-94A24	-1.411988	0.9551991	-6.152046	1.99E-09	1.72E-08	10.961036
MRPS27	-0.095693	6.4548919	-6.151986	1.99E-09	1.72E-08	10.960705
GLIPR1	0.2762538	5.4272744	6.1504385	2.00E-09	1.73E-08	10.952136
PRKCA	-0.127448	6.2900873	-6.14993	2.01E-09	1.74E-08	10.949322
DGKZP1	0.5272582	3.1519607	6.1499127	2.01E-09	1.74E-08	10.949225
ZNF43	0.430236	4.8942311	6.1494545	2.02E-09	1.74E-08	10.946688
ABCF1	-0.085751	6.6368556	-6.148942	2.02E-09	1.75E-08	10.943849
TMSB4XP8	0.6186974	3.7528993	6.1488266	2.02E-09	1.75E-08	10.943211
KIAA1045	1.1403659	1.7955712	6.1487054	2.02E-09	1.75E-08	10.942541
MEIS3P2	0.9437076	-0.315051	6.1481052	2.03E-09	1.75E-08	10.939218
AIP	-0.108809	6.3927077	-6.147326	2.04E-09	1.76E-08	10.934903
HP	-0.390112	7.6239289	-6.147188	2.04E-09	1.76E-08	10.934143
UBA52	-0.118236	7.1163243	-6.146639	2.05E-09	1.77E-08	10.9311
PPP1R14C	1.3371038	0.7388688	6.1464209	2.05E-09	1.77E-08	10.929895
CSF1R	0.2279888	6.0222917	6.1452659	2.06E-09	1.78E-08	10.923504
TAF6L	-0.113182	5.9151367	-6.14499	2.07E-09	1.78E-08	10.921977
RP11-274E7	-0.325172	4.1538898	-6.144483	2.07E-09	1.79E-08	10.919172

EBF4	0.4550388	5.3062753	6.1443235	2.08E-09	1.79E-08	10.918289
AMFR	-0.118554	6.7710865	-6.144197	2.08E-09	1.79E-08	10.917591
FCGR2B	0.7276765	4.5544849	6.1439941	2.08E-09	1.79E-08	10.916467
RP11-312B8	-0.812333	2.5241402	-6.143728	2.08E-09	1.79E-08	10.914992
SGSM1	0.7684553	3.9341131	6.1430465	2.09E-09	1.80E-08	10.911224
TREM1	1.0982271	2.9902033	6.1419995	2.10E-09	1.81E-08	10.905432
SMIM7	-0.108123	6.3739445	-6.141628	2.11E-09	1.81E-08	10.903378
WBSCR22	-0.099533	6.5186314	-6.140964	2.12E-09	1.82E-08	10.899708
SH2D7	0.9582583	-0.417787	6.1409432	2.12E-09	1.82E-08	10.89959
USP5	-0.087887	6.5798925	-6.140172	2.12E-09	1.83E-08	10.895326
SH3YL1	0.276899	5.4978269	6.1395137	2.13E-09	1.84E-08	10.891685
CTD-2588E2	0.9778685	0.0355927	6.1392633	2.14E-09	1.84E-08	10.890301
LMCD1	0.1771837	5.8707696	6.1391617	2.14E-09	1.84E-08	10.889739
PKP1	1.3535338	1.8774276	6.1385155	2.15E-09	1.84E-08	10.886166
RP11-686D2	0.7301481	3.0553613	6.1385141	2.15E-09	1.84E-08	10.886159
RP11-23P13	1.065012	2.5354204	6.1382309	2.15E-09	1.85E-08	10.884593
GPIHBP1	-0.352904	5.0294817	-6.138131	2.15E-09	1.85E-08	10.884041
PDAP1	-0.085441	6.6226554	-6.138083	2.15E-09	1.85E-08	10.883773
PEPD	-0.135686	6.7840381	-6.137799	2.15E-09	1.85E-08	10.882208
CTSV	1.0999841	3.8463122	6.1373427	2.16E-09	1.85E-08	10.879683
CDC42SE1	0.0973347	6.3834897	6.1370473	2.16E-09	1.86E-08	10.87805
S100A11	0.2224855	6.3315758	6.135073	2.19E-09	1.88E-08	10.867139
CHSY1	0.1873254	5.831137	6.1339834	2.20E-09	1.89E-08	10.861118
LRRC1	0.526819	5.3920285	6.133608	2.21E-09	1.89E-08	10.859044
RP11-317M2	-0.611679	-1.163991	-6.133095	2.21E-09	1.90E-08	10.85621
AMBP	-0.267792	7.7158947	-6.131739	2.23E-09	1.91E-08	10.848718
HCFC1R1	-0.142872	6.1690122	-6.131336	2.23E-09	1.92E-08	10.846493
XXbac-B444	-0.937348	1.9482119	-6.131311	2.24E-09	1.92E-08	10.846355
ABAT	-0.25669	6.8478866	-6.130894	2.24E-09	1.92E-08	10.844053
PDE1B	0.2372457	4.788872	6.1304716	2.25E-09	1.92E-08	10.841718
AC144652.1	-0.429966	4.6755933	-6.130135	2.25E-09	1.93E-08	10.839859
RP11-422P2	-0.269264	4.4433742	-6.129646	2.26E-09	1.93E-08	10.83716
URB1-AS1	-0.234188	5.0912864	-6.129537	2.26E-09	1.93E-08	10.836557
IGLV3-12	0.9104131	-0.77701	6.1294816	2.26E-09	1.93E-08	10.836252
FAM225B	1.0460573	-0.026176	6.1290375	2.26E-09	1.94E-08	10.833799
KIAA1614	0.4066122	4.5760452	6.1289477	2.27E-09	1.94E-08	10.833303
ARHGEF38	0.8604791	3.1176753	6.1274968	2.28E-09	1.95E-08	10.825293
IGHV5-78	1.0701364	-0.178643	6.1272295	2.29E-09	1.96E-08	10.823817
DPEP2	0.3203505	4.563457	6.1271992	2.29E-09	1.96E-08	10.82365
RP5-1028K7	1.1581846	2.2782688	6.1270233	2.29E-09	1.96E-08	10.822679
RYR2	1.2513288	2.872946	6.126315	2.30E-09	1.96E-08	10.818769
CARD14	0.7001323	3.4111558	6.1261567	2.30E-09	1.97E-08	10.817895
AC125238.2	-0.783103	-0.962637	-6.125356	2.31E-09	1.97E-08	10.813474
MIF4GD	-0.121909	6.0167883	-6.125344	2.31E-09	1.97E-08	10.813409
CIRBP	-0.108493	6.7825522	-6.125154	2.32E-09	1.98E-08	10.812363
IFNG-AS1	1.107513	-0.176646	6.1237889	2.33E-09	1.99E-08	10.804828
TRGV5	1.1439922	1.7252886	6.122723	2.35E-09	2.00E-08	10.798947
SLC28A1	-0.664327	5.7529537	-6.122317	2.35E-09	2.01E-08	10.796705
RP11-124N1	0.9346385	-0.262959	6.1222693	2.35E-09	2.01E-08	10.796444
MMP14	0.1645469	6.4771556	6.1210711	2.37E-09	2.02E-08	10.789834
IGHV3-63	0.962241	-0.72262	6.1199177	2.39E-09	2.03E-08	10.783473

UFSP2	-0.115007	5.8463076	-6.119691	2.39E-09	2.04E-08	10.782224
THAP3	-0.133485	5.7605713	-6.118953	2.40E-09	2.04E-08	10.778154
PTGR1	-0.262037	6.8312776	-6.118575	2.40E-09	2.05E-08	10.776069
SLC25A13	-0.142237	6.6719069	-6.118292	2.41E-09	2.05E-08	10.774506
RTFDC1	-0.075295	6.5618184	-6.118108	2.41E-09	2.05E-08	10.773493
DDRGK1	-0.120591	6.6364464	-6.118072	2.41E-09	2.05E-08	10.773297
SNHG23	1.1657698	-0.435681	6.1165757	2.43E-09	2.07E-08	10.765046
ZNF622	-0.114849	6.2564172	-6.116025	2.44E-09	2.07E-08	10.762012
DHTKD1	-0.181954	6.7146607	-6.115969	2.44E-09	2.07E-08	10.761702
NAGA	-0.130251	6.4440219	-6.115	2.45E-09	2.09E-08	10.756363
ASB4	-0.98428	3.847774	-6.114165	2.46E-09	2.10E-08	10.751761
SNRPD3	-0.104326	6.6231511	-6.113373	2.48E-09	2.10E-08	10.747396
AC104088.1	-1.213788	-0.294446	-6.113294	2.48E-09	2.10E-08	10.746958
ADD3	0.2048132	6.0388696	6.1129284	2.48E-09	2.11E-08	10.744945
AC145110.1	1.0593408	0.2708149	6.1127296	2.48E-09	2.11E-08	10.743849
ACTG1P3	1.0302498	1.7629751	6.1109711	2.51E-09	2.13E-08	10.734162
HSPB6	0.4645406	5.3470807	6.1108862	2.51E-09	2.13E-08	10.733694
RMND1	-0.123449	6.0694911	-6.110185	2.52E-09	2.14E-08	10.729834
RP11-288C1	0.6909054	3.6792388	6.1095256	2.53E-09	2.15E-08	10.7262
RNF103	-0.107794	6.468957	-6.109289	2.53E-09	2.15E-08	10.724895
C11orf21	0.6865157	3.5949882	6.108483	2.55E-09	2.16E-08	10.720459
TVP23A	0.3565938	3.7429848	6.1084258	2.55E-09	2.16E-08	10.720144
SPIN1	0.1252031	6.2426717	6.1075376	2.56E-09	2.17E-08	10.715253
CRYZ	-0.161569	6.7396254	-6.107485	2.56E-09	2.17E-08	10.714966
IL12A	1.092249	2.0345885	6.1074222	2.56E-09	2.17E-08	10.714618
WSCD1	0.4657127	4.9395219	6.1068707	2.57E-09	2.18E-08	10.711581
LINC01150	1.0551297	0.3103118	6.1061958	2.58E-09	2.18E-08	10.707866
CDHR5	-0.50274	6.7084064	-6.106175	2.58E-09	2.18E-08	10.707749
ZBTB18	-0.155157	6.2993139	-6.105602	2.59E-09	2.19E-08	10.704596
LRP8	0.5171975	4.1627248	6.1051309	2.59E-09	2.20E-08	10.702004
ZFX	0.1283968	6.0436778	6.1050503	2.60E-09	2.20E-08	10.701561
URAHP	-0.329215	5.0936594	-6.104904	2.60E-09	2.20E-08	10.700755
KCNMB1	0.4486308	4.1424261	6.1044468	2.60E-09	2.20E-08	10.698239
ITIH4	-0.340713	6.2003253	-6.104313	2.61E-09	2.20E-08	10.697505
RP11-573D1	-0.436721	5.2233593	-6.103978	2.61E-09	2.21E-08	10.69566
DNASE2	-0.154969	6.4527779	-6.103599	2.62E-09	2.21E-08	10.693576
HIST1H1C	-0.255059	6.607218	-6.102851	2.63E-09	2.22E-08	10.689458
KB-1460A1.	1.3576065	1.0022715	6.1019607	2.64E-09	2.23E-08	10.684558
ZKSCAN2	-0.159499	5.3339686	-6.101738	2.65E-09	2.23E-08	10.683335
FABP5	0.2658204	5.3270351	6.1016706	2.65E-09	2.23E-08	10.682962
P4HA3	0.6515625	3.8552339	6.1014215	2.65E-09	2.24E-08	10.681592
AC023137.2	-1.017781	-0.218575	-6.100775	2.66E-09	2.24E-08	10.678035
ZNF296	0.5658875	4.0950319	6.1007648	2.66E-09	2.24E-08	10.677979
ARHGAP8	1.2263096	0.5803395	6.0999702	2.67E-09	2.25E-08	10.673608
ZNF385A	0.1871616	5.7503597	6.0998447	2.67E-09	2.26E-08	10.672918
VARS2	-0.101739	6.3792223	-6.09883	2.69E-09	2.27E-08	10.66734
BATF3	0.4313553	4.1906643	6.0983784	2.70E-09	2.27E-08	10.664854
AC108463.2	0.7903987	2.6308738	6.0980237	2.70E-09	2.28E-08	10.662904
SULT1B1	-0.541655	5.471529	-6.096453	2.73E-09	2.30E-08	10.654266
FAM155A	1.081486	2.5568175	6.0964215	2.73E-09	2.30E-08	10.654094
C1orf122	-0.165773	6.1547734	-6.095519	2.74E-09	2.31E-08	10.649134

MIR222HG	0.5797181	3.7165111	6.095208	2.75E-09	2.31E-08	10.647423
SERAC1	0.1477903	5.4200615	6.094724	2.75E-09	2.32E-08	10.644763
DCST2	-0.419843	4.4554289	-6.094336	2.76E-09	2.32E-08	10.642629
OLFML2B	0.2817676	5.4987839	6.0942024	2.76E-09	2.32E-08	10.641896
C19orf53	-0.1302	6.573289	-6.094082	2.76E-09	2.32E-08	10.641235
RP11-46C24	-0.265725	4.8141621	-6.093133	2.78E-09	2.34E-08	10.636016
SGCD	1.0916323	3.8615822	6.0929685	2.78E-09	2.34E-08	10.635115
RP5-906C1.	1.0449656	1.5495171	6.0926638	2.79E-09	2.34E-08	10.633441
RP1-244F24	1.0688791	0.0786893	6.0910034	2.81E-09	2.36E-08	10.624318
C19orf43	-0.123975	6.6771372	-6.090331	2.82E-09	2.37E-08	10.620622
MSH3	-0.121667	5.9107089	-6.090126	2.83E-09	2.37E-08	10.619498
RP11-764K9	1.0034773	1.1883675	6.0897209	2.83E-09	2.38E-08	10.617273
MARC2	-0.18257	6.5982887	-6.089426	2.84E-09	2.38E-08	10.615653
TP53TG5	0.8682242	2.4889417	6.0861369	2.89E-09	2.43E-08	10.597591
TEX30	-0.165997	5.9079536	-6.086089	2.89E-09	2.43E-08	10.59733
PCDHAC2	1.4578243	1.4800312	6.084971	2.91E-09	2.44E-08	10.591191
ARL9	1.1107186	0.8387881	6.0848819	2.91E-09	2.44E-08	10.590702
NINJ1	-0.107997	6.5833266	-6.083825	2.93E-09	2.46E-08	10.584899
AP1M1	-0.095732	6.637141	-6.083319	2.94E-09	2.46E-08	10.582125
COL8A1	0.4947363	5.1429616	6.0826476	2.95E-09	2.47E-08	10.578439
ACSL4	0.3861632	6.6600465	6.0825999	2.95E-09	2.47E-08	10.578177
DAB2	0.2133397	6.2227735	6.0825972	2.95E-09	2.47E-08	10.578162
KLHL29	0.4675029	5.3767631	6.0821047	2.96E-09	2.48E-08	10.57546
RP11-363E6	0.9662931	-0.30389	6.0812045	2.97E-09	2.49E-08	10.57052
RP11-160H2	-1.032683	0.5860398	-6.081194	2.97E-09	2.49E-08	10.570461
TRAV41	0.9637125	-0.404255	6.0801574	2.99E-09	2.50E-08	10.564776
ETV6	0.1058403	6.0183203	6.0801562	2.99E-09	2.50E-08	10.564769
SPECC1	0.3390235	5.3089181	6.079989	2.99E-09	2.50E-08	10.563852
C10orf32	-0.151432	6.373261	-6.0799	2.99E-09	2.51E-08	10.563364
RP3-525N10	0.969997	-0.261666	6.0784787	3.02E-09	2.53E-08	10.555568
CEACAM3	1.1279765	0.7942016	6.0774058	3.04E-09	2.54E-08	10.549684
TNFAIP8L2	0.3241206	4.8831978	6.0769534	3.04E-09	2.55E-08	10.547203
FAM20A	-0.2066	6.5237823	-6.076742	3.05E-09	2.55E-08	10.546044
CTBP1-AS	0.7413263	3.765153	6.0767362	3.05E-09	2.55E-08	10.546012
MECOM	0.2722496	5.3122236	6.0765227	3.05E-09	2.55E-08	10.544841
BAIAP3	-0.259177	5.7078837	-6.075787	3.07E-09	2.56E-08	10.54081
MBLAC1	-0.225128	4.9295671	-6.075696	3.07E-09	2.56E-08	10.540307
CCDC28A	-0.132144	6.0944185	-6.075651	3.07E-09	2.56E-08	10.540063
SPA17P1	-0.810909	-0.867538	-6.075402	3.07E-09	2.56E-08	10.538697
DHX58	-0.191312	5.9329738	-6.075376	3.07E-09	2.56E-08	10.538556
LINC00963	-0.132026	6.5142285	-6.074146	3.09E-09	2.58E-08	10.531814
GRPEL1	-0.117827	6.3976213	-6.072834	3.12E-09	2.60E-08	10.524622
DNM1P46	1.2261435	0.661781	6.0726357	3.12E-09	2.60E-08	10.523534
PSMA7	-0.101692	6.82236	-6.071793	3.14E-09	2.61E-08	10.518916
HIGD2A	-0.130747	6.5243935	-6.071587	3.14E-09	2.62E-08	10.517788
AC016907.3	-0.971529	0.8704498	-6.070583	3.16E-09	2.63E-08	10.512286
ACSS3	-0.370953	6.3493358	-6.070152	3.16E-09	2.64E-08	10.509924
FAM118A	0.1726582	5.7932123	6.0696126	3.17E-09	2.64E-08	10.506971
IGFBP2	-0.335079	6.8868348	-6.068556	3.19E-09	2.66E-08	10.501181
SRP14-AS1	-0.209611	5.2657515	-6.068063	3.20E-09	2.67E-08	10.498483
LIMD1	-0.109378	6.3458188	-6.067824	3.21E-09	2.67E-08	10.497175

GRM6	1.1804701	0.6600871	6.0674229	3.21E-09	2.67E-08	10.494977
RPF1	-0.096144	6.0605046	-6.066644	3.23E-09	2.69E-08	10.490712
NFKBID	0.2817809	4.6546118	6.0665687	3.23E-09	2.69E-08	10.4903
ARHGAP30	0.2218539	5.6458069	6.0662976	3.23E-09	2.69E-08	10.488815
ZNF286A	0.302855	4.521445	6.0662824	3.23E-09	2.69E-08	10.488732
SERF2	-0.124858	7.167227	-6.065954	3.24E-09	2.69E-08	10.486932
MLLT3	0.4544767	4.7889954	6.0651304	3.26E-09	2.71E-08	10.482425
POLR1D	-0.098189	6.4484283	-6.064917	3.26E-09	2.71E-08	10.481259
SNHG14	0.4010969	5.7023013	6.0647923	3.26E-09	2.71E-08	10.480574
ZNF793	0.656392	4.0992948	6.0636193	3.28E-09	2.73E-08	10.474153
SIGLEC22P	0.8937907	-0.382794	6.0634307	3.29E-09	2.73E-08	10.47312
RP11-403I1	-0.658817	-0.999696	-6.062891	3.30E-09	2.74E-08	10.470166
RP11-958N2	1.0580919	3.5848864	6.0621831	3.31E-09	2.75E-08	10.466292
CTC-281M2C	-0.725411	-1.219532	-6.061746	3.32E-09	2.75E-08	10.463902
ZPLD1	1.5908414	1.0439294	6.0612095	3.33E-09	2.76E-08	10.460964
GPR133	0.7717395	5.1138629	6.0609847	3.33E-09	2.76E-08	10.459735
LRRC47	-0.089288	6.398316	-6.060696	3.34E-09	2.77E-08	10.458152
ZNF576	-0.108003	5.8305444	-6.058138	3.39E-09	2.81E-08	10.444162
ACOT11	0.8338495	3.5791582	6.057454	3.40E-09	2.82E-08	10.440421
ST7	-0.117887	6.2542876	-6.057349	3.40E-09	2.82E-08	10.439847
NFKBIA	-0.117851	6.6951352	-6.05678	3.41E-09	2.83E-08	10.436733
CACHD1	0.4010824	5.0961813	6.0566361	3.42E-09	2.83E-08	10.435948
SPRY4	0.1728638	5.9797939	6.0565055	3.42E-09	2.83E-08	10.435233
NRIP3	0.8202089	2.6964753	6.0559178	3.43E-09	2.84E-08	10.43202
GSPT2	0.6978121	4.9464572	6.0555099	3.44E-09	2.85E-08	10.429789
NCKAP5L	0.1799714	5.859064	6.055434	3.44E-09	2.85E-08	10.429375
SEC62	-0.087456	6.7349002	-6.055215	3.44E-09	2.85E-08	10.428178
AIMP1	-0.098772	6.3334573	-6.054235	3.46E-09	2.86E-08	10.422818
KCNJ16	1.6063574	1.2610382	6.0540362	3.47E-09	2.87E-08	10.421733
RIN2	0.1997536	5.7012855	6.0535561	3.48E-09	2.87E-08	10.419108
BCKDK	-0.114327	6.5109378	-6.052627	3.49E-09	2.89E-08	10.414033
C4BPB	-0.313765	6.8818091	-6.052382	3.50E-09	2.89E-08	10.412689
FBXO4	-0.12797	5.6823218	-6.050868	3.53E-09	2.92E-08	10.404419
HRASLS	1.0691089	-0.170139	6.0494557	3.56E-09	2.94E-08	10.396701
RBM4	0.1410435	5.484133	6.0492666	3.56E-09	2.94E-08	10.395668
RP11-321A1	-0.533111	3.8768899	-6.049171	3.56E-09	2.94E-08	10.395145
SEMA6D	0.4768042	4.5156953	6.0473044	3.60E-09	2.97E-08	10.38495
ELOF1	-0.108459	6.3176017	-6.047186	3.60E-09	2.97E-08	10.384301
SKIL	0.132583	6.1399202	6.046867	3.61E-09	2.98E-08	10.382561
BAK1	0.171739	5.8695562	6.0461565	3.62E-09	2.99E-08	10.378682
RSPH1	1.1520672	2.9869278	6.04597	3.63E-09	2.99E-08	10.377663
KLRK1	1.1902988	1.3092354	6.0456924	3.63E-09	3.00E-08	10.376147
RP11-143J1	1.0173742	1.5395248	6.0453863	3.64E-09	3.00E-08	10.374476
EMC9	-0.150706	5.8432061	-6.045271	3.64E-09	3.00E-08	10.373847
HGD	-0.335827	6.9364568	-6.044315	3.66E-09	3.02E-08	10.368625
MRPS31P4	1.0392957	2.5018702	6.0438339	3.67E-09	3.03E-08	10.366001
RP11-329B9	1.0390595	0.4748945	6.0437054	3.67E-09	3.03E-08	10.365299
PTX3	0.9634026	2.9827096	6.0431119	3.69E-09	3.04E-08	10.362059
RPS15A	-0.117757	6.9985719	-6.042764	3.69E-09	3.04E-08	10.360162
RP1-292L2C	-0.53908	3.6063327	-6.042629	3.70E-09	3.04E-08	10.359421
AC016745.3	-1.127263	0.2140843	-6.042173	3.71E-09	3.05E-08	10.356935

ITGB4	0.2690437	5.7337424	6.0420429	3.71E-09	3.05E-08	10.356225
RP11-76E17	0.9981143	-0.174777	6.0403114	3.75E-09	3.08E-08	10.346776
SLCO4A1-AS	1.2945482	-0.041542	6.0380376	3.79E-09	3.12E-08	10.334371
UBE2D1	0.114758	5.7786845	6.0378541	3.80E-09	3.12E-08	10.333371
SIRT7	-0.123465	6.040268	-6.03774	3.80E-09	3.12E-08	10.332747
PAG1	0.2532369	5.8938517	6.0354962	3.85E-09	3.16E-08	10.320511
PAF1	-0.095238	6.5271099	-6.035034	3.86E-09	3.17E-08	10.317993
RP5-908M14	-0.319906	4.6013152	-6.034931	3.86E-09	3.17E-08	10.317429
PSME2P2	-0.271697	4.5085691	-6.033507	3.89E-09	3.20E-08	10.309665
GDPD3	0.350679	4.3705936	6.0326052	3.91E-09	3.21E-08	10.30475
ABCC10	0.1259908	5.8652611	6.0322058	3.92E-09	3.22E-08	10.302573
ZHX3	-0.131506	6.1733295	-6.031824	3.93E-09	3.22E-08	10.300491
RP1-127D3.	-1.25199	-0.170835	-6.0318	3.93E-09	3.22E-08	10.300363
MVD	-0.176129	6.4452316	-6.0311	3.95E-09	3.24E-08	10.296548
TRAV22	0.9257549	-0.581775	6.0307102	3.95E-09	3.24E-08	10.294423
WWOX	-0.153735	5.7584792	-6.030332	3.96E-09	3.25E-08	10.292361
HOMEZ	-0.10451	5.7995005	-6.029485	3.98E-09	3.26E-08	10.287747
PGBD4	-0.158187	4.9168897	-6.02938	3.98E-09	3.27E-08	10.287175
PNPLA2	-0.117027	6.6374058	-6.029011	3.99E-09	3.27E-08	10.285166
MGAT4A	0.1324787	6.0944629	6.0286102	4.00E-09	3.28E-08	10.282981
TMEM214	-0.074488	6.6720877	-6.028483	4.00E-09	3.28E-08	10.282288
ATP6V0A1	-0.112902	6.5819115	-6.027635	4.02E-09	3.29E-08	10.27767
PLXDC1	0.2767355	5.3636121	6.0274356	4.03E-09	3.30E-08	10.276582
NECAB3	-0.145064	6.3281763	-6.027308	4.03E-09	3.30E-08	10.275886
CYCSP34	1.0990377	1.4618757	6.0262154	4.06E-09	3.32E-08	10.269937
HAO1	-0.508251	6.6154871	-6.025565	4.07E-09	3.33E-08	10.266393
TMEM56	-0.221097	6.5692962	-6.025493	4.07E-09	3.33E-08	10.266004
ZSCAN23	1.1084205	-0.157439	6.0246555	4.09E-09	3.35E-08	10.261443
TTLL3	0.1928449	5.3686805	6.024497	4.10E-09	3.35E-08	10.26058
HMOX2	-0.13661	6.4546081	-6.022631	4.14E-09	3.38E-08	10.250423
IQGAP2	-0.159088	6.7334408	-6.022007	4.15E-09	3.39E-08	10.247023
RP11-80H5.	-0.938922	1.5886944	-6.021881	4.16E-09	3.40E-08	10.246337
AL845472.1	0.8876482	-0.571686	6.0207268	4.18E-09	3.42E-08	10.240057
SYCE2	-0.339563	4.1436645	-6.020709	4.18E-09	3.42E-08	10.239959
SLC12A8	0.4045802	5.6961904	6.0206476	4.18E-09	3.42E-08	10.239627
C7	0.7032392	5.7583107	6.020457	4.19E-09	3.42E-08	10.23859
DDN	1.2036561	0.6921365	6.0198038	4.20E-09	3.43E-08	10.235035
ERGIC3	-0.094559	6.8974806	-6.018915	4.23E-09	3.45E-08	10.2302
TRAV1-1	0.9369571	-0.476594	6.018433	4.24E-09	3.46E-08	10.227577
IFT43	-0.134464	6.0268975	-6.018375	4.24E-09	3.46E-08	10.227263
SLCO5A1	1.203985	2.4679438	6.0172627	4.27E-09	3.48E-08	10.221211
MRPL45	-0.104199	6.296914	-6.016702	4.28E-09	3.49E-08	10.21816
SLC5A6	-0.170613	6.5468448	-6.016693	4.28E-09	3.49E-08	10.21811
IGLV3-16	1.0456076	-0.570572	6.0165788	4.28E-09	3.49E-08	10.217491
NCF4	0.2736103	5.2671189	6.0163208	4.29E-09	3.49E-08	10.216088
SEPN1	0.0900907	6.4520864	6.0158914	4.30E-09	3.50E-08	10.213753
BCAT2	-0.158499	6.2471441	-6.015775	4.30E-09	3.50E-08	10.213117
TAPBP	-0.106486	7.0234806	-6.015172	4.32E-09	3.51E-08	10.20984
TOM1	-0.110267	6.6768261	-6.013419	4.36E-09	3.55E-08	10.200311
RP11-145E1	-0.860134	-0.864286	-6.012831	4.37E-09	3.56E-08	10.19711
RP3-523K23	1.3777861	-0.001469	6.0121975	4.39E-09	3.57E-08	10.193669

GCHFR	-0.243048	5.8549606	-6.012004	4.39E-09	3.57E-08	10.192616
DOCK9-AS1	-0.816539	-0.705737	-6.011081	4.42E-09	3.59E-08	10.187603
RP3-414A15	-0.56471	3.9236043	-6.010838	4.42E-09	3.60E-08	10.186283
DMPK	0.1472381	5.7895612	6.0104998	4.43E-09	3.60E-08	10.184443
LINC00926	0.4261425	4.0460995	6.0096651	4.45E-09	3.62E-08	10.179907
FRMD8	0.0769611	6.1822825	6.0093268	4.46E-09	3.62E-08	10.178069
PARP15	0.5389348	4.2360727	6.0089599	4.47E-09	3.63E-08	10.176075
ECH1	-0.143568	7.00224	-6.008353	4.48E-09	3.64E-08	10.17278
IL34	0.4255617	4.9982663	6.0082386	4.49E-09	3.64E-08	10.172156
ARHGDIB	0.1365316	6.418375	6.0079458	4.49E-09	3.65E-08	10.170566
ANKRD36C	0.6240884	3.5455803	6.0075053	4.51E-09	3.66E-08	10.168173
CPA3	1.2290287	3.2370642	6.0072664	4.51E-09	3.66E-08	10.166875
RP11-354K1	-1.161025	0.1571319	-6.007213	4.51E-09	3.66E-08	10.166586
RP11-582J1	0.7424303	3.6864992	6.0063854	4.53E-09	3.68E-08	10.16209
AC004862.6	-1.215866	4.3810006	-6.006134	4.54E-09	3.68E-08	10.160724
RP11-42801	-0.948573	2.4912941	-6.005878	4.55E-09	3.69E-08	10.159333
RAD23B	-0.084191	6.8785191	-6.00574	4.55E-09	3.69E-08	10.158586
ZBTB8OS	-0.122762	5.9612209	-6.00562	4.55E-09	3.69E-08	10.157934
METTL7B	-0.20723	6.7171709	-6.005465	4.56E-09	3.69E-08	10.157089
RP3-399L15	0.8237505	2.0117788	6.0045935	4.58E-09	3.71E-08	10.152359
RRAD	0.6737636	4.4084825	6.0045318	4.58E-09	3.71E-08	10.152024
RP11-111M2	-0.27172	4.9588641	-6.003995	4.60E-09	3.72E-08	10.149112
LINC01073	-1.020992	-0.106413	-6.003641	4.60E-09	3.73E-08	10.14719
ANKRD18A	1.5620969	1.8026615	6.0029345	4.62E-09	3.74E-08	10.143352
RNF44	0.1084882	6.1943411	6.001953	4.65E-09	3.76E-08	10.138024
CYP2D6	-0.439317	6.6371069	-6.001592	4.66E-09	3.77E-08	10.136067
LRRC16A	0.4214778	5.3966082	6.0015801	4.66E-09	3.77E-08	10.136
TMEM61	1.3756324	1.6349913	6.0015448	4.66E-09	3.77E-08	10.135808
SLC8A2	1.1316424	0.760165	6.0015292	4.66E-09	3.77E-08	10.135724
LY75	0.3542849	4.5921234	6.0013674	4.66E-09	3.77E-08	10.134845
CRIPT	-0.100961	5.9154084	-6.000992	4.67E-09	3.78E-08	10.13281
PCBP3-OT1	0.7322921	-0.976552	5.9999271	4.70E-09	3.80E-08	10.127029
RP11-70C1.	-1.153367	0.9757271	-5.997921	4.75E-09	3.84E-08	10.116142
ABI3	0.1985127	5.5067955	5.9978502	4.76E-09	3.84E-08	10.115761
AC093616.4	0.8786816	2.6876773	5.9973211	4.77E-09	3.85E-08	10.112891
BRSK2	1.1960212	2.528149	5.9968364	4.78E-09	3.86E-08	10.110261
EMR1	0.797476	3.2377603	5.9967592	4.79E-09	3.86E-08	10.109843
RP11-252E2	-1.449562	1.3362186	-5.996714	4.79E-09	3.86E-08	10.109599
KLK1	1.1365748	0.0187767	5.995954	4.81E-09	3.88E-08	10.105475
RP11-390E2	-0.175586	5.2324849	-5.995442	4.82E-09	3.89E-08	10.102698
AP000783.1	1.0125795	0.2468547	5.9942442	4.85E-09	3.91E-08	10.096203
PRAP1	-0.454329	6.944576	-5.994135	4.86E-09	3.91E-08	10.095613
BHMT	-0.539918	6.5588691	-5.993261	4.88E-09	3.93E-08	10.090873
FBXW5	-0.106586	6.7970981	-5.992929	4.89E-09	3.94E-08	10.089071
COX7B	-0.129422	6.7500129	-5.99291	4.89E-09	3.94E-08	10.088968
GPT2	-0.263441	6.7055662	-5.992741	4.89E-09	3.94E-08	10.088053
GPATCH2L	0.1130848	5.7872113	5.9926827	4.90E-09	3.94E-08	10.087738
RASGRP4	0.3551885	4.194745	5.9924595	4.90E-09	3.95E-08	10.086528
MYOCD	1.1234214	3.0083207	5.9919003	4.92E-09	3.96E-08	10.083496
BDKRB1	1.0171915	3.0471582	5.9914117	4.93E-09	3.97E-08	10.080848
TMEM151B	0.930503	-0.019004	5.9911765	4.94E-09	3.97E-08	10.079573

RP11-502I4	-0.358462	4.1422487	-5.991022	4.94E-09	3.97E-08	10.078736
C3orf70	0.365106	4.3419265	5.9904877	4.96E-09	3.99E-08	10.07584
CMKLR1	0.2635057	5.4065544	5.9890781	5.00E-09	4.02E-08	10.068202
HFE2	-0.568669	6.603085	-5.988923	5.00E-09	4.02E-08	10.067361
FP325317.1	-1.325475	0.7823171	-5.988789	5.00E-09	4.02E-08	10.066636
TP53I11	0.1552135	6.1519446	5.9878826	5.03E-09	4.04E-08	10.061725
CTB-31N19.	0.6829289	2.540916	5.9875606	5.04E-09	4.05E-08	10.05998
AC142472.6	-0.312408	4.3167598	-5.987448	5.04E-09	4.05E-08	10.059371
PHOSPHO1	0.8369887	2.39024	5.9871442	5.05E-09	4.05E-08	10.057725
CLPB	-0.133868	6.0282688	-5.986643	5.07E-09	4.07E-08	10.055007
CCDC36	1.0114263	1.4830203	5.986318	5.07E-09	4.07E-08	10.053249
EREG	1.4872725	1.0940905	5.9855311	5.10E-09	4.09E-08	10.048988
RP11-378A1	0.2644203	4.2004408	5.9852199	5.11E-09	4.10E-08	10.047302
COL5A3	-0.301821	6.2443281	-5.984937	5.11E-09	4.10E-08	10.045772
RP11-767I2	1.2753063	0.4808036	5.9848842	5.12E-09	4.10E-08	10.045484
RPL37	-0.126482	7.2047154	-5.984876	5.12E-09	4.10E-08	10.045438
CADM3-AS1	1.0156485	-0.381423	5.9829653	5.17E-09	4.14E-08	10.035094
TRPC4	0.9017759	3.0660187	5.981512	5.21E-09	4.18E-08	10.027227
FAM227A	1.0432969	2.4360208	5.9812852	5.22E-09	4.18E-08	10.025999
ZNF212	-0.097838	5.8411257	-5.97954	5.27E-09	4.22E-08	10.016555
TNIP3	1.2374663	1.0288077	5.979012	5.29E-09	4.23E-08	10.013698
TMEM97	-0.184928	6.6001893	-5.978763	5.29E-09	4.24E-08	10.012349
HSPA7	0.5907505	4.4608559	5.9786514	5.30E-09	4.24E-08	10.011747
PKD1L1	0.6105059	3.2893385	5.9782169	5.31E-09	4.25E-08	10.009396
CNPY4	0.1371871	5.5206553	5.9780415	5.32E-09	4.25E-08	10.008447
RNASE6	0.2999562	5.2893465	5.9769483	5.35E-09	4.28E-08	10.002533
TNFRSF11B	0.8763681	4.7977853	5.9768383	5.35E-09	4.28E-08	10.001938
C8A	-0.451936	6.685839	-5.976774	5.35E-09	4.28E-08	10.001593
RP13-977J1	0.8982987	2.7733252	5.9764976	5.36E-09	4.29E-08	10.000095
SETD7	-0.136673	6.5458698	-5.976394	5.36E-09	4.29E-08	9.9995321
RP11-701H2	1.250967	0.8929125	5.9756931	5.39E-09	4.30E-08	9.9957434
LHFPL3-AS2	1.5463723	1.2558834	5.9752092	5.40E-09	4.31E-08	9.9931263
CDC34	-0.119764	6.5576414	-5.974848	5.41E-09	4.32E-08	9.9911726
FDX1	-0.142923	6.3592026	-5.974795	5.41E-09	4.32E-08	9.990887
C7orf60	0.1430317	5.2007231	5.9747053	5.42E-09	4.32E-08	9.9904016
HDAC8	-0.09854	5.940601	-5.974574	5.42E-09	4.32E-08	9.9896916
GPM6B	0.6011396	3.5334173	5.9740723	5.43E-09	4.34E-08	9.9869786
RP11-119F7	0.5984117	3.9735577	5.9739068	5.44E-09	4.34E-08	9.9860838
IGHV1OR15-	1.0499379	-0.565779	5.9738942	5.44E-09	4.34E-08	9.9860156
KIF3B	-0.084954	6.4877806	-5.973818	5.44E-09	4.34E-08	9.9856043
APOA5	-0.529901	6.5993573	-5.973247	5.46E-09	4.35E-08	9.9825189
BEGAIN	1.1736328	2.8062249	5.9724905	5.48E-09	4.37E-08	9.9784263
ASGR1	-0.266271	7.090349	-5.972284	5.49E-09	4.37E-08	9.9773079
RP11-545A1	1.0831964	-0.290982	5.9720644	5.50E-09	4.38E-08	9.9761229
STARD3	-0.086609	6.3930939	-5.97181	5.50E-09	4.38E-08	9.974749
TUB	1.0941484	4.0041616	5.9715424	5.51E-09	4.39E-08	9.9733013
AP002954.4	1.0751811	0.4143305	5.970785	5.54E-09	4.41E-08	9.9692076
EHBP1L1	0.1083773	6.202741	5.9707386	5.54E-09	4.41E-08	9.9689568
EXOSC5	-0.143095	6.1101196	-5.970501	5.54E-09	4.41E-08	9.9676735
GPR25	0.8725784	-0.551685	5.9704546	5.55E-09	4.41E-08	9.9674219
BLK	1.1659721	2.3881042	5.9694667	5.58E-09	4.44E-08	9.9620829

MED8	-0.087788	6.3509971	-5.96926	5.58E-09	4.44E-08	9.9609643
INTS1	-0.085685	6.6967073	-5.969105	5.59E-09	4.44E-08	9.9601314
RP11-320M2	-0.285462	4.3667155	-5.968031	5.62E-09	4.47E-08	9.9543272
RAP1GDS1	-0.134971	6.0957997	-5.967647	5.63E-09	4.48E-08	9.9522524
NDUFV3	-0.116351	6.4021975	-5.967218	5.65E-09	4.49E-08	9.9499339
ANKRD10-IT	0.496094	4.3958161	5.9654748	5.70E-09	4.53E-08	9.9405175
PLXNC1	0.3247995	5.7443761	5.9622564	5.81E-09	4.61E-08	9.9231395
EIF4EBP2	-0.109724	6.8954743	-5.961496	5.83E-09	4.63E-08	9.9190374
UFD1L	-0.100662	6.4969689	-5.960269	5.87E-09	4.66E-08	9.9124148
TTR	-0.361847	7.3187183	-5.960197	5.87E-09	4.66E-08	9.9120216
CC2D2A	0.2252703	5.1351915	5.9599239	5.88E-09	4.67E-08	9.9105501
NCR3	0.7511123	3.2042067	5.9589676	5.91E-09	4.69E-08	9.9053896
GPD2	0.1444253	5.7547851	5.9569493	5.98E-09	4.74E-08	9.8945002
CDHR3	-0.229172	5.245938	-5.956947	5.98E-09	4.74E-08	9.8944868
IGFBPL1	1.508654	1.2580834	5.956523	5.99E-09	4.75E-08	9.8922006
RNH1	-0.091703	6.7564722	-5.956234	6.00E-09	4.76E-08	9.8906406
SAPCD2	0.4716046	4.648329	5.9557456	6.02E-09	4.77E-08	9.8880077
DERL1	-0.110875	6.6881138	-5.955287	6.04E-09	4.78E-08	9.8855355
IKZF3	0.3683656	5.1445789	5.954971	6.05E-09	4.79E-08	9.88383
LINC00272	-0.955191	-0.798565	-5.954716	6.06E-09	4.80E-08	9.8824534
ZNF571-AS1	0.9500892	2.1128085	5.9543338	6.07E-09	4.80E-08	9.8803938
C3orf14	0.7076737	4.2792874	5.9540576	6.08E-09	4.81E-08	9.8789046
RP11-66602	-0.535547	3.2814345	-5.954008	6.08E-09	4.81E-08	9.8786354
TNFSF9	1.0131231	3.1618915	5.9539484	6.08E-09	4.81E-08	9.8783158
REEP1	0.8817379	3.9658265	5.9538404	6.08E-09	4.81E-08	9.877733
RAPGEF5	0.1833773	6.0119853	5.9536584	6.09E-09	4.82E-08	9.8767519
RP4-60503.	-0.367779	3.9545089	-5.952861	6.12E-09	4.84E-08	9.8724529
PI3	1.4989419	2.7698102	5.9521638	6.14E-09	4.86E-08	9.8686938
LINC00908	1.1894524	0.4848996	5.9513303	6.17E-09	4.88E-08	9.8642008
DUSP3	-0.089194	6.7728467	-5.950486	6.20E-09	4.90E-08	9.8596506
DNAH10OS	0.6348394	4.1129796	5.9503983	6.20E-09	4.90E-08	9.8591776
BCL2L14	0.8571398	3.6140273	5.950313	6.21E-09	4.90E-08	9.8587179
ZNF8	0.2004554	5.2068534	5.9501391	6.21E-09	4.91E-08	9.8577805
SP100	-0.111523	6.4259112	-5.949224	6.24E-09	4.93E-08	9.8528465
ESM1	-0.421339	5.419725	-5.948861	6.26E-09	4.94E-08	9.8508937
C11orf74	-0.134827	5.6349542	-5.948701	6.26E-09	4.94E-08	9.8500318
CUBN	0.4971375	3.7153015	5.9485634	6.27E-09	4.94E-08	9.8492894
NCAM2	1.398264	2.4087932	5.9484196	6.27E-09	4.95E-08	9.8485144
CHRNA6	0.9862	-0.351779	5.9477646	6.29E-09	4.96E-08	9.8449858
RP11-334E6	-0.912238	-0.497725	-5.947748	6.29E-09	4.96E-08	9.8448971
TNFSF18	0.9954988	2.3715751	5.9475693	6.30E-09	4.97E-08	9.8439335
C6orf62	-0.084361	6.8014982	-5.947482	6.30E-09	4.97E-08	9.8434622
FMO3	-0.374933	6.804389	-5.946802	6.33E-09	4.99E-08	9.8397996
CADM1	-0.213319	6.534798	-5.94656	6.34E-09	4.99E-08	9.8384985
RP11-354I1	-0.718804	-0.94511	-5.945151	6.39E-09	5.03E-08	9.8309096
PDP2	-0.129715	5.8267891	-5.945049	6.39E-09	5.03E-08	9.8303578
NKAIN1	1.2438122	0.3527525	5.9448591	6.40E-09	5.04E-08	9.8293351
RP11-231P2	-0.851141	4.8625951	-5.944446	6.41E-09	5.05E-08	9.8271124
DAXX	-0.084963	6.4397726	-5.944304	6.42E-09	5.05E-08	9.8263467
RP11-598P2	1.1080721	2.3641191	5.9441812	6.42E-09	5.05E-08	9.8256846
MRPL10	-0.088349	6.3055556	-5.943899	6.43E-09	5.06E-08	9.8241638

PSAPL1	1.2618706	-0.112043	5.943214	6.46E-09	5.08E-08	9.8204768
FOXS1	0.5540816	4.5403045	5.9420125	6.50E-09	5.11E-08	9.8140077
CDH4	0.9811509	2.7189098	5.9418672	6.50E-09	5.11E-08	9.8132256
MRPL23	-0.150899	6.298086	-5.940905	6.54E-09	5.14E-08	9.8080464
CD7	0.3737592	5.250664	5.9403663	6.56E-09	5.15E-08	9.805147
LINC01342	0.747429	-0.898312	5.938797	6.62E-09	5.20E-08	9.7967017
RP4-671014	0.9595697	0.1584611	5.9386094	6.62E-09	5.20E-08	9.7956922
GSS	-0.100341	6.462407	-5.937437	6.67E-09	5.24E-08	9.7893841
IGHJ3P	0.883904	-0.795017	5.9364663	6.70E-09	5.26E-08	9.7841627
ENTPD3	1.287211	1.0581674	5.9354987	6.74E-09	5.29E-08	9.778958
PRDM11	0.2178066	5.1714365	5.9343167	6.78E-09	5.32E-08	9.7726011
CDC42EP4	-0.100851	6.4724622	-5.933881	6.80E-09	5.34E-08	9.770258
SMUG1	-0.100762	6.1889506	-5.933466	6.82E-09	5.35E-08	9.7680243
LEFTY1	1.1915695	2.8701875	5.9324434	6.85E-09	5.38E-08	9.7625289
GLE1	-0.091799	6.2001656	-5.93222	6.86E-09	5.38E-08	9.7613283
HS1BP3-IT1	-0.636567	4.6139685	-5.931651	6.89E-09	5.40E-08	9.7582679
RP11-848P1	0.4271043	3.7865682	5.9314425	6.89E-09	5.40E-08	9.7571483
CTD-3064M3	0.9919922	2.3243139	5.9311905	6.90E-09	5.41E-08	9.7557934
RP11-57H14	0.3271769	4.4737778	5.9310473	6.91E-09	5.41E-08	9.755024
IL24	0.6533148	3.4973145	5.930973	6.91E-09	5.41E-08	9.7546248
RP6-109B7.	-0.816746	2.6272432	-5.930915	6.91E-09	5.41E-08	9.7543128
TCF21	0.8335032	3.5819724	5.9301754	6.94E-09	5.44E-08	9.7503378
IDI1	-0.145583	6.7447531	-5.928912	6.99E-09	5.47E-08	9.7435474
PHYHD1	-0.522038	5.5674511	-5.928781	7.00E-09	5.48E-08	9.7428448
C6	-0.40404	6.6892052	-5.928625	7.00E-09	5.48E-08	9.7420072
UBE2D3	-0.079088	6.8332362	-5.928043	7.02E-09	5.50E-08	9.7388795
ZNF320	0.4811469	5.179052	5.9274719	7.05E-09	5.51E-08	9.7358107
CEACAM6	1.5431975	0.6172695	5.9273637	7.05E-09	5.51E-08	9.7352293
PCDHGB8P	1.0520047	0.0846256	5.9268936	7.07E-09	5.53E-08	9.7327039
CYSRT1	-0.389566	4.1333977	-5.926566	7.08E-09	5.54E-08	9.7309456
FABP3	0.3403533	4.9803212	5.9265056	7.09E-09	5.54E-08	9.7306197
USMG5	-0.142112	6.5893209	-5.926089	7.10E-09	5.55E-08	9.7283818
LINC00279	-0.480348	-1.3239	-5.925847	7.11E-09	5.55E-08	9.7270827
RPL34	-0.135447	7.0126059	-5.923824	7.19E-09	5.62E-08	9.7162179
RSPH4A	0.8147749	2.4098765	5.9235444	7.20E-09	5.62E-08	9.7147163
THEM6	-0.155164	6.5623165	-5.923057	7.22E-09	5.64E-08	9.7120975
AC005592.2	1.1382517	0.6751642	5.9217499	7.27E-09	5.68E-08	9.7050818
LRRC70	0.9222425	2.45615	5.9216425	7.28E-09	5.68E-08	9.7045052
CROCCP2	-0.149034	6.062391	-5.921427	7.29E-09	5.69E-08	9.7033463
ARHGAP5-AS	-0.278334	4.6142085	-5.921065	7.30E-09	5.70E-08	9.7014054
DECR1	-0.149819	6.866978	-5.920809	7.31E-09	5.70E-08	9.7000328
CTC-350I8.	-0.885011	-0.31739	-5.920765	7.31E-09	5.70E-08	9.6997937
GCC2	0.1268648	5.9922745	5.9207122	7.32E-09	5.70E-08	9.6995117
CTD-2006M2	0.9109413	-0.606283	5.9205389	7.32E-09	5.71E-08	9.6985817
SLC35E4	0.2974204	4.6635671	5.9199513	7.35E-09	5.73E-08	9.6954279
CACNA1F	1.123265	1.5659339	5.9178472	7.43E-09	5.79E-08	9.6841374
ADK	-0.151041	6.3463474	-5.917577	7.45E-09	5.80E-08	9.6826852
TRBV28	0.5019436	4.6523381	5.9173963	7.45E-09	5.80E-08	9.6817183
RP11-308D1	0.8322809	2.5390831	5.917373	7.45E-09	5.80E-08	9.6815929
BCL7B	-0.092951	6.3408612	-5.917293	7.46E-09	5.80E-08	9.6811655
NFS1	-0.091577	6.2793224	-5.917101	7.47E-09	5.81E-08	9.6801313

MTHFD1L	0.2055974	5.7309668	5.9166519	7.48E-09	5.82E-08	9.6777244
RP11-528A4	1.2709125	1.1766807	5.9164504	7.49E-09	5.83E-08	9.6766436
TINAGL1	0.1808307	6.055121	5.9148411	7.56E-09	5.88E-08	9.6680122
RP11-598F7	1.068791	0.1123113	5.9146352	7.57E-09	5.88E-08	9.6669077
HLA-DRB9	1.0924141	0.1824196	5.91454	7.57E-09	5.89E-08	9.6663974
NANS	-0.113943	6.3073069	-5.914496	7.57E-09	5.89E-08	9.666162
RP4-794H19	-0.441137	3.7982868	-5.914281	7.58E-09	5.89E-08	9.6650082
ZNF563	-0.2465	5.5813376	-5.913981	7.60E-09	5.90E-08	9.6633978
EPN1	-0.09573	6.8735911	-5.913883	7.60E-09	5.90E-08	9.6628755
GINS4	0.4224157	4.767846	5.9136253	7.61E-09	5.91E-08	9.6614926
ZNF398	-0.103108	5.7949008	-5.913348	7.62E-09	5.92E-08	9.6600062
HYI	-0.133826	5.8617474	-5.91318	7.63E-09	5.92E-08	9.6591037
GLIS3	0.4134495	5.3330354	5.9130159	7.64E-09	5.93E-08	9.658225
PRKCD	0.1493456	5.9922085	5.9119313	7.68E-09	5.96E-08	9.6524103
C1orf86	-0.121111	6.2503466	-5.911704	7.69E-09	5.97E-08	9.6511928
FOXJ1	1.4271937	2.5552178	5.9114681	7.70E-09	5.97E-08	9.6499272
HECTD2	0.2064089	5.3151906	5.9104633	7.75E-09	6.01E-08	9.6445411
FAM27C	1.1390876	0.3002355	5.9098391	7.77E-09	6.02E-08	9.6411961
TSSC4	-0.110415	6.2453391	-5.909515	7.79E-09	6.03E-08	9.639458
POP4	-0.093989	6.2209387	-5.9095	7.79E-09	6.03E-08	9.63938
GPR137B	0.2879883	5.7335022	5.9092537	7.80E-09	6.04E-08	9.6380587
TMEM161A	-0.11655	6.4286074	-5.909068	7.81E-09	6.05E-08	9.6370631
SMIM24	1.338866	3.9027498	5.9077611	7.86E-09	6.09E-08	9.6300608
TBRG4	-0.09004	6.5105277	-5.907587	7.87E-09	6.09E-08	9.629127
LILRB3	0.3166985	4.4765491	5.9074787	7.87E-09	6.10E-08	9.6285482
N4BP3	0.4272115	4.5609543	5.9071836	7.89E-09	6.10E-08	9.6269669
CEP85L	0.2598543	4.8738569	5.9068757	7.90E-09	6.11E-08	9.6253176
ZDHHC15	1.1096275	2.0509054	5.9062389	7.93E-09	6.13E-08	9.6219066
FZD10-AS1	1.1768768	1.6055832	5.9042951	8.01E-09	6.20E-08	9.6114957
NDUFB5	-0.09015	6.5436584	-5.904242	8.02E-09	6.20E-08	9.6112089
PHGR1	-1.215259	0.0554195	-5.904148	8.02E-09	6.20E-08	9.6107106
PLRG1	-0.083568	6.314275	-5.904131	8.02E-09	6.20E-08	9.6106149
NFIX	-0.150795	6.4274663	-5.904061	8.03E-09	6.20E-08	9.610244
ZMYND12	-0.571076	4.5993099	-5.903914	8.03E-09	6.21E-08	9.6094526
KLHL30	0.96021	2.7789092	5.9037377	8.04E-09	6.21E-08	9.6085111
C3	-0.168592	7.8903545	-5.903611	8.05E-09	6.21E-08	9.6078341
RPS27L	-0.129901	6.5643449	-5.903611	8.05E-09	6.21E-08	9.6078331
MEFV	0.6475342	3.1215312	5.9029972	8.07E-09	6.23E-08	9.6045459
MCOLN1	-0.105537	6.2202545	-5.901914	8.12E-09	6.27E-08	9.5987456
TAZ	-0.111416	6.1477374	-5.901709	8.13E-09	6.27E-08	9.5976516
PTRH2	-0.127657	6.1440509	-5.900309	8.19E-09	6.32E-08	9.5901544
LIFR	0.3775426	5.4597839	5.9002365	8.20E-09	6.32E-08	9.5897678
TXNDC12	-0.079587	6.5116877	-5.899973	8.21E-09	6.33E-08	9.5883551
THAP7-AS1	-0.319396	4.8604337	-5.899718	8.22E-09	6.34E-08	9.5869931
ATP5O	-0.131267	6.562207	-5.899494	8.23E-09	6.34E-08	9.5857922
RP11-554I8	1.0592515	-0.69165	5.8987957	8.26E-09	6.37E-08	9.5820573
RP13-1032I	-0.211164	4.9932113	-5.898315	8.28E-09	6.38E-08	9.5794836
LPXN	0.1218657	5.6336671	5.8981323	8.29E-09	6.39E-08	9.5785078
TTLL9	0.9488267	0.8913209	5.8980069	8.30E-09	6.39E-08	9.5778365
BPIFB1	1.4440466	0.6062029	5.897784	8.31E-09	6.40E-08	9.5766444
DRAP1	-0.109196	6.5651885	-5.897675	8.31E-09	6.40E-08	9.5760584

NCRNA0025C	-1.09418	0.4746792	-5.897336	8.33E-09	6.41E-08	9.5742465
MIR143HG	0.6719278	3.1281126	5.8972357	8.33E-09	6.41E-08	9.5737106
TRAV6	0.861001	-0.608956	5.8958487	8.40E-09	6.46E-08	9.5662916
MAPT-IT1	-1.189689	0.5560351	-5.895831	8.40E-09	6.46E-08	9.5661976
GLDCP1	1.154655	2.5291221	5.8956048	8.41E-09	6.47E-08	9.5649869
GLRX2	-0.157184	5.7811542	-5.895314	8.42E-09	6.48E-08	9.5634304
CCDC3	0.302688	5.6550845	5.894951	8.44E-09	6.49E-08	9.5614901
ECEL1	1.4688602	1.9270925	5.8947584	8.45E-09	6.49E-08	9.5604599
C9orf3	-0.109615	6.2753091	-5.893979	8.49E-09	6.52E-08	9.5562916
RPS2P32	0.8999538	2.5818526	5.8937644	8.50E-09	6.53E-08	9.5551445
NEFL	1.2185831	-0.013107	5.893617	8.50E-09	6.53E-08	9.5543563
BIRC7	1.2757404	1.4401579	5.8935019	8.51E-09	6.53E-08	9.553741
ACSL1	-0.219689	7.0436386	-5.893215	8.52E-09	6.54E-08	9.5522082
CYP17A1	-0.78219	5.3824942	-5.893078	8.53E-09	6.54E-08	9.551476
SAMD9	0.2483326	5.3439311	5.8930712	8.53E-09	6.54E-08	9.5514378
CTD-2015G9	1.3181359	0.7606342	5.8929576	8.53E-09	6.55E-08	9.5508308
RNF34	0.0816794	5.9659521	5.8927854	8.54E-09	6.55E-08	9.5499102
UGP2	-0.143111	6.9336877	-5.890761	8.64E-09	6.62E-08	9.5390884
PKD1P6	0.70761	4.9966105	5.8906408	8.64E-09	6.63E-08	9.5384456
SHROOM1	-0.167074	6.5256942	-5.889406	8.70E-09	6.67E-08	9.5318485
ALG14	-0.114074	5.944667	-5.889293	8.71E-09	6.67E-08	9.5312411
CHIC1	0.4823591	4.625693	5.8892614	8.71E-09	6.67E-08	9.531074
MED12L	1.0066751	1.8771846	5.8887605	8.73E-09	6.69E-08	9.5283971
KBTBD3	-0.142835	5.2087681	-5.888065	8.77E-09	6.72E-08	9.5246811
MXRA5Y	1.0759472	-0.212104	5.8878601	8.78E-09	6.72E-08	9.523586
KCNS3	0.5783859	4.8439832	5.887307	8.81E-09	6.74E-08	9.5206313
TRAV25	0.8119836	-0.765854	5.8866473	8.84E-09	6.76E-08	9.5171069
RNLS	-0.256412	5.5757478	-5.886187	8.86E-09	6.78E-08	9.5146496
CHRNA7	1.0118673	-0.278967	5.8853721	8.90E-09	6.81E-08	9.5102958
ADORA2BP1	-1.386153	3.1693299	-5.885332	8.90E-09	6.81E-08	9.5100791
CTD-2050E2	-0.594403	-1.173165	-5.884404	8.95E-09	6.84E-08	9.5051247
PON3	-0.28667	6.610597	-5.883797	8.98E-09	6.86E-08	9.5018816
HNRNPCP1	0.5502907	3.0211951	5.8830561	9.01E-09	6.89E-08	9.4979285
MMP17	1.0220127	3.3660229	5.8829149	9.02E-09	6.90E-08	9.4971743
DIO3OS	1.3960333	3.0763223	5.8823534	9.05E-09	6.92E-08	9.4941766
ISCA1	-0.098023	6.2077311	-5.882316	9.05E-09	6.92E-08	9.4939743
ALG13	-0.100058	6.0790419	-5.882121	9.06E-09	6.92E-08	9.4929347
PPP1R7	-0.09542	6.4387877	-5.880442	9.15E-09	6.98E-08	9.4839757
SLC39A3	-0.117788	6.0840095	-5.88025	9.16E-09	6.99E-08	9.4829503
PNPLA4	-0.142943	6.0003679	-5.879516	9.19E-09	7.02E-08	9.4790336
AKR1C2	-0.387972	6.7821342	-5.879117	9.21E-09	7.03E-08	9.4769018
EIF3F	-0.091586	6.6914353	-5.87903	9.22E-09	7.03E-08	9.4764374
HNRNPA1P15	-0.773902	-0.790516	-5.879004	9.22E-09	7.03E-08	9.4763018
PIK3CD-AS2	1.1532721	2.4803489	5.8789392	9.22E-09	7.03E-08	9.4759541
ARHGAP11B	0.3743229	4.5456842	5.8788643	9.23E-09	7.04E-08	9.475554
ENDOG	-0.187452	5.6079519	-5.878791	9.23E-09	7.04E-08	9.475163
STK24P1	0.9429008	1.0153524	5.8787905	9.23E-09	7.04E-08	9.4751605
KDM6A	0.1493544	5.9295646	5.87856	9.24E-09	7.04E-08	9.4739304
IL6	1.0906346	2.439205	5.8778323	9.28E-09	7.07E-08	9.4700476
RIMBP3	1.0771526	0.5585991	5.8776006	9.29E-09	7.08E-08	9.4688115
C12orf29	-0.095536	5.7866952	-5.877284	9.31E-09	7.09E-08	9.4671229

SH3RF2	-0.408673	5.6815815	-5.877214	9.31E-09	7.09E-08	9.4667504
CCDC147-AS	0.9387093	0.0553567	5.8770537	9.32E-09	7.09E-08	9.4658943
RP11-61102	-1.112087	3.8260539	-5.877	9.32E-09	7.09E-08	9.465609
CD38	0.482204	4.7966569	5.8765613	9.34E-09	7.11E-08	9.4632674
FRMD4A	0.191896	5.7428674	5.8764767	9.35E-09	7.11E-08	9.4628163
CCDC39	0.5384877	3.9742088	5.8764153	9.35E-09	7.11E-08	9.4624885
SDHAF1	-0.149486	5.8807107	-5.876319	9.36E-09	7.12E-08	9.461977
RRAGA	-0.090372	6.4759025	-5.875965	9.37E-09	7.13E-08	9.460085
MGAT4B	-0.117527	6.9538858	-5.875844	9.38E-09	7.13E-08	9.4594407
RP11-680F8	-0.459342	3.7495457	-5.875746	9.39E-09	7.13E-08	9.4589199
ESPN	-0.298001	6.4985024	-5.87564	9.39E-09	7.14E-08	9.458355
MAPK15	1.185256	2.1205863	5.8755855	9.39E-09	7.14E-08	9.4580625
YEATS2-AS1	0.8076065	2.6850968	5.8752036	9.41E-09	7.15E-08	9.4560258
KCNE5	1.2766147	2.924437	5.8751086	9.42E-09	7.15E-08	9.4555191
HSD17B6	-0.414579	6.8620449	-5.87496	9.43E-09	7.16E-08	9.4547281
GS1-21A4.2	-1.126427	2.4604449	-5.874846	9.43E-09	7.16E-08	9.4541166
SPG20	0.3194302	5.3321846	5.8748068	9.43E-09	7.16E-08	9.4539094
ACKR2	-0.457127	5.3895789	-5.874147	9.47E-09	7.18E-08	9.4503924
MMP7	1.4427792	3.8191494	5.8740127	9.48E-09	7.19E-08	9.4496748
NHSL2	1.0405535	2.5977558	5.8738704	9.48E-09	7.19E-08	9.4489159
SLC22A18	-0.260491	6.5780258	-5.873684	9.49E-09	7.20E-08	9.4479245
RP11-689B2	0.8518239	-0.709108	5.8732707	9.52E-09	7.21E-08	9.4457187
PIGBOS1	-0.118171	5.5808169	-5.872586	9.55E-09	7.24E-08	9.4420675
HORMAD2	-1.47014	2.6095039	-5.870836	9.64E-09	7.31E-08	9.4327418
NUDC	-0.11438	6.7460363	-5.870835	9.64E-09	7.31E-08	9.4327319
RP11-112L6	-0.875968	2.7617987	-5.870532	9.66E-09	7.32E-08	9.4311204
ZC3H12B	0.6898399	4.0499062	5.8694047	9.72E-09	7.36E-08	9.4251114
ANAPC15	-0.114933	5.6339416	-5.867124	9.84E-09	7.45E-08	9.4129598
MPRIPP1	1.0389343	0.6626454	5.866994	9.85E-09	7.46E-08	9.4122674
STOML2	-0.103238	6.6279874	-5.866883	9.86E-09	7.46E-08	9.4116784
GS1-279B7.	0.8589023	1.7834832	5.8661322	9.90E-09	7.49E-08	9.4076767
SLC1A2	-0.679483	5.8239965	-5.866007	9.90E-09	7.49E-08	9.4070122
PAN3	0.1127103	5.8530249	5.8656146	9.93E-09	7.51E-08	9.4049194
LIPC	-0.322161	6.5186917	-5.865503	9.93E-09	7.51E-08	9.4043277
KLRAP1	0.5195117	3.7830418	5.865445	9.93E-09	7.51E-08	9.4040161
UBQLN1	-0.077327	6.6732539	-5.865259	9.95E-09	7.52E-08	9.4030276
NME9	0.9563897	1.7348618	5.8648158	9.97E-09	7.53E-08	9.4006654
SOX11	1.3468079	0.7562053	5.8647559	9.97E-09	7.54E-08	9.4003465
SHISA3	1.2456471	3.0848393	5.8645298	9.99E-09	7.54E-08	9.3991423
LRCH2	0.7448259	3.3128812	5.8640033	1.00E-08	7.56E-08	9.396339
YIPF4	-0.081736	6.4169488	-5.863935	1.00E-08	7.57E-08	9.3959768
RP11-673E1	0.9262035	-0.138363	5.8638164	1.00E-08	7.57E-08	9.3953434
DGKG	0.6605225	4.4403324	5.8634578	1.00E-08	7.58E-08	9.3934341
SCARB2	-0.084344	6.9225952	-5.863448	1.00E-08	7.58E-08	9.3933809
PSMB1	-0.11942	6.7889474	-5.863076	1.01E-08	7.59E-08	9.3913999
SMIM3	0.2849265	5.2961011	5.8629547	1.01E-08	7.60E-08	9.3907557
RPLP0P2	0.8893952	2.8718423	5.8626739	1.01E-08	7.61E-08	9.3892607
RP11-290F5	-0.660315	3.6825079	-5.862524	1.01E-08	7.61E-08	9.3884648
FAM20C	-0.150088	6.6014433	-5.862449	1.01E-08	7.61E-08	9.3880627
NUDT16L1	-0.114401	6.2175484	-5.86205	1.01E-08	7.63E-08	9.3859392
RP11-176H8	-0.45825	3.4534557	-5.86144	1.02E-08	7.65E-08	9.3826927

IGLC3	1.0793632	4.6162825	5.8612333	1.02E-08	7.66E-08	9.3815917
PAICS	-0.08789	6.7267992	-5.860704	1.02E-08	7.68E-08	9.378776
RP11-498C9	-0.795838	-0.884332	-5.860654	1.02E-08	7.68E-08	9.3785098
ANKRD54	-0.103239	6.0673485	-5.860641	1.02E-08	7.68E-08	9.3784392
MUL1	-0.094642	6.2218013	-5.860607	1.02E-08	7.68E-08	9.3782604
PPIEL	1.0723307	1.1744765	5.8603507	1.02E-08	7.69E-08	9.3768942
INPP5F	0.1510872	5.4492723	5.8601969	1.02E-08	7.70E-08	9.3760756
PPAP2C	0.8219308	4.9818634	5.8598719	1.02E-08	7.71E-08	9.3743464
LILRA4	1.0595811	2.0830068	5.8596028	1.03E-08	7.72E-08	9.372914
AC005775.2	-0.983245	1.3306384	-5.859174	1.03E-08	7.73E-08	9.3706301
RP11-223A3	-0.536916	2.6964912	-5.858807	1.03E-08	7.75E-08	9.3686801
KIAA1731NL	0.8858327	-0.419333	5.85875	1.03E-08	7.75E-08	9.368376
NDUFA10	-0.090858	6.6577315	-5.858297	1.03E-08	7.77E-08	9.365967
TXNRD3	-0.124925	5.6840937	-5.858149	1.03E-08	7.77E-08	9.3651791
NDUFS3	-0.106207	6.5629752	-5.858126	1.03E-08	7.77E-08	9.365057
RP11-104N1	-0.549319	3.0870102	-5.85805	1.03E-08	7.77E-08	9.364654
MFAP5	1.1739175	1.917262	5.8577428	1.04E-08	7.78E-08	9.3630175
PSMA5	-0.102849	6.6769043	-5.856227	1.05E-08	7.85E-08	9.3549523
AC008592.4	-1.304534	1.980464	-5.856069	1.05E-08	7.85E-08	9.3541129
UCP2	0.2007913	6.0539757	5.8559335	1.05E-08	7.86E-08	9.353393
ITGA10	0.4595699	4.2395257	5.8558194	1.05E-08	7.86E-08	9.3527862
TTC39A-AS1	1.226487	1.0731749	5.8554691	1.05E-08	7.87E-08	9.3509231
C9orf9	-0.244974	5.1738449	-5.855432	1.05E-08	7.87E-08	9.3507266
LRCH4	0.2831058	5.5417788	5.8552811	1.05E-08	7.88E-08	9.3499231
ZNF267	0.1294389	5.3903804	5.8551201	1.05E-08	7.88E-08	9.3490671
LA16c-429E	-0.931063	2.1177387	-5.854643	1.05E-08	7.90E-08	9.3465282
OSTN-AS1	-1.452352	1.9412262	-5.85426	1.06E-08	7.92E-08	9.3444951
SLC38A7	-0.10518	6.224877	-5.853545	1.06E-08	7.95E-08	9.3406937
CTA-293F17	1.2996281	0.6900171	5.8525964	1.07E-08	7.99E-08	9.3356481
CTD-2350C1	-0.495484	4.6896284	-5.852577	1.07E-08	7.99E-08	9.3355449
GUSB	-0.109431	6.797467	-5.852532	1.07E-08	7.99E-08	9.3353053
RAP1GAP	0.2630292	6.202768	5.8522288	1.07E-08	8.00E-08	9.3336937
RAD23A	-0.07466	6.5992895	-5.852167	1.07E-08	8.00E-08	9.3333676
KIAA0922	-0.153189	6.0981577	-5.851556	1.07E-08	8.03E-08	9.3301157
C1orf56	-0.155385	5.8311292	-5.85151	1.07E-08	8.03E-08	9.3298734
FKBP8	-0.096679	6.9654893	-5.851159	1.07E-08	8.04E-08	9.3280096
ARID3B	0.1930192	5.2554366	5.8501079	1.08E-08	8.09E-08	9.3224207
SESN2	-0.131559	6.1498333	-5.849699	1.08E-08	8.10E-08	9.3202482
GYG1	0.1160731	5.9862698	5.8486427	1.09E-08	8.15E-08	9.3146349
TCEAL7	0.6514871	3.230167	5.8485295	1.09E-08	8.15E-08	9.3140335
CD3D	0.4951881	4.883961	5.8484284	1.09E-08	8.15E-08	9.3134964
COX11	-0.081715	6.3325493	-5.848351	1.09E-08	8.16E-08	9.3130874
AC068134.1	0.9658585	1.6380468	5.8482689	1.09E-08	8.16E-08	9.312649
NOD2	0.471227	4.4860429	5.848207	1.09E-08	8.16E-08	9.3123201
RP11-883G1	-1.16331	2.1912941	-5.847986	1.09E-08	8.17E-08	9.3111441
FASLG	0.7839811	3.1514037	5.8472848	1.10E-08	8.20E-08	9.3074206
ALLC	1.2352478	1.022643	5.846517	1.10E-08	8.23E-08	9.3033422
SMC4	0.2861273	5.8149714	5.8464616	1.10E-08	8.23E-08	9.303048
USP2-AS1	-0.747583	4.0458525	-5.845983	1.11E-08	8.25E-08	9.3005053
CTC-338M12	-0.845324	-0.545589	-5.845732	1.11E-08	8.26E-08	9.299172
C1orf162	0.2345542	5.4147067	5.8444963	1.11E-08	8.31E-08	9.2926102

TANGO6	-0.144976	5.9393185	-5.843781	1.12E-08	8.35E-08	9.2888144
MT-CO3	-0.117934	7.8779654	-5.842399	1.13E-08	8.41E-08	9.2814736
AKAP10	0.114056	5.7030838	5.841237	1.14E-08	8.46E-08	9.2753072
RNASEH2C	-0.140803	6.1400216	-5.841125	1.14E-08	8.46E-08	9.2747108
IQCH-AS1	-0.157701	5.4075545	-5.840507	1.14E-08	8.49E-08	9.2714344
LLOXNC01-1	0.8066713	-0.652285	5.8399966	1.14E-08	8.51E-08	9.268724
ZNF37BP	0.1975798	5.3128593	5.8396658	1.14E-08	8.53E-08	9.2669687
TGFB2	0.5806065	4.6346342	5.8395907	1.15E-08	8.53E-08	9.2665698
ZNF454	1.0842854	1.8094801	5.83939	1.15E-08	8.54E-08	9.2655049
PAPOLG	0.1200774	5.5665226	5.8382649	1.15E-08	8.59E-08	9.2595356
SPATA33	-0.162063	5.1575475	-5.838043	1.16E-08	8.60E-08	9.2583589
RP11-725G5	-1.023589	2.2865847	-5.837485	1.16E-08	8.62E-08	9.2553991
SNHG8	-0.16883	6.2385546	-5.83699	1.16E-08	8.64E-08	9.2527713
RP11-536K7	0.2867774	4.5164092	5.8366334	1.16E-08	8.66E-08	9.2508806
TCF25	-0.102765	6.5833463	-5.836245	1.17E-08	8.67E-08	9.2488191
C1orf127	0.6798212	2.8316527	5.8357527	1.17E-08	8.70E-08	9.2462092
CTC-297N7.	-0.604288	4.4557991	-5.834481	1.18E-08	8.75E-08	9.2394675
RP5-1139B1	0.8045683	-0.743455	5.8340099	1.18E-08	8.78E-08	9.2369676
ZBTB16	-0.551084	5.2484291	-5.833539	1.18E-08	8.80E-08	9.2344714
RP5-1057J7	-0.50833	4.1046031	-5.833157	1.19E-08	8.81E-08	9.232443
DCHS2	1.2685236	0.6525441	5.8326702	1.19E-08	8.84E-08	9.2298649
SSTR5-AS1	1.7669747	1.3469705	5.8319956	1.19E-08	8.87E-08	9.2262885
PILRA	0.2453105	5.0447969	5.8314986	1.20E-08	8.89E-08	9.2236545
RP11-375N1	-0.844159	-0.716476	-5.830785	1.20E-08	8.92E-08	9.219872
CTD-2537I9	0.6402249	3.8130905	5.8301607	1.21E-08	8.95E-08	9.2165636
E2F3	0.168704	5.8007309	5.8294431	1.21E-08	8.98E-08	9.2127609
ATP10B	1.467795	1.2652948	5.8290578	1.21E-08	9.00E-08	9.2107196
PPP3CB-AS1	-0.179721	5.0151257	-5.828378	1.22E-08	9.03E-08	9.2071178
TRAF3	0.1205951	5.8878414	5.8271768	1.23E-08	9.09E-08	9.2007542
MALL	1.0106024	2.9121131	5.8269229	1.23E-08	9.10E-08	9.1994095
TMEM255A	0.8427198	3.4012335	5.8267526	1.23E-08	9.11E-08	9.1985074
MRPL48	-0.100262	6.0509584	-5.82559	1.24E-08	9.16E-08	9.1923493
PLGRKT	-0.127009	5.8505269	-5.825482	1.24E-08	9.17E-08	9.1917789
FZD8	0.4088496	4.9876862	5.8253942	1.24E-08	9.17E-08	9.1913129
RP13-188A5	0.922649	1.4588461	5.8253126	1.24E-08	9.17E-08	9.190881
SP8	1.4640887	0.3955981	5.8252603	1.24E-08	9.17E-08	9.1906038
FGF7	1.2265681	3.0404166	5.8245275	1.24E-08	9.21E-08	9.1867233
CLN3	-0.139631	6.2784266	-5.823985	1.25E-08	9.23E-08	9.1838494
ADORA3	0.3766749	4.7157754	5.8232749	1.25E-08	9.27E-08	9.1800912
STK11IP	0.1147658	5.783746	5.823203	1.25E-08	9.27E-08	9.1797106
CTB-186H2.	-1.1726	1.2455898	-5.822646	1.26E-08	9.30E-08	9.176764
CD99	-0.107415	6.8498484	-5.822139	1.26E-08	9.32E-08	9.1740789
PDK4	-0.283501	6.5192729	-5.820706	1.27E-08	9.39E-08	9.1664959
RP11-1094H	0.9743621	-0.504634	5.820433	1.27E-08	9.40E-08	9.1650489
RP11-521B2	0.4007514	3.7640092	5.8200212	1.28E-08	9.42E-08	9.1628699
GNE	-0.18829	6.4663235	-5.819517	1.28E-08	9.45E-08	9.1602014
CLRN2	-0.83441	-0.765628	-5.819081	1.28E-08	9.47E-08	9.1578941
NRK	1.2152229	1.4400329	5.8189334	1.28E-08	9.47E-08	9.1571139
AATF	-0.087495	6.4359645	-5.818403	1.29E-08	9.50E-08	9.1543082
IL13RA1	-0.114205	6.7723104	-5.817747	1.29E-08	9.53E-08	9.1508397
PIGM	-0.102701	6.0459792	-5.817597	1.29E-08	9.54E-08	9.150044

SLC39A6	0.1240388	6.1295661	5.8168075	1.30E-08	9.58E-08	9.1458677
RP11-13P5.	0.9481967	-0.388454	5.8167643	1.30E-08	9.58E-08	9.1456394
CCNK	0.7542441	4.8965917	5.8163649	1.30E-08	9.60E-08	9.1435271
IGHV1-45	1.0528436	-0.300315	5.8161068	1.30E-08	9.61E-08	9.1421619
LAPTM5	0.1668852	6.4955606	5.8160004	1.30E-08	9.61E-08	9.1415989
NOS3	0.2367995	5.4717858	5.8158966	1.30E-08	9.62E-08	9.1410501
TMSB15A	0.9925121	1.3758139	5.8157916	1.31E-08	9.62E-08	9.1404947
C2orf15	0.3675594	4.4565126	5.8155234	1.31E-08	9.63E-08	9.1390766
PXMP4	-0.136986	6.1648569	-5.815459	1.31E-08	9.63E-08	9.1387355
AL161668.5	-0.846472	3.8446377	-5.815331	1.31E-08	9.64E-08	9.13806
DOLK	-0.099758	6.0838375	-5.815204	1.31E-08	9.64E-08	9.1373877
LINC00630	0.6899384	3.4095559	5.814511	1.31E-08	9.68E-08	9.1337231
S100Z	0.958111	0.3863109	5.8143862	1.32E-08	9.68E-08	9.133063
MRPL49	-0.084746	6.4414006	-5.814363	1.32E-08	9.68E-08	9.1329397
CDO1	-0.436481	6.6206081	-5.813601	1.32E-08	9.72E-08	9.1289136
USP7	-0.069146	6.5763745	-5.8129	1.33E-08	9.76E-08	9.1252083
ZNF792	0.1746686	5.1401412	5.812017	1.33E-08	9.80E-08	9.1205381
LAMTOR5	-0.099257	6.4404569	-5.81147	1.34E-08	9.83E-08	9.1176471
SGIP1	0.706321	4.0192841	5.8112122	1.34E-08	9.84E-08	9.1162845
HSP90AA2P	0.9634862	2.3533497	5.8110568	1.34E-08	9.85E-08	9.1154633
SMPD3	0.3818268	4.4100312	5.8110345	1.34E-08	9.85E-08	9.1153458
LY6E	0.3172405	6.4188048	5.809795	1.35E-08	9.91E-08	9.1087954
TBX2	0.2204235	5.706683	5.8097383	1.35E-08	9.91E-08	9.1084957
FCRL3	1.0640867	2.9375656	5.8095539	1.35E-08	9.92E-08	9.1075217
RP11-476H1	-0.80876	2.065407	-5.809473	1.35E-08	9.92E-08	9.1070957
ZNF818P	1.1792965	1.9527805	5.8090605	1.35E-08	9.94E-08	9.1049148
HAUS4	-0.183866	5.9395369	-5.807117	1.37E-08	1.00E-07	9.0946487
SPACA1	-0.968887	-0.586668	-5.806556	1.37E-08	1.01E-07	9.0916837
TCHH	0.9737755	1.9837698	5.8064414	1.37E-08	1.01E-07	9.0910796
SLC51A	-0.41321	6.2588917	-5.805769	1.38E-08	1.01E-07	9.0875282
CD247	0.2987337	4.9623438	5.8057155	1.38E-08	1.01E-07	9.0872461
B3GAT3	-0.124372	6.3969994	-5.805564	1.38E-08	1.01E-07	9.0864458
SNIP1	-0.096837	5.8475463	-5.805108	1.38E-08	1.01E-07	9.0840373
APOL4	0.3773561	4.9193872	5.8049728	1.38E-08	1.02E-07	9.083324
AP001347.6	1.0607458	0.6116413	5.8046133	1.39E-08	1.02E-07	9.081426
GNAS-AS1	1.2130382	0.5741269	5.8025337	1.40E-08	1.03E-07	9.0704472
P3H2	0.3382639	4.8759682	5.8018338	1.41E-08	1.03E-07	9.0667531
ROGDI	-0.134544	6.1016072	-5.801587	1.41E-08	1.03E-07	9.0654504
EMR3	1.1498457	0.5646588	5.8012332	1.41E-08	1.04E-07	9.063583
RP11-20I23	0.8622438	2.7000306	5.8010713	1.41E-08	1.04E-07	9.0627286
B4GALNT2	1.8024846	1.7720299	5.8007314	1.42E-08	1.04E-07	9.0609347
HMGB1	-0.072873	6.7340864	-5.800365	1.42E-08	1.04E-07	9.0590036
MFAP3L	-0.506782	5.5604944	-5.800143	1.42E-08	1.04E-07	9.0578308
CYP4F23P	1.2618116	0.8316216	5.7984519	1.44E-08	1.05E-07	9.0489079
MT-ATP6	-0.122689	7.7278969	-5.798118	1.44E-08	1.05E-07	9.0471468
CRACR2B	0.3569104	5.2077696	5.7980747	1.44E-08	1.05E-07	9.0469177
BRINP1	1.3176743	1.0765125	5.797992	1.44E-08	1.05E-07	9.0464814
TBX10	-0.86348	3.6875702	-5.797811	1.44E-08	1.05E-07	9.0455295
TRAFD1	0.0873726	6.0828928	5.797585	1.44E-08	1.05E-07	9.0443348
IL33	0.3792343	5.3391906	5.7974216	1.44E-08	1.05E-07	9.0434731
RP4-794I6.	0.9758023	1.8881966	5.7971742	1.45E-08	1.06E-07	9.0421679

NPBWR1	1.5423898	1.2240967	5.7971694	1.45E-08	1.06E-07	9.0421429
LRRN2	0.6047122	4.5845263	5.7965466	1.45E-08	1.06E-07	9.0388578
SEC16B	-0.38779	4.9716444	-5.796301	1.45E-08	1.06E-07	9.0375633
UGT2B10	-0.553309	6.4838187	-5.795981	1.45E-08	1.06E-07	9.0358761
MYBPC3	1.019196	1.2083913	5.7954645	1.46E-08	1.06E-07	9.0331516
AC005578.3	-0.980392	0.8037869	-5.795154	1.46E-08	1.07E-07	9.0315134
PSMD13	-0.095076	6.6813148	-5.794977	1.46E-08	1.07E-07	9.0305825
FGF11	1.0411849	0.1944277	5.7943827	1.47E-08	1.07E-07	9.0274472
IGHV3-71	0.958453	-0.657925	5.7933766	1.48E-08	1.08E-07	9.0221434
SLC16A3	0.269464	5.8036221	5.7922695	1.48E-08	1.08E-07	9.0163076
RDH5	-0.373283	5.3089436	-5.792028	1.49E-08	1.08E-07	9.0150366
SNHG4	0.8057597	3.4820351	5.791438	1.49E-08	1.09E-07	9.0119252
ADIPOR2	-0.108437	6.7161122	-5.791286	1.49E-08	1.09E-07	9.0111237
RP11-452C1	1.02019	0.3050373	5.7911846	1.49E-08	1.09E-07	9.0105902
RP11-397G1	1.4553176	3.8108881	5.7911444	1.49E-08	1.09E-07	9.0103782
RP11-7M8.2	-1.432675	2.0313956	-5.79081	1.50E-08	1.09E-07	9.0086134
AMICA1	0.2702354	5.66001	5.7898738	1.50E-08	1.09E-07	9.0036827
SNORD116-4	0.8575943	-0.679305	5.7894331	1.51E-08	1.10E-07	9.0013607
RP11-1080G	-1.046427	-0.473608	-5.788505	1.52E-08	1.10E-07	8.9964725
RP11-264M1	-0.555629	3.5928184	-5.788244	1.52E-08	1.10E-07	8.9950968
RP11-238F2	-1.775433	1.6821682	-5.788109	1.52E-08	1.10E-07	8.9943863
PSMD6	-0.074332	6.3980248	-5.787265	1.53E-08	1.11E-07	8.9899382
CD300E	0.6130831	3.8132007	5.7871434	1.53E-08	1.11E-07	8.9892995
KIAA1211L	0.3047347	4.800611	5.7862464	1.53E-08	1.12E-07	8.9845753
GRAP	0.6576843	3.7213839	5.7855502	1.54E-08	1.12E-07	8.9809094
RP11-85A1.	-0.91016	1.5774624	-5.785465	1.54E-08	1.12E-07	8.98046
ERMAP	-0.145287	5.828341	-5.784221	1.55E-08	1.13E-07	8.9739119
PHF14	-0.080141	6.2492494	-5.784183	1.55E-08	1.13E-07	8.973709
ZNF160	0.2637466	5.441859	5.7840458	1.55E-08	1.13E-07	8.9729887
GFER	-0.128856	6.0629199	-5.783232	1.56E-08	1.13E-07	8.9687058
ANKHD1	-0.127791	5.324032	-5.783019	1.56E-08	1.13E-07	8.9675812
C4orf48	0.5937077	4.1029559	5.7818343	1.57E-08	1.14E-07	8.9613483
DDX28	-0.12987	5.8380051	-5.780661	1.58E-08	1.15E-07	8.9551733
GPBP1L1	-0.075506	6.6037145	-5.780434	1.58E-08	1.15E-07	8.953982
FBXL13	0.7713143	2.2702684	5.7803195	1.58E-08	1.15E-07	8.9533772
CYP4F2	-0.65746	6.2429241	-5.779332	1.59E-08	1.16E-07	8.9481815
RPS28	-0.137376	6.945395	-5.778072	1.60E-08	1.16E-07	8.9415539
G3BP1	-0.078011	6.724004	-5.777692	1.61E-08	1.17E-07	8.9395525
SAMM50	-0.114498	6.451264	-5.777455	1.61E-08	1.17E-07	8.9383099
RP4-545K15	1.0073077	0.0445097	5.7774218	1.61E-08	1.17E-07	8.9381335
MRPS5	-0.094952	6.5000989	-5.776961	1.61E-08	1.17E-07	8.9357108
SLC26A6	-0.173487	6.0472493	-5.776926	1.61E-08	1.17E-07	8.9355237
NAIF1	-0.083651	5.6735628	-5.776754	1.62E-08	1.17E-07	8.9346235
CLEC1B	1.3604322	0.6277438	5.7760775	1.62E-08	1.17E-07	8.9310636
RP11-102K1	1.0484551	0.9228097	5.7759323	1.62E-08	1.17E-07	8.9303005
STAP2	-0.181131	6.4905927	-5.775559	1.63E-08	1.18E-07	8.9283399
ANKDD1A	0.2205642	4.76136	5.774976	1.63E-08	1.18E-07	8.9252722
ACO1	-0.129851	6.7634579	-5.774107	1.64E-08	1.19E-07	8.9207029
TMUB1	-0.115753	6.466293	-5.773817	1.64E-08	1.19E-07	8.9191769
ANKRD13D	0.1366939	5.8718493	5.7735648	1.64E-08	1.19E-07	8.9178536
RP11-185E8	1.1408011	2.3967675	5.7732269	1.65E-08	1.19E-07	8.9160773

RP11-541N1	0.2983184	4.2110353	5.7729789	1.65E-08	1.19E-07	8.9147738
PQBP1	-0.114474	6.4605973	-5.772801	1.65E-08	1.19E-07	8.9138376
RP11-473M2	-0.829204	1.9450058	-5.772773	1.65E-08	1.19E-07	8.9136918
NBPF2P	1.0646551	1.1453596	5.7724428	1.65E-08	1.20E-07	8.9119558
ZNF652	-0.090226	6.2348064	-5.771766	1.66E-08	1.20E-07	8.9083991
ATG9A	-0.081325	6.6025011	-5.771522	1.66E-08	1.20E-07	8.9071179
NUCB1	-0.100988	7.1443488	-5.771427	1.66E-08	1.20E-07	8.9066166
ETV3	0.1237943	5.9264189	5.7710337	1.67E-08	1.20E-07	8.9045509
LILRB2	0.2568762	5.2305659	5.769892	1.68E-08	1.21E-07	8.8985523
SCN3A	1.0994373	1.3172441	5.769421	1.68E-08	1.21E-07	8.8960777
GATM-AS1	-0.996757	1.6991226	-5.768874	1.69E-08	1.22E-07	8.8932039
OR13A1	1.0203702	-0.470497	5.7685965	1.69E-08	1.22E-07	8.8917468
RP11-395L1	0.6787205	2.7897386	5.7673987	1.70E-08	1.23E-07	8.8854555
C1orf233	0.4948944	4.4673362	5.7669787	1.70E-08	1.23E-07	8.88325
RP11-90P13	1.096218	0.9545464	5.7666118	1.71E-08	1.23E-07	8.8813234
RP11-18H21	1.0859087	1.8241523	5.7660835	1.71E-08	1.23E-07	8.8785489
KIRREL3	1.0013544	1.9584832	5.7659019	1.71E-08	1.24E-07	8.8775957
NFE2L1	-0.094429	7.0143531	-5.765799	1.71E-08	1.24E-07	8.877055
AKR7A3	-0.489761	6.1241534	-5.765773	1.72E-08	1.24E-07	8.8769171
MGST3	-0.115078	6.596526	-5.765035	1.72E-08	1.24E-07	8.8730455
L1TD1	1.0051953	-0.106915	5.7649866	1.72E-08	1.24E-07	8.8727897
SLC31A1	-0.123555	6.6475649	-5.764253	1.73E-08	1.25E-07	8.8689379
TMEM223	-0.111021	6.0392713	-5.763744	1.73E-08	1.25E-07	8.8662656
SH3RF3-AS1	1.1122762	2.2058815	5.7633506	1.74E-08	1.25E-07	8.8642019
CTF1	0.7259733	3.5636307	5.7623406	1.75E-08	1.26E-07	8.8589011
CTD-3222D1	0.5095394	3.3326006	5.7621149	1.75E-08	1.26E-07	8.8577163
NKAP	-0.095896	6.0713301	-5.762029	1.75E-08	1.26E-07	8.8572631
ING4	-0.106976	6.0753371	-5.760091	1.77E-08	1.27E-07	8.8470975
C3orf18	-0.226382	5.4283629	-5.759578	1.77E-08	1.28E-07	8.8444048
GS1-306C12	-0.756448	-0.965354	-5.759237	1.78E-08	1.28E-07	8.8426174
TRGV7	0.9699868	0.0779629	5.7590313	1.78E-08	1.28E-07	8.8415379
CTC-260E6.	1.0241456	2.6980202	5.7560545	1.81E-08	1.30E-07	8.8259266
CKMT2-AS1	-0.173091	5.5871254	-5.756038	1.81E-08	1.30E-07	8.8258393
ETV4	0.6632495	5.3838319	5.7557881	1.81E-08	1.30E-07	8.8245296
ZNF3	-0.086498	6.1498677	-5.755024	1.82E-08	1.31E-07	8.8205236
DDB1	-0.061905	6.8723878	-5.754459	1.82E-08	1.31E-07	8.8175613
NAA10	-0.13874	6.2890277	-5.754214	1.83E-08	1.31E-07	8.8162801
MARCO	1.1049378	3.4777802	5.7540028	1.83E-08	1.31E-07	8.8151707
HSPA9	-0.091087	7.1115956	-5.753635	1.83E-08	1.31E-07	8.8132443
AKR1C4	-0.473292	6.6411088	-5.75328	1.84E-08	1.32E-07	8.8113823
CHCHD10	-0.18315	6.661402	-5.752878	1.84E-08	1.32E-07	8.8092745
WT1	1.4276743	1.0002647	5.7520761	1.85E-08	1.33E-07	8.805073
PRPH	1.0417711	-0.317832	5.7508892	1.86E-08	1.33E-07	8.7988538
RP11-295P2	0.8823483	-0.83548	5.7506929	1.86E-08	1.33E-07	8.7978253
LA16c-312E	1.2312846	1.8707062	5.7504759	1.86E-08	1.34E-07	8.7966885
CST6	1.0143672	-0.09345	5.7495545	1.87E-08	1.34E-07	8.7918616
RP11-539L1	-0.485816	4.3969192	-5.749156	1.88E-08	1.34E-07	8.7897728
TSPAN32	0.4742935	3.8342722	5.7491432	1.88E-08	1.34E-07	8.7897071
RPL14	-0.120935	7.1205742	-5.748525	1.88E-08	1.35E-07	8.7864683
SACS	0.2828948	5.2642761	5.7480455	1.89E-08	1.35E-07	8.7839581
LYPLAL1	-0.13852	6.2336179	-5.74788	1.89E-08	1.35E-07	8.783089

ATG13	-0.070959	6.5488973	-5.747563	1.89E-08	1.36E-07	8.7814291
MGAT1	-0.087926	6.9022704	-5.747327	1.90E-08	1.36E-07	8.7801958
GAS2L3	0.2470977	5.2826692	5.7464989	1.90E-08	1.36E-07	8.7758587
FAM65A	0.0969893	6.2532971	5.7457498	1.91E-08	1.37E-07	8.771937
FAM8A1	-0.142458	6.6451979	-5.745544	1.91E-08	1.37E-07	8.7708585
SNX11	-0.086847	6.0489378	-5.745376	1.92E-08	1.37E-07	8.769981
ARNTL2	0.3898445	5.4609131	5.7453459	1.92E-08	1.37E-07	8.7698225
CCL20	0.7947673	5.5644575	5.7452337	1.92E-08	1.37E-07	8.7692349
RP11-500C1	-0.29826	4.21506	-5.744912	1.92E-08	1.37E-07	8.7675511
RP11-420L9	-0.196269	5.4645344	-5.74446	1.93E-08	1.38E-07	8.765186
CTD-2017D1	0.9483065	2.0934088	5.7428923	1.94E-08	1.39E-07	8.75698
TRAV39	0.8129225	-0.579172	5.7426345	1.94E-08	1.39E-07	8.7556306
SRSF12	0.7142947	3.8345982	5.7419397	1.95E-08	1.39E-07	8.7519953
PIN1	-0.100921	6.268185	-5.74188	1.95E-08	1.39E-07	8.7516845
ZDHHC12	-0.117247	6.2999951	-5.74181	1.95E-08	1.39E-07	8.751315
ZNF567	0.1958336	4.9979915	5.7407324	1.96E-08	1.40E-07	8.7456784
MRPS9	-0.099302	6.1352222	-5.739194	1.98E-08	1.41E-07	8.7376321
LINC01012	1.1207482	0.0385157	5.7374621	2.00E-08	1.43E-07	8.7285735
FRZB	0.3748221	5.3733328	5.7371027	2.00E-08	1.43E-07	8.7266945
NMT2	-0.150809	6.1420023	-5.736579	2.01E-08	1.43E-07	8.7239555
RP3-355L5.	0.9762959	0.9069645	5.7365195	2.01E-08	1.43E-07	8.7236452
DAPK2	-0.247791	5.6886005	-5.734434	2.03E-08	1.45E-07	8.7127409
TTC16	0.9334386	1.9363879	5.7339953	2.04E-08	1.45E-07	8.7104497
RP4-616B8.	-0.859675	2.4532312	-5.733904	2.04E-08	1.45E-07	8.7099732
C2	-0.195959	7.1484582	-5.73366	2.04E-08	1.45E-07	8.708699
RP11-488L1	1.0670011	1.0361419	5.7323374	2.06E-08	1.46E-07	8.7017861
MYEF2	0.8336286	3.9579986	5.7317529	2.06E-08	1.47E-07	8.6987319
ATP7A	0.168549	5.3434998	5.731634	2.06E-08	1.47E-07	8.698111
SMAP2	-0.095785	6.5782691	-5.731283	2.07E-08	1.47E-07	8.6962768
CTD-2541M1	0.7064348	2.5349989	5.7312333	2.07E-08	1.47E-07	8.6960175
M6PR	0.161665	6.1481903	5.7303104	2.08E-08	1.48E-07	8.691196
LINC01152	1.3860489	2.2268905	5.7302942	2.08E-08	1.48E-07	8.6911115
IGFBP5	0.2519597	6.3507158	5.7295751	2.09E-08	1.48E-07	8.6873553
RNF167	-0.089408	6.636479	-5.728585	2.10E-08	1.49E-07	8.6821822
MAN1B1	-0.092795	6.5601397	-5.728563	2.10E-08	1.49E-07	8.6820684
RP11-1221G	-1.174727	0.4218987	-5.728459	2.10E-08	1.49E-07	8.6815265
AHCYL1	-0.079385	6.6716362	-5.728363	2.10E-08	1.49E-07	8.6810225
RP11-13K12	0.8637749	-0.596266	5.7279163	2.11E-08	1.50E-07	8.6786924
PWWP2B	0.2358387	5.7877919	5.7276468	2.11E-08	1.50E-07	8.6772851
EIF1AD	-0.071995	6.0461627	-5.726285	2.12E-08	1.51E-07	8.6701763
ZBED4	0.1167145	5.8791473	5.726238	2.13E-08	1.51E-07	8.6699299
TEX43	-0.768	-0.704857	-5.725802	2.13E-08	1.51E-07	8.6676527
IGKV1OR22-	0.7817785	-0.92412	5.7255406	2.13E-08	1.51E-07	8.666289
HACE1	0.2915096	5.0853594	5.7253535	2.14E-08	1.52E-07	8.6653126
COL11A2	0.5669346	3.8632625	5.7253527	2.14E-08	1.52E-07	8.6653082
INO80C	-0.144878	5.8133956	-5.725106	2.14E-08	1.52E-07	8.6640194
SERPIND1	-0.350848	7.1917128	-5.724835	2.14E-08	1.52E-07	8.6626067
DYNC2H1	0.5037218	4.3862676	5.7247195	2.14E-08	1.52E-07	8.6620031
RDH16	-0.496894	6.3827715	-5.724562	2.14E-08	1.52E-07	8.6611802
NBAT1	0.583433	-1.130373	5.7244585	2.15E-08	1.52E-07	8.6606411
RP11-510I6	-0.652259	-1.183729	-5.724458	2.15E-08	1.52E-07	8.6606392

B4GALT7	-0.11116	6.2287609	-5.724102	2.15E-08	1.52E-07	8.6587789
SPR	-0.117215	6.5564818	-5.723803	2.15E-08	1.53E-07	8.6572174
MKS1	-0.10672	5.6242839	-5.723429	2.16E-08	1.53E-07	8.6552705
CEACAM4	0.8349012	2.2436255	5.722972	2.16E-08	1.53E-07	8.6528834
AC091729.9	-0.185895	5.3821221	-5.722132	2.17E-08	1.54E-07	8.6484981
RNASE2	1.1450364	1.9118564	5.7215922	2.18E-08	1.54E-07	8.6456843
LHX6	0.6158501	3.802146	5.7205842	2.19E-08	1.55E-07	8.6404262
AP000997.2	-1.133176	0.2102212	-5.720487	2.19E-08	1.55E-07	8.6399188
AC069282.6	0.8026575	3.9829211	5.7204547	2.19E-08	1.55E-07	8.6397508
DIP2A	0.1044624	5.9142694	5.7202381	2.20E-08	1.55E-07	8.6386207
SHD	-0.905289	4.7799234	-5.71915	2.21E-08	1.56E-07	8.6329462
ANKRD36	0.59559	3.7667351	5.7190646	2.21E-08	1.56E-07	8.6325002
RP11-424M2	0.9465419	-0.112468	5.7187067	2.21E-08	1.57E-07	8.6306339
RFX3	0.1957289	5.0922001	5.7183214	2.22E-08	1.57E-07	8.6286247
PCDHGC5	1.1276173	0.378018	5.7181551	2.22E-08	1.57E-07	8.6277576
WFDC1	0.605176	4.2300541	5.7180418	2.22E-08	1.57E-07	8.6271666
TMEM243	0.2741068	5.1572896	5.7178211	2.22E-08	1.57E-07	8.6260159
GZMM	0.4605837	4.2611676	5.7172779	2.23E-08	1.58E-07	8.6231837
CTB-78F1.2	-1.423769	1.3255967	-5.717032	2.23E-08	1.58E-07	8.6219041
DENND2D	0.2487915	5.6957029	5.7165271	2.24E-08	1.58E-07	8.6192696
IL1RL1	1.0921908	2.7045382	5.7164787	2.24E-08	1.58E-07	8.619017
STARD4-AS1	0.7085217	3.9534595	5.71607	2.25E-08	1.58E-07	8.6168866
CCDC58	-0.11786	5.9699758	-5.716068	2.25E-08	1.58E-07	8.6168785
XXbac-B562	1.0392232	2.0456348	5.715666	2.25E-08	1.59E-07	8.6147808
ST5	0.1713114	6.1786133	5.7154135	2.25E-08	1.59E-07	8.6134648
UBE2B	-0.093759	6.4547425	-5.714615	2.26E-08	1.60E-07	8.6093004
IL10RA	0.2250045	5.7386224	5.7145436	2.26E-08	1.60E-07	8.6089309
NKAIN4	1.0338739	0.0354724	5.7143111	2.27E-08	1.60E-07	8.6077192
ATP13A2	0.1243159	5.9646991	5.7140865	2.27E-08	1.60E-07	8.6065485
BFSP2	1.2733252	1.5225047	5.714056	2.27E-08	1.60E-07	8.6063897
RP5-1057I2	0.8032857	-0.643767	5.7140069	2.27E-08	1.60E-07	8.6061336
VNN2	0.5375017	5.3123811	5.7138469	2.27E-08	1.60E-07	8.6053001
MYO3A	1.1316583	-0.490603	5.7138407	2.27E-08	1.60E-07	8.6052677
GOLGA2P7	0.8557946	2.6575408	5.7134276	2.28E-08	1.60E-07	8.603115
RGS13	1.2208682	0.6836423	5.7133908	2.28E-08	1.60E-07	8.6029235
RILPL1	0.1374161	5.4418366	5.7124644	2.29E-08	1.61E-07	8.5980961
WDR86	0.6059301	3.4779771	5.7119698	2.30E-08	1.62E-07	8.5955195
FCER1A	0.9475436	3.3590581	5.7115733	2.30E-08	1.62E-07	8.5934542
GPR85	0.6395822	3.3702764	5.7113912	2.30E-08	1.62E-07	8.5925052
POF1B	1.4144719	3.088875	5.7112678	2.30E-08	1.62E-07	8.5918623
GLYATL1	-0.542864	6.2234674	-5.711058	2.31E-08	1.62E-07	8.5907709
C14orf119	-0.080208	6.2243955	-5.709659	2.32E-08	1.63E-07	8.5834829
GIMAP4	0.1671174	5.9082314	5.7096556	2.32E-08	1.63E-07	8.5834653
LRRC8D	-0.10452	6.3010411	-5.709625	2.33E-08	1.63E-07	8.5833081
SFT2D1	0.1120008	5.7754286	5.7094054	2.33E-08	1.64E-07	8.5821625
RP11-465B2	0.7978818	4.1825766	5.7092128	2.33E-08	1.64E-07	8.5811593
RP11-15B24	-1.307118	1.5797749	-5.709064	2.33E-08	1.64E-07	8.5803848
BMPER	1.1135227	2.3127205	5.7088953	2.33E-08	1.64E-07	8.5795059
DNAJC8	-0.072258	6.4966404	-5.70866	2.34E-08	1.64E-07	8.5782805
NOV	0.4864251	4.6445215	5.7086346	2.34E-08	1.64E-07	8.5781484
BHMT2	-0.319251	6.8388838	-5.70835	2.34E-08	1.64E-07	8.5766652

CRYM-AS1	0.8968869	-0.057049	5.7081738	2.34E-08	1.64E-07	8.5757493
RP11-707P1	0.9926464	2.2262406	5.7072526	2.36E-08	1.65E-07	8.5709531
NMD3	-0.085399	6.4153366	-5.706667	2.36E-08	1.66E-07	8.5679054
MRPS10	-0.080438	6.3524675	-5.706452	2.37E-08	1.66E-07	8.5667839
CTD-2554C2	1.0516394	-0.035958	5.7056137	2.38E-08	1.67E-07	8.5624217
B3GNT3	1.1329433	4.8065165	5.7050916	2.38E-08	1.67E-07	8.5597044
NCLN	-0.097839	6.6536039	-5.705044	2.38E-08	1.67E-07	8.559456
C3AR1	0.2817292	5.2862952	5.7049952	2.38E-08	1.67E-07	8.5592029
GPR162	0.4219741	4.7327025	5.7049657	2.38E-08	1.67E-07	8.5590491
IGHV3-35	0.9920662	-0.515129	5.7044265	2.39E-08	1.67E-07	8.556243
RP11-393N2	-0.459796	-1.330354	-5.704405	2.39E-08	1.67E-07	8.55613
TMEM185B	0.1319284	5.7726815	5.7039268	2.40E-08	1.68E-07	8.553643
CDC37	-0.077385	6.7501303	-5.70383	2.40E-08	1.68E-07	8.5531405
CDX1	1.0702385	1.1969018	5.7037777	2.40E-08	1.68E-07	8.5528671
HK3	0.3756906	4.8146001	5.703308	2.41E-08	1.68E-07	8.5504229
ABCB8	-0.113552	6.3870968	-5.703173	2.41E-08	1.68E-07	8.5497193
FDPS	-0.140444	6.8633047	-5.702398	2.42E-08	1.69E-07	8.5456888
RELL1	0.4264939	4.3733992	5.7022967	2.42E-08	1.69E-07	8.5451616
RP11-550F7	0.8780877	2.6437266	5.7022078	2.42E-08	1.69E-07	8.544699
COL6A6	1.0148678	1.5010342	5.7011496	2.43E-08	1.70E-07	8.5391944
TEKT2	1.0610965	0.6870678	5.7010603	2.44E-08	1.70E-07	8.5387303
AC012368.1	0.8133515	-0.546315	5.7006041	2.44E-08	1.71E-07	8.5363571
RP11-449P1	0.6781309	4.3548584	5.7005062	2.44E-08	1.71E-07	8.5358483
C10orf11	-0.164127	5.9049621	-5.70041	2.44E-08	1.71E-07	8.5353456
PCDHGB6	0.6612776	4.0090151	5.7001112	2.45E-08	1.71E-07	8.5337937
DTHD1	1.1069988	0.9244202	5.6998232	2.45E-08	1.71E-07	8.532296
DNTTIP2	-0.075738	6.3475478	-5.699045	2.46E-08	1.72E-07	8.5282483
PKHD1L1	1.3153335	1.7724182	5.6990223	2.46E-08	1.72E-07	8.5281314
CDK17	0.1078349	5.9134931	5.6987119	2.47E-08	1.72E-07	8.5265174
NDST3	1.0706401	-0.03914	5.6976541	2.48E-08	1.73E-07	8.5210179
AC144831.3	1.0700803	0.772365	5.6975929	2.48E-08	1.73E-07	8.5206998
TRBC2	0.3735066	5.4115813	5.6975929	2.48E-08	1.73E-07	8.5206994
RP5-1142A6	0.8124115	3.5380514	5.6975179	2.48E-08	1.73E-07	8.5203095
PHKB	-0.108808	6.3559135	-5.696134	2.50E-08	1.74E-07	8.5131143
PLA2G4F	1.2956977	0.4341637	5.6954424	2.51E-08	1.75E-07	8.5095216
LLGL1	0.1510829	5.7166012	5.6952821	2.51E-08	1.75E-07	8.5086886
RP11-452L6	-0.195552	5.1666052	-5.695213	2.51E-08	1.75E-07	8.5083298
LGALS3BP	0.235641	6.9410728	5.695021	2.52E-08	1.75E-07	8.5073316
RP11-480C1	-0.81205	-0.628756	-5.694684	2.52E-08	1.76E-07	8.5055824
CHST6	1.1375516	1.5328929	5.694416	2.52E-08	1.76E-07	8.5041878
PPP1R16B	0.2768863	5.2083384	5.6943453	2.52E-08	1.76E-07	8.5038204
XXbac-BPGE	-0.855119	-0.617303	-5.694173	2.53E-08	1.76E-07	8.5029239
FCGRT	-0.125838	6.9522888	-5.693977	2.53E-08	1.76E-07	8.5019057
CCDC12	-0.119031	6.2497108	-5.693474	2.54E-08	1.77E-07	8.4992936
RIOK2	-0.084276	5.9700672	-5.693335	2.54E-08	1.77E-07	8.4985734
AC109642.1	0.914513	1.4350942	5.6929961	2.54E-08	1.77E-07	8.4968103
TAX1BP1	-0.074825	6.7131606	-5.692234	2.55E-08	1.78E-07	8.4928519
AC005593.2	-0.831627	-0.24971	-5.691893	2.56E-08	1.78E-07	8.4910775
DTX2P1	0.4002313	3.9277647	5.6914265	2.56E-08	1.78E-07	8.4886566
FAM104B	-0.111405	5.6421003	-5.689266	2.59E-08	1.80E-07	8.4774356
ATP2B1	0.119103	6.1885363	5.6887102	2.60E-08	1.81E-07	8.4745514

STK17A	0.1283567	5.9537079	5.6884475	2.61E-08	1.81E-07	8.4731872
STYXL1	-0.148397	6.1321262	-5.688429	2.61E-08	1.81E-07	8.4730919
PCCB	-0.141842	6.6725844	-5.687676	2.62E-08	1.82E-07	8.4691829
MFRP	0.8784148	-0.224386	5.687165	2.62E-08	1.82E-07	8.4665299
SNORD12B	0.9020168	-0.137853	5.6870532	2.63E-08	1.82E-07	8.4659492
TSKS	1.0631287	1.3519431	5.6869548	2.63E-08	1.82E-07	8.4654387
FBXL5	-0.109667	6.5261659	-5.686933	2.63E-08	1.82E-07	8.4653245
MIIP	-0.133447	6.09847	-5.686836	2.63E-08	1.82E-07	8.4648235
FJX1	0.2553533	5.0244825	5.6864148	2.63E-08	1.83E-07	8.4626358
RP11-71E19	-0.821372	3.2349608	-5.68585	2.64E-08	1.83E-07	8.4597053
LINC01422	1.1159601	1.0250328	5.6849123	2.66E-08	1.84E-07	8.4548383
THSD7B	1.0744264	2.4354359	5.6838935	2.67E-08	1.85E-07	8.4495522
CYP4F3	-0.356069	6.7145153	-5.68348	2.68E-08	1.86E-07	8.4474092
PARD6A	-0.269568	5.1084497	-5.682906	2.69E-08	1.86E-07	8.4444281
AF127577.1	-0.738455	-0.780028	-5.68237	2.69E-08	1.87E-07	8.4416511
RP13-58209	-0.463266	3.6241544	-5.682268	2.69E-08	1.87E-07	8.4411223
PFKM	0.2177852	5.7426396	5.6821718	2.70E-08	1.87E-07	8.4406208
CTD-2165H1	-0.54875	3.3223103	-5.68176	2.70E-08	1.87E-07	8.4384838
CCDC160	0.8605567	-0.780132	5.6803849	2.72E-08	1.89E-07	8.4313536
CA9	1.4100064	3.0978534	5.6802759	2.72E-08	1.89E-07	8.4307886
TMIGD2	0.8174379	2.5642573	5.6795583	2.73E-08	1.89E-07	8.4270677
CSGALNACT1	0.2107717	5.4433706	5.6793563	2.74E-08	1.89E-07	8.4260203
TET1	0.5546123	4.1899519	5.6792299	2.74E-08	1.90E-07	8.425365
ZPR1	-0.092793	6.3247592	-5.678434	2.75E-08	1.90E-07	8.4212366
RP4-669P10	-1.190638	2.3092986	-5.677932	2.76E-08	1.91E-07	8.4186374
SLC4A5	0.7511864	2.7275457	5.6778793	2.76E-08	1.91E-07	8.4183631
KCP	0.632441	3.7484874	5.677708	2.76E-08	1.91E-07	8.4174753
ALG3	-0.101699	6.5849225	-5.67744	2.77E-08	1.91E-07	8.4160883
MYH11	0.3732464	5.7396387	5.6765737	2.78E-08	1.92E-07	8.4115959
CBR4	-0.160279	6.1336595	-5.676131	2.78E-08	1.92E-07	8.4093034
TTPAL	-0.162006	6.1399932	-5.676043	2.79E-08	1.93E-07	8.4088454
GYS2	-0.856319	5.6236208	-5.67552	2.79E-08	1.93E-07	8.4061355
SNX6	0.0978573	6.1542871	5.675003	2.80E-08	1.94E-07	8.4034565
AC006129.1	0.9773164	0.0719917	5.6749558	2.80E-08	1.94E-07	8.4032119
RPTOR	-0.08242	6.2814709	-5.67456	2.81E-08	1.94E-07	8.4011593
RP11-861E2	1.0203846	0.3347687	5.6742465	2.81E-08	1.94E-07	8.3995371
KISS1R	1.3526423	0.451292	5.6741813	2.81E-08	1.94E-07	8.3991995
XPA	-0.102807	5.7991887	-5.674097	2.82E-08	1.94E-07	8.3987616
TMEM35	1.0832161	0.410624	5.6739051	2.82E-08	1.94E-07	8.3977685
LINC00892	0.9306964	2.3719546	5.6737711	2.82E-08	1.95E-07	8.3970741
MANEAL	-0.249532	5.8882355	-5.673653	2.82E-08	1.95E-07	8.3964604
RABGEF1	-0.151719	5.4779354	-5.673343	2.83E-08	1.95E-07	8.3948548
GREM1	1.0605061	4.1121784	5.6729129	2.83E-08	1.95E-07	8.3926285
AC009133.1	0.9857382	0.0148435	5.6727444	2.84E-08	1.95E-07	8.3917556
RP5-907D15	0.9088918	-0.636054	5.6723929	2.84E-08	1.96E-07	8.3899354
PARPG1	0.7721442	3.5772975	5.6721857	2.84E-08	1.96E-07	8.3888622
GATC	-0.082172	6.0488104	-5.671864	2.85E-08	1.96E-07	8.387196
RAMP2-AS1	0.7892203	3.5210295	5.6715534	2.85E-08	1.97E-07	8.3855874
TGFBR3L	-0.785341	4.2556691	-5.671545	2.85E-08	1.97E-07	8.3855437
IGKV1-39	1.1096266	-0.444315	5.6715105	2.85E-08	1.97E-07	8.3853654
RP11-785H5	-0.107035	6.1786323	-5.671132	2.86E-08	1.97E-07	8.3834029

RP11-462L8	0.868114	3.706405	5.6701743	2.88E-08	1.98E-07	8.3784461
SERPINF1	-0.187756	7.2292566	-5.670012	2.88E-08	1.98E-07	8.377607
HSD3B7	-0.170468	6.4478593	-5.669852	2.88E-08	1.98E-07	8.3767779
TF	-0.268089	7.7305863	-5.669556	2.88E-08	1.98E-07	8.3752449
RP4-724E16	0.9160379	2.5518321	5.6694177	2.89E-08	1.99E-07	8.3745289
CYTH1	-0.08323	6.4471258	-5.669082	2.89E-08	1.99E-07	8.372792
NPB	-1.00762	0.8800178	-5.668884	2.90E-08	1.99E-07	8.3717665
RP11-486I1	-0.51321	3.4535745	-5.668745	2.90E-08	1.99E-07	8.371046
CCDC174	-0.072828	5.9136258	-5.66871	2.90E-08	1.99E-07	8.3708644
CLEC9A	1.0503139	2.1314582	5.6675052	2.92E-08	2.00E-07	8.3646288
ZNF683	0.964441	3.1400273	5.6672215	2.92E-08	2.01E-07	8.3631606
TMED5	-0.107247	6.7043749	-5.666913	2.93E-08	2.01E-07	8.3615653
WIPF3	0.8724737	4.527385	5.6668846	2.93E-08	2.01E-07	8.361417
RTN3	-0.075778	6.7656412	-5.666043	2.94E-08	2.02E-07	8.3570639
MYZAP	0.6713297	3.2286294	5.6651975	2.95E-08	2.03E-07	8.3526871
MRFAP1	-0.072815	6.9458134	-5.665126	2.95E-08	2.03E-07	8.3523195
GS1-24F4.2	0.8453423	-0.787134	5.6649371	2.96E-08	2.03E-07	8.3513397
LINGO1-AS1	0.8762898	-0.834096	5.6639866	2.97E-08	2.04E-07	8.3464225
SLC6A3	1.2124908	0.1629336	5.6625045	3.00E-08	2.06E-07	8.3387563
CTD-2240J1	-0.997218	1.5470626	-5.662108	3.00E-08	2.06E-07	8.3367042
RP11-1277A	1.1133014	1.7199895	5.6616602	3.01E-08	2.06E-07	8.3343904
NXF3	1.1604979	0.8955837	5.6610697	3.02E-08	2.07E-07	8.3313367
PRKCH	0.1840082	5.6211736	5.6608605	3.02E-08	2.07E-07	8.3302552
LINC00251	-0.512227	-1.306269	-5.660491	3.03E-08	2.08E-07	8.3283447
MOV10	-0.090218	6.581997	-5.660008	3.04E-08	2.08E-07	8.3258455
TNFSF4	0.3093179	4.933549	5.6591962	3.05E-08	2.09E-07	8.3216507
RP11-766F1	-0.906141	2.7678572	-5.658779	3.06E-08	2.09E-07	8.3194953
TMSB15B	0.8992858	-0.347403	5.6581966	3.07E-08	2.10E-07	8.3164838
PPP4C	-0.094167	6.6186779	-5.65803	3.07E-08	2.10E-07	8.3156223
CTD-2186M1	-0.2113	4.8750394	-5.655977	3.10E-08	2.12E-07	8.3050157
ENDOD1	0.2569444	5.604377	5.6541539	3.13E-08	2.14E-07	8.2955953
ZNF831	0.8284246	3.3143368	5.6537876	3.14E-08	2.15E-07	8.2937032
DCAF6	-0.0929	6.6915375	-5.653737	3.14E-08	2.15E-07	8.2934397
ZNF211	0.1871416	5.2320107	5.6536372	3.14E-08	2.15E-07	8.2929264
CD24	0.6419024	6.2016793	5.6533834	3.15E-08	2.15E-07	8.2916155
RP11-178L8	-1.097039	1.6218703	-5.653139	3.15E-08	2.15E-07	8.2903535
RP11-271K1	0.9528184	0.2431912	5.6517103	3.17E-08	2.17E-07	8.2829752
CFAP46	1.3493346	1.4814413	5.6515659	3.18E-08	2.17E-07	8.2822297
CYP4F24P	0.9762817	-0.154645	5.6507051	3.19E-08	2.18E-07	8.2777852
DHDDS	-0.098931	6.15551	-5.6503	3.20E-08	2.19E-07	8.2756917
PRR27	-0.92072	-0.733273	-5.650265	3.20E-08	2.19E-07	8.2755148
IGFL2	1.0695409	-0.138105	5.6496362	3.21E-08	2.19E-07	8.272267
C2orf47	-0.100446	6.1461506	-5.648242	3.23E-08	2.21E-07	8.2650719
PPM1H	0.397346	5.41098	5.647348	3.25E-08	2.22E-07	8.2604576
ADCK5	-0.151629	5.8855968	-5.646851	3.26E-08	2.22E-07	8.257893
CTD-2033C1	-0.584842	2.6356063	-5.646456	3.27E-08	2.23E-07	8.2558557
RP11-815I9	0.6586745	2.6290514	5.6460203	3.27E-08	2.23E-07	8.2536071
RP11-8L8.2	0.9573503	1.3675131	5.6454945	3.28E-08	2.24E-07	8.2508942
FCER1G	0.2110075	5.8719826	5.644996	3.29E-08	2.24E-07	8.2483225
CPNE7	0.8250164	4.0895295	5.6442823	3.30E-08	2.25E-07	8.2446411
NBPF15	0.362158	5.3258648	5.6434265	3.32E-08	2.26E-07	8.2402274

APAF1	0.2617611	5.5326067	5.6433965	3.32E-08	2.26E-07	8.240073
DHODH	-0.203156	6.2988548	-5.643068	3.33E-08	2.27E-07	8.2383809
AP4B1-AS1	0.8991023	1.7947886	5.642409	3.34E-08	2.27E-07	8.2349806
APOM	-0.296827	6.8522246	-5.642196	3.34E-08	2.28E-07	8.2338807
PIANP	1.010261	0.7543231	5.6407897	3.37E-08	2.29E-07	8.2266314
RP11-95P2.	-0.692795	2.6572642	-5.640562	3.37E-08	2.30E-07	8.2254589
PHLDA2	0.4546584	5.1386384	5.6403841	3.37E-08	2.30E-07	8.224541
UBBP4	-0.157414	5.4950222	-5.640382	3.37E-08	2.30E-07	8.2245325
CNRIP1	0.217016	5.0354921	5.6401558	3.38E-08	2.30E-07	8.2233642
FER1L5	1.0045318	1.857021	5.6395212	3.39E-08	2.31E-07	8.220093
RCCD1	-0.106772	5.8297426	-5.639188	3.39E-08	2.31E-07	8.2183735
MSS51	0.3221756	4.0463304	5.6379073	3.42E-08	2.33E-07	8.2117753
PNPO	-0.164704	6.4012292	-5.637166	3.43E-08	2.33E-07	8.2079578
HOXA4	0.5980181	3.3040514	5.6361883	3.45E-08	2.35E-07	8.2029188
MVK	-0.150871	6.3476521	-5.635771	3.46E-08	2.35E-07	8.2007708
ZNF140	0.1726073	5.5192997	5.6354873	3.46E-08	2.35E-07	8.1993074
GSTA1	-0.601313	6.8408334	-5.634889	3.47E-08	2.36E-07	8.196226
RP1-78014.	1.0917894	0.7224248	5.6347503	3.48E-08	2.36E-07	8.1955115
CTD-2561B2	1.049131	0.4907224	5.6347289	3.48E-08	2.36E-07	8.195401
TGFB2-AS1	1.1769469	0.8095453	5.6342059	3.49E-08	2.37E-07	8.1927074
RP5-1004I9	-0.936868	-0.31936	-5.634181	3.49E-08	2.37E-07	8.1925804
APOA2	-0.341587	7.7819785	-5.634165	3.49E-08	2.37E-07	8.1924958
SMCHD1	0.1054688	6.0779712	5.6340684	3.49E-08	2.37E-07	8.1919995
ART3	1.1653952	0.6687674	5.6339637	3.49E-08	2.37E-07	8.1914602
CATSPERB	1.1626538	2.3141395	5.6337089	3.50E-08	2.37E-07	8.1901479
ZBTB1	0.152326	5.8083074	5.6336484	3.50E-08	2.37E-07	8.1898363
FAM63A	-0.125122	6.1747137	-5.633347	3.50E-08	2.38E-07	8.1882857
YPEL3	-0.120724	6.2845385	-5.633239	3.50E-08	2.38E-07	8.1877296
PTPN14	0.2510854	5.6000055	5.6328272	3.51E-08	2.38E-07	8.1856079
CTD-2357A8	0.9767959	-0.292837	5.6324128	3.52E-08	2.39E-07	8.183474
GBAS	0.1224745	5.9882736	5.6322698	3.52E-08	2.39E-07	8.182738
SLC25A39	-0.090299	6.8488925	-5.632132	3.53E-08	2.39E-07	8.1820296
RAB37	-0.223879	5.7659074	-5.63181	3.53E-08	2.39E-07	8.1803717
RFT1	-0.083347	6.0401797	-5.631522	3.54E-08	2.40E-07	8.1788898
LINC01344	-1.126592	2.5981557	-5.631477	3.54E-08	2.40E-07	8.1786543
MTFMT	-0.088459	5.6964308	-5.631318	3.54E-08	2.40E-07	8.1778401
CALM2P2	0.965368	1.8742453	5.63129	3.54E-08	2.40E-07	8.1776937
KAT2B	-0.171735	6.1645914	-5.630151	3.56E-08	2.41E-07	8.1718314
FCER2	0.9717669	2.1337754	5.6296165	3.57E-08	2.42E-07	8.1690799
RP11-146F1	-0.711154	-0.949997	-5.629154	3.58E-08	2.42E-07	8.166698
LINC00853	-0.566705	4.1939342	-5.629025	3.58E-08	2.43E-07	8.1660364
RP11-158L1	0.7549647	3.0618507	5.6279028	3.61E-08	2.44E-07	8.1602613
MAP2K7	-0.076457	6.2788517	-5.62781	3.61E-08	2.44E-07	8.1597842
LYRM9	-0.185009	5.3977991	-5.627737	3.61E-08	2.44E-07	8.1594105
PARP10	-0.138233	6.6687921	-5.627357	3.62E-08	2.45E-07	8.1574551
IL18RAP	0.4988335	3.8534399	5.6268258	3.63E-08	2.45E-07	8.15472
PHAX	-0.080833	6.2330354	-5.625969	3.64E-08	2.46E-07	8.1503151
ALDH3B2	1.2090069	-0.033232	5.6253625	3.65E-08	2.47E-07	8.1471931
MCCC2	-0.110442	6.7114752	-5.625227	3.66E-08	2.47E-07	8.1464937
PPM1G	-0.073487	6.6469937	-5.625164	3.66E-08	2.47E-07	8.1461715
SRGN	0.1863551	6.11179	5.6243195	3.68E-08	2.48E-07	8.1418291

GALT	-0.131806	6.5294072	-5.624199	3.68E-08	2.48E-07	8.141207
APCDD1L	1.1271548	0.0752893	5.6238244	3.69E-08	2.49E-07	8.1392828
CTD-255501	-0.661632	-0.868683	-5.623446	3.69E-08	2.49E-07	8.1373374
HARS	-0.086007	6.3970052	-5.623031	3.70E-08	2.50E-07	8.1352016
SULT1C2	0.9816435	5.0339327	5.6227792	3.71E-08	2.50E-07	8.1339085
GPAM	-0.265456	6.7254279	-5.621858	3.72E-08	2.51E-07	8.1291705
ADORA2A	-0.397468	4.6240657	-5.621536	3.73E-08	2.52E-07	8.1275175
FOXK1	0.1206849	6.0253264	5.6213881	3.73E-08	2.52E-07	8.1267571
PLSCR1	0.1680716	6.0725813	5.6209841	3.74E-08	2.52E-07	8.1246805
XXYLT1-AS2	0.892511	-0.460161	5.6207602	3.75E-08	2.53E-07	8.1235298
FNBP4	0.0837272	6.1637275	5.6206338	3.75E-08	2.53E-07	8.1228804
RP11-736K2	-0.494011	3.9490422	-5.620525	3.75E-08	2.53E-07	8.1223191
LAGE3	-0.168956	6.0662394	-5.620503	3.75E-08	2.53E-07	8.1222084
TRGC2	0.7162178	3.517139	5.6202822	3.76E-08	2.53E-07	8.1210732
FRA10AC1	-0.106962	5.9799894	-5.620101	3.76E-08	2.53E-07	8.1201437
ABCG4	0.9031321	1.2671184	5.6200859	3.76E-08	2.53E-07	8.1200641
STX3	0.1626216	5.8608002	5.619881	3.76E-08	2.53E-07	8.119011
IGKV2-29	1.366205	0.2234043	5.619874	3.76E-08	2.53E-07	8.1189755
USP44	0.9925028	1.7385149	5.6193029	3.78E-08	2.54E-07	8.1160403
LINC01197	0.5073768	3.9800767	5.6192108	3.78E-08	2.54E-07	8.1155672
ABHD15	-0.212817	6.0401863	-5.619118	3.78E-08	2.54E-07	8.1150916
MROH8	-0.303674	4.5094248	-5.618282	3.80E-08	2.55E-07	8.1107939
RP11-468E2	0.8228581	2.3255455	5.6174165	3.81E-08	2.56E-07	8.1063485
RP11-972P1	0.7140511	2.9123079	5.6172506	3.82E-08	2.57E-07	8.1054958
AHNAK2	0.9284629	4.1465921	5.6170721	3.82E-08	2.57E-07	8.1045789
SULT2A1	-0.587003	6.8253518	-5.615998	3.84E-08	2.58E-07	8.0990601
DKK2	1.1156296	3.1251249	5.6157115	3.85E-08	2.59E-07	8.0975904
RP11-317B7	-0.873745	-0.789668	-5.61559	3.85E-08	2.59E-07	8.0969675
CXCL6	1.6528248	2.9483935	5.6147888	3.87E-08	2.60E-07	8.0928519
ZNF525	0.5319133	4.7068607	5.6144559	3.87E-08	2.60E-07	8.0911423
KRT80	1.1460192	3.5624597	5.6139638	3.88E-08	2.61E-07	8.0886153
SIRPAP1	1.0155934	0.851013	5.613806	3.89E-08	2.61E-07	8.0878054
PARM1	0.3390539	5.2130181	5.6136188	3.89E-08	2.61E-07	8.0868443
HAP1	1.0114669	2.0899033	5.6135905	3.89E-08	2.61E-07	8.086699
EDRF1	0.1115047	5.5338135	5.612833	3.91E-08	2.62E-07	8.0828098
KLK10	1.2374081	0.592357	5.6126186	3.91E-08	2.62E-07	8.0817092
PHACTR2	0.1884442	5.7821496	5.6118669	3.93E-08	2.63E-07	8.0778508
CTC-50503.	-1.099986	4.4374493	-5.611283	3.94E-08	2.64E-07	8.0748532
DUSP15	0.8526897	3.0590049	5.6112756	3.94E-08	2.64E-07	8.0748158
RP11-24M17	-0.755543	-0.93341	-5.611043	3.94E-08	2.64E-07	8.0736228
AP000253.1	-1.001071	1.6544352	-5.610848	3.95E-08	2.65E-07	8.0726193
CTD-3162L1	1.1231024	1.0028681	5.6102365	3.96E-08	2.66E-07	8.0694828
TRAV26-2	0.7377891	-0.825374	5.6093095	3.98E-08	2.67E-07	8.0647258
CNNM3	-0.099157	6.349151	-5.60856	4.00E-08	2.68E-07	8.06088
RPP25	0.2861663	5.3504794	5.6083753	4.00E-08	2.68E-07	8.0599329
TGFBRAP1	0.0858575	5.85625	5.6076988	4.02E-08	2.69E-07	8.0564626
ADIPOR1	-0.084642	6.7860919	-5.607497	4.02E-08	2.69E-07	8.055428
PEX5	-0.100132	6.4452791	-5.605856	4.06E-08	2.71E-07	8.0470082
ZDHHC21	0.2040782	5.451136	5.605676	4.06E-08	2.72E-07	8.0460877
RP11-314N1	0.9788676	0.6324135	5.6047815	4.08E-08	2.73E-07	8.0415009
MLF2	-0.087597	6.9172892	-5.604465	4.09E-08	2.73E-07	8.039879

NDUFAF2	-0.147117	5.8447164	-5.60304	4.12E-08	2.75E-07	8.0325735
ZNF468	0.5067212	5.2506339	5.6012902	4.16E-08	2.78E-07	8.0236041
RP11-800A3	-0.938378	0.2015468	-5.601284	4.16E-08	2.78E-07	8.0235735
SMR3A	-0.842688	-1.144515	-5.601228	4.16E-08	2.78E-07	8.0232862
OGN	1.3201653	3.1382447	5.6011208	4.16E-08	2.78E-07	8.0227357
PDE6G	0.4269902	4.0585724	5.6008779	4.16E-08	2.78E-07	8.0214908
PWAR5	1.2371394	1.5778724	5.6007826	4.17E-08	2.78E-07	8.0210025
C2orf50	0.9640589	-0.045034	5.6007816	4.17E-08	2.78E-07	8.0209974
LAIR1	0.2437524	5.5931343	5.6004162	4.17E-08	2.79E-07	8.0191249
RP11-1166F	0.9098748	-0.731983	5.5998872	4.19E-08	2.80E-07	8.0164146
CTD-2012K1	-0.404956	4.9553177	-5.599854	4.19E-08	2.80E-07	8.0162465
CDHR2	0.9203465	5.0589134	5.5997731	4.19E-08	2.80E-07	8.01583
MMP28	0.8611466	3.54671	5.5994131	4.20E-08	2.80E-07	8.0139853
CTC-205M6.	0.3248144	4.1668976	5.5991758	4.20E-08	2.80E-07	8.0127699
CSTF1	-0.079768	6.1654111	-5.599111	4.20E-08	2.80E-07	8.0124354
TRPV2	0.200635	5.5657478	5.5989072	4.21E-08	2.81E-07	8.0113937
HIST1H2BD	-0.23786	6.0191646	-5.597921	4.23E-08	2.82E-07	8.0063393
BANF1	-0.094336	6.671431	-5.597392	4.24E-08	2.83E-07	8.0036321
RP11-89C3.	1.0397039	0.393823	5.5973329	4.24E-08	2.83E-07	8.0033298
RP11-67L2.	0.1904419	5.0332824	5.5973235	4.24E-08	2.83E-07	8.0032816
FUCA1	-0.121536	6.5405549	-5.596557	4.26E-08	2.84E-07	7.9993583
JPH4	0.5890796	3.0934564	5.5957668	4.28E-08	2.85E-07	7.9953093
CYP20A1	-0.074684	6.1050844	-5.595522	4.28E-08	2.85E-07	7.9940569
SLC46A1	-0.141128	6.1984672	-5.595499	4.28E-08	2.85E-07	7.9939371
KRT5	1.4587558	1.3690619	5.5954606	4.29E-08	2.85E-07	7.9937413
NDUFS2	-0.096906	6.8881755	-5.594849	4.30E-08	2.86E-07	7.9906116
RP11-104J2	-0.903476	2.3355272	-5.594768	4.30E-08	2.86E-07	7.9901971
TMEM72	1.3814137	1.6309405	5.5938682	4.32E-08	2.88E-07	7.985589
KIF19	0.8638898	2.6012503	5.5933121	4.34E-08	2.89E-07	7.9827422
CTC-260E6.	1.0130656	1.7493496	5.5928428	4.35E-08	2.89E-07	7.9803399
GPR35	0.6060094	4.5233444	5.5920302	4.36E-08	2.90E-07	7.9761811
PITX1	1.5229155	3.268045	5.5919757	4.37E-08	2.90E-07	7.9759023
ZFP1	-0.175788	5.4513916	-5.591641	4.37E-08	2.91E-07	7.9741912
SDHD	-0.107016	6.607243	-5.5914	4.38E-08	2.91E-07	7.9729586
NT5C3B	-0.132628	6.2776934	-5.591224	4.38E-08	2.91E-07	7.9720567
HIST1H4PS1	-0.898989	-0.446967	-5.590807	4.39E-08	2.92E-07	7.9699199
CSRP2	-0.215973	6.0010917	-5.59075	4.39E-08	2.92E-07	7.9696315
RP11-804H8	0.7243611	2.6987258	5.5900599	4.41E-08	2.93E-07	7.9660993
RP3-522D1.	1.0346634	0.849048	5.5899418	4.41E-08	2.93E-07	7.9654952
CYP2D7	-0.465774	5.5521415	-5.589812	4.42E-08	2.93E-07	7.9648295
RTL1	1.061557	-0.42577	5.589393	4.43E-08	2.94E-07	7.9626878
THRB	-0.156042	6.1673006	-5.588482	4.45E-08	2.95E-07	7.9580283
TUBA1B	0.1630338	6.557185	5.5881822	4.45E-08	2.96E-07	7.9564942
INCENP	0.1982757	5.5930979	5.5879783	4.46E-08	2.96E-07	7.9554513
HTRA4	1.1768032	1.3665372	5.5870494	4.48E-08	2.97E-07	7.9507007
IDH1-AS1	-0.474075	3.8692719	-5.586629	4.49E-08	2.98E-07	7.9485485
AC010524.4	0.8163638	-0.560951	5.5860394	4.51E-08	2.99E-07	7.9455361
DLL3	1.0453766	-0.183493	5.584503	4.54E-08	3.01E-07	7.9376811
TSFM	-0.095834	6.2625159	-5.584124	4.55E-08	3.02E-07	7.9357451
KIAA1524	0.3464072	4.8857719	5.5839243	4.56E-08	3.02E-07	7.9347231
RP11-404F1	0.8759075	-0.232469	5.5838609	4.56E-08	3.02E-07	7.9343991

NR1H2	-0.084323	6.5500682	-5.582039	4.60E-08	3.05E-07	7.9250877
LAMTOR4	-0.135208	6.4894842	-5.581154	4.62E-08	3.06E-07	7.9205639
GGH	-0.234852	6.7406283	-5.579624	4.66E-08	3.09E-07	7.9127491
RP11-20E24	-0.685443	2.3745324	-5.579445	4.67E-08	3.09E-07	7.9118377
FAM49A	0.2340596	5.4305857	5.5788834	4.68E-08	3.10E-07	7.908967
RP11-329B9	0.7052784	-0.770356	5.5783209	4.69E-08	3.11E-07	7.9060939
WDR47	0.1845428	5.2464892	5.577911	4.70E-08	3.11E-07	7.904001
TXNRD2	-0.145477	6.4737244	-5.577677	4.71E-08	3.12E-07	7.9028067
PHEX	1.1716149	2.6718709	5.5773293	4.72E-08	3.12E-07	7.9010302
TREML1	0.9953813	1.2921334	5.5773014	4.72E-08	3.12E-07	7.9008878
RP4-681N20	0.9562103	1.3297923	5.5758291	4.76E-08	3.15E-07	7.8933707
ITGB1P1	0.8732722	2.692982	5.5756183	4.76E-08	3.15E-07	7.8922945
ANKRD37	-0.298707	5.0712343	-5.575587	4.76E-08	3.15E-07	7.8921324
ERVMER34-1	1.3945628	1.7585042	5.5754356	4.77E-08	3.15E-07	7.891362
MEMO1P1	0.6193017	3.7997715	5.5750527	4.78E-08	3.16E-07	7.8894072
SIAH3	0.7784537	-0.796576	5.5746952	4.79E-08	3.16E-07	7.8875825
TUSC8	-1.000816	4.3918043	-5.573966	4.80E-08	3.17E-07	7.8838603
CSDE1	-0.069268	7.032988	-5.572751	4.83E-08	3.19E-07	7.8776608
NECAB2	-0.666447	5.2957319	-5.572684	4.84E-08	3.19E-07	7.8773159
DNM1	0.4203076	5.0222738	5.5717573	4.86E-08	3.21E-07	7.8725898
RP11-219G1	1.1445508	0.3608114	5.5715927	4.86E-08	3.21E-07	7.8717502
CTD-2547L2	0.8633821	2.2005781	5.5715052	4.87E-08	3.21E-07	7.8713034
ZNF80	1.0240292	0.0106334	5.5713195	4.87E-08	3.22E-07	7.8703564
DNAJC9	0.1575624	5.6708512	5.5709561	4.88E-08	3.22E-07	7.8685025
LIMK2	0.1607856	6.1533218	5.5708129	4.88E-08	3.22E-07	7.8677719
MARCKSL1	0.1720154	6.4213467	5.5708118	4.88E-08	3.22E-07	7.8677663
CINP	-0.11286	6.1224158	-5.570657	4.89E-08	3.22E-07	7.8669774
SNORD89	0.8587883	1.0143588	5.5703899	4.90E-08	3.23E-07	7.8656139
FAM83B	1.1946683	-0.287899	5.5703257	4.90E-08	3.23E-07	7.8652867
PCDHB10	0.8135406	3.2102721	5.5701283	4.90E-08	3.23E-07	7.8642795
BAG1	-0.113847	6.4007102	-5.569946	4.91E-08	3.23E-07	7.8633521
TRIM26	-0.085826	6.6228277	-5.569859	4.91E-08	3.24E-07	7.8629065
RP11-881M1	-0.63774	-1.122435	-5.569596	4.92E-08	3.24E-07	7.8615661
TLL2	1.0008931	2.3757874	5.5692102	4.93E-08	3.24E-07	7.8595968
RP13-516M1	-0.911783	1.8310221	-5.569188	4.93E-08	3.24E-07	7.8594858
CTD-2240J1	-1.000596	1.3538786	-5.569164	4.93E-08	3.24E-07	7.859359
S100A4	0.2638315	5.8424358	5.5688251	4.94E-08	3.25E-07	7.8576329
SELM	0.3188712	5.9949807	5.5685595	4.94E-08	3.25E-07	7.8562784
RRP9	-0.11725	6.0348499	-5.568535	4.94E-08	3.25E-07	7.8561552
MTMR8	0.9362356	2.5062406	5.5682889	4.95E-08	3.26E-07	7.8548984
RP11-1017G	0.9211297	0.8734209	5.5679318	4.96E-08	3.26E-07	7.8530777
RP11-78002	-0.89738	-0.382702	-5.567907	4.96E-08	3.26E-07	7.8529503
PCSK4	-0.27643	5.0916666	-5.567801	4.96E-08	3.26E-07	7.8524101
SLC16A1-AS	-0.274108	4.8976713	-5.56779	4.96E-08	3.26E-07	7.8523533
CTC-523E23	0.9867307	1.1878569	5.5676988	4.97E-08	3.26E-07	7.8518897
RILP	-0.179541	6.0120185	-5.567081	4.98E-08	3.28E-07	7.8487388
POLR3H	-0.096681	6.229904	-5.566002	5.01E-08	3.29E-07	7.8432396
RP11-416N2	1.0189586	0.4985411	5.5659201	5.01E-08	3.29E-07	7.8428214
NDUFB4	-0.087949	6.6134851	-5.565919	5.01E-08	3.29E-07	7.8428179
RIPK1	-0.074505	6.3207277	-5.565561	5.02E-08	3.30E-07	7.840993
LLNLR-260G	0.5724952	-1.113803	5.5653707	5.03E-08	3.30E-07	7.8400212

LINC00877	0.9717097	1.6414439	5.5649941	5.04E-08	3.31E-07	7.8381016
PGF	0.2611228	5.4589113	5.5639915	5.06E-08	3.32E-07	7.8329919
PRDX2	-0.105876	6.8768498	-5.563764	5.07E-08	3.33E-07	7.8318333
LDLRAD2	0.435452	3.772692	5.5636625	5.07E-08	3.33E-07	7.8313154
RP11-210M1	1.4139971	1.2328834	5.5635408	5.08E-08	3.33E-07	7.830695
TOB1	-0.156631	6.6776555	-5.563409	5.08E-08	3.33E-07	7.8300218
MYB	0.8157394	3.2306798	5.563322	5.08E-08	3.33E-07	7.8295803
SENP3-EIF4	0.8421103	-0.245498	5.5630468	5.09E-08	3.34E-07	7.8281777
SLC10A5	-0.283026	4.8804499	-5.562821	5.10E-08	3.34E-07	7.8270263
SPRYD4	-0.149685	6.3957352	-5.562761	5.10E-08	3.34E-07	7.8267196
MAOA	-0.182747	6.7338026	-5.562728	5.10E-08	3.34E-07	7.8265559
EVA1A	-0.303103	6.2639038	-5.562493	5.10E-08	3.35E-07	7.8253563
IFITM3	-0.125072	7.3606397	-5.561753	5.12E-08	3.36E-07	7.8215873
EBI3	0.3703549	4.6881972	5.5616592	5.13E-08	3.36E-07	7.8211087
F8A1	-0.181909	5.6765948	-5.561524	5.13E-08	3.36E-07	7.8204175
SLC17A3	-0.554084	5.4363698	-5.561344	5.14E-08	3.36E-07	7.8195046
CASP8AP2	0.2001491	5.5065978	5.5613177	5.14E-08	3.36E-07	7.8193687
AP003385.2	-0.629242	-0.996963	-5.561035	5.14E-08	3.37E-07	7.8179313
AP000344.4	1.0599795	-0.291293	5.5608785	5.15E-08	3.37E-07	7.8171318
DENND1A	-0.087361	6.237564	-5.560542	5.16E-08	3.37E-07	7.8154168
C9orf78	-0.082579	6.4362606	-5.560534	5.16E-08	3.37E-07	7.8153765
MAP1B	0.2185182	5.746544	5.5604805	5.16E-08	3.38E-07	7.8151047
RBM22	-0.073005	6.2740955	-5.560285	5.16E-08	3.38E-07	7.8141082
PIGCP1	0.8858497	3.4578686	5.5602574	5.17E-08	3.38E-07	7.813968
AC104076.3	-0.853219	-0.430908	-5.559849	5.18E-08	3.38E-07	7.811889
TMEM104	-0.104813	6.2371437	-5.559565	5.18E-08	3.39E-07	7.8104425
TMEM151A	1.0293657	3.3555939	5.5594114	5.19E-08	3.39E-07	7.8096594
TMC4	0.4560007	5.0778354	5.559308	5.19E-08	3.39E-07	7.8091332
OPN1SW	0.3530738	4.4527008	5.5591945	5.19E-08	3.39E-07	7.8085549
AGPAT1	-0.08645	6.5423873	-5.558965	5.20E-08	3.40E-07	7.8073857
SWAP70	0.1094325	6.1106499	5.5586421	5.21E-08	3.40E-07	7.8057425
RPL27	-0.111166	7.1264143	-5.558534	5.21E-08	3.40E-07	7.8051935
LINC00886	-0.445252	4.9224528	-5.557987	5.23E-08	3.41E-07	7.8024063
TBC1D32	0.5629099	3.8536788	5.5579116	5.23E-08	3.41E-07	7.8020229
SPX	-0.784018	4.6991484	-5.556215	5.28E-08	3.44E-07	7.7933873
PMM1	-0.129273	6.40389	-5.556159	5.28E-08	3.44E-07	7.7931004
ZNF675	0.3352657	4.8243328	5.5552534	5.30E-08	3.46E-07	7.788492
TMED10P2	0.8150458	-0.751826	5.5548971	5.31E-08	3.47E-07	7.7866787
RP11-883G1	-1.109101	0.792004	-5.554142	5.33E-08	3.48E-07	7.7828382
SPACA6P-AS	0.7261552	-0.921302	5.5538312	5.34E-08	3.48E-07	7.7812548
CCDC124	-0.106146	6.4439753	-5.553704	5.35E-08	3.49E-07	7.7806065
MIR325HG	-1.38046	1.5425487	-5.55315	5.36E-08	3.50E-07	7.7777889
GOLGA6A	-1.152904	0.1462031	-5.553023	5.37E-08	3.50E-07	7.7771426
CD93	0.1444659	6.2396327	5.5523274	5.39E-08	3.51E-07	7.7736041
MS4A14	0.6252297	3.574343	5.5521006	5.39E-08	3.51E-07	7.7724501
AC008982.2	0.9551738	1.1167007	5.5506464	5.43E-08	3.54E-07	7.765054
OTUD3	0.2033859	5.0719881	5.549614	5.46E-08	3.56E-07	7.7598038
RPL24	-0.115442	7.0995292	-5.549029	5.48E-08	3.57E-07	7.7568311
IAH1	-0.090188	6.2458921	-5.54751	5.52E-08	3.60E-07	7.7491077
TNNC2	-0.646856	2.9979678	-5.547459	5.53E-08	3.60E-07	7.7488475
GGCX	-0.108855	6.8473146	-5.546564	5.55E-08	3.61E-07	7.7442995

HABP4	-0.174318	5.8359167	-5.546207	5.56E-08	3.62E-07	7.7424857
RP11-72M17	-0.893325	2.168315	-5.544537	5.61E-08	3.65E-07	7.7339966
C3P1	-0.673584	6.0938065	-5.543968	5.63E-08	3.66E-07	7.7311063
ATP12A	0.9984386	-0.714228	5.5432814	5.65E-08	3.67E-07	7.7276185
LINC00693	0.9670639	0.0394739	5.543127	5.65E-08	3.68E-07	7.7268344
TFG	-0.069029	6.7508652	-5.54222	5.68E-08	3.69E-07	7.7222249
RRP36	-0.087224	6.2688804	-5.542188	5.68E-08	3.69E-07	7.7220663
CAST	-0.081539	6.7449987	-5.541244	5.71E-08	3.71E-07	7.7172701
SIGLEC6	0.9779809	-0.094309	5.5406267	5.73E-08	3.72E-07	7.7141356
PAQR5	0.8603072	4.8913141	5.540515	5.73E-08	3.72E-07	7.7135683
SEC24C	0.3300146	6.0185696	5.5397031	5.76E-08	3.74E-07	7.7094459
ALS2CR11	0.9595938	1.6431891	5.5396535	5.76E-08	3.74E-07	7.7091938
RP11-539L1	0.5272568	3.2596972	5.5393823	5.77E-08	3.74E-07	7.7078172
TNR	1.3195947	1.1684683	5.5392535	5.77E-08	3.75E-07	7.7071634
ENO1-AS1	-1.011345	1.3569806	-5.539137	5.77E-08	3.75E-07	7.706573
AC074289.1	0.4828519	4.017641	5.539064	5.78E-08	3.75E-07	7.7062013
FAM63B	0.1491268	5.7003801	5.5382651	5.80E-08	3.76E-07	7.7021454
RP1-317E23	-0.358764	3.9582899	-5.538255	5.80E-08	3.76E-07	7.7020943
STX4	-0.085147	6.355848	-5.53816	5.80E-08	3.76E-07	7.7016145
TIMM13	-0.123561	6.5387445	-5.53703	5.84E-08	3.79E-07	7.6958773
ARFIP2	-0.079876	6.3538462	-5.536428	5.86E-08	3.80E-07	7.6928193
LEAP2	-0.314753	6.4709176	-5.536328	5.86E-08	3.80E-07	7.6923131
RP11-456H1	-0.629412	-1.004335	-5.536045	5.87E-08	3.80E-07	7.6908756
LCN12	-0.275237	5.5201558	-5.536022	5.87E-08	3.80E-07	7.6907599
IGHV3-19	0.8350788	-0.820136	5.5340997	5.93E-08	3.84E-07	7.6810084
PDHB	-0.08869	6.5312708	-5.533821	5.94E-08	3.85E-07	7.6795923
HMSD	1.2000726	1.1811706	5.5331219	5.96E-08	3.86E-07	7.6760483
ZNF648	-1.273585	3.0430905	-5.533047	5.96E-08	3.86E-07	7.6756701
ZNF695	1.3469817	0.7982769	5.5329337	5.97E-08	3.86E-07	7.6750941
ITGA1	-0.108056	6.6870764	-5.532529	5.98E-08	3.87E-07	7.6730421
SLFN12	0.409662	4.3007676	5.531463	6.01E-08	3.89E-07	7.6676356
SMPDL3B	0.9467606	3.6008145	5.5306569	6.04E-08	3.91E-07	7.6635481
ATL3	-0.095899	6.4675012	-5.529737	6.07E-08	3.93E-07	7.6588836
RP11-54C4.	-0.888449	-0.667024	-5.529415	6.08E-08	3.93E-07	7.6572514
RP11-295K3	0.7729138	3.0960819	5.5288818	6.10E-08	3.94E-07	7.654549
SBK1	0.5503423	4.0699283	5.5285028	6.11E-08	3.95E-07	7.6526282
AL162759.1	1.264835	0.9530891	5.5281302	6.12E-08	3.96E-07	7.6507395
RP11-88E10	0.952173	2.038338	5.5276365	6.14E-08	3.97E-07	7.6482374
LINC00243	0.9360821	0.0596419	5.5274023	6.14E-08	3.97E-07	7.6470508
RP5-1142A6	0.8046554	1.935575	5.5270422	6.15E-08	3.98E-07	7.6452256
RP11-109D9	-1.130223	0.9709419	-5.526904	6.16E-08	3.98E-07	7.6445278
COL18A1	-0.122517	7.2900381	-5.526896	6.16E-08	3.98E-07	7.6444837
FAM150B	1.2596989	1.8093097	5.526611	6.17E-08	3.98E-07	7.6430408
MAP3K11	-0.087228	6.7823126	-5.526557	6.17E-08	3.98E-07	7.6427686
RP11-519G1	-0.934984	0.4980139	-5.526415	6.17E-08	3.99E-07	7.6420476
FKBP9P1	0.8206164	2.9756003	5.5250586	6.22E-08	4.01E-07	7.6351756
SNAP29	-0.084203	6.4218429	-5.524817	6.23E-08	4.02E-07	7.6339509
LAMP1	-0.101435	7.1032299	-5.524195	6.25E-08	4.03E-07	7.6308032
GIN1	-0.101574	5.2711844	-5.5241	6.25E-08	4.03E-07	7.6303181
ARHGEF34P	0.7474953	4.2757039	5.5232405	6.28E-08	4.05E-07	7.6259667
S1PR5	0.3763267	4.0670974	5.5230151	6.29E-08	4.05E-07	7.624825

SNHG22	-0.34624	4.2015986	-5.522573	6.30E-08	4.06E-07	7.6225867
CES1P2	-1.16295	1.3729826	-5.52245	6.30E-08	4.06E-07	7.6219642
MTF2	0.1066217	5.7239068	5.5223396	6.31E-08	4.07E-07	7.6214043
EHF	0.739125	4.7926728	5.522193	6.31E-08	4.07E-07	7.6206619
NRSN1	0.9774474	-0.529258	5.5220538	6.32E-08	4.07E-07	7.619957
RP11-309L2	0.538825	3.5110042	5.5218191	6.33E-08	4.08E-07	7.6187687
OSMR	0.2908759	5.9407928	5.521754	6.33E-08	4.08E-07	7.6184392
ZBTB22	-0.095832	6.0387897	-5.521653	6.33E-08	4.08E-07	7.617929
CICP14	0.6098816	4.3061647	5.5210285	6.35E-08	4.09E-07	7.614766
TRBV10-2	0.7617444	-0.761581	5.5205478	6.37E-08	4.10E-07	7.6123326
MT-ND1	-0.114499	7.7209734	-5.52034	6.38E-08	4.10E-07	7.6112804
LHX2	0.8340507	3.4194542	5.5199727	6.39E-08	4.11E-07	7.6094214
WRN	0.1983905	5.3180821	5.5185677	6.43E-08	4.14E-07	7.60231
LINC01123	1.1104636	1.3582117	5.518293	6.44E-08	4.15E-07	7.6009199
CEP192	0.1447289	5.7305057	5.5181687	6.45E-08	4.15E-07	7.6002906
DBT	-0.12642	6.2149644	-5.518159	6.45E-08	4.15E-07	7.6002395
ZCWPW1	-0.214909	5.2811918	-5.51798	6.45E-08	4.15E-07	7.5993362
PNMAL2	0.498324	3.7396188	5.5176667	6.47E-08	4.16E-07	7.5977507
AC131056.3	0.9820644	2.1290752	5.5171304	6.48E-08	4.17E-07	7.5950368
SERPINB6	-0.106275	6.5020702	-5.517063	6.49E-08	4.17E-07	7.5946977
MME-AS1	-0.574917	-1.176769	-5.516966	6.49E-08	4.17E-07	7.5942026
EIF3I	-0.096103	6.800226	-5.516921	6.49E-08	4.17E-07	7.5939763
DPH5	-0.098767	5.9615657	-5.516783	6.50E-08	4.17E-07	7.5932783
HMCN1	0.4587659	5.2454538	5.5166061	6.50E-08	4.18E-07	7.5923842
C1orf115	-0.142453	6.7617771	-5.515385	6.54E-08	4.20E-07	7.5862054
ASTL	0.7548536	-0.760169	5.5152039	6.55E-08	4.20E-07	7.585291
CPB2-AS1	-0.500417	4.0176975	-5.514712	6.57E-08	4.21E-07	7.5828025
CELF4	0.9922207	1.9210361	5.5144413	6.58E-08	4.22E-07	7.5814337
AGGF1P2	0.7361378	-0.858857	5.5142555	6.58E-08	4.22E-07	7.5804941
GS1-124K5.	0.2423828	4.9358623	5.5137057	6.60E-08	4.24E-07	7.5777133
PSTK	-0.15733	5.3071511	-5.513501	6.61E-08	4.24E-07	7.5766769
UBA7	-0.131716	6.3711906	-5.513416	6.61E-08	4.24E-07	7.5762474
RP11-963H4	-1.089976	2.1657693	-5.512518	6.64E-08	4.26E-07	7.5717091
PPIA	-0.088774	7.0275716	-5.512486	6.64E-08	4.26E-07	7.5715461
TRAV27	0.8138089	-0.551502	5.5123761	6.65E-08	4.26E-07	7.5709902
PTCHD2	1.1354884	0.9089718	5.5112588	6.69E-08	4.29E-07	7.5653414
FMO5	-0.250603	6.8546598	-5.511097	6.69E-08	4.29E-07	7.564523
CACTIN	-0.075812	5.9625374	-5.511089	6.69E-08	4.29E-07	7.5644842
RP11-479G2	0.2573721	5.1508346	5.5106888	6.71E-08	4.30E-07	7.5624602
TNFRSF1A	-0.09079	6.7662737	-5.510229	6.72E-08	4.31E-07	7.5601348
GTF2H5	-0.121988	6.2325643	-5.509858	6.74E-08	4.31E-07	7.5582593
RP11-205M3	-1.349003	0.8937919	-5.509434	6.75E-08	4.32E-07	7.5561196
MYDGF	-0.106901	6.7688878	-5.509407	6.75E-08	4.32E-07	7.5559797
GPKOW	-0.089146	6.139583	-5.508769	6.77E-08	4.34E-07	7.5527579
INPP4B	0.2798087	4.9878266	5.5085768	6.78E-08	4.34E-07	7.5517865
LL22NC03-1	-1.271598	2.1477221	-5.508379	6.79E-08	4.34E-07	7.5507878
SLC16A12-A	-0.426467	-1.332333	-5.508239	6.79E-08	4.35E-07	7.5500804
MTIF2	-0.075316	6.3287441	-5.507707	6.81E-08	4.36E-07	7.5473902
SKP1	-0.081485	6.8586458	-5.507695	6.81E-08	4.36E-07	7.5473324
LINC00116	-0.196721	5.8719793	-5.507686	6.81E-08	4.36E-07	7.5472861
PAN2	-0.114252	6.261719	-5.507534	6.82E-08	4.36E-07	7.5465183

C12orf79	0.8671903	2.7618947	5.5074884	6.82E-08	4.36E-07	7.5462873
TLDC2	0.4692856	4.646045	5.5071047	6.83E-08	4.37E-07	7.5443489
FAM53B	0.1291739	5.895002	5.5070364	6.84E-08	4.37E-07	7.5440035
ASIC2	1.0959896	0.4025091	5.5052207	6.90E-08	4.41E-07	7.5348321
TNN	0.6558052	3.9500072	5.5049613	6.91E-08	4.41E-07	7.5335224
PDE4B	0.3069779	5.3666996	5.5048856	6.91E-08	4.41E-07	7.5331399
EIF4E3	0.3096562	5.4794266	5.5041802	6.94E-08	4.43E-07	7.5295776
SCN5A	1.0449407	0.7875578	5.5036561	6.96E-08	4.44E-07	7.5269311
RIT1	0.0934823	5.8746625	5.5036237	6.96E-08	4.44E-07	7.5267675
ENKD1	-0.155397	5.7000925	-5.50361	6.96E-08	4.44E-07	7.5266984
ASNSD1	-0.070188	6.2897835	-5.503458	6.97E-08	4.44E-07	7.5259291
IGLV2-28	0.7572268	-0.892858	5.5025542	7.00E-08	4.46E-07	7.5213678
YBX1P10	0.4114151	4.6344771	5.5021169	7.02E-08	4.47E-07	7.5191604
CEBPA-AS1	-0.264379	5.6008399	-5.501631	7.03E-08	4.48E-07	7.5167052
SF3A3	-0.072859	6.4441695	-5.501341	7.04E-08	4.49E-07	7.5152459
RP5-836J3.	-1.031676	0.0809966	-5.500661	7.07E-08	4.51E-07	7.5118132
UBE2G2	-0.070414	6.4716521	-5.500193	7.09E-08	4.52E-07	7.5094491
APOH	-0.316463	7.5866751	-5.499809	7.10E-08	4.52E-07	7.5075118
RP1-198K11	-0.276096	4.4099098	-5.499447	7.11E-08	4.53E-07	7.5056853
FDFT1	-0.125001	6.646382	-5.498724	7.14E-08	4.55E-07	7.5020353
BOK-AS1	-1.240112	1.7929886	-5.498236	7.16E-08	4.56E-07	7.4995775
AC009014.3	1.5295073	2.3779748	5.4975288	7.19E-08	4.58E-07	7.4960077
STK39	0.353384	5.5077605	5.4971954	7.20E-08	4.58E-07	7.4943257
COA7	-0.096697	6.1587579	-5.497146	7.20E-08	4.58E-07	7.4940742
ANKMY1	-0.110602	5.6699978	-5.496703	7.22E-08	4.59E-07	7.4918424
MYLK2	0.9737314	1.5716867	5.496002	7.24E-08	4.61E-07	7.4883067
EPN3	1.0870261	2.1063506	5.4956996	7.26E-08	4.62E-07	7.4867817
RP11-325N1	-0.910137	-0.067679	-5.495125	7.28E-08	4.63E-07	7.4838815
RP11-510N1	1.013886	2.57896	5.4948445	7.29E-08	4.63E-07	7.4824696
RP11-61L23	1.0408786	1.3873316	5.4947452	7.29E-08	4.64E-07	7.4819688
RP11-552F3	0.7099616	3.2471915	5.4944138	7.30E-08	4.64E-07	7.4802976
RP11-424I1	0.8761439	0.0594991	5.4942812	7.31E-08	4.65E-07	7.4796292
RBM15	-0.087022	5.6943016	-5.49423	7.31E-08	4.65E-07	7.4793701
FIBP	-0.090226	6.3253592	-5.494226	7.31E-08	4.65E-07	7.4793515
ARHGAP4	0.1957913	6.0713143	5.4938763	7.32E-08	4.65E-07	7.4775877
SRSF5	-0.076399	6.7298559	-5.493552	7.34E-08	4.66E-07	7.4759529
TSTD1	-0.210827	6.2157122	-5.492759	7.37E-08	4.68E-07	7.471957
HNRNPUL2-B	0.7591783	3.2448479	5.492489	7.38E-08	4.68E-07	7.4705942
TSSK1A	-0.899467	0.4791381	-5.49244	7.38E-08	4.69E-07	7.4703487
CTD-2292M1	0.6638578	3.0700266	5.4922289	7.39E-08	4.69E-07	7.4692831
SLC26A1	-0.210067	6.024645	-5.491951	7.40E-08	4.70E-07	7.4678806
LRRC3-AS1	-1.047196	1.4684446	-5.49186	7.40E-08	4.70E-07	7.4674223
LYRM5	-0.138352	6.0940103	-5.491817	7.40E-08	4.70E-07	7.4672061
RP11-230L2	-0.466043	-1.285291	-5.490871	7.44E-08	4.72E-07	7.4624387
ANGPTL3	-0.38979	6.8566868	-5.490729	7.45E-08	4.72E-07	7.4617254
CTC-523E23	0.3088237	4.3209634	5.4904394	7.46E-08	4.73E-07	7.4602647
STYX	-0.098085	6.1914961	-5.489658	7.49E-08	4.75E-07	7.4563292
NACA	-0.082418	7.1705973	-5.488985	7.51E-08	4.76E-07	7.4529392
LA16c-390H	0.9577025	0.5021942	5.4886015	7.53E-08	4.77E-07	7.4510052
RP4-647C14	0.8038479	2.4337318	5.4885155	7.53E-08	4.77E-07	7.4505718
SETD3	-0.075886	6.3860276	-5.487408	7.58E-08	4.80E-07	7.4449941

SMIM2	-0.969817	-0.031529	-5.487213	7.58E-08	4.80E-07	7.4440133
GRAP2	0.5011337	4.4949512	5.4867911	7.60E-08	4.81E-07	7.4418864
PEX12	-0.147273	5.6421387	-5.486685	7.61E-08	4.82E-07	7.4413516
AQP7P2	-1.156928	0.6001352	-5.486203	7.63E-08	4.83E-07	7.438925
CYP3A4	-0.80099	6.1849813	-5.48615	7.63E-08	4.83E-07	7.4386559
LMX1B	1.3482846	0.4390935	5.4860033	7.63E-08	4.83E-07	7.4379191
ATP6V0D2	1.1213936	2.8377276	5.4855822	7.65E-08	4.84E-07	7.4357988
ZNF101	0.0929891	5.4856996	5.4843942	7.70E-08	4.87E-07	7.4298174
COL6A1	0.1784209	6.6455893	5.4841721	7.71E-08	4.87E-07	7.4286992
CA12	0.810148	5.0238489	5.483236	7.74E-08	4.90E-07	7.4239873
AIFM1	-0.116987	6.7057105	-5.483056	7.75E-08	4.90E-07	7.4230793
TRAV23DV6	0.8749368	-0.442754	5.482591	7.77E-08	4.91E-07	7.4207409
CPSF3L	-0.084052	6.5032794	-5.482269	7.78E-08	4.92E-07	7.4191227
RASL12	0.2538769	5.0478464	5.4818787	7.80E-08	4.93E-07	7.4171564
CYP2B6	-0.545677	6.1927825	-5.481785	7.80E-08	4.93E-07	7.416684
RPS25	-0.097618	7.0424484	-5.481776	7.80E-08	4.93E-07	7.4166393
HYAL4	0.9176531	-0.540044	5.4815616	7.81E-08	4.93E-07	7.4155605
SLC33A1	-0.083412	6.4337381	-5.481555	7.81E-08	4.93E-07	7.4155249
OGG1	-0.108949	6.0881742	-5.481189	7.83E-08	4.94E-07	7.4136871
ATP5S	-0.096046	5.9112717	-5.48103	7.83E-08	4.95E-07	7.4128867
AFAP1L1	0.1528663	5.6630858	5.4806313	7.85E-08	4.96E-07	7.4108797
MKRN1	-0.065826	6.5230137	-5.480214	7.87E-08	4.97E-07	7.4087804
SMIM22	1.2596045	1.8746176	5.4799316	7.88E-08	4.97E-07	7.4073595
RP11-473M2	0.3408006	3.6352525	5.4792553	7.91E-08	4.99E-07	7.4039574
CTD-2002H8	0.6982049	3.0298369	5.4792339	7.91E-08	4.99E-07	7.4038495
EPHA10	1.4125517	2.1710003	5.4791489	7.91E-08	4.99E-07	7.4034218
TMEM155	1.1083471	0.3789693	5.4790104	7.92E-08	4.99E-07	7.4027252
CTA-14H9.5	0.7577209	2.1569917	5.4788121	7.93E-08	5.00E-07	7.4017279
RPS4XP5	-1.169857	0.7597198	-5.478303	7.95E-08	5.01E-07	7.3991654
ZNF205	-0.109411	5.8290107	-5.478262	7.95E-08	5.01E-07	7.3989589
RHOA	-0.062524	7.0208659	-5.47797	7.96E-08	5.02E-07	7.397492
IMP4	-0.090851	6.4725227	-5.476198	8.03E-08	5.06E-07	7.3885804
15-Sep	-0.073413	6.6932633	-5.476106	8.04E-08	5.06E-07	7.3881185
S1PR4	0.3993987	4.3265403	5.475577	8.06E-08	5.08E-07	7.3854594
ARSK	-0.16486	5.402095	-5.47519	8.08E-08	5.09E-07	7.3835118
STX1B	-0.349029	5.0580802	-5.475091	8.08E-08	5.09E-07	7.3830168
HMGCLL1	1.1473173	0.5290303	5.474462	8.11E-08	5.10E-07	7.3798546
TOMM22	-0.087892	6.5271034	-5.474287	8.12E-08	5.11E-07	7.3789746
PODXL2	0.8215167	4.3718456	5.4737541	8.14E-08	5.12E-07	7.3762964
LRRC37A5P	-0.766595	3.0054464	-5.47331	8.16E-08	5.13E-07	7.374065
NADSYN1	-0.086459	6.3499435	-5.473309	8.16E-08	5.13E-07	7.3740585
ADIRF-AS1	0.6563411	3.3326059	5.4729139	8.17E-08	5.14E-07	7.3720739
GDPGP1	-0.116496	5.4037121	-5.472397	8.20E-08	5.15E-07	7.3694758
SLCO4A1	0.7248584	3.7835946	5.4720411	8.21E-08	5.16E-07	7.3676881
NTPCR	-0.116215	6.3411194	-5.47104	8.25E-08	5.19E-07	7.362658
CTB-39G8.2	-1.023325	1.4837217	-5.470788	8.26E-08	5.19E-07	7.3613911
ZNF366	0.8469989	3.4364462	5.4703244	8.28E-08	5.21E-07	7.3590636
MYL12B	-0.09295	7.0290412	-5.469882	8.30E-08	5.22E-07	7.3568403
LRRC37B	0.1800406	4.9894654	5.4696556	8.31E-08	5.22E-07	7.3557042
LINC01569	-0.269113	4.9361173	-5.469292	8.33E-08	5.23E-07	7.3538766
SPG21	-0.074493	6.6409529	-5.468571	8.36E-08	5.25E-07	7.350256

AC010980.2	1.0469204	-0.177346	5.4683908	8.37E-08	5.25E-07	7.349352
LINC01281	0.8666303	-0.575217	5.4683304	8.37E-08	5.26E-07	7.3490484
RP11-452F1	-0.195861	5.4212985	-5.46812	8.38E-08	5.26E-07	7.3479939
MRPL4	-0.10523	6.4325749	-5.467895	8.39E-08	5.27E-07	7.3468605
ZNF888	1.1347835	1.0771951	5.4678013	8.39E-08	5.27E-07	7.3463917
LA16c-380F	0.765602	1.9995471	5.4671159	8.42E-08	5.29E-07	7.3429501
GDAP1	0.3586738	5.1877195	5.4663725	8.46E-08	5.30E-07	7.3392182
RP1-27K12.	-0.91727	2.3461349	-5.466167	8.47E-08	5.31E-07	7.3381882
GPR83	0.9738223	1.0626135	5.4659044	8.48E-08	5.32E-07	7.3368683
CDKN2C	-0.192448	5.875201	-5.465765	8.48E-08	5.32E-07	7.3361686
SORCS2	0.8421464	4.6604764	5.4656992	8.49E-08	5.32E-07	7.3358381
TMEM202	-0.605029	-1.205193	-5.464906	8.52E-08	5.34E-07	7.3318578
ZNF713	0.2528498	4.3664484	5.4646466	8.53E-08	5.35E-07	7.3305546
CYP2A7P1	-1.027162	-0.465793	-5.464406	8.54E-08	5.35E-07	7.3293485
ZSWIM6	0.1849969	5.487056	5.4637538	8.57E-08	5.37E-07	7.3260742
RP11-84A19	-1.062653	0.3301408	-5.463623	8.58E-08	5.37E-07	7.3254173
TAS2R19	0.8723322	0.0674422	5.4628892	8.61E-08	5.39E-07	7.3217357
VPS29	-0.070318	6.4571769	-5.462241	8.64E-08	5.41E-07	7.3184811
MIR483	0.9641749	-0.752522	5.4607525	8.71E-08	5.45E-07	7.3110168
MSR1	0.2348158	5.5864835	5.460504	8.72E-08	5.46E-07	7.3097701
SLC16A2	-0.256679	6.3553041	-5.460246	8.73E-08	5.46E-07	7.3084769
AC007750.5	1.0106513	0.3765845	5.4589392	8.79E-08	5.50E-07	7.3019227
SMIM13	-0.097719	6.0636436	-5.458002	8.83E-08	5.53E-07	7.2972214
NDUFB2	-0.114133	6.6032735	-5.457508	8.86E-08	5.54E-07	7.2947488
NELL2	0.8854296	3.8341048	5.4569121	8.89E-08	5.56E-07	7.2917598
TMEM259	-0.078722	6.7527557	-5.456371	8.91E-08	5.57E-07	7.2890468
CABP4	0.5207006	3.6121179	5.4557986	8.94E-08	5.59E-07	7.2861783
MRPS2	-0.111751	6.4042545	-5.454967	8.98E-08	5.61E-07	7.2820115
JAKMIP2-AS	-1.214031	0.4462951	-5.454716	8.99E-08	5.62E-07	7.2807548
RAB27B	0.7552561	4.2684662	5.4536383	9.04E-08	5.65E-07	7.2753529
MFSD3	-0.169671	6.3524988	-5.452674	9.08E-08	5.67E-07	7.2705228
ICAM1	0.2037123	6.3019257	5.4521073	9.11E-08	5.69E-07	7.2676829
PITHD1	-0.092215	6.2339746	-5.452079	9.11E-08	5.69E-07	7.2675397
PTCD1	-0.123774	5.6412292	-5.451885	9.12E-08	5.69E-07	7.2665693
CXCL8	0.7236088	5.0037175	5.4518767	9.12E-08	5.69E-07	7.2665279
C10orf12	0.2302665	4.6660386	5.4517593	9.13E-08	5.70E-07	7.26594
ARHGAP44	0.4079666	4.7406755	5.4516668	9.13E-08	5.70E-07	7.2654764
ALG1	-0.099879	6.0880582	-5.450874	9.17E-08	5.72E-07	7.2615066
AC026202.3	-0.488265	3.5291578	-5.45084	9.17E-08	5.72E-07	7.2613374
VWA2	1.0550066	-0.020762	5.450682	9.18E-08	5.72E-07	7.2605445
10-Mar	0.9013077	-0.607862	5.4500093	9.21E-08	5.74E-07	7.2571759
MST1R	0.6977266	3.9857476	5.4495718	9.23E-08	5.76E-07	7.2549849
RP11-770E5	-1.103654	0.7505954	-5.449078	9.26E-08	5.77E-07	7.2525138
RP11-447D1	0.9084843	2.6684812	5.4489642	9.26E-08	5.77E-07	7.2519427
RP5-858L17	0.8837608	2.6008066	5.4488554	9.27E-08	5.77E-07	7.2513982
WASF1	0.1897867	5.5894422	5.4485846	9.28E-08	5.78E-07	7.2500423
ZNF618	0.1531896	5.8551524	5.448504	9.28E-08	5.78E-07	7.2496387
LONP1	-0.091658	6.7648289	-5.447834	9.32E-08	5.80E-07	7.2462842
BBS5	0.3968076	4.6553514	5.447384	9.34E-08	5.81E-07	7.2440322
RP11-936I5	-1.046147	2.006198	-5.447118	9.35E-08	5.82E-07	7.2427009
ANXA2P2	0.3463148	4.7028368	5.4469558	9.36E-08	5.83E-07	7.2418892

S100A6	0.2455233	6.3255861	5.4465857	9.38E-08	5.84E-07	7.2400369
TRAPPC9	-0.113238	6.2934737	-5.444638	9.47E-08	5.89E-07	7.2302927
CHP1	-0.110213	6.8743798	-5.444509	9.48E-08	5.90E-07	7.229645
RP5-1054A2	1.0733331	0.6464612	5.4441101	9.50E-08	5.91E-07	7.2276493
PTPRT	1.254355	2.1049581	5.4439552	9.50E-08	5.91E-07	7.2268743
GTF2B	-0.089111	6.0764397	-5.443451	9.53E-08	5.93E-07	7.2243538
ASS1	-0.182255	7.1832106	-5.443313	9.54E-08	5.93E-07	7.2236604
RP11-452L6	-0.295613	3.9689043	-5.443307	9.54E-08	5.93E-07	7.2236324
RP11-1102F	0.8273037	-0.223409	5.4429451	9.56E-08	5.94E-07	7.2218217
CR1L	1.0735696	0.3673407	5.4417876	9.61E-08	5.97E-07	7.2160323
ITGA2B	0.8693366	2.2083021	5.4412395	9.64E-08	5.99E-07	7.2132913
PUS3	-0.112814	6.0056545	-5.440974	9.65E-08	6.00E-07	7.2119633
LMNB2	0.1226884	6.1024183	5.4407884	9.66E-08	6.00E-07	7.2110357
PDE12	-0.078437	6.1818224	-5.440766	9.66E-08	6.00E-07	7.2109246
RIC8B	0.1095664	5.8191901	5.4398674	9.71E-08	6.03E-07	7.2064308
FAM65B	0.3267087	4.9045366	5.4396218	9.72E-08	6.04E-07	7.2052031
NEDD1	0.1248442	5.5919853	5.4391112	9.75E-08	6.05E-07	7.2026502
TRAV10	0.7914744	-0.656345	5.4389508	9.76E-08	6.05E-07	7.2018486
LONRF2	1.1215977	3.9674684	5.4377138	9.82E-08	6.09E-07	7.1956656
RP11-360F5	0.910509	1.5760638	5.4372044	9.84E-08	6.11E-07	7.1931198
ETS1	0.1462064	6.2581485	5.4371522	9.85E-08	6.11E-07	7.1928591
DFFA	-0.082625	6.2410597	-5.43567	9.92E-08	6.15E-07	7.1854518
SGTA	-0.078659	6.5773385	-5.435635	9.92E-08	6.15E-07	7.1852786
TRAV29DV5	0.9998826	0.0922431	5.4355021	9.93E-08	6.16E-07	7.1846136
MRPL1	-0.113088	5.9716135	-5.435494	9.93E-08	6.16E-07	7.1845751
GSTA10P	-0.4511	-1.254748	-5.435145	9.95E-08	6.17E-07	7.1828295
MASP2	-0.449859	6.583545	-5.43498	9.96E-08	6.17E-07	7.1820074
RP11-342I1	-0.634106	2.934676	-5.434461	9.99E-08	6.19E-07	7.1794114
TACSTD2	0.8790059	4.0149437	5.4341925	1.00E-07	6.19E-07	7.1780717
ELN	0.4382154	5.7116742	5.4338528	1.00E-07	6.20E-07	7.1763749
C6orf222	1.2233872	0.3973312	5.4338475	1.00E-07	6.20E-07	7.1763483
RP11-815J4	0.9508673	0.4227258	5.4329586	1.01E-07	6.23E-07	7.1719086
ABHD3	0.127155	6.2517705	5.4327099	1.01E-07	6.24E-07	7.1706668
KLHL33	0.7821949	-0.492103	5.4318202	1.01E-07	6.27E-07	7.1662237
CLN6	-0.095074	6.3829654	-5.431276	1.02E-07	6.28E-07	7.163505
AC004988.1	0.9910926	0.4108751	5.4312737	1.02E-07	6.28E-07	7.1634954
ZBTB3	-0.115747	5.2142831	-5.43006	1.02E-07	6.32E-07	7.1574365
INSM1	0.9257649	-0.671024	5.4298649	1.02E-07	6.33E-07	7.1564623
S1PR3	0.3251741	5.556759	5.4297075	1.02E-07	6.33E-07	7.1556765
DISP1	-0.19481	5.7910094	-5.429313	1.03E-07	6.34E-07	7.1537071
KLHL2	-0.157708	5.938963	-5.429163	1.03E-07	6.35E-07	7.1529591
KIF2A	0.147507	5.5568666	5.4289633	1.03E-07	6.35E-07	7.1519621
PURG	0.849033	-0.482266	5.428823	1.03E-07	6.35E-07	7.1512619
LINC00869	-0.248884	5.5712799	-5.428698	1.03E-07	6.36E-07	7.1506358
KLRB1	0.3701015	4.8263357	5.4286903	1.03E-07	6.36E-07	7.1505994
SDF2L1	-0.150634	6.4022931	-5.428075	1.03E-07	6.38E-07	7.1475275
RPL11	-0.101236	7.1667101	-5.427977	1.03E-07	6.38E-07	7.1470382
RP11-474I1	-0.966244	0.6183379	-5.42781	1.03E-07	6.38E-07	7.1462076
APBB3	-0.167633	5.9631588	-5.427687	1.03E-07	6.39E-07	7.1455911
NABP1	0.179824	5.6298631	5.427124	1.04E-07	6.40E-07	7.1427831
LPCAT3	0.3278644	5.888575	5.4270587	1.04E-07	6.40E-07	7.1424576

GTSE1	0.3672051	5.091492	5.4269653	1.04E-07	6.41E-07	7.1419915
TOP3B	0.7892419	2.9368483	5.4267608	1.04E-07	6.41E-07	7.1409711
CASC3	-0.069168	6.4662584	-5.426189	1.04E-07	6.43E-07	7.138118
RP11-676M6	0.6488663	4.3922268	5.4255009	1.05E-07	6.45E-07	7.134686
ZBED9	1.0023037	4.3812361	5.4252714	1.05E-07	6.46E-07	7.133541
ROMO1	-0.138796	6.5275023	-5.425231	1.05E-07	6.46E-07	7.1333409
CYSLTR1	0.3781096	4.201147	5.4251881	1.05E-07	6.46E-07	7.1331253
ZNF790-AS1	0.6385024	3.1988162	5.4248269	1.05E-07	6.47E-07	7.1313236
TRAPPC6B	-0.080184	6.0477165	-5.424432	1.05E-07	6.48E-07	7.1293522
RP11-468N1	-1.086983	3.6756477	-5.423586	1.06E-07	6.51E-07	7.1251365
CLDN10	1.5017598	1.8402982	5.4235342	1.06E-07	6.51E-07	7.1248767
RP11-43505	-0.808096	1.8490488	-5.423508	1.06E-07	6.51E-07	7.1247478
RP11-729I1	-0.921803	-0.068476	-5.423461	1.06E-07	6.51E-07	7.124513
AP002954.3	0.841366	-0.382365	5.4232112	1.06E-07	6.52E-07	7.1232661
SLC15A2	0.4188533	3.8035894	5.4230231	1.06E-07	6.52E-07	7.1223284
LZTS2	0.1149289	6.2110497	5.4226285	1.06E-07	6.54E-07	7.1203607
SNUPN	-0.084837	5.855111	-5.422343	1.06E-07	6.54E-07	7.1189387
PSMB3P2	-0.459638	-1.241118	-5.421896	1.07E-07	6.56E-07	7.116709
TBC1D10C	0.2801282	5.3115844	5.421765	1.07E-07	6.56E-07	7.1160555
CREB1	0.0720141	5.9714061	5.4216576	1.07E-07	6.56E-07	7.1155203
VEGFB	0.2661223	6.166367	5.4214395	1.07E-07	6.57E-07	7.1144326
RIN1	0.2679009	4.9772215	5.4208103	1.07E-07	6.59E-07	7.1112964
NEBL	0.7921865	4.4281952	5.4204436	1.07E-07	6.60E-07	7.1094687
NPNT	0.3762747	5.4357202	5.4201438	1.08E-07	6.61E-07	7.1079745
BCAP31	-0.108565	7.0231035	-5.419375	1.08E-07	6.64E-07	7.1041403
SUSD4	1.0518622	4.8167706	5.4191257	1.08E-07	6.64E-07	7.1029002
RELA	-0.060135	6.5506486	-5.418968	1.08E-07	6.65E-07	7.1021159
AP001189.4	1.0796936	1.4557145	5.4188001	1.08E-07	6.65E-07	7.1012777
HDDC3	-0.119433	5.7855422	-5.418666	1.08E-07	6.66E-07	7.1006094
APEX1	-0.071124	6.6893879	-5.418253	1.09E-07	6.67E-07	7.0985532
RP11-342M1	-0.587651	3.6420808	-5.418127	1.09E-07	6.67E-07	7.0979237
LINC00323	0.9482343	-0.286864	5.4171106	1.09E-07	6.71E-07	7.0928601
TRIM6	0.6216889	4.3593066	5.4170454	1.09E-07	6.71E-07	7.0925352
CYB5R1	-0.112339	6.5612144	-5.416943	1.09E-07	6.71E-07	7.0920258
MAT2B	-0.099051	6.4238105	-5.416402	1.10E-07	6.73E-07	7.089332
CLSTN2	0.8140431	3.8575994	5.4163754	1.10E-07	6.73E-07	7.0891976
U91324.1	-0.750051	3.9960261	-5.416333	1.10E-07	6.73E-07	7.0889873
RP13-297E1	0.9780487	0.2211832	5.415822	1.10E-07	6.74E-07	7.0864412
PPFIBP1	0.1222291	6.0268504	5.415745	1.10E-07	6.75E-07	7.0860578
RP11-467H1	-0.490166	3.0698332	-5.414517	1.11E-07	6.79E-07	7.0799434
MAGEB17	-1.47703	1.5878533	-5.414492	1.11E-07	6.79E-07	7.0798181
TSPAN1	-0.322803	5.1011474	-5.414231	1.11E-07	6.80E-07	7.0785202
ACCS	-0.171199	5.7636623	-5.414216	1.11E-07	6.80E-07	7.0784442
MAP6	0.5502181	4.4152851	5.4141258	1.11E-07	6.80E-07	7.0779942
RP11-545E1	-0.25963	4.6466437	-5.413256	1.11E-07	6.83E-07	7.0736635
ARL6IP6	0.1659743	5.408524	5.4126782	1.12E-07	6.85E-07	7.0707867
KIF25-AS1	0.9994181	-0.486598	5.4124902	1.12E-07	6.85E-07	7.0698508
LA16c-359F	-0.951774	0.4750886	-5.411585	1.12E-07	6.88E-07	7.0653434
CTC-205M6.	-0.538096	3.2695035	-5.411401	1.13E-07	6.89E-07	7.0644293
GNAZ	0.5431478	5.3261227	5.4113947	1.13E-07	6.89E-07	7.064398
AP000688.1	-0.724742	2.5431707	-5.410486	1.13E-07	6.92E-07	7.059877

SSUH2	0.860301	4.3203385	5.4096838	1.14E-07	6.95E-07	7.0558836
ZNF667	0.709076	3.7158969	5.4092665	1.14E-07	6.96E-07	7.0538072
ELAVL4	0.9730713	0.97763	5.4089775	1.14E-07	6.97E-07	7.0523694
ZNF155	0.5145485	4.2546974	5.4078437	1.15E-07	7.01E-07	7.0467286
WDR86-AS1	0.9701822	1.9463868	5.4077615	1.15E-07	7.01E-07	7.0463196
HLA-DPA1	0.2130454	6.524181	5.4077044	1.15E-07	7.01E-07	7.0460355
TCAF1P1	1.1355937	2.6729001	5.4072313	1.15E-07	7.03E-07	7.0436822
RAB8B	0.1189111	5.9267218	5.407179	1.15E-07	7.03E-07	7.0434226
FBXL15	-0.138864	5.9195119	-5.406727	1.15E-07	7.04E-07	7.0411735
CTD-2541J1	1.1677629	1.0432333	5.4062892	1.16E-07	7.06E-07	7.0389971
ADAM9	0.1812585	6.1264669	5.4060835	1.16E-07	7.06E-07	7.0379736
GCFC2	-0.094302	6.0012899	-5.40578	1.16E-07	7.07E-07	7.0364653
RBM42	-0.098614	6.6029816	-5.40561	1.16E-07	7.08E-07	7.0356203
MURC	0.5958443	3.0122194	5.4053479	1.16E-07	7.09E-07	7.0343159
ZDHHC7	0.09657	6.0707006	5.4053056	1.16E-07	7.09E-07	7.0341056
MCF2L2	0.9753085	3.0167147	5.4050962	1.16E-07	7.10E-07	7.0330642
PLEKHG4B	1.1817774	1.998761	5.4046871	1.17E-07	7.11E-07	7.0310303
SOCS5P4	0.8745814	0.9554239	5.4041372	1.17E-07	7.13E-07	7.0282963
MIR99AHG	-0.493604	4.7463739	-5.404075	1.17E-07	7.13E-07	7.0279866
RP11-508N2	0.8616781	1.7781149	5.4040742	1.17E-07	7.13E-07	7.0279832
UCN	-0.408222	4.2575175	-5.403905	1.17E-07	7.13E-07	7.0271407
CTA-390C10	0.72724	3.2311359	5.4038002	1.17E-07	7.14E-07	7.0266209
HIST3H2BB	1.2620311	0.6138721	5.4022302	1.18E-07	7.19E-07	7.018817
CTD-2530H1	0.880699	0.3957724	5.4018867	1.18E-07	7.20E-07	7.0171098
SLC39A9	-0.084825	6.6074255	-5.40168	1.18E-07	7.21E-07	7.0160847
PEX11B	-0.088385	6.2588194	-5.401141	1.19E-07	7.23E-07	7.013406
RP3-47704.	0.8107938	1.6771271	5.4009419	1.19E-07	7.24E-07	7.0124149
NTNG1	0.7692782	-0.853805	5.400755	1.19E-07	7.24E-07	7.011486
AFM	-0.543161	6.432933	-5.400488	1.19E-07	7.25E-07	7.0101608
LDHB	0.1818114	6.0802298	5.3996648	1.20E-07	7.28E-07	7.0060696
TBKBP1	0.191863	5.4992395	5.399598	1.20E-07	7.28E-07	7.0057374
RP5-1139B1	0.731398	-0.906342	5.3993876	1.20E-07	7.29E-07	7.0046923
TAX1BP3	0.1962827	5.7343522	5.3993116	1.20E-07	7.29E-07	7.0043147
UBE2Q1	-0.077145	6.6490278	-5.398931	1.20E-07	7.30E-07	7.0024241
TCTN2	0.2599609	5.0891549	5.3988373	1.20E-07	7.31E-07	7.0019587
CXCR5	0.7705757	-0.746521	5.398768	1.20E-07	7.31E-07	7.0016143
PPP2CA	-0.065189	6.6649375	-5.398678	1.20E-07	7.31E-07	7.0011668
TRPV5	0.834585	-0.804679	5.3984868	1.20E-07	7.31E-07	7.0002175
MT-ND4	-0.106545	7.9473003	-5.398366	1.20E-07	7.32E-07	6.9996188
SUPV3L1	-0.087972	6.2001115	-5.397684	1.21E-07	7.34E-07	6.996231
RP11-166N6	-1.079958	0.9239684	-5.396879	1.21E-07	7.37E-07	6.9922345
NT5M	-0.306892	4.8891336	-5.396819	1.21E-07	7.37E-07	6.9919365
AKR1C6P	-0.963891	4.5096882	-5.396136	1.22E-07	7.40E-07	6.9885438
ECE2	-0.144351	5.9239677	-5.396078	1.22E-07	7.40E-07	6.9882543
RP11-47304	0.9227827	0.2824199	5.3959678	1.22E-07	7.40E-07	6.987708
RP11-1081L	0.9405241	1.4033517	5.3953769	1.22E-07	7.42E-07	6.9847739
PAXIP1-AS1	-0.14514	5.5886715	-5.395192	1.22E-07	7.43E-07	6.9838568
COX5A	-0.104909	6.686507	-5.394847	1.23E-07	7.44E-07	6.9821454
LRRC34	0.4545084	3.3621923	5.394367	1.23E-07	7.46E-07	6.9797606
VHL	0.1232301	6.0342622	5.3937692	1.23E-07	7.48E-07	6.9767933
LYZL2	-0.906974	-0.547035	-5.393736	1.23E-07	7.48E-07	6.9766267

ACAT1	-0.146491	6.9408717	-5.393378	1.24E-07	7.49E-07	6.9748502
DDX12P	0.401308	4.4489068	5.3933067	1.24E-07	7.49E-07	6.9744978
RAI2	0.3576433	4.9133543	5.3932201	1.24E-07	7.50E-07	6.974068
MED19	-0.097788	5.6558802	-5.392538	1.24E-07	7.52E-07	6.9706825
PITPNC1	-0.147687	5.9830018	-5.392449	1.24E-07	7.52E-07	6.9702403
HULC	-0.584576	6.4482846	-5.392342	1.24E-07	7.53E-07	6.9697087
AGAP1	0.1187796	5.9288118	5.3923041	1.24E-07	7.53E-07	6.9695225
XXbac-BPG2	0.7116788	2.2090387	5.3923035	1.24E-07	7.53E-07	6.9695195
RP11-226L1	-0.143327	5.4955864	-5.392235	1.24E-07	7.53E-07	6.9691795
CTD-2530H1	0.8770538	0.2955484	5.3922116	1.24E-07	7.53E-07	6.9690632
AC159540.1	0.9841624	1.2203636	5.3920691	1.24E-07	7.53E-07	6.9683564
GP2	1.5728021	1.21302	5.3919706	1.24E-07	7.53E-07	6.9678674
KCTD9	0.1458443	5.6524968	5.391939	1.24E-07	7.53E-07	6.9677108
CYBRD1	0.2594587	5.8798648	5.3909915	1.25E-07	7.57E-07	6.9630094
COX6C	-0.144666	6.9174352	-5.3904	1.25E-07	7.59E-07	6.9600772
RP4-671014	0.8138496	-0.416277	5.3902198	1.26E-07	7.60E-07	6.959181
SLC38A5	0.482979	4.1615869	5.3896717	1.26E-07	7.62E-07	6.9564623
R3HCC1L	0.0866485	5.7059545	5.3896469	1.26E-07	7.62E-07	6.9563395
AC026471.6	-0.979708	1.3176371	-5.389556	1.26E-07	7.62E-07	6.9558895
CASP2	0.1031386	5.9392607	5.3885573	1.27E-07	7.66E-07	6.950935
VENTX	0.6241299	3.2111209	5.388093	1.27E-07	7.67E-07	6.9486329
TRIP10	0.1292619	5.9908899	5.3875639	1.27E-07	7.69E-07	6.9460091
FBXO11	0.1835464	5.7817587	5.3875364	1.27E-07	7.69E-07	6.9458724
FSCN2	0.9780985	1.2454272	5.3874614	1.27E-07	7.70E-07	6.9455008
ARAF	-0.091657	6.5591469	-5.386718	1.28E-07	7.72E-07	6.9418147
AC005519.4	0.7947022	2.602655	5.3865777	1.28E-07	7.73E-07	6.9411192
SLC18B1	0.1602352	5.6841311	5.3854432	1.29E-07	7.77E-07	6.935495
TRMT1L	-0.09906	6.115036	-5.384854	1.29E-07	7.79E-07	6.9325743
CLYBL	-0.186942	6.1775016	-5.38472	1.29E-07	7.80E-07	6.9319124
ZNF32-AS2	0.8421753	2.4356461	5.3844402	1.29E-07	7.81E-07	6.930524
MIR1254-1	0.918488	0.5395345	5.3843332	1.29E-07	7.81E-07	6.9299935
ASTN2	-0.19065	5.2023383	-5.384135	1.30E-07	7.82E-07	6.9290128
CYB5B	-0.101646	6.4026354	-5.384073	1.30E-07	7.82E-07	6.9287024
ATP10D	0.2161586	5.4751629	5.3840636	1.30E-07	7.82E-07	6.9286574
KCNJ10	0.8780963	3.1673027	5.3838769	1.30E-07	7.82E-07	6.927732
LCN10	0.9821335	-0.142143	5.3837061	1.30E-07	7.83E-07	6.9268856
HAMP	0.8681425	4.8973186	5.3833339	1.30E-07	7.84E-07	6.9250412
LINC01133	1.0595135	0.4804038	5.3832771	1.30E-07	7.85E-07	6.92476
GGT1	0.3709026	6.0850447	5.3826706	1.31E-07	7.87E-07	6.9217547
RNF113A	-0.116751	5.8901123	-5.382235	1.31E-07	7.88E-07	6.9195988
COPZ1	-0.072174	6.7707436	-5.381327	1.31E-07	7.92E-07	6.9150972
8-Mar	-0.1045	6.3243905	-5.381269	1.31E-07	7.92E-07	6.9148106
DLL1	0.3512858	5.0964394	5.3811832	1.32E-07	7.92E-07	6.9143859
IGHV1-67	0.9544884	-0.543218	5.3800103	1.32E-07	7.97E-07	6.9085764
RP5-940F7.	-0.742399	-0.71722	-5.379233	1.33E-07	8.00E-07	6.9047256
CHST8	1.2176638	0.9876989	5.3791486	1.33E-07	8.00E-07	6.9043091
MPZL1	0.0938023	6.4997538	5.3789822	1.33E-07	8.01E-07	6.903485
TVP23C	0.2567682	4.1324787	5.3785869	1.33E-07	8.02E-07	6.9015279
PRKAR2A	-0.079229	6.3973537	-5.378137	1.34E-07	8.04E-07	6.899298
CDKN1C	0.3760242	5.0798018	5.3768079	1.35E-07	8.09E-07	6.8927205
TOPORS-AS1	-0.190289	5.1305673	-5.376706	1.35E-07	8.10E-07	6.8922156

WDR33	-0.053582	6.4267482	-5.376231	1.35E-07	8.12E-07	6.8898666
TUSC3	0.4293968	5.1954336	5.3756237	1.35E-07	8.14E-07	6.8868591
RTP4	-0.251512	5.5618956	-5.375037	1.36E-07	8.16E-07	6.8839541
C11orf54	-0.141638	6.5614805	-5.374229	1.36E-07	8.20E-07	6.8799589
C14orf159	-0.147681	6.1287587	-5.374219	1.36E-07	8.20E-07	6.8799078
GTF2F1	-0.071151	6.460902	-5.374091	1.36E-07	8.20E-07	6.8792742
KNDC1	1.3375748	3.921417	5.3726627	1.37E-07	8.26E-07	6.8722077
AC093110.3	1.01806	3.0945672	5.372632	1.37E-07	8.26E-07	6.872056
AC068138.1	-0.970869	-0.84583	-5.372309	1.38E-07	8.27E-07	6.8704564
PLK3	0.1686206	5.4723082	5.3722897	1.38E-07	8.27E-07	6.8703627
FAM189A2	0.4901527	3.5079418	5.37186	1.38E-07	8.29E-07	6.8682375
DRD1	-1.180941	2.3850298	-5.369622	1.40E-07	8.38E-07	6.8571684
GUCD1	-0.101359	6.8340807	-5.368226	1.41E-07	8.44E-07	6.8502701
SECISBP2	-0.07465	6.3053473	-5.368106	1.41E-07	8.44E-07	6.8496737
ARHGEF6	0.2023316	5.5181344	5.3678575	1.41E-07	8.45E-07	6.8484463
BMP7	1.345854	0.7444129	5.3676731	1.41E-07	8.46E-07	6.8475348
GPER1	-0.287942	5.8598758	-5.366729	1.42E-07	8.50E-07	6.8428697
PABPC4L	0.6101172	3.3375881	5.3661251	1.42E-07	8.53E-07	6.8398842
MMP19	0.3483954	5.1739691	5.3652738	1.43E-07	8.56E-07	6.8356777
RNF128	-0.140194	6.5931709	-5.365087	1.43E-07	8.57E-07	6.8347556
ACP6	-0.13218	6.0371597	-5.36477	1.43E-07	8.58E-07	6.8331877
GTF3A	-0.114147	6.6565811	-5.364166	1.44E-07	8.61E-07	6.8302037
RP11-2E11.	0.7300981	2.2436717	5.3625801	1.45E-07	8.68E-07	6.822371
LST1	0.248018	5.4088757	5.3622494	1.45E-07	8.69E-07	6.8207378
ATP5J2	-0.117076	6.7153948	-5.361811	1.45E-07	8.71E-07	6.8185715
FER	0.1647862	5.5391897	5.3605774	1.46E-07	8.76E-07	6.8124813
SLC22A31	1.5963579	2.6473201	5.3597592	1.47E-07	8.80E-07	6.8084421
CSNK1E	0.0785389	6.3847123	5.3597261	1.47E-07	8.80E-07	6.8082782
ENY2	-0.116186	6.5253596	-5.359592	1.47E-07	8.80E-07	6.8076154
RP1-140A9.	-0.7648	3.0842384	-5.35886	1.48E-07	8.83E-07	6.8040018
RP11-206P5	0.9010972	-0.767508	5.3587822	1.48E-07	8.83E-07	6.8036189
ZNF608	0.2381448	5.3378062	5.3586159	1.48E-07	8.84E-07	6.8027984
RORA	-0.17982	6.1439119	-5.357917	1.48E-07	8.87E-07	6.7993502
PPP2R3A	0.2928417	4.8792932	5.3577484	1.48E-07	8.88E-07	6.7985165
AD000864.6	0.6590775	2.8791373	5.357682	1.48E-07	8.88E-07	6.798189
CCIN	0.9031907	-0.093847	5.3574601	1.49E-07	8.89E-07	6.7970941
GSTA3	-0.890921	-0.410483	-5.35669	1.49E-07	8.92E-07	6.7932938
CTD-3098H1	-1.220723	3.4939134	-5.355824	1.50E-07	8.96E-07	6.7890231
LRRC32	0.1890583	6.0364455	5.3551015	1.50E-07	8.99E-07	6.7854569
MYO6	0.1424026	6.2498721	5.354822	1.51E-07	9.00E-07	6.784078
AC068282.3	-0.27623	4.8434256	-5.354749	1.51E-07	9.00E-07	6.7837186
PLEKHA3P1	0.8805629	0.2218218	5.3540319	1.51E-07	9.04E-07	6.7801809
OLFM4	1.3289363	0.382376	5.3536072	1.52E-07	9.05E-07	6.7780861
PCDHA4	1.1323309	0.5532326	5.3528848	1.52E-07	9.09E-07	6.7745235
RP11-632K2	0.4755871	4.1283162	5.3527655	1.52E-07	9.09E-07	6.7739352
RNU1-75P	-0.951089	-0.046216	-5.352441	1.52E-07	9.10E-07	6.7723374
LRP12	0.3431265	4.9849636	5.3497268	1.55E-07	9.23E-07	6.7589547
FAM181B	0.8931019	-0.398722	5.3491743	1.55E-07	9.26E-07	6.7562318
POLR1C	-0.102461	6.036351	-5.348933	1.55E-07	9.27E-07	6.7550423
C2orf68	-0.086827	6.1928791	-5.348863	1.55E-07	9.27E-07	6.7546985
CFHR2	-0.740653	5.2940447	-5.348788	1.55E-07	9.27E-07	6.7543301

HPDL	0.8732071	2.6511868	5.348634	1.55E-07	9.27E-07	6.753569
SOAT1	0.0908182	6.2358537	5.348367	1.56E-07	9.29E-07	6.7522532
RNF185	-0.09056	6.3497551	-5.347921	1.56E-07	9.31E-07	6.7500577
LINC00937	0.8403428	1.7658763	5.3475682	1.56E-07	9.32E-07	6.7483171
RP11-31601	-0.785187	2.3064722	-5.347519	1.56E-07	9.32E-07	6.7480742
MATN3	1.0338475	3.0864244	5.3474915	1.56E-07	9.32E-07	6.7479394
NUS1P1	0.8768995	3.2856693	5.3467837	1.57E-07	9.35E-07	6.744452
RASGEF1C	1.0068837	0.0785132	5.3466884	1.57E-07	9.36E-07	6.7439826
KRT15	1.3289806	0.62922	5.3466755	1.57E-07	9.36E-07	6.7439192
RP11-1293J	-0.448303	4.047923	-5.346259	1.57E-07	9.37E-07	6.7418657
CCNB1IP1	-0.200481	6.1441539	-5.345945	1.58E-07	9.39E-07	6.7403221
TACR2	0.8748497	2.4907353	5.3458505	1.58E-07	9.39E-07	6.7398549
LPP-AS2	-0.187624	4.9187855	-5.345738	1.58E-07	9.40E-07	6.7392995
HES4	0.2998416	4.9571323	5.3454511	1.58E-07	9.41E-07	6.7378878
OLA1P1	0.4845762	3.5418721	5.3449067	1.58E-07	9.43E-07	6.7352064
PPP2R5C	-0.0818	6.5399241	-5.344359	1.59E-07	9.46E-07	6.7325071
RP11-713P1	0.8014624	-0.401234	5.3441022	1.59E-07	9.47E-07	6.7312445
NRL	-0.171647	4.8813141	-5.343899	1.59E-07	9.48E-07	6.7302454
ABCA5	-0.141565	6.2809909	-5.343586	1.60E-07	9.49E-07	6.7287043
RN7SL184P	-0.30979	-1.42092	-5.343043	1.60E-07	9.51E-07	6.7260301
PALM	0.2702265	5.6625495	5.342744	1.60E-07	9.53E-07	6.7245572
GPR34	0.3905684	4.7266288	5.3426864	1.60E-07	9.53E-07	6.7242737
ANKRD36B	0.8160259	2.8288096	5.3419458	1.61E-07	9.56E-07	6.7206275
AC128709.3	-0.742254	-0.834049	-5.341684	1.61E-07	9.58E-07	6.7193397
PDIK1L	-0.112713	5.7642648	-5.341171	1.62E-07	9.60E-07	6.7168137
H1F0	-0.10342	7.0428739	-5.340288	1.62E-07	9.64E-07	6.7124673
TRIM58	0.9368526	0.080616	5.3401429	1.62E-07	9.65E-07	6.711754
EIF4E2	-0.081997	6.5156873	-5.340028	1.63E-07	9.65E-07	6.7111882
TCF4	0.1648655	5.9186401	5.3395919	1.63E-07	9.67E-07	6.7090424
COL4A4	0.4729233	4.7681621	5.3389446	1.63E-07	9.70E-07	6.705857
MOB1A	0.0605835	6.4646149	5.3387742	1.64E-07	9.71E-07	6.7050188
DNAJB12	-0.077142	6.4055172	-5.338279	1.64E-07	9.73E-07	6.7025817
ABTB1	-0.11025	6.1369599	-5.338184	1.64E-07	9.73E-07	6.7021161
FIGF	-0.58555	4.2049943	-5.33791	1.64E-07	9.75E-07	6.7007679
MUM1	-0.095205	6.2742025	-5.337898	1.64E-07	9.75E-07	6.7007083
CPVL	0.2635281	5.891061	5.3375974	1.65E-07	9.76E-07	6.6992295
GNPDA2	0.1809109	5.2484637	5.3373587	1.65E-07	9.77E-07	6.6980554
AKR1C5P	-1.191852	1.7364716	-5.336883	1.65E-07	9.79E-07	6.6957132
CCDC71	-0.091233	5.8989628	-5.336224	1.66E-07	9.82E-07	6.6924759
RP11-426C2	0.9081683	1.3475629	5.3362109	1.66E-07	9.82E-07	6.6924094
DNMT3B	0.2840931	4.8873855	5.3360399	1.66E-07	9.83E-07	6.6915686
OST4	-0.092721	6.6980835	-5.336004	1.66E-07	9.83E-07	6.6913935
RP11-528G1	0.8834203	-0.595464	5.3357156	1.66E-07	9.84E-07	6.6899736
AC007391.2	-0.770439	-0.45096	-5.33562	1.66E-07	9.85E-07	6.6895021
MYL6	-0.088387	7.1892617	-5.335496	1.66E-07	9.85E-07	6.6888937
SYT5	1.093867	1.550963	5.3351615	1.67E-07	9.86E-07	6.6872489
RP11-706C1	-0.885101	4.6053905	-5.335108	1.67E-07	9.87E-07	6.6869868
CTD-2583A1	0.9486154	0.7116083	5.3350364	1.67E-07	9.87E-07	6.6866336
RP5-1142J1	0.7539782	-0.77836	5.3343364	1.67E-07	9.90E-07	6.6831917
RP13-516M1	0.682551	3.0835592	5.3340506	1.68E-07	9.91E-07	6.6817869
FLAD1	-0.097558	6.4389346	-5.334015	1.68E-07	9.91E-07	6.6816101

THNSL1	-0.144778	5.8935313	-5.333814	1.68E-07	9.92E-07	6.6806243
HNRNPK	-0.049011	7.0732223	-5.332914	1.69E-07	9.97E-07	6.6762015
CTD-2326C4	-0.229576	-1.480822	-5.332693	1.69E-07	9.98E-07	6.675114
BCS1L	-0.092629	6.096755	-5.332655	1.69E-07	9.98E-07	6.6749261
RPP38	-0.085668	5.77288	-5.331883	1.69E-07	1.00E-06	6.6711305
TRBJ2-7	0.7627966	-0.641588	5.3318063	1.69E-07	1.00E-06	6.6707549
RP5-837I24	0.9195192	0.5061062	5.331733	1.70E-07	1.00E-06	6.6703946
RPL15	-0.085066	7.1618624	-5.330982	1.70E-07	1.01E-06	6.666702
CSN2	-0.933855	-0.855142	-5.33096	1.70E-07	1.01E-06	6.6665978
ZNF384	0.1608293	5.6545768	5.3303467	1.71E-07	1.01E-06	6.663582
VAC14	-0.092096	6.3710047	-5.329898	1.71E-07	1.01E-06	6.6613754
TPPP2	-0.81277	3.7287266	-5.327534	1.73E-07	1.02E-06	6.6497663
CTC-429P9.	0.8082155	3.1040197	5.3275172	1.73E-07	1.02E-06	6.6496828
RNU2-17P	-0.742607	-0.561494	-5.327339	1.73E-07	1.02E-06	6.6488094
SLC38A3	-0.289328	7.0667763	-5.326928	1.74E-07	1.03E-06	6.6467866
C1orf204	0.8880007	2.3248753	5.3260311	1.75E-07	1.03E-06	6.642385
PPP6R2	-0.103142	6.7527167	-5.325337	1.75E-07	1.03E-06	6.6389787
ANTXRLP1	0.8173564	-0.5172	5.3249692	1.76E-07	1.04E-06	6.6371712
GOLGA6B	-1.140546	0.0824356	-5.323623	1.77E-07	1.04E-06	6.6305643
SGCE	0.3624971	5.6905338	5.3228098	1.77E-07	1.05E-06	6.626572
ATP8A1	0.2627974	5.1450427	5.322389	1.78E-07	1.05E-06	6.6245072
RPL7AP64	0.792689	-0.391303	5.3221579	1.78E-07	1.05E-06	6.6233732
GCSH	-0.166019	5.6546068	-5.322065	1.78E-07	1.05E-06	6.6229166
KCNK15-AS1	0.8661897	-0.583214	5.3218012	1.78E-07	1.05E-06	6.6216229
GFM2	-0.098784	6.2733333	-5.321529	1.79E-07	1.05E-06	6.6202849
RP11-356M2	1.1107769	2.8963975	5.3213378	1.79E-07	1.05E-06	6.619349
RP11-576I2	0.8308542	2.0299938	5.3212728	1.79E-07	1.05E-06	6.6190298
TRAPPC13	-0.08705	5.900938	-5.321087	1.79E-07	1.05E-06	6.6181165
CISD1	-0.113601	6.1869689	-5.321085	1.79E-07	1.05E-06	6.6181069
FGF18	0.9942994	2.1718688	5.3203834	1.80E-07	1.06E-06	6.6146665
HSPB2	0.7777312	2.7716787	5.320014	1.80E-07	1.06E-06	6.6128544
FAM47E-STE	-0.20221	5.8305001	-5.31807	1.82E-07	1.07E-06	6.6033213
MTSS1	-0.123739	6.7399094	-5.318058	1.82E-07	1.07E-06	6.6032635
BX842568.1	-1.253518	1.631842	-5.317887	1.82E-07	1.07E-06	6.6024221
IFITM10	0.341605	5.5015067	5.317757	1.82E-07	1.07E-06	6.6017851
AKR1C1	-0.262816	7.0213294	-5.317395	1.82E-07	1.07E-06	6.6000125
PPP4R2	-0.09402	6.4764119	-5.317043	1.83E-07	1.07E-06	6.5982858
IGHV1OR21-	0.7374683	-0.905562	5.3170025	1.83E-07	1.07E-06	6.5980854
PCDH17	0.2277858	5.4108875	5.3169293	1.83E-07	1.08E-06	6.5977266
GABRB3	1.4752192	2.277691	5.3167794	1.83E-07	1.08E-06	6.5969916
RP11-802E1	0.4199049	3.9944258	5.3164593	1.83E-07	1.08E-06	6.5954226
DCLRE1C	0.185618	5.1763126	5.3162744	1.83E-07	1.08E-06	6.594516
RP11-87501	1.108196	2.3050491	5.3162117	1.84E-07	1.08E-06	6.5942084
AC005932.1	0.8950189	0.9266863	5.3161435	1.84E-07	1.08E-06	6.5938744
FAM184A	-0.447074	5.2575418	-5.316067	1.84E-07	1.08E-06	6.5934987
MOCOS	-0.162866	6.2097739	-5.315902	1.84E-07	1.08E-06	6.5926881
CUL1	-0.077549	6.5255323	-5.315558	1.84E-07	1.08E-06	6.5910019
GPR115	1.0672342	-0.328583	5.3152644	1.84E-07	1.08E-06	6.5895652
WDR18	-0.102742	6.2934528	-5.314815	1.85E-07	1.09E-06	6.587362
RP11-452L6	-0.676129	2.5513697	-5.314753	1.85E-07	1.09E-06	6.5870583
PTGES3L	0.8026499	2.1328031	5.3139048	1.86E-07	1.09E-06	6.5829017

RP11-265N7	1.0971569	0.9026881	5.3138307	1.86E-07	1.09E-06	6.5825385
DNM1P51	0.9811848	0.5093349	5.3127245	1.87E-07	1.10E-06	6.5771181
NDUFA3	-0.142565	6.4839596	-5.312353	1.87E-07	1.10E-06	6.5752977
USP34	0.2166552	5.9632816	5.3122186	1.87E-07	1.10E-06	6.5746393
DYNLRB1	-0.104092	6.6038265	-5.311639	1.88E-07	1.10E-06	6.5718003
WNT7A	1.0086431	-0.206171	5.3115404	1.88E-07	1.10E-06	6.5713167
NCBP2-AS2	-0.126966	5.8078584	-5.311216	1.88E-07	1.10E-06	6.5697272
CYP2A6	-0.857848	6.2626213	-5.30971	1.90E-07	1.11E-06	6.5623533
TUBA1C	0.1293348	6.2568797	5.3090636	1.90E-07	1.12E-06	6.5591864
OGDH	-0.094713	6.7277086	-5.308929	1.90E-07	1.12E-06	6.5585283
RAB11FIP1	0.3411309	5.5959528	5.3088513	1.91E-07	1.12E-06	6.5581468
TMEM135	-0.117486	6.1667677	-5.308081	1.91E-07	1.12E-06	6.5543746
APTR	-0.121802	5.6095844	-5.307708	1.92E-07	1.12E-06	6.552549
AC064829.1	-0.372261	-1.393472	-5.306819	1.93E-07	1.13E-06	6.5481948
DPP4	-0.235073	6.4797899	-5.306756	1.93E-07	1.13E-06	6.5478905
CYP2C9	-0.449319	6.7203961	-5.306706	1.93E-07	1.13E-06	6.5476427
RP11-686D2	1.0099615	0.8276804	5.3052254	1.94E-07	1.14E-06	6.5403978
AEBP2	0.0956549	5.8299297	5.3049356	1.94E-07	1.14E-06	6.5389793
GUCY2C	1.1391538	3.4296374	5.3041166	1.95E-07	1.14E-06	6.5349719
RP11-467D6	-0.291132	3.8345507	-5.303841	1.95E-07	1.14E-06	6.5336215
UCKL1-AS1	0.7507538	2.6609312	5.3031931	1.96E-07	1.15E-06	6.5304538
TMCO1	-0.083273	6.8288752	-5.302742	1.97E-07	1.15E-06	6.5282488
RP11-218E2	0.9975809	-0.244716	5.302431	1.97E-07	1.15E-06	6.526726
TFPI	-0.198808	6.8655196	-5.302104	1.97E-07	1.15E-06	6.5251253
DSEL	0.44793	4.9979226	5.3019838	1.97E-07	1.15E-06	6.5245388
ANKRD22	1.0520071	3.3003606	5.3018772	1.97E-07	1.15E-06	6.524017
CTC-559E9.	0.5119154	3.864613	5.3018508	1.97E-07	1.15E-06	6.5238882
KDM5B	0.1550397	6.1618331	5.3016994	1.98E-07	1.15E-06	6.5231475
C12orf57	-0.133841	6.4275989	-5.301656	1.98E-07	1.15E-06	6.5229362
MCTS1	-0.106342	6.2685582	-5.300717	1.99E-07	1.16E-06	6.5183417
CES1P1	-0.694466	5.1847939	-5.300705	1.99E-07	1.16E-06	6.5182828
AP000476.1	0.9845212	0.3714461	5.3003789	1.99E-07	1.16E-06	6.51669
JUND	-0.129198	6.817193	-5.298569	2.01E-07	1.17E-06	6.5078422
RDH11	-0.114276	6.6827179	-5.298456	2.01E-07	1.17E-06	6.5072892
DUS3L	-0.099573	6.0880312	-5.297257	2.02E-07	1.18E-06	6.50143
PPP1R9A	0.6207377	4.7022667	5.2965991	2.03E-07	1.18E-06	6.498213
C15orf27	0.4300657	4.1774014	5.2957208	2.04E-07	1.19E-06	6.4939213
C14orf2	-0.121233	6.4920796	-5.294407	2.05E-07	1.20E-06	6.4875006
AC007191.4	0.647031	3.824742	5.2941441	2.05E-07	1.20E-06	6.4862183
RGMA	0.5545474	3.9834021	5.2938447	2.06E-07	1.20E-06	6.4847555
ACTR5	-0.118312	5.6276155	-5.293659	2.06E-07	1.20E-06	6.4838503
ZKSCAN1	-0.100339	6.6797592	-5.293342	2.06E-07	1.20E-06	6.482298
UTP15	-0.081895	5.8267305	-5.293012	2.07E-07	1.20E-06	6.4806891
TRAV35	0.7171312	-0.783434	5.2917238	2.08E-07	1.21E-06	6.4743977
SLC22A20	0.7956972	2.4690325	5.2916734	2.08E-07	1.21E-06	6.4741515
CYP3A5	-0.287292	6.7782737	-5.291432	2.08E-07	1.21E-06	6.4729708
IGLJ2	0.6700633	-0.991424	5.2913339	2.08E-07	1.21E-06	6.4724939
RP11-303E1	-0.186798	5.3274908	-5.291048	2.09E-07	1.21E-06	6.4710993
HLA-C	-0.128352	7.3536598	-5.290589	2.09E-07	1.22E-06	6.468857
C19orf66	-0.126635	6.4926298	-5.290559	2.09E-07	1.22E-06	6.4687118
ARHGAP40	1.2962916	0.5844712	5.2894794	2.10E-07	1.22E-06	6.4634404

RP11-117D2	0.677943	-0.806486	5.2886816	2.11E-07	1.23E-06	6.4595464
RP11-526D8	0.7301389	2.2135751	5.2886582	2.11E-07	1.23E-06	6.4594319
STAT5B	-0.077551	6.4239002	-5.288569	2.11E-07	1.23E-06	6.4589979
MOGAT2	-0.914356	5.2536954	-5.288069	2.12E-07	1.23E-06	6.4565547
ELAC1	-0.116245	5.4842947	-5.287714	2.12E-07	1.23E-06	6.4548228
ATP5E	-0.115247	6.9435689	-5.287028	2.13E-07	1.24E-06	6.451476
PPAPDC2	-0.134458	6.0114974	-5.286534	2.13E-07	1.24E-06	6.4490666
PWAR6	0.644224	4.5172153	5.2863276	2.14E-07	1.24E-06	6.4480593
RP4-680D5.	-0.594851	3.9498049	-5.285734	2.14E-07	1.25E-06	6.4451623
WNT1	0.8212035	-0.526122	5.2852928	2.15E-07	1.25E-06	6.4430114
TAPBPL	-0.152729	6.1990568	-5.285035	2.15E-07	1.25E-06	6.4417537
DNAJC16	-0.098164	6.1608815	-5.284704	2.15E-07	1.25E-06	6.4401417
RP11-1057E	-0.568354	-1.11302	-5.284243	2.16E-07	1.25E-06	6.4378922
GNG8	0.9620838	-0.066152	5.2837806	2.16E-07	1.26E-06	6.4356359
AP000472.3	-0.815145	-0.580651	-5.283704	2.17E-07	1.26E-06	6.4352647
KRT36	0.8224337	-0.416741	5.283011	2.17E-07	1.26E-06	6.4318831
SELP	0.6981954	4.0767653	5.2827589	2.18E-07	1.26E-06	6.4306541
AP000354.4	0.7043652	-0.804364	5.2826722	2.18E-07	1.26E-06	6.4302313
TRAV20	0.8217776	-0.540921	5.2815085	2.19E-07	1.27E-06	6.4245576
LINC01058	-0.99782	0.230678	-5.280797	2.20E-07	1.28E-06	6.4210885
CRB2	0.9889818	1.3431766	5.2806374	2.20E-07	1.28E-06	6.4203117
VCP	-0.065997	7.0109449	-5.27952	2.21E-07	1.28E-06	6.4148648
CCM2	-0.104803	6.5276305	-5.279254	2.22E-07	1.28E-06	6.413569
TMEM129	-0.101681	6.4618254	-5.278218	2.23E-07	1.29E-06	6.4085229
POMP	-0.104488	6.5866144	-5.278168	2.23E-07	1.29E-06	6.4082772
C12orf43	-0.082855	5.8420761	-5.277773	2.23E-07	1.29E-06	6.4063519
DDX42	-0.050954	6.5677297	-5.277724	2.23E-07	1.29E-06	6.4061163
ITPK1	-0.115214	6.6580896	-5.277643	2.23E-07	1.29E-06	6.405718
RP11-726G1	0.6943794	2.944414	5.2775749	2.23E-07	1.29E-06	6.4053879
RHNO1	0.1140548	5.7834837	5.2772227	2.24E-07	1.30E-06	6.4036723
C2CD3	0.09857	5.760532	5.276742	2.24E-07	1.30E-06	6.4013307
APOB	-0.21355	7.6747853	-5.275367	2.26E-07	1.31E-06	6.3946356
SPATA2L	-0.136632	5.9148004	-5.275344	2.26E-07	1.31E-06	6.3945208
RNFT2	0.5134514	4.4029109	5.274452	2.27E-07	1.31E-06	6.3901777
SNRNP35	-0.114133	6.0787236	-5.274419	2.27E-07	1.31E-06	6.3900172
GJB6	1.246008	0.480873	5.2743819	2.27E-07	1.31E-06	6.3898366
N4BP2L1	-0.159291	6.0301211	-5.274275	2.27E-07	1.31E-06	6.3893156
UHMK1	-0.085927	6.7101419	-5.274112	2.27E-07	1.32E-06	6.3885199
DDX49	-0.09278	6.4060442	-5.273017	2.29E-07	1.32E-06	6.3831933
SNORA71C	0.8949746	0.17252	5.2723272	2.29E-07	1.33E-06	6.3798332
RP11-1148L	0.4548442	4.6801505	5.2722374	2.30E-07	1.33E-06	6.379396
RP11-354K1	-0.730357	-0.946711	-5.27191	2.30E-07	1.33E-06	6.3778014
FKBP5	-0.16554	6.6618339	-5.271718	2.30E-07	1.33E-06	6.3768657
RP11-347P5	0.7822985	-0.334593	5.2714351	2.30E-07	1.33E-06	6.3754912
RP11-434H6	-0.26953	4.7523417	-5.271274	2.31E-07	1.33E-06	6.3747066
EIF3K	-0.105778	6.8021331	-5.271265	2.31E-07	1.33E-06	6.3746618
CLUH	-0.100627	6.9118021	-5.270947	2.31E-07	1.33E-06	6.3731167
UMODL1	1.2482589	0.7445798	5.2709256	2.31E-07	1.33E-06	6.3730115
ULK2	0.3150816	5.2680357	5.2699427	2.32E-07	1.34E-06	6.368229
FAM86EP	-0.16536	5.0126744	-5.269514	2.33E-07	1.34E-06	6.3661411
IGSF1	0.807776	4.5866776	5.2691202	2.33E-07	1.35E-06	6.3642268

OMA1	-0.110165	5.936819	-5.268939	2.33E-07	1.35E-06	6.3633436
SUGCT	-0.20299	5.9472832	-5.268882	2.33E-07	1.35E-06	6.3630679
GPR89B	-0.161074	5.2052268	-5.267877	2.35E-07	1.35E-06	6.3581801
SWSAP1	-0.140557	5.2535513	-5.267681	2.35E-07	1.36E-06	6.3572279
AC061992.2	0.9848525	0.15567	5.2676243	2.35E-07	1.36E-06	6.3569504
LINC00657	-0.063196	6.8763471	-5.267282	2.35E-07	1.36E-06	6.3552863
CTC-338M12	-0.965974	0.3164641	-5.267177	2.36E-07	1.36E-06	6.3547756
RP11-757A1	-1.106673	0.0632736	-5.266953	2.36E-07	1.36E-06	6.3536858
RP11-25705	-0.728746	2.6386753	-5.266652	2.36E-07	1.36E-06	6.3522228
ANXA7	-0.074999	6.7498186	-5.26611	2.37E-07	1.36E-06	6.3495876
CCZ1B	0.167135	5.1230698	5.2657335	2.37E-07	1.37E-06	6.3477552
ARFRP1	-0.093303	6.3644222	-5.265606	2.37E-07	1.37E-06	6.347137
CTD-2325A1	-0.968284	2.468036	-5.264977	2.38E-07	1.37E-06	6.3440781
FAM50A	-0.125049	6.5780334	-5.264523	2.39E-07	1.37E-06	6.3418707
MUSK	1.0684386	-0.23283	5.2638405	2.40E-07	1.38E-06	6.3385521
AC078941.1	0.8247683	-0.622832	5.2637502	2.40E-07	1.38E-06	6.338113
MED16	-0.088436	6.4577408	-5.26335	2.40E-07	1.38E-06	6.3361671
MDH2	-0.103474	7.0025942	-5.263237	2.40E-07	1.38E-06	6.3356176
HOXB6	0.4692366	3.8847595	5.2632008	2.40E-07	1.38E-06	6.3354427
TMPRSS4	1.1462338	1.575353	5.262989	2.41E-07	1.38E-06	6.3344134
SSU72P3	-0.248763	-1.472392	-5.262539	2.41E-07	1.39E-06	6.3322245
RP11-166N6	-0.697785	-0.891766	-5.262133	2.42E-07	1.39E-06	6.3302516
RP11-347I1	0.7416149	3.4400527	5.2620209	2.42E-07	1.39E-06	6.3297083
MYRIP	-0.600116	5.6715497	-5.26182	2.42E-07	1.39E-06	6.3287339
GAS8	-0.133732	5.8095928	-5.261344	2.43E-07	1.39E-06	6.3264203
TMSB4XP1	0.8493809	0.1220731	5.2610237	2.43E-07	1.40E-06	6.3248627
FASN	-0.14953	7.1788913	-5.260677	2.43E-07	1.40E-06	6.3231779
SLC25A3	-0.077776	7.0836019	-5.260622	2.43E-07	1.40E-06	6.3229092
CLDN4	0.7047328	5.2415928	5.2602663	2.44E-07	1.40E-06	6.3211834
LEF1	0.5467717	5.0069288	5.2602455	2.44E-07	1.40E-06	6.3210823
RP11-178C3	-0.571262	3.4185654	-5.25913	2.45E-07	1.41E-06	6.3156637
COL4A2	0.1315633	6.8678561	5.2588366	2.46E-07	1.41E-06	6.3142385
AK4P2	-0.840329	-0.864909	-5.258382	2.46E-07	1.41E-06	6.3120305
KLF12	-0.169122	6.0250675	-5.25783	2.47E-07	1.42E-06	6.3093489
CTC-786C10	1.0600107	1.4769645	5.2576367	2.47E-07	1.42E-06	6.3084113
ZNF100	0.2592603	4.8891825	5.2575839	2.47E-07	1.42E-06	6.3081547
CYP17A1-AS	-0.924384	0.5017514	-5.25752	2.47E-07	1.42E-06	6.3078466
ZNHIT3	-0.08044	6.0876955	-5.257415	2.47E-07	1.42E-06	6.3073337
EID1	-0.090861	6.6093157	-5.257402	2.47E-07	1.42E-06	6.3072694
MYOZ1	0.9985809	1.8081705	5.2569887	2.48E-07	1.42E-06	6.3052648
RP11-338C1	0.8442669	0.5769038	5.2569558	2.48E-07	1.42E-06	6.3051051
RUNX1	0.1793797	5.9280584	5.2569309	2.48E-07	1.42E-06	6.3049841
LILRA2	0.4667498	4.0031874	5.2567068	2.48E-07	1.42E-06	6.303896
GPX7	0.3722519	5.2051585	5.2565651	2.49E-07	1.42E-06	6.3032082
RP13-514E2	0.8500369	-0.350242	5.2564653	2.49E-07	1.43E-06	6.3027236
AL928768.3	0.9268406	-0.328973	5.2564057	2.49E-07	1.43E-06	6.3024341
TMEM245	-0.122622	6.5697174	-5.256282	2.49E-07	1.43E-06	6.3018357
BPESC1	-0.833339	-0.638019	-5.25611	2.49E-07	1.43E-06	6.3009967
WHSC1	0.1033278	6.2241841	5.2552288	2.50E-07	1.43E-06	6.2967211
RP13-870H1	1.1602399	0.8145702	5.2548925	2.51E-07	1.44E-06	6.2950886
SP5	-0.578519	5.1042604	-5.254845	2.51E-07	1.44E-06	6.2948593

TRAPPC12-A	0.8568701	2.6834974	5.2545389	2.51E-07	1.44E-06	6.2933724
RP11-548L2	-1.30409	2.5544356	-5.254084	2.52E-07	1.44E-06	6.2911665
RP11-283G6	-0.347904	4.343177	-5.253888	2.52E-07	1.44E-06	6.2902122
ZNF76	-0.07876	6.2165743	-5.253611	2.52E-07	1.44E-06	6.2888698
MGAM	1.2256181	2.4904142	5.2532059	2.53E-07	1.45E-06	6.2869032
RP11-14101	-0.830744	-0.565636	-5.25313	2.53E-07	1.45E-06	6.2865337
IGSF5	0.9351145	-0.430961	5.2531125	2.53E-07	1.45E-06	6.2864503
CDADC1	-0.140311	5.5855342	-5.252891	2.53E-07	1.45E-06	6.2853762
ARAP2	0.2181984	5.5243971	5.2523147	2.54E-07	1.45E-06	6.282579
RP4-545C24	0.9278117	2.2053129	5.2520065	2.54E-07	1.45E-06	6.2810836
BMS1P20	0.3410181	4.3992068	5.2510954	2.55E-07	1.46E-06	6.276664
RBM22P3	-0.411583	-1.389693	-5.250646	2.56E-07	1.46E-06	6.2744857
POMK	0.7295987	3.1660793	5.2505965	2.56E-07	1.46E-06	6.2742443
RP11-701H2	0.9383083	-0.282579	5.2503959	2.56E-07	1.46E-06	6.273271
TERF2	-0.072929	6.1136283	-5.250379	2.56E-07	1.46E-06	6.2731882
TAF15	-0.061393	6.7066205	-5.250003	2.57E-07	1.47E-06	6.2713649
APBB1IP	0.2770245	5.9017225	5.2479663	2.60E-07	1.48E-06	6.2614893
RP11-535M1	0.9548829	0.4434163	5.2473096	2.60E-07	1.49E-06	6.2583055
ISCA1P3	-0.384295	-1.372945	-5.246222	2.62E-07	1.50E-06	6.2530349
RP11-7K24.	1.0708675	0.4962157	5.2456011	2.63E-07	1.50E-06	6.2500243
RP11-13N13	0.9381677	0.3146914	5.245362	2.63E-07	1.50E-06	6.2488656
NDUFA9	-0.104188	6.3469012	-5.244772	2.64E-07	1.51E-06	6.2460066
RP11-419C5	0.7804736	2.3091804	5.2447289	2.64E-07	1.51E-06	6.2457977
PHLDB3	-0.125073	6.057655	-5.244158	2.65E-07	1.51E-06	6.2430314
AMZ2	-0.078335	6.2133748	-5.244076	2.65E-07	1.51E-06	6.2426355
IGHV1-12	0.8523941	-0.724449	5.2438485	2.65E-07	1.51E-06	6.2415318
TCEA2	-0.171574	6.3125707	-5.242421	2.67E-07	1.52E-06	6.2346156
MT-CO1	-0.104185	8.0062005	-5.242313	2.67E-07	1.52E-06	6.2340912
LYRM7	-0.098661	5.8904719	-5.24217	2.67E-07	1.52E-06	6.2334022
HIST1H1A	-0.835402	-0.476046	-5.241996	2.67E-07	1.52E-06	6.2325596
TAS2R5	0.9445982	1.4738494	5.241683	2.68E-07	1.53E-06	6.2310421
IPPK	0.5657572	4.4393946	5.241438	2.68E-07	1.53E-06	6.2298552
UQCC3	-0.132391	6.0942598	-5.241156	2.69E-07	1.53E-06	6.2284899
BAZ2A	0.0735835	6.4063672	5.2408838	2.69E-07	1.53E-06	6.2271715
NOG	0.8553818	-0.532522	5.2407534	2.69E-07	1.53E-06	6.2265399
CH17-431G2	0.7876987	2.6528156	5.2401545	2.70E-07	1.54E-06	6.2236398
PCDHGB4	1.070963	3.1599073	5.2390945	2.71E-07	1.55E-06	6.2185077
NCAM1	0.6816751	3.7837085	5.2387766	2.72E-07	1.55E-06	6.2169688
GSDMA	1.0395283	2.1609396	5.2382845	2.73E-07	1.55E-06	6.2145865
BX322559.3	-0.748486	-1.142317	-5.238245	2.73E-07	1.55E-06	6.2143956
ZNF402P	-0.463338	-1.320309	-5.237808	2.73E-07	1.55E-06	6.2122803
PTH1R	0.5113212	4.5934248	5.2373714	2.74E-07	1.56E-06	6.210167
ANKRD18B	1.1975454	0.4696856	5.237001	2.74E-07	1.56E-06	6.2083745
RP11-4K16.	0.8994584	0.1505691	5.2365014	2.75E-07	1.56E-06	6.2059569
RP11-867G2	-0.248747	5.2437273	-5.236432	2.75E-07	1.56E-06	6.2056203
ALG5	-0.103844	6.2727191	-5.236288	2.75E-07	1.57E-06	6.2049238
FNBP1P1	0.9518452	2.0872478	5.2359329	2.76E-07	1.57E-06	6.2032056
ZNF524	-0.127312	5.9870378	-5.235707	2.76E-07	1.57E-06	6.2021142
RP11-121A8	0.7752336	2.0533617	5.2356868	2.76E-07	1.57E-06	6.202015
CTNNBIP1	-0.106333	6.0779008	-5.234231	2.78E-07	1.58E-06	6.1949735
AC090181.1	0.7125611	-0.630908	5.2337739	2.79E-07	1.58E-06	6.1927607

TCAP	-0.3953	4.357179	-5.233385	2.79E-07	1.59E-06	6.1908795
RP11-839D1	-0.998123	1.3155253	-5.233362	2.79E-07	1.59E-06	6.1907673
LAMC3	0.4456203	5.575035	5.2330343	2.80E-07	1.59E-06	6.1891838
LL09NC01-1	-0.709395	2.5273137	-5.232926	2.80E-07	1.59E-06	6.1886608
AKAP1	-0.084963	6.6250656	-5.23287	2.80E-07	1.59E-06	6.1883897
EEF1B2P5	-0.44645	-1.266745	-5.232628	2.80E-07	1.59E-06	6.1872172
TRAPPC12	-0.077563	6.2914522	-5.232466	2.81E-07	1.59E-06	6.1864347
MASP1	-0.261271	6.5575842	-5.231893	2.81E-07	1.60E-06	6.1836654
HIST1H4J	-0.876502	2.1863485	-5.231701	2.82E-07	1.60E-06	6.1827378
RNF121	-0.081108	6.0379622	-5.23146	2.82E-07	1.60E-06	6.18157
RHOQP2	0.8384084	0.213933	5.2313831	2.82E-07	1.60E-06	6.1811988
RP11-200A1	-1.139424	1.6275185	-5.23097	2.83E-07	1.60E-06	6.1792027
H19	0.4253435	6.2476666	5.2305912	2.83E-07	1.61E-06	6.1773701
SRRM3	0.8261699	3.8283927	5.2302102	2.84E-07	1.61E-06	6.1755282
DYX1C1	0.9687969	1.0428395	5.2297673	2.84E-07	1.61E-06	6.1733872
EIF4A2P1	0.711311	-0.645275	5.2296926	2.85E-07	1.61E-06	6.1730262
AATBC	0.6027564	3.10949	5.2292448	2.85E-07	1.62E-06	6.1708617
IGHV3-38	0.860121	-0.558639	5.2292372	2.85E-07	1.62E-06	6.170825
CLEC4E	0.7525265	3.499641	5.229076	2.85E-07	1.62E-06	6.1700458
RP11-327L3	-0.885318	0.0056187	-5.228819	2.86E-07	1.62E-06	6.168806
TIMM50	-0.094069	6.4767982	-5.228171	2.87E-07	1.62E-06	6.1656705
HSPA2	0.195498	5.5706406	5.22817	2.87E-07	1.62E-06	6.1656674
TRPC7-AS1	0.9428393	-0.385456	5.228143	2.87E-07	1.62E-06	6.1655367
AMHR2	1.0152626	0.0018312	5.2281193	2.87E-07	1.62E-06	6.1654225
QTRTD1	0.0919657	5.7677015	5.2281096	2.87E-07	1.62E-06	6.1653755
CTD-2227E1	-0.442363	4.3459262	-5.227979	2.87E-07	1.62E-06	6.1647464
ITGB1	0.1278274	6.8148996	5.2275839	2.88E-07	1.63E-06	6.162835
RP11-5407.	0.6806258	3.3758288	5.2274174	2.88E-07	1.63E-06	6.1620306
AC003075.4	-0.415701	3.8297177	-5.227271	2.88E-07	1.63E-06	6.1613235
PDZD11	-0.088808	6.1982868	-5.226986	2.88E-07	1.63E-06	6.1599484
SLC39A10	0.1979291	5.6295696	5.2264585	2.89E-07	1.64E-06	6.1573976
RP11-263F1	-0.978774	-0.529164	-5.226282	2.90E-07	1.64E-06	6.1565453
HLA-DQB1-A	0.8888425	2.4912707	5.2262395	2.90E-07	1.64E-06	6.1563398
IQSEC1	-0.096874	6.6394812	-5.225962	2.90E-07	1.64E-06	6.1549988
C21orf62	0.9305389	0.2625199	5.2253329	2.91E-07	1.64E-06	6.15196
IFNL3P1	0.7676999	-0.732658	5.2244034	2.92E-07	1.65E-06	6.1474706
HEXIM2	-0.166724	4.9747094	-5.223464	2.94E-07	1.66E-06	6.142932
RP11-285F1	-0.964393	1.4470844	-5.222532	2.95E-07	1.67E-06	6.1384351
MARK2	0.0726439	6.161755	5.2224334	2.95E-07	1.67E-06	6.137958
MIR331	0.8825848	0.2263093	5.2224267	2.95E-07	1.67E-06	6.1379257
HM13-AS1	-0.709224	2.417571	-5.222362	2.95E-07	1.67E-06	6.1376124
C2orf91	0.9255843	0.0968492	5.2214896	2.97E-07	1.67E-06	6.133402
AVEN	-0.125309	5.8353933	-5.221053	2.97E-07	1.68E-06	6.1312945
GSPT1	-0.061986	6.5930599	-5.220548	2.98E-07	1.68E-06	6.128855
RP11-4C20.	0.8590438	-0.063911	5.2204794	2.98E-07	1.68E-06	6.128526
ERICH5	0.9461754	5.5525803	5.2192796	3.00E-07	1.69E-06	6.1227356
AC005253.2	0.4459307	3.6043364	5.2178291	3.02E-07	1.70E-06	6.1157372
RP11-73M18	0.6643316	2.8707716	5.2166922	3.04E-07	1.71E-06	6.1102529
GPD1	-0.359999	6.2322948	-5.216385	3.04E-07	1.72E-06	6.1087698
USP20	-0.07554	6.1523683	-5.216273	3.04E-07	1.72E-06	6.1082291
RP11-49002	-0.666595	-1.038309	-5.216024	3.05E-07	1.72E-06	6.1070281

NBR1	-0.08423	6.7732786	-5.215672	3.05E-07	1.72E-06	6.1053335
ANXA3	0.7001374	3.8883376	5.2156717	3.05E-07	1.72E-06	6.1053312
CTD-2526A2	-0.869161	0.9653399	-5.215621	3.05E-07	1.72E-06	6.1050879
COPZ2	-0.224322	5.9172204	-5.215151	3.06E-07	1.72E-06	6.1028213
GTF2IRD2	-0.211353	4.7609013	-5.215036	3.06E-07	1.73E-06	6.1022633
IGLV2-34	0.7304989	-0.911191	5.2150324	3.06E-07	1.73E-06	6.1022481
EPT1	-0.090997	6.4034781	-5.214679	3.07E-07	1.73E-06	6.1005431
CTD-2636A2	-0.414578	4.3276105	-5.214624	3.07E-07	1.73E-06	6.1002795
AC079305.1	-0.703733	2.0649524	-5.214416	3.07E-07	1.73E-06	6.0992763
ERP29	-0.085896	6.8983885	-5.213711	3.08E-07	1.74E-06	6.0958765
CXCL16	0.1782903	6.3957067	5.2129131	3.10E-07	1.74E-06	6.0920308
SNRPGP15	0.9235284	0.8823999	5.2128752	3.10E-07	1.74E-06	6.0918479
RNF220	-0.067429	6.3734579	-5.212282	3.11E-07	1.75E-06	6.0889891
ABCG5	-0.461625	6.1231019	-5.210937	3.13E-07	1.76E-06	6.0825043
FOXO3B	0.8854542	2.1929646	5.2104887	3.13E-07	1.76E-06	6.0803466
PVALB	1.0473445	0.4064468	5.2101021	3.14E-07	1.77E-06	6.0784837
EGFR-AS1	1.3666106	2.3526325	5.2094603	3.15E-07	1.77E-06	6.0753916
BCYRN1	0.7963182	2.8047852	5.2091423	3.16E-07	1.77E-06	6.0738596
KB-68A7.1	-0.843507	4.0868643	-5.2089	3.16E-07	1.78E-06	6.0726909
LENG1	-0.116718	5.7162439	-5.208898	3.16E-07	1.78E-06	6.0726832
HNRNPA3P12	0.7397299	1.6453858	5.2081418	3.17E-07	1.78E-06	6.0690401
RP11-401.2	0.3388923	4.6636182	5.2081237	3.17E-07	1.78E-06	6.0689531
GIMAP6	0.1920506	5.6267391	5.2068439	3.19E-07	1.79E-06	6.0627895
OTUD7B	-0.089311	6.3045022	-5.206456	3.20E-07	1.80E-06	6.0609212
MIR429	0.6653648	-0.993766	5.2059546	3.21E-07	1.80E-06	6.0585075
PRR13P5	0.4145988	3.9376564	5.2057385	3.21E-07	1.80E-06	6.0574667
RP11-820I1	-0.884865	-0.159003	-5.204846	3.22E-07	1.81E-06	6.0531696
RETN	1.0122321	-0.023239	5.2045724	3.23E-07	1.81E-06	6.0518529
LINC00167	-0.890121	0.4787564	-5.203887	3.24E-07	1.82E-06	6.0485556
RP11-589N1	-0.50861	-1.139802	-5.203572	3.25E-07	1.82E-06	6.0470361
LAYN	0.2784146	4.5749427	5.2035564	3.25E-07	1.82E-06	6.0469629
FAM76A	-0.097905	5.5233295	-5.203201	3.25E-07	1.82E-06	6.0452533
CLDN14	-0.405803	5.8693359	-5.203014	3.25E-07	1.83E-06	6.0443513
RP11-1319K	0.9005165	1.9616784	5.2027467	3.26E-07	1.83E-06	6.0430658
GLULP3	-0.945027	-0.463414	-5.202404	3.26E-07	1.83E-06	6.0414185
HDAC3	-0.074011	6.3354439	-5.202311	3.27E-07	1.83E-06	6.0409699
PEMT	-0.18059	6.3611183	-5.201972	3.27E-07	1.83E-06	6.0393377
C14orf166	-0.082751	6.5622747	-5.201744	3.28E-07	1.84E-06	6.0382415
SLC3A1	0.9733487	4.1226035	5.201632	3.28E-07	1.84E-06	6.037702
PRPS1P2	0.8264109	2.7103484	5.2011068	3.29E-07	1.84E-06	6.0351752
NKIRAS2	-0.065892	6.3394145	-5.20093	3.29E-07	1.84E-06	6.0343247
AP000357.4	0.7757799	-0.308127	5.20079	3.29E-07	1.84E-06	6.033651
SVILP1	0.9087725	1.9878364	5.1998754	3.31E-07	1.85E-06	6.0292517
S100A14	0.6729913	5.3434616	5.1994172	3.31E-07	1.86E-06	6.0270478
GOT2	-0.128408	6.9231705	-5.199295	3.32E-07	1.86E-06	6.0264606
SORBS2	-0.185864	6.5258053	-5.198832	3.32E-07	1.86E-06	6.024235
AC017104.6	0.8601433	2.0174812	5.198761	3.32E-07	1.86E-06	6.0238918
PIAS3	0.1692603	5.7472365	5.1984835	3.33E-07	1.86E-06	6.0225576
AC114812.1	-1.051531	0.2905433	-5.198399	3.33E-07	1.86E-06	6.0221533
LINC00861	0.5756612	3.8386698	5.1974328	3.35E-07	1.87E-06	6.0175052
SAMD12-AS1	0.8723201	-0.579012	5.1966658	3.36E-07	1.88E-06	6.0138176

NXN	0.3714729	5.4378993	5.1965052	3.36E-07	1.88E-06	6.0130457
RP11-10J21	0.8426187	-0.430212	5.196409	3.36E-07	1.88E-06	6.0125831
PLA2G4D	0.8575432	-0.61319	5.1959443	3.37E-07	1.88E-06	6.0103495
SLC25A33	-0.183296	6.0804503	-5.194804	3.39E-07	1.90E-06	6.004869
CC2D1B	-0.067879	6.2760052	-5.194705	3.39E-07	1.90E-06	6.0043915
NEURL2	-0.22447	4.4775011	-5.19465	3.39E-07	1.90E-06	6.0041267
CTD-2280E9	-1.232192	1.4518896	-5.194225	3.40E-07	1.90E-06	6.0020884
HAGHL	0.4146286	4.378845	5.1940157	3.40E-07	1.90E-06	6.0010806
C10orf90	1.224597	0.2583456	5.1939196	3.41E-07	1.90E-06	6.0006186
NDUFAF1	-0.114898	6.0859192	-5.193911	3.41E-07	1.90E-06	6.0005751
ZNF826P	0.834535	3.784671	5.1937301	3.41E-07	1.90E-06	5.999708
BEND5	0.7926293	3.1967187	5.1936112	3.41E-07	1.90E-06	5.9991365
RNPEP	-0.092708	6.572284	-5.193361	3.42E-07	1.91E-06	5.9979325
ZNF331	0.3639281	5.6630703	5.1933515	3.42E-07	1.91E-06	5.9978889
CBX6	0.2433427	5.91725	5.1932532	3.42E-07	1.91E-06	5.9974164
STAT4	0.3190219	5.0147324	5.1931701	3.42E-07	1.91E-06	5.9970171
DNAJC5B	0.7481112	2.9646385	5.1929719	3.42E-07	1.91E-06	5.9960653
VRK2	0.1562935	5.5026653	5.1928991	3.42E-07	1.91E-06	5.9957155
JAK2	0.1798682	5.3649331	5.1923517	3.43E-07	1.91E-06	5.9930854
SFRP4	0.6588585	4.5917058	5.1919622	3.44E-07	1.92E-06	5.9912142
SF1	-0.04836	6.7676061	-5.191921	3.44E-07	1.92E-06	5.9910144
RP3-329A5.	0.5914893	3.0633744	5.1912441	3.45E-07	1.92E-06	5.9877652
MT1X	-0.376186	6.2880723	-5.191139	3.45E-07	1.92E-06	5.98726
RP3-428L16	0.5361746	3.165151	5.1906764	3.46E-07	1.93E-06	5.9850384
AKR1C3	-0.141436	7.0266764	-5.190527	3.46E-07	1.93E-06	5.9843224
SMR3B	-0.70707	-1.197599	-5.19023	3.47E-07	1.93E-06	5.9828962
GALR3	-1.103107	1.821638	-5.190149	3.47E-07	1.93E-06	5.9825056
RP11-320L1	0.6349725	2.9393825	5.1900535	3.47E-07	1.93E-06	5.9820471
IGLV5-37	0.8537283	-0.733354	5.1900109	3.47E-07	1.93E-06	5.9818427
RP11-432J2	1.0981932	0.0442627	5.1897448	3.48E-07	1.94E-06	5.9805649
RP11-291B2	1.0954327	1.1032649	5.1892994	3.49E-07	1.94E-06	5.9784259
CTD-2245F1	0.9778001	1.9027895	5.1892162	3.49E-07	1.94E-06	5.9780267
ZMAT5	-0.102108	5.9400082	-5.188821	3.49E-07	1.94E-06	5.9761296
RP11-357H1	0.8696199	-0.514313	5.1888189	3.49E-07	1.94E-06	5.9761192
ZNF462	0.4290316	4.3584114	5.1885856	3.50E-07	1.95E-06	5.9749991
THOC6	-0.099378	6.0514986	-5.188465	3.50E-07	1.95E-06	5.9744199
ITPKB	0.1525695	5.6529518	5.1884335	3.50E-07	1.95E-06	5.9742684
CTB-25B13.	-0.155326	5.5734412	-5.187999	3.51E-07	1.95E-06	5.9721827
MCTS2P	0.7993023	2.7438965	5.1874081	3.52E-07	1.96E-06	5.9693462
GATSL2	0.5334073	4.0377006	5.1865098	3.53E-07	1.96E-06	5.9650349
RPL4P1	0.8880784	0.3051873	5.1865096	3.53E-07	1.96E-06	5.9650339
IER5L	0.2708651	5.4921177	5.1857035	3.55E-07	1.97E-06	5.9611654
NUPR1	-0.168436	6.8972089	-5.185354	3.56E-07	1.98E-06	5.9594886
DCLK2	0.335858	4.5485567	5.1851501	3.56E-07	1.98E-06	5.95851
CCL4	0.3107979	5.0313008	5.1849447	3.56E-07	1.98E-06	5.9575244
FAM98C	-0.098809	5.9319533	-5.18478	3.57E-07	1.98E-06	5.9567336
CNTN4	0.5864713	4.1004101	5.1845153	3.57E-07	1.98E-06	5.9554642
KCNQ3	0.7709577	3.0442121	5.1843788	3.57E-07	1.98E-06	5.9548092
KIAA1456	0.7974593	4.0202715	5.1839623	3.58E-07	1.99E-06	5.952811
ETNPPL	-0.669183	5.966724	-5.183922	3.58E-07	1.99E-06	5.952619
AACS	0.1533319	5.863275	5.1837239	3.58E-07	1.99E-06	5.9516675

RP1-30M3.6	-0.303158	3.6848946	-5.183719	3.58E-07	1.99E-06	5.9516418
SUCLG2	-0.122497	6.7114504	-5.183407	3.59E-07	1.99E-06	5.9501485
RP11-1149C	0.4738808	3.5836445	5.1833913	3.59E-07	1.99E-06	5.9500721
PLEKHF1	-0.240255	5.8281995	-5.183261	3.59E-07	1.99E-06	5.9494483
CTD-255501	0.6594735	2.5670559	5.1828681	3.60E-07	2.00E-06	5.9475622
ABCC9	-0.342297	5.8160255	-5.182826	3.60E-07	2.00E-06	5.9473595
UBIAD1	-0.096029	6.1367861	-5.180745	3.64E-07	2.02E-06	5.9373823
LPHN1	0.3186334	5.3852779	5.1804403	3.64E-07	2.02E-06	5.9359202
AF121897.4	-0.725251	-0.791012	-5.180132	3.65E-07	2.02E-06	5.9344411
PRRX1	0.4997937	4.5967114	5.1799215	3.65E-07	2.02E-06	5.9334328
RAB17	-0.265366	6.4671149	-5.179615	3.66E-07	2.03E-06	5.9319623
CIITA	0.2119866	5.5875925	5.1794232	3.66E-07	2.03E-06	5.9310442
PRDM6	0.6835436	3.3708705	5.1787939	3.67E-07	2.03E-06	5.9280274
LINC00924	0.5433768	4.0146358	5.1781667	3.69E-07	2.04E-06	5.9250217
BDKRB2	0.4154748	5.0265514	5.1774013	3.70E-07	2.05E-06	5.9213536
CTB-180A7.	-0.613666	-1.072374	-5.176796	3.71E-07	2.05E-06	5.9184541
PRR29	0.2433636	4.3328315	5.1760881	3.72E-07	2.06E-06	5.9150612
Z95114.5	-0.206598	-1.490917	-5.175536	3.73E-07	2.07E-06	5.9124154
TFPI2	0.554114	4.1394652	5.1752237	3.74E-07	2.07E-06	5.9109203
GNAI2	-0.065427	6.9059718	-5.174979	3.74E-07	2.07E-06	5.9097494
TMEM220-AS	-0.43966	5.1167925	-5.174712	3.75E-07	2.07E-06	5.9084712
RP11-138H1	-0.98629	-0.399533	-5.174086	3.76E-07	2.08E-06	5.9054703
SRPK3	0.5745848	3.2720377	5.1740156	3.76E-07	2.08E-06	5.9051335
TMED4	-0.064831	6.5798042	-5.173919	3.76E-07	2.08E-06	5.9046694
ABCG1	0.218236	5.7811432	5.172978	3.78E-07	2.09E-06	5.9001645
JAG2	0.1980916	5.6949064	5.1720888	3.80E-07	2.10E-06	5.895907
FBXO30	0.1327851	5.6601129	5.1717295	3.81E-07	2.10E-06	5.8941867
RP11-477H2	1.1425855	0.9400472	5.1706208	3.83E-07	2.11E-06	5.8888793
GOSR1	-0.058598	6.3069631	-5.17039	3.83E-07	2.12E-06	5.887777
RP11-396F2	-0.511156	3.9231115	-5.169174	3.85E-07	2.13E-06	5.8819535
DNAJB9	-0.12648	6.5453345	-5.168347	3.87E-07	2.14E-06	5.8779991
RP11-234K2	0.9539604	0.1264167	5.1679567	3.88E-07	2.14E-06	5.8761302
NUCKS1	-0.076481	7.0349837	-5.167557	3.89E-07	2.15E-06	5.8742162
WFDC2	1.2761048	3.2648369	5.1672153	3.89E-07	2.15E-06	5.8725833
HLA-DMB	0.2177123	5.871414	5.1670701	3.90E-07	2.15E-06	5.8718885
NPIPB5	0.7166816	3.4450758	5.1670532	3.90E-07	2.15E-06	5.8718077
RHO	1.0960363	0.8282434	5.1668646	3.90E-07	2.15E-06	5.8709053
PGBD4P3	-0.83654	-0.024316	-5.16683	3.90E-07	2.15E-06	5.8707405
EDIL3	0.3440372	5.140625	5.1663052	3.91E-07	2.16E-06	5.8682298
MT1CP	-1.017748	-0.021451	-5.166163	3.91E-07	2.16E-06	5.8675508
FAM135A	0.179549	5.4423283	5.1657608	3.92E-07	2.16E-06	5.8656257
LRRC3	-0.320717	5.9533805	-5.165707	3.92E-07	2.16E-06	5.8653682
MPO	1.0243912	1.0211993	5.1646447	3.94E-07	2.17E-06	5.8602885
LPAR4	0.9144982	0.2516295	5.1637511	3.96E-07	2.18E-06	5.8560159
TMEM165	0.1000982	6.1315901	5.1636744	3.96E-07	2.18E-06	5.8556491
BPIFB2	1.4877916	2.7892467	5.1631095	3.97E-07	2.19E-06	5.8529483
ST7L	-0.089783	5.5329111	-5.162695	3.98E-07	2.19E-06	5.850967
NGF	0.581184	3.6876549	5.1615647	4.00E-07	2.21E-06	5.8455642
RP11-37N22	0.8408562	-0.356907	5.1615436	4.00E-07	2.21E-06	5.8454633
AC012074.2	0.895469	0.975641	5.1615112	4.00E-07	2.21E-06	5.8453086
RP11-118K6	0.8237928	-0.392239	5.1611379	4.01E-07	2.21E-06	5.8435243

KIAA1161	-0.150074	6.5756132	-5.159707	4.04E-07	2.22E-06	5.8366861
SUSD2	0.3710376	5.3298272	5.1596158	4.04E-07	2.23E-06	5.836251
PRPS1	-0.097534	6.3250202	-5.15919	4.05E-07	2.23E-06	5.8342191
DNAJC7	-0.067637	6.4554917	-5.15918	4.05E-07	2.23E-06	5.8341697
FAM45A	-0.125507	5.9844443	-5.158919	4.06E-07	2.23E-06	5.8329202
CTD-2207P1	0.9541655	-0.050105	5.1576637	4.08E-07	2.25E-06	5.8269261
LMF1	-0.182067	5.9941037	-5.156902	4.10E-07	2.25E-06	5.8232886
OCIAD1	-0.073084	6.7169235	-5.155869	4.12E-07	2.27E-06	5.8183561
ENOX1-AS2	-0.365665	-1.42103	-5.155687	4.12E-07	2.27E-06	5.8174862
SLC46A3	-0.329606	6.0429805	-5.154919	4.14E-07	2.28E-06	5.8138219
TRAPPC3L	0.8597993	0.149939	5.1539798	4.16E-07	2.29E-06	5.8093361
MMRN1	0.4523412	4.6366768	5.153412	4.17E-07	2.29E-06	5.806626
REEP6	-0.235004	6.8671545	-5.153143	4.18E-07	2.29E-06	5.8053403
APLP1	0.6292248	4.1775657	5.1530432	4.18E-07	2.30E-06	5.8048659
AKT1S1	-0.090033	6.5197481	-5.151871	4.20E-07	2.31E-06	5.7992735
HPN	-0.210229	7.0759728	-5.151845	4.20E-07	2.31E-06	5.7991474
CUZD1	0.7692168	2.7817266	5.1511484	4.22E-07	2.32E-06	5.7958245
GSDMD	-0.112808	6.7988491	-5.150306	4.23E-07	2.33E-06	5.7918041
C8G	-0.24875	6.8882904	-5.149361	4.25E-07	2.34E-06	5.7872976
DYNC1I2	0.0936843	6.2192423	5.1491226	4.26E-07	2.34E-06	5.7861608
PCDHB15	0.4965481	4.0171688	5.1484698	4.27E-07	2.35E-06	5.7830474
IGHV3-52	0.7556267	-0.849337	5.1479538	4.28E-07	2.35E-06	5.780587
CALR4P	-0.918748	0.2680069	-5.147907	4.29E-07	2.35E-06	5.780362
TMEM254	-0.107267	6.239418	-5.147182	4.30E-07	2.36E-06	5.7769056
RPL13	-0.121461	7.2406583	-5.146778	4.31E-07	2.36E-06	5.774982
GSG2	0.567133	3.7470585	5.146554	4.31E-07	2.37E-06	5.7739127
PRPF38B	0.071701	6.1643179	5.1463239	4.32E-07	2.37E-06	5.7728158
TAF12	-0.097819	5.94694	-5.145717	4.33E-07	2.38E-06	5.7699249
ENPP1	-0.15844	6.4410787	-5.145447	4.34E-07	2.38E-06	5.7686371
RP5-1171I1	0.6982285	3.6312377	5.1448543	4.35E-07	2.39E-06	5.7658109
GRTP1	-0.165516	6.1870612	-5.144794	4.35E-07	2.39E-06	5.7655242
CTB-31020.	0.7720531	3.5399166	5.1447078	4.35E-07	2.39E-06	5.7651129
ACP1	-0.063116	6.5764217	-5.144507	4.36E-07	2.39E-06	5.7641581
CEP63	-0.0597	5.9819671	-5.144312	4.36E-07	2.39E-06	5.7632272
RP4-753P9.	0.8371943	0.5334538	5.1440458	4.37E-07	2.39E-06	5.761958
RP11-649A1	0.7740337	-0.296087	5.1432406	4.39E-07	2.40E-06	5.7581209
CDC20B	1.2017139	1.0442103	5.1431101	4.39E-07	2.40E-06	5.7574993
IGHV3OR16-	0.8017592	-0.723137	5.142636	4.40E-07	2.41E-06	5.7552403
RP11-812E1	0.7746211	-0.840152	5.1425286	4.40E-07	2.41E-06	5.7547286
ASLP1	0.850536	1.8887452	5.1424669	4.40E-07	2.41E-06	5.7544348
RP5-963E22	-0.612155	2.7237173	-5.142395	4.40E-07	2.41E-06	5.7540905
PKD1P5	0.8508982	3.0516233	5.1413194	4.43E-07	2.42E-06	5.7489686
THYN1	-0.10049	6.1226872	-5.139805	4.46E-07	2.44E-06	5.7417557
MMAA	-0.140808	5.9320891	-5.139126	4.48E-07	2.45E-06	5.7385252
PRPF31	-0.093922	6.4315805	-5.138917	4.48E-07	2.45E-06	5.7375261
FSD1L	0.2739651	4.7339635	5.1383961	4.49E-07	2.46E-06	5.7350479
BCORL1	0.1563167	5.7759922	5.1381865	4.50E-07	2.46E-06	5.7340503
RP11-712B9	0.6208688	-0.998511	5.1376445	4.51E-07	2.47E-06	5.7314701
SDHC	-0.083407	6.7696417	-5.136899	4.53E-07	2.47E-06	5.7279221
PLD5	1.0505058	0.9550238	5.1364077	4.54E-07	2.48E-06	5.7255832
LA16c-325D	0.6540058	-1.082349	5.136279	4.54E-07	2.48E-06	5.7249707

BTC	0.8857864	3.7597789	5.1361745	4.54E-07	2.48E-06	5.7244732
RP11-10N23	0.6605938	2.9372777	5.1361167	4.54E-07	2.48E-06	5.7241982
FUT8-AS1	0.8801074	0.3468047	5.1356906	4.55E-07	2.49E-06	5.7221706
RP11-12A20	0.9903032	0.241846	5.1354784	4.56E-07	2.49E-06	5.7211608
RP11-813N2	-0.892301	4.3103563	-5.135143	4.57E-07	2.49E-06	5.7195662
CTB-5506.1	-0.830965	-0.235152	-5.135042	4.57E-07	2.49E-06	5.7190864
TMEM110-MU	-0.291675	4.1082008	-5.134815	4.57E-07	2.50E-06	5.7180048
DACT3-AS1	0.7946345	0.0916763	5.1343714	4.58E-07	2.50E-06	5.7158937
MRTO4	-0.095652	6.2295306	-5.132894	4.62E-07	2.52E-06	5.7088641
BX470102.3	0.9169746	-0.123722	5.1325584	4.62E-07	2.52E-06	5.7072694
P2RX5-TAX1	0.398016	3.1992774	5.1324243	4.63E-07	2.52E-06	5.7066317
RP11-544M2	0.821642	2.5927709	5.1324093	4.63E-07	2.52E-06	5.7065602
TLE3	0.1748301	6.0963111	5.1322184	4.63E-07	2.53E-06	5.7056522
RP11-505K9	-0.588896	3.4241582	-5.131946	4.64E-07	2.53E-06	5.7043576
ITGA2	0.3645946	5.3221615	5.1318383	4.64E-07	2.53E-06	5.7038449
AADAC	-0.394879	6.7639969	-5.131744	4.64E-07	2.53E-06	5.7033985
ZNF28	0.5016521	5.1024807	5.1316649	4.65E-07	2.53E-06	5.7030204
VPS51	-0.080926	6.5215436	-5.131222	4.66E-07	2.54E-06	5.7009155
PRKAG1	-0.055902	6.343796	-5.130949	4.66E-07	2.54E-06	5.699614
PTP4A3	0.2387892	5.9517432	5.1307119	4.67E-07	2.54E-06	5.6984886
SYS1-DBNDD	0.7787504	1.390148	5.1299122	4.69E-07	2.55E-06	5.6946865
PEG3	0.7665994	4.5252987	5.1295867	4.69E-07	2.56E-06	5.6931392
CASC8	1.0834878	0.283856	5.1293613	4.70E-07	2.56E-06	5.6920677
RPS6KA6	1.2892195	1.0276611	5.1290778	4.71E-07	2.56E-06	5.6907203
ADAMTS5	0.2862933	5.0850439	5.1283449	4.72E-07	2.57E-06	5.6872369
CASP16	-0.938419	3.9588073	-5.128061	4.73E-07	2.57E-06	5.6858888
NEURL3	0.6968857	4.6838809	5.1277021	4.74E-07	2.58E-06	5.6841818
NUDT10	1.0238205	0.1876609	5.1276555	4.74E-07	2.58E-06	5.6839605
TRGV2	0.8721709	-0.154672	5.1275834	4.74E-07	2.58E-06	5.6836176
GPR114	0.5857556	4.7579427	5.1275509	4.74E-07	2.58E-06	5.6834634
RP11-373D7	-0.67418	-1.052478	-5.127435	4.74E-07	2.58E-06	5.6829111
IDH3B	-0.081171	6.5482579	-5.126781	4.76E-07	2.59E-06	5.6798048
RBMS1P1	0.9135332	0.7322673	5.1267427	4.76E-07	2.59E-06	5.6796231
RP11-538D1	-1.193523	2.66415	-5.126729	4.76E-07	2.59E-06	5.6795568
CASQ1	0.8872506	1.6615702	5.1259264	4.78E-07	2.60E-06	5.6757447
EIF5A2	0.4779862	5.0653039	5.1253279	4.79E-07	2.61E-06	5.6729014
SELL	0.290942	5.0934701	5.124717	4.81E-07	2.61E-06	5.6699995
PMPCB	-0.084209	6.5201985	-5.124523	4.81E-07	2.62E-06	5.6690772
KRT6C	1.2899399	0.2039257	5.1240015	4.83E-07	2.62E-06	5.6666011
NRSN2-AS1	0.3261125	3.9795115	5.1238416	4.83E-07	2.62E-06	5.6658419
C5orf30	0.4513924	4.7046012	5.1237996	4.83E-07	2.62E-06	5.6656426
ATP5L	-0.089386	6.7569766	-5.123711	4.83E-07	2.62E-06	5.6652237
PARP3	-0.11595	5.9613819	-5.123631	4.83E-07	2.62E-06	5.6648427
RP11-480I1	-0.390709	5.1570478	-5.123112	4.85E-07	2.63E-06	5.6623752
AC097662.2	0.4897632	3.0914414	5.123109	4.85E-07	2.63E-06	5.6623627
PMPCA	-0.106129	6.5695028	-5.122913	4.85E-07	2.63E-06	5.6614343
CTC-1337H2	-0.785401	-0.102466	-5.122818	4.85E-07	2.63E-06	5.6609797
MNT	0.088823	5.7740266	5.1224292	4.86E-07	2.64E-06	5.6591351
RASSF8	0.2348853	5.4358376	5.1219476	4.87E-07	2.64E-06	5.6568483
RLN2	0.7271771	-0.714384	5.1219156	4.88E-07	2.64E-06	5.6566967
RPS27	-0.106753	7.151629	-5.121909	4.88E-07	2.64E-06	5.6566629

BLACAT1	0.8846043	-0.552836	5.1215667	4.88E-07	2.65E-06	5.6550403
SYTL4	-0.233652	5.8579383	-5.121123	4.89E-07	2.65E-06	5.6529354
KIF9	-0.144233	5.1842296	-5.121113	4.89E-07	2.65E-06	5.6528869
RECQL	0.2158015	5.6030223	5.1210551	4.90E-07	2.65E-06	5.6526115
RP11-977G1	-0.289494	3.9480471	-5.120553	4.91E-07	2.66E-06	5.650228
SEMA5B	0.2526792	5.1557037	5.1204908	4.91E-07	2.66E-06	5.6499329
GCKR	-0.378626	6.2014296	-5.120004	4.92E-07	2.67E-06	5.6476246
PCK1	-0.461976	6.8360794	-5.119171	4.94E-07	2.68E-06	5.6436717
PCAT14	1.0138473	-0.276854	5.119132	4.94E-07	2.68E-06	5.6434843
AADACP1	-0.565694	4.7090826	-5.118741	4.95E-07	2.68E-06	5.6416302
RGS12	-0.104715	6.2623023	-5.11845	4.96E-07	2.69E-06	5.6402499
RP11-450I1	-0.308697	-1.434897	-5.118374	4.96E-07	2.69E-06	5.639887
TEDDM1	-0.93233	-0.445226	-5.117667	4.98E-07	2.70E-06	5.6365342
CD244	0.5156724	3.8540135	5.1176262	4.98E-07	2.70E-06	5.6363395
ZNF205-AS1	-0.342618	3.6650909	-5.117227	4.99E-07	2.70E-06	5.6344443
MSANTD2	0.1393731	5.1809728	5.1172001	4.99E-07	2.70E-06	5.6343185
PLCE1	0.1497329	5.533744	5.1164315	5.01E-07	2.71E-06	5.6306726
SF3B2	-0.052844	6.827768	-5.11618	5.02E-07	2.71E-06	5.6294809
LMTK3	0.9550874	3.5272149	5.1161745	5.02E-07	2.71E-06	5.6294535
RP11-186N1	0.5390452	3.2740768	5.1157869	5.03E-07	2.72E-06	5.6276152
STIM1	-0.082815	6.6200318	-5.115784	5.03E-07	2.72E-06	5.6276012
RPL32	-0.110403	7.2092335	-5.115768	5.03E-07	2.72E-06	5.627524
BTN1A1	0.7832606	-0.61672	5.1152697	5.04E-07	2.72E-06	5.6251624
RP5-881L22	-0.968794	2.5068199	-5.114727	5.05E-07	2.73E-06	5.6225905
CTD-2576D5	0.9356487	0.0226804	5.1145733	5.06E-07	2.73E-06	5.6218603
EPM2AIP1	-0.104312	6.0662023	-5.11445	5.06E-07	2.73E-06	5.6212754
SEMA3E	1.4142288	1.0776446	5.1143545	5.06E-07	2.73E-06	5.6208223
RP5-1050D4	0.8913106	0.8386293	5.1142859	5.06E-07	2.73E-06	5.6204974
LINC00310	0.8064039	1.7920531	5.1142178	5.06E-07	2.73E-06	5.6201745
RP3-500L14	-0.999084	1.3230618	-5.112743	5.10E-07	2.75E-06	5.6131849
IGLL1	0.7667833	-0.723151	5.1127376	5.10E-07	2.75E-06	5.6131571
DIO1	-0.434027	6.3316222	-5.111844	5.12E-07	2.77E-06	5.6089224
RP11-38408	0.9564587	-0.325956	5.1115246	5.13E-07	2.77E-06	5.6074077
RP11-696D2	0.7241472	-0.543191	5.1108986	5.15E-07	2.78E-06	5.6044411
ARRB1	0.2033289	5.7499448	5.1108499	5.15E-07	2.78E-06	5.6042104
TBCAP1	-0.213277	4.5484474	-5.110674	5.15E-07	2.78E-06	5.6033782
MMP10	1.2412038	1.6097254	5.1105307	5.16E-07	2.78E-06	5.602698
DDX19B	-0.109416	5.817595	-5.110475	5.16E-07	2.78E-06	5.6024344
ZBTB2	0.0902679	5.7194769	5.1102019	5.17E-07	2.79E-06	5.6011401
LA16c-313D	0.6131385	2.6309206	5.1096583	5.18E-07	2.79E-06	5.5985642
CYP1D1P	-0.572845	-1.127675	-5.109247	5.19E-07	2.80E-06	5.5966136
EIF5B	-0.078827	6.6274099	-5.108843	5.20E-07	2.80E-06	5.5947009
NAT8L	0.9116135	2.8331361	5.1084773	5.21E-07	2.81E-06	5.5929697
S1PR2	0.2441542	5.7484963	5.107894	5.23E-07	2.82E-06	5.590207
PPP3R1	-0.062987	6.3546643	-5.107836	5.23E-07	2.82E-06	5.5899321
PDE4D	0.3046614	5.0935414	5.1070469	5.25E-07	2.83E-06	5.586195
GHRHR	1.3519579	2.280493	5.1064686	5.26E-07	2.83E-06	5.5834567
RUNX1T1	0.4536059	4.2050854	5.1063253	5.27E-07	2.84E-06	5.5827781
KCNC4	0.2171862	5.0778017	5.106293	5.27E-07	2.84E-06	5.5826249
COL4A1	0.128968	6.8335052	5.1057325	5.28E-07	2.84E-06	5.5799714
CLTCL1	-0.196639	5.7568751	-5.105588	5.29E-07	2.84E-06	5.5792893

FOXN3-AS1	-0.273852	4.767473	-5.105447	5.29E-07	2.85E-06	5.5786194
GNL1	-0.083413	6.4526244	-5.104714	5.31E-07	2.86E-06	5.5751491
JPH2	0.6745956	3.4910175	5.1045688	5.31E-07	2.86E-06	5.5744621
CHN1	0.2613298	4.8843691	5.1039413	5.33E-07	2.87E-06	5.5714917
FSTL1	0.1714299	6.3275548	5.1035927	5.34E-07	2.87E-06	5.5698418
LDB3	0.4872717	3.5844981	5.1035854	5.34E-07	2.87E-06	5.5698076
CRTC2	-0.072614	6.4172371	-5.103528	5.34E-07	2.87E-06	5.5695373
RP11-756G2	0.819025	0.0247805	5.1031208	5.35E-07	2.88E-06	5.5676087
CTD-3220F1	0.9466666	0.3361642	5.1029078	5.36E-07	2.88E-06	5.5666008
ZNRF2	-0.107842	6.1351795	-5.102658	5.36E-07	2.88E-06	5.5654204
NAA16	0.1205074	5.4919959	5.1025592	5.37E-07	2.88E-06	5.5649508
RP3-434P1.	-1.202266	3.1680723	-5.102334	5.37E-07	2.89E-06	5.5638832
RP11-894P9	0.5906769	3.4444811	5.1022017	5.37E-07	2.89E-06	5.5632596
RASSF9	0.7927237	3.8561874	5.1020747	5.38E-07	2.89E-06	5.5626587
DBH-AS1	-0.289339	5.7305713	-5.101513	5.39E-07	2.90E-06	5.5600004
RP4-785G19	0.7716298	2.3859899	5.1014616	5.39E-07	2.90E-06	5.5597574
POM121B	0.9451101	1.9304693	5.1009812	5.41E-07	2.90E-06	5.557485
SSX2IP	-0.20179	5.9521898	-5.10056	5.42E-07	2.91E-06	5.555493
AL133247.2	-0.486064	-1.18223	-5.100439	5.42E-07	2.91E-06	5.5549202
ELOVL6	-0.20944	6.276052	-5.100164	5.43E-07	2.91E-06	5.5536189
HAGLR	0.6686374	4.3403766	5.0995192	5.45E-07	2.92E-06	5.5505695
FAM65C	0.5122616	4.2870384	5.0991836	5.46E-07	2.93E-06	5.5489823
PRND	1.1630112	2.5901961	5.0990005	5.46E-07	2.93E-06	5.5481165
AC004067.5	0.908659	1.2531259	5.098515	5.47E-07	2.94E-06	5.5458206
RP11-483G2	0.7642983	-0.749607	5.0983726	5.48E-07	2.94E-06	5.5451468
AICDA	0.716501	-0.88617	5.097981	5.49E-07	2.94E-06	5.5432954
CHMP1A	-0.090285	6.5044705	-5.09792	5.49E-07	2.94E-06	5.5430078
ZSCAN20	0.3290658	4.1146387	5.0972851	5.51E-07	2.95E-06	5.5400051
NFASC	0.2896219	5.4582864	5.0972395	5.51E-07	2.95E-06	5.5397896
CDC14B	-0.202632	6.1218563	-5.097042	5.51E-07	2.95E-06	5.5388572
LA16c-390E	0.8913841	2.0407427	5.0961099	5.54E-07	2.97E-06	5.5344495
ADAMTS9-AS	0.8923597	1.7731593	5.0955063	5.56E-07	2.98E-06	5.5315965
BRMS1	-0.101471	6.3569333	-5.095466	5.56E-07	2.98E-06	5.5314064
SCAMP5	0.2529005	5.7682833	5.0951945	5.56E-07	2.98E-06	5.5301232
DBR1	0.0976613	5.6604287	5.0950138	5.57E-07	2.98E-06	5.5292687
RN7SK	-0.340985	4.967336	-5.094573	5.58E-07	2.99E-06	5.5271848
LY6G5B	0.6190215	4.072645	5.0943991	5.59E-07	2.99E-06	5.5263641
THSD7A	0.3354809	5.0789223	5.0936643	5.61E-07	3.00E-06	5.522892
RARB	0.3265517	4.7801363	5.0932741	5.62E-07	3.01E-06	5.5210485
RP11-284H1	-1.115677	0.3766292	-5.092975	5.62E-07	3.01E-06	5.519634
GBA	-0.107889	6.5606613	-5.092846	5.63E-07	3.01E-06	5.5190241
COBLL1	-0.162291	6.4634482	-5.092728	5.63E-07	3.01E-06	5.5184667
MFSD11	-0.092823	5.9753187	-5.092708	5.63E-07	3.01E-06	5.5183746
HTATIP2	-0.134732	6.6137965	-5.092459	5.64E-07	3.02E-06	5.5171998
AC000032.2	-0.962265	-0.436934	-5.091938	5.65E-07	3.02E-06	5.5147388
EEF1B2	-0.101509	6.9247673	-5.091491	5.67E-07	3.03E-06	5.5126256
EFR3B	0.3755764	3.8140326	5.0909971	5.68E-07	3.04E-06	5.510293
ITGB7	0.5432723	4.4749696	5.0900277	5.71E-07	3.05E-06	5.505715
TIMM10B	-0.068911	6.1188098	-5.08989	5.71E-07	3.05E-06	5.5050647
NFYC	-0.066444	6.3665674	-5.089808	5.71E-07	3.05E-06	5.5046784
CENPV	-0.179729	6.1357046	-5.089068	5.73E-07	3.06E-06	5.5011837

CEBPE	0.8173456	-0.158297	5.0886145	5.75E-07	3.07E-06	5.4990427
CTD-2547L2	0.8107105	-0.241046	5.0878887	5.77E-07	3.08E-06	5.4956163
RP11-701H2	1.0037954	0.2417651	5.0865943	5.80E-07	3.10E-06	5.4895075
RP11-321G1	0.8899273	-0.435631	5.0865784	5.81E-07	3.10E-06	5.4894321
SLC6A8	0.3645908	5.6961475	5.0861707	5.82E-07	3.11E-06	5.4875085
LINC01003	-0.185914	5.1042931	-5.085528	5.84E-07	3.11E-06	5.484476
CPN2	-0.50537	6.6980689	-5.085477	5.84E-07	3.11E-06	5.4842332
RP4-673D20	0.7594861	-0.520637	5.0854613	5.84E-07	3.11E-06	5.4841612
DSC3	1.1466745	0.5490931	5.0852082	5.84E-07	3.12E-06	5.4829669
ZC3H10	-0.079933	5.6400249	-5.08427	5.87E-07	3.13E-06	5.4785394
IMPAD1	-0.105557	6.6817329	-5.084192	5.87E-07	3.13E-06	5.4781743
CPB2	-0.326652	7.0478274	-5.084147	5.87E-07	3.13E-06	5.477959
TNFRSF25	0.1988642	5.3572171	5.0840183	5.88E-07	3.13E-06	5.4773534
TMEM106A	0.2689262	5.0899198	5.0839554	5.88E-07	3.14E-06	5.4770569
IMMP1L	-0.094975	5.6142184	-5.083141	5.90E-07	3.15E-06	5.4732151
SLC5A10	0.793058	3.1712054	5.0829619	5.91E-07	3.15E-06	5.4723706
CEND1	0.8372292	2.1638332	5.0829136	5.91E-07	3.15E-06	5.4721431
ANGPT1	0.5111249	4.737515	5.0828722	5.91E-07	3.15E-06	5.4719476
PCDHGC4	0.9043364	-0.26558	5.0823057	5.93E-07	3.16E-06	5.4692761
ZBTB47	0.1671021	5.542623	5.0819546	5.94E-07	3.16E-06	5.4676203
RP11-517A5	-0.376387	-1.338046	-5.080801	5.97E-07	3.18E-06	5.4621805
NEK10	0.7332544	3.7703397	5.0795256	6.01E-07	3.20E-06	5.4561691
GDPD2	0.8283454	0.1602984	5.0794965	6.01E-07	3.20E-06	5.4560319
TRRAP	0.0813238	6.3173268	5.0793611	6.01E-07	3.20E-06	5.4553936
MPV17L2	-0.117017	5.9533461	-5.078637	6.04E-07	3.21E-06	5.4519815
GNGT2	0.3596327	4.0334261	5.0784779	6.04E-07	3.22E-06	5.4512311
ACTG1P14	0.7353854	1.7644069	5.0783651	6.04E-07	3.22E-06	5.4506995
LINC01503	0.4413406	4.3987775	5.0778759	6.06E-07	3.22E-06	5.4483944
DHRS4-AS1	-0.159673	5.9923857	-5.077673	6.07E-07	3.23E-06	5.4474373
CTC-490E21	-1.029517	-0.203692	-5.076706	6.09E-07	3.24E-06	5.4428818
ASCL1	-1.393552	1.5865742	-5.076667	6.10E-07	3.24E-06	5.4426971
RHOT2	-0.072067	6.4718727	-5.076364	6.10E-07	3.25E-06	5.4412701
DCAF16	0.1219005	5.7880906	5.0753972	6.13E-07	3.26E-06	5.4367166
KNG1	-0.362037	7.4298497	-5.075373	6.13E-07	3.26E-06	5.4366036
THAP11	-0.079791	5.9769527	-5.074955	6.15E-07	3.27E-06	5.4346353
MRPS30	-0.075396	6.2584759	-5.074889	6.15E-07	3.27E-06	5.434325
RP1-167F1.	0.6548388	-0.923753	5.0747862	6.15E-07	3.27E-06	5.4338392
AL031587.1	0.7208059	1.8492121	5.0743545	6.16E-07	3.28E-06	5.4318059
LRPAP1	-0.083254	6.7836127	-5.073924	6.18E-07	3.28E-06	5.4297801
CCND3P1	-0.656509	-0.841183	-5.073924	6.18E-07	3.28E-06	5.4297786
NDUFA13	-0.142543	6.2929727	-5.073863	6.18E-07	3.28E-06	5.4294937
OGFR	-0.092135	6.5604781	-5.073384	6.19E-07	3.29E-06	5.4272372
ATP11C	-0.194988	6.0219876	-5.073023	6.21E-07	3.29E-06	5.4255349
NLRP7	0.9152916	-0.231686	5.0727885	6.21E-07	3.30E-06	5.4244321
COQ6	-0.099875	5.8468632	-5.072238	6.23E-07	3.31E-06	5.4218408
CIAO1	-0.052866	6.5043895	-5.072179	6.23E-07	3.31E-06	5.4215633
AL158801.1	0.4517238	3.0371855	5.072044	6.24E-07	3.31E-06	5.4209275
ZNF90	0.6525056	3.0637228	5.0713403	6.26E-07	3.32E-06	5.417615
ADH4	-0.614502	6.6120103	-5.070931	6.27E-07	3.33E-06	5.4156867
VANGL1	0.1072562	5.7250295	5.0708323	6.27E-07	3.33E-06	5.4152238
RP11-168F9	0.9731785	0.2208122	5.0708205	6.27E-07	3.33E-06	5.4151684

NFAT5	0.2629981	5.577519	5.070432	6.28E-07	3.33E-06	5.4133399
FSTL3	0.2791785	5.8801496	5.0678387	6.37E-07	3.37E-06	5.401138
POLL	-0.085745	6.0768461	-5.06708	6.39E-07	3.39E-06	5.3975691
VPS28	-0.123323	6.8062029	-5.066763	6.40E-07	3.39E-06	5.3960791
AC108938.5	0.8860428	1.8265898	5.0664205	6.41E-07	3.40E-06	5.3944675
RPL23	-0.099814	7.1608559	-5.066162	6.42E-07	3.40E-06	5.3932537
HMGXB4	0.1087208	5.8244599	5.0659612	6.42E-07	3.40E-06	5.392308
ZNF641	0.0777543	5.7579608	5.0656944	6.43E-07	3.41E-06	5.3910532
HKDC1	0.5474865	5.8883506	5.0656634	6.43E-07	3.41E-06	5.3909072
CYHR1	-0.107534	6.5125895	-5.065554	6.44E-07	3.41E-06	5.3903952
IGKV2OR22-	0.7912791	-0.746244	5.0654334	6.44E-07	3.41E-06	5.3898257
JADE1	-0.101045	6.1331321	-5.065135	6.45E-07	3.41E-06	5.388422
RP11-148L2	0.7999875	-0.493922	5.0645572	6.47E-07	3.42E-06	5.3857064
RP11-283G6	0.9599434	-0.1138	5.0635382	6.50E-07	3.44E-06	5.3809158
REC8	0.2214698	5.0367655	5.0634059	6.51E-07	3.44E-06	5.3802938
RP13-516M1	0.8420117	2.0675291	5.0631899	6.51E-07	3.45E-06	5.3792785
PTPRR	0.9274987	2.1497751	5.0627616	6.53E-07	3.45E-06	5.3772658
MRPS26	-0.100103	6.4408478	-5.061556	6.56E-07	3.47E-06	5.3716022
C11orf84	0.1626985	5.5710002	5.0614812	6.57E-07	3.47E-06	5.3712484
CTC-441N14	-0.761427	-0.899366	-5.061031	6.58E-07	3.48E-06	5.3691324
NYAP1	0.5651008	3.2821829	5.0608291	6.59E-07	3.48E-06	5.3681844
C7orf55-LU	-0.05639	6.153791	-5.060778	6.59E-07	3.48E-06	5.3679444
ADAMTS7P4	0.8461139	1.4443696	5.0604942	6.60E-07	3.49E-06	5.3666109
ZFHX2	0.424653	4.0892487	5.0602142	6.61E-07	3.49E-06	5.3652954
RPS10	-0.120214	6.7245276	-5.059941	6.62E-07	3.50E-06	5.3640102
ZC3H12A	0.1876517	5.7610219	5.059634	6.63E-07	3.50E-06	5.36257
LIAS	-0.109224	5.7727032	-5.059424	6.63E-07	3.50E-06	5.3615834
LINC00240	-0.343153	4.4711798	-5.058956	6.65E-07	3.51E-06	5.3593838
RP11-46H11	0.8191443	0.7523632	5.0589046	6.65E-07	3.51E-06	5.3591439
R3HCC1	-0.098727	6.1242515	-5.058463	6.67E-07	3.52E-06	5.3570715
NPIPB4	0.8248741	3.4135916	5.0582035	6.67E-07	3.52E-06	5.3558512
LILRA6	0.3996373	3.9710559	5.0579186	6.68E-07	3.53E-06	5.354513
ABCC2	-0.283418	6.6487371	-5.056586	6.73E-07	3.55E-06	5.3482539
RP11-231E4	-0.352906	-1.404888	-5.05657	6.73E-07	3.55E-06	5.3481804
MGC32805	-0.886046	4.2785774	-5.056439	6.73E-07	3.55E-06	5.3475639
TEC	-0.291943	4.7512109	-5.056408	6.73E-07	3.55E-06	5.347421
DLGAP1-AS3	0.8728249	-0.681619	5.0562204	6.74E-07	3.55E-06	5.3465397
NLGN2	0.2645386	5.3717822	5.055659	6.76E-07	3.56E-06	5.3439039
PITPNM2	-0.151145	6.11892	-5.054318	6.80E-07	3.59E-06	5.3376109
RP11-283I3	-1.199463	2.2259965	-5.054048	6.81E-07	3.59E-06	5.3363444
TFR2	-0.253506	7.1555352	-5.053509	6.83E-07	3.60E-06	5.3338148
NPTX2	1.1466381	3.4931075	5.0534707	6.83E-07	3.60E-06	5.3336336
SFRP2	1.3384221	1.1071283	5.0534299	6.83E-07	3.60E-06	5.333442
IL11	1.0053047	1.2138074	5.0534181	6.83E-07	3.60E-06	5.333387
EPHA1-AS1	1.0563783	1.5809442	5.0531341	6.84E-07	3.60E-06	5.3320541
RHBDF1	0.1577742	5.7436076	5.0529817	6.85E-07	3.61E-06	5.3313391
OVOL1	1.2676536	1.7380441	5.052567	6.86E-07	3.61E-06	5.3293935
CMTR1	-0.071533	6.3890327	-5.052295	6.87E-07	3.62E-06	5.3281184
CROCCP3	0.2997084	4.2971916	5.0519741	6.88E-07	3.62E-06	5.3266118
TNFRSF9	0.7283367	3.826629	5.0515596	6.89E-07	3.63E-06	5.3246675
NDUFB9	-0.125913	6.9191672	-5.05125	6.91E-07	3.63E-06	5.3232147

PRR15L	0.8504496	4.3918022	5.050827	6.92E-07	3.64E-06	5.3212312
ADH1C	-0.52077	6.6407206	-5.050744	6.92E-07	3.64E-06	5.3208428
TWSG1	0.1905698	5.6389547	5.0505626	6.93E-07	3.65E-06	5.3199911
TRBV14	0.8225583	-0.219546	5.050386	6.93E-07	3.65E-06	5.3191627
SLC30A1	-0.141484	6.6061559	-5.04962	6.96E-07	3.66E-06	5.3155695
BCL7C	-0.118543	6.1851975	-5.049321	6.97E-07	3.67E-06	5.3141705
MAML1	0.0838379	6.0654345	5.0492463	6.97E-07	3.67E-06	5.3138178
NLRP12	0.8373652	1.4818542	5.0490659	6.98E-07	3.67E-06	5.3129719
ECHDC3	-0.353727	6.3745902	-5.047923	7.02E-07	3.69E-06	5.3076115
AC007792.1	-0.767716	-0.508838	-5.047703	7.03E-07	3.69E-06	5.3065846
ATN1	0.0727683	6.468346	5.0476562	7.03E-07	3.69E-06	5.3063628
FAM207A	-0.126884	5.8061863	-5.047619	7.03E-07	3.69E-06	5.3061892
CCND2P1	-1.0935	4.4715219	-5.047116	7.05E-07	3.70E-06	5.303833
MRPL35	-0.07097	6.269203	-5.046194	7.08E-07	3.72E-06	5.2995079
CRBN	-0.073299	6.1436607	-5.045925	7.09E-07	3.72E-06	5.2982485
ITM2A	0.3264174	5.2773837	5.0450775	7.12E-07	3.74E-06	5.2942773
CYP7B1	-0.394499	5.3420826	-5.044251	7.15E-07	3.75E-06	5.2904039
AC062017.1	-0.390537	3.6924548	-5.04405	7.15E-07	3.76E-06	5.2894655
PGP	-0.133431	6.0460702	-5.043996	7.15E-07	3.76E-06	5.2892102
DOK2	0.235335	5.3313417	5.0437758	7.16E-07	3.76E-06	5.2881789
ESD	-0.100801	6.612776	-5.043775	7.16E-07	3.76E-06	5.288175
AC034243.1	-1.000226	2.3102941	-5.043566	7.17E-07	3.76E-06	5.287195
RP1-149A16	0.8282295	0.0853885	5.0434066	7.18E-07	3.76E-06	5.286449
SLC35D1	-0.129336	6.4811917	-5.04312	7.19E-07	3.77E-06	5.2851048
CYP7A1	-0.96979	5.5672251	-5.042664	7.20E-07	3.78E-06	5.28297
UPK1B	0.9918841	-0.230209	5.0426614	7.20E-07	3.78E-06	5.2829587
PSMA1	-0.069841	6.4528899	-5.042038	7.22E-07	3.79E-06	5.2800399
EIF2S3L	0.7810941	2.1451912	5.0419779	7.23E-07	3.79E-06	5.2797573
FAM3C2	0.646821	3.874528	5.0418899	7.23E-07	3.79E-06	5.2793455
RITA1	-0.096873	6.2183108	-5.041673	7.24E-07	3.79E-06	5.2783289
PTPN18	-0.069622	6.388552	-5.041386	7.25E-07	3.80E-06	5.2769875
CTD-2331H1	1.1656048	2.2358389	5.0396044	7.31E-07	3.83E-06	5.2686443
AC108448.2	0.6471215	-0.823198	5.0388782	7.34E-07	3.84E-06	5.2652451
PPP1R11	-0.074784	6.5232385	-5.038046	7.37E-07	3.86E-06	5.2613477
FZD4	-0.139781	6.2085787	-5.037784	7.38E-07	3.86E-06	5.2601217
LRSAM1	-0.111778	6.0848347	-5.037397	7.39E-07	3.87E-06	5.2583132
STXBP4	0.1592671	5.4793859	5.0370372	7.40E-07	3.88E-06	5.2566293
AP1S3	0.3069475	4.4455215	5.0364032	7.43E-07	3.89E-06	5.2536627
RP11-45M22	0.7701689	3.0468111	5.0363765	7.43E-07	3.89E-06	5.2535379
SBDS	-0.078871	6.5359531	-5.036371	7.43E-07	3.89E-06	5.2535135
STXBP6	-0.239867	5.6913722	-5.036261	7.43E-07	3.89E-06	5.2529964
UBXN10	-0.601519	5.1878275	-5.036144	7.43E-07	3.89E-06	5.2524517
UBL7	-0.084132	6.272913	-5.036046	7.44E-07	3.89E-06	5.2519895
RP1-152L7.	-0.421597	-1.300567	-5.035401	7.46E-07	3.90E-06	5.2489751
AC007278.3	0.8327008	-0.255898	5.0342876	7.50E-07	3.92E-06	5.2437664
RPL36	-0.121264	7.0075706	-5.034158	7.51E-07	3.93E-06	5.2431605
RNF217	-0.197076	5.8533318	-5.033744	7.52E-07	3.93E-06	5.2412264
ZFYVE19	-0.101762	6.2166948	-5.033677	7.52E-07	3.93E-06	5.2409118
PRKAR1A	-0.080467	6.8465503	-5.032849	7.56E-07	3.95E-06	5.2370379
PLAC1	0.8813419	-0.428757	5.032576	7.57E-07	3.95E-06	5.2357625
KIAA0226	0.0716237	5.8210199	5.0323963	7.57E-07	3.96E-06	5.2349224

COL17A1	0.9013968	2.4618268	5.0314307	7.61E-07	3.97E-06	5.2304082
UQCRHL	-0.186705	4.8014486	-5.030816	7.63E-07	3.99E-06	5.2275354
RPS26	-0.118432	6.7182624	-5.030815	7.63E-07	3.99E-06	5.227529
RPS13	-0.092348	6.9805467	-5.030732	7.63E-07	3.99E-06	5.2271411
PCDHGA10	0.6038091	4.2783826	5.0307248	7.63E-07	3.99E-06	5.2271086
KLRC2	0.8609593	-0.242624	5.0307105	7.63E-07	3.99E-06	5.2270414
FNDC3B	0.0912796	6.5354955	5.0283067	7.72E-07	4.03E-06	5.2158084
DEPDC1B	0.4946585	4.9579103	5.0282432	7.73E-07	4.03E-06	5.2155116
HMCES	-0.076723	6.4398474	-5.027272	7.76E-07	4.05E-06	5.2109754
VGLL1	0.8273878	-0.788151	5.026553	7.79E-07	4.07E-06	5.2076164
FCGR2A	0.2059581	5.6591517	5.0263619	7.80E-07	4.07E-06	5.2067238
AC006042.6	0.84668	2.4451851	5.0263543	7.80E-07	4.07E-06	5.2066882
PPIE	-0.087173	6.1885914	-5.026303	7.80E-07	4.07E-06	5.206448
GABRR2	0.9370392	1.0270755	5.0262719	7.80E-07	4.07E-06	5.2063032
ITFG3	-0.122193	6.4316389	-5.02558	7.83E-07	4.08E-06	5.2030743
RCVRN	0.6446969	-0.806617	5.0255403	7.83E-07	4.08E-06	5.2028868
GALNT4	1.0667912	1.9575715	5.025358	7.84E-07	4.08E-06	5.2020351
AC091849.1	0.903033	0.5312322	5.0253153	7.84E-07	4.09E-06	5.2018358
RP11-286E1	-0.91603	1.5450623	-5.025281	7.84E-07	4.09E-06	5.2016748
ERCC2	-0.09929	6.1901306	-5.024933	7.85E-07	4.09E-06	5.2000484
LINC00925	0.9536984	2.711555	5.0241146	7.88E-07	4.11E-06	5.1962294
CHKB-CPT1B	0.7803207	3.4043423	5.0239217	7.89E-07	4.11E-06	5.1953288
CREBL2	-0.098243	6.3943927	-5.023806	7.90E-07	4.11E-06	5.194789
ADAMTS7P1	0.8833411	0.4706091	5.0236722	7.90E-07	4.11E-06	5.1941643
RP11-3304.	0.9273508	1.2021694	5.0236245	7.90E-07	4.11E-06	5.1939414
KCNK10	1.0662418	0.5669575	5.0234398	7.91E-07	4.12E-06	5.1930794
CYB5D1	0.5875882	3.7736742	5.023277	7.92E-07	4.12E-06	5.1923192
PDLIM3	0.2706074	5.566877	5.023071	7.92E-07	4.12E-06	5.1913577
BZRAP1-AS1	0.7172482	2.6978439	5.023042	7.93E-07	4.12E-06	5.1912223
CH17-264L2	0.6501849	3.4871032	5.0226604	7.94E-07	4.13E-06	5.1894414
TMEM216	0.1219072	5.2447078	5.0225653	7.94E-07	4.13E-06	5.1889975
RP11-551L1	0.9603584	0.4263284	5.0224098	7.95E-07	4.14E-06	5.1882716
LSS	-0.131538	6.6428962	-5.022117	7.96E-07	4.14E-06	5.1869035
RP11-627G1	0.880919	0.5966528	5.0221017	7.96E-07	4.14E-06	5.1868337
THOC7	-0.083773	6.2745573	-5.021332	7.99E-07	4.16E-06	5.1832419
AC010642.1	0.7619675	1.8780117	5.021047	8.00E-07	4.16E-06	5.1819115
ATP6V0D1	-0.089582	6.597967	-5.02101	8.00E-07	4.16E-06	5.1817405
GATSL3	-0.252156	4.4653144	-5.020625	8.02E-07	4.17E-06	5.1799409
XKR4	0.9044679	-0.434348	5.0206235	8.02E-07	4.17E-06	5.1799355
SDF4	-0.077347	6.8126928	-5.0197	8.06E-07	4.19E-06	5.1756281
SNORD63	0.881333	1.6662233	5.0196878	8.06E-07	4.19E-06	5.1755699
ARF5	-0.080107	6.6650368	-5.018883	8.09E-07	4.20E-06	5.1718155
ULBP2	0.7528176	3.1915983	5.0187567	8.09E-07	4.20E-06	5.1712265
ASB12	0.9361961	0.6097079	5.0186739	8.10E-07	4.20E-06	5.1708405
RP11-278C7	0.2687561	3.8178133	5.01864	8.10E-07	4.20E-06	5.1706825
YWHABP2	0.78368	0.0438173	5.0184688	8.10E-07	4.21E-06	5.1698837
UNC5A	0.8480036	2.8317347	5.0182631	8.11E-07	4.21E-06	5.1689243
CCDC51	-0.096231	5.8823659	-5.018198	8.11E-07	4.21E-06	5.1686231
MS4A7	0.2105635	5.7166443	5.0179875	8.12E-07	4.22E-06	5.1676391
NPIPB3	0.7793149	2.9616252	5.0179129	8.13E-07	4.22E-06	5.1672911
RP11-114J1	-0.787749	-0.40509	-5.017597	8.14E-07	4.22E-06	5.1658166

RP11-342C2	-0.503197	-1.248428	-5.016285	8.19E-07	4.25E-06	5.1597009
PNPLA1	0.7426097	-0.574815	5.0160292	8.20E-07	4.25E-06	5.1585074
RP11-327J1	-0.856441	3.1082765	-5.015637	8.22E-07	4.26E-06	5.1566786
RP11-774D1	-1.543965	1.5102489	-5.015265	8.23E-07	4.27E-06	5.154943
ZNF471	0.6298269	4.1861244	5.0152264	8.23E-07	4.27E-06	5.1547651
AC004832.1	-0.597512	-0.998609	-5.01506	8.24E-07	4.27E-06	5.1539897
COX7A2L	-0.070099	6.6167442	-5.013939	8.28E-07	4.29E-06	5.1487664
ARMC6	-0.118917	6.3325088	-5.013842	8.29E-07	4.29E-06	5.1483136
RNF169	0.2184802	5.3150405	5.0138	8.29E-07	4.30E-06	5.1481166
NR2F2-AS1	0.3693043	3.759411	5.0135602	8.30E-07	4.30E-06	5.1469993
FGF1	0.5677239	3.8648397	5.0134218	8.31E-07	4.30E-06	5.146354
CTD-2583P5	-0.871223	-0.055492	-5.013413	8.31E-07	4.30E-06	5.1463154
SPATA3-AS1	1.0342081	0.9078324	5.0122407	8.35E-07	4.33E-06	5.1408507
VPS26B	-0.078197	6.4614209	-5.011831	8.37E-07	4.33E-06	5.1389399
RP1-16A9.1	0.8389769	-0.009198	5.0115262	8.38E-07	4.34E-06	5.1375222
RRN3P3	0.1796337	4.5858213	5.0111894	8.40E-07	4.35E-06	5.1359529
RP11-547D1	0.9052503	1.4283063	5.0111179	8.40E-07	4.35E-06	5.1356202
CTD-2014B1	-0.71088	-0.795668	-5.011079	8.40E-07	4.35E-06	5.1354369
CTD-2001C1	-0.84047	0.4137437	-5.011048	8.40E-07	4.35E-06	5.1352948
RP11-314C1	-0.868235	1.9164878	-5.010636	8.42E-07	4.36E-06	5.1333741
RP11-76E12	-0.248747	-1.472399	-5.010566	8.42E-07	4.36E-06	5.1330516
APOBEC3A	0.8869645	2.0570431	5.0104697	8.43E-07	4.36E-06	5.1326005
CAPZB	-0.065127	6.7730307	-5.009082	8.48E-07	4.39E-06	5.1261388
RP11-274B2	0.9402817	1.5728821	5.0087848	8.50E-07	4.39E-06	5.1247543
CTD-2313J1	-0.375803	3.4282745	-5.008766	8.50E-07	4.39E-06	5.1246666
BCAS4	0.2916925	5.0929364	5.0086637	8.50E-07	4.39E-06	5.1241903
DPP10-AS1	1.1386592	-0.494889	5.008622	8.50E-07	4.39E-06	5.1239962
S100P	0.9593568	4.753415	5.0084099	8.51E-07	4.40E-06	5.1230085
ZNF775	-0.154797	5.9251346	-5.007997	8.53E-07	4.41E-06	5.1210842
F13B	-0.496557	6.4444966	-5.007824	8.54E-07	4.41E-06	5.1202795
RBBP8	0.1309356	5.8309621	5.0076486	8.54E-07	4.41E-06	5.1194643
FOXE1	0.96214	-0.522505	5.0072491	8.56E-07	4.42E-06	5.1176046
MIR6772	0.8831805	0.1554573	5.006858	8.58E-07	4.43E-06	5.1157839
MIR3649	-0.775398	-0.48291	-5.006346	8.60E-07	4.44E-06	5.1133997
MUM1L1	1.3828376	2.1051892	5.006256	8.60E-07	4.44E-06	5.1129821
XYLB	-0.188499	6.2042048	-5.005915	8.61E-07	4.45E-06	5.1113941
FBXO18	0.064979	6.2784217	5.0058985	8.62E-07	4.45E-06	5.1113182
RP11-66B24	0.7838384	-0.798404	5.0058862	8.62E-07	4.45E-06	5.1112607
IGKV1OR2-1	1.106555	0.9679702	5.0055097	8.63E-07	4.45E-06	5.1095088
SLC25A20	-0.141147	6.4495089	-5.00493	8.66E-07	4.46E-06	5.106811
CNIH1	-0.092334	6.6656443	-5.004796	8.66E-07	4.47E-06	5.1061873
EIF3M	-0.072161	6.6554296	-5.003997	8.70E-07	4.48E-06	5.1024687
XRCC6BP1	-0.1647	5.3075049	-5.003857	8.70E-07	4.49E-06	5.1018175
LINC00632	1.0069685	-0.357619	5.0036769	8.71E-07	4.49E-06	5.1009808
RP11-132N1	0.9932094	0.4007589	5.0026724	8.75E-07	4.51E-06	5.096308
PON1	-0.443941	6.7146822	-5.002433	8.76E-07	4.52E-06	5.0951966
AC079325.6	0.8785092	0.2697115	5.0020524	8.78E-07	4.52E-06	5.0934246
RCN3	0.1475773	5.8034141	5.002051	8.78E-07	4.52E-06	5.0934178
ERBB4	0.9576227	-0.265057	5.0019124	8.78E-07	4.53E-06	5.0927731
RP11-38H17	-0.958139	-0.095686	-5.001543	8.80E-07	4.53E-06	5.0910559
SCN11A	0.9144128	1.5749575	5.0015152	8.80E-07	4.53E-06	5.0909262

LINC00665	0.4845444	5.0574388	5.0012612	8.81E-07	4.54E-06	5.0897451
U2AF1	0.4657402	3.7871325	5.0012451	8.81E-07	4.54E-06	5.0896703
GALNT8	0.8624944	1.6327885	5.0009366	8.83E-07	4.54E-06	5.0882356
CLPX	-0.08869	6.4796249	-5.000285	8.85E-07	4.56E-06	5.0852035
RP11-480I1	-0.574163	3.9991785	-5.000212	8.86E-07	4.56E-06	5.0848678
MCTP1	0.2991487	5.2489941	5.0001673	8.86E-07	4.56E-06	5.0846583
CDK5RAP1	-0.06718	6.1205375	-4.999971	8.87E-07	4.56E-06	5.0837478
NEIL1	-0.159043	5.7762175	-4.999961	8.87E-07	4.56E-06	5.0837
MTBP	0.2453466	4.8767908	4.9996815	8.88E-07	4.57E-06	5.0824002
GFRA1	-0.756319	5.5195625	-4.999635	8.88E-07	4.57E-06	5.0821815
UROS	-0.104948	6.3216222	-4.99887	8.91E-07	4.58E-06	5.0786271
RP11-261C1	0.832904	0.0343628	4.9985304	8.93E-07	4.59E-06	5.0770491
RP11-227H1	0.9852106	0.3617333	4.9983991	8.94E-07	4.59E-06	5.0764389
LIMCH1	0.2682116	5.5774274	4.9981535	8.95E-07	4.60E-06	5.0752972
CTD-2231H1	0.8374948	-0.717288	4.9978426	8.96E-07	4.60E-06	5.0738525
TRPC7	0.6596813	-0.950794	4.9975756	8.97E-07	4.61E-06	5.0726117
GUCY1A2	0.3084944	5.0979071	4.9973503	8.98E-07	4.61E-06	5.0715645
RP11-274B2	0.6072834	4.5702286	4.9971934	8.99E-07	4.62E-06	5.0708354
KLHL4	0.6553938	3.2071058	4.9968114	9.00E-07	4.62E-06	5.0690605
RAB3C	1.1384086	3.2045816	4.9961966	9.03E-07	4.64E-06	5.0662038
DRG1	-0.080701	6.3912238	-4.995906	9.04E-07	4.64E-06	5.0648536
EMC8	-0.080431	5.9986323	-4.995883	9.05E-07	4.64E-06	5.0647468
ENTHD2	-0.09071	5.7889857	-4.995585	9.06E-07	4.65E-06	5.0633629
LINC00707	0.7801453	-0.834764	4.9950122	9.08E-07	4.66E-06	5.0607015
AGXT	-0.39916	7.1878234	-4.994692	9.10E-07	4.67E-06	5.0592139
TMEM164	0.1786822	5.8479526	4.9945354	9.10E-07	4.67E-06	5.058487
KB-431C1.5	0.7184801	2.36408	4.9945091	9.11E-07	4.67E-06	5.0583648
RPP40	-0.147249	5.5526886	-4.994311	9.11E-07	4.68E-06	5.0574451
LINC01272	0.5208235	4.0506326	4.9942267	9.12E-07	4.68E-06	5.0570529
OSGEP	-0.073045	6.0642051	-4.993791	9.14E-07	4.69E-06	5.0550301
PTOV1-AS2	0.3026659	4.4972712	4.9934187	9.15E-07	4.69E-06	5.0533008
GUK1	-0.10562	6.8869264	-4.992984	9.17E-07	4.70E-06	5.0512804
NKD2	0.8557571	3.991797	4.9927501	9.18E-07	4.71E-06	5.0501961
RP4-782L23	-0.739854	3.2937202	-4.992693	9.19E-07	4.71E-06	5.0499306
LRRIQ1	0.9846197	-0.245629	4.9923175	9.20E-07	4.72E-06	5.0481875
TBX20	0.7408466	-0.688509	4.9922297	9.21E-07	4.72E-06	5.0477798
C16orf52	-0.122306	5.7399118	-4.992073	9.21E-07	4.72E-06	5.0470523
AKR1E2	0.699469	2.8886034	4.9910863	9.26E-07	4.74E-06	5.0424714
PCDHGA7	0.7665664	3.1193033	4.9910405	9.26E-07	4.74E-06	5.0422589
AC006126.4	0.723401	3.6278417	4.9909932	9.26E-07	4.74E-06	5.0420394
C1QTNF1-AS	1.1958836	1.6423408	4.9909842	9.26E-07	4.74E-06	5.0419976
DSTNP5	-0.348648	-1.403847	-4.990084	9.30E-07	4.76E-06	5.037818
RP11-278H7	-0.722877	-0.488153	-4.988783	9.36E-07	4.79E-06	5.0317798
AKR7L	-0.326254	5.3337085	-4.987481	9.42E-07	4.82E-06	5.0257417
PLCD3	0.2113283	5.3109101	4.9873887	9.43E-07	4.82E-06	5.0253132
IGKV1D-43	0.862228	-0.662338	4.9871958	9.43E-07	4.83E-06	5.0244183
RABGGTB	-0.082674	6.4332351	-4.98694	9.45E-07	4.83E-06	5.0232293
TRAF3IP2-A	0.2002315	4.3616413	4.9866816	9.46E-07	4.84E-06	5.022033
RP11-10J5.	0.7019152	-0.856444	4.9865977	9.46E-07	4.84E-06	5.0216438
PSMD2	-0.063886	6.8044284	-4.985592	9.51E-07	4.86E-06	5.0169803
ATP11A	0.1263604	6.2611038	4.9851415	9.53E-07	4.87E-06	5.0148902

RAB4A	-0.098485	6.4605787	-4.98509	9.53E-07	4.87E-06	5.0146521
CX3CR1	0.399736	4.2333739	4.9850519	9.53E-07	4.87E-06	5.0144751
CTA-384D8.	0.94762	0.3459591	4.98502	9.53E-07	4.87E-06	5.0143269
C19orf12	-0.104718	6.3526483	-4.984774	9.55E-07	4.88E-06	5.0131871
ESYT1	-0.074379	6.7590033	-4.984664	9.55E-07	4.88E-06	5.0126748
WRB	-0.09214	6.0941603	-4.984246	9.57E-07	4.89E-06	5.0107374
ABCD2	0.7883554	2.6148534	4.9838954	9.59E-07	4.90E-06	5.0091125
SBK3	1.0582652	1.0881017	4.9826187	9.65E-07	4.93E-06	5.0031945
RPL7AP10	0.78132	-0.209334	4.9825418	9.65E-07	4.93E-06	5.0028378
SLC24A4	0.5330528	2.796285	4.9820708	9.67E-07	4.94E-06	5.000655
COX7A2	-0.110359	6.6732349	-4.982066	9.67E-07	4.94E-06	5.0006346
NT5DC1	-0.123186	6.203066	-4.981976	9.68E-07	4.94E-06	5.0002145
ZNF594	0.3962158	4.3407566	4.9813992	9.70E-07	4.95E-06	4.9975427
STX6	0.0816443	6.0092889	4.9811651	9.71E-07	4.96E-06	4.9964578
ZNF461	0.1830022	4.8949367	4.9789792	9.82E-07	5.01E-06	4.9863305
SUSD6	0.1120012	6.1164983	4.9788891	9.82E-07	5.01E-06	4.9859133
RTEL1	0.6937597	3.5673201	4.9786527	9.83E-07	5.02E-06	4.9848181
FOXQ1	1.1203558	4.0448017	4.9780571	9.86E-07	5.03E-06	4.9820596
MPDZ	-0.207804	6.2436549	-4.977943	9.87E-07	5.03E-06	4.9815327
AC093818.1	0.4468632	3.2989797	4.977373	9.89E-07	5.05E-06	4.9788916
CTB-131K11	0.2416572	5.2124719	4.976733	9.92E-07	5.06E-06	4.9759276
MIR4489	-0.816716	1.2728891	-4.975923	9.96E-07	5.08E-06	4.9721766
SRP54-AS1	-0.23449	4.1813285	-4.975572	9.98E-07	5.09E-06	4.9705547
CA5A	-0.674451	5.5223319	-4.97507	1.00E-06	5.10E-06	4.968229
AC007308.6	0.8920841	0.5851115	4.9746607	1.00E-06	5.11E-06	4.9663341
AC108463.1	0.7898664	1.3178639	4.9745648	1.00E-06	5.11E-06	4.9658904
ADAMTS1	0.2039812	5.9561657	4.9745471	1.00E-06	5.11E-06	4.9658085
OR5H8	-0.805012	-0.783083	-4.974479	1.00E-06	5.11E-06	4.9654941
ANKZF1	-0.081347	6.1949741	-4.973864	1.01E-06	5.13E-06	4.9626461
IL11RA	-0.139632	5.9689889	-4.973832	1.01E-06	5.13E-06	4.9624982
CTD-2313F1	0.7552631	-0.560938	4.9733715	1.01E-06	5.14E-06	4.9603678
TRIM39	-0.08665	5.8139657	-4.973064	1.01E-06	5.14E-06	4.9589461
VPS4A	-0.086498	6.2994202	-4.972063	1.02E-06	5.17E-06	4.9543111
RTN4	-0.088643	6.9400085	-4.971918	1.02E-06	5.17E-06	4.9536407
NDUFA12	-0.084396	6.3341848	-4.971877	1.02E-06	5.17E-06	4.9534536
NKX3-2	1.1493902	0.5240889	4.9712997	1.02E-06	5.18E-06	4.950782
SYBU	-0.27247	6.2414343	-4.970668	1.02E-06	5.20E-06	4.9478591
VEGFC	0.1964092	5.2453008	4.9706518	1.02E-06	5.20E-06	4.947785
INMT	0.449402	5.138663	4.9705988	1.02E-06	5.20E-06	4.9475399
ARHGEF18	0.2660049	5.3047168	4.9705044	1.02E-06	5.20E-06	4.9471034
POU5F1P3	0.8156252	0.972844	4.9704329	1.02E-06	5.20E-06	4.9467726
SPRYD7	-0.122983	5.9164385	-4.968969	1.03E-06	5.24E-06	4.9400004
C8orf46	-0.469347	4.8562275	-4.968177	1.03E-06	5.26E-06	4.936342
SOX30	0.85595	0.2730804	4.9679966	1.04E-06	5.26E-06	4.935507
ABCB6	-0.181386	5.7445749	-4.967665	1.04E-06	5.27E-06	4.9339728
RP11-434D9	-0.982024	3.8718666	-4.967405	1.04E-06	5.28E-06	4.9327715
EPO	1.3061152	2.662776	4.9671482	1.04E-06	5.28E-06	4.9315845
CDK15	0.91672	0.0206561	4.9666467	1.04E-06	5.29E-06	4.9292668
RPL10	-0.091057	7.3387115	-4.966506	1.04E-06	5.30E-06	4.9286165
TNNT3	0.920515	1.104846	4.9664471	1.04E-06	5.30E-06	4.928344
RP11-776A1	-0.587851	-1.096512	-4.966091	1.04E-06	5.30E-06	4.9266995

IMMP2L	-0.152553	5.9352897	-4.966025	1.05E-06	5.31E-06	4.9263911
GORASP1	-0.057116	6.2618722	-4.965601	1.05E-06	5.32E-06	4.9244342
RP11-11N7.	0.6158617	3.1258716	4.9652626	1.05E-06	5.32E-06	4.9228702
6-Mar	-0.065852	6.6676055	-4.964818	1.05E-06	5.33E-06	4.9208152
GNRH1	0.3787472	3.5774672	4.9648105	1.05E-06	5.33E-06	4.9207811
OR51E1	0.5805944	3.5126298	4.964803	1.05E-06	5.33E-06	4.9207461
SLC38A1	0.3177919	6.0088166	4.9639922	1.06E-06	5.35E-06	4.9170003
AC068499.1	-0.91521	0.7546508	-4.963598	1.06E-06	5.36E-06	4.9151795
HES2	0.914864	2.647465	4.9629424	1.06E-06	5.38E-06	4.9121506
SPATA41	-0.487386	4.6783982	-4.960815	1.07E-06	5.43E-06	4.9023248
LINC01270	-0.270218	5.1946144	-4.959763	1.08E-06	5.46E-06	4.8974694
MEAF6	-0.091812	6.3522006	-4.959442	1.08E-06	5.47E-06	4.8959882
PLA2G4E	1.0204365	0.4217343	4.9589538	1.08E-06	5.48E-06	4.8937335
CCL4L1	0.4892226	4.6127387	4.9580697	1.09E-06	5.50E-06	4.8896528
CTD-2313J1	-0.640204	-0.766448	-4.95774	1.09E-06	5.51E-06	4.8881307
HEATR1	0.1370796	6.0762737	4.9572732	1.09E-06	5.52E-06	4.8859773
GTF2A2	-0.071009	6.1710283	-4.956843	1.09E-06	5.53E-06	4.8839925
RP11-1017G	-0.617171	2.7184091	-4.956679	1.09E-06	5.54E-06	4.8832333
DCLRE1B	0.1274496	5.367038	4.9566427	1.09E-06	5.54E-06	4.8830678
KRT23	1.240385	4.461163	4.9563465	1.10E-06	5.55E-06	4.8817011
RP11-235E1	0.5993984	3.1960316	4.9551733	1.10E-06	5.58E-06	4.8762889
CYP4A11	-0.425617	6.7134308	-4.955165	1.10E-06	5.58E-06	4.8762488
PTOV1	-0.092313	6.7185242	-4.95471	1.10E-06	5.59E-06	4.8741499
REL	0.1883201	5.028786	4.9545718	1.10E-06	5.59E-06	4.8735147
AC002128.5	0.8706243	0.4378068	4.9533172	1.11E-06	5.62E-06	4.8677286
HOXC13	0.9805782	-0.388	4.9530495	1.11E-06	5.63E-06	4.8664941
AC074011.2	0.6192131	-0.844192	4.9523917	1.12E-06	5.65E-06	4.8634613
MEF2C-AS1	0.8115139	1.3220621	4.9518058	1.12E-06	5.66E-06	4.86076
ISLR2	0.6998504	3.2328104	4.9513999	1.12E-06	5.67E-06	4.8588889
SERPINF2	-0.234069	7.2811196	-4.951199	1.12E-06	5.68E-06	4.8579643
C9orf114	-0.081713	6.1837949	-4.950797	1.12E-06	5.69E-06	4.8561079
AZGP1P2	-1.078847	1.6804233	-4.950758	1.13E-06	5.69E-06	4.8559297
LINC00982	1.0669589	2.6955299	4.9501694	1.13E-06	5.70E-06	4.8532174
TNNT2	1.0327307	2.3607348	4.950046	1.13E-06	5.71E-06	4.8526488
PTGES3L-AA	0.6568383	-0.811378	4.9496299	1.13E-06	5.72E-06	4.8507314
FNIP1	-0.093004	6.1292951	-4.94956	1.13E-06	5.72E-06	4.8504073
RP11-222K1	0.8364831	2.2318171	4.9492753	1.13E-06	5.72E-06	4.8490973
CFL1P6	-0.641718	-0.822189	-4.948845	1.14E-06	5.73E-06	4.8471162
PBLD	-0.214675	6.4747406	-4.948482	1.14E-06	5.74E-06	4.8454419
RP11-405M1	0.7601037	1.9350373	4.9480327	1.14E-06	5.76E-06	4.8433719
SOWAHC	-0.121649	6.2577053	-4.947792	1.14E-06	5.76E-06	4.8422638
RP11-687F6	0.828664	0.429176	4.9475447	1.14E-06	5.77E-06	4.841124
GEMIN8P4	-0.219435	4.4792525	-4.947459	1.14E-06	5.77E-06	4.8407307
EML1	0.2380437	5.2322914	4.9468439	1.15E-06	5.79E-06	4.8378957
RP11-167H9	-1.309722	1.2658224	-4.946837	1.15E-06	5.79E-06	4.8378622
ALDH1B1	-0.147529	6.6535442	-4.946715	1.15E-06	5.79E-06	4.8373028
RP11-405A1	1.059953	0.2045558	4.9465437	1.15E-06	5.79E-06	4.8365131
PLN	0.8192217	3.3922947	4.9464409	1.15E-06	5.79E-06	4.8360396
ACAP1	0.2120435	5.3530049	4.9452371	1.16E-06	5.83E-06	4.8304957
SCRG1	0.8975664	0.6278566	4.9445514	1.16E-06	5.85E-06	4.8273386
RP11-39602	-1.315111	0.7440541	-4.943912	1.16E-06	5.86E-06	4.8243939

ANXA5	0.1215808	6.7820193	4.9436985	1.16E-06	5.87E-06	4.8234123
USF1	-0.08436	6.4372056	-4.94314	1.17E-06	5.88E-06	4.8208397
RP11-353N1	1.1328305	1.3446886	4.9428119	1.17E-06	5.89E-06	4.8193312
PSMB8	-0.145265	6.5805995	-4.942671	1.17E-06	5.89E-06	4.8186821
POT1-AS1	0.6510699	2.7302427	4.9425438	1.17E-06	5.90E-06	4.8180969
RP5-1024N4	0.8541243	1.418428	4.9424261	1.17E-06	5.90E-06	4.8175555
NAA25	0.0803146	5.8524927	4.9423429	1.17E-06	5.90E-06	4.8171723
IQCK	0.3195668	4.7313319	4.941951	1.17E-06	5.91E-06	4.8153688
CLN8	0.1427878	5.7560611	4.9413805	1.18E-06	5.93E-06	4.8127434
ATAD3A	-0.114498	6.0741023	-4.941357	1.18E-06	5.93E-06	4.8126358
FAM169B	1.3797173	1.5095403	4.9411644	1.18E-06	5.93E-06	4.8117493
GTPBP10	-0.088029	5.9995311	-4.941091	1.18E-06	5.93E-06	4.8114125
NCR3LG1	0.9028396	2.9508426	4.9405648	1.18E-06	5.95E-06	4.8089903
RP11-45F15	-0.155373	-1.513423	-4.940479	1.18E-06	5.95E-06	4.8085963
LINC00672	0.4895559	3.4498083	4.940057	1.18E-06	5.96E-06	4.806654
GKAP1	-0.107965	5.5395133	-4.939624	1.19E-06	5.97E-06	4.8046635
RP11-20J15	0.8991735	-0.059994	4.9391672	1.19E-06	5.98E-06	4.8025612
SSR4	-0.111708	6.8381954	-4.93906	1.19E-06	5.99E-06	4.8020682
CCDC107	-0.131795	5.6943511	-4.938082	1.20E-06	6.01E-06	4.7975678
SEMA4F	0.4676136	5.1206461	4.9377454	1.20E-06	6.02E-06	4.796022
SOX21	0.8713555	-0.486289	4.9373874	1.20E-06	6.03E-06	4.7943756
SCAANT1	-0.856286	1.3758998	-4.936952	1.20E-06	6.04E-06	4.7923756
HIF1A-AS2	0.7431545	-0.498211	4.9369077	1.20E-06	6.04E-06	4.7921701
SLC9C1	0.8169666	-0.403435	4.9363493	1.21E-06	6.06E-06	4.7896025
GLULP6	-0.314287	-1.398951	-4.936302	1.21E-06	6.06E-06	4.789385
MVB12A	-0.115493	6.1530212	-4.93616	1.21E-06	6.06E-06	4.7887327
KDM4B	-0.100815	6.2719657	-4.936041	1.21E-06	6.07E-06	4.7881847
ZNF311	0.9440114	3.1000299	4.9358771	1.21E-06	6.07E-06	4.7874317
CATIP	-0.509293	3.0561472	-4.935484	1.21E-06	6.08E-06	4.7856252
GFOD1	-0.156872	5.7222896	-4.935244	1.21E-06	6.09E-06	4.784521
ARNT	-0.084669	6.5150447	-4.934913	1.21E-06	6.10E-06	4.783001
POLD4	-0.113114	6.2906661	-4.934834	1.21E-06	6.10E-06	4.7826389
MORN1	-0.166459	5.1029895	-4.934817	1.21E-06	6.10E-06	4.782559
PACERR	0.5184023	-1.11564	4.933642	1.22E-06	6.13E-06	4.777159
SLC25A17	-0.070909	6.0497842	-4.933405	1.22E-06	6.14E-06	4.7760689
ZNF768	-0.07717	6.3564239	-4.933343	1.22E-06	6.14E-06	4.7757872
RNU1-138P	-0.811774	-0.615259	-4.932872	1.23E-06	6.15E-06	4.7736196
RP1-15005.	0.8121262	-0.394467	4.9324928	1.23E-06	6.16E-06	4.7718789
SLC9A2	1.0175489	0.8583832	4.9323328	1.23E-06	6.16E-06	4.7711439
RP2	0.2051319	5.6695654	4.9313125	1.24E-06	6.19E-06	4.7664567
MIR1249	0.6583955	-0.666905	4.9303983	1.24E-06	6.22E-06	4.7622578
RP11-173B1	0.8241591	0.4137	4.9303557	1.24E-06	6.22E-06	4.7620622
RP11-338N1	1.0313845	-0.05559	4.9299855	1.24E-06	6.23E-06	4.7603621
ICAM2	0.1792274	5.3768875	4.9293441	1.25E-06	6.25E-06	4.7574169
LINC01426	0.8468051	4.0162462	4.9287558	1.25E-06	6.27E-06	4.7547157
DESI1	-0.093516	6.5721216	-4.928728	1.25E-06	6.27E-06	4.7545893
CTD-2506J1	0.704751	-0.647839	4.9287155	1.25E-06	6.27E-06	4.7545305
ATG4C	-0.088343	5.7034857	-4.928672	1.25E-06	6.27E-06	4.754333
UBE2F	-0.086889	6.1570063	-4.928344	1.25E-06	6.28E-06	4.752827
AMDHD2	-0.114925	6.0315419	-4.928245	1.25E-06	6.28E-06	4.7523705
RSL24D1	-0.069296	6.425988	-4.927751	1.26E-06	6.29E-06	4.7501022

C21orf2	-0.121964	5.9819248	-4.927647	1.26E-06	6.29E-06	4.7496252
FUT11	0.1042578	5.5683659	4.9271447	1.26E-06	6.31E-06	4.74732
RP11-121M2	-0.482995	3.1329799	-4.92518	1.27E-06	6.37E-06	4.7383035
MYCBPAP	0.8026387	2.1424323	4.9251724	1.27E-06	6.37E-06	4.7382687
LINC01499	-0.229881	-1.469525	-4.925041	1.27E-06	6.37E-06	4.7376643
ARMC9	0.2501052	4.9460427	4.9248285	1.27E-06	6.38E-06	4.736691
TEX26	0.6109526	-1.047559	4.9244179	1.28E-06	6.39E-06	4.734807
SYT16	0.7018963	-0.876764	4.9244177	1.28E-06	6.39E-06	4.7348061
POU3F1	1.0049717	0.9003382	4.924125	1.28E-06	6.39E-06	4.7334635
LINC00342	0.2437021	4.8767144	4.9236378	1.28E-06	6.41E-06	4.7312284
RP11-181C3	-0.396575	4.4376362	-4.923245	1.28E-06	6.42E-06	4.7294261
ZEB2-AS1	0.7906278	1.3185455	4.9231978	1.28E-06	6.42E-06	4.72921
RIBC2	0.7812817	3.6476998	4.9229797	1.29E-06	6.43E-06	4.7282098
STARD3NL	0.1210861	5.8216723	4.9229123	1.29E-06	6.43E-06	4.7279005
CDC42EP5	0.243783	4.6574124	4.9228031	1.29E-06	6.43E-06	4.7273997
STT3B	-0.065735	6.7824937	-4.92236	1.29E-06	6.44E-06	4.7253681
CTBS	-0.116955	6.0804023	-4.921801	1.29E-06	6.46E-06	4.7228036
CTB-129P6.	-0.320951	5.0217854	-4.92158	1.29E-06	6.46E-06	4.7217931
SLIRP	-0.122077	6.2676535	-4.921261	1.30E-06	6.47E-06	4.7203261
NOTCH2NL	0.7881247	2.111233	4.920206	1.30E-06	6.51E-06	4.7154915
COL24A1	1.117969	2.4706772	4.9201473	1.30E-06	6.51E-06	4.7152223
SENCR	0.3910932	3.3495282	4.9197314	1.31E-06	6.52E-06	4.7133155
MINOS1	-0.117619	5.9962185	-4.919702	1.31E-06	6.52E-06	4.7131832
HIGD1C	-0.801088	-0.209403	-4.919693	1.31E-06	6.52E-06	4.7131419
C4BPA	-0.294357	7.1924766	-4.919525	1.31E-06	6.52E-06	4.712368
ZNF681	0.680602	4.016142	4.9190126	1.31E-06	6.54E-06	4.7100209
AC130469.1	0.8752699	1.2295751	4.9176366	1.32E-06	6.58E-06	4.7037154
EIF4HP2	0.7387672	2.3676579	4.9175546	1.32E-06	6.58E-06	4.7033397
RP4-569M23	-0.883966	0.692737	-4.916354	1.33E-06	6.62E-06	4.6978372
CAPN14	0.9596754	0.6243978	4.9161432	1.33E-06	6.62E-06	4.6968733
DUOXA2	1.3657728	2.1988362	4.915687	1.33E-06	6.64E-06	4.6947834
RP4-580N22	-1.09741	1.6919019	-4.915655	1.33E-06	6.64E-06	4.6946386
GLDN	0.8731368	3.9377181	4.9155195	1.33E-06	6.64E-06	4.694016
TAL1	0.3031533	4.4640404	4.9145924	1.34E-06	6.67E-06	4.6897698
IGHV3-47	0.9008077	-0.258056	4.9142843	1.34E-06	6.68E-06	4.6883589
RP11-153K1	-1.050312	1.7418709	-4.914137	1.34E-06	6.68E-06	4.6876854
AC012456.3	-0.583339	-0.974191	-4.913246	1.35E-06	6.71E-06	4.6836042
ABCD3	-0.088184	6.6293114	-4.913144	1.35E-06	6.71E-06	4.6831393
LGR6	0.6939321	4.2057787	4.9126539	1.35E-06	6.73E-06	4.6808939
IGSF6	0.2026364	5.5548291	4.9125663	1.35E-06	6.73E-06	4.6804928
BEST3	0.9266486	-0.413916	4.9121174	1.35E-06	6.74E-06	4.6784378
RP11-524F1	-0.847491	1.7118856	-4.91207	1.35E-06	6.74E-06	4.6782212
HNF4A	-0.292211	6.9336368	-4.911621	1.36E-06	6.76E-06	4.6761661
RP13-314C1	-0.807039	2.4633073	-4.911201	1.36E-06	6.77E-06	4.6742426
EMP1	0.1968846	5.9901322	4.9110704	1.36E-06	6.77E-06	4.6736453
PFN2	0.3134704	5.4678481	4.9107682	1.36E-06	6.78E-06	4.6722625
KIAA0368	-0.063402	6.5916082	-4.910606	1.36E-06	6.79E-06	4.6715196
EFHC2	1.0723928	0.4984916	4.910453	1.37E-06	6.79E-06	4.6708197
HERC5	-0.280656	5.4300969	-4.910268	1.37E-06	6.80E-06	4.6699736
EXOSC6	-0.086983	6.0552724	-4.910035	1.37E-06	6.80E-06	4.6689077
ZCCHC10	-0.084276	5.75464	-4.909939	1.37E-06	6.80E-06	4.6684668

LYRM4	-0.111282	6.0744477	-4.909454	1.37E-06	6.82E-06	4.6662494
ALDH1L1-AS	-0.930427	0.5513318	-4.909151	1.37E-06	6.83E-06	4.6648601
SEPT7	0.0520191	6.5097825	4.9090851	1.37E-06	6.83E-06	4.6645607
RP11-1094M	-0.166335	5.3002736	-4.908663	1.38E-06	6.84E-06	4.6626296
PRKAB1	-0.075968	6.2321043	-4.9086	1.38E-06	6.84E-06	4.6623405
SLC25A19	0.1547909	5.3354358	4.9080156	1.38E-06	6.86E-06	4.659668
AHSG	-0.380352	7.403768	-4.907232	1.39E-06	6.89E-06	4.6560817
RP5-1099D1	0.8124284	0.3864429	4.9059142	1.40E-06	6.93E-06	4.6500575
NNAT	0.9365106	2.0395208	4.9054573	1.40E-06	6.94E-06	4.6479681
CHCHD2P7	-0.816271	-0.572684	-4.905007	1.40E-06	6.96E-06	4.645908
RP11-325L1	-0.929624	1.4685902	-4.904806	1.40E-06	6.96E-06	4.6449924
PDCD4	0.0937893	6.3046309	4.9045941	1.40E-06	6.97E-06	4.6440219
EEF2	-0.072263	7.4767849	-4.904445	1.41E-06	6.97E-06	4.643341
SLC43A2	0.1681425	5.9251303	4.9043625	1.41E-06	6.98E-06	4.6429632
UBAC2	-0.080405	6.5366503	-4.904024	1.41E-06	6.99E-06	4.6414167
HOMER2	-0.241947	6.2416263	-4.903713	1.41E-06	7.00E-06	4.6399956
LPIN1	-0.15166	6.2263257	-4.903671	1.41E-06	7.00E-06	4.6398032
CFHR4	-0.677312	5.6927567	-4.903604	1.41E-06	7.00E-06	4.6394971
PELI3	-0.174831	5.471969	-4.902826	1.42E-06	7.02E-06	4.6359397
RP11-191L1	-0.690532	2.1461301	-4.902549	1.42E-06	7.03E-06	4.6346746
KCND3-IT1	-0.379299	-1.370729	-4.902407	1.42E-06	7.03E-06	4.6340261
SDHA	-0.095971	6.890563	-4.902214	1.42E-06	7.04E-06	4.6331414
RP11-775A3	-0.712678	-0.462462	-4.90163	1.42E-06	7.06E-06	4.6304753
MCM3AP-AS1	0.2344543	4.4935985	4.9015143	1.43E-06	7.06E-06	4.6299462
AP000859.4	-0.217701	-1.474876	-4.900952	1.43E-06	7.08E-06	4.6273785
KLF5	0.4780507	5.095757	4.9007029	1.43E-06	7.09E-06	4.6262393
CTD-2554C2	1.0221114	0.7458115	4.9003849	1.43E-06	7.10E-06	4.6247866
XIRP1	0.9341245	1.0243061	4.900263	1.43E-06	7.10E-06	4.6242296
FOLH1	-0.294264	5.5362216	-4.899826	1.44E-06	7.11E-06	4.6222355
RP11-712C1	-0.386079	-1.280523	-4.899419	1.44E-06	7.13E-06	4.6203769
RP11-3304.	-0.236756	4.4851376	-4.899001	1.44E-06	7.14E-06	4.6184638
IL2	0.5941287	-0.936394	4.8988766	1.44E-06	7.14E-06	4.6178976
RPS8	-0.095177	7.2343319	-4.898646	1.44E-06	7.15E-06	4.6168423
RP4-737E23	0.8033186	0.40022	4.8985497	1.45E-06	7.15E-06	4.6164049
SELT	-0.061475	6.4829254	-4.898506	1.45E-06	7.15E-06	4.6162046
RP11-521B2	0.6764	3.0840514	4.8982055	1.45E-06	7.16E-06	4.6148329
COX6B1	-0.116051	6.9152551	-4.897759	1.45E-06	7.18E-06	4.6127946
RP11-31506	-0.40647	4.1402592	-4.897586	1.45E-06	7.18E-06	4.612006
CH17-13I23	-0.28504	-1.44529	-4.897365	1.45E-06	7.19E-06	4.6109931
RP11-133K1	0.8351936	0.4677725	4.8973266	1.45E-06	7.19E-06	4.6108199
ZBED1	-0.081386	6.5197905	-4.89732	1.45E-06	7.19E-06	4.6107912
CYP4F36P	-0.377182	-1.324332	-4.897166	1.46E-06	7.19E-06	4.6100866
ARL2BP	0.5443987	4.8183405	4.896499	1.46E-06	7.21E-06	4.6070416
CYP4A22	-0.546784	6.1759555	-4.896448	1.46E-06	7.22E-06	4.6068082
PPM1A	-0.082384	6.4505401	-4.896197	1.46E-06	7.22E-06	4.6056649
IGHV3OR16-	0.7416639	-0.814303	4.8958907	1.46E-06	7.23E-06	4.6042651
C17orf62	-0.083934	6.4630849	-4.895702	1.47E-06	7.24E-06	4.603404
F2	-0.298952	7.3274866	-4.895357	1.47E-06	7.25E-06	4.6018314
DMTF1	0.0938671	5.9931588	4.8950625	1.47E-06	7.26E-06	4.600485
C2CD4B	0.6582125	3.2738193	4.8948162	1.47E-06	7.27E-06	4.5993613
RP1-197B17	-0.261338	4.2610815	-4.894672	1.47E-06	7.27E-06	4.5987012

C14orf142	-0.099879	5.6852121	-4.894668	1.47E-06	7.27E-06	4.5986835
PLCXD2	-0.292226	5.2422889	-4.894271	1.48E-06	7.28E-06	4.596875
RP11-21B23	-0.509975	-1.128187	-4.89401	1.48E-06	7.29E-06	4.5956843
CHTF8	-0.067647	6.4646916	-4.893336	1.48E-06	7.31E-06	4.5926058
JRKL	0.1522487	5.4095784	4.8929742	1.48E-06	7.32E-06	4.5909567
MPC2	-0.098191	6.9660357	-4.892519	1.49E-06	7.34E-06	4.5888821
TEKT3	0.8912103	0.4978258	4.8921851	1.49E-06	7.35E-06	4.5873567
LINC00336	0.7284057	-0.835296	4.8916933	1.49E-06	7.37E-06	4.5851138
CREG2	0.9367605	2.0134564	4.8913653	1.50E-06	7.38E-06	4.5836176
RP11-7I15.	-0.926807	0.4452383	-4.891358	1.50E-06	7.38E-06	4.5835839
CDCA4	0.1468327	5.4521864	4.891336	1.50E-06	7.38E-06	4.5834843
CDK2AP2	-0.103231	6.57051	-4.889617	1.51E-06	7.44E-06	4.5756437
MAP3K14-AS	0.5977578	3.2800137	4.8895459	1.51E-06	7.44E-06	4.5753213
PARP8	0.2025618	5.6928788	4.8879136	1.52E-06	7.49E-06	4.5678803
RP11-863P1	-0.788604	-0.515327	-4.887891	1.52E-06	7.49E-06	4.5677767
RP11-15I20	-0.452307	-1.210344	-4.887199	1.53E-06	7.52E-06	4.5646223
F13A1	0.3892414	5.252659	4.8868443	1.53E-06	7.53E-06	4.5630074
CTD-3023L1	-0.329345	-1.412328	-4.886441	1.53E-06	7.54E-06	4.5611697
AC083843.2	-0.829119	0.0440126	-4.886373	1.53E-06	7.54E-06	4.5608618
VRK3	-0.085442	6.2843943	-4.886157	1.53E-06	7.55E-06	4.5598742
CTD-3128G1	0.853207	1.2446094	4.8858973	1.54E-06	7.56E-06	4.5586921
RP11-399K2	-0.379822	-1.364442	-4.885706	1.54E-06	7.56E-06	4.5578217
RP11-999E2	0.9327221	1.4988238	4.8854776	1.54E-06	7.57E-06	4.55678
SUCLG1	-0.090056	6.7137713	-4.885417	1.54E-06	7.57E-06	4.5565024
AC092835.2	0.8782636	0.5377321	4.8845208	1.55E-06	7.60E-06	4.5524211
ORM2	-0.274649	7.1083823	-4.884007	1.55E-06	7.62E-06	4.550081
FZD9	1.1169408	1.8318095	4.8837195	1.55E-06	7.63E-06	4.5487715
SKAP2	0.2008142	5.8998478	4.8837113	1.55E-06	7.63E-06	4.5487341
PLEKHA4	0.2564105	5.8781452	4.8833892	1.55E-06	7.64E-06	4.5472672
SSU72P5	-0.224278	-1.471986	-4.882976	1.56E-06	7.66E-06	4.5453865
CLHC1	0.2587828	4.2140107	4.8822342	1.56E-06	7.68E-06	4.5420076
CTB-12A17.	0.6193772	-0.745102	4.8821199	1.56E-06	7.68E-06	4.5414873
MYSM1	0.1545988	5.7012384	4.8816811	1.57E-06	7.70E-06	4.5394893
IP6K3	1.1669481	3.7498666	4.881265	1.57E-06	7.71E-06	4.537595
PTPMT1	-0.083655	5.8323859	-4.881188	1.57E-06	7.72E-06	4.5372451
PF4V1	1.2021591	0.6665191	4.8811355	1.57E-06	7.72E-06	4.5370055
CCER2	-0.602332	3.2526769	-4.881001	1.57E-06	7.72E-06	4.536392
ALCAM	-0.11541	6.6422049	-4.880997	1.57E-06	7.72E-06	4.5363732
AQP7P1	-0.949406	3.6743289	-4.880864	1.57E-06	7.72E-06	4.5357684
SMTN	0.1014319	6.1593473	4.88071	1.57E-06	7.73E-06	4.5350686
RP11-359I1	-0.745869	-0.495828	-4.880467	1.58E-06	7.74E-06	4.5339622
KIAA1462	0.1691006	5.9727392	4.8798591	1.58E-06	7.76E-06	4.5311956
RP11-108M9	-0.396871	4.3410883	-4.879793	1.58E-06	7.76E-06	4.5308941
ID3	0.1669469	5.9521525	4.8793022	1.58E-06	7.78E-06	4.5286612
CDK19	0.1147108	5.8451955	4.8790929	1.59E-06	7.78E-06	4.5277086
VPS13A-AS1	-0.365666	-1.318904	-4.878773	1.59E-06	7.79E-06	4.5262543
PFN1P7	1.0174896	0.9461596	4.8786394	1.59E-06	7.80E-06	4.525645
VPS9D1	-0.103744	5.9673511	-4.878091	1.59E-06	7.82E-06	4.5231519
MAOB	-0.186705	6.8701949	-4.877436	1.60E-06	7.84E-06	4.5201698
RHOU	-0.123441	6.5605017	-4.876987	1.60E-06	7.86E-06	4.5181284
RPL22	-0.081584	6.8240146	-4.876469	1.61E-06	7.87E-06	4.5157694

LINC00840	0.974549	0.5479699	4.8761945	1.61E-06	7.88E-06	4.5145227
CPQ	-0.109734	6.6033095	-4.876153	1.61E-06	7.88E-06	4.5143346
RAB8A	-0.076697	6.3203615	-4.875498	1.61E-06	7.91E-06	4.5113546
RP11-478P1	-0.925993	0.1262216	-4.875477	1.61E-06	7.91E-06	4.5112614
ISCA2	-0.093299	5.9031848	-4.875112	1.62E-06	7.92E-06	4.5095991
ESRRB	0.9307269	1.0260087	4.8749323	1.62E-06	7.93E-06	4.5087823
PABPN1	-0.07392	6.5265488	-4.874264	1.62E-06	7.95E-06	4.5057454
EDEM1	-0.100234	6.5119405	-4.873131	1.63E-06	7.99E-06	4.5005951
EPHX4	0.7766582	2.4414013	4.8730785	1.63E-06	7.99E-06	4.5003541
RP13-631K1	-0.962583	-0.066399	-4.872646	1.64E-06	8.01E-06	4.4983872
SLC25A30-A	-0.53152	3.1188645	-4.871894	1.64E-06	8.04E-06	4.4949681
RPL23A	-0.105271	7.0278184	-4.871718	1.64E-06	8.04E-06	4.4941695
HLA-E	-0.099323	7.2073985	-4.871238	1.65E-06	8.06E-06	4.4919886
HYMAI	0.616469	-0.912611	4.8711721	1.65E-06	8.06E-06	4.4916898
RP4-742C19	0.7133872	3.0204018	4.871134	1.65E-06	8.06E-06	4.4915167
UBE2R2	-0.064617	6.5938269	-4.871069	1.65E-06	8.06E-06	4.4912225
RP11-155G1	-0.560408	-1.040478	-4.870828	1.65E-06	8.07E-06	4.4901259
GSN-AS1	0.81356	1.1899349	4.8707791	1.65E-06	8.07E-06	4.4899038
ZZEF1	0.0883412	6.1213574	4.870266	1.65E-06	8.09E-06	4.4875727
AC109333.1	0.7202301	1.7385838	4.8698796	1.66E-06	8.10E-06	4.4858173
RP4-728D4.	0.740497	1.9567093	4.869717	1.66E-06	8.11E-06	4.4850783
AC091177.1	0.608992	-0.832723	4.8692946	1.66E-06	8.12E-06	4.4831596
METTL14	-0.088992	5.9874009	-4.868829	1.67E-06	8.14E-06	4.4810447
LINC01137	-0.192661	5.4023636	-4.868401	1.67E-06	8.15E-06	4.479102
RP11-483P2	0.7805316	1.6571436	4.8683846	1.67E-06	8.15E-06	4.4790262
TRIM34	0.7064971	2.3291663	4.8683839	1.67E-06	8.15E-06	4.4790227
RP11-472N1	0.8405717	0.8246646	4.8681201	1.67E-06	8.16E-06	4.4778245
RP11-35N6.	-0.409862	5.6664507	-4.867726	1.67E-06	8.18E-06	4.476033
RP11-392E2	1.0584488	0.431213	4.8676284	1.67E-06	8.18E-06	4.4755917
CTD-226502	-0.811048	-0.543496	-4.867034	1.68E-06	8.20E-06	4.4728936
INHA	1.1469595	2.0332821	4.8669903	1.68E-06	8.20E-06	4.4726939
SGK3	-0.138381	5.7896159	-4.866713	1.68E-06	8.21E-06	4.4714368
RP11-102M1	-0.706538	-0.582418	-4.866516	1.68E-06	8.22E-06	4.4705384
AGR3	1.01969	-0.308622	4.8663343	1.69E-06	8.23E-06	4.4697155
RP1-45I4.3	-0.766191	3.0436293	-4.866229	1.69E-06	8.23E-06	4.4692371
SDCCAG3P2	0.7082758	-0.630465	4.8661348	1.69E-06	8.23E-06	4.4688096
WDR54	0.222195	5.1258717	4.8661013	1.69E-06	8.23E-06	4.4686578
ZFAND6	-0.060921	6.4832397	-4.86504	1.70E-06	8.27E-06	4.4638398
SH3TC1	0.11202	6.1207041	4.8649438	1.70E-06	8.27E-06	4.4634033
HORMAD1	0.9019969	0.1259086	4.8644776	1.70E-06	8.29E-06	4.4612872
CFAP54	0.7227574	2.2682572	4.8642193	1.70E-06	8.30E-06	4.460115
RP11-1166F	-0.229127	-1.469856	-4.864076	1.70E-06	8.31E-06	4.4594666
RSL1D1	-0.070451	6.6677459	-4.86402	1.70E-06	8.31E-06	4.4592115
EXOC6B	0.0952115	5.800184	4.8638095	1.71E-06	8.31E-06	4.4582551
RP11-386M2	-0.747193	-0.537842	-4.863548	1.71E-06	8.32E-06	4.4570671
RP1-153P14	0.686675	-0.6509	4.8633523	1.71E-06	8.33E-06	4.4561802
RP11-521I2	0.8180383	1.1894727	4.8632074	1.71E-06	8.33E-06	4.455523
CCBL2	-0.10255	5.924364	-4.861963	1.72E-06	8.38E-06	4.449877
LINC00661	0.9794615	-0.22665	4.8607774	1.73E-06	8.43E-06	4.4444987
PRNP	0.1959487	6.231279	4.8603358	1.73E-06	8.44E-06	4.4424959
PIH1D1	-0.0915	6.4717409	-4.860326	1.73E-06	8.44E-06	4.4424497

HNRNPU-AS1	0.2607845	5.1282995	4.8599437	1.74E-06	8.46E-06	4.4407175
Z95114.3	-0.262868	-1.432705	-4.859795	1.74E-06	8.46E-06	4.4400422
RP11-294C1	0.7932935	-0.688864	4.8597488	1.74E-06	8.46E-06	4.4398337
ZNF773	0.3271942	4.751658	4.8596357	1.74E-06	8.47E-06	4.4393208
RPS20	-0.113884	7.2727459	-4.859109	1.74E-06	8.49E-06	4.4369324
AKAP2	1.0013159	1.4061538	4.8585713	1.75E-06	8.51E-06	4.4344946
FBLN5	0.2979681	5.8368752	4.8584098	1.75E-06	8.51E-06	4.4337625
PANK1	-0.152708	6.2914848	-4.857131	1.76E-06	8.56E-06	4.4279673
CRYGN	0.7102966	-0.634075	4.8569077	1.76E-06	8.57E-06	4.4269536
PHYKPL	-0.100361	6.2692772	-4.856659	1.76E-06	8.58E-06	4.4258247
MAGIX	-0.235849	5.7376084	-4.856222	1.77E-06	8.60E-06	4.4238465
B3GALT6	-0.10203	6.0645182	-4.855862	1.77E-06	8.61E-06	4.4222144
ESCO1	0.0779721	5.8944479	4.8555939	1.77E-06	8.62E-06	4.4209991
RP1-288H2.	-0.966787	-0.033648	-4.85484	1.78E-06	8.65E-06	4.4175821
TMEM150C	-0.238737	5.8303354	-4.85443	1.78E-06	8.67E-06	4.4157279
RP11-247A1	0.7949114	1.3164593	4.8542255	1.79E-06	8.67E-06	4.4147991
ZDHHC3	-0.057494	6.3455433	-4.85407	1.79E-06	8.68E-06	4.4140962
SCCPDH	-0.139667	6.8673732	-4.853372	1.79E-06	8.71E-06	4.4109351
MYH14	-0.134997	6.7593031	-4.8531	1.79E-06	8.72E-06	4.4097019
SLC2A3	0.2076855	5.6524563	4.8527678	1.80E-06	8.73E-06	4.4081963
GSTA8P	-0.957956	0.8057297	-4.852529	1.80E-06	8.74E-06	4.4071166
OR52K2	-0.208413	-1.478957	-4.851806	1.81E-06	8.77E-06	4.4038387
AARS	-0.081329	6.8243949	-4.85167	1.81E-06	8.77E-06	4.403223
ZNF142	0.0805292	5.7936696	4.8514786	1.81E-06	8.78E-06	4.4023584
TMEM43	0.0692049	6.2590212	4.8513949	1.81E-06	8.78E-06	4.4019793
RP1-140K8.	-0.884061	-0.07491	-4.851316	1.81E-06	8.78E-06	4.4016217
RP11-448G1	0.4407984	3.792448	4.8503879	1.82E-06	8.82E-06	4.3974202
WAS	0.1589196	5.5199866	4.8500662	1.82E-06	8.83E-06	4.3959637
CCDC120	0.11089	5.6976483	4.8500182	1.82E-06	8.83E-06	4.3957466
AF186192.5	0.9451511	0.2663447	4.8492265	1.83E-06	8.87E-06	4.392163
LATS1	0.1205109	5.8509455	4.8483414	1.84E-06	8.90E-06	4.388157
RP4-730K3.	0.7322981	2.0890551	4.8482303	1.84E-06	8.91E-06	4.3876544
NDUFV2	-0.128995	6.0595931	-4.847895	1.84E-06	8.92E-06	4.3861352
RP11-76908	0.7718344	0.0688766	4.8474712	1.84E-06	8.93E-06	4.3842191
APOA1BP	-0.09688	6.7348934	-4.84734	1.84E-06	8.94E-06	4.3836238
NOL10	-0.056785	6.1660688	-4.847097	1.85E-06	8.95E-06	4.3825258
CLEC4D	0.8295695	-0.193924	4.8468707	1.85E-06	8.96E-06	4.3815025
NAA40	0.1018355	5.7746984	4.8466447	1.85E-06	8.97E-06	4.38048
NUDT9	-0.103698	6.2589979	-4.846521	1.85E-06	8.97E-06	4.3799204
RP11-219E7	0.8306692	1.2500497	4.8456844	1.86E-06	9.00E-06	4.376136
NCDN	0.09683	5.8906144	4.8456239	1.86E-06	9.01E-06	4.375862
CLPTM1	-0.070887	6.8754468	-4.844586	1.87E-06	9.05E-06	4.3711679
HSCB	-0.115233	5.8978899	-4.844311	1.87E-06	9.06E-06	4.369925
MBD3	-0.096815	6.5485447	-4.843457	1.88E-06	9.09E-06	4.3660641
MAP3K6	0.1473371	5.5777702	4.8430117	1.88E-06	9.11E-06	4.3640494
MRS2	-0.090131	6.3039032	-4.842948	1.88E-06	9.11E-06	4.3637617
RASSF10	0.9033962	-0.483905	4.8427913	1.88E-06	9.12E-06	4.3630533
PDGFB	0.1345013	5.8684529	4.8423315	1.89E-06	9.14E-06	4.3609744
VAMP7	-0.101119	6.3809322	-4.841933	1.89E-06	9.15E-06	4.3591723
RP11-731J8	0.8292774	-0.365121	4.8419245	1.89E-06	9.15E-06	4.3591348
GIMAP7	0.1932756	5.5351055	4.8416449	1.89E-06	9.16E-06	4.3578708

ECT2	0.2103305	5.681864	4.8414721	1.90E-06	9.17E-06	4.3570897
LINC01522	0.5247454	-1.149031	4.8410762	1.90E-06	9.19E-06	4.3553007
FRMD5	0.9236779	0.1598022	4.8406408	1.90E-06	9.20E-06	4.3533328
PSMC4	-0.079458	6.6762595	-4.840317	1.91E-06	9.22E-06	4.3518693
RP11-634H2	-0.523562	3.0683081	-4.840071	1.91E-06	9.23E-06	4.3507573
HNRNPFP1	-0.159014	-1.511823	-4.839432	1.91E-06	9.25E-06	4.3478727
AC012360.6	0.8255558	-0.046414	4.8393646	1.92E-06	9.26E-06	4.3475663
RP11-46301	0.50861	3.6315557	4.8393378	1.92E-06	9.26E-06	4.347445
C5orf15	-0.070335	6.4769251	-4.839083	1.92E-06	9.26E-06	4.3462928
AC138969.4	0.7314498	2.2595479	4.8390679	1.92E-06	9.26E-06	4.3462257
AP003774.6	0.7375677	-0.247163	4.838713	1.92E-06	9.28E-06	4.3446226
FAM26E	0.2907178	4.7501888	4.8378332	1.93E-06	9.32E-06	4.340648
TSPAN18	0.1900273	5.6607921	4.8377271	1.93E-06	9.32E-06	4.3401688
RP11-427L1	0.8329374	1.2595111	4.8368834	1.94E-06	9.36E-06	4.3363585
LIN7A	-0.166913	6.1556807	-4.836278	1.94E-06	9.38E-06	4.333624
CRYAA	-1.044563	0.0698896	-4.836205	1.94E-06	9.38E-06	4.3332929
AMN1	0.2667629	4.8413949	4.8358764	1.95E-06	9.40E-06	4.3318111
MIR503HG	0.3530345	3.8284563	4.8356609	1.95E-06	9.40E-06	4.3308381
KCNA5	0.8921348	2.6229295	4.8354362	1.95E-06	9.41E-06	4.3298237
PIP5K1C	0.079164	6.1047375	4.8352536	1.95E-06	9.42E-06	4.3289995
SAT2	-0.13218	6.6995472	-4.834631	1.96E-06	9.45E-06	4.3261896
MPRIP	0.072503	6.485257	4.8335513	1.97E-06	9.49E-06	4.3213154
TPSAB1	0.9674971	3.353679	4.8334576	1.97E-06	9.50E-06	4.3208923
TXLNA	-0.069607	6.5486789	-4.833024	1.97E-06	9.52E-06	4.318936
UGT2B4	-0.344373	7.1404851	-4.832986	1.97E-06	9.52E-06	4.318762
LINC01561	0.7076475	-0.808403	4.8329028	1.97E-06	9.52E-06	4.3183886
LINC00854	0.5552158	3.151132	4.8326498	1.98E-06	9.53E-06	4.3172467
CDK5R1	0.2908833	4.6849584	4.8322763	1.98E-06	9.54E-06	4.3155617
NR2F6	-0.106555	6.6422108	-4.831932	1.98E-06	9.56E-06	4.314009
CXADR	-0.144896	6.41814	-4.83133	1.99E-06	9.58E-06	4.3112901
LA16c-380H	-0.374296	4.8941109	-4.831137	1.99E-06	9.59E-06	4.3104201
SPDYE3	-0.109148	5.5264365	-4.830721	2.00E-06	9.61E-06	4.3085431
CTD-2647L4	0.6578577	2.8806031	4.8304736	2.00E-06	9.62E-06	4.3074287
FDX1L	-0.176826	4.9235265	-4.830393	2.00E-06	9.62E-06	4.3070666
C17orf70	-0.075181	6.3345182	-4.830243	2.00E-06	9.63E-06	4.3063891
KIAA2022	0.9784181	0.0837146	4.8294852	2.01E-06	9.66E-06	4.3029703
EXOSC7	-0.085196	6.0901744	-4.829373	2.01E-06	9.66E-06	4.3024659
LINC01128	-0.145568	5.5051828	-4.829228	2.01E-06	9.67E-06	4.3018119
RP11-96K19	-0.751896	2.0950465	-4.828375	2.02E-06	9.71E-06	4.2979651
RP11-142C4	-0.993998	0.608894	-4.827906	2.02E-06	9.73E-06	4.2958472
CTC-265F19	-0.619962	-0.903838	-4.827868	2.02E-06	9.73E-06	4.2956799
BCL9	0.1380695	5.9792688	4.8278591	2.02E-06	9.73E-06	4.2956379
TRGV4	0.8583753	0.2923575	4.827678	2.02E-06	9.73E-06	4.2948216
RP11-324D1	-0.234756	-1.467383	-4.826756	2.03E-06	9.78E-06	4.2906639
GPR149	-0.253817	-1.459008	-4.825842	2.04E-06	9.82E-06	4.2865455
RP11-369K1	0.9295276	-0.006244	4.8257252	2.04E-06	9.82E-06	4.2860188
SPCS2	-0.075074	6.364346	-4.825505	2.04E-06	9.83E-06	4.2850261
MXRA7	0.1632112	6.0267704	4.8252398	2.05E-06	9.84E-06	4.2838314
UBXN10-AS1	-0.979926	1.8715078	-4.825212	2.05E-06	9.84E-06	4.2837066
ZC3H12D	0.3750335	4.0908411	4.8248774	2.05E-06	9.85E-06	4.2821984
ENTHD1	0.7301756	-0.676851	4.8248435	2.05E-06	9.86E-06	4.2820457

MZT2B	-0.113469	6.4809952	-4.824729	2.05E-06	9.86E-06	4.281528
HNF4A-AS1	-0.892265	4.6485767	-4.824717	2.05E-06	9.86E-06	4.2814735
MDM2	-0.090096	6.5427925	-4.824274	2.06E-06	9.88E-06	4.2794776
RP11-524O1	-0.778307	0.2318816	-4.823925	2.06E-06	9.89E-06	4.2779061
RHOC	-0.096494	6.8132606	-4.823405	2.07E-06	9.92E-06	4.2755639
RNF11	-0.082632	6.4235593	-4.823187	2.07E-06	9.92E-06	4.2745834
FOXD1	0.9063848	-0.268647	4.8230017	2.07E-06	9.93E-06	4.2737476
NONOP2	0.5886198	3.1420041	4.8229411	2.07E-06	9.93E-06	4.2734746
PPIB	-0.082911	7.0478513	-4.82267	2.07E-06	9.94E-06	4.2722534
RASA4B	0.7771942	-0.154524	4.8224647	2.07E-06	9.95E-06	4.2713285
DOK5	0.533718	3.9696834	4.8221058	2.08E-06	9.97E-06	4.2697124
RP3-468K18	0.8901282	0.9901068	4.8220765	2.08E-06	9.97E-06	4.2695804
ZNF30-AS1	-0.787224	-0.072254	-4.821411	2.08E-06	1.00E-05	4.2665846
GOLGB1	-0.056732	6.5991291	-4.820672	2.09E-06	1.00E-05	4.2632568
C14orf37	0.6237845	3.6931685	4.8202812	2.10E-06	1.00E-05	4.2614959
MESDC2	-0.06448	6.4756582	-4.820281	2.10E-06	1.00E-05	4.2614956
IL22RA2	0.7252848	-0.779229	4.8200618	2.10E-06	1.01E-05	4.2605079
ATP5F1	-0.072192	6.7146908	-4.819122	2.11E-06	1.01E-05	4.2562791
ZNF503-AS2	-0.189993	5.0840535	-4.819078	2.11E-06	1.01E-05	4.25608
RP11-384K6	0.7390823	4.1309999	4.8190654	2.11E-06	1.01E-05	4.2560224
TMCO3	0.1323175	6.2507113	4.8187783	2.11E-06	1.01E-05	4.2547303
DSCR3	-0.073832	6.2933113	-4.81875	2.11E-06	1.01E-05	4.254603
MIR3653	0.7451715	0.019604	4.8184339	2.11E-06	1.01E-05	4.2531799
RP11-173C1	0.8322099	0.8037815	4.8179777	2.12E-06	1.01E-05	4.2511266
CLEC2B	0.2040009	5.2748802	4.8177971	2.12E-06	1.02E-05	4.250314
ZBTB32	0.6801382	2.7497346	4.8177032	2.12E-06	1.02E-05	4.2498914
HIATL1	0.0868591	6.0902134	4.8174058	2.12E-06	1.02E-05	4.2485532
ZNF586	0.1524435	5.0104899	4.8172907	2.13E-06	1.02E-05	4.2480351
RP1-97G4.1	-0.252473	-1.457265	-4.81721	2.13E-06	1.02E-05	4.2476725
RP11-355B1	-0.178264	4.6922717	-4.816173	2.14E-06	1.02E-05	4.243006
PTGES3	-0.059452	6.9603843	-4.815979	2.14E-06	1.02E-05	4.2421349
ZNF528	0.3310715	4.9102891	4.8154066	2.14E-06	1.03E-05	4.2395583
CMPK1	-0.07136	6.8297567	-4.815365	2.15E-06	1.03E-05	4.2393697
SYT8	1.1709945	2.6477991	4.8149016	2.15E-06	1.03E-05	4.2372868
RP11-401P9	-0.939213	-0.345357	-4.814709	2.15E-06	1.03E-05	4.2364186
APITD1	-0.188268	4.8779672	-4.814555	2.15E-06	1.03E-05	4.2357276
NUGGC	-0.563457	5.4161683	-4.814541	2.15E-06	1.03E-05	4.2356628
SNAPIN	-0.072051	6.2109308	-4.81436	2.16E-06	1.03E-05	4.2348486
DAG1	-0.074714	6.7805816	-4.814287	2.16E-06	1.03E-05	4.2345238
NAGPA	-0.100242	6.064247	-4.814187	2.16E-06	1.03E-05	4.2340707
LRRC63	0.9725809	1.223947	4.813976	2.16E-06	1.03E-05	4.233124
RCAN1	-0.147785	6.300986	-4.813216	2.17E-06	1.04E-05	4.2297049
MITF	0.2225006	5.0321184	4.8127657	2.17E-06	1.04E-05	4.2276816
CNST	-0.098276	6.2191066	-4.812545	2.17E-06	1.04E-05	4.2266875
FAM221B	-0.729214	2.1436302	-4.812356	2.18E-06	1.04E-05	4.2258385
BRINP2	0.9428767	0.1342143	4.8122649	2.18E-06	1.04E-05	4.2254299
RNA5SP46	-0.757005	-0.605808	-4.812188	2.18E-06	1.04E-05	4.2250862
SLAIN1	0.7855083	3.9721253	4.8118297	2.18E-06	1.04E-05	4.2234735
PALM2	-0.584203	3.8482871	-4.811491	2.18E-06	1.04E-05	4.2219509
APOL1	-0.13905	6.839008	-4.810943	2.19E-06	1.05E-05	4.2194867
OPHN1	0.4069623	4.4792481	4.8108448	2.19E-06	1.05E-05	4.2190463

COLEC11	0.3405524	5.9577199	4.8101972	2.20E-06	1.05E-05	4.2161359
LDLRAD4-AS	-1.066887	0.2598034	-4.810182	2.20E-06	1.05E-05	4.2160654
STARD7	-0.056837	6.743237	-4.810071	2.20E-06	1.05E-05	4.2155681
SLC3A2	-0.100998	6.6643161	-4.809926	2.20E-06	1.05E-05	4.2149166
VWA5B2	0.7463414	2.4669749	4.8096938	2.20E-06	1.05E-05	4.2138735
FKBP3	-0.073757	6.3422828	-4.809691	2.20E-06	1.05E-05	4.2138608
XPNPEP3	-0.084677	6.1084242	-4.809471	2.21E-06	1.05E-05	4.2128726
ST6GALNAC6	-0.113238	6.3690683	-4.808927	2.21E-06	1.05E-05	4.2104302
GPR62	-0.998922	1.9666766	-4.808587	2.21E-06	1.06E-05	4.2089016
CYP4F35P	0.8571788	-0.221456	4.8079486	2.22E-06	1.06E-05	4.2060325
WDR53	-0.076548	5.4785623	-4.807691	2.22E-06	1.06E-05	4.2048755
LMCD1-AS1	0.5278844	2.7727641	4.8066003	2.24E-06	1.07E-05	4.1999764
DACH1	0.7183101	2.9843029	4.8062671	2.24E-06	1.07E-05	4.1984798
LAMB2	-0.110239	6.8174887	-4.80624	2.24E-06	1.07E-05	4.198357
COL6A5	0.7280163	-0.703886	4.8058169	2.24E-06	1.07E-05	4.1964584
AZGP1P1	-0.608124	5.3192371	-4.805174	2.25E-06	1.07E-05	4.1935693
ELMO2	0.0754848	6.0347211	4.804425	2.26E-06	1.08E-05	4.1902087
FAM172BP	0.6404721	-0.832322	4.8041568	2.26E-06	1.08E-05	4.1890049
SLC13A5	-0.501377	6.582201	-4.803877	2.26E-06	1.08E-05	4.1877472
RASAL2	0.1441151	5.8486471	4.8036637	2.27E-06	1.08E-05	4.1867913
KRAS	0.0924291	6.0778301	4.8033737	2.27E-06	1.08E-05	4.1854895
SIAH2-AS1	-0.800921	2.2951539	-4.803004	2.27E-06	1.08E-05	4.1838284
GABARAPL1	-0.164378	6.5192232	-4.802909	2.27E-06	1.08E-05	4.183402
RPL17P11	-1.073093	1.3671708	-4.80274	2.28E-06	1.08E-05	4.1826476
DDX24	-0.06877	6.6265942	-4.802708	2.28E-06	1.08E-05	4.1825
RP11-482D2	0.8263595	0.0607631	4.8021296	2.28E-06	1.09E-05	4.1799063
RP11-253M7	0.7992494	1.671517	4.8017237	2.29E-06	1.09E-05	4.1780847
CTB-147N14	0.9555028	1.2311834	4.8015657	2.29E-06	1.09E-05	4.1773759
GS1-388B5.	-0.544251	-1.088779	-4.801019	2.29E-06	1.09E-05	4.1749208
RP11-133N2	-0.775808	0.1874815	-4.800604	2.30E-06	1.09E-05	4.1730617
NHLRC1	-0.22816	5.1189494	-4.800504	2.30E-06	1.09E-05	4.1726117
C11orf96	0.2573051	5.6410335	4.8004749	2.30E-06	1.09E-05	4.1724819
SF3B5	-0.107768	6.5363638	-4.80022	2.30E-06	1.09E-05	4.1713401
RP11-405A1	0.9000396	-0.39789	4.8000482	2.31E-06	1.10E-05	4.1705677
LYPLA2	-0.083538	6.5647365	-4.80003	2.31E-06	1.10E-05	4.1704856
HRK	0.8750432	-0.337707	4.8000154	2.31E-06	1.10E-05	4.1704208
RP11-400N9	0.5234024	-0.996175	4.7999645	2.31E-06	1.10E-05	4.1701922
ZNF804A	1.0152646	1.5255771	4.7997733	2.31E-06	1.10E-05	4.1693345
TXNDC5	0.5760042	5.0873365	4.7995446	2.31E-06	1.10E-05	4.1683089
NABP2	-0.086618	6.183417	-4.799391	2.31E-06	1.10E-05	4.1676186
HMGB3P32	0.7831363	-0.205766	4.7989353	2.32E-06	1.10E-05	4.1655762
RAP2A	0.1114345	6.2076553	4.7987769	2.32E-06	1.10E-05	4.1648655
C9orf16	-0.135901	6.235364	-4.79858	2.32E-06	1.10E-05	4.1639825
KRTCAP2	-0.109894	6.2159706	-4.798177	2.33E-06	1.10E-05	4.1621767
WARS2-IT1	0.8595611	-0.01328	4.797979	2.33E-06	1.10E-05	4.1612875
CTD-2583A1	0.165034	4.7661422	4.7976362	2.33E-06	1.11E-05	4.1597507
ITGA5	0.092306	6.643679	4.7974957	2.33E-06	1.11E-05	4.1591204
CTA-217C2.	0.8014251	1.6548768	4.7972285	2.34E-06	1.11E-05	4.1579226
ZFC3H1	0.0916248	5.9077104	4.7968594	2.34E-06	1.11E-05	4.1562677
RBMS1	0.1416178	6.0614075	4.7962534	2.35E-06	1.11E-05	4.1535511
CPTP	-0.118351	6.3750457	-4.796207	2.35E-06	1.11E-05	4.153342

TPM3P9	0.2588	5.1031344	4.7959422	2.35E-06	1.11E-05	4.1521565
SFTA2	0.891307	-0.442517	4.7956562	2.35E-06	1.12E-05	4.1508742
CNOT4	-0.064009	5.958791	-4.795633	2.35E-06	1.12E-05	4.1507722
CCDC149	0.2223129	5.5282763	4.7955676	2.35E-06	1.12E-05	4.1504772
UGT2B15	-0.477657	6.6061986	-4.795568	2.35E-06	1.12E-05	4.150477
DISC1	0.2838885	4.7899161	4.7951713	2.36E-06	1.12E-05	4.1487011
RP11-452L6	-0.816787	-0.100379	-4.795145	2.36E-06	1.12E-05	4.148584
TBX21	0.4038761	3.9374613	4.7947882	2.36E-06	1.12E-05	4.1469842
OR10J5	-0.526258	-1.066994	-4.794255	2.37E-06	1.12E-05	4.1445965
RP11-433P1	-0.383551	-1.281633	-4.793891	2.37E-06	1.12E-05	4.1429624
KIAA0232	-0.07766	6.37743	-4.793428	2.38E-06	1.13E-05	4.1408884
FKBP9	0.0740053	6.3297	4.7930546	2.38E-06	1.13E-05	4.1392164
RP11-208N2	-0.213746	-1.487777	-4.792532	2.39E-06	1.13E-05	4.1368772
SIGLEC5	0.8209545	1.9083749	4.7924039	2.39E-06	1.13E-05	4.1363015
RP11-290F5	-0.567366	4.9629075	-4.791163	2.40E-06	1.14E-05	4.1307429
RP11-273G1	1.1006437	2.5432847	4.7900719	2.42E-06	1.14E-05	4.1258579
ZNF112	0.3175959	4.5344634	4.7898962	2.42E-06	1.14E-05	4.1250713
TMEM175	-0.102924	6.1764452	-4.789865	2.42E-06	1.14E-05	4.1249324
SHPK	-0.158635	5.4492853	-4.789645	2.42E-06	1.14E-05	4.123948
RP11-434P1	0.7020832	-0.341441	4.7889733	2.43E-06	1.15E-05	4.1209392
CACTIN-AS1	0.6876685	1.9150356	4.7884782	2.43E-06	1.15E-05	4.1187231
TNNT1	1.0931587	2.06414	4.7883562	2.44E-06	1.15E-05	4.118177
AMDHD1	-0.285887	6.3057189	-4.788119	2.44E-06	1.15E-05	4.1171157
RP11-2E11.	0.7053751	-0.286319	4.7876004	2.44E-06	1.16E-05	4.1147941
RPL9P29	-0.507674	2.9883115	-4.786915	2.45E-06	1.16E-05	4.1117254
OFCC1	0.9014091	-0.621315	4.7867463	2.45E-06	1.16E-05	4.1109722
F11	-0.373391	6.2766837	-4.785926	2.46E-06	1.16E-05	4.1073014
CCT6B	-0.360063	4.5108943	-4.784753	2.48E-06	1.17E-05	4.1020559
NUAK1	0.1206229	5.9049288	4.7844419	2.48E-06	1.17E-05	4.1006627
RP11-264B1	0.8087936	2.5357187	4.7835581	2.49E-06	1.18E-05	4.0967097
RP11-59N23	-0.850721	0.3387145	-4.783452	2.49E-06	1.18E-05	4.0962359
LRRC4B	0.3308011	4.431854	4.7833199	2.49E-06	1.18E-05	4.0956447
RP11-142G1	-0.2698	-1.438489	-4.783192	2.50E-06	1.18E-05	4.0950724
VN1R25P	-0.197094	-1.495093	-4.782936	2.50E-06	1.18E-05	4.0939265
RTKN2	0.6879745	4.0524878	4.7826563	2.50E-06	1.18E-05	4.0926774
SLC25A46	-0.086051	6.2779824	-4.782545	2.50E-06	1.18E-05	4.0921789
TMUB2	-0.066326	6.2704852	-4.781944	2.51E-06	1.18E-05	4.0894916
AC008074.1	0.7600499	0.3921684	4.7818371	2.51E-06	1.18E-05	4.0890146
CHORDC1	0.1190563	5.8417805	4.7817186	2.51E-06	1.19E-05	4.0884848
RP4-584D14	0.6644087	3.291645	4.7805894	2.53E-06	1.19E-05	4.0834369
HES6	-0.1707	5.5192638	-4.780356	2.53E-06	1.19E-05	4.0823927
RHCE	-0.678786	3.7245036	-4.780254	2.53E-06	1.19E-05	4.0819361
IGKJ5	0.664813	-0.895735	4.7800823	2.53E-06	1.19E-05	4.0811704
FAM163A	0.941282	1.3470736	4.7797875	2.54E-06	1.20E-05	4.0798531
C1orf186	1.0176554	3.6873571	4.7787741	2.55E-06	1.20E-05	4.0753246
STAC2	0.8606316	-0.26356	4.7780562	2.56E-06	1.20E-05	4.0721171
ASL	-0.156557	6.7568215	-4.777923	2.56E-06	1.21E-05	4.0715204
ALOX12	0.4887355	3.7989179	4.7776476	2.56E-06	1.21E-05	4.0702918
GPR31	0.6985891	-0.708576	4.7776266	2.56E-06	1.21E-05	4.0701978
SCMH1	0.0933748	5.9229733	4.7774637	2.56E-06	1.21E-05	4.0694701
RN7SL608P	0.7492681	1.2324569	4.7769746	2.57E-06	1.21E-05	4.0672854

TTC9	-0.320062	5.7531549	-4.776919	2.57E-06	1.21E-05	4.0670376
RP11-147L1	0.8432638	0.8247964	4.776776	2.57E-06	1.21E-05	4.0663984
CCNA1	0.6815709	-0.77238	4.7767007	2.57E-06	1.21E-05	4.0660623
ZNF408	-0.083999	5.9737334	-4.77657	2.57E-06	1.21E-05	4.0654775
RN7SL832P	-0.482922	3.1037195	-4.776343	2.58E-06	1.21E-05	4.0644661
TMEM140	-0.119105	6.4116415	-4.776342	2.58E-06	1.21E-05	4.0644622
RAPGEF1	0.0676149	6.4428214	4.7762967	2.58E-06	1.21E-05	4.0642577
IMPG2	0.7021829	2.4010171	4.7759438	2.58E-06	1.21E-05	4.0626815
C14orf28	-0.128981	5.2731732	-4.775885	2.58E-06	1.21E-05	4.0624184
HNRNPA2B1	-0.044773	7.1669789	-4.775798	2.58E-06	1.21E-05	4.0620306
AC002451.3	-0.700123	2.6603081	-4.775553	2.59E-06	1.22E-05	4.0609355
CTA-268H5.	0.8917677	0.3141378	4.7752459	2.59E-06	1.22E-05	4.0595652
HP09025	-0.920194	1.0162965	-4.775246	2.59E-06	1.22E-05	4.0595652
RP11-311F1	-0.902314	-0.580977	-4.774769	2.60E-06	1.22E-05	4.057435
PNMA3	0.7846968	3.9875021	4.7745075	2.60E-06	1.22E-05	4.0562681
SORL1	-0.177014	6.498823	-4.774365	2.60E-06	1.22E-05	4.0556311
COX15	-0.070849	6.2989086	-4.772495	2.62E-06	1.23E-05	4.0472853
AHCY	-0.092679	6.9681763	-4.772423	2.62E-06	1.23E-05	4.0469646
PCDHB4	0.6212039	3.5122269	4.7720849	2.63E-06	1.23E-05	4.0454544
DFFB	0.1549915	4.9844582	4.771235	2.64E-06	1.24E-05	4.0416621
WASIR2	0.8566484	-0.177834	4.7710775	2.64E-06	1.24E-05	4.0409595
FAM83C-AS1	-0.826421	1.1069282	-4.770646	2.65E-06	1.24E-05	4.0390321
RP11-451N1	-0.241266	-1.457895	-4.769904	2.66E-06	1.25E-05	4.0357255
TAS1R3	0.6069926	3.4976463	4.7697177	2.66E-06	1.25E-05	4.0348929
RP11-798K2	0.5100166	3.0769285	4.7696393	2.66E-06	1.25E-05	4.0345432
F7	-0.302446	6.6864472	-4.76928	2.66E-06	1.25E-05	4.0329396
MOGAT1	-0.924161	2.4107911	-4.769113	2.67E-06	1.25E-05	4.0321979
EMILIN3	0.852626	1.7310972	4.7690832	2.67E-06	1.25E-05	4.0320629
MPP7	0.3563533	5.250312	4.7683275	2.67E-06	1.26E-05	4.0286925
SMYD2	-0.124026	6.4104171	-4.768206	2.68E-06	1.26E-05	4.028152
RP11-302M6	-0.764835	0.4612161	-4.767339	2.69E-06	1.26E-05	4.024286
RP11-412D9	-0.233086	4.5305671	-4.766931	2.69E-06	1.26E-05	4.0224667
SIK2	-0.108379	6.6164984	-4.766545	2.70E-06	1.26E-05	4.020745
AMY2B	-0.257889	5.3480438	-4.766528	2.70E-06	1.26E-05	4.0206687
TIGD2	-0.201736	5.8217383	-4.765547	2.71E-06	1.27E-05	4.0162976
SLC25A52	-0.850551	-0.232442	-4.765488	2.71E-06	1.27E-05	4.0160348
RMND5A	-0.089886	6.5718958	-4.765239	2.71E-06	1.27E-05	4.0149232
SH2D3A	0.2939224	4.9191736	4.7651908	2.71E-06	1.27E-05	4.0147081
TECRP1	-0.258822	4.3713782	-4.764568	2.72E-06	1.28E-05	4.0119325
LINC01448	-0.980057	-0.345981	-4.764432	2.72E-06	1.28E-05	4.011328
ZNF282	-0.07207	6.1982667	-4.763844	2.73E-06	1.28E-05	4.0087067
AC002979.1	-0.430077	-1.267069	-4.763175	2.74E-06	1.28E-05	4.0057242
YTHDF2	-0.055197	6.3868103	-4.762688	2.75E-06	1.29E-05	4.0035541
RP11-138I1	0.7949075	1.6047714	4.76263	2.75E-06	1.29E-05	4.0032975
PCDHGB5	0.7987986	3.8531989	4.7624716	2.75E-06	1.29E-05	4.0025921
KB-1269D1.	0.5395348	-1.106293	4.7620403	2.75E-06	1.29E-05	4.0006706
PSMB6	-0.099824	6.6059544	-4.760986	2.77E-06	1.30E-05	3.9959748
C6orf1	-0.121304	5.976825	-4.760696	2.77E-06	1.30E-05	3.9946819
AC002511.3	0.7366644	-0.731789	4.7598654	2.78E-06	1.30E-05	3.9909851
CSGALNACT2	0.1039032	5.8511117	4.7594751	2.79E-06	1.30E-05	3.9892473
PCDHB7	0.6698346	3.4641445	4.7586724	2.80E-06	1.31E-05	3.9856737

RP11-410L1	-0.345473	4.0041283	-4.758566	2.80E-06	1.31E-05	3.9851983
AP2M1	-0.063937	6.9193202	-4.758547	2.80E-06	1.31E-05	3.985115
RP11-295D4	0.7340593	3.3408968	4.75842	2.80E-06	1.31E-05	3.9845502
LINC00967	-0.795548	-0.608705	-4.75726	2.82E-06	1.32E-05	3.979386
SLC6A1	-0.301007	6.5050729	-4.757084	2.82E-06	1.32E-05	3.9786053
SLC13A2	1.0544885	3.7091366	4.756955	2.82E-06	1.32E-05	3.9780296
LRRC37A17F	0.3182801	4.5094611	4.7567511	2.82E-06	1.32E-05	3.9771222
SENP7	0.1138791	5.6765632	4.7567348	2.82E-06	1.32E-05	3.97705
SLC25A53	-0.141424	4.9028277	-4.756607	2.83E-06	1.32E-05	3.9764808
PSMD9	-0.078935	5.9927225	-4.756539	2.83E-06	1.32E-05	3.9761778
LSM12P1	0.5305036	3.8072442	4.7564161	2.83E-06	1.32E-05	3.9756314
GRID1	0.878263	3.9576796	4.7559575	2.83E-06	1.32E-05	3.9735913
TPSG1	1.1688693	1.5657182	4.7556817	2.84E-06	1.33E-05	3.9723641
LINC00683	0.9133334	-0.349079	4.7556097	2.84E-06	1.33E-05	3.9720438
RCBTB2	0.1291664	5.3982951	4.7553918	2.84E-06	1.33E-05	3.9710741
MRE11A	0.0791351	5.7921563	4.7551047	2.85E-06	1.33E-05	3.9697969
RNU7-154P	-0.590129	-0.96767	-4.755038	2.85E-06	1.33E-05	3.9694982
FBLN7	-0.233581	5.7293369	-4.754848	2.85E-06	1.33E-05	3.9686542
CST5	1.0421263	-0.046825	4.7547362	2.85E-06	1.33E-05	3.9681579
CMTM4	0.1627597	5.7224657	4.7541031	2.86E-06	1.33E-05	3.9653416
RBM8A	-0.069194	6.584956	-4.753782	2.86E-06	1.34E-05	3.963912
RP13-516M1	-0.79148	1.8315457	-4.752637	2.88E-06	1.34E-05	3.958822
PAK6	0.6719842	-0.829841	4.7524998	2.88E-06	1.34E-05	3.9582114
LINC00968	0.5850285	-0.946831	4.7523459	2.88E-06	1.34E-05	3.9575275
KB-1980E6.	0.6980324	-0.844267	4.7523354	2.88E-06	1.34E-05	3.9574806
RP11-531F1	0.5864914	2.8073865	4.7519515	2.89E-06	1.35E-05	3.9557737
RP11-423E7	0.8254173	0.5085761	4.7519121	2.89E-06	1.35E-05	3.9555985
AC005082.1	0.8441898	0.8641775	4.7517525	2.89E-06	1.35E-05	3.9548888
AC093850.2	0.9531497	1.1310832	4.7515938	2.89E-06	1.35E-05	3.9541835
FAM35A	-0.091893	6.1972104	-4.750631	2.91E-06	1.35E-05	3.9499054
SDCBP	-0.101411	6.7589933	-4.750397	2.91E-06	1.36E-05	3.9488643
FAM156A	0.7361008	1.0719542	4.7502634	2.91E-06	1.36E-05	3.9482694
CKMT1B	1.2047296	0.5612739	4.7497828	2.92E-06	1.36E-05	3.9461337
MYO1D	0.1873682	6.3069741	4.7493902	2.92E-06	1.36E-05	3.944389
RP11-230F1	0.7867006	-0.05624	4.7490955	2.93E-06	1.36E-05	3.9430793
GSTA5	-0.702914	-0.615288	-4.748418	2.94E-06	1.37E-05	3.9400676
RP3-402G11	-0.317828	4.1118806	-4.747711	2.95E-06	1.37E-05	3.9369284
RP11-115J1	-0.8382	4.2646049	-4.747632	2.95E-06	1.37E-05	3.936575
LINC00870	-0.500779	4.2161241	-4.747483	2.95E-06	1.37E-05	3.9359137
NETO2	0.2843646	4.7976644	4.7470753	2.95E-06	1.37E-05	3.9341039
CFD	0.2638369	5.1628619	4.746927	2.96E-06	1.38E-05	3.9334449
LRRC61	-0.12849	6.1572134	-4.746298	2.96E-06	1.38E-05	3.9306511
NOL7	-0.076679	6.3700275	-4.745759	2.97E-06	1.38E-05	3.9282558
CH17-385C1	0.8128873	0.2437436	4.7451146	2.98E-06	1.39E-05	3.9253961
TTC3P1	0.682286	4.0281878	4.7449171	2.98E-06	1.39E-05	3.9245192
PIK3C2B	0.123122	6.0190402	4.7448991	2.98E-06	1.39E-05	3.924439
SLC38A2	-0.114548	6.8401151	-4.744886	2.98E-06	1.39E-05	3.9243816
RP11-343P9	-0.479929	-1.111696	-4.744352	2.99E-06	1.39E-05	3.9220108
PGAP2	-0.084794	6.2319211	-4.74423	2.99E-06	1.39E-05	3.9214667
VPS37C	0.0686079	5.9104272	4.7441592	2.99E-06	1.39E-05	3.921154
PCDHGB3	0.9856211	1.8655732	4.7441142	2.99E-06	1.39E-05	3.9209543

SBK2	0.5682891	-1.09315	4.7424212	3.02E-06	1.40E-05	3.9134395
RP11-122K1	-0.851451	-0.002011	-4.742248	3.02E-06	1.40E-05	3.9126696
ADTRP	0.9010287	3.3170422	4.7419925	3.02E-06	1.41E-05	3.911537
RP11-674P1	0.7531109	-0.269471	4.7419022	3.03E-06	1.41E-05	3.911136
RP11-488L1	0.2136537	5.1837543	4.7414653	3.03E-06	1.41E-05	3.9091975
SMC3	0.1100028	6.2402602	4.7413236	3.03E-06	1.41E-05	3.9085686
RNF215	-0.090158	5.885983	-4.741188	3.04E-06	1.41E-05	3.907968
PLG	-0.360155	7.2177175	-4.741104	3.04E-06	1.41E-05	3.9075924
PABPC1P4	0.8070671	2.3225171	4.7409598	3.04E-06	1.41E-05	3.9069545
SMTNL2	0.881519	1.3374904	4.7401209	3.05E-06	1.42E-05	3.9032326
RP11-216N1	0.8223117	0.7150024	4.7399607	3.05E-06	1.42E-05	3.902522
PLEKHS1	1.0946514	1.5256166	4.7397234	3.06E-06	1.42E-05	3.9014691
LPP-AS1	-0.636162	-0.951797	-4.739398	3.06E-06	1.42E-05	3.900028
RP11-123K1	0.6425487	-1.014476	4.7393974	3.06E-06	1.42E-05	3.900023
TMBIM4	-0.083231	6.405326	-4.737973	3.08E-06	1.43E-05	3.8937073
TRBV5-5	0.6238087	-0.832327	4.7368465	3.10E-06	1.44E-05	3.8887111
IQCB1	0.0987358	5.6407183	4.7363899	3.10E-06	1.44E-05	3.8866868
AC003988.1	-0.564313	-1.055578	-4.736277	3.11E-06	1.44E-05	3.8861872
MS4A2	1.0631272	1.1736889	4.7360322	3.11E-06	1.44E-05	3.8851013
JMJD4	-0.10858	6.0672133	-4.735968	3.11E-06	1.44E-05	3.8848159
CLTC	-0.054085	7.040846	-4.735886	3.11E-06	1.44E-05	3.884451
TMEM26-AS1	0.6541714	-0.594239	4.7356802	3.11E-06	1.44E-05	3.8835409
IGHVII-78-	0.6277202	-0.861576	4.7351936	3.12E-06	1.45E-05	3.8813842
AC004160.4	-1.014991	0.1726032	-4.735021	3.12E-06	1.45E-05	3.8806191
IFT88	-0.107927	5.8207751	-4.735015	3.12E-06	1.45E-05	3.8805938
VIM	0.0993496	6.8965467	4.734525	3.13E-06	1.45E-05	3.8784208
RP11-504G3	0.7673319	0.185494	4.7344898	3.13E-06	1.45E-05	3.878265
AC114812.9	-0.749515	-0.423483	-4.73417	3.14E-06	1.45E-05	3.8768497
FAM72B	0.7376485	3.0223388	4.7341491	3.14E-06	1.45E-05	3.876755
AC008440.1	0.7296534	-0.453163	4.7333119	3.15E-06	1.46E-05	3.8730453
RP11-402D2	-0.826021	1.0252947	-4.73288	3.16E-06	1.46E-05	3.8711321
RP4-800G7.	0.7169518	-0.256408	4.7328462	3.16E-06	1.46E-05	3.8709824
RP9	-0.111078	5.4404971	-4.73227	3.16E-06	1.46E-05	3.8684304
PPP6R1	0.0709895	6.5207979	4.7308695	3.19E-06	1.47E-05	3.8622267
RP11-132A1	-0.991679	3.3170735	-4.730748	3.19E-06	1.47E-05	3.8616876
GREM2	-1.020228	4.5809027	-4.730669	3.19E-06	1.48E-05	3.8613372
MIR3646	-0.788175	2.1084769	-4.730358	3.19E-06	1.48E-05	3.859961
CHRNA1	0.9991338	0.7104368	4.7300966	3.20E-06	1.48E-05	3.858804
RP5-1050E1	-1.067559	0.1661313	-4.729922	3.20E-06	1.48E-05	3.8580317
CD4	0.1608847	6.2577385	4.7298615	3.20E-06	1.48E-05	3.8577633
LL22NC03-2	0.644558	-0.932771	4.7297329	3.20E-06	1.48E-05	3.8571938
DDX1	-0.055291	6.5897632	-4.729464	3.21E-06	1.48E-05	3.8560017
AC245100.1	-0.261367	4.8440159	-4.729184	3.21E-06	1.48E-05	3.8547644
AAR2	-0.05506	6.3341857	-4.729123	3.21E-06	1.48E-05	3.8544943
CTB-31N19.	-0.523345	-1.101944	-4.729106	3.21E-06	1.48E-05	3.8544172
OR11Q1P	1.1811898	0.1821393	4.7288292	3.22E-06	1.49E-05	3.8531929
HOXB5	0.3695486	3.8533648	4.7283237	3.22E-06	1.49E-05	3.850955
CFAP221	0.9519566	3.5998748	4.7279997	3.23E-06	1.49E-05	3.8495212
CDC42EP3	0.1715714	5.5490527	4.7277518	3.23E-06	1.49E-05	3.8484239
TMEM230	-0.072084	6.7120041	-4.727595	3.23E-06	1.49E-05	3.8477283
ERAP1	-0.113255	6.4524422	-4.72732	3.24E-06	1.50E-05	3.846512

PSMA3	-0.076752	6.5693493	-4.726807	3.25E-06	1.50E-05	3.8442432
LINC00845	-0.679035	-0.953964	-4.726801	3.25E-06	1.50E-05	3.8442157
LRRN4CL	0.9839776	1.06548	4.7265423	3.25E-06	1.50E-05	3.843071
RP11-388P9	0.6331951	-0.842369	4.7265098	3.25E-06	1.50E-05	3.8429275
GUSBP9	0.828862	0.3757812	4.7263236	3.25E-06	1.50E-05	3.8421033
ZNF141	0.3769076	4.7990178	4.7257302	3.26E-06	1.51E-05	3.8394779
FAM213B	0.1444503	5.9805319	4.7255325	3.26E-06	1.51E-05	3.8386031
SCAMP1-AS1	-0.115613	5.2621851	-4.725404	3.27E-06	1.51E-05	3.8380329
AC007919.2	-0.385538	-1.332666	-4.725329	3.27E-06	1.51E-05	3.8377031
LINC00961	-0.305718	4.0860139	-4.724624	3.28E-06	1.51E-05	3.8345828
TMED7	-0.084144	6.6513269	-4.723645	3.29E-06	1.52E-05	3.8302547
RP11-881M1	-0.62158	2.8977072	-4.723378	3.30E-06	1.52E-05	3.8290754
TAF1B	0.1082445	5.4528703	4.7223561	3.31E-06	1.53E-05	3.8245548
LHFPL3	0.992746	-0.145995	4.7223105	3.31E-06	1.53E-05	3.8243528
LLOXNC01-2	0.7813241	2.1372526	4.7221743	3.32E-06	1.53E-05	3.8237507
ZNF784	-0.118691	5.7157199	-4.722156	3.32E-06	1.53E-05	3.8236695
RP11-154H2	0.866701	1.1601708	4.7201655	3.35E-06	1.54E-05	3.814871
APOC2	-0.341366	6.3202999	-4.719497	3.36E-06	1.55E-05	3.8119146
TAF1A	0.2294032	4.8967868	4.7192134	3.36E-06	1.55E-05	3.8106631
NREP	0.1654638	6.1826916	4.7191294	3.36E-06	1.55E-05	3.810292
SGTB	0.1498095	5.2370213	4.7184508	3.37E-06	1.55E-05	3.8072934
MEF2C	0.1343935	5.7703659	4.7181544	3.38E-06	1.56E-05	3.8059841
PCDHGA6	0.4450317	4.0285588	4.7181257	3.38E-06	1.56E-05	3.8058572
DUS2	-0.10872	5.7229156	-4.717952	3.38E-06	1.56E-05	3.8050889
ST8SIA6-AS	-1.388558	3.1210003	-4.717763	3.38E-06	1.56E-05	3.8042535
AC005754.8	0.9634465	0.4029241	4.7174375	3.39E-06	1.56E-05	3.8028169
TFF3	0.4888642	4.847891	4.7170825	3.40E-06	1.56E-05	3.8012487
CARHSP1	-0.091361	6.6682293	-4.717028	3.40E-06	1.56E-05	3.8010066
KBTBD4	0.2906847	5.2479908	4.7166787	3.40E-06	1.57E-05	3.7994651
RP13-672B3	-0.788292	2.0116812	-4.716325	3.41E-06	1.57E-05	3.7979021
KARS	-0.077191	6.6446192	-4.71588	3.41E-06	1.57E-05	3.7959358
AC007163.2	-0.899811	-0.771337	-4.715712	3.42E-06	1.57E-05	3.7951965
IGLC6	1.0080955	0.1303185	4.7152918	3.42E-06	1.57E-05	3.7933405
GSTA2	-0.715322	5.941096	-4.714996	3.43E-06	1.58E-05	3.7920321
RP11-156K1	1.0443543	1.1004341	4.7139622	3.44E-06	1.58E-05	3.7874702
GNL2	-0.068979	6.3809024	-4.713772	3.45E-06	1.59E-05	3.786629
FAM72A	0.4822208	3.4843183	4.7137432	3.45E-06	1.59E-05	3.7865034
ZSCAN12P1	0.3824017	3.8715599	4.7135923	3.45E-06	1.59E-05	3.7858371
PKLR	-0.481535	6.4743008	-4.713372	3.45E-06	1.59E-05	3.7848626
LCOR	0.1130103	5.8660551	4.7131933	3.46E-06	1.59E-05	3.7840757
SH3GL1P1	0.7131891	2.0437793	4.7129554	3.46E-06	1.59E-05	3.783026
ZNF680	-0.179834	5.6032013	-4.712936	3.46E-06	1.59E-05	3.7829409
RP11-4B16.	0.819928	-0.130131	4.7127133	3.46E-06	1.59E-05	3.7819575
MATK	0.2360819	4.8583023	4.7122232	3.47E-06	1.59E-05	3.7797945
RP11-1275H	-0.298091	4.4082554	-4.712034	3.48E-06	1.60E-05	3.7789574
PSPN	-0.145144	4.9591596	-4.71189	3.48E-06	1.60E-05	3.778322
RP11-408H2	-0.897528	0.8092324	-4.711377	3.49E-06	1.60E-05	3.7760615
PLA2G16	-0.193856	6.2967645	-4.710928	3.49E-06	1.60E-05	3.7740809
ZNF92	0.1550283	5.2399737	4.7107169	3.50E-06	1.61E-05	3.7731477
RASA2	0.1360763	5.579168	4.7101551	3.51E-06	1.61E-05	3.7706692
Z95114.4	-0.345054	-1.360774	-4.710075	3.51E-06	1.61E-05	3.7703174

RP11-235G2	1.000072	0.1483229	4.7098606	3.51E-06	1.61E-05	3.7693703
AC141586.5	0.2876382	4.1964513	4.7094061	3.52E-06	1.61E-05	3.7673653
RP11-274B2	0.9037758	2.8574878	4.7093131	3.52E-06	1.61E-05	3.7669552
C16orf91	-0.103837	5.713678	-4.708761	3.53E-06	1.62E-05	3.7645221
PRELID1	-0.083502	6.443209	-4.70839	3.53E-06	1.62E-05	3.7628847
RP4-816N1.	-0.5699	2.481754	-4.707805	3.54E-06	1.63E-05	3.7603026
CTD-2162K1	0.9745799	0.7568639	4.7072018	3.55E-06	1.63E-05	3.7576448
C1QC	0.1634489	6.480714	4.706812	3.56E-06	1.63E-05	3.7559262
CTD-2650P2	-0.669553	2.7059815	-4.706711	3.56E-06	1.63E-05	3.7554822
DLK1	1.5348339	1.8706755	4.7065424	3.57E-06	1.63E-05	3.754738
MYCN	0.8562264	3.6132203	4.7065297	3.57E-06	1.63E-05	3.7546819
RP11-432J2	0.9308497	-0.387073	4.7061825	3.57E-06	1.64E-05	3.7531511
SOX15	0.4597412	3.0719176	4.7051042	3.59E-06	1.64E-05	3.7483984
NRCAM	0.9132556	4.3080711	4.7049382	3.59E-06	1.65E-05	3.7476668
PAIP2	-0.067462	6.4118123	-4.704644	3.60E-06	1.65E-05	3.7463706
TEX15	1.0155101	-0.337926	4.7045065	3.60E-06	1.65E-05	3.7457645
CACNG4	1.2549964	2.7185159	4.704472	3.60E-06	1.65E-05	3.7456124
RP11-325B2	-0.30879	-1.380944	-4.704236	3.60E-06	1.65E-05	3.7445745
RP11-83A24	-0.143345	4.9397855	-4.704101	3.61E-06	1.65E-05	3.7439757
TMEM160	-0.168256	5.469203	-4.703107	3.62E-06	1.66E-05	3.7395986
ZUFSP	0.1027352	5.3288739	4.7029096	3.63E-06	1.66E-05	3.7387284
ALDOB	-0.298683	7.4364188	-4.70274	3.63E-06	1.66E-05	3.7379813
RP11-104H1	-0.781334	-0.103091	-4.702268	3.64E-06	1.66E-05	3.735901
AC112497.1	0.337516	4.2400165	4.7021189	3.64E-06	1.66E-05	3.7352455
HSPB8	0.4408363	5.3875519	4.7020951	3.64E-06	1.66E-05	3.7351404
MTG2	-0.086494	6.3541718	-4.701981	3.64E-06	1.67E-05	3.7346358
FCHSD2	0.0807171	5.9938721	4.7014977	3.65E-06	1.67E-05	3.7325093
SYT3	1.1509202	1.8255717	4.7014652	3.65E-06	1.67E-05	3.732366
PCYOX1	-0.095378	6.7396643	-4.701173	3.65E-06	1.67E-05	3.73108
C6orf3	0.3284333	3.6390157	4.701062	3.66E-06	1.67E-05	3.7305903
ZNF484	0.176645	4.6415108	4.7001277	3.67E-06	1.68E-05	3.7264763
PSMD7	-0.077086	6.3846503	-4.698557	3.70E-06	1.69E-05	3.7195611
KCNG3	0.7719176	-0.696302	4.6985187	3.70E-06	1.69E-05	3.719393
RP11-701P1	-0.930452	0.6864492	-4.698139	3.71E-06	1.69E-05	3.717723
FAM96A	-0.089956	6.5583566	-4.697745	3.71E-06	1.70E-05	3.7159892
MAP2K4P1	0.7866819	0.7708886	4.6976221	3.72E-06	1.70E-05	3.7154467
NSUN4	-0.073337	5.9702233	-4.697195	3.72E-06	1.70E-05	3.7135685
CLEC12B	0.7450115	-0.319657	4.6971517	3.72E-06	1.70E-05	3.7133763
RP11-715J2	-0.533764	3.0197369	-4.697143	3.72E-06	1.70E-05	3.7133376
HCG23	0.6798676	-0.516185	4.6970959	3.72E-06	1.70E-05	3.7131308
AC116366.6	0.5298259	3.7557355	4.6968846	3.73E-06	1.70E-05	3.712201
RP11-428K3	-0.453334	4.242924	-4.696459	3.74E-06	1.71E-05	3.7103264
USP51	0.4329878	4.1753049	4.6963393	3.74E-06	1.71E-05	3.7098017
NOXA1	-0.146121	5.8088307	-4.695994	3.74E-06	1.71E-05	3.7082819
RP11-7306.	0.7421282	-0.154773	4.6956397	3.75E-06	1.71E-05	3.7067238
GATM	-0.170149	7.2278283	-4.695521	3.75E-06	1.71E-05	3.7062029
RNF187	-0.087052	6.8232511	-4.695404	3.75E-06	1.71E-05	3.7056864
EVPL	1.062143	3.1594169	4.6951978	3.76E-06	1.71E-05	3.7047796
IGKV2-28	0.7791783	-0.816228	4.6951458	3.76E-06	1.71E-05	3.7045508
FBXW4	-0.084435	6.3765458	-4.69447	3.77E-06	1.72E-05	3.7015763
HPX	-0.26987	7.4012254	-4.694232	3.77E-06	1.72E-05	3.7005317

RP11-457I1	-0.278826	-1.415741	-4.694158	3.78E-06	1.72E-05	3.7002061
ZNF263	-0.064166	6.106111	-4.693507	3.79E-06	1.73E-05	3.697342
ABHD2	-0.118185	6.852382	-4.69341	3.79E-06	1.73E-05	3.6969146
THRSP	-0.98315	4.8588517	-4.693072	3.79E-06	1.73E-05	3.6954307
MAP7D1	0.0789617	6.2699243	4.6930044	3.80E-06	1.73E-05	3.6951329
CDK9	-0.079212	6.4170473	-4.692908	3.80E-06	1.73E-05	3.6947085
CD160	0.5272192	3.352243	4.692543	3.80E-06	1.73E-05	3.6931041
RPS20P22	0.7146761	-0.671773	4.6924009	3.81E-06	1.73E-05	3.6924793
HMGN1P36	0.7315382	1.6771692	4.6923614	3.81E-06	1.73E-05	3.6923058
APOC1P1	-0.454931	5.920581	-4.691988	3.81E-06	1.74E-05	3.6906659
TBC1D7	-0.098509	5.9615078	-4.691954	3.81E-06	1.74E-05	3.6905148
FAHD2B	0.5339837	4.5916081	4.691954	3.81E-06	1.74E-05	3.6905147
DOHH	-0.092744	5.86293	-4.691633	3.82E-06	1.74E-05	3.6891043
MPP3	0.4133026	4.5426133	4.6916034	3.82E-06	1.74E-05	3.6889735
RGS22	0.83119	-0.17612	4.6912673	3.83E-06	1.74E-05	3.6874957
SHPRH	0.3427737	4.9222881	4.6909752	3.83E-06	1.74E-05	3.6862119
TENM3	1.1290268	1.0743794	4.6909414	3.83E-06	1.74E-05	3.6860634
RP11-713M1	-0.257659	5.691775	-4.690317	3.84E-06	1.75E-05	3.6833204
ZNF703	0.2583365	5.6817134	4.6900142	3.85E-06	1.75E-05	3.6819885
RP11-600L4	-0.321562	-1.373513	-4.6899	3.85E-06	1.75E-05	3.6814866
SGK494	0.4484148	4.0974474	4.6895353	3.86E-06	1.75E-05	3.6798838
CTTNBP2	0.9074046	3.5167142	4.6894912	3.86E-06	1.75E-05	3.67969
DTX3L	-0.07817	6.591966	-4.689467	3.86E-06	1.75E-05	3.679585
LPCAT1	0.1568307	6.2015758	4.6894581	3.86E-06	1.75E-05	3.6795444
AGRN	0.1000739	6.7659727	4.689277	3.86E-06	1.76E-05	3.6787487
AP000692.1	-0.729546	2.1625762	-4.689042	3.87E-06	1.76E-05	3.6777179
NME1	-0.124233	6.4297674	-4.688978	3.87E-06	1.76E-05	3.6774334
AC004231.2	1.0423545	0.4607449	4.6889224	3.87E-06	1.76E-05	3.6771905
NCS1	0.3151803	5.4052467	4.6888051	3.87E-06	1.76E-05	3.6766753
PAK7	0.8926613	-0.501463	4.6880282	3.88E-06	1.76E-05	3.673262
CTB-66B24.	-0.287912	-1.408206	-4.687275	3.90E-06	1.77E-05	3.6699526
CCNE2	0.3340969	4.6031483	4.6870197	3.90E-06	1.77E-05	3.6688322
RP11-53B2.	0.7965058	0.9855929	4.6868813	3.90E-06	1.77E-05	3.6682244
BAG2	-0.178385	5.8938558	-4.686855	3.90E-06	1.77E-05	3.6681091
TNNI2	0.4999161	3.7996522	4.6864742	3.91E-06	1.78E-05	3.6664363
RP1-266L20	0.6574928	2.3396345	4.6864662	3.91E-06	1.78E-05	3.6664011
FAM114A1	-0.080378	6.4905385	-4.686316	3.91E-06	1.78E-05	3.6657422
SNTG1	-1.272497	1.4945747	-4.68616	3.92E-06	1.78E-05	3.6650582
ANXA10	-0.564141	5.3320476	-4.685987	3.92E-06	1.78E-05	3.6642965
PPP6C	-0.057515	6.3411307	-4.685906	3.92E-06	1.78E-05	3.6639417
CCDC181	0.4478466	2.8906159	4.6858162	3.92E-06	1.78E-05	3.663547
CD72	0.2748046	4.9346205	4.6857435	3.92E-06	1.78E-05	3.6632274
CCL24	0.8740398	2.9309906	4.6853567	3.93E-06	1.78E-05	3.6615293
HEIH	-0.092718	6.05833	-4.685118	3.94E-06	1.78E-05	3.6604826
PROZ	-0.53024	5.6578759	-4.684386	3.95E-06	1.79E-05	3.6572672
CBX8	-0.117857	5.7548513	-4.684186	3.95E-06	1.79E-05	3.6563874
RPS29	-0.110651	6.8908984	-4.684117	3.95E-06	1.79E-05	3.6560886
SNTB1	-0.133546	6.7636913	-4.684076	3.95E-06	1.79E-05	3.6559085
MAD2L1BP	-0.082788	6.0376015	-4.683826	3.96E-06	1.79E-05	3.6548102
IGHV3-62	0.617024	-0.951769	4.6833244	3.97E-06	1.80E-05	3.6526076
RP11-517H2	0.8722411	0.6512719	4.6833184	3.97E-06	1.80E-05	3.6525809

PTCHD3P2	1.012836	0.578299	4.6833042	3.97E-06	1.80E-05	3.6525186
SP2-AS1	-0.23824	4.4725058	-4.683231	3.97E-06	1.80E-05	3.6521991
SCGB2A1	1.1266174	0.8607995	4.6830125	3.97E-06	1.80E-05	3.6512383
PPP1R14D	1.106001	1.8899886	4.6825863	3.98E-06	1.80E-05	3.649368
RP11-225H2	0.7218323	-0.251313	4.6823243	3.99E-06	1.81E-05	3.6482183
RP11-25E2.	-0.860951	0.0703826	-4.681781	4.00E-06	1.81E-05	3.6458333
IFI35	-0.128911	6.3473576	-4.681725	4.00E-06	1.81E-05	3.6455872
CTD-2095E4	0.6593906	2.8749467	4.6815497	4.00E-06	1.81E-05	3.6448193
CEP295	0.1349621	5.3791299	4.6815254	4.00E-06	1.81E-05	3.6447129
MT-ND5	-0.119405	7.5728912	-4.681435	4.00E-06	1.81E-05	3.6443156
RP11-396K3	0.2474499	5.2535894	4.6814273	4.00E-06	1.81E-05	3.6442824
SSR4P1	-0.218858	4.5200495	-4.680304	4.02E-06	1.82E-05	3.6393547
RPGR	0.2280289	5.23949	4.6796353	4.04E-06	1.83E-05	3.636421
AC011290.5	0.7017554	2.2718263	4.6790164	4.05E-06	1.83E-05	3.6337069
ZNF850	0.2606128	4.5758358	4.6778981	4.07E-06	1.84E-05	3.628803
ACSM1	-0.626493	5.5555028	-4.677878	4.07E-06	1.84E-05	3.6287132
RP11-372E1	-0.76119	5.2722512	-4.677301	4.08E-06	1.84E-05	3.6261832
TJP3	0.4643738	5.5861516	4.6771723	4.08E-06	1.85E-05	3.625621
ZNF236	0.1583713	5.4422279	4.6770669	4.08E-06	1.85E-05	3.6251587
AC093382.1	-0.803255	0.5723825	-4.676573	4.09E-06	1.85E-05	3.6229925
DSTYK	0.1091594	5.7677975	4.6763423	4.10E-06	1.85E-05	3.6219823
APOC4-APOC	-0.457739	5.8104938	-4.676145	4.10E-06	1.85E-05	3.6211175
EIF3B	-0.076354	6.8747847	-4.676091	4.10E-06	1.85E-05	3.6208813
GALM	-0.118069	6.5207551	-4.675984	4.10E-06	1.85E-05	3.6204128
EFCAB10	0.4973696	2.804239	4.6758264	4.11E-06	1.86E-05	3.6197214
VPS16	-0.070986	6.2605868	-4.67566	4.11E-06	1.86E-05	3.6189904
LINC01232	-0.17288	5.1851038	-4.675284	4.12E-06	1.86E-05	3.6173428
FATE1	-0.808055	2.7891872	-4.675127	4.12E-06	1.86E-05	3.6166546
FCAR	0.8698733	0.4444902	4.6743648	4.14E-06	1.87E-05	3.6133162
CTD-228701	-0.220405	4.2268699	-4.674315	4.14E-06	1.87E-05	3.6130983
DPYS	-0.402851	6.65983	-4.673683	4.15E-06	1.87E-05	3.6103272
TMEM37	-0.15308	6.5278177	-4.673669	4.15E-06	1.87E-05	3.6102675
ZNF506	0.2155343	5.2534385	4.6734598	4.15E-06	1.87E-05	3.609351
NANOS1	0.6184036	3.9425528	4.6729615	4.16E-06	1.88E-05	3.6071681
ST3GAL1	-0.161713	6.6791761	-4.672028	4.18E-06	1.89E-05	3.6030774
AC137723.1	-0.424036	-1.196953	-4.671994	4.18E-06	1.89E-05	3.6029292
SMKR1	1.0181552	1.754734	4.6702139	4.22E-06	1.90E-05	3.5951357
EEF2KMT	-0.103944	5.6452666	-4.669907	4.22E-06	1.90E-05	3.5937918
RP11-102L1	0.7144517	-0.286982	4.6698839	4.22E-06	1.90E-05	3.5936907
SUMO2	-0.062484	6.6398676	-4.669862	4.22E-06	1.90E-05	3.5935946
SLC5A5	0.9249443	0.4137069	4.6696504	4.23E-06	1.91E-05	3.5926686
RP11-656D1	0.7923141	0.1676839	4.669236	4.23E-06	1.91E-05	3.5908544
BCOR	0.1097808	6.0721828	4.669221	4.23E-06	1.91E-05	3.5907885
RP11-626G1	0.8103145	0.4876009	4.6691523	4.24E-06	1.91E-05	3.5904879
MUT	-0.119606	6.6337611	-4.668906	4.24E-06	1.91E-05	3.5894078
WDR63	0.8067813	0.1094163	4.6685698	4.25E-06	1.91E-05	3.5879384
RP11-346J1	-0.347313	-1.319881	-4.66837	4.25E-06	1.91E-05	3.5870643
PUF60	-0.09685	6.8818991	-4.6677	4.26E-06	1.92E-05	3.5841297
HMGCS1	-0.144587	6.7744976	-4.667385	4.27E-06	1.92E-05	3.5827553
GAL	1.0782995	0.3189935	4.6672	4.27E-06	1.92E-05	3.5819436
ANKRD6	0.1374437	5.4657146	4.6669145	4.28E-06	1.93E-05	3.5806947

FBXO2	-0.291387	6.0313201	-4.665352	4.31E-06	1.94E-05	3.5738593
DSG1	-0.929001	4.5080864	-4.66528	4.31E-06	1.94E-05	3.5735427
DND1P1	1.0793097	0.9340821	4.6648643	4.32E-06	1.94E-05	3.5717258
DNA2	0.1962711	5.1172934	4.6646155	4.33E-06	1.95E-05	3.5706377
SYT2	0.7210244	2.3998344	4.6635231	4.35E-06	1.96E-05	3.5658602
HOOK3	0.1196713	6.1122943	4.6633966	4.35E-06	1.96E-05	3.5653074
UBE2D2	-0.060069	6.4736933	-4.663171	4.35E-06	1.96E-05	3.5643221
TEK	0.3926126	5.1055399	4.6629567	4.36E-06	1.96E-05	3.5633838
CA14	-0.375469	5.1153047	-4.662923	4.36E-06	1.96E-05	3.5632352
STMN4	-0.820556	-0.398895	-4.662819	4.36E-06	1.96E-05	3.5627799
ARPC5	-0.066171	6.8110466	-4.662719	4.36E-06	1.96E-05	3.5623459
RP11-195F1	-0.306407	3.9536071	-4.66256	4.37E-06	1.96E-05	3.5616483
HIST1H4I	-0.184833	5.7965291	-4.662115	4.38E-06	1.97E-05	3.5597043
PPP1R12B	0.1046043	5.9936389	4.6616894	4.38E-06	1.97E-05	3.5578436
RP11-325K4	0.682284	2.5499698	4.6614638	4.39E-06	1.97E-05	3.5568577
TSPAN3	0.0928968	6.513809	4.6612353	4.39E-06	1.97E-05	3.5558587
DNAJB11	-0.077063	6.6989947	-4.661041	4.40E-06	1.98E-05	3.5550107
PARP2	-0.072905	5.9573464	-4.660912	4.40E-06	1.98E-05	3.5544446
ATP6V0B	-0.094312	6.7461199	-4.660456	4.41E-06	1.98E-05	3.5524525
ISCU	-0.073578	6.5308459	-4.659774	4.42E-06	1.99E-05	3.5494722
SURF6	-0.073157	6.2374582	-4.659644	4.43E-06	1.99E-05	3.5489068
EPB41L1	0.235326	5.9886813	4.6593105	4.43E-06	1.99E-05	3.5474477
SH2D3C	0.1279179	5.7639964	4.6588541	4.44E-06	1.99E-05	3.5454537
ANXA13	0.7305997	5.3225449	4.6585986	4.45E-06	2.00E-05	3.5443375
SFMBT1	-0.097097	5.7813018	-4.658525	4.45E-06	2.00E-05	3.5440145
RP11-471B2	0.8291016	0.9803608	4.6579437	4.46E-06	2.00E-05	3.5414766
NR1D2	-0.096732	6.2167093	-4.657679	4.47E-06	2.00E-05	3.5403203
MIR1273F	-0.453715	-1.180153	-4.657157	4.48E-06	2.01E-05	3.5380418
MMP8	0.6044157	-1.05047	4.6569707	4.48E-06	2.01E-05	3.537227
ZBTB12	0.2753169	4.9351954	4.6563782	4.49E-06	2.01E-05	3.5346394
GOLGA6L10	0.6791496	2.5497636	4.6560036	4.50E-06	2.02E-05	3.533004
FLJ22447	0.7582853	1.4134495	4.6555349	4.51E-06	2.02E-05	3.5309572
IL5RA	0.817436	0.1898177	4.6554834	4.51E-06	2.02E-05	3.5307328
BEAN1	0.6283389	3.3109633	4.6554279	4.51E-06	2.02E-05	3.5304901
CAMK1G	0.8402278	2.3163447	4.6549011	4.52E-06	2.03E-05	3.5281905
PPP1CA	-0.068946	6.7809482	-4.654732	4.53E-06	2.03E-05	3.5274531
AC007064.2	-0.279043	-1.414436	-4.654685	4.53E-06	2.03E-05	3.5272473
ITLN1	1.0876668	1.4677072	4.6546212	4.53E-06	2.03E-05	3.5269686
MUC1	0.5558964	4.322038	4.65451	4.53E-06	2.03E-05	3.5264831
RBM38	0.1156547	5.9829791	4.6540722	4.54E-06	2.03E-05	3.5245724
RP11-848G1	0.6667391	-0.488316	4.653825	4.54E-06	2.04E-05	3.5234934
TCEA1P2	0.6992651	3.9841778	4.6536543	4.55E-06	2.04E-05	3.5227483
KDM7A	0.1150027	5.8694565	4.6530553	4.56E-06	2.04E-05	3.5201339
CFI	-0.142944	7.0037906	-4.65288	4.56E-06	2.04E-05	3.51937
LINC01091	0.8017019	2.8406143	4.652876	4.56E-06	2.04E-05	3.5193515
FOXN3	-0.097239	6.4135438	-4.652796	4.57E-06	2.04E-05	3.5190007
RPL34P33	-0.78141	-0.266926	-4.652317	4.58E-06	2.05E-05	3.5169116
CTD-3203P2	0.7241981	2.7357124	4.6522404	4.58E-06	2.05E-05	3.5165784
AC021188.4	0.7801947	0.9392352	4.6521415	4.58E-06	2.05E-05	3.5161465
PPAPDC3	0.5388514	3.3502285	4.651573	4.59E-06	2.05E-05	3.5136662
ACAD10	-0.092001	6.3739243	-4.651012	4.60E-06	2.06E-05	3.5112192

PSPC1P1	0.7917898	0.8143603	4.6503855	4.62E-06	2.07E-05	3.5084863
ZNF724P	0.7507102	2.863338	4.6499264	4.63E-06	2.07E-05	3.5064841
EHBP1	-0.101189	6.3846707	-4.649508	4.64E-06	2.07E-05	3.5046586
RP11-466A1	0.8303235	0.2508201	4.6494074	4.64E-06	2.07E-05	3.5042203
RP11-554A1	0.8533481	-0.007842	4.6488208	4.65E-06	2.08E-05	3.5016626
RPL30	-0.103547	7.2326516	-4.648508	4.66E-06	2.08E-05	3.5002978
FAM131B	0.5387194	3.097562	4.6478173	4.67E-06	2.09E-05	3.4972874
ISL2	1.0533014	1.6576501	4.6470111	4.69E-06	2.10E-05	3.4937729
JSRP1	0.5799445	3.32177	4.6469205	4.69E-06	2.10E-05	3.4933779
APOF	-0.612963	5.9006207	-4.646917	4.69E-06	2.10E-05	3.4933614
TMEM143	-0.119166	5.7991724	-4.646177	4.71E-06	2.10E-05	3.490137
C6orf183	1.0519979	2.3333336	4.6461761	4.71E-06	2.10E-05	3.4901335
RP11-297K7	-0.817682	0.0052282	-4.645821	4.71E-06	2.11E-05	3.488585
TNFAIP8L1	-0.117379	6.4613072	-4.645702	4.72E-06	2.11E-05	3.4880694
C14orf93	-0.084747	5.6560684	-4.645696	4.72E-06	2.11E-05	3.4880422
HDAC5	-0.082964	6.4629714	-4.645252	4.73E-06	2.11E-05	3.4861064
SERPINA6	-0.219133	7.0420644	-4.645051	4.73E-06	2.11E-05	3.4852309
SEC14L1P1	0.7738317	2.0978331	4.6447765	4.74E-06	2.11E-05	3.4840347
SLC43A1	-0.120522	6.798832	-4.644191	4.75E-06	2.12E-05	3.4814816
PDX1-AS1	0.8912033	-0.32887	4.644167	4.75E-06	2.12E-05	3.481379
IGKV1D-39	0.7326189	-0.794875	4.6440256	4.75E-06	2.12E-05	3.4807631
GPR182	0.9528975	2.6486429	4.6438767	4.76E-06	2.12E-05	3.4801144
RP11-353K1	-0.368935	3.4471368	-4.643071	4.77E-06	2.13E-05	3.4766058
GDNF-AS1	-1.204242	1.7981337	-4.642888	4.78E-06	2.13E-05	3.4758063
FAM46B	0.7042355	3.1009697	4.6428646	4.78E-06	2.13E-05	3.4757059
PPP1R3D	0.2774533	4.675826	4.6425502	4.79E-06	2.13E-05	3.4743369
HLA-DPA3	0.7622527	-0.51082	4.6424174	4.79E-06	2.14E-05	3.4737582
KHDRBS2	0.7751076	-0.496386	4.6419445	4.80E-06	2.14E-05	3.4716988
RP11-467I2	-0.483876	-1.092388	-4.641687	4.80E-06	2.14E-05	3.4705766
AF196972.9	-0.668111	2.2868188	-4.641655	4.81E-06	2.14E-05	3.4704401
PROK1	-1.005496	0.6856402	-4.641613	4.81E-06	2.14E-05	3.4702541
LINC00402	-1.035919	1.4438605	-4.641262	4.81E-06	2.15E-05	3.4687281
TRIM61	0.8739765	-0.020125	4.6411426	4.82E-06	2.15E-05	3.4682072
RP11-6E9.4	-0.263435	-1.430123	-4.641135	4.82E-06	2.15E-05	3.4681747
IGF1	-0.438501	5.1097402	-4.641123	4.82E-06	2.15E-05	3.4681222
UTP11L	-0.064434	6.0430222	-4.640919	4.82E-06	2.15E-05	3.4672346
TLR6	0.3717435	4.4534355	4.6403537	4.83E-06	2.15E-05	3.4647724
MOCS3	-0.078393	5.7096059	-4.639773	4.85E-06	2.16E-05	3.4622465
RPL5	-0.083786	7.1953298	-4.639493	4.85E-06	2.16E-05	3.4610262
CTD-2192J1	0.4725358	3.5126568	4.6394891	4.85E-06	2.16E-05	3.4610092
LINC01504	0.7220113	2.5064779	4.6394248	4.85E-06	2.16E-05	3.4607291
TMEM176A	-0.169382	7.2175352	-4.639264	4.86E-06	2.16E-05	3.4600271
LINC00582	0.6681773	-0.818412	4.6388454	4.87E-06	2.17E-05	3.4582074
PPP2R5A	-0.08946	6.6772445	-4.638597	4.87E-06	2.17E-05	3.4571253
HMGN1P37	0.7002528	2.1955856	4.6385684	4.87E-06	2.17E-05	3.4570018
TMPPE	0.2847529	4.090363	4.6382661	4.88E-06	2.17E-05	3.4556864
RP11-574K1	0.7032038	1.5764161	4.6378445	4.89E-06	2.18E-05	3.453852
RP1-118J21	-0.788035	-0.140756	-4.637066	4.91E-06	2.18E-05	3.4504636
RP11-290H9	-0.760062	-0.368841	-4.637013	4.91E-06	2.18E-05	3.450235
AC004540.5	0.9387913	2.1998662	4.6366525	4.92E-06	2.19E-05	3.4486658
FN3KRP	-0.084982	6.3336812	-4.636412	4.92E-06	2.19E-05	3.447621

TMEM52	-0.384346	4.9353708	-4.636138	4.93E-06	2.19E-05	3.4464275
MIR570	0.5582365	3.0218185	4.6360395	4.93E-06	2.19E-05	3.4459994
OR2H2	0.5361424	-1.14507	4.6358663	4.93E-06	2.19E-05	3.4452457
RNF208	-0.161247	5.6237307	-4.635794	4.94E-06	2.19E-05	3.44493
HTR2A-AS1	-0.645665	-0.764201	-4.635682	4.94E-06	2.19E-05	3.4444436
TPT1	-0.086724	7.5463767	-4.63528	4.95E-06	2.20E-05	3.442696
CCT7	-0.064118	6.8508766	-4.63475	4.96E-06	2.20E-05	3.440389
UQCRB	-0.109717	6.9438129	-4.63369	4.98E-06	2.21E-05	3.4357825
P2RY1	0.2282904	4.6869161	4.633159	5.00E-06	2.22E-05	3.4334732
PA2G4	-0.069534	6.6861903	-4.632302	5.01E-06	2.23E-05	3.4297499
ASRGL1	0.278584	5.2554959	4.6320367	5.02E-06	2.23E-05	3.4285948
ZNF500	-0.076269	5.6400827	-4.631968	5.02E-06	2.23E-05	3.4282946
RP11-624L4	1.048677	2.0132479	4.6315028	5.03E-06	2.23E-05	3.4262745
RREB1	-0.081213	6.4830285	-4.630723	5.05E-06	2.24E-05	3.422886
COL4A5	0.7235431	4.5399291	4.6304792	5.06E-06	2.24E-05	3.4218261
ANKRD13A	0.071173	6.2329055	4.6301358	5.06E-06	2.25E-05	3.4203343
TMEM38B	-0.098745	6.1118815	-4.63005	5.07E-06	2.25E-05	3.4199629
RN7SL251P	-0.39956	-1.328082	-4.6296	5.08E-06	2.25E-05	3.418006
FETUB	-0.583938	6.0570729	-4.629285	5.08E-06	2.26E-05	3.416636
TMX3	0.0841653	5.9755461	4.6291838	5.09E-06	2.26E-05	3.4161984
RP11-727F1	-0.201063	4.2242599	-4.628603	5.10E-06	2.26E-05	3.4136742
OIP5-AS1	-0.071393	6.5812614	-4.628596	5.10E-06	2.26E-05	3.4136461
AK3	-0.101518	6.671319	-4.628012	5.11E-06	2.27E-05	3.411109
PVR	-0.092474	6.4991451	-4.627786	5.12E-06	2.27E-05	3.4101282
BLVRA	0.1889452	5.9395554	4.6277335	5.12E-06	2.27E-05	3.409899
ETV2	-0.207657	4.7871195	-4.627427	5.13E-06	2.27E-05	3.4085677
CCDC150P1	0.8378012	1.9229018	4.6270958	5.14E-06	2.28E-05	3.4071301
NES	0.126441	6.2282426	4.6268409	5.14E-06	2.28E-05	3.4060232
AC006272.2	0.6992065	-0.308435	4.6267403	5.14E-06	2.28E-05	3.4055861
PHTF2	0.0985638	5.7495332	4.6266804	5.15E-06	2.28E-05	3.4053264
ITPR1	0.1838402	5.6583584	4.6265531	5.15E-06	2.28E-05	3.4047735
TRMT2A	-0.077888	6.3200622	-4.626551	5.15E-06	2.28E-05	3.4047623
SIPA1L3	0.117581	6.1080174	4.6264379	5.15E-06	2.28E-05	3.4042734
ZNF668	0.3183147	4.8064037	4.6261807	5.16E-06	2.28E-05	3.4031567
CTD-2583P5	-0.396723	-1.242357	-4.626095	5.16E-06	2.28E-05	3.4027827
DBF4	0.1266368	5.4062436	4.6260939	5.16E-06	2.28E-05	3.4027796
SKIDA1	-0.310431	5.1384354	-4.62553	5.17E-06	2.29E-05	3.4003303
VASP	0.0797136	6.4649293	4.6254772	5.17E-06	2.29E-05	3.4001025
STEAP1B	0.8720526	1.6317129	4.6246042	5.19E-06	2.30E-05	3.3963135
RP11-789C1	0.8916801	1.3532691	4.6242943	5.20E-06	2.30E-05	3.3949682
ENOX1	0.6290623	3.3436473	4.6242939	5.20E-06	2.30E-05	3.3949665
SCGB1B2P	-0.394101	3.4433507	-4.62404	5.21E-06	2.30E-05	3.3938648
TUBB8	0.825509	-0.215881	4.623927	5.21E-06	2.30E-05	3.3933745
LAMP2	-0.089425	7.005503	-4.623067	5.23E-06	2.31E-05	3.3896447
RP11-21K12	0.8887783	1.1942142	4.6229857	5.23E-06	2.31E-05	3.3892897
AC008810.1	-0.425036	-1.209925	-4.622249	5.25E-06	2.32E-05	3.3860933
AC073636.1	-0.319733	-1.350504	-4.621847	5.26E-06	2.33E-05	3.3843515
RPS6	-0.090677	7.3422678	-4.621685	5.26E-06	2.33E-05	3.3836469
CDKN1B	-0.075273	6.3469977	-4.621645	5.26E-06	2.33E-05	3.383475
RP11-28201	0.2799819	3.7364354	4.6212178	5.28E-06	2.33E-05	3.3816204
C14orf180	1.0958122	0.404824	4.6211684	5.28E-06	2.33E-05	3.3814062

DUOXA1	0.9930393	0.8789025	4.6208031	5.29E-06	2.34E-05	3.3798218
RP11-106M3	-0.836473	1.2498257	-4.620553	5.29E-06	2.34E-05	3.3787378
LRRC29	-0.180505	4.8832968	-4.620424	5.29E-06	2.34E-05	3.378178
OTOP3	0.8827926	-0.660099	4.6201629	5.30E-06	2.34E-05	3.3770456
FCGBP	0.3189188	5.2727973	4.6197615	5.31E-06	2.34E-05	3.3753049
RP11-259K5	-0.180187	4.8967863	-4.619744	5.31E-06	2.34E-05	3.3752274
RP5-1125A1	-0.237577	4.3385315	-4.61836	5.34E-06	2.36E-05	3.3692281
AC140912.1	0.7809733	-0.483665	4.617162	5.37E-06	2.37E-05	3.3640358
KIAA1683	-0.30278	4.9729332	-4.616968	5.38E-06	2.37E-05	3.3631972
RPL35	-0.111941	7.0465435	-4.616846	5.38E-06	2.37E-05	3.362666
SAR1A	-0.059844	6.624192	-4.616665	5.39E-06	2.38E-05	3.3618806
SAT1	-0.10759	6.9776367	-4.616159	5.40E-06	2.38E-05	3.3596913
GID8	-0.067567	6.523034	-4.615572	5.41E-06	2.39E-05	3.3571445
LIPJ	-0.815221	0.3064031	-4.615494	5.41E-06	2.39E-05	3.3568098
RP4-625H18	0.8148079	-0.408446	4.6154805	5.41E-06	2.39E-05	3.3567495
MEGF11	0.8307165	0.8722701	4.6150998	5.42E-06	2.39E-05	3.3551002
EIF4G1	-0.057724	7.0999539	-4.614913	5.43E-06	2.39E-05	3.3542894
RIPPLY1	-0.819859	4.0502648	-4.614333	5.44E-06	2.40E-05	3.3517773
VPS33A	-0.082342	6.0509362	-4.613815	5.46E-06	2.41E-05	3.3495337
GPA33	0.7883129	-0.085555	4.6137306	5.46E-06	2.41E-05	3.3491693
KLHDC4	-0.092725	6.0643054	-4.613537	5.46E-06	2.41E-05	3.3483315
SNORA59B	-0.896298	1.8688794	-4.613534	5.46E-06	2.41E-05	3.348316
RP11-529E1	0.5419892	3.3047646	4.6133104	5.47E-06	2.41E-05	3.3473494
RHOBTB2	0.1483351	5.6500084	4.6131295	5.47E-06	2.41E-05	3.3465657
ZKSCAN7	0.5318569	4.0037771	4.6130146	5.48E-06	2.41E-05	3.3460683
RP3-465N24	-0.674154	1.7970602	-4.612404	5.49E-06	2.42E-05	3.3434251
SV2B	0.8134098	3.6313172	4.6121181	5.50E-06	2.42E-05	3.3421865
TMEM52B	1.0593317	1.1479748	4.6118997	5.50E-06	2.42E-05	3.3412407
ASCL5	0.8472	0.1092913	4.6118011	5.51E-06	2.42E-05	3.3408138
HLA-DPB2	0.934627	3.0700611	4.611426	5.52E-06	2.43E-05	3.3391898
MIR4653	0.8309722	0.571326	4.61128	5.52E-06	2.43E-05	3.338558
S100A3	0.736591	2.7925203	4.6104957	5.54E-06	2.44E-05	3.3351629
ABCB11	-0.768335	5.485466	-4.609286	5.57E-06	2.45E-05	3.3299261
RP11-161D1	-0.748914	-0.998023	-4.60815	5.60E-06	2.46E-05	3.3250115
PAM16	-0.10439	5.692528	-4.606591	5.64E-06	2.48E-05	3.3182667
RP13-554M1	0.4061653	3.9150042	4.6062117	5.65E-06	2.48E-05	3.3166277
SNHG18	0.6166579	4.1244081	4.6061892	5.65E-06	2.48E-05	3.3165301
SLC34A1	-0.826186	3.0707641	-4.6061	5.65E-06	2.49E-05	3.3161434
RPL37A	-0.103059	7.1890046	-4.605715	5.66E-06	2.49E-05	3.3144797
NCAPD2	0.1085036	6.1393281	4.6052759	5.67E-06	2.49E-05	3.3125807
MPDU1	-0.099974	6.5503989	-4.605065	5.68E-06	2.50E-05	3.3116688
RP11-783K1	0.7530577	0.068494	4.6041912	5.70E-06	2.51E-05	3.307891
APOBEC3H	0.5681761	3.3061459	4.6040818	5.70E-06	2.51E-05	3.3074179
RPS17	-0.094273	7.1839716	-4.603356	5.72E-06	2.51E-05	3.3042793
TMEM79	-0.101186	5.5106591	-4.6033	5.72E-06	2.52E-05	3.3040399
RP4-61404.	0.3218386	4.0424793	4.6031907	5.73E-06	2.52E-05	3.303566
RP11-283I3	0.1490058	5.1184954	4.6030167	5.73E-06	2.52E-05	3.302814
AC005042.2	0.6155474	-0.820291	4.6026159	5.74E-06	2.52E-05	3.3010817
CTC-378H22	0.7544422	0.130539	4.602568	5.74E-06	2.52E-05	3.300875
SNAPC5	-0.072695	5.9647628	-4.601931	5.76E-06	2.53E-05	3.2981209
RP11-579D7	-0.515932	3.5945363	-4.601823	5.76E-06	2.53E-05	3.2976552

HMGN5	-0.411748	4.9551403	-4.601228	5.78E-06	2.54E-05	3.2950855
TRAV24	0.676063	-0.758441	4.6010995	5.78E-06	2.54E-05	3.2945294
PTCD2	-0.091666	5.6053926	-4.600971	5.78E-06	2.54E-05	3.2939749
IGLV4-3	0.6370162	-0.982221	4.6007121	5.79E-06	2.54E-05	3.2928557
RP11-89K11	0.811841	1.1341914	4.6003157	5.80E-06	2.55E-05	3.2911432
RP11-567M1	0.6660867	-0.540066	4.5998385	5.81E-06	2.55E-05	3.2890818
UQCRBP2	-0.258637	-1.432231	-4.599324	5.83E-06	2.56E-05	3.2868606
ZNF649	0.2630634	4.902235	4.5992192	5.83E-06	2.56E-05	3.2864069
TMEM220	-0.207348	6.1824301	-4.599129	5.83E-06	2.56E-05	3.2860176
RP11-849N1	0.8125852	0.2436752	4.5989332	5.84E-06	2.56E-05	3.2851714
CH17-13I23	-0.422742	-1.341347	-4.597712	5.87E-06	2.57E-05	3.2798982
TPSB2	0.8762377	3.5779424	4.5976756	5.87E-06	2.57E-05	3.2797412
GAL3ST1	0.6006817	5.0866408	4.5976727	5.87E-06	2.57E-05	3.2797284
TVP23C-CDR	0.7590025	0.3160475	4.5974999	5.88E-06	2.58E-05	3.2789823
SRP14	-0.065088	6.8594046	-4.597165	5.88E-06	2.58E-05	3.2775352
CASP1P2	0.7967703	0.0973263	4.5963927	5.91E-06	2.59E-05	3.2742024
RP11-547D2	-0.650132	-0.738299	-4.596384	5.91E-06	2.59E-05	3.274164
GNB2L1	-0.085914	7.2842438	-4.595874	5.92E-06	2.59E-05	3.2719615
SNORA71A	0.7737971	0.3406163	4.5956093	5.93E-06	2.60E-05	3.2708211
RP11-575G1	0.4646803	-1.090599	4.5955644	5.93E-06	2.60E-05	3.2706276
GJB1	-0.222325	7.0155032	-4.595149	5.94E-06	2.60E-05	3.2688341
RP1-149A16	0.6939745	0.6976485	4.5945557	5.95E-06	2.61E-05	3.2662745
EIF2S2P2	-0.716099	-0.365943	-4.594456	5.96E-06	2.61E-05	3.2658447
RP11-62F24	0.6604919	-0.781052	4.5939161	5.97E-06	2.62E-05	3.2635146
MEST	0.1506405	6.1742809	4.5938998	5.97E-06	2.62E-05	3.2634444
RPLP2	-0.100532	7.1731184	-4.59384	5.97E-06	2.62E-05	3.2631863
RP11-283I3	-0.647817	-0.638248	-4.593753	5.98E-06	2.62E-05	3.2628108
NTMT1	-0.10318	5.9754574	-4.593477	5.98E-06	2.62E-05	3.2616187
TCFL5	-0.110134	5.6930504	-4.593384	5.99E-06	2.62E-05	3.2612198
RP11-367J1	-0.437342	3.5466138	-4.592922	6.00E-06	2.63E-05	3.2592254
SYMPK	-0.069037	6.6480826	-4.592554	6.01E-06	2.63E-05	3.2576397
ADSS	0.0783086	6.2460056	4.5918945	6.03E-06	2.64E-05	3.2547943
SCAMP2	-0.05603	6.58861	-4.591795	6.03E-06	2.64E-05	3.2543635
GBE1	-0.133468	6.4300498	-4.591516	6.04E-06	2.64E-05	3.2531627
SYNGR1	0.3935465	5.4303148	4.5914432	6.04E-06	2.64E-05	3.2528478
RP11-383J2	1.0306299	0.3440168	4.5907616	6.06E-06	2.65E-05	3.2499085
UQCC1	-0.069059	6.2245129	-4.590644	6.06E-06	2.65E-05	3.2494025
SAMD5	0.6979976	4.6171167	4.590122	6.08E-06	2.66E-05	3.2471507
MMP25	0.3040207	4.3804041	4.5897637	6.09E-06	2.66E-05	3.2456061
GPR123	0.9268574	-0.371857	4.5897104	6.09E-06	2.66E-05	3.2453764
CTD-2373J6	-0.760886	0.6604742	-4.589179	6.10E-06	2.67E-05	3.2430843
CTSW	0.358914	4.8087288	4.5890967	6.10E-06	2.67E-05	3.242731
AP5S1	-0.080614	5.9442958	-4.587497	6.15E-06	2.69E-05	3.2358363
ASAH1	0.0973286	6.5020855	4.5874291	6.15E-06	2.69E-05	3.235544
ABCC11	-0.321723	5.0207525	-4.586766	6.17E-06	2.69E-05	3.2326887
AC079584.2	-0.292828	-1.437792	-4.586729	6.17E-06	2.69E-05	3.232527
MIR135A1	-0.469917	4.3236569	-4.586059	6.19E-06	2.70E-05	3.2296419
BEND3P3	0.9501013	1.3098798	4.5859391	6.19E-06	2.70E-05	3.2291242
SNORD6	0.7858538	1.3901828	4.5858886	6.19E-06	2.70E-05	3.2289067
SZT2	-0.071895	6.1051386	-4.585788	6.20E-06	2.70E-05	3.2284716
MGAT4C	1.0701554	2.3101169	4.585755	6.20E-06	2.71E-05	3.228331

KLK11	1.0130225	-0.02021	4.5857053	6.20E-06	2.71E-05	3.2281171
MTX1	-0.083005	6.033534	-4.584587	6.23E-06	2.72E-05	3.2233001
WBSCR16	-0.074487	6.5148694	-4.584476	6.23E-06	2.72E-05	3.2228239
RP11-497H1	0.7908457	0.663827	4.5841743	6.24E-06	2.72E-05	3.2215229
VN1R83P	0.9074148	0.9604622	4.5833112	6.27E-06	2.73E-05	3.2178065
GRASP	0.1818945	5.3428511	4.5830232	6.27E-06	2.74E-05	3.2165667
CGNL1	-0.178059	6.5202766	-4.582498	6.29E-06	2.74E-05	3.2143051
ENPEP	-0.202698	6.1874989	-4.582231	6.30E-06	2.75E-05	3.2131555
ALOXE3	0.7852097	-0.55569	4.5822102	6.30E-06	2.75E-05	3.2130665
CBFA2T3	0.2914931	4.7919523	4.5821034	6.30E-06	2.75E-05	3.2126068
BRD8	-0.067054	6.2170749	-4.581816	6.31E-06	2.75E-05	3.2113711
SUMO2P13	-0.423739	-1.257392	-4.581624	6.31E-06	2.75E-05	3.2105451
ARHGDIA	-0.064441	6.8406934	-4.581487	6.32E-06	2.75E-05	3.2099524
ATL1	0.4853405	3.8783427	4.5814834	6.32E-06	2.75E-05	3.2099382
LMF2	-0.071265	6.6624818	-4.580954	6.33E-06	2.76E-05	3.2076585
RP11-613D1	0.8741133	0.8923744	4.5808523	6.34E-06	2.76E-05	3.2072218
FAM92B	0.7498239	-0.585198	4.5805576	6.34E-06	2.76E-05	3.2059538
RP11-365N1	0.6735464	2.1638911	4.5805507	6.34E-06	2.76E-05	3.2059237
RP5-1039K5	0.2410181	4.0483134	4.5801127	6.36E-06	2.77E-05	3.2040389
RP11-468N1	0.9120782	0.1178086	4.5799716	6.36E-06	2.77E-05	3.2034321
GPR156	0.8014792	0.2342175	4.5799669	6.36E-06	2.77E-05	3.2034117
U47924.31	-0.255869	4.2274533	-4.579782	6.37E-06	2.77E-05	3.2026174
GGT3P	0.7586149	-0.253979	4.5792655	6.38E-06	2.78E-05	3.2003941
TLX1	-0.724426	4.4461901	-4.578687	6.40E-06	2.78E-05	3.1979033
SDHAF2	-0.101113	5.9406035	-4.578497	6.40E-06	2.79E-05	3.1970885
RP11-69E11	0.3061838	4.2910491	4.5781714	6.41E-06	2.79E-05	3.1956872
SRRM2-AS1	-0.251706	4.4477164	-4.578028	6.42E-06	2.79E-05	3.1950711
ABT1	-0.074038	6.2392024	-4.577864	6.42E-06	2.79E-05	3.1943633
HSPBP1	-0.092187	6.3362177	-4.576941	6.45E-06	2.81E-05	3.1903937
TMEM206	0.1269403	5.2835806	4.5763604	6.47E-06	2.81E-05	3.1878988
RP11-180D2	-1.11472	1.9370064	-4.5754	6.49E-06	2.82E-05	3.1837717
ZP2	-0.411852	-1.309775	-4.575377	6.50E-06	2.82E-05	3.1836692
MIOX	1.0364146	2.3531547	4.5748016	6.51E-06	2.83E-05	3.1811974
NIPAL4	0.7937726	-0.22512	4.5746127	6.52E-06	2.83E-05	3.1803853
CEP19	0.2743192	4.2734762	4.5743654	6.52E-06	2.84E-05	3.1793225
DOC2A	0.7329887	2.2533414	4.5720853	6.59E-06	2.87E-05	3.1695243
RALB	0.0681353	6.2017809	4.5715623	6.61E-06	2.87E-05	3.1672774
AP001625.6	0.8111331	0.2841893	4.5712668	6.62E-06	2.88E-05	3.166008
IGFBP7-AS1	0.7549159	-0.044242	4.5710743	6.62E-06	2.88E-05	3.1651812
ARPC4-TTLL	0.6696748	1.5717535	4.5706792	6.63E-06	2.88E-05	3.1634843
RP11-1151E	-0.470033	5.629492	-4.570633	6.64E-06	2.88E-05	3.1632875
SLC17A1	-0.469309	5.6116673	-4.570622	6.64E-06	2.88E-05	3.1632402
HIST1H2BN	-0.33725	4.6565173	-4.570541	6.64E-06	2.88E-05	3.1628903
ZNF71	0.2045567	5.1807061	4.5701464	6.65E-06	2.89E-05	3.161196
RP11-809N8	0.4925186	-1.043247	4.5698604	6.66E-06	2.89E-05	3.1599679
ALKBH6	-0.150086	5.0071128	-4.569737	6.66E-06	2.89E-05	3.1594365
PHIP	0.0900039	6.045383	4.5696318	6.67E-06	2.89E-05	3.1589863
STK11	-0.074071	6.4278826	-4.569454	6.67E-06	2.90E-05	3.1582217
SNX25P1	0.6018196	3.193285	4.569196	6.68E-06	2.90E-05	3.1571149
PCDHA10	0.9851502	1.495795	4.5691255	6.68E-06	2.90E-05	3.1568119
CYP26C1	0.7803695	-0.038591	4.5686257	6.70E-06	2.91E-05	3.1546663

ABCG8	-0.589254	6.1093161	-4.568199	6.71E-06	2.91E-05	3.1528336
RP13-216E2	0.7230646	1.9046547	4.5680407	6.71E-06	2.91E-05	3.1521546
RP5-1153D9	0.6241691	-0.59493	4.5676089	6.73E-06	2.92E-05	3.1503011
TTC32	-0.153937	5.394283	-4.567027	6.74E-06	2.92E-05	3.1478047
AC072052.7	0.7824707	-0.404088	4.56685	6.75E-06	2.93E-05	3.1470437
AL357515.1	0.7009331	1.2112322	4.5666451	6.76E-06	2.93E-05	3.1461646
RP11-21L23	-0.924474	2.6929628	-4.566384	6.76E-06	2.93E-05	3.1450453
CHRFAM7A	0.8104787	-0.258744	4.5660337	6.78E-06	2.94E-05	3.1435404
PMM2	0.1776312	5.6625435	4.5651769	6.80E-06	2.95E-05	3.1398644
SMG1P5	0.645006	2.7227747	4.5651296	6.80E-06	2.95E-05	3.1396614
C12orf5	0.1694939	5.3325377	4.5651252	6.80E-06	2.95E-05	3.1396424
RAB11B	-0.072036	6.6711443	-4.565094	6.80E-06	2.95E-05	3.1395086
SLC20A2	-0.126716	6.3801241	-4.564942	6.81E-06	2.95E-05	3.1388572
RP11-586D1	0.6679292	-0.79962	4.5645942	6.82E-06	2.95E-05	3.1373645
KCNIP3	0.734903	4.2602702	4.5643258	6.83E-06	2.96E-05	3.1362129
CTC-548K16	0.6221584	-0.697581	4.5642646	6.83E-06	2.96E-05	3.1359504
RP11-276H1	0.8594489	0.464758	4.5641851	6.83E-06	2.96E-05	3.1356093
DUSP18	0.1863801	4.5673411	4.5640138	6.84E-06	2.96E-05	3.1348745
SLC6A12	-0.321753	6.1086164	-4.563597	6.85E-06	2.97E-05	3.1330868
RP11-474P2	0.7819338	0.4233049	4.5632941	6.86E-06	2.97E-05	3.1317877
AC092580.4	0.7030573	2.7878445	4.5630813	6.87E-06	2.97E-05	3.1308751
LGALS3	0.2072044	6.2482471	4.5619938	6.90E-06	2.99E-05	3.1262114
APLNR	0.2945055	5.8108829	4.5619822	6.90E-06	2.99E-05	3.1261619
SSBP1	-0.077923	6.4396776	-4.560977	6.93E-06	3.00E-05	3.1218533
RP11-352M1	-0.458796	-1.22845	-4.560148	6.96E-06	3.01E-05	3.1182972
ASB9P1	-0.997387	1.6536421	-4.559569	6.98E-06	3.02E-05	3.1158156
SMARCE1P5	0.708895	0.1641695	4.5587835	7.00E-06	3.03E-05	3.1124509
RP11-43303	0.4824234	-1.130023	4.5582287	7.02E-06	3.03E-05	3.1100736
THY1	0.144825	6.3257842	4.5580093	7.02E-06	3.04E-05	3.1091337
CCL15	-0.463525	5.8056367	-4.557738	7.03E-06	3.04E-05	3.10797
AC005104.3	0.7610202	1.9204558	4.5577083	7.03E-06	3.04E-05	3.1078439
CCDC173	0.7648371	2.2072151	4.5575655	7.04E-06	3.04E-05	3.1072322
DLEU7-AS1	0.8923799	0.8170465	4.5575049	7.04E-06	3.04E-05	3.1069726
CHRND	1.0013836	0.1352056	4.5574586	7.04E-06	3.04E-05	3.106774
SLC25A51	0.1048702	5.430467	4.557186	7.05E-06	3.05E-05	3.1056062
AC002467.7	-0.215707	4.4760222	-4.556825	7.06E-06	3.05E-05	3.1040595
TTC39C	-0.145305	6.7088974	-4.556637	7.07E-06	3.05E-05	3.1032551
TRIM60P18	0.5673225	3.4336187	4.556257	7.08E-06	3.06E-05	3.1016271
GALNT16	0.4676946	4.5152921	4.5561046	7.09E-06	3.06E-05	3.1009746
ARHGAP20	0.6411497	3.6756818	4.5559833	7.09E-06	3.06E-05	3.1004551
DKFZp779MC	-0.437989	4.7018591	-4.555718	7.10E-06	3.06E-05	3.0993199
WDHD1	0.2064485	5.1495883	4.555653	7.10E-06	3.06E-05	3.0990404
RP11-1008C	0.8933522	0.309821	4.5552206	7.11E-06	3.07E-05	3.0971889
MEOX2-AS1	-1.021141	0.0761417	-4.554431	7.14E-06	3.08E-05	3.0938097
MFN2	-0.07509	6.6870831	-4.55426	7.14E-06	3.08E-05	3.0930752
SH3PXD2A	0.0969364	6.2201565	4.5541816	7.15E-06	3.08E-05	3.09274
ASPG	1.0032603	4.9625052	4.5540819	7.15E-06	3.08E-05	3.0923134
OR7E7P	0.7988619	1.2615728	4.5528038	7.19E-06	3.10E-05	3.0868424
PARL	-0.067559	6.2976192	-4.552453	7.20E-06	3.11E-05	3.0853417
KLK14	0.8364335	0.7230059	4.5521775	7.21E-06	3.11E-05	3.0841617
TSC2	-0.088607	6.4777335	-4.551308	7.24E-06	3.12E-05	3.0804413

ABHD8	-0.105871	6.03256	-4.551243	7.24E-06	3.12E-05	3.0801621
WHSC1L1	0.0957543	6.1099607	4.5511988	7.24E-06	3.12E-05	3.0799736
RP11-437L7	-0.74143	-0.633015	-4.551186	7.24E-06	3.12E-05	3.0799171
ATP11B	0.1068229	6.1152132	4.5508463	7.26E-06	3.13E-05	3.0784654
RP11-737F9	-0.370301	-1.345604	-4.550695	7.26E-06	3.13E-05	3.0778178
OR2I1P	0.4507207	6.0763627	4.5506078	7.26E-06	3.13E-05	3.0774451
KIF6	0.840302	2.9100631	4.5502472	7.27E-06	3.13E-05	3.0759023
AC128709.2	-0.341554	-1.313365	-4.550157	7.28E-06	3.14E-05	3.0755149
CFHR5	-0.610001	6.2009759	-4.550045	7.28E-06	3.14E-05	3.0750357
TMEM88B	0.9238406	-0.084166	4.5496237	7.30E-06	3.14E-05	3.0732353
AC092155.4	-0.767147	-0.584071	-4.549428	7.30E-06	3.14E-05	3.0723993
CLNS1A	-0.078434	6.5190634	-4.548665	7.33E-06	3.16E-05	3.0691361
CTD-2013N2	0.4079587	3.253967	4.5481037	7.35E-06	3.16E-05	3.0667348
GPR89A	-0.114522	5.4023377	-4.547761	7.36E-06	3.17E-05	3.0652674
RP5-1184F4	0.7914883	0.3633724	4.5474576	7.37E-06	3.17E-05	3.0639721
CTC-325H20	0.5766811	2.9608525	4.547403	7.37E-06	3.17E-05	3.0637388
PRR3	-0.090186	5.7708696	-4.547258	7.37E-06	3.17E-05	3.0631202
DENND1C	0.132609	5.8880686	4.5472558	7.37E-06	3.17E-05	3.0631094
KIAA1257	0.7928379	1.9926289	4.5470131	7.38E-06	3.18E-05	3.0620715
HOXA6	0.9001075	-0.206047	4.5464125	7.40E-06	3.18E-05	3.0595039
VSIG4	0.2931578	5.3043618	4.5459594	7.42E-06	3.19E-05	3.0575671
FAIM	0.1305842	5.5557195	4.545781	7.42E-06	3.19E-05	3.0568048
SMYD1	-0.856317	-0.475346	-4.545759	7.42E-06	3.19E-05	3.0567088
AKNAD1	0.6545305	-0.681435	4.5456934	7.43E-06	3.19E-05	3.0564302
RP11-157F2	0.6122708	-0.903371	4.5449362	7.45E-06	3.20E-05	3.0531941
RP11-4204.	-0.816134	1.4048156	-4.544904	7.45E-06	3.20E-05	3.0530576
PGGT1B	-0.064042	6.0116279	-4.544603	7.46E-06	3.21E-05	3.0517683
AAMP	-0.056343	6.6621785	-4.544517	7.46E-06	3.21E-05	3.0514043
KCNE2	-0.520372	3.1055794	-4.544426	7.47E-06	3.21E-05	3.051015
RP1-120G22	-0.59725	2.8827303	-4.544363	7.47E-06	3.21E-05	3.0507453
RP11-216B9	0.2576993	3.3389151	4.5439414	7.48E-06	3.22E-05	3.0489434
TAF13	-0.081387	6.0348639	-4.543884	7.49E-06	3.22E-05	3.0486987
MRPL32	-0.072774	6.2962063	-4.543796	7.49E-06	3.22E-05	3.0483222
RP11-170N1	-0.651119	2.6786984	-4.543454	7.50E-06	3.22E-05	3.0468607
MLIP	-0.429265	5.4992715	-4.543069	7.51E-06	3.23E-05	3.0452176
FIGNL2	0.8114789	3.2039937	4.5427719	7.52E-06	3.23E-05	3.0439471
B3GNT2	0.1031318	5.9010945	4.5426819	7.53E-06	3.23E-05	3.0435629
RP11-495P1	0.8378769	1.2100292	4.5425654	7.53E-06	3.23E-05	3.0430651
DYNC1LI2	0.15713	6.0253623	4.5425349	7.53E-06	3.23E-05	3.0429348
RP11-1259L	-1.151937	2.2091842	-4.542218	7.54E-06	3.24E-05	3.0415819
UCK1	-0.088418	6.294953	-4.541409	7.57E-06	3.25E-05	3.0381263
ITGAD	0.5730319	3.7725327	4.5412931	7.57E-06	3.25E-05	3.0376313
AC092301.3	0.7535016	0.9092186	4.5411223	7.58E-06	3.25E-05	3.0369016
CTD-2530N2	0.7641331	1.247739	4.5404782	7.60E-06	3.26E-05	3.0341515
NCAPD3	0.1055925	5.8204909	4.5404585	7.60E-06	3.26E-05	3.0340676
CEACAM7	1.0534677	-0.339183	4.5402623	7.61E-06	3.26E-05	3.0332298
LONRF3	-0.204506	5.3823674	-4.539851	7.62E-06	3.27E-05	3.0314748
NISCH	-0.057863	6.4633728	-4.539764	7.63E-06	3.27E-05	3.0311038
RP6-91H8.3	0.5223564	-1.029605	4.5395576	7.63E-06	3.27E-05	3.030221
DFFBP1	0.7521136	0.6146725	4.5392518	7.64E-06	3.28E-05	3.0289159
NOL11	-0.062721	6.3069511	-4.539207	7.64E-06	3.28E-05	3.028724

CELF6	-0.770699	-0.341202	-4.538282	7.68E-06	3.29E-05	3.0247747
PDCD1LG2	0.4256864	4.0501127	4.5381497	7.68E-06	3.29E-05	3.0242117
RP11-28001	-0.712764	-0.883025	-4.537825	7.69E-06	3.30E-05	3.022826
RP11-339B2	0.8181166	0.0658095	4.5376519	7.70E-06	3.30E-05	3.0220873
METTL5	-0.081231	6.1124629	-4.537065	7.72E-06	3.31E-05	3.0195819
MARS2	0.1502501	5.5847573	4.5370566	7.72E-06	3.31E-05	3.0195473
NGFRAP1	0.2471593	6.3352389	4.536485	7.74E-06	3.31E-05	3.0171083
AC099668.5	-0.730244	1.9633833	-4.53636	7.74E-06	3.32E-05	3.0165757
RP11-298H2	-0.611519	-0.95481	-4.536171	7.75E-06	3.32E-05	3.0157675
RBM11	0.8164172	-0.29128	4.5358675	7.76E-06	3.32E-05	3.0144739
CTD-2134A5	-0.39052	4.4428335	-4.535497	7.77E-06	3.33E-05	3.0128917
RP11-57902	-0.212603	-1.465953	-4.534472	7.81E-06	3.34E-05	3.0085209
LRRC43	0.839585	1.7547838	4.5341868	7.82E-06	3.35E-05	3.0073056
CSF1	0.1328506	6.1391189	4.5339904	7.83E-06	3.35E-05	3.0064682
RP11-58E21	-0.438467	-1.184241	-4.533967	7.83E-06	3.35E-05	3.0063665
CDH16	1.1281119	2.8675698	4.5338041	7.83E-06	3.35E-05	3.0056738
CTD-2503I6	-0.212241	-1.477275	-4.533487	7.84E-06	3.36E-05	3.0043216
RP11-38L15	-0.508962	4.4065378	-4.533049	7.86E-06	3.36E-05	3.0024558
ANKRD39	-0.095174	5.8734113	-4.532849	7.87E-06	3.36E-05	3.0016003
AL132709.8	0.6631329	-1.017274	4.5319744	7.90E-06	3.38E-05	2.997873
SLC2A2	-0.363639	6.8650778	-4.531718	7.91E-06	3.38E-05	2.9967793
SNORD14E	0.7832895	1.6532497	4.5314548	7.92E-06	3.38E-05	2.9956585
LINC01018	-1.020801	5.0454252	-4.530964	7.93E-06	3.39E-05	2.9935664
CCDC73	-0.331059	3.7597065	-4.530889	7.94E-06	3.39E-05	2.9932448
AP2S1	-0.091201	6.5808351	-4.530431	7.95E-06	3.40E-05	2.9912962
RP11-121G2	-0.625695	-0.760493	-4.530139	7.96E-06	3.40E-05	2.9900513
RP11-406A2	-1.037699	0.4190424	-4.529727	7.98E-06	3.41E-05	2.9882953
ZNF300P1	0.8719804	2.8330056	4.5295123	7.98E-06	3.41E-05	2.9873807
KCNK17	0.8888639	2.6253538	4.5289587	8.00E-06	3.42E-05	2.985022
C2orf42	-0.080379	5.6827962	-4.528802	8.01E-06	3.42E-05	2.9843535
MIR3189	0.9435859	0.5045279	4.5287677	8.01E-06	3.42E-05	2.9842083
ADAM24P	-0.246899	-1.450885	-4.52846	8.02E-06	3.43E-05	2.9828985
HK1	0.1552238	5.8985631	4.5283861	8.03E-06	3.43E-05	2.9825831
CORO1C	0.0623172	6.5228684	4.5283437	8.03E-06	3.43E-05	2.9824026
SLC15A4	0.0934362	6.1085001	4.5282996	8.03E-06	3.43E-05	2.9822146
BCL2	0.1921371	5.272958	4.5281499	8.03E-06	3.43E-05	2.981577
GSTCD	0.1607285	5.2426942	4.5281225	8.03E-06	3.43E-05	2.9814602
MLLT1	-0.068741	6.3889206	-4.528055	8.04E-06	3.43E-05	2.9811743
SLC25A11	-0.093229	6.4957783	-4.527935	8.04E-06	3.43E-05	2.9806602
GRIN1	0.9701051	0.5653475	4.5274915	8.06E-06	3.44E-05	2.9787729
AC007064.2	-0.726615	-0.668565	-4.527283	8.06E-06	3.44E-05	2.9778842
SARS	-0.074643	6.7222191	-4.52712	8.07E-06	3.44E-05	2.9771894
ANXA2P1	0.718747	0.8569702	4.526951	8.08E-06	3.44E-05	2.976471
RP11-573G6	0.7198938	-0.201999	4.5266623	8.09E-06	3.45E-05	2.9752415
CTA-85E5.7	-0.339433	-1.334506	-4.526391	8.10E-06	3.45E-05	2.9740864
DGCR6	-0.260753	5.3288988	-4.525794	8.12E-06	3.46E-05	2.9715462
RP11-757F1	0.5252375	-1.048872	4.5255954	8.13E-06	3.46E-05	2.9706992
LAMB4	0.7290377	2.1262976	4.5255621	8.13E-06	3.46E-05	2.9705574
RP11-775A1	-0.203502	-1.492277	-4.525132	8.14E-06	3.47E-05	2.968728
RP11-22B23	0.2278678	4.9501339	4.5246768	8.16E-06	3.48E-05	2.9667889
CETN3	-0.080762	5.6018142	-4.523742	8.19E-06	3.49E-05	2.9628099

IGLV3-29	0.6017962	-1.010116	4.5235306	8.20E-06	3.49E-05	2.9619104
FBXO34	0.0896827	5.8315941	4.5231598	8.22E-06	3.50E-05	2.9603326
RP11-386M2	-0.462459	-1.157802	-4.523146	8.22E-06	3.50E-05	2.9602754
RP11-153M7	0.8817101	0.3318717	4.5231145	8.22E-06	3.50E-05	2.9601401
U3	-0.447142	3.95725	-4.523102	8.22E-06	3.50E-05	2.9600882
KRT8P37	0.7112258	-0.131859	4.5230771	8.22E-06	3.50E-05	2.9599808
HLA-DRA	0.1483497	6.8888675	4.523022	8.22E-06	3.50E-05	2.9597464
SURF2	-0.130844	5.887305	-4.522721	8.23E-06	3.50E-05	2.9584654
NR2C2	0.1470482	5.9043058	4.5225218	8.24E-06	3.51E-05	2.9576183
RPS16	-0.09382	7.2161037	-4.522453	8.24E-06	3.51E-05	2.9573258
CTD-2299I2	-0.864111	0.1772819	-4.52215	8.25E-06	3.51E-05	2.9560377
MAL2	0.2809039	6.362931	4.5201824	8.33E-06	3.54E-05	2.947667
EPHA2	0.1697458	5.9689707	4.5197279	8.34E-06	3.55E-05	2.9457345
C20orf202	0.567214	2.5500194	4.519097	8.37E-06	3.56E-05	2.9430519
CYTH3	0.1373409	5.7151194	4.518904	8.37E-06	3.56E-05	2.9422313
CCDC167	-0.125612	6.1137952	-4.51857	8.39E-06	3.57E-05	2.9408118
RP11-274B1	-0.606909	-1.065918	-4.518503	8.39E-06	3.57E-05	2.9405245
AC005562.1	0.4031777	4.0667253	4.5184469	8.39E-06	3.57E-05	2.9402879
CYP2C8	-0.396705	6.567959	-4.518303	8.40E-06	3.57E-05	2.9396778
PCDHB6	0.7705149	2.6002947	4.5182296	8.40E-06	3.57E-05	2.9393643
EI24P1	-0.19519	-1.484766	-4.518198	8.40E-06	3.57E-05	2.9392316
COPE	-0.082245	6.7738109	-4.518025	8.41E-06	3.57E-05	2.9384962
RP11-338N1	0.7669239	-0.656831	4.5170755	8.44E-06	3.59E-05	2.9344587
MOB2	-0.073478	6.1244483	-4.516809	8.45E-06	3.59E-05	2.9333268
KCTD15	0.2342655	5.5107061	4.5166549	8.46E-06	3.59E-05	2.9326712
RB1	0.1379729	5.9527808	4.5165169	8.46E-06	3.60E-05	2.9320845
RPS18	-0.092008	7.3385866	-4.516386	8.47E-06	3.60E-05	2.931527
GOLGA6L5P	0.7626794	2.2565965	4.5163371	8.47E-06	3.60E-05	2.9313207
MLLT10	0.1015441	6.0357587	4.5158637	8.49E-06	3.60E-05	2.9293091
NDE1	0.1275363	5.5894517	4.5156131	8.50E-06	3.61E-05	2.9282441
CNPPD1	-0.065214	6.4880418	-4.515587	8.50E-06	3.61E-05	2.9281322
AC005550.3	-1.242505	0.678817	-4.515264	8.51E-06	3.61E-05	2.9267597
TLE1	-0.094436	6.596422	-4.515122	8.52E-06	3.61E-05	2.9261599
LINC00315	0.7589502	1.0002387	4.514655	8.53E-06	3.62E-05	2.9241739
ALB	-0.183853	8.1641226	-4.513554	8.58E-06	3.64E-05	2.9194983
RP3-465N24	0.832528	0.5241289	4.5130031	8.60E-06	3.65E-05	2.9171575
KIF23	0.2926002	5.2284928	4.5128439	8.60E-06	3.65E-05	2.9164817
RP11-675F6	0.6190728	-0.985858	4.5118745	8.64E-06	3.67E-05	2.9123655
RNF7	-0.065202	6.3847908	-4.511764	8.64E-06	3.67E-05	2.9118972
RP11-57802	0.7554274	1.5276141	4.5112874	8.66E-06	3.67E-05	2.909873
MMS22L	0.2092586	5.1579678	4.5111846	8.67E-06	3.68E-05	2.9094368
ZBTB12P1	0.8196237	0.6788946	4.5103148	8.70E-06	3.69E-05	2.9057446
GLYATL1P4	-1.079106	1.157512	-4.509317	8.74E-06	3.71E-05	2.9015087
GLULP4	-0.969666	0.6268058	-4.509309	8.74E-06	3.71E-05	2.9014761
CRYBG3	0.5954996	4.658874	4.5089102	8.76E-06	3.71E-05	2.8997839
STAG1	0.179129	5.795716	4.5087831	8.76E-06	3.71E-05	2.8992445
CTB-161C1.	-0.82517	0.2171256	-4.508656	8.77E-06	3.71E-05	2.8987056
RP11-550P1	-0.894242	0.244963	-4.508349	8.78E-06	3.72E-05	2.8974032
BTN2A1	0.0880859	5.9073988	4.5074251	8.81E-06	3.73E-05	2.8934833
AF131215.8	0.5858565	-0.901275	4.5070994	8.83E-06	3.74E-05	2.8921015
PMS2P10	-0.679563	1.9786972	-4.506764	8.84E-06	3.74E-05	2.8906773

RP11-79E3.	-1.001596	0.5747818	-4.505099	8.91E-06	3.77E-05	2.8836202
RPL6P9	-0.570299	-0.906867	-4.504666	8.92E-06	3.78E-05	2.8817807
URM1	-0.081583	6.4342981	-4.504608	8.93E-06	3.78E-05	2.8815359
RP11-338N1	0.8893479	-0.280541	4.5044737	8.93E-06	3.78E-05	2.8809673
ROPN1L	0.733699	1.3265144	4.5040714	8.95E-06	3.79E-05	2.8792617
RP3-323A16	0.9677785	1.2905843	4.5037602	8.96E-06	3.79E-05	2.8779426
CDKN2A-AS1	-0.835411	0.2912788	-4.502458	9.01E-06	3.81E-05	2.8724251
EBAG9	-0.098076	6.2012208	-4.502373	9.02E-06	3.82E-05	2.8720616
CDS2	-0.074251	6.4432927	-4.502072	9.03E-06	3.82E-05	2.8707872
RPL7L1P3	-0.769905	1.6582263	-4.501411	9.05E-06	3.83E-05	2.8679871
MATR3	0.2251622	5.3476474	4.5009622	9.07E-06	3.84E-05	2.8660857
RAD51AP2	-0.843602	2.0838864	-4.500465	9.09E-06	3.85E-05	2.8639813
RNU6-796P	-0.660165	-0.617687	-4.500303	9.10E-06	3.85E-05	2.8632934
SPOPL	0.087289	5.9846941	4.499975	9.11E-06	3.85E-05	2.861904
PTPRN2	0.2325768	4.9140719	4.4999395	9.11E-06	3.85E-05	2.8617533
TAGLN	0.1579823	6.3668605	4.4996307	9.13E-06	3.86E-05	2.8604458
SCNN1D	-0.209867	5.3192942	-4.499311	9.14E-06	3.86E-05	2.8590915
HIST1H3H	-0.486925	4.7013209	-4.49888	9.16E-06	3.87E-05	2.8572648
FAM185A	-0.105666	5.3320081	-4.498595	9.17E-06	3.87E-05	2.8560605
TECRL	-0.836593	-0.767262	-4.498417	9.18E-06	3.88E-05	2.8553054
FTL	-0.114865	7.8901445	-4.498341	9.18E-06	3.88E-05	2.854982
TGM1	0.4220582	3.360417	4.4981243	9.19E-06	3.88E-05	2.8540665
SYCE1L	0.4979429	3.2743337	4.4980092	9.19E-06	3.88E-05	2.8535792
DSG2	0.3676243	5.9742073	4.4977481	9.20E-06	3.89E-05	2.8524738
SPATA24	-0.173224	4.9651364	-4.497633	9.21E-06	3.89E-05	2.8519871
PSMG3	-0.109741	6.1197771	-4.497362	9.22E-06	3.89E-05	2.850838
EXOSC4	-0.142579	6.2303746	-4.496648	9.25E-06	3.91E-05	2.8478183
CYC1	-0.099616	6.8787253	-4.496623	9.25E-06	3.91E-05	2.8477091
TSSK4	0.6244664	2.8056368	4.4964517	9.26E-06	3.91E-05	2.8469855
IL17B	0.8115158	0.2218684	4.4958488	9.28E-06	3.92E-05	2.8444338
FTCD	-0.342807	6.7815703	-4.495837	9.28E-06	3.92E-05	2.8443834
FUNDC1	0.0919677	5.5872006	4.4955735	9.29E-06	3.92E-05	2.8432691
CCL13	0.9292827	2.2330654	4.495405	9.30E-06	3.92E-05	2.8425558
ATG2A	-0.079433	6.3961934	-4.49537	9.30E-06	3.92E-05	2.8424085
RP11-296P7	-0.778461	-0.153269	-4.495305	9.30E-06	3.92E-05	2.8421346
CDKL1	0.5603665	3.3432514	4.4949484	9.32E-06	3.93E-05	2.8406237
RFWD3	0.0817427	5.8811284	4.4948929	9.32E-06	3.93E-05	2.8403888
CPEB3	-0.196165	5.6070437	-4.494832	9.32E-06	3.93E-05	2.8401322
MTCH1	-0.059273	6.7743572	-4.49455	9.34E-06	3.94E-05	2.8389393
DKK3	0.2466466	5.8584174	4.4934754	9.38E-06	3.95E-05	2.8343917
RP11-105N1	-0.441159	-1.181163	-4.493404	9.38E-06	3.96E-05	2.8340895
IGLV1-41	0.7972222	-0.546969	4.4932044	9.39E-06	3.96E-05	2.8332456
CSPP1	0.1221132	5.7806135	4.4929576	9.40E-06	3.96E-05	2.8322016
ZNF750	0.7957283	0.9850571	4.492872	9.41E-06	3.96E-05	2.8318393
RP11-791G1	0.6286284	2.7379507	4.4924632	9.42E-06	3.97E-05	2.8301103
GAPDHP62	0.7256493	0.0557468	4.4920479	9.44E-06	3.98E-05	2.8283539
MFSD4	0.2789217	4.4345188	4.4918465	9.45E-06	3.98E-05	2.8275025
RP11-106D4	0.6824626	2.2577592	4.4912571	9.47E-06	3.99E-05	2.8250101
EGFL6	0.9333202	2.0390147	4.4911897	9.48E-06	3.99E-05	2.8247252
WDR41	-0.074466	6.0031882	-4.491116	9.48E-06	3.99E-05	2.8244122
NUBP1	-0.079022	5.8859348	-4.490556	9.50E-06	4.00E-05	2.822047

EHD2	0.1333142	6.2114109	4.4905213	9.50E-06	4.00E-05	2.8218989
MUC5B	1.2300325	2.8503798	4.490203	9.52E-06	4.01E-05	2.8205535
ZNF667-AS1	0.4349375	4.6414314	4.4898516	9.53E-06	4.01E-05	2.819068
RMDN1	-0.096221	6.424452	-4.48938	9.55E-06	4.02E-05	2.8170757
PAMR1	0.3188751	4.7645632	4.4892661	9.56E-06	4.02E-05	2.8165932
TXNL1	-0.084534	6.5229758	-4.488112	9.61E-06	4.04E-05	2.8117167
ABL1	0.0743938	6.3070868	4.4872353	9.64E-06	4.06E-05	2.8080116
AC022182.1	0.712149	-0.311347	4.4869682	9.66E-06	4.06E-05	2.806883
TIMM17B	-0.084743	6.3429312	-4.486625	9.67E-06	4.07E-05	2.8054347
PYGL	-0.126156	6.7177263	-4.486597	9.67E-06	4.07E-05	2.8053138
CHKB-AS1	-0.239875	4.009779	-4.486572	9.67E-06	4.07E-05	2.8052074
RP11-89K21	1.2061219	0.7545706	4.4856905	9.71E-06	4.08E-05	2.8014857
HSF4	-0.171364	5.6239977	-4.485651	9.71E-06	4.08E-05	2.8013173
SPATA6	0.3324155	4.6674141	4.4851884	9.73E-06	4.09E-05	2.7993653
RP11-394I1	0.6705603	-0.585882	4.4847918	9.75E-06	4.10E-05	2.7976904
COG6	0.1266439	5.7005094	4.4843063	9.77E-06	4.11E-05	2.7956403
UHRF1BP1	0.0958882	5.9566358	4.4842632	9.77E-06	4.11E-05	2.7954585
ANGEL1	0.1095767	6.0196433	4.4840866	9.78E-06	4.11E-05	2.7947129
RP11-560J1	-0.517399	3.1087458	-4.483839	9.79E-06	4.11E-05	2.7936689
RP11-401F2	-0.247202	-1.437255	-4.48379	9.79E-06	4.11E-05	2.7934594
PFDN2	-0.092675	6.4464952	-4.483781	9.79E-06	4.11E-05	2.7934211
RP11-797A1	0.8369486	0.5875899	4.4832382	9.82E-06	4.12E-05	2.791131
LINC00704	0.9593289	0.6374686	4.4831612	9.82E-06	4.12E-05	2.7908059
RP11-767N6	-0.126561	4.6770837	-4.483048	9.83E-06	4.13E-05	2.7903292
RP11-527J8	0.4822156	2.9595354	4.4827253	9.84E-06	4.13E-05	2.7889657
PXN-AS1	-0.142325	5.2760513	-4.482559	9.85E-06	4.13E-05	2.7882622
OR12D2	0.5527357	-1.116115	4.482307	9.86E-06	4.14E-05	2.7872003
RPS9	-0.092705	7.2125714	-4.482217	9.86E-06	4.14E-05	2.7868202
LSM4	-0.091227	6.6502986	-4.482087	9.87E-06	4.14E-05	2.7862717
RHBDL2	0.4846898	3.3285921	4.4815741	9.89E-06	4.15E-05	2.784107
PRPF6	-0.072779	6.7464317	-4.481072	9.91E-06	4.16E-05	2.7819897
RP11-36B15	0.7063319	1.1685848	4.4809173	9.92E-06	4.16E-05	2.7813354
XPC	-0.089886	6.2349508	-4.480498	9.94E-06	4.17E-05	2.7795683
EPB41L5	-0.09668	6.2551807	-4.479805	9.97E-06	4.18E-05	2.7766417
APBA3	-0.081403	5.9562015	-4.479354	9.99E-06	4.19E-05	2.7747403
VTI1B	-0.068808	6.4291917	-4.479201	9.99E-06	4.19E-05	2.7740937
RP1-102K2.	1.0102869	0.9084523	4.4787291	1.00E-05	4.20E-05	2.7721041
FAM120AOS	-0.061027	6.1379236	-4.478085	1.00E-05	4.21E-05	2.7693887
RP1-40E16.	-0.422786	3.3801165	-4.477811	1.01E-05	4.22E-05	2.7682329
KLHL41	0.7819837	1.7468534	4.4775539	1.01E-05	4.22E-05	2.7671482
AC053503.4	-0.712364	2.6649922	-4.47737	1.01E-05	4.22E-05	2.7663735
RP11-835E1	-0.726003	0.026455	-4.47728	1.01E-05	4.22E-05	2.7659915
CLTA	-0.079486	6.7055355	-4.477209	1.01E-05	4.23E-05	2.7656935
RP5-1158E1	0.7549489	-0.23611	4.4770615	1.01E-05	4.23E-05	2.7650718
RHBDD2	-0.090042	6.6067691	-4.477009	1.01E-05	4.23E-05	2.7648526
CTD-2666L2	0.7523392	1.4896219	4.4765986	1.01E-05	4.24E-05	2.76312
RP11-464F9	-0.504833	2.9319358	-4.4762	1.01E-05	4.24E-05	2.7614388
RN7SL843P	-0.179169	-1.491805	-4.475934	1.01E-05	4.25E-05	2.7603164
RP11-1D12.	-0.835195	0.848778	-4.475828	1.01E-05	4.25E-05	2.7598729
RP11-342D1	0.5007442	-1.065858	4.4752155	1.02E-05	4.26E-05	2.7572899
ZNF883	0.8132759	3.1260508	4.4742991	1.02E-05	4.28E-05	2.753428

TMEM170B	-0.159362	5.900588	-4.474027	1.02E-05	4.28E-05	2.7522816
HLA-DPB1	0.1511249	6.6177809	4.4736174	1.02E-05	4.29E-05	2.7505556
C19orf33	0.7729926	3.0362697	4.4735382	1.02E-05	4.29E-05	2.7502218
PIK3R1	-0.126637	6.547056	-4.473507	1.03E-05	4.29E-05	2.7500919
ZNF48	-0.087501	5.6738996	-4.473378	1.03E-05	4.29E-05	2.7495469
CFP	0.295018	4.9186119	4.4730349	1.03E-05	4.30E-05	2.7481014
RP11-16402	-0.774456	2.5769612	-4.472484	1.03E-05	4.31E-05	2.7457804
PRPF40B	0.205062	5.1922062	4.4723632	1.03E-05	4.31E-05	2.7452717
RP11-105N1	-0.909032	1.5377108	-4.472134	1.03E-05	4.31E-05	2.7443047
SERHL2	0.3367642	4.1547115	4.4719894	1.03E-05	4.32E-05	2.7436972
RP11-266K4	0.7370943	1.619606	4.4719588	1.03E-05	4.32E-05	2.7435682
CLMN	-0.120054	6.365535	-4.471721	1.03E-05	4.32E-05	2.7425665
RPL4P6	0.5327638	2.5677842	4.4716662	1.03E-05	4.32E-05	2.7423359
CSPG4	0.1658901	5.690019	4.4710632	1.04E-05	4.33E-05	2.7397964
NIPSNAP1	-0.096916	6.9056978	-4.471023	1.04E-05	4.33E-05	2.7396287
CACFD1	-0.092106	6.5598245	-4.471013	1.04E-05	4.33E-05	2.7395854
AC253576.2	0.8130855	0.71187	4.4708658	1.04E-05	4.33E-05	2.738965
RP11-1166F	0.6994137	-0.222434	4.4708295	1.04E-05	4.33E-05	2.7388122
RPL4	-0.081741	7.3370417	-4.469987	1.04E-05	4.35E-05	2.7352627
UBE2V1	0.1352661	5.3572812	4.4699787	1.04E-05	4.35E-05	2.7352298
AP003774.1	0.7943767	0.2540751	4.4695332	1.04E-05	4.36E-05	2.733354
RNU2-71P	-0.285765	-1.422646	-4.4694	1.04E-05	4.36E-05	2.7327941
RP5-850E9.	-0.758306	1.5135605	-4.469131	1.05E-05	4.36E-05	2.7316625
RP5-978I12	-0.815038	-0.771974	-4.468713	1.05E-05	4.37E-05	2.7299006
FTSJ2	-0.05764	6.1429372	-4.468423	1.05E-05	4.38E-05	2.7286813
RPL35AP26	-0.621061	-0.620151	-4.467748	1.05E-05	4.39E-05	2.7258379
FAM189A1	0.9037921	1.4759177	4.467734	1.05E-05	4.39E-05	2.7257811
GATA2	0.2808555	4.7517423	4.4676767	1.05E-05	4.39E-05	2.7255397
LYPD6	1.0482402	2.4923475	4.4670724	1.05E-05	4.40E-05	2.7229966
CTC-359D24	0.497559	3.8408141	4.4665292	1.06E-05	4.41E-05	2.720711
PDZD2	0.2604854	4.7486528	4.4662427	1.06E-05	4.42E-05	2.7195057
REEP2	0.6516939	3.5170809	4.4659502	1.06E-05	4.42E-05	2.7182751
RP11-522N1	0.5999896	-0.662598	4.4658812	1.06E-05	4.42E-05	2.7179849
RP11-766N7	0.646794	-0.813862	4.4658559	1.06E-05	4.42E-05	2.7178785
SLC26A2	0.1333302	5.6366997	4.4655667	1.06E-05	4.43E-05	2.7166617
CIART	-0.282749	5.3745887	-4.465345	1.06E-05	4.43E-05	2.7157293
AC009948.5	0.2116621	4.6706463	4.4651097	1.06E-05	4.44E-05	2.7147394
CALR	-0.075896	7.4502639	-4.465045	1.06E-05	4.44E-05	2.7144693
NR1D1	-0.134551	6.1784914	-4.464907	1.06E-05	4.44E-05	2.7138882
RP11-783L4	-0.196896	-1.484017	-4.464854	1.07E-05	4.44E-05	2.7136649
PHPT1	-0.111701	6.525542	-4.464815	1.07E-05	4.44E-05	2.7135019
CAMP	0.684702	-0.547017	4.4642957	1.07E-05	4.45E-05	2.7113161
RPS19BP1	-0.096985	6.4795345	-4.463473	1.07E-05	4.46E-05	2.7078562
BDH2	-0.141736	6.0131353	-4.463331	1.07E-05	4.47E-05	2.7072581
PLEKHH1	0.2583329	5.1319898	4.4632773	1.07E-05	4.47E-05	2.7070334
LTF	0.5146071	4.2312327	4.4631254	1.07E-05	4.47E-05	2.7063949
CECR2	-0.428247	5.4647765	-4.462845	1.07E-05	4.47E-05	2.7052157
TMEM238	-0.245293	5.2473716	-4.462379	1.08E-05	4.48E-05	2.7032585
RPS7	-0.086035	7.1041324	-4.462106	1.08E-05	4.49E-05	2.7021108
CWC25	-0.056495	5.9307886	-4.462062	1.08E-05	4.49E-05	2.7019262
TRADD	-0.104226	6.0018951	-4.461795	1.08E-05	4.49E-05	2.7008025

EIF2D	-0.071757	6.4795873	-4.461395	1.08E-05	4.50E-05	2.6991193
GPX3	-0.166564	7.1717649	-4.461288	1.08E-05	4.50E-05	2.698669
RPSAP52	0.6197843	-0.838033	4.4609826	1.08E-05	4.51E-05	2.6973874
PRR7-AS1	0.8318975	1.5850673	4.4609142	1.08E-05	4.51E-05	2.6970996
ASB8	-0.062967	6.1825289	-4.460786	1.08E-05	4.51E-05	2.6965602
CTB-52I2.3	-0.502616	-1.090622	-4.460769	1.08E-05	4.51E-05	2.6964908
CETN2	-0.085564	6.2231984	-4.460321	1.09E-05	4.52E-05	2.6946083
RP11-396C2	-0.671255	1.6904078	-4.460217	1.09E-05	4.52E-05	2.6941687
MLEC	-0.07676	6.9305529	-4.46013	1.09E-05	4.52E-05	2.6938029
TRMT5	-0.076571	5.8325922	-4.459796	1.09E-05	4.53E-05	2.6923997
CALCR	0.939087	0.5512232	4.4588596	1.09E-05	4.55E-05	2.6884665
CXCR2	0.7928732	2.1271635	4.458659	1.09E-05	4.55E-05	2.6876242
SOCS1	0.284131	5.0521593	4.4582427	1.10E-05	4.56E-05	2.6858755
KCNT1	0.7456793	1.4171437	4.4577451	1.10E-05	4.57E-05	2.6837854
RP11-240E2	-0.362136	-1.313959	-4.457597	1.10E-05	4.57E-05	2.6831642
RPL3	-0.080332	7.4784112	-4.457582	1.10E-05	4.57E-05	2.6831019
WDR5B	-0.084557	5.5490323	-4.457557	1.10E-05	4.57E-05	2.6829936
AGMAT	-0.192909	6.4201672	-4.457357	1.10E-05	4.57E-05	2.6821546
TMEM121	0.6492577	3.3638551	4.456663	1.10E-05	4.59E-05	2.679241
RP11-723J4	0.7110817	1.1838558	4.4564864	1.11E-05	4.59E-05	2.6784994
ZNF551	0.2932928	4.7950572	4.4562466	1.11E-05	4.59E-05	2.6774927
AC007461.2	0.8223793	0.3342172	4.4560155	1.11E-05	4.60E-05	2.6765222
ATL2	-0.072803	6.3851431	-4.455954	1.11E-05	4.60E-05	2.6762633
SAMD15	0.7591484	1.834775	4.4554846	1.11E-05	4.61E-05	2.6742932
RP11-181C2	0.7589144	2.4530667	4.4542696	1.12E-05	4.63E-05	2.6691931
NOC2L	-0.077045	6.5092152	-4.454243	1.12E-05	4.63E-05	2.6690808
RP3-512B11	-0.317146	5.0275377	-4.454122	1.12E-05	4.63E-05	2.6685745
PLEKHO1	0.1429148	6.0328547	4.4540983	1.12E-05	4.63E-05	2.6684745
TNMD	-0.827092	-0.135134	-4.454044	1.12E-05	4.64E-05	2.6682477
FSCN3	0.6921241	0.0622041	4.4538399	1.12E-05	4.64E-05	2.66739
SH2D4A	-0.137568	6.1162221	-4.453365	1.12E-05	4.65E-05	2.6653972
RP11-672F9	-0.243003	-1.45614	-4.453087	1.12E-05	4.65E-05	2.6642293
SEC11A	-0.063759	6.6177243	-4.452493	1.13E-05	4.66E-05	2.6617377
AC024937.2	0.6471393	-0.420872	4.4524445	1.13E-05	4.67E-05	2.6615348
CXCR2P1	0.6203385	4.0953435	4.4523603	1.13E-05	4.67E-05	2.6611816
CCDC42B	0.6421146	1.7237638	4.452073	1.13E-05	4.67E-05	2.6599762
TES	0.1611173	5.9880431	4.4520234	1.13E-05	4.67E-05	2.6597682
ETFDH	-0.131529	6.3535842	-4.451751	1.13E-05	4.68E-05	2.6586252
SSNA1	-0.09444	6.3698104	-4.451168	1.13E-05	4.69E-05	2.6561788
PRAMEF18	-0.238671	-1.463329	-4.450814	1.13E-05	4.70E-05	2.6546968
SOCS5	0.072999	5.8490999	4.4505583	1.13E-05	4.70E-05	2.6536228
AGGF1	-0.060876	6.1708613	-4.450551	1.13E-05	4.70E-05	2.6535927
GPR75	0.6743214	3.6191648	4.4502527	1.14E-05	4.71E-05	2.652341
FAM196B	0.6305225	-0.774924	4.4500405	1.14E-05	4.71E-05	2.6514512
CYP1A2	-1.218984	4.0649124	-4.4494	1.14E-05	4.72E-05	2.6487663
HS6ST3	0.8570711	-0.31016	4.4489041	1.14E-05	4.73E-05	2.6466861
CNTD2	0.7225527	4.2832597	4.4487665	1.14E-05	4.73E-05	2.6461091
C5orf49	1.007231	3.1277075	4.4487398	1.14E-05	4.73E-05	2.6459973
EXOSC2	-0.078633	5.9325932	-4.448658	1.14E-05	4.74E-05	2.6456552
PCDHGB1	0.9515403	2.3081777	4.4482647	1.15E-05	4.74E-05	2.6440056
CTD-3023L1	-0.204697	-1.480589	-4.446961	1.15E-05	4.77E-05	2.6385394

AC093627.1	0.6253624	3.7304275	4.4469332	1.15E-05	4.77E-05	2.6384248
RP11-362K1	0.7947911	1.6404108	4.4460806	1.16E-05	4.79E-05	2.6348519
MNX1-AS2	0.8188453	-0.647558	4.4453637	1.16E-05	4.80E-05	2.6318481
RP11-79P5.	-0.444092	3.2036359	-4.444894	1.16E-05	4.81E-05	2.629881
RP11-424C2	0.9259713	1.7126823	4.4444599	1.17E-05	4.82E-05	2.6280621
ACAT2	-0.126426	6.4596651	-4.44388	1.17E-05	4.83E-05	2.6256332
APLP2	-0.063718	7.1919174	-4.443786	1.17E-05	4.83E-05	2.6252389
RPUSD1	-0.089426	5.9728281	-4.443371	1.17E-05	4.84E-05	2.6235008
TAF2	0.1374772	5.9519338	4.4430662	1.17E-05	4.85E-05	2.6222249
RP11-981P6	0.719473	1.6388911	4.4428085	1.17E-05	4.85E-05	2.6211459
LYPD1	0.6849852	4.4860175	4.4422264	1.18E-05	4.87E-05	2.6187088
NPAS2	0.1585675	5.9319765	4.442159	1.18E-05	4.87E-05	2.6184265
ATP5G3	-0.08231	6.9179904	-4.442132	1.18E-05	4.87E-05	2.6183147
AL022341.3	-0.731391	1.412671	-4.441367	1.18E-05	4.88E-05	2.6151127
ACTR1B	-0.057349	6.457568	-4.441148	1.18E-05	4.89E-05	2.6141959
SLC2A13	0.1726945	5.6364542	4.4408892	1.18E-05	4.89E-05	2.6131108
ELFN1-AS1	1.1354884	1.1987504	4.4404054	1.19E-05	4.90E-05	2.6110859
MRPS23	-0.073695	6.2879197	-4.440183	1.19E-05	4.91E-05	2.6101542
TTL	-0.072575	6.1707419	-4.439021	1.19E-05	4.93E-05	2.6052938
KLF2	0.1710528	5.6469938	4.4379842	1.20E-05	4.95E-05	2.6009545
RP11-87501	0.4700386	2.6609667	4.4376296	1.20E-05	4.96E-05	2.5994713
EFR3A	-0.101702	6.5351042	-4.43748	1.20E-05	4.96E-05	2.5988469
RP11-100M1	-0.223849	-1.472175	-4.437446	1.20E-05	4.96E-05	2.5987033
RHOQ	0.1081546	5.9718515	4.4372009	1.20E-05	4.97E-05	2.5976783
PRKAG2	-0.142185	6.0180912	-4.436611	1.21E-05	4.98E-05	2.5952103
RP11-87H9.	0.8167583	0.2634734	4.4364521	1.21E-05	4.98E-05	2.5945465
MTND6P3	-0.776365	0.0262397	-4.436318	1.21E-05	4.98E-05	2.5939862
RP11-1149M	0.9550494	2.2875383	4.4357082	1.21E-05	5.00E-05	2.5914358
UNC13B	-0.108792	6.3728976	-4.435468	1.21E-05	5.00E-05	2.5904319
PRCP	-0.10327	6.5499142	-4.435012	1.22E-05	5.01E-05	2.5885232
NT5DC2	0.2075983	5.9160122	4.4349297	1.22E-05	5.01E-05	2.5881807
WDR61	-0.061559	6.257114	-4.434643	1.22E-05	5.02E-05	2.5869836
ADORA1	0.7361419	3.885921	4.4343416	1.22E-05	5.02E-05	2.5857223
LRMP	0.1986626	4.8330289	4.4341701	1.22E-05	5.03E-05	2.5850054
MIR4442	0.8726725	1.708032	4.4337704	1.22E-05	5.04E-05	2.5833345
SALL1	-0.270761	6.2651179	-4.433764	1.22E-05	5.04E-05	2.5833059
CHRNB2	0.8186158	1.54669	4.4333386	1.22E-05	5.04E-05	2.58153
SDCBP2-AS1	-0.166494	4.9512679	-4.433296	1.22E-05	5.05E-05	2.5813505
ASAP3	-0.165194	5.955559	-4.43325	1.22E-05	5.05E-05	2.5811596
GAPDHP37	-0.731891	-0.472631	-4.433082	1.23E-05	5.05E-05	2.580457
TMEM144	0.3626872	4.8615869	4.4323117	1.23E-05	5.07E-05	2.5772386
IFNAR1	-0.069399	6.5916565	-4.432156	1.23E-05	5.07E-05	2.5765884
SLCO2B1	-0.159103	6.7908864	-4.431984	1.23E-05	5.07E-05	2.5758706
RP13-766D2	0.7209349	-0.208367	4.4318551	1.23E-05	5.07E-05	2.5753308
PHBP8	-0.579424	-0.782962	-4.431203	1.24E-05	5.09E-05	2.5726064
SPNS3	0.5061842	3.8289191	4.4311547	1.24E-05	5.09E-05	2.5724047
GNAI1	-0.135969	6.1583177	-4.43062	1.24E-05	5.10E-05	2.5701711
UPK1A-AS1	1.0575055	0.5965868	4.4305933	1.24E-05	5.10E-05	2.5700594
LMNA	-0.077383	7.099788	-4.430581	1.24E-05	5.10E-05	2.5700088
SLC35G1	0.1966921	5.3813695	4.4305338	1.24E-05	5.10E-05	2.5698109
RP11-568N6	0.4510475	3.340472	4.4305298	1.24E-05	5.10E-05	2.5697941

MTMR9	0.1161582	5.4153393	4.4303436	1.24E-05	5.10E-05	2.5690167
BMP2K	0.1454086	5.1375326	4.4303046	1.24E-05	5.10E-05	2.5688536
SLC22A1	-0.485404	6.1578348	-4.429673	1.24E-05	5.12E-05	2.5662178
CCDC102A	0.2155859	5.0713308	4.4294575	1.25E-05	5.12E-05	2.5653157
FILIP1	0.2528056	5.0345319	4.4292903	1.25E-05	5.12E-05	2.5646176
NPM1P9	0.7346396	0.1939245	4.429268	1.25E-05	5.12E-05	2.5645243
PNLDC1	0.8023891	1.0758403	4.428407	1.25E-05	5.14E-05	2.5609292
RP11-599J1	0.789701	2.101414	4.428099	1.25E-05	5.15E-05	2.5596436
HSBP1P2	-0.881609	-0.000704	-4.427935	1.25E-05	5.15E-05	2.5589567
BNIP3P1	-0.490819	3.0965471	-4.427488	1.26E-05	5.16E-05	2.5570946
DZANK1	0.2345356	4.2510318	4.4272335	1.26E-05	5.17E-05	2.5560304
RP11-879F1	0.698875	1.8052887	4.4270494	1.26E-05	5.17E-05	2.5552618
ADSSL1	-0.208529	5.7640646	-4.426693	1.26E-05	5.18E-05	2.5537749
RP11-864J1	0.7279139	0.8591936	4.4266349	1.26E-05	5.18E-05	2.5535317
TK1	-0.158263	6.1549084	-4.4266	1.26E-05	5.18E-05	2.5533851
ERP44	-0.071197	6.616534	-4.426555	1.26E-05	5.18E-05	2.5531985
IL1RAP	0.1641237	6.0752003	4.4265105	1.26E-05	5.18E-05	2.5530127
PCDHB2	0.810732	3.1254319	4.4264201	1.26E-05	5.18E-05	2.5526355
RNU6-713P	-0.16083	-1.499863	-4.426344	1.26E-05	5.18E-05	2.5523158
SDCBP2	0.3139967	5.4232852	4.4262595	1.26E-05	5.18E-05	2.5519649
KANSL2	0.0641483	5.8795809	4.4258411	1.27E-05	5.19E-05	2.5502192
COL15A1	0.2133047	5.9290094	4.4248065	1.27E-05	5.22E-05	2.545902
NUDT6	-0.265806	5.1057309	-4.424795	1.27E-05	5.22E-05	2.5458533
RPS15	-0.100203	6.8984761	-4.423916	1.28E-05	5.24E-05	2.5421875
RP11-344P1	-0.596961	4.0006111	-4.423822	1.28E-05	5.24E-05	2.5417958
SH3BP5	0.3872504	4.9538295	4.4234234	1.28E-05	5.25E-05	2.5401323
RP6-201G1C	0.7909735	0.7971738	4.4226448	1.28E-05	5.26E-05	2.536885
SPICE1	0.1230662	5.4237193	4.4225901	1.28E-05	5.26E-05	2.5366571
TSPAN9	-0.097537	6.5509022	-4.422482	1.28E-05	5.27E-05	2.5362052
DIRC3	0.6796318	1.7156751	4.4224029	1.28E-05	5.27E-05	2.5358766
CDKL2	0.6912167	-0.568253	4.422166	1.29E-05	5.27E-05	2.5348885
LIN7B	-0.169506	5.0700809	-4.422159	1.29E-05	5.27E-05	2.5348608
PCDHB8	1.0009273	1.8153463	4.4220185	1.29E-05	5.27E-05	2.5342735
LGALS14	0.7510894	-0.906612	4.4219217	1.29E-05	5.28E-05	2.5338697
ZNF32	-0.088024	6.026747	-4.421699	1.29E-05	5.28E-05	2.5329426
C8B	-0.329278	6.8538641	-4.421669	1.29E-05	5.28E-05	2.5328164
E2F3P1	0.5582818	-0.905704	4.4212905	1.29E-05	5.29E-05	2.5312379
RP11-498E2	0.5546889	-0.902946	4.4208988	1.29E-05	5.30E-05	2.5296052
GTF2H2C	0.2506879	4.902859	4.4206562	1.29E-05	5.30E-05	2.5285936
RP11-115L1	0.7971795	0.5580868	4.4205753	1.30E-05	5.30E-05	2.5282567
ARL4D	-0.180468	6.1187075	-4.420124	1.30E-05	5.31E-05	2.5263754
SQRDL	-0.096218	6.3676814	-4.419615	1.30E-05	5.33E-05	2.5242544
RNF182	0.8371831	0.1395664	4.4195479	1.30E-05	5.33E-05	2.5239743
RP11-692P1	-0.494495	-0.918159	-4.419234	1.30E-05	5.33E-05	2.5226677
ITFG1	-0.099475	6.4022529	-4.41876	1.31E-05	5.34E-05	2.5206908
SNORD101	0.7456392	0.2134702	4.4180756	1.31E-05	5.36E-05	2.5178392
SNORD116-2	0.5446419	-0.918554	4.418062	1.31E-05	5.36E-05	2.5177825
GPRIN1	0.3456611	4.9632428	4.418033	1.31E-05	5.36E-05	2.5176615
MUTYH	-0.093396	5.7093162	-4.417886	1.31E-05	5.36E-05	2.5170477
RP6-43L17.	-0.184695	-1.500541	-4.41744	1.31E-05	5.37E-05	2.5151902
PCBD2	-0.091439	5.6533836	-4.417273	1.31E-05	5.37E-05	2.514495

RP11-139I1	-0.929624	0.2264866	-4.41703	1.32E-05	5.38E-05	2.5134825
HLA-DQA2	0.6706369	5.0444235	4.4170252	1.32E-05	5.38E-05	2.513463
C5AR1	0.1834346	5.4722716	4.4170092	1.32E-05	5.38E-05	2.5133966
RP11-113I2	-0.860072	-0.573292	-4.416922	1.32E-05	5.38E-05	2.513032
RP11-384L8	-0.303214	5.388664	-4.416756	1.32E-05	5.38E-05	2.5123435
MSRA	-0.143743	6.0950316	-4.416663	1.32E-05	5.39E-05	2.5119557
AC133785.1	0.7648009	-0.657491	4.4161472	1.32E-05	5.40E-05	2.5098063
BX842568.2	-1.011197	3.1890145	-4.41535	1.33E-05	5.42E-05	2.506488
RP11-46H11	0.7446199	1.1751061	4.4153014	1.33E-05	5.42E-05	2.5062839
FARP2	0.088671	5.8966878	4.4152827	1.33E-05	5.42E-05	2.5062062
RP11-1008C	0.6116015	3.1889692	4.4148484	1.33E-05	5.43E-05	2.5043978
FAM47E	-0.230255	4.8189738	-4.414633	1.33E-05	5.43E-05	2.5035001
AC005152.3	0.8244213	-0.288959	4.4143883	1.33E-05	5.44E-05	2.5024821
RP11-530C5	0.7357734	2.0918699	4.4142095	1.33E-05	5.44E-05	2.5017376
LCE2D	-0.627841	-0.954173	-4.413919	1.33E-05	5.45E-05	2.500529
CTD-2256P1	-0.727673	-0.060966	-4.413403	1.34E-05	5.46E-05	2.4983795
RP11-131L2	-0.734858	1.9743315	-4.413247	1.34E-05	5.46E-05	2.4977294
TGIF2	0.1186134	6.0231147	4.4131048	1.34E-05	5.46E-05	2.497139
AGR2	1.3088218	2.3679265	4.4126987	1.34E-05	5.47E-05	2.495449
TMEM63B	-0.075257	6.5674066	-4.412245	1.34E-05	5.48E-05	2.4935607
RP11-59H7.	0.7798363	0.9046367	4.4121774	1.34E-05	5.48E-05	2.4932794
MIR559	0.4496567	-1.205374	4.4117187	1.35E-05	5.49E-05	2.4913707
SLC45A2	-0.860324	3.0658033	-4.411507	1.35E-05	5.50E-05	2.4904891
CTD-3195I5	0.6453637	1.8442408	4.4114554	1.35E-05	5.50E-05	2.4902752
RP11-401F2	-0.475109	3.370132	-4.410817	1.35E-05	5.51E-05	2.4876188
STX10	-0.087404	6.3076377	-4.410565	1.35E-05	5.52E-05	2.4865698
PMFBP1	0.4844069	3.8277966	4.4094199	1.36E-05	5.55E-05	2.4818074
RP11-417F2	0.7931317	0.3899111	4.4092502	1.36E-05	5.55E-05	2.4811017
AP000347.4	0.6256704	3.010815	4.4090441	1.36E-05	5.55E-05	2.4802444
KIAA2026	0.1130549	5.7355936	4.4089033	1.36E-05	5.56E-05	2.4796591
CRLF2	1.0337921	0.9879899	4.4082837	1.37E-05	5.57E-05	2.4770825
ACADL	-0.827557	4.8354967	-4.406923	1.38E-05	5.60E-05	2.4714271
EP400	0.0627483	6.1936418	4.4068265	1.38E-05	5.61E-05	2.4710243
TAF6	0.140954	5.9769402	4.4064116	1.38E-05	5.62E-05	2.4692995
MAP2K5	-0.081011	5.8860336	-4.406334	1.38E-05	5.62E-05	2.4689767
TIMM23	-0.064409	6.3873487	-4.406328	1.38E-05	5.62E-05	2.4689524
TIMM9	-0.083792	6.0354911	-4.406315	1.38E-05	5.62E-05	2.4688971
RP11-529F4	0.6212046	2.6738141	4.4056854	1.38E-05	5.63E-05	2.4662814
SPAG17	0.9003713	0.4299566	4.4054703	1.38E-05	5.64E-05	2.4653876
DDAH1	-0.127708	6.5801522	-4.404578	1.39E-05	5.66E-05	2.461678
OTOF	0.7697786	2.1317436	4.4043635	1.39E-05	5.66E-05	2.4607883
IGHV3-41	0.7266032	-0.562149	4.4041276	1.39E-05	5.67E-05	2.4598084
PPARD	0.0943362	6.2329217	4.4039456	1.39E-05	5.67E-05	2.4590521
AC104650.2	0.5866626	-0.79562	4.4035373	1.40E-05	5.68E-05	2.4573561
GS1-39E22.	-0.230048	-1.467118	-4.403475	1.40E-05	5.68E-05	2.4570978
PREX2	0.3296281	4.8969475	4.4033407	1.40E-05	5.68E-05	2.4565394
FDCSP	1.2352285	1.2310233	4.4027655	1.40E-05	5.70E-05	2.4541501
FXN	-0.119517	5.7765452	-4.402359	1.40E-05	5.71E-05	2.4524629
RP11-449J2	0.6664688	3.7427086	4.4023491	1.40E-05	5.71E-05	2.4524206
RP11-122K1	0.5874659	3.3744166	4.4018539	1.41E-05	5.72E-05	2.4503642
AP001476.2	0.7850398	-0.453133	4.4017971	1.41E-05	5.72E-05	2.4501282

KCTD5	0.0688574	6.0498988	4.4017889	1.41E-05	5.72E-05	2.4500942
RP13-20L14	0.7819488	1.7567738	4.4015899	1.41E-05	5.72E-05	2.4492679
CRNKL1	-0.062723	6.1921841	-4.401563	1.41E-05	5.72E-05	2.4491548
LRRC56	0.256356	4.3874114	4.4008857	1.41E-05	5.74E-05	2.446344
CD24P4	0.7054874	-0.422723	4.4005094	1.41E-05	5.75E-05	2.4447817
USP37	0.2286768	5.0308117	4.4000259	1.42E-05	5.76E-05	2.4427744
RAB11FIP5	0.1348513	5.9114816	4.3999401	1.42E-05	5.76E-05	2.4424183
BCL2L1	-0.082667	6.6739312	-4.399463	1.42E-05	5.77E-05	2.4404361
NFATC2IP	-0.066939	6.098498	-4.399373	1.42E-05	5.78E-05	2.440063
LENG8	0.0807991	6.4746265	4.3992561	1.42E-05	5.78E-05	2.4395793
EZR	0.1133269	6.5275394	4.3983009	1.43E-05	5.80E-05	2.435615
PDXDC1	-0.074058	6.7376163	-4.397984	1.43E-05	5.81E-05	2.4342992
RP5-1030M6	-0.380776	-1.400895	-4.397528	1.43E-05	5.82E-05	2.4324088
TBC1D30	0.3316538	4.9452425	4.3967723	1.44E-05	5.84E-05	2.4292731
RFESD	-0.174231	4.554362	-4.396432	1.44E-05	5.85E-05	2.4278594
TBC1D9B	-0.065958	6.7274601	-4.396222	1.44E-05	5.85E-05	2.4269905
TMEM38A	-0.14973	5.7577762	-4.395807	1.44E-05	5.86E-05	2.4252694
DDB2	-0.106807	6.1223245	-4.395637	1.44E-05	5.86E-05	2.4245632
NFKBIZ	0.1502438	5.8687229	4.3953833	1.45E-05	5.87E-05	2.4235116
LINC00173	0.8348482	1.3924437	4.3951633	1.45E-05	5.88E-05	2.4225996
SLC18A1	0.670399	-0.620186	4.3950664	1.45E-05	5.88E-05	2.4221975
IPO11	-0.061522	5.8982751	-4.394553	1.45E-05	5.89E-05	2.4200682
ZNF169	0.2453812	4.2081941	4.3945324	1.45E-05	5.89E-05	2.4199833
SULT1C2P1	0.9579544	0.7918557	4.3942409	1.45E-05	5.90E-05	2.4187745
AC005682.5	0.4499949	3.6139889	4.3942089	1.45E-05	5.90E-05	2.4186421
CCDC186	0.116941	5.5498966	4.3941992	1.45E-05	5.90E-05	2.4186015
NRGN	0.220147	4.9672587	4.3928088	1.46E-05	5.93E-05	2.4128378
HDAC2	0.0695185	6.3014331	4.3925998	1.46E-05	5.94E-05	2.4119713
VAMP3	-0.073611	6.3928406	-4.392486	1.46E-05	5.94E-05	2.4114988
COL28A1	1.0787059	1.5152831	4.3919493	1.47E-05	5.95E-05	2.4092755
TSTD2	0.4783748	5.0333145	4.3917894	1.47E-05	5.96E-05	2.4086129
FERMT1	0.8787374	4.256804	4.3907445	1.48E-05	5.98E-05	2.4042832
TYMSOS	-0.295525	4.7890566	-4.390366	1.48E-05	5.99E-05	2.4027135
COG8	-0.07161	5.9897619	-4.389954	1.48E-05	6.00E-05	2.4010064
RP11-1398P	0.4815844	3.1900435	4.3898791	1.48E-05	6.00E-05	2.4006978
GULP1	0.8704276	3.5365976	4.3891619	1.49E-05	6.02E-05	2.3977269
HID1-AS1	0.767421	1.0506116	4.3891352	1.49E-05	6.02E-05	2.3976163
LAMA5	0.124382	6.4082222	4.389046	1.49E-05	6.02E-05	2.3972469
PSME4	-0.055491	6.5440353	-4.388909	1.49E-05	6.03E-05	2.3966812
RP3-508I15	0.6033998	2.572247	4.388879	1.49E-05	6.03E-05	2.3965554
FOXRED2	-0.115297	6.2449442	-4.388728	1.49E-05	6.03E-05	2.3959284
CTD-2523D1	0.8271646	0.8825639	4.3882665	1.49E-05	6.04E-05	2.3940188
MTUS2	1.0873118	1.4244933	4.3879361	1.49E-05	6.05E-05	2.3926505
PCDHA7	0.8968168	0.4899248	4.3877269	1.50E-05	6.05E-05	2.3917843
CDC5L	-0.069248	6.4048823	-4.387709	1.50E-05	6.05E-05	2.3917088
POFUT2	0.0757742	5.9986458	4.3876553	1.50E-05	6.05E-05	2.3914879
SCOC-AS1	-0.336074	3.7954899	-4.387629	1.50E-05	6.05E-05	2.391381
RP11-61K9.	-0.191744	4.7824664	-4.387401	1.50E-05	6.06E-05	2.3904343
SCAMP3	-0.080844	6.6741176	-4.387299	1.50E-05	6.06E-05	2.3900128
LINC00158	0.6884617	-0.631192	4.3866909	1.50E-05	6.08E-05	2.3874952
AP2B1	-0.059871	6.6529298	-4.385786	1.51E-05	6.10E-05	2.3837497

NPTN-IT1	0.9123007	1.5272157	4.3857272	1.51E-05	6.10E-05	2.383506
BMP6	0.519766	3.9611967	4.3851048	1.51E-05	6.12E-05	2.3809298
C2CD4C	0.6439143	3.0553542	4.3848986	1.51E-05	6.12E-05	2.3800766
DDX39B-AS1	-0.730369	1.2143969	-4.384746	1.52E-05	6.12E-05	2.3794472
C1orf100	0.8003865	0.5754874	4.3844162	1.52E-05	6.13E-05	2.3780803
AC093159.1	-0.820785	0.3057096	-4.383463	1.52E-05	6.16E-05	2.3741359
SLC27A4	-0.098477	6.3648835	-4.38323	1.53E-05	6.16E-05	2.3731726
RP11-5407.	0.812643	-0.560401	4.382896	1.53E-05	6.17E-05	2.3717909
DMTN	0.1844578	6.0362716	4.3825948	1.53E-05	6.18E-05	2.3705452
UBE4A	-0.082419	6.1288461	-4.382487	1.53E-05	6.18E-05	2.3700989
HOXC6	1.0852335	0.9566277	4.3824781	1.53E-05	6.18E-05	2.3700623
SPARC	0.0950161	7.0585253	4.3823373	1.53E-05	6.18E-05	2.3694801
CC2D1A	-0.085089	6.3364043	-4.381499	1.54E-05	6.21E-05	2.3660117
XX-C00717C	0.6647811	-0.332335	4.3810845	1.54E-05	6.22E-05	2.3642991
PWAR1	0.7064072	-0.436539	4.3810131	1.54E-05	6.22E-05	2.3640039
RP11-676J1	0.6621065	1.2928923	4.3809278	1.54E-05	6.22E-05	2.3636511
POFUT1	-0.072414	6.6260423	-4.380605	1.54E-05	6.23E-05	2.3623158
PARN	-0.059774	6.2408338	-4.380334	1.54E-05	6.23E-05	2.3611972
RP11-493L1	0.712911	-0.239945	4.3800351	1.55E-05	6.24E-05	2.35996
ZNF614	0.2492616	5.0356262	4.3798789	1.55E-05	6.25E-05	2.3593143
NDUFS1	-0.066464	6.6872829	-4.37948	1.55E-05	6.26E-05	2.357667
CD36	-0.224913	6.2873492	-4.37914	1.55E-05	6.26E-05	2.3562596
MAP4K3	0.0955908	6.0564676	4.3790226	1.55E-05	6.27E-05	2.355775
GAS2	-0.24549	5.7682474	-4.378456	1.56E-05	6.28E-05	2.353433
MYO1E	0.1341929	6.2968199	4.3784473	1.56E-05	6.28E-05	2.3533972
SSR3	-0.061048	6.8202884	-4.378181	1.56E-05	6.29E-05	2.3522961
RP11-478K1	-0.301091	-1.402415	-4.378175	1.56E-05	6.29E-05	2.35227
MPEG1	0.1581227	5.9954552	4.3778092	1.56E-05	6.30E-05	2.3507601
PIK3CD-AS1	0.6765268	-0.346201	4.3777609	1.56E-05	6.30E-05	2.3505605
DUSP7	0.0850182	5.7951433	4.3770264	1.57E-05	6.32E-05	2.3475257
RP11-923I1	0.7787553	0.0677542	4.3765036	1.57E-05	6.33E-05	2.3453657
SLC5A2	-0.60318	2.9157716	-4.376493	1.57E-05	6.33E-05	2.3453219
TRHDE	1.2119189	2.3262273	4.376341	1.57E-05	6.33E-05	2.344694
ZNF788	0.3893044	4.2507327	4.3760567	1.57E-05	6.34E-05	2.3435196
CTC-492K19	0.7153237	0.6890183	4.3756974	1.58E-05	6.35E-05	2.3420356
OAZ2	-0.062679	6.5383908	-4.375568	1.58E-05	6.35E-05	2.3415006
RBP2	1.0450345	1.0710759	4.3751377	1.58E-05	6.36E-05	2.3397241
SLC7A5P1	0.6047916	-0.744214	4.3746681	1.58E-05	6.38E-05	2.3377848
RP11-588H2	0.7666667	2.6319647	4.3743502	1.59E-05	6.38E-05	2.3364718
TCERG1	0.0729872	6.110128	4.3742999	1.59E-05	6.39E-05	2.3362642
MAMDC2	0.7279357	2.9428588	4.3738781	1.59E-05	6.40E-05	2.3345226
SPAG4	-0.250036	5.2566689	-4.373103	1.59E-05	6.42E-05	2.3313229
FBXL14	0.2053533	5.3071772	4.3730859	1.59E-05	6.42E-05	2.3312516
GNMT	-0.404127	6.0243149	-4.373059	1.59E-05	6.42E-05	2.3311389
RP11-390K5	0.5302813	3.264242	4.3727737	1.60E-05	6.42E-05	2.3299632
KIAA0195	-0.058438	6.4770782	-4.372736	1.60E-05	6.42E-05	2.3298084
RP11-545A1	0.6298641	-0.777654	4.3724315	1.60E-05	6.43E-05	2.3285505
IL32	-0.162754	6.9879459	-4.372161	1.60E-05	6.44E-05	2.327435
ZNF202	0.0805303	5.4638554	4.3719093	1.60E-05	6.45E-05	2.3263951
KCNJ1	0.6397206	-0.515199	4.3715524	1.60E-05	6.45E-05	2.3249221
PINX1	-0.117256	5.434296	-4.371246	1.61E-05	6.46E-05	2.3236571

SERPINB8	0.2342194	5.5241961	4.3711648	1.61E-05	6.46E-05	2.3233227
DGKZ	0.057618	6.2582128	4.3706056	1.61E-05	6.48E-05	2.321015
PAK3	1.1261333	2.0702604	4.3704813	1.61E-05	6.48E-05	2.3205022
RP11-700H6	-0.743004	3.0246108	-4.370341	1.61E-05	6.49E-05	2.3199232
TAS2R20	0.7191164	1.8104714	4.3700246	1.62E-05	6.49E-05	2.3186182
REPIN1	-0.06726	6.7525687	-4.369786	1.62E-05	6.50E-05	2.3176355
FOSL1	0.3420348	4.1851326	4.3697664	1.62E-05	6.50E-05	2.3175528
RFX4	0.7274957	-0.597748	4.3693458	1.62E-05	6.51E-05	2.3158176
R3HDM2	-0.063815	6.2856808	-4.368938	1.62E-05	6.52E-05	2.3141365
PIGS	0.0859061	6.0122632	4.368728	1.62E-05	6.53E-05	2.3132694
DEPDC5	-0.106215	5.9378771	-4.3684	1.63E-05	6.54E-05	2.3119149
RPS18P13	-0.720035	-0.14732	-4.36798	1.63E-05	6.55E-05	2.3101843
VIP	0.651514	2.5633148	4.3678555	1.63E-05	6.55E-05	2.3096712
ANKRD33B	0.5385587	4.0983938	4.3672398	1.64E-05	6.57E-05	2.3071323
VDAC1P2	0.7118569	1.1499572	4.3671994	1.64E-05	6.57E-05	2.3069657
DLG5-AS1	-0.440044	3.6030696	-4.367052	1.64E-05	6.57E-05	2.3063596
PNPLA7	-0.191667	5.6080264	-4.366698	1.64E-05	6.58E-05	2.3048976
ACTR3B	-0.106485	5.5099885	-4.366563	1.64E-05	6.58E-05	2.3043419
TUBB1	0.6862442	1.9496274	4.36652	1.64E-05	6.58E-05	2.3041646
IGKV1D-27	0.6213165	-1.007788	4.366333	1.64E-05	6.59E-05	2.3033939
PHF1	-0.075071	6.3331757	-4.365585	1.65E-05	6.61E-05	2.3003112
DIRAS2	1.1581128	2.0373992	4.3649128	1.65E-05	6.63E-05	2.2975399
IL12A-AS1	0.690215	-0.517874	4.3648829	1.65E-05	6.63E-05	2.2974167
WNK4	0.5901156	4.5570985	4.3636984	1.66E-05	6.66E-05	2.2925358
CDKN2A	-0.303036	5.6340771	-4.362848	1.67E-05	6.68E-05	2.2890325
RP11-161D1	-0.544384	-1.148211	-4.362827	1.67E-05	6.68E-05	2.2889435
ERBB3	-0.152114	6.8781704	-4.362592	1.67E-05	6.69E-05	2.2879785
EIF2A	-0.059386	6.5169023	-4.362187	1.67E-05	6.70E-05	2.2863101
ATF6	-0.080452	6.5786983	-4.362128	1.67E-05	6.70E-05	2.2860657
GRAMD2	0.8056825	0.8149195	4.362078	1.67E-05	6.70E-05	2.2858604
MRFAP1L1	-0.05672	6.4210547	-4.361774	1.67E-05	6.71E-05	2.2846076
MAK	0.5195881	2.7162693	4.3616298	1.68E-05	6.72E-05	2.2840142
RP11-304L1	-0.302106	3.7541012	-4.361316	1.68E-05	6.72E-05	2.2827227
CES3	-0.392545	5.9548716	-4.361285	1.68E-05	6.72E-05	2.2825962
SYNPO2L	0.7353446	0.051872	4.3609989	1.68E-05	6.73E-05	2.2814163
RP11-483H2	0.6464249	-0.503114	4.3609242	1.68E-05	6.73E-05	2.2811085
RP11-15A1.	0.7479452	1.8200474	4.3607392	1.68E-05	6.74E-05	2.2803468
GTF3C2-AS1	0.7249037	1.5024461	4.3605118	1.68E-05	6.74E-05	2.2794103
TSACC	-0.293802	4.3417757	-4.35897	1.70E-05	6.79E-05	2.2730631
ZNF584	-0.093355	5.5058365	-4.358901	1.70E-05	6.79E-05	2.272781
SRSF9P1	0.6862742	1.8488726	4.3588646	1.70E-05	6.79E-05	2.2726292
RP11-290H9	-0.514766	2.7681047	-4.358453	1.70E-05	6.80E-05	2.2709356
CDC7	0.2039665	5.1337699	4.3584341	1.70E-05	6.80E-05	2.2708572
CFH	-0.150869	7.3442167	-4.357917	1.70E-05	6.82E-05	2.2687294
GK-AS1	0.8084367	2.31852	4.3576991	1.70E-05	6.82E-05	2.2678324
THG1L	-0.094037	5.7132846	-4.357535	1.71E-05	6.82E-05	2.2671571
PSORS1C2	0.5747346	-0.87597	4.3575347	1.71E-05	6.82E-05	2.2671558
CCT8P1	-0.208272	5.820261	-4.357156	1.71E-05	6.84E-05	2.2655977
NBL1	0.2668133	5.1595069	4.3571292	1.71E-05	6.84E-05	2.2654873
RP11-417E7	0.8691384	0.7737088	4.3570007	1.71E-05	6.84E-05	2.2649584
SCTR	0.6915514	3.9045895	4.3568166	1.71E-05	6.84E-05	2.2642013

AQP10	1.1310403	0.6183458	4.3564615	1.71E-05	6.85E-05	2.2627402
DDX11L1	-0.253868	-1.458986	-4.356345	1.71E-05	6.86E-05	2.2622614
ZNF277	-0.086191	6.0811838	-4.356082	1.72E-05	6.86E-05	2.2611787
C3orf67-AS	-0.837992	0.166711	-4.355742	1.72E-05	6.87E-05	2.2597814
TTC39A	0.3728181	5.3053722	4.3550934	1.72E-05	6.89E-05	2.2571126
LRRC25	0.1996828	5.243358	4.3549021	1.73E-05	6.90E-05	2.2563255
CTD-2154B1	-0.7083	-0.161622	-4.354851	1.73E-05	6.90E-05	2.2561146
BLMH	0.1939256	5.9779136	4.3543891	1.73E-05	6.91E-05	2.2542158
PCDHB3	0.8134022	2.6767006	4.3541745	1.73E-05	6.92E-05	2.2533333
CEP72	0.1636822	5.3499953	4.354066	1.73E-05	6.92E-05	2.2528872
MAP3K1	0.0868544	5.9681117	4.353598	1.74E-05	6.93E-05	2.2509629
CH17-125A1	-0.396993	-1.36149	-4.35354	1.74E-05	6.93E-05	2.2507261
MCAM	0.1054013	6.379939	4.3533802	1.74E-05	6.94E-05	2.2500675
MAPKBP1	0.0880145	5.6340089	4.3533238	1.74E-05	6.94E-05	2.2498355
ZBTB4	0.0807907	6.1218613	4.3531254	1.74E-05	6.94E-05	2.24902
ZSCAN1	0.8039076	0.0250724	4.3530597	1.74E-05	6.94E-05	2.2487496
HOXA5	0.5181602	3.7660317	4.3529459	1.74E-05	6.95E-05	2.248282
NBPF3	0.4798336	4.3181769	4.3520827	1.75E-05	6.97E-05	2.2447334
HILPDA	0.1671762	5.3038946	4.3520069	1.75E-05	6.97E-05	2.244422
AC005077.5	-0.384849	-1.248783	-4.351813	1.75E-05	6.98E-05	2.2436257
RP11-876N2	0.2403342	4.5451135	4.351801	1.75E-05	6.98E-05	2.2435755
MT-RNR1	-0.114149	7.3981357	-4.351669	1.75E-05	6.98E-05	2.2430328
RP11-65L3.	0.7689012	1.7848624	4.3516034	1.75E-05	6.98E-05	2.2427634
RP11-296E7	0.7581074	1.7491097	4.3513719	1.75E-05	6.99E-05	2.2418123
GDF9	-0.339958	3.7323547	-4.350641	1.76E-05	7.01E-05	2.2388078
FAM73B	-0.081371	6.0115394	-4.350415	1.76E-05	7.02E-05	2.2378789
AC026700.1	-0.176105	-1.486524	-4.350251	1.76E-05	7.02E-05	2.2372063
RAB38	0.6098285	3.3220822	4.3500861	1.76E-05	7.02E-05	2.2365284
ZMYM5	0.1081598	5.5057952	4.3493895	1.77E-05	7.04E-05	2.2336668
RP11-353N1	0.9091896	2.0347349	4.3493249	1.77E-05	7.05E-05	2.2334014
C9orf106	0.9400582	1.5721889	4.3490413	1.77E-05	7.05E-05	2.2322363
BARD1	0.2129696	5.0798751	4.3485402	1.77E-05	7.07E-05	2.230178
NR0B1	1.111241	0.1126716	4.3482749	1.78E-05	7.08E-05	2.2290882
PRKCI	0.0764181	5.9162788	4.348209	1.78E-05	7.08E-05	2.2288175
ASGR2	-0.194194	7.1088587	-4.348193	1.78E-05	7.08E-05	2.2287529
CTD-2655K5	-0.738025	2.1253931	-4.347862	1.78E-05	7.09E-05	2.2273942
LONRF1	0.145761	5.5268104	4.3478115	1.78E-05	7.09E-05	2.2271854
KMT2E	0.0873519	6.1761269	4.3477022	1.78E-05	7.09E-05	2.2267362
TMEM169	-0.265897	4.9075515	-4.347557	1.78E-05	7.09E-05	2.2261417
SPPL2B	-0.074007	6.2763553	-4.347403	1.78E-05	7.10E-05	2.2255068
FXR1	-0.053534	6.599259	-4.345391	1.80E-05	7.16E-05	2.2172489
HOXB7	0.4344731	4.0173627	4.345373	1.80E-05	7.16E-05	2.2171737
RP11-843B1	0.7235143	-0.179216	4.344698	1.80E-05	7.18E-05	2.214403
RP11-181G1	0.4018502	3.7243836	4.3445876	1.80E-05	7.18E-05	2.21395
TMEM141	-0.111594	6.459552	-4.344272	1.81E-05	7.19E-05	2.2126532
COPS7A	-0.063352	6.4995834	-4.344131	1.81E-05	7.19E-05	2.2120765
ACVR1	0.0892354	6.0710542	4.343839	1.81E-05	7.20E-05	2.2108781
MIB1	0.0885416	6.2293366	4.343621	1.81E-05	7.21E-05	2.2099837
AC016738.4	0.8207447	1.9932386	4.3429068	1.82E-05	7.23E-05	2.2070531
AC004471.1	-0.712596	0.924176	-4.342162	1.82E-05	7.25E-05	2.2039985
MALRD1	0.907457	0.6529304	4.342139	1.82E-05	7.25E-05	2.2039036

RP5-881L22	-0.891573	1.3368249	-4.341597	1.83E-05	7.27E-05	2.2016801
RP11-307E1	0.7741988	0.8301251	4.341271	1.83E-05	7.28E-05	2.2003435
HMGCR	-0.115402	6.4844299	-4.340647	1.84E-05	7.30E-05	2.1977853
RP11-327P2	-0.200334	4.135062	-4.340532	1.84E-05	7.30E-05	2.1973126
RP11-669E1	0.7627768	0.570784	4.3403419	1.84E-05	7.30E-05	2.1965333
FP325331.1	0.6890343	-0.303733	4.3398445	1.84E-05	7.32E-05	2.1944939
FAM154A	-0.770543	0.9189637	-4.339264	1.85E-05	7.34E-05	2.1921137
FAM170A	-0.76358	-0.157038	-4.3383	1.85E-05	7.37E-05	2.1881622
RP11-159G9	0.5189981	3.7140248	4.3377594	1.86E-05	7.38E-05	2.1859472
KSR2	0.9477899	1.6133348	4.3375043	1.86E-05	7.39E-05	2.1849016
UCKL1	-0.070012	6.2582904	-4.337465	1.86E-05	7.39E-05	2.1847411
UGT2B29P	-0.552683	-0.963744	-4.337332	1.86E-05	7.40E-05	2.1841951
TMEM161B-A	-0.159014	5.2958284	-4.337302	1.86E-05	7.40E-05	2.1840734
LACTB	-0.083494	6.2295673	-4.337212	1.86E-05	7.40E-05	2.1837036
AC009236.2	0.5609142	-0.90168	4.3367283	1.87E-05	7.41E-05	2.181722
GJA8	-0.31537	-1.384228	-4.336669	1.87E-05	7.41E-05	2.1814808
RP11-274B2	0.3072183	4.7753832	4.3365941	1.87E-05	7.41E-05	2.181172
RAB1B	-0.053329	6.7085638	-4.335991	1.87E-05	7.43E-05	2.1787012
CA4	-0.970119	2.7216384	-4.335495	1.88E-05	7.45E-05	2.1766715
CFAP70	-0.331737	4.4932152	-4.335164	1.88E-05	7.46E-05	2.1753122
CLCF1	0.2695411	5.0304133	4.3350971	1.88E-05	7.46E-05	2.1750397
CEP70	-0.102771	5.9408696	-4.334446	1.89E-05	7.48E-05	2.1723714
CREG1	-0.113371	6.9160522	-4.334356	1.89E-05	7.48E-05	2.172006
DDX51	-0.070949	6.0177167	-4.334004	1.89E-05	7.49E-05	2.1705635
CCDC62	0.7029421	0.8167396	4.3339215	1.89E-05	7.49E-05	2.1702252
RP11-848P1	-0.580914	-0.863768	-4.333794	1.89E-05	7.50E-05	2.1697028
EPS15	-0.057569	6.3471007	-4.333343	1.89E-05	7.51E-05	2.1678575
RP11-110I1	0.724078	2.1240843	4.3332592	1.90E-05	7.51E-05	2.1675132
GGNBP1	0.6195793	-0.579737	4.3328007	1.90E-05	7.53E-05	2.1656363
TXNL4A	-0.084701	6.3904573	-4.332688	1.90E-05	7.53E-05	2.1651736
HELQ	-0.074414	5.5372086	-4.33227	1.90E-05	7.54E-05	2.163463
PEX11A	-0.1241	6.0130741	-4.332225	1.90E-05	7.54E-05	2.1632792
FOXN1	0.7256766	-0.657308	4.3320577	1.90E-05	7.55E-05	2.1625945
CARM1	0.1136466	6.1475268	4.3317555	1.91E-05	7.56E-05	2.1613575
DNAJC14	0.1551643	5.7999622	4.3315882	1.91E-05	7.56E-05	2.1606729
ALKBH5	-0.071639	6.5599436	-4.331114	1.91E-05	7.58E-05	2.158733
AC005042.4	0.7448992	1.3972049	4.3309148	1.91E-05	7.58E-05	2.157917
PER1	-0.126307	6.4317061	-4.330533	1.92E-05	7.59E-05	2.1563532
HIST1H2AC	-0.160719	6.47845	-4.330447	1.92E-05	7.60E-05	2.1560018
TNFAIP3	0.1407381	6.1970902	4.3303021	1.92E-05	7.60E-05	2.1554096
PFN3	-0.531846	-0.966394	-4.329723	1.92E-05	7.62E-05	2.1530406
RNASEH1-AS	-0.142524	5.1064253	-4.329677	1.92E-05	7.62E-05	2.1528527
IL5	-0.633138	-0.228198	-4.329606	1.93E-05	7.62E-05	2.1525598
CTC-340D7.	-0.601852	-0.713677	-4.329225	1.93E-05	7.63E-05	2.1510025
RALGAPA1P	0.5793853	3.654704	4.3290541	1.93E-05	7.64E-05	2.1503041
RP11-55L3.	-0.672443	-1.059444	-4.32785	1.94E-05	7.68E-05	2.14538
RP11-699A5	-0.832952	1.2539827	-4.327671	1.94E-05	7.68E-05	2.1446454
RP11-667F1	0.6797651	0.9132108	4.3275599	1.94E-05	7.68E-05	2.1441924
FBXO42	-0.063278	5.9736196	-4.327219	1.95E-05	7.69E-05	2.1427992
PRAMEF19	-0.276789	-1.431341	-4.326323	1.95E-05	7.72E-05	2.1391348
SFI1	0.1260798	5.8420898	4.3250944	1.96E-05	7.76E-05	2.1341124

RP11-89B16	0.5626323	-0.819261	4.3248507	1.97E-05	7.77E-05	2.1331164
SETD6	-0.070093	5.6977054	-4.324849	1.97E-05	7.77E-05	2.1331096
DYDC2	0.9722553	3.4143235	4.3248483	1.97E-05	7.77E-05	2.1331067
KLHL11	0.6806639	3.3744905	4.3247453	1.97E-05	7.77E-05	2.1326856
HIGD1AP10	-0.288919	-1.3966	-4.324673	1.97E-05	7.77E-05	2.1323897
FIG4	0.0822121	5.6958894	4.324406	1.97E-05	7.78E-05	2.1312988
FBXL8	-0.156996	5.1738159	-4.324329	1.97E-05	7.78E-05	2.1309836
CATSPER1	0.6380128	2.1902417	4.3241679	1.97E-05	7.79E-05	2.1303261
FAM182B	0.7930215	2.0967121	4.3239721	1.97E-05	7.79E-05	2.1295257
KLC2	0.1054545	5.8609546	4.3235614	1.98E-05	7.81E-05	2.1278478
ZNF208	0.875253	2.0428324	4.3235197	1.98E-05	7.81E-05	2.1276772
RP11-685B1	-0.877582	0.1742605	-4.323129	1.98E-05	7.82E-05	2.1260822
METTL2B	-0.073075	6.0294633	-4.322992	1.98E-05	7.82E-05	2.1255207
ALDOAP1	-0.60109	-0.734086	-4.322829	1.98E-05	7.83E-05	2.1248545
RP11-631N1	0.2345856	4.3771231	4.322807	1.98E-05	7.83E-05	2.1247653
IFI44L	0.297072	5.2827807	4.3224907	1.99E-05	7.84E-05	2.123473
TECTA	0.3878766	3.8595843	4.3220195	1.99E-05	7.85E-05	2.1215485
SENP1	0.076598	5.6779629	4.3219988	1.99E-05	7.85E-05	2.1214639
IGF2	0.4269284	6.52845	4.3218564	1.99E-05	7.86E-05	2.1208822
QRICH1	-0.045405	6.3452035	-4.321783	1.99E-05	7.86E-05	2.1205832
MKNK2	-0.071229	6.6971322	-4.321559	1.99E-05	7.87E-05	2.119669
TYROBP	0.1494912	6.1274513	4.3214556	1.99E-05	7.87E-05	2.1192452
CTD-3187F8	0.7350254	0.026815	4.3210463	2.00E-05	7.88E-05	2.1175736
AC008592.3	-0.386188	3.674021	-4.320533	2.00E-05	7.90E-05	2.1154778
RP11-45506	0.7157108	1.0959956	4.320413	2.00E-05	7.90E-05	2.1149873
AFF4	-0.082754	6.5794501	-4.31969	2.01E-05	7.92E-05	2.1120347
JARID2-AS1	-0.698282	0.965157	-4.31959	2.01E-05	7.93E-05	2.1116275
PVRL1	0.1450438	5.8991867	4.3190007	2.02E-05	7.95E-05	2.1092214
C16orf72	-0.062959	6.286139	-4.318675	2.02E-05	7.96E-05	2.1078929
RPS11	-0.087199	7.2907407	-4.318557	2.02E-05	7.96E-05	2.1074091
HOXB9	0.9578847	0.3200879	4.3178149	2.03E-05	7.98E-05	2.1043812
RPS23	-0.082716	7.1012011	-4.317507	2.03E-05	7.99E-05	2.1031239
FAM86JP	-0.187546	4.7897514	-4.317264	2.03E-05	8.00E-05	2.1021314
ZNF283	0.227659	4.7479391	4.3172316	2.03E-05	8.00E-05	2.1020009
NIPSNAP3A	-0.110237	6.005359	-4.316889	2.03E-05	8.01E-05	2.100603
NIPA2	0.0899709	5.9936054	4.3157874	2.04E-05	8.05E-05	2.0961087
RBMS2P1	0.7295343	0.1976654	4.3157664	2.04E-05	8.05E-05	2.0960231
NCOA3	0.0808755	6.2298277	4.3154244	2.05E-05	8.06E-05	2.0946278
AC145343.2	-0.52989	3.2932644	-4.315256	2.05E-05	8.07E-05	2.0939411
CEP162	0.1184251	5.2515985	4.3152116	2.05E-05	8.07E-05	2.0937597
ELP2	-0.067818	6.4717586	-4.315094	2.05E-05	8.07E-05	2.0932789
AC118754.4	-0.887283	1.1657891	-4.314972	2.05E-05	8.07E-05	2.0927835
RAB11FIP1F	0.8234012	2.6843891	4.3143835	2.06E-05	8.09E-05	2.0903824
RP11-612B6	-0.360969	4.4662133	-4.314093	2.06E-05	8.10E-05	2.0891973
SND1	-0.059053	6.9600019	-4.314072	2.06E-05	8.10E-05	2.0891132
NKX2-8	0.611504	-1.015028	4.3140285	2.06E-05	8.10E-05	2.0889347
AC080008.1	-0.835402	1.1554369	-4.313973	2.06E-05	8.10E-05	2.0887091
AC137932.4	0.4433868	3.5359884	4.3137646	2.06E-05	8.11E-05	2.0878586
MYO16	-0.469541	4.5047246	-4.313743	2.06E-05	8.11E-05	2.0877711
CH17-140K2	0.5555109	-0.943959	4.3136074	2.06E-05	8.11E-05	2.0872176
TAS2R14	0.3642359	3.5381283	4.3135057	2.06E-05	8.12E-05	2.0868028

NANOGP6	-0.660931	-0.521396	-4.313468	2.06E-05	8.12E-05	2.0866501
FOS	0.2172441	6.219484	4.3133484	2.07E-05	8.12E-05	2.0861614
SLED1	-0.801906	1.9616962	-4.313146	2.07E-05	8.13E-05	2.0853355
RP11-274B2	0.4239996	3.5253658	4.3128907	2.07E-05	8.13E-05	2.0842952
ONECUT2	-0.216586	6.4177552	-4.312784	2.07E-05	8.14E-05	2.0838583
GHITM	-0.072941	6.8962499	-4.312265	2.07E-05	8.15E-05	2.0817466
UTP20	0.0801638	5.9150201	4.312166	2.08E-05	8.16E-05	2.0813412
RP11-261P9	0.6863566	2.2890724	4.3114863	2.08E-05	8.18E-05	2.0785706
TCEB3-AS1	-0.242082	4.3707152	-4.3113	2.08E-05	8.19E-05	2.0778114
IGHV7-56	0.5366109	-1.0427	4.3109026	2.09E-05	8.20E-05	2.0761918
MAP1LC3A	-0.183898	6.02125	-4.310609	2.09E-05	8.21E-05	2.0749945
EPPK1	0.634739	4.8724952	4.3102325	2.09E-05	8.22E-05	2.073461
AP000320.6	-0.75199	0.5815149	-4.310085	2.09E-05	8.23E-05	2.0728607
G2E3	0.1006976	5.5233834	4.3098485	2.10E-05	8.23E-05	2.0718963
NOC4L	-0.082439	6.0710344	-4.309406	2.10E-05	8.25E-05	2.0700956
RP11-48501	-1.063323	0.7676952	-4.309344	2.10E-05	8.25E-05	2.0698407
RP11-605F2	-0.417469	-1.277206	-4.30851	2.11E-05	8.28E-05	2.0664455
ARHGAP35	-0.068877	6.6768072	-4.30818	2.11E-05	8.29E-05	2.0650978
CTD-2369P2	0.7532088	2.5041989	4.3080203	2.11E-05	8.29E-05	2.0644491
AC016682.1	-0.814021	-0.18999	-4.307869	2.11E-05	8.30E-05	2.0638331
UBE2O	-0.062139	6.1842734	-4.307392	2.12E-05	8.31E-05	2.0618915
DBNDD1	-0.20646	6.0714041	-4.307154	2.12E-05	8.32E-05	2.0609201
IGLV1-50	0.7518932	-0.318131	4.3071406	2.12E-05	8.32E-05	2.0608669
RP11-1134I	0.6253297	-0.743703	4.3071228	2.12E-05	8.32E-05	2.0607943
DNAJB2	-0.080236	6.6090847	-4.306976	2.12E-05	8.33E-05	2.0601975
FAM58A	-0.100073	5.9020422	-4.306971	2.12E-05	8.33E-05	2.0601757
RP11-145B3	-0.250942	-1.415619	-4.306905	2.12E-05	8.33E-05	2.0599079
RAD51C	-0.089915	5.7508499	-4.306597	2.13E-05	8.34E-05	2.0586531
GGNBP2	-0.047254	6.4313506	-4.306517	2.13E-05	8.34E-05	2.0583291
C15orf52	0.2613533	5.4013702	4.3059253	2.13E-05	8.36E-05	2.0559188
SH3RF3	0.3173995	5.0896434	4.3056705	2.13E-05	8.37E-05	2.0548815
SYNE1	0.1279283	6.0101484	4.3054508	2.14E-05	8.37E-05	2.0539872
C15orf43	-1.070878	1.03108	-4.305346	2.14E-05	8.38E-05	2.0535587
RIMS3	0.3030649	4.5315136	4.3049959	2.14E-05	8.39E-05	2.0521353
MGARP	0.706628	1.0118272	4.3047039	2.14E-05	8.40E-05	2.050947
DHCR7	-0.134385	6.8392608	-4.304559	2.14E-05	8.40E-05	2.0503573
TAT-AS1	-0.631756	3.252153	-4.304286	2.15E-05	8.41E-05	2.0492477
RGS14	-0.113331	6.416678	-4.303141	2.16E-05	8.45E-05	2.0445873
ST3GAL6-AS	-0.510903	3.2964379	-4.302461	2.16E-05	8.48E-05	2.0418199
SOCS7	0.1230793	5.6372835	4.3024439	2.16E-05	8.48E-05	2.041751
RP11-815J2	0.5677972	-0.795216	4.3019136	2.17E-05	8.49E-05	2.039594
TRIM8	-0.071805	6.6449814	-4.301409	2.17E-05	8.51E-05	2.0375423
RP11-982M1	0.7371097	-0.335735	4.3008628	2.18E-05	8.53E-05	2.0353203
KLHL42	0.0893434	5.8625389	4.2999021	2.19E-05	8.57E-05	2.031414
ALG6	-0.071126	5.8610246	-4.299059	2.20E-05	8.60E-05	2.0279859
ZNF418	0.4525363	4.3069819	4.2987162	2.20E-05	8.61E-05	2.0265926
PQLC2L	0.9440595	0.0095407	4.298637	2.20E-05	8.61E-05	2.026271
RP11-582E3	-0.112234	5.3271108	-4.298552	2.20E-05	8.61E-05	2.0259273
AC000068.1	-0.568202	2.2532418	-4.298383	2.20E-05	8.62E-05	2.0252377
SLC7A6OS	0.1953893	4.9005779	4.2979441	2.21E-05	8.63E-05	2.0234547
ARL10	0.2086585	4.8843387	4.2974834	2.21E-05	8.65E-05	2.0215823

RFXANK	-0.095857	6.1915041	-4.296171	2.22E-05	8.70E-05	2.0162503
CTD-3116E2	0.6781882	-0.330973	4.2961094	2.22E-05	8.70E-05	2.0159994
MTND4P9	0.4038883	-1.216789	4.295716	2.23E-05	8.71E-05	2.0144015
LINC00299	0.7486042	2.7065488	4.2950232	2.23E-05	8.74E-05	2.0115873
RP11-358D1	0.6353892	-0.477328	4.2949273	2.24E-05	8.74E-05	2.0111978
FAM160B1	-0.078615	6.1679441	-4.294715	2.24E-05	8.75E-05	2.010337
MROH2A	-0.973217	3.5317167	-4.294662	2.24E-05	8.75E-05	2.0101191
CASKIN1	0.9377233	1.4418093	4.2945736	2.24E-05	8.75E-05	2.009761
TUBGCP2	-0.064853	6.4524973	-4.294184	2.24E-05	8.76E-05	2.0081805
RP11-15B24	-0.517179	-1.139636	-4.293798	2.25E-05	8.78E-05	2.006612
RP11-11N5.	-0.719931	-0.709974	-4.293621	2.25E-05	8.78E-05	2.0058944
RP11-64B16	0.7283837	2.1910784	4.2934792	2.25E-05	8.79E-05	2.0053171
CDC20P1	0.813657	0.6782116	4.2931911	2.25E-05	8.80E-05	2.0041471
EFNB2	0.142281	5.8085452	4.292992	2.25E-05	8.80E-05	2.0033388
MIR4664	0.8631401	2.5655439	4.2928636	2.26E-05	8.81E-05	2.0028175
MAP2K2	-0.07716	6.717486	-4.29248	2.26E-05	8.82E-05	2.0012617
WI2-2118C2	-0.880132	-0.122348	-4.292262	2.26E-05	8.83E-05	2.000376
CTD-2649C1	-0.768859	0.5828298	-4.291936	2.26E-05	8.84E-05	1.9990508
RP11-837J7	0.6973149	0.8496177	4.2919115	2.26E-05	8.84E-05	1.9989527
RP11-238I1	-0.302185	-1.370647	-4.291899	2.26E-05	8.84E-05	1.9989
AP002954.6	-0.908147	0.1912358	-4.291383	2.27E-05	8.86E-05	1.9968094
RP11-178H8	0.3204768	3.9412991	4.2912951	2.27E-05	8.86E-05	1.9964508
RPL10A	-0.08188	7.0815276	-4.290991	2.27E-05	8.87E-05	1.9952183
SLC25A23	-0.09289	6.4221956	-4.29046	2.28E-05	8.89E-05	1.9930601
AL133247.3	-0.465423	-1.124247	-4.289763	2.29E-05	8.92E-05	1.9902346
CTD-2349P2	0.696632	1.1487147	4.2897062	2.29E-05	8.92E-05	1.9900035
TMEM64	-0.157456	6.2107478	-4.289305	2.29E-05	8.93E-05	1.988377
ANGPTL4	-0.231854	6.5399402	-4.289284	2.29E-05	8.93E-05	1.9882888
RP11-352D1	0.945305	0.5405616	4.2890103	2.29E-05	8.94E-05	1.9871804
LINC00476	-0.154937	4.8492207	-4.28775	2.31E-05	8.99E-05	1.9820699
RABL3	-0.064314	6.0140774	-4.28703	2.31E-05	9.02E-05	1.9791475
BTNL9	-0.280673	5.3696995	-4.286871	2.31E-05	9.02E-05	1.9785055
MORF4L1P1	0.3258396	5.0141515	4.2866575	2.32E-05	9.03E-05	1.9776387
C11orf24	-0.088106	6.4285339	-4.286444	2.32E-05	9.03E-05	1.9767723
GDF2	1.0890604	0.0057088	4.2863396	2.32E-05	9.04E-05	1.9763498
FAM83H-AS1	0.3852025	4.1273063	4.2857233	2.33E-05	9.06E-05	1.9738511
CCND2-AS1	0.6769232	-0.4249	4.285704	2.33E-05	9.06E-05	1.9737732
A1BG	-0.373663	6.3401285	-4.285644	2.33E-05	9.06E-05	1.9735303
PET117	-0.111026	5.330718	-4.285618	2.33E-05	9.06E-05	1.9734236
CTC-367J11	-0.834708	2.2709511	-4.285502	2.33E-05	9.06E-05	1.9729551
PPM1M	0.1271054	5.742736	4.2855015	2.33E-05	9.06E-05	1.9729523
ZNF337-AS1	-0.175462	4.882928	-4.285267	2.33E-05	9.07E-05	1.9719996
BANF1P2	-0.553879	4.0508267	-4.284993	2.33E-05	9.08E-05	1.9708926
RP11-625I7	-0.591811	-1.058074	-4.28473	2.34E-05	9.09E-05	1.9698254
LPPR3	0.8648058	0.1432887	4.2845484	2.34E-05	9.10E-05	1.9690889
RASSF6	0.5730113	4.2746983	4.2842635	2.34E-05	9.11E-05	1.9679346
NEU3	-0.098053	5.6884193	-4.283988	2.34E-05	9.12E-05	1.9668199
UQCRH	-0.083197	6.6297498	-4.283724	2.35E-05	9.13E-05	1.9657499
KCTD2	-0.070713	6.1473678	-4.283663	2.35E-05	9.13E-05	1.9654994
AC073254.1	0.3666747	3.9234858	4.2829321	2.35E-05	9.16E-05	1.9625398
RP11-457P1	-0.254825	-1.406293	-4.28198	2.36E-05	9.19E-05	1.9586848

EPM2A	-0.134071	5.3226201	-4.281883	2.36E-05	9.20E-05	1.9582887
ZNF561-AS1	-0.107659	5.0575997	-4.281592	2.37E-05	9.21E-05	1.9571131
RP11-212I2	0.7406317	0.2419528	4.281306	2.37E-05	9.22E-05	1.955953
ATP5G2	-0.071828	6.9282007	-4.280735	2.38E-05	9.24E-05	1.9536413
AGPAT4	0.2686569	5.0149804	4.280735	2.38E-05	9.24E-05	1.9536406
RP11-730G2	-0.519178	-1.197796	-4.280623	2.38E-05	9.24E-05	1.953186
STK4	0.0563376	6.1242893	4.2805834	2.38E-05	9.24E-05	1.9530268
RP11-119F1	0.835825	1.3557419	4.280132	2.38E-05	9.26E-05	1.9511991
NFX1	-0.064103	6.3347831	-4.279999	2.38E-05	9.26E-05	1.9506591
TP53I3	-0.162288	6.1038646	-4.279774	2.39E-05	9.27E-05	1.9497494
IGHJ1	0.5589982	-1.038631	4.2794636	2.39E-05	9.28E-05	1.9484928
CTD-2192J1	-0.693015	1.0334086	-4.279401	2.39E-05	9.28E-05	1.9482402
C8orf34	0.6691941	-0.674894	4.2793773	2.39E-05	9.28E-05	1.9481434
CTD-2008P7	0.7849228	-0.639055	4.2793011	2.39E-05	9.28E-05	1.947835
RP11-255H2	-0.935802	-0.0029	-4.277872	2.40E-05	9.34E-05	1.9420517
RP11-380O2	-0.539947	2.5561698	-4.276684	2.42E-05	9.39E-05	1.937242
CTB-102L5.	0.7839297	-0.499163	4.2766597	2.42E-05	9.39E-05	1.9371451
SLC16A6	0.3962152	4.1476256	4.2763589	2.42E-05	9.40E-05	1.9359281
R3HDML	-0.832459	0.1729593	-4.276145	2.42E-05	9.41E-05	1.9350635
TRPM1	-0.870858	1.2559877	-4.27597	2.42E-05	9.41E-05	1.9343537
RP11-169K1	-0.84956	3.2157958	-4.27586	2.43E-05	9.42E-05	1.9339092
UBE2MP1	0.4087144	3.0429045	4.275704	2.43E-05	9.42E-05	1.933279
ATP6V1G1	-0.075615	6.5749863	-4.275484	2.43E-05	9.43E-05	1.9323894
RPL35A	-0.086021	7.0148245	-4.275356	2.43E-05	9.43E-05	1.93187
FARSA	-0.064846	6.429148	-4.27411	2.44E-05	9.48E-05	1.9268302
LAMA4	0.114587	6.0785464	4.2732637	2.45E-05	9.52E-05	1.9234101
FUT5	-0.761168	0.1805224	-4.273139	2.45E-05	9.52E-05	1.9229041
NDUFB3	-0.075249	6.2956221	-4.272498	2.46E-05	9.54E-05	1.9203158
CASZ1	0.2082115	5.0047307	4.2724731	2.46E-05	9.54E-05	1.9202137
STX2	0.1090641	5.7124674	4.2723641	2.46E-05	9.55E-05	1.9197732
PCDHGB9P	0.8144728	0.6744703	4.2721817	2.46E-05	9.55E-05	1.9190361
EXOSC9	-0.069834	5.9908443	-4.271819	2.47E-05	9.57E-05	1.9175707
DSCAS	-0.770853	0.8915518	-4.271253	2.47E-05	9.59E-05	1.9152819
CLEC4C	0.5216747	-0.937772	4.2704877	2.48E-05	9.62E-05	1.9121898
RP11-426C2	-1.010576	1.5013262	-4.27026	2.48E-05	9.63E-05	1.9112702
PAH	-0.303635	7.1090127	-4.270194	2.49E-05	9.63E-05	1.911004
F11-AS1	-0.397174	5.2070494	-4.269834	2.49E-05	9.64E-05	1.9095489
TNS1	0.0860795	6.5801971	4.2694169	2.49E-05	9.66E-05	1.9078637
ZNF280A	0.657063	-0.929759	4.2687284	2.50E-05	9.69E-05	1.9050824
LRRC14	-0.088982	6.175128	-4.267988	2.51E-05	9.72E-05	1.9020924
SPSB2	-0.125445	5.6829359	-4.267502	2.51E-05	9.74E-05	1.9001282
MIDN	0.0884117	6.4331827	4.2674645	2.51E-05	9.74E-05	1.899978
RPL27A	-0.092855	7.1864855	-4.266813	2.52E-05	9.76E-05	1.8973466
FBXW12	0.8121318	1.1707359	4.2660572	2.53E-05	9.79E-05	1.8942963
RP11-398G2	-0.360207	-1.273163	-4.266023	2.53E-05	9.79E-05	1.8941592
HMGN4	0.1239599	5.9551951	4.2652383	2.54E-05	9.83E-05	1.8909905
FLNB	-0.065876	6.8974727	-4.264587	2.55E-05	9.85E-05	1.8883619
LINC00934	-0.289806	-1.400963	-4.264524	2.55E-05	9.85E-05	1.8881075
SNHG9	-0.228113	5.1994996	-4.26358	2.56E-05	9.89E-05	1.8842975
AC026167.1	-0.427572	-1.300016	-4.262313	2.57E-05	9.95E-05	1.8791886
SETP22	0.4919365	-0.903102	4.2622582	2.57E-05	9.95E-05	1.8789665

VKORC1L1	-0.058523	6.3495042	-4.262015	2.57E-05	9.96E-05	1.8779835
PSCA	0.9101766	1.4699106	4.2611156	2.58E-05	9.99E-05	1.874358
EGFL8	0.318771	3.9826241	4.2610077	2.58E-05	1.00E-04	1.873923
METAP1D	-0.139276	5.5084408	-4.260282	2.59E-05	0.0001003	1.8709983
RP11-13K12	0.6488296	-0.666916	4.259994	2.60E-05	0.0001004	1.8698356
RPL21	-0.098156	6.7665998	-4.259821	2.60E-05	0.0001004	1.8691388
HSD17B3	-0.403073	4.5808881	-4.259816	2.60E-05	0.0001004	1.8691174
SERPINA10	-0.2856	6.8151105	-4.259532	2.60E-05	0.0001005	1.8679714
ZFP30	0.3511632	4.9559486	4.2591903	2.60E-05	0.0001007	1.8665955
ADAMTS8	0.5261028	2.7411772	4.2589355	2.61E-05	0.0001008	1.8655685
RP11-434I1	0.7081375	2.2721377	4.2588301	2.61E-05	0.0001008	1.8651435
RP11-649A1	0.7017869	0.2221384	4.2580655	2.62E-05	0.0001011	1.862062
JMJD1C-AS1	0.5683167	2.5135496	4.257171	2.63E-05	0.0001015	1.8584576
ASB16	0.411311	3.9743195	4.2569709	2.63E-05	0.0001016	1.8576511
SNAP91	0.7669745	-0.668751	4.2568621	2.63E-05	0.0001016	1.8572128
HMGCS2	-0.243288	7.2513464	-4.256791	2.63E-05	0.0001016	1.856926
KLRC4	0.5481419	-0.920945	4.2566625	2.63E-05	0.0001017	1.8564089
RP11-267J2	0.4297406	2.8620393	4.2562174	2.64E-05	0.0001019	1.8546157
RP11-568J2	0.5895878	-0.8058	4.256104	2.64E-05	0.0001019	1.854159
RNF144B	-0.144306	5.8774068	-4.255814	2.64E-05	0.000102	1.8529888
PMS2P1	-0.09501	5.5801504	-4.25559	2.64E-05	0.0001021	1.8520865
PCNAP1	-0.209956	-1.478279	-4.254918	2.65E-05	0.0001024	1.8493811
MYH13	0.8736442	-0.287956	4.2548943	2.65E-05	0.0001024	1.8492863
AC005785.5	0.7094664	1.0667314	4.254769	2.65E-05	0.0001024	1.8487816
SLC29A4	0.4319495	5.3703412	4.2545194	2.66E-05	0.0001025	1.8477766
SYNE3	0.1527341	5.4443637	4.2543026	2.66E-05	0.0001026	1.8469037
HIST1H4K	-0.75154	1.8720779	-4.254298	2.66E-05	0.0001026	1.8468856
ZDHHC17	0.0773092	5.865794	4.2540951	2.66E-05	0.0001027	1.8460682
FAM86C2P	-0.209496	4.9714641	-4.254036	2.66E-05	0.0001027	1.8458321
RP11-259G1	-0.582127	2.6100312	-4.253852	2.66E-05	0.0001028	1.8450911
THADA	-0.072823	6.174747	-4.253587	2.67E-05	0.0001029	1.8440224
ST7-AS1	-0.291743	4.1041938	-4.25332	2.67E-05	0.000103	1.8429467
AC011343.1	-0.726944	-0.189235	-4.252999	2.67E-05	0.0001031	1.8416547
TMEM236	0.5237559	2.6675932	4.2519669	2.69E-05	0.0001036	1.8375005
RP11-478K1	-0.928691	0.870647	-4.251824	2.69E-05	0.0001036	1.8369241
HAO2-IT1	-0.887124	0.1252384	-4.251731	2.69E-05	0.0001036	1.8365523
RP11-545M1	-0.610052	-0.462085	-4.251482	2.69E-05	0.0001037	1.8355489
ROR1-AS1	0.6073996	-0.821974	4.2514095	2.69E-05	0.0001038	1.8352573
LINC01484	-0.4635	4.2917103	-4.251308	2.69E-05	0.0001038	1.8348495
RP11-554J4	-0.319084	3.4726146	-4.25101	2.70E-05	0.0001039	1.8336493
PRRC2B	0.1049005	6.3699981	4.2507834	2.70E-05	0.000104	1.8327377
PSORS1C1	0.6987252	3.025182	4.2507558	2.70E-05	0.000104	1.8326268
RP11-203M5	0.4745612	-1.103399	4.2507199	2.70E-05	0.000104	1.8324824
CTD-2330J2	-0.17876	-1.480822	-4.250168	2.71E-05	0.0001042	1.8302619
ZNF835	0.6353906	3.1957919	4.2501162	2.71E-05	0.0001043	1.8300531
BOC	0.4595755	4.2993106	4.2500276	2.71E-05	0.0001043	1.8296967
LARP1B	-0.09841	6.1116549	-4.249827	2.71E-05	0.0001044	1.8288917
RP1-257A7.	0.4174023	3.3043275	4.2495121	2.71E-05	0.0001045	1.8276232
NIPAL1	0.3702619	5.1599679	4.2491776	2.72E-05	0.0001046	1.8262776
AC005281.1	0.6966659	-0.612256	4.2489961	2.72E-05	0.0001047	1.8255473
RP11-314N2	-0.743071	-0.652445	-4.248803	2.72E-05	0.0001048	1.8247689

XXbac-BPG2	-0.207973	4.4896004	-4.248724	2.72E-05	0.0001048	1.8244531
DBF4P1	0.6409179	-0.211235	4.248493	2.73E-05	0.0001049	1.8235239
SENP2	-0.063703	6.1910429	-4.248043	2.73E-05	0.0001051	1.8217154
RP11-20J15	0.895069	0.9976857	4.2478261	2.73E-05	0.0001052	1.820842
FAM131C	1.0386285	1.4035678	4.2477077	2.74E-05	0.0001052	1.820366
IGF2-AS	1.2172031	1.2567861	4.2473936	2.74E-05	0.0001053	1.819103
CES5A	-0.86711	3.8232129	-4.247368	2.74E-05	0.0001053	1.8189999
KLF7-IT1	0.5498865	-0.781794	4.2471984	2.74E-05	0.0001054	1.818318
ABCB1	-0.324361	6.1748977	-4.246917	2.74E-05	0.0001055	1.8171863
NEDD4L	-0.101863	6.4316853	-4.245333	2.76E-05	0.0001062	1.8108208
MCAT	-0.101529	5.9648218	-4.245282	2.76E-05	0.0001062	1.8106147
SCARF1	0.1050003	5.6467502	4.2444201	2.77E-05	0.0001066	1.8071508
ANXA4	0.0984963	6.7865394	4.2443873	2.77E-05	0.0001066	1.8070188
AC020951.1	0.5456281	-0.838863	4.2438093	2.78E-05	0.0001069	1.8046965
MON1B	-0.064199	6.2051206	-4.243189	2.79E-05	0.0001071	1.8022027
RP11-763B2	-0.414353	-1.292227	-4.243181	2.79E-05	0.0001071	1.8021723
EIF4A3	-0.063508	6.4579271	-4.242326	2.80E-05	0.0001075	1.7987369
CLSPN	0.4366032	4.3006037	4.2416422	2.81E-05	0.0001078	1.7959915
LPHN3	0.5409577	3.5332851	4.241638	2.81E-05	0.0001078	1.7959745
MRPS14	-0.071496	6.1856858	-4.241626	2.81E-05	0.0001078	1.7959278
LYNX1	-0.235739	6.2202315	-4.241299	2.81E-05	0.0001079	1.7946127
ATPAF2	-0.09235	5.9144105	-4.241227	2.81E-05	0.000108	1.7943253
RP5-1050D4	0.6035847	-0.532241	4.2411754	2.81E-05	0.000108	1.794117
MED17	0.0749502	5.842543	4.2410432	2.81E-05	0.000108	1.793586
TDRD1	0.8819169	0.0211504	4.2407818	2.82E-05	0.0001081	1.7925366
SMARCC2	0.1360216	6.2187937	4.2404672	2.82E-05	0.0001083	1.7912734
GRIK5	0.6355118	2.7677162	4.2404536	2.82E-05	0.0001083	1.7912187
APOOL	-0.09662	6.2061957	-4.240282	2.82E-05	0.0001083	1.7905288
WDR59	-0.079377	6.3349731	-4.239766	2.83E-05	0.0001085	1.7884584
SSSCA1-AS1	-0.151155	4.2936588	-4.239665	2.83E-05	0.0001086	1.7880539
EIF5AP4	0.6835236	0.8524703	4.2391531	2.84E-05	0.0001088	1.7859982
LA16c-313D	-0.798037	1.1370388	-4.239084	2.84E-05	0.0001088	1.785719
KRT8P5	0.6327831	-0.387738	4.2387959	2.84E-05	0.0001089	1.7845645
SENP6	0.0650843	6.2616773	4.2386517	2.84E-05	0.000109	1.7839857
DAP3	-0.067571	6.7229398	-4.238479	2.84E-05	0.0001091	1.7832914
RP1-151F17	-0.203606	5.1399293	-4.238401	2.85E-05	0.0001091	1.7829778
DHRSX	-0.10846	6.2476512	-4.238007	2.85E-05	0.0001093	1.7813996
AC006994.2	-0.69018	-0.121959	-4.23753	2.86E-05	0.0001095	1.779483
XCL2	0.6292585	3.0861781	4.2374446	2.86E-05	0.0001095	1.7791419
PAPSS2	-0.139899	6.436422	-4.237104	2.86E-05	0.0001097	1.7777769
RP11-712B9	0.4184233	-1.188439	4.2364619	2.87E-05	0.0001099	1.7751993
DNM3	0.2549463	4.5582099	4.2363266	2.87E-05	0.00011	1.7746567
ROCK2	-0.074322	6.3526252	-4.235948	2.88E-05	0.0001102	1.7731382
AC087650.1	-0.709428	0.0818223	-4.234956	2.89E-05	0.0001106	1.7691597
THUMPD3	-0.048146	6.180335	-4.2349	2.89E-05	0.0001106	1.7689344
COG1	-0.0589	6.1688775	-4.234719	2.89E-05	0.0001107	1.7682084
UBE2Q2P1	0.4669658	3.717757	4.2346698	2.89E-05	0.0001107	1.7680118
SDCCAG8	0.0731572	5.8482634	4.2346348	2.89E-05	0.0001107	1.7678715
RPS6KB2	-0.065813	6.3837175	-4.234475	2.89E-05	0.0001108	1.7672294
FIP1L1	0.0599561	6.0493236	4.2343527	2.89E-05	0.0001108	1.7667404
RP11-2C24.	0.7186813	0.3750902	4.2343416	2.89E-05	0.0001108	1.7666956

RP11-756J1	0.5252272	-0.944672	4.2341234	2.90E-05	0.0001109	1.7658207
LINC01116	0.7104783	2.891216	4.2336181	2.90E-05	0.0001111	1.763795
MICALL2	0.1208369	5.9731311	4.233597	2.90E-05	0.0001111	1.7637103
DNAH7	0.7760988	2.3041188	4.232563	2.92E-05	0.0001116	1.7595654
RP5-1065P1	-0.805196	1.9087174	-4.23253	2.92E-05	0.0001116	1.7594342
CSNK2A2	-0.057627	6.3640894	-4.232349	2.92E-05	0.0001117	1.7587078
GFRA3	0.9402676	0.4536768	4.2310264	2.94E-05	0.0001123	1.7534074
BZW1P2	0.6292013	2.428352	4.2308183	2.94E-05	0.0001124	1.752574
RP11-36501	0.7948805	1.3769123	4.2307659	2.94E-05	0.0001124	1.7523638
DDX5	-0.047268	7.0383935	-4.230659	2.94E-05	0.0001124	1.7519337
MED13	-0.069708	6.4215496	-4.230298	2.94E-05	0.0001126	1.7504875
RN7SKP16	0.7480101	0.373138	4.2301207	2.95E-05	0.0001127	1.7497789
SEC61A1	-0.057338	7.0932043	-4.230041	2.95E-05	0.0001127	1.7494582
LINC01290	-0.716887	0.7049843	-4.229812	2.95E-05	0.0001128	1.7485404
PPIL3	-0.089662	5.8460901	-4.22934	2.96E-05	0.000113	1.7466509
OFD1P2Y	-0.365949	-1.284956	-4.228831	2.96E-05	0.0001132	1.7446131
AF127936.3	0.677471	-0.095891	4.2285608	2.97E-05	0.0001134	1.7435308
G6PC	-0.339374	6.8835354	-4.228469	2.97E-05	0.0001134	1.7431618
RP11-120E1	-0.423304	2.8281446	-4.22831	2.97E-05	0.0001135	1.7425257
LINC00622	0.5567399	2.8850741	4.2280926	2.97E-05	0.0001136	1.7416559
CPSF4	-0.078933	6.0993612	-4.22806	2.97E-05	0.0001136	1.741524
UBE2Q1-AS1	0.7381188	0.9265384	4.2277502	2.98E-05	0.0001137	1.7402851
OSBP	-0.063793	6.6227186	-4.227681	2.98E-05	0.0001137	1.7400093
RP11-1081M	-0.521371	-1.181641	-4.227617	2.98E-05	0.0001137	1.7397509
CTC-523E23	0.7071225	-0.174041	4.2272738	2.98E-05	0.0001139	1.7383778
C19orf52	-0.068985	5.8039623	-4.227231	2.98E-05	0.0001139	1.7382067
BMP8B	0.5749321	4.2836269	4.2272182	2.98E-05	0.0001139	1.7381549
CAP2	-0.161351	6.0992628	-4.227051	2.99E-05	0.000114	1.737484
SAMD13	0.5887723	3.1091373	4.2268431	2.99E-05	0.0001141	1.7366533
BDNF	0.7450341	1.9438511	4.2262163	3.00E-05	0.0001143	1.7341443
FAM66D	0.6254094	-0.59934	4.225963	3.00E-05	0.0001145	1.7331303
RP11-946P6	0.683619	0.9439371	4.2258085	3.00E-05	0.0001145	1.7325121
TOE1	-0.080184	5.6400072	-4.225701	3.00E-05	0.0001146	1.7320837
RP11-423H2	0.9661358	2.0075061	4.2245892	3.02E-05	0.0001151	1.7276328
C17orf104	0.5227656	2.5100618	4.2241511	3.02E-05	0.0001153	1.7258796
GXYLT1P6	-0.878696	0.0161147	-4.223239	3.03E-05	0.0001157	1.722232
AC093326.1	-0.184168	-1.489609	-4.221321	3.06E-05	0.0001167	1.7145605
BRCC3P1	0.6699001	0.1857442	4.2212416	3.06E-05	0.0001167	1.7142426
LINC01006	-0.145067	5.5711427	-4.221122	3.06E-05	0.0001167	1.7137644
AC006128.2	0.3648473	4.3830326	4.2210176	3.06E-05	0.0001168	1.7133472
LINC00694	0.7040012	3.3506953	4.2206423	3.07E-05	0.0001169	1.7118468
CTB-113P19	-0.345206	3.9159181	-4.220195	3.07E-05	0.0001171	1.7100572
RP11-492D6	0.9818161	0.9528148	4.2201503	3.07E-05	0.0001172	1.7098798
FGA	-0.210771	7.813971	-4.21998	3.08E-05	0.0001172	1.709198
AC246787.3	0.4720104	-1.132883	4.2194412	3.08E-05	0.0001175	1.7070453
TREM2	0.363734	5.0171691	4.2191067	3.09E-05	0.0001176	1.7057086
RP11-31F19	0.6708857	-0.497193	4.2189545	3.09E-05	0.0001177	1.7051001
RP11-14101	-0.69675	3.0019689	-4.21867	3.09E-05	0.0001178	1.7039616
RARS	-0.062405	6.4253827	-4.218546	3.10E-05	0.0001179	1.7034663
AC134882.3	-0.199039	-1.474587	-4.218524	3.10E-05	0.0001179	1.7033785
VSIG10L	0.2987228	5.267342	4.2184263	3.10E-05	0.0001179	1.7029894

FCRL4	0.5112453	-1.091439	4.2174502	3.11E-05	0.0001184	1.6990892
FAM153C	0.7404885	-0.346305	4.21708	3.11E-05	0.0001186	1.6976104
RP11-43402	-0.634379	-0.325014	-4.216759	3.12E-05	0.0001187	1.6963289
ACTR3	0.0448719	6.6275266	4.2166718	3.12E-05	0.0001187	1.69598
ZNF444	-0.090608	6.454623	-4.215858	3.13E-05	0.0001191	1.6927299
LINC00322	0.6321786	-0.655669	4.2154765	3.14E-05	0.0001193	1.691206
RP11-50C13	0.2356151	4.4109157	4.2153361	3.14E-05	0.0001194	1.6906452
RP11-468E2	-0.27304	3.403063	-4.214713	3.15E-05	0.0001197	1.6881571
LTBP1	0.2205949	5.8576819	4.2146957	3.15E-05	0.0001197	1.6880878
TLR1	0.2605376	5.0200708	4.2145811	3.15E-05	0.0001197	1.6876306
CCDC64	0.2884695	5.5771811	4.2145467	3.15E-05	0.0001197	1.6874932
CSRP2BP	-0.081654	5.9615331	-4.214277	3.15E-05	0.0001199	1.6864165
RP11-77K12	-0.529617	-1.061633	-4.213312	3.16E-05	0.0001203	1.682565
ZNF23	0.5560408	3.5682494	4.2132001	3.17E-05	0.0001204	1.6821173
MSI2	0.129994	6.1337608	4.2131557	3.17E-05	0.0001204	1.68194
MT1A	-0.587021	4.9075736	-4.212765	3.17E-05	0.0001206	1.6803791
RPL39L	0.4914444	4.8823857	4.2124853	3.18E-05	0.0001207	1.6792642
TRAF4	-0.078892	6.4388756	-4.212193	3.18E-05	0.0001208	1.6780985
AC004490.1	0.5800504	-0.655284	4.2119756	3.18E-05	0.0001209	1.6772305
FES	0.1928644	5.9739159	4.2115805	3.19E-05	0.0001211	1.6756537
KCNH4	0.6857594	2.8635817	4.2114839	3.19E-05	0.0001212	1.6752685
RP4-584D14	-0.467496	3.2966316	-4.210895	3.20E-05	0.0001214	1.6729173
EXOC3	-0.059583	6.3831184	-4.210888	3.20E-05	0.0001214	1.6728903
ZBTB17	-0.062878	6.0124127	-4.210855	3.20E-05	0.0001214	1.6727611
TMEM191C	0.5986247	2.5237826	4.2107018	3.20E-05	0.0001215	1.672148
ELOVL5	-0.090785	6.6502351	-4.210597	3.20E-05	0.0001216	1.6717315
RN7SL138P	0.580581	2.2224855	4.2105045	3.20E-05	0.0001216	1.6713609
CTD-2026K1	-0.530071	2.8849139	-4.210483	3.20E-05	0.0001216	1.6712736
RP1-140K8.	-0.551557	-0.820777	-4.210417	3.20E-05	0.0001216	1.6710123
KLHL21	-0.113749	6.3149396	-4.209747	3.21E-05	0.0001219	1.6683389
NUDT17	0.2465818	4.6396945	4.2096534	3.21E-05	0.000122	1.667966
PNCK	1.1832207	1.3835021	4.2094874	3.22E-05	0.000122	1.6673039
NOSIP	-0.093225	6.3507867	-4.208749	3.23E-05	0.0001224	1.6643607
PRTFDC1	0.2924563	4.7812039	4.208489	3.23E-05	0.0001225	1.6633226
NASP	-0.073412	6.4802257	-4.208473	3.23E-05	0.0001225	1.6632592
GPAA1	-0.09494	6.9060971	-4.20837	3.23E-05	0.0001226	1.6628461
AC090616.2	-0.425922	3.0937495	-4.208271	3.23E-05	0.0001226	1.6624519
LINC00524	0.9310674	-0.230963	4.2080865	3.24E-05	0.0001227	1.6617178
RIMKLA	0.6581223	3.0019367	4.208027	3.24E-05	0.0001227	1.6614806
RP11-797H7	0.8508183	1.0353289	4.207995	3.24E-05	0.0001227	1.6613528
POLR1E	-0.094864	5.9540414	-4.207937	3.24E-05	0.0001227	1.6611208
FAM86C1	-0.099228	5.2637565	-4.207509	3.24E-05	0.0001229	1.6594167
OLR1	0.8901437	3.1245117	4.2073668	3.24E-05	0.000123	1.6588483
ALKBH3	-0.139377	5.7154019	-4.207257	3.25E-05	0.000123	1.6584123
RP11-182J1	0.783668	0.8015729	4.2072488	3.25E-05	0.000123	1.6583778
CLTB	-0.089731	6.4876561	-4.206853	3.25E-05	0.0001232	1.656802
HNF1B	0.3656559	5.7654445	4.2065378	3.26E-05	0.0001234	1.655544
RP11-274B2	0.7815453	2.0654422	4.2060511	3.26E-05	0.0001236	1.6536041
FAM195B	-0.112118	6.5499581	-4.20569	3.27E-05	0.0001238	1.6521643
SLC16A11	-0.354526	5.6002814	-4.205237	3.27E-05	0.000124	1.6503596
QRFP	0.7357339	1.4003812	4.2051176	3.28E-05	0.0001241	1.6498841

ATP1A4	0.7791488	0.2524834	4.2050044	3.28E-05	0.0001241	1.6494332
NIPA1	0.1251601	5.8881783	4.2044852	3.28E-05	0.0001244	1.6473643
NSL1	-0.069587	6.1260796	-4.203646	3.30E-05	0.0001248	1.6440221
CBFB	0.0739357	6.0132142	4.2035383	3.30E-05	0.0001249	1.6435922
HLA-DQB1	0.1894759	6.2763493	4.2030624	3.30E-05	0.0001251	1.6416968
ARAP1-AS2	0.7628365	0.9396815	4.2027189	3.31E-05	0.0001253	1.6403288
TPP1	-0.067288	6.9434824	-4.202448	3.31E-05	0.0001254	1.6392496
RP11-301M1	-0.327965	-1.344775	-4.202206	3.32E-05	0.0001255	1.638288
STON1-GTF2	0.5486264	-0.954129	4.202177	3.32E-05	0.0001255	1.6381709
RP11-541M1	0.5842227	-0.595011	4.2021126	3.32E-05	0.0001255	1.6379145
TFF2	1.1464372	0.73101	4.2019792	3.32E-05	0.0001256	1.637383
MST1L	-0.256855	5.5634083	-4.201299	3.33E-05	0.0001259	1.6346756
RP11-762H8	0.6969291	2.0259792	4.2007689	3.34E-05	0.0001262	1.6325645
RP5-902P8.	-0.349972	3.4329471	-4.200731	3.34E-05	0.0001262	1.6324142
EPC2	0.0830295	5.8045468	4.200647	3.34E-05	0.0001262	1.6320795
PRR4	0.3265196	3.4075522	4.2005661	3.34E-05	0.0001263	1.6317572
HRAS	-0.10606	6.0966897	-4.200407	3.34E-05	0.0001263	1.6311227
TELO2	-0.067195	6.2362596	-4.200214	3.34E-05	0.0001264	1.6303554
AC084082.3	-0.782586	0.8720274	-4.199982	3.35E-05	0.0001265	1.6294324
CACNA2D3	0.6725495	2.9477111	4.1999021	3.35E-05	0.0001266	1.6291145
SIRT2	-0.070494	6.4331286	-4.19988	3.35E-05	0.0001266	1.6290278
CLU	-0.114176	7.6663132	-4.199747	3.35E-05	0.0001266	1.6284979
MAPK14	-0.061395	6.4929524	-4.199449	3.36E-05	0.0001268	1.6273096
RP4-734G22	0.4767748	3.1725156	4.1993282	3.36E-05	0.0001268	1.6268305
RP4-781B1.	0.5685291	-0.96047	4.198839	3.36E-05	0.0001271	1.6248835
RP11-61F12	0.4640333	-1.057578	4.1980671	3.37E-05	0.0001275	1.6218126
MICALL1	0.0712427	6.0404439	4.1980462	3.38E-05	0.0001275	1.6217294
LLNLR-268E	0.6772304	1.6603648	4.1979075	3.38E-05	0.0001275	1.6211776
SEC14L1	0.0743497	6.2611153	4.1965263	3.40E-05	0.0001283	1.6156833
AGAP4	0.2386524	4.1755672	4.1961546	3.40E-05	0.0001285	1.6142051
ZNF609	0.07689	6.1220002	4.1960143	3.40E-05	0.0001285	1.6136472
CTD-2325B1	-0.146585	-1.506121	-4.195323	3.41E-05	0.0001289	1.6109
SH3BGRL	0.1334125	6.1019123	4.1948416	3.42E-05	0.0001291	1.6089843
AC141928.1	0.9891727	1.9700601	4.1947334	3.42E-05	0.0001292	1.6085541
ASTN2-AS1	-0.534693	-0.882398	-4.194604	3.42E-05	0.0001292	1.6080378
SGMS1-AS1	-0.137233	5.1589366	-4.194449	3.43E-05	0.0001293	1.6074242
AL117187.1	-0.638033	-0.491232	-4.194318	3.43E-05	0.0001294	1.606904
PRDM10	0.0847697	5.4871566	4.1936737	3.44E-05	0.0001297	1.6043417
RP11-559N1	0.668221	-0.506129	4.1934865	3.44E-05	0.0001298	1.6035975
TRMT112P4	-0.758372	2.6752066	-4.193435	3.44E-05	0.0001298	1.6033916
STAB1	0.110822	6.3599679	4.1934324	3.44E-05	0.0001298	1.6033825
CPNE9	0.7654485	0.8219585	4.1934135	3.44E-05	0.0001298	1.6033075
AC129778.2	-0.324415	-1.322855	-4.193145	3.45E-05	0.0001299	1.6022416
JPH3	0.7913925	0.9144662	4.1931021	3.45E-05	0.0001299	1.6020699
PGLS	-0.093813	6.3953932	-4.192916	3.45E-05	0.00013	1.6013307
VTN	-0.168197	7.7268212	-4.192644	3.45E-05	0.0001302	1.6002487
CECR5-AS1	-0.512571	2.6893301	-4.192437	3.46E-05	0.0001303	1.5994259
RP11-652L8	0.7531981	1.0481023	4.1918892	3.46E-05	0.0001305	1.5972501
LA16c-380F	0.4584344	-1.026535	4.1915648	3.47E-05	0.0001307	1.5959613
RP11-20J15	0.9026731	0.0841778	4.1911687	3.47E-05	0.0001309	1.5943878
OASL	-0.231572	5.8632884	-4.190626	3.48E-05	0.0001312	1.5922331

AGAP2	0.1633048	5.0321813	4.190028	3.49E-05	0.0001315	1.589857
HDLBP	-0.054558	7.1891888	-4.189751	3.50E-05	0.0001317	1.5887563
RP11-716H6	-0.413821	-1.161025	-4.189381	3.50E-05	0.0001318	1.5872893
AADAT	-0.329606	5.2286664	-4.189036	3.51E-05	0.000132	1.5859157
IGKV1-33	0.6736505	-0.82557	4.1884228	3.51E-05	0.0001323	1.5834829
RBM24	-0.386105	4.9989279	-4.187944	3.52E-05	0.0001326	1.5815833
TAF8	-0.06924	6.0740683	-4.187786	3.52E-05	0.0001327	1.5809547
TMEM167A	-0.056053	6.5188803	-4.187522	3.53E-05	0.0001328	1.5799064
AC012462.3	-0.163966	-1.498484	-4.187419	3.53E-05	0.0001328	1.5794973
COMMD8	0.1058861	5.63265	4.1874017	3.53E-05	0.0001328	1.5794293
LETMD1	-0.062089	6.360684	-4.187387	3.53E-05	0.0001328	1.5793695
KRCC1	-0.090096	6.0843012	-4.18726	3.53E-05	0.0001329	1.5788653
CASC10	-0.206596	5.8866496	-4.186795	3.54E-05	0.0001331	1.577021
SSR1	-0.056806	6.8594138	-4.186637	3.54E-05	0.0001332	1.5763937
RP3-483K16	-0.544574	3.3996242	-4.186598	3.54E-05	0.0001332	1.5762382
SNX18P3	0.8995658	0.5172457	4.1863276	3.55E-05	0.0001334	1.5751666
PRSS12	0.9728056	2.9376654	4.1859216	3.55E-05	0.0001336	1.5735554
RP11-416N4	0.581255	-0.737299	4.1853862	3.56E-05	0.0001339	1.5714309
STC2	0.2245779	5.4445232	4.1852757	3.56E-05	0.0001339	1.5709924
GZMA	0.2684036	4.9989781	4.1852686	3.56E-05	0.0001339	1.5709642
EXOSC10	-0.053899	6.3183324	-4.185006	3.57E-05	0.000134	1.5699217
PARS2	-0.093946	5.45583	-4.184895	3.57E-05	0.0001341	1.5694817
CTC-429P9.	0.6882178	2.5732321	4.184505	3.57E-05	0.0001343	1.5679352
CTC-308K20	0.5093279	2.5313433	4.1844485	3.57E-05	0.0001343	1.5677109
C1orf105	-0.636088	3.2235651	-4.184324	3.58E-05	0.0001344	1.5672177
FRAT1	-0.130098	5.8416577	-4.18431	3.58E-05	0.0001344	1.5671598
RP11-111F1	0.6128147	2.6917615	4.1842174	3.58E-05	0.0001344	1.5667944
CHL1	1.179605	1.829632	4.1839411	3.58E-05	0.0001345	1.5656983
XAGE3	-0.821847	1.1162583	-4.183408	3.59E-05	0.0001348	1.5635823
AC005071.3	0.6726576	-0.162189	4.183322	3.59E-05	0.0001349	1.5632431
CNNM1	0.9196589	3.9037473	4.1833147	3.59E-05	0.0001349	1.5632141
IGFBP1	-0.322659	6.9580268	-4.182908	3.60E-05	0.0001351	1.5616004
DHH	0.8467663	1.8976596	4.1826416	3.60E-05	0.0001352	1.5605449
LTBP3	0.1096092	6.5640471	4.1824851	3.60E-05	0.0001353	1.5599242
CANX	-0.05913	7.2965059	-4.182023	3.61E-05	0.0001355	1.5580938
PRPF39	0.0719769	5.8222052	4.1819034	3.61E-05	0.0001356	1.557618
AC092066.1	-0.115318	5.8289814	-4.181648	3.62E-05	0.0001357	1.5566072
RP11-53019	0.5069609	2.8991665	4.1815723	3.62E-05	0.0001357	1.5563054
PSMD1	-0.049864	6.6402723	-4.181427	3.62E-05	0.0001358	1.5557295
RP11-70702	0.6958537	-0.176557	4.1813979	3.62E-05	0.0001358	1.5556138
LINC01314	-0.350447	5.6788836	-4.181347	3.62E-05	0.0001358	1.5554103
CTB-175E5.	0.6903281	0.4678002	4.1811093	3.62E-05	0.000136	1.5544698
EDEM2	-0.063022	6.3720597	-4.181063	3.62E-05	0.000136	1.5542867
GRWD1	-0.067951	6.311582	-4.181031	3.63E-05	0.000136	1.5541614
CXCL13	1.0230522	3.2035377	4.1810243	3.63E-05	0.000136	1.554133
SPRY4-IT1	0.7173972	-0.219193	4.1808743	3.63E-05	0.000136	1.5535386
IGF2BP3	0.9487297	3.441103	4.1807656	3.63E-05	0.0001361	1.5531077
KLHDC7B	0.4011362	4.3824361	4.1803681	3.64E-05	0.0001363	1.551532
AC005550.5	-0.546264	-0.987661	-4.17973	3.65E-05	0.0001366	1.5490018
CALD1	-0.069372	6.9363683	-4.179531	3.65E-05	0.0001367	1.5482158
BBS4	-0.086638	5.8412312	-4.179493	3.65E-05	0.0001368	1.5480633

CCT3	-0.071999	7.058484	-4.179456	3.65E-05	0.0001368	1.5479185
C19orf71	0.4325361	3.9203825	4.1786421	3.66E-05	0.0001372	1.544693
RP13-15201	0.7238312	1.3631973	4.1786054	3.66E-05	0.0001372	1.5445474
RP11-755F1	0.7206049	0.9805184	4.1784126	3.67E-05	0.0001373	1.5437838
RP11-452C8	-0.47508	-1.228829	-4.178314	3.67E-05	0.0001374	1.5433916
ZNF878	0.813528	1.1854315	4.1782533	3.67E-05	0.0001374	1.5431525
KRTAP12-3	-0.24606	-1.437756	-4.178044	3.67E-05	0.0001375	1.5423237
RP11-562L8	-0.164332	-1.509487	-4.177964	3.67E-05	0.0001375	1.5420078
TTC28-AS1	-0.106188	5.6897443	-4.177838	3.67E-05	0.0001376	1.5415057
TBC1D14	0.2063093	5.6108254	4.1770597	3.69E-05	0.000138	1.5384251
ENC1	0.1606913	5.9948043	4.1770012	3.69E-05	0.000138	1.5381934
GDPD5	0.1528728	5.6069521	4.1764941	3.70E-05	0.0001383	1.5361852
KIF15	0.3311713	4.8356527	4.1762021	3.70E-05	0.0001385	1.5350287
IGSF11	0.8566546	0.1304515	4.1752844	3.71E-05	0.000139	1.5313952
XXbac-B135	0.5496086	3.2529614	4.1752836	3.71E-05	0.000139	1.5313922
EIF1B	-0.06151	6.2501748	-4.175229	3.71E-05	0.000139	1.5311745
GS1-21A4.1	-0.757976	0.5918996	-4.175172	3.72E-05	0.000139	1.5309494
PKHD1	0.6718601	4.4725597	4.1751098	3.72E-05	0.000139	1.5307044
AC103563.5	0.4976101	-1.093763	4.175017	3.72E-05	0.0001391	1.5303368
SPNS1	0.2887167	4.7945901	4.1748599	3.72E-05	0.0001391	1.5297151
ILK	0.3174905	5.4193072	4.1748511	3.72E-05	0.0001391	1.5296801
RPL18	-0.090569	7.1664682	-4.174835	3.72E-05	0.0001391	1.5296179
CPSF6	0.0445474	6.3201996	4.1745944	3.72E-05	0.0001393	1.528664
RPL12	-0.092786	7.0839219	-4.174005	3.73E-05	0.0001396	1.5263296
RP11-84C13	-0.616466	3.0476409	-4.17352	3.74E-05	0.0001399	1.5244109
DSC1	-0.759791	0.5649663	-4.173371	3.74E-05	0.0001399	1.523822
ACTL6A	0.0773991	6.0932268	4.1729209	3.75E-05	0.0001402	1.522041
HELLS	0.2085679	5.3906598	4.1728111	3.75E-05	0.0001402	1.5216065
PGM5P2	0.704791	1.149592	4.1724272	3.76E-05	0.0001405	1.5200877
FUK	-0.081747	6.0836689	-4.171767	3.77E-05	0.0001408	1.5174771
PABPC3	0.5203744	2.9554642	4.1716896	3.77E-05	0.0001409	1.5171695
LINC01096	0.6341435	-0.928058	4.1712622	3.78E-05	0.0001411	1.515479
VARS	-0.074054	6.6874967	-4.171188	3.78E-05	0.0001411	1.5151873
HSPE1	-0.085995	6.8112034	-4.170885	3.78E-05	0.0001413	1.5139881
KRTAP5-1	0.8536322	0.9411004	4.1707645	3.79E-05	0.0001413	1.5135105
RP11-373E1	-0.729418	-0.567294	-4.170621	3.79E-05	0.0001414	1.5129418
LIPM	0.9177497	0.2176995	4.1702222	3.79E-05	0.0001416	1.5113658
RP11-92C4.	0.7928816	0.8329999	4.17003	3.80E-05	0.0001417	1.5106059
CKLF-CMTM1	0.6943809	1.1243702	4.1698835	3.80E-05	0.0001418	1.5100268
RP11-437J2	-0.286244	-1.389621	-4.169659	3.80E-05	0.0001419	1.5091379
CTD-2325M2	-0.353333	3.4849314	-4.169508	3.81E-05	0.000142	1.508542
ZNF280D	-0.064511	6.1322235	-4.169226	3.81E-05	0.0001422	1.5074268
NXPH4	0.5516534	4.2913554	4.1691475	3.81E-05	0.0001422	1.5071166
FER1L4	0.5807922	4.1504085	4.1689711	3.81E-05	0.0001423	1.5064192
ORMDL2	-0.081501	6.2120025	-4.16872	3.82E-05	0.0001424	1.5054283
VWF	-0.163579	6.4624209	-4.1687	3.82E-05	0.0001424	1.5053466
RP11-424D1	-0.202806	-1.470257	-4.168568	3.82E-05	0.0001425	1.5048265
SIX5	0.1909961	5.5940936	4.1672977	3.84E-05	0.0001432	1.4998046
CTD-3035D6	-0.690047	2.8813092	-4.167176	3.84E-05	0.0001433	1.4993228
SMURF2	0.0995062	5.7315564	4.1670661	3.84E-05	0.0001433	1.4988896
FUT1	0.2288228	4.919146	4.1665336	3.85E-05	0.0001436	1.4967851

SDR42E2	-0.700177	0.8631578	-4.166344	3.86E-05	0.0001437	1.4960377
RP11-1376F	0.6988354	1.7427935	4.1662059	3.86E-05	0.0001438	1.4954905
ZNF350-AS1	0.7151855	0.7891069	4.1657316	3.87E-05	0.0001441	1.4936167
LIMS2	-0.151252	6.1964153	-4.165541	3.87E-05	0.0001442	1.4928638
RP1-276N6.	-1.1197	1.5872283	-4.164599	3.88E-05	0.0001447	1.4891424
YTHDF1	-0.043667	6.4241043	-4.16402	3.89E-05	0.0001451	1.4868562
RP11-111A2	-0.576724	1.8258219	-4.163774	3.90E-05	0.0001452	1.485886
UNC119B	0.118353	5.8435025	4.1634829	3.90E-05	0.0001454	1.4847351
VNN1	0.4256078	6.2157707	4.1632561	3.91E-05	0.0001455	1.4838393
RP11-504P2	-0.148352	5.2919124	-4.163203	3.91E-05	0.0001455	1.4836304
EYA2	0.7170386	3.222329	4.162893	3.91E-05	0.0001457	1.4824058
S100A5	0.5665329	-0.749722	4.1626303	3.92E-05	0.0001458	1.4813686
MS4A4A	0.2036896	5.4231071	4.1625028	3.92E-05	0.0001459	1.4808652
OSBP2	0.269995	5.039112	4.1624134	3.92E-05	0.0001459	1.4805124
C1orf21	-0.198438	6.0364612	-4.162375	3.92E-05	0.0001459	1.4803621
RP1-93H18.	0.6792789	0.0635162	4.1622758	3.92E-05	0.000146	1.4799692
MLIP-AS1	-0.933533	1.2008293	-4.162099	3.92E-05	0.000146	1.4792719
NPM1P26	0.6584776	0.3094215	4.1620938	3.92E-05	0.000146	1.4792506
F2RL1	0.4606964	5.3933544	4.162082	3.93E-05	0.000146	1.4792043
RP11-469J4	0.6280655	-0.360941	4.1620783	3.93E-05	0.000146	1.4791895
CDC42EP1	0.1003626	6.7310555	4.1619609	3.93E-05	0.0001461	1.478726
RP11-424N2	-0.696424	1.073075	-4.161855	3.93E-05	0.0001462	1.4783062
HM13	-0.072399	6.9159807	-4.161721	3.93E-05	0.0001462	1.4777804
PRR15	1.0353587	2.6831551	4.160371	3.95E-05	0.000147	1.4724512
TYK2	-0.051394	6.5060005	-4.160346	3.95E-05	0.000147	1.4723508
VSX1	0.9726347	1.8456796	4.1603383	3.95E-05	0.000147	1.4723222
RP11-123K3	-0.744782	1.2995701	-4.160268	3.96E-05	0.0001471	1.4720436
YWHAZ	0.072079	7.0116409	4.1599725	3.96E-05	0.0001472	1.4708789
RP11-15F12	0.7780029	0.5672347	4.1599606	3.96E-05	0.0001472	1.470832
LL21NC02-1	-0.593575	2.5259429	-4.159641	3.97E-05	0.0001474	1.4695697
GUSBP11	0.3010317	4.2502858	4.1584304	3.99E-05	0.0001481	1.4647952
PPIL4	0.0858565	5.7963048	4.1583425	3.99E-05	0.0001482	1.4644485
RNA5SP283	0.5662263	-0.650537	4.1582442	3.99E-05	0.0001482	1.4640608
AL445183.1	-0.397643	-1.185688	-4.157861	4.00E-05	0.0001484	1.4625503
ARHGAP39	0.1766723	5.1810639	4.1575348	4.00E-05	0.0001486	1.4612631
RP11-998D1	-0.347221	-1.277944	-4.157476	4.00E-05	0.0001486	1.4610329
C6orf165	0.6737614	1.6015962	4.1573759	4.00E-05	0.0001487	1.4606366
RP11-276E1	-0.878894	0.4025279	-4.157259	4.01E-05	0.0001487	1.4601747
PFAS	0.0859956	5.9482195	4.1570316	4.01E-05	0.0001489	1.4592788
RP11-455B2	-0.592636	-0.603195	-4.156662	4.02E-05	0.0001491	1.4578196
RP11-71H17	0.6226591	-0.317701	4.1565449	4.02E-05	0.0001491	1.4573597
RNU6-968P	-0.459759	-1.056849	-4.155782	4.03E-05	0.0001496	1.4543529
ZNF385D-AS	-0.218225	-1.485809	-4.155485	4.03E-05	0.0001498	1.4531823
SH3TC2	0.5492423	3.012849	4.1550021	4.04E-05	0.0001501	1.451278
FAM220CP	0.7296094	1.0250002	4.1543127	4.05E-05	0.0001505	1.448561
HNRNPLP2	0.4617809	2.9167713	4.1541505	4.06E-05	0.0001506	1.4479218
CDS1	0.6568828	4.367719	4.1534276	4.07E-05	0.000151	1.4450734
ALPL	-0.3045	6.011419	-4.153156	4.07E-05	0.0001512	1.4440039
GNL3LP1	0.6464028	-0.317172	4.1518353	4.10E-05	0.000152	1.4388011
RP11-68I18	-0.480508	3.5556824	-4.151354	4.11E-05	0.0001523	1.4369046
C16orf45	-0.204075	5.7730752	-4.151268	4.11E-05	0.0001523	1.4365663

OR7E102P	-0.845232	0.6684363	-4.150785	4.11E-05	0.0001526	1.4346663
ZNF829	0.4657844	4.36714	4.1502884	4.12E-05	0.0001529	1.4327094
TMCC1	-0.11457	6.2581986	-4.15002	4.13E-05	0.0001531	1.431652
CDHR1	0.6974691	2.6435067	4.1494628	4.14E-05	0.0001534	1.4294588
ANKRD7	0.725613	-0.524362	4.1492588	4.14E-05	0.0001535	1.4286559
CCDC141	0.818285	2.151188	4.1488906	4.15E-05	0.0001537	1.4272068
AC104777.4	-0.607001	-0.694648	-4.148681	4.15E-05	0.0001539	1.4263829
ZNF451	-0.064968	6.1007421	-4.148594	4.15E-05	0.0001539	1.4260375
RP11-180I4	0.6273366	-0.658718	4.1484815	4.15E-05	0.000154	1.4255965
DPP6	0.8075049	0.5532995	4.148241	4.16E-05	0.0001541	1.4246498
GC	-0.189266	7.5177618	-4.147557	4.17E-05	0.0001545	1.4219575
RP11-803D5	0.7250081	0.8805477	4.1472439	4.18E-05	0.0001547	1.4207261
SPARCL1	-0.208468	6.4336151	-4.146737	4.19E-05	0.000155	1.4187325
LINC00229	-0.465328	-1.291965	-4.146413	4.19E-05	0.0001552	1.4174565
RP4-541C22	0.898943	0.0211887	4.146216	4.19E-05	0.0001553	1.4166821
AKR1B1	0.1827545	5.8943915	4.1462149	4.19E-05	0.0001553	1.4166778
ID1	0.198951	5.9047626	4.1461742	4.19E-05	0.0001553	1.4165177
TAOK2	-0.054334	6.4071673	-4.145521	4.21E-05	0.0001557	1.4139471
RP11-961A1	-0.750485	0.9514632	-4.144992	4.22E-05	0.0001561	1.4118668
COMMD10	-0.086746	5.8929906	-4.144765	4.22E-05	0.0001562	1.4109744
FIGN	0.3844804	4.98557	4.144756	4.22E-05	0.0001562	1.4109397
SLC35A5	-0.072637	6.0072113	-4.14451	4.22E-05	0.0001563	1.4099728
PFKL	-0.06744	6.8379112	-4.14432	4.23E-05	0.0001564	1.4092249
PTENP1	0.4573591	3.7553667	4.1440563	4.23E-05	0.0001566	1.4081886
NAA20	-0.075018	6.5317644	-4.143601	4.24E-05	0.0001569	1.4063996
SEMA3F-AS1	0.3079653	3.8726549	4.1434836	4.24E-05	0.0001569	1.405937
EPHA6	0.8902083	-0.268605	4.1429827	4.25E-05	0.0001572	1.4039678
RP11-480D4	-0.202435	-1.466343	-4.142711	4.26E-05	0.0001574	1.4028993
RP11-517A5	-0.623938	-0.444432	-4.142506	4.26E-05	0.0001575	1.4020936
HNRNPA1P16	0.367214	3.593263	4.1425005	4.26E-05	0.0001575	1.4020724
RP11-561C5	0.6033207	-0.524282	4.1416592	4.27E-05	0.000158	1.398766
RNPEPL1	-0.070807	6.6538831	-4.141602	4.28E-05	0.0001581	1.3985424
ZBTB26	0.1234154	5.1826313	4.1413475	4.28E-05	0.0001582	1.3975409
RP11-159K7	-1.010799	-0.021507	-4.140952	4.29E-05	0.0001585	1.3959849
ADAR	-0.055251	6.9864198	-4.140761	4.29E-05	0.0001586	1.3952378
L3MBTL4-AS	0.6615267	1.8460387	4.1407568	4.29E-05	0.0001586	1.3952199
AK1	-0.129128	5.7115069	-4.140693	4.29E-05	0.0001586	1.394971
XXbac-B476	-0.216946	4.2159908	-4.140339	4.30E-05	0.0001588	1.3935782
ACKR4	0.6780682	1.9757048	4.1400201	4.30E-05	0.000159	1.3923256
POC1B	0.0811152	5.6669386	4.1400164	4.30E-05	0.000159	1.392311
SENP8	-0.185572	4.5510627	-4.139915	4.31E-05	0.000159	1.3919138
RP11-25H12	-1.023757	1.6964225	-4.139592	4.31E-05	0.0001592	1.3906438
C21orf91	0.1496275	5.4012262	4.1395332	4.31E-05	0.0001593	1.3904129
CTC-529I10	0.4411276	3.1174114	4.1394334	4.31E-05	0.0001593	1.3900208
TRPV3	0.7785716	2.9848187	4.1387635	4.33E-05	0.0001597	1.3873899
REXO4	-0.076519	6.1826105	-4.138338	4.33E-05	0.00016	1.3857193
FAM19A3	0.5957111	-0.561419	4.1376445	4.35E-05	0.0001605	1.3829958
C8orf4	0.1803274	6.088	4.1372469	4.35E-05	0.0001607	1.3814345
PRAMENP	-1.017492	1.3223831	-4.137235	4.35E-05	0.0001607	1.3813859
STAC3	0.173081	4.9705439	4.137206	4.35E-05	0.0001607	1.381274
MIR647	0.6155937	2.8551105	4.1367838	4.36E-05	0.000161	1.3796165

ZNF26	0.0883126	5.6116919	4.1366831	4.36E-05	0.000161	1.3792213
H1FX	-0.084003	6.5582992	-4.136547	4.37E-05	0.0001611	1.3786859
SMAD5	0.0714211	6.3813857	4.1349245	4.40E-05	0.0001622	1.3723192
RAD17	-0.054422	6.0022893	-4.134803	4.40E-05	0.0001622	1.3718438
SLC9A3	0.7837687	3.3988658	4.1344388	4.41E-05	0.0001625	1.3704133
FOXA2	-0.325278	6.2981918	-4.134169	4.41E-05	0.0001626	1.3693565
UGDH-AS1	-0.222036	4.6556518	-4.134052	4.41E-05	0.0001627	1.368897
EMG1	-0.096911	5.9152483	-4.133847	4.42E-05	0.0001628	1.3680905
C1RL-AS1	-0.163689	5.4410712	-4.133696	4.42E-05	0.0001629	1.3674998
IFNGR2	0.0810903	6.3657396	4.1336027	4.42E-05	0.0001629	1.3671329
IGHV7-27	0.4820294	-1.146032	4.1335181	4.42E-05	0.000163	1.3668011
RELL2	0.318644	4.3427996	4.1332046	4.43E-05	0.0001632	1.3655714
POP7	-0.080241	6.2052666	-4.132972	4.43E-05	0.0001633	1.3646577
RP11-329B9	0.6682177	1.8209274	4.1323189	4.44E-05	0.0001637	1.3620978
RP11-452K1	-0.611007	2.6435553	-4.132271	4.44E-05	0.0001638	1.3619115
RP13-130D2	-0.465131	-1.063178	-4.132254	4.45E-05	0.0001638	1.3618439
SUMF1	-0.070339	6.2808789	-4.131931	4.45E-05	0.000164	1.3605775
FNBP1	0.0653062	6.2471363	4.1318981	4.45E-05	0.000164	1.3604474
PSMC3IP	0.2610739	4.6249115	4.1316775	4.46E-05	0.0001641	1.3595825
SDCCAG3	-0.076181	6.3153005	-4.131512	4.46E-05	0.0001642	1.3589353
RP1-286D6.	0.4498078	3.4374971	4.1313144	4.46E-05	0.0001643	1.3581587
FAM169A	-0.38769	4.7954883	-4.131039	4.47E-05	0.0001645	1.3570794
OTC	-0.575206	6.166754	-4.130953	4.47E-05	0.0001645	1.3567399
CHRM2	0.8480963	-0.260929	4.1308743	4.47E-05	0.0001646	1.3564331
FSIP1	0.7164551	2.2586329	4.1307158	4.47E-05	0.0001647	1.3558118
THRB-AS1	-0.362963	3.7529441	-4.130681	4.47E-05	0.0001647	1.3556765
RP11-196G1	-0.087589	5.4586101	-4.130671	4.47E-05	0.0001647	1.3556344
ACE	0.1732237	5.4180329	4.1306573	4.47E-05	0.0001647	1.3555826
CTD-324401	-0.310914	-1.333539	-4.130489	4.48E-05	0.0001647	1.3549231
ZNF300	0.4478466	4.4911872	4.1302763	4.48E-05	0.0001649	1.3540887
RP1-90L6.3	0.5440821	-0.788932	4.1298094	4.49E-05	0.0001652	1.3522587
BUB1B	0.2870881	5.1981343	4.1293577	4.50E-05	0.0001655	1.3504882
B3GALNT2	0.1662153	5.2606878	4.1292216	4.50E-05	0.0001656	1.3499549
TTLL1	0.1245447	5.1415556	4.1290478	4.50E-05	0.0001657	1.3492736
CTD-2235C1	-0.621761	2.8475488	-4.128217	4.52E-05	0.0001662	1.3460182
THBS4	-0.417929	5.3855641	-4.127758	4.53E-05	0.0001665	1.3442183
NPIPB11	0.4891922	3.1257114	4.1273302	4.54E-05	0.0001668	1.3425438
NUP205	0.0729235	6.1336665	4.1272313	4.54E-05	0.0001668	1.3421566
ANGPTL2	0.1635588	5.8119177	4.1255129	4.57E-05	0.000168	1.335426
SLC27A2	-0.251035	6.590125	-4.124998	4.58E-05	0.0001684	1.3334089
DPF1	0.6977097	1.669418	4.1248969	4.58E-05	0.0001684	1.3330142
MROH3P	0.9235437	0.1920017	4.1248575	4.58E-05	0.0001684	1.33286
RP11-872J2	-0.190212	4.3703026	-4.124427	4.59E-05	0.0001687	1.3311745
LMBRD2	-0.105571	6.0211418	-4.124233	4.60E-05	0.0001688	1.3304148
PSENEN	-0.092097	6.2860133	-4.123679	4.61E-05	0.0001692	1.3282458
ATP5B	-0.062547	7.2227367	-4.123634	4.61E-05	0.0001692	1.3280696
CTD-2588C8	-0.716138	0.160548	-4.123137	4.62E-05	0.0001696	1.326123
UAP1	-0.094752	6.6214669	-4.122559	4.63E-05	0.0001699	1.3238622
SMURF2P1	0.7077994	0.6183477	4.1219488	4.64E-05	0.0001704	1.321475
PLA2G5	0.3562725	4.6058663	4.1218307	4.64E-05	0.0001704	1.321013
PCDHGA5	0.705995	3.0077904	4.1218227	4.64E-05	0.0001704	1.3209817

NEK11	0.276644	4.3834765	4.121758	4.64E-05	0.0001704	1.3207287
RP11-9L18.	0.5614864	2.4408465	4.1213721	4.65E-05	0.0001707	1.3192189
RPL31	-0.083283	7.1452045	-4.121356	4.65E-05	0.0001707	1.319156
TPTE2P1	0.7775537	0.6132278	4.120926	4.66E-05	0.000171	1.3174734
GABRR1	0.6715283	-0.569843	4.1207881	4.66E-05	0.0001711	1.316934
LINC01431	-0.4348	3.268462	-4.120729	4.66E-05	0.0001711	1.3167028
CCDC90B	-0.06199	6.1336468	-4.120703	4.66E-05	0.0001711	1.3165996
TNFAIP2	0.1235706	6.3471825	4.1203184	4.67E-05	0.0001713	1.3150969
PTPN5	0.8740191	1.0838447	4.1193451	4.69E-05	0.000172	1.3112902
CYP2C60P	-0.207522	-1.457022	-4.119306	4.69E-05	0.000172	1.3111361
RP11-136H1	0.580096	-0.296205	4.1189923	4.70E-05	0.0001722	1.3099105
NAP1L1P1	0.3937703	2.873946	4.1188933	4.70E-05	0.0001723	1.3095236
RP11-241F1	-0.710303	-0.520503	-4.11885	4.70E-05	0.0001723	1.3093555
SPTA1	0.9316817	1.1637711	4.1185703	4.71E-05	0.0001725	1.3082605
SPTLC1	0.1026924	5.9193435	4.1180616	4.72E-05	0.0001728	1.3062719
PLSCR2	0.7598055	-0.022031	4.1174794	4.73E-05	0.0001732	1.3039959
SYNPO2	0.2109227	5.4577931	4.1173793	4.73E-05	0.0001733	1.3036044
FAM89B	-0.086163	5.8633003	-4.117346	4.73E-05	0.0001733	1.3034742
RP11-54A9.	0.7824948	0.3096674	4.1173141	4.73E-05	0.0001733	1.3033499
FAM180A	0.9756863	2.9208785	4.1168095	4.74E-05	0.0001736	1.3013776
RP11-170L3	0.5108315	-1.076745	4.1165161	4.75E-05	0.0001738	1.3002307
RPS6KB1	-0.05896	6.0911006	-4.116214	4.75E-05	0.000174	1.2990493
RP11-34E5.	0.584999	-0.603146	4.1159582	4.76E-05	0.0001742	1.2980507
UBAP2	0.0699955	6.0572612	4.1159394	4.76E-05	0.0001742	1.2979773
KIZ	-0.099666	5.8783963	-4.115665	4.76E-05	0.0001744	1.2969032
SURF4	-0.056971	7.0493975	-4.11534	4.77E-05	0.0001746	1.2956347
C12orf66	-0.093114	5.6002951	-4.115302	4.77E-05	0.0001746	1.2954872
RP11-468E2	-0.743106	0.8616071	-4.114678	4.78E-05	0.000175	1.2930493
IRGM	-0.855815	0.2441186	-4.114622	4.78E-05	0.0001751	1.2928292
CCAR1	0.0555143	6.298166	4.113865	4.80E-05	0.0001756	1.289873
TBX19	0.1648196	4.7142929	4.1137349	4.80E-05	0.0001757	1.289365
CTA-125H2.	-0.197394	-1.459138	-4.113258	4.81E-05	0.000176	1.2875027
AL022326.1	0.5902259	-0.531582	4.1131902	4.81E-05	0.000176	1.2872377
VWA3B	0.904665	1.2455882	4.1126386	4.82E-05	0.0001764	1.2850838
AC013264.2	0.7377518	0.0498523	4.1122052	4.83E-05	0.0001767	1.2833916
SCHIP1	0.5032455	3.2056936	4.1121528	4.83E-05	0.0001767	1.283187
ZCCHC24	-0.100746	6.2554823	-4.111425	4.85E-05	0.0001773	1.2803438
RP11-1081M	-0.480272	-1.237222	-4.111075	4.85E-05	0.0001775	1.2789812
ATM	0.0808254	6.0714965	4.1109703	4.86E-05	0.0001776	1.2785707
APOPT1	-0.094975	5.887769	-4.110551	4.86E-05	0.0001778	1.2769324
RP11-1280N	0.6870608	1.995218	4.1104724	4.87E-05	0.0001779	1.2766272
NCAPH2	-0.079013	6.3419975	-4.109353	4.89E-05	0.0001787	1.2722587
DYNC1I2P1	0.6114682	2.0958961	4.1091359	4.89E-05	0.0001788	1.2714117
WBP1L	-0.064041	6.428921	-4.109091	4.89E-05	0.0001789	1.2712374
CTAGE8	0.7804794	1.2927331	4.1088934	4.90E-05	0.000179	1.2704657
UBA5	-0.055866	6.2570579	-4.108392	4.91E-05	0.0001793	1.2685111
TSC22D3	-0.167854	6.4774391	-4.107777	4.92E-05	0.0001798	1.2661095
SYCP2L	0.7745694	1.5151908	4.107583	4.92E-05	0.0001799	1.2653538
AC093824.1	-0.231903	-1.435147	-4.107395	4.93E-05	0.00018	1.2646198
MESTIT1	0.6805342	0.3561383	4.1068877	4.94E-05	0.0001804	1.2626422
SLITRK2	0.6737215	-0.522036	4.106834	4.94E-05	0.0001804	1.262433

RP11-96D1.	0.7696596	1.772686	4.1067987	4.94E-05	0.0001804	1.2622952
CTD-3126B1	0.6392755	-0.174637	4.1066439	4.94E-05	0.0001805	1.2616917
SHISA9	0.9659007	0.3603754	4.1066155	4.94E-05	0.0001805	1.2615809
KRT19P4	-0.270189	-1.380169	-4.106462	4.95E-05	0.0001806	1.2609805
FLJ42969	0.5576335	-0.943424	4.1062631	4.95E-05	0.0001807	1.2602067
RAET1K	0.8001104	0.9463951	4.1061665	4.95E-05	0.0001808	1.2598298
RP11-326K1	-0.318691	-1.305378	-4.105411	4.97E-05	0.0001813	1.2568833
TSNAX	-0.066579	6.2632473	-4.10518	4.97E-05	0.0001815	1.2559841
ZNF117	0.1717888	5.3270323	4.1050402	4.98E-05	0.0001816	1.2554388
CTD-2033D1	0.8008585	0.2264507	4.1047693	4.98E-05	0.0001818	1.2543831
SETD9	-0.133529	5.4392364	-4.104587	4.99E-05	0.0001819	1.2536727
HMGB1P42	-0.149722	-1.504743	-4.104396	4.99E-05	0.000182	1.2529292
PLEKHD1	0.7791254	1.2420279	4.1040384	5.00E-05	0.0001823	1.2515341
NR1I2	-0.656298	5.6052077	-4.103849	5.00E-05	0.0001824	1.2507979
ZNF503-AS1	0.5223658	2.6165126	4.1037802	5.00E-05	0.0001824	1.2505279
C10orf35	0.5704095	4.8257906	4.1035863	5.01E-05	0.0001826	1.2497721
ZNF502	0.4242783	4.3622972	4.1034581	5.01E-05	0.0001826	1.2492728
PTBP3	0.0580156	6.3899215	4.1034381	5.01E-05	0.0001826	1.2491946
CSNK2A3	0.7063972	0.9960442	4.1034029	5.01E-05	0.0001826	1.2490575
SERINC2	-0.112273	6.9094703	-4.103127	5.02E-05	0.0001828	1.2479836
GMPPB	-0.074192	6.1832923	-4.102794	5.02E-05	0.0001831	1.2466866
GRIK1	0.5871179	2.1428489	4.1023328	5.03E-05	0.0001834	1.2448884
RP11-1072A	-0.696658	1.5698876	-4.102106	5.04E-05	0.0001835	1.2440063
RP11-760D2	-0.278405	-1.410049	-4.101207	5.06E-05	0.0001842	1.2405024
ABCD1P2	-0.317597	-1.355047	-4.101103	5.06E-05	0.0001843	1.240097
HOXB-AS2	0.5444253	-0.782507	4.1006584	5.07E-05	0.0001846	1.2383664
LINC00473	0.8726146	-0.015925	4.1002283	5.08E-05	0.0001849	1.2366913
RP11-567G1	0.9987729	0.8379372	4.0993457	5.09E-05	0.0001856	1.2332547
CCT6P3	0.1458158	4.7262353	4.0988563	5.11E-05	0.0001859	1.2313497
SGOL2	0.2048428	5.1283915	4.0980253	5.12E-05	0.0001865	1.228115
RP11-632C1	-0.236445	5.1202308	-4.097562	5.13E-05	0.0001869	1.2263113
CCRN4L	0.2217966	5.1792262	4.0975268	5.13E-05	0.0001869	1.2261751
CTNNA1	-0.049729	6.9650246	-4.09736	5.14E-05	0.000187	1.2255275
RPL7A	-0.080623	7.2499602	-4.097219	5.14E-05	0.0001871	1.2249789
RP11-498C9	-0.464422	3.078643	-4.097101	5.14E-05	0.0001872	1.2245195
RP11-542B1	0.5433425	-0.95176	4.0964949	5.16E-05	0.0001876	1.2221596
RP11-390F4	-0.736837	0.2890673	-4.096431	5.16E-05	0.0001876	1.2219102
RP11-30K9.	-0.501122	2.4563331	-4.096254	5.16E-05	0.0001878	1.2212221
RP11-257A2	-0.193698	-1.477802	-4.096201	5.16E-05	0.0001878	1.221016
RBBP8NL	0.8496747	-0.339706	4.0961206	5.16E-05	0.0001878	1.2207032
RP11-166N1	0.6449511	1.443385	4.0956297	5.17E-05	0.0001882	1.2187936
ACVR2B-AS1	-0.209768	4.224499	-4.095552	5.18E-05	0.0001882	1.2184915
CACNA1A	0.4676836	3.0343768	4.0954373	5.18E-05	0.0001883	1.2180451
AMOTL1	0.2218152	5.9450422	4.0950948	5.19E-05	0.0001885	1.216713
RP11-114M5	-0.296852	-1.359625	-4.094935	5.19E-05	0.0001887	1.2160928
AP000640.2	-0.648557	-0.16073	-4.094793	5.19E-05	0.0001887	1.2155396
HAUS6	0.0915713	5.7335882	4.0947379	5.19E-05	0.0001888	1.2153248
NOTCH2	0.1032647	6.4449343	4.0945188	5.20E-05	0.0001889	1.2144729
RP11-513G1	-0.82214	3.7204735	-4.094381	5.20E-05	0.000189	1.2139381
MYL9	0.1497741	6.3641341	4.0939264	5.21E-05	0.0001893	1.2121689
CNN3	-0.084907	6.7171704	-4.093707	5.22E-05	0.0001895	1.2113148

LOX	0.2478797	5.3304454	4.093554	5.22E-05	0.0001896	1.2107211
RP11-977G1	-0.163211	4.8854668	-4.093407	5.22E-05	0.0001897	1.2101496
XXbac-BPG1	0.6313733	-0.606526	4.0933607	5.22E-05	0.0001897	1.2099696
C1QL2	-0.437717	-1.151782	-4.093151	5.23E-05	0.0001899	1.209154
RP11-264B1	0.444732	3.3709044	4.0931152	5.23E-05	0.0001899	1.209015
SH3BP4	-0.110351	6.4881143	-4.092779	5.24E-05	0.0001901	1.2077095
ANKS1A	0.0744964	5.9790146	4.0925825	5.24E-05	0.0001902	1.206944
DAP	-0.069211	6.8546911	-4.092113	5.25E-05	0.0001906	1.2051184
HMGN2P47	-0.766992	2.0890801	-4.091623	5.26E-05	0.000191	1.2032158
RP5-875H18	-0.500641	3.9434631	-4.091446	5.26E-05	0.0001911	1.2025273
CCDC78	0.4700462	3.5603978	4.0912991	5.27E-05	0.0001912	1.2019559
RBMX2	-0.075935	5.9072655	-4.091011	5.27E-05	0.0001914	1.2008355
RUSC2	0.0868806	6.0414822	4.0906837	5.28E-05	0.0001916	1.1995644
XYLT2	-0.068845	6.2135794	-4.090648	5.28E-05	0.0001916	1.1994238
UPK1A	0.8921325	1.8261595	4.0901734	5.29E-05	0.000192	1.1975816
UBAC1	-0.063425	6.1849333	-4.089999	5.30E-05	0.0001921	1.1969038
GSTM2P1	-0.381398	-1.236052	-4.089978	5.30E-05	0.0001921	1.1968232
IGHE	0.9598356	0.7288288	4.0899333	5.30E-05	0.0001921	1.1966486
RP11-48G14	-0.588193	-0.804934	-4.089867	5.30E-05	0.0001922	1.1963914
LMAN2L	-0.06242	6.1040141	-4.089763	5.30E-05	0.0001922	1.1959881
HLA-A	-0.095545	7.3718749	-4.089702	5.30E-05	0.0001922	1.1957505
RP11-1C1.4	0.7151247	-0.823359	4.0896932	5.30E-05	0.0001922	1.1957158
KAAG1	0.9181095	2.466464	4.0888973	5.32E-05	0.0001928	1.1926242
TMEM237	0.1154251	5.5095763	4.0888963	5.32E-05	0.0001928	1.1926203
RP11-602.3	0.6614021	2.5513099	4.0881527	5.34E-05	0.0001934	1.1897323
MCM8-AS1	-0.701943	0.0415488	-4.08812	5.34E-05	0.0001934	1.1896058
PDCD6	-0.058137	6.5169573	-4.087716	5.35E-05	0.0001937	1.1880363
LINGO4	-0.670853	3.7250147	-4.086888	5.36E-05	0.0001944	1.1848235
GPR119	0.626963	-0.936331	4.0868005	5.37E-05	0.0001944	1.184482
ZNF394	-0.065495	5.9332973	-4.086349	5.38E-05	0.0001948	1.1827276
RP11-431K2	-0.611751	2.1710572	-4.085609	5.39E-05	0.0001953	1.1798579
LRRC74A	0.6723791	-0.448639	4.0853468	5.40E-05	0.0001955	1.1788389
CTD-2003C8	0.5179153	-0.913028	4.0850626	5.40E-05	0.0001957	1.1777362
KLHDC9	-0.294063	5.2914986	-4.083984	5.43E-05	0.0001966	1.1735498
MIR100HG	-0.811865	2.695947	-4.083758	5.43E-05	0.0001967	1.172673
KRBA2	0.7173828	1.6830186	4.0834955	5.44E-05	0.0001969	1.1716554
PIF1	0.2898984	4.7580786	4.0831544	5.45E-05	0.0001972	1.1703322
RPS3A	-0.08276	6.8690107	-4.083104	5.45E-05	0.0001972	1.1701381
RP11-19P22	-0.179447	-1.483195	-4.082935	5.45E-05	0.0001973	1.1694796
SCML1	-0.115853	6.1151327	-4.082771	5.46E-05	0.0001975	1.1688446
KB-208E9.1	0.7778799	1.7581071	4.0822335	5.47E-05	0.0001979	1.1667601
RP11-126K1	0.4911692	2.4650283	4.0821044	5.47E-05	0.000198	1.1662597
WASL	-0.061513	6.5337784	-4.080808	5.50E-05	0.000199	1.1612305
HEBP2	-0.095452	6.4528792	-4.080748	5.50E-05	0.000199	1.1610012
CISH	-0.142892	6.1484617	-4.080654	5.50E-05	0.0001991	1.1606356
RP11-345M2	-0.751311	2.6660209	-4.080611	5.50E-05	0.0001991	1.1604684
SH3BGRL2	-0.124312	6.4352943	-4.080546	5.51E-05	0.0001991	1.1602161
RP11-794M8	-0.769644	-0.590185	-4.079871	5.52E-05	0.0001997	1.1575986
RP11-71E19	-0.778538	2.3445245	-4.0797	5.52E-05	0.0001998	1.1569354
KAT2A	-0.079769	6.4263627	-4.079671	5.53E-05	0.0001998	1.1568262
FXYD3	0.844903	3.7066708	4.0796653	5.53E-05	0.0001998	1.1568025

AP003068.9	-0.399117	3.1255477	-4.079533	5.53E-05	0.0001999	1.1562917
TMEM50A	-0.059919	6.5263233	-4.079036	5.54E-05	0.0002003	1.1543645
ATXN7L3	0.0619788	6.3520205	4.0789119	5.54E-05	0.0002003	1.1538825
CCDC61	-0.093777	5.7758209	-4.07876	5.55E-05	0.0002004	1.1532942
RIOK3	-0.061733	6.4750268	-4.078326	5.56E-05	0.0002008	1.1516123
RP11-791G1	-0.682623	0.8665271	-4.077433	5.58E-05	0.0002015	1.1481509
RP5-1057I2	0.61196	-0.487436	4.0772719	5.58E-05	0.0002016	1.1475279
MRPS31	-0.091755	5.9396341	-4.077095	5.58E-05	0.0002017	1.1468418
C16orf71	-0.262653	4.1021208	-4.076865	5.59E-05	0.0002019	1.1459502
PGLYRP4	0.6745368	-0.584323	4.0768089	5.59E-05	0.0002019	1.1457343
AL592549.1	-0.191017	-1.475437	-4.076794	5.59E-05	0.0002019	1.1456778
RP1-13D10.	0.6200133	-0.602798	4.0765577	5.60E-05	0.0002021	1.1447611
RP11-12A20	0.4320167	-1.095348	4.0764415	5.60E-05	0.0002022	1.144311
E2F5	0.1626647	5.3119821	4.0758307	5.61E-05	0.0002027	1.1419454
CPA6	0.9483169	0.9150772	4.075571	5.62E-05	0.0002029	1.1409397
RP4-613A2.	-0.573144	-0.674951	-4.075047	5.63E-05	0.0002033	1.1389089
PTGES3P3	0.4236933	3.6441511	4.0748877	5.64E-05	0.0002034	1.1382939
RP5-1050E1	-0.864363	0.0236098	-4.074473	5.65E-05	0.0002037	1.1366874
UHRF1	0.2757068	5.1623013	4.0742454	5.65E-05	0.0002039	1.1358067
GLTSCR2	-0.0849	6.923165	-4.074226	5.65E-05	0.0002039	1.1357322
SRSF8	-0.099086	6.0971618	-4.074028	5.66E-05	0.000204	1.1349665
MZF1-AS1	-0.175251	4.6912814	-4.07399	5.66E-05	0.000204	1.1348186
TRAV36DV7	0.6114116	-0.557651	4.0738714	5.66E-05	0.0002041	1.1343591
ZNF256	0.3297582	4.6119625	4.073776	5.66E-05	0.0002042	1.1339898
CCDC23	-0.1181	5.65587	-4.07303	5.68E-05	0.0002048	1.1311036
ROPN1B	-0.846667	2.4797088	-4.072689	5.69E-05	0.000205	1.1297823
UGT1A3	-0.798472	4.4563359	-4.072685	5.69E-05	0.000205	1.1297686
SBNO2	0.0688159	6.3192423	4.0715958	5.71E-05	0.0002059	1.1255521
TRPC3	0.8960988	0.4956674	4.0715146	5.71E-05	0.000206	1.1252378
TMSB4XP2	0.6465896	-0.314191	4.0714777	5.71E-05	0.000206	1.1250951
MIR8071-1	0.5916924	-0.850693	4.0709501	5.73E-05	0.0002064	1.1230539
SIAE	-0.139489	6.3089301	-4.070872	5.73E-05	0.0002065	1.1227507
IZUMO1R	0.5866425	-0.808604	4.0705898	5.74E-05	0.0002067	1.1216601
DPY19L3	0.0735353	5.8559749	4.0702859	5.74E-05	0.0002069	1.1204848
IMPDH2	-0.086233	6.6299937	-4.07019	5.75E-05	0.000207	1.1201119
DDHD2	0.1047929	5.8063442	4.0700832	5.75E-05	0.0002071	1.1197005
CCDC185	0.8857949	-0.037418	4.069769	5.76E-05	0.0002073	1.1184854
ERICH6	-0.685044	1.1537772	-4.069705	5.76E-05	0.0002073	1.1182368
PFKFB2	0.1316283	5.7778912	4.069554	5.76E-05	0.0002075	1.117654
C16orf47	0.7818705	0.9201267	4.0695001	5.76E-05	0.0002075	1.1174454
FUT2	0.487899	4.1608644	4.0693668	5.76E-05	0.0002076	1.1169301
PCYT1A	-0.0473	6.3653037	-4.06914	5.77E-05	0.0002077	1.1160528
NIN	0.0907135	6.0433895	4.0690482	5.77E-05	0.0002078	1.1156978
REP15	0.5973228	2.4359291	4.0684609	5.79E-05	0.0002083	1.1134268
AC093901.1	-0.576113	2.9800566	-4.068319	5.79E-05	0.0002084	1.1128798
SNORA5C	0.6568645	1.4989988	4.068126	5.79E-05	0.0002085	1.112132
PGBD4P7	0.402554	-1.168846	4.0679528	5.80E-05	0.0002087	1.1114625
RP11-293A2	-0.579011	2.7737067	-4.067882	5.80E-05	0.0002087	1.1111897
COMMD6	-0.097017	6.3589556	-4.067808	5.80E-05	0.0002087	1.1109012
CNTFR-AS1	0.7317972	-0.479173	4.0677976	5.80E-05	0.0002087	1.1108625
RP13-616I3	-0.614208	3.9479946	-4.067643	5.81E-05	0.0002088	1.1102667

TCTN3	-0.058693	6.263546	-4.067519	5.81E-05	0.0002089	1.1097845
RP11-160H2	-0.606618	2.1349562	-4.066592	5.83E-05	0.0002097	1.1062009
RP11-651P2	0.4615718	2.7330426	4.0662347	5.84E-05	0.00021	1.1048217
RNF10	-0.048021	6.7631261	-4.06606	5.84E-05	0.0002101	1.1041467
AREG	0.7133825	3.3068672	4.0659209	5.85E-05	0.0002102	1.103609
MKRN2OS	0.766019	2.4751431	4.0652327	5.86E-05	0.0002108	1.1009499
RTP5	0.8209983	-0.069589	4.0652299	5.86E-05	0.0002108	1.1009391
SPATC1L	0.4081198	5.004915	4.064858	5.87E-05	0.0002111	1.0995022
EFCAB5	0.6245854	1.5742694	4.0645311	5.88E-05	0.0002113	1.0982392
RP11-445P1	-0.855712	3.163212	-4.064471	5.88E-05	0.0002114	1.0980073
RP1-92014.	0.5475661	2.5343138	4.0644531	5.88E-05	0.0002114	1.0979381
MAGI1	-0.08197	6.1902813	-4.064411	5.88E-05	0.0002114	1.0977754
RP11-75C1C	0.6884969	1.154419	4.0641226	5.89E-05	0.0002116	1.0966614
RP11-66A2.	-0.898913	0.996457	-4.064024	5.89E-05	0.0002117	1.0962796
LINC01436	1.0365893	2.0157522	4.0636933	5.90E-05	0.0002119	1.0950032
SNURF	0.7581459	1.5733734	4.0634571	5.91E-05	0.0002121	1.0940909
TPST2	-0.086022	6.5649883	-4.063259	5.91E-05	0.0002123	1.0933278
CTD-2553L1	-0.38106	-1.166382	-4.062308	5.93E-05	0.0002131	1.0896535
CCDC93	0.0686543	6.0313403	4.0622774	5.93E-05	0.0002131	1.0895356
FKBP4	-0.076428	6.7315662	-4.061832	5.95E-05	0.0002135	1.087815
SPRY1	0.1298425	5.9032024	4.0617016	5.95E-05	0.0002136	1.0873126
NUP85	-0.060803	6.2517014	-4.06153	5.95E-05	0.0002137	1.0866491
TRBV13	0.649188	-0.622705	4.0613393	5.96E-05	0.0002138	1.0859139
RP1-309I22	0.6569433	3.111787	4.0612152	5.96E-05	0.0002139	1.0854348
RP11-131M6	-0.276796	-1.373189	-4.060944	5.97E-05	0.0002141	1.0843868
SNORD83A	0.6607766	0.4499205	4.0609285	5.97E-05	0.0002141	1.084328
NFKBIB	-0.083869	6.2548827	-4.060899	5.97E-05	0.0002141	1.0842145
PODXL	0.1278006	6.2300567	4.0602985	5.98E-05	0.0002146	1.0818966
RP1-506.6	0.6497142	-0.071298	4.059614	6.00E-05	0.0002152	1.0792551
SMU1	-0.04907	6.3665795	-4.058503	6.03E-05	0.0002162	1.0749675
DDX54	-0.05205	6.5471266	-4.057314	6.06E-05	0.0002172	1.0703841
LRRC37A15F	0.7469835	0.5395613	4.0568619	6.07E-05	0.0002176	1.0686386
GNPTAB	0.0742477	6.0518417	4.0563903	6.08E-05	0.000218	1.0668202
RP11-800A1	1.0097241	0.6087331	4.0560394	6.09E-05	0.0002183	1.065467
PCDHA11	0.957091	0.3770841	4.0559269	6.09E-05	0.0002184	1.0650332
C11orf80	0.1758812	5.298709	4.0550426	6.11E-05	0.0002191	1.0616241
GIMAP8	0.1365777	5.6517106	4.0550399	6.11E-05	0.0002191	1.0616139
TBXA2R	0.190626	4.9583841	4.0549235	6.12E-05	0.0002192	1.0611652
RP1-47A17.	0.6542783	-0.2367	4.0546476	6.12E-05	0.0002194	1.0601016
ALG1L6P	0.410809	3.1989313	4.054626	6.12E-05	0.0002194	1.0600181
IGSF22	0.3731483	3.5924791	4.054244	6.13E-05	0.0002197	1.0585459
AC011899.1	-0.363327	-1.376872	-4.053409	6.15E-05	0.0002205	1.0553277
HAR1A	0.8417261	1.4118321	4.0531072	6.16E-05	0.0002207	1.0541654
NBPF25P	0.3020572	4.1222269	4.0529143	6.17E-05	0.0002209	1.0534221
BCAS2	-0.062024	6.1609127	-4.052572	6.18E-05	0.0002211	1.0521032
AC013275.2	0.7803754	3.3899693	4.0525712	6.18E-05	0.0002211	1.0521001
RP11-491F9	-0.739916	-0.139903	-4.051584	6.20E-05	0.000222	1.0482985
PPP4R1L	0.2378333	4.5941622	4.0503375	6.23E-05	0.0002231	1.0434968
RP11-573G6	-0.568242	-0.641579	-4.050299	6.23E-05	0.0002231	1.0433475
CTC-459F4.	0.6087849	2.300647	4.0502653	6.23E-05	0.0002232	1.0432187
RP1-293L6.	-0.205261	-1.491505	-4.049778	6.25E-05	0.0002236	1.0413429

NGLY1	-0.05539	6.0974012	-4.049658	6.25E-05	0.0002237	1.0408797
OR2A1-AS1	0.4432902	4.2905011	4.0495191	6.25E-05	0.0002238	1.0403454
CNNM4	0.092015	5.7031086	4.0486312	6.28E-05	0.0002246	1.0369273
PDLIM1	-0.090999	6.7653459	-4.048537	6.28E-05	0.0002246	1.0365644
BATF	0.3627129	4.7868075	4.0482183	6.29E-05	0.0002249	1.0353383
COLCA1	0.4595645	4.4674919	4.0479508	6.29E-05	0.0002251	1.0343086
RP3-407E4.	-0.62409	-0.625818	-4.047785	6.30E-05	0.0002253	1.0336716
CTD-2269F5	0.71197	-0.012573	4.0475932	6.30E-05	0.0002254	1.0329326
TPSD1	0.9046238	0.5573639	4.0475567	6.30E-05	0.0002254	1.0327921
GALNT14	0.4473875	3.6286834	4.0464555	6.33E-05	0.0002264	1.0285547
NBPF14	0.1629566	5.3410946	4.046362	6.33E-05	0.0002265	1.028195
RP11-44F14	0.4170208	3.624615	4.046027	6.34E-05	0.0002268	1.0269062
PDILT	-0.708285	0.0125861	-4.045825	6.35E-05	0.0002269	1.0261297
RP11-350E1	0.8945012	0.828488	4.0456351	6.35E-05	0.0002271	1.0253989
LAD1	0.4059295	6.1320401	4.0454741	6.36E-05	0.0002272	1.0247795
RP11-123J1	-0.768742	0.5782499	-4.044738	6.38E-05	0.0002279	1.0219478
IFFO1	-0.082616	5.9931302	-4.043922	6.40E-05	0.0002286	1.018811
AF064858.1	0.7412903	0.5849523	4.0438655	6.40E-05	0.0002286	1.0185931
RP11-1267H	-0.555934	-0.891876	-4.043328	6.41E-05	0.0002291	1.0165272
PCDHA3	0.8905435	0.6300311	4.0424681	6.44E-05	0.0002299	1.013221
BRF2	0.2299864	5.2036929	4.0424578	6.44E-05	0.0002299	1.0131815
JAKMIP3	0.7856615	2.6288716	4.0424489	6.44E-05	0.0002299	1.013147
AC002310.1	-0.238769	4.0870071	-4.042316	6.44E-05	0.00023	1.0126361
LINC00888	0.218179	5.0470475	4.0419998	6.45E-05	0.0002303	1.0114209
PPP1R36	0.7212232	2.0019579	4.0418474	6.45E-05	0.0002304	1.0108352
FFAR4	0.4111118	3.5360948	4.0415405	6.46E-05	0.0002306	1.0096556
GLUD1	-0.108806	7.0844933	-4.041366	6.47E-05	0.0002308	1.0089862
EMID1	0.2752877	5.4346089	4.0412187	6.47E-05	0.0002309	1.008419
RP11-15B24	-0.229549	-1.43739	-4.0412	6.47E-05	0.0002309	1.0083478
C4orf22	-0.454795	-1.089294	-4.04107	6.47E-05	0.000231	1.0078478
AC246787.4	0.5234664	-1.01915	4.0408696	6.48E-05	0.0002312	1.0070778
RPL32P1	0.6072514	-0.428578	4.0400372	6.50E-05	0.0002319	1.0038795
DNAH17	0.2663459	4.3507395	4.0396124	6.51E-05	0.0002323	1.0022476
RP11-257I8	-0.151814	-1.503824	-4.03928	6.52E-05	0.0002326	1.0009692
SCARNA15	0.6602805	2.2761269	4.039264	6.52E-05	0.0002326	1.0009093
RP11-211G2	-0.301458	-1.379928	-4.039107	6.53E-05	0.0002327	1.0003049
GCA	0.1533508	5.6083288	4.0389098	6.53E-05	0.0002329	0.9995489
SEPW1	0.1457832	6.4240583	4.0388228	6.53E-05	0.0002329	0.9992146
CTD-3222D1	0.6820842	0.1812193	4.0382461	6.55E-05	0.0002335	0.9969999
RP11-732A1	0.5450561	-0.677128	4.0375505	6.57E-05	0.0002341	0.994329
RP11-307C1	-0.257749	3.7547479	-4.037215	6.58E-05	0.0002344	0.9930402
RP11-70D24	-0.479674	-0.959167	-4.036975	6.58E-05	0.0002346	0.9921182
ZNF845	0.298371	4.9273377	4.0369076	6.58E-05	0.0002347	0.9918606
TDGF1P4	-0.409587	-1.182024	-4.036513	6.59E-05	0.000235	0.990345
ZNF334	0.5819664	3.8774978	4.0364459	6.60E-05	0.0002351	0.9900884
RIC8A	-0.042786	6.5712819	-4.036132	6.60E-05	0.0002353	0.9888831
ABCC5	0.0776903	5.9272375	4.0360677	6.61E-05	0.0002354	0.9886366
A2ML1	0.7320179	-0.308664	4.0359889	6.61E-05	0.0002354	0.9883341
SLC25A34	-0.295527	4.641454	-4.035893	6.61E-05	0.0002355	0.9879676
CSNK1G3	-0.064747	6.0172104	-4.035493	6.62E-05	0.0002359	0.9864322
DTNB	-0.076334	5.7255895	-4.035362	6.63E-05	0.000236	0.98593

ARRDC3-AS1	0.5655157	2.2911761	4.0351245	6.63E-05	0.0002362	0.9850167
CTB-129P6.	0.6129343	1.2676975	4.0340074	6.66E-05	0.0002372	0.9807303
UBA2	0.0514938	6.4485389	4.0336257	6.67E-05	0.0002376	0.9792663
RP4-738P15	0.5471783	-0.573484	4.0333895	6.68E-05	0.0002378	0.97836
KCNAB1	0.1610838	4.6765752	4.0331904	6.68E-05	0.0002379	0.9775961
ZNF286B	0.4205228	3.2725253	4.0325994	6.70E-05	0.0002385	0.9753293
BCL10	0.0689406	5.7321812	4.0325286	6.70E-05	0.0002385	0.9750581
FAM114A2	-0.066242	5.9715074	-4.032515	6.70E-05	0.0002385	0.9750042
HLX-AS1	-0.728047	0.0403278	-4.031572	6.73E-05	0.0002394	0.9713879
FBXO9	-0.07038	6.3823213	-4.031541	6.73E-05	0.0002394	0.9712703
AC002511.2	0.6934625	-0.602708	4.0314969	6.73E-05	0.0002395	0.9711018
AC005077.8	-0.544842	-0.973103	-4.03131	6.74E-05	0.0002396	0.9703849
PCDHB5	0.6511855	3.8415147	4.0311772	6.74E-05	0.0002397	0.9698759
PDK3	0.1952386	5.0719742	4.030945	6.75E-05	0.0002399	0.9689856
SCAF1	-0.05312	6.5538791	-4.030826	6.75E-05	0.00024	0.9685313
CCL3L3	0.4401019	4.242228	4.029306	6.79E-05	0.0002415	0.962703
SIGLEC1	0.1864744	5.5671815	4.0292861	6.79E-05	0.0002415	0.9626269
CEP128	0.2447484	4.6980682	4.0290403	6.80E-05	0.0002417	0.9616851
ZGRF1	0.1742725	4.793023	4.0289921	6.80E-05	0.0002417	0.9615003
ANAPC1P1	0.5908606	-0.619466	4.0289454	6.80E-05	0.0002417	0.9613214
SLC25A5	-0.07003	7.0001853	-4.028872	6.80E-05	0.0002418	0.9610417
NLRP11	-0.882654	3.4863422	-4.028682	6.81E-05	0.000242	0.9603123
PM20D2	0.1534124	5.6863531	4.0282641	6.82E-05	0.0002423	0.9587108
PLAC9	0.2662526	4.8573572	4.0274821	6.84E-05	0.0002431	0.9557145
AF186192.6	0.6464946	-0.525969	4.027175	6.85E-05	0.0002434	0.9545381
RPS21	-0.096999	6.9668449	-4.026539	6.87E-05	0.000244	0.9521038
AC000123.2	0.1962859	4.4470142	4.026278	6.88E-05	0.0002442	0.9511026
C2orf88	0.2898746	4.6579155	4.0262308	6.88E-05	0.0002442	0.9509221
SNORD36C	0.5069565	-0.813129	4.026096	6.88E-05	0.0002444	0.9504054
CTC-512J14	0.4181067	2.4434508	4.0256648	6.89E-05	0.0002448	0.9487545
ZNF382	0.3920893	4.3120835	4.0256174	6.89E-05	0.0002448	0.9485729
TNFSF12-TN	0.4841287	-0.879743	4.0245297	6.92E-05	0.0002458	0.9444083
ABCC6P2	-0.23673	5.0994995	-4.024476	6.93E-05	0.0002459	0.944203
BRI3	-0.089743	6.7168749	-4.024438	6.93E-05	0.0002459	0.9440569
RP11-383J2	0.4101907	-1.151073	4.0241154	6.94E-05	0.0002462	0.9428222
MYH3	-0.262025	4.7592267	-4.023545	6.95E-05	0.0002467	0.9406397
ACOT1	-0.241354	5.5511195	-4.023363	6.96E-05	0.0002469	0.9399445
GDE1	-0.06412	6.2653442	-4.023319	6.96E-05	0.0002469	0.9397728
RP5-907C10	-0.952734	0.2847411	-4.023057	6.97E-05	0.0002471	0.9387712
POLR3G	-0.182169	4.8756178	-4.023045	6.97E-05	0.0002471	0.9387256
SCOC	-0.061771	6.3138113	-4.022804	6.97E-05	0.0002473	0.9378026
MFI2-AS1	0.4211437	3.8435116	4.0228018	6.97E-05	0.0002473	0.937795
LILRA5	0.3424905	4.0676632	4.0227434	6.98E-05	0.0002474	0.9375714
LIPH	0.8012006	3.4754483	4.022548	6.98E-05	0.0002475	0.9368237
GPN2	-0.059096	6.0862311	-4.022426	6.98E-05	0.0002476	0.9363571
GSTM5P1	-0.245893	-1.424055	-4.02224	6.99E-05	0.0002478	0.9356466
ADAMTS9	0.1503925	5.681282	4.0218812	7.00E-05	0.0002481	0.9342723
ST8SIA2	0.5951318	-0.777153	4.0218799	7.00E-05	0.0002481	0.9342673
C1RL	-0.098497	6.6946973	-4.021725	7.00E-05	0.0002483	0.9336753
PSMG2	-0.05539	6.3071246	-4.021462	7.01E-05	0.0002485	0.932669
MSLN	0.7125157	2.1167097	4.0213588	7.01E-05	0.0002486	0.9322737

FRMD6	0.1916941	5.5402414	4.0212364	7.02E-05	0.0002487	0.9318056
ZNF625	0.7240098	0.9201258	4.0210825	7.02E-05	0.0002488	0.9312168
YIF1B	-0.085224	6.3894101	-4.02088	7.03E-05	0.000249	0.930442
RP11-707A1	-0.71724	-0.726827	-4.02019	7.05E-05	0.0002497	0.9278028
RHEBP2	-0.723671	3.4877464	-4.01986	7.06E-05	0.00025	0.9265397
ACADSB	-0.138309	6.8087917	-4.019812	7.06E-05	0.00025	0.9263562
IRF9	0.1989516	5.0375022	4.0196861	7.06E-05	0.0002501	0.925876
TPRKB	-0.070226	5.9230931	-4.01955	7.07E-05	0.0002502	0.9253558
RP11-303E1	-0.623918	-0.116136	-4.019142	7.08E-05	0.0002506	0.9237965
RP11-80A15	-0.604772	2.8495262	-4.018606	7.09E-05	0.0002511	0.9217454
LINC00441	-0.619512	1.8638867	-4.018581	7.09E-05	0.0002511	0.9216493
RP11-466P2	-0.944232	1.2708859	-4.018329	7.10E-05	0.0002514	0.9206886
CLUAP1	-0.085217	5.678305	-4.018077	7.11E-05	0.0002516	0.9197258
CTA-228A9.	0.4182085	3.6113438	4.0179769	7.11E-05	0.0002517	0.9193413
TSG101	-0.047485	6.4579025	-4.017933	7.11E-05	0.0002517	0.919175
PAPPA2	-0.860744	4.2154394	-4.01724	7.13E-05	0.0002524	0.9165256
RP11-490D1	-0.397127	-1.196861	-4.017196	7.13E-05	0.0002524	0.9163548
CXCL9	0.274293	5.7681292	4.0171871	7.13E-05	0.0002524	0.9163227
OBSL1	0.2650588	6.1929608	4.0170847	7.14E-05	0.0002525	0.9159313
RNU1-133P	-0.14926	-1.504946	-4.016746	7.15E-05	0.0002528	0.9146387
PTGDR	0.5931772	2.687588	4.0162407	7.16E-05	0.0002533	0.912706
ACOXL	0.7497053	1.260606	4.0162378	7.16E-05	0.0002533	0.9126951
LAT	0.4201127	3.0313601	4.0160714	7.17E-05	0.0002534	0.9120591
COX4I2	-0.295013	4.6288448	-4.015783	7.18E-05	0.0002537	0.9109564
TMSB4X	0.0855234	7.1319466	4.0156627	7.18E-05	0.0002538	0.9104978
VMP1	-0.060748	6.7717088	-4.015572	7.18E-05	0.0002539	0.910151
DDX47	0.2140739	4.1112803	4.0154486	7.19E-05	0.000254	0.9096798
MIR378A	-0.61436	-0.472118	-4.015101	7.20E-05	0.0002543	0.9083532
TMX4	-0.070465	6.5279675	-4.014894	7.20E-05	0.0002545	0.9075618
CUTC	-0.088308	5.9269843	-4.014704	7.21E-05	0.0002546	0.9068359
FAM13B	0.0876707	5.7358122	4.0147036	7.21E-05	0.0002546	0.9068337
CTD-2537I9	0.6368056	1.9936311	4.01426	7.22E-05	0.0002551	0.9051396
GSK3B	-0.048953	6.3146891	-4.013839	7.23E-05	0.0002555	0.9035326
TRAM1L1	0.9277647	2.6947546	4.0137216	7.24E-05	0.0002556	0.9030831
LINC01559	0.9379398	0.1705131	4.0132659	7.25E-05	0.000256	0.9013432
RP11-326C3	0.7129474	1.9269695	4.0122027	7.28E-05	0.0002571	0.8972837
FBP1	-0.215792	6.7356094	-4.012165	7.28E-05	0.0002571	0.8971417
SEPT3	0.6036877	3.7394965	4.0121095	7.28E-05	0.0002571	0.8969279
TFAP2C	0.9751756	0.5905853	4.0120696	7.28E-05	0.0002572	0.8967756
SLC38A9	-0.081027	5.9790117	-4.011976	7.29E-05	0.0002572	0.8964176
PYY	-0.73027	1.236288	-4.011387	7.30E-05	0.0002578	0.8941713
CAPNS1	-0.067485	6.934407	-4.011323	7.31E-05	0.0002579	0.8939263
SNX30	0.0881965	5.9068099	4.0110533	7.31E-05	0.0002581	0.8928965
FASTKD2	-0.057144	6.1300513	-4.011024	7.32E-05	0.0002581	0.8927856
LEFTY2	0.6964111	-0.122647	4.0109762	7.32E-05	0.0002582	0.8926025
SAFB	-0.044316	6.5462743	-4.010258	7.34E-05	0.0002589	0.8898633
RN7SL381P	0.7094297	0.2337341	4.0098619	7.35E-05	0.0002593	0.8883501
AGAP5	0.6539068	1.9252315	4.0097938	7.35E-05	0.0002593	0.8880902
FAM208B	0.0820933	6.1204893	4.0095007	7.36E-05	0.0002596	0.8869723
SPCS3	-0.066477	6.5691711	-4.009315	7.37E-05	0.0002598	0.8862625
FAT2	0.7225792	1.935801	4.009106	7.37E-05	0.00026	0.8854663

PHYH	-0.122309	6.9092804	-4.008703	7.38E-05	0.0002604	0.8839284
TMED10	-0.059436	6.9371675	-4.008685	7.39E-05	0.0002604	0.8838607
SMLR1	-0.29513	6.1685177	-4.007692	7.41E-05	0.0002614	0.8800744
RP11-313A2	-0.584567	-0.431519	-4.007645	7.42E-05	0.0002614	0.8798954
AC003973.3	0.7941154	2.6335801	4.0074795	7.42E-05	0.0002616	0.8792627
COX5BP6	0.6758564	0.8333244	4.0074431	7.42E-05	0.0002616	0.879124
STRADA	0.1144698	5.3069592	4.0067218	7.44E-05	0.0002623	0.8763738
GLUD1P7	-0.345759	3.476921	-4.006542	7.45E-05	0.0002625	0.8756866
RP11-2B6.2	0.6414184	-0.347682	4.0063504	7.46E-05	0.0002627	0.8749575
GTF2H2B	0.6138797	2.8486578	4.005727	7.47E-05	0.0002633	0.872581
RP11-3B7.1	0.7309586	0.3590525	4.0052706	7.49E-05	0.0002638	0.8708416
IGHV1-17	0.4375332	-1.177725	4.0047726	7.50E-05	0.0002643	0.8689436
CTNNB1	-0.058914	6.9041173	-4.004215	7.52E-05	0.0002648	0.8668199
AC011893.3	0.445951	-1.079387	4.0040893	7.52E-05	0.000265	0.8663396
ZNF432	0.1350997	5.3547329	4.0038528	7.53E-05	0.0002652	0.8654385
RBL1	0.1463063	5.3470044	4.0030775	7.55E-05	0.000266	0.8624847
DLX5	0.9355617	0.9274091	4.0030291	7.56E-05	0.000266	0.8623004
SCARA3	0.2790459	5.7374576	4.0029327	7.56E-05	0.0002661	0.8619331
RAB2A	-0.071048	6.7586438	-4.002928	7.56E-05	0.0002661	0.8619151
C2orf69	-0.088523	5.8228721	-4.002916	7.56E-05	0.0002661	0.861871
ACER2	0.6346227	3.3909958	4.0028366	7.56E-05	0.0002661	0.8615669
DUS4L	-0.078364	5.5010628	-4.002232	7.58E-05	0.0002668	0.859264
CACNA2D4	0.241126	4.5404708	4.0020688	7.59E-05	0.0002669	0.8586423
DCAF10	-0.059887	6.2469478	-4.001932	7.59E-05	0.000267	0.858121
MED23	0.0898978	5.8549228	4.001766	7.60E-05	0.0002672	0.857489
ARL16	-0.094435	6.0335435	-4.001696	7.60E-05	0.0002672	0.857221
RP11-4C20.	0.4842615	-0.983982	4.0015524	7.60E-05	0.0002674	0.8566758
SAPCD2P1	-0.384512	-1.322996	-4.001148	7.61E-05	0.0002678	0.8551374
ACOT7	-0.084038	6.1683198	-4.000919	7.62E-05	0.000268	0.8542629
MTERF1	0.0847611	5.5802385	4.0007868	7.63E-05	0.0002681	0.8537603
MBD5	0.0827083	5.5890149	4.0006751	7.63E-05	0.0002682	0.853335
RP5-855D21	0.6695012	1.6552466	4.0006714	7.63E-05	0.0002682	0.8533212
KLHL26	-0.136901	5.5116819	-3.999957	7.65E-05	0.0002689	0.8506007
RP11-712B9	0.5478618	2.8479929	3.9997682	7.66E-05	0.0002691	0.8498823
AC011524.2	-0.213452	-1.454416	-3.999252	7.67E-05	0.0002697	0.8479178
RP11-231I1	0.6945208	0.1223411	3.998496	7.70E-05	0.0002705	0.8450404
ZFY-AS1	-0.994537	0.8890601	-3.998451	7.70E-05	0.0002705	0.8448709
RP11-306I1	-0.565164	-0.629622	-3.998352	7.70E-05	0.0002706	0.8444933
USP32P3	0.7478193	0.2101719	3.9982357	7.70E-05	0.0002707	0.8440497
MCCD1	1.1329253	0.99057	3.9980638	7.71E-05	0.0002708	0.8433959
CLP1	-0.061535	5.7289334	-3.997846	7.72E-05	0.000271	0.842566
ST6GAL1	-0.115968	7.195187	-3.996939	7.75E-05	0.000272	0.8391178
MIR3176	0.6392741	-0.202884	3.9968689	7.75E-05	0.000272	0.8388494
RNF31	0.1704378	5.6810496	3.9968686	7.75E-05	0.000272	0.8388485
RP11-676J1	1.0216473	1.605568	3.9966995	7.75E-05	0.0002722	0.8382051
RP11-602.2	0.4132045	-1.174895	3.9966902	7.75E-05	0.0002722	0.8381699
AC002551.1	-0.342312	-1.255099	-3.996473	7.76E-05	0.0002724	0.8373424
MED12	0.0716784	6.156814	3.9961796	7.77E-05	0.0002727	0.8362274
SYT6	0.7975239	0.5765427	3.9959223	7.78E-05	0.0002729	0.8352488
DAZAP2	-0.047515	6.853564	-3.995815	7.78E-05	0.000273	0.8348422
RP11-414C2	-0.795028	0.4413292	-3.995807	7.78E-05	0.000273	0.8348094

APOA1	-0.254471	7.6304322	-3.995739	7.78E-05	0.0002731	0.8345534
OSTC	-0.065186	6.5932076	-3.99543	7.79E-05	0.0002734	0.8333768
CTC-527H23	-0.67082	1.5302735	-3.995412	7.79E-05	0.0002734	0.8333081
bP-21264C1	-0.512142	2.8707611	-3.995353	7.79E-05	0.0002734	0.8330856
RP13-92302	0.3912149	3.160172	3.995235	7.80E-05	0.0002735	0.8326352
CHML	0.1763261	5.7177555	3.995081	7.80E-05	0.0002737	0.8320494
IRF2BP1	-0.07929	6.2840539	-3.994784	7.81E-05	0.000274	0.8309191
RP11-525G1	0.7481017	1.0760739	3.9947522	7.81E-05	0.000274	0.8307991
U82695.9	0.5553063	-0.938871	3.9946182	7.82E-05	0.0002741	0.8302898
CHFR	0.1010753	5.9169095	3.9945131	7.82E-05	0.0002742	0.82989
FOLR1	0.9981657	2.2868492	3.9944836	7.82E-05	0.0002742	0.8297781
COLCA2	0.4550501	4.4965919	3.9942713	7.83E-05	0.0002744	0.8289708
CLIP2	0.1927511	5.8032562	3.9939288	7.84E-05	0.0002748	0.8276687
AMD1	0.0660161	6.2857215	3.9935454	7.85E-05	0.0002752	0.8262112
RP11-701H1	0.6138674	-0.10654	3.9931285	7.87E-05	0.0002756	0.8246268
RP11-108K3	0.5342493	-0.973784	3.9929137	7.87E-05	0.0002758	0.8238101
RHOJ	0.1432588	5.3304466	3.992833	7.87E-05	0.0002759	0.8235035
RP5-1007F2	0.6916696	-0.449122	3.9926881	7.88E-05	0.000276	0.8229527
AGBL5	-0.080791	6.0577129	-3.992658	7.88E-05	0.000276	0.8228371
RAB7A	-0.042336	6.8823391	-3.992646	7.88E-05	0.000276	0.8227914
AC016716.2	-0.323816	-1.374857	-3.991548	7.92E-05	0.0002772	0.8186191
EDC4	0.1508402	5.9855543	3.9914521	7.92E-05	0.0002773	0.8182561
SEMA6C	-0.149944	5.9062437	-3.991384	7.92E-05	0.0002773	0.8179957
FUT3	0.7398546	2.8367386	3.9911842	7.93E-05	0.0002775	0.8172383
KDSR	-0.06357	6.4624718	-3.990679	7.94E-05	0.0002781	0.8153186
NPHP1	0.2625685	4.2371352	3.9906126	7.95E-05	0.0002781	0.8150668
DCDC2	0.687749	5.3240614	3.9904824	7.95E-05	0.0002782	0.8145721
RP11-455G1	-0.58226	-0.638245	-3.99048	7.95E-05	0.0002782	0.8145614
CTD-2313J1	-0.653898	0.0377277	-3.99026	7.96E-05	0.0002784	0.8137268
NAT9	-0.080903	6.1627251	-3.990208	7.96E-05	0.0002785	0.8135282
RASGRP2	0.2015462	5.2278959	3.990182	7.96E-05	0.0002785	0.8134311
CTD-2013N1	0.3918829	3.8533986	3.9899558	7.97E-05	0.0002787	0.812572
TPT1P8	-0.659047	-0.785004	-3.989812	7.97E-05	0.0002788	0.8120246
PRDX3P1	0.6989528	0.6457245	3.9894294	7.98E-05	0.0002792	0.8105728
KIF21B	0.243136	5.5086723	3.9892792	7.99E-05	0.0002794	0.8100024
EIF3KP1	0.5286581	-0.835471	3.9890453	8.00E-05	0.0002796	0.8091144
RP11-486G1	-0.415999	3.8986636	-3.988877	8.00E-05	0.0002798	0.8084743
RP11-1267H	-0.838312	2.3501054	-3.988835	8.00E-05	0.0002798	0.8083144
ARMC10	-0.054225	6.0509975	-3.988704	8.01E-05	0.0002799	0.8078189
PCDHGB2	0.7240528	3.4693363	3.9886658	8.01E-05	0.0002799	0.8076733
ABCB7	-0.072433	5.9993148	-3.98852	8.01E-05	0.0002801	0.8071186
HMGA1P8	0.4762602	-0.932723	3.9879221	8.03E-05	0.0002807	0.8048494
IL21R-AS1	0.5531693	-0.66778	3.9876299	8.04E-05	0.000281	0.8037403
ZNF607	0.3220556	4.6785997	3.9871593	8.06E-05	0.0002815	0.8019541
UBAC2-AS1	-0.23404	4.2783683	-3.987085	8.06E-05	0.0002816	0.8016708
C5orf60	-0.663881	0.10373	-3.98662	8.08E-05	0.0002821	0.7999069
OPN4	-0.654427	-0.592337	-3.986617	8.08E-05	0.0002821	0.7998977
ANKS4B	-0.413235	5.8942977	-3.986351	8.08E-05	0.0002824	0.7988869
SREK1IP1	-0.069521	6.0327869	-3.986185	8.09E-05	0.0002825	0.7982576
CST7	0.2537524	4.9201278	3.9861726	8.09E-05	0.0002825	0.7982092
CFAP74	0.5348752	2.7053744	3.9858755	8.10E-05	0.0002828	0.797082

RP11-806H1	0.8444557	1.8352713	3.9855903	8.11E-05	0.0002831	0.7959997
RP11-725G5	-0.514656	-0.775264	-3.985029	8.13E-05	0.0002837	0.7938689
TRIM23	-0.089052	5.7473919	-3.984715	8.14E-05	0.0002841	0.7926803
C17orf75	-0.060227	5.8907447	-3.984681	8.14E-05	0.0002841	0.7925501
PIGO	-0.068266	6.2523355	-3.98401	8.16E-05	0.0002848	0.7900064
RNF20	-0.057583	6.2505756	-3.983944	8.16E-05	0.0002849	0.7897531
GABRG1	0.7739878	-0.033038	3.9837172	8.17E-05	0.0002851	0.7888941
KXD1	-0.056849	6.4225644	-3.983651	8.17E-05	0.0002852	0.7886421
AK4	-0.205788	6.5853262	-3.983475	8.18E-05	0.0002853	0.7879747
KRTAP5-AS1	0.8673977	1.9372449	3.9833281	8.18E-05	0.0002854	0.7874183
SNORA66	0.6400094	1.1413984	3.9833269	8.18E-05	0.0002854	0.787414
TTC34	0.7478358	2.1804601	3.983228	8.19E-05	0.0002855	0.7870391
CTD-2012J1	0.6773426	0.0516586	3.9828958	8.20E-05	0.0002859	0.7857791
SIGLEC17P	0.634273	2.1173766	3.9825549	8.21E-05	0.0002863	0.7844866
LRRC37A2	0.4099759	4.3702435	3.9824489	8.21E-05	0.0002863	0.7840847
PMEL	-0.194674	5.3023278	-3.982362	8.22E-05	0.0002864	0.7837569
ZBED6	0.7634203	3.9448384	3.9816945	8.24E-05	0.0002872	0.7812244
RP11-377H2	-0.462164	-1.093801	-3.981205	8.25E-05	0.0002877	0.7793707
PTGFRN	0.1452157	6.2489896	3.9808142	8.27E-05	0.0002881	0.7778876
RP11-894J1	-0.496748	2.7146196	-3.980389	8.28E-05	0.0002886	0.7762751
OXER1	-0.197282	5.9117267	-3.980165	8.29E-05	0.0002888	0.7754279
MYH4	1.4018131	2.4093001	3.9799681	8.29E-05	0.000289	0.7746813
WSCD2	0.746974	0.5788652	3.979814	8.30E-05	0.0002892	0.7740975
AP000473.5	-0.710508	2.5479616	-3.979191	8.32E-05	0.0002899	0.7717384
LMOD2	-0.753805	-0.030444	-3.978483	8.34E-05	0.0002907	0.7690562
IGSF10	0.7894604	2.3701225	3.9783374	8.35E-05	0.0002908	0.768503
CARD17	0.688004	-0.142669	3.9782774	8.35E-05	0.0002909	0.7682759
RP11-1275H	-0.223627	4.697927	-3.978253	8.35E-05	0.0002909	0.7681838
KRT19P1	0.3919837	-1.194973	3.9781651	8.36E-05	0.0002909	0.7678504
RP11-730G2	0.3363899	4.7379745	3.9780135	8.36E-05	0.0002911	0.7672763
DTWD1	-0.063883	5.7231181	-3.977646	8.37E-05	0.0002915	0.7658856
IFI27	-0.249653	6.5621287	-3.977588	8.37E-05	0.0002915	0.7656646
RP11-118B1	-1.010052	1.9110997	-3.977323	8.38E-05	0.0002918	0.7646619
CACNA1C-AS	0.678603	0.2483402	3.9768859	8.40E-05	0.0002923	0.7630058
ADAMTSL1	0.3691189	4.6739041	3.9765397	8.41E-05	0.0002927	0.761695
MYPN	0.6708233	-0.204167	3.9760793	8.43E-05	0.0002932	0.7599517
RP11-53B2.	0.6683146	0.1821863	3.9755604	8.44E-05	0.0002938	0.7579871
TCEB3	-0.062046	6.3684704	-3.975094	8.46E-05	0.0002943	0.7562224
RP13-392I1	-0.695786	3.4291861	-3.974407	8.48E-05	0.0002951	0.7536227
GPS2P1	0.8808379	1.9800614	3.974222	8.49E-05	0.0002953	0.7529216
HS6ST1P1	0.7770286	2.5123396	3.9740887	8.49E-05	0.0002954	0.7524172
TMEM194A	0.1097643	5.7914838	3.973745	8.51E-05	0.0002958	0.7511166
PLP2	0.1698078	6.1247656	3.9736119	8.51E-05	0.0002959	0.7506127
KRT18P5	0.692113	1.0576589	3.9735065	8.51E-05	0.000296	0.7502139
PPIAP25	-0.451873	-0.999353	-3.973353	8.52E-05	0.0002962	0.749632
PLA2G12B	-0.453856	6.1120181	-3.973254	8.52E-05	0.0002963	0.7492582
CTD-2619J1	0.4677376	2.6694692	3.973189	8.52E-05	0.0002963	0.7490128
ZNF716	-1.059333	0.4125535	-3.97317	8.53E-05	0.0002963	0.7489419
TMEM78	-0.478177	-0.983264	-3.973145	8.53E-05	0.0002963	0.7488469
ZNF528-AS1	0.3259361	4.2175191	3.9726003	8.54E-05	0.000297	0.7467857
GOLGA8VP	0.6257963	-0.442529	3.97197	8.57E-05	0.0002977	0.7444012

ZNF776	0.113769	5.5628838	3.9719115	8.57E-05	0.0002977	0.7441799
HS3ST6	0.729883	-0.411865	3.9716975	8.58E-05	0.0002979	0.7433705
LINC01366	0.5711536	-0.625507	3.9712736	8.59E-05	0.0002984	0.7417674
WDR73	0.1245592	4.9033688	3.9710841	8.60E-05	0.0002986	0.7410509
RAB40B	-0.097577	5.9908812	-3.970766	8.61E-05	0.000299	0.7398469
KIF3A	0.1092259	5.3459745	3.9707544	8.61E-05	0.000299	0.739804
RP11-199F1	0.2991629	3.5930868	3.9707149	8.61E-05	0.000299	0.7396546
RP11-136F1	-0.349136	-1.287398	-3.970445	8.62E-05	0.0002993	0.7386357
RP1L1	0.6983593	0.5231622	3.9704401	8.62E-05	0.0002993	0.7386158
RP11-132A1	-0.41512	-1.184879	-3.969785	8.64E-05	0.0003	0.7361389
XRCC6	-0.052141	6.910653	-3.969776	8.64E-05	0.0003	0.7361035
IFNG	0.8589861	1.1747842	3.9697044	8.64E-05	0.0003001	0.735834
ZNF808	0.2774456	4.9609026	3.9696326	8.65E-05	0.0003001	0.7355627
AIF1	0.1587662	5.8192045	3.9693585	8.66E-05	0.0003004	0.7345267
AC016734.2	-0.312946	3.5922337	-3.969117	8.67E-05	0.0003007	0.7336132
RP4-694B14	-0.300411	4.0195649	-3.969069	8.67E-05	0.0003007	0.7334334
FOXO4	-0.091283	5.8966953	-3.968794	8.68E-05	0.000301	0.7323938
PSMG1	-0.078738	6.0407408	-3.968442	8.69E-05	0.0003014	0.7310628
CTD-2639E6	-0.298643	3.4429662	-3.968154	8.70E-05	0.0003017	0.729975
PLA2G4B	0.6520505	2.1625468	3.9678626	8.71E-05	0.0003021	0.728873
TMEM109	-0.05944	6.563237	-3.967674	8.72E-05	0.0003023	0.7281607
EIF5	-0.063715	6.837588	-3.967494	8.72E-05	0.0003025	0.7274793
AC098830.1	-0.847057	-0.161079	-3.967151	8.73E-05	0.0003028	0.7261859
SCEL	0.6286	-0.743534	3.9670584	8.74E-05	0.0003029	0.7258342
LINC01573	-0.415922	3.8552554	-3.967025	8.74E-05	0.0003029	0.7257066
SH2D5	0.8366154	1.3774281	3.9670231	8.74E-05	0.0003029	0.7257008
HARS2	-0.059884	6.1736408	-3.966413	8.76E-05	0.0003036	0.7233948
POLR2A	0.0640654	6.5707022	3.9662801	8.76E-05	0.0003038	0.7228942
AC092614.2	0.6068424	1.8710725	3.9654134	8.80E-05	0.0003048	0.7196204
FST	-0.249215	6.5006438	-3.965186	8.80E-05	0.000305	0.7187622
NCOA4	-0.070939	6.9413374	-3.965155	8.80E-05	0.0003051	0.7186458
KCNJ11	0.4775097	4.535637	3.9647412	8.82E-05	0.0003055	0.717082
ADH1A	-0.315923	6.8205345	-3.964469	8.83E-05	0.0003058	0.7160542
RN7SL255P	-0.432702	-1.097851	-3.964383	8.83E-05	0.0003059	0.7157299
ZNF503	-0.168471	5.8227314	-3.96402	8.84E-05	0.0003063	0.7143602
KRT40	0.4149666	-1.196333	3.9638681	8.85E-05	0.0003065	0.7137854
CAB39L	0.1161563	5.3332428	3.9637059	8.86E-05	0.0003067	0.7131732
C7orf50	-0.089393	6.460413	-3.963131	8.88E-05	0.0003073	0.7110016
ZNF736	0.2038239	5.1276323	3.9630711	8.88E-05	0.0003074	0.7107768
LINC01566	-0.205832	-1.473463	-3.962651	8.89E-05	0.0003079	0.7091918
KANTR	0.5585633	2.8630664	3.9617096	8.93E-05	0.000309	0.7056384
AMZ1	0.7765318	2.4332208	3.9615894	8.93E-05	0.0003091	0.7051849
RP11-713N1	0.5892686	-0.519434	3.961398	8.94E-05	0.0003093	0.7044625
LL22NC03-7	0.4823298	-0.927981	3.9609596	8.95E-05	0.0003099	0.7028085
SGPL1	-0.070071	6.4993835	-3.960325	8.98E-05	0.0003106	0.700413
TRIM47	0.1406658	6.110446	3.9601907	8.98E-05	0.0003108	0.6999081
RP3-380B4.	-0.471786	-1.171169	-3.959654	9.00E-05	0.0003114	0.6978834
SLC12A6	0.1078764	5.6403866	3.9596069	9.00E-05	0.0003114	0.697706
FAM13A	-0.203614	5.7504287	-3.959469	9.01E-05	0.0003116	0.6971868
HDHD1	0.0954474	5.8586157	3.9590678	9.02E-05	0.000312	0.6956728
DNAH8	0.82372	1.7352502	3.9588824	9.03E-05	0.0003122	0.6949736

PDGFD	0.2059681	5.4191476	3.9584658	9.04E-05	0.0003127	0.6934026
ALG1L13P	0.7734763	2.1656734	3.9583239	9.05E-05	0.0003129	0.6928679
C16orf74	0.4268172	3.6223883	3.9582815	9.05E-05	0.0003129	0.6927078
IGHV3-60	0.5360356	-0.96087	3.9577942	9.07E-05	0.0003135	0.6908707
PRODH2	-0.476255	6.2565025	-3.957777	9.07E-05	0.0003135	0.6908046
GATA1	0.6486357	-0.232019	3.9577081	9.07E-05	0.0003135	0.6905459
AC007318.5	0.2748852	4.5293487	3.9575442	9.08E-05	0.0003137	0.6899284
RNF43	-0.268631	5.9717994	-3.957363	9.08E-05	0.0003139	0.6892433
PTPRG-AS1	0.7779991	1.9427674	3.9572255	9.09E-05	0.0003141	0.6887266
C10orf54	0.1147504	6.0537566	3.9571842	9.09E-05	0.0003141	0.688571
PCDHA12	0.9019832	0.0616881	3.9561187	9.13E-05	0.0003154	0.6845551
RP1-80N2.3	-0.385263	4.0242971	-3.955948	9.14E-05	0.0003156	0.6839108
BPIFA2	0.9729309	0.3240498	3.9558529	9.14E-05	0.0003157	0.6835534
HERC2P3	0.7286003	3.660626	3.9556995	9.15E-05	0.0003158	0.6829756
RP11-287D1	0.6220786	-0.203656	3.9554735	9.15E-05	0.0003161	0.6821238
RP11-611L7	0.689217	0.7937464	3.9552621	9.16E-05	0.0003163	0.6813273
CTD-2228K2	-0.211589	-1.466398	-3.955142	9.17E-05	0.0003165	0.6808765
CTD-2240J1	-0.703316	0.4639564	-3.954911	9.17E-05	0.0003167	0.6800052
AC096579.1	0.4849804	-1.072472	3.9546222	9.19E-05	0.0003171	0.6789167
U47924.6	0.2658609	4.3802795	3.9542389	9.20E-05	0.0003175	0.6774729
AC083900.1	-0.494814	3.4250222	-3.954099	9.20E-05	0.0003177	0.6769452
CTD-301801	0.6763113	-0.082589	3.9539548	9.21E-05	0.0003178	0.6764025
PCGF3	0.0588721	6.1763525	3.9528427	9.25E-05	0.0003192	0.672214
NNT	-0.111109	6.7041568	-3.952639	9.26E-05	0.0003194	0.6714478
TRIM44	-0.05092	6.4636561	-3.952509	9.26E-05	0.0003196	0.6709563
LRRC3C	-0.692943	-0.226885	-3.952482	9.26E-05	0.0003196	0.670856
AC011515.2	0.5468011	-0.719161	3.95197	9.28E-05	0.0003202	0.6689283
TTN	0.2830925	4.7528066	3.9515428	9.30E-05	0.0003207	0.6673198
CTD-2256P1	-0.783399	0.2238524	-3.951387	9.30E-05	0.0003209	0.6667349
MTND6P4	-0.554482	3.1438976	-3.951192	9.31E-05	0.0003211	0.666001
RP11-115J1	-0.347328	-1.244066	-3.950741	9.33E-05	0.0003217	0.6643027
CCDC125	-0.082721	6.1422315	-3.949912	9.36E-05	0.0003227	0.6611832
GGTLC1	0.7550053	0.2466622	3.9495248	9.37E-05	0.0003232	0.6597249
CTD-2506P8	-0.379386	3.2720336	-3.949517	9.38E-05	0.0003232	0.6596952
RP11-248B2	0.7161604	-0.41227	3.9493731	9.38E-05	0.0003233	0.6591542
AARD	0.6566016	-0.392499	3.9492949	9.38E-05	0.0003234	0.6588599
RP11-400F1	0.6957335	2.1801331	3.949245	9.39E-05	0.0003234	0.6586721
TUBA8	0.6433873	2.2835399	3.9491357	9.39E-05	0.0003235	0.6582607
LINC01001	0.660634	2.2474379	3.9489508	9.40E-05	0.0003237	0.657565
RP11-379F4	-0.655019	0.9630218	-3.948636	9.41E-05	0.0003241	0.6563816
AL133243.2	0.2619238	3.3464684	3.9485735	9.41E-05	0.0003242	0.6561458
POLR2K	-0.080911	6.4095405	-3.948165	9.43E-05	0.0003247	0.6546074
RP11-521M1	-0.901594	3.2253055	-3.94806	9.43E-05	0.0003247	0.6542147
POLR2C	-0.073411	6.4429007	-3.948058	9.43E-05	0.0003247	0.6542069
ZFYVE1	-0.073749	6.0425027	-3.947781	9.44E-05	0.0003251	0.6531642
SPRED2	0.0899156	6.1373937	3.9475665	9.45E-05	0.0003253	0.6523579
CBLN2	0.6381281	-0.777677	3.9474308	9.45E-05	0.0003255	0.6518477
RP11-676J1	0.5594225	-0.460999	3.9474117	9.45E-05	0.0003255	0.6517756
EDARADD	0.6292871	3.5158151	3.9473982	9.45E-05	0.0003255	0.651725
SLC52A3	0.5809004	3.9177738	3.9472505	9.46E-05	0.0003256	0.6511693
GP1BA	0.3351599	3.8171726	3.9471802	9.46E-05	0.0003257	0.650905

PDZK1	-0.198259	6.4221579	-3.947056	9.47E-05	0.0003258	0.6504392
PRCC	-0.062926	6.5461067	-3.946895	9.47E-05	0.000326	0.6498326
GS1-600G8.	0.7394867	-0.501211	3.9468924	9.47E-05	0.000326	0.6498229
RN7SL23P	0.5791352	-0.317707	3.9466702	9.48E-05	0.0003262	0.6489874
FAM219B	0.1205974	5.2929611	3.9464695	9.49E-05	0.0003265	0.6482327
RP11-506H2	-0.678929	0.7226851	-3.946277	9.50E-05	0.0003267	0.6475076
KIAA1715	-0.079782	6.155567	-3.946085	9.50E-05	0.0003269	0.6467883
GFOD1-AS1	-0.4289	-1.038934	-3.94532	9.53E-05	0.0003279	0.6439118
AC137932.5	-0.491603	3.1684078	-3.944649	9.56E-05	0.0003287	0.6413871
RP6-22P16.	-0.21759	-1.436768	-3.944559	9.56E-05	0.0003288	0.6410489
RP11-6B6.3	0.6118901	0.1975315	3.9444648	9.57E-05	0.0003289	0.6406964
BCL2L2-PAE	0.557734	2.3700527	3.944422	9.57E-05	0.0003289	0.6405357
OXCT2	0.7864162	2.4576327	3.9441681	9.58E-05	0.0003292	0.6395816
RN7SKP44	-0.293455	-1.34687	-3.944089	9.58E-05	0.0003293	0.6392855
LINC00702	0.4972775	3.4588875	3.9437925	9.59E-05	0.0003297	0.63817
PLEK2	0.5182593	4.9442669	3.9437593	9.59E-05	0.0003297	0.6380452
EARS2	-0.077689	6.1928333	-3.943702	9.60E-05	0.0003297	0.6378294
RP5-1182A1	-0.946814	1.9186179	-3.943634	9.60E-05	0.0003298	0.6375732
ENTPD4	0.090372	5.943353	3.943119	9.62E-05	0.0003304	0.6356391
CTD-2047H1	0.7103827	0.4136341	3.9430516	9.62E-05	0.0003305	0.635386
TRBJ2-2P	0.4854711	-0.915184	3.9427053	9.63E-05	0.0003309	0.634085
FAM84A	0.3750974	4.584085	3.9418659	9.67E-05	0.000332	0.6309318
RP11-875H7	-0.526203	-0.977856	-3.941705	9.67E-05	0.0003322	0.6303278
C6orf163	0.5279614	2.6434601	3.941418	9.68E-05	0.0003325	0.6292496
RP11-77K12	-0.462687	3.6095327	-3.941328	9.69E-05	0.0003326	0.6289114
C4orf45	-0.497706	-0.863915	-3.940615	9.72E-05	0.0003335	0.6262341
LGALSL	0.0863734	5.8390431	3.9404981	9.72E-05	0.0003337	0.6257951
LEPR	-0.254007	6.4847345	-3.940279	9.73E-05	0.0003339	0.6249709
KLHL10	-0.648465	0.8409386	-3.940217	9.73E-05	0.000334	0.6247396
RP11-496H1	0.7192061	2.3196972	3.9400175	9.74E-05	0.0003342	0.6239907
RP11-77H9.	-0.714717	0.8865697	-3.939915	9.74E-05	0.0003343	0.6236071
RDH12	0.4068628	4.7431155	3.9385438	9.80E-05	0.0003361	0.6184587
CLEC6A	0.5089631	-0.981188	3.9384932	9.80E-05	0.0003362	0.6182687
EMC1	-0.061447	6.3417986	-3.938353	9.80E-05	0.0003363	0.617743
FHIT	-0.12895	5.7573529	-3.937941	9.82E-05	0.0003368	0.6161963
CNP	-0.052642	6.6304388	-3.937603	9.83E-05	0.0003373	0.614928
PCNXL4	0.0805786	5.9831605	3.937145	9.85E-05	0.0003378	0.6132096
GRIPAP1	-0.047449	6.3503995	-3.937076	9.85E-05	0.0003379	0.6129489
RP11-126L1	0.681548	1.8013718	3.9370258	9.86E-05	0.0003379	0.6127624
JUNB	0.1166795	6.5606795	3.936885	9.86E-05	0.0003381	0.6122343
EGR4	0.6991003	-0.278854	3.9361511	9.89E-05	0.0003391	0.6094811
PRKG1-AS1	0.7505395	-0.389921	3.9357948	9.90E-05	0.0003395	0.6081444
USP47	-0.051049	6.4884735	-3.93576	9.91E-05	0.0003395	0.6080154
RBP1	0.3208721	5.9332096	3.9356873	9.91E-05	0.0003396	0.6077414
PQLC2	-0.086211	6.084198	-3.93533	9.92E-05	0.00034	0.6064018
WASF3	0.4861796	4.9545061	3.9353285	9.92E-05	0.00034	0.6063958
NOTUM	-0.579047	5.3895931	-3.934869	9.94E-05	0.0003406	0.6046731
GTF2IRD1P1	-0.699326	1.6088301	-3.934101	9.97E-05	0.0003416	0.6017921
POM121	0.1318739	6.0024431	3.9340637	9.97E-05	0.0003416	0.601653
IGKV2-26	0.4874301	-1.121333	3.9339711	9.98E-05	0.0003417	0.6013058
RASD2	0.3288248	4.3734218	3.9338274	9.98E-05	0.0003419	0.600767

RP11-925D8	-0.41076	-1.118016	-3.933401	1.00E-04	0.0003425	0.5991679
RP3-407E4.	-0.732751	-0.284435	-3.933375	0.0001	0.0003425	0.5990716
RP11-248E9	-0.780743	-0.165945	-3.932975	0.0001002	0.000343	0.5975729
RP3-510D11	-0.29608	4.0333068	-3.932621	0.0001003	0.0003434	0.596243
C4orf50	0.5748889	-0.593488	3.9321424	0.0001005	0.000344	0.594451
EXD2	0.0789502	5.7832242	3.9321084	0.0001005	0.0003441	0.5943235
COQ3	-0.098652	5.6473778	-3.931977	0.0001006	0.0003442	0.5938302
SIX3	0.7064964	-0.74092	3.9317527	0.0001007	0.0003445	0.5929906
RN7SL578P	-0.17595	-1.477979	-3.931059	0.0001009	0.0003454	0.5903926
SIGLEC12	0.6839254	3.2136192	3.9306911	0.0001011	0.0003459	0.5890131
OR2H1	0.5715691	-0.991633	3.9306627	0.0001011	0.0003459	0.5889067
RP11-284B1	-0.751254	1.1912465	-3.930389	0.0001012	0.0003462	0.5878806
RP11-313D6	0.6316472	0.4876184	3.9301359	0.0001013	0.0003466	0.5869332
ZNF223	0.3113112	3.7600762	3.9300064	0.0001014	0.0003467	0.5864482
HCAR1	0.7629528	1.3576522	3.9299179	0.0001014	0.0003468	0.586117
RP11-405F3	-0.586339	-0.661715	-3.929787	0.0001014	0.0003469	0.5856262
CTD-260009	0.5621145	2.9445747	3.9294555	0.0001016	0.0003474	0.584385
AC083899.3	0.2192121	4.2723425	3.9291833	0.0001017	0.0003477	0.5833655
IER3IP1	-0.058793	6.3500084	-3.928942	0.0001018	0.000348	0.5824613
LINC01521	0.5041834	3.336497	3.9285819	0.0001019	0.0003485	0.5811133
SNX18P26	-0.641675	-0.668195	-3.928545	0.000102	0.0003485	0.5809735
AC011747.4	1.0095255	1.4057632	3.926953	0.0001026	0.0003507	0.5750155
ZMYM2	0.0690793	6.1650655	3.9264648	0.0001028	0.0003513	0.5731879
LINC00390	-0.728816	0.288926	-3.926458	0.0001028	0.0003513	0.5731642
RP4-533D7.	-0.754895	0.8188833	-3.926155	0.0001029	0.0003517	0.572028
ATP6V1C2	0.1692985	4.540279	3.9258809	0.000103	0.0003521	0.5710029
WDR13	-0.094788	6.6701422	-3.925853	0.0001031	0.0003521	0.5708976
C2orf71	-0.586235	-0.974482	-3.925593	0.0001032	0.0003524	0.5699248
RP11-12K6.	-0.445095	-1.228563	-3.925555	0.0001032	0.0003524	0.569785
RP11-661A1	-0.438841	-0.968576	-3.924986	0.0001034	0.0003532	0.5676554
CSN1S2AP	-0.230955	-1.41037	-3.924261	0.0001037	0.0003542	0.5649431
RTF1	-0.043892	6.3295325	-3.924163	0.0001037	0.0003543	0.564574
RPS27A	-0.076858	7.0688592	-3.924035	0.0001038	0.0003544	0.5640988
PPAN	-0.118426	5.425654	-3.923942	0.0001038	0.0003545	0.5637501
DRAM1	0.1015445	5.8503267	3.923566	0.000104	0.000355	0.5623428
UBXN2B	-0.094272	6.2228999	-3.923473	0.000104	0.0003551	0.5619968
CCL15-CCL1	-0.467094	4.5656989	-3.923371	0.0001041	0.0003552	0.5616129
ADM5	0.3538251	3.3674652	3.9230061	0.0001042	0.0003557	0.5602489
RP11-124G5	-0.14926	-1.504946	-3.922624	0.0001044	0.0003562	0.5588199
ZNF550	0.2364031	5.1969809	3.9223267	0.0001045	0.0003566	0.5577082
TLR4	0.1652598	5.4164902	3.9223176	0.0001045	0.0003566	0.5576744
RP13-270P1	-0.206052	4.5881748	-3.921962	0.0001047	0.0003571	0.5563461
NSUN6	-0.104881	6.0399695	-3.921466	0.0001049	0.0003577	0.5544903
NOP16	-0.105621	6.0428492	-3.921154	0.000105	0.0003582	0.5533261
GBP7	-0.552577	5.5055375	-3.920815	0.0001051	0.0003586	0.552056
TRAK1	0.0612206	6.1946012	3.9206976	0.0001052	0.0003587	0.5516185
AC006273.5	0.7157748	2.9435466	3.9204091	0.0001053	0.0003591	0.5505403
NPRL3	-0.070718	6.2027029	-3.919937	0.0001055	0.0003598	0.5487772
RORA-AS1	0.6397614	1.3903103	3.9198331	0.0001056	0.0003599	0.5483875
DEFB132	-1.039042	2.3146225	-3.919794	0.0001056	0.0003599	0.5482398
TOMM20	-0.06565	6.8991458	-3.919693	0.0001056	0.00036	0.5478625

RP11-572M1	0.6212353	0.8003778	3.9195963	0.0001057	0.0003601	0.5475028
MIR4524B	-0.456264	-0.994471	-3.919277	0.0001058	0.0003605	0.5463111
ITGB6	0.8315985	2.8998541	3.9188954	0.0001059	0.0003611	0.544884
RNF180	0.3726167	4.7505182	3.9185439	0.0001061	0.0003615	0.5435708
LINC00511	0.706215	4.1543693	3.9177933	0.0001064	0.0003626	0.5407674
RP11-696N1	-0.270314	4.602763	-3.917728	0.0001064	0.0003626	0.5405236
RP1-68D18.	-0.332516	-1.287016	-3.917651	0.0001065	0.0003627	0.5402339
STIL	0.1844249	5.1584932	3.9173852	0.0001066	0.0003631	0.539243
C1QL4	0.923442	1.4377566	3.917367	0.0001066	0.0003631	0.5391752
GANC	-0.090848	5.7489791	-3.916047	0.0001072	0.0003649	0.5342453
RP1-232L22	0.5604026	3.2774814	3.9150018	0.0001076	0.0003664	0.5303443
MYBPHL	0.9403856	0.7785135	3.9145278	0.0001078	0.0003671	0.5285754
RBM14	-0.037459	6.2563298	-3.914426	0.0001079	0.0003672	0.5281966
RP11-886P1	-0.529449	-0.655121	-3.91437	0.0001079	0.0003672	0.5279869
FHL3	0.1180078	5.755063	3.9143508	0.0001079	0.0003672	0.5279147
RBBP9	-0.097132	6.1629919	-3.91434	0.0001079	0.0003672	0.5278747
RP11-114G1	-0.91063	0.6444012	-3.914064	0.000108	0.0003676	0.5268442
ZNRD1	-0.097137	5.931075	-3.913984	0.000108	0.0003677	0.5265441
AC083949.1	0.6828505	0.3771411	3.9139646	0.000108	0.0003677	0.5264737
CTC-451A6.	-0.467163	-1.086964	-3.913302	0.0001083	0.0003686	0.5240005
TIMMDC1	-0.049883	6.3602632	-3.912574	0.0001086	0.0003696	0.521284
RP11-783K1	0.351637	3.4718394	3.9118434	0.000109	0.0003707	0.5185598
DCLRE1CP1	0.5388558	-0.70937	3.9114076	0.0001092	0.0003713	0.5169348
RP11-574K1	0.5207642	-0.674593	3.9113495	0.0001092	0.0003713	0.516718
LCLAT1	-0.090199	5.8465962	-3.910232	0.0001097	0.0003729	0.51255
PNKD	-0.076011	6.8378106	-3.909818	0.0001098	0.0003735	0.5110076
RP11-1084J	-0.429441	-1.135461	-3.909461	0.00011	0.000374	0.5096754
OLMALINC	-0.18599	5.5457072	-3.909389	0.00011	0.0003741	0.5094071
SERPINA4	-0.404411	6.6704311	-3.908715	0.0001103	0.0003751	0.5068956
EOGT	0.0860873	5.5774126	3.9086534	0.0001104	0.0003751	0.5066664
HMGB3P24	0.6199049	1.1419161	3.9084016	0.0001105	0.0003754	0.5057282
RP11-1020A	-0.108199	4.776614	-3.908394	0.0001105	0.0003754	0.5056999
RP5-1065J2	-0.218488	4.3377961	-3.908103	0.0001106	0.0003758	0.5046139
CTA-253N17	0.5198212	2.8021642	3.907985	0.0001106	0.000376	0.5041756
APCS	-0.276631	7.0632105	-3.907973	0.0001107	0.000376	0.5041326
RP11-159H1	0.6925448	-0.309019	3.9073871	0.0001109	0.0003768	0.5019478
PTBP2	0.0923371	5.6313532	3.9072822	0.000111	0.0003769	0.5015568
MS4A8	0.9512139	0.0476755	3.9072784	0.000111	0.0003769	0.5015428
EWSAT1	0.7027144	2.4263433	3.9072583	0.000111	0.0003769	0.5014678
MTND1P15	-0.239303	-1.416065	-3.906191	0.0001114	0.0003785	0.4974933
CTD-319301	-0.628938	0.8479719	-3.906117	0.0001115	0.0003785	0.4972159
RP11-420K1	0.6136015	0.0938393	3.9060477	0.0001115	0.0003786	0.4969583
RP11-675F6	0.5524342	-0.954058	3.9056896	0.0001117	0.0003791	0.4956243
BRD7	-0.064952	6.2651114	-3.904511	0.0001122	0.0003808	0.4912337
HORMAD2-AS	-1.11857	3.7371252	-3.904165	0.0001123	0.0003813	0.4899471
CTD-2116N2	-0.240602	-1.43586	-3.90409	0.0001124	0.0003814	0.4896695
SNX1	-0.053096	6.5773506	-3.903767	0.0001125	0.0003819	0.488466
DNMT3L	-0.956175	2.0224306	-3.903493	0.0001126	0.0003822	0.4874465
TTF1	0.0676371	5.5134637	3.9031938	0.0001128	0.0003827	0.4863319
RP11-122C5	0.4125199	-1.105597	3.9028333	0.0001129	0.0003832	0.4849899
RP11-830F9	0.5181233	-0.853816	3.9025177	0.0001131	0.0003836	0.4838153

RP11-400K9	0.8694365	1.5104495	3.9023517	0.0001132	0.0003838	0.4831977
KB-1507C5.	0.7053236	0.5442114	3.9019932	0.0001133	0.0003844	0.4818636
ZNF497	-0.132701	4.7480817	-3.90192	0.0001133	0.0003844	0.4815902
SLC39A7	-0.062608	6.8773784	-3.900156	0.0001141	0.0003871	0.4750275
C19orf25	-0.084643	6.1299403	-3.900055	0.0001142	0.0003872	0.4746533
SEPT8	0.0645969	6.2133846	3.8998273	0.0001143	0.0003875	0.4738062
SLC17A4	-0.316169	6.1941542	-3.899698	0.0001143	0.0003877	0.4733259
INSIG2	-0.104562	6.2512994	-3.899083	0.0001146	0.0003886	0.4710384
CSN3	-0.552433	-1.058634	-3.898865	0.0001147	0.0003889	0.4702259
MDM1	0.1300223	5.2615905	3.8986638	0.0001148	0.0003892	0.4694795
RP11-3B12.	0.3615895	-1.180546	3.8979418	0.0001151	0.0003903	0.4667949
RP11-448A1	-0.134247	4.7620514	-3.897891	0.0001152	0.0003903	0.466606
RP11-480A1	0.402112	3.4926372	3.8976797	0.0001153	0.0003906	0.4658207
CTC-236F12	0.7867334	1.0792996	3.8969884	0.0001156	0.0003916	0.4632512
LSM2	-0.087489	6.1670447	-3.896977	0.0001156	0.0003916	0.4632081
GALC	0.185272	5.6295795	3.8968069	0.0001157	0.0003918	0.4625766
TCEAL1	-0.113931	5.7766644	-3.896539	0.0001158	0.0003922	0.4615825
RP1-167G20	-0.237807	-1.409102	-3.896442	0.0001158	0.0003923	0.4612206
SUPT7L	-0.038463	6.2052877	-3.896076	0.000116	0.0003929	0.4598587
KRI1	-0.053455	6.1274113	-3.895982	0.000116	0.000393	0.4595123
RP11-218D6	-0.149327	-1.498289	-3.89593	0.0001161	0.000393	0.4593182
PPP1R27	-0.739778	1.3266949	-3.895897	0.0001161	0.000393	0.4591957
MT-RNR2	-0.086386	7.8552253	-3.89588	0.0001161	0.000393	0.4591308
ZMYND15	0.2149367	5.3227736	3.8954007	0.0001163	0.0003937	0.4573512
IL23R	0.7319748	0.3322879	3.8951028	0.0001165	0.0003942	0.4562445
LPA	-0.522445	5.6234737	-3.8948	0.0001166	0.0003946	0.455119
AC004012.1	-0.735611	2.3231462	-3.893856	0.000117	0.000396	0.4516131
OSGEPL1	-0.074292	5.5850843	-3.893286	0.0001173	0.0003969	0.4494953
ELMOD3	-0.059711	5.9011387	-3.892836	0.0001175	0.0003976	0.4478246
UBE2L2	-0.22592	-1.460102	-3.892675	0.0001176	0.0003978	0.4472265
FMR1	-0.083564	6.4139725	-3.892381	0.0001177	0.0003982	0.446137
TDRD3	-0.076892	5.8094231	-3.89236	0.0001177	0.0003982	0.4460594
KCNAB2	0.1927563	5.8962071	3.8922809	0.0001178	0.0003983	0.4457645
SCIN	0.6896637	3.0668792	3.8916083	0.0001181	0.0003993	0.4432674
ZNF460	0.3884767	3.7176836	3.8913246	0.0001182	0.0003997	0.4422146
RBFA	-0.086749	6.058104	-3.891157	0.0001183	0.0004	0.4415917
PPID	-0.071587	6.2582873	-3.890577	0.0001186	0.0004008	0.4394406
AC012499.1	-0.727795	-0.406507	-3.890385	0.0001186	0.0004011	0.4387271
RP11-63E5.	-0.208305	-1.468419	-3.890361	0.0001187	0.0004011	0.4386367
RALGAPB	0.0470891	6.2536992	3.8903344	0.0001187	0.0004011	0.4385394
RP11-326A1	-0.640194	0.1157326	-3.890129	0.0001188	0.0004014	0.4377789
RP11-317J1	-0.444393	3.9991883	-3.889845	0.0001189	0.0004018	0.4367246
SCN2B	0.6800022	-0.194632	3.8897901	0.0001189	0.0004019	0.4365197
RP11-295P2	-0.328242	-1.38538	-3.889555	0.000119	0.0004022	0.4356479
TGFB1I1	0.1260272	5.5708393	3.8891106	0.0001192	0.0004029	0.4339987
HSPA1B	-0.156419	6.3680057	-3.888888	0.0001194	0.0004032	0.4331714
SYNDIG1L	0.6682331	-0.033669	3.8882396	0.0001197	0.0004042	0.4307679
AC092839.3	-0.545094	-0.671761	-3.888199	0.0001197	0.0004042	0.4306176
GCC1	-0.069489	6.0761942	-3.888164	0.0001197	0.0004042	0.4304885
HNRNPA1P70	0.4476729	-0.976282	3.8879667	0.0001198	0.0004045	0.4297558
AQP7P4	-0.668868	-0.413622	-3.887697	0.0001199	0.0004049	0.4287566

AC008268.1	-0.431543	-1.140973	-3.88758	0.00012	0.0004051	0.4283217
ZNF174	-0.063157	5.5778891	-3.887322	0.0001201	0.0004054	0.4273661
RP11-603J2	0.7086358	1.0917177	3.8871468	0.0001202	0.0004057	0.426715
HSPA4	-0.06366	6.7390815	-3.887068	0.0001202	0.0004058	0.4264229
RP11-993B2	0.4327055	-1.128727	3.8869064	0.0001203	0.000406	0.4258237
RP11-15G16	-0.196512	-1.473022	-3.886782	0.0001204	0.0004061	0.4253629
WFDC21P	0.6549005	3.3074517	3.8854049	0.000121	0.0004083	0.4202573
TIMP3	0.5134748	4.6154815	3.8853958	0.000121	0.0004083	0.4202236
AE000661.3	0.6505173	-0.165694	3.88519	0.0001211	0.0004086	0.4194609
HECTD3	-0.063717	6.3435928	-3.885125	0.0001211	0.0004087	0.4192213
POLR2B	0.0656641	6.3342642	3.8850594	0.0001212	0.0004087	0.4189768
RP11-519G1	0.8078817	0.0637776	3.8850413	0.0001212	0.0004087	0.4189097
TRIM9	0.7543241	3.3023818	3.8848579	0.0001213	0.000409	0.4182301
KRTAP4-1	0.5024862	-1.096554	3.8848303	0.0001213	0.000409	0.4181275
OPN3	0.2506779	5.5674575	3.883947	0.0001217	0.0004104	0.4148546
APOD	0.4077158	4.2923085	3.8837287	0.0001218	0.0004107	0.4140455
IL4R	0.0929655	6.4521481	3.8835911	0.0001219	0.0004109	0.4135359
ZNF330	-0.07848	6.043699	-3.883171	0.0001221	0.0004115	0.4119809
RP11-560A1	-0.290261	-1.3896	-3.883113	0.0001221	0.0004116	0.4117655
RP11-376P6	0.6700622	0.504183	3.8827334	0.0001223	0.0004122	0.4103583
RPL13A	-0.078855	7.4011872	-3.882683	0.0001223	0.0004122	0.4101725
PDE1C	0.5032361	3.3969279	3.8826068	0.0001224	0.0004123	0.4098894
RP11-407N1	0.310126	3.7766159	3.8820313	0.0001226	0.0004132	0.4077578
RP4-530I15	0.7541714	1.3540914	3.8818554	0.0001227	0.0004134	0.4071065
SPRY2	0.1178487	5.723385	3.8818389	0.0001227	0.0004134	0.4070454
MAGT1	-0.062923	6.7274322	-3.881463	0.0001229	0.000414	0.4056518
SNORA71B	0.6844104	0.5468765	3.8811553	0.0001231	0.0004145	0.4045139
ANKLE1	0.4606542	3.021922	3.8810561	0.0001231	0.0004146	0.4041468
C20orf27	-0.091259	6.1792262	-3.880986	0.0001231	0.0004147	0.4038875
CCDC34	-0.128331	5.6987519	-3.880635	0.0001233	0.0004152	0.4025865
FAM209A	0.6533056	0.2603039	3.8805263	0.0001234	0.0004154	0.402185
RP4-712E4.	-0.242355	-1.420601	-3.87955	0.0001238	0.0004169	0.3985704
RP11-79H23	0.602646	2.8101022	3.8795492	0.0001238	0.0004169	0.3985679
RP11-327F2	0.6526263	0.8611883	3.8792751	0.000124	0.0004173	0.3975535
AC092198.1	0.7322803	-0.215345	3.8791454	0.000124	0.0004175	0.3970732
ATP1B3	0.1257038	6.1111406	3.8790773	0.0001241	0.0004175	0.3968215
GPR143	0.6187028	4.1913696	3.878811	0.0001242	0.000418	0.3958357
A1CF	-0.247185	6.7706973	-3.878594	0.0001243	0.0004183	0.3950336
LGMNP1	0.638	1.8238649	3.8784634	0.0001244	0.0004184	0.3945496
RBM12	0.0456042	6.2642888	3.8783496	0.0001244	0.0004186	0.3941283
RNA5SP335	-0.379833	-1.1679	-3.87745	0.0001249	0.00042	0.3908008
SNX33	-0.060634	6.3310589	-3.876897	0.0001251	0.0004209	0.3887529
RP11-973H7	0.8426691	0.2525206	3.8768127	0.0001252	0.000421	0.3884423
LINC00936	0.2077147	3.9131059	3.8766925	0.0001252	0.0004212	0.3879978
COX16	-0.071831	5.983631	-3.8764	0.0001254	0.0004216	0.3869155
ZSCAN21	-0.089612	5.4690939	-3.875828	0.0001257	0.0004225	0.384802
ACN9	-0.129155	5.7376547	-3.875425	0.0001259	0.0004232	0.383311
IFI27L2	0.209306	5.3465275	3.8744703	0.0001263	0.0004247	0.3797801
RP4-612B15	0.4478966	2.4287703	3.8742403	0.0001265	0.0004251	0.37893
YY1	-0.042198	6.5996159	-3.874192	0.0001265	0.0004251	0.3787506
SLC4A1AP	-0.044016	6.0920788	-3.873948	0.0001266	0.0004255	0.37785

SLC1A3	0.2133497	5.3366101	3.8733832	0.0001269	0.0004264	0.3757617
ASCC3	0.0851958	6.2526194	3.8732265	0.000127	0.0004266	0.3751825
ASMTL-AS1	-0.198593	5.0423539	-3.873155	0.000127	0.0004267	0.3749182
MYL7	-0.541981	-0.958503	-3.873119	0.000127	0.0004267	0.3747836
IDH3A	0.1493263	5.6955645	3.8729913	0.0001271	0.0004269	0.3743133
MIB2	-0.076307	6.1057586	-3.872749	0.0001272	0.0004273	0.3734181
RP11-431J2	-0.949734	1.7539632	-3.872416	0.0001274	0.0004278	0.3721891
PTPRJ	-0.0656	6.3591994	-3.872276	0.0001274	0.000428	0.3716712
PPY2	0.6279395	-0.935247	3.8720487	0.0001276	0.0004283	0.3708301
EBAG9P1	0.6451125	0.1361855	3.8720055	0.0001276	0.0004284	0.3706705
AC125238.3	-0.321263	-1.369351	-3.871674	0.0001278	0.0004289	0.3694445
C6orf57	-0.090628	5.5718666	-3.870936	0.0001281	0.0004301	0.3667195
RRP1	-0.078125	6.2288751	-3.869975	0.0001286	0.0004317	0.3631707
LINC00540	0.8696493	0.1615646	3.8698462	0.0001287	0.0004319	0.3626944
RP11-290M5	-0.69698	0.2718731	-3.86953	0.0001288	0.0004324	0.3615285
RP5-903E17	0.4360381	-1.105114	3.869136	0.000129	0.000433	0.3600722
DOK4	-0.092624	6.322512	-3.869094	0.0001291	0.000433	0.3599165
RP1-13D10.	0.52425	-0.772986	3.8685532	0.0001293	0.0004339	0.3579205
MED24	-0.049538	6.490134	-3.868376	0.0001294	0.0004342	0.3572652
RP11-100M1	-0.338721	-1.326331	-3.868371	0.0001294	0.0004342	0.3572482
MRPL39	-0.074847	5.957368	-3.86822	0.0001295	0.0004344	0.3566894
AHDC1	0.0884922	5.8742326	3.86757	0.0001298	0.0004355	0.3542909
UTP6	-0.050363	6.2584192	-3.867412	0.0001299	0.0004357	0.3537062
LTC4S	0.516444	-0.720074	3.8668362	0.0001302	0.0004366	0.3515829
COPG1	-0.045245	6.8808526	-3.866751	0.0001303	0.0004367	0.3512682
C9orf117	0.2063514	4.1759186	3.8664791	0.0001304	0.0004372	0.3502653
STAT1	0.091028	6.832266	3.8662038	0.0001305	0.0004376	0.3492494
BSND	0.8291479	0.1186321	3.8661186	0.0001306	0.0004377	0.348935
AC007326.1	-0.802663	-0.259183	-3.865835	0.0001307	0.0004382	0.3478876
IFIT1	-0.18116	6.1322599	-3.865562	0.0001309	0.0004386	0.3468815
RASEF	0.7311342	4.7523738	3.8655413	0.0001309	0.0004386	0.3468053
AC013444.1	0.6043544	-0.804706	3.8650327	0.0001311	0.0004394	0.344929
RP3-522J7.	0.6925597	0.7516545	3.8650062	0.0001312	0.0004394	0.3448314
TAS2R15P	0.6317618	1.2986722	3.864534	0.0001314	0.0004402	0.3430897
RP11-219G1	-0.45717	-0.954579	-3.864355	0.0001315	0.0004405	0.3424299
PDCL	0.0597277	5.7014609	3.864288	0.0001315	0.0004406	0.3421823
LINC00398	-0.783015	0.5755397	-3.864044	0.0001316	0.0004409	0.3412825
C2CD4D	0.5946601	2.602715	3.8640281	0.0001317	0.0004409	0.341224
RP11-12M9.	0.5760177	-0.231101	3.8639566	0.0001317	0.000441	0.3409601
RP11-248E9	-0.858573	0.1899683	-3.863916	0.0001317	0.000441	0.340812
AC010649.1	-0.355899	-1.260724	-3.863813	0.0001318	0.0004412	0.3404288
POLR2H	-0.072799	6.3086984	-3.863658	0.0001318	0.0004414	0.3398586
SRD5A3	-0.090515	6.0300541	-3.863432	0.000132	0.0004418	0.3390259
SLC22A2	-0.856474	1.1086321	-3.863407	0.000132	0.0004418	0.3389341
ARHGEF19	0.2719425	4.902977	3.8631002	0.0001321	0.0004422	0.3378024
LRRC7	0.3516529	3.5831351	3.8630391	0.0001322	0.0004423	0.337577
ACADM	-0.115099	6.5776044	-3.862965	0.0001322	0.0004424	0.3373024
CTD-3138F1	-0.64541	-0.913604	-3.862886	0.0001323	0.0004425	0.3370117
BCL2L13	-0.055867	6.4111212	-3.862538	0.0001324	0.0004431	0.3357293
CRPP1	0.7915392	-0.112282	3.8621766	0.0001326	0.0004437	0.3343973
RP1-265C24	-0.636995	0.6720144	-3.862088	0.0001327	0.0004438	0.3340707

RP11-175K6	0.5583978	2.4187665	3.8618181	0.0001328	0.0004442	0.3330761
MYH7	0.5081619	-0.947776	3.8613178	0.0001331	0.000445	0.331232
AC011294.3	0.8637375	2.8308243	3.8610318	0.0001332	0.0004455	0.330178
RP4-601P9.	-0.941216	2.2136059	-3.86103	0.0001332	0.0004455	0.3301731
FGG	-0.219716	7.663174	-3.860593	0.0001334	0.0004462	0.3285599
AC064850.4	0.5423989	-0.644482	3.8605538	0.0001335	0.0004462	0.3284166
CDKL5	0.147172	5.4460105	3.8598089	0.0001339	0.0004475	0.3256721
RP11-686G8	-0.366551	2.9177967	-3.859386	0.0001341	0.0004482	0.3241161
ZBTB43	0.1055757	5.6731804	3.8589045	0.0001343	0.000449	0.3223409
ERICH3	-0.788174	1.843378	-3.85873	0.0001344	0.0004493	0.3216963
AGT	-0.102063	7.5813653	-3.858599	0.0001345	0.0004494	0.321217
CTPS1	-0.101563	6.3196276	-3.858444	0.0001346	0.0004497	0.3206437
LINC01119	0.4961982	2.9955478	3.8584119	0.0001346	0.0004497	0.3205265
AC019100.3	0.4722405	-1.011953	3.8582704	0.0001347	0.0004499	0.3200055
DDO	-0.145784	5.6916852	-3.858082	0.0001348	0.0004502	0.3193102
ARL3	-0.079801	5.8970877	-3.857958	0.0001348	0.0004504	0.3188548
RP1-253P7.	0.6886944	1.1819247	3.8575553	0.0001351	0.000451	0.3173723
LDB2	0.1559344	5.6653488	3.8575157	0.0001351	0.0004511	0.3172261
MICA	-0.137666	5.8423481	-3.857292	0.0001352	0.0004514	0.3164026
RP11-723G8	-0.250752	-1.433361	-3.856954	0.0001354	0.000452	0.3151591
IFT74	-0.088793	5.6267829	-3.856953	0.0001354	0.000452	0.3151552
RAD50	-0.062336	6.3287471	-3.856603	0.0001356	0.0004525	0.3138662
GRHL1	0.1837054	5.3787423	3.8564179	0.0001357	0.0004528	0.3131847
CENPE	0.224711	5.1730794	3.8562621	0.0001357	0.0004531	0.3126112
LEF1-AS1	0.7755942	1.3233839	3.8561842	0.0001358	0.0004532	0.3123244
RBM15B	-0.04586	6.3724298	-3.855725	0.000136	0.0004539	0.3106345
ROPN1	-0.528028	-0.869572	-3.855379	0.0001362	0.0004545	0.3093593
RP11-98G7.	-0.632025	3.1983836	-3.85524	0.0001363	0.0004547	0.3088503
GRIK3	0.7100416	-0.384217	3.854975	0.0001364	0.0004551	0.3078744
LHFPL5	0.5736362	-0.858674	3.8549055	0.0001365	0.0004552	0.3076185
CENPK	0.284869	4.7448677	3.8545711	0.0001366	0.0004558	0.3063879
RP11-88E10	-0.156266	5.1841383	-3.853716	0.0001371	0.0004573	0.3032418
RP11-161H2	0.6160827	-0.188361	3.8535985	0.0001372	0.0004574	0.3028097
ARTN	0.4936065	3.0445573	3.8531706	0.0001374	0.0004582	0.3012358
GDF3	0.5637846	-0.58428	3.8530654	0.0001375	0.0004583	0.3008487
TRAV18	0.3445755	-1.235797	3.8525108	0.0001378	0.0004593	0.298809
RP11-443B2	0.4002599	3.3772176	3.8524862	0.0001378	0.0004593	0.2987185
AC067945.4	0.6700019	1.1006611	3.8524001	0.0001378	0.0004594	0.2984019
ARL6IP1	-0.067924	6.8575874	-3.852363	0.0001378	0.0004594	0.2982663
FRMD7	-0.880688	0.3097898	-3.851926	0.0001381	0.0004602	0.2966584
C12orf4	0.0600623	5.6455136	3.8518574	0.0001381	0.0004602	0.2964063
PTBP1	-0.039105	6.8940457	-3.851448	0.0001383	0.0004609	0.2949008
RP11-73M7.	0.6397542	0.2884142	3.8513405	0.0001384	0.0004611	0.2945057
RP11-212I2	0.8228853	1.1210113	3.8512285	0.0001385	0.0004613	0.2940941
OSR1	0.6805522	3.4704254	3.8504791	0.0001389	0.0004626	0.2913392
FBXO6	-0.103376	5.9908688	-3.850209	0.000139	0.000463	0.2903457
TINCR	0.6997733	1.203102	3.8501923	0.000139	0.000463	0.290285
SLC22A18AS	-0.324787	5.1719034	-3.850105	0.0001391	0.0004631	0.2899634
ZNF354A	0.2088967	5.1998825	3.8493396	0.0001395	0.0004645	0.2871512
RP11-643C9	0.6557825	-0.165184	3.8486158	0.0001399	0.0004658	0.2844914
RP11-466P2	0.6843317	1.8540362	3.8484362	0.00014	0.000466	0.2838317

SRR	-0.115271	5.2170622	-3.847647	0.0001404	0.0004674	0.2809309
EDRF1-AS1	0.649935	0.3671278	3.8476076	0.0001404	0.0004675	0.2807876
CTD-2537I9	0.6774898	1.1103312	3.8475381	0.0001405	0.0004676	0.2805321
RP11-395I1	0.6738656	0.5804737	3.846899	0.0001408	0.0004687	0.2781848
ZBTB7A	-0.061635	6.2598082	-3.846628	0.000141	0.0004691	0.2771902
GCNT2	-0.173491	5.7977338	-3.845991	0.0001413	0.0004703	0.2748499
INTS6	0.075085	6.081485	3.8458947	0.0001414	0.0004704	0.2744968
TRAF1	0.10286	5.6563937	3.8457639	0.0001415	0.0004706	0.2740165
RP11-44N11	-0.3962	3.5399103	-3.845077	0.0001418	0.0004718	0.2714948
ANXA8	0.908338	1.1356411	3.8447938	0.000142	0.0004723	0.2704549
TBC1D22B	0.0798849	5.6617141	3.8439848	0.0001424	0.0004738	0.2674855
C19orf57	-0.221318	4.782086	-3.843818	0.0001425	0.000474	0.2668717
LINC00261	-0.286902	6.6218697	-3.843134	0.0001429	0.0004753	0.2643643
FLOT1	-0.062723	6.8191599	-3.842783	0.0001431	0.0004759	0.2630766
CSPG4P8	0.3537585	4.0536986	3.8421826	0.0001435	0.000477	0.2608726
DGAT2	-0.192819	6.6669842	-3.84156	0.0001438	0.0004781	0.2585902
SDK1	0.5870847	4.1997764	3.8413062	0.0001439	0.0004785	0.2576579
RP4-796I8.	-0.409244	-1.275563	-3.840834	0.0001442	0.0004794	0.2559268
CALM3	-0.058369	6.9018321	-3.839862	0.0001448	0.0004811	0.252362
ACTBP7	0.6122312	0.018797	3.839834	0.0001448	0.0004811	0.2522593
ACAP2	0.0720274	6.0822348	3.839829	0.0001448	0.0004811	0.2522411
AIFM2	-0.093125	6.3647709	-3.8396	0.0001449	0.0004815	0.2514013
CTD-2373H9	-0.578739	1.0530753	-3.838662	0.0001454	0.0004832	0.2479645
RP11-484L8	0.4782137	-0.843252	3.8383604	0.0001456	0.0004838	0.2468575
AKT2	-0.056609	6.7114809	-3.83826	0.0001457	0.0004839	0.2464889
KLK4	-0.94351	-0.121991	-3.837923	0.0001459	0.0004845	0.2452537
SEC23IP	0.055232	6.2083467	3.8379053	0.0001459	0.0004845	0.2451895
KIAA0196-A	0.6395282	0.8465262	3.8378457	0.0001459	0.0004846	0.2449711
RPS7P3	0.6503941	0.8863687	3.8378139	0.0001459	0.0004846	0.2448544
RARA-AS1	-0.134326	5.0316093	-3.837464	0.0001461	0.0004852	0.2435713
GPS2	0.1281611	4.8864392	3.8373136	0.0001462	0.0004854	0.243021
MIR548AA1	0.5921421	-0.411421	3.8366529	0.0001466	0.0004866	0.2406005
RP11-798G7	-0.609642	1.5127924	-3.83658	0.0001466	0.0004867	0.2403342
TCF12	0.0670747	6.2069934	3.8365065	0.0001467	0.0004868	0.2400641
DMXL2	0.0987882	5.8826204	3.8361251	0.0001469	0.0004875	0.2386667
MT-TE	-0.596312	-0.448999	-3.836023	0.000147	0.0004877	0.2382941
UBALD2	-0.097531	6.2860329	-3.83595	0.000147	0.0004877	0.2380244
RP11-353N1	0.7860978	1.4366059	3.8359491	0.000147	0.0004877	0.238022
RP5-951N9.	-0.376173	-1.22936	-3.835793	0.0001471	0.000488	0.2374505
CTA-941F9.	0.8571744	0.2413032	3.835381	0.0001473	0.0004887	0.2359413
GRB10	-0.065857	6.4571422	-3.835308	0.0001474	0.0004888	0.2356735
ACHE	0.3635785	4.8244771	3.8351823	0.0001474	0.000489	0.2352134
EIF1B-AS1	-0.196654	4.0650568	-3.834598	0.0001478	0.0004901	0.2330743
CNNM2	-0.082352	5.9009166	-3.834351	0.0001479	0.0004905	0.2321699
UMAD1	-0.079106	5.9159816	-3.833864	0.0001482	0.0004914	0.2303866
MORC2-AS1	0.6028015	1.6213154	3.8337634	0.0001483	0.0004916	0.2300179
CEACAMP1	-0.153468	-1.503097	-3.833089	0.0001487	0.0004928	0.2275482
RP11-496N1	0.5574766	-0.444827	3.833058	0.0001487	0.0004928	0.2274357
TCF24	0.705886	-0.553673	3.8330475	0.0001487	0.0004928	0.227397
RPL7P50	0.5572042	-0.513317	3.8326033	0.0001489	0.0004936	0.2257712
AC124789.1	0.4588	2.6194266	3.8325287	0.000149	0.0004937	0.2254982

AC002480.5	0.5377524	-0.912571	3.8321066	0.0001492	0.0004944	0.2239533
LINC00337	-0.459628	3.3026645	-3.832103	0.0001492	0.0004944	0.223941
RN7SKP214	0.4618737	-1.06785	3.8312303	0.0001497	0.0004961	0.2207468
RP5-890E16	-0.325031	3.6708962	-3.831213	0.0001497	0.0004961	0.2206842
Z82214.2	-0.370851	-1.198527	-3.830994	0.0001499	0.0004965	0.2198814
DNM1P47	0.508611	-0.897726	3.830599	0.0001501	0.0004971	0.2184371
STK25	-0.049481	6.5412415	-3.830595	0.0001501	0.0004971	0.2184222
uc_338	0.6522008	2.2942039	3.8305604	0.0001501	0.0004972	0.2182957
GDI1	-0.059077	6.6229181	-3.83034	0.0001503	0.0004975	0.2174894
CTC-526N19	0.354161	3.3168556	3.8303339	0.0001503	0.0004975	0.2174672
RANBP17	0.6557872	4.1992695	3.8302374	0.0001503	0.0004977	0.2171144
GNG4	0.9723454	3.4416708	3.829587	0.0001507	0.0004989	0.2147352
RP11-489E7	0.6315891	0.1694203	3.8292867	0.0001509	0.0004994	0.213637
FLG-AS1	0.8704378	2.1438128	3.829049	0.000151	0.0004998	0.2127676
FBXW8	0.0758921	5.704291	3.8288591	0.0001511	0.0005001	0.2120732
RP11-227F1	0.3619897	-1.191588	3.8288483	0.0001511	0.0005001	0.2120337
CTD-2534I2	-0.222132	-1.434022	-3.828823	0.0001511	0.0005001	0.2119401
LTB4R	-0.151372	5.4195076	-3.828306	0.0001515	0.0005011	0.2100493
LINC00479	0.796033	1.6918025	3.8281984	0.0001515	0.0005013	0.2096575
RP11-664H1	0.6047399	-0.81745	3.8276977	0.0001518	0.0005022	0.2078268
RP1-205F14	-0.244467	-1.420367	-3.827487	0.0001519	0.0005026	0.2070571
CEP120	-0.07634	5.8030657	-3.826778	0.0001524	0.0005039	0.2044645
SLC9B2	-0.157803	6.0024069	-3.826534	0.0001525	0.0005044	0.2035737
MRPS12	-0.090986	6.3405568	-3.826419	0.0001526	0.0005045	0.2031522
KCNMB3	0.3397568	4.1947331	3.826375	0.0001526	0.0005046	0.202992
RP11-727A2	0.6007194	1.7036738	3.8263185	0.0001526	0.0005046	0.2027857
DFNB59	-0.21819	4.3245075	-3.825899	0.0001529	0.0005054	0.2012511
JMJD7-PLA2	0.3751389	4.4793203	3.82527	0.0001533	0.0005066	0.1989545
LINC00899	-0.243532	4.4575085	-3.825117	0.0001534	0.0005069	0.1983943
IGLV2-33	0.4354684	-1.148008	3.8247714	0.0001536	0.0005075	0.1971326
ZDHHC2	0.1904359	5.406856	3.8247452	0.0001536	0.0005075	0.197037
YIPF6	-0.058774	6.4243463	-3.82438	0.0001538	0.0005082	0.1957038
L29074.3	-0.531619	-0.674483	-3.823942	0.0001541	0.000509	0.1941022
GADL1	-0.666176	-0.558101	-3.823731	0.0001542	0.0005094	0.1933341
GOLGA2P5	-0.154472	5.7850858	-3.823177	0.0001545	0.0005105	0.191308
NCOR1P3	-0.173226	-1.483253	-3.823107	0.0001546	0.0005106	0.1910525
ADAMTS7P3	0.6890058	0.8897938	3.8228094	0.0001547	0.0005111	0.1899671
SYNJ1	0.1032936	5.527043	3.8227558	0.0001548	0.0005112	0.1897713
MYCBP2	0.0923878	6.0179025	3.8224498	0.000155	0.0005117	0.1886539
NCBP2L	-0.584824	-0.643393	-3.821965	0.0001553	0.0005126	0.1868856
ZNF540	-0.205169	4.2792042	-3.821593	0.0001555	0.0005133	0.1855268
ADRB3	0.4076371	-1.115081	3.8215642	0.0001555	0.0005134	0.1854208
RNU6-250P	-0.266513	-1.402367	-3.821484	0.0001555	0.0005135	0.1851282
AC093642.5	0.2659565	4.229167	3.8213623	0.0001556	0.0005137	0.1846837
MFSD2A	-0.473224	5.4548861	-3.821107	0.0001558	0.0005141	0.1837527
AC067956.1	-0.213729	-1.447884	-3.820865	0.0001559	0.0005145	0.182869
FAM66A	0.4577306	-0.967153	3.8208599	0.0001559	0.0005145	0.1828501
FAXDC2	-0.150481	6.4446284	-3.820672	0.000156	0.0005149	0.1821651
C4orf46	0.1196287	5.1450464	3.8197568	0.0001566	0.0005167	0.1788247
ZNF671	0.2321192	4.9388223	3.8193822	0.0001568	0.0005173	0.1774579
FRMD1	0.8962785	1.6438387	3.8193732	0.0001568	0.0005173	0.1774249

AC002064.5	-0.348494	-1.213561	-3.819261	0.0001569	0.0005175	0.1770148
RP11-311P8	0.5917377	1.9796746	3.8184801	0.0001574	0.000519	0.174167
NELFCD	-0.05556	6.594891	-3.818409	0.0001574	0.0005191	0.1739068
PROX2	0.6575056	0.6847945	3.818374	0.0001574	0.0005192	0.17378
TBC1D8	0.0881263	6.1661845	3.8182663	0.0001575	0.0005193	0.1733869
TPST1	0.1496731	5.9655771	3.818209	0.0001575	0.0005194	0.1731779
RP11-431K2	-0.808917	1.2749799	-3.817586	0.0001579	0.0005206	0.1709041
RP11-15I11	-0.66979	4.1431784	-3.817379	0.0001581	0.000521	0.1701518
NPAS1	0.507381	3.3771778	3.8171595	0.0001582	0.0005214	0.1693505
RP11-701H2	0.7937522	0.7554103	3.8168774	0.0001584	0.0005219	0.168322
ABHD4	-0.083079	6.3279236	-3.816847	0.0001584	0.0005219	0.1682129
RNF135	0.1898175	5.3224788	3.8166766	0.0001585	0.0005222	0.1675897
MIR659	-0.59882	-0.097002	-3.816598	0.0001585	0.0005224	0.1673027
AC006126.3	-0.584757	-0.323183	-3.816172	0.0001588	0.0005232	0.1657504
NARF-IT1	0.6475755	1.1223737	3.8156627	0.0001591	0.0005242	0.1638937
VWA7	0.2585842	5.0698696	3.8155852	0.0001592	0.0005243	0.1636112
UGT1A4	-0.734113	5.4100939	-3.815336	0.0001593	0.0005247	0.1627011
CTD-2302E2	0.6203736	1.1264778	3.8150888	0.0001595	0.0005252	0.1618017
VPS18	-0.067126	6.2313806	-3.815001	0.0001595	0.0005253	0.1614812
TMPRSS6	-0.253176	6.7147581	-3.814521	0.0001598	0.0005263	0.1597339
PRKXP1	0.7412901	1.5062035	3.8143974	0.0001599	0.0005265	0.1592821
AC096558.1	0.4661898	-0.963232	3.8142934	0.00016	0.0005266	0.158903
RP1-159A19	0.4924626	-0.844291	3.8141908	0.00016	0.0005268	0.1585292
RP5-1148A2	-0.191049	5.23866	-3.814079	0.0001601	0.000527	0.1581232
PDCD5	-0.07668	6.3917851	-3.814062	0.0001601	0.000527	0.1580609
RP11-1082L	-0.409848	-1.069254	-3.813972	0.0001602	0.0005271	0.1577319
NEDD4	-0.135643	6.2415035	-3.813583	0.0001604	0.0005279	0.1563149
RP1-20N2.8	0.6097384	1.2598747	3.8131581	0.0001607	0.0005287	0.1547666
RP11-73301	0.6129144	2.1518568	3.8127732	0.0001609	0.0005294	0.1533645
ADCK1	-0.083812	5.6537336	-3.812758	0.0001609	0.0005294	0.153309
PDDC1	-0.066995	6.369051	-3.812668	0.000161	0.0005296	0.1529797
U91328.19	-0.153384	5.2724638	-3.81239	0.0001612	0.0005301	0.1519675
ACPT	0.8832664	1.4539621	3.8123363	0.0001612	0.0005301	0.1517732
RP3-337018	0.6206562	0.728929	3.8120467	0.0001614	0.0005307	0.1507183
RP11-63701	-0.222833	-1.445628	-3.811476	0.0001617	0.0005318	0.1486415
SRP9	-0.05883	6.7846463	-3.811407	0.0001618	0.0005319	0.1483885
SRP68P1	-0.188609	-1.456587	-3.81127	0.0001619	0.0005322	0.1478885
KIAA1407	0.1357179	4.9686239	3.8111193	0.000162	0.0005324	0.1473412
NPM1P6	0.4189109	2.7328039	3.8109678	0.000162	0.0005327	0.1467897
MIR762HG	-0.178123	3.9778086	-3.810937	0.0001621	0.0005327	0.1466764
LPIN2	-0.104468	6.7721758	-3.810714	0.0001622	0.0005331	0.1458674
RP11-571L1	-0.356138	-1.199488	-3.810392	0.0001624	0.0005337	0.1446948
ZNF449	0.1044347	5.0743074	3.8102267	0.0001625	0.000534	0.1440914
ATP5HP2	-0.618252	-0.024159	-3.810157	0.0001626	0.0005341	0.1438366
SNX2	-0.054979	6.4086219	-3.810133	0.0001626	0.0005341	0.1437501
RPL26P30	-0.510652	2.1228872	-3.809811	0.0001628	0.0005347	0.1425778
RP11-1281K	-0.485489	-0.995992	-3.809732	0.0001628	0.0005348	0.1422911
CTD-2008P7	0.3971723	-1.129866	3.809728	0.0001628	0.0005348	0.1422762
KIF1B	-0.074008	6.3226667	-3.809666	0.0001629	0.0005349	0.1420513
RP11-173P1	0.669787	0.7917326	3.8091379	0.0001632	0.000536	0.1401284
BRE	-0.065054	6.1967213	-3.808995	0.0001633	0.0005362	0.1396068

AC000123.4	0.3027508	3.584677	3.808868	0.0001634	0.0005364	0.1391462
MCFD2	-0.063536	6.7829052	-3.808826	0.0001634	0.0005364	0.1389949
CELF3	0.6524024	-0.080411	3.8088152	0.0001634	0.0005364	0.1389541
INO80D	0.0820594	5.7757005	3.8081932	0.0001638	0.0005377	0.1366905
NRADDP	0.5972166	0.1875164	3.8080085	0.0001639	0.000538	0.1360185
RP1-69D17.	0.483651	-0.756036	3.8077495	0.0001641	0.0005385	0.1350763
GTF2IP1	0.2995383	4.0767386	3.8063873	0.000165	0.0005413	0.1301213
CARKD	-0.082105	6.443969	-3.806203	0.0001651	0.0005417	0.1294513
RP11-181K3	0.7022704	0.6023518	3.8059022	0.0001653	0.0005423	0.1283572
RPL7L1	-0.05097	6.6224756	-3.805667	0.0001654	0.0005427	0.1275014
RPL39P36	0.603407	1.3913051	3.8052482	0.0001657	0.0005435	0.125979
HNRNPA3P6	0.2542795	3.8467985	3.8050722	0.0001658	0.0005439	0.125339
DIP2B	0.0588156	6.2658331	3.8048189	0.000166	0.0005443	0.124418
SLC22A16	0.6886794	0.1263022	3.8047205	0.000166	0.0005445	0.1240603
CDK5R2	0.5662042	-0.912991	3.8046811	0.0001661	0.0005445	0.1239172
MED13L	0.0784246	6.1716756	3.8045264	0.0001662	0.0005448	0.1233548
AC099552.4	0.6077979	-0.883678	3.8037432	0.0001667	0.0005464	0.120508
AC093802.1	-0.223583	-1.436469	-3.803584	0.0001668	0.0005467	0.1199307
PRR36	0.4451519	4.1799797	3.8034925	0.0001668	0.0005468	0.1195965
AC019097.7	0.6183512	0.7190331	3.8034652	0.0001668	0.0005468	0.1194974
CTD-2240J1	-0.626895	2.3428956	-3.803463	0.0001668	0.0005468	0.1194908
PES1	-0.066016	6.5598607	-3.803401	0.0001669	0.0005469	0.1192644
RP11-740C1	-0.575886	3.5500214	-3.803084	0.0001671	0.0005475	0.1181138
ARMC12	-0.359217	3.4838728	-3.803031	0.0001671	0.0005476	0.1179201
MAST3	-0.083502	6.1369597	-3.802388	0.0001675	0.0005489	0.1155828
PITPNM1	0.1179789	5.9836346	3.8022249	0.0001677	0.0005492	0.1149905
CHDH	-0.113024	6.5732328	-3.802158	0.0001677	0.0005493	0.1147458
AC092338.5	0.632356	0.4798233	3.8013321	0.0001682	0.000551	0.1117468
RP5-965G21	0.619378	1.7811604	3.8012655	0.0001683	0.0005511	0.1115052
RP11-49501	0.3715706	-1.17495	3.8010219	0.0001684	0.0005516	0.1106202
PRAMEF2	-0.779669	-0.405165	-3.800914	0.0001685	0.0005518	0.1102279
CTC1	0.0745268	5.8481828	3.8005456	0.0001687	0.0005525	0.1088901
RP11-21401	0.5076776	3.6733138	3.8004805	0.0001688	0.0005526	0.108654
RP11-48901	0.6942104	1.7399291	3.7999994	0.0001691	0.0005536	0.1069066
RP11-254F7	-0.122996	5.2905436	-3.799798	0.0001692	0.000554	0.106174
IGF2BP1	1.0357921	4.0625876	3.7991852	0.0001696	0.0005552	0.1039504
FABP5P14	-0.155048	-1.502403	-3.799098	0.0001697	0.0005554	0.1036332
RP11-505P4	-0.6224	1.5792513	-3.798836	0.0001699	0.0005559	0.1026826
BCDIN3D	-0.071259	5.3105856	-3.798596	0.00017	0.0005564	0.1018112
RP11-107E5	0.3556331	-1.166662	3.7983771	0.0001702	0.0005568	0.1010166
RN7SKP296	0.6718204	-0.055018	3.7983565	0.0001702	0.0005568	0.1009419
AC063976.7	0.5663371	-0.541003	3.7982878	0.0001702	0.0005569	0.1006927
TAB1	-0.05468	6.2119542	-3.798254	0.0001703	0.0005569	0.1005705
LRIG3	0.1477237	5.7691147	3.7977188	0.0001706	0.000558	0.0986273
CCL5	0.1971811	5.8027054	3.7976281	0.0001707	0.0005582	0.0982981
SIX4	0.6929943	3.6139886	3.7973421	0.0001709	0.0005587	0.0972604
CTC-276P9.	0.3967523	-1.095671	3.7971276	0.000171	0.0005591	0.0964819
C5orf24	-0.068184	6.3447521	-3.797054	0.0001711	0.0005593	0.0962141
RP11-567M2	-0.359098	-1.221606	-3.796698	0.0001713	0.00056	0.0949226
MED15	0.0487056	6.4039154	3.7966561	0.0001713	0.00056	0.0947708
AC114812.5	-0.742024	0.104816	-3.796012	0.0001717	0.0005614	0.0924344

ATP6AP1L	-0.164064	4.9854063	-3.795356	0.0001722	0.0005628	0.0900531
MYEOV2	-0.098403	6.1542495	-3.795036	0.0001724	0.0005634	0.0888951
RP3-467K16	-0.473707	-1.06737	-3.794713	0.0001726	0.0005641	0.0877214
ZNF385B	-0.499644	5.0759538	-3.794601	0.0001727	0.0005643	0.0873163
P2RY14	0.405539	3.6254475	3.7944508	0.0001728	0.0005645	0.0867714
HSP90AB2P	0.5097189	3.460677	3.7939519	0.0001731	0.0005656	0.0849622
DAPK1	0.0941081	6.3192083	3.7938561	0.0001732	0.0005657	0.084615
SNAP47	-0.070643	6.2780462	-3.793799	0.0001732	0.0005658	0.0844078
DGKD	0.0691864	5.9715002	3.7936078	0.0001734	0.0005662	0.0837148
NPY1R	0.4689291	4.2538142	3.7933489	0.0001735	0.0005667	0.0827763
AP000442.4	-0.654243	0.436327	-3.792861	0.0001739	0.0005677	0.0810083
TRIM31-AS1	0.7023461	0.0787385	3.7928227	0.0001739	0.0005677	0.0808685
CTD-2600H1	0.5293649	2.9926715	3.7927478	0.0001739	0.0005679	0.0805969
UMPS	-0.058093	6.2901485	-3.792512	0.0001741	0.0005683	0.0797413
LINC00642	0.3455813	-1.203765	3.7924009	0.0001742	0.0005685	0.0793396
NRG4	0.5431277	3.578237	3.7922927	0.0001742	0.0005687	0.0789475
PDXK	-0.070886	6.602031	-3.791948	0.0001745	0.0005694	0.0776992
RSPO1	0.6031907	-0.767312	3.791517	0.0001748	0.0005703	0.0761361
CECR9	-0.60159	-0.628447	-3.791004	0.0001751	0.0005714	0.0742764
ACTA2-AS1	0.4219838	3.3268056	3.7908177	0.0001752	0.0005718	0.0736021
C16orf62	-0.124898	5.9741008	-3.790802	0.0001753	0.0005718	0.0735448
RP11-15A1.	0.7515652	1.7979359	3.790734	0.0001753	0.0005719	0.0732989
SYAP1	-0.064151	6.5671692	-3.790528	0.0001754	0.0005723	0.0725523
KREMEN2	0.7035424	2.3469281	3.7902117	0.0001757	0.0005729	0.0714065
BANF1P1	-0.566866	-0.767162	-3.789264	0.0001763	0.000575	0.0679728
TOP1	0.0517012	6.4518648	3.7888808	0.0001766	0.0005758	0.066586
OPLAH	-0.109017	6.4996146	-3.78877	0.0001766	0.000576	0.0661832
GPSM1	0.2492486	5.6044755	3.7880333	0.0001771	0.0005776	0.0635172
DENND5A	0.0577417	6.251704	3.7875798	0.0001775	0.0005785	0.0618751
UBE3A	-0.050764	6.3926781	-3.787468	0.0001775	0.0005787	0.0614687
GS1-358P8.	0.1542455	5.5884299	3.7874587	0.0001775	0.0005787	0.0614368
NDUFA6-AS1	-0.250864	5.2210105	-3.786932	0.0001779	0.0005798	0.0595291
KIF14	0.2566086	5.0592758	3.7867893	0.000178	0.0005801	0.0590135
TACC2	-0.085019	6.2329932	-3.786642	0.0001781	0.0005804	0.0584789
RP11-753A2	-0.725976	-0.372607	-3.786521	0.0001782	0.0005806	0.0580438
CALCRL	0.1749569	5.8112716	3.7864853	0.0001782	0.0005806	0.0579131
TMEM246	0.3900152	5.1315897	3.7864019	0.0001783	0.0005807	0.0576115
RPS5	-0.079424	7.1290335	-3.786125	0.0001785	0.0005813	0.0566083
FYCO1	-0.077323	6.2338197	-3.785972	0.0001786	0.0005816	0.056055
RP11-616M2	-0.427005	4.1718914	-3.785641	0.0001788	0.0005823	0.0548587
RNY4P34	-0.172972	-1.479288	-3.785454	0.0001789	0.0005827	0.0541817
HNRNPCP2	0.1448022	5.4364195	3.785258	0.0001791	0.0005831	0.0534717
USP31	-0.089989	5.8822938	-3.785153	0.0001791	0.0005832	0.0530909
CTC-479C5.	0.1934409	4.3094606	3.785135	0.0001791	0.0005832	0.0530269
RNU6-30P	0.5269982	-0.701735	3.7843061	0.0001797	0.0005851	0.0500281
RP11-638I2	0.6157782	0.0251105	3.7840281	0.0001799	0.0005856	0.0490223
ALDH18A1	0.074978	6.4375111	3.7839922	0.0001799	0.0005857	0.0488926
MAU2	0.0498963	6.2307093	3.7831013	0.0001806	0.0005876	0.0456705
RP11-498E1	-0.29731	-1.298407	-3.7831	0.0001806	0.0005876	0.0456668
AC104772.1	-0.250624	-1.378185	-3.783071	0.0001806	0.0005876	0.045562
RP4-635A23	0.5359149	2.2582835	3.7825207	0.000181	0.0005888	0.0435706

ZNF518A	0.0958503	5.8610948	3.7824124	0.000181	0.000589	0.0431792
NDOR1	0.0910101	5.6248882	3.7823213	0.0001811	0.0005892	0.0428496
HCG25	-0.232276	3.758173	-3.781424	0.0001817	0.0005911	0.0396062
PDK2	-0.100424	6.2272574	-3.781178	0.0001819	0.0005917	0.0387171
ZP1	0.6487668	-0.208201	3.781039	0.000182	0.0005919	0.038214
IRF8	0.1591763	5.7830144	3.7806132	0.0001823	0.0005928	0.036675
TRPC6	0.3315904	4.0252048	3.7805641	0.0001823	0.0005929	0.0364976
STAT2	-0.066763	6.6206633	-3.780132	0.0001826	0.0005938	0.0349351
TSC22D1	-0.096743	6.6783551	-3.779873	0.0001828	0.0005944	0.0340007
CTD-2132N1	0.6639328	2.2963539	3.77967	0.000183	0.0005948	0.0332664
RP11-413M3	0.5464166	-0.51714	3.7795909	0.000183	0.0005949	0.0329809
LNP1	-0.187593	4.9393699	-3.779492	0.0001831	0.0005951	0.0326243
AC005822.1	0.6336778	0.6062126	3.7791913	0.0001833	0.0005957	0.0315369
KIF18A	0.3190697	4.6560287	3.7790088	0.0001834	0.0005961	0.0308776
TMSB4XP4	0.381537	3.2475719	3.7785978	0.0001837	0.000597	0.0293927
RP11-288L9	0.6917597	0.1456956	3.7785609	0.0001838	0.000597	0.0292595
RP11-363N2	0.6378967	1.8331247	3.7784146	0.0001839	0.0005973	0.0287309
MIR3648-2	-0.152976	-1.503313	-3.77828	0.000184	0.0005976	0.0282449
RP1-55C23.	0.5232787	-0.812666	3.7782498	0.000184	0.0005976	0.0281359
CNN2P9	0.6160989	0.7409077	3.7782409	0.000184	0.0005976	0.0281035
TRAPPC10	0.1163059	5.7008027	3.7777103	0.0001844	0.0005987	0.0261871
RN7SL526P	0.4759497	-0.941459	3.7775413	0.0001845	0.0005991	0.0255769
AGXT2	-0.461287	5.8864168	-3.777526	0.0001845	0.0005991	0.0255215
RP4-613B23	0.6483497	0.6428449	3.7769657	0.0001849	0.0006003	0.0234982
ORC6	0.2287443	4.8784248	3.7768516	0.000185	0.0006005	0.0230861
AC073657.1	0.4774551	-0.771627	3.7767382	0.0001851	0.0006007	0.0226768
SHISA4	-0.199312	5.7059059	-3.776616	0.0001851	0.000601	0.0222357
AC034228.3	-0.638927	-0.876777	-3.776424	0.0001853	0.0006013	0.021543
AC013460.1	0.5126238	-0.885461	3.7763338	0.0001853	0.0006015	0.0212167
RP11-4104.	-0.224584	4.8322992	-3.776204	0.0001854	0.0006017	0.0207493
RP11-720L2	-0.172527	-1.48356	-3.775977	0.0001856	0.0006022	0.0199267
RP11-64D22	-0.810148	1.040473	-3.775638	0.0001858	0.000603	0.0187062
ZNF841	0.1567674	5.3087474	3.7754774	0.000186	0.0006033	0.0181251
PROK2	0.6120589	-0.281785	3.7753891	0.000186	0.0006034	0.0178061
PLA2G4E-AS	-0.646284	0.3575706	-3.775306	0.0001861	0.0006036	0.0175079
CTD-2310F1	-0.32493	3.2265396	-3.774997	0.0001863	0.0006042	0.0163902
PEG10	0.6390361	5.3691404	3.7744463	0.0001867	0.0006055	0.0144037
PCBP1-AS1	-0.071073	5.7621334	-3.774217	0.0001869	0.000606	0.0135748
INTS9	0.0861913	5.5781835	3.7735266	0.0001874	0.0006075	0.011085
GAPDHP33	0.5254855	-0.671867	3.7728875	0.0001878	0.000609	0.0087793
USP12	-0.082373	6.1103098	-3.772819	0.0001879	0.0006091	0.0085325
RP11-65N13	-0.547563	-0.488085	-3.772758	0.0001879	0.0006091	0.0083139
MPP5	-0.075282	6.082339	-3.772741	0.0001879	0.0006091	0.0082498
RP11-309N2	-0.187054	-1.463681	-3.77238	0.0001882	0.0006099	0.0069477
RBM23	-0.076444	6.3270407	-3.772374	0.0001882	0.0006099	0.0069262
BPIFA1	0.6939927	-0.748867	3.7721724	0.0001883	0.0006103	0.0062
NEU1	-0.076252	6.7067212	-3.771901	0.0001885	0.0006109	0.0052216
RP11-342K6	-0.689356	1.6023605	-3.771842	0.0001886	0.000611	0.0050081
PABPC1P5	-0.303241	-1.386592	-3.771151	0.0001891	0.0006126	0.002515
RP11-6I2.3	0.6529318	0.1225226	3.7710899	0.0001891	0.0006127	0.0022962
ARIH2OS	-0.186719	4.4628089	-3.770503	0.0001896	0.000614	0.0001791

RP1-153G14	0.6418373	1.2061448	3.7704764	0.0001896	0.000614	8.43E-05
BTBD7	0.0693331	5.8772359	3.7704392	0.0001896	0.000614	-5.01E-05
ZNF143	0.0596721	5.736846	3.7701166	0.0001898	0.0006147	-0.001213
PAPD7	-0.068116	6.0930795	-3.770106	0.0001899	0.0006147	-0.00125
RP11-414H2	-0.265942	-1.353081	-3.76993	0.00019	0.0006151	-0.001886
UGT8	0.9380397	1.1622419	3.7697978	0.0001901	0.0006153	-0.002362
CCDC22	-0.064979	6.077895	-3.769538	0.0001903	0.0006159	-0.003299
MRPS28	-0.092023	6.077648	-3.769533	0.0001903	0.0006159	-0.003316
YRDC	-0.063508	5.8713144	-3.769368	0.0001904	0.0006162	-0.003912
MED11	-0.088517	5.9216785	-3.769321	0.0001904	0.0006163	-0.004079
SNX32	0.365245	3.1763411	3.7691956	0.0001905	0.0006165	-0.004533
RP11-1246C	0.2511092	4.4186042	3.7684979	0.000191	0.0006181	-0.007047
TARBP2	-0.067619	6.0818078	-3.768358	0.0001911	0.0006184	-0.007553
RP11-87C12	-0.398771	-1.097471	-3.768322	0.0001912	0.0006184	-0.007682
RNMTL1	-0.100104	5.9265953	-3.767949	0.0001914	0.0006192	-0.009024
SLC35F1	0.64872	2.0557885	3.7679321	0.0001914	0.0006192	-0.009086
RP11-542A1	-0.362923	-1.247441	-3.767846	0.0001915	0.0006194	-0.009395
EXD3	-0.086893	5.6836024	-3.767437	0.0001918	0.0006203	-0.010871
TMEM261	-0.129103	5.9420594	-3.766306	0.0001927	0.000623	-0.014942
CTD-3179P9	0.4584168	-1.031196	3.7660955	0.0001928	0.0006234	-0.015702
RARRES1	0.2531235	5.2394864	3.7659963	0.0001929	0.0006236	-0.016059
AF196779.1	-0.446075	-0.996856	-3.765829	0.000193	0.0006239	-0.016661
RHEB	-0.055484	6.4543293	-3.76581	0.000193	0.0006239	-0.016731
SMO	-0.137305	6.4364048	-3.765297	0.0001934	0.0006251	-0.018575
N6AMT2	-0.114645	5.4483721	-3.764792	0.0001938	0.0006263	-0.020395
TRPV1	0.6966032	3.0562306	3.7645675	0.0001939	0.0006268	-0.021203
AC005616.2	-0.415328	-1.202453	-3.764446	0.000194	0.000627	-0.02164
RP11-1081M	-0.433499	-1.233961	-3.764081	0.0001943	0.0006278	-0.022955
CDC42BPA	-0.098922	6.3983507	-3.763961	0.0001944	0.0006281	-0.023386
MARK2P9	0.5274829	-0.666915	3.7638971	0.0001944	0.0006282	-0.023616
OR2A13P	0.533083	-0.644627	3.763572	0.0001947	0.0006289	-0.024787
ZNF280C	0.2003941	4.850314	3.7635189	0.0001947	0.000629	-0.024978
WHAMML1	0.1427411	4.5959747	3.763205	0.000195	0.0006297	-0.026107
ATP5G1P4	0.7121716	1.6287998	3.7629231	0.0001952	0.0006303	-0.027122
LINC01264	-0.214438	-1.455193	-3.762915	0.0001952	0.0006303	-0.027153
PLEKHN1	0.5921588	3.6105818	3.7626596	0.0001954	0.0006308	-0.02807
RP11-205K6	-0.251608	-1.404453	-3.762476	0.0001955	0.0006312	-0.028731
HNRNPKP1	0.360874	3.3723457	3.7619025	0.0001959	0.0006326	-0.030794
SHARPIN	-0.085316	6.5707045	-3.761637	0.0001962	0.0006331	-0.03175
SLC25A30	-0.130482	6.2846408	-3.761602	0.0001962	0.0006332	-0.031874
CTB-171A8.	-0.210158	4.7307231	-3.761506	0.0001962	0.0006334	-0.03222
GNS	-0.052399	6.837626	-3.761221	0.0001965	0.000634	-0.033245
RARRES3	-0.152404	6.2501968	-3.761175	0.0001965	0.0006341	-0.033412
BET1	-0.057704	6.0197104	-3.761082	0.0001966	0.0006342	-0.033745
RGAG4	0.2637833	4.9930033	3.7610188	0.0001966	0.0006343	-0.033973
CTD-2574D2	0.4043033	3.4750533	3.7607517	0.0001968	0.0006349	-0.034934
RP1-267L14	0.5294522	2.053973	3.760374	0.0001971	0.0006358	-0.036292
FAM160A1	0.784611	2.4972541	3.760166	0.0001973	0.0006362	-0.03704
RP11-571L1	-0.205652	4.1367963	-3.75985	0.0001975	0.000637	-0.038177
PRKAB2	-0.095269	6.5206889	-3.759812	0.0001975	0.000637	-0.038314
RP11-215P8	-0.942755	1.6525024	-3.759697	0.0001976	0.0006372	-0.038727

RP11-326C3	-0.507598	3.3246532	-3.759452	0.0001978	0.0006378	-0.039606
ADA	0.1245589	5.5055433	3.7590798	0.0001981	0.0006386	-0.040946
RRN3	-0.067632	6.2259175	-3.758306	0.0001987	0.0006405	-0.043728
IQGAP3	0.2049142	5.7918847	3.7577645	0.0001991	0.0006417	-0.045674
LL22NC03-2	0.3338935	3.6887934	3.7577594	0.0001991	0.0006417	-0.045692
GOLGA6C	-0.289502	-1.343786	-3.75774	0.0001991	0.0006417	-0.045761
RP3-395M2C	0.2054215	5.2205302	3.7577065	0.0001991	0.0006417	-0.045882
ZNF664	0.0514566	6.590403	3.7573994	0.0001994	0.0006424	-0.046986
RP11-707M3	0.4234587	2.9234098	3.7571408	0.0001996	0.000643	-0.047915
RP11-289I1	0.2680118	4.1960619	3.7563499	0.0002002	0.0006449	-0.050757
AC000078.5	0.7308887	0.6912796	3.7561777	0.0002003	0.0006453	-0.051376
CCNE1	0.2635955	4.9848093	3.7559089	0.0002005	0.0006459	-0.052341
GCLM	-0.10422	6.4116247	-3.75579	0.0002006	0.0006461	-0.052767
TAF4	0.0647279	5.9572082	3.7556554	0.0002007	0.0006464	-0.053252
PPA2	-0.080018	6.3547625	-3.755384	0.0002009	0.000647	-0.054228
C15orf57	0.0839624	5.4751672	3.75524	0.000201	0.0006473	-0.054744
PDSS1	0.1035099	5.472739	3.7550129	0.0002012	0.0006478	-0.055559
SCART1	0.3768079	4.2290305	3.7548946	0.0002013	0.0006481	-0.055984
CTD-2213F2	-0.288583	2.9608667	-3.754785	0.0002014	0.0006483	-0.056379
LINC01278	-0.075536	5.8769899	-3.754654	0.0002015	0.0006486	-0.056847
NANOGP4	-0.458412	-0.926107	-3.754524	0.0002016	0.0006488	-0.057314
ZMIZ1-AS1	0.3943577	3.8625771	3.754287	0.0002018	0.0006494	-0.058166
BCKDHB	-0.127926	6.0954523	-3.754239	0.0002018	0.0006494	-0.058339
CARD6	0.183823	5.3920523	3.7541172	0.0002019	0.0006497	-0.058776
RP11-359J6	-0.414117	-1.09309	-3.753849	0.0002021	0.0006503	-0.059741
DHX29	-0.056826	6.229432	-3.753517	0.0002024	0.0006511	-0.060932
RP11-15E1.	-0.255613	-1.379945	-3.753169	0.0002026	0.0006519	-0.062179
ARL8B	-0.043561	6.480839	-3.752847	0.0002029	0.0006526	-0.063336
AC010880.1	-0.485072	-1.153586	-3.752835	0.0002029	0.0006526	-0.063381
RP11-298I3	-0.177685	4.2932972	-3.752397	0.0002032	0.0006536	-0.064952
PTPLAD1	-0.072251	6.8048449	-3.752352	0.0002033	0.0006537	-0.065113
LINC00898	-0.685426	-0.565522	-3.752317	0.0002033	0.0006537	-0.06524
ZNF600	0.2435719	5.2320257	3.7522105	0.0002034	0.0006539	-0.065621
RP11-223I1	-0.220498	5.2900627	-3.752178	0.0002034	0.0006539	-0.065738
VN1R42P	0.5028663	-0.651173	3.7520236	0.0002035	0.0006543	-0.066292
TBC1D28	-0.1737	-1.480711	-3.751958	0.0002036	0.0006544	-0.066527
CCDC137	-0.075159	6.0725302	-3.751876	0.0002037	0.0006545	-0.066822
NAT2	-0.811357	4.2916569	-3.751843	0.0002037	0.0006546	-0.066939
RP11-650L1	0.7434428	0.7774371	3.7514509	0.000204	0.0006555	-0.068347
CTD-2187J2	-0.361867	-1.187021	-3.751304	0.0002041	0.0006558	-0.068873
NLGN4X	0.6061818	3.9812956	3.7502103	0.000205	0.0006585	-0.072798
ERO1LB	-0.135654	6.2224463	-3.750079	0.0002051	0.0006588	-0.073269
ZNF790	-0.095541	5.3426879	-3.74991	0.0002052	0.0006591	-0.073874
WDFY2	0.0781649	5.7894803	3.7498861	0.0002052	0.0006592	-0.073961
GRM3	0.7305744	-0.205565	3.7496118	0.0002054	0.0006598	-0.074945
RBSN	-0.049475	6.0599975	-3.749588	0.0002055	0.0006598	-0.07503
RP11-17501	0.1018941	5.3756037	3.7494971	0.0002055	0.00066	-0.075356
RP5-858B6.	-0.805598	0.7452972	-3.74937	0.0002056	0.0006602	-0.075812
RP11-304L1	-0.621117	-0.42869	-3.749201	0.0002058	0.0006606	-0.076419
RP11-972P1	0.6056375	1.2079947	3.7491617	0.0002058	0.0006606	-0.076559
DOPEY2	0.1223036	6.0553851	3.7486029	0.0002062	0.000662	-0.078563

BTF3L4P2	0.288018	3.5567621	3.7485915	0.0002062	0.000662	-0.078604
SETP14	0.6032364	1.6844686	3.7483633	0.0002064	0.0006625	-0.079422
ATP5C1	-0.060313	6.9099198	-3.748117	0.0002066	0.0006631	-0.080307
AC018766.4	0.315666	3.5336473	3.7475656	0.0002071	0.0006644	-0.082282
GIMAP1	0.1436256	5.3305531	3.7472967	0.0002073	0.000665	-0.083246
GET4	0.1125588	5.2042743	3.747248	0.0002073	0.0006651	-0.083421
AC073052.1	0.5487638	-0.425534	3.7467741	0.0002077	0.0006663	-0.08512
CTC-360J11	0.6613891	-0.015275	3.7467336	0.0002077	0.0006663	-0.085265
KMT2A	0.060092	6.1699148	3.7467246	0.0002077	0.0006663	-0.085297
RP11-72304	0.5290137	3.0460807	3.7465245	0.0002079	0.0006667	-0.086014
DDIT4L	0.5540694	2.9359576	3.746475	0.0002079	0.0006668	-0.086191
ZNF213	0.1044769	5.5121486	3.7464386	0.000208	0.0006668	-0.086322
EDN1	0.2776489	4.6917447	3.7464336	0.000208	0.0006668	-0.08634
RP11-142A2	0.6304353	-0.59338	3.7454247	0.0002088	0.0006693	-0.089955
DLEU2L	0.7077767	0.5780748	3.745327	0.0002088	0.0006695	-0.090305
CMSS1	-0.076265	5.8897603	-3.745218	0.0002089	0.0006697	-0.090695
FAM98A	-0.054263	6.2326068	-3.744724	0.0002093	0.0006709	-0.092466
RP4-742J24	0.5813247	-0.457035	3.7446741	0.0002094	0.000671	-0.092644
RP5-894A10	0.2201508	4.2565539	3.7446371	0.0002094	0.000671	-0.092777
GJA5	0.2195443	5.3943215	3.7439788	0.0002099	0.0006727	-0.095135
TCTN1	-0.104771	5.8563191	-3.743861	0.00021	0.0006729	-0.095556
HERPUD1	-0.0754	6.7029371	-3.74344	0.0002104	0.0006739	-0.097065
PLBD1-AS1	0.8366026	1.1876256	3.7430667	0.0002107	0.0006749	-0.098402
PPP2R5D	-0.058318	6.3702598	-3.742804	0.0002109	0.0006754	-0.099341
SMIM15	-0.04931	6.3702844	-3.7428	0.0002109	0.0006754	-0.099357
AC009263.2	-0.497774	-0.903269	-3.742679	0.000211	0.0006757	-0.099789
CCT6P1	0.1197604	4.9105817	3.7426264	0.000211	0.0006758	-0.099978
RP11-158H5	0.6786066	0.9152843	3.7422541	0.0002113	0.0006767	-0.101311
AARS2	-0.062285	6.0582371	-3.741479	0.0002119	0.0006786	-0.104085
FAM222B	0.0632964	6.0493622	3.7408685	0.0002124	0.0006801	-0.106271
AC004053.1	-0.253942	-1.376144	-3.740578	0.0002127	0.0006808	-0.10731
XCL1	0.5878333	3.2769003	3.7401587	0.000213	0.0006819	-0.108811
GOLGA2P8	0.6218456	-0.362777	3.7395041	0.0002136	0.0006835	-0.111154
PSMC6	-0.048698	6.4308403	-3.73937	0.0002137	0.0006838	-0.111632
RP11-161M6	0.4314385	-1.001064	3.738969	0.000214	0.0006848	-0.113068
TRIM71	1.1612894	2.8722112	3.7389647	0.000214	0.0006848	-0.113083
LINC01142	-0.432191	-1.064127	-3.738858	0.0002141	0.000685	-0.113466
ZNF45	0.1021248	5.4635612	3.738526	0.0002144	0.0006858	-0.114653
CTC-444N24	0.4053667	2.797659	3.7377957	0.000215	0.0006877	-0.117264
CFAP45	0.5408849	2.7739239	3.7377217	0.000215	0.0006878	-0.117529
HSPD1P1	0.4542198	3.1575272	3.7376496	0.0002151	0.0006879	-0.117787
LGALS9B	0.8290397	0.2292848	3.7375646	0.0002151	0.0006881	-0.118091
TRIM31	0.5799791	4.9473738	3.737553	0.0002152	0.0006881	-0.118133
CEACAM22P	-0.703623	2.3482897	-3.737163	0.0002155	0.000689	-0.119527
AOX1	-0.246332	6.9940246	-3.736228	0.0002163	0.0006914	-0.122869
RP11-319G6	-0.131667	4.5908101	-3.734935	0.0002173	0.0006948	-0.12749
PIP5K1A	0.0789985	6.2680455	3.7340601	0.0002181	0.0006971	-0.130618
FERP1	0.627453	0.1835755	3.7337116	0.0002183	0.0006979	-0.131863
WWP2	-0.057547	6.3283	-3.733548	0.0002185	0.0006983	-0.132447
CCDC81	0.360505	3.0083293	3.7334505	0.0002186	0.0006985	-0.132796
CHIT1	0.801198	3.2777191	3.7331462	0.0002188	0.0006993	-0.133883

GPR61	-0.640887	1.6092809	-3.732768	0.0002191	0.0007002	-0.135235
RPL9	-0.081389	7.0299834	-3.732362	0.0002195	0.0007013	-0.136683
RP11-345J4	0.1927965	4.941169	3.7321097	0.0002197	0.0007019	-0.137585
RP11-368L1	0.4525025	-1.194055	3.732063	0.0002197	0.0007019	-0.137752
RP4-800G7.	0.3985982	4.2248126	3.7318658	0.0002199	0.0007024	-0.138456
MCF2L	0.1221316	5.8385409	3.7308551	0.0002207	0.0007051	-0.142065
RP1-310013	0.6033734	0.9302875	3.7306248	0.0002209	0.0007056	-0.142887
SEMA6A-AS1	0.3356309	3.1606197	3.7301335	0.0002214	0.0007069	-0.144641
MAT2A	0.0863291	6.5032122	3.7300435	0.0002214	0.0007071	-0.144962
RPSA	-0.081981	7.0804396	-3.729414	0.000222	0.0007087	-0.14721
LINC01093	-0.79928	3.4704804	-3.728862	0.0002224	0.0007101	-0.149177
C7orf66	-0.166226	-1.482252	-3.728823	0.0002225	0.0007102	-0.149319
SLC9A3P3	-0.553361	-0.827985	-3.728496	0.0002227	0.000711	-0.150483
NBPF9	0.1840934	5.4358718	3.7283094	0.0002229	0.0007115	-0.15115
OTUB1	-0.053995	6.4955044	-3.728191	0.000223	0.0007117	-0.151574
PLIN4	-0.185114	6.1615418	-3.727948	0.0002232	0.0007123	-0.15244
GPR108	-0.056227	6.4796967	-3.72742	0.0002237	0.0007137	-0.154325
RFPL2	0.7248007	1.0161613	3.7271079	0.0002239	0.0007145	-0.155437
AC010975.1	-0.342996	-1.338187	-3.726565	0.0002244	0.0007159	-0.157373
RP11-22C8.	-0.219535	-1.413587	-3.72607	0.0002248	0.0007172	-0.159137
COLEC10	0.6600582	3.5373577	3.7258571	0.000225	0.0007177	-0.159897
EFHC1	0.1126265	5.312265	3.7258558	0.000225	0.0007177	-0.159902
ALOX12B	0.6519459	-0.521946	3.7257414	0.0002251	0.0007179	-0.16031
AGPAT3	-0.067001	6.7435049	-3.725512	0.0002253	0.0007185	-0.161126
EML4	-0.057122	6.6430038	-3.725393	0.0002254	0.0007187	-0.161551
U82670.9	0.3542208	-1.162251	3.7253262	0.0002255	0.0007189	-0.16179
RP11-697E2	0.4198545	-0.991293	3.724762	0.0002259	0.0007204	-0.163801
PALMD	-0.155038	6.1984462	-3.724707	0.000226	0.0007204	-0.163999
HSDL1	-0.082348	5.840203	-3.724423	0.0002262	0.0007212	-0.165011
RP11-248M1	-0.365224	2.8701997	-3.723965	0.0002266	0.0007224	-0.166644
ANKFN1	-1.010934	0.4302063	-3.723846	0.0002267	0.0007226	-0.167067
CTD-2278I1	0.6306861	0.713701	3.7237711	0.0002268	0.0007228	-0.167333
ACADVL	-0.080412	7.1242682	-3.723669	0.0002269	0.000723	-0.167699
MTFR2	0.3028603	4.3669147	3.7236251	0.0002269	0.000723	-0.167853
METTL13	-0.058994	6.2857809	-3.723542	0.000227	0.0007231	-0.168151
RP11-557H1	0.7931687	0.6791685	3.7235368	0.000227	0.0007231	-0.168168
BFAR	-0.04177	6.3291912	-3.723525	0.000227	0.0007231	-0.168212
LIPG	-0.219701	6.0967669	-3.723289	0.0002272	0.0007237	-0.169052
CRISP3	0.943672	0.3090088	3.7231732	0.0002273	0.000724	-0.169464
RP11-16L14	-0.23262	-1.430755	-3.722873	0.0002276	0.0007247	-0.170534
CCDC25	-0.08521	6.1864388	-3.722276	0.0002281	0.0007263	-0.172659
HLA-DRB5	0.1875636	6.2909904	3.7222679	0.0002281	0.0007263	-0.172689
CHRNG	0.5387175	-0.470514	3.7220631	0.0002283	0.0007268	-0.173419
RP11-297J2	0.3978609	-1.125837	3.7217252	0.0002286	0.0007277	-0.174623
S100A10	-0.115178	6.7040643	-3.7217	0.0002286	0.0007277	-0.174713
PAX9	0.72855	0.0313471	3.7215524	0.0002287	0.000728	-0.175238
AC013463.2	-0.443351	3.6787475	-3.721508	0.0002288	0.0007281	-0.175397
ZNF271P	-0.054662	6.0218273	-3.721397	0.0002289	0.0007283	-0.175791
TGFBR3	-0.158215	5.9384338	-3.720915	0.0002293	0.0007296	-0.17751
CSPG4P13	0.6001409	-0.345048	3.7209067	0.0002293	0.0007296	-0.177538
CDNF	-0.197609	4.7937223	-3.720716	0.0002295	0.00073	-0.178217

PHF2	0.0830267	6.0038239	3.7202955	0.0002298	0.0007311	-0.179714
RP11-156K1	0.7228077	1.4680396	3.7200151	0.0002301	0.0007319	-0.180713
RP11-405M1	-0.198558	-1.458627	-3.719754	0.0002303	0.0007325	-0.181641
RNASEH1	-0.065733	5.8017287	-3.719553	0.0002305	0.000733	-0.18236
RP11-526I2	0.4240883	3.6507262	3.7193461	0.0002307	0.0007335	-0.183094
RP4-612B18	-0.329448	-1.242961	-3.719304	0.0002307	0.0007336	-0.183245
AC006272.1	0.6254452	0.1926905	3.7192124	0.0002308	0.0007338	-0.183571
TPBGL	0.5608892	3.2690505	3.7190098	0.000231	0.0007343	-0.184292
C11orf65	-0.28413	3.6471336	-3.718958	0.000231	0.0007344	-0.184476
RP11-7F17.	-0.180963	-1.475777	-3.718519	0.0002314	0.0007355	-0.186037
RP11-460N2	0.5840131	-0.324621	3.7179174	0.0002319	0.0007372	-0.18818
RP11-278H7	-0.41501	-1.156781	-3.717898	0.0002319	0.0007372	-0.188249
ZNF157	0.4748403	-0.958008	3.717832	0.000232	0.0007373	-0.188484
SP3	0.0595041	6.3554144	3.7172348	0.0002325	0.0007389	-0.190609
LINC01347	0.5788315	2.7011093	3.7170034	0.0002327	0.0007395	-0.191432
AGAP1-IT1	0.6518077	2.9538784	3.7161198	0.0002335	0.0007419	-0.194575
NOX5	0.7376954	1.0585441	3.7157491	0.0002339	0.0007429	-0.195894
SNHG5	-0.135138	6.3890301	-3.715516	0.0002341	0.0007435	-0.196724
PRKCG	0.5824232	-0.649422	3.7154368	0.0002341	0.0007437	-0.197005
BMP1	-0.093406	6.2084893	-3.714501	0.000235	0.0007462	-0.200332
LCP1	0.1228909	6.4181899	3.7144689	0.000235	0.0007463	-0.200446
ZNF483	0.629424	2.2521356	3.7143241	0.0002351	0.0007466	-0.200961
NARS2	-0.079275	6.0280977	-3.714149	0.0002353	0.0007471	-0.201586
PLIN3	0.0696433	6.2635469	3.714036	0.0002354	0.0007473	-0.201985
NR2F1-AS1	0.2547933	4.7366639	3.7140318	0.0002354	0.0007473	-0.202
CBFA2T2	0.0720136	5.9895473	3.7139612	0.0002355	0.0007474	-0.202251
RP11-184M1	0.6732948	0.0230667	3.7119051	0.0002373	0.0007532	-0.209559
CRB3P1	-0.161088	-1.477423	-3.711789	0.0002374	0.0007535	-0.209973
KDM5C	0.04778	6.4912832	3.7117427	0.0002375	0.0007535	-0.210136
NAV3	0.4774248	4.5626918	3.7115019	0.0002377	0.0007542	-0.210992
TBC1D3B	0.6502861	0.2676973	3.711431	0.0002377	0.0007543	-0.211244
PPT1	0.0728719	6.4345255	3.711059	0.0002381	0.0007553	-0.212566
IGLVI-70	0.6442781	-0.786207	3.7107159	0.0002384	0.0007562	-0.213784
POU3F2	0.7836079	0.6298652	3.7106566	0.0002384	0.0007563	-0.213995
NAA38	-0.100568	6.2006527	-3.710506	0.0002386	0.0007567	-0.214528
KL	0.4473159	3.6647604	3.7102125	0.0002388	0.0007575	-0.215572
TMEM258	-0.070637	6.5214718	-3.710005	0.000239	0.000758	-0.21631
APCDD1L-AS	0.5986814	-0.48656	3.7099287	0.0002391	0.0007581	-0.21658
TCP11	-0.693036	1.2139486	-3.709916	0.0002391	0.0007581	-0.216624
RN7SL28P	-0.331457	-1.27905	-3.709552	0.0002394	0.0007591	-0.217919
CNPY3	-0.083793	6.5171081	-3.709504	0.0002395	0.0007592	-0.218089
ST13P20	0.5198959	-0.973375	3.7094069	0.0002396	0.0007594	-0.218433
ANP32A	-0.049877	6.6666505	-3.709155	0.0002398	0.0007601	-0.219329
ANXA8L1	0.8716334	0.5700896	3.7091391	0.0002398	0.0007601	-0.219384
FXYD6	0.1598572	5.6773818	3.7088848	0.0002401	0.0007607	-0.220287
COX18	-0.079118	5.9446834	-3.708647	0.0002403	0.0007613	-0.221131
LINC00482	-0.277041	5.01506	-3.708467	0.0002404	0.0007618	-0.221772
RP1-163G9.	0.5307001	-0.938033	3.7082825	0.0002406	0.0007623	-0.222426
AOC3	0.1146526	5.8910663	3.7082385	0.0002406	0.0007623	-0.222582
TPRG1-AS2	-0.442849	-0.978138	-3.708115	0.0002408	0.0007626	-0.223019
DNAJA3	-0.062489	6.5331813	-3.708068	0.0002408	0.0007627	-0.223188

HPCA	0.6677057	0.0803881	3.7079389	0.0002409	0.000763	-0.223645
DTX4	0.2053271	5.9804585	3.707815	0.000241	0.0007633	-0.224085
IGSF3	0.2747995	5.5516723	3.7072473	0.0002416	0.0007649	-0.2261
FECH	-0.086454	6.1438939	-3.707155	0.0002416	0.0007651	-0.226427
KLF4	0.2161144	5.4900487	3.7071415	0.0002416	0.0007651	-0.226476
TRIM67	0.5628334	2.743989	3.7071185	0.0002417	0.0007651	-0.226557
ERAS	-0.656896	0.7980429	-3.706991	0.0002418	0.0007654	-0.227012
TMPRSS7	0.5887759	-0.694142	3.7066249	0.0002421	0.0007664	-0.228309
RP11-15H2C	-0.194394	5.0399329	-3.706344	0.0002424	0.0007671	-0.229305
MSTO2P	0.2130547	4.5068911	3.7062885	0.0002424	0.0007672	-0.229503
GYPC	0.1241513	5.8298366	3.7062846	0.0002424	0.0007672	-0.229517
RP11-271K1	-0.288333	-1.323468	-3.706182	0.0002425	0.0007674	-0.22988
RWDD1	-0.070308	6.2528639	-3.705482	0.0002432	0.0007694	-0.232366
HCG4P11	0.6566561	1.8495366	3.7054293	0.0002432	0.0007695	-0.232551
RP11-347C1	-0.58539	2.5406324	-3.705387	0.0002433	0.0007695	-0.232702
HILS1	0.4669498	-1.069017	3.7050174	0.0002436	0.0007705	-0.234013
RP11-187A9	0.6176251	0.6645644	3.7050113	0.0002436	0.0007705	-0.234034
MOB3A	0.0655519	6.0533783	3.7049575	0.0002437	0.0007706	-0.234225
BOD1L1	0.0979562	6.009764	3.7049002	0.0002437	0.0007707	-0.234428
RP11-795A2	-0.312656	-1.34568	-3.704463	0.0002441	0.0007718	-0.235978
GGACT	-0.148568	5.6330952	-3.704458	0.0002441	0.0007718	-0.235995
IGKV1-13	0.540741	-0.995418	3.7040526	0.0002445	0.000773	-0.237434
P2RY8	0.1846698	5.3631689	3.7039463	0.0002446	0.0007732	-0.237811
RP11-426K3	0.5713021	-0.295176	3.7035835	0.0002449	0.0007742	-0.239098
AUH	-0.088339	5.9300195	-3.703518	0.000245	0.0007743	-0.23933
HNRNPH2	-0.053168	6.5348675	-3.703426	0.0002451	0.0007745	-0.239657
HMX3	0.5315184	-1.055423	3.7028557	0.0002456	0.0007762	-0.241678
HOXD10	-0.91121	1.438606	-3.702436	0.000246	0.0007773	-0.243165
TSPAN7	0.1543328	5.6759244	3.7023919	0.0002461	0.0007774	-0.243322
AC006960.7	-0.358033	-1.286004	-3.702099	0.0002463	0.0007782	-0.244359
C11orf53	0.7819946	-0.510361	3.7014605	0.0002469	0.00078	-0.246624
DOCK3	0.4394471	3.1424316	3.7011259	0.0002472	0.0007809	-0.24781
SPP2	-0.619907	5.8646978	-3.701116	0.0002472	0.0007809	-0.247844
CTD-2589M5	0.6571618	-0.68485	3.700926	0.0002474	0.0007814	-0.248518
DPRXP4	0.5571137	-0.393842	3.7007456	0.0002476	0.0007819	-0.249157
PRSS3	1.0564039	3.4666616	3.7005723	0.0002478	0.0007823	-0.249771
AC079610.2	-0.59154	-0.421738	-3.699871	0.0002484	0.0007843	-0.252254
ZNF599	-0.13605	5.0720746	-3.699577	0.0002487	0.0007851	-0.253298
RP11-345P4	0.6203782	2.575109	3.6984443	0.0002498	0.0007885	-0.257309
MTND1P34	-0.214607	-1.426915	-3.698233	0.00025	0.000789	-0.258058
SUGT1	-0.067469	6.2431306	-3.698075	0.0002501	0.0007894	-0.258618
RP11-52L5.	-0.706633	-0.376648	-3.698058	0.0002501	0.0007894	-0.258678
DUSP8P5	0.3986535	3.3440036	3.698014	0.0002502	0.0007895	-0.258833
PPP4R1	0.0503831	6.3050183	3.6979603	0.0002502	0.0007896	-0.259023
RP11-402J7	0.4526325	-0.94267	3.6977938	0.0002504	0.00079	-0.259613
FANCB	0.4549318	3.2202323	3.697693	0.0002505	0.0007902	-0.25997
AC009542.2	0.611291	0.665092	3.6976516	0.0002505	0.0007903	-0.260116
CHST12	0.0679715	5.7716892	3.6973458	0.0002508	0.0007911	-0.261199
ARID1B	0.0631684	6.1820797	3.6972919	0.0002509	0.0007912	-0.26139
ADCY6	0.0682141	6.2011793	3.6971431	0.000251	0.0007916	-0.261916
C16orf70	-0.099087	6.1854947	-3.697136	0.000251	0.0007916	-0.261942

LINC00649	0.4073139	4.8334446	3.6969384	0.0002512	0.0007921	-0.262641
SLC10A4	0.6745183	0.0685474	3.6969152	0.0002512	0.0007921	-0.262723
CCL7	0.5289794	-0.908069	3.6967636	0.0002514	0.0007925	-0.26326
FUBP1	0.0570545	6.2681082	3.696292	0.0002518	0.0007938	-0.264929
RTEL1-TNFR	0.3210131	4.3062461	3.6962569	0.0002519	0.0007939	-0.265054
SIDT2	-0.064737	6.358858	-3.696152	0.000252	0.0007941	-0.265426
CTC-471F3.	0.439745	3.4985568	3.6960985	0.000252	0.0007942	-0.265614
RP11-71L14	0.5632341	-0.62029	3.6960665	0.000252	0.0007942	-0.265727
RP11-304L1	-0.184674	4.2006406	-3.695782	0.0002523	0.000795	-0.266733
SPOCK1	0.6798216	3.8336713	3.6957265	0.0002524	0.0007951	-0.266931
RETSAT	-0.092919	6.7616516	-3.694555	0.0002535	0.0007986	-0.271077
RP11-310P5	0.4515998	-0.893228	3.6943456	0.0002537	0.0007992	-0.271817
TRO	-0.248202	5.4001897	-3.694213	0.0002538	0.0007995	-0.272285
LSM7	-0.086042	6.1992593	-3.693981	0.000254	0.0008001	-0.273108
RP1-199J3.	0.5645681	1.5559863	3.6938916	0.0002541	0.0008003	-0.273423
RP4-710M16	-0.37529	4.9361754	-3.693527	0.0002545	0.0008014	-0.274713
TMC3-AS1	-0.402675	3.8161343	-3.69326	0.0002547	0.0008021	-0.275657
CYTL1	0.6515169	2.2249454	3.6928753	0.0002551	0.0008032	-0.277017
MRPL23-AS1	0.9029131	0.5122821	3.6928327	0.0002551	0.0008033	-0.277168
ZBTB49	0.106842	5.0642817	3.6928005	0.0002552	0.0008033	-0.277282
ZNF700	0.0835672	5.5480825	3.6925928	0.0002554	0.0008039	-0.278016
RP11-334J6	-0.687042	1.065533	-3.692103	0.0002559	0.0008053	-0.279747
FLJ38122	0.6987303	0.61202	3.6920434	0.0002559	0.0008054	-0.279959
DSCR8	-1.070941	0.8509598	-3.691823	0.0002561	0.000806	-0.280739
GABRA4	0.413751	-1.146099	3.6917741	0.0002562	0.0008061	-0.280911
AP001205.1	0.6038313	0.0876815	3.6917589	0.0002562	0.0008061	-0.280965
RP4-742C19	0.6600175	0.8267431	3.6917006	0.0002562	0.0008062	-0.281171
UQCRHP4	-0.184062	-1.463966	-3.691193	0.0002567	0.0008077	-0.282965
CFHR3	-0.409955	6.1298174	-3.690749	0.0002572	0.0008089	-0.284536
CTD-2566J3	0.8201068	0.0116332	3.6902578	0.0002577	0.0008104	-0.286271
RNF138	0.074232	5.8292189	3.6902228	0.0002577	0.0008104	-0.286395
FZR1	-0.061414	6.3778855	-3.689918	0.000258	0.0008113	-0.287472
RP11-341D1	0.7762298	0.6057667	3.6896933	0.0002582	0.0008119	-0.288266
CDH20	0.5981671	0.0438866	3.6894649	0.0002584	0.0008125	-0.289073
RP11-295K2	0.562227	1.7463695	3.6893934	0.0002585	0.0008127	-0.289325
CTD-2184D3	-0.625836	-0.469176	-3.689323	0.0002586	0.0008128	-0.289575
RP11-262D1	0.4838362	2.1011978	3.6890798	0.0002588	0.0008135	-0.290433
RRM2P3	0.4540316	-0.999207	3.6886483	0.0002592	0.0008148	-0.291958
BRWD1	-0.063074	6.29087	-3.688443	0.0002594	0.0008153	-0.292683
POLR3D	0.0831975	5.6333507	3.68836	0.0002595	0.0008155	-0.292976
BRPF1	0.0512704	5.8429058	3.6880687	0.0002598	0.0008164	-0.294005
LINC00562	0.5114286	2.2553799	3.688049	0.0002598	0.0008164	-0.294075
PIGT	-0.052985	6.7691335	-3.6879	0.00026	0.0008167	-0.294601
POLE4	-0.09579	6.0624602	-3.687639	0.0002602	0.0008175	-0.295524
GHR	-0.21059	6.1703909	-3.687512	0.0002603	0.0008178	-0.29597
OR51B2	-0.144037	-1.496077	-3.687392	0.0002605	0.0008181	-0.296394
CLDN15	-0.187517	6.1755861	-3.687182	0.0002607	0.0008187	-0.297135
FBXL16	0.6558334	3.8371635	3.68716	0.0002607	0.0008187	-0.297215
TRDV1	0.6021428	-0.332371	3.6869728	0.0002609	0.0008192	-0.297875
KANK4	0.9399754	2.4594931	3.6868709	0.000261	0.0008194	-0.298235
LMF1-AS1	0.5839934	0.3245838	3.6868293	0.000261	0.0008195	-0.298382

OSBPL8	0.0748146	6.1009078	3.686821	0.000261	0.0008195	-0.298412
CLGN	0.5638844	5.0940828	3.6864056	0.0002614	0.0008207	-0.299878
RP11-477N3	-0.238294	4.116283	-3.685891	0.0002619	0.0008222	-0.301695
AC018738.2	-0.179477	5.3050652	-3.685409	0.0002624	0.0008236	-0.303396
PSMG3-AS1	-0.149683	5.3497602	-3.683857	0.000264	0.0008284	-0.308874
CLLU1OS	0.6153185	-0.766512	3.6835372	0.0002643	0.0008293	-0.310002
MIR4458HG	-0.341106	5.2381172	-3.683205	0.0002646	0.0008303	-0.311173
RP11-337C1	-0.153634	5.0027345	-3.682955	0.0002649	0.000831	-0.312054
RP11-612B6	-0.244307	-1.399751	-3.682849	0.000265	0.0008313	-0.312429
RNU2-23P	-0.200343	-1.478425	-3.682684	0.0002651	0.0008317	-0.31301
AP000347.2	0.3427387	3.5924919	3.6826355	0.0002652	0.0008318	-0.313183
HLCS	-0.06606	6.0764972	-3.682411	0.0002654	0.0008325	-0.313973
TKTL1	0.8460034	1.1951202	3.682192	0.0002656	0.0008331	-0.314747
RP11-400N1	-0.605886	-0.400246	-3.682157	0.0002657	0.0008331	-0.314871
GPS2P2	0.3826213	-1.146329	3.6815024	0.0002663	0.0008351	-0.317179
AC055733.1	-0.480711	-1.190297	-3.681181	0.0002667	0.0008361	-0.318314
MTA3	0.0651442	6.0228079	3.6810029	0.0002668	0.0008365	-0.31894
BOD1	-0.058772	6.2075557	-3.68097	0.0002669	0.0008366	-0.319056
PTGER1	0.8244184	1.9555622	3.6805715	0.0002673	0.0008378	-0.320461
NYNRIN	0.2021539	5.6691858	3.6804597	0.0002674	0.0008381	-0.320855
THRAP3	-0.036854	6.6183135	-3.680401	0.0002674	0.0008382	-0.321063
GDI2	-0.048136	6.9079532	-3.680323	0.0002675	0.0008383	-0.321338
RP3-522D1.	-0.481036	-0.85758	-3.68024	0.0002676	0.0008385	-0.32163
RNF212B	0.647746	1.2576943	3.6801175	0.0002677	0.0008389	-0.322062
CTD-2525P1	0.6543395	-0.05267	3.6795328	0.0002683	0.0008406	-0.324123
C8orf59	-0.097761	6.193608	-3.679459	0.0002684	0.0008408	-0.324381
RP11-236F9	-0.587549	-0.521079	-3.679443	0.0002684	0.0008408	-0.324439
AC087073.1	-0.255596	-1.420785	-3.679041	0.0002688	0.000842	-0.325858
AC005517.3	0.6119817	1.1568626	3.6780037	0.0002699	0.0008452	-0.329511
RP11-76908	0.5969934	-0.118292	3.6776685	0.0002702	0.0008462	-0.330692
UNC5B-AS1	0.6680361	-0.041634	3.6773819	0.0002705	0.000847	-0.331702
C10orf126	-0.869423	1.1164949	-3.677379	0.0002705	0.000847	-0.331712
CERS2	-0.070978	7.1889998	-3.677232	0.0002707	0.0008474	-0.332231
CD300LG	-0.83561	2.6380286	-3.677151	0.0002708	0.0008476	-0.332515
ORM1	-0.220584	7.4257086	-3.67709	0.0002708	0.0008477	-0.33273
RHBDD1	-0.05524	6.1429091	-3.676767	0.0002711	0.0008487	-0.333867
UGT1A2P	-0.995064	2.5136317	-3.676678	0.0002712	0.0008489	-0.334182
SPDEF	0.9079562	1.709054	3.676447	0.0002715	0.0008496	-0.334995
CTB-5506.1	0.1459112	4.2689771	3.6755902	0.0002724	0.0008522	-0.338012
XXbac-B444	0.5206123	2.2621022	3.6752514	0.0002727	0.0008533	-0.339205
RP11-90D4.	-0.364616	-1.258597	-3.674669	0.0002733	0.0008551	-0.341255
ATP6V1D	-0.057086	6.3322466	-3.674491	0.0002735	0.0008556	-0.341882
DUSP16	-0.089699	6.5181628	-3.674039	0.000274	0.0008569	-0.343471
RP11-101E1	-0.082348	5.7284123	-3.673356	0.0002747	0.0008591	-0.345876
BET1L	-0.044359	6.4824684	-3.672889	0.0002752	0.0008605	-0.34752
RIMS4	0.5550604	-0.824706	3.6723437	0.0002757	0.0008622	-0.349438
RP11-93K22	0.4189479	-0.980535	3.6722196	0.0002758	0.0008626	-0.349875
PLK4	0.1892597	4.8494057	3.6718782	0.0002762	0.0008635	-0.351076
ANKRD52	0.0720146	6.2113172	3.6718737	0.0002762	0.0008635	-0.351091
RP11-80I3.	-0.175347	-1.475694	-3.671132	0.000277	0.0008659	-0.353702
NFE2	0.6931852	2.2238933	3.6707994	0.0002773	0.0008669	-0.35487

RP11-138J2	-1.028929	1.7538568	-3.670703	0.0002774	0.0008671	-0.355209
CELSR3	-0.239321	5.2583366	-3.670188	0.000278	0.0008688	-0.35702
RP11-116D2	-0.359335	6.1367208	-3.669977	0.0002782	0.0008694	-0.357763
HPCAL1	-0.061628	6.3686408	-3.669757	0.0002784	0.00087	-0.358535
CDRT15P1	0.6527252	1.6597949	3.6696223	0.0002786	0.0008704	-0.359009
NFKB1	0.0672446	6.2091195	3.6695056	0.0002787	0.0008707	-0.359419
FEM1C	-0.075982	6.1396394	-3.669451	0.0002787	0.0008708	-0.359613
IGLVIVOR22	0.3734758	-1.167925	3.66895	0.0002793	0.0008724	-0.361372
CWC22	0.0519897	5.9493523	3.6689153	0.0002793	0.0008724	-0.361494
CNOT10	-0.054618	6.0919803	-3.668906	0.0002793	0.0008724	-0.361526
RP11-752D2	0.7042002	0.0207774	3.6686569	0.0002796	0.0008731	-0.362403
DAGLB	-0.05692	6.0510838	-3.668266	0.00028	0.0008743	-0.363776
VSNL1	-0.505461	5.4988354	-3.668009	0.0002803	0.0008751	-0.364681
RP11-231C1	0.6597003	0.691746	3.6679521	0.0002803	0.0008752	-0.36488
ST20	-0.149942	5.1141401	-3.667933	0.0002803	0.0008752	-0.364946
FAM3D	0.684704	0.9010581	3.6678379	0.0002804	0.0008754	-0.365281
AP000696.2	0.4923462	-1.109677	3.6673765	0.0002809	0.0008769	-0.366903
RP11-380M2	-0.504693	2.43039	-3.66709	0.0002812	0.0008777	-0.367908
RHEBP1	-0.657408	1.9624929	-3.667075	0.0002813	0.0008777	-0.367962
ABLIM1	-0.072495	6.4654495	-3.666483	0.0002819	0.0008796	-0.37004
RP11-457D2	-0.13337	-1.505299	-3.666309	0.0002821	0.0008801	-0.370654
UBE2J2	-0.060037	6.2524135	-3.666287	0.0002821	0.0008801	-0.370731
SCNM1	-0.08635	6.1297077	-3.665721	0.0002827	0.0008819	-0.372719
LRRC36	0.7811838	1.117694	3.6657161	0.0002827	0.0008819	-0.372735
EGR1	0.1587367	6.4604206	3.6656336	0.0002828	0.0008821	-0.373025
SIRPG-AS1	0.4266282	-1.010783	3.6655864	0.0002828	0.0008821	-0.373191
CENPO	0.1377846	5.4118856	3.6655787	0.0002828	0.0008821	-0.373218
RP11-108B1	-0.587775	-0.283977	-3.664941	0.0002835	0.0008842	-0.375459
ENO4	0.6103796	0.8721702	3.6645388	0.000284	0.0008854	-0.37687
LINC00535	-0.695808	3.3427398	-3.664284	0.0002842	0.0008862	-0.377766
VIT	0.4735463	-1.036677	3.6641841	0.0002843	0.0008864	-0.378115
RP11-755B1	-0.457195	2.6623609	-3.664169	0.0002844	0.0008864	-0.378167
AP001055.6	0.6149205	0.1761921	3.6634658	0.0002851	0.0008887	-0.380637
IQCF6	-0.267027	-1.366994	-3.663391	0.0002852	0.0008889	-0.380898
HEBP1	-0.076835	6.4610215	-3.663303	0.0002853	0.0008891	-0.381208
ABCD4	-0.065392	6.2527898	-3.663261	0.0002853	0.0008892	-0.381355
SLC2A4	-0.302303	5.1296801	-3.661991	0.0002867	0.0008933	-0.385813
CKMT2	0.45587	3.9802237	3.6617529	0.000287	0.0008941	-0.386648
PLAC4	0.6834203	-0.424897	3.6616833	0.000287	0.0008942	-0.386892
MAP4	-0.043888	6.8253024	-3.660882	0.0002879	0.0008969	-0.389702
GMEB1	0.0509533	5.74017	3.6607427	0.000288	0.0008973	-0.390192
FAR2P2	0.7310031	0.3333476	3.6607193	0.0002881	0.0008973	-0.390274
TMEM116	-0.080688	5.6440731	-3.660594	0.0002882	0.0008976	-0.390713
TUBA8P2	-0.159232	-1.482774	-3.660103	0.0002887	0.0008991	-0.392437
C12orf73	-0.078399	5.5959905	-3.660101	0.0002887	0.0008991	-0.392444
RP11-492I2	0.5153108	1.8681364	3.6599962	0.0002889	0.0008994	-0.39281
RP11-264I1	-0.243245	4.0923075	-3.659362	0.0002895	0.0009015	-0.395035
AP1S2	0.0875171	5.6270694	3.659083	0.0002899	0.0009023	-0.396013
ATAD5	0.1691522	4.9747871	3.6587821	0.0002902	0.0009033	-0.397068
RP3-405J10	0.5954524	0.0746256	3.6586802	0.0002903	0.0009036	-0.397425
RNF213	-0.064813	6.8459619	-3.658608	0.0002904	0.0009037	-0.397677

FHL2	0.1887993	5.5655149	3.6584253	0.0002906	0.0009043	-0.398319
CDH26	0.6020808	2.222664	3.6583374	0.0002907	0.0009045	-0.398627
RP1-134E15	0.5194797	-0.700872	3.6579427	0.0002911	0.0009058	-0.400011
PRSS3P2	0.7911761	-0.141636	3.6576336	0.0002914	0.0009067	-0.401094
RP11-203M5	0.6674595	1.0702541	3.6575035	0.0002916	0.000907	-0.40155
ARHGEF33	0.551143	1.7617832	3.6574969	0.0002916	0.000907	-0.401573
SRPK2	0.0684492	6.0275495	3.6573791	0.0002917	0.0009074	-0.401986
FLJ16779	0.5725654	-0.728217	3.6572815	0.0002918	0.0009076	-0.402328
AC103563.2	0.5708299	-0.848974	3.6571216	0.000292	0.0009081	-0.402888
FOXM1	0.1950972	5.7407742	3.6568734	0.0002923	0.0009089	-0.403758
COX6B2	0.6776821	0.5576597	3.6568466	0.0002923	0.0009089	-0.403852
DNMT3A	0.0907122	6.0825021	3.656757	0.0002924	0.0009091	-0.404166
GAR1	-0.069135	5.8051036	-3.656732	0.0002924	0.0009091	-0.404253
MAP3K15	0.7254174	1.8352246	3.6564921	0.0002927	0.0009099	-0.405094
XDH	-0.325923	6.1360563	-3.655885	0.0002934	0.0009119	-0.407223
HMGN2P5	0.383451	3.1125235	3.6557036	0.0002936	0.0009124	-0.407857
MAP6D1	-0.176535	4.3279788	-3.655256	0.0002941	0.0009139	-0.409426
C6orf136	-0.077392	6.0727391	-3.654816	0.0002945	0.0009153	-0.410967
COMMD5	-0.08476	6.2210262	-3.654565	0.0002948	0.0009161	-0.411845
KRTAP5-5	0.7935734	-0.231661	3.6541144	0.0002953	0.0009175	-0.413423
RP11-181E1	0.6707556	0.3667038	3.6541035	0.0002953	0.0009175	-0.413461
RP11-692D1	-0.354249	3.0470309	-3.653611	0.0002959	0.0009191	-0.415185
FNDC5	-0.489672	5.2193555	-3.653507	0.000296	0.0009194	-0.415551
DVL1	-0.075137	6.4629442	-3.653433	0.0002961	0.0009196	-0.41581
RP4-777L9.	-0.428459	-0.950608	-3.653243	0.0002963	0.0009202	-0.416476
RP11-847H1	-0.402663	-1.22615	-3.65304	0.0002965	0.0009208	-0.417186
CASP4	0.0865775	6.2533663	3.6529234	0.0002966	0.0009211	-0.417593
IGKV7-3	0.4622648	-1.070148	3.6521883	0.0002975	0.0009236	-0.420167
NRXN1	0.6630731	-0.5112	3.6520245	0.0002977	0.0009241	-0.42074
CCDC85B	-0.11917	5.8115285	-3.651793	0.0002979	0.0009248	-0.421549
C12orf75	0.312929	5.3159358	3.6514134	0.0002983	0.0009261	-0.422879
OXCT2P1	0.6364034	2.1937012	3.6513112	0.0002985	0.0009263	-0.423236
LINC00216	0.6297361	0.6409094	3.6512904	0.0002985	0.0009263	-0.423309
RP11-155D1	-0.599318	2.7509094	-3.651265	0.0002985	0.0009263	-0.423399
HERC2P2	0.1460301	6.0255318	3.6511822	0.0002986	0.0009265	-0.423688
RP11-678G1	0.8927133	0.9866596	3.6511608	0.0002986	0.0009265	-0.423763
BACH1-IT1	0.5485338	1.7657032	3.6510854	0.0002987	0.0009267	-0.424027
KCNIP4	0.2829167	3.5132934	3.6509759	0.0002988	0.000927	-0.42441
CTD-2010I1	0.3880505	2.9764513	3.65085	0.000299	0.0009274	-0.42485
IQCF3	-0.189905	-1.465971	-3.650728	0.0002991	0.0009277	-0.425278
RP11-568J2	0.634116	0.4508984	3.6506953	0.0002991	0.0009277	-0.425391
RP11-321E8	-0.116147	-1.512866	-3.650687	0.0002992	0.0009277	-0.425422
AC007682.1	-0.509339	-1.100016	-3.650575	0.0002993	0.000928	-0.425813
PYCARD-AS1	0.6247854	0.0121536	3.6502422	0.0002997	0.0009291	-0.426977
CALU	0.0568621	6.5988903	3.6501139	0.0002998	0.0009295	-0.427426
TMEM229B	0.1948528	4.9697402	3.6500754	0.0002998	0.0009295	-0.42756
LRRC18	0.4266062	-1.103423	3.6497315	0.0003002	0.0009307	-0.428763
AC139099.5	-0.424858	-1.003391	-3.649466	0.0003005	0.0009315	-0.429692
EML3	-0.054264	6.2640356	-3.648932	0.0003011	0.0009333	-0.43156
PDCD6IPP2	0.5587922	2.326039	3.6486083	0.0003015	0.0009344	-0.432692
PPRC1	0.0676653	6.137492	3.6485856	0.0003015	0.0009344	-0.432771

RP11-660L1	-0.221788	4.8902028	-3.648534	0.0003016	0.0009344	-0.432953
NBPF8P	0.2658004	4.9893172	3.6485249	0.0003016	0.0009344	-0.432983
RP11-622A1	-0.617815	4.7152324	-3.648193	0.000302	0.0009355	-0.434144
CD163	0.1645528	5.9725466	3.6476988	0.0003025	0.0009372	-0.435872
SLC38A4	-0.237382	6.7747791	-3.647414	0.0003029	0.0009381	-0.436866
ANLN	0.2123023	5.5478992	3.647274	0.000303	0.0009385	-0.437357
ENTPD5	-0.122116	6.7892509	-3.647248	0.000303	0.0009385	-0.437449
CYP2A7	-0.910397	4.4141778	-3.647173	0.0003031	0.0009387	-0.437709
HIST1H2AE	-0.4604	4.3693594	-3.64715	0.0003032	0.0009387	-0.437789
RP11-467K1	-0.250245	-1.397601	-3.646161	0.0003043	0.0009421	-0.441248
ZBTB20-AS1	0.5961396	-0.06856	3.6455133	0.000305	0.0009443	-0.443511
C9orf173	-0.50905	3.8058042	-3.645499	0.000305	0.0009443	-0.443562
UCP3	0.251454	3.7197393	3.6452799	0.0003053	0.000945	-0.444326
GRB2	-0.037481	6.7353139	-3.645171	0.0003054	0.0009453	-0.444707
DUSP28	-0.070966	5.0698723	-3.645141	0.0003054	0.0009453	-0.444812
RP11-322E1	0.4352392	-0.936105	3.6450909	0.0003055	0.0009454	-0.444987
TPSP2	-0.548586	4.0208931	-3.644817	0.0003058	0.0009463	-0.445943
SNAPC1	0.0892919	5.2143591	3.6444245	0.0003063	0.0009476	-0.447315
HLA-B	-0.104299	7.3975655	-3.644005	0.0003068	0.000949	-0.448781
TREX2	0.3872243	2.9898824	3.6438338	0.0003069	0.0009496	-0.449378
EBF1	0.2033774	5.0092937	3.6430162	0.0003079	0.0009524	-0.452233
CTC-327F10	0.5923094	-0.511935	3.6429951	0.0003079	0.0009524	-0.452307
RP11-77801	-0.219379	-1.423075	-3.642317	0.0003087	0.0009547	-0.454675
GON4L	0.0612863	6.082364	3.6419576	0.0003091	0.0009559	-0.455929
EXPH5	-0.551808	4.6017646	-3.641881	0.0003092	0.0009561	-0.456197
TTC23	-0.085137	5.9233507	-3.641668	0.0003095	0.0009568	-0.456942
MYBL2	0.2526723	5.7049103	3.641567	0.0003096	0.0009571	-0.457293
HIPK2	-0.094686	6.4821424	-3.64155	0.0003096	0.0009571	-0.457352
PCDHB11	0.6023728	3.384785	3.6413922	0.0003098	0.0009576	-0.457903
HMOX1	0.1286634	6.3368959	3.6412814	0.0003099	0.0009579	-0.45829
ABCB9	0.189594	4.9296793	3.640883	0.0003104	0.0009592	-0.45968
KCNIP2-AS1	-0.492039	2.1423428	-3.640787	0.0003105	0.0009595	-0.460015
DTX1	0.3396387	5.5037264	3.6407461	0.0003105	0.0009595	-0.460158
RP4-781K5.	-0.265599	-1.394281	-3.640732	0.0003105	0.0009595	-0.460207
CTA-292E10	-0.250399	4.2945552	-3.640608	0.0003107	0.0009599	-0.46064
QSER1	0.0659037	5.9675761	3.6403678	0.000311	0.0009607	-0.461478
RP11-347C1	0.5929145	-0.367518	3.6403332	0.000311	0.0009607	-0.461599
CLDN23	-0.1267	5.6803773	-3.640152	0.0003112	0.0009613	-0.462231
ANO7P1	-0.392959	4.1518099	-3.640142	0.0003112	0.0009613	-0.462267
SLCO1C1	0.5943758	2.4225675	3.6395176	0.000312	0.0009634	-0.464445
POU4F1	0.6394858	-0.597335	3.6393175	0.0003122	0.0009641	-0.465143
CTC-447K7.	-0.311425	-1.319965	-3.639252	0.0003123	0.0009642	-0.465372
CFLAR-AS1	0.5149983	2.2267217	3.6389799	0.0003126	0.0009651	-0.466321
RP11-348N5	0.5236105	2.3624816	3.6388408	0.0003128	0.0009655	-0.466806
RP11-147L1	0.6133768	0.1813067	3.6387353	0.0003129	0.0009658	-0.467174
RP11-100G1	0.3263907	-1.288727	3.6386075	0.000313	0.0009662	-0.46762
GAPDHP20	-0.382986	-1.125322	-3.638144	0.0003136	0.0009678	-0.469237
LIN37	-0.079602	5.1961309	-3.63808	0.0003136	0.000968	-0.469461
ETV5-AS1	0.3627445	-1.114738	3.6378692	0.0003139	0.0009687	-0.470195
RP11-84D1.	0.3594535	-1.144796	3.6373228	0.0003145	0.0009705	-0.4721
RP11-867G2	0.4528843	2.5532084	3.6373123	0.0003145	0.0009705	-0.472137

USP38	-0.081305	6.0954323	-3.637216	0.0003147	0.0009708	-0.472473
C2orf40	0.7129489	2.0180372	3.6368566	0.0003151	0.000972	-0.473726
MAN2B1	0.0640806	6.5138623	3.6365924	0.0003154	0.0009729	-0.474647
PITX2	0.8561372	0.0050197	3.6359812	0.0003161	0.000975	-0.476778
WRAP73	-0.061644	5.8449195	-3.635957	0.0003161	0.000975	-0.476861
SQSTM1	-0.089195	7.202002	-3.635685	0.0003165	0.000976	-0.47781
CPS1	-0.328425	7.0533476	-3.635472	0.0003167	0.0009767	-0.478552
RP11-470P2	-0.60445	-0.567096	-3.634843	0.0003175	0.0009789	-0.480745
ATP6V1E1	-0.051776	6.6129186	-3.634821	0.0003175	0.0009789	-0.480821
RPS26P4	-0.243048	-1.424155	-3.634764	0.0003176	0.000979	-0.481022
CDC14A	0.1824693	4.9017427	3.6342354	0.0003182	0.0009809	-0.482862
TYW1	-0.055802	5.9526017	-3.634047	0.0003184	0.0009815	-0.483518
CFAP44	0.1534733	5.186722	3.6335799	0.000319	0.0009831	-0.485145
ZNF821	0.2057498	4.6647811	3.6332972	0.0003193	0.0009841	-0.48613
SLC39A5	-0.386149	6.2245212	-3.633182	0.0003194	0.0009844	-0.486531
SERPINB9P1	0.4646713	3.9199852	3.6329963	0.0003197	0.000985	-0.487178
TUBAP2	0.4984068	2.2907753	3.6329492	0.0003197	0.0009851	-0.487342
RAB44	0.6306357	0.1486068	3.6329029	0.0003198	0.0009852	-0.487504
RP11-216B9	0.6141002	1.1500671	3.632661	0.0003201	0.000986	-0.488346
CYP2A13	-0.977332	2.6563289	-3.63223	0.0003206	0.0009875	-0.489849
GRK5	0.0903508	5.8386541	3.6321536	0.0003207	0.0009877	-0.490113
NKAIN1P1	-0.190215	-1.464626	-3.632083	0.0003208	0.0009879	-0.490361
RP11-404O1	0.4142563	-0.983165	3.6319241	0.000321	0.0009884	-0.490912
AC008984.2	0.3626603	-1.117009	3.6317463	0.0003212	0.000989	-0.491531
EPYC	0.3872839	-1.164705	3.6316359	0.0003213	0.0009893	-0.491916
AC007365.1	-0.481028	-0.795232	-3.631016	0.000322	0.0009915	-0.494073
MUC3A	0.5625596	5.0473289	3.6308218	0.0003223	0.0009921	-0.49475
RP11-40E6.	-0.495897	-0.711874	-3.630691	0.0003224	0.0009925	-0.495206
N4BP2L2-IT	0.2333254	4.1051491	3.6302884	0.0003229	0.0009939	-0.496607
LKAAEAR1	0.6690596	-0.429631	3.6302468	0.000323	0.000994	-0.496752
PRSS30P	0.6478252	1.8129487	3.6301968	0.000323	0.0009941	-0.496926
RP11-188P2	0.5411719	2.0641163	3.6300485	0.0003232	0.0009946	-0.497442
RP11-162P2	-0.471346	-0.751472	-3.62982	0.0003235	0.0009953	-0.498236
PCNPP1	0.5196695	1.6208355	3.6295373	0.0003238	0.0009963	-0.499221
ANKRD45	0.6761867	2.14288	3.6293303	0.0003241	0.000997	-0.499941
DNAAF2	-0.077933	5.8234274	-3.629205	0.0003242	0.0009974	-0.500379
ZNF467	0.1920935	5.5711105	3.6290427	0.0003244	0.0009979	-0.500942
DNASE1L1	0.1109621	5.7354188	3.6289196	0.0003246	0.0009983	-0.50137
IRAK4	0.0501693	5.7808662	3.6287007	0.0003248	0.000999	-0.502132
SNAI3-AS1	-0.165653	4.8371307	-3.628668	0.0003249	0.0009991	-0.502244
TMEM255B	0.1246951	5.2193518	3.6283348	0.0003253	0.0010002	-0.503405
TRMT61A	-0.075321	6.0951508	-3.628088	0.0003256	0.0010011	-0.504264
BVES-AS1	0.4992855	-0.783248	3.6280348	0.0003257	0.0010012	-0.504449
AL022344.7	0.3906932	-1.113313	3.6278161	0.0003259	0.0010019	-0.505209
ELAVL1	-0.041461	6.4628042	-3.627523	0.0003263	0.0010029	-0.50623
AJ239318.1	-0.19201	-1.465047	-3.627502	0.0003263	0.0010029	-0.506302
RP11-422N1	-0.791115	2.7369943	-3.627471	0.0003263	0.0010029	-0.506409
COPS5	-0.064422	6.4977543	-3.627245	0.0003266	0.0010037	-0.507195
HM13-IT1	0.5465822	1.8595715	3.6265888	0.0003274	0.0010061	-0.509478
CACNG8	0.6082495	2.0755844	3.6265348	0.0003275	0.0010062	-0.509666
RP11-230G5	0.3798432	-1.042692	3.6264751	0.0003276	0.0010063	-0.509873

DYNLRB2	-0.674156	1.6842252	-3.626453	0.0003276	0.0010063	-0.509951
GPATCH8	0.0708882	6.0925458	3.6263088	0.0003278	0.0010067	-0.510451
HOXC10	0.9136806	0.0977219	3.6263067	0.0003278	0.0010067	-0.510459
C17orf99	0.681844	-0.113526	3.626128	0.000328	0.0010073	-0.51108
TRPC2	0.592603	1.2926997	3.6257463	0.0003285	0.0010087	-0.512407
EZH1	-0.054241	6.0589712	-3.625346	0.0003289	0.0010101	-0.513799
RAC1	-0.052829	6.8880166	-3.625277	0.000329	0.0010103	-0.51404
ERICH6B	0.6581261	1.9866187	3.6251665	0.0003292	0.0010106	-0.514423
PEX13	-0.05256	6.2404707	-3.625102	0.0003292	0.0010107	-0.514648
INTS12	-0.061812	5.8744777	-3.625086	0.0003293	0.0010107	-0.514703
TMEM62	-0.072885	6.0154885	-3.624252	0.0003303	0.0010138	-0.517602
RP4-601P9.	-0.494766	-0.900334	-3.623946	0.0003307	0.0010149	-0.518665
EPB41L4A-A	-0.109244	5.7599734	-3.623719	0.000331	0.0010156	-0.519453
PAPL	0.616654	-0.374728	3.6233209	0.0003314	0.0010171	-0.520837
RAB28	0.0636384	5.5678121	3.6231554	0.0003317	0.0010176	-0.521412
ZMYND19	-0.073659	5.9109441	-3.622849	0.000332	0.0010187	-0.522477
PLAC8L1	-0.483947	2.8085128	-3.622778	0.0003321	0.0010189	-0.522721
ZBED1P1	-0.315784	-1.276043	-3.622564	0.0003324	0.0010196	-0.523468
SERTAD2	-0.065421	6.146497	-3.62232	0.0003327	0.0010204	-0.524314
PSORS1C3	-0.715373	2.6120752	-3.622314	0.0003327	0.0010204	-0.524336
ACADS	-0.101211	6.5584341	-3.621849	0.0003333	0.0010221	-0.525951
RP11-165F2	0.7136094	0.1806131	3.6217915	0.0003333	0.0010222	-0.52615
CCDC162P	0.7922483	3.3423601	3.6214317	0.0003338	0.0010234	-0.527399
RP11-332H1	-0.478744	2.8007944	-3.621424	0.0003338	0.0010234	-0.527426
KCNN3	0.1819187	5.0243738	3.6213412	0.0003339	0.0010237	-0.527714
IP6K1	-0.050877	6.3492539	-3.621299	0.000334	0.0010237	-0.52786
ZSCAN30	0.0817275	5.5535284	3.6212875	0.000334	0.0010237	-0.5279
RTCB	-0.054961	6.5655609	-3.620957	0.0003344	0.0010249	-0.529048
RP11-202G1	0.4407181	-1.025949	3.6207911	0.0003346	0.0010254	-0.529624
FLJ42102	-0.455677	-0.911098	-3.620074	0.0003355	0.0010281	-0.532114
MAGEA10	0.7196459	-0.635134	3.6199884	0.0003356	0.0010283	-0.53241
CHRNA5	0.6212358	2.6429487	3.6197199	0.0003359	0.0010293	-0.533343
RP11-211N1	0.5422159	-0.448003	3.6196613	0.000336	0.0010294	-0.533546
ELL	0.0509228	5.9481393	3.6191262	0.0003367	0.0010314	-0.535403
CREB5	0.2131318	4.8469339	3.6190904	0.0003367	0.0010314	-0.535528
MIR6753	0.5425987	-0.413943	3.6189146	0.0003369	0.001032	-0.536138
LARP7	-0.055053	6.1436527	-3.618482	0.0003375	0.0010336	-0.537639
RP11-5C23.	-0.124059	4.9790941	-3.618476	0.0003375	0.0010336	-0.537661
SNHG12	0.11934	5.3749085	3.6184135	0.0003376	0.0010337	-0.537877
SNX18P7	0.4931975	-0.919416	3.6183785	0.0003376	0.0010338	-0.537998
SEPT11	0.0658736	6.4092026	3.6181103	0.000338	0.0010347	-0.538928
ZDHHC24	-0.071581	6.0174827	-3.618061	0.000338	0.0010348	-0.5391
METTL22	-0.064722	5.6303019	-3.618029	0.0003381	0.0010348	-0.539211
ZNF37A	0.0725582	5.8152351	3.6179603	0.0003381	0.001035	-0.539449
CTD-2515H2	-0.399012	-1.001621	-3.617676	0.0003385	0.001036	-0.540435
PTCHD1-AS	-0.312031	-1.320116	-3.617495	0.0003387	0.0010366	-0.541062
RP11-439K3	0.5114922	-0.577753	3.6173595	0.0003389	0.0010371	-0.541533
RP11-64C12	-0.170742	-1.470847	-3.617042	0.0003393	0.0010382	-0.542634
RP11-385F7	-0.286534	3.8297644	-3.616728	0.0003397	0.0010393	-0.543723
LINC01322	-0.811661	0.290204	-3.616265	0.0003403	0.0010411	-0.545329
SLC8A1-AS1	0.5204168	-0.660243	3.6162339	0.0003403	0.0010411	-0.545438

PCDHAC1	0.8467648	0.3894092	3.6161428	0.0003404	0.0010414	-0.545754
NPDC1	0.1244738	5.8648373	3.6159169	0.0003407	0.0010421	-0.546537
PEX2	-0.063668	6.2886874	-3.615775	0.0003409	0.0010426	-0.54703
FGD1	0.1464157	5.4833198	3.6156865	0.000341	0.0010429	-0.547336
NEXN-AS1	0.6434726	0.5384109	3.6154848	0.0003413	0.0010436	-0.548036
ANKIB1	0.0732044	6.0525728	3.6151552	0.0003417	0.0010448	-0.549178
TNFSF12	0.1168382	5.7699504	3.6147743	0.0003422	0.0010462	-0.550499
SETD1A	-0.044852	6.1934184	-3.614486	0.0003426	0.0010472	-0.551497
CTD-3065J1	-0.199951	4.6261276	-3.61447	0.0003426	0.0010472	-0.551552
DGUOK-AS1	0.3696847	3.0438308	3.614317	0.0003428	0.0010477	-0.552084
ORMDL1	-0.047784	6.1419585	-3.61417	0.000343	0.0010482	-0.552594
RP11-533E1	-0.294037	3.332349	-3.614068	0.0003431	0.0010485	-0.552947
AP003068.2	-0.20156	5.2732667	-3.613454	0.0003439	0.0010508	-0.555076
FN1	-0.066039	7.6837426	-3.612651	0.0003449	0.0010538	-0.557858
VAMP2	-0.075279	6.230911	-3.612618	0.0003449	0.0010538	-0.557973
RP11-411B1	0.5709837	-0.341656	3.6126133	0.000345	0.0010538	-0.557989
CTD-2044J1	-0.626316	0.5715971	-3.612451	0.0003452	0.0010544	-0.558551
RP11-423F2	0.4993492	-0.541037	3.6121802	0.0003455	0.0010553	-0.559489
RP11-167N2	-0.168622	-1.471779	-3.611679	0.0003462	0.0010572	-0.561224
RP11-466A1	0.4904019	-0.670069	3.6116482	0.0003462	0.0010573	-0.561332
AC091114.1	-0.286508	-1.281951	-3.611533	0.0003463	0.0010576	-0.561732
HRC	0.2849954	4.4658944	3.6113273	0.0003466	0.0010584	-0.562444
COA6	-0.087471	6.2314251	-3.610945	0.0003471	0.0010598	-0.563768
OR2A7	0.6990282	2.3986135	3.6104658	0.0003477	0.0010616	-0.565427
CTA-390C10	0.3383637	-1.207812	3.6102504	0.000348	0.0010623	-0.566173
MBOAT1	0.4637448	4.8017641	3.6098049	0.0003486	0.001064	-0.567716
KCMF1	-0.041737	6.4016353	-3.609548	0.0003489	0.0010649	-0.568605
RP1-283E3.	0.3100868	4.3216436	3.6094302	0.0003491	0.0010653	-0.569013
METTL8	0.0648971	5.6066443	3.6094059	0.0003491	0.0010653	-0.569097
CXXC4	0.4559508	4.2792259	3.6092879	0.0003493	0.0010657	-0.569506
RP11-513G1	-0.577562	2.1851981	-3.609191	0.0003494	0.001066	-0.569842
CTD-3222D1	-0.19648	4.3262488	-3.609043	0.0003496	0.0010665	-0.570354
RASGRF1	0.616097	3.1850271	3.6088862	0.0003498	0.001067	-0.570896
RP11-165J3	0.4773526	2.3436069	3.6085879	0.0003502	0.001068	-0.571929
RP11-74C13	-0.751893	1.4117405	-3.608576	0.0003502	0.001068	-0.571969
TICRR	0.3129247	4.6324005	3.6085673	0.0003502	0.001068	-0.572
RP11-299M1	-0.62388	0.697456	-3.608488	0.0003503	0.0010683	-0.572273
WDSUB1	0.0956304	5.4962013	3.6081232	0.0003508	0.0010696	-0.573537
ZNF230	0.1185398	4.9467731	3.6080653	0.0003508	0.0010698	-0.573738
RP11-433B3	-0.293668	-1.310893	-3.606824	0.0003525	0.0010746	-0.578033
RP11-66B24	0.6443536	-0.498175	3.6067879	0.0003525	0.0010747	-0.578158
RP11-686D2	0.4386236	2.9112667	3.6065195	0.0003529	0.0010757	-0.579086
PAQR4	0.1888058	5.3575346	3.6063091	0.0003531	0.0010764	-0.579814
TMEM102	0.1356697	5.1869586	3.6058903	0.0003537	0.001078	-0.581263
RP11-730K1	-0.625861	-0.002364	-3.605179	0.0003546	0.0010808	-0.583724
RP13-467H1	-0.697332	1.7491878	-3.603878	0.0003564	0.0010859	-0.588221
CLEC4G	0.7303009	2.5274316	3.6038442	0.0003564	0.001086	-0.588338
ACTBP2	0.5628558	1.8170027	3.6034125	0.000357	0.0010876	-0.58983
RAB3B	0.5243851	5.1661563	3.6031246	0.0003574	0.0010887	-0.590826
DUSP11	0.0552143	5.8050561	3.603033	0.0003575	0.001089	-0.591142
GIGYF1	-0.051845	6.4013512	-3.603005	0.0003575	0.001089	-0.59124

ZFAND2A	-0.096064	6.2693591	-3.60292	0.0003576	0.0010892	-0.591531
FLT4	0.1214038	5.8006785	3.6024413	0.0003583	0.0010911	-0.593187
BMP10	0.5374484	-0.979678	3.6024208	0.0003583	0.0010911	-0.593258
AC007163.3	-0.520592	-0.805942	-3.602028	0.0003588	0.0010926	-0.594615
IL1A	0.5972111	-0.119808	3.6018675	0.000359	0.0010932	-0.59517
CD320	-0.094853	6.1377083	-3.601575	0.0003594	0.0010942	-0.596182
PPP1R3F	-0.165843	5.2847109	-3.601573	0.0003594	0.0010942	-0.596187
RP11-16E23	0.5348746	-0.381252	3.6013547	0.0003597	0.001095	-0.596942
EIF4HP1	0.6074019	1.9350344	3.6012211	0.0003599	0.0010954	-0.597403
SH3GL1P2	0.6184934	1.0178673	3.6011403	0.00036	0.0010957	-0.597682
RP3-467L1.	-0.477635	2.6040975	-3.601009	0.0003602	0.0010961	-0.598136
MED14P1	-0.140349	-1.502233	-3.600758	0.0003605	0.001097	-0.599004
LINC00881	-0.700841	1.0848539	-3.600317	0.0003611	0.0010987	-0.600525
ATE1	-0.063968	6.1742705	-3.600119	0.0003614	0.0010995	-0.601209
CDK4	0.0738614	6.3110932	3.5999684	0.0003616	0.0011	-0.60173
TFF1	0.9773713	0.6976671	3.5997505	0.0003619	0.0011008	-0.602483
GTDC1	0.0779433	5.661392	3.5993023	0.0003625	0.0011025	-0.604031
FLG	0.8215845	0.7837704	3.5991853	0.0003626	0.0011029	-0.604434
RP11-351E7	-0.195187	-1.45603	-3.599011	0.0003629	0.0011035	-0.605037
CTC-436P18	0.5632777	0.0963023	3.5988633	0.0003631	0.001104	-0.605546
RP11-108A1	-0.405156	-1.080119	-3.598702	0.0003633	0.0011046	-0.606103
LRRC2	-0.324544	5.4742639	-3.598664	0.0003633	0.0011047	-0.606234
CCDC6	0.0481821	6.2247675	3.5979686	0.0003643	0.0011074	-0.608635
MOBP	0.5855861	-0.286216	3.5979363	0.0003643	0.0011075	-0.608747
GPLD1	-0.330675	5.7472624	-3.597665	0.0003647	0.0011085	-0.609684
RPL6	-0.06405	7.1554978	-3.597635	0.0003647	0.0011085	-0.609785
TRBJ2-1	0.4690927	-0.820389	3.5974918	0.0003649	0.001109	-0.610281
FCGR3A	0.1562099	5.999747	3.5974262	0.000365	0.0011092	-0.610507
RP11-37C7.	0.5765729	2.3075082	3.5972685	0.0003652	0.0011097	-0.611051
RAB35	0.0413726	6.2234024	3.597254	0.0003652	0.0011097	-0.611101
UFC1	-0.063472	6.6322229	-3.596792	0.0003659	0.0011115	-0.612695
NSUN2	-0.047808	6.4652949	-3.596777	0.0003659	0.0011115	-0.612748
ARMC1	-0.064064	6.2761384	-3.596701	0.000366	0.0011117	-0.613009
TMEM200B	0.3317214	5.1408942	3.5964306	0.0003663	0.0011128	-0.613943
PANX2	-0.307686	5.6864124	-3.596156	0.0003667	0.0011138	-0.614891
CCZ1	0.1290013	5.2136847	3.5959961	0.0003669	0.0011144	-0.615442
RANBP1	-0.063933	6.4074982	-3.595886	0.0003671	0.0011147	-0.615822
TMEM127	-0.042044	6.6546248	-3.595769	0.0003672	0.0011151	-0.616227
ADO	0.0504512	5.8215654	3.5956151	0.0003675	0.0011157	-0.616757
IFNGR1	-0.094986	6.4964538	-3.595446	0.0003677	0.0011163	-0.61734
CYP11B1	-0.267144	-1.403833	-3.595318	0.0003679	0.0011167	-0.617781
LINC01207	1.0378766	0.8715849	3.595285	0.0003679	0.0011168	-0.617895
C15orf38-A	0.6111741	1.5204262	3.594718	0.0003687	0.001119	-0.619851
AC097468.4	0.7582312	0.8471688	3.5946453	0.0003688	0.0011192	-0.620102
RP11-70D24	-0.390496	-1.103685	-3.594549	0.0003689	0.0011195	-0.620434
MIR5582	-0.264999	-1.307316	-3.594453	0.000369	0.0011198	-0.620765
USH1C	1.0517739	3.379234	3.5941673	0.0003694	0.0011209	-0.62175
MSTO1	-0.088661	5.8340724	-3.594149	0.0003695	0.0011209	-0.621812
DLX6	0.9091319	0.0347592	3.5935906	0.0003702	0.0011231	-0.623738
CTB-78F1.1	-0.33169	-1.284885	-3.593291	0.0003706	0.0011243	-0.624773
RP11-562A8	0.585461	2.2001906	3.5926166	0.0003716	0.001127	-0.627096

JMY	-0.081265	5.9981276	-3.59134	0.0003733	0.0011323	-0.631496
LINC00943	0.7289942	0.5933794	3.5911577	0.0003736	0.0011329	-0.632124
GPR39	-0.219553	5.1353187	-3.59055	0.0003744	0.0011354	-0.634216
RP11-407A1	0.828362	-0.158596	3.5904381	0.0003746	0.0011358	-0.634604
RNA5SP40	-0.520012	-0.865079	-3.589945	0.0003753	0.0011378	-0.636301
GPN3	-0.061906	6.0403015	-3.589082	0.0003765	0.0011413	-0.639274
ARMCX4	0.1230549	5.1339804	3.5879593	0.000378	0.001146	-0.64314
BMX	0.5054647	3.2803404	3.5879118	0.0003781	0.0011461	-0.643304
MUC17	0.493681	-0.987374	3.5878611	0.0003782	0.0011462	-0.643479
PDCD6IP	-0.04209	6.6335377	-3.587555	0.0003786	0.0011474	-0.644531
MAPK12	0.2052979	5.4610247	3.587419	0.0003788	0.0011479	-0.645
FAM227B	-0.156342	4.742374	-3.587134	0.0003792	0.001149	-0.64598
CGB7	0.5036581	-0.646017	3.5871119	0.0003792	0.001149	-0.646057
LA16c-380H	-0.459025	3.6980473	-3.586805	0.0003797	0.0011502	-0.647115
ERMP1	0.0841243	6.1689792	3.5859334	0.0003809	0.0011538	-0.650113
CYP3A52P	-0.358377	-1.190567	-3.585219	0.0003819	0.0011568	-0.652572
PNMT	-0.871121	0.922009	-3.584838	0.0003824	0.0011583	-0.653882
HIST1H3A	-0.641265	0.587359	-3.584765	0.0003825	0.0011586	-0.654133
POPDC3	0.9528433	0.4872467	3.5834363	0.0003844	0.0011642	-0.658703
ARPC1A	-0.056569	6.691636	-3.583402	0.0003845	0.0011642	-0.658822
LINC01562	-0.60745	0.1721747	-3.583264	0.0003847	0.0011647	-0.659296
XXbac-BPG1	-0.287206	4.5617008	-3.583124	0.0003849	0.0011652	-0.659776
SNX18P23	-0.230966	-1.386239	-3.582918	0.0003852	0.001166	-0.660485
SNRPD2	-0.081854	6.673404	-3.582536	0.0003857	0.0011676	-0.661799
DPM2	-0.078224	6.3460609	-3.58247	0.0003858	0.0011678	-0.662026
ABCB5	0.9237129	0.8744923	3.5822696	0.0003861	0.0011685	-0.662715
RP11-247I1	0.6275985	0.9353212	3.5822359	0.0003861	0.0011686	-0.662831
SNTB2	0.0700806	5.8374076	3.5820112	0.0003865	0.0011695	-0.663603
KAT6B	0.0828848	5.7852575	3.5818769	0.0003866	0.0011699	-0.664065
NXF5	-0.615209	-0.37623	-3.58179	0.0003868	0.0011702	-0.664363
DUOX1	0.2591483	4.7566213	3.5816512	0.000387	0.0011707	-0.66484
HNRNPL	-0.032531	6.7533773	-3.580975	0.0003879	0.0011736	-0.667164
RP1-27K12.	-0.74843	4.2594347	-3.580819	0.0003882	0.0011741	-0.667701
CIR1	-0.050262	6.1658984	-3.580462	0.0003887	0.0011756	-0.668927
RP11-730B2	0.4855625	-0.626773	3.5803563	0.0003888	0.001176	-0.669291
RPUSD4	-0.066016	6.0030482	-3.580187	0.0003891	0.0011766	-0.669871
DLGAP1-AS1	-0.173341	5.3694686	-3.580139	0.0003891	0.0011767	-0.670038
CTD-2256P1	-0.519607	2.390195	-3.579773	0.0003897	0.0011782	-0.671295
SMCO2	0.7284064	1.6065431	3.5792285	0.0003905	0.0011805	-0.673165
MAN2B2	-0.069559	6.4745023	-3.578994	0.0003908	0.0011814	-0.673971
RP5-1142A6	0.6118668	1.569258	3.5788946	0.0003909	0.0011817	-0.674312
AF131216.6	0.6091951	0.5159903	3.5787096	0.0003912	0.0011825	-0.674947
ENGASE	-0.071614	6.0550482	-3.578535	0.0003915	0.0011831	-0.675548
RPL39	-0.089206	6.6220288	-3.578237	0.0003919	0.0011843	-0.67657
TMEM150A	-0.077936	6.2503558	-3.577868	0.0003924	0.0011858	-0.677837
INMT-FAM18	0.4211409	-1.013859	3.5778592	0.0003924	0.0011858	-0.677868
LINC01106	0.6426139	2.3545212	3.5777943	0.0003925	0.001186	-0.678091
PCBP2-OT1	0.6054355	0.116869	3.5777457	0.0003926	0.0011861	-0.678258
SNCB	0.5675879	-0.618873	3.5774929	0.000393	0.0011871	-0.679126
TPTE2P5	0.5431361	2.1089422	3.5773744	0.0003931	0.0011875	-0.679532
CTB-60B18.	0.5351899	-0.770939	3.5767264	0.0003941	0.0011903	-0.681757

CENPF	0.1767898	5.9115773	3.5759729	0.0003952	0.0011935	-0.684343
RP11-893F2	0.5793946	1.6288659	3.5758371	0.0003954	0.001194	-0.68481
AGAP7P	0.7111379	1.1270634	3.5755114	0.0003959	0.0011953	-0.685928
CTD-2006K2	0.6405088	0.6952486	3.5752935	0.0003962	0.0011962	-0.686675
ANXA2P3	0.4347903	-0.820777	3.5751846	0.0003963	0.0011966	-0.687049
RP11-224P1	-0.200159	-1.449094	-3.575043	0.0003965	0.0011971	-0.687536
NDUFB2-AS1	0.1576102	3.878323	3.5750086	0.0003966	0.0011971	-0.687653
TRIM38	-0.079371	6.0811924	-3.574464	0.0003974	0.0011994	-0.689521
ZMAT4	0.577779	-0.847528	3.5744501	0.0003974	0.0011994	-0.689569
EFCAB7	0.1392925	4.881758	3.5744433	0.0003974	0.0011994	-0.689592
AASS	-0.334051	5.5287403	-3.574114	0.0003979	0.0012007	-0.690721
CYB561A3	-0.065822	6.2635642	-3.573693	0.0003985	0.0012025	-0.692165
PCCA	-0.095022	6.3741155	-3.573659	0.0003986	0.0012025	-0.692282
RP11-285E9	0.6340896	0.1150294	3.5734258	0.0003989	0.0012035	-0.693082
RP11-426C2	-0.762688	0.2600431	-3.573184	0.0003993	0.0012044	-0.693911
MGAT4EP	-0.564038	-0.533682	-3.572904	0.0003997	0.0012056	-0.694873
RP11-380G5	0.6224111	0.6148836	3.5728772	0.0003997	0.0012056	-0.694964
BBS12	0.2111193	4.3174969	3.5728662	0.0003997	0.0012056	-0.695002
APELA	0.6291512	-0.758837	3.5725286	0.0004002	0.001207	-0.69616
RP11-40401	0.4438133	-0.88939	3.5721379	0.0004008	0.0012086	-0.697499
RP11-800A3	0.6373181	1.4786057	3.5718088	0.0004013	0.00121	-0.698628
RCC2P6	0.490675	-0.780039	3.5717771	0.0004014	0.00121	-0.698736
CTB-61M7.2	0.7015555	0.3059028	3.5716798	0.0004015	0.0012104	-0.69907
RP4-569M23	-0.536518	3.0315741	-3.571399	0.0004019	0.0012115	-0.700032
KIAA1841	0.1160165	5.2186116	3.5705488	0.0004032	0.0012152	-0.702946
MIR3134	-0.276037	-1.31108	-3.57036	0.0004035	0.001216	-0.703594
SERPINA1	-0.106698	8.0204727	-3.570332	0.0004035	0.001216	-0.703689
AC027601.1	0.3137636	3.9369649	3.5702944	0.0004036	0.0012161	-0.703819
BAZ1A	0.0777647	5.992883	3.5701323	0.0004038	0.0012167	-0.704374
C6orf201	-0.395739	2.9107147	-3.569964	0.000404	0.0012173	-0.704949
RP11-1113L	-0.492472	3.4695005	-3.569837	0.0004042	0.0012178	-0.705386
RP5-1139I1	-0.520366	-0.551457	-3.569736	0.0004044	0.0012182	-0.705733
MYRF	0.1204533	6.3605521	3.5697062	0.0004044	0.0012182	-0.705834
RHOBTB1	0.2376073	5.6620016	3.5687424	0.0004059	0.0012224	-0.709136
AC022182.3	0.6560282	0.839334	3.5684446	0.0004063	0.0012237	-0.710156
RP5-119802	0.6586771	2.0600741	3.5683274	0.0004065	0.0012241	-0.710558
KATNB1	-0.068461	5.9643951	-3.568119	0.0004068	0.0012249	-0.711271
RP11-212I2	0.410889	-1.027511	3.5676757	0.0004075	0.0012268	-0.71279
MIER2	0.104894	5.74887	3.5670229	0.0004085	0.0012296	-0.715025
PHBP9	0.586131	1.7915156	3.5670175	0.0004085	0.0012296	-0.715044
RP4-61404.	0.6178134	1.2573593	3.5670058	0.0004085	0.0012296	-0.715084
MARVELD2	-0.121213	6.2392174	-3.566814	0.0004088	0.0012303	-0.715742
TFDP3	-0.945706	-0.062797	-3.566579	0.0004091	0.0012313	-0.716545
LINC00643	0.3100628	-1.283637	3.5662908	0.0004096	0.0012325	-0.717532
RP11-618I1	0.9523728	0.9052548	3.5660907	0.0004099	0.0012333	-0.718217
Z99756.1	-0.200299	-1.430868	-3.565968	0.00041	0.0012338	-0.718636
NEMF	0.0946212	5.7685041	3.565889	0.0004102	0.001234	-0.718907
ATP6AP1	-0.056049	6.779201	-3.56564	0.0004105	0.001235	-0.719759
C7orf61	0.5724304	1.6102872	3.5656382	0.0004105	0.001235	-0.719766
CTD-2382E5	0.6800704	0.7847568	3.5650556	0.0004114	0.0012375	-0.72176
ABCA13	0.8033524	1.9898983	3.5648908	0.0004117	0.0012382	-0.722324

CKMT1A	0.9122546	0.8546784	3.5648226	0.0004118	0.0012383	-0.722557
CPS1-IT1	-0.868419	1.3623909	-3.564816	0.0004118	0.0012383	-0.722581
DRICH1	0.5940971	1.9535678	3.5644559	0.0004123	0.0012399	-0.723812
ZNF18	-0.081503	5.5111192	-3.56442	0.0004124	0.0012399	-0.723934
COG7	-0.056504	5.9649054	-3.563709	0.0004135	0.0012431	-0.726369
TSPAN6	-0.087223	6.6482616	-3.563634	0.0004136	0.0012433	-0.726624
SLCO1B1	-0.345699	6.4316013	-3.563421	0.0004139	0.0012442	-0.727354
NACAP3	-0.642823	0.9299402	-3.562925	0.0004147	0.0012464	-0.72905
RP11-132N1	0.7062275	0.4782585	3.562605	0.0004152	0.0012477	-0.730144
LINC00960	0.6151981	3.0179358	3.5625188	0.0004153	0.001248	-0.730439
STOML1	-0.079252	6.0094366	-3.561872	0.0004163	0.0012509	-0.732649
BRD3	0.0903783	5.9470877	3.5617593	0.0004165	0.0012513	-0.733036
RP4-621N11	-0.555504	-0.395637	-3.56133	0.0004171	0.0012532	-0.734503
ACTN3	0.6533965	0.290121	3.561264	0.0004172	0.0012534	-0.73473
TMEM180	-0.110446	5.6899942	-3.561247	0.0004172	0.0012534	-0.734787
SLC39A11	-0.081557	6.4095592	-3.560871	0.0004178	0.001255	-0.736073
RP11-57C13	-0.182213	-1.465808	-3.560695	0.0004181	0.0012557	-0.736673
ADNP2	0.0655678	5.8700719	3.5601786	0.0004189	0.001258	-0.73844
SHISA8	0.6697979	-0.151499	3.5598865	0.0004193	0.0012592	-0.739438
DDAH2	-0.090919	6.4487452	-3.559797	0.0004195	0.0012595	-0.739743
KLRC4-KLRK	0.603505	-0.333475	3.559672	0.0004197	0.00126	-0.740171
RP11-955H2	-0.150124	-1.494612	-3.559174	0.0004204	0.0012621	-0.741874
TOR1A	-0.05662	6.2750556	-3.559161	0.0004205	0.0012621	-0.741919
CTD-2653D5	0.4723934	-0.849493	3.5591603	0.0004205	0.0012621	-0.74192
TCAF2	0.2961668	4.7145456	3.5591369	0.0004205	0.0012621	-0.742
RNMT	0.0544055	6.0779584	3.5590035	0.0004207	0.0012626	-0.742456
HSD3BP5	0.6784426	0.8863648	3.5589325	0.0004208	0.0012628	-0.742698
TTPA	-0.311301	6.1272548	-3.558829	0.000421	0.0012632	-0.743051
ARHGDIG	0.6721867	-0.085953	3.5587091	0.0004212	0.0012637	-0.743462
BYSL	-0.075679	6.037457	-3.558161	0.000422	0.001266	-0.745335
ADH7	-0.819279	1.1119251	-3.558157	0.000422	0.001266	-0.745348
CD302	-0.139609	6.0281645	-3.557648	0.0004228	0.0012683	-0.747087
LINC00235	-0.386276	3.4669456	-3.557609	0.0004229	0.0012684	-0.747221
HSD3BP1	-0.49183	-0.881189	-3.557275	0.0004234	0.0012698	-0.748362
TBCC	-0.064788	5.9814501	-3.557161	0.0004236	0.0012703	-0.748751
TMA16	-0.064228	5.7651002	-3.556608	0.0004244	0.0012727	-0.750638
YPEL5	-0.04784	6.5357326	-3.556339	0.0004248	0.0012739	-0.751556
RP11-15H7.	-0.508769	-0.796297	-3.556001	0.0004254	0.0012754	-0.75271
TMEM217	0.3543838	3.7528149	3.5558545	0.0004256	0.001276	-0.753211
AP001469.7	0.6074586	0.2728059	3.5555426	0.0004261	0.0012773	-0.754276
HRG	-0.355037	7.179594	-3.555236	0.0004266	0.0012787	-0.755322
RP11-416N4	0.5553387	-0.510614	3.5542394	0.0004281	0.0012832	-0.758724
TNRC6A	-0.064067	6.2718607	-3.55421	0.0004282	0.0012832	-0.758824
RP11-307C1	0.425473	-0.987867	3.5541954	0.0004282	0.0012832	-0.758874
SLC17A7	0.528881	2.6081068	3.5541167	0.0004283	0.0012835	-0.759143
AC002463.3	-0.443964	-1.125329	-3.553435	0.0004294	0.0012866	-0.761467
TAF9P3	0.5438137	-0.006685	3.5533094	0.0004296	0.0012871	-0.761898
MYLK	-0.117292	6.4686393	-3.553098	0.0004299	0.001288	-0.762619
RP11-1109M	-0.329401	-1.277089	-3.552665	0.0004306	0.0012899	-0.764096
EIF2AK3	0.0710181	5.8556716	3.5526598	0.0004306	0.0012899	-0.764114
RP4-576H24	0.3354584	-1.18924	3.5525943	0.0004307	0.0012901	-0.764337

KCNJ2	0.2100032	4.7356426	3.5523025	0.0004312	0.0012913	-0.765333
SEC24A	-0.075777	6.383285	-3.55222	0.0004313	0.0012916	-0.765614
IFT20	-0.054116	6.016732	-3.552178	0.0004314	0.0012917	-0.765759
RP11-588L1	0.675418	0.8882545	3.5521161	0.0004315	0.0012919	-0.765968
PPM1J	0.3386794	3.5912347	3.5517926	0.000432	0.0012933	-0.767071
GP6	0.6608931	0.5686136	3.5517802	0.000432	0.0012933	-0.767114
RP11-258F1	-0.741731	3.5352059	-3.551683	0.0004322	0.0012937	-0.767447
RP11-66101	0.5930836	0.4518782	3.5516424	0.0004322	0.0012937	-0.767584
AC099344.3	-0.23289	-1.38365	-3.551603	0.0004323	0.0012938	-0.767719
KRT18P63	0.5683725	-0.124478	3.5514844	0.0004325	0.0012943	-0.768123
ADAM11	0.4422222	3.4183113	3.5511174	0.0004331	0.0012959	-0.769374
ARIH2	-0.047933	6.3935232	-3.550748	0.0004337	0.0012976	-0.770635
NCOR1P1	-0.137815	-1.496477	-3.550491	0.0004341	0.0012987	-0.771509
UBE4B	-0.064094	6.2721817	-3.55041	0.0004342	0.001299	-0.771786
CDH8	0.7856636	1.5207916	3.5502719	0.0004344	0.0012995	-0.772257
PPP2R2A	0.0689682	6.0095075	3.5501759	0.0004346	0.0012999	-0.772584
DPYSL5	0.5811354	-0.741097	3.5501442	0.0004346	0.0012999	-0.772692
RP11-582E3	-0.44614	-0.993669	-3.550085	0.0004347	0.0013001	-0.772896
RP3-425P12	0.5712962	-0.162446	3.5499087	0.000435	0.0013008	-0.773495
METTL1	-0.081689	5.8886416	-3.54982	0.0004351	0.0013011	-0.773796
ASAP2	0.3534361	4.9245308	3.5497791	0.0004352	0.0013012	-0.773937
RP11-282I1	0.5471924	-0.673101	3.549756	0.0004352	0.0013012	-0.774015
AC007292.6	-0.229969	3.9336858	-3.549194	0.0004361	0.0013038	-0.775933
RP11-62901	-0.336415	3.9428648	-3.547886	0.0004382	0.00131	-0.780389
CTD-2288F1	-0.54142	2.1030223	-3.547844	0.0004383	0.0013101	-0.780533
C20orf144	0.5977016	1.5788602	3.5477127	0.0004385	0.0013106	-0.780978
UBE2U	0.8311529	-0.103511	3.5475617	0.0004388	0.0013112	-0.781493
C22orf34	0.2812207	4.0046655	3.547457	0.0004389	0.0013116	-0.78185
B4GALNT3	0.5369892	3.825273	3.5472691	0.0004392	0.0013124	-0.78249
RP11-109D2	-0.402812	-1.010578	-3.547121	0.0004395	0.001313	-0.782996
CEP170P1	0.602207	0.0647043	3.5469336	0.0004398	0.0013138	-0.783632
LINC01571	-0.426045	-1.040172	-3.546787	0.00044	0.0013144	-0.78413
RP11-467I2	-0.294783	-1.306387	-3.546726	0.0004401	0.0013146	-0.784339
ZNF669	0.2179356	4.8481653	3.5466886	0.0004402	0.0013147	-0.784467
RP3-466P17	-0.524323	1.9750543	-3.546482	0.0004405	0.0013156	-0.785172
MERTK	-0.157477	5.8504412	-3.546407	0.0004406	0.0013158	-0.785426
TRABD2A	0.4258447	4.633481	3.5463391	0.0004407	0.001316	-0.785657
CTD-2515H2	-0.466174	-0.792907	-3.546322	0.0004408	0.001316	-0.785716
AF064860.7	0.3777262	-1.231456	3.546065	0.0004412	0.0013171	-0.786591
RP11-701I2	-0.139123	-1.501779	-3.546058	0.0004412	0.0013171	-0.786613
EIF3L	0.1056208	6.5336031	3.5459225	0.0004414	0.0013176	-0.787076
RP11-522B1	0.4356518	-1.131596	3.5457403	0.0004417	0.0013184	-0.787696
CD2BP2	-0.058779	6.3965475	-3.545538	0.000442	0.0013193	-0.788384
RP11-387A1	0.4687371	-0.943891	3.5452912	0.0004424	0.0013204	-0.789226
RP11-47A8.	0.2263185	4.3654877	3.5449922	0.0004429	0.0013217	-0.790243
MRGPRF	0.4148785	4.7678289	3.5444854	0.0004437	0.0013241	-0.791969
RP11-35G9.	0.2755423	3.6142871	3.5439478	0.0004446	0.0013266	-0.793799
CTC-490E21	-0.372188	4.5827074	-3.543877	0.0004447	0.0013268	-0.794041
GUCA2A	0.9081086	0.8543349	3.5436766	0.0004451	0.0013277	-0.794721
C9orf43	-0.195981	4.1758777	-3.543622	0.0004452	0.0013278	-0.794908
RFPL4A	-0.76302	0.6890677	-3.543583	0.0004452	0.0013279	-0.79504

POLD3	0.0748913	5.7315858	3.5435608	0.0004453	0.0013279	-0.795116
PLCD4	0.157137	4.7656957	3.5432822	0.0004457	0.0013292	-0.796063
DNAJC21	-0.045091	6.3646577	-3.543243	0.0004458	0.0013292	-0.796196
HCG17	-0.585502	2.1896275	-3.543022	0.0004461	0.0013301	-0.796949
LYZ	0.2232632	6.578016	3.5430132	0.0004461	0.0013301	-0.796979
TM9SF2	-0.05578	6.8882617	-3.542873	0.0004464	0.0013307	-0.797454
Six3os1_1	0.5281976	-0.900174	3.5428608	0.0004464	0.0013307	-0.797497
EDDM3B	-0.240757	-1.457126	-3.542628	0.0004468	0.0013316	-0.798288
RP11-773H2	0.6182063	1.2715881	3.5426162	0.0004468	0.0013316	-0.79833
TMEM132C	0.6144566	-0.750009	3.5419973	0.0004478	0.0013346	-0.800435
RSG1	-0.187806	4.7616629	-3.541923	0.0004479	0.0013348	-0.800689
CTB-58E17.	-0.462019	2.8902806	-3.541778	0.0004482	0.0013354	-0.801181
GOLGA1	-0.056629	6.0618075	-3.541265	0.000449	0.0013378	-0.802927
SPACA7	-0.787328	0.0117796	-3.541245	0.000449	0.0013378	-0.802994
ALK	0.6281337	0.2587796	3.5409667	0.0004495	0.0013391	-0.803941
PHBP11	-0.598267	2.8981903	-3.540685	0.00045	0.0013403	-0.804898
TEX40	-0.342579	-1.146645	-3.540517	0.0004502	0.0013411	-0.80547
NFYA	0.0691647	6.0585824	3.5403589	0.0004505	0.0013417	-0.806007
AC091814.3	0.4255989	-0.907305	3.5395979	0.0004518	0.0013454	-0.808595
RP11-1069G	-0.635721	4.3329046	-3.539131	0.0004525	0.0013475	-0.810182
TAS2R6P	0.5698878	-0.323103	3.5389252	0.0004529	0.0013483	-0.810881
LINC01476	-0.2302	-1.403081	-3.538925	0.0004529	0.0013483	-0.810883
KIFAP3	-0.076929	6.1614408	-3.538751	0.0004532	0.0013491	-0.811475
SH3GL3	-0.85902	-0.197734	-3.538622	0.0004534	0.0013496	-0.811911
KLRC1	0.6994693	1.5653223	3.538349	0.0004538	0.0013508	-0.81284
RP5-884C9.	-0.591703	-0.551336	-3.538347	0.0004538	0.0013508	-0.812848
ODF3B	-0.116683	5.9611878	-3.538301	0.0004539	0.0013509	-0.813001
AC004009.2	-0.196331	-1.439481	-3.537802	0.0004548	0.0013532	-0.814699
NRBP2	-0.110949	6.602654	-3.53758	0.0004551	0.0013542	-0.815451
DHX15	0.0418953	6.4422146	3.5374382	0.0004554	0.0013548	-0.815935
RP11-328C8	0.3558581	-1.152123	3.5372885	0.0004556	0.0013555	-0.816443
ZDHHC5	-0.039005	6.5945438	-3.53724	0.0004557	0.0013556	-0.816607
OR7E38P	-0.152369	5.2987864	-3.536861	0.0004563	0.0013574	-0.817895
RASA1	0.113587	5.8399499	3.5356045	0.0004584	0.0013635	-0.822163
SH2B3	0.0794095	6.2105135	3.5351898	0.0004591	0.0013655	-0.823571
SLC23A2	-0.117041	6.4668707	-3.534663	0.00046	0.001368	-0.825361
KITLG	0.1800343	5.3912265	3.5344656	0.0004603	0.0013689	-0.82603
PPP1R2P1	0.4111409	-0.942177	3.5343103	0.0004606	0.0013695	-0.826557
EXOSC1	-0.068282	6.0094604	-3.53419	0.0004608	0.00137	-0.826965
LINC01117	0.6563457	-0.065177	3.5340061	0.0004611	0.0013708	-0.82759
COL18A1-AS	0.4194826	-1.016659	3.5339235	0.0004613	0.0013711	-0.82787
AC073842.1	-0.65594	2.8691796	-3.533679	0.0004617	0.0013723	-0.828699
RP11-846F4	-0.395488	-1.168325	-3.533143	0.0004626	0.0013748	-0.830521
CTC-459F4.	0.3652647	3.3209707	3.5331327	0.0004626	0.0013748	-0.830555
RP11-278L1	-0.569251	2.819763	-3.532717	0.0004633	0.0013768	-0.831967
EPB41L4B	-0.178757	6.2634411	-3.532395	0.0004638	0.0013783	-0.833057
DEDD	-0.050182	6.2969729	-3.532271	0.000464	0.0013786	-0.833477
CTD-2589H1	0.439835	3.6380265	3.5322624	0.0004641	0.0013786	-0.833508
GNG7	-0.183822	5.5648223	-3.532254	0.0004641	0.0013786	-0.833537
CTD-3092A1	0.4498823	2.9541808	3.5322376	0.0004641	0.0013786	-0.833592
TRIM24	-0.072559	6.4486001	-3.532219	0.0004641	0.0013786	-0.833655

RAB23	0.2842838	4.7486657	3.5320795	0.0004644	0.0013792	-0.834128
CTB-161M19	-0.583648	0.6471311	-3.532031	0.0004645	0.0013793	-0.834292
C5orf64	0.7011175	0.2123833	3.531941	0.0004646	0.0013797	-0.834598
CTD-2561J2	0.7082043	2.4272931	3.5316268	0.0004651	0.0013811	-0.835664
PHF21B	0.528925	-0.837332	3.5314834	0.0004654	0.0013817	-0.836151
FAM13C	0.3804756	4.4063032	3.5313819	0.0004656	0.0013821	-0.836495
RP4-683M8.	-0.162703	-1.481466	-3.531122	0.000466	0.0013833	-0.837378
NXPH2	0.4112323	-1.164053	3.5308809	0.0004664	0.0013845	-0.838194
CELSR3-AS1	-0.567137	2.0458118	-3.530682	0.0004668	0.0013853	-0.83887
GATA2-AS1	0.5361426	3.2132552	3.5305885	0.0004669	0.0013857	-0.839186
GABRA2	0.9422691	0.1500619	3.5305475	0.000467	0.0013857	-0.839325
ZNF653	-0.081064	5.3438592	-3.530546	0.000467	0.0013857	-0.83933
AC112721.2	0.3928566	-1.124157	3.5305048	0.0004671	0.0013857	-0.83947
SARM1	0.1764819	4.9978977	3.5305006	0.0004671	0.0013857	-0.839484
TECTB	-0.711403	2.5909774	-3.530438	0.0004672	0.0013859	-0.839697
RPS4XP12	-0.182818	-1.461248	-3.530027	0.0004679	0.0013879	-0.841089
RP11-996F1	0.4259162	3.1051309	3.5297906	0.0004683	0.001389	-0.841892
MMP21	0.6097733	0.6285415	3.5296818	0.0004685	0.0013894	-0.842261
PIM2	0.1070771	5.8443532	3.5295297	0.0004687	0.0013901	-0.842776
TMEM201	0.0873978	5.7236651	3.5294658	0.0004688	0.0013903	-0.842993
SLC38A8	0.7941164	0.0101426	3.5291059	0.0004695	0.001392	-0.844213
ACOT12	-0.426477	5.851807	-3.529046	0.0004696	0.0013922	-0.844416
UNGP1	-0.335171	-1.162606	-3.528999	0.0004696	0.0013923	-0.844576
PGAM1P3	-0.315587	-1.224391	-3.528755	0.0004701	0.0013934	-0.845402
LINC01465	-0.233466	3.8117969	-3.528167	0.0004711	0.0013963	-0.847396
RP11-680N2	-0.130044	-1.503435	-3.528134	0.0004711	0.0013964	-0.847509
CDKN2B-AS1	-0.485944	3.6127117	-3.527911	0.0004715	0.0013974	-0.848265
LINC01497	-0.344678	-1.279817	-3.527892	0.0004715	0.0013974	-0.848326
RP11-620J1	-0.176939	4.8226189	-3.527831	0.0004716	0.0013976	-0.848533
ZSCAN12	0.1421587	5.0466495	3.5274941	0.0004722	0.0013992	-0.849676
RP1-80N2.2	0.3820672	-1.061908	3.5271813	0.0004728	0.0014007	-0.850736
ACTA1	0.4727236	2.4962571	3.527116	0.0004729	0.0014009	-0.850957
AC011591.1	-0.145247	-1.496755	-3.527037	0.000473	0.0014011	-0.851227
CCDC154	0.3636387	3.8169045	3.5270205	0.000473	0.0014011	-0.851281
RP11-433A1	-0.426389	-0.991409	-3.527009	0.0004731	0.0014011	-0.85132
RP11-554D1	-0.142762	-1.49001	-3.526911	0.0004732	0.0014015	-0.851651
TATDN2	-0.062788	6.0312139	-3.526629	0.0004737	0.0014028	-0.852606
RP11-455F5	0.5928552	1.1552844	3.5263242	0.0004742	0.0014043	-0.85364
CTD-2562J1	-0.624849	-0.234383	-3.52629	0.0004743	0.0014043	-0.853755
AC102953.4	-0.609218	1.3474649	-3.525919	0.0004749	0.0014061	-0.855011
WDR55	-0.054393	6.2258157	-3.525896	0.000475	0.0014061	-0.85509
RP11-596C2	-0.544665	1.3772167	-3.525564	0.0004756	0.0014077	-0.856216
MYT1	0.7458884	2.1949143	3.5255331	0.0004756	0.0014078	-0.856319
PRR7	0.2327824	4.8049886	3.5255061	0.0004757	0.0014078	-0.856411
CENPJ	0.1118056	5.3390988	3.5254852	0.0004757	0.0014078	-0.856482
RP11-23N2.	0.7086308	1.8591816	3.5251474	0.0004763	0.0014094	-0.857626
ZFP69B	0.2192347	4.1428192	3.5251352	0.0004763	0.0014094	-0.857667
ME3	-0.144481	5.3294329	-3.525076	0.0004764	0.0014095	-0.857866
SPINK8	-0.258042	-1.423663	-3.524843	0.0004768	0.0014106	-0.858656
RP11-307N1	-0.335521	-1.151205	-3.524721	0.000477	0.0014111	-0.859068
CLCN3	-0.078796	6.3013366	-3.524686	0.0004771	0.0014112	-0.859189

RP11-439A1	0.3733636	-1.073549	3.5246752	0.0004771	0.0014112	-0.859225
HES7	0.5194256	-0.733257	3.5242747	0.0004778	0.0014131	-0.860581
RIPK2	0.1068514	5.8881763	3.5238795	0.0004785	0.001415	-0.861919
CCDC68	0.2646071	5.2368656	3.523613	0.000479	0.0014163	-0.862821
LINC01136	0.6856389	1.8757815	3.5235749	0.000479	0.0014164	-0.86295
ABCA11P	0.2349156	3.9076079	3.5233513	0.0004794	0.0014174	-0.863707
TAS1R1	-0.605482	0.8346747	-3.523341	0.0004794	0.0014174	-0.863743
NPHP3	0.0774818	5.307104	3.5232945	0.0004795	0.0014175	-0.863899
RP11-145P1	0.5870073	-0.491413	3.5228319	0.0004803	0.0014198	-0.865465
MVB12B	0.0939635	5.840086	3.5226715	0.0004806	0.0014204	-0.866008
KDM4A-AS1	-0.25398	3.9754028	-3.52266	0.0004806	0.0014204	-0.866048
CABYR	0.4909943	4.3891239	3.5223089	0.0004812	0.0014221	-0.867235
RP11-599B1	-0.52657	-0.493959	-3.522017	0.0004818	0.0014235	-0.868224
BEND3P1	0.6912095	3.0505563	3.5216169	0.0004825	0.0014255	-0.869577
FAM138E	-0.283103	-1.345949	-3.521454	0.0004828	0.0014262	-0.870127
KRT8P33	0.3513605	3.1535899	3.5206826	0.0004841	0.0014301	-0.872737
MAZ	0.0989532	6.2658464	3.5205754	0.0004843	0.0014306	-0.8731
DUSP27	0.6230995	-0.264514	3.5205339	0.0004844	0.0014307	-0.87324
RP11-124G5	-0.362686	-1.249221	-3.520272	0.0004848	0.0014319	-0.874124
AC007038.7	0.445818	2.8264225	3.5200849	0.0004852	0.0014328	-0.874759
RP11-91I20	-0.528137	-0.463487	-3.519759	0.0004857	0.0014344	-0.875861
RNU6-725P	-0.547239	-0.828358	-3.519334	0.0004865	0.0014365	-0.877297
LRRIQ4	0.7341279	0.9977243	3.5183677	0.0004882	0.0014414	-0.880565
CGRRF1	-0.069271	5.549894	-3.518266	0.0004884	0.0014419	-0.880909
HLA-DOB	0.3472991	4.2842052	3.5182073	0.0004885	0.001442	-0.881107
SMARCA5	0.0525889	6.3648619	3.518108	0.0004887	0.0014425	-0.881443
ZNF730	0.7779481	0.4356629	3.5180489	0.0004888	0.0014426	-0.881643
RP11-679B1	0.5293421	2.2375407	3.5179864	0.0004889	0.0014429	-0.881854
KCNJ2-AS1	0.4717642	2.2615841	3.517739	0.0004893	0.001444	-0.88269
SLC44A3	0.3264555	5.6731886	3.5173335	0.0004901	0.0014461	-0.884061
CFLAR	-0.039716	6.5720585	-3.517083	0.0004905	0.0014473	-0.884909
PRAMEF4	-0.965783	0.61179	-3.516877	0.0004909	0.0014482	-0.885604
C1orf95	0.4913423	3.5967442	3.5168155	0.000491	0.0014484	-0.885811
GLI4	-0.101427	5.8706352	-3.516792	0.000491	0.0014484	-0.885889
DBF4B	0.1183581	5.2750596	3.5166111	0.0004914	0.0014493	-0.886502
CAP1P2	0.5970516	0.8673651	3.5165746	0.0004914	0.0014494	-0.886625
BNIP3P17	0.716638	0.0566033	3.5163891	0.0004918	0.0014502	-0.887252
TTYH3	0.094632	6.4751093	3.5162719	0.000492	0.0014507	-0.887648
KB-1615E4.	0.7344124	0.5642031	3.5159999	0.0004924	0.001452	-0.888567
RP11-436K8	0.7243616	0.0769323	3.5159617	0.0004925	0.0014521	-0.888696
NHP2L1	-0.059387	6.6113841	-3.515674	0.000493	0.0014535	-0.889668
CHMP5	-0.04915	6.3667961	-3.515426	0.0004935	0.0014547	-0.890506
DNASE1L2	-0.452793	3.4899881	-3.515363	0.0004936	0.001455	-0.890718
PDGFA	0.168638	5.9645628	3.5152181	0.0004939	0.0014556	-0.891208
RPS7P6	-0.255973	-1.352925	-3.515174	0.0004939	0.0014557	-0.891358
CTA-212A2.	-0.561269	-0.052402	-3.515112	0.000494	0.0014559	-0.891565
RP11-502F1	-0.301032	-1.37045	-3.514942	0.0004944	0.0014567	-0.892139
IL17D	0.4800492	3.8871694	3.5148626	0.0004945	0.001457	-0.892409
TBC1D15	-0.043745	6.2273749	-3.514764	0.0004947	0.0014574	-0.89274
KRT8P36	0.5914332	1.8884798	3.5144595	0.0004952	0.0014589	-0.89377
RP11-656G2	0.6247923	-0.338691	3.5142954	0.0004955	0.0014597	-0.894324

IL15RA	0.1225229	5.9211351	3.5141798	0.0004957	0.0014602	-0.894714
TCP10L2	0.7818537	0.2825676	3.5139422	0.0004962	0.0014613	-0.895517
SMNDC1	0.0445012	5.980738	3.5137897	0.0004964	0.001462	-0.896032
RP11-407G2	0.6404096	0.4090891	3.5137632	0.0004965	0.001462	-0.896121
ITCH	-0.059448	6.4300499	-3.51354	0.0004969	0.0014631	-0.896876
RP11-760D2	-0.307867	-1.360663	-3.513314	0.0004973	0.0014642	-0.897639
UBE2CP3	-0.129639	-1.505078	-3.513278	0.0004974	0.0014643	-0.897759
GM2A	-0.062342	6.5367889	-3.513228	0.0004974	0.0014644	-0.897929
AC010733.5	0.4924264	2.275263	3.5131007	0.0004977	0.001465	-0.898358
EPHA4	0.3007339	4.5041807	3.5129461	0.000498	0.0014657	-0.898879
RNU6-1297F	-0.146453	-1.488389	-3.51287	0.0004981	0.001466	-0.899136
AC024937.6	0.6563788	1.3746695	3.5126014	0.0004986	0.0014673	-0.900043
CTD-2586B1	-0.25832	-1.338988	-3.511498	0.0005006	0.0014731	-0.903768
IRGQ	-0.063135	6.1124689	-3.511469	0.0005006	0.0014731	-0.903863
IMMT	-0.044722	6.5849021	-3.511371	0.0005008	0.0014735	-0.904194
RP11-216L1	0.6224909	0.2553226	3.5113181	0.0005009	0.0014737	-0.904374
AC027612.6	0.4314669	3.9013349	3.5109675	0.0005016	0.0014754	-0.905557
HSD17B1	-0.131656	4.7280879	-3.510962	0.0005016	0.0014754	-0.905577
RP11-843P1	0.6940359	1.8134344	3.5106302	0.0005022	0.001477	-0.906695
ARPC4	-0.062442	6.6090947	-3.510426	0.0005025	0.001478	-0.907384
TP53I13	-0.123999	6.142395	-3.509981	0.0005034	0.0014803	-0.908885
ANKRD1	0.6820077	3.682161	3.5098262	0.0005036	0.001481	-0.909407
HTR7P1	0.2121243	4.7299424	3.5098091	0.0005037	0.001481	-0.909465
TMED8	0.0705949	5.6623214	3.5094621	0.0005043	0.0014827	-0.910635
MIR193A	-0.481281	-0.615817	-3.509117	0.0005049	0.0014845	-0.911798
TRIQK	-0.089246	6.2366686	-3.508875	0.0005054	0.0014856	-0.912614
RP11-72I8.	-0.57896	0.6794791	-3.508473	0.0005061	0.0014877	-0.913969
PID1	-0.12631	6.2471262	-3.508331	0.0005064	0.0014884	-0.914449
AVPR1B	0.3443026	-1.218727	3.5081463	0.0005067	0.0014892	-0.915071
EPB42	0.5715375	0.1601364	3.5078845	0.0005072	0.0014905	-0.915954
GSTA4	-0.127783	6.0731221	-3.5077	0.0005076	0.0014914	-0.916577
AOX3P	-0.785641	2.3952509	-3.507158	0.0005086	0.0014942	-0.918403
RP11-690P1	0.5188013	-0.351056	3.5070379	0.0005088	0.0014948	-0.918808
LINC00624	0.5593936	2.9712966	3.5069352	0.000509	0.0014952	-0.919154
RP11-96H17	0.5289498	-0.972864	3.5068361	0.0005092	0.0014956	-0.919488
RP4-809F18	0.4615044	-1.127984	3.5067275	0.0005094	0.0014961	-0.919854
MALT1	0.073195	6.0158445	3.5065743	0.0005096	0.0014968	-0.92037
RASSF5	0.1273402	5.8087128	3.506311	0.0005101	0.0014981	-0.921257
THBS3	-0.090933	5.8975629	-3.506071	0.0005106	0.0014993	-0.922066
ATP1A2	0.4867833	4.0651219	3.505961	0.0005108	0.0014998	-0.922436
NR1H5P	-0.133921	-1.504065	-3.505642	0.0005114	0.0015014	-0.923512
CITF22-1A6	0.2268503	3.7746157	3.5054601	0.0005117	0.0015023	-0.924124
TEX33	-0.308118	-1.252417	-3.505371	0.0005119	0.0015026	-0.924423
ERV3-1	0.1774301	4.9994679	3.5049079	0.0005127	0.0015051	-0.925984
RP11-684B2	-0.526665	-0.63272	-3.504616	0.0005133	0.0015065	-0.926967
NRBP1	-0.046943	6.6535481	-3.504335	0.0005138	0.0015079	-0.927915
PLCB3	0.0552108	5.9972927	3.5042018	0.0005141	0.0015086	-0.928363
MAP7D2	0.8326301	2.9666391	3.5040367	0.0005144	0.0015093	-0.928919
MAP3K14	-0.10547	6.009002	-3.503989	0.0005144	0.0015095	-0.929079
HSF2BP	0.4712598	3.0886333	3.5035498	0.0005153	0.0015118	-0.930558
JPX	-0.068995	5.8577196	-3.503503	0.0005154	0.0015119	-0.930716

MEP1A	0.9599477	2.8480768	3.5033723	0.0005156	0.0015125	-0.931156
HIST1H2BL	-0.662416	0.458884	-3.502652	0.000517	0.0015163	-0.933582
RP13-554M1	0.658293	1.7513997	3.5024156	0.0005174	0.0015175	-0.934377
CEACAM5	0.7831362	-0.223463	3.5016311	0.0005189	0.0015217	-0.937017
AIMP2	-0.076746	6.1024015	-3.501187	0.0005197	0.0015241	-0.938513
CNTNAP3B	-0.617852	3.6861283	-3.500997	0.0005201	0.001525	-0.939149
SNX16	0.1092264	5.3496295	3.5004713	0.0005211	0.0015278	-0.94092
RP1-80N2.4	-0.497762	2.6951878	-3.499559	0.0005228	0.0015327	-0.943989
SSH2	0.0593808	5.8682419	3.4994442	0.000523	0.0015332	-0.944375
CEACAMP8	-0.154304	-1.49347	-3.499336	0.0005232	0.0015337	-0.944738
HSPA6	0.2382197	4.9913486	3.4992147	0.0005234	0.0015343	-0.945147
RP11-720D4	-0.346974	-1.215363	-3.49835	0.0005251	0.001539	-0.948055
AMMECR1	0.087507	5.5854789	3.4982863	0.0005252	0.0015392	-0.948269
SYNJ2BP-CC	0.4427929	2.1511915	3.4982571	0.0005253	0.0015392	-0.948367
MTMR7	0.2682064	4.6473743	3.4979295	0.0005259	0.001541	-0.949468
SBF1	0.0687234	6.3816376	3.4978362	0.0005261	0.0015414	-0.949782
DOCK1	0.1155361	6.1503938	3.4977543	0.0005262	0.0015417	-0.950057
CSNK1D	-0.037682	6.6946233	-3.497157	0.0005274	0.0015449	-0.952065
EFTUD2	-0.043233	6.5698327	-3.496951	0.0005278	0.0015459	-0.952759
AC142528.1	-0.526345	2.8655763	-3.496936	0.0005278	0.0015459	-0.952808
HPGDS	0.4425167	3.4682041	3.4968445	0.000528	0.0015462	-0.953116
GLMP	-0.076415	6.6360283	-3.496829	0.000528	0.0015462	-0.953168
NRBF2P5	0.623977	0.5394167	3.4968229	0.000528	0.0015462	-0.953188
MMP15	-0.086175	6.6500627	-3.496529	0.0005286	0.0015477	-0.954175
RP11-763B2	-0.435145	-1.16131	-3.496148	0.0005293	0.0015496	-0.955457
IGHV3OR16-	0.4048251	-1.124157	3.4961386	0.0005293	0.0015496	-0.955488
RP11-120K2	0.5761452	-0.759043	3.4960919	0.0005294	0.0015498	-0.955645
CACNA1I	0.8201838	1.9574796	3.4960374	0.0005295	0.00155	-0.955828
AC131263.1	0.5944403	1.7198234	3.4957473	0.0005301	0.0015515	-0.956803
RP11-434I1	0.9139603	2.1422134	3.4955887	0.0005304	0.0015522	-0.957336
AC011247.3	0.528632	-0.366004	3.4954697	0.0005306	0.0015528	-0.957736
TLL1	0.607949	2.2893239	3.4952452	0.000531	0.0015539	-0.95849
MGA	0.0747085	5.9956218	3.4951627	0.0005312	0.0015543	-0.958767
FAM179A	0.4511524	3.197024	3.4951045	0.0005313	0.0015545	-0.958963
OR2B2	-0.235778	-1.386917	-3.494869	0.0005318	0.0015557	-0.959754
UCHL5	-0.06229	6.2989662	-3.494512	0.0005325	0.0015576	-0.960954
MANF	-0.085411	6.6212803	-3.493876	0.0005337	0.001561	-0.963089
AGO2	0.0786953	6.1543358	3.4933404	0.0005347	0.0015639	-0.964888
MATN1	0.5894376	1.0553858	3.4932671	0.0005349	0.0015642	-0.965134
ANKRD34B	0.6791453	-0.455569	3.4929373	0.0005355	0.001566	-0.966242
XKR5	0.5619821	-0.637781	3.4928643	0.0005356	0.0015663	-0.966487
ESCO2	0.2852937	4.5815559	3.492377	0.0005366	0.0015689	-0.968123
ILVBL	-0.075822	6.6060729	-3.492192	0.0005369	0.0015698	-0.968744
BUD13	-0.046279	5.8638543	-3.49165	0.000538	0.0015728	-0.970562
AC062028.1	0.5106506	-0.66051	3.4911348	0.000539	0.0015756	-0.972292
TAS2R64P	0.5298139	-0.539353	3.4902832	0.0005407	0.0015803	-0.97515
RNPS1P1	0.5990615	0.6271127	3.4900169	0.0005412	0.0015817	-0.976043
FOXI3	0.3721066	-1.245874	3.4897844	0.0005417	0.0015829	-0.976823
HOXA-AS3	0.7378232	0.0501812	3.4895678	0.0005421	0.0015841	-0.97755
ABCC6P1	-0.284296	5.8524407	-3.489405	0.0005424	0.0015849	-0.978097
CPSF7	-0.044823	6.4645596	-3.488616	0.0005439	0.0015893	-0.980742

RP11-457I1	-0.326695	-1.149607	-3.488251	0.0005447	0.0015912	-0.981965
RP11-109E2	0.5572254	-0.746525	3.488128	0.0005449	0.0015918	-0.982379
RPL37AP1	-0.497294	2.4346802	-3.487744	0.0005457	0.0015939	-0.983666
TMEM191B	0.7150489	1.3425829	3.4876091	0.0005459	0.0015946	-0.984119
YWHAEP7	0.8571615	0.5229225	3.4870568	0.000547	0.0015976	-0.98597
GOLT1A	-0.243517	6.5277956	-3.486749	0.0005476	0.0015993	-0.987003
PLAA	-0.057421	6.1939797	-3.486539	0.0005481	0.0016004	-0.987707
HIST1H2BH	0.7059257	0.3234376	3.4864754	0.0005482	0.0016005	-0.987919
SZRD1	-0.045533	6.568181	-3.486472	0.0005482	0.0016005	-0.987929
AC246787.1	0.3447526	-1.219213	3.486329	0.0005485	0.0016012	-0.98841
MKL1	0.0470002	6.1506445	3.4857741	0.0005496	0.0016043	-0.99027
VAT1L	0.6507153	3.5802362	3.4856589	0.0005498	0.0016048	-0.990656
DCK	0.1139747	5.7141061	3.4855672	0.00055	0.0016052	-0.990963
IL1F10	-0.156999	-1.482762	-3.485427	0.0005503	0.0016059	-0.991434
RP11-566K1	0.477951	-0.896263	3.485284	0.0005505	0.0016066	-0.991912
RP11-616M2	0.752788	0.1934326	3.4852181	0.0005507	0.0016069	-0.992133
C15orf53	0.4630849	-0.735009	3.4851518	0.0005508	0.0016071	-0.992355
CCL17	0.5531802	2.4385607	3.4838805	0.0005534	0.0016144	-0.996614
C3orf36	0.724639	1.6076212	3.4837877	0.0005535	0.0016148	-0.996925
RP11-861E2	0.3850208	-1.091417	3.4836337	0.0005538	0.0016156	-0.997441
PACRGL	0.0792812	5.2307559	3.4834956	0.0005541	0.0016163	-0.997903
ALG2	-0.059614	6.2921363	-3.483339	0.0005544	0.0016171	-0.998427
HOTTIP	-0.924106	2.7193071	-3.4833	0.0005545	0.0016172	-0.998558
AC004510.3	-0.640974	-0.018671	-3.482772	0.0005556	0.0016201	-1.000328
VPS72	-0.061757	6.3176151	-3.482708	0.0005557	0.0016204	-1.000539
YTHDF1P1	-0.14546	-1.487832	-3.482555	0.000556	0.0016211	-1.001054
ACACA	-0.075624	6.3689296	-3.482452	0.0005562	0.0016216	-1.001397
CDHR4	0.6722373	0.2743262	3.4823811	0.0005564	0.0016218	-1.001635
CCPG1	-0.081394	6.0891541	-3.482371	0.0005564	0.0016218	-1.00167
HIF1A-AS1	0.4932033	-0.668574	3.482336	0.0005565	0.0016219	-1.001786
RP5-1024G6	-0.363349	3.3323984	-3.481611	0.0005579	0.001626	-1.004214
TNFRSF10C	0.2625771	4.5097592	3.4812241	0.0005587	0.001628	-1.005508
SLC5A4	0.6043661	2.0857452	3.4812232	0.0005587	0.001628	-1.005511
AC145343.1	0.5044748	-0.397783	3.4810991	0.0005589	0.0016286	-1.005927
GML	-0.22473	-1.448337	-3.48096	0.0005592	0.0016293	-1.006391
GTF2I	0.1579682	5.9221834	3.4807768	0.0005596	0.0016303	-1.007006
CCDC26	0.4090879	-1.015382	3.4805354	0.0005601	0.0016316	-1.007814
PRR13	-0.059645	6.4918527	-3.48016	0.0005608	0.0016337	-1.009069
RP11-813B8	-0.261676	-1.361627	-3.479571	0.000562	0.001637	-1.011039
FCN1	0.2758153	4.7724009	3.4792475	0.0005627	0.0016387	-1.012123
TTC12	-0.086139	5.4790978	-3.479243	0.0005627	0.0016387	-1.012138
DDX43	0.7744816	1.8722621	3.479121	0.000563	0.0016393	-1.012546
VWA8-AS1	-0.619224	0.74677	-3.477932	0.0005654	0.0016462	-1.016522
RP11-522M2	0.3594233	-1.108143	3.477728	0.0005658	0.0016473	-1.017205
LINC01288	-0.179982	-1.448997	-3.47767	0.0005659	0.0016475	-1.0174
RAB11FIP3	-0.069858	6.2785682	-3.47761	0.000566	0.0016477	-1.0176
CTC-453G23	0.5620675	-0.096088	3.4773344	0.0005666	0.0016492	-1.018521
CTC-303L1.	0.5267906	-0.533436	3.477232	0.0005668	0.0016497	-1.018863
RAP2CP1	-0.741015	0.579423	-3.476583	0.0005681	0.0016535	-1.021033
AC073046.2	0.6226189	1.626443	3.4763313	0.0005687	0.0016548	-1.021874
CSE1L-AS1	-0.571483	-0.082632	-3.475527	0.0005703	0.0016595	-1.024561

AL359771.1	-0.279331	-1.305097	-3.475214	0.000571	0.0016612	-1.025609
AC073072.5	0.5054354	-0.726288	3.4749751	0.0005714	0.0016625	-1.026407
RNU6-1031F	-0.245816	-1.378222	-3.474861	0.0005717	0.0016631	-1.026787
NAALAD2	0.3379407	3.9059584	3.4748058	0.0005718	0.0016633	-1.026972
TTC26	0.1952763	4.8421858	3.4747655	0.0005719	0.0016633	-1.027107
RP13-34902	0.5803508	-0.188916	3.4747586	0.0005719	0.0016633	-1.02713
LRWD1	-0.06747	6.0191066	-3.474215	0.000573	0.0016665	-1.028948
YWHAE	-0.051132	6.9609111	-3.473973	0.0005735	0.0016678	-1.029756
ALOX15B	0.6915195	3.2017832	3.4738207	0.0005738	0.0016686	-1.030264
CTD-2501B8	-0.527962	-0.866958	-3.473774	0.0005739	0.0016687	-1.030419
FGF23	0.5364364	-0.936393	3.473721	0.000574	0.0016689	-1.030597
DUX4L19	-0.301059	-1.351074	-3.47318	0.0005752	0.001672	-1.032403
POLA2	-0.08259	5.8552767	-3.473014	0.0005755	0.0016729	-1.032957
CTD-2555K7	-0.509868	2.0408251	-3.472913	0.0005757	0.0016734	-1.033296
S100A9	0.2423333	5.4303762	3.4728138	0.0005759	0.0016738	-1.033627
VN1R88P	-0.273425	-1.373746	-3.472543	0.0005765	0.0016753	-1.034531
MAPRE3	-0.09532	5.9395679	-3.47245	0.0005767	0.0016758	-1.034842
RFX2	0.143562	5.0588545	3.4718008	0.000578	0.0016795	-1.037009
IGFBP7	0.0897636	6.8560794	3.4717844	0.0005781	0.0016795	-1.037064
RPL7P28	0.420685	-1.016252	3.4714119	0.0005788	0.0016816	-1.038307
PPP1R2	-0.0524	6.1597032	-3.470812	0.0005801	0.0016852	-1.040309
CEBPG	-0.062665	6.6056391	-3.47078	0.0005802	0.0016852	-1.040416
MIR339	0.4407747	-0.74223	3.4705761	0.0005806	0.0016863	-1.041097
CTD-2083E4	0.5846711	0.5603873	3.4701908	0.0005814	0.0016885	-1.042383
GADD45B	-0.12671	6.5976814	-3.47009	0.0005816	0.0016889	-1.042719
WDR34	-0.092336	6.2828721	-3.47009	0.0005816	0.0016889	-1.042721
RP11-175P1	-0.368133	3.2933954	-3.469789	0.0005822	0.0016906	-1.043722
RP11-90P16	-0.160213	-1.470187	-3.469423	0.000583	0.0016926	-1.044945
AC097724.3	-0.277251	3.339477	-3.46941	0.000583	0.0016926	-1.044988
RP11-29B9.	-0.215461	-1.420045	-3.469371	0.0005831	0.0016927	-1.04512
POLB	-0.106755	5.9517438	-3.469154	0.0005836	0.0016939	-1.045842
PMCH	0.4640723	-0.783928	3.4687744	0.0005844	0.0016961	-1.047109
MTND5P28	0.3600801	-1.051071	3.4686455	0.0005846	0.0016967	-1.047539
KMT2D	0.0661536	6.3189074	3.4680539	0.0005859	0.0017002	-1.049512
AGK	0.0693158	5.983671	3.4678471	0.0005863	0.0017014	-1.050202
KRT8P39	0.5730282	0.8758801	3.4673293	0.0005874	0.0017044	-1.051929
LAG3	0.2343991	4.918797	3.4670566	0.000588	0.0017059	-1.052838
TBL2	-0.054359	6.2762305	-3.466969	0.0005882	0.0017063	-1.05313
MRPL19	-0.052465	6.3335959	-3.466886	0.0005884	0.0017067	-1.053406
RPIA	0.0572894	5.7359328	3.4667735	0.0005886	0.0017073	-1.053782
RP11-401P9	-0.685406	2.4720146	-3.466523	0.0005891	0.0017087	-1.054616
BRSK1	0.2110937	4.8120123	3.4657869	0.0005907	0.0017131	-1.057071
CTD-3214H1	0.6233136	1.7543072	3.4656363	0.000591	0.0017139	-1.057573
ANKRD29	-0.29859	5.3347776	-3.465522	0.0005913	0.0017144	-1.057953
IAPP	-0.721298	1.4671405	-3.465514	0.0005913	0.0017144	-1.057981
SRP9P1	0.5727611	1.8372971	3.4653591	0.0005916	0.0017152	-1.058497
RP11-231E1	0.3276757	-1.139601	3.4650659	0.0005922	0.0017169	-1.059474
ASB7	-0.061143	5.787773	-3.465006	0.0005924	0.0017171	-1.059673
RP11-328J2	-1.085559	1.5688667	-3.464943	0.0005925	0.0017173	-1.059884
RPS23P3	-0.30071	-1.255672	-3.464644	0.0005931	0.0017191	-1.060879
GYLTL1B	0.4942254	4.8181267	3.4645848	0.0005933	0.0017193	-1.061077

C20orf203	0.5025328	-0.608693	3.4641082	0.0005943	0.0017221	-1.062665
RP11-787B4	-0.252932	-1.354937	-3.463918	0.0005947	0.0017231	-1.063299
OR8B9P	-0.150008	-1.49112	-3.463901	0.0005947	0.0017231	-1.063356
RP11-356N1	0.4506481	-0.792793	3.4637858	0.000595	0.0017237	-1.063739
F2RL3	0.3627897	4.7787057	3.4636557	0.0005952	0.0017244	-1.064173
RPL36A	-0.104659	6.3655703	-3.463114	0.0005964	0.0017276	-1.065975
LY86-AS1	-0.735695	-0.079245	-3.462465	0.0005978	0.0017315	-1.068137
RP11-760D2	-0.486514	-1.149542	-3.462232	0.0005983	0.0017328	-1.068913
LRP2	0.9322708	2.4439857	3.4619042	0.000599	0.0017347	-1.070006
RNF219-AS1	0.549755	-0.208169	3.4618141	0.0005992	0.0017351	-1.070306
XRCC5	-0.038053	6.8838972	-3.461756	0.0005993	0.0017353	-1.070498
RAB4B-EGLN	0.3726433	-1.008038	3.4617372	0.0005994	0.0017353	-1.070562
HNRNPA1P22	-0.573617	-0.254485	-3.461625	0.0005996	0.0017359	-1.070934
TOM1L1	-0.100953	6.2922063	-3.461469	0.0005999	0.0017367	-1.071453
AP001059.5	-0.640725	-0.154576	-3.460833	0.0006013	0.0017406	-1.073574
WNT3A	0.8861045	0.8041908	3.4605651	0.0006019	0.0017421	-1.074464
RP11-433C9	0.4738042	-0.883053	3.460259	0.0006026	0.0017439	-1.075482
RP11-767N6	0.608139	1.3266104	3.4601943	0.0006027	0.0017442	-1.075698
KRT17P8	-0.76614	4.4041865	-3.460134	0.0006028	0.0017444	-1.075898
RP11-356I2	0.1748841	4.1611358	3.4598019	0.0006036	0.0017464	-1.077004
RNU6-222P	-0.114881	-1.513423	-3.459628	0.0006039	0.0017473	-1.077582
ATP6V0A4	0.563596	-0.552572	3.4590791	0.0006051	0.0017506	-1.079409
CTD-2528L1	0.2823267	4.2525762	3.4583654	0.0006067	0.001755	-1.081783
TBP	0.0591293	5.6500915	3.45732	0.000609	0.0017614	-1.08526
ZNF428	-0.079411	6.1216838	-3.457287	0.000609	0.0017615	-1.085371
RP11-744I2	-0.158742	-1.478454	-3.457084	0.0006095	0.0017627	-1.086044
RP11-461F1	-0.582936	-0.812177	-3.456416	0.0006109	0.0017668	-1.088266
SUGP2	0.0641678	6.1301455	3.4558143	0.0006123	0.0017704	-1.090267
LINC01507	0.462027	-1.073684	3.4557171	0.0006125	0.0017709	-1.09059
CTAGE15	0.756059	0.7995111	3.4555022	0.000613	0.0017722	-1.091304
RP11-807E1	-0.188451	-1.440741	-3.455446	0.0006131	0.0017724	-1.091489
HSPB1	-0.089975	7.1341611	-3.455075	0.0006139	0.0017746	-1.092723
KLHL8	-0.086731	5.8328064	-3.455008	0.000614	0.0017749	-1.092947
EGLN1	-0.061468	6.6329058	-3.454595	0.000615	0.0017774	-1.09432
IL26	0.4526501	-0.832642	3.4540216	0.0006162	0.0017809	-1.096224
ARID3C	-0.438661	4.6528433	-3.453893	0.0006165	0.0017816	-1.096651
TMEM72-AS1	0.6126962	0.3801547	3.4536135	0.0006171	0.0017832	-1.09758
RP11-493E1	0.5548492	0.051941	3.4529171	0.0006187	0.0017875	-1.099894
LINC00629	0.373933	-1.050527	3.4528945	0.0006187	0.0017876	-1.099969
SPINK1	0.627147	5.9272787	3.4525984	0.0006194	0.0017893	-1.100952
G0S2	-0.221528	6.2272286	-3.452406	0.0006198	0.0017904	-1.10159
CDH10	0.6847075	-0.617543	3.4523332	0.00062	0.0017907	-1.101833
C5AR2	0.2601418	5.2026443	3.4509411	0.0006231	0.0017996	-1.106455
RP11-351M8	-0.336913	-1.150995	-3.450554	0.0006239	0.0018019	-1.10774
DUSP1	-0.115464	6.7938015	-3.450415	0.0006242	0.0018027	-1.108202
IQCA1	0.5204223	2.9122979	3.4502217	0.0006247	0.0018038	-1.108843
ZNF541	-0.467191	3.7571674	-3.449882	0.0006254	0.0018058	-1.109971
HMG20A	0.1260376	5.8145906	3.4497335	0.0006258	0.0018067	-1.110464
KB-176G8.1	-0.565744	0.8368708	-3.449556	0.0006262	0.0018077	-1.111053
BCCIP	-0.052814	6.3699764	-3.449182	0.000627	0.0018099	-1.112292
PRKG2	0.6891402	-0.037746	3.4491242	0.0006271	0.0018102	-1.112486

PCSK7	0.1015555	5.7700212	3.4489774	0.0006275	0.001811	-1.112973
OR5H2	-0.38334	-1.17557	-3.448847	0.0006278	0.0018117	-1.113405
ROCK1	0.0632748	6.1894567	3.4486099	0.0006283	0.0018131	-1.114192
RP4-798A10	0.3252681	3.1211543	3.448565	0.0006284	0.0018132	-1.114341
LINC00343	-0.184445	-1.44937	-3.448396	0.0006288	0.0018142	-1.114901
ISM2	0.6733425	-0.57892	3.4482527	0.0006291	0.001815	-1.115377
S100A2	0.6278623	2.8804802	3.4479112	0.0006299	0.0018171	-1.11651
RP11-683L2	0.7228998	0.048298	3.4478234	0.0006301	0.0018175	-1.116801
RP11-442P1	-0.17058	-1.480339	-3.447304	0.0006313	0.0018207	-1.118525
AFF3	0.3547856	5.4368375	3.4468148	0.0006324	0.0018238	-1.120146
AC022431.2	0.5436	-0.541554	3.4467187	0.0006326	0.0018243	-1.120465
PWP2	0.7534904	2.7595888	3.4466629	0.0006327	0.0018245	-1.12065
CAMKV	0.6675958	-0.361394	3.4466299	0.0006328	0.0018246	-1.120759
HYI-AS1	-0.44725	2.6799417	-3.446585	0.0006329	0.0018247	-1.120909
TRNAU1AP	-0.072553	5.7763052	-3.446212	0.0006337	0.001827	-1.122146
RASL10A	0.3571681	3.0249287	3.4461103	0.000634	0.0018275	-1.122482
RP1-28H20.	-0.399291	3.0547179	-3.446007	0.0006342	0.0018281	-1.122825
LINC01474	-0.544884	3.1432463	-3.445243	0.0006359	0.0018329	-1.125359
RP11-77K12	-0.662051	1.054473	-3.445099	0.0006363	0.0018337	-1.125836
DLGAP2	0.7559884	0.3042073	3.4449842	0.0006365	0.0018344	-1.126215
RP11-79P5.	0.4667301	2.459733	3.4448119	0.0006369	0.0018353	-1.126786
LRRC66	0.6807	2.0830111	3.4446724	0.0006372	0.0018361	-1.127249
RP11-106M7	-0.379958	-1.057267	-3.444626	0.0006373	0.0018363	-1.127401
CTC-661I16	-0.155218	-1.484537	-3.444605	0.0006374	0.0018363	-1.127473
AC090154.1	0.6071943	1.0225223	3.444326	0.000638	0.001838	-1.128396
CASP9	-0.085995	5.8231777	-3.443975	0.0006388	0.0018401	-1.12956
RP11-108F1	0.5843742	0.1592337	3.4431086	0.0006408	0.0018457	-1.13243
DIS3L2P1	-0.57324	-0.423123	-3.443055	0.0006409	0.0018459	-1.132606
RP11-136K7	0.941744	2.2759266	3.4413746	0.0006448	0.0018569	-1.138173
AF064858.6	-0.576939	3.9849892	-3.441215	0.0006452	0.0018578	-1.138702
ZNF587B	0.0870466	5.5874014	3.4410136	0.0006456	0.001859	-1.139368
ACAP2-IT1	0.571431	0.4131052	3.4408627	0.000646	0.0018599	-1.139868
TRBV21-1	0.3543352	-1.133421	3.4406305	0.0006465	0.0018613	-1.140637
RP4-761J14	0.5858731	0.5042461	3.4404063	0.000647	0.0018626	-1.141379
PICK1	-0.066046	6.1197497	-3.440182	0.0006476	0.001864	-1.142122
TMCC3	0.1256985	5.6068141	3.4401192	0.0006477	0.0018642	-1.142329
RP11-594N1	0.4608318	-0.943083	3.4400797	0.0006478	0.0018644	-1.14246
RBMY2FP	-0.785447	-0.50463	-3.439875	0.0006483	0.0018656	-1.143137
WNK3	-0.329836	5.5186294	-3.438309	0.0006519	0.0018759	-1.148319
IGHV3-6	0.2879834	-1.31026	3.4382132	0.0006521	0.0018764	-1.148637
CTD-2206G1	-0.550882	-0.105013	-3.438048	0.0006525	0.0018774	-1.149184
AC243945.1	-0.245126	-1.380017	-3.437819	0.0006531	0.0018788	-1.149939
KDELR3	0.1784631	5.9167686	3.4376842	0.0006534	0.0018794	-1.150387
C18orf32	-0.07022	5.5934632	-3.437678	0.0006534	0.0018794	-1.150406
PTP4A2	-0.045276	6.6985448	-3.437555	0.0006537	0.0018801	-1.150814
RP11-390F4	-0.309982	4.587226	-3.437404	0.000654	0.001881	-1.151313
KIRREL2	0.7255755	0.1292403	3.4369973	0.000655	0.0018835	-1.152658
RP11-663P9	-0.50308	-0.630721	-3.436605	0.0006559	0.001886	-1.153956
IGFBP3	0.112952	6.6960433	3.4359649	0.0006574	0.0018902	-1.156073
C19orf81	0.8495539	0.4209998	3.435627	0.0006582	0.0018924	-1.15719
AC104653.1	0.5993811	-0.044055	3.4349585	0.0006598	0.0018967	-1.159399

C18orf42	-0.640985	-0.38501	-3.434825	0.0006601	0.0018975	-1.159839
LINC00880	-0.710745	1.8199207	-3.434775	0.0006602	0.0018977	-1.160008
NCOA7	0.1073269	6.1842777	3.43472	0.0006603	0.0018979	-1.160188
RPS3AP2	-0.436902	-0.864373	-3.434432	0.000661	0.0018997	-1.161141
XXyac-YM21	0.8612666	1.8177814	3.4342136	0.0006615	0.001901	-1.161861
POMGNT2	-0.072164	5.865227	-3.434173	0.0006616	0.0019012	-1.161994
MRPL17	-0.066249	6.2582137	-3.433567	0.0006631	0.0019051	-1.163997
C9orf69	-0.062542	6.2920861	-3.433463	0.0006633	0.0019057	-1.164342
CTB-32H22.	-0.201618	-1.417639	-3.433329	0.0006636	0.0019065	-1.164786
AC108676.1	0.6051878	2.0751765	3.4329307	0.0006646	0.0019089	-1.1661
PAFAH2	-0.078329	5.9827783	-3.432924	0.0006646	0.0019089	-1.166122
RP4-568C11	0.7780548	3.6616466	3.4328708	0.0006647	0.0019091	-1.166298
RP11-925D8	-0.419088	-0.988093	-3.432839	0.0006648	0.0019092	-1.166402
IYD	-0.397208	5.5680935	-3.432808	0.0006649	0.0019093	-1.166507
SCAF8	0.0567136	5.967571	3.4325539	0.0006655	0.0019107	-1.167345
AC004895.4	-0.556643	0.9006352	-3.432551	0.0006655	0.0019107	-1.167356
TRBV11-1	0.3706648	-1.09903	3.4321693	0.0006664	0.0019132	-1.168615
RP11-361L1	-0.739213	1.7326135	-3.432073	0.0006666	0.0019137	-1.168934
RP11-475J5	-0.609338	0.0844194	-3.432047	0.0006667	0.0019137	-1.16902
CTD-2587H2	0.1689559	4.2368377	3.4317894	0.0006673	0.0019153	-1.16987
AC012531.2	0.4141849	-1.123701	3.4313563	0.0006683	0.0019181	-1.1713
MRPS18A	-0.066332	6.0959371	-3.43127	0.0006685	0.0019185	-1.171585
RP11-505P4	0.5256732	-0.075068	3.431253	0.0006686	0.0019185	-1.171641
MLKL	0.1040331	5.4936437	3.430585	0.0006702	0.001923	-1.173847
SLC25A35	0.1106801	5.1093338	3.4301115	0.0006713	0.0019261	-1.17541
RAC1P2	0.1386357	4.4216794	3.4299975	0.0006716	0.0019267	-1.175786
CRIP3	-0.319437	5.3225695	-3.429622	0.0006725	0.0019291	-1.177024
LDHAL6B	0.5609432	-0.14469	3.4294309	0.0006729	0.0019303	-1.177657
AP001056.1	0.3710065	-1.060307	3.4292471	0.0006734	0.0019314	-1.178263
ACSL6	-0.319259	5.2756746	-3.42913	0.0006737	0.0019321	-1.178648
CTC-325H20	0.5724758	0.292519	3.4289847	0.000674	0.0019329	-1.179129
RP11-299G2	-0.281177	5.0803813	-3.428696	0.0006747	0.0019348	-1.180083
RBX1	-0.060473	6.3118153	-3.428488	0.0006752	0.0019361	-1.180769
RP3-394A18	-0.246436	5.011968	-3.428439	0.0006753	0.0019362	-1.180931
UBTFL10	-0.492565	-0.877227	-3.427875	0.0006767	0.00194	-1.18279
SMC1B	0.6907086	2.8366154	3.4277236	0.0006771	0.0019409	-1.18329
MAFK	0.1405109	5.954122	3.4273969	0.0006778	0.001943	-1.184368
KCNN2	0.4823834	3.5870299	3.4269082	0.000679	0.0019462	-1.18598
SPC24	-0.175538	5.6177894	-3.426467	0.0006801	0.0019492	-1.187434
RP11-963H4	-0.31942	-1.203016	-3.426291	0.0006805	0.0019502	-1.188016
AC092684.1	-0.181079	-1.472183	-3.42625	0.0006806	0.0019503	-1.188151
RP11-381K2	0.6435371	0.1802564	3.4262403	0.0006806	0.0019503	-1.188182
GPR158	-0.819111	3.8823201	-3.425514	0.0006824	0.0019552	-1.190578
RP11-830F9	0.4938314	-0.536302	3.4253763	0.0006828	0.001956	-1.191031
RP11-475B2	0.3789051	-1.100475	3.4247791	0.0006842	0.00196	-1.193
KB-1125A3.	-0.684018	1.317499	-3.424191	0.0006856	0.001964	-1.194937
IGHV3-22	0.410487	-1.115013	3.4239728	0.0006862	0.0019653	-1.195657
NCAPH	0.1915314	5.2351903	3.4238877	0.0006864	0.0019658	-1.195938
GLB1	-0.052384	6.5721039	-3.423836	0.0006865	0.001966	-1.196107
C6orf25	0.5886852	0.7336503	3.4237209	0.0006868	0.0019666	-1.196487
CTSD	-0.069487	7.4392937	-3.423282	0.0006879	0.0019696	-1.197932

CTD-3010D2	0.625905	-0.570089	3.4232563	0.0006879	0.0019696	-1.198018
MAP3K2	-0.050128	6.3495211	-3.423145	0.0006882	0.0019702	-1.198385
LINC01552	-0.345763	-1.244839	-3.423125	0.0006883	0.0019702	-1.198452
RP11-11N9.	0.5879012	3.1180854	3.4230439	0.0006885	0.0019706	-1.198718
MRPS17	-0.086052	5.8929687	-3.42288	0.0006889	0.0019716	-1.199259
RP11-120K1	0.408446	-0.895582	3.422664	0.0006894	0.001973	-1.199969
RP11-214K3	0.5188107	-0.349788	3.4223551	0.0006901	0.001975	-1.200987
SIRPD	0.3766216	-1.037957	3.4218228	0.0006915	0.0019786	-1.20274
CALCOCO1	-0.05877	6.3607108	-3.421589	0.000692	0.0019801	-1.20351
RP11-248B2	-0.30098	-1.28522	-3.421391	0.0006925	0.0019813	-1.204163
NLK	-0.049366	6.0720052	-3.421129	0.0006932	0.001983	-1.205024
AC008079.1	0.3337432	3.3547016	3.4210627	0.0006933	0.0019833	-1.205244
COL23A1	0.4149439	3.8452807	3.4207817	0.000694	0.0019852	-1.206169
EDN3	0.6801424	-0.348778	3.4207168	0.0006942	0.0019855	-1.206382
RP11-381E2	0.6437341	0.4123893	3.4203931	0.000695	0.0019876	-1.207448
CTC-463A16	-0.167904	4.3039466	-3.419804	0.0006964	0.0019916	-1.209386
LLNLR-470E	0.7164037	1.4234371	3.4194148	0.0006974	0.0019942	-1.210669
DBNL	-0.046095	6.5989171	-3.419179	0.000698	0.0019957	-1.211446
DDX3P1	-0.560742	0.023389	-3.419171	0.000698	0.0019957	-1.211472
CTD-2349P2	0.3185787	3.2132606	3.419146	0.0006981	0.0019957	-1.211553
MIR616	0.6026296	0.2804499	3.4190563	0.0006983	0.0019962	-1.211849
C9orf24	-0.442548	3.0650741	-3.418865	0.0006988	0.0019974	-1.212479
RP11-131N1	0.3106423	-1.246365	3.4188343	0.0006989	0.0019974	-1.212579
C18orf8	-0.055817	6.0486077	-3.418302	0.0007002	0.0020011	-1.21433
RP11-327F2	0.39242	-0.966041	3.4179869	0.000701	0.0020031	-1.215367
HSP90B1	-0.054441	7.4020023	-3.417941	0.0007011	0.0020033	-1.215518
TFB1M	-0.089024	5.6387287	-3.417671	0.0007018	0.0020051	-1.216408
RP11-169E6	-0.56715	0.7017011	-3.417649	0.0007018	0.0020051	-1.216479
ANKRD13B	0.2267281	4.7221284	3.4174205	0.0007024	0.0020066	-1.217231
TRIM2	-0.143935	5.8833716	-3.417362	0.0007025	0.0020068	-1.217424
CD79B	0.1559099	4.9849196	3.4172637	0.0007028	0.0020074	-1.217746
RP11-264L1	-0.632449	-0.018685	-3.417067	0.0007033	0.0020086	-1.218392
LRRC37A7P	-0.710323	3.9486655	-3.416615	0.0007044	0.0020117	-1.219882
CTB-26E19.	-0.547082	-0.773191	-3.416427	0.0007049	0.0020129	-1.220497
RHBDF2	0.0940065	6.0273201	3.4160886	0.0007057	0.0020152	-1.221611
SHCBP1	0.2581755	4.9015171	3.4158999	0.0007062	0.0020163	-1.222232
SMPX	-0.906941	2.5016174	-3.415672	0.0007068	0.0020178	-1.22298
UBE2V2	-0.064299	6.4013204	-3.415239	0.0007079	0.0020208	-1.224406
IPO4	-0.107996	5.3950372	-3.415188	0.000708	0.002021	-1.224572
SHROOM4	0.1615707	5.0700009	3.4150681	0.0007083	0.0020217	-1.224967
ATG4A	-0.073987	5.9748298	-3.415042	0.0007084	0.0020217	-1.225051
RP11-248E9	-0.318985	-1.34253	-3.414866	0.0007088	0.0020226	-1.225632
KCNA2	0.5720084	-0.525403	3.4148531	0.0007088	0.0020226	-1.225673
C5orf38	0.5011917	-0.896122	3.4148485	0.0007088	0.0020226	-1.225688
LGSN	-0.704579	3.4561374	-3.414586	0.0007095	0.0020244	-1.226552
CABP5	-0.216197	-1.420397	-3.414444	0.0007099	0.0020252	-1.227017
ZDHHC16	-0.05806	6.1778963	-3.414257	0.0007103	0.0020264	-1.227633
RP11-38M8.	0.6865121	2.8997407	3.4141703	0.0007106	0.0020269	-1.227918
RP11-386G1	0.6342628	1.475331	3.4140281	0.0007109	0.0020277	-1.228385
RSL24D1P3	-0.188705	-1.425173	-3.414011	0.000711	0.0020277	-1.228442
CHRNA3	0.7292202	0.2398974	3.4136626	0.0007118	0.0020301	-1.229586

RP11-179G5	-0.372702	3.038305	-3.413559	0.0007121	0.0020307	-1.229927
AP003068.1	-0.251781	3.9606867	-3.413506	0.0007122	0.0020309	-1.230102
RP11-2N1.2	0.3925452	-1.152104	3.4132362	0.0007129	0.0020327	-1.230987
RP11-26F2.	0.685213	0.3227786	3.4132012	0.000713	0.0020328	-1.231102
CASC22	-0.759463	0.1860353	-3.413031	0.0007134	0.0020339	-1.231662
SYNJ2BP	-0.063633	6.2525632	-3.412925	0.0007137	0.0020345	-1.232011
RP1-152L7.	-0.196229	5.6357215	-3.412587	0.0007146	0.0020368	-1.233121
AC138623.1	-0.22619	-1.411595	-3.412539	0.0007147	0.0020369	-1.233277
RP11-804H8	-0.457758	-0.756746	-3.412453	0.0007149	0.0020374	-1.233561
PCAT7	-0.364547	4.2392637	-3.412403	0.000715	0.0020376	-1.233726
AKIP1	-0.056396	5.9208387	-3.411671	0.0007169	0.0020428	-1.236131
PRAMEF11	-0.526687	-0.850326	-3.411574	0.0007171	0.0020433	-1.236449
AC004980.1	0.5449949	-0.324953	3.4114114	0.0007176	0.0020443	-1.236982
TRMT12	-0.078534	5.6988611	-3.410908	0.0007188	0.0020478	-1.238635
UNC45B	0.5677404	0.806262	3.4100502	0.000721	0.0020539	-1.241451
RP11-545M1	-0.48175	1.8037738	-3.409718	0.0007219	0.0020562	-1.242541
PCED1A	-0.070109	6.1864039	-3.40968	0.000722	0.0020563	-1.242665
DHX8	-0.040062	6.2769215	-3.409503	0.0007224	0.0020573	-1.243248
HOXC5	0.3983824	-1.162617	3.4094948	0.0007225	0.0020573	-1.243274
RP1-29C18.	0.6457357	0.570882	3.4092656	0.0007231	0.0020589	-1.244027
MGAT5	0.0888943	6.0251822	3.4087315	0.0007244	0.0020626	-1.24578
RP5-98107.	0.5303268	0.1226316	3.4086222	0.0007247	0.0020632	-1.246138
RP11-587P2	-0.445379	-1.004374	-3.408031	0.0007262	0.0020673	-1.248078
CTD-2201I1	-0.422454	3.4825422	-3.408019	0.0007263	0.0020673	-1.248119
FAM216B	0.4291377	-1.084967	3.4079885	0.0007263	0.0020674	-1.248218
RP11-348J1	-0.519363	-0.678364	-3.407891	0.0007266	0.002068	-1.248537
MLIP-IT1	-0.641562	0.1384225	-3.407813	0.0007268	0.0020684	-1.248792
HLA-F	-0.122594	6.5494465	-3.407753	0.000727	0.0020687	-1.248992
PPP2R3B	-0.081206	5.6803079	-3.406815	0.0007294	0.0020754	-1.252068
SHROOM2	0.3098034	5.4901129	3.4064787	0.0007302	0.0020776	-1.25317
CTD-2531D1	0.6782703	-0.082848	3.4064738	0.0007303	0.0020776	-1.253187
USP6NL	0.0691136	6.0501002	3.4062167	0.0007309	0.0020793	-1.25403
ADAT2	0.1125265	5.2472868	3.4060752	0.0007313	0.0020802	-1.254494
CTD-2116N2	0.6458808	0.3869368	3.4056973	0.0007323	0.0020828	-1.255733
AC007204.2	0.3248555	-1.261098	3.4056267	0.0007325	0.0020832	-1.255964
CTD-2192J1	0.5798915	0.8789504	3.4053557	0.0007332	0.002085	-1.256853
ESRRA	-0.064148	6.539938	-3.405273	0.0007334	0.0020855	-1.257123
RP11-622C2	0.603363	0.2105008	3.4051591	0.0007337	0.0020862	-1.257497
AC006547.1	0.1421876	4.4820462	3.4046659	0.000735	0.0020897	-1.259114
SEC22A	-0.052482	5.8097162	-3.404361	0.0007358	0.0020918	-1.260114
TUBB	-0.053825	7.159427	-3.404094	0.0007365	0.0020936	-1.26099
TFDP2	-0.054208	6.1501689	-3.403992	0.0007367	0.0020942	-1.261325
TEX37	-0.668277	-0.699026	-3.403592	0.0007378	0.002097	-1.262636
PNPLA6	-0.056329	6.4105245	-3.403518	0.000738	0.0020973	-1.262876
PIP4K2B	-0.043335	6.3849906	-3.403504	0.000738	0.0020973	-1.262923
NANOGP7	0.3297849	-1.255357	3.4033149	0.0007385	0.0020986	-1.263542
HNRNPAB	-0.050982	6.6855502	-3.403241	0.0007387	0.0020989	-1.263784
FIBCD1	0.8208101	1.0705263	3.4029791	0.0007394	0.0021007	-1.264643
BUD31	-0.059117	6.2639975	-3.402918	0.0007395	0.002101	-1.264843
PLEKHA7	0.1321581	5.8337419	3.4028873	0.0007396	0.0021011	-1.264943
ZNF725P	-0.48682	-1.050889	-3.401464	0.0007434	0.0021116	-1.269604

RP11-9E17.	0.1783918	4.6454267	3.4012143	0.000744	0.0021133	-1.270423
TNNI3	0.8256476	0.3941769	3.4011664	0.0007441	0.0021135	-1.27058
WDFY3	0.0955007	5.8852142	3.4011336	0.0007442	0.0021135	-1.270688
RP11-1101K	-0.304012	-1.264902	-3.401105	0.0007443	0.0021136	-1.270782
PIP5KL1	0.5066415	2.5627935	3.4007513	0.0007452	0.0021161	-1.27194
CTB-140J7.	-0.556371	-0.386009	-3.40051	0.0007459	0.0021177	-1.27273
TKT	-0.099076	6.9313214	-3.40042	0.0007461	0.0021182	-1.273025
RBM12B	0.0873397	5.5683241	3.4002211	0.0007466	0.0021195	-1.273676
RP11-611L7	-0.332224	-1.220965	-3.400207	0.0007467	0.0021195	-1.27372
RP11-315I2	-0.22373	3.7601373	-3.399945	0.0007474	0.0021213	-1.274579
PLEKHA8P1	0.1197421	4.9084134	3.3999317	0.0007474	0.0021213	-1.274623
RPL28	-0.078893	7.1907761	-3.39974	0.0007479	0.0021225	-1.275251
AP000705.7	-0.550609	-0.631615	-3.399697	0.000748	0.0021227	-1.275392
RP11-305L7	0.5739978	1.5331278	3.3991725	0.0007494	0.0021265	-1.277108
FAM83C	-0.601663	0.1236296	-3.398757	0.0007505	0.0021294	-1.278468
AP001628.6	0.570052	0.2742956	3.398544	0.0007511	0.0021309	-1.279165
RP4-566L20	-0.144999	-1.490987	-3.398448	0.0007514	0.0021313	-1.27948
RP11-158H5	0.3330363	3.5341718	3.3984389	0.0007514	0.0021313	-1.279509
UTS2	0.8180022	1.1935479	3.3983049	0.0007517	0.0021322	-1.279948
RP11-1006G	-0.501711	-0.405741	-3.398254	0.0007519	0.0021324	-1.280114
ACTR2	0.0388181	6.7464208	3.3981842	0.0007521	0.0021328	-1.280343
CARS2	-0.065629	6.3008067	-3.397692	0.0007534	0.0021363	-1.281954
CTD-2270L9	0.5808968	1.3329206	3.3971168	0.0007549	0.0021405	-1.283835
AC027119.1	-0.453417	-1.084649	-3.396805	0.0007557	0.0021427	-1.284855
FBXO25	-0.086123	6.2537401	-3.396393	0.0007568	0.0021457	-1.286204
RP11-142C4	-0.569293	0.0342373	-3.39631	0.0007571	0.0021462	-1.286474
RP11-57J16	-0.178877	-1.440653	-3.396051	0.0007578	0.002148	-1.28732
HOXD9	-0.672174	3.672755	-3.395199	0.0007601	0.0021541	-1.290108
RP4-806M20	0.6311726	-0.305721	3.3951872	0.0007601	0.0021541	-1.290145
C1QB	0.1269099	6.5463928	3.3951771	0.0007601	0.0021541	-1.290178
GAPDH	-0.062049	7.5172848	-3.395076	0.0007604	0.0021547	-1.290507
EIF3E	-0.077018	6.9148156	-3.394866	0.0007609	0.0021561	-1.291195
MAP4K5	0.0706026	5.9001408	3.3945679	0.0007618	0.0021583	-1.29217
ENTPD7	0.1118314	5.5221602	3.3945121	0.0007619	0.0021585	-1.292352
INE1	0.2958864	3.4439407	3.3943617	0.0007623	0.0021595	-1.292844
SRSF9	0.0490854	6.4050091	3.3940262	0.0007632	0.0021619	-1.29394
B2M	-0.069131	7.5328601	-3.393106	0.0007657	0.0021688	-1.296947
RP3-391022	-0.253979	-1.340895	-3.392968	0.0007661	0.0021697	-1.2974
PMF1-BGLAP	-0.178753	4.516026	-3.392904	0.0007663	0.0021699	-1.297608
ZBED3	-0.093349	6.1561937	-3.392901	0.0007663	0.0021699	-1.297618
SBDSP1	0.1256447	5.5887309	3.3927555	0.0007667	0.0021708	-1.298093
BMP3	0.5073045	-0.86857	3.3926364	0.000767	0.0021715	-1.298482
CENPT	-0.052979	6.0423296	-3.392625	0.000767	0.0021715	-1.298519
RP11-672A2	0.3650105	-1.025497	3.3925039	0.0007673	0.0021722	-1.298915
SLC35B2	-0.056867	6.5224542	-3.392473	0.0007674	0.0021723	-1.299018
RP11-1399P	0.7266655	1.3055012	3.3922592	0.000768	0.0021738	-1.299714
PGBD4P8	-0.200597	-1.449775	-3.392215	0.0007681	0.002174	-1.29986
RP11-927P2	-0.270925	-1.297737	-3.391625	0.0007697	0.0021783	-1.301786
RP11-11N7.	-0.477457	-0.567591	-3.391332	0.0007705	0.0021804	-1.302743
RP11-848P1	0.6756487	0.8652132	3.3908953	0.0007717	0.0021836	-1.304169
PURA	-0.061766	6.0746353	-3.390531	0.0007727	0.0021863	-1.305359

RP11-470M1	0.4498082	-0.86719	3.3903046	0.0007733	0.0021878	-1.306098
RP11-691N7	0.6175621	0.5004508	3.3900891	0.0007739	0.0021893	-1.306802
RP11-481F2	-0.897601	0.0693223	-3.390027	0.0007741	0.0021897	-1.307006
FGF21	-0.590131	5.4332198	-3.389535	0.0007754	0.0021933	-1.30861
GTSF1L	0.4483402	-0.947527	3.389517	0.0007755	0.0021933	-1.308669
CCDC88B	0.1411642	5.6648895	3.3894822	0.0007756	0.0021934	-1.308783
RP11-763E3	-0.572268	-0.043184	-3.389391	0.0007758	0.0021939	-1.30908
VMAC	-0.077408	5.2038616	-3.388811	0.0007774	0.0021982	-1.310972
RPSAP9	-0.201985	3.9568986	-3.388604	0.000778	0.0021997	-1.311648
PFN1P11	-0.863368	2.1345068	-3.388552	0.0007781	0.0021999	-1.31182
HERPUD2	-0.049263	6.1687462	-3.388481	0.0007783	0.0022003	-1.31205
POLE3	-0.046578	6.3356711	-3.388171	0.0007792	0.0022025	-1.313062
SLC25A5-AS	-0.18005	4.59761	-3.388152	0.0007792	0.0022025	-1.313123
PCDHA5	0.6445615	-0.197138	3.3881318	0.0007793	0.0022025	-1.31319
SPDL1	0.1069932	5.4651616	3.3880173	0.0007796	0.0022032	-1.313564
RP11-517M2	-0.219285	-1.411363	-3.387995	0.0007797	0.0022032	-1.313636
ITGAM	0.1876469	5.4286607	3.3874587	0.0007812	0.0022072	-1.315386
AL353662.3	-0.481551	-0.369206	-3.387253	0.0007817	0.0022087	-1.316058
LINC01273	-0.346121	4.027076	-3.387063	0.0007822	0.00221	-1.316676
AC010518.2	0.4275889	-0.983189	3.3869995	0.0007824	0.0022103	-1.316884
RP11-47I22	0.7206712	0.7130564	3.3866722	0.0007833	0.0022127	-1.317952
ZNF780B	0.1009336	5.4306793	3.3866483	0.0007834	0.0022127	-1.31803
TLK2P1	0.4773799	1.9226222	3.3865944	0.0007835	0.002213	-1.318206
CLRN1	-0.301196	-1.263801	-3.386326	0.0007843	0.0022149	-1.319083
TMEM167B	-0.044335	6.2691179	-3.386022	0.0007851	0.0022171	-1.320072
AC005740.6	0.4934656	1.6712739	3.3859935	0.0007852	0.0022171	-1.320166
ERG	0.1281367	5.4946298	3.385977	0.0007853	0.0022171	-1.320219
CLK3	-0.037452	6.2116663	-3.385953	0.0007853	0.0022171	-1.320297
TSEN15	-0.059491	6.0683539	-3.385898	0.0007855	0.0022174	-1.320477
CYP4F60P	-0.909129	0.9902244	-3.385715	0.000786	0.0022184	-1.321074
NNT-AS1	-0.083014	5.7926282	-3.385711	0.000786	0.0022184	-1.321087
RP11-142E9	0.4032618	3.6173232	3.3857067	0.000786	0.0022184	-1.321101
PIGK	-0.066052	6.0829583	-3.385176	0.0007875	0.0022224	-1.322832
CST3	-0.078576	7.0058334	-3.385104	0.0007877	0.0022228	-1.323067
ANKH	-0.068733	6.5709729	-3.384424	0.0007896	0.0022279	-1.325284
RP3-402G11	0.3586025	3.1852961	3.3841734	0.0007903	0.0022297	-1.3261
HS3ST5	0.5694872	-0.853485	3.3841318	0.0007904	0.0022299	-1.326235
RP11-108K3	0.3935755	-1.134231	3.3840918	0.0007905	0.00223	-1.326366
SLC7A5	0.176287	5.86603	3.3839568	0.0007909	0.0022309	-1.326806
ACOT2	-0.108692	6.1683192	-3.383855	0.0007912	0.0022315	-1.327138
RAB5C	-0.043018	6.5739131	-3.383782	0.0007914	0.0022319	-1.327375
RP11-19E11	0.8607539	0.1799223	3.3836455	0.0007918	0.0022329	-1.32782
TSGA13	-0.233452	-1.361077	-3.383462	0.0007923	0.0022341	-1.32842
HMGB1P3	0.5405472	-0.357795	3.3833906	0.0007925	0.0022345	-1.328651
NOMO3	0.5545447	4.2470018	3.3831287	0.0007932	0.0022364	-1.329504
RPL5P3	0.5706601	0.3008332	3.3825883	0.0007947	0.0022405	-1.331265
RP11-142C4	-0.532541	3.2423088	-3.38257	0.0007948	0.0022405	-1.331324
AKAP6	-0.216122	5.0427061	-3.382372	0.0007953	0.0022419	-1.33197
RP11-504G3	0.5504862	0.1503383	3.382353	0.0007954	0.0022419	-1.332032
RAB43P1	0.3583	3.958867	3.3821157	0.000796	0.0022434	-1.332805
KRT222	0.7018325	3.4101321	3.3820981	0.0007961	0.0022434	-1.332862

PCSK6	-0.1561	6.5441016	-3.382086	0.0007961	0.0022434	-1.332901
CHCHD6	-0.079678	5.5912448	-3.381461	0.0007979	0.0022482	-1.334938
NMRK2	0.5640457	-0.647744	3.3808816	0.0007995	0.0022527	-1.336825
PLCB1	0.1830135	5.7357482	3.3807992	0.0007997	0.0022531	-1.337093
VNN3	0.450587	5.3990779	3.3807684	0.0007998	0.0022532	-1.337193
RP11-150C1	-0.630442	1.0959879	-3.379695	0.0008029	0.0022616	-1.34069
OFD1	0.0561511	5.9359708	3.3794068	0.0008037	0.0022637	-1.341626
PP14571	0.5969313	0.0989401	3.379149	0.0008044	0.0022655	-1.342465
ABTB2	-0.115851	5.9754026	-3.37914	0.0008044	0.0022655	-1.342494
CTD-2541M1	0.6484522	0.6312105	3.3790983	0.0008046	0.0022657	-1.34263
PLA2G2A	0.625347	5.6834399	3.3789825	0.0008049	0.0022664	-1.343007
RPLP0P1	-0.377074	-1.020202	-3.378282	0.0008069	0.0022718	-1.345287
DDOST	-0.046109	6.866807	-3.377906	0.000808	0.0022747	-1.346512
MTMR9LP	0.1307155	5.023491	3.3778698	0.0008081	0.0022748	-1.346629
POLI	0.0951522	5.5344163	3.3776984	0.0008085	0.002276	-1.347186
AC005264.2	0.552669	0.745298	3.3773179	0.0008096	0.0022789	-1.348424
CMTM2	0.5625831	1.4725744	3.3772069	0.0008099	0.0022794	-1.348785
ACSM3	-0.205202	5.8597789	-3.377203	0.00081	0.0022794	-1.348797
AQP7	-0.229256	5.5841424	-3.377179	0.00081	0.0022795	-1.348877
CCAT1	0.618523	4.0710176	3.3771167	0.0008102	0.0022797	-1.349079
FAM198B	0.1259825	5.7912722	3.3771093	0.0008102	0.0022797	-1.349103
CTD-2252P2	0.3628654	-1.048648	3.3767589	0.0008112	0.0022823	-1.350243
RP11-795F1	0.509214	2.7041282	3.3767006	0.0008114	0.0022826	-1.350432
SAR1P1	-0.15308	-1.492104	-3.376503	0.000812	0.0022839	-1.351075
SFXN4	-0.074729	6.2106627	-3.376493	0.000812	0.0022839	-1.351108
IGHV1-14	0.3906161	-1.139622	3.3762091	0.0008128	0.002286	-1.352031
RNU1-70P	-0.395748	5.7428888	-3.375344	0.0008153	0.0022928	-1.354843
RSPO2	-0.896781	1.0483635	-3.375106	0.000816	0.0022946	-1.355616
RP11-9D8.1	-0.575082	0.0250688	-3.375003	0.0008163	0.0022952	-1.355953
OAZ1	-0.061759	7.0607247	-3.374576	0.0008175	0.0022985	-1.35734
RP1-12208.	0.4032539	-0.822547	3.3745396	0.0008176	0.0022986	-1.357459
RP11-110I1	0.5957225	1.684998	3.3739846	0.0008192	0.0023029	-1.359263
TXNL4B	-0.068394	5.8843004	-3.373974	0.0008192	0.0023029	-1.359298
TRBV7-4	0.4273999	-0.931626	3.3737694	0.0008198	0.0023044	-1.359963
CDPF1	-0.081646	5.6634816	-3.373562	0.0008204	0.0023059	-1.360636
MCIDAS	0.7212538	-0.005052	3.3726996	0.0008229	0.0023127	-1.363439
RP11-121J2	-0.165727	-1.468757	-3.371871	0.0008253	0.0023193	-1.366132
RPA2	-0.053688	6.1367662	-3.370918	0.0008281	0.0023269	-1.369225
TCTEX1D4	-0.643061	1.5106554	-3.370782	0.0008285	0.0023278	-1.369669
ISG15	-0.132952	6.369836	-3.3707	0.0008287	0.0023283	-1.369936
ADGB	0.3964325	-1.095359	3.3703696	0.0008297	0.0023308	-1.371007
F9	-0.470029	6.3515314	-3.370349	0.0008298	0.0023308	-1.371073
KCNG2	0.6062858	1.4949529	3.3702477	0.00083	0.0023315	-1.371403
CTD-2203K1	-0.349954	3.2737051	-3.370143	0.0008304	0.0023322	-1.371744
PKI55	0.1732065	4.8439324	3.369713	0.0008316	0.0023355	-1.373139
TRAK2	0.0545188	6.2005556	3.3696359	0.0008318	0.002336	-1.373389
RP11-248J2	0.477198	-0.487747	3.3695369	0.0008321	0.0023366	-1.373711
NUP107	0.0550785	6.0927301	3.3691268	0.0008333	0.0023398	-1.375042
PLOD3	-0.058715	6.7453296	-3.368918	0.0008339	0.0023413	-1.375718
YTHDF3	-0.056897	6.5047413	-3.368666	0.0008347	0.0023433	-1.376539
CRYBB2	0.6475034	1.1881213	3.3686193	0.0008348	0.0023435	-1.376689

RP11-923I1	0.4030193	4.6039024	3.3682897	0.0008358	0.002346	-1.377759
SLAIN2	-0.050267	6.2569896	-3.36796	0.0008368	0.0023485	-1.378829
HOXC11	0.5261337	-0.920534	3.3679011	0.0008369	0.0023488	-1.37902
CDH22	-0.892015	0.3909955	-3.367864	0.000837	0.002349	-1.379139
RP11-273B2	-0.194173	4.8622169	-3.367808	0.0008372	0.0023493	-1.379322
RP13-12804	0.5863179	2.3483376	3.3674468	0.0008383	0.0023521	-1.380494
IQUB	0.5727905	1.2957899	3.367206	0.000839	0.0023539	-1.381275
SLCO1A2	-0.331594	4.951595	-3.367106	0.0008393	0.0023545	-1.3816
CATSPER3	-0.200211	3.8860266	-3.36682	0.0008401	0.0023567	-1.382526
TMEM204	0.1201384	5.761763	3.366724	0.0008404	0.0023573	-1.382839
CTB-158E9.	-0.115155	-1.51231	-3.366671	0.0008406	0.0023576	-1.383011
NOVA2	0.1294076	5.0148064	3.366555	0.0008409	0.0023584	-1.383387
RP11-861L1	0.410662	-0.953616	3.3662399	0.0008418	0.0023606	-1.384409
LRRC48	0.1343928	4.7669683	3.3662253	0.0008419	0.0023606	-1.384456
CERS5	0.052773	6.014353	3.3662253	0.0008419	0.0023606	-1.384456
LDLR	-0.085816	6.5685245	-3.36617	0.000842	0.0023608	-1.384634
SSR2	-0.065845	6.9715604	-3.36613	0.0008422	0.002361	-1.384766
HCN1	0.7287593	-0.259024	3.3659143	0.0008428	0.0023626	-1.385465
FAM83H	-0.101652	6.6119574	-3.365834	0.000843	0.0023631	-1.385726
CR848007.2	-0.827677	0.5699593	-3.365808	0.0008431	0.0023631	-1.385809
RP5-1061H2	-0.343172	-1.134431	-3.365427	0.0008442	0.0023661	-1.387044
MPZL3	0.1123643	5.767368	3.3652732	0.0008447	0.0023672	-1.387544
LINC00520	0.5116803	-0.75067	3.3651846	0.000845	0.0023677	-1.387831
RP11-428G5	-0.181015	-1.449351	-3.365105	0.0008452	0.0023682	-1.388088
RP11-548P2	-0.52915	1.4606878	-3.364962	0.0008456	0.0023692	-1.388553
HBEGF	0.1178965	5.4179243	3.3647674	0.0008462	0.0023707	-1.389183
FPGT	-0.064524	5.7693797	-3.364674	0.0008465	0.0023713	-1.389485
SNRPA	-0.060193	6.3565227	-3.364562	0.0008468	0.002372	-1.389849
NATP	-0.216767	-1.415394	-3.364486	0.000847	0.0023725	-1.390097
NOMO2	0.2074178	5.3662532	3.3642952	0.0008476	0.0023739	-1.390714
RP11-477D1	0.2490335	3.80494	3.3641994	0.0008479	0.0023745	-1.391025
CTC-492K19	-0.552052	-0.210326	-3.364066	0.0008483	0.0023754	-1.391456
RP11-700H6	-0.464727	-0.625185	-3.364031	0.0008484	0.0023756	-1.391572
CTD-2005H7	-0.506534	-0.50783	-3.363529	0.0008499	0.0023796	-1.393197
UGT1A10	0.9204809	0.6715085	3.3632543	0.0008507	0.0023817	-1.394088
RNU6-1160P	-0.590418	0.0661946	-3.362804	0.0008521	0.0023853	-1.395548
RP11-711C1	-0.390083	-1.183476	-3.362581	0.0008527	0.0023869	-1.39627
HOXA7	0.6046148	-0.360947	3.3625679	0.0008528	0.0023869	-1.396312
PRDX3	-0.063736	6.9026084	-3.362482	0.000853	0.0023874	-1.396591
DENND5B-AS	0.5272456	-0.033534	3.3623491	0.0008534	0.0023883	-1.397021
GLOD5	-0.541921	3.9341797	-3.36233	0.0008535	0.0023883	-1.397083
UBE2E1-AS1	0.4294135	-0.871329	3.3623012	0.0008536	0.0023884	-1.397176
URB2	0.0810912	5.6430893	3.3619205	0.0008547	0.0023914	-1.398409
RP11-278L1	0.4804273	4.3098489	3.3617563	0.0008552	0.0023926	-1.398941
KRT17	0.5120913	3.952868	3.361456	0.0008561	0.0023949	-1.399914
RP11-46802	-0.506104	-0.164081	-3.361323	0.0008565	0.0023959	-1.400343
TP53TG1	-0.106789	6.0400249	-3.361154	0.000857	0.0023971	-1.400891
RP11-79N23	0.6984157	0.8267239	3.3607608	0.0008582	0.0024002	-1.402165
RP11-483P2	-0.787444	0.682559	-3.360254	0.0008597	0.0024043	-1.403807
LEPROT	-0.052173	6.5946961	-3.360206	0.0008599	0.0024045	-1.403961
RP1-40E16.	-0.493084	-0.457751	-3.359914	0.0008608	0.0024067	-1.404908

RP11-83B20	-0.475269	-0.914424	-3.359902	0.0008608	0.0024067	-1.404945
ZNF699	0.5054321	3.3821113	3.3594756	0.0008621	0.0024102	-1.406326
RP11-212E4	0.4137554	-0.818402	3.3594068	0.0008623	0.0024106	-1.406549
DPPA3P2	-0.169724	-1.475831	-3.359157	0.0008631	0.0024125	-1.407359
SPINT3	-0.388681	-1.240551	-3.359119	0.0008632	0.0024126	-1.407482
PPM1B	-0.05239	6.3486934	-3.358877	0.0008639	0.0024145	-1.408265
RPS4X	-0.064361	7.2676387	-3.358672	0.0008645	0.0024159	-1.408928
TRIM55	-0.661281	5.3811027	-3.358667	0.0008645	0.0024159	-1.408945
POLR2G	-0.056358	6.3184499	-3.358463	0.0008652	0.0024175	-1.409603
CUX2	-0.386382	5.8322377	-3.358089	0.0008663	0.0024204	-1.410813
TH	0.6711338	-0.139137	3.3580738	0.0008663	0.0024204	-1.410864
OSBPL9	0.065546	6.5612566	3.3580302	0.0008665	0.0024206	-1.411005
OLFML1	0.2559107	5.3390353	3.3575971	0.0008678	0.0024239	-1.412406
RP4-610C12	0.5065647	-0.455301	3.3575948	0.0008678	0.0024239	-1.412413
SDR16C5	0.5653883	-0.678983	3.3575744	0.0008679	0.0024239	-1.412479
TMEM41A	-0.058143	6.1494183	-3.35751	0.0008681	0.0024243	-1.412686
GS1-166A23	-0.516317	-0.425698	-3.357287	0.0008687	0.0024259	-1.413409
CCDC50	-0.042538	6.5774656	-3.357275	0.0008688	0.0024259	-1.413448
RP11-80F22	-0.441846	-1.124005	-3.357249	0.0008689	0.0024259	-1.413531
DGCR14	-0.054943	5.9249833	-3.356897	0.0008699	0.0024287	-1.41467
AC104777.2	-0.252566	-1.35147	-3.356737	0.0008704	0.0024299	-1.41519
CTIF	-0.066363	6.2785196	-3.356515	0.0008711	0.0024316	-1.415905
RFC1	-0.052734	6.4534332	-3.356263	0.0008719	0.0024336	-1.416722
SLC17A6	-0.221374	-1.421665	-3.355627	0.0008738	0.0024388	-1.418779
OCIAD1-AS1	0.5487381	1.0984605	3.3556069	0.0008739	0.0024388	-1.418843
RP11-564P9	-0.201267	-1.425747	-3.355465	0.0008743	0.0024399	-1.419302
KB-1836B5.	-0.602386	0.6285767	-3.355305	0.0008748	0.002441	-1.41982
PSMD8P1	-0.55612	0.1085767	-3.355271	0.0008749	0.0024411	-1.419928
NCOA5	0.0451881	6.1099087	3.3552388	0.000875	0.0024412	-1.420033
SUCLA2-AS1	-0.376765	2.5182915	-3.35522	0.0008751	0.0024412	-1.420093
FCRL6	0.3710106	3.6256795	3.3549969	0.0008757	0.0024429	-1.420816
C1orf226	-0.136706	5.9274546	-3.354962	0.0008759	0.002443	-1.420929
CTB-193M12	0.5250499	-0.141603	3.3548386	0.0008762	0.0024439	-1.421327
MEGF6	0.2643286	5.4445412	3.354685	0.0008767	0.002445	-1.421824
DGCR11	0.1614813	4.536738	3.3545329	0.0008772	0.0024462	-1.422316
SLC35F6	-0.062482	6.5391748	-3.3544	0.0008776	0.0024471	-1.422746
RP11-10A14	-0.568651	2.7436608	-3.354076	0.0008786	0.0024497	-1.423793
RP11-63B13	-0.22349	-1.389523	-3.354059	0.0008786	0.0024497	-1.423848
RAF1	-0.04594	6.6911688	-3.354013	0.0008788	0.0024499	-1.423996
CCDC85A	0.66043	1.6303596	3.3539152	0.0008791	0.0024505	-1.424312
PAPD5	-0.071148	5.8826054	-3.35348	0.0008804	0.0024541	-1.425719
RP11-540N6	-0.183418	-1.449504	-3.353253	0.0008811	0.0024558	-1.426451
DTD2	-0.065925	5.7116495	-3.353207	0.0008813	0.0024561	-1.4266
CDC42P6	0.5569297	1.7138787	3.3531738	0.0008814	0.0024562	-1.426708
RP11-68I3.	0.6230263	1.139753	3.353152	0.0008814	0.0024562	-1.426779
RP11-39602	-0.432125	-1.089296	-3.352548	0.0008833	0.0024612	-1.42873
KRTAP10-3	-0.183326	-1.438699	-3.35249	0.0008835	0.0024615	-1.428918
AP000593.7	-0.787847	0.9314663	-3.352437	0.0008836	0.0024618	-1.429089
IMPA1	-0.075846	6.1816514	-3.352158	0.0008845	0.002464	-1.42999
PTGES2	-0.073043	6.3968985	-3.35172	0.0008859	0.0024676	-1.431405
PYY2	0.5230221	2.5412569	3.3515615	0.0008864	0.0024688	-1.431917

ZNF264	0.0824248	5.8395801	3.3514907	0.0008866	0.0024692	-1.432146
CYB561D1	0.1082375	5.3671855	3.3511776	0.0008875	0.0024717	-1.433157
AEN	-0.080899	6.1257146	-3.351087	0.0008878	0.0024722	-1.433449
SFTPA2	0.6565277	-0.421014	3.351072	0.0008879	0.0024722	-1.433498
EIF4EBP1	-0.097971	6.2174468	-3.350843	0.0008886	0.002474	-1.434238
AC009498.1	-0.210267	-1.408399	-3.350388	0.00089	0.0024778	-1.435708
SLC17A2	-0.439989	5.8823294	-3.350044	0.0008911	0.0024806	-1.436817
HIST1H2AM	-0.566284	2.1465138	-3.3495	0.0008928	0.0024851	-1.438574
MPRIP-AS1	-0.463715	-0.653343	-3.348862	0.0008948	0.0024905	-1.440632
U4	0.5372004	0.0200658	3.348467	0.000896	0.0024938	-1.441908
RABL2B	0.0681581	5.5037614	3.3479132	0.0008978	0.0024984	-1.443695
RP11-59H7.	0.6023579	0.9318871	3.3477447	0.0008983	0.0024997	-1.444239
RP11-574F2	-0.248523	3.5951987	-3.347572	0.0008988	0.002501	-1.444795
RP11-204C2	-0.383403	-1.209001	-3.347517	0.000899	0.0025013	-1.444974
RP11-754N2	-0.677063	0.0202385	-3.347472	0.0008991	0.0025015	-1.445118
RP11-11N5.	-0.449036	-1.058349	-3.347305	0.0008997	0.0025028	-1.445659
ARSJ	0.4392602	4.3516055	3.3468877	0.000901	0.0025062	-1.447003
GPRC5B	0.1672003	5.7302302	3.3466521	0.0009017	0.0025081	-1.447763
KMT2B	0.0602791	6.346645	3.3465938	0.0009019	0.0025084	-1.447951
EVA1B	0.124289	5.4342259	3.3464991	0.0009022	0.0025091	-1.448257
RP11-196E1	-0.632198	-0.271812	-3.346416	0.0009025	0.0025096	-1.448525
ANAPC5	-0.038097	6.6355488	-3.346341	0.0009027	0.0025101	-1.448766
RP11-864N7	0.6057645	0.02053	3.3462563	0.000903	0.0025106	-1.44904
RP11-30015	0.4218315	-0.929775	3.3462225	0.0009031	0.0025107	-1.449149
LAP3	-0.085485	6.667586	-3.346203	0.0009031	0.0025107	-1.449213
ARFGAP1	-0.05161	6.5143217	-3.346143	0.0009033	0.0025111	-1.449407
bP-218909.	-0.782497	0.4050515	-3.346091	0.0009035	0.0025113	-1.449574
NUPL1P1	-0.148467	-1.474007	-3.345494	0.0009054	0.0025164	-1.451499
RP5-915N17	-0.184379	-1.447065	-3.345356	0.0009058	0.0025174	-1.451944
SLC29A2	-0.123022	5.8649464	-3.344788	0.0009076	0.0025222	-1.453774
IFT80	0.1167801	5.3725742	3.3440551	0.00091	0.0025285	-1.456137
RP11-75A9.	0.5255603	0.2220334	3.3434283	0.000912	0.0025339	-1.458157
LTBR	-0.058224	6.7524198	-3.343335	0.0009123	0.0025345	-1.458458
SMYD4	0.0671642	5.5594811	3.3426539	0.0009144	0.00254	-1.460653
FAT4	0.3466238	5.0684017	3.3426437	0.0009145	0.00254	-1.460685
GACAT3	0.4032058	-1.14936	3.34263	0.0009145	0.00254	-1.46073
TP53INP2	-0.086768	6.4674519	-3.34263	0.0009145	0.00254	-1.46073
AP005901.1	-0.679787	-0.489668	-3.342242	0.0009157	0.0025432	-1.461979
RP11-1055B	-0.254055	4.5606741	-3.341939	0.0009167	0.0025457	-1.462956
PHACTR4	-0.050875	6.2918748	-3.341773	0.0009172	0.002547	-1.46349
OR10Y1P	-0.140321	-1.496218	-3.341699	0.0009175	0.0025475	-1.463729
LTA4H	-0.045469	6.4421098	-3.341633	0.0009177	0.0025479	-1.463943
RP11-120K1	-0.481794	1.7775177	-3.341028	0.0009196	0.0025531	-1.46589
RP11-329A1	0.5360941	2.4476964	3.3405471	0.0009212	0.0025572	-1.467439
TBC1D2	-0.098482	5.8495331	-3.340432	0.0009216	0.0025579	-1.467808
RYK	0.0426489	6.1201615	3.3404282	0.0009216	0.0025579	-1.467821
PTS	-0.071428	5.9661266	-3.340402	0.0009217	0.0025579	-1.467905
NOX4	0.2100959	4.3808246	3.3402479	0.0009222	0.0025591	-1.468402
RAB40C	-0.05719	6.0154897	-3.340109	0.0009226	0.0025601	-1.468849
CMTM8	-0.102196	6.1013154	-3.340019	0.0009229	0.0025607	-1.469138
RP11-672A2	-0.280321	-1.318283	-3.339963	0.0009231	0.0025611	-1.46932

GNPAT	-0.061609	6.5439472	-3.339917	0.0009232	0.0025613	-1.469468
AC093822.1	-0.277942	-1.30104	-3.339896	0.0009233	0.0025613	-1.469535
RP11-162G1	-0.60071	1.3673577	-3.339802	0.0009236	0.0025619	-1.469839
ZNF217	0.091338	6.2053356	3.3395217	0.0009245	0.0025642	-1.47074
RP4-657D16	0.4338088	2.5224487	3.339485	0.0009246	0.0025643	-1.470858
CTD-2582M2	-0.231259	-1.390243	-3.339473	0.0009247	0.0025643	-1.470896
PDHX	-0.052084	6.1811705	-3.339431	0.0009248	0.0025645	-1.471032
PTMAP4	0.2717146	3.0872182	3.3393059	0.0009252	0.0025654	-1.471434
CTD-3105H1	-0.400779	2.723173	-3.339204	0.0009255	0.0025661	-1.471763
NRP1	0.0772744	6.5390813	3.3391726	0.0009256	0.0025662	-1.471864
RP11-354E1	0.5217173	-0.561933	3.3391534	0.0009257	0.0025662	-1.471925
RASA4CP	0.2122003	4.5127353	3.3390055	0.0009262	0.0025673	-1.472401
FEM1AP4	-0.172659	-1.479425	-3.338732	0.000927	0.0025695	-1.473281
STX1A	0.1277245	4.8091709	3.3386763	0.0009272	0.0025699	-1.473461
HSBP1	-0.051953	6.4617656	-3.338211	0.0009287	0.0025738	-1.474957
RP11-730A1	0.4104278	2.4455845	3.3381494	0.0009289	0.0025742	-1.475156
AKIRIN2	-0.066839	6.2561534	-3.338008	0.0009294	0.0025753	-1.475611
GTPBP4	0.0462867	6.2788251	3.3369738	0.0009328	0.0025844	-1.478939
GUCY2EP	-0.781291	1.4693714	-3.336826	0.0009332	0.0025855	-1.479415
RP11-179K3	-0.684207	0.290699	-3.336543	0.0009342	0.0025879	-1.480325
NBPF8	0.1455839	5.0426051	3.3360943	0.0009356	0.0025918	-1.481767
MYH1	0.8206214	0.2023585	3.3359816	0.000936	0.0025926	-1.48213
RP11-240L7	-0.654106	1.6152999	-3.335959	0.0009361	0.0025926	-1.482202
RP11-386I1	-0.620156	1.1786836	-3.335807	0.0009366	0.0025938	-1.482692
ERCC1	-0.066352	6.312495	-3.335564	0.0009374	0.0025958	-1.483474
B3GALT1	-0.825589	0.6252523	-3.335542	0.0009374	0.0025958	-1.483543
LRRC6	0.368568	3.6386294	3.335456	0.0009377	0.0025964	-1.48382
TRIM40	0.8066116	1.3369713	3.3350733	0.000939	0.0025996	-1.48505
FMR1-AS1	-0.623254	0.7743284	-3.335016	0.0009392	0.0025999	-1.485233
CTD-2008P7	-0.183417	-1.430665	-3.334751	0.00094	0.0026022	-1.486087
AP000432.1	0.5050715	1.6998971	3.3346195	0.0009405	0.0026032	-1.486509
RP13-631K1	-0.639593	0.1731642	-3.333949	0.0009427	0.0026091	-1.488666
LINC00682	-0.303233	-1.247851	-3.333892	0.0009428	0.0026092	-1.488847
PLP1	0.651306	-0.532889	3.333886	0.0009429	0.0026092	-1.488867
RP4-583P15	-0.673033	1.6727151	-3.333507	0.0009441	0.0026125	-1.490084
ACTC1	0.7601945	0.5931914	3.3328287	0.0009464	0.0026184	-1.492265
CHST13	-0.134471	6.2730945	-3.332811	0.0009464	0.0026184	-1.492321
IDH3G	-0.066047	6.4028587	-3.332696	0.0009468	0.0026193	-1.492691
LL22NC03-6	0.4134395	-1.103539	3.3323365	0.000948	0.0026222	-1.493846
HMGA1P3	0.4597359	-0.565574	3.3323306	0.000948	0.0026222	-1.493865
AC018495.3	-0.177741	-1.492433	-3.332313	0.0009481	0.0026222	-1.493923
RP11-488L1	0.6016638	0.1136641	3.3319982	0.0009491	0.0026249	-1.494933
KNCN	-0.138138	-1.496335	-3.331691	0.0009501	0.0026275	-1.49592
AURKAPS1	0.5006402	-0.481692	3.331586	0.0009505	0.0026283	-1.496257
YWHAQP7	-0.313895	-1.157838	-3.331326	0.0009513	0.0026304	-1.497092
SPINK2	0.450543	-0.832425	3.3312382	0.0009516	0.002631	-1.497374
RP11-157B1	-0.194716	-1.4295	-3.331161	0.0009519	0.0026316	-1.497622
LRCH3	0.0410835	6.039661	3.3305606	0.0009539	0.0026369	-1.49955
RP11-323J4	-0.509298	-0.388082	-3.330404	0.0009544	0.0026381	-1.500051
SNRNP70	-0.050523	6.7756817	-3.330293	0.0009548	0.0026389	-1.500408
CTC-498J12	-0.548256	3.0684004	-3.329952	0.0009559	0.0026418	-1.501505

TRMT10A	-0.08112	5.4394337	-3.329937	0.0009559	0.0026418	-1.501553
XXbac-BPG2	0.1355923	5.1257791	3.3293155	0.000958	0.0026473	-1.503547
ELP3	-0.066868	6.1512636	-3.329022	0.000959	0.0026498	-1.504489
SH3BGR	-0.119584	5.1299797	-3.328859	0.0009595	0.0026512	-1.505013
CCL18	0.3976041	4.5591434	3.3285752	0.0009605	0.0026536	-1.505923
RNU1-120P	-0.313304	-1.206696	-3.328495	0.0009608	0.002654	-1.506179
ELAVL3	0.5616728	-0.135181	3.3284915	0.0009608	0.002654	-1.506191
RP11-430B1	0.5825198	2.9102265	3.328332	0.0009613	0.0026552	-1.506703
MDM4	0.0658106	6.118752	3.328033	0.0009623	0.0026578	-1.507663
TMEM182	0.2303123	4.7872782	3.3277253	0.0009633	0.0026604	-1.50865
LINC00371	-0.47717	-0.860744	-3.32762	0.0009637	0.0026612	-1.508987
RP11-468N1	-0.174745	-1.449772	-3.327435	0.0009643	0.0026627	-1.509582
AC112198.1	0.6517332	0.2065433	3.3274057	0.0009644	0.0026628	-1.509675
DEF8	-0.063626	6.1541339	-3.327259	0.0009649	0.002664	-1.510145
RP11-542K2	0.4896325	-0.782567	3.3269607	0.0009659	0.0026665	-1.511103
DTNBP1	0.0954837	5.7306865	3.3268603	0.0009662	0.0026671	-1.511425
FAM178B	0.8817576	0.9036461	3.3268566	0.0009663	0.0026671	-1.511437
UBXN7	0.0572284	6.0211888	3.3266132	0.0009671	0.0026691	-1.512217
FOXP1	-0.063834	6.408838	-3.326593	0.0009671	0.0026691	-1.512282
TCEAL2	0.6526192	-0.27982	3.3264588	0.0009676	0.0026702	-1.512713
IQCH	-0.319691	4.2254272	-3.326194	0.0009685	0.0026723	-1.513562
IGKV2D-28	0.3289252	-1.255839	3.3261926	0.0009685	0.0026723	-1.513566
SEZ6	0.8420581	2.2166482	3.3261046	0.0009688	0.0026729	-1.513849
RP11-146F1	-0.215194	3.927459	-3.326029	0.000969	0.0026734	-1.514091
AC093906.1	-0.182227	-1.435105	-3.325678	0.0009702	0.0026764	-1.515215
SLITRK5	0.7390666	0.9776004	3.3255287	0.0009707	0.0026776	-1.515695
RUFY2	0.0659317	5.5441855	3.3254383	0.000971	0.0026783	-1.515985
HDHD1P2	-0.189274	-1.451542	-3.325271	0.0009716	0.0026796	-1.516522
PISD	0.051182	6.0646127	3.3249595	0.0009727	0.0026823	-1.51752
RP11-665E1	-0.277614	-1.330436	-3.324521	0.0009741	0.0026862	-1.518926
THUMPD2	0.0756758	5.4322672	3.3244073	0.0009745	0.0026871	-1.51929
STK32B	0.4242933	3.2617783	3.3243014	0.0009749	0.0026879	-1.51963
CCER1	-0.148089	-1.48767	-3.32407	0.0009757	0.0026898	-1.520373
SMARCA2	-0.100266	6.3701357	-3.323523	0.0009775	0.0026948	-1.522123
TNFSF10	-0.106566	6.5675266	-3.323494	0.0009776	0.0026948	-1.522217
KLKP1	-0.420286	-1.170527	-3.323267	0.0009784	0.0026968	-1.522945
CYB5R2	0.2124678	4.9964403	3.3231296	0.0009789	0.0026978	-1.523385
RP11-359J1	0.616575	0.4264355	3.3230199	0.0009793	0.0026985	-1.523736
RP11-131H2	0.2999882	-1.24655	3.3230052	0.0009793	0.0026985	-1.523784
CYP24A1	0.6172581	-0.603768	3.3229962	0.0009793	0.0026985	-1.523812
RP11-650L1	-0.517927	-0.091142	-3.322883	0.0009797	0.0026993	-1.524175
RP11-480D4	-0.615954	2.3274931	-3.322701	0.0009803	0.0027009	-1.524759
CACNB4	0.5382032	3.0691966	3.3219644	0.0009829	0.0027076	-1.527118
MKNK1-AS1	0.5923291	1.1481157	3.3219252	0.000983	0.0027077	-1.527243
ZNF221	0.3445131	3.4170922	3.3218524	0.0009832	0.0027082	-1.527477
ASS1P5	-0.56818	-0.36786	-3.321777	0.0009835	0.0027087	-1.527717
NT5C1A	0.2677894	-1.321582	3.3215946	0.0009841	0.0027102	-1.528302
LINC01044	-0.486645	-0.895315	-3.320965	0.0009863	0.002716	-1.530318
RP11-12D16	-0.15487	-1.468458	-3.320538	0.0009877	0.0027198	-1.531687
CTA-242H14	-0.519224	-0.355519	-3.320354	0.0009884	0.0027214	-1.532275
RP11-649G1	-0.525768	1.4441173	-3.320155	0.0009891	0.002723	-1.532911

SETP20	0.4420402	-0.660864	3.3201058	0.0009892	0.0027233	-1.53307
KCNB1	-0.563967	4.6224249	-3.319802	0.0009903	0.002726	-1.534043
RP11-360F5	0.3553534	3.3021867	3.3196409	0.0009908	0.0027273	-1.534558
RP11-537H1	0.5642505	0.2244124	3.319581	0.000991	0.0027276	-1.53475
C11orf73	-0.063885	5.9507059	-3.319445	0.0009915	0.0027287	-1.535186
SYT15	0.5794825	3.1906996	3.3190786	0.0009928	0.002732	-1.536358
LRRC28	-0.087957	6.1791788	-3.318926	0.0009933	0.0027332	-1.536846
HNRNPUL2	-0.046497	6.347761	-3.318902	0.0009934	0.0027333	-1.536922
TTLL5	0.0639704	5.7054407	3.3188288	0.0009936	0.0027338	-1.537157
RP11-452I5	0.3204807	4.8852434	3.3187697	0.0009938	0.0027341	-1.537346
RP11-44901	-0.314004	-1.177373	-3.318027	0.0009964	0.002741	-1.539722
LECT2	-0.550142	5.858247	-3.317777	0.0009973	0.0027432	-1.540523
CSTF3	-0.041527	6.0965681	-3.317728	0.0009974	0.0027434	-1.540678
STRBP	0.0567172	5.9848985	3.3175527	0.000998	0.0027449	-1.54124
RN7SKP272	-0.278986	-1.228935	-3.317292	0.000999	0.0027471	-1.542075
RNU6-33P	-0.290528	-1.232608	-3.317275	0.000999	0.0027471	-1.54213
RP4-631H13	-0.338282	-1.136953	-3.317084	0.0009997	0.0027487	-1.542739
RP4-760C5.	0.605164	0.180026	3.3170626	0.0009998	0.0027487	-1.542808
HECW2	0.1365993	5.4810312	3.3170203	0.0009999	0.0027489	-1.542943
MS4A6E	0.4146879	-0.946084	3.3169221	0.0010002	0.0027497	-1.543258
CTB-152G17	0.3418844	2.922709	3.316741	0.0010009	0.0027512	-1.543837
POLR2J4	-0.082966	5.8523623	-3.31671	0.001001	0.0027513	-1.543937
HNRNPC	-0.033841	6.9758143	-3.316504	0.0010017	0.002753	-1.544594
CPN1	-0.325253	6.1697208	-3.316205	0.0010027	0.0027557	-1.545551
RP5-1021I2	-0.493836	-0.718183	-3.316136	0.001003	0.0027561	-1.545771
SGK1	0.1262325	6.3398747	3.3160521	0.0010033	0.0027567	-1.54604
AC017079.4	-0.345569	-1.097039	-3.315938	0.0010037	0.0027576	-1.546405
KCNJ4	-0.685396	4.0239365	-3.315881	0.0010039	0.002758	-1.546587
RP11-407B7	-0.666466	1.2054223	-3.31577	0.0010042	0.0027588	-1.546941
AC016717.1	-0.402051	-1.215411	-3.31573	0.0010044	0.002759	-1.547069
KB-1517D11	0.4468468	-0.923341	3.3155801	0.0010049	0.0027602	-1.547549
RP11-162A1	-0.300583	3.5382288	-3.315532	0.0010051	0.0027605	-1.547704
FLYWCH1	-0.060829	6.0981319	-3.315491	0.0010052	0.0027607	-1.547833
EPHA5	0.5732904	-0.790808	3.315149	0.0010064	0.0027637	-1.548927
PMS2CL	0.104205	5.0485656	3.3149691	0.001007	0.0027651	-1.549502
RPN2	-0.044454	7.0908494	-3.31496	0.0010071	0.0027651	-1.549531
CTD-228701	-0.139265	5.3621973	-3.314686	0.001008	0.0027676	-1.550409
GM2AP2	-0.263991	-1.30655	-3.314371	0.0010091	0.0027704	-1.551415
HECW1	0.6715334	2.5805219	3.3143213	0.0010093	0.0027706	-1.551573
AC018735.1	-0.304663	-1.24956	-3.314116	0.00101	0.0027724	-1.552228
LINC00942	0.8719699	0.9375081	3.3140536	0.0010103	0.0027727	-1.552429
AC006373.1	-0.443398	-0.986696	-3.314043	0.0010103	0.0027727	-1.552462
AGPAT6	-0.069989	6.5399694	-3.313907	0.0010108	0.0027738	-1.552897
EWSR1	-0.032476	6.8127583	-3.313686	0.0010115	0.0027757	-1.553604
CTC-327F10	0.5087846	-0.662965	3.3132321	0.0010131	0.0027799	-1.555054
GRPR	-0.806459	1.9902324	-3.313206	0.0010132	0.0027799	-1.555137
CTD-3094K1	0.4222298	-0.857067	3.3131034	0.0010136	0.0027807	-1.555465
RP13-131K1	0.4972276	-0.394859	3.3126944	0.001015	0.0027844	-1.556772
NKX1-1	-0.234092	-1.421437	-3.312679	0.0010151	0.0027844	-1.556822
RP11-266L9	-0.172647	3.8099414	-3.312568	0.0010155	0.0027853	-1.557177
SNORD94	0.519074	1.6178508	3.3121268	0.001017	0.0027893	-1.558585

SEC14L3	-0.820831	2.5917668	-3.311567	0.001019	0.0027945	-1.560373
RP11-130C6	-0.588671	1.0365766	-3.311203	0.0010203	0.0027979	-1.561534
CENPI	0.285113	4.5622016	3.3109732	0.0010211	0.0027999	-1.562269
RP11-1376F	-0.330728	-1.215106	-3.310707	0.0010221	0.0028023	-1.56312
RP11-6J21.	-0.222468	-1.367806	-3.310425	0.0010231	0.0028048	-1.564021
SCFD1	-0.042391	6.2904544	-3.310315	0.0010234	0.0028056	-1.564369
RP11-4B16.	0.4383414	-0.730247	3.3096053	0.001026	0.0028124	-1.566636
RP11-47502	-0.558402	-0.344918	-3.309479	0.0010264	0.0028133	-1.567039
KRT8P43	0.3393158	-1.118281	3.3094649	0.0010265	0.0028133	-1.567085
SPAG1	0.1420999	5.4420405	3.3094329	0.0010266	0.0028134	-1.567186
FAM110A	0.1144533	5.4240857	3.3089767	0.0010282	0.0028176	-1.568642
AC016712.2	-0.499822	1.6682428	-3.308941	0.0010283	0.0028178	-1.568757
MYO5A	0.1219163	5.6825455	3.3088171	0.0010288	0.0028188	-1.569152
ENAH	0.0872117	6.4913243	3.3087446	0.001029	0.0028193	-1.569383
RP11-187C1	0.5218432	2.1131784	3.3084505	0.0010301	0.0028219	-1.570322
RP11-44D5.	-0.536859	-0.166639	-3.308222	0.0010309	0.002824	-1.57105
RP11-493L1	0.5486244	-0.579413	3.3080929	0.0010314	0.0028249	-1.571463
RP11-5017.	-0.234699	4.332236	-3.308061	0.0010315	0.0028249	-1.571566
GPR3	0.2884831	3.5788045	3.3080578	0.0010315	0.0028249	-1.571575
SSX8	-0.417119	-1.139077	-3.307833	0.0010323	0.0028269	-1.572292
CEBPZOS	-0.05169	6.2807735	-3.307779	0.0010325	0.0028272	-1.572463
RP11-274B2	0.6751077	1.9977049	3.3074091	0.0010338	0.0028307	-1.573644
EBLN2	0.6065278	2.4958266	3.3073193	0.0010341	0.0028313	-1.573931
RP11-430H1	0.6200219	-0.169691	3.3072989	0.0010342	0.0028313	-1.573996
SPDYE5	0.5043434	1.991589	3.3069676	0.0010354	0.0028344	-1.575052
GRN	-0.049377	7.0482069	-3.306836	0.0010359	0.0028354	-1.575472
SARAF	-0.061206	6.852115	-3.306816	0.0010359	0.0028354	-1.575537
OTUD6B-AS1	-0.071235	6.0203607	-3.306612	0.0010367	0.0028372	-1.576186
MYOZ2	0.5473727	-0.206519	3.3052649	0.0010415	0.0028503	-1.580482
RP11-29G8.	0.4018104	3.4733611	3.3050714	0.0010422	0.002852	-1.581099
RP11-90B9.	0.4969309	-0.292396	3.3050469	0.0010423	0.002852	-1.581177
TCEB1	-0.074597	6.4024364	-3.304951	0.0010427	0.0028527	-1.581483
CREBRF	-0.068711	5.9841947	-3.304632	0.0010438	0.0028557	-1.582498
EIF4A2	-0.049522	6.8929316	-3.303588	0.0010476	0.0028658	-1.585828
FRYL	0.058017	5.9926975	3.3035459	0.0010477	0.002866	-1.585961
FAM27E3	0.6017309	0.6492402	3.3031607	0.0010491	0.0028696	-1.587188
WI2-1959D1	-0.223967	-1.37995	-3.302914	0.00105	0.0028719	-1.587975
CRY1	0.0900668	5.8382253	3.3017978	0.0010541	0.0028828	-1.59153
AC093702.1	0.4816621	-0.936533	3.3015143	0.0010551	0.0028854	-1.592432
CTD-2008P7	0.7433571	-0.287533	3.301063	0.0010568	0.0028897	-1.593869
COL9A1	0.5587638	3.0801938	3.3008638	0.0010575	0.0028915	-1.594504
USP8	-0.045723	6.2598957	-3.300835	0.0010576	0.0028915	-1.594594
RCN1P2	0.2815676	4.6266607	3.300411	0.0010592	0.0028956	-1.595945
MIR5195	0.3345183	-1.150872	3.3003382	0.0010594	0.0028961	-1.596177
RPF2	-0.069307	6.1825949	-3.300221	0.0010599	0.002897	-1.59655
MSH4	0.6671924	1.0485452	3.3000732	0.0010604	0.0028983	-1.59702
TRAV33	-0.223332	-1.405508	-3.30001	0.0010607	0.0028987	-1.597221
RP4-655L22	-0.342128	-1.136206	-3.299931	0.0010609	0.0028993	-1.597472
PTRHD1	-0.080749	5.8712883	-3.299493	0.0010626	0.0029035	-1.598868
RP11-7F17.	-0.623381	1.1484024	-3.299457	0.0010627	0.0029036	-1.598983
RP11-434H6	-0.196998	4.037439	-3.299263	0.0010634	0.0029053	-1.5996

RP11-4N23.	-0.302349	-1.292457	-3.299107	0.001064	0.0029067	-1.600096
TM4SF19-TC	0.3739933	-1.077249	3.2990868	0.001064	0.0029067	-1.60016
MGEA5	-0.044675	6.5907377	-3.298841	0.001065	0.0029089	-1.600942
WDR92	-0.110601	4.4555333	-3.298763	0.0010652	0.0029095	-1.601192
OR12D1	0.3420109	-1.25148	3.2986108	0.0010658	0.0029108	-1.601675
SERPINE3	0.5207029	1.6829755	3.2984194	0.0010665	0.0029125	-1.602284
BCORP1	0.6487493	0.1562352	3.2983299	0.0010668	0.0029132	-1.602569
TPGS1	-0.145413	5.1856316	-3.297778	0.0010689	0.0029186	-1.604326
RPSAP47	-0.53351	2.1897648	-3.297716	0.0010691	0.002919	-1.604522
SLC14A2-AS	0.3694682	-1.042553	3.2975104	0.0010699	0.0029208	-1.605176
RASGRP3	0.1070099	5.7040704	3.2974837	0.00107	0.0029209	-1.60526
LINC01233	-0.332448	-1.26732	-3.296919	0.0010721	0.0029264	-1.607058
KRT8P12	-0.096501	5.3363026	-3.296457	0.0010738	0.0029308	-1.608526
POLA1	0.08304	5.6353889	3.2963347	0.0010742	0.0029318	-1.608915
RP11-10N16	-0.583696	-0.144256	-3.29606	0.0010752	0.0029344	-1.609788
RP11-522B1	0.3816634	-1.146866	3.2955764	0.001077	0.0029391	-1.611326
SALL4P1	-0.114623	-1.510904	-3.295411	0.0010777	0.0029406	-1.61185
RP11-60802	-0.828393	0.8781102	-3.295305	0.0010781	0.0029414	-1.612187
ZNF92P3	-0.692982	0.7912848	-3.29514	0.0010787	0.0029427	-1.612714
ITGAE	0.113508	5.5153842	3.295135	0.0010787	0.0029427	-1.612729
ARL13B	0.1341274	5.2775187	3.2947871	0.00108	0.002946	-1.613835
PNP	0.0835139	6.1771542	3.2946925	0.0010803	0.0029468	-1.614136
SCD	-0.133868	7.3461537	-3.294608	0.0010807	0.0029474	-1.614404
DDTL	-0.176163	5.282898	-3.294381	0.0010815	0.0029495	-1.615127
SMARCE1P2	-0.357931	-1.088921	-3.294101	0.0010825	0.0029521	-1.616016
C17orf49	0.1286593	4.4467536	3.2940829	0.0010826	0.0029521	-1.616073
C1QL3	0.6419127	2.9666749	3.2937498	0.0010839	0.0029553	-1.617131
RP11-423H2	-0.144939	5.4866924	-3.293217	0.0010859	0.0029605	-1.618825
PABPC1P1	0.5008177	-0.255876	3.2930752	0.0010864	0.0029617	-1.619275
NSMCE2	-0.084761	6.0624622	-3.292831	0.0010873	0.002964	-1.620051
RP4-764022	-0.43374	-0.816392	-3.292	0.0010904	0.0029723	-1.62269
PCDHGA4	0.5971271	3.3319363	3.2916935	0.0010916	0.0029752	-1.623663
SVOPL	0.571128	-0.455388	3.2916605	0.0010917	0.0029752	-1.623768
ZCCHC11	0.1013482	6.0395003	3.2916568	0.0010917	0.0029752	-1.62378
DNHD1	-0.116175	5.5362868	-3.291413	0.0010926	0.0029775	-1.624554
RP5-1039K5	0.108299	4.7176463	3.2913194	0.001093	0.0029782	-1.624851
SYN3	0.5100278	3.0883383	3.2912073	0.0010934	0.0029791	-1.625207
MIR6797	0.4493836	-0.632226	3.2911163	0.0010938	0.0029798	-1.625496
RAB40A	0.5249876	1.8393209	3.2907244	0.0010952	0.0029836	-1.62674
NAPA-AS1	-0.178048	4.3461759	-3.290499	0.0010961	0.0029858	-1.627457
BTBD8	0.5466505	2.0347322	3.2903765	0.0010966	0.0029868	-1.627845
GRIN3B	0.638818	1.3814503	3.2903179	0.0010968	0.0029872	-1.628031
RNVU1-20	0.4207701	-0.954767	3.2892062	0.001101	0.0029984	-1.631559
BCRP2	0.5364816	-0.704121	3.2891407	0.0011013	0.0029989	-1.631767
RP11-342D1	0.5699782	0.9898448	3.2891099	0.0011014	0.002999	-1.631865
BMI1	-0.054816	6.3099819	-3.289071	0.0011015	0.0029992	-1.631987
LINC00639	0.4860214	2.6808996	3.2889541	0.001102	0.0030001	-1.632359
KCNIP2	0.3036053	3.7513881	3.2884316	0.0011039	0.0030053	-1.634017
ATMIN	-0.04833	6.2183296	-3.288376	0.0011042	0.0030055	-1.634194
SAMD1	-0.066969	6.2441563	-3.288375	0.0011042	0.0030055	-1.634197
ALDH8A1	-0.243445	6.3384937	-3.288344	0.0011043	0.0030056	-1.634295

AC114494.1	0.4949215	3.0264204	3.2881219	0.0011051	0.0030077	-1.634999
C17orf85	0.0527891	6.0354911	3.288004	0.0011056	0.0030087	-1.635374
PLEKHM1P	0.0960861	5.3534526	3.2878624	0.0011061	0.0030099	-1.635823
SETD5	0.0489328	6.3804099	3.2878185	0.0011063	0.0030101	-1.635962
AC087294.2	0.5292998	1.3940802	3.2877308	0.0011066	0.0030108	-1.63624
RP11-93001	0.3755756	-1.041939	3.2873797	0.001108	0.0030142	-1.637354
CYP11B2	-0.4079	-1.27098	-3.287342	0.0011081	0.0030144	-1.637472
RP11-472G2	0.5516794	-0.328253	3.2872087	0.0011086	0.0030156	-1.637896
CYP4F26P	0.5859123	-0.484646	3.2866861	0.0011106	0.0030208	-1.639553
ITGB3BP	-0.08473	5.5889079	-3.286095	0.0011129	0.0030266	-1.641429
CHRNA10	0.2522923	3.4047835	3.2860874	0.0011129	0.0030266	-1.641452
LYZL6	-0.270689	-1.302049	-3.285859	0.0011138	0.0030287	-1.642175
RP11-298J2	0.6316338	1.9856102	3.2858199	0.0011139	0.0030289	-1.6423
TRPM3	-0.547263	3.0130703	-3.285198	0.0011163	0.0030352	-1.64427
RP11-214K3	0.5607754	0.3081907	3.2851411	0.0011165	0.0030356	-1.644452
COPS2	-0.042065	6.3716955	-3.28508	0.0011168	0.003036	-1.644645
RPL8	-0.079888	7.4143213	-3.284546	0.0011188	0.0030413	-1.646336
FAM218A	0.6408102	0.0363172	3.2844139	0.0011194	0.0030425	-1.646757
SLCO1B7	-0.72321	0.1014916	-3.28393	0.0011212	0.0030474	-1.64829
PLCE1-AS1	0.4933123	-0.592191	3.2837998	0.0011217	0.0030485	-1.648702
ENOSF1	-0.06649	6.3385734	-3.283627	0.0011224	0.0030501	-1.649251
FKSG52	-0.490052	-0.350187	-3.283508	0.0011229	0.0030511	-1.649627
PVRIG	0.5697012	0.3675005	3.2832428	0.0011239	0.0030537	-1.650467
CTB-119C2.	0.4823358	2.6574177	3.2826115	0.0011263	0.0030601	-1.652467
LINC01483	-0.527883	-0.70044	-3.282524	0.0011267	0.0030608	-1.652745
LINC01191	0.6247446	-0.069126	3.282093	0.0011283	0.0030651	-1.654109
LINC00940	-0.716471	0.1800542	-3.281907	0.0011291	0.0030668	-1.654697
RP11-1086F	-0.321698	-1.143781	-3.281845	0.0011293	0.0030673	-1.654895
LOH12CR2	-0.195859	4.1051974	-3.281215	0.0011318	0.0030737	-1.656889
GLIS3-AS1	0.3663169	-1.055206	3.2810096	0.0011326	0.0030757	-1.657539
AC114776.3	-0.155539	-1.47345	-3.280796	0.0011334	0.0030777	-1.658217
RP5-1042K1	0.5167441	-0.189066	3.2804422	0.0011348	0.0030812	-1.659336
RP11-194G1	-0.329029	-1.259229	-3.280221	0.0011356	0.0030833	-1.660036
LHX4	0.3999574	3.8394963	3.2801807	0.0011358	0.0030835	-1.660163
POMC	0.4423578	2.9047573	3.2801281	0.001136	0.0030839	-1.66033
RP11-428J1	0.3802636	2.4605449	3.2799348	0.0011368	0.0030857	-1.660942
AC005306.3	0.5316679	1.4828335	3.2798685	0.001137	0.0030862	-1.661151
RP11-80F22	-0.139871	-1.487954	-3.279534	0.0011383	0.0030895	-1.66221
OR51B8P	-0.11697	-1.500349	-3.27941	0.0011388	0.0030906	-1.662604
AC093484.4	0.2705653	-1.277291	3.2793744	0.001139	0.0030907	-1.662715
CASC6	-0.354504	-1.18039	-3.278752	0.0011414	0.0030971	-1.664684
G6PD	0.1329561	6.1223854	3.2785183	0.0011423	0.0030994	-1.665424
AC013268.3	0.3776036	-1.136767	3.2778803	0.0011448	0.003106	-1.667442
ADAT3	0.2130167	4.5375433	3.2777868	0.0011452	0.0031067	-1.667738
SMAD1	0.310391	5.4042867	3.2774887	0.0011464	0.0031097	-1.668681
RP11-925D8	-0.328989	-1.159602	-3.277237	0.0011474	0.0031122	-1.669476
LCN15	0.6038091	-0.532967	3.2770199	0.0011482	0.0031143	-1.670163
VPS45	-0.04671	6.2048156	-3.276984	0.0011484	0.0031144	-1.670277
YOD1	0.0780432	5.742214	3.2768293	0.001149	0.0031158	-1.670766
FGB	-0.177327	7.6806858	-3.276582	0.00115	0.0031183	-1.671547
ZIM2-AS1	0.7207721	0.6045364	3.276452	0.0011505	0.0031194	-1.671959

RP11-68D16	-0.201834	-1.42463	-3.276299	0.0011511	0.0031208	-1.672444
SLC39A4	0.3419385	5.3369177	3.276115	0.0011518	0.0031226	-1.673024
OR7E128P	0.5883801	0.3151428	3.275472	0.0011544	0.0031293	-1.675057
RNASE11	-0.17057	-1.470923	-3.275296	0.0011551	0.0031309	-1.675613
TDRD6	0.2578155	4.2670647	3.2751767	0.0011555	0.003132	-1.67599
TLX2	0.6760469	-0.113223	3.274913	0.0011566	0.0031346	-1.676824
HAGLROS	0.5652597	2.0112906	3.2747364	0.0011573	0.0031362	-1.677382
AP001107.1	0.5047043	-0.281442	3.2747134	0.0011574	0.0031363	-1.677455
RP11-30101	-0.336037	3.2046212	-3.274512	0.0011582	0.0031382	-1.67809
AC005197.2	0.3965473	-0.986069	3.2740806	0.0011599	0.0031426	-1.679454
TCEA1	-0.058031	6.4469246	-3.274036	0.0011601	0.0031429	-1.679593
PJA2	-0.058762	6.5842246	-3.273349	0.0011628	0.0031501	-1.681765
TCERG1L	0.5777799	-0.670673	3.2732379	0.0011633	0.003151	-1.682116
C6orf223	0.8961735	2.5619547	3.2731059	0.0011638	0.0031522	-1.682533
RP11-293M1	-0.337112	4.1442274	-3.272875	0.0011647	0.0031545	-1.683262
LA16c-349E	0.5592955	-0.360155	3.2728066	0.001165	0.003155	-1.683479
RP5-1172N1	0.5104639	-0.456276	3.272715	0.0011653	0.0031558	-1.683768
BECN1	0.0807004	6.2005829	3.2724977	0.0011662	0.0031579	-1.684454
KIF13A	-0.062671	6.2888706	-3.27241	0.0011666	0.0031586	-1.68473
PRF1	0.1702522	5.2916321	3.2722983	0.001167	0.0031596	-1.685084
AC004076.7	0.4512896	-0.615827	3.2721201	0.0011677	0.0031613	-1.685647
ERH	-0.04756	6.5092602	-3.27195	0.0011684	0.0031629	-1.686184
LNX1	0.1551677	5.3714175	3.2718138	0.001169	0.0031642	-1.686614
BUB1	0.1957266	5.3926437	3.2717383	0.0011693	0.0031647	-1.686852
COL21A1	0.3140877	4.6412961	3.2716044	0.0011698	0.003166	-1.687275
RNU6-485P	-0.591026	0.1064151	-3.271412	0.0011706	0.0031678	-1.687882
SNRPCP16	-0.218309	-1.38297	-3.27114	0.0011717	0.0031706	-1.688742
PPP1R26-AS	0.3033419	3.7802098	3.2709909	0.0011723	0.0031719	-1.689212
KIDINS220	0.0718444	6.2475768	3.2707089	0.0011734	0.0031748	-1.690102
RP11-465N4	0.2295272	4.2892581	3.2706818	0.0011735	0.0031748	-1.690188
TEX26-AS1	0.3341415	-1.141932	3.2700397	0.0011761	0.0031816	-1.692214
GRSF1	-0.04536	6.4559821	-3.269925	0.0011766	0.0031826	-1.692576
CFL1P8	-0.185123	-1.426746	-3.269837	0.0011769	0.0031834	-1.692855
RP11-12601	-0.403147	2.7384989	-3.269657	0.0011777	0.003185	-1.693423
AC011322.1	0.5651784	-0.356135	3.2696438	0.0011777	0.003185	-1.693464
RNU6-1231F	-0.438651	-0.852684	-3.269153	0.0011797	0.0031901	-1.695014
NFIA-AS2	0.6174167	0.0026267	3.2684244	0.0011827	0.0031977	-1.697311
PJA1	0.091862	5.7898764	3.2684192	0.0011827	0.0031977	-1.697327
AC104667.3	-0.419544	4.172741	-3.268251	0.0011834	0.0031993	-1.697856
BTN3A2	-0.09697	6.205297	-3.268123	0.0011839	0.0032005	-1.69826
BNIP3P27	0.4466385	-0.770327	3.2679714	0.0011845	0.0032019	-1.69874
RP11-14N9.	-0.360027	-1.092729	-3.267945	0.0011846	0.003202	-1.698824
NMNAT1	-0.091441	5.6173041	-3.26771	0.0011856	0.0032042	-1.699565
RP3-470B24	0.3698218	-1.161685	3.2677005	0.0011856	0.0032042	-1.699594
PDCD4-AS1	0.1737938	4.1018788	3.2676295	0.0011859	0.0032047	-1.699818
AC009505.2	0.3829422	-0.954439	3.2673599	0.001187	0.0032072	-1.700668
ZFR2	0.6763957	0.7654188	3.2673514	0.001187	0.0032072	-1.700695
RPLP0P11	-0.339457	-1.199072	-3.267337	0.0011871	0.0032072	-1.70074
CTD-2006C1	0.5870168	1.2179408	3.2671161	0.001188	0.0032094	-1.701437
RPLP0	-0.060618	7.3816311	-3.26703	0.0011883	0.00321	-1.701709
MEPE	-0.631045	-0.094833	-3.267021	0.0011884	0.00321	-1.701738

DYNLT3	-0.074901	6.2069963	-3.266791	0.0011893	0.0032123	-1.702464
GBA3	-0.482056	5.4798771	-3.266491	0.0011905	0.0032154	-1.703408
KCNJ13	0.4479802	-0.902938	3.2660636	0.0011923	0.0032197	-1.704755
RP11-268G1	0.5395029	-0.657404	3.2660603	0.0011923	0.0032197	-1.704766
ARHGAP11A	0.1546029	5.4646321	3.2659241	0.0011928	0.0032209	-1.705195
RP5-999L4.	0.5356472	-0.123239	3.2656249	0.0011941	0.003224	-1.706138
KIF18B	0.2332772	5.171124	3.265525	0.0011945	0.0032249	-1.706453
RP11-60L3.	0.4371644	-0.980685	3.2648483	0.0011973	0.0032319	-1.708586
REV1	0.0674299	5.8944984	3.2648295	0.0011973	0.0032319	-1.708645
LINC00519	0.590033	-0.296834	3.2648213	0.0011974	0.0032319	-1.708671
DNAJA1	-0.05791	6.8723537	-3.264228	0.0011998	0.0032383	-1.71054
RP11-108L7	0.5795568	0.3408552	3.263985	0.0012008	0.0032407	-1.711306
RP11-320N7	-0.215144	-1.396102	-3.263119	0.0012044	0.0032501	-1.714034
RP11-506M1	0.5863216	1.8011079	3.2628832	0.0012054	0.0032525	-1.714776
SLC35E3	-0.055846	6.0002927	-3.262161	0.0012083	0.0032603	-1.717052
GS1-122H1.	-0.242865	-1.320527	-3.262149	0.0012084	0.0032603	-1.71709
DRD2	0.4193423	-0.986676	3.261778	0.0012099	0.0032642	-1.718257
DGKI	0.3000854	3.3954419	3.2617344	0.0012101	0.0032644	-1.718394
CHSY3	0.2841032	4.3788895	3.261481	0.0012112	0.003267	-1.719192
PI4K2A	-0.056009	6.1649229	-3.261351	0.0012117	0.0032682	-1.719602
CTD-2095E4	0.5384325	0.9969588	3.2612437	0.0012121	0.0032692	-1.719939
XXbac-BPG2	-0.546087	1.6447769	-3.26088	0.0012137	0.003273	-1.721085
UBAP1L	-0.150372	4.7796656	-3.260797	0.001214	0.0032737	-1.721345
SHROOM2P1	-0.589697	-0.719911	-3.260736	0.0012143	0.0032741	-1.721537
ZNF543	0.124105	4.92525	3.2602738	0.0012162	0.0032791	-1.722991
CLCNKB	0.6221024	-0.065708	3.2600558	0.0012171	0.0032813	-1.723677
C1orf54	0.1031525	5.3012365	3.2597235	0.0012185	0.0032848	-1.724723
PGAM1P7	0.4977261	-0.392852	3.2596846	0.0012186	0.003285	-1.724845
AC005387.2	0.5519254	0.6273445	3.2591042	0.0012211	0.0032911	-1.726671
ABC7-42418	-0.20802	-1.439923	-3.259102	0.0012211	0.0032911	-1.72668
AC022384.1	0.6810891	0.94467	3.2588441	0.0012222	0.0032937	-1.72749
ILKAP	-0.051052	6.0391593	-3.258756	0.0012225	0.0032945	-1.727765
AP000619.5	0.4281446	-1.069118	3.2585707	0.0012233	0.0032963	-1.72835
RNU6-339P	-0.515518	-0.429885	-3.258296	0.0012245	0.0032992	-1.729213
MGAT2	0.240695	4.9109291	3.2582449	0.0012247	0.0032995	-1.729374
ZNF285B	0.5378325	0.3258784	3.2581766	0.001225	0.0033	-1.729589
MANBAL	-0.05356	6.3327281	-3.257683	0.001227	0.0033054	-1.731141
WHAMMP2	0.1543234	4.3677349	3.257574	0.0012275	0.0033064	-1.731484
DSCAM	0.6485195	0.1564681	3.2573543	0.0012284	0.0033086	-1.732175
XX-DJ76P10	-0.225158	-1.39942	-3.257196	0.0012291	0.0033102	-1.732672
RP11-234K2	0.4428412	-0.760196	3.2570191	0.0012298	0.0033119	-1.733229
TM9SF3	-0.039428	6.804716	-3.256409	0.0012324	0.0033186	-1.735148
SIRPA	0.0862959	6.4540356	3.2557812	0.001235	0.0033255	-1.73712
KCNN1	0.6516665	2.2034134	3.2555045	0.0012362	0.0033284	-1.73799
RP11-77901	-0.601173	0.3692053	-3.255375	0.0012368	0.0033296	-1.738397
RP11-1260E	0.4636538	-0.626812	3.2549625	0.0012385	0.0033341	-1.739693
RP11-338K1	0.5340709	0.6207435	3.2548551	0.001239	0.0033351	-1.740031
CTD-2616J1	0.5068729	1.9704991	3.2542679	0.0012415	0.0033415	-1.741876
FZD3	0.3191225	4.6475694	3.2541048	0.0012421	0.0033432	-1.742388
LIMS1	0.0588994	6.2093775	3.2540627	0.0012423	0.0033434	-1.74252
IRF6	-0.196002	6.4749134	-3.254017	0.0012425	0.0033437	-1.742664

TP53	0.1047554	6.1593053	3.2535344	0.0012446	0.0033489	-1.74418
RP11-50I19	-0.246269	3.7643721	-3.253197	0.001246	0.0033526	-1.74524
FAM217B	0.1955372	5.2240451	3.2528043	0.0012477	0.0033568	-1.746473
CCNL2	-0.067952	6.5403345	-3.25262	0.0012485	0.0033587	-1.74705
RP13-20L14	-0.632778	1.0775388	-3.252551	0.0012488	0.0033592	-1.747269
RBBP5	-0.049169	6.1346985	-3.252394	0.0012494	0.0033608	-1.74776
RP11-13N13	0.4637117	-0.610488	3.2520676	0.0012508	0.0033643	-1.748786
MIER3	-0.060728	5.918159	-3.252046	0.0012509	0.0033643	-1.748855
CALM1	-0.048128	6.8625844	-3.251856	0.0012517	0.0033662	-1.74945
DNAJB3	-0.672621	1.0714062	-3.251525	0.0012532	0.0033698	-1.750489
RABAC1	-0.082677	6.4527595	-3.251125	0.0012549	0.0033742	-1.751746
COLGALT2	0.4665063	3.2989278	3.2503041	0.0012584	0.0033832	-1.754321
TTC23L	-0.43369	2.5204465	-3.250299	0.0012584	0.0033832	-1.754338
RP11-701H2	0.5044158	-0.62781	3.2502169	0.0012588	0.0033839	-1.754595
ARRB2	0.0714458	6.3040186	3.2501299	0.0012591	0.0033847	-1.754867
SLC9C2	-0.734049	1.0449666	-3.249515	0.0012618	0.0033915	-1.756797
RNA5SP141	-0.134602	-1.503766	-3.249325	0.0012626	0.0033935	-1.757392
AL356289.1	-0.229927	-1.374464	-3.249248	0.0012629	0.0033941	-1.757636
RP13-104F2	0.1470961	4.7743665	3.2488551	0.0012646	0.0033984	-1.758867
AC098820.3	-0.306657	3.4479713	-3.248808	0.0012648	0.0033987	-1.759014
RP11-429E1	0.2810499	-1.239945	3.2487151	0.0012652	0.0033996	-1.759306
RP11-809H1	0.4238125	-0.984333	3.2486032	0.0012657	0.0034006	-1.759657
KIAA2012	-0.573382	3.0097602	-3.248457	0.0012664	0.0034021	-1.760115
SPATS2	0.0704391	5.9419199	3.2482181	0.0012674	0.0034045	-1.760865
RP11-21B21	0.5905129	0.8758947	3.2482032	0.0012675	0.0034045	-1.760911
AC012456.4	-0.539385	-0.30008	-3.248157	0.0012677	0.0034048	-1.761055
NONO	-0.040342	6.9733268	-3.248029	0.0012682	0.003406	-1.761458
TAS2R62P	-0.361308	-1.20409	-3.247861	0.0012689	0.0034077	-1.761983
CTD-2545G1	0.3940976	-0.855689	3.2476721	0.0012698	0.0034097	-1.762577
RP11-492E3	0.8076683	2.201309	3.247622	0.00127	0.00341	-1.762734
AC011286.1	-0.370455	-1.096632	-3.247497	0.0012705	0.0034112	-1.763126
CH17-373J2	0.5269869	0.0859418	3.2474333	0.0012708	0.0034117	-1.763325
RP11-359D2	-0.258289	-1.373804	-3.247009	0.0012726	0.0034164	-1.764655
CPOX	-0.062822	6.0178578	-3.24693	0.001273	0.0034171	-1.764903
RP11-150O1	0.6174552	0.8185706	3.2468567	0.0012733	0.0034177	-1.765133
MSX2P1	0.3844486	-1.011452	3.2465608	0.0012746	0.0034209	-1.766061
Six3os1_2	0.3835852	-1.126866	3.2461722	0.0012763	0.0034252	-1.767279
GSDMB	-0.113136	5.9613724	-3.245785	0.001278	0.0034295	-1.768492
GSTA9P	-0.41102	-1.111361	-3.245765	0.0012781	0.0034295	-1.768555
LINC00992	0.8454739	1.4324693	3.245491	0.0012793	0.0034324	-1.769414
CARD10	0.0752028	6.273292	3.2454583	0.0012794	0.0034325	-1.769516
COL2A1	0.987298	1.7147392	3.245089	0.001281	0.0034366	-1.770673
RNF19B	0.085247	5.8515066	3.2449362	0.0012817	0.0034381	-1.771152
RP11-823E8	0.5462968	2.28879	3.2449169	0.0012818	0.0034381	-1.771213
HCRTR1	0.4635042	-0.43093	3.2448289	0.0012822	0.0034389	-1.771488
RP4-536B24	-0.233973	-1.37829	-3.244761	0.0012824	0.0034395	-1.7717
FGF14-AS2	-0.312399	4.3460093	-3.244597	0.0012832	0.003441	-1.772213
TREH	-0.541391	4.2548366	-3.244591	0.0012832	0.003441	-1.772234
RP11-235D1	-0.467591	-0.676001	-3.244451	0.0012838	0.0034423	-1.772672
RP11-627K1	0.4912222	-0.42402	3.2443279	0.0012843	0.0034435	-1.773058
ANKRD26	0.1066826	5.2571614	3.2438712	0.0012863	0.0034487	-1.774488

HSPA14	0.0486616	5.8959098	3.2435718	0.0012877	0.0034519	-1.775426
MCCC1-AS1	0.5079464	2.2626309	3.2434347	0.0012883	0.0034533	-1.775855
CTD-2547L1	-0.456386	2.6612272	-3.243052	0.0012899	0.0034575	-1.777054
NEIL3	0.3643967	4.2520996	3.2427588	0.0012912	0.0034608	-1.777972
RP11-566K1	0.6584998	0.5929961	3.242612	0.0012919	0.0034622	-1.778431
PTER	0.0950554	5.8173912	3.2425435	0.0012922	0.0034628	-1.778646
RP11-69H7.	0.3826774	-0.937252	3.242345	0.0012931	0.0034649	-1.779267
LINC01127	-0.394986	4.9898055	-3.242204	0.0012937	0.0034663	-1.77971
PPP2R4	-0.057893	6.7066232	-3.242085	0.0012942	0.0034674	-1.780082
RP11-536C1	0.5937112	0.9284887	3.2420696	0.0012943	0.0034674	-1.780129
SNORD100	0.5308375	1.5784894	3.2419974	0.0012946	0.003468	-1.780356
FAM107B	-0.062581	6.624014	-3.241948	0.0012948	0.0034683	-1.780511
RP11-10A14	-0.467575	4.290172	-3.241862	0.0012952	0.0034691	-1.780778
SLC23A1	-0.225683	5.9684842	-3.241803	0.0012955	0.0034694	-1.780963
NPPC	0.6108775	-0.10294	3.2417877	0.0012955	0.0034694	-1.781012
STAMBPL1	0.1774007	5.1567111	3.2413107	0.0012976	0.0034748	-1.782505
RP3-439F8.	0.6027994	2.0886691	3.2412053	0.0012981	0.0034758	-1.782835
RP11-667M1	0.5114216	-0.183963	3.2406728	0.0013005	0.0034819	-1.784501
LINC00705	0.434908	-0.937633	3.2401987	0.0013026	0.0034873	-1.785984
GUCY2D	0.641273	2.8262487	3.2400114	0.0013034	0.0034892	-1.786571
FAF2	-0.036081	6.4089237	-3.239746	0.0013046	0.0034921	-1.787402
STRA8	0.7402322	0.7294155	3.2396989	0.0013048	0.0034924	-1.787548
HEPACAM	-1.033888	2.1333612	-3.239244	0.0013068	0.0034976	-1.788971
RP11-640N2	0.3740283	2.5767477	3.2391868	0.0013071	0.003498	-1.78915
UBE2L6	-0.093129	6.5063084	-3.238785	0.0013089	0.0035026	-1.790408
UBOX5	-0.061984	5.7513085	-3.23861	0.0013096	0.0035044	-1.790954
RP11-274B2	0.4099358	3.1023914	3.2385587	0.0013099	0.0035046	-1.791114
AP3D1	-0.049746	6.7827992	-3.238546	0.0013099	0.0035046	-1.791153
MRPL30	-0.042075	6.1657892	-3.238456	0.0013103	0.0035055	-1.791435
RP11-255P5	-0.487462	-0.77892	-3.238354	0.0013108	0.0035064	-1.791755
RP11-510I2	-0.129011	-1.496268	-3.237881	0.0013129	0.0035118	-1.793234
KCTD11	0.0778124	5.5183476	3.2377658	0.0013134	0.003513	-1.793594
TRBV11-3	0.31896	-1.186265	3.2377169	0.0013136	0.0035133	-1.793747
SYNGR4	-0.578118	1.9026992	-3.237591	0.0013142	0.0035144	-1.794139
PRPF38A	-0.043495	6.1550122	-3.237576	0.0013143	0.0035144	-1.794186
RRP7A	-0.071372	6.3534683	-3.237497	0.0013146	0.0035151	-1.794433
RP1-68D18.	0.4514559	-0.816734	3.2370766	0.0013165	0.0035198	-1.795748
RP11-164O2	-0.536976	-0.287821	-3.237067	0.0013165	0.0035198	-1.795778
ACMSD	-0.326124	6.2185555	-3.23684	0.0013176	0.0035222	-1.796487
ZNF554	-0.078824	5.3163174	-3.236748	0.001318	0.0035231	-1.796775
AC004158.1	-0.355981	-1.08144	-3.23669	0.0013182	0.0035235	-1.796958
AC025627.4	-0.391563	-0.92139	-3.23655	0.0013189	0.0035248	-1.797396
RP11-266K4	0.3016085	-1.165483	3.2365413	0.0013189	0.0035248	-1.797421
ETF1	-0.040679	6.5717995	-3.236435	0.0013194	0.0035258	-1.797753
IGLV5-48	0.5032889	-0.833039	3.2361388	0.0013207	0.0035291	-1.798679
CTD-2026K1	0.4433153	2.4203641	3.235875	0.0013219	0.003532	-1.799504
CCL3	0.2049017	4.9207365	3.2354897	0.0013236	0.0035363	-1.800708
OR1F12	-0.261477	-1.283397	-3.235475	0.0013237	0.0035363	-1.800755
CNOT11	0.0496589	6.3623668	3.2350658	0.0013255	0.003541	-1.802032
ALDH7A1P1	-0.501428	2.2797936	-3.23489	0.0013263	0.0035429	-1.802582
RP11-1012A	-0.274057	-1.255169	-3.234277	0.0013291	0.00355	-1.804495

TMEM70	-0.081685	6.3051726	-3.233974	0.0013305	0.0035534	-1.805442
SMCR8	0.0699133	5.9450474	3.2337166	0.0013316	0.0035563	-1.806246
LINC00824	0.5730974	-0.195692	3.2336422	0.001332	0.0035569	-1.806479
C5orf58	0.7675911	2.0781884	3.2335606	0.0013324	0.0035576	-1.806733
STAM	0.0550431	5.823017	3.2331525	0.0013342	0.0035623	-1.808008
CACNA1B	0.6015349	-0.313905	3.2329156	0.0013353	0.0035649	-1.808747
SIN3B	0.0455905	6.1348667	3.2328121	0.0013358	0.0035659	-1.80907
RP11-686D2	0.4527075	-0.668699	3.2326173	0.0013366	0.003568	-1.809678
RNASE8	-0.197226	-1.409236	-3.23256	0.0013369	0.0035685	-1.809858
STXBP1	0.1610551	5.5127233	3.2324106	0.0013376	0.00357	-1.810324
CCKBR	0.4854107	-0.946267	3.2320871	0.0013391	0.0035737	-1.811334
AJ006998.2	-0.739523	2.7851718	-3.232009	0.0013394	0.0035744	-1.811579
LINC01352	-0.608446	2.1809554	-3.231742	0.0013406	0.0035774	-1.812409
FAM87A	0.6937062	0.6935072	3.2316861	0.0013409	0.0035778	-1.812585
RP11-20I2C	0.2832979	4.5992983	3.2308741	0.0013446	0.0035874	-1.815119
TIAF1	0.1805908	4.6934565	3.2308523	0.0013447	0.0035874	-1.815187
SHH	-0.219127	5.786779	-3.230548	0.0013461	0.0035909	-1.816135
HMHA1	0.0864653	6.0990748	3.2304223	0.0013467	0.0035921	-1.816529
CYP4F29P	0.6636088	0.2375377	3.2302383	0.0013475	0.0035941	-1.817103
FABP4	-0.384926	5.0311349	-3.230038	0.0013484	0.0035963	-1.817729
NIFK-AS1	-0.086998	5.0213965	-3.229863	0.0013492	0.0035982	-1.818275
TRIM4	-0.059835	6.1727404	-3.229707	0.0013499	0.0035998	-1.81876
SUMO2P19	0.4208252	-0.737111	3.2288126	0.0013541	0.0036105	-1.821549
ACTL8	0.8982176	0.7376892	3.2284917	0.0013555	0.0036142	-1.82255
SULT1A3	0.5032419	-0.02043	3.227902	0.0013582	0.0036212	-1.824389
BBS1	0.1583822	4.1371625	3.2275881	0.0013597	0.0036248	-1.825367
AC025627.7	-0.504295	2.66412	-3.227359	0.0013608	0.0036274	-1.826081
RP11-47L3.	0.4575522	2.1744751	3.227113	0.0013619	0.0036301	-1.826848
ZFP37	0.3824175	3.97335	3.2265732	0.0013644	0.0036365	-1.828531
CASD1	0.0742603	5.8121932	3.2264457	0.001365	0.0036378	-1.828928
RPS3	-0.063697	7.1994442	-3.226239	0.0013659	0.0036401	-1.829572
RP11-57K17	0.4585338	-0.61052	3.2258468	0.0013678	0.0036447	-1.830794
TRMT1	-0.060357	6.2355656	-3.225542	0.0013692	0.0036482	-1.831743
GOLGA2P10	0.3170553	4.327511	3.2253888	0.0013699	0.0036499	-1.832221
CYP2G1P	-0.676762	0.6028003	-3.225293	0.0013703	0.0036508	-1.83252
SPATA13	0.0728906	6.2168085	3.225137	0.0013711	0.0036525	-1.833005
RNU6-821P	-0.16712	-1.464068	-3.225091	0.0013713	0.0036528	-1.833149
RP11-127I2	-0.268485	-1.254387	-3.224868	0.0013723	0.0036553	-1.833844
CTB-50L17.	0.2636925	3.6438507	3.2244602	0.0013742	0.0036601	-1.835113
LL21NC02-2	0.4032253	3.7594653	3.2242222	0.0013753	0.0036625	-1.835854
CTB-50L17.	0.500201	1.6192589	3.2242188	0.0013754	0.0036625	-1.835865
RP11-456K2	0.3114268	3.6937112	3.2241112	0.0013759	0.0036635	-1.8362
RP11-283G6	0.4241275	-1.027697	3.2240844	0.001376	0.0036635	-1.836284
IRG1	0.3549723	-1.141956	3.2240787	0.001376	0.0036635	-1.836301
SAV1	-0.08222	5.8865178	-3.223991	0.0013764	0.0036643	-1.836574
PWP1	-0.039683	6.3046407	-3.223653	0.001378	0.0036682	-1.837626
POT1	-0.064729	5.8562467	-3.223265	0.0013798	0.0036728	-1.838833
RMDN2	-0.107129	5.6390195	-3.223234	0.00138	0.0036729	-1.838932
SUCLA2	0.0688509	5.9121412	3.2230519	0.0013808	0.0036749	-1.839498
TMEM55B	-0.048737	6.1241833	-3.222945	0.0013813	0.003676	-1.839831
RP11-390K5	-0.509191	2.1490582	-3.222361	0.0013841	0.003683	-1.841648

CTD-2021H9	0.7122339	0.3553492	3.2218853	0.0013863	0.0036887	-1.843129
RP11-638I8	-0.151599	4.3299568	-3.221814	0.0013866	0.0036893	-1.84335
RP11-29H23	0.551885	0.7978418	3.2217556	0.0013869	0.0036898	-1.843533
RP11-68D16	-0.15967	-1.479255	-3.221567	0.0013878	0.0036913	-1.844121
THNSL2	-0.265287	6.1976973	-3.221561	0.0013878	0.0036913	-1.844137
RP11-242C1	0.5607805	2.0697148	3.221551	0.0013879	0.0036913	-1.84417
RP11-461M2	0.3424269	-1.177906	3.2215445	0.0013879	0.0036913	-1.84419
GOLGA6L9	0.2008636	4.4348201	3.2213203	0.001389	0.0036939	-1.844887
RP3-455J7.	0.6104372	0.3505598	3.2210998	0.00139	0.0036964	-1.845574
SLC25A25-A	0.1257903	5.6525601	3.2210059	0.0013904	0.0036973	-1.845866
RP11-28101	0.5752936	-0.085778	3.2207401	0.0013917	0.0037004	-1.846692
RP11-486L1	-0.189079	-1.411137	-3.220636	0.0013922	0.0037014	-1.847016
ACKR1	0.6912569	3.560511	3.2204542	0.001393	0.0037034	-1.847582
RP11-366M4	-0.160317	-1.473094	-3.220352	0.0013935	0.0037044	-1.8479
AC005027.3	-0.268517	-1.28412	-3.220315	0.0013937	0.0037046	-1.848016
CPT1C	0.1771707	4.5861038	3.2201131	0.0013947	0.0037067	-1.848643
RP11-6J24.	0.340827	-1.052654	3.2201058	0.0013947	0.0037067	-1.848666
CRAMP1L	0.0753144	5.5760494	3.2197915	0.0013962	0.0037104	-1.849643
FAM71F2	0.3439973	3.6975308	3.2194866	0.0013976	0.003714	-1.850592
CNOT6LP1	0.503264	-0.612585	3.2193202	0.0013984	0.0037158	-1.851109
RP11-144G6	0.5370665	1.0979827	3.2190508	0.0013997	0.0037189	-1.851947
AC016735.2	0.5035138	-0.623538	3.2188	0.0014009	0.0037218	-1.852726
ZNF568	0.2561707	4.9779803	3.218745	0.0014011	0.0037222	-1.852898
TMEM240	0.3684516	2.9949375	3.2186795	0.0014014	0.0037228	-1.853101
LRRC2-AS1	-0.561419	1.5417639	-3.218537	0.0014021	0.0037243	-1.853543
CPA1	0.6038622	-0.396115	3.2184179	0.0014027	0.0037255	-1.853914
RP3-406P24	-0.507987	0.152675	-3.218304	0.0014032	0.0037267	-1.854268
ANKRD55	0.5417569	2.9910862	3.2182779	0.0014034	0.0037268	-1.85435
AC113607.2	-0.151161	-1.47706	-3.217958	0.0014049	0.0037305	-1.855343
FAM132B	0.2577703	4.2347991	3.2176173	0.0014065	0.0037346	-1.856403
FAM86B1	0.520779	2.6240921	3.2171093	0.0014089	0.0037408	-1.857982
RP11-542A1	-0.221038	-1.394255	-3.217011	0.0014094	0.0037417	-1.858287
CNOT3	0.0401989	6.230347	3.2169366	0.0014098	0.0037424	-1.858519
CTD-2015H6	-0.230393	-1.331884	-3.216763	0.0014106	0.0037443	-1.859059
AC131097.4	-0.731461	0.5528769	-3.216718	0.0014108	0.0037445	-1.859199
PLK1	0.183365	5.5457823	3.2167101	0.0014108	0.0037445	-1.859222
KDF1	0.4297342	4.8322112	3.2166697	0.001411	0.0037447	-1.859348
FAM96AP2	0.6262375	1.4881974	3.2165999	0.0014114	0.0037453	-1.859565
RP4-635E18	-0.439387	2.2103633	-3.216479	0.0014119	0.0037466	-1.85994
SMARCD1	0.0415917	6.2767469	3.2163252	0.0014127	0.0037483	-1.860418
GOLGA6L4	0.6025565	0.7153592	3.2162654	0.001413	0.0037487	-1.860604
RP11-10J21	0.4179393	-0.86177	3.2162142	0.0014132	0.003749	-1.860763
NPM1P27	0.2002855	4.4690629	3.2161987	0.0014133	0.003749	-1.860812
RP11-155G1	0.5745468	0.2072411	3.216136	0.0014136	0.0037496	-1.861006
MAD1L1	-0.076527	6.2023037	-3.216075	0.0014139	0.0037501	-1.861196
NKTR	0.0755313	6.1612641	3.2156972	0.0014157	0.0037546	-1.862369
RP11-1379J	0.4475348	2.1533481	3.2155795	0.0014163	0.0037558	-1.862735
JAM2	0.1783634	5.237951	3.2155157	0.0014166	0.0037564	-1.862933
UQCRC2	-0.057442	6.764864	-3.215404	0.0014171	0.0037575	-1.863281
RP11-143K1	0.4025756	-0.99224	3.2153435	0.0014174	0.003758	-1.863468
GJA1	0.1145386	5.8194002	3.215024	0.0014189	0.0037618	-1.86446

ODCP	0.5666696	0.4268791	3.2149781	0.0014191	0.0037621	-1.864603
SP6	0.3286694	3.9297716	3.2147032	0.0014205	0.0037654	-1.865457
RP11-19G24	-0.203166	-1.392416	-3.214676	0.0014206	0.0037654	-1.86554
HADHB	-0.057271	6.8728005	-3.214288	0.0014225	0.0037701	-1.866747
CDK20	0.1364508	5.1708329	3.2142166	0.0014228	0.0037707	-1.866967
EN2	0.8043931	0.3726564	3.2140712	0.0014235	0.0037723	-1.867419
EIF1AX	-0.057683	6.4206982	-3.213897	0.0014243	0.0037743	-1.867959
EIF1P7	-0.460682	-0.46331	-3.213859	0.0014245	0.0037745	-1.868078
SHMT1P1	-0.413409	-0.837392	-3.213768	0.001425	0.0037754	-1.868359
PTH	-0.164513	-1.483004	-3.213466	0.0014264	0.003779	-1.869298
RP11-74M13	-0.695504	1.4771526	-3.213156	0.0014279	0.0037824	-1.870261
B3GALTL	-0.076396	5.562686	-3.213156	0.0014279	0.0037824	-1.870262
SYPL1	-0.047304	6.592763	-3.212976	0.0014288	0.0037844	-1.870819
FBXO48	-0.07928	5.1919116	-3.212866	0.0014293	0.0037856	-1.871161
AL928761.1	0.4225088	-0.987507	3.2125571	0.0014308	0.0037892	-1.872119
RP11-461F1	-0.580479	-0.609804	-3.212402	0.0014316	0.003791	-1.8726
APOL6	-0.085611	6.6148773	-3.212343	0.0014319	0.0037914	-1.872785
PUS7L	0.0582386	5.775981	3.2122617	0.0014323	0.0037922	-1.873036
TGM3	-0.419258	5.1845207	-3.212191	0.0014326	0.0037928	-1.873254
LRP3	0.1086557	6.3597231	3.2121634	0.0014327	0.0037929	-1.873341
RP11-731D1	-0.496511	-0.523816	-3.212071	0.0014332	0.0037938	-1.873628
TSPY5P	-0.159372	-1.485908	-3.211772	0.0014346	0.0037974	-1.874556
BST1	0.1915283	4.8047853	3.2115943	0.0014355	0.0037989	-1.875107
MTHFSD	-0.057718	5.7371861	-3.211593	0.0014355	0.0037989	-1.87511
RP11-1114A	0.2651664	3.7525613	3.2115905	0.0014355	0.0037989	-1.875118
FBXO24	-0.185347	4.1685826	-3.210521	0.0014407	0.0038124	-1.878437
PSKH1	-0.064409	6.1599261	-3.210368	0.0014415	0.003814	-1.878911
CTC-524C5.	0.1717301	4.4935067	3.210356	0.0014415	0.003814	-1.878948
TAS2R31	0.4639064	-0.560595	3.2100322	0.0014431	0.0038179	-1.879952
RP11-34P13	0.635046	2.2007269	3.2099738	0.0014434	0.0038184	-1.880133
VSTM4	-0.18572	5.7423771	-3.209908	0.0014437	0.0038189	-1.880337
SLC38A6	-0.083398	5.6424975	-3.209576	0.0014453	0.003823	-1.881367
CACNA2D3-A	-0.24564	-1.376647	-3.209397	0.0014462	0.003825	-1.881922
CTC-248019	-0.588215	-0.17336	-3.209357	0.0014464	0.0038252	-1.882046
SLC19A1	-0.089596	6.1818721	-3.209314	0.0014466	0.0038255	-1.882179
FAUP1	-0.331834	2.8430781	-3.209211	0.0014471	0.0038266	-1.8825
TBC1D20	-0.043607	6.2634669	-3.208924	0.0014485	0.00383	-1.88339
RP11-261N1	-0.928185	1.6696703	-3.208856	0.0014489	0.0038306	-1.883598
ERI2	-0.067585	5.7494347	-3.208729	0.0014495	0.003832	-1.883993
SUB1	-0.049852	6.7804947	-3.208609	0.0014501	0.0038333	-1.884364
CTD-2308L2	0.5154465	-0.504314	3.2084087	0.0014511	0.0038355	-1.884986
RP11-214K3	0.5157382	-0.134045	3.2083912	0.0014511	0.0038355	-1.88504
SNTG2	0.4591069	-0.891337	3.2076394	0.0014548	0.003845	-1.88737
RP11-45L9.	0.3726602	-0.908446	3.2074941	0.0014556	0.0038466	-1.88782
CDH24	0.1512364	4.9956497	3.2069823	0.0014581	0.0038529	-1.889406
GPSM2	0.1227001	5.2741704	3.2069663	0.0014582	0.0038529	-1.889456
TNKS2-AS1	-0.525636	2.2974575	-3.206933	0.0014583	0.0038531	-1.88956
PER3	-0.124117	5.911785	-3.20674	0.0014593	0.0038553	-1.890157
TRAV34	0.3359652	-1.149199	3.2062312	0.0014618	0.0038617	-1.891733
CDCA7L	0.2028276	5.2526294	3.2061568	0.0014621	0.0038624	-1.891964
RP5-1049N1	-0.25362	-1.307154	-3.206062	0.0014626	0.0038631	-1.892256

U40455.1	-0.533168	0.3591106	-3.206058	0.0014626	0.0038631	-1.892271
RP11-291L2	0.3506982	3.1474433	3.2059827	0.001463	0.0038638	-1.892503
MBLAC2	-0.110656	5.4158135	-3.205096	0.0014674	0.0038751	-1.89525
IQCC	-0.122892	4.8203907	-3.205048	0.0014676	0.0038755	-1.895399
RP11-6B19.	-0.175828	-1.437457	-3.204652	0.0014696	0.0038804	-1.896624
NUPL1	0.0588162	6.0474933	3.204559	0.0014701	0.0038813	-1.896912
BPIFB4	0.7579682	0.7508371	3.2045158	0.0014703	0.0038816	-1.897046
VDAC1P1	0.4938848	1.6414294	3.204465	0.0014705	0.003882	-1.897203
ERI1	0.080944	5.5786671	3.2044286	0.0014707	0.0038822	-1.897316
CTC-820M8.	0.5800972	0.6988406	3.20427	0.0014715	0.003884	-1.897807
ZNF235	0.1071023	4.8003735	3.204235	0.0014717	0.0038842	-1.897915
SNRPB2	-0.049988	6.3060313	-3.203883	0.0014734	0.0038885	-1.899004
RP11-421M1	0.5214817	1.6965485	3.2037637	0.001474	0.0038898	-1.899374
RND2	0.4849435	4.3833569	3.2034762	0.0014755	0.003893	-1.900264
PCDH20	-0.737148	0.2539439	-3.203475	0.0014755	0.003893	-1.900267
NT5C	-0.081752	5.9664183	-3.203187	0.0014769	0.0038965	-1.901158
AC016292.3	0.359484	3.7622932	3.2030535	0.0014776	0.003898	-1.901572
LLGL2	-0.067038	6.561612	-3.202845	0.0014786	0.0039005	-1.902218
RP11-385N2	0.4208761	-1.040678	3.2026103	0.0014798	0.0039033	-1.902944
DPRX	-0.21616	-1.41507	-3.202573	0.00148	0.0039035	-1.903059
SH3GL2	0.6438228	0.2378248	3.2024386	0.0014806	0.003905	-1.903475
RP11-452K1	-0.513228	1.708393	-3.202302	0.0014813	0.0039065	-1.903899
KRT16	0.8300123	0.4096911	3.2022772	0.0014814	0.0039066	-1.903975
CSNK1G1	0.0558453	5.6645589	3.2021291	0.0014822	0.0039082	-1.904433
ARL6IP4	-0.121759	4.9435398	-3.201947	0.0014831	0.0039104	-1.904996
CTB-13F3.1	0.4099816	2.6539851	3.2018268	0.0014837	0.0039117	-1.905368
RP11-807H2	0.535003	0.0106333	3.2017929	0.0014839	0.0039118	-1.905473
TIMM8AP1	-0.537258	2.0026827	-3.201705	0.0014843	0.0039127	-1.905743
ZNF81	0.137491	4.6776054	3.2009704	0.001488	0.0039221	-1.908017
RP11-669I1	-0.468484	-0.866746	-3.200792	0.0014889	0.0039242	-1.90857
RP11-167N2	-0.574634	-0.54938	-3.200567	0.00149	0.0039269	-1.909265
KIR2DL4	0.6515419	1.2536	3.2004304	0.0014907	0.0039284	-1.909687
ZNF285	0.3847018	3.9665609	3.2000375	0.0014927	0.0039334	-1.910902
RP11-464F9	0.462295	-0.364323	3.1999421	0.0014932	0.0039344	-1.911197
RBM47	-0.07001	6.4772024	-3.199831	0.0014937	0.0039356	-1.911542
ABL2	0.0595522	6.0370367	3.1996705	0.0014945	0.0039374	-1.912037
AP000431.2	-0.343168	-1.263516	-3.199649	0.0014946	0.0039374	-1.912105
ABCA3	0.1748341	5.4023124	3.1994259	0.0014958	0.0039401	-1.912793
HPS6	-0.05546	5.9091545	-3.19927	0.0014966	0.0039417	-1.913275
MBTPS1	-0.043778	6.5234535	-3.19926	0.0014966	0.0039417	-1.913307
ATP5A1	-0.049841	7.118107	-3.199107	0.0014974	0.0039435	-1.913779
PPP4R4	-0.200155	5.1524275	-3.198808	0.0014989	0.003947	-1.914704
RP11-540K1	-0.389046	-0.978182	-3.1988	0.0014989	0.003947	-1.914728
CNTNAP5	0.5039176	-0.832855	3.1987595	0.0014991	0.0039472	-1.914853
RP11-697E2	-0.315093	-1.207941	-3.198528	0.0015003	0.00395	-1.91557
UQCRFS1	-0.063211	6.5570626	-3.198446	0.0015007	0.0039508	-1.915822
AC105398.3	-0.189273	-1.44649	-3.198238	0.0015018	0.0039533	-1.916464
MDH1	-0.060885	6.8236842	-3.197947	0.0015033	0.0039569	-1.917365
NINL	0.1562446	5.7827485	3.1979183	0.0015034	0.003957	-1.917453
ARHGEF16	0.3572454	5.4808528	3.1978686	0.0015036	0.0039574	-1.917607
RP4-671O14	0.4901769	2.4951063	3.1972048	0.001507	0.003966	-1.919658

AC092765.1	-0.185876	-1.440129	-3.196976	0.0015082	0.0039688	-1.920364
RP11-136C2	-0.28523	-1.215402	-3.196675	0.0015097	0.0039725	-1.921295
UXS1	0.0530526	6.2289696	3.1963866	0.0015112	0.0039761	-1.922185
HNRNPA1P1C	0.4319196	2.5878532	3.1963372	0.0015114	0.0039762	-1.922338
LY96	0.2304423	5.2116839	3.1963326	0.0015115	0.0039762	-1.922352
OR11H7	0.3244762	-1.168927	3.1959742	0.0015133	0.0039808	-1.923459
RP11-31I22	-0.135578	-1.485546	-3.195824	0.0015141	0.0039825	-1.923921
TLK2	-0.039009	6.0148772	-3.195783	0.0015143	0.0039828	-1.924049
KRT8P10	0.4586871	-0.478279	3.1954194	0.0015161	0.0039874	-1.925172
MUC7	-0.199948	-1.454932	-3.195369	0.0015164	0.0039877	-1.925328
RP11-671J1	-0.246115	-1.305603	-3.194923	0.0015187	0.0039935	-1.926704
AP001187.9	-0.287805	4.4956999	-3.194448	0.0015211	0.0039996	-1.928172
KB-1572G7.	0.3340995	3.345778	3.1940839	0.001523	0.0040042	-1.929295
HSPB3	0.4539371	-1.025151	3.1933356	0.0015268	0.004014	-1.931604
OLAH	0.5714933	-0.016987	3.1932993	0.001527	0.0040142	-1.931717
UBE2E3	-0.046609	6.247606	-3.192971	0.0015287	0.0040184	-1.932731
CP	-0.15944	7.1169299	-3.192836	0.0015294	0.0040199	-1.933147
RP11-467J1	-0.371859	-0.870372	-3.192461	0.0015313	0.0040247	-1.934303
FGF9	0.6411788	-0.160297	3.1920861	0.0015332	0.0040293	-1.93546
TSGA10IP	0.6049355	0.8944902	3.1920806	0.0015333	0.0040293	-1.935476
ABCA8	-0.309541	5.6402266	-3.191881	0.0015343	0.0040317	-1.936093
RGS9	0.3654424	4.2067597	3.1918147	0.0015346	0.0040323	-1.936297
HNRNPCP4	0.4610312	-0.383676	3.1914003	0.0015368	0.0040376	-1.937575
RP11-241F1	-0.635941	-0.070727	-3.191363	0.001537	0.0040379	-1.937689
RP11-248N2	0.2860419	-1.274678	3.1904334	0.0015418	0.0040502	-1.940557
CXCL17	0.7519022	2.3895163	3.1901226	0.0015434	0.0040542	-1.941515
TOPBP1	0.0620049	6.0533435	3.1900941	0.0015436	0.0040543	-1.941603
RP11-772C9	-0.70637	0.8734707	-3.18966	0.0015458	0.0040599	-1.942939
ZNF793-AS1	0.6529523	2.7004276	3.1893376	0.0015475	0.004064	-1.943935
CTSS	0.0986624	6.5580442	3.1892446	0.001548	0.004065	-1.944222
GJC3	-0.396981	3.4046796	-3.189137	0.0015485	0.0040662	-1.944553
CECR5	-0.059112	6.3329845	-3.188986	0.0015493	0.004068	-1.945017
RP11-451G4	-0.346215	-1.244362	-3.188945	0.0015495	0.0040681	-1.945145
RP11-15L13	0.457073	1.9008271	3.1889348	0.0015496	0.0040681	-1.945176
RP11-249C2	-0.513833	-0.718256	-3.188679	0.0015509	0.0040713	-1.945966
ZNF501	0.2921141	4.1732159	3.1885824	0.0015514	0.0040723	-1.946262
IGHMBP2	-0.067338	5.974472	-3.188419	0.0015523	0.0040743	-1.946765
NEK8	-0.087074	5.531712	-3.188384	0.0015525	0.0040745	-1.946875
NR2F1	0.1935218	5.7833633	3.188125	0.0015538	0.0040777	-1.947672
RP11-1026M	-0.143442	-1.493254	-3.188026	0.0015544	0.0040788	-1.947978
MIR4500HG	-0.666807	0.0916515	-3.187969	0.0015546	0.0040793	-1.948153
RP11-91P24	0.4628675	-0.466889	3.1868952	0.0015603	0.0040937	-1.95146
FBLL1	0.7288638	3.0744152	3.1868385	0.0015606	0.0040941	-1.951635
CTD-3216D2	0.5072332	1.7808551	3.1868261	0.0015606	0.0040941	-1.951673
FBRS	-0.035549	6.389426	-3.186685	0.0015614	0.0040958	-1.952108
AC124944.3	0.5279538	-0.231276	3.1864116	0.0015628	0.0040992	-1.95295
RP11-380L1	0.3948232	3.7650776	3.1861391	0.0015642	0.0041027	-1.953789
AC069213.1	0.6091615	3.5060787	3.1860571	0.0015647	0.0041035	-1.954041
CDKN1A	-0.107641	6.6915179	-3.185878	0.0015656	0.0041057	-1.954592
TMEM126B	-0.051343	6.0662978	-3.185615	0.001567	0.004109	-1.955404
SNORA75	0.5497317	0.4678776	3.18553	0.0015675	0.0041099	-1.955664

RP11-757G1	0.732612	3.4294737	3.1853782	0.0015683	0.0041117	-1.956132
RP11-532F6	0.4620514	2.6448789	3.1852691	0.0015688	0.0041128	-1.956467
SECTM1	0.1369386	5.7860968	3.1852589	0.0015689	0.0041128	-1.956499
RP11-762I7	0.5611142	0.6237032	3.1851223	0.0015696	0.0041144	-1.95692
TPTE2	0.4089383	-0.845269	3.1849016	0.0015708	0.0041171	-1.957599
SEC14L4	0.4090683	5.2052828	3.1847562	0.0015715	0.0041189	-1.958046
SHOX2	0.6384787	2.0682155	3.1847004	0.0015718	0.0041193	-1.958218
CTD-2561B2	0.3230445	3.440343	3.1841777	0.0015746	0.0041263	-1.959827
MT1H	0.7010577	3.5392303	3.183807	0.0015766	0.0041311	-1.960968
TTTY14	-0.949795	2.2733749	-3.183769	0.0015768	0.0041314	-1.961085
CTD-2083E4	0.3848685	2.5776094	3.1835725	0.0015778	0.0041338	-1.961689
VCAN-AS1	0.2995837	-1.289512	3.1834445	0.0015785	0.0041353	-1.962083
CCR10	0.3666513	3.1628761	3.183395	0.0015787	0.0041357	-1.962236
RP11-535M1	0.5411387	-0.101849	3.183297	0.0015793	0.0041367	-1.962537
ANP32B	-0.056241	6.6425467	-3.183149	0.0015801	0.0041385	-1.962992
RP11-46E17	-0.171595	-1.44407	-3.183109	0.0015803	0.0041388	-1.963116
MEF2BNB	-0.062788	5.8171795	-3.182901	0.0015814	0.0041412	-1.963756
AC006378.2	0.3645485	2.47893	3.1828872	0.0015814	0.0041412	-1.963798
CLDN20	0.5513878	0.2108894	3.1826233	0.0015828	0.0041446	-1.96461
CTD-2012J1	0.5578532	0.7609682	3.1825627	0.0015832	0.0041452	-1.964796
CLUL1	0.6377651	1.7329644	3.1824762	0.0015836	0.0041461	-1.965062
GPN1	-0.039363	6.1196641	-3.182416	0.0015839	0.0041466	-1.965247
PIM1	-0.09267	6.2239616	-3.182277	0.0015847	0.0041483	-1.965674
C5orf42	0.1293637	5.4145597	3.1817908	0.0015873	0.0041547	-1.96717
CRYZL1	-0.049631	5.7567546	-3.181642	0.0015881	0.0041565	-1.967628
CTD-3075F1	-0.489764	-0.341544	-3.181399	0.0015894	0.0041594	-1.968375
HSD3BP3	-0.32832	-1.16517	-3.181396	0.0015894	0.0041594	-1.968386
XPO7	0.0545988	6.2118609	3.1809774	0.0015916	0.0041647	-1.969671
RP11-574K1	0.1672963	3.8579537	3.1809694	0.0015917	0.0041647	-1.969696
ZFP91-CNTF	-0.307719	-1.1391	-3.180858	0.0015923	0.004166	-1.970038
AC005740.3	-0.492977	-0.539373	-3.180122	0.0015962	0.004176	-1.972301
RP5-1085F1	0.4218751	-0.662575	3.18008	0.0015964	0.004176	-1.97243
RP11-177H1	0.4777313	1.7575749	3.1800787	0.0015964	0.004176	-1.972434
MIR4719	-0.156969	-1.463402	-3.179765	0.0015981	0.0041798	-1.973398
CNTN3	0.9454493	2.3694831	3.1797628	0.0015981	0.0041798	-1.973405
WI2-87327E	0.70155	0.0532373	3.1797138	0.0015984	0.0041802	-1.973556
RP11-1006G	-0.187461	-1.441176	-3.178429	0.0016053	0.004198	-1.977505
PTMAP2	0.3173981	3.3953606	3.1779837	0.0016077	0.004204	-1.978872
URGCP	-0.05058	6.2735218	-3.177905	0.0016081	0.0042048	-1.979113
TCTE3	0.2155688	4.3808162	3.1776346	0.0016096	0.0042083	-1.979944
TCEAL4	-0.058071	6.488793	-3.17716	0.0016122	0.0042147	-1.981403
SUN2	-0.065962	6.7716144	-3.176695	0.0016147	0.004221	-1.98283
WFDC12	-0.413223	-1.047833	-3.176627	0.001615	0.0042216	-1.983038
CHRM1	0.5561313	-0.632651	3.176437	0.0016161	0.004224	-1.983622
RP11-597D1	0.525481	0.0210367	3.1761743	0.0016175	0.0042275	-1.984429
RP11-433J2	0.3404227	-1.088081	3.1760899	0.001618	0.0042284	-1.984688
KRTAP5-6	-0.580619	3.0246828	-3.176039	0.0016182	0.0042288	-1.984843
CKBP1	-0.410358	-1.005466	-3.175175	0.0016229	0.0042407	-1.987496
RP11-440G5	-0.603727	1.7759022	-3.175105	0.0016233	0.0042414	-1.987711
LINC01143	0.4521202	-0.953887	3.1743339	0.0016275	0.0042521	-1.990078
RP11-33504	0.4469275	-0.406278	3.1734834	0.0016322	0.004264	-1.992687

LCTL	0.5051425	2.1239772	3.1730763	0.0016344	0.0042695	-1.993936
METTL15	-0.054183	5.7122136	-3.172865	0.0016355	0.0042721	-1.994584
STK10	0.0672541	6.0310729	3.1728512	0.0016356	0.0042721	-1.994627
AC003991.3	0.3941504	-0.914755	3.1725334	0.0016374	0.0042763	-1.995601
AC005077.7	-0.702434	0.2845345	-3.172418	0.001638	0.0042776	-1.995954
KANSL3	0.042196	6.3436555	3.1724052	0.0016381	0.0042776	-1.995994
FBXO21	0.0698474	6.1343214	3.1721603	0.0016394	0.0042808	-1.996745
GATAD2A	0.0398598	6.4326396	3.1717405	0.0016417	0.0042865	-1.998033
RP11-243M5	0.4935063	-0.689372	3.1712399	0.0016445	0.0042934	-1.999568
SOGA3	0.5810253	0.1151308	3.1711482	0.001645	0.0042944	-1.999848
DMRTA1	-0.349813	5.3860118	-3.170932	0.0016462	0.0042972	-2.000513
RP11-10L12	-0.15521	4.0432217	-3.170661	0.0016477	0.0043008	-2.001342
SLC7A9	-0.283107	5.781004	-3.170546	0.0016483	0.0043021	-2.001693
AC106706.1	-0.120679	-1.497379	-3.169997	0.0016513	0.0043097	-2.003377
RP11-564D1	0.6976774	1.2597621	3.1699199	0.0016518	0.0043105	-2.003613
SEC23A	-0.065554	6.4822233	-3.169886	0.001652	0.0043107	-2.003718
E2F8	0.3258738	4.7278537	3.1693606	0.0016549	0.004318	-2.005327
C9orf147	-0.309005	2.5592521	-3.169208	0.0016557	0.0043199	-2.005794
SEMA7A	0.1995905	5.4556328	3.1688451	0.0016577	0.0043249	-2.006907
ZNF791	-0.045399	5.9096091	-3.168802	0.001658	0.0043252	-2.007039
TMEM231	0.2579953	4.5147745	3.1682608	0.001661	0.0043327	-2.008696
IGLV5-52	0.3877497	-0.98107	3.1682151	0.0016612	0.0043331	-2.008837
ZNF689	-0.055867	5.7022141	-3.168046	0.0016622	0.0043352	-2.009355
RP5-908D6.	-0.365208	-1.008834	-3.167993	0.0016625	0.0043356	-2.009517
INTS3	0.0803882	6.3206613	3.1679733	0.0016626	0.0043356	-2.009577
CTD-2335A1	-0.147352	-1.483458	-3.167134	0.0016672	0.0043475	-2.012148
RP11-26N15	-0.176375	-1.439419	-3.166978	0.0016681	0.0043495	-2.012626
MUS81	-0.036833	6.1034525	-3.166561	0.0016704	0.0043553	-2.013903
ACTG2	-0.207795	5.210685	-3.166466	0.001671	0.0043563	-2.014192
UGT1A8	-0.679041	1.1254411	-3.166375	0.0016715	0.0043573	-2.014471
LINC01395	-0.305442	-1.304082	-3.166276	0.001672	0.0043585	-2.014774
RNF144A-AS	0.6307225	1.8142648	3.1660374	0.0016734	0.0043616	-2.015505
PCDHA8	0.4835338	-0.919515	3.1651481	0.0016784	0.0043741	-2.018226
KANK1	-0.093006	6.5648969	-3.165144	0.0016784	0.0043741	-2.018238
RP11-214K3	0.5252633	0.0651048	3.165038	0.001679	0.0043753	-2.018563
MAPK8IP3	0.0776031	6.0883491	3.1645921	0.0016815	0.0043815	-2.019928
SPEF2	0.1392528	5.0833856	3.1644655	0.0016822	0.0043831	-2.020315
ADPRM	-0.088242	5.4842761	-3.1643	0.0016831	0.0043852	-2.020821
FRMPD1	0.5746429	4.0153121	3.1642563	0.0016834	0.0043855	-2.020955
TAGLN2P1	0.4619796	2.2393761	3.1640714	0.0016844	0.0043879	-2.021521
HNF1A-AS1	-0.242246	5.6699355	-3.163896	0.0016854	0.0043902	-2.022057
DYRK4	-0.081592	5.6426306	-3.163617	0.001687	0.004394	-2.02291
AC093620.5	0.5234925	0.8523944	3.1634789	0.0016878	0.0043954	-2.023333
SPTSSA	-0.084635	6.5167488	-3.163478	0.0016878	0.0043954	-2.023335
SUMF2	-0.054826	6.7560706	-3.163437	0.001688	0.0043957	-2.023462
PCDHGA2	0.5403012	3.9588213	3.1633999	0.0016882	0.0043959	-2.023575
SMAGP	0.1117165	5.6236999	3.1632643	0.001689	0.0043976	-2.02399
ABHD12	-0.060926	6.5478909	-3.163029	0.0016903	0.0044007	-2.024708
FANCD2	0.1366862	5.4435671	3.1627639	0.0016918	0.0044043	-2.02552
GCNT3	0.5399255	4.4055973	3.1626271	0.0016926	0.004406	-2.025938
WDR62	0.1574063	5.3623585	3.1625768	0.0016929	0.0044064	-2.026092

ZNRF2P2	0.563075	0.3995936	3.1625401	0.0016931	0.0044067	-2.026204
CTA-407F11	-0.286189	-1.300521	-3.161915	0.0016966	0.0044156	-2.028114
RP11-22N19	-0.441138	2.3341471	-3.161727	0.0016977	0.004418	-2.02869
EFNA1	-0.085396	6.9833845	-3.161517	0.0016989	0.0044208	-2.029331
RP11-401P9	-0.272457	-1.308819	-3.161322	0.0017	0.0044234	-2.029928
LRRC37A14F	-0.547249	-0.042011	-3.160922	0.0017022	0.004429	-2.03115
VPS9D1-AS1	0.3079117	4.1024369	3.1608133	0.0017029	0.0044303	-2.031483
RP11-299H2	0.8401498	1.74692	3.1607856	0.001703	0.0044304	-2.031568
AL163953.3	0.5890243	2.0122232	3.1606205	0.001704	0.0044325	-2.032072
TEKT5	-0.559792	2.7842698	-3.159849	0.0017084	0.0044436	-2.03443
ZNF25	0.0929547	5.3034733	3.1596881	0.0017093	0.0044457	-2.034921
RP11-362L2	-0.214937	-1.359258	-3.158865	0.001714	0.0044576	-2.037436
RP11-366M4	-0.210443	-1.430518	-3.158786	0.0017144	0.0044585	-2.037677
ATR	0.0546243	5.9260823	3.1586903	0.001715	0.0044596	-2.037969
WDR60	0.082816	5.5200968	3.1584612	0.0017163	0.0044627	-2.038669
RPS24	-0.070623	7.2286603	-3.158419	0.0017165	0.004463	-2.038797
METTL4	0.0618764	5.4128946	3.1581276	0.0017182	0.004467	-2.039688
HIST1H2APS	-0.548903	-0.143411	-3.158057	0.0017186	0.0044678	-2.039902
KLHL31	-0.24622	4.2049535	-3.15764	0.001721	0.0044737	-2.041178
RP11-379F4	0.2973705	3.4769129	3.1575699	0.0017214	0.0044744	-2.04139
RP11-104J2	-0.480148	3.4401118	-3.157474	0.001722	0.0044755	-2.041684
RP11-106J2	-0.191438	-1.471174	-3.157359	0.0017226	0.0044769	-2.042035
IFNA22P	-0.231559	-1.346537	-3.156978	0.0017248	0.0044823	-2.043198
CDH1	0.1390432	6.7217112	3.1567474	0.0017261	0.0044854	-2.043902
RP11-153M3	0.3822679	2.7655125	3.1566844	0.0017265	0.004486	-2.044094
NAPEPLD	-0.061746	5.8459615	-3.156471	0.0017277	0.0044889	-2.044746
LINC00205	0.1765634	4.6012382	3.156395	0.0017282	0.0044897	-2.044977
RP11-45901	-0.398505	-0.999962	-3.156356	0.0017284	0.00449	-2.045095
OR11A1	0.3276308	-1.23684	3.1561757	0.0017294	0.0044924	-2.045647
EPS15L1	0.050047	6.047749	3.1558272	0.0017315	0.0044973	-2.04671
RP11-366M4	-0.132491	-1.489453	-3.155798	0.0017316	0.0044974	-2.046799
GOLGA8R	0.6020715	1.5827853	3.1555046	0.0017333	0.0045015	-2.047694
POP1	0.0714995	5.386075	3.1554383	0.0017337	0.0045022	-2.047897
PDIA3	-0.047975	7.2111291	-3.155092	0.0017357	0.0045071	-2.048954
PAFAH1B3	0.1552623	5.784753	3.1545233	0.001739	0.0045153	-2.050688
PTPRB	0.1315988	5.859951	3.1541813	0.001741	0.0045201	-2.051731
TNKS2	0.0589967	6.214403	3.154041	0.0017418	0.0045219	-2.052159
USP10	-0.04269	6.3550513	-3.15397	0.0017422	0.0045227	-2.052376
CDK3	0.3868602	3.5326355	3.1538073	0.0017432	0.0045248	-2.052872
AC016700.4	-0.199544	-1.39771	-3.153598	0.0017444	0.0045277	-2.05351
RP11-437B1	0.3702569	2.6350452	3.1535012	0.0017449	0.0045288	-2.053806
CRABP1	0.4990516	-0.865066	3.1534105	0.0017455	0.0045298	-2.054082
RP11-421L2	-0.116621	5.0677899	-3.152939	0.0017482	0.0045367	-2.055521
POLM	-0.048149	5.9614882	-3.152776	0.0017492	0.0045386	-2.056016
PDZD8	0.0684859	6.2483269	3.1527674	0.0017492	0.0045386	-2.056043
RP11-850A1	0.4798456	-0.291166	3.1527215	0.0017495	0.004539	-2.056183
UNC5CL	0.1771451	5.9486181	3.1524766	0.0017509	0.0045424	-2.05693
BLOC1S5	-0.057264	5.8947234	-3.152254	0.0017522	0.0045454	-2.057608
RP11-334C1	-0.46206	2.3611515	-3.151847	0.0017546	0.0045513	-2.058848
AC004066.3	0.2776958	-1.316028	3.151764	0.0017551	0.0045522	-2.059102
AVPI1	-0.099526	6.0526129	-3.151712	0.0017554	0.0045527	-2.059261

AC009095.4	0.5179304	0.3553384	3.1515218	0.0017565	0.0045552	-2.05984
ARHGAP17	0.0411378	6.2146994	3.1513356	0.0017576	0.0045577	-2.060408
KB-1552D7.	-0.603007	1.7726099	-3.150569	0.0017621	0.0045691	-2.062742
ZNF195	0.0549572	5.7577178	3.1503182	0.0017636	0.0045726	-2.063507
FAM133B	0.0842193	5.2794028	3.1500699	0.001765	0.004576	-2.064264
PPM1K	-0.150346	5.4717924	-3.150021	0.0017653	0.0045764	-2.064413
RP5-956018	0.5426044	0.16068	3.1494018	0.001769	0.0045856	-2.066299
PAQR9-AS1	-0.281712	5.2163158	-3.149145	0.0017705	0.0045892	-2.06708
RP11-275I1	0.5544254	0.7256522	3.1488886	0.001772	0.0045928	-2.067862
RP11-28101	-0.21733	-1.428053	-3.148778	0.0017726	0.0045941	-2.068197
ETV5	0.112957	5.8173568	3.148749	0.0017728	0.0045942	-2.068287
HOXB13	0.8405563	0.3875875	3.1485386	0.0017741	0.0045971	-2.068928
DPH6-AS1	-0.585314	2.3501411	-3.148204	0.001776	0.0046019	-2.069948
KB-1674E1.	-0.490077	-0.115692	-3.147899	0.0017778	0.0046063	-2.070874
CDC42	-0.037894	6.7556423	-3.146816	0.0017843	0.0046226	-2.074173
RP11-425L1	-0.145501	5.2892731	-3.146746	0.0017847	0.0046234	-2.074386
MTO1	-0.059033	6.0909731	-3.146275	0.0017875	0.0046303	-2.075819
RBM12B-AS1	0.4804742	2.0783184	3.1460724	0.0017887	0.0046331	-2.076434
HOXC9	0.8431349	0.8086009	3.145916	0.0017896	0.0046352	-2.07691
PLEKHG6	0.2376908	5.351855	3.1456996	0.0017909	0.0046382	-2.077569
RP11-861A1	0.5214488	0.0100768	3.1456214	0.0017914	0.0046391	-2.077807
TADA1	-0.079385	6.0622763	-3.145306	0.0017932	0.0046436	-2.078766
AC093668.2	0.5302536	0.9335703	3.145073	0.0017946	0.0046469	-2.079475
PKD2	0.1224854	5.7013211	3.1447777	0.0017964	0.0046511	-2.080373
EPSTI1	0.1906704	5.4824935	3.1447002	0.0017969	0.004652	-2.080609
RNF26	-0.049824	6.3105337	-3.144349	0.001799	0.0046571	-2.081678
ARPC1B	0.0724857	6.6858789	3.1439446	0.0018014	0.004663	-2.082907
TMEM132D	0.7181619	1.2022319	3.1438435	0.001802	0.0046643	-2.083214
CLDN9	-0.312318	4.5356367	-3.143709	0.0018028	0.0046658	-2.083624
RP11-488C1	0.1953075	4.1653719	3.1437034	0.0018028	0.0046658	-2.08364
RAD51AP1	0.196327	5.0270147	3.1434994	0.0018041	0.0046686	-2.08426
OR10V3P	-0.151097	-1.469525	-3.143035	0.0018069	0.0046755	-2.085673
CTC-550B14	0.4033767	-0.82885	3.142965	0.0018073	0.0046763	-2.085885
PPP1R35	-0.080005	5.9478719	-3.142805	0.0018082	0.0046784	-2.086371
RNF32	-0.134857	4.6939363	-3.142715	0.0018088	0.0046795	-2.086645
AC145676.2	0.6100793	0.8484445	3.1426237	0.0018093	0.0046804	-2.086922
FLJ31104	-0.375461	3.0194569	-3.142613	0.0018094	0.0046804	-2.086956
RAI1-AS1	-0.218276	-1.392656	-3.142533	0.0018099	0.0046813	-2.087197
CTD-2014B1	-0.408259	-0.953713	-3.142394	0.0018107	0.0046832	-2.087621
RP11-120K2	0.3499659	-1.155594	3.1421803	0.001812	0.0046862	-2.08827
AC010878.3	-0.311252	-1.118452	-3.142083	0.0018126	0.0046873	-2.088567
AC144568.4	-0.467609	-0.474229	-3.141997	0.0018131	0.0046884	-2.088828
PDIA4	-0.05379	7.1180317	-3.141734	0.0018147	0.0046921	-2.089626
ACBD5	-0.06935	6.4441812	-3.141632	0.0018153	0.0046934	-2.089937
RP11-603B2	0.7407481	2.2503249	3.141577	0.0018156	0.0046939	-2.090103
MSANTD3	0.0637405	5.8093229	3.1415465	0.0018158	0.004694	-2.090196
KBTBD2	0.0346355	6.2176494	3.1415263	0.0018159	0.004694	-2.090257
AP001601.2	-0.401857	-1.125905	-3.141379	0.0018168	0.004696	-2.090704
SLC22A9	-0.323739	6.0757027	-3.141293	0.0018173	0.004697	-2.090965
TTC30A	-0.114677	5.3585044	-3.141107	0.0018185	0.0046996	-2.091531
TMED9	-0.052578	6.9665045	-3.141012	0.0018191	0.0047007	-2.09182

CTAGE3P	0.5658456	1.0003645	3.1399832	0.0018253	0.0047165	-2.094945
AC104699.1	0.4960324	-0.676352	3.1399279	0.0018256	0.004717	-2.095113
RP11-128N1	0.4859112	-0.386115	3.1399002	0.0018258	0.0047171	-2.095197
LINC00462	0.4024198	-1.100783	3.1392204	0.0018299	0.0047275	-2.097262
AC004076.5	0.3694589	2.9778272	3.139109	0.0018306	0.0047289	-2.0976
RP11-47502	-0.687347	1.6818218	-3.139089	0.0018307	0.0047289	-2.097662
NDRG3	0.059324	6.1074026	3.1390102	0.0018312	0.0047298	-2.0979
LRP6	-0.071368	6.3456057	-3.138657	0.0018333	0.004735	-2.098972
LOH12CR1	-0.07835	5.5457096	-3.138526	0.0018341	0.0047366	-2.099371
PLA2G12A	-0.062539	6.1752237	-3.138513	0.0018342	0.0047366	-2.099411
RP11-94D20	-0.364605	-1.118663	-3.138492	0.0018344	0.0047366	-2.099474
CTD-2235C1	0.5239166	0.8114742	3.1384411	0.0018347	0.004737	-2.099627
TBC1D3J	-0.195518	-1.435302	-3.138195	0.0018362	0.0047406	-2.100374
RP11-446H1	0.4626747	-0.569842	3.1380797	0.0018369	0.0047421	-2.100724
RANBP3	-0.039938	6.3437236	-3.137723	0.001839	0.0047474	-2.101808
LRRC59	-0.041686	6.7492437	-3.137641	0.0018395	0.0047483	-2.102057
RP11-1023L	0.1846171	4.2461656	3.1371729	0.0018424	0.0047554	-2.103477
RP11-69L16	-0.489716	0.1221781	-3.136903	0.0018441	0.0047593	-2.104297
DDX11L16	-0.176034	-1.454969	-3.13665	0.0018456	0.004763	-2.105062
NUDT13	-0.138286	5.2461851	-3.136627	0.0018457	0.004763	-2.105133
OR1L8	0.3009244	-1.151621	3.1364332	0.0018469	0.0047656	-2.105721
FABP6	0.7613741	0.4825754	3.1364173	0.001847	0.0047656	-2.105769
MATN4	0.6027357	0.7531798	3.1363728	0.0018473	0.004766	-2.105904
CLEC2L	0.8522639	0.6723696	3.1361827	0.0018485	0.0047685	-2.106481
NOP14-AS1	-0.068465	5.5923162	-3.136169	0.0018486	0.0047685	-2.106522
LINC01268	-0.244842	4.5652927	-3.136122	0.0018488	0.004769	-2.106664
OTOL1	-0.263942	-1.316755	-3.135967	0.0018498	0.0047711	-2.107135
LSG1	-0.035084	6.230831	-3.135229	0.0018543	0.0047824	-2.109373
ACOX3	-0.071305	6.0808728	-3.135199	0.0018545	0.0047826	-2.109465
RP11-236L1	0.5397264	1.5122194	3.1351528	0.0018548	0.004783	-2.109605
CASP10	0.059451	5.8336783	3.1349196	0.0018562	0.0047863	-2.110312
AC133528.2	-0.104669	5.1812999	-3.134836	0.0018568	0.0047873	-2.110566
PI4KAP1	0.2497191	4.7080342	3.1347007	0.0018576	0.0047888	-2.110976
SPTAN1	-0.036754	6.9425346	-3.1347	0.0018576	0.0047888	-2.110978
CTD-2235C1	-0.38393	-0.919618	-3.134426	0.0018593	0.0047928	-2.111807
RBM44	0.3965244	2.6661097	3.1340178	0.0018618	0.004799	-2.113046
RP11-746P2	0.4968767	-0.618447	3.1339221	0.0018624	0.0048002	-2.113336
MNAT1	-0.060839	5.7240295	-3.133469	0.0018652	0.0048071	-2.114711
SOX21-AS1	0.5370401	-0.532211	3.1333357	0.001866	0.0048089	-2.115114
RP11-19D2.	-0.659089	3.6613217	-3.133285	0.0018663	0.0048093	-2.115266
RPA4	0.5322904	0.4023384	3.1331047	0.0018675	0.0048119	-2.115814
TAC1	0.4115327	-1.108448	3.1329253	0.0018686	0.0048144	-2.116358
PCDHA6	0.6927859	0.0570507	3.1323878	0.0018719	0.0048226	-2.117987
LGALS9C	0.7465947	1.6778584	3.1321215	0.0018736	0.0048266	-2.118794
KISS1	-0.333127	4.3974089	-3.132055	0.001874	0.0048273	-2.118994
CTD-250301	-0.586381	0.8868127	-3.131404	0.001878	0.0048374	-2.120967
AC073321.4	-0.667451	0.1037514	-3.131202	0.0018793	0.0048401	-2.121578
RNA5SP18	0.5088887	-0.619671	3.1311906	0.0018794	0.0048401	-2.121614
THPO	-0.239033	5.821392	-3.131117	0.0018798	0.0048408	-2.121837
ZMYM6	-0.045314	5.6055272	-3.131084	0.00188	0.0048408	-2.121937
EXOC6	0.0514968	6.0114541	3.1310815	0.00188	0.0048408	-2.121944

RP11-128N1	0.4922885	-0.334237	3.1310541	0.0018802	0.0048409	-2.122027
CTD-2368P2	0.2408147	4.4343874	3.1309454	0.0018809	0.0048419	-2.122356
AMZ2P1	-0.080933	5.3488381	-3.130938	0.0018809	0.0048419	-2.122378
CNN2P1	-0.544562	2.1289237	-3.130928	0.001881	0.0048419	-2.122409
LPAL2	-0.243986	4.9472311	-3.130714	0.0018823	0.0048449	-2.123056
CYP3A43	-0.539295	4.1678106	-3.130699	0.0018824	0.0048449	-2.123102
ST3GAL4-AS	0.1955948	4.5898224	3.1303845	0.0018844	0.0048497	-2.124055
PRMT3	0.0703715	5.6986241	3.1301401	0.0018859	0.0048532	-2.124795
FGF10	0.5172731	-0.69088	3.1298963	0.0018874	0.0048568	-2.125534
CALN1	0.6103767	-0.277446	3.1298763	0.0018876	0.0048568	-2.125594
TRBV12-5	0.3486739	-1.126394	3.1298412	0.0018878	0.004857	-2.1257
AC007879.7	0.4846945	-0.284292	3.1293309	0.001891	0.0048649	-2.127245
RP11-573M3	0.4628804	-0.449244	3.1283812	0.0018969	0.0048799	-2.13012
SGSM2	0.0692211	6.0766926	3.1282062	0.001898	0.0048824	-2.13065
RP11-156K1	0.407221	4.6504443	3.1281471	0.0018984	0.004883	-2.130828
ZNF35	0.1355104	4.9310814	3.1275184	0.0019024	0.0048928	-2.132731
VSTM1	0.4163973	-0.833447	3.1272624	0.001904	0.0048966	-2.133505
ECHDC1	-0.093892	6.3448245	-3.126893	0.0019063	0.0049023	-2.134623
REM2	0.4322496	1.9473632	3.1267008	0.0019075	0.0049051	-2.135204
AL590762.7	-0.498126	-0.411578	-3.12668	0.0019077	0.0049051	-2.135267
CTC-542B22	0.4683471	2.1188764	3.1262918	0.0019101	0.004911	-2.136441
LEPROTL1	0.0626123	6.1864395	3.1259858	0.0019121	0.0049157	-2.137367
SLC34A3	0.6235668	1.004293	3.1259535	0.0019123	0.0049158	-2.137465
SVOP	-0.564167	2.8875245	-3.125635	0.0019143	0.0049207	-2.138428
CTD-3064H1	0.5468617	-0.40888	3.1256132	0.0019144	0.0049207	-2.138494
AC073115.6	0.3402959	-1.192592	3.1255469	0.0019148	0.0049214	-2.138694
ZNF787	-0.065624	6.4118635	-3.125417	0.0019157	0.0049232	-2.139086
ELP5	-0.064793	6.1771773	-3.124691	0.0019203	0.0049347	-2.141283
AC090587.2	0.4477684	2.9614013	3.1246498	0.0019205	0.004935	-2.141407
RCN2	0.0614374	6.1090126	3.1245289	0.0019213	0.0049367	-2.141772
AC017104.3	0.4778532	-0.265862	3.1244293	0.0019219	0.0049379	-2.142073
CTB-36H16.	-0.174189	4.4909309	-3.124308	0.0019227	0.0049396	-2.142438
CTD-3195I5	0.3555575	-1.049839	3.1242694	0.0019229	0.0049399	-2.142557
OR1E1	-0.132495	-1.490564	-3.124221	0.0019233	0.0049401	-2.142704
RP11-750B1	-0.261286	4.7999186	-3.12421	0.0019233	0.0049401	-2.142737
MAST1	-0.335391	4.3541962	-3.12403	0.0019245	0.0049427	-2.143281
EYS	0.2270297	4.5943468	3.1239231	0.0019252	0.0049441	-2.143603
SERPINA11	-0.337796	6.4035476	-3.123753	0.0019262	0.0049466	-2.144117
RP11-336A1	-0.611793	1.3273134	-3.123643	0.0019269	0.004948	-2.14445
TTC7B	0.110989	5.7359559	3.123459	0.0019281	0.0049507	-2.145006
RP11-83B20	0.4466217	-0.593832	3.1234225	0.0019283	0.0049509	-2.145116
ACTR1A	-0.046044	6.5869832	-3.123367	0.0019287	0.0049515	-2.145283
RP11-506B6	0.5047995	-0.206877	3.1231971	0.0019298	0.0049539	-2.145797
STAR	0.6807366	0.9713822	3.1231779	0.0019299	0.0049539	-2.145855
RP11-88H10	-0.199198	-1.402398	-3.123099	0.0019304	0.0049548	-2.146094
VPRBP	-0.04436	6.1183988	-3.122771	0.0019325	0.0049599	-2.147085
RP1-53C18.	-0.395831	-1.057044	-3.122493	0.0019343	0.0049641	-2.147924
RP11-338K1	0.5556224	0.417784	3.1222342	0.0019359	0.004968	-2.148706
RP11-181K1	-0.266859	-1.276795	-3.122215	0.0019361	0.004968	-2.148763
PYCARD	0.174617	5.6676449	3.1221469	0.0019365	0.0049687	-2.14897
RP11-342M1	-0.316593	-1.191679	-3.1221	0.0019368	0.0049692	-2.149112

RPL18A	-0.074081	6.923815	-3.122025	0.0019373	0.00497	-2.149337
RP11-429P3	0.5179845	0.0995853	3.1220014	0.0019374	0.0049701	-2.14941
NPPB	0.6130358	-0.334422	3.1213887	0.0019414	0.0049798	-2.15126
KCTD8	0.449178	-0.998299	3.1210019	0.0019439	0.0049858	-2.152428
CDRT15	0.5193447	-0.144547	3.1207505	0.0019455	0.0049896	-2.153187
RPS28P1	-0.223955	-1.356288	-3.120707	0.0019458	0.0049897	-2.153319
FKSG62	-0.730237	1.2579163	-3.120703	0.0019458	0.0049897	-2.15333
LINC01280	-0.233867	-1.351933	-3.120465	0.0019473	0.0049933	-2.154048
TRIP13	0.1841664	5.1631254	3.1203133	0.0019483	0.0049955	-2.154507
GSTM5	0.7047864	2.8763226	3.1196441	0.0019526	0.0050062	-2.156527
HMHB1	-0.374631	-0.968228	-3.119481	0.0019537	0.0050085	-2.157019
SKA2	-0.06384	6.035951	-3.119375	0.0019543	0.0050099	-2.157339
AC079807.2	-0.145664	4.0058115	-3.119182	0.0019556	0.0050126	-2.157922
C19orf44	-0.07257	5.4662228	-3.119169	0.0019557	0.0050126	-2.157962
MT2A	-0.159744	6.8260577	-3.119004	0.0019567	0.0050148	-2.15846
RP11-305D1	0.4396732	-0.596575	3.1189957	0.0019568	0.0050148	-2.158485
DUSP10	-0.098233	6.2014092	-3.118975	0.0019569	0.0050148	-2.158547
ASIC5	-0.630948	0.8421436	-3.118624	0.0019592	0.0050203	-2.159606
RP11-16E18	-0.304876	-1.23934	-3.118549	0.0019597	0.0050212	-2.159833
AC008592.8	-0.635342	1.4105282	-3.118437	0.0019604	0.0050227	-2.16017
POLR3K	-0.07067	5.7300594	-3.118413	0.0019606	0.0050227	-2.160241
FAM149B1	-0.048895	6.0860432	-3.118057	0.0019629	0.0050283	-2.161316
RP5-1074L1	-0.188014	3.9123691	-3.117683	0.0019653	0.0050341	-2.162446
UNK	-0.049707	6.0812715	-3.117556	0.0019661	0.0050359	-2.162827
RPRD1A	0.0443252	6.2670579	3.1174889	0.0019666	0.0050367	-2.163031
FARSA-AS1	-0.380808	-0.98804	-3.117359	0.0019674	0.0050384	-2.163423
C8orf89	0.4111831	-0.858507	3.1173442	0.0019675	0.0050384	-2.163467
AP000345.1	0.3251418	-1.210022	3.1171205	0.001969	0.0050417	-2.164142
CTD-2228K2	-0.138144	-1.474007	-3.115754	0.0019779	0.0050642	-2.168263
CTD-2383M3	-0.523565	0.8779683	-3.115604	0.0019788	0.0050663	-2.168714
IGLV2-5	0.3989684	-1.105725	3.1155216	0.0019794	0.0050674	-2.168963
AC138472.4	-0.45194	-0.814547	-3.115401	0.0019802	0.005069	-2.169326
NR1H4	-0.149431	6.4615032	-3.115376	0.0019803	0.0050691	-2.169402
LURAP1	0.489347	2.4214042	3.1149563	0.0019831	0.0050758	-2.170667
RFXAP	0.1262665	4.6299289	3.1146393	0.0019852	0.0050807	-2.171623
DNAI1	0.6949443	1.2743323	3.1145748	0.0019856	0.0050815	-2.171817
ARHGAP26	0.0790502	5.8019968	3.1142568	0.0019877	0.0050864	-2.172776
SMIM6	0.449646	4.5117091	3.1142192	0.0019879	0.0050867	-2.172889
KIAA0430	-0.044028	6.2997271	-3.11389	0.0019901	0.0050919	-2.17388
RP11-539I5	0.4727631	4.1604933	3.1138061	0.0019906	0.0050929	-2.174134
SAFB2	-0.037726	6.3727093	-3.113775	0.0019908	0.0050931	-2.174228
WDR27	0.1138191	5.4044722	3.1136142	0.0019919	0.0050955	-2.174712
ERICH2	-0.265711	4.3568213	-3.113436	0.0019931	0.0050981	-2.175249
ZBTB42	-0.080935	5.7566066	-3.113036	0.0019957	0.0051043	-2.176454
ST14	0.1843517	6.1474829	3.1130264	0.0019958	0.0051043	-2.176483
RP11-96H17	0.574156	-0.73271	3.1128547	0.0019969	0.0051068	-2.177
RP11-94B19	-0.492744	-0.843884	-3.112662	0.0019982	0.0051097	-2.177579
FAM101B	0.0912136	5.5931989	3.1124175	0.0019998	0.0051135	-2.178317
OXCT1-AS1	0.5446527	-0.397116	3.1121913	0.0020013	0.0051169	-2.178998
RP5-857K21	-0.281982	-1.22118	-3.111835	0.0020036	0.0051226	-2.180072
CA1	0.707326	0.3072862	3.1115889	0.0020053	0.0051264	-2.180812

RP1-111B22	0.5277847	0.3311263	3.1112291	0.0020076	0.0051321	-2.181895
CSNK2B-LY6	0.5130526	1.0089088	3.1111292	0.0020083	0.0051335	-2.182196
FAM21EP	-0.262003	3.0102261	-3.11101	0.0020091	0.0051351	-2.182555
RP11-672L1	-0.326472	3.5689069	-3.110712	0.0020111	0.0051398	-2.183453
RP1-228H13	0.1378122	4.629719	3.1105674	0.002012	0.0051419	-2.183887
DAND5	0.6137563	1.3526885	3.1102341	0.0020142	0.0051472	-2.18489
STX17-AS1	-0.265693	3.7752748	-3.109959	0.0020161	0.0051515	-2.185718
RP11-752G1	-0.344713	2.9493573	-3.10994	0.0020162	0.0051515	-2.185774
HERC2P10	0.4907568	-0.270218	3.1095248	0.002019	0.0051582	-2.187025
AF127577.1	-0.323734	-1.090108	-3.109465	0.0020194	0.0051588	-2.187205
PBX2P1	0.5271164	0.3804562	3.1094361	0.0020195	0.005159	-2.187292
RP11-358H9	-0.413289	-0.786506	-3.109368	0.00202	0.0051598	-2.187497
KLRD1	0.2441793	4.3516684	3.1091505	0.0020214	0.0051631	-2.188152
EEF1DP3	0.5743538	0.9703989	3.1090566	0.0020221	0.005164	-2.188434
GHRL	0.2995765	3.2400687	3.1090524	0.0020221	0.005164	-2.188447
RP11-506M1	0.5320521	2.0879085	3.108738	0.0020242	0.005169	-2.189392
SUPT16H	-0.035811	6.4878126	-3.1086	0.0020251	0.005171	-2.189809
ZBED5	0.0505006	5.9017792	3.1082235	0.0020276	0.0051771	-2.19094
CSTB	-0.066564	6.7590116	-3.108155	0.0020281	0.0051779	-2.191145
CTD-2015H3	-0.546326	-0.334619	-3.108079	0.0020286	0.0051788	-2.191374
NUDT15	0.060055	5.8313015	3.1079594	0.0020294	0.0051805	-2.191735
DGAT1	-0.077174	6.5652089	-3.107935	0.0020296	0.0051806	-2.191808
RP11-146I2	0.4888008	-0.609807	3.1079088	0.0020297	0.0051806	-2.191887
AC012593.1	-0.150282	-1.462046	-3.107818	0.0020303	0.0051816	-2.19216
C19orf54	-0.059252	5.7576943	-3.107811	0.0020304	0.0051816	-2.192181
LRRC37A16F	0.1651299	5.0218489	3.1073847	0.0020332	0.0051885	-2.193463
RP11-711C1	-0.45134	-0.96867	-3.107321	0.0020337	0.0051892	-2.193654
PPIAP17	-0.182927	-1.454331	-3.107218	0.0020344	0.0051906	-2.193964
RP11-700A2	0.5110799	0.0900674	3.1072014	0.0020345	0.0051906	-2.194014
AP000688.1	-0.331136	-1.181231	-3.10714	0.0020349	0.0051913	-2.1942
MPV17	0.0549692	6.1375174	3.1069281	0.0020363	0.0051945	-2.194836
DMRT1	0.3371062	-1.198112	3.1068239	0.002037	0.0051959	-2.195149
TULP1	0.4086941	-0.860366	3.1065368	0.0020389	0.0052005	-2.196012
RNF217-AS1	-0.509224	2.4439535	-3.106399	0.0020399	0.0052025	-2.196426
RP11-760H2	-0.13269	5.135435	-3.106379	0.00204	0.0052025	-2.196487
SLC6A15	0.6423283	-0.576133	3.1061487	0.0020415	0.0052061	-2.197179
C10orf25	-0.103012	5.1042537	-3.10593	0.002043	0.0052094	-2.197837
RP3-433F14	-0.22836	-1.390115	-3.105906	0.0020432	0.0052095	-2.19791
MIR17HG	0.341427	3.7540062	3.1058253	0.0020437	0.0052105	-2.198152
FOXN3P1	-0.15878	-1.43832	-3.105279	0.0020474	0.0052195	-2.199792
ZNF136	0.0840005	5.295069	3.105043	0.002049	0.0052232	-2.200503
RNU6-1053F	0.4025095	-0.704696	3.1043928	0.0020534	0.0052341	-2.202456
RNU2-28P	-0.238027	-1.290964	-3.103813	0.0020573	0.0052437	-2.204198
MTX1P1	0.3294335	4.618857	3.1035405	0.0020592	0.005248	-2.205017
VN1R87P	-0.452289	-0.871602	-3.103358	0.0020604	0.0052508	-2.205564
RBM25	0.0420593	6.3312285	3.1032257	0.0020613	0.0052528	-2.205962
PMS2	0.1145909	5.3204777	3.1031361	0.0020619	0.0052539	-2.206231
ACTR3C	-0.126346	5.3377651	-3.10291	0.0020634	0.0052575	-2.206909
CALML6	-0.443495	3.1511343	-3.102852	0.0020638	0.0052581	-2.207083
RP4-755D9.	0.6074702	2.2190512	3.1027898	0.0020643	0.0052588	-2.207271
RP11-67L3.	0.3840883	3.0241821	3.102642	0.0020653	0.005261	-2.207715

MAPKAPK2	-0.05284	6.7086173	-3.102525	0.0020661	0.0052627	-2.208066
FAM173B	-0.058724	5.732282	-3.102271	0.0020678	0.0052667	-2.20883
AC078899.1	0.5239949	0.2399797	3.1022016	0.0020683	0.0052675	-2.209037
ATAD1	-0.053142	6.2287667	-3.102159	0.0020685	0.0052679	-2.209165
PAGE5	-0.790543	2.1118427	-3.10164	0.0020721	0.0052766	-2.210723
AC074212.5	-0.162777	4.5352113	-3.101302	0.0020744	0.0052821	-2.211739
AP000695.4	0.5487557	1.6451752	3.1011761	0.0020753	0.0052839	-2.212116
TTLL11	-0.094626	5.0341379	-3.100899	0.0020771	0.0052883	-2.212946
RHOD	-0.107982	6.2726743	-3.100651	0.0020788	0.0052923	-2.213691
CLCC1	-0.058111	5.9796057	-3.100444	0.0020803	0.0052955	-2.214313
RP11-770E5	-0.270801	-1.30669	-3.100397	0.0020806	0.005296	-2.214453
CBX5	0.0580948	6.3963438	3.1001533	0.0020822	0.0052998	-2.215185
SORCS3	0.5638272	-0.764338	3.1000705	0.0020828	0.0053009	-2.215434
METTL25	0.0682911	5.068188	3.1000175	0.0020832	0.0053015	-2.215593
RP11-72L22	-0.164601	-1.47316	-3.099981	0.0020834	0.0053017	-2.215701
LINC01415	0.5385716	1.0603699	3.0997902	0.0020847	0.0053047	-2.216275
KPNA1	-0.042381	6.3110459	-3.099603	0.002086	0.0053076	-2.216836
SIPA1L1	-0.051224	6.2832579	-3.099002	0.0020901	0.0053177	-2.218639
OTUD1	0.0656948	5.6512147	3.0989846	0.0020903	0.0053177	-2.218691
ICAM5	0.7241525	1.7869874	3.0987003	0.0020922	0.0053219	-2.219544
TNNI1	0.5223219	4.750553	3.0986992	0.0020922	0.0053219	-2.219547
TCF3	0.0555913	6.2456722	3.0983061	0.0020949	0.0053284	-2.220726
MIR4292	0.5295857	1.5357886	3.0982024	0.0020957	0.0053298	-2.221037
PLEKHG1	0.1114174	5.5901273	3.0981866	0.0020958	0.0053298	-2.221085
ABHD17C	0.1087347	5.6144532	3.0981066	0.0020963	0.0053308	-2.221325
AC011330.1	0.5351578	0.8018185	3.0980827	0.0020965	0.0053309	-2.221396
FHAD1	0.4592751	3.4373709	3.0980343	0.0020968	0.0053313	-2.221542
MPHOSPH10	-0.045767	6.2219066	-3.097967	0.0020973	0.0053321	-2.221742
KLHDC2	-0.072696	6.3319884	-3.097822	0.0020983	0.0053343	-2.222177
IGHV3OR16-	0.3139785	-1.215323	3.0972499	0.0021022	0.005344	-2.223893
TMEM254-AS	-0.188683	4.407943	-3.097136	0.002103	0.0053456	-2.224233
CTC-465D4.	0.3932836	-1.109292	3.0967507	0.0021057	0.005352	-2.22539
SCLT1	0.0941676	5.1616741	3.0965355	0.0021072	0.0053554	-2.226035
FAM196A	0.5816772	0.6215922	3.0965152	0.0021073	0.0053554	-2.226096
SYT1	0.6369379	3.8648065	3.0963603	0.0021084	0.0053578	-2.22656
RP11-356B1	-0.380316	3.0422734	-3.096118	0.0021101	0.0053617	-2.227285
PDLIM2	-0.088083	5.9142947	-3.095361	0.0021153	0.0053747	-2.229556
AC073333.8	0.4514594	-0.550727	3.0949471	0.0021182	0.0053816	-2.230795
AC005682.6	0.4147865	2.9190825	3.0949105	0.0021184	0.0053819	-2.230904
IL6ST	-0.05747	6.8696285	-3.094872	0.0021187	0.0053822	-2.23102
RP1-151B14	0.484371	-0.14079	3.0946935	0.00212	0.0053849	-2.231554
TMEM39B	-0.05026	5.7656123	-3.09467	0.0021201	0.005385	-2.231626
LRRFIP1	-0.050241	6.5145356	-3.094632	0.0021204	0.0053853	-2.23174
SLPI	0.4253139	6.0732207	3.0942673	0.0021229	0.0053914	-2.232831
RP11-1033H	-0.185133	-1.443408	-3.093828	0.002126	0.0053988	-2.234145
NUTF2	-0.059374	6.491916	-3.093613	0.0021275	0.0054022	-2.23479
RALYL	0.7193286	0.1141078	3.0934497	0.0021286	0.0054047	-2.235279
C9orf47	0.6196787	1.2729978	3.0933766	0.0021292	0.0054053	-2.235498
RP11-45901	-0.569497	-0.344581	-3.093373	0.0021292	0.0054053	-2.235509
AC113192.1	0.2955106	-1.160064	3.0932815	0.0021298	0.0054066	-2.235783
MTL5	0.2971167	4.6513716	3.0927084	0.0021338	0.0054164	-2.237499

RP11-91A18	-0.149186	-1.474164	-3.09263	0.0021344	0.0054174	-2.237733
AC134882.2	-0.137795	-1.492192	-3.092487	0.0021354	0.0054195	-2.238161
GZMB	0.2447355	4.3498824	3.0924702	0.0021355	0.0054195	-2.238212
CTD-2260A1	0.3723713	2.7893044	3.0924471	0.0021357	0.0054195	-2.238281
RP11-66N11	-0.314845	-1.1552	-3.091979	0.0021389	0.0054275	-2.239681
RP11-454P7	-0.282386	-1.304889	-3.091935	0.0021393	0.0054279	-2.239814
LAPTM4B	0.120569	6.5756007	3.09154	0.002142	0.0054346	-2.240996
LLNLR-304A	0.4427199	-0.572672	3.0913914	0.0021431	0.0054368	-2.241441
LMO4	-0.086366	6.1032774	-3.091196	0.0021444	0.00544	-2.242026
DLK2	0.2184322	4.3909043	3.0907844	0.0021473	0.0054469	-2.243257
TADA2A	-0.052153	5.6566816	-3.090665	0.0021482	0.0054487	-2.243613
RP11-501C1	0.5298867	-0.798169	3.0904503	0.0021497	0.0054521	-2.244257
TUFT1	0.0939583	5.8282583	3.0903488	0.0021504	0.0054536	-2.24456
ZSCAN32	0.1599748	4.3318943	3.090098	0.0021522	0.0054574	-2.245311
RP5-849H19	-0.387695	5.1696173	-3.090091	0.0021522	0.0054574	-2.245331
CDK16	0.0507482	6.3912305	3.0899297	0.0021534	0.0054597	-2.245814
SFTA1P	-0.526901	2.4880438	-3.089922	0.0021534	0.0054597	-2.245836
ANO6	-0.05707	6.6627419	-3.089552	0.0021561	0.005466	-2.246945
PRH1-PRR4	0.5153765	0.89966	3.0894843	0.0021565	0.0054668	-2.247146
SPIC	0.6401996	1.2763528	3.0894635	0.0021567	0.0054668	-2.247208
ARIH1	-0.042799	6.3508924	-3.08893	0.0021605	0.005476	-2.248803
SPESP1	0.6883176	2.2585543	3.0887657	0.0021616	0.0054786	-2.249295
DIABLO	0.1463184	5.4195325	3.0885093	0.0021634	0.0054828	-2.250062
FAM86LP	-0.229445	-1.340379	-3.088486	0.0021636	0.0054828	-2.250131
AES	-0.055019	6.9131088	-3.087778	0.0021686	0.0054952	-2.252248
ARF1	-0.044849	7.1558431	-3.087717	0.0021691	0.0054959	-2.252431
RP11-295G2	0.5887624	1.3418382	3.0875176	0.0021705	0.0054991	-2.253026
NUDT18	-0.108011	5.341132	-3.087072	0.0021737	0.0055068	-2.254359
FLOT2	-0.056884	6.692576	-3.086952	0.0021745	0.0055086	-2.254716
CCDC54	-0.465738	-0.416177	-3.086714	0.0021762	0.0055125	-2.255428
RP11-144L1	0.5467476	0.714518	3.0864825	0.0021779	0.0055163	-2.25612
RP1-197B17	-0.435761	2.4671809	-3.086402	0.0021784	0.0055174	-2.256361
GNAQ	-0.049557	6.3599781	-3.086313	0.0021791	0.0055186	-2.256627
KRT8P13	0.5000423	0.0035576	3.0862784	0.0021793	0.0055188	-2.25673
CTD-2535L2	-0.225753	-1.356491	-3.085956	0.0021816	0.0055238	-2.257692
ST3GAL2	-0.048397	6.16996	-3.08595	0.0021817	0.0055238	-2.257709
FAM107A	-0.159363	5.3534597	-3.085943	0.0021817	0.0055238	-2.257733
RP11-530C5	-0.308795	-1.183169	-3.085438	0.0021853	0.0055325	-2.259239
LRP5	-0.065232	6.8045821	-3.085387	0.0021857	0.0055331	-2.259393
RP11-195L1	-0.260601	-1.259414	-3.085172	0.0021872	0.0055366	-2.260034
SEMA3G	0.1434783	5.6044868	3.0851312	0.0021875	0.0055369	-2.260157
FAM171A1	0.2375394	5.9438975	3.0848775	0.0021893	0.0055411	-2.260914
GSG1	0.4096976	-0.807898	3.0847819	0.00219	0.0055425	-2.2612
AC092574.1	0.3920911	-0.801305	3.0841267	0.0021947	0.005554	-2.263156
ALG9	-0.066566	5.6547787	-3.083872	0.0021966	0.0055583	-2.263917
PRDM12	-0.524529	1.9469163	-3.083585	0.0021986	0.0055631	-2.264774
RP11-43N5.	0.5173612	-0.299018	3.0835252	0.0021991	0.0055638	-2.264952
ELANE	0.5901853	0.1129506	3.0833194	0.0022006	0.0055672	-2.265566
KCTD1	0.1226566	5.2336781	3.0831795	0.0022016	0.005569	-2.265984
RP11-242F2	-0.1577	-1.467748	-3.083176	0.0022016	0.005569	-2.265994
AC016831.3	-0.124258	-1.493604	-3.08292	0.0022034	0.0055733	-2.266758

CTD-220702	0.2694488	-1.260694	3.082752	0.0022046	0.0055757	-2.26726
AC007228.9	-0.346733	4.2121092	-3.082731	0.0022048	0.0055757	-2.267322
DICER1	0.061708	6.2388286	3.0827275	0.0022048	0.0055757	-2.267333
RP11-66N11	-0.216519	4.1446583	-3.082689	0.0022051	0.005576	-2.267448
PUM2	-0.03432	6.6194151	-3.082596	0.0022058	0.0055769	-2.267725
KRT4	0.582816	-0.666587	3.0825957	0.0022058	0.0055769	-2.267726
RP11-545D2	-0.455024	-0.240486	-3.082391	0.0022073	0.0055803	-2.268336
MCM10	0.2438044	4.8886806	3.0817204	0.0022121	0.0055922	-2.270338
RP11-262I2	0.5031452	-0.627689	3.0816644	0.0022125	0.0055928	-2.270505
RP11-61I13	-0.293845	3.7674994	-3.081373	0.0022146	0.0055977	-2.271374
MIR200A	0.2416679	-1.336227	3.0812679	0.0022154	0.0055993	-2.271688
LA16c-395F	0.4137492	-0.898395	3.0810655	0.0022169	0.0056026	-2.272292
RP11-217E1	-0.24682	-1.387053	-3.080679	0.0022197	0.0056093	-2.273445
RP11-566E1	0.2301064	3.9410456	3.0801406	0.0022236	0.0056188	-2.275051
CNN1	0.2209885	5.0438344	3.0799135	0.0022252	0.0056226	-2.275728
HSD17B12	-0.061346	6.5304058	-3.079695	0.0022268	0.0056262	-2.276378
TGM5	0.4104336	-0.998427	3.0789818	0.002232	0.005639	-2.278506
DDX50P1	0.5313454	1.1398116	3.0787647	0.0022336	0.0056426	-2.279153
CHD6	0.0571957	6.07896	3.0786336	0.0022346	0.0056442	-2.279544
RP11-540D1	-0.240172	-1.313557	-3.078631	0.0022346	0.0056442	-2.279553
RP11-627G1	0.3959329	-0.871546	3.0786157	0.0022347	0.0056442	-2.279597
MAVS	-0.051	6.545571	-3.078532	0.0022353	0.0056453	-2.279846
RP11-322E1	0.5060799	2.0773476	3.0779108	0.0022399	0.0056564	-2.281698
NCOA6	0.0461899	6.2764033	3.0776958	0.0022414	0.0056597	-2.282339
AP4B1	-0.054259	5.7277913	-3.077689	0.0022415	0.0056597	-2.282359
RP11-1060J	0.5611252	2.4669861	3.0771972	0.0022451	0.0056685	-2.283825
C5orf47	0.516743	0.1612951	3.0771384	0.0022455	0.0056692	-2.284
AC008746.5	0.5531464	1.108389	3.0771142	0.0022457	0.0056692	-2.284072
AC092652.1	-0.393084	-1.142865	-3.076803	0.002248	0.0056746	-2.285
HHAT	-0.123457	5.5847004	-3.076737	0.0022485	0.0056754	-2.285197
AC067945.3	0.4014976	-0.74877	3.0766692	0.002249	0.0056763	-2.285397
CDH15	0.7754905	2.9303003	3.0765471	0.0022499	0.0056782	-2.285761
AC069257.6	0.3011185	-1.141789	3.0764896	0.0022503	0.0056787	-2.285933
UPF3B	0.0630876	5.7230281	3.0764789	0.0022504	0.0056787	-2.285964
ACTBL2	0.4639957	-0.787749	3.0762818	0.0022518	0.0056819	-2.286552
RP11-2E11.	0.3714209	-1.021257	3.0759645	0.0022542	0.0056874	-2.287497
CDR1	0.3279075	-1.234311	3.0755492	0.0022572	0.0056946	-2.288733
RP11-147L1	-0.05203	6.0960711	-3.07554	0.0022573	0.0056946	-2.288762
AP000362.1	-0.383002	-1.013331	-3.075375	0.0022585	0.0056972	-2.289251
LINC01048	-0.390344	-1.182727	-3.074995	0.0022613	0.0057039	-2.290383
AHSA1	-0.049309	6.5551235	-3.074614	0.0022641	0.0057106	-2.291517
ZBTB37	-0.064695	5.9017659	-3.074505	0.0022649	0.0057123	-2.291844
RNY3P16	-0.556655	1.2569195	-3.074385	0.0022658	0.0057141	-2.292199
KLK6	0.5884998	-0.513185	3.0742782	0.0022666	0.0057156	-2.292518
CDC26	-0.073795	5.4286632	-3.074261	0.0022667	0.0057156	-2.29257
SIM2	0.2211821	4.2631951	3.0736416	0.0022713	0.0057268	-2.294413
EIF4EP2	0.5148984	0.9793362	3.0736024	0.0022716	0.0057272	-2.294529
CNN2P3	-0.462249	-0.846393	-3.073514	0.0022723	0.0057284	-2.294792
KCNU1	-0.964633	1.0066521	-3.073172	0.0022748	0.0057344	-2.29581
COL7A1	-0.238257	5.358881	-3.073092	0.0022754	0.0057355	-2.296048
BAATP1	-0.604808	2.9756569	-3.072732	0.0022781	0.0057417	-2.297118

RP11-173M1	-0.210004	4.651595	-3.072718	0.0022782	0.0057417	-2.297161
GBX2	0.5265201	-0.298009	3.0721194	0.0022827	0.0057526	-2.298942
CTD-2540B1	-0.54798	-0.113013	-3.071518	0.0022871	0.0057635	-2.30073
USP41	0.413617	-0.872609	3.0711955	0.0022896	0.0057692	-2.30169
CABS1	-0.168181	-1.445569	-3.070873	0.002292	0.0057748	-2.302649
LINC00304	0.6042386	0.1869127	3.0708015	0.0022925	0.0057758	-2.302861
PCNA	-0.058708	6.4908704	-3.070698	0.0022933	0.0057773	-2.303169
OR2C1	-0.497462	-0.189502	-3.070661	0.0022936	0.0057776	-2.303278
LINC00574	-0.34402	4.1075293	-3.070305	0.0022962	0.005784	-2.304338
LMAN1	-0.057434	6.8152584	-3.070203	0.002297	0.0057851	-2.304642
RALGPS2	-0.078248	6.4116008	-3.070201	0.002297	0.0057851	-2.304647
CEP112	0.1067216	5.2334794	3.070034	0.0022983	0.0057879	-2.305143
CTD-2210P2	-0.308612	-1.21522	-3.069796	0.0023	0.0057916	-2.305851
ACSF3	-0.092243	6.0979937	-3.069793	0.0023001	0.0057916	-2.305861
GP5	0.6397484	0.3624286	3.0688445	0.0023072	0.0058092	-2.308679
RP11-1079K	0.437075	-0.405028	3.0687831	0.0023077	0.00581	-2.308862
SEZ6L2	0.4436168	5.3434521	3.0686667	0.0023085	0.0058118	-2.309208
CCNC	-0.057401	6.3935094	-3.068405	0.0023105	0.0058163	-2.309985
PDSS2	-0.063388	6.1027804	-3.068321	0.0023111	0.0058175	-2.310235
SCUBE2	0.3660317	4.1834257	3.0682985	0.0023113	0.0058175	-2.310302
LEMD1	0.4425139	-0.965222	3.0679174	0.0023142	0.0058244	-2.311434
SNORA34	0.4279166	-0.475796	3.0678546	0.0023147	0.0058252	-2.31162
AC098828.2	-0.589802	1.5783022	-3.067214	0.0023195	0.005837	-2.313523
AC062029.1	-0.133653	4.644835	-3.06714	0.0023201	0.005838	-2.313744
EIF2S2P4	0.3912057	2.285284	3.0670541	0.0023207	0.0058392	-2.313998
HMGN1P17	-0.31377	-1.27167	-3.066402	0.0023256	0.0058512	-2.315936
CCDC96	0.1350577	4.3658811	3.0662083	0.0023271	0.0058545	-2.31651
AC008154.5	-0.435955	-0.913642	-3.06577	0.0023304	0.0058625	-2.317813
RP11-671M2	0.3709343	-0.899666	3.0650925	0.0023356	0.005875	-2.319823
RPL13P12	-0.150499	5.4678336	-3.065011	0.0023362	0.0058761	-2.320064
AC005324.6	-0.19406	-1.447106	-3.064996	0.0023363	0.0058761	-2.320108
RP11-359E8	-0.244678	-1.353594	-3.064752	0.0023382	0.0058804	-2.320834
SNX18P13	-0.227637	-1.341764	-3.064706	0.0023385	0.0058808	-2.32097
PCDHA2	0.5738181	-0.463896	3.0645419	0.0023398	0.0058836	-2.321457
ATP1A1-AS1	-0.115913	4.7043269	-3.064135	0.0023429	0.005891	-2.322664
CTD-2124B8	-0.138182	4.3963686	-3.063757	0.0023458	0.0058976	-2.323787
SNHG19	-0.15599	5.4401526	-3.063748	0.0023459	0.0058976	-2.323812
PAXIP1	0.0655258	5.7730575	3.0636427	0.0023467	0.0058992	-2.324125
CYP27B1	0.3377964	3.935493	3.0631456	0.0023505	0.0059084	-2.3256
CALHM3	0.3591397	-1.113985	3.062276	0.0023571	0.0059247	-2.32818
CD28	0.2103463	4.6030101	3.0618472	0.0023604	0.0059326	-2.329451
RP11-421E1	-0.260062	-1.245861	-3.061649	0.002362	0.005936	-2.33004
TCAF2P1	0.5166192	-0.294134	3.0616311	0.0023621	0.005936	-2.330092
ANO7	0.1977478	4.8188223	3.0615597	0.0023626	0.0059366	-2.330304
CHCHD7	-0.078082	6.0984922	-3.061555	0.0023627	0.0059366	-2.330317
RAB33B	-0.080587	5.6880375	-3.061096	0.0023662	0.0059451	-2.331677
RP11-309L2	0.4913734	0.980363	3.0609995	0.002367	0.0059465	-2.331964
TPD52L1	-0.150954	5.9696592	-3.060863	0.002368	0.0059488	-2.33237
LCORL	0.0756348	5.4931582	3.0607369	0.002369	0.0059508	-2.332743
GULOP	-0.609931	0.6381612	-3.060525	0.0023706	0.0059545	-2.333372
ABHD5	-0.076622	5.7461567	-3.060315	0.0023722	0.0059582	-2.333995

ARPC3P1	0.4268447	1.2562183	3.0601244	0.0023737	0.0059615	-2.334558
SSX5	-0.674023	-0.314516	-3.060053	0.0023743	0.0059624	-2.33477
MTATP6P3	-0.18118	-1.437781	-3.059905	0.0023754	0.0059649	-2.335209
RP13-977J1	0.504364	-0.090879	3.0592741	0.0023803	0.0059767	-2.337078
TUBB4B	-0.059816	6.8559349	-3.059214	0.0023807	0.0059775	-2.337255
SLC39A14	-0.078025	6.9587307	-3.059135	0.0023814	0.0059786	-2.33749
RP11-454E5	0.1866531	3.7587771	3.0589709	0.0023826	0.0059814	-2.337977
REST	0.0501126	6.020263	3.0581736	0.0023888	0.0059965	-2.340338
RP11-314B1	-0.70105	-0.269729	-3.057987	0.0023903	0.0059998	-2.340891
RP11-206L1	0.5853192	1.2934495	3.0579562	0.0023905	0.006	-2.340982
LINC01204	-0.380037	-1.101247	-3.057769	0.002392	0.0060032	-2.341537
RNF141	-0.045358	6.1292618	-3.057662	0.0023928	0.0060049	-2.341852
CTD-255008	-0.5516	0.7783084	-3.057627	0.0023931	0.006005	-2.341957
PPM1N	0.3100731	3.8494833	3.0576114	0.0023932	0.006005	-2.342003
LZTR1	-0.046078	6.3625046	-3.057508	0.002394	0.0060067	-2.34231
SLC22A25	-0.396844	5.3751608	-3.057309	0.0023956	0.0060101	-2.3429
RP11-588G2	-0.493731	-0.314624	-3.057041	0.0023976	0.006015	-2.343693
SORD2P	-0.280398	5.2515321	-3.057011	0.0023979	0.0060151	-2.34378
RP1-12803.	0.3170155	-1.0854	3.0564286	0.0024024	0.0060261	-2.345506
AKR7A2P1	-0.573117	0.4705389	-3.056366	0.0024029	0.0060269	-2.34569
RP11-326C3	-0.217252	-1.377615	-3.056019	0.0024056	0.0060333	-2.346718
MRPL53	-0.078434	5.0774177	-3.055905	0.0024065	0.0060351	-2.347055
RP11-67L3.	0.4353275	2.3295143	3.0555522	0.0024093	0.0060417	-2.3481
RP11-685B1	-0.633621	1.6340247	-3.055373	0.0024107	0.0060448	-2.34863
NOM1	-0.050603	5.9189384	-3.055112	0.0024127	0.0060495	-2.349402
RPS12	-0.068467	7.1752935	-3.055091	0.0024129	0.0060495	-2.349463
RP11-12M5.	0.5015082	-0.47003	3.0549383	0.0024141	0.0060521	-2.349916
SNORA73B	0.5588543	1.8313209	3.0545123	0.0024174	0.00606	-2.351177
TCTEX1D1	0.4994148	3.1909355	3.0540208	0.0024213	0.0060693	-2.352631
PRDM2	0.0565345	5.8471512	3.0539133	0.0024222	0.006071	-2.352949
LSMEM2	0.5014308	0.0785727	3.0537217	0.0024237	0.0060744	-2.353516
INTU	0.1989527	4.8484185	3.0531365	0.0024283	0.0060855	-2.355247
ZRANB1	0.1431109	5.7999574	3.0527747	0.0024311	0.0060923	-2.356316
HEY2	-0.168922	5.2194639	-3.05179	0.0024389	0.0061114	-2.359226
ULK4	-0.145723	5.2959002	-3.051713	0.0024395	0.0061125	-2.359455
ZNF436	0.0750189	5.5335246	3.051115	0.0024443	0.0061239	-2.361223
RAET1L	0.4057418	-0.954586	3.0506542	0.0024479	0.0061325	-2.362585
RHAG	0.4195701	-1.020255	3.050644	0.002448	0.0061325	-2.362615
FOLH1B	-0.777224	3.1977016	-3.050558	0.0024487	0.0061338	-2.362868
HMGA1P2	0.5077751	0.1069919	3.0504861	0.0024493	0.0061348	-2.363081
KNTC1	0.114137	5.6993342	3.0495846	0.0024565	0.0061523	-2.365745
CH17-13I23	0.627355	0.7731651	3.0489415	0.0024616	0.0061648	-2.367644
LNX1-AS2	0.2481735	-1.318335	3.0487045	0.0024635	0.0061691	-2.368344
STK4-AS1	-0.265464	3.4009337	-3.048543	0.0024648	0.0061719	-2.368821
ZFP14	0.084952	5.3724089	3.0484158	0.0024658	0.006174	-2.369197
USP18	0.2297667	5.2709032	3.04803	0.0024689	0.0061813	-2.370336
AAED1	-0.077142	5.6245662	-3.047771	0.002471	0.0061861	-2.371102
RP5-899E9.	0.4260924	2.2494577	3.047732	0.0024713	0.0061865	-2.371216
RP11-153F1	-0.259965	-1.278241	-3.047661	0.0024718	0.0061875	-2.371425
RP1-68D18.	0.4710311	-0.596614	3.0474417	0.0024736	0.0061915	-2.372073
SERINC5	-0.072694	6.6436677	-3.047349	0.0024743	0.0061929	-2.372348

HOXB-AS3	0.577808	1.0948993	3.0470311	0.0024769	0.0061989	-2.373285
POLQ	0.2552194	4.7318586	3.0463884	0.0024821	0.0062112	-2.375182
IGHV3OR16-	0.3298861	-1.177438	3.0463783	0.0024821	0.0062112	-2.375212
RP11-285A1	0.494034	-0.04688	3.0459694	0.0024854	0.006219	-2.376418
RP1-20208.	-0.408253	-0.800117	-3.045923	0.0024858	0.0062195	-2.376556
RP11-243M5	0.585326	-0.149989	3.0455963	0.0024885	0.0062256	-2.377519
AP000235.2	-0.216599	-1.402429	-3.045523	0.002489	0.0062267	-2.377735
FAS	-0.146586	5.6852985	-3.045421	0.0024899	0.0062283	-2.378038
HHLA2	0.7021391	0.2914472	3.0452001	0.0024917	0.0062324	-2.378688
RP11-1082L	0.3561238	-0.970044	3.0450796	0.0024926	0.0062344	-2.379043
XXbac-BPG1	0.3003453	-1.157902	3.0450165	0.0024931	0.0062352	-2.37923
RP11-13402	0.3340777	-1.097339	3.0448139	0.0024948	0.0062387	-2.379827
NR2C2AP	-0.068149	5.952291	-3.044801	0.0024949	0.0062387	-2.379865
RP11-479J7	0.4231875	-0.780924	3.0446785	0.0024959	0.0062408	-2.380227
CXorf56	-0.053549	5.9110626	-3.044524	0.0024971	0.0062428	-2.380683
RHBDD3	-0.064369	6.1521231	-3.044516	0.0024972	0.0062428	-2.380706
ACTN1	0.0543345	6.7306683	3.0445143	0.0024972	0.0062428	-2.380711
MIR770	0.296432	-1.250973	3.0443764	0.0024983	0.006245	-2.381118
KAT6A	0.0702935	6.0331066	3.0443657	0.0024984	0.006245	-2.381149
RP11-1101H	0.4942922	-0.559879	3.04417	0.0025	0.0062485	-2.381726
DPEP3	0.5771932	0.712429	3.0441134	0.0025004	0.0062492	-2.381893
RP11-285F7	0.1231355	5.0502648	3.0440855	0.0025007	0.0062494	-2.381976
RNF133	0.5073974	-0.061226	3.0439598	0.0025017	0.0062515	-2.382346
USP13	-0.065253	5.9372567	-3.043781	0.0025031	0.0062547	-2.382874
PIGH	-0.055496	5.8667834	-3.043469	0.0025057	0.0062606	-2.383794
RP11-81K2.	0.2792671	-1.252211	3.0431543	0.0025082	0.0062665	-2.384721
HEPACAM2	0.5636543	-0.365343	3.0430615	0.002509	0.006268	-2.384995
ENO1	-0.059576	7.2911863	-3.042601	0.0025127	0.0062769	-2.386352
EDDM3A	-0.444456	-1.160539	-3.042582	0.0025129	0.0062769	-2.386408
HAUS2	0.0505122	5.8036805	3.0425477	0.0025132	0.0062772	-2.38651
CD276	0.0521087	6.4506378	3.0424934	0.0025136	0.0062778	-2.38667
FOXC1	0.2306318	4.8412499	3.0423245	0.002515	0.0062808	-2.387167
TPM3P6	0.5212798	2.4369771	3.0420248	0.0025174	0.0062865	-2.388051
WBP2NL	-0.239801	3.8279635	-3.041674	0.0025203	0.0062932	-2.389084
POGLUT1	-0.068409	5.7320897	-3.041493	0.0025218	0.0062963	-2.389618
SNORD46	0.4944432	0.2134026	3.0414824	0.0025219	0.0062963	-2.389649
AC000095.1	-0.572052	0.1110889	-3.041441	0.0025222	0.0062967	-2.38977
RP11-550I2	-0.184523	3.9297618	-3.040968	0.0025261	0.0063059	-2.391165
ERN1	-0.071991	6.2743279	-3.040807	0.0025274	0.0063088	-2.391638
RP11-489D6	-0.168799	-1.477578	-3.040633	0.0025288	0.0063119	-2.39215
GIT1	0.0487652	6.2138323	3.0399262	0.0025346	0.0063259	-2.394233
RP11-295P9	0.0843427	5.3467201	3.039762	0.0025359	0.0063289	-2.394717
AC026271.5	-0.079906	4.9920693	-3.039305	0.0025397	0.0063378	-2.396063
RP11-48F14	-0.173448	-1.443255	-3.039032	0.0025419	0.006343	-2.396866
MAGEC1	-0.9753	1.1368595	-3.038909	0.002543	0.0063451	-2.397228
RP11-443C1	0.588141	0.4588205	3.0388432	0.0025435	0.006346	-2.397423
SPDYE2	0.5331158	0.3086323	3.0387305	0.0025444	0.0063479	-2.397754
IGHV3-76	0.3483358	-1.145749	3.0380328	0.0025502	0.0063618	-2.399808
CTC-471J1.	0.4830149	-0.146041	3.0379526	0.0025508	0.006363	-2.400044
KIR3DL2	0.4500291	-0.588607	3.0376724	0.0025532	0.0063683	-2.400869
RAB6C-AS1	0.487696	-0.600401	3.0374525	0.002555	0.0063724	-2.401516

CICP16	0.5723476	0.3802446	3.0372929	0.0025563	0.0063753	-2.401986
AZI2	0.0819788	5.7273144	3.0369265	0.0025593	0.0063824	-2.403064
COL6A4P1	0.6166398	0.2011867	3.0368455	0.00256	0.0063837	-2.403302
MFAP1	-0.037318	6.0842045	-3.036689	0.0025613	0.0063865	-2.403762
RP11-740P5	-0.173169	-1.455119	-3.036389	0.0025638	0.0063922	-2.404646
CEACAM1	-0.116196	6.4765786	-3.036025	0.0025668	0.0063993	-2.405715
SERHL	0.3819511	2.8751775	3.0358322	0.0025684	0.0064029	-2.406283
AC004019.1	-0.336589	-1.019349	-3.035657	0.0025699	0.0064061	-2.406798
CXorf40A	-0.069951	5.6674658	-3.035325	0.0025726	0.0064125	-2.407774
JADE2	0.0655325	6.007067	3.0351125	0.0025744	0.0064165	-2.4084
SSX1	-1.055749	1.9100944	-3.034495	0.0025795	0.0064284	-2.410216
SNX21	-0.065224	5.7028046	-3.034494	0.0025795	0.0064284	-2.410218
RNU4-48P	-0.184399	-1.395535	-3.034388	0.0025804	0.0064302	-2.410531
RPP21	-0.079684	5.5325328	-3.033959	0.002584	0.0064387	-2.411791
MANSC1	0.0758593	5.8553179	3.0338639	0.0025848	0.0064402	-2.412071
RP3-492J12	0.344305	-1.076417	3.0336802	0.0025863	0.0064436	-2.412611
RP11-502M1	-0.209299	-1.367021	-3.033459	0.0025882	0.0064478	-2.41326
BST2	-0.13821	6.6979153	-3.03305	0.0025916	0.0064559	-2.414463
NAV2	0.1105881	6.2084876	3.0327499	0.0025941	0.0064617	-2.415345
PCDHB12	0.4269641	3.2956239	3.0324407	0.0025967	0.0064677	-2.416254
CCR3	0.7473103	0.8448891	3.0324064	0.002597	0.006468	-2.416354
TMPO-AS1	-0.141074	5.0648681	-3.03224	0.0025984	0.006471	-2.416844
EDC3	-0.04229	6.074568	-3.031755	0.0026025	0.0064807	-2.418267
RP11-214N9	-0.301089	3.0927104	-3.031732	0.0026027	0.0064808	-2.418335
NFYB	-0.066474	6.2115009	-3.031293	0.0026064	0.0064895	-2.419626
SGSM3	-0.043949	6.3958922	-3.03113	0.0026077	0.0064925	-2.420103
CKAP2P1	-0.117368	-1.499306	-3.031092	0.0026081	0.0064929	-2.420217
RP11-316N2	-0.272459	-1.275245	-3.031067	0.0026083	0.006493	-2.420288
AK8	0.5225031	2.8584996	3.0308866	0.0026098	0.0064959	-2.420819
SNORD62B	0.4907172	-0.055983	3.0308824	0.0026098	0.0064959	-2.420831
ADCY4	0.1190364	5.376672	3.0307892	0.0026106	0.0064975	-2.421105
LPPR2	-0.071505	6.1685021	-3.03036	0.0026142	0.006506	-2.422364
SNX18P25	-0.151602	-1.4638	-3.030062	0.0026168	0.0065118	-2.42324
ZNF385C	0.3095994	4.0436621	3.0296687	0.0026201	0.0065197	-2.424395
KRTDAP	0.5582425	-0.470519	3.0294275	0.0026221	0.0065243	-2.425103
NHLH1	0.5461794	0.8788155	3.0291892	0.0026242	0.0065289	-2.425803
RP11-129M6	-0.856763	0.9689532	-3.02899	0.0026258	0.0065327	-2.426388
HSF2	0.0742774	5.5025265	3.0289264	0.0026264	0.0065335	-2.426574
SNORA11	0.478991	2.0256584	3.0287117	0.0026282	0.0065376	-2.427204
RP13-463N1	-0.516674	-0.322808	-3.028562	0.0026295	0.0065404	-2.427644
RNU6-1161F	0.4344133	-0.921022	3.0272578	0.0026406	0.0065675	-2.43147
PAIP1P1	0.4736784	2.369228	3.0270916	0.002642	0.0065706	-2.431958
SLC1A1	-0.307597	5.851095	-3.026945	0.0026432	0.0065732	-2.432389
NCKAP1	0.0451363	6.562601	3.02688	0.0026438	0.0065742	-2.432578
RP11-626G1	0.5317027	0.0374857	3.0266333	0.0026459	0.006579	-2.433302
AC234582.1	0.2717136	-1.290892	3.0264898	0.0026471	0.0065816	-2.433723
CTA-228A9.	0.4360487	1.8975396	3.0263693	0.0026481	0.0065837	-2.434076
DNAH9	0.5944608	0.1948718	3.0262772	0.0026489	0.0065852	-2.434346
SLC40A1	-0.065254	6.8677906	-3.026217	0.0026494	0.006586	-2.434522
ASIC4	0.5343288	-0.256118	3.0255933	0.0026548	0.0065988	-2.436352
OR5BA1P	0.3769719	-1.041982	3.0253291	0.002657	0.006604	-2.437126

HTT	-0.046377	6.5257056	-3.025267	0.0026576	0.0066049	-2.437309
AC003985.1	-0.299813	-1.302835	-3.025219	0.002658	0.0066054	-2.437448
PTGES3P1	0.1258608	4.9098755	3.024583	0.0026634	0.0066186	-2.439313
CDCA2	0.2639167	4.57344	3.0244419	0.0026647	0.0066211	-2.439727
RP1-101A2.	-0.242719	3.7525437	-3.024079	0.0026678	0.0066281	-2.44079
MTATP6P1	-0.09047	7.1728264	-3.024071	0.0026679	0.0066281	-2.440814
SERTAD4	0.5781255	2.8029463	3.0239193	0.0026692	0.0066309	-2.441258
FAM193A	0.0417438	6.0500163	3.0238866	0.0026694	0.0066312	-2.441354
RP11-407N1	0.5280846	3.2228608	3.0238656	0.0026696	0.0066312	-2.441416
AC016768.1	-0.566235	3.8733686	-3.02366	0.0026714	0.0066351	-2.442017
CLPTM1L	-0.049958	6.754138	-3.023537	0.0026724	0.0066373	-2.442379
RP11-66N24	-0.468458	0.3864029	-3.023022	0.0026769	0.0066479	-2.443888
MXD1	0.081755	5.6932374	3.0229201	0.0026778	0.0066496	-2.444186
RP11-124G5	-0.225982	-1.395332	-3.022819	0.0026786	0.0066513	-2.444482
RP11-574M7	-0.159164	-1.469307	-3.022462	0.0026817	0.0066585	-2.445527
APBB2	-0.069686	6.1687473	-3.021641	0.0026888	0.0066757	-2.447933
NUCB2	-0.07521	6.4899243	-3.021463	0.0026904	0.0066791	-2.448454
PARD3-AS1	-0.463895	1.9993426	-3.021273	0.002692	0.0066827	-2.449009
CTD-2540B1	-0.556302	1.7823002	-3.020964	0.0026947	0.0066889	-2.449915
DXO	-0.060387	6.0046506	-3.020888	0.0026954	0.0066901	-2.450138
NXPH3	0.266426	3.7722667	3.0205687	0.0026981	0.0066966	-2.451072
XIST	1.0322233	2.0236348	3.0202533	0.0027009	0.0067029	-2.451995
EHMT2-AS1	0.3210872	-1.057934	3.0201508	0.0027018	0.0067047	-2.452295
RP11-640N1	0.6024007	-0.351383	3.0201237	0.002702	0.0067048	-2.452374
MT1XP1	-0.644071	1.5895861	-3.020033	0.0027028	0.0067063	-2.45264
ZWILCH	0.0714716	5.5815794	3.0198317	0.0027046	0.0067102	-2.453229
AC007285.6	0.3732906	-0.921524	3.0196377	0.0027062	0.0067139	-2.453797
RP1-125I3.	-0.343774	-1.005493	-3.01957	0.0027068	0.0067149	-2.453994
DAZAP2P1	0.4768012	2.99509	3.0195452	0.002707	0.006715	-2.454068
GAREM	0.1127342	5.6385147	3.0195035	0.0027074	0.0067155	-2.45419
CEBPD	-0.10121	6.6026026	-3.019328	0.0027089	0.0067188	-2.454703
RP5-855F14	-0.406851	-0.800754	-3.019232	0.0027098	0.0067204	-2.454984
ACD	-0.062303	5.7965436	-3.01869	0.0027145	0.0067317	-2.456571
PI15	0.7227293	3.0699025	3.0182276	0.0027186	0.0067413	-2.457923
SOWAHA	-0.2518	5.5787466	-3.018148	0.0027193	0.0067426	-2.458157
AC244230.1	0.6143086	1.2118403	3.0179734	0.0027208	0.0067455	-2.458666
ST7-AS2	-0.365189	-0.994621	-3.017969	0.0027208	0.0067455	-2.458678
RP11-48B3.	0.5198198	2.6575837	3.0178228	0.0027221	0.0067482	-2.459107
ZNF544	-0.095054	5.9348229	-3.017637	0.0027237	0.0067518	-2.459649
U47924.27	0.4279783	-1.04745	3.0176124	0.0027239	0.0067519	-2.459722
FAM86MP	-0.21264	-1.358709	-3.017085	0.0027286	0.0067629	-2.461265
AC006159.4	-0.293202	-1.212582	-3.017064	0.0027288	0.0067629	-2.461327
LINC01587	0.7689124	0.9976064	3.0159026	0.002739	0.0067874	-2.464721
RP11-17M16	-0.195076	3.840115	-3.015898	0.002739	0.0067874	-2.464734
KIAA0825	0.3473867	3.4980916	3.015858	0.0027394	0.0067878	-2.464852
RP11-466C2	0.4291989	-0.572976	3.0155024	0.0027425	0.0067948	-2.465891
DNM1P34	-0.136037	-1.468304	-3.015497	0.0027426	0.0067948	-2.465907
DDX21	0.0606026	6.3756344	3.0151035	0.002746	0.0068029	-2.467057
AC013401.2	-0.260616	-1.291326	-3.01495	0.0027474	0.0068058	-2.467504
SEMA3B	-0.201017	5.5249321	-3.014783	0.0027489	0.006809	-2.467993
LL22NC01-1	-0.289472	-1.182467	-3.014743	0.0027492	0.0068094	-2.46811

AC114730.7	-0.369143	-0.852134	-3.014682	0.0027498	0.0068103	-2.468289
LCMT2	-0.061219	5.5992511	-3.014603	0.0027505	0.0068116	-2.468518
ZNF526	-0.048712	5.7161507	-3.014455	0.0027518	0.0068144	-2.468952
ABCA12	0.6126914	0.867996	3.0144078	0.0027522	0.0068145	-2.469089
HMGN2	-0.050476	6.6694477	-3.014401	0.0027522	0.0068145	-2.469108
PPFIA3	-0.103937	5.6026377	-3.014383	0.0027524	0.0068145	-2.469161
AC098823.3	0.4159397	-0.616635	3.0141208	0.0027547	0.0068198	-2.469928
SLC48A1	-0.060316	6.1547118	-3.014023	0.0027556	0.0068215	-2.470213
RP11-286E1	-0.499552	-0.054938	-3.013988	0.0027559	0.0068218	-2.470315
RP11-554I8	0.3019509	-1.157002	3.0139053	0.0027566	0.0068232	-2.470557
RP6-109B7.	0.4585443	-0.443207	3.0138406	0.0027572	0.0068241	-2.470746
ASPHD1	0.537238	4.2754361	3.0138081	0.0027575	0.0068244	-2.470841
SPHKAP	-0.14051	-1.462046	-3.013722	0.0027583	0.0068258	-2.471092
LINC00864	-0.704331	3.6097007	-3.013649	0.0027589	0.0068269	-2.471305
RN7SL368P	0.2761912	-1.223221	3.0134561	0.0027606	0.0068307	-2.471869
KCNQ2	0.5339547	-0.484506	3.013035	0.0027644	0.0068395	-2.473099
SSH3	0.062465	6.0556386	3.0128734	0.0027658	0.0068426	-2.473571
RP11-467J1	-0.509387	-0.075625	-3.012778	0.0027666	0.0068442	-2.473851
RP11-574K1	0.2820071	3.0202072	3.01276	0.0027668	0.0068442	-2.473902
CPNE6	-0.66956	1.1640823	-3.011969	0.0027739	0.0068611	-2.476212
SEMA3B-AS1	-0.475288	2.8896727	-3.011176	0.0027809	0.0068782	-2.478526
RPS19	-0.071684	7.2322107	-3.011122	0.0027814	0.0068786	-2.478686
AC002059.1	0.4985951	0.863663	3.0111127	0.0027815	0.0068786	-2.478711
RP11-64D22	-0.23251	-1.330021	-3.010937	0.0027831	0.0068821	-2.479225
RP11-1212A	0.4438521	-0.212894	3.0107687	0.0027846	0.0068853	-2.479715
ZNF414	-0.058965	5.7359097	-3.009786	0.0027934	0.0069066	-2.482582
RP11-356J5	0.3445169	4.4761048	3.0097383	0.0027938	0.006907	-2.482722
FGF12-AS1	-0.150555	-1.461926	-3.009729	0.0027939	0.006907	-2.482748
CXorf58	-0.496915	0.8455964	-3.009606	0.002795	0.0069092	-2.483107
TDRD12	0.5181027	1.1284651	3.0095619	0.0027954	0.0069097	-2.483236
CUEDC1	0.0796644	5.9746063	3.0094068	0.0027968	0.0069127	-2.483689
MIR4701	0.3392649	-0.93139	3.0091614	0.002799	0.0069177	-2.484405
NR4A1	0.1651771	5.8457329	3.0089488	0.0028009	0.0069219	-2.485025
RP11-580J4	-0.128832	-1.48851	-3.008806	0.0028022	0.0069247	-2.485443
ERGIC1	-0.052361	6.9292688	-3.00809	0.0028086	0.0069401	-2.487531
UBD	0.2326799	6.2811449	3.0080465	0.002809	0.0069406	-2.487656
CKAP2	0.0914094	5.7218568	3.0074539	0.0028144	0.0069534	-2.489384
CTD-3116E2	0.5434406	0.7323345	3.0072537	0.0028162	0.0069574	-2.489967
SERPINA9	-0.605558	1.7486553	-3.006785	0.0028204	0.0069674	-2.491332
RP11-1072A	0.2906077	4.474054	3.0066777	0.0028214	0.0069693	-2.491646
FGD5	0.1138057	5.682864	3.0064825	0.0028232	0.0069732	-2.492215
RP1-124C6.	0.4494126	-0.676894	3.0061663	0.002826	0.0069798	-2.493137
CARS	-0.049955	6.3218206	-3.005741	0.0028299	0.0069889	-2.494376
RP11-141C7	0.564927	0.9516073	3.0054971	0.0028321	0.0069939	-2.495087
KLK2	-0.724205	-0.068897	-3.005452	0.0028325	0.0069944	-2.495219
GID4	-0.071446	5.6553024	-3.005375	0.0028332	0.0069957	-2.495443
RP11-195F1	-0.289324	3.913974	-3.005078	0.0028359	0.0070019	-2.496308
NFIA-AS1	-0.371261	-0.950413	-3.004747	0.0028389	0.0070088	-2.497272
AJ011932.1	0.4672426	-0.737224	3.0042575	0.0028434	0.0070194	-2.498698
REXO1	0.0612624	6.0949625	3.0040037	0.0028457	0.0070246	-2.499437
HOXC8	0.6590709	-0.259422	3.0039209	0.0028465	0.007026	-2.499678

FGFBP3	-0.192349	4.2051252	-3.003671	0.0028488	0.0070312	-2.500405
GNAT1	-0.478421	3.3287484	-3.003318	0.002852	0.0070386	-2.501433
MIR7152	-0.33402	-1.142578	-3.003191	0.0028531	0.007041	-2.501803
SLC41A2	-0.118997	6.2807743	-3.003081	0.0028541	0.007043	-2.502122
PRDM9	-0.64692	0.4239749	-3.002819	0.0028565	0.0070485	-2.502887
DCP1B	-0.05814	5.7259041	-3.002648	0.0028581	0.0070519	-2.503383
CCDC130	-0.054781	6.0263342	-3.002578	0.0028587	0.007053	-2.503586
RP11-264J4	-0.208516	-1.373821	-3.00246	0.0028598	0.0070552	-2.50393
FAM127B	0.126581	6.0069954	3.0023793	0.0028606	0.0070565	-2.504166
RP11-739B2	0.4892519	0.0492218	3.0018319	0.0028656	0.0070684	-2.505759
WFDC10A	0.4016066	-0.96163	3.0017925	0.002866	0.0070689	-2.505873
LINC01182	-0.73499	1.0112114	-3.00112	0.0028721	0.0070836	-2.507829
ZCCHC7	-0.045775	5.8446207	-3.000871	0.0028744	0.0070888	-2.508554
RP5-875H18	0.4249812	-0.798689	3.0006176	0.0028768	0.0070941	-2.509292
EIF3J	-0.039157	6.4203777	-3.000305	0.0028797	0.0071007	-2.510201
CPEB2-AS1	-0.535455	0.6384278	-3.000235	0.0028803	0.0071018	-2.510404
SLC16A10	-0.262321	5.209351	-3.000188	0.0028807	0.0071024	-2.510541
RPL9P31	-0.201604	-1.406225	-2.999576	0.0028864	0.0071159	-2.512322
GLTP	0.0467183	6.1065969	2.9995255	0.0028869	0.0071165	-2.512468
TMEM82	-0.411192	5.2502647	-2.999324	0.0028887	0.0071207	-2.513055
PPP1R3B	-0.108436	6.4501814	-2.999239	0.0028895	0.0071221	-2.513302
LINC00964	-0.505718	0.0168928	-2.999168	0.0028902	0.0071229	-2.513508
AC009961.2	0.384644	-0.732166	2.9991609	0.0028902	0.0071229	-2.513528
GBA2	-0.043758	6.4050714	-2.999025	0.0028915	0.0071256	-2.513922
PER2	-0.076406	5.8937676	-2.998976	0.0028919	0.0071262	-2.514067
LINC00266-	-0.201831	-1.409725	-2.99882	0.0028934	0.0071293	-2.51452
AC093323.3	-0.087278	5.7838994	-2.99842	0.0028971	0.0071379	-2.515681
RP11-497K1	-0.228482	-1.343077	-2.998102	0.0029	0.0071447	-2.516606
COLEC12	0.3271868	4.4251344	2.9979272	0.0029017	0.0071482	-2.517115
LYN	0.0721558	6.1753131	2.997717	0.0029036	0.0071526	-2.517725
CAPRIN2	0.0707558	5.7539717	2.9973643	0.0029069	0.0071602	-2.51875
AC069394.1	-0.382453	-0.983853	-2.997307	0.0029074	0.007161	-2.518916
ITM2B	-0.054621	7.099483	-2.997045	0.0029099	0.0071665	-2.519679
RP11-466F5	-0.460164	-0.307136	-2.996959	0.0029107	0.007168	-2.519929
ARHGEF38-I	0.3572884	-1.076989	2.996929	0.002911	0.0071682	-2.520015
KIAA1468	0.0483744	5.9305323	2.9968714	0.0029115	0.0071691	-2.520183
POLR3GP1	0.2555043	-1.297002	2.9965947	0.0029141	0.0071749	-2.520986
ZNF876P	0.3585181	3.0511359	2.9965619	0.0029144	0.0071752	-2.521082
PDCL2P2	-0.200507	-1.404157	-2.996307	0.0029168	0.0071806	-2.521824
RP11-178L8	-0.364165	2.9168361	-2.996261	0.0029172	0.0071812	-2.521956
RP11-507K2	0.4505914	1.8921395	2.9955512	0.0029238	0.007197	-2.524018
LINC00676	-0.441015	-0.87451	-2.995461	0.0029247	0.0071986	-2.524281
BTBD2	-0.050452	6.4747148	-2.994321	0.0029354	0.0072244	-2.527589
WASF2	0.0470215	6.456332	2.9941981	0.0029365	0.0072268	-2.527947
CRYBA2	0.5735592	-0.413773	2.9938352	0.0029399	0.0072347	-2.529
CDK14	-0.092895	6.0613733	-2.993644	0.0029417	0.0072386	-2.529555
RP11-325K4	0.3787584	2.8016067	2.9934841	0.0029432	0.0072418	-2.530019
UBR2	-0.045197	6.3501471	-2.993374	0.0029443	0.0072439	-2.53034
GGT7	-0.074647	5.9126698	-2.993067	0.0029472	0.0072505	-2.53123
ARHGEF5	0.225101	5.6340232	2.9928197	0.0029495	0.0072558	-2.531947
AATK-AS1	0.5092359	-0.777373	2.9926359	0.0029512	0.0072592	-2.532481

LINC01428	-0.638023	1.7471333	-2.992627	0.0029513	0.0072592	-2.532505
PARD6B	0.0896316	5.5760746	2.9925654	0.0029519	0.0072602	-2.532685
RP11-370I1	-0.341608	-1.121426	-2.99243	0.0029532	0.0072624	-2.533077
ZNF404	0.2579861	4.247142	2.9924294	0.0029532	0.0072624	-2.53308
P2RX4	0.0790752	6.1477538	2.9921635	0.0029557	0.0072681	-2.533851
CTD-2201E1	-0.147645	-1.461003	-2.992112	0.0029562	0.0072687	-2.534
DYNLL1	-0.04964	6.7492172	-2.992095	0.0029563	0.0072687	-2.534049
RP11-305A4	-0.121454	-1.497511	-2.991861	0.0029585	0.0072736	-2.534728
FAM160A2	-0.047776	6.2456463	-2.991704	0.00296	0.0072768	-2.535184
CITF22-49E	0.6317582	1.3573068	2.9913442	0.0029634	0.0072847	-2.536228
PHF19	0.0936729	5.7389048	2.9912759	0.0029641	0.0072858	-2.536426
SNORA7	0.4373146	-0.26707	2.9911466	0.0029653	0.0072883	-2.536801
FAM134B	-0.373715	5.1425468	-2.990967	0.002967	0.007292	-2.537322
KIF11	0.1262697	5.4901518	2.9905635	0.0029708	0.0073009	-2.538492
CRHR1	0.3766683	-1.174389	2.990406	0.0029723	0.007304	-2.538948
RP11-48601	-0.109406	4.7714881	-2.990388	0.0029725	0.007304	-2.539
C9orf72	-0.115053	5.5157723	-2.990014	0.0029761	0.0073123	-2.540084
RP3-340N1.	-0.437946	4.1065009	-2.989765	0.0029784	0.0073175	-2.540807
RP11-62I21	0.3294323	-1.196883	2.9897494	0.0029786	0.0073175	-2.540852
RP11-73E17	-0.112311	4.6248715	-2.989595	0.0029801	0.0073206	-2.541299
CALML3	-0.67295	1.4535508	-2.989498	0.002981	0.0073224	-2.54158
EFCAB8	0.56024	0.8633588	2.9892182	0.0029836	0.0073284	-2.542392
IRF3	-0.059441	6.4652509	-2.98875	0.0029881	0.0073389	-2.543749
FAM224B	-0.309116	-1.207105	-2.988246	0.0029929	0.0073502	-2.54521
SNRPN	0.1472933	6.201995	2.9880987	0.0029943	0.0073532	-2.545636
NCOR2	0.0406335	6.5821753	2.9880747	0.0029946	0.0073533	-2.545706
HAL	0.348383	6.1417641	2.9879781	0.0029955	0.007355	-2.545985
TMEM33	-0.049093	6.5631487	-2.987895	0.0029963	0.0073565	-2.546226
FRG1B	0.1492433	5.311677	2.9878311	0.0029969	0.0073575	-2.546411
VPS33B	-0.040016	5.8164391	-2.987734	0.0029978	0.0073593	-2.546691
ZNF385D	-0.542524	3.5048893	-2.987334	0.0030017	0.0073682	-2.547852
FAM131A	0.0718175	5.6377552	2.9871592	0.0030033	0.0073715	-2.548358
INAFM2	0.118172	5.0418186	2.9871501	0.0030034	0.0073715	-2.548384
KIAA1549	0.3427441	4.8113303	2.9870574	0.0030043	0.0073732	-2.548652
ZNF22	-0.058849	6.0867273	-2.986754	0.0030072	0.0073795	-2.549531
RP11-632F7	0.4712527	-0.688122	2.9867477	0.0030073	0.0073795	-2.549549
ADM2	0.2228241	5.7043966	2.9865748	0.0030089	0.0073831	-2.55005
CTD-2135D7	0.5496077	0.1907839	2.9861556	0.003013	0.0073925	-2.551264
RP11-35G9.	0.1336744	4.5403519	2.9861333	0.0030132	0.0073925	-2.551328
ABC7-48172	-0.330403	-1.168104	-2.985855	0.0030159	0.0073986	-2.552135
KRT6A	0.7909335	0.1179556	2.9857829	0.0030166	0.0073998	-2.552343
AC078883.3	0.5236043	0.8621119	2.9857354	0.003017	0.0074004	-2.552481
SCARNA7	-0.545394	1.4475364	-2.985037	0.0030237	0.0074164	-2.554502
ATP9A	0.1053012	6.3150095	2.9848862	0.0030252	0.0074195	-2.554939
RP13-204A1	-0.408378	-0.751558	-2.984592	0.003028	0.007426	-2.555791
RP11-16C1.	-0.454788	-0.418366	-2.984556	0.0030284	0.0074263	-2.555894
RP11-157G2	0.4504161	-0.584536	2.9840381	0.0030334	0.0074381	-2.557393
POLN	-0.215108	4.4493307	-2.983947	0.0030343	0.0074398	-2.557658
AC022154.7	0.4871186	1.5138711	2.983805	0.0030357	0.0074426	-2.558068
DNASE1	0.0813375	5.4542615	2.9836069	0.0030376	0.0074468	-2.558641
RP4-663N10	0.2754971	-1.261824	2.9834666	0.0030389	0.0074496	-2.559047

RP11-430E1	-0.530075	0.8186057	-2.98345	0.0030391	0.0074496	-2.559094
LYZL1	-0.563852	0.8363255	-2.983366	0.0030399	0.0074511	-2.559337
DNAJC6	0.2212032	5.0393116	2.9832776	0.0030408	0.0074527	-2.559594
RP4-673D2C	-0.178253	-1.422895	-2.983191	0.0030416	0.0074542	-2.559845
DEFA4	0.4455558	-0.849482	2.9828981	0.0030444	0.0074607	-2.560691
SLC50A1	-0.069178	6.5548728	-2.982727	0.0030461	0.0074643	-2.561186
TXLNG	0.0522883	5.9132498	2.982696	0.0030464	0.0074645	-2.561276
AGAP6	0.0982158	5.1967725	2.9823568	0.0030497	0.0074721	-2.562257
FOXL2NB	0.3976445	-1.04762	2.9817707	0.0030554	0.0074856	-2.563952
RP11-256I2	0.5281552	-0.161941	2.9809096	0.0030638	0.0075056	-2.566441
HK2P1	0.2580283	-1.281342	2.9806783	0.0030661	0.0075107	-2.56711
AC006942.4	-0.321079	3.0674629	-2.980205	0.0030707	0.0075215	-2.568477
HOXC-AS1	0.6228277	-0.438078	2.9801154	0.0030716	0.0075231	-2.568737
RP11-137J7	0.3712981	-0.735151	2.9795203	0.0030774	0.0075369	-2.570457
IPP	-0.059247	5.6286993	-2.978835	0.0030841	0.0075529	-2.572437
ZMYND10	-0.25812	3.894843	-2.978734	0.0030851	0.0075548	-2.57273
LRRC10B	0.3987337	3.2194315	2.9783112	0.0030893	0.0075645	-2.57395
MLH1	-0.042647	6.1345114	-2.977881	0.0030935	0.0075744	-2.575192
KB-1615E4.	0.6876179	2.1030034	2.9776058	0.0030962	0.0075805	-2.575987
RP11-888D1	0.4714947	-0.494559	2.9775214	0.0030971	0.007582	-2.576231
CICP13	-0.254548	-1.278483	-2.977455	0.0030977	0.0075831	-2.576422
PATL1	0.0524364	6.2827752	2.9774135	0.0030981	0.0075836	-2.576542
RP11-94H18	0.3396495	-1.083516	2.9772372	0.0030999	0.0075874	-2.577051
NUP50	0.0463147	6.3228618	2.9772086	0.0031002	0.0075875	-2.577134
GNB2	-0.044938	6.7975337	-2.977091	0.0031013	0.0075899	-2.577472
RP11-84N19	-0.708323	0.3279175	-2.97701	0.0031021	0.0075913	-2.577707
BICD2	0.0451124	5.9983688	2.9769591	0.0031026	0.0075916	-2.577854
HMGA1P1	0.3525403	-0.941924	2.9769548	0.0031027	0.0075916	-2.577866
CTA-384D8.	0.5626015	0.1519115	2.9768764	0.0031034	0.0075924	-2.578093
GABRB1	0.5314753	-0.601998	2.9768642	0.0031036	0.0075924	-2.578128
RP11-78F17	0.4988515	-0.607437	2.9768618	0.0031036	0.0075924	-2.578135
TRBJ2-2	0.3066687	-1.1162	2.9765861	0.0031063	0.0075985	-2.578931
WWC1	0.097451	6.2440293	2.9764342	0.0031078	0.0076017	-2.57937
LINC00475	0.5596455	0.6823896	2.9763209	0.0031089	0.0076039	-2.579696
EXOC7	-0.045529	6.3400118	-2.976303	0.0031091	0.0076039	-2.579749
CTD-2036A1	-0.174222	-1.425342	-2.976172	0.0031104	0.0076065	-2.580127
C16orf93	0.4228852	2.6227581	2.9757188	0.0031149	0.007617	-2.581434
RP11-203J2	0.3228992	3.2666904	2.9746873	0.0031251	0.0076411	-2.584411
PCDHGA3	0.6566643	2.4768666	2.9746848	0.0031252	0.0076411	-2.584418
SYNPR-AS1	0.3301786	-1.241965	2.9744507	0.0031275	0.0076458	-2.585093
RP11-160H1	-0.3016	-1.234988	-2.974448	0.0031275	0.0076458	-2.585102
NANOS3	0.5457864	0.6077165	2.9743363	0.0031286	0.007648	-2.585423
ACSBG2	0.5363441	0.4349152	2.9741682	0.0031303	0.0076516	-2.585908
TADA2B	-0.044673	6.1848785	-2.973522	0.0031368	0.0076668	-2.587772
GDI2P1	-0.233797	-1.32677	-2.973472	0.0031372	0.0076675	-2.587915
HLA-DRB1	0.1075958	6.7694012	2.9732555	0.0031394	0.0076723	-2.58854
TMEM75	0.3299779	-1.054457	2.9729427	0.0031425	0.0076794	-2.589442
MRRF	-0.05241	6.0906354	-2.972513	0.0031468	0.0076893	-2.590682
CNFN	0.4968324	3.2160276	2.9724956	0.003147	0.0076893	-2.590731
L3MBTL1	0.1465962	4.8647505	2.972124	0.0031507	0.0076979	-2.591803
SMAP1	0.0696404	5.5806843	2.9720164	0.0031518	0.0077	-2.592113

EYA4	0.7765072	0.5700949	2.9719011	0.003153	0.0077023	-2.592445
DKFZp434JC	0.5858545	-0.280109	2.9717923	0.0031541	0.0077045	-2.592759
ACTB	-0.043629	7.6129513	-2.971737	0.0031546	0.0077053	-2.592918
GPC6	0.1579842	6.1353394	2.9713379	0.0031586	0.0077146	-2.594068
SEC61G	-0.067614	6.4295526	-2.971258	0.0031594	0.007716	-2.594297
MFAP3	0.1482909	5.4067915	2.9707803	0.0031642	0.0077272	-2.595675
GPM6A	0.5649765	3.2398834	2.9707288	0.0031647	0.007728	-2.595823
CTD-2623N2	0.3387925	2.7772707	2.9705216	0.0031668	0.0077323	-2.59642
CCDC33	0.3123496	-1.163982	2.9705113	0.0031669	0.0077323	-2.59645
SMPDL3A	-0.088009	6.235886	-2.970422	0.0031678	0.007734	-2.596708
MOSPD2	0.0727281	5.7303833	2.9703338	0.0031687	0.0077356	-2.596962
ARHGEF35	0.4893244	4.4612456	2.9702932	0.0031691	0.007736	-2.597078
GMFG	0.0986042	5.7500637	2.9702746	0.0031693	0.007736	-2.597132
C11orf57	-0.039706	6.0369846	-2.969555	0.0031766	0.0077532	-2.599206
RP11-46J23	-0.426267	2.1439647	-2.969262	0.0031795	0.00776	-2.60005
POLR3A	-0.045662	6.0617511	-2.969153	0.0031806	0.0077621	-2.600363
RP11-27P7.	-0.155319	-1.484493	-2.968831	0.0031839	0.0077695	-2.60129
TSEN54	-0.058547	6.1565478	-2.968723	0.003185	0.0077716	-2.601602
SPAG7	-0.067902	6.2749017	-2.968705	0.0031852	0.0077716	-2.601654
LINC01210	-0.197088	-1.448915	-2.968403	0.0031882	0.0077786	-2.602523
SRGAP2B	0.2681342	4.0559999	2.9682149	0.0031901	0.0077827	-2.603064
TNK2-AS1	0.5725337	1.2158382	2.9681639	0.0031906	0.0077834	-2.603211
UBQLN4	-0.055025	6.350412	-2.968112	0.0031912	0.0077842	-2.603361
CITF22-92A	0.4075675	-0.891436	2.9679874	0.0031924	0.0077867	-2.603719
KRT18	-0.068208	7.2183514	-2.967597	0.0031964	0.0077959	-2.604844
CYP2W1	0.6043767	2.1073714	2.9671921	0.0032005	0.0078054	-2.606008
ADAMTS13	0.1215253	5.3894602	2.9671316	0.0032011	0.0078064	-2.606182
RP11-66H6.	0.5586431	0.9583537	2.9670928	0.0032015	0.0078065	-2.606294
ADCY9	-0.066436	6.341916	-2.967083	0.0032016	0.0078065	-2.606321
MIR4645	0.4688406	-0.542717	2.9669091	0.0032034	0.0078103	-2.606823
RP11-288H1	0.2247009	4.2868626	2.9666812	0.0032057	0.0078155	-2.607478
AC021087.1	0.5295565	-0.038191	2.9662163	0.0032104	0.0078265	-2.608816
DNAH1	-0.106793	5.8526912	-2.96614	0.0032112	0.0078277	-2.609036
POLE	-0.053409	6.2667158	-2.966125	0.0032114	0.0078277	-2.609079
A4GALT	0.1349629	5.349093	2.9660054	0.0032126	0.0078302	-2.609423
LINC00327	0.5832753	0.1872023	2.9658094	0.0032146	0.007834	-2.609987
C18orf21	-0.055747	5.7729488	-2.965809	0.0032146	0.007834	-2.609989
LIN9	0.1199071	5.184255	2.9657712	0.003215	0.0078344	-2.610097
EIF4ENIF1	0.0427366	5.9625093	2.9656392	0.0032163	0.0078372	-2.610476
CTD-2291D1	-0.189025	-1.406569	-2.965346	0.0032193	0.007844	-2.611321
RP11-515E2	0.3056828	-1.091293	2.9652699	0.0032201	0.0078453	-2.611539
MYRFL	0.624731	1.88857	2.9650267	0.0032226	0.00785	-2.612238
LINC00843	0.5297846	0.8000922	2.9650211	0.0032226	0.00785	-2.612254
RP11-173A1	0.4208396	-0.551433	2.9650177	0.0032227	0.00785	-2.612264
TDGF1P3	-0.56176	-0.476143	-2.964972	0.0032231	0.0078506	-2.612394
RP11-77K12	-0.250235	-1.389067	-2.964766	0.0032252	0.0078552	-2.612987
CTD-3105H1	-0.460707	-0.468257	-2.964696	0.003226	0.0078565	-2.613189
RP11-157F2	-0.287969	-1.152274	-2.964456	0.0032284	0.0078619	-2.61388
ADIG	-0.645611	0.2830834	-2.963895	0.0032342	0.0078754	-2.615492
RP11-6D1.3	-0.337389	-1.033074	-2.96351	0.0032381	0.0078843	-2.616598
KLF17	0.3987119	-0.966708	2.9634917	0.0032383	0.0078843	-2.616652

AP000897.1	-0.14086	-1.470479	-2.963476	0.0032385	0.0078843	-2.616696
TCEA1P3	0.3864235	-0.882947	2.9634146	0.0032391	0.0078853	-2.616873
GAS5-AS1	-0.1217	4.1630201	-2.962998	0.0032434	0.0078952	-2.61807
CNR1	0.6766147	2.3537322	2.9618452	0.0032553	0.0079236	-2.621383
C4orf17	-0.32625	-1.10414	-2.961476	0.0032591	0.0079324	-2.622444
CTD-2286N8	0.351837	2.6298604	2.9612783	0.0032611	0.0079368	-2.623012
RP11-133K1	0.4618362	-0.460618	2.9611536	0.0032624	0.0079394	-2.62337
RP11-59D5_	0.5618655	3.5056584	2.9611181	0.0032628	0.0079398	-2.623472
UTP3	-0.046047	6.0725244	-2.960802	0.0032661	0.0079472	-2.624381
CREB3L3	-0.205849	6.7907382	-2.960711	0.003267	0.0079486	-2.624642
RP11-620J1	0.3827417	-0.94015	2.9607056	0.0032671	0.0079486	-2.624657
SAMD4A	-0.088402	5.9571929	-2.960624	0.0032679	0.0079501	-2.624892
RANP1	0.4856348	1.4747172	2.9605849	0.0032683	0.0079506	-2.625004
MAP3K4	0.0493305	5.930656	2.9605066	0.0032691	0.007952	-2.625229
LINC01293	0.4487553	-0.755545	2.9597734	0.0032767	0.00797	-2.627334
AC005498.3	0.5666459	0.2476001	2.9595369	0.0032792	0.0079755	-2.628013
RP11-327J1	0.5239305	0.3956713	2.9592532	0.0032821	0.0079821	-2.628827
RP11-292E2	0.3680566	-0.965599	2.9591407	0.0032833	0.0079844	-2.62915
COX6A2	-0.872348	2.6411583	-2.958692	0.003288	0.0079952	-2.630438
AC002331.1	0.5508832	0.128218	2.9586716	0.0032882	0.0079952	-2.630497
AC079305.8	-0.316056	-1.045855	-2.958547	0.0032895	0.0079978	-2.630854
CTC-510F12	0.2906185	3.1878833	2.9579175	0.0032961	0.0080133	-2.632661
RP11-1074C	0.4826514	0.5138464	2.9575824	0.0032996	0.0080213	-2.633623
IGHV3-25	0.3374044	-1.180299	2.9570927	0.0033047	0.0080332	-2.635027
CHAF1B	0.1691433	5.267098	2.9561909	0.0033141	0.0080556	-2.637614
RP11-178L8	-0.575986	1.3608888	-2.956169	0.0033144	0.0080556	-2.637676
TASP1	0.0801207	5.3098882	2.9560932	0.0033152	0.008057	-2.637894
RP11-291C6	-0.195671	-1.407964	-2.955889	0.0033173	0.0080617	-2.63848
B3GNT4	0.307012	3.1101331	2.955697	0.0033193	0.0080658	-2.63903
RP11-473N1	0.4960747	1.4893127	2.9556883	0.0033194	0.0080658	-2.639055
SOX7	0.1986015	4.9020856	2.9554641	0.0033218	0.0080709	-2.639698
KHDRBS1	-0.029643	6.6314489	-2.955386	0.0033226	0.0080724	-2.639922
TMTC2	0.236545	4.4867393	2.9549918	0.0033267	0.0080819	-2.641052
RP11-996F1	0.265668	2.8725329	2.9544296	0.0033327	0.0080958	-2.642664
RP11-351J2	0.4633208	3.3919355	2.954406	0.0033329	0.0080959	-2.642732
BSN-AS2	-0.584134	1.5099371	-2.954317	0.0033339	0.0080976	-2.642986
MAPK8IP1	-0.14349	5.4212916	-2.953931	0.0033379	0.008107	-2.644092
VIMP	-0.052339	6.4746188	-2.953277	0.0033448	0.0081232	-2.645967
MKRN2	-0.042123	6.0558121	-2.95324	0.0033452	0.0081236	-2.646074
GNL3L	0.0986948	5.2592951	2.9532181	0.0033455	0.0081237	-2.646136
RP11-65L3.	0.505435	1.9037779	2.9530837	0.0033469	0.0081264	-2.646521
AC009495.2	0.3969432	-0.786097	2.9530698	0.003347	0.0081264	-2.64656
FCRLB	0.3643862	3.7569415	2.9524882	0.0033532	0.0081408	-2.648227
SMAD6	0.1357326	5.3476433	2.9520371	0.003358	0.0081519	-2.649519
RP11-175P1	0.3505948	-0.861445	2.9519933	0.0033585	0.0081522	-2.649644
HDHD2	0.0810473	5.9306728	2.9519823	0.0033586	0.0081522	-2.649676
PDPK2P	0.5052168	2.2021712	2.9519275	0.0033592	0.0081531	-2.649832
RP11-165F2	0.3952182	-0.86954	2.9517411	0.0033611	0.0081574	-2.650366
PIK3C2A	0.0581892	6.1754194	2.9514491	0.0033642	0.0081644	-2.651203
RP11-135A1	-0.610043	0.9622316	-2.951336	0.0033654	0.0081667	-2.651525
METTL17	-0.041257	6.1816649	-2.951162	0.0033673	0.0081707	-2.652024

DAB1	-0.324838	4.8022542	-2.950982	0.0033692	0.0081748	-2.652539
SLC35G2	0.1777452	5.0255727	2.9504681	0.0033747	0.0081875	-2.654011
AC006014.8	-0.227403	-1.285593	-2.950359	0.0033759	0.0081898	-2.654323
JMJD7	0.4821568	0.6888587	2.9500052	0.0033796	0.0081984	-2.655336
PIGX	-0.050012	5.8769253	-2.949913	0.0033806	0.0082003	-2.6556
RP11-370I1	0.5732393	0.4179861	2.9498086	0.0033817	0.0082025	-2.655899
HNRNPA1P7	0.448318	-0.201515	2.9493804	0.0033863	0.008213	-2.657124
RP3-327A19	0.4987042	0.5017024	2.9493221	0.0033869	0.008214	-2.657291
ALOX12P1	-0.212243	-1.347937	-2.949101	0.0033893	0.0082188	-2.657924
RP11-893F2	0.3242058	-1.091493	2.9490947	0.0033894	0.0082188	-2.657942
LINC00954	0.5003368	1.6290385	2.9489747	0.0033907	0.0082214	-2.658285
NCLP1	0.480274	0.0665509	2.9485755	0.0033949	0.0082312	-2.659427
RP1-60019.	-0.466687	-0.502128	-2.948543	0.0033953	0.0082315	-2.659519
ZFYVE21	-0.053408	6.0687451	-2.948394	0.0033969	0.0082348	-2.659947
SLC6A6P1	-0.159472	-1.462893	-2.948281	0.0033981	0.0082372	-2.660269
AC005534.8	-0.486646	1.7949231	-2.948087	0.0034002	0.0082417	-2.660824
RSF1	0.047248	6.0409268	2.9477388	0.0034039	0.0082502	-2.66182
MTND4P11	-0.3183	-1.133904	-2.946956	0.0034124	0.0082701	-2.664059
RP11-167N5	0.5189238	0.7175802	2.9468707	0.0034133	0.0082715	-2.664303
ZNF490	0.2430327	3.5905433	2.9468621	0.0034134	0.0082715	-2.664328
RP11-1281K	-0.256602	-1.240811	-2.946787	0.0034142	0.0082729	-2.664542
RHOBTB3	-0.091166	6.3647847	-2.946404	0.0034183	0.0082824	-2.665638
YY2	0.3424224	3.6059056	2.9462977	0.0034195	0.0082844	-2.665941
BX322557.1	0.2510118	3.8485191	2.9462827	0.0034196	0.0082844	-2.665984
RP11-93209	-0.40083	-0.677672	-2.945994	0.0034227	0.008291	-2.666808
RP11-128A6	-0.114005	-1.498687	-2.94599	0.0034228	0.008291	-2.666821
KANSL1	0.0515408	6.1537577	2.9457691	0.0034252	0.0082962	-2.667452
NR2F2	0.0563141	6.3153066	2.9449899	0.0034336	0.0083161	-2.669679
RP11-2501C	-0.328667	-1.07344	-2.944711	0.0034366	0.0083229	-2.670477
TMEM74B	0.1879258	4.9790285	2.9444688	0.0034393	0.0083282	-2.671168
RP1-72A23.	-0.345593	-0.965973	-2.944466	0.0034393	0.0083282	-2.671177
GSC	0.5559246	-0.089646	2.944127	0.003443	0.0083366	-2.672145
RP3-369A17	0.358138	-0.90537	2.9440774	0.0034435	0.0083373	-2.672286
RP11-80H8.	0.4070245	-0.592967	2.9437041	0.0034476	0.0083466	-2.673353
KLF2P1	0.6410529	-0.371498	2.9435845	0.0034489	0.0083492	-2.673694
PRR22	-0.146535	4.7712732	-2.943553	0.0034492	0.0083495	-2.673784
BRF1	-0.044179	6.1309415	-2.943243	0.0034526	0.0083571	-2.67467
ST20-AS1	-0.106923	5.1855252	-2.943075	0.0034544	0.008361	-2.675148
RP11-354I1	-0.149791	-1.468356	-2.94289	0.0034564	0.0083653	-2.675678
C9orf116	-0.195593	4.4133943	-2.942692	0.0034586	0.0083695	-2.676243
CBWD1	-0.079124	5.5030449	-2.942682	0.0034587	0.0083695	-2.676271
LINC01354	-0.647398	2.0510549	-2.942634	0.0034592	0.0083695	-2.676408
PEBP4	-0.751394	1.1707531	-2.94263	0.0034593	0.0083695	-2.676419
AB015752.3	-0.526418	0.1654643	-2.942625	0.0034593	0.0083695	-2.676434
MAP3K8	0.1520971	5.4428797	2.942535	0.0034603	0.0083713	-2.676691
RP11-499P2	-0.148813	5.0749005	-2.942452	0.0034612	0.008373	-2.676928
OPN5	-0.198388	-1.38501	-2.942305	0.0034628	0.0083763	-2.677347
IGDCC3	0.7723622	0.9895954	2.9422048	0.0034639	0.0083784	-2.677634
RP11-166P1	0.6073108	1.2732342	2.9421094	0.003465	0.0083804	-2.677906
TOR1AIP1	-0.060672	6.366619	-2.9413	0.0034738	0.0084013	-2.680218
RP1-257I20	0.4494891	1.8244206	2.9412699	0.0034741	0.0084015	-2.680303

CTD-3080P1	0.4961552	3.8902652	2.9412318	0.0034746	0.0084019	-2.680411
LINC00664	0.6594971	0.9694858	2.9410975	0.003476	0.0084049	-2.680795
AC016747.3	-0.063116	5.6499402	-2.941062	0.0034764	0.0084053	-2.680896
CTBP1	-0.037168	6.688052	-2.940442	0.0034832	0.0084212	-2.682667
ARL6IP5	-0.048553	6.4487877	-2.940088	0.0034871	0.00843	-2.683674
LINC01135	-0.571498	0.9045231	-2.939617	0.0034923	0.008442	-2.685018
SERPINA7P1	-0.152113	-1.477414	-2.939589	0.0034926	0.0084422	-2.6851
CTD-2555C1	0.6949688	0.7493024	2.9392185	0.0034967	0.0084515	-2.686155
RNU6ATAC32	-0.365147	-0.895557	-2.939013	0.003499	0.0084564	-2.686741
IGKV1OR2-3	0.3083507	-1.204025	2.9389486	0.0034997	0.0084576	-2.686925
RP11-568K1	-0.08529	6.2191019	-2.93889	0.0035003	0.0084586	-2.687092
MTDH	-0.054251	6.787009	-2.938635	0.0035031	0.0084648	-2.68782
WT1-AS	0.6659613	0.0147486	2.938251	0.0035074	0.0084745	-2.688915
CTB-51J22.	0.6687786	1.5916224	2.9381285	0.0035087	0.0084772	-2.689264
TULP2	0.5478216	0.3187236	2.9380522	0.0035096	0.0084786	-2.689481
LEP	0.5045881	-0.545355	2.9380368	0.0035097	0.0084786	-2.689525
UVSSA	0.0924603	5.4081625	2.9378752	0.0035115	0.0084823	-2.689986
RP1-142L7.	0.3568097	-0.913778	2.9378021	0.0035123	0.0084837	-2.690195
LHFPL4	0.7946367	0.6257416	2.9376892	0.0035136	0.0084862	-2.690516
RP11-1C1.6	0.410509	-1.122125	2.9372186	0.0035188	0.0084982	-2.691858
RP11-254F1	0.5167647	-0.294913	2.9370224	0.003521	0.0085029	-2.692417
FAM184B	0.3594341	2.5180349	2.9370023	0.0035212	0.0085029	-2.692474
RP11-977G1	0.3636144	2.7253021	2.9368942	0.0035224	0.0085052	-2.692782
CTD-2517O1	-0.201583	3.9818152	-2.936837	0.003523	0.0085062	-2.692946
FAM187B	-0.328759	-1.036745	-2.936526	0.0035265	0.0085134	-2.693831
PRAMEF17	-0.400493	-0.997096	-2.936525	0.0035265	0.0085134	-2.693834
RP11-1191J	0.3581133	-0.95551	2.936185	0.0035303	0.008522	-2.694803
CTD-2534I2	-0.211741	-1.353757	-2.936142	0.0035308	0.0085226	-2.694926
VWA9	-0.034551	6.1502928	-2.935736	0.0035353	0.0085327	-2.696084
SLC22A3	-0.221136	6.0202539	-2.935724	0.0035354	0.0085327	-2.696118
ACP2	-0.053143	6.6091676	-2.935311	0.00354	0.0085432	-2.697293
CXXC1	-0.043601	6.2530525	-2.935223	0.003541	0.008545	-2.697543
IGKV1D-33	0.3459958	-1.16524	2.9342292	0.0035521	0.0085712	-2.700374
RP3-337H4.	-0.500755	0.7927768	-2.934179	0.0035526	0.008572	-2.700518
CFL1	-0.041799	7.1701889	-2.934148	0.003553	0.0085722	-2.700604
ABHD17B	-0.060257	5.8168956	-2.9339	0.0035558	0.0085784	-2.701311
BIN3	0.0760738	5.4672257	2.9331789	0.0035638	0.0085973	-2.703365
CDC40	0.0747351	5.7452421	2.9320765	0.0035762	0.0086266	-2.706502
SLC25A22	-0.076489	6.2375668	-2.931324	0.0035847	0.0086465	-2.708643
RP11-354P1	-0.229422	-1.282372	-2.931234	0.0035857	0.0086484	-2.708899
LRRTM4	-0.779342	0.9611009	-2.93098	0.0035886	0.0086547	-2.709623
MZT2A	-0.077943	6.2087339	-2.930792	0.0035907	0.0086592	-2.710155
SEMA6B	0.0877527	5.6745898	2.9305997	0.0035929	0.0086639	-2.710703
CUX1	-0.049199	6.5068646	-2.930434	0.0035947	0.0086678	-2.711174
RP11-363N2	-0.504769	0.1337549	-2.930411	0.003595	0.0086679	-2.711241
PPP1R13B	0.0526629	6.0188864	2.9302018	0.0035974	0.008673	-2.711835
ZXDC	-0.044724	6.1267054	-2.930159	0.0035979	0.0086736	-2.711958
MT1P1	-0.51238	0.9457046	-2.929808	0.0036018	0.0086826	-2.712955
SLC4A4	-0.244813	5.7872494	-2.929488	0.0036055	0.0086908	-2.713865
FAM187B2P	0.4768236	0.0649371	2.9288778	0.0036124	0.0087069	-2.7156
PRSS1	0.5590287	-0.646436	2.9282704	0.0036193	0.008723	-2.717326

MCM4	0.0766276	6.320893	2.9282032	0.00362	0.0087242	-2.717517
RP11-222K1	0.517296	-0.105281	2.9281249	0.0036209	0.0087258	-2.717739
BCL6	-0.070213	6.2944077	-2.927812	0.0036245	0.0087338	-2.718628
MTND2P21	-0.186964	-1.401494	-2.927773	0.0036249	0.0087343	-2.718739
KIAA1551	-0.077297	6.1973114	-2.927543	0.0036276	0.00874	-2.719392
NCOA2	-0.066572	6.4965074	-2.927331	0.00363	0.0087452	-2.719996
PTGER3	0.3591041	3.7546423	2.9273137	0.0036302	0.0087452	-2.720044
TAF1	0.0774641	5.9689507	2.9269538	0.0036343	0.0087545	-2.721067
FTH1P1	-0.488514	0.4606493	-2.926836	0.0036356	0.0087572	-2.721401
RP11-380I1	0.3792698	-0.963591	2.9264317	0.0036402	0.0087677	-2.72255
RP11-1055E	-0.302136	4.0217386	-2.926399	0.0036406	0.0087681	-2.722643
CEBPZ	-0.03959	6.2481891	-2.926358	0.0036411	0.0087686	-2.72276
RPS19P3	0.4320186	1.6515743	2.9257926	0.0036476	0.0087836	-2.724365
RP11-72304	0.3808609	-0.746544	2.9255571	0.0036503	0.0087893	-2.725034
AC096559.1	-0.653644	0.6948553	-2.925543	0.0036504	0.0087893	-2.725073
RN7SL689P	0.3756391	2.9837708	2.9252544	0.0036537	0.0087967	-2.725893
RP11-63N3.	-0.280844	-1.309791	-2.925154	0.0036549	0.0087989	-2.726179
GS1-124K5.	-0.270154	-1.195089	-2.925068	0.0036559	0.0088007	-2.726421
HN1L	-0.049151	6.5700912	-2.924887	0.003658	0.0088051	-2.726936
MTMR3	0.0851993	5.238897	2.9247159	0.0036599	0.0088093	-2.727422
PLGLA	-0.683603	3.4245874	-2.924633	0.0036609	0.008811	-2.727658
LINC00671	-0.297254	4.7236311	-2.924557	0.0036617	0.0088125	-2.727873
XXbac-BPG1	0.3063645	-1.100412	2.9244685	0.0036628	0.0088144	-2.728124
EIF4EP1	0.4968375	-0.09556	2.9240518	0.0036676	0.0088253	-2.729307
KHDRBS3	-0.111566	5.818377	-2.923886	0.0036695	0.0088293	-2.729776
PTP4A1	-0.058823	7.1619491	-2.923824	0.0036702	0.0088305	-2.729954
RP3-420J14	-0.754028	1.6313017	-2.923453	0.0036744	0.0088402	-2.731005
DNAJC10	0.0464715	6.3733214	2.9232543	0.0036767	0.008845	-2.73157
HIST4H4	0.3058611	3.1513323	2.9232368	0.0036769	0.008845	-2.73162
KB-1507C5.	-0.158288	4.7851969	-2.923012	0.0036795	0.0088507	-2.732257
IFIT5	-0.068676	5.9681246	-2.922935	0.0036804	0.0088522	-2.732476
PCDHB14	0.2659924	4.6533766	2.9213821	0.0036984	0.0088949	-2.736881
RP4-568B10	-0.529202	2.0975125	-2.92103	0.0037025	0.0089041	-2.73788
FRMD6-AS1	0.5135543	1.3934404	2.92089	0.0037041	0.0089071	-2.738276
LINC00087	0.3406034	4.1373757	2.9208832	0.0037042	0.0089071	-2.738295
RP11-461F1	-0.254079	-1.217925	-2.920324	0.0037107	0.0089221	-2.739879
RNASEH2A	-0.08774	5.9841483	-2.920104	0.0037133	0.0089277	-2.740504
RP11-175P1	0.5420016	2.6177242	2.9198234	0.0037165	0.0089344	-2.7413
RP11-673P1	-0.237649	-1.350562	-2.919822	0.0037166	0.0089344	-2.741305
RP11-182J1	0.4652159	0.2889147	2.9197236	0.0037177	0.0089366	-2.741582
POTEF	0.5264772	-0.225647	2.9195684	0.0037195	0.0089402	-2.742022
AC008592.5	-0.55797	0.4256056	-2.919531	0.0037199	0.0089402	-2.742129
FAM208A	0.0395641	6.2818171	2.9195298	0.00372	0.0089402	-2.742131
RP11-316M1	-0.327962	2.8531285	-2.919403	0.0037214	0.0089432	-2.74249
CTC-351M12	-0.16285	4.3872409	-2.919351	0.003722	0.0089441	-2.742639
BAIAP2L2	0.2202856	5.4103142	2.9189542	0.0037267	0.0089546	-2.743763
RFPL4AP1	-0.164827	-1.430003	-2.918875	0.0037276	0.0089562	-2.743986
RP11-118A3	-0.155164	-1.459901	-2.91879	0.0037286	0.0089581	-2.744229
PYGO2	-0.04717	6.4282121	-2.918327	0.003734	0.0089705	-2.74554
CHRDL2	0.4180252	5.0211889	2.9180342	0.0037374	0.0089781	-2.746369
LINC00395	-0.171709	-1.463211	-2.917105	0.0037483	0.0090037	-2.749

FOXG1	0.3710029	-1.178246	2.9162038	0.0037589	0.0090286	-2.751552
GRIA1	0.5000639	-0.431971	2.915903	0.0037625	0.0090365	-2.752403
RP11-25G10	0.616141	1.0940039	2.91588	0.0037627	0.0090365	-2.752469
HNRNPA1P59	0.5232579	1.1261378	2.9157077	0.0037648	0.0090408	-2.752956
UTF1	0.3069396	-1.098523	2.9155755	0.0037663	0.0090436	-2.753331
HOOK2	0.0777181	5.9605957	2.9155653	0.0037665	0.0090436	-2.753359
RP11-151H2	-0.215681	-1.330213	-2.914725	0.0037764	0.0090667	-2.755737
AP2A2	-0.033334	6.564122	-2.914698	0.0037767	0.0090667	-2.755813
RP11-320P7	-0.154443	-1.454932	-2.914689	0.0037768	0.0090667	-2.755839
CTD-2547L1	-0.431578	-0.514518	-2.914595	0.0037779	0.0090688	-2.756106
ZBTB39	0.0886457	5.4010171	2.9145171	0.0037788	0.0090704	-2.756326
DLGAP5	0.1984169	5.1991258	2.9142992	0.0037814	0.009076	-2.756942
RP11-350N1	0.2833364	-1.213259	2.9140313	0.0037846	0.009083	-2.7577
LDHAP5	0.4699118	1.0682903	2.9136915	0.0037886	0.0090915	-2.758661
AC108925.1	-0.30086	-1.096699	-2.913691	0.0037886	0.0090915	-2.758663
IL36A	-0.176381	-1.414756	-2.91366	0.003789	0.0090918	-2.75875
ATP6V1G1P3	-0.131215	-1.494091	-2.913489	0.003791	0.009096	-2.759234
UBE2E2	0.1283864	5.7825518	2.9129575	0.0037973	0.0091106	-2.760737
ZIC5	0.8134398	2.9101111	2.9125876	0.0038017	0.0091202	-2.761783
DCTN2	-0.035992	6.5639696	-2.91258	0.0038018	0.0091202	-2.761804
ABCA2	-0.078192	6.4558064	-2.912497	0.0038028	0.0091219	-2.762039
RP11-486M2	0.2626951	-1.267688	2.9124342	0.0038036	0.0091231	-2.762217
BIN2P1	0.2279866	-1.292982	2.9123494	0.0038046	0.0091248	-2.762456
RP11-203J2	0.4687941	3.2438549	2.9123333	0.0038048	0.0091248	-2.762502
ERRFI1	-0.105076	6.8721965	-2.912226	0.003806	0.0091273	-2.762805
RP4-553F4.	-0.149963	-1.483134	-2.912141	0.003807	0.0091291	-2.763046
CHORDC2P	-0.276755	-1.258595	-2.911871	0.0038103	0.0091362	-2.763808
AC010148.1	-0.556041	2.2944351	-2.911775	0.0038114	0.0091384	-2.764081
RP11-587P2	-0.845528	0.5576915	-2.911641	0.003813	0.0091413	-2.764458
RP13-131K1	0.4059103	-0.570944	2.9116287	0.0038131	0.0091413	-2.764494
CH507-3904	-0.178781	-1.418927	-2.911573	0.0038138	0.0091423	-2.764651
CYP2C18	-0.300627	6.0379292	-2.911002	0.0038206	0.0091581	-2.766264
CKAP4	0.0610986	6.6788934	2.9107272	0.0038239	0.0091653	-2.767042
AL672183.2	-0.479587	0.3665182	-2.910466	0.003827	0.0091722	-2.767779
RP11-113C1	-0.418588	-0.703449	-2.910308	0.0038289	0.0091762	-2.768227
EMC3-AS1	0.2650264	4.0402726	2.9098807	0.0038341	0.0091878	-2.769433
MEIKIN	-0.309576	-1.16562	-2.909843	0.0038345	0.0091883	-2.769539
RP13-516M1	-0.117202	4.7944192	-2.908884	0.003846	0.0092153	-2.772248
RP11-83M16	-0.29249	-1.123366	-2.90885	0.0038464	0.0092157	-2.772346
OR7E126P	0.516584	0.3823547	2.9086539	0.0038488	0.0092207	-2.772898
TJAP1	-0.041261	6.190576	-2.908524	0.0038504	0.0092239	-2.773265
SLC16A8	0.4116789	2.8655232	2.9084888	0.0038508	0.0092243	-2.773365
POC1B-GALN	0.4425752	1.2182439	2.9083526	0.0038524	0.0092276	-2.773749
RNF224	0.5731962	0.7378626	2.9080299	0.0038563	0.0092363	-2.77466
RP11-285J1	0.4687451	1.7220382	2.9077837	0.0038593	0.0092422	-2.775355
TRIT1	-0.047938	5.7460363	-2.907782	0.0038593	0.0092422	-2.775359
HCG15	0.5486473	2.1642844	2.9077499	0.0038597	0.0092426	-2.775451
ODAM	-0.904566	1.2777655	-2.907721	0.00386	0.0092428	-2.775531
HERC2P9	0.1345925	5.0041741	2.9074181	0.0038637	0.0092509	-2.776387
LINC00161	-0.236457	-1.2817	-2.907342	0.0038646	0.0092525	-2.776603
STX16-NPEF	0.3753604	3.5496091	2.9069685	0.0038691	0.0092627	-2.777656

AP001630.5	-0.372092	-0.772067	-2.906946	0.0038694	0.0092628	-2.77772
RP11-29B2.	-0.491274	1.2710486	-2.906907	0.0038699	0.0092633	-2.777829
NLRC5	0.0905532	5.9803657	2.9060071	0.0038807	0.0092888	-2.780369
FBXL19-AS1	0.2385718	4.2373972	2.9059467	0.0038815	0.0092899	-2.78054
RP11-126H7	-0.301174	-1.186554	-2.905674	0.0038848	0.0092972	-2.781308
AC083884.8	0.4819666	-0.044121	2.9051452	0.0038912	0.009312	-2.782801
PCOLCE2	-0.26338	5.7118144	-2.904955	0.0038935	0.0093169	-2.783336
MAPKAPK5-A	-0.065843	5.6553939	-2.904813	0.0038953	0.0093204	-2.783739
RP11-54H7.	0.4399584	-0.971637	2.9045952	0.0038979	0.0093261	-2.784352
FOXD4	0.501551	1.9386071	2.904495	0.0038991	0.0093284	-2.784635
ATXN7L3B	-0.048157	6.5371244	-2.904315	0.0039013	0.0093331	-2.785142
CTD-2007L1	0.4951791	0.8815536	2.9038282	0.0039072	0.0093467	-2.786515
DGCR2	-0.043431	6.6496525	-2.903342	0.0039132	0.0093598	-2.787886
PRDM7	-0.627529	1.2267465	-2.903327	0.0039134	0.0093598	-2.787927
CHODL-AS1	-0.15158	-1.455197	-2.903314	0.0039135	0.0093598	-2.787963
CASC19	0.6440832	2.1054605	2.9032055	0.0039148	0.0093624	-2.78827
SMAD4	-0.04548	6.3920184	-2.903168	0.0039153	0.0093629	-2.788377
KCNC1	0.6499741	0.780145	2.9027465	0.0039205	0.0093746	-2.789564
RP5-888M10	-0.441123	3.1744495	-2.902704	0.003921	0.0093752	-2.789685
TUBB8P11	-0.140577	-1.452945	-2.902127	0.003928	0.0093915	-2.791309
AC098824.6	0.3959186	-0.64172	2.902091	0.0039285	0.0093919	-2.791411
LINC01482	-0.487368	2.5097991	-2.901838	0.0039316	0.0093987	-2.792124
MOGAT3	-0.359783	5.7037053	-2.901493	0.0039358	0.0094082	-2.793095
UPF3A	-0.049876	6.0001625	-2.90143	0.0039366	0.0094095	-2.793272
RP11-190A1	0.5840804	1.2379448	2.9013519	0.0039376	0.0094112	-2.793493
AC012358.4	-0.156335	3.9426415	-2.90085	0.0039437	0.0094253	-2.794908
RP11-1277A	-0.374428	2.8447709	-2.90081	0.0039442	0.0094258	-2.795021
EPHA7	0.56723	-0.390987	2.900702	0.0039455	0.0094284	-2.795324
ERMARD	-0.082475	5.9305311	-2.900164	0.0039522	0.0094436	-2.79684
RP5-908M14	0.3186136	-1.278843	2.9000027	0.0039542	0.0094473	-2.797293
RP11-700J1	0.4823617	1.0625559	2.8999969	0.0039542	0.0094473	-2.79731
TPM2	0.096369	6.2455175	2.8998989	0.0039554	0.0094496	-2.797585
SNORA67	0.3437007	-0.945745	2.8998656	0.0039558	0.0094499	-2.797679
MTFR1	-0.068276	6.3224329	-2.899793	0.0039567	0.0094515	-2.797885
AC159540.2	-0.119212	-1.481574	-2.899611	0.003959	0.0094562	-2.798398
LENG8-AS1	0.1491455	4.2666633	2.8994704	0.0039607	0.0094597	-2.798792
CLEC4M	0.7073425	0.2478911	2.8992869	0.003963	0.009464	-2.799309
SFR1	0.0785561	5.0615515	2.8992802	0.0039631	0.009464	-2.799328
EID2	-0.053528	5.7262297	-2.899264	0.0039633	0.009464	-2.799374
RBMY1F	-0.192369	-1.448257	-2.898875	0.0039681	0.0094748	-2.800469
GRAMD4P8	0.4077169	-0.732535	2.8988458	0.0039684	0.0094751	-2.80055
RP11-10N23	-0.500435	0.680386	-2.898662	0.0039707	0.0094799	-2.801067
RP1-41C23.	-0.451444	1.8928853	-2.89831	0.0039751	0.0094897	-2.802058
STX8	-0.071144	5.9837041	-2.89806	0.0039782	0.0094964	-2.802762
RP11-1143G	0.4928264	0.175447	2.8977783	0.0039817	0.0095042	-2.803555
PPP1CB	-0.031602	6.6899954	-2.897572	0.0039842	0.0095097	-2.804136
RP5-966M1.	0.5141522	0.921241	2.8974417	0.0039859	0.0095129	-2.804502
DHRS2	-0.43213	5.4472145	-2.897378	0.0039867	0.0095142	-2.804682
BRDT	0.5407106	-0.713235	2.8971269	0.0039898	0.009521	-2.805387
OR51E2	0.5727631	0.8079068	2.8969613	0.0039918	0.0095253	-2.805853
LACTB2	-0.084178	6.2263287	-2.89677	0.0039942	0.0095296	-2.806391

RP11-599B1	0.562164	-0.526443	2.896768	0.0039942	0.0095296	-2.806397
AC008697.1	0.3017344	-1.17973	2.8967509	0.0039944	0.0095296	-2.806445
AC018892.9	0.437411	-0.616622	2.8963589	0.0039993	0.0095407	-2.807548
RP11-384P7	0.459831	-0.503184	2.895504	0.00401	0.0095653	-2.809951
RP6-149D17	-0.119208	-1.496401	-2.895489	0.0040102	0.0095653	-2.809994
PIWIL2	-0.269363	4.2687472	-2.895308	0.0040124	0.0095701	-2.810504
TOMM20P3	-0.152604	-1.463402	-2.89511	0.0040149	0.0095753	-2.811059
CTA-276F8.	0.3897772	2.4548057	2.8950393	0.0040158	0.0095768	-2.811258
RP11-4K3__	0.4938002	1.1008691	2.894668	0.0040204	0.0095873	-2.812302
RPL35P2	-0.281858	2.9270209	-2.894208	0.0040262	0.0096001	-2.813595
FAM3B	0.6521061	4.3719705	2.8941977	0.0040263	0.0096001	-2.813624
KLHL5	0.0637272	6.3397611	2.893789	0.0040315	0.0096117	-2.814772
ALYREF	-0.065208	6.3048861	-2.893761	0.0040318	0.0096119	-2.814851
TMED2	-0.037192	7.0010046	-2.893376	0.0040366	0.0096224	-2.815931
DIS3L	-0.053446	6.0611815	-2.893359	0.0040369	0.0096224	-2.815981
SAA4	-0.33986	6.2097003	-2.893346	0.004037	0.0096224	-2.816018
RP11-84C10	0.2338136	-1.292693	2.893295	0.0040377	0.0096233	-2.81616
NAPG	0.0449009	6.0592263	2.8932523	0.0040382	0.009624	-2.81628
KCNT2	0.2767153	5.1107623	2.8927358	0.0040447	0.0096388	-2.817731
ELAVL2	-0.568652	0.6530314	-2.892311	0.00405	0.0096509	-2.818924
CYB561D2	-0.068061	6.3048909	-2.891945	0.0040547	0.0096613	-2.819953
RBMS3-AS3	0.4832306	-0.204423	2.8918062	0.0040564	0.0096649	-2.820342
HTR3B	0.5005807	-0.700638	2.8916099	0.0040589	0.0096701	-2.820893
TGS1	0.0595618	5.8206312	2.8914346	0.0040611	0.0096748	-2.821385
RP11-311H1	-0.432632	-0.482265	-2.891164	0.0040645	0.0096823	-2.822145
RPL23AP64	0.5352171	1.7456562	2.8910926	0.0040654	0.0096838	-2.822346
RP5-965F6.	0.3984691	-0.789621	2.8910185	0.0040664	0.0096854	-2.822554
LCN1	0.3532358	-0.978965	2.8909734	0.0040669	0.0096861	-2.822681
SENP5	-0.034876	6.1218139	-2.890881	0.0040681	0.0096882	-2.822939
CCDC169	0.5331348	-0.567955	2.8908623	0.0040683	0.0096882	-2.822992
RNF126	-0.052361	6.2859327	-2.89017	0.0040771	0.0097085	-2.824936
TMEM56-RWD	-0.185856	4.3357238	-2.889943	0.00408	0.0097147	-2.825572
DRAXIN	0.4035967	2.258264	2.8892923	0.0040883	0.0097337	-2.827399
AC009166.5	-0.441447	3.7308287	-2.889224	0.0040891	0.0097352	-2.827592
CTC-441N14	-0.198475	-1.372235	-2.88885	0.0040939	0.0097458	-2.828639
RP11-578F2	0.4524379	-0.221095	2.8887281	0.0040954	0.0097489	-2.828982
RP11-70L8.	0.5395117	1.1576662	2.8884707	0.0040987	0.0097561	-2.829704
LINC00989	0.5085294	0.2120072	2.8883956	0.0040997	0.0097577	-2.829915
RP11-968A1	0.4215097	-0.462228	2.8883151	0.0041007	0.0097595	-2.83014
RP11-706P1	-0.356533	2.8921316	-2.887892	0.0041061	0.0097717	-2.831328
RP3-461P17	0.4178631	2.128673	2.8875385	0.0041106	0.0097818	-2.832318
RP11-307C1	-0.4997	1.6537792	-2.887213	0.0041148	0.0097911	-2.83323
DNAJC24	0.0998032	5.2794581	2.8871222	0.0041159	0.0097932	-2.833486
TCF7	-0.14532	5.81625	-2.886889	0.0041189	0.0097997	-2.834138
INPP5A	0.0502606	5.9797986	2.8865476	0.0041233	0.0098094	-2.835097
IL31	-0.366884	-1.0992	-2.886489	0.004124	0.0098106	-2.83526
TEX19	0.5972188	1.5397766	2.886296	0.0041265	0.0098158	-2.835802
SPAG11B	-0.153359	-1.468569	-2.886195	0.0041278	0.0098183	-2.836086
PTGFR	0.667472	3.9682508	2.8859616	0.0041308	0.0098248	-2.836739
RP11-371E8	-0.332901	-0.971682	-2.885879	0.0041319	0.0098266	-2.83697
GS1-309P15	-0.910874	0.7739596	-2.8856	0.0041354	0.0098345	-2.837754

PERM1	0.5133236	3.2056253	2.8855385	0.0041362	0.0098358	-2.837925
EXTL1	0.5766776	1.8432124	2.8854477	0.0041374	0.0098379	-2.838179
CSF2	0.4236048	-0.556313	2.8852042	0.0041405	0.0098447	-2.838862
RP11-1114I	0.3853585	-0.961259	2.8851304	0.0041415	0.0098461	-2.839069
CTC-255N2C	0.5035867	-0.680079	2.8851178	0.0041416	0.0098461	-2.839104
FANCM	0.0967849	5.0184303	2.8849903	0.0041433	0.0098493	-2.839461
CTR9	-0.052717	6.4473457	-2.884134	0.0041543	0.0098749	-2.841861
AC009784.3	0.2824454	-1.190471	2.8839828	0.0041563	0.0098789	-2.842284
RP11-407H1	-0.309196	-1.247569	-2.883956	0.0041566	0.0098791	-2.842359
CTGLF12P	0.5882895	1.9128899	2.8837383	0.0041594	0.0098851	-2.842969
AC006077.3	-0.309365	3.1187154	-2.883588	0.0041613	0.0098891	-2.843389
MIR210	0.4479536	-0.458055	2.8835408	0.004162	0.0098899	-2.843521
CTD-2514C3	0.5623561	1.9910524	2.8833742	0.0041641	0.0098943	-2.843988
RPS6KA5	0.1364047	4.8930771	2.8831413	0.0041671	0.0099006	-2.84464
NUAK2	0.1277633	5.6264173	2.8831289	0.0041673	0.0099006	-2.844675
RP11-148B1	0.4904282	-0.36877	2.8829223	0.00417	0.0099063	-2.845254
RP11-430C7	0.5402329	0.3562632	2.8827225	0.0041725	0.0099118	-2.845813
CLCNKA	0.5860189	2.8528792	2.8826953	0.0041729	0.009912	-2.845889
PPP1R15A	0.0678864	6.2450749	2.88243	0.0041763	0.0099195	-2.846632
CTD-2540L5	-0.451528	-0.65642	-2.882347	0.0041774	0.0099214	-2.846864
BATF2	0.1238141	5.2936274	2.8817278	0.0041854	0.0099399	-2.848598
RP11-342K6	0.3327726	3.0223311	2.8816375	0.0041866	0.009942	-2.84885
EIF2AK4	-0.047306	6.353621	-2.88125	0.0041917	0.0099533	-2.849934
AC074117.1	-0.089269	5.2457188	-2.88091	0.0041961	0.0099631	-2.850885
LA16c-381G	0.3039074	-1.18498	2.8807633	0.004198	0.009967	-2.851297
SPAM1	-0.154424	-1.453947	-2.880465	0.0042019	0.0099756	-2.852131
SRM	-0.064671	6.45574	-2.880222	0.004205	0.009982	-2.852812
CTD-2622I1	0.4977465	0.5790353	2.8802185	0.0042051	0.009982	-2.852821
LINC01534	-0.174063	4.0719413	-2.88016	0.0042058	0.0099831	-2.852984
ZNF663P	0.4025898	-0.865722	2.8800231	0.0042076	0.0099867	-2.853367
GPR142	0.3219892	-1.061595	2.8799553	0.0042085	0.0099882	-2.853557
ZNF200	-0.055204	5.5916966	-2.879683	0.0042121	0.009996	-2.854319
CTD-2135D7	-0.457922	0.1236088	-2.879582	0.0042134	0.0099983	-2.854601
BNIP3P37	-0.194561	-1.373496	-2.879564	0.0042136	0.0099983	-2.854651
MSH2	0.0645664	5.9357932	2.8792555	0.0042177	0.0100073	-2.855514
LINC00472	0.5117168	1.8849351	2.8789735	0.0042214	0.0100154	-2.856303
LZIC	-0.066927	5.9344469	-2.878938	0.0042218	0.0100158	-2.856403
ZNF735	-0.527376	-0.841076	-2.878825	0.0042233	0.0100187	-2.856718
PANO1	-0.259701	3.1596432	-2.878584	0.0042265	0.0100255	-2.857392
RP11-849H4	0.3415404	2.7754824	2.8784599	0.0042281	0.0100287	-2.857739
CTD-2521M2	0.3986261	2.2117567	2.8782061	0.0042314	0.0100359	-2.858448
YY1AP1	-0.041214	6.3649793	-2.877844	0.0042362	0.0100466	-2.85946
MAG	0.5974271	0.018088	2.8777439	0.0042375	0.0100488	-2.859741
LINC00533	0.3502426	-1.087454	2.8777298	0.0042377	0.0100488	-2.85978
RP11-564A8	0.2606731	-1.243188	2.8772177	0.0042444	0.0100641	-2.861211
PPP1R3G	-0.285529	5.5455545	-2.876799	0.0042499	0.0100761	-2.86238
PTPDC1	0.1230961	5.0579252	2.8767839	0.0042501	0.0100761	-2.862424
HMGN3-AS1	0.1836478	4.134669	2.8767724	0.0042502	0.0100761	-2.862456
GINS3	0.1084351	5.1093741	2.8766865	0.0042514	0.0100776	-2.862696
IL17F	0.3218535	-1.109173	2.8766634	0.0042517	0.0100776	-2.86276
RP11-153I2	-0.460624	0.9395846	-2.876661	0.0042517	0.0100776	-2.862768

RN7SL614P	-0.237086	-1.261833	-2.876241	0.0042573	0.0100901	-2.863942
MASTL	0.0681321	5.6008297	2.8756741	0.0042647	0.0101071	-2.865524
PMS2P5	-0.250729	3.9017593	-2.875549	0.0042664	0.0101104	-2.865872
FRG2FP	-0.143751	-1.483114	-2.874928	0.0042746	0.0101292	-2.867609
LMTK2	0.0556001	6.1955903	2.8748127	0.0042761	0.0101321	-2.86793
MAGED1	0.0841015	6.7692687	2.8745844	0.0042792	0.0101387	-2.868567
RP11-18B16	0.6109089	1.4488268	2.874298	0.0042829	0.010147	-2.869367
RP11-314A2	0.418513	-0.394231	2.8741878	0.0042844	0.0101496	-2.869675
CTSB	-0.058883	7.3339267	-2.874174	0.0042846	0.0101496	-2.869714
FRMPD4	0.3731497	-1.050343	2.8741064	0.0042855	0.010151	-2.869902
RP1-171K16	-0.126806	-1.480788	-2.873697	0.0042909	0.0101632	-2.871044
SMIM17	0.5265658	0.228723	2.8736633	0.0042914	0.0101637	-2.871139
EGFLAM	0.1265046	5.0952337	2.873547	0.0042929	0.0101665	-2.871464
ENPP7P7	0.3502164	-1.004047	2.8735308	0.0042931	0.0101665	-2.871509
SULT1A1	-0.124326	6.4701473	-2.873299	0.0042962	0.0101725	-2.872156
AL358852.1	-0.394156	2.0972546	-2.873298	0.0042962	0.0101725	-2.872157
RP5-933K21	-0.235001	-1.275253	-2.873098	0.0042989	0.0101781	-2.872715
RN7SL416P	-0.181126	-1.395688	-2.872606	0.0043054	0.010193	-2.87409
SMG7	-0.03769	6.5676052	-2.872456	0.0043074	0.0101971	-2.874509
CCT7P2	0.512793	-0.709731	2.8724315	0.0043078	0.0101971	-2.874576
ZNF585B	0.1494736	5.2985658	2.8724146	0.004308	0.0101971	-2.874624
MSANTD4	0.1880031	5.2973392	2.872004	0.0043135	0.0102094	-2.875769
MRPL18	-0.050572	6.364262	-2.871813	0.004316	0.0102147	-2.876302
RP5-1112D6	0.1869563	4.2680674	2.8715424	0.0043196	0.0102226	-2.877057
TRIM35	-0.09408	5.9177954	-2.871384	0.0043218	0.010227	-2.877499
GPR4	0.0910648	5.4522154	2.8713055	0.0043228	0.0102288	-2.877718
YARS	-0.054065	6.5499981	-2.871283	0.0043231	0.0102288	-2.87778
RP11-478C6	0.5102903	0.7423427	2.8709505	0.0043276	0.0102387	-2.878708
MIR4539	0.2274994	-1.344171	2.8704811	0.0043338	0.0102529	-2.880017
AL050310.1	-0.338451	-0.914245	-2.870205	0.0043375	0.0102607	-2.880788
VPS13B	0.0610493	6.0473662	2.8701948	0.0043377	0.0102607	-2.880815
MEX3A	0.1467543	5.2642726	2.870063	0.0043394	0.0102639	-2.881183
ELMO3	0.1927359	5.4705688	2.8700508	0.0043396	0.0102639	-2.881217
IFT22	-0.068683	5.7301079	-2.870029	0.0043399	0.0102639	-2.881277
SIX2	0.6571109	2.0818382	2.8698528	0.0043423	0.0102689	-2.881769
GSDMC	0.6221898	2.3435499	2.8697398	0.0043438	0.0102718	-2.882084
FMO2	0.4012186	4.2671709	2.8695323	0.0043466	0.0102777	-2.882662
MAB21L2	0.6201627	3.4273956	2.8691742	0.0043514	0.0102884	-2.88366
CASK	-0.065781	6.2356732	-2.869033	0.0043533	0.0102916	-2.884054
TMCO5B	-0.125054	-1.495158	-2.869017	0.0043535	0.0102916	-2.884099
NHLH2	0.410658	-1.05361	2.8690113	0.0043536	0.0102916	-2.884114
AL122127.2	0.5104267	-0.512719	2.868736	0.0043573	0.0102997	-2.884882
TAF7	-0.052407	6.5269336	-2.868436	0.0043613	0.0103086	-2.885717
RPS10L	-0.484991	1.7980072	-2.868413	0.0043616	0.0103087	-2.885782
ELP4	-0.046516	5.7050939	-2.868169	0.0043649	0.0103158	-2.886461
BACE1	-0.056055	6.2950451	-2.867797	0.00437	0.010327	-2.887498
KLB	-0.193119	6.363737	-2.867451	0.0043746	0.0103374	-2.888463
EN1	0.5549361	-0.507898	2.8673851	0.0043755	0.0103388	-2.888645
SLC12A7	-0.049535	6.7481473	-2.86732	0.0043764	0.0103402	-2.888827
RBM27	-0.038416	5.94054	-2.867128	0.004379	0.0103457	-2.889363
RP11-582J1	0.1744609	3.6377294	2.8670925	0.0043795	0.0103461	-2.88946

RP11-521M1	-0.311807	-1.176103	-2.866911	0.0043819	0.0103513	-2.889966
BCL7A	0.0617773	5.9444353	2.8668797	0.0043824	0.0103516	-2.890053
LRG1	-0.136073	7.0406768	-2.866807	0.0043833	0.0103533	-2.890255
C6orf203	-0.066808	5.8955078	-2.866241	0.004391	0.0103707	-2.891831
RP1-278022	0.4620612	-0.024666	2.8660237	0.004394	0.010377	-2.892437
SNHG1	0.0706093	6.014102	2.8658419	0.0043964	0.0103822	-2.892943
RP11-395B7	0.6469793	1.3761297	2.86544	0.0044019	0.0103944	-2.894062
BPIFB9P	0.3842908	-0.649768	2.8653857	0.0044026	0.0103955	-2.894213
MRPL40P1	0.5241412	0.3215869	2.8652585	0.0044044	0.0103989	-2.894567
RP1-315G1.	-0.466462	1.6005761	-2.865136	0.004406	0.0104021	-2.894908
CTB-189B5.	-0.367829	-0.794513	-2.865109	0.0044064	0.0104023	-2.894982
RP11-863P1	0.5085294	-0.148993	2.8650288	0.0044075	0.0104042	-2.895206
RP11-341G2	0.4581454	-0.67564	2.864928	0.0044089	0.0104068	-2.895487
LDHAL6A	0.494083	0.2161417	2.8648459	0.00441	0.0104088	-2.895716
ERCC6	0.0593911	5.4096895	2.8647781	0.0044109	0.0104097	-2.895904
ANKFY1	0.0638414	6.1595157	2.8647758	0.0044109	0.0104097	-2.895911
DUX4L18	-0.346853	-1.244553	-2.864678	0.0044123	0.0104121	-2.896182
SREBF2	-0.055859	6.8034933	-2.86422	0.0044185	0.0104259	-2.897456
RP11-402J6	0.3914352	2.0270165	2.8642092	0.0044187	0.0104259	-2.897487
OR7E47P	-0.638093	1.1396883	-2.863894	0.004423	0.0104354	-2.898364
RP11-80P2C	0.4604023	2.2731041	2.8635247	0.004428	0.0104466	-2.899392
RP11-99E15	0.4120595	-0.538439	2.8632452	0.0044318	0.0104549	-2.900169
ZBTB40	0.0513644	5.9008983	2.8630812	0.0044341	0.0104595	-2.900626
DNTTIP1	-0.045439	6.0519238	-2.862691	0.0044394	0.0104715	-2.901711
IPMK	0.0703327	5.6064717	2.8624716	0.0044424	0.0104779	-2.902321
SRD5A3-AS1	0.1583596	3.5618901	2.8620347	0.0044484	0.0104909	-2.903536
RP11-14501	-0.119111	-1.507029	-2.862027	0.0044485	0.0104909	-2.903557
ITGA6	-0.068216	6.4490638	-2.862003	0.0044489	0.010491	-2.903625
UHRF2	0.0522839	5.9548151	2.8617834	0.0044519	0.0104975	-2.904235
RP11-352G9	0.5007356	0.1413589	2.8617388	0.0044525	0.0104982	-2.904359
MYCT1	0.1156915	5.371442	2.8615834	0.0044546	0.0105026	-2.904791
CDX2	0.630595	0.1172588	2.8612116	0.0044597	0.010514	-2.905824
XRCC2	0.2119126	4.6778064	2.8611205	0.004461	0.0105162	-2.906078
RP3-335N17	0.3919337	-0.958126	2.8611008	0.0044613	0.0105162	-2.906132
LOC1004211	-0.558082	0.3643551	-2.860868	0.0044645	0.0105231	-2.906781
CTC-412M14	0.2746758	-1.192603	2.8607061	0.0044667	0.0105276	-2.90723
AP005135.2	-0.107076	-1.505689	-2.860306	0.0044722	0.0105397	-2.908341
ZIM3	-0.130818	-1.4799	-2.860294	0.0044724	0.0105397	-2.908376
RP4-647C14	0.4475668	1.5646028	2.8602061	0.0044736	0.0105418	-2.908619
ADCK2	-0.057637	6.1893832	-2.859965	0.0044769	0.010549	-2.90929
RP11-443B7	-0.180654	4.840016	-2.859854	0.0044784	0.0105519	-2.909597
RP11-164P1	0.4690142	-0.380087	2.8596621	0.0044811	0.0105575	-2.910131
EIF3EP2	-0.113768	-1.498554	-2.859472	0.0044837	0.0105625	-2.910659
MYO7B	0.2614046	4.8994415	2.8594669	0.0044838	0.0105625	-2.910673
CBLN1	-0.499617	4.174267	-2.859423	0.0044844	0.0105632	-2.910795
VN1R2	-0.111044	-1.492782	-2.859337	0.0044856	0.0105654	-2.911035
C3orf79	-0.181581	-1.423635	-2.859072	0.0044893	0.0105733	-2.911769
P2RY2	-0.184149	4.9281925	-2.858964	0.0044908	0.0105762	-2.91207
RP11-309M7	0.3170533	-1.147033	2.858037	0.0045036	0.0106057	-2.914645
FAM53A	-0.200427	4.6975772	-2.85787	0.0045059	0.0106105	-2.915108
FABP5P7	0.495431	0.1241334	2.8575777	0.00451	0.0106194	-2.91592

MLLT10P1	0.4582638	0.0168663	2.8572315	0.0045148	0.0106296	-2.916881
AC104532.4	0.4727709	1.9217782	2.8572231	0.0045149	0.0106296	-2.916904
RP11-3J1.1	-0.653295	0.4761451	-2.857058	0.0045172	0.0106343	-2.917361
RPL6P2	-0.256205	-1.195557	-2.85689	0.0045196	0.0106392	-2.917829
RP11-474D1	-0.349159	-1.14759	-2.856866	0.0045199	0.0106393	-2.917896
ITGA7	-0.087019	6.0505977	-2.856498	0.004525	0.0106507	-2.918918
C15orf48	0.3633406	4.6628441	2.8564585	0.0045256	0.0106509	-2.919027
ADAMTS18	0.6703349	1.3628477	2.8564484	0.0045257	0.0106509	-2.919055
GLS2	0.5834333	4.1029536	2.8562956	0.0045278	0.0106552	-2.919479
RN7SL445P	-0.182412	-1.395283	-2.855987	0.0045322	0.0106647	-2.920336
GSE1	0.054618	6.2856802	2.8558277	0.0045344	0.0106692	-2.920777
LHX1	0.4110024	-1.079518	2.8557977	0.0045348	0.0106695	-2.92086
GRM2	0.4553274	2.7495644	2.8557686	0.0045352	0.0106695	-2.920941
C1orf27	-0.057361	6.2976598	-2.855758	0.0045353	0.0106695	-2.920972
RP11-297A1	-0.489616	0.3786384	-2.855627	0.0045372	0.0106731	-2.921334
C17orf80	-0.045153	5.9165535	-2.855569	0.004538	0.0106743	-2.921495
AL022476.2	0.4429203	1.4225397	2.85532	0.0045415	0.0106818	-2.922186
RP11-54A4.	0.374971	2.6406353	2.8552673	0.0045422	0.0106829	-2.922332
MIR5187	-0.282482	-1.155084	-2.855105	0.0045445	0.0106875	-2.922782
SCARNA21	0.4967158	-0.252338	2.8549969	0.004546	0.0106898	-2.923082
SEMA5A	0.1678387	5.6516333	2.8549933	0.004546	0.0106898	-2.923092
DCAF4L2	-1.012026	1.5483844	-2.854725	0.0045498	0.010698	-2.923838
ZNF672	-0.049363	6.345081	-2.854154	0.0045578	0.0107161	-2.92542
RPL31P35	-0.224528	-1.309076	-2.854017	0.0045597	0.0107199	-2.925801
RP11-736K2	-0.339442	3.879316	-2.853704	0.0045641	0.0107296	-2.926668
RP11-48B3.	-0.142075	4.2592613	-2.853607	0.0045655	0.0107321	-2.926937
AGER	0.0995438	4.9153732	2.8533902	0.0045685	0.0107386	-2.927538
SMIM5	0.40336	2.8491171	2.8532541	0.0045704	0.0107424	-2.927915
ANAPC1	0.0523066	5.6607304	2.8531701	0.0045716	0.0107444	-2.928148
STX12	-0.039203	6.1682239	-2.852681	0.0045785	0.0107599	-2.929504
BDH2P1	-0.486807	-0.123127	-2.852638	0.0045791	0.0107607	-2.929624
AC092687.4	-0.130082	-1.473255	-2.852556	0.0045803	0.0107625	-2.929851
RP11-5P18.	0.4879951	-0.632157	2.8525409	0.0045805	0.0107625	-2.929892
ZSCAN5A	0.0797831	5.0580533	2.8524851	0.0045813	0.010763	-2.930047
RP11-757O6	-0.592444	-0.044312	-2.852465	0.0045816	0.010763	-2.930103
FTLP14	0.3106144	4.010887	2.8524636	0.0045816	0.010763	-2.930107
LL22NC03-2	0.5535349	0.4739831	2.8519588	0.0045887	0.010779	-2.931506
PF4	0.5708342	0.244127	2.8518592	0.0045901	0.0107816	-2.931782
DHRS7B	-0.066902	6.091511	-2.851763	0.0045915	0.0107841	-2.932048
NUP93	0.0481735	5.9982303	2.8512891	0.0045982	0.0107992	-2.933361
RP11-457M1	0.3697039	-0.771266	2.8512028	0.0045994	0.0108013	-2.933601
PRIMPOL	-0.064262	5.5127879	-2.851071	0.0046013	0.0108044	-2.933967
PPP5C	0.0361551	6.3721561	2.8510637	0.0046014	0.0108044	-2.933986
NT5C3A	0.0616227	5.8596405	2.8510476	0.0046016	0.0108044	-2.93403
CTC-329D1.	-0.181967	3.559612	-2.850989	0.0046024	0.0108057	-2.934194
ZRANB2	-0.038407	6.2874561	-2.850935	0.0046032	0.0108068	-2.934343
ARHGEF37	-0.190039	5.4181615	-2.850823	0.0046048	0.0108098	-2.934654
RP11-1228E	0.5078224	0.1370428	2.8506433	0.0046073	0.0108151	-2.93515
SIKE1	-0.043049	6.0740805	-2.850602	0.0046079	0.0108158	-2.935264
RP3-329E20	0.3174016	-1.139405	2.8504918	0.0046095	0.0108187	-2.93557
IGKV1OR22-	0.3631742	-1.06843	2.8502685	0.0046126	0.0108255	-2.936189

GFM1	-0.044525	6.4208767	-2.85019	0.0046137	0.0108273	-2.936407
TRAV8-5	0.3315235	-1.057967	2.8501727	0.004614	0.0108273	-2.936454
ARV1	-0.059796	6.0574442	-2.850067	0.0046155	0.0108301	-2.936746
SNAPC2	-0.055702	5.7930279	-2.849987	0.0046166	0.0108321	-2.936968
AC010731.2	-0.224558	-1.359199	-2.849939	0.0046173	0.0108324	-2.937102
AC020550.7	-0.195354	-1.380017	-2.849936	0.0046174	0.0108324	-2.93711
CEBPA	-0.095863	6.7740761	-2.849334	0.0046259	0.0108517	-2.938777
LINC01473	-0.293762	2.9594746	-2.849139	0.0046287	0.0108575	-2.939315
VPS52	-0.052052	6.3761522	-2.848922	0.0046318	0.0108641	-2.939918
C12orf60	-0.109327	4.8951744	-2.848737	0.0046344	0.0108693	-2.940428
DEFB1	0.4811743	5.8400914	2.8487258	0.0046346	0.0108693	-2.94046
P2RX2	0.5927397	-0.22071	2.848484	0.004638	0.0108766	-2.94113
LINC01067	0.5725001	-0.073333	2.8483549	0.0046399	0.0108803	-2.941487
TMEM81	-0.087538	5.1418709	-2.848069	0.0046439	0.0108891	-2.942279
PRKDC	0.0532612	6.629185	2.847729	0.0046488	0.0108998	-2.943219
RP11-572O6	-0.501589	1.1189727	-2.847676	0.0046495	0.0109009	-2.943365
PFN1P1	0.3731793	2.7109617	2.8473788	0.0046538	0.01091	-2.944188
RP11-662G2	-0.496752	-0.740721	-2.847361	0.004654	0.01091	-2.944236
SART3	0.031464	6.2402379	2.8473049	0.0046548	0.0109112	-2.944393
DDX39B	0.0716518	6.3110936	2.8465906	0.0046651	0.0109345	-2.946369
CCDC27	0.2952481	-1.138661	2.8464868	0.0046666	0.0109373	-2.946656
RP4-612B18	-0.473154	-0.00426	-2.846012	0.0046734	0.0109526	-2.947969
SPDYA	0.2827297	3.1927695	2.8456978	0.0046779	0.0109624	-2.948838
RP4-775C13	0.4591429	-0.133636	2.8454889	0.0046809	0.0109688	-2.949416
SH2B1	-0.040488	6.2954857	-2.845434	0.0046817	0.0109699	-2.949567
C1QTNF9	-0.535646	1.090626	-2.845373	0.0046826	0.0109708	-2.949737
C17orf59	-0.079089	5.6227621	-2.845366	0.0046827	0.0109708	-2.949757
CTD-2571L2	0.4326903	1.549931	2.845273	0.004684	0.0109732	-2.950013
CA7	0.3939899	-0.917967	2.8451653	0.0046855	0.0109761	-2.95031
C10orf82	0.3002704	-1.195477	2.8450965	0.0046865	0.0109778	-2.950501
H1FX-AS1	-0.154645	4.5252393	-2.845013	0.0046877	0.0109799	-2.950732
OR2B8P	-0.174943	-1.406776	-2.844573	0.0046941	0.010994	-2.951947
CAPN9	0.5911455	2.4078324	2.8442916	0.0046981	0.0110028	-2.952726
ZNF385D-AS	-0.593291	-0.4413	-2.8442	0.0046995	0.0110052	-2.952979
ADRB1	-0.580016	2.4011449	-2.844172	0.0046999	0.0110054	-2.953056
AIM1	-0.142093	5.7306658	-2.843467	0.0047101	0.0110286	-2.955006
RP11-158K1	0.1323184	4.7070238	2.8430181	0.0047165	0.0110431	-2.956245
AC074286.1	0.128265	4.3019048	2.8428557	0.0047189	0.0110479	-2.956694
RP11-661A1	-0.562163	3.2892819	-2.842798	0.0047197	0.0110491	-2.956853
SRSF4	-0.034441	6.4614253	-2.842776	0.00472	0.0110492	-2.956914
RP11-16E18	-0.116419	4.496288	-2.842691	0.0047213	0.0110513	-2.95715
RP11-12A2.	-0.729431	1.3115223	-2.842617	0.0047224	0.0110528	-2.957353
RP11-248J1	0.2625144	2.9358326	2.8426065	0.0047225	0.0110528	-2.957382
SNORA26	0.5201008	1.1973353	2.8425521	0.0047233	0.0110539	-2.957533
GPR116	0.0932505	5.9247261	2.842338	0.0047264	0.0110605	-2.958124
PROX1	-0.107813	6.6917531	-2.842233	0.0047279	0.011063	-2.958415
RP11-497G1	0.5322187	-0.478596	2.8422226	0.0047281	0.011063	-2.958443
RP1-95L4.4	-0.130984	5.0136638	-2.841742	0.004735	0.0110786	-2.95977
MDH1B	0.4795368	2.1734095	2.8415653	0.0047376	0.0110839	-2.960258
VRK1	0.0695625	5.5150183	2.84143	0.0047396	0.0110878	-2.960632
LRRC75B	-0.094636	5.9956083	-2.84116	0.0047435	0.0110962	-2.961377

TYW3	-0.05061	5.8708445	-2.841105	0.0047443	0.0110974	-2.961528
RP11-177F1	-0.567444	-0.393107	-2.840964	0.0047464	0.0111015	-2.961918
RP11-231G3	-0.192507	3.6463026	-2.840819	0.0047485	0.0111057	-2.96232
SLC2A1	0.177629	5.395927	2.8404304	0.0047541	0.0111183	-2.963392
ACTG1P1	0.448387	1.9294029	2.8403744	0.004755	0.0111195	-2.963546
NOMO1	-0.049749	6.2782886	-2.840156	0.0047581	0.0111262	-2.964149
BTG1	0.0558004	6.7849634	2.8399875	0.0047606	0.0111312	-2.964614
KRR1P1	0.4614112	-0.186368	2.839918	0.0047616	0.0111329	-2.964806
RP11-394B2	0.5001705	0.5525355	2.8397242	0.0047644	0.0111388	-2.965341
DIAPH2	-0.059157	6.1121961	-2.839634	0.0047658	0.0111412	-2.96559
RP11-795A2	-0.202515	-1.408159	-2.83902	0.0047747	0.0111614	-2.967283
COMT	-0.075743	6.7033933	-2.838866	0.004777	0.011166	-2.96771
CALR3	-0.510773	0.3752931	-2.838443	0.0047832	0.0111798	-2.968875
SLC7A15P	-0.24737	-1.323642	-2.83818	0.0047871	0.0111878	-2.969602
AADACL2-AS	-0.458567	-0.444273	-2.838168	0.0047872	0.0111878	-2.969636
RP1-137D17	0.4939177	0.7205176	2.8379956	0.0047898	0.011193	-2.97011
TDGF1	-0.494071	4.5898132	-2.837948	0.0047905	0.0111939	-2.970242
ZNF180	0.0626804	5.4195335	2.8378091	0.0047925	0.0111979	-2.970625
CTD-2315E1	-0.537199	-0.152642	-2.83776	0.0047932	0.0111989	-2.970759
MBD4	-0.0431	6.1893245	-2.8377	0.0047941	0.0112002	-2.970925
RP11-16E12	0.4305743	3.8607416	2.8375245	0.0047967	0.0112048	-2.97141
RP11-104L2	-0.491091	2.0018981	-2.837511	0.0047969	0.0112048	-2.971446
TM4SF5	-0.232475	6.6630675	-2.837505	0.004797	0.0112048	-2.971464
CTC-441N14	-0.25771	-1.237824	-2.837333	0.0047995	0.0112095	-2.971939
RP4-738P11	0.5361305	-0.512682	2.8372965	0.0048	0.0112095	-2.972038
GRPEL2-AS1	0.3951179	-0.588895	2.8372906	0.0048001	0.0112095	-2.972055
USP27X-AS1	0.3567858	3.804116	2.8372856	0.0048002	0.0112095	-2.972068
CTD-2008P7	-0.206987	-1.372412	-2.837244	0.0048008	0.0112102	-2.972184
TAB2	-0.056519	6.3626212	-2.837081	0.0048032	0.0112151	-2.972632
KRT8P24	0.5343217	0.507888	2.8370297	0.004804	0.0112161	-2.972774
RP11-655C2	0.2370136	-1.278879	2.8360104	0.004819	0.0112505	-2.975584
Z84812.4	0.2997308	-1.050351	2.835823	0.0048218	0.0112562	-2.976101
RP4-756G23	0.3535132	3.0366374	2.8357445	0.0048229	0.0112582	-2.976317
LL0XNC01-2	0.4189961	-0.583912	2.8356404	0.0048245	0.0112609	-2.976604
SNRPE	-0.062234	6.436867	-2.835626	0.0048247	0.0112609	-2.976644
RP11-478C1	0.4979677	0.6879478	2.8355369	0.004826	0.0112632	-2.976889
MPHOSPH8	-0.039371	6.1901055	-2.835308	0.0048294	0.0112704	-2.97752
DNAH3	0.6069367	1.3351161	2.8348025	0.0048369	0.0112871	-2.978913
CYP4Z1	-0.607891	1.1227856	-2.834341	0.0048437	0.0113017	-2.980186
DNAJC28	-0.119225	4.552106	-2.834339	0.0048437	0.0113017	-2.980189
ATP5G1P3	-0.205614	-1.34398	-2.834207	0.0048457	0.0113055	-2.980553
RP1-257C22	0.6131267	1.2500792	2.834163	0.0048463	0.0113064	-2.980675
RUSC1	0.0654876	6.0490305	2.8341358	0.0048467	0.0113066	-2.980749
RP11-438F1	-0.349379	-1.152919	-2.834057	0.0048479	0.0113078	-2.980968
RNF183	0.6269177	1.1358991	2.834055	0.0048479	0.0113078	-2.980972
PPP5D1	0.2904682	2.4316101	2.8340368	0.0048482	0.0113078	-2.981022
C5orf66-AS	0.3873324	-1.072852	2.8339832	0.004849	0.011309	-2.98117
TFCP2	0.0622989	5.9885907	2.8336664	0.0048537	0.0113192	-2.982043
HBS1L	-0.046081	6.2700853	-2.833436	0.0048571	0.0113265	-2.982677
RP11-159D1	0.1352951	5.1428523	2.8333599	0.0048583	0.0113284	-2.982887
SRGAP1	0.1606309	5.2565687	2.8332268	0.0048602	0.0113323	-2.983253

C22orf39	-0.04937	5.9215764	-2.833007	0.0048635	0.0113392	-2.983859
AC099552.2	-0.439464	-0.849667	-2.832639	0.004869	0.0113513	-2.984872
RP11-326A1	0.3822297	-0.905414	2.8322916	0.0048742	0.0113626	-2.985828
RP1-102D24	0.3885888	-0.850357	2.8322422	0.0048749	0.0113636	-2.985964
SNORD113-3	0.2937762	-1.254554	2.8322049	0.0048755	0.0113642	-2.986067
RP11-407N1	0.4560992	0.6909991	2.832161	0.0048761	0.011365	-2.986188
RP11-433J8	0.2747232	-1.140799	2.8320817	0.0048773	0.011367	-2.986406
SPIRE2	-0.086772	5.9356111	-2.832038	0.004878	0.0113678	-2.986527
ACSBG1	0.4917575	1.6763978	2.8319505	0.0048793	0.0113688	-2.986767
RP11-449J2	0.6046254	2.5096236	2.8319476	0.0048793	0.0113688	-2.986775
BPI	0.5474543	2.3457123	2.8319461	0.0048793	0.0113688	-2.986779
GRID1-AS1	0.2940298	-1.181752	2.8318298	0.0048811	0.0113721	-2.987099
FABP2	-0.584606	-0.355963	-2.831629	0.0048841	0.0113784	-2.987652
A2MP1	0.4684559	2.5915223	2.8313316	0.0048885	0.011388	-2.988471
FAM86B2	0.4140015	-0.594028	2.8311555	0.0048911	0.0113934	-2.988955
MAGOHB	-0.057511	5.8086536	-2.831024	0.0048931	0.0113973	-2.989317
RP5-997D16	0.1209533	4.3945489	2.8309634	0.004894	0.0113987	-2.989484
CTB-3601.3	-0.345294	-0.894451	-2.830438	0.0049019	0.0114163	-2.99093
ALG12	0.0878557	5.9171515	2.8303677	0.004903	0.011418	-2.991123
RP11-404E1	-0.25409	-1.274486	-2.830257	0.0049046	0.0114212	-2.991428
SPTBN1	-0.043322	7.1149059	-2.830197	0.0049055	0.0114225	-2.991593
ABCC8	0.6568134	1.7281204	2.829965	0.004909	0.0114299	-2.992231
PRKAA2	0.4169848	5.2641891	2.8293373	0.0049184	0.0114511	-2.993957
PKD2L2	0.4657305	0.0159144	2.8292805	0.0049193	0.0114524	-2.994113
CPEB2	-0.084254	6.0141159	-2.829119	0.0049217	0.0114573	-2.994558
C2orf72	-0.077413	6.7756895	-2.829014	0.0049233	0.0114602	-2.994845
RP11-1217F	-0.182106	-1.419099	-2.828856	0.0049257	0.011465	-2.99528
RP11-466F5	-0.458878	-0.017452	-2.828714	0.0049278	0.0114693	-2.995672
EFNB1	0.0693684	6.0840623	2.8286659	0.0049285	0.0114703	-2.995803
PHF8	-0.070985	6.2650371	-2.828467	0.0049315	0.0114765	-2.99635
MTOR	-0.056726	6.4645578	-2.828302	0.004934	0.0114816	-2.996803
SETBP1	0.1341527	5.5441678	2.8280878	0.0049372	0.0114884	-2.997393
SDC4	-0.069421	7.055245	-2.827861	0.0049407	0.0114956	-2.998015
AC092667.2	0.5910248	1.9888695	2.8277019	0.0049431	0.0115004	-2.998453
RHOQP3	0.2940229	-1.067796	2.827668	0.0049436	0.0115009	-2.998547
RP11-320N2	0.4746813	-0.277371	2.8273583	0.0049483	0.0115111	-2.999398
WDR48	-0.034461	6.1691034	-2.827332	0.0049487	0.0115112	-2.99947
AC096649.2	-0.450234	0.1267442	-2.827302	0.0049491	0.0115116	-2.999552
PRR5	-0.085729	5.8489373	-2.826799	0.0049567	0.0115286	-3.000936
RP5-857K21	-0.136546	4.987194	-2.826466	0.0049618	0.0115396	-3.00185
TGIF2-C20c	0.486948	0.3386384	2.8262972	0.0049643	0.0115448	-3.002314
RAD51D	0.0578662	5.6254636	2.8259698	0.0049693	0.0115556	-3.003213
RBAK	0.0585173	5.7506668	2.8258721	0.0049708	0.0115583	-3.003481
AC073957.1	-0.329307	-0.998623	-2.82583	0.0049714	0.011559	-3.003596
STEAP3-AS1	0.5182566	1.573965	2.8258066	0.0049718	0.0115591	-3.003661
FBXW4P1	0.2699841	3.3343323	2.8255074	0.0049763	0.0115687	-3.004483
NEK3	-0.118289	5.5510283	-2.825494	0.0049765	0.0115687	-3.004519
LINC00174	-0.098027	5.3679181	-2.825449	0.0049772	0.0115696	-3.004644
CTC-338M12	-0.372476	-0.762266	-2.825377	0.0049783	0.0115714	-3.00484
ZSCAN22	-0.050901	5.4389418	-2.825095	0.0049826	0.0115806	-3.005617
CTA-941F9.	0.2909108	3.4040214	2.8250536	0.0049832	0.0115813	-3.00573

ZNF605	0.0699354	5.6795268	2.8248534	0.0049863	0.0115877	-3.006279
ZBTB11-AS1	-0.095519	4.7364109	-2.824699	0.0049886	0.0115924	-3.006703
ARL14EP	-0.034831	5.9421993	-2.824383	0.0049934	0.0116027	-3.007571
C2orf76	-0.064607	5.294782	-2.824365	0.0049937	0.0116027	-3.007619
RNU6-863P	-0.191045	-1.37625	-2.824346	0.004994	0.0116027	-3.007673
RP11-212P7	-0.083579	5.1924591	-2.824302	0.0049947	0.0116035	-3.007792
AC104024.1	0.2637782	-1.175425	2.8241766	0.0049966	0.0116072	-3.008137
C5	-0.100244	7.0904645	-2.823928	0.0050004	0.0116153	-3.008819
DENND5B	-0.066882	6.0926775	-2.82349	0.0050071	0.0116301	-3.010023
DEFA10P	0.3080401	-1.211153	2.8233396	0.0050094	0.0116347	-3.010435
RP11-451G4	-0.64572	-0.204863	-2.823244	0.0050108	0.0116373	-3.010696
ZNF252P	-0.064922	6.157235	-2.823221	0.0050112	0.0116374	-3.01076
RP11-136C2	-0.194181	-1.355788	-2.823054	0.0050137	0.0116426	-3.011218
CTD-2410N1	-0.250289	3.6713438	-2.822789	0.0050178	0.0116513	-3.011946
KCNJ6-AS1	-0.171823	-1.431188	-2.822735	0.0050186	0.0116525	-3.012095
DDX23	-0.027577	6.5649398	-2.822503	0.0050222	0.01166	-3.01273
BAALC	0.2552188	4.6146971	2.8224028	0.0050237	0.0116628	-3.013006
UBL4A	-0.053522	6.386576	-2.82226	0.0050259	0.0116671	-3.013397
TUBA4B	-0.54021	2.9810984	-2.82214	0.0050277	0.0116707	-3.013726
DDC	-0.325264	6.0141417	-2.821915	0.0050312	0.011678	-3.014344
C9orf142	-0.075465	6.1359019	-2.821865	0.005032	0.011679	-3.014482
POGK	0.0493853	6.3575566	2.8214573	0.0050382	0.0116925	-3.0156
RNU6-415P	0.4158518	0.9225333	2.8214446	0.0050384	0.0116925	-3.015635
EIF2B2	-0.045647	6.0652747	-2.821421	0.0050388	0.0116926	-3.015699
AC010136.2	0.5602937	1.2399023	2.8210827	0.005044	0.0117039	-3.016627
ACKR3	0.1536386	5.7553851	2.820919	0.0050465	0.011709	-3.017076
MIR219A1	-0.381205	-0.617058	-2.820599	0.0050515	0.0117197	-3.017954
CAMTA1-IT1	-0.348069	-1.113833	-2.81993	0.0050618	0.0117429	-3.019789
TENM2	-0.691959	3.5927472	-2.819881	0.0050625	0.0117439	-3.019921
INTS8	0.0537737	6.0960614	2.8197976	0.0050638	0.0117462	-3.020151
RP11-328K4	-0.656519	3.9504737	-2.819651	0.0050661	0.0117507	-3.020553
HSPD1	-0.044486	7.1748109	-2.819438	0.0050694	0.0117575	-3.021136
AC000124.1	-0.354983	-0.896805	-2.819083	0.0050749	0.0117695	-3.022109
ZNF670	0.2247571	4.8321373	2.8178898	0.0050933	0.0118117	-3.025379
RP11-190J1	0.4279032	-0.977025	2.8178414	0.0050941	0.0118127	-3.025511
AL136419.6	0.2433468	3.3070823	2.8178135	0.0050945	0.0118129	-3.025588
RP4-547N15	-0.132183	-1.465462	-2.817756	0.0050954	0.0118142	-3.025744
TACC1	0.0786524	6.4085432	2.817733	0.0050958	0.0118143	-3.025808
RP11-44501	0.441791	0.2001415	2.8176639	0.0050969	0.0118161	-3.025998
PSIP1	-0.060886	6.2005821	-2.817639	0.0050972	0.0118162	-3.026067
RP11-10A14	-0.211128	-1.324574	-2.81742	0.0051006	0.0118233	-3.026665
MSTN	0.5493249	0.9988361	2.8173658	0.0051015	0.0118245	-3.026814
RP11-252K2	-0.424826	2.0416008	-2.816906	0.0051086	0.0118404	-3.028073
RIPPLY3	0.6101755	2.5076994	2.816461	0.0051156	0.0118557	-3.029292
ZNF41	0.0862169	5.3171921	2.816293	0.0051182	0.011861	-3.029752
RP11-248G5	-0.156265	-1.440954	-2.81606	0.0051218	0.0118687	-3.030389
WDR45B	-0.036061	6.5280048	-2.81584	0.0051253	0.0118759	-3.030993
DLX6-AS1	0.739193	0.3320531	2.8157682	0.0051264	0.0118777	-3.031189
FAM74A7	-0.101085	-1.505689	-2.815648	0.0051283	0.0118813	-3.031519
RP11-256L6	0.6488839	2.2573575	2.8155478	0.0051298	0.0118842	-3.031792
CYP2R1	-0.050484	5.7443795	-2.815107	0.0051367	0.0118994	-3.032999

ZNF517	-0.082947	5.887009	-2.814946	0.0051392	0.0119045	-3.03344
RP4-736H5.	0.3395624	-1.179388	2.8148887	0.0051401	0.0119058	-3.033596
UBE2SP1	0.4890456	2.2387667	2.81463	0.0051442	0.0119142	-3.034304
CLK4	-0.056294	5.7961844	-2.814615	0.0051444	0.0119142	-3.034346
ADCYAP1	0.6411942	1.1107671	2.8145794	0.005145	0.0119147	-3.034443
FADS3	-0.070654	6.0792583	-2.814303	0.0051493	0.011924	-3.035199
ZNF493	0.1501386	5.0283843	2.813674	0.0051592	0.0119461	-3.03692
LDHAP4	0.4017078	4.0456883	2.8133377	0.0051645	0.0119576	-3.03784
AP000351.4	-0.32839	-1.006965	-2.813259	0.0051657	0.0119597	-3.038056
NEBL-AS1	0.4690581	-0.703019	2.8132037	0.0051666	0.011961	-3.038206
MREG	-0.090806	5.5265167	-2.813096	0.0051683	0.0119641	-3.0385
NAP1L4P1	0.5485893	2.9885226	2.812564	0.0051766	0.0119828	-3.039956
ING5	-0.051929	5.9152162	-2.812461	0.0051783	0.0119857	-3.040237
RFPL3S	0.4619895	1.6366401	2.8123338	0.0051803	0.0119896	-3.040585
PLCL2	-0.121344	5.7095093	-2.812276	0.0051812	0.0119905	-3.040744
RNU6-577P	-0.411374	-0.812334	-2.812259	0.0051815	0.0119905	-3.04079
KPNA5	0.1125039	5.0873631	2.812248	0.0051816	0.0119905	-3.04082
FAM24B	0.4440772	3.1210335	2.8121429	0.0051833	0.0119936	-3.041107
SH2B2	0.1172996	4.906214	2.8118727	0.0051875	0.0120027	-3.041846
ZNF134	0.0890012	5.6160055	2.8117926	0.0051888	0.0120048	-3.042065
SPATA17	0.6379845	1.935629	2.8117731	0.0051891	0.0120048	-3.042118
AC087163.2	0.468683	0.1543593	2.8117091	0.0051901	0.0120064	-3.042293
RPL32P3	0.0912873	5.1608971	2.8114843	0.0051937	0.0120138	-3.042908
RP11-121L1	0.4866659	1.417367	2.8114296	0.0051945	0.012015	-3.043057
IGHD3-3	0.2516764	-1.316371	2.8113131	0.0051964	0.0120185	-3.043376
PRPSAP2	-0.055461	6.0116192	-2.811153	0.0051989	0.0120236	-3.043814
RP11-347E1	-0.713513	1.8489374	-2.810968	0.0052018	0.0120296	-3.044319
RP11-585P4	-0.455773	2.2799497	-2.810332	0.0052119	0.0120522	-3.046056
CIT	0.125282	5.4485769	2.81027	0.0052129	0.0120537	-3.046226
F8	-0.12902	5.7095062	-2.810022	0.0052168	0.012062	-3.046903
CCDC144CP	0.4171634	-0.617302	2.8093157	0.005228	0.0120872	-3.048834
PPIH	-0.061761	5.8184618	-2.809199	0.0052299	0.0120907	-3.049151
CTD-2047H1	0.3506877	2.9636712	2.8090258	0.0052327	0.0120963	-3.049625
ZNF234	0.0783248	5.2923196	2.8083964	0.0052427	0.0121187	-3.051344
LINC01021	-0.69131	3.2799018	-2.808333	0.0052437	0.0121203	-3.051517
TTBK2	0.0623922	5.4743336	2.8078795	0.0052509	0.0121362	-3.052756
MT-TL2	-0.451471	-0.378655	-2.80746	0.0052576	0.0121504	-3.053902
RP11-21K12	0.5232336	1.0930824	2.8074383	0.005258	0.0121504	-3.05396
RP11-7908.	-0.468298	0.131316	-2.807434	0.005258	0.0121504	-3.053972
CD44	0.1339305	6.1755218	2.8074009	0.0052586	0.0121508	-3.054062
UBR5	0.0480209	6.425772	2.8073395	0.0052595	0.0121523	-3.05423
RP5-1021I2	0.3887047	-0.494859	2.807294	0.0052603	0.0121532	-3.054354
PAXBP1	0.0509166	5.9038733	2.8069864	0.0052652	0.0121638	-3.055194
TNFRSF10A	0.1206778	5.3461867	2.80693	0.0052661	0.0121651	-3.055348
TRPC4AP	-0.031104	6.6082631	-2.806724	0.0052694	0.012172	-3.055911
NMRAL1	-0.125801	6.2742922	-2.806617	0.0052711	0.0121752	-3.056201
RP11-303E1	0.4703327	1.5983653	2.8064097	0.0052744	0.0121821	-3.056768
RP1-30E17.	0.3718698	-0.877629	2.8062084	0.0052777	0.0121887	-3.057317
SEL1L	-0.055034	6.7588261	-2.806104	0.0052793	0.0121914	-3.057601
TEX9	0.2562978	4.0073099	2.8060943	0.0052795	0.0121914	-3.057628
RP11-63G10	-0.119461	-1.485127	-2.806057	0.0052801	0.0121921	-3.057731

NTF4	0.3096325	-1.203719	2.8060139	0.0052808	0.0121929	-3.057848
HNRNPA3P5	0.4001636	2.0933751	2.8059866	0.0052812	0.0121931	-3.057922
RNU6-1011F	0.4114447	-0.358224	2.8056266	0.005287	0.0122057	-3.058904
RP11-26P13	0.2191198	-1.306134	2.8053431	0.0052915	0.0122154	-3.059678
RP11-403P1	0.2936479	3.0388283	2.8047397	0.0053012	0.012237	-3.061324
RP11-295G2	-0.13331	5.6411326	-2.804708	0.0053018	0.0122374	-3.061409
CTD-3032H1	-0.210414	-1.374054	-2.804588	0.0053037	0.0122411	-3.061738
PPP2R2D	-0.044138	6.064546	-2.80453	0.0053046	0.0122425	-3.061895
GACAT2	0.4104775	-0.994032	2.8043408	0.0053077	0.0122488	-3.062411
RP11-561B1	-0.265683	-1.260114	-2.804104	0.0053115	0.0122568	-3.063056
VDAC1P10	-0.111972	-1.501204	-2.80385	0.0053156	0.0122655	-3.063751
VPS11	-0.037419	6.2629249	-2.803782	0.0053167	0.0122673	-3.063935
RN7SL521P	-0.476623	1.3107418	-2.803381	0.0053232	0.0122814	-3.065028
ADAMTS4	0.1045377	5.8216849	2.8033434	0.0053238	0.012282	-3.065131
RNASEL	-0.146252	5.2447543	-2.803004	0.0053293	0.0122939	-3.066056
ACVRL1	0.0754047	5.8168924	2.8029069	0.0053308	0.0122968	-3.06632
SLC52A2	0.0785973	6.1367773	2.8025183	0.0053371	0.0123105	-3.06738
RP11-475J5	-0.503056	1.5482794	-2.802473	0.0053379	0.0123114	-3.067503
SCN1B	0.1078416	5.5247462	2.8020454	0.0053448	0.0123266	-3.068668
AC007405.6	-0.155714	4.7881332	-2.801941	0.0053465	0.0123298	-3.068953
LINC00324	-0.122308	5.0495711	-2.801656	0.0053511	0.0123397	-3.06973
MPP4	0.4729319	0.7814252	2.8012794	0.0053572	0.012353	-3.070755
RP11-329N1	0.2568289	3.5591586	2.8011329	0.0053596	0.0123577	-3.071154
RP11-795F1	0.2186283	3.7748357	2.8009052	0.0053633	0.0123654	-3.071774
TRBV10-1	0.2878195	-1.216702	2.8006237	0.0053679	0.0123752	-3.072541
RP11-390G1	0.4908704	0.6067738	2.8005688	0.0053688	0.0123765	-3.072691
RP1-34B20.	-0.531524	2.3790218	-2.800536	0.0053693	0.0123769	-3.072779
HMGN2P3	0.367408	2.6897308	2.8003728	0.005372	0.0123823	-3.073224
CTC-241N9.	-0.153456	4.253461	-2.799888	0.0053799	0.0123997	-3.074545
RP11-30L15	0.5106509	1.4247996	2.7989937	0.0053945	0.0124326	-3.076979
SUCLG2P2	0.4549756	2.8278755	2.798809	0.0053975	0.0124388	-3.077482
CTC-281F24	0.4779622	1.4973705	2.7986537	0.0054	0.0124432	-3.077905
DHRSX-IT1	0.472961	1.1032025	2.7986494	0.0054001	0.0124432	-3.077916
GRK6	-0.049295	6.1786993	-2.798525	0.0054022	0.0124471	-3.078255
ZNF837	-0.114827	5.0316825	-2.798252	0.0054066	0.0124566	-3.078996
RP11-193M2	0.2654144	-1.288468	2.7980819	0.0054094	0.0124621	-3.079461
FAM49B	0.0605196	6.2350861	2.7980663	0.0054097	0.0124621	-3.079503
PTBP1P	0.364434	-0.842952	2.7979976	0.0054108	0.0124639	-3.07969
RP11-855A2	0.4766004	0.4656338	2.7976709	0.0054162	0.0124755	-3.080579
LMNB1	0.1007236	5.9928859	2.797615	0.0054171	0.0124768	-3.080731
PKN3	0.0889602	5.6697302	2.7974811	0.0054193	0.0124811	-3.081095
KRT86	0.5692167	3.0790818	2.7972899	0.0054224	0.0124875	-3.081615
RP11-85I17	0.4898651	0.0803922	2.7970404	0.0054265	0.0124962	-3.082294
RP11-595B2	-0.376497	3.6326159	-2.796979	0.0054275	0.0124977	-3.08246
SLC36A4	0.1070714	5.4844139	2.7969517	0.005428	0.0124978	-3.082535
RAD51	0.162826	4.9397331	2.7969293	0.0054283	0.0124978	-3.082596
DPH1	0.0737002	5.8161107	2.796914	0.0054286	0.0124978	-3.082638
CTNNA2	-0.863344	2.1123711	-2.796746	0.0054314	0.0125034	-3.083095
RP11-49014	0.5571707	1.8860359	2.7960551	0.0054427	0.0125288	-3.084974
NGEF	-0.16932	6.2057053	-2.796022	0.0054433	0.0125292	-3.085063
RP11-110H1	0.4826071	0.649499	2.7959146	0.0054451	0.0125321	-3.085356

PRR19	0.3605165	3.8770477	2.7958978	0.0054453	0.0125321	-3.085401
RP11-150D5	-0.390711	-0.893946	-2.795884	0.0054455	0.0125321	-3.085438
FARP1	0.0596468	6.4719047	2.7958322	0.0054464	0.0125333	-3.08558
U51561.1	-0.271596	-1.130055	-2.795776	0.0054473	0.0125346	-3.085732
CACNA1E	0.7093798	1.5128088	2.7952276	0.0054564	0.0125547	-3.087224
TFE3	0.0355163	6.3611548	2.7951073	0.0054584	0.0125585	-3.08755
CBX2	0.1722662	5.1133071	2.7947079	0.005465	0.0125729	-3.088636
LINC01099	0.3554628	-1.029301	2.7946208	0.0054664	0.0125754	-3.088873
CH17-437K3	-0.476685	3.1576081	-2.794566	0.0054673	0.0125767	-3.089023
HSPA8P11	0.4163914	-0.426989	2.794507	0.0054683	0.0125781	-3.089182
RP11-167N2	-0.173958	-1.427791	-2.794265	0.0054723	0.0125866	-3.089841
YBX1P1	0.3059182	3.2779937	2.7942169	0.0054731	0.0125876	-3.089971
RP11-328N1	-0.731486	2.0660512	-2.794118	0.0054748	0.0125906	-3.09024
RNU4-86P	-0.130768	-1.484116	-2.79378	0.0054804	0.0126027	-3.091157
DNAJB13	0.5652979	0.9304328	2.7934615	0.0054856	0.012614	-3.092023
CTD-2331H1	0.511439	-0.093165	2.793252	0.0054891	0.0126213	-3.092592
MYH7B	-0.196588	4.660328	-2.792884	0.0054952	0.0126345	-3.093592
WDR91	0.0577635	5.9891849	2.7927608	0.0054973	0.0126384	-3.093926
RP3-388N13	0.3375538	-1.017261	2.7927301	0.0054978	0.0126388	-3.09401
UBE2M	-0.052012	6.450307	-2.792368	0.0055038	0.0126515	-3.094993
STK35	0.0515455	6.1104285	2.792357	0.005504	0.0126515	-3.095023
PPP1R21	0.0485195	5.968774	2.7916428	0.0055159	0.0126781	-3.096963
RP11-80H5.	-0.227218	-1.28455	-2.791388	0.0055202	0.012687	-3.097654
BAMBI	0.2026281	6.0160005	2.7912776	0.005522	0.0126905	-3.097954
ATP1A1	0.0485047	7.1188042	2.7912529	0.0055224	0.0126906	-3.098021
IGBP1-AS1	0.452194	0.6788057	2.7912168	0.005523	0.0126912	-3.098119
MYO5B	-0.0827	6.2204544	-2.790814	0.0055298	0.0127059	-3.099213
RP11-210M1	0.3695412	-0.880406	2.7903601	0.0055374	0.0127222	-3.100444
APLN	-0.179075	5.3373169	-2.79035	0.0055375	0.0127222	-3.100473
MUC13	0.4553493	5.5873404	2.7900326	0.0055429	0.0127336	-3.101333
CTD-3224K1	0.4497998	-0.241305	2.7897005	0.0055484	0.0127455	-3.102234
CH25H	0.3605611	3.8930078	2.7896227	0.0055497	0.0127477	-3.102445
KLHL30-AS1	0.4122963	-0.679175	2.7894563	0.0055525	0.0127534	-3.102897
RP5-1052M9	-0.128166	-1.474096	-2.789433	0.0055529	0.0127535	-3.102961
HSD17B1P1	0.3465044	3.0248004	2.7893064	0.005555	0.0127575	-3.103303
LARGE	-0.0801	6.0340927	-2.789207	0.0055567	0.0127606	-3.103573
CCDC67	0.4945103	0.166411	2.7887251	0.0055648	0.0127784	-3.10488
SHC2	-0.102644	6.482222	-2.788329	0.0055715	0.0127929	-3.105956
RP11-98D18	-0.309623	-1.102747	-2.788212	0.0055734	0.0127966	-3.106272
RP11-227H1	0.4162122	-0.595565	2.7880934	0.0055754	0.0128004	-3.106594
PTCRA	0.5302113	1.3989163	2.7878115	0.0055802	0.0128097	-3.107358
LINC00852	0.2181917	3.4373235	2.7878099	0.0055802	0.0128097	-3.107362
PTGDR2	-0.254916	4.5214625	-2.787771	0.0055809	0.0128104	-3.107468
RP11-16N11	-0.260311	3.4133399	-2.787726	0.0055816	0.0128114	-3.107591
EPB41L4A-A	0.3707975	3.0661667	2.7874567	0.0055862	0.012821	-3.10832
RP11-313P2	0.4980804	1.0551564	2.7872827	0.0055891	0.0128269	-3.108792
KLHL32	-0.422853	3.2764022	-2.786718	0.0055987	0.0128477	-3.110324
MATN2	0.1359435	6.091802	2.7866994	0.005599	0.0128477	-3.110373
LINC00621	-0.263487	-1.261861	-2.786684	0.0055992	0.0128477	-3.110415
TBC1D10A	-0.063975	5.7801778	-2.786655	0.0055997	0.012848	-3.110493
CTA-221G9.	0.4325897	-0.386863	2.7864119	0.0056038	0.0128567	-3.111152

PARD3	-0.044025	6.3699229	-2.786044	0.0056101	0.0128702	-3.11215
KIF20A	0.1682895	5.4969086	2.7860055	0.0056107	0.0128709	-3.112253
CPA2	0.5821326	-0.366908	2.7858766	0.0056129	0.0128751	-3.112603
DAPL1	0.5247252	-0.482928	2.7850807	0.0056264	0.0129052	-3.114759
PARP14	-0.050876	6.5954613	-2.784942	0.0056288	0.0129098	-3.115133
ELMO1	-0.072578	6.2381942	-2.784716	0.0056326	0.0129178	-3.115746
RNF168	-0.047269	5.8754845	-2.784627	0.0056341	0.0129202	-3.115987
CPED1	-0.21164	5.4820032	-2.784613	0.0056344	0.0129202	-3.116026
RP11-325O2	-0.109505	-1.504621	-2.784437	0.0056374	0.0129263	-3.116502
TDRP	0.170004	5.5237508	2.7841674	0.005642	0.012936	-3.117233
RP11-863K1	-0.456537	2.9561017	-2.784075	0.0056435	0.0129388	-3.117483
SH2D1B	0.354851	2.8403256	2.7839188	0.0056462	0.0129434	-3.117906
CLEC16A	-0.050916	6.1120946	-2.783916	0.0056462	0.0129434	-3.117915
AC068858.1	0.547104	0.7387434	2.7838378	0.0056476	0.0129456	-3.118125
RN7SL8P	0.5435923	-0.456596	2.7836703	0.0056504	0.0129513	-3.118579
PRAC2	0.4854297	-0.784103	2.7834472	0.0056542	0.0129593	-3.119183
ADAMTS16	0.6465028	3.1032431	2.7833645	0.0056556	0.0129617	-3.119407
RP11-20I2C	0.2439717	-1.280765	2.7831842	0.0056587	0.0129679	-3.119895
KCTD6	-0.085558	5.7285391	-2.782817	0.005665	0.0129815	-3.12089
PTPN1	0.035394	6.2863115	2.7827435	0.0056663	0.0129836	-3.121088
RP11-182N2	-0.370309	-0.718039	-2.782571	0.0056692	0.0129895	-3.121555
OR10Q1	0.2439528	-1.320701	2.7824723	0.0056709	0.0129925	-3.121822
PLEKHA3	-0.046251	5.8834698	-2.782416	0.0056719	0.0129932	-3.121974
RFPL1S	0.5726834	1.8914625	2.7823948	0.0056722	0.0129932	-3.122032
PKMP1	0.3289253	-0.966431	2.782393	0.0056723	0.0129932	-3.122037
SNX29	0.0659945	5.7361785	2.7819311	0.0056802	0.0130105	-3.123287
RP11-336K2	0.251348	-1.239374	2.7813932	0.0056894	0.0130308	-3.124742
DUX4L16	-0.109076	-1.502476	-2.781027	0.0056957	0.0130444	-3.125733
RBMXL2	-0.418543	-0.670387	-2.780991	0.0056963	0.013045	-3.12583
CTC-251D13	0.3861984	2.111135	2.7806762	0.0057017	0.0130566	-3.126682
ZNF572	-0.173726	4.6795897	-2.780181	0.0057102	0.0130753	-3.128021
SLC25A15	-0.142411	6.0420704	-2.779827	0.0057163	0.0130884	-3.12898
AL133493.2	0.6639649	1.4054499	2.7794907	0.0057221	0.0131009	-3.129888
UBXN11	-0.07644	5.8336621	-2.779364	0.0057243	0.0131051	-3.13023
TAS2R60	-0.35161	-1.051073	-2.779276	0.0057258	0.0131077	-3.130467
LA16c-361A	0.4731476	0.297827	2.7791376	0.0057282	0.0131124	-3.130842
RP11-3M1.3	-0.10265	-1.505299	-2.778963	0.0057312	0.0131185	-3.131315
LGALS17A	0.613966	0.6478048	2.7787728	0.0057345	0.0131244	-3.131828
GPATCH2	0.0545574	5.6927585	2.7787706	0.0057346	0.0131244	-3.131834
OGFRP1	0.3419481	3.1909474	2.7784492	0.0057401	0.0131363	-3.132703
RP4-669K1C	0.2963631	3.1773759	2.778226	0.005744	0.0131438	-3.133306
CTD-3199J2	0.3083798	3.1429032	2.7782187	0.0057441	0.0131438	-3.133326
LINC01277	-0.396869	2.9128642	-2.778168	0.005745	0.013145	-3.133464
PRMT1P1	0.3367458	-0.849053	2.7779745	0.0057483	0.0131518	-3.133986
DGKK	0.6779292	0.2661821	2.7778917	0.0057498	0.0131542	-3.13421
FMR1-IT1	0.5086916	1.2103351	2.7778724	0.0057501	0.0131542	-3.134262
AP001043.1	-0.133228	-1.469539	-2.777852	0.0057505	0.0131542	-3.134318
HRNR	0.5640466	0.5819012	2.7775636	0.0057555	0.0131648	-3.135096
ZNF345	0.0949836	4.7605606	2.7774989	0.0057566	0.0131666	-3.135271
GS1-594A7.	-0.147144	-1.443649	-2.777411	0.0057581	0.0131692	-3.135509
ZMYM4	0.0404845	6.1353346	2.7769329	0.0057664	0.0131874	-3.1368

TOMM20P2	0.4937243	0.3661685	2.7765688	0.0057727	0.013201	-3.137784
MRPL15	-0.060774	6.4358268	-2.776439	0.005775	0.0132044	-3.138135
PRSS56	0.651934	-0.202602	2.7764228	0.0057753	0.0132044	-3.138178
WDR5	-0.046576	6.3610831	-2.776422	0.0057753	0.0132044	-3.138181
TRIM16L	-0.182699	5.742778	-2.776333	0.0057768	0.0132068	-3.13842
IGKV2OR22-	0.3066736	-1.170451	2.7763196	0.0057771	0.0132068	-3.138457
TLCD1	-0.104725	5.752212	-2.776223	0.0057788	0.0132098	-3.138719
RP1-67A8.3	0.4102178	-0.753622	2.7752512	0.0057957	0.0132477	-3.141342
RP11-33001	0.2468029	3.1205216	2.7751124	0.0057981	0.0132524	-3.141717
LINC01231	-0.419474	-1.082645	-2.774872	0.0058023	0.0132612	-3.142364
AADACL2	-0.205916	-1.355324	-2.774559	0.0058078	0.0132729	-3.143211
CGGBP1	-0.030705	6.4343101	-2.774338	0.0058116	0.0132809	-3.143807
GOLM1	0.1216182	6.4536064	2.7740763	0.0058162	0.0132905	-3.144513
RP3-368A4.	0.1923447	4.3954917	2.7739595	0.0058183	0.0132943	-3.144828
SASS6	0.1099028	5.0945819	2.7736485	0.0058237	0.0133059	-3.145668
SLC25A15P2	-0.111409	-1.503785	-2.773446	0.0058273	0.0133132	-3.146214
NIPAL3	0.0580894	5.7108229	2.7729948	0.0058352	0.0133305	-3.147431
AL161645.1	-0.442438	-0.664784	-2.772881	0.0058372	0.0133342	-3.147738
FNDC8	0.3467281	-0.866771	2.7727101	0.0058402	0.01334	-3.148199
RP11-361H1	0.4663248	0.3021691	2.7726959	0.0058404	0.01334	-3.148238
RP11-333A2	-0.121332	-1.484305	-2.772568	0.0058427	0.0133443	-3.148582
MTCYBP4	-0.187062	-1.375048	-2.77253	0.0058433	0.0133449	-3.148684
CTA-315H11	-0.296899	3.8730573	-2.772454	0.0058447	0.0133472	-3.148889
AC083843.1	0.1381167	5.1549056	2.7724107	0.0058455	0.0133481	-3.149007
PAPSS1	0.0585648	5.9651424	2.7722371	0.0058485	0.0133542	-3.149475
CBLB	0.0564014	5.8821118	2.7721908	0.0058493	0.0133552	-3.1496
GINS1	0.1500348	5.4589458	2.7718401	0.0058555	0.0133685	-3.150546
RN7SKP71	-0.111335	-1.49719	-2.771684	0.0058583	0.0133734	-3.150967
BEND3P2	0.2364039	-1.291241	2.7716767	0.0058584	0.0133734	-3.150986
RP4-715N11	-0.423693	-0.816293	-2.771618	0.0058594	0.0133749	-3.151145
PIK3IP1	0.0934508	5.9871965	2.7714331	0.0058627	0.0133815	-3.151643
CTD-3060P2	0.299675	-1.186489	2.7713806	0.0058636	0.0133828	-3.151785
TBX18	0.5599559	2.2831055	2.7712444	0.005866	0.0133875	-3.152152
ITGAV	0.0768704	6.4211061	2.7711803	0.0058671	0.0133892	-3.152325
WDPCP	-0.129387	4.9045864	-2.770962	0.005871	0.0133972	-3.152913
RP11-250B2	-0.217754	4.1373358	-2.77076	0.0058746	0.0134045	-3.153458
ATP13A5	0.2202683	-1.313779	2.77052	0.0058788	0.0134133	-3.154105
STAG3L3	0.2229265	4.1120733	2.7700873	0.0058864	0.0134299	-3.155271
ZNF626	0.2624266	4.6468562	2.7700445	0.0058872	0.0134308	-3.155386
WDR81	-0.052447	6.3723926	-2.769318	0.0059001	0.0134594	-3.157345
AC007952.5	0.5123094	-0.33195	2.7692751	0.0059008	0.0134602	-3.157459
GTSF1	0.7040125	2.6781079	2.7682252	0.0059195	0.0135019	-3.160288
AC073410.1	0.4860109	0.2068812	2.7681112	0.0059215	0.0135057	-3.160595
ABCC3	-0.089894	6.7554177	-2.767491	0.0059326	0.0135301	-3.162264
HIST2H2AC	-0.203502	4.0655258	-2.767449	0.0059333	0.0135309	-3.162379
RP11-514F8	-0.118953	-1.484238	-2.767285	0.0059362	0.0135368	-3.162819
MOK	0.0903755	5.1193517	2.7670676	0.0059401	0.0135448	-3.163405
GLB1L3	0.6896543	0.809921	2.7667623	0.0059456	0.0135563	-3.164227
LHX3	-0.779562	1.1292029	-2.766697	0.0059467	0.0135582	-3.164402
TIMM44	-0.046793	6.2991926	-2.766614	0.0059482	0.0135607	-3.164626
BTN3A1	-0.079643	6.1343892	-2.76647	0.0059508	0.0135657	-3.165012

AREL1	0.0467494	6.091201	2.7663316	0.0059533	0.0135705	-3.165386
AKIRIN1	0.0499162	6.2683501	2.7655435	0.0059674	0.0136018	-3.167507
RP11-402P6	-0.688749	-0.198047	-2.765515	0.0059679	0.0136021	-3.167582
RP3-449M8.	0.4697094	0.2020578	2.7654656	0.0059688	0.0136033	-3.167716
RP11-325I2	0.4856577	0.469182	2.7652406	0.0059728	0.0136116	-3.168322
RP11-254I2	-0.463355	1.8520903	-2.765099	0.0059753	0.0136166	-3.168703
RP11-180M1	0.4232605	2.1716915	2.7650566	0.0059761	0.0136175	-3.168817
FNDC7	0.3137536	-1.092913	2.7647768	0.0059811	0.013628	-3.169569
REG1P	-0.350413	-1.161855	-2.764483	0.0059864	0.0136392	-3.170359
XPOTP1	0.4639614	0.0603519	2.7640791	0.0059937	0.0136549	-3.171446
RP11-570L1	0.2773335	-1.119443	2.7636695	0.006001	0.0136709	-3.172547
MIR5695	-0.165219	-1.411048	-2.763615	0.006002	0.0136723	-3.172694
TESC-AS1	0.591917	0.2887432	2.7635776	0.0060027	0.0136729	-3.172795
CTD-2330K9	0.4154948	-0.491334	2.7633685	0.0060065	0.0136807	-3.173357
LINC00677	0.4367479	-0.289555	2.7631499	0.0060104	0.0136888	-3.173944
ZBTB14	-0.047168	5.7430147	-2.762984	0.0060134	0.0136948	-3.174392
RP11-332J1	-0.65094	-0.137997	-2.762873	0.0060154	0.0136985	-3.174689
RP4-555D2C	0.293529	-1.225095	2.7627684	0.0060173	0.0137019	-3.17497
CTD-2350J1	-0.271304	-1.277155	-2.762584	0.0060206	0.0137086	-3.175467
SLC14A1	0.3107618	3.8075876	2.7624089	0.0060238	0.013715	-3.175937
RP11-522B1	0.389404	-0.655698	2.762303	0.0060257	0.0137185	-3.176221
XXbac-BPG3	-0.264778	-1.219422	-2.762041	0.0060304	0.0137284	-3.176926
TRGVB	-0.196531	-1.392005	-2.761953	0.006032	0.0137312	-3.177161
RP11-543E8	-0.130595	-1.473029	-2.761622	0.006038	0.013744	-3.178052
EGLN2	-0.071459	6.0935346	-2.761335	0.0060432	0.0137549	-3.178823
WWP1	-0.058568	6.7068526	-2.760997	0.0060493	0.013768	-3.179729
AC012146.7	-0.164095	4.8643683	-2.760862	0.0060518	0.0137719	-3.180094
SPACA4	0.2476476	-1.298428	2.7608616	0.0060518	0.0137719	-3.180094
TMSB10	0.0864565	7.040843	2.7607149	0.0060545	0.0137771	-3.180488
CTA-268H5.	0.4029855	-0.415527	2.7601774	0.0060642	0.0137985	-3.181932
MARK3P3	-0.104939	-1.493604	-2.760149	0.0060648	0.0137988	-3.182009
HEATR6	0.0575938	5.6364951	2.7598552	0.0060701	0.0138101	-3.182797
CKS1B	-0.065254	6.1015845	-2.759733	0.0060723	0.0138143	-3.183126
RP11-371E8	0.3831833	-0.527786	2.7596051	0.0060746	0.0138181	-3.183469
PHEX-AS1	-0.251053	-1.29803	-2.7596	0.0060747	0.0138181	-3.183483
IFNE	0.2610272	-1.293905	2.7592637	0.0060809	0.0138311	-3.184386
FAM32B	-0.264647	-1.162219	-2.759066	0.0060845	0.0138385	-3.184915
NMU	0.491503	-0.301594	2.7589984	0.0060857	0.0138404	-3.185098
LRFN3	-0.081172	5.8000729	-2.758958	0.0060865	0.0138412	-3.185208
PCDHGA8	0.5517707	1.7615585	2.7589376	0.0060868	0.0138412	-3.185261
HMGN1P38	0.4817778	0.2747395	2.7587752	0.0060898	0.0138471	-3.185697
RP11-343L5	0.2879055	3.2600317	2.7586648	0.0060918	0.0138508	-3.185993
RP11-1136G	-0.155989	-1.444516	-2.758438	0.0060959	0.0138594	-3.186601
RP11-46A1C	0.379994	-0.753397	2.7583326	0.0060979	0.0138629	-3.186885
STAM-AS1	0.4610123	2.0893114	2.7576286	0.0061107	0.0138913	-3.188774
RP11-104N1	0.1682209	4.4895079	2.7575064	0.006113	0.0138955	-3.189102
RP11-325L1	0.4811552	0.4638908	2.7574843	0.0061134	0.0138956	-3.189161
CORO1B	-0.044742	6.6021412	-2.757396	0.006115	0.0138984	-3.189399
SLC6A10P	0.3723597	-1.161896	2.7570294	0.0061217	0.0139128	-3.190382
AC006483.5	0.2440881	-1.246731	2.7561308	0.0061382	0.0139494	-3.192792
GPR143P	-0.207392	-1.339246	-2.75582	0.0061439	0.0139615	-3.193626

SLC6A9	0.2032275	5.0058362	2.7552842	0.0061538	0.0139831	-3.195063
ANO3	0.6621033	2.4628865	2.7551995	0.0061554	0.0139847	-3.19529
UGT2B17	-0.762302	3.5953863	-2.755199	0.0061554	0.0139847	-3.195291
RPL7P25	-0.163528	-1.408248	-2.755184	0.0061557	0.0139847	-3.195333
GAN	0.0678117	5.2591506	2.7551204	0.0061568	0.0139853	-3.195502
CCR9	0.4380717	-0.432971	2.7551097	0.006157	0.0139853	-3.19553
RP4-616B8.	-0.470614	0.1152388	-2.755095	0.0061573	0.0139853	-3.195571
RP11-567J2	-0.242087	-1.269056	-2.755086	0.0061575	0.0139853	-3.195594
RP11-690I2	0.487633	0.4703675	2.7549749	0.0061595	0.0139891	-3.195892
RP11-517B1	0.119265	4.4409403	2.7548687	0.0061615	0.0139927	-3.196177
RP11-119J1	-0.284605	-1.243464	-2.754768	0.0061633	0.013996	-3.196445
AC079305.1	-0.36693	-0.907995	-2.754598	0.0061665	0.0140023	-3.196902
ZNF781	0.3601424	3.4358097	2.7545638	0.0061671	0.0140028	-3.196994
RP4-785G19	-0.477035	1.8124887	-2.754533	0.0061677	0.0140032	-3.197077
RP11-1252D	0.4717962	2.0375087	2.753805	0.0061811	0.0140329	-3.199028
NR4A3	0.247954	4.653151	2.7530095	0.0061958	0.0140655	-3.201159
CASP7	0.0588028	5.9770104	2.7529351	0.0061972	0.0140677	-3.201358
RP11-114H2	-0.733229	0.4499404	-2.752868	0.0061985	0.0140697	-3.201537
RP11-168K1	0.3654859	-0.686591	2.7524281	0.0062066	0.0140873	-3.202717
BEX5	0.4089756	4.0564986	2.7524032	0.0062071	0.0140875	-3.202783
SAA1	-0.342694	6.4757768	-2.752382	0.0062075	0.0140875	-3.202841
RP11-331F9	0.3834144	-0.672675	2.752338	0.0062083	0.0140885	-3.202958
IGBP1	-0.048092	6.2664408	-2.752306	0.0062089	0.014089	-3.203043
RP11-307B6	-0.517838	0.0744861	-2.75207	0.0062133	0.014098	-3.203675
AP001627.1	0.5249141	0.897298	2.7517543	0.0062191	0.0141105	-3.204521
RP11-127I2	0.4694631	0.7163849	2.7516383	0.0062213	0.0141145	-3.204832
RP11-63A11	-0.150549	-1.462554	-2.751549	0.006223	0.0141174	-3.205072
CTD-2337A1	-0.521327	1.1876179	-2.751284	0.0062279	0.0141277	-3.205782
AC241377.2	0.5984925	1.5132234	2.7512639	0.0062283	0.0141277	-3.205834
RP11-114H7	-0.204238	-1.413555	-2.751014	0.0062329	0.0141374	-3.206504
PPP1R1B	0.692317	1.7268524	2.7509673	0.0062338	0.0141385	-3.206628
ZNF32-AS1	0.4532796	1.1284091	2.7508013	0.0062369	0.0141446	-3.207073
RP11-572O1	0.3036121	3.2108862	2.7507564	0.0062377	0.0141456	-3.207193
USF2	-0.043419	6.6797704	-2.75057	0.0062412	0.0141526	-3.207691
TMEM87A	0.0406692	6.1503756	2.7501599	0.0062489	0.0141691	-3.208789
TXNRD1	-0.082018	6.7417235	-2.749616	0.006259	0.0141913	-3.210244
LINC01055	0.3090233	-1.055301	2.7493191	0.0062646	0.014203	-3.211039
AP000577.2	0.346237	-0.805072	2.7487587	0.0062751	0.0142259	-3.212539
TRPS1	0.1855315	5.0907756	2.7485695	0.0062786	0.0142331	-3.213045
RP11-181G1	-0.533974	0.9145332	-2.7484	0.0062818	0.0142395	-3.213499
KRT8P42	0.5244518	0.4694692	2.7483193	0.0062833	0.014242	-3.213714
SLC24A1	0.0881446	5.4455705	2.7482268	0.0062851	0.014245	-3.213961
IFNL4P1	-0.17209	-1.450109	-2.748161	0.0062863	0.014247	-3.214137
SLC5A3	0.1023967	5.6956283	2.748072	0.006288	0.0142499	-3.214375
RNU11	-0.356275	-0.793282	-2.748034	0.0062887	0.0142506	-3.214478
CTD-3105H1	0.3655421	-0.808774	2.7479085	0.0062911	0.0142551	-3.214813
RNPS1	-0.034807	6.4493959	-2.747743	0.0062942	0.0142612	-3.215255
STK24-AS1	-0.360994	2.7582547	-2.747558	0.0062976	0.0142682	-3.215749
RP11-54O01	0.4418728	1.4894947	2.7472124	0.0063042	0.0142821	-3.216674
AC010492.2	0.5152248	0.202149	2.7467748	0.0063124	0.0142999	-3.217844
AC024560.3	0.0730183	5.4923863	2.7466529	0.0063147	0.0143042	-3.21817

MSH5-SAPCD	-0.180993	4.0664938	-2.746609	0.0063155	0.0143052	-3.218286
ZNF233	0.2645427	3.8903939	2.7465465	0.0063167	0.014307	-3.218454
ZBTB24	0.0530165	5.5104887	2.7464311	0.0063189	0.014311	-3.218763
POLR3E	-0.037912	6.1684313	-2.746264	0.006322	0.0143173	-3.21921
AK4P3	0.439225	-0.322065	2.7461815	0.0063236	0.0143199	-3.21943
MTNR1A	0.3018143	-1.14779	2.7458974	0.006329	0.0143312	-3.220189
GMDS	-0.081047	6.1762886	-2.745869	0.0063295	0.0143315	-3.220265
RP11-276H7	0.4882335	0.2348529	2.7454442	0.0063375	0.0143488	-3.2214
CTD-252902	0.5381025	1.2900143	2.7450768	0.0063445	0.0143636	-3.222382
CTC-55802.	0.3627545	-0.811832	2.7447364	0.0063509	0.0143774	-3.223291
AC093609.1	0.5159574	1.7423247	2.7446765	0.0063521	0.0143779	-3.223451
DLG1	-0.042013	6.2920643	-2.744665	0.0063523	0.0143779	-3.223483
FLJ21408	-0.310598	2.8043303	-2.744661	0.0063524	0.0143779	-3.223492
RP11-804A2	-0.360346	-0.825676	-2.744565	0.0063542	0.0143812	-3.223749
PTP4A2P2	0.4051205	1.9666899	2.7440402	0.0063642	0.0144028	-3.225151
FGFR3	0.1050674	6.4197505	2.7439833	0.0063652	0.0144029	-3.225303
CTD-2527I2	-0.274898	-1.185761	-2.743972	0.0063654	0.0144029	-3.225333
MYH10	0.08248	6.2901707	2.743957	0.0063657	0.0144029	-3.225373
RP11-230C9	-0.369758	2.6154736	-2.743955	0.0063658	0.0144029	-3.225378
RTN4R	0.1635194	5.4727875	2.7438686	0.0063674	0.0144057	-3.225609
RNU6-481P	-0.448591	1.3603549	-2.743762	0.0063694	0.0144094	-3.225894
KRT17P2	0.2158099	-1.331277	2.7436648	0.0063713	0.0144127	-3.226153
RP11-14802	0.2364705	-1.296406	2.743344	0.0063774	0.0144256	-3.22701
FPGT-TNNI3	0.4848239	1.8324401	2.7426336	0.0063909	0.0144553	-3.228906
XG	0.6412644	2.4501721	2.7424218	0.006395	0.0144635	-3.229472
OR2B6	-0.499202	2.0771611	-2.742294	0.0063974	0.0144682	-3.229814
Z98750.1	0.4540479	0.0090067	2.7421728	0.0063997	0.0144725	-3.230136
PDLIM1P4	-0.442711	2.2722638	-2.742079	0.0064015	0.0144757	-3.230387
RP11-266L9	-0.385935	-0.663637	-2.742041	0.0064022	0.0144764	-3.230487
AC019172.2	0.3272576	-1.15724	2.7419469	0.006404	0.0144796	-3.230739
ASS1P12	0.3988904	2.2296545	2.7418692	0.0064055	0.014482	-3.230946
RP11-505K9	-0.339671	-0.827112	-2.741336	0.0064157	0.0145042	-3.232368
PGC	0.8909558	2.6760236	2.7409513	0.0064231	0.0145196	-3.233396
SLC35A3	-0.074109	6.168946	-2.740937	0.0064233	0.0145196	-3.233433
BTBD19	0.1150195	5.0555886	2.7408047	0.0064259	0.0145245	-3.233787
RP11-1193F	0.263796	-1.215704	2.740744	0.006427	0.0145262	-3.233948
RP11-93B14	0.5339013	2.2553755	2.7406884	0.0064281	0.0145277	-3.234097
GAL3ST2	0.6416308	1.1941323	2.7402405	0.0064367	0.0145462	-3.235292
CTD-2022H1	-0.278665	-1.254069	-2.740027	0.0064408	0.0145546	-3.23586
SIK3	-0.051672	6.2437566	-2.739917	0.0064429	0.0145585	-3.236155
LINC01214	-0.531731	1.9705201	-2.739617	0.0064487	0.0145706	-3.236956
TMCO2	-0.40732	-0.641944	-2.739531	0.0064503	0.0145734	-3.237183
RP11-693N9	0.1328535	4.735379	2.739503	0.0064509	0.0145737	-3.237258
FBL	-0.065212	6.6419111	-2.739468	0.0064515	0.0145743	-3.237351
KRT8P46	0.4920606	1.1150549	2.7394115	0.0064526	0.0145759	-3.237502
FAM92A1	-0.089996	5.6879483	-2.739307	0.0064546	0.0145795	-3.237781
PTEN	-0.055183	6.5282041	-2.739081	0.006459	0.0145885	-3.238384
DEFB124	0.2879883	-1.108712	2.7389266	0.006462	0.0145943	-3.238795
CKAP2L	0.1910962	5.0846624	2.7388482	0.0064635	0.0145968	-3.239004
RP11-277B1	-0.359926	2.4056363	-2.738717	0.006466	0.0146016	-3.239353
C1D	-0.049114	5.8478589	-2.738409	0.0064719	0.0146141	-3.240174

RP1-92014.	-0.302035	3.0008459	-2.738287	0.0064743	0.0146185	-3.2405
RP11-384M2	0.4620753	0.9622148	2.738238	0.0064752	0.0146197	-3.240631
TATDN3	-0.060379	5.9106284	-2.737841	0.0064829	0.0146361	-3.24169
RP11-519G1	0.4804652	-0.056803	2.7376135	0.0064873	0.0146451	-3.242295
LINC00316	0.4466732	-0.646798	2.7364264	0.0065103	0.0146953	-3.245457
PPM1L	-0.101102	5.7807996	-2.736424	0.0065103	0.0146953	-3.245464
FASTKD5	0.0550301	5.9378912	2.7360873	0.0065168	0.0147088	-3.246361
EIF3LP3	0.4246144	-0.116582	2.7360741	0.0065171	0.0147088	-3.246396
RP11-20D14	-0.775516	2.0030559	-2.735993	0.0065187	0.0147115	-3.246613
P2RX3	-0.556938	2.5110761	-2.735893	0.0065206	0.0147149	-3.246877
RP11-278C7	0.3854151	1.7853054	2.7357977	0.0065225	0.0147182	-3.247132
RALGPS1	0.1595557	5.1719154	2.7357253	0.0065239	0.0147204	-3.247325
AC016757.3	0.5191345	1.8355824	2.7356144	0.006526	0.0147244	-3.24762
HOXC13-AS	0.3160402	-1.208538	2.7353365	0.0065314	0.0147357	-3.24836
PRR14L	0.0504315	6.0902184	2.7353137	0.0065319	0.0147358	-3.24842
UBL5P2	-0.423195	0.1589939	-2.735187	0.0065343	0.0147404	-3.248759
RP5-908M14	0.3968239	2.80672	2.7351067	0.0065359	0.014743	-3.248972
RP11-1415C	0.321817	-0.990299	2.7350514	0.006537	0.0147445	-3.249119
RGS17P1	0.40945	-0.279556	2.7349835	0.0065383	0.0147466	-3.2493
RP11-156E6	-0.053057	5.5964481	-2.734554	0.0065467	0.0147646	-3.250443
SACM1L	-0.037174	6.1874024	-2.73433	0.006551	0.0147735	-3.25104
RP11-845M1	0.4713196	-0.548303	2.7340834	0.0065558	0.0147834	-3.251696
MIR3197	0.2373247	-1.307194	2.7335874	0.0065655	0.0148043	-3.253015
CTC-293G12	-0.196543	-1.404372	-2.733062	0.0065758	0.0148266	-3.254414
RP11-744D1	-0.302978	3.0207701	-2.732765	0.0065816	0.0148388	-3.255204
PTCHD3	0.5351319	-0.545374	2.7326919	0.006583	0.0148411	-3.255398
SQLE	-0.110663	6.5166091	-2.732665	0.0065835	0.0148411	-3.255468
TRIOBP	-0.055264	6.095538	-2.732636	0.0065841	0.0148411	-3.255545
SNN	0.0553741	5.9689256	2.7326282	0.0065843	0.0148411	-3.255567
FCN3	0.2488819	4.904644	2.7325359	0.0065861	0.0148443	-3.255813
CTD-2619J1	-0.084769	5.2716123	-2.732142	0.0065938	0.0148608	-3.256862
LMX1A	0.591099	0.3152792	2.7320898	0.0065948	0.0148622	-3.256999
PCDHB16	0.3167875	4.1584887	2.7319914	0.0065968	0.0148656	-3.257261
RP11-1041F	-0.707536	0.0607631	-2.731903	0.0065985	0.0148686	-3.257497
CCNYL2	0.377865	-1.074812	2.7318691	0.0065992	0.0148692	-3.257586
CSNK2A1	0.0349706	6.4430099	2.7314352	0.0066077	0.0148875	-3.25874
RP11-834C1	0.4163558	-0.620499	2.7307841	0.0066205	0.0149152	-3.260471
CRYAB	0.2034721	5.3706071	2.7307682	0.0066208	0.0149152	-3.260513
RP11-554D1	0.3576742	-0.920255	2.7304797	0.0066265	0.0149271	-3.26128
RXFP4	0.4539124	-0.596123	2.7299304	0.0066373	0.0149505	-3.26274
GGTLC2	0.3934103	-0.635295	2.7297557	0.0066408	0.0149574	-3.263204
SHFM1P1	-0.496038	0.3875145	-2.729634	0.0066432	0.0149619	-3.263528
LRP4	0.2821618	4.476113	2.729475	0.0066463	0.014968	-3.26395
PSD3	-0.115187	6.0423841	-2.729171	0.0066523	0.0149806	-3.264758
PPIG	-0.035078	6.3881792	-2.729121	0.0066533	0.0149819	-3.264891
BCAS2P2	0.3226038	-0.949846	2.7290378	0.0066549	0.0149846	-3.265112
RP11-426A6	0.3898519	1.9449101	2.7290192	0.0066553	0.0149846	-3.265161
CEBPB-AS1	-0.157363	3.9018879	-2.728753	0.0066606	0.0149955	-3.265867
RP11-1267H	-0.405965	-0.75998	-2.728646	0.0066627	0.0149994	-3.266152
MRPL38	-0.053561	5.586411	-2.72857	0.0066642	0.0150019	-3.266356
RP4-616B8.	-0.426723	2.7470866	-2.728244	0.0066707	0.0150155	-3.267221

RP11-666A2	-0.462696	0.9809352	-2.727904	0.0066774	0.0150297	-3.268124
KIAA0556	0.0609961	5.7884714	2.727668	0.0066821	0.0150393	-3.26875
RP4-669B10	0.3768546	-0.979518	2.727259	0.0066902	0.0150567	-3.269836
LRRD1	0.3385136	-0.888227	2.7271904	0.0066916	0.0150588	-3.270018
RMST	0.6822553	1.2889165	2.7270543	0.0066943	0.015064	-3.270379
ARMC4	0.6457106	0.4759824	2.7268808	0.0066977	0.0150708	-3.27084
CICP3	-0.356791	-0.798483	-2.726804	0.0066992	0.0150733	-3.271043
MIR186	0.4808399	0.0445717	2.7266994	0.0067013	0.0150771	-3.271322
RP4-539M6.	0.5583784	0.8774593	2.7265984	0.0067033	0.0150807	-3.27159
MORF4	0.3519528	-0.713797	2.7264237	0.0067068	0.0150875	-3.272053
RP11-363H1	-0.281033	-1.140715	-2.726218	0.0067109	0.0150958	-3.2726
CTD-2078B5	0.5101304	0.6120893	2.7260304	0.0067147	0.0151033	-3.273097
CTD-2129N1	0.2305186	-1.285498	2.7259762	0.0067157	0.0151048	-3.273241
JAZF1	-0.094958	5.9535809	-2.725937	0.0067165	0.0151056	-3.273344
STK32C	0.0944062	5.2919784	2.725722	0.0067208	0.0151144	-3.273916
UCP1	0.2910627	-1.169909	2.7255827	0.0067236	0.0151188	-3.274285
RP11-67K19	-0.513721	2.0051305	-2.725572	0.0067238	0.0151188	-3.274314
ATP6V0CP3	0.2935694	-1.048435	2.7255612	0.006724	0.0151188	-3.274342
HMGN2P15	0.4522229	2.5222999	2.7248568	0.0067381	0.0151495	-3.276211
DNM1L	0.0359114	6.2672339	2.7245913	0.0067434	0.0151598	-3.276915
UBA1	-0.032059	6.9389277	-2.724586	0.0067435	0.0151598	-3.276928
TOMM40	-0.054333	6.5052651	-2.72454	0.0067444	0.0151609	-3.277052
LINC00929	-0.135005	-1.486015	-2.724244	0.0067504	0.0151733	-3.277838
RP11-316M2	-0.143058	-1.466561	-2.724147	0.0067523	0.0151768	-3.278094
OAS3	0.0917731	6.1137956	2.7239412	0.0067564	0.0151851	-3.27864
MRPS36P1	-0.281925	-1.083601	-2.723806	0.0067591	0.0151902	-3.278997
RP11-138B4	0.5387335	-0.31477	2.7237478	0.0067603	0.0151919	-3.279152
CTD-2532D1	-0.405955	-0.814588	-2.723687	0.0067615	0.0151938	-3.279315
SNCAIP	0.2407452	4.5500137	2.7236303	0.0067627	0.0151954	-3.279464
MPZL2	0.0819522	6.0643516	2.7235918	0.0067634	0.0151962	-3.279566
RPS26P55	-0.221977	-1.265992	-2.723467	0.0067659	0.0152005	-3.279896
RP11-408H1	-0.492025	2.615234	-2.723455	0.0067662	0.0152005	-3.279928
MRM1	-0.066888	5.6403455	-2.723396	0.0067674	0.0152022	-3.280085
CTC-428H11	0.2797402	2.6365159	2.7232381	0.0067705	0.0152084	-3.280504
RP11-580P2	-0.323151	-0.973878	-2.723151	0.0067723	0.0152114	-3.280735
CBX3P6	-0.259611	-1.239995	-2.723041	0.0067745	0.0152146	-3.281027
GINS2	-0.117309	5.5129058	-2.723039	0.0067745	0.0152146	-3.281032
PROX1-AS1	-0.195049	5.528138	-2.723017	0.006775	0.0152146	-3.28109
IGHV3-42	0.2803707	-1.159377	2.7228274	0.0067788	0.0152222	-3.281593
NEFM	0.4632521	-0.628174	2.7227269	0.0067808	0.0152258	-3.281859
VAV2	-0.04825	6.576104	-2.722666	0.006782	0.0152276	-3.28202
PSMB9	-0.09403	6.263445	-2.722355	0.0067883	0.0152407	-3.282844
UNC45A	-0.041322	6.4647605	-2.722333	0.0067887	0.0152408	-3.282903
RP11-338K1	0.2468194	-1.220922	2.7222127	0.0067912	0.0152453	-3.283222
RP11-408A1	0.3792192	-0.580487	2.7221352	0.0067927	0.0152473	-3.283427
SAGE2P	-0.154827	-1.468477	-2.722128	0.0067928	0.0152473	-3.283445
RP11-583F2	-0.497986	0.856328	-2.722015	0.0067951	0.0152509	-3.283746
BTBD6	-0.081111	6.2016662	-2.722006	0.0067953	0.0152509	-3.283771
C3orf62	-0.065061	5.5638432	-2.721915	0.0067972	0.0152541	-3.284011
NXF1	-0.028424	6.4376591	-2.721846	0.0067985	0.0152563	-3.284194
AC024937.1	0.4507035	1.0847381	2.7216367	0.0068028	0.0152648	-3.284748

RP11-118F1	0.2781089	-1.120039	2.7212409	0.0068108	0.0152818	-3.285797
PDE3B	-0.110276	6.1108555	-2.72087	0.0068183	0.0152977	-3.286781
TLK1	-0.041641	6.36589	-2.720585	0.006824	0.0153096	-3.287534
GOLGA2P9	-0.178253	-1.391608	-2.720331	0.0068291	0.0153202	-3.288207
AP000866.1	0.446517	1.2092879	2.7201092	0.0068336	0.0153294	-3.288795
RP11-274B1	-0.151996	-1.467085	-2.719834	0.0068392	0.0153409	-3.289523
CTD-3064M3	0.4661463	-0.647892	2.7196788	0.0068424	0.0153471	-3.289934
HOXA10	0.6916436	2.9767568	2.719624	0.0068435	0.0153486	-3.29008
RN7SL751P	0.4843989	-0.178895	2.7195682	0.0068446	0.0153502	-3.290227
RP11-467P9	-0.476063	0.6384849	-2.719191	0.0068523	0.0153664	-3.291227
RP11-196G1	-0.367969	2.3611279	-2.719134	0.0068534	0.0153676	-3.291377
CDC23	-0.042076	6.1906185	-2.719123	0.0068536	0.0153676	-3.291406
ITGB3	0.3603802	3.9869902	2.7184977	0.0068663	0.0153952	-3.293061
OR7E91P	0.232753	-1.341225	2.7180213	0.006876	0.015416	-3.294322
HAO2	-0.440412	5.4489371	-2.717933	0.0068778	0.0154191	-3.294555
CTD-2621I1	0.4558593	2.4332016	2.7178163	0.0068802	0.0154235	-3.294864
LINC00544	0.2279526	-1.283959	2.7177677	0.0068812	0.0154247	-3.294993
ZNF132	0.1770823	4.4791124	2.7177067	0.0068825	0.0154259	-3.295155
DLG5	0.1278002	5.7714785	2.717701	0.0068826	0.0154259	-3.295169
CTC-297N7.	-0.171784	-1.390964	-2.717617	0.0068843	0.0154288	-3.295391
RP11-187C1	-0.487457	1.8918975	-2.717472	0.0068872	0.0154345	-3.295776
P3H1	-0.047648	6.3274504	-2.717232	0.0068921	0.0154445	-3.296411
DDX53	-0.758888	0.4641905	-2.716898	0.006899	0.0154588	-3.297293
SEC63P1	0.2875213	3.7535895	2.7163955	0.0069092	0.0154809	-3.298623
USH1G	0.5404605	-0.373871	2.7159687	0.006918	0.0154995	-3.299752
RP11-404P2	0.5028239	-0.017188	2.7157476	0.0069225	0.0155087	-3.300337
SEC31B	-0.127477	5.1898026	-2.715527	0.006927	0.0155179	-3.300921
RP11-350N1	0.2747656	-1.23152	2.7149815	0.0069382	0.015542	-3.302362
CTD-2272D1	-0.10684	-1.492296	-2.71486	0.0069407	0.0155466	-3.302682
RP11-643A5	-0.486797	-0.676406	-2.714498	0.0069481	0.0155623	-3.30364
RP11-758P1	-0.444419	1.2204436	-2.71446	0.0069489	0.0155631	-3.30374
AC073115.7	0.412097	-0.689414	2.7137792	0.0069629	0.0155936	-3.30554
CTC-537E7.	-0.618752	1.7277687	-2.713582	0.006967	0.0156017	-3.306061
CMTM6	0.0533533	6.5508145	2.7134061	0.0069706	0.0156089	-3.306526
PLEKHA1	0.0478596	6.2733662	2.7133774	0.0069712	0.0156092	-3.306601
ARX	0.4647924	-0.742771	2.713324	0.0069723	0.0156107	-3.306742
RP11-291L2	0.3421883	3.6542711	2.7129005	0.0069811	0.0156294	-3.307861
CTD-2616J1	0.253083	-1.198644	2.7127053	0.0069851	0.0156374	-3.308377
RP11-289F5	-0.434408	-0.961189	-2.712612	0.006987	0.0156408	-3.308624
RNA5-8S5	-0.139607	-1.492812	-2.712431	0.0069908	0.0156482	-3.309102
TRAM2	0.0579292	6.1184414	2.7121197	0.0069972	0.0156616	-3.309923
FDPSP1	-0.431083	-0.217397	-2.711974	0.0070002	0.0156665	-3.310308
RP11-555J4	-0.124474	-1.499204	-2.711972	0.0070002	0.0156665	-3.310312
RPL5P18	0.3714598	-0.637033	2.7118267	0.0070033	0.0156723	-3.310697
RNU6-1069P	-0.145573	-1.470743	-2.711287	0.0070144	0.0156964	-3.312122
AP001062.7	0.1530204	4.3810008	2.7112122	0.007016	0.0156987	-3.31232
RP11-366M4	-0.130036	-1.475826	-2.711197	0.0070163	0.0156987	-3.31236
RP11-651P2	0.289261	-1.026954	2.7108197	0.0070241	0.0157152	-3.313356
RP11-299H2	0.4690377	-0.479893	2.7106192	0.0070283	0.0157236	-3.313885
RP11-532L1	0.486278	-0.019161	2.7105419	0.0070299	0.0157262	-3.314089
RP11-553L6	0.2893424	-1.087752	2.7104759	0.0070313	0.0157283	-3.314263

ATP1B3-AS1	0.4207225	-0.542454	2.7099836	0.0070415	0.0157502	-3.315562
LIN52	-0.061649	5.6549759	-2.709927	0.0070427	0.0157519	-3.315712
HCG20	0.541437	1.3836216	2.7096926	0.0070476	0.0157618	-3.31633
HYALP1	-0.113095	-1.487345	-2.709249	0.0070568	0.0157815	-3.3175
C1orf198	0.0677313	6.3913262	2.7092155	0.0070575	0.0157821	-3.317589
SAPCD1-AS1	0.460357	1.5487659	2.7087664	0.0070669	0.0158021	-3.318774
RP4-594I10	-0.582524	1.5022856	-2.708486	0.0070728	0.0158143	-3.319513
MIR3180-1	-0.471862	-0.097548	-2.70841	0.0070743	0.0158169	-3.319714
CSAD	-0.101343	6.0903847	-2.708337	0.0070759	0.0158193	-3.319907
RNU6-137P	0.4269785	-0.184988	2.7082804	0.007077	0.015821	-3.320055
RPL3P4	-0.117159	5.7053428	-2.70816	0.0070796	0.0158256	-3.320374
AP000472.2	-0.594652	2.5328346	-2.707963	0.0070837	0.0158339	-3.320892
CYP4F22	0.4589832	5.1355349	2.7078042	0.007087	0.0158403	-3.321311
ALKBH8	0.0589837	5.33397	2.7077282	0.0070886	0.0158429	-3.321511
DEDD2	-0.047119	6.1201872	-2.707699	0.0070892	0.0158433	-3.321588
CENPBD1P1	0.0439424	5.8187876	2.7075548	0.0070922	0.0158491	-3.321969
RP11-521C2	-0.347513	-0.839928	-2.707519	0.007093	0.0158498	-3.322063
RP11-91J19	0.3178563	2.7968668	2.7074137	0.0070952	0.015853	-3.322341
MESTP1	0.3022353	-1.006906	2.7074092	0.0070953	0.015853	-3.322353
ESF1	0.063548	5.9296679	2.7073122	0.0070973	0.0158566	-3.322608
TMEM185A	0.1015052	5.2972194	2.7072417	0.0070988	0.0158588	-3.322794
LINC01285	-0.388112	2.5804778	-2.707222	0.0070992	0.0158588	-3.322845
CTC-537E7.	-0.512985	-0.240043	-2.707154	0.0071006	0.0158611	-3.323027
GRM1	0.4775513	-0.105741	2.7070254	0.0071033	0.0158661	-3.323364
AP001063.1	0.4992007	-0.498319	2.7069438	0.007105	0.015869	-3.323579
RP11-133F8	0.3561261	-1.009745	2.7068494	0.007107	0.0158716	-3.323828
RP1-167O22	-0.647769	0.1866946	-2.706845	0.0071071	0.0158716	-3.32384
ZNF34	-0.069218	5.4325735	-2.706571	0.0071129	0.0158835	-3.324561
RP11-158I2	-0.216615	-1.336641	-2.706369	0.0071171	0.015892	-3.325094
PRPF38AP1	-0.270997	-1.276971	-2.706193	0.0071208	0.0158993	-3.325558
LINC00347	-0.1417	-1.454654	-2.705998	0.0071249	0.0159075	-3.326073
RP11-673C5	-0.070773	5.3502371	-2.705903	0.0071269	0.015911	-3.326321
CXorf23	0.0669429	5.4504689	2.7057646	0.0071298	0.0159165	-3.326687
VN1R28P	-0.295406	-1.214797	-2.705583	0.0071336	0.0159241	-3.327166
CSMD2	0.4225603	3.7298072	2.7054777	0.0071359	0.015928	-3.327443
PLA2G2F	0.3356784	-1.034318	2.7053707	0.0071381	0.0159321	-3.327725
TM7SF3	-0.063248	6.4990616	-2.705123	0.0071433	0.0159428	-3.328377
RP11-135F9	-0.057685	5.6576909	-2.704831	0.0071495	0.0159556	-3.329147
HCN2	0.3839317	3.754426	2.7047373	0.0071515	0.015959	-3.329394
ZNF101P2	0.3166521	-0.953792	2.7046156	0.007154	0.0159637	-3.329714
LINC00997	-0.085791	5.0702533	-2.704541	0.0071556	0.0159663	-3.32991
RP11-554D1	0.361018	-0.925237	2.7043469	0.0071597	0.0159744	-3.330422
PTPRM	0.0996613	6.1376272	2.7043203	0.0071603	0.0159747	-3.330492
APITD1-COR	-0.259066	3.1829406	-2.704092	0.0071651	0.0159845	-3.331093
ADD3-AS1	0.4575963	1.4245555	2.7038687	0.0071698	0.0159941	-3.331681
TMEM263	0.0431165	6.3110651	2.7037559	0.0071722	0.0159984	-3.331978
BUB3	-0.033277	6.4627481	-2.703227	0.0071834	0.0160224	-3.33337
PSD4	-0.06168	6.4789295	-2.703175	0.0071845	0.0160239	-3.333506
ARG1	-0.232116	6.7501018	-2.703055	0.007187	0.0160286	-3.333823
RP11-587P2	-0.235788	-1.301085	-2.702948	0.0071893	0.0160327	-3.334105
RNASEK	0.0663315	5.4203073	2.7028494	0.0071914	0.0160363	-3.334364

DEK	-0.05026	6.5563091	-2.702672	0.0071952	0.0160437	-3.334831
ATP6V0E1P4	-0.1513	-1.435946	-2.702625	0.0071962	0.016045	-3.334955
CFAP61	0.5500628	0.5589779	2.7025384	0.007198	0.0160481	-3.335183
LINC01224	0.7291813	1.864587	2.7024692	0.0071995	0.01605	-3.335365
RP11-462G1	0.4221318	1.7310782	2.7024571	0.0071997	0.01605	-3.335397
SAP30L-AS1	0.3579534	2.1695562	2.7021937	0.0072053	0.0160615	-3.33609
PRR11	0.1517846	5.24834	2.7021009	0.0072073	0.0160649	-3.336334
RP1-27C22.	-0.244414	-1.329582	-2.701935	0.0072108	0.0160718	-3.33677
CTD-2233K9	0.2351337	3.3897149	2.7018495	0.0072126	0.0160749	-3.336996
GALNT18	0.1213492	5.8488599	2.7018201	0.0072133	0.0160753	-3.337073
NCKAP5	0.2681785	4.5275919	2.7014625	0.0072209	0.0160912	-3.338014
DNAJC1	-0.051951	6.3909515	-2.701382	0.0072226	0.016094	-3.338225
RP11-490O6	-0.293796	2.8181722	-2.701237	0.0072257	0.0161	-3.338607
PPL	-0.142733	5.9238613	-2.701202	0.0072264	0.0161006	-3.338698
TMEM31	0.4599319	-0.287099	2.701024	0.0072302	0.0161081	-3.339168
RP11-359G2	0.4168965	-0.131641	2.7008213	0.0072345	0.0161168	-3.339701
CTD-2616J1	0.3232432	-0.922042	2.7007667	0.0072357	0.0161184	-3.339844
IPO7P2	0.2938096	-1.045491	2.7006468	0.0072382	0.0161224	-3.34016
RP11-481J2	0.3306254	-1.012553	2.7006412	0.0072384	0.0161224	-3.340175
C15orf41	-0.075746	5.619422	-2.700601	0.0072392	0.0161233	-3.340279
AP000350.7	-0.447601	-0.177049	-2.700465	0.0072421	0.0161287	-3.340637
SERPINI2	0.3879668	-0.878614	2.7001152	0.0072496	0.0161444	-3.341558
SPANXB2	0.3694253	-1.000816	2.6999597	0.0072529	0.0161508	-3.341967
KIAA1217	0.0576397	6.2078246	2.6990973	0.0072714	0.0161909	-3.344234
ABBA01017E	0.3862374	-0.422227	2.6990321	0.0072728	0.0161931	-3.344405
TRIM26BP	-0.203623	-1.314375	-2.698599	0.0072821	0.0162127	-3.345544
RP11-521C2	0.4456162	-0.108618	2.6984592	0.0072851	0.0162184	-3.345911
DACT2	0.6473411	4.0653899	2.6979806	0.0072953	0.0162403	-3.347169
RP11-157D2	0.2210771	-1.314079	2.6975739	0.0073041	0.0162588	-3.348237
AC100830.5	0.4173	-0.154748	2.6973755	0.0073083	0.0162673	-3.348759
KBTBD8	0.2439971	4.1347346	2.6970781	0.0073147	0.0162806	-3.34954
EIF1AXP1	0.3946712	2.2267653	2.6970419	0.0073155	0.0162813	-3.349635
DDX55	0.0447233	5.8685962	2.697012	0.0073162	0.0162817	-3.349713
TEX11	0.6870628	1.9015125	2.696632	0.0073244	0.016299	-3.350711
ADAT1	-0.05764	5.7699629	-2.696383	0.0073297	0.0163099	-3.351366
RAB21	-0.032162	6.3956171	-2.696337	0.0073307	0.0163111	-3.351487
RP11-849F2	0.4160006	-0.322557	2.6959213	0.0073397	0.0163301	-3.352578
RP11-589P1	-0.146527	4.2513782	-2.69576	0.0073432	0.0163369	-3.353001
TMEM67	0.1443238	4.7540956	2.6953752	0.0073515	0.0163544	-3.354012
GAPDHP74	-0.568979	0.3506935	-2.695099	0.0073575	0.0163667	-3.354736
ARHGAP19	0.0605964	5.5793329	2.6950714	0.0073581	0.016367	-3.354809
LINC01549	-0.64542	2.2993237	-2.694797	0.007364	0.0163792	-3.355529
TSN	-0.033458	6.494987	-2.694298	0.0073748	0.0164023	-3.356839
RP11-413E1	0.2981377	-1.027108	2.6942445	0.007376	0.0164039	-3.356979
RP11-401E5	-0.682034	0.6785238	-2.6935	0.0073922	0.0164389	-3.358933
DCTN1	-0.032121	6.594454	-2.693367	0.007395	0.0164443	-3.359281
RP11-53B2.	0.4102369	-0.417925	2.6928682	0.0074059	0.0164674	-3.36059
GLYATL1P1	-0.488622	0.5555389	-2.69226	0.0074192	0.0164959	-3.362185
QRFPR	0.6007991	0.4981014	2.6922184	0.0074201	0.0164969	-3.362294
RP11-578F2	-0.4232	-0.33143	-2.691926	0.0074265	0.0165101	-3.363061
CLRN1-AS1	-0.567885	0.1017108	-2.691675	0.0074319	0.0165212	-3.363718

PHRF1	-0.037232	6.3838602	-2.691556	0.0074345	0.016526	-3.364032
GNB1L	-0.082894	5.3946127	-2.691505	0.0074357	0.0165275	-3.364165
OACYLP	0.4706872	0.5030656	2.6912946	0.0074403	0.0165367	-3.364716
RP11-368I7	-0.38893	2.3475953	-2.690819	0.0074507	0.0165588	-3.365963
GRIK2	0.6407706	1.243084	2.6907457	0.0074523	0.0165614	-3.366155
RBMY2OP	-0.1432	-1.464743	-2.69066	0.0074541	0.0165628	-3.366378
RP11-95M15	0.4996261	-0.219517	2.6906589	0.0074542	0.0165628	-3.366382
AC034228.4	-0.164788	-1.414265	-2.690654	0.0074543	0.0165628	-3.366395
ENDOU	0.4727272	0.5254849	2.6905775	0.0074559	0.0165655	-3.366595
KIAA0355	0.0515911	5.9927193	2.6904551	0.0074586	0.0165705	-3.366916
CDC16	-0.039983	6.3736328	-2.690234	0.0074635	0.0165802	-3.367494
RP13-492C1	-0.129087	-1.458452	-2.690013	0.0074683	0.01659	-3.368074
RPL23AP12	-0.257199	-1.166757	-2.689695	0.0074753	0.0166045	-3.368907
RP11-402G3	-0.273395	-1.111359	-2.689577	0.0074779	0.0166092	-3.369216
RP5-1185K9	-0.170874	-1.448463	-2.689423	0.0074813	0.0166158	-3.36962
ASPRV1	-0.145393	4.1114869	-2.689311	0.0074837	0.0166202	-3.369914
SNRPG	-0.047439	6.2244916	-2.689271	0.0074846	0.0166212	-3.370019
NCBP2	0.0315116	6.3571441	2.6885964	0.0074995	0.0166531	-3.371785
SPANXN1	-0.202393	-1.388027	-2.688437	0.007503	0.0166599	-3.372203
BIRC3	0.1305997	6.2381933	2.6878084	0.0075168	0.0166892	-3.373849
ALDOAP2	0.3149072	-0.954938	2.6877975	0.0075171	0.0166892	-3.373877
NEURL4	0.0455815	5.7537088	2.6876073	0.0075213	0.0166975	-3.374375
INSRR	0.5947795	0.7265361	2.6870472	0.0075337	0.016724	-3.375841
LINC00891	0.3131207	-0.959571	2.6869794	0.0075352	0.0167263	-3.376019
RP11-430H1	-0.551004	0.5418492	-2.686869	0.0075376	0.0167307	-3.376308
AKTIP	-0.06628	5.9358754	-2.686772	0.0075397	0.0167344	-3.376561
SYS1	-0.044364	6.3392631	-2.686425	0.0075474	0.0167504	-3.377469
AC005363.1	-0.427414	-0.303064	-2.686235	0.0075516	0.0167588	-3.377966
KPNA6	-0.037583	6.3511912	-2.686135	0.0075539	0.0167627	-3.378229
AF131215.3	0.227854	-1.284014	2.6858106	0.0075611	0.0167776	-3.379077
ADAMTS10	0.1600788	5.5384133	2.685782	0.0075617	0.016778	-3.379152
TUBB2A	-0.091897	6.2943627	-2.68572	0.0075631	0.01678	-3.379315
CTD-2020K1	0.4723167	2.085503	2.6856882	0.0075638	0.0167806	-3.379397
CTDSPL2	0.0475844	5.9435641	2.6855972	0.0075658	0.016784	-3.379636
AC112229.1	0.5439183	0.5987304	2.6851794	0.0075751	0.0168036	-3.380728
RP11-83B20	-0.188158	-1.360972	-2.684976	0.0075796	0.0168126	-3.381259
RBCK1	-0.048569	6.703078	-2.68473	0.0075851	0.0168237	-3.381904
AC106876.2	-0.229943	4.6380629	-2.684465	0.007591	0.0168358	-3.382597
RP11-430H1	0.2711786	-1.196861	2.6843319	0.0075939	0.0168413	-3.382945
DENND4B	0.0401012	6.2976733	2.6838343	0.007605	0.0168649	-3.384246
DIO2	0.6154458	2.9450452	2.6836369	0.0076094	0.0168737	-3.384762
CTD-2201E1	0.16071	4.562693	2.6836081	0.0076101	0.0168741	-3.384837
AP001891.1	-0.256893	-1.21317	-2.683485	0.0076128	0.0168781	-3.385159
RP11-77901	-0.463772	-0.090513	-2.683485	0.0076128	0.0168781	-3.38516
MRPS35	-0.03917	6.5301969	-2.683323	0.0076164	0.0168851	-3.385581
RN7SL132P	-0.186192	-1.364391	-2.683144	0.0076204	0.0168929	-3.38605
MAGEC2	-0.911483	1.9110344	-2.682937	0.007625	0.0169022	-3.386591
MBP	0.075241	6.18653	2.6828672	0.0076266	0.0169046	-3.386774
RP11-736N1	-0.348399	3.9799038	-2.682783	0.0076285	0.0169077	-3.386993
SLC22A12	-0.858624	2.5875952	-2.682523	0.0076343	0.0169196	-3.387672
EHMT2	-0.04888	6.4216475	-2.682438	0.0076362	0.0169228	-3.387895

RP11-385F5	0.3877831	1.9619829	2.6818816	0.0076487	0.0169494	-3.389349
RP11-815N9	-0.429086	2.2124135	-2.681135	0.0076654	0.0169855	-3.3913
USP46	0.0627979	5.5487771	2.68097	0.0076691	0.0169927	-3.39173
RNVU1-3	0.4638808	0.0697264	2.6808062	0.0076728	0.0169998	-3.392158
GOLGA5	-0.042819	6.4128635	-2.680686	0.0076755	0.0170048	-3.392472
RP5-988G15	-0.177811	-1.371809	-2.680214	0.0076861	0.0170273	-3.393705
RP11-156K2	-0.14399	4.3175105	-2.679982	0.0076914	0.0170378	-3.39431
RP11-849F2	0.2667968	2.7191191	2.6799188	0.0076928	0.0170399	-3.394475
DUSP5P1	0.58885	0.2727491	2.6797532	0.0076965	0.0170472	-3.394907
CAND1	0.0308904	6.4788556	2.6796291	0.0076993	0.0170523	-3.395231
UBE2H	-0.043757	6.6400859	-2.679282	0.0077072	0.0170686	-3.396138
CTD-2015H3	-0.636893	1.0294457	-2.678974	0.0077141	0.017083	-3.396942
GIPC2	-0.200486	5.5780876	-2.678731	0.0077196	0.0170941	-3.397576
DIAPH3	0.1966298	4.8341169	2.6781971	0.0077317	0.0171198	-3.398969
AC005722.4	-0.223366	-1.365078	-2.678145	0.0077329	0.0171214	-3.399106
FOXD4L1	0.4703963	0.3162707	2.6781058	0.0077338	0.0171223	-3.399207
TEX38	-0.454174	0.208126	-2.67802	0.0077357	0.0171256	-3.399431
CTD-204901	0.5088679	-0.006627	2.6779405	0.0077375	0.0171285	-3.399638
MAP3K7	0.0415214	6.1109953	2.6777865	0.007741	0.0171352	-3.40004
RP11-345P4	-0.425651	-0.037049	-2.677756	0.0077417	0.0171357	-3.400119
DSP	-0.069194	6.8799866	-2.677719	0.0077425	0.0171365	-3.400216
RP11-395D3	-0.150155	-1.447773	-2.677643	0.0077442	0.0171393	-3.400415
GRM8	0.7022872	1.866122	2.6774189	0.0077493	0.0171495	-3.400999
C1QTNF6	0.0831275	5.9311082	2.6770262	0.0077582	0.017168	-3.402023
SSX4B	-0.355009	-1.049847	-2.677009	0.0077586	0.017168	-3.402069
RP11-434H1	-0.221756	-1.323391	-2.676924	0.0077606	0.0171712	-3.402289
RP11-123J1	-0.200712	-1.317095	-2.676788	0.0077637	0.0171771	-3.402645
SPINK13	0.4481404	-0.509792	2.6764568	0.0077712	0.0171927	-3.403508
ANKRD27	0.0617591	5.9552092	2.6761678	0.0077777	0.0172049	-3.404261
RN7SKP80	0.4640023	0.4855751	2.6761537	0.0077781	0.0172049	-3.404298
CHRM3-AS1	-0.45005	-0.736016	-2.676152	0.0077781	0.0172049	-3.404304
RP11-221N1	0.2641694	-1.166749	2.6758489	0.007785	0.0172191	-3.405093
GPR113	0.2984766	2.742357	2.6757328	0.0077877	0.0172239	-3.405395
PPP1R8	-0.031543	6.1268893	-2.675479	0.0077934	0.0172356	-3.406056
PLD3	-0.038832	6.8494264	-2.675399	0.0077953	0.0172384	-3.406265
RP11-955H2	-0.112516	-1.490275	-2.675384	0.0077956	0.0172384	-3.406305
LSM6	-0.051439	5.68975	-2.675264	0.0077983	0.0172434	-3.406618
RP11-1022E	-0.14921	-1.45373	-2.675047	0.0078033	0.0172532	-3.407182
RP11-1084E	0.3894721	-0.838364	2.674841	0.007808	0.0172626	-3.40772
RP11-331F4	0.3816702	-0.614943	2.6747521	0.00781	0.0172661	-3.407951
RP11-265N6	0.3198528	1.9087107	2.6746907	0.0078114	0.0172681	-3.408111
BANP	-0.055799	5.7944717	-2.674311	0.0078201	0.0172857	-3.4091
ALG1L	-0.402531	4.8313768	-2.674289	0.0078206	0.0172857	-3.409159
DNAJC9-AS1	-0.439045	1.0887143	-2.674281	0.0078208	0.0172857	-3.409179
RP11-495P1	-0.729576	0.427178	-2.67412	0.0078245	0.0172928	-3.409599
RP11-1084J	-0.494531	-0.323501	-2.673999	0.0078272	0.0172971	-3.409913
FTH1	-0.048467	7.1896413	-2.673988	0.0078275	0.0172971	-3.409942
RP11-867G2	0.2690929	-1.209978	2.6739727	0.0078278	0.0172971	-3.409982
PCAT4	-0.205095	-1.39644	-2.673718	0.0078337	0.0173089	-3.410646
LRRC53	-0.120434	-1.482997	-2.67344	0.0078401	0.017322	-3.41137
ATRN	-0.058384	6.8512795	-2.673064	0.0078487	0.01734	-3.41235

GSKIP	0.0630889	6.0000873	2.6728741	0.007853	0.0173485	-3.412843
RP11-367F2	0.5055476	0.2895845	2.6725134	0.0078613	0.0173658	-3.413783
CTA-984G1.	0.3172843	2.5804842	2.6720916	0.007871	0.0173861	-3.414881
AC137932.6	0.4498561	1.2692483	2.6720638	0.0078717	0.0173865	-3.414953
RP4-535B20	-0.295657	-0.98966	-2.672029	0.0078725	0.0173872	-3.415044
GZMAP1	0.2087088	-1.317758	2.671894	0.0078756	0.017393	-3.415395
CIAPIN1	-0.045282	6.2344327	-2.671783	0.0078781	0.0173976	-3.415684
EEF1D	-0.055259	6.7376415	-2.670725	0.0079025	0.0174504	-3.418438
AC093495.4	0.1988281	3.9361062	2.670638	0.0079045	0.0174538	-3.418664
RP5-1086K1	0.3299404	3.3296337	2.6703756	0.0079106	0.017466	-3.419347
FAM166A	-0.488141	2.2442151	-2.670359	0.007911	0.017466	-3.419391
PTCHD3P3	0.4353269	-0.613768	2.6701213	0.0079165	0.017477	-3.420008
DCAF4L1	0.39022	2.8944848	2.6696746	0.0079268	0.0174979	-3.42117
MRPL50	-0.05143	6.0546947	-2.669671	0.0079269	0.0174979	-3.42118
DCAF17	0.0605537	5.618238	2.6696198	0.0079281	0.0174995	-3.421313
STOX1	0.4666596	3.5426189	2.6695806	0.007929	0.0175004	-3.421415
RP11-474P2	-0.362697	-0.637082	-2.669297	0.0079355	0.017513	-3.422152
RP4-736L20	-0.296807	-1.074289	-2.669278	0.007936	0.017513	-3.422203
RP11-40G16	-0.294005	-1.180571	-2.669271	0.0079361	0.017513	-3.42222
CTD-307407	-0.181428	-1.382198	-2.669238	0.0079369	0.0175137	-3.422306
RBM4B	-0.043424	5.7519848	-2.669008	0.0079422	0.0175244	-3.422905
COX20P1	-0.587273	1.5562132	-2.66872	0.0079489	0.0175381	-3.423652
RP11-491F9	-0.522686	0.732074	-2.668675	0.00795	0.0175393	-3.423771
NEUROD6	-0.180001	-1.424737	-2.668406	0.0079562	0.017552	-3.42447
RP11-370B1	-0.412506	-0.525229	-2.66817	0.0079617	0.0175631	-3.425082
MAP3K3	0.0421695	6.0195254	2.6681488	0.0079622	0.0175631	-3.425138
RP11-12G12	-0.084285	5.7218684	-2.667976	0.0079662	0.0175709	-3.425587
RP11-511B2	-0.500332	1.4432592	-2.667893	0.0079681	0.0175741	-3.425803
H2AFY	0.0345846	6.6664845	2.6678485	0.0079692	0.0175753	-3.425919
ZGPAT	-0.077614	5.7191705	-2.667825	0.0079697	0.0175755	-3.425981
NLRP1	0.1069269	5.6985914	2.6674893	0.0079775	0.0175916	-3.426852
UBE2G1	0.0458295	6.2028858	2.6673629	0.0079805	0.0175971	-3.427181
ERCC6L	0.2386699	4.2973528	2.6669518	0.0079901	0.0176171	-3.428249
AC007743.1	0.4963068	1.7155896	2.6669253	0.0079907	0.0176174	-3.428318
ZNF559-ZNF	0.4832472	1.4721874	2.6667383	0.007995	0.017626	-3.428804
PCIF1	-0.032508	6.2321012	-2.666239	0.0080067	0.0176502	-3.430102
LLOXNC01-3	0.4436436	-0.007307	2.6662269	0.008007	0.0176502	-3.430133
ATP8B4	0.1369168	4.8528512	2.6660513	0.0080111	0.0176581	-3.430589
APP	0.0678603	7.0381607	2.6660169	0.0080119	0.0176583	-3.430678
MED7	-0.046635	5.6879333	-2.666007	0.0080121	0.0176583	-3.430704
IP6K2	-0.040989	6.3250325	-2.665925	0.008014	0.0176614	-3.430917
NMI	-0.058864	5.9944762	-2.665437	0.0080254	0.0176855	-3.432183
RP11-700E2	-0.150994	-1.431253	-2.665391	0.0080265	0.0176868	-3.432303
LINC00619	0.413479	-0.55348	2.6651055	0.0080332	0.0177005	-3.433045
VDAC1	-0.040779	6.9491891	-2.665083	0.0080337	0.0177006	-3.433104
RP11-305L7	0.3588354	-0.891061	2.6650074	0.0080355	0.0177034	-3.4333
4-Mar	0.4743907	2.0002802	2.6649806	0.0080361	0.0177037	-3.433369
SRL	0.2896649	3.2876053	2.66488	0.0080385	0.0177079	-3.433631
TIA1	0.0447807	6.2447543	2.6646301	0.0080444	0.0177197	-3.434279
RP11-944L7	0.4381563	-0.218294	2.6642513	0.0080532	0.0177382	-3.435263
WDR77	-0.046779	6.020268	-2.66409	0.008057	0.0177455	-3.435681

RP11-313I2	-0.121167	-1.471234	-2.664067	0.0080576	0.0177456	-3.435742
SF3A1	-0.032731	6.5892231	-2.663966	0.00806	0.0177498	-3.436004
AC092168.2	0.3365025	-0.78451	2.6637815	0.0080643	0.0177583	-3.436482
KCNQ1OT1	0.1754496	4.707873	2.6633685	0.008074	0.0177786	-3.437554
DSPP	-0.194086	-1.340931	-2.663297	0.0080757	0.0177812	-3.437741
RP3-337H4.	0.2732381	3.0624064	2.6630852	0.0080807	0.0177911	-3.438289
RP4-798A10	-0.508586	0.9841854	-2.663043	0.0080817	0.0177922	-3.438399
RCC2P3	-0.296226	-1.04858	-2.662489	0.0080947	0.0178199	-3.439837
COG5	0.0549622	5.9056056	2.6623517	0.008098	0.017826	-3.440193
CLASRP	-0.045059	6.203396	-2.6621	0.0081039	0.0178374	-3.440846
TRIM66	-0.081971	5.5808289	-2.662091	0.0081041	0.0178374	-3.440869
RP11-529H2	0.3804363	2.0867653	2.6616773	0.0081139	0.0178578	-3.441942
RNU6-37P	-0.286355	-1.060669	-2.661474	0.0081187	0.0178673	-3.442469
CTD-2396E7	-0.229158	5.7201848	-2.661129	0.0081269	0.0178842	-3.443365
RP11-84A19	0.3686981	-0.604403	2.6610604	0.0081285	0.0178867	-3.443542
TEX261	-0.034136	6.5482278	-2.660998	0.00813	0.0178889	-3.443705
RP11-302L1	0.4224851	2.3792644	2.6608728	0.0081329	0.0178943	-3.444028
KDELR1	-0.039995	6.8569162	-2.660779	0.0081352	0.0178981	-3.444271
PDHA1	-0.04273	6.6092001	-2.660599	0.0081394	0.0179064	-3.444738
RP11-195M1	0.3932898	-0.771781	2.6605034	0.0081417	0.0179103	-3.444986
RP11-593F2	0.4513672	-0.165278	2.6604532	0.0081429	0.0179119	-3.445116
CECR1	0.0987733	6.0929115	2.6602337	0.0081481	0.0179223	-3.445685
GNPDA1	0.0566654	6.1439482	2.6600156	0.0081533	0.0179326	-3.44625
GUCY1B2	0.71168	1.6377784	2.659924	0.0081554	0.0179363	-3.446488
DIS3	-0.047239	6.1271969	-2.659498	0.0081656	0.0179574	-3.447592
NLGN1	0.5775829	0.4453593	2.6594381	0.008167	0.0179595	-3.447747
RP11-14N7.	0.5150826	1.7269396	2.6592185	0.0081722	0.0179699	-3.448316
TET2	0.0774958	5.5173139	2.6591276	0.0081744	0.0179736	-3.448552
RP11-1360M	-0.105732	-1.492782	-2.65909	0.0081753	0.0179744	-3.448649
RP11-1094M	0.2370786	-1.203	2.6589987	0.0081774	0.0179782	-3.448886
RP11-304L1	0.4889816	1.1371649	2.6588603	0.0081807	0.0179843	-3.449244
RP11-481H1	0.5471386	0.706395	2.6588061	0.008182	0.0179861	-3.449385
RP11-66N24	-0.551645	2.5955813	-2.658685	0.0081849	0.0179913	-3.449698
AC023283.1	0.3957438	-0.532705	2.6584841	0.0081897	0.0180008	-3.450219
RP4-591C20	0.1685874	4.6560385	2.6583962	0.0081918	0.0180043	-3.450447
TBC1D13	-0.054057	6.2289905	-2.658196	0.0081966	0.0180129	-3.450965
PLEKHM1	-0.042747	5.9567881	-2.658192	0.0081967	0.0180129	-3.450976
RP11-322D1	0.3783374	-0.614438	2.6578814	0.0082041	0.018028	-3.45178
FAM166B	-0.249092	3.3709053	-2.65786	0.0082046	0.0180281	-3.451835
CTD-2170G1	0.5578667	-0.063794	2.6578361	0.0082052	0.0180282	-3.451897
POM121L2	0.2762361	-1.250728	2.6577534	0.0082071	0.0180315	-3.452112
RP11-712L6	0.3328208	-0.792893	2.657709	0.0082082	0.0180327	-3.452227
RP11-47I22	0.4238225	-0.505773	2.6574853	0.0082135	0.0180434	-3.452806
RP11-218M2	-0.102426	5.6844548	-2.657429	0.0082149	0.0180453	-3.452953
TBL1X	-0.058489	6.2951221	-2.657383	0.008216	0.0180466	-3.45307
AP000692.9	0.4617048	1.3808745	2.6572151	0.00822	0.0180543	-3.453506
HSPA5	-0.045237	7.3287475	-2.656479	0.0082376	0.018092	-3.455412
RP11-799M1	-0.391715	1.9972762	-2.656411	0.0082393	0.0180944	-3.455587
CTPS2	0.0497379	5.9733647	2.6563492	0.0082407	0.0180966	-3.455748
RP13-235E2	-0.183249	-1.369505	-2.655845	0.0082528	0.0181221	-3.457052
RP4-665J23	-0.333547	-0.934105	-2.655408	0.0082633	0.018144	-3.458183

CTD-2017D1	0.2440829	4.1336006	2.6553558	0.0082646	0.0181457	-3.458319
RP1-283E3.	0.2167303	3.8747764	2.6553183	0.0082655	0.0181466	-3.458416
VPS37B	0.0669074	5.7825844	2.6550927	0.0082709	0.0181574	-3.459
NFE2L2	-0.050618	6.5541327	-2.65503	0.0082724	0.0181596	-3.459161
CTD-2033D2	-0.43334	-0.23031	-2.655006	0.008273	0.0181598	-3.459223
CLCN7	-0.049372	6.4893612	-2.654959	0.0082741	0.0181612	-3.459345
DNAJC3	-0.049247	6.6536281	-2.654924	0.008275	0.018162	-3.459436
RP11-494K3	0.2721533	-1.105401	2.6548505	0.0082767	0.0181647	-3.459626
AC008067.2	-0.118237	-1.499793	-2.654672	0.008281	0.0181731	-3.460088
LRRC37A6P	0.4883464	3.0336126	2.6544646	0.008286	0.0181829	-3.460624
LEMD3	0.0447218	5.8645871	2.6541195	0.0082944	0.0182001	-3.461517
EIF3H	-0.051989	6.8239455	-2.654044	0.0082962	0.018203	-3.461711
RP11-1260E	-0.373496	3.7311988	-2.653576	0.0083075	0.0182267	-3.462923
ZNF592	-0.036159	6.2623356	-2.653371	0.0083124	0.0182365	-3.463453
TTLL12	-0.053248	6.4133518	-2.653319	0.0083137	0.0182374	-3.463588
UBE2Q2P6	-0.118431	6.4471453	-2.653312	0.0083138	0.0182374	-3.463605
RP11-263K1	0.33313	3.0204056	2.6531179	0.0083185	0.0182466	-3.464107
PLEKHG3	0.0591297	6.23572	2.6530536	0.0083201	0.0182489	-3.464274
PTRF	0.0641053	6.5284881	2.652986	0.0083217	0.0182514	-3.464448
GCNT7	0.4483466	0.5176058	2.6527263	0.008328	0.0182641	-3.46512
RP11-7F18.	0.4600443	1.5266432	2.6526223	0.0083305	0.0182677	-3.465389
RP11-258C1	0.5217722	0.6349746	2.652616	0.0083307	0.0182677	-3.465405
TXNDC2	0.2964678	-1.013394	2.6524234	0.0083353	0.0182769	-3.465903
OSGIN2	0.0666283	6.0956427	2.6523124	0.008338	0.0182817	-3.46619
RP11-330C7	-0.125714	-1.468304	-2.651894	0.0083482	0.0183028	-3.467271
RP11-70J12	0.2422199	-1.231436	2.6518214	0.0083499	0.0183056	-3.467458
TNK2	0.0591831	6.2106497	2.6514561	0.0083588	0.0183239	-3.468402
LL09NC01-2	-0.419445	1.5930324	-2.651165	0.0083659	0.0183383	-3.469156
ATP5HP1	-0.192731	-1.345347	-2.650949	0.0083711	0.0183487	-3.469713
SFN	-0.336231	5.4836248	-2.650892	0.0083725	0.0183506	-3.469859
RP11-543P1	-0.131962	5.4229267	-2.650832	0.008374	0.0183527	-3.470014
OBP2A	0.33548	-0.991522	2.6505073	0.0083819	0.018369	-3.470854
HAUS3	0.0455995	5.6478395	2.6504553	0.0083831	0.0183706	-3.470988
RP11-317J9	0.5180796	1.3500592	2.6502286	0.0083887	0.0183816	-3.471574
NKX6-3	0.3300638	-1.161902	2.6500799	0.0083923	0.0183885	-3.471958
TCEB1P28	-0.390511	-0.538477	-2.649858	0.0083977	0.0183992	-3.47253
RP11-767C1	0.4670036	0.5922124	2.6494005	0.0084088	0.0184225	-3.473712
LLPH	-0.03681	5.9648546	-2.649344	0.0084102	0.0184243	-3.473857
DPRXP6	-0.211715	-1.299572	-2.649327	0.0084106	0.0184243	-3.473902
MEF2A	0.0653146	6.10557	2.6488642	0.0084219	0.0184479	-3.475097
AC006116.1	0.2874584	-1.020561	2.6488442	0.0084224	0.0184479	-3.475149
PTGES3P4	-0.473073	0.218683	-2.648618	0.008428	0.0184589	-3.475732
RP11-173P1	0.4708672	0.3403399	2.6484441	0.0084322	0.0184671	-3.476181
UGT1A1	-0.349632	5.9539049	-2.648378	0.0084338	0.0184696	-3.476352
RP11-686O6	-0.173328	3.8999126	-2.648352	0.0084345	0.0184698	-3.476419
GAD1	0.6546284	2.1003618	2.6481985	0.0084382	0.018477	-3.476815
SNORA55	0.4341562	-0.094934	2.6475734	0.0084536	0.0185094	-3.478428
RP11-63H19	-0.257614	-1.170915	-2.647256	0.0084613	0.0185253	-3.479247
ALG11	0.0794592	5.1617385	2.6472009	0.0084627	0.0185272	-3.47939
AC013439.4	-0.510927	0.1943765	-2.647154	0.0084638	0.0185286	-3.47951
AP006621.9	0.5191726	1.4967904	2.6471235	0.0084646	0.0185291	-3.479589

YES1P1	0.4525627	0.1314818	2.6467561	0.0084736	0.0185478	-3.480537
CTD-2353F2	0.4104841	-0.521166	2.646728	0.0084743	0.0185482	-3.48061
LRRC46	-0.137744	4.3716547	-2.646649	0.0084762	0.0185513	-3.480812
TXNP1	-0.291514	-1.065416	-2.646469	0.0084807	0.0185599	-3.481277
AC145124.2	0.4858451	1.2632794	2.6463644	0.0084833	0.0185644	-3.481547
MIR646HG	0.6571606	1.6906756	2.6462305	0.0084866	0.0185705	-3.481893
SPTBN5	0.1754976	4.7584241	2.6461297	0.008489	0.0185748	-3.482153
UNC80	0.4928057	1.1504861	2.6457992	0.0084972	0.0185915	-3.483005
LRRC9	0.487994	0.4916466	2.6457234	0.008499	0.0185945	-3.483201
RP5-965G21	-0.187253	-1.368337	-2.645702	0.0084996	0.0185945	-3.483256
XXyac-YX15	-0.537232	0.0846572	-2.645226	0.0085113	0.0186191	-3.484483
KTN1	-0.03912	6.7675502	-2.645144	0.0085133	0.0186214	-3.484694
FKBP1C	0.2023108	3.1345302	2.6451424	0.0085134	0.0186214	-3.484698
PCDHB13	0.3611451	4.0304	2.6450897	0.0085147	0.0186231	-3.484835
RP11-64K12	0.4293536	0.0502105	2.6450126	0.0085166	0.0186262	-3.485033
RP11-98J23	0.4443052	-0.115394	2.6448944	0.0085195	0.0186314	-3.485338
TMC2	0.4784711	0.4074684	2.6443704	0.0085324	0.0186586	-3.486688
TRIM43B	-0.125855	-1.490977	-2.644316	0.0085338	0.0186605	-3.486829
RP11-47506	-0.392066	3.3568997	-2.644284	0.0085346	0.0186611	-3.486911
AC051649.1	-0.138905	-1.473138	-2.644239	0.0085357	0.0186624	-3.487028
RP11-83B2C	-0.462836	-0.548017	-2.644063	0.0085401	0.0186708	-3.48748
ACTBP11	0.4464923	2.0918998	2.643956	0.0085427	0.0186755	-3.487757
PROL1	-0.740515	2.1507662	-2.643768	0.0085474	0.0186845	-3.488241
AC003088.1	-0.457327	-0.425592	-2.643692	0.0085492	0.0186875	-3.488437
SMG1P2	0.1610489	3.8766112	2.6436679	0.0085498	0.0186877	-3.488499
RSL24D1P6	0.414012	1.2794917	2.6431028	0.0085639	0.0187172	-3.489955
TTK	0.1947681	5.0760351	2.6430716	0.0085646	0.0187178	-3.490035
EVPLL	-0.640497	1.3626668	-2.642808	0.0085712	0.018731	-3.490714
RP11-799B1	-0.145813	5.4202496	-2.642632	0.0085755	0.0187394	-3.491167
RP1-117P2C	-0.541971	-0.084974	-2.642033	0.0085904	0.0187708	-3.492709
MESP2	-0.31116	4.032974	-2.641978	0.0085918	0.0187727	-3.492852
RP11-34P13	0.4810858	0.341675	2.6419318	0.008593	0.0187731	-3.492971
OAT	0.1917405	5.9609549	2.6419298	0.008593	0.0187731	-3.492976
B3GNT5	0.1922077	5.3391861	2.6417026	0.0085987	0.0187843	-3.493561
AF196970.3	-0.338135	-0.738325	-2.641511	0.0086034	0.0187936	-3.494053
ERVH48-1	0.4941607	0.5216075	2.6413257	0.0086081	0.0188026	-3.494531
MCF2	0.4762867	0.7155962	2.6407302	0.0086229	0.0188339	-3.496065
COPS4	-0.043484	6.0176309	-2.640612	0.0086259	0.0188392	-3.49637
RP11-158D2	-0.170174	-1.418074	-2.64048	0.0086292	0.0188451	-3.496708
KIAA1467	0.0934243	5.2668055	2.6404636	0.0086296	0.0188451	-3.496751
AC024896.1	-0.335929	-0.898196	-2.64028	0.0086342	0.0188539	-3.497223
RP11-680A1	0.2099382	3.4796988	2.639901	0.0086436	0.0188735	-3.498198
UGT2A3	0.3212959	5.6492499	2.6397756	0.0086468	0.0188792	-3.498521
LYVE1	0.1999374	4.8463542	2.6397342	0.0086478	0.0188804	-3.498628
LARP4B	0.0303984	6.3447852	2.6395551	0.0086523	0.018889	-3.499089
RP11-459I1	-0.371031	3.4928066	-2.639517	0.0086533	0.01889	-3.499187
EEF1A1	-0.039723	7.5872989	-2.639096	0.0086638	0.0189119	-3.500268
PRKCDBP	0.1233006	5.4580514	2.6390271	0.0086655	0.0189145	-3.500447
C4orf36	-0.110617	4.5586757	-2.638896	0.0086688	0.0189206	-3.500785
HYLS1	-0.080278	5.3079649	-2.638795	0.0086714	0.018925	-3.501043
ZNF529	0.064942	5.5634477	2.6385198	0.0086783	0.0189384	-3.501751

SMG8	0.0844929	5.7431903	2.6385097	0.0086785	0.0189384	-3.501778
RP11-81802	0.4530338	0.4306705	2.6384329	0.0086805	0.0189414	-3.501975
RP11-33N14	0.3942693	1.6054826	2.6384035	0.0086812	0.0189419	-3.502051
PLGLB2	0.4405036	4.4032363	2.638172	0.008687	0.0189533	-3.502646
EIF1P5	-0.460336	0.757378	-2.638147	0.0086876	0.0189533	-3.502709
APOL2	-0.059306	6.526316	-2.63813	0.0086881	0.0189533	-3.502754
MIR4520-1	-0.359048	-0.736442	-2.638113	0.0086885	0.0189533	-3.502799
FBXO3	-0.041611	6.2263106	-2.637684	0.0086993	0.0189757	-3.503902
RP11-495P1	-0.426351	-0.722859	-2.6376	0.0087014	0.0189792	-3.504116
MFSD12	-0.05566	6.2982736	-2.637433	0.0087056	0.0189872	-3.504547
ITIH2	-0.125031	7.469333	-2.637365	0.0087073	0.0189898	-3.504722
RP11-91A15	-0.15892	-1.413599	-2.637171	0.0087122	0.0189994	-3.50522
ZC3H7A	0.0414736	6.1576914	2.6369838	0.0087169	0.0190085	-3.505701
FAM229A	-0.118596	4.8024532	-2.636736	0.0087232	0.0190199	-3.506339
RP3-425C14	0.1198964	5.2329964	2.636735	0.0087232	0.0190199	-3.50634
PSG7	-0.17757	-1.418902	-2.636556	0.0087277	0.019028	-3.5068
ITPKA	0.2371051	5.2785846	2.6365476	0.0087279	0.019028	-3.506822
UFM1	-0.044033	6.4054395	-2.636482	0.0087296	0.0190304	-3.50699
AC012363.8	0.2485283	-1.183975	2.6360984	0.0087393	0.0190504	-3.507976
MSL3	0.0341318	5.8662617	2.6360684	0.00874	0.0190509	-3.508053
RN7SL263P	0.3546801	-0.621596	2.6356985	0.0087494	0.0190702	-3.509004
AKAP11	0.0608815	6.0158824	2.6351109	0.0087643	0.0191015	-3.510513
PRG4	0.2426994	6.3181707	2.6349052	0.0087695	0.0191117	-3.511042
RP13-228J1	0.3536532	-0.780562	2.6348344	0.0087713	0.0191145	-3.511224
RP11-158C2	-0.223988	-1.275666	-2.634775	0.0087728	0.0191166	-3.511376
LAMC1	0.059957	6.7307243	2.634691	0.0087749	0.0191201	-3.511592
RP11-381N2	-0.334639	-1.122203	-2.63466	0.0087757	0.0191202	-3.511672
AC093375.1	0.5897029	1.9979708	2.6346474	0.008776	0.0191202	-3.511704
ARHGEF7-IT	-0.165764	-1.397553	-2.634447	0.0087811	0.0191302	-3.512219
LRGUK	0.414646	2.6736621	2.6336223	0.008802	0.0191747	-3.514336
KIAA1279	0.0477867	5.8592523	2.6334953	0.0088053	0.0191806	-3.514662
XAF1	0.2147592	5.1480724	2.6332704	0.008811	0.0191919	-3.515239
ADRA1A	-0.486888	4.323691	-2.633227	0.0088121	0.0191931	-3.515351
CTD-2036P1	0.4234447	1.1522832	2.6328111	0.0088227	0.0192151	-3.516418
DNAJC19P9	-0.446111	0.4129503	-2.632681	0.008826	0.0192211	-3.516752
RP4-630A11	-0.431133	0.0645612	-2.632599	0.0088281	0.0192245	-3.516962
ZFX-AS1	-0.307678	-0.956566	-2.632499	0.0088307	0.019229	-3.51722
RP11-74E22	0.4393192	-0.592825	2.6322467	0.0088371	0.0192418	-3.517867
HSPD1P10	0.3666794	-0.567023	2.6321504	0.0088396	0.019246	-3.518114
ING3	0.0548388	5.4350082	2.6321034	0.0088408	0.019247	-3.518234
TCEANC	-0.062644	5.0593509	-2.632091	0.0088411	0.019247	-3.518266
SMARCE1P6	0.4627964	-0.009704	2.632001	0.0088434	0.0192509	-3.518497
TCF15	-0.27287	3.7195342	-2.631892	0.0088462	0.0192558	-3.518778
BCL6B	0.0859673	5.7151752	2.6317382	0.0088501	0.0192627	-3.519171
DRC7	-0.322057	3.8091793	-2.631727	0.0088504	0.0192627	-3.5192
RNF103-CHM	0.4831846	0.2655682	2.6314551	0.0088573	0.0192767	-3.519898
GK5	0.0680558	5.6765383	2.6313881	0.008859	0.0192793	-3.52007
PTPN6	0.0448072	6.2518401	2.6313244	0.0088607	0.0192817	-3.520233
SAPCD2P4	-0.120419	-1.490185	-2.630858	0.0088726	0.0193065	-3.521429
IFITM2	-0.080956	6.7880092	-2.630748	0.0088754	0.0193115	-3.521711
RP11-84C10	-0.410533	-0.257335	-2.630698	0.0088767	0.0193131	-3.521839

MPPED2	0.3370037	3.9289555	2.6305188	0.0088813	0.0193219	-3.522299
ZNF638	-0.026587	6.5161613	-2.630421	0.0088838	0.0193262	-3.52255
RP11-15B24	-0.344896	-1.035724	-2.630045	0.0088935	0.0193461	-3.523515
RN7SKP9	-0.123703	-1.47139	-2.629738	0.0089013	0.0193621	-3.5243
AQP1	0.1157544	6.3121586	2.6295746	0.0089055	0.0193701	-3.52472
MYLK3	0.3834319	2.6352071	2.6294421	0.0089089	0.0193763	-3.525059
AC018832.1	-0.158508	-1.416454	-2.629029	0.0089196	0.0193983	-3.526118
FAM188B	0.1649152	4.4155427	2.6289278	0.0089222	0.0194028	-3.526378
CTAGE7P	0.4521012	2.1207933	2.6288801	0.0089234	0.0194043	-3.5265
METRN	-0.149906	5.8317814	-2.62875	0.0089267	0.0194104	-3.526833
RP11-397P1	0.3878158	-0.494355	2.6283178	0.0089379	0.0194335	-3.527941
RP11-344N1	-0.266053	-1.259301	-2.628212	0.0089406	0.0194382	-3.528211
EPS8L3	0.5147373	5.0149917	2.6276448	0.0089553	0.0194689	-3.529665
MAS1L	0.343065	-0.959359	2.6275098	0.0089587	0.0194754	-3.530011
RP11-766F1	-0.604978	0.4495672	-2.627474	0.0089597	0.0194762	-3.530102
ISY1	-0.051931	5.8536785	-2.62739	0.0089619	0.0194798	-3.530319
RP11-28F1.	0.4743426	0.965509	2.6273601	0.0089626	0.0194803	-3.530394
RPS4Y1	-0.784023	4.8588921	-2.627067	0.0089702	0.0194956	-3.531144
USP2	-0.15376	5.4872011	-2.626363	0.0089884	0.0195334	-3.532948
KIF5B	0.0346299	6.5544761	2.6263538	0.0089887	0.0195334	-3.532971
AL356585.3	0.2965469	-0.920785	2.6262044	0.0089925	0.0195407	-3.533354
CASC5	-0.095426	5.8135039	-2.626131	0.0089944	0.0195436	-3.533541
AC005795.1	0.4371519	0.4663247	2.6259596	0.0089989	0.0195521	-3.533981
GLYR1	-0.040331	6.5398263	-2.625827	0.0090023	0.0195585	-3.534321
CPEB4	-0.073221	6.374826	-2.625571	0.009009	0.0195717	-3.534975
MIR137HG	0.394565	-0.967428	2.6254736	0.0090115	0.019576	-3.535225
NEUROG3	0.2928744	-1.103748	2.6254519	0.0090121	0.0195761	-3.53528
ZSCAN18	0.166034	5.5845154	2.6253854	0.0090138	0.0195787	-3.535451
FAM47C	-0.273819	-1.181809	-2.624872	0.0090271	0.0196065	-3.536765
BX255923.3	0.2487151	-1.229583	2.6247555	0.0090302	0.0196119	-3.537063
RP4-610C12	0.498251	0.5984751	2.6245628	0.0090352	0.0196217	-3.537556
AC092597.3	0.3621631	-0.598979	2.6241208	0.0090467	0.0196455	-3.538687
RP11-504P2	0.4679721	2.3629851	2.6239049	0.0090523	0.0196566	-3.539239
HEXA-AS1	-0.410255	2.6160287	-2.623806	0.0090549	0.019661	-3.539491
RP11-78022	-0.100391	-1.505299	-2.623623	0.0090597	0.0196702	-3.539961
LA16c-380H	-0.472106	0.6476774	-2.623457	0.009064	0.0196784	-3.540384
RP11-167P2	-0.157553	-1.41675	-2.622591	0.0090867	0.0197264	-3.5426
BIVM	-0.057869	5.9670575	-2.622443	0.0090905	0.0197337	-3.542978
LINC00572	-0.263178	-1.171911	-2.622307	0.0090941	0.0197402	-3.543324
SDHCP4	-0.281701	-1.039491	-2.62221	0.0090966	0.0197445	-3.543573
RP11-554F2	-0.442306	0.2080659	-2.622128	0.0090988	0.019748	-3.543782
CH17-26001	0.4019498	-0.327267	2.6220326	0.0091013	0.0197523	-3.544027
AC099344.2	-0.322988	-1.104988	-2.621956	0.0091033	0.0197555	-3.544222
PTCSC3	-0.119393	-1.486196	-2.621885	0.0091052	0.0197583	-3.544404
TMEM106B	-0.045618	6.440999	-2.621555	0.0091138	0.0197759	-3.545247
RNF2	0.0794313	5.67369	2.6212866	0.0091209	0.01979	-3.545934
TBX2-AS1	0.3061959	3.6545997	2.6206449	0.0091377	0.0198255	-3.547573
RP11-8J23.	-0.13029	-1.450837	-2.620465	0.0091425	0.0198346	-3.548034
C9orf152	-0.48615	3.7875383	-2.620103	0.009152	0.0198541	-3.548958
CTD-2370N5	0.2585529	-1.16411	2.6199035	0.0091572	0.0198643	-3.549467
RPGRIP1L	0.0886178	4.883754	2.6196006	0.0091652	0.0198804	-3.550241

RP11-505E2	0.4722516	0.5281127	2.6195699	0.009166	0.019881	-3.55032
KRT27	0.5107435	0.0632298	2.6195491	0.0091666	0.019881	-3.550373
AC021451.1	-0.244767	-1.243282	-2.618962	0.0091821	0.0199134	-3.551871
RP11-225N1	-0.407605	-0.458307	-2.618914	0.0091833	0.019915	-3.551994
RPS20P1	-0.141758	-1.442473	-2.618591	0.0091919	0.0199323	-3.552819
AC022210.2	0.4306217	1.6623203	2.6182692	0.0092004	0.0199496	-3.553641
RP5-963E22	-0.141552	-1.434726	-2.618213	0.0092019	0.0199516	-3.553784
SSX3	-0.562881	-0.507562	-2.618134	0.009204	0.019955	-3.553987
C1orf234	-0.426538	-0.14231	-2.618058	0.009206	0.0199581	-3.554179
VN1R1	0.3575635	3.3673184	2.6177232	0.0092149	0.0199762	-3.555035
TSR1	0.0475438	6.0446763	2.6175983	0.0092182	0.0199822	-3.555354
SPINK14	-0.439161	-0.306722	-2.617168	0.0092296	0.0200057	-3.556453
ADH5P3	-0.255169	-1.124149	-2.617147	0.0092301	0.0200057	-3.556504
MRPL44	-0.041238	6.2234574	-2.616781	0.0092399	0.0200256	-3.557438
CTSC	0.0849279	6.4642901	2.6163789	0.0092505	0.0200476	-3.558465
RP4-724E13	0.4792052	1.3426674	2.6162982	0.0092527	0.0200511	-3.558671
SLC35D2	-0.056496	6.3972534	-2.615895	0.0092634	0.0200731	-3.559698
ATP2B4	0.0729132	6.4110946	2.6158613	0.0092643	0.0200739	-3.559785
ZNF678	0.0983557	5.3382524	2.6152369	0.009281	0.0201088	-3.561378
OR7E46P	-0.120267	-1.487738	-2.614622	0.0092974	0.0201431	-3.562947
ZNF395	-0.060024	6.09785	-2.614434	0.0093024	0.0201528	-3.563424
CTA-212A2.	-0.351263	-0.638709	-2.614182	0.0093091	0.0201662	-3.564067
FEM1B	-0.040284	6.3487789	-2.613642	0.0093236	0.0201963	-3.565443
HNRNPKP4	0.3611468	2.6335857	2.6135567	0.0093259	0.0202001	-3.565661
FABP12	-0.205991	-1.326564	-2.613323	0.0093321	0.0202124	-3.566255
AC012512.1	0.4372979	-0.741513	2.6130722	0.0093389	0.0202258	-3.566895
FOXD2-AS1	0.2095951	4.9306488	2.6129762	0.0093414	0.0202302	-3.56714
DDA1	-0.039947	6.2174165	-2.612788	0.0093465	0.0202399	-3.56762
AC011298.2	-0.189777	-1.380941	-2.612672	0.0093496	0.0202454	-3.567914
RP1-167A14	0.4299402	0.8577063	2.612531	0.0093534	0.0202524	-3.568274
ATP6V1A	-0.034066	6.5081724	-2.611952	0.0093689	0.0202849	-3.569748
RP11-255M6	-0.528014	0.3482582	-2.611921	0.0093698	0.0202855	-3.569828
GEMIN8P2	-0.133857	-1.458316	-2.611631	0.0093776	0.0203012	-3.570567
RP5-965G21	0.4378853	2.2298511	2.6114038	0.0093837	0.0203133	-3.571145
CTC-325H20	0.4125209	-0.318214	2.6109682	0.0093954	0.0203375	-3.572254
CDH2	-0.098511	6.5363551	-2.610237	0.0094152	0.020379	-3.574116
SUDS3P1	0.4235034	1.2520479	2.6099415	0.0094232	0.0203951	-3.574868
AC012358.7	0.3552879	-0.719519	2.609833	0.0094261	0.0204002	-3.575144
CTD-3105H1	-0.339321	-0.723765	-2.609697	0.0094298	0.0204069	-3.575489
OMP	0.5107198	1.1076308	2.6092441	0.009442	0.0204323	-3.576642
RP11-244M2	0.6385062	2.0901292	2.6091069	0.0094458	0.0204391	-3.576991
SLC30A8	0.5251494	-0.396123	2.6089206	0.0094508	0.0204487	-3.577465
MIR3193	-0.110857	-1.491872	-2.608901	0.0094513	0.0204487	-3.577514
RP13-150K1	-0.286697	-1.17715	-2.60878	0.0094546	0.0204546	-3.577822
DLX2-AS1	-0.404035	-0.842256	-2.60856	0.0094606	0.0204663	-3.578382
HMGB1P10	-0.131816	4.4344833	-2.608446	0.0094637	0.0204718	-3.578673
TWF2	-0.0691	6.3425494	-2.608397	0.009465	0.0204735	-3.578798
AC110611.1	0.2099075	-1.282809	2.6083358	0.0094667	0.0204759	-3.578953
TMEM128	0.0455608	5.7235592	2.6082838	0.0094681	0.0204777	-3.579085
PIP	0.4401324	-0.73828	2.6079905	0.009476	0.0204937	-3.579831
IKBKB	0.0490578	6.0771336	2.6078211	0.0094807	0.0205018	-3.580262

UBE2FP1	0.2947286	3.647158	2.6078113	0.0094809	0.0205018	-3.580287
CTD-2376I4	-0.20843	3.1358738	-2.607401	0.0094921	0.0205248	-3.581331
MYL6P1	-0.236065	-1.196214	-2.607293	0.009495	0.0205299	-3.581604
OR6U2P	-0.149934	-1.457129	-2.607095	0.0095004	0.0205403	-3.582109
TMEM145	0.4821987	3.2129297	2.6069183	0.0095052	0.0205495	-3.582558
ZBTB7B	-0.054508	6.5429773	-2.606727	0.0095104	0.0205596	-3.583043
HIGD2B	-0.322927	-0.908722	-2.606581	0.0095144	0.020567	-3.583415
RP11-35L17	-0.279054	-1.243715	-2.606551	0.0095152	0.0205675	-3.583492
AC127383.1	-0.139088	-1.458352	-2.605894	0.0095332	0.0206051	-3.585162
RPL7AP34	-0.351146	2.9713504	-2.605851	0.0095343	0.0206064	-3.58527
KRT8P8	0.5019668	1.6388141	2.605751	0.0095371	0.0206111	-3.585525
SLTM	-0.028226	6.4238796	-2.605286	0.0095498	0.0206373	-3.586706
FAM120C	-0.077278	5.5793027	-2.60517	0.009553	0.020643	-3.587001
DERL2	-0.049952	6.2942884	-2.605011	0.0095573	0.0206511	-3.587404
DOCK6	0.0417066	6.2297773	2.6049236	0.0095597	0.0206551	-3.587627
P2RY11	-0.083014	5.2507227	-2.604681	0.0095664	0.0206669	-3.588242
ZFP69	0.1126889	4.9945224	2.6046739	0.0095666	0.0206669	-3.588261
RP11-395N3	-0.508984	0.3365262	-2.604662	0.0095669	0.0206669	-3.588292
NKX2-5	0.4470176	-0.845209	2.6045697	0.0095694	0.0206712	-3.588526
KDM8	-0.131919	5.7309663	-2.604372	0.0095748	0.0206816	-3.589028
XXyac-YR29	-0.153697	-1.446056	-2.604108	0.0095821	0.0206961	-3.589698
PRPF3	-0.044432	6.247001	-2.604035	0.0095841	0.0206992	-3.589885
C6orf47	-0.061646	6.0889672	-2.603928	0.009587	0.0207043	-3.590156
MAPKAPK3	-0.049054	6.2374181	-2.603756	0.0095917	0.0207133	-3.590592
RP11-692E1	-0.13446	-1.466262	-2.603284	0.0096047	0.02074	-3.59179
KLF14	0.3483316	-1.012718	2.6030578	0.0096109	0.0207523	-3.592365
ZCCHC12	0.5823535	0.7780972	2.6024236	0.0096284	0.0207887	-3.593975
RP11-56B16	0.3969693	1.6875154	2.6023419	0.0096307	0.0207924	-3.594182
C2CD2L	-0.047528	5.7960369	-2.6022	0.0096346	0.0207995	-3.594542
RP11-14D22	0.2265691	-1.287712	2.6021811	0.0096351	0.0207995	-3.59459
RP11-359E3	-0.136202	4.3411495	-2.602049	0.0096387	0.0208061	-3.594925
CYP4F10P	-0.286788	-1.06983	-2.601739	0.0096473	0.0208234	-3.595713
AC004967.7	0.0974001	4.7325178	2.6014492	0.0096553	0.0208394	-3.596447
RP11-927P2	0.4026616	1.6721655	2.6013934	0.0096568	0.0208415	-3.596589
PLAG1	0.3376477	4.3312936	2.6012658	0.0096604	0.0208479	-3.596912
CHMP4C	0.0981743	5.7009481	2.6012198	0.0096616	0.0208494	-3.597029
C1orf64	-0.725125	0.9993031	-2.601078	0.0096656	0.0208566	-3.597388
RP11-737O2	0.4585905	1.3300833	2.6006971	0.0096761	0.0208781	-3.598355
RP11-401P9	-0.561889	-0.084715	-2.600552	0.0096801	0.0208856	-3.598723
MFSD1	-0.041306	6.4295739	-2.600392	0.0096846	0.0208939	-3.59913
SOX2	0.6795221	0.6811363	2.6003434	0.0096859	0.0208956	-3.599252
ARPP19	-0.034167	6.5642361	-2.600283	0.0096876	0.0208977	-3.599404
GMDS-AS1	-0.06255	5.1454426	-2.600267	0.009688	0.0208977	-3.599445
SLC30A10	-0.200239	6.1827166	-2.599953	0.0096967	0.0209152	-3.600242
CTC-429L19	0.4381216	1.1611621	2.5992915	0.0097151	0.0209536	-3.601919
RBMY2QP	-0.278612	-1.304025	-2.598907	0.0097258	0.0209754	-3.602895
AC079779.4	0.4032134	-0.664506	2.5988458	0.0097275	0.0209774	-3.603049
RNF214	0.0515771	5.6437681	2.5988317	0.0097279	0.0209774	-3.603084
ZNF214	-0.329071	3.9097384	-2.598744	0.0097303	0.0209815	-3.603307
CYP11A1	-0.563528	4.5859577	-2.59869	0.0097318	0.0209823	-3.603444
PIGR	-0.308838	6.1574946	-2.598689	0.0097318	0.0209823	-3.603446

COX5AP2	-0.214269	-1.264153	-2.598483	0.0097376	0.0209934	-3.603969
STAU1	-0.03761	6.8492664	-2.598195	0.0097456	0.0210094	-3.604697
RP11-517I3	0.1360686	4.3336447	2.5981491	0.0097469	0.021011	-3.604815
FAM127A	0.0918785	6.3302668	2.5977436	0.0097582	0.0210341	-3.605842
RP11-157J2	0.3435503	-0.941993	2.5973655	0.0097687	0.0210556	-3.6068
RP1-35C21.	0.3967843	-0.313298	2.5973334	0.0097696	0.021056	-3.606881
ZNF322	0.1046291	4.9646696	2.5973186	0.00977	0.021056	-3.606919
RP11-20J1.	-0.240991	-1.310413	-2.597211	0.0097731	0.0210612	-3.607192
TLE4	0.1132333	5.3326968	2.597078	0.0097768	0.0210679	-3.607528
THAP4	-0.039728	6.3601222	-2.596898	0.0097818	0.0210775	-3.607983
ZCCHC9	-0.05329	6.1525986	-2.596554	0.0097914	0.021097	-3.608855
MEI4	0.7251401	1.4271567	2.5960663	0.0098051	0.0211252	-3.61009
FAM20B	-0.042414	6.4719509	-2.595636	0.0098171	0.0211499	-3.611179
TTLL11-IT1	0.3352033	-0.809455	2.595346	0.0098253	0.0211662	-3.611914
RP4-562J12	-0.33575	-0.840588	-2.59483	0.0098398	0.0211962	-3.61322
RP1-149A16	0.2738377	-1.033986	2.5942283	0.0098567	0.0212314	-3.614742
IRAK1BP1	0.1560465	4.608536	2.5941828	0.009858	0.0212329	-3.614857
RP5-965G21	0.2939754	3.3050857	2.5940905	0.0098606	0.0212364	-3.615091
CTC-203F4.	-0.246601	2.85836	-2.594084	0.0098608	0.0212364	-3.615108
TCP10L	-0.327561	4.6286248	-2.593949	0.0098645	0.0212433	-3.615449
LA16c-329F	0.4558998	0.0957634	2.5933928	0.0098802	0.0212758	-3.616856
LRRN4	0.5829657	1.3570928	2.5931775	0.0098863	0.0212873	-3.617401
RP11-44F14	0.3216996	-0.939863	2.5931636	0.0098867	0.0212873	-3.617436
AC007279.2	0.4385466	0.1199021	2.5930504	0.0098899	0.0212929	-3.617722
FAM228A	0.5083216	0.8398953	2.5915191	0.0099332	0.0213849	-3.621594
MCM9	0.0604383	5.5578053	2.5914517	0.0099351	0.0213877	-3.621764
AIFM3	0.216891	4.556703	2.5914233	0.0099359	0.0213877	-3.621836
TMEM50B	-0.042614	6.1300839	-2.591408	0.0099364	0.0213877	-3.621876
RP11-624C2	0.2211124	-1.265368	2.59139	0.0099369	0.0213877	-3.62192
CTC-461F20	-0.278935	-1.17883	-2.591184	0.0099427	0.021399	-3.62244
PRAMEF1	-0.216493	-1.351899	-2.591054	0.0099464	0.0214057	-3.62277
RARRES2P7	-0.318821	-1.173191	-2.590836	0.0099526	0.0214178	-3.623322
LYSMD4	-0.069045	5.579722	-2.590553	0.0099606	0.021433	-3.624035
NR3C2	-0.1425	5.3649638	-2.590545	0.0099609	0.021433	-3.624056
ZZZ3	-0.036617	6.1304888	-2.590408	0.0099648	0.0214401	-3.624402
AP001610.5	0.4414882	-0.255473	2.5900508	0.0099749	0.0214607	-3.625304
ASH2L	-0.051411	6.0967191	-2.589843	0.0099808	0.0214722	-3.625829
ANKRD26P4	-0.170944	-1.40378	-2.589684	0.0099853	0.0214806	-3.626231
ANKRD20A17	-0.451035	-0.603439	-2.589606	0.0099876	0.0214842	-3.626429
TBX3	-0.189685	6.1185913	-2.589564	0.0099888	0.0214855	-3.626534
TMEM107	0.0889059	5.0476387	2.5895222	0.00999	0.0214868	-3.62664
RP11-389C8	0.1221847	4.8106885	2.589497	0.0099907	0.021487	-3.626703
AC024704.2	0.281936	-1.043089	2.5894354	0.0099924	0.0214885	-3.626859
RP11-430L3	-0.147053	-1.460833	-2.589432	0.0099925	0.0214885	-3.626866
COL19A1	0.4925151	-0.224023	2.5894061	0.0099933	0.0214888	-3.626933
KRT7	0.430161	5.2182527	2.5893474	0.0099949	0.0214911	-3.627081
LINC01219	0.3895411	-0.825377	2.589012	0.0100045	0.0215104	-3.627928
TEX41	0.5123418	3.5921757	2.5887746	0.0100113	0.0215237	-3.628528
MED14	0.0475403	6.1751072	2.5885645	0.0100173	0.0215354	-3.629058
HTR1B	0.441789	-0.300809	2.5881293	0.0100297	0.0215608	-3.630157
ZC4H2	0.1613029	5.353152	2.5872503	0.0100549	0.0216136	-3.632376

TMPRSS11B	-0.128867	-1.47659	-2.587056	0.0100604	0.0216243	-3.632866
RP3-416H24	0.6536091	1.5132665	2.5869774	0.0100627	0.0216279	-3.633064
RP11-563D1	-0.659501	1.6420028	-2.586276	0.0100828	0.0216699	-3.634834
AC010095.5	0.3329716	-0.762853	2.5860339	0.0100898	0.0216836	-3.635445
RP11-94B19	-0.102605	-1.492296	-2.585963	0.0100918	0.0216867	-3.635623
UCMA	-0.215649	-1.36162	-2.585851	0.010095	0.0216923	-3.635905
ITFG2	0.0473568	5.8077419	2.585643	0.010101	0.0217039	-3.636431
RP11-55L4.	-0.212813	-1.324425	-2.584788	0.0101256	0.0217555	-3.638587
USP40	-0.040334	6.3227125	-2.584554	0.0101324	0.0217688	-3.639178
CENPN	-0.07321	5.5978967	-2.584524	0.0101332	0.0217693	-3.639252
LINC00659	-0.662038	3.0447371	-2.584354	0.0101382	0.0217786	-3.639681
AC021224.1	0.4316255	0.5956639	2.5842049	0.0101425	0.0217866	-3.640057
LINC00635	-0.428233	-0.740756	-2.58398	0.010149	0.0217992	-3.640625
PHOX2A	-0.209469	-1.400809	-2.58369	0.0101573	0.0218159	-3.641355
RAP1B	0.035223	6.1054822	2.5833822	0.0101662	0.0218338	-3.642131
APEX2	-0.040619	6.1679355	-2.583294	0.0101688	0.021838	-3.642354
TMEM190	0.3659941	-0.803941	2.5832427	0.0101703	0.0218399	-3.642482
ADH5P2	-0.177896	-1.34491	-2.583144	0.0101731	0.0218447	-3.642731
SS18	-0.033913	6.3922352	-2.582742	0.0101848	0.0218678	-3.643743
CTD-2574D2	0.3811915	-0.33486	2.582716	0.0101855	0.0218678	-3.643809
RP11-18005	-0.389952	-0.597296	-2.582711	0.0101857	0.0218678	-3.643823
P2RX7	0.1256786	5.4766752	2.5826361	0.0101879	0.0218712	-3.64401
TM4SF19	0.5314862	1.6638377	2.5824304	0.0101938	0.0218827	-3.644529
MXD3	-0.093382	5.6549801	-2.582233	0.0101996	0.0218937	-3.645027
CTC-523E23	-0.119817	-1.492688	-2.581781	0.0102127	0.0219206	-3.646165
CYP4X1	-0.211996	4.835222	-2.581683	0.0102155	0.0219244	-3.646411
NT5C3AP1	0.4566942	0.7828171	2.581678	0.0102157	0.0219244	-3.646424
RP11-333E1	0.3965171	-0.24175	2.5813951	0.0102239	0.0219408	-3.647136
MORF4L2	-0.035333	6.8381948	-2.581329	0.0102258	0.0219436	-3.647302
ZC3H6	-0.074807	5.4411714	-2.5808	0.0102412	0.0219751	-3.648635
TWISTNB	-0.044933	5.8370925	-2.580784	0.0102417	0.0219751	-3.648676
LINC01267	0.3639847	-0.826541	2.5807237	0.0102434	0.0219776	-3.648827
RP11-17403	0.3422311	-0.70675	2.5807008	0.0102441	0.0219777	-3.648884
GATAD1	-0.042225	6.2202967	-2.580505	0.0102498	0.0219886	-3.649376
PAQR9	-0.174062	5.9822657	-2.580239	0.0102576	0.0220032	-3.650046
U73166.2	-0.303778	3.3039516	-2.580231	0.0102578	0.0220032	-3.650067
RNASE3	0.255303	-1.178111	2.5797123	0.0102729	0.0220344	-3.651373
KRT81	0.5714247	1.8474179	2.5795814	0.0102768	0.0220413	-3.651702
EIF3D	-0.041267	6.7889758	-2.579534	0.0102781	0.022043	-3.65182
PLA2G4C	-0.10441	5.9759182	-2.57938	0.0102827	0.0220508	-3.652209
RP11-108M9	-0.43414	0.0835413	-2.579369	0.010283	0.0220508	-3.652237
RP11-514P8	0.3115037	-0.847516	2.5790563	0.0102921	0.0220691	-3.653023
ZFAND1	-0.052455	6.0946171	-2.578832	0.0102987	0.0220818	-3.653587
RDH13	0.1034598	5.6935861	2.5780906	0.0103204	0.0221271	-3.655453
STXBP5L	0.4182626	-0.751504	2.577115	0.0103491	0.0221872	-3.657906
TFDP1	0.0615911	6.2133227	2.5769155	0.0103549	0.0221985	-3.658407
GPR101	-0.2974	-1.061141	-2.576634	0.0103632	0.0222149	-3.659114
RP11-799B1	0.4398372	2.5042003	2.5765776	0.0103649	0.0222172	-3.659257
CTD-2284J1	-0.137477	5.1521402	-2.576514	0.0103667	0.0222199	-3.659416
AC128709.4	-0.485788	0.3461468	-2.576408	0.0103699	0.0222253	-3.659683
RP11-401L1	-0.346935	-0.674864	-2.576239	0.0103748	0.0222347	-3.660107

RP11-100E1	0.314151	-1.123119	2.5760644	0.01038	0.0222444	-3.660547
TAF9B	-0.060731	5.9233851	-2.575528	0.0103958	0.022277	-3.661893
RPL12P33	-0.405685	-0.204771	-2.575449	0.0103982	0.0222807	-3.662094
PRSS53	0.2721957	4.1271653	2.5754101	0.0103993	0.0222818	-3.662191
PYROXD1	-0.052352	5.8058276	-2.575377	0.0104003	0.0222827	-3.662275
RNA5SP82	0.4112293	-0.366604	2.5752629	0.0104036	0.0222885	-3.662561
RP11-138I1	0.2465291	-1.21434	2.5751583	0.0104067	0.0222939	-3.662823
ZFP36L1	-0.044141	6.8930677	-2.575029	0.0104105	0.0223007	-3.663147
LINC01266	0.4048361	-0.579424	2.5748411	0.0104161	0.0223113	-3.66362
RP11-514D2	0.2895751	-1.093941	2.5747435	0.010419	0.0223162	-3.663865
AC011523.2	-0.112624	-1.489456	-2.574474	0.010427	0.022332	-3.664542
RP11-864I4	-0.076182	5.0394188	-2.574442	0.0104279	0.0223327	-3.664623
EIF5A	-0.048734	6.9948189	-2.574368	0.0104301	0.0223361	-3.664809
RP11-810P1	-0.181191	3.9226748	-2.574091	0.0104383	0.0223523	-3.665505
RP11-6B4.1	0.7666329	1.83108	2.5740317	0.0104401	0.0223548	-3.665653
ZNF684	-0.099392	5.4536533	-2.573878	0.0104446	0.0223626	-3.666039
RP11-357N1	-0.208485	-1.27574	-2.573867	0.010445	0.0223626	-3.666068
TPM4P1	-0.117469	-1.489959	-2.573644	0.0104516	0.0223754	-3.666626
DUSP13	0.602844	0.5125287	2.5735745	0.0104536	0.0223786	-3.666801
LYSMD3	-0.059417	6.0590315	-2.573378	0.0104595	0.0223897	-3.667294
RP11-305F1	0.4502077	-0.765289	2.5733222	0.0104611	0.022392	-3.667435
DKK4	-0.771037	2.0645848	-2.572726	0.0104788	0.0224285	-3.668931
CELA1	0.2352309	-1.214718	2.5726923	0.0104798	0.0224294	-3.669016
HIRA	-0.047931	5.9710474	-2.572277	0.0104922	0.0224545	-3.670057
ZIC1	-0.816561	3.4382095	-2.572161	0.0104956	0.0224606	-3.670349
DSG2-AS1	0.4583587	2.4092711	2.5720406	0.0104992	0.0224669	-3.670652
UBE2FP3	0.3769203	1.6985473	2.5717754	0.0105071	0.0224825	-3.671317
RSPO4	0.5826227	0.9925013	2.5716212	0.0105117	0.0224911	-3.671704
AC093690.1	0.4378698	0.4815957	2.571532	0.0105144	0.0224954	-3.671928
CYCSP45	0.3558154	-0.639221	2.5715076	0.0105151	0.0224957	-3.671989
RP11-130F1	0.3625284	2.3702975	2.5711385	0.0105261	0.0225179	-3.672915
RPRM	0.4394523	-0.669765	2.5709252	0.0105325	0.0225302	-3.67345
KIAA0040	0.0827434	5.9387899	2.5707905	0.0105365	0.0225375	-3.673788
SLC25A14	-0.064685	5.2488226	-2.570604	0.0105421	0.0225481	-3.674255
CHCHD3P3	0.3890901	1.9190568	2.5703851	0.0105486	0.0225608	-3.674805
LINC01031	0.2659757	-1.114235	2.5703568	0.0105495	0.0225613	-3.674876
RNASE13	-0.485243	1.1220777	-2.570189	0.0105545	0.0225706	-3.675295
MSANTD3-TM	0.3704032	-0.404138	2.5699978	0.0105602	0.0225816	-3.675776
ZNF510	0.0785918	5.3804664	2.5699604	0.0105613	0.0225827	-3.67587
VASH2	0.2548899	4.433263	2.5698262	0.0105654	0.0225899	-3.676206
GOLGA6L7P	0.3055771	-1.11813	2.5697953	0.0105663	0.0225906	-3.676283
RP11-310E2	0.4115098	-0.715334	2.5696679	0.0105701	0.0225964	-3.676603
DPYD-IT1	0.2457852	-1.165994	2.5696634	0.0105702	0.0225964	-3.676614
ZFP57	0.6097815	0.6094914	2.5691397	0.0105859	0.0226286	-3.677927
ZNF487	-0.067327	4.8890813	-2.569011	0.0105898	0.0226347	-3.678251
SLC9A4	0.3291854	-1.109211	2.569004	0.01059	0.0226347	-3.678267
KDM3B	0.0381827	6.3511829	2.568921	0.0105925	0.0226376	-3.678475
AOX2P	-0.522381	1.0201365	-2.568917	0.0105926	0.0226376	-3.678486
RSPH14	0.4382369	2.6170429	2.5682581	0.0106124	0.022678	-3.680137
RP11-1084A	0.4177014	0.1698762	2.5682468	0.0106128	0.022678	-3.680165
ZBTB8OSP2	-0.499269	0.2320229	-2.567973	0.010621	0.0226942	-3.68085

RP11-145M4	-0.479036	-0.371162	-2.567906	0.010623	0.0226972	-3.681019
AC007238.1	-0.197869	3.4092865	-2.567837	0.0106251	0.0227004	-3.681192
HCG18	0.062023	5.7820317	2.5675452	0.0106339	0.0227178	-3.681923
FBXO38	-0.043773	6.1771394	-2.567341	0.01064	0.0227296	-3.682435
NAT16	-0.515264	1.0354002	-2.567273	0.0106421	0.0227327	-3.682605
RP11-667K1	-0.482546	0.1503146	-2.567181	0.0106449	0.0227373	-3.682836
RNU6-1178F	-0.125768	-1.476576	-2.567129	0.0106464	0.0227392	-3.682964
CTD-2024I7	-0.281919	-1.055915	-2.567098	0.0106473	0.0227394	-3.683043
RP11-846F4	-0.170987	-1.395515	-2.567085	0.0106477	0.0227394	-3.683076
ARL17B	0.3817091	2.8752924	2.5665551	0.0106637	0.0227723	-3.684403
SLC25A18	-0.173689	6.1967273	-2.566082	0.010678	0.0228014	-3.685586
RP11-10J18	0.1936414	-1.320418	2.5652095	0.0107044	0.0228565	-3.687772
RP11-862L9	0.3053661	-0.873383	2.5651681	0.0107057	0.0228579	-3.687875
WFDC10B	0.2275308	-1.281217	2.5646087	0.0107227	0.0228927	-3.689275
RBBP8P1	-0.246516	-1.199167	-2.564366	0.01073	0.0229071	-3.689883
AC011239.1	0.2136772	-1.32343	2.5642282	0.0107342	0.0229147	-3.690227
IBTK	-0.048167	6.5184267	-2.564158	0.0107364	0.0229179	-3.690403
RP11-11302	-0.193296	-1.341278	-2.564089	0.0107384	0.022921	-3.690574
KB-1980E6.	0.2504746	-1.154394	2.5639673	0.0107421	0.0229276	-3.69088
RNF139	-0.044765	6.2373392	-2.563923	0.0107435	0.0229291	-3.69099
INAFM1	-0.080295	5.6617525	-2.563709	0.01075	0.0229417	-3.691527
LINC00691	0.2379682	-1.175651	2.5636839	0.0107508	0.022942	-3.691589
RP11-571O6	-0.243778	-1.260812	-2.563606	0.0107531	0.0229457	-3.691783
CHRNB3	0.3063264	-1.15276	2.5634513	0.0107578	0.0229544	-3.692171
RP11-61E11	-0.346974	-0.711759	-2.563273	0.0107633	0.0229634	-3.692618
ATOH8	-0.223686	5.4146787	-2.563264	0.0107635	0.0229634	-3.692638
RNF2P1	0.4131555	2.3088443	2.5632503	0.010764	0.0229634	-3.692674
PSMA8	0.4203517	-0.743503	2.5629925	0.0107718	0.0229779	-3.693318
RP11-517P1	0.3131106	3.8026237	2.5629869	0.010772	0.0229779	-3.693332
RP1-240B8.	-0.148124	-1.434474	-2.5628	0.0107777	0.0229887	-3.693801
RP11-101E7	0.4200757	0.2334465	2.5626954	0.0107809	0.0229941	-3.694061
RP11-803B1	-0.129241	-1.455933	-2.562371	0.0107908	0.0230139	-3.694873
NEK9	-0.035471	6.4776776	-2.561824	0.0108075	0.0230482	-3.696241
ACTN2	-0.497724	3.9488977	-2.561645	0.0108129	0.0230585	-3.696688
VMO1	0.1134572	4.9125109	2.5614424	0.0108191	0.0230704	-3.697194
SIRT4	-0.090743	4.9734327	-2.561406	0.0108203	0.0230714	-3.697285
RP11-826N1	-0.122843	-1.468815	-2.56119	0.0108269	0.0230842	-3.697825
CLDN2	-0.276881	5.8735244	-2.561136	0.0108285	0.0230864	-3.697961
NAV2-AS3	-0.415293	-0.581909	-2.561103	0.0108295	0.0230871	-3.698042
N4BP2L2	-0.040829	6.2933495	-2.560843	0.0108375	0.0231027	-3.698691
KB-68A7.2	-0.584823	1.1934498	-2.560363	0.0108522	0.0231328	-3.699892
DDX10P1	0.4155059	-0.266882	2.5601536	0.0108586	0.0231451	-3.700414
GS1-257G1.	0.4125298	1.8478066	2.5599867	0.0108638	0.0231546	-3.700831
PAPPA-AS2	-0.142678	-1.443061	-2.559934	0.0108654	0.0231546	-3.700962
PDZK1IP1	-0.415482	5.4060768	-2.55993	0.0108655	0.0231546	-3.700973
AC015849.1	-0.508072	2.1984437	-2.559926	0.0108656	0.0231546	-3.700983
INSL5	-0.156332	-1.423565	-2.559715	0.0108721	0.023167	-3.701509
RP11-40109	0.277409	-1.192337	2.5596923	0.0108728	0.0231672	-3.701567
TRMT10C	-0.043048	6.0735813	-2.55945	0.0108802	0.0231794	-3.702171
CELSR2	0.1158338	5.6220824	2.5594449	0.0108804	0.0231794	-3.702185
DDX60L	-0.093019	5.7216625	-2.559445	0.0108804	0.0231794	-3.702185

TMEM218	-0.045002	5.7887238	-2.559394	0.010882	0.0231813	-3.702311
RP11-536G4	-0.229844	-1.264396	-2.559274	0.0108857	0.0231878	-3.702611
PREP	0.0430983	6.1069143	2.5591948	0.0108881	0.0231917	-3.702809
NAP1L1P3	0.4169284	1.0379454	2.5590805	0.0108916	0.0231978	-3.703094
PCOLCE	0.1456591	6.1950021	2.5584927	0.0109097	0.023235	-3.704562
LINC00469	-0.176357	-1.385515	-2.55843	0.0109116	0.0232378	-3.704718
C5orf66	0.3672616	3.4425726	2.5583835	0.0109131	0.0232395	-3.704835
SSX2B	-0.391074	-0.927435	-2.558288	0.010916	0.0232444	-3.705074
C1orf35	-0.059346	6.0176184	-2.558076	0.0109226	0.023257	-3.705603
RP11-324I2	-0.156691	4.2741856	-2.557786	0.0109315	0.0232746	-3.706326
RP11-480G7	-0.439223	-0.246048	-2.557744	0.0109328	0.0232761	-3.706432
SLC2A11	-0.071803	5.3877704	-2.557198	0.0109497	0.0233097	-3.707794
CTD-2058B2	-0.200951	-1.29789	-2.557191	0.0109499	0.0233097	-3.707811
RP11-122K1	-0.453824	2.8904108	-2.556981	0.0109564	0.0233222	-3.708335
AC112721.1	0.2600111	-1.191233	2.5566637	0.0109662	0.0233417	-3.709127
RP11-92J19	-0.116029	-1.481332	-2.55649	0.0109716	0.0233518	-3.709561
LGI4	0.1641461	5.0562666	2.556199	0.0109806	0.0233696	-3.710286
CFHR1	-0.259287	6.7929633	-2.555941	0.0109886	0.0233853	-3.71093
BTAF1	0.0689796	6.0413795	2.5559211	0.0109892	0.0233853	-3.71098
GLUD2	0.158762	5.4347734	2.5551854	0.0110121	0.0234325	-3.712814
MIR568	0.4169384	3.4990587	2.5551351	0.0110136	0.0234345	-3.71294
RBM48	-0.043042	5.5664949	-2.554911	0.0110206	0.0234479	-3.713499
TEPP	0.3765107	-0.702309	2.5544048	0.0110364	0.0234801	-3.71476
RP11-335L2	-0.375207	-0.518454	-2.554281	0.0110402	0.023486	-3.71507
IGLVIV-64	-0.145725	-1.429566	-2.554275	0.0110404	0.023486	-3.715085
GS1-124K5.	0.4094882	2.4303227	2.5538422	0.0110539	0.0235133	-3.716163
RP11-849I1	-0.181756	-1.387584	-2.553684	0.0110588	0.0235224	-3.716557
FEZF1	-0.590362	0.1482977	-2.553245	0.0110725	0.0235501	-3.717651
TBC1D3K	-0.158897	-1.430866	-2.553132	0.0110761	0.0235563	-3.717933
MIR30C2	0.3004315	-0.989755	2.5530749	0.0110778	0.0235587	-3.718075
WASH6P	-0.067146	5.3893977	-2.55297	0.0110811	0.0235643	-3.718336
RP11-88I21	0.3618291	-0.996885	2.5527708	0.0110873	0.0235762	-3.718832
AC083862.6	0.3338062	-0.805892	2.5522101	0.0111049	0.0236121	-3.720229
CEP350	-0.043465	6.2797776	-2.552	0.0111115	0.0236247	-3.720753
RP11-507K2	-0.279862	-0.998926	-2.551821	0.0111171	0.0236353	-3.721199
SDR42E1	0.4120564	4.6483571	2.5516574	0.0111222	0.0236448	-3.721605
AC007249.3	-0.280532	2.7307668	-2.551626	0.0111232	0.0236455	-3.721683
CTD-2028E8	-0.167159	-1.400241	-2.551451	0.0111287	0.0236558	-3.722118
ZDHHC18	-0.043477	6.2153441	-2.551411	0.0111299	0.0236571	-3.722218
MIR3944	-0.274418	-1.061234	-2.551059	0.011141	0.0236787	-3.723095
FLRT1	0.3929714	2.8452658	2.551046	0.0111414	0.0236787	-3.723128
MYL12A	-0.044109	6.9029393	-2.550578	0.0111561	0.0237086	-3.724294
RPL31P52	0.3750659	-0.361877	2.5505418	0.0111572	0.0237096	-3.724383
RP11-787I2	0.4357381	0.8014063	2.5504009	0.0111617	0.0237176	-3.724734
RP1-21018.	0.2752073	-1.158444	2.5503566	0.0111631	0.0237192	-3.724844
TRMT11	0.0825757	5.6784622	2.550148	0.0111696	0.0237318	-3.725363
REPS2	-0.113946	5.8128829	-2.550062	0.0111723	0.0237362	-3.725577
GOSR2	-0.035839	5.9969822	-2.550027	0.0111734	0.0237371	-3.725664
SNORD88A	0.2712846	-1.054714	2.5499262	0.0111766	0.0237414	-3.725915
CTC-575I10	-0.281172	-1.065546	-2.549922	0.0111767	0.0237414	-3.725926
PCDH10	0.4338494	-0.426794	2.5492626	0.0111975	0.0237841	-3.727566

RP11-903H1	-0.30804	3.3738186	-2.54868	0.0112159	0.0238218	-3.729015
RNF223	0.36566	-0.879842	2.5486247	0.0112177	0.0238241	-3.729153
RP11-114M1	-0.120677	-1.479937	-2.548177	0.0112318	0.0238528	-3.730267
RP11-61L19	0.193325	3.591656	2.5480389	0.0112362	0.0238601	-3.73061
EML6	-0.209059	4.6703186	-2.548028	0.0112365	0.0238601	-3.730638
ZNF598	-0.045609	6.2387647	-2.547952	0.0112389	0.0238638	-3.730826
PRORSD1P	-0.114404	4.2705243	-2.547787	0.0112442	0.0238735	-3.731237
C10orf95	0.3670672	2.0733979	2.5477203	0.0112463	0.0238766	-3.731402
CTD-3187F8	0.2201923	-1.225373	2.5476704	0.0112478	0.0238767	-3.731526
RP11-250B2	-0.313275	3.0450608	-2.547648	0.0112485	0.0238767	-3.731581
RP11-603J2	-0.390956	-0.323597	-2.547645	0.0112486	0.0238767	-3.73159
DAB2IP	0.0645519	6.2763173	2.5476357	0.0112489	0.0238767	-3.731613
RP11-297L1	-0.289549	4.8043187	-2.547372	0.0112573	0.023893	-3.732268
ZBTB10	-0.060451	6.1685407	-2.547076	0.0112667	0.0239116	-3.733004
RP11-395A1	-0.102612	4.2910192	-2.547045	0.0112677	0.0239123	-3.733082
SOCS4	0.0467283	5.7456012	2.5469375	0.0112711	0.0239181	-3.733348
XXyac-YX65	0.4373108	-0.592095	2.5467826	0.011276	0.0239271	-3.733733
RP11-211A1	-0.17897	-1.350991	-2.546748	0.0112771	0.0239281	-3.73382
RP11-299J3	-0.15684	3.8843627	-2.546667	0.0112796	0.023931	-3.73402
UPF3AP2	0.4008045	1.383508	2.5466636	0.0112798	0.023931	-3.734029
CAMSAP3	-0.103896	6.2996231	-2.546547	0.0112835	0.0239375	-3.73432
RNF19A	0.0539503	6.4009666	2.5464568	0.0112863	0.0239421	-3.734543
RP11-883A1	0.4300179	-0.557908	2.5464297	0.0112872	0.0239426	-3.734611
CALCA	0.6184914	2.4630483	2.5464052	0.011288	0.0239428	-3.734672
LINC01348	-0.324058	5.2492538	-2.546075	0.0112985	0.0239637	-3.735492
AC092159.1	-0.157123	-1.414605	-2.54593	0.0113031	0.0239721	-3.735853
SALRNA2	0.2169428	-1.265205	2.5457013	0.0113104	0.0239856	-3.736421
POU6F2	0.6565929	0.5392098	2.5456893	0.0113107	0.0239856	-3.73645
RP11-418I2	-0.385019	-0.742837	-2.545653	0.0113119	0.0239866	-3.736541
AK4P1	0.4253044	3.1642845	2.545597	0.0113137	0.023989	-3.73668
RP1-293L8.	-0.193147	-1.341621	-2.545295	0.0113233	0.024008	-3.737431
RP11-677I1	0.4255946	1.1383994	2.5452582	0.0113245	0.0240091	-3.737522
CTD-2562G1	-0.36163	2.2032188	-2.5452	0.0113263	0.0240116	-3.737665
RP11-197K3	-0.19461	-1.313365	-2.544634	0.0113444	0.0240485	-3.739072
CTC-497E21	0.2181786	-1.349396	2.544538	0.0113474	0.0240536	-3.73931
ZNF304	0.0959226	5.3535289	2.5443721	0.0113527	0.0240634	-3.739722
RN7SL219P	-0.264348	-1.118156	-2.543723	0.0113735	0.024106	-3.741333
RBPMS2	-0.128964	5.7269024	-2.543571	0.0113783	0.0241149	-3.741711
RP11-10K17	-0.291978	-1.030536	-2.543549	0.0113791	0.024115	-3.741766
MUC2	0.5285187	-0.288256	2.543467	0.0113817	0.0241192	-3.74197
LMO7-AS1	0.5228577	1.2645272	2.5433739	0.0113847	0.0241241	-3.742201
RP11-336A1	-0.18857	-1.337812	-2.543351	0.0113854	0.0241242	-3.742257
RP11-879F1	-0.369946	-0.54875	-2.543036	0.0113955	0.0241442	-3.743041
RP11-794C2	-0.252383	-1.183523	-2.542965	0.0113977	0.0241476	-3.743215
LINC00426	0.2703394	3.3938581	2.5424109	0.0114155	0.0241822	-3.744591
RPS26P34	-0.209451	-1.361983	-2.542409	0.0114156	0.0241822	-3.744596
RP5-1061H2	-0.399046	-0.789528	-2.542395	0.011416	0.0241822	-3.744632
AC104534.2	0.4431293	2.3859737	2.5421692	0.0114233	0.0241961	-3.745191
NAT8	-0.225722	6.0773871	-2.542106	0.0114253	0.024199	-3.745347
FAM110C	0.4117324	5.3531797	2.5417812	0.0114357	0.0242197	-3.746153
ENPP7P14	-0.272944	-1.210312	-2.541676	0.0114391	0.0242255	-3.746416

RP11-475J5	-0.186178	-1.365205	-2.541078	0.0114584	0.0242648	-3.747897
CHKA	-0.062472	6.2991133	-2.540906	0.0114639	0.0242751	-3.748325
KBTBD6	-0.057397	5.57134	-2.540821	0.0114666	0.0242795	-3.748535
AE000662.9	0.225693	-1.283001	2.5405496	0.0114754	0.0242966	-3.749208
MED27	-0.054356	5.7038629	-2.540506	0.0114768	0.0242982	-3.749317
CTC-457E21	-0.438287	-0.694672	-2.540099	0.0114899	0.0243242	-3.750325
CTD-2196E1	0.2054179	-1.251633	2.540083	0.0114904	0.0243242	-3.750365
AC007326.1	-0.191155	-1.386464	-2.539898	0.0114964	0.0243354	-3.750823
CCNO	0.6355551	3.1081279	2.5398206	0.0114989	0.0243394	-3.751015
RP11-334E6	-0.190536	-1.332934	-2.53967	0.0115038	0.0243483	-3.75139
TSPEAR-AS1	-0.495443	3.8432072	-2.539594	0.0115062	0.024352	-3.751578
HNRNPU	-0.024497	6.9880391	-2.539517	0.0115087	0.0243559	-3.751769
IMPDH1P6	0.4820115	2.020279	2.5394681	0.0115103	0.0243578	-3.751889
SRSF1	-0.025052	6.6633961	-2.539416	0.011512	0.02436	-3.752019
WDR88	0.2017868	3.6854178	2.5392619	0.011517	0.0243691	-3.7524
ZNF225	0.0721897	5.0399417	2.5391971	0.0115191	0.0243721	-3.752561
IGFLR1	-0.136279	4.7575924	-2.539059	0.0115235	0.0243801	-3.752902
SLC25A48	0.6608791	1.4307272	2.538901	0.0115287	0.0243896	-3.753295
RP11-401N1	-0.15682	-1.437655	-2.538695	0.0115353	0.0244023	-3.753805
LRRK2	0.1815669	5.2049207	2.5382881	0.0115485	0.0244287	-3.754813
EMC6	0.1136298	4.9540153	2.5382619	0.0115494	0.0244291	-3.754878
WDR49	0.3975375	-0.634889	2.5378487	0.0115628	0.0244561	-3.755902
MKI67	0.1172537	6.0390351	2.5375973	0.0115709	0.0244719	-3.756525
AC064875.2	0.4562903	0.7628881	2.5374297	0.0115764	0.0244813	-3.75694
AP001257.1	0.1921079	-1.307033	2.5374187	0.0115767	0.0244813	-3.756967
IFITM4P	-0.466551	1.8840487	-2.537105	0.0115869	0.0245015	-3.757745
RAB14	-0.035424	6.6268263	-2.536635	0.0116022	0.0245324	-3.758909
PELP1	-0.049212	6.4037383	-2.536492	0.0116069	0.0245409	-3.759263
CTD-2616J1	0.41157	1.6122924	2.5363798	0.0116105	0.0245471	-3.75954
UGCG	0.0597008	6.3011705	2.5361544	0.0116179	0.0245605	-3.760098
GALNT15	-0.198563	4.8276825	-2.536145	0.0116182	0.0245605	-3.760122
SLC19A2	-0.076512	6.1498585	-2.535982	0.0116235	0.0245703	-3.760525
RP11-618L2	-0.386825	-0.54338	-2.535918	0.0116256	0.0245733	-3.760684
ACBD3	0.0420896	6.3616081	2.5355558	0.0116374	0.0245968	-3.76158
RP11-174B4	-0.135254	-1.447664	-2.535237	0.0116478	0.0246174	-3.762368
TEX35	-0.347621	-0.674794	-2.535142	0.0116509	0.0246213	-3.762603
PPP3CC	0.0527487	5.6472038	2.5351398	0.011651	0.0246213	-3.762609
CTB-43E15.	-0.318796	3.6868436	-2.535111	0.011652	0.0246217	-3.762681
GPR125	-0.083379	6.2898056	-2.535092	0.0116526	0.0246217	-3.762728
SLC22A7	-0.272732	6.5604635	-2.535062	0.0116535	0.0246223	-3.762801
RP11-93209	-0.386436	-0.009407	-2.534746	0.0116639	0.0246428	-3.763583
CTC-360P9.	-0.126248	-1.480751	-2.534652	0.011667	0.0246479	-3.763817
UBR3	-0.047615	6.2887473	-2.534396	0.0116753	0.0246641	-3.764449
CRHBP	0.3453054	4.0459048	2.5342131	0.0116813	0.0246754	-3.764902
ZNF548	0.0704586	5.466907	2.5341806	0.0116824	0.0246762	-3.764982
EIF3C	0.1366278	5.15883	2.5337577	0.0116962	0.024704	-3.766028
RP11-826F1	0.437422	1.6257872	2.5334431	0.0117066	0.0247244	-3.766806
ARHGEF40	-0.049873	6.5471219	-2.533255	0.0117127	0.024736	-3.767272
TBX5-AS1	0.2573241	-1.239365	2.5329209	0.0117237	0.0247577	-3.768098
CTD-2616J1	0.5287279	0.5918591	2.5327934	0.0117279	0.0247652	-3.768413
TAS2R43	0.2557419	-1.109123	2.5326473	0.0117327	0.0247739	-3.768774

AP001596.6	0.4288331	-0.245895	2.53254	0.0117362	0.0247799	-3.769039
RP11-317P1	0.4864519	1.3163065	2.5321237	0.0117499	0.0248074	-3.770069
RP11-68I3.	0.3937835	-0.39399	2.5320971	0.0117508	0.0248078	-3.770134
C19orf80	-0.251161	6.2428577	-2.532046	0.0117525	0.0248099	-3.770261
RP11-563M4	-0.157319	-1.415511	-2.531874	0.0117581	0.0248204	-3.770685
HNRNPA1	-0.033074	7.0145161	-2.531713	0.0117635	0.0248302	-3.771084
CTC-459M5.	0.2124048	-1.25275	2.5316758	0.0117647	0.0248314	-3.771176
TXNDC8	-0.199264	-1.315422	-2.531466	0.0117716	0.0248446	-3.771695
RP11-74C1.	0.3434782	-0.618588	2.5310823	0.0117843	0.0248698	-3.772642
AC011524.3	-0.126646	-1.468827	-2.530952	0.0117886	0.0248775	-3.772965
RP11-559M2	-0.324742	-0.833541	-2.530789	0.011794	0.0248874	-3.773367
KRT8P48	0.4855481	1.9877472	2.530686	0.0117974	0.0248931	-3.773621
LINC01399	0.2685412	-1.210011	2.5303916	0.0118071	0.0249122	-3.774349
HBD	0.4668595	-0.454236	2.529792	0.0118269	0.0249526	-3.77583
ACTR8	-0.036515	5.8475993	-2.529742	0.0118286	0.0249547	-3.775952
RP11-74D7.	-0.43432	1.4611735	-2.52926	0.0118446	0.0249869	-3.777143
CTB-113P19	0.281633	-1.007594	2.5292394	0.0118453	0.0249869	-3.777194
ZEB1-AS1	-0.082908	5.0256802	-2.529006	0.011853	0.0250016	-3.777772
FRS3	-0.058672	5.3079642	-2.528988	0.0118536	0.0250016	-3.777814
RP5-867C24	0.3173142	-0.783574	2.5288099	0.0118595	0.0250127	-3.778255
BRMS1L	0.0563304	5.3341731	2.5286555	0.0118646	0.025022	-3.778636
AC090043.1	-0.231757	-1.213702	-2.528483	0.0118704	0.0250327	-3.779061
RP11-499E1	0.3493055	-0.840827	2.5280759	0.0118839	0.0250598	-3.780066
C3orf35	0.2286218	3.4081863	2.5279411	0.0118884	0.0250678	-3.780399
TUBGCP5	-0.052196	5.7871483	-2.527401	0.0119064	0.0251043	-3.781732
RPL7P13	-0.310829	-0.971814	-2.527286	0.0119102	0.0251109	-3.782015
ZNF772	0.1663474	4.8743433	2.5268298	0.0119254	0.0251415	-3.783141
RP11-297H3	-0.658926	0.8232014	-2.526775	0.0119273	0.0251439	-3.783277
RNA5SP37	0.3741593	-0.476771	2.5266123	0.0119327	0.0251539	-3.783677
RP4-669H2.	0.4688473	0.8405069	2.5262947	0.0119433	0.0251748	-3.784461
LINC01028	-0.126836	-1.456809	-2.52627	0.0119441	0.0251751	-3.784522
SLC12A9	0.0495277	6.2622152	2.5261668	0.0119475	0.0251809	-3.784776
CDK1	0.1239241	5.7243754	2.5257453	0.0119616	0.0252092	-3.785816
ESPL1	0.1459606	5.5436755	2.5254316	0.0119721	0.0252298	-3.786589
AC005229.7	-0.368222	-0.595618	-2.525122	0.0119825	0.0252502	-3.787352
HCCS	-0.040435	6.0460389	-2.524951	0.0119882	0.0252608	-3.787773
PDLIM5	-0.050979	6.5502697	-2.524749	0.011995	0.0252737	-3.788272
BOP1	-0.076103	6.5091377	-2.524479	0.0120041	0.0252913	-3.788936
LINC00334	0.3581961	-0.673236	2.5242261	0.0120126	0.0253077	-3.789561
RALY	-0.039459	6.6869413	-2.523961	0.0120215	0.025325	-3.790215
DPP9-AS1	0.3150753	2.5951749	2.5231178	0.0120498	0.0253833	-3.792291
SETD8	0.0389786	5.9048554	2.5227866	0.012061	0.0254053	-3.793107
RP11-462G1	0.4275971	0.2693981	2.5224449	0.0120725	0.0254281	-3.793948
CD109	0.167603	5.4943029	2.5221129	0.0120837	0.0254503	-3.794766
RP11-1036E	0.6920356	1.4010099	2.5219191	0.0120903	0.0254626	-3.795243
WNT9B	0.5089414	0.6454754	2.521533	0.0121033	0.0254886	-3.796194
COPG2	0.0507407	6.0059826	2.5214671	0.0121055	0.0254918	-3.796356
MMP24-AS1	-0.094877	6.1531886	-2.521339	0.0121099	0.0254995	-3.796673
AC073130.3	0.4489609	0.19399	2.5209026	0.0121246	0.0255278	-3.797746
C1QA	0.0932471	6.5593146	2.5209005	0.0121247	0.0255278	-3.797751
IFITM1	0.115955	6.0820043	2.5208393	0.0121268	0.0255297	-3.797901

RNU6-967P	-0.119588	-1.484951	-2.520832	0.012127	0.0255297	-3.797919
TMEM98	0.1953461	6.1579397	2.520423	0.0121409	0.0255574	-3.798926
ZNF770	0.1570916	5.6765146	2.5203991	0.0121417	0.0255576	-3.798985
SEC11C	-0.064541	6.6744097	-2.520375	0.0121425	0.0255579	-3.799043
RP11-573D1	0.2908701	2.893022	2.5202777	0.0121458	0.0255634	-3.799283
ZNF74	0.0573044	5.5901535	2.5202304	0.0121474	0.0255653	-3.7994
CTA-29F11.	-0.106312	4.8471861	-2.51971	0.0121651	0.0256009	-3.800679
SAA2-SAA4	-0.581994	4.680735	-2.519394	0.0121758	0.0256221	-3.801458
UTP14A	-0.045279	5.9814671	-2.519338	0.0121777	0.0256246	-3.801594
SNORA22	0.3375062	-0.647011	2.5191815	0.012183	0.0256343	-3.80198
RP11-138I1	-0.223031	-1.245682	-2.518862	0.0121939	0.0256557	-3.802766
CTD-2303H2	0.5724303	1.1531253	2.5188217	0.0121953	0.0256571	-3.802865
LETM1	-0.041658	6.3864129	-2.518549	0.0122046	0.0256752	-3.803535
RNF126P1	-0.520315	1.4554383	-2.518355	0.0122112	0.0256876	-3.804012
HECTD1	-0.04513	6.6209462	-2.518206	0.0122162	0.0256968	-3.804378
RP11-453N1	0.2912028	-0.976589	2.51805	0.0122216	0.0257065	-3.804763
MFSD7	0.1621816	5.3319754	2.5171986	0.0122506	0.0257661	-3.806855
RP11-370A5	-0.253734	4.0595129	-2.517168	0.0122517	0.0257669	-3.806931
SERGEF	-0.066318	5.8410995	-2.517084	0.0122545	0.0257708	-3.807137
PIDD1	-0.048933	5.931541	-2.517072	0.012255	0.0257708	-3.807167
RPS2	-0.053404	7.162344	-2.516837	0.012263	0.0257862	-3.807745
TSIX	0.4812688	0.0707251	2.5165567	0.0122726	0.0258049	-3.808433
CLDN3	-0.200824	6.0369899	-2.516454	0.0122761	0.0258108	-3.808686
HSP90AA5P	-0.414733	0.2077511	-2.516369	0.012279	0.0258154	-3.808894
GAST	0.5496871	-0.453695	2.5162597	0.0122828	0.0258218	-3.809163
RP11-10C24	0.2303873	3.1089931	2.5156118	0.012305	0.025867	-3.810754
ZC3H18	-0.03361	6.1950024	-2.515319	0.012315	0.0258867	-3.811474
ATG7	-0.035035	5.968355	-2.515169	0.0123202	0.025896	-3.811842
ATG3	-0.032839	6.2696693	-2.514628	0.0123388	0.0259336	-3.813171
MIATNB	0.1271808	4.3331415	2.514371	0.0123476	0.0259507	-3.813801
PRAMEF5	-0.137958	-1.451228	-2.514108	0.0123567	0.0259682	-3.814446
TEX10	0.0416236	5.8677047	2.514003	0.0123603	0.0259743	-3.814705
RTP2	-0.249978	-1.196065	-2.513826	0.0123664	0.0259856	-3.815139
KBTBD13	-0.144992	-1.426345	-2.513662	0.012372	0.025996	-3.815542
RPL21P28	-0.177114	4.1374578	-2.513421	0.0123803	0.0260119	-3.816132
COX6B1P5	0.3154518	-0.791871	2.5132973	0.0123846	0.0260194	-3.816437
LINC01192	-0.485681	-0.412238	-2.513182	0.0123886	0.0260263	-3.816721
ANKRD46	-0.067087	6.0912799	-2.512966	0.012396	0.0260404	-3.817249
DLG1-AS1	-0.473036	0.60115	-2.512867	0.0123995	0.0260462	-3.817493
RP3-42906.	-0.119328	-1.477979	-2.512787	0.0124022	0.0260504	-3.817688
AC007950.1	-0.318868	-0.883739	-2.512734	0.0124041	0.0260528	-3.817819
ZYX	0.0518704	6.7372345	2.5124532	0.0124138	0.0260717	-3.818508
RP11-64C12	-0.102538	-1.488899	-2.512394	0.0124158	0.0260745	-3.818653
RP1-100J12	-0.407327	1.2667492	-2.512246	0.0124209	0.0260837	-3.819015
LINC01105	0.326762	-1.154168	2.511903	0.0124328	0.0261072	-3.819857
RP11-395I6	0.2520611	3.5766128	2.5117375	0.0124385	0.0261177	-3.820263
RP11-521D1	0.3379173	-0.870654	2.511599	0.0124433	0.0261263	-3.820603
AC068196.1	0.3188724	-0.848896	2.5115225	0.012446	0.0261303	-3.820791
KLHL40	-0.177209	-1.360259	-2.511469	0.0124478	0.0261327	-3.820921
MTND1P14	-0.314228	-0.871525	-2.511171	0.0124582	0.0261529	-3.821652
PLA2G10	0.4372843	0.1189929	2.5110109	0.0124637	0.0261631	-3.822045

CTD-2022H1	0.3841659	-0.182901	2.5109816	0.0124647	0.0261637	-3.822117
SEC16A	-0.040068	6.71614	-2.510752	0.0124727	0.0261789	-3.82268
RP11-522B1	0.3228727	3.3107804	2.5104785	0.0124822	0.0261965	-3.82335
ZNF682	0.2574386	4.6317342	2.5104692	0.0124825	0.0261965	-3.823373
RP11-644A7	0.2165883	-1.247046	2.5104106	0.0124846	0.0261993	-3.823517
GJB7	0.3949696	-0.745781	2.5100018	0.0124988	0.0262276	-3.824519
RP11-168K1	0.4904845	1.7803455	2.509697	0.0125094	0.0262484	-3.825266
PRCD	0.2115986	3.5391554	2.5095956	0.0125129	0.0262543	-3.825514
NPM1P39	0.3499664	2.2349379	2.5095562	0.0125143	0.0262556	-3.825611
RP11-832A4	0.4844117	0.5148765	2.509523	0.0125155	0.0262566	-3.825692
KLK13	0.503356	-0.418107	2.5093873	0.0125202	0.026265	-3.826025
KIAA1107	0.1650065	4.6920028	2.5093136	0.0125227	0.0262688	-3.826205
ZRANB2-AS2	0.415921	1.3284232	2.5092322	0.0125256	0.0262733	-3.826405
RRN3P2	0.202462	3.5530973	2.5089783	0.0125344	0.0262903	-3.827027
CH507-513H	-0.311277	2.8633724	-2.508753	0.0125423	0.0263053	-3.827579
PEX7	-0.062764	5.5393468	-2.507643	0.0125811	0.0263851	-3.830296
ATP5HP3	-0.181976	-1.373739	-2.507579	0.0125833	0.0263883	-3.830454
RP11-15001	0.4797837	-0.003354	2.5072403	0.0125952	0.0264117	-3.831284
U62631.5	0.2315259	-1.23104	2.5070271	0.0126027	0.0264258	-3.831805
RP11-6N13.	-0.113014	-1.492779	-2.506888	0.0126075	0.0264346	-3.832146
RLF	0.059702	5.7321023	2.5067612	0.012612	0.026441	-3.832456
VAMP4	-0.050154	5.8930719	-2.506759	0.0126121	0.026441	-3.832462
SETMAR	-0.054402	5.6514836	-2.506724	0.0126133	0.026442	-3.832547
ETS2	-0.060373	6.787877	-2.506235	0.0126304	0.0264765	-3.833745
ARHGAP29	0.0610152	6.2138683	2.5060203	0.012638	0.0264908	-3.83427
CH507-42P1	0.4932059	-0.068255	2.5059744	0.0126396	0.0264926	-3.834382
RABEP2	0.0562207	5.9118841	2.5058885	0.0126426	0.0264974	-3.834592
ESR2	0.2605145	2.8793386	2.5056951	0.0126494	0.0265102	-3.835065
RP5-837M10	-0.192255	-1.347758	-2.505532	0.0126551	0.0265207	-3.835464
PTPN23	-0.033405	6.4099318	-2.505461	0.0126576	0.0265244	-3.835637
CSTF3-AS1	0.4307007	0.1871128	2.5051133	0.0126699	0.0265485	-3.836489
RAPGEF2	-0.067162	6.1910508	-2.504982	0.0126745	0.0265566	-3.836809
CTD-2311B1	0.2635189	-1.193584	2.5046315	0.0126869	0.026581	-3.837667
PIGA	0.061599	5.5688513	2.5044658	0.0126927	0.0265917	-3.838073
LINC01194	-0.735477	0.7990119	-2.503537	0.0127255	0.0266589	-3.840344
MKLN1	-0.050367	6.0984206	-2.503473	0.0127277	0.0266621	-3.840499
AC073283.7	0.3131192	-0.772103	2.5033156	0.0127333	0.0266723	-3.840885
KRT8P49	0.3645069	-0.429054	2.5032536	0.0127355	0.0266753	-3.841037
NAPA	-0.045086	6.5943035	-2.503143	0.0127394	0.026682	-3.841306
DSCC1	0.0993925	5.1481337	2.5030694	0.012742	0.0266859	-3.841487
RP11-739L1	-0.228041	4.0093129	-2.502857	0.0127496	0.0267001	-3.842007
CD59	-0.045958	6.9294954	-2.502828	0.0127506	0.0267007	-3.842076
SMIM8	-0.064471	5.3976501	-2.502678	0.0127559	0.0267103	-3.842444
HYDIN	0.616978	1.1554071	2.5025102	0.0127618	0.0267212	-3.842854
AC006538.1	0.3762016	2.6384173	2.5024483	0.012764	0.0267243	-3.843005
IGKV2-18	0.2054872	-1.328022	2.5023963	0.0127659	0.0267263	-3.843132
C5orf63	0.2998556	4.7210665	2.50238	0.0127665	0.0267263	-3.843172
FAM21A	-0.042443	6.1935952	-2.502248	0.0127711	0.0267339	-3.843494
RP11-434H6	-0.110608	-1.479179	-2.502237	0.0127715	0.0267339	-3.843522
ETNK1	0.0409577	6.328861	2.5021829	0.0127734	0.0267363	-3.843654
RP11-178L8	0.3546332	-0.586627	2.5021225	0.0127756	0.0267393	-3.843801

RP11-744K1	-0.415676	-0.728418	-2.502061	0.0127778	0.0267423	-3.84395
PLCG2	-0.085117	6.1188	-2.501929	0.0127825	0.0267506	-3.844274
RP11-204L2	0.3995092	-0.137317	2.5018388	0.0127857	0.0267558	-3.844494
TRIM52	-0.059011	5.518971	-2.501682	0.0127912	0.0267659	-3.844878
RP11-87G24	0.2076248	-1.341068	2.5015116	0.0127973	0.026777	-3.845294
RP11-458F8	0.4473776	2.1304727	2.5010011	0.0128154	0.0268134	-3.846541
RP11-145A3	0.5378988	1.6178537	2.5008626	0.0128203	0.0268222	-3.846879
BNIP3P42	-0.198389	-1.284928	-2.500598	0.0128298	0.0268404	-3.847525
AC018816.3	-0.327307	2.9068545	-2.500296	0.0128405	0.0268614	-3.848264
ABLIM2	0.1837096	5.0976518	2.5002458	0.0128423	0.0268635	-3.848385
LEO1	-0.039077	6.1674436	-2.500159	0.0128454	0.0268685	-3.848598
GNB1	-0.029904	6.8910409	-2.500079	0.0128483	0.0268728	-3.848793
VN1R35P	-0.166994	-1.400371	-2.50006	0.0128489	0.0268728	-3.84884
AP4E1	0.0477486	5.6427863	2.4998106	0.0128578	0.0268898	-3.849448
RP11-885N1	-0.145766	-1.446456	-2.49962	0.0128646	0.0269025	-3.849913
UBE2Q2P2	0.2091196	3.6066803	2.4994664	0.0128701	0.0269124	-3.850288
SNORA65	0.3078251	2.1528675	2.4994393	0.0128711	0.0269129	-3.850354
RP11-392O1	0.3318537	2.8684901	2.499219	0.012879	0.0269278	-3.850892
RAB5A	-0.032841	6.3082735	-2.498934	0.0128891	0.0269476	-3.851588
RP11-219B4	-0.399704	-0.245871	-2.498689	0.0128979	0.0269641	-3.852185
RNU6-1209F	0.2514901	-1.08541	2.4986725	0.0128985	0.0269641	-3.852226
RNU1-122P	-0.420641	-0.26561	-2.498431	0.0129071	0.0269806	-3.852816
RP11-313J2	0.5569564	2.7013323	2.4981862	0.0129159	0.0269974	-3.853412
L3HYPDH	-0.079599	5.6524064	-2.498053	0.0129207	0.0270058	-3.853736
RP11-304L1	0.4929865	1.2498405	2.4979043	0.012926	0.0270149	-3.8541
AC084219.3	0.4708657	0.8964639	2.4978906	0.0129265	0.0270149	-3.854133
AC009501.4	0.3449055	3.555437	2.4970397	0.012957	0.0270771	-3.856209
EGFEM1P	0.6354667	0.671307	2.4969097	0.0129617	0.0270853	-3.856526
CTD-233602	-0.081956	5.4511198	-2.496484	0.012977	0.0271157	-3.857563
RP11-331F4	0.4044898	1.908538	2.4962471	0.0129855	0.027132	-3.858141
LINC00488	-0.641128	0.5222942	-2.496091	0.0129911	0.0271422	-3.858522
ZNF182	0.0516613	5.3796847	2.495953	0.0129961	0.027151	-3.858858
CNTF	0.3568856	2.7524056	2.4955361	0.0130111	0.0271809	-3.859874
MED10	0.0448583	5.9890133	2.4954335	0.0130148	0.027187	-3.860125
MIR590	0.378827	-0.395402	2.495204	0.0130231	0.0272028	-3.860684
DGKB	0.5047727	1.4899149	2.4950757	0.0130277	0.0272101	-3.860997
DDX19A	-0.036449	6.2009925	-2.495065	0.0130281	0.0272101	-3.861022
CTD-2574D2	0.1081458	4.518019	2.4949891	0.0130309	0.0272143	-3.861208
RRAGB	0.05885	5.5137759	2.4948577	0.0130356	0.0272226	-3.861528
STON2	-0.128725	5.2033278	-2.494793	0.0130379	0.0272259	-3.861684
C6orf211	-0.060942	6.1172934	-2.494655	0.0130429	0.0272341	-3.862022
PURB	-0.029327	6.2610984	-2.494644	0.0130433	0.0272341	-3.862049
APOBEC3F	0.145014	5.1383	2.4945318	0.0130474	0.027241	-3.862322
FCN2	-0.597034	2.823094	-2.494395	0.0130523	0.0272498	-3.862656
RP11-216L1	0.2448119	3.6065016	2.4943344	0.0130545	0.0272528	-3.862803
RP11-718B1	-0.388987	-0.591007	-2.493972	0.0130676	0.0272783	-3.863686
NAT8B	0.3663945	4.3040722	2.493955	0.0130682	0.0272783	-3.863727
HPGD	0.3902321	5.6960201	2.4937902	0.0130742	0.0272877	-3.864128
RPS6KL1	-0.160307	5.2207968	-2.49379	0.0130742	0.0272877	-3.864129
EPN2-AS1	0.2117535	-1.252603	2.4937266	0.0130765	0.0272909	-3.864283
KCNK2	0.6200564	0.252251	2.4933084	0.0130917	0.0273195	-3.865302

MYLKP1	-0.21862	-1.248455	-2.493307	0.0130917	0.0273195	-3.865306
SASH1	0.0724668	5.8591418	2.4932038	0.0130955	0.0273257	-3.865556
TOP2A	0.1183133	6.1446056	2.4931458	0.0130976	0.0273286	-3.865698
MIR1295A	-0.524845	1.0854367	-2.492941	0.013105	0.0273425	-3.866196
SCNN1B	0.5048049	2.6898963	2.4926221	0.0131166	0.0273651	-3.866973
DEFA6	0.2803257	-1.202573	2.4923728	0.0131256	0.0273824	-3.86758
RP11-44N11	-0.449765	1.0807218	-2.491914	0.0131423	0.0274127	-3.868697
RPL36AP29	-0.31633	-0.900985	-2.491908	0.0131425	0.0274127	-3.868711
EIF2S1	-0.036117	6.372977	-2.491902	0.0131427	0.0274127	-3.868726
RP3-412A9.	-0.194625	-1.298375	-2.491892	0.0131431	0.0274127	-3.868751
RP4-765H13	0.2467772	-1.285425	2.4918108	0.0131461	0.0274172	-3.868948
CAPS	-0.074562	5.7574187	-2.491765	0.0131477	0.0274191	-3.869058
RNU6-817P	-0.428049	0.1879731	-2.491618	0.0131531	0.0274287	-3.869417
RP11-162J8	-0.351305	-0.83897	-2.491397	0.0131611	0.027444	-3.869955
C4orf51	0.2337559	-1.234992	2.4913664	0.0131622	0.0274447	-3.870029
CYP4B1	0.5144722	0.7512001	2.4913075	0.0131644	0.0274476	-3.870172
EIF4A1P4	0.3807882	1.525552	2.4911717	0.0131693	0.0274563	-3.870503
TSPEAR-AS2	-0.504092	3.8778318	-2.490775	0.0131838	0.0274849	-3.871468
OAS1	-0.084764	6.2439597	-2.490576	0.0131911	0.0274985	-3.871953
AC064853.2	-0.39863	-0.888421	-2.490527	0.0131928	0.0275007	-3.872071
RP11-728K2	0.4430789	0.7377171	2.490355	0.0131991	0.0275122	-3.87249
HHATL	-0.526337	0.421036	-2.490175	0.0132057	0.0275243	-3.872928
PRAMEF9	-0.626996	0.1799959	-2.490132	0.0132073	0.027526	-3.873033
MIR23A	0.2048145	-1.282686	2.4901072	0.0132082	0.0275263	-3.873093
CLK3P2	-0.109458	-1.477648	-2.490047	0.0132104	0.0275293	-3.873239
LINC01125	-0.133196	4.4791136	-2.489957	0.0132136	0.0275333	-3.873457
CREBBP	-0.036656	6.450479	-2.489954	0.0132138	0.0275333	-3.873465
PTAR1	-0.050965	6.0990599	-2.489832	0.0132182	0.027541	-3.873763
RPL21P13	-0.329384	-0.883124	-2.489795	0.0132196	0.0275423	-3.873852
RP11-196I1	0.41461	1.0141675	2.4896516	0.0132248	0.0275516	-3.8742
CH17-12M21	0.2950227	-0.88131	2.4894915	0.0132307	0.0275622	-3.87459
MTND5P10	-0.376492	-0.583536	-2.48944	0.0132326	0.0275645	-3.874714
DIO2-AS1	-0.096611	-1.500806	-2.48916	0.0132428	0.0275843	-3.875395
OSCP1	-0.146257	4.7954494	-2.488589	0.0132637	0.0276264	-3.876784
BECN1P1	0.399615	-0.656115	2.4885609	0.0132648	0.0276269	-3.876852
SETD4	0.0435831	5.6357273	2.4882338	0.0132768	0.0276504	-3.877647
BRAP	-0.027433	5.9919308	-2.488197	0.0132781	0.0276516	-3.877737
RP5-1142A6	-0.279406	-1.007527	-2.487958	0.0132869	0.0276683	-3.878317
CORT	-0.380768	1.9623031	-2.487908	0.0132887	0.0276705	-3.878439
HSP90AB3P	0.2343046	3.7372019	2.4876963	0.0132965	0.0276852	-3.878953
LPPR4	0.5014024	2.6596986	2.4875422	0.0133022	0.0276954	-3.879328
C5orf34	0.1328166	4.5502064	2.4872903	0.0133115	0.0277131	-3.87994
ZDHHC22	0.3424806	-0.928541	2.4872463	0.0133131	0.0277149	-3.880047
LINC00365	0.5095517	2.9074566	2.4871791	0.0133155	0.0277184	-3.88021
RP11-1B20.	-0.178078	-1.345289	-2.487086	0.013319	0.027724	-3.880435
RP13-895J2	-0.31089	-1.146735	-2.486977	0.013323	0.0277308	-3.880701
WDFY3-AS2	0.2400152	3.7264406	2.4869318	0.0133246	0.0277326	-3.880811
ACSM6	-0.474819	0.236079	-2.486847	0.0133278	0.0277376	-3.881017
EPG5	0.0485937	5.972498	2.4867692	0.0133306	0.0277419	-3.881206
MAN1A2	-0.042583	6.3409191	-2.48644	0.0133428	0.0277656	-3.882006
RP11-92K15	0.3629214	2.7947565	2.4860803	0.013356	0.0277916	-3.882879

ZHX2	-0.053257	6.334774	-2.485672	0.0133711	0.0278214	-3.88387
DAW1	0.257589	-1.181192	2.4855083	0.0133772	0.0278313	-3.884268
LRPPRC	-0.03219	6.7385161	-2.485502	0.0133774	0.0278313	-3.884282
ATAD2B	0.0564516	5.6565411	2.4852651	0.0133861	0.0278479	-3.884858
RP11-229P1	0.3656179	2.1377955	2.4852106	0.0133882	0.0278505	-3.88499
CCDC108	0.4720324	1.7894269	2.4851485	0.0133905	0.0278537	-3.885141
AC091633.3	-0.441273	0.4790614	-2.48504	0.0133945	0.0278605	-3.885404
BAIAP2L1	0.1010128	6.4348417	2.4849599	0.0133974	0.0278651	-3.885599
RP11-130C1	-0.329864	-0.776786	-2.484821	0.0134026	0.0278742	-3.885937
SNRK-AS1	0.3798265	-0.478573	2.4845053	0.0134143	0.0278964	-3.886702
CH17-140K2	0.3127309	-0.994514	2.4844906	0.0134148	0.0278964	-3.886738
GAPDHP31	-0.278181	-1.042151	-2.484431	0.013417	0.0278994	-3.886882
ARFGAP3	0.0428288	6.3869973	2.4843024	0.0134218	0.0279078	-3.887195
AC012462.1	-0.34296	-0.716884	-2.484256	0.0134235	0.0279098	-3.887308
RP11-39404	0.3061985	-0.897543	2.4841428	0.0134277	0.0279169	-3.887582
ADARB1	0.0648325	5.7019638	2.483635	0.0134465	0.0279545	-3.888814
MFSD5	-0.044646	6.1535505	-2.483559	0.0134493	0.0279587	-3.888998
MTND4LP1	-0.1626	-1.392206	-2.483438	0.0134539	0.0279665	-3.889292
C8orf17	-0.160734	-1.394769	-2.483276	0.0134599	0.0279774	-3.889686
DLEU2	0.1032988	4.8308985	2.4832123	0.0134622	0.0279807	-3.88984
AUNIP	0.1861272	3.9512407	2.4830987	0.0134665	0.0279879	-3.890115
TSPYL1	-0.047594	6.4218689	-2.483039	0.0134687	0.0279904	-3.890261
RP3-508I15	0.4132082	0.3500827	2.4830248	0.0134692	0.0279904	-3.890294
APMAP	-0.057157	6.9785243	-2.482164	0.0135012	0.0280554	-3.892382
VSIG10	-0.051907	6.1883871	-2.48212	0.0135029	0.0280572	-3.892488
RP11-159D1	0.2786872	-1.096508	2.4819309	0.0135099	0.0280703	-3.892947
AP2A1	-0.035052	6.6934903	-2.481114	0.0135404	0.028132	-3.894927
ZDHHC20P1	0.4447323	0.6841391	2.4810461	0.0135429	0.0281357	-3.895092
MCHR1	-0.31955	4.6068463	-2.480889	0.0135488	0.0281462	-3.895472
KCNH7	0.5639624	0.5013944	2.4807859	0.0135527	0.0281527	-3.895722
PRMT6	0.1869843	5.4330805	2.4804742	0.0135643	0.0281753	-3.896478
PARD6G-AS1	-0.173511	4.6916943	-2.480444	0.0135655	0.028176	-3.89655
FNDC4	-0.127965	6.2029372	-2.480401	0.0135671	0.0281778	-3.896656
MSANTD1	0.3009133	2.6497002	2.4803759	0.013568	0.0281781	-3.896716
TAF3	0.0387147	5.6893108	2.4802433	0.013573	0.0281868	-3.897037
MTND2P12	-0.331874	-0.783977	-2.479866	0.0135871	0.0282146	-3.897952
ALX1	0.4632451	-0.760026	2.4788474	0.0136253	0.0282923	-3.900418
LINC01291	0.747342	2.3098551	2.478703	0.0136307	0.0283019	-3.900768
AC108142.1	0.2924476	-1.18466	2.4786104	0.0136342	0.0283075	-3.900992
RP5-1029K1	0.2203375	-1.22827	2.4782743	0.0136468	0.0283322	-3.901806
RP11-345P4	0.4657437	0.7798268	2.4781005	0.0136534	0.0283441	-3.902226
PRPF18	-0.038469	5.6396528	-2.477651	0.0136703	0.0283768	-3.903314
DAZAP1	-0.030033	6.589285	-2.47763	0.0136711	0.0283768	-3.903366
INHBE	0.1593218	6.3297887	2.4776208	0.0136714	0.0283768	-3.903388
EGF	0.7056424	1.3594053	2.4775396	0.0136745	0.0283815	-3.903584
RP11-46C24	-0.183311	-1.3681	-2.477416	0.0136792	0.0283896	-3.903883
RP11-129K1	0.3293912	-0.683711	2.4773019	0.0136835	0.0283969	-3.904159
RP11-267M2	0.1717375	4.1628603	2.4770398	0.0136933	0.0284158	-3.904794
AC005758.1	-0.203571	-1.371224	-2.477001	0.0136948	0.0284172	-3.904888
RTN4IP1	-0.055125	5.639188	-2.476939	0.0136971	0.0284204	-3.905037
RP11-677M1	0.3723124	2.8224218	2.4769116	0.0136982	0.028421	-3.905104

C1orf143	0.2636237	-1.173288	2.4767198	0.0137054	0.0284344	-3.905568
CITED4	-0.118765	6.0869923	-2.476271	0.0137224	0.0284679	-3.906653
ERCC6-PGBD	0.4102859	0.408553	2.4760449	0.0137309	0.0284841	-3.907201
NOP14	-0.037837	6.3213777	-2.475918	0.0137357	0.0284923	-3.907507
RP4-669L17	0.4282196	1.5995189	2.4755628	0.0137492	0.0285187	-3.908367
TTN-AS1	0.2209079	4.2109378	2.4755015	0.0137515	0.0285218	-3.908515
ASIC3	-0.18487	4.3946515	-2.475445	0.0137536	0.0285247	-3.908651
OPRD1	0.3730696	2.3952859	2.4753012	0.0137591	0.0285343	-3.908999
CTD-2012K1	0.3966301	0.844191	2.4752072	0.0137626	0.0285401	-3.909227
AC015849.1	-0.233734	-1.166249	-2.474791	0.0137784	0.0285712	-3.910233
USP6	0.325946	2.9927083	2.4747638	0.0137795	0.0285717	-3.910299
RP11-972P1	0.4611019	1.0992638	2.4745778	0.0137865	0.0285847	-3.910748
PGS1	0.0359668	5.8670482	2.4744344	0.013792	0.0285944	-3.911095
GUCA1A	0.2427888	-1.204248	2.474352	0.0137951	0.0285993	-3.911294
IL1RN	-0.128252	6.2287807	-2.474004	0.0138083	0.028625	-3.912135
EEF1A1P4	0.3133906	2.8296518	2.4739253	0.0138113	0.0286296	-3.912326
CTB-58E17.	0.3976539	1.3713482	2.4736623	0.0138213	0.0286487	-3.912961
MAGEB3	-0.231702	-1.277322	-2.473471	0.0138286	0.0286622	-3.913424
FAM157C	0.494087	1.6118298	2.4733949	0.0138315	0.0286666	-3.913607
DGKE	0.1447211	5.1207211	2.4731541	0.0138406	0.0286839	-3.914189
UBTFL9	-0.19865	-1.293682	-2.472589	0.0138622	0.0287256	-3.915555
GUCA1C	-0.115909	-1.473765	-2.472585	0.0138623	0.0287256	-3.915564
AC226118.1	0.4590047	-0.423669	2.4724204	0.0138686	0.028737	-3.915962
KLRG2	0.3361584	-0.752204	2.4722613	0.0138747	0.0287479	-3.916346
SPINK6	-0.325139	-0.8894	-2.472204	0.0138769	0.0287499	-3.916485
RP11-461A8	-0.322419	-0.920698	-2.472195	0.0138772	0.0287499	-3.916505
RP11-475J5	-0.179491	-1.359776	-2.472166	0.0138783	0.0287506	-3.916576
WDR12	-0.03904	6.0934651	-2.472034	0.0138833	0.0287594	-3.916894
NPAP1	0.2945933	-1.046072	2.471893	0.0138887	0.0287689	-3.917235
SYDE2	0.2520512	4.5283148	2.4716517	0.013898	0.0287864	-3.917818
RP6-91H8.2	0.2195105	-1.290989	2.4716123	0.0138995	0.0287879	-3.917913
RP4-543J13	-0.244871	-1.218604	-2.47153	0.0139026	0.0287928	-3.918112
AC005256.1	0.3232153	-1.082106	2.4714085	0.0139073	0.0288007	-3.918405
NCAPG2	0.0756068	5.7513862	2.4712439	0.0139136	0.0288121	-3.918803
C16orf89	0.395299	2.5559625	2.4711409	0.0139175	0.0288187	-3.919052
ZACN	-0.187408	3.3704459	-2.471057	0.0139207	0.0288221	-3.919254
CTD-3030D2	0.3674915	-0.717534	2.4710568	0.0139207	0.0288221	-3.919255
UTP18	-0.039889	6.1463221	-2.471016	0.0139223	0.0288237	-3.919353
RP5-1042K1	0.4056795	0.0994866	2.4707137	0.0139339	0.028846	-3.920083
RP11-176N1	0.2141482	-1.323166	2.4701981	0.0139536	0.0288853	-3.921327
RERE	0.0440612	6.3845031	2.4701214	0.0139566	0.0288897	-3.921512
AC006262.5	0.4317821	-0.593731	2.4700825	0.0139581	0.0288912	-3.921606
LINC01239	-0.423224	3.0376255	-2.469614	0.0139761	0.0289268	-3.922737
RP11-274H2	0.2700652	3.8232268	2.4694833	0.0139811	0.0289355	-3.923052
AC007246.3	-0.056291	5.6328194	-2.469445	0.0139826	0.028937	-3.923145
LINC00326	-0.119936	-1.473045	-2.469309	0.0139878	0.0289461	-3.923473
RP11-83C7.	-0.292636	-1.043843	-2.469141	0.0139942	0.0289579	-3.923878
RP11-245D1	-0.334991	2.775251	-2.468685	0.0140118	0.0289925	-3.924977
KRTAP1-1	0.3439357	-0.959489	2.4686152	0.0140145	0.0289964	-3.925146
SOX5P	-0.158475	-1.405523	-2.468416	0.0140221	0.0290107	-3.925627
RP11-510M2	-0.12199	-1.478331	-2.468392	0.0140231	0.0290109	-3.925685

RP11-1000E	-0.288092	-0.998021	-2.467761	0.0140474	0.0290596	-3.927206
HCST	0.1374073	4.9924593	2.4677292	0.0140486	0.0290605	-3.927283
POM121L4P	0.3871379	-0.571367	2.4676401	0.0140521	0.029066	-3.927498
RP11-810P1	-0.116619	-1.48293	-2.467595	0.0140538	0.0290679	-3.927607
CD40	-0.108805	5.8702645	-2.467007	0.0140765	0.0291133	-3.929024
ANKRD62P1	-0.106967	-1.490497	-2.466802	0.0140844	0.0291267	-3.929517
AC004386.4	-0.317493	-0.822168	-2.466798	0.0140846	0.0291267	-3.929527
RP11-203L2	0.2206812	-1.213832	2.4667257	0.0140874	0.0291308	-3.929702
RP4-738P11	0.3849063	-0.678881	2.4665784	0.0140931	0.029141	-3.930057
OLFM2	-0.118852	6.3986759	-2.466461	0.0140977	0.0291487	-3.930341
RP11-15N24	0.3431032	2.4737123	2.4658761	0.0141203	0.0291939	-3.931749
FAM122A	-0.047111	5.8668189	-2.465775	0.0141242	0.0292004	-3.931993
LAMB3	-0.131862	5.8853309	-2.465636	0.0141296	0.0292099	-3.932328
AC005624.2	0.3941905	-0.293764	2.4655352	0.0141335	0.0292153	-3.93257
RP5-1024G6	-0.301221	-0.870856	-2.465526	0.0141339	0.0292153	-3.932591
UBAP1	-0.033038	6.4273143	-2.46547	0.0141361	0.0292182	-3.932727
GTPBP2	0.0444722	6.2460096	2.4654062	0.0141385	0.0292216	-3.932881
LINC01423	-0.238913	-1.226096	-2.465144	0.0141487	0.029241	-3.933512
AC098820.4	-0.412035	1.2351333	-2.465073	0.0141515	0.0292451	-3.933683
AC104135.2	0.379539	-0.577191	2.4649036	0.014158	0.029257	-3.934092
AC008277.1	-0.230759	-1.199676	-2.464814	0.0141615	0.0292626	-3.934307
PIWIL1	0.4165544	-0.175006	2.4647619	0.0141636	0.0292651	-3.934433
RP11-809F4	-0.136916	-1.435105	-2.464731	0.0141648	0.0292659	-3.934508
TMEFF1	0.1994372	-1.302924	2.4645898	0.0141702	0.0292756	-3.934847
DAAM2	0.1876336	5.3408915	2.4644441	0.0141759	0.0292856	-3.935198
USP43	0.465958	4.2482618	2.4638878	0.0141976	0.0293287	-3.936537
RP11-231E4	0.4216392	0.3826348	2.463852	0.014199	0.0293299	-3.936624
RP11-1C8.6	0.4338893	1.3794194	2.463815	0.0142004	0.0293313	-3.936713
AC093063.2	0.3789765	-0.878985	2.4637276	0.0142038	0.0293366	-3.936923
AC093585.6	-0.294048	-0.970587	-2.463696	0.014205	0.0293375	-3.937
RP5-933B4.	-0.129682	-1.464469	-2.463514	0.0142121	0.0293505	-3.937438
ITPA	-0.0544	6.33014	-2.463335	0.0142191	0.0293633	-3.937868
AL008723.1	0.2636301	-1.075017	2.4631631	0.0142258	0.0293755	-3.938282
MCC	0.153564	5.6135924	2.4630216	0.0142313	0.0293852	-3.938622
LINC01235	-0.49049	2.5545312	-2.462863	0.0142375	0.0293962	-3.939004
AP1G1	-0.038916	6.4765994	-2.462844	0.0142383	0.0293962	-3.939051
KLHL12	-0.039277	6.2609884	-2.462774	0.014241	0.0294002	-3.939219
NME6	-0.042813	5.8038012	-2.462708	0.0142436	0.0294038	-3.939377
ACRBP	0.1653992	4.400656	2.4626475	0.014246	0.029407	-3.939523
RP11-503N1	-0.25218	-1.119052	-2.462459	0.0142533	0.0294206	-3.939977
WDR7	-0.051725	5.8489849	-2.46243	0.0142544	0.0294213	-3.940045
CTB-43E15.	-0.483628	-0.141863	-2.462138	0.0142659	0.0294431	-3.940747
ALX4	0.4094005	-0.443658	2.4619335	0.0142739	0.029458	-3.94124
ZCCHC16	-0.710622	2.0054045	-2.461899	0.0142752	0.0294591	-3.941323
RP4-583K8.	-0.322182	-0.964947	-2.461709	0.0142827	0.0294713	-3.94178
CTSZ	-0.050692	6.999071	-2.461707	0.0142827	0.0294713	-3.941785
ADCY10	-0.201678	5.0082939	-2.461625	0.0142859	0.0294762	-3.941981
GHRH	0.2606074	-1.22898	2.4612444	0.0143009	0.0295042	-3.942898
E2F2	0.202	4.7175071	2.4612387	0.0143011	0.0295042	-3.942912
RP11-571M6	-0.133311	3.9570743	-2.461188	0.0143031	0.0295065	-3.943033
IGKV2D-30	0.3120619	-1.155703	2.4611694	0.0143038	0.0295065	-3.943078

BTN2A3P	0.1158608	4.5357986	2.4611272	0.0143055	0.0295082	-3.94318
ASIC1	0.3613318	4.1540571	2.4607489	0.0143203	0.0295372	-3.944089
DNAJC5	0.0383564	6.4659979	2.4605706	0.0143273	0.0295483	-3.944518
KRIT1	0.0557558	5.9230029	2.4605698	0.0143273	0.0295483	-3.94452
LSAMP-AS1	0.2991118	-1.133965	2.4605483	0.0143282	0.0295484	-3.944572
TRIM39-RPF	-0.26031	-1.073538	-2.460464	0.0143315	0.0295535	-3.944773
ZW10	-0.038137	5.9028122	-2.460325	0.014337	0.0295631	-3.945108
GALNT1	-0.042015	6.55342	-2.460075	0.0143468	0.0295817	-3.945708
AC010761.8	0.1880698	3.8064198	2.4598237	0.0143567	0.0296004	-3.946313
DNAH11	-0.393584	4.0534418	-2.459797	0.0143577	0.0296009	-3.946378
snoU109	0.3817856	1.7525298	2.4597164	0.0143609	0.0296058	-3.946571
RP11-177C1	0.4619165	2.6536681	2.4593334	0.014376	0.0296352	-3.947492
BSG	-0.046325	7.1146661	-2.459024	0.0143882	0.0296586	-3.948236
RP11-17E13	-0.213826	4.1747043	-2.459005	0.0143889	0.0296586	-3.948282
RP11-2H3.6	0.4342148	0.9371384	2.4589069	0.0143928	0.0296648	-3.948517
CTC-425023	0.4921562	0.8132455	2.4588793	0.0143939	0.029665	-3.948583
AC024937.4	0.4034237	-0.452805	2.4588639	0.0143945	0.029665	-3.94862
SIRT6	-0.051155	5.9311222	-2.458842	0.0143953	0.0296651	-3.948672
CBR3-AS1	0.1657954	4.0848557	2.4586616	0.0144024	0.0296781	-3.949106
SRSF2	-0.025597	6.5911462	-2.458314	0.0144162	0.0297047	-3.949941
OPA1	-0.035371	6.4113765	-2.458193	0.0144209	0.0297128	-3.950232
NAP1L4	-0.027446	6.6181477	-2.458171	0.0144218	0.0297129	-3.950284
STAG3L5P-F	-0.095363	4.8935847	-2.458083	0.0144253	0.0297184	-3.950496
RNF139-AS1	0.1448748	4.2623806	2.4579839	0.0144292	0.0297248	-3.950734
GSTTP2	-0.2927	-0.983593	-2.457941	0.0144309	0.0297267	-3.950838
BNIP3P9	-0.448247	-0.502381	-2.457879	0.0144333	0.02973	-3.950986
RP11-108L7	0.460836	1.5640373	2.4578099	0.0144361	0.0297339	-3.951152
RP5-874C20	0.4550067	0.0160447	2.457587	0.0144449	0.0297504	-3.951687
RP11-557C1	-0.300584	-1.013091	-2.457106	0.0144639	0.0297871	-3.952841
LMBR1	-0.041729	6.1586889	-2.457096	0.0144643	0.0297871	-3.952865
RP11-561N1	-0.17158	-1.369346	-2.457072	0.0144653	0.0297873	-3.952923
NDNF	0.5127228	0.7607605	2.4569831	0.0144688	0.0297929	-3.953137
AC092171.2	0.3747489	-0.22692	2.4566467	0.0144821	0.0298187	-3.953945
JRK	0.0723598	5.6196446	2.4562457	0.014498	0.0298498	-3.954907
TMEM9	-0.047195	6.6971082	-2.455989	0.0145082	0.029869	-3.955522
CWH43	-0.54444	0.0337131	-2.45591	0.0145114	0.0298738	-3.955712
C20orf24	-0.054891	6.2071833	-2.455648	0.0145218	0.0298936	-3.956341
RP11-13601	-0.483933	2.2151339	-2.455514	0.0145271	0.0299028	-3.956663
RP11-229P1	-0.207949	-1.247154	-2.45534	0.014534	0.0299154	-3.957081
RP11-24C3.	-0.267516	-1.031031	-2.45532	0.0145348	0.0299154	-3.957129
CTC-260E6.	0.3986879	-0.550628	2.4548324	0.0145542	0.0299536	-3.958299
AC007349.5	-0.16186	-1.383452	-2.454609	0.0145631	0.0299702	-3.958834
RP11-355N1	-0.449119	1.0115204	-2.454239	0.0145779	0.0299989	-3.959721
SNRNP200	-0.02701	6.8152247	-2.453685	0.0146	0.0300427	-3.961049
AC015849.1	0.3611612	2.5529991	2.4535828	0.0146041	0.0300494	-3.961295
LINC01370	0.7715675	2.6497141	2.4533706	0.0146125	0.0300652	-3.961804
HARBI1	-0.059439	5.0563852	-2.452827	0.0146343	0.0301082	-3.963107
AC107057.1	-0.107751	-1.492806	-2.452529	0.0146462	0.0301311	-3.963822
RP11-1336C	0.3145338	-0.996093	2.4523942	0.0146516	0.030138	-3.964144
RP5-106502	-0.381144	-0.865439	-2.452392	0.0146517	0.030138	-3.964149
REV3L	0.0730739	5.7288581	2.4523824	0.0146521	0.030138	-3.964173

DLX3	0.5291014	-0.182025	2.452352	0.0146533	0.0301388	-3.964246
RP11-65M17	0.3228699	-0.952602	2.4521366	0.0146619	0.0301549	-3.964762
IGBP1P3	-0.097313	-1.497786	-2.451963	0.0146689	0.0301674	-3.965177
RP11-554A1	0.5826532	1.6548125	2.4516642	0.0146809	0.0301904	-3.965894
AC005052.1	-0.406533	0.665246	-2.4513	0.0146955	0.0302164	-3.966767
AC090627.1	0.2417461	-1.219831	2.4512902	0.0146959	0.0302164	-3.96679
BZRAP1	0.1472829	5.2356762	2.4512874	0.014696	0.0302164	-3.966796
RP11-520H1	0.4117662	2.2863676	2.4510754	0.0147045	0.0302322	-3.967304
RP11-262H1	0.1982258	-1.289358	2.450735	0.0147182	0.0302584	-3.968119
NPR2	-0.132962	5.8695658	-2.450716	0.014719	0.0302584	-3.968166
RP11-227G1	-0.262219	-1.073934	-2.450697	0.0147197	0.0302584	-3.96821
RP11-177B4	0.4695883	0.6397684	2.4503971	0.0147318	0.0302815	-3.968929
AL513327.1	0.374007	-0.294591	2.4503517	0.0147336	0.0302835	-3.969037
RP11-434P1	0.2648107	-0.990079	2.4502549	0.0147375	0.0302899	-3.969269
GGT2	0.4502479	-0.098705	2.4500569	0.0147455	0.0303034	-3.969743
ZNF146	0.0336438	6.3745712	2.4500506	0.0147457	0.0303034	-3.969759
AC010243.1	-0.195588	-1.292569	-2.449765	0.0147573	0.0303253	-3.970443
PLEKHJ1	-0.05384	6.3773889	-2.449644	0.0147621	0.030332	-3.970731
CTB-5506.8	-0.129138	5.5319038	-2.449642	0.0147622	0.030332	-3.970736
RP11-143E2	-0.314632	-1.204045	-2.449417	0.0147713	0.030349	-3.971274
RP11-13E1.	-0.141508	-1.428913	-2.449242	0.0147783	0.0303618	-3.971694
RP11-789C1	0.5369757	-0.370061	2.4491851	0.0147806	0.0303648	-3.97183
RP11-16K12	0.4920336	0.3963935	2.4491347	0.0147827	0.0303673	-3.971951
RAD54L	0.2140759	4.5420446	2.4489662	0.0147895	0.0303796	-3.972354
C2CD2	0.0687567	5.88701	2.4481628	0.014822	0.0304446	-3.974277
RP11-168P8	-0.091562	-1.500349	-2.448044	0.0148267	0.0304527	-3.97456
TFAP2A	0.4154297	3.5890483	2.4479734	0.0148296	0.0304569	-3.97473
TMEM91	0.1170837	5.1423254	2.4473405	0.0148553	0.0305078	-3.976244
RP11-1198D	-0.13128	-1.445436	-2.447299	0.014857	0.0305096	-3.976344
RP11-680F2	0.2364814	-1.230486	2.4471299	0.0148638	0.0305205	-3.976747
RP1-209A6.	-0.538926	0.1949827	-2.447126	0.014864	0.0305205	-3.976756
DPP9	-0.036385	6.5960077	-2.4468	0.0148772	0.030546	-3.977536
RP4-778K6.	0.4054583	0.6245244	2.4460421	0.014908	0.0306075	-3.979348
RP11-85501	0.3092901	-1.033665	2.4456377	0.0149245	0.0306396	-3.980315
TRAM1	-0.04367	6.9596573	-2.445128	0.0149452	0.0306799	-3.981533
RP11-775D2	0.3188168	-0.883554	2.4451143	0.0149458	0.0306799	-3.981566
HIST2H4A	-0.338787	2.9367637	-2.44503	0.0149492	0.0306853	-3.981768
APOA4	0.5253675	4.8578822	2.4448333	0.0149572	0.0307	-3.982237
RP4-754E20	-0.643607	1.161271	-2.444796	0.0149588	0.0307014	-3.982326
SRRM5	0.2126997	3.5036602	2.4447738	0.0149597	0.0307015	-3.98238
TUB-AS1	-0.129223	-1.448972	-2.444689	0.0149631	0.0307069	-3.982582
IGHV3OR16-	0.2223871	-1.29347	2.444523	0.0149699	0.0307175	-3.982979
CA8	0.4357358	2.5628054	2.4445207	0.01497	0.0307175	-3.982984
C8orf58	0.0838472	4.9045479	2.4442866	0.0149796	0.0307354	-3.983543
RP11-495P1	-0.67207	1.9813515	-2.444198	0.0149832	0.0307411	-3.983755
FAM13A-AS1	0.1842491	3.8986002	2.444166	0.0149845	0.0307421	-3.983832
TXNDC12-AS	-0.217348	-1.200442	-2.444121	0.0149863	0.0307441	-3.983939
ZNF319	0.0480579	5.637565	2.4440858	0.0149878	0.0307453	-3.984023
RP11-81H14	-0.608726	1.0229238	-2.443689	0.015004	0.0307768	-3.98497
OR51Q1	-0.166204	-1.38299	-2.443593	0.0150079	0.0307832	-3.985201
UBOX5-AS1	0.3495095	2.0064156	2.4434379	0.0150143	0.0307945	-3.985571

AC007405.8	0.2354437	-1.228924	2.4431061	0.0150278	0.0308206	-3.986363
RP11-248J1	-0.196585	3.5480131	-2.442433	0.0150555	0.0308755	-3.987971
CAPN10-AS1	0.1727245	4.3310123	2.4422428	0.0150632	0.0308898	-3.988424
TRIO	0.0524879	6.2279066	2.4420315	0.0150719	0.0309058	-3.988928
CRYBB1	0.2555733	3.095683	2.4415997	0.0150897	0.0309405	-3.989959
RP11-108K1	0.3994476	0.2248904	2.4410101	0.0151139	0.0309885	-3.991365
KIF13B	0.0577776	6.1852975	2.4409843	0.015115	0.0309889	-3.991427
IQCE	0.0561524	5.8855521	2.4409268	0.0151174	0.030992	-3.991564
hsa-mir-45	0.2216112	-1.27966	2.4404567	0.0151367	0.03103	-3.992686
RPL31P28	-0.201792	-1.265614	-2.439804	0.0151637	0.0310834	-3.994242
RP11-717F1	0.4704407	0.8478357	2.4396444	0.0151702	0.0310952	-3.994623
CTSA	-0.046911	7.0198469	-2.439506	0.015176	0.0311052	-3.994953
NDUFAF7	-0.037069	5.7861553	-2.438647	0.0152115	0.0311763	-3.997001
RAB27A	0.0703649	6.1024393	2.4383598	0.0152234	0.0311989	-3.997685
ANGEL2	-0.041844	6.0432544	-2.438231	0.0152287	0.0312081	-3.997992
AC009120.6	0.1042752	4.6257568	2.4375782	0.0152558	0.0312618	-3.999548
RP5-1009E2	0.4676486	1.2854027	2.4374644	0.0152605	0.0312697	-3.999819
FAM219A	0.0401262	5.8506245	2.4374389	0.0152616	0.0312702	-3.999879
CTD-3220F1	0.3691561	-0.359403	2.437354	0.0152651	0.0312756	-4.000082
ENTPD1-AS1	0.1309777	4.0739236	2.4372263	0.0152704	0.0312832	-4.000386
IGLJ3	0.1945741	-1.348385	2.4372234	0.0152705	0.0312832	-4.000393
TPT1P12	-0.425823	-0.053881	-2.437179	0.0152724	0.0312852	-4.000498
GOLPH3L	-0.047135	6.1124702	-2.437126	0.0152746	0.031288	-4.000625
VWCE	-0.223473	5.5881342	-2.436908	0.0152837	0.0313032	-4.001144
RP4-545L17	-0.270372	2.697515	-2.436906	0.0152837	0.0313032	-4.001149
ZBTB20	-0.125861	4.3498268	-2.436733	0.015291	0.0313163	-4.001562
NPAT	0.056548	5.618665	2.4366062	0.0152962	0.0313253	-4.001863
DEGS2	0.3443721	3.7257543	2.436321	0.0153081	0.0313478	-4.002542
RP11-99J16	0.3573789	-0.626193	2.4362277	0.015312	0.031354	-4.002764
COCH	0.4764266	4.2240016	2.4361451	0.0153154	0.0313593	-4.002961
KRT39	0.5299141	0.3274813	2.4359749	0.0153225	0.0313721	-4.003366
SLC25A25	-0.097647	6.4366673	-2.435638	0.0153365	0.031399	-4.004167
HPSE2	0.4645666	0.1533294	2.4351708	0.015356	0.0314372	-4.005281
PSAP	-0.035568	7.4249336	-2.435145	0.0153571	0.0314376	-4.005341
CPE	0.1104124	6.2787795	2.4350237	0.0153622	0.0314463	-4.005631
CTD-2374C2	-0.603261	0.6553103	-2.434901	0.0153673	0.031455	-4.005922
CRYGD	-0.10457	-1.506376	-2.434685	0.0153763	0.0314717	-4.006436
RP11-727A2	-0.125109	4.2948767	-2.434412	0.0153878	0.0314933	-4.007087
RP11-346C1	-0.232146	-1.192546	-2.434385	0.0153889	0.0314935	-4.00715
H2AFZ	-0.046963	6.6271887	-2.434369	0.0153896	0.0314935	-4.007189
AE000658.2	0.3563122	-1.070259	2.4343117	0.0153919	0.0314966	-4.007325
TAC4	0.425003	1.2092964	2.4341724	0.0153978	0.0315067	-4.007656
AC019129.2	-0.490334	0.517854	-2.434108	0.0154005	0.0315094	-4.007809
RP11-5809.	0.3711373	-0.479108	2.4341007	0.0154008	0.0315094	-4.007827
TRPM8	-0.302719	5.5542259	-2.433924	0.0154082	0.0315227	-4.008248
MAF	-0.055663	6.3223917	-2.433875	0.0154102	0.0315251	-4.008364
RP11-419C1	-0.426471	-0.738788	-2.43379	0.0154138	0.0315307	-4.008567
AF131217.1	0.4746669	1.3229803	2.4334894	0.0154264	0.0315542	-4.009281
DTYMK	-0.057633	6.1283256	-2.433474	0.015427	0.0315542	-4.009317
RAD52	0.0643424	5.3162276	2.433431	0.0154288	0.0315561	-4.00942
MIRLET7D	0.4304321	0.3052065	2.4332267	0.0154374	0.0315719	-4.009906

RP11-57G10	-0.156771	-1.386279	-2.432815	0.0154547	0.0316055	-4.010886
RP11-753H1	0.4156939	0.0159029	2.4325916	0.0154641	0.0316229	-4.011416
TAPT1-AS1	0.1404337	4.1842932	2.4324028	0.015472	0.0316362	-4.011865
LRRC37A	0.3514785	2.9581696	2.4323955	0.0154723	0.0316362	-4.011883
RP11-378E1	0.3246911	-0.662578	2.4319822	0.0154897	0.03167	-4.012865
RP11-177J6	0.4001685	0.238942	2.4316602	0.0155032	0.0316959	-4.013631
RP11-318A1	-0.406829	1.7390416	-2.431552	0.0155078	0.0317034	-4.013887
GPX1P2	-0.350661	2.2807134	-2.431489	0.0155104	0.0317071	-4.014037
GABRA5	0.3971805	-0.908307	2.4314194	0.0155134	0.0317113	-4.014203
ARRDC1	-0.041241	6.2005252	-2.430993	0.0155314	0.0317463	-4.015216
KRT8P14	0.3872926	-0.181	2.4309648	0.0155325	0.0317469	-4.015283
EMBP1	0.2119504	4.5929421	2.4308226	0.0155385	0.0317558	-4.015621
RP11-963H1	-0.148333	-1.408017	-2.430821	0.0155386	0.0317558	-4.015625
LINGO2	0.3331597	-1.001715	2.4307642	0.015541	0.0317589	-4.01576
ABCF2	-0.037307	6.2894215	-2.430527	0.015551	0.0317776	-4.016324
ASS1P6	-0.211827	-1.255489	-2.430455	0.0155541	0.0317817	-4.016495
ESR1	-0.266685	4.9001298	-2.430438	0.0155548	0.0317817	-4.016536
RGS3	0.0602343	6.3199132	2.4300423	0.0155715	0.0318141	-4.017475
DISC1-IT1	-0.114992	-1.45976	-2.429936	0.015576	0.0318215	-4.017728
LIN28A	0.363629	-0.925691	2.4298728	0.0155787	0.0318252	-4.017878
RP11-92A5.	-0.16668	-1.426383	-2.429838	0.0155801	0.0318264	-4.017961
PARPBP	0.1445405	4.9500185	2.4296988	0.015586	0.0318366	-4.018291
MIR320A	-0.178823	-1.36642	-2.429414	0.0155981	0.0318595	-4.018968
RP11-109N2	-0.348589	1.7243693	-2.429373	0.0155998	0.0318612	-4.019065
UGDH	-0.067005	6.7502481	-2.429292	0.0156033	0.0318665	-4.019258
GALNTL6	0.5935521	0.1576933	2.4290335	0.0156142	0.031887	-4.019871
PLCXD1	0.096534	6.0728224	2.4290103	0.0156152	0.0318872	-4.019926
RP6-191P20	0.239653	-1.22494	2.4286654	0.0156298	0.0319153	-4.020745
RP11-453E1	0.2350446	-1.12415	2.4279403	0.0156606	0.0319764	-4.022466
CD209	0.1548487	5.0551501	2.4274787	0.0156802	0.0320131	-4.023561
CTD-2325A1	-0.388343	-0.563221	-2.427476	0.0156803	0.0320131	-4.023567
CHD4	-0.024975	6.7843388	-2.427245	0.0156901	0.0320313	-4.024115
RP11-89K10	0.4141546	2.0113999	2.4272018	0.015692	0.0320333	-4.024218
RBMXP1	-0.484349	-0.061696	-2.427167	0.0156935	0.0320346	-4.024302
RP11-14C10	-0.301271	3.77642	-2.42681	0.0157086	0.0320638	-4.025147
ENPP2	0.1216772	6.256179	2.426609	0.0157172	0.0320795	-4.025625
RP11-831H9	0.224505	-1.165976	2.4265464	0.0157199	0.0320831	-4.025773
TBC1D19	0.0964896	4.8458588	2.4257504	0.0157539	0.0321507	-4.027661
SSH1	0.0391248	6.1745966	2.4254953	0.0157648	0.0321711	-4.028266
ADRA2C	0.3381761	4.5334444	2.4253657	0.0157703	0.0321806	-4.028573
IFNAR2	0.0427836	5.8819229	2.424806	0.0157942	0.0322277	-4.0299
SLC9A3P2	-0.219419	-1.31775	-2.424758	0.0157963	0.03223	-4.030013
RP11-1080G	0.3767854	-0.133158	2.4247316	0.0157974	0.0322306	-4.030077
RN7SKP97	0.4010546	-0.197478	2.4244294	0.0158104	0.0322552	-4.030793
RPS15AP1	-0.138977	4.1351657	-2.424186	0.0158208	0.0322746	-4.03137
RPH3A	0.4278907	0.2829106	2.4241094	0.0158241	0.0322795	-4.031551
NPM1P10	-0.115335	-1.480943	-2.424069	0.0158258	0.0322813	-4.031647
PIGC	-0.042182	6.2385735	-2.424021	0.0158279	0.0322836	-4.03176
RP11-383C5	0.3962055	0.0978978	2.4238997	0.0158331	0.0322925	-4.032048
FREM2	0.7217383	1.973158	2.4238538	0.015835	0.0322947	-4.032157
ZNF84	0.0440191	5.8632796	2.4237302	0.0158403	0.0323037	-4.03245

FRY	-0.072423	6.1011349	-2.423672	0.0158429	0.032307	-4.032588
MDFIC	0.1200523	5.7946673	2.4235202	0.0158494	0.0323185	-4.032947
DPH6	-0.054629	5.4580057	-2.423342	0.015857	0.0323323	-4.033369
CLIP1-AS1	-0.382678	-0.165947	-2.423206	0.0158629	0.0323424	-4.033693
RP11-829H1	0.5170493	0.7537325	2.4226722	0.0158858	0.0323874	-4.034956
RP11-1072C	-0.397151	1.0728375	-2.422582	0.0158897	0.0323935	-4.03517
TAMM41	-0.050738	5.3567442	-2.422557	0.0158908	0.0323939	-4.03523
HIST1H4B	-0.417037	-0.220238	-2.422479	0.0158941	0.0323989	-4.035413
TRUB1	0.0583045	5.7953998	2.4219722	0.015916	0.0324416	-4.036614
XXbac-BPG2	-0.119892	-1.473774	-2.421848	0.0159213	0.0324507	-4.036909
LRRC19	0.5182627	1.8911059	2.4218006	0.0159234	0.032453	-4.03702
AC005355.2	-0.222371	4.7910301	-2.421756	0.0159253	0.0324552	-4.037127
DVL2	0.0484907	6.0117485	2.4217322	0.0159263	0.0324554	-4.037182
PAEP	0.7207623	1.1202135	2.421578	0.015933	0.0324671	-4.037547
PITX3	0.2664505	-1.097572	2.4215233	0.0159353	0.0324701	-4.037676
RP4-568F9.	0.3215239	-1.019114	2.4214183	0.0159398	0.0324776	-4.037925
NET1	-0.043618	6.3817871	-2.421364	0.0159422	0.0324805	-4.038052
CASP1	0.0949914	5.5985048	2.4208936	0.0159625	0.0325201	-4.039167
RP11-45A16	-0.302184	-1.064677	-2.420541	0.0159777	0.0325493	-4.040001
FAT1	-0.08231	6.6322826	-2.419998	0.0160012	0.0325954	-4.041286
PPIF	-0.051331	6.6901347	-2.419843	0.0160079	0.0326072	-4.041652
RP11-431M7	-0.126237	-1.461447	-2.419352	0.0160292	0.0326482	-4.042814
AF015720.3	-0.106449	-1.477227	-2.419338	0.0160298	0.0326482	-4.042848
LINC01043	-0.232482	-1.221164	-2.418852	0.0160509	0.0326893	-4.043996
AC009120.3	0.3673695	2.0683279	2.4188002	0.0160532	0.0326921	-4.044119
SOX9-AS1	0.34525	4.9723621	2.4186092	0.0160615	0.0327071	-4.044571
RP11-139H1	-0.467911	1.6097739	-2.418542	0.0160644	0.0327113	-4.04473
FOXD2	0.2022541	4.6849265	2.4185032	0.0160661	0.0327129	-4.044822
RP11-15A1.	-0.234172	3.4566164	-2.418414	0.0160699	0.0327189	-4.045032
RP11-379B1	-0.213914	-1.258143	-2.418316	0.0160742	0.032724	-4.045263
SULT1E1	-0.448633	4.5292198	-2.418316	0.0160742	0.032724	-4.045265
NPM1P32	0.3158321	-0.718587	2.4181596	0.016081	0.032736	-4.045634
GCNT1P4	0.327257	-0.899385	2.4179786	0.0160889	0.0327502	-4.046062
RP11-545N8	0.3052419	-0.852897	2.4176027	0.0161053	0.0327817	-4.04695
BAG3	0.0569697	6.2407442	2.4174681	0.0161111	0.0327918	-4.047269
SSXP9	-0.131517	-1.444303	-2.417417	0.0161133	0.0327944	-4.047389
RP1-96H9.5	-0.403021	-0.05379	-2.417188	0.0161233	0.032813	-4.047932
LIMD1-AS1	-0.387589	1.3526394	-2.417161	0.0161245	0.0328135	-4.047994
NDST2	0.0611877	4.5823194	2.4170614	0.0161288	0.0328205	-4.04823
GS1-72M22.	0.2631194	-1.192336	2.4168491	0.0161381	0.0328375	-4.048731
RP5-1052I5	-0.46155	0.0933706	-2.416576	0.01615	0.0328599	-4.049377
VAPA	-0.033218	6.6864188	-2.416515	0.0161527	0.0328635	-4.04952
TTC21A	0.0939103	4.6253108	2.4164944	0.0161536	0.0328635	-4.049569
CTD-3193K9	0.274564	-1.036665	2.4164486	0.0161556	0.0328658	-4.049678
RP11-739P1	0.2851444	-1.022721	2.4160084	0.0161748	0.0329024	-4.050717
TMEM88	-0.122788	4.9197151	-2.415996	0.0161754	0.0329024	-4.050747
RP11-655G2	0.2689298	-1.174162	2.4159152	0.0161789	0.0329077	-4.050938
RN7SL566P	0.323739	-0.684374	2.4155263	0.0161959	0.0329405	-4.051856
LINC00313	0.5201832	1.685485	2.4154521	0.0161991	0.0329452	-4.052031
ACBD7	0.4831703	1.7752609	2.4150879	0.0162151	0.0329741	-4.052891
YWHAEP5	0.3985863	0.4404794	2.4150869	0.0162151	0.0329741	-4.052894

CPSF1P1	0.5985084	2.1572773	2.4150149	0.0162183	0.0329786	-4.053064
RP5-1039K5	0.1774296	-1.315262	2.4148522	0.0162254	0.0329913	-4.053448
SLC25A47	-0.350487	5.7575841	-2.414534	0.0162394	0.0330177	-4.054199
RAB6C	0.3597672	-0.345379	2.4145153	0.0162402	0.0330177	-4.054243
COX6A1P2	-0.113698	4.3931033	-2.414474	0.016242	0.0330195	-4.05434
SLC28A2	-0.428631	3.0127551	-2.414306	0.0162494	0.0330327	-4.054738
IGKV1OR10-	0.2381371	-1.253599	2.4142403	0.0162523	0.0330367	-4.054892
KRTAP5-8	-0.394329	-0.318462	-2.413709	0.0162756	0.0330823	-4.056147
DPH2	-0.046685	6.0514121	-2.413572	0.0162816	0.0330927	-4.05647
MIR1302-3	-0.2732	-1.034338	-2.413186	0.0162986	0.0331254	-4.057381
C2orf44	-0.044817	5.6328635	-2.412904	0.016311	0.0331487	-4.058046
ZNF257	0.4559195	2.9970633	2.4122941	0.0163379	0.0332015	-4.059484
CTC-490E21	-0.276497	3.0762173	-2.411831	0.0163583	0.0332412	-4.060576
AC008746.3	0.2263154	-1.22944	2.4117915	0.0163601	0.0332429	-4.06067
CRB3	-0.179216	5.7957257	-2.411708	0.0163638	0.0332485	-4.060866
KRTAP5-7	-0.50767	0.1802361	-2.411652	0.0163662	0.0332517	-4.060998
RP11-794M8	-0.225215	-1.176808	-2.411227	0.016385	0.033288	-4.062
AP000439.5	-0.253075	-1.189597	-2.410976	0.0163961	0.0333087	-4.062593
TBC1D26	-0.211247	-1.347407	-2.410895	0.0163997	0.0333141	-4.062783
SIPA1	-0.044095	6.3506484	-2.41081	0.0164035	0.0333199	-4.062984
TBCEL	0.0575964	5.961875	2.409936	0.0164422	0.0333967	-4.065044
RFX7	0.0505333	5.6847895	2.4097994	0.0164483	0.0334072	-4.065366
RP11-736N1	0.3179486	-0.897722	2.4095085	0.0164612	0.0334315	-4.066051
ANGPT2	-0.107386	5.4208067	-2.409442	0.0164641	0.0334357	-4.066208
RP11-395L1	0.0879761	4.6537687	2.4093195	0.0164696	0.0334449	-4.066496
LINC01419	-0.856313	2.1066917	-2.409266	0.0164719	0.0334463	-4.066623
TSPAN4	-0.062654	6.3882508	-2.409262	0.0164721	0.0334463	-4.066631
IL13RA2	0.4460071	2.6707361	2.4091934	0.0164752	0.0334507	-4.066793
RP11-90B9.	0.3881854	0.7081132	2.4090732	0.0164805	0.0334596	-4.067076
FAM212A	0.1221277	4.5338859	2.4089076	0.0164879	0.0334727	-4.067466
FDPSP5	-0.320525	-0.669745	-2.408782	0.0164935	0.0334822	-4.067762
RP11-323I1	-0.308668	-0.904619	-2.408566	0.0165031	0.0334999	-4.068272
RP11-28002	-0.433898	0.7624775	-2.408187	0.0165199	0.0335322	-4.069163
SLC25A37	-0.065757	5.9094817	-2.408152	0.0165215	0.0335329	-4.069247
CTB-5409.9	-0.372973	-0.029321	-2.408138	0.0165221	0.0335329	-4.069278
LARS	-0.031842	6.5120981	-2.407796	0.0165373	0.033562	-4.070083
SNORD114-1	-0.092843	-1.504322	-2.407662	0.0165433	0.0335716	-4.070398
SCN4A	-0.219148	4.4569033	-2.407648	0.016544	0.0335716	-4.070432
ERN2	0.4879318	-0.229422	2.407485	0.0165512	0.0335845	-4.070816
ACVR2A	0.0469792	5.6626109	2.4073622	0.0165567	0.0335938	-4.071105
TMF1	0.0447149	6.0380936	2.4072909	0.0165599	0.0335984	-4.071273
CTD-2003D5	0.3456366	-0.67789	2.4070174	0.0165721	0.0336213	-4.071917
RP13-131K1	-0.395994	1.6534418	-2.406693	0.0165866	0.0336488	-4.07268
SH3GLB1	0.0292614	6.4187973	2.4066369	0.0165891	0.033652	-4.072812
RP11-644F5	-0.187481	-1.293581	-2.406464	0.0165968	0.0336658	-4.073219
RP11-527L4	0.2988235	-1.123083	2.4055428	0.0166381	0.0337475	-4.075386
ZNF446	-0.048258	5.4949424	-2.405523	0.0166389	0.0337475	-4.075433
EEF1A1P14	0.3502986	2.6383498	2.405448	0.0166423	0.0337525	-4.075609
HSP90AB1	-0.037491	7.3600691	-2.405298	0.016649	0.0337642	-4.075961
PTCHD4	-0.459198	3.2900914	-2.405155	0.0166555	0.0337754	-4.076299
AC011290.4	-0.347876	-0.685091	-2.404731	0.0166745	0.0338121	-4.077295

DNAJB5-AS1	-0.436898	-0.018608	-2.404679	0.0166768	0.033815	-4.077418
TSEN2	-0.051509	5.4811662	-2.404357	0.0166913	0.0338424	-4.078175
OR52N3P	-0.125261	-1.455006	-2.404211	0.0166978	0.0338538	-4.078518
ZNF846	-0.069649	5.2695473	-2.403684	0.0167215	0.0339	-4.079757
RP11-377K2	-0.244919	-1.182725	-2.403556	0.0167273	0.0339098	-4.080058
PRC1	0.0994838	5.8563806	2.4032078	0.016743	0.0339397	-4.080876
SLC9B1	0.170725	3.3173889	2.4030066	0.0167521	0.0339562	-4.081349
AC004538.3	-0.581516	2.0904794	-2.402661	0.0167677	0.0339859	-4.08216
AC002398.9	0.3103535	-0.785686	2.4024072	0.0167791	0.0340073	-4.082757
CIDECP	-0.057944	5.5279839	-2.402151	0.0167907	0.0340289	-4.08336
SMPD4	0.0337717	6.3068114	2.4017373	0.0168094	0.0340648	-4.084331
RN7SKP68	-0.117394	-1.46452	-2.401647	0.0168135	0.0340712	-4.084542
PLXNB2	-0.04219	7.0084015	-2.401554	0.0168177	0.0340772	-4.08476
LLNLF-18A1	-0.136973	-1.451661	-2.40154	0.0168183	0.0340772	-4.084794
RPL41	-0.048203	7.3018999	-2.40152	0.0168192	0.0340772	-4.084841
ZKSCAN3	0.0763425	5.5376442	2.4012995	0.0168292	0.0340955	-4.085359
RP11-344P1	-0.301006	3.865306	-2.401273	0.0168304	0.034096	-4.08542
RP11-753B1	-0.531809	1.4804908	-2.401019	0.0168419	0.0341174	-4.086017
NEK5	-0.143321	3.9251477	-2.400429	0.0168686	0.0341697	-4.087402
RP11-128B1	-0.188534	-1.309841	-2.40018	0.0168799	0.0341907	-4.087986
EFCAB14	-0.032784	6.5177977	-2.40013	0.0168822	0.0341934	-4.088103
ZRANB2-AS1	0.3425418	-0.457863	2.4001019	0.0168835	0.0341941	-4.08817
CTD-2536I1	0.3640794	-0.484549	2.3998977	0.0168928	0.034211	-4.088649
MRAS	0.0939247	5.7998379	2.3998699	0.016894	0.0342117	-4.088715
RP11-681L4	-0.282413	-0.906942	-2.399697	0.0169019	0.0342257	-4.089121
SIGLEC14	0.56451	3.0704462	2.3988198	0.0169418	0.0343046	-4.091178
EFCC1	0.1514486	4.3465615	2.398412	0.0169604	0.0343403	-4.092135
RP3-496C20	-0.385667	-0.16314	-2.398303	0.0169653	0.0343485	-4.092391
KATNA1	0.0433842	5.6696383	2.3981923	0.0169704	0.034356	-4.09265
RP11-400L8	0.3841386	0.1937185	2.3981796	0.016971	0.034356	-4.09268
HNRNPA3P10	0.382199	1.0083118	2.3979444	0.0169817	0.0343755	-4.093232
ANP32E	-0.03828	6.3861219	-2.397927	0.0169825	0.0343755	-4.093272
RP11-530N7	-0.481484	1.7838038	-2.39763	0.0169961	0.0344006	-4.093969
AL021918.1	-0.08578	-1.505565	-2.397615	0.0169967	0.0344006	-4.094004
GAGE13	-0.103972	-1.484296	-2.39713	0.0170189	0.0344435	-4.09514
RP11-35J10	-0.125493	-1.447875	-2.397046	0.0170227	0.0344494	-4.095339
AF064858.8	0.423229	1.6645637	2.3966291	0.0170418	0.0344861	-4.096315
CTSG	0.5755815	2.1314268	2.3963952	0.0170525	0.0345058	-4.096863
DCAF15	0.0406849	5.9975359	2.396371	0.0170536	0.0345061	-4.09692
FAHD2P1	0.2242024	-1.266614	2.396318	0.017056	0.0345088	-4.097044
RP11-465K1	0.2151049	-1.234182	2.3963014	0.0170568	0.0345088	-4.097083
DLGAP3	0.2226265	3.396176	2.3959201	0.0170743	0.0345422	-4.097977
RP11-571I1	0.3794914	1.1391634	2.395822	0.0170788	0.0345494	-4.098207
RP3-388E23	0.4390218	0.9306042	2.3956511	0.0170866	0.0345633	-4.098607
LUC7L3	-0.028706	6.4655932	-2.395534	0.017092	0.0345723	-4.09888
FKBP7	-0.07493	5.3802077	-2.395413	0.0170975	0.0345816	-4.099165
C10orf76	-0.033674	6.0952177	-2.39539	0.0170986	0.0345819	-4.09922
RP11-44503	0.2945357	-1.146956	2.3952985	0.0171028	0.0345876	-4.099433
IER5	0.0568664	6.0382947	2.3952868	0.0171033	0.0345876	-4.09946
RP11-324C1	-0.26288	-1.148	-2.39526	0.0171046	0.0345882	-4.099524
RP11-818F2	0.3958766	-0.558995	2.395083	0.0171127	0.0346027	-4.099938

FBXW9	-0.058763	5.473823	-2.394066	0.0171595	0.0346953	-4.102319
CDH5	0.0756665	6.1716446	2.3938723	0.0171684	0.0347114	-4.102773
CBY3	-0.40272	0.8843173	-2.393852	0.0171693	0.0347114	-4.102821
BASP1P1	-0.201057	-1.336091	-2.393734	0.0171748	0.0347205	-4.103096
AC069294.1	0.4040767	-0.446509	2.3935023	0.0171855	0.0347402	-4.103639
ARHGAP21	0.0480004	6.2121696	2.3933958	0.0171904	0.0347469	-4.103888
CTD-3032J1	0.2132432	-1.203076	2.3933883	0.0171907	0.0347469	-4.103906
CLEC1A	0.1591502	4.373024	2.3926636	0.0172242	0.0348126	-4.105602
RP11-462G2	-0.307995	-0.879953	-2.392609	0.0172267	0.0348149	-4.10573
ZCCHC17	-0.038784	5.9644874	-2.392599	0.0172272	0.0348149	-4.105754
RN7SKP51	-0.095636	-1.488389	-2.392457	0.0172337	0.0348262	-4.106085
TPT1P3	-0.161747	-1.378867	-2.392299	0.017241	0.034839	-4.106455
PRR5-ARHGA	0.3929073	-0.392096	2.3921415	0.0172483	0.0348499	-4.106823
RP11-419C1	-0.201358	-1.353651	-2.392141	0.0172483	0.0348499	-4.106824
TFPT	-0.058194	5.8020803	-2.391934	0.0172579	0.0348673	-4.107308
RP11-507B1	-0.44889	-0.231754	-2.391898	0.0172596	0.0348688	-4.107393
RIN3	0.0659048	6.118475	2.3917803	0.017265	0.0348778	-4.107668
HNRNPA1P49	0.4359761	0.5776719	2.3916581	0.0172707	0.0348873	-4.107954
SNX4	-0.038527	6.3280388	-2.391489	0.0172785	0.0349012	-4.108349
WI2-89031E	0.4018751	-0.256641	2.3913437	0.0172853	0.0349129	-4.108689
PROSER2	-0.087588	6.1300255	-2.391104	0.0172964	0.0349334	-4.109249
AC140542.2	-0.115052	-1.456539	-2.390825	0.0173093	0.0349564	-4.109903
ASUN	0.0443567	5.9174256	2.3908172	0.0173097	0.0349564	-4.109921
TOPORSLP	-0.218789	-1.253881	-2.390698	0.0173152	0.0349657	-4.1102
STRIP1	-0.044376	5.9689932	-2.390445	0.017327	0.0349875	-4.110791
CTD-2015A6	-0.24841	-1.189763	-2.390268	0.0173352	0.0350021	-4.111204
FARSB	-0.039462	6.2533367	-2.390192	0.0173387	0.0350073	-4.111383
CNTNAP2	0.3894329	4.9841624	2.3900651	0.0173446	0.0350173	-4.111679
RP11-521C2	0.1473351	4.1382645	2.3899559	0.0173497	0.0350256	-4.111934
PCSK5	0.140731	5.3876307	2.3899079	0.0173519	0.0350282	-4.112046
GPR111	0.2717976	-1.164184	2.389856	0.0173543	0.0350311	-4.112168
ZMAT1	-0.152072	5.3236422	-2.389801	0.0173569	0.0350343	-4.112296
PCGF5	-0.040819	6.4241339	-2.389419	0.0173747	0.0350682	-4.113188
CTB-31020.	0.3162683	-0.749951	2.3890492	0.0173919	0.0351011	-4.114053
SERPINB11	0.3363527	-1.060045	2.3890269	0.0173929	0.0351013	-4.114105
TTTY16	-0.150876	-1.431187	-2.388843	0.0174015	0.0351166	-4.114535
RP11-466G1	-0.326804	-0.684182	-2.388796	0.0174037	0.0351177	-4.114645
AC104809.4	0.5407469	2.2639368	2.3887904	0.017404	0.0351177	-4.114658
PHC3	0.0486714	5.8982798	2.3884896	0.017418	0.0351441	-4.115361
AL122127.2	0.2605387	-1.187827	2.3880707	0.0174375	0.0351816	-4.116339
SMIM23	0.2657476	-1.091112	2.3879892	0.0174414	0.0351868	-4.116529
NLRP14	-0.512117	1.1156233	-2.387974	0.0174421	0.0351868	-4.116566
RP11-334L9	0.2855017	2.829244	2.3879486	0.0174433	0.0351872	-4.116624
RP11-557H1	0.4213734	2.2939173	2.3878718	0.0174468	0.0351919	-4.116804
RNU6-450P	-0.183792	-1.325205	-2.387858	0.0174475	0.0351919	-4.116835
NELL1	0.396171	-0.863594	2.3875021	0.0174641	0.0352235	-4.117667
PSMC1P10	-0.457078	-0.125414	-2.387289	0.0174741	0.0352416	-4.118164
MIR5707	-0.212386	-1.355711	-2.38689	0.0174928	0.0352774	-4.119097
PUS7	0.0577646	5.7694474	2.386101	0.0175298	0.03535	-4.120938
IGHV4OR15-	0.2163803	-1.269456	2.3860371	0.0175328	0.0353541	-4.121087
ITIH6	0.3521757	-0.829281	2.3858285	0.0175426	0.0353719	-4.121574

CPD	0.0508985	6.8081864	2.3856899	0.0175491	0.0353831	-4.121897
RP3-412A9.	0.3233677	2.7995977	2.3856541	0.0175507	0.0353845	-4.121981
MAATS1	-0.314083	4.1377876	-2.385584	0.017554	0.0353892	-4.122143
AP001048.4	-0.17759	-1.357876	-2.385543	0.017556	0.0353912	-4.12224
RP11-685M7	0.3445474	1.8940223	2.384615	0.0175996	0.0354772	-4.124404
MAD2L1	0.10248	5.487493	2.3844206	0.0176088	0.0354933	-4.124858
RP11-723D2	0.4180381	0.0060334	2.3844041	0.0176095	0.0354933	-4.124896
KIAA1958	-0.066327	5.7518878	-2.384354	0.0176119	0.0354961	-4.125012
TIPARP	-0.056621	6.0312723	-2.384311	0.017614	0.0354983	-4.125114
RP5-837J1.	0.3385829	-0.576401	2.3842144	0.0176185	0.0355054	-4.125339
LY6K	0.515566	1.4563324	2.3839973	0.0176287	0.0355241	-4.125845
MIPEPP3	0.2581056	2.9707728	2.3837698	0.0176395	0.0355438	-4.126375
RP11-360O1	0.2152294	-1.30102	2.3837292	0.0176414	0.0355457	-4.12647
FBF1	-0.091188	5.0007632	-2.383535	0.0176506	0.0355622	-4.126923
RP11-110I1	-0.302852	1.9875234	-2.383186	0.017667	0.0355934	-4.127736
EIF4A1P6	-0.151694	-1.395656	-2.383072	0.0176724	0.0356023	-4.128003
SNX18P14	-0.182916	-1.361447	-2.383042	0.0176738	0.0356032	-4.128071
CIC	0.033204	6.4627107	2.3827259	0.0176888	0.0356304	-4.128809
RP5-1014D1	0.080368	4.5774559	2.3827147	0.0176893	0.0356304	-4.128835
RABL6	0.0447369	6.2112416	2.3825589	0.0176967	0.0356433	-4.129198
MPV17L	-0.149601	6.1411038	-2.382301	0.0177089	0.0356659	-4.129798
SUMO1P1	0.225224	-1.268338	2.3820049	0.0177229	0.0356922	-4.130489
NUDT3	-0.037877	6.001036	-2.381953	0.0177254	0.0356952	-4.13061
ANGPTL5	0.2023806	-1.321306	2.3812119	0.0177605	0.035763	-4.132336
KCNK9	0.6688036	1.5238468	2.3812023	0.017761	0.035763	-4.132359
PLCH2	0.27186	4.8717368	2.3809907	0.017771	0.0357812	-4.132851
TREML4	0.504909	-0.1767	2.380925	0.0177741	0.0357855	-4.133004
ANKRD61	0.2790808	2.5754572	2.380743	0.0177828	0.035801	-4.133428
HNRNPA1P53	0.3237136	-0.624973	2.3806986	0.0177849	0.0358032	-4.133532
FUT10	0.0912351	4.9288064	2.3805835	0.0177904	0.0358115	-4.1338
RGS20	0.486029	1.2501075	2.3805542	0.0177918	0.0358115	-4.133868
RP11-217B1	-0.283253	2.5927715	-2.380551	0.0177919	0.0358115	-4.133876
C1orf110	0.2814235	-1.033669	2.3804873	0.0177949	0.0358155	-4.134024
MYO3B	-0.519844	2.0135601	-2.380235	0.0178069	0.0358377	-4.134611
MSH5	0.191827	4.6095011	2.3799613	0.01782	0.035862	-4.135248
MMD2	-0.151153	-1.418437	-2.379903	0.0178227	0.0358655	-4.135383
SDAD1	0.0648475	5.8776474	2.3797477	0.0178301	0.0358785	-4.135745
RP4-744I24	-0.178004	-1.340654	-2.379698	0.0178325	0.0358813	-4.135861
RP1-239B22	0.3215683	3.8145678	2.379061	0.0178629	0.0359404	-4.137344
ZNF547	0.1463558	4.3734962	2.3790258	0.0178646	0.0359418	-4.137426
RP11-442G2	0.3994143	0.2500007	2.3789391	0.0178687	0.0359481	-4.137627
EPHA5-AS1	0.3508254	-1.059094	2.3786111	0.0178844	0.0359777	-4.138391
FOXD3-AS1	0.3874651	-0.81563	2.3783643	0.0178961	0.0359994	-4.138965
RP11-113K2	-0.415162	1.5854043	-2.378152	0.0179063	0.0360178	-4.139458
RP11-1055B	-0.385225	-0.055768	-2.377975	0.0179147	0.0360328	-4.13987
RP11-768B2	0.2283836	-1.240024	2.3779332	0.0179168	0.0360349	-4.139968
LRRC26	0.5344865	0.2540715	2.3775436	0.0179354	0.0360704	-4.140874
RP11-93209	0.4317847	1.5558994	2.3774236	0.0179412	0.03608	-4.141153
RP11-379F1	-0.147696	-1.412502	-2.377401	0.0179422	0.0360801	-4.141205
KLHL28	0.0701408	5.507183	2.3772794	0.0179481	0.0360899	-4.141488
CKS1BP6	-0.239599	-1.138549	-2.377224	0.0179507	0.0360921	-4.141616

IGSF9	0.3615784	5.1380143	2.3772156	0.0179511	0.0360921	-4.141636
AC079466.1	0.7431671	2.8860325	2.3770509	0.017959	0.0361059	-4.142019
RBBP4P1	0.3959387	1.7329328	2.3767615	0.0179729	0.0361318	-4.142692
YPEL2	0.0590779	6.0785348	2.3765405	0.0179835	0.0361512	-4.143206
FRMD3	-0.156794	4.888668	-2.376454	0.0179876	0.0361573	-4.143407
CTD-2527I2	0.3902997	-0.329114	2.3764355	0.0179885	0.0361573	-4.14345
RP11-45I20	-0.152879	-1.418803	-2.376392	0.0179906	0.0361595	-4.14355
HIVEP2	0.0602908	5.7920064	2.3762249	0.0179986	0.0361737	-4.14394
PRSS44	0.4430708	-0.342311	2.3760045	0.0180092	0.036193	-4.144452
RNU6V	0.1773883	-1.298062	2.3751949	0.0180482	0.0362692	-4.146333
RP11-112J1	0.451705	1.3380344	2.3748479	0.0180649	0.0363008	-4.147139
RP1-39G22.	-0.074932	5.0636896	-2.374636	0.0180751	0.0363179	-4.147633
MYD88	-0.043292	6.3557819	-2.374631	0.0180754	0.0363179	-4.147644
CERK	-0.050242	6.4244935	-2.374335	0.0180896	0.0363445	-4.148331
RP11-806L2	0.3624827	-0.429922	2.3740239	0.0181046	0.0363727	-4.149053
CTB-113P19	0.3164395	-0.801954	2.3739153	0.0181099	0.0363812	-4.149306
METTL18	-0.057394	5.5071929	-2.373822	0.0181144	0.0363883	-4.149523
RELB	0.0670668	6.0772322	2.3735042	0.0181297	0.0364171	-4.15026
BBS2	-0.06185	5.9329279	-2.373264	0.0181413	0.036437	-4.150817
AURKB	0.1413807	5.3772979	2.3732583	0.0181416	0.036437	-4.150831
RPL37P2	-0.32062	2.7978537	-2.3732	0.0181444	0.0364406	-4.150966
LINC01355	0.1206798	4.7431894	2.3731152	0.0181485	0.0364468	-4.151163
HRASLS2	-0.572388	2.8511013	-2.372966	0.0181558	0.0364594	-4.15151
AKAP5	0.1604332	4.3723448	2.3729238	0.0181578	0.0364614	-4.151608
CXorf57	-0.180628	4.7291818	-2.372826	0.0181625	0.0364689	-4.151835
RP11-793H1	0.399768	0.3601078	2.372791	0.0181642	0.0364703	-4.151916
SPINK4	0.4861275	-0.392287	2.3721607	0.0181948	0.0365296	-4.153379
GFPT1	0.0391481	6.5310874	2.3716816	0.018218	0.0365743	-4.15449
POTENP	0.3644684	-0.315526	2.3716527	0.0182194	0.0365751	-4.154557
LINC00504	-0.422553	2.7976798	-2.371582	0.0182229	0.03658	-4.154721
RP11-452N1	-0.269188	2.8393207	-2.370974	0.0182524	0.0366373	-4.156132
IQCG	-0.07935	5.2228686	-2.37094	0.0182541	0.0366386	-4.156211
RP11-75N6.	-0.175796	-1.337145	-2.370747	0.0182634	0.0366554	-4.156658
ZNF585A	0.1043816	5.0101439	2.3706198	0.0182696	0.0366658	-4.156953
RP11-63L7.	-0.342048	-0.582866	-2.370469	0.018277	0.0366772	-4.157304
PLCH1	0.4217238	3.0877214	2.3704414	0.0182783	0.0366772	-4.157367
PEBP1P1	-0.143999	-1.399169	-2.370441	0.0182783	0.0366772	-4.157367
XRRA1	-0.074362	5.519668	-2.370203	0.0182899	0.0366984	-4.157919
ZNF346	-0.04118	5.6212724	-2.370003	0.0182996	0.036716	-4.158382
LGR5	0.6788066	3.4722641	2.3695892	0.0183198	0.0367545	-4.159343
RP11-56M3.	0.3911524	1.2917653	2.3692906	0.0183344	0.0367817	-4.160035
RP11-814P5	-0.124177	-1.459026	-2.369117	0.0183429	0.0367967	-4.160438
RBM19	-0.03246	6.1720527	-2.368772	0.0183597	0.0368285	-4.161237
RP5-1063M2	0.2288016	-1.2213	2.3687027	0.0183631	0.0368332	-4.161397
TTC9C	-0.03073	5.8117216	-2.368663	0.0183651	0.0368351	-4.161489
RP11-206L1	0.4122573	1.2905799	2.3685775	0.0183692	0.0368414	-4.161687
RYBP	-0.038773	6.2066403	-2.368166	0.0183894	0.0368798	-4.16264
MGRN1	-0.038659	6.5484714	-2.367183	0.0184376	0.0369744	-4.164919
MIR92B	-0.120916	-1.470412	-2.367114	0.0184409	0.0369791	-4.165078
RP11-255H2	0.5861448	1.9679744	2.3669842	0.0184473	0.0369899	-4.165378
HNRNPA1P33	0.2918796	-0.980274	2.3668911	0.0184519	0.036997	-4.165594

SLC31A2	0.2377777	4.1027481	2.3668144	0.0184556	0.0370025	-4.165771
LINC00242	-0.191966	4.649514	-2.36654	0.0184691	0.0370275	-4.166406
ATP8B1	0.0681284	6.2485062	2.3663967	0.0184762	0.0370396	-4.166738
CAV3	0.1969791	-1.284101	2.3657924	0.0185059	0.0370971	-4.168137
SIAH1	-0.038984	5.7292157	-2.365711	0.0185099	0.0371031	-4.168325
EEF1A1P33	0.3368092	-0.576576	2.3656341	0.0185137	0.0371087	-4.168504
FLRT3	0.1735678	5.5088431	2.3656057	0.0185151	0.0371094	-4.168569
ZDHHC9	-0.047375	6.6266266	-2.365456	0.0185224	0.0371221	-4.168915
TMEM138	-0.040561	6.0449185	-2.365191	0.0185355	0.0371463	-4.169528
AC004854.4	-0.420347	0.1971308	-2.364966	0.0185466	0.0371649	-4.17005
UBFD1	-0.036375	6.3178361	-2.364961	0.0185468	0.0371649	-4.170061
RFPL4AL1	-0.345857	-0.826213	-2.364674	0.018561	0.0371913	-4.170725
ADRA1D	0.5435299	2.0132273	2.3644714	0.018571	0.0372093	-4.171194
RP11-794P6	-0.117743	-1.462845	-2.364324	0.0185783	0.0372218	-4.171534
OR55B1P	-0.134914	-1.435525	-2.364075	0.0185906	0.0372444	-4.172111
ZNF615	0.0975379	5.1446519	2.3639632	0.0185961	0.0372534	-4.172369
G6PC2	-0.352244	-0.625961	-2.363643	0.018612	0.0372831	-4.173111
RIF1	0.0378473	6.1679691	2.3636188	0.0186131	0.0372835	-4.173166
NPM1P24	0.3893142	0.6747821	2.363447	0.0186216	0.0372984	-4.173563
C9orf64	-0.052566	6.003812	-2.36309	0.0186393	0.0373318	-4.174389
SNX7	0.197605	5.5987161	2.3629265	0.0186474	0.037346	-4.174766
HMP19	0.460518	0.5859903	2.362522	0.0186675	0.0373805	-4.175701
RP11-482M8	-0.115802	-1.474459	-2.362522	0.0186675	0.0373805	-4.175702
CTD-2062F1	0.2393862	-1.232421	2.3625167	0.0186677	0.0373805	-4.175714
PALM2-AKAF	0.5107178	2.3848551	2.3624949	0.0186688	0.0373806	-4.175764
RP1-278C19	-0.390304	1.8593293	-2.362319	0.0186775	0.0373961	-4.176172
MRPS6	-0.053058	6.0484029	-2.362249	0.018681	0.0374009	-4.176331
RP11-345N1	0.4892197	-0.271303	2.3621213	0.0186873	0.0374116	-4.176627
SPDYE1	0.3821296	1.3724955	2.361987	0.018694	0.0374229	-4.176938
RP11-95I16	0.2693224	-1.170955	2.3619546	0.0186956	0.037424	-4.177013
LINC01363	-0.168034	-1.370167	-2.361634	0.0187115	0.0374538	-4.177753
YARS2	0.037074	5.7765588	2.3615394	0.0187162	0.0374612	-4.177972
FZD6	0.1305368	5.5868444	2.3614927	0.0187186	0.0374638	-4.17808
SUPT5H	-0.030089	6.7245531	-2.361	0.0187431	0.0375108	-4.179218
CTB-3M24.2	-0.267134	-1.098277	-2.36077	0.0187545	0.0375305	-4.17975
RP11-383C5	0.3698856	-0.232768	2.3607614	0.0187549	0.0375305	-4.179769
KRT41P	-0.233689	-1.324441	-2.360692	0.0187584	0.0375353	-4.179929
ZKSCAN5	-0.034828	5.8091794	-2.360519	0.018767	0.0375505	-4.18033
AC073283.4	0.4428136	1.4012657	2.3602513	0.0187804	0.0375751	-4.180947
TSPAN17	-0.042827	6.2993409	-2.360191	0.0187834	0.0375791	-4.181086
RP11-483E1	0.3941458	-0.518432	2.3598637	0.0187997	0.0376097	-4.181842
ZNF438	0.0527352	5.5131803	2.3596856	0.0188086	0.0376254	-4.182253
MCMDC2	-0.130109	4.5697603	-2.35961	0.0188124	0.0376309	-4.182429
RNU6-665P	-0.189589	-1.297464	-2.3591	0.0188378	0.0376798	-4.183605
RP11-468E2	-0.095053	4.578958	-2.359071	0.0188393	0.0376807	-4.183673
RNU6ATAC	-0.340372	-0.619505	-2.358449	0.0188704	0.0377408	-4.185108
RP3-406P24	-0.324265	-0.609676	-2.358219	0.0188819	0.0377618	-4.185637
MAGEB6	-0.436212	-0.562452	-2.358176	0.0188841	0.0377641	-4.185738
AMBRA1	-0.032892	6.1777493	-2.358134	0.0188862	0.0377643	-4.185835
FABP5P1	0.2444509	-1.124991	2.3581317	0.0188863	0.0377643	-4.18584
TCEB2P1	-0.216226	-1.19012	-2.357579	0.018914	0.0378177	-4.187115

CES5AP1	0.1918224	-1.292948	2.3575261	0.0189167	0.0378209	-4.187237
FAM83A-AS1	-0.710893	2.7748152	-2.357426	0.0189217	0.0378269	-4.187467
NPM1P21	0.3702139	-0.109418	2.3574254	0.0189217	0.0378269	-4.187469
RP11-152N1	0.1582331	3.9104832	2.3572276	0.0189317	0.0378447	-4.187925
CTD-2576D5	-0.096417	-1.497283	-2.357093	0.0189384	0.037856	-4.188235
HIST2H2BE	-0.095605	5.7558321	-2.357074	0.0189394	0.037856	-4.188281
C7orf26	-0.03533	6.0626746	-2.35652	0.0189672	0.0379095	-4.189558
AC016712.1	-0.296451	-0.894156	-2.356098	0.0189884	0.0379499	-4.19053
RN7SL425P	-0.136731	-1.417193	-2.35577	0.019005	0.0379808	-4.191287
SNHG16	-0.056667	5.9027503	-2.355352	0.019026	0.0380208	-4.192249
CTA-992D9.	0.3858679	-0.430594	2.3552754	0.0190299	0.0380265	-4.192426
CTC-265F19	-0.230116	-1.180197	-2.355026	0.0190425	0.0380495	-4.193001
SNORD17	0.3907089	1.946703	2.3549677	0.0190454	0.0380533	-4.193135
PTCH1	0.1399547	5.3263831	2.3548691	0.0190504	0.0380612	-4.193362
RP11-286H1	0.3294108	2.2668712	2.3548133	0.0190532	0.0380647	-4.193491
RP11-280G9	0.286096	-1.132892	2.3547365	0.0190571	0.0380704	-4.193668
PKMP3	0.34284	2.212031	2.3545258	0.0190677	0.0380896	-4.194153
RP11-323F2	0.3285025	-0.51991	2.3540054	0.019094	0.03814	-4.195352
RP11-123K3	0.3979126	-0.047326	2.3538749	0.0191007	0.0381511	-4.195653
VAPB	-0.032384	6.4098058	-2.35376	0.0191065	0.0381607	-4.195918
AC099342.1	-0.102304	-1.489753	-2.353275	0.019131	0.0382076	-4.197034
PBX3	0.0675303	5.9850615	2.3530736	0.0191412	0.0382259	-4.197498
ZDHHC20-IT	0.3435649	-0.371766	2.3528432	0.0191529	0.0382472	-4.198028
SCO2	-0.053799	6.041355	-2.352818	0.0191542	0.0382477	-4.198087
CDCA4P4	0.3038551	-0.833305	2.3524342	0.0191737	0.0382825	-4.19897
CTC-431G16	-0.510806	0.344343	-2.352432	0.0191738	0.0382825	-4.198974
AC253572.1	0.3486646	3.1355052	2.3523303	0.019179	0.0382908	-4.199209
UQCRFS1P1	0.359828	1.7771004	2.3522889	0.0191811	0.0382929	-4.199304
UBQLN2	-0.039823	6.2581426	-2.351966	0.0191974	0.0383217	-4.200047
RP11-617F2	0.1284702	5.1742205	2.3519639	0.0191976	0.0383217	-4.200052
PRUNE	-0.048015	6.1704976	-2.351913	0.0192001	0.0383247	-4.200168
WEE2-AS1	-0.146543	4.1670275	-2.351652	0.0192134	0.0383491	-4.20077
C11orf58	-0.030586	6.5774121	-2.351595	0.0192163	0.0383528	-4.200902
CEACAMP6	-0.094514	-1.502005	-2.351282	0.0192322	0.0383825	-4.20162
AL590431.1	0.3716342	1.7918193	2.3505656	0.0192688	0.0384532	-4.203269
AC006116.2	0.3528835	1.5487124	2.3502195	0.0192864	0.0384864	-4.204065
TREML3P	0.6267415	0.8586339	2.3500319	0.019296	0.0385034	-4.204497
PLVAP	-0.062787	6.6226509	-2.349972	0.019299	0.0385057	-4.204633
RP11-132A1	-0.336175	4.0230326	-2.349968	0.0192993	0.0385057	-4.204644
CMB9-22P13	-0.26074	4.0863661	-2.349681	0.0193139	0.0385328	-4.205303
ACER1	-0.459738	-0.268251	-2.349162	0.0193404	0.0385822	-4.206496
PTMA	-0.029492	7.0801232	-2.349156	0.0193408	0.0385822	-4.20651
AC096664.2	-0.213295	-1.255435	-2.34903	0.0193472	0.0385929	-4.206799
STEAP3	-0.107293	6.528194	-2.348606	0.0193689	0.0386341	-4.207775
SUV39H2	0.0519386	5.4925108	2.3483865	0.0193802	0.0386544	-4.208279
RP11-36I17	0.3829059	-0.206008	2.348247	0.0193873	0.0386666	-4.2086
PILRB	0.1979189	4.9646715	2.347928	0.0194037	0.0386971	-4.209332
INSM2	-0.273928	-1.039219	-2.347741	0.0194133	0.0387117	-4.209762
CTD-2145A2	0.33346	2.0597091	2.3477289	0.0194139	0.0387117	-4.20979
RP11-501C1	0.5528109	0.7737276	2.347723	0.0194142	0.0387117	-4.209804
RP11-540A2	0.3143417	2.9754902	2.3472779	0.0194371	0.0387552	-4.210826

RP4-737E23	0.3998564	-0.490704	2.347058	0.0194484	0.038774	-4.211331
LINC00449	-0.426059	0.5174836	-2.347053	0.0194486	0.038774	-4.211343
LLOXNC01-7	0.1470469	4.0937911	2.3469335	0.0194548	0.0387842	-4.211617
MCM8	0.0807403	5.5419974	2.3468026	0.0194615	0.0387954	-4.211917
ARRDC3	-0.063911	6.3820333	-2.346647	0.0194695	0.0388092	-4.212274
ENO3	-0.12233	5.8500824	-2.346438	0.0194803	0.0388287	-4.212756
CTD-2509G1	-0.099881	-1.486524	-2.346395	0.0194825	0.0388309	-4.212853
RP11-45101	-0.311758	-1.057991	-2.346159	0.0194946	0.038853	-4.213394
IGLVI-63	-0.138938	-1.415508	-2.345791	0.0195136	0.0388852	-4.214241
RP11-20B24	0.2184674	-1.192648	2.3457906	0.0195136	0.0388852	-4.214241
RP11-48F14	-0.210618	-1.338123	-2.345783	0.019514	0.0388852	-4.214258
RP5-828K20	-0.382368	0.0618513	-2.345762	0.0195151	0.0388853	-4.214307
RP4-737E23	0.2775906	-0.985319	2.3455698	0.019525	0.0389029	-4.214748
RP13-714J1	0.5253306	0.4860724	2.3454175	0.0195329	0.0389165	-4.215097
MBNL2	-0.067154	6.3484002	-2.345349	0.0195364	0.0389214	-4.215255
GPR98	-0.203976	5.6653755	-2.345302	0.0195388	0.0389241	-4.215364
SLC7A14	-0.57987	1.1166927	-2.344767	0.0195664	0.038977	-4.216589
LRP1B	0.6501881	1.8089473	2.3447435	0.0195677	0.0389773	-4.216644
ERLIN1	-0.053323	6.4743664	-2.344466	0.019582	0.0390038	-4.217281
RP11-399B1	0.4160617	3.5429611	2.3442736	0.019592	0.0390215	-4.217722
TMEM232	-0.347073	2.8838157	-2.343998	0.0196062	0.0390477	-4.218354
RNF115	-0.033746	6.3430016	-2.343894	0.0196116	0.0390564	-4.218593
HOXC-AS3	0.294539	-1.120756	2.3437931	0.0196168	0.0390628	-4.218825
RP11-467L1	-0.200127	-1.257086	-2.343771	0.019618	0.0390628	-4.218874
RP11-4L24.	0.2322665	-1.138922	2.3437603	0.0196185	0.0390628	-4.2189
SPZ1	-0.147536	-1.43259	-2.343749	0.0196191	0.0390628	-4.218926
SRSF3	-0.026083	6.7787656	-2.343397	0.0196374	0.0390969	-4.219732
TGIF2LX	-0.489357	-0.389488	-2.343237	0.0196457	0.0391114	-4.2201
SMARCAD1	0.0694765	5.788286	2.3431387	0.0196508	0.0391194	-4.220325
RP3-508I15	0.1893697	3.6461924	2.3431146	0.019652	0.0391197	-4.220381
LA16c-352F	0.2247864	-1.249063	2.3430211	0.0196569	0.0391272	-4.220595
FTLP2	-0.170664	4.7103682	-2.342501	0.0196839	0.0391788	-4.221786
PSEN1	-0.03228	6.4012281	-2.342283	0.0196952	0.0391981	-4.222287
TMEM132B	0.4221464	2.1976273	2.3422736	0.0196957	0.0391981	-4.222308
AC007277.3	-0.553178	0.9869953	-2.342098	0.0197048	0.0392141	-4.222711
TMEM92-AS1	0.5977361	1.3908525	2.3418037	0.0197201	0.0392425	-4.223385
GIPC3	0.0874041	5.2351427	2.341764	0.0197222	0.0392444	-4.223476
AL109763.2	-0.62255	0.1781603	-2.341373	0.0197426	0.0392828	-4.224373
AC108004.2	0.394568	-0.308657	2.3408504	0.0197698	0.0393349	-4.22557
LA16c-60H5	0.4918641	1.2331036	2.3407404	0.0197755	0.0393441	-4.225822
TBCE	-0.045586	6.2585516	-2.34055	0.0197855	0.0393617	-4.226257
GTSE1-AS1	0.3079317	2.1856847	2.34011	0.0198084	0.0394053	-4.227265
CITF22-92A	-0.098738	4.6512855	-2.339827	0.0198232	0.0394326	-4.227913
AC007950.2	-0.398645	0.9185121	-2.339614	0.0198344	0.0394526	-4.228401
SLC44A1	-0.044056	6.5347512	-2.339546	0.0198379	0.0394575	-4.228557
AC144449.1	-0.269758	3.0376473	-2.339415	0.0198448	0.0394675	-4.228857
ZNF571	0.1197268	4.7235839	2.339409	0.0198451	0.0394675	-4.228871
AC098973.2	-0.758737	1.4018959	-2.339184	0.0198569	0.0394888	-4.229387
SIGLEC11	0.2857068	3.4741677	2.3391508	0.0198586	0.0394901	-4.229462
SUZ12P1	0.0757654	5.1099838	2.3391075	0.0198609	0.0394924	-4.229561
AC006978.6	-0.050704	5.3702033	-2.339004	0.0198663	0.0395011	-4.229799

GOLGA8IP	0.4439895	0.2385197	2.3387985	0.0198771	0.0395203	-4.230268
CTSH	-0.058264	6.7670701	-2.338652	0.0198847	0.0395334	-4.230603
ENPP4	0.1148135	5.8316117	2.3385752	0.0198888	0.0395393	-4.230779
AC003006.1	0.2674951	-1.092795	2.3383436	0.0199009	0.0395613	-4.231309
MME	-0.535665	4.5830964	-2.3382	0.0199084	0.0395741	-4.231637
CTD-2026D2	-0.088213	4.5907156	-2.338056	0.019916	0.0395869	-4.231966
BPIFA4P	0.286307	-1.127056	2.3380237	0.0199177	0.0395882	-4.232041
JOSD2	-0.06343	6.0240962	-2.337903	0.0199241	0.0395986	-4.232318
STRN4	0.028605	6.4350433	2.3377849	0.0199303	0.0396088	-4.232588
TNFSF11	0.5120312	3.1340895	2.3377184	0.0199337	0.0396136	-4.23274
RP11-100E5	-0.421534	0.2277161	-2.337552	0.0199425	0.0396288	-4.233121
RP11-511I1	-0.177745	-1.336573	-2.33723	0.0199594	0.0396603	-4.233857
RP11-209M4	-0.353245	-0.449209	-2.337159	0.0199631	0.0396655	-4.234019
SNORA2	0.2825529	2.1819699	2.3368767	0.019978	0.0396929	-4.234665
CAPN7	-0.035452	6.0807276	-2.336742	0.0199851	0.0397048	-4.234973
RP11-512F2	0.354953	1.6542381	2.336307	0.020008	0.0397482	-4.235968
OR2B7P	-0.139712	-1.417502	-2.336122	0.0200178	0.0397654	-4.23639
RP11-112J3	0.3521496	2.3348814	2.3360392	0.0200221	0.0397719	-4.23658
RP11-715J2	-0.443552	0.7028445	-2.335897	0.0200296	0.0397847	-4.236906
RP4-673M15	-0.212801	3.4644087	-2.335651	0.0200426	0.0398061	-4.237467
BMPR1A	0.0422593	5.8503461	2.3356511	0.0200426	0.0398061	-4.237467
RP11-317N8	0.399014	0.0143351	2.3354215	0.0200547	0.039828	-4.237992
AC130469.2	0.4252448	0.298319	2.3353181	0.0200602	0.0398366	-4.238228
C12orf54	0.2895501	-0.901253	2.3352649	0.020063	0.0398401	-4.23835
IGKV1OR2-1	0.2532869	-1.232808	2.3351671	0.0200682	0.0398482	-4.238574
RP11-98D18	0.2895456	-0.84427	2.334701	0.0200928	0.0398949	-4.239639
RP11-959F1	-0.427914	3.2414798	-2.334664	0.0200948	0.0398966	-4.239723
HMGN2P25	-0.19207	-1.293007	-2.334282	0.020115	0.0399346	-4.240596
RP11-278C7	0.1439	3.961631	2.3342372	0.0201174	0.0399371	-4.240698
CTC-420A11	-0.208881	-1.311201	-2.334169	0.020121	0.0399422	-4.240855
RP5-837J1.	0.3891686	0.5484177	2.3341308	0.020123	0.039944	-4.240942
IGLV3-7	-0.086772	-1.506121	-2.333994	0.0201303	0.0399562	-4.241255
TCP10	0.5240796	0.1325334	2.333677	0.0201471	0.0399874	-4.241978
ZNF559	0.0948901	5.3353182	2.3336448	0.0201488	0.0399886	-4.242052
NAA30	-0.036678	6.0761136	-2.333507	0.0201561	0.0400009	-4.242366
NUP88	-0.042677	6.2342183	-2.333071	0.0201792	0.0400447	-4.243362
ZNRF3	0.4635688	2.2289389	2.3329279	0.0201868	0.0400576	-4.243689
NCAN	-0.492278	0.8134721	-2.332751	0.0201962	0.040074	-4.244092
AC008073.7	0.3060619	-0.678352	2.3326814	0.0201999	0.0400792	-4.244251
SNORA5A	0.373385	0.1100763	2.3326091	0.0202037	0.0400847	-4.244417
AC005307.1	0.2995786	-1.131218	2.3323438	0.0202179	0.0401105	-4.245022
RPL7L1P1	-0.424206	-0.430263	-2.332292	0.0202206	0.0401138	-4.245141
CDCA8	0.1182041	5.4563846	2.3320036	0.020236	0.040142	-4.245799
RP11-94B19	-0.194689	-1.292673	-2.331792	0.0202472	0.0401622	-4.246282
SMG1P3	0.2965313	3.2354962	2.3311815	0.0202798	0.0402245	-4.247675
CALHM1	0.2567318	-1.066791	2.3310937	0.0202844	0.0402316	-4.247875
IDUA	-0.065667	5.9256119	-2.331068	0.0202858	0.0402322	-4.247934
EIF1AY	-0.79865	3.5812896	-2.330672	0.0203069	0.0402705	-4.248837
RP11-1217F	-0.124186	-1.464899	-2.330665	0.0203073	0.0402705	-4.248854
RP11-203I1	-0.24087	-1.125362	-2.330615	0.02031	0.040272	-4.248968
AC011648.1	-0.207675	-1.228679	-2.330609	0.0203103	0.040272	-4.24898

SPIN4	0.1329469	4.8008049	2.3305439	0.0203138	0.0402765	-4.249129
RP11-386M2	0.2461635	-1.15613	2.3305253	0.0203148	0.0402765	-4.249172
RP11-674N2	0.3308448	-0.864962	2.3304798	0.0203172	0.0402791	-4.249276
TLX1NB	-0.420608	-0.305281	-2.330394	0.0203218	0.040286	-4.249471
RP11-805I2	-0.146435	-1.405719	-2.330253	0.0203293	0.0402988	-4.249793
CH17-11806	-0.138947	-1.412552	-2.329891	0.0203487	0.040335	-4.250619
RP11-527N2	0.3719404	2.740996	2.3298003	0.0203535	0.0403424	-4.250825
SLFN5	0.0839083	5.8237991	2.3297767	0.0203548	0.0403427	-4.250879
SMG1	0.0410776	6.3618947	2.3296004	0.0203642	0.0403592	-4.251281
FCGR3B	0.4505579	2.9878636	2.3294825	0.0203705	0.0403695	-4.25155
WIF1	0.5414789	-0.199898	2.3292801	0.0203814	0.0403888	-4.252011
FLJ38576	-0.282393	3.048035	-2.329188	0.0203863	0.0403964	-4.252222
RP11-758P1	-0.205038	-1.225974	-2.329036	0.0203945	0.0404103	-4.252568
FAM19A1	0.4130793	0.0858165	2.3288524	0.0204043	0.0404276	-4.252986
PRDM15	0.0695006	5.5354577	2.3287729	0.0204086	0.0404339	-4.253168
ADCK4	-0.044787	6.1393377	-2.328728	0.020411	0.0404357	-4.253269
RNU4-1	-0.443005	0.4981581	-2.328715	0.0204117	0.0404357	-4.2533
EFNA4	-0.074173	5.6763838	-2.32853	0.0204216	0.040453	-4.253722
FTLP3	-0.123408	5.4240559	-2.328511	0.0204226	0.040453	-4.253765
RP11-5407.	0.3530632	-0.469275	2.3282585	0.0204362	0.0404776	-4.25434
BMPR2	0.0461173	6.2504375	2.327823	0.0204596	0.0405217	-4.255332
ETV7	0.1879903	4.8602706	2.3275765	0.0204728	0.0405441	-4.255894
AP000355.2	-0.468915	3.791359	-2.327572	0.0204731	0.0405441	-4.255905
RP11-712L6	0.1017885	4.5838361	2.3275327	0.0204752	0.0405461	-4.255994
RP11-436I9	-0.437162	2.9664908	-2.327415	0.0204815	0.0405564	-4.256262
LINC01132	-0.231831	3.5358301	-2.327387	0.020483	0.0405572	-4.256326
RP5-855F16	-0.273069	-0.933671	-2.327363	0.0204843	0.0405575	-4.256381
RP1-203P18	0.3078837	-0.633002	2.3269885	0.0205045	0.0405952	-4.257233
CTD-2105E1	0.3315945	-0.648153	2.3269319	0.0205075	0.0405991	-4.257362
NID1	0.055514	6.748945	2.326771	0.0205162	0.0406126	-4.257729
CSNK1A1P1	-0.401615	1.606503	-2.326764	0.0205166	0.0406126	-4.257745
RIMKLBP2	0.4228681	1.5891572	2.3264687	0.0205325	0.0406419	-4.258417
QKI	0.0396221	6.4537076	2.3263789	0.0205373	0.0406493	-4.258622
UTY	-0.783449	3.2492563	-2.326257	0.0205439	0.0406601	-4.2589
PPAPDC1B	0.0631688	6.0251558	2.3259891	0.0205583	0.0406865	-4.259509
SMIM1	-0.189288	4.8506029	-2.325964	0.0205597	0.0406869	-4.259565
ART1	0.2013197	-1.276188	2.325789	0.0205691	0.0407034	-4.259965
LMOD3	0.3780315	1.1194719	2.3257013	0.0205739	0.0407106	-4.260164
CNOT8	-0.02827	6.2016015	-2.325634	0.0205775	0.0407156	-4.260317
LYPLA1	-0.048331	6.486727	-2.32529	0.0205961	0.0407502	-4.261101
CTBP1-AS2	-0.044878	5.8632815	-2.325184	0.0206018	0.0407592	-4.261341
RNU6-8	-0.311384	2.8402268	-2.325038	0.0206097	0.0407727	-4.261673
CTD-3234P1	0.4119567	0.9903418	2.3243175	0.0206487	0.0408476	-4.263313
FAM175B	-0.033914	5.9340448	-2.324184	0.020656	0.0408597	-4.263617
HIST2H2BF	0.32531	3.244188	2.3235359	0.0206911	0.0409271	-4.265091
RP13-452N2	-0.471784	1.4644633	-2.323237	0.0207074	0.040957	-4.265772
C17orf102	0.2151068	-1.189053	2.3230766	0.0207161	0.040972	-4.266136
TNFRSF14	-0.050199	6.4424917	-2.323024	0.0207189	0.0409754	-4.266255
WWTR1-IT1	0.2371972	-1.119211	2.3226924	0.020737	0.0410088	-4.26701
BPIFB6	0.4056693	-0.793586	2.3225313	0.0207457	0.0410239	-4.267376
TIAL1	-0.027398	6.4085012	-2.322405	0.0207526	0.0410353	-4.267664

RP11-305A4	-0.218604	-1.283848	-2.322278	0.0207595	0.0410446	-4.267951
SPTLC2	0.0323072	6.1293023	2.3222773	0.0207595	0.0410446	-4.267953
TUBG1	-0.047001	6.2635743	-2.322118	0.0207682	0.0410595	-4.268315
HIST1H2BE	-0.406029	1.4611591	-2.322074	0.0207706	0.041062	-4.268415
MTMR1	0.0443407	5.9985777	2.3219485	0.0207774	0.0410733	-4.268701
AC005224.2	0.2346558	3.0357698	2.3218872	0.0207808	0.0410777	-4.26884
RP11-314N1	-0.646541	0.5672919	-2.321841	0.0207833	0.0410804	-4.268945
RP11-460N2	0.3889023	1.7674049	2.321819	0.0207845	0.0410806	-4.268995
ICA1	0.1380229	5.6553052	2.3217404	0.0207888	0.0410868	-4.269173
RP5-1092A1	-0.541306	0.6213279	-2.321657	0.0207933	0.0410936	-4.269364
RP11-188C1	-0.139432	-1.469587	-2.321402	0.0208072	0.0411189	-4.269943
RIMKLBP1	0.2876832	-0.868219	2.3213771	0.0208086	0.0411193	-4.269999
RP11-497H1	0.4321813	0.6807521	2.3208913	0.0208351	0.0411695	-4.271103
UNC93B3	0.3845826	-0.295391	2.3208055	0.0208398	0.0411765	-4.271298
HBA2	-0.20638	5.0143302	-2.320763	0.0208421	0.0411789	-4.271395
GCOM1	0.4250747	0.0663115	2.3206072	0.0208506	0.0411934	-4.271748
AC079776.1	0.2652284	-1.085873	2.3203964	0.0208621	0.041214	-4.272227
AC108868.5	-0.350064	-1.023965	-2.320046	0.0208813	0.0412496	-4.273023
BTG4	-0.131414	-1.425683	-2.319808	0.0208943	0.0412731	-4.273563
RP5-1057I2	0.3725082	0.1988794	2.3197511	0.0208975	0.041277	-4.273692
AGBL3	0.0849135	5.1041145	2.3196132	0.020905	0.0412897	-4.274006
SLC7A6	0.1141362	5.0847467	2.319377	0.0209179	0.041313	-4.274542
C9orf85	-0.041464	5.5002425	-2.319327	0.0209207	0.0413162	-4.274655
RP5-1042I8	0.1610692	4.3442169	2.3190483	0.020936	0.0413441	-4.275288
VPS39	-0.027344	6.3511461	-2.318942	0.0209418	0.0413534	-4.275529
HIST1H2BPS	-0.381249	-0.580633	-2.31885	0.0209468	0.0413611	-4.275737
C7orf57	0.3139591	-0.782754	2.3186371	0.0209585	0.041382	-4.276222
COX7CP1	-0.363906	1.5603945	-2.318564	0.0209625	0.0413877	-4.276387
MT-TH	-0.261879	-1.051053	-2.31815	0.0209853	0.0414303	-4.277327
RNASEH2B-A	0.3305792	1.9338297	2.3177373	0.021008	0.0414714	-4.278263
Z83851.4	-0.112594	4.4500389	-2.317721	0.0210089	0.0414714	-4.278301
MAB21L3	-0.432854	3.4025491	-2.31771	0.0210095	0.0414714	-4.278326
AC006273.4	0.4709335	1.028099	2.3176555	0.0210125	0.041475	-4.278449
XPO5	-0.040406	6.2827156	-2.317611	0.0210149	0.0414776	-4.27855
NDFIP2	-0.052683	6.1497248	-2.317538	0.0210189	0.0414833	-4.278715
RP11-349J5	-0.295442	-0.84638	-2.317488	0.0210217	0.0414865	-4.278829
YWHAG	-0.029339	6.7807226	-2.317451	0.0210237	0.0414883	-4.278913
BANCR	0.2958136	-0.963595	2.3169921	0.021049	0.0415359	-4.279954
AC004004.2	-0.107026	-1.466284	-2.316935	0.0210522	0.0415399	-4.280084
WNT16	0.436038	0.3992283	2.3167685	0.0210613	0.0415557	-4.280461
MKLN1-AS	0.1618767	4.3520665	2.3166219	0.0210694	0.0415694	-4.280794
RP1-278E11	-0.225769	3.3396359	-2.316443	0.0210793	0.0415867	-4.2812
IL36G	-0.408432	-0.303977	-2.316132	0.0210964	0.0416182	-4.281904
CLCA2	0.4399611	-0.559038	2.3158577	0.0211116	0.0416459	-4.282526
RP5-1102E8	-0.152259	-1.389663	-2.315616	0.0211249	0.0416699	-4.283073
DNALI1	-0.199849	5.499078	-2.315212	0.0211473	0.0417118	-4.283989
RP11-735A1	-0.231149	-1.129359	-2.315173	0.0211494	0.0417138	-4.284078
LCN8	0.2110467	-1.270939	2.3147535	0.0211727	0.0417573	-4.285029
STAT6	-0.048801	6.7154939	-2.314691	0.0211761	0.041762	-4.285172
RP11-810K2	-0.131894	-1.461309	-2.314535	0.0211847	0.0417767	-4.285523
LRCOL1	-0.483855	4.065647	-2.31433	0.0211961	0.0417969	-4.285989

LA16c-329F	-0.172402	-1.368248	-2.314204	0.0212031	0.0418083	-4.286273
MIR4263	0.3856235	1.2856094	2.3139814	0.0212155	0.0418305	-4.286778
CCDC24	-0.08833	5.4281407	-2.313903	0.0212198	0.0418368	-4.286957
ATG101	-0.039808	6.0321906	-2.313879	0.0212212	0.0418372	-4.287011
RP11-62J1.	-0.243957	-1.092071	-2.313447	0.0212451	0.0418815	-4.287989
RP5-912I13	0.394562	-0.885084	2.3134326	0.0212459	0.0418815	-4.288021
RP11-179B2	-0.264946	2.7293184	-2.313399	0.0212478	0.0418829	-4.288097
TRIM56	-0.033325	6.3826978	-2.313161	0.021261	0.0419067	-4.288635
TMEM123	-0.045013	7.004854	-2.313015	0.0212691	0.0419204	-4.288966
PLK5	-0.475653	1.1526937	-2.312877	0.0212768	0.0419333	-4.28928
SAP25	0.3970673	1.293244	2.3121901	0.021315	0.0420064	-4.290835
RGL1	0.072795	5.7881165	2.311934	0.0213293	0.0420322	-4.291414
RP11-274B2	0.2340822	-1.135203	2.3118748	0.0213326	0.0420365	-4.291548
RP11-535A1	0.4523414	1.8677482	2.3115806	0.021349	0.0420665	-4.292214
RP11-435F1	0.2920344	2.7980882	2.3115472	0.0213509	0.0420679	-4.29229
AC079448.1	-0.167357	-1.367256	-2.311428	0.0213576	0.0420788	-4.29256
CAPN5	-0.075006	6.508241	-2.311284	0.0213656	0.0420913	-4.292884
PMPCAP1	-0.494828	-0.311185	-2.311273	0.0213662	0.0420913	-4.292911
RANGRF	0.1079744	5.4055349	2.3107828	0.0213936	0.0421429	-4.294019
RP11-867G2	0.4247245	0.309917	2.3106041	0.0214036	0.0421603	-4.294423
FOXD3	0.2957542	-1.09154	2.3104802	0.0214105	0.0421717	-4.294704
RP11-493L1	0.4322885	-0.242428	2.3103727	0.0214165	0.0421813	-4.294947
CTB-138E5.	0.3693343	-0.651266	2.310247	0.0214235	0.0421928	-4.295231
ANKRD9	-0.069298	5.8914906	-2.30991	0.0214424	0.0422277	-4.295992
RP11-484D2	0.265504	-0.92541	2.3097253	0.0214527	0.0422458	-4.296411
CAPZA1	0.0289499	6.559122	2.3095961	0.02146	0.0422578	-4.296703
RP11-131M6	-0.240097	-1.112504	-2.309224	0.0214808	0.0422966	-4.297545
TMEM260	0.0483378	5.8820862	2.3091549	0.0214847	0.0423014	-4.297701
AHRR	0.244224	3.6093732	2.309139	0.0214856	0.0423014	-4.297736
PPIL1	-0.046988	6.1331997	-2.309054	0.0214904	0.0423086	-4.29793
AC007405.4	-0.27212	2.9679688	-2.308963	0.0214955	0.0423154	-4.298135
AC092071.1	-0.600231	0.8241967	-2.308951	0.0214962	0.0423154	-4.298163
RP11-545I5	-0.146283	4.3953846	-2.308609	0.0215154	0.0423509	-4.298935
UBR1	0.0483186	5.8722064	2.3077197	0.0215654	0.042447	-4.300944
ISPD	-0.110583	4.9633047	-2.30758	0.0215732	0.0424601	-4.301259
IRX1	-0.160496	-1.405068	-2.307048	0.0216032	0.0425168	-4.30246
RARRES2P9	-0.119531	-1.46461	-2.30684	0.0216149	0.0425377	-4.302932
RP11-70601	0.5826196	1.6786999	2.3066226	0.0216272	0.0425595	-4.303422
RPS6KC1	0.0439584	5.9926441	2.3065217	0.0216329	0.0425684	-4.30365
VPS13C	0.0505161	6.1630612	2.3061935	0.0216514	0.0426026	-4.304391
MCMBP	0.0319927	6.2818836	2.3060071	0.0216619	0.042621	-4.304812
RP11-819C2	0.1099919	4.3196243	2.30595	0.0216651	0.042625	-4.304941
FAM111A	-0.045107	6.0560497	-2.305762	0.0216758	0.0426437	-4.305366
RP11-409K2	0.2226227	-1.161039	2.3056051	0.0216846	0.0426588	-4.30572
CASC18	0.4046468	-0.137694	2.3054337	0.0216943	0.0426756	-4.306106
HSPE1P18	0.2382848	-1.101102	2.3053315	0.0217001	0.0426846	-4.306337
DLEU1	-0.071023	5.1850297	-2.304952	0.0217216	0.0427246	-4.307194
EP300	0.0463086	6.3678061	2.3048199	0.0217291	0.042737	-4.307492
LYSMD1	-0.048978	5.7319651	-2.304707	0.0217355	0.0427472	-4.307745
MAK16	0.0565018	5.6621135	2.3046869	0.0217366	0.0427472	-4.307792
RP11-545P7	0.4201927	0.32877	2.3045354	0.0217452	0.0427612	-4.308134

RPL24P2	-0.148761	4.0483314	-2.30452	0.0217461	0.0427612	-4.308167
MIR4457	-0.101357	-1.490329	-2.304372	0.0217545	0.0427755	-4.308503
RP11-50D16	0.3880757	0.4023395	2.3043334	0.0217567	0.0427774	-4.308589
RP3-333H23	0.409435	-0.154115	2.3040555	0.0217724	0.042804	-4.309216
AKR1D1	-0.329411	5.7988069	-2.304053	0.0217726	0.042804	-4.309221
BLOC1S5-TX	0.4353217	1.6812851	2.3039714	0.0217772	0.0428109	-4.309406
RNU6-899P	-0.099912	-1.489519	-2.303852	0.021784	0.0428219	-4.309676
RP11-872D1	-0.369284	0.3182799	-2.303627	0.0217968	0.0428447	-4.310182
RP11-372K1	0.3527163	2.0639285	2.3033139	0.0218146	0.0428774	-4.310889
SPIN2A	0.3550775	-0.378096	2.3032214	0.0218198	0.0428854	-4.311098
CTD-2201G3	0.455305	0.4884816	2.3032006	0.021821	0.0428854	-4.311144
MKX-AS1	-0.11971	-1.461579	-2.303149	0.0218239	0.0428888	-4.31126
CTA-445C9.	-0.129594	4.6257302	-2.302484	0.0218618	0.0429609	-4.31276
MLK4	0.2306036	5.2814396	2.3023639	0.0218686	0.0429721	-4.313031
RP11-73K9.	-0.242181	3.3806589	-2.302311	0.0218716	0.0429757	-4.31315
TMEM45A	0.1944492	5.7836665	2.3021362	0.0218816	0.0429929	-4.313544
CTC-458G6.	0.40469	-0.767416	2.3020046	0.0218891	0.0430054	-4.313841
PGGT1BP1	-0.212673	-1.181478	-2.301827	0.0218992	0.043023	-4.314242
RP11-270C1	-0.317161	2.3506989	-2.301637	0.0219101	0.043042	-4.31467
ANPEP	-0.072142	7.1412786	-2.301601	0.0219121	0.0430436	-4.31475
RP11-51401	0.3895431	2.0274897	2.3015776	0.0219135	0.043044	-4.314803
RP11-64C12	0.4194039	-0.175394	2.3014752	0.0219193	0.0430531	-4.315034
RP11-73M18	0.3658383	0.8974855	2.3011878	0.0219357	0.043083	-4.315681
RP11-65903	-0.159332	-1.404974	-2.301091	0.0219413	0.0430916	-4.3159
SNRPCP2	-0.260601	-0.950046	-2.301052	0.0219435	0.0430937	-4.315988
RP11-446H1	-0.174421	-1.324195	-2.300841	0.0219555	0.0431132	-4.316462
DUXA	-0.27232	-1.221229	-2.300837	0.0219558	0.0431132	-4.316473
ZNF805	0.0893429	4.4254944	2.3005149	0.0219742	0.043147	-4.317197
MYBL1	0.096374	5.1316554	2.3000063	0.0220033	0.0432018	-4.318343
DRG2	-0.043204	6.0943184	-2.299777	0.0220164	0.0432253	-4.318858
NOXRED1	0.249083	3.0658881	2.2992878	0.0220445	0.043278	-4.319961
GORASP2	-0.028894	6.6065724	-2.299196	0.0220497	0.043286	-4.320167
RP11-69J7.	-0.384196	-0.194254	-2.299074	0.0220568	0.0432975	-4.320442
SYNM	0.1127496	5.6802552	2.2989061	0.0220664	0.0433141	-4.32082
PRDM13	0.2386356	-1.280947	2.298489	0.0220904	0.0433588	-4.321759
CTD-2342N2	-0.427486	3.3852225	-2.298398	0.0220956	0.0433667	-4.321964
ZSCAN25	0.0395587	5.6835905	2.2979945	0.0221188	0.0434099	-4.322871
CMYA5	0.1899537	4.8864968	2.2975406	0.0221449	0.0434589	-4.323893
COG3	-0.054294	6.2637126	-2.296906	0.0221815	0.0435283	-4.325321
RP11-309J1	-0.126397	-1.462219	-2.296809	0.0221871	0.043537	-4.325539
RP11-243A1	-0.410637	3.2676901	-2.296733	0.0221915	0.0435432	-4.32571
CTD-2349P2	0.2269423	-1.128416	2.2963743	0.0222122	0.0435815	-4.326516
DPY19L1	-0.054861	6.1114443	-2.296159	0.0222246	0.0436036	-4.327
GJA10	0.5646306	-0.057401	2.2960269	0.0222323	0.0436162	-4.327297
RP11-460I1	-0.306461	-0.8498	-2.295951	0.0222367	0.0436225	-4.327468
ANGPTL6	-0.191779	4.8174029	-2.295749	0.0222483	0.043643	-4.327921
RP13-270P1	0.2005314	3.0835193	2.2955222	0.0222614	0.0436664	-4.328431
ZCCHC5	0.2566111	-1.085998	2.2953692	0.0222703	0.0436814	-4.328775
LINC00032	0.2939727	-0.88874	2.2952698	0.0222761	0.0436904	-4.328999
RP11-249C2	-0.173665	-1.379725	-2.295076	0.0222873	0.04371	-4.329435
RP11-468N1	-0.351276	-0.814242	-2.295018	0.0222907	0.0437143	-4.329565

AC079807.4	-0.410811	0.084026	-2.294925	0.022296	0.0437225	-4.329774
IL9RP3	0.4263753	0.7189106	2.2945212	0.0223194	0.043766	-4.330681
RP11-350J2	0.215207	-1.242413	2.2941732	0.0223396	0.0438033	-4.331463
ZNF552	0.0662216	5.3538335	2.2939406	0.0223531	0.0438274	-4.331985
MT-TL1	0.3854352	2.4616485	2.2938654	0.0223575	0.0438323	-4.332154
EEF1B2P3	-0.116539	4.5090299	-2.293856	0.022358	0.0438323	-4.332175
GINM1	-0.038285	6.2105824	-2.293622	0.0223716	0.0438566	-4.332701
RP5-943J3.	0.3055423	-0.780931	2.2935446	0.0223761	0.0438631	-4.332875
RP11-111I1	0.2742563	-1.040557	2.2934667	0.0223807	0.0438696	-4.333049
BTRC	0.0382527	5.8736672	2.2929082	0.0224131	0.043929	-4.334304
ZFHX4	-0.148417	5.5774299	-2.292904	0.0224134	0.043929	-4.334313
ZNF492	0.5414185	0.747387	2.2927135	0.0224245	0.0439484	-4.334741
LINC01141	0.3941303	0.0946854	2.2926221	0.0224298	0.0439565	-4.334946
TMX1	-0.0355	6.1936924	-2.292386	0.0224436	0.0439811	-4.335476
LINC01546	0.2550366	-1.03754	2.2922688	0.0224504	0.0439908	-4.335739
SLMO2P11	-0.33861	-0.532547	-2.292259	0.0224509	0.0439908	-4.33576
CTB-92J24.	-0.572881	0.8670635	-2.29205	0.0224631	0.0440124	-4.33623
RNU6ATAC2F	-0.093764	-1.501584	-2.291775	0.0224792	0.0440415	-4.336847
MTX2	0.0319514	6.1103476	2.2908676	0.0225322	0.0441429	-4.338883
ZNF557	-0.041868	5.4028486	-2.290712	0.0225413	0.0441572	-4.339233
CTD-2026C7	0.1716359	-1.307075	2.2907019	0.0225419	0.0441572	-4.339255
FLJ44511	0.3994092	3.3653733	2.2905826	0.0225489	0.0441685	-4.339523
MIP	-0.395825	2.5600859	-2.290435	0.0225575	0.0441828	-4.339853
NF1P8	0.3548716	-1.012066	2.2904163	0.0225586	0.0441828	-4.339896
HAUS7	-0.105998	4.7840809	-2.29033	0.0225637	0.0441904	-4.34009
BTBD7P1	0.4775276	0.3784692	2.2902527	0.0225682	0.0441968	-4.340262
RP11-753D2	-0.433318	0.0587899	-2.290092	0.0225776	0.0442129	-4.340623
PRR23B	-0.113339	-1.489752	-2.289887	0.0225896	0.0442341	-4.341083
NKRF	-0.042162	5.6324308	-2.289767	0.0225967	0.0442455	-4.341353
RP11-88I18	0.4136564	0.7945753	2.2897044	0.0226003	0.0442502	-4.341492
CT83	0.4545047	-0.70117	2.2894271	0.0226166	0.0442797	-4.342114
PDE7B	0.1503285	4.7409464	2.2890547	0.0226384	0.0443201	-4.342948
AGBL4	0.5291849	1.0907768	2.2888515	0.0226504	0.0443411	-4.343404
RNU7-49P	0.3389005	-0.459007	2.2887815	0.0226545	0.0443468	-4.343561
TBC1D23	-0.039954	6.102769	-2.288452	0.0226739	0.0443824	-4.3443
MAPK4	0.5383328	3.4412973	2.2882563	0.0226854	0.0444025	-4.344738
TTC14	0.0484208	5.8370135	2.28808	0.0226957	0.0444204	-4.345133
BNIP3P3	-0.098992	-1.474759	-2.28788	0.0227075	0.0444411	-4.345582
CCNT2-AS1	0.1742826	3.4747576	2.2878082	0.0227117	0.0444469	-4.345742
RP11-338I2	0.3962606	1.7429261	2.2877191	0.022717	0.0444548	-4.345941
RNU6-1223F	0.213524	-1.144228	2.2873128	0.0227409	0.0444993	-4.346852
RP11-394B2	-0.362353	1.9333751	-2.287159	0.02275	0.0445147	-4.347197
RP11-793I1	-0.306266	-0.771229	-2.287045	0.0227567	0.0445254	-4.347452
RPAP3	0.0260085	5.9845598	2.2867357	0.022775	0.0445587	-4.348144
KRT8P7	0.4184281	1.1950651	2.2865597	0.0227853	0.0445767	-4.348538
MICF	-0.250638	-1.058987	-2.28636	0.0227971	0.0445973	-4.348984
NELFB	-0.032129	6.4493076	-2.286333	0.0227988	0.0445981	-4.349046
SRI	0.078593	6.1037115	2.2862887	0.0228014	0.0446008	-4.349145
SNORD99	0.3894399	1.7268733	2.2862025	0.0228064	0.0446084	-4.349338
CMTM5	0.2996317	-0.868453	2.2860019	0.0228183	0.0446292	-4.349787
RNU6-96P	-0.222302	-1.119216	-2.285653	0.0228389	0.0446671	-4.350567

CCDC126	-0.062417	5.5000449	-2.285356	0.0228565	0.0446992	-4.351233
RP11-208N1	-0.106807	-1.488182	-2.285017	0.0228766	0.0447354	-4.351991
RP11-485M7	-0.103789	-1.488715	-2.285002	0.0228775	0.0447354	-4.352025
LLNLR-304A	0.3987311	0.1306981	2.2847928	0.0228899	0.0447572	-4.352493
RP11-300M2	0.2119031	-1.321447	2.2846996	0.0228954	0.0447656	-4.352702
RP11-631N1	0.1979244	3.5041222	2.2845546	0.022904	0.0447795	-4.353026
SCARNA22	0.2073345	-1.19889	2.2845388	0.022905	0.0447795	-4.353061
RP11-241K1	-0.31193	-0.722687	-2.284052	0.0229339	0.0448336	-4.354151
RP4-781L3.	0.2367658	-1.1051	2.2840291	0.0229352	0.0448338	-4.354202
RP11-893F2	0.2179101	-1.186896	2.2837906	0.0229494	0.0448591	-4.354735
HEATR5B	0.0464919	5.941773	2.2835186	0.0229655	0.0448883	-4.355344
PCDH12	0.065792	5.8790419	2.283167	0.0229865	0.0449268	-4.35613
RP11-222O2	-0.107734	-1.483073	-2.282964	0.0229985	0.044948	-4.356583
AXDND1	0.4275264	1.6178889	2.2827423	0.0230117	0.0449714	-4.357079
CTC-542B22	0.3797524	-0.420339	2.282663	0.0230165	0.0449782	-4.357257
RP11-697E2	0.2320293	-1.131628	2.282211	0.0230434	0.0450282	-4.358267
CAPN10	0.0410501	5.8083234	2.2821927	0.0230445	0.0450282	-4.358308
CHCHD2P2	0.2848491	3.93752	2.2820008	0.0230559	0.0450465	-4.358737
SCRT1	-0.403715	0.0415968	-2.281994	0.0230564	0.0450465	-4.358752
RP11-326L1	-0.324086	-0.767892	-2.281737	0.0230717	0.045074	-4.359325
FLJ31356	-0.460445	0.7355494	-2.281615	0.023079	0.0450859	-4.359599
AC092620.3	0.3525556	-0.27959	2.2814523	0.0230887	0.0451024	-4.359962
RP11-115C2	0.0883881	4.5145848	2.2808125	0.0231269	0.0451734	-4.361392
CTC-270D5.	-0.215047	-1.335899	-2.280803	0.0231275	0.0451734	-4.361412
ADCY8	-0.614459	0.5922192	-2.280621	0.0231384	0.0451923	-4.361819
ZNF530	0.1310614	4.7178236	2.2805139	0.0231448	0.0452024	-4.362059
TEN1-CDK3	0.1735274	4.417818	2.2803385	0.0231553	0.0452205	-4.36245
ZBTB8B	0.3514738	-0.306552	2.2800363	0.0231734	0.0452502	-4.363125
RP11-135N5	0.3937339	0.6159545	2.280028	0.0231739	0.0452502	-4.363144
RP1-159M24	0.3680113	0.2416399	2.2800221	0.0231743	0.0452502	-4.363157
CNEP1R1	0.0476787	5.5456385	2.279867	0.0231836	0.045266	-4.363503
HSP90AA4P	0.336462	-0.459947	2.279549	0.0232026	0.0453008	-4.364213
ZNF266	0.0841174	5.809664	2.2795173	0.0232045	0.0453014	-4.364284
snoU13	0.4029288	-0.037557	2.2795023	0.0232054	0.0453014	-4.364317
CLVS1	-0.37553	2.9059883	-2.27948	0.0232068	0.0453016	-4.364368
RP11-1029J	0.3269072	-0.890493	2.2792457	0.0232208	0.0453266	-4.36489
RNY1P16	-0.341193	-0.481553	-2.278987	0.0232364	0.0453545	-4.365468
NDUFV2P1	0.220216	4.4649387	2.2788928	0.023242	0.0453554	-4.365678
RP11-163E9	-0.227329	3.2979683	-2.27889	0.0232422	0.0453554	-4.365684
RP11-327E2	-0.11485	4.4539044	-2.278888	0.0232423	0.0453554	-4.365689
CTA-963H5.	0.1444652	3.9848901	2.2788799	0.0232428	0.0453554	-4.365707
RP11-956J1	-0.224195	-1.279275	-2.278876	0.023243	0.0453554	-4.365714
TNKS1BP1	-0.034402	6.6823541	-2.278688	0.0232543	0.045375	-4.366134
MIS18A	0.0625321	5.5522525	2.2785828	0.0232606	0.0453849	-4.36637
TM4SF4	-0.127832	7.0600698	-2.278552	0.0232625	0.0453861	-4.366439
MBNL3	-0.097005	6.6645839	-2.27848	0.0232668	0.0453911	-4.366599
RP11-71L14	0.2358442	-1.186469	2.2784537	0.0232684	0.0453911	-4.366658
RP11-303G3	0.3571793	-0.329017	2.2784473	0.0232688	0.0453911	-4.366672
FOXP2	0.6095412	3.1787802	2.278398	0.0232718	0.0453926	-4.366782
RP11-615I2	-0.113711	-1.455787	-2.278393	0.023272	0.0453926	-4.366792
LHX4-AS1	-0.045125	6.216302	-2.278292	0.0232781	0.0454021	-4.367019

RP4-534N18	0.2176991	-1.133875	2.2781853	0.0232846	0.0454122	-4.367257
OPTN	-0.041362	6.7198479	-2.27814	0.0232873	0.045415	-4.367357
KIF17	0.123046	4.6877808	2.2777583	0.0233103	0.0454574	-4.368209
DAP3P1	-0.301097	-0.814161	-2.277717	0.0233128	0.0454599	-4.368303
EPHB2	0.2345747	5.0530265	2.2776829	0.0233148	0.0454611	-4.368378
ZC3HAV1	-0.03317	6.2374212	-2.277665	0.0233159	0.0454611	-4.368417
IGHVII-33-	-0.167461	-1.339132	-2.27742	0.0233306	0.0454875	-4.368964
RP11-33B1.	0.4446821	2.2647601	2.2773244	0.0233364	0.0454963	-4.369177
PIGB	0.0598078	5.5177969	2.2773002	0.0233379	0.0454967	-4.369231
NINJ2	-0.141289	5.41894	-2.277138	0.0233477	0.0455134	-4.369594
CCDC77	-0.054571	5.3745832	-2.276602	0.02338	0.045574	-4.370788
RP11-748L1	-0.215131	-1.245606	-2.276365	0.0233943	0.0455979	-4.371317
RP11-180M1	-0.254839	2.7427179	-2.276357	0.0233948	0.0455979	-4.371334
VIL1	0.2632556	5.9081297	2.2762498	0.0234013	0.0456066	-4.371573
MIR140	-0.104748	-1.481753	-2.276242	0.0234017	0.0456066	-4.37159
MIR101-2	-0.131663	-1.418704	-2.276156	0.023407	0.0456143	-4.371782
SYNGR2	-0.039495	6.7334892	-2.27611	0.0234097	0.0456173	-4.371885
NLN	-0.059251	6.245924	-2.275928	0.0234207	0.0456363	-4.372291
RP11-161H2	0.3089515	-0.778797	2.2757421	0.023432	0.0456558	-4.372705
SLX1A-SULT	0.3733517	0.3085049	2.2755072	0.0234462	0.0456811	-4.373229
SNRPF	-0.050907	6.2804445	-2.275169	0.0234667	0.0457185	-4.373983
HPRT1	-0.05585	6.1564042	-2.274614	0.0235003	0.0457816	-4.375218
WWC2-AS2	-0.189637	3.6430227	-2.274211	0.0235248	0.0458268	-4.376116
MKRNP2	-0.177071	-1.347933	-2.273927	0.023542	0.045858	-4.376749
VN1R78P	-0.226497	-1.242663	-2.273888	0.0235444	0.0458602	-4.376837
LGALS4	-0.217012	6.6248313	-2.273825	0.0235482	0.0458652	-4.376976
ARHGEF28	-0.101029	5.9177477	-2.273604	0.0235617	0.0458889	-4.377469
RP11-7F17.	-0.109254	4.0316917	-2.273413	0.0235733	0.0459091	-4.377894
NEO1	0.0508893	6.1376638	2.2732574	0.0235828	0.0459244	-4.37824
RAB3IP	0.0625691	5.9260854	2.2732418	0.0235837	0.0459244	-4.378275
ALMS1P	0.366075	1.9350749	2.2732039	0.023586	0.0459265	-4.378359
CAP2P1	0.3040606	-0.61145	2.2728223	0.0236092	0.0459693	-4.379209
AC006116.2	0.3095828	-0.737897	2.272277	0.0236425	0.0460315	-4.380423
F8A3	-0.24107	-1.109978	-2.272099	0.0236534	0.0460502	-4.38082
RP11-1042E	-0.153578	-1.369766	-2.272005	0.0236591	0.0460589	-4.381029
AC006037.2	-0.484122	0.1964047	-2.271074	0.023716	0.0461672	-4.3831
AC003002.6	0.3519871	-0.204533	2.2710417	0.0237179	0.0461685	-4.383171
ZNF184	0.0709495	5.2439874	2.2707233	0.0237374	0.046204	-4.383879
RYR3	0.1637091	3.7651262	2.2705594	0.0237474	0.046221	-4.384244
RP11-540B6	-0.066268	5.0667153	-2.27027	0.0237652	0.0462531	-4.384888
RP11-14K3.	-0.389602	-0.869588	-2.270155	0.0237722	0.0462643	-4.385144
LINC01515	-0.18288	4.32025	-2.269945	0.0237851	0.0462869	-4.385611
CNTD1	0.1241464	4.0274989	2.2697924	0.0237944	0.0463009	-4.38595
TDGF1P6	-0.192827	-1.257611	-2.269786	0.0237948	0.0463009	-4.385964
RAD21	0.0366779	6.7479906	2.2695232	0.0238109	0.0463298	-4.386548
RNU5B-2P	-0.261136	2.5424455	-2.269018	0.0238419	0.0463877	-4.387671
RP11-789C2	-0.523429	0.3109761	-2.268887	0.02385	0.0464004	-4.387962
TICAM2	0.3778513	0.9402989	2.2688709	0.023851	0.0464004	-4.387998
RP13-415G1	0.4011818	-0.113418	2.2688218	0.023854	0.0464038	-4.388107
LA16c-306A	0.3644948	1.2734301	2.2687641	0.0238575	0.0464082	-4.388235
ZNHIT6	0.043494	5.6675839	2.2686296	0.0238658	0.0464212	-4.388534

VENTXP2	-0.16824	-1.392817	-2.268614	0.0238668	0.0464212	-4.388569
BBOX1-AS1	0.551272	0.3847595	2.2684638	0.023876	0.0464367	-4.388903
SMEK1	-0.03179	6.3947721	-2.268427	0.0238783	0.0464386	-4.388984
TMEM60	-0.042354	5.8403862	-2.268147	0.0238955	0.0464696	-4.389606
RP11-367G1	0.4160611	1.4676253	2.2680734	0.0239	0.046476	-4.38977
FGF19	0.6709732	2.3772607	2.2679551	0.0239073	0.0464876	-4.390033
RP11-418J1	0.096953	4.7809843	2.2677186	0.0239219	0.0465135	-4.390558
CARD8-AS1	0.1095199	4.709264	2.2669461	0.0239695	0.0466036	-4.392274
HPS1	-0.041193	6.4602356	-2.266849	0.0239755	0.0466128	-4.392488
MYOM2	0.2806102	3.9670208	2.2666505	0.0239878	0.0466341	-4.39293
RP11-351I2	-0.299233	-0.71114	-2.266418	0.0240021	0.0466582	-4.393446
RNU1-67P	-0.274643	-0.994007	-2.266388	0.024004	0.0466582	-4.393512
FANCC	-0.073843	5.8376701	-2.266388	0.024004	0.0466582	-4.393513
RPL29P14	0.3927013	0.1258826	2.2663289	0.0240076	0.0466628	-4.393644
RP11-214K3	0.3693206	1.0957751	2.2662716	0.0240112	0.0466672	-4.393771
FAM209B	0.3927392	1.0437428	2.2657317	0.0240446	0.0467285	-4.394969
RP11-753D2	-0.114776	-1.473884	-2.26572	0.0240453	0.0467285	-4.394995
SBSN	0.4293971	-0.343709	2.2655073	0.0240585	0.0467517	-4.395467
HNF4G	0.14606	5.72586	2.2651758	0.024079	0.0467891	-4.396203
RP11-51J9.	-0.166548	3.8926994	-2.264741	0.0241059	0.0468389	-4.397168
LRRC38	0.3336549	-1.017449	2.2644484	0.0241241	0.0468717	-4.397817
CTC-756D1.	-0.395411	0.0677353	-2.263859	0.0241607	0.0469404	-4.399125
RP11-165D6	-0.286075	-0.874998	-2.263432	0.0241873	0.046987	-4.400072
ISL1	0.2905753	-1.099018	2.2634312	0.0241873	0.046987	-4.400073
MT-TG	-0.257318	-1.001498	-2.263159	0.0242042	0.0470174	-4.400676
PRY	-0.1207	-1.45319	-2.263107	0.0242075	0.0470212	-4.400791
GEMIN7	-0.053808	5.8892565	-2.263064	0.0242101	0.0470239	-4.400887
ABCA9	-0.181457	5.1463771	-2.26296	0.0242166	0.0470339	-4.401116
AC004593.3	-0.496019	1.6596048	-2.262845	0.0242238	0.0470454	-4.401372
RP1-240K6.	-0.277105	3.1341687	-2.262712	0.0242321	0.047059	-4.401668
ARMC7	-0.049151	5.936668	-2.262609	0.0242385	0.0470689	-4.401894
POLR1A	-0.032079	6.276005	-2.262121	0.0242689	0.0471255	-4.402977
CHMP1B2P	0.5374952	-0.019077	2.2619308	0.0242808	0.047146	-4.403398
NDUFAF5	-0.042233	5.7728885	-2.261557	0.0243041	0.0471889	-4.404227
RFWD2	-0.03698	6.409573	-2.261511	0.024307	0.0471919	-4.404328
RPA1	0.0312173	6.3488993	2.2612186	0.0243253	0.0472249	-4.404976
LINC00917	-0.23575	-1.271812	-2.261075	0.0243342	0.0472398	-4.405293
RP11-203B9	-0.195914	3.2902948	-2.260936	0.0243429	0.0472542	-4.405602
BTN3A3	-0.07176	5.93935	-2.260296	0.024383	0.0473294	-4.407019
CLDND2	-0.146018	4.5735054	-2.260169	0.024391	0.0473424	-4.407301
VN1R51P	0.4263538	-0.158329	2.2600358	0.0243993	0.047356	-4.407595
RP11-394G3	-0.349631	-0.767175	-2.259958	0.0244042	0.047363	-4.407768
DHFRP1	0.3719482	2.3736689	2.2598248	0.0244125	0.0473767	-4.408062
RP6-7406.2	0.3086584	2.553251	2.2595885	0.0244274	0.0474029	-4.408585
TPR	0.0358025	6.5862992	2.2595181	0.0244318	0.0474068	-4.408741
PPTC7	-0.04446	6.1579637	-2.259515	0.0244319	0.0474068	-4.408747
FAM178A	0.0462326	6.0175085	2.2593397	0.024443	0.0474257	-4.409136
RP11-196G1	0.449433	0.4353551	2.259289	0.0244462	0.0474293	-4.409248
RP4-738P15	0.2107722	-1.274143	2.2591758	0.0244533	0.0474395	-4.409499
AF127936.9	-0.10427	4.9675974	-2.259132	0.024456	0.0474395	-4.409595
GAPDHP76	-0.258363	-0.975565	-2.259115	0.0244571	0.0474395	-4.409633

COBL	-0.091238	6.268731	-2.259097	0.0244582	0.0474395	-4.409673
MAP1S	0.0368952	6.1514698	2.2590877	0.0244588	0.0474395	-4.409694
TIMM23B	0.0689349	5.2189451	2.2590781	0.0244594	0.0474395	-4.409715
RP3-406A7.	-0.263441	4.4630874	-2.259061	0.0244605	0.0474395	-4.409753
CTD-2357A8	-0.188557	-1.27065	-2.259026	0.0244627	0.0474412	-4.409831
RP11-251M1	0.3505923	2.5664585	2.2588963	0.0244708	0.0474545	-4.410117
HMGN2P40	-0.308685	-0.800135	-2.258789	0.0244776	0.0474651	-4.410355
UPF3AP1	0.281257	-0.736399	2.2586658	0.0244853	0.0474775	-4.410627
WEE2	0.377832	0.3704433	2.2583478	0.0245053	0.0475138	-4.411331
MTND4P21	-0.127164	-1.457346	-2.258326	0.0245067	0.0475139	-4.411378
RP11-693M3	0.3706314	-0.145504	2.2581768	0.0245161	0.0475296	-4.411709
AC009120.5	0.3963693	1.1564808	2.2581143	0.02452	0.0475347	-4.411848
AC007919.1	-0.193516	-1.237664	-2.258093	0.0245214	0.0475348	-4.411895
RP11-164H1	0.2403997	-1.198841	2.2580378	0.0245248	0.047539	-4.412017
CCL23	0.3258933	2.6510166	2.2579614	0.0245296	0.0475458	-4.412186
NAP1L4P2	-0.132307	-1.426717	-2.257831	0.0245378	0.0475591	-4.412473
RP4-806M20	0.1673268	-1.333736	2.2574907	0.0245593	0.0475982	-4.413227
ANKLE2	0.0263681	6.3228345	2.2572354	0.0245754	0.0476257	-4.413791
RP11-432M8	-0.122645	-1.473774	-2.257224	0.0245761	0.0476257	-4.413817
ZNF222	0.1095902	4.6618589	2.256708	0.0246087	0.0476863	-4.414958
CTD-2410N1	0.2336423	-1.058745	2.256668	0.0246112	0.0476882	-4.415046
LINC00454	-0.150238	-1.434117	-2.256651	0.0246122	0.0476882	-4.415083
DTWD2	-0.072323	5.4312413	-2.25661	0.0246149	0.0476908	-4.415175
PSMD7P1	-0.366311	0.0403731	-2.256371	0.0246299	0.0477174	-4.415702
ZNF625-ZNF	0.3098731	-0.553456	2.2561741	0.0246424	0.047739	-4.416138
CICP20	-0.179374	-1.311774	-2.256	0.0246534	0.0477578	-4.416523
ORAI1	-0.049617	6.0764467	-2.255928	0.024658	0.0477641	-4.416682
RP11-804N1	-0.170811	-1.364738	-2.255485	0.024686	0.0478144	-4.417661
RP11-203I2	-0.50097	2.5779955	-2.255476	0.0246866	0.0478144	-4.41768
bP-21264C1	-0.212393	-1.206671	-2.25531	0.0246971	0.0478322	-4.418046
PSMB8-AS1	-0.086285	5.5541659	-2.255266	0.0246999	0.0478351	-4.418144
HEATR5A	0.0488608	5.8357323	2.2549064	0.0247227	0.0478763	-4.418939
LINC00636	0.2305963	-1.223708	2.2548892	0.0247237	0.0478763	-4.418977
RP11-654C2	-0.213611	-1.191728	-2.254799	0.0247295	0.0478848	-4.419176
MIR6859-3	0.2942443	-0.791542	2.254493	0.0247489	0.047919	-4.419852
AC079613.1	-0.45587	-0.457538	-2.254454	0.0247514	0.047919	-4.419939
RP11-297L6	-0.126528	-1.457242	-2.25444	0.0247522	0.047919	-4.419969
RP1-267D11	0.0969325	4.2871206	2.2544378	0.0247524	0.047919	-4.419974
MYLK4	0.2362651	3.812275	2.2541755	0.024769	0.0479487	-4.420553
NTN1	-0.176325	5.6501437	-2.253942	0.0247839	0.0479749	-4.421068
IKZF2	0.160903	4.6453613	2.2538664	0.0247887	0.0479816	-4.421236
RP11-353N4	-0.222619	4.1264146	-2.253648	0.0248025	0.0480059	-4.421717
HFE	-0.066067	6.0422416	-2.253547	0.024809	0.0480159	-4.421942
GLTPD2	-0.147139	5.9352173	-2.253151	0.0248341	0.048062	-4.422814
RP11-680E1	-0.285305	-1.173146	-2.252976	0.0248453	0.048081	-4.423201
KRT17P7	-0.305311	-0.918678	-2.252542	0.024873	0.0481321	-4.42416
ZNF19	-0.061648	4.8348102	-2.252424	0.0248805	0.0481441	-4.424421
LIME1	-0.112593	4.9994837	-2.252076	0.0249027	0.0481844	-4.425187
OAZ3	-0.09402	4.6542099	-2.251947	0.0249109	0.0481978	-4.425472
CTD-260009	0.2664231	2.5756176	2.2517334	0.0249245	0.0482216	-4.425943
YJEFN3	0.1615746	4.5340146	2.2516708	0.0249285	0.0482268	-4.426081

C22orf15	0.3264901	2.2697002	2.2510858	0.0249659	0.0482966	-4.427372
MYO1H	0.3882029	0.9638564	2.2509924	0.0249719	0.0483055	-4.427578
RP11-379B1	-0.239052	-1.129248	-2.250931	0.0249758	0.0483106	-4.427714
FAM214B	-0.042972	5.7814161	-2.25059	0.0249977	0.0483503	-4.428466
RP11-286B1	0.4298056	-0.360521	2.2504427	0.0250071	0.0483659	-4.42879
AC004129.7	-0.111247	-1.459204	-2.250179	0.0250239	0.048396	-4.429371
WDFY1	0.0350797	6.1417816	2.2501457	0.0250261	0.0483976	-4.429445
HNRNPD	-0.02579	6.6815425	-2.250106	0.0250287	0.0484	-4.429533
NFRKB	-0.033858	6.1043018	-2.249956	0.0250383	0.048416	-4.429863
CTB-25B13.	0.3586473	1.722656	2.2495752	0.0250627	0.0484606	-4.430702
RP11-433A1	-0.437522	1.099713	-2.249317	0.0250792	0.0484901	-4.431271
AC016739.2	-0.121157	4.6709619	-2.249159	0.0250894	0.0485065	-4.43162
LHB	0.415823	-0.112825	2.2491433	0.0250904	0.0485065	-4.431654
TBC1D29	-0.358558	-0.396237	-2.248976	0.0251012	0.0485247	-4.432023
TMEM181	0.0455987	6.2188685	2.2487535	0.0251154	0.0485498	-4.432513
LL0XNC01-2	0.1520026	-1.386498	2.2486866	0.0251197	0.0485555	-4.43266
RP11-555M1	-0.297745	3.0943625	-2.248633	0.0251232	0.0485596	-4.432779
LRP4-AS1	-0.409002	0.7249644	-2.248509	0.0251312	0.0485725	-4.433052
OSBPL2	-0.025481	6.2761725	-2.248397	0.0251384	0.0485838	-4.433298
BCRP1	0.2297062	-1.169878	2.2481333	0.0251553	0.048614	-4.433879
RP11-215P8	-0.345476	-0.245047	-2.248101	0.0251574	0.0486155	-4.43395
RP11-295D4	-0.190317	-1.256512	-2.248005	0.0251636	0.0486225	-4.434161
ASTN1	0.417723	2.5024859	2.2480029	0.0251637	0.0486225	-4.434166
INTS6P1	0.3329123	-0.26113	2.2477606	0.0251793	0.0486501	-4.4347
RP11-343H5	-0.382668	0.2081329	-2.247694	0.0251836	0.0486553	-4.434846
RPL7P56	-0.192687	-1.260945	-2.247678	0.0251847	0.0486553	-4.434883
SPATA8	0.2665811	-1.169237	2.2474256	0.0252009	0.0486841	-4.435438
ZFYVE27	-0.037547	6.1853776	-2.24724	0.0252129	0.0487046	-4.435846
COX7A2P2	-0.221549	-1.127082	-2.246968	0.0252304	0.0487343	-4.436445
RP11-544I2	0.3773474	0.2631467	2.24696	0.0252309	0.0487343	-4.436463
GJA1P1	-0.184788	-1.270911	-2.2466	0.0252542	0.0487766	-4.437254
CTB-33018.	0.2862268	-0.864863	2.246318	0.0252724	0.0488093	-4.437876
AP000892.4	-0.285349	-0.82734	-2.246117	0.0252854	0.0488318	-4.438318
AC073869.1	0.3090485	-0.82128	2.2456528	0.0253154	0.0488847	-4.43934
UBE2A	-0.033818	6.3597609	-2.245635	0.0253166	0.0488847	-4.439379
FHL1	0.0953434	5.8327601	2.2456312	0.0253168	0.0488847	-4.439387
JUP	-0.039744	6.8339288	-2.245599	0.0253189	0.0488862	-4.439458
RP11-431K2	-0.26047	-1.100605	-2.245407	0.0253314	0.0489076	-4.439881
AC012075.2	-0.135217	-1.46828	-2.245333	0.0253361	0.0489142	-4.440042
C2CD4A	0.4386742	4.0717318	2.2450809	0.0253525	0.0489423	-4.440598
LDHAP2	0.3461993	0.1923709	2.2450674	0.0253533	0.0489423	-4.440627
RP11-354K4	-0.111548	-1.464575	-2.24499	0.0253584	0.0489494	-4.440799
POLR2D	-0.028401	6.2304706	-2.244924	0.0253626	0.048955	-4.440943
TMEM221	0.3232217	2.5651559	2.2447899	0.0253713	0.0489692	-4.441238
RP11-394B2	0.2020851	-1.230214	2.2447029	0.025377	0.0489775	-4.441429
OCLM	0.4278766	1.2236983	2.2445599	0.0253862	0.0489928	-4.441744
RP11-354G1	-0.136577	-1.41785	-2.244531	0.0253881	0.0489939	-4.441808
RP3-508I15	0.3688735	0.1328899	2.2441558	0.0254125	0.0490383	-4.442632
GAD2	0.2368938	-1.311152	2.2441204	0.0254148	0.0490401	-4.44271
WASH1	-0.070288	5.3092627	-2.244046	0.0254196	0.0490468	-4.442874
C16orf96	-0.391893	2.2801235	-2.243855	0.025432	0.0490682	-4.443293

ZNF723P	-0.484235	-0.448424	-2.243383	0.0254627	0.0491248	-4.444332
CTD-2553C6	-0.430717	0.2362064	-2.243106	0.0254807	0.049157	-4.44494
RSRC2	0.0274417	6.3066436	2.2430802	0.0254824	0.0491576	-4.444997
HIST2H4B	-0.122148	-1.443294	-2.242977	0.0254891	0.0491679	-4.445223
TXLNGY	-0.75826	3.4968161	-2.242874	0.0254958	0.0491783	-4.445449
RP11-11601	0.2541958	-1.0742	2.2426094	0.0255131	0.049209	-4.446031
TM9SF4	-0.025792	6.6068488	-2.242555	0.0255166	0.0492132	-4.446152
MTND1P23	-0.201331	4.2972359	-2.242336	0.0255308	0.0492381	-4.446631
ZSCAN31	-0.10332	5.6323853	-2.242283	0.0255343	0.0492408	-4.446749
STK25P1	0.1628806	-1.355322	2.2422729	0.025535	0.0492408	-4.446771
AC023669.1	-0.125995	-1.437271	-2.242072	0.0255481	0.0492635	-4.447212
HINT3	-0.037453	6.1975083	-2.241348	0.0255954	0.0493521	-4.448803
RP11-284J1	-0.171207	-1.361746	-2.241206	0.0256047	0.0493674	-4.449115
EXOC5P1	-0.151544	-1.363901	-2.240939	0.0256221	0.0493984	-4.449701
PDE11A	-0.281172	4.8365395	-2.240903	0.0256245	0.0494003	-4.44978
SFTPC	-0.369322	-0.676629	-2.240871	0.0256265	0.0494017	-4.449849
NSD1	0.0325757	6.3079219	2.2406986	0.0256378	0.0494209	-4.450228
CDC42P4	0.2956211	-0.749609	2.2406188	0.0256431	0.0494283	-4.450404
INF2	0.0453623	6.6266949	2.2402541	0.0256669	0.0494718	-4.451204
MAN2A2	0.0619468	6.3690535	2.2400507	0.0256803	0.0494948	-4.451651
SLC35B3	-0.038973	6.1295589	-2.239491	0.0257169	0.0495629	-4.452878
CYP2B7P	-0.312608	5.5322632	-2.239149	0.0257394	0.0496036	-4.45363
RNF114	-0.028959	6.494417	-2.238962	0.0257517	0.0496246	-4.454039
ZNF197-AS1	0.3921982	0.0473012	2.2388876	0.0257566	0.0496304	-4.454203
KLF3-AS1	0.2193126	4.0097877	2.2388753	0.0257574	0.0496304	-4.45423
SHC3	0.2603792	3.8076841	2.238771	0.0257642	0.0496409	-4.454458
LINC01013	0.3904166	0.2500443	2.2387283	0.025767	0.0496437	-4.454552
RPL15P3	-0.094725	4.9766512	-2.238622	0.025774	0.0496546	-4.454786
RP11-44201	0.41791	1.8735827	2.238492	0.0257826	0.0496684	-4.45507
CTD-2147F2	-0.254019	-1.159852	-2.238418	0.0257874	0.0496751	-4.455232
BET1P1	0.2059005	-1.185172	2.2383312	0.0257931	0.0496835	-4.455423
IKBIP	0.0530344	5.845664	2.2377641	0.0258305	0.0497528	-4.456667
RP11-118E1	-0.331925	-0.61266	-2.237573	0.0258431	0.0497744	-4.457086
AC005251.3	-0.301053	2.2385295	-2.237201	0.0258676	0.049819	-4.457901
TMOD1	-0.189103	5.3367742	-2.236426	0.0259187	0.0499149	-4.4596
RP11-677M1	0.2822307	2.5452616	2.2362415	0.0259309	0.0499358	-4.460004
UNC50	-0.027939	6.1110224	-2.236031	0.0259448	0.0499599	-4.460465
Vax2os1_3	0.2378992	-1.204749	2.2360017	0.0259468	0.049961	-4.46053
RP11-323P1	0.3316535	-0.648707	2.2359196	0.0259522	0.0499688	-4.46071
ABHD11-AS1	0.4538879	0.9382247	2.2356343	0.0259711	0.0500024	-4.461335
RNA5SP494	-0.18104	-1.312458	-2.235615	0.0259723	0.0500024	-4.461377
AC005336.4	-0.345237	4.7261028	-2.235584	0.0259744	0.0500036	-4.461444
B4GALT6	0.1229548	4.9591643	2.2354658	0.0259822	0.0500161	-4.461704
XKR3	-0.396491	-0.617927	-2.235373	0.0259884	0.0500253	-4.461907
UBE2L5P	0.3501881	1.0644928	2.2351156	0.0260054	0.0500555	-4.462471
DUSP8P3	-0.379411	0.8874315	-2.234935	0.0260174	0.0500759	-4.462867
CTC-559E9.	0.2258359	3.4679835	2.2348281	0.0260245	0.0500868	-4.4631
IDI2-AS1	0.3871095	0.9054308	2.2342515	0.0260627	0.0501578	-4.464363
CDKL4	0.3903962	-0.154	2.2342215	0.0260647	0.050159	-4.464429
RP11-389G6	0.2569254	-1.209049	2.2340994	0.0260728	0.050172	-4.464696
RP11-589M4	-0.206556	-1.200732	-2.233903	0.0260859	0.0501945	-4.465127

MED28P4	-0.074388	-1.513423	-2.233695	0.0260997	0.0502183	-4.465581
RFTN1P1	-0.172278	-1.380243	-2.233623	0.0261044	0.0502249	-4.465738
USP45	0.0638443	5.2673807	2.2335503	0.0261093	0.0502316	-4.465898
SAMD8	0.0411726	5.8268839	2.233298	0.0261261	0.0502612	-4.46645
RP11-109J4	0.5476131	0.4983646	2.2332419	0.0261298	0.0502657	-4.466573
HOXA3	0.3012325	4.1879197	2.2332095	0.026132	0.0502672	-4.466644
CATSPERG	-0.127606	4.3577018	-2.233177	0.0261341	0.0502687	-4.466715
EAF1-AS1	-0.31471	2.2101252	-2.232606	0.0261721	0.0503392	-4.467964
RP5-1041C1	0.3396936	-0.246003	2.2325711	0.0261745	0.050341	-4.468041
MANSC4	0.3166924	-0.541158	2.2325202	0.0261779	0.0503449	-4.468152
RP4-799G3.	-0.187761	-1.287638	-2.232451	0.0261825	0.0503511	-4.468304
RP11-73K9.	-0.289005	2.4761558	-2.232342	0.0261897	0.0503624	-4.468542
NLE1	-0.055946	5.8944474	-2.232246	0.0261961	0.0503721	-4.468752
ACTG1P9	0.3472166	-0.243804	2.2320818	0.0262071	0.0503905	-4.469111
MBTPS2	-0.042837	6.1391997	-2.231999	0.0262126	0.0503984	-4.469292
AC007566.1	0.2454197	3.1349189	2.231914	0.0262183	0.0504067	-4.469478
RNFT1P2	-0.254556	-1.029514	-2.231827	0.0262241	0.0504152	-4.469669
RP11-556N2	0.2485944	-1.137696	2.2315658	0.0262415	0.0504461	-4.47024
RGR	0.1828025	-1.326495	2.2314458	0.0262495	0.0504588	-4.470502
LINC00202-	0.3563174	2.6634098	2.2312074	0.0262654	0.0504868	-4.471023
RP11-46H11	-0.391772	1.5802458	-2.231074	0.0262744	0.0505012	-4.471314
MFSD8	-0.040636	5.7800904	-2.23104	0.0262767	0.0505029	-4.47139
CHERP	-0.036199	6.3377427	-2.23102	0.026278	0.0505029	-4.471433
HNRNPUP1	0.3550379	1.0030327	2.2309513	0.0262826	0.050509	-4.471583
SSTR1	-0.319519	5.0314504	-2.230661	0.026302	0.0505437	-4.472218
RP11-69M1.	-0.154113	3.8291669	-2.230499	0.0263129	0.0505619	-4.472573
LL22NC03-3	0.337058	-0.512974	2.2303057	0.0263258	0.050584	-4.472994
TMPRSS11A	-0.192619	-1.356616	-2.230142	0.0263367	0.0506024	-4.473352
ZNF621	0.0471643	5.7930574	2.2301086	0.026339	0.0506041	-4.473425
CTD-2066L2	-0.119989	-1.435338	-2.229751	0.0263629	0.0506475	-4.474207
RP11-435P2	-0.346357	-0.377449	-2.229643	0.0263702	0.0506587	-4.474442
RP3-354N19	0.3305705	-0.376655	2.2289694	0.0264154	0.0507429	-4.475914
RP11-730B2	0.2253604	-1.083698	2.2289125	0.0264192	0.0507475	-4.476038
RP11-157P1	0.1373414	4.1317577	2.2288669	0.0264223	0.0507507	-4.476138
RP11-557L1	0.3044055	-0.815738	2.2285485	0.0264437	0.0507892	-4.476833
DPP8	0.0349506	5.9862075	2.2285191	0.0264456	0.0507903	-4.476898
ZSWIM1	-0.041078	5.6301831	-2.228465	0.0264493	0.0507934	-4.477016
KRT18P20	-0.334155	-0.434053	-2.228454	0.02645	0.0507934	-4.47704
ZNF692	-0.055555	6.0322916	-2.228205	0.0264667	0.0508228	-4.477583
ATP4A	0.4673334	-0.254732	2.2281671	0.0264693	0.0508251	-4.477666
B4GALT4	0.0353355	5.9933346	2.2281206	0.0264724	0.0508284	-4.477768
RP11-84A19	0.2279065	-1.204579	2.2280815	0.0264751	0.0508308	-4.477853
AC004932.1	0.369737	0.7897216	2.2280432	0.0264776	0.050833	-4.477937
SEPHS1P6	-0.432388	1.2284446	-2.22789	0.026488	0.0508502	-4.478272
RP4-809F18	0.2001348	-1.345268	2.2277727	0.0264958	0.0508627	-4.478527
RPS12P31	0.2765069	-0.884006	2.2276078	0.0265069	0.050881	-4.478887
AC009237.1	0.448965	1.3403005	2.2275895	0.0265082	0.050881	-4.478927
PACRG	-0.316054	4.082649	-2.227414	0.02652	0.050901	-4.479311
WBSCR27	-0.155867	5.0030801	-2.227337	0.0265252	0.0509082	-4.479478
CTC-471J1.	0.1599543	3.9049844	2.2271346	0.0265388	0.0509318	-4.47992
RP11-545J1	-0.546436	0.8009851	-2.227093	0.0265416	0.0509345	-4.480011

PP2D1	0.3077751	2.0684193	2.22694	0.026552	0.0509516	-4.480345
FOCAD-AS1	-0.350545	-0.228764	-2.226726	0.0265664	0.0509766	-4.480811
CCDC58P1	-0.184328	-1.253323	-2.226532	0.0265795	0.050999	-4.481234
GNRHR	0.3524222	-0.255228	2.2264537	0.0265848	0.0510042	-4.481406
RNU6-705P	-0.160297	-1.359597	-2.226451	0.026585	0.0510042	-4.481412
SAGE1	0.4134174	-0.792219	2.225766	0.0266313	0.0510904	-4.482907
OVOL3	0.3466648	-0.357078	2.2256503	0.0266391	0.0511027	-4.483159
THUMPD3-AS	-0.06036	5.6188868	-2.225395	0.0266564	0.0511332	-4.483716
RP11-661A1	-0.126783	4.4375372	-2.225185	0.0266706	0.0511569	-4.484175
RP11-551L1	0.3846872	-0.115798	2.2251706	0.0266715	0.0511569	-4.484205
RP11-481J2	-0.103146	4.1017128	-2.22486	0.0266926	0.0511946	-4.484882
HS6ST1	-0.060324	6.4343538	-2.224801	0.0266966	0.0511995	-4.485011
RP11-430B1	-0.266113	-0.996452	-2.224582	0.0267115	0.0512254	-4.485489
AC006461.1	0.2207548	-1.116373	2.2244501	0.0267204	0.0512398	-4.485776
RP11-434D1	0.3682703	1.3964738	2.2241831	0.0267385	0.0512718	-4.486358
TSPAN14	0.0348827	6.428435	2.2238322	0.0267623	0.0513148	-4.487123
UST	0.2543171	4.1836645	2.2233807	0.026793	0.051371	-4.488107
RHOXF1	-0.458952	0.7776818	-2.223247	0.0268021	0.0513857	-4.488398
FAM46C	0.1288172	5.5311357	2.2230948	0.0268124	0.0514028	-4.48873
ARL13A	0.3604878	0.1609359	2.2230513	0.0268154	0.0514058	-4.488824
RIPPLY2	0.4497137	-0.573526	2.2229695	0.026821	0.0514138	-4.489003
RP11-167B3	0.3415588	-0.921374	2.2228419	0.0268296	0.0514277	-4.489281
SBNO1	0.0319206	6.1534698	2.2228026	0.0268323	0.0514301	-4.489366
CTD-3051D2	-0.400276	0.412849	-2.222712	0.0268385	0.0514392	-4.489563
DYDC1	0.43129	-0.115055	2.2225085	0.0268524	0.0514631	-4.490007
RP11-352D1	0.3738066	-0.473401	2.2224145	0.0268588	0.0514727	-4.490211
AC083864.4	-0.127556	-1.440743	-2.222385	0.0268607	0.0514738	-4.490275
LINC00944	0.5156985	2.5274286	2.2222592	0.0268693	0.0514876	-4.49055
RNU7-128P	-0.08414	-1.504645	-2.222167	0.0268756	0.0514969	-4.490751
RP3-393E18	0.4062803	2.5831014	2.2219887	0.0268878	0.0515175	-4.491139
AC098828.3	-0.365649	-0.445223	-2.221642	0.0269114	0.0515601	-4.491894
LINC01433	0.2588562	-1.020953	2.2215656	0.0269166	0.051564	-4.49206
CTAGE4	0.4703384	1.4353043	2.2215505	0.0269177	0.051564	-4.492093
RP11-218C1	0.2860377	2.3721331	2.2215503	0.0269177	0.051564	-4.492094
AC114765.1	-0.127619	-1.436987	-2.221392	0.0269285	0.051582	-4.492438
FAM9C	0.2964817	-0.922677	2.2211844	0.0269427	0.0516064	-4.49289
AL121893.2	-0.151566	-1.34285	-2.221115	0.0269474	0.0516129	-4.493042
RP11-57902	-0.186614	-1.281614	-2.221027	0.0269534	0.0516216	-4.493233
CTXN3	-0.105063	-1.479429	-2.220982	0.0269565	0.0516248	-4.49333
RP11-556I1	-0.222655	-1.226451	-2.220433	0.0269941	0.051694	-4.494526
SLX4	0.0575368	5.454124	2.2202887	0.0270039	0.0517102	-4.49484
MEMO1	0.0567489	4.9087372	2.2200425	0.0270208	0.0517398	-4.495375
MEP1B	-0.547706	1.1806518	-2.219973	0.0270256	0.0517462	-4.495527
KIAA0087	0.2149628	-1.237348	2.219732	0.0270421	0.0517751	-4.496051
RP11-560A1	-0.353937	-0.877888	-2.219683	0.0270454	0.0517788	-4.496158
SNORA16B	0.326993	-0.487019	2.2194897	0.0270587	0.0518014	-4.496578
RP11-520P1	-0.124833	-1.466926	-2.21933	0.0270696	0.0518197	-4.496926
RP11-223C2	0.2640251	-1.01051	2.219255	0.0270748	0.0518268	-4.497088
COX20	-0.057308	5.5911514	-2.219169	0.0270807	0.0518354	-4.497276
RP1-81D8.3	-0.428962	-0.031278	-2.219103	0.0270852	0.0518413	-4.497419
LINC01068	0.2964123	-0.814499	2.2187947	0.0271064	0.0518791	-4.498089

TRIB1	-0.058891	6.5703544	-2.218609	0.0271191	0.0519007	-4.498492
MAEA	0.0288358	6.3282461	2.2185377	0.027124	0.0519075	-4.498648
RP11-95G6.	0.369372	1.1325921	2.2183455	0.0271372	0.05193	-4.499066
RNF38	0.0447111	6.0041455	2.2179036	0.0271676	0.0519855	-4.500027
SIGLEC18P	0.2091753	-1.161079	2.2174698	0.0271975	0.0520399	-4.50097
CCDC168	-0.375921	1.9255129	-2.217284	0.0272103	0.0520617	-4.501374
TSPAN19	0.2733592	-1.12447	2.2172392	0.0272134	0.0520648	-4.501471
CTD-2246P4	0.3463832	-0.221621	2.2172002	0.027216	0.0520672	-4.501556
CDKL3	-0.106391	3.7853865	-2.2171	0.0272229	0.0520777	-4.501773
RP11-403P1	-0.206233	-1.194595	-2.216727	0.0272487	0.0521242	-4.502584
FAM83G	0.0690138	6.279575	2.2166193	0.0272561	0.0521341	-4.502818
CABIN1	0.0320892	6.2783842	2.2166107	0.0272567	0.0521341	-4.502836
LINC00163	0.2581183	-1.1838	2.2162101	0.0272844	0.0521842	-4.503707
RNF216P1	0.0418536	5.8353045	2.2161148	0.0272909	0.0521941	-4.503914
RP11-563N6	0.2195678	-1.177786	2.2160575	0.0272949	0.0521989	-4.504038
PRSS50	-0.434505	2.9988307	-2.215793	0.0273131	0.0522311	-4.504612
MIR4450	-0.156681	-1.361506	-2.215464	0.027336	0.052272	-4.505328
MAFF	0.0787103	6.0223849	2.2151432	0.0273581	0.0523116	-4.506024
RP11-115D1	0.5771166	0.7841215	2.2150859	0.0273621	0.0523165	-4.506148
LRRC3B	0.4438909	1.0008557	2.2149839	0.0273692	0.0523272	-4.50637
MCCC1	-0.05081	6.3235318	-2.214837	0.0273793	0.0523439	-4.506688
RP11-310P5	0.2413062	-1.23512	2.214529	0.0274007	0.052382	-4.507357
ADM	-0.08215	5.8537163	-2.21446	0.0274055	0.0523884	-4.507507
ZFY	-0.729721	3.0498751	-2.213327	0.0274841	0.0525359	-4.509964
RP11-98I9.	0.1708164	4.0153746	2.2132183	0.0274917	0.0525477	-4.510201
RP11-138I1	-0.182271	-1.286723	-2.213183	0.0274942	0.0525497	-4.510279
LRIG2	0.043485	5.6468854	2.2131178	0.0274987	0.0525555	-4.510419
AC002398.1	0.417806	-0.215429	2.2130953	0.0275002	0.0525558	-4.510468
AC012362.3	-0.182843	-1.307831	-2.213047	0.0275036	0.0525595	-4.510573
RP11-432M8	-0.097512	-1.484238	-2.212911	0.027513	0.0525747	-4.510867
ASS1P7	-0.325432	-0.633453	-2.212309	0.0275549	0.052652	-4.512172
RP3-395M20	0.3235515	2.2922886	2.2122501	0.027559	0.0526572	-4.512301
SP4	0.0698227	5.2562289	2.2120622	0.0275721	0.0526794	-4.512708
AKAP8P1	-0.20734	-1.237392	-2.211872	0.0275854	0.052702	-4.513121
LINC00921	-0.117158	4.2136592	-2.211049	0.0276428	0.052809	-4.514905
HPS5	0.0655169	6.1138897	2.2109591	0.0276491	0.0528182	-4.515099
RP11-276H7	0.3636831	1.9573529	2.2107712	0.0276622	0.0528405	-4.515507
TGOLN2	-0.036129	6.9664915	-2.210477	0.0276828	0.0528771	-4.516145
MAN1A2P1	-0.152639	-1.415707	-2.210334	0.0276928	0.0528934	-4.516454
COX17P1	-0.269143	2.2487038	-2.210083	0.0277104	0.0529241	-4.516997
AC008268.2	0.3901327	-0.527695	2.2098817	0.0277245	0.0529483	-4.517434
LGALS12	0.4353305	0.4688688	2.2097351	0.0277347	0.0529652	-4.517751
AC002056.3	0.351945	-0.009275	2.2095766	0.0277458	0.0529836	-4.518095
EEF1A1P7	0.3148257	2.1700973	2.2089355	0.0277908	0.0530667	-4.519483
LINC01330	-0.408435	1.9896339	-2.208506	0.027821	0.0531215	-4.520413
RP11-360L9	-0.407838	0.8145204	-2.208399	0.0278285	0.053133	-4.520644
MAP2K3	-0.046766	6.4284202	-2.20834	0.0278326	0.0531382	-4.520772
C5orf51	-0.038032	6.1162167	-2.208207	0.0278419	0.0531532	-4.521059
RSPRY1	-0.038319	6.0874542	-2.208084	0.0278506	0.053167	-4.521327
RP11-505K9	-0.286259	1.835316	-2.208041	0.0278537	0.05317	-4.52142
AC016995.3	-0.406305	1.4647793	-2.208017	0.0278553	0.0531704	-4.521472

NAMPT	-0.074429	6.6856094	-2.2078	0.0278706	0.0531967	-4.521941
SCAF11	0.0288223	6.4716241	2.2076126	0.0278838	0.0532191	-4.522347
PTRH1	0.3609098	0.4644339	2.2069163	0.0279328	0.0533099	-4.523853
SPACA3	-0.177855	-1.33385	-2.20667	0.0279502	0.0533403	-4.524386
LINC01046	-0.101564	-1.474489	-2.206649	0.0279517	0.0533403	-4.524431
RP11-66N24	-0.180194	3.7392867	-2.206614	0.0279541	0.0533422	-4.524506
RP11-735G4	0.1909956	-1.225582	2.2065524	0.0279585	0.0533478	-4.52464
IGHV3-33-2	-0.145	-1.378412	-2.206422	0.0279677	0.0533626	-4.524923
UBBP5	-0.233924	-1.122444	-2.206239	0.0279806	0.0533821	-4.525317
RAPH1	0.1331077	5.1443445	2.2062353	0.0279808	0.0533821	-4.525326
LMLN-AS1	-0.182791	-1.328813	-2.206148	0.027987	0.0533899	-4.525514
AC016894.1	-0.192591	-1.262816	-2.206136	0.0279879	0.0533899	-4.525541
RP11-138H8	0.309151	-0.803261	2.2056731	0.0280206	0.0534495	-4.526542
RP11-361A2	-0.084097	-1.504621	-2.205532	0.0280305	0.0534657	-4.526847
RP11-370I1	-0.321399	-0.495481	-2.205225	0.0280522	0.0535043	-4.527511
RP11-1035H	-0.239283	-1.065589	-2.205178	0.0280555	0.0535078	-4.527611
TUBBP9	-0.434261	0.0409271	-2.204901	0.0280752	0.0535424	-4.528211
U47924.32	0.3862013	0.8201352	2.2046231	0.0280948	0.0535772	-4.528811
RP3-414A15	-0.297092	-0.562069	-2.204379	0.0281121	0.0536073	-4.529338
SNRPB	-0.042309	6.7619887	-2.203989	0.0281398	0.0536573	-4.530182
TRIM75P	-0.222615	-1.129373	-2.203963	0.0281416	0.053658	-4.530237
TMSB4Y	-0.608612	2.1957889	-2.203915	0.028145	0.0536617	-4.530341
RP4-570O12	0.3801975	-0.717734	2.2036053	0.028167	0.0537008	-4.53101
AQP3	0.0862909	6.3262647	2.2034617	0.0281772	0.0537174	-4.53132
RP5-933K21	-0.323761	2.0364139	-2.203119	0.0282016	0.0537611	-4.532061
MESDC1	0.0482278	5.8696575	2.2024955	0.0282459	0.0538428	-4.533407
CD55	-0.106318	5.9165587	-2.202442	0.0282497	0.0538472	-4.533522
RP11-65J3.	-0.243761	-0.993376	-2.202374	0.0282545	0.0538536	-4.533668
HNRNPA1P14	0.3010959	-0.607836	2.2019381	0.0282856	0.05391	-4.53461
MAP2K4	0.0403951	6.0001357	2.2017976	0.0282956	0.0539263	-4.534914
RPL5P28	0.2319484	-1.110887	2.2013871	0.0283249	0.0539792	-4.5358
RP11-756A2	0.5360466	2.4611017	2.2013528	0.0283273	0.0539811	-4.535874
LPCAT2	0.116812	5.4062768	2.2011266	0.0283435	0.054009	-4.536362
RP3-445O1C	-0.180061	-1.27655	-2.200289	0.0284033	0.0541202	-4.538169
WFIKKN2	-0.449479	0.1249155	-2.200066	0.0284193	0.0541477	-4.538649
TPX2	0.089843	6.049909	2.1998632	0.0284338	0.0541724	-4.539087
MIR320E	0.3210904	-0.386051	2.1998441	0.0284352	0.0541724	-4.539128
LRCH1	0.0571817	5.8349868	2.1998128	0.0284374	0.0541738	-4.539196
VLDLR	0.2909945	4.739012	2.1997175	0.0284442	0.054184	-4.539401
TMED1	-0.049075	5.8263172	-2.199657	0.0284486	0.0541894	-4.539532
FOXI2	0.4048236	0.1068286	2.1995625	0.0284553	0.0541995	-4.539735
AL390877.1	0.5029008	1.4736758	2.1994804	0.0284612	0.0542079	-4.539913
RP11-19J5.	-0.119422	-1.442115	-2.199242	0.0284783	0.0542376	-4.540427
CCDC66	0.0472542	5.4347838	2.1984232	0.028537	0.0543466	-4.542191
RP11-528I4	-0.351972	-0.218667	-2.198287	0.0285468	0.0543624	-4.542486
RUFY1	-0.032527	6.3558031	-2.198116	0.0285591	0.0543829	-4.542853
HTRA2	-0.033811	6.0072854	-2.198015	0.0285663	0.0543939	-4.54307
LINC01506	-0.509805	1.342934	-2.19795	0.028571	0.0544	-4.543212
APBA1	-0.145112	5.6420393	-2.197844	0.0285786	0.0544116	-4.543439
RP5-901A4.	0.3499246	1.7833235	2.1977956	0.0285821	0.0544154	-4.543544
FANCE	0.0971505	5.1113195	2.1977572	0.0285849	0.0544179	-4.543627

ZFYVE9	-0.046202	5.9281471	-2.197637	0.0285935	0.0544287	-4.543886
HOTAIR	0.279903	-1.166289	2.1976164	0.028595	0.0544287	-4.54393
LINC01166	0.2680268	-1.076415	2.1976156	0.0285951	0.0544287	-4.543932
CYP2C58P	-0.311253	-0.797876	-2.197509	0.0286027	0.0544405	-4.544161
RP11-736K2	-0.152918	4.4507984	-2.197344	0.0286146	0.0544602	-4.544516
RP11-595B2	-0.506507	1.2726981	-2.19686	0.0286494	0.0545237	-4.545559
RAB43	0.1138039	5.2012728	2.1967523	0.0286572	0.0545356	-4.545791
CHCHD2P6	-0.195517	3.3471887	-2.196651	0.0286645	0.0545467	-4.54601
RP11-486A1	-0.22195	4.2415165	-2.19654	0.0286725	0.0545591	-4.546249
AC010761.1	0.4058034	0.8007613	2.1961879	0.0286979	0.0546046	-4.547006
DKFZP434L1	0.2029779	-1.286694	2.1960834	0.0287055	0.0546161	-4.547231
ALDH3A1	-0.464974	4.8817154	-2.196051	0.0287078	0.0546176	-4.5473
FAR2P1	0.6440262	0.6945936	2.1959816	0.0287128	0.0546244	-4.547451
OGFR-AS1	-0.262206	3.0493392	-2.195295	0.0287624	0.0547158	-4.548928
PHBP7	-0.194293	-1.212719	-2.194414	0.0288262	0.0548343	-4.550825
RP11-240G2	0.2784866	-0.808711	2.1943925	0.0288277	0.0548344	-4.55087
WDR11	0.0400181	6.2289846	2.1941946	0.028842	0.0548588	-4.551296
PMCHL2	0.2001357	-1.208128	2.1940513	0.0288524	0.0548713	-4.551604
AC079776.2	0.3497596	-0.519302	2.1940507	0.0288525	0.0548713	-4.551606
CTD-2026K1	-0.229423	-1.07035	-2.194039	0.0288533	0.0548713	-4.551631
TMEM80	-0.046142	5.8321744	-2.194021	0.0288546	0.0548713	-4.55167
PRDX4	-0.049506	6.7087594	-2.193855	0.0288666	0.0548897	-4.552026
RP11-307P5	0.4000339	-0.412282	2.193846	0.0288673	0.0548897	-4.552046
RP11-546B8	0.2682979	-0.93171	2.1937304	0.0288757	0.0549028	-4.552295
CCK	0.3651119	-0.768675	2.1935577	0.0288882	0.0549237	-4.552666
SLCO1B3	0.634047	3.8742618	2.1933815	0.028901	0.0549442	-4.553045
HIPK1	0.045493	6.2511702	2.1933679	0.028902	0.0549442	-4.553074
AL031768.1	-0.156066	-1.334968	-2.193243	0.0289111	0.0549586	-4.553343
PPAP2A	-0.067237	6.4208526	-2.193129	0.0289193	0.0549689	-4.553588
RP13-465B1	-0.382632	0.5711422	-2.193127	0.0289195	0.0549689	-4.553593
LINC00592	0.2535529	-1.104491	2.193051	0.028925	0.0549765	-4.553756
ANKUB1	0.291178	-0.782778	2.1929115	0.0289351	0.0549929	-4.554055
EIF4B	-0.032024	6.9160843	-2.192845	0.02894	0.0549975	-4.554198
HOGA1	-0.239541	5.6624203	-2.192835	0.0289407	0.0549975	-4.55422
EIF5AP3	0.1832551	-1.252038	2.1928162	0.0289421	0.0549975	-4.55426
FAM26D	-0.222225	-1.221878	-2.192716	0.0289494	0.0550085	-4.554476
RP11-18707	0.3905533	1.1874076	2.1925033	0.0289648	0.055035	-4.554933
HNRNPM	-0.023691	6.7891048	-2.19221	0.0289861	0.0550724	-4.555563
RP13-494C2	0.2046752	-1.201816	2.1921915	0.0289875	0.0550724	-4.555603
RAB26	-0.152951	5.5985771	-2.191966	0.0290039	0.0551007	-4.556088
SLC2A3P2	-0.27213	-0.942758	-2.191875	0.0290106	0.0551104	-4.556283
RP11-180N1	-0.526516	0.9900171	-2.19181	0.0290153	0.0551166	-4.556424
FANCI	0.081711	5.7251946	2.1917223	0.0290217	0.0551258	-4.556612
RP11-302F1	0.2319849	-1.214313	2.1916621	0.0290261	0.0551312	-4.556741
LINC00652	0.3991193	0.5304426	2.1915331	0.0290355	0.0551462	-4.557018
RP11-533E1	-0.233798	3.1978042	-2.191277	0.0290541	0.0551788	-4.557567
OR7E4P	-0.118262	-1.459882	-2.190983	0.0290756	0.0552136	-4.558201
KLK3	-0.487481	-0.634866	-2.190969	0.0290766	0.0552136	-4.558229
DNAJC3-AS1	-0.094289	4.9587162	-2.190964	0.029077	0.0552136	-4.558241
KARSP2	-0.280087	3.9586735	-2.190909	0.029081	0.0552183	-4.558359
XAGE2B	0.3192964	-1.024071	2.1907831	0.0290902	0.0552329	-4.558629

XXyac-YM21	0.5837873	0.9605159	2.1907372	0.0290935	0.0552364	-4.558728
RPL34P34	-0.374101	0.4110668	-2.190644	0.0291003	0.0552465	-4.558929
DPH3	-0.035119	6.2010725	-2.190592	0.0291041	0.0552508	-4.55904
ENPP3	0.336481	4.4318257	2.1904786	0.0291124	0.0552636	-4.559283
DDX39AP1	-0.103643	-1.476258	-2.190324	0.0291237	0.0552821	-4.559614
VBP1	-0.03423	6.1855036	-2.190081	0.0291414	0.055313	-4.560136
MEF2B	0.232832	2.9705437	2.1900563	0.0291433	0.0553136	-4.56019
AC007000.1	0.3671683	0.6217474	2.1899727	0.0291494	0.0553223	-4.56037
KB-1043D8.	-0.382911	-0.09808	-2.189852	0.0291582	0.0553362	-4.560629
GRAMD3	-0.044721	5.8984105	-2.189686	0.0291704	0.0553564	-4.560986
RNU7-3P	-0.158431	-1.33554	-2.189555	0.02918	0.0553717	-4.561267
FABP7	-0.340868	-0.650383	-2.189317	0.0291974	0.0554004	-4.561778
SIPA1L2	0.0978136	6.0382569	2.1893064	0.0291981	0.0554004	-4.5618
UNCX	-0.104607	-1.48763	-2.189224	0.0292042	0.055409	-4.561976
ZNF17	0.0567572	4.9945862	2.1887117	0.0292417	0.0554774	-4.563076
AC073415.2	0.3413952	-0.374434	2.1886759	0.0292444	0.0554795	-4.563153
CENPL	0.0905683	5.1987188	2.1882231	0.0292776	0.0555396	-4.564125
AC114808.3	-0.104146	-1.49603	-2.188102	0.0292864	0.0555535	-4.564384
RP11-10801	-0.274854	4.0747719	-2.188022	0.0292923	0.0555618	-4.564556
ANKRD44	0.0649949	5.5308592	2.1877848	0.0293098	0.055591	-4.565065
OXT	0.6169876	1.3216999	2.1877716	0.0293107	0.055591	-4.565093
AMY2A	-0.238873	-1.190526	-2.187674	0.0293179	0.0556017	-4.565302
TSNAXIP1	-0.136196	3.9673049	-2.187585	0.0293244	0.0556086	-4.565492
RP11-495P1	-0.604306	1.4709944	-2.187583	0.0293246	0.0556086	-4.565498
RP11-316M2	0.3621562	1.5723271	2.1875096	0.02933	0.0556159	-4.565655
HIVEP1	0.0681422	5.8742387	2.18734	0.0293425	0.0556367	-4.566019
PIKFYVE	0.0505706	5.9988066	2.187248	0.0293492	0.0556466	-4.566216
RP11-227D1	0.3874505	0.0960137	2.186998	0.0293676	0.0556786	-4.566752
RP1-90L14.	-0.576212	0.4375823	-2.186879	0.0293764	0.0556924	-4.567008
RP11-1267H	-0.484749	0.9034619	-2.186715	0.0293885	0.0557124	-4.56736
TTC27	-0.03281	5.9126964	-2.186519	0.0294029	0.0557368	-4.567779
RN7SKP230	-0.254132	-0.981336	-2.186479	0.0294059	0.0557395	-4.567865
ASCL4	0.2239677	-1.152786	2.1863356	0.0294164	0.0557539	-4.568172
ILF2	-0.03103	6.7362838	-2.186326	0.0294171	0.0557539	-4.568193
ARPC2	0.0253227	6.7202089	2.1863138	0.029418	0.0557539	-4.568219
RP11-333E1	0.2123834	3.5710265	2.186225	0.0294246	0.0557634	-4.56841
RPL18P13	-0.556732	0.8960339	-2.186133	0.0294313	0.0557733	-4.568606
GAS2L1P1	0.1469122	-1.389352	2.1859864	0.0294422	0.055791	-4.568921
ZNF720P1	-0.36891	-0.864046	-2.184967	0.0295174	0.0559307	-4.571105
RN7SL39P	-0.096973	-1.48335	-2.184805	0.0295295	0.0559505	-4.571453
RP11-823P9	-0.108602	-1.469669	-2.184775	0.0295316	0.0559518	-4.571516
HNRNPCL3	-0.272141	-1.189782	-2.184727	0.0295352	0.0559557	-4.57162
CSH2	-0.132747	-1.423044	-2.184615	0.0295435	0.0559671	-4.571859
RP11-5C23.	-0.155192	3.6104163	-2.184604	0.0295443	0.0559671	-4.571884
CYB5R4	0.0477977	5.7039275	2.1844346	0.0295568	0.0559831	-4.572246
RP11-946P6	0.2845763	-0.939975	2.1844337	0.0295569	0.0559831	-4.572248
RP11-94B19	-0.239794	-1.129133	-2.184428	0.0295573	0.0559831	-4.572261
TCL6	0.5550486	1.1876171	2.184193	0.0295747	0.0560131	-4.572763
RP11-210K2	-0.273219	-0.768485	-2.184161	0.0295771	0.0560147	-4.572833
RP11-258C1	0.0809165	5.069863	2.1836703	0.0296135	0.0560806	-4.573882
TP73	-0.231676	4.612023	-2.183463	0.0296289	0.0561048	-4.574327

SH3GL1	-0.034491	6.4766022	-2.183456	0.0296293	0.0561048	-4.574341
AANAT	0.406384	0.9381543	2.1828681	0.029673	0.0561846	-4.5756
RP11-490H2	0.3714043	0.4530731	2.1828319	0.0296757	0.0561867	-4.575677
PAQR3	0.1052655	5.388734	2.1828114	0.0296772	0.0561867	-4.575721
RP11-439M1	0.380853	-0.57253	2.1827383	0.0296826	0.0561941	-4.575877
AC012370.3	-0.225668	-1.077395	-2.182439	0.0297048	0.0562325	-4.576518
NANOGP1	-0.485775	0.4424705	-2.182423	0.029706	0.0562325	-4.576551
RP11-431D1	-0.111821	-1.475618	-2.182309	0.0297145	0.0562458	-4.576797
RP11-317G6	-0.201607	-1.221822	-2.182146	0.0297266	0.0562646	-4.577145
RP5-884M6.	0.3498029	-0.794992	2.1821332	0.0297276	0.0562646	-4.577172
CTC-297N7.	0.2849017	-0.713821	2.1821124	0.0297291	0.0562647	-4.577217
SRSF1P1	-0.113066	-1.456288	-2.181885	0.0297461	0.0562938	-4.577704
CTA-212A2.	0.3322669	2.6547479	2.1816467	0.0297638	0.0563244	-4.578213
PVRL4	0.4142622	2.7417407	2.1815543	0.0297707	0.0563335	-4.578411
RP11-348N5	0.3668935	1.0543217	2.1815407	0.0297717	0.0563335	-4.57844
LMO7	-0.057206	6.4004274	-2.181023	0.0298103	0.0564036	-4.579547
RP11-114G1	-0.605217	2.279824	-2.180987	0.029813	0.0564058	-4.579625
RP11-63A1.	-0.379801	0.3108647	-2.180955	0.0298153	0.0564073	-4.579692
KCTD13	-0.046569	5.6693156	-2.180934	0.0298169	0.0564074	-4.579738
TMEM191A	0.1639915	4.7379722	2.1807493	0.0298307	0.0564305	-4.580133
CBY1	-0.040473	5.9342423	-2.18058	0.0298433	0.0564514	-4.580493
ERCC4	-0.066963	5.3524919	-2.180375	0.0298586	0.0564774	-4.580932
CYP2AB1P	0.3986024	-0.538006	2.1801905	0.0298724	0.0565006	-4.581327
RPL7	-0.051843	7.140737	-2.180111	0.0298783	0.0565089	-4.581496
DNAJC12	-0.167582	5.4995136	-2.179976	0.0298884	0.0565251	-4.581786
TSEN34	-0.036075	6.3126316	-2.179806	0.0299011	0.0565462	-4.582149
PCSK2	0.3190589	-0.930861	2.1797537	0.029905	0.0565507	-4.582261
ZNF830	-0.042249	5.7194046	-2.17952	0.0299225	0.0565764	-4.58276
RP3-468B3.	-0.39822	1.5182434	-2.17951	0.0299232	0.0565764	-4.582781
KCTD20	-0.031845	6.5506014	-2.17951	0.0299233	0.0565764	-4.582783
RP11-490K7	0.379767	0.5209622	2.1791685	0.0299488	0.0566217	-4.583511
HOMER2P2	-0.173937	-1.367377	-2.179103	0.0299537	0.056628	-4.583651
CAMK2A	0.5060861	1.9076946	2.178733	0.0299814	0.0566775	-4.584442
CYP2AC1P	-0.16854	-1.360727	-2.178694	0.0299844	0.0566801	-4.584525
RNA5SP490	0.2557746	-0.95991	2.1785883	0.0299923	0.0566921	-4.584751
GGTA1P	0.0849397	5.438399	2.1784185	0.030005	0.0567133	-4.585114
TRPM2-AS	0.4089167	0.4542387	2.1783729	0.0300084	0.0567168	-4.585211
ZNF350	0.0830965	5.3016339	2.1782504	0.0300176	0.0567312	-4.585473
SERPINB12	-0.102446	-1.492483	-2.177929	0.0300418	0.0567739	-4.58616
CTD-2227I1	-0.333289	-0.67607	-2.177781	0.0300529	0.056792	-4.586476
RP1-85F18.	-0.226147	-1.086839	-2.177711	0.0300581	0.0567989	-4.586624
RP11-710E1	-0.197066	-1.231637	-2.177351	0.0300852	0.0568471	-4.587394
RP1-225E12	0.3076185	-0.586447	2.1771712	0.0300987	0.0568697	-4.587777
VWA3A	0.3781335	0.2563525	2.1771355	0.0301014	0.0568718	-4.587854
PINK1	-0.075183	6.1822012	-2.176985	0.0301126	0.0568902	-4.588174
KDM1A	-0.03184	6.2977287	-2.176674	0.0301361	0.0569314	-4.588838
RP11-21L19	0.266535	-0.91781	2.1765878	0.0301426	0.0569408	-4.589023
C5orf56	-0.068918	5.2419478	-2.176351	0.0301604	0.0569716	-4.589529
RNU6-476P	0.2469372	-0.901415	2.1762417	0.0301686	0.0569841	-4.589761
DBH	0.2200054	4.8201022	2.1762149	0.0301706	0.056985	-4.589818
RP1-79C4.4	0.3085096	-0.805457	2.1759304	0.0301921	0.0570225	-4.590426

KB-1125A3.	-0.365726	1.3847614	-2.175881	0.0301958	0.0570267	-4.590532
RP11-7F17.	-0.337472	3.2579611	-2.175487	0.0302255	0.0570798	-4.591372
ANKRD40	-0.027861	6.2066287	-2.17512	0.0302532	0.0571292	-4.592155
DR1	0.0275558	6.2776451	2.1749919	0.0302629	0.0571445	-4.592427
CTD-3131K8	0.3650003	0.8850652	2.1748108	0.0302766	0.0571674	-4.592814
RP4-644L1.	0.3304237	-0.650742	2.174611	0.0302917	0.0571929	-4.59324
HPS3	0.0477909	6.0073474	2.1741023	0.0303302	0.0572626	-4.594325
HNRNPCP8	-0.255102	-0.873405	-2.173917	0.0303442	0.0572861	-4.594719
LILRP1	0.1802333	-1.285032	2.1738086	0.0303524	0.0572986	-4.594951
RPL29P19	0.3760796	1.0837644	2.1737712	0.0303552	0.057301	-4.59503
RP11-129B9	-0.375694	1.4904528	-2.173542	0.0303726	0.0573299	-4.595519
OR8K2P	-0.071535	-1.511823	-2.173528	0.0303736	0.0573299	-4.595549
LINC00453	-0.094167	-1.497565	-2.1735	0.0303758	0.057331	-4.595609
AC114498.1	-0.382341	2.3739574	-2.173459	0.0303789	0.0573316	-4.595696
ANKRD36P1	-0.143508	-1.406651	-2.173454	0.0303793	0.0573316	-4.595707
GAFA3	0.417109	1.46269	2.1733549	0.0303868	0.0573428	-4.595918
IL17RB	-0.082661	6.3467243	-2.1732	0.0303985	0.057362	-4.596248
RP11-234A1	-0.104945	4.9935638	-2.172826	0.0304269	0.0574125	-4.597044
ATG4D	0.0568987	5.7087734	2.1726941	0.0304369	0.0574285	-4.597326
ZNF556	0.4560254	0.787763	2.1723314	0.0304644	0.0574775	-4.598098
AC015971.2	0.4278257	1.3558151	2.1722714	0.030469	0.0574809	-4.598226
AP001372.2	-0.067225	5.0379503	-2.172264	0.0304695	0.0574809	-4.598242
CD63	-0.038186	7.1745454	-2.172245	0.030471	0.0574809	-4.598282
PSMC1P4	-0.1652	-1.344025	-2.172173	0.0304765	0.0574855	-4.598436
IRF2	-0.04243	6.1502483	-2.172172	0.0304766	0.0574855	-4.598438
WI2-85898F	0.2454442	-1.069352	2.1719884	0.0304905	0.0575088	-4.598829
STRC	0.4528501	1.0071107	2.1718944	0.0304977	0.0575193	-4.599029
IQCJ-SCHIF	0.2635661	4.0967997	2.1717625	0.0305077	0.0575353	-4.59931
AC073343.1	0.4131103	1.0044259	2.1715875	0.030521	0.0575574	-4.599683
TOP1MT	-0.058816	6.3334054	-2.171429	0.0305331	0.0575772	-4.600021
FDPSP4	-0.170271	-1.295322	-2.171366	0.0305379	0.0575832	-4.600154
RP11-352G1	0.3650993	1.3314554	2.1713452	0.0305394	0.0575833	-4.600199
CTC-246B18	-0.308747	2.8779035	-2.171201	0.0305504	0.0575991	-4.600505
RP3-422G23	-0.184876	-1.318915	-2.171193	0.030551	0.0575991	-4.600523
GDNF	-0.56136	2.9332445	-2.171171	0.0305527	0.0575994	-4.600571
HIST2H2AA4	-0.25474	-1.090299	-2.171028	0.0305636	0.0576169	-4.600873
RP13-216E2	0.269723	-0.864343	2.1705244	0.030602	0.0576863	-4.601946
PRELID1P4	0.3141311	1.3674921	2.1703711	0.0306137	0.0577054	-4.602273
CTD-2033A1	0.3279114	2.2690989	2.1702738	0.0306211	0.0577164	-4.60248
ATP1B4	-0.196492	-1.229536	-2.170212	0.0306258	0.0577223	-4.602612
HNRNPLL	0.0248801	5.9784364	2.1701396	0.0306313	0.0577297	-4.602765
CH507-9B2.	-0.193215	4.7336856	-2.16998	0.0306435	0.0577497	-4.603105
GPR176	0.0977687	5.3504412	2.1699289	0.0306474	0.0577541	-4.603214
RP13-638C3	0.3746962	1.0995401	2.1698524	0.0306533	0.0577621	-4.603376
EIF5AP2	0.3487664	0.137229	2.1697549	0.0306607	0.0577731	-4.603584
RP11-399J1	-0.357734	3.4477081	-2.169615	0.0306714	0.0577903	-4.603882
RP1-20C7.6	-0.243729	2.7740151	-2.169534	0.0306776	0.057799	-4.604055
RP11-24M17	0.2159401	-1.126254	2.169107	0.0307103	0.0578575	-4.604962
L2HGDH	-0.071608	5.6477837	-2.168936	0.0307234	0.057878	-4.605326
RP11-332K1	0.2657013	-1.20109	2.1689232	0.0307243	0.057878	-4.605353
RP11-267C1	-0.52142	0.033102	-2.168836	0.030731	0.0578876	-4.605539

LINC00543	-0.181184	4.9569758	-2.168431	0.030762	0.0579431	-4.606401
RP11-66602	-0.295233	-0.75517	-2.168154	0.0307832	0.05798	-4.606989
TLN1	0.0292008	6.9146003	2.1680494	0.0307913	0.0579922	-4.607211
CLIC5	0.1853189	4.6528703	2.1676358	0.030823	0.0580489	-4.608091
RP11-669C1	-0.206229	-1.181474	-2.167398	0.0308412	0.0580773	-4.608595
RP11-217B7	-0.223033	-1.135413	-2.167398	0.0308412	0.0580773	-4.608596
BNIP3P34	-0.217484	-1.220469	-2.167153	0.03086	0.0581097	-4.609116
IGLV3-22	0.2012378	-1.317052	2.1670603	0.0308672	0.0581201	-4.609314
LINC01258	-0.262605	-1.17136	-2.166853	0.0308831	0.0581466	-4.609755
RP11-284G1	-0.602649	0.597218	-2.166836	0.0308844	0.0581466	-4.609791
ORC1	0.1319739	5.0073563	2.1663149	0.0309245	0.0582191	-4.610898
RP11-561B1	-0.354583	0.0504617	-2.166175	0.0309353	0.0582357	-4.611195
RP11-36N2C	0.2997024	-1.016196	2.166156	0.0309367	0.0582357	-4.611235
SEMA4B	0.0525813	6.2555982	2.1661378	0.0309381	0.0582357	-4.611274
SLC14A2	-0.509762	1.3988829	-2.165923	0.0309547	0.0582639	-4.611731
ZNF367	-0.086042	5.5490138	-2.165864	0.0309592	0.0582694	-4.611856
TAS2R4	0.314608	2.1438555	2.1655433	0.0309839	0.0583129	-4.612537
RP11-35501	0.1360206	3.982244	2.1653166	0.0310014	0.0583428	-4.613018
PGM5P4	0.2623814	-0.963555	2.1652638	0.0310055	0.0583475	-4.61313
RP11-418H1	-0.396809	1.6755227	-2.164876	0.0310354	0.0584007	-4.613953
RP11-483C6	0.1925317	-1.229614	2.1647142	0.0310479	0.0584212	-4.614297
RP11-416A1	0.2879174	-0.669543	2.1644535	0.031068	0.0584561	-4.614851
AC090804.1	0.3637589	0.2670102	2.1643596	0.0310753	0.0584668	-4.61505
RP11-205K6	-0.245965	-1.09995	-2.163996	0.0311034	0.0585167	-4.615822
RP11-799D4	-0.206205	3.919344	-2.163635	0.0311313	0.0585662	-4.616588
CREM	-0.040612	6.0897588	-2.163021	0.0311789	0.0586527	-4.617891
FGF5	0.2853035	-1.113615	2.1622895	0.0312356	0.0587552	-4.619442
RP11-76E17	0.2449458	-1.154171	2.1622765	0.0312366	0.0587552	-4.619469
TMEM92	0.5244249	4.3412602	2.1622515	0.0312386	0.0587558	-4.619522
GS1-124K5.	0.2533089	3.3852809	2.1622147	0.0312414	0.0587582	-4.619601
AZU1	0.4000874	0.4134275	2.1620526	0.031254	0.0587788	-4.619944
RP11-484N1	-0.400601	3.9164306	-2.16199	0.0312589	0.0587849	-4.620077
MAGEB10	-0.285134	-1.083282	-2.161793	0.0312742	0.0588092	-4.620495
RP13-926M1	-0.215116	-1.208782	-2.161782	0.031275	0.0588092	-4.620518
PPBPP2	-0.122514	-1.443307	-2.161382	0.0313061	0.0588647	-4.621366
ERC2	0.4975225	0.9099773	2.1611144	0.031327	0.0589008	-4.621933
RP11-624J1	-0.088631	-1.48994	-2.161043	0.0313326	0.0589053	-4.622086
HOXA13	-0.546416	4.0367059	-2.161042	0.0313326	0.0589053	-4.622086
RPS15AP6	-0.183939	-1.277862	-2.16102	0.0313343	0.0589055	-4.622133
MAGOH2P	0.3296804	2.6654975	2.1609055	0.0313432	0.0589183	-4.622376
RP11-815J4	0.3654526	0.9231946	2.1608915	0.0313443	0.0589183	-4.622406
AC090954.5	0.3556256	-0.646741	2.1608333	0.0313488	0.0589237	-4.622529
RNU6-833P	-0.30131	-0.67104	-2.160607	0.0313664	0.0589538	-4.623008
ZNF429	-0.078354	5.2381067	-2.160465	0.0313776	0.0589717	-4.62331
RPL32P34	-0.158821	-1.33826	-2.160191	0.0313989	0.0590087	-4.62389
KRT16P2	0.3337825	-0.931203	2.1600101	0.031413	0.0590322	-4.624273
RP11-671J1	-0.253695	-0.901487	-2.159878	0.0314233	0.0590485	-4.624553
RP1-20N18.	-0.20343	-1.281247	-2.159845	0.0314259	0.0590501	-4.624623
AC104809.3	0.4652051	1.711747	2.1598255	0.0314274	0.0590501	-4.624664
PRMT1	0.0376303	6.4752062	2.1597668	0.031432	0.0590557	-4.624789
COX10	0.0493911	5.7508302	2.1596363	0.0314422	0.0590718	-4.625065

EXO5	-0.051987	5.3638073	-2.159614	0.0314439	0.059072	-4.625112
RP11-283I3	-0.406353	0.1293203	-2.159591	0.0314457	0.0590724	-4.625161
RP11-109P6	-0.395695	-0.822695	-2.159554	0.0314486	0.0590747	-4.625239
GGTA2P	-0.297436	-0.63587	-2.159381	0.0314621	0.0590971	-4.625605
KRTAP5-10	-0.428118	1.424312	-2.159289	0.0314693	0.0591076	-4.625801
RP11-439K3	0.2221989	-1.127947	2.1592629	0.0314713	0.0591083	-4.625856
TAAR6	0.1683519	-1.360108	2.15911	0.0314833	0.0591277	-4.62618
AC109829.1	-0.377134	1.3503059	-2.159051	0.0314878	0.0591333	-4.626304
RRP1B	-0.035234	6.2231303	-2.158894	0.0315002	0.0591534	-4.626638
FAM106A	0.3459631	-0.637315	2.1586993	0.0315154	0.0591789	-4.627049
RP1-122P22	0.4141453	1.5231228	2.1586184	0.0315217	0.0591877	-4.627221
UBXN7-AS1	-0.187267	-1.239876	-2.15852	0.0315294	0.0591991	-4.627428
RP11-165M1	-0.174101	-1.264227	-2.158475	0.0315329	0.0592027	-4.627524
NUTM2G	0.3621239	1.0933726	2.1582324	0.0315519	0.0592353	-4.628038
RP5-1085F1	0.0792328	5.1867943	2.1581946	0.0315549	0.0592378	-4.628118
HJURP	0.1358239	5.3739793	2.1580816	0.0315637	0.0592514	-4.628357
RP9P	0.0899712	4.84469	2.1579925	0.0315707	0.0592598	-4.628545
ST8SIA3	0.5181536	1.4530003	2.1579827	0.0315715	0.0592598	-4.628566
RP11-531H8	-0.103971	-1.469369	-2.157628	0.0315993	0.059309	-4.629317
RP11-30J20	-0.65736	1.5395681	-2.157416	0.0316159	0.0593371	-4.629765
RP1-34H18.	-0.34645	-0.679055	-2.157337	0.0316221	0.0593457	-4.629933
TGIF2LY	-0.245155	-1.23355	-2.156692	0.0316727	0.0594377	-4.631297
AP002381.2	0.2778408	-0.709154	2.1566687	0.0316746	0.0594381	-4.631347
MAGEB16	-0.400504	-0.7742	-2.156647	0.0316763	0.0594382	-4.631392
RNU6ATAC9F	-0.118548	-1.431553	-2.156535	0.0316851	0.0594492	-4.631629
AC067969.2	-0.209997	-1.31832	-2.156531	0.0316854	0.0594492	-4.631638
RP11-567P1	-0.139664	-1.399975	-2.156389	0.0316965	0.0594608	-4.631937
ZFP41	-0.076021	5.7578923	-2.156378	0.0316974	0.0594608	-4.63196
ZNF12	0.0382411	5.9062505	2.156372	0.0316979	0.0594608	-4.631974
ADPGK-AS1	0.3502016	0.6287213	2.1563548	0.0316992	0.0594608	-4.63201
RP11-33E12	0.240343	4.2941765	2.1563485	0.0316997	0.0594608	-4.632024
USP36	-0.031727	6.2890316	-2.156269	0.031706	0.0594695	-4.632192
HMGB1P39	-0.138779	-1.379755	-2.156178	0.0317131	0.0594798	-4.632384
RRP7B	0.0710373	4.9761562	2.1553272	0.0317801	0.0596024	-4.634183
HNRNPA1P35	0.338039	1.3031393	2.1552903	0.031783	0.0596048	-4.634261
ARHGAP42	-0.092181	5.4853959	-2.155161	0.0317932	0.0596209	-4.634535
RP11-618I1	0.3637566	-0.488936	2.1551222	0.0317963	0.0596235	-4.634617
AACSP1	0.4669543	-0.294516	2.1549062	0.0318133	0.0596524	-4.635073
RBFOX3	0.3739847	0.2952347	2.153957	0.0318882	0.0597898	-4.637079
ZDHHC23	0.0831482	5.5377574	2.1539037	0.0318925	0.0597947	-4.637192
RNA5SP78	0.2736736	-0.795896	2.1538319	0.0318981	0.0598022	-4.637343
CDK2	0.0475106	5.9796166	2.1537724	0.0319028	0.059808	-4.637469
BPTF	0.0373019	6.2336591	2.1536568	0.031912	0.059822	-4.637713
TMEM234	-0.051147	5.4876698	-2.153381	0.0319338	0.0598598	-4.638296
MIR6812	0.2069804	-1.158578	2.1532542	0.0319438	0.0598756	-4.638563
LRP2BP	-0.105658	4.2759135	-2.152766	0.0319824	0.0599449	-4.639594
DMRT2	0.3742496	-0.794422	2.1526728	0.0319899	0.0599557	-4.639791
AC104532.2	-0.357986	0.1680406	-2.152644	0.0319921	0.0599569	-4.639851
TTC37	-0.043259	6.3935165	-2.152607	0.0319951	0.0599593	-4.63993
RP11-344N1	-0.204637	-1.286589	-2.152337	0.0320165	0.0599964	-4.640501
SOD2	-0.057414	7.230687	-2.151911	0.0320503	0.0600566	-4.641399

LINC01189	-0.252186	-1.174716	-2.151769	0.0320615	0.0600747	-4.641699
KATNAL1	-0.077843	5.486045	-2.151728	0.0320648	0.0600776	-4.641785
RP11-474J1	-0.241381	-1.075511	-2.151611	0.032074	0.0600919	-4.642032
FNTB	-0.036272	5.615622	-2.151434	0.0320881	0.0601152	-4.642405
GBP1	-0.075891	6.2895211	-2.151356	0.0320943	0.0601238	-4.642571
AL132709.1	0.175356	-1.35916	2.1512307	0.0321043	0.0601343	-4.642835
CTC-360P9.	-0.223018	-1.268959	-2.151228	0.0321045	0.0601343	-4.642841
CDYL2	0.1162328	5.2966623	2.1512228	0.0321049	0.0601343	-4.642852
SLC22A4	-0.159266	4.4184427	-2.151162	0.0321097	0.0601403	-4.64298
CTA-407F11	-0.273979	-1.033378	-2.150793	0.0321391	0.0601921	-4.643758
RN7SL551P	-0.175347	-1.288798	-2.150712	0.0321455	0.0602012	-4.643929
LLNLR-304G	0.2553011	-1.050705	2.1506234	0.0321526	0.0602113	-4.644116
IQCF1	-0.099395	-1.469914	-2.150456	0.0321659	0.0602306	-4.644469
BTNL10	0.3818606	0.6879887	2.150452	0.0321662	0.0602306	-4.644478
AC073869.7	-0.168306	-1.332788	-2.150335	0.0321756	0.060245	-4.644725
PVRL3-AS1	-0.365474	1.5609363	-2.1502	0.0321863	0.060262	-4.645009
HCP5B	-0.439255	0.9403814	-2.149877	0.0322121	0.0603072	-4.645691
AC008547.1	-0.230656	-1.075485	-2.149749	0.0322223	0.0603232	-4.645961
RP11-365D2	0.3247935	1.7658126	2.1493262	0.032256	0.0603832	-4.646852
AC016582.2	0.3491414	-0.668726	2.1489949	0.0322824	0.0604296	-4.64755
RP5-1185H1	0.1685914	-1.334462	2.1488381	0.032295	0.0604479	-4.647881
RPL14P1	-0.08093	4.9702779	-2.148831	0.0322955	0.0604479	-4.647896
FAM57B	-0.399377	2.0820082	-2.148031	0.0323595	0.0605645	-4.649582
RP11-556N4	-0.099726	-1.477979	-2.147803	0.0323778	0.0605941	-4.650063
RP11-552C1	-0.44603	-0.230801	-2.147792	0.0323786	0.0605941	-4.650085
GABRG3	0.5266428	0.6201275	2.1476245	0.032392	0.0606161	-4.650438
MRPL13	-0.052605	6.3738038	-2.147423	0.0324082	0.0606432	-4.650862
MOSPD1	0.0734081	5.6513827	2.1473729	0.0324122	0.0606476	-4.650968
RP11-26J3.	0.3157818	1.6571035	2.1472393	0.0324229	0.0606645	-4.65125
TOB2	-0.042244	6.3542041	-2.147137	0.0324311	0.0606768	-4.651466
RP11-466M2	-0.181983	-1.271795	-2.14701	0.0324412	0.0606927	-4.651732
AP001092.4	-0.126126	-1.425673	-2.146847	0.0324544	0.0607141	-4.652077
CFAP36	-0.045064	5.8225874	-2.146786	0.0324592	0.0607201	-4.652204
CTA-363E6.	0.1886067	-1.275889	2.1466877	0.0324671	0.0607318	-4.652411
ZNF24	-0.025902	6.4225566	-2.146523	0.0324804	0.0607534	-4.652758
RP11-391L3	0.2682862	-0.749714	2.1464861	0.0324833	0.0607544	-4.652836
RANBP9	-0.035699	6.2817938	-2.146475	0.0324842	0.0607544	-4.652859
MICU3	-0.175309	4.9795473	-2.146177	0.0325081	0.060796	-4.653487
PRSS51	0.4655515	1.9795685	2.146139	0.0325112	0.0607986	-4.653567
HP1BP3	-0.027542	6.5870367	-2.146112	0.0325134	0.0607996	-4.653624
ASS1P9	-0.401784	1.0085988	-2.14608	0.0325159	0.0608012	-4.653691
CTD-2251F1	-0.322655	-0.966254	-2.145998	0.0325226	0.0608105	-4.653864
HSPD1P5	0.4056138	1.1055621	2.1459298	0.032528	0.0608176	-4.654007
RP11-33I11	-0.31525	-0.820167	-2.145489	0.0325634	0.0608807	-4.654934
CTD-2342N2	0.3585768	-0.668569	2.1448244	0.032617	0.0609762	-4.656333
RP11-360L9	-0.147962	-1.353234	-2.144814	0.0326178	0.0609762	-4.656356
RP11-192H2	0.1371309	4.0480655	2.1447696	0.0326214	0.0609797	-4.656448
RP11-615J4	-0.301666	-1.102098	-2.144694	0.0326275	0.0609879	-4.656607
RP11-119H1	-0.144109	-1.361956	-2.144614	0.0326339	0.0609968	-4.656775
SH3RF1	0.0764027	5.7189792	2.1445655	0.0326378	0.0610011	-4.656878
PAK4	0.0384014	6.3245454	2.1441427	0.0326719	0.0610617	-4.657767

PFDN1	-0.030888	6.1781995	-2.143737	0.0327047	0.0611197	-4.65862
ETV3L	0.234014	-1.051996	2.1436196	0.0327142	0.0611343	-4.658867
ZSWIM7	-0.056202	5.7713309	-2.143468	0.0327264	0.0611541	-4.659187
RP11-227D1	-0.22311	-1.067292	-2.143332	0.0327374	0.0611715	-4.659473
DCAF12L1	0.3422448	-0.976666	2.1432393	0.0327449	0.0611824	-4.659667
RP11-383F6	0.2438711	-0.993711	2.1425801	0.0327982	0.0612789	-4.661053
RP11-758N1	-0.56085	1.9849934	-2.142473	0.0328069	0.061292	-4.661279
LINC01387	-0.082571	-1.503766	-2.142404	0.0328125	0.0612986	-4.661422
AGBL5-AS1	-0.351195	0.0750546	-2.142388	0.0328138	0.0612986	-4.661457
LA16c-431H	0.3929285	0.546641	2.1419845	0.0328465	0.0613564	-4.662304
LSM1	0.0496892	5.8032277	2.1418832	0.0328547	0.0613686	-4.662517
RNU1-59P	-0.192015	-1.220557	-2.141649	0.0328737	0.061401	-4.66301
MAP3K10	0.0561044	5.6338183	2.1415888	0.0328786	0.061407	-4.663136
ZNF536	0.3097059	-0.932563	2.1413214	0.0329003	0.0614443	-4.663697
C1orf195	0.2544535	-1.046124	2.1412731	0.0329042	0.0614474	-4.663799
GREB1	-0.183751	5.445133	-2.14126	0.0329053	0.0614474	-4.663827
RP11-567C2	0.5562174	0.9613999	2.1409477	0.0329306	0.0614916	-4.664482
CPSF2	-0.027057	6.2673302	-2.140848	0.0329387	0.0615035	-4.664692
RP11-95I16	0.3556685	-0.260235	2.1405173	0.0329656	0.0615506	-4.665386
C16orf87	-0.057616	5.7411611	-2.140299	0.0329833	0.0615805	-4.665844
LINC01088	0.425121	0.2214319	2.1401271	0.0329973	0.0616036	-4.666205
SNHG10	-0.078155	4.9892942	-2.139952	0.0330116	0.061627	-4.666573
DPCR1	0.4572177	-0.105966	2.1398598	0.0330191	0.0616379	-4.666767
CCNT2	0.0376195	6.0575761	2.139395	0.033057	0.0617054	-4.667742
AP000697.6	0.153349	-1.36852	2.1393739	0.0330587	0.0617055	-4.667787
RP11-392A1	-0.211381	-1.088724	-2.139241	0.0330695	0.0617225	-4.668066
RP11-300G2	-0.123627	-1.412655	-2.139218	0.0330714	0.0617229	-4.668115
RP11-632L2	0.3208769	3.3510268	2.1388631	0.0331003	0.0617737	-4.668858
RPL5P11	-0.212531	-1.113338	-2.138813	0.0331044	0.0617781	-4.668963
FCAMR	-0.487104	4.0962478	-2.13878	0.0331071	0.0617801	-4.669032
PIGUP1	-0.141068	-1.372122	-2.13874	0.0331103	0.061783	-4.669116
DPYD	0.083919	6.27136	2.1386526	0.0331175	0.0617932	-4.6693
TPGS2	0.046669	6.2100225	2.1383823	0.0331396	0.0618264	-4.669867
CTD-2152M2	0.2483931	2.8311562	2.1383753	0.0331402	0.0618264	-4.669882
RP11-380M2	-0.128821	-1.395535	-2.138372	0.0331404	0.0618264	-4.669888
OSTCP1	-0.149596	-1.351392	-2.138089	0.0331636	0.0618648	-4.670483
ST6GALNAC3	0.1442861	4.5155074	2.1380788	0.0331644	0.0618648	-4.670504
AC108868.6	-0.298616	-1.075092	-2.13794	0.0331757	0.0618828	-4.670795
KRTAP19-1	0.2454991	-1.165312	2.1375818	0.033205	0.0619314	-4.671546
NPY4R	0.2030942	-1.290042	2.13758	0.0332052	0.0619314	-4.67155
RP13-379O2	-0.109719	-1.481771	-2.136995	0.033253	0.0620175	-4.672775
RP3-414A15	-0.168233	4.2529419	-2.136932	0.0332583	0.062024	-4.672909
RP11-114F3	0.3564954	0.0348412	2.1369112	0.0332599	0.062024	-4.672952
RP5-836N17	0.3596774	0.0267389	2.136827	0.0332668	0.0620337	-4.673129
AC007163.1	-0.303813	-0.694328	-2.136768	0.0332716	0.0620374	-4.673251
LINC01237	0.3839818	2.0353427	2.1367443	0.0332736	0.0620374	-4.673302
FTO	-0.042011	6.1060582	-2.136741	0.0332739	0.0620374	-4.67331
RP11-117L5	-0.218893	-1.069135	-2.136505	0.0332932	0.0620702	-4.673803
RP3-355L5.	0.3473644	1.7141801	2.1364685	0.0332962	0.0620727	-4.67388
AC004980.7	0.0819525	4.4813801	2.1364352	0.0332989	0.0620746	-4.67395
LINC00598	0.3583366	1.3484466	2.1364129	0.0333008	0.0620748	-4.673997

RP11-241F1	-0.377304	-0.560434	-2.136343	0.0333065	0.0620824	-4.674144
PHKA1P1	0.3205294	-0.357233	2.135947	0.033339	0.0621398	-4.674973
ICE1	0.0368098	6.0945542	2.1357367	0.0333563	0.0621688	-4.675414
RP11-17J14	-0.321366	-0.289924	-2.135656	0.0333629	0.062178	-4.675583
RP11-813F2	-0.337171	-0.752966	-2.135581	0.033369	0.0621862	-4.675739
PYROXD2	0.1876123	5.098772	2.1355347	0.0333729	0.0621902	-4.675837
DENND6B	0.0678924	5.3216945	2.1352987	0.0333923	0.0622232	-4.676331
ZSCAN2	-0.050224	5.6400658	-2.134904	0.0334248	0.0622805	-4.677158
FBXO36P1	-0.250398	-1.069811	-2.13484	0.03343	0.0622872	-4.677293
AC024592.1	-0.19796	-1.194094	-2.134736	0.0334386	0.0622986	-4.677511
OGFOD1P1	-0.361613	-0.110772	-2.134722	0.0334397	0.0622986	-4.677539
RP13-554M1	-0.376676	0.8138376	-2.134703	0.0334413	0.0622986	-4.677579
RP11-394I1	0.3400502	2.1606238	2.1346104	0.0334489	0.0623096	-4.677773
RP11-79M19	0.2857556	-0.655966	2.1345419	0.0334545	0.0623164	-4.677916
CRCT1	0.3150718	-1.000905	2.1345245	0.033456	0.0623164	-4.677953
AFG3L1P	0.0473318	5.4299743	2.1344912	0.0334587	0.0623183	-4.678023
RP11-467I2	-0.190006	-1.219082	-2.134329	0.0334721	0.06234	-4.678362
AC005013.5	-0.219514	-1.13418	-2.133969	0.0335018	0.0623921	-4.679116
PCGF2	0.0580905	5.8941169	2.1336653	0.0335268	0.0624355	-4.679752
RP11-297L1	0.3637091	0.1939886	2.133645	0.0335285	0.0624355	-4.679794
RP11-293B2	0.1936227	-1.271738	2.1333971	0.0335489	0.0624698	-4.680313
RP4-777O23	0.2375273	-0.982256	2.1333801	0.0335503	0.0624698	-4.680349
NDNL2	-0.03891	5.8343712	-2.133324	0.033555	0.0624754	-4.680467
PRAMEF14	-0.212874	-1.257009	-2.133279	0.0335587	0.0624774	-4.680561
KDM3A	0.0377305	6.1620202	2.133269	0.0335595	0.0624774	-4.680581
RP11-107N1	0.3250095	-0.711952	2.133081	0.033575	0.0625031	-4.680975
MRVI1-AS1	0.3535362	-0.370637	2.1330351	0.0335788	0.0625058	-4.681071
RP11-317N8	-0.20768	-1.132858	-2.133022	0.0335799	0.0625058	-4.681099
RELN	0.3897682	5.4111303	2.1329132	0.0335889	0.0625193	-4.681326
DNAI2	0.2964273	-0.632747	2.1325238	0.0336211	0.0625761	-4.682141
AC025442.3	-0.170338	-1.279003	-2.13242	0.0336297	0.0625889	-4.682359
AC009166.7	-0.380346	0.9476889	-2.132378	0.0336332	0.0625921	-4.682446
RP11-571M6	0.3036231	-0.536033	2.1322972	0.0336398	0.0626012	-4.682615
CCDC40	-0.092148	4.9815435	-2.132278	0.0336414	0.0626012	-4.682655
FAM21FP	-0.163485	3.2107932	-2.132131	0.0336536	0.0626206	-4.682962
MUC15	-0.625342	0.6379817	-2.131779	0.0336827	0.0626716	-4.683699
DEPTOR	-0.061087	6.1211982	-2.131674	0.0336915	0.0626847	-4.683919
C2orf16	-0.126341	4.5917087	-2.131397	0.0337144	0.0627242	-4.684498
PIP4K2C	0.0340635	6.1869409	2.1313227	0.0337205	0.0627324	-4.684653
MS4A15	0.2555963	-1.108619	2.1311563	0.0337343	0.0627548	-4.685001
KLRC3	0.2061963	-1.218186	2.131132	0.0337364	0.0627554	-4.685051
AC084809.2	0.3521797	0.0001078	2.1310632	0.0337421	0.0627599	-4.685195
RP11-6N13.	-0.490877	0.4539913	-2.131061	0.0337422	0.0627599	-4.6852
GAPDHP28	-0.216561	-1.114869	-2.131002	0.0337472	0.0627659	-4.685324
CTC-448F2.	0.1427103	3.5601683	2.1309607	0.0337506	0.062769	-4.68541
GP9	-0.273773	-0.954453	-2.130934	0.0337528	0.06277	-4.685466
TCN2	0.0620865	5.997445	2.1308588	0.033759	0.0627784	-4.685623
SEMA4G	-0.073294	6.5048479	-2.130784	0.0337653	0.0627868	-4.68578
LUZP2	-0.503774	1.048362	-2.130514	0.0337877	0.0628252	-4.686344
LAIR2	0.4326646	1.9184989	2.1303942	0.0337976	0.0628379	-4.686594
SNX18P24	-0.216202	-1.287063	-2.13039	0.0337979	0.0628379	-4.686602

RP11-367B6	-0.361974	0.4039039	-2.129895	0.0338391	0.0629112	-4.687637
MYH6	0.2758985	-1.003201	2.1298671	0.0338414	0.0629124	-4.687696
NEK4	0.0738318	5.860508	2.1294947	0.0338724	0.0629667	-4.688474
RP11-69G7.	-0.175371	-1.330997	-2.12945	0.0338761	0.0629696	-4.688566
APOBEC1	0.2026462	-1.324412	2.1294283	0.0338779	0.0629696	-4.688612
ARL5B	0.0423362	6.3315599	2.129414	0.0338791	0.0629696	-4.688642
AC005009.1	0.2091567	-1.210651	2.1289776	0.0339155	0.0630339	-4.689554
ADAMTSL4	-0.092969	6.1243312	-2.128924	0.0339199	0.063039	-4.689665
UGT1A9	-0.386258	5.4344291	-2.128874	0.0339241	0.0630436	-4.689771
SLC9A6	-0.042431	5.8742821	-2.128758	0.0339338	0.0630583	-4.690013
RRN3P1	0.1271165	4.7683386	2.1285037	0.033955	0.0630945	-4.690544
CAMKK2	-0.030498	6.4012379	-2.128482	0.0339567	0.0630946	-4.690588
PLA2G2C	-0.385888	0.275314	-2.128364	0.0339666	0.0631097	-4.690835
RP5-1125A1	-0.280873	2.4730077	-2.128181	0.0339819	0.0631349	-4.691218
MKX	0.519203	2.3413974	2.1281447	0.0339849	0.0631373	-4.691293
CTD-2342J1	-0.185176	3.2400623	-2.128098	0.0339888	0.0631414	-4.691392
CTD-2297D1	0.5860945	0.8694352	2.1280179	0.0339955	0.063148	-4.691558
DDX50	0.0303264	6.0699741	2.1280139	0.0339958	0.063148	-4.691566
RP11-958N2	0.5527285	1.146709	2.1278281	0.0340113	0.0631736	-4.691954
AC114730.1	0.3356567	2.0309092	2.127799	0.0340138	0.0631749	-4.692015
PUS10	-0.049358	5.6960036	-2.127201	0.0340637	0.063262	-4.693263
PROSER2-AS	0.2649771	3.0716783	2.1271962	0.0340642	0.063262	-4.693273
SOX5	-0.131808	5.465675	-2.126916	0.0340876	0.0633023	-4.693858
CTA-351J1.	0.3540511	1.4407534	2.1268774	0.0340908	0.0633051	-4.693939
RP11-138P2	-0.177728	-1.254348	-2.126801	0.0340973	0.0633138	-4.694099
KRT8P50	-0.21442	-1.105319	-2.126719	0.0341041	0.0633233	-4.694269
INS	-0.243318	-1.25326	-2.126639	0.0341108	0.0633301	-4.694436
RP5-854E16	-0.35052	0.1744174	-2.126634	0.0341112	0.0633301	-4.694447
DEFA8P	0.2701955	-1.092443	2.126525	0.0341203	0.0633438	-4.694674
PSMD6-AS2	0.3426354	2.3871205	2.1263901	0.0341316	0.0633615	-4.694955
TNFRSF19	0.2697404	4.7366576	2.1258938	0.0341732	0.0634332	-4.695991
RP11-1003J	-0.087485	-1.498064	-2.125888	0.0341737	0.0634332	-4.696003
AC092159.3	-0.189956	-1.358591	-2.125811	0.0341802	0.0634411	-4.696164
LA16c-316G	0.3670866	0.8920306	2.1257959	0.0341814	0.0634411	-4.696195
CTD-3099C6	0.2349425	-1.144549	2.1256162	0.0341965	0.0634659	-4.69657
KRT85	0.5368854	0.9074192	2.1255224	0.0342044	0.0634772	-4.696765
RP11-118F2	-0.153452	-1.348705	-2.125203	0.0342312	0.0635238	-4.697432
IPO5P1	-0.108233	5.4119213	-2.125004	0.0342479	0.0635515	-4.697845
IDO1	0.2538673	4.5150669	2.1249638	0.0342513	0.0635546	-4.69793
RP11-76C10	-0.180873	-1.289426	-2.124409	0.0342979	0.0636379	-4.699087
LINC00484	0.3442683	-0.320373	2.124364	0.0343017	0.0636417	-4.69918
AC016722.4	0.3683608	1.8154934	2.124262	0.0343103	0.0636544	-4.699393
AC087350.1	0.39794	0.7990923	2.1241799	0.0343172	0.063664	-4.699564
TOMM40L	-0.046534	6.2471657	-2.12404	0.034329	0.0636825	-4.699855
RP3-380B8.	-0.233754	-1.130156	-2.123968	0.0343351	0.0636906	-4.700006
QRSL1P3	-0.133333	-1.389608	-2.123634	0.0343632	0.0637396	-4.700702
RP11-108P2	0.2552092	-0.932599	2.1235324	0.0343718	0.0637522	-4.700913
TNPO3	-0.028744	6.3731846	-2.123227	0.0343975	0.0637966	-4.701549
POLR3F	-0.03294	5.6492961	-2.122885	0.0344264	0.0638441	-4.702262
KLHL23	0.0691637	5.8393948	2.1228824	0.0344266	0.0638441	-4.702267
FKRP	0.0377642	5.7046695	2.1227095	0.0344412	0.063868	-4.702628

CTA-363E6.	0.4325571	-0.17977	2.1226767	0.0344439	0.0638698	-4.702696
SLC30A5	-0.029023	6.2660836	-2.121948	0.0345055	0.0639807	-4.704213
FAM95B1	-0.229261	-1.067112	-2.12187	0.0345121	0.0639898	-4.704377
PTCD3	-0.029682	6.3941747	-2.121701	0.0345264	0.064013	-4.704728
SSX2	-0.264446	-1.070654	-2.121597	0.0345352	0.0640246	-4.704943
PCDHB17P	0.3731622	-0.407711	2.121583	0.0345364	0.0640246	-4.704973
CFC1	-0.105651	-1.472657	-2.121565	0.0345379	0.0640246	-4.705011
RP11-81H3.	-0.678283	1.5130773	-2.121357	0.0345555	0.0640536	-4.705444
AC007881.4	-0.123751	-1.429267	-2.121338	0.0345571	0.0640536	-4.705483
C11orf88	0.3043866	-0.634912	2.121318	0.0345588	0.0640536	-4.705525
SUGT1P	-0.386994	1.9004899	-2.121227	0.0345665	0.0640621	-4.705715
PCDHA13	0.4913527	0.0542556	2.1212225	0.0345669	0.0640621	-4.705724
RP11-20B24	-0.082672	-1.49862	-2.1211	0.0345773	0.0640781	-4.705979
RP11-1277A	0.1158462	4.3828846	2.1208981	0.0345944	0.0641065	-4.706399
LDLRAD4	0.071946	6.0146151	2.1208608	0.0345976	0.0641091	-4.706476
HYPK	-0.065766	4.5807053	-2.120817	0.0346012	0.0641127	-4.706567
U82695.5	-0.166187	-1.412526	-2.120496	0.0346285	0.0641599	-4.707236
SPP1	0.2206667	6.4748036	2.1203951	0.0346371	0.0641725	-4.707446
NADK	-0.044445	6.5077673	-2.120318	0.0346436	0.0641813	-4.707605
TRIM62	0.0673439	5.1073986	2.1202365	0.0346505	0.0641909	-4.707775
PCDHB9	0.3918071	2.6916634	2.1201446	0.0346583	0.0642021	-4.707967
RP11-884K1	0.0526944	5.1035837	2.1198333	0.0346847	0.0642478	-4.708614
VN2R3P	-0.114987	-1.439556	-2.119737	0.0346929	0.0642583	-4.708815
MTRNR2L13	-0.182496	-1.276336	-2.119725	0.0346939	0.0642583	-4.708838
DNER	0.563559	0.7041852	2.1195048	0.0347127	0.0642897	-4.709297
TRGV9	0.343149	-0.436838	2.1192867	0.0347312	0.0643208	-4.709751
CTB-109A12	0.3595077	0.0116162	2.11923	0.034736	0.0643265	-4.709869
RPS2P7	0.2154521	3.1982667	2.1191969	0.0347388	0.0643284	-4.709938
CTD-2193P3	-0.17037	-1.315186	-2.118886	0.0347653	0.0643729	-4.710584
CRIPAK	-0.051334	5.5593658	-2.118873	0.0347664	0.0643729	-4.710611
AC004837.5	0.3566592	-0.105139	2.1182406	0.0348202	0.0644694	-4.711926
GPR146	0.2550237	4.3503003	2.1180404	0.0348373	0.0644977	-4.712342
RNU6-1099F	0.2732326	-0.718441	2.1179746	0.0348429	0.0645048	-4.712479
NPM1P37	0.3293784	0.3173428	2.1179543	0.0348447	0.0645048	-4.712521
RP4-565E6.	-0.455926	2.4131816	-2.117908	0.0348486	0.0645071	-4.712617
RP5-89003.	0.099198	4.2666766	2.1178981	0.0348495	0.0645071	-4.712638
SRGAP3-AS2	0.3023877	-0.986649	2.1176995	0.0348664	0.0645352	-4.71305
GAPDHP52	0.2104197	-1.112054	2.1174844	0.0348847	0.0645659	-4.713497
MIR217HG	-0.542594	0.4592677	-2.117455	0.0348872	0.0645672	-4.713558
RP11-322L2	-0.159029	-1.381643	-2.117334	0.0348976	0.0645831	-4.71381
CTD-2290P7	0.2273927	-1.027218	2.1172419	0.0349055	0.0645944	-4.714001
AC023115.4	0.1733998	-1.317522	2.1165841	0.0349617	0.0646951	-4.715368
ZNF470	0.1577849	4.9877256	2.1165528	0.0349643	0.0646952	-4.715432
AC138035.2	0.3857814	1.7196061	2.1165424	0.0349652	0.0646952	-4.715454
RP11-982M1	0.3386835	-0.622474	2.1163519	0.0349815	0.064722	-4.71585
AC068641.1	0.2175249	-1.071195	2.1162266	0.0349923	0.0647386	-4.71611
WAC-AS1	-0.037066	5.954503	-2.116166	0.0349974	0.0647449	-4.716236
AC112518.3	-0.099122	-1.478245	-2.116041	0.0350082	0.0647615	-4.716496
PNISR	0.0417487	6.2463985	2.1158177	0.0350273	0.0647908	-4.716959
RP11-886D1	-0.164305	-1.332364	-2.115814	0.0350276	0.0647908	-4.716966
CTC-457L16	0.2366846	-1.04549	2.1157908	0.0350296	0.0647912	-4.717015

LINC00680	0.0732165	5.1172145	2.1157334	0.0350345	0.064797	-4.717134
ISM1	0.2765391	4.7335338	2.115711	0.0350364	0.0647973	-4.717181
RP11-645C2	0.3700396	1.5581744	2.1153673	0.0350659	0.0648485	-4.717894
AD000092.3	-0.198638	2.86953	-2.114866	0.0351089	0.0649247	-4.718934
CTD-2034I2	-0.257239	-1.15584	-2.114494	0.0351408	0.0649775	-4.719707
MYO5C	0.0684306	5.8229845	2.114492	0.035141	0.0649775	-4.71971
ATF3	-0.072854	6.1717926	-2.114446	0.035145	0.0649816	-4.719806
RP11-452D1	0.254596	-0.985986	2.1143107	0.0351566	0.0649997	-4.720087
AC104655.2	0.3325744	1.1246295	2.1142214	0.0351642	0.0650106	-4.720272
RPL26	-0.052617	6.821843	-2.114173	0.0351684	0.0650151	-4.720373
INHBA-AS1	-0.379216	2.4021167	-2.114018	0.0351818	0.0650364	-4.720695
SLC12A5	0.2409095	3.9212076	2.1139293	0.0351893	0.0650443	-4.720878
OR52P1P	-0.132848	-1.409813	-2.113927	0.0351896	0.0650443	-4.720884
RP11-247L2	-0.321644	-0.463597	-2.113455	0.0352301	0.0651159	-4.721861
PGBD5	0.1391224	5.5657629	2.1131785	0.035254	0.0651567	-4.722435
SYP	0.1384078	4.6342946	2.1129034	0.0352777	0.0651972	-4.723005
SAP30	-0.067545	5.316389	-2.112881	0.0352796	0.0651975	-4.723052
DEAF1	-0.038927	6.0460854	-2.112712	0.0352942	0.0652211	-4.723402
RP11-807H1	-0.143905	-1.393418	-2.112427	0.0353187	0.0652632	-4.723992
CCDC86	-0.042663	6.0119095	-2.112191	0.0353391	0.0652975	-4.724482
IL23A	0.126417	3.997272	2.1121411	0.0353434	0.0653004	-4.724586
RP11-583F2	-0.109449	-1.473707	-2.112132	0.0353442	0.0653004	-4.724606
RP11-643A5	-0.09493	-1.477002	-2.112087	0.0353481	0.0653042	-4.724697
ACTG1P10	0.3320915	-0.112572	2.1117386	0.0353782	0.0653565	-4.72542
ATXN3	-0.03692	5.8516994	-2.111697	0.0353817	0.0653598	-4.725506
NUP188	0.0352798	6.1741459	2.1116765	0.0353835	0.0653598	-4.725549
TMED7-TICA	0.3012185	2.5519421	2.1114664	0.0354017	0.06539	-4.725984
MRGPRD	-0.204431	-1.282585	-2.111354	0.0354114	0.0654046	-4.726216
STT3A	-0.029685	6.8005027	-2.111331	0.0354134	0.065405	-4.726264
HNRNPDL	-0.023614	6.6667109	-2.111204	0.0354244	0.065422	-4.726528
GS1-166A23	0.3682098	1.3401784	2.1111539	0.0354287	0.0654267	-4.726631
LL0XNC01-1	0.1653631	-1.296624	2.1108469	0.0354553	0.0654725	-4.727267
AC007969.5	-0.099878	5.0996618	-2.110774	0.0354616	0.0654808	-4.727418
RNU6-312P	0.343182	0.0806572	2.1107326	0.0354652	0.0654841	-4.727504
RP11-366O2	-0.26506	-0.799655	-2.110697	0.0354683	0.0654865	-4.727579
SPCS2P4	0.1340303	3.9666165	2.1105675	0.0354795	0.0655039	-4.727846
RP11-31H5.	-0.219708	-1.109554	-2.110323	0.0355007	0.0655397	-4.728353
RPL27AP5	-0.391875	0.3139243	-2.109934	0.0355344	0.0655986	-4.729158
AC025171.1	-0.15469	4.3032732	-2.109752	0.0355501	0.0656244	-4.729535
AC005682.7	0.3390535	-0.244715	2.1095985	0.0355635	0.0656457	-4.729853
FAM210CP	-0.239814	-1.083023	-2.109392	0.0355814	0.0656755	-4.730281
RP11-408O1	0.3420751	0.0582772	2.1093429	0.0355857	0.06568	-4.730382
RNF138P1	0.3385915	0.0179166	2.1092962	0.0355897	0.0656841	-4.730478
TTC5	0.048575	5.4451106	2.1090665	0.0356097	0.0657177	-4.730954
CNGB3	0.4405759	0.3309055	2.1087392	0.0356381	0.0657668	-4.731631
AC091199.1	-0.168871	-1.332423	-2.108591	0.035651	0.0657873	-4.731938
NKPD1	0.4298118	0.864882	2.1084512	0.0356632	0.0658041	-4.732227
CSPG4P12	0.335449	2.2484542	2.1084446	0.0356637	0.0658041	-4.732241
RP11-1070N	-0.345594	2.622524	-2.10797	0.035705	0.065877	-4.733223
NLRP6	-0.308775	4.382095	-2.107896	0.0357114	0.0658855	-4.733375
CCNF	0.0813089	5.5435043	2.1078721	0.0357136	0.0658861	-4.733425

ARGLU1	0.0359626	6.2326806	2.1077545	0.0357238	0.0659016	-4.733668
RP11-231C1	-0.213467	-1.162998	-2.107733	0.0357257	0.0659017	-4.733713
CRISP2	0.423973	-0.592967	2.1076388	0.0357339	0.0659136	-4.733908
LTN1	0.0562059	5.9110922	2.1072424	0.0357684	0.065974	-4.734728
LIG3	-0.034539	6.030543	-2.107018	0.035788	0.0660067	-4.735191
RPS20P15	-0.343903	-0.651907	-2.106943	0.0357945	0.0660154	-4.735346
RP13-578N3	-0.133147	-1.378068	-2.106914	0.0357971	0.0660163	-4.735408
RP11-219B4	0.2615821	-0.927609	2.1068963	0.0357986	0.0660163	-4.735443
KLF6	-0.05481	6.6650956	-2.106844	0.0358032	0.0660215	-4.735552
AC106873.4	-0.118552	-1.433668	-2.106688	0.0358168	0.0660431	-4.735873
AC090051.1	-0.183683	-1.298987	-2.106578	0.0358264	0.0660575	-4.7361
GPATCH4	-0.044238	6.2731213	-2.106504	0.0358329	0.0660662	-4.736255
CD58	0.0543288	5.5456407	2.1062746	0.0358529	0.0660998	-4.736728
RP11-156E8	-0.264475	2.6905145	-2.106208	0.0358588	0.0661072	-4.736867
RP11-31F15	-0.241587	2.8488478	-2.106158	0.0358631	0.0661118	-4.736969
SLK	0.0328314	6.3002617	2.1061181	0.0358666	0.066115	-4.737052
FAM90A2P	0.1652786	-1.290607	2.1056098	0.0359111	0.0661909	-4.738102
AC002056.5	-0.245213	-0.968863	-2.105606	0.0359114	0.0661909	-4.738111
RP11-799N1	-0.241333	-1.188308	-2.105339	0.0359347	0.0662305	-4.738661
UBE2R2-AS1	0.310078	-0.317857	2.1043978	0.0360172	0.0663792	-4.740606
CDC25A	0.1327676	5.0116465	2.1042669	0.0360287	0.0663971	-4.740877
COX7B2	-0.736894	1.7185622	-2.103981	0.0360538	0.0664399	-4.741466
AGAP11	0.2761263	-0.838178	2.1039383	0.0360576	0.0664435	-4.741555
C1orf94	0.2010272	-1.322065	2.1039176	0.0360594	0.0664435	-4.741598
DHX40	-0.039223	6.0021356	-2.103822	0.0360678	0.0664557	-4.741796
CTC-756D1.	-0.365132	-0.031506	-2.103713	0.0360773	0.0664677	-4.74202
RP11-609D2	0.4074177	1.4058915	2.1037061	0.036078	0.0664677	-4.742035
RP11-12N13	-0.097726	-1.475315	-2.103662	0.0360819	0.0664698	-4.742126
FFAR3	0.3219045	-0.624189	2.1036513	0.0360828	0.0664698	-4.742148
RP11-148B1	0.4015969	0.5127403	2.1034346	0.0361018	0.0665015	-4.742595
PGDP1	0.260198	-0.836905	2.1033775	0.0361068	0.0665074	-4.742713
SLCO2A1	0.1967379	5.4506402	2.1032499	0.0361181	0.0665248	-4.742976
ZCCHC8	0.035646	5.9748182	2.1029473	0.0361447	0.0665704	-4.743601
RP11-366M4	-0.349008	-0.000148	-2.102877	0.0361508	0.0665784	-4.743745
TTLL6	0.4153477	2.0316641	2.1027243	0.0361643	0.0665999	-4.744061
APPL2	0.03656	6.0843468	2.1026921	0.0361671	0.0666	-4.744127
CADM2	0.5640726	1.2525132	2.1026794	0.0361683	0.0666	-4.744154
RP11-49I11	0.3497279	-0.141774	2.1026611	0.0361699	0.0666	-4.744191
RP11-384M1	0.3465126	0.1398019	2.1026374	0.036172	0.0666005	-4.74424
TMEM11	-0.043437	5.9651194	-2.102513	0.0361829	0.0666173	-4.744497
XXbac-BPG3	0.3332637	-0.043106	2.1022195	0.0362088	0.0666616	-4.745103
DKFZp434P2	-0.309994	-0.616622	-2.102023	0.0362261	0.0666901	-4.745508
RP11-479J7	-0.114272	-1.447921	-2.101797	0.036246	0.0667234	-4.745975
RP11-173E2	-0.113356	-1.462731	-2.101668	0.0362574	0.066741	-4.746241
XPO1	-0.022106	6.6561162	-2.101487	0.0362734	0.066767	-4.746614
LINC01578	-0.042457	6.1217833	-2.101377	0.0362831	0.0667815	-4.74684
SMUG1P1	-0.552174	0.5934608	-2.10058	0.0363535	0.0669077	-4.748483
RP1-10C16.	-0.21099	-1.18988	-2.100541	0.036357	0.0669108	-4.748565
BMS1P17	-0.159535	-1.315496	-2.100295	0.0363787	0.0669474	-4.749072
RP11-405K6	-0.244856	-1.175612	-2.100207	0.0363865	0.0669583	-4.749252
RP11-139H1	0.2601195	2.9427043	2.1000998	0.036396	0.0669724	-4.749474

RP13-884E1	-0.245692	-1.03279	-2.10003	0.0364022	0.0669805	-4.749618
RP11-274A1	0.2467117	-0.908804	2.0997921	0.0364232	0.0670158	-4.750108
RP11-574K1	0.3073709	1.447445	2.0997288	0.0364288	0.0670228	-4.750238
COX11P1	-0.141382	-1.364364	-2.099674	0.0364337	0.0670284	-4.750352
AC144835.1	-0.244736	-1.142362	-2.099455	0.0364531	0.0670607	-4.750804
INPP5B	-0.044181	5.7358653	-2.099334	0.0364638	0.067077	-4.751052
AC022007.5	-0.114512	4.4254355	-2.099266	0.0364698	0.0670847	-4.751191
C21orf59	-0.038931	5.844306	-2.099191	0.0364765	0.0670935	-4.751346
TRIM72	0.423487	1.1771205	2.098726	0.0365178	0.0671661	-4.752304
RP3-334F4.	-0.098765	-1.464154	-2.098683	0.0365216	0.0671698	-4.752394
MAFIP	-0.399749	2.1121818	-2.098557	0.0365328	0.0671869	-4.752653
TMEM241	-0.066844	5.2744633	-2.09842	0.0365449	0.0672058	-4.752934
STAG3L2	0.086918	4.7727136	2.0978483	0.0365958	0.0672959	-4.754112
RNU6-50P	0.2460447	-0.882258	2.097669	0.0366117	0.0673219	-4.754481
ORAOV1P1	0.3873476	0.5226053	2.0972442	0.0366495	0.067388	-4.755356
LINC01535	0.4914306	1.8643799	2.0968511	0.0366845	0.067449	-4.756165
RP11-1010.	0.2830878	-0.982817	2.0963372	0.0367303	0.0675298	-4.757222
RP6-218J18	0.3043463	-0.39118	2.096193	0.0367432	0.0675501	-4.757519
RP1-117B12	0.3693077	2.4076821	2.0961709	0.0367452	0.0675503	-4.757564
RP11-368I2	0.4352663	-0.169543	2.0961503	0.036747	0.0675503	-4.757607
CTD-2337I7	0.2564676	-1.122044	2.0960207	0.0367586	0.0675681	-4.757873
ZIC4	-0.638817	2.5502308	-2.095757	0.0367821	0.067608	-4.758415
RP11-114M5	-0.349986	-0.224407	-2.095478	0.036807	0.0676489	-4.758989
ATP6V0C	0.0708587	6.1348446	2.0954667	0.0368081	0.0676489	-4.759013
ASB1	0.0443308	5.9001555	2.0950541	0.036845	0.0677108	-4.759861
AC084117.3	0.3726817	0.6558946	2.0950483	0.0368455	0.0677108	-4.759873
RD3L	0.1743548	-1.359721	2.0950107	0.0368489	0.0677136	-4.759951
RP11-495P1	-0.370555	-0.075569	-2.094939	0.0368553	0.067722	-4.760099
DCAF5	-0.029238	6.3219697	-2.094848	0.0368634	0.0677315	-4.760284
C1orf109	0.0572488	5.6181169	2.0948372	0.0368644	0.0677315	-4.760307
RP1-181J22	-0.361291	0.0520737	-2.094819	0.036866	0.0677315	-4.760345
RP11-259O2	0.183387	-1.354657	2.0947549	0.0368717	0.0677386	-4.760476
FBN3	0.4659896	3.1934436	2.0945905	0.0368865	0.0677622	-4.760814
AK3P3	0.330091	-0.001122	2.0945105	0.0368936	0.067772	-4.760979
RPS23P8	-0.142803	4.1147534	-2.094482	0.0368962	0.0677732	-4.761037
DNASE2B	0.4203224	1.6603374	2.0938593	0.036952	0.0678723	-4.762318
CABLES1	-0.08777	5.8779336	-2.093399	0.0369932	0.0679435	-4.763263
GNA11	-0.026995	6.4167725	-2.093386	0.0369945	0.0679435	-4.763291
CASP3	0.0392709	6.1061495	2.093107	0.0370195	0.0679861	-4.763863
RP13-631K1	-0.141798	-1.365863	-2.093037	0.0370257	0.0679942	-4.764006
AP000620.1	-0.366559	0.4969852	-2.092889	0.0370391	0.0680152	-4.764312
RP11-431M7	0.2537956	-0.983829	2.0927909	0.0370479	0.0680275	-4.764513
MBIP	-0.052353	5.7852642	-2.09276	0.0370506	0.0680275	-4.764575
LINC01513	-0.134801	-1.413249	-2.092753	0.0370513	0.0680275	-4.764591
RGPD3	-0.354794	1.9947387	-2.09264	0.0370614	0.0680426	-4.764822
STARD6	0.3251814	-0.759742	2.0922162	0.0370996	0.0681092	-4.765693
GRIN2B	0.5226053	2.3030652	2.0921257	0.0371077	0.0681207	-4.765879
DDX18P1	0.2789138	-0.682568	2.0921015	0.0371099	0.0681213	-4.765928
STMN1P1	0.3074424	-0.391003	2.0920251	0.0371168	0.0681305	-4.766086
RTP1	-0.252716	-1.072303	-2.091676	0.0371482	0.0681848	-4.766803
MRPL47	-0.040834	6.189146	-2.09158	0.0371569	0.0681972	-4.767

RP11-529H2	-0.146793	-1.420143	-2.09147	0.0371668	0.0682119	-4.767225
NUSAP1	0.0780318	5.9695101	2.0911177	0.0371985	0.0682662	-4.767948
RP11-333E1	-0.361957	0.8542628	-2.091101	0.0372001	0.0682662	-4.767984
CHMP2B	0.0316264	6.2134914	2.0910525	0.0372044	0.0682707	-4.768082
RP11-234B2	0.3230405	-0.637791	2.0909242	0.037216	0.0682885	-4.768345
CTA-398F10	-0.31104	-0.769348	-2.090803	0.037227	0.0683052	-4.768595
FAM71D	-0.318468	1.4689576	-2.090552	0.0372496	0.0683409	-4.76911
IGFL4	0.3301745	-0.567045	2.0905459	0.0372501	0.0683409	-4.769122
PNPLA3	-0.109214	5.7878567	-2.090323	0.0372702	0.0683744	-4.769579
RP11-524N5	0.1869753	-1.295116	2.0902876	0.0372734	0.0683768	-4.769652
LPPR5	0.2404389	-1.243266	2.090025	0.0372972	0.0684142	-4.77019
EHD4	-0.03854	6.4043378	-2.09002	0.0372976	0.0684142	-4.7702
PRR32	-0.208158	-1.28374	-2.089733	0.0373236	0.0684585	-4.77079
FOXP1-AS1	0.2695217	-0.812762	2.0895271	0.0373422	0.0684892	-4.771212
NKD1	-0.288685	4.9594329	-2.089503	0.0373444	0.0684898	-4.771262
CBX1	0.0338138	6.2115689	2.0894738	0.037347	0.0684911	-4.771321
OGFOD2	0.0895311	4.7252968	2.089419	0.037352	0.0684968	-4.771433
RP11-157L3	-0.107921	-1.457339	-2.089239	0.0373683	0.0685232	-4.771803
ZHX1	-0.048916	6.4366912	-2.089129	0.0373782	0.068538	-4.772028
RP11-49K24	0.2728935	-0.70382	2.0889518	0.0373943	0.0685632	-4.772392
LINC00626	0.2120018	-1.221677	2.0889362	0.0373957	0.0685632	-4.772423
IGLV3-13	0.1522353	-1.372122	2.0888879	0.0374001	0.0685678	-4.772523
HHATL-AS1	-0.365091	-0.521219	-2.0888	0.037408	0.0685777	-4.772702
CXorf38	0.0328759	5.9330462	2.0887863	0.0374093	0.0685777	-4.772731
ZG16	-0.404637	4.9054226	-2.088721	0.0374152	0.0685852	-4.772866
TCF7L1	0.0725575	5.939653	2.0886017	0.037426	0.0686015	-4.773109
TISP43	-0.114373	-1.465667	-2.088471	0.0374379	0.0686198	-4.773378
RPS27P29	-0.352727	0.2788518	-2.088187	0.0374636	0.0686635	-4.773959
NUF2	0.1417856	5.2243398	2.0880627	0.0374749	0.0686808	-4.774214
AL662800.1	-0.24232	2.5951516	-2.087879	0.0374916	0.068708	-4.774591
IBA57	-0.050369	5.8217342	-2.087785	0.0375001	0.0687202	-4.774784
AP000343.2	-0.186498	-1.253953	-2.087757	0.0375027	0.0687213	-4.774841
AF129075.5	0.2493002	2.5306034	2.0874982	0.0375262	0.0687609	-4.775371
RP13-726E6	0.2046669	-1.219965	2.0873785	0.037537	0.0687774	-4.775616
IKZF5	-0.045069	5.8950421	-2.087072	0.0375649	0.068825	-4.776244
R3HDM4	-0.036498	6.4026811	-2.086732	0.0375958	0.0688781	-4.77694
RP11-1113L	-0.305393	-0.549313	-2.086637	0.0376044	0.0688905	-4.777134
RP11-445F6	0.2134246	-1.15127	2.0864974	0.0376172	0.0689104	-4.777421
TRMT13	0.0466972	5.5203889	2.0862078	0.0376436	0.0689553	-4.778014
RPL18AP7	0.2981342	-0.387517	2.0860202	0.0376607	0.0689831	-4.778398
RTN4RL1	0.2110836	5.1135516	2.0859734	0.0376649	0.0689875	-4.778494
AGO1	-0.034063	6.0610629	-2.085877	0.0376737	0.0690001	-4.778691
COPA	-0.02986	6.9395182	-2.085818	0.0376791	0.0690065	-4.778812
HSPA8P8	0.4296617	0.7842523	2.0854891	0.0377091	0.069058	-4.779486
CTD-2245E1	-0.381676	-0.01237	-2.085425	0.0377149	0.0690652	-4.779617
RP11-416I2	-0.411997	0.6005625	-2.085383	0.0377188	0.0690688	-4.779703
TMEM86A	0.0695102	5.4064364	2.0853553	0.0377213	0.0690699	-4.77976
RP11-725P1	-0.368512	0.1052775	-2.085006	0.0377532	0.0691249	-4.780474
PDCL3P5	0.3401786	0.6436443	2.0849624	0.0377572	0.0691287	-4.780564
FURIN	0.0618576	7.0764328	2.0848637	0.0377662	0.0691417	-4.780766
PDE4DIP1	-0.31916	-0.584941	-2.084806	0.0377714	0.0691457	-4.780884

RP11-542K2	0.3248138	-0.603578	2.0847984	0.0377722	0.0691457	-4.7809
HEY1	0.0796699	5.3584757	2.0847097	0.0377803	0.0691571	-4.781081
TIGD3	-0.227382	3.8275637	-2.084402	0.0378084	0.0692036	-4.781712
C9orf57	0.4962273	0.5603601	2.0843903	0.0378095	0.0692036	-4.781735
RP6-65G23.	-0.229149	3.6340644	-2.084296	0.0378181	0.0692159	-4.781927
RP1-170019	-0.533645	1.2379474	-2.08425	0.0378223	0.0692202	-4.782023
RP11-229M1	-0.197138	-1.202448	-2.084144	0.037832	0.0692345	-4.782239
CNN2P2	-0.151369	-1.368528	-2.084028	0.0378426	0.0692503	-4.782475
CEP170B	0.0419108	6.2520573	2.0838798	0.0378562	0.0692717	-4.782779
RP11-681N2	-0.22326	-1.063712	-2.083465	0.0378941	0.0693377	-4.783626
CTD-2376I4	-0.25765	2.1709081	-2.08338	0.037902	0.0693486	-4.783802
MIR23B	-0.174888	-1.27271	-2.083337	0.0379059	0.0693523	-4.78389
RP1-89D4.1	0.3419812	1.4517361	2.0832343	0.0379153	0.069366	-4.784099
GPI	-0.039912	7.071851	-2.08308	0.0379295	0.0693884	-4.784415
POTEA	-0.079559	-1.487832	-2.082964	0.0379401	0.0694017	-4.784653
RP11-305E6	0.1159393	4.4511413	2.0829593	0.0379405	0.0694017	-4.784662
AC092881.1	0.3586392	-0.293096	2.0827591	0.0379589	0.0694318	-4.785071
RP11-220I1	-0.276141	-0.880203	-2.082736	0.037961	0.0694322	-4.785118
RP11-736E3	-0.215181	-1.222457	-2.082665	0.0379675	0.0694407	-4.785263
MIR4537	0.1630248	-1.337583	2.0825872	0.0379747	0.0694502	-4.785422
RP4-569D19	-0.127282	-1.420937	-2.0824	0.0379918	0.0694781	-4.785804
AP001271.3	0.3675248	-0.084359	2.0822363	0.0380069	0.0695022	-4.78614
PRPF8	-0.028702	6.7857393	-2.082187	0.0380114	0.0695048	-4.786241
RP4-791C19	0.3569538	0.3413747	2.0821793	0.0380121	0.0695048	-4.786256
PABPC1P7	0.2102444	-1.114013	2.0821239	0.0380172	0.0695106	-4.786369
AL365181.2	0.2511358	2.3020703	2.0815435	0.0380706	0.0696047	-4.787555
AC125421.1	-0.337789	-0.533932	-2.081519	0.0380728	0.0696053	-4.787605
RP11-159F2	0.2891572	2.0048499	2.0813459	0.0380887	0.069631	-4.787959
COPB1	0.0276809	6.654764	2.081188	0.0381033	0.0696541	-4.788282
NFKBIE	0.0645862	6.0007085	2.0811654	0.0381054	0.0696544	-4.788328
MIR6503	0.159895	-1.302667	2.0811008	0.0381113	0.0696618	-4.78846
LILRP2	0.2619032	-0.947267	2.0810798	0.0381132	0.0696618	-4.788503
RP11-148B3	-0.23058	-1.155806	-2.080996	0.038121	0.0696725	-4.788675
ZNF181	-0.043258	5.6936625	-2.08095	0.0381252	0.0696766	-4.788768
AC017002.2	0.2681612	-0.757688	2.0807478	0.0381438	0.0697072	-4.789181
AC079610.1	-0.138724	-1.408622	-2.08041	0.038175	0.0697606	-4.789871
ADAD1	-0.238891	-1.280295	-2.080272	0.0381877	0.0697804	-4.790153
RP4-563E14	0.1852519	3.2819764	2.0795291	0.0382562	0.0699022	-4.79167
CRP	0.2660812	6.7606324	2.079442	0.0382643	0.0699134	-4.791847
BRD7P2	0.2954637	1.8912574	2.0794127	0.038267	0.0699148	-4.791907
LINC01089	-0.075327	5.2969652	-2.079108	0.0382952	0.0699628	-4.792529
XXbac-BPGE	-0.358559	-0.352722	-2.079037	0.0383017	0.0699712	-4.792674
RP11-47402	0.2806509	2.9143897	2.0788512	0.0383189	0.0699992	-4.793053
RP11-475J5	-0.366157	2.3755598	-2.078803	0.0383234	0.0700039	-4.793153
RP11-145M4	-0.120991	-1.414432	-2.078742	0.038329	0.0700082	-4.793276
ZNF717	-0.130725	5.1288441	-2.07872	0.0383311	0.0700082	-4.793322
RP11-613D1	-0.345044	1.2599849	-2.078699	0.038333	0.0700082	-4.793363
KIAA0930	0.0463736	6.4974643	2.0786939	0.0383335	0.0700082	-4.793374
SERPINB2	0.3726352	-0.58122	2.0786708	0.0383356	0.0700086	-4.793422
UBBP3	-0.183441	-1.254705	-2.078516	0.03835	0.0700314	-4.793738
AC244250.3	0.1882573	-1.301011	2.0784069	0.03836	0.0700462	-4.79396

ZNF577	0.1227063	5.0111619	2.0783486	0.0383654	0.0700526	-4.794079
RP11-111M2	0.3230607	-0.351214	2.0783219	0.0383679	0.0700536	-4.794133
AC009120.1	-0.349763	0.1628879	-2.078286	0.0383712	0.0700561	-4.794206
RNU6-944P	-0.162855	-1.296997	-2.07821	0.0383783	0.0700656	-4.794362
TRDV3	0.1573151	-1.340784	2.0781866	0.0383804	0.0700659	-4.794409
MUC5AC	0.4590879	-0.238602	2.0781403	0.0383847	0.0700703	-4.794504
DCAF12L2	-0.284212	-0.986933	-2.078045	0.0383935	0.0700829	-4.794698
RP11-63K6.	-0.107369	-1.461124	-2.077791	0.0384171	0.0701213	-4.795216
ALMS1-IT1	0.2643711	3.0805958	2.0777766	0.0384184	0.0701213	-4.795246
RP11-407N1	0.2622961	3.1684736	2.0775799	0.0384366	0.0701511	-4.795647
RP11-356C4	0.479053	2.3313755	2.0772707	0.0384653	0.0701999	-4.796278
HCG22	0.2838156	-0.779138	2.0769987	0.0384906	0.0702425	-4.796832
YLPM1	0.0311334	6.2112398	2.0765313	0.038534	0.0703169	-4.797785
RP11-80F22	-0.266385	-1.088413	-2.076518	0.0385352	0.0703169	-4.797811
FAM122B	0.0430121	5.9569008	2.0764899	0.0385379	0.0703182	-4.797869
RP11-554E2	0.3452255	1.1852904	2.0764482	0.0385417	0.0703213	-4.797954
SLFNL1-AS1	0.2198136	3.8521971	2.0764303	0.0385434	0.0703213	-4.797991
RP11-73B2.	-0.304172	-0.440656	-2.076094	0.0385747	0.0703748	-4.798677
AP001437.1	-0.337237	1.2979726	-2.076029	0.0385807	0.0703824	-4.79881
RP11-156G1	-0.345321	0.2802124	-2.075953	0.0385878	0.0703918	-4.798964
BCRP4	0.2076529	-1.221041	2.0755347	0.0386267	0.0704592	-4.799816
RP11-33109	-0.072403	-1.51231	-2.075358	0.0386432	0.0704857	-4.800176
AC005229.1	0.198237	3.478538	2.0752494	0.0386533	0.0705007	-4.800398
RP1-118J21	0.4310521	0.4091788	2.0752134	0.0386567	0.0705032	-4.800471
RP11-284F2	-0.118509	-1.428721	-2.075096	0.0386676	0.0705197	-4.80071
NCOA7-AS1	-0.375988	-0.382483	-2.074595	0.0387143	0.0706013	-4.80173
RP11-420C9	-0.356282	0.8725138	-2.074475	0.0387255	0.0706183	-4.801975
RPL34-AS1	-0.358875	1.2557294	-2.074138	0.038757	0.0706722	-4.802662
TTC4	-0.059386	4.7743046	-2.074073	0.0387631	0.0706797	-4.802794
TYRO3P	0.3069744	-0.428303	2.0738256	0.0387862	0.0707183	-4.803297
CTA-363E6.	-0.183507	-1.291037	-2.073795	0.0387891	0.07072	-4.80336
RP11-186B7	-0.355484	0.5651048	-2.073309	0.0388345	0.0707967	-4.804349
CEP131	-0.052704	5.8649517	-2.073304	0.038835	0.0707967	-4.80436
CPNE2	0.051672	5.9408161	2.0731604	0.0388484	0.0708175	-4.804651
TUBD1	-0.04609	5.4153525	-2.072582	0.0389026	0.0709114	-4.805829
DLGAP4	0.0323323	6.4720485	2.0725694	0.0389037	0.0709114	-4.805854
RP11-143E2	-0.161171	-1.381545	-2.072419	0.0389178	0.0709313	-4.80616
RPL34P27	-0.346784	-0.053855	-2.072411	0.0389185	0.0709313	-4.806175
GLT1D1	0.3263513	5.4988695	2.0723593	0.0389234	0.0709366	-4.806282
TNRC6C	-0.052425	5.7864955	-2.072263	0.0389325	0.0709495	-4.806477
RP3-449017	0.0911381	4.4561279	2.0722354	0.038935	0.0709507	-4.806534
DOCK9	0.0611751	5.9886454	2.0719392	0.0389628	0.0709978	-4.807136
VTI1A	-0.029151	5.8133564	-2.07183	0.038973	0.0710129	-4.807358
RPL21P54	-0.196253	-1.209432	-2.071717	0.0389836	0.0710286	-4.807587
RP11-893F2	0.1911094	-1.223666	2.071649	0.0389901	0.0710368	-4.807726
LYPD2	0.4384564	-0.250526	2.0716001	0.0389946	0.0710407	-4.807826
CASP12	-0.355935	1.7502127	-2.071585	0.0389961	0.0710407	-4.807857
MIR4677	0.2004257	-1.120103	2.0713557	0.0390176	0.0710763	-4.808323
AC114776.1	0.2158044	-1.048713	2.0711086	0.0390408	0.0711151	-4.808825
RP11-816J6	-0.367845	1.0738732	-2.07103	0.0390482	0.071125	-4.808985
ARHGAP36	0.3723271	-0.914572	2.0709945	0.0390515	0.0711275	-4.809057

DDX3Y	-0.693334	3.9515335	-2.07071	0.0390783	0.0711726	-4.809635
GPALPP1	-0.038767	5.8426694	-2.070626	0.0390862	0.0711836	-4.809807
TEX21P	-0.339743	1.6100328	-2.070259	0.0391208	0.0712429	-4.810553
AC005786.3	-0.168406	-1.288596	-2.069971	0.0391479	0.0712887	-4.811138
TMPRSS11F	-0.10734	-1.466056	-2.069779	0.039166	0.0713182	-4.811529
AP1B1	-0.026869	6.6531577	-2.069562	0.0391864	0.0713517	-4.811968
RP11-263G2	-0.509584	2.1243604	-2.069384	0.0392032	0.0713788	-4.812331
RP11-443B7	0.3735771	0.5201197	2.0691974	0.0392208	0.0714073	-4.81271
ZNF440	0.0597874	5.2447659	2.0691356	0.0392266	0.0714143	-4.812835
GSTA6P	-0.352759	0.0835099	-2.069101	0.0392299	0.0714167	-4.812905
IGHV7-34-1	0.2097861	-1.251248	2.068972	0.0392421	0.0714353	-4.813168
AC008753.6	0.3118135	-0.515258	2.0687817	0.03926	0.0714644	-4.813554
KRT10	-0.053937	6.0057798	-2.068691	0.0392685	0.0714764	-4.813737
RN7SL481P	0.2836147	-0.624983	2.0677505	0.0393575	0.0716348	-4.815648
SPNS2	0.1197193	5.4174431	2.0677197	0.0393604	0.0716365	-4.81571
C22orf46	-0.038522	5.8855052	-2.067568	0.0393748	0.071659	-4.816018
COX10-AS1	0.0742942	4.8009521	2.0673854	0.0393921	0.0716869	-4.816389
BNIP3P18	-0.282898	-1.041702	-2.066792	0.0394483	0.0717857	-4.817594
KRT13	0.4981689	-0.271389	2.066703	0.0394567	0.0717975	-4.817774
MACROD2	0.12506	5.0320345	2.0664366	0.039482	0.0718399	-4.818315
TKTL2	0.2470383	-1.166212	2.0663929	0.0394862	0.0718438	-4.818403
GPR88	-0.435193	4.7206026	-2.066279	0.0394969	0.0718599	-4.818634
RP11-1042B	-0.137287	-1.447446	-2.06603	0.0395206	0.0718994	-4.81914
CTB-118N6.	0.2579003	-0.896356	2.0658563	0.0395371	0.0719258	-4.819492
BRD2	-0.027893	6.8158275	-2.065826	0.03954	0.0719264	-4.819554
IRF2BPL	0.04665	6.0639411	2.065811	0.0395414	0.0719264	-4.819583
CNN2	0.0539172	6.4107995	2.0656566	0.0395561	0.0719495	-4.819897
RP11-429B1	-0.150045	-1.401133	-2.065592	0.0395622	0.0719571	-4.820028
TUBBP1	0.1361855	3.7571699	2.0654818	0.0395727	0.0719726	-4.820251
MAP2	-0.128733	5.8025422	-2.065423	0.0395783	0.0719792	-4.820371
RP4-641G12	0.3082724	2.3694405	2.0649845	0.03962	0.0720514	-4.82126
CTC-137K3.	0.2564553	-0.826995	2.0649237	0.0396258	0.0720584	-4.821383
TUBB4A	0.3947895	4.1509729	2.0647072	0.0396464	0.0720923	-4.821822
LL21NC02-1	0.3207879	1.8883491	2.0644328	0.0396725	0.0721362	-4.822378
TDP1	0.0388893	5.6754372	2.0641446	0.0397	0.0721768	-4.822962
RP11-156P1	-0.066216	4.8001581	-2.06414	0.0397005	0.0721768	-4.822972
RP11-197N1	-0.309963	-0.445677	-2.064137	0.0397008	0.0721768	-4.822979
RPSAP63	0.2137524	-1.122704	2.0639695	0.0397167	0.0722012	-4.823317
DRGX	-0.501531	0.1878298	-2.063954	0.0397182	0.0722012	-4.823349
PPP1CC	-0.026523	6.5991626	-2.063863	0.0397268	0.0722134	-4.823532
GTSCR1	0.1854808	-1.2711	2.063687	0.0397437	0.0722403	-4.823889
FLJ35934	0.2036958	-1.1431	2.0636084	0.0397512	0.0722503	-4.824049
PRKRIRP7	0.3654945	0.8992679	2.0634106	0.03977	0.0722811	-4.824449
RP11-454L9	0.3516122	-0.110799	2.0633764	0.0397733	0.0722834	-4.824519
LINC01524	-0.447304	0.0277301	-2.063178	0.0397922	0.0723142	-4.824921
CYP1B1	0.139729	5.7062528	2.0631098	0.0397988	0.0723225	-4.825059
RP11-342A2	-0.417145	1.5322308	-2.062746	0.0398336	0.0723821	-4.825796
RP11-324D1	-0.176909	-1.276173	-2.062501	0.039857	0.0724211	-4.826293
KLHL36	0.0403411	6.0519055	2.0624623	0.0398607	0.0724241	-4.82637
RPS15AP17	-0.35387	0.7057768	-2.062242	0.0398817	0.0724588	-4.826816
CCT4	-0.030052	6.7084324	-2.062142	0.0398913	0.0724726	-4.827019

RP11-314N1	0.3394985	-0.159473	2.0618699	0.0399174	0.0725164	-4.82757
NOL4	-0.519391	0.7205017	-2.061823	0.0399219	0.0725209	-4.827665
BMS1P16	0.2086293	-1.143717	2.0616711	0.0399364	0.0725437	-4.827972
TYW1B	-0.117739	4.8955337	-2.061633	0.0399401	0.0725468	-4.82805
RP11-843P1	0.3943268	1.2849439	2.0615868	0.0399445	0.0725512	-4.828143
RP11-65D17	0.196239	-1.261814	2.0605045	0.0400483	0.0727362	-4.830333
RPL7AP60	-0.337679	0.308968	-2.060325	0.0400656	0.0727639	-4.830696
RN7SL417P	0.1847477	-1.245999	2.0603026	0.0400677	0.0727641	-4.830741
RP11-328P2	-0.273639	-0.939156	-2.05999	0.0400978	0.0728151	-4.831374
RP1-207H1.	0.1967954	-1.227224	2.0598716	0.0401092	0.0728321	-4.831613
HSPA8P7	0.210864	-1.081825	2.0598411	0.0401121	0.0728338	-4.831675
NIP7	-0.036503	5.9563174	-2.059666	0.0401289	0.0728607	-4.832028
RP11-244K5	-0.129218	-1.402149	-2.059556	0.0401395	0.0728763	-4.832251
ZNF623	0.0420212	6.036681	2.0591898	0.0401748	0.0729368	-4.832992
SMCO4	-0.05386	6.2740818	-2.058978	0.0401951	0.0729701	-4.83342
NSUN5P1	-0.064017	5.553286	-2.058805	0.0402119	0.0729968	-4.83377
AC008278.3	-0.148482	-1.369206	-2.058308	0.0402597	0.0730801	-4.834774
IFIT3	-0.070769	6.0921751	-2.058205	0.0402697	0.0730944	-4.834984
RP11-354E2	0.3120624	-0.399887	2.0581854	0.0402716	0.0730944	-4.835022
CEP57	-0.040885	6.3321356	-2.0579	0.0402992	0.0731408	-4.8356
GCSHP5	-0.340338	1.6878909	-2.057839	0.040305	0.0731478	-4.835722
DEFA3	0.2649611	-1.017339	2.0577393	0.0403147	0.0731616	-4.835924
ERP29P1	0.323742	0.5836519	2.0574461	0.040343	0.0732094	-4.836516
MIR25	0.2922475	2.4021009	2.057373	0.0403501	0.0732186	-4.836664
PIK3R3	-0.052528	5.7967149	-2.057339	0.0403533	0.0732209	-4.836732
DNAJC27	0.0672964	4.8978036	2.0568454	0.0404011	0.0733039	-4.837729
RP11-5809.	-0.277614	-0.758049	-2.056769	0.0404085	0.0733137	-4.837885
LAMP5-AS1	0.1815296	-1.369943	2.0563898	0.0404452	0.0733766	-4.838649
RPS4XP13	0.3512033	0.4578163	2.0562988	0.040454	0.073389	-4.838833
ZMYM1	0.0546085	5.3997422	2.05604	0.0404791	0.0734308	-4.839356
C11orf44	-0.092965	-1.487168	-2.055983	0.0404846	0.0734372	-4.839471
C10orf131	0.3393659	0.3751731	2.0559527	0.0404875	0.0734389	-4.839532
RP13-638C3	-0.34418	-0.163076	-2.055871	0.0404954	0.0734496	-4.839697
ENTPD2	0.1713993	4.9367121	2.0556894	0.0405131	0.0734779	-4.840064
CTD-230905	-0.091243	-1.484659	-2.055519	0.0405296	0.0735042	-4.840408
RP11-566K1	0.3069572	-0.346111	2.0550037	0.0405796	0.073588	-4.841448
CCDC184	0.2530072	2.991619	2.0550014	0.0405799	0.073588	-4.841452
TGIF1	-0.048684	6.278984	-2.054904	0.0405893	0.0736016	-4.841649
OR56B3P	-0.093045	-1.484561	-2.054755	0.0406038	0.0736215	-4.841949
NDUFB1P1	-0.314887	-0.241557	-2.05473	0.0406063	0.0736215	-4.842001
PLEKHA6	-0.064311	6.4265744	-2.054728	0.0406064	0.0736215	-4.842003
RP11-30504	-0.339241	1.0388924	-2.054701	0.0406091	0.0736227	-4.842058
RP11-561N1	-0.35813	-0.848649	-2.053348	0.0407407	0.0738576	-4.844786
RP4-61404.	0.3365301	0.5493876	2.0532864	0.0407467	0.0738586	-4.844911
VASN	-0.053505	6.3381829	-2.053282	0.0407472	0.0738586	-4.844921
CTD-2021J1	-0.197583	-1.169976	-2.053281	0.0407473	0.0738586	-4.844923
AF146191.4	0.3505416	0.6612578	2.0530972	0.0407652	0.0738874	-4.845293
MIR5091	-0.145155	-1.339255	-2.052933	0.0407812	0.0739127	-4.845624
AC007364.1	-0.286302	-0.832238	-2.052581	0.0408156	0.0739702	-4.846334
C9orf135	0.2647578	-1.029275	2.0525665	0.0408169	0.0739702	-4.846362
SLFNL1	0.3354089	2.6187545	2.0522796	0.040845	0.0740173	-4.846941

DMD	-0.090669	6.1093054	-2.052258	0.040847	0.0740174	-4.846984
RP11-281A2	-0.218802	-1.207869	-2.051909	0.0408811	0.0740755	-4.847687
ZNF589	0.0574967	5.2955764	2.0517818	0.0408936	0.0740918	-4.847944
STRADB	-0.038014	6.3295459	-2.051776	0.0408942	0.0740918	-4.847956
ZNF473	0.0388187	5.6055395	2.0517496	0.0408968	0.0740928	-4.848009
RP11-108L7	-0.356063	0.8701803	-2.051699	0.0409017	0.074098	-4.848111
RP11-817J1	0.56244	0.8672006	2.0512287	0.0409477	0.0741777	-4.849058
SEPT1	0.0737395	5.4579728	2.0511651	0.0409539	0.0741853	-4.849186
LINC01411	0.2096957	-1.22242	2.0509627	0.0409738	0.0742142	-4.849594
LINC00264	0.3148279	-0.46645	2.0509606	0.040974	0.0742142	-4.849598
SEMA3F	-0.041395	6.0468317	-2.050784	0.0409913	0.0742419	-4.849954
CCDC146	0.1115433	5.1168918	2.0506305	0.0410063	0.0742654	-4.850263
RP11-64C12	-0.085527	-1.490497	-2.050549	0.0410143	0.0742763	-4.850428
RP11-304L1	-0.143001	-1.378615	-2.05043	0.041026	0.0742937	-4.850667
RPL21P110	0.249482	-0.882724	2.0501378	0.0410546	0.0743418	-4.851255
RP1	0.3250666	-0.692003	2.0501091	0.0410574	0.0743432	-4.851313
CLEC4GP1	0.3498051	-0.586509	2.0498869	0.0410792	0.074379	-4.85176
RPL35AP32	0.1999298	-1.124698	2.0494219	0.0411249	0.074458	-4.852696
RP11-567C2	0.1864485	-1.305003	2.0491212	0.0411544	0.0745078	-4.853301
SLC5A8	0.2189761	-1.21098	2.0490832	0.0411582	0.0745109	-4.853377
RP11-621L6	0.2460432	-0.939353	2.0489932	0.041167	0.0745232	-4.853558
AC084357.1	-0.207761	-1.214536	-2.048795	0.0411866	0.0745549	-4.853958
HMGN1P4	0.2823353	-0.813929	2.0485931	0.0412064	0.0745871	-4.854363
USP42	0.0374923	5.7225216	2.0485626	0.0412094	0.0745888	-4.854425
TRIM60	-0.276125	-1.139602	-2.048435	0.0412219	0.0746078	-4.854681
CST9L	-0.090261	-1.479587	-2.048305	0.0412347	0.0746273	-4.854943
DNMBP	-0.050385	5.9947864	-2.048224	0.0412427	0.074638	-4.855106
RP11-298O2	-0.244031	-1.150972	-2.048075	0.0412574	0.0746609	-4.855406
RP11-202G1	-0.135512	-1.393403	-2.047913	0.0412733	0.0746861	-4.855731
RP13-444K1	-0.212375	-1.075037	-2.047649	0.0412994	0.0747295	-4.856263
RP11-49K24	0.3604604	1.7641982	2.04737	0.0413269	0.0747755	-4.856823
DUX4	-0.098981	-1.483162	-2.047121	0.0413514	0.0748162	-4.857323
SNORA74	-0.173922	-1.259713	-2.047009	0.0413625	0.074828	-4.85755
SATL1	0.1922137	-1.227889	2.047	0.0413634	0.074828	-4.857567
RP11-220D1	-0.340587	-0.163377	-2.046993	0.0413641	0.074828	-4.857581
RNU6-875P	0.2171532	-1.096075	2.0469352	0.0413698	0.0748346	-4.857697
C17orf96	-0.164691	5.2546583	-2.046511	0.0414117	0.0749066	-4.858549
ANKRD53	0.3026729	3.0365148	2.0464453	0.0414182	0.0749147	-4.858682
SNX24	-0.041025	5.7288709	-2.0464	0.0414227	0.0749192	-4.858774
ADPRHL2	-0.036021	6.1220071	-2.046157	0.0414467	0.0749589	-4.859262
CTB-70G10.	-0.071062	-1.511558	-2.046056	0.0414567	0.0749733	-4.859465
AP001062.9	-0.311122	-0.37525	-2.04567	0.0414948	0.0750385	-4.860239
ALOX15	0.3977053	1.894211	2.0455481	0.0415069	0.0750567	-4.860485
VPS8	0.0243403	5.9460493	2.0454121	0.0415204	0.0750773	-4.860758
AC012123.1	0.152787	-1.386058	2.0449865	0.0415626	0.0751466	-4.861613
BLOC1S2	-0.033096	6.1624739	-2.044984	0.0415629	0.0751466	-4.861618
RP11-1114A	0.3415887	1.0792694	2.0448876	0.0415724	0.0751601	-4.861811
FAM21C	-0.03175	6.1219152	-2.04483	0.0415781	0.0751668	-4.861928
OR7E41P	-0.134187	-1.396319	-2.044646	0.0415963	0.0751959	-4.862296
ZNRF2P1	0.3290039	1.5906905	2.0443539	0.0416253	0.0752447	-4.862883
RP11-1029J	0.2913011	-1.063853	2.0441195	0.0416486	0.075283	-4.863354

ST6GALNAC4	0.3231751	1.4725068	2.0440931	0.0416512	0.075284	-4.863406
RP11-71602	-0.316434	-0.642932	-2.044008	0.0416597	0.0752956	-4.863577
FAM109A	0.04657	6.0201521	2.0437074	0.0416895	0.0753431	-4.864181
GTF2E2	0.0473264	5.9771516	2.0437015	0.0416901	0.0753431	-4.864193
EFCAB12	-0.212113	4.7962296	-2.043675	0.0416927	0.0753441	-4.864245
IGLJ1	0.1659988	-1.344205	2.0435349	0.0417067	0.0753639	-4.864527
IGHV1OR16-	0.2093303	-1.257237	2.0435235	0.0417078	0.0753639	-4.86455
FLJ31662	-0.070431	-1.511204	-2.043374	0.0417227	0.0753871	-4.86485
ZNF865	-0.038984	5.8928308	-2.042905	0.0417694	0.0754677	-4.865792
KAT5	0.0416788	5.9747611	2.0428631	0.0417735	0.0754714	-4.865875
MTMR11	-0.125552	5.5708973	-2.042499	0.0418097	0.0755331	-4.866604
RP5-1009N1	-0.256266	-0.815568	-2.042407	0.0418189	0.075546	-4.866789
CTD-2291D1	-0.10745	-1.47726	-2.042313	0.0418283	0.0755592	-4.866978
NEURL1B	0.0620693	5.9931919	2.0422227	0.0418373	0.0755715	-4.86716
CERS3-AS1	-0.392134	-0.378028	-2.042204	0.0418392	0.0755715	-4.867198
AC079354.5	-0.353677	0.0928693	-2.041989	0.0418606	0.0756063	-4.867627
RAB40AL	0.2802297	-0.59125	2.0418896	0.0418705	0.0756205	-4.867828
FOCAD	0.0402971	5.9266285	2.0418085	0.0418786	0.0756314	-4.86799
NECAB1	0.2850133	3.6165005	2.0416712	0.0418923	0.0756524	-4.868265
UBA6-AS1	-0.047164	5.3986744	-2.041505	0.041909	0.0756787	-4.8686
AP000662.4	0.3837552	1.4963651	2.0414636	0.041913	0.0756823	-4.868682
AC093388.3	-0.259083	2.3719013	-2.041391	0.0419203	0.0756888	-4.868827
B4GALT3	-0.03447	6.2967047	-2.041386	0.0419208	0.0756888	-4.868837
HSD17B13	-0.337053	5.5261419	-2.040932	0.0419661	0.0757669	-4.869747
AP000705.6	-0.204979	-1.19385	-2.04085	0.0419743	0.075778	-4.869912
AC010884.1	-0.184355	-1.204314	-2.040579	0.0420014	0.0758232	-4.870455
MIR4477B	0.2682404	-0.859778	2.0404595	0.0420134	0.075841	-4.870694
RP11-164P1	0.3545775	0.1610849	2.0401912	0.0420402	0.0758857	-4.871232
RP11-15B17	0.2911356	-0.602213	2.0401363	0.0420457	0.0758888	-4.871342
DNAL1	0.045583	5.3823938	2.0401328	0.0420461	0.0758888	-4.871349
RP11-94C24	-0.171229	-1.263745	-2.040077	0.0420517	0.0758951	-4.871461
CACYBP	-0.039958	6.4307863	-2.039853	0.0420741	0.0759319	-4.87191
PSG8	0.2491881	-1.119357	2.0397946	0.04208	0.0759386	-4.872026
CTD-2525I3	0.2301097	-0.962282	2.0396627	0.0420932	0.0759587	-4.872291
RP11-328C8	0.2813528	-0.507582	2.0396076	0.0420987	0.0759649	-4.872401
RP11-143N1	-0.116402	-1.432496	-2.039574	0.0421021	0.0759673	-4.872469
CMIP	0.0327485	6.237801	2.0392498	0.0421346	0.0760221	-4.873118
RP11-9502.	0.2358075	-0.981159	2.0391577	0.0421438	0.0760351	-4.873302
SCARNA12	0.3300805	1.1232402	2.0390722	0.0421524	0.0760433	-4.873473
ZNF679	-0.334891	-0.94837	-2.03906	0.0421536	0.0760433	-4.873497
RBMY3AP	-0.185873	-1.346985	-2.03905	0.0421546	0.0760433	-4.873518
ITPR1-AS1	0.3356159	1.9872918	2.0389702	0.0421626	0.076054	-4.873678
RBFOX2	0.0346918	6.3720856	2.0389215	0.0421675	0.076059	-4.873775
KIF2C	0.1236221	5.4596413	2.0387252	0.0421872	0.0760908	-4.874168
FOXO1	-0.062872	6.0710978	-2.0387	0.0421897	0.0760915	-4.874218
ZRANB3	-0.052208	5.2682244	-2.038561	0.0422037	0.076113	-4.874497
ABHD14A-AC	-0.211528	3.5172169	-2.03853	0.0422068	0.0761149	-4.874559
RNU5B-1	-0.147131	-1.372417	-2.038501	0.0422097	0.0761163	-4.874617
POLR3C	-0.038871	6.0304171	-2.038334	0.0422265	0.0761429	-4.874952
RP11-252A2	0.101828	4.2135171	2.0382484	0.0422351	0.0761546	-4.875123
CTC-425023	0.2238226	3.8206588	2.0380938	0.0422506	0.0761788	-4.875432

AC015987.1	0.3590342	-0.272451	2.0378349	0.0422767	0.076222	-4.875951
NAA50	-0.023658	6.592823	-2.037716	0.0422886	0.0762399	-4.876189
RP11-159D8	-0.098304	-1.462557	-2.037576	0.0423027	0.0762581	-4.876468
CTB-58E17.	-0.30787	-0.483286	-2.037574	0.0423029	0.0762581	-4.876473
RP11-25D10	-0.190933	-1.324268	-2.037295	0.042331	0.0763049	-4.877031
RP11-789C1	0.2783685	-0.915388	2.0372573	0.0423348	0.076308	-4.877106
MAN1B1-AS1	-0.118365	4.2899672	-2.037056	0.0423551	0.0763407	-4.877509
LINC00856	0.3598025	-0.431971	2.0369464	0.0423661	0.0763554	-4.877728
RP11-537A6	0.2759873	-0.657678	2.0369336	0.0423674	0.0763554	-4.877754
UBE2CP2	0.3268529	-0.127655	2.0368965	0.0423711	0.0763584	-4.877828
PIGW	-0.046399	5.49511	-2.036858	0.042375	0.0763616	-4.877905
LINC01340	-0.142249	-1.362457	-2.036828	0.042378	0.0763633	-4.877965
SLC25A24P1	0.3898932	-0.636916	2.0367321	0.0423877	0.0763769	-4.878157
MGLL	0.0710487	6.6339808	2.0364822	0.0424129	0.0764186	-4.878657
DPM1	-0.030886	6.1651243	-2.03616	0.0424454	0.0764734	-4.879302
GVQW1	0.136423	3.5432549	2.036111	0.0424503	0.0764785	-4.879399
ZNF655	-0.036244	6.224414	-2.035996	0.042462	0.0764957	-4.87963
CCDC59	-0.030448	5.9319813	-2.035968	0.0424648	0.0764969	-4.879685
SMARCE1	-0.026194	6.3731829	-2.035847	0.042477	0.0765152	-4.879927
MUC19	0.3663699	-0.542474	2.0358184	0.0424799	0.0765166	-4.879984
RP11-438L1	0.2106536	3.0521257	2.0347096	0.042592	0.0767147	-4.882201
TSPO2	0.3187473	3.4014581	2.0343097	0.0426325	0.0767839	-4.883
AC007182.6	-0.448594	1.7554422	-2.034108	0.0426529	0.076817	-4.883404
RP11-983P1	-0.06365	5.5725736	-2.034069	0.0426568	0.0768201	-4.88348
EFHD1	-0.142817	5.5991318	-2.03397	0.0426669	0.0768345	-4.883678
RP1-90K10.	-0.09358	-1.47847	-2.033874	0.0426766	0.0768448	-4.88387
MIR574	-0.233435	-0.910015	-2.033872	0.0426768	0.0768448	-4.883875
KRTAP19-5	0.2913841	-1.067666	2.0338423	0.0426798	0.0768464	-4.883934
IGKV1OR2-2	-0.071756	-1.511947	-2.033591	0.0427053	0.0768885	-4.884435
RP11-519M1	-0.450836	0.0430757	-2.033531	0.0427114	0.0768958	-4.884556
FAM60CP	0.1630066	-1.306125	2.0334802	0.0427166	0.0769012	-4.884657
RP11-407G2	0.2912076	-0.481512	2.0333392	0.0427309	0.0769231	-4.884938
ZNF485	0.0774167	4.6968965	2.0328703	0.0427785	0.0770051	-4.885875
VPS13D	-0.035048	6.2874337	-2.032684	0.0427974	0.0770353	-4.886247
SAG	0.3256164	-0.308399	2.0326537	0.0428005	0.0770371	-4.886307
RP11-379B8	-0.248085	4.6918027	-2.032265	0.04284	0.0771044	-4.887083
RAMP2	0.0767143	5.7442975	2.0321332	0.0428534	0.0771247	-4.887346
OR2W3	0.2095343	-1.14321	2.0319653	0.0428705	0.0771517	-4.887681
RN7SL809P	0.320783	-0.248211	2.0317654	0.0428908	0.0771845	-4.88808
NPM1P12	0.2233208	-1.021978	2.0315742	0.0429103	0.0772157	-4.888462
LINC01492	-0.136719	-1.379834	-2.031425	0.0429255	0.0772374	-4.888759
TIPRL	-0.028641	6.3583899	-2.031395	0.0429285	0.0772374	-4.888819
LRRC37BP1	0.054191	5.2465753	2.0313936	0.0429287	0.0772374	-4.888822
HNRNPA3P1	0.3126972	-0.26655	2.0312645	0.0429419	0.0772573	-4.88908
LINC00113	0.2475332	-1.232651	2.0311541	0.0429531	0.0772737	-4.8893
PRSS41	0.2279238	-1.235592	2.0309998	0.0429689	0.0772982	-4.889608
MIR1307	0.1840603	-1.195192	2.0309621	0.0429727	0.0773013	-4.889683
IGHV1OR15-	-0.150209	-1.359493	-2.030927	0.0429763	0.0773039	-4.889753
RP11-455P2	-0.138224	-1.362425	-2.030869	0.0429821	0.0773076	-4.889868
C7orf49	0.0360942	6.1067599	2.0308654	0.0429826	0.0773076	-4.889876
RP11-285G1	0.2035262	-1.115555	2.0308343	0.0429857	0.077309	-4.889938

COPS3	-0.036318	6.2132704	-2.030816	0.0429876	0.077309	-4.889974
TMED6	-0.174081	4.6192302	-2.030745	0.0429949	0.0773183	-4.890116
AP004782.1	0.2726962	-0.892792	2.0306994	0.0429995	0.0773228	-4.890207
C4B	-0.081864	6.8326172	-2.030583	0.0430114	0.0773404	-4.890439
RPL35AP21	-0.151799	-1.339044	-2.030429	0.0430271	0.0773648	-4.890746
ITPKC	0.038464	6.2341389	2.0302897	0.0430413	0.0773866	-4.891024
CTD-2555A7	0.3138185	-0.155598	2.0302164	0.0430488	0.0773962	-4.89117
RP11-5407.	0.2260315	-1.160503	2.0301923	0.0430513	0.0773968	-4.891218
AC008154.4	-0.156242	-1.346334	-2.030139	0.0430567	0.0774029	-4.891326
LINC00919	-0.180786	-1.307358	-2.029961	0.0430749	0.0774317	-4.891679
RSPH3	0.0501483	5.5047813	2.0294439	0.0431278	0.0775229	-4.892711
IRX2	0.3515399	-0.411913	2.0293819	0.0431341	0.0775305	-4.892834
CTD-2619J1	-0.164746	5.3428741	-2.029228	0.0431499	0.077555	-4.893141
RP11-162D1	0.4117206	0.3252956	2.0291269	0.0431602	0.0775698	-4.893342
KIAA0586	0.0396102	5.633816	2.0289545	0.0431778	0.0775977	-4.893686
RP11-46A1C	0.165748	3.862313	2.0287818	0.0431955	0.0776234	-4.89403
NLGN4Y-AS1	-0.304239	-0.727263	-2.028764	0.0431973	0.0776234	-4.894066
ZNF197	-0.04431	5.7646721	-2.028752	0.0431985	0.0776234	-4.894089
TTC39C-AS1	0.3775886	2.2688571	2.0285283	0.0432215	0.0776586	-4.894535
LINC00847	-0.043159	5.7183569	-2.028519	0.0432224	0.0776586	-4.894553
CASQ2	0.2829684	3.8815525	2.0282897	0.043246	0.0776971	-4.895011
TMPRSS4-AS	-0.08574	-1.481574	-2.028215	0.0432536	0.077707	-4.895159
RP11-761I4	-0.3319	-0.157262	-2.027977	0.043278	0.0777471	-4.895634
RP11-16801	0.3507064	-0.31775	2.0277527	0.043301	0.0777846	-4.89608
BCL2L11	0.0511542	6.1189332	2.0276007	0.0433166	0.0778088	-4.896383
HSD11B1L	-0.077943	5.1455237	-2.027432	0.043334	0.0778361	-4.896719
RP11-339B2	0.1697302	-1.308144	2.0273136	0.0433461	0.0778541	-4.896955
CTD-2140B2	-0.195917	-1.192099	-2.027249	0.0433528	0.0778622	-4.897083
COPS8	-0.023994	6.1902064	-2.027223	0.0433555	0.0778633	-4.897136
DPY19L2P2	0.2985886	2.9648913	2.0271827	0.0433596	0.0778668	-4.897215
GNAO1	0.2670438	4.8314285	2.0270352	0.0433747	0.0778902	-4.897509
LINC01176	-0.107469	4.1997828	-2.026874	0.0433913	0.077916	-4.897829
RP11-690C2	-0.153377	-1.34712	-2.02666	0.0434133	0.0779518	-4.898256
CTD-2270P1	-0.209226	3.4723746	-2.026536	0.0434261	0.0779709	-4.898504
RANP8	-0.18905	-1.220674	-2.026498	0.04343	0.0779741	-4.898579
MORF4L2-AS	0.2451896	2.889149	2.0264325	0.0434367	0.0779823	-4.898709
VN1R107P	0.310817	-0.459345	2.026312	0.0434491	0.0780007	-4.898949
LINC01510	-0.432209	-0.041523	-2.026148	0.043466	0.0780272	-4.899275
KB-1410C5.	-0.147564	-1.379029	-2.026077	0.0434734	0.0780366	-4.899417
NME2P1	-0.212817	3.1818677	-2.026048	0.0434763	0.0780381	-4.899475
RP5-107303	0.1575404	-1.303988	2.0260158	0.0434796	0.0780401	-4.899538
RP11-332J1	0.175059	-1.299573	2.0258404	0.0434977	0.0780687	-4.899887
RP3-475N16	0.1120654	4.1304145	2.025748	0.0435072	0.078082	-4.900071
RP11-430L1	-0.133538	-1.423131	-2.025719	0.0435102	0.0780834	-4.900128
RP11-114F3	0.2455013	-0.910064	2.025512	0.0435315	0.0781179	-4.900541
SMG1P6	0.3470962	0.8013547	2.0254089	0.0435422	0.0781332	-4.900746
RP11-242D8	0.1789341	3.8288992	2.0252774	0.0435557	0.0781537	-4.901007
SECISBP2L	-0.044191	6.1579716	-2.025082	0.0435759	0.0781861	-4.901397
SEC13P1	0.2105905	-1.005755	2.0250303	0.0435812	0.0781917	-4.901499
AP000640.1	0.3388355	0.3662227	2.0247739	0.0436077	0.0782354	-4.902009
AC004985.1	0.2638236	2.4460014	2.0247519	0.04361	0.0782356	-4.902053

AOC4P	-0.26032	5.1880587	-2.024135	0.0436737	0.0783461	-4.90328
XIRP2	0.2883236	-1.014571	2.0240736	0.0436801	0.0783536	-4.903402
COL4A3BP	-0.032817	6.1554734	-2.023924	0.0436956	0.0783776	-4.9037
TERF2IP	-0.032629	6.3284978	-2.023873	0.0437007	0.0783823	-4.903799
PZP	0.2332616	4.1774025	2.0238566	0.0437025	0.0783823	-4.903833
CTD-226502	-0.199401	-1.128819	-2.023786	0.0437098	0.0783917	-4.903974
SPAG8	0.1516716	3.5656988	2.0237587	0.0437126	0.0783928	-4.904027
LIN28B	0.6314916	0.5822494	2.02324	0.0437663	0.0784852	-4.905058
AC007879.5	0.3429658	0.965574	2.0230802	0.0437829	0.0785098	-4.905376
CTD-2410N1	0.3364866	0.1461333	2.0230665	0.0437843	0.0785098	-4.905403
RPL5P30	0.3619512	1.2383805	2.0227811	0.0438139	0.0785589	-4.90597
RP11-347H1	-0.255959	-1.12499	-2.022599	0.0438327	0.0785889	-4.906332
ATP6V1B1-A	0.1652697	-1.33691	2.0225784	0.0438349	0.0785889	-4.906373
RP4-575N6.	-0.359646	0.3621326	-2.022525	0.0438404	0.0785949	-4.906479
RP11-805L2	-0.101486	-1.474445	-2.022471	0.0438461	0.0786012	-4.906587
RP11-124N1	-0.286959	-0.990198	-2.022359	0.0438576	0.0786181	-4.906808
GPR123-AS1	0.230054	-1.217779	2.02224	0.04387	0.0786364	-4.907045
RP1-81D8.6	-0.104884	-1.471636	-2.022205	0.0438736	0.078639	-4.907114
AC079922.2	-0.138031	4.4145611	-2.022156	0.0438787	0.0786442	-4.907212
ITGB1BP1	-0.037685	6.1486835	-2.021692	0.0439269	0.0787236	-4.908134
ADRBK2	0.1137513	5.6518407	2.0216884	0.0439273	0.0787236	-4.908141
LINC00595	0.1879504	-1.263651	2.0216186	0.0439345	0.0787327	-4.90828
RP11-317B1	0.1853453	-1.197727	2.0214718	0.0439498	0.0787562	-4.908571
RP3-359N14	-0.11048	-1.477113	-2.021363	0.0439611	0.0787727	-4.908788
TTC39B	-0.092619	5.5273243	-2.021103	0.0439881	0.0788172	-4.909303
NRM	0.0729409	5.7513443	2.0210112	0.0439977	0.0788304	-4.909486
LINC01398	-0.14415	-1.359605	-2.020855	0.044014	0.0788531	-4.909797
SERP2	0.1809653	4.4860101	2.0208481	0.0440146	0.0788531	-4.909809
RP11-88H9.	-0.482368	2.28682	-2.020699	0.0440301	0.078877	-4.910105
RP11-16802	-0.109899	-1.464839	-2.020187	0.0440835	0.0789687	-4.911122
ANTXR2	0.0593938	6.1203616	2.0201219	0.0440903	0.078977	-4.911251
FAM204A	-0.027097	6.0662479	-2.019908	0.0441125	0.079013	-4.911674
RP11-12601	-0.408051	-0.279524	-2.019834	0.0441203	0.0790229	-4.911821
RP11-145H9	0.2449793	-0.906594	2.0197955	0.0441243	0.0790263	-4.911898
RP11-323F2	-0.343215	0.7580691	-2.019615	0.0441431	0.0790526	-4.912256
PLSCR4	0.0872449	5.8469117	2.0196133	0.0441433	0.0790526	-4.91226
FAM215A	0.1511111	-1.315773	2.0195419	0.0441508	0.079062	-4.912401
LINC00618	0.2489239	-0.954312	2.0195033	0.0441548	0.0790654	-4.912478
CSRP1	-0.057501	6.6482974	-2.019342	0.0441716	0.0790915	-4.912797
RP11-75L1.	0.1685543	-1.243057	2.0188881	0.044219	0.0791726	-4.913698
OGDHL	-0.185372	6.2836405	-2.018348	0.0442755	0.0792698	-4.914769
AC004540.4	0.4593229	1.567065	2.0182733	0.0442833	0.0792799	-4.914917
AC004947.2	0.2843107	-0.794781	2.0180709	0.0443045	0.0793102	-4.915319
C1orf141	-0.150755	-1.391733	-2.018051	0.0443065	0.0793102	-4.915357
GCN1L1	-0.025063	6.6088941	-2.01805	0.0443067	0.0793102	-4.915361
DDIT4	0.0760619	6.3381603	2.0178218	0.0443306	0.079349	-4.915812
LINC00987	0.1856512	4.5233317	2.0174083	0.0443739	0.0794226	-4.916632
RP11-474D1	-0.205328	-1.312939	-2.017286	0.0443866	0.0794416	-4.916874
AP006216.1	-0.213245	-1.044164	-2.016862	0.0444311	0.0795173	-4.917714
MUCL1	-0.405506	-0.354474	-2.016825	0.0444351	0.0795204	-4.917788
RP11-110A1	0.2986425	-1.005506	2.0167215	0.0444459	0.0795359	-4.917993

MCM7	-0.04359	6.5564491	-2.01655	0.0444639	0.0795642	-4.918332
ST13P15	0.3275212	1.8526998	2.0163692	0.0444829	0.0795928	-4.918691
ZFP36	0.0537247	6.6355017	2.0163561	0.0444842	0.0795928	-4.918717
TSSC1-IT1	-0.337757	1.6477373	-2.015547	0.0445693	0.079741	-4.920319
UGT2B11	-0.224444	5.5249903	-2.015336	0.0445915	0.0797733	-4.920737
RP11-134N1	0.3815354	-0.200204	2.0153343	0.0445916	0.0797733	-4.92074
RP4-536B24	-0.331448	-0.298239	-2.015297	0.0445955	0.0797763	-4.920813
HLA-F-AS1	-0.108958	4.6827789	-2.014958	0.0446313	0.0798359	-4.921486
CTD-2501M5	-0.193051	-1.282776	-2.01494	0.0446332	0.0798359	-4.921521
CTD-219904	0.3659416	1.2674606	2.0145522	0.044674	0.0799029	-4.922288
CTD-3107M8	-0.186435	-1.235165	-2.014524	0.044677	0.0799029	-4.922344
CTD-2323K1	0.2117969	2.788407	2.0145218	0.0446772	0.0799029	-4.922348
RP11-764K9	0.2635661	-0.958238	2.0142454	0.0447064	0.0799511	-4.922895
DSC2	0.0999828	5.7063282	2.0141437	0.0447171	0.0799663	-4.923097
RP1-191J18	-0.107977	4.1929901	-2.01386	0.044747	0.0800159	-4.923658
RP1-228P16	0.2330205	2.6637396	2.0136714	0.0447669	0.0800476	-4.924031
RP11-326C3	-0.153801	3.988832	-2.013625	0.0447719	0.0800525	-4.924123
STS	0.083179	5.8927626	2.0135858	0.044776	0.0800559	-4.9242
CTC-422A18	-0.240308	-0.901898	-2.013413	0.0447942	0.0800846	-4.924542
AP000295.9	0.349058	0.0003865	2.0133701	0.0447987	0.0800888	-4.924627
CTD-2262B2	0.3674925	1.4462944	2.0130494	0.0448326	0.0801455	-4.925261
AC073316.2	-0.162828	-1.373191	-2.01269	0.0448706	0.0802094	-4.925971
RPS4XP1	0.3393353	0.8880601	2.0126213	0.0448779	0.0802185	-4.926108
RP11-327J1	0.16916	-1.263504	2.0123481	0.0449068	0.0802637	-4.926648
KRT18P27	-0.19525	-1.167543	-2.012341	0.0449076	0.0802637	-4.926662
RP11-96K19	0.1940625	3.5296202	2.012205	0.044922	0.0802855	-4.926931
RP11-361C1	-0.195253	-1.257122	-2.01215	0.0449278	0.0802921	-4.92704
RP11-185B1	-0.13183	-1.383182	-2.012055	0.0449379	0.0803061	-4.927228
RP11-23J9.	0.2267668	-1.08604	2.0113318	0.0450145	0.0804326	-4.928657
RP11-407N8	-0.199513	-1.114896	-2.011332	0.0450145	0.0804326	-4.928657
LMO2	0.0662513	5.641671	2.0113246	0.0450153	0.0804326	-4.928671
RP11-1348G	0.198222	3.1005857	2.0112654	0.0450215	0.0804399	-4.928788
EIF3FP3	-0.085118	5.0769196	-2.010947	0.0450554	0.0804964	-4.929418
PRSS45	-0.428443	2.2286916	-2.01074	0.0450774	0.0805317	-4.929827
N4BP1	-0.026059	6.1307132	-2.010548	0.0450977	0.0805641	-4.930205
KRT8P11	0.3762505	2.7812736	2.0104824	0.0451047	0.0805724	-4.930334
RP11-819M1	0.3160317	-0.390306	2.0104627	0.0451068	0.0805724	-4.930373
AC116366.5	0.2530876	-0.848349	2.0101734	0.0451375	0.0806234	-4.930945
OTP	0.2817007	-0.969839	2.0100418	0.0451515	0.0806444	-4.931205
RP11-139H1	0.2958013	1.9094771	2.009923	0.0451641	0.080663	-4.931439
ENOX2	0.048669	5.6785972	2.0098399	0.045173	0.0806749	-4.931603
RP11-640N2	-0.071062	-1.511558	-2.00976	0.0451815	0.0806861	-4.93176
TMSB10P1	0.3679961	0.1996867	2.009601	0.0451984	0.0807124	-4.932075
RBM48P1	-0.12324	-1.427041	-2.009563	0.0452024	0.0807156	-4.932149
FAM81B	0.3140108	-0.703361	2.0094896	0.0452103	0.0807257	-4.932295
RP11-164P1	0.3387174	3.1029364	2.0093593	0.0452241	0.0807465	-4.932552
RP5-1119A7	-0.271145	-0.713754	-2.009301	0.0452304	0.0807537	-4.932668
UBP1	-0.02864	6.3980827	-2.009111	0.0452506	0.0807858	-4.933042
SLC35E2	0.1780377	3.7665674	2.0088976	0.0452733	0.0808224	-4.933463
HS3ST2	0.1782484	4.3154085	2.0087447	0.0452896	0.0808476	-4.933765
RP1-178F15	-0.224418	3.1070143	-2.008564	0.0453089	0.0808781	-4.934122

DSN1	-0.045421	5.953598	-2.008521	0.0453134	0.0808822	-4.934206
RGS5	0.0592556	6.8337625	2.008229	0.0453446	0.0809339	-4.934783
RP11-57H14	0.3241832	0.803027	2.008197	0.0453481	0.0809349	-4.934846
TUNAR	0.2729385	-1.017636	2.0081825	0.0453496	0.0809349	-4.934875
CLVS2	-0.507216	-0.155089	-2.008109	0.0453575	0.080945	-4.93502
ZFP90	-0.039164	5.694244	-2.008019	0.0453671	0.0809582	-4.935198
SLC35C1	-0.064241	6.5912432	-2.007738	0.045397	0.0810014	-4.935751
WFDC5	-0.203438	-1.21635	-2.007732	0.0453977	0.0810014	-4.935763
BMPR1APS1	0.3501552	0.0986812	2.0077293	0.045398	0.0810014	-4.935769
CTD-2267D1	-0.321587	0.1098294	-2.007674	0.0454039	0.081008	-4.935877
HMX2	0.3007565	-1.001304	2.0071702	0.0454578	0.0811001	-4.936871
ROS1	-0.510915	1.3666808	-2.006855	0.0454915	0.0811529	-4.937493
MIR573	0.3288784	-0.150559	2.0068521	0.0454918	0.0811529	-4.937498
DKKL1	0.4063251	1.98425	2.0064084	0.0455393	0.0812337	-4.938373
RP11-513G1	-0.381295	0.9864362	-2.005924	0.0455912	0.0813224	-4.939328
NUDT12	-0.133533	5.8782417	-2.005875	0.0455964	0.0813277	-4.939424
KRT1	0.4114483	-0.203534	2.0057621	0.0456086	0.0813413	-4.939647
KRT8P45	0.2385862	3.8240854	2.0057442	0.0456105	0.0813413	-4.939682
SGK2	-0.095503	6.2429466	-2.005742	0.0456108	0.0813413	-4.939688
AC008060.7	0.3746044	-0.738107	2.005661	0.0456194	0.0813528	-4.939846
RP11-887P2	-0.334806	1.3160832	-2.005597	0.0456263	0.081361	-4.939973
RP5-1031D4	0.1977159	-1.169836	2.0055729	0.0456289	0.081361	-4.94002
RABIF	-0.039475	5.944772	-2.005556	0.0456307	0.081361	-4.940054
P3H4	0.07931	5.731267	2.0054416	0.045643	0.0813788	-4.940278
RP11-34H11	-0.07943	-1.502005	-2.005354	0.0456523	0.0813916	-4.940451
JKAMP	-0.033114	6.1258712	-2.005322	0.0456558	0.0813937	-4.940514
C2orf49	0.0290968	5.755007	2.0050273	0.0456874	0.0814462	-4.941095
GLRX3P2	0.3132407	-0.267813	2.0050037	0.04569	0.0814468	-4.941141
AD000684.2	0.269739	2.7766813	2.0047241	0.04572	0.0814963	-4.941692
RP11-431K2	-0.13334	-1.417991	-2.004577	0.0457358	0.0815205	-4.941981
GABARAPL3	-0.181693	-1.203951	-2.004512	0.0457428	0.081529	-4.942109
RP11-374A4	-0.382855	0.1075461	-2.00439	0.0457559	0.0815484	-4.942349
ARF6	-0.025396	6.6106018	-2.004285	0.0457672	0.0815645	-4.942556
HNRNPA1P12	0.3210612	1.1364819	2.0041239	0.0457846	0.0815915	-4.942874
SNHG3	0.0755828	5.6829509	2.0038937	0.0458093	0.0816316	-4.943327
GPR87	0.2683143	-1.004647	2.0037652	0.0458232	0.0816523	-4.94358
LINC00862	0.3053526	2.9654379	2.0035194	0.0458496	0.0816918	-4.944064
AC092610.1	-0.285205	-0.535984	-2.003513	0.0458503	0.0816918	-4.944076
RP11-51J9.	-0.158241	-1.289444	-2.003497	0.0458521	0.0816918	-4.944108
RP11-326C3	-0.178713	-1.231589	-2.003349	0.045868	0.0817162	-4.944399
METTL21EP	-0.344847	0.7549178	-2.003312	0.045872	0.0817194	-4.944473
RERG-IT1	0.149372	-1.356504	2.0032573	0.0458779	0.0817259	-4.94458
CYP51A1	-0.091115	5.612022	-2.002946	0.0459115	0.0817807	-4.945193
HNRNPA1P61	0.1845901	-1.16582	2.0029304	0.0459131	0.0817807	-4.945223
RP11-64D24	-0.154547	-1.306307	-2.002743	0.0459334	0.0818127	-4.945592
AF124730.4	-0.430532	1.9323826	-2.002645	0.0459439	0.0818257	-4.945785
RP11-572C1	-0.134203	-1.378147	-2.002634	0.0459452	0.0818257	-4.945807
RP1-101G11	-0.176856	-1.223145	-2.002173	0.0459949	0.0819104	-4.946714
QRICH2	-0.089073	5.2559734	-2.002079	0.0460051	0.0819245	-4.946899
CTD-2619J1	0.305003	-0.703138	2.0019716	0.0460167	0.0819411	-4.94711
AURKC	0.1124768	4.1313862	2.0018311	0.0460318	0.0819606	-4.947386

RP11-523H2	0.2677412	2.0635569	2.0018286	0.0460321	0.0819606	-4.947391
AC002064.4	-0.263127	-0.77796	-2.001725	0.0460433	0.0819747	-4.947595
REEP3	-0.031265	6.2466573	-2.001709	0.046045	0.0819747	-4.947625
LPP	0.0357041	6.4777945	2.001693	0.0460468	0.0819747	-4.947658
ACAD11	-0.181072	4.4198338	-2.00155	0.0460622	0.0819982	-4.947938
AC145123.2	-0.208737	-1.232504	-2.00145	0.046073	0.0820134	-4.948135
SRP54	-0.031804	6.4029265	-2.001342	0.0460847	0.0820302	-4.948347
HIST1H2AG	-0.377407	4.1920694	-2.001291	0.0460903	0.0820361	-4.948448
AC093326.3	0.277716	-0.886538	2.0012135	0.0460986	0.0820432	-4.9486
PPP2R3C	-0.032623	5.8124765	-2.001213	0.0460987	0.0820432	-4.948602
RP11-1060G	0.3444805	0.6262044	2.0011274	0.046108	0.0820556	-4.94877
RP5-940J5.	0.3487194	0.3252384	2.0010368	0.0461178	0.0820691	-4.948948
AP001258.4	0.0791023	4.6790977	2.0006478	0.0461599	0.08214	-4.949713
RP11-349N1	0.2304911	2.2461611	2.0004053	0.0461862	0.0821828	-4.950189
SMG9	0.040433	6.0611187	2.0003366	0.0461936	0.0821921	-4.950324
CTB-79E8.3	-0.23445	3.3179107	-2.000178	0.0462109	0.0822149	-4.950637
AC009960.1	-0.128499	-1.402304	-2.000177	0.046211	0.0822149	-4.950638
TTTY25P	-0.153843	-1.370667	-2.000116	0.0462176	0.0822227	-4.950758
HCG27	-0.128785	4.1598218	-1.999859	0.0462455	0.0822682	-4.951263
PLBD2	0.0287643	6.5821692	1.999545	0.0462795	0.0823234	-4.95188
CYCS	-0.040564	6.6802353	-1.999532	0.046281	0.0823234	-4.951906
NBEAP1	0.3613802	-0.119672	1.999139	0.0463236	0.0823952	-4.952677
LINC00638	-0.146532	4.1542153	-1.999111	0.0463267	0.0823966	-4.952732
RP11-305L7	0.3916985	1.0547237	1.9990275	0.0463357	0.0824088	-4.952896
LARP1	-0.026852	6.8217026	-1.998757	0.0463651	0.082457	-4.953427
AC017079.3	-0.147086	-1.337415	-1.998595	0.0463827	0.0824843	-4.953745
ANKRD12	0.0384118	6.0720733	1.9982155	0.0464241	0.0825538	-4.954491
ARHGEF1	0.0261689	6.4948306	1.9977977	0.0464696	0.0826282	-4.955311
FABP1	-0.194513	7.0525438	-1.99779	0.0464704	0.0826282	-4.955327
RP11-366L5	0.2333325	2.8274474	1.9976286	0.046488	0.0826554	-4.955643
RP11-201A3	0.2045501	-1.187589	1.997267	0.0465274	0.0827215	-4.956353
TMEM183A	-0.029299	6.1905539	-1.99705	0.0465511	0.0827589	-4.956779
VWA8	-0.056398	6.1793436	-1.997033	0.046553	0.0827589	-4.956812
LINC01170	0.2883875	-0.861363	1.996984	0.0465583	0.0827643	-4.956908
MYADML2	0.3625484	-0.076219	1.9968423	0.0465738	0.0827878	-4.957186
LCMT1	-0.03974	5.9030544	-1.99682	0.0465763	0.0827881	-4.957231
STAG2	-0.032256	6.4251072	-1.996694	0.04659	0.0828085	-4.957478
TRIM63	0.429158	0.1444491	1.9963406	0.0466286	0.0828731	-4.95817
SNORD15B	0.3275948	-0.186377	1.9961643	0.0466478	0.0829032	-4.958516
CD164L2	0.2743011	-1.048108	1.9961199	0.0466527	0.0829066	-4.958603
AC006946.1	0.2586804	-0.838954	1.9960979	0.0466551	0.0829066	-4.958646
EEF1A1P3	0.3271471	1.337866	1.9960849	0.0466565	0.0829066	-4.958672
RP11-485F1	-0.496581	0.1206643	-1.995886	0.0466782	0.0829411	-4.959061
ASB16-AS1	-0.048681	5.4745679	-1.995861	0.046681	0.0829419	-4.95911
GABRB2	0.3919504	-0.106143	1.9957999	0.0466877	0.0829498	-4.959231
RNF8	-0.03803	5.8985634	-1.995699	0.0466987	0.0829654	-4.959429
MESP1	-0.122322	4.8576139	-1.995453	0.0467256	0.0830091	-4.95991
TPT1P10	-0.175002	-1.225083	-1.99538	0.0467336	0.0830193	-4.960053
DNTT	-0.3276	-0.82711	-1.995358	0.046736	0.0830196	-4.960097
C14orf178	-0.215	-1.072921	-1.995013	0.0467739	0.0830828	-4.960774
DEFB118	-0.095405	-1.471036	-1.99495	0.0467808	0.0830892	-4.960898

RP11-57H12	0.2885645	2.2328087	1.9949378	0.0467821	0.0830892	-4.960921
NPIPA1	0.0844827	4.8466736	1.9948648	0.0467901	0.0830994	-4.961064
SCARNA2	-0.123589	-1.404622	-1.994778	0.0467996	0.0831123	-4.961235
RP11-883G1	-0.499164	1.9480828	-1.994165	0.0468669	0.0832277	-4.962436
RP11-76H14	-0.182245	-1.19743	-1.994121	0.0468716	0.083232	-4.962521
RP5-857K21	-0.240057	-1.04269	-1.993723	0.0469153	0.0833056	-4.963301
ZNF33A	-0.026321	6.1988	-1.99345	0.0469453	0.0833549	-4.963836
RP11-153M7	0.2659923	-0.632019	1.9934057	0.0469502	0.0833595	-4.963923
AC011754.1	-0.286635	-1.090922	-1.993293	0.0469626	0.0833774	-4.964143
RNA5SP118	-0.229335	-1.052338	-1.992693	0.0470286	0.0834881	-4.965318
SYT17	0.1543678	5.7315899	1.9926848	0.0470295	0.0834881	-4.965335
MLK7-AS1	-0.269342	2.7523901	-1.99249	0.0470509	0.083522	-4.965715
RHBDL1	-0.133075	4.7179042	-1.992378	0.0470633	0.0835399	-4.965935
NGRN	0.0380449	6.0706109	1.9923104	0.0470707	0.0835491	-4.966068
ENHO	0.2378355	5.2592299	1.9922172	0.047081	0.0835632	-4.96625
RP11-77403	-0.097935	4.9747378	-1.992068	0.0470975	0.0835884	-4.966542
AC007036.4	-0.144195	-1.36344	-1.991852	0.0471213	0.0836266	-4.966966
SNAI2	-0.125647	5.5205841	-1.991776	0.0471296	0.0836373	-4.967113
ZBED6CL	-0.067598	5.9564644	-1.991715	0.0471364	0.0836449	-4.967233
PKD1L2	0.3821056	3.5557846	1.991696	0.0471385	0.0836449	-4.96727
MC2R	-0.125428	-1.436769	-1.991521	0.0471578	0.0836751	-4.967613
WWTR1	0.0562771	6.1896966	1.9912184	0.0471912	0.0837303	-4.968205
RP11-116D1	-0.205978	-1.199731	-1.991117	0.0472023	0.083746	-4.968403
SRP72	-0.02519	6.5227496	-1.991013	0.0472138	0.0837623	-4.968607
GTF3C1	-0.024732	6.4403343	-1.990909	0.0472253	0.0837775	-4.968811
NCK1-AS1	-0.053259	4.8241084	-1.990894	0.0472269	0.0837775	-4.968839
AC078852.1	-0.175938	-1.212225	-1.990814	0.0472358	0.0837891	-4.968996
RPL13P6	-0.236558	-0.940826	-1.990553	0.0472647	0.0838363	-4.969507
SORBS3	-0.042748	6.556984	-1.990504	0.0472701	0.0838414	-4.969602
IGHV3OR16-	0.2704803	-0.929026	1.990485	0.0472722	0.0838414	-4.96964
TSSC1	-0.03444	6.0021121	-1.990241	0.0472992	0.0838826	-4.970118
U6	-0.308048	1.7324256	-1.990233	0.0473	0.0838826	-4.970132
RARRES2P8	-0.526914	0.1174078	-1.990121	0.0473125	0.0839006	-4.970352
PATZ1	-0.036306	6.2775604	-1.990072	0.0473179	0.0839061	-4.970448
ESRP2	-0.123738	6.316092	-1.989996	0.0473263	0.083917	-4.970597
DPY19L2	0.3984975	1.891121	1.9898027	0.0473477	0.0839508	-4.970974
CTD-3203P2	0.2818203	2.2873512	1.9896493	0.0473646	0.0839768	-4.971274
HNRNPCP3	0.2818282	-0.460066	1.9890129	0.0474352	0.0840978	-4.972518
RPL7AP26	-0.300718	-0.431671	-1.988221	0.047523	0.0842494	-4.974065
DCTPP1	-0.041196	6.0874785	-1.98803	0.0475443	0.084283	-4.974439
RP11-775C2	-0.303703	-0.230304	-1.987924	0.047556	0.0842997	-4.974645
ABRACL	-0.09264	5.6288118	-1.987806	0.0475692	0.0843156	-4.974877
C2orf43	0.033078	5.8807617	1.9878021	0.0475696	0.0843156	-4.974884
RP11-648L3	-0.180284	-1.243186	-1.987601	0.047592	0.0843506	-4.975277
ZBTB20-AS4	-0.284858	-0.684716	-1.987583	0.0475939	0.0843506	-4.975312
STK40	-0.035985	6.3733454	-1.987204	0.047636	0.084421	-4.976051
RP11-328C8	-0.095137	-1.479864	-1.987184	0.0476383	0.084421	-4.97609
RP11-431N1	-0.219173	-1.041166	-1.986621	0.047701	0.0845281	-4.977191
FAM19A2	0.1450165	3.5960621	1.9864337	0.0477219	0.0845609	-4.977556
CPSF4L	0.3965349	0.7014857	1.9863286	0.0477336	0.0845775	-4.977761
HIST1H4A	-0.235961	-0.941978	-1.986152	0.0477532	0.0846083	-4.978105

RP11-796G6	0.3355741	0.9834962	1.9861006	0.047759	0.0846143	-4.978206
NPY2R	-0.156812	-1.394054	-1.98608	0.0477612	0.0846143	-4.978246
AC091814.2	0.3039513	-0.740468	1.9859066	0.0477806	0.0846445	-4.978585
KCNK5	0.1741574	5.3799262	1.9857562	0.0477974	0.0846701	-4.978878
NAB2	0.0427025	6.029388	1.985629	0.0478116	0.0846911	-4.979126
VAX1	0.2570304	-1.160795	1.9854676	0.0478296	0.0847189	-4.979441
RP11-343J3	0.2207746	-1.135441	1.9854363	0.0478331	0.084721	-4.979502
MED6	-0.03082	5.8371572	-1.985022	0.0478794	0.0847958	-4.980311
AGO3	0.0382356	5.7086	1.9850026	0.0478816	0.0847958	-4.980348
RP11-524F1	0.2390855	3.5056852	1.9849958	0.0478823	0.0847958	-4.980361
KB-1572G7.	0.3516615	0.2114814	1.9849043	0.0478925	0.0848098	-4.98054
RP11-324I2	0.2339193	2.5521054	1.9848229	0.0479016	0.0848218	-4.980699
RP11-66B24	0.1548094	-1.336987	1.9845298	0.0479344	0.0848757	-4.98127
RN7SL356P	-0.092493	-1.480289	-1.984324	0.0479575	0.0849103	-4.981673
RP11-347C1	0.3546234	0.5397345	1.9843137	0.0479586	0.0849103	-4.981692
FXYD7	0.3774307	1.2229074	1.9841986	0.0479715	0.084927	-4.981916
RP1-121G13	-0.129745	-1.4071	-1.984177	0.0479739	0.084927	-4.981959
AC124861.1	-0.297822	-0.767977	-1.984167	0.047975	0.084927	-4.981978
RPS20P14	-0.145026	4.1146785	-1.984109	0.0479815	0.0849344	-4.982091
CTC-435M10	0.1599454	-1.28233	1.9840452	0.0479886	0.0849429	-4.982215
ATF7	0.0286968	6.0243075	1.9838595	0.0480095	0.0849756	-4.982578
CTD-2619J1	0.2338795	3.3516241	1.9835483	0.0480443	0.0850332	-4.983184
RP11-127B2	0.2458431	3.1054267	1.9835132	0.0480482	0.085036	-4.983253
CTTN	-0.025952	6.7635868	-1.983488	0.0480511	0.0850364	-4.983302
AC012506.4	-0.199355	-1.160634	-1.98347	0.0480531	0.0850364	-4.983338
LPO	0.3572467	-0.35857	1.9829721	0.0481089	0.0851273	-4.984307
COX7BP2	-0.106652	-1.443649	-1.98297	0.0481091	0.0851273	-4.984311
RP11-88I18	-0.155476	-1.327414	-1.982862	0.0481213	0.0851446	-4.984521
NEAT1	-0.048328	6.9213878	-1.982789	0.0481295	0.0851551	-4.984665
RP11-162A1	-0.119262	4.3369884	-1.982659	0.0481441	0.0851767	-4.984917
RP11-32K4.	0.2627511	-1.09315	1.982448	0.0481678	0.0852145	-4.985328
KRTAP10-2	-0.10573	-1.448938	-1.982387	0.0481746	0.0852225	-4.985447
SLC16A7	0.1661405	5.5528196	1.9822971	0.0481847	0.0852362	-4.985622
BOLA3P2	-0.095566	-1.476605	-1.982148	0.0482015	0.0852618	-4.985913
ARHGEF26	-0.124168	5.9242413	-1.981979	0.0482205	0.0852908	-4.986242
RP11-400N1	-0.459493	0.8127768	-1.98196	0.0482226	0.0852908	-4.986278
RP11-57C13	-0.351421	1.1460935	-1.981846	0.0482354	0.0853038	-4.9865
MIR4324	0.2407432	-0.903764	1.9818326	0.0482369	0.0853038	-4.986527
EFCAB2	-0.056089	5.6610144	-1.981826	0.0482377	0.0853038	-4.986541
RP11-644F5	-0.052028	5.3821087	-1.981794	0.0482412	0.0853038	-4.986601
CYP21A1P	0.1522889	5.1097764	1.9817908	0.0482416	0.0853038	-4.986608
C6orf229	-0.341762	-0.426914	-1.98165	0.0482574	0.0853276	-4.986882
RP11-508N1	-0.159892	-1.308178	-1.98155	0.0482687	0.0853434	-4.987077
SNORA14B	0.3263922	0.4971103	1.9814567	0.0482792	0.0853579	-4.987259
HCG16	0.3930278	1.2390767	1.9808069	0.0483524	0.0854831	-4.988524
PRKACB	-0.047763	6.0453699	-1.980529	0.0483838	0.0855344	-4.989066
PKDREJ	-0.282954	2.8191617	-1.980397	0.0483986	0.0855565	-4.989321
RP11-320H1	0.3884635	-0.745546	1.9803219	0.0484071	0.0855673	-4.989468
LINC00444	0.224363	-0.978863	1.9802408	0.0484162	0.0855794	-4.989626
TMEM105	0.4115894	3.9610604	1.9799496	0.0484491	0.085633	-4.990193
CYCSP10	0.3165271	-0.185081	1.9799111	0.0484534	0.085633	-4.990267

COIL	-0.028989	5.9758283	-1.97991	0.0484536	0.085633	-4.99027
RP11-120M1	-0.333128	1.6521512	-1.979875	0.0484575	0.0856358	-4.990339
GDA	-0.283349	5.8801234	-1.979811	0.0484647	0.0856443	-4.990462
LINC01555	-0.127916	-1.396366	-1.97965	0.0484829	0.0856723	-4.990775
CIR1P2	-0.09433	-1.476987	-1.978878	0.0485701	0.0858223	-4.992277
TTC3-AS1	0.3022208	-0.025387	1.9788474	0.0485736	0.0858233	-4.992337
PGM5P3-AS1	0.2913134	-0.732102	1.9788318	0.0485754	0.0858233	-4.992367
AC009473.1	-0.338721	0.276756	-1.978796	0.0485795	0.0858264	-4.992437
HIST1H1PS1	0.4178513	0.929631	1.9787097	0.0485892	0.0858394	-4.992604
CYCSP24	0.2642296	-0.70657	1.9786666	0.048594	0.0858438	-4.992688
CASC2	0.2596123	3.0208761	1.9783997	0.0486243	0.0858931	-4.993207
CHPF2	0.0314267	6.3571693	1.9782332	0.0486431	0.0859222	-4.993531
ZFP64	0.0321697	5.8277569	1.9781628	0.0486511	0.0859321	-4.993668
CTD-2525I3	0.3115193	-0.143011	1.9779685	0.0486731	0.0859668	-4.994045
ELF3	0.0928665	6.4929328	1.9778464	0.0486869	0.0859871	-4.994283
CH17-360D5	0.5266103	1.8480754	1.9777501	0.0486979	0.0860022	-4.99447
LINC01446	-0.581364	1.1943953	-1.977575	0.0487177	0.0860331	-4.99481
RP11-425M5	0.3585605	0.5532133	1.9775369	0.048722	0.0860366	-4.994884
SRCIN1	-0.114608	5.6885581	-1.977231	0.0487568	0.0860938	-4.995479
SUMO4	-0.408726	1.1546544	-1.97707	0.048775	0.0861219	-4.995792
VWA5B1	-0.295256	-0.854726	-1.977041	0.0487783	0.0861235	-4.995848
RP11-307L3	-0.088108	-1.482901	-1.976827	0.0488025	0.0861621	-4.996263
NPHS2	0.2303514	-1.206888	1.9767111	0.0488157	0.0861813	-4.996489
RP11-122L4	-0.076823	-1.497565	-1.976679	0.0488194	0.0861822	-4.996551
HIST1H3I	-0.260423	-0.856004	-1.976665	0.048821	0.0861822	-4.996578
RP11-128P1	-0.12133	-1.449654	-1.976548	0.0488343	0.086198	-4.996806
MATN1-AS1	0.1192846	4.1841104	1.9765444	0.0488347	0.086198	-4.996813
FGFBP1	0.3504003	-0.677476	1.975762	0.0489237	0.0863509	-4.998332
FLVCR2	-0.075357	5.8507198	-1.975729	0.0489274	0.0863532	-4.998395
FBXO16	0.2853426	3.9862258	1.9756718	0.0489339	0.0863584	-4.998507
CTD-2013N1	-0.173674	-1.239754	-1.975662	0.048935	0.0863584	-4.998526
RP11-714G1	0.3026441	-0.321385	1.9752676	0.0489799	0.0864335	-4.999292
FANCL	0.0339228	5.7096119	1.9752471	0.0489823	0.0864335	-4.999332
RP11-326C3	-0.337433	3.1162419	-1.975055	0.0490042	0.0864679	-4.999705
ALG1L5P	-0.195201	-1.176616	-1.974827	0.0490301	0.0865095	-5.000147
RP11-44M6.	-0.274315	3.4426221	-1.974771	0.0490365	0.0865166	-5.000256
RN7SKP239	0.1852184	-1.227963	1.9746492	0.0490504	0.086537	-5.000492
RP11-631M6	0.1378501	-1.348719	1.9745832	0.049058	0.0865461	-5.00062
GTF2H4	-0.063486	4.9312258	-1.974492	0.0490683	0.0865602	-5.000797
RP11-600F2	0.1708575	3.879976	1.9743019	0.0490901	0.0865944	-5.001166
CTB-89H12.	0.0550475	5.5045498	1.9742354	0.0490976	0.0866035	-5.001295
GTF3C4	0.0324707	5.9632802	1.9741321	0.0491094	0.0866202	-5.001496
APOBEC2	0.3356359	1.4823237	1.9740393	0.04912	0.0866347	-5.001676
BNIPL	-0.146982	3.8630761	-1.973889	0.0491372	0.0866608	-5.001968
AC097713.5	-0.214875	-1.080242	-1.973854	0.0491411	0.0866635	-5.002035
SVIL	0.061531	6.1960482	1.9737696	0.0491508	0.0866764	-5.002199
AC073465.1	-0.130803	-1.436581	-1.973749	0.0491532	0.0866765	-5.00224
PTDSS1	-0.041659	6.5682298	-1.973719	0.0491566	0.0866783	-5.002297
ROBO1	0.1112664	6.2776585	1.9735775	0.0491728	0.0867026	-5.002572
RNU6-20P	-0.140126	-1.374926	-1.973106	0.0492267	0.0867904	-5.003486
GK2	-0.112843	-1.458384	-1.9731	0.0492273	0.0867904	-5.003497

HMGN3	-0.042663	6.3182488	-1.972901	0.0492501	0.0868264	-5.003884
RP13-122B2	0.186707	-1.197885	1.9726938	0.0492739	0.086864	-5.004285
PCMT1	-0.032752	6.329141	-1.972592	0.0492856	0.0868805	-5.004483
GPATCH1	-0.033293	5.7278696	-1.972438	0.0493032	0.0869069	-5.004782
LINC00670	0.2273166	-1.028005	1.9724189	0.0493054	0.0869069	-5.004818
TTTY10	-0.320913	-0.301887	-1.97224	0.0493258	0.0869388	-5.005165
CHRNA4	-0.571867	3.6619492	-1.972093	0.0493426	0.0869643	-5.005449
DNAAF3	0.3494533	2.8350119	1.9716349	0.0493952	0.0870527	-5.006338
ACRC	0.1184759	3.9892285	1.9715845	0.049401	0.0870587	-5.006435
AC091320.1	-0.245701	-0.974809	-1.971438	0.0494179	0.0870832	-5.00672
MAPK8IP2	0.2048826	5.2092516	1.9714217	0.0494197	0.0870832	-5.006751
HINT1P1	-0.279152	-0.528101	-1.970829	0.0494877	0.087199	-5.007899
TUBA3FP	-0.257274	2.382606	-1.97058	0.0495163	0.0872451	-5.008381
AC068533.7	0.3971256	1.5395634	1.9705348	0.0495216	0.0872501	-5.008469
CTD-2651B2	-0.406333	1.0055205	-1.970468	0.0495292	0.0872594	-5.008598
CT55	0.3427252	-0.876982	1.9704116	0.0495357	0.0872667	-5.008708
RPL31P11	0.3461321	-0.140496	1.970383	0.049539	0.0872682	-5.008763
FTSJ1	-0.030697	6.0326898	-1.970089	0.0495729	0.0873237	-5.009333
ZSCAN4	0.2610824	-0.902377	1.9700391	0.0495786	0.0873295	-5.009429
EBF3	0.2193015	3.833745	1.9700005	0.049583	0.0873331	-5.009504
RP11-255G1	-0.159879	-1.316179	-1.969978	0.0495856	0.0873334	-5.009546
PIGU	-0.039593	6.1675966	-1.969859	0.0495994	0.0873534	-5.009778
RP4-758J18	-0.17463	4.3809671	-1.969785	0.0496078	0.087362	-5.009921
RP11-2C24.	-0.314726	-0.216902	-1.969775	0.049609	0.087362	-5.00994
GALNT2	0.0372993	6.7750723	1.9694558	0.0496458	0.0874225	-5.010558
CYR61	0.0719477	6.2477904	1.9694178	0.0496501	0.0874255	-5.010632
RP11-87H9.	0.0640835	4.9178887	1.9693993	0.0496523	0.0874255	-5.010668
FAM102A	0.0462395	6.4206877	1.9690577	0.0496916	0.0874879	-5.011329
AC073987.1	-0.151595	-1.391035	-1.96905	0.0496925	0.0874879	-5.011343
TRIM22	0.0972628	5.9052905	1.9688756	0.0497126	0.0875192	-5.011681
AC005014.5	0.2042341	-1.109992	1.968841	0.0497166	0.087522	-5.011748
AMMECR1L	0.0278513	6.012901	1.9688038	0.0497209	0.0875253	-5.01182
RP11-1149C	0.1738539	4.2987371	1.9686646	0.049737	0.0875494	-5.012089
PSPC1	0.0323228	5.9943777	1.9685912	0.0497454	0.08756	-5.012231
RP11-227D1	-0.295497	-0.485436	-1.968258	0.0497839	0.0876207	-5.012875
HNRNPF	-0.023189	6.8508704	-1.968251	0.0497847	0.0876207	-5.012889
AC087380.1	0.359416	0.3827485	1.9681914	0.0497916	0.0876241	-5.013005
RP11-616K2	0.3134108	-0.334703	1.9681783	0.0497931	0.0876241	-5.01303
RP3-437I16	-0.194888	-1.17749	-1.968171	0.049794	0.0876241	-5.013044
RPS27P25	0.3466939	-0.410368	1.9681514	0.0497962	0.0876241	-5.013082
ZNF227	0.0458929	5.4753036	1.9681121	0.0498008	0.0876278	-5.013158
TRIML1	-0.142927	-1.392762	-1.967889	0.0498266	0.0876652	-5.01359
KRTAP5-4	0.2062752	-1.154248	1.9678869	0.0498268	0.0876652	-5.013594
APOL3	-0.077186	6.3008103	-1.967831	0.0498333	0.0876724	-5.013703
FOLR3	0.3331284	-0.512759	1.9677096	0.0498473	0.0876928	-5.013937
RP11-406A2	-0.170845	-1.280581	-1.96751	0.0498704	0.0877292	-5.014323
HLA-V	0.4285187	2.8190801	1.9673779	0.0498856	0.0877518	-5.014578
RP11-485G7	0.334135	-0.273773	1.9673479	0.0498891	0.0877527	-5.014636
RP11-111E1	-0.25261	-0.950289	-1.967332	0.049891	0.0877527	-5.014667
RP11-82L7.	-0.133814	-1.418848	-1.966993	0.0499301	0.0878174	-5.015321
RNU4-78P	-0.202423	-1.115605	-1.966745	0.0499589	0.0878637	-5.015801

AC006116.2	-0.297334	-0.569172	-1.966717	0.0499621	0.0878651	-5.015855
TMEM25	-0.148262	5.2530998	-1.96642	0.0499965	0.0879214	-5.016429
SNORD88	0.186407	-1.200791	1.9663364	0.0500062	0.0879343	-5.016592
AC007041.2	-0.317619	0.7953222	-1.966204	0.0500216	0.0879571	-5.016848
BCHE	-0.213945	5.8176054	-1.965904	0.0500564	0.088014	-5.017427
BCAM	0.0611763	6.7586429	1.9657678	0.0500722	0.0880375	-5.01769
DHDH	0.3491011	2.7650382	1.9656648	0.0500841	0.0880543	-5.017889
TOR1B	-0.032915	6.1882872	-1.965603	0.0500913	0.0880627	-5.018008
RP11-336N8	-0.143634	-1.337722	-1.965144	0.0501446	0.0881522	-5.018896
YBX1	-0.029951	6.9636441	-1.964367	0.050235	0.0883067	-5.020396
AC244250.4	0.1532779	-1.330346	1.9640521	0.0502716	0.0883669	-5.021004
RANBP3L	-0.351518	3.4083592	-1.964002	0.0502774	0.0883728	-5.0211
FAM89A	0.0844333	5.5369854	1.963866	0.0502933	0.0883923	-5.021363
C3orf56	-0.082016	-1.494998	-1.963866	0.0502933	0.0883923	-5.021363
TRAJ24	-0.074617	-1.499306	-1.963639	0.0503197	0.0884344	-5.021801
CTC-343N3.	0.313866	-0.236232	1.9633822	0.0503497	0.0884828	-5.022297
RP1-18D14.	0.3657716	1.2167997	1.963205	0.0503703	0.0885149	-5.022639
SNORA59A	0.3267192	0.7236588	1.9628656	0.0504099	0.0885802	-5.023294
MED9	-0.042172	5.9833705	-1.962772	0.0504209	0.0885952	-5.023475
RP11-128A1	-0.249094	-0.879541	-1.962554	0.0504463	0.0886355	-5.023894
CRELD2	-0.045578	6.4170276	-1.962323	0.0504733	0.0886786	-5.02434
CTB-102L5.	0.2731538	-0.608294	1.9621556	0.0504929	0.088707	-5.024663
KIAA0895L	0.0645637	5.3794075	1.9621436	0.0504943	0.088707	-5.024686
PPIAP22	-0.036887	6.0749895	-1.962085	0.0505011	0.0887148	-5.0248
RETNLB	-0.341354	-0.664338	-1.961901	0.0505227	0.0887445	-5.025155
RP11-1105G	-0.310247	-0.559527	-1.961899	0.0505229	0.0887445	-5.025159
MARK2P13	-0.188832	-1.211519	-1.961779	0.0505369	0.0887647	-5.025389
C4orf29	-0.044093	5.6308001	-1.961674	0.0505492	0.0887821	-5.025592
FAM222A	0.082436	5.788902	1.9616275	0.0505546	0.0887874	-5.025682
RP1-63M2.5	-0.238291	-0.961612	-1.961471	0.0505729	0.0888153	-5.025984
RNF41	0.0202293	6.0885047	1.9613089	0.0505919	0.0888413	-5.026296
RP11-159D1	0.1881423	-1.121202	1.9613026	0.0505926	0.0888413	-5.026308
TOPORS	-0.039666	5.9211282	-1.961027	0.0506249	0.0888938	-5.02684
PRR14	-0.025587	6.1402559	-1.960773	0.0506547	0.0889418	-5.02733
RP11-29H23	-0.330539	0.3245181	-1.960679	0.0506656	0.0889567	-5.027509
CTD-2647E9	-0.130118	-1.388313	-1.960424	0.0506956	0.089005	-5.028002
NADK2-AS1	0.4338057	2.0515703	1.9598094	0.0507677	0.0891273	-5.029186
RP11-640L9	0.293615	2.5644635	1.9596239	0.0507895	0.0891591	-5.029543
RP11-121E1	0.1617832	-1.295544	1.9596135	0.0507907	0.0891591	-5.029563
URI1	-0.025271	6.3692043	-1.959565	0.0507963	0.0891647	-5.029655
IGLV8OR8-1	0.1677434	-1.340726	1.9591759	0.0508421	0.0892407	-5.030406
RP11-384J4	-0.361296	-0.563914	-1.959082	0.0508532	0.0892559	-5.030587
NUS1P2	-0.295488	-0.914911	-1.958994	0.0508634	0.0892696	-5.030755
RP11-364B1	-0.161186	-1.375505	-1.958693	0.0508989	0.0893275	-5.031335
LRRTM1	0.4215444	0.2120891	1.9586257	0.0509068	0.0893371	-5.031465
RP11-599B1	0.2802841	-0.950311	1.9585172	0.0509196	0.0893553	-5.031674
RP11-256I2	0.1983083	-1.277045	1.9584761	0.0509244	0.0893594	-5.031753
RP11-403.1	-0.163901	-1.349822	-1.958449	0.0509276	0.0893607	-5.031805
HIST1H3B	-0.412401	2.0222175	-1.958123	0.0509659	0.0894237	-5.032432
RP11-543D5	-0.28611	-0.993627	-1.958038	0.050976	0.0894371	-5.032597
GFI1B	0.2892701	-0.820663	1.9577918	0.051005	0.0894837	-5.03307

SMCR5	0.1563042	-1.267148	1.9577337	0.0510119	0.0894914	-5.033182
SCAI	-0.049048	5.3716411	-1.957678	0.0510185	0.0894987	-5.03329
CTD-2595P9	0.2718035	-0.729453	1.9576425	0.0510226	0.0895016	-5.033358
RP11-111F5	-0.156256	-1.357204	-1.957606	0.051027	0.089505	-5.033429
BDNF-AS	-0.112133	4.7121451	-1.957557	0.0510327	0.0895107	-5.033522
RP11-432M8	-0.097147	-1.490093	-1.957319	0.0510608	0.0895557	-5.033981
SNORA54	-0.151632	-1.285262	-1.957292	0.051064	0.089557	-5.034033
IGFL1P1	0.251656	-1.057539	1.9572321	0.051071	0.089565	-5.034147
C6orf10	0.1479562	-1.331786	1.9569488	0.0511044	0.0896194	-5.034692
IFNA20P	0.3419771	0.3480875	1.9567768	0.0511247	0.0896507	-5.035023
PTMAP3	0.22536	-0.959643	1.9567499	0.0511279	0.0896519	-5.035075
PHKA2	-0.034821	6.5005578	-1.956576	0.0511484	0.0896836	-5.035409
SPAG6	0.3896743	0.0712107	1.9565196	0.0511551	0.089691	-5.035518
LGALS9DP	0.1994834	-1.225108	1.9563648	0.0511734	0.0897188	-5.035816
MIR133A1HG	0.196589	-1.104446	1.9562856	0.0511828	0.0897309	-5.035968
PSMA6P2	-0.212285	-1.089046	-1.955791	0.0512413	0.0898291	-5.036918
HMGB1P11	0.2177317	-1.051232	1.9555463	0.0512702	0.0898756	-5.037389
THAP6	-0.036326	5.6446341	-1.955225	0.0513083	0.089938	-5.038007
RP11-134K1	0.2077162	-1.025175	1.9551632	0.0513156	0.0899465	-5.038126
FGF20	0.3182926	-0.779574	1.9551085	0.0513221	0.0899535	-5.038231
AMELY	-0.189029	-1.27287	-1.954854	0.0513523	0.0900021	-5.038721
AC002310.1	0.2844762	-0.622606	1.9545554	0.0513877	0.0900594	-5.039294
UBL4B	-0.368575	-0.42171	-1.954521	0.0513917	0.0900594	-5.039359
HOXB8	0.395282	0.1144092	1.9545155	0.0513924	0.0900594	-5.03937
TGFBR2	0.045547	6.5375367	1.9544707	0.0513977	0.0900644	-5.039456
CNTN2	0.4014934	1.5010307	1.953606	0.0515004	0.09024	-5.041117
RP11-96D1.	-0.171325	2.8701873	-1.953446	0.0515193	0.0902689	-5.041424
BRIP1	-0.100736	5.1923341	-1.953396	0.0515253	0.090275	-5.04152
TSPAN16	0.1488826	-1.296821	1.9531532	0.0515542	0.0903213	-5.041987
RP11-659E9	-0.32735	-0.243326	-1.952709	0.051607	0.0904095	-5.042839
IFT81	0.0760972	5.4801839	1.9526823	0.0516102	0.0904108	-5.042891
RNA5SP194	-0.1266	-1.440865	-1.952525	0.051629	0.0904371	-5.043193
RPL37P15	0.2046604	-1.256579	1.9525144	0.0516302	0.0904371	-5.043213
OR11H13P	-0.142374	-1.353761	-1.952365	0.051648	0.0904639	-5.043499
RP11-244H3	0.101005	4.4970134	1.9523105	0.0516545	0.090471	-5.043604
RP11-152N1	-0.123766	3.8456107	-1.951984	0.0516934	0.0905347	-5.04423
RP1-140K8.	-0.355575	3.4370059	-1.951735	0.0517231	0.0905824	-5.044709
CHCHD2P9	0.1714598	4.8194551	1.9513398	0.0517702	0.0906606	-5.045467
FAM149A	-0.105964	5.7941681	-1.951139	0.0517942	0.0906983	-5.045852
RP11-977B1	-0.252817	2.1060972	-1.951049	0.0518049	0.0907126	-5.046024
PYCR2	-0.034353	6.4389221	-1.950927	0.0518195	0.0907339	-5.046259
CTC-551A13	-0.139643	-1.368795	-1.950709	0.0518456	0.0907752	-5.046677
RBMY2EP	-0.168237	-1.360878	-1.950227	0.0519032	0.0908716	-5.047601
SNORD114-1	0.1479573	-1.371543	1.9500982	0.0519186	0.0908943	-5.047848
RP5-1107A1	-0.130566	-1.386629	-1.949925	0.0519393	0.090925	-5.04818
KRT18P59	-0.343761	1.3641753	-1.94991	0.0519411	0.090925	-5.048209
RP11-360D2	-0.308686	0.2558498	-1.949858	0.0519473	0.0909314	-5.048308
SHISA6	-0.416293	0.9114708	-1.949494	0.051991	0.0910035	-5.049007
RP11-382A2	0.1612687	-1.268205	1.9493713	0.0520056	0.0910248	-5.049241
RP3-414A15	-0.272176	-0.61828	-1.949226	0.0520231	0.0910486	-5.04952
EIF4H	-0.022658	6.7781347	-1.949216	0.0520242	0.0910486	-5.049538

QRSL1P1	0.3892194	0.0933804	1.9490549	0.0520435	0.0910748	-5.049847
XPO4	0.0351717	5.8916766	1.9490496	0.0520442	0.0910748	-5.049857
RP11-463I2	-0.116646	-1.446878	-1.94884	0.0520693	0.0911145	-5.05026
SH3KBP1	0.0499858	6.1483816	1.9487055	0.0520854	0.0911383	-5.050517
VN1R20P	0.3168164	-0.290504	1.9483314	0.0521303	0.0912125	-5.051233
KCNJ6	0.4959489	1.1466227	1.948234	0.052142	0.0912285	-5.05142
PRLR	-0.115916	6.0756358	-1.948199	0.0521462	0.0912315	-5.051486
CCDC148	0.2082255	3.2044838	1.9481514	0.0521519	0.0912371	-5.051578
XPO6	-0.024601	6.4540241	-1.948049	0.0521642	0.0912543	-5.051774
KDM5D	-0.655573	3.7859716	-1.947872	0.0521855	0.0912872	-5.052113
RP11-1151B	-0.248411	4.2775679	-1.947824	0.0521912	0.0912928	-5.052205
PDYN	-0.2064	-1.189082	-1.947452	0.0522359	0.0913666	-5.052916
HHIPL2	-0.427013	3.0556547	-1.947337	0.0522499	0.0913829	-5.053138
RP11-78J21	-0.082413	-1.479709	-1.947323	0.0522515	0.0913829	-5.053164
NUDT19	-0.065835	5.8167795	-1.947312	0.0522528	0.0913829	-5.053185
C3orf58	-0.081666	5.9459654	-1.947152	0.052272	0.0914122	-5.053491
RP11-237N1	-0.090782	-1.491146	-1.947067	0.0522823	0.0914258	-5.053655
GPR97	0.2259736	4.1432593	1.9469467	0.0522968	0.0914467	-5.053884
ZNF284	0.1107658	4.4930461	1.9467648	0.0523187	0.0914806	-5.054233
ZNF229	0.2792378	4.0885296	1.9467085	0.0523254	0.0914881	-5.05434
NTAN1P3	0.2001628	-1.209777	1.9466034	0.0523381	0.0915058	-5.054541
RP11-203B7	0.1404213	-1.378746	1.9465755	0.0523415	0.0915073	-5.054595
ZNF570	0.0857023	5.0890551	1.9465359	0.0523462	0.0915113	-5.054671
RNF170	-0.050522	6.0233142	-1.946201	0.0523866	0.0915774	-5.055311
HMGB1P16	-0.106035	-1.434973	-1.946176	0.0523896	0.0915782	-5.055358
FP671120.5	-0.11923	-1.449286	-1.945853	0.0524286	0.0916421	-5.055978
MRPL42	-0.023183	6.284868	-1.945276	0.0524982	0.0917594	-5.057081
KDM2A	-0.025925	6.5957083	-1.945003	0.0525311	0.0918125	-5.057602
RP11-10B2.	-0.322998	2.2187865	-1.944843	0.0525505	0.0918419	-5.057908
SSBP4	0.0400088	6.1380579	1.9446206	0.0525774	0.0918811	-5.058334
HMGB1P38	0.2237109	-1.182603	1.9446161	0.0525779	0.0918811	-5.058343
ATP9B	0.0432511	5.8819183	1.9444437	0.0525988	0.0919132	-5.058672
RP11-407P1	-0.337646	-0.383565	-1.944367	0.052608	0.0919249	-5.058818
RP1-90J20.	-0.436212	0.3005617	-1.944345	0.0526108	0.0919253	-5.058862
KCNH8	0.4922564	1.0029351	1.9441816	0.0526305	0.0919553	-5.059173
USP3-AS1	-0.091778	4.2567228	-1.944072	0.0526437	0.0919741	-5.059382
MYC	0.0995749	6.2192823	1.9439127	0.052663	0.0920034	-5.059687
RP11-672A2	-0.454346	1.1856589	-1.943878	0.0526673	0.0920064	-5.059754
RP1-228P16	0.3020466	2.179502	1.9436516	0.0526946	0.0920498	-5.060186
RP11-25C19	-0.131633	-1.391966	-1.943546	0.0527075	0.0920678	-5.060388
CDC6	0.0969393	5.6617482	1.9433494	0.0527313	0.092105	-5.060763
RP11-195F1	-0.293781	1.8881145	-1.943264	0.0527417	0.0921187	-5.060927
AC069368.3	0.1508829	-1.277687	1.9432364	0.052745	0.0921201	-5.060979
RP11-757G1	-0.221534	-0.972457	-1.943121	0.0527589	0.09214	-5.061199
RN7SL145P	0.2592494	-0.674009	1.9430578	0.0527666	0.0921491	-5.061321
DCAF13P2	-0.069438	-1.510648	-1.94301	0.0527724	0.0921547	-5.061411
NXT2	-0.051108	5.7459955	-1.942934	0.0527816	0.0921665	-5.061557
PPP1R3A	0.2180774	-1.306718	1.9427557	0.0528033	0.0921999	-5.061898
PARP4P1	0.4239064	0.4629471	1.9427334	0.052806	0.0922002	-5.06194
MLLT11	0.1224856	5.0669388	1.9425187	0.052832	0.0922413	-5.06235
MTND6P21	-0.380585	0.1946845	-1.942478	0.052837	0.0922455	-5.062428

WASF4P	0.1554991	-1.274418	1.9419294	0.0529036	0.0923574	-5.063476
FAM19A4	0.5036248	0.1648216	1.9417997	0.0529194	0.0923805	-5.063723
AC140725.4	-0.171753	-1.240424	-1.941517	0.0529537	0.092436	-5.064262
CTB-46B19.	-0.168727	-1.244735	-1.941436	0.0529636	0.0924489	-5.064418
WI2-3308P1	-0.25384	-1.170456	-1.941356	0.0529733	0.0924614	-5.064569
OSTF1	-0.031044	6.138486	-1.94092	0.0530264	0.0925496	-5.065402
RAB30	-0.057966	5.5677202	-1.940817	0.0530389	0.0925632	-5.065598
MTHFS	-0.077293	6.2500703	-1.940815	0.0530392	0.0925632	-5.065603
AL138726.1	-0.238842	-0.980136	-1.94064	0.0530605	0.0925959	-5.065936
ATIC	-0.034906	6.5340859	-1.940606	0.0530647	0.0925976	-5.066002
RP11-46B11	0.3390717	-0.478912	1.9405904	0.0530666	0.0925976	-5.066031
RP11-1136G	0.2632273	-0.959333	1.9402245	0.0531112	0.092671	-5.066729
OLIG1	0.331204	-0.615625	1.9401248	0.0531233	0.0926878	-5.066919
FAM8A6P	-0.21431	-1.158135	-1.940078	0.053129	0.0926898	-5.067008
PTPRF	-0.034893	7.0248221	-1.940073	0.0531296	0.0926898	-5.067017
CYP4F62P	-0.085666	-1.488278	-1.939983	0.0531406	0.0927047	-5.06719
SLC25A6P1	-0.082512	-1.489487	-1.939955	0.0531441	0.0927063	-5.067244
INIP	0.0323721	5.9361485	1.9396954	0.0531757	0.092757	-5.067738
MTCO1P3	-0.167648	-1.280345	-1.939613	0.0531858	0.0927702	-5.067896
RP11-61K12	0.15113	-1.298539	1.9394518	0.0532055	0.0928	-5.068203
CCNDBP1	-0.032562	6.150433	-1.939151	0.0532422	0.0928596	-5.068776
KRTCAP3	0.2332858	4.5253195	1.9389334	0.0532688	0.0929016	-5.069191
CTD-2380F2	0.25387	-0.835895	1.9388132	0.0532835	0.0929226	-5.06942
RP11-390F4	-0.301214	4.6843895	-1.938794	0.0532859	0.0929226	-5.069458
CD68	0.1393935	4.4538477	1.9385986	0.0533097	0.0929597	-5.069829
DKFZP434IC	0.0851295	4.5474351	1.9384561	0.0533272	0.0929857	-5.070101
RP11-543B1	-0.302983	2.5926805	-1.938287	0.0533479	0.0930174	-5.070424
TMEM27	-0.220274	4.632735	-1.938192	0.0533594	0.0930331	-5.070604
RP11-89H19	0.3626859	2.8958668	1.9379939	0.0533837	0.093071	-5.070982
PHKG1	-0.093526	4.0345959	-1.937847	0.0534018	0.0930979	-5.071262
RP11-69L16	0.2694916	-1.041062	1.9376561	0.0534251	0.0931321	-5.071625
RP3-413H6.	-0.335447	-0.472717	-1.937645	0.0534265	0.0931321	-5.071646
IGKV6D-41	0.1515405	-1.367309	1.937595	0.0534326	0.0931384	-5.071742
RP11-466H1	-0.090144	5.3310659	-1.937546	0.0534386	0.0931438	-5.071835
OR51I1	-0.103086	-1.448564	-1.937528	0.0534408	0.0931438	-5.071869
SH3BGRL3	0.0450292	6.5874807	1.9374011	0.0534564	0.0931665	-5.072111
RPL6P27	-0.079254	5.3957792	-1.937379	0.0534591	0.0931668	-5.072154
RP11-20G13	0.2155902	-1.171564	1.9372213	0.0534784	0.093196	-5.072453
RP11-216N2	-0.175536	-1.230267	-1.937058	0.0534985	0.0932265	-5.072765
POLR2J3	-0.076599	4.6750448	-1.936855	0.0535233	0.0932654	-5.073151
KLK15	-0.274057	-0.999594	-1.936814	0.0535285	0.0932698	-5.07323
AC093590.1	-0.281082	-0.602747	-1.936705	0.0535417	0.0932885	-5.073436
ANKRD11	0.0286118	6.3405715	1.9366561	0.0535478	0.0932943	-5.07353
TRIM60P14	-0.119838	-1.414247	-1.936636	0.0535502	0.0932943	-5.073567
LDHAP7	0.388242	2.8416658	1.9364848	0.0535688	0.0933223	-5.073856
HSPE1-MOB4	0.3273656	0.4466107	1.9364613	0.0535717	0.0933229	-5.073901
CTA-38K21.	0.3086353	-0.128446	1.9364325	0.0535752	0.0933246	-5.073955
RICTOR	0.033632	6.0327506	1.9362716	0.053595	0.0933546	-5.074262
KIRREL3-AS	-0.112565	-1.4509	-1.936112	0.0536146	0.0933842	-5.074565
SERBP1P6	0.2795181	-0.396756	1.9360759	0.053619	0.0933876	-5.074634
RP11-171I2	0.2969504	2.3953394	1.9360491	0.0536223	0.0933888	-5.074685

ARPP21	-0.489896	2.0274418	-1.935621	0.053675	0.0934761	-5.0755
RP11-967K2	-0.115598	4.1754651	-1.935571	0.0536812	0.0934789	-5.075596
RP11-5A1.1	-0.15658	-1.394328	-1.935566	0.0536818	0.0934789	-5.075605
INHBC	-0.177286	6.0192441	-1.93553	0.0536862	0.0934822	-5.075674
RP11-30K9.	0.5025253	1.2014591	1.9354495	0.0536961	0.093495	-5.075827
RP11-16F15	-0.248374	2.7129999	-1.935416	0.0537002	0.0934977	-5.07589
VWC2	0.1814651	-1.281159	1.9348622	0.0537684	0.093612	-5.076944
Z83826.1	0.3050314	-0.208305	1.934624	0.0537978	0.0936586	-5.077397
CH17-302M2	-0.347906	1.6551298	-1.934579	0.0538033	0.0936637	-5.077482
KCNC4-AS1	0.3310841	0.2664493	1.9345342	0.0538088	0.0936689	-5.077568
LRRC42	-0.039417	6.1781744	-1.934483	0.0538152	0.0936755	-5.077665
AC069154.4	-0.212006	-1.000808	-1.934419	0.0538231	0.0936848	-5.077788
MTND2P9	-0.208611	-1.088881	-1.934232	0.0538461	0.0937205	-5.078143
RP11-122C2	-0.106585	-1.462678	-1.934174	0.0538533	0.0937284	-5.078254
RP11-210H1	-0.147709	-1.332942	-1.933989	0.0538761	0.0937637	-5.078605
RNU7-123P	0.2178564	-0.965969	1.9336767	0.0539146	0.0938263	-5.079199
ABRA	0.213764	-1.066762	1.933592	0.0539251	0.09384	-5.07936
EEF1A1P10	0.2710164	2.2259252	1.933148	0.0539799	0.093931	-5.080204
CT47B1	-0.302861	-0.918861	-1.932983	0.0540003	0.093962	-5.080517
NDUFA5P11	-0.274709	-0.498805	-1.932937	0.054006	0.0939674	-5.080604
ERCC8	-0.038027	5.3136391	-1.932747	0.0540296	0.0940039	-5.080967
CTD-3154N5	-0.186471	-1.173588	-1.932682	0.0540376	0.0940134	-5.08109
RP11-321E2	-0.114703	-1.449121	-1.932589	0.0540491	0.0940288	-5.081266
RP4-790G17	-0.189791	-1.166238	-1.932506	0.0540594	0.0940423	-5.081424
HUS1B	0.3347072	1.1334	1.9323423	0.0540796	0.094073	-5.081735
ALG1L3P	-0.201472	-1.143307	-1.932305	0.0540842	0.0940765	-5.081805
RP11-1136G	-0.391746	0.9621548	-1.93211	0.0541083	0.094114	-5.082175
RP11-19J5.	-0.186872	-1.223035	-1.931934	0.0541301	0.0941448	-5.08251
GJA4	0.0787814	5.7667482	1.9319258	0.0541312	0.0941448	-5.082526
RP11-770E5	-0.135885	-1.373815	-1.931874	0.0541376	0.0941515	-5.082625
RP11-651L5	-0.185981	3.4869205	-1.93177	0.0541505	0.0941694	-5.082822
LINC01015	-0.14382	-1.374019	-1.931519	0.0541817	0.0942142	-5.083299
AF213884.2	0.2277055	-0.91922	1.931512	0.0541825	0.0942142	-5.083312
CLIP4	0.1666091	5.3228558	1.9314996	0.054184	0.0942142	-5.083335
TCEA1P1	-0.093818	-1.467772	-1.93141	0.0541951	0.0942291	-5.083506
HMGA1	0.058554	6.4997386	1.9312215	0.0542185	0.0942652	-5.083864
MIR6739	0.2370552	-0.846107	1.9309118	0.0542569	0.0943276	-5.084452
AF127577.8	0.3189643	-0.972888	1.9305728	0.054299	0.0943939	-5.085095
ANKRD16	-0.041367	5.5574342	-1.93055	0.0543019	0.0943939	-5.085138
CTD-2542C2	0.1903703	-1.299827	1.9305418	0.0543029	0.0943939	-5.085154
RP11-359I1	-0.351737	2.2426208	-1.930369	0.0543243	0.0944267	-5.085481
AP001471.1	0.1522786	-1.31214	1.9302671	0.054337	0.0944443	-5.085675
RP11-392O1	0.3146888	-0.444422	1.9301024	0.0543575	0.0944754	-5.085988
ERICH4	0.3573798	-0.343875	1.9300048	0.0543696	0.094492	-5.086173
SPG20-AS1	0.2846506	-0.731334	1.9297915	0.0543962	0.0945336	-5.086578
C12orf65	-0.028222	5.9383165	-1.929726	0.0544043	0.0945433	-5.086702
PRAMEF10	-0.578369	1.8266396	-1.929642	0.0544147	0.0945569	-5.086861
RP11-383I2	-0.352741	1.1435869	-1.929155	0.0544754	0.0946578	-5.087786
CTD-2576F9	0.284711	2.20105	1.9290702	0.054486	0.0946716	-5.087946
RP11-757O6	-0.379377	-0.280862	-1.928988	0.0544961	0.0946848	-5.088101
MIR31HG	0.181232	-1.298204	1.9287167	0.05453	0.0947391	-5.088617

ZNF442	-0.068742	4.7990589	-1.928474	0.0545603	0.0947866	-5.089077
RP11-584P2	0.3034941	-0.396178	1.9284559	0.0545625	0.0947866	-5.089111
CABP7	0.191108	3.0330041	1.9282873	0.0545836	0.0948186	-5.089431
BIK	0.302377	3.7594	1.9281269	0.0546036	0.0948489	-5.089735
RP11-454K7	-0.32305	0.7527223	-1.927685	0.0546588	0.0949403	-5.090574
CTD-255501	-0.11731	4.0009186	-1.927605	0.0546688	0.0949497	-5.090725
SLC11A2	-0.035988	6.3197989	-1.9276	0.0546694	0.0949497	-5.090735
RP11-565F1	0.1122721	3.8412856	1.9274144	0.0546926	0.0949854	-5.091086
KBTBD11	0.2517681	4.6394958	1.9273002	0.0547068	0.094999	-5.091302
KRT14	0.4918138	0.1776607	1.9272941	0.0547076	0.094999	-5.091314
RP11-855A2	0.3185925	-0.432407	1.9272892	0.0547082	0.094999	-5.091323
UG0898H09	0.2629368	-1.061041	1.9271846	0.0547213	0.0950172	-5.091521
VSTM2B	-0.101754	-1.475116	-1.927081	0.0547343	0.0950352	-5.091718
AC010890.1	0.3346539	-0.205135	1.9267455	0.0547762	0.0951035	-5.092353
RP5-856G1.	0.3319774	-0.564089	1.9266133	0.0547928	0.0951277	-5.092604
MIR554	-0.102478	-1.437745	-1.926127	0.0548537	0.0952257	-5.093525
FOXP4-AS1	0.3899423	2.2791575	1.9261209	0.0548544	0.0952257	-5.093536
DPF3	-0.212521	4.1792471	-1.926057	0.0548624	0.0952351	-5.093658
SH3BP5L	-0.031579	6.2889154	-1.92574	0.0549021	0.0952994	-5.094257
CREBZF	0.0377186	6.1428893	1.925605	0.0549191	0.0953243	-5.094513
HIST1H2APS	-0.403283	0.5731746	-1.92557	0.0549234	0.0953273	-5.094579
RP11-428G5	0.3573105	0.0411158	1.9251369	0.0549778	0.0954172	-5.095399
S100A1	-0.142021	5.0091689	-1.925062	0.0549872	0.0954289	-5.095541
CENPQ	0.0702403	5.2304351	1.9250291	0.0549913	0.0954316	-5.095604
N6AMT1	-0.052965	5.5089747	-1.924895	0.0550082	0.0954563	-5.095857
BACE1-AS	0.0688472	4.8244655	1.9246601	0.0550377	0.0955029	-5.096302
RP11-478H1	0.3237724	-0.795839	1.9243895	0.0550717	0.0955574	-5.096814
ERLEC1	-0.025436	6.4538441	-1.924293	0.0550838	0.0955739	-5.096996
RGPD1	0.3730545	0.6530279	1.9242672	0.0550871	0.095575	-5.097045
CBWD3	0.1462129	3.3293523	1.9240173	0.0551185	0.095625	-5.097518
RP11-140A1	0.1768123	-1.283459	1.9238851	0.0551351	0.0956493	-5.097768
HIST1H4L	-0.089379	-1.476115	-1.923839	0.0551409	0.0956547	-5.097855
PCNXL3	-0.028708	6.433433	-1.923581	0.0551734	0.0957066	-5.098344
RP11-689K5	-0.404489	0.6369454	-1.922951	0.0552528	0.0958397	-5.099535
AL023806.1	0.3223988	1.3986523	1.9227963	0.0552722	0.095869	-5.099828
CTC-543D15	0.172863	-1.181437	1.9223561	0.0553278	0.0959607	-5.10066
PHLDA1	0.0726564	6.3455107	1.9221318	0.0553561	0.0960052	-5.101084
TYW5	0.037338	5.4507693	1.9221039	0.0553596	0.0960068	-5.101137
IL4	0.2038563	-1.16469	1.9220037	0.0553723	0.0960242	-5.101326
EEF1A1P12	0.1251815	4.1532763	1.9218882	0.0553868	0.0960449	-5.101544
WFDC3	0.3134687	2.384501	1.9216632	0.0554153	0.0960858	-5.101969
RP11-923I1	0.350901	1.7336882	1.9216495	0.055417	0.0960858	-5.101995
RP11-148B6	-0.073513	-1.498687	-1.921639	0.0554183	0.0960858	-5.102015
NUPR1L	-0.236574	-1.131244	-1.921612	0.0554217	0.0960871	-5.102066
C2orf27AP2	-0.096839	-1.479339	-1.92151	0.0554346	0.0961048	-5.102258
ACSM4	0.3670028	0.3645329	1.9213982	0.0554488	0.0961249	-5.10247
RP11-254F7	-0.351335	0.8427048	-1.921236	0.0554692	0.0961558	-5.102776
KRT18P28	0.2869203	1.5875921	1.9211678	0.0554779	0.0961662	-5.102905
NACA3P	-0.26627	4.3363987	-1.920998	0.0554993	0.0961948	-5.103225
RP11-275I4	0.2120654	-1.106355	1.9209961	0.0554996	0.0961948	-5.10323
TRIM36-IT1	-0.083122	-1.484627	-1.920936	0.0555072	0.0961993	-5.103344

TCEANC2	-0.037762	5.5315677	-1.920934	0.0555075	0.0961993	-5.103347
RP11-250B2	-0.190113	-1.121043	-1.920626	0.0555464	0.0962622	-5.103928
ANAPC7	-0.028063	6.255023	-1.920448	0.055569	0.0962968	-5.104266
CCDC17	-0.121469	4.3520015	-1.920143	0.0556077	0.0963592	-5.104841
NAPB	-0.037862	5.6559587	-1.920048	0.0556197	0.0963714	-5.105021
RP11-787D1	-0.157064	-1.329571	-1.920045	0.05562	0.0963714	-5.105025
RP11-54D18	-0.388627	0.040469	-1.919745	0.0556581	0.0964328	-5.105593
RP11-44K6.	-0.285598	-0.91092	-1.919467	0.0556934	0.0964893	-5.106117
LAMA3	-0.147336	5.7543654	-1.919433	0.0556977	0.0964922	-5.106181
ESPNP	-0.360464	2.0197056	-1.919379	0.0557045	0.0964995	-5.106283
ZNF812	0.4036392	2.280522	1.9193348	0.0557101	0.0965046	-5.106367
CCSAP	0.0622729	5.5609097	1.919024	0.0557496	0.096564	-5.106953
HEMGN	0.2806712	-0.787598	1.919003	0.0557522	0.096564	-5.106993
LINC01563	0.1722359	-1.276506	1.9190022	0.0557523	0.096564	-5.106994
ZIM2	0.3794175	-0.256878	1.9187893	0.0557794	0.0966063	-5.107396
CCDC117	0.1409297	4.8291623	1.9183742	0.0558321	0.0966931	-5.108179
CCDC30	0.1157129	4.0388977	1.9181171	0.0558648	0.0967451	-5.108664
RP11-428G5	-0.247271	-0.644526	-1.918059	0.0558723	0.0967534	-5.108775
RP11-528A1	-0.104254	-1.446445	-1.918003	0.0558794	0.0967611	-5.10888
RPL21P4	-0.299791	-0.181599	-1.917869	0.0558964	0.096786	-5.109132
AC109631.1	-0.139815	-1.335857	-1.917838	0.0559003	0.0967882	-5.10919
TRAPPC2B	-0.080707	5.3728478	-1.917782	0.0559075	0.0967961	-5.109297
MYO15B	-0.061471	6.3353384	-1.917483	0.0559455	0.0968573	-5.10986
RP3-486I3.	-0.299558	1.4721993	-1.917305	0.0559682	0.096892	-5.110196
ANXA9	-0.062423	6.2991325	-1.917251	0.0559751	0.0968992	-5.110297
VSIG8	0.3245453	-0.412132	1.9172162	0.0559795	0.0969024	-5.110363
RNU4-40P	-0.079819	-1.49702	-1.917088	0.0559958	0.096926	-5.110604
ZNF734P	-0.091566	-1.482748	-1.91686	0.0560249	0.0969718	-5.111035
CAMSAP2	0.0413968	6.0352905	1.9168204	0.05603	0.0969739	-5.111109
FBXW10	-0.479807	2.3844259	-1.916809	0.0560315	0.0969739	-5.111131
CTC-548K16	0.3415795	-0.245013	1.9166573	0.0560508	0.0969996	-5.111417
AC023590.1	0.3110459	0.9425852	1.9166508	0.0560516	0.0969996	-5.111429
AHR	-0.05742	6.477872	-1.916629	0.0560544	0.0969998	-5.11147
RP11-229P1	0.2601042	-0.749637	1.9163865	0.0560854	0.0970487	-5.111927
CTA-299D3.	0.2785783	-1.052832	1.9163644	0.0560882	0.097049	-5.111969
RP11-439L1	0.2554874	-0.661856	1.9160388	0.0561298	0.0971163	-5.112582
PDIA3P2	0.153433	-1.2778	1.9159159	0.0561454	0.0971389	-5.112814
RP5-1184F4	0.276744	-0.438777	1.9156557	0.0561787	0.0971918	-5.113304
RPL7P60	-0.389836	1.0296138	-1.91561	0.0561845	0.0971973	-5.11339
RP13-580F1	0.3544634	2.0726539	1.9154306	0.0562075	0.0972324	-5.113728
TEKT4P2	-0.144437	4.4852215	-1.915408	0.0562103	0.0972327	-5.11377
ERBB2IP	-0.031348	6.4846256	-1.914755	0.056294	0.0973728	-5.115001
GLIS2-AS1	0.3557637	2.9278125	1.9144368	0.0563347	0.0974385	-5.1156
CH17-125A1	-0.384126	-0.555801	-1.914217	0.0563629	0.0974827	-5.116014
SEMG1	0.1688849	-1.317075	1.9141564	0.0563706	0.0974914	-5.116127
CAPNS2	0.2145822	-1.051014	1.9140949	0.0563785	0.0975005	-5.116243
SPOCK3	-0.471892	0.051471	-1.913918	0.0564011	0.097535	-5.116576
GOLGA8B	-0.069133	5.9393125	-1.913769	0.0564203	0.0975635	-5.116857
TGM2	0.0583786	6.9335338	1.9136344	0.0564375	0.0975887	-5.11711
ADAM7	-0.130104	-1.413381	-1.913613	0.0564403	0.0975889	-5.117151
LINC00559	-0.240564	-1.055619	-1.913499	0.0564548	0.0976094	-5.117364

LINC00994	0.1600848	-1.276296	1.913453	0.0564608	0.0976151	-5.117451
RP11-229P1	0.3298563	1.2726828	1.91338	0.0564702	0.0976267	-5.117589
AC064836.3	0.3049579	-0.112755	1.9133024	0.0564801	0.0976393	-5.117735
MDGA1	0.1736877	4.8037907	1.913242	0.0564879	0.0976481	-5.117848
RNF157	-0.129646	5.6526335	-1.91289	0.0565331	0.0977205	-5.11851
NAV2-IT1	-0.180954	-1.172184	-1.912874	0.0565351	0.0977205	-5.11854
OR1F2P	-0.150683	-1.342704	-1.912803	0.0565442	0.0977248	-5.118674
AC008265.2	-0.172955	-1.308882	-1.912791	0.0565459	0.0977248	-5.118697
LCE3D	-0.162242	-1.371394	-1.912791	0.0565459	0.0977248	-5.118698
AC104134.2	-0.361498	-0.465526	-1.912772	0.0565483	0.0977248	-5.118733
KIAA1522	0.0459652	6.5113478	1.9122621	0.0566138	0.0978333	-5.119691
RP11-840I1	0.2855005	-0.453924	1.9121835	0.0566239	0.0978462	-5.119839
RP11-351I2	0.2361953	-0.973096	1.9120717	0.0566383	0.0978664	-5.12005
R3HDM1	0.0291089	5.998856	1.9119936	0.0566484	0.0978791	-5.120196
CARTPT	-0.171682	-1.337605	-1.911762	0.0566782	0.097926	-5.120632
RP11-160A9	-0.196119	-1.090876	-1.911662	0.0566911	0.0979419	-5.12082
AC009120.4	-0.284538	-0.607154	-1.911649	0.0566927	0.0979419	-5.120845
PTPRQ	0.4332278	0.2136988	1.9115849	0.056701	0.0979515	-5.120965
NDUFAF4	-0.04899	5.9467605	-1.911374	0.0567281	0.0979938	-5.121361
RP11-104L2	-0.311341	2.7458831	-1.911209	0.0567494	0.0980258	-5.121671
RP11-10017	0.2985066	0.2958635	1.9110758	0.0567666	0.0980509	-5.121922
OR7A5	0.2459242	-1.070548	1.9110063	0.0567756	0.0980615	-5.122052
RP11-720L8	0.3782359	-0.22611	1.9109864	0.0567781	0.0980615	-5.12209
AIF1L	0.1122264	5.4532679	1.9108772	0.0567922	0.0980812	-5.122295
NFE4	0.3286089	-0.588636	1.9106784	0.0568178	0.0981209	-5.122669
CHTOP	-0.022295	6.5165507	-1.910642	0.0568226	0.0981243	-5.122737
AF165138.7	0.1982128	-1.143601	1.9104626	0.0568457	0.0981597	-5.123074
SNORD53_SN	-0.153668	-1.263869	-1.910332	0.0568625	0.098184	-5.123319
AC003090.1	-0.460443	0.7192411	-1.910296	0.0568672	0.0981876	-5.123388
CCL14	-0.150705	4.7827816	-1.910059	0.0568979	0.0982358	-5.123833
PPP1R1C	0.3437408	4.1345348	1.9098757	0.0569215	0.0982719	-5.124177
SLC25A6P5	-0.132433	-1.3826	-1.90927	0.0569998	0.0984025	-5.125314
RNASEK-C17	0.1334471	3.3310068	1.9091759	0.057012	0.0984189	-5.125491
RP11-475I2	-0.22548	-1.16905	-1.909153	0.0570149	0.0984193	-5.125533
RP11-70D24	-0.244852	-0.755894	-1.908896	0.0570482	0.0984721	-5.126016
RP11-271K2	-0.17572	-1.17738	-1.908852	0.0570539	0.0984772	-5.126098
HGFAC	0.3291577	5.502012	1.9083875	0.0571141	0.0985764	-5.126971
AC011477.2	-0.257394	-0.817359	-1.908305	0.0571248	0.0985902	-5.127126
AC027319.1	0.1734541	-1.218362	1.908255	0.0571312	0.0985968	-5.127219
RP11-506E9	-0.137502	-1.371666	-1.908196	0.0571389	0.0986034	-5.127331
MESTP3	0.2299454	-0.960858	1.9081834	0.0571405	0.0986034	-5.127354
ERICH1	0.0463113	5.5729285	1.9081584	0.0571438	0.0986044	-5.127401
CCDC172	-0.136223	-1.409792	-1.907925	0.057174	0.0986519	-5.127838
LIPT2	-0.085604	4.480244	-1.907824	0.0571871	0.0986626	-5.128028
ACAD8	-0.037215	6.1176813	-1.907821	0.0571876	0.0986626	-5.128034
AC139099.4	-0.136924	-1.374159	-1.907815	0.0571883	0.0986626	-5.128045
ZFAND4	-0.046671	5.4766399	-1.907747	0.0571971	0.0986732	-5.128173
RP11-100G1	-0.159285	-1.279782	-1.9077	0.0572033	0.0986778	-5.128262
FCF1P2	0.044116	5.071489	1.9076847	0.0572052	0.0986778	-5.12829
CYP21A2	-0.161094	5.281434	-1.90763	0.0572123	0.0986853	-5.128392
LINC00930	0.340199	-0.351292	1.9075828	0.0572184	0.0986912	-5.128481

RBM18	-0.031986	5.9892317	-1.907305	0.0572545	0.0987487	-5.129002
TUBA3D	0.3479283	2.3365647	1.9070415	0.0572887	0.0987997	-5.129496
LINC01226	-0.406586	1.7665104	-1.907036	0.0572894	0.0987997	-5.129507
SGMS2	-0.069553	5.7676135	-1.906896	0.0573076	0.0988191	-5.12977
RSPH10B	0.2999342	-0.014182	1.9068944	0.0573078	0.0988191	-5.129772
IHH	-0.235073	4.9308672	-1.906873	0.0573106	0.0988191	-5.129812
RP11-977G1	-0.269854	-0.637454	-1.906866	0.0573115	0.0988191	-5.129825
KCNE3	0.0744894	5.3725076	1.9067043	0.0573325	0.0988507	-5.130128
C1orf140	-0.487089	0.6365527	-1.906657	0.0573387	0.0988566	-5.130217
RP5-849H19	-0.222621	-0.959286	-1.906555	0.057352	0.0988749	-5.130409
CTD-2006C1	-0.095482	4.5408004	-1.906483	0.0573613	0.0988862	-5.130543
CCDC158	-0.221331	4.0955138	-1.905616	0.0574742	0.0990762	-5.132169
RP11-366M4	-0.20151	-1.270508	-1.905408	0.0575013	0.0991182	-5.132559
CDKN2B	-0.083838	5.498851	-1.905219	0.0575259	0.0991559	-5.132912
RP11-649A1	0.1992928	3.8364466	1.9051285	0.0575377	0.0991716	-5.133082
RP11-378J1	-0.224739	-1.135642	-1.905016	0.0575524	0.0991923	-5.133294
KLHL25	-0.047168	5.8618369	-1.904705	0.0575929	0.0992574	-5.133875
RP11-727F1	-0.180557	3.0788337	-1.904632	0.0576024	0.0992692	-5.134012
PRAMEF6	-0.187676	-1.259888	-1.904055	0.0576778	0.0993858	-5.135093
CTD-208902	-0.115597	-1.424695	-1.904052	0.0576782	0.0993858	-5.135098
RP11-83J16	0.2455116	-0.734057	1.9040514	0.0576783	0.0993858	-5.1351
AC004009.3	-0.135733	-1.393293	-1.903956	0.0576907	0.0994026	-5.135279
AC093788.1	-0.175032	-1.179218	-1.903849	0.0577048	0.099422	-5.13548
SCO1	-0.035576	6.2035936	-1.903819	0.0577086	0.099424	-5.135535
RP11-9502.	0.3513731	1.0475336	1.9037892	0.0577125	0.099426	-5.135591
RP11-575L7	0.3466926	2.0682865	1.903722	0.0577213	0.0994365	-5.135717
FAF2P1	0.1240327	-1.387934	1.9036911	0.0577254	0.0994387	-5.135775
RP11-159N1	-0.149926	3.4724575	-1.903583	0.0577394	0.0994583	-5.135976
RRBP1	-0.034246	7.1925991	-1.903236	0.0577849	0.099532	-5.136627
RANBP6	-0.044431	5.92363	-1.902995	0.0578164	0.0995815	-5.137078
RNF165	0.2685433	3.8228814	1.902858	0.0578344	0.0996077	-5.137334
RP11-446H1	-0.209476	-1.096865	-1.902663	0.0578599	0.0996466	-5.1377
PRSS33	0.2022646	-1.228632	1.9026441	0.0578624	0.0996466	-5.137734
RP11-689C9	0.3045033	-0.732851	1.9021987	0.0579207	0.0997369	-5.138568
RP11-191N8	-0.219708	-1.158212	-1.902185	0.0579225	0.0997369	-5.138594
LINC01317	-0.205963	-1.145734	-1.902181	0.057923	0.0997369	-5.138601
DOT1L	0.0412316	6.1559503	1.9020302	0.0579428	0.0997663	-5.138883
RP11-131L1	0.2785658	2.2861844	1.9014789	0.0580152	0.0998862	-5.139914
ZNF546	-0.049898	5.16927	-1.901376	0.0580287	0.0999047	-5.140107
SLC24A2	0.290619	-0.727498	1.9012258	0.0580484	0.099933	-5.140388
NEK2P4	0.1878617	-1.206172	1.9012091	0.0580506	0.099933	-5.140419
RP11-541P9	-0.342593	1.3476413	-1.900987	0.0580798	0.0999712	-5.140834
LINC00658	-0.109298	-1.428399	-1.900987	0.0580798	0.0999712	-5.140834
RP11-452B1	-0.08835	-1.470059	-1.900978	0.058081	0.0999712	-5.140851
NFYC-AS1	0.203377	3.5349557	1.9008177	0.058102	0.1000027	-5.141151
POU5F1	-0.169183	4.478313	-1.900782	0.0581068	0.1000061	-5.141218
UCHL3	-0.062923	5.487363	-1.900711	0.058116	0.1000173	-5.141349
PRSS42	-0.362875	0.9028009	-1.900513	0.0581421	0.1000576	-5.141721
CYP2F1	-0.26826	-0.750376	-1.900419	0.0581545	0.1000741	-5.141897
RN7SL471P	-0.078065	-1.492782	-1.900385	0.0581589	0.1000762	-5.141959
BTNL8	0.443167	3.1974268	1.9003676	0.0581612	0.1000762	-5.141992

AC093106.5	-0.315474	-0.030936	-1.899697	0.0582494	0.1002209	-5.143244
RPL31P12	-0.158595	-1.304315	-1.899687	0.0582508	0.1002209	-5.143263
PAIP2B	0.2262812	5.4589285	1.8996446	0.0582564	0.1002259	-5.143343
RP11-65B7.	0.2435676	-0.776455	1.8995404	0.0582701	0.1002447	-5.143538
RP11-644F5	-0.085192	4.730633	-1.899465	0.0582801	0.1002572	-5.143679
RP11-411K7	-0.40501	0.7747408	-1.899419	0.0582862	0.1002629	-5.143765
AC012363.7	0.2148923	-1.001119	1.8993126	0.0583002	0.1002822	-5.143963
FBXO3-AS1	-0.125217	-1.396801	-1.899264	0.0583066	0.1002885	-5.144054
HELT	0.1611963	-1.31848	1.8988717	0.0583583	0.1003728	-5.144787
CTD-237103	0.2530162	-0.733603	1.8986548	0.0583869	0.1004173	-5.145192
REG3A	-0.668021	2.6806504	-1.898394	0.0584214	0.1004717	-5.145679
SAA2	-0.304761	5.882849	-1.898359	0.058426	0.100475	-5.145745
ATAD2	0.0592324	6.3040726	1.8983107	0.0584324	0.1004812	-5.145834
ANKRD32	0.0546913	5.1648012	1.8982567	0.0584395	0.1004887	-5.145935
RP11-388C1	0.3238935	0.293448	1.8981383	0.0584551	0.1005109	-5.146156
CTBP2P8	0.2649035	-0.701766	1.8980469	0.0584672	0.1005269	-5.146327
TBC1D3L	0.2745181	3.3118948	1.897542	0.058534	0.1006357	-5.147269
HLA-H	-0.064211	6.2068843	-1.897527	0.058536	0.1006357	-5.147298
C19orf40	0.0663342	4.9334849	1.8971345	0.0585879	0.1007202	-5.14803
RP11-70601	0.1313999	4.9837844	1.8969658	0.0586102	0.1007538	-5.148345
ZNF638-IT1	0.3669392	1.4461571	1.8969325	0.0586146	0.1007567	-5.148407
AC073109.2	0.2234438	-0.99471	1.896877	0.058622	0.1007645	-5.14851
CHIC2	0.0360163	5.5811897	1.8966962	0.0586459	0.100801	-5.148848
RP11-445F1	0.2632565	-1.14024	1.896659	0.0586508	0.1008047	-5.148917
RP11-308B1	-0.452423	0.1012967	-1.89622	0.0587091	0.1009	-5.149736
SMC6	-0.036181	6.1276247	-1.895951	0.0587448	0.1009566	-5.150238
RP11-715F3	0.2904046	1.5990379	1.8958856	0.0587534	0.1009667	-5.15036
AC018766.6	0.317244	0.4329005	1.8958279	0.058761	0.1009751	-5.150467
SULT6B1	-0.214643	-1.118515	-1.895643	0.0587857	0.1010126	-5.150813
CTD-2385L2	-0.179388	-1.282177	-1.895589	0.0587928	0.1010201	-5.150913
FAM87B	0.3011955	0.1643559	1.895522	0.0588017	0.1010306	-5.151038
RTBDN	-0.503699	1.1981756	-1.895494	0.0588053	0.1010321	-5.151089
PSPH	0.0581406	6.0212776	1.8952372	0.0588395	0.101086	-5.151569
MYL6B	-0.05543	6.0628177	-1.895122	0.0588548	0.1011076	-5.151783
STARD8	0.0634977	5.7844068	1.8947542	0.0589037	0.1011868	-5.152469
RP11-471M2	-0.428961	0.1001076	-1.894444	0.0589449	0.1012529	-5.153047
LINC01467	-0.120874	-1.435053	-1.89426	0.0589694	0.1012903	-5.15339
DIRC3-AS1	-0.119674	-1.40173	-1.894187	0.0589791	0.1013021	-5.153526
RP11-119H1	-0.068326	-1.510024	-1.893977	0.059007	0.1013453	-5.153916
RP11-20G13	0.3157956	-0.537269	1.8939392	0.0590121	0.1013492	-5.153987
RP11-174G6	0.1316321	4.0983644	1.8937386	0.0590388	0.1013904	-5.154361
CTA-280A3.	-0.10345	-1.427023	-1.893437	0.059079	0.1014546	-5.154923
NKG7	0.1123848	5.2844942	1.8932534	0.0591035	0.1014919	-5.155265
SPRN	-0.07245	5.3287043	-1.893165	0.0591153	0.1015073	-5.155429
RP11-390P2	-0.094905	5.0093293	-1.892997	0.0591377	0.101541	-5.155742
FNDC1-IT1	0.126192	-1.386723	1.8929701	0.0591413	0.1015424	-5.155792
RP3-395C13	0.3270073	0.6202866	1.8927208	0.0591745	0.1015947	-5.156256
RP11-17P16	0.2331894	-0.902023	1.8926929	0.0591783	0.1015964	-5.156308
RP11-1105C	0.2647256	-1.049785	1.8923513	0.0592239	0.1016699	-5.156944
RPL36AP39	-0.16371	-1.244264	-1.8923	0.0592307	0.1016768	-5.157039
SRRD	0.033299	5.7993694	1.8922576	0.0592364	0.1016818	-5.157119

ANGPTL7	-0.376247	-0.577292	-1.892195	0.0592448	0.1016915	-5.157236
ALG13-AS1	0.3093557	-0.07889	1.892077	0.0592605	0.1017137	-5.157455
ALPK3	0.1234546	5.6652805	1.891934	0.0592796	0.1017417	-5.157721
UMOD	-0.153244	-1.339898	-1.891867	0.0592886	0.1017522	-5.157845
IFNA5	-0.097929	-1.441736	-1.891812	0.059296	0.1017602	-5.157948
HSP90AA6P	0.3223201	0.2338701	1.891762	0.0593026	0.1017668	-5.158041
CROCC	0.0428098	5.7881096	1.8916239	0.0593211	0.1017937	-5.158298
SELE	0.2686111	4.132549	1.8913029	0.059364	0.1018626	-5.158895
RP11-154D3	-0.200667	-1.07352	-1.891253	0.0593707	0.1018679	-5.158987
TAS2R18	-0.172162	-1.188363	-1.891238	0.0593727	0.1018679	-5.159016
AC005336.5	-0.222364	-1.036162	-1.891013	0.0594029	0.1019149	-5.159435
RP11-44N12	-0.380212	-0.154075	-1.890702	0.0594445	0.101979	-5.160013
LOC440461	0.2108036	-1.053361	1.8906921	0.0594458	0.101979	-5.160031
AC018442.1	-0.160648	-1.262304	-1.890482	0.059474	0.1020225	-5.160422
RP11-465L1	0.3723442	2.2324148	1.8902655	0.059503	0.1020675	-5.160825
UBTD1	-0.046496	5.8411422	-1.890228	0.0595081	0.1020679	-5.160895
EEF1A1P19	0.0949641	4.4686412	1.8902219	0.0595089	0.1020679	-5.160906
AC079586.1	0.2850839	-0.964672	1.8901941	0.0595126	0.102068	-5.160958
KRT8P35	0.2635975	-0.659815	1.89018	0.0595145	0.102068	-5.160984
RND3	0.0675698	6.1296252	1.8897642	0.0595703	0.1021588	-5.161757
AL035610.1	0.4596316	-0.049705	1.8895991	0.0595924	0.1021921	-5.162064
STARD4	-0.065358	5.9482565	-1.889524	0.0596025	0.1022045	-5.162203
AC073621.2	0.2367577	-0.940169	1.8894891	0.0596072	0.1022078	-5.162268
TLE2	-0.084833	5.7526901	-1.889153	0.0596524	0.1022804	-5.162893
U2AF1L4	-0.051062	5.5047337	-1.889029	0.059669	0.1023042	-5.163123
ISY1-RAB43	-0.197488	-1.068265	-1.88874	0.0597079	0.1023647	-5.16366
RP5-894A10	-0.084979	4.9214469	-1.888725	0.0597099	0.1023647	-5.163689
RP11-44F14	0.2092081	-1.127635	1.8883199	0.0597644	0.1024532	-5.164441
RP11-15M15	-0.237625	-0.988972	-1.887972	0.0598113	0.1025288	-5.165087
RP11-440G9	-0.454259	0.3854379	-1.88786	0.0598263	0.1025484	-5.165295
RP11-288I2	0.2722289	1.8705939	1.8878447	0.0598284	0.1025484	-5.165323
ANKRD26P3	0.1422029	-1.377747	1.8877428	0.0598421	0.1025672	-5.165512
RP11-491H1	-0.257016	-0.93375	-1.887602	0.0598611	0.1025949	-5.165774
CTD-3185P2	-0.076975	4.303065	-1.887554	0.0598675	0.1026011	-5.165863
KRT8	-0.046034	7.2461894	-1.887282	0.0599042	0.1026591	-5.166368
AC016727.3	-0.073513	-1.498687	-1.887229	0.0599113	0.1026665	-5.166466
COL9A3	0.2430908	4.6536318	1.887098	0.059929	0.102692	-5.166709
CEP97	0.0635806	5.2913412	1.8869508	0.0599489	0.1027208	-5.166983
FAM25A	-0.095343	-1.469734	-1.886932	0.0599514	0.1027208	-5.167017
RP11-365P1	0.3270957	1.3934842	1.8867702	0.0599732	0.1027513	-5.167318
RP11-57G10	-0.268077	1.9197937	-1.886758	0.0599749	0.1027513	-5.16734
MCPH1	0.0410904	5.6234299	1.8866775	0.0599858	0.1027652	-5.16749
CEP83	0.0397306	5.4330135	1.8864031	0.0600228	0.1028238	-5.167999
RP11-826N1	0.1741069	-1.274222	1.8863681	0.0600275	0.1028271	-5.168064
AP5M1	-0.036773	6.0896778	-1.886318	0.0600343	0.1028339	-5.168158
LL22NC03-8	0.1962088	-1.187796	1.8862195	0.0600476	0.1028518	-5.16834
MIR572	-0.098991	-1.46288	-1.886042	0.0600716	0.102888	-5.168669
RP11-253M7	0.2056777	-1.06854	1.885936	0.0600859	0.1029078	-5.168866
CT45A1	0.2792311	-1.082044	1.8856458	0.0601252	0.1029702	-5.169404
PCDHB19P	0.3259324	0.2353087	1.8855687	0.0601356	0.1029832	-5.169547
RARRES2P1	0.2986942	-0.704546	1.8854116	0.0601568	0.1030147	-5.169839

KRT8P32	0.3105549	0.1325121	1.8852973	0.0601723	0.1030364	-5.170051
CORIN	0.2879474	3.128108	1.8849279	0.0602223	0.1031172	-5.170736
ATRX	0.0345094	6.1802641	1.8847235	0.06025	0.103157	-5.171115
SLC26A8	-0.291791	2.2298964	-1.884715	0.0602512	0.103157	-5.171131
GLYATL2	0.3139081	-0.493447	1.8844841	0.0602825	0.1032056	-5.171558
CHD7	0.0491603	5.9591619	1.8844538	0.0602866	0.1032078	-5.171615
RP11-84C10	0.2854306	1.7549	1.8843655	0.0602985	0.1032235	-5.171778
JRKL-AS1	-0.063225	-1.507164	-1.883592	0.0604035	0.1033983	-5.173212
LINC01311	-0.139819	3.7670448	-1.88349	0.0604173	0.1034171	-5.1734
RP11-467L1	0.306955	-0.616181	1.8832889	0.0604446	0.103459	-5.173773
SERPINB3	0.3489497	-0.759004	1.8831894	0.0604582	0.1034774	-5.173958
XRCC3	0.0549947	5.547082	1.8831671	0.0604612	0.1034777	-5.173999
TOP2B	0.0227307	6.5293454	1.8831208	0.0604675	0.1034836	-5.174085
TMEM120B	-0.036294	5.7240285	-1.88293	0.0604935	0.1035232	-5.174439
MTRNR2L5	-0.104564	-1.432944	-1.882458	0.0605576	0.1036281	-5.175312
SEC31A	-0.025431	6.7728177	-1.882324	0.0605758	0.1036544	-5.17556
KLRF1	0.252133	2.7580295	1.8819362	0.0606286	0.1037399	-5.176278
LEKR1	-0.131935	3.7271236	-1.881879	0.0606364	0.1037483	-5.176384
AC013404.1	-0.164587	-1.251883	-1.881837	0.0606422	0.1037492	-5.176463
LINC00222	0.4025361	1.6106347	1.8818338	0.0606426	0.1037492	-5.176468
RP5-1103B4	-0.328985	2.6269769	-1.881757	0.060653	0.1037622	-5.176609
RP11-265E1	-0.331278	0.8004015	-1.881684	0.060663	0.1037744	-5.176745
OVAAL	-0.314513	-0.720349	-1.881606	0.0606736	0.1037876	-5.176889
RSRC1	0.0330426	5.9007541	1.8815062	0.0606872	0.1038061	-5.177075
AC092839.4	-0.224989	-0.906696	-1.88134	0.0607098	0.1038399	-5.177381
AC008069.2	-0.387796	0.3175962	-1.881064	0.0607476	0.1038996	-5.177894
AC008391.1	0.2906678	-0.599902	1.8810283	0.0607524	0.103903	-5.177959
RP11-63M22	-0.123665	-1.399462	-1.88094	0.0607644	0.1039186	-5.178121
RP11-314A2	-0.310794	2.0447917	-1.880894	0.0607707	0.1039246	-5.178207
AC008063.2	-0.331414	-0.004099	-1.880866	0.0607746	0.1039263	-5.178259
LDHAP3	0.355895	2.9177214	1.8806248	0.0608075	0.1039777	-5.178706
HSPA8P14	0.231331	-0.881393	1.880585	0.0608129	0.1039821	-5.178779
RP3-366N23	0.3476279	-0.198539	1.880462	0.0608297	0.104006	-5.179007
ZNF77	0.0762695	4.8910435	1.8800692	0.0608834	0.1040928	-5.179733
FAM103A2P	0.3178299	-0.241837	1.8799482	0.0608999	0.1041163	-5.179957
SP140L	-0.050298	5.8443207	-1.879879	0.0609094	0.1041276	-5.180085
RPL12P9	-0.172925	-1.1814	-1.879712	0.0609322	0.1041572	-5.180394
NDUFAF6	-0.04835	6.0126856	-1.87969	0.0609353	0.1041572	-5.180435
LINC01412	0.1554623	-1.302928	1.8796894	0.0609353	0.1041572	-5.180436
LIN54	-0.036261	5.733394	-1.879652	0.0609405	0.1041612	-5.180506
LINC00564	0.1838796	-1.291966	1.8796261	0.060944	0.1041623	-5.180553
RP11-634B7	-0.127515	-1.373131	-1.878975	0.061033	0.1043096	-5.181756
AC105399.2	-0.206683	-1.052607	-1.878722	0.0610677	0.104364	-5.182224
UBR7	-0.027608	6.1044734	-1.878699	0.0610708	0.1043645	-5.182266
SPERT	0.4222932	-0.089183	1.8777478	0.0612013	0.1045826	-5.184024
KIAA1429	-0.031642	6.4132531	-1.877668	0.0612123	0.1045964	-5.184172
RP11-219E7	0.1820012	-1.22133	1.8774643	0.0612403	0.1046393	-5.184548
CTB-5506.1	-0.323708	1.0756637	-1.877362	0.0612543	0.1046583	-5.184736
MAGEB6P1	-0.146812	-1.343629	-1.877313	0.0612611	0.1046651	-5.184828
EXT1	0.0420592	6.6616716	1.877288	0.0612645	0.104666	-5.184873
RP1-52D1.1	-0.184851	-1.144798	-1.877045	0.0612979	0.1047181	-5.185322

RP4-603I14	-0.143397	-1.320843	-1.877023	0.0613009	0.1047185	-5.185364
PTP4A2P1	-0.213719	3.2902217	-1.876076	0.0614312	0.1049326	-5.187112
NTAN1	0.045283	6.0436422	1.8760699	0.061432	0.1049326	-5.187122
TOR3A	0.0433777	6.3771443	1.8759092	0.0614541	0.1049655	-5.187419
TSGA10	0.071802	4.652544	1.8758614	0.0614607	0.1049718	-5.187507
RP11-407N8	-0.114121	-1.410581	-1.875838	0.061464	0.1049725	-5.187551
RP11-363J2	0.2335914	-0.790786	1.8757334	0.0614784	0.1049921	-5.187743
GUCY2GP	-0.269157	-0.870475	-1.875705	0.0614823	0.1049939	-5.187796
ELFN2	0.4281923	4.2948927	1.8755384	0.0615052	0.1050282	-5.188103
SMARCA1	0.0484199	6.5565634	1.8751365	0.0615606	0.1051179	-5.188845
RNVU1-19	0.1864789	-1.142656	1.8750911	0.0615669	0.1051237	-5.188929
RP11-234O6	-0.16032	-1.349294	-1.874816	0.0616048	0.1051835	-5.189435
TMEM68	-0.039986	5.8965902	-1.874665	0.0616257	0.1052142	-5.189715
EEF1A1P16	0.2570236	2.255824	1.8746433	0.0616287	0.1052144	-5.189755
HEATR9	0.3508471	0.4129312	1.8746217	0.0616317	0.1052146	-5.189794
RP3-323P24	0.2481954	1.5491788	1.8743461	0.0616698	0.1052746	-5.190303
RP11-575F1	-0.480493	1.3898234	-1.874157	0.0616958	0.1053142	-5.190651
GAPDHP22	-0.213274	-1.051911	-1.874085	0.0617058	0.1053264	-5.190784
RP11-333I1	0.2987168	1.6035308	1.8740541	0.0617101	0.1053287	-5.190841
RP4-535B2C	-0.300538	-0.210142	-1.873854	0.0617378	0.1053711	-5.191211
RHD	0.2927463	2.8556196	1.8736662	0.0617637	0.1054104	-5.191556
CTB-75G16.	0.28067	1.5897377	1.8733534	0.061807	0.1054793	-5.192133
VN1R84P	0.2069212	-1.041886	1.8732376	0.061823	0.1055018	-5.192346
RNU5E-7P	-0.146172	-1.358773	-1.873146	0.0618357	0.1055184	-5.192515
LINC01481	-0.296821	-0.210849	-1.87296	0.0618615	0.1055576	-5.192859
AC010761.9	0.3306966	0.3180657	1.8728368	0.0618785	0.1055802	-5.193085
RP11-329J1	0.1431716	-1.326012	1.8728219	0.0618806	0.1055802	-5.193112
AC007381.2	-0.09796	-1.455999	-1.872454	0.0619316	0.1056623	-5.19379
HGH1	-0.05023	6.3652355	-1.872196	0.0619674	0.1057161	-5.194266
IGLV3-30	0.1415858	-1.392338	1.8721792	0.0619697	0.1057161	-5.194296
RP11-662I1	0.1726093	-1.302251	1.8721638	0.0619718	0.1057161	-5.194325
RP11-397E7	0.2754833	-0.349638	1.8720933	0.0619816	0.1057269	-5.194454
BACH2	0.1925167	4.5992061	1.8720765	0.0619839	0.1057269	-5.194485
LINC01413	-0.162229	-1.30271	-1.871926	0.0620048	0.1057575	-5.194762
EPHA1	0.1463861	5.9747891	1.8717569	0.0620283	0.1057927	-5.195074
RP11-347C1	0.2675629	2.0053559	1.8717005	0.0620361	0.1058011	-5.195178
RP11-452J6	-0.213854	-0.995842	-1.87167	0.0620403	0.1058034	-5.195234
RP11-718B1	0.2779635	-0.564087	1.871607	0.0620491	0.1058133	-5.19535
RPL37P6	-0.208176	3.2746846	-1.871528	0.06206	0.105827	-5.195495
BBIP1	-0.032022	5.5697036	-1.871323	0.0620885	0.1058706	-5.195872
RP11-324L1	0.2314135	-1.048482	1.8711354	0.0621146	0.1059102	-5.196218
RP11-241F1	-0.133001	-1.347548	-1.870967	0.062138	0.1059452	-5.196528
RP11-407G2	-0.31627	0.4316603	-1.87087	0.0621514	0.1059601	-5.196706
MEIS1	0.0539197	5.3069291	1.8708622	0.0621526	0.1059601	-5.196721
FAM71F1	0.2783602	-0.670296	1.870648	0.0621824	0.1060059	-5.197115
RP11-745A2	0.1466577	-1.304762	1.870592	0.0621902	0.1060128	-5.197218
ASS1P4	-0.182907	-1.192525	-1.870577	0.0621922	0.1060128	-5.197245
DUX4L27	0.3592889	1.0194721	1.8702558	0.0622369	0.1060841	-5.197837
TMEM69	-0.032172	6.0105068	-1.87009	0.06226	0.1061184	-5.198141
ATOH1	0.231226	-1.18406	1.8699979	0.0622728	0.1061354	-5.198312
TRAV38-1	0.206245	-1.099058	1.8699733	0.0622763	0.1061363	-5.198357

RNU6-1337F	-0.139044	-1.360147	-1.869942	0.0622806	0.1061388	-5.198415
NPM1	-0.032235	7.021311	-1.869761	0.0623059	0.1061769	-5.198748
RP11-402G3	0.2134907	-1.067803	1.8695157	0.06234	0.1062301	-5.199199
AC007362.3	0.1752853	-1.174123	1.8688975	0.0624262	0.106372	-5.200335
ERCC6L2	0.0433706	5.4582772	1.8688126	0.0624381	0.1063872	-5.200492
CNPY1	0.2535082	-0.943766	1.8687263	0.0624501	0.1064028	-5.20065
CTB-113I20	-0.139242	-1.364158	-1.868686	0.0624558	0.1064075	-5.200725
GAB2	0.0519707	5.908862	1.8679829	0.062554	0.1065698	-5.202017
CASC9	-0.628597	2.1573989	-1.867834	0.0625748	0.1066003	-5.202291
CDC14C	-0.16191	-1.285451	-1.867124	0.0626741	0.1067645	-5.203595
NUPL2	-0.026104	5.9963278	-1.866477	0.0627648	0.106914	-5.204784
AC108488.4	-0.083854	4.4495023	-1.866306	0.0627887	0.1069497	-5.205097
AC096670.3	-0.331529	0.425308	-1.86608	0.0628204	0.1069987	-5.205512
SLC12A4	-0.036813	6.2667177	-1.86568	0.0628766	0.1070894	-5.206247
TCIRG1	0.0446359	6.5751753	1.865412	0.0629142	0.1071485	-5.206739
TFIP11	0.0455769	6.1718787	1.8653543	0.0629223	0.1071571	-5.206845
TRPC5	-0.496808	1.7159778	-1.865334	0.0629251	0.1071571	-5.206881
WNT5A	0.2549434	4.9779133	1.8652696	0.0629342	0.1071675	-5.207
KRT79	0.2485457	-0.99118	1.8650772	0.0629612	0.1072086	-5.207353
RP11-95P13	-0.315412	-0.803718	-1.865049	0.0629652	0.1072102	-5.207404
CTD-2333K2	-0.14091	-1.345358	-1.864987	0.0629739	0.1072202	-5.207519
S100PBP	0.0351684	5.7502569	1.8649604	0.0629777	0.1072215	-5.207567
AC092669.3	-0.102068	-1.430089	-1.864933	0.0629816	0.1072232	-5.207618
C5orf66-AS	-0.08817	-1.475437	-1.864825	0.0629967	0.1072439	-5.207816
KRT8P15	0.1723251	-1.181679	1.8646596	0.06302	0.1072785	-5.208119
LYPD3	0.1746963	4.1055232	1.8645673	0.0630329	0.1072956	-5.208289
CYP2E1	-0.216819	6.906073	-1.864152	0.0630914	0.1073901	-5.209051
RP11-10017	0.2610367	2.0935957	1.8639792	0.0631157	0.1074265	-5.209367
ANKRD19P	0.1281517	3.845286	1.8638311	0.0631366	0.107457	-5.209639
CTC-339F2.	0.2954877	-0.214944	1.8638077	0.0631399	0.1074576	-5.209682
RP5-839B4.	0.3797789	-0.049324	1.8637116	0.0631534	0.1074748	-5.209858
AC002117.1	-0.323033	0.8009926	-1.863694	0.0631559	0.1074748	-5.20989
DNAJC13	0.0277778	6.2281103	1.8634388	0.0631918	0.107531	-5.210358
CTD-3014M2	-0.272499	2.5820426	-1.863407	0.0631964	0.1075337	-5.210418
RP11-981G7	0.2770889	-0.442754	1.8630979	0.0632399	0.1076028	-5.210983
ZNF44	0.0370941	5.6948197	1.8630329	0.0632491	0.1076108	-5.211103
WDR36	-0.030291	6.1207024	-1.863023	0.0632505	0.1076108	-5.211121
PRIM1	-0.069535	5.5638235	-1.862968	0.0632582	0.1076155	-5.211221
RP11-17A1.	-0.306232	1.5287239	-1.862961	0.0632592	0.1076155	-5.211234
AC005534.6	0.3151143	0.3069776	1.862727	0.0632923	0.1076667	-5.211663
RNA5SP111	0.3753711	1.3508085	1.8627056	0.0632953	0.1076669	-5.211703
HOXC-AS2	0.4024485	-0.033563	1.8621815	0.0633693	0.1077861	-5.212663
CTD-2034I4	-0.288855	-0.52461	-1.862167	0.0633713	0.1077861	-5.212689
MIR5188	-0.184033	-1.167832	-1.862032	0.0633904	0.1078136	-5.212937
IARS	0.0303799	6.4615695	1.8618696	0.0634134	0.1078476	-5.213234
SRMS	0.3514207	2.0802703	1.8616953	0.063438	0.1078786	-5.213554
TBX6	-0.10985	4.2522229	-1.861685	0.0634394	0.1078786	-5.213572
C1orf131	0.0432027	5.8223161	1.861678	0.0634405	0.1078786	-5.213585
WNK1	0.0281995	6.6658877	1.8614347	0.0634749	0.1079301	-5.214031
RP11-69E11	0.3047656	-0.067324	1.8614067	0.0634788	0.1079301	-5.214082
AC009362.2	-0.275522	-0.551576	-1.861401	0.0634796	0.1079301	-5.214092

RP11-81K2.	0.2359846	-0.740841	1.8611878	0.0635098	0.1079727	-5.214483
THEM4	-0.038422	5.9866121	-1.861183	0.0635105	0.1079727	-5.214492
RP3-426I6.	-0.264317	-0.645951	-1.861055	0.0635287	0.1079985	-5.214727
RP5-1120P1	0.4286686	2.7801105	1.8609153	0.0635484	0.1080268	-5.214982
RP11-430C7	0.2595658	3.5470045	1.8608949	0.0635513	0.1080268	-5.215019
LINC01059	0.1823402	-1.279679	1.8608439	0.0635585	0.10803	-5.215113
KIAA1586	-0.046485	5.425153	-1.86084	0.0635591	0.10803	-5.21512
BOLA3	-0.043844	5.9101349	-1.86081	0.0635633	0.1080321	-5.215174
AC073257.2	-0.295867	2.6773067	-1.860777	0.0635679	0.108035	-5.215235
PLCG1	0.0386431	6.2521525	1.8606614	0.0635844	0.1080579	-5.215447
RP11-1060J	0.2846387	1.8429693	1.8604336	0.0636167	0.1081077	-5.215864
RP11-305K5	-0.106729	4.6012244	-1.860261	0.0636412	0.1081433	-5.216181
RP11-13E5.	-0.225133	-1.112645	-1.860234	0.063645	0.1081433	-5.216229
RP11-131M1	0.2680352	1.998426	1.8602232	0.0636465	0.1081433	-5.216249
FSD2	-0.317166	0.1340114	-1.860181	0.0636525	0.1081485	-5.216327
RP11-677M1	0.3323446	2.1080417	1.8601094	0.0636626	0.1081567	-5.216457
CHMP7	-0.030673	6.1449842	-1.860105	0.0636633	0.1081567	-5.216466
NOX3	0.1689393	-1.308206	1.8599453	0.0636859	0.1081902	-5.216758
NACAP5	-0.268722	-0.929459	-1.859749	0.0637137	0.1082324	-5.217117
TMEM5	-0.029645	5.829632	-1.859579	0.0637378	0.1082683	-5.217427
AC005487.2	-0.282315	-0.660668	-1.859248	0.0637848	0.1083431	-5.218033
RP11-47909	-0.325909	0.3648248	-1.859195	0.0637924	0.1083509	-5.21813
AC097461.4	0.317665	0.579435	1.8590805	0.0638087	0.1083736	-5.21834
MARC1	-0.077995	6.3285097	-1.858693	0.0638638	0.1084621	-5.219049
RAP2C	-0.039827	6.3529881	-1.858579	0.06388	0.1084845	-5.219257
TTC7A	-0.04221	6.186931	-1.858536	0.0638862	0.10849	-5.219337
RN7SL272P	-0.129653	-1.406659	-1.858357	0.0639116	0.1085255	-5.219664
MTATP8P1	0.3144446	2.5141227	1.858347	0.063913	0.1085255	-5.219682
RP11-680F2	0.2504486	-0.836386	1.8583121	0.063918	0.1085288	-5.219745
RP11-483I1	0.2914161	-0.379125	1.8582485	0.063927	0.1085392	-5.219862
AC026799.1	-0.079969	-1.493849	-1.858155	0.0639403	0.1085567	-5.220033
CCNYL1	0.0381722	5.9356598	1.8578193	0.0639881	0.1086328	-5.220646
MT-TP	-0.058184	6.2932489	-1.857667	0.0640098	0.1086646	-5.220925
PCBP1	0.0459036	6.7629912	1.8574159	0.0640456	0.1087203	-5.221384
CYP2U1	-0.088846	5.4343488	-1.857198	0.0640766	0.1087679	-5.221781
IGHV3-75	0.204611	-1.222899	1.8570006	0.0641048	0.1088107	-5.222143
RP11-100L2	0.3554172	1.3958267	1.8566712	0.0641518	0.1088854	-5.222745
WASF3-AS1	-0.145783	-1.336834	-1.856558	0.064168	0.1089078	-5.222952
NPW	0.2614921	4.7348677	1.8563306	0.0642005	0.1089578	-5.223367
SLC6A16	-0.152582	4.9799652	-1.856203	0.0642187	0.1089836	-5.2236
RP11-751K2	0.1414611	-1.292693	1.8560596	0.0642392	0.1090134	-5.223862
RP11-546K2	-0.249623	2.7310296	-1.855855	0.0642684	0.1090579	-5.224235
RP11-523H2	-0.204644	3.402686	-1.855818	0.0642738	0.1090619	-5.224303
EEF1B2P6	-0.134013	4.1175671	-1.855211	0.0643605	0.1092041	-5.225411
RP3-370M22	0.2924827	1.5594576	1.8550378	0.0643853	0.1092411	-5.225727
RP11-183I6	-0.10862	-1.423008	-1.854985	0.0643929	0.1092489	-5.225824
RP11-477J2	0.1516287	-1.301237	1.8548554	0.0644115	0.1092753	-5.22606
RP11-641D5	-0.131464	3.8444124	-1.854815	0.0644172	0.10928	-5.226134
TULP3P1	-0.158979	-1.323386	-1.854439	0.0644711	0.1093662	-5.22682
BIRC6-AS2	0.2255602	-0.881051	1.8544067	0.0644757	0.1093691	-5.226879
C3orf22	0.3205848	0.039541	1.8541739	0.0645091	0.1094206	-5.227304

MEGF9	-0.0458	6.3569735	-1.854126	0.0645159	0.1094271	-5.227391
USP1	0.0353745	6.0565287	1.8539599	0.0645398	0.1094625	-5.227694
ARL17A	0.1830243	3.2910544	1.8538637	0.0645536	0.1094808	-5.22787
RP11-291I6	0.1955152	-1.118733	1.8533315	0.06463	0.1096053	-5.228841
RP11-445L6	-0.346123	-0.459812	-1.853065	0.0646683	0.1096652	-5.229327
RP13-487K5	0.1487512	-1.2912	1.852666	0.0647257	0.1097573	-5.230054
CUL5	-0.026469	6.1389535	-1.852497	0.06475	0.1097935	-5.230363
LINC01498	-0.115843	-1.406122	-1.85243	0.0647596	0.1098016	-5.230485
EPGN	0.1948877	-1.136018	1.8524215	0.0647608	0.1098016	-5.2305
TMEM233	0.1446201	4.0304432	1.8523481	0.0647714	0.1098144	-5.230634
AC019181.2	-0.347968	2.4272648	-1.852238	0.0647872	0.1098362	-5.230834
ZFAT	0.0386474	5.6123517	1.852213	0.0647908	0.1098372	-5.23088
MIR4999	-0.193188	-1.136675	-1.852189	0.0647943	0.109838	-5.230924
FKSG54	-0.202223	-1.04536	-1.852013	0.0648196	0.1098757	-5.231244
RP11-790I1	-0.368431	-0.023433	-1.851829	0.0648461	0.1099155	-5.231579
AGTPBP1	0.0432182	5.5178668	1.8516837	0.064867	0.109946	-5.231844
LINC00698	0.1881822	-1.244443	1.8516083	0.0648779	0.1099593	-5.231982
RP11-330M2	0.2336128	-0.768078	1.8514523	0.0649004	0.1099923	-5.232266
C9orf172	0.0729276	5.2965351	1.8514181	0.0649053	0.1099955	-5.232328
PPP2R1A	-0.029267	6.8989103	-1.850818	0.0649918	0.1101323	-5.233421
RN7SL513P	-0.173319	-1.190892	-1.850816	0.0649921	0.1101323	-5.233424
PPT2-EGFL8	0.0908278	4.0951714	1.8505305	0.0650333	0.1101971	-5.233945
AP000936.4	-0.107822	-1.441495	-1.850464	0.065043	0.1102051	-5.234067
PRKRIP1	-0.030003	6.0686535	-1.850456	0.0650441	0.1102051	-5.234081
RGPD8	0.1981951	3.890741	1.8504015	0.065052	0.1102133	-5.23418
PPP1R12BP1	-0.094135	-1.463855	-1.850307	0.0650657	0.1102315	-5.234353
IFIT1B	-0.248969	-0.830517	-1.849766	0.0651438	0.1103587	-5.235337
KIF4A	0.1109304	5.5285146	1.8496925	0.0651544	0.1103715	-5.235471
GAK	-0.025026	6.572727	-1.849609	0.0651665	0.1103869	-5.235623
RP11-49907	-0.138357	-1.376231	-1.849406	0.0651958	0.1104314	-5.235992
CTA-243E7.	0.31906	0.6827275	1.8492373	0.0652202	0.1104677	-5.236299
RP11-405F3	0.2278745	-0.972405	1.8492032	0.0652252	0.1104709	-5.236361
RP11-676J1	-0.307625	0.823716	-1.849104	0.0652396	0.1104902	-5.236542
C1orf220	0.1998409	3.6784488	1.8485513	0.0653196	0.1106205	-5.237547
PSMA6P1	-0.205962	2.6045338	-1.848515	0.0653248	0.1106242	-5.237613
MSNP1	0.3076754	-0.146082	1.8484707	0.0653312	0.11063	-5.237694
MIR4428	-0.116275	-1.419749	-1.847888	0.0654157	0.1107679	-5.238754
DEGS1	0.0357731	6.3227584	1.8478009	0.0654284	0.1107842	-5.238912
CTD-2224J9	0.4103768	1.027399	1.847704	0.0654424	0.1108029	-5.239088
COL4A2-AS2	0.2305415	-0.91726	1.8476133	0.0654556	0.11082	-5.239253
EEF1A2	0.3770008	5.231074	1.8475602	0.0654633	0.110828	-5.23935
TMEM2	-0.042648	6.6545812	-1.847178	0.0655188	0.1109167	-5.240044
RP5-998N21	0.1827876	-1.248641	1.8471484	0.0655231	0.1109189	-5.240098
ASB6	-0.028755	5.9434861	-1.847013	0.0655428	0.1109471	-5.240345
PBDC1	-0.037251	5.9348046	-1.846891	0.0655604	0.11097	-5.240566
RP11-374M1	0.1833844	-1.176471	1.8468779	0.0655624	0.11097	-5.24059
RP11-1260E	0.28769	2.4336771	1.8467606	0.0655794	0.1109937	-5.240803
TTBK1	-0.183485	4.9944066	-1.846569	0.0656073	0.1110262	-5.241152
AGO4	0.034513	5.8742647	1.8465506	0.06561	0.1110262	-5.241185
RP11-259A2	-0.108089	-1.428311	-1.846544	0.065611	0.1110262	-5.241197
RP11-1085N	-0.106803	-1.436701	-1.84654	0.0656115	0.1110262	-5.241204

ZNF613	0.067925	5.0074266	1.846524	0.0656138	0.1110262	-5.241233
CHMP4BP1	0.2463598	2.2391925	1.8463668	0.0656367	0.1110597	-5.241519
GOLGA8N	0.179939	3.8673874	1.8463175	0.0656439	0.111064	-5.241609
ORC5	-0.02958	5.9170386	-1.846308	0.0656453	0.111064	-5.241626
CTD-3064H1	0.2057145	-1.055753	1.8462667	0.0656513	0.1110689	-5.241701
RP11-214C8	0.2394225	-0.706129	1.8462405	0.0656551	0.1110702	-5.241748
KIF9-AS1	-0.076854	4.3577101	-1.846066	0.0656805	0.1111081	-5.242066
MAML3	-0.064026	5.5331402	-1.845981	0.0656929	0.1111206	-5.24222
CTC-429P9.	0.2042529	2.753609	1.8459733	0.065694	0.1111206	-5.242234
AP001432.1	0.3329527	1.1119601	1.8458856	0.0657067	0.111137	-5.242393
DOCK5	0.0562787	5.9819166	1.8458123	0.0657174	0.1111499	-5.242526
AC015922.6	-0.249942	-1.080012	-1.845761	0.0657249	0.1111575	-5.24262
RP11-472F1	0.315922	-0.316327	1.8455546	0.065755	0.1112031	-5.242994
RP11-70601	0.3162506	-0.609853	1.8455092	0.0657616	0.1112091	-5.243077
FBXO8	-0.045693	6.0153797	-1.845459	0.0657689	0.1112163	-5.243168
LINC01193	-0.242152	-1.140236	-1.845054	0.0658279	0.111311	-5.243903
CTD-2366F1	-0.251319	2.5560039	-1.845015	0.0658336	0.1113155	-5.243974
CEP164P1	0.312371	1.2672189	1.8449017	0.0658501	0.1113383	-5.24418
RHPN1	0.0899555	5.3589775	1.8448587	0.0658564	0.1113437	-5.244258
NACC2	-0.037371	6.1812449	-1.844553	0.065901	0.111414	-5.244813
GLDC	0.1146564	6.3541602	1.8444832	0.0659112	0.111426	-5.24494
CEP152	0.0804566	4.9046693	1.8443056	0.0659371	0.1114647	-5.245262
ARHGAP5	-0.035187	6.28906	-1.84421	0.0659511	0.1114832	-5.245436
RP11-372E1	0.3786808	1.9071905	1.8438677	0.0660011	0.1115625	-5.246057
RP11-57C13	-0.223107	-1.079555	-1.843723	0.0660222	0.1115903	-5.246319
CHPF	-0.048866	6.6282783	-1.843713	0.0660237	0.1115903	-5.246337
RP11-459F6	0.2686026	1.2762413	1.8436816	0.0660283	0.111593	-5.246394
AC024162.2	-0.121783	-1.391343	-1.843594	0.0660411	0.1116095	-5.246554
RP11-756P1	0.3847442	1.6800374	1.8434701	0.0660592	0.1116349	-5.246778
CUL4B	-0.025822	6.4333247	-1.843352	0.0660764	0.1116589	-5.246992
TLE1P1	0.1708479	4.2583027	1.8431773	0.066102	0.1116969	-5.247309
CTD-3088G3	-0.17567	-1.170955	-1.843147	0.0661064	0.1116992	-5.247363
RP11-613F7	0.3487616	1.1453148	1.8428432	0.0661509	0.1117692	-5.247915
RASSF1	-0.030413	6.0458108	-1.842533	0.0661964	0.1118409	-5.248478
LINC01526	-0.253093	-0.756329	-1.842284	0.0662329	0.1118973	-5.24893
RP11-272B1	-0.088379	-1.495206	-1.842096	0.0662604	0.1119386	-5.24927
RP11-613F2	-0.15444	-1.279577	-1.84172	0.0663156	0.1120266	-5.249952
LCE1E	-0.164302	-1.322847	-1.841303	0.0663767	0.1121207	-5.250707
HNRNPA1P50	0.2564463	-0.590614	1.8412968	0.0663777	0.1121207	-5.250719
CPSF1	-0.03396	6.6053281	-1.841276	0.0663807	0.1121207	-5.250756
RP11-347C1	-0.13737	4.0402015	-1.841257	0.0663836	0.1121207	-5.250791
CTC-43909.	0.1983812	-1.08629	1.8411357	0.0664013	0.1121456	-5.251011
OGFOD1	-0.027436	6.1165436	-1.840956	0.0664277	0.112185	-5.251336
RP11-363G1	-0.161047	-1.336012	-1.840895	0.0664367	0.112195	-5.251447
RP11-373E1	-0.15902	-1.282606	-1.840644	0.0664736	0.1122521	-5.251902
C10orf111	0.2382463	2.054198	1.8406069	0.066479	0.112256	-5.251969
RP11-728K2	-0.223315	-0.927424	-1.840581	0.0664828	0.1122572	-5.252015
RP11-415C1	0.1622516	-1.315591	1.8402116	0.0665372	0.1123438	-5.252685
NF1P2	-0.101776	-1.450446	-1.839999	0.0665685	0.1123914	-5.25307
WDR26	0.027326	6.6307882	1.8396794	0.0666155	0.1124657	-5.253648
RNU6-1016F	0.2818476	-0.520775	1.8394744	0.0666457	0.1125115	-5.254019

RP11-184D1	-0.177397	-1.288738	-1.839129	0.0666965	0.112592	-5.254644
RP11-468E2	-0.188439	2.9430632	-1.839044	0.0667091	0.112608	-5.254798
AF064858.7	-0.14447	-1.296245	-1.839017	0.0667131	0.1126095	-5.254847
RP11-154D6	0.1690471	-1.231346	1.838871	0.0667347	0.1126408	-5.255112
GRAMD4	0.0615523	6.3611854	1.8387902	0.0667466	0.1126557	-5.255258
FAM201B	0.1728719	-1.208619	1.8387496	0.0667526	0.1126606	-5.255331
RP11-44N22	-0.302485	1.9799882	-1.838524	0.0667858	0.1127115	-5.255739
UPK3BP1	0.2224836	-0.901147	1.8384081	0.0668029	0.1127352	-5.255949
SLC13A1	0.2602199	-0.988592	1.8383514	0.0668113	0.1127441	-5.256052
UBL3	-0.040004	6.3260423	-1.838032	0.0668585	0.1128184	-5.256629
CD274	0.1160754	4.5364351	1.837989	0.0668648	0.112824	-5.256707
RP11-887P2	0.2935824	0.5263018	1.8379053	0.0668772	0.1128396	-5.256859
RP11-654E1	-0.109123	-1.433768	-1.837614	0.0669202	0.112907	-5.257386
CTB-133G6.	0.2856458	0.3377494	1.8375012	0.0669369	0.1129299	-5.25759
TEX29	-0.27886	2.1746644	-1.837473	0.0669411	0.1129319	-5.257641
CTD-2332E1	-0.155734	-1.325486	-1.837391	0.0669532	0.112947	-5.257789
AL122003.1	-0.117669	-1.434835	-1.836928	0.0670217	0.1130573	-5.258626
RP11-342K6	-0.25931	-0.506882	-1.836746	0.0670486	0.1130975	-5.258955
MED22	0.0355186	5.9918374	1.836697	0.0670559	0.1131004	-5.259044
TRPM5	0.3747641	0.2131382	1.8366925	0.0670566	0.1131004	-5.259052
AC000067.1	0.2896879	-0.223252	1.8364801	0.067088	0.1131483	-5.259436
ZNF565	0.0460644	4.8974392	1.8363838	0.0671023	0.1131671	-5.25961
RP11-242J7	0.5080856	2.99488	1.8363408	0.0671087	0.1131726	-5.259687
ZNF354B	0.052197	5.1785973	1.836296	0.0671153	0.1131786	-5.259769
PYGB	0.0405108	6.5268279	1.8361297	0.06714	0.1132149	-5.260069
RP11-245J2	-0.072977	-1.498387	-1.836046	0.0671523	0.1132306	-5.26022
TRIM64FP	-0.230872	-0.890156	-1.835998	0.0671595	0.1132374	-5.260307
AP000721.4	-0.196255	-1.074637	-1.83568	0.0672066	0.1133117	-5.260882
RP11-384F7	-0.157542	-1.334802	-1.835532	0.0672285	0.1133433	-5.261148
BCKDHA	0.1023837	5.2823916	1.8352977	0.0672634	0.1133968	-5.261572
RP11-439H9	-0.436344	0.3153788	-1.834728	0.067348	0.1135344	-5.262602
CTC-444N24	-0.154547	3.077156	-1.834501	0.0673818	0.113586	-5.263012
RMI1	-0.052737	5.6068975	-1.834438	0.067391	0.1135963	-5.263124
ABLIM3	-0.061108	6.4126085	-1.834388	0.0673985	0.1136036	-5.263215
RP11-219D1	-0.278741	1.6118099	-1.83419	0.067428	0.1136466	-5.263573
MIR5010	0.2792089	-0.369051	1.834175	0.0674302	0.1136466	-5.2636
RP11-111K1	0.2975694	1.1351876	1.8337681	0.0674907	0.1137434	-5.264334
RP5-855D21	0.2911787	1.6349399	1.8336828	0.0675034	0.1137583	-5.264488
PANX3	0.2882529	-0.806233	1.8336667	0.0675058	0.1137583	-5.264517
UNC93B2	0.3277719	-0.483517	1.833566	0.0675208	0.1137784	-5.264699
MUC16	0.3446187	0.4695182	1.8334488	0.0675383	0.1138025	-5.26491
TBC1D16	-0.040192	6.341815	-1.833291	0.0675619	0.113837	-5.265196
RP1-224A6.	0.3051709	0.3082679	1.8332284	0.0675711	0.1138473	-5.265308
KCNJ3	0.4108019	3.9495293	1.8329444	0.0676134	0.1139134	-5.26582
SPATS2L	0.0282186	6.7368985	1.832872	0.0676242	0.1139258	-5.265951
CIDEA	-0.423438	-0.315841	-1.832835	0.0676297	0.1139258	-5.266017
RP11-629B1	-0.286162	1.836344	-1.832817	0.0676324	0.1139258	-5.26605
IDH2	-0.042318	6.9384415	-1.832811	0.0676333	0.1139258	-5.266061
RP11-437J2	-0.17748	-1.255455	-1.83277	0.0676394	0.1139261	-5.266134
RFX3-AS1	0.2743261	2.6070059	1.8327681	0.0676397	0.1139261	-5.266138
CTB-102L5.	0.2588336	-0.541135	1.8326001	0.0676648	0.1139631	-5.266441

EIF4A1P10	0.1822551	3.1744326	1.8319945	0.0677552	0.1141057	-5.267533
RP3-322G13	0.2204928	-0.792281	1.8319909	0.0677557	0.1141057	-5.26754
MINPP1	-0.046981	6.1445741	-1.831928	0.0677651	0.1141162	-5.267653
AC231657.1	-0.109591	-1.427488	-1.831865	0.0677745	0.1141268	-5.267767
AC011516.2	-0.211846	-1.217571	-1.831811	0.0677826	0.1141352	-5.267865
YIPF5	-0.025354	6.2567888	-1.831636	0.0678087	0.1141739	-5.26818
RP11-295H2	-0.268177	2.0552128	-1.831513	0.0678271	0.1141952	-5.268401
RP11-135F9	0.1764993	3.3996263	1.8315094	0.0678277	0.1141952	-5.268408
RP11-266L9	-0.328415	0.0910605	-1.831465	0.0678343	0.1142012	-5.268489
AC005481.5	0.2169621	-0.990049	1.8313063	0.067858	0.1142358	-5.268774
CEACAMP3	0.2108246	-1.056921	1.8312548	0.0678657	0.1142435	-5.268867
RP11-53B2.	0.2174827	-0.935791	1.8311731	0.0678779	0.1142588	-5.269014
RP11-197K6	0.2635935	-1.123243	1.8311303	0.0678843	0.1142643	-5.269091
SLC16A13	-0.077882	6.0696602	-1.830966	0.0679089	0.1142977	-5.269387
RP11-21401	-0.19105	-1.07442	-1.830956	0.0679104	0.1142977	-5.269406
RP11-313P1	-0.288609	1.0912421	-1.830911	0.0679172	0.1143037	-5.269487
RBM20	0.259352	3.2296664	1.8308177	0.0679311	0.114322	-5.269655
RP11-598F7	-0.169981	3.6099449	-1.830784	0.0679362	0.1143252	-5.269716
PPFIBP2	-0.040526	6.2360464	-1.83069	0.0679503	0.1143431	-5.269885
RP11-151N1	0.2956052	0.6181933	1.8306709	0.0679531	0.1143431	-5.269919
TJP1	-0.034625	6.454212	-1.830576	0.0679673	0.1143617	-5.27009
FEV	0.2049289	-1.124259	1.8303802	0.0679966	0.1144024	-5.270443
RP11-526P5	-0.066934	-1.509244	-1.830373	0.0679977	0.1144024	-5.270457
AC241585.2	0.2784693	2.9307102	1.8302476	0.0680165	0.1144287	-5.270682
AF131216.1	-0.301439	1.135819	-1.830105	0.0680379	0.1144594	-5.270939
LINC01134	-0.149075	4.2845893	-1.829874	0.0680725	0.1145123	-5.271355
RP11-197N1	0.195205	3.1153848	1.8298346	0.0680784	0.114517	-5.271426
JMJD6	-0.037171	6.0941746	-1.829788	0.0680853	0.1145234	-5.271509
RP1-56K13.	0.2119834	-0.945125	1.8294401	0.0681376	0.114606	-5.272136
ABC14-108C	-0.14339	-1.309939	-1.829299	0.0681587	0.114633	-5.272391
AC092839.1	-0.249765	-0.791529	-1.829291	0.0681599	0.114633	-5.272405
KIAA0907	0.0348349	6.1389109	1.8292157	0.0681712	0.1146451	-5.27254
AKAP17BP	-0.206293	-1.055196	-1.829201	0.0681734	0.1146451	-5.272566
RP11-720L2	-0.109448	-1.432683	-1.829164	0.068179	0.1146493	-5.272634
IVNS1ABP	0.0329123	6.5094799	1.8290518	0.0681958	0.1146723	-5.272835
RSBN1	0.0360529	5.6665713	1.8287659	0.0682388	0.1147392	-5.27335
SLC16A14	0.149196	4.8764047	1.8286276	0.0682595	0.1147688	-5.273599
SLC7A2	-0.080683	6.6551021	-1.828318	0.0683061	0.1148418	-5.274156
NOP56P1	-0.461402	0.8361102	-1.828281	0.0683117	0.1148459	-5.274223
ZNF479	-0.132135	-1.396189	-1.828116	0.0683364	0.1148823	-5.27452
SLC35G5	0.2426623	-0.656697	1.8278912	0.0683703	0.1149338	-5.274924
ITPKB-IT1	0.1865043	-1.119896	1.8271653	0.0684796	0.1151123	-5.27623
KIN	0.0294438	5.854269	1.8269529	0.0685116	0.1151608	-5.276612
EMC2	-0.035786	6.2904714	-1.826907	0.0685185	0.115163	-5.276694
C2orf66	0.308031	-0.312204	1.8269021	0.0685192	0.115163	-5.276703
CTC-459F4.	0.0678624	4.7272958	1.8268623	0.0685252	0.1151678	-5.276775
PPP1R1A	0.2130161	6.0114369	1.8265143	0.0685777	0.1152507	-5.277401
NEK7	0.043559	6.2905506	1.8261209	0.0686371	0.1153451	-5.278108
SCG5	0.1331265	5.0604647	1.8258402	0.0686794	0.115403	-5.278612
SMG1P1	0.3334979	1.2780389	1.8258321	0.0686807	0.115403	-5.278627
RP11-74E24	0.1981666	-1.035654	1.8258301	0.068681	0.115403	-5.27863

SPAG11A	-0.107803	-1.45664	-1.825777	0.0686889	0.115411	-5.278725
CTD-2521M2	-0.128533	5.2878128	-1.825434	0.0687408	0.115493	-5.279343
CTC-277H1.	-0.300352	0.1578765	-1.825055	0.0687981	0.1155838	-5.280022
RP1-228P16	-0.098498	-1.437811	-1.825025	0.0688027	0.1155863	-5.280078
LITAF	-0.038687	6.758569	-1.824969	0.0688112	0.1155952	-5.280178
CTD-2024I7	0.2840623	-0.415911	1.8247439	0.0688452	0.1156421	-5.280582
RNU6-322P	0.3161213	0.4627591	1.8247422	0.0688454	0.1156421	-5.280585
RP11-269F2	0.1746026	-1.308667	1.8242236	0.0689239	0.1157686	-5.281516
CTD-2116N1	0.2417529	-0.647757	1.8241907	0.0689289	0.1157717	-5.281575
CNTLN	-0.058765	5.8061806	-1.824149	0.0689352	0.1157762	-5.28165
RP11-57C13	-0.194624	-1.068267	-1.824127	0.0689386	0.1157762	-5.281689
RP11-38107	-0.236381	-0.901648	-1.82411	0.0689411	0.1157762	-5.28172
SNRPGP10	0.2504657	2.106792	1.8238884	0.0689747	0.1158273	-5.282118
CYP51A1-AS	-0.277179	2.203603	-1.823725	0.0689995	0.1158635	-5.282411
RNU6-925P	0.3097153	0.2998287	1.8233201	0.0690609	0.1159613	-5.283138
RP4-740C4.	-0.147728	4.0136782	-1.822816	0.0691374	0.1160845	-5.284043
DOCK7	0.0390149	6.0137157	1.8226823	0.0691577	0.1161132	-5.284283
CTC-518B2.	-0.072977	-1.498387	-1.822213	0.0692291	0.1162277	-5.285125
RP11-797A1	0.2775881	-0.463686	1.8221784	0.0692343	0.1162305	-5.285187
FLJ33581	-0.400398	-0.053427	-1.822146	0.0692391	0.1162305	-5.285244
AJ003147.9	0.2125058	-1.115114	1.8221389	0.0692403	0.1162305	-5.285257
LSM12	0.0369855	5.4577373	1.8220832	0.0692488	0.1162393	-5.285357
FBXL18	0.0610166	5.1206656	1.8220391	0.0692555	0.1162452	-5.285436
SGOL1-AS1	-0.293043	0.0035663	-1.821866	0.0692818	0.1162841	-5.285747
BIRC2	-0.024149	6.3200573	-1.82135	0.0693603	0.1164105	-5.286672
SLC25A43	-0.049543	5.9449109	-1.82109	0.0693999	0.1164716	-5.287138
BNIP3P5	-0.312718	1.5207231	-1.820964	0.0694192	0.1164986	-5.287365
TBX5	0.2649696	-0.927587	1.8206996	0.0694594	0.1165607	-5.287838
PHTF1	0.0484138	5.5915339	1.8206056	0.0694737	0.1165794	-5.288006
UPF3AP3	0.3142978	1.0818372	1.8204618	0.0694957	0.1166109	-5.288264
RP5-915N17	-0.288037	-0.200484	-1.820051	0.0695584	0.1167107	-5.289001
RP11-893F2	0.1620577	-1.311179	1.8197987	0.0695968	0.1167699	-5.289452
LINC00359	-0.151926	-1.310593	-1.819767	0.0696017	0.1167728	-5.289509
SPAG9	-0.028781	6.3986508	-1.819587	0.0696291	0.1168133	-5.289831
BCL2L15	0.2535882	2.6906283	1.8193942	0.0696586	0.1168575	-5.290177
RNU6-383P	-0.211503	-1.081729	-1.819216	0.0696858	0.1168977	-5.290495
RP13-991F5	0.2735067	-0.441958	1.8187542	0.0697565	0.1170108	-5.291322
SGCG	0.2597774	-0.821039	1.8186449	0.0697732	0.1170335	-5.291518
SERINC1	-0.032238	6.7893266	-1.818618	0.0697773	0.117035	-5.291566
CTA-796E4.	-0.404838	-0.088693	-1.818508	0.0697941	0.1170579	-5.291763
HNRNPCP7	0.2801518	2.7070101	1.8184094	0.0698092	0.1170771	-5.29194
HIAT1	0.0333889	6.128368	1.8183913	0.069812	0.1170771	-5.291972
FAM197Y9	-0.147788	-1.368295	-1.818236	0.0698358	0.1171116	-5.29225
RN7SL208P	0.2524438	-0.747328	1.8182022	0.069841	0.1171117	-5.292311
RP11-149I2	0.3258283	0.2773729	1.8181938	0.0698422	0.1171117	-5.292326
DHX9	0.0214815	6.6387346	1.8181626	0.069847	0.1171143	-5.292382
DCAF8L2	-0.520965	0.4601483	-1.818014	0.0698697	0.1171421	-5.292647
LINC01077	0.124365	-1.401993	1.8180127	0.06987	0.1171421	-5.29265
COX7A1	0.0999536	4.9527208	1.8178597	0.0698934	0.117176	-5.292924
AC010872.1	-0.260176	-0.515622	-1.81783	0.069898	0.1171783	-5.292977
GUCA2B	-0.388812	3.5100636	-1.8176	0.0699332	0.1172318	-5.293388

TRIM36	0.232249	3.471572	1.8173411	0.0699729	0.1172931	-5.293852
RNU4-8P	-0.134737	-1.314552	-1.817096	0.0700105	0.1173508	-5.29429
LINC00538	0.3108349	0.1458315	1.8168407	0.0700497	0.117411	-5.294747
C8orf44	-0.064777	4.8572555	-1.816748	0.070064	0.1174295	-5.294913
RP11-94B19	-0.35408	-0.395606	-1.816545	0.0700951	0.1174763	-5.295275
ALPI	0.5202074	1.1337953	1.8164672	0.070107	0.1174909	-5.295414
RP11-403A2	0.3266894	0.290472	1.8163722	0.0701216	0.11751	-5.295584
CLC	0.2081342	-1.180465	1.8163374	0.070127	0.1175136	-5.295647
CXorf22	0.4364132	0.9836796	1.8162621	0.0701386	0.1175276	-5.295781
THUMPD1	-0.027833	6.1343514	-1.816233	0.0701431	0.1175298	-5.295834
DNMBP-AS1	-0.272607	3.8206099	-1.816116	0.070161	0.1175543	-5.296042
AC018685.1	-0.100969	-1.45642	-1.815823	0.070206	0.1176244	-5.296566
JARID2	0.0367212	5.829234	1.8157797	0.0702127	0.1176302	-5.296644
KDELC1P1	0.1509994	-1.264166	1.8156064	0.0702394	0.1176695	-5.296953
FAM167A-AS	-0.115896	-1.423597	-1.815474	0.0702598	0.1176983	-5.29719
LINC00907	0.3648133	1.3174592	1.8152884	0.0702883	0.1177407	-5.297522
WDR31	-0.087869	4.9744373	-1.814958	0.0703391	0.1178204	-5.298111
MAPK1IP1L	-0.021609	6.4501253	-1.814847	0.0703562	0.1178436	-5.29831
SERPINA5	-0.106408	6.9871822	-1.814656	0.0703857	0.1178875	-5.298651
RP1-34L19.	-0.267717	-0.64194	-1.814629	0.0703898	0.1178891	-5.298699
RP11-1137G	-0.188841	-1.211981	-1.814342	0.0704341	0.1179578	-5.299212
EXOSC8	-0.033747	5.8742019	-1.814312	0.0704388	0.1179602	-5.299267
OR11K1P	-0.067458	-1.509538	-1.814283	0.0704433	0.1179622	-5.299318
AC118344.1	0.2573624	-0.455962	1.8142621	0.0704464	0.1179622	-5.299355
RP11-380J1	0.2095044	-1.145355	1.8141946	0.0704568	0.1179743	-5.299476
PRKCE	0.063484	5.6858451	1.8140833	0.070474	0.1179976	-5.299674
RP11-51301	0.3159258	-0.093017	1.8139044	0.0705016	0.1180317	-5.299994
DUPD1	-0.099336	-1.450983	-1.813895	0.0705031	0.1180317	-5.300011
AC107622.1	-0.162258	-1.259992	-1.813888	0.0705041	0.1180317	-5.300022
AC112715.2	0.1695525	-1.257932	1.8136837	0.0705357	0.1180792	-5.300388
MRPL45P1	-0.373807	0.0080253	-1.813444	0.0705727	0.1181357	-5.300815
CTA-397H3.	-0.240329	-0.812199	-1.813274	0.070599	0.1181743	-5.30112
RP3-333A15	0.2149067	-1.077625	1.8131629	0.0706161	0.1181976	-5.301318
CTD-2161F6	-0.10014	-1.458179	-1.813087	0.0706279	0.1182119	-5.301454
CCDC122	0.0805427	4.6773496	1.8129617	0.0706472	0.1182388	-5.301677
AP000487.6	-0.268436	1.7656746	-1.812829	0.0706677	0.1182676	-5.301913
RNU6-418P	0.3024883	-0.079223	1.8125716	0.0707075	0.1183289	-5.302373
RPL5P17	-0.302693	0.2092205	-1.812439	0.070728	0.1183578	-5.302609
DLEU1-AS1	-0.17256	-1.275774	-1.81236	0.0707403	0.1183729	-5.302751
BCL2L2	0.0357294	5.9775776	1.8120136	0.0707939	0.1184571	-5.303368
RP3-347M6.	-0.109995	-1.405766	-1.811963	0.0708017	0.1184648	-5.303458
RP11-154D6	0.3326825	0.6482292	1.8119404	0.0708052	0.1184652	-5.303499
SIRPB3P	0.1384609	-1.317663	1.8118585	0.0708179	0.118481	-5.303645
RP11-231D2	0.1958131	-1.04643	1.8117852	0.0708293	0.1184946	-5.303776
HOMER2P1	-0.452156	-0.073803	-1.8117	0.0708424	0.1185018	-5.303927
RP11-536K7	0.2985371	-0.471471	1.8116824	0.0708452	0.1185018	-5.303959
SPAG16	-0.092271	5.5126006	-1.811678	0.0708459	0.1185018	-5.303968
XRCC6P2	0.247925	2.1231901	1.8116734	0.0708466	0.1185018	-5.303975
RP11-662I1	0.14958	-1.256673	1.811597	0.0708584	0.1185146	-5.304111
VPS37A	0.0433151	6.0838842	1.8115822	0.0708607	0.1185146	-5.304138
RPS10-NUDT	0.1839314	-1.148285	1.8113355	0.070899	0.1185731	-5.304578

LINC00685	-0.204628	3.0300188	-1.81129	0.070906	0.1185794	-5.304658
RP11-458D2	-0.1941	4.1144257	-1.811201	0.0709198	0.1185971	-5.304817
AC090945.1	0.3355983	0.2186116	1.8111284	0.0709311	0.1186105	-5.304947
CTD-2542C2	0.159824	-1.326676	1.8101271	0.0710865	0.118865	-5.306732
RP11-467L1	-0.330533	3.7154563	-1.809767	0.0711425	0.1189505	-5.307373
RP5-1057J7	-0.252989	-0.588042	-1.809756	0.0711442	0.1189505	-5.307393
AC004383.3	0.230815	-0.835601	1.8097343	0.0711476	0.1189507	-5.307432
LINC01438	-0.096292	-1.462276	-1.809691	0.0711543	0.1189513	-5.307509
FEN1	-0.044065	6.1556595	-1.80969	0.0711544	0.1189513	-5.30751
RP11-710F7	0.2714243	-0.757578	1.8090259	0.0712578	0.1191186	-5.308693
CDK8	0.0308678	5.7810314	1.8088901	0.0712789	0.1191485	-5.308935
RP11-344N1	-0.204503	-1.197909	-1.808808	0.0712916	0.1191643	-5.309081
RANP4	0.3198668	0.9007841	1.8084996	0.0713397	0.1192393	-5.309631
LYPLA1P2	-0.073031	-1.484172	-1.808321	0.0713675	0.1192803	-5.309948
PARD6G	0.0563455	5.3844601	1.8081142	0.0713998	0.1193287	-5.310317
TM4SF20	0.5714983	1.7208196	1.8078409	0.0714424	0.1193945	-5.310803
CSNK1G2-AS	0.2003779	-0.986405	1.8076657	0.0714698	0.1194348	-5.311115
HOXB-AS4	0.1783818	-1.308772	1.8075717	0.0714844	0.1194532	-5.311282
TCEB3B	0.2217566	-0.947543	1.8075529	0.0714874	0.1194532	-5.311316
RP11-12302	-0.079817	-1.482774	-1.807454	0.0715028	0.1194735	-5.311491
RNU6-1280F	0.1688897	-1.186751	1.8071821	0.0715453	0.1195377	-5.311976
RN7SL452P	-0.12035	-1.379954	-1.807166	0.0715478	0.1195377	-5.312004
RP11-211C9	0.2703838	-0.687898	1.8068614	0.0715954	0.1196108	-5.312546
ITPK1-AS1	-0.182586	-1.117131	-1.806844	0.071598	0.1196108	-5.312576
TERC	-0.149202	-1.288012	-1.80678	0.0716081	0.1196221	-5.312691
OSMR-AS1	0.3512611	1.5357292	1.8062392	0.0716927	0.1197579	-5.313653
USP32P2	0.2832865	-0.706844	1.8062048	0.0716981	0.1197614	-5.313714
TRBV7-7	0.1776839	-1.21983	1.8061564	0.0717056	0.1197686	-5.3138
RBM14-RBM4	0.0890285	4.0752603	1.8061051	0.0717137	0.1197765	-5.313891
LINC01558	-0.216524	4.8705686	-1.80556	0.0717989	0.1199135	-5.314859
CTC-458A3.	0.3885976	0.4364521	1.8053433	0.071833	0.1199648	-5.315245
RP4-753D1C	-0.087786	-1.474264	-1.805311	0.0718381	0.1199679	-5.315304
AC012485.2	0.3428006	1.1394038	1.8051432	0.0718643	0.1200062	-5.315601
ARFGEF1	0.0312414	6.3172682	1.8048498	0.0719103	0.1200775	-5.316122
LINC00028	0.1310617	-1.347323	1.8045148	0.0719629	0.1201598	-5.316718
GRB14	-0.069597	6.1245781	-1.804377	0.0719846	0.1201905	-5.316963
CTRC	0.3086524	-0.090027	1.804337	0.0719908	0.1201954	-5.317033
RP11-650K2	-0.328224	1.2532013	-1.80428	0.0719998	0.1202049	-5.317135
DISP2	0.1990199	4.4541876	1.8041242	0.0720242	0.1202402	-5.317411
LINC00303	0.1975822	-1.06871	1.8040807	0.0720311	0.1202461	-5.317489
DYNLL1-AS1	0.0557774	5.1873661	1.8039726	0.072048	0.120269	-5.317681
RP11-23D24	0.3969294	0.4328945	1.8039325	0.0720543	0.120274	-5.317752
PPP2R5B	-0.033302	5.8634041	-1.803849	0.0720675	0.1202905	-5.317901
RNU6-510P	0.2789713	-0.104137	1.8036384	0.0721006	0.1203401	-5.318274
GPR17	0.3577673	2.7314708	1.803399	0.0721382	0.1203974	-5.318699
RBMY1E	-0.246457	-1.080583	-1.803323	0.0721502	0.120412	-5.318835
PGBD1	0.085973	5.2297174	1.8031312	0.0721803	0.1204534	-5.319175
MCRS1	-0.024622	6.3483969	-1.803123	0.0721816	0.1204534	-5.31919
CCNB2	0.1063491	5.5097954	1.8030204	0.0721978	0.1204748	-5.319371
HSPA13	0.0389724	6.0101802	1.8029093	0.0722152	0.1204985	-5.319569
SKA3	0.1262145	5.0227703	1.8026294	0.0722593	0.1205665	-5.320065

RP11-668G1	0.2749897	-0.401096	1.802605	0.0722631	0.1205674	-5.320109
XIAP	-0.030861	6.3666745	-1.8024	0.0722954	0.1206157	-5.320472
RXRG	-0.430267	2.6849116	-1.802286	0.0723134	0.1206402	-5.320674
IL21	0.140076	-1.356514	1.8010881	0.0725024	0.1209494	-5.3228
LINC01338	-0.315339	-0.676084	-1.801069	0.0725054	0.1209494	-5.322833
ZMYM6NB	0.1047917	5.1945361	1.8010236	0.0725125	0.12095	-5.322914
MT-TV	-0.221719	-0.956005	-1.801008	0.0725151	0.12095	-5.322942
AC000120.7	0.3215995	0.2079683	1.8010037	0.0725157	0.12095	-5.322949
RP11-318C2	0.3049133	1.5447724	1.8007413	0.0725571	0.1210136	-5.323414
ARR3	0.2726863	1.8902398	1.8006488	0.0725718	0.1210325	-5.323578
EXO1	0.1239639	5.0949994	1.800457	0.0726021	0.1210775	-5.323918
HAR1B	0.3513149	0.0056275	1.8003134	0.0726248	0.1211098	-5.324173
RPS11P6	-0.213481	2.9668807	-1.800257	0.0726336	0.1211191	-5.324272
HTR1D	0.4838032	2.3407796	1.8002	0.0726427	0.1211287	-5.324374
FXYD1	-0.257215	5.1982695	-1.800175	0.0726466	0.1211297	-5.324418
RP11-777B9	-0.285318	-0.398081	-1.7998	0.072706	0.1212232	-5.325083
MCCD1P1	-0.199106	-1.052097	-1.799721	0.0727184	0.1212383	-5.325222
ARPP21-AS1	-0.114449	-1.415286	-1.799605	0.0727368	0.1212635	-5.325428
FKBP1A	-0.029707	6.7376435	-1.799489	0.0727552	0.1212886	-5.325634
ZNF566	0.0435191	5.3265403	1.799138	0.0728108	0.1213758	-5.326256
RP11-575F1	-0.30604	4.3414939	-1.799088	0.0728188	0.1213819	-5.326345
RP11-54G14	0.2825832	0.8289327	1.7990728	0.0728212	0.1213819	-5.326371
AC097468.7	-0.076059	-1.48587	-1.798992	0.0728339	0.1213977	-5.326514
EED	0.0283771	5.7969723	1.7983633	0.0729337	0.1215584	-5.327628
AK3P5	0.1941682	-1.057076	1.7980422	0.0729846	0.1216377	-5.328196
ATG4B	-0.026734	6.3455004	-1.797717	0.0730363	0.1217183	-5.328772
RP11-331D5	-0.083285	-1.472698	-1.797613	0.0730528	0.1217402	-5.328956
CENPA	0.1629026	4.7317714	1.7975702	0.0730596	0.121746	-5.329032
ATXN7	-0.033506	5.9197333	-1.797519	0.0730678	0.1217541	-5.329123
MED4-AS1	0.2665413	-0.145912	1.7973261	0.0730984	0.1217996	-5.329464
RP13-455A7	-0.155164	-1.263268	-1.797078	0.0731379	0.1218596	-5.329903
EEPD1	-0.044151	6.1303391	-1.797057	0.0731411	0.1218596	-5.329939
CSE1L	-0.026233	6.5266692	-1.796871	0.0731708	0.1219035	-5.330269
RP6-159A1.	-0.328272	0.5722315	-1.796683	0.0732007	0.1219478	-5.330602
RP11-384C1	-0.123819	-1.3815	-1.796619	0.073211	0.1219569	-5.330715
RBMXL1	-0.039738	5.7869232	-1.796605	0.0732132	0.1219569	-5.33074
PIGHP1	0.3160058	0.7146683	1.7965856	0.0732162	0.1219569	-5.330774
HNRNPA1P52	0.2182193	-0.822944	1.7965221	0.0732263	0.1219681	-5.330886
RP11-436D1	0.1929288	-1.01479	1.7961953	0.0732784	0.1220493	-5.331464
AC093642.6	-0.145814	-1.308742	-1.796023	0.0733058	0.1220894	-5.331768
PEX5L	0.324287	1.2575181	1.7959348	0.0733199	0.1221073	-5.331925
RP11-313E1	0.2059682	-1.05149	1.7958635	0.0733312	0.1221206	-5.332051
DNAJC11	0.0541126	5.9864616	1.7954888	0.073391	0.1222145	-5.332713
RP11-120J1	0.3147787	-0.056133	1.7950609	0.0734593	0.1223226	-5.33347
RAD1	-0.026072	5.9481237	-1.795038	0.0734629	0.1223231	-5.33351
AC108868.3	-0.098534	-1.459708	-1.794833	0.0734957	0.1223721	-5.333873
RP11-177A2	-0.19895	-1.021956	-1.794616	0.0735303	0.1224168	-5.334255
bP-21264C1	-0.355223	0.4067595	-1.794592	0.0735342	0.1224168	-5.334299
RP11-400K9	-0.214015	-0.93335	-1.794581	0.0735359	0.1224168	-5.334317
RP11-563N4	-0.263929	2.0111306	-1.794581	0.0735359	0.1224168	-5.334318
ATG2B	0.0386369	6.1578083	1.7945496	0.0735409	0.122419	-5.334373

KRBOX1	0.3324695	1.4590053	1.7945195	0.0735457	0.122419	-5.334426
AC132008.1	0.3221989	0.6591548	1.7945095	0.0735473	0.122419	-5.334444
FAM200A	-0.033774	5.4014595	-1.794462	0.0735549	0.122426	-5.334527
SNORA14A	-0.275507	-0.41495	-1.794351	0.0735727	0.1224501	-5.334725
SPRR3	0.3338261	-0.679766	1.7941833	0.0735995	0.122489	-5.33502
FAM193B	-0.034389	6.1627868	-1.794061	0.0736191	0.1225161	-5.335237
GPR19	0.2396687	3.207763	1.7938787	0.0736482	0.1225589	-5.335558
RP11-713M1	0.3165369	-0.103794	1.7935034	0.0737082	0.1226533	-5.336221
SLC26A7	0.3395213	2.3097811	1.7932632	0.0737467	0.1227117	-5.336645
AC008065.1	-0.242675	-0.720276	-1.793038	0.0737827	0.122766	-5.337042
GNG12	-0.05402	6.3286696	-1.792952	0.0737965	0.1227834	-5.337194
HMGB1P1	0.2698085	1.9178228	1.7928659	0.0738103	0.1228008	-5.337347
PRICKLE2-A	0.1587299	-1.240181	1.7927702	0.0738257	0.1228207	-5.337516
AF241725.6	-0.095939	-1.48209	-1.792744	0.0738299	0.1228222	-5.337562
AC034220.3	-0.102412	4.6731264	-1.792675	0.0738409	0.1228349	-5.337684
AC005077.1	-0.222006	4.6529563	-1.792496	0.0738695	0.1228769	-5.337999
LRIT2	-0.109249	-1.44239	-1.792468	0.073874	0.1228788	-5.338048
LHX5	0.192309	-1.185401	1.7922741	0.0739052	0.122925	-5.338391
RP11-329L6	-0.141435	4.1514825	-1.792207	0.073916	0.1229375	-5.33851
IRF2BP2	-0.028006	6.8267738	-1.792156	0.0739242	0.1229455	-5.3386
INTS2	0.0561693	5.547786	1.7920074	0.073948	0.1229794	-5.338862
RP3-473L9.	-0.347772	0.8239584	-1.791909	0.0739638	0.1230001	-5.339036
RP11-255M2	-0.07389	-1.495921	-1.79184	0.0739748	0.1230128	-5.339157
TTC6	-0.158467	4.7864801	-1.791755	0.0739886	0.12303	-5.339308
LINC01004	0.1110843	4.1184546	1.7916051	0.0740126	0.1230644	-5.339572
RP11-573D1	-0.31827	1.7559112	-1.791552	0.0740211	0.123073	-5.339665
C11orf30	0.0357317	5.6026102	1.7913015	0.0740614	0.1231343	-5.340107
GDF5	0.2838495	-0.57737	1.79085	0.0741339	0.1232493	-5.340903
AC009517.2	-0.11761	-1.389987	-1.79075	0.07415	0.1232704	-5.34108
RP11-85G21	-0.23867	-1.040089	-1.79038	0.0742096	0.1233637	-5.341732
AC005779.2	-0.233154	-0.756974	-1.790359	0.0742129	0.1233637	-5.341768
LINC01320	-0.499013	2.7642692	-1.790006	0.0742698	0.1234527	-5.342392
RP11-13P5.	0.1286216	-1.351669	1.7896618	0.0743252	0.1235392	-5.342998
RP11-24H1.	-0.171725	-1.171757	-1.789637	0.0743292	0.1235402	-5.343041
CYLD	-0.032326	6.1785277	-1.789443	0.0743606	0.1235867	-5.343384
PHYHIP	0.2251038	3.349508	1.7893603	0.0743738	0.1236031	-5.343529
ST13P5	0.2445108	2.6751796	1.7892668	0.0743889	0.1236225	-5.343694
RP11-1430C	-0.403727	0.6104057	-1.78911	0.0744142	0.1236589	-5.34397
RP11-778H2	-0.187352	-1.177868	-1.788973	0.0744363	0.12369	-5.344211
RP11-600K1	0.137611	-1.328652	1.7888682	0.0744532	0.1237125	-5.344396
RP11-703I1	0.2024546	-1.094112	1.7886877	0.0744824	0.1237553	-5.344714
RP11-58E21	-0.193638	-1.022782	-1.787831	0.0746208	0.1239797	-5.346223
MIR3186	0.1983404	-1.023987	1.7875805	0.0746613	0.1240413	-5.346663
RP11-368J2	0.2092181	3.053811	1.7873315	0.0747016	0.1241026	-5.347102
CH17-360D5	0.4111077	0.2317352	1.7870943	0.07474	0.1241607	-5.347519
MAP7D3	0.0981023	5.2984821	1.786898	0.0747718	0.1242079	-5.347864
SOSTDC1	0.4398301	1.8973256	1.7868749	0.0747755	0.1242085	-5.347905
TCP11L1	-0.042901	5.3744362	-1.786727	0.0747994	0.1242425	-5.348165
RP11-171I2	0.2930225	-0.15421	1.7864567	0.0748433	0.1243097	-5.348641
GS1-25M2.1	-0.21875	-0.948996	-1.786362	0.0748587	0.1243247	-5.348808
RPS6KA4	0.0263434	6.117208	1.7863589	0.0748591	0.1243247	-5.348813

NDST1	-0.034773	6.7150471	-1.786185	0.0748872	0.1243657	-5.349118
JMJD1C	0.0436404	6.2024834	1.7859123	0.0749315	0.1244337	-5.349598
RP11-507E2	0.2384901	2.3830126	1.7858221	0.0749462	0.12445	-5.349757
CLCN5	-0.050697	6.190139	-1.78581	0.0749482	0.12445	-5.349779
RP3-454B23	-0.25753	-0.657824	-1.785652	0.0749738	0.1244869	-5.350057
PTPN20A	0.4728673	1.65819	1.7855561	0.0749893	0.1245037	-5.350225
AL121657.4	-0.13152	-1.338633	-1.785547	0.0749908	0.1245037	-5.35024
GNAQP1	0.3144693	0.2275898	1.7853177	0.0750281	0.12456	-5.350644
HMGB2P1	0.3487888	0.4228563	1.7852315	0.0750421	0.1245775	-5.350795
AC010987.6	0.1467994	-1.360925	1.7851462	0.0750559	0.1245949	-5.350945
PHLDA3	0.1315706	5.6956696	1.7850839	0.075066	0.124606	-5.351055
TMEM106C	-0.04361	6.4709657	-1.784978	0.0750833	0.1246289	-5.351241
RP11-37B2.	0.0746601	4.970757	1.7849136	0.0750937	0.1246368	-5.351354
MARCKSL1P1	0.2356504	-0.814096	1.7849066	0.0750948	0.1246368	-5.351366
CRYBB3	0.2645897	2.806717	1.7848068	0.0751111	0.124658	-5.351542
SH3GLB2	-0.031239	6.3099356	-1.784786	0.0751144	0.124658	-5.351578
RP11-368M1	-0.18357	-1.246716	-1.784503	0.0751605	0.1247287	-5.352076
TENM1	0.4237061	3.8363673	1.7844091	0.0751757	0.1247484	-5.352241
SERPINE1	0.0857816	6.5051051	1.7842372	0.0752037	0.1247868	-5.352543
S100A8	0.1699936	4.5058004	1.784225	0.0752057	0.1247868	-5.352564
RP6-109B7.	0.3096655	1.1431839	1.784155	0.0752171	0.1248	-5.352687
RP11-638F5	0.2739567	-0.92693	1.7838269	0.0752705	0.1248829	-5.353263
FAM161B	0.0494085	5.0733332	1.7834348	0.0753344	0.1249832	-5.353952
AC006156.2	-0.169083	-1.307415	-1.783117	0.0753861	0.1250634	-5.35451
TIGD5	-0.052923	5.8295825	-1.782531	0.0754818	0.1252128	-5.35554
DNAJB1	-0.036731	6.6846054	-1.782519	0.0754837	0.1252128	-5.35556
VTRNA1-1	-0.163148	-1.249941	-1.782502	0.0754865	0.1252128	-5.355589
AP001171.1	-0.083984	-1.480589	-1.782389	0.0755049	0.1252377	-5.355788
ASS1P11	-0.376826	0.6861536	-1.782032	0.0755633	0.1253289	-5.356416
GRAPL	0.252561	-0.656494	1.7816702	0.0756224	0.1254211	-5.357049
RP4-781K5.	-0.414739	2.5816948	-1.781605	0.0756331	0.1254332	-5.357164
ZBTB45	0.030644	5.7253087	1.7815819	0.0756368	0.1254337	-5.357204
AC006042.8	0.2567001	-0.627615	1.7815465	0.0756426	0.1254376	-5.357267
TPM3P7	-0.122432	-1.357984	-1.781428	0.0756619	0.1254639	-5.357474
SALL3	0.1743091	-1.29951	1.7813263	0.0756786	0.1254859	-5.357653
RP11-383M4	-0.283705	0.4327875	-1.781136	0.0757097	0.1255318	-5.357986
IL31RA	0.3845141	0.3557588	1.7810684	0.0757208	0.1255445	-5.358105
AC093642.1	0.3604156	0.3216888	1.7810327	0.0757266	0.1255484	-5.358168
RP1-102K2.	0.1476116	-1.297534	1.7802106	0.0758613	0.1257659	-5.359609
RP11-706D8	-0.094649	-1.4451	-1.780167	0.0758684	0.125772	-5.359685
ASPM	0.0932786	5.8305143	1.7799749	0.0758999	0.1258186	-5.360022
LINC01289	-0.102175	-1.447416	-1.779584	0.075964	0.1259191	-5.360707
ST8SIA5	0.3367176	1.5763295	1.7795306	0.0759728	0.1259279	-5.360801
MIR378J	0.2542409	-0.529055	1.7793648	0.076	0.1259673	-5.361092
ENG	0.0514684	6.5239145	1.779237	0.076021	0.1259964	-5.361316
RP11-647P1	-0.066934	-1.509244	-1.778678	0.0761128	0.1261429	-5.362295
RYKP1	0.2103891	-0.838015	1.778605	0.0761248	0.126157	-5.362423
ZNF706	-0.036753	6.5063075	-1.778533	0.0761367	0.1261655	-5.362549
SF3B4	-0.029686	6.565894	-1.778532	0.0761369	0.1261655	-5.362551
VN1R90P	-0.080651	-1.488444	-1.778498	0.0761425	0.1261691	-5.362611
HNRNPCL1	-0.244466	-0.838122	-1.778392	0.0761599	0.1261922	-5.362796

NPC2	-0.037425	6.6725624	-1.778275	0.0761791	0.1262183	-5.363001
CTD-2215E1	0.2735897	-0.396577	1.7779921	0.0762256	0.1262896	-5.363496
RP11-332J1	-0.133572	-1.322207	-1.777835	0.0762514	0.1263266	-5.36377
FBXL20	-0.030793	5.919341	-1.777706	0.0762727	0.1263563	-5.363998
PCAT1	0.3129329	1.3247675	1.7774955	0.0763073	0.1264078	-5.364365
RP11-14C10	-0.093665	-1.451571	-1.77728	0.0763428	0.1264609	-5.364743
LINC00977	-0.17296	-1.261868	-1.777021	0.0763855	0.1265215	-5.365196
GLYATL1P2	-0.171505	-1.244091	-1.777016	0.0763863	0.1265215	-5.365205
FOXE3	0.2913722	-0.423824	1.7767706	0.0764268	0.1265788	-5.365634
CS	0.0267996	6.586951	1.7767639	0.0764279	0.1265788	-5.365646
SETP4	-0.136019	-1.313323	-1.776655	0.0764458	0.1265989	-5.365835
MMADHC	-0.027691	6.5179237	-1.776648	0.076447	0.1265989	-5.365848
CTGLF8P	0.33146	0.7851379	1.7766236	0.076451	0.1265999	-5.365891
NFU1	-0.030532	6.0425503	-1.776505	0.0764706	0.1266265	-5.366099
RP11-359P5	0.2999893	0.4318053	1.7764611	0.0764778	0.1266328	-5.366175
GAPDHP48	0.1239264	-1.373749	1.7764075	0.0764867	0.1266417	-5.366269
EEF1A1P42	-0.147557	-1.278148	-1.776358	0.0764949	0.1266496	-5.366357
RP11-154H2	0.2152622	-0.972759	1.7762623	0.0765106	0.1266699	-5.366523
RP1-249I4.	0.2653457	-0.346646	1.7760487	0.0765459	0.1267225	-5.366897
FLJ33360	0.3656947	1.0552742	1.775659	0.0766103	0.1268233	-5.367578
RP11-371I2	0.3171372	0.3381057	1.7756036	0.0766194	0.1268327	-5.367675
P3H2-AS1	0.1712893	-1.264426	1.7755804	0.0766232	0.1268333	-5.367716
CTD-2171N6	0.3863736	0.6310129	1.7754135	0.0766508	0.1268732	-5.368008
RNU5F-1	-0.218731	-0.903088	-1.775199	0.0766862	0.1269211	-5.368382
RNU6ATAC35	-0.285275	2.4905197	-1.775196	0.0766867	0.1269211	-5.368387
SPRR2B	-0.108254	-1.463779	-1.774829	0.0767474	0.1270158	-5.369029
RP11-687E1	0.272786	-0.298773	1.7747245	0.0767648	0.1270388	-5.369212
KB-1742H10	0.3669191	1.3730751	1.7744627	0.0768081	0.1271047	-5.36967
RP11-44F14	0.1763108	-1.22124	1.7744396	0.0768119	0.1271053	-5.36971
AC022816.2	-0.353813	3.4022727	-1.774368	0.0768237	0.127119	-5.369834
THRB-IT1	-0.327208	0.1995296	-1.774333	0.0768296	0.1271231	-5.369897
INGX	0.2861709	-0.42729	1.774285	0.0768375	0.1271304	-5.36998
RP11-619A1	-0.367482	1.8970516	-1.774202	0.0768514	0.1271475	-5.370126
CTD-2313J2	-0.138302	-1.328599	-1.774152	0.0768596	0.1271553	-5.370212
SNORD56	0.286904	0.2687789	1.7740716	0.0768729	0.1271716	-5.370353
FAM205A	-0.177808	-1.176507	-1.773948	0.0768933	0.1271996	-5.370568
IGKV2-4	0.1655276	-1.291052	1.7738503	0.0769096	0.1272207	-5.37074
AL162151.3	0.2454122	2.7026128	1.7731416	0.0770271	0.1274093	-5.371977
PLA2G15	-0.039713	5.996365	-1.773074	0.0770383	0.1274221	-5.372095
NBPF4	0.2570938	-0.982338	1.7730223	0.0770469	0.1274305	-5.372185
EYA1	0.4005799	0.4432609	1.7729334	0.0770616	0.1274492	-5.372341
AF127936.7	0.292711	1.5762813	1.7727928	0.077085	0.1274802	-5.372586
C6orf141	0.3945943	3.0599337	1.7727783	0.0770874	0.1274802	-5.372612
RPL7AP3	-0.162753	-1.206612	-1.772424	0.0771462	0.1275717	-5.37323
ASMT	0.3086779	-0.091394	1.7723531	0.077158	0.1275855	-5.373354
AC005255.3	0.1914908	2.9689908	1.7722138	0.0771812	0.127618	-5.373597
RP11-394A1	-0.299369	-0.661809	-1.771946	0.0772258	0.1276859	-5.374065
RP4-713B5.	0.3037645	1.0963558	1.7713605	0.0773231	0.1278304	-5.375086
RP13-39P12	0.3339626	1.4639711	1.771358	0.0773235	0.1278304	-5.37509
CNGA3	0.1568985	-1.338778	1.7713572	0.0773237	0.1278304	-5.375092
ERICD	0.1486461	3.7073982	1.7712348	0.0773441	0.1278538	-5.375305

RP4-635E18	0.29495	1.4050442	1.7712301	0.0773448	0.1278538	-5.375313
RP11-314P1	-0.110348	-1.436733	-1.771164	0.0773558	0.1278662	-5.375428
PARP4P2	0.3294134	0.5402516	1.7710292	0.0773783	0.1278975	-5.375664
C10orf62	-0.333846	0.916372	-1.770916	0.0773972	0.127923	-5.375861
RP1-200K18	0.3128263	-0.795675	1.770782	0.0774195	0.127954	-5.376095
AC092625.1	-0.361119	-0.389875	-1.770757	0.0774237	0.1279551	-5.376139
RP11-136L2	-0.185186	-1.112549	-1.770306	0.0774988	0.1280735	-5.376924
IGHD3-9	0.1326831	-1.365861	1.7702352	0.0775106	0.1280873	-5.377048
PSRC1	0.0863061	5.2390428	1.7701687	0.0775217	0.1280998	-5.377164
AC007099.1	0.4128863	0.5360657	1.7700863	0.0775355	0.1281168	-5.377308
RP11-407A1	0.1941139	-1.249281	1.7697106	0.0775982	0.1282146	-5.377963
RPS4XP18	-0.190579	-1.04726	-1.769454	0.0776411	0.1282757	-5.37841
AC006539.3	0.2740488	-0.503054	1.7694358	0.0776441	0.1282757	-5.378442
POPDC2	0.0920124	4.3277473	1.7694258	0.0776457	0.1282757	-5.378459
LINC00384	0.1244917	-1.356209	1.7691011	0.0777	0.1283595	-5.379025
TDRKH	-0.06624	5.713167	-1.768802	0.07775	0.1284364	-5.379546
EML5	0.4015096	1.0558792	1.7685981	0.0777841	0.1284868	-5.379901
RP11-403D1	-0.082239	-1.492748	-1.7685	0.0778005	0.1285081	-5.380072
RP13-395E1	-0.111143	-1.411889	-1.768473	0.077805	0.1285097	-5.380118
RP1-153P14	0.1554155	-1.238988	1.7683939	0.0778183	0.1285258	-5.380257
MBD2	-0.025103	6.3398832	-1.768227	0.0778461	0.1285645	-5.380547
B4GALT2	-0.034442	6.3405496	-1.768212	0.0778487	0.1285645	-5.380573
RP11-96D1.	0.1693703	-1.173708	1.7681856	0.0778531	0.128566	-5.38062
RP11-53I6.	0.2800871	-0.356869	1.7681523	0.0778587	0.1285694	-5.380678
GNG12-AS1	-0.245895	3.0844573	-1.767665	0.0779403	0.1286983	-5.381526
LINC01425	-0.128722	-1.402297	-1.767062	0.0780415	0.1288595	-5.382576
AC005307.3	0.3911259	-0.104254	1.7670057	0.0780509	0.1288692	-5.382673
RNF222	0.1942314	-1.003828	1.7666131	0.0781167	0.1289721	-5.383357
RUVBL1-AS1	-0.114992	-1.379633	-1.766303	0.0781688	0.12905	-5.383896
RP11-73702	0.3204813	0.8881076	1.7662901	0.078171	0.12905	-5.383919
SRY	-0.138528	-1.394201	-1.76626	0.078176	0.1290525	-5.383971
MRPS18AP1	0.2935728	-0.147474	1.7662363	0.07818	0.1290532	-5.384012
RP11-434E6	-0.451039	2.3808359	-1.765996	0.0782204	0.129114	-5.38443
MAF1	-0.037345	6.6364878	-1.765628	0.0782823	0.1292103	-5.38507
EMC10	-0.032869	6.7674536	-1.765587	0.0782891	0.1292157	-5.385141
AP006621.8	0.2764671	-0.080497	1.7651131	0.0783689	0.1293416	-5.385965
RP11-656D1	-0.223702	3.1657162	-1.764709	0.0784369	0.129448	-5.386667
MRPS31P5	0.337768	1.958635	1.7645257	0.0784678	0.1294873	-5.386986
ZNF362	-0.030489	5.9883771	-1.764526	0.0784678	0.1294873	-5.386987
ID2	-0.044727	6.7333289	-1.764244	0.0785152	0.1295595	-5.387475
RP11-321P1	0.1919774	-1.11227	1.7642239	0.0785186	0.1295595	-5.387511
TOMM20L	-0.247609	1.8462239	-1.763858	0.0785803	0.1296554	-5.388146
AC091878.1	0.2213042	-1.150266	1.7636857	0.0786094	0.1296976	-5.388446
LINC01479	-0.250987	-0.911723	-1.763584	0.0786266	0.129716	-5.388623
LGALS8	-0.037575	6.5079905	-1.763577	0.0786277	0.129716	-5.388634
FTH1P21	-0.191228	-1.029693	-1.763555	0.0786314	0.1297162	-5.388672
SLC22A10	-0.241783	5.4274799	-1.763512	0.0786387	0.1297171	-5.388747
LA16c-313F	0.2527135	1.9502088	1.76351	0.0786391	0.1297171	-5.388751
RNY1P9	0.1616355	-1.1915	1.7633193	0.0786713	0.1297644	-5.389082
IGHVII-40-	-0.173673	-1.229541	-1.76321	0.0786897	0.129789	-5.389272
RP11-380N8	-0.297648	0.4785301	-1.763136	0.0787022	0.1298027	-5.3894

RP11-170K4	0.2815175	-0.281744	1.7631073	0.0787071	0.1298027	-5.38945
RP11-611E1	-0.072945	4.5853486	-1.763097	0.0787087	0.1298027	-5.389467
KRT17P5	-0.201378	-1.139733	-1.762896	0.0787428	0.129853	-5.389817
C8orf37-AS	0.3461959	1.2770781	1.7627084	0.0787745	0.1298994	-5.390143
PGAM1	0.0442187	6.0947291	1.7625571	0.0788	0.1299357	-5.390406
RPS6P22	-0.147341	-1.249331	-1.762323	0.0788397	0.1299952	-5.390812
RP11-278A2	0.2385496	-0.771915	1.7619007	0.0789111	0.1301022	-5.391545
RP11-243M5	-0.141281	-1.303668	-1.761897	0.0789117	0.1301022	-5.391551
RP11-191L9	-0.444188	0.5154925	-1.761747	0.078937	0.1301381	-5.391811
RP11-589C2	0.2202647	-0.763073	1.7612919	0.0790142	0.1302594	-5.392601
TM4SF19-AS	-0.215822	2.8744155	-1.760956	0.0790711	0.1303474	-5.393184
SNORA12	0.2827239	-0.204527	1.7608312	0.0790923	0.1303763	-5.3934
KRTAP5-9	-0.318572	1.1880004	-1.76072	0.0791112	0.1304017	-5.393594
ASB5	0.2367926	-0.980312	1.7606672	0.0791201	0.1304064	-5.393685
GPX6	-0.081606	-1.480522	-1.760661	0.0791212	0.1304064	-5.393696
RP11-384E2	-0.062232	-1.506608	-1.760607	0.0791303	0.1304156	-5.393789
AC092107.1	-0.092032	-1.446134	-1.760523	0.0791446	0.1304331	-5.393935
GAPDHP71	0.3061551	0.088072	1.7602611	0.079189	0.1305004	-5.394389
ZMIZ2	-0.024166	6.4705683	-1.760037	0.079227	0.1305572	-5.394777
ASF1B	0.0929594	5.4828394	1.7600136	0.079231	0.1305579	-5.394818
UPK2	0.3407604	0.3472268	1.7597283	0.0792795	0.1306319	-5.395312
TACC3	0.0575634	6.0820913	1.7595932	0.0793024	0.1306638	-5.395546
CTD-2265M8	-0.293371	1.83318	-1.759534	0.0793125	0.1306744	-5.395649
PRKD1	0.143633	4.8217719	1.7592678	0.0793577	0.1307431	-5.39611
RP1-111C20	0.1963971	2.8286071	1.7592415	0.0793622	0.1307446	-5.396156
C9orf41-AS	0.2253286	-0.773617	1.75909	0.079388	0.1307811	-5.396418
ANKRA2	-0.043354	5.7830675	-1.759014	0.0794009	0.1307966	-5.39655
AL161752.1	-0.082878	-1.470245	-1.758856	0.0794279	0.1308297	-5.396825
CTDSP2	0.0235921	6.6984115	1.7588534	0.0794282	0.1308297	-5.396828
SOD1P3	-0.24064	-0.734046	-1.758764	0.0794434	0.1308489	-5.396983
RP11-434D9	0.3252051	-0.193762	1.7586522	0.0794625	0.1308743	-5.397177
KRT8P40	-0.164433	-1.237105	-1.758425	0.0795011	0.1309294	-5.39757
C19orf68	-0.033509	5.6304227	-1.758414	0.0795031	0.1309294	-5.39759
INADL	-0.045118	6.2149538	-1.758328	0.0795177	0.1309476	-5.397739
RP11-461G1	-0.316504	0.0802352	-1.758295	0.0795233	0.1309509	-5.397795
SMAD3	-0.034417	6.3746045	-1.758065	0.0795625	0.131004	-5.398194
ZNF709	0.2141627	2.3118858	1.7580635	0.0795627	0.131004	-5.398196
PCDHGA1	0.3246561	2.9984293	1.7578851	0.0795931	0.1310481	-5.398505
TLN2	-0.069109	5.8364983	-1.757699	0.0796249	0.1310945	-5.398828
KIFC3	0.0361791	6.4121507	1.7576562	0.0796322	0.1311006	-5.398902
CTD-2026J2	-0.146708	-1.286155	-1.757559	0.0796487	0.1311219	-5.39907
PES1P1	-0.425568	0.2202058	-1.757536	0.0796527	0.1311226	-5.39911
RP11-164J1	0.2085696	4.0783083	1.7574525	0.0796669	0.13114	-5.399254
RNASET2	0.0486406	6.2326022	1.7572713	0.0796978	0.131185	-5.399568
AC124312.1	0.1459792	-1.282138	1.7571949	0.0797109	0.1312006	-5.3997
C11orf49	0.0651637	5.5987714	1.7571286	0.0797222	0.1312132	-5.399815
SEC63	-0.02649	6.6415854	-1.757058	0.0797342	0.1312272	-5.399937
RP11-452D2	-0.103842	-1.425605	-1.756976	0.0797483	0.1312444	-5.40008
AC105760.2	-0.156732	3.1736781	-1.756892	0.0797626	0.131262	-5.400225
AQP6	-0.467237	1.895301	-1.756767	0.079784	0.1312913	-5.400441
ASIP	-0.283666	2.5889019	-1.756693	0.0797966	0.1313061	-5.400569

RPL5P34	-0.128955	3.6274027	-1.755795	0.0799501	0.1315525	-5.402122
SUMO1P3	0.2118193	2.5494316	1.7557748	0.0799535	0.1315525	-5.402157
RPL21P33	-0.094853	-1.443948	-1.755753	0.0799573	0.1315527	-5.402195
AC078899.4	-0.104811	-1.422882	-1.75561	0.0799817	0.131587	-5.402442
AC009120.8	0.1862613	-1.034244	1.7555426	0.0799933	0.1316001	-5.402559
RP11-252I1	0.1540807	-1.220181	1.7553297	0.0800297	0.1316541	-5.402927
TMEM168	0.0366832	5.929172	1.7550275	0.0800815	0.1317298	-5.403449
RP11-243J1	0.1980755	-0.992812	1.7550188	0.080083	0.1317298	-5.403464
RP11-242D8	-0.264912	-0.335568	-1.754747	0.0801296	0.1318006	-5.403934
SPATA20P1	0.123823	-1.385073	1.75451	0.0801701	0.1318614	-5.404343
POMZP3	-0.057933	5.0159088	-1.754305	0.0802053	0.1319132	-5.404698
LUC7L2	-0.208984	-0.914027	-1.75421	0.0802216	0.1319342	-5.404862
PLCG1-AS1	0.2711636	1.3430536	1.7538833	0.0802777	0.1320204	-5.405426
RP11-536C5	0.2295674	2.1387319	1.7537595	0.0802989	0.1320494	-5.40564
MMP1	0.3398376	3.4526839	1.7535205	0.08034	0.1321109	-5.406053
CTD-2530H1	-0.091172	-1.452193	-1.753449	0.0803523	0.1321252	-5.406177
RN7SL2	-0.060572	5.9247887	-1.75339	0.0803624	0.132136	-5.406279
FAM126A	0.0513646	5.7074337	1.7533632	0.080367	0.1321375	-5.406325
RP13-436F1	-0.117954	-1.440436	-1.753243	0.0803876	0.1321654	-5.406532
ZNF852	0.0711777	4.4384587	1.7531501	0.0804036	0.1321855	-5.406693
RNU2-51P	0.2330281	-0.772272	1.7531295	0.0804071	0.1321855	-5.406728
TBX15	0.1825834	5.3361865	1.7531088	0.0804107	0.1321855	-5.406764
TYSND1	-0.042459	6.136986	-1.75301	0.0804277	0.1322075	-5.406934
RP11-303E1	-0.291231	1.3388152	-1.752887	0.0804489	0.1322364	-5.407147
RP11-76C10	-0.369572	-0.556608	-1.752853	0.0804546	0.1322399	-5.407205
RP11-73G16	0.1175371	-1.391576	1.7525228	0.0805115	0.1323274	-5.407776
RP4-758J24	0.1508565	2.8885999	1.7523758	0.0805368	0.132363	-5.40803
SPTBN4	-0.157503	4.2711295	-1.751825	0.0806315	0.1325128	-5.408979
RP11-96801	0.2937127	2.9973743	1.7518008	0.0806358	0.1325132	-5.409022
SLC10A6	0.3499754	1.4559325	1.7517818	0.080639	0.1325132	-5.409055
AP000936.1	-0.277755	1.6048637	-1.751473	0.0806922	0.1325946	-5.409587
LINC00678	-0.118157	-1.404236	-1.751338	0.0807156	0.132627	-5.409821
MUSTN1	-0.348119	1.390659	-1.751199	0.0807394	0.1326595	-5.41006
7SK	-0.154766	-1.223534	-1.751181	0.0807426	0.1326595	-5.410092
MYEOV	0.4024042	2.6492684	1.7511078	0.0807552	0.1326743	-5.410217
CTD-2666L2	0.3084712	0.5028806	1.7509789	0.0807775	0.1327048	-5.41044
FAM43A	-0.084519	5.4881665	-1.750716	0.0808229	0.1327734	-5.410894
SLC22A24	-0.329004	0.3995573	-1.750549	0.0808516	0.1328097	-5.41118
EFCAB1	-0.385433	1.67768	-1.750546	0.0808522	0.1328097	-5.411187
HMGN2P46	0.2933959	2.1307818	1.7504999	0.0808601	0.1328167	-5.411266
FLNB-AS1	0.1947483	3.7761475	1.7502781	0.0808984	0.1328737	-5.411648
CTD-2081C1	0.1918302	3.0173589	1.7496693	0.0810036	0.1330365	-5.412697
FOXO3	0.0357432	6.2328671	1.7496624	0.0810049	0.1330365	-5.412709
RP11-244B2	-0.093608	-1.435751	-1.749499	0.081033	0.1330768	-5.41299
RP11-428L2	-0.159141	-1.223522	-1.749352	0.0810585	0.1331107	-5.413244
OR7E43P	-0.10325	-1.432976	-1.749338	0.081061	0.1331107	-5.413269
NPM1P35	0.2014906	-0.926418	1.7492657	0.0810735	0.1331252	-5.413393
RP11-744H1	-0.103028	-1.414085	-1.74894	0.0811299	0.1332118	-5.413955
RNA5-8SP2	-0.080687	-1.497507	-1.748765	0.0811601	0.1332555	-5.414256
HS3ST3B1	-0.103295	5.9966046	-1.748679	0.081175	0.1332685	-5.414403
RP11-114H2	0.3390537	-0.153406	1.7486773	0.0811753	0.1332685	-5.414407

RP11-113E2	0.1636354	-1.27958	1.7482026	0.0812576	0.1333975	-5.415224
RP11-556E1	0.4641206	3.280451	1.7480473	0.0812845	0.1334357	-5.415492
NFATC3	-0.04139	5.8600313	-1.747889	0.081312	0.1334749	-5.415765
LINC00487	0.3380256	0.144409	1.7478132	0.0813251	0.1334904	-5.415895
RP11-182L2	-0.103957	4.0283838	-1.747669	0.0813501	0.1335253	-5.416143
RP4-710M16	0.2236536	-0.839684	1.7473059	0.0814132	0.1336229	-5.416768
RP11-631M2	0.1921256	-1.010797	1.7470548	0.0814568	0.1336884	-5.4172
TRIM3	-0.035798	5.6557478	-1.746839	0.0814943	0.133744	-5.417572
SOX2-OT	-0.329055	2.1613277	-1.74673	0.0815132	0.1337691	-5.417759
RPS17P1	-0.168552	-1.228065	-1.746514	0.0815507	0.1338245	-5.41813
MYOM1	0.1108104	6.114463	1.7464292	0.0815655	0.1338376	-5.418277
RP11-444D1	-0.09367	-1.468796	-1.746426	0.081566	0.1338376	-5.418282
MTND5P1	-0.399851	1.7986781	-1.746177	0.0816094	0.1339027	-5.418711
AC084219.4	0.3312033	0.9965489	1.7461562	0.081613	0.1339027	-5.418746
LINC00404	-0.13677	-1.384905	-1.74587	0.0816628	0.1339784	-5.419239
RPL24P8	-0.20264	3.036911	-1.745773	0.0816797	0.134	-5.419405
RP11-787D1	-0.102674	-1.431899	-1.745635	0.0817036	0.1340333	-5.419642
LCE5A	-0.096658	-1.436503	-1.745517	0.0817242	0.1340611	-5.419845
RP11-306B9	-0.189042	-1.119521	-1.745407	0.0817434	0.1340865	-5.420035
KLF10	0.0502041	6.3190427	1.7452656	0.081768	0.1341209	-5.420278
UBE2C	0.1072171	5.6434117	1.7452289	0.0817744	0.1341253	-5.420341
LINC01186	-0.365378	2.5233297	-1.745082	0.0818	0.1341613	-5.420593
HCG4	0.391175	2.2864252	1.7448047	0.0818484	0.1342346	-5.42107
ZRSR1	0.2450197	-0.44151	1.7444818	0.0819047	0.1343209	-5.421625
RP11-871F6	-0.075828	-1.482762	-1.744032	0.0819832	0.1344436	-5.422398
C15orf59-A	0.160307	-1.291154	1.7438615	0.082013	0.1344864	-5.422691
IFNL1	0.1816349	-1.149404	1.7436448	0.0820509	0.1345396	-5.423063
TULP3	0.0370726	5.8059115	1.7436338	0.0820528	0.1345396	-5.423082
STARD9	0.0682838	5.0813235	1.7435903	0.0820604	0.134546	-5.423157
AC024619.2	-0.097817	-1.43811	-1.743082	0.0821493	0.1346857	-5.42403
RPL29P12	-0.264552	1.3185105	-1.743031	0.0821581	0.1346941	-5.424117
LINC01560	0.0779923	4.2121721	1.7428693	0.0821864	0.1347345	-5.424395
LINC00607	0.2518534	3.5482221	1.7428145	0.082196	0.1347442	-5.424489
C2orf74	-0.069458	4.9562782	-1.742788	0.0822006	0.1347457	-5.424534
RP11-1348G	-0.114643	3.7389023	-1.742563	0.08224	0.1348023	-5.42492
LRRC37A11P	0.3234147	-0.044785	1.7425486	0.0822426	0.1348023	-5.424945
CTC-453G23	-0.131579	-1.310176	-1.742496	0.0822518	0.1348114	-5.425036
SMARCA4	0.0253991	6.562614	1.7424627	0.0822576	0.1348149	-5.425093
LOC124685	-0.198723	-0.963712	-1.74238	0.0822722	0.1348281	-5.425235
bP-218909.	-0.342581	0.0948814	-1.742361	0.0822753	0.1348281	-5.425266
RP11-447H1	0.2002939	-0.948665	1.7423533	0.0822768	0.1348281	-5.42528
RP11-400D2	-0.405623	0.1311537	-1.742279	0.0822898	0.1348434	-5.425408
RP11-162D9	-0.196001	-1.304867	-1.741899	0.0823563	0.1349463	-5.42606
RP11-190C2	-0.228686	-0.715881	-1.741844	0.082366	0.1349525	-5.426154
RP11-781A6	-0.130741	-1.371721	-1.741836	0.0823674	0.1349525	-5.426169
RP11-267D1	-0.293649	-0.222331	-1.741751	0.0823823	0.1349708	-5.426314
SIMC1	-0.063478	5.7102426	-1.741566	0.0824148	0.135018	-5.426632
RP11-2A4.4	-0.140183	-1.41507	-1.741485	0.082429	0.1350351	-5.426771
AL021546.6	-0.13216	-1.316894	-1.741141	0.0824892	0.1351248	-5.42736
RP11-89C3.	0.1821509	-1.114048	1.7411162	0.0824937	0.1351248	-5.427403
RP11-474L2	0.2012102	-0.936298	1.7411094	0.0824948	0.1351248	-5.427415

RP13-16H11	0.2826515	0.1354283	1.7410081	0.0825126	0.1351479	-5.427588
RP11-640A1	-0.082589	-1.466829	-1.740954	0.0825221	0.1351573	-5.427681
C1QTNF9B-A	-0.287902	1.7344725	-1.740696	0.0825674	0.1352254	-5.428123
RP5-875013	-0.391361	0.7315786	-1.74066	0.0825737	0.1352297	-5.428185
MYO15A	0.1969689	3.6948238	1.7405712	0.0825893	0.1352493	-5.428338
SMAD9-IT1	0.1628675	-1.192352	1.7403746	0.0826239	0.1352998	-5.428675
RBM39	-0.017924	6.7490526	-1.740126	0.0826676	0.1353653	-5.429101
RP11-107I1	0.1800112	-1.236474	1.739998	0.0826901	0.1353914	-5.42932
RP11-339D2	-0.112509	-1.421641	-1.739993	0.082691	0.1353914	-5.429329
AP001505.1	-0.113093	4.481591	-1.739692	0.0827439	0.135472	-5.429845
RNU6-5P	0.1592314	-1.165792	1.7394548	0.0827856	0.1355342	-5.430251
JAK1	-0.027614	6.6851618	-1.739102	0.0828477	0.1356297	-5.430855
FICD	-0.047007	5.6551695	-1.738553	0.0829444	0.135782	-5.431795
CTD-2058B2	-0.109282	-1.40863	-1.738326	0.0829845	0.1358416	-5.432185
ARC	0.3054758	2.1443882	1.7382853	0.0829917	0.1358472	-5.432255
SULT1D1P	-0.130958	-1.357997	-1.737989	0.083044	0.1359268	-5.432763
RP11-546J1	0.2506205	2.5679811	1.7378327	0.0830715	0.1359657	-5.43303
RP11-299J3	-0.171086	-1.109694	-1.737739	0.0830881	0.1359867	-5.43319
RUVBL1	-0.028134	6.2107635	-1.737497	0.0831308	0.1360505	-5.433604
CCNB1	0.0756687	5.9221217	1.7374754	0.0831346	0.1360507	-5.433641
RP1-65J11.	-0.3669	1.1839832	-1.737393	0.0831491	0.1360683	-5.433781
RNU6-1055P	-0.078307	-1.492918	-1.737351	0.0831566	0.1360696	-5.433854
PUM1	-0.022635	6.4024418	-1.737347	0.0831574	0.1360696	-5.433862
AC007381.1	-0.103066	-1.425851	-1.737292	0.083167	0.1360792	-5.433955
FTX	0.123016	3.9830315	1.7372712	0.0831707	0.1360792	-5.433991
RP11-309L2	-0.102875	-1.422765	-1.737201	0.0831831	0.1360933	-5.43411
IL18R1	0.1350058	4.974582	1.7371022	0.0832006	0.1361159	-5.43428
CTD-237103	-0.110159	4.2257329	-1.737043	0.0832111	0.136127	-5.434382
CTD-3138B1	0.2906832	1.6644354	1.736968	0.0832243	0.1361424	-5.43451
CCDC177	-0.466583	1.9175965	-1.736947	0.083228	0.1361424	-5.434545
SLC29A3	-0.042648	5.775932	-1.736602	0.0832891	0.1362362	-5.435136
UGT1A6	-0.2051	5.8148144	-1.736507	0.0833057	0.1362574	-5.435298
RP11-113I2	-0.222646	-1.042863	-1.736171	0.0833653	0.1363488	-5.435874
ACTA2	0.052883	6.5683062	1.7359605	0.0834025	0.1364035	-5.436233
BAIAP2-AS1	-0.046741	6.0291123	-1.735917	0.0834102	0.1364099	-5.436307
RP11-3P17.	-0.102184	4.5151636	-1.735854	0.0834214	0.1364221	-5.436415
CTD-2135J3	0.2719979	1.2347395	1.7356657	0.0834547	0.1364705	-5.436737
HSPA8P1	0.2840594	2.7876895	1.7355252	0.0834796	0.1365051	-5.436977
AC009955.8	0.1111358	-1.409409	1.7354903	0.0834858	0.1365091	-5.437037
BAZ2B	0.0438161	5.8348401	1.7354444	0.0834939	0.1365163	-5.437115
GUCA1B	0.2119705	3.2425643	1.7354064	0.0835007	0.1365205	-5.437181
HINFP	-0.023887	5.782636	-1.735388	0.083504	0.1365205	-5.437212
CRK	-0.029149	6.4497956	-1.735359	0.0835091	0.1365228	-5.437262
TFAP2A-AS1	-0.408159	1.8976606	-1.735291	0.0835211	0.1365362	-5.437377
AP000436.4	-0.18003	-1.076771	-1.735208	0.0835358	0.1365542	-5.43752
LNPEP	-0.034531	6.106849	-1.735093	0.0835562	0.1365813	-5.437715
RPL4P3	0.2878924	0.7451812	1.7350662	0.083561	0.1365831	-5.437762
PALLD	0.0695564	6.3341989	1.7344884	0.0836635	0.1367446	-5.438749
LINC01243	-0.103793	-1.419035	-1.73443	0.0836739	0.1367538	-5.438849
PHKA1	0.1645886	4.7693062	1.7344142	0.0836767	0.1367538	-5.438876
BCL2L12	-0.043018	5.9787721	-1.73404	0.0837431	0.1368562	-5.439515

RP11-20B24	-0.077228	-1.50077	-1.733867	0.083774	0.1369006	-5.439812
LINC01019	-0.465352	0.0350808	-1.733766	0.0837919	0.1369237	-5.439984
MMP13	0.315011	-0.647227	1.733504	0.0838384	0.1369936	-5.440431
RP1-142L7.	0.3417833	0.4336313	1.7334274	0.083852	0.1370098	-5.440562
MBL2	-0.20427	6.1325569	-1.733317	0.0838716	0.1370356	-5.44075
CTC-448F2.	-0.132345	-1.435335	-1.733275	0.0838791	0.1370417	-5.440822
RP11-77P6.	-0.104627	3.9564888	-1.733019	0.0839247	0.1371101	-5.44126
LA16c-360A	0.2409703	-0.68063	1.7326454	0.0839912	0.1372126	-5.441897
DYSF	-0.05313	6.3056967	-1.73224	0.0840635	0.1373245	-5.442589
RP1-20N2.7	-0.122817	-1.351286	-1.732139	0.0840815	0.1373478	-5.442762
CH507-154E	0.4390262	1.3227637	1.7317758	0.0841462	0.1374473	-5.443381
TBC1D5	-0.025199	6.2781315	-1.731464	0.0842019	0.1375321	-5.443914
CTD-2139B1	0.154416	-1.362443	1.7313196	0.0842276	0.137568	-5.444159
KRT18P16	0.2869275	0.1942022	1.7312674	0.0842369	0.1375747	-5.444248
PHF20L1	-0.030537	6.1792309	-1.731254	0.0842392	0.1375747	-5.44427
RP5-963E22	-0.188354	-1.027195	-1.731125	0.0842624	0.1376064	-5.444492
HS2ST1	-0.026385	6.2225546	-1.730928	0.0842974	0.1376574	-5.444826
COX8C	-0.248773	-0.908842	-1.730875	0.0843071	0.1376627	-5.444918
LINC01300	0.136737	-1.323831	1.730868	0.0843082	0.1376627	-5.44493
PVRL3	-0.10335	5.7615506	-1.730569	0.0843616	0.1377437	-5.445439
RP11-552F3	0.2816886	0.0001641	1.7305034	0.0843734	0.1377528	-5.445551
HTR2A	-0.338772	0.8481361	-1.730496	0.0843748	0.1377528	-5.445564
AC009506.1	-0.07378	4.5147619	-1.730412	0.0843897	0.137771	-5.445707
RNY1P10	-0.06124	-1.506051	-1.729982	0.0844666	0.1378904	-5.44644
RP11-598D1	0.1668703	-1.27859	1.7299522	0.0844719	0.137893	-5.446491
RNU6-1061F	0.1744979	-1.149053	1.7297048	0.0845162	0.1379592	-5.446912
RP11-837J7	-0.301825	1.7176356	-1.729633	0.084529	0.1379738	-5.447034
C11orf42	0.3184761	0.360161	1.7293389	0.0845817	0.1380537	-5.447536
DUSP9	0.2015243	5.354605	1.729247	0.0845982	0.1380744	-5.447693
H1FNT	-0.313491	-0.561049	-1.728939	0.0846534	0.1381584	-5.448218
NLGN4Y	-0.537055	2.7547958	-1.728892	0.0846618	0.1381659	-5.448297
RNU6-570P	0.1890152	-1.051031	1.7287931	0.0846795	0.1381839	-5.448466
ARL2	0.0521582	5.963685	1.7287881	0.0846804	0.1381839	-5.448474
TXNDC17	-0.045267	6.3214398	-1.728656	0.084704	0.1382163	-5.448698
FAM210A	-0.028427	5.9066856	-1.728451	0.0847409	0.1382703	-5.449048
ADSL	-0.028677	6.3778437	-1.728371	0.0847552	0.1382875	-5.449184
RP11-114H2	-0.251124	-0.584563	-1.728293	0.0847692	0.1383041	-5.449317
STAG3L1	0.2714871	2.1101192	1.7282058	0.0847849	0.1383234	-5.449466
MTRNR2L2	-0.208145	-0.941926	-1.728023	0.0848176	0.1383707	-5.449776
GLIPR1L1	-0.324776	1.3799487	-1.727874	0.0848444	0.1384064	-5.45003
RP11-524H1	0.1755308	-1.271254	1.7278593	0.0848471	0.1384064	-5.450056
MOB4	0.0237749	5.9248575	1.7278136	0.0848553	0.1384136	-5.450133
TMEM40	-0.401172	1.9630396	-1.727644	0.0848858	0.1384552	-5.450423
RP11-429A2	-0.065474	-1.508425	-1.727629	0.0848884	0.1384552	-5.450447
FADS2	-0.093895	6.6517291	-1.727559	0.084901	0.1384695	-5.450566
RAB3GAP1	0.0203425	6.2521626	1.7274972	0.0849121	0.1384815	-5.450672
AC006000.5	0.1783907	-1.2265	1.7274728	0.0849165	0.1384825	-5.450714
RP4-814D15	0.2805675	0.4918341	1.7269617	0.0850084	0.1386261	-5.451583
MIR4728	-0.145166	-1.284242	-1.72646	0.0850986	0.1387671	-5.452437
RP11-746B8	-0.080196	-1.476754	-1.726363	0.0851162	0.1387895	-5.452603
RP4-584D14	0.2950415	0.3551303	1.7263272	0.0851225	0.1387937	-5.452663

CTB-1048E9	-0.156938	-1.201572	-1.725781	0.085221	0.1389468	-5.453592
LIPI	0.1499387	-1.270036	1.7257636	0.0852241	0.1389468	-5.453621
RP11-266J6	-0.26751	-0.125666	-1.725693	0.0852367	0.1389612	-5.453741
RP11-498C9	0.3497238	1.5196671	1.7254609	0.0852786	0.1390223	-5.454136
LINC01183	0.3258787	1.5456291	1.7254434	0.0852818	0.1390223	-5.454166
CTC-436P18	0.2729741	2.0026024	1.725346	0.0852993	0.1390447	-5.454331
RHOA-IT1	0.283758	-0.063741	1.7252349	0.0853194	0.1390712	-5.45452
RP11-90L1.	-0.282526	3.2691536	-1.725062	0.0853507	0.1391081	-5.454815
CDA	-0.133851	5.6106608	-1.725047	0.0853533	0.1391081	-5.45484
NAF1	0.0421047	5.3112999	1.7250458	0.0853535	0.1391081	-5.454841
RP11-14J7.	-0.157729	-1.301788	-1.725023	0.0853575	0.1391085	-5.454879
FAM157A	0.25793	-0.900952	1.7249084	0.0853783	0.1391361	-5.455075
RP11-411B1	-0.387446	0.78417	-1.72472	0.0854123	0.1391854	-5.455395
FAM212B	0.0611868	5.3030102	1.7246149	0.0854313	0.13921	-5.455574
CTC-450M9.	0.1791882	-1.080836	1.7244776	0.0854561	0.1392442	-5.455807
RP11-192H2	0.1912002	2.9205755	1.7243104	0.0854863	0.1392872	-5.456091
HNRNPA3	-0.017732	6.8314919	-1.723973	0.0855473	0.1393804	-5.456665
RP11-175B9	-0.103752	4.8864549	-1.723821	0.0855747	0.1394189	-5.456922
FAM46A	-0.054957	6.0496105	-1.723766	0.0855846	0.1394255	-5.457015
RP4-622L5.	0.3061527	0.9064372	1.7237563	0.0855864	0.1394255	-5.457032
LL22NC03-3	-0.081016	-1.478925	-1.723675	0.0856012	0.1394433	-5.457171
RP11-399K2	-0.168087	3.3113306	-1.723583	0.0856177	0.139464	-5.457326
LINC01213	0.1481103	-1.285987	1.7233989	0.0856511	0.1395121	-5.457639
RP11-43N16	0.1162162	4.1357834	1.7233743	0.0856555	0.1395132	-5.457681
RP11-453E1	0.1735316	2.7707249	1.7232211	0.0856832	0.1395521	-5.457941
CTNS	-0.032206	5.9844895	-1.723182	0.0856903	0.1395574	-5.458007
KCNC2	0.4742763	0.4565327	1.7229659	0.0857294	0.1396149	-5.458374
CYP2G2P	-0.240106	-0.929659	-1.722895	0.0857422	0.1396279	-5.458494
NELFA	0.0259927	6.0747489	1.7228795	0.0857451	0.1396279	-5.458521
ZNF729	-0.307356	-0.734952	-1.722838	0.0857525	0.1396338	-5.458591
CTB-160022	-0.104078	-1.42087	-1.722132	0.0858805	0.1398337	-5.459789
TAOK1	0.0269893	6.2733297	1.7221186	0.085883	0.1398337	-5.459812
AB019440.5	-0.089345	-1.476095	-1.722042	0.0858968	0.13985	-5.459942
AIDA	0.0379609	5.9198694	1.7216588	0.0859664	0.1399505	-5.460592
PACSIN3	-0.068885	6.1670536	-1.721637	0.0859703	0.1399505	-5.460629
RIMBP3C	0.2375569	-0.797821	1.7216213	0.0859732	0.1399505	-5.460656
DPPA2P4	-0.153354	-1.294843	-1.721618	0.0859738	0.1399505	-5.460662
AP003025.2	-0.078564	-1.473029	-1.721586	0.0859797	0.1399537	-5.460717
FTOP1	-0.176084	4.3413869	-1.721311	0.0860296	0.1400287	-5.461183
AC006116.1	0.1815563	-1.079695	1.7212172	0.0860466	0.1400501	-5.461341
CAHM	-0.176424	3.2288293	-1.721151	0.0860586	0.1400634	-5.461453
RP11-20G13	0.2573976	-0.746465	1.7210451	0.0860778	0.1400885	-5.461633
FKBP11	-0.052865	6.4074721	-1.720724	0.0861362	0.1401773	-5.462178
CLPSL2	0.2937739	-0.696485	1.7206787	0.0861444	0.14018	-5.462254
CDYL	0.031032	5.7744659	1.7206535	0.086149	0.14018	-5.462297
TTI2	0.0448829	5.40843	1.720651	0.0861494	0.14018	-5.462301
AKAP12	0.1068129	5.8662103	1.7205906	0.0861604	0.1401917	-5.462404
UPF2	0.0267959	6.1896544	1.720407	0.0861938	0.1402397	-5.462715
CCDC28B	0.0953228	5.1793802	1.7203789	0.0861989	0.1402418	-5.462763
NME1P1	-0.061364	-1.506121	-1.720214	0.086229	0.1402822	-5.463043
RP11-6L6.4	0.278165	-0.074015	1.7202	0.0862315	0.1402822	-5.463066

RNA5SP383	0.2961979	0.4831396	1.7199648	0.0862743	0.1403412	-5.463465
GS1-304P7.	0.261878	-0.530559	1.7199586	0.0862754	0.1403412	-5.463475
MND1	-0.102688	4.8147067	-1.719786	0.0863067	0.1403859	-5.463767
PPP2R1B	-0.047486	6.6123781	-1.719581	0.0863442	0.1404406	-5.464115
RP3-393E18	0.1635554	-1.18454	1.7193863	0.0863796	0.1404919	-5.464445
AC090602.2	-0.2629	-0.272772	-1.719244	0.0864056	0.140528	-5.464686
HMGB1P4	-0.160829	-1.166807	-1.71916	0.0864209	0.1405466	-5.464829
GZF1	-0.024197	5.807506	-1.718953	0.0864585	0.1405959	-5.465178
GABRQ	0.3324414	2.1960811	1.7189511	0.0864589	0.1405959	-5.465182
ARHGEF9	-0.04408	5.7427823	-1.718728	0.0864995	0.1406557	-5.465559
HTR5BP	0.2480932	-0.363198	1.7184614	0.0865483	0.1407286	-5.466011
MT-TY	-0.247225	2.9631409	-1.718276	0.086582	0.1407772	-5.466324
WDR45BP1	0.2683611	-0.634969	1.718232	0.0865901	0.1407841	-5.4664
RP1-81D8.5	-0.360179	1.2686415	-1.718119	0.0866108	0.1408115	-5.466591
DENND4C	0.0551908	5.9097974	1.7180803	0.0866178	0.1408166	-5.466656
AP000695.6	-0.212908	3.5188474	-1.718034	0.0866263	0.1408242	-5.466736
RP11-386I8	-0.20324	-0.908916	-1.717684	0.0866901	0.1409216	-5.467326
CLOCK	-0.034229	6.0309986	-1.717064	0.0868035	0.1410997	-5.468376
RBMX	-0.01772	6.5783808	-1.716955	0.0868234	0.1411257	-5.46856
RP11-104C4	-0.180143	-1.187396	-1.716486	0.0869093	0.1412591	-5.469354
RP11-335G2	0.1576646	-1.175545	1.7164277	0.08692	0.1412701	-5.469453
RP11-513G1	-0.361665	0.0023027	-1.716321	0.0869395	0.1412956	-5.469633
KIF1C	-0.032234	6.8135282	-1.716225	0.086957	0.1413178	-5.469795
SLC16A4	0.1693109	4.6040262	1.7161471	0.0869714	0.1413348	-5.469927
TP53RK	-0.035061	5.8623744	-1.715882	0.0870199	0.1414074	-5.470375
POTEM	0.1511376	-1.281312	1.7158294	0.0870296	0.1414168	-5.470464
CXCR1	0.3135177	2.1089889	1.7156689	0.087059	0.1414583	-5.470736
RP11-862L9	0.1821638	-1.096853	1.71558	0.0870753	0.1414785	-5.470886
RP11-25I15	-0.27161	-0.727469	-1.715454	0.0870984	0.1415051	-5.471099
SNAP23P	-0.214597	-0.787772	-1.715448	0.0870995	0.1415051	-5.471108
LCAT	-0.096722	6.1421092	-1.715417	0.0871052	0.1415081	-5.471161
EIF2S2	-0.025787	6.5546993	-1.715136	0.0871567	0.1415806	-5.471636
RBPMS	0.0419142	6.3513958	1.7151317	0.0871576	0.1415806	-5.471644
RP11-655G2	0.2683498	-0.931289	1.7149408	0.0871926	0.1416312	-5.471966
RP11-231E6	-0.11667	-1.431844	-1.714913	0.0871977	0.1416332	-5.472013
AC116035.1	-0.300465	0.1215236	-1.71425	0.0873195	0.1418247	-5.473133
RP11-214J9	0.2027734	-0.951955	1.7141103	0.0873452	0.1418601	-5.473369
HIST2H2BD	0.3082595	0.846955	1.7137313	0.0874149	0.141967	-5.474009
CTC-204F22	0.2389402	2.0190862	1.7136098	0.0874372	0.141997	-5.474214
COX4I1P1	-0.115561	-1.371631	-1.713403	0.0874753	0.1420524	-5.474564
CTB-1I21.1	-0.32024	-0.556921	-1.71322	0.0875089	0.1421008	-5.474872
RP13-476E2	0.3314763	0.6622046	1.7130827	0.0875342	0.1421356	-5.475104
RP11-135J2	0.2666745	-0.166961	1.7129771	0.0875537	0.1421608	-5.475283
RNU6-1257P	-0.115328	-1.384106	-1.712664	0.0876114	0.1422483	-5.475811
RP11-605F2	-0.075052	-1.494348	-1.712421	0.0876562	0.1423146	-5.476221
TOMM34	-0.029118	6.1800795	-1.712307	0.0876773	0.1423425	-5.476414
RP1-309H15	-0.117379	-1.395493	-1.712221	0.0876931	0.1423619	-5.476559
AP000867.1	-0.095994	-1.452997	-1.712099	0.0877156	0.1423921	-5.476765
TMEM8C	-0.346261	-0.19728	-1.712001	0.0877337	0.1424152	-5.476931
RP11-981G7	0.3274526	2.3925428	1.7118836	0.0877553	0.1424438	-5.477128
RP11-405L1	0.2695618	-0.248389	1.7114901	0.0878279	0.1425554	-5.477791

BRCA2	0.0809373	4.9824916	1.7113871	0.087847	0.1425799	-5.477965
RLN1	0.2312805	-0.845552	1.7111378	0.087893	0.1426483	-5.478385
AC092431.3	-0.218455	-0.809311	-1.710994	0.0879196	0.1426852	-5.478628
SNX18P27	-0.087118	-1.460602	-1.710956	0.0879266	0.1426902	-5.478692
RBM6	-0.023805	6.3488568	-1.710849	0.0879465	0.1427161	-5.478873
SNORA2A	0.2706834	-0.15029	1.7108131	0.087953	0.1427204	-5.478933
UPK3B	-0.193293	3.6402341	-1.710722	0.0879698	0.1427413	-5.479086
ZEB2P1	-0.374986	0.1013314	-1.710496	0.0880117	0.1428028	-5.479468
NCL	-0.019167	7.0795914	-1.710436	0.0880228	0.1428146	-5.479569
RP11-105C1	-0.12876	-1.358308	-1.710341	0.0880403	0.1428366	-5.479728
RP11-320N2	0.1859926	-1.060225	1.7102205	0.0880626	0.1428665	-5.479932
RP11-197M2	0.2725873	-0.205069	1.7094558	0.0882042	0.1430898	-5.48122
RP11-567J2	0.1880007	-1.058931	1.7094282	0.0882094	0.1430918	-5.481267
PLEKHH3	-0.041887	6.0186648	-1.70938	0.0882182	0.1430998	-5.481347
RP11-285C1	-0.087236	-1.453922	-1.709273	0.0882382	0.1431259	-5.481529
RP11-10N16	-0.117183	-1.386617	-1.709015	0.0882859	0.1431952	-5.481962
RP11-168A1	-0.330442	0.4667009	-1.708984	0.0882916	0.1431952	-5.482014
ACR	-0.276368	2.6380428	-1.708978	0.0882927	0.1431952	-5.482024
RP1-232P2C	-0.492107	1.368801	-1.708559	0.0883706	0.1433152	-5.482731
CCND2-AS2	0.1350582	-1.310528	1.7084083	0.0883985	0.143354	-5.482984
AC009274.6	-0.119908	-1.348388	-1.708128	0.0884505	0.143432	-5.483455
BID	-0.040159	6.3679809	-1.708052	0.0884648	0.1434487	-5.483585
RP11-752G1	-0.302777	0.6279203	-1.707843	0.0885035	0.1435052	-5.483936
MT1DP	-0.283381	3.1924494	-1.70766	0.0885375	0.1435539	-5.484243
RP11-264B1	0.2205808	-0.825478	1.7076167	0.0885456	0.1435606	-5.484316
ABI1	0.0215131	6.3045234	1.7074991	0.0885674	0.143584	-5.484514
DGUOK	-0.033749	6.1748136	-1.707497	0.0885679	0.143584	-5.484518
KDM2B	0.0316349	6.0061625	1.7073482	0.0885955	0.1436224	-5.484768
CTA-246H3.	0.2785915	-0.673292	1.7072123	0.0886208	0.143657	-5.484997
RP11-458F8	-0.060965	5.3304565	-1.707078	0.0886457	0.143691	-5.485222
bP-21201H5	0.3614792	-0.100593	1.7070456	0.0886518	0.1436945	-5.485277
RP11-241G9	-0.245518	-0.836831	-1.706749	0.0887069	0.1437776	-5.485776
RP11-11601	0.206045	-1.004132	1.706544	0.0887451	0.1438331	-5.486121
LINC01374	0.2650964	-0.466584	1.7064059	0.0887709	0.1438645	-5.486353
RP4-758J18	-0.072385	4.5997243	-1.706377	0.0887763	0.1438645	-5.486402
AC074121.4	-0.126683	-1.337791	-1.706376	0.0887764	0.1438645	-5.486403
HMGN2P41	0.2589303	1.9157437	1.7059512	0.0888556	0.1439865	-5.487118
GRIA4	0.2759582	-0.555114	1.7059131	0.0888627	0.1439916	-5.487182
RP11-506B6	0.2493555	-0.39165	1.7058115	0.0888816	0.1440159	-5.487352
RP11-432I5	-0.10421	-1.420812	-1.705784	0.0888867	0.1440178	-5.487398
RP11-7807.	-0.183817	-1.043418	-1.705748	0.0888934	0.1440223	-5.487459
AC000068.5	-0.203278	3.0624504	-1.705423	0.0889542	0.1441143	-5.488006
CTD-2046J7	-0.205105	-0.852573	-1.705154	0.0890043	0.14418	-5.488457
AC009531.2	-0.060593	-1.505689	-1.705145	0.089006	0.14418	-5.488473
IGHV1-68	0.1393005	-1.319416	1.7051387	0.0890071	0.14418	-5.488483
AP001065.7	-0.389381	0.9539929	-1.70512	0.0890106	0.14418	-5.488514
HAUS5	-0.031978	5.8765505	-1.704985	0.0890358	0.1442145	-5.488741
RP4-803A2.	0.2304227	-0.780376	1.704963	0.0890399	0.1442148	-5.488778
RP11-40C6.	-0.160308	3.4478555	-1.704853	0.0890604	0.1442376	-5.488963
KCTD16	0.3142552	0.157332	1.7048454	0.0890619	0.1442376	-5.488976
RP1-40G4P.	0.264387	1.109005	1.7046027	0.0891072	0.1443046	-5.489384

FAM229B	-0.08913	5.183177	-1.704327	0.0891588	0.1443818	-5.489847
SHKBP1	0.030507	6.3036561	1.704256	0.089172	0.1443967	-5.489966
RPL7AP4	-0.156324	-1.214551	-1.704219	0.089179	0.144398	-5.490029
PRRC1	-0.024816	6.4535029	-1.704209	0.0891807	0.144398	-5.490044
LACC1	0.059304	5.4036404	1.7039344	0.0892322	0.1444749	-5.490506
RNU4-47P	0.2829894	1.1158876	1.7034925	0.0893149	0.1446024	-5.491248
LY6D	0.3445555	-0.521168	1.7034391	0.0893249	0.1446052	-5.491338
SCAPER	-0.044765	5.6897119	-1.703431	0.0893264	0.1446052	-5.491352
RP11-416H1	-0.160806	-1.174221	-1.703399	0.0893323	0.1446052	-5.491405
DLG2	-0.143779	4.2116682	-1.703398	0.0893325	0.1446052	-5.491406
CTC-436P18	0.1298444	-1.356186	1.7030797	0.0893922	0.144691	-5.491941
KYNU	0.0971152	6.1104793	1.7030731	0.0893934	0.144691	-5.491952
DMRTA2	0.2403595	-0.945854	1.702764	0.0894513	0.1447783	-5.492471
RP11-3N13.	-0.077223	-1.472277	-1.702671	0.0894687	0.1448001	-5.492626
RP11-494M8	-0.09402	-1.445024	-1.702617	0.0894789	0.1448102	-5.492718
DPCD	-0.054086	5.4777536	-1.702499	0.0895009	0.1448394	-5.492915
GCGR	-0.375576	4.9636798	-1.702446	0.0895109	0.1448491	-5.493004
P2RX6	0.2925195	2.6887614	1.7023335	0.089532	0.1448769	-5.493193
PGM5P4-AS1	0.1899385	-1.107289	1.7021393	0.0895685	0.1449294	-5.493519
AC107983.4	-0.156578	3.7583816	-1.702053	0.0895847	0.1449492	-5.493664
RP11-55801	-0.166649	-1.133399	-1.701407	0.089706	0.145131	-5.494747
RP11-274H2	0.2983202	2.0106806	1.7014022	0.0897068	0.145131	-5.494755
AC006277.2	-0.255603	1.8006035	-1.701391	0.089709	0.145131	-5.494774
HSP90AA1	-0.026672	7.2710951	-1.701366	0.0897136	0.1451321	-5.494815
RPL7P8	-0.255369	-0.403562	-1.701312	0.0897238	0.1451365	-5.494906
METTL15P1	0.2653258	2.9894334	1.7013093	0.0897243	0.1451365	-5.494911
RP11-856M7	0.2250686	-0.770576	1.7011411	0.0897559	0.1451812	-5.495193
C5orf28	0.0378928	5.6209462	1.70094	0.0897937	0.1452359	-5.49553
AC018755.1	-0.29479	2.3233898	-1.700792	0.0898215	0.1452745	-5.495778
SLC25A12	0.0891964	5.1841595	1.7003195	0.0899104	0.1454118	-5.49657
GABPB2	0.0390851	5.7681151	1.7002652	0.0899206	0.1454218	-5.496661
RP11-219C2	-0.488675	1.1126132	-1.700085	0.0899546	0.1454704	-5.496963
YBX1P2	0.1907066	2.7593711	1.7000606	0.0899591	0.1454712	-5.497003
RP11-649E7	0.2876984	0.1590176	1.7000008	0.0899704	0.145483	-5.497104
AC017074.2	0.2613646	2.4011293	1.6999147	0.0899866	0.1455028	-5.497248
XXbac-BPG1	0.1105185	-1.395511	1.6995131	0.0900623	0.1456187	-5.497921
RP1-12G14.	0.2236391	-0.710299	1.6991978	0.0901217	0.1457083	-5.498449
RPS7P1	-0.074151	5.1382794	-1.699019	0.0901555	0.1457531	-5.498748
RP11-894J1	-0.15485	2.6920438	-1.699009	0.0901574	0.1457531	-5.498765
RP11-538D1	-0.199055	-1.144199	-1.69896	0.0901664	0.1457613	-5.498846
RP11-544D2	-0.104587	-1.43181	-1.698837	0.0901898	0.1457926	-5.499053
ZNF91	0.0409127	5.7253951	1.6987818	0.0902002	0.1458029	-5.499145
PSMC1P11	-0.117313	-1.381726	-1.698651	0.0902248	0.1458363	-5.499364
Six3os1_5	0.1736553	-1.203276	1.6981918	0.0903115	0.14597	-5.500133
SLC25A1P5	0.2337761	-0.533856	1.6981604	0.0903175	0.1459731	-5.500185
RP11-556H2	-0.253493	-0.790972	-1.698126	0.090324	0.1459772	-5.500243
GNAI3	-0.019207	6.4797553	-1.697602	0.0904229	0.1461306	-5.501119
ARHGEF11	0.0353696	6.4101731	1.6974287	0.0904558	0.1461772	-5.501409
FLCN	0.0314569	6.1171072	1.6973956	0.090462	0.1461809	-5.501465
RP11-359K1	-0.158577	-1.185427	-1.696812	0.0905725	0.1463529	-5.502442
DLD	-0.030745	6.565898	-1.696558	0.0906206	0.1464212	-5.502866

SDHCP2	-0.146295	-1.211798	-1.696546	0.0906228	0.1464212	-5.502885
RP13-22502	0.3194455	0.1554931	1.6964354	0.0906438	0.1464486	-5.503071
MORC2	-0.02304	6.3019743	-1.696182	0.0906918	0.1465198	-5.503495
GOT1	-0.055972	6.9104385	-1.695932	0.0907391	0.1465896	-5.503911
RP11-593P2	-0.149167	-1.29546	-1.695865	0.0907518	0.1466037	-5.504023
RP11-182J2	0.2907137	-0.053211	1.6957826	0.0907675	0.1466226	-5.504162
NXPE4	0.1561008	-1.29055	1.6952822	0.0908624	0.1467694	-5.504998
AC013474.4	0.1287361	-1.308116	1.6952025	0.0908775	0.1467831	-5.505131
RP11-385F5	-0.101748	4.1354902	-1.695195	0.0908789	0.1467831	-5.505143
RP11-325K1	-0.070016	-1.496727	-1.695004	0.0909152	0.1468351	-5.505462
SEPT4-AS1	-0.172236	2.9352076	-1.694769	0.0909599	0.1469009	-5.505856
CTD-2532N2	0.2823424	0.0522164	1.694564	0.0909988	0.1469572	-5.506198
AC013262.1	-0.133619	-1.310966	-1.694271	0.0910545	0.1470407	-5.506687
PFN4	-0.243815	3.382764	-1.694242	0.0910599	0.1470429	-5.506735
RP11-476K1	0.4867808	1.5740828	1.6942211	0.091064	0.147043	-5.50677
HLA-DRB6	0.1875787	5.535131	1.6941778	0.0910722	0.1470498	-5.506842
RP11-218C1	-0.087268	-1.451848	-1.694146	0.0910783	0.1470517	-5.506895
PSMC1	0.0555847	5.7496539	1.6941292	0.0910815	0.1470517	-5.506924
RP13-57D9.	-0.089303	-1.458357	-1.694093	0.0910883	0.1470562	-5.506983
RP11-407P1	-0.334852	1.7771838	-1.694048	0.0910969	0.1470635	-5.507059
RP11-116B1	-0.349315	1.2966723	-1.69399	0.091108	0.147075	-5.507157
HERC1	-0.036183	6.2516346	-1.693689	0.0911652	0.1471608	-5.507658
RGS7	0.2896774	-0.650601	1.6934961	0.0912019	0.1472136	-5.50798
RP11-278L1	-0.138099	-1.324675	-1.693472	0.0912065	0.1472144	-5.50802
RP1-197B17	0.2135301	2.755811	1.6933379	0.0912321	0.1472492	-5.508244
DDX46	-0.021877	6.2988871	-1.693192	0.0912599	0.147284	-5.508488
RP3-324017	0.1950941	-0.932537	1.6931823	0.0912617	0.147284	-5.508504
TPM3P8	0.2325036	-0.636937	1.6930926	0.0912788	0.1473051	-5.508654
GNRHR2	-0.120122	3.9494801	-1.692169	0.0914548	0.147577	-5.510194
RP11-497G1	0.4022891	1.0701475	1.6921665	0.0914553	0.147577	-5.510199
RP11-217B7	-0.320024	1.5236782	-1.69214	0.0914604	0.1475786	-5.510243
AC083867.4	-0.115766	-1.373875	-1.691882	0.0915096	0.1476514	-5.510672
HMBOX1	0.0385474	5.5327206	1.6915318	0.0915765	0.1477529	-5.511257
H3F3AP4	0.0815545	4.7671087	1.6912548	0.0916294	0.1478309	-5.511719
RP11-424M2	0.2498196	-0.602629	1.6912364	0.0916329	0.1478309	-5.511749
YAP1	0.0354803	6.4103112	1.6911627	0.091647	0.1478471	-5.511872
UNC13C	0.2862262	-0.56705	1.690911	0.0916951	0.1479151	-5.512292
LDLRAD1	0.4013106	3.4731876	1.6908996	0.0916973	0.1479151	-5.512311
RP11-509J2	0.2225717	-0.814082	1.6908413	0.0917085	0.1479266	-5.512408
FOXJ2	-0.038171	5.835594	-1.690788	0.0917187	0.1479365	-5.512497
NCR1	0.3188822	1.4185148	1.6903175	0.0918087	0.1480751	-5.513281
RP11-274H2	0.3132721	0.4505101	1.6902774	0.0918164	0.148081	-5.513347
AC007228.1	0.2850591	2.1852346	1.6900322	0.0918633	0.1481501	-5.513756
MSL1	0.0210615	6.4154823	1.6897417	0.0919189	0.1482333	-5.51424
RP11-305P2	0.1219837	-1.374838	1.6894783	0.0919694	0.1483081	-5.514678
RP11-889D3	-0.085389	-1.464154	-1.689443	0.0919761	0.1483124	-5.514737
SKINTL	0.2150165	-0.856201	1.6894003	0.0919844	0.1483192	-5.514808
RP11-39H3.	0.3994404	2.6227828	1.6892692	0.0920095	0.1483531	-5.515027
RP11-46107	-0.429649	0.9570087	-1.68892	0.0920764	0.1484545	-5.515607
PXK	0.0414031	5.7887376	1.6888042	0.0920987	0.1484789	-5.5158
CTD-2571L2	0.1876649	-0.997895	1.6887988	0.0920997	0.1484789	-5.515809

CACNG6	0.1980673	-1.147475	1.6885863	0.0921405	0.1485381	-5.516163
HBG2	0.2925124	-0.188519	1.688068	0.0922401	0.148692	-5.517026
RP11-416N2	0.1891742	-1.166335	1.6877793	0.0922955	0.1487749	-5.517506
MFSD2B	0.2419907	3.1224137	1.6876289	0.0923244	0.1488149	-5.517756
GUCY2F	0.2112258	-1.027225	1.6873418	0.0923797	0.1488973	-5.518234
NMTRQ-TTG1	-0.086577	-1.47826	-1.687238	0.0923996	0.1489229	-5.518406
SCGB3A1	0.2398625	2.6607204	1.6870408	0.0924376	0.1489775	-5.518734
PPP3R2	0.1936023	-1.151982	1.6869486	0.0924553	0.1489995	-5.518887
MT2P1	-0.340106	2.9178673	-1.686673	0.0925084	0.1490785	-5.519346
LINC01585	0.2292005	-0.720756	1.6866211	0.0925184	0.149088	-5.519432
ABHD16A	0.051679	5.4323791	1.6865673	0.0925287	0.1490981	-5.519521
TIAM1	0.1052933	5.0967339	1.6863463	0.0925713	0.1491601	-5.519889
TPTE2P3	0.3112843	-0.680767	1.6862806	0.092584	0.1491739	-5.519998
GUSBP4	0.2452074	1.9351969	1.6858918	0.0926589	0.1492881	-5.520644
AC099539.1	-0.144348	-1.223685	-1.685851	0.0926668	0.1492943	-5.520712
ITCH-IT1	-0.123338	-1.336066	-1.685741	0.0926879	0.1493216	-5.520894
RP11-363D1	0.2240914	-0.939006	1.6851752	0.0927972	0.1494911	-5.521834
PPIAP23	-0.123224	-1.333193	-1.685035	0.0928243	0.1495282	-5.522068
SPSB3	0.1252853	4.6592317	1.6849557	0.0928395	0.1495461	-5.522199
AP000593.5	-0.239809	-1.072626	-1.684736	0.0928821	0.149608	-5.522564
ZNF30	-0.046248	5.1319011	-1.68439	0.0929488	0.1497089	-5.523138
RP11-48402	-0.153324	-1.218471	-1.684295	0.0929672	0.1497319	-5.523296
TMEM215	-0.098914	-1.446056	-1.684175	0.0929904	0.1497627	-5.523495
GRK7	0.2320107	-0.718141	1.683938	0.0930363	0.1498292	-5.523888
EIF4A2P2	0.269156	0.1188475	1.6839192	0.0930399	0.1498292	-5.52392
RP11-57B24	0.1037598	-1.413545	1.6838206	0.093059	0.1498419	-5.524083
RP4-784A16	0.1408931	-1.273564	1.6838203	0.093059	0.1498419	-5.524084
C4BPAP1	-0.318145	0.3822663	-1.683815	0.0930601	0.1498419	-5.524093
RP11-762I7	0.1648353	2.379196	1.6837494	0.0930728	0.1498557	-5.524202
CHST7	0.0874301	5.2671135	1.6837168	0.0930791	0.1498592	-5.524256
RP11-58A11	-0.232718	-0.58049	-1.683691	0.0930841	0.1498607	-5.524299
EPOR	0.049701	5.4553631	1.6834443	0.0931318	0.1499309	-5.524708
RP11-50D9.	-0.27268	2.2403037	-1.683362	0.0931478	0.1499452	-5.524845
AC006539.1	0.1905383	-1.01503	1.6833559	0.0931489	0.1499452	-5.524854
DLGAP1-AS5	0.2402873	-1.080278	1.6832349	0.0931724	0.1499764	-5.525055
SEPHS1P1	0.1915858	-0.995747	1.6831952	0.0931801	0.1499821	-5.525121
MRPL48P1	-0.074985	-1.499513	-1.683106	0.0931974	0.1499981	-5.52527
RAB3GAP2	0.0284928	6.225528	1.6831015	0.0931982	0.1499981	-5.525277
RP11-685G9	0.2150273	-1.051242	1.6829361	0.0932303	0.1500431	-5.525551
KPTN	-0.042012	5.7276306	-1.682876	0.0932418	0.1500551	-5.52565
TBC1D10B	0.0235614	6.1604355	1.6828134	0.093254	0.1500681	-5.525754
PPIAP21	0.3137997	1.5634503	1.6827604	0.0932643	0.150078	-5.525842
AFG3L2	-0.02715	6.4139297	-1.682563	0.0933026	0.1501331	-5.52617
ZNF7	-0.034458	5.9626463	-1.68246	0.0933226	0.1501585	-5.526341
LINC00284	0.2725026	-0.581573	1.6824099	0.0933323	0.1501675	-5.526424
RP11-147G7	0.1472426	-1.226824	1.6823736	0.0933393	0.1501722	-5.526484
DOC2GP	-0.224176	2.9821271	-1.682351	0.0933437	0.1501727	-5.526522
INO80	0.0232163	6.0807432	1.6823268	0.0933484	0.1501736	-5.526561
PGLYRP2	-0.174211	6.3438569	-1.682289	0.0933558	0.1501789	-5.526625
CCDC37	-0.280222	0.8155969	-1.681977	0.0934162	0.1502695	-5.527141
RP11-284F2	0.336735	3.7788766	1.6818994	0.0934313	0.1502872	-5.52727

CYFIP2	0.0832609	6.1526018	1.6818549	0.09344	0.1502944	-5.527344
TTC3	0.0242355	6.6172224	1.6817527	0.0934598	0.1503197	-5.527513
RP11-161D1	-0.140514	-1.304353	-1.68165	0.0934797	0.1503451	-5.527683
NWD2	0.4590231	1.1520096	1.6815319	0.0935027	0.1503754	-5.527879
KERA	0.118516	-1.362537	1.6814117	0.093526	0.1504064	-5.528078
SNTN	0.1254863	-1.384141	1.6813312	0.0935417	0.1504249	-5.528212
HIST1H2APS	-0.187969	-1.115755	-1.680888	0.0936278	0.1505517	-5.528946
CLUHP4	-0.069074	-1.496199	-1.680883	0.0936288	0.1505517	-5.528954
LA16c-444G	0.2358593	-0.980092	1.6807977	0.0936454	0.1505718	-5.529096
DESI2	0.029833	6.2169748	1.6806262	0.0936787	0.1506188	-5.52938
RP11-167B3	0.2056553	-1.130882	1.6803126	0.0937397	0.1507102	-5.529899
RP5-89003.	-0.201649	-0.876937	-1.680145	0.0937724	0.1507551	-5.530177
RP11-752D2	-0.066307	-1.494647	-1.680127	0.0937759	0.1507551	-5.530207
KRT8P28	-0.116538	-1.389084	-1.680098	0.0937815	0.1507575	-5.530255
RPL7P59	0.1866118	-1.009367	1.679961	0.0938082	0.1507937	-5.530482
RP11-15001	0.4562872	2.0272094	1.6796703	0.0938648	0.1508781	-5.530963
FGFR1OP	-0.037962	5.8854765	-1.679552	0.0938878	0.1509084	-5.531158
RP11-23E1C	-0.060372	-1.505565	-1.679373	0.0939227	0.1509456	-5.531454
CTD-2102P2	0.1744625	-1.048604	1.6793716	0.0939231	0.1509456	-5.531457
FAM221A	-0.117635	5.1040163	-1.67937	0.0939233	0.1509456	-5.53146
RP11-7807.	0.1242082	3.4746037	1.6793087	0.0939353	0.1509582	-5.531562
RP11-98D18	-0.181588	3.1281406	-1.679163	0.0939637	0.1509971	-5.531802
NPHS1	0.3715877	0.233613	1.6791166	0.0939728	0.1509999	-5.53188
ZNF426	0.0866102	5.3328205	1.6790925	0.0939775	0.1509999	-5.531919
CTD-2007H1	-0.208947	2.706563	-1.67909	0.093978	0.1509999	-5.531924
OR2A9P	0.3029648	2.2144021	1.6790693	0.093982	0.1509999	-5.531958
RP11-80H18	-0.26697	-0.144793	-1.67845	0.0941028	0.1511758	-5.532982
BBC3	0.0471765	5.7218147	1.6784454	0.0941037	0.1511758	-5.53299
U2SURP	0.0228663	6.4016541	1.6784448	0.0941038	0.1511758	-5.532991
KCNMA1-AS3	-0.163748	-1.200139	-1.677817	0.0942265	0.1513662	-5.53403
NIPAL2	0.0656749	6.1336446	1.6777487	0.0942398	0.1513809	-5.534142
RP11-1E11.	0.2458317	-0.704802	1.6775383	0.094281	0.1514403	-5.53449
TEAD3	0.0312185	6.0672563	1.6773685	0.0943142	0.1514837	-5.534771
AC012668.2	-0.341392	2.0158488	-1.677358	0.0943163	0.1514837	-5.534789
MAGEA4	0.3486104	-0.659666	1.6772378	0.0943398	0.1515147	-5.534987
GS1-124K5.	-0.044011	5.2387241	-1.677208	0.0943456	0.1515174	-5.535036
RP11-209K1	-0.217088	4.6510084	-1.677125	0.0943618	0.1515368	-5.535173
LEUTX	-0.082198	-1.474385	-1.677093	0.094368	0.1515402	-5.535226
PUS1	-0.037522	6.1470018	-1.676868	0.0944121	0.1516043	-5.535599
ZSCAN29	0.0306227	5.8424997	1.676702	0.0944446	0.1516429	-5.535873
PPEF1	0.3409662	1.5129387	1.6767018	0.0944447	0.1516429	-5.535873
LINC00353	-0.113871	-1.367429	-1.676678	0.0944493	0.1516429	-5.535912
RP11-321C2	0.2751872	-0.214298	1.6766604	0.0944528	0.1516429	-5.535942
NAP1L1	0.0292621	6.8262168	1.6764976	0.0944846	0.1516874	-5.536211
TSPY11P	-0.106177	-1.450055	-1.67562	0.0946567	0.1519569	-5.537661
CTD-207306	-0.212692	-0.861507	-1.675358	0.0947081	0.1520328	-5.538094
CTD-2298J1	-0.26736	-0.866661	-1.675198	0.0947395	0.1520765	-5.538358
CHAMP1	0.0408983	5.8774138	1.6751374	0.0947514	0.1520889	-5.538458
INHBA	0.127383	5.5056442	1.674625	0.094852	0.1522413	-5.539303
TSHZ2	-0.102555	5.9124968	-1.674611	0.0948547	0.1522413	-5.539326
CUL4A	-0.02589	6.4979706	-1.674577	0.0948614	0.1522454	-5.539382

AC115617.2	-0.211886	2.7899851	-1.674525	0.0948716	0.1522551	-5.539468
RP4-694A7.	0.2169376	-0.942467	1.6744314	0.0948901	0.152278	-5.539623
LCMT1-AS2	0.2768393	0.0043067	1.6743287	0.0949103	0.1523037	-5.539793
MYCNOS	0.3790558	0.4927936	1.6738912	0.0949963	0.152435	-5.540515
SNRPGP14	0.2723722	0.2504496	1.6735772	0.0950581	0.1525246	-5.541032
INPPL1	-0.026153	6.4789309	-1.673552	0.0950631	0.1525246	-5.541074
SLC25A38P1	-0.153785	-1.20021	-1.673544	0.0950647	0.1525246	-5.541088
AF186192.1	0.272326	-0.394111	1.6733279	0.0951072	0.1525861	-5.541444
RP11-192M2	0.2997055	0.0251422	1.6733042	0.0951118	0.1525869	-5.541483
RPL15P18	0.265929	0.1152833	1.6731738	0.0951375	0.1526195	-5.541698
KCNIP4-IT1	-0.063973	-1.493339	-1.673159	0.0951405	0.1526195	-5.541723
GRTP1-AS1	0.2512719	2.7322047	1.6730399	0.0951639	0.1526503	-5.541919
HIST1H2BK	-0.067183	6.1343824	-1.672888	0.0951937	0.1526914	-5.542168
FBXW2	0.0275023	6.4171455	1.6728588	0.0951996	0.1526941	-5.542217
RP11-392O1	0.2452245	-0.797493	1.6726386	0.095243	0.152757	-5.54258
MT1F	0.1711016	5.2211061	1.6725354	0.0952633	0.1527798	-5.542751
RP11-420A2	-0.104526	4.3260037	-1.672524	0.0952656	0.1527798	-5.542769
RP11-775H9	-0.095241	-1.452571	-1.672307	0.0953084	0.1528418	-5.543127
TNPO1	-0.021917	6.4668029	-1.672091	0.095351	0.1529034	-5.543483
VIPAS39	-0.027742	5.96564	-1.672016	0.0953659	0.1529205	-5.543607
CTC-573N18	0.4180669	0.6238917	1.671986	0.0953717	0.1529231	-5.543656
RP11-145A3	-0.14849	-1.233302	-1.671469	0.0954739	0.1530803	-5.544509
RP11-148K1	0.2778363	0.1014855	1.6712039	0.0955262	0.1531574	-5.544945
ZNF177	0.161719	-1.188325	1.6711124	0.0955442	0.1531796	-5.545096
PVT1	0.1218048	5.0436823	1.6708973	0.0955868	0.1532411	-5.54545
RP11-180I4	-0.182589	-1.171487	-1.670619	0.0956417	0.1533225	-5.545908
SMEK2	-0.021486	6.5255099	-1.67044	0.0956772	0.1533725	-5.546203
LRRC8A	-0.027119	6.5339823	-1.670336	0.0956977	0.1533988	-5.546374
RNF40	0.0473157	6.2709398	1.6700148	0.0957614	0.153494	-5.546903
RP3-331H24	0.2535795	-0.55479	1.669765	0.0958108	0.1535666	-5.547314
AC093627.8	0.1723442	-1.164303	1.6696028	0.095843	0.1536113	-5.547581
RP11-93O14	0.2549598	-0.161675	1.6693179	0.0958994	0.1536951	-5.54805
ENPP7P11	0.1604325	-1.178514	1.6692493	0.095913	0.1537044	-5.548163
PKD1P1	0.189592	-0.937369	1.669246	0.0959137	0.1537044	-5.548168
RP11-4104.	0.1713778	-1.212912	1.6692121	0.0959204	0.1537085	-5.548224
RP11-576C2	-0.288559	-0.434803	-1.669106	0.0959415	0.153734	-5.548399
ZXDB	-0.061137	5.9470987	-1.669089	0.0959447	0.153734	-5.548426
GBP2	-0.053011	6.5450458	-1.668804	0.0960013	0.1538178	-5.548895
ZNF268	-0.049485	5.8796837	-1.668775	0.096007	0.1538202	-5.548943
SH2D6	0.2495675	3.157411	1.6685263	0.0960565	0.1538927	-5.549352
RPS27P23	-0.249623	-0.392847	-1.668168	0.0961275	0.1539998	-5.549941
RP11-78L16	-0.428843	0.3508964	-1.667936	0.0961736	0.1540669	-5.550323
CTC-471J1.	0.214855	-0.792603	1.6677277	0.0962151	0.1541265	-5.550666
VAMP1	-0.059923	5.3856159	-1.667432	0.0962739	0.154214	-5.551152
TRAPPC2	-0.028327	5.7614894	-1.667409	0.0962784	0.1542145	-5.551189
CRYZP1	0.2761498	-0.128667	1.6670359	0.0963527	0.1543266	-5.551803
EMX2OS	-0.295708	-0.223667	-1.666794	0.0964007	0.1543968	-5.552199
RN7SL605P	-0.190376	-0.983551	-1.666556	0.0964481	0.154466	-5.552591
MIR6730	-0.251405	-0.280448	-1.666529	0.0964535	0.1544678	-5.552635
AC011551.3	-0.27274	0.2733664	-1.666427	0.0964738	0.1544915	-5.552803
TSPEAR	-0.336048	3.1140123	-1.666413	0.0964768	0.1544915	-5.552827

ANKRD30BP2	-0.239841	-0.967542	-1.666275	0.0965042	0.154528	-5.553053
RASGRF2	-0.068737	5.3769762	-1.666236	0.096512	0.154528	-5.553117
GTF2H3	0.0312392	5.9716353	1.6662202	0.0965151	0.154528	-5.553143
RP11-88G17	0.1710817	-1.172821	1.6662131	0.0965165	0.154528	-5.553154
RP11-17G12	0.2705209	-0.099554	1.6661458	0.0965299	0.1545427	-5.553265
RBM26-AS1	-0.114477	4.2837272	-1.666121	0.0965349	0.1545439	-5.553306
SNORA81	0.3278265	1.3767025	1.6660313	0.0965527	0.1545625	-5.553453
GCSAML-AS1	0.2894411	-0.528097	1.6660201	0.096555	0.1545625	-5.553471
RPS19P1	-0.295439	1.1332138	-1.665958	0.0965673	0.1545755	-5.553573
RP11-297N6	-0.320808	1.3177455	-1.665828	0.0965933	0.1546103	-5.553787
RP11-473M1	-0.074608	-1.489577	-1.665734	0.096612	0.1546303	-5.553941
NUP214	-0.020135	6.4069613	-1.665722	0.0966143	0.1546303	-5.55396
RP1-84015.	-0.102083	-1.418662	-1.665537	0.0966514	0.1546828	-5.554265
FMO8P	-0.267358	-0.853577	-1.665483	0.096662	0.1546924	-5.554353
RP11-196G1	0.3054863	2.1160451	1.6654644	0.0966658	0.1546924	-5.554384
EFTUD1P1	0.1372288	-1.283631	1.665102	0.0967381	0.1548013	-5.554979
RP11-22B23	0.290844	-0.131044	1.6650774	0.096743	0.1548024	-5.555019
PAUPAR	-0.0986	-1.430092	-1.664154	0.0969274	0.1550906	-5.556534
LINC00237	0.1938635	-1.246062	1.6641322	0.0969318	0.155091	-5.55657
RP5-1096D1	-0.20103	-0.928977	-1.663937	0.096971	0.1551468	-5.556891
IGKV1OR2-1	0.140805	-1.312262	1.6638919	0.0969799	0.1551543	-5.556965
PPIAP3	-0.278384	0.3107222	-1.663798	0.0969986	0.1551774	-5.557118
MIR3145	0.1576621	-1.2001	1.6635946	0.0970394	0.1552354	-5.557452
UBE2K	-0.022399	6.5581432	-1.663575	0.0970434	0.1552354	-5.557485
TUBAL3	0.2957153	-0.699035	1.6635102	0.0970563	0.1552492	-5.557591
DCC	-0.407442	0.9589333	-1.663489	0.0970606	0.1552493	-5.557626
C9orf91	-0.048028	5.6905545	-1.663435	0.0970714	0.1552598	-5.557714
RP11-361M1	-0.264157	-0.064185	-1.663255	0.0971073	0.1553105	-5.558009
FMO6P	-0.27058	-0.371329	-1.663229	0.0971125	0.155312	-5.558051
GCK	-0.444024	2.6703946	-1.66314	0.0971304	0.1553338	-5.558198
VN1R108P	0.2910068	0.9702957	1.6630454	0.0971494	0.1553573	-5.558353
NLRP4	0.3084568	-0.60495	1.6629859	0.0971613	0.1553695	-5.55845
CTD-2385L2	-0.076537	-1.497405	-1.662672	0.0972242	0.1554633	-5.558965
SOX12	0.0577289	6.0769914	1.6625572	0.0972472	0.1554933	-5.559153
AC005154.7	0.2060343	2.9480638	1.661915	0.097376	0.1556924	-5.560205
AC010907.2	0.3515789	0.191041	1.6618635	0.0973863	0.1557021	-5.56029
RP1-102E24	-0.174779	-1.010356	-1.661822	0.0973946	0.1557085	-5.560357
KCNJ14	-0.112895	4.1387051	-1.661716	0.0974159	0.1557314	-5.560531
VDAC3	-0.035441	6.4948822	-1.661708	0.0974174	0.1557314	-5.560544
STARD5	-0.117587	5.1452807	-1.661684	0.0974224	0.1557324	-5.560584
RP11-383B4	0.3173429	1.0818906	1.6612477	0.09751	0.1558657	-5.561298
LINC00957	0.0964219	4.6173035	1.6612196	0.0975156	0.1558679	-5.561344
SNX8	-0.032885	6.2107615	-1.661153	0.097529	0.1558825	-5.561454
ZNF252P-AS	0.1907883	3.091116	1.6603788	0.0976847	0.1561244	-5.562721
MAP3K5	0.0994219	5.6623383	1.6601623	0.0977282	0.1561872	-5.563075
RNU6-438P	-0.119969	-1.36156	-1.660112	0.0977383	0.1561965	-5.563157
RNU2-11P	0.2570364	-0.351287	1.6600146	0.097758	0.1562194	-5.563317
RP11-423P1	0.10857	3.80346	1.6599985	0.0977612	0.1562194	-5.563343
IFT46	-0.051265	5.5469684	-1.659956	0.0977698	0.1562262	-5.563413
CTD-2184D3	-0.210971	-0.791587	-1.659852	0.0977907	0.1562528	-5.563583
MIR765	0.2168908	-0.738009	1.6598264	0.0977959	0.1562542	-5.563625

ZNF662	0.1561571	4.464877	1.6597297	0.0978153	0.1562785	-5.563783
PTMAP1	0.2141318	-0.782422	1.6596205	0.0978373	0.1563068	-5.563962
RP11-603K1	-0.119703	-1.35057	-1.659579	0.0978457	0.1563133	-5.56403
NAGK	0.0244953	6.2231887	1.6592199	0.0979181	0.1564221	-5.564617
EVL	0.0547813	6.2713322	1.6591046	0.0979413	0.1564523	-5.564806
PAGR1	-0.038309	5.4792798	-1.658954	0.0979717	0.156494	-5.565052
CCDC142	-0.040419	5.4637799	-1.658639	0.0980353	0.1565888	-5.565568
BMS1	-0.018736	6.2401399	-1.658405	0.0980825	0.1566574	-5.565951
PRSS8	0.4199494	4.3014909	1.6583688	0.0980897	0.1566621	-5.566009
AC108479.2	-0.251574	-0.252212	-1.658286	0.0981064	0.1566819	-5.566144
RP11-99L13	-0.260294	-0.737074	-1.658186	0.0981267	0.1567074	-5.566308
PTPN3	-0.059722	6.3110549	-1.658009	0.0981623	0.1567574	-5.566597
RP11-572M1	0.2810657	0.1427027	1.6578619	0.0981921	0.1567981	-5.566838
RP11-48G14	0.2143243	-0.796497	1.65773	0.0982187	0.1568338	-5.567053
BSN	-0.116963	4.9594561	-1.657443	0.0982768	0.1569196	-5.567523
RP11-420K1	0.2820072	-0.683907	1.6574102	0.0982834	0.1569233	-5.567576
RP11-403A3	0.4566908	1.2409857	1.6571351	0.098339	0.1570052	-5.568025
TMEM194B	0.0415592	5.3513905	1.6570584	0.0983545	0.1570216	-5.568151
XX-FYM637E	-0.158515	-1.330576	-1.657042	0.0983579	0.1570216	-5.568178
RP11-122M1	0.2906278	-0.059808	1.6569374	0.098379	0.1570434	-5.568348
DPYSL2	0.0458242	6.2171766	1.6569217	0.0983822	0.1570434	-5.568374
CMAS	-0.02906	6.3222571	-1.656911	0.0983844	0.1570434	-5.568392
RP3-448I9.	-0.127706	-1.33427	-1.656711	0.0984248	0.1571009	-5.568717
CTD-2113L7	-0.118295	-1.369221	-1.656618	0.0984436	0.1571241	-5.568869
RPL26P19	0.1423376	3.6542059	1.6563236	0.0985033	0.1572063	-5.569351
RPS4XP16	0.2081714	2.6378287	1.6563216	0.0985037	0.1572063	-5.569354
GPRASP2	-0.062648	5.5440095	-1.65627	0.0985141	0.157216	-5.569438
XXbac-BPG1	-0.214825	2.2338464	-1.656024	0.0985639	0.1572886	-5.569839
RP11-498C9	0.0875912	4.6110885	1.6559779	0.0985733	0.1572904	-5.569915
NCAPG	0.0958416	5.4839381	1.6559764	0.0985736	0.1572904	-5.569918
RP11-23F23	-0.364052	1.783652	-1.655855	0.0985982	0.1573227	-5.570115
CNIH2	0.1608805	3.4380224	1.655744	0.0986207	0.1573518	-5.570297
C4orf26	0.1864264	-1.08395	1.6556664	0.0986365	0.15737	-5.570424
CPT1B	0.1164916	4.4462695	1.655596	0.0986507	0.1573859	-5.570539
UBE2W	-0.028538	6.115959	-1.65483	0.0988061	0.1576228	-5.571788
FBXO45	-0.02655	5.9110952	-1.654822	0.0988079	0.1576228	-5.571802
SULT4A1	-0.499225	1.5076127	-1.654755	0.0988215	0.1576376	-5.571911
PNLIPRP2	0.2517571	-0.987132	1.6547007	0.0988324	0.1576482	-5.571999
RP11-617F2	0.2082989	-0.902027	1.6546517	0.0988424	0.1576572	-5.572079
DPY19L1P2	-0.220935	2.2052354	-1.654391	0.0988954	0.1577272	-5.572505
AC068831.1	-0.220386	-0.820074	-1.654382	0.0988972	0.1577272	-5.57252
ZNF697	0.0517819	5.5875882	1.6543717	0.0988993	0.1577272	-5.572536
CTC-487M23	0.1989187	3.0423937	1.6542701	0.0989199	0.1577533	-5.572702
RS1	0.3034604	0.3707109	1.6541787	0.0989385	0.157776	-5.572851
RP3-417L20	-0.325317	-0.592317	-1.654035	0.0989676	0.1578156	-5.573085
INVS	-0.032468	5.6745526	-1.653999	0.098975	0.1578204	-5.573144
AC104395.1	-0.167136	-1.225625	-1.653965	0.098982	0.1578247	-5.5732
CTC-453G23	0.2747798	0.2178259	1.6536411	0.0990478	0.1579227	-5.573727
AC007292.7	-0.292837	0.928916	-1.653307	0.0991158	0.1580243	-5.574272
RP11-400K9	-0.160219	-1.253574	-1.653261	0.0991251	0.1580266	-5.574347
AC006116.2	0.2952493	0.328175	1.6532571	0.099126	0.1580266	-5.574353

RP11-945A1	-0.176487	-1.209267	-1.653095	0.0991589	0.1580722	-5.574617
PTTG1IP	-0.022955	6.8523619	-1.653061	0.0991658	0.1580764	-5.574673
RP11-424M2	-0.098027	-1.438813	-1.653035	0.0991712	0.158078	-5.574715
GAPDHP23	-0.356685	0.2093546	-1.653011	0.099176	0.1580788	-5.574754
ZNF763	0.1089664	4.1644865	1.6529525	0.099188	0.1580905	-5.57485
CSTF2T	-0.030594	6.1117573	-1.652933	0.099192	0.1580905	-5.574882
CTD-2302E2	0.2790732	0.9070735	1.6527438	0.0992305	0.1581449	-5.57519
RP11-474D1	0.2973587	0.2395598	1.6526069	0.0992584	0.1581825	-5.575413
AP000438.4	-0.141786	-1.293632	-1.652103	0.0993611	0.1583392	-5.576234
FAM133DP	0.1878749	-0.916348	1.6520395	0.0993741	0.1583523	-5.576337
AC010967.2	0.3015667	-0.219496	1.6520203	0.099378	0.1583523	-5.576369
RP11-1217F	-0.127482	-1.369901	-1.651985	0.0993852	0.1583569	-5.576426
RP11-873E2	0.1643113	3.3406886	1.6519254	0.0993973	0.1583693	-5.576523
TLR3	-0.085453	5.4210047	-1.651808	0.0994213	0.1584006	-5.576715
HOXA11-AS	-0.384247	0.7682277	-1.651647	0.0994542	0.1584461	-5.576977
PPAT	0.0409578	5.6590532	1.6515297	0.0994781	0.1584773	-5.577168
KCNMB2	0.3122015	-0.053305	1.651211	0.0995432	0.1585705	-5.577686
BACH1-IT2	0.256277	1.7008226	1.6512005	0.0995453	0.1585705	-5.577704
RP11-580I1	-0.132114	3.3369788	-1.651093	0.0995673	0.1585986	-5.577879
ARID5A	0.0449756	6.0868695	1.6510361	0.0995789	0.1586102	-5.577971
RP11-319E1	-0.180279	-1.121512	-1.650955	0.0995955	0.1586297	-5.578103
AC005009.2	0.1600557	-1.216939	1.650831	0.0996208	0.1586631	-5.578305
RNU6-574P	0.2714497	0.3110838	1.6507864	0.0996299	0.1586707	-5.578378
SUGT1P3	0.1765149	2.8856498	1.6505775	0.0996726	0.1587318	-5.578718
RAPGEF4-AS	0.3014596	0.1068018	1.6505312	0.0996821	0.1587399	-5.578793
COL20A1	0.2727866	-0.516941	1.6504867	0.0996912	0.1587475	-5.578865
CTD-2554C2	0.2792595	1.0560196	1.6504532	0.099698	0.1587515	-5.57892
AC092566.1	-0.084009	-1.45338	-1.650349	0.0997195	0.1587786	-5.57909
MTA1	0.0283559	6.1760392	1.6502307	0.0997436	0.1588101	-5.579282
PTMAP8	0.2591323	-0.010793	1.6499794	0.099795	0.1588837	-5.579691
TMEM229A	0.1680205	-1.283867	1.6499621	0.0997985	0.1588837	-5.579719
RP6-186E3.	-0.089713	-1.449355	-1.649447	0.099904	0.1590447	-5.580557
LINC00909	-0.036744	5.3907861	-1.649258	0.0999428	0.1590996	-5.580865
UACA	0.0287007	6.2176774	1.6490587	0.0999836	0.1591544	-5.581188
AFAP1-AS1	0.4356108	2.6554931	1.649047	0.099986	0.1591544	-5.581207
GHSR	-0.129987	-1.38553	-1.648939	0.1000081	0.1591828	-5.581383
E2F1	-0.082846	5.8439307	-1.648876	0.100021	0.1591963	-5.581485
RP1-39G22.	-0.128231	-1.311458	-1.64883	0.1000305	0.1592045	-5.58156
CACNB2	0.1071276	4.7433365	1.648707	0.1000557	0.1592376	-5.58176
STOM	-0.04105	6.9481177	-1.648367	0.1001255	0.1593417	-5.582313
RP11-778J1	-0.184339	-1.005349	-1.648186	0.1001626	0.1593938	-5.582607
RP11-190P1	-0.133211	-1.306678	-1.648008	0.1001991	0.159445	-5.582896
RPL12P2	-0.159966	-1.150812	-1.647851	0.1002314	0.1594895	-5.583152
RP11-709D2	0.2578538	-0.142577	1.6477418	0.1002538	0.1595181	-5.583328
MIR4326	0.1645564	-1.111892	1.6475521	0.1002928	0.1595732	-5.583636
TEKT1	0.3228916	1.403559	1.6474205	0.1003198	0.1596092	-5.58385
RP11-330M1	0.3172624	0.1006275	1.6471658	0.1003722	0.1596856	-5.584264
VSTM2L	0.3438915	3.8632022	1.646824	0.1004425	0.1597905	-5.584819
DNAH14	0.0462914	5.504402	1.64671	0.1004659	0.1598158	-5.585004
EIF2S2P3	-0.245871	1.5761246	-1.646704	0.1004672	0.1598158	-5.585014
POLR2F	0.289423	0.3118533	1.64657	0.1004947	0.1598527	-5.585231

RP11-107P7	-0.131721	-1.402497	-1.646297	0.100551	0.1599352	-5.585675
LMLN	-0.080027	5.1787151	-1.64618	0.1005751	0.1599666	-5.585865
SUV420H2	0.0440445	5.625979	1.6460888	0.1005938	0.1599884	-5.586012
RP11-540B6	0.1686497	-1.111821	1.6460686	0.100598	0.1599884	-5.586045
OIP5	0.125256	4.6283456	1.6460322	0.1006055	0.1599884	-5.586104
FAM135B	0.3515336	2.7127763	1.6460279	0.1006064	0.1599884	-5.586111
PPP1R14A	0.084403	5.1777131	1.6460068	0.1006107	0.1599884	-5.586145
DENND2C	-0.067511	5.5011103	-1.645966	0.1006191	0.1599947	-5.586211
RP11-699L2	-0.253996	2.4877103	-1.645589	0.1006967	0.1601112	-5.586823
SETP17	0.266833	0.4048194	1.645285	0.1007595	0.160204	-5.587317
RP4-813D12	0.154553	-1.313294	1.6452156	0.1007738	0.1602198	-5.587429
AC006277.3	-0.178003	3.2295302	-1.645179	0.1007814	0.1602249	-5.587489
IMPDH1P10	0.2023284	-0.836927	1.6450405	0.1008099	0.1602633	-5.587713
BANF2	-0.39415	1.0139117	-1.645006	0.1008171	0.1602676	-5.587769
TBC1D22A	-0.02885	6.2639185	-1.644977	0.100823	0.1602701	-5.587816
PRRG3	0.2718177	2.6620184	1.6449187	0.1008351	0.1602823	-5.587911
KDR	0.0676374	6.0339393	1.6446173	0.1008973	0.1603742	-5.588399
RP11-186B7	0.0882087	4.2198303	1.6442581	0.1009715	0.1604851	-5.588982
CTD-2510F5	-0.084728	-1.459262	-1.644149	0.1009941	0.1605122	-5.589159
OFD1P6Y	-0.068376	-1.495808	-1.644133	0.1009973	0.1605122	-5.589184
RP13-977J1	-0.225523	-1.048085	-1.643869	0.101052	0.1605921	-5.589613
H3F3BP1	0.2786991	0.679813	1.6437143	0.1010839	0.1606358	-5.589863
RPL13AP25	-0.083464	5.1826964	-1.64341	0.1011469	0.160729	-5.590357
RP11-609N1	-0.305724	2.139702	-1.643332	0.101163	0.1607475	-5.590483
KRT17P3	-0.119111	-1.354964	-1.642944	0.1012433	0.1608682	-5.591112
RCOR1	0.0296152	6.0374772	1.6428542	0.1012618	0.1608906	-5.591257
CH17-76K2.	-0.141179	-1.245877	-1.642567	0.1013212	0.160977	-5.591721
E2F6	0.0303236	5.5729435	1.6425491	0.101325	0.160977	-5.591751
AC010969.1	0.3962915	0.871239	1.6422628	0.1013844	0.1610643	-5.592215
RP11-3K24.	0.2694452	-0.281125	1.6422103	0.1013952	0.1610745	-5.5923
AC026954.6	-0.146848	-1.207695	-1.641995	0.1014399	0.1611385	-5.592649
CAV1	0.0448348	6.1919413	1.641746	0.1014915	0.1612135	-5.593051
PRMT5-AS1	0.221429	2.027325	1.6415557	0.101531	0.1612692	-5.593359
NR0B2	-0.163767	6.215874	-1.641335	0.1015769	0.1613314	-5.593717
WNT6	0.2853934	2.1560204	1.6413243	0.101579	0.1613314	-5.593734
RBPJ	-0.022082	6.2683601	-1.641017	0.1016429	0.1614258	-5.594232
NUDT22	-0.041815	6.0204839	-1.640858	0.1016759	0.1614712	-5.594489
BDP1	0.0348348	5.9201991	1.6407118	0.1017062	0.1615124	-5.594725
GPR107	0.0239215	6.4513752	1.6406598	0.1017171	0.1615225	-5.594809
GBP6	0.3309296	0.7321462	1.6405015	0.10175	0.1615677	-5.595065
USP32	-0.031654	6.0863193	-1.640426	0.1017656	0.1615856	-5.595187
NTSR1	0.3770869	1.4047577	1.6402865	0.1017947	0.1616246	-5.595413
RP11-1C8.5	0.2753689	-0.191039	1.6401904	0.1018146	0.1616493	-5.595568
TRAF3IP1	-0.026857	5.845225	-1.640039	0.1018462	0.1616915	-5.595814
AC015922.5	-0.223373	-1.058225	-1.64002	0.10185	0.1616915	-5.595844
RP11-51J9.	0.2522802	1.7880149	1.6397121	0.1019142	0.1617862	-5.596342
RP11-211G3	-0.225918	-0.759367	-1.639593	0.101939	0.1618186	-5.596534
UBA52P6	-0.281574	1.3776902	-1.639551	0.1019476	0.1618253	-5.596602
RP11-661C8	-0.102984	-1.438676	-1.639374	0.1019845	0.1618767	-5.596888
IGFL3	0.1210569	-1.386624	1.6392238	0.1020158	0.1619195	-5.597131
CCDC113	-0.165688	4.615238	-1.639011	0.1020602	0.1619829	-5.597475

KPRP	0.2012977	-1.139626	1.63877	0.1021104	0.1620555	-5.597864
ZCCHC3	0.030534	6.0758253	1.6386723	0.1021308	0.1620808	-5.598022
LINC01324	-0.165877	-1.297818	-1.63858	0.10215	0.1621043	-5.598172
MIR5572	0.2248085	-0.892202	1.6384104	0.1021854	0.1621533	-5.598446
RP11-659P1	0.1139694	-1.373375	1.6380857	0.1022531	0.1622538	-5.59897
HAS3	0.0858316	4.4963101	1.6375438	0.1023662	0.1624262	-5.599845
CST1	0.4882327	1.3116298	1.6374401	0.1023879	0.1624535	-5.600013
ARSEP1	-0.460149	2.9289407	-1.637364	0.1024038	0.1624717	-5.600136
OTOA	0.1449593	3.4257494	1.6372801	0.1024214	0.1624925	-5.600271
KIF12	0.1469888	5.8389402	1.6370314	0.1024733	0.1625678	-5.600672
LINC01337	-0.250379	-0.348959	-1.636997	0.1024806	0.1625723	-5.600728
PBK	0.1059304	5.2001738	1.6368399	0.1025134	0.1626154	-5.600981
HTR4	0.379128	1.7743568	1.6368241	0.1025167	0.1626154	-5.601007
TMBIM7P	-0.160562	-1.224266	-1.636796	0.1025226	0.1626178	-5.601053
FERMT2	-0.035955	6.3976298	-1.636711	0.1025402	0.1626386	-5.601189
TAS2R2P	0.2008207	-0.986403	1.6362975	0.1026268	0.162769	-5.601857
FAR2P3	0.1349433	-1.348125	1.6362566	0.1026354	0.1627755	-5.601923
SKI	0.0322868	6.3520771	1.636202	0.1026468	0.1627865	-5.602011
INPP1	-0.052157	5.9543976	-1.635781	0.1027351	0.1629194	-5.602691
AC018866.1	-0.063754	-1.507461	-1.635735	0.1027446	0.1629275	-5.602764
ARHGEF7-AS	-0.294618	-0.412733	-1.635416	0.1028115	0.1630263	-5.603278
RP11-428J1	-0.088293	4.3719175	-1.635388	0.1028173	0.1630285	-5.603323
OR2N1P	-0.234541	-1.100473	-1.634954	0.1029083	0.1631657	-5.604023
RAB4B	-0.04289	5.7488367	-1.634862	0.1029276	0.1631892	-5.604171
CCDC69	-0.059727	6.132373	-1.634548	0.1029936	0.1632868	-5.604679
LCE3A	-0.097498	-1.423686	-1.634454	0.1030133	0.1633109	-5.604829
RARA	-0.030837	6.4480555	-1.633927	0.103124	0.1634792	-5.605679
PES1P2	-0.094848	-1.410934	-1.633862	0.1031376	0.1634938	-5.605784
RP11-378A1	-0.273872	1.4913009	-1.633802	0.1031503	0.1635068	-5.605881
AC244157.1	0.1216829	-1.389251	1.6337615	0.1031587	0.1635131	-5.605945
KBTBD11-OT	-0.327532	-0.060862	-1.633556	0.1032019	0.1635744	-5.606276
RP1-131F15	-0.252724	2.1876322	-1.633499	0.103214	0.1635853	-5.606369
RP11-724N1	-0.314495	1.4926236	-1.633481	0.1032177	0.1635853	-5.606397
NDUFB8P2	-0.26504	0.9672394	-1.63342	0.1032306	0.1635985	-5.606496
GGA1	-0.023997	6.3702954	-1.633337	0.1032479	0.1636189	-5.606628
CETN4P	-0.28994	-0.07318	-1.63327	0.103262	0.1636342	-5.606737
PPP1R12C	-0.022514	6.4610051	-1.633168	0.1032836	0.1636612	-5.606902
LL22NC03-3	-0.065314	-1.494091	-1.632887	0.1033427	0.1637478	-5.607354
CLLU1	0.1963764	-1.120885	1.6328271	0.1033553	0.1637606	-5.60745
BACH1-AS1	0.279804	0.4509683	1.6328037	0.1033602	0.1637613	-5.607488
MIR3972	-0.065295	-1.508325	-1.632765	0.1033684	0.1637671	-5.607551
PSAT1	-0.077314	6.4399643	-1.632504	0.1034232	0.1638469	-5.60797
RP11-760D2	-0.326395	-0.470343	-1.632357	0.1034542	0.163889	-5.608207
RP5-1112D6	0.188647	3.1161417	1.632026	0.103524	0.1639923	-5.60874
RP11-166B2	0.2089174	-0.731258	1.6318273	0.1035659	0.1640516	-5.60906
RP3-43804.	0.2583793	-0.620441	1.6310943	0.1037205	0.1642894	-5.610239
FOXK2	-0.026848	6.2618169	-1.631018	0.1037367	0.1643078	-5.610362
RP11-373N2	-0.250596	2.1256136	-1.630989	0.1037427	0.1643102	-5.610408
CTD-2522E6	0.1894407	-0.892771	1.6302125	0.1039068	0.164563	-5.611657
OAS2	0.0705296	6.0623737	1.630098	0.103931	0.1645942	-5.611841
RP11-45P15	-0.174762	2.9159335	-1.630041	0.1039431	0.1646062	-5.611933

RP11-889L3	-0.148078	3.4215689	-1.630002	0.1039513	0.1646087	-5.611995
CHODL	0.3680285	1.0734473	1.6299906	0.1039537	0.1646087	-5.612013
ZNF433	-0.077036	4.8986734	-1.629909	0.1039709	0.1646288	-5.612144
ARID2	0.0284875	5.8475736	1.6297132	0.1040124	0.1646874	-5.612459
RP11-775C2	-0.117708	3.7609326	-1.629564	0.104044	0.1647303	-5.612699
RP11-49K24	0.2984759	0.8096584	1.6294416	0.1040698	0.1647641	-5.612896
AC116609.2	0.1975922	-1.158177	1.6291653	0.1041283	0.1648495	-5.61334
RPS20P33	0.2573961	1.159011	1.6290686	0.1041488	0.1648697	-5.613495
UNC93B1	0.0340798	6.1391676	1.6290625	0.1041501	0.1648697	-5.613505
DAAM1	-0.048731	6.0221539	-1.628736	0.1042192	0.1649697	-5.614029
RP4-593H12	-0.247043	-0.258807	-1.628722	0.1042223	0.1649697	-5.614052
RP11-79C23	0.2405852	-0.836479	1.6286575	0.1042359	0.164984	-5.614155
KCNMB2-AS1	-0.420333	2.5183413	-1.628544	0.10426	0.1650098	-5.614338
ZSCAN5D	0.2563682	-0.371746	1.6285379	0.1042612	0.1650098	-5.614347
GZMH	0.1228211	4.5146815	1.6283307	0.1043051	0.1650722	-5.61468
RP3-461F17	0.2075899	2.2667598	1.6282994	0.1043118	0.1650755	-5.61473
NT5CP2	-0.161208	-1.191433	-1.628244	0.1043234	0.1650868	-5.614819
RP11-91K8.	-0.287738	-0.034248	-1.628103	0.1043534	0.165127	-5.615045
TTLL13P	-0.220804	2.0737109	-1.62786	0.104405	0.1652016	-5.615436
MAST4	0.0553252	5.9599296	1.6278234	0.1044128	0.1652067	-5.615495
OSR2	-0.370556	2.9492579	-1.627798	0.1044182	0.1652081	-5.615536
RP3-412A9.	0.2650555	0.6746427	1.6273737	0.1045082	0.1653391	-5.616216
ZNF823	0.0418185	5.2436552	1.6273652	0.10451	0.1653391	-5.61623
DCUN1D3	0.035434	5.6546022	1.62719	0.1045472	0.1653908	-5.616511
IPO8P1	0.2325223	-0.531222	1.6269435	0.1045996	0.1654664	-5.616907
RP11-406A9	-0.197718	-1.090099	-1.626779	0.1046345	0.1655145	-5.61717
SEPT7P6	-0.148949	-1.194115	-1.626563	0.1046805	0.1655801	-5.617517
KLF2P3	-0.064783	-1.508038	-1.625832	0.1048361	0.165819	-5.61869
RP11-182J1	-0.282779	0.2488613	-1.625731	0.1048576	0.1658459	-5.618852
GOLGA2P11	0.3437957	-0.056088	1.6256822	0.1048679	0.1658549	-5.618929
PGD	-0.036369	6.6621863	-1.625647	0.1048754	0.1658596	-5.618986
MBD1	-0.019394	6.3303392	-1.625552	0.1048956	0.1658824	-5.619138
PRDX2P1	-0.135232	-1.266031	-1.625537	0.1048989	0.1658824	-5.619162
MTERF2	-0.030397	5.8184737	-1.625509	0.1049047	0.1658845	-5.619207
CTD-213801	-0.19033	-1.132101	-1.625422	0.1049233	0.1659065	-5.619346
RP11-218M2	-0.164439	-1.176918	-1.625213	0.104968	0.16597	-5.619682
AC009228.1	0.1475832	-1.243269	1.6246445	0.1050891	0.1661543	-5.620592
NCAM1-AS1	-0.322666	-0.207035	-1.624571	0.1051047	0.1661718	-5.62071
GYS1	0.0297538	5.9650785	1.6244882	0.1051224	0.1661927	-5.620843
ZNF555	0.0778258	5.1554878	1.624203	0.1051833	0.1662816	-5.6213
GS1-293C5.	-0.279908	0.8724053	-1.624098	0.1052057	0.16631	-5.621468
RP4-651E10	-0.104391	-1.414044	-1.623989	0.1052289	0.1663395	-5.621642
CTD-2623N2	0.226744	-0.732883	1.6239595	0.1052352	0.1663422	-5.62169
TMPRSS2	-0.118014	6.1145652	-1.623914	0.1052449	0.1663503	-5.621762
TMEM213	0.2173513	-0.868206	1.6235353	0.1053259	0.1664711	-5.622369
HIST1H2BB	-0.176158	-1.074861	-1.623214	0.1053946	0.1665681	-5.622884
RP11-93H24	0.1433233	3.3434805	1.6232054	0.1053964	0.1665681	-5.622897
GPR179	0.2924115	0.2840017	1.6230897	0.1054211	0.1665969	-5.623082
CTC-529G1.	0.2030013	-1.151813	1.6230774	0.1054237	0.1665969	-5.623102
RP11-806L2	0.3101328	0.8277472	1.6230193	0.1054362	0.1666093	-5.623195
CTD-2200P1	-0.119684	-1.383723	-1.622886	0.1054646	0.1666471	-5.623408

AC010127.3	-0.403232	0.5215904	-1.622691	0.1055063	0.1667057	-5.62372
RP11-274M1	-0.137555	-1.284292	-1.622587	0.1055287	0.1667339	-5.623887
HSD3B2	-0.361375	0.9735815	-1.622294	0.1055914	0.1668258	-5.624356
RP1-300G12	-0.149848	-1.214484	-1.622244	0.105602	0.1668353	-5.624435
AP3M1	0.0223857	6.2736269	1.6221788	0.105616	0.1668502	-5.62454
DBIL5P2	-0.256697	-0.234853	-1.621714	0.1057155	0.1670002	-5.625283
AC074117.1	0.1636578	-1.12127	1.6215773	0.1057449	0.1670393	-5.625502
KDELC2	0.0524577	5.8550846	1.6213729	0.1057887	0.1671013	-5.625829
AC012506.3	0.3097086	0.0357626	1.6212242	0.1058206	0.1671445	-5.626066
PRKY	-0.489953	2.9171129	-1.621037	0.1058607	0.1672006	-5.626366
OSBPL1A	0.0602188	5.8859829	1.6207866	0.1059145	0.1672783	-5.626766
KIF27	0.0861636	4.3522916	1.6207226	0.1059282	0.1672927	-5.626868
RP11-304M2	-0.075252	-1.475694	-1.620301	0.1060188	0.1674255	-5.627542
C8orf76	-0.047896	5.6950773	-1.620288	0.1060215	0.1674255	-5.627562
RP11-264J4	0.2120352	-0.830578	1.6198658	0.1061123	0.1675617	-5.628237
CTD-2377D2	0.1237178	-1.402355	1.6197446	0.1061383	0.1675956	-5.628431
RPL31P61	0.2291994	-0.515131	1.6196142	0.1061664	0.1676326	-5.628639
AC005532.5	-0.305774	0.4931527	-1.619493	0.1061925	0.1676647	-5.628834
CEACAMP5	-0.091859	-1.445079	-1.619477	0.1061959	0.1676647	-5.628859
TOMM5	-0.033097	5.9527815	-1.619206	0.1062541	0.1677493	-5.629291
CNBD2	-0.136879	3.1763578	-1.619152	0.1062659	0.1677606	-5.629378
GOLIM4	-0.03982	6.4607821	-1.618692	0.1063648	0.1679095	-5.630112
AC008269.2	-0.151091	-1.188126	-1.618453	0.1064163	0.1679835	-5.630493
ZDHHC6	-0.024639	6.1857513	-1.618182	0.1064748	0.1680687	-5.630927
CTC-260F20	-0.070281	4.1957381	-1.617919	0.1065315	0.1681509	-5.631346
RP4-549F15	-0.13807	-1.270604	-1.617848	0.1065468	0.1681677	-5.631459
UBA52P3	-0.239034	-0.485989	-1.617771	0.1065632	0.1681865	-5.631581
ZNF343	-0.033097	5.576205	-1.617643	0.1065909	0.1682229	-5.631786
AC006042.7	-0.152939	-1.171603	-1.617555	0.10661	0.1682419	-5.631927
RP11-805F1	0.1303937	-1.265206	1.6175446	0.1066122	0.1682419	-5.631943
AC004158.3	-0.106721	-1.403575	-1.617268	0.1066718	0.1683287	-5.632384
RP11-133K1	-0.153392	-1.182085	-1.616984	0.1067333	0.1684184	-5.632838
LLNLF-65H9	-0.170476	-1.278501	-1.6166	0.1068161	0.1685419	-5.63345
PCMTD1	-0.03139	6.2497402	-1.616573	0.1068219	0.1685438	-5.633492
RP13-52K8.	0.1350508	-1.299097	1.6165106	0.1068354	0.1685578	-5.633592
LYPD8	-0.442949	2.2969885	-1.616462	0.106846	0.1685671	-5.63367
GOLGA8UP	0.1557693	-1.132606	1.6164306	0.1068527	0.1685705	-5.633719
RP11-159M1	-0.12577	-1.332504	-1.616275	0.1068864	0.1686164	-5.633968
LGI3	-0.411858	1.7680255	-1.615763	0.1069972	0.1687837	-5.634784
AC007365.3	0.212018	-0.806928	1.6155891	0.1070347	0.1688357	-5.63506
CH17-140K2	0.1428709	-1.276454	1.615557	0.1070417	0.1688394	-5.635112
RP11-65N13	-0.212007	-0.79285	-1.615511	0.1070517	0.1688479	-5.635185
UBTFL11	-0.112514	-1.36551	-1.615292	0.107099	0.1689152	-5.635533
RP11-1007C	-0.213809	2.4344211	-1.615036	0.1071546	0.1689955	-5.635942
CTD-2311M2	-0.342642	1.2336165	-1.615006	0.107161	0.1689984	-5.63599
RP11-379F4	-0.139431	2.9681746	-1.614931	0.1071772	0.1690096	-5.636108
RP4-604G5.	-0.358039	0.7429976	-1.614919	0.1071798	0.1690096	-5.636128
RP11-440L1	0.0965275	4.2640113	1.6149091	0.107182	0.1690096	-5.636144
FAM133A	-0.513591	1.9667497	-1.614736	0.1072196	0.1690615	-5.63642
CCDC38	-0.211835	3.8780546	-1.614711	0.107225	0.1690627	-5.63646
RP11-427P5	0.2521794	-0.032818	1.6146224	0.1072441	0.1690856	-5.6366

KDELC1	0.0563603	5.5877169	1.6145799	0.1072533	0.1690889	-5.636668
FOXA1	-0.082903	6.186054	-1.61457	0.1072554	0.1690889	-5.636683
RP1-65P5.5	0.2526324	-0.811921	1.6143773	0.1072973	0.1691475	-5.636991
PGAM5P1	-0.104108	-1.384657	-1.614291	0.1073161	0.1691699	-5.637129
RP11-594N1	0.2700768	-0.725789	1.6142598	0.1073227	0.1691731	-5.637178
AC007131.2	-0.103166	-1.408468	-1.614125	0.107352	0.1692119	-5.637393
RP13-81N3.	-0.06298	-1.492782	-1.614086	0.1073605	0.169218	-5.637455
CHRAC1	-0.03169	6.1697671	-1.613659	0.1074532	0.1693568	-5.638135
RP11-219E7	0.1049334	-1.398642	1.613606	0.1074646	0.1693675	-5.638218
RHOQP1	0.2816413	1.8030381	1.6135319	0.1074807	0.1693855	-5.638336
RP11-81A22	-0.06627	5.2680569	-1.613463	0.1074956	0.1694017	-5.638446
USP27X	0.0980165	5.0208629	1.6130911	0.1075765	0.1695218	-5.639038
NRAP	0.2488557	4.7656604	1.6129781	0.107601	0.1695477	-5.639217
WRNIP1	-0.025477	6.3515476	-1.612969	0.1076031	0.1695477	-5.639232
CDC37P1	0.2821463	-0.254304	1.6129512	0.1076069	0.1695477	-5.63926
RP11-403A3	-0.109527	-1.406779	-1.612874	0.1076236	0.1695608	-5.639383
EEF1A1P34	-0.145223	-1.205887	-1.612856	0.1076276	0.1695608	-5.639412
RP11-1228E	-0.108015	-1.367124	-1.612849	0.1076291	0.1695608	-5.639423
RP11-195C7	-0.094943	-1.420188	-1.612621	0.1076787	0.1696317	-5.639786
LA16c-380A	0.2076714	-0.862091	1.6125418	0.1076959	0.1696514	-5.639911
TMSB10P2	-0.112063	-1.362756	-1.612398	0.1077272	0.1696906	-5.640141
HSPA8P15	0.2835652	0.4637196	1.6123846	0.1077301	0.1696906	-5.640161
AP5Z1	-0.027491	6.2970412	-1.612334	0.107741	0.1697004	-5.640241
MIR148A	-0.298267	0.6780858	-1.61218	0.1077745	0.1697459	-5.640486
RNASEH2CP1	-0.069605	-1.482252	-1.611565	0.1079084	0.1699495	-5.641464
SEPHS1	-0.019743	6.4399509	-1.611106	0.1080086	0.1700999	-5.642194
PNPLA5	-0.314371	-0.357959	-1.610953	0.1080419	0.170145	-5.642437
UBXN8	-0.047809	5.8620705	-1.610925	0.1080479	0.1701471	-5.642481
RPL36AP21	-0.216246	-0.683144	-1.610609	0.108117	0.1702453	-5.642984
CHD9	-0.036823	6.0259536	-1.610597	0.1081196	0.1702453	-5.643003
RP11-419C1	-0.136977	-1.326736	-1.610203	0.1082055	0.1703733	-5.643628
SESTD1	0.0419513	6.0673646	1.6100368	0.1082418	0.1704207	-5.643892
C2orf48	0.3062187	2.1238884	1.6100225	0.1082449	0.1704207	-5.643915
RP4-533D7.	0.186731	-0.976524	1.6099287	0.1082654	0.1704455	-5.644064
LINC01220	0.2978074	0.8100796	1.6097278	0.1083093	0.1705057	-5.644383
RP13-766D2	0.2040005	-1.026453	1.6097111	0.108313	0.1705057	-5.644409
AKAP17A	0.0253557	6.2439341	1.609587	0.1083401	0.170541	-5.644606
RP11-567F1	0.2647736	1.1441319	1.6095567	0.1083467	0.1705441	-5.644654
TM9SF1	-0.034522	5.4731976	-1.609435	0.1083733	0.1705779	-5.644848
EXOC1	0.0265219	5.8814763	1.6094158	0.1083775	0.1705779	-5.644878
TMEM262	0.0791763	4.0656291	1.6088774	0.1084952	0.1707558	-5.645732
CABP2	-0.081459	-1.448972	-1.608519	0.1085736	0.1708718	-5.646301
AC010976.2	0.1603666	3.6694335	1.6079719	0.1086935	0.1710531	-5.647169
TSPAN13	0.1013469	5.9672145	1.6079054	0.1087081	0.1710686	-5.647274
RP11-573N1	-0.069984	-1.474007	-1.607862	0.1087175	0.1710761	-5.647343
C14orf79	-0.04631	5.3352843	-1.607814	0.1087281	0.1710854	-5.647419
DYNLT3P2	-0.241512	-0.439865	-1.607302	0.1088403	0.1712522	-5.648231
GXYLT1P5	-0.311013	-0.274454	-1.607288	0.1088435	0.1712522	-5.648254
MCM5	-0.037973	6.4215258	-1.607003	0.1089059	0.1713318	-5.648705
RP11-304F1	-0.091393	-1.445284	-1.606995	0.1089076	0.1713318	-5.648717
RP11-177G2	0.2086713	-0.97294	1.606993	0.1089081	0.1713318	-5.648721

PPP4R1-AS1	-0.277681	0.1106075	-1.606798	0.1089509	0.1713877	-5.64903
RP11-368I2	0.2598776	-0.696419	1.6067882	0.1089531	0.1713877	-5.649046
AD000671.6	0.2573352	0.029867	1.6067127	0.1089696	0.1714064	-5.649165
AC009336.1	0.2334394	-0.698508	1.6064363	0.1090303	0.1714945	-5.649603
RP11-356C4	-0.12999	-1.32826	-1.606104	0.1091034	0.1716019	-5.65013
HPS4	-0.027841	6.1072524	-1.605746	0.1091821	0.1717184	-5.650697
C7orf33	-0.195074	-1.077198	-1.605323	0.109275	0.1718571	-5.651366
TBR1	0.15076	-1.238789	1.6051558	0.1093119	0.1719077	-5.651631
STK38	-0.027389	6.2713594	-1.60495	0.1093572	0.1719715	-5.651957
TVP23B	0.0401028	5.8379051	1.6047603	0.109399	0.1720212	-5.652257
RP4-555D20	0.1557194	-1.32155	1.6047536	0.1094004	0.1720212	-5.652268
RP11-701I2	-0.123904	-1.303481	-1.604742	0.1094029	0.1720212	-5.652286
RP11-755J8	-0.2074	-0.921406	-1.604709	0.1094103	0.1720254	-5.652339
RP11-1000B	0.2746081	1.9102889	1.6044192	0.1094741	0.1721183	-5.652797
HNRNPA1P4	0.2577258	1.5354212	1.6043033	0.1094996	0.172151	-5.652981
PSG1	-0.101856	-1.416311	-1.604195	0.1095234	0.172181	-5.653151
STOX2	0.1134986	5.0953221	1.6036541	0.1096428	0.1723612	-5.654008
CTD-2201E1	0.2862732	0.9753076	1.6036281	0.1096485	0.1723628	-5.654049
RP11-962G1	-0.42823	0.4193785	-1.603559	0.1096639	0.1723795	-5.654159
CTU1	-0.056039	5.1412613	-1.603476	0.109682	0.1723915	-5.654289
AC079780.3	0.1555564	-1.219614	1.6034683	0.1096838	0.1723915	-5.654302
AC009974.1	-0.271963	-0.216026	-1.60346	0.1096856	0.1723915	-5.654315
RP11-237D3	-0.172144	-1.207449	-1.603341	0.1097118	0.1724198	-5.654503
RP5-857K21	-0.055445	6.6353402	-1.603336	0.1097131	0.1724198	-5.654511
RP11-1094M	-0.210989	-0.8273	-1.603153	0.1097534	0.1724757	-5.6548
APOLD1	-0.059109	6.0352159	-1.602999	0.1097873	0.1725216	-5.655043
PKN2-AS1	-0.300389	0.4216443	-1.602899	0.1098094	0.1725488	-5.655201
LINC01234	0.4752182	1.4425993	1.602697	0.1098541	0.1726117	-5.655521
IL1R2	-0.075165	5.5643427	-1.602657	0.1098629	0.172618	-5.655584
AC092155.1	-0.265895	1.2507501	-1.60224	0.1099551	0.1727555	-5.656244
CHAF1A	-0.038715	5.9379985	-1.602198	0.1099644	0.1727627	-5.65631
CASC20	-0.409667	0.0734661	-1.602091	0.109988	0.1727923	-5.656478
KRT83	0.262261	-0.785658	1.6019918	0.11001	0.1728194	-5.656636
APOO	-0.037574	5.8015819	-1.60191	0.110028	0.1728403	-5.656764
COX6B1P4	-0.300625	0.08028	-1.601599	0.110097	0.1729412	-5.657257
WDR37	0.0288694	5.8163964	1.6015021	0.1101184	0.1729673	-5.657409
SLC25A32	-0.033518	6.0515041	-1.60142	0.1101364	0.1729883	-5.657538
RPS16P9	-0.143827	-1.284372	-1.601224	0.1101799	0.1730485	-5.657848
AC004549.6	-0.211007	-0.681945	-1.601204	0.1101843	0.1730485	-5.65788
ZNF443	0.0497264	4.8666179	1.6011286	0.1102011	0.1730674	-5.657999
SCARNA24	0.2541622	-0.065837	1.6010939	0.1102088	0.1730721	-5.658054
TSSC2	0.2914485	2.3120561	1.6009633	0.1102377	0.1731101	-5.65826
TMPRSS11E	0.2219948	-0.944787	1.6008441	0.1102641	0.1731441	-5.658448
RPSAP55	-0.265064	0.3841429	-1.600647	0.1103077	0.1732051	-5.658759
AC009299.4	-0.090194	-1.453792	-1.600597	0.1103189	0.1732152	-5.658839
AP001172.2	-0.129176	-1.31469	-1.600344	0.1103751	0.173296	-5.659239
RNA5SP334	0.2259676	-0.965898	1.6002931	0.1103863	0.1733061	-5.659318
RP11-335L2	-0.2676	1.1207847	-1.600175	0.1104125	0.1733398	-5.659505
SLC2A1-AS1	0.3212985	1.4193956	1.5995767	0.1105452	0.1735408	-5.660449
RP13-347D8	-0.222543	-0.74477	-1.599353	0.110595	0.1736114	-5.660802
RP11-429D1	0.2184068	-0.729624	1.5988921	0.1106974	0.1737646	-5.661529

TCP1P1	0.2291673	-0.495283	1.598699	0.1107403	0.1738245	-5.661833
MT1M	0.2603471	4.360727	1.5985625	0.1107706	0.1738647	-5.662049
RP11-47909	0.2672371	0.0177771	1.598513	0.1107816	0.1738745	-5.662127
UEVLD	0.0272937	5.8794453	1.5984619	0.110793	0.1738849	-5.662207
ZSWIM3	-0.032584	5.1785916	-1.598147	0.110863	0.1739872	-5.662703
RP11-347C1	-0.188981	2.8122166	-1.597681	0.1109668	0.1741427	-5.663438
RP11-496I9	0.1198069	4.4245026	1.5972716	0.1110581	0.1742784	-5.664083
CTD-2369P2	0.197544	2.465493	1.596944	0.1111311	0.1743855	-5.664599
AP000477.3	-0.095956	-1.405599	-1.596556	0.1112176	0.1745137	-5.66521
YAP1P1	0.128934	-1.310983	1.5964857	0.1112333	0.1745309	-5.665321
AC004448.5	0.1837092	-1.068179	1.59621	0.1112948	0.1746199	-5.665755
RP11-725M2	-0.079976	-1.462989	-1.596121	0.1113148	0.1746437	-5.665896
TOP3A	-0.028737	5.9816679	-1.596089	0.1113218	0.1746472	-5.665945
RP11-404P2	0.2113408	-0.984625	1.5959568	0.1113514	0.1746843	-5.666154
RP11-290M5	-0.0977	-1.39531	-1.59594	0.1113551	0.1746843	-5.66618
REXO2	-0.029177	6.1592523	-1.595843	0.1113769	0.1747111	-5.666333
C9orf171	0.1651526	-1.083176	1.5956508	0.1114197	0.1747708	-5.666636
RP11-37606	0.2235795	-0.895511	1.5955411	0.1114442	0.1748017	-5.666808
FP236383.6	-0.12117	-1.355052	-1.595152	0.1115312	0.1749307	-5.66742
RP3-406P24	0.2090797	3.0564116	1.5949112	0.1115851	0.1750012	-5.667799
RP11-619A1	-0.249366	-0.699807	-1.594908	0.1115858	0.1750012	-5.667804
RP11-111J6	-0.177106	2.4680113	-1.594796	0.1116109	0.175033	-5.66798
GTF2A1	-0.028211	6.1598688	-1.594471	0.1116836	0.1751396	-5.668492
RP11-391H1	0.3002892	1.5896161	1.5944284	0.1116931	0.175147	-5.668559
RP11-424G1	-0.40189	1.5500421	-1.594406	0.1116981	0.1751473	-5.668594
RP11-314D7	-0.218526	-0.985587	-1.594297	0.1117225	0.1751779	-5.668765
RP11-480C2	0.1789383	-1.1171	1.5940924	0.1117684	0.1752424	-5.669087
RP11-1079K	0.1762652	-0.963775	1.5936093	0.1118767	0.1754046	-5.669847
RP11-65I12	0.3919196	1.9794719	1.5934146	0.1119203	0.1754597	-5.670153
RP11-206F1	0.234755	-0.179914	1.5934097	0.1119214	0.1754597	-5.67016
NKX2-3	0.3410781	0.2944947	1.5933439	0.1119361	0.1754753	-5.670264
KRTAP20-2	0.1946166	-1.184274	1.5932593	0.1119551	0.1754975	-5.670397
RP11-27504	0.116364	-1.363743	1.5932033	0.1119677	0.1755096	-5.670485
SEPT14	0.3221917	-0.690336	1.5931666	0.1119759	0.175515	-5.670542
ZNF425	-0.116362	4.6106019	-1.592901	0.1120356	0.175601	-5.67096
LRRTM2	-0.278201	1.6895885	-1.592772	0.1120644	0.1756386	-5.671162
MADCAM1	-0.145609	3.7441918	-1.592572	0.1121094	0.1756942	-5.671477
FOXC2-AS1	0.1290144	-1.316726	1.5925714	0.1121095	0.1756942	-5.671478
AC004453.8	-0.095718	4.4216057	-1.592547	0.112115	0.1756952	-5.671516
MGC50722	0.4517332	2.7225149	1.5924782	0.1121304	0.1757068	-5.671624
GPAT2	0.1386377	4.3601317	1.5924713	0.112132	0.1757068	-5.671635
RP11-326N1	0.2134654	-0.931488	1.5921941	0.1121943	0.1757969	-5.67207
LCT	0.2377712	-0.631985	1.5920329	0.1122305	0.1758461	-5.672323
RP11-742B1	-0.417349	1.0088931	-1.591897	0.1122611	0.1758833	-5.672537
GTF2H1	0.0187655	6.1468402	1.5918841	0.1122639	0.1758833	-5.672557
RP11-27K13	0.2518708	1.6614952	1.5918267	0.1122768	0.175896	-5.672647
RP1-206D15	-0.263201	2.8708935	-1.591109	0.1124382	0.1761413	-5.673774
RP11-680G1	-0.257632	-0.310497	-1.590757	0.1125175	0.176258	-5.674327
RP11-155D1	0.1709525	-1.070128	1.5905989	0.1125531	0.1763061	-5.674574
RPL12P38	-0.256389	0.9491401	-1.590396	0.1125989	0.1763704	-5.674894
CDK11A	0.0570132	5.235153	1.5902714	0.1126269	0.1764043	-5.675088

EEF1E1P1	-0.282444	1.1713627	-1.590256	0.1126303	0.1764043	-5.675112
SYT12	0.1195061	5.870465	1.5901117	0.1126629	0.1764478	-5.675339
AC010149.4	-0.283587	-0.106691	-1.590078	0.1126704	0.176452	-5.675391
AC246787.5	0.0987719	-1.416341	1.5900175	0.1126841	0.176466	-5.675487
HIST2H2BA	0.1512385	-1.252393	1.5898499	0.1127219	0.1765176	-5.675749
PPIAP33	0.2195952	-0.612197	1.5898196	0.1127287	0.1765201	-5.675797
RP1-78B3.1	0.2663272	1.2604215	1.5897997	0.1127332	0.1765201	-5.675828
CACNA1S	0.3141921	0.7104402	1.5895639	0.1127864	0.1765928	-5.676198
TM4SF1	0.0568196	6.5694873	1.589551	0.1127893	0.1765928	-5.676218
CTD-2026G2	-0.154406	-1.206971	-1.589312	0.1128434	0.1766698	-5.676593
RP11-445P1	-0.291754	1.9011926	-1.589131	0.1128842	0.1767261	-5.676877
RP11-429J1	0.3049115	-0.039268	1.5890033	0.112913	0.1767637	-5.677077
PTPRH	0.133968	6.0198002	1.5887784	0.1129638	0.1768356	-5.677429
GDPD1	0.0906458	4.8216696	1.5885938	0.1130055	0.1768933	-5.677719
CAMTA2	0.0298701	6.0758055	1.5884513	0.1130377	0.1769361	-5.677942
CTC-366B18	0.1009282	3.4981012	1.5883063	0.1130704	0.1769798	-5.678169
CTAGE9	0.2669744	-0.051515	1.5881275	0.1131109	0.1770355	-5.678449
AK9	0.0627127	4.9162788	1.5880452	0.1131295	0.177057	-5.678578
XXbac-BPG3	-0.264692	-0.342661	-1.587963	0.113148	0.1770785	-5.678706
STPG1	0.0510952	5.1887993	1.5878167	0.1131812	0.1771227	-5.678936
CTD-2132N1	-0.211501	2.846264	-1.587761	0.1131938	0.1771349	-5.679023
PLEKHM3	0.0930563	4.6530913	1.5876646	0.1132156	0.1771614	-5.679174
SMYD3	0.0612136	5.4517969	1.5875483	0.1132419	0.177195	-5.679356
AC010970.2	-0.365409	0.2361379	-1.587495	0.1132539	0.1772061	-5.679439
BNIP3P30	-0.304903	-0.40107	-1.587441	0.1132662	0.1772135	-5.679525
RP11-72304	0.2261743	1.9189505	1.5874318	0.1132683	0.1772135	-5.679539
NIPSNAP3B	0.0892078	4.0394304	1.5874103	0.1132731	0.1772135	-5.679572
ASCC1	0.0293111	5.8932001	1.587375	0.1132811	0.1772184	-5.679628
LINC00645	-0.264326	-0.767798	-1.587209	0.1133188	0.1772623	-5.679888
RNU6-1017F	-0.120425	-1.396853	-1.587192	0.1133225	0.1772623	-5.679914
BLOC1S4	-0.03149	5.8007562	-1.587187	0.1133238	0.1772623	-5.679923
HYPM	-0.096286	-1.426094	-1.586842	0.1134018	0.1773768	-5.680462
AP000807.1	-0.256523	-0.547932	-1.586696	0.1134349	0.1774209	-5.680691
NDC80	0.0808821	5.4261794	1.5866465	0.1134461	0.1774309	-5.680768
ZBTB33	-0.037013	6.2597794	-1.58659	0.1134589	0.1774433	-5.680856
RP11-300J1	-0.179172	-1.035955	-1.586439	0.1134932	0.1774894	-5.681093
FAN1	-0.047691	5.7367493	-1.586406	0.1135007	0.1774934	-5.681145
MBNL1-AS1	0.11362	4.0156675	1.5863477	0.1135139	0.1775065	-5.681236
RP1-155D22	-0.082394	-1.446687	-1.586274	0.1135306	0.1775232	-5.681351
RN7SL130P	-0.253536	1.8099864	-1.586258	0.1135343	0.1775232	-5.681377
MIR6835	0.2211177	-0.752145	1.5861778	0.1135524	0.1775439	-5.681502
RP11-235E1	0.271481	0.4387493	1.5857637	0.1136464	0.1776832	-5.68215
RP11-288G3	-0.170223	-1.086526	-1.585723	0.1136556	0.17769	-5.682213
TSPY6P	-0.190288	-1.233116	-1.585689	0.1136632	0.1776904	-5.682266
GMCL1	0.0302694	6.0208927	1.5856791	0.1136656	0.1776904	-5.682282
RP11-369E1	-0.169041	-1.232475	-1.585317	0.1137478	0.1778112	-5.682848
RBP3	0.1024545	-1.414277	1.5851798	0.113779	0.1778524	-5.683063
CDON	0.066876	5.2283991	1.5851127	0.1137942	0.1778679	-5.683168
CTD-2328D6	-0.227807	2.8820188	-1.585093	0.1137986	0.1778679	-5.683198
RP1-257I9.	-0.069276	-1.485481	-1.58494	0.1138334	0.1779147	-5.683438
AP000647.3	0.1318471	-1.351593	1.5848924	0.1138443	0.177924	-5.683512

U91328.2	-0.187583	-0.984658	-1.584793	0.1138669	0.1779517	-5.683668
MTND2P5	-0.164119	-1.221799	-1.584711	0.1138855	0.1779732	-5.683796
RPL23AP82	0.0434492	5.2285956	1.5844099	0.113954	0.1780726	-5.684266
AC007040.1	0.2357464	-0.48401	1.5843598	0.1139654	0.178079	-5.684345
RP11-620J1	-0.20146	-0.707221	-1.584349	0.1139678	0.178079	-5.684361
AFP	0.2191254	5.6806371	1.5843015	0.1139787	0.1780883	-5.684436
CRTC1	0.0324042	5.8095323	1.584026	0.1140413	0.1781786	-5.684867
RP11-375I2	0.2827005	0.6181867	1.5839615	0.114056	0.1781939	-5.684967
TTC17	-0.023556	6.4888548	-1.583912	0.1140672	0.1782012	-5.685044
FAM206A	0.0322734	5.7432265	1.5838981	0.1140704	0.1782012	-5.685066
RP11-572N2	-0.079563	-1.471132	-1.583801	0.1140925	0.1782281	-5.685218
ANO5	0.2966262	4.6333537	1.5835748	0.1141441	0.178301	-5.685571
RP11-536L3	0.2814454	0.1587536	1.5835136	0.114158	0.1783151	-5.685667
RP11-401O9	0.1941596	-1.05506	1.5834877	0.1141639	0.1783167	-5.685707
LA16c-306E	-0.304382	0.9192239	-1.583401	0.1141836	0.1783398	-5.685843
DKC1	0.0257048	6.3103709	1.5833312	0.1141996	0.1783571	-5.685952
LINC00563	0.3642669	0.6874633	1.5831032	0.1142515	0.1784306	-5.686308
VN1R110P	0.221395	-0.910127	1.5827416	0.114334	0.1785458	-5.686873
MYBPC1	-0.317812	2.3190798	-1.582737	0.1143351	0.1785458	-5.68688
RP5-991G20	0.1837212	-1.010892	1.5826445	0.1143561	0.178571	-5.687024
LINC00640	-0.287144	-0.111218	-1.582582	0.1143704	0.1785857	-5.687122
MIR200B	0.0937496	-1.419157	1.5824834	0.1143928	0.1786131	-5.687276
RP11-686G8	-0.221608	-0.628808	-1.582361	0.1144208	0.1786492	-5.687467
C1orf52	-0.024635	5.6739321	-1.582315	0.1144314	0.178658	-5.687539
RP11-179B1	0.1047548	-1.398742	1.5821401	0.1144712	0.1787126	-5.687812
BRCC3	0.0329025	5.8480364	1.5817819	0.114553	0.1788326	-5.688371
CTD-2515C1	0.2361479	-0.937575	1.5815681	0.1146018	0.1788963	-5.688704
TRPM7	-0.037478	6.2463096	-1.58156	0.1146036	0.1788963	-5.688716
EPS8L2	-0.034541	6.6925343	-1.581467	0.1146249	0.1789188	-5.688862
GAL3ST3	0.1520266	-1.274954	1.5814343	0.1146324	0.1789188	-5.688913
CCDC178	-0.271026	-0.532444	-1.581433	0.1146327	0.1789188	-5.688915
GBP3	-0.085448	5.8837028	-1.581347	0.1146523	0.1789417	-5.689049
C1orf177	-0.22899	2.8083544	-1.581216	0.1146823	0.1789808	-5.689253
RP11-38J22	0.1251317	-1.345168	1.5811909	0.114688	0.1789822	-5.689293
CTA-246H3.	0.2926017	1.4050358	1.5811505	0.1146973	0.1789889	-5.689356
CTD-2193G5	0.1484141	-1.180146	1.5809794	0.1147364	0.1790423	-5.689623
GOPC	0.0272214	6.0584557	1.5808785	0.1147595	0.1790707	-5.68978
CTD-2568P8	0.2435919	-0.555548	1.5808493	0.1147662	0.1790735	-5.689826
CXorf67	0.3409711	-0.46523	1.5807012	0.1148	0.1791187	-5.690057
RP11-60A24	-0.250137	-0.534622	-1.580587	0.1148263	0.1791519	-5.690235
RP11-96C21	0.2814614	-0.130259	1.5805426	0.1148363	0.17916	-5.690304
EEF1A1P25	0.2345596	1.7943735	1.5804438	0.1148589	0.1791876	-5.690458
RP11-90H3.	0.1819854	-0.940676	1.5804002	0.1148689	0.1791955	-5.690526
AC008746.1	0.1361731	-1.25633	1.580342	0.1148822	0.1792086	-5.690617
RP11-102N1	0.188859	3.1275423	1.5801765	0.1149201	0.1792601	-5.690875
RP11-1016E	0.1705175	-1.148379	1.5801521	0.1149257	0.1792611	-5.690913
TIGD1	0.0692831	4.9827367	1.5800653	0.1149456	0.1792788	-5.691048
AP006285.2	0.3101233	2.4093745	1.5800599	0.1149468	0.1792788	-5.691056
CTD-2309H9	-0.185367	-1.178616	-1.580009	0.1149586	0.1792894	-5.691136
RP11-661C3	0.2283511	-0.520084	1.5797607	0.1150154	0.1793703	-5.691523
C10orf67	-0.317548	-0.271052	-1.579472	0.1150816	0.1794596	-5.691973

RP11-10102	-0.098559	-1.439047	-1.579468	0.1150825	0.1794596	-5.691979
RP11-110G2	-0.072808	4.5147335	-1.579247	0.115133	0.1795297	-5.692323
TARBP1	0.0412352	6.1789491	1.579229	0.1151372	0.1795297	-5.692351
RP11-39C10	0.2657164	0.9352762	1.5790292	0.1151831	0.1795935	-5.692662
CPB1	0.3497469	1.2037061	1.578982	0.1151939	0.1796027	-5.692736
GAPDHP73	0.2454472	-0.385312	1.578721	0.1152538	0.1796884	-5.693143
AC006480.1	-0.164581	-1.048241	-1.578621	0.1152768	0.1797119	-5.693298
AC116614.1	0.165631	-1.242071	1.5786125	0.1152787	0.1797119	-5.693311
RP11-106A1	-0.273137	0.5108871	-1.578571	0.1152881	0.1797189	-5.693375
FARP1-AS1	0.219728	-0.759949	1.5782333	0.1153658	0.1798323	-5.693902
RP5-991C6.	-0.177006	-1.061476	-1.578162	0.1153822	0.1798429	-5.694014
NOTCH4	0.0410271	6.0050856	1.5781607	0.1153824	0.1798429	-5.694015
RP11-442J2	0.1233657	-1.388308	1.5780434	0.1154094	0.1798772	-5.694198
FAM183DP	0.1553045	-1.196592	1.5779171	0.1154384	0.1799148	-5.694394
ZBTB8A	-0.056496	5.1273411	-1.577816	0.1154617	0.1799433	-5.694552
DSG3	0.3202786	-0.588098	1.5777179	0.1154842	0.1799672	-5.694704
RP11-70502	0.2080663	-1.013806	1.5777064	0.1154868	0.1799672	-5.694722
RP11-349K1	0.1579204	-1.328052	1.57766	0.1154975	0.1799761	-5.694794
GRAMD4P2	0.1328989	-1.327526	1.5775753	0.115517	0.1799988	-5.694926
AP000240.9	0.2223175	2.6469517	1.5772184	0.115599	0.180119	-5.695482
RPL34P31	-0.224897	-0.597315	-1.577145	0.1156159	0.1801376	-5.695596
ASF1A	-0.03395	5.9026321	-1.576667	0.1157259	0.1803012	-5.696339
STXBP2	0.042684	6.1831791	1.5766273	0.1157351	0.1803079	-5.696401
RNU6-117P	0.1935961	-0.898331	1.576533	0.1157568	0.180334	-5.696548
KCNQ1-AS1	0.1658907	-1.185178	1.5763889	0.11579	0.180378	-5.696772
CTD-2143L2	0.1184536	-1.359594	1.5762905	0.1158127	0.1804056	-5.696925
RP11-667M1	-0.191638	-0.877906	-1.576265	0.1158185	0.180407	-5.696964
RP5-952N6.	0.1501274	-1.290536	1.5759831	0.1158835	0.1804849	-5.697403
PTPN4	-0.030778	5.7355876	-1.575977	0.1158849	0.1804849	-5.697413
XXbac-BPG2	0.1697517	-1.038775	1.5759696	0.1158866	0.1804849	-5.697424
RN7SL535P	0.2050399	-0.788043	1.5759625	0.1158883	0.1804849	-5.697435
BPNT1	-0.027254	6.2601428	-1.575927	0.1158964	0.1804898	-5.69749
RP11-381E2	0.1775958	-0.999604	1.5757716	0.1159323	0.180538	-5.697732
RP11-127L2	0.1856993	-0.951464	1.5756433	0.1159619	0.1805764	-5.697931
GSTM1	-0.357199	4.2559961	-1.575595	0.115973	0.1805861	-5.698007
HOXD4	0.3519303	0.7600033	1.5755703	0.1159787	0.1805872	-5.698045
RP11-302F1	0.0972952	-1.402924	1.5755188	0.1159906	0.180598	-5.698125
IRAK1	-0.032511	6.6920789	-1.575287	0.116044	0.1806735	-5.698485
UBE2D3P1	0.2643106	1.2462637	1.5752423	0.1160544	0.1806797	-5.698555
GAGE12H	-0.077044	-1.477965	-1.575227	0.1160579	0.1806797	-5.698578
AC006133.7	-0.062765	-1.506906	-1.574962	0.1161189	0.180767	-5.698989
C17orf47	-0.256635	-0.106611	-1.574727	0.1161733	0.180844	-5.699355
TMPRSS11CF	0.2125858	-0.710314	1.574644	0.1161925	0.1808661	-5.699484
TTLL10-AS1	-0.166335	-1.106454	-1.574333	0.1162644	0.1809703	-5.699968
RP11-77H9.	0.0675487	4.2415619	1.57416	0.1163043	0.1810225	-5.700236
CD24P2	-0.062682	-1.492615	-1.574145	0.1163079	0.1810225	-5.70026
LINC00866	-0.18004	-1.150227	-1.573971	0.1163479	0.1810772	-5.700529
GS1-124K5.	0.1868331	2.5339848	1.5737104	0.1164083	0.1811634	-5.700934
KIF24	0.0904032	4.7460803	1.5736506	0.1164221	0.1811772	-5.701027
RP11-1C8.4	0.1326124	-1.357444	1.5735751	0.1164396	0.1811966	-5.701144
IGHD3-22	0.1085264	-1.410872	1.5735491	0.1164456	0.1811983	-5.701184

CTB-3204.2	-0.26456	1.1747816	-1.573417	0.1164763	0.1812383	-5.70139
RP11-626H1	-0.155002	-1.225751	-1.573306	0.1165019	0.1812703	-5.701562
HOXA9	0.3292115	0.7628228	1.5730929	0.1165512	0.1813394	-5.701893
HCG4P3	0.2087343	3.1901817	1.5730709	0.1165563	0.1813396	-5.701927
RPL36AP43	0.2672593	1.8477152	1.57302	0.1165681	0.1813502	-5.702006
RP11-322E1	-0.22442	-0.642158	-1.572843	0.116609	0.1814061	-5.70228
RP11-115N1	0.1689462	-1.058238	1.5727841	0.1166227	0.1814198	-5.702372
CXADRP3	0.1901025	-1.016919	1.5725969	0.1166661	0.1814719	-5.702662
HTR2B	0.2067833	4.0289448	1.5725966	0.1166662	0.1814719	-5.702663
ADAM20	0.2288835	-0.404907	1.5725621	0.1166742	0.1814766	-5.702716
RP4-569M23	0.187641	-0.956857	1.5725314	0.1166813	0.1814799	-5.702764
ARL6IP1P2	-0.123084	-1.30234	-1.572419	0.1167074	0.1815127	-5.702939
RNU6-346P	-0.059268	-1.504946	-1.572173	0.1167644	0.1815937	-5.70332
RNU4-23P	0.0997128	-1.396366	1.5720629	0.1167899	0.1816256	-5.703491
HSP90B3P	0.2714996	0.7252198	1.571765	0.116859	0.1817254	-5.703953
GS1-204I12	0.1512935	-1.212609	1.5717352	0.1168659	0.1817284	-5.703999
MIR6819	0.1901671	-0.869939	1.5716882	0.1168768	0.1817376	-5.704072
KRT12	-0.376161	-0.008451	-1.571579	0.1169021	0.1817691	-5.704241
LRRC39	0.1220751	3.7380194	1.5715204	0.1169158	0.1817827	-5.704332
RP11-881M1	0.1619712	-1.218463	1.5714552	0.1169309	0.1817984	-5.704433
SERPINB4	0.2419333	-1.00589	1.5713969	0.1169444	0.1818117	-5.704523
RP11-597G2	0.1509855	-1.258014	1.5713398	0.1169577	0.1818246	-5.704612
RP11-78C3.	-0.128028	-1.314322	-1.571207	0.1169885	0.1818648	-5.704818
RPL10AP6	-0.088817	4.5439226	-1.571141	0.1170038	0.1818808	-5.70492
RP11-368I2	0.1541341	-1.198339	1.571074	0.1170194	0.1818973	-5.705024
ACAP3	-0.032876	6.1700134	-1.571024	0.117031	0.1819077	-5.705102
TBPL2	0.1220824	-1.34703	1.5708839	0.1170636	0.1819505	-5.705319
ENTPD8	-0.130593	5.4468391	-1.570186	0.1172257	0.1821947	-5.706399
RP11-753H1	0.2472358	-0.494597	1.5700791	0.1172507	0.1822258	-5.706566
SSX7	-0.125545	-1.367239	-1.569966	0.117277	0.1822589	-5.706741
PTGES3P2	-0.2374	3.6614664	-1.569891	0.1172945	0.1822784	-5.706858
RP11-343C2	-0.139606	-1.262014	-1.569833	0.1173079	0.1822914	-5.706946
DFNB31	-0.061391	5.5403515	-1.569792	0.1173174	0.1822985	-5.70701
RP11-655B2	-0.159084	-1.139413	-1.569595	0.1173633	0.182362	-5.707315
KLHL15	-0.046139	5.7284101	-1.569499	0.1173858	0.1823891	-5.707465
PCM1	0.0335842	6.4827643	1.5692792	0.1174369	0.1824608	-5.707804
GPR128	-0.237213	4.4241355	-1.569113	0.1174757	0.1825133	-5.708062
EIF3IP1	-0.123608	-1.288962	-1.568628	0.1175887	0.1826811	-5.708813
MIR621	0.1855818	5.2042517	1.568544	0.1176082	0.1827037	-5.708942
RP11-713P1	0.1723711	-1.088884	1.5684473	0.1176308	0.1827236	-5.709092
BLOC1S6	-0.02081	6.2693999	-1.568442	0.117632	0.1827236	-5.7091
AF254982.3	-0.106725	-1.411114	-1.568425	0.1176361	0.1827236	-5.709127
ZNF29P	-0.080222	-1.437012	-1.56829	0.1176674	0.1827645	-5.709335
TANGO2	-0.035823	5.9298672	-1.56821	0.1176861	0.1827858	-5.709459
RP11-168L7	0.3777652	2.5536409	1.5681381	0.1177029	0.1828041	-5.709571
RP11-134D3	-0.127503	-1.291935	-1.567946	0.1177477	0.1828597	-5.709867
RP11-661C8	-0.150514	-1.26576	-1.567942	0.1177488	0.1828597	-5.709874
FNTA	0.0320809	6.2380216	1.5678657	0.1177665	0.1828795	-5.709992
RP11-807H7	0.1181383	-1.376994	1.5677854	0.1177852	0.1829008	-5.710116
LL22NC03-8	0.0444968	5.2504311	1.5675971	0.1178292	0.1829613	-5.710407
CTC-261N6.	-0.088668	-1.439046	-1.567513	0.1178489	0.182984	-5.710537

CCDC43	0.0266288	5.735631	1.5673631	0.1178839	0.1830306	-5.710769
SMC2	0.0431039	5.7937989	1.5672993	0.1178988	0.183046	-5.710868
ANKRD26P1	0.3334237	-0.586426	1.5671911	0.1179241	0.1830774	-5.711035
HOXD-AS2	-0.356621	1.1436329	-1.566607	0.1180607	0.1832818	-5.711939
WASH4P	-0.088252	3.8357366	-1.566485	0.1180892	0.1833183	-5.712127
ADH5P4	-0.236198	2.315731	-1.566401	0.1181089	0.183341	-5.712257
ZDHHC4	-0.040636	6.1879192	-1.566355	0.1181196	0.1833498	-5.712328
RP11-905K4	-0.157309	-1.159943	-1.566202	0.1181553	0.1833975	-5.712564
NRTN	-0.080842	5.3504662	-1.566179	0.1181606	0.1833979	-5.712599
AC114302.1	-0.090623	-1.432012	-1.566147	0.1181683	0.183402	-5.712649
C1QTNF8	0.1537421	-1.251722	1.5654029	0.1183425	0.1836646	-5.713799
RP11-574H6	-0.06298	-1.492782	-1.565348	0.1183554	0.1836768	-5.713884
AGPAT9	-0.09352	5.64281	-1.565296	0.1183675	0.1836878	-5.713963
PTF1A	0.2576873	-0.89694	1.5652238	0.1183845	0.1837063	-5.714075
LA16c-444G	0.159689	-1.221589	1.5651272	0.1184071	0.1837337	-5.714224
GSX1	-0.081561	-1.471731	-1.564991	0.118439	0.1837754	-5.714435
HDAC4	0.0385826	5.6826648	1.5649661	0.1184449	0.1837766	-5.714473
TMCO4	-0.03145	5.8664018	-1.564772	0.1184904	0.1838394	-5.714773
DHX57	-0.025064	5.9084957	-1.564409	0.1185757	0.1839639	-5.715334
RBM5	-0.017844	6.3601248	-1.564346	0.1185904	0.183979	-5.715431
RP11-77E14	-0.148484	-1.213302	-1.56426	0.1186106	0.1840025	-5.715563
CTC-459F4.	-0.113158	-1.395821	-1.564102	0.1186477	0.1840522	-5.715807
RP11-81H14	-0.34118	3.4826351	-1.563989	0.1186741	0.1840854	-5.715981
PRRC2C	0.0231572	6.6630077	1.5635836	0.1187695	0.1842254	-5.716607
C10orf105	0.2752078	0.890308	1.5631546	0.1188703	0.184374	-5.717269
RP11-299A1	-0.110854	-1.343162	-1.563121	0.1188782	0.1843783	-5.71732
RP11-474N2	-0.27381	0.121061	-1.563037	0.1188979	0.1844011	-5.71745
USP21	0.0292193	5.9290461	1.5627009	0.118977	0.184516	-5.717968
RP11-521L9	-0.264001	1.3896795	-1.562542	0.1190145	0.1845663	-5.718214
AC068279.3	-0.143272	-1.214894	-1.562465	0.1190325	0.1845863	-5.718332
AC015969.3	0.1911824	-1.084809	1.5624424	0.1190379	0.1845868	-5.718367
RWDD3	-0.0367	5.3589011	-1.562313	0.1190684	0.1846264	-5.718567
AL590226.1	-0.293751	0.0030736	-1.562215	0.1190914	0.1846535	-5.718717
RP4-594A5.	0.2498736	-0.774369	1.5621798	0.1190997	0.1846535	-5.718772
AKR1C7P	-0.279847	2.6088627	-1.562147	0.1191075	0.1846535	-5.718823
MAP3K7CL	-0.056302	5.3842519	-1.562135	0.1191102	0.1846535	-5.71884
IGHD2-15	-0.058732	-1.504645	-1.562131	0.1191112	0.1846535	-5.718847
RP11-159N1	-0.073584	-1.474759	-1.562021	0.1191372	0.1846859	-5.719017
SLC22A8	0.3609695	0.0324444	1.5616574	0.1192228	0.184805	-5.719577
RP11-46F15	0.2305758	2.2855371	1.5616519	0.1192241	0.184805	-5.719585
OR7E36P	-0.090301	-1.42263	-1.561234	0.1193228	0.18495	-5.72023
RP11-846F4	-0.282073	-0.018723	-1.56105	0.1193661	0.1850093	-5.720513
CCDC144B	0.3586371	1.212586	1.5607623	0.119434	0.1851067	-5.720956
MTRNR2L11	-0.1498	-1.224057	-1.560497	0.1194965	0.1851958	-5.721364
RPL7AP15	-0.28359	0.4043636	-1.560439	0.1195102	0.1852091	-5.721453
RP11-561I1	0.1611233	-1.247208	1.5603555	0.11953	0.1852319	-5.721582
PIN4P1	-0.250999	1.3161243	-1.560225	0.1195607	0.1852717	-5.721782
AC005062.2	0.2029919	-0.923279	1.5599401	0.1196281	0.1853683	-5.722221
RHOT1P1	0.1140648	-1.344831	1.5598355	0.1196529	0.1853987	-5.722382
PTPRU	0.0903477	5.9089787	1.5597642	0.1196697	0.1854113	-5.722492
AC069363.1	0.3073262	0.3412898	1.5597583	0.1196711	0.1854113	-5.722501

CTD-2616J1	-0.278315	0.2440821	-1.559712	0.119682	0.1854203	-5.722572
RP11-65L19	-0.145386	-1.324913	-1.559574	0.1197148	0.1854613	-5.722785
KCTD3	-0.028305	6.6740231	-1.559557	0.1197186	0.1854613	-5.72281
CTC-459F4.	-0.078794	-1.47018	-1.559467	0.11974	0.1854865	-5.72295
PANK2	-0.020501	6.0220223	-1.559412	0.1197531	0.1854989	-5.723035
SPON2	-0.046586	6.8087793	-1.559308	0.1197775	0.1855289	-5.723194
AF064858.1	0.1976931	-0.9552	1.5592667	0.1197874	0.1855363	-5.723258
AC108456.1	0.1922855	-0.875902	1.5586955	0.1199226	0.1857378	-5.724136
AC005150.1	-0.38497	-0.214953	-1.558434	0.1199845	0.1858258	-5.724538
ASTE1	-0.026538	5.4003717	-1.558406	0.1199911	0.1858271	-5.724581
RP11-996F1	0.0956598	3.8883133	1.5583874	0.1199956	0.1858271	-5.72461
RP11-293M1	-0.131399	-1.317298	-1.558339	0.120007	0.185837	-5.724684
CBX3P9	0.2646645	0.3817871	1.5582541	0.1200271	0.1858603	-5.724815
CRYBB2P1	0.0469365	5.4819548	1.5580927	0.1200654	0.1859116	-5.725063
PRMT8	0.110213	-1.367192	1.5578146	0.1201313	0.1860059	-5.725491
IFI6	-0.066179	6.5600879	-1.557171	0.120284	0.1862343	-5.72648
CTD-2555A7	0.1457261	-1.276065	1.557077	0.1203063	0.1862587	-5.726624
RP13-753N3	-0.259029	1.65896	-1.557062	0.1203099	0.1862587	-5.726647
RP11-944C7	-0.250755	2.5643411	-1.556983	0.1203288	0.1862799	-5.726769
WAPAL	0.0193614	6.3089458	1.5568521	0.1203597	0.18632	-5.72697
AMH	0.3278873	2.4321744	1.5567184	0.1203915	0.1863612	-5.727175
CRTC3-AS1	-0.204447	2.2947879	-1.55613	0.1205313	0.1865669	-5.728079
PSMB10	-0.046213	6.0339905	-1.556116	0.1205346	0.1865669	-5.7281
RP11-25L3.	-0.087613	-1.454134	-1.555532	0.1206736	0.1867742	-5.728997
GNLY	0.0993703	4.8613878	1.5554449	0.1206943	0.1867982	-5.72913
IL17RA	0.0239416	6.082001	1.5554167	0.120701	0.1868007	-5.729174
UBBP2	-0.213591	-0.605342	-1.555319	0.1207243	0.1868244	-5.729324
ATRIP	0.2491411	0.63822	1.5553095	0.1207265	0.1868244	-5.729338
FYTTD1	-0.021318	6.2600731	-1.555178	0.1207578	0.1868645	-5.72954
STXBP3	0.0291919	6.1098581	1.5551575	0.1207627	0.1868645	-5.729572
GALNTL5	-0.116337	-1.372865	-1.554978	0.1208054	0.1869211	-5.729846
SCGB1D2	0.3678325	-0.059454	1.5549609	0.1208095	0.1869211	-5.729873
POTEJ	0.136637	-1.192813	1.5548374	0.1208389	0.1869587	-5.730063
CARNS1	0.1167207	4.2832467	1.5547307	0.1208644	0.1869901	-5.730226
PPP1R11P1	-0.098396	-1.382041	-1.554684	0.1208756	0.1869995	-5.730299
RP11-73B8.	-0.132658	-1.242535	-1.554543	0.1209092	0.1870371	-5.730515
PLXNB1	-0.031579	6.7086684	-1.554539	0.1209101	0.1870371	-5.730521
RP11-351C2	0.2523013	1.7168732	1.5544501	0.1209312	0.1870619	-5.730657
RP11-308D1	-0.28744	-0.351582	-1.55438	0.120948	0.1870798	-5.730765
RP11-425A2	-0.097602	-1.389608	-1.554324	0.1209613	0.1870926	-5.73085
CCDC18	0.0608903	4.9965502	1.5542347	0.1209826	0.1871175	-5.730987
ACVR1B	0.0313181	6.3676168	1.5541931	0.1209925	0.1871249	-5.731051
SLC25A16	-0.032411	6.1382696	-1.553903	0.1210616	0.187224	-5.731496
RPS15AP40	-0.223291	-0.518388	-1.553861	0.1210717	0.1872316	-5.73156
RP11-288E1	0.2373512	1.7050044	1.553685	0.1211137	0.1872886	-5.73183
RP11-618M2	0.179256	-1.048214	1.5535483	0.1211463	0.1873242	-5.73204
XXbac-B444	-0.198574	3.3697443	-1.553545	0.121147	0.1873242	-5.732044
RP1-313L4.	0.2710079	-0.004668	1.5534987	0.1211582	0.1873336	-5.732116
MAN2C1	-0.035873	6.3530546	-1.553464	0.1211665	0.1873385	-5.732169
MARS	-0.022157	6.541992	-1.553314	0.1212023	0.1873859	-5.732399
KB-1440D3.	-0.160173	-1.087904	-1.553235	0.121221	0.1874036	-5.732519

TTC25	0.1545534	3.4869236	1.5532228	0.121224	0.1874036	-5.732539
AC139100.3	-0.202946	3.5535779	-1.553077	0.1212589	0.1874496	-5.732762
KB-1732A1.	-0.099159	4.469967	-1.552961	0.1212866	0.1874845	-5.73294
TIMP4	0.236664	2.9834757	1.5528433	0.1213147	0.18752	-5.73312
MROH9	-0.145599	-1.194615	-1.552795	0.1213263	0.1875299	-5.733194
RP11-92C4.	0.1389747	-1.248206	1.5526802	0.1213537	0.1875644	-5.73337
RP13-735L2	0.2605016	2.4722478	1.5524997	0.1213968	0.1876231	-5.733647
FER1L6-AS2	0.2521104	-0.903139	1.5523032	0.1214438	0.1876878	-5.733948
OTX2-AS1	-0.15733	-1.234009	-1.552274	0.1214508	0.1876906	-5.733992
RP1-154K9.	-0.222877	-0.961424	-1.551767	0.1215721	0.1878701	-5.734769
CT45A10	0.2554291	-1.000141	1.5517328	0.1215803	0.1878748	-5.734821
RQCD1	0.0200334	6.2801005	1.5516562	0.1215986	0.1878952	-5.734938
RP11-12505	-0.326175	1.1877882	-1.551629	0.1216051	0.1878973	-5.73498
KRT24	-0.168249	-1.229958	-1.551605	0.1216108	0.1878982	-5.735017
RP11-714M2	-0.403639	2.8551633	-1.551373	0.1216665	0.1879763	-5.735373
SNX25	0.0457182	5.6459364	1.5513186	0.1216795	0.1879883	-5.735455
RP11-48001	-0.073119	-1.469427	-1.551286	0.1216874	0.1879926	-5.735506
RP11-547D2	0.2461227	2.2724903	1.551086	0.1217352	0.1880513	-5.735811
CRB1	0.3266484	0.5680149	1.5510837	0.1217357	0.1880513	-5.735815
RP11-568A7	0.3252866	-0.079952	1.5509197	0.121775	0.1881041	-5.736066
RP3-473B4.	0.1509358	-1.229922	1.5505921	0.1218536	0.1882175	-5.736567
OBFC1	-0.030652	5.7692049	-1.550562	0.1218609	0.1882208	-5.736614
AAK1	0.0289199	6.0841567	1.5501447	0.1219609	0.1883616	-5.737252
RP11-415I1	0.253154	0.5758273	1.5501387	0.1219624	0.1883616	-5.737261
IL10RB	-0.027018	6.2393059	-1.550029	0.1219888	0.1883944	-5.737429
PRICKLE3	0.0354607	5.3398716	1.5499952	0.1219968	0.1883988	-5.73748
CTD-2291D1	0.3382786	0.0524784	1.5494351	0.1221313	0.1885934	-5.738337
RP11-386G1	-0.260719	0.8829081	-1.549427	0.1221331	0.1885934	-5.738349
NRG2	0.2548204	3.5414171	1.5493136	0.1221605	0.1886276	-5.738523
RP1-79C4.1	-0.102302	-1.388752	-1.548916	0.1222561	0.1887674	-5.739131
RP11-702C7	-0.058156	-1.504322	-1.548884	0.1222636	0.188771	-5.739179
SPATA4	0.1473237	-1.21158	1.5488351	0.1222755	0.1887813	-5.739254
RP1-128M12	-0.147203	-1.180912	-1.548749	0.1222961	0.1888052	-5.739385
RP11-466L1	0.1531599	-1.120614	1.5486461	0.1223209	0.1888283	-5.739543
CCDC114	0.2310511	2.6099272	1.5486257	0.1223258	0.1888283	-5.739574
CAMSAP1	0.0267751	5.9485172	1.5486224	0.1223266	0.1888283	-5.739579
IGKV3D-7	0.1531265	-1.208014	1.5483084	0.1224022	0.1889352	-5.740059
IL13	-0.294839	-0.227303	-1.548291	0.1224063	0.1889352	-5.740085
TMEM9B	-0.024404	6.2757731	-1.548086	0.1224556	0.1890034	-5.740398
RP11-271C2	-0.178403	-0.919569	-1.547918	0.1224962	0.1890581	-5.740656
GPX5	-0.162787	-1.251089	-1.54747	0.122604	0.1892164	-5.741339
RP4-665N4.	0.1480662	-1.224138	1.5471134	0.12269	0.1893412	-5.741884
RP11-526I2	0.1943077	-0.922183	1.5469775	0.1227228	0.1893837	-5.742091
CTB-33018.	-0.219427	2.9153841	-1.546912	0.1227385	0.1893995	-5.742191
ENO1-IT1	0.2504388	-0.213129	1.546892	0.1227434	0.1893995	-5.742222
AC092798.2	-0.209376	-0.656776	-1.546845	0.1227548	0.1894091	-5.742294
PRDX3P2	0.2027372	-0.738409	1.5467707	0.1227726	0.1894286	-5.742407
RASSF7	-0.04168	6.3972553	-1.546321	0.1228811	0.1895881	-5.743093
RP11-462D1	0.2002455	-0.943658	1.5460471	0.1229473	0.1896821	-5.743511
RCHY1	0.0300506	5.7109574	1.5458248	0.123001	0.1897569	-5.74385
FAM50B	-0.097914	5.7035493	-1.545772	0.1230138	0.1897686	-5.743931

PRL	0.1642925	-1.241805	1.5456529	0.1230425	0.1898049	-5.744113
SSBP3-AS1	0.230399	2.6729396	1.5455222	0.1230741	0.1898456	-5.744312
SLC43A3	-0.057795	6.5032432	-1.545475	0.1230854	0.189855	-5.744383
RP11-1151E	0.2486613	-0.546863	1.5454008	0.1231034	0.1898748	-5.744497
C2-AS1	0.2413305	3.1214613	1.5452065	0.1231504	0.1899392	-5.744793
SIK3-IT1	0.226449	-0.545472	1.5450285	0.1231934	0.1899976	-5.745065
AC087430.1	-0.068742	-1.478789	-1.544676	0.1232787	0.1901211	-5.745602
RP11-602M1	0.2151229	-0.591555	1.5444378	0.1233364	0.1902009	-5.745965
ATP5G2P4	-0.230255	-0.420388	-1.544419	0.1233409	0.1902009	-5.745994
RPL29P30	-0.179183	-0.93849	-1.54431	0.1233673	0.1902336	-5.74616
PDZK1P2	-0.141468	-1.205953	-1.544162	0.1234031	0.1902807	-5.746385
RP11-255P5	-0.153034	-1.116452	-1.544052	0.1234298	0.190314	-5.746554
NCK1	0.0321201	5.9819687	1.5432446	0.1236255	0.190604	-5.747783
GCOM2	0.2383059	-0.270042	1.5432324	0.1236284	0.190604	-5.747802
CHRM4	0.279752	-0.160705	1.5430435	0.1236742	0.190665	-5.748089
SLC2A3P4	0.2530222	0.2416084	1.5430264	0.1236784	0.190665	-5.748115
RP11-101E1	0.4201176	0.8696072	1.5429601	0.1236945	0.1906818	-5.748216
HNRNPR	-0.017456	6.5396783	-1.542762	0.1237425	0.1907478	-5.748518
LLNLR-246C	0.2017981	2.3734393	1.5425695	0.1237893	0.1908118	-5.748811
RP11-248J2	-0.153352	-1.13837	-1.542272	0.1238616	0.1909141	-5.749264
SNORD114-3	0.1263325	-1.347323	1.542253	0.1238661	0.1909141	-5.749293
RP11-617B3	0.1178061	-1.345712	1.5422194	0.1238743	0.1909186	-5.749344
CTD-2318O1	0.2988927	0.8538684	1.5415924	0.1240267	0.1911454	-5.750298
RP11-876N2	0.117222	3.8903178	1.5413192	0.1240931	0.1912397	-5.750714
STRAP	-0.020571	6.558743	-1.541221	0.1241169	0.1912683	-5.750862
EXTL3	0.0328096	6.135352	1.5407258	0.1242375	0.1914461	-5.751616
PHACTR2P1	0.1307009	-1.250239	1.5406873	0.1242469	0.1914525	-5.751675
RP11-26608	0.1315809	-1.325287	1.5405391	0.124283	0.1915	-5.7519
RP11-294C1	0.1146776	-1.393179	1.540396	0.1243178	0.1915456	-5.752117
AC091492.2	0.2467821	-0.076005	1.5400866	0.1243932	0.1916537	-5.752588
KB-1460A1.	0.1515411	-1.181275	1.5398288	0.1244561	0.1917424	-5.752979
CTSL3P	-0.093793	-1.440376	-1.539711	0.1244847	0.1917785	-5.753158
THOC3	-0.035178	5.5958383	-1.539547	0.1245249	0.1918322	-5.753408
SLC6A20	0.3676438	0.8190175	1.5395073	0.1245345	0.1918389	-5.753468
RP11-91I20	-0.15725	-1.151123	-1.539415	0.1245571	0.1918656	-5.753609
FAM83D	-0.072912	5.6761797	-1.539242	0.1245993	0.1919206	-5.753872
YWHAB	-0.016383	6.8300252	-1.539225	0.1246033	0.1919206	-5.753896
RP11-1180F	0.2541764	0.1353251	1.5389601	0.124668	0.192007	-5.754299
CCDC82	0.0330371	5.8148942	1.5389524	0.1246699	0.192007	-5.754311
MTND5P12	-0.163606	-1.10142	-1.538766	0.1247154	0.192069	-5.754594
MIEF2	-0.033769	5.7717208	-1.538299	0.1248294	0.1922365	-5.755303
IL3RA	-0.047342	5.4912547	-1.538165	0.1248621	0.1922787	-5.755506
ITGAL	0.055922	6.1262208	1.5381396	0.1248684	0.1922803	-5.755545
RP5-1170K4	0.3624505	0.861248	1.5380498	0.1248904	0.192306	-5.755681
RP4-803A2.	0.2016802	-0.76297	1.5378248	0.1249454	0.1923826	-5.756023
RP11-768F2	-0.152101	3.2227044	-1.537562	0.1250098	0.1924736	-5.756422
RP11-321C2	-0.093609	-1.436489	-1.536817	0.125192	0.192746	-5.757551
SRSF7	-0.016968	6.4847227	-1.536697	0.1252214	0.1927832	-5.757734
ZNF324	-0.028635	5.4609803	-1.53664	0.1252354	0.1927966	-5.75782
SNORA70F	-0.207838	-0.708421	-1.536529	0.1252625	0.1928251	-5.757988
PITPNA-AS1	-0.067556	4.8523572	-1.536521	0.1252645	0.1928251	-5.758

LINC00355	-0.441954	1.2124312	-1.536481	0.1252745	0.1928323	-5.758062
AC087793.1	0.2504228	1.2918171	1.536303	0.125318	0.1928912	-5.758331
FMO7P	-0.231569	-0.778007	-1.536279	0.1253238	0.1928919	-5.758367
RP11-597A1	-0.065454	-1.488967	-1.535808	0.1254394	0.1930618	-5.759082
ANO10	-0.034909	6.1241297	-1.535498	0.1255155	0.1931708	-5.759552
RGPD2	0.2770057	2.583614	1.535425	0.1255333	0.1931901	-5.759662
RAB41	0.13439	3.3658078	1.5353009	0.1255638	0.1932257	-5.75985
RP11-680C2	-0.207038	-0.862663	-1.535288	0.125567	0.1932257	-5.75987
ZC3H3	-0.034467	6.2585584	-1.535018	0.1256333	0.1933195	-5.760279
RP11-733D4	-0.188255	-0.9183	-1.534811	0.1256841	0.1933895	-5.760592
RP11-299H2	0.1239427	3.5888797	1.5346796	0.1257164	0.193431	-5.760791
SNORD104	-0.131053	4.55823	-1.534587	0.125739	0.1934577	-5.760931
CTC-512J14	0.2321061	0.3335859	1.534506	0.1257591	0.1934804	-5.761054
PMS2P3	0.0548047	4.6487241	1.5343524	0.1257968	0.1935303	-5.761287
KRT18P32	-0.111148	-1.342949	-1.534022	0.1258782	0.1936473	-5.761788
ELOVL2	-0.085753	6.4460069	-1.533816	0.1259287	0.1937169	-5.762099
POLG2	-0.034883	5.5162354	-1.533582	0.1259864	0.1937974	-5.762453
FOXJ3	0.0219875	6.1071736	1.5334111	0.1260284	0.1938499	-5.762712
CTB-113D17	-0.29783	1.6164718	-1.5334	0.1260311	0.1938499	-5.762728
RP11-515O1	-0.172389	3.6772517	-1.533178	0.1260859	0.1939259	-5.763065
RALGAPA2	0.0360808	6.4436155	1.5328171	0.1261747	0.1940544	-5.76361
ARHGEF10L	-0.030723	6.6378763	-1.532464	0.1262617	0.19418	-5.764144
AC005071.4	-0.193691	-0.869224	-1.532145	0.1263404	0.1942929	-5.764627
TSPAN8	0.1807605	6.143524	1.5319174	0.1263966	0.1943711	-5.764971
NCBP2-AS1	0.2680229	0.4533023	1.5318794	0.126406	0.1943762	-5.765029
CTC-321K16	-0.151586	-1.257202	-1.531861	0.1264105	0.1943762	-5.765057
AC090505.4	-0.107003	-1.372898	-1.53176	0.1264355	0.1944064	-5.76521
AC007880.1	0.1169971	-1.343187	1.5315219	0.1264942	0.1944884	-5.765569
OR7C1	0.2101941	-1.106075	1.531456	0.1265105	0.1945053	-5.765669
RPL23AP81	0.2022087	-0.668088	1.5313763	0.1265302	0.1945273	-5.765789
RP11-157H4	-0.194395	-0.911956	-1.531307	0.1265473	0.1945373	-5.765894
ITGB1BP2	-0.115104	3.9506343	-1.531307	0.1265473	0.1945373	-5.765894
MYOT	0.3022841	0.1210016	1.5312466	0.1265622	0.194552	-5.765985
RP11-388M2	-0.144157	-1.192372	-1.531099	0.1265987	0.1945999	-5.766208
PSAT1P1	-0.093839	-1.413178	-1.530598	0.1267224	0.1947818	-5.766964
WNT8A	-0.081991	-1.479472	-1.530368	0.1267793	0.1948611	-5.767312
DENND1B	0.0364269	5.8642827	1.5303282	0.1267892	0.1948651	-5.767373
BZW1P1	-0.153582	-1.154659	-1.530315	0.1267926	0.1948651	-5.767393
RP11-104F1	-0.24286	2.3007299	-1.530055	0.1268568	0.1949557	-5.767785
PIK3C3	0.0218432	6.0192852	1.5298236	0.1269141	0.1950354	-5.768135
RP11-399E6	0.262078	-0.503104	1.5298003	0.1269198	0.1950361	-5.76817
RNF4	0.0211257	6.2860473	1.5296114	0.1269666	0.1950997	-5.768455
ANKRD20A7F	-0.394513	0.6499586	-1.529344	0.1270329	0.1951934	-5.768859
RP11-761E2	-0.281301	0.289845	-1.529068	0.1271011	0.19529	-5.769274
SNX3	-0.022884	6.7093437	-1.52904	0.1271082	0.1952927	-5.769318
RP11-109E1	0.2128352	-0.634249	1.5286797	0.1271975	0.1954097	-5.769861
RP11-351M1	-0.095441	-1.388564	-1.528677	0.1271981	0.1954097	-5.769865
TMC3	-0.255207	1.879031	-1.528668	0.1272004	0.1954097	-5.769879
SLC13A4	0.1452995	3.7325094	1.5284659	0.1272505	0.1954784	-5.770184
OTUD7A	0.1003503	4.2525272	1.528389	0.1272696	0.1954995	-5.7703
ZNF727	0.3730538	0.9718408	1.5281995	0.1273167	0.195557	-5.770585

MB	0.2802564	3.3957898	1.528195	0.1273178	0.195557	-5.770592
RP11-428P1	0.2822036	0.2884806	1.5281017	0.1273409	0.1955843	-5.770733
IMPA2	-0.041382	6.2357811	-1.52799	0.1273685	0.1956185	-5.770901
RPL7P58	-0.172257	-1.031963	-1.527857	0.1274017	0.1956613	-5.771102
XXcos-LUCA	0.1550066	-1.08664	1.527752	0.1274277	0.195693	-5.77126
CH17-189H2	0.1342573	4.583725	1.5276893	0.1274433	0.1957087	-5.771355
RP11-45P15	-0.119625	-1.340117	-1.527499	0.1274906	0.1957731	-5.771642
RP1-287H17	0.2224389	-0.931695	1.527401	0.1275149	0.1958021	-5.771789
ODF2	0.0268331	6.084296	1.5273427	0.1275294	0.1958162	-5.771877
RP11-128P1	-0.059504	-1.505078	-1.527288	0.127543	0.1958288	-5.77196
DHX32	0.0639348	5.833726	1.5269722	0.1276215	0.1959411	-5.772436
USP49	0.0406802	5.2831791	1.5269432	0.1276287	0.1959439	-5.772479
LINC00973	0.1226092	-1.328774	1.5268828	0.1276437	0.1959588	-5.772571
RP11-17403	0.1900945	-1.047956	1.5268454	0.127653	0.1959648	-5.772627
RP11-10A14	0.3059428	1.3376194	1.5267367	0.1276801	0.195998	-5.772791
RP11-30506	0.2329251	1.7440728	1.5266495	0.1277017	0.1960231	-5.772922
RNA5SP284	-0.065893	-1.494415	-1.526603	0.1277133	0.1960326	-5.772992
DBET	-0.31983	0.324709	-1.52654	0.127729	0.1960484	-5.773087
RP11-276E1	-0.258378	0.5841063	-1.526179	0.127819	0.1961782	-5.773632
TBC1D1	0.0344342	6.2803849	1.5259347	0.1278797	0.1962632	-5.773999
LINC01252	0.2624978	3.0305267	1.5257564	0.1279241	0.196323	-5.774267
RP11-589M4	-0.236131	-0.459614	-1.525473	0.1279947	0.1964232	-5.774694
UBQLN4P1	0.2694649	0.9527613	1.5252694	0.1280454	0.1964928	-5.775001
MTND4P4	-0.089978	-1.445736	-1.524938	0.1281281	0.1966114	-5.7755
RP11-95M15	0.3777022	1.8680345	1.5244432	0.1282515	0.1967896	-5.776244
KIAA0319L	-0.021732	6.5836028	-1.524429	0.128255	0.1967896	-5.776265
FLJ46284	0.1899376	3.0816655	1.5241041	0.1283362	0.1969058	-5.776754
LINC00412	-0.212838	-0.653028	-1.523841	0.1284018	0.1969983	-5.77715
SNORD116-2	0.1158144	-1.326627	1.5237987	0.1284125	0.1970063	-5.777214
CCDC180	0.2002152	3.3804466	1.5237604	0.128422	0.1970127	-5.777271
FLJ37035	0.1535051	3.0529782	1.523669	0.1284449	0.1970395	-5.777409
AC010894.5	0.1056291	-1.349561	1.523409	0.1285098	0.1971309	-5.7778
ZNF774	0.0749159	4.4747026	1.5230194	0.1286073	0.197272	-5.778385
CTD-2231E1	0.2617292	0.4923697	1.5229811	0.1286169	0.1972784	-5.778443
TOR2A	-0.028952	5.88028	-1.522646	0.1287007	0.1973988	-5.778947
NPM2	0.2151982	4.4201408	1.5224939	0.1287388	0.1974488	-5.779175
RP11-407G2	-0.271911	0.5525456	-1.522356	0.1287734	0.1974937	-5.779383
RP11-84A14	-0.196752	1.8070073	-1.522302	0.1287869	0.1975061	-5.779464
MITD1	0.0229583	5.7635923	1.5222562	0.1287983	0.1975152	-5.779532
PCNT	0.0283489	6.0792555	1.5220475	0.1288506	0.1975871	-5.779846
PXYLP1	0.0724082	5.4524654	1.5219966	0.1288633	0.1975983	-5.779922
PRAMEF7	-0.301597	-0.300711	-1.521951	0.1288748	0.1976077	-5.779991
CDKN2D	0.0453119	5.2160736	1.5218794	0.1288927	0.1976267	-5.780098
KIF1A	0.3599776	3.1058264	1.5217507	0.1289249	0.1976679	-5.780292
DLG3-AS1	0.1651833	-1.1193	1.5211943	0.1290644	0.1978735	-5.781127
FAM177B	0.1848866	3.6773305	1.5211132	0.1290848	0.1978963	-5.781249
NSFP1	0.2985794	0.1255968	1.5208291	0.129156	0.1979973	-5.781675
SLC41A3	-0.023986	6.2755243	-1.520537	0.1292294	0.1981014	-5.782114
RP11-157L3	-0.083516	-1.442434	-1.520481	0.1292434	0.1981147	-5.782198
POLR2J2	0.3218138	0.7262085	1.5203225	0.1292832	0.1981593	-5.782436
CTA-292E10	-0.207189	1.91018	-1.520322	0.1292834	0.1981593	-5.782437

AP006222.2	0.2714872	2.1807729	1.520112	0.1293361	0.1982317	-5.782752
COG2	-0.026638	6.1251453	-1.519933	0.1293811	0.1982923	-5.78302
RP1-63M2.7	-0.181081	3.2465566	-1.519451	0.1295021	0.1984696	-5.783742
ATP2A2	0.0229992	6.9081068	1.5191062	0.129589	0.1985943	-5.78426
RP11-829H1	-0.176361	-0.965983	-1.518921	0.1296355	0.1986573	-5.784537
RP5-827C21	0.2035912	-0.869027	1.5188877	0.129644	0.1986619	-5.784588
RP11-708L7	-0.139712	-1.204448	-1.518307	0.1297903	0.1988778	-5.785459
AC090810.1	0.1756566	-0.983945	1.5181801	0.1298222	0.1989183	-5.785648
CCDC169-SC	-0.060647	-1.491474	-1.517643	0.1299575	0.1991173	-5.786453
CTB-174021	0.1675747	-0.978335	1.5174583	0.1300042	0.1991804	-5.78673
CLN5	-0.035653	5.7328566	-1.517316	0.13004	0.1992269	-5.786942
PRKRIRP6	-0.132113	-1.338792	-1.517067	0.130103	0.199315	-5.787316
HBQ1	0.218052	-1.00597	1.5168284	0.1301631	0.1993988	-5.787673
CTA-972D3.	-0.15169	-1.088547	-1.516507	0.1302442	0.1995147	-5.788154
SHANK2-AS2	0.1201917	-1.298661	1.5163859	0.1302749	0.1995533	-5.788335
RP11-157E2	-0.256882	-0.75277	-1.516264	0.1303057	0.1995921	-5.788518
LA16c-380H	-0.228771	2.314023	-1.516102	0.1303465	0.1996463	-5.78876
RP11-378J1	0.0724197	4.1952043	1.5158369	0.1304136	0.1997407	-5.789157
RP11-321M2	-0.069727	-1.484749	-1.515768	0.130431	0.1997588	-5.789259
EIF4A1P7	0.2284399	-0.376986	1.5154392	0.1305142	0.199878	-5.789752
RP11-359B1	0.0809293	4.245633	1.515188	0.1305778	0.199967	-5.790128
AC011747.6	-0.316672	0.3187548	-1.514984	0.1306294	0.2000376	-5.790432
LINC00523	0.1643819	-1.214037	1.5148882	0.1306537	0.2000664	-5.790576
NEK4P2	-0.071829	-1.473774	-1.514638	0.1307171	0.200155	-5.79095
RP11-1109F	0.2809005	0.8749694	1.5145516	0.130739	0.2001802	-5.791079
SLC4A1APP1	-0.235207	-0.069357	-1.51449	0.1307545	0.2001955	-5.791171
SPTBN2	-0.104511	5.9459871	-1.514449	0.1307649	0.200203	-5.791232
LINC00601	0.2462843	-0.945681	1.5143167	0.1307985	0.2002461	-5.79143
CLEC18A	0.2734495	0.9710823	1.5140695	0.1308612	0.2003337	-5.7918
RP11-166B2	0.2517281	0.614835	1.5137931	0.1309313	0.2004326	-5.792213
PRELID1P1	-0.187573	3.5489761	-1.513726	0.1309484	0.2004503	-5.792314
AC062021.1	-0.100156	-1.385529	-1.513453	0.1310175	0.2005466	-5.792721
RP11-509A1	0.3278545	0.3956254	1.5134345	0.1310222	0.2005466	-5.792749
CRIM1	0.0635281	6.1737303	1.5130922	0.1311091	0.2006663	-5.79326
FLYWCH1P1	-0.065808	-1.491389	-1.513083	0.1311114	0.2006663	-5.793273
RP11-513I1	-0.1625	2.8917849	-1.512838	0.1311738	0.2007534	-5.79364
RP5-1107A1	0.2121255	-0.542795	1.5126907	0.1312111	0.200802	-5.79386
RP11-550H2	-0.083907	-1.437811	-1.512517	0.1312552	0.2008611	-5.794119
GTF2H2	0.1247986	4.5468365	1.5122389	0.1313259	0.2009609	-5.794534
FLJ26245	0.1720418	-1.189963	1.5121733	0.1313426	0.2009744	-5.794632
RP11-425D1	-0.248958	1.1392655	-1.512161	0.1313458	0.2009744	-5.794651
RP11-343J3	-0.061988	-1.492226	-1.512058	0.131372	0.2010061	-5.794805
TSPAN12	0.0692963	5.8203783	1.5120213	0.1313813	0.2010119	-5.794859
RP11-503N1	0.199438	-0.653985	1.5116583	0.1314736	0.2011447	-5.795401
RP13-56501	0.1238488	-1.284663	1.511616	0.1314844	0.2011528	-5.795464
RP11-176D1	0.2142059	-0.674169	1.5115935	0.1314901	0.2011531	-5.795497
TTC21B-AS1	0.1740919	-0.961226	1.5115524	0.1315006	0.2011607	-5.795559
BAHCC1	0.0563216	5.7463578	1.5113018	0.1315644	0.2012498	-5.795932
EIF4A1P5	0.1709023	-0.959791	1.5112334	0.1315818	0.201268	-5.796034
MIR1915	-0.181348	-1.001637	-1.511124	0.1316097	0.2013022	-5.796198
KRTAP5-2	0.2368627	-0.833394	1.5110106	0.1316385	0.2013379	-5.796367

RP11-613E4	0.2407455	-0.450679	1.5107309	0.1317098	0.2014385	-5.796784
AC234917.1	0.2597324	-0.594814	1.5105276	0.1317616	0.2015009	-5.797087
RPL7AP58	-0.163879	-1.119615	-1.510527	0.1317616	0.2015009	-5.797087
EPHB4	0.0390914	6.5833815	1.5104968	0.1317694	0.2015044	-5.797133
ZHX1-C8orf	-0.082979	4.3325849	-1.510468	0.1317769	0.2015073	-5.797176
SIGLEC20P	-0.075176	-1.459694	-1.510356	0.1318055	0.2015426	-5.797344
MCCD1P2	-0.199306	-0.831744	-1.510074	0.1318772	0.2016439	-5.797763
RP5-1115A1	-0.119868	-1.297342	-1.510019	0.1318913	0.2016559	-5.797846
RP11-316M2	0.235833	1.4095844	1.5100001	0.1318961	0.2016559	-5.797874
GIP	-0.271544	-0.670669	-1.509889	0.1319245	0.2016908	-5.798039
AC139143.2	0.1453253	-1.139728	1.5097193	0.1319678	0.2017482	-5.798292
CAPN2	0.0325868	6.6257312	1.5096984	0.1319731	0.2017482	-5.798323
AC005281.2	0.1141789	-1.36199	1.5096113	0.1319953	0.2017738	-5.798453
RP11-260M2	0.119992	3.4415075	1.5094944	0.1320252	0.2018109	-5.798627
CEACAM19	-0.066468	5.4685016	-1.509418	0.1320446	0.2018322	-5.798741
TRAPPC8	0.0360045	6.2007771	1.5087461	0.1322163	0.2020862	-5.799742
AL590762.1	0.2614459	0.2564056	1.5086364	0.1322443	0.2021206	-5.799905
RP11-46D6.	0.1023669	5.0798338	1.5085924	0.1322556	0.2021293	-5.799971
RP11-385D1	0.1304842	-1.25357	1.5084589	0.1322897	0.20217	-5.80017
LINC01341	-0.129578	4.526852	-1.508445	0.1322933	0.20217	-5.80019
SLC25A29	-0.037467	6.0064467	-1.508259	0.1323409	0.2022342	-5.800467
RP11-809C1	0.1594636	-1.168067	1.5081853	0.1323597	0.2022545	-5.800577
SCARNA8	0.1383578	-1.194826	1.508018	0.1324025	0.2023115	-5.800826
RP11-16L21	-0.276362	-0.219826	-1.507976	0.1324132	0.2023193	-5.800888
RP11-480N2	-0.159248	-1.126359	-1.507872	0.1324399	0.2023517	-5.801043
NAPSA	-0.099529	3.8100854	-1.507848	0.1324459	0.2023524	-5.801079
TRAF2	-0.032388	6.1555511	-1.507576	0.1325156	0.2024504	-5.801484
MIR548D2	-0.151681	-1.163825	-1.507168	0.1326201	0.2026016	-5.802091
RP11-255G1	0.1002712	-1.387011	1.5070656	0.1326463	0.2026332	-5.802243
RP11-490G8	-0.217729	2.0883073	-1.506979	0.1326685	0.2026585	-5.802372
FLJ37505	0.1478382	-1.332591	1.5067407	0.1327296	0.2027434	-5.802727
ORAOV1	0.0440677	5.6720226	1.5067186	0.1327353	0.2027436	-5.802759
RPL7AP28	0.2754019	-0.502631	1.506517	0.132787	0.2028141	-5.803059
GTF2IRD2P1	-0.224301	1.922316	-1.506349	0.1328302	0.2028715	-5.80331
RP11-530A1	0.2486439	0.7746499	1.5062376	0.1328587	0.2029065	-5.803475
IBSP	0.4013255	0.9814566	1.506124	0.1328878	0.2029425	-5.803644
WWC3	0.0379522	5.9608934	1.5060262	0.1329129	0.2029724	-5.803789
CNTNAP4	-0.474891	1.4907199	-1.505833	0.1329626	0.2030398	-5.804077
RP11-678G1	-0.066425	-1.48499	-1.505122	0.133145	0.2033099	-5.805133
RP11-196G1	-0.064755	5.339653	-1.505041	0.1331658	0.2033331	-5.805252
CTD-2154I1	-0.249663	0.2555687	-1.504989	0.1331794	0.2033453	-5.805331
PRKAG3	-0.286392	0.9248015	-1.504917	0.1331978	0.203365	-5.805438
LRRC27	-0.038509	5.359085	-1.504855	0.1332138	0.2033809	-5.80553
LINC00381	-0.113562	-1.362791	-1.504523	0.1332992	0.2034943	-5.806023
SNORA79	-0.214441	-0.619199	-1.504522	0.1332993	0.2034943	-5.806024
LATS2	0.0419042	5.8663082	1.504364	0.13334	0.203548	-5.806259
PHLPP2	0.0456169	5.5716215	1.5042866	0.1333599	0.2035699	-5.806374
AC007163.6	-0.160508	-1.116969	-1.504119	0.1334029	0.203627	-5.806622
RP11-346C4	0.169468	-1.097884	1.5039929	0.1334355	0.2036682	-5.80681
SHANK2	-0.098601	6.039276	-1.503913	0.1334561	0.2036912	-5.806929
WBSCR28	0.3559967	1.322463	1.5034436	0.133577	0.2038671	-5.807625

FGFR4	-0.07028	6.8086615	-1.503183	0.133644	0.203961	-5.808012
AC025811.3	-0.160862	-1.244655	-1.503008	0.1336892	0.2040214	-5.808272
RPL21P5	-0.188233	-0.792266	-1.502678	0.1337743	0.2041427	-5.808761
TMEM178A	0.1157302	4.4628373	1.502447	0.1338339	0.2042204	-5.809104
TCOF1	-0.027313	6.3066298	-1.502437	0.1338364	0.2042204	-5.809118
DYNLL1P1	0.2299691	0.0526534	1.5023491	0.1338591	0.2042465	-5.809249
LSM8	-0.029811	5.9759294	-1.501879	0.1339805	0.2044185	-5.809946
HNRNPA1P8	0.250783	1.2301175	1.501869	0.1339831	0.2044185	-5.809961
SPINK7	-0.135243	-1.241211	-1.501763	0.1340105	0.2044518	-5.810118
LINC00401	-0.157706	-1.178656	-1.501731	0.1340188	0.2044535	-5.810166
PQLC3	0.0511663	5.6904039	1.5017153	0.1340228	0.2044535	-5.810189
RP11-1069G	-0.327654	0.8243385	-1.501687	0.1340301	0.204456	-5.810231
AC012065.5	0.1607084	-1.034784	1.5015595	0.134063	0.204495	-5.81042
POLRMTP1	-0.210495	4.179015	-1.501545	0.1340668	0.204495	-5.810441
DHX35	0.0294486	5.7277855	1.5013532	0.1341163	0.204562	-5.810725
[truncated: 1,069,803 more chars]
