# Supplementary material for: Analysis of the Heterogeneity of the Tumor Microenvironment and the Prognosis and Immunotherapy Response of Different Immune Subtypes in Hepatocellular Carcinoma
Source: J Oncol. 2022 Mar 29;2022:1087399. doi: 10.1155/2022/1087399 (PMC8984740; doi:10.1155/2022/1087399)
Supplement: Supplementary 2 — Table S2: significantly differently expressed genes between stroma and nonstroma subtypes. [file 1087399.f2.pdf]

ID	logFC	AveExpr	t	P.Value	adj.P.Val	B
ARID3A	-0.738375	5.8137493	-13.42168	5.69E-30	2.08E-25	57.019315
DQX1	-3.659615	2.4480001	-12.90309	2.39E-28	4.37E-24	53.393331
RP11-497G1	-3.235234	1.2468945	-12.79555	5.19E-28	6.32E-24	52.64271
COL6A4P1	-2.515479	0.4721093	-10.89628	3.76E-22	3.44E-18	39.540991
HIC2	-0.465682	5.5230298	-10.78259	8.32E-22	6.07E-18	38.770609
AL163953.2	-2.645615	1.1521817	-10.53491	4.65E-21	2.83E-17	37.099796
ACPT	-2.677693	1.8420279	-10.45311	8.18E-21	4.26E-17	36.550407
ZIM2	-2.416066	-0.090179	-10.3815	1.34E-20	6.12E-17	36.070453
MFSD2A	1.4757618	5.2469738	10.25954	3.10E-20	1.26E-16	35.255398
SLC46A3	0.7108861	5.8981673	10.206564	4.46E-20	1.63E-16	34.902273
POLE2	-0.514326	5.1717163	-10.19092	4.97E-20	1.65E-16	34.798082
DNMT3A	-0.275247	6.1223568	-10.09288	9.71E-20	2.95E-16	34.14643
ESR1	1.1337578	4.7829609	10.075184	1.10E-19	3.08E-16	34.029036
CHKA	-0.29994	6.271666	-10.04782	1.32E-19	3.44E-16	33.847623
SIAE	0.391098	6.2476453	10.029249	1.50E-19	3.65E-16	33.724592
CAV2	0.4538359	6.2350929	9.9220208	3.11E-19	7.10E-16	33.015718
NNMT	0.7683303	6.6706739	9.8610306	4.71E-19	1.01E-15	32.613674
COIL	-0.167919	5.9630918	-9.796038	7.31E-19	1.48E-15	32.186193
CD59	0.2054353	6.9093035	9.7733638	8.52E-19	1.64E-15	32.037292
HECTD3	0.1899233	6.3155984	9.7391973	1.07E-18	1.88E-15	31.81315
MRPL53	-0.294062	5.0429575	-9.737689	1.08E-18	1.88E-15	31.80326
FHL2	0.534469	5.6484644	9.7302623	1.14E-18	1.89E-15	31.75458
TMPO-AS1	-0.53566	5.0028869	-9.671874	1.69E-18	2.64E-15	31.372316
ARID3B	-0.340196	5.3402402	-9.667878	1.74E-18	2.64E-15	31.346181
AKTIP	0.2546513	5.906755	9.5180902	4.74E-18	6.93E-15	30.369551
STS	0.4855849	5.9293075	9.4824159	6.02E-18	8.46E-15	30.137794
PTGDR2	-1.160189	4.4094643	-9.466648	6.69E-18	9.05E-15	30.035466
TK2	0.2080723	6.1876905	9.4384486	8.08E-18	1.05E-14	29.852619
NAMPT	0.3549932	6.6529086	9.416294	9.36E-18	1.18E-14	29.709115
SKP2	-0.347731	5.9652647	-9.400057	1.04E-17	1.25E-14	29.604023
MSN	0.2479384	6.8273029	9.3975504	1.06E-17	1.25E-14	29.587808
BCO2	1.3772038	4.7520225	9.3679894	1.29E-17	1.47E-14	29.396684
ACVR2B	-0.310921	5.6340941	-9.246759	2.89E-17	3.20E-14	28.615369
TROAP	-0.7183	5.4367631	-9.204507	3.82E-17	4.10E-14	28.344017
SLC1A1	1.1758014	5.7159514	9.1457274	5.63E-17	5.87E-14	27.967364
CD55	0.4475946	5.8698477	9.1395476	5.86E-17	5.95E-14	27.927822
ARHGEF39	-0.49477	5.2760963	-9.111873	7.04E-17	6.62E-14	27.750884
C1QTNF1	0.6107118	5.9827394	9.1113526	7.06E-17	6.62E-14	27.747555
TUBD1	-0.233718	5.3951028	-9.109165	7.16E-17	6.62E-14	27.733578
CNOT11	-0.163217	6.3841846	-9.107342	7.25E-17	6.62E-14	27.721929
MEP1A	-2.761351	3.2698328	-9.097368	7.74E-17	6.76E-14	27.658227
TICRR	-0.84702	4.7698849	-9.096178	7.80E-17	6.76E-14	27.650634
FLVCR1	-0.35371	5.8488569	-9.093142	7.96E-17	6.76E-14	27.631246
TET1	-0.836435	4.4336225	-9.089515	8.15E-17	6.76E-14	27.608094
TMEM47	0.5368073	5.708561	9.0821845	8.56E-17	6.94E-14	27.561311
LIN9	-0.39122	5.2369366	-9.064172	9.63E-17	7.64E-14	27.446422
ZCCHC3	-0.200385	6.0892405	-9.042354	1.11E-16	8.63E-14	27.307391
KIF15	-0.743159	4.9811539	-9.007286	1.40E-16	1.06E-13	27.084216
AP001469.9	-0.869437	4.034988	-8.96238	1.88E-16	1.40E-13	26.798973
WLS	0.6128738	5.7861482	8.899282	2.83E-16	2.05E-13	26.399215

GAS6	0.3872512	6.3661259	8.8972678	2.87E-16	2.05E-13	26.386474
AURKB	-0.623813	5.439414	-8.879325	3.23E-16	2.24E-13	26.273032
ZFP1	0.313087	5.3741587	8.8781648	3.25E-16	2.24E-13	26.2657
MYBL2	-0.687156	5.8159226	-8.855904	3.76E-16	2.54E-13	26.125106
FAM134B	1.230101	4.9783541	8.8520694	3.85E-16	2.54E-13	26.100902
MVP	0.2422101	6.7837672	8.8501945	3.90E-16	2.54E-13	26.08907
ZHX3	0.2235227	6.1155518	8.8466445	3.99E-16	2.56E-13	26.066669
PIF1	-0.680901	4.8854463	-8.82899	4.48E-16	2.82E-13	25.955328
AC118754.4	2.0996164	0.7759587	8.8087682	5.10E-16	3.16E-13	25.827916
SPSB1	0.3317802	6.2614561	8.8024375	5.32E-16	3.23E-13	25.788054
TTK	-0.762675	5.161607	-8.790412	5.75E-16	3.39E-13	25.712372
KIF18B	-0.716427	5.2736151	-8.79038	5.75E-16	3.39E-13	25.712167
SRPX	0.9158095	4.7979402	8.7827175	6.04E-16	3.47E-13	25.663969
MYO1A	-1.548913	4.2715774	-8.781636	6.08E-16	3.47E-13	25.657169
CDC25A	-0.636094	5.0699784	-8.777894	6.23E-16	3.50E-13	25.633634
LIMCH1	0.560196	5.6952669	8.7662187	6.72E-16	3.71E-13	25.560247
CDCA4	-0.320944	5.5166978	-8.764262	6.81E-16	3.71E-13	25.547952
MFAP3L	0.9500518	5.3378382	8.7609143	6.96E-16	3.73E-13	25.526919
VPS33A	-0.185102	6.0147591	-8.758812	7.05E-16	3.73E-13	25.513713
SERPINB8	0.4248967	5.6271011	8.7525454	7.35E-16	3.83E-13	25.474355
CPED1	0.6854467	5.3890187	8.7365109	8.15E-16	4.14E-13	25.373707
AFP	-1.666982	5.7769105	-8.736303	8.16E-16	4.14E-13	25.372405
KIFC1	-0.561427	5.7329377	-8.727587	8.63E-16	4.32E-13	25.317726
RP5-1112D6	-0.636237	4.3502073	-8.718555	9.15E-16	4.51E-13	25.261095
CDKN3	-0.62106	5.4711522	-8.711402	9.58E-16	4.66E-13	25.216267
RNF11	0.1698668	6.3872548	8.7079909	9.80E-16	4.71E-13	25.194892
DCN	0.746448	6.2673072	8.6853514	1.13E-15	5.38E-13	25.053136
DNMT3B	-0.498322	5.0122027	-8.682922	1.15E-15	5.39E-13	25.037936
NREP	-0.339646	6.2553886	-8.680105	1.17E-15	5.42E-13	25.020309
MAP3K5	0.5900049	5.7060196	8.6647633	1.29E-15	5.91E-13	24.924371
SPNS2	0.5827215	5.4700422	8.6562693	1.37E-15	6.16E-13	24.871287
FAM136A	-0.167563	6.2914985	-8.636193	1.56E-15	6.93E-13	24.745911
NPR3	0.8892393	5.1086645	8.6165515	1.77E-15	7.77E-13	24.623383
SYT9	2.7921526	0.8643753	8.603964	1.91E-15	8.32E-13	24.544926
TRIM24	-0.219839	6.4167213	-8.586444	2.14E-15	9.20E-13	24.435815
HJURP	-0.637275	5.433654	-8.580857	2.22E-15	9.43E-13	24.401042
SUV39H2	-0.224197	5.5153302	-8.574717	2.31E-15	9.69E-13	24.362838
ENDOD1	0.4564718	5.7172663	8.5608441	2.52E-15	1.05E-12	24.276567
ARHGAP10	0.4905254	5.2794091	8.5529722	2.66E-15	1.09E-12	24.227643
NEK2	-0.737397	5.4034106	-8.548857	2.73E-15	1.11E-12	24.202074
SYN3	-1.463272	3.3124206	-8.541021	2.87E-15	1.15E-12	24.153407
SKA3	-0.676358	5.078223	-8.535265	2.97E-15	1.18E-12	24.117668
CDK1	-0.485879	5.7788218	-8.49722	3.79E-15	1.48E-12	23.881752
WDR44	0.3058674	5.7959762	8.4967061	3.81E-15	1.48E-12	23.878566
STX12	0.1490884	6.151	8.4920849	3.92E-15	1.51E-12	23.849944
GGA3	-0.131054	6.1398094	-8.481596	4.19E-15	1.59E-12	23.785005
GTSE1	-0.637872	5.2528247	-8.47159	4.47E-15	1.68E-12	23.723095
TRIM71	-2.948251	3.3824274	-8.452893	5.03E-15	1.87E-12	23.607499
CTB-167G5.	-1.786666	-0.245415	-8.442951	5.36E-15	1.98E-12	23.546086
PSPH	-0.326249	6.0468219	-8.440366	5.45E-15	1.99E-12	23.530119
CD81	0.2438824	7.0395509	8.415842	6.37E-15	2.30E-12	23.378795

CCNB2	-0.589098	5.5565202	-8.40277	6.92E-15	2.48E-12	23.298218
SLC7A10	-2.641228	2.0283949	-8.399811	7.05E-15	2.49E-12	23.279986
GINS1	-0.517794	5.524864	-8.399185	7.08E-15	2.49E-12	23.27613
ATAD5	-0.402617	5.0491047	-8.383431	7.83E-15	2.72E-12	23.179125
MCCD1	-2.888995	1.4883243	-8.379709	8.01E-15	2.73E-12	23.156216
SLC26A11	-0.189928	5.9628278	-8.377222	8.14E-15	2.73E-12	23.140913
FBXO43	-0.973912	4.1800463	-8.376895	8.16E-15	2.73E-12	23.138906
IGF2BP1	-2.434485	4.5176661	-8.376855	8.16E-15	2.73E-12	23.138659
GFOD2	0.2256452	6.0970917	8.3539166	9.44E-15	3.11E-12	22.997623
TLX2	-2.188437	0.1838007	-8.353484	9.46E-15	3.11E-12	22.994967
BMF	-0.396039	5.8851548	-8.350765	9.63E-15	3.14E-12	22.978263
SNRPA1	-0.179015	6.0631097	-8.341019	1.02E-14	3.29E-12	22.918404
MIR483	-2.128072	-0.328909	-8.340203	1.03E-14	3.29E-12	22.913396
FANCG	-0.27261	5.7030838	-8.339096	1.04E-14	3.29E-12	22.906598
TOP2A	-0.452758	6.196587	-8.323546	1.14E-14	3.54E-12	22.811182
AC004538.3	2.183997	1.8349885	8.3235334	1.14E-14	3.54E-12	22.811103
PARPBP	-0.603618	5.0135228	-8.323297	1.15E-14	3.54E-12	22.809652
PARP3	0.2236774	5.910439	8.3167509	1.19E-14	3.66E-12	22.769511
TBC1D2B	0.2402792	6.2438484	8.3090579	1.25E-14	3.81E-12	22.722356
GUCY1B2	-2.514941	1.9504573	-8.298792	1.34E-14	4.04E-12	22.659465
TAF5	-0.209857	5.3164603	-8.286257	1.45E-14	4.33E-12	22.58272
FAM222A	-0.398221	5.8251205	-8.284024	1.47E-14	4.36E-12	22.569055
KCNC1	-2.285596	1.0657132	-8.278761	1.52E-14	4.47E-12	22.536857
SOD2	0.269649	7.2054618	8.2758617	1.55E-14	4.50E-12	22.519122
MCC	0.5161789	5.6810612	8.2751561	1.55E-14	4.50E-12	22.514806
PTS	0.2144694	5.9347447	8.2698079	1.61E-14	4.62E-12	22.482102
RP11-497G1	-1.97722	-0.244765	-8.260331	1.71E-14	4.86E-12	22.424176
PLAGL2	-0.24877	5.7610964	-8.258381	1.73E-14	4.88E-12	22.412261
UPK1A-AS1	-2.394993	1.0612051	-8.25612	1.75E-14	4.92E-12	22.398448
ALPL	0.8140503	5.8776359	8.241749	1.92E-14	5.34E-12	22.310694
CENPF	-0.483164	5.9892505	-8.238569	1.96E-14	5.41E-12	22.291286
CDCA3	-0.556595	5.4048626	-8.224329	2.14E-14	5.87E-12	22.204423
NUF2	-0.701249	5.2866337	-8.219447	2.21E-14	6.01E-12	22.174657
DLK2	-0.5808	4.4868731	-8.19038	2.65E-14	7.16E-12	21.997639
TTLL4	-0.311435	6.0679992	-8.186839	2.71E-14	7.27E-12	21.976097
SPDL1	-0.28839	5.5121694	-8.184365	2.75E-14	7.33E-12	21.961046
LSM11	-0.26308	5.4257692	-8.164335	3.12E-14	8.25E-12	21.839287
FCN3	0.932911	5.013991	8.1430026	3.56E-14	9.36E-12	21.709777
RP11-132N1	-1.933646	0.7885418	-8.138468	3.67E-14	9.56E-12	21.68227
RP11-329L6	-0.704103	4.0893425	-8.130858	3.84E-14	9.95E-12	21.636125
P3H2	0.5805649	5.0245855	8.1290678	3.89E-14	9.99E-12	21.625271
ZBTB12	-0.480371	5.0561567	-8.10499	4.52E-14	1.15E-11	21.479428
RANBP10	0.204256	6.1117978	8.0892432	4.99E-14	1.26E-11	21.384164
RPS6KA2	0.3715633	5.8136508	8.0843549	5.14E-14	1.29E-11	21.35461
LMNB1	-0.332209	6.0371391	-8.084233	5.14E-14	1.29E-11	21.353876
ASXL1	-0.125959	6.4307048	-8.082009	5.22E-14	1.30E-11	21.340429
PINK1	0.3074109	6.1491693	8.0726849	5.53E-14	1.36E-11	21.284094
ENPP3	-1.414844	4.5796597	-8.067824	5.70E-14	1.40E-11	21.254738
ATXN7L3	-0.125017	6.3792511	-8.066257	5.75E-14	1.40E-11	21.245275
BIRC5	-0.519655	5.8844923	-8.061722	5.92E-14	1.43E-11	21.217895
MBNL2	0.2821944	6.3188959	8.0507503	6.34E-14	1.52E-11	21.151693

RP11-726G1	-0.948041	3.2494917	-8.045631	6.54E-14	1.56E-11	21.120818
ADAMTS2	0.5081689	5.8593778	8.038981	6.82E-14	1.61E-11	21.080728
FOXM1	-0.497766	5.8264907	-8.038448	6.84E-14	1.61E-11	21.077516
ATP11C	0.3830664	5.9363192	8.037033	6.90E-14	1.61E-11	21.068988
PHLPP2	0.2764972	5.5916634	8.0289842	7.26E-14	1.69E-11	21.020493
CCNF	-0.377555	5.5792276	-8.019398	7.70E-14	1.77E-11	20.962767
SMUG1P1	2.4180909	0.3508615	8.0190994	7.71E-14	1.77E-11	20.960971
MAP3K6	0.3058627	5.6425033	8.017816	7.78E-14	1.77E-11	20.953245
HELLS	-0.463078	5.4822948	-7.996631	8.87E-14	2.01E-11	20.825815
RP11-599J1	-1.542095	2.4483716	-7.989538	9.27E-14	2.09E-11	20.783189
SMIM8	-0.232464	5.3693245	-7.98459	9.56E-14	2.14E-11	20.753467
GPC3	-0.789996	6.8408563	-7.982387	9.69E-14	2.16E-11	20.740237
GAS1	0.9871994	4.3580468	7.9726692	1.03E-13	2.27E-11	20.681894
ZNF530	-0.503114	4.7754059	-7.972222	1.03E-13	2.27E-11	20.679211
BUB1B	-0.605509	5.3242674	-7.966709	1.07E-13	2.33E-11	20.646129
ACYP2	0.2399169	5.6849541	7.9607649	1.11E-13	2.41E-11	20.610477
ASPM	-0.502881	5.8714965	-7.959172	1.12E-13	2.42E-11	20.600923
ALPK1	0.3423531	5.5350861	7.9536837	1.16E-13	2.48E-11	20.568021
HDGFRP3	0.4170988	5.3237169	7.9523505	1.17E-13	2.49E-11	20.56003
SGOL2	-0.459611	5.2183898	-7.929035	1.35E-13	2.86E-11	20.420391
CCNB1	-0.418383	5.955367	-7.92423	1.39E-13	2.93E-11	20.391642
EME1	-0.679414	4.6996977	-7.92236	1.40E-13	2.95E-11	20.380457
PM20D2	-0.320091	5.7537553	-7.913598	1.48E-13	3.09E-11	20.328062
ESPL1	-0.50245	5.6078038	-7.899326	1.62E-13	3.36E-11	20.242775
ZNF233	-0.88581	4.0066215	-7.889229	1.72E-13	3.55E-11	20.182492
RP11-498C9	-0.474006	4.6495719	-7.884312	1.78E-13	3.64E-11	20.153147
BEND3	-0.390326	5.2167137	-7.875595	1.87E-13	3.82E-11	20.101155
FOSL2	0.260512	6.3244512	7.8616991	2.04E-13	4.14E-11	20.01833
XRCC3	-0.258931	5.5712441	-7.857761	2.09E-13	4.21E-11	19.994874
GGT5	0.4830012	6.015362	7.8569603	2.10E-13	4.21E-11	19.990103
PEG10	-1.462092	5.6499029	-7.849718	2.20E-13	4.38E-11	19.946983
GNG4	-2.100656	3.8688738	-7.847935	2.22E-13	4.39E-11	19.936367
UBE2C	-0.586927	5.6905179	-7.847649	2.23E-13	4.39E-11	19.934667
KIF20B	-0.314095	5.447599	-7.840421	2.33E-13	4.57E-11	19.891658
KNTC1	-0.328569	5.7494807	-7.83492	2.41E-13	4.70E-11	19.858937
PRAMEF10	2.7691994	1.5725313	7.8334051	2.43E-13	4.72E-11	19.849929
RTKN2	-1.115131	4.3547515	-7.832143	2.45E-13	4.73E-11	19.842425
CEP250	-0.160896	6.122669	-7.831053	2.46E-13	4.73E-11	19.835944
SLC35C1	0.3055329	6.5630188	7.8167364	2.69E-13	5.14E-11	19.750872
SGOL1	-0.689896	4.8381542	-7.81455	2.73E-13	5.18E-11	19.737889
RAI2	0.5777992	5.070486	7.8092332	2.82E-13	5.33E-11	19.706319
EVA1C	0.6011399	5.1048902	7.8039421	2.91E-13	5.47E-11	19.674915
BLM	-0.502446	5.0911381	-7.803074	2.93E-13	5.48E-11	19.669766
EPHA2	0.3088215	6.0435491	7.7911747	3.15E-13	5.86E-11	19.599185
MDFIC	0.4040689	5.8474127	7.7866422	3.23E-13	5.99E-11	19.572316
PRAME	-2.87098	2.9717143	-7.780595	3.36E-13	6.15E-11	19.536482
ITGAV	0.2667494	6.4548793	7.7803207	3.36E-13	6.15E-11	19.534856
DNAH8	-1.849329	2.0971541	-7.778299	3.40E-13	6.15E-11	19.52288
SPC24	-0.492269	5.5406664	-7.77818	3.41E-13	6.15E-11	19.522175
COL2A1	-2.895546	2.1485116	-7.77783	3.41E-13	6.15E-11	19.520103
TPX2	-0.372052	6.0893818	-7.777565	3.42E-13	6.15E-11	19.518531

CSRP1	0.2379747	6.623034	7.769044	3.60E-13	6.45E-11	19.468074
TMEM100	1.4257332	3.9649123	7.759419	3.82E-13	6.80E-11	19.411115
C19orf48	-0.229314	6.3579854	-7.75865	3.84E-13	6.80E-11	19.406564
IFITM2	0.267895	6.7524407	7.7577626	3.86E-13	6.81E-11	19.401316
SH3BGRL	0.2822565	6.1605274	7.7541393	3.95E-13	6.90E-11	19.379886
CCNA2	-0.469169	5.6135356	-7.753893	3.95E-13	6.90E-11	19.378429
ECM1	0.4360024	5.5715516	7.7512098	4.02E-13	6.96E-11	19.362564
KLHL8	0.2179665	5.794701	7.7509299	4.02E-13	6.96E-11	19.360909
PARVA	0.201613	6.1836862	7.7484101	4.09E-13	7.04E-11	19.346012
JAK1	0.1517613	6.6730295	7.7462769	4.14E-13	7.09E-11	19.333403
TNIP1	0.1656246	6.7973024	7.7424185	4.24E-13	7.23E-11	19.310602
ZIM2-AS1	-2.093839	0.9212099	-7.740772	4.28E-13	7.27E-11	19.300871
C1orf131	-0.209474	5.8412973	-7.739416	4.32E-13	7.30E-11	19.292862
RP11-119F7	-0.673864	4.2364718	-7.729787	4.58E-13	7.70E-11	19.235997
MIMT1	-1.926285	-0.142245	-7.728485	4.61E-13	7.73E-11	19.22831
THBS2	0.6095957	6.1629173	7.7180437	4.92E-13	8.20E-11	19.166697
ANTXR1	0.4612314	5.8903894	7.7097214	5.17E-13	8.58E-11	19.11762
EGFR-AS1	-2.194989	2.9530571	-7.708278	5.22E-13	8.62E-11	19.109109
DIRAS3	1.5113885	3.2499771	7.7067048	5.27E-13	8.64E-11	19.099839
CENPO	-0.321795	5.4724217	-7.706432	5.28E-13	8.64E-11	19.098233
SH3GLB1	0.1166124	6.4316533	7.6997651	5.50E-13	8.96E-11	19.058946
DBF4B	-0.2871	5.3270606	-7.698838	5.53E-13	8.96E-11	19.053484
PXN-AS1	-0.2946	5.2135205	-7.698206	5.55E-13	8.96E-11	19.04976
DSE	0.3717675	5.5115149	7.6932998	5.72E-13	9.19E-11	19.020867
C9orf40	-0.303784	5.3048171	-7.683752	6.06E-13	9.70E-11	18.964663
EFCAB14	0.1196236	6.5033938	7.6735459	6.45E-13	1.03E-10	18.904629
CLIC4	0.1777415	6.4496269	7.669425	6.61E-13	1.05E-10	18.880401
NEXN	0.5270514	5.1103971	7.6652098	6.78E-13	1.07E-10	18.855626
BNIPL	-0.69886	3.798499	-7.663376	6.86E-13	1.08E-10	18.844852
RP11-128M1	-1.033375	4.0146289	-7.658818	7.05E-13	1.10E-10	18.818071
HAND2-AS1	2.051108	3.0904041	7.6514547	7.37E-13	1.15E-10	18.774833
SPC25	-0.634765	4.9353344	-7.648556	7.50E-13	1.17E-10	18.757816
HERC6	0.4485475	5.3942873	7.6394141	7.93E-13	1.23E-10	18.704175
COQ9	0.2194362	6.3875957	7.6335967	8.21E-13	1.27E-10	18.670058
RP11-25705	-0.251199	5.2111646	-7.624115	8.70E-13	1.33E-10	18.614485
CDCA5	-0.44392	5.6742237	-7.623768	8.72E-13	1.33E-10	18.612453
PRC1	-0.363024	5.9000892	-7.622916	8.76E-13	1.33E-10	18.607456
TTC39B	0.4369782	5.4866318	7.6173781	9.06E-13	1.37E-10	18.57502
CENPA	-0.822758	4.8033431	-7.616132	9.13E-13	1.38E-10	18.567724
NCAPG	-0.539987	5.5260464	-7.615256	9.18E-13	1.38E-10	18.562592
ZWINT	-0.368625	5.848417	-7.614187	9.24E-13	1.38E-10	18.556335
AC004160.4	1.8463726	-0.273336	7.6141738	9.24E-13	1.38E-10	18.556256
SGMS2	0.3464598	5.737055	7.6133876	9.28E-13	1.38E-10	18.551653
CMPK1	0.1433494	6.7984045	7.6128126	9.31E-13	1.38E-10	18.548287
MAPRE3	0.26878	5.897689	7.6052492	9.75E-13	1.44E-10	18.504019
STAT3	0.1471866	6.8064446	7.6005385	1.00E-12	1.47E-10	18.476461
ATL3	0.147203	6.4253676	7.5964935	1.03E-12	1.50E-10	18.452805
ENG	0.2687669	6.5465273	7.5813241	1.13E-12	1.64E-10	18.364151
LUM	0.9578955	5.9747836	7.5802424	1.13E-12	1.64E-10	18.357833
CDR2	0.1804175	6.0992516	7.5767945	1.16E-12	1.67E-10	18.337698
GSTO2	-0.632384	5.3414658	-7.575895	1.16E-12	1.67E-10	18.332446

CKAP2L	-0.593713	5.1686211	-7.573127	1.18E-12	1.69E-10	18.316283
LINC01224	-2.445759	2.184955	-7.567717	1.22E-12	1.74E-10	18.284713
AKT3	0.3776512	5.5924991	7.5645905	1.25E-12	1.77E-10	18.26647
pk	0.2008962	6.2966122	7.5575718	1.30E-12	1.84E-10	18.225535
KIF18A	-0.701723	4.796213	-7.556587	1.31E-12	1.84E-10	18.219795
PRKAG2	0.2883557	5.9556216	7.5527989	1.34E-12	1.88E-10	18.19771
RP3-323A16	-2.033162	1.7157807	-7.551338	1.35E-12	1.88E-10	18.189197
ADORA2BP1	2.1122313	2.5603194	7.5510322	1.35E-12	1.88E-10	18.187414
BDKRB2	0.6230698	5.2090916	7.5480664	1.38E-12	1.90E-10	18.170131
SVEP1	0.8404451	5.1824019	7.5480294	1.38E-12	1.90E-10	18.169915
AC011997.1	-1.607603	-0.407951	-7.540716	1.44E-12	1.98E-10	18.127312
FANCD2	-0.369786	5.5036207	-7.539096	1.45E-12	1.99E-10	18.117878
COL3A1	0.3596139	6.9247867	7.5373504	1.47E-12	2.01E-10	18.107717
RP11-219C2	2.5736169	0.8979125	7.5351122	1.49E-12	2.03E-10	18.094688
FAM83A-AS1	2.7979815	2.4624821	7.5331711	1.51E-12	2.04E-10	18.083389
LAMA5-AS1	2.2515997	3.5607407	7.5293095	1.54E-12	2.08E-10	18.060917
ANAPC7	-0.13233	6.2426934	-7.528997	1.54E-12	2.08E-10	18.059096
CDC25C	-0.724183	5.0029924	-7.528316	1.55E-12	2.08E-10	18.055137
PRIM1	-0.363535	5.5332732	-7.524381	1.59E-12	2.12E-10	18.032243
C14orf159	0.2842481	6.0638744	7.5160114	1.67E-12	2.22E-10	17.98358
DDX55	-0.140028	5.8882455	-7.509786	1.73E-12	2.30E-10	17.947402
SLC4A4	0.7878172	5.6796901	7.5091383	1.74E-12	2.30E-10	17.943638
MIS18A	-0.247036	5.5797262	-7.50879	1.74E-12	2.30E-10	17.941618
CNDP2	0.1616481	6.8238878	7.5044087	1.79E-12	2.35E-10	17.916165
KIF11	-0.3992	5.5456287	-7.496505	1.88E-12	2.46E-10	17.870276
CYP2W1	-1.864836	2.3729062	-7.494432	1.90E-12	2.48E-10	17.858244
PLAT	0.4180032	5.7804384	7.4918851	1.93E-12	2.51E-10	17.843466
PKD2	0.3216105	5.7551354	7.4882943	1.97E-12	2.55E-10	17.822634
AC133528.2	-0.301364	5.1353131	-7.483353	2.03E-12	2.59E-10	17.793976
MAP1LC3B	0.1911701	6.3504192	7.4828227	2.04E-12	2.59E-10	17.790902
CTSO	0.2932545	6.3012519	7.4827077	2.04E-12	2.59E-10	17.790235
CCDC71L	0.3674303	5.9569615	7.4826252	2.04E-12	2.59E-10	17.789757
NDC80	-0.46667	5.4617152	-7.48259	2.04E-12	2.59E-10	17.789555
C1R	0.2749884	7.2317242	7.4694297	2.21E-12	2.80E-10	17.713285
TIGD1	-0.400876	5.0131764	-7.465646	2.26E-12	2.85E-10	17.691371
CBFA2T2	-0.170545	6.0211867	-7.464411	2.27E-12	2.85E-10	17.684218
ATMIN	0.1375013	6.1970956	7.4643849	2.27E-12	2.85E-10	17.684069
JDP2	0.2915119	5.6772149	7.4639913	2.28E-12	2.85E-10	17.681791
RACGAP1	-0.308892	5.8389138	-7.462155	2.31E-12	2.87E-10	17.671159
SOCS2	0.4845509	5.6689914	7.4604534	2.33E-12	2.89E-10	17.661309
DUSP9	-1.154419	5.4431453	-7.454816	2.41E-12	2.98E-10	17.628683
MT1X	0.6738715	6.1227938	7.4492393	2.49E-12	3.07E-10	17.596425
KIF20A	-0.548636	5.5708471	-7.444469	2.56E-12	3.15E-10	17.568841
CCDC163P	-0.426883	4.8238381	-7.438135	2.66E-12	3.25E-10	17.532228
BRIP1	-0.460116	5.1480753	-7.438118	2.66E-12	3.25E-10	17.532133
PDE2A	0.4428935	5.5008782	7.4374545	2.67E-12	3.25E-10	17.528298
MCM10	-0.6451	4.9957968	-7.435174	2.71E-12	3.29E-10	17.51512
AC084219.4	-1.721935	1.1420641	-7.406253	3.22E-12	3.89E-10	17.348222
RCAN2	0.5350273	5.4560231	7.4009436	3.32E-12	4.00E-10	17.317624
RP4-747G18	-1.591106	0.6502759	-7.399073	3.36E-12	4.03E-10	17.306845
PRIM2	-0.204854	5.6186541	-7.39595	3.42E-12	4.10E-10	17.288859

SSC5D	0.7012565	4.8618441	7.3922638	3.50E-12	4.17E-10	17.267628
MT2A	0.4452155	6.7558736	7.3917842	3.51E-12	4.17E-10	17.264866
RFC4	-0.247779	5.8824708	-7.390888	3.53E-12	4.18E-10	17.259706
RP11-588K2	0.4586378	5.2651103	7.3791215	3.78E-12	4.47E-10	17.191991
ZNF620	-0.350958	4.758247	-7.374685	3.88E-12	4.57E-10	17.166474
PITPNA	0.1339893	6.3505015	7.3708592	3.97E-12	4.66E-10	17.144479
GUCY1B3	0.3445683	5.5378822	7.369451	4.01E-12	4.69E-10	17.136385
IGDCC3	-2.336788	1.3289351	-7.36166	4.20E-12	4.89E-10	17.091614
CNTN3	2.4059132	2.7848692	7.3593633	4.25E-12	4.95E-10	17.078424
CAST	0.1318376	6.7091745	7.3579695	4.29E-12	4.97E-10	17.07042
IL1RAPL2	2.09116	1.5921818	7.3509768	4.47E-12	5.15E-10	17.030274
BTN3A3	0.2850733	5.9078218	7.3507115	4.48E-12	5.15E-10	17.028751
SCML2	-0.611369	4.9798872	-7.35041	4.49E-12	5.15E-10	17.027017
SKA1	-0.712607	5.1101486	-7.342419	4.70E-12	5.38E-10	16.981169
GEM	0.5372841	5.5374061	7.3420135	4.72E-12	5.38E-10	16.978846
TCF24	-1.842723	-0.24354	-7.338142	4.83E-12	5.49E-10	16.956645
PTTG1	-0.504753	5.7283691	-7.330844	5.04E-12	5.71E-10	16.914812
LIG1	-0.187507	6.1896654	-7.32941	5.08E-12	5.74E-10	16.906591
RP11-466F5	-1.164715	2.8201955	-7.324662	5.23E-12	5.89E-10	16.879393
RP5-1158E1	-1.611846	0.0955795	-7.32221	5.30E-12	5.94E-10	16.865348
E2F8	-0.713852	4.8710274	-7.322037	5.31E-12	5.94E-10	16.864362
PRR11	-0.489803	5.3150271	-7.321182	5.34E-12	5.96E-10	16.859466
C10orf88	-0.165361	5.4552355	-7.317507	5.45E-12	6.04E-10	16.838424
ZBTB7A	0.1460377	6.2327289	7.317335	5.46E-12	6.04E-10	16.837439
HAND2	1.7808194	3.9317305	7.3170718	5.47E-12	6.04E-10	16.835932
SAPCD2	-0.67661	4.8555299	-7.316723	5.48E-12	6.04E-10	16.833938
ASB13	0.2419948	6.2694483	7.3128526	5.61E-12	6.16E-10	16.811785
FAM3B	-1.736807	4.6584753	-7.309397	5.72E-12	6.27E-10	16.792015
NAT2	1.9408224	3.9351848	7.3053536	5.86E-12	6.40E-10	16.768885
KIF4A	-0.539775	5.5772522	-7.301696	5.99E-12	6.52E-10	16.747971
SERPINI1	-0.495173	5.2869869	-7.301018	6.01E-12	6.52E-10	16.744092
VWA5A	0.5126297	5.4148707	7.3006886	6.02E-12	6.52E-10	16.742212
PNPLA7	0.4065926	5.5238168	7.2965895	6.17E-12	6.66E-10	16.718782
BSND	-2.013378	0.4829209	-7.28893	6.46E-12	6.95E-10	16.675023
XPO5	-0.150466	6.2649633	-7.286461	6.55E-12	7.03E-10	16.660921
NAALADL1	-0.596453	5.0513851	-7.285938	6.57E-12	7.04E-10	16.657937
SDS	0.8586909	6.3752896	7.285148	6.60E-12	7.05E-10	16.653425
ASAH1	0.1942515	6.5448471	7.2843868	6.63E-12	7.06E-10	16.649079
ITPRIP	0.2796155	5.8976215	7.2806457	6.78E-12	7.20E-10	16.627722
C11orf96	0.4147514	5.7540813	7.2780999	6.88E-12	7.28E-10	16.613194
AXL	0.300871	5.7892725	7.2749651	7.01E-12	7.40E-10	16.595307
C17orf53	-0.450402	4.9683969	-7.273385	7.08E-12	7.45E-10	16.586292
PQLC3	0.2911884	5.712884	7.2727874	7.10E-12	7.45E-10	16.582885
RP11-713M1	-0.550618	5.578572	-7.27216	7.13E-12	7.46E-10	16.579305
WBSCR17	1.9815478	2.6554683	7.2704001	7.20E-12	7.51E-10	16.569269
LINC00634	-1.748756	0.8894847	-7.267527	7.33E-12	7.60E-10	16.552886
MCM2	-0.319621	6.1291727	-7.267513	7.33E-12	7.60E-10	16.552807
NRG1	1.8158927	3.7837561	7.2664646	7.37E-12	7.62E-10	16.546828
EXO1	-0.605407	5.1494633	-7.264641	7.45E-12	7.68E-10	16.536434
FBXO46	-0.18854	5.9703563	-7.263242	7.51E-12	7.68E-10	16.528461
MKI67	-0.394922	6.0905509	-7.263052	7.52E-12	7.68E-10	16.527377

B2M	0.1876478	7.5024872	7.2628304	7.53E-12	7.68E-10	16.526113
STRBP	-0.152934	6.0098173	-7.262737	7.54E-12	7.68E-10	16.525579
RP11-142E9	-0.685956	3.7944976	-7.260481	7.64E-12	7.77E-10	16.512725
GABARAPL1	0.3067972	6.447003	7.2548351	7.90E-12	8.01E-10	16.480558
CYLD	0.163507	6.1643253	7.2475267	8.24E-12	8.33E-10	16.438944
RP11-542K2	-1.43526	-0.46087	-7.246191	8.31E-12	8.38E-10	16.431342
RP4-694B14	-0.682005	3.8875784	-7.244335	8.40E-12	8.41E-10	16.420777
UQCRC2	0.1720354	6.7396265	7.2442622	8.40E-12	8.41E-10	16.420363
TBC1D10A	0.1873547	5.7520702	7.2440793	8.41E-12	8.41E-10	16.419322
ARHGEF12	0.1579135	6.7433709	7.2433858	8.45E-12	8.42E-10	16.415376
COL8A1	0.6732773	5.3603255	7.2428479	8.47E-12	8.43E-10	16.412315
CLK2	-0.147848	6.2226201	-7.241097	8.56E-12	8.49E-10	16.402352
FSTL1	0.2947541	6.4028731	7.2397783	8.63E-12	8.51E-10	16.394851
ST6GAL2	1.6715389	3.5179529	7.2397367	8.63E-12	8.51E-10	16.394614
RP4-570012	-1.631642	-0.550693	-7.237975	8.72E-12	8.58E-10	16.384595
CTD-2224J9	-1.940501	1.2076993	-7.235454	8.85E-12	8.68E-10	16.370256
SLIT2	0.7537611	4.9723944	7.2333898	8.96E-12	8.77E-10	16.358517
HIST2H2AC	-0.665763	3.9761165	-7.23275	8.99E-12	8.78E-10	16.354882
CTC-458A3.	-1.904728	0.6071836	-7.23106	9.08E-12	8.84E-10	16.345271
DEPDC4	-0.466212	3.9493585	-7.223731	9.48E-12	9.20E-10	16.303619
IL6ST	0.1674617	6.8443789	7.2232905	9.51E-12	9.20E-10	16.301118
MSI1	-1.21401	4.8715585	-7.217567	9.83E-12	9.49E-10	16.268608
TEK	0.6358529	5.2780354	7.2164149	9.90E-12	9.53E-10	16.262067
MYLK	0.2609253	6.4171067	7.2133636	1.01E-11	9.67E-10	16.244744
C16orf62	0.2950527	5.9192266	7.2130816	1.01E-11	9.67E-10	16.243143
RP11-73M7.	-1.87177	1.6318338	-7.205805	1.05E-11	1.01E-09	16.201851
FBN1	0.3713358	6.1115252	7.1966662	1.11E-11	1.06E-09	16.150025
GNA14	0.7212553	4.512507	7.1935097	1.13E-11	1.08E-09	16.132134
PRR3	-0.172814	5.731246	-7.192283	1.14E-11	1.08E-09	16.12518
IKBKAP	-0.203508	6.2865099	-7.189857	1.16E-11	1.09E-09	16.111433
POLD1	-0.207646	6.0517494	-7.189244	1.16E-11	1.09E-09	16.107965
DLGAP5	-0.604934	5.2863009	-7.18715	1.18E-11	1.11E-09	16.096098
LINC01424	-1.546168	0.9894852	-7.185579	1.19E-11	1.11E-09	16.0872
SALL2	-0.721036	5.0548566	-7.185268	1.19E-11	1.11E-09	16.085443
PHYKPL	0.1882345	6.2251834	7.1850967	1.19E-11	1.11E-09	16.084471
EZH2	-0.314877	5.751892	-7.184691	1.19E-11	1.11E-09	16.082172
AMFR	0.1676013	6.7189994	7.1844987	1.19E-11	1.11E-09	16.081084
ATP8B4	0.4595269	4.913006	7.183778	1.20E-11	1.11E-09	16.077003
OLFM1	0.8171865	4.6404119	7.1833118	1.20E-11	1.11E-09	16.074363
LINC01354	1.877675	1.7666185	7.1829453	1.20E-11	1.11E-09	16.072288
ASF1B	-0.460566	5.5236814	-7.182216	1.21E-11	1.11E-09	16.068161
FEN1	-0.218594	6.1362992	-7.175678	1.26E-11	1.15E-09	16.031151
COL14A1	0.5600522	5.9472366	7.1688191	1.31E-11	1.20E-09	15.992347
MITF	0.4283547	5.1298748	7.1665904	1.33E-11	1.21E-09	15.979743
CTD-2540B1	-1.618468	1.5378873	-7.166392	1.33E-11	1.21E-09	15.97862
RP11-15A1.	-1.955415	1.193932	-7.164846	1.34E-11	1.22E-09	15.969877
OIP5	-0.680749	4.6833772	-7.163582	1.35E-11	1.22E-09	15.962733
SATB1	0.476481	5.5597347	7.16145	1.37E-11	1.23E-09	15.950681
POLG2	-0.202553	5.5009092	-7.159785	1.38E-11	1.24E-09	15.941269
CCDC184	1.0766123	3.1027785	7.1585606	1.39E-11	1.25E-09	15.934352
RPS27P25	-1.583031	-0.258047	-7.156031	1.41E-11	1.26E-09	15.920061

SALL4	-1.582614	3.7838415	-7.153888	1.43E-11	1.28E-09	15.907954
ZNF330	0.1637265	6.0092184	7.1531057	1.43E-11	1.28E-09	15.903533
COL1A2	0.3231191	6.8796494	7.1502563	1.46E-11	1.30E-09	15.88744
GNG12-AS1	1.1808014	2.9764226	7.1499521	1.46E-11	1.30E-09	15.885723
THOC1	-0.142292	5.9484551	-7.149239	1.47E-11	1.30E-09	15.881695
AC090954.5	-1.51538	-0.490495	-7.148543	1.47E-11	1.30E-09	15.877766
DDX12P	-0.419103	4.6252227	-7.148337	1.47E-11	1.30E-09	15.876601
FILIP1L	0.3201378	5.7960755	7.1432017	1.52E-11	1.34E-09	15.847615
RP11-133K1	-1.482588	-0.257709	-7.142273	1.53E-11	1.34E-09	15.842375
PTRF	0.2091848	6.556653	7.1414387	1.54E-11	1.34E-09	15.837665
SYTL4	0.4034856	5.7552828	7.1408052	1.54E-11	1.34E-09	15.83409
SGCE	-0.502131	5.849798	-7.140753	1.54E-11	1.34E-09	15.833796
RAD51AP1	-0.485576	5.1132715	-7.135808	1.59E-11	1.38E-09	15.805897
RNVU1-6	-1.620241	1.3325477	-7.130998	1.63E-11	1.41E-09	15.778778
USP21	-0.158893	5.9418837	-7.130193	1.64E-11	1.42E-09	15.774237
PLXDC2	0.3847526	5.7572929	7.1264466	1.68E-11	1.45E-09	15.753119
PRELP	0.6828343	5.7375033	7.1223319	1.72E-11	1.47E-09	15.729934
NFIB	0.2443501	6.2627644	7.1222968	1.72E-11	1.47E-09	15.729736
GAS2L3	-0.353121	5.3912323	-7.119271	1.75E-11	1.50E-09	15.712689
RP4-616B8.	-1.415615	2.5596048	-7.119027	1.75E-11	1.50E-09	15.711318
PODN	0.6982106	5.4534329	7.1112541	1.83E-11	1.56E-09	15.667552
NUP37	-0.168541	5.7724049	-7.110043	1.84E-11	1.57E-09	15.660735
ISLR	0.8780159	5.4822588	7.1086153	1.86E-11	1.58E-09	15.652701
NR6A1	-0.389942	5.42163	-7.108414	1.86E-11	1.58E-09	15.651568
MAN1C1	0.4673425	5.7168351	7.1072341	1.87E-11	1.58E-09	15.644929
HSD11B1	0.8288593	6.1157597	7.1056604	1.89E-11	1.59E-09	15.636075
CBX1	-0.138547	6.2264251	-7.102173	1.93E-11	1.62E-09	15.616456
FAM65A	0.1338834	6.2959096	7.1004948	1.95E-11	1.64E-09	15.60702
ALYREF	-0.171076	6.2762367	-7.093165	2.03E-11	1.70E-09	15.565812
MIER1	0.1415531	6.1139545	7.089635	2.08E-11	1.73E-09	15.545978
MRPL23-AS1	-2.220859	0.9089797	-7.08772	2.10E-11	1.75E-09	15.535221
SFSWAP	-0.112695	6.1835818	-7.084311	2.14E-11	1.78E-09	15.516073
LRRFIP1	0.1367415	6.492462	7.0822247	2.17E-11	1.80E-09	15.504358
TCF25	0.1426692	6.5381962	7.081924	2.17E-11	1.80E-09	15.502669
FAM72A	-0.754515	3.6961835	-7.080202	2.19E-11	1.81E-09	15.493004
XRCC2	-0.623837	4.7709109	-7.079485	2.20E-11	1.81E-09	15.488976
PLCXD3	1.7127064	3.7332388	7.0784783	2.22E-11	1.82E-09	15.483326
CDC6	-0.413771	5.7043388	-7.078367	2.22E-11	1.82E-09	15.482699
RP1-232P20	2.3970309	1.1525923	7.0780761	2.22E-11	1.82E-09	15.481068
TNXB	0.5453591	5.4717816	7.071671	2.30E-11	1.88E-09	15.445127
MED28	-0.12833	6.1802597	-7.066274	2.38E-11	1.94E-09	15.414856
BUB1	-0.490106	5.4786368	-7.066015	2.38E-11	1.94E-09	15.413407
PMP22	0.3237729	5.7988263	7.0655856	2.39E-11	1.94E-09	15.410999
AQP1	0.3945779	6.3630157	7.0651209	2.39E-11	1.94E-09	15.408394
COLCA2	-0.99711	4.6965195	-7.061785	2.44E-11	1.97E-09	15.389695
RP11-48601	-0.334527	4.7234201	-7.058622	2.49E-11	2.00E-09	15.371964
ZSCAN2	-0.21686	5.6179998	-7.050078	2.61E-11	2.10E-09	15.324108
HLA-B	0.2389162	7.3517414	7.0410572	2.75E-11	2.21E-09	15.273617
GLT8D2	0.5604454	4.6199128	7.0374031	2.81E-11	2.25E-09	15.253176
WDR62	-0.418292	5.4315155	-7.036815	2.82E-11	2.25E-09	15.249887
TAF11	-0.124104	6.1197151	-7.033176	2.88E-11	2.30E-09	15.229538

SOCS3	0.3871352	6.1372041	7.0319494	2.90E-11	2.31E-09	15.222679
CLCN3	0.1832657	6.2667175	7.0279796	2.97E-11	2.36E-09	15.200489
CDC20	-0.525731	5.7540656	-7.023251	3.05E-11	2.42E-09	15.174068
PRRG1	0.3187358	5.497622	7.0195025	3.12E-11	2.46E-09	15.15313
SH3RF1	0.3154685	5.7525469	7.0132155	3.23E-11	2.55E-09	15.118029
GSG2	-0.877941	3.9962302	-7.012966	3.24E-11	2.55E-09	15.116636
CCDC80	0.6232516	5.9491218	7.0124335	3.25E-11	2.55E-09	15.113664
CDC37L1	0.2060144	6.0548702	7.0072912	3.35E-11	2.62E-09	15.084971
DTL	-0.457472	5.5910847	-7.006231	3.37E-11	2.63E-09	15.079056
TAGLN	0.2902463	6.4362706	7.0058796	3.37E-11	2.63E-09	15.077096
AURKA	-0.33923	5.9163872	-7.002511	3.44E-11	2.68E-09	15.058307
ARHGAP11A	-0.382849	5.5325573	-7.000743	3.47E-11	2.70E-09	15.048453
WASF1	-0.295907	5.6728256	-6.999669	3.50E-11	2.71E-09	15.04246
SYNPO	0.1960991	6.6621662	6.9988028	3.51E-11	2.71E-09	15.037634
TMEM159	0.4842863	4.9477443	6.9987797	3.51E-11	2.71E-09	15.037505
CCDC142	-0.201846	5.4460217	-6.998679	3.52E-11	2.71E-09	15.036946
BORA	-0.394998	4.7256145	-6.99824	3.53E-11	2.71E-09	15.034499
RP5-821D11	-0.354629	4.9555597	-6.996192	3.57E-11	2.74E-09	15.023083
RP11-216L1	-0.859104	3.7140605	-6.995144	3.59E-11	2.74E-09	15.01724
EIF4E3	0.399017	5.615475	6.995042	3.59E-11	2.74E-09	15.016672
RP11-806L2	-1.630389	0.964005	-6.993641	3.62E-11	2.76E-09	15.008867
ZNF337-AS1	-0.37117	4.8058382	-6.990755	3.68E-11	2.80E-09	14.992785
CTBS	0.2117953	6.0290179	6.9897314	3.70E-11	2.81E-09	14.987084
SNHG10	-0.316291	4.9549564	-6.989082	3.72E-11	2.81E-09	14.983469
MELK	-0.530855	5.3485364	-6.987027	3.76E-11	2.84E-09	14.972022
INMT	0.6932789	5.3361092	6.9867734	3.77E-11	2.84E-09	14.97061
TACC3	-0.264129	6.107382	-6.984391	3.82E-11	2.87E-09	14.957344
PLK1	-0.497593	5.6263443	-6.980402	3.91E-11	2.93E-09	14.935139
AC125421.1	-1.247326	-0.68234	-6.979835	3.92E-11	2.94E-09	14.931985
FANCI	-0.304357	5.7610946	-6.978555	3.95E-11	2.95E-09	14.924861
TSLP	1.6651442	3.5189646	6.9753086	4.02E-11	3.00E-09	14.906798
ARFIP1	0.1368605	6.0166075	6.9748619	4.03E-11	3.01E-09	14.904313
IGHVII-78-	-1.329761	-0.585785	-6.972809	4.08E-11	3.03E-09	14.892892
PRSS23	0.3030034	6.1332984	6.9719925	4.10E-11	3.04E-09	14.888352
SP100	0.1726586	6.3769133	6.9705359	4.14E-11	3.06E-09	14.880252
RP11-599B1	-1.775736	-0.279455	-6.969656	4.16E-11	3.07E-09	14.875359
H2AFX	-0.246484	6.0973989	-6.969522	4.16E-11	3.07E-09	14.874616
PAFAH1B3	-0.338633	5.852968	-6.968443	4.19E-11	3.08E-09	14.868613
BCL2	0.3673175	5.357374	6.966691	4.23E-11	3.10E-09	14.858875
TLR3	0.4597474	5.3834607	6.9661405	4.24E-11	3.10E-09	14.855815
EPS15	0.117537	6.3218077	6.9660831	4.24E-11	3.10E-09	14.855496
KCTD12	0.2912506	6.0756424	6.9619754	4.34E-11	3.17E-09	14.832667
NCAM1	0.9741691	4.0832046	6.9609134	4.37E-11	3.19E-09	14.826766
SARAF	0.1571012	6.8252238	6.960005	4.39E-11	3.20E-09	14.821719
SRD5A1	0.2859449	6.1575034	6.9593502	4.41E-11	3.20E-09	14.818081
KIF23	-0.49696	5.3570476	-6.958948	4.42E-11	3.20E-09	14.815846
MTA3	-0.147901	6.0514293	-6.956142	4.49E-11	3.25E-09	14.800263
LA16c-312E	-1.844336	2.4116749	-6.955636	4.51E-11	3.25E-09	14.797453
RP11-749I1	-1.590034	1.0482518	-6.953783	4.55E-11	3.28E-09	14.787164
RP5-1120P1	-1.917993	2.9684474	-6.952482	4.59E-11	3.30E-09	14.779939
LAMA2	0.7614102	5.4387796	6.94866	4.69E-11	3.36E-09	14.758721

RP13-735L2	-1.372559	2.5867	-6.948084	4.71E-11	3.37E-09	14.755526
PBX2	-0.148326	6.3877879	-6.944229	4.81E-11	3.44E-09	14.734136
CTD-2561B2	-0.674085	3.5822736	-6.943095	4.84E-11	3.45E-09	14.72784
CENPW	-0.428082	5.3292073	-6.942681	4.85E-11	3.45E-09	14.725548
EMILIN1	0.3869954	6.1485183	6.9423523	4.86E-11	3.45E-09	14.723723
PAFAH2	0.193062	5.9483643	6.9410059	4.90E-11	3.47E-09	14.716255
EPS8	0.1700347	6.3101253	6.9388214	4.96E-11	3.51E-09	14.70414
RP11-736K2	0.5505611	4.3836133	6.9360006	5.04E-11	3.56E-09	14.6885
DHODH	0.3430823	6.2095978	6.9318505	5.17E-11	3.64E-09	14.665497
CTA-246H3.	-1.480772	-0.550892	-6.931755	5.17E-11	3.64E-09	14.664965
KIAA0430	0.1248583	6.2803834	6.9278496	5.29E-11	3.71E-09	14.643328
BCAR1	0.145568	6.4252716	6.9275985	5.29E-11	3.71E-09	14.641937
RP11-486A1	-0.935345	4.1440022	-6.925791	5.35E-11	3.74E-09	14.631924
DVL2	-0.160845	6.033053	-6.92473	5.38E-11	3.75E-09	14.626047
VPS13D	0.1515443	6.2720351	6.9246632	5.38E-11	3.75E-09	14.625678
IFI16	0.2830048	6.0866008	6.9200079	5.53E-11	3.84E-09	14.599901
ZFYVE28	0.3367563	5.3952752	6.9195406	5.54E-11	3.85E-09	14.597315
HMGN5	0.7680635	4.7742377	6.9185672	5.57E-11	3.86E-09	14.591926
SNHG1	-0.215889	6.0451244	-6.917277	5.62E-11	3.88E-09	14.584784
FER1L6	-2.400249	2.3116521	-6.916462	5.64E-11	3.89E-09	14.580273
NVL	-0.144097	5.8581369	-6.915881	5.66E-11	3.90E-09	14.577057
CCBE1	1.4224689	3.4211873	6.9154364	5.68E-11	3.90E-09	14.574599
SLC29A4	-0.733349	5.5601196	-6.914911	5.69E-11	3.91E-09	14.571693
DBF4	-0.235253	5.4618819	-6.914533	5.71E-11	3.91E-09	14.569602
C2orf68	-0.132183	6.1547314	-6.914301	5.71E-11	3.91E-09	14.568317
CASKIN1	-1.859114	1.8538009	-6.913981	5.72E-11	3.91E-09	14.566546
CREBL2	0.1773878	6.3512293	6.9119303	5.79E-11	3.94E-09	14.5552
AEBP1	0.4584891	6.4153621	6.9064389	5.98E-11	4.06E-09	14.524829
EFEMP1	0.8645769	5.8572909	6.9025895	6.11E-11	4.15E-09	14.503548
COL5A1	0.3915743	6.349046	6.90205	6.13E-11	4.15E-09	14.500566
RRM2	-0.35916	6.0588514	-6.900937	6.17E-11	4.17E-09	14.494415
FAM180A	1.8393153	3.3495493	6.8990958	6.23E-11	4.20E-09	14.48424
ZNF462	0.6289795	4.5469077	6.8989632	6.24E-11	4.20E-09	14.483508
PDE1A	0.6128424	4.9222553	6.8980049	6.27E-11	4.22E-09	14.478213
TGM2	0.2453432	6.9591826	6.8951861	6.37E-11	4.28E-09	14.46264
DPYSL2	0.2162002	6.2373096	6.893851	6.42E-11	4.30E-09	14.455266
GAS6-AS1	0.7310586	4.6783244	6.8907378	6.54E-11	4.37E-09	14.438074
PNMAL2	0.739129	3.958559	6.8884903	6.62E-11	4.42E-09	14.425666
ENTHD2	-0.142002	5.749132	-6.885721	6.73E-11	4.48E-09	14.410382
RFTN1	0.322922	6.1851646	6.8851876	6.75E-11	4.49E-09	14.407437
PPA2	0.1692801	6.3196061	6.8841813	6.79E-11	4.51E-09	14.401884
ADAMTS12	0.4704136	5.1817661	6.882259	6.86E-11	4.54E-09	14.391276
RP11-672L1	-0.92597	3.4254705	-6.881834	6.88E-11	4.54E-09	14.388931
UBE2T	-0.401374	5.5429568	-6.881709	6.88E-11	4.54E-09	14.38824
AHCYL1	0.1147908	6.6367583	6.8801183	6.95E-11	4.58E-09	14.379467
GPRC5B	0.3977421	5.8036902	6.8796444	6.97E-11	4.58E-09	14.376853
AF064858.6	1.5064724	3.7315091	6.87906	6.99E-11	4.59E-09	14.373629
ZNF445	-0.154211	5.8953304	-6.874315	7.18E-11	4.71E-09	14.347464
CD44	0.3767895	6.2343646	6.8726545	7.25E-11	4.74E-09	14.338309
CDCA8	-0.429981	5.5083179	-6.871714	7.29E-11	4.76E-09	14.333123
U2SURP	-0.108286	6.4117005	-6.869974	7.36E-11	4.80E-09	14.323535

PDE7B	0.4900599	4.8069937	6.8692437	7.39E-11	4.80E-09	14.31951
BEX2	-1.342834	4.7730869	-6.86915	7.40E-11	4.80E-09	14.318992
CYTH3	0.2486696	5.7754606	6.8662349	7.52E-11	4.88E-09	14.302931
KLHL23	-0.263968	5.8697821	-6.865418	7.56E-11	4.89E-09	14.29843
MYOF	0.3455994	5.8571548	6.8647983	7.58E-11	4.90E-09	14.295017
ATP2B1	-0.179141	6.2408645	-6.859603	7.81E-11	5.04E-09	14.266406
COL6A2	0.2591103	6.7519875	6.8567426	7.94E-11	5.11E-09	14.250658
APOL6	0.2371025	6.5772638	6.8553225	8.00E-11	5.14E-09	14.242841
IGSF1	-1.108969	4.9415765	-6.853257	8.10E-11	5.20E-09	14.231476
CCDC150	-0.426524	4.9409921	-6.852352	8.14E-11	5.21E-09	14.226496
BPIFB6	-1.605662	-0.615354	-6.850814	8.21E-11	5.25E-09	14.21803
KCNB1	1.3004099	4.3746445	6.8498354	8.26E-11	5.27E-09	14.212649
LTBP4	0.3394448	6.2409388	6.8489186	8.30E-11	5.29E-09	14.207605
KIAA1586	-0.192355	5.4047296	-6.839819	8.74E-11	5.55E-09	14.15757
SRGAP1	0.4462923	5.3271424	6.8396762	8.75E-11	5.55E-09	14.156788
CENPM	-0.545486	5.2754343	-6.838857	8.79E-11	5.57E-09	14.152286
AC004854.4	-1.448053	0.01245	-6.832851	9.10E-11	5.75E-09	14.119289
RP11-560J1	-1.043035	2.8814251	-6.830979	9.19E-11	5.81E-09	14.109007
HSPB8	0.6410464	5.5812347	6.8302189	9.23E-11	5.82E-09	14.104832
SYNE2	0.1898766	6.3369002	6.8293172	9.28E-11	5.84E-09	14.099882
ARL6IP5	0.1394146	6.427456	6.8282442	9.34E-11	5.87E-09	14.09399
MCM5	-0.19346	6.4048423	-6.825762	9.47E-11	5.94E-09	14.080362
CYBRD1	0.3336277	5.9938588	6.8255595	9.48E-11	5.94E-09	14.079252
PDZD4	0.5851877	4.7497113	6.8241974	9.56E-11	5.97E-09	14.071776
ADCK4	0.1568331	6.1196605	6.8240588	9.56E-11	5.97E-09	14.071015
SOX12	-0.270649	6.1023548	-6.820767	9.74E-11	6.07E-09	14.052955
CMTR2	0.2015606	5.7996764	6.8201669	9.78E-11	6.08E-09	14.04966
RP3-420J14	2.1073669	1.3000172	6.8154774	1.00E-10	6.23E-09	14.023938
KLHL2	0.2387554	5.8696736	6.8116709	1.03E-10	6.36E-09	14.003068
IL1RL1	1.4138499	3.1843956	6.8092922	1.04E-10	6.43E-09	13.990029
ABRACL	0.3868764	5.5881103	6.8032569	1.08E-10	6.65E-09	13.956961
ME3	0.3807577	5.2659545	6.7992223	1.10E-10	6.79E-09	13.934865
SRPX2	0.555843	5.0908258	6.7971985	1.11E-10	6.86E-09	13.923784
RP11-655M1	-1.762586	0.9323274	-6.796094	1.12E-10	6.89E-09	13.917737
RP11-152N1	-0.511537	3.9800034	-6.79581	1.12E-10	6.89E-09	13.916183
MTFR2	-0.639553	4.4999773	-6.795096	1.13E-10	6.90E-09	13.912275
RRN3	0.1356047	6.1962034	6.7946836	1.13E-10	6.91E-09	13.910017
RPIA	-0.144665	5.7611031	-6.79334	1.14E-10	6.95E-09	13.902665
AP1G1	0.1240436	6.4595016	6.7914115	1.15E-10	7.01E-09	13.892111
CH17-472G2	1.2722156	-0.614516	6.7890208	1.17E-10	7.10E-09	13.879031
PMF1-BGLAF	-0.404825	4.4374904	-6.78795	1.17E-10	7.13E-09	13.873176
TTC9	0.5249152	5.6125345	6.785877	1.19E-10	7.20E-09	13.861836
C16orf45	0.4044194	5.6834142	6.7853842	1.19E-10	7.21E-09	13.859141
DHX34	-0.157621	6.1117695	-6.78465	1.20E-10	7.23E-09	13.855127
USP38	0.1836433	6.0597105	6.7822334	1.21E-10	7.32E-09	13.841913
EMP1	0.3253188	6.0766341	6.7803317	1.23E-10	7.38E-09	13.831517
NAV1	0.2879753	5.622509	6.7738705	1.27E-10	7.65E-09	13.796211
SMYD3	-0.291855	5.4786913	-6.771837	1.29E-10	7.72E-09	13.785101
F8	0.3574152	5.6528207	6.7713188	1.29E-10	7.73E-09	13.782273
CENPL	-0.336002	5.2385103	-6.770241	1.30E-10	7.76E-09	13.776387
RNASEL	0.418701	5.1804981	6.7697869	1.30E-10	7.76E-09	13.773907

ZNF879	-0.584369	4.2334727	-6.769593	1.30E-10	7.76E-09	13.77285
CXCL6	2.4075769	3.6745672	6.7695373	1.30E-10	7.76E-09	13.772544
LINC01554	1.5035419	4.3281368	6.7693047	1.30E-10	7.76E-09	13.771274
RP13-580F1	1.4635428	2.2283885	6.7690065	1.31E-10	7.76E-09	13.769646
SAT1	0.1890552	6.9303666	6.7687387	1.31E-10	7.76E-09	13.768183
A4GALT	0.3588376	5.4083893	6.7676455	1.32E-10	7.79E-09	13.762214
RP11-973H7	-0.320478	4.1376556	-6.765704	1.33E-10	7.86E-09	13.751615
TMCO4	0.1709286	5.8525842	6.7649429	1.34E-10	7.89E-09	13.747461
ADAMTS1	0.3412379	6.0457855	6.7643667	1.34E-10	7.90E-09	13.744316
SNAI1	0.5530825	4.9978682	6.7634281	1.35E-10	7.93E-09	13.739194
DNAJC9	-0.195821	5.7400768	-6.76031	1.37E-10	8.06E-09	13.722178
CRPP1	1.8594044	0.2354833	6.7589478	1.38E-10	8.11E-09	13.714748
AC087294.2	-1.222882	1.6266297	-6.757381	1.40E-10	8.16E-09	13.706204
KIF14	-0.526262	5.1720176	-6.755878	1.41E-10	8.22E-09	13.698006
C7	0.8232434	6.067281	6.755697	1.41E-10	8.22E-09	13.697017
RCAN1	0.2589968	6.2360563	6.7539453	1.42E-10	8.28E-09	13.687465
ARHGAP11B	-0.502524	4.7101441	-6.751305	1.44E-10	8.40E-09	13.673071
RP11-46A10	-0.585396	3.9351349	-6.750287	1.45E-10	8.43E-09	13.667523
MRC2	0.3871714	5.8541655	6.7495565	1.46E-10	8.45E-09	13.663539
TDP1	-0.149136	5.6925233	-6.749247	1.46E-10	8.45E-09	13.661855
TRIM59	-0.443487	4.8602147	-6.749079	1.46E-10	8.45E-09	13.660938
ERCC6L	-0.739831	4.4022131	-6.745943	1.49E-10	8.59E-09	13.64385
MFAP4	0.8525699	5.5561885	6.7424331	1.52E-10	8.74E-09	13.624727
CEBPA-AS1	-0.446847	5.484684	-6.73962	1.54E-10	8.87E-09	13.609409
NUSAP1	-0.293955	6.0037936	-6.734449	1.59E-10	9.10E-09	13.581258
RP5-1112D6	-0.364967	4.5374714	-6.734394	1.59E-10	9.10E-09	13.580959
C5orf34	-0.427582	4.6085598	-6.734223	1.59E-10	9.10E-09	13.580028
TSPEAR	-1.692117	2.9663686	-6.733601	1.60E-10	9.12E-09	13.576639
WISP2	1.5256942	3.8057337	6.7320942	1.61E-10	9.18E-09	13.568441
FLNA	0.2126262	6.8512963	6.7305241	1.62E-10	9.25E-09	13.559898
FRMD6	0.3940847	5.6244628	6.7299619	1.63E-10	9.26E-09	13.556839
JMJD8	0.1522504	6.230526	6.7278084	1.65E-10	9.36E-09	13.545125
ZNF850	-0.422249	4.6903368	-6.726635	1.66E-10	9.40E-09	13.53874
TBXA2R	0.3944774	5.0421363	6.7265892	1.66E-10	9.40E-09	13.538493
METRN	0.4663972	5.7659197	6.7257462	1.67E-10	9.43E-09	13.533909
HIVEP3	0.3326709	5.274012	6.7234212	1.69E-10	9.54E-09	13.521267
ASPN	0.4947956	5.7964499	6.7231212	1.69E-10	9.54E-09	13.519636
CTSV	-1.20207	4.3295937	-6.72198	1.70E-10	9.59E-09	13.513431
SGCD	1.1920001	4.3411942	6.7206585	1.72E-10	9.63E-09	13.506248
ZNF605	-0.192564	5.7102532	-6.720636	1.72E-10	9.63E-09	13.506126
AP000892.6	0.6309096	4.2641188	6.7198348	1.72E-10	9.66E-09	13.501771
RP5-967N21	-0.653794	4.0382499	-6.718738	1.74E-10	9.70E-09	13.495811
CNOT10	-0.123638	6.0679835	-6.718237	1.74E-10	9.72E-09	13.493088
GUCY1A3	0.4113262	5.4339621	6.7176392	1.75E-10	9.73E-09	13.489838
RP11-359E1	1.234184	2.9390481	6.7174063	1.75E-10	9.73E-09	13.488573
TCF19	-0.328861	5.8559673	-6.716663	1.76E-10	9.76E-09	13.484534
MICAL2	0.2229315	6.166023	6.7121236	1.80E-10	9.99E-09	13.459874
TMEM173	0.2736982	5.8746069	6.7112798	1.81E-10	1.00E-08	13.455292
CBR4	0.2407623	6.0632405	6.7109336	1.81E-10	1.00E-08	13.453412
KIAA0040	0.2485091	5.9751435	6.7076611	1.85E-10	1.02E-08	13.435643
NOL11	-0.11188	6.2793945	-6.70744	1.85E-10	1.02E-08	13.434445

MAGI2-AS3	0.6363999	5.1238335	6.7071906	1.85E-10	1.02E-08	13.433089
SERPINA13F	-1.332455	-0.610145	-6.705968	1.87E-10	1.03E-08	13.42645
RAB20	0.2698201	6.089115	6.7050034	1.88E-10	1.03E-08	13.421216
RP11-390F4	-1.428528	2.0774537	-6.703736	1.89E-10	1.04E-08	13.414338
RAPGEF4-AS	-1.533297	0.239249	-6.700361	1.92E-10	1.05E-08	13.396023
ZKSCAN3	-0.260178	5.5711855	-6.700064	1.93E-10	1.05E-08	13.394414
CSRNP1	0.2300035	6.12538	6.6970434	1.96E-10	1.07E-08	13.37803
TRIM17	-1.651768	2.9314739	-6.694476	1.99E-10	1.08E-08	13.364109
NCAPD2	-0.199683	6.1869995	-6.691953	2.02E-10	1.10E-08	13.350428
RP11-433A1	-1.593905	0.9074862	-6.689532	2.05E-10	1.11E-08	13.337305
ZMYM3	-0.136082	6.2124602	-6.683572	2.12E-10	1.15E-08	13.305015
PRICKLE1	0.8868897	4.1784528	6.6812601	2.14E-10	1.16E-08	13.292494
LPAR1	0.9583901	4.3645208	6.6810475	2.15E-10	1.16E-08	13.291343
CCDC155	-1.985968	0.8238764	-6.680118	2.16E-10	1.16E-08	13.286309
CDA	0.6463893	5.5518532	6.6785724	2.18E-10	1.17E-08	13.277942
GRM4	-1.833451	0.8436153	-6.676213	2.20E-10	1.19E-08	13.265171
WHSC1	-0.15801	6.2695815	-6.672044	2.26E-10	1.21E-08	13.24261
TRIM2	0.3637755	5.8201334	6.6712988	2.27E-10	1.22E-08	13.238577
JARID2	-0.155008	5.8453676	-6.670392	2.28E-10	1.22E-08	13.233673
KCND3	1.2081265	4.90782	6.6701347	2.28E-10	1.22E-08	13.23228
MAFK	0.2306891	6.0158559	6.6685709	2.30E-10	1.23E-08	13.223821
H19	-0.696705	6.4345425	-6.666299	2.33E-10	1.24E-08	13.211536
TRANK1	0.2888497	5.9977605	6.6660833	2.33E-10	1.24E-08	13.210368
RBMS3	0.4154911	5.1278154	6.6602296	2.41E-10	1.28E-08	13.178724
AP001626.2	-1.963534	0.877316	-6.656854	2.46E-10	1.31E-08	13.160483
TIMP1	0.31477	6.906714	6.6550578	2.48E-10	1.32E-08	13.15078
AC093375.1	-1.79155	2.2570586	-6.65201	2.52E-10	1.34E-08	13.13432
ARMCX2	0.4464541	5.0499225	6.6503346	2.55E-10	1.35E-08	13.125273
DSCR9	-1.372824	1.1292534	-6.647261	2.59E-10	1.37E-08	13.10868
TMEM59	0.1216533	6.9360993	6.6463818	2.61E-10	1.37E-08	13.103935
TERF2IP	0.1210696	6.3141623	6.6454208	2.62E-10	1.38E-08	13.098749
KISS1R	-2.060404	1.0455796	-6.644879	2.63E-10	1.38E-08	13.095825
MSH2	-0.161547	5.9641607	-6.644458	2.63E-10	1.38E-08	13.093551
CNBD2	-0.70338	3.1162198	-6.64191	2.67E-10	1.40E-08	13.079803
RP11-353N1	-1.900966	1.8424012	-6.640198	2.70E-10	1.41E-08	13.070571
WARS2-IT1	1.5757002	0.3643708	6.6401977	2.70E-10	1.41E-08	13.070568
STAB1	0.207457	6.4086579	6.6397078	2.70E-10	1.41E-08	13.067926
SIRT4	-0.304489	4.9335646	-6.636718	2.75E-10	1.43E-08	13.051801
RARB	0.4601073	4.9236079	6.6361078	2.76E-10	1.44E-08	13.048512
MYO5A	0.2713696	5.7361098	6.6357104	2.77E-10	1.44E-08	13.046369
IKZF2	0.5211334	4.7160545	6.6345067	2.78E-10	1.45E-08	13.03988
RP11-434D9	-1.548429	-0.050882	-6.634082	2.79E-10	1.45E-08	13.037591
ETS1	0.2294269	6.3223848	6.6328493	2.81E-10	1.46E-08	13.030946
ANLN	-0.469495	5.6411749	-6.631691	2.83E-10	1.46E-08	13.024705
BTN3A2	0.2359129	6.1626931	6.6304498	2.85E-10	1.47E-08	13.018014
TMEM204	0.2820731	5.8145462	6.6272258	2.90E-10	1.50E-08	13.000643
FXYD5	0.2333108	6.1869576	6.6266105	2.91E-10	1.50E-08	12.997328
NFIC	0.1894691	6.7158589	6.622439	2.98E-10	1.53E-08	12.974862
TLCD1	-0.288542	5.7062008	-6.622214	2.98E-10	1.53E-08	12.97365
RP5-1125A1	-0.4105	4.2341514	-6.621617	2.99E-10	1.53E-08	12.970438
SPERT	-1.859888	0.0963531	-6.62092	3.00E-10	1.54E-08	12.966685

GPATCH1	-0.1409	5.713242	-6.620653	3.01E-10	1.54E-08	12.965244
PSMB8	0.2585722	6.5167768	6.6195435	3.03E-10	1.54E-08	12.959273
CPNE1	-0.15525	6.5487037	-6.619483	3.03E-10	1.54E-08	12.958948
TRIM38	0.175437	6.0463204	6.6147459	3.11E-10	1.58E-08	12.933453
GBE1	0.2346233	6.3714103	6.6131612	3.14E-10	1.59E-08	12.924927
LEPROT	0.117415	6.5717738	6.6124741	3.15E-10	1.60E-08	12.92123
RORB	1.7105372	1.6230956	6.6113716	3.17E-10	1.61E-08	12.9153
CLUL1	-1.525444	2.0131684	-6.611155	3.17E-10	1.61E-08	12.914135
MMP2	0.3944467	6.1763311	6.607547	3.24E-10	1.64E-08	12.894732
MMAA	0.2350587	5.8702249	6.6071348	3.24E-10	1.64E-08	12.892516
ARHGAP31	0.284899	5.6598306	6.6060179	3.26E-10	1.65E-08	12.886511
ZNF521	0.5656252	4.7086973	6.6055991	3.27E-10	1.65E-08	12.884259
CSTL1	-1.621198	-0.202412	-6.603809	3.31E-10	1.66E-08	12.874639
CRHBP	1.0940147	4.1976158	6.6033752	3.31E-10	1.66E-08	12.872305
RP11-501C1	-1.896663	1.0166068	-6.603343	3.31E-10	1.66E-08	12.872134
CRISPLD2	0.441214	5.8542891	6.6018498	3.34E-10	1.67E-08	12.864107
IL32	0.2899304	6.9164394	6.6013656	3.35E-10	1.68E-08	12.861506
MCM4	-0.206993	6.3545596	-6.599526	3.39E-10	1.69E-08	12.851623
DLX6-AS1	-2.292655	0.6568198	-6.598818	3.40E-10	1.69E-08	12.847818
GPR119	-1.509609	-0.660873	-6.598795	3.40E-10	1.69E-08	12.847695
LAMP2	0.1558642	6.9662139	6.598305	3.41E-10	1.69E-08	12.845062
DYRK3	0.4267777	5.039255	6.5977506	3.42E-10	1.70E-08	12.842084
HEPH	0.4606843	5.2524063	6.5970528	3.43E-10	1.70E-08	12.838335
SPATA3-AS1	-1.705882	1.3622149	-6.594468	3.48E-10	1.72E-08	12.824451
ANGPTL1	0.8727566	4.7992037	6.5939522	3.49E-10	1.73E-08	12.821684
ZBTB34	-0.183253	5.5656602	-6.592665	3.52E-10	1.74E-08	12.814774
MEGF6	0.560855	5.5606748	6.5917468	3.53E-10	1.74E-08	12.809842
COL1A1	0.3178634	7.0223614	6.5872509	3.62E-10	1.79E-08	12.785712
AC004076.5	-0.928361	3.1401501	-6.585503	3.66E-10	1.80E-08	12.776334
MRVI1	0.4119042	5.3374323	6.5820516	3.73E-10	1.83E-08	12.757818
LRRC32	0.2933512	6.1195088	6.5802231	3.77E-10	1.85E-08	12.748012
RP11-79N23	-1.66171	1.1335751	-6.580171	3.77E-10	1.85E-08	12.747731
CTPS1	0.2096095	6.2750055	6.5785976	3.80E-10	1.86E-08	12.739296
ITFG1	0.1883448	6.3585484	6.5782274	3.81E-10	1.86E-08	12.737312
FNDC4	0.4382088	6.1467154	6.5768959	3.84E-10	1.87E-08	12.730173
SAMD5	1.0136068	4.9237841	6.5761766	3.85E-10	1.88E-08	12.726317
CDR2L	0.3744105	5.3762627	6.5748006	3.88E-10	1.89E-08	12.718942
LINC00840	1.6694169	0.976141	6.5744033	3.89E-10	1.89E-08	12.716813
ING5	-0.156219	5.8924011	-6.573394	3.91E-10	1.90E-08	12.711402
IGFBP7	0.2185591	6.8955174	6.5730531	3.92E-10	1.90E-08	12.709577
CFAP57	1.3236608	3.2860869	6.5665051	4.07E-10	1.97E-08	12.6745
CWF19L1	-0.125058	5.9463247	-6.566154	4.08E-10	1.97E-08	12.672618
DEPDC1B	-0.682351	5.1752401	-6.562916	4.15E-10	2.00E-08	12.655285
SETD7	0.2013542	6.4858222	6.5621789	4.17E-10	2.01E-08	12.651337
C12orf73	-0.173727	5.5615456	-6.560103	4.22E-10	2.03E-08	12.640227
RHNO1	-0.170943	5.8335941	-6.560076	4.22E-10	2.03E-08	12.640082
GAL3ST1	-1.04365	5.3505521	-6.558077	4.26E-10	2.05E-08	12.629387
RP11-1055E	-1.525818	2.1380715	-6.555464	4.32E-10	2.07E-08	12.615408
RBMS2	0.2471516	5.7064871	6.553843	4.36E-10	2.09E-08	12.606734
GLP1R	-2.253203	1.1866781	-6.550217	4.45E-10	2.13E-08	12.587343
TANGO2	0.1784487	5.9141281	6.5489655	4.48E-10	2.14E-08	12.580653

RND3	0.3033977	6.1593122	6.5477946	4.51E-10	2.15E-08	12.574394
MT1E	0.6762344	5.9487082	6.5472448	4.53E-10	2.16E-08	12.571455
ELOVL2-AS1	-1.378582	3.2684159	-6.5464	4.55E-10	2.16E-08	12.566941
NT5DC3	-0.223393	5.7590592	-6.543384	4.62E-10	2.20E-08	12.550823
WDHD1	-0.334507	5.2402921	-6.543029	4.63E-10	2.20E-08	12.548925
GNAO1	0.8403704	4.948755	6.5425387	4.65E-10	2.20E-08	12.546307
SUZ12P1	-0.254941	5.1432716	-6.540151	4.71E-10	2.23E-08	12.533554
RP11-563N4	-1.249492	1.8951727	-6.540032	4.71E-10	2.23E-08	12.532917
C1QTNF7	0.9636905	3.6597094	6.5389315	4.74E-10	2.24E-08	12.527039
CHST7	0.3756694	5.3055262	6.538718	4.75E-10	2.24E-08	12.525899
NCAPH	-0.462487	5.3193402	-6.538274	4.76E-10	2.24E-08	12.523531
FAT4	0.5520945	5.2206919	6.5380869	4.76E-10	2.24E-08	12.522529
ZNF296	-0.637155	4.3436563	-6.534645	4.85E-10	2.28E-08	12.504151
CLEC16A	0.1570049	6.0897245	6.5331284	4.90E-10	2.30E-08	12.496056
CTC-463A16	0.4196791	4.2301776	6.5310637	4.95E-10	2.32E-08	12.485037
LOXL1	0.5646812	5.2673371	6.5280671	5.03E-10	2.36E-08	12.469049
PRDM9	-1.728122	0.1397484	-6.527046	5.06E-10	2.37E-08	12.463603
TPRKB	-0.13189	5.892239	-6.52687	5.07E-10	2.37E-08	12.462665
ZNF552	-0.233948	5.3829281	-6.526318	5.08E-10	2.37E-08	12.459716
ZWILCH	-0.178833	5.6129807	-6.522342	5.20E-10	2.42E-08	12.438516
GAS8	0.1922026	5.7508373	6.5223395	5.20E-10	2.42E-08	12.438502
NFYA	-0.150008	6.0889701	-6.521336	5.23E-10	2.43E-08	12.433151
ADAT1	0.1571277	5.7446385	6.5204758	5.25E-10	2.44E-08	12.428566
THBD	0.3695457	5.6869987	6.5187632	5.30E-10	2.46E-08	12.419437
QRSL1P1	-1.628145	0.2643852	-6.51855	5.31E-10	2.46E-08	12.4183
PPAPDC1A	1.8238933	2.2948163	6.518183	5.32E-10	2.46E-08	12.416345
RP11-43505	-0.641685	3.9704477	-6.514802	5.42E-10	2.50E-08	12.398327
GLUD2	-0.481396	5.504526	-6.514692	5.42E-10	2.50E-08	12.397741
FAM64A	-0.883139	4.3316265	-6.513802	5.45E-10	2.51E-08	12.393001
UPK3A	-1.910902	3.6681501	-6.513755	5.45E-10	2.51E-08	12.392749
SSBP3	0.1659541	6.4096676	6.5132968	5.46E-10	2.51E-08	12.39031
GALNT10	0.1783005	6.0148153	6.5112019	5.53E-10	2.53E-08	12.379152
RP11-883G1	1.982055	1.7287734	6.5075221	5.64E-10	2.58E-08	12.359558
RP11-863K1	1.2570282	2.7555209	6.5070225	5.66E-10	2.59E-08	12.356899
RSPO3	1.8121203	2.9005387	6.5058279	5.69E-10	2.60E-08	12.350539
PIM3	0.1639698	6.4195042	6.504085	5.75E-10	2.62E-08	12.341263
CCDC34	-0.264629	5.6423694	-6.502849	5.79E-10	2.64E-08	12.334685
MMS22L	-0.321079	5.2499062	-6.501855	5.82E-10	2.65E-08	12.329398
KIAA1191	0.1305752	6.5289407	6.5011211	5.84E-10	2.66E-08	12.325492
SMIM12	0.1541557	6.200615	6.4922328	6.14E-10	2.79E-08	12.278224
DGAT2L7P	-1.563006	1.5970674	-6.491475	6.16E-10	2.79E-08	12.274195
CAP1	0.0916788	6.8085966	6.4908308	6.18E-10	2.80E-08	12.270771
CENPE	-0.448484	5.2718069	-6.488879	6.25E-10	2.83E-08	12.260397
CPT2	0.2337652	6.342361	6.4876404	6.29E-10	2.84E-08	12.253818
TYSND1	-0.194793	6.1183317	-6.486607	6.33E-10	2.86E-08	12.248328
RP11-337C1	-0.345919	4.935235	-6.484103	6.42E-10	2.89E-08	12.235025
DONSON	-0.195361	5.8479805	-6.482965	6.46E-10	2.91E-08	12.228983
ARHGAP24	0.3111084	5.4432112	6.4818886	6.50E-10	2.92E-08	12.223267
PTPRM	0.2702423	6.1814137	6.4815248	6.51E-10	2.92E-08	12.221335
LINC01136	-1.438617	2.1770191	-6.480309	6.55E-10	2.94E-08	12.214878
SERTAD1	0.2174071	5.857942	6.4797997	6.57E-10	2.94E-08	12.212176

SMIM10	0.549088	4.5304254	6.4786583	6.61E-10	2.96E-08	12.206116
HTR2B	0.8831497	4.1197957	6.4786379	6.61E-10	2.96E-08	12.206008
COL6A1	0.2601347	6.723979	6.4770048	6.67E-10	2.98E-08	12.19734
AC002116.7	-0.978452	2.7683266	-6.474409	6.77E-10	3.02E-08	12.183563
CNTN4	0.7494521	4.3580781	6.473917	6.79E-10	3.02E-08	12.180954
STOM	0.1897786	6.9300824	6.4738687	6.79E-10	3.02E-08	12.180698
ZBTB11-AS1	-0.264303	4.6944442	-6.473638	6.80E-10	3.02E-08	12.179472
CTD-252902	-1.581533	1.5264314	-6.473561	6.80E-10	3.02E-08	12.179063
UPK1A	-1.693781	2.2181207	-6.472706	6.83E-10	3.03E-08	12.174531
RAD9A	-0.165372	5.8280529	-6.470923	6.90E-10	3.05E-08	12.16507
LRRC4C	1.4271923	2.9312067	6.469811	6.94E-10	3.07E-08	12.159173
PLXNA4	1.523697	3.3318375	6.4657745	7.10E-10	3.13E-08	12.13777
CCL2	0.4122308	5.7327506	6.4647803	7.14E-10	3.15E-08	12.132499
ATOH8	0.6914278	5.3164016	6.463178	7.20E-10	3.17E-08	12.124006
SF3A2	-0.147512	6.4002682	-6.462255	7.24E-10	3.18E-08	12.119115
TAF13	0.1484566	5.9991063	6.4605993	7.30E-10	3.21E-08	12.110341
BTBD1	0.1204288	6.2535563	6.4603729	7.31E-10	3.21E-08	12.109142
MCM3AP-AS1	-0.327989	4.5966067	-6.459353	7.35E-10	3.22E-08	12.103739
SFRP1	1.4186946	3.6830921	6.4552112	7.52E-10	3.29E-08	12.0818
TSHZ3	0.3939314	4.8404074	6.4521695	7.65E-10	3.34E-08	12.065694
CD99L2	0.2089978	6.5144942	6.4517579	7.67E-10	3.35E-08	12.063515
GMPR	0.5914941	4.9923249	6.4512903	7.69E-10	3.35E-08	12.06104
FIBIN	1.064471	4.4628937	6.4511695	7.69E-10	3.35E-08	12.0604
ADCY10P1	-0.411774	4.8295677	-6.449773	7.75E-10	3.37E-08	12.053007
C21orf58	-0.365134	5.0978387	-6.448356	7.81E-10	3.40E-08	12.045511
STXBP6	-0.420127	5.5859861	-6.446892	7.88E-10	3.42E-08	12.037762
PARP2	-0.12339	5.9253155	-6.445469	7.94E-10	3.44E-08	12.030236
ARSJ	0.8914663	4.5445958	6.4454516	7.94E-10	3.44E-08	12.030142
POLR2D	-0.100717	6.2179925	-6.435009	8.41E-10	3.64E-08	11.974925
PPP1CC	-0.099176	6.5875095	-6.43457	8.43E-10	3.64E-08	11.972604
COMMD2	-0.108919	6.04904	-6.431425	8.57E-10	3.70E-08	11.955987
DEPDC1	-0.582334	5.0801686	-6.430559	8.62E-10	3.71E-08	11.951414
KIF2C	-0.496582	5.513955	-6.429437	8.67E-10	3.73E-08	11.945489
USP39	-0.083736	6.2501687	-6.428954	8.69E-10	3.73E-08	11.942935
TRIM22	0.3692913	5.9480232	6.4288726	8.70E-10	3.73E-08	11.942507
PAPPA	0.8864344	3.7277975	6.4281029	8.73E-10	3.75E-08	11.938442
MACF1	0.1172497	6.630629	6.4270148	8.78E-10	3.76E-08	11.932695
CYR61	0.3048908	6.2794008	6.4269902	8.79E-10	3.76E-08	11.932566
CCSAP	-0.232353	5.5882695	-6.426114	8.83E-10	3.77E-08	11.927939
MAP7D3	0.3950892	5.3415836	6.4245882	8.90E-10	3.80E-08	11.919884
FGF7	1.5409474	3.5793131	6.4235762	8.95E-10	3.82E-08	11.914542
CDT1	-0.441892	5.6090094	-6.421596	9.05E-10	3.85E-08	11.904091
RP11-325P1	-1.478991	1.2978269	-6.421123	9.07E-10	3.86E-08	11.901592
MBTPS1	0.1033974	6.5042196	6.4198345	9.14E-10	3.88E-08	11.894795
RFC5	-0.158515	5.8319508	-6.418793	9.19E-10	3.90E-08	11.889302
TDG	-0.135417	5.8041077	-6.418729	9.19E-10	3.90E-08	11.888961
RP11-314A2	-1.319217	1.9082436	-6.416477	9.31E-10	3.94E-08	11.877083
ZBTB21	0.2101906	5.7619571	6.4098835	9.65E-10	4.08E-08	11.842315
SPTBN2	0.5232609	5.9000699	6.4098552	9.65E-10	4.08E-08	11.842166
TIGD2	0.3199904	5.7331052	6.4095452	9.67E-10	4.08E-08	11.840532
POMGNT1	0.1258255	6.4406197	6.4081937	9.74E-10	4.10E-08	11.833409

HIST1H3C	-1.433193	1.1355412	-6.405929	9.86E-10	4.15E-08	11.821474
RP11-46501	-1.43314	-0.458324	-6.404754	9.92E-10	4.17E-08	11.815286
ADIPOR1P2	-1.207502	-0.691071	-6.404341	9.95E-10	4.18E-08	11.813111
MCM3	-0.169981	6.4851864	-6.40198	1.01E-09	4.23E-08	11.800673
BX842568.1	1.9389386	1.0811051	6.4003236	1.02E-09	4.26E-08	11.791949
RP11-637A1	-0.839023	2.8787458	-6.398799	1.03E-09	4.29E-08	11.78392
NOS3	0.3123263	5.5758245	6.3986879	1.03E-09	4.29E-08	11.783337
HAUS5	-0.160706	5.862501	-6.398429	1.03E-09	4.29E-08	11.781975
ITGAM	0.4147894	5.5111039	6.3955266	1.04E-09	4.35E-08	11.766695
RN7SL751P	-1.497157	0.0339269	-6.39512	1.05E-09	4.36E-08	11.764555
CCDC138	-0.325478	5.0315026	-6.394645	1.05E-09	4.36E-08	11.762054
FBXL12	-0.130267	5.8563562	-6.394249	1.05E-09	4.37E-08	11.759969
ZFP36	0.208536	6.6591058	6.3941037	1.05E-09	4.37E-08	11.759207
RP11-810P1	-0.609016	3.8430679	-6.392945	1.06E-09	4.39E-08	11.753109
TYMS	-0.294481	5.9298536	-6.392804	1.06E-09	4.39E-08	11.752368
PIGC	-0.143878	6.2200407	-6.390506	1.07E-09	4.44E-08	11.740278
CCDC113	0.7712171	4.5424423	6.390305	1.07E-09	4.44E-08	11.73922
ODF3B	0.2529393	5.9099226	6.3895255	1.08E-09	4.45E-08	11.73512
UCKL1	-0.127979	6.2275306	-6.383975	1.11E-09	4.59E-08	11.70593
DOT1L	-0.178168	6.1740656	-6.382823	1.12E-09	4.60E-08	11.699876
SASH1	0.2248722	5.8909803	6.38281	1.12E-09	4.60E-08	11.699809
CTD-2555C1	-1.908795	1.0546391	-6.379912	1.14E-09	4.67E-08	11.684577
RAPGEFL1	-0.343208	5.4666104	-6.379557	1.14E-09	4.67E-08	11.682712
GOLGA2P5	-0.358616	5.717218	-6.379501	1.14E-09	4.67E-08	11.682418
TNNC1	-1.182509	3.8139029	-6.379338	1.14E-09	4.67E-08	11.681564
SYNPO2	0.403161	5.5504626	6.3791744	1.14E-09	4.67E-08	11.680703
CALHM2	0.2592842	5.4706473	6.3789323	1.14E-09	4.67E-08	11.679431
BX842568.2	1.9087569	2.7447417	6.3787769	1.14E-09	4.67E-08	11.678614
ARHGEF10	0.3807696	5.2821675	6.3771074	1.15E-09	4.71E-08	11.669843
ZBED8	-0.276952	5.030375	-6.376598	1.16E-09	4.71E-08	11.667169
KIF26A	0.6092268	4.7475463	6.3756164	1.16E-09	4.73E-08	11.662012
RFC3	-0.206586	5.5910289	-6.371982	1.19E-09	4.82E-08	11.642926
BRE	0.1304801	6.1681396	6.369789	1.20E-09	4.87E-08	11.631413
SAYSD1	-0.155368	5.9801059	-6.369677	1.20E-09	4.87E-08	11.630828
COL4A2	0.1877694	6.9256588	6.3684226	1.21E-09	4.90E-08	11.624241
RP11-385F5	-0.47903	4.0907869	-6.3683	1.21E-09	4.90E-08	11.6236
EPB41L4A	0.614946	4.683998	6.3681574	1.21E-09	4.90E-08	11.622849
NCAPH2	-0.145856	6.3072829	-6.367513	1.22E-09	4.91E-08	11.619465
CD1D	0.4799958	5.100538	6.3672357	1.22E-09	4.91E-08	11.618012
RP11-35501	-0.545475	4.042005	-6.367182	1.22E-09	4.91E-08	11.617731
TBXAS1	0.2927078	5.5295428	6.3666054	1.22E-09	4.92E-08	11.614705
KIAA0101	-0.385333	5.5255675	-6.36608	1.23E-09	4.93E-08	11.611949
RP11-108L7	-1.469809	1.766507	-6.364865	1.23E-09	4.95E-08	11.605575
C1orf112	-0.25513	5.4274904	-6.364778	1.23E-09	4.95E-08	11.605118
RP3-342P20	1.3764484	2.0044098	6.3628431	1.25E-09	5.00E-08	11.594966
RP4-738P11	-1.608105	-0.277131	-6.36255	1.25E-09	5.00E-08	11.593426
TTC13	-0.176354	5.9334059	-6.3623	1.25E-09	5.00E-08	11.592118
RP11-1069G	1.5246087	4.0535987	6.3622051	1.25E-09	5.00E-08	11.59162
FGF1	0.8861167	4.114271	6.3607796	1.26E-09	5.03E-08	11.584144
FAM43B	1.5651553	3.0948018	6.3578583	1.28E-09	5.11E-08	11.568826
MIXL1	-1.695202	1.1674986	-6.355779	1.30E-09	5.16E-08	11.557929

RP11-45901	-1.632128	-0.536005	-6.353964	1.31E-09	5.21E-08	11.548414
UHRF1	-0.501499	5.283434	-6.353038	1.32E-09	5.22E-08	11.543561
DLEU2	-0.308062	4.8762831	-6.352911	1.32E-09	5.22E-08	11.542898
ABCC10	-0.161381	5.9206155	-6.352148	1.32E-09	5.24E-08	11.538899
TYMSOS	-0.523566	4.6592167	-6.351503	1.33E-09	5.25E-08	11.535521
PPT2	-0.241937	5.7031592	-6.349681	1.34E-09	5.30E-08	11.525972
RP11-341G2	-0.953889	3.4458687	-6.347982	1.35E-09	5.34E-08	11.517077
VGLL3	0.7771248	4.4949498	6.3474421	1.36E-09	5.35E-08	11.514248
CHEK1	-0.317769	5.5012944	-6.344793	1.38E-09	5.42E-08	11.500376
DPH7	-0.161147	5.8248448	-6.34405	1.38E-09	5.44E-08	11.496485
TSPO	0.2425805	6.3045139	6.3439958	1.38E-09	5.44E-08	11.496204
REXO2	0.140344	6.1464332	6.3430975	1.39E-09	5.46E-08	11.491501
RAD51	-0.436905	5.0112712	-6.342962	1.39E-09	5.46E-08	11.490794
SLC28A1	0.9168077	5.4610796	6.3426698	1.39E-09	5.46E-08	11.489263
ANKRD35	1.1933564	4.0794314	6.3402958	1.41E-09	5.52E-08	11.476838
SRSF8	0.2056562	6.0536281	6.3396623	1.42E-09	5.54E-08	11.473524
HTRA1	0.2922707	6.6626868	6.3393095	1.42E-09	5.54E-08	11.471677
FER1L5	-1.382999	2.2983651	-6.337016	1.44E-09	5.60E-08	11.45968
BCAS1	-1.117167	4.3526421	-6.333629	1.46E-09	5.70E-08	11.441961
RP11-295H2	-0.481609	4.0066243	-6.331137	1.48E-09	5.77E-08	11.428936
LINC00702	0.8899685	3.6773679	6.3310275	1.48E-09	5.77E-08	11.428362
PLCG2	0.2598332	6.0814038	6.329966	1.49E-09	5.80E-08	11.422813
METTL21A	-0.13408	5.8236461	-6.328128	1.51E-09	5.85E-08	11.413206
AKR7A3	0.747046	5.9089755	6.3247249	1.53E-09	5.95E-08	11.395424
ATAD2	-0.233814	6.3300965	-6.321992	1.56E-09	6.04E-08	11.381149
SNHG20	-0.233262	5.1693353	-6.319768	1.58E-09	6.10E-08	11.369534
RRAS	0.2025186	6.0913767	6.3186671	1.59E-09	6.13E-08	11.363787
RP11-141C7	-1.47709	1.1998098	-6.317708	1.59E-09	6.16E-08	11.35878
GPR182	1.5554362	3.0673013	6.3161775	1.61E-09	6.20E-08	11.35079
RP11-553L6	0.2181634	5.9986975	6.315698	1.61E-09	6.21E-08	11.348288
ILF3	-0.086748	6.7690822	-6.314893	1.62E-09	6.23E-08	11.344086
HNRNPA1P1C	-0.953309	2.7776184	-6.313667	1.63E-09	6.27E-08	11.337687
RPL36AP43	-1.266677	1.9651364	-6.313403	1.63E-09	6.27E-08	11.336309
HNRNPA1P16	-0.586218	3.7545996	-6.312998	1.64E-09	6.28E-08	11.334199
TARBP1	-0.186386	6.197066	-6.312864	1.64E-09	6.28E-08	11.3335
ARRDC2	0.2757066	6.3207997	6.3119653	1.64E-09	6.30E-08	11.328809
FGGY	0.4338168	6.2273357	6.3115737	1.65E-09	6.31E-08	11.326766
KRT86	1.3437529	3.3291689	6.3106246	1.66E-09	6.33E-08	11.321815
FAM169B	2.2226314	2.1157234	6.3095998	1.67E-09	6.36E-08	11.31647
HECTD2	-0.242404	5.405877	-6.309222	1.67E-09	6.37E-08	11.314497
CCPG1	0.1999654	6.0533932	6.3073026	1.69E-09	6.43E-08	11.304489
MYLK-AS1	-0.608545	3.7958034	-6.306824	1.69E-09	6.44E-08	11.301994
DNAL1	0.1592014	5.4024208	6.3064424	1.69E-09	6.44E-08	11.300003
GAS6-AS2	1.2259315	3.2330863	6.3034648	1.72E-09	6.54E-08	11.28448
TRIM35	0.2048304	5.8764609	6.3022446	1.73E-09	6.58E-08	11.278121
CTD-2162K1	-1.655693	1.1850485	-6.301581	1.74E-09	6.60E-08	11.274663
CDCA7	-0.865686	4.6986157	-6.300986	1.75E-09	6.60E-08	11.271563
CCDC183	-0.490931	4.3141581	-6.300936	1.75E-09	6.60E-08	11.271302
CTD-2008L1	-2.033433	0.6595083	-6.29922	1.76E-09	6.66E-08	11.262358
RBP2	-1.889307	1.530215	-6.29835	1.77E-09	6.68E-08	11.257827
TMEM45A	0.5406685	5.8690984	6.2969656	1.78E-09	6.73E-08	11.250615

ERG	0.2964485	5.5509271	6.2942136	1.81E-09	6.82E-08	11.236282
CDCA2	-0.692436	4.6893926	-6.293852	1.81E-09	6.83E-08	11.2344
GPC1	0.4197071	6.2970159	6.2930817	1.82E-09	6.85E-08	11.230388
PTGIR	0.4357766	4.9948449	6.2922745	1.83E-09	6.86E-08	11.226186
PAMR1	0.5398353	4.904662	6.2922696	1.83E-09	6.86E-08	11.22616
PRORSD1P	-0.336083	4.2202606	-6.292102	1.83E-09	6.86E-08	11.225287
SMPD4	-0.104379	6.3216491	-6.29156	1.84E-09	6.88E-08	11.222465
ABT1	-0.125905	6.2066734	-6.289867	1.85E-09	6.93E-08	11.213651
MTF1	0.15578	5.6719461	6.2892494	1.86E-09	6.95E-08	11.210439
LINC01451	-1.040049	4.413883	-6.287841	1.87E-09	7.00E-08	11.203108
CACNA1C	0.4685279	5.0607786	6.2873893	1.88E-09	7.01E-08	11.200759
CTD-2078B5	1.4919258	0.8362166	6.2869055	1.88E-09	7.02E-08	11.198241
TRPC5OS	1.5773787	0.0053443	6.285408	1.90E-09	7.07E-08	11.19045
PGA5	-1.425711	-0.09718	-6.28288	1.93E-09	7.16E-08	11.1773
RNU1-47P	1.7628409	0.8581362	6.2819104	1.94E-09	7.18E-08	11.172257
BRCA2	-0.340176	5.0180517	-6.281762	1.94E-09	7.18E-08	11.171484
ZBTB7C	0.8499854	4.1401364	6.2814165	1.94E-09	7.19E-08	11.169689
GSN	0.1848262	6.7953517	6.2807148	1.95E-09	7.21E-08	11.16604
RP11-564D1	-1.67621	1.5662888	-6.278764	1.97E-09	7.28E-08	11.155894
CACNA2D1	0.5999185	4.6496414	6.2773605	1.98E-09	7.32E-08	11.148601
UBE2O	-0.111751	6.1569726	-6.27731	1.98E-09	7.32E-08	11.148341
RP11-533E1	-0.897827	3.0950843	-6.276931	1.99E-09	7.33E-08	11.146367
CHAF1A	-0.188217	5.920989	-6.276454	1.99E-09	7.34E-08	11.143891
SLC27A4	0.1673586	6.3216175	6.2760828	2.00E-09	7.35E-08	11.14196
RP11-68I18	-1.028243	3.3445699	-6.27502	2.01E-09	7.38E-08	11.136439
CDH11	0.5881507	5.3868189	6.2747427	2.01E-09	7.39E-08	11.134996
ADAMTS15	0.6082133	4.4961497	6.2728965	2.03E-09	7.45E-08	11.125403
CTD-3138B1	-0.314409	5.0498647	-6.272542	2.04E-09	7.46E-08	11.12356
AC006116.2	-1.123237	-0.699807	-6.26594	2.11E-09	7.72E-08	11.089276
MXRA5	0.5203525	5.4315121	6.2639326	2.13E-09	7.80E-08	11.078854
PYGO2	-0.131554	6.4074878	-6.263647	2.14E-09	7.80E-08	11.077371
VIL1	-0.921637	6.0237918	-6.263638	2.14E-09	7.80E-08	11.077327
NCOA5	-0.1109	6.1297622	-6.262313	2.15E-09	7.84E-08	11.070447
SRR	0.2382329	5.1664173	6.2603673	2.17E-09	7.92E-08	11.060352
CDC45	-0.443668	5.2952404	-6.260215	2.18E-09	7.92E-08	11.059562
HMOX2	0.1683495	6.3945882	6.2590818	2.19E-09	7.96E-08	11.053682
LINC01320	2.0898542	2.5450264	6.2584174	2.20E-09	7.98E-08	11.050236
ATXN7L3B	-0.100848	6.5159663	-6.255775	2.23E-09	8.09E-08	11.036531
PSMD5-AS1	-0.263335	5.7648846	-6.254885	2.24E-09	8.12E-08	11.031916
BGN	0.2682777	6.8736963	6.2544119	2.24E-09	8.13E-08	11.029462
OSBPL1A	0.2800588	5.9124402	6.2536178	2.25E-09	8.16E-08	11.025345
DDX11-AS1	-0.930906	3.3043863	-6.247876	2.33E-09	8.40E-08	10.995583
CTD-2349P2	-0.674099	3.3532291	-6.247718	2.33E-09	8.40E-08	10.994766
KCTD6	-0.248972	5.690949	-6.247082	2.34E-09	8.42E-08	10.991471
CDYL2	0.3643613	5.3477295	6.2464525	2.34E-09	8.44E-08	10.98821
CH17-360D5	2.0452546	2.0794433	6.2444129	2.37E-09	8.53E-08	10.977644
RP11-317J1	0.9563922	3.803943	6.2436059	2.38E-09	8.55E-08	10.973465
CTC-537E7.	1.6803447	1.4559179	6.2435072	2.38E-09	8.55E-08	10.972954
BPIFB2	-2.185163	3.4429126	-6.240413	2.42E-09	8.68E-08	10.95693
ZNFX1	0.1280938	6.2959512	6.240346	2.42E-09	8.68E-08	10.956585
SUCLG2	0.2053681	6.657631	6.2398456	2.43E-09	8.70E-08	10.953994

NAT1	0.282613	5.2306326	6.2374284	2.46E-09	8.80E-08	10.941482
AKAP7	0.296657	5.7300879	6.2305932	2.55E-09	9.12E-08	10.906119
RP11-522B1	0.8159746	3.4526355	6.2299656	2.56E-09	9.15E-08	10.902873
JUNB	0.206457	6.611943	6.2279001	2.59E-09	9.24E-08	10.892193
GPR124	0.3460312	5.8090494	6.2272729	2.60E-09	9.26E-08	10.88895
GADD45A	0.2206382	6.2958233	6.2269104	2.60E-09	9.27E-08	10.887076
ITGA9	0.3254556	5.7321408	6.2244126	2.64E-09	9.39E-08	10.874165
IQGAP1	0.177976	6.3308004	6.223024	2.66E-09	9.45E-08	10.866989
PAWRP1	-1.13013	-0.699533	-6.221625	2.68E-09	9.51E-08	10.859758
TSPEAR-AS2	-1.605254	3.6563574	-6.220907	2.69E-09	9.54E-08	10.856052
CCDC3	0.3398772	5.7880714	6.2192312	2.71E-09	9.61E-08	10.847393
AC009961.3	-0.754416	3.2869292	-6.218666	2.72E-09	9.63E-08	10.844472
INCENP	-0.223482	5.6802109	-6.21808	2.73E-09	9.65E-08	10.84145
THSD4	0.5180873	5.6351366	6.2173236	2.74E-09	9.68E-08	10.837541
PLSCR1	0.2231546	6.1464241	6.2136845	2.79E-09	9.86E-08	10.818752
MYL9	0.2658383	6.4299378	6.2114553	2.83E-09	9.97E-08	10.807246
SLC12A4	0.1489091	6.2505438	6.2088617	2.87E-09	1.01E-07	10.793862
BFSP1	-0.503477	4.3260887	-6.208415	2.87E-09	1.01E-07	10.791559
CPSF4L	-1.552402	0.8757045	-6.206829	2.90E-09	1.02E-07	10.783374
RP11-295M3	1.3395063	2.48141	6.2059795	2.91E-09	1.02E-07	10.778994
IQCK	0.3328141	4.8717346	6.2052894	2.92E-09	1.03E-07	10.775435
LHFP	0.2869073	5.8348475	6.2046615	2.93E-09	1.03E-07	10.772196
NEK1	0.2144585	5.4471452	6.204415	2.94E-09	1.03E-07	10.770925
NRP2	0.2591672	5.9054773	6.2034434	2.95E-09	1.03E-07	10.765915
MIR600HG	-0.408227	4.8801378	-6.203401	2.95E-09	1.03E-07	10.765699
CRIP2	0.2161422	6.347991	6.2033849	2.95E-09	1.03E-07	10.765613
STEAP3	0.3062526	6.4810544	6.2011886	2.99E-09	1.04E-07	10.75429
LINC01219	-1.313986	-0.654231	-6.200693	3.00E-09	1.04E-07	10.751735
MOXD1	0.905446	4.9606043	6.2002066	3.00E-09	1.05E-07	10.749227
CKAP2	-0.222364	5.7620178	-6.199515	3.01E-09	1.05E-07	10.745662
MAP1B	0.2868655	5.8425507	6.1991687	3.02E-09	1.05E-07	10.743878
CENPH	-0.310602	5.2184466	-6.198454	3.03E-09	1.05E-07	10.740196
HERC5	0.4696822	5.30679	6.198191	3.04E-09	1.05E-07	10.738839
ZYG11B	0.1541389	6.1621703	6.1976281	3.05E-09	1.06E-07	10.735938
DDX39A	-0.165862	6.4308187	-6.197365	3.05E-09	1.06E-07	10.734583
ISX	-2.287012	3.162143	-6.197081	3.05E-09	1.06E-07	10.733119
RP11-1094M	-0.265575	5.2271937	-6.195401	3.08E-09	1.07E-07	10.724463
CHST13	-0.366775	6.2140142	-6.193257	3.12E-09	1.08E-07	10.713419
CTD-2201G3	1.4832681	0.6885213	6.1927417	3.13E-09	1.08E-07	10.710765
MSH5-SAPCD	-0.494441	3.986974	-6.192485	3.13E-09	1.08E-07	10.709442
KDSR	0.1274508	6.434542	6.1909498	3.16E-09	1.09E-07	10.701537
RP11-578B1	-1.324827	-0.634387	-6.187579	3.21E-09	1.11E-07	10.684181
CRIM1	0.267626	6.2016416	6.1873472	3.22E-09	1.11E-07	10.68299
DHDDS	0.144206	6.1120444	6.1862946	3.24E-09	1.11E-07	10.677572
FOXN3-AS1	-0.380314	4.6471553	-6.185876	3.24E-09	1.11E-07	10.675416
PCDH7	1.2040295	3.9136496	6.1849376	3.26E-09	1.12E-07	10.670589
AC097639.8	-1.288258	0.0692964	-6.184155	3.27E-09	1.12E-07	10.666562
AC005077.7	1.6002438	-0.024082	6.1837103	3.28E-09	1.12E-07	10.664273
TET3	-0.178795	5.8972228	-6.183261	3.29E-09	1.12E-07	10.661959
RP11-968A1	-1.051181	2.9011193	-6.181872	3.31E-09	1.13E-07	10.654816
IGFBP5	0.3243469	6.4614151	6.1795694	3.35E-09	1.14E-07	10.642972

PRKCD	-0.190617	6.057824	-6.176249	3.41E-09	1.16E-07	10.625899
PTH1R	0.6145857	4.8180753	6.1731719	3.47E-09	1.18E-07	10.610082
OFCC1	-1.707575	-0.225278	-6.173171	3.47E-09	1.18E-07	10.610079
IL15	0.5469295	4.7520396	6.1716785	3.50E-09	1.19E-07	10.602408
GPR19	-0.96963	3.3130621	-6.171487	3.50E-09	1.19E-07	10.601425
KIRREL	0.3370598	5.7250461	6.1708015	3.52E-09	1.19E-07	10.597902
LINC01121	-1.748652	1.8308041	-6.1703	3.52E-09	1.19E-07	10.595324
SNRPD1	-0.135655	6.2162008	-6.170279	3.53E-09	1.19E-07	10.595215
RP11-44N11	-0.799653	3.3658388	-6.169349	3.54E-09	1.20E-07	10.59044
ABHD2	0.2048603	6.8004571	6.1672803	3.58E-09	1.21E-07	10.579814
AL590226.1	-1.425598	-0.125987	-6.16708	3.59E-09	1.21E-07	10.578784
ID1	0.3294162	5.9921723	6.1661822	3.60E-09	1.22E-07	10.574175
PHYHD1	0.6858403	5.3380923	6.1628502	3.67E-09	1.24E-07	10.557067
RP11-465N4	-0.465784	4.3901016	-6.162825	3.67E-09	1.24E-07	10.55694
C1orf21	0.3391419	5.9492767	6.1602354	3.72E-09	1.25E-07	10.543646
MAST4	0.226393	5.9842369	6.1591587	3.74E-09	1.26E-07	10.538121
ZNF25	0.2001525	5.3443132	6.1586465	3.75E-09	1.26E-07	10.535493
DNAJB4	0.2595311	5.7717035	6.1579147	3.77E-09	1.26E-07	10.531738
FAM83D	-0.367334	5.6441456	-6.155728	3.81E-09	1.28E-07	10.52052
RPLP0P6	-0.360939	5.1007392	-6.155632	3.81E-09	1.28E-07	10.520026
TAF4	-0.130517	5.9856466	-6.155416	3.82E-09	1.28E-07	10.518922
OR13Z2P	-1.054806	-0.797674	-6.152347	3.88E-09	1.30E-07	10.503183
JADE1	0.1524486	6.0887378	6.1502183	3.92E-09	1.31E-07	10.492269
TCF3	-0.143521	6.2700964	-6.149829	3.93E-09	1.31E-07	10.490273
MAP2K1	0.1061459	6.2995416	6.1497857	3.93E-09	1.31E-07	10.490051
LITAF	0.1646759	6.7415718	6.1496422	3.94E-09	1.31E-07	10.489315
RASGEF1B	0.3323123	5.8348763	6.148579	3.96E-09	1.32E-07	10.483866
RP11-583F2	-1.420577	0.6375363	-6.148303	3.96E-09	1.32E-07	10.482453
TMOD2	0.2846811	5.3979751	6.1471548	3.99E-09	1.32E-07	10.476567
DUSP1	0.2574376	6.7430722	6.1402747	4.14E-09	1.37E-07	10.441324
SYP	-0.457601	4.6951046	-6.138879	4.17E-09	1.38E-07	10.434178
BHLHE22	1.5676286	3.0874369	6.1377817	4.19E-09	1.39E-07	10.428561
RAD54B	-0.384955	4.6904885	-6.137268	4.20E-09	1.39E-07	10.425931
RGS11	1.1051329	3.510717	6.1369313	4.21E-09	1.39E-07	10.424208
PDLIM5	0.1645245	6.5278718	6.1368347	4.21E-09	1.39E-07	10.423714
RP11-468N1	-1.877486	0.8604445	-6.136098	4.23E-09	1.40E-07	10.419944
ZNF26	-0.149899	5.6504923	-6.135666	4.24E-09	1.40E-07	10.417731
KCNE1	1.539594	1.5630839	6.1344044	4.27E-09	1.41E-07	10.411276
SULF1	0.5938339	5.6920603	6.1318928	4.33E-09	1.42E-07	10.398425
SAC3D1	-0.188834	5.8328914	-6.130614	4.36E-09	1.43E-07	10.391884
NCOA7	0.2382574	6.2314321	6.1305215	4.36E-09	1.43E-07	10.391411
RPS3AP46	-1.159767	-0.750129	-6.129948	4.37E-09	1.43E-07	10.388479
RHOQ	0.1788677	6.0193696	6.129471	4.38E-09	1.44E-07	10.386038
SLC9A1	0.2114801	5.7227647	6.1282804	4.41E-09	1.45E-07	10.379949
MAMSTR	-0.534049	4.3256113	-6.127696	4.42E-09	1.45E-07	10.376963
STK24-AS1	-1.030682	2.5996508	-6.125483	4.48E-09	1.46E-07	10.365649
RIN2	0.2078068	5.7890479	6.1248145	4.49E-09	1.47E-07	10.362229
TMEM92-AS1	1.9210195	1.6534697	6.1240242	4.51E-09	1.47E-07	10.35819
LDB2	0.294199	5.733859	6.122848	4.54E-09	1.48E-07	10.352179
UST	0.6160026	4.2953995	6.1209335	4.59E-09	1.49E-07	10.342396
MYO1B	0.1939395	6.8032722	6.1198637	4.61E-09	1.50E-07	10.33693

ZFP14	-0.209575	5.4097328	-6.11844	4.65E-09	1.51E-07	10.329658
SLC35D2	-0.171174	6.3724319	-6.117904	4.66E-09	1.51E-07	10.326922
H2AFY2	-0.463751	5.7775889	-6.116945	4.68E-09	1.52E-07	10.322021
DUSP12	-0.152144	5.7403916	-6.116697	4.69E-09	1.52E-07	10.320753
CTD-2510F5	-1.003194	3.5448666	-6.115081	4.73E-09	1.53E-07	10.3125
CNOT3	-0.095154	6.2480085	-6.113676	4.77E-09	1.54E-07	10.305328
OSMR	0.3661579	6.06859	6.1134606	4.77E-09	1.54E-07	10.30423
POLD2P1	-1.589272	-0.05207	-6.113183	4.78E-09	1.54E-07	10.302814
AC007128.1	-1.718276	-0.111684	-6.110639	4.84E-09	1.56E-07	10.289827
EIF4G3	0.1337539	6.3314189	6.1058501	4.97E-09	1.60E-07	10.265394
KRT18P5	-1.256967	1.3617409	-6.10516	4.99E-09	1.61E-07	10.261874
SLFN5	0.2663503	5.8606644	6.1045021	5.00E-09	1.61E-07	10.258519
SNHG18	0.7030189	4.3953387	6.1042035	5.01E-09	1.61E-07	10.256996
MGME1	-0.119864	5.9530393	-6.102608	5.05E-09	1.63E-07	10.248863
PIEZO1	0.1436721	6.4988185	6.1024324	5.06E-09	1.63E-07	10.247965
CSF1	0.2257181	6.1974872	6.1020985	5.07E-09	1.63E-07	10.246262
LSM14B	-0.121108	6.18665	-6.101959	5.07E-09	1.63E-07	10.245551
TSPAN1	0.5249764	4.9593229	6.1016338	5.08E-09	1.63E-07	10.243894
ERMAP	0.1910681	5.7645086	6.1010757	5.10E-09	1.63E-07	10.241048
PRDM2	0.1406887	5.8719898	6.098878	5.16E-09	1.65E-07	10.229846
OMD	1.8232066	2.5340289	6.0980032	5.18E-09	1.66E-07	10.225387
BCL9	-0.208023	6.03993	-6.095814	5.24E-09	1.67E-07	10.214232
MTBP	-0.335838	4.9845846	-6.093808	5.30E-09	1.69E-07	10.204011
RMI2	-0.377434	5.3352009	-6.09375	5.30E-09	1.69E-07	10.203719
ZNF891	-0.247946	4.9224627	-6.093402	5.31E-09	1.69E-07	10.201944
CPXM2	0.6315168	5.0413719	6.0908837	5.38E-09	1.71E-07	10.18912
SMARCA2	0.2398175	6.3260834	6.0899859	5.40E-09	1.72E-07	10.184548
PLCB1	-0.351211	5.8161558	-6.087146	5.49E-09	1.74E-07	10.17009
PALLD	0.2551661	6.3647587	6.0855887	5.53E-09	1.76E-07	10.162165
ZNF790-AS1	-0.817379	3.4793442	-6.082335	5.63E-09	1.78E-07	10.14561
SPAG8	-0.5297	3.6323362	-6.081653	5.65E-09	1.79E-07	10.14214
POLQ	-0.590286	4.84399	-6.081467	5.65E-09	1.79E-07	10.141193
PIGU	-0.141118	6.1502012	-6.08117	5.66E-09	1.79E-07	10.139685
FLYWCH1	0.1405352	6.0714064	6.0798274	5.70E-09	1.80E-07	10.132856
PRC1-AS1	-1.345693	1.1578579	-6.078296	5.75E-09	1.82E-07	10.125068
ZBED4	-0.145357	5.9304262	-6.075417	5.84E-09	1.84E-07	10.110429
RP11-580I1	-0.600163	3.278934	-6.07517	5.84E-09	1.84E-07	10.109175
BLVRB	0.1952243	6.6768847	6.0734628	5.90E-09	1.86E-07	10.100499
SLC38A2	0.1872755	6.7897881	6.0731787	5.91E-09	1.86E-07	10.099055
VCL	0.135097	6.3618825	6.0717659	5.95E-09	1.87E-07	10.091876
LINC01137	0.2966249	5.3177174	6.0683637	6.06E-09	1.90E-07	10.074593
PITPNA-AS1	-0.302456	4.8226762	-6.06807	6.07E-09	1.90E-07	10.073101
DGKE	0.3724118	5.1843047	6.067881	6.07E-09	1.90E-07	10.072142
CALD1	0.132405	6.9058896	6.0655839	6.15E-09	1.93E-07	10.060476
DNAJC3-AS1	-0.332983	4.9172902	-6.06347	6.22E-09	1.95E-07	10.049744
MAD2L1	-0.320047	5.5325179	-6.062375	6.25E-09	1.96E-07	10.044187
CTD-2583A1	-0.244284	4.8386504	-6.061694	6.28E-09	1.96E-07	10.040732
DAB2	0.2434667	6.3165049	6.061174	6.29E-09	1.97E-07	10.038091
FAM72D	-1.313846	2.9011999	-6.061017	6.30E-09	1.97E-07	10.037296
RAB31	0.2551269	5.8927678	6.0604162	6.32E-09	1.97E-07	10.034246
UBE3D	-0.296683	4.8172826	-6.058748	6.37E-09	1.99E-07	10.025782

ARSD	0.2046418	6.3248914	6.0584116	6.39E-09	1.99E-07	10.024075
UNK	-0.112233	6.0594325	-6.05798	6.40E-09	1.99E-07	10.021885
ALDH1A3	0.8525412	4.6158863	6.0570324	6.43E-09	2.00E-07	10.017078
MAMDC2	1.2231842	3.2626796	6.0563137	6.46E-09	2.00E-07	10.013433
SDHB	0.1793516	6.5980619	6.0548704	6.51E-09	2.02E-07	10.006113
RUNDC3B	0.6610107	4.9990229	6.0543214	6.53E-09	2.02E-07	10.003329
ID3	0.2313404	6.0255011	6.0506145	6.65E-09	2.06E-07	9.9845355
RS1	-1.362905	0.5040372	-6.049832	6.68E-09	2.07E-07	9.9805701
MYH11	0.4709914	5.9036257	6.0489913	6.71E-09	2.07E-07	9.9763083
DLL1	0.4612601	5.2507779	6.0473353	6.77E-09	2.09E-07	9.9679173
RP11-169K1	1.4737109	2.8425392	6.0461538	6.81E-09	2.10E-07	9.9619309
F2R	0.2569163	6.0620823	6.0433956	6.91E-09	2.13E-07	9.9479599
SETDB1	-0.117433	6.2323768	-6.042392	6.95E-09	2.14E-07	9.942876
AC074117.1	-0.224183	5.2064982	-6.041726	6.97E-09	2.15E-07	9.9395035
PLK2	0.1977527	6.1491271	6.0416371	6.98E-09	2.15E-07	9.9390547
TNFSF4	-0.408165	5.0694488	-6.038788	7.08E-09	2.18E-07	9.9246305
FAM189B	-0.163755	6.1774357	-6.038586	7.09E-09	2.18E-07	9.923606
RP11-35N6.	-0.759198	5.4863767	-6.038508	7.09E-09	2.18E-07	9.923214
RP11-16P6.	-0.785779	3.4786138	-6.038025	7.11E-09	2.18E-07	9.9207688
AKR1B1	0.3139561	5.9746852	6.037749	7.12E-09	2.18E-07	9.9193718
BRCA1	-0.267975	5.530182	-6.037507	7.13E-09	2.18E-07	9.9181456
FKBP5	0.2440388	6.5891037	6.0369783	7.15E-09	2.19E-07	9.915471
GNG12	0.2580313	6.3049358	6.0354531	7.21E-09	2.20E-07	9.9077533
RPL10L	-1.760688	0.0216845	-6.034832	7.23E-09	2.21E-07	9.9046094
DSCR3	0.1158857	6.2608729	6.0346889	7.24E-09	2.21E-07	9.9038864
TAC3	-1.630243	0.807757	-6.033714	7.27E-09	2.22E-07	9.8989522
COPZ2	0.3323706	5.8186638	6.0316166	7.35E-09	2.24E-07	9.8883453
BCL2L12	-0.181003	5.9598718	-6.031222	7.37E-09	2.24E-07	9.8863488
RP11-38L15	-1.335006	-0.546639	-6.030131	7.41E-09	2.25E-07	9.8808306
MAPRE2	0.1859648	6.2537093	6.0291703	7.45E-09	2.26E-07	9.8759744
ARHGAP23	0.2573189	5.73542	6.0279465	7.50E-09	2.28E-07	9.8697867
PER1	0.2351144	6.3762127	6.027744	7.51E-09	2.28E-07	9.8687633
FRRS1L	-2.114389	1.4879786	-6.024588	7.63E-09	2.31E-07	9.8528128
C1S	0.2394097	7.3853069	6.0244163	7.64E-09	2.31E-07	9.8519435
ZBTB4	0.1399598	6.1573569	6.02329	7.68E-09	2.32E-07	9.846252
REEP5	0.1267898	6.7357782	6.0215407	7.76E-09	2.34E-07	9.837414
E2F1	-0.371959	5.8075319	-6.02128	7.77E-09	2.34E-07	9.8360976
TRAIP	-0.418385	5.0427511	-6.021197	7.77E-09	2.34E-07	9.835677
LINC00282	-1.440108	1.8526127	-6.02105	7.78E-09	2.34E-07	9.8349366
RP11-30015	-1.064543	-0.744442	-6.020896	7.78E-09	2.34E-07	9.8341597
CENPK	-0.473427	4.8700258	-6.019356	7.84E-09	2.36E-07	9.8263799
HLA-C	0.1872651	7.2972679	6.0183667	7.89E-09	2.37E-07	9.8213828
KB-431C1.4	-0.193726	5.2758439	-6.017888	7.91E-09	2.38E-07	9.8189674
RP11-110A1	-1.298265	-0.874296	-6.017018	7.94E-09	2.38E-07	9.8145704
PML	0.122804	6.3093973	6.0160721	7.98E-09	2.39E-07	9.8097968
PF4V1	1.9290182	1.1946914	6.0118167	8.16E-09	2.45E-07	9.7883183
WI2-189601	0.9540831	3.6625243	6.0104675	8.22E-09	2.46E-07	9.7815106
DYDC1	-1.520169	0.0744339	-6.009122	8.28E-09	2.48E-07	9.7747233
CTD-2583A1	-0.286646	4.8670932	-6.007476	8.35E-09	2.50E-07	9.7664203
AMPH	1.1183165	3.0562748	6.0056628	8.43E-09	2.52E-07	9.7572761
SLC41A2	0.3275999	6.2284926	6.0055584	8.43E-09	2.52E-07	9.7567496

MYO1E	0.220996	6.355778	6.0031189	8.54E-09	2.55E-07	9.7444502
ZFPM2	0.5065305	4.6490325	6.0028808	8.55E-09	2.55E-07	9.7432501
ZNF133	-0.147411	5.64499	-6.00256	8.57E-09	2.55E-07	9.7416311
WDFY3	0.2091636	5.9271727	6.0023806	8.58E-09	2.55E-07	9.7407286
SCP2	0.2017809	6.9230773	6.0015835	8.61E-09	2.56E-07	9.7367108
VSIG4	0.4997321	5.4331616	5.9992384	8.72E-09	2.59E-07	9.7248933
SUV420H2	-0.193515	5.6453301	-5.997969	8.78E-09	2.60E-07	9.7184994
FTOP1	-0.631972	4.2640241	-5.997718	8.79E-09	2.61E-07	9.7172318
CIPC	0.1535065	5.8412165	5.997143	8.82E-09	2.61E-07	9.7143363
AC062029.1	-0.315036	4.5861139	-5.993259	9.00E-09	2.66E-07	9.6947751
LINC00176	-0.857626	4.4251003	-5.99292	9.01E-09	2.67E-07	9.693067
INTS4	-0.097087	5.8795849	-5.992528	9.03E-09	2.67E-07	9.691092
KAT2A	-0.141514	6.391316	-5.990329	9.14E-09	2.70E-07	9.6800226
LINC01184	0.3233713	5.5855709	5.9901823	9.14E-09	2.70E-07	9.6792858
MMP24	0.6300264	4.6436478	5.9891593	9.19E-09	2.71E-07	9.6741371
SPG20	0.3681793	5.4725272	5.9885499	9.22E-09	2.72E-07	9.6710702
MS4A6A	0.2857384	6.0029089	5.9878375	9.26E-09	2.72E-07	9.6674849
GDF2	2.160757	0.4841909	5.9877234	9.26E-09	2.72E-07	9.6669112
SASS6	-0.281241	5.1428681	-5.987285	9.28E-09	2.73E-07	9.6647038
C2CD5	-0.123612	6.0464302	-5.986658	9.31E-09	2.74E-07	9.6615489
COLEC10	1.3021845	3.8273563	5.9859824	9.35E-09	2.74E-07	9.6581513
LGALS14	-1.58372	-0.576619	-5.985921	9.35E-09	2.74E-07	9.6578415
SMC3	-0.131125	6.2885903	-5.985184	9.39E-09	2.75E-07	9.654134
COL6A3	0.2681416	6.5066773	5.9847244	9.41E-09	2.75E-07	9.6518229
RP5-905G11	-1.482262	-0.341281	-5.981736	9.56E-09	2.79E-07	9.636793
ACAT1	0.2320686	6.8765105	5.9816411	9.56E-09	2.79E-07	9.6363159
CCND3	0.1579985	6.1699418	5.9808023	9.60E-09	2.80E-07	9.6320985
TPTE2P2	-1.042931	-0.947698	-5.980306	9.63E-09	2.81E-07	9.6296033
LINC00858	-1.495218	-0.423328	-5.979831	9.65E-09	2.81E-07	9.6272169
CAMK1D	0.3460128	6.0160922	5.9785928	9.72E-09	2.83E-07	9.6209905
OCIAD2	0.4604049	6.169477	5.9779473	9.75E-09	2.84E-07	9.6177461
NELFE	-0.149616	6.4526228	-5.976147	9.84E-09	2.86E-07	9.6086984
PPT2-EGFL8	-0.336598	4.1350769	-5.976142	9.84E-09	2.86E-07	9.6086728
MKL2	0.1678571	5.9278445	5.9739216	9.96E-09	2.89E-07	9.5975168
STMN1	-0.192304	6.4842085	-5.973841	9.96E-09	2.89E-07	9.5971129
KPNA6	0.1069704	6.3346791	5.9735902	9.97E-09	2.89E-07	9.5958521
METTL6	-0.11924	5.5279674	-5.971549	1.01E-08	2.92E-07	9.5855965
DCUN1D3	0.1575429	5.6701702	5.9710848	1.01E-08	2.93E-07	9.5832675
CEP95	-0.120995	5.9444088	-5.970849	1.01E-08	2.93E-07	9.5820838
RPL31P11	-1.346299	0.0115787	-5.970607	1.01E-08	2.93E-07	9.5808695
POLR3F	-0.113873	5.6348239	-5.969191	1.02E-08	2.95E-07	9.5737564
ABR	0.2528298	5.9399976	5.968835	1.02E-08	2.95E-07	9.5719699
NDUFV2-AS1	-0.256484	4.3497444	-5.967571	1.03E-08	2.97E-07	9.5656227
LINC00470	-1.947116	2.2255793	-5.965828	1.04E-08	2.99E-07	9.5568747
ZNF473	-0.151721	5.6225946	-5.965214	1.04E-08	3.00E-07	9.5537942
RP11-16F15	-0.966952	2.6038761	-5.963897	1.05E-08	3.01E-07	9.5471865
C1orf122	0.1940735	6.0819403	5.963765	1.05E-08	3.01E-07	9.5465219
MSMB	-1.776805	1.3395713	-5.963585	1.05E-08	3.01E-07	9.5456164
ABCC1	0.328882	5.8381632	5.9635289	1.05E-08	3.01E-07	9.5453371
PPP1R18	0.1663594	6.3360879	5.9635112	1.05E-08	3.01E-07	9.5452485
STX1B	0.4962625	4.9047333	5.9613335	1.06E-08	3.05E-07	9.5343227

CERS1	-1.743649	2.3992624	-5.957055	1.09E-08	3.11E-07	9.5128636
RP11-398C1	-0.484978	3.7446204	-5.956779	1.09E-08	3.11E-07	9.5114798
NUP107	-0.110338	6.116929	-5.956554	1.09E-08	3.11E-07	9.5103514
STRA8	-1.695759	1.0546389	-5.956533	1.09E-08	3.11E-07	9.510247
RNFT2	-0.551404	4.6284973	-5.954016	1.10E-08	3.15E-07	9.4976315
CLEC11A	0.347331	5.3741153	5.9508397	1.12E-08	3.20E-07	9.481714
ZNF385C	-0.680715	4.1796856	-5.949174	1.13E-08	3.23E-07	9.4733701
ZNF761	-0.280182	5.6195813	-5.948226	1.14E-08	3.24E-07	9.4686186
MRC1	0.408315	5.7936168	5.9478664	1.14E-08	3.25E-07	9.46682
PTPRB	0.2976676	5.9177693	5.9470022	1.15E-08	3.26E-07	9.4624917
MCM7	-0.16326	6.5372975	-5.946822	1.15E-08	3.26E-07	9.4615905
AD000092.3	-0.691839	2.7822577	-5.944217	1.16E-08	3.30E-07	9.4485475
RP11-454H1	1.3741624	0.1803804	5.9433219	1.17E-08	3.31E-07	9.444065
NUP85	-0.107674	6.2249873	-5.942223	1.17E-08	3.33E-07	9.4385642
UBAC2-AS1	-0.421544	4.1755423	-5.941689	1.18E-08	3.34E-07	9.4358918
PDIA3P1	-0.221622	5.3746686	-5.940951	1.18E-08	3.35E-07	9.4322002
ZNF74	-0.167249	5.6153303	-5.940338	1.19E-08	3.35E-07	9.4291329
ZNF256	-0.470859	4.7568428	-5.940283	1.19E-08	3.35E-07	9.428855
FANCE	-0.312223	5.1540029	-5.939704	1.19E-08	3.36E-07	9.4259561
IGF2BP2	-0.582298	5.7190501	-5.939587	1.19E-08	3.36E-07	9.4253732
GPM6A	1.3493238	3.4881075	5.9391175	1.19E-08	3.37E-07	9.423024
FXYD6	0.2853276	5.7476156	5.9380348	1.20E-08	3.38E-07	9.4176074
FATE1	-1.421504	2.4341656	-5.937604	1.20E-08	3.39E-07	9.4154533
CRYAB	0.4935338	5.4600032	5.9370023	1.21E-08	3.39E-07	9.4124425
HSD11B2	-0.325884	5.4722611	-5.93628	1.21E-08	3.41E-07	9.408831
VN1R1	-0.876974	3.524415	-5.934615	1.22E-08	3.43E-07	9.400501
RP11-511B2	-1.64013	-0.291855	-5.933975	1.23E-08	3.44E-07	9.3973046
AC008440.1	-1.287135	-0.132587	-5.93315	1.23E-08	3.45E-07	9.3931772
RP11-49401	-1.286767	0.86926	-5.932947	1.23E-08	3.45E-07	9.3921612
PGBD1	-0.330142	5.2674899	-5.932868	1.23E-08	3.45E-07	9.3917696
RP11-383H1	0.7715179	4.6917025	5.9326211	1.24E-08	3.45E-07	9.3905329
LINC00205	-0.394924	4.6788119	-5.931344	1.24E-08	3.48E-07	9.384148
EPHA3	0.636756	5.1241318	5.931177	1.24E-08	3.48E-07	9.3833143
RP11-568A7	-1.507178	-0.174475	-5.93067	1.25E-08	3.48E-07	9.3807819
CYP2U1	0.2780333	5.3953141	5.9302497	1.25E-08	3.49E-07	9.378679
ZNF572	-0.501344	4.6032624	-5.929768	1.25E-08	3.49E-07	9.3762727
USB1	0.1107747	6.2605775	5.9294863	1.26E-08	3.50E-07	9.3748638
LZTS2	-0.156032	6.2615441	-5.927913	1.27E-08	3.52E-07	9.3670017
ARHGEF6	0.2719763	5.6070294	5.9264601	1.28E-08	3.55E-07	9.3597427
FGD4	0.2645878	5.9157228	5.9253391	1.28E-08	3.56E-07	9.354143
IRAK3	0.4338799	4.9598349	5.9231949	1.30E-08	3.60E-07	9.3434338
ANKRD49	-0.127127	5.5963338	-5.921727	1.31E-08	3.63E-07	9.3361063
CENPQ	-0.25323	5.2612954	-5.92141	1.31E-08	3.63E-07	9.3345189
CTA-390C10	-0.860324	-1.059151	-5.916378	1.34E-08	3.72E-07	9.3094061
NELFA	-0.107443	6.0861689	-5.916261	1.34E-08	3.72E-07	9.3088215
RP11-651L5	-0.69794	3.4052092	-5.916141	1.35E-08	3.72E-07	9.3082207
PLK3	0.2183119	5.5463922	5.9149444	1.35E-08	3.74E-07	9.3022526
HERC3	0.2290176	5.7017337	5.9136817	1.36E-08	3.76E-07	9.2959536
LBR	-0.133641	6.578701	-5.913251	1.37E-08	3.77E-07	9.293803
VPS4B	0.111362	6.1991316	5.912087	1.37E-08	3.79E-07	9.2879999
PLCL2	0.3088974	5.6561964	5.9106819	1.38E-08	3.81E-07	9.2809929

FBLL1	-1.543394	3.3946437	-5.910218	1.39E-08	3.82E-07	9.2786776
BSN-AS2	-1.430701	1.2532961	-5.909648	1.39E-08	3.83E-07	9.2758364
FBLN2	0.5456255	5.8342342	5.9093853	1.39E-08	3.83E-07	9.2745279
AGAP2-AS1	0.406602	4.711993	5.9088848	1.40E-08	3.84E-07	9.2720326
ORC6	-0.428746	4.9789243	-5.907968	1.40E-08	3.85E-07	9.2674606
RP11-278C7	-0.42692	4.0248539	-5.907838	1.40E-08	3.85E-07	9.2668146
CDC42BPA	0.2030232	6.354889	5.906817	1.41E-08	3.87E-07	9.2617254
CPNE2	0.1632723	5.9635183	5.9067337	1.41E-08	3.87E-07	9.26131
WDR7	0.1547478	5.8262595	5.9045174	1.43E-08	3.91E-07	9.2502656
BNC2	0.7999533	3.9563843	5.9028294	1.44E-08	3.94E-07	9.2418555
PARN	0.1039865	6.2145717	5.9022155	1.45E-08	3.95E-07	9.2387975
TCF21	0.8707489	3.9481746	5.9010352	1.46E-08	3.97E-07	9.2329183
BMPER	1.3825884	2.8019502	5.9009709	1.46E-08	3.97E-07	9.232598
ARHGAP42	0.3260652	5.4448957	5.9008658	1.46E-08	3.97E-07	9.2320745
CTD-237103	-0.457951	4.1773343	-5.899458	1.47E-08	4.00E-07	9.225065
TMPO	-0.1296	6.5471204	-5.8986	1.47E-08	4.01E-07	9.2207903
OSBPL5	0.3038168	5.4211429	5.896092	1.49E-08	4.06E-07	9.2083053
RP11-132N1	-1.519786	0.8371286	-5.895868	1.49E-08	4.06E-07	9.207191
RP1-200K18	-1.392329	-0.658234	-5.895484	1.50E-08	4.07E-07	9.2052813
RP11-46107	1.7530684	0.7682412	5.8945134	1.51E-08	4.09E-07	9.2004485
UBXN10	0.9012422	4.9235484	5.8939181	1.51E-08	4.10E-07	9.197486
SOCS2-AS1	0.6802206	4.2393261	5.8924026	1.52E-08	4.12E-07	9.1899449
RBL1	-0.260808	5.4112845	-5.891383	1.53E-08	4.14E-07	9.1848728
FOXC1	0.4705573	4.9425786	5.8907899	1.53E-08	4.15E-07	9.1819215
TPM3P9	-0.346761	5.216839	-5.889479	1.55E-08	4.18E-07	9.1754
PELI2	0.5452165	4.6474874	5.889463	1.55E-08	4.18E-07	9.1753215
RP11-44F14	0.4915947	3.8078344	5.8892611	1.55E-08	4.18E-07	9.1743174
RP11-443B2	-0.708309	3.553073	-5.88857	1.55E-08	4.19E-07	9.1708816
PLA2R1	0.6530186	4.452094	5.8879861	1.56E-08	4.20E-07	9.1679765
DLD	0.1287846	6.5523901	5.8876204	1.56E-08	4.20E-07	9.1661578
SGCB	0.3397937	5.6914595	5.8873529	1.56E-08	4.21E-07	9.1648279
RP11-404G1	-1.39025	3.8435578	-5.885482	1.58E-08	4.24E-07	9.1555279
RP11-872J2	-0.419702	3.950088	-5.88506	1.58E-08	4.25E-07	9.1534262
MGP	0.37121	6.2048485	5.8848856	1.58E-08	4.25E-07	9.1525611
SPARC	0.1575992	7.1002709	5.8844283	1.59E-08	4.26E-07	9.1502877
CNRIP1	0.2764944	5.1308387	5.8842673	1.59E-08	4.26E-07	9.1494877
CPTP	0.1842047	6.323048	5.8837067	1.59E-08	4.27E-07	9.146701
ITGA11	0.4370404	5.2647586	5.8832689	1.60E-08	4.27E-07	9.1445252
PDPN	1.4238761	3.7252889	5.8822611	1.60E-08	4.29E-07	9.139517
RP11-394I1	-1.113497	2.3100259	-5.881824	1.61E-08	4.30E-07	9.1373457
EGR1	0.2973196	6.5301621	5.8817846	1.61E-08	4.30E-07	9.1371489
UBE2E1-AS1	-1.064244	-0.682665	-5.881343	1.61E-08	4.30E-07	9.1349526
RP11-293M1	0.6387624	3.996116	5.8798571	1.62E-08	4.33E-07	9.1275719
BSDC1	0.1243121	6.5353387	5.8784014	1.64E-08	4.36E-07	9.120341
MBTD1	-0.139481	5.9087218	-5.876532	1.65E-08	4.40E-07	9.1110555
RP11-457M1	-1.030755	-0.608836	-5.875973	1.66E-08	4.41E-07	9.1082787
ACTA1	-0.954701	2.7039497	-5.874703	1.67E-08	4.44E-07	9.1019726
CTD-3157E1	0.675042	3.6247895	5.8739532	1.67E-08	4.45E-07	9.0982523
RPS6KA1	0.2168078	6.037916	5.8720413	1.69E-08	4.49E-07	9.0887619
TANC1	0.1757666	5.8912444	5.8688363	1.72E-08	4.57E-07	9.0728575
IL18R1	0.5243562	5.0338973	5.8686877	1.72E-08	4.57E-07	9.0721204

MGLL	0.2365568	6.6651962	5.8659712	1.75E-08	4.63E-07	9.0586452
PFKFB3	0.3285452	6.1165847	5.8646361	1.76E-08	4.66E-07	9.052024
RP11-83M16	-1.685169	0.5513403	-5.86403	1.76E-08	4.67E-07	9.0490173
KHSRP	-0.092226	6.7166717	-5.86342	1.77E-08	4.68E-07	9.0459936
GYLTL1B	-0.856605	5.0352662	-5.863289	1.77E-08	4.68E-07	9.0453457
MTG1	-0.200882	5.6069018	-5.862965	1.77E-08	4.68E-07	9.0437376
HEY2	0.361688	5.1452474	5.8626477	1.78E-08	4.69E-07	9.0421652
DCAF15	-0.118345	6.015411	-5.862127	1.78E-08	4.70E-07	9.039582
RP11-320G1	-1.751524	0.125889	-5.861735	1.78E-08	4.70E-07	9.0376384
KIAA1211L	0.3726274	4.9344971	5.860231	1.80E-08	4.73E-07	9.0301857
ZNF331	-0.40995	5.8229632	-5.859692	1.80E-08	4.74E-07	9.0275148
RP11-529E1	-0.801217	3.5428893	-5.859114	1.81E-08	4.76E-07	9.0246481
GLI3	0.5638381	4.2914267	5.8585201	1.81E-08	4.77E-07	9.0217071
MDC1	-0.137597	6.1945659	-5.858086	1.82E-08	4.77E-07	9.0195541
NNT	0.2014385	6.6553406	5.8580208	1.82E-08	4.77E-07	9.0192328
TMOD1	0.605335	5.2536911	5.8569465	1.83E-08	4.80E-07	9.0139101
C6orf163	-0.882999	2.8754216	-5.855776	1.84E-08	4.82E-07	9.0081134
NAP1L4P1	-1.334307	3.229547	-5.855478	1.84E-08	4.82E-07	9.0066334
HDX	0.6691202	4.3014099	5.8550737	1.85E-08	4.83E-07	9.004633
ZNF143	-0.106709	5.7630632	-5.85213	1.87E-08	4.90E-07	8.9900561
HECA	0.1821174	5.9577644	5.8503713	1.89E-08	4.94E-07	8.9813483
HMGB2	-0.167226	6.3188975	-5.849962	1.90E-08	4.95E-07	8.9793217
PDZK1IP1	1.2816503	5.2235335	5.8499204	1.90E-08	4.95E-07	8.9791159
PTGFR	1.5361354	4.2615067	5.8498372	1.90E-08	4.95E-07	8.9787046
SH3RF3	0.4505679	5.2290939	5.8496264	1.90E-08	4.95E-07	8.977661
GLUD1P3	-0.477501	3.8960407	-5.848167	1.91E-08	4.98E-07	8.9704383
PRKAG2-AS1	0.6221892	4.8358362	5.847927	1.92E-08	4.98E-07	8.9692502
DSN1	-0.159827	5.9336423	-5.846364	1.93E-08	5.02E-07	8.961516
KAL1	0.7660104	4.3295776	5.844944	1.95E-08	5.05E-07	8.9544903
NT5DC2	-0.310422	6.0072212	-5.841715	1.98E-08	5.14E-07	8.9385221
BANF2	-1.713788	0.8407407	-5.841218	1.98E-08	5.14E-07	8.9360645
PPP2CB	0.1338746	6.3639364	5.8401578	1.99E-08	5.17E-07	8.9308201
KIAA0513	0.2399722	5.5416629	5.8386087	2.01E-08	5.21E-07	8.9231616
FAP	0.8387152	4.6692647	5.8383146	2.01E-08	5.21E-07	8.9217082
CDKN1A	0.2551022	6.6442256	5.836762	2.03E-08	5.25E-07	8.9140341
CA2	0.3801902	6.2427766	5.8348877	2.05E-08	5.30E-07	8.9047723
RUSC1-AS1	-0.303223	5.3026451	-5.834314	2.05E-08	5.31E-07	8.901936
ZEB2	0.243915	5.8243488	5.8342001	2.06E-08	5.31E-07	8.9013754
ZBTB26	-0.194433	5.2368542	-5.83319	2.07E-08	5.33E-07	8.8963844
TTC36	1.3869349	4.0138673	5.8320342	2.08E-08	5.36E-07	8.8906759
RP11-15A1.	-1.13115	2.1486594	-5.831231	2.09E-08	5.38E-07	8.8867092
TMEM101	-0.133157	6.2277575	-5.830189	2.10E-08	5.40E-07	8.8815607
ANXA1	0.2255564	6.1238737	5.8291407	2.11E-08	5.43E-07	8.8763866
PRR36	-0.806295	4.3755586	-5.828784	2.11E-08	5.43E-07	8.8746255
ECM2	0.3431682	5.7941902	5.8285755	2.12E-08	5.44E-07	8.8735963
BCL7B	0.1102542	6.3000231	5.8283207	2.12E-08	5.44E-07	8.8723383
CTB-5506.1	-1.249098	0.9334416	-5.827554	2.13E-08	5.46E-07	8.8685523
RPAP1	-0.098802	6.1591532	-5.82751	2.13E-08	5.46E-07	8.868335
KIAA0907	-0.140832	6.1542157	-5.827079	2.13E-08	5.46E-07	8.8662067
HECW2	0.30397	5.5410465	5.8269494	2.13E-08	5.46E-07	8.8655689
REG4	-1.517837	1.9726185	-5.824694	2.16E-08	5.52E-07	8.8544372

RP11-88E10	-0.31464	5.1154825	-5.824111	2.17E-08	5.54E-07	8.8515612
C17orf51	0.5703243	4.7125718	5.8230647	2.18E-08	5.56E-07	8.8463978
CNN1	0.4850969	5.1409264	5.8225803	2.18E-08	5.57E-07	8.8440081
MIR22HG	0.2319843	5.6887747	5.8211391	2.20E-08	5.61E-07	8.8368984
RPS6KC1	-0.132712	6.0119574	-5.820837	2.20E-08	5.61E-07	8.8354093
IMPDH1P6	-1.303775	2.2320522	-5.820725	2.20E-08	5.61E-07	8.8348573
PGR	1.2770062	3.20549	5.8183674	2.23E-08	5.68E-07	8.8232291
CTIF	0.1356647	6.2493626	5.8176361	2.24E-08	5.70E-07	8.8196232
LINC01197	0.5520692	4.2029942	5.8167528	2.25E-08	5.72E-07	8.8152684
CAMKK1	0.3483501	4.7701594	5.8157999	2.26E-08	5.74E-07	8.8105709
CCDC146	0.3176388	5.1658987	5.8157051	2.26E-08	5.74E-07	8.8101034
TMEM200C	0.9801373	3.7662217	5.8149571	2.27E-08	5.76E-07	8.8064165
SAAL1	-0.145444	5.6728654	-5.814084	2.28E-08	5.78E-07	8.8021124
TMEM145	-1.202712	3.4247852	-5.81264	2.30E-08	5.82E-07	8.7949995
RGS4	0.6737586	4.702876	5.8121331	2.30E-08	5.83E-07	8.7924995
RP11-552D8	-1.222675	-0.236272	-5.811938	2.31E-08	5.83E-07	8.7915391
PYGO1	1.2012277	3.2144752	5.8107337	2.32E-08	5.86E-07	8.7856052
DHX58	0.2563711	5.8489201	5.807915	2.35E-08	5.95E-07	8.7717217
TRPC7-AS1	-1.489443	0.0287836	-5.807714	2.36E-08	5.95E-07	8.7707315
ZNF789	-0.1655	5.4073452	-5.806615	2.37E-08	5.98E-07	8.7653219
RNF130	0.1380072	6.6450378	5.8055504	2.38E-08	6.00E-07	8.7600785
LINC00890	2.2066606	2.2816864	5.8054464	2.38E-08	6.00E-07	8.7595666
IGHV3-79	-1.230943	-0.304478	-5.80476	2.39E-08	6.02E-07	8.7561868
MCM8	-0.241572	5.5774709	-5.803977	2.40E-08	6.04E-07	8.7523341
CTC-523E23	-0.330837	4.456646	-5.802246	2.42E-08	6.09E-07	8.7438136
AP001432.1	-1.346526	1.2582439	-5.801326	2.43E-08	6.12E-07	8.7392884
RP11-5407.	1.4894136	2.7355446	5.8003953	2.45E-08	6.14E-07	8.7347069
IQSEC2	0.3215002	5.0559955	5.7996201	2.46E-08	6.16E-07	8.7308934
SNHG3	-0.283433	5.7161585	-5.799051	2.46E-08	6.18E-07	8.728094
ABI3BP	0.53857	5.1074373	5.7984997	2.47E-08	6.19E-07	8.7253816
NMNAT1	0.2049317	5.5771291	5.7980417	2.48E-08	6.20E-07	8.7231291
IL11RA	-0.216647	5.9076413	-5.797735	2.48E-08	6.20E-07	8.721619
ACADL	1.4419742	4.471907	5.7975732	2.48E-08	6.21E-07	8.7208246
PNMA3	-1.056318	4.332261	-5.795062	2.51E-08	6.28E-07	8.7084734
ADAM33	0.9115997	3.8754818	5.7941812	2.53E-08	6.31E-07	8.7041452
C5AR1	0.3012717	5.5528642	5.7923721	2.55E-08	6.36E-07	8.6952522
KIAA0922	0.2001931	6.0308539	5.7912124	2.56E-08	6.39E-07	8.6895524
NUP155	-0.121258	6.0974844	-5.788272	2.60E-08	6.49E-07	8.6751058
TMEM132E	1.158045	3.4619182	5.7850618	2.65E-08	6.59E-07	8.6593367
FMOD	0.4871734	5.7169811	5.7848079	2.65E-08	6.59E-07	8.6580898
EXPH5	1.0306303	4.3593258	5.7834854	2.67E-08	6.63E-07	8.6515962
CTTNBP2NL	0.1960469	5.6982666	5.7820443	2.69E-08	6.68E-07	8.6445211
ADNP	-0.092136	6.481903	-5.78195	2.69E-08	6.68E-07	8.6440582
TCF4	0.2161288	5.9910743	5.7810241	2.70E-08	6.70E-07	8.6395136
RAB11FIP4	-0.247671	6.2246792	-5.780776	2.70E-08	6.71E-07	8.6382935
HNRNPA1P48	-0.266992	4.6258842	-5.777624	2.75E-08	6.81E-07	8.6228274
MDK	-0.337949	6.6026546	-5.777285	2.75E-08	6.82E-07	8.6211675
SMARCC1	-0.105335	6.4764785	-5.776575	2.76E-08	6.84E-07	8.6176806
VPS18	0.1183939	6.2018884	5.7764925	2.76E-08	6.84E-07	8.6172772
IDS	0.1776308	6.454616	5.7764193	2.77E-08	6.84E-07	8.6169182
FZD4	0.2037625	6.1471653	5.7756921	2.78E-08	6.86E-07	8.6133509

RP11-134G8	1.0850454	2.9047292	5.775286	2.78E-08	6.87E-07	8.6113594
EHD2	0.2081043	6.2699829	5.7749658	2.79E-08	6.87E-07	8.6097888
PRKG1	0.4121391	4.9045958	5.7723887	2.82E-08	6.96E-07	8.5971511
RP11-488C1	-0.405679	4.2511808	-5.771546	2.84E-08	6.98E-07	8.593018
DSCC1	-0.297447	5.1918021	-5.770314	2.85E-08	7.02E-07	8.5869785
CEBPB	0.1830688	6.5783505	5.7700259	2.86E-08	7.03E-07	8.5855678
TCHH	1.1649461	2.4116011	5.7699207	2.86E-08	7.03E-07	8.5850522
PPM1J	0.6575222	3.7400346	5.769401	2.87E-08	7.04E-07	8.5825049
RP11-647K1	-0.675763	3.0033112	-5.769006	2.87E-08	7.05E-07	8.5805677
EDN1	0.4789426	4.8137306	5.768656	2.88E-08	7.06E-07	8.5788538
MAP7D1	0.1244115	6.3046163	5.7685989	2.88E-08	7.06E-07	8.5785738
SPRN	-0.25029	5.2968732	-5.767261	2.90E-08	7.10E-07	8.5720187
POU2F1	-0.112453	5.9551056	-5.765602	2.92E-08	7.16E-07	8.563891
SOX13	-0.141755	6.3725108	-5.765221	2.93E-08	7.17E-07	8.5620244
SPTSSA	0.1826916	6.4795641	5.7644794	2.94E-08	7.19E-07	8.5583905
RHOC	0.1304257	6.7708659	5.7623664	2.97E-08	7.26E-07	8.548042
RP11-118B2	1.4588861	0.5959759	5.7623171	2.97E-08	7.26E-07	8.5478005
GNE	0.2566178	6.3835976	5.7621349	2.98E-08	7.26E-07	8.5469083
NTN4	0.284239	5.7263016	5.760719	3.00E-08	7.31E-07	8.5399759
MAPK10	0.6537579	4.285959	5.7601555	3.01E-08	7.32E-07	8.5372168
AC004980.7	-0.247012	4.5173862	-5.759519	3.02E-08	7.34E-07	8.5341027
TOR3A	-0.167139	6.3962025	-5.7573	3.05E-08	7.42E-07	8.5232419
AC010761.8	-0.529856	3.8890488	-5.756863	3.06E-08	7.43E-07	8.5211038
EFCAB11	-0.159219	5.4146257	-5.75677	3.06E-08	7.43E-07	8.5206487
SNRPB	-0.131995	6.7434	-5.756483	3.06E-08	7.44E-07	8.519242
ZFYVE21	0.11791	6.0452801	5.7546716	3.09E-08	7.50E-07	8.5103796
NXN	0.4537373	5.6011071	5.7513912	3.14E-08	7.62E-07	8.4943342
MS4A7	0.3058611	5.809156	5.7500871	3.16E-08	7.67E-07	8.4879572
MAGEA8	-1.794999	0.0849605	-5.750065	3.16E-08	7.67E-07	8.4878477
RP11-83M16	-1.591637	0.0943889	-5.749914	3.17E-08	7.67E-07	8.4871104
ABCA2	0.2002462	6.4214524	5.7493719	3.18E-08	7.68E-07	8.4844606
RPS7P11	-0.524705	3.9458997	-5.749262	3.18E-08	7.68E-07	8.4839257
PBK	-0.472058	5.2467147	-5.749195	3.18E-08	7.68E-07	8.483598
CCNYL1	-0.133026	5.9524308	-5.749058	3.18E-08	7.68E-07	8.4829281
KDM8	0.3589091	5.6730073	5.7483974	3.19E-08	7.70E-07	8.4796967
CAMKV	-1.494643	-0.068084	-5.748311	3.19E-08	7.70E-07	8.4792741
MMP28	1.0748589	3.9250574	5.7478637	3.20E-08	7.71E-07	8.4770878
PGC	-2.251166	3.0674678	-5.747114	3.21E-08	7.74E-07	8.4734251
ZNF529-AS1	-0.305821	4.6226281	-5.746742	3.22E-08	7.74E-07	8.4716036
ESM1	-0.55179	5.2346084	-5.746727	3.22E-08	7.74E-07	8.4715321
RGL1	0.2254183	5.8200992	5.7450937	3.25E-08	7.80E-07	8.4635503
TMEM255B	0.227764	5.274137	5.74391	3.27E-08	7.84E-07	8.4577666
VCAN	0.4863427	6.0272643	5.7423365	3.29E-08	7.89E-07	8.4500801
RP11-727A2	-0.38916	4.2399097	-5.742331	3.29E-08	7.89E-07	8.4500536
RNF152	0.2454546	6.106604	5.7419428	3.30E-08	7.90E-07	8.4481571
ZNF792	-0.211893	5.2168824	-5.741841	3.30E-08	7.90E-07	8.4476579
NPDC1	0.2403213	5.9195252	5.7416159	3.30E-08	7.91E-07	8.44656
GATA2	0.443078	4.875137	5.741161	3.31E-08	7.92E-07	8.4443385
LEPROTL1	0.1308464	6.2139484	5.7409821	3.31E-08	7.92E-07	8.4434648
GLYR1	0.1020106	6.5221068	5.7405081	3.32E-08	7.94E-07	8.4411499
MCM6	-0.199904	6.1592024	-5.739994	3.33E-08	7.95E-07	8.438637

DCLK2	0.4118484	4.6961169	5.7398026	3.33E-08	7.96E-07	8.4377049
DLX6	-2.006705	0.4341891	-5.738421	3.36E-08	8.01E-07	8.4309601
DHX57	-0.116917	5.8974839	-5.737905	3.37E-08	8.02E-07	8.4284381
ELK3	0.1681586	6.0692184	5.7370324	3.38E-08	8.05E-07	8.42418
CHD9	0.1611523	6.0097752	5.7365149	3.39E-08	8.07E-07	8.4216539
ZNF692	-0.172214	6.0078832	-5.736361	3.39E-08	8.07E-07	8.4209003
AK2	0.1135692	6.6144434	5.7359421	3.40E-08	8.08E-07	8.4188581
PLXNB2	0.1275294	6.9898651	5.7356064	3.41E-08	8.09E-07	8.4172197
YAE1D1	-0.13848	5.4637841	-5.734679	3.42E-08	8.12E-07	8.4126913
RIMKLB	0.3488383	5.1435601	5.7345991	3.42E-08	8.12E-07	8.4123038
DNAJB1P1	-1.233133	-0.569828	-5.732391	3.46E-08	8.21E-07	8.4015315
NCOA3	-0.135965	6.2653607	-5.730765	3.49E-08	8.27E-07	8.3935983
PEAR1	0.3029018	5.3146659	5.730617	3.49E-08	8.27E-07	8.3928762
DKK2	1.3041491	3.6152802	5.7297446	3.51E-08	8.30E-07	8.3886216
COL12A1	0.3482808	5.7803145	5.7295705	3.51E-08	8.30E-07	8.3877725
CTTNBP2	1.0918829	3.9153852	5.7280898	3.54E-08	8.36E-07	8.380552
TGFB3	0.2926686	5.7084403	5.7274482	3.55E-08	8.38E-07	8.3774235
NTRK2	1.2064859	4.1286739	5.7267798	3.56E-08	8.41E-07	8.3741651
RP11-235G2	-1.630629	0.5877077	-5.725857	3.58E-08	8.44E-07	8.3696669
FHL1	0.2996745	5.8746495	5.7251669	3.59E-08	8.47E-07	8.3663028
TNFSF13	0.2949794	5.0465512	5.7248697	3.60E-08	8.47E-07	8.364854
CTD-2568P8	-1.182666	-0.448525	-5.724709	3.60E-08	8.47E-07	8.3640723
BCL2L14	-0.903838	3.9906143	-5.723297	3.63E-08	8.53E-07	8.3571917
SFR1	-0.188696	5.0960654	-5.722931	3.63E-08	8.54E-07	8.3554051
GPR34	0.4158069	4.8982262	5.7214515	3.66E-08	8.60E-07	8.3481975
RP11-83M16	-1.192486	-0.584177	-5.721004	3.67E-08	8.61E-07	8.346018
RP11-245D1	-0.990763	2.6280715	-5.718465	3.72E-08	8.72E-07	8.3336497
RP11-776H1	-1.953512	0.7825173	-5.717886	3.73E-08	8.74E-07	8.3308325
RP3-417G15	-0.76289	3.3383222	-5.717856	3.73E-08	8.74E-07	8.3306857
CSF1R	0.252847	6.1224593	5.7148529	3.79E-08	8.86E-07	8.3160638
LHX4	-0.620785	4.0152188	-5.714686	3.79E-08	8.87E-07	8.3152504
RP11-154H2	-1.333533	1.5409586	-5.713628	3.81E-08	8.91E-07	8.3101019
ADRA1B	0.6388156	4.6836199	5.7132702	3.82E-08	8.92E-07	8.3083602
LINC01524	-1.471602	-0.168794	-5.71285	3.82E-08	8.93E-07	8.3063135
DCTN6	0.1474849	5.8329083	5.7123652	3.83E-08	8.95E-07	8.3039557
KLF9	0.2544728	6.4263677	5.7118927	3.84E-08	8.96E-07	8.3016567
RP11-503N1	1.3556067	0.8197533	5.7113076	3.85E-08	8.98E-07	8.2988096
FGFR3	-0.294517	6.4659121	-5.711139	3.86E-08	8.99E-07	8.2979889
ETFDH	0.2393613	6.2957967	5.7107191	3.87E-08	9.00E-07	8.2959464
ADCY3	0.2641285	5.492102	5.7103394	3.87E-08	9.01E-07	8.2940992
GATB	0.162047	6.0294768	5.7097645	3.88E-08	9.03E-07	8.2913023
C17orf80	-0.107565	5.8967153	-5.708093	3.92E-08	9.10E-07	8.2831723
RP11-999E2	1.2443129	1.9086182	5.7078943	3.92E-08	9.11E-07	8.2822054
SEC22B	0.1250608	6.2661033	5.7077347	3.92E-08	9.11E-07	8.2814293
SLC9A7	0.2334991	5.8109577	5.7076015	3.93E-08	9.11E-07	8.2807815
FGFBP3	-0.452674	4.1206162	-5.706888	3.94E-08	9.14E-07	8.2773124
TNFAIP2	0.1993378	6.4014737	5.7058487	3.96E-08	9.18E-07	8.272258
CLEC1B	1.835039	1.2254539	5.7057607	3.96E-08	9.18E-07	8.27183
NOL7	-0.109088	6.3363384	-5.704625	3.99E-08	9.22E-07	8.2663075
CTD-2587H2	-0.797406	3.376637	-5.703804	4.00E-08	9.26E-07	8.262319
COL13A1	0.5892052	4.1489071	5.7033914	4.01E-08	9.27E-07	8.260312

RP1-152L7.	-0.388308	5.5495078	-5.702299	4.03E-08	9.32E-07	8.255002
DOK5	0.6155034	4.2041741	5.7021497	4.04E-08	9.32E-07	8.2542767
SERPING1	0.1973061	7.443557	5.7016908	4.05E-08	9.33E-07	8.2520467
EPB41L4A-A	0.9190551	3.2290777	5.7009187	4.06E-08	9.36E-07	8.2482947
RECQL4	-0.285975	5.9279112	-5.700837	4.06E-08	9.36E-07	8.2478992
SPAG5	-0.215281	6.1144588	-5.700483	4.07E-08	9.37E-07	8.246176
SEC24D	0.1808124	6.3161733	5.7004517	4.07E-08	9.37E-07	8.2460256
HTR2A	1.4379994	0.6992954	5.7002002	4.08E-08	9.37E-07	8.2448036
MT1XP1	1.5195595	1.3066114	5.700071	4.08E-08	9.37E-07	8.2441757
RP11-166D1	0.5483762	4.7378931	5.6988572	4.11E-08	9.43E-07	8.2382789
BMP6	0.7453623	4.1895575	5.6985495	4.11E-08	9.43E-07	8.2367838
RNF150	0.9923898	4.0607647	5.6979219	4.13E-08	9.46E-07	8.2337357
RP11-849H4	-0.287575	4.1781224	-5.696634	4.15E-08	9.51E-07	8.2274781
PLK4	-0.344271	4.9325575	-5.693535	4.22E-08	9.66E-07	8.2124325
SNCA	0.9952978	3.3918127	5.6930042	4.23E-08	9.68E-07	8.2098561
LINC00885	1.8754676	1.6655683	5.6906297	4.28E-08	9.79E-07	8.1983312
FEZ1	0.3227868	5.2137614	5.6903035	4.29E-08	9.80E-07	8.1967481
RNF180	0.5533636	4.9142285	5.6893293	4.31E-08	9.84E-07	8.192021
ACOT9	0.1571805	6.0164843	5.6892397	4.31E-08	9.84E-07	8.191586
PAQR3	0.242527	5.4349827	5.6890193	4.32E-08	9.85E-07	8.1905168
RP11-568K1	-1.3327	0.9535407	-5.688439	4.33E-08	9.87E-07	8.1877018
CYYR1	0.2825846	5.5639463	5.688126	4.33E-08	9.88E-07	8.186183
RP11-832A4	-1.396474	0.7277043	-5.68733	4.35E-08	9.91E-07	8.1823212
SNORD104	-0.638624	4.5006514	-5.687068	4.36E-08	9.92E-07	8.18105
DPT	1.3033585	4.4889316	5.686444	4.37E-08	9.94E-07	8.1780238
KIAA1524	-0.385448	5.0379669	-5.684622	4.41E-08	1.00E-06	8.1691891
AIFM3	-0.518269	4.6519947	-5.684408	4.42E-08	1.00E-06	8.1681477
KIAA1958	-0.212727	5.7227468	-5.681448	4.48E-08	1.02E-06	8.1537978
RGS3	0.1544909	6.3463773	5.6810984	4.49E-08	1.02E-06	8.1521053
CTD-2227E1	-0.690107	4.1515728	-5.679993	4.52E-08	1.02E-06	8.146746
WDFY3-AS2	0.6007465	3.8318921	5.6789729	4.54E-08	1.03E-06	8.1418045
DGCR5	0.9831382	4.808642	5.6779834	4.56E-08	1.03E-06	8.1370099
PNMA2	0.8301837	4.0613236	5.6763686	4.60E-08	1.04E-06	8.1291872
FBL	-0.148458	6.6132598	-5.675944	4.61E-08	1.04E-06	8.1271315
EIF4HP2	-0.963158	2.6922375	-5.675904	4.61E-08	1.04E-06	8.1269362
ARMC5	0.2031662	5.8435823	5.6745378	4.64E-08	1.05E-06	8.1203197
RP5-1112D6	-0.797386	3.1990244	-5.673149	4.68E-08	1.06E-06	8.1135936
DKK1	-1.422238	4.1603017	-5.672902	4.68E-08	1.06E-06	8.1124007
PLEKHM1	0.1064696	5.9380072	5.6728314	4.68E-08	1.06E-06	8.1120569
RP11-98I9.	-0.487389	4.0904233	-5.672407	4.69E-08	1.06E-06	8.1100034
FNDC1	0.9602359	4.8612257	5.6718576	4.71E-08	1.06E-06	8.1073422
RFX6	-1.714973	0.0355372	-5.671842	4.71E-08	1.06E-06	8.107267
HHIP	1.7006564	3.1515549	5.6708992	4.73E-08	1.06E-06	8.1027024
VPS4A	0.1088636	6.2614172	5.6703136	4.74E-08	1.07E-06	8.0998679
RP4-738P11	-1.205008	-0.509771	-5.670289	4.74E-08	1.07E-06	8.0997505
GREB1	-0.51571	5.3644013	-5.669598	4.76E-08	1.07E-06	8.0964026
PCBP3	1.0246724	3.6666638	5.6693803	4.77E-08	1.07E-06	8.0953508
MST02P	-0.351454	4.6004973	-5.668769	4.78E-08	1.07E-06	8.0923905
DNM3OS	0.852432	3.8339198	5.6682728	4.79E-08	1.07E-06	8.0899913
DBNDD1	0.3205677	5.9806951	5.6677043	4.81E-08	1.08E-06	8.0872403
EMC3-AS1	-0.515521	4.1567128	-5.667398	4.81E-08	1.08E-06	8.085757

MT1M	0.9908171	4.4751114	5.6672987	4.82E-08	1.08E-06	8.0852779
USP53	0.2179361	5.5980961	5.6670808	4.82E-08	1.08E-06	8.0842237
CHAF1B	-0.388214	5.3414117	-5.665154	4.87E-08	1.09E-06	8.0749024
RP11-179H1	-1.196553	-0.590614	-5.663468	4.91E-08	1.10E-06	8.0667462
KCNAB2	0.2965772	5.9808951	5.6622662	4.94E-08	1.10E-06	8.0609364
CRHR1-IT1	-0.380343	5.1113096	-5.659076	5.02E-08	1.12E-06	8.0455125
RP11-408P1	-0.205597	5.0721064	-5.658667	5.03E-08	1.12E-06	8.0435385
MUL1	0.1210721	6.18022	5.6583828	5.04E-08	1.12E-06	8.0421637
TWSG1	0.2227934	5.7226821	5.6564217	5.09E-08	1.13E-06	8.0326873
FAM72C	-1.331013	2.3298489	-5.655016	5.12E-08	1.14E-06	8.025897
LONP2	0.1680285	6.634947	5.6541502	5.15E-08	1.14E-06	8.0217138
SLC22A18	0.3340283	6.4635781	5.6526939	5.18E-08	1.15E-06	8.0146801
FAR2P1	-2.177356	0.9775485	-5.651446	5.22E-08	1.16E-06	8.0086527
PABPC1L	-0.255869	5.9847049	-5.650067	5.25E-08	1.17E-06	8.0019981
PRDM15	-0.213555	5.565993	-5.647128	5.33E-08	1.18E-06	7.9878102
FAM72B	-0.971411	3.346427	-5.646265	5.36E-08	1.19E-06	7.983644
ARHGAP6	0.414425	4.7056703	5.6462026	5.36E-08	1.19E-06	7.9833445
CMTM8	-0.248502	6.056415	-5.646195	5.36E-08	1.19E-06	7.9833059
GMEB2	-0.114319	6.0091693	-5.645212	5.38E-08	1.19E-06	7.9785647
C10orf95	-0.949528	2.23467	-5.644142	5.41E-08	1.20E-06	7.9734044
CTC-232P5.	-0.876026	2.5003196	-5.642856	5.45E-08	1.20E-06	7.9672018
HCG11	0.5063092	3.9429496	5.6427847	5.45E-08	1.20E-06	7.9668561
VRK1	-0.164296	5.5455808	-5.64235	5.46E-08	1.21E-06	7.9647573
NCAPD2P1	-1.852431	1.4907365	-5.637727	5.59E-08	1.23E-06	7.9424716
ELN	0.4601399	5.9042055	5.6362364	5.63E-08	1.24E-06	7.9352861
SLC25A19	-0.190337	5.4034436	-5.635626	5.65E-08	1.24E-06	7.9323461
ISG20L2	-0.105741	6.265929	-5.635387	5.66E-08	1.25E-06	7.9311923
SH3RF3-AS1	1.1322032	2.6945635	5.6351103	5.66E-08	1.25E-06	7.9298594
SNHG7	-0.21077	6.0823809	-5.634829	5.67E-08	1.25E-06	7.9285035
MXD3	-0.252097	5.6139525	-5.633846	5.70E-08	1.25E-06	7.9237685
NAT8B	-0.8485	4.4650487	-5.633061	5.72E-08	1.26E-06	7.9199873
ATP6V0D1	0.1230142	6.5586088	5.6326099	5.74E-08	1.26E-06	7.9178138
AKAP6	0.3918929	4.9477521	5.6308785	5.79E-08	1.27E-06	7.9094746
TMPOP2	-1.357588	0.8803533	-5.630807	5.79E-08	1.27E-06	7.9091324
UNG	-0.105577	6.3067822	-5.629347	5.83E-08	1.28E-06	7.9021013
STAG3L4	-0.233048	5.3468606	-5.628874	5.85E-08	1.28E-06	7.8998228
PDLIM3	0.340561	5.6857692	5.6278844	5.87E-08	1.29E-06	7.8950586
COPS7B	-0.097414	6.121441	-5.627445	5.89E-08	1.29E-06	7.8929437
GNAT1	-1.215027	3.1185528	-5.62739	5.89E-08	1.29E-06	7.8926772
NANOS1	-0.815411	4.2142503	-5.627093	5.90E-08	1.29E-06	7.8912494
HAMP	0.979499	5.2787397	5.6265475	5.91E-08	1.29E-06	7.8886235
FANCB	-0.784588	3.420108	-5.625819	5.94E-08	1.30E-06	7.8851158
OVGP1	-0.434645	5.3295834	-5.625583	5.94E-08	1.30E-06	7.8839803
POU3F2	-1.537934	0.9741458	-5.625341	5.95E-08	1.30E-06	7.8828174
IL13RA1	0.1388953	6.7221342	5.6249969	5.96E-08	1.30E-06	7.8811612
WNT2	1.8295205	1.9812666	5.6247441	5.97E-08	1.30E-06	7.8799449
FOS	0.3274984	6.3149309	5.6246405	5.97E-08	1.30E-06	7.8794463
CTD-2207A1	-1.666305	-0.098038	-5.624146	5.99E-08	1.30E-06	7.8770664
PASK	-0.195417	5.4591847	-5.623965	5.99E-08	1.30E-06	7.8761969
GLI2	0.9623618	3.7831321	5.6226552	6.03E-08	1.31E-06	7.8698944
ZNF419	-0.252098	5.3073313	-5.622472	6.04E-08	1.31E-06	7.8690157

DTYMK	-0.159992	6.1030046	-5.622261	6.04E-08	1.31E-06	7.8679982
AC008592.8	1.4992152	1.1313885	5.6192454	6.13E-08	1.33E-06	7.8534949
ZNF273	-0.315719	4.7537295	-5.61909	6.14E-08	1.33E-06	7.8527471
EHMT2	-0.129894	6.400172	-5.618499	6.16E-08	1.33E-06	7.8499078
HSF2BP	-0.893737	3.2956828	-5.618276	6.16E-08	1.33E-06	7.8488334
RAD1	-0.105879	5.9366691	-5.617651	6.18E-08	1.34E-06	7.8458272
ST8SIA6	1.4821571	1.532552	5.6170186	6.20E-08	1.34E-06	7.842789
PLEKHG1	0.2371793	5.6390789	5.6140898	6.30E-08	1.36E-06	7.828713
GPR1-AS	-1.654569	-0.40607	-5.612728	6.34E-08	1.37E-06	7.8221702
LARP7	0.0981778	6.1194648	5.6124881	6.35E-08	1.37E-06	7.8210172
ACTA2	0.203747	6.5915405	5.6118763	6.37E-08	1.37E-06	7.8180779
MT2P1	1.4381254	2.7684407	5.6093095	6.45E-08	1.39E-06	7.8057497
FBLN5	0.4000459	5.9677884	5.609276	6.45E-08	1.39E-06	7.8055887
AXDND1	-1.315447	1.8057239	-5.609186	6.45E-08	1.39E-06	7.8051581
RP1-240K6.	-0.871733	3.0124215	-5.608131	6.49E-08	1.40E-06	7.800091
PCAT19	0.3432741	4.8918237	5.6075072	6.51E-08	1.40E-06	7.7970951
SEC24B	0.163849	6.2202925	5.6066295	6.54E-08	1.40E-06	7.7928816
UNC5C	0.5190978	4.5383358	5.6063309	6.55E-08	1.41E-06	7.7914482
DACT3	0.5656073	4.7214248	5.6061349	6.55E-08	1.41E-06	7.7905076
PSRC1	-0.338585	5.2769617	-5.605439	6.57E-08	1.41E-06	7.7871684
CYP7B1	0.6220322	5.1687582	5.6044081	6.61E-08	1.42E-06	7.7822192
SPEF2	-0.321294	5.1445667	-5.603066	6.65E-08	1.43E-06	7.7757797
ZBTB47	0.1978151	5.6160399	5.6004324	6.74E-08	1.44E-06	7.7631439
TPK1	0.3360098	4.9701391	5.6002103	6.75E-08	1.44E-06	7.7620789
TSC22D3	0.275168	6.4036919	5.6001833	6.75E-08	1.44E-06	7.7619495
GABBR1	0.4090944	5.2092696	5.5993436	6.78E-08	1.45E-06	7.7579219
LINC01010	1.3493224	2.3829273	5.5967417	6.87E-08	1.47E-06	7.7454451
VPS72	-0.124022	6.2904822	-5.59167	7.04E-08	1.50E-06	7.7211369
C10orf128	0.3237785	5.2808157	5.5908629	7.07E-08	1.51E-06	7.7172707
LGALS9	0.2132714	6.1743896	5.5905159	7.09E-08	1.51E-06	7.7156083
ZNF687	-0.133727	6.2791902	-5.590064	7.10E-08	1.51E-06	7.7134443
CFI	0.2280796	6.9409879	5.5898035	7.11E-08	1.51E-06	7.7121958
WFDC1	0.6470337	4.4959401	5.5887494	7.15E-08	1.52E-06	7.7071471
ZBTB5	-0.134156	5.861554	-5.588322	7.16E-08	1.52E-06	7.7050989
ALG1L	-1.081367	4.6545234	-5.588101	7.17E-08	1.52E-06	7.7040438
CTD-2207P1	-1.420897	0.3691108	-5.587545	7.19E-08	1.53E-06	7.7013808
RPL14P1	-0.277057	4.934721	-5.586711	7.22E-08	1.53E-06	7.6973867
ADTRP	1.027807	3.712912	5.5847169	7.29E-08	1.55E-06	7.687839
KATNAL1	0.2495648	5.4518445	5.5844949	7.30E-08	1.55E-06	7.6867768
RP5-968D22	-1.107277	3.9360255	-5.580978	7.43E-08	1.58E-06	7.6699474
PALM2	0.9744668	3.5916159	5.5787233	7.52E-08	1.59E-06	7.6591604
RP11-761N2	-0.786536	2.8813557	-5.578451	7.53E-08	1.59E-06	7.6578568
RP13-1032I	-0.237482	4.9004357	-5.578138	7.54E-08	1.60E-06	7.6563623
RP11-61J19	-0.333396	4.4938804	-5.577847	7.55E-08	1.60E-06	7.6549678
SEMA6C	-0.269145	5.8403652	-5.577677	7.55E-08	1.60E-06	7.6541547
BCORL1	-0.201342	5.8446704	-5.576871	7.59E-08	1.60E-06	7.6503015
STEAP3-AS1	1.1479265	1.8016626	5.5768314	7.59E-08	1.60E-06	7.6501125
IGLVIVOR22	-0.881418	-1.003838	-5.576317	7.61E-08	1.60E-06	7.6476516
ZNF606	-0.289061	5.4363632	-5.57565	7.63E-08	1.61E-06	7.6444641
RP11-252E2	1.7098785	0.6993492	5.5755568	7.64E-08	1.61E-06	7.644018
C4orf33	0.2982749	5.2403906	5.5754868	7.64E-08	1.61E-06	7.6436831

SLC16A7	0.4844992	5.6258139	5.5751728	7.65E-08	1.61E-06	7.6421821
APLNR	0.3956124	5.9402748	5.5747048	7.67E-08	1.61E-06	7.6399448
CEP131	-0.178452	5.8417961	-5.574677	7.67E-08	1.61E-06	7.6398119
CEP55	-0.46389	5.1097087	-5.574533	7.67E-08	1.61E-06	7.6391242
ITGA3	0.3541673	5.8409185	5.5745098	7.68E-08	1.61E-06	7.6390125
SLCO2A1	0.6478574	5.5370776	5.5739852	7.70E-08	1.62E-06	7.6365046
LINC01430	1.5612199	0.7472491	5.573653	7.71E-08	1.62E-06	7.6349166
FBXW12	-1.334734	1.5275485	-5.573433	7.72E-08	1.62E-06	7.6338666
RP11-426C2	1.6120818	1.0573267	5.5681743	7.92E-08	1.66E-06	7.608739
C6orf183	-1.567083	2.7955321	-5.56756	7.95E-08	1.66E-06	7.6058035
PIGQ	0.1370795	6.2418142	5.5668292	7.98E-08	1.67E-06	7.6023147
CLTB	0.1260473	6.4482325	5.5661374	8.00E-08	1.67E-06	7.5990111
RP11-501C1	-1.458447	0.0114937	-5.566111	8.00E-08	1.67E-06	7.5988841
TNFRSF1B	0.2074305	6.3390505	5.565134	8.04E-08	1.68E-06	7.5942202
CACNG4	-1.685221	3.2699025	-5.564231	8.08E-08	1.69E-06	7.589907
GID8	-0.10714	6.4933483	-5.564021	8.09E-08	1.69E-06	7.5889069
WDR78	0.4719012	4.2302964	5.5623625	8.15E-08	1.70E-06	7.5809902
TAL1	0.4149473	4.5972318	5.5607776	8.22E-08	1.71E-06	7.5734267
PDE10A	0.5472003	4.5254655	5.5605111	8.23E-08	1.71E-06	7.5721549
PMM2	0.1792151	5.7405864	5.5597567	8.26E-08	1.72E-06	7.5685554
ODF2L	0.3105869	5.2420495	5.5582126	8.33E-08	1.73E-06	7.561189
PRR15L	-1.108893	4.7654499	-5.557573	8.35E-08	1.74E-06	7.5581375
RERG	0.4333977	4.8607322	5.5535924	8.52E-08	1.77E-06	7.5391568
MKX	1.5798712	2.5695109	5.5528062	8.55E-08	1.78E-06	7.5354091
RPSAP54	-0.98106	2.4826571	-5.551884	8.59E-08	1.78E-06	7.5310118
OR1J1	-0.883799	-0.979718	-5.551449	8.61E-08	1.79E-06	7.5289396
AC098820.3	-0.735071	3.3132407	-5.550696	8.64E-08	1.79E-06	7.5253527
TMEM86B	-0.3851	5.8731598	-5.550582	8.65E-08	1.79E-06	7.5248066
NLN	0.1816891	6.2198917	5.5503561	8.66E-08	1.79E-06	7.5237322
ZNF345	-0.227908	4.8022919	-5.549488	8.70E-08	1.80E-06	7.519596
C5orf56	0.2252749	5.2116683	5.547163	8.80E-08	1.82E-06	7.5085196
RP11-314A2	-0.662519	3.2398681	-5.546011	8.85E-08	1.83E-06	7.5030318
MIOX	-1.507295	2.8085067	-5.545733	8.86E-08	1.83E-06	7.5017074
SLC22A23	-0.17417	6.2681164	-5.545445	8.87E-08	1.83E-06	7.5003393
ZNF555	-0.223645	5.1896808	-5.545382	8.87E-08	1.83E-06	7.5000387
RP11-258C1	-0.237209	5.105414	-5.544973	8.89E-08	1.84E-06	7.4980904
ZNF519	-0.432606	4.4155933	-5.544393	8.92E-08	1.84E-06	7.4953291
GDAP1	-0.315103	5.3453039	-5.542684	8.99E-08	1.85E-06	7.4871934
CNN3	0.1434071	6.679866	5.5421718	9.02E-08	1.86E-06	7.484754
ZFAND5	0.1760852	6.7547281	5.5421128	9.02E-08	1.86E-06	7.4844731
RPS7P4	-0.979708	2.0545356	-5.541482	9.05E-08	1.86E-06	7.4814699
ANTXR2	0.1985241	6.1464565	5.5404261	9.10E-08	1.87E-06	7.4764454
UBAP2	-0.122956	6.0880139	-5.54034	9.10E-08	1.87E-06	7.4760367
RP11-568A7	-1.546372	0.0629632	-5.539892	9.12E-08	1.87E-06	7.4739025
SPON1	0.7838726	4.7666928	5.53845	9.19E-08	1.89E-06	7.4670427
GEMIN7	-0.142112	5.8656157	-5.538097	9.20E-08	1.89E-06	7.4653631
FAHD2A	0.1689846	6.1203587	5.5378567	9.21E-08	1.89E-06	7.4642201
CTD-219904	-1.30137	1.4282382	-5.537786	9.22E-08	1.89E-06	7.4638856
CTAGE3P	-1.246327	1.2489705	-5.536681	9.27E-08	1.90E-06	7.4586288
NIPSNAP3A	0.1597038	5.9569261	5.5366548	9.27E-08	1.90E-06	7.458503
SNORA12	-1.156285	-0.080311	-5.536025	9.30E-08	1.90E-06	7.4555052

DHH	1.4175739	2.269689	5.5334804	9.42E-08	1.93E-06	7.443407
ZNF404	-0.540822	4.360489	-5.533238	9.43E-08	1.93E-06	7.4422527
FAS	0.3118758	5.6208955	5.5318565	9.49E-08	1.94E-06	7.435687
RP3-337H4.	-0.621458	3.1824544	-5.530421	9.56E-08	1.95E-06	7.4288633
HMMR	-0.373424	5.4815747	-5.529835	9.59E-08	1.96E-06	7.4260789
C3orf22	-1.229592	0.1803909	-5.52901	9.63E-08	1.96E-06	7.4221567
APP	0.1709719	7.0679753	5.5277114	9.69E-08	1.97E-06	7.4159889
CTBP2P8	1.0484091	-0.58538	5.5256194	9.79E-08	1.99E-06	7.4060515
NPM3	-0.285223	5.7363692	-5.525421	9.80E-08	2.00E-06	7.4051106
RP11-229P1	-0.936753	2.2984308	-5.524847	9.83E-08	2.00E-06	7.4023815
RP11-411K7	1.3871831	0.5967983	5.5247763	9.83E-08	2.00E-06	7.4020474
RP11-1103G	-1.793518	0.1682085	-5.523918	9.87E-08	2.01E-06	7.3979709
RAMP3	0.4348119	5.6383997	5.5233513	9.90E-08	2.01E-06	7.395281
AP000704.5	-1.065956	1.8376456	-5.522573	9.94E-08	2.02E-06	7.3915852
RP11-89H19	-1.160099	3.055214	-5.521267	1.00E-07	2.03E-06	7.385386
LINC00992	-1.806008	1.8039309	-5.520417	1.00E-07	2.04E-06	7.3813534
RP11-381N2	-1.308461	0.2266248	-5.520278	1.01E-07	2.04E-06	7.3806898
MAT2B	0.108249	6.3802923	5.5201956	1.01E-07	2.04E-06	7.3803009
PTGS2	0.794714	3.976048	5.5200867	1.01E-07	2.04E-06	7.3797839
MFI2-AS1	-0.652328	4.0285424	-5.519982	1.01E-07	2.04E-06	7.3792854
FOXP4-AS1	-1.355992	2.4504799	-5.519683	1.01E-07	2.04E-06	7.3778688
GPR116	0.2124695	5.965696	5.5190218	1.01E-07	2.04E-06	7.3747302
STK10	0.1502515	6.0606212	5.5182219	1.02E-07	2.05E-06	7.3709348
ZNF93	-0.561956	4.4801194	-5.517366	1.02E-07	2.06E-06	7.3668736
KCNMA1	0.4064642	5.2852051	5.5173635	1.02E-07	2.06E-06	7.366862
COL4A1	0.1644101	6.8901677	5.5172082	1.02E-07	2.06E-06	7.3661253
RP11-802D6	-1.343047	0.6065906	-5.515121	1.03E-07	2.08E-06	7.3562244
C3orf36	1.435832	1.9259936	5.5144893	1.03E-07	2.08E-06	7.3532285
RP5-837I24	-1.223358	0.9100998	-5.514375	1.04E-07	2.08E-06	7.3526864
MEIS3	0.3846087	4.6707011	5.5140867	1.04E-07	2.09E-06	7.3513197
LINC01506	1.4968668	1.1189498	5.5136323	1.04E-07	2.09E-06	7.3491648
RUNX1T1	0.5478647	4.4043786	5.5132899	1.04E-07	2.09E-06	7.3475411
CLIP4	0.5126848	5.396056	5.5119169	1.05E-07	2.11E-06	7.3410316
MXRA8	0.3168966	5.7935519	5.5112505	1.05E-07	2.11E-06	7.3378723
RP11-501C1	-1.360848	-0.565361	-5.510975	1.05E-07	2.11E-06	7.3365658
PPP1R12B	0.1613768	6.0395971	5.5109529	1.05E-07	2.11E-06	7.3364617
SFI1	-0.201935	5.8974833	-5.51058	1.05E-07	2.11E-06	7.3346932
TRPM4	0.2601864	5.9073441	5.5101884	1.06E-07	2.12E-06	7.3328378
PRKCH	0.2338557	5.7020182	5.5098255	1.06E-07	2.12E-06	7.3311179
HOXB2	0.2944332	4.8182889	5.5095799	1.06E-07	2.12E-06	7.329954
C11orf98	-0.175967	5.0459666	-5.5095	1.06E-07	2.12E-06	7.3295765
SCN1B	0.2371086	5.5721268	5.5094775	1.06E-07	2.12E-06	7.3294688
RP11-557H1	1.2605815	2.479049	5.5079077	1.07E-07	2.14E-06	7.3220299
IDO2	1.4683554	2.596915	5.5069451	1.07E-07	2.14E-06	7.3174691
ABHD3	-0.1564	6.3076365	-5.505166	1.08E-07	2.16E-06	7.3090432
SPATA6L	0.3845113	4.8654124	5.5039039	1.09E-07	2.17E-06	7.3030643
MBL1P	1.3879001	2.77297	5.5037901	1.09E-07	2.17E-06	7.3025252
GAS2L1	0.1456134	6.0552282	5.5035893	1.09E-07	2.18E-06	7.3015743
NPY1R	0.7623189	4.4598397	5.5034051	1.09E-07	2.18E-06	7.3007019
ABLIM3	0.2207225	6.3857604	5.5024437	1.10E-07	2.19E-06	7.2961498
FAM13A	0.3441242	5.6609703	5.5015929	1.10E-07	2.19E-06	7.2921218

CPSF4	-0.120949	6.0646817	-5.499997	1.11E-07	2.21E-06	7.2845653
GRIK4	-1.278339	3.3368662	-5.499682	1.11E-07	2.21E-06	7.283074
RILP	0.2271456	5.9331367	5.4934846	1.15E-07	2.28E-06	7.2537563
SAA1	0.929205	6.3252134	5.4926468	1.15E-07	2.29E-06	7.2497942
RP11-757G1	-1.246693	3.7513491	-5.491182	1.16E-07	2.30E-06	7.24287
RP11-10017	-1.069794	0.4270133	-5.491048	1.16E-07	2.30E-06	7.2422357
GMPS	-0.096477	6.3591285	-5.491007	1.16E-07	2.30E-06	7.2420438
ADORA3	0.4296138	4.8812687	5.4880965	1.18E-07	2.34E-06	7.2282849
RP5-1014D1	-0.227714	4.6127659	-5.487755	1.18E-07	2.34E-06	7.2266713
LINC01537	1.0175462	2.4169246	5.4877426	1.18E-07	2.34E-06	7.2266128
TSPEAR-AS1	-1.380801	3.6255328	-5.486972	1.19E-07	2.34E-06	7.2229705
MIR17HG	-0.668494	3.9040132	-5.485811	1.19E-07	2.36E-06	7.2174846
FOXF1	0.5146304	4.6889003	5.4853368	1.20E-07	2.36E-06	7.215246
GRB10	0.1177632	6.4282077	5.4844929	1.20E-07	2.37E-06	7.2112597
COL4A4	0.5607696	4.9759424	5.4844539	1.20E-07	2.37E-06	7.2110756
MEX3A	-0.394906	5.3287496	-5.483655	1.21E-07	2.38E-06	7.2073012
SPNS3	0.6664692	4.0513126	5.4829654	1.21E-07	2.38E-06	7.204046
RP11-285J1	-1.068007	1.9279828	-5.482935	1.21E-07	2.38E-06	7.2039042
MT1JP	1.4510223	-0.199221	5.4825272	1.21E-07	2.39E-06	7.2019765
NR4A1	0.325231	5.918304	5.4817196	1.22E-07	2.39E-06	7.1981632
C8orf44	-0.247311	4.8287955	-5.481695	1.22E-07	2.39E-06	7.1980492
KRBA1	-0.336242	5.2702067	-5.48152	1.22E-07	2.39E-06	7.1972221
FAM26E	0.31382	4.8779166	5.4801452	1.23E-07	2.41E-06	7.1907303
KDM2B	-0.11099	6.0200614	-5.479921	1.23E-07	2.41E-06	7.1896726
RP11-90L1.	1.1236413	3.145025	5.4797908	1.23E-07	2.41E-06	7.1890574
STX11	0.4633404	4.8052307	5.4784801	1.24E-07	2.43E-06	7.1828713
IFI44	0.3250384	5.7455052	5.4756332	1.25E-07	2.46E-06	7.169438
FLT4	0.2255218	5.8540176	5.474333	1.26E-07	2.47E-06	7.1633047
SDHD	0.1386085	6.5602254	5.47326	1.27E-07	2.48E-06	7.1582439
C14orf37	0.6765435	3.9672302	5.4731509	1.27E-07	2.48E-06	7.1577294
SPRY3	0.3009602	4.3613567	5.472144	1.28E-07	2.50E-06	7.1529812
CENPI	-0.545526	4.6874669	-5.471863	1.28E-07	2.50E-06	7.1516567
TSKU	0.3666531	6.5801226	5.4717571	1.28E-07	2.50E-06	7.1511569
LINC01561	1.1894547	-0.497496	5.4714558	1.28E-07	2.50E-06	7.1497362
RP11-356J5	0.605775	4.6274693	5.4699666	1.29E-07	2.52E-06	7.1427155
TUBA1A	0.1934046	6.1408896	5.4692045	1.29E-07	2.52E-06	7.1391232
RP11-15E18	-1.02679	1.812843	-5.468056	1.30E-07	2.54E-06	7.1337123
KANK3	0.3063237	5.3040697	5.4674768	1.31E-07	2.54E-06	7.1309807
ZNF764	-0.138243	5.600145	-5.466262	1.31E-07	2.56E-06	7.1252584
ALDH1L2	0.5481436	4.3078316	5.465422	1.32E-07	2.57E-06	7.1212988
ISLR2	0.9050059	3.5402918	5.4645344	1.32E-07	2.58E-06	7.1171172
WDR13	0.1598995	6.6284968	5.4638185	1.33E-07	2.58E-06	7.1137455
NOVA2	0.2555513	5.071662	5.4636035	1.33E-07	2.59E-06	7.1127325
PRAMEF9	1.6171767	-0.095477	5.4630869	1.33E-07	2.59E-06	7.1102997
HPS5	0.19183	6.1426748	5.4625974	1.34E-07	2.60E-06	7.1079945
RNASEH2A	-0.207385	5.9455994	-5.462185	1.34E-07	2.60E-06	7.1060516
HTR2A-AS1	0.8533984	-1.047876	5.4620317	1.34E-07	2.60E-06	7.1053304
RP11-329N1	0.5435796	3.6719972	5.4600438	1.35E-07	2.62E-06	7.095971
FBXO10	-0.209822	5.4822082	-5.459597	1.36E-07	2.63E-06	7.0938655
TGFB1I1	0.2198948	5.6262097	5.4589636	1.36E-07	2.64E-06	7.0908858
GCK	1.5618508	2.4753114	5.4581584	1.37E-07	2.64E-06	7.0870961

TRIP13	-0.421627	5.2440394	-5.458104	1.37E-07	2.64E-06	7.0868418
TLR4	0.2720935	5.4890976	5.4563846	1.38E-07	2.67E-06	7.078749
GINM1	0.1079949	6.1937617	5.4561456	1.38E-07	2.67E-06	7.0776242
APOL1	0.2272185	6.7779159	5.4561422	1.38E-07	2.67E-06	7.0776086
ANGEL1	-0.15366	6.0677861	-5.452797	1.40E-07	2.71E-06	7.0618711
NR3C2	0.3711988	5.302356	5.4524587	1.41E-07	2.71E-06	7.0602814
FOXJ1	-1.597267	3.1822598	-5.450633	1.42E-07	2.73E-06	7.0516966
C11orf63	0.6462862	3.8133267	5.4497882	1.42E-07	2.74E-06	7.0477247
SRRT	-0.075797	6.5603969	-5.444878	1.46E-07	2.81E-06	7.0246476
MEDAG	1.0128292	3.7985145	5.4446083	1.46E-07	2.81E-06	7.0233825
KRT87P	1.7532427	1.8075454	5.4442682	1.46E-07	2.82E-06	7.0217846
AC092415.1	-1.198145	-0.696743	-5.444194	1.46E-07	2.82E-06	7.0214376
CTD-2235C1	-1.083975	2.5743764	-5.44341	1.47E-07	2.82E-06	7.0177557
ZNF211	-0.192583	5.3142319	-5.442745	1.47E-07	2.83E-06	7.0146289
NSL1	-0.118046	6.0955065	-5.442106	1.48E-07	2.84E-06	7.0116294
HMCN1	0.4228554	5.4470141	5.4413207	1.48E-07	2.85E-06	7.0079418
UGP2	0.1852053	6.8708116	5.4412386	1.49E-07	2.85E-06	7.0075563
ITPR1	0.2518385	5.7391291	5.4393149	1.50E-07	2.87E-06	6.9985247
OGN	1.5109378	3.7182634	5.4383309	1.51E-07	2.89E-06	6.9939057
ZNF599	-0.272396	5.0123004	-5.436828	1.52E-07	2.91E-06	6.986853
SUSD6	0.1263593	6.1657063	5.4364936	1.52E-07	2.91E-06	6.985283
FBXL3	0.129725	6.0973545	5.4345749	1.53E-07	2.94E-06	6.9762804
RAD54L	-0.582175	4.6360995	-5.433647	1.54E-07	2.95E-06	6.9719256
SOAT2	-1.390658	4.36463	-5.433416	1.54E-07	2.95E-06	6.9708445
LURAP1L	0.2847253	6.0198934	5.4327356	1.55E-07	2.96E-06	6.9676531
CYP1B1	0.4171222	5.7676432	5.4324868	1.55E-07	2.96E-06	6.9664863
XXbac-BPG2	0.6239534	4.4749271	5.4318785	1.55E-07	2.97E-06	6.9636335
ZNF84	-0.120601	5.8826196	-5.431582	1.56E-07	2.97E-06	6.9622425
FN3KRP	-0.118404	6.296344	-5.430839	1.56E-07	2.98E-06	6.9587591
FBXO5	-0.236618	5.2233673	-5.430516	1.57E-07	2.98E-06	6.9572464
ETS2	0.1714153	6.7613521	5.4303298	1.57E-07	2.98E-06	6.9563714
RP11-977G1	-0.724065	2.8850572	-5.429863	1.57E-07	2.99E-06	6.9541841
CKS2	-0.228028	5.9013912	-5.429009	1.58E-07	3.00E-06	6.9501802
NOP56	-0.128287	6.4980202	-5.428651	1.58E-07	3.00E-06	6.9485009
PDGFRA	0.668399	5.4390575	5.4284719	1.58E-07	3.00E-06	6.9476616
ARPP21	1.6633628	1.8122044	5.4275944	1.59E-07	3.02E-06	6.9435487
NFE2L1	0.1103484	6.9728654	5.4259047	1.60E-07	3.04E-06	6.9356305
CTD-2267D1	-0.484154	3.8243221	-5.425834	1.60E-07	3.04E-06	6.9352972
AC012146.7	-0.372571	4.7922727	-5.425707	1.60E-07	3.04E-06	6.9347036
KCNK6	0.2546242	5.4010282	5.4252999	1.61E-07	3.04E-06	6.9327966
TEFM	-0.112469	5.3411814	-5.424148	1.61E-07	3.06E-06	6.9274013
RP11-54A9.	1.3377579	0.6534589	5.4234274	1.62E-07	3.07E-06	6.9240246
TPRG1	0.4667162	4.7199585	5.4233419	1.62E-07	3.07E-06	6.9236239
SAA2	1.2216979	5.7489511	5.4232326	1.62E-07	3.07E-06	6.9231121
KRTAP10-4	-0.917115	-0.941567	-5.422926	1.62E-07	3.07E-06	6.9216738
ARL6IP6	-0.191587	5.4814453	-5.422458	1.63E-07	3.08E-06	6.919486
WNK2	-0.998182	5.1248159	-5.422309	1.63E-07	3.08E-06	6.9187875
LSM2	-0.135423	6.1286062	-5.421851	1.63E-07	3.08E-06	6.9166413
BMP1	0.1554779	6.167451	5.4191388	1.66E-07	3.12E-06	6.9039422
EOGT	0.1452762	5.6152353	5.418814	1.66E-07	3.13E-06	6.9024218
PHACTR3	1.3675032	1.1869946	5.4184926	1.66E-07	3.13E-06	6.9009174

AHCY	-0.13217	6.9274577	-5.418292	1.66E-07	3.13E-06	6.8999777
RP11-363N2	-1.110026	2.1133866	-5.417061	1.67E-07	3.15E-06	6.8942175
NFATC1	0.3044201	5.4447785	5.4169668	1.67E-07	3.15E-06	6.893776
CCDC97	-0.10575	6.1433651	-5.416407	1.68E-07	3.15E-06	6.8911556
BEND6	1.3596136	2.1633479	5.41631	1.68E-07	3.15E-06	6.8907023
GALNT18	0.2903463	5.9021751	5.4159261	1.68E-07	3.16E-06	6.8889057
MR1	0.219513	5.9271518	5.4158715	1.68E-07	3.16E-06	6.8886507
CXCL2	0.4669024	5.9395738	5.4157272	1.68E-07	3.16E-06	6.8879754
RP11-422N1	1.4937253	2.3894154	5.4157126	1.68E-07	3.16E-06	6.8879069
LHX4-AS1	-0.131625	6.1964764	-5.414403	1.69E-07	3.18E-06	6.8817795
PNCK	-1.987028	1.9033538	-5.41424	1.70E-07	3.18E-06	6.8810164
ACTR3C	0.3101057	5.2822546	5.4134264	1.70E-07	3.19E-06	6.877211
HIST1H2AL	-1.243996	0.0240591	-5.411389	1.72E-07	3.22E-06	6.8676811
TTI1	-0.094956	6.1057797	-5.411127	1.72E-07	3.22E-06	6.8664566
SLC24A3	1.0832306	3.8113399	5.4107015	1.72E-07	3.23E-06	6.8644678
INS-IGF2	1.8039727	0.154418	5.4103171	1.73E-07	3.23E-06	6.8626702
RCBTB2	0.1823325	5.4550447	5.4096501	1.73E-07	3.24E-06	6.8595516
SOBP	-0.478217	5.318384	-5.409396	1.74E-07	3.24E-06	6.8583627
COL16A1	0.430574	5.6184186	5.4076948	1.75E-07	3.27E-06	6.8504117
ZNF780B	-0.189826	5.4750248	-5.407354	1.75E-07	3.27E-06	6.8488166
ZNF581	-0.165611	5.8097134	-5.405562	1.77E-07	3.30E-06	6.8404437
TNRC6A	0.1161585	6.2437127	5.405174	1.77E-07	3.30E-06	6.8386314
ITGBL1	0.9709043	4.7206306	5.4044389	1.78E-07	3.31E-06	6.8351973
GPX8	0.3300676	5.2704259	5.4035542	1.79E-07	3.32E-06	6.8310644
SUSD2	0.3916547	5.4928437	5.4035536	1.79E-07	3.32E-06	6.8310614
ZNF582	-0.466512	4.6227208	-5.402373	1.80E-07	3.34E-06	6.8255471
RAP1B	0.0897467	6.1209575	5.4018613	1.80E-07	3.35E-06	6.823157
MTERF2	-0.132179	5.8051189	-5.401814	1.80E-07	3.35E-06	6.8229378
LINC00680	-0.210195	5.1493824	-5.401128	1.81E-07	3.35E-06	6.8197327
WDR76	-0.316467	5.3482346	-5.401121	1.81E-07	3.35E-06	6.8196989
DEXI	0.1959795	5.4902715	5.4001139	1.82E-07	3.37E-06	6.8149973
MT-TP	0.2165896	6.2676855	5.3993409	1.82E-07	3.38E-06	6.8113887
HK1	0.2116256	5.9667611	5.3981468	1.83E-07	3.40E-06	6.8058141
SEMA5A	0.3264177	5.7253738	5.3979699	1.84E-07	3.40E-06	6.8049887
SCARF2	0.3189384	5.1804628	5.3968205	1.85E-07	3.42E-06	6.7996241
CASP1	0.2472057	5.6402396	5.3962291	1.85E-07	3.43E-06	6.7968643
ZSWIM1	-0.122633	5.6121353	-5.395835	1.85E-07	3.43E-06	6.7950255
PLA2G1B	-1.08169	3.68114	-5.39489	1.86E-07	3.44E-06	6.7906165
FAM222B	-0.118135	6.0771716	-5.394721	1.86E-07	3.45E-06	6.7898261
RP3-512B11	-0.468835	4.8881986	-5.394323	1.87E-07	3.45E-06	6.787969
PDZRN4	1.6358224	1.5727775	5.3942428	1.87E-07	3.45E-06	6.7875961
STEAP4	0.5795797	5.453754	5.3909949	1.90E-07	3.50E-06	6.7724474
KPNA2	-0.15931	6.4343501	-5.390982	1.90E-07	3.50E-06	6.7723896
REP15	-0.958904	2.6983647	-5.390794	1.90E-07	3.50E-06	6.7715125
RGS7BP	1.4696525	2.9024362	5.3882253	1.93E-07	3.55E-06	6.7595353
FBN3	-1.49489	3.3981776	-5.387778	1.93E-07	3.55E-06	6.7574493
SSTR3	-1.577504	1.5628126	-5.385638	1.95E-07	3.59E-06	6.7474758
CTD-2008L1	-1.561624	-0.172681	-5.385386	1.95E-07	3.59E-06	6.7463027
SLC15A3	0.239997	5.9754057	5.384636	1.96E-07	3.60E-06	6.7428083
CSE1L	-0.09909	6.5151435	-5.384114	1.96E-07	3.61E-06	6.7403778
RP11-672A2	1.2596372	2.4742169	5.3835213	1.97E-07	3.62E-06	6.7376156

PPP1CB	0.0696354	6.676111	5.3833949	1.97E-07	3.62E-06	6.7370267
RP11-76C10	-1.511017	-0.298773	-5.383099	1.97E-07	3.62E-06	6.735647
RPL7P19	-1.055279	-0.21805	-5.382378	1.98E-07	3.63E-06	6.7322892
KCNMB3	-0.56948	4.3440062	-5.381902	1.99E-07	3.64E-06	6.7300723
AARSD1	-0.146997	5.2023717	-5.381712	1.99E-07	3.64E-06	6.7291901
HEG1	0.1806497	6.2197229	5.3816359	1.99E-07	3.64E-06	6.7288338
AC006994.3	1.2326523	-0.135213	5.3810381	1.99E-07	3.65E-06	6.7260502
PRNP	0.2153156	6.3173696	5.3802712	2.00E-07	3.66E-06	6.722479
RP11-83M16	-1.73476	1.1201659	-5.378774	2.02E-07	3.68E-06	6.715507
RP11-34F20	-0.955312	2.2570681	-5.37762	2.03E-07	3.70E-06	6.7101354
NELFCD	-0.099259	6.5704804	-5.377185	2.03E-07	3.71E-06	6.7081127
PSMB8-AS1	0.2614976	5.5162564	5.3771793	2.03E-07	3.71E-06	6.7080857
ZNF496	-0.223224	5.9378283	-5.376922	2.03E-07	3.71E-06	6.7068866
KRT17P4	1.5144389	0.3582974	5.37654	2.04E-07	3.72E-06	6.7051103
RHOB	0.1828971	7.003603	5.3763945	2.04E-07	3.72E-06	6.7044333
C1orf35	-0.153338	5.9915447	-5.375648	2.05E-07	3.73E-06	6.7009619
CBLB	0.1293109	5.9068919	5.3753147	2.05E-07	3.73E-06	6.6994089
HOXA10	-1.597105	3.2806325	-5.374147	2.06E-07	3.75E-06	6.6939763
TMEM167B	0.088749	6.249639	5.3738819	2.06E-07	3.75E-06	6.6927426
COL8A2	0.5453064	4.7484814	5.3734092	2.07E-07	3.76E-06	6.6905437
FAM111B	-0.371591	5.3371294	-5.372865	2.07E-07	3.77E-06	6.6880107
IGFBP4	0.1695696	7.2171699	5.3715835	2.09E-07	3.79E-06	6.6820524
PPP1R15A	0.1589505	6.274901	5.3712416	2.09E-07	3.80E-06	6.6804623
RP1-266L20	-0.467945	4.0309973	-5.37014	2.10E-07	3.81E-06	6.6753414
ZMPSTE24	0.0966797	6.4415239	5.3694817	2.11E-07	3.82E-06	6.6722791
GALNS	0.1619726	5.869732	5.3691447	2.11E-07	3.83E-06	6.6707127
RP11-231G3	-0.493129	3.5617238	-5.369041	2.11E-07	3.83E-06	6.6702303
RP11-36N20	-1.080061	-0.884521	-5.367937	2.13E-07	3.85E-06	6.665099
CCDC59	-0.099384	5.9186039	-5.367929	2.13E-07	3.85E-06	6.6650595
PLEKHB2	0.1229472	6.3191922	5.3677364	2.13E-07	3.85E-06	6.6641659
ZNF782	-0.1836	4.940107	-5.365826	2.15E-07	3.88E-06	6.6552857
RP11-634H2	-0.806692	2.8382793	-5.364822	2.16E-07	3.90E-06	6.650623
ORC1	-0.39528	5.0653395	-5.364628	2.16E-07	3.90E-06	6.649722
RP11-126K1	-0.413888	4.0611518	-5.364604	2.16E-07	3.90E-06	6.6496119
MSH5	-0.397581	4.6937809	-5.364226	2.16E-07	3.90E-06	6.6478557
SPATA18	0.7147913	4.5364511	5.3629505	2.18E-07	3.93E-06	6.6419284
LIFR	0.3862652	5.6256584	5.3622181	2.18E-07	3.94E-06	6.6385268
RP11-111M2	-1.156123	2.0498538	-5.361369	2.19E-07	3.95E-06	6.6345838
NPY5R	1.4987864	1.470139	5.3597463	2.21E-07	3.98E-06	6.6270488
PIK3R2	-0.416645	4.4932168	-5.359536	2.21E-07	3.98E-06	6.6260739
PCID2	-0.116946	6.097379	-5.359432	2.21E-07	3.98E-06	6.6255906
EFNB3	0.8699702	3.6865683	5.3579728	2.23E-07	4.01E-06	6.6188157
RCE1	-0.121339	5.8026475	-5.35571	2.26E-07	4.05E-06	6.6083132
GFRA3	-1.625478	0.8667863	-5.355293	2.26E-07	4.06E-06	6.6063798
RP11-511H2	-0.816825	2.7375996	-5.354963	2.26E-07	4.06E-06	6.6048482
PIGK	0.1243221	6.0539383	5.3548574	2.26E-07	4.06E-06	6.6043583
NME6	-0.113315	5.784991	-5.353538	2.28E-07	4.09E-06	6.598237
RP13-476E2	-1.270208	0.8078397	-5.352715	2.29E-07	4.10E-06	6.5944197
NDRG1	-0.191695	6.8222264	-5.352428	2.29E-07	4.11E-06	6.5930895
CTB-31020.	-0.27467	4.79261	-5.350944	2.31E-07	4.13E-06	6.5862049
PXYLP1	-0.268677	5.4842781	-5.350936	2.31E-07	4.13E-06	6.586169

TIE1	0.2295825	6.0487934	5.3500956	2.32E-07	4.15E-06	6.5822728
CXCL12	0.3388329	6.2746979	5.3495536	2.32E-07	4.16E-06	6.5797599
RP11-535A1	-0.808545	2.8517633	-5.349078	2.33E-07	4.16E-06	6.5775569
CALCRL	0.2898339	5.8881394	5.3487817	2.33E-07	4.17E-06	6.5761812
RAD51C	-0.133755	5.7113457	-5.347652	2.35E-07	4.19E-06	6.5709442
LINC00472	1.1071913	2.1097595	5.3474797	2.35E-07	4.19E-06	6.5701465
RP1-30E17.	-0.998844	-0.714247	-5.346408	2.36E-07	4.21E-06	6.5651776
FIGNL1	-0.201624	5.5051938	-5.346316	2.36E-07	4.21E-06	6.5647517
CFLAR	0.0772562	6.5546092	5.3456336	2.37E-07	4.22E-06	6.5615908
FAM178A	-0.128828	6.0378209	-5.34201	2.41E-07	4.29E-06	6.5448062
ZNF682	-0.560705	4.7448406	-5.34144	2.42E-07	4.30E-06	6.5421663
DDX60L	0.2509975	5.6807944	5.3410033	2.42E-07	4.31E-06	6.5401429
REEP2	-0.813619	3.8034046	-5.340677	2.43E-07	4.32E-06	6.5386302
AC016682.1	1.1712446	-0.547632	5.3396007	2.44E-07	4.34E-06	6.5336487
TAGLN2	0.1133051	7.0740136	5.3384886	2.45E-07	4.36E-06	6.5285005
KCNS3	0.5441235	5.0980989	5.3381461	2.46E-07	4.36E-06	6.5269153
CASC10	-0.356998	5.7958808	-5.337713	2.46E-07	4.37E-06	6.5249089
SCUBE1	1.0372245	4.867842	5.3367822	2.47E-07	4.39E-06	6.5206026
AC138472.4	-0.895369	-1.013108	-5.336754	2.47E-07	4.39E-06	6.5204713
MT1F	0.6048237	5.2962801	5.3362072	2.48E-07	4.40E-06	6.5179417
KCNK10	-1.529825	1.0354142	-5.335572	2.49E-07	4.41E-06	6.5150031
KNDC1	1.4534676	4.5090847	5.3348835	2.49E-07	4.42E-06	6.511817
RP11-251G2	-1.111647	2.1271364	-5.333997	2.51E-07	4.44E-06	6.5077168
CT45A1	-1.169465	-0.959363	-5.333117	2.52E-07	4.45E-06	6.5036458
TRIQK	0.1644701	6.197458	5.3327086	2.52E-07	4.46E-06	6.5017561
FLI1	0.2074222	5.7001932	5.3312177	2.54E-07	4.49E-06	6.4948611
GJB6	-1.671705	1.0283105	-5.330226	2.55E-07	4.51E-06	6.4902781
TRPM5	-1.379615	0.377792	-5.328778	2.57E-07	4.54E-06	6.4835824
PHYHIPL	-0.713388	5.0783482	-5.326574	2.60E-07	4.58E-06	6.4733968
CAPN2	0.1543792	6.6400483	5.3259284	2.61E-07	4.60E-06	6.4704119
BCL3	0.1685924	6.4412509	5.3255563	2.61E-07	4.60E-06	6.4686924
RNF8	-0.12901	5.8818546	-5.324386	2.63E-07	4.63E-06	6.463285
KIF13B	0.1491516	6.2106823	5.3242912	2.63E-07	4.63E-06	6.4628476
TIGD7	-0.310433	4.5750524	-5.321274	2.66E-07	4.69E-06	6.4489131
URGCP	0.1052121	6.2512994	5.321167	2.67E-07	4.69E-06	6.448418
PXDN	0.2198806	6.1086019	5.3209628	2.67E-07	4.70E-06	6.4474751
ID4	0.4971526	4.9791909	5.3206635	2.67E-07	4.70E-06	6.4460933
CT45A10	-1.254176	-0.887917	-5.320203	2.68E-07	4.71E-06	6.443967
RP11-302I1	-1.120353	1.0943836	-5.320011	2.68E-07	4.71E-06	6.44308
HSPG2	0.2389541	6.5446885	5.3198303	2.68E-07	4.71E-06	6.4422464
COL27A1	-0.187777	6.3777522	-5.31924	2.69E-07	4.72E-06	6.4395191
REPS2	0.2924425	5.7628206	5.3183187	2.70E-07	4.74E-06	6.4352686
NFYAP1	-1.258586	-0.423051	-5.318071	2.71E-07	4.75E-06	6.4341241
RP11-276E1	-1.150681	0.4705869	-5.317461	2.71E-07	4.76E-06	6.4313084
PIK3R3	0.1955418	5.7736366	5.3170552	2.72E-07	4.76E-06	6.4294371
KIN	-0.108414	5.8672052	-5.316328	2.73E-07	4.78E-06	6.4260831
TLK2	-0.077273	5.9977385	-5.316041	2.73E-07	4.78E-06	6.4247589
IFIT2	0.2691831	5.8854368	5.3159244	2.73E-07	4.78E-06	6.4242187
BTN3A1	0.2004983	6.099398	5.3155731	2.74E-07	4.79E-06	6.4225982
PAM	0.206991	6.0741645	5.3141549	2.76E-07	4.82E-06	6.4160551
H6PD	0.1761584	6.7943474	5.3138519	2.76E-07	4.82E-06	6.4146573

NEIL3	-0.692381	4.4121984	-5.311989	2.79E-07	4.86E-06	6.4060658
ZNF740	-0.106209	6.0764312	-5.311931	2.79E-07	4.86E-06	6.4057965
AC004381.6	-0.265516	5.2657265	-5.311016	2.80E-07	4.88E-06	6.4015804
RP4-734P14	-1.060573	0.1849337	-5.310562	2.81E-07	4.89E-06	6.3994864
RP11-513G1	1.376974	-0.156596	5.3097119	2.82E-07	4.91E-06	6.3955659
AP001505.1	-0.45233	4.4319034	-5.309424	2.82E-07	4.91E-06	6.3942406
TMEM133	0.2507251	5.2041254	5.3092637	2.82E-07	4.92E-06	6.3934998
RNF19A	0.1347924	6.4246699	5.3088501	2.83E-07	4.92E-06	6.3915934
RP11-711D1	-1.062454	-0.351276	-5.308099	2.84E-07	4.94E-06	6.3881309
KB-1208A12	-0.278922	4.9717896	-5.307543	2.85E-07	4.95E-06	6.3855679
RP11-195F1	0.7061686	3.7868586	5.3050257	2.88E-07	5.01E-06	6.3739695
TMEM194A	-0.147382	5.8397091	-5.304128	2.89E-07	5.03E-06	6.3698318
CTA-315H11	-0.816184	3.742614	-5.304054	2.90E-07	5.03E-06	6.3694944
SAMD11	0.7814174	4.3278043	5.3039093	2.90E-07	5.03E-06	6.3688267
RAB33B	0.1722833	5.6526312	5.303516	2.90E-07	5.04E-06	6.3670148
ACSM5	0.5941548	5.9666358	5.3031407	2.91E-07	5.04E-06	6.3652866
CRTAC1	1.5388448	2.4148076	5.3030784	2.91E-07	5.04E-06	6.3649994
PTCHD3P3	-1.195265	-0.422506	-5.302817	2.91E-07	5.04E-06	6.3637961
HLA-A	0.1471792	7.329897	5.3025436	2.92E-07	5.05E-06	6.3625366
RNF2P1	-0.97178	2.4903655	-5.301742	2.93E-07	5.06E-06	6.3588439
AE000658.2	-1.163881	-0.913712	-5.301724	2.93E-07	5.06E-06	6.35876
CAV1	0.1675963	6.2116396	5.3007075	2.94E-07	5.09E-06	6.3540816
MBNL1	0.0826956	6.502582	5.2992559	2.96E-07	5.12E-06	6.347399
ERVK3-1	-0.114556	5.7167243	-5.299141	2.97E-07	5.12E-06	6.3468696
CMB9-94B1.	-1.032227	1.3279647	-5.298412	2.98E-07	5.14E-06	6.3435152
CTD-2118P1	-1.719688	0.2881877	-5.298025	2.98E-07	5.14E-06	6.3417313
EFNB1	0.1710709	6.1145396	5.2977759	2.98E-07	5.15E-06	6.3405867
HIST1H1E	-1.131017	2.3416581	-5.295843	3.01E-07	5.19E-06	6.3316901
LINC01018	1.6012928	4.5969332	5.2947709	3.03E-07	5.22E-06	6.32676
LIPT2	-0.303245	4.4426336	-5.294299	3.04E-07	5.23E-06	6.3245911
RP11-1109F	-0.844115	3.1286707	-5.294233	3.04E-07	5.23E-06	6.3242856
LYAR	-0.14592	5.7802586	-5.293978	3.04E-07	5.23E-06	6.3231114
FGD5	0.2592813	5.7328649	5.2928626	3.06E-07	5.26E-06	6.3179825
RP11-276H1	-1.722267	0.4394629	-5.292624	3.06E-07	5.26E-06	6.3168862
BCAS4	-0.326728	5.2210924	-5.291088	3.08E-07	5.30E-06	6.3098205
CDC42EP3	0.2371475	5.6244332	5.2904345	3.09E-07	5.31E-06	6.3068179
GPR137C	-0.378029	4.6003356	-5.290044	3.10E-07	5.32E-06	6.3050247
PODXL	0.2208983	6.2862063	5.2886765	3.12E-07	5.35E-06	6.2987367
LINC01205	-1.351372	-0.33797	-5.288213	3.13E-07	5.36E-06	6.2966083
FAM177B	0.7021639	3.7585611	5.2876065	3.13E-07	5.37E-06	6.2938188
SAA2-SAA4	1.7362744	4.4250342	5.2875945	3.14E-07	5.37E-06	6.2937635
GABRQ	-1.244447	2.3421402	-5.286905	3.15E-07	5.39E-06	6.2905948
RP5-1007F2	1.3125138	-0.145234	5.2866062	3.15E-07	5.39E-06	6.289222
EDNRB	0.2509425	5.9510848	5.2857003	3.16E-07	5.41E-06	6.2850599
MMRN2	0.2214156	6.024565	5.2853625	3.17E-07	5.42E-06	6.283508
CTB-5506.1	-0.242825	4.3330836	-5.285093	3.17E-07	5.42E-06	6.2822685
RP11-96C21	-1.225328	-0.006598	-5.284738	3.18E-07	5.43E-06	6.2806379
OXNAD1	0.1523315	5.7962014	5.2845668	3.18E-07	5.43E-06	6.2798527
GAA	0.1313737	6.774389	5.2840831	3.19E-07	5.44E-06	6.2776308
MIR4783	-1.252734	0.9379752	-5.283993	3.19E-07	5.44E-06	6.2772171
MECR	0.1328825	5.9551953	5.2832478	3.20E-07	5.46E-06	6.2737946

PLS3	0.1321415	6.6643355	5.2827053	3.21E-07	5.47E-06	6.2713028
LACC1	0.2181128	5.4296958	5.28181	3.22E-07	5.49E-06	6.2671918
PTCHD4	1.132272	3.0883413	5.2813761	3.23E-07	5.50E-06	6.2651996
LINGO1-AS1	-1.245266	-0.449095	-5.280892	3.24E-07	5.51E-06	6.2629778
KIAA1456	0.9186987	4.3706378	5.2795541	3.26E-07	5.54E-06	6.2568348
PHIP	-0.125214	6.0849264	-5.279418	3.26E-07	5.54E-06	6.2562104
NCK1	0.1019515	5.9960808	5.277159	3.30E-07	5.60E-06	6.2458429
RP11-95D17	-0.166343	5.1882011	-5.277033	3.30E-07	5.60E-06	6.2452665
MRGBP	-0.120454	6.0134282	-5.27675	3.30E-07	5.61E-06	6.2439655
ADRA1A	1.3122582	4.1097754	5.2763045	3.31E-07	5.62E-06	6.241922
CLMP	0.6162527	4.1007292	5.2757852	3.32E-07	5.63E-06	6.2395397
CLEC2L	-1.902581	1.0468144	-5.274699	3.34E-07	5.66E-06	6.2345588
STK35	-0.121264	6.1330752	-5.274495	3.34E-07	5.66E-06	6.2336226
CNIH2	-0.600661	3.5087058	-5.272512	3.37E-07	5.71E-06	6.2245249
CXCL17	-1.489483	2.7198669	-5.272474	3.37E-07	5.71E-06	6.2243525
OCA2	-1.55843	3.1313583	-5.272195	3.38E-07	5.71E-06	6.2230726
ADCY4	0.2656879	5.428971	5.2695221	3.42E-07	5.78E-06	6.210819
LLNLR-260G	-0.872231	-0.862275	-5.268871	3.43E-07	5.80E-06	6.2078339
KNSTRN	-0.146456	5.7742521	-5.26879	3.43E-07	5.80E-06	6.2074629
RP11-288C1	-1.437625	0.0225146	-5.268764	3.43E-07	5.80E-06	6.2073458
CIT	-0.271715	5.5036199	-5.268422	3.44E-07	5.80E-06	6.2057756
RBMX	-0.068539	6.5705954	-5.26831	3.44E-07	5.80E-06	6.2052652
DNA2	-0.258179	5.2035257	-5.268024	3.44E-07	5.81E-06	6.2039521
RP11-45601	-1.4576	-0.181274	-5.266554	3.47E-07	5.85E-06	6.1972172
RP11-111M2	-0.303341	4.8394831	-5.266351	3.47E-07	5.85E-06	6.196289
MYCT1	0.2678899	5.4222714	5.2659046	3.48E-07	5.86E-06	6.194242
PRKG1-AS1	-1.383341	-0.060169	-5.265822	3.48E-07	5.86E-06	6.1938643
TNFRSF1A	0.1106349	6.7263851	5.2657817	3.48E-07	5.86E-06	6.1936787
PDCD2L	-0.198112	5.4038666	-5.265208	3.49E-07	5.87E-06	6.1910529
RP3-337H4.	-0.770521	2.8982791	-5.264569	3.50E-07	5.89E-06	6.1881257
FAM24B	-0.967781	3.3161402	-5.264558	3.50E-07	5.89E-06	6.1880754
EDEM1	0.1459208	6.4679024	5.2639822	3.51E-07	5.90E-06	6.185436
PCAT18	1.3410437	0.1488235	5.2629289	3.53E-07	5.93E-06	6.1806125
SDCBP	0.1399415	6.714438	5.2627651	3.53E-07	5.93E-06	6.1798626
ZNF684	0.2368844	5.4099852	5.2625968	3.54E-07	5.93E-06	6.1790919
RP11-1036E	1.725067	1.7050579	5.2623794	3.54E-07	5.93E-06	6.1780961
CTD-2561J2	-1.230771	2.7384448	-5.261995	3.55E-07	5.94E-06	6.1763343
RP11-375N1	-1.102141	2.0145804	-5.261459	3.55E-07	5.95E-06	6.173883
PRAMEF4	1.7517479	0.18747	5.2599483	3.58E-07	5.99E-06	6.1669663
ATG4B	-0.09642	6.3337549	-5.259917	3.58E-07	5.99E-06	6.1668231
PHF19	-0.190236	5.7800603	-5.25982	3.58E-07	5.99E-06	6.1663773
HLA-F	0.2361303	6.4955844	5.2577939	3.62E-07	6.05E-06	6.1571067
LXN	0.3430161	5.0772398	5.2571386	3.63E-07	6.06E-06	6.1541083
BACE1-AS	-0.237516	4.8547137	-5.257126	3.63E-07	6.06E-06	6.1540494
CHST4	1.906718	2.5141241	5.2566661	3.64E-07	6.07E-06	6.1519465
RP11-626G1	-1.16885	0.8436151	-5.256468	3.64E-07	6.08E-06	6.1510422
RP11-583F2	-1.195018	0.7550619	-5.255937	3.65E-07	6.09E-06	6.1486114
TMEM156	0.6133745	4.7966279	5.2544706	3.68E-07	6.13E-06	6.1419031
CRYBG3	0.6501967	4.9205086	5.2540242	3.68E-07	6.14E-06	6.1398618
MCCC1	0.163626	6.3012085	5.2534255	3.69E-07	6.15E-06	6.1371239
FAM198A	0.8298771	4.2151943	5.2531903	3.70E-07	6.16E-06	6.1360484

PPAP2B	0.1788323	6.543614	5.251332	3.73E-07	6.21E-06	6.1275518
RNU6-945P	-0.917731	-0.697076	-5.251114	3.74E-07	6.21E-06	6.1265567
CXorf66	1.1801969	-0.571529	5.2504327	3.75E-07	6.23E-06	6.1234407
TSACC	-0.441204	4.2126928	-5.249758	3.76E-07	6.25E-06	6.1203562
TIMELESS	-0.139003	6.2498157	-5.248767	3.78E-07	6.28E-06	6.1158271
IL1R1	0.1500491	6.5309394	5.2487141	3.78E-07	6.28E-06	6.1155862
CIDEB	0.8468215	3.3589871	5.2485133	3.78E-07	6.28E-06	6.1146684
LCOR	-0.146957	5.9157065	-5.248485	3.78E-07	6.28E-06	6.1145368
ANXA3	0.8550858	4.1959451	5.2484157	3.78E-07	6.28E-06	6.1142223
RP11-127B2	-0.712204	3.2134386	-5.24827	3.79E-07	6.28E-06	6.1135561
PLEKHB1	-0.831291	4.7031273	-5.247162	3.81E-07	6.31E-06	6.108495
RP11-22B23	-0.221942	5.0502484	-5.246995	3.81E-07	6.31E-06	6.1077318
MORF4L2-AS	-0.683431	2.9968738	-5.245661	3.83E-07	6.35E-06	6.1016377
ACTL6A	-0.109614	6.1272324	-5.245433	3.84E-07	6.35E-06	6.1005964
KLF2P1	-1.581852	-0.089849	-5.245245	3.84E-07	6.35E-06	6.0997367
TBC1D15	0.0844054	6.2081553	5.2452082	3.84E-07	6.35E-06	6.0995684
SYTL5	0.9794021	3.9905361	5.2429499	3.88E-07	6.42E-06	6.0892556
TMEM192	0.1571561	6.2240098	5.242046	3.90E-07	6.44E-06	6.0851283
RUFY3	0.1492506	5.8581835	5.2416768	3.91E-07	6.45E-06	6.083443
DRC7	-0.844745	3.6676825	-5.240524	3.93E-07	6.48E-06	6.0781806
ZNF852	-0.250248	4.4697308	-5.240169	3.94E-07	6.49E-06	6.0765594
MBD4	0.0992504	6.1703884	5.2401061	3.94E-07	6.49E-06	6.0762731
ZNF813	-0.509386	5.0167049	-5.239297	3.95E-07	6.51E-06	6.072581
MYCNOS	-1.508757	0.659333	-5.237886	3.98E-07	6.56E-06	6.0661409
PCDHGA12	1.0448007	3.6880301	5.2376557	3.98E-07	6.56E-06	6.0650911
SPATA25	-0.466442	4.075668	-5.237441	3.99E-07	6.56E-06	6.0641128
NUDT17	-0.351694	4.748031	-5.237421	3.99E-07	6.56E-06	6.064019
NUDT12	0.3771118	5.8195737	5.2373048	3.99E-07	6.56E-06	6.0634905
GPER1	-0.41227	5.7333676	-5.236761	4.00E-07	6.58E-06	6.0610104
NLRP3	0.4167745	4.5016143	5.2357784	4.02E-07	6.60E-06	6.056527
RP11-165F2	-1.356986	0.4941396	-5.23486	4.04E-07	6.63E-06	6.0523392
CPSF6	-0.072299	6.3397717	-5.234479	4.04E-07	6.64E-06	6.0506
LRRC37BP1	-0.162068	5.2703843	-5.23442	4.05E-07	6.64E-06	6.0503321
CTD-2314B2	-1.238472	-0.428342	-5.233985	4.05E-07	6.65E-06	6.0483501
ALKBH3	0.2284062	5.6541664	5.2333052	4.07E-07	6.67E-06	6.0452483
DSTN	0.1042641	6.8065906	5.232753	4.08E-07	6.68E-06	6.0427306
MRPS23	-0.105788	6.2555418	-5.23147	4.10E-07	6.72E-06	6.0368831
TM6SF1	0.69189	3.9122708	5.2303309	4.13E-07	6.75E-06	6.0316896
TMEM170B	-0.252081	5.8305717	-5.230308	4.13E-07	6.75E-06	6.0315868
CD93	0.1779172	6.3031042	5.2299489	4.13E-07	6.76E-06	6.0299484
RNASEK	0.1473569	5.4494502	5.2297242	4.14E-07	6.76E-06	6.0289246
ECHDC2	0.1989349	6.6606782	5.2286817	4.16E-07	6.80E-06	6.0241738
RP11-649A1	-0.5087	3.9240065	-5.228413	4.16E-07	6.80E-06	6.0229491
NUP210	-0.154296	6.4332401	-5.228367	4.16E-07	6.80E-06	6.0227377
TARID	1.2789126	0.9353134	5.2282044	4.17E-07	6.80E-06	6.021999
ST3GAL4	0.1824711	6.0051331	5.2270853	4.19E-07	6.83E-06	6.0169007
RAPGEF2	0.1609465	6.1615428	5.2267457	4.20E-07	6.84E-06	6.0153536
CORO2B	0.6138533	4.3770938	5.2265347	4.20E-07	6.85E-06	6.0143923
SLC25A25-A	-0.2594	5.7078265	-5.226478	4.20E-07	6.85E-06	6.0141351
ZNF514	-0.191616	5.4791102	-5.225397	4.22E-07	6.88E-06	6.0092107
TLR2	0.3771419	5.3474022	5.224934	4.23E-07	6.89E-06	6.0071021

PTF1A	-1.177807	-0.783724	-5.224243	4.25E-07	6.91E-06	6.0039566
RP11-530N7	1.3793914	1.5722624	5.2238465	4.26E-07	6.92E-06	6.0021496
TNFAIP8	0.2383807	5.6079185	5.2234272	4.26E-07	6.93E-06	6.0002406
ACAA2	0.1944889	6.8365657	5.2225749	4.28E-07	6.96E-06	5.9963604
RP1-89D4.1	-1.019328	1.6019866	-5.222466	4.28E-07	6.96E-06	5.9958634
NUDT1	-0.202884	5.6486916	-5.222161	4.29E-07	6.96E-06	5.9944769
RP11-118N2	-1.325006	-0.670526	-5.220798	4.32E-07	7.01E-06	5.9882711
ERCC4	0.1730496	5.3230716	5.219004	4.36E-07	7.06E-06	5.9801078
AP5M1	0.1208155	6.0735216	5.2189381	4.36E-07	7.06E-06	5.9798082
KRTAP19-5	-1.080505	-0.939645	-5.218909	4.36E-07	7.06E-06	5.9796762
ZNF765	-0.155401	5.3889271	-5.218801	4.36E-07	7.06E-06	5.9791834
PSMG3-AS1	0.2693462	5.2839966	5.2184285	4.37E-07	7.07E-06	5.9774895
C6	0.5394106	6.511689	5.2182453	4.37E-07	7.07E-06	5.976656
CH17-360D5	1.556234	0.4123567	5.2181602	4.37E-07	7.07E-06	5.9762687
ADRB1	1.3736801	2.1463131	5.2180747	4.37E-07	7.07E-06	5.97588
ELOVL2	-0.386234	6.4083312	-5.217673	4.38E-07	7.08E-06	5.974054
KLHDC10	0.1318782	6.3821143	5.2175262	4.39E-07	7.08E-06	5.9733847
PPP3CA	0.1145596	6.0163361	5.2164802	4.41E-07	7.12E-06	5.9686263
RP11-475C1	-0.28646	5.1354503	-5.215822	4.42E-07	7.14E-06	5.9656335
HMGA2	-1.786436	2.0694403	-5.215564	4.43E-07	7.14E-06	5.964458
TGFBR3	0.2613883	5.8689214	5.215512	4.43E-07	7.14E-06	5.9642232
DYNLRB2	1.215996	1.3880328	5.2147274	4.44E-07	7.16E-06	5.9606548
ABHD5	0.1516511	5.7124927	5.2142892	4.45E-07	7.18E-06	5.9586625
KRTAP20-2	-0.929855	-1.098769	-5.213441	4.47E-07	7.20E-06	5.9548057
LYSMD1	-0.141231	5.7104462	-5.21171	4.51E-07	7.26E-06	5.9469363
LINC01101	-1.457983	0.3669432	-5.211356	4.52E-07	7.27E-06	5.9453296
GRIA2	-1.38067	-0.245993	-5.211191	4.52E-07	7.27E-06	5.9445783
HERPUD1	0.1315342	6.66981	5.2109961	4.52E-07	7.27E-06	5.9436924
HELLPAR	-0.484463	4.0823748	-5.21047	4.54E-07	7.29E-06	5.9413025
TMEM98	-0.27312	6.2437656	-5.210067	4.54E-07	7.30E-06	5.9394696
ARAP3	0.1977532	5.6995168	5.2097277	4.55E-07	7.31E-06	5.9379285
NFATC2	-0.200218	5.8819517	-5.209666	4.55E-07	7.31E-06	5.9376471
SLIT3	0.3553236	5.5576815	5.209603	4.55E-07	7.31E-06	5.9373616
SNIP1	0.1101252	5.8050006	5.2089831	4.57E-07	7.32E-06	5.9345452
LINC01534	-0.359092	3.9954662	-5.208577	4.58E-07	7.33E-06	5.9326998
E2F7	-0.645504	4.3088099	-5.208565	4.58E-07	7.33E-06	5.9326442
NAT9	-0.122082	6.1271803	-5.208253	4.58E-07	7.34E-06	5.9312273
NKIRAS2	-0.078904	6.3104645	-5.207162	4.61E-07	7.37E-06	5.9262735
AC010136.2	-1.25856	1.486069	-5.206972	4.61E-07	7.38E-06	5.9254083
15-Sep	0.0923385	6.661009	5.2067236	4.62E-07	7.38E-06	5.9242807
HNRNPA3	-0.064698	6.8237015	-5.206636	4.62E-07	7.38E-06	5.9238814
RPS7P1	-0.280871	5.1057009	-5.206404	4.62E-07	7.39E-06	5.9228291
ZNF529	-0.155413	5.5919801	-5.205039	4.65E-07	7.43E-06	5.9166299
RP11-108K1	-1.104887	0.4003889	-5.203943	4.68E-07	7.47E-06	5.911655
CUX1	0.1014947	6.485249	5.2037567	4.68E-07	7.47E-06	5.9108084
NDNF	1.3918942	0.9860269	5.2002491	4.76E-07	7.59E-06	5.894888
HLA-E	0.1364826	7.1637608	5.2001276	4.76E-07	7.60E-06	5.8943366
PTPN13	0.60522	4.5341498	5.1999217	4.77E-07	7.60E-06	5.8934027
CTSS	0.1995818	6.6013918	5.1992654	4.78E-07	7.62E-06	5.8904249
RP11-21C4.	1.2806458	0.4196979	5.1986206	4.80E-07	7.64E-06	5.8874995
AP5B1	0.1373535	5.8855497	5.1984933	4.80E-07	7.64E-06	5.8869219

CLSTN2	0.9375436	4.2152518	5.198425	4.80E-07	7.64E-06	5.886612
RPARP-AS1	-0.1842	5.5081509	-5.19835	4.81E-07	7.64E-06	5.8862727
RPSAP15	-1.059296	1.6274141	-5.19833	4.81E-07	7.64E-06	5.8861805
IPO9	-0.096642	6.5164346	-5.197693	4.82E-07	7.66E-06	5.883292
ARHGAP19	-0.146531	5.6059562	-5.197418	4.83E-07	7.66E-06	5.8820436
RPS24P8	-0.583027	3.1709102	-5.197291	4.83E-07	7.67E-06	5.8814665
CTD-3234P1	-1.16202	1.1713362	-5.194834	4.89E-07	7.75E-06	5.8703262
TWIST2	1.2029114	2.6515557	5.1946874	4.89E-07	7.75E-06	5.8696615
PDGFC	0.2706719	5.7238733	5.1946712	4.89E-07	7.75E-06	5.8695882
AP000344.4	-1.43434	0.1744127	-5.194546	4.89E-07	7.75E-06	5.8690204
ZDHHC7	0.1200615	6.113129	5.1943854	4.90E-07	7.76E-06	5.8682923
RP5-1148A2	-0.357178	5.1547221	-5.194048	4.90E-07	7.76E-06	5.8667645
ZNF189	-0.144324	6.0165255	-5.193907	4.91E-07	7.77E-06	5.8661252
PDXDC1	0.1170074	6.7050788	5.193169	4.93E-07	7.79E-06	5.8627778
AC099850.1	-0.525373	3.2861074	-5.191852	4.96E-07	7.84E-06	5.8568086
GHR	0.4227548	6.0778675	5.1914762	4.97E-07	7.85E-06	5.8551058
FTO	0.1199201	6.0876006	5.1907791	4.98E-07	7.87E-06	5.8519467
PAFAH1B1	0.0839783	6.4496005	5.187778	5.05E-07	7.98E-06	5.838351
CCT6P3	-0.21023	4.7903	-5.187379	5.06E-07	7.99E-06	5.8365454
MCL1	0.0973606	7.0159848	5.1854728	5.11E-07	8.06E-06	5.8279118
SH3BGR	0.2427226	5.0774403	5.1831094	5.17E-07	8.15E-06	5.8172127
GATM-AS1	-1.242387	1.2611945	-5.182812	5.17E-07	8.15E-06	5.8158673
ADORA2A	-0.534727	4.4494369	-5.182632	5.18E-07	8.16E-06	5.8150505
NDUFAF1	0.1562496	6.0354385	5.1821963	5.19E-07	8.17E-06	5.8130803
SH2B3	0.1300718	6.2454023	5.1821039	5.19E-07	8.17E-06	5.8126619
ZNF577	-0.332934	5.0650733	-5.181939	5.20E-07	8.17E-06	5.8119152
EMCN	0.3020392	5.5804681	5.1812886	5.21E-07	8.20E-06	5.8089724
TBX4	-1.626025	1.463933	-5.180372	5.23E-07	8.23E-06	5.8048233
SBDS	0.0956489	6.501301	5.1800898	5.24E-07	8.24E-06	5.8035484
DDX19B	0.127427	5.7695227	5.1780796	5.29E-07	8.31E-06	5.7944556
SLC25A46	0.1261285	6.2401755	5.1766717	5.33E-07	8.36E-06	5.7880885
GPR155	0.2218796	5.7155638	5.176428	5.33E-07	8.37E-06	5.7869864
TRIM21	0.166027	5.9417542	5.1756645	5.35E-07	8.40E-06	5.7835343
CTC-360J11	-1.22415	0.2753087	-5.174923	5.37E-07	8.42E-06	5.7801837
ZCCHC16	1.8832478	1.6931907	5.1744972	5.38E-07	8.44E-06	5.7782572
NR4A3	0.5209374	4.7620904	5.173974	5.40E-07	8.45E-06	5.7758921
C14orf93	-0.119649	5.6188347	-5.173353	5.41E-07	8.47E-06	5.7730873
AC104088.1	-1.091856	-0.827728	-5.172768	5.43E-07	8.49E-06	5.7704427
AC007131.1	-1.457908	-0.520782	-5.17163	5.46E-07	8.54E-06	5.765301
POM121L9P	1.0470776	2.6940327	5.1711645	5.47E-07	8.55E-06	5.7631958
ABCA11P	-0.3975	4.0108188	-5.168967	5.52E-07	8.64E-06	5.7532673
ERBB2	0.2342057	6.5069787	5.168871	5.53E-07	8.64E-06	5.7528354
MT1L	0.6619666	4.6004012	5.1687474	5.53E-07	8.64E-06	5.7522769
EPHB6	0.4692372	5.1467774	5.1683933	5.54E-07	8.65E-06	5.7506775
THRSP	1.4706992	4.4269017	5.1681365	5.55E-07	8.66E-06	5.749518
CPEB2	0.1812816	5.9770986	5.1675132	5.56E-07	8.68E-06	5.7467031
E2F6	-0.119262	5.5862663	-5.167347	5.57E-07	8.68E-06	5.7459506
SNRPGP2	-0.206099	4.5939719	-5.166389	5.59E-07	8.71E-06	5.7416255
FAM163B	1.5832982	2.6449242	5.1663665	5.59E-07	8.71E-06	5.7415253
ASXL3	1.5699371	2.1255882	5.1662874	5.60E-07	8.71E-06	5.7411684
CYP1A2	1.8721576	3.5293481	5.1651122	5.63E-07	8.76E-06	5.7358629

EFHD1	0.3685825	5.5363847	5.1643884	5.65E-07	8.78E-06	5.7325955
CTD-2623N2	-0.973448	-0.633263	-5.164217	5.65E-07	8.79E-06	5.731821
RP11-624L4	-1.412755	2.4739874	-5.164173	5.65E-07	8.79E-06	5.7316229
KCTD1	0.261651	5.2875677	5.1637853	5.66E-07	8.80E-06	5.7298736
RP11-227L6	-0.932816	-0.70794	-5.163544	5.67E-07	8.81E-06	5.7287828
PSMC3IP	-0.300791	4.7396152	-5.16234	5.70E-07	8.85E-06	5.723353
AC008592.4	1.4612486	1.4073129	5.1621026	5.71E-07	8.86E-06	5.7222801
STXBP3	0.1040169	6.1226836	5.1615121	5.72E-07	8.88E-06	5.7196159
AC004593.3	-1.489537	1.4416775	-5.160668	5.75E-07	8.91E-06	5.715808
RP11-13N13	-1.221704	0.7268783	-5.158836	5.80E-07	8.98E-06	5.7075463
KARSP2	-0.895281	3.8356164	-5.158807	5.80E-07	8.98E-06	5.7074151
FBXO32	0.2939862	5.6041384	5.1577857	5.83E-07	9.02E-06	5.7028083
CTC-338M12	-1.121688	-0.744377	-5.157259	5.84E-07	9.04E-06	5.7004347
DENR	-0.075156	6.3645495	-5.157118	5.84E-07	9.04E-06	5.6997987
RP1-257A7.	-0.523234	3.4877145	-5.156495	5.86E-07	9.06E-06	5.6969867
SCARB2	0.0973235	6.8855386	5.1563813	5.86E-07	9.06E-06	5.6964761
ROBO4	0.2113876	5.9165514	5.1563451	5.86E-07	9.06E-06	5.696313
PLEKHG4	-0.529473	4.7375757	-5.155257	5.90E-07	9.11E-06	5.691408
MGRN1	0.1019617	6.5314862	5.1540771	5.93E-07	9.15E-06	5.6860901
RP11-88H9.	1.480161	2.0748901	5.1534772	5.94E-07	9.18E-06	5.6833867
CTD-3199J2	-0.578028	3.2783909	-5.153388	5.95E-07	9.18E-06	5.6829839
VAMP3	0.104378	6.3604996	5.1532585	5.95E-07	9.18E-06	5.6824009
COL23A1	0.6267324	4.0275876	5.152726	5.97E-07	9.20E-06	5.6800018
CKAP4	-0.124945	6.7057373	-5.152364	5.98E-07	9.21E-06	5.6783695
RP1-62D2.4	-0.837436	-0.938945	-5.151437	6.00E-07	9.25E-06	5.6741948
LINC01353	-1.101192	1.7125044	-5.150868	6.02E-07	9.27E-06	5.6716316
RP11-112J1	-1.152901	1.5364924	-5.14996	6.04E-07	9.30E-06	5.667541
HIST1H2AH	-0.944688	-0.643653	-5.149898	6.05E-07	9.30E-06	5.6672602
TRAF3IP2	0.2259558	5.7181675	5.1494098	6.06E-07	9.32E-06	5.6650631
SUV39H1	-0.150027	5.8403045	-5.148709	6.08E-07	9.35E-06	5.6619094
HERC2P5	-1.149379	-0.442458	-5.147365	6.12E-07	9.40E-06	5.6558559
TMEM233	0.4193426	4.0939825	5.1469455	6.13E-07	9.42E-06	5.6539672
AC068134.1	-0.985533	2.0623997	-5.146172	6.15E-07	9.45E-06	5.6504846
INHBA	0.3805592	5.5616104	5.1457648	6.17E-07	9.46E-06	5.6486521
OLMALINC	-0.272786	5.4639919	-5.145243	6.18E-07	9.48E-06	5.6463058
TLN2	0.2441889	5.806135	5.1452207	6.18E-07	9.48E-06	5.6462034
RTN4	0.1049607	6.9010629	5.144446	6.20E-07	9.51E-06	5.6427169
MAP4	0.0731813	6.8060201	5.1436213	6.23E-07	9.54E-06	5.6390055
UBC	0.0828369	7.1080904	5.1435698	6.23E-07	9.54E-06	5.6387739
RP11-265N7	-1.313955	1.3847274	-5.143054	6.25E-07	9.56E-06	5.6364542
FCHO2	0.1185243	6.0481243	5.1417255	6.28E-07	9.61E-06	5.6304766
C11orf1	0.2109564	5.5304082	5.1416325	6.29E-07	9.61E-06	5.6300583
LYSMD4	-0.181597	5.549387	-5.141271	6.30E-07	9.63E-06	5.6284315
FKBP6	-1.147035	-0.26475	-5.14077	6.31E-07	9.65E-06	5.6261799
AC142472.6	-0.351626	4.1795022	-5.140327	6.33E-07	9.66E-06	5.6241866
RP1-63M2.7	-0.734753	3.1669981	-5.139448	6.35E-07	9.70E-06	5.6202334
ABLIM2	0.4542769	5.1783652	5.1392383	6.36E-07	9.70E-06	5.61929
PLEKHH1	-0.299263	5.2454891	-5.138893	6.37E-07	9.72E-06	5.6177391
GSTZ1	0.2612497	6.0358394	5.1371381	6.42E-07	9.79E-06	5.6098477
JAZF1	0.2013617	5.9118608	5.1356494	6.47E-07	9.86E-06	5.603156
ANKLE1	-0.735569	3.2243118	-5.135191	6.48E-07	9.87E-06	5.6010953

UBA2	-0.083214	6.4711628	-5.133815	6.52E-07	9.93E-06	5.5949151
MS4A4E	0.8801067	2.6488651	5.13363	6.53E-07	9.94E-06	5.5940817
CTA-204B4.	-0.297047	4.5881913	-5.132875	6.55E-07	9.97E-06	5.5906911
GPC5	-1.26471	3.7540812	-5.132575	6.56E-07	9.98E-06	5.5893401
MYH9	0.0812131	7.2506193	5.1321195	6.58E-07	1.00E-05	5.587296
RAB9A	0.1181039	5.9333183	5.1313261	6.60E-07	1.00E-05	5.5837322
AC012360.6	1.19023	0.3162965	5.1303993	6.63E-07	1.01E-05	5.5795699
RP11-64C12	-1.195623	0.0088722	-5.129197	6.67E-07	1.01E-05	5.5741725
R3HDM1	-0.093852	6.0116451	-5.128353	6.69E-07	1.02E-05	5.5703817
SERPINA12	-1.170536	3.7004136	-5.128017	6.70E-07	1.02E-05	5.5688736
RP11-535A1	-1.214341	2.0664858	-5.127772	6.71E-07	1.02E-05	5.567775
BNIP3L	0.1324887	6.2223385	5.1276411	6.72E-07	1.02E-05	5.567186
MAGEA10	-1.517165	-0.318955	-5.127499	6.72E-07	1.02E-05	5.5665499
ZNF551	-0.333076	4.9239162	-5.127249	6.73E-07	1.02E-05	5.5654275
PHKB	0.1252248	6.3081082	5.1269712	6.74E-07	1.02E-05	5.5641794
MYOCD	1.0378002	3.5018994	5.1265489	6.75E-07	1.02E-05	5.5622837
PLA2G5	0.4759237	4.7623957	5.126121	6.76E-07	1.02E-05	5.5603633
RSPRY1	0.0995416	6.0706188	5.1256131	6.78E-07	1.03E-05	5.5580841
RP11-408B1	-1.515891	0.0075264	-5.125288	6.79E-07	1.03E-05	5.5566239
KDELC2	0.1964271	5.8781321	5.1249109	6.80E-07	1.03E-05	5.554933
NEIL2	0.1609921	5.713087	5.1248565	6.80E-07	1.03E-05	5.5546892
PNPLA8	0.1266505	6.1309566	5.1246995	6.81E-07	1.03E-05	5.5539847
RP1-267L14	-0.784401	2.2865895	-5.123782	6.84E-07	1.03E-05	5.5498672
C16orf46	0.3879728	3.8917498	5.123534	6.85E-07	1.03E-05	5.5487555
RP11-242J7	-1.639934	3.218109	-5.122534	6.88E-07	1.04E-05	5.5442683
RP11-17403	-0.893076	-0.964437	-5.120777	6.94E-07	1.05E-05	5.5363908
ATXN7L1	0.1880296	5.3948721	5.1183946	7.01E-07	1.06E-05	5.5257085
METTL2A	-0.102485	5.8146657	-5.118093	7.02E-07	1.06E-05	5.5243574
SHROOM3	0.2627678	6.0883281	5.1180658	7.03E-07	1.06E-05	5.5242346
RNF125	0.377942	5.4626484	5.1178095	7.03E-07	1.06E-05	5.5230856
PIAS4	-0.100032	6.0749655	-5.117326	7.05E-07	1.06E-05	5.520917
CYP4V2	0.220025	6.4441405	5.1170076	7.06E-07	1.06E-05	5.5194918
TTPAL	0.1677683	6.0688152	5.1158032	7.10E-07	1.07E-05	5.5140942
POLR3A	-0.096293	6.0416895	-5.114974	7.13E-07	1.07E-05	5.5103776
SUMO2	-0.079564	6.612415	-5.114453	7.15E-07	1.07E-05	5.5080443
IL6	1.163691	2.9183786	5.1144397	7.15E-07	1.07E-05	5.5079853
ANKRD44	0.197318	5.5594149	5.1138247	7.17E-07	1.08E-05	5.5052303
PSMB10	0.184986	6.0136869	5.1130549	7.19E-07	1.08E-05	5.5017821
LAPTM4A	0.0768969	6.9195251	5.1125169	7.21E-07	1.08E-05	5.4993726
TMEM231	0.4127109	4.6281256	5.1117461	7.24E-07	1.08E-05	5.4959203
MAPK13	-0.41014	5.8008068	-5.110789	7.27E-07	1.09E-05	5.4916341
FO538757.2	-0.24126	4.7468283	-5.110623	7.28E-07	1.09E-05	5.4908934
SELE	0.7672352	4.2505641	5.1101991	7.29E-07	1.09E-05	5.4889936
RP11-234A1	-0.30241	4.947456	-5.109247	7.32E-07	1.10E-05	5.4847328
WDR83	-0.139426	5.4843122	-5.108708	7.34E-07	1.10E-05	5.4823178
RPSAP4	-1.142005	0.6926122	-5.108086	7.36E-07	1.10E-05	5.4795361
RARG	0.2154758	5.4777877	5.1072079	7.39E-07	1.10E-05	5.4756047
SOX8	1.0756553	2.6736238	5.1066963	7.41E-07	1.11E-05	5.4733155
TEAD1	0.1424902	6.2686568	5.1059475	7.44E-07	1.11E-05	5.4699647
DPYSL3	0.298075	5.8446919	5.1058248	7.44E-07	1.11E-05	5.469416
POC1A	-0.201684	5.6088023	-5.105489	7.45E-07	1.11E-05	5.4679144

ZNF550	-0.290335	5.3008453	-5.10522	7.46E-07	1.11E-05	5.4667091
ADAM11	-0.774745	3.612603	-5.103397	7.53E-07	1.12E-05	5.4585572
CTC-453G23	-1.071838	0.3385512	-5.10321	7.53E-07	1.12E-05	5.4577181
NEBL	0.841498	4.7762448	5.103054	7.54E-07	1.12E-05	5.4570214
RASD1	0.4293222	6.0431898	5.102478	7.56E-07	1.13E-05	5.4544456
CLEC2B	0.2558206	5.3645087	5.1023839	7.56E-07	1.13E-05	5.4540246
PITPNM1	0.1707998	6.0354689	5.1010861	7.61E-07	1.13E-05	5.4482217
NR3C1	0.12328	6.4052921	5.1002982	7.64E-07	1.14E-05	5.4446995
ADAMTSL2	0.410377	5.8702778	5.0995837	7.66E-07	1.14E-05	5.4415054
PLCXD1	-0.240727	6.1152349	-5.099553	7.66E-07	1.14E-05	5.4413682
PCED1A	-0.12365	6.1556014	-5.099195	7.68E-07	1.14E-05	5.4397677
RP11-173M1	-1.169524	1.1989839	-5.098157	7.71E-07	1.15E-05	5.4351298
WWP2	0.0972276	6.3030165	5.0976648	7.73E-07	1.15E-05	5.4329292
RP5-1033H2	-0.528556	4.8796285	-5.097469	7.74E-07	1.15E-05	5.4320558
RP11-397A1	-1.373673	-0.454545	-5.097009	7.76E-07	1.15E-05	5.4300009
TMEM39A	-0.091906	6.0452366	-5.096615	7.77E-07	1.15E-05	5.4282372
EDC3	-0.090507	6.0559879	-5.096411	7.78E-07	1.15E-05	5.4273267
CLEC3B	0.4412777	5.377221	5.0959794	7.79E-07	1.15E-05	5.4253991
SQLE	-0.268721	6.4679891	-5.095403	7.81E-07	1.16E-05	5.4228253
AC098820.4	-1.111462	1.0541043	-5.095378	7.82E-07	1.16E-05	5.4227106
C1RL	0.1728936	6.6514225	5.0947307	7.84E-07	1.16E-05	5.4198209
CLOCK	0.1201195	6.0159599	5.0947281	7.84E-07	1.16E-05	5.4198091
COX7A1	0.3382938	4.9966357	5.0926497	7.92E-07	1.17E-05	5.4105272
CASC20	-1.588973	-0.106522	-5.092529	7.92E-07	1.17E-05	5.4099866
MLKL	0.1854112	5.5393509	5.0920314	7.94E-07	1.17E-05	5.4077667
CDC14B	0.2156659	6.0328293	5.0918255	7.95E-07	1.17E-05	5.4068473
LDHD	0.3594092	6.2862807	5.0901178	8.01E-07	1.18E-05	5.3992239
CASC4	0.1087769	6.4907211	5.0877506	8.10E-07	1.19E-05	5.3886598
RP11-1143G	-1.242767	3.62737	-5.084664	8.22E-07	1.21E-05	5.3748894
MFSD12	0.1211541	6.2738193	5.0840785	8.24E-07	1.21E-05	5.3722799
CTD-2116N2	-1.246808	0.6707066	-5.083913	8.25E-07	1.21E-05	5.3715397
HTR3B	-1.24944	-0.480707	-5.082583	8.30E-07	1.22E-05	5.3656119
TBC1D9	0.1758692	6.0489735	5.0811531	8.35E-07	1.23E-05	5.3592373
RP11-182J1	-1.040747	0.4933088	-5.081067	8.36E-07	1.23E-05	5.3588548
SNRPGP10	-0.816941	2.2168348	-5.080488	8.38E-07	1.23E-05	5.356271
ROR2	0.7946329	4.4946276	5.0799279	8.40E-07	1.24E-05	5.3537763
BHLHE40	0.1604787	6.6897096	5.0790476	8.44E-07	1.24E-05	5.3498535
RP1-167A14	-1.008099	1.0466019	-5.077602	8.49E-07	1.25E-05	5.3434128
MMS19	-0.084852	6.2848479	-5.076489	8.54E-07	1.25E-05	5.3384534
MSRB3	0.2691907	5.6084521	5.0756673	8.57E-07	1.26E-05	5.334795
GS1-166A23	-1.120621	1.5019525	-5.07541	8.58E-07	1.26E-05	5.3336479
RP11-367F2	-1.229324	0.5116984	-5.075006	8.60E-07	1.26E-05	5.3318497
KIF24	-0.331931	4.7857992	-5.07451	8.62E-07	1.26E-05	5.3296398
PPP2R2A	0.115892	6.0398089	5.074039	8.64E-07	1.27E-05	5.3275439
SNRPF	-0.131448	6.2580784	-5.073324	8.67E-07	1.27E-05	5.3243597
CTB-43E15.	1.2611393	-0.354347	5.07226	8.71E-07	1.28E-05	5.3196239
PCDHGC3	0.6353307	4.8597491	5.0721033	8.72E-07	1.28E-05	5.3189264
SLC22A18AS	0.5926625	5.0292072	5.0712664	8.75E-07	1.28E-05	5.3152012
SPG7	0.1032751	6.4090046	5.0702157	8.79E-07	1.29E-05	5.3105255
MAP3K14	0.1907799	5.9626636	5.0698338	8.81E-07	1.29E-05	5.3088261
RP11-424C2	-1.315957	2.1195107	-5.069515	8.82E-07	1.29E-05	5.3074083

NEU3	-0.143084	5.6453393	-5.069097	8.84E-07	1.29E-05	5.3055465
CHSY3	0.4450925	4.5037111	5.0690431	8.84E-07	1.29E-05	5.3053079
ZDHHC16	-0.114254	6.1523874	-5.068132	8.88E-07	1.30E-05	5.3012558
SGK1	0.2192492	6.3953353	5.0678152	8.89E-07	1.30E-05	5.2998452
ZNF37BP	-0.182128	5.3996666	-5.066754	8.94E-07	1.30E-05	5.2951234
RP11-395N3	-0.675331	3.3251328	-5.066618	8.94E-07	1.30E-05	5.2945195
RP5-908M14	-0.354191	4.4607634	-5.065436	8.99E-07	1.31E-05	5.2892656
EGR2	0.5079144	4.940518	5.0651576	9.00E-07	1.31E-05	5.2880255
RP11-480I1	-0.748651	3.7469182	-5.063833	9.06E-07	1.32E-05	5.2821346
AC090616.2	0.6630555	2.9066194	5.0638222	9.06E-07	1.32E-05	5.2820884
SDHA	0.1297093	6.848398	5.0622347	9.13E-07	1.33E-05	5.2750317
IFITM1	0.2814773	6.1329495	5.0608559	9.19E-07	1.34E-05	5.2689043
TAPBP	0.1136791	6.9766956	5.0606214	9.20E-07	1.34E-05	5.2678624
RPL7AP6	-0.380864	4.3070437	-5.059648	9.24E-07	1.34E-05	5.2635354
C2orf44	-0.128268	5.6131729	-5.059302	9.25E-07	1.34E-05	5.262002
NUP160	-0.099319	6.169213	-5.05901	9.27E-07	1.35E-05	5.260704
RP11-121C2	-0.253604	4.6372885	-5.057066	9.35E-07	1.36E-05	5.2520691
NCAM2	1.2762938	3.0231248	5.0568196	9.36E-07	1.36E-05	5.2509739
GTF2H4	-0.171328	4.9033329	-5.056818	9.36E-07	1.36E-05	5.2509686
PPFIBP1	0.1453573	6.0805521	5.0567811	9.36E-07	1.36E-05	5.2508029
TRIM11	-0.12419	6.0620471	-5.056074	9.39E-07	1.36E-05	5.2476647
GSPT1	0.0785942	6.565826	5.0557404	9.41E-07	1.36E-05	5.2461819
BMPR2	0.1219788	6.2706993	5.0554277	9.42E-07	1.36E-05	5.2447936
CTC-559E9.	-0.344142	4.0801263	-5.054275	9.47E-07	1.37E-05	5.2396753
KIAA1614-A	-1.158284	0.9899721	-5.053877	9.49E-07	1.37E-05	5.2379113
RPL39P36	-0.957573	1.6564138	-5.052876	9.53E-07	1.38E-05	5.2334671
ZNF800	0.106114	5.8066848	5.0523288	9.56E-07	1.38E-05	5.2310378
KMT2B	-0.112199	6.3731288	-5.051914	9.58E-07	1.38E-05	5.2291978
ZNF71	-0.252374	5.2705787	-5.049826	9.67E-07	1.40E-05	5.2199316
FAM103A1	-0.123017	5.7289545	-5.047189	9.79E-07	1.41E-05	5.2082392
AC002066.1	1.2310371	0.4656189	5.0434388	9.96E-07	1.44E-05	5.1916131
RNU6-817P	-1.065962	-9.15E-05	-5.042277	1.00E-06	1.45E-05	5.186465
GADD45B	0.2381369	6.5420108	5.0419053	1.00E-06	1.45E-05	5.1848177
GACAT3	-0.962454	-0.97221	-5.040736	1.01E-06	1.45E-05	5.1796361
DCAF16	-0.14805	5.841648	-5.040539	1.01E-06	1.45E-05	5.1787663
RP11-122G1	-0.976682	1.6380837	-5.040534	1.01E-06	1.45E-05	5.1787406
NUDT16P1	0.6756594	4.981399	5.0402695	1.01E-06	1.46E-05	5.1775707
NKX3-2	-1.582314	1.029077	-5.03985	1.01E-06	1.46E-05	5.1757133
GFPT2	0.7201685	4.5781284	5.0395304	1.01E-06	1.46E-05	5.1742972
SLC7A8	0.2959136	5.4030706	5.038285	1.02E-06	1.47E-05	5.1687819
RP11-20G13	-1.217411	0.8210069	-5.037799	1.02E-06	1.47E-05	5.1666287
RP11-77P6.	-0.419979	3.9105206	-5.03715	1.03E-06	1.47E-05	5.1637556
RPS18P12	-0.856001	2.7818097	-5.037017	1.03E-06	1.47E-05	5.163168
RP11-15N24	-0.831078	2.6244558	-5.035066	1.04E-06	1.49E-05	5.1545293
ADAMTSL1	0.526262	4.8360776	5.0347258	1.04E-06	1.49E-05	5.1530253
PLEC	0.1044377	6.9293821	5.0343541	1.04E-06	1.49E-05	5.1513803
CITF22-1A6	-0.421929	3.8742831	-5.034176	1.04E-06	1.49E-05	5.1505923
POSTN	0.6527488	5.6601643	5.0336768	1.04E-06	1.49E-05	5.1483829
PAPSS2	0.2357996	6.3749568	5.0335402	1.04E-06	1.50E-05	5.1477786
PALM2-AKAP	1.2415389	2.6092406	5.0331155	1.05E-06	1.50E-05	5.1458993
RPS10P16	-1.068813	0.6169262	-5.033017	1.05E-06	1.50E-05	5.1454631

RAP1GDS1	0.128655	6.0364996	5.0328348	1.05E-06	1.50E-05	5.1446573
CEP78	-0.140342	5.7733485	-5.032786	1.05E-06	1.50E-05	5.1444413
TPPP	0.5115767	5.3656912	5.0319826	1.05E-06	1.50E-05	5.140887
DAZAP2	0.080094	6.8326883	5.0315169	1.05E-06	1.51E-05	5.1388266
NBR2	-0.176852	5.2894222	-5.031412	1.05E-06	1.51E-05	5.1383634
COL10A1	1.5222075	3.1550195	5.0313762	1.05E-06	1.51E-05	5.1382043
FAM195B	-0.150334	6.5006987	-5.029938	1.06E-06	1.52E-05	5.1318438
FAM188A	0.1346566	5.6934567	5.0296723	1.06E-06	1.52E-05	5.1306684
LMOD1	0.3799744	5.3860323	5.0289201	1.07E-06	1.52E-05	5.1273421
RP11-342K2	-0.296806	4.3878093	-5.027771	1.07E-06	1.53E-05	5.1222596
VPS54	-0.097324	6.2298319	-5.027434	1.07E-06	1.53E-05	5.120772
TRPC5	1.6601471	1.4977037	5.0267015	1.08E-06	1.53E-05	5.1175333
MBNL3	-0.296384	6.6219644	-5.026337	1.08E-06	1.54E-05	5.1159239
TTC21B	-0.112285	5.7376848	-5.025492	1.08E-06	1.54E-05	5.1121857
DIRC2	0.128496	5.6842498	5.025434	1.08E-06	1.54E-05	5.1119312
FRAT2	-0.155781	5.9567346	-5.02445	1.09E-06	1.55E-05	5.1075819
CTC-329D1.	-0.383309	3.479664	-5.023852	1.09E-06	1.55E-05	5.1049396
MNX1-AS1	-1.841377	0.69578	-5.023752	1.09E-06	1.55E-05	5.1044971
AMOTL2	0.2044205	6.047754	5.0232133	1.09E-06	1.56E-05	5.1021188
IPO9-AS1	-0.975421	1.7684162	-5.022402	1.10E-06	1.56E-05	5.0985353
LIMS2	0.2505223	6.1299623	5.0214549	1.10E-06	1.57E-05	5.0943516
LINC00167	-1.057714	0.0876788	-5.02083	1.11E-06	1.57E-05	5.0915912
RP11-184A2	-1.229827	-0.275062	-5.020208	1.11E-06	1.58E-05	5.0888456
PROCA1	-0.339759	4.4619864	-5.019961	1.11E-06	1.58E-05	5.0877558
C4orf36	0.2699201	4.5100759	5.0195728	1.11E-06	1.58E-05	5.0860402
C4orf46	-0.194787	5.1976056	-5.019069	1.12E-06	1.58E-05	5.0838147
C15orf27	-0.480149	4.3663521	-5.018329	1.12E-06	1.59E-05	5.0805479
WSCD1	-0.400325	5.1441342	-5.017675	1.12E-06	1.59E-05	5.0776619
ZDHHC15	1.0397963	2.5384237	5.0171604	1.13E-06	1.59E-05	5.0753903
SNHG21	-0.388029	3.8926304	-5.016941	1.13E-06	1.59E-05	5.0744201
VIM	0.1291549	6.9401962	5.016755	1.13E-06	1.60E-05	5.0736012
POLA2	-0.148883	5.8189906	-5.016409	1.13E-06	1.60E-05	5.0720742
C6orf48	-0.15397	6.4302276	-5.015845	1.13E-06	1.60E-05	5.0695837
AQP4	1.6050949	2.7635473	5.0156355	1.13E-06	1.60E-05	5.0686605
ZNF695	-1.644086	1.3900774	-5.01457	1.14E-06	1.61E-05	5.0639589
PCDH12	0.1789626	5.9079478	5.0142946	1.14E-06	1.61E-05	5.0627442
SULF2	0.2559055	6.3603305	5.0142456	1.14E-06	1.61E-05	5.0625283
BCL7A	-0.137697	5.9715773	-5.013726	1.14E-06	1.61E-05	5.0602363
GRSF1	0.0842543	6.4360533	5.0135407	1.14E-06	1.61E-05	5.0594184
SCAI	-0.149887	5.3500918	-5.012949	1.15E-06	1.62E-05	5.0568088
AP001628.6	-1.125845	0.5247497	-5.012875	1.15E-06	1.62E-05	5.0564803
KRTAP20-4	-1.039672	-0.976995	-5.012592	1.15E-06	1.62E-05	5.0552357
SMUG1	-0.101821	6.1446805	-5.011937	1.15E-06	1.62E-05	5.0523449
CTD-2233K9	-0.407995	3.4930216	-5.011541	1.16E-06	1.63E-05	5.0505981
MAB21L2	1.3662889	3.699866	5.010299	1.16E-06	1.64E-05	5.0451222
TMEM140	0.1685534	6.3593126	5.0101574	1.16E-06	1.64E-05	5.0444976
DNAJB14	0.120708	5.8936777	5.0097847	1.16E-06	1.64E-05	5.0428545
FHOD1	0.1701857	5.7181768	5.009442	1.17E-06	1.64E-05	5.0413437
KCNN2	0.7030535	3.7989665	5.0093971	1.17E-06	1.64E-05	5.0411459
LTBP2	0.2885711	6.1456699	5.0092458	1.17E-06	1.64E-05	5.040479
CYP19A1	-1.517431	2.3742533	-5.009027	1.17E-06	1.64E-05	5.0395145

PPP1R32	0.3495489	4.5202326	5.0089901	1.17E-06	1.64E-05	5.0393516
EDRF1	-0.11048	5.5828035	-5.008759	1.17E-06	1.64E-05	5.0383349
RP11-152N1	-0.436493	3.7912338	-5.008657	1.17E-06	1.64E-05	5.0378847
HIST1H2AJ	-1.015141	-0.495421	-5.008169	1.17E-06	1.65E-05	5.0357322
KCNE4	0.4901507	5.0963167	5.0076636	1.18E-06	1.65E-05	5.0335048
GNPTG	0.1247085	6.5629166	5.0073123	1.18E-06	1.65E-05	5.0319567
ARHGEF25	0.3248918	5.1296064	5.0068207	1.18E-06	1.65E-05	5.0297905
XYLT1	0.4192164	4.7330693	5.0065666	1.18E-06	1.65E-05	5.0286704
DEFB132	1.7440193	1.8581164	5.0064665	1.18E-06	1.65E-05	5.0282297
TMEM120B	-0.120447	5.7080826	-5.006261	1.18E-06	1.66E-05	5.027325
RP11-485M7	-1.13421	1.1500106	-5.005187	1.19E-06	1.66E-05	5.0225912
SMN1	-0.140509	5.2886537	-5.005064	1.19E-06	1.66E-05	5.0220506
LIME1	-0.289639	4.9500155	-5.002781	1.20E-06	1.68E-05	5.0119926
CCT7P2	-1.276154	-0.484434	-5.002629	1.20E-06	1.68E-05	5.011326
CTC-820M8.	-1.133672	0.9537081	-5.001573	1.21E-06	1.69E-05	5.0066766
ASPH	0.1717634	6.7593843	5.0015569	1.21E-06	1.69E-05	5.006604
GDNF	1.519618	2.6866094	5.0013974	1.21E-06	1.69E-05	5.005902
ZC3H13	0.1359663	6.2736508	5.0002406	1.22E-06	1.70E-05	5.0008088
TMEM154	0.5742787	4.7022082	4.9985153	1.23E-06	1.71E-05	4.9932148
FAM183A	-1.569007	0.910765	-4.998405	1.23E-06	1.71E-05	4.9927294
RP11-37L2.	-0.856902	3.0137532	-4.998148	1.23E-06	1.71E-05	4.9915985
AL133243.1	-0.323791	4.1143361	-4.997415	1.23E-06	1.72E-05	4.9883742
RP11-1020A	-0.943954	2.2352442	-4.997127	1.24E-06	1.72E-05	4.9871064
VWCE	-0.549134	5.4899504	-4.997009	1.24E-06	1.72E-05	4.9865877
SLC45A1	0.3666613	4.0196955	4.9965483	1.24E-06	1.72E-05	4.9845599
IFIT3	0.2179029	6.0610826	4.9965409	1.24E-06	1.72E-05	4.9845269
SLC25A30	0.2145744	6.227313	4.9964943	1.24E-06	1.72E-05	4.984322
API5	0.0791968	6.4388747	4.9964146	1.24E-06	1.72E-05	4.9839715
PCNPP1	-0.875004	1.8491539	-4.995939	1.24E-06	1.72E-05	4.9818785
RP11-893F2	-0.961231	1.8834247	-4.995223	1.25E-06	1.73E-05	4.9787298
GPD1	0.5022737	6.0741281	4.9938412	1.25E-06	1.74E-05	4.9726521
AKR1A1	0.1288885	6.7781276	4.9936335	1.26E-06	1.74E-05	4.9717388
GLRX	0.2413665	6.241904	4.9928422	1.26E-06	1.75E-05	4.9682593
CASP4	0.1354446	6.2914043	4.9927028	1.26E-06	1.75E-05	4.9676462
ZNF512B	-0.138469	6.2919912	-4.991589	1.27E-06	1.76E-05	4.9627513
RP11-778D9	-0.634351	3.4076477	-4.990978	1.27E-06	1.76E-05	4.9600621
OTOP3	-1.463084	-0.272242	-4.990608	1.27E-06	1.76E-05	4.9584371
RP11-712B9	0.9538171	-0.72573	4.9904607	1.27E-06	1.76E-05	4.9577894
TTC32	-0.205756	5.3266504	-4.989886	1.28E-06	1.77E-05	4.9552653
MTHFD1	0.197393	6.720049	4.9897574	1.28E-06	1.77E-05	4.9546985
TSPYL5	0.4183418	4.6353185	4.9889677	1.28E-06	1.77E-05	4.951228
CTD-2591A6	-1.631053	0.4309074	-4.988956	1.28E-06	1.77E-05	4.9511753
SCNN1A	0.474375	5.2329848	4.9888008	1.28E-06	1.77E-05	4.9504947
ABLIM1	0.1146717	6.4335988	4.9881514	1.29E-06	1.78E-05	4.9476411
DYNC2H1	0.4296763	4.6075793	4.987492	1.29E-06	1.78E-05	4.9447439
DPF3	0.6099483	4.0858754	4.9856485	1.30E-06	1.80E-05	4.9366459
CD74	0.1708814	7.345939	4.985559	1.30E-06	1.80E-05	4.9362527
ST6GALNAC1	1.2188863	2.2169455	4.9849897	1.31E-06	1.80E-05	4.9337526
C1orf220	-0.592745	3.7662495	-4.984676	1.31E-06	1.80E-05	4.9323731
SLC25A37	0.1738825	5.8805912	4.984395	1.31E-06	1.80E-05	4.9311409
SLCO3A1	0.2507464	5.7945876	4.9841699	1.31E-06	1.80E-05	4.9301527

CCDC37	-1.081448	0.6924805	-4.984168	1.31E-06	1.80E-05	4.9301435
C1QTNF2	0.7159808	3.7526872	4.9830749	1.32E-06	1.81E-05	4.9253447
CTB-50L17.	-0.422528	3.7597048	-4.98225	1.32E-06	1.82E-05	4.9217228
RBL2	0.1648587	6.2428457	4.9818279	1.33E-06	1.82E-05	4.9198704
TJAP1	-0.087562	6.1724481	-4.981718	1.33E-06	1.82E-05	4.9193882
KLHL36	0.116508	6.0696295	4.9815404	1.33E-06	1.82E-05	4.9186089
MKLN1	0.1168938	6.0762917	4.9791698	1.34E-06	1.84E-05	4.9082056
JPH2	0.767453	3.7874031	4.9790858	1.34E-06	1.84E-05	4.907837
KRT7	1.0317481	5.4072453	4.9785878	1.35E-06	1.85E-05	4.9056522
CDAN1	-0.106625	5.8212647	-4.978198	1.35E-06	1.85E-05	4.9039402
YOD1	-0.152212	5.7765025	-4.978179	1.35E-06	1.85E-05	4.9038598
ZMYND12	0.6516624	4.3484059	4.9771006	1.35E-06	1.86E-05	4.8991281
SNHG11	-0.191598	5.8090745	-4.976661	1.36E-06	1.86E-05	4.8972018
MAPKAPK2	0.1059048	6.6854018	4.9754105	1.37E-06	1.87E-05	4.8917159
MISP	-1.56658	3.7542241	-4.975382	1.37E-06	1.87E-05	4.8915892
CD47	0.1342664	6.3635484	4.975296	1.37E-06	1.87E-05	4.891214
MFSD1	0.1044	6.4114262	4.9750373	1.37E-06	1.87E-05	4.8900796
HADH	0.1682098	6.5639206	4.9745184	1.37E-06	1.88E-05	4.8878044
RP11-274M1	-1.367022	-0.442258	-4.973889	1.37E-06	1.88E-05	4.8850446
LINC00959	0.3782506	4.0321726	4.9738696	1.38E-06	1.88E-05	4.8849598
PEBP4	1.541609	0.8406258	4.9738201	1.38E-06	1.88E-05	4.884743
METTL3	-0.087219	6.0368904	-4.973636	1.38E-06	1.88E-05	4.8839343
TMEM260	-0.127603	5.9033236	-4.97271	1.38E-06	1.89E-05	4.8798771
IL18	0.3479942	5.2635355	4.9723083	1.39E-06	1.89E-05	4.8781159
RNF34	-0.084685	6.0018382	-4.972024	1.39E-06	1.89E-05	4.8768693
RP11-488L1	-0.249643	5.2776237	-4.970934	1.39E-06	1.90E-05	4.8720913
KAZALD1	-0.419101	4.7613531	-4.970841	1.39E-06	1.90E-05	4.871684
CTU1	-0.215017	5.1166406	-4.969608	1.40E-06	1.91E-05	4.8662822
SCARF1	0.1559851	5.6928824	4.9694101	1.40E-06	1.91E-05	4.8654165
ZC3H12A	0.2227577	5.8434673	4.9694062	1.40E-06	1.91E-05	4.8653994
CCNDBP1	0.1075198	6.1361266	4.9654892	1.43E-06	1.95E-05	4.8482449
THUMPD3-AS	-0.169234	5.5923674	-4.964171	1.44E-06	1.96E-05	4.8424756
RP11-96P7.	-1.141193	-0.626115	-4.963677	1.44E-06	1.96E-05	4.8403128
RP11-472I2	-0.997805	-0.259871	-4.963564	1.44E-06	1.96E-05	4.839817
RGS6	1.230389	2.0644065	4.9634579	1.44E-06	1.96E-05	4.8393528
NRXN2	0.7028094	4.8788578	4.9631513	1.44E-06	1.96E-05	4.838011
RP4-789D17	-1.0917	1.2063577	-4.963104	1.45E-06	1.96E-05	4.8378035
LILRA2	0.5379587	4.2082554	4.9627226	1.45E-06	1.97E-05	4.8361348
FAM98A	0.0859263	6.208766	4.9597295	1.47E-06	1.99E-05	4.8230393
ACTBP8	-1.550792	0.6496765	-4.959029	1.47E-06	2.00E-05	4.8199756
RP3-375P9.	-0.786347	2.703127	-4.958978	1.47E-06	2.00E-05	4.819753
SNRPG	-0.100749	6.2036493	-4.95896	1.47E-06	2.00E-05	4.8196717
SNTB2	0.107582	5.8681977	4.9573949	1.48E-06	2.01E-05	4.8128292
KCTD9P4	1.2233688	-0.049906	4.9572734	1.48E-06	2.01E-05	4.8122981
CCL11	1.4149858	2.3273615	4.9565043	1.49E-06	2.02E-05	4.8089354
C2orf82	-0.418283	5.7401446	-4.956468	1.49E-06	2.02E-05	4.8087745
PROCR	0.2934567	5.3228659	4.9557649	1.49E-06	2.02E-05	4.8057026
GNB4	0.2031984	5.5548235	4.9525068	1.52E-06	2.05E-05	4.791464
XXbac-BPG2	-0.247566	5.185352	-4.952231	1.52E-06	2.05E-05	4.7902581
RMST	-1.560415	1.5886675	-4.951797	1.52E-06	2.06E-05	4.7883623
RNY4P10	-0.812175	2.0899923	-4.950841	1.53E-06	2.07E-05	4.7841885

STAM-AS1	-0.99046	2.2918586	-4.950702	1.53E-06	2.07E-05	4.7835786
GIMAP8	0.2137264	5.7117164	4.9502797	1.53E-06	2.07E-05	4.781735
CPO	1.2442559	1.1608715	4.9494828	1.54E-06	2.08E-05	4.7782548
COLCA1	-0.66895	4.669403	-4.949375	1.54E-06	2.08E-05	4.7777823
BOLL	-0.798056	-0.956839	-4.949305	1.54E-06	2.08E-05	4.7774773
MFN2	0.1123098	6.6540921	4.949137	1.54E-06	2.08E-05	4.7767446
GSTA6P	-1.059597	-0.071476	-4.94879	1.54E-06	2.08E-05	4.7752311
WRNIP1	-0.09168	6.3403543	-4.94866	1.54E-06	2.08E-05	4.7746635
CTD-2034I2	1.5060952	3.1416316	4.9481286	1.55E-06	2.08E-05	4.7723414
MYH13	-1.419476	0.0958819	-4.947254	1.55E-06	2.09E-05	4.768521
C8orf4	0.2632435	6.1672274	4.946923	1.56E-06	2.09E-05	4.7670779
ANGPTL6	0.5301637	4.7331444	4.9468177	1.56E-06	2.09E-05	4.7666184
CDK3	-0.54409	3.7026038	-4.946285	1.56E-06	2.10E-05	4.7642925
IGF2-AS	-1.921743	1.7915681	-4.945366	1.57E-06	2.11E-05	4.7602803
DNASE2	0.1763711	6.3846917	4.9450717	1.57E-06	2.11E-05	4.7589975
RP11-146N2	-1.048156	1.2772597	-4.943744	1.58E-06	2.12E-05	4.7532054
SLC39A5	-0.749266	6.0548654	-4.943597	1.58E-06	2.12E-05	4.7525641
RP11-308B1	-1.478246	-0.097477	-4.943595	1.58E-06	2.12E-05	4.7525556
AC006277.3	-0.661768	3.1513241	-4.942614	1.59E-06	2.13E-05	4.7482758
FAM129B	0.1686257	6.452058	4.9426028	1.59E-06	2.13E-05	4.7482254
LINC00879	-1.420053	-0.492362	-4.942177	1.59E-06	2.13E-05	4.7463687
AGBL2	-0.426471	4.6625273	-4.942052	1.59E-06	2.13E-05	4.7458214
MYL6B	-0.162877	6.0384644	-4.941318	1.60E-06	2.14E-05	4.742622
SLC22A1	0.7203343	5.9445711	4.9410571	1.60E-06	2.14E-05	4.7414832
PLB1	0.3785571	4.4625874	4.9408492	1.60E-06	2.14E-05	4.7405764
ART3	-1.364693	1.1807874	-4.940464	1.60E-06	2.15E-05	4.7388965
HSPA14	-0.091261	5.9172894	-4.940419	1.60E-06	2.15E-05	4.7386998
CTD-2568A1	-1.048662	-0.222999	-4.940346	1.60E-06	2.15E-05	4.7383808
GRAP	0.4698087	4.0103396	4.9398452	1.61E-06	2.15E-05	4.7361984
PRR19	-0.6771	4.0354418	-4.939246	1.61E-06	2.15E-05	4.7335846
CTC-471F3.	-0.635561	3.6917602	-4.938358	1.62E-06	2.16E-05	4.7297122
GAL3ST4	0.4262103	4.895642	4.9373743	1.63E-06	2.17E-05	4.7254261
USP49	-0.14921	5.301052	-4.936685	1.63E-06	2.18E-05	4.722421
RP11-390F4	-0.674222	4.4510344	-4.936444	1.63E-06	2.18E-05	4.7213698
FSCN1	0.1659197	6.2352429	4.9362331	1.63E-06	2.18E-05	4.7204524
LYG2	-1.165095	1.2390458	-4.936207	1.64E-06	2.18E-05	4.7203386
PPP1R14A	0.3266479	5.2147959	4.9361173	1.64E-06	2.18E-05	4.719948
ZNF420	-0.154924	5.3217923	-4.93526	1.64E-06	2.19E-05	4.7162117
RP11-63A1.	-1.062051	0.143998	-4.935079	1.64E-06	2.19E-05	4.7154231
GABRP	1.4422647	2.6754621	4.9348762	1.65E-06	2.19E-05	4.7145402
RP11-384C4	-0.981133	1.1783288	-4.934712	1.65E-06	2.19E-05	4.7138256
KAZN	0.4334279	4.7122646	4.9345494	1.65E-06	2.19E-05	4.713116
RP11-47I22	1.3623514	1.0296855	4.9341408	1.65E-06	2.20E-05	4.711336
EML3	0.0881405	6.2401944	4.9340316	1.65E-06	2.20E-05	4.7108605
ADSL	-0.106739	6.3652444	-4.933852	1.65E-06	2.20E-05	4.7100784
GSTM5	1.2331158	3.1859727	4.9337609	1.65E-06	2.20E-05	4.7096812
RP11-485G7	-1.150431	0.9543149	-4.933534	1.66E-06	2.20E-05	4.7086914
HSPB6	0.3999241	5.551178	4.9320903	1.67E-06	2.21E-05	4.7024046
RP11-923I1	-0.621183	4.7809701	-4.932075	1.67E-06	2.21E-05	4.7023367
SLC9A9	0.2872818	5.214514	4.9310118	1.67E-06	2.22E-05	4.697708
EYA2	0.9852387	3.5373621	4.9303588	1.68E-06	2.23E-05	4.6948647

RP11-400N1	1.3932251	0.6108973	4.9299666	1.68E-06	2.23E-05	4.6931572
ECT2	-0.249172	5.7742733	-4.929846	1.68E-06	2.23E-05	4.6926315
PTGS1	0.3315327	5.2545624	4.9289109	1.69E-06	2.24E-05	4.6885616
GATSL3	0.3082657	4.3545291	4.9289058	1.69E-06	2.24E-05	4.6885393
HSF2	-0.141277	5.5351605	-4.928728	1.69E-06	2.24E-05	4.6877642
RP11-567M1	-0.225788	4.8555897	-4.927044	1.71E-06	2.26E-05	4.6804384
APBB2	0.1307126	6.1381306	4.9269734	1.71E-06	2.26E-05	4.6801293
CES2	0.2706937	6.9072811	4.9268508	1.71E-06	2.26E-05	4.6795956
RP11-5017.	-0.392156	4.2291201	-4.926701	1.71E-06	2.26E-05	4.6789453
KCP	-0.623091	4.0263523	-4.926355	1.71E-06	2.26E-05	4.6774364
NMB	-0.313509	5.1303624	-4.92583	1.71E-06	2.26E-05	4.6751557
GYPC	0.2088064	5.8843829	4.9247166	1.72E-06	2.28E-05	4.6703105
TYMP	0.1985088	6.5967138	4.9228887	1.74E-06	2.29E-05	4.6623605
RP11-383I2	-1.162878	0.9886089	-4.921661	1.75E-06	2.31E-05	4.6570205
PGK1	0.1095438	6.9570046	4.9212964	1.75E-06	2.31E-05	4.6554371
PANX3	-1.081416	-0.679588	-4.921126	1.75E-06	2.31E-05	4.6546978
SLC16A4	0.4640453	4.6784134	4.9204313	1.76E-06	2.32E-05	4.6516762
MAP7D2	-1.480532	3.3324577	-4.919558	1.76E-06	2.32E-05	4.6478805
POTEKP	-1.226372	-0.280935	-4.918028	1.78E-06	2.34E-05	4.6412331
GPR125	0.1971673	6.2531727	4.9179788	1.78E-06	2.34E-05	4.6410173
P4HA3	0.5615788	4.1414999	4.9173824	1.78E-06	2.35E-05	4.638426
ADH1C	0.7390723	6.4119186	4.9170277	1.79E-06	2.35E-05	4.6368849
SAPCD1	-0.879253	3.109166	-4.91603	1.79E-06	2.36E-05	4.632552
SNRPE	-0.126844	6.4095243	-4.91487	1.80E-06	2.37E-05	4.6275118
CAMTA2	0.1102023	6.0889291	4.9148283	1.80E-06	2.37E-05	4.6273312
ZNF285	-0.583232	4.1355809	-4.914607	1.80E-06	2.37E-05	4.6263687
RP11-384L8	-0.512342	5.2554459	-4.914005	1.81E-06	2.38E-05	4.6237552
ZNF772	-0.324733	4.9474286	-4.912907	1.82E-06	2.39E-05	4.6189895
HEATR6	-0.116527	5.6617991	-4.912593	1.82E-06	2.39E-05	4.6176267
DDX60	0.2927326	5.8624062	4.9123913	1.82E-06	2.39E-05	4.6167492
NPAS3	1.0775312	2.4470078	4.9120887	1.83E-06	2.39E-05	4.6154358
FLJ39080	-1.531223	0.5122461	-4.911616	1.83E-06	2.40E-05	4.6133835
RP11-158L1	-0.748981	3.3935468	-4.911281	1.83E-06	2.40E-05	4.6119315
RP11-7F18.	-1.087742	1.7287651	-4.910417	1.84E-06	2.41E-05	4.6081794
SIRPB2	0.6156391	4.2094949	4.9103464	1.84E-06	2.41E-05	4.607873
RDM1	-0.984007	3.542903	-4.910251	1.84E-06	2.41E-05	4.6074598
TCEAL8	-0.215352	6.1525488	-4.909824	1.84E-06	2.41E-05	4.6056043
ZNF232	-0.150142	5.4426063	-4.90918	1.85E-06	2.42E-05	4.6028134
KLF6	0.1599919	6.6410147	4.90866	1.85E-06	2.42E-05	4.6005551
RP11-251M1	0.9633344	2.7204923	4.9082613	1.86E-06	2.43E-05	4.5988256
RP1-67A8.3	-1.034208	-0.573392	-4.908115	1.86E-06	2.43E-05	4.5981897
SNORA13	-1.004442	0.3918449	-4.907377	1.87E-06	2.44E-05	4.5949884
UBA7	0.1687017	6.3133209	4.9065752	1.87E-06	2.44E-05	4.5915114
ZC3H12C	0.2004159	5.4510787	4.9063959	1.87E-06	2.45E-05	4.5907337
RP11-474G2	-0.938958	2.1786256	-4.90602	1.88E-06	2.45E-05	4.5891016
CAPS2	0.4175413	4.2885154	4.9058842	1.88E-06	2.45E-05	4.5885145
VWA5B2	-0.903962	2.7948823	-4.905788	1.88E-06	2.45E-05	4.5880973
ATP5F1P3	-1.005047	-0.895982	-4.905764	1.88E-06	2.45E-05	4.587993
FAR2P4	-1.485693	-0.185188	-4.903876	1.90E-06	2.47E-05	4.5798047
C1QL4	-1.484409	1.8434737	-4.903789	1.90E-06	2.47E-05	4.5794302
RP11-43F13	1.3319998	2.6720736	4.9031634	1.90E-06	2.48E-05	4.5767175

NUBP1	0.0996083	5.8512163	4.9030557	1.90E-06	2.48E-05	4.5762504
FBXL17	0.1249097	5.9823756	4.9021412	1.91E-06	2.49E-05	4.5722865
LENG8-AS1	-0.288746	4.3321909	-4.900254	1.93E-06	2.51E-05	4.5641086
PTBP1	-0.061801	6.8768649	-4.898693	1.94E-06	2.52E-05	4.5573449
STRADA	-0.125023	5.3572518	-4.89819	1.95E-06	2.53E-05	4.5551687
SMIM14	0.1726235	6.7307192	4.8980131	1.95E-06	2.53E-05	4.5544003
ITGB3	0.6372894	4.1453243	4.8970548	1.96E-06	2.54E-05	4.5502501
RNU6-77P	-0.757041	-1.153609	-4.896795	1.96E-06	2.54E-05	4.5491259
SCD5	0.4820967	4.8933725	4.8956582	1.97E-06	2.55E-05	4.5442028
GHITM	0.1089711	6.8642031	4.8955631	1.97E-06	2.55E-05	4.5437908
BMP4	-0.325937	5.3705981	-4.894922	1.97E-06	2.56E-05	4.5410147
WASH3P	-0.155158	5.3312534	-4.894514	1.98E-06	2.56E-05	4.5392493
RP11-399K2	-1.11679	0.0137424	-4.894211	1.98E-06	2.57E-05	4.5379385
CTD-2008P7	-1.577683	0.0390633	-4.894099	1.98E-06	2.57E-05	4.5374511
MTUS1	0.156106	6.4663233	4.8937403	1.99E-06	2.57E-05	4.5359
NFAM1	0.301261	5.2739912	4.8929723	1.99E-06	2.58E-05	4.5325759
AC005307.3	-1.471927	0.0675887	-4.892903	1.99E-06	2.58E-05	4.532278
COX19	-0.132865	5.7763369	-4.890905	2.01E-06	2.60E-05	4.523631
DNAH14	-0.180652	5.5247403	-4.890455	2.02E-06	2.61E-05	4.5216822
RP11-106A1	-1.055875	0.3908836	-4.88962	2.02E-06	2.61E-05	4.5180732
MSRA	0.2077856	6.0318777	4.8893904	2.02E-06	2.62E-05	4.5170788
ROM1	0.2260912	4.8858892	4.8885012	2.03E-06	2.63E-05	4.513233
TRPC4	0.7954921	3.4622167	4.8882504	2.04E-06	2.63E-05	4.5121483
C16orf59	-0.350776	5.1554263	-4.888034	2.04E-06	2.63E-05	4.5112135
ZNF512	-0.12513	5.8521987	-4.887771	2.04E-06	2.63E-05	4.5100748
RP11-115C1	1.8032984	2.2379806	4.8873965	2.04E-06	2.64E-05	4.5084556
RP11-169F1	-1.822442	0.7665905	-4.886157	2.06E-06	2.65E-05	4.503099
PRSS36	0.3391606	4.4342877	4.8860541	2.06E-06	2.65E-05	4.5026519
ZNF547	-0.345866	4.4377981	-4.885654	2.06E-06	2.65E-05	4.5009207
KLHL4	0.7461007	3.4950552	4.884209	2.07E-06	2.67E-05	4.4946768
CCDC158	0.67402	3.9982714	4.8837393	2.08E-06	2.68E-05	4.4926472
RP11-567N4	-0.858839	-0.93686	-4.882882	2.09E-06	2.68E-05	4.4889428
AC139452.2	-1.070697	0.516198	-4.882097	2.09E-06	2.69E-05	4.4855522
CCT6P1	-0.184398	4.9631988	-4.881665	2.10E-06	2.70E-05	4.4836844
PDZRN3	0.8775383	4.2101937	4.8815791	2.10E-06	2.70E-05	4.4833136
RP11-3P17.	-0.351893	4.4702687	-4.881573	2.10E-06	2.70E-05	4.4832876
BLCAP	0.111143	6.4728396	4.8814457	2.10E-06	2.70E-05	4.4827374
TMEM198	-0.308075	5.3300908	-4.881218	2.10E-06	2.70E-05	4.4817533
PRSS50	-1.254872	2.8079298	-4.880972	2.10E-06	2.70E-05	4.4806919
ANO7	-0.277833	4.9057034	-4.880674	2.11E-06	2.70E-05	4.4794027
CTD-2091N2	-1.166535	-0.458035	-4.880571	2.11E-06	2.70E-05	4.4789595
RP11-1275H	-0.398723	4.2772883	-4.880556	2.11E-06	2.70E-05	4.4788924
RPS3AP54	-1.053604	1.2664198	-4.88028	2.11E-06	2.71E-05	4.4777044
ZSCAN16	-0.214507	5.1560014	-4.880257	2.11E-06	2.71E-05	4.4776016
EFS	0.5349541	4.270644	4.8797749	2.12E-06	2.71E-05	4.4755211
ACADM	0.1914987	6.5270351	4.8795215	2.12E-06	2.71E-05	4.4744266
AC006460.2	1.1949825	0.2925245	4.879514	2.12E-06	2.71E-05	4.4743942
NDFIP1	0.1047908	6.7969409	4.8794366	2.12E-06	2.71E-05	4.47406
DYSF	0.1963775	6.282354	4.8788653	2.12E-06	2.72E-05	4.4715928
AC005281.1	-1.161239	-0.306174	-4.878431	2.13E-06	2.72E-05	4.4697178
POLA1	-0.152409	5.6718728	-4.877081	2.14E-06	2.74E-05	4.4638881

SHE	0.3126888	5.6196605	4.876956	2.14E-06	2.74E-05	4.4633498
bP-21201H5	-1.370239	0.0582238	-4.876176	2.15E-06	2.75E-05	4.4599837
RNF43	-0.484456	5.8537756	-4.875586	2.16E-06	2.75E-05	4.457437
CTB-63M22.	-0.380592	4.6669614	-4.875517	2.16E-06	2.75E-05	4.4571406
CAPN10-AS1	-0.411684	4.4068993	-4.874297	2.17E-06	2.77E-05	4.4518738
BCAP29	0.0810417	6.2673468	4.8739089	2.17E-06	2.77E-05	4.4502
RIPPLY3	-1.272792	2.7757819	-4.873144	2.18E-06	2.78E-05	4.4469021
RIBC2	-0.832142	3.9909583	-4.872603	2.19E-06	2.79E-05	4.4445674
RPS7P10	-0.444314	3.6462953	-4.871952	2.19E-06	2.79E-05	4.4417563
SLC6A19	-1.78082	1.8926712	-4.870749	2.20E-06	2.81E-05	4.4365685
SPATS2	-0.141327	5.9728675	-4.870594	2.21E-06	2.81E-05	4.4359002
SLC8A1	0.2995383	5.0693448	4.8705612	2.21E-06	2.81E-05	4.4357602
PPARG	-0.209819	5.9526056	-4.870418	2.21E-06	2.81E-05	4.4351422
KLF2	0.2357381	5.7221464	4.8703561	2.21E-06	2.81E-05	4.434876
AF127577.8	-1.125751	-0.83275	-4.869822	2.21E-06	2.82E-05	4.4325736
ABHD4	0.1345674	6.2914224	4.8686937	2.22E-06	2.83E-05	4.4277086
ORC2	-0.103092	5.8437006	-4.867496	2.24E-06	2.84E-05	4.4225454
GYPE	1.1514997	0.9806067	4.8666662	2.25E-06	2.85E-05	4.41897
TUBG1	-0.124656	6.2429241	-4.865402	2.26E-06	2.87E-05	4.4135224
TSPAN7	-0.27775	5.7437311	-4.864982	2.26E-06	2.87E-05	4.4117141
FAT1	0.1990103	6.5961193	4.8649381	2.26E-06	2.87E-05	4.4115239
LBH	0.1761994	6.1540281	4.8636229	2.28E-06	2.89E-05	4.4058582
RPS3AP53	-0.625144	-1.18248	-4.862957	2.28E-06	2.90E-05	4.4029919
SEMA4G	-0.248095	6.4726462	-4.862908	2.28E-06	2.90E-05	4.4027772
GABARAPL2	0.0919504	6.3785472	4.8627033	2.29E-06	2.90E-05	4.4018977
TUSC1	0.3819415	5.3398055	4.8625241	2.29E-06	2.90E-05	4.4011257
CTC-471J1.	-0.395425	3.9752608	-4.862194	2.29E-06	2.90E-05	4.3997042
HPDL	-0.946159	3.034833	-4.861756	2.30E-06	2.91E-05	4.3978201
LUZP1	0.1173607	5.848887	4.8616807	2.30E-06	2.91E-05	4.3974941
RBPMS	0.1354538	6.369811	4.8614773	2.30E-06	2.91E-05	4.3966182
THOC5	-0.107477	6.085222	-4.861035	2.30E-06	2.91E-05	4.3947157
YTHDF2	0.0717201	6.3625592	4.8609166	2.30E-06	2.91E-05	4.3942038
USP2	0.3614621	5.4196462	4.8608331	2.31E-06	2.91E-05	4.3938444
EHD4	0.1137595	6.3874051	4.860728	2.31E-06	2.91E-05	4.393392
TBCCD1	-0.10675	5.7345579	-4.859366	2.32E-06	2.93E-05	4.3875305
SEPT14	-1.358073	-0.548781	-4.858858	2.33E-06	2.94E-05	4.3853432
KIAA1683	0.3867898	4.8399057	4.8586172	2.33E-06	2.94E-05	4.384306
RP11-274H2	-0.606683	3.9418808	-4.857988	2.34E-06	2.95E-05	4.3815967
CCDC85A	1.1476464	1.9205216	4.8574356	2.34E-06	2.95E-05	4.3792213
RP11-187C1	-1.14273	1.6777319	-4.857217	2.34E-06	2.96E-05	4.3782816
KLF4	0.3583461	5.5849992	4.8571499	2.34E-06	2.96E-05	4.377992
CACYBPP2	-0.876498	1.8077154	-4.856905	2.35E-06	2.96E-05	4.3769394
MPDZ	0.2247703	6.1523557	4.8561814	2.35E-06	2.97E-05	4.3738252
ITPKB	0.1876084	5.7199837	4.8560649	2.36E-06	2.97E-05	4.3733239
ZNF569	-0.281421	5.1533245	-4.855712	2.36E-06	2.97E-05	4.3718065
TMEM191C	-0.743304	2.7867902	-4.854451	2.37E-06	2.99E-05	4.3663834
SORCS1	-1.656823	0.9560813	-4.853599	2.38E-06	3.00E-05	4.3627164
SLC40A1	0.1330502	6.8391212	4.8529789	2.39E-06	3.00E-05	4.3600512
AKAP13	0.115996	6.4926917	4.8527972	2.39E-06	3.01E-05	4.3592699
NANOGP1	-1.360286	0.2290435	-4.852746	2.39E-06	3.01E-05	4.3590497
AC093162.3	-1.084666	1.249779	-4.851919	2.40E-06	3.02E-05	4.3554933

AC000403.4	0.9750176	2.133553	4.8511309	2.41E-06	3.02E-05	4.3521065
AC012314.8	-0.345608	3.9509887	-4.851107	2.41E-06	3.02E-05	4.3520015
NUDT10	1.344452	0.6374796	4.8508683	2.41E-06	3.03E-05	4.3509776
PTP4A2	0.0817761	6.6786528	4.8504444	2.42E-06	3.03E-05	4.3491556
LINC00637	-1.056597	-0.027566	-4.849798	2.42E-06	3.04E-05	4.3463791
HCG18	-0.136113	5.8092817	-4.849304	2.43E-06	3.05E-05	4.3442552
ITIH6	-1.003402	-0.674552	-4.848141	2.44E-06	3.06E-05	4.339257
HNRNPA3P6	-0.326216	3.958517	-4.846545	2.46E-06	3.08E-05	4.3324017
BCO1	1.3885251	2.539382	4.8464811	2.46E-06	3.08E-05	4.3321267
AC090945.1	-1.192003	0.3660577	-4.846037	2.47E-06	3.09E-05	4.3302184
CTC-458G6.	-1.191611	-0.589614	-4.845658	2.47E-06	3.09E-05	4.3285908
RP11-403I1	1.5491001	3.1539274	4.8455517	2.47E-06	3.09E-05	4.3281348
MAPKAPK5	-0.073276	6.1027864	-4.845039	2.48E-06	3.10E-05	4.3259347
MAPK11	0.2392114	5.5554078	4.8447202	2.48E-06	3.10E-05	4.3245642
RET	0.898517	3.5689892	4.8441716	2.49E-06	3.11E-05	4.3222085
CPEB3	0.2568642	5.5208582	4.8428995	2.50E-06	3.12E-05	4.3167474
RP11-47P18	-1.133531	-0.530151	-4.841153	2.52E-06	3.15E-05	4.30925
SNAI2	0.3361697	5.4653807	4.8408465	2.52E-06	3.15E-05	4.3079357
F2RL1	0.5442835	5.5957628	4.8407332	2.53E-06	3.15E-05	4.3074497
ABCA8	0.5613499	5.5042288	4.8406215	2.53E-06	3.15E-05	4.3069703
KDR	0.2283612	6.063656	4.8401019	2.53E-06	3.16E-05	4.3047408
LAP3	0.1616057	6.6300278	4.8398742	2.53E-06	3.16E-05	4.3037637
WASH7P	-0.212562	5.8677268	-4.839794	2.54E-06	3.16E-05	4.303418
FAM198B	0.2199226	5.846623	4.839791	2.54E-06	3.16E-05	4.3034066
CCNI	0.0994085	6.6774911	4.8397719	2.54E-06	3.16E-05	4.3033247
TRIM69	0.1452861	6.0595901	4.8394315	2.54E-06	3.16E-05	4.3018642
S1PR3	0.2883847	5.6996252	4.8394118	2.54E-06	3.16E-05	4.3017799
LAMC3	0.3977067	5.7708197	4.8388168	2.55E-06	3.17E-05	4.2992273
UBAP2L	-0.083291	6.6797361	-4.838727	2.55E-06	3.17E-05	4.2988419
RP11-848P1	-0.782773	2.3817164	-4.838015	2.56E-06	3.18E-05	4.2957896
BEAN1-AS1	1.0118735	-0.309632	4.8376391	2.56E-06	3.18E-05	4.2941754
TMEM86A	0.2173482	5.436976	4.8376038	2.56E-06	3.18E-05	4.2940242
FRMD3	0.3087053	4.8197802	4.8373494	2.56E-06	3.18E-05	4.2929331
RP11-126L1	-0.982816	2.100812	-4.837118	2.57E-06	3.19E-05	4.2919398
OR8A1	-0.991714	-0.823299	-4.836801	2.57E-06	3.19E-05	4.2905793
GPR176	0.2594718	5.3933961	4.8365444	2.57E-06	3.19E-05	4.2894807
COQ10B	0.0959507	6.1907163	4.8357959	2.58E-06	3.20E-05	4.2862711
LINC00987	0.4771774	4.6048982	4.834845	2.59E-06	3.22E-05	4.2821944
RP11-506M1	-1.030205	2.0587101	-4.832326	2.62E-06	3.25E-05	4.2713979
GDPD3	-0.339708	4.5246655	-4.831977	2.63E-06	3.26E-05	4.2699029
ANKFY1	0.1130851	6.1875646	4.831266	2.64E-06	3.26E-05	4.2668555
CASC3	-0.073529	6.4358693	-4.831264	2.64E-06	3.26E-05	4.2668459
WDR88	-0.374018	3.7740734	-4.831053	2.64E-06	3.27E-05	4.2659409
RP11-510M2	1.6472217	0.6873439	4.8308809	2.64E-06	3.27E-05	4.2652052
KCTD15	0.2932639	5.6136313	4.8303258	2.65E-06	3.27E-05	4.2628271
MAFA-AS1	-1.489393	0.62433	-4.83031	2.65E-06	3.27E-05	4.2627609
CENPV	-0.23843	6.0567403	-4.829463	2.66E-06	3.28E-05	4.2591323
ADAM15	0.1390238	6.5544891	4.8290161	2.66E-06	3.29E-05	4.2572172
CLASRP	-0.099888	6.1835991	-4.828755	2.67E-06	3.29E-05	4.2561007
MEP1AP4	-0.824795	-1.039716	-4.828671	2.67E-06	3.29E-05	4.2557385
KIF17	0.296673	4.7418414	4.8285516	2.67E-06	3.29E-05	4.255228

LL09NC01-1	-0.932486	2.2156387	-4.828342	2.67E-06	3.30E-05	4.2543285
LAT2	0.2063511	5.5264402	4.8272955	2.68E-06	3.31E-05	4.249849
ASB16	-0.46263	4.1550303	-4.8257	2.70E-06	3.33E-05	4.2430189
KCNK17	1.0707602	3.0158789	4.8256526	2.70E-06	3.33E-05	4.2428153
AC016735.1	-1.462399	2.1599487	-4.825242	2.71E-06	3.34E-05	4.2410585
CD14	0.2399469	6.8387552	4.8249905	2.71E-06	3.34E-05	4.2399816
BEAN1	0.7275822	3.5870259	4.82414	2.72E-06	3.35E-05	4.2363416
ZNF582-AS1	-0.554882	4.0484669	-4.824068	2.72E-06	3.35E-05	4.236035
CERS4	-0.213177	6.4120662	-4.823592	2.73E-06	3.36E-05	4.2339984
IDH3G	0.1135016	6.3738407	4.8206373	2.76E-06	3.40E-05	4.2213557
RP1-18D14.	1.1598551	1.3775026	4.8204125	2.77E-06	3.40E-05	4.2203945
ZNF469	0.4193522	4.6615675	4.8179143	2.80E-06	3.44E-05	4.2097122
MMGT1	0.1262922	5.9018253	4.8167707	2.81E-06	3.46E-05	4.2048234
HBEGF	0.2011831	5.4697225	4.816716	2.81E-06	3.46E-05	4.2045894
LINC01093	1.412502	3.119314	4.8157165	2.83E-06	3.47E-05	4.2003179
CTB-179K24	-0.584062	2.7434685	-4.815592	2.83E-06	3.47E-05	4.1997856
RP11-47P18	-1.449923	-0.073907	-4.814722	2.84E-06	3.49E-05	4.1960664
JMJD1C-AS1	-0.769622	2.7632413	-4.813712	2.85E-06	3.50E-05	4.1917511
ZSCAN5B	-0.987089	-0.23329	-4.813364	2.86E-06	3.50E-05	4.190264
ADCY9	0.142631	6.3127272	4.8118454	2.88E-06	3.53E-05	4.1837797
ARHGEF2	-0.147131	6.211451	-4.811608	2.88E-06	3.53E-05	4.1827642
SNORD83A	-0.980755	0.7402347	-4.811425	2.88E-06	3.53E-05	4.181983
SIRPA	0.1406705	6.49195	4.8113666	2.88E-06	3.53E-05	4.1817348
RP11-15A1.	-0.707261	3.3515834	-4.810921	2.89E-06	3.54E-05	4.1798309
EXOC3L2	0.2170975	5.4732284	4.8097223	2.90E-06	3.56E-05	4.1747137
DPY19L1	0.1420243	6.0873409	4.8087387	2.92E-06	3.57E-05	4.1705149
RP11-476K1	-1.717672	1.7879515	-4.807717	2.93E-06	3.58E-05	4.1661541
TGM4	-1.121985	0.1903178	-4.806831	2.94E-06	3.60E-05	4.1623744
RNLS	0.2754011	5.4630924	4.8065475	2.95E-06	3.60E-05	4.1611628
CTD-2292M1	-0.602993	3.3616946	-4.806007	2.95E-06	3.61E-05	4.1588552
TCF7	-0.282675	5.7524033	-4.805332	2.96E-06	3.62E-05	4.1559771
LINC01485	0.8702653	5.4387561	4.8036135	2.99E-06	3.65E-05	4.1486463
RP11-317N1	-1.669523	0.1723044	-4.803285	2.99E-06	3.65E-05	4.1472464
RP11-554J4	-0.489837	3.332424	-4.803264	2.99E-06	3.65E-05	4.1471563
RP11-483P2	1.3922116	0.3365929	4.8030251	2.99E-06	3.65E-05	4.1461366
GPR89A	-0.14818	5.3520222	-4.802795	3.00E-06	3.65E-05	4.1451559
MFSD2B	-0.80204	3.228733	-4.802781	3.00E-06	3.65E-05	4.1450951
TNFRSF12A	0.2012478	6.3163828	4.8023765	3.00E-06	3.66E-05	4.1433707
JAM2	0.3359781	5.3163155	4.8007583	3.02E-06	3.68E-05	4.1364717
RP11-277B1	-0.836004	2.2475016	-4.800582	3.03E-06	3.69E-05	4.1357184
RP11-614F1	-1.363266	-0.48114	-4.799798	3.04E-06	3.70E-05	4.1323763
TNKS1BP1	0.0922932	6.6672396	4.7988327	3.05E-06	3.71E-05	4.1282638
RP11-38L15	-0.774621	4.1829237	-4.798584	3.05E-06	3.72E-05	4.1272035
ST6GALNAC3	0.3843159	4.5788999	4.7971217	3.07E-06	3.74E-05	4.1209731
IL15RA	0.2031296	5.9749659	4.7965072	3.08E-06	3.75E-05	4.1183552
AAAS	-0.074739	6.2516756	-4.796051	3.09E-06	3.75E-05	4.1164099
RP11-416I2	1.1816965	0.4195501	4.7959507	3.09E-06	3.75E-05	4.1159849
SWAP70	0.1157814	6.1587294	4.7949508	3.10E-06	3.77E-05	4.1117257
FGFR4	-0.292135	6.7777838	-4.794603	3.11E-06	3.77E-05	4.1102459
HGF	0.4448214	5.3350187	4.7942245	3.11E-06	3.78E-05	4.1086329
ABCB1	0.5067141	6.0323888	4.7941647	3.11E-06	3.78E-05	4.1083782

CCL25	-1.491559	3.3760981	-4.793943	3.12E-06	3.78E-05	4.1074335
ABTB1	0.1201826	6.088521	4.7939123	3.12E-06	3.78E-05	4.1073034
ADAMTS9-AS	1.0293684	2.1652203	4.7938444	3.12E-06	3.78E-05	4.1070144
GINS2	-0.256384	5.4613658	-4.793828	3.12E-06	3.78E-05	4.1069433
KBTBD11	0.63433	4.7501109	4.7927904	3.13E-06	3.80E-05	4.1025267
CTD-3157E1	1.091047	2.1960679	4.7920023	3.14E-06	3.81E-05	4.0991713
PLEKHG5	0.1944489	5.384117	4.7917503	3.15E-06	3.81E-05	4.0980988
GPRC5A	1.0120099	3.6932522	4.7898935	3.17E-06	3.84E-05	4.0901963
CKAP5	-0.073295	6.5049251	-4.789332	3.18E-06	3.85E-05	4.0878061
CHRNA2	-1.215554	-0.267631	-4.789112	3.19E-06	3.85E-05	4.086871
AGL	0.1703191	6.1015445	4.7890924	3.19E-06	3.85E-05	4.0867877
FAM221B	-0.972951	1.8232478	-4.787079	3.22E-06	3.89E-05	4.0782204
MYOM2	0.7132221	4.0903078	4.786279	3.23E-06	3.90E-05	4.0748201
NOTCH4	0.1606566	6.0231109	4.7860777	3.23E-06	3.90E-05	4.0739637
GRIA3	1.3631383	3.5828755	4.7859176	3.23E-06	3.90E-05	4.0732833
MYRIP	0.7599434	5.4078866	4.7843532	3.25E-06	3.93E-05	4.0666312
TSEN54	-0.120625	6.1308249	-4.784227	3.26E-06	3.93E-05	4.0660952
ZFY-AS1	1.4308107	0.4521072	4.7841243	3.26E-06	3.93E-05	4.0656582
STARD5	0.3431017	5.0936187	4.7840787	3.26E-06	3.93E-05	4.0654644
MEIS3P1	1.0819232	2.4689861	4.7837503	3.26E-06	3.93E-05	4.0640683
MSS51	-0.323868	4.1878792	-4.783666	3.26E-06	3.93E-05	4.0637118
RP11-1275H	-0.338146	4.5996756	-4.782796	3.28E-06	3.95E-05	4.0600103
PGS1	-0.084479	5.8828503	-4.78277	3.28E-06	3.95E-05	4.0599001
RP4-655J12	-1.188759	1.8015833	-4.782407	3.28E-06	3.95E-05	4.0583564
DENND4B	-0.093633	6.3152919	-4.782142	3.29E-06	3.96E-05	4.0572318
RP5-943J3.	-0.722766	2.871564	-4.78199	3.29E-06	3.96E-05	4.0565864
PYCR1	-0.486543	5.6990048	-4.781849	3.29E-06	3.96E-05	4.055986
SDSL	0.2270277	6.3058174	4.781662	3.29E-06	3.96E-05	4.0551923
BST2	0.2634087	6.6371923	4.7802331	3.32E-06	3.98E-05	4.0491209
WDTC1	0.0901815	6.3751581	4.7797802	3.32E-06	3.99E-05	4.0471968
REEP3	0.0944072	6.2329209	4.7794021	3.33E-06	4.00E-05	4.0455907
ZNF600	-0.305894	5.3390398	-4.779376	3.33E-06	4.00E-05	4.0454797
FBXO33	0.1137079	5.6427784	4.778917	3.33E-06	4.00E-05	4.0435304
SLC25A5P1	-0.858377	2.1704486	-4.778428	3.34E-06	4.01E-05	4.0414534
ZNF816	-0.423838	4.7748919	-4.777837	3.35E-06	4.02E-05	4.0389451
SCHLAP1	-0.947254	-1.037267	-4.776713	3.37E-06	4.04E-05	4.0341687
CADM2-AS1	-0.580124	-1.248366	-4.776709	3.37E-06	4.04E-05	4.0341527
RP4-758J18	-1.167135	1.0836008	-4.776126	3.38E-06	4.05E-05	4.0316768
LINC01297	-1.143654	-0.751661	-4.776053	3.38E-06	4.05E-05	4.0313682
CACYBP	-0.121828	6.4132307	-4.77595	3.38E-06	4.05E-05	4.0309294
SNX25P1	-0.731041	3.4576963	-4.775856	3.38E-06	4.05E-05	4.0305322
HAUS8	-0.14616	5.4001347	-4.775524	3.39E-06	4.05E-05	4.0291203
SENP1	-0.098024	5.7116165	-4.775314	3.39E-06	4.05E-05	4.028232
NGFR	0.4620724	5.4946329	4.7744571	3.40E-06	4.07E-05	4.0245934
ACBD5	0.1343702	6.4137119	4.774419	3.40E-06	4.07E-05	4.0244319
RTTN	-0.13984	5.4637667	-4.773863	3.41E-06	4.08E-05	4.022074
SPAG1	0.2470746	5.5044726	4.7737961	3.41E-06	4.08E-05	4.0217883
RP11-295M1	1.1415847	1.660723	4.7737446	3.41E-06	4.08E-05	4.0215697
HOXD1	-1.477043	1.1493288	-4.773318	3.42E-06	4.08E-05	4.0197599
RP11-23J9.	-0.790104	-0.98641	-4.772831	3.43E-06	4.09E-05	4.0176942
MTHFD1P1	1.0565941	1.2275263	4.7727076	3.43E-06	4.09E-05	4.017169

NKX3-1	0.642021	4.3415637	4.771717	3.44E-06	4.11E-05	4.0129661
OMA1	0.1329513	5.8884178	4.7712393	3.45E-06	4.12E-05	4.0109398
GALNT16	0.5727033	4.7207752	4.7707492	3.46E-06	4.12E-05	4.0088606
BACE2	0.3699888	5.9728719	4.7698928	3.47E-06	4.14E-05	4.0052282
BAGE5	-1.053392	-0.821567	-4.769178	3.48E-06	4.15E-05	4.0021958
DCP2	-0.102268	6.0796327	-4.76903	3.49E-06	4.15E-05	4.001568
LINC00351	-0.911643	-0.959355	-4.768122	3.50E-06	4.17E-05	3.9977176
TRIM28	-0.089645	6.8484967	-4.767704	3.51E-06	4.17E-05	3.995947
ZNF583	-0.267223	5.025804	-4.767377	3.51E-06	4.18E-05	3.9945606
ZNF329	-0.204531	5.6616156	-4.767023	3.52E-06	4.18E-05	3.9930589
RP11-95P2.	0.8231375	2.3528827	4.7662716	3.53E-06	4.20E-05	3.9898747
HSPB2	0.8196149	3.1133773	4.7658026	3.54E-06	4.20E-05	3.987887
CTC-448F2.	-0.363745	3.6228685	-4.765475	3.54E-06	4.21E-05	3.9865002
TRIM67	-0.846223	2.9912715	-4.765253	3.54E-06	4.21E-05	3.9855589
CDIPT-AS1	-1.209427	1.3927253	-4.765135	3.55E-06	4.21E-05	3.9850592
EPPK1	-0.708191	5.1513697	-4.764511	3.56E-06	4.22E-05	3.9824119
ATF3	0.2109	6.139784	4.7645043	3.56E-06	4.22E-05	3.982385
AGO2	-0.133227	6.1889109	-4.76443	3.56E-06	4.22E-05	3.9820702
ZNF468	-0.382625	5.4732634	-4.764229	3.56E-06	4.22E-05	3.9812173
RAMP1	0.3845454	6.2703029	4.7640652	3.56E-06	4.23E-05	3.9805243
RP11-15A1.	-1.176207	1.0052017	-4.763925	3.57E-06	4.23E-05	3.9799323
RP11-50102	-1.118725	-0.801283	-4.763382	3.57E-06	4.23E-05	3.9776305
TGIF2	-0.148542	6.0752279	-4.763375	3.57E-06	4.23E-05	3.9775993
ZNF724P	-0.836273	3.1931649	-4.762646	3.59E-06	4.25E-05	3.9745139
L3MBTL1	-0.291298	4.929158	-4.762638	3.59E-06	4.25E-05	3.9744788
FZD5	-0.15904	6.4168397	-4.761627	3.60E-06	4.26E-05	3.9701964
SUZ12	-0.089365	6.1908385	-4.761444	3.61E-06	4.27E-05	3.9694198
BCAR3	0.1504332	5.9476755	4.7612323	3.61E-06	4.27E-05	3.9685242
GPX1	0.1337268	6.9406959	4.7609362	3.61E-06	4.27E-05	3.9672705
LINC00519	1.1926568	-0.037601	4.7596428	3.63E-06	4.30E-05	3.9617935
C12orf76	-0.132466	5.6087608	-4.759509	3.64E-06	4.30E-05	3.9612253
DSCR4	-1.528438	-0.283777	-4.759024	3.64E-06	4.30E-05	3.9591743
PEX11G	0.2653556	5.32165	4.7573517	3.67E-06	4.34E-05	3.9520955
RASA4CP	-0.344905	4.6059661	-4.756875	3.68E-06	4.34E-05	3.9500783
RP11-302L1	0.9176256	2.5648846	4.7566821	3.68E-06	4.35E-05	3.9492616
RPS21	-0.126554	6.9242282	-4.75624	3.69E-06	4.35E-05	3.94739
DNAH5	0.9210347	4.5105191	4.7562266	3.69E-06	4.35E-05	3.9473341
MAP3K4	-0.098511	5.9523295	-4.755988	3.69E-06	4.35E-05	3.9463262
IGIP	0.1378717	5.7018558	4.7555801	3.70E-06	4.36E-05	3.9445986
KIF7	-0.322601	5.0203	-4.754967	3.71E-06	4.37E-05	3.9420039
RP11-173M1	0.3991	4.5593291	4.7547029	3.72E-06	4.38E-05	3.9408874
MS4A4A	0.3035096	5.5125987	4.7545188	3.72E-06	4.38E-05	3.9401085
SOX7	0.3144251	4.9893417	4.7539881	3.73E-06	4.39E-05	3.9378637
SLC22A16	-1.170213	0.4288756	-4.753551	3.74E-06	4.39E-05	3.9360154
RP11-802E1	-0.420139	4.1789123	-4.749739	3.80E-06	4.47E-05	3.9198979
MTURN	0.2167706	5.503419	4.7494631	3.80E-06	4.47E-05	3.9187312
ZNF607	-0.4318	4.8200958	-4.749282	3.81E-06	4.47E-05	3.9179647
MSC	0.4937521	5.3629074	4.7485431	3.82E-06	4.49E-05	3.9148426
APBB1IP	0.2578859	6.0234341	4.748442	3.82E-06	4.49E-05	3.9144156
TMEM201	-0.149076	5.7620636	-4.748035	3.83E-06	4.49E-05	3.9126963
EXOSC2	-0.10684	5.8980457	-4.748002	3.83E-06	4.49E-05	3.9125565

RP11-92K2.	-0.473781	3.5266575	-4.747785	3.83E-06	4.50E-05	3.9116384
PTPLB	-0.123414	6.5849056	-4.747693	3.83E-06	4.50E-05	3.9112518
ERAP1	0.1503894	6.4026833	4.7475564	3.84E-06	4.50E-05	3.9106735
RP11-287D1	-1.020235	0.0696563	-4.746814	3.85E-06	4.51E-05	3.9075384
HDAC9	0.4244866	4.6564863	4.7465028	3.85E-06	4.52E-05	3.9062221
RP11-286H1	-1.106256	2.0353217	-4.745103	3.88E-06	4.54E-05	3.9003112
RP11-154D1	-1.071854	0.9386258	-4.745079	3.88E-06	4.54E-05	3.9002068
PITRM1	0.1011031	6.4543525	4.7446268	3.89E-06	4.55E-05	3.8982982
RP11-139I1	-1.157435	-0.181946	-4.7446	3.89E-06	4.55E-05	3.8981832
PLAU	0.2146509	5.6427688	4.743583	3.90E-06	4.57E-05	3.8938904
TMEM194B	-0.143514	5.3696497	-4.742572	3.92E-06	4.59E-05	3.8896207
RNASE7	1.0648102	2.6974391	4.742386	3.93E-06	4.59E-05	3.8888364
PA2G4P4	-0.853781	2.1296256	-4.742043	3.93E-06	4.60E-05	3.8873885
ITGA10	0.3831853	4.4414392	4.741799	3.94E-06	4.60E-05	3.8863584
CTC-246B18	-0.873089	2.7422545	-4.741243	3.95E-06	4.61E-05	3.8840099
RPLP0	-0.096813	7.3549984	-4.741069	3.95E-06	4.61E-05	3.8832758
FAM43A	0.2319846	5.451033	4.7401135	3.97E-06	4.63E-05	3.8792448
HCG25	-0.366322	3.6561218	-4.739729	3.97E-06	4.64E-05	3.8776219
TOPBP1	-0.10094	6.0805856	-4.739584	3.97E-06	4.64E-05	3.877011
PATZ1	-0.115746	6.2616092	-4.739499	3.98E-06	4.64E-05	3.8766508
BMP10	1.1015254	-0.743549	4.7385152	3.99E-06	4.66E-05	3.8725007
ZSCAN5A	-0.162935	5.0931063	-4.738275	4.00E-06	4.66E-05	3.8714855
CDK2	-0.120666	6.0004905	-4.738206	4.00E-06	4.66E-05	3.8711981
RP4-583P15	-0.909723	2.6819123	-4.73756	4.01E-06	4.67E-05	3.8684722
PTGIS	0.6293691	5.1613376	4.7364088	4.03E-06	4.69E-05	3.863616
PRAMEF8	1.3224726	-0.07627	4.7348579	4.06E-06	4.73E-05	3.8570764
ICAM1	0.2275865	6.3914273	4.7337271	4.08E-06	4.75E-05	3.8523089
NIM1K	0.6607246	2.9054268	4.7333581	4.09E-06	4.75E-05	3.8507537
CHRM2	1.411133	0.1116847	4.7323926	4.10E-06	4.77E-05	3.8466843
RP11-351J2	-0.853043	3.7334298	-4.732046	4.11E-06	4.78E-05	3.8452223
ENPEP	0.276033	6.098443	4.7314251	4.12E-06	4.79E-05	3.8426071
EXOC3L1	0.1644676	5.3516834	4.7313813	4.12E-06	4.79E-05	3.8424226
OGDH	0.1111702	6.686096	4.7304338	4.14E-06	4.81E-05	3.8384304
GDF6	1.2069995	2.4676728	4.7301021	4.15E-06	4.81E-05	3.8370327
ZNF518A	-0.138012	5.9032069	-4.729967	4.15E-06	4.82E-05	3.8364651
PLEKHA4	0.2145072	5.9907999	4.7295524	4.16E-06	4.82E-05	3.8347171
MZT1	-0.137831	5.6393232	-4.728634	4.17E-06	4.84E-05	3.8308502
BCKDK	0.1188232	6.4607078	4.7282727	4.18E-06	4.85E-05	3.829327
SAMD9	0.2385942	5.4530368	4.7281867	4.18E-06	4.85E-05	3.8289648
EFCAB6	0.712948	3.4553193	4.7276989	4.19E-06	4.86E-05	3.8269106
LENG9	0.2768358	5.2056264	4.7274017	4.20E-06	4.86E-05	3.8256591
FIZ1	-0.086884	5.8349341	-4.727023	4.20E-06	4.87E-05	3.8240648
RP11-574K1	-0.881414	1.5824893	-4.726826	4.21E-06	4.87E-05	3.8232341
STXBP4	-0.18161	5.5493604	-4.726381	4.22E-06	4.88E-05	3.8213624
MYO1C	0.0993792	6.7505524	4.7260779	4.22E-06	4.88E-05	3.8200851
ZNF841	-0.21354	5.3776236	-4.725684	4.23E-06	4.89E-05	3.8184257
RP11-17501	-0.145197	5.4203712	-4.725344	4.23E-06	4.90E-05	3.8169975
ZSCAN22	-0.108314	5.4165781	-4.724955	4.24E-06	4.90E-05	3.8153599
ESAM	0.1551606	6.0857518	4.7246838	4.25E-06	4.91E-05	3.8142169
EFNA4	-0.192709	5.6437958	-4.7236	4.27E-06	4.93E-05	3.8096577
NFIA	0.1499955	6.3196705	4.7227467	4.28E-06	4.95E-05	3.8060652

B4GALT3	-0.0971	6.2815602	-4.722539	4.29E-06	4.95E-05	3.8051925
HOXD3	-1.203056	0.9813243	-4.721776	4.30E-06	4.97E-05	3.8019801
ANAPC5	-0.071968	6.6188108	-4.721377	4.31E-06	4.97E-05	3.8003043
LMO4	0.1861638	6.0653321	4.7210959	4.32E-06	4.98E-05	3.7991201
KIAA1598	0.1396942	6.3784874	4.720752	4.32E-06	4.98E-05	3.7976735
NFASC	0.311524	5.5855327	4.7201937	4.33E-06	4.99E-05	3.7953256
SLC16A14	0.4457839	4.9419545	4.7196007	4.34E-06	5.01E-05	3.7928318
ARHGAP29	0.1448948	6.2406755	4.7195133	4.35E-06	5.01E-05	3.7924639
SVIL	0.1642529	6.223082	4.719429	4.35E-06	5.01E-05	3.7921095
ZNF284	-0.298389	4.5417114	-4.719119	4.35E-06	5.01E-05	3.7908046
HSPB7	0.8134594	3.9959297	4.7184025	4.37E-06	5.03E-05	3.7877931
NXF3	1.2958493	1.405452	4.7183484	4.37E-06	5.03E-05	3.7875659
SP8	-1.695408	1.0388501	-4.718184	4.37E-06	5.03E-05	3.7868752
CENPJ	-0.171587	5.3882209	-4.716962	4.40E-06	5.05E-05	3.7817396
EMG1	-0.125305	5.8726702	-4.716962	4.40E-06	5.05E-05	3.7817363
QRICH2	-0.274616	5.216839	-4.716878	4.40E-06	5.05E-05	3.7813864
INTS8	-0.115186	6.119687	-4.716825	4.40E-06	5.05E-05	3.7811596
HPSE2	-1.213997	0.3574382	-4.716111	4.41E-06	5.07E-05	3.7781616
LINC01352	1.1347951	1.9136329	4.7160769	4.41E-06	5.07E-05	3.7780174
CPAMD8	0.390416	4.5325282	4.715159	4.43E-06	5.08E-05	3.7741597
IMMP1L	-0.111804	5.5724907	-4.715109	4.43E-06	5.08E-05	3.7739504
GOLM1	0.2449378	6.5070398	4.7145719	4.44E-06	5.09E-05	3.771693
ZNF416	-0.204659	5.0232514	-4.713824	4.46E-06	5.11E-05	3.7685515
CDK4	-0.119531	6.3435445	-4.71277	4.48E-06	5.13E-05	3.7641235
ZNF32-AS1	-0.981346	1.327559	-4.712717	4.48E-06	5.13E-05	3.7638997
CUZD1	-0.772765	3.1196843	-4.712503	4.48E-06	5.13E-05	3.7630026
ZNF28	-0.407657	5.3228831	-4.712255	4.49E-06	5.14E-05	3.7619592
DCAF7	-0.070391	6.4757028	-4.711404	4.50E-06	5.16E-05	3.7583862
FLJ22447	0.9864352	1.7466045	4.7108088	4.52E-06	5.17E-05	3.7558863
NRM	-0.207116	5.7833911	-4.710672	4.52E-06	5.17E-05	3.7553139
ZNF542P	-0.274583	5.5959655	-4.709832	4.54E-06	5.19E-05	3.7517873
ANKH	0.120025	6.5407748	4.7095278	4.54E-06	5.19E-05	3.7505083
DSCR4-IT1	-1.119648	-0.767691	-4.708713	4.56E-06	5.21E-05	3.747086
DNAJA2	0.103124	6.4159029	4.7083766	4.57E-06	5.22E-05	3.7456756
ZGLP1	-0.335072	4.1335148	-4.706194	4.61E-06	5.27E-05	3.736518
PITPNM3	0.7096451	3.7978084	4.7047672	4.64E-06	5.30E-05	3.7305308
PPL	0.3136273	5.861151	4.7044354	4.65E-06	5.30E-05	3.7291388
IL20	1.0339484	-0.619606	4.7041705	4.65E-06	5.31E-05	3.7280277
RP11-162A1	0.3527966	4.2845903	4.7037838	4.66E-06	5.32E-05	3.7264058
SAP130	-0.080217	6.0581893	-4.70331	4.67E-06	5.32E-05	3.7244169
RNF144B	0.2053494	5.8140057	4.7025938	4.68E-06	5.34E-05	3.7214154
ANXA8	1.4552249	1.5347222	4.7014304	4.71E-06	5.37E-05	3.7165376
DNAAF1	1.1423533	2.9474118	4.7008627	4.72E-06	5.38E-05	3.7141579
DNMT1	-0.114963	6.3248704	-4.700856	4.72E-06	5.38E-05	3.7141286
LMX1A	-1.371807	0.5749804	-4.700618	4.73E-06	5.38E-05	3.7131305
RP1-228P16	-0.634666	2.7661179	-4.700515	4.73E-06	5.38E-05	3.7126996
RP11-467J1	0.9647948	-0.299426	4.7004869	4.73E-06	5.38E-05	3.7125828
PLA2G12A	0.1197832	6.1477468	4.7004556	4.73E-06	5.38E-05	3.7124514
FBXO8	0.1539205	5.9953044	4.6991983	4.76E-06	5.41E-05	3.7071817
RP11-448G1	-1.186385	1.107811	-4.69895	4.76E-06	5.41E-05	3.7061421
SIX2	-1.433084	2.3705419	-4.698785	4.76E-06	5.41E-05	3.7054479

TSPYL2	-0.174276	5.9467367	-4.697548	4.79E-06	5.44E-05	3.7002687
SEZ6	-1.599295	2.5866091	-4.69712	4.80E-06	5.45E-05	3.6984754
UBE2L6	0.1876775	6.4653918	4.696343	4.82E-06	5.47E-05	3.6952191
PPM1L	0.2057872	5.7363803	4.695817	4.83E-06	5.48E-05	3.6930157
PCSK7	0.1316327	5.81464	4.6949786	4.84E-06	5.50E-05	3.6895048
PTPRE	0.2137024	5.5952331	4.6946081	4.85E-06	5.50E-05	3.6879534
RALGPS2	-0.160408	6.3772221	-4.694422	4.86E-06	5.51E-05	3.6871758
FGF18	0.9945991	2.6087173	4.6943144	4.86E-06	5.51E-05	3.6867233
NODAL	-1.050661	0.7113049	-4.694121	4.86E-06	5.51E-05	3.6859132
SH3BP1	-0.225665	5.6198999	-4.693899	4.87E-06	5.52E-05	3.684985
KIAA1109	0.1254548	6.1697513	4.6936776	4.87E-06	5.52E-05	3.6840573
PPM1B	0.0984601	6.3256757	4.6931588	4.88E-06	5.53E-05	3.6818851
TONSL	-0.189259	5.8797355	-4.692483	4.90E-06	5.54E-05	3.6790578
SOSTDC1	-1.417075	2.0905663	-4.69177	4.91E-06	5.56E-05	3.6760717
SPAST	-0.085947	5.9246476	-4.691466	4.92E-06	5.57E-05	3.674799
RP11-14C1C	-1.252663	2.0760135	-4.691433	4.92E-06	5.57E-05	3.6746624
NEURL2	-0.259741	4.3788798	-4.691298	4.92E-06	5.57E-05	3.6740948
RP11-291L2	-0.300604	4.4984757	-4.690777	4.94E-06	5.58E-05	3.6719173
IL4R	0.1174339	6.4929928	4.6902032	4.95E-06	5.59E-05	3.6695148
CEL	-0.878463	3.2834688	-4.690138	4.95E-06	5.59E-05	3.6692431
HNRNPA1	-0.078482	6.9999848	-4.689914	4.95E-06	5.59E-05	3.6683046
POGZ	-0.095581	6.4445549	-4.689413	4.97E-06	5.60E-05	3.6662093
DECR1	0.1552547	6.8011545	4.6893631	4.97E-06	5.60E-05	3.6659999
C15orf65	0.4566146	3.7634234	4.6888691	4.98E-06	5.61E-05	3.6639335
C6orf223	-1.53422	2.9556913	-4.68868	4.98E-06	5.62E-05	3.6631433
RRAD	0.6054948	4.7045026	4.6877949	5.00E-06	5.64E-05	3.65944
RP11-321F8	-0.849463	1.9500793	-4.687648	5.00E-06	5.64E-05	3.6588278
RP1-193H18	-0.353846	4.9579351	-4.687476	5.01E-06	5.64E-05	3.6581076
PRPF39	-0.099139	5.8538285	-4.687408	5.01E-06	5.64E-05	3.6578206
THSD1	0.3002344	5.0093081	4.6874008	5.01E-06	5.64E-05	3.657792
DNAJB13	-1.240626	1.1787982	-4.686982	5.02E-06	5.65E-05	3.6560398
LINC01338	-0.946132	-0.814629	-4.686513	5.03E-06	5.66E-05	3.6540794
FANCF	-0.098843	5.8596146	-4.686133	5.04E-06	5.67E-05	3.6524897
FAM167B	0.2380219	5.2924736	4.6860977	5.04E-06	5.67E-05	3.6523425
PSMD7	0.1010338	6.3507826	4.6859754	5.04E-06	5.67E-05	3.651831
ZMAT3	0.1361759	6.0833642	4.6845693	5.07E-06	5.70E-05	3.6459527
CBX8	-0.140778	5.7030703	-4.68396	5.09E-06	5.71E-05	3.6434047
CEP162	-0.145672	5.303629	-4.683955	5.09E-06	5.71E-05	3.6433851
UTS2R	-1.023096	-0.526186	-4.683929	5.09E-06	5.71E-05	3.643275
TPR	-0.082791	6.6020291	-4.683918	5.09E-06	5.71E-05	3.6432286
RP11-323F2	-0.994249	0.6072764	-4.683649	5.09E-06	5.72E-05	3.6421047
FOXP4	-0.111041	6.5153366	-4.68333	5.10E-06	5.72E-05	3.6407716
SNX29P2	1.0526772	2.4606108	4.6831229	5.11E-06	5.72E-05	3.6399071
FOXP1-AS1	-0.846514	-0.694346	-4.683077	5.11E-06	5.72E-05	3.6397134
NAA25	-0.089735	5.8877792	-4.683032	5.11E-06	5.72E-05	3.6395284
MLIP-IT1	1.0673201	-0.14345	4.6829653	5.11E-06	5.72E-05	3.6392487
DIP2C	0.2853264	5.9553405	4.6829018	5.11E-06	5.72E-05	3.6389831
RP11-758M4	-1.600922	0.1802565	-4.681781	5.14E-06	5.75E-05	3.6342987
RP11-428G2	-0.843277	-1.037606	-4.681066	5.15E-06	5.77E-05	3.6313106
STK38L	0.1217984	5.8707048	4.6809941	5.15E-06	5.77E-05	3.6310124
RCOR3	-0.107717	6.1112016	-4.680352	5.17E-06	5.78E-05	3.6283284

RHEBP1	-1.141356	1.6736587	-4.680049	5.17E-06	5.79E-05	3.6270638
FKBP7	0.1862642	5.3472869	4.6794098	5.19E-06	5.80E-05	3.6243943
SVOPL	-1.153469	-0.204461	-4.678763	5.20E-06	5.82E-05	3.6216914
IGHV7-81	-1.211495	0.5423102	-4.677158	5.24E-06	5.86E-05	3.6149918
RP11-595B2	-0.860293	3.4672009	-4.676254	5.26E-06	5.88E-05	3.6112183
NUP133	-0.088511	6.2564885	-4.675499	5.28E-06	5.89E-05	3.608068
LINC00924	0.5412757	4.2533701	4.6754847	5.28E-06	5.89E-05	3.6080069
SLC30A4	0.3505052	5.0345447	4.6753995	5.28E-06	5.89E-05	3.607651
OSGEPL1	-0.109863	5.552444	-4.675291	5.28E-06	5.90E-05	3.6071967
ADM2	-0.351739	5.8022951	-4.675113	5.29E-06	5.90E-05	3.6064569
GNB1L	-0.178367	5.3581928	-4.674971	5.29E-06	5.90E-05	3.6058628
RP11-16B13	-1.077341	0.1215751	-4.674779	5.30E-06	5.90E-05	3.6050611
RP11-48501	1.3848602	0.3005211	4.6746069	5.30E-06	5.91E-05	3.6043434
EFNB2	0.1935581	5.8710568	4.6742055	5.31E-06	5.91E-05	3.6026685
ATAT1	-0.186087	5.5077497	-4.672955	5.34E-06	5.95E-05	3.5974507
SLC6A14	-1.602979	0.7559385	-4.672464	5.35E-06	5.96E-05	3.5954014
RPS3P6	-0.960358	0.5018574	-4.671329	5.38E-06	5.98E-05	3.5906701
STK25	-0.079083	6.5195019	-4.671203	5.38E-06	5.99E-05	3.5901417
SLC6A11	-1.674348	3.8658061	-4.670823	5.39E-06	5.99E-05	3.5885571
CPNE8	0.2076736	5.7108386	4.6704218	5.40E-06	6.00E-05	3.586885
GTF3C2-AS1	-0.910772	1.8209347	-4.670043	5.41E-06	6.01E-05	3.5853039
PLEKHF1	0.2950662	5.7226427	4.6695658	5.42E-06	6.02E-05	3.5833158
MON1B	0.0894539	6.1769144	4.6693022	5.43E-06	6.03E-05	3.5822166
TRIM50	-1.338561	3.7057135	-4.669295	5.43E-06	6.03E-05	3.582186
KIAA1644	1.1628432	2.4873576	4.6681683	5.45E-06	6.05E-05	3.5774897
YEATS4	-0.109635	5.7108913	-4.667432	5.47E-06	6.07E-05	3.5744198
RP11-496H1	-0.970009	2.6356827	-4.667046	5.48E-06	6.08E-05	3.5728128
MYEOV	1.3259676	2.8260659	4.6665008	5.49E-06	6.09E-05	3.57054
HS6ST2	-1.536575	2.6424053	-4.6662	5.50E-06	6.10E-05	3.5692872
SHISA3	1.2381751	3.6321182	4.6655374	5.52E-06	6.12E-05	3.5665255
PLSCR4	0.2363842	5.885243	4.6650381	5.53E-06	6.13E-05	3.5644451
RASL11B	1.0841285	3.1253872	4.6650164	5.53E-06	6.13E-05	3.5643547
PPIL1	-0.113927	6.1125553	-4.664914	5.53E-06	6.13E-05	3.5639292
CTA-941F9.	-0.552728	3.531834	-4.664682	5.54E-06	6.13E-05	3.56296
RP4-594A5.	-1.009701	-0.664586	-4.663772	5.56E-06	6.15E-05	3.5591712
MN1	0.4788648	4.8213925	4.6635347	5.57E-06	6.16E-05	3.5581827
OR8Q1P	-0.597149	-1.240018	-4.663098	5.58E-06	6.17E-05	3.5563621
CDPF1	-0.136802	5.6276102	-4.663041	5.58E-06	6.17E-05	3.5561264
CELA3A	-1.211369	-0.374942	-4.663002	5.58E-06	6.17E-05	3.5559644
DNAH3	-1.260455	1.6017757	-4.662395	5.59E-06	6.18E-05	3.5534378
RP11-74C1.	-0.953495	0.975133	-4.661604	5.61E-06	6.20E-05	3.5501437
RP4-604A21	-0.982374	1.0037528	-4.661541	5.61E-06	6.20E-05	3.5498822
LINC00844	1.6521549	2.9894969	4.6607912	5.63E-06	6.22E-05	3.5467583
ACRV1	-1.129302	1.3682202	-4.66077	5.63E-06	6.22E-05	3.5466715
INPP4B	0.3053373	5.1107615	4.6607035	5.63E-06	6.22E-05	3.5463933
MLYCD	0.1869133	6.0207049	4.6604116	5.64E-06	6.22E-05	3.5451783
RNF182	1.2321354	0.5073854	4.660152	5.65E-06	6.23E-05	3.5440977
AVPR1A	0.9484698	4.7770482	4.6598341	5.66E-06	6.24E-05	3.5427744
FCHSD2	0.097383	6.0293354	4.6577009	5.71E-06	6.29E-05	3.5338968
TRMT6	-0.115715	5.8680547	-4.657694	5.71E-06	6.29E-05	3.5338666
RBM14	-0.058666	6.239872	-4.65691	5.73E-06	6.31E-05	3.5306057

DAZAP1	-0.067474	6.57609	-4.656821	5.73E-06	6.31E-05	3.5302364
AC093063.2	-1.061373	-0.712481	-4.656501	5.74E-06	6.32E-05	3.528906
PMS2P3	-0.201929	4.6728027	-4.656293	5.75E-06	6.32E-05	3.5280391
RP11-40C6.	-0.552168	3.3774235	-4.656271	5.75E-06	6.32E-05	3.5279463
RNF19B	0.1467411	5.8889601	4.6562152	5.75E-06	6.32E-05	3.5277157
C1orf123	0.0996182	6.1222169	4.6555404	5.76E-06	6.34E-05	3.5249087
SNRPEP4	-0.620654	2.6935287	-4.655399	5.77E-06	6.34E-05	3.5243187
LINC01234	-1.742012	1.6513879	-4.655114	5.77E-06	6.35E-05	3.5231358
RP11-796E1	-1.370671	0.6935594	-4.655048	5.78E-06	6.35E-05	3.5228606
CTD-2666L2	-0.978974	1.4373531	-4.654723	5.78E-06	6.35E-05	3.5215074
P2RX4	-0.142324	6.1824957	-4.654	5.80E-06	6.37E-05	3.5185044
SECTM1	-0.242177	5.8462611	-4.653804	5.81E-06	6.37E-05	3.5176857
C17orf75	-0.086906	5.8642839	-4.653774	5.81E-06	6.37E-05	3.5175617
ITGB1	0.1152343	6.8710609	4.6526546	5.84E-06	6.40E-05	3.5129089
MT-ATP6	0.1308077	7.6739931	4.6522009	5.85E-06	6.41E-05	3.5110226
LMO2	0.1936484	5.6707787	4.6521193	5.85E-06	6.41E-05	3.5106836
RP11-357P1	1.1399117	0.4578985	4.6520752	5.85E-06	6.41E-05	3.5105003
RP11-247I1	-0.989172	1.2110586	-4.649883	5.91E-06	6.47E-05	3.5013908
RNU6-8	-0.795301	2.7034195	-4.649829	5.91E-06	6.47E-05	3.5011664
TMEM249	-1.169096	2.281761	-4.649326	5.92E-06	6.49E-05	3.4990752
TNS2	0.1418593	6.5275283	4.6489181	5.93E-06	6.50E-05	3.4973801
FECH	0.1507588	6.1059102	4.6485559	5.94E-06	6.50E-05	3.4958756
SNORA11	-0.925814	2.2361045	-4.64831	5.95E-06	6.51E-05	3.4948532
SYT8	-1.429313	3.1622792	-4.647999	5.96E-06	6.52E-05	3.4935602
RNF135	0.26288	5.4058757	4.647622	5.97E-06	6.52E-05	3.4919963
MT1A	0.8963266	4.6496641	4.6472369	5.98E-06	6.53E-05	3.4903966
RP11-295H2	-0.879513	1.9373884	-4.647073	5.98E-06	6.54E-05	3.4897176
UBE2E2	0.2124077	5.8389588	4.646949	5.99E-06	6.54E-05	3.4892008
RP11-70C1.	1.2994345	1.7241865	4.6465003	6.00E-06	6.55E-05	3.4873374
SLC2A4RG	-0.135966	6.6563216	-4.64586	6.01E-06	6.57E-05	3.4846778
XXyac-YM21	-1.490137	1.2170047	-4.645161	6.03E-06	6.58E-05	3.4817748
RP11-168K1	1.0951114	1.9958413	4.6442267	6.06E-06	6.61E-05	3.4778977
STK40	0.1034826	6.3575353	4.6441914	6.06E-06	6.61E-05	3.477751
TMC2	-1.134121	0.6176862	-4.643888	6.07E-06	6.61E-05	3.4764903
CCDC68	0.2971275	5.3531215	4.6433765	6.08E-06	6.63E-05	3.4743686
RPSAP8	-1.001695	0.2889354	-4.643363	6.08E-06	6.63E-05	3.4743116
PEG3	-0.787713	4.8621065	-4.643301	6.08E-06	6.63E-05	3.4740571
BRPF3	-0.122959	6.3143546	-4.643149	6.09E-06	6.63E-05	3.4734234
PEX14	0.136243	6.0446297	4.6424646	6.10E-06	6.65E-05	3.4705839
PDK4	0.263066	6.3947158	4.6422419	6.11E-06	6.65E-05	3.4696597
RP11-154D6	1.1014495	0.7943943	4.6419727	6.12E-06	6.66E-05	3.4685428
NFKB1	0.0981916	6.2386636	4.6417321	6.12E-06	6.66E-05	3.4675445
RBM8A	-0.082419	6.5545555	-4.64158	6.13E-06	6.66E-05	3.4669147
TNRC18	-0.08335	6.6265582	-4.6414	6.13E-06	6.67E-05	3.4661667
UGT1A2P	1.5377624	2.0764474	4.6393816	6.19E-06	6.72E-05	3.4577934
CCL21	0.7636214	5.5133792	4.6391139	6.19E-06	6.73E-05	3.4566832
PLA2G16	0.2377719	6.2115934	4.6390179	6.20E-06	6.73E-05	3.4562851
SORL1	0.2167337	6.4210513	4.6382494	6.22E-06	6.75E-05	3.4530978
LDLRAP1	0.1313271	6.1778441	4.6381959	6.22E-06	6.75E-05	3.4528759
RP11-599B1	-0.920232	-0.827168	-4.637767	6.23E-06	6.76E-05	3.451098
RAD18	-0.10531	5.727967	-4.637738	6.23E-06	6.76E-05	3.4509776

ATP5F1P5	1.0824044	-0.069825	4.6376274	6.24E-06	6.76E-05	3.4505187
RP1-292B18	-0.650965	2.7283175	-4.63701	6.25E-06	6.78E-05	3.4479594
FAM200A	-0.104425	5.3866208	-4.636913	6.25E-06	6.78E-05	3.4475548
PRMT9	0.1240919	5.3729326	4.6367332	6.26E-06	6.78E-05	3.4468112
ANKRD33B	0.6214004	4.3350113	4.6363033	6.27E-06	6.79E-05	3.445029
CHTF18	-0.173527	5.6756749	-4.635061	6.31E-06	6.83E-05	3.4398799
RP11-428J1	-0.861228	-0.616551	-4.634632	6.32E-06	6.84E-05	3.4381033
TRIM62	0.1745338	5.1369864	4.633925	6.34E-06	6.86E-05	3.4351722
NEURL1	-0.573898	4.2383708	-4.633692	6.34E-06	6.86E-05	3.4342081
TERT	-1.292953	3.3706459	-4.63353	6.35E-06	6.87E-05	3.4335364
TCL6	-1.466711	1.4314794	-4.63329	6.35E-06	6.87E-05	3.4325413
ESYT2	0.0904149	6.4648172	4.6330864	6.36E-06	6.88E-05	3.4316975
TLN1	0.0780412	6.9274298	4.633036	6.36E-06	6.88E-05	3.4314886
ZNF335	-0.078416	6.0564506	-4.630796	6.42E-06	6.94E-05	3.4222095
MED15P4	-0.887733	-1.057445	-4.630793	6.42E-06	6.94E-05	3.422197
LY96	0.3862192	5.3129294	4.6304148	6.44E-06	6.95E-05	3.4206311
PGM1	0.1539994	6.6948986	4.6302002	6.44E-06	6.95E-05	3.4197424
ZNF517	-0.168839	5.8505657	-4.630105	6.44E-06	6.95E-05	3.4193504
FEM1C	0.120717	6.1062566	4.6300722	6.45E-06	6.95E-05	3.4192125
CLPB	0.1336354	5.9694534	4.6295945	6.46E-06	6.96E-05	3.4172343
FLVCR1-AS1	-0.416836	4.9034322	-4.629551	6.46E-06	6.96E-05	3.4170525
TCERG1	-0.087571	6.1421951	-4.629485	6.46E-06	6.96E-05	3.4167813
RAP2C-AS1	0.4284675	3.8510366	4.6293959	6.46E-06	6.96E-05	3.416412
ZCCHC8	-0.087964	5.9904794	-4.629173	6.47E-06	6.97E-05	3.4154896
RP11-713M1	-1.087775	0.0352773	-4.629119	6.47E-06	6.97E-05	3.4152664
MAML2	0.2372548	5.3660784	4.6285451	6.49E-06	6.98E-05	3.4128898
GLIPR1	0.2353123	5.5486474	4.6284741	6.49E-06	6.98E-05	3.4125957
RP1-80N2.3	-0.617198	3.8550304	-4.62842	6.49E-06	6.98E-05	3.4123727
SRSF9	-0.078885	6.4265749	-4.628303	6.50E-06	6.99E-05	3.4118891
FAM177A1	0.1115615	6.2869574	4.6262776	6.55E-06	7.05E-05	3.4035048
RP4-568C11	1.1746804	4.0034873	4.6255201	6.57E-06	7.07E-05	3.4003701
CDC23	-0.087403	6.1721324	-4.625412	6.58E-06	7.07E-05	3.3999238
RP11-627K1	-0.608811	3.0296964	-4.624509	6.60E-06	7.09E-05	3.3961884
FGFR1	0.25607	5.8091701	4.6211783	6.70E-06	7.20E-05	3.3824123
DVL3	-0.076389	6.499248	-4.619727	6.74E-06	7.24E-05	3.3764111
RP11-519M1	-1.265525	-0.155	-4.619377	6.75E-06	7.25E-05	3.3749677
HAO2-IT1	1.1445895	-0.264522	4.6187575	6.77E-06	7.27E-05	3.3724055
AC004540.5	1.1479097	2.6123271	4.6186338	6.78E-06	7.27E-05	3.3718946
DNAH2	0.7497207	3.5631201	4.6177277	6.80E-06	7.29E-05	3.3681503
C15orf52	0.351105	5.5161966	4.6170645	6.82E-06	7.31E-05	3.36541
U91328.22	-1.064423	1.3015657	-4.616739	6.83E-06	7.32E-05	3.3640667
HID1-AS1	1.0117471	1.3877804	4.6166154	6.84E-06	7.32E-05	3.3635546
CTD-2134P3	-0.856853	-0.949689	-4.616364	6.84E-06	7.33E-05	3.3625171
ZNF589	-0.16083	5.3208378	-4.615245	6.88E-06	7.36E-05	3.3578923
RP13-941N1	-0.931289	1.2645036	-4.614896	6.89E-06	7.37E-05	3.3564521
STIL	-0.262428	5.2395209	-4.614703	6.89E-06	7.38E-05	3.3556559
RBMS3-AS3	1.0604553	0.0078861	4.6136399	6.92E-06	7.41E-05	3.3512654
CARM1P1	-0.992909	-0.956117	-4.613326	6.93E-06	7.42E-05	3.3499697
TLX1NB	-1.033721	-0.490077	-4.613239	6.94E-06	7.42E-05	3.3496109
GDE1	0.1010533	6.2371727	4.6128876	6.95E-06	7.43E-05	3.3481596
BNIP3P10	-0.956092	-0.260198	-4.612071	6.97E-06	7.45E-05	3.3447881

KLHL20	0.1412721	5.8190119	4.6120613	6.97E-06	7.45E-05	3.3447483
PPCS	0.0938364	6.314725	4.6116807	6.98E-06	7.46E-05	3.3431773
LRRC49	0.4752361	3.8994131	4.6111638	7.00E-06	7.47E-05	3.341044
CTD-2134P3	-1.229086	-0.352029	-4.611141	7.00E-06	7.47E-05	3.3409507
RP11-696N1	-0.446704	4.4839999	-4.6101	7.03E-06	7.50E-05	3.3366532
PAK4	-0.114305	6.3414172	-4.609237	7.06E-06	7.53E-05	3.3330925
RP11-727A2	-0.98695	0.18305	-4.608656	7.08E-06	7.55E-05	3.3306944
ZCCHC9	-0.126297	6.1291855	-4.608479	7.08E-06	7.55E-05	3.3299667
PTAFR	0.2836713	5.4089631	4.6083511	7.09E-06	7.55E-05	3.329438
ACP6	-0.158267	5.9790862	-4.607103	7.13E-06	7.59E-05	3.3242885
RP11-128N1	-0.982536	-0.172629	-4.607016	7.13E-06	7.59E-05	3.3239298
LPAR5	0.413844	4.5068104	4.6067386	7.14E-06	7.60E-05	3.322787
HIST1H1B	-1.097629	0.3927241	-4.606467	7.14E-06	7.61E-05	3.3216656
EFR3A	0.1210018	6.4904209	4.6061933	7.15E-06	7.61E-05	3.3205385
GBP3	0.3037523	5.8461608	4.606079	7.16E-06	7.61E-05	3.3200671
NACAP5	-0.846343	-1.047522	-4.605568	7.17E-06	7.63E-05	3.3179607
NHS	0.4838005	3.9968842	4.605253	7.18E-06	7.64E-05	3.3166615
SYNDIG1	1.3893886	1.7522099	4.6050536	7.19E-06	7.64E-05	3.3158392
GPSM2	-0.21962	5.328079	-4.604835	7.20E-06	7.65E-05	3.31494
TCAF2	0.4315881	4.8446673	4.6047317	7.20E-06	7.65E-05	3.3145123
RP11-10G12	-0.531939	3.2723225	-4.604546	7.21E-06	7.65E-05	3.3137472
AKR1C5P	1.3542728	1.2128279	4.6036269	7.23E-06	7.68E-05	3.309958
CCDC162P	-1.19326	3.6904369	-4.603412	7.24E-06	7.69E-05	3.309071
PELO	0.1223306	6.0416688	4.6026502	7.26E-06	7.71E-05	3.3059327
TCP10	-1.336396	0.3627894	-4.602161	7.28E-06	7.72E-05	3.3039151
SMC2	-0.137383	5.8127367	-4.601106	7.31E-06	7.75E-05	3.2995685
HAGH	0.1864562	6.6428108	4.6011016	7.31E-06	7.75E-05	3.2995519
EPDR1	0.2905038	5.9246824	4.6001244	7.35E-06	7.79E-05	3.295526
GATC	-0.081558	6.0127079	-4.600056	7.35E-06	7.79E-05	3.2952458
AC004453.8	-0.320796	4.3795517	-4.599931	7.35E-06	7.79E-05	3.29473
C16orf52	0.1225529	5.6861762	4.599044	7.38E-06	7.82E-05	3.2910761
RAD51D	-0.114879	5.6508873	-4.598814	7.39E-06	7.82E-05	3.2901279
RPH3AL	0.1843486	5.7581846	4.5980666	7.41E-06	7.84E-05	3.2870512
SLC16A2	0.3038281	6.2425312	4.5960197	7.48E-06	7.91E-05	3.2786241
MAGI2	0.3506094	4.7491888	4.5951323	7.51E-06	7.94E-05	3.2749717
RELL1	0.3347276	4.5607807	4.5950199	7.51E-06	7.94E-05	3.274509
SLC30A7	0.0976292	6.1961836	4.5950022	7.51E-06	7.94E-05	3.2744364
RP11-75C10	-0.962877	1.4569122	-4.594947	7.51E-06	7.94E-05	3.2742097
COL25A1	1.2659453	1.5227151	4.5948668	7.52E-06	7.94E-05	3.2738789
NCOA6	-0.080756	6.2966969	-4.594765	7.52E-06	7.94E-05	3.2734585
RP11-624L1	-1.031691	1.4421806	-4.594698	7.52E-06	7.94E-05	3.2731837
CSGALNACT1	0.2324942	5.5359738	4.5939721	7.54E-06	7.96E-05	3.2701975
RP11-69L16	-0.961571	2.1810024	-4.591483	7.63E-06	8.05E-05	3.2599589
ACCS	0.1883014	5.6884457	4.5909237	7.65E-06	8.06E-05	3.2576575
RP11-507E2	-0.715171	2.487794	-4.590851	7.65E-06	8.06E-05	3.2573595
RP11-452K1	-0.990548	1.4829046	-4.590849	7.65E-06	8.06E-05	3.2573503
HMGCL	0.1759011	6.6469086	4.5907613	7.65E-06	8.06E-05	3.2569894
NAA11	-1.622568	0.0465872	-4.59058	7.66E-06	8.07E-05	3.2562431
MCCC2	0.1231281	6.6629524	4.5905326	7.66E-06	8.07E-05	3.2560489
CTD-2630F2	-0.292715	4.0350892	-4.59003	7.67E-06	8.08E-05	3.2539826
HNRNPA1P46	-0.889349	-0.927035	-4.589622	7.69E-06	8.10E-05	3.2523043

VEGFC	0.2273176	5.3315938	4.5891319	7.70E-06	8.11E-05	3.2502897
CNIH4	-0.119513	6.2700565	-4.589032	7.71E-06	8.11E-05	3.2498782
CTC-490E21	0.5483453	4.4191854	4.5888759	7.71E-06	8.11E-05	3.2492372
PKHD1L1	-1.320271	2.350314	-4.588127	7.74E-06	8.14E-05	3.2461608
PI4KAP2	-0.188323	5.1624972	-4.587673	7.75E-06	8.15E-05	3.2442926
RP11-397E7	-0.878841	-0.228603	-4.587299	7.77E-06	8.16E-05	3.2427563
HLA-DMA	0.2187724	6.3266502	4.5871869	7.77E-06	8.16E-05	3.2422948
RP3-325F22	1.1140899	0.1780782	4.5869531	7.78E-06	8.17E-05	3.241334
C8orf49	-0.455057	4.3213829	-4.586163	7.81E-06	8.19E-05	3.2380851
RP11-1430C	1.2480514	0.4330269	4.5860855	7.81E-06	8.19E-05	3.2377688
SPIRE1	0.5160803	5.069241	4.5860766	7.81E-06	8.19E-05	3.2377321
SGCA	0.8424208	4.1415396	4.5860304	7.81E-06	8.19E-05	3.2375422
PMEPA1	0.3312479	5.8346943	4.5859874	7.81E-06	8.19E-05	3.2373656
SVIP	-0.133726	5.9179036	-4.585618	7.82E-06	8.20E-05	3.2358462
OR10J6P	1.2683439	3.361561	4.5854985	7.83E-06	8.21E-05	3.2353568
STUB1	0.1038091	6.2122228	4.5853915	7.83E-06	8.21E-05	3.2349171
E2F2	-0.433785	4.8062564	-4.585077	7.84E-06	8.22E-05	3.2336247
ITGAE	-0.199208	5.5652543	-4.585041	7.84E-06	8.22E-05	3.2334786
ZNF318	-0.103469	6.1264575	-4.584548	7.86E-06	8.23E-05	3.2314518
DIAPH2	0.1203423	6.0862055	4.5841522	7.87E-06	8.24E-05	3.2298261
TFPT	-0.117525	5.7765125	-4.583995	7.88E-06	8.25E-05	3.2291795
CCDC137	-0.111819	6.0395091	-4.583831	7.88E-06	8.25E-05	3.2285063
CYP11A1	1.2746203	4.3383698	4.5837879	7.89E-06	8.25E-05	3.2283297
EARS2	0.1271499	6.1587005	4.5837696	7.89E-06	8.25E-05	3.2282547
HNRNPA1L2	-0.213936	4.7514644	-4.582994	7.91E-06	8.27E-05	3.225068
BNIP2	0.08654	6.2359515	4.5827628	7.92E-06	8.28E-05	3.22412
SNX30	-0.122901	5.9455593	-4.582667	7.92E-06	8.28E-05	3.223725
PCCA	0.1667564	6.3323672	4.5824347	7.93E-06	8.29E-05	3.2227722
RP11-372B4	-0.841694	2.0571376	-4.581816	7.95E-06	8.31E-05	3.2202325
SNRK	0.1201343	6.1856706	4.5814718	7.97E-06	8.32E-05	3.2188189
CTD-3001H1	-0.606187	3.0048123	-4.581024	7.98E-06	8.33E-05	3.216979
BFSP2	-1.255095	2.0819441	-4.580731	7.99E-06	8.34E-05	3.2157761
AKR7L	0.4239113	5.1903678	4.5806538	7.99E-06	8.34E-05	3.2154605
PLN	0.8881081	3.7522223	4.580628	7.99E-06	8.34E-05	3.2153549
RP11-505K9	0.7871447	3.165425	4.5804414	8.00E-06	8.34E-05	3.2145888
PACSIN1	-0.817069	4.2646682	-4.579709	8.03E-06	8.36E-05	3.2115819
KCTD13	0.1285602	5.6488552	4.5794156	8.04E-06	8.37E-05	3.2103784
ZNF283	-0.267567	4.8479618	-4.579296	8.04E-06	8.37E-05	3.2098894
RPP30	-0.091963	5.9522354	-4.579211	8.04E-06	8.37E-05	3.2095378
ZFP82	-0.420141	4.6821188	-4.579178	8.05E-06	8.37E-05	3.2094039
CPZ	1.3441427	1.1217847	4.5791085	8.05E-06	8.37E-05	3.209118
RP11-424M2	-1.022841	0.3033982	-4.579093	8.05E-06	8.37E-05	3.2090537
HOXB4	0.377901	4.3711658	4.5783893	8.07E-06	8.40E-05	3.2061666
STMN2	1.5385878	3.4345843	4.5771516	8.12E-06	8.44E-05	3.2010884
RP11-332M2	-0.138982	5.3165961	-4.57704	8.12E-06	8.44E-05	3.2006298
ZNF491	-0.368464	4.2048588	-4.576756	8.13E-06	8.45E-05	3.199465
TNC	0.308213	5.9043333	4.5765048	8.14E-06	8.45E-05	3.1984353
MGAT4B	0.1217199	6.9022498	4.5765	8.14E-06	8.45E-05	3.1984155
RP1-120G22	0.7718121	2.6203267	4.576014	8.16E-06	8.47E-05	3.1964218
YWHAEP7	-1.494537	0.8995191	-4.575803	8.16E-06	8.47E-05	3.1955573
GATS	0.2118676	5.4789009	4.5754752	8.18E-06	8.48E-05	3.1942119

CDH5	0.1758758	6.2048889	4.5750152	8.19E-06	8.50E-05	3.1923254
RP11-261P1	-1.015004	0.5914583	-4.57364	8.24E-06	8.55E-05	3.1866853
RHOJ	0.2140646	5.3933878	4.5735203	8.25E-06	8.55E-05	3.1861957
LA16c-359F	0.991709	0.0569236	4.5731342	8.26E-06	8.56E-05	3.184613
LRP10	0.1120391	6.676964	4.5727541	8.27E-06	8.57E-05	3.1830548
CTNNAL1	-0.158919	6.0552234	-4.572621	8.28E-06	8.57E-05	3.1825085
C20orf96	-0.160547	5.3551785	-4.572544	8.28E-06	8.57E-05	3.1821926
SLC35E3	-0.099549	5.9757568	-4.571748	8.31E-06	8.60E-05	3.1789307
RP11-498C9	-0.738643	2.8745979	-4.57083	8.34E-06	8.63E-05	3.1751681
GIMAP6	0.2218099	5.7111171	4.5704353	8.36E-06	8.65E-05	3.1735513
AC104534.2	-0.922903	2.580664	-4.570393	8.36E-06	8.65E-05	3.1733767
ALMS1P	-0.923599	2.095911	-4.570178	8.37E-06	8.65E-05	3.1724966
DKFZp779MC	0.620062	4.5094272	4.569918	8.38E-06	8.66E-05	3.1714318
RPS11P5	-0.451465	3.6885349	-4.569894	8.38E-06	8.66E-05	3.1713339
SRSF12	-0.630569	4.1484258	-4.569613	8.39E-06	8.66E-05	3.1701829
RP1-234P15	-0.448552	3.3351448	-4.569363	8.40E-06	8.67E-05	3.1691573
CTD-3224K1	-0.966235	-0.043684	-4.568983	8.41E-06	8.68E-05	3.1676007
RP11-574K1	-0.291376	3.9314558	-4.568855	8.41E-06	8.69E-05	3.1670756
APOBEC2	-0.979629	1.6297863	-4.56881	8.42E-06	8.69E-05	3.166891
RP11-746P2	-0.976658	-0.400143	-4.568339	8.43E-06	8.70E-05	3.1649623
CHRNG	-0.933383	-0.233827	-4.568184	8.44E-06	8.70E-05	3.1643281
RP5-1157M2	-1.020719	1.8762554	-4.566599	8.50E-06	8.76E-05	3.157837
BGLAP	-0.588156	3.6990308	-4.566432	8.50E-06	8.77E-05	3.1571523
RNF216P1	-0.108364	5.853693	-4.565897	8.52E-06	8.78E-05	3.1549615
FAM57B	-1.105276	1.9065409	-4.565232	8.55E-06	8.80E-05	3.1522416
FNDC5	0.6966783	5.0042167	4.5652153	8.55E-06	8.80E-05	3.1521717
ENPP7P13	-1.273937	-0.182446	-4.564732	8.57E-06	8.82E-05	3.1501927
PGM5	0.563661	4.8763298	4.5645963	8.57E-06	8.82E-05	3.149638
TPST1	0.1750952	6.0313364	4.5644617	8.58E-06	8.83E-05	3.1490868
RP11-499F3	-1.286685	-0.084884	-4.562779	8.64E-06	8.89E-05	3.1422011
MNX1	-1.695112	1.1767436	-4.562635	8.64E-06	8.89E-05	3.1416115
EPS8L3	-1.054324	5.2411431	-4.562043	8.67E-06	8.91E-05	3.1391893
CLEC14A	0.205752	5.9393804	4.5620294	8.67E-06	8.91E-05	3.1391333
TPH1	-0.92237	2.7338954	-4.561822	8.67E-06	8.91E-05	3.138286
LINC01128	0.1419807	5.4412269	4.5615512	8.68E-06	8.92E-05	3.1371769
MEN1	-0.080638	6.2691573	-4.561226	8.70E-06	8.93E-05	3.1358464
AC000068.1	-0.798269	2.0036004	-4.561182	8.70E-06	8.93E-05	3.1356651
ZNF485	-0.207425	4.7309097	-4.560915	8.71E-06	8.94E-05	3.1345735
AC006116.2	-1.058696	0.4578936	-4.560058	8.74E-06	8.97E-05	3.1310695
ZNF134	-0.168599	5.6551084	-4.558677	8.79E-06	9.02E-05	3.1254216
RP11-314N1	-0.7126	2.550278	-4.5586	8.80E-06	9.02E-05	3.1251066
PTPRR	0.972196	2.5572745	4.5583689	8.80E-06	9.03E-05	3.1241613
CCNT2-AS1	-0.42363	3.5513292	-4.557044	8.86E-06	9.08E-05	3.1187454
RP11-163F1	1.0661693	-0.763416	4.5567192	8.87E-06	9.09E-05	3.1174169
FAM120A	0.065966	6.7395823	4.5565804	8.87E-06	9.09E-05	3.1168496
P2RY12	1.0600538	2.3929194	4.5564252	8.88E-06	9.09E-05	3.1162154
EGR3	0.4921318	4.2957515	4.5557107	8.91E-06	9.12E-05	3.1132951
GTF2IRD2B	0.2050358	5.0524035	4.5556064	8.91E-06	9.12E-05	3.1128687
COQ4	0.1053534	6.3153176	4.5552601	8.92E-06	9.13E-05	3.1114538
KCNJ9	-1.00416	-0.48646	-4.554415	8.96E-06	9.16E-05	3.1080019
RP11-387A1	-0.918621	-0.73795	-4.553762	8.98E-06	9.19E-05	3.1053329

CTB-50L17.	-0.813747	1.8390237	-4.553026	9.01E-06	9.21E-05	3.1023263
HDAC11	-0.15331	6.0588504	-4.552671	9.02E-06	9.22E-05	3.1008755
RP11-109P6	-0.977785	-0.996545	-4.552615	9.03E-06	9.22E-05	3.1006474
RPS5	-0.103013	7.0941381	-4.551795	9.06E-06	9.25E-05	3.0972962
ARNTL2	0.3202583	5.6321925	4.5513138	9.08E-06	9.27E-05	3.0953329
TUBB6	0.1990497	5.9086414	4.5503534	9.11E-06	9.31E-05	3.0914112
POLL	-0.092622	6.0391739	-4.550114	9.12E-06	9.31E-05	3.0904325
RP11-114H2	-1.494414	0.1277939	-4.550014	9.13E-06	9.32E-05	3.0900245
RP11-565J7	-0.992718	1.2136115	-4.549581	9.15E-06	9.33E-05	3.0882584
PMS1	-0.091326	5.6278258	-4.548942	9.17E-06	9.35E-05	3.0856489
AC104809.4	-1.288703	2.5015156	-4.548457	9.19E-06	9.37E-05	3.083669
PRCC	-0.090131	6.5184601	-4.548352	9.19E-06	9.37E-05	3.0832426
RBM4B	-0.094705	5.7329066	-4.54752	9.23E-06	9.40E-05	3.079846
CERCAM	0.3255174	5.5426107	4.5470914	9.24E-06	9.42E-05	3.0780966
RP11-200A1	1.2811986	1.1269092	4.5465885	9.26E-06	9.44E-05	3.0760447
FDPSP5	-0.737372	-0.810568	-4.54648	9.27E-06	9.44E-05	3.0756024
OR8G5	-1.194493	-0.521432	-4.546281	9.28E-06	9.44E-05	3.0747916
RPS7	-0.09389	7.0663328	-4.545854	9.29E-06	9.46E-05	3.0730468
AR	0.5308253	6.0236032	4.5456713	9.30E-06	9.46E-05	3.0723028
RP3-333B15	-0.616468	-1.217539	-4.545626	9.30E-06	9.46E-05	3.0721179
TSPAN4	0.1345417	6.3607234	4.5454517	9.31E-06	9.47E-05	3.0714069
KRT6C	-1.604854	0.7706648	-4.545295	9.32E-06	9.47E-05	3.0707689
KLF2P4	-1.033435	-0.776221	-4.544816	9.34E-06	9.49E-05	3.068815
RNU6-850P	-1.091595	1.1951134	-4.544233	9.36E-06	9.51E-05	3.0664377
GSTK1	0.1192537	6.8971117	4.5438921	9.37E-06	9.52E-05	3.0650454
MYO10	0.2976206	5.7384364	4.5429573	9.41E-06	9.55E-05	3.0612337
CKMT1A	-1.539114	1.2554803	-4.542907	9.41E-06	9.55E-05	3.0610286
RP11-179B2	-0.701855	2.6129138	-4.542517	9.43E-06	9.57E-05	3.0594397
IGF1	0.5210437	4.9170836	4.5422239	9.44E-06	9.58E-05	3.0582434
ZNF259P1	-1.047644	1.7541495	-4.54186	9.46E-06	9.59E-05	3.05676
HOXC9	-1.653225	1.1790348	-4.541601	9.47E-06	9.60E-05	3.0557031
CFAP44	-0.206793	5.254151	-4.541577	9.47E-06	9.60E-05	3.055606
COL15A1	0.2710308	6.0227255	4.5413796	9.47E-06	9.60E-05	3.0548014
ZNF678	-0.209224	5.3814653	-4.541241	9.48E-06	9.61E-05	3.0542373
CYP2C8	0.5702515	6.3936654	4.5406781	9.50E-06	9.63E-05	3.0519421
SSPN	0.5570635	4.6744754	4.5401622	9.52E-06	9.64E-05	3.0498395
RP11-262I2	-1.068799	-0.406631	-4.539979	9.53E-06	9.65E-05	3.0490948
RPL7P13	-0.668238	-1.108378	-4.53942	9.56E-06	9.67E-05	3.0468129
RP11-395L1	-0.968155	-0.023593	-4.538634	9.59E-06	9.70E-05	3.0436123
POLR2J4	-0.126843	5.8159112	-4.538603	9.59E-06	9.70E-05	3.0434853
KIAA1671	0.1426805	6.4871002	4.5380681	9.61E-06	9.72E-05	3.0413064
ZNF710	-0.162391	5.7946747	-4.537685	9.63E-06	9.73E-05	3.0397439
BACH2	0.5558466	4.6837889	4.5376306	9.63E-06	9.73E-05	3.0395242
AGPAT6	0.106635	6.5092196	4.5375441	9.63E-06	9.73E-05	3.0391718
TARBP2	-0.105394	6.0520991	-4.536488	9.68E-06	9.77E-05	3.0348687
ELP4	-0.094904	5.684657	-4.53634	9.68E-06	9.78E-05	3.0342691
ZNF587	-0.148355	5.6350183	-4.53633	9.68E-06	9.78E-05	3.0342252
TNFSF13B	0.2789292	5.2080999	4.5362591	9.69E-06	9.78E-05	3.033938
RP11-539E1	0.7883221	-1.025639	4.5360816	9.69E-06	9.78E-05	3.033215
KRTAP19-1	-0.795605	-1.057451	-4.535902	9.70E-06	9.79E-05	3.032485
FGF14-AS2	0.4990175	4.2087558	4.5349265	9.74E-06	9.82E-05	3.0285113

RPL29P24	-0.911618	0.4654247	-4.534661	9.75E-06	9.83E-05	3.0274324
THUMPD2	-0.119617	5.4655156	-4.534433	9.76E-06	9.84E-05	3.0265006
ZNF587B	-0.14802	5.6256457	-4.534427	9.76E-06	9.84E-05	3.0264779
OGDHL	0.488632	6.2021967	4.5343854	9.76E-06	9.84E-05	3.0263085
TIMM9	-0.095533	5.9986769	-4.534126	9.78E-06	9.84E-05	3.0252521
MLXIP	0.1025251	6.340714	4.5336557	9.80E-06	9.86E-05	3.0233378
LINC01036	-0.877931	-1.000968	-4.531447	9.89E-06	9.95E-05	3.0143505
PRDM6	0.63201	3.6711875	4.5307344	9.92E-06	9.98E-05	3.0114491
PAQR9	-0.499777	5.9057909	-4.530531	9.93E-06	9.99E-05	3.0106214
RP11-61L19	-0.410639	3.6765939	-4.530238	9.94E-06	1.00E-04	3.0094283
AC114752.2	-0.875281	-0.532265	-4.530094	9.95E-06	0.0001	3.0088453
RP11-458D2	-0.609553	4.0291475	-4.529563	9.97E-06	0.0001002	3.0066848
FLRT2	0.6435797	4.8491004	4.5295603	9.97E-06	0.0001002	3.0066728
RP11-446J8	-1.238882	0.235478	-4.529442	9.97E-06	0.0001002	3.0061918
HIST1H4E	-1.10487	1.7859967	-4.529046	9.99E-06	0.0001003	3.0045815
TESC-AS1	1.262158	0.5488038	4.5290011	9.99E-06	0.0001003	3.0043986
PINK1-AS	0.1802058	4.9185557	4.5289605	1.00E-05	0.0001003	3.0042331
SKIDA1	-0.439221	5.0020465	-4.528684	1.00E-05	0.0001004	3.0031078
TBC1D9B	0.089021	6.6984811	4.5282093	1.00E-05	0.0001006	3.0011783
CLEC4M	1.5379046	0.5586643	4.5279051	1.00E-05	0.0001007	2.9999413
ZRANB3	-0.147725	5.2452866	-4.527597	1.01E-05	0.0001008	2.9986891
AC018641.7	-1.24475	-0.109388	-4.527595	1.01E-05	0.0001008	2.998682
UTP18	-0.092073	6.1287968	-4.527169	1.01E-05	0.0001009	2.9969469
NFKBIA	0.1157177	6.6433569	4.5270247	1.01E-05	0.000101	2.9963611
KLHL21	0.1481482	6.2649637	4.5263236	1.01E-05	0.0001012	2.993511
RP11-1259L	1.5329347	1.703077	4.5255879	1.01E-05	0.0001015	2.9905203
RP11-546J1	-0.778307	2.6780919	-4.525504	1.01E-05	0.0001015	2.9901794
RP11-190C2	-1.006098	1.0786138	-4.525345	1.02E-05	0.0001016	2.9895332
CCNH	0.1053495	5.9572803	4.5249743	1.02E-05	0.0001017	2.9880263
MED20	-0.093295	5.9028542	-4.524638	1.02E-05	0.0001018	2.9866603
FAM149A	0.3294082	5.7476123	4.5238315	1.02E-05	0.0001022	2.9833818
METTL10	-0.116034	5.6056604	-4.523762	1.02E-05	0.0001022	2.9831013
BRDT	-1.271054	-0.475673	-4.523367	1.02E-05	0.0001023	2.9814964
PTGES3L	-0.858222	2.4854498	-4.523352	1.02E-05	0.0001023	2.9814355
RP11-98D18	-0.679228	3.0483592	-4.522628	1.03E-05	0.0001026	2.9784903
DDR2	0.2474948	5.5660005	4.5224579	1.03E-05	0.0001026	2.977801
CRK	0.0953579	6.4369889	4.5211416	1.03E-05	0.0001032	2.9724545
WIPF3	0.6532071	4.910709	4.5208586	1.04E-05	0.0001033	2.971305
TUBB8	-1.137034	0.1468088	-4.520764	1.04E-05	0.0001033	2.9709191
RP11-425L1	-0.259967	5.2253466	-4.520669	1.04E-05	0.0001033	2.9705368
CTD-255501	0.6244813	2.8567976	4.5201737	1.04E-05	0.0001035	2.9685236
UBR4	0.0849147	6.631156	4.5200079	1.04E-05	0.0001036	2.9678501
MRE11A	-0.096521	5.8269245	-4.519521	1.04E-05	0.0001037	2.9658718
SNRPEP2	-0.376071	3.8677016	-4.519276	1.04E-05	0.0001038	2.9648797
TCP11L1	0.141854	5.3555876	4.5189223	1.04E-05	0.000104	2.9634422
HIPK3	0.0893317	6.3730968	4.5182803	1.05E-05	0.0001042	2.960836
BEST4	-0.810176	2.9097074	-4.51786	1.05E-05	0.0001044	2.9591294
FAHD1	0.1348543	6.4036446	4.5178349	1.05E-05	0.0001044	2.9590282
PPM1K	0.263278	5.4057374	4.5175168	1.05E-05	0.0001045	2.957737
PRAM1	0.4018	4.3541869	4.5172077	1.05E-05	0.0001046	2.9564823
DLG1-AS1	-1.047168	0.3933203	-4.517134	1.05E-05	0.0001046	2.9561821

SAMD9L	0.2573702	5.5770747	4.5167838	1.05E-05	0.0001047	2.9547619
RNH1	0.0826406	6.7161823	4.5166338	1.05E-05	0.0001047	2.9541532
STKLD1	-0.288401	4.1518356	-4.516571	1.05E-05	0.0001047	2.9538997
RN7SKP214	-0.844507	-0.864924	-4.516571	1.05E-05	0.0001047	2.9538976
RASGRP3	0.1713513	5.7510856	4.5160721	1.06E-05	0.0001049	2.9518739
RPS10P2	-0.910686	2.0744455	-4.515727	1.06E-05	0.000105	2.950475
FGF14	0.9933123	3.9669632	4.5153025	1.06E-05	0.0001052	2.9487513
RP11-93B14	1.0422001	-0.558226	4.51501	1.06E-05	0.0001053	2.9475645
GATA2-AS1	0.8577679	3.4488111	4.514798	1.06E-05	0.0001054	2.9467044
RP11-119B1	-0.530614	2.9476051	-4.514402	1.06E-05	0.0001055	2.9450987
KLF8	0.437738	4.0628213	4.5143778	1.06E-05	0.0001055	2.9449998
C2CD4C	0.7890385	3.33826	4.5138327	1.07E-05	0.0001057	2.9427887
MID1IP1	-0.139698	6.5343071	-4.513417	1.07E-05	0.0001059	2.9411036
CTD-2368P2	-0.348603	4.54019	-4.513296	1.07E-05	0.0001059	2.9406124
SHROOM4	0.2650674	5.1409875	4.51283	1.07E-05	0.0001061	2.9387222
LINC01420	0.2105243	5.5445711	4.5123608	1.07E-05	0.0001063	2.9368194
RP11-278C7	-0.274473	3.9358921	-4.512269	1.07E-05	0.0001063	2.9364469
AC064836.3	-0.96395	0.0212289	-4.512228	1.07E-05	0.0001063	2.936282
SNX29	0.1430161	5.7651734	4.5119921	1.08E-05	0.0001064	2.9353241
MAFF	0.1856393	6.0569665	4.5113913	1.08E-05	0.0001066	2.9328882
PALD1	0.1975988	5.5718652	4.5110306	1.08E-05	0.0001067	2.9314257
AGO4	0.1016834	5.8894281	4.5110267	1.08E-05	0.0001067	2.93141
ARHGEF15	0.2051227	5.6126665	4.5108298	1.08E-05	0.0001068	2.9306117
AQP10	-1.580636	1.1152719	-4.510321	1.08E-05	0.000107	2.9285473
LHX6	0.5324173	4.0727216	4.5100296	1.08E-05	0.0001071	2.9273683
RP11-433C9	-0.884891	-0.674885	-4.509621	1.09E-05	0.0001073	2.9257117
S100A1	-0.426212	4.9467716	-4.509439	1.09E-05	0.0001073	2.9249735
RP11-108L7	-1.036434	0.5954852	-4.508871	1.09E-05	0.0001075	2.9226717
RNF17	-1.448903	-0.014203	-4.508816	1.09E-05	0.0001075	2.9224478
TMEM168	-0.116336	5.9452889	-4.507749	1.09E-05	0.000108	2.9181254
CTSLP8	-1.021484	-0.607431	-4.507668	1.10E-05	0.000108	2.917797
FMO2	0.6857958	4.4434475	4.5076137	1.10E-05	0.000108	2.9175776
ME1	0.3494624	5.8679791	4.5072848	1.10E-05	0.0001081	2.9162453
DHX35	-0.086265	5.7407239	-4.507023	1.10E-05	0.0001082	2.9151861
MKKS	-0.09794	6.4780425	-4.50687	1.10E-05	0.0001083	2.9145653
BMS1P8	-1.700227	3.1683494	-4.505975	1.10E-05	0.0001086	2.9109392
MOCS3	-0.100199	5.6751637	-4.505624	1.10E-05	0.0001088	2.9095159
MED18	0.1035016	5.8292441	4.5055778	1.11E-05	0.0001088	2.9093307
MCRS1	-0.074955	6.3375792	-4.505397	1.11E-05	0.0001088	2.9085981
ZNRD1	-0.125193	5.8883976	-4.504802	1.11E-05	0.0001091	2.9061888
TFAP2E	-0.647457	3.3340756	-4.504556	1.11E-05	0.0001092	2.9051937
XXyac-YX65	0.8808733	2.2911409	4.5040062	1.11E-05	0.0001094	2.9029664
MAPKAPK5-A	-0.117305	5.6264658	-4.502729	1.12E-05	0.00011	2.8977955
ZNF8	-0.15683	5.2949241	-4.502617	1.12E-05	0.00011	2.8973417
PLA2G4A	0.6016052	4.0748056	4.5020509	1.12E-05	0.0001102	2.895051
RP11-186F1	-0.879815	-0.686251	-4.50205	1.12E-05	0.0001102	2.8950477
SLC29A2	-0.219949	5.8108963	-4.502022	1.12E-05	0.0001102	2.8949332
SH3BP5L	-0.0924	6.275041	-4.499983	1.13E-05	0.0001111	2.8866827
TMEM44-AS1	-0.196111	5.2942721	-4.499535	1.13E-05	0.0001113	2.8848683
RP11-412D9	-0.309229	4.4281598	-4.499274	1.14E-05	0.0001114	2.8838152
SLC2A12	0.6797349	4.7484485	4.4989494	1.14E-05	0.0001115	2.8825012

RP11-12A2.	1.402413	0.9910443	4.4978393	1.14E-05	0.000112	2.8780113
CCNI2	-1.035968	2.4718739	-4.497569	1.14E-05	0.0001121	2.8769188
BAK1P1	-0.898898	1.6214462	-4.497377	1.14E-05	0.0001122	2.8761431
RP11-81H14	1.1847081	3.3327368	4.4972165	1.15E-05	0.0001122	2.8754927
TRIM5	0.1317325	6.0240503	4.4968895	1.15E-05	0.0001124	2.8741702
MEAF6	0.09605	6.3118629	4.4964173	1.15E-05	0.0001126	2.8722609
RP11-572O1	-0.6069	3.3442791	-4.495222	1.16E-05	0.0001131	2.8674301
FAM83A	1.2884292	2.3510311	4.4949773	1.16E-05	0.0001132	2.8664391
CHRNB2	-1.044656	1.9063514	-4.493354	1.16E-05	0.0001139	2.8598783
AC007038.7	-0.626503	3.0222941	-4.492675	1.17E-05	0.0001142	2.857133
RP11-824M1	-0.84485	-0.534289	-4.492299	1.17E-05	0.0001144	2.8556152
LMF1	0.2009428	5.9141122	4.4922353	1.17E-05	0.0001144	2.8553582
MIPEP	0.1435074	5.7311338	4.4921849	1.17E-05	0.0001144	2.8551544
RP11-94A24	1.3256771	0.3348379	4.492136	1.17E-05	0.0001144	2.8549568
RP11-214K3	-0.95603	0.092546	-4.491437	1.17E-05	0.0001147	2.852131
LEMD2	-0.082403	6.3394733	-4.490908	1.18E-05	0.0001149	2.8499942
ATG4A	0.127973	5.9423232	4.4904841	1.18E-05	0.0001151	2.848284
EML1	0.2598414	5.3368766	4.49047	1.18E-05	0.0001151	2.8482271
LL22NC03-2	-0.431968	3.8354905	-4.48943	1.18E-05	0.0001156	2.8440251
XXyac-YM21	-0.891834	-0.859788	-4.488837	1.19E-05	0.0001158	2.8416326
CTD-319301	-0.924721	0.5716461	-4.488532	1.19E-05	0.0001159	2.840401
RGS9	0.4849292	4.367318	4.4882618	1.19E-05	0.0001161	2.8393098
SLC12A1	1.4921174	1.7180888	4.4876161	1.19E-05	0.0001163	2.8367034
NCOA7-AS1	0.9787428	-0.547674	4.4872827	1.20E-05	0.0001165	2.8353575
SNRPC	-0.098018	6.5359325	-4.486984	1.20E-05	0.0001166	2.8341502
GLIS3	0.4020716	5.5146857	4.4868273	1.20E-05	0.0001166	2.833519
ELTD1	0.1720919	5.774795	4.4865917	1.20E-05	0.0001167	2.8325683
C10orf62	-1.093639	0.7696959	-4.486397	1.20E-05	0.0001168	2.8317833
RP11-483G2	-1.041778	-0.41381	-4.485954	1.20E-05	0.000117	2.8299964
MROH2A	1.3038647	3.1041307	4.4857553	1.20E-05	0.0001171	2.8291926
AC012074.2	-1.01987	1.3690681	-4.484961	1.21E-05	0.0001174	2.8259883
DYNC1LI2	0.1558101	6.0943978	4.4844313	1.21E-05	0.0001176	2.8238506
CCR6	-1.174913	1.6374142	-4.484412	1.21E-05	0.0001176	2.8237744
RP11-358L2	-0.298517	3.999823	-4.484142	1.21E-05	0.0001177	2.822683
RP11-574M7	-0.908591	-1.023949	-4.483782	1.21E-05	0.0001179	2.8212306
CHTF8	0.0762393	6.4349706	4.4836603	1.21E-05	0.0001179	2.8207402
ZBTB25	0.0994912	5.6286556	4.483653	1.21E-05	0.0001179	2.8207106
AC012360.4	-0.969211	0.2488517	-4.48256	1.22E-05	0.0001184	2.8163003
MIR548AC	-0.638974	-1.131887	-4.482407	1.22E-05	0.0001185	2.8156863
DNASE1L1	0.1308801	5.7841703	4.4809205	1.23E-05	0.0001192	2.8096905
SYNC	0.9090562	2.7635998	4.4805043	1.23E-05	0.0001194	2.8080125
HHLA3	0.2269906	5.46887	4.4798457	1.23E-05	0.0001197	2.8053577
FRMD4B	0.1356009	5.9508487	4.4794788	1.24E-05	0.0001198	2.8038785
PPY2	-1.152517	-0.65936	-4.479344	1.24E-05	0.0001198	2.8033336
RP11-303E1	0.9048786	2.4980235	4.4793303	1.24E-05	0.0001198	2.8032799
ADAMTSL3	0.4179815	5.5401849	4.4791626	1.24E-05	0.0001199	2.8026043
ADAM28	0.5156803	4.7017742	4.4781767	1.24E-05	0.0001204	2.7986307
CXCL1	1.0072326	4.6022862	4.4776749	1.25E-05	0.0001206	2.7966085
FSTL3	0.3045932	6.0028075	4.4775518	1.25E-05	0.0001206	2.7961127
AC093627.9	-0.453974	3.4999026	-4.475975	1.25E-05	0.0001214	2.7897586
SEPSECS-AS	-0.232005	4.8677723	-4.475886	1.25E-05	0.0001214	2.7894014

PCDH18	0.3352548	4.8320481	4.4751685	1.26E-05	0.0001218	2.7865115
SLC22A20	-0.768066	2.8186245	-4.474405	1.26E-05	0.0001221	2.7834369
EXOSC3	-0.102223	5.779678	-4.474329	1.26E-05	0.0001221	2.783131
ALG8	-0.098758	6.3529024	-4.474142	1.26E-05	0.0001222	2.7823793
FBXL22	-0.346089	3.800414	-4.473693	1.27E-05	0.0001224	2.7805704
GUCY1A2	0.2642244	5.233445	4.4731671	1.27E-05	0.0001226	2.7784517
BTF3P4	-0.949297	-0.062837	-4.472566	1.27E-05	0.0001229	2.7760328
C16orf95	0.2695219	4.4182254	4.47198	1.28E-05	0.0001232	2.7736729
UGT2B17	1.5587854	3.2604665	4.4719467	1.28E-05	0.0001232	2.773539
RP11-676J1	1.3559946	2.054432	4.4705383	1.28E-05	0.0001239	2.7678704
GPRASP2	0.2044376	5.5164849	4.4704928	1.28E-05	0.0001239	2.7676872
DLX4	-1.266757	2.1445135	-4.47044	1.28E-05	0.0001239	2.7674733
AC016710.1	-1.500182	-0.026527	-4.47031	1.29E-05	0.0001239	2.7669526
ZNF749	-0.203174	4.8656483	-4.470037	1.29E-05	0.000124	2.765855
GTF3C1	0.0766914	6.4294681	4.4696778	1.29E-05	0.0001242	2.764408
EPHB1	0.5091936	3.9001822	4.469429	1.29E-05	0.0001243	2.7634069
CD151	0.1125574	6.9127974	4.4693481	1.29E-05	0.0001243	2.7630813
RP11-685F1	0.8070966	-1.114534	4.4691131	1.29E-05	0.0001244	2.7621359
RP11-29601	-0.271502	4.3109347	-4.468947	1.29E-05	0.0001244	2.7614695
RP11-758H9	-0.287677	4.3541401	-4.46786	1.30E-05	0.000125	2.7570953
RP11-830F9	1.3341676	1.6644239	4.4673975	1.30E-05	0.0001252	2.7552347
MT1G	0.7927084	5.4847698	4.4673296	1.30E-05	0.0001252	2.7549616
CLN5	0.1214376	5.7171925	4.4671078	1.30E-05	0.0001253	2.7540698
PCOLCE2	-0.497463	5.5960975	-4.466089	1.31E-05	0.0001258	2.7499722
C16orf72	0.0919793	6.2584777	4.46534	1.31E-05	0.0001262	2.7469611
RP11-104F1	-0.901113	2.1940286	-4.465019	1.31E-05	0.0001263	2.7456694
CBX4	-0.110772	6.250346	-4.464882	1.32E-05	0.0001263	2.745118
RP11-602.4	-0.970281	2.5527554	-4.464299	1.32E-05	0.0001266	2.742776
CWH43	1.2074115	-0.205488	4.4640332	1.32E-05	0.0001267	2.741708
RP11-977G1	-0.780772	-0.756015	-4.463689	1.32E-05	0.0001269	2.7403239
TOPORS-AS1	-0.199591	5.0469633	-4.463641	1.32E-05	0.0001269	2.7401323
PLEKHO2	0.1296248	6.0800222	4.4625691	1.33E-05	0.0001274	2.7358239
FAM124B	0.4484312	4.4375395	4.4624237	1.33E-05	0.0001275	2.7352396
MID2	0.2933897	5.3226119	4.4617866	1.33E-05	0.0001278	2.7326798
CDKAL1	-0.096772	5.8501093	-4.461008	1.34E-05	0.0001282	2.7295503
TRPC1	-0.377697	4.6709878	-4.460916	1.34E-05	0.0001282	2.7291805
ARAP2	0.2301352	5.6202632	4.4608194	1.34E-05	0.0001282	2.7287943
DSEL	0.419165	5.1947221	4.4607676	1.34E-05	0.0001282	2.7285862
SPPL3	-0.075096	6.3007304	-4.459926	1.34E-05	0.0001286	2.7252057
JRK	-0.166546	5.6514361	-4.459751	1.34E-05	0.0001287	2.7245008
PARP6	0.246765	5.7182167	4.4596281	1.34E-05	0.0001287	2.7240093
JUN	0.1412016	6.6856048	4.4594223	1.35E-05	0.0001288	2.7231829
MIR548AA1	-0.936736	-0.151262	-4.458163	1.35E-05	0.0001295	2.7181259
LINC00659	1.4782501	2.7538686	4.4570782	1.36E-05	0.00013	2.7137709
FLJ22763	1.5010198	2.0937686	4.4568653	1.36E-05	0.0001301	2.7129161
POLK	0.126652	5.7784268	4.4561269	1.37E-05	0.0001305	2.7099522
DUSP10	0.1911339	6.1582504	4.4560406	1.37E-05	0.0001305	2.7096059
RP11-255M6	1.1106546	0.1162734	4.4553281	1.37E-05	0.0001309	2.7067466
LINC00383	-1.207748	-0.392345	-4.454693	1.37E-05	0.0001312	2.7041973
OR13A1	-1.176451	-0.022194	-4.454547	1.37E-05	0.0001312	2.7036111
GALNT15	0.448265	4.7404433	4.454507	1.37E-05	0.0001312	2.7034518

YY1AP1	-0.082878	6.3468718	-4.45329	1.38E-05	0.0001319	2.6985685
FAAH	0.2056083	6.0270416	4.4532032	1.38E-05	0.0001319	2.6982209
MPZ	0.5085415	5.1632067	4.4530897	1.38E-05	0.0001319	2.6977655
RP3-331H24	0.7603016	3.470324	4.452953	1.38E-05	0.0001319	2.6972174
KCND2	1.2982308	1.9906746	4.4529472	1.38E-05	0.0001319	2.6971939
RP11-386G1	-1.070294	1.7539963	-4.452888	1.38E-05	0.0001319	2.6969573
FAM189A2	0.4774713	3.7232919	4.4525322	1.39E-05	0.0001321	2.6955293
RP11-166P1	-1.095342	1.540058	-4.452272	1.39E-05	0.0001322	2.6944861
ACTB	0.071813	7.5937829	4.4521432	1.39E-05	0.0001322	2.6939693
OGFRL1	0.1560424	5.7598309	4.4510928	1.39E-05	0.0001328	2.6897571
ANO7P1	0.5561138	3.979162	4.4501133	1.40E-05	0.0001333	2.6858295
RP11-666A8	-0.682085	2.7200819	-4.45002	1.40E-05	0.0001333	2.6854538
PRKCDBP	0.2418004	5.5122239	4.4490592	1.41E-05	0.0001338	2.6816042
UFSP2	0.1031301	5.7957791	4.4484165	1.41E-05	0.0001342	2.679028
RP11-248E9	1.1699636	-0.187248	4.4482245	1.41E-05	0.0001342	2.6782587
UTP6	-0.073666	6.2362922	-4.447916	1.41E-05	0.0001344	2.6770229
HLA-H	0.1917831	6.1786728	4.4475804	1.42E-05	0.0001345	2.6756774
RN7SL521P	-0.974274	1.1013361	-4.447488	1.42E-05	0.0001346	2.6753089
HCLS1	0.1712537	6.1305617	4.4472647	1.42E-05	0.0001346	2.6744121
RP11-816J6	-1.052375	0.9122595	-4.446605	1.42E-05	0.000135	2.6717678
ZNF586	-0.171353	5.0774665	-4.446089	1.42E-05	0.0001352	2.6697025
RP5-965G21	-0.907949	2.053286	-4.445373	1.43E-05	0.0001356	2.6668343
SYTL2	0.2412672	5.3519504	4.4448141	1.43E-05	0.0001359	2.6645948
ATP5G1P4	-1.047425	1.9416946	-4.444462	1.43E-05	0.0001361	2.663183
RSG1	0.3085317	4.6791495	4.4443049	1.44E-05	0.0001361	2.6625555
PFKP	0.3085565	5.7731595	4.4439444	1.44E-05	0.0001363	2.6611121
SART3	-0.058607	6.2540617	-4.443758	1.44E-05	0.0001363	2.6603655
BDH2	0.1715064	5.9508632	4.4437301	1.44E-05	0.0001363	2.6602537
MSR1	0.2411945	5.6896506	4.443728	1.44E-05	0.0001363	2.6602455
NAPA	0.0971929	6.5744948	4.4434651	1.44E-05	0.0001364	2.6591928
CTD-2073O6	-0.683565	-0.954954	-4.442722	1.45E-05	0.0001368	2.6562159
UBR1	0.1004986	5.8934353	4.4424727	1.45E-05	0.0001369	2.6552194
PRPF3	-0.09493	6.2274796	-4.442128	1.45E-05	0.0001371	2.6538385
AC011322.1	-1.058541	-0.107822	-4.441985	1.45E-05	0.0001372	2.6532651
PIP4K2B	-0.066384	6.365951	-4.441853	1.45E-05	0.0001372	2.6527396
USP33	0.0798496	6.2168708	4.440966	1.46E-05	0.0001377	2.6491882
CFAP221	1.0185651	4.0181199	4.4407454	1.46E-05	0.0001378	2.6483051
DLX2	-1.26725	-0.052742	-4.44057	1.46E-05	0.0001378	2.6476036
LGALS8-AS1	-0.831822	2.619971	-4.440262	1.46E-05	0.000138	2.6463689
RP11-385F5	-0.755372	2.1323567	-4.44007	1.46E-05	0.0001381	2.6456005
RP11-170M1	-0.355722	3.9692954	-4.439965	1.46E-05	0.0001381	2.6451835
CFAP58	0.868483	1.2988678	4.4389205	1.47E-05	0.0001387	2.6410029
AC129492.6	-1.228578	0.7236325	-4.438768	1.47E-05	0.0001387	2.6403922
PKNOX2	0.72812	3.8231787	4.4379712	1.47E-05	0.0001392	2.637205
RP11-95P9.	-0.799524	-1.092701	-4.437754	1.48E-05	0.0001392	2.6363361
RPS18P6	-0.932469	-0.929658	-4.437679	1.48E-05	0.0001393	2.6360369
RP11-78J21	-0.764426	-0.986649	-4.437415	1.48E-05	0.0001394	2.6349799
GJA4	0.2164277	5.801361	4.4372961	1.48E-05	0.0001394	2.6345049
FOXQ1	-1.089602	4.5370335	-4.436936	1.48E-05	0.0001396	2.6330638
ANKZF1	-0.088535	6.1592338	-4.436807	1.48E-05	0.0001396	2.6325482
AC007383.3	-0.231307	4.6051601	-4.436787	1.48E-05	0.0001396	2.6324702

RP11-655G2	-0.976843	-0.813389	-4.436672	1.48E-05	0.0001396	2.6320093
TEN1-CDK3	-0.291989	4.4940578	-4.436212	1.49E-05	0.0001399	2.6301703
SCARA5	1.184905	3.2350399	4.435802	1.49E-05	0.0001401	2.6285298
LINC01192	-1.053194	-0.625623	-4.435278	1.49E-05	0.0001404	2.6264357
RP11-145G2	-1.304751	-0.326091	-4.435151	1.49E-05	0.0001404	2.6259273
RP11-31E13	0.646244	-1.189282	4.4345396	1.50E-05	0.0001407	2.6234826
NCAPG2	-0.164473	5.7846043	-4.433857	1.50E-05	0.0001411	2.6207545
AC099552.2	0.7733956	-1.042747	4.4335843	1.50E-05	0.0001412	2.6196643
SPAG6	-1.183107	0.2424153	-4.433224	1.50E-05	0.0001414	2.6182233
MAP1LC3B2	0.2804485	4.0874665	4.4329919	1.51E-05	0.0001415	2.6172966
FAM118A	-0.150349	5.8690703	-4.432966	1.51E-05	0.0001415	2.6171916
TSPAN2	0.7141238	3.8276024	4.432841	1.51E-05	0.0001415	2.6166934
RP1-27K12.	1.2830298	3.9306096	4.4325359	1.51E-05	0.0001416	2.6154742
RP11-736K2	0.4979744	3.7319968	4.4325182	1.51E-05	0.0001416	2.6154034
IFNGR1	0.1261868	6.4547216	4.432316	1.51E-05	0.0001417	2.6145957
VN1R48P	-1.227966	1.2503286	-4.431248	1.52E-05	0.0001423	2.6103263
WASIR2	-1.104258	0.1985366	-4.430412	1.52E-05	0.0001428	2.6069885
CIB1	0.0970996	6.6374504	4.4279048	1.54E-05	0.0001443	2.5969761
ZNF556	-1.207906	0.9881192	-4.427606	1.54E-05	0.0001444	2.5957846
RP3-412A9.	-0.710704	2.9416703	-4.427347	1.54E-05	0.0001445	2.5947472
DNAJC27-AS	-0.272689	4.3534523	-4.427324	1.54E-05	0.0001445	2.5946572
FRS3	-0.11752	5.2821863	-4.427174	1.54E-05	0.0001446	2.59406
AHDC1	0.1235447	5.9131119	4.4268616	1.55E-05	0.0001447	2.5928114
IL17F	-0.76793	-0.967766	-4.425894	1.55E-05	0.0001453	2.5889502
CKS1B	-0.131865	6.0729148	-4.424488	1.56E-05	0.0001461	2.5833383
TMEM26	0.4342168	4.0028323	4.4244722	1.56E-05	0.0001461	2.5832752
MESP2	-0.70576	3.896265	-4.423769	1.57E-05	0.0001465	2.5804676
FPGS	0.1073739	6.4114896	4.4235068	1.57E-05	0.0001466	2.5794234
TACC1	0.148753	6.4430994	4.4232968	1.57E-05	0.0001467	2.5785858
RNASE6	0.2626031	5.4211332	4.4231555	1.57E-05	0.0001468	2.578022
ZNF534	-1.241655	0.1914076	-4.422836	1.57E-05	0.0001469	2.5767468
ZNF44	-0.111764	5.7111171	-4.42182	1.58E-05	0.0001474	2.5726956
CELF2	0.2553326	5.4852293	4.4218018	1.58E-05	0.0001474	2.5726226
RP11-1109F	-1.027408	0.9983839	-4.42178	1.58E-05	0.0001474	2.5725359
DNM1P35	-0.843531	3.0247603	-4.421776	1.58E-05	0.0001474	2.5725215
RP11-1035H	0.9470583	1.6243113	4.4217573	1.58E-05	0.0001474	2.5724453
RWDD4	0.1109971	5.4560296	4.4215425	1.58E-05	0.0001475	2.5715887
RP11-876N2	0.4731595	3.7429751	4.4212889	1.58E-05	0.0001476	2.5705773
ACO2	0.1155767	6.559358	4.4188284	1.60E-05	0.0001491	2.5607679
GUSBP11	-0.368101	4.3825451	-4.41872	1.60E-05	0.0001492	2.5603346
HMGA1	-0.168203	6.5254645	-4.418291	1.60E-05	0.0001494	2.5586272
ZDHHC2	0.2519142	5.4905246	4.4179913	1.60E-05	0.0001495	2.5574314
WISP1	0.5131173	4.494915	4.4177003	1.61E-05	0.0001496	2.5562718
RPS4XP6	-0.892873	1.6175096	-4.417668	1.61E-05	0.0001496	2.5561428
ADAT2	-0.192011	5.2967256	-4.417663	1.61E-05	0.0001496	2.5561233
HLA-DPA1	0.2192956	6.6177832	4.4171228	1.61E-05	0.0001499	2.5539706
RP11-15J22	-0.642016	-1.041931	-4.416903	1.61E-05	0.00015	2.5530963
RP11-762L8	0.8756577	2.374454	4.4166641	1.61E-05	0.0001502	2.5521427
S100A11	0.1910659	6.4293255	4.4164568	1.62E-05	0.0001502	2.5513169
KRI1	-0.078129	6.1039258	-4.416106	1.62E-05	0.0001504	2.5499207
NUDT9	0.1214549	6.2134378	4.4157405	1.62E-05	0.0001506	2.5484633

ELFN1-AS1	-1.440317	1.6976307	-4.415717	1.62E-05	0.0001506	2.5483702
ABCC6P2	-0.413298	4.9954913	-4.415651	1.62E-05	0.0001506	2.5481072
CD82	0.1445362	6.3016291	4.4153006	1.62E-05	0.0001508	2.5467109
BAALC-AS1	0.8712106	3.3123045	4.4152802	1.62E-05	0.0001508	2.5466299
CDC7	-0.245338	5.2233833	-4.41336	1.64E-05	0.000152	2.5389815
VSIG2	0.6465192	4.0425307	4.4129658	1.64E-05	0.0001521	2.5374129
TDO2	0.4610825	6.4496344	4.412953	1.64E-05	0.0001521	2.537362
MAP2K3	0.1075546	6.4078734	4.4128973	1.64E-05	0.0001521	2.53714
ICMT	0.0798098	6.3810073	4.4126975	1.64E-05	0.0001522	2.5363448
ZBED6CL	-0.209438	5.9267649	-4.412281	1.64E-05	0.0001525	2.5346858
RP11-761I4	-0.918099	-0.303083	-4.412083	1.65E-05	0.0001525	2.5338965
RNA5SP219	0.9242911	-0.480008	4.4111649	1.65E-05	0.0001531	2.5302436
NDUFS5P2	-0.606821	-1.25718	-4.410872	1.65E-05	0.0001532	2.529078
CTD-2026D2	-0.209468	4.5519591	-4.410646	1.66E-05	0.0001534	2.5281767
RP11-245P1	-1.167168	0.7188107	-4.410563	1.66E-05	0.0001534	2.5278491
PGAP2	-0.116003	6.1946668	-4.410074	1.66E-05	0.0001536	2.5259007
RP11-706C1	-1.138737	4.2165188	-4.40978	1.66E-05	0.0001538	2.5247342
NOTCH3	0.1916971	6.3530337	4.4093416	1.66E-05	0.000154	2.5229878
HOXD10	-1.388793	1.038263	-4.408623	1.67E-05	0.0001545	2.5201299
CCNE1	-0.358874	5.1006208	-4.408218	1.67E-05	0.0001547	2.5185169
LINC00665	-0.493243	5.2703249	-4.407336	1.68E-05	0.0001552	2.5150085
RAE1	-0.087926	6.1182151	-4.407326	1.68E-05	0.0001552	2.5149707
AKAP9	0.1342287	6.3487192	4.4072408	1.68E-05	0.0001552	2.5146309
C5orf60	-0.916956	-0.187948	-4.406648	1.68E-05	0.0001556	2.5122726
PABPC1P1	-0.916477	-0.03584	-4.40653	1.68E-05	0.0001556	2.5118041
THUMPD1	0.0792073	6.1221229	4.4061361	1.69E-05	0.0001558	2.5102374
THEMIS2	0.2220928	5.7162198	4.4060787	1.69E-05	0.0001558	2.5100092
RP11-399K2	-0.55653	3.237481	-4.405457	1.69E-05	0.0001562	2.5075368
RRP7B	-0.193938	5.0073667	-4.405355	1.69E-05	0.0001562	2.5071302
DDIT4	0.1996065	6.3715783	4.4048906	1.70E-05	0.0001565	2.5052855
SLC25A5P5	-1.185691	0.3629935	-4.403624	1.70E-05	0.0001573	2.5002496
HAVCR2	0.2635681	5.4623161	4.4016512	1.72E-05	0.0001586	2.4924112
ATP11AUN	-0.822896	-0.721521	-4.401153	1.72E-05	0.0001589	2.4904334
LA16c-83F1	-1.142249	-0.400633	-4.401075	1.72E-05	0.0001589	2.4901237
AP1S2	0.1453656	5.6655203	4.4006044	1.73E-05	0.0001591	2.4882524
MIR5010	-0.904197	-0.24638	-4.400437	1.73E-05	0.0001592	2.4875869
RP11-796E2	-0.715763	3.1744712	-4.400243	1.73E-05	0.0001593	2.486816
FNIP2	0.1656581	6.2252469	4.4001546	1.73E-05	0.0001593	2.4864657
PDLIM1P4	0.8468515	2.0777573	4.3997142	1.73E-05	0.0001596	2.4847164
LINC00958	-1.354288	-0.073951	-4.39959	1.73E-05	0.0001596	2.484224
RP11-973F1	-1.231058	0.5998276	-4.397782	1.75E-05	0.0001608	2.4770449
HSD17B13	0.9142603	5.3780567	4.397683	1.75E-05	0.0001608	2.4766509
CTB-111H14	0.9980762	-0.295393	4.3975353	1.75E-05	0.0001609	2.4760643
HS3ST2	0.4800307	4.3937225	4.3968274	1.75E-05	0.0001613	2.4732542
HCG15	-0.972912	2.4053343	-4.396474	1.76E-05	0.0001615	2.4718524
AQP11	-0.254506	5.6325827	-4.396235	1.76E-05	0.0001616	2.4709022
KDELC1	-0.199358	5.612479	-4.396091	1.76E-05	0.0001617	2.4703302
LARP4	0.0979592	6.4703563	4.3960084	1.76E-05	0.0001617	2.4700034
ST8SIA4	0.2148674	5.3091201	4.3950355	1.77E-05	0.0001623	2.4661425
LINC01150	1.0199932	0.7738863	4.3946639	1.77E-05	0.0001626	2.464668
RP11-439L1	-0.792856	-0.598129	-4.394598	1.77E-05	0.0001626	2.4644062

WASH6P	-0.135671	5.3598971	-4.394272	1.77E-05	0.0001627	2.4631136
TANC2	0.337856	5.2678379	4.3940389	1.78E-05	0.0001628	2.4621882
PTGER3	0.6443902	3.9124158	4.394015	1.78E-05	0.0001628	2.4620933
CTD-3222D1	-0.269161	4.2399246	-4.393952	1.78E-05	0.0001628	2.4618447
CTD-3162L1	-1.050856	1.4108962	-4.39223	1.79E-05	0.000164	2.4550141
RP5-823G15	-0.761515	-0.966675	-4.392061	1.79E-05	0.0001641	2.4543409
HIF1A	0.1308494	6.5013996	4.3916342	1.79E-05	0.0001643	2.4526499
RP11-134G8	0.4785688	4.1676591	4.3910153	1.80E-05	0.0001647	2.4501958
DBIL5P	-0.803269	2.0281944	-4.390876	1.80E-05	0.0001648	2.4496433
TEC	0.3119548	4.6229447	4.3901276	1.80E-05	0.0001652	2.4466763
NF1P8	-1.011173	-0.856153	-4.38962	1.81E-05	0.0001655	2.4446656
XRCC6BP1	-0.193279	5.2351436	-4.389603	1.81E-05	0.0001655	2.4445947
ZNF2	-0.10659	5.1449894	-4.389293	1.81E-05	0.0001657	2.4433674
ZNF559-ZNF	-0.997031	1.6845035	-4.388521	1.82E-05	0.0001662	2.4403085
KLHL26	0.198296	5.4515339	4.3879539	1.82E-05	0.0001665	2.4380603
RP11-557H1	1.2200782	1.0276496	4.3874029	1.83E-05	0.0001669	2.4358768
STT3A-AS1	-0.851367	-0.388039	-4.386733	1.83E-05	0.0001673	2.4332228
FOXRED2	-0.137802	6.1942882	-4.386687	1.83E-05	0.0001673	2.433039
RP11-452F1	-0.205129	5.3352464	-4.386196	1.83E-05	0.0001676	2.4310946
ISCU	0.0846128	6.4985193	4.3861663	1.83E-05	0.0001676	2.4309774
RP11-19G24	-0.947482	0.2391267	-4.386128	1.84E-05	0.0001676	2.4308248
SMARCB1	-0.088428	6.4064006	-4.385924	1.84E-05	0.0001677	2.4300192
EEF1E1P1	-1.016074	1.0472702	-4.385802	1.84E-05	0.0001677	2.4295339
NEGR1	1.0418352	2.310924	4.3856923	1.84E-05	0.0001678	2.4290999
OTUD4	0.1046548	6.0685948	4.3850661	1.84E-05	0.0001682	2.4266194
EPHA4	0.4310787	4.6363091	4.3846124	1.85E-05	0.0001684	2.4248224
AC104389.2	-1.189009	-0.044669	-4.384604	1.85E-05	0.0001684	2.4247908
TDRKH	-0.236287	5.6840642	-4.384301	1.85E-05	0.0001686	2.4235905
SCNM1	-0.122841	6.0917697	-4.383385	1.86E-05	0.0001692	2.4199625
PPARGC1A	0.4519134	6.0378105	4.382721	1.86E-05	0.0001696	2.4173332
ACSF3	0.1262206	6.0574665	4.3823858	1.86E-05	0.0001698	2.4160061
RP11-396C2	-0.65515	3.7257297	-4.382191	1.87E-05	0.0001699	2.4152335
SLC30A8	-1.251427	-0.165397	-4.382132	1.87E-05	0.0001699	2.4150031
SH3D19	0.1279926	6.3782724	4.3819261	1.87E-05	0.00017	2.4141866
SMARCD3	0.2623414	5.5325025	4.3816197	1.87E-05	0.0001702	2.4129736
OGFOD1	0.0795736	6.1044893	4.3807378	1.88E-05	0.0001708	2.4094834
RP11-475B2	0.7526929	-0.934002	4.3806664	1.88E-05	0.0001708	2.4092006
RPL7P28	-0.789404	-0.831422	-4.380558	1.88E-05	0.0001708	2.4087712
SMAD1	0.2819248	5.540658	4.3798421	1.88E-05	0.0001713	2.4059388
HHIP-AS1	1.1174144	2.162355	4.379331	1.89E-05	0.0001716	2.4039166
OAZ2	0.0775443	6.5108524	4.3792197	1.89E-05	0.0001716	2.4034763
RP11-632F7	-0.96583	-0.481075	-4.379151	1.89E-05	0.0001717	2.403204
CTD-2588E2	0.9422884	0.4652223	4.3789158	1.89E-05	0.0001718	2.4022741
COX4I1	0.1110551	6.9646917	4.3783497	1.90E-05	0.0001721	2.4000346
ANKAR	-0.165232	4.6832854	-4.378283	1.90E-05	0.0001721	2.39977
RP11-286E1	1.0440529	1.1426018	4.3782603	1.90E-05	0.0001721	2.3996809
CTNND2	-1.333359	4.3214404	-4.378245	1.90E-05	0.0001721	2.3996208
RP4-713B5.	-0.950081	1.2298156	-4.378118	1.90E-05	0.0001721	2.3991192
SPATA5L1	-0.107598	5.7263128	-4.377854	1.90E-05	0.0001723	2.3980744
SFMBT2	0.2615282	5.1784632	4.3778071	1.90E-05	0.0001723	2.3978883
TBP	-0.087664	5.6760701	-4.377702	1.90E-05	0.0001723	2.3974706

MMP14	0.1566493	6.5494498	4.3776446	1.90E-05	0.0001723	2.3972456
PACSIN3	-0.1908	6.1367886	-4.376697	1.91E-05	0.000173	2.3934996
TSSK6	-0.25922	4.7396066	-4.375918	1.92E-05	0.0001735	2.3904157
HLA-DRB5	0.2719581	6.373397	4.3756937	1.92E-05	0.0001736	2.3895307
UBE2S	-0.195903	5.8289745	-4.375693	1.92E-05	0.0001736	2.3895289
RP11-517H2	-1.034087	1.0344938	-4.373256	1.94E-05	0.0001753	2.379894
SFTPD	1.086648	2.6738194	4.373205	1.94E-05	0.0001753	2.3796936
RP11-214K3	-0.984973	0.5545691	-4.372984	1.94E-05	0.0001754	2.3788213
USP30-AS1	0.5135623	4.6598163	4.3728639	1.94E-05	0.0001755	2.3783457
AC005154.7	-0.663322	3.0385857	-4.371191	1.95E-05	0.0001766	2.3717369
RP11-462L8	0.695805	4.0878136	4.3711692	1.95E-05	0.0001766	2.3716499
RP4-777L9.	-0.963907	1.321766	-4.371145	1.95E-05	0.0001766	2.3715559
KIZ	-0.144021	5.8346079	-4.369658	1.97E-05	0.0001777	2.3656802
SLC27A1	0.1878867	5.858211	4.3695485	1.97E-05	0.0001777	2.3652485
CREM	0.104651	6.0719159	4.3693564	1.97E-05	0.0001778	2.36449
COX11	-0.080392	6.2966473	-4.369202	1.97E-05	0.0001779	2.3638805
CTD-231801	-1.059183	0.9851878	-4.367967	1.98E-05	0.0001787	2.3590057
RP11-708J1	-0.365191	3.5962828	-4.367933	1.98E-05	0.0001787	2.3588704
LINC01311	-0.440221	3.705615	-4.367497	1.98E-05	0.000179	2.3571475
RP1-191J18	-0.288291	4.14555	-4.367106	1.99E-05	0.0001793	2.3556041
ATP2C1	0.0642941	6.4020989	4.366599	1.99E-05	0.0001796	2.3536038
ANAPC1	-0.104729	5.6837115	-4.366014	2.00E-05	0.00018	2.351295
ADAMTSL5	0.5575141	4.8684999	4.3659723	2.00E-05	0.00018	2.3511306
CCRN4L	0.2792337	5.2766733	4.3657834	2.00E-05	0.0001801	2.3503848
MB	0.9405675	3.5189213	4.3656792	2.00E-05	0.0001801	2.3499736
ZC3H6	0.1382809	5.4083048	4.3656186	2.00E-05	0.0001801	2.3497349
SS18	0.0713037	6.3773354	4.3654591	2.00E-05	0.0001802	2.3491052
MOB3C	0.0971813	5.7298649	4.3650839	2.00E-05	0.0001804	2.3476246
RP3-508I15	-0.41654	3.9693321	-4.364241	2.01E-05	0.000181	2.3442985
APOBEC3C	0.2400699	5.6146063	4.3641199	2.01E-05	0.0001811	2.3438215
GNG11	0.1623507	5.9889074	4.3640068	2.01E-05	0.0001811	2.3433751
RP11-20J15	-1.284942	0.4807701	-4.363354	2.02E-05	0.0001816	2.3407996
CYP3A4	0.8587531	5.833064	4.3632809	2.02E-05	0.0001816	2.3405118
VCX	-1.466314	0.3631667	-4.363238	2.02E-05	0.0001816	2.3403423
RP11-347H1	-0.714829	-1.144405	-4.362899	2.02E-05	0.0001818	2.3390066
GPR17	1.0338284	2.8886569	4.362519	2.03E-05	0.000182	2.3375069
BOC	0.4448115	4.5012265	4.3623732	2.03E-05	0.0001821	2.3369318
GPBAR1	0.4012688	4.637699	4.3622734	2.03E-05	0.0001821	2.3365379
PDGFA	0.242434	6.0386544	4.3619879	2.03E-05	0.0001823	2.3354122
RP11-813F2	0.8265877	-0.901103	4.3619323	2.03E-05	0.0001823	2.3351932
RP11-66B24	0.7024383	2.8806537	4.3616157	2.03E-05	0.0001825	2.3339445
RP11-380B4	-0.565092	2.7824207	-4.361375	2.04E-05	0.0001826	2.3329937
OSBP	0.0942272	6.594691	4.3605549	2.04E-05	0.0001832	2.3297621
CHST15	0.1528314	6.1569977	4.3604102	2.04E-05	0.0001833	2.3291916
SIRT7	-0.105576	5.9860231	-4.359543	2.05E-05	0.0001839	2.3257746
MEF2C	0.156102	5.8294121	4.359264	2.05E-05	0.000184	2.3246733
AC024560.3	-0.140249	5.5244671	-4.359259	2.05E-05	0.000184	2.3246549
FAM124A	0.3806137	4.3948941	4.3587246	2.06E-05	0.0001844	2.3225477
UBE2H	0.0826376	6.620861	4.358507	2.06E-05	0.0001845	2.3216898
ALDH9A1	0.1248914	6.6757341	4.3581714	2.06E-05	0.0001847	2.3203673
GTF3C5	-0.084583	6.313763	-4.357894	2.07E-05	0.0001849	2.3192737

ADAMTS10	0.2965579	5.6087444	4.3576844	2.07E-05	0.000185	2.3184483
LACTB2	0.1603392	6.1893448	4.3570262	2.07E-05	0.0001855	2.3158553
RP11-126K1	-0.832651	-0.493191	-4.35604	2.08E-05	0.0001862	2.3119699
CTD-2562G1	-0.847542	2.0443356	-4.356006	2.08E-05	0.0001862	2.3118371
LINC01518	-1.135449	-0.65844	-4.355018	2.09E-05	0.0001869	2.3079439
NEK8	-0.147056	5.4934558	-4.354883	2.09E-05	0.0001869	2.3074142
RP11-65J21	0.5271774	3.6434537	4.3548694	2.09E-05	0.0001869	2.3073598
AP001187.9	-0.60246	4.3692521	-4.354108	2.10E-05	0.0001875	2.3043623
AKAP8L	-0.082316	6.2656937	-4.353934	2.10E-05	0.0001876	2.3036745
IL17RE	-0.320373	5.5324535	-4.353471	2.10E-05	0.0001879	2.3018515
GNB1	0.067777	6.8779026	4.3523946	2.11E-05	0.0001887	2.2976159
SLC22A9	-0.728257	5.9334672	-4.35185	2.12E-05	0.0001891	2.2954732
UPF3A	-0.101756	5.9782495	-4.350145	2.13E-05	0.0001903	2.2887611
FBXL5	0.0988456	6.4779834	4.3501285	2.13E-05	0.0001903	2.288698
RPL7AP30	-0.511838	3.2746785	-4.349927	2.14E-05	0.0001904	2.2879038
SLPI	0.5192569	6.2600837	4.3498786	2.14E-05	0.0001904	2.2877148
RN7SL221P	-0.986382	0.1153579	-4.349761	2.14E-05	0.0001905	2.2872501
SYBU	0.3550081	6.1217236	4.3487615	2.15E-05	0.0001912	2.2833203
RP11-621K7	-0.862308	-0.378576	-4.348465	2.15E-05	0.0001914	2.2821555
LRRC15	1.21406	2.9412579	4.3480496	2.15E-05	0.0001917	2.28052
CBFA2T3	0.3213853	4.9200207	4.3479187	2.15E-05	0.0001918	2.280005
TAT	0.5542895	6.5627252	4.3472033	2.16E-05	0.0001923	2.2771917
CD40	0.2470097	5.8224605	4.3468695	2.16E-05	0.0001925	2.2758791
RP3-475N16	-0.252878	4.1796507	-4.346478	2.17E-05	0.0001928	2.2743403
DCBLD1	0.1182409	5.7409244	4.3463788	2.17E-05	0.0001928	2.2739496
NAIF1	-0.078519	5.6368105	-4.346166	2.17E-05	0.0001929	2.2731115
RP11-21L23	0.6234258	4.9970879	4.3455745	2.18E-05	0.0001934	2.2707877
CTC-455F18	1.1204269	0.6626099	4.3450471	2.18E-05	0.0001937	2.2687147
RP11-715H1	1.3431579	0.6613645	4.345027	2.18E-05	0.0001937	2.2686356
NABP1	0.1660263	5.7088694	4.3445607	2.18E-05	0.000194	2.2668027
C18orf21	-0.089978	5.7484561	-4.344417	2.19E-05	0.0001941	2.2662382
GLIPR2	0.2001667	5.6037126	4.3443052	2.19E-05	0.0001942	2.2657984
PNPLA4	0.1461085	5.9375656	4.3440388	2.19E-05	0.0001943	2.2647514
AFF1	0.1088052	6.3083019	4.3439049	2.19E-05	0.0001944	2.2642254
MT-RNR2	0.1185997	7.8172713	4.3434403	2.19E-05	0.0001947	2.2623997
LATS2	0.135995	5.884719	4.3430443	2.20E-05	0.000195	2.2608437
GPR4	0.1659742	5.4922249	4.3422211	2.21E-05	0.0001956	2.2576093
DCLRE1C	-0.172526	5.2578644	-4.341505	2.21E-05	0.0001962	2.2547956
AC006116.1	-0.880168	-0.412936	-4.341352	2.21E-05	0.0001962	2.2541967
MBP	0.1354153	6.2195873	4.3404808	2.22E-05	0.0001969	2.2507735
KLHL7-AS1	-0.876064	2.8545152	-4.340401	2.22E-05	0.0001969	2.250459
RP11-179A1	-0.966096	-0.826807	-4.339915	2.23E-05	0.0001973	2.2485512
RPSA	-0.10438	7.0444211	-4.339803	2.23E-05	0.0001973	2.2481124
TCOF1	-0.097518	6.2946298	-4.339556	2.23E-05	0.0001975	2.247142
SNORA60	-0.864489	1.6266194	-4.339298	2.23E-05	0.0001976	2.246128
EVPL	-1.223663	3.6260727	-4.339234	2.23E-05	0.0001976	2.2458773
MAF	0.1365439	6.2979359	4.3390858	2.23E-05	0.0001977	2.2452957
RP5-890E16	-0.51584	3.528093	-4.338901	2.24E-05	0.0001978	2.244569
PARP8	0.1939863	5.781875	4.3388633	2.24E-05	0.0001978	2.2444219
CLEC4G	1.1298162	2.8482916	4.3387494	2.24E-05	0.0001978	2.2439747
PDZK1	-0.352377	6.3350522	-4.338307	2.24E-05	0.0001982	2.2422367

RP11-1023L	-0.301608	4.3272777	-4.338052	2.24E-05	0.0001983	2.2412355
CENPU	-0.223643	5.5568732	-4.338046	2.24E-05	0.0001983	2.2412136
AC092620.3	-0.87543	-0.124694	-4.337588	2.25E-05	0.0001986	2.2394155
RP11-332J1	0.5855864	-1.22266	4.3374492	2.25E-05	0.0001987	2.2388707
MDM4	-0.109543	6.1476661	-4.336775	2.26E-05	0.0001992	2.2362236
IFIH1	0.1581795	5.9904792	4.3364737	2.26E-05	0.0001994	2.2350425
SNHG17	-0.146891	5.8653855	-4.336147	2.26E-05	0.0001996	2.2337618
EXOSC8	-0.097003	5.8593749	-4.335224	2.27E-05	0.0002003	2.2301369
AC007966.1	-1.17524	0.5928941	-4.334994	2.27E-05	0.0002005	2.229237
TMEM8A	0.0930522	6.5715919	4.3346781	2.28E-05	0.0002007	2.2279972
FAM3C	0.1154078	6.0353377	4.3340717	2.28E-05	0.0002012	2.2256185
GCC1	0.0955738	6.0456638	4.3336925	2.29E-05	0.0002014	2.2241311
U2AF2	-0.056858	6.6735005	-4.333657	2.29E-05	0.0002014	2.2239917
COX18	0.1213214	5.9099227	4.333382	2.29E-05	0.0002016	2.2229133
SNURF	-1.05411	1.9064672	-4.333236	2.29E-05	0.0002017	2.2223389
RP11-848P1	0.4030632	3.548953	4.333079	2.29E-05	0.0002017	2.2217248
ADRA2A	0.7000232	4.5329862	4.3330409	2.29E-05	0.0002017	2.2215752
AC092071.1	1.3442634	0.5604832	4.3329364	2.29E-05	0.0002018	2.2211654
RBM34	-0.174195	4.595779	-4.33168	2.30E-05	0.0002027	2.2162392
POU6F1	0.1747487	5.1822294	4.331679	2.30E-05	0.0002027	2.2162352
SYPL1	0.0775312	6.57198	4.3313702	2.31E-05	0.0002029	2.2150245
NDST2	-0.131906	4.6092024	-4.331347	2.31E-05	0.0002029	2.2149327
RP11-85G20	-0.89523	0.0436872	-4.330891	2.31E-05	0.0002033	2.2131463
MUC6	1.3272129	3.4044182	4.3306947	2.31E-05	0.0002034	2.2123764
OSTM1	0.129045	6.0758177	4.3303087	2.32E-05	0.0002036	2.2108632
MAGEA4	-1.270875	-0.506503	-4.330157	2.32E-05	0.0002037	2.2102699
IMMP2L	0.1882416	5.8682651	4.3294296	2.33E-05	0.0002043	2.2074177
RP1-148H17	-1.140736	0.7040759	-4.329335	2.33E-05	0.0002043	2.207046
OR7E102P	1.0961699	0.297081	4.3291347	2.33E-05	0.0002044	2.2062619
RP11-439C1	1.2514166	0.4682849	4.3287088	2.33E-05	0.0002048	2.2045931
RP11-573J2	-0.718307	-1.110624	-4.327984	2.34E-05	0.0002053	2.201752
SH3TC2	0.6016553	3.2541603	4.3274874	2.35E-05	0.0002057	2.1998078
MPLKIP	-0.12947	6.2893999	-4.327127	2.35E-05	0.000206	2.1983961
RP11-138A9	-1.068878	0.4680046	-4.326549	2.35E-05	0.0002064	2.1961299
VPS16	-0.080069	6.2293989	-4.325676	2.36E-05	0.0002071	2.1927109
RP11-524D1	0.8803645	2.5196535	4.3255601	2.36E-05	0.0002072	2.192259
GNAS-AS1	-1.206064	1.107079	-4.325068	2.37E-05	0.0002075	2.1903307
FAM229B	0.2664525	5.1440173	4.3250319	2.37E-05	0.0002075	2.1901906
SNED1	0.232378	5.6281162	4.323484	2.38E-05	0.0002088	2.1841302
TMEM258	-0.089578	6.4904372	-4.323397	2.39E-05	0.0002088	2.183791
C19orf40	-0.197922	4.962629	-4.322311	2.40E-05	0.0002097	2.1795394
NR2C2AP	-0.127061	5.9223496	-4.321878	2.40E-05	0.0002101	2.177845
AP000344.3	-1.205325	0.1110937	-4.32181	2.40E-05	0.0002101	2.1775769
MYH3	-0.285122	4.6441053	-4.321574	2.40E-05	0.0002102	2.1766554
KIF1B	0.1089563	6.2901509	4.3212884	2.41E-05	0.0002104	2.1755371
HLA-DRA	0.1800611	6.9540454	4.3211555	2.41E-05	0.0002105	2.1750171
PRAMEF7	1.0806189	-0.433218	4.3208477	2.41E-05	0.0002107	2.1738128
RP4-782L23	0.9534925	2.9686629	4.3206765	2.41E-05	0.0002108	2.1731432
C22orf29	-0.12209	5.881696	-4.320367	2.42E-05	0.000211	2.1719329
PRMT3	-0.112097	5.729542	-4.319338	2.43E-05	0.0002119	2.1679067
KIAA1841	-0.172025	5.2695838	-4.319241	2.43E-05	0.0002119	2.1675294

SLC14A1	0.5936592	3.9441217	4.3189775	2.43E-05	0.0002121	2.166497
RP11-535A5	0.9553317	0.1463189	4.3181842	2.44E-05	0.0002127	2.1633945
TIGD3	-0.535067	3.7276626	-4.317991	2.44E-05	0.0002129	2.1626407
VLDLR	0.6317674	4.8668613	4.3178014	2.44E-05	0.000213	2.1618977
RP11-968A1	-0.866718	-0.277037	-4.317747	2.44E-05	0.000213	2.1616856
PDCD11	-0.090982	6.2970064	-4.317591	2.44E-05	0.0002131	2.1610738
ANO1	0.3546019	6.1037358	4.3174528	2.45E-05	0.0002131	2.1605348
RQCD1	-0.071407	6.2889022	-4.316844	2.45E-05	0.0002136	2.158154
AC000111.6	0.6760471	-1.192099	4.316785	2.45E-05	0.0002136	2.1579235
NPSR1-AS1	-1.559959	1.826885	-4.316775	2.45E-05	0.0002136	2.1578826
NLRC5	0.1734668	6.0201506	4.3162017	2.46E-05	0.000214	2.1556432
MAP9	0.6095226	4.0027894	4.3157132	2.46E-05	0.0002144	2.1537341
DDX11	-0.189575	5.7248258	-4.315192	2.47E-05	0.0002148	2.1516975
CABP7	-0.517413	3.1169681	-4.314923	2.47E-05	0.000215	2.150647
TMEM54	0.3292416	5.5608764	4.3146913	2.47E-05	0.0002152	2.1497402
BRSK2	-1.059009	3.0536246	-4.314004	2.48E-05	0.0002157	2.1470525
SBF2-AS1	-0.205239	4.9901423	-4.314002	2.48E-05	0.0002157	2.1470458
MAGEA9	-0.768829	-1.177733	-4.313545	2.49E-05	0.0002161	2.1452602
RAB3D	-0.364977	5.3555875	-4.313277	2.49E-05	0.0002162	2.1442137
RP11-837J7	-0.947343	1.5850279	-4.3129	2.49E-05	0.0002165	2.1427424
RP4-806M20	-1.099227	-0.028413	-4.312666	2.49E-05	0.0002167	2.1418251
RFWD2	-0.090058	6.3933255	-4.310596	2.52E-05	0.0002185	2.1337432
TMEM251	-0.105019	5.6033212	-4.310097	2.52E-05	0.0002189	2.1317921
ERF	-0.097269	6.4412698	-4.309802	2.52E-05	0.0002191	2.1306438
RP11-465N4	-0.41255	4.714401	-4.309208	2.53E-05	0.0002196	2.1283224
ZNF614	-0.265872	5.1451401	-4.309142	2.53E-05	0.0002196	2.1280638
CHEK2	-0.187709	5.6752745	-4.308589	2.54E-05	0.0002201	2.1259075
COL4A3BP	0.0901418	6.1410552	4.3084971	2.54E-05	0.0002201	2.1255482
BBOX1-AS1	-1.434663	0.6269626	-4.308	2.54E-05	0.0002205	2.1236086
RP11-80H5.	-0.946184	0.8338186	-4.307881	2.54E-05	0.0002205	2.1231435
CELA3B	-0.947827	-0.629898	-4.307813	2.55E-05	0.0002205	2.1228777
MIS18A-AS1	-0.930864	1.1041829	-4.307781	2.55E-05	0.0002205	2.1227549
CTC-242N15	-0.651638	-1.213297	-4.306796	2.56E-05	0.0002214	2.1189078
UCK2	-0.143454	6.1927754	-4.306451	2.56E-05	0.0002217	2.1175633
RP11-343B5	-0.766183	1.74539	-4.304052	2.59E-05	0.0002238	2.1082051
RP11-307C1	-1.098467	1.9956658	-4.303661	2.59E-05	0.0002241	2.1066786
RD3L	0.5961208	-1.283118	4.3033015	2.59E-05	0.0002244	2.1052789
HIC1	0.2143301	5.3667657	4.3031728	2.59E-05	0.0002245	2.1047768
TMEM119	0.4984396	5.0051429	4.3027346	2.60E-05	0.0002248	2.1030682
RP11-215P8	-0.633951	2.2949508	-4.302475	2.60E-05	0.000225	2.1020544
MIR148A	-1.024474	0.5470413	-4.302298	2.60E-05	0.0002251	2.1013652
KRT81	1.1589243	2.0984751	4.302069	2.61E-05	0.0002253	2.1004734
CCDC177	1.44187	1.712602	4.3017591	2.61E-05	0.0002255	2.0992653
NR2F2	0.1088749	6.3400484	4.300362	2.62E-05	0.0002268	2.0938199
LNX2	0.1473212	5.9888306	4.2993252	2.64E-05	0.0002277	2.0897798
ARPC1B	0.1058206	6.7177257	4.2979205	2.65E-05	0.000229	2.0843073
FAM65C	0.4698024	4.5121021	4.2973995	2.66E-05	0.0002294	2.082278
RPS10L	-0.932835	1.5849249	-4.297355	2.66E-05	0.0002294	2.0821053
TMEM50A	0.0761626	6.4999978	4.2972427	2.66E-05	0.0002295	2.0816675
TRAM1L1	-1.134969	3.1023709	-4.29712	2.66E-05	0.0002295	2.0811891
RP11-93H24	-0.471129	3.40645	-4.296727	2.66E-05	0.0002298	2.0796603

CEP72	-0.200082	5.4219095	-4.29624	2.67E-05	0.0002303	2.0777639
ECHDC1	0.1825007	6.3035728	4.2961539	2.67E-05	0.0002303	2.0774272
RP11-161M6	0.6317736	3.5413044	4.2950459	2.68E-05	0.0002313	2.0731134
LINC00668	-1.543495	0.63629	-4.29482	2.69E-05	0.0002314	2.0722352
KCNMB2-AS1	-1.450282	2.3336666	-4.294011	2.69E-05	0.0002322	2.0690842
RAPGEF5	0.1580689	6.0925527	4.2938439	2.70E-05	0.0002323	2.0684342
CYS1	0.989239	3.8117439	4.2934885	2.70E-05	0.0002326	2.067051
CRYZ	0.1380999	6.6686395	4.2932154	2.70E-05	0.0002328	2.0659881
SELP	0.6486464	4.3835196	4.2929943	2.71E-05	0.0002329	2.0651279
VPS9D1	0.1177172	5.921771	4.2926271	2.71E-05	0.0002332	2.063699
RP11-260E1	0.8704557	-0.639214	4.2925917	2.71E-05	0.0002332	2.0635611
LRBA	0.1049573	6.2203631	4.2924722	2.71E-05	0.0002333	2.0630961
RP11-298I3	-0.708544	2.2566305	-4.292381	2.71E-05	0.0002333	2.0627416
RP1-90L14.	-1.369391	0.1844216	-4.292354	2.71E-05	0.0002333	2.0626355
CSNK2A1	-0.073768	6.4583744	-4.291973	2.72E-05	0.0002336	2.0611538
RP11-478J1	-0.596355	4.2026223	-4.291345	2.72E-05	0.0002341	2.0587116
SAFB2	-0.062685	6.3561343	-4.291302	2.73E-05	0.0002341	2.0585424
TAF1A	-0.198048	4.9975758	-4.291137	2.73E-05	0.0002342	2.0579002
DPP7	0.1595104	6.5661893	4.2908253	2.73E-05	0.0002344	2.0566888
RP11-283C2	-1.109663	0.6449963	-4.290807	2.73E-05	0.0002344	2.0566182
AC005077.1	1.0060228	2.7584966	4.2904849	2.73E-05	0.0002347	2.0553647
CYFIP1	0.0688652	6.5789149	4.2900353	2.74E-05	0.0002351	2.0536161
PRCP	0.1456966	6.5045424	4.2883202	2.76E-05	0.0002367	2.0469465
AC060834.3	-0.922681	-0.968965	-4.288183	2.76E-05	0.0002368	2.0464128
CRNKL1	-0.076409	6.1646266	-4.287772	2.77E-05	0.0002371	2.0448134
MPP7	-0.320992	5.4068769	-4.286446	2.78E-05	0.0002384	2.0396615
COL5A3	0.2856874	6.1117222	4.2857127	2.79E-05	0.000239	2.0368112
LINC00870	-0.666872	3.9961054	-4.285649	2.79E-05	0.000239	2.036563
TBC1D13	-0.106337	6.2052405	-4.285151	2.80E-05	0.0002395	2.0346282
DDX5	0.059861	7.0176263	4.2846147	2.80E-05	0.00024	2.0325448
UBASH3B	0.3157405	5.0984193	4.2839445	2.81E-05	0.0002406	2.0299413
RP11-173M1	-0.983334	1.7561826	-4.28288	2.82E-05	0.0002416	2.0258077
CNDP1	1.11812	3.7475043	4.2827857	2.82E-05	0.0002416	2.02544
ANXA2	0.1174827	6.9993086	4.2824051	2.83E-05	0.0002419	2.0239619
WWTR1	0.1515488	6.2144222	4.2823958	2.83E-05	0.0002419	2.0239258
RP11-1105G	-0.626977	2.905127	-4.281701	2.84E-05	0.0002425	2.0212281
DDAH2	-0.138554	6.4087996	-4.281609	2.84E-05	0.0002425	2.0208719
KBTBD11-OT	1.0545479	-0.204764	4.2805299	2.85E-05	0.0002436	2.0166811
GRIK5	-0.71263	3.0469302	-4.280475	2.85E-05	0.0002436	2.0164697
LRRC69	-0.524167	3.6921619	-4.279718	2.86E-05	0.0002443	2.0135289
RP11-395I6	-0.530087	3.6873566	-4.279146	2.87E-05	0.0002448	2.0113085
MGA	-0.092626	6.0284453	-4.278358	2.87E-05	0.0002455	2.0082517
AC002511.2	-1.069159	-0.298033	-4.278066	2.88E-05	0.0002458	2.007118
C8orf46	0.5418792	4.6500184	4.2777831	2.88E-05	0.000246	2.0060204
TUFM	0.0936042	6.9069209	4.2774352	2.89E-05	0.0002463	2.0046707
SYNE1	0.1396223	6.0663541	4.2771566	2.89E-05	0.0002465	2.0035898
CCDC25	0.1224088	6.1490018	4.2766513	2.90E-05	0.000247	2.0016296
PRSS42	-1.065473	0.7433706	-4.276511	2.90E-05	0.000247	2.0010865
RP11-127B1	-0.823816	-1.10318	-4.276485	2.90E-05	0.000247	2.0009852
DSG1-AS1	-0.816424	-1.005805	-4.276471	2.90E-05	0.000247	2.0009308
CTD-3020H1	-0.919091	-0.277635	-4.275807	2.91E-05	0.0002476	1.9983542

SHMT1	0.2331531	6.7276339	4.2754155	2.91E-05	0.000248	1.9968364
ZNF571	-0.182412	4.7761862	-4.274998	2.91E-05	0.0002482	1.9952188
HOXD4	-1.25924	0.914625	-4.274994	2.91E-05	0.0002482	1.9952022
RPS12P23	-0.855613	-0.506169	-4.274933	2.92E-05	0.0002482	1.9949656
CEBPA	-0.215719	6.7319584	-4.274922	2.92E-05	0.0002482	1.9949228
AP001626.1	-1.05499	3.5554595	-4.274649	2.92E-05	0.0002484	1.9938642
SNX22	-0.250448	5.5370865	-4.274021	2.93E-05	0.000249	1.9914292
CTD-2623N2	-0.732658	-0.837947	-4.273747	2.93E-05	0.0002492	1.9903659
RARRES3	0.234742	6.1832378	4.2736519	2.93E-05	0.0002492	1.9899977
CTD-2134A5	-0.529449	4.2712573	-4.273646	2.93E-05	0.0002492	1.9899742
BHLHE41	0.5119948	4.7996725	4.272494	2.95E-05	0.0002503	1.9855092
ATP5G2	-0.081442	6.896643	-4.272465	2.95E-05	0.0002503	1.9853951
ZNF207	-0.048344	6.572278	-4.272228	2.95E-05	0.0002505	1.9844796
GBP4	0.2210488	6.0383963	4.2721735	2.95E-05	0.0002505	1.9842672
RP11-537H1	-0.959161	0.4723176	-4.271755	2.95E-05	0.0002509	1.9826437
CILP	0.6460123	4.0236375	4.2702532	2.97E-05	0.0002524	1.9768256
CTD-2293H3	-0.915446	0.1070921	-4.269806	2.98E-05	0.0002528	1.9750934
SNRPEP5	-0.631814	-0.97423	-4.269215	2.99E-05	0.0002534	1.9728029
C1orf234	-0.869656	-0.329711	-4.268936	2.99E-05	0.0002536	1.9717244
LRRC4	0.4234137	3.9596222	4.2688561	2.99E-05	0.0002536	1.9714135
CLUAP1	0.1132555	5.6408645	4.2688033	2.99E-05	0.0002536	1.9712092
METTL5	-0.08721	6.0767737	-4.268584	2.99E-05	0.0002538	1.9703592
GIMAP1	0.2145924	5.3936554	4.2682236	3.00E-05	0.0002541	1.9689638
LMNB2	-0.130649	6.1563219	-4.267867	3.00E-05	0.0002544	1.9675817
MAVS	-0.088233	6.5231639	-4.267676	3.00E-05	0.0002546	1.9668434
AC093609.1	0.9431956	1.9690122	4.2675245	3.01E-05	0.0002546	1.9662567
CREB3L1	0.5722766	5.0960136	4.2675079	3.01E-05	0.0002546	1.9661923
CCDC88B	0.1888354	5.7269104	4.2672332	3.01E-05	0.0002548	1.9651289
SNX19	0.128148	6.3449431	4.2672141	3.01E-05	0.0002548	1.9650547
NR2F6	-0.121709	6.5953956	-4.266846	3.01E-05	0.0002551	1.9636313
C19orf67	-1.046436	0.8104384	-4.266828	3.01E-05	0.0002551	1.9635613
RP4-594I10	-1.178322	1.2463519	-4.266791	3.02E-05	0.0002551	1.9634151
RP4-541C22	1.3238809	0.4161421	4.2663058	3.02E-05	0.0002555	1.9615383
KAT2B	0.1601062	6.0891392	4.2654418	3.03E-05	0.0002564	1.9581938
AKR1C8P	1.3468881	2.8557033	4.2648381	3.04E-05	0.000257	1.9558569
SEC11C	-0.146731	6.6460534	-4.264283	3.05E-05	0.0002575	1.9537088
RP11-228B1	0.291286	4.3673374	4.263699	3.05E-05	0.000258	1.951449
FYCO1	0.106083	6.1998474	4.2631518	3.06E-05	0.0002586	1.9493318
RP11-395N3	-0.997208	0.1129023	-4.262936	3.06E-05	0.0002587	1.9484961
GAB3	0.2254716	4.8929848	4.2629129	3.06E-05	0.0002587	1.9484073
RALY	-0.080757	6.6696049	-4.262881	3.06E-05	0.0002587	1.948286
POTENP	-0.888859	-0.155396	-4.261749	3.08E-05	0.0002598	1.9439063
FANCL	-0.08902	5.724516	-4.261154	3.09E-05	0.0002604	1.9416035
PCSK5	0.2941257	5.4494613	4.2599753	3.10E-05	0.0002616	1.9370459
UNC119B	-0.146938	5.8955013	-4.25956	3.11E-05	0.000262	1.9354396
AK3	0.1206739	6.6267166	4.258687	3.12E-05	0.0002629	1.9320653
TPTE2P3	-1.077401	-0.544003	-4.258234	3.12E-05	0.0002633	1.9303145
PDE6C	-0.570543	3.130635	-4.258037	3.13E-05	0.0002635	1.9295518
CNPY2	-0.101197	6.2340871	-4.2575	3.13E-05	0.000264	1.9274762
ST3GAL6	0.2482394	5.8260879	4.2573807	3.13E-05	0.000264	1.9270165
RP11-178H8	-0.339212	4.0821015	-4.257184	3.14E-05	0.0002642	1.9262573

AP001627.1	-1.052849	1.1279206	-4.255617	3.16E-05	0.0002658	1.9202035
S100A6	0.2190527	6.4334576	4.2552377	3.16E-05	0.0002661	1.9187366
RP11-143K1	-0.724249	2.0296988	-4.255206	3.16E-05	0.0002661	1.9186145
HS3ST1	0.360169	4.6944971	4.2551933	3.16E-05	0.0002661	1.9185648
INTS7	-0.099028	6.1779753	-4.25517	3.16E-05	0.0002661	1.9184741
EMB	0.3019665	5.3040386	4.254658	3.17E-05	0.0002666	1.9164972
FAM102A	0.1261274	6.4410032	4.2541549	3.18E-05	0.0002671	1.9145543
RAB6A	0.0743358	6.5668289	4.2528724	3.19E-05	0.0002684	1.9096015
LINC01063	-1.00887	0.9196663	-4.252608	3.20E-05	0.0002687	1.9085797
BX322557.1	-0.385029	3.9588019	-4.252546	3.20E-05	0.0002687	1.9083396
ANKRD42	0.1148353	5.3148259	4.2523063	3.20E-05	0.0002689	1.907416
SRPR	0.0820514	6.8416745	4.2518493	3.21E-05	0.0002692	1.9056518
RP11-129M6	-1.531539	0.5925318	-4.251824	3.21E-05	0.0002692	1.9055523
UNC93A	-1.038923	4.8605487	-4.251806	3.21E-05	0.0002692	1.9054839
CTC-231011	-0.75231	3.2474776	-4.251332	3.21E-05	0.0002697	1.9036561
GPRC5D	-0.654477	2.9024366	-4.251277	3.21E-05	0.0002697	1.9034406
SCRT1	-0.944455	-0.135777	-4.251116	3.22E-05	0.0002698	1.9028208
FAM216A	-0.179485	5.2959245	-4.250929	3.22E-05	0.00027	1.9021007
FOSB	0.4669258	5.4555807	4.2507503	3.22E-05	0.0002701	1.9014097
WFDC10A	-0.858115	-0.785182	-4.248759	3.25E-05	0.0002722	1.8937238
PCDHGB7	0.7371928	3.8280492	4.2487133	3.25E-05	0.0002722	1.8935495
C12orf66	0.1118648	5.5593852	4.2480868	3.26E-05	0.0002729	1.8911324
SEC31A	0.0683367	6.7616444	4.2480168	3.26E-05	0.0002729	1.8908623
GPR153	0.2849844	5.3525886	4.2470855	3.27E-05	0.0002739	1.8872702
SAMD12	0.6646419	4.441036	4.24685	3.27E-05	0.0002741	1.886362
ACACA	-0.117775	6.3357039	-4.24622	3.28E-05	0.0002747	1.8839345
RP11-111M2	-0.883759	-0.209277	-4.246189	3.28E-05	0.0002747	1.8838149
SYDE1	0.1863717	5.6138813	4.2455278	3.29E-05	0.0002754	1.8812637
RP11-143I2	-0.839145	0.7392449	-4.245294	3.29E-05	0.0002756	1.8803638
RP11-745L1	-0.661756	-1.076906	-4.244511	3.30E-05	0.0002764	1.8773418
HIF1AN	-0.091311	6.1893439	-4.244207	3.31E-05	0.0002767	1.8761711
FPR3	0.256394	5.5535868	4.2440806	3.31E-05	0.0002767	1.8756846
ZC3H10	-0.086402	5.6049059	-4.244021	3.31E-05	0.0002767	1.8754568
ANKRD13C	0.0976735	5.7793672	4.2439785	3.31E-05	0.0002767	1.8752911
CD163	0.2643148	6.0448433	4.2437855	3.31E-05	0.0002769	1.8745473
RP11-493L1	-1.09102	-0.052501	-4.243727	3.31E-05	0.0002769	1.874321
ZNF516	-0.103975	5.9370064	-4.243598	3.32E-05	0.000277	1.8738258
BBS2	0.1215902	5.905754	4.243217	3.32E-05	0.0002774	1.8723561
HSD3B7	0.1659738	6.3729638	4.2428411	3.33E-05	0.0002777	1.8709076
IGFBP6	0.3065408	5.046113	4.2426064	3.33E-05	0.0002779	1.8700032
CDH9	-1.433911	-0.104012	-4.242121	3.34E-05	0.0002784	1.8681316
CTD-3035K2	-0.90998	-0.411379	-4.242033	3.34E-05	0.0002785	1.8677949
NOS2	0.469569	4.2534174	4.2418745	3.34E-05	0.0002785	1.8671833
AZIN2	0.2894609	4.6221731	4.2418697	3.34E-05	0.0002785	1.8671649
RP11-28F1.	0.962605	1.1739129	4.241846	3.34E-05	0.0002785	1.8670736
RN7SL812P	-0.786186	-0.681705	-4.241694	3.34E-05	0.0002786	1.8664863
DACT1	0.3746963	4.9330882	4.2415532	3.34E-05	0.0002787	1.8659456
ESCO2	-0.39946	4.7069006	-4.241419	3.35E-05	0.0002788	1.8654266
RMDN3	0.0913344	6.3656513	4.2409272	3.35E-05	0.0002793	1.8635339
RP11-316M2	-0.860285	1.5131983	-4.240856	3.35E-05	0.0002793	1.8632599
RNU6-322P	-0.949578	0.6016479	-4.239655	3.37E-05	0.0002806	1.8586354

ZNF423	0.3481004	4.7252052	4.2395809	3.37E-05	0.0002806	1.8583484
KANSL2	-0.078274	5.9077646	-4.239546	3.37E-05	0.0002806	1.8582153
RGCC	0.1697037	5.7169533	4.2393304	3.38E-05	0.0002808	1.8573838
AGBL5	-0.112259	6.0222173	-4.239251	3.38E-05	0.0002808	1.8570765
RP11-324E6	-1.013508	0.7714469	-4.239123	3.38E-05	0.0002809	1.8565871
P2RY13	0.3984727	4.6478991	4.2386776	3.38E-05	0.0002813	1.8548703
FENDRR	1.1235778	1.869574	4.2383098	3.39E-05	0.0002817	1.8534541
AC104777.4	0.6965512	-0.961335	4.2381205	3.39E-05	0.0002819	1.8527252
TIPARP-AS1	-0.300301	4.4325253	-4.23802	3.39E-05	0.0002819	1.8523386
TMED6	-0.45842	4.542747	-4.237406	3.40E-05	0.0002826	1.8499733
DSCR10	-0.766221	-1.07644	-4.237033	3.41E-05	0.0002829	1.8485383
FYN	0.1549747	5.9633134	4.2368663	3.41E-05	0.0002831	1.8478974
NPL	0.2398754	5.9410951	4.2365738	3.41E-05	0.0002833	1.8467714
ANKRD37	0.2605694	4.9399965	4.2353134	3.43E-05	0.0002847	1.8419211
MRPL50	-0.099515	6.0320988	-4.235222	3.43E-05	0.0002848	1.8415689
TM4SF18	0.1976004	5.539714	4.2349982	3.44E-05	0.000285	1.8407084
RP1-45C12.	-1.197183	0.9489813	-4.234742	3.44E-05	0.0002852	1.8397214
RPL23A	-0.098555	6.9815674	-4.233935	3.45E-05	0.0002861	1.8366188
GLUD1P8	-0.916609	2.0523182	-4.233887	3.45E-05	0.0002861	1.8364318
CCM2L	0.2409777	4.9710643	4.2336764	3.45E-05	0.0002862	1.8356234
ZIC5	-1.477648	3.2674984	-4.233675	3.45E-05	0.0002862	1.8356191
AL132709.8	-1.011222	-0.725925	-4.233416	3.46E-05	0.0002864	1.8346216
RP11-437L7	0.7956902	-0.958764	4.2333093	3.46E-05	0.0002864	1.8342114
PLA2G4C	0.2177919	5.9300453	4.2333075	3.46E-05	0.0002864	1.8342046
SGPP1	0.1120125	6.0105087	4.2315576	3.48E-05	0.0002884	1.827475
DES	0.9116232	3.5605522	4.2313467	3.49E-05	0.0002886	1.8266644
ZNF444	-0.101543	6.4148141	-4.231252	3.49E-05	0.0002886	1.826299
SOCS1	0.3243549	5.1769932	4.229489	3.51E-05	0.0002907	1.8195233
CCDC167	-0.128444	6.0586073	-4.229182	3.52E-05	0.000291	1.8183448
RPL7P48	-0.783913	-0.62262	-4.22909	3.52E-05	0.000291	1.8179885
CTD-3184A7	-0.215019	4.9781543	-4.22819	3.53E-05	0.000292	1.8145315
CTD-2336H1	-0.818297	-0.991572	-4.228166	3.53E-05	0.000292	1.8144408
MCTP2	0.6296804	4.5799132	4.2278437	3.54E-05	0.0002923	1.8132009
AC009005.2	-0.582563	4.0156067	-4.22781	3.54E-05	0.0002923	1.8130731
RPP21	-0.142779	5.4975236	-4.227712	3.54E-05	0.0002923	1.8126944
LDLRAD3	0.2538092	5.1367744	4.2274383	3.54E-05	0.0002926	1.811643
C3orf20	-0.995963	0.6245625	-4.227365	3.54E-05	0.0002926	1.8113625
ZNF266	-0.161967	5.8466212	-4.227195	3.55E-05	0.0002927	1.8107081
ATAD3C	0.5040608	4.3250354	4.2270575	3.55E-05	0.0002928	1.8101802
AC007191.4	-0.549345	4.1090171	-4.226741	3.55E-05	0.0002931	1.8089645
AK5	0.9979769	1.7436672	4.2266937	3.55E-05	0.0002931	1.8087827
SESN2	0.1178248	6.0920324	4.2263356	3.56E-05	0.0002935	1.8074075
GBGT1	0.2527796	4.907045	4.2262442	3.56E-05	0.0002936	1.8070562
ACTC1	1.2763677	0.9271853	4.225592	3.57E-05	0.0002943	1.8045515
TM7SF3	0.1227776	6.4712736	4.2254249	3.57E-05	0.0002944	1.8039098
SNX9	0.0905847	6.4401689	4.2251182	3.58E-05	0.0002947	1.8027317
RP11-449J2	-1.057466	2.7752676	-4.224372	3.59E-05	0.0002955	1.7998672
AQP6	-1.264279	1.6900191	-4.224355	3.59E-05	0.0002955	1.7998014
RBM43	0.1502381	5.5196655	4.2241748	3.59E-05	0.0002956	1.7991096
KB-1440D3.	-1.080254	0.8655794	-4.223974	3.59E-05	0.0002958	1.7983397
ADPRH	0.156329	5.4618	4.2238496	3.60E-05	0.0002959	1.7978609

KLB	-0.459398	6.2788895	-4.223657	3.60E-05	0.0002961	1.7971215
RP11-467P9	-0.936258	0.4293253	-4.223232	3.60E-05	0.0002965	1.7954891
UBALD1	0.1222567	6.0828598	4.2230629	3.61E-05	0.0002966	1.794841
TCP10L2	-1.218124	0.6260774	-4.223036	3.61E-05	0.0002966	1.7947369
MAP3K2	0.0751242	6.327497	4.2222701	3.62E-05	0.0002975	1.7917984
RP4-782L23	1.036965	0.4343208	4.2220202	3.62E-05	0.0002977	1.7908391
CDIPT	0.1050845	6.6306833	4.2215654	3.63E-05	0.0002982	1.7890941
NASP	-0.097056	6.447972	-4.220814	3.64E-05	0.0002991	1.7862124
PITPNM2	0.1713695	6.0525137	4.2205897	3.64E-05	0.0002993	1.7853504
FMNL3	0.1297156	5.9520667	4.2200021	3.65E-05	0.0002999	1.7830959
SENP5	-0.06378	6.106491	-4.218509	3.68E-05	0.0003017	1.7773677
ZNF30	-0.150424	5.1115818	-4.218325	3.68E-05	0.0003018	1.7766627
GPR126	0.288931	6.2833118	4.2182237	3.68E-05	0.0003019	1.7762751
HOXA-AS3	-1.246824	0.3743461	-4.217676	3.69E-05	0.0003025	1.774176
RP11-98D18	-0.909483	0.3448708	-4.217618	3.69E-05	0.0003025	1.7739524
C2CD4A	0.9773888	4.2644646	4.2171447	3.70E-05	0.000303	1.7721378
LINC01549	-1.229226	2.0157563	-4.216624	3.70E-05	0.0003036	1.7701402
H2AFZ	-0.099958	6.6065554	-4.216431	3.71E-05	0.0003038	1.7694029
HLX-AS1	0.9433564	-0.279542	4.2150477	3.73E-05	0.0003054	1.7640998
PIDD1	-0.106874	5.9100419	-4.214878	3.73E-05	0.0003056	1.76345
FAM110C	0.6831023	5.5340756	4.214521	3.74E-05	0.0003059	1.7620815
CCDC8	0.583162	4.2526987	4.2142731	3.74E-05	0.0003062	1.7611315
ZNF66	-0.866438	2.9010436	-4.213872	3.75E-05	0.0003066	1.7595934
VAMP2	0.1111499	6.1978367	4.2138188	3.75E-05	0.0003066	1.7593909
RP11-248G5	-0.944335	0.0696392	-4.212772	3.76E-05	0.0003079	1.7553793
RP11-393I2	-0.612711	2.7095953	-4.212349	3.77E-05	0.0003083	1.753762
MAN2B2	0.1130984	6.4439414	4.2123018	3.77E-05	0.0003083	1.7535794
BARD1	-0.230039	5.1734439	-4.212247	3.77E-05	0.0003083	1.7533712
PAXIP1-AS2	0.2075436	5.0921687	4.2117126	3.78E-05	0.0003089	1.751323
MILR1	0.2803303	4.6558393	4.2115042	3.78E-05	0.0003091	1.7505247
GCN1L1	-0.066269	6.5978826	-4.211348	3.78E-05	0.0003092	1.7499251
PGGT1B	0.0755822	5.9834908	4.2104949	3.80E-05	0.0003102	1.74666
LINC01087	-1.014982	-0.645669	-4.209946	3.81E-05	0.0003109	1.7445598
BIRC3	0.2658303	6.2955727	4.2094862	3.81E-05	0.0003114	1.7427979
FAM110D	0.4345765	4.2744049	4.2092263	3.82E-05	0.0003116	1.7418033
SLC34A3	-1.08318	1.278259	-4.209091	3.82E-05	0.0003117	1.741287
ZCCHC6	0.1309956	6.1471993	4.2087732	3.82E-05	0.000312	1.740069
RP11-256I2	-0.997884	0.0701059	-4.208772	3.82E-05	0.000312	1.7400627
STYK1	1.1214953	1.399768	4.2086995	3.83E-05	0.000312	1.7397866
PIGBOS1	-0.106112	5.5288981	-4.208065	3.84E-05	0.0003128	1.7373565
WDSUB1	-0.121468	5.5382168	-4.207637	3.84E-05	0.0003132	1.7357217
C10orf35	-0.525074	5.0764019	-4.207513	3.84E-05	0.0003133	1.7352471
ZAR1L	-0.699033	-0.876108	-4.20747	3.84E-05	0.0003133	1.735081
RP11-542K2	-0.899122	-0.567445	-4.207383	3.85E-05	0.0003133	1.7347475
LDHB	0.1839341	6.1601092	4.2073658	3.85E-05	0.0003133	1.7346827
ABCG2	0.3197776	5.6356688	4.2073245	3.85E-05	0.0003133	1.7345248
SNX10	0.2191111	6.1185345	4.2072253	3.85E-05	0.0003134	1.7341453
HCG16	-1.022295	1.4117546	-4.206232	3.86E-05	0.0003146	1.7303449
AC004932.1	-0.889608	0.9521667	-4.205835	3.87E-05	0.000315	1.7288252
MYO9A	0.1681677	5.4786932	4.2057413	3.87E-05	0.000315	1.7284678
GTPBP3	-0.09735	5.9057193	-4.205655	3.87E-05	0.0003151	1.7281383

ZNF234	-0.14158	5.3267319	-4.205198	3.88E-05	0.0003156	1.7263894
CCM2	0.0958896	6.4815848	4.2050243	3.88E-05	0.0003157	1.7257251
COL4A3	0.6384657	4.0071761	4.2050133	3.88E-05	0.0003157	1.7256829
NRF1	-0.067185	5.7574939	-4.204626	3.89E-05	0.0003161	1.7242032
RN7SL541P	-0.825221	-0.558885	-4.204462	3.89E-05	0.0003163	1.723573
SLC17A5	-0.132521	6.28079	-4.203944	3.90E-05	0.0003169	1.7215928
AP1B1	0.069102	6.6413528	4.2025363	3.92E-05	0.0003186	1.7162116
GSTA4	-0.202116	6.0169804	-4.202283	3.93E-05	0.0003189	1.7152421
NRP1	0.1075216	6.5730321	4.2022257	3.93E-05	0.0003189	1.7150243
B4GALT1-AS	0.3321867	4.1579094	4.2018135	3.93E-05	0.0003193	1.7134488
RP11-545D1	-0.752097	-1.057235	-4.201687	3.94E-05	0.0003194	1.7129666
SREBF2	-0.105354	6.7789516	-4.201495	3.94E-05	0.0003196	1.7122313
ZMYND11	0.0765303	6.3556107	4.2006305	3.95E-05	0.0003207	1.7089277
SPCS3	0.0907356	6.5399642	4.2004627	3.96E-05	0.0003208	1.7082866
CARD11	0.3049594	5.1573225	4.1991495	3.98E-05	0.0003224	1.7032693
HTRA3	0.4124826	5.3666892	4.1991227	3.98E-05	0.0003224	1.7031668
RUSC2	0.1171723	6.0796535	4.1982888	3.99E-05	0.0003234	1.6999814
RP1-265C24	-0.850146	0.3921485	-4.197799	4.00E-05	0.000324	1.6981122
RP11-902B1	0.8291479	2.0721168	4.1977801	4.00E-05	0.000324	1.6980384
RER1	0.0963392	6.6370924	4.1963176	4.02E-05	0.0003258	1.6924537
BTRC	-0.085805	5.8904736	-4.196223	4.02E-05	0.0003259	1.6920917
ZEB2P1	-1.171287	-0.06342	-4.196002	4.03E-05	0.0003261	1.691247
RP11-884K1	-0.905633	1.97414	-4.19566	4.03E-05	0.0003265	1.6899417
CYFIP2	0.2210845	6.1891827	4.1954091	4.04E-05	0.0003268	1.6889855
AC006000.5	-0.648812	-1.148124	-4.19496	4.05E-05	0.0003273	1.6872703
UACA	0.0901721	6.2302872	4.1941709	4.06E-05	0.0003283	1.6842593
CTD-2095E4	-0.652251	2.5937463	-4.193569	4.07E-05	0.0003289	1.6819642
MYL12A	0.0836931	6.8835601	4.193556	4.07E-05	0.0003289	1.6819128
XXbac-BPG1	-0.708631	2.1394621	-4.193491	4.07E-05	0.0003289	1.681665
DOCK5	0.1573661	6.0066428	4.1934603	4.07E-05	0.0003289	1.6815478
TAPT1	0.1197951	6.136182	4.1930594	4.08E-05	0.0003294	1.6800181
RP11-553N1	-0.911611	0.3949295	-4.192797	4.08E-05	0.0003296	1.6790186
FUNDC2P2	-1.316975	0.9367584	-4.192784	4.08E-05	0.0003296	1.6789673
RP11-98F14	-1.013141	0.6479943	-4.191931	4.10E-05	0.0003307	1.6757145
ENKD1	0.1420451	5.6318182	4.191801	4.10E-05	0.0003308	1.6752173
CD248	0.1918409	5.8145615	4.1907025	4.12E-05	0.0003322	1.6710272
VCX3A	-1.224603	-0.213841	-4.188956	4.15E-05	0.0003345	1.664366
LYN	0.1235119	6.207015	4.1880143	4.16E-05	0.0003357	1.6607782
SPA17	0.228329	4.9863467	4.1879211	4.16E-05	0.0003357	1.6604226
HPGD	0.6861213	5.8674698	4.1878775	4.16E-05	0.0003357	1.6602565
RP11-305L7	-1.010856	1.2268176	-4.187472	4.17E-05	0.0003361	1.6587095
HIP1	0.1257304	6.2769182	4.1873865	4.17E-05	0.0003361	1.6583853
UBE2SP1	-0.903993	2.4536304	-4.187351	4.17E-05	0.0003361	1.6582501
RP4-715N11	-0.75209	-1.002443	-4.18734	4.17E-05	0.0003361	1.6582084
RP11-876N2	0.2983242	4.6507051	4.1868468	4.18E-05	0.0003367	1.6563285
PEPD	0.1212274	6.7244241	4.1864445	4.19E-05	0.0003372	1.6547955
RP11-484K9	-0.933788	0.2502799	-4.185914	4.20E-05	0.0003379	1.6527736
TTC27	-0.0775	5.8982812	-4.185819	4.20E-05	0.0003379	1.652411
RP11-344P1	-0.984863	0.8839884	-4.185333	4.21E-05	0.0003385	1.6505617
AC005534.6	-0.938981	0.445424	-4.185194	4.21E-05	0.0003386	1.6500295
MTL5	-0.437821	4.7819108	-4.184662	4.22E-05	0.0003393	1.6480047

AC022816.2	-1.178855	3.2468236	-4.184488	4.22E-05	0.0003394	1.6473403
RP3-329A5.	-0.462669	3.323247	-4.184476	4.22E-05	0.0003394	1.6472943
RP11-147L1	-0.190801	4.7409491	-4.184427	4.22E-05	0.0003394	1.647109
MED15P9	-0.953256	-0.808983	-4.184325	4.22E-05	0.0003394	1.646719
RP11-884K1	-0.131775	5.1267352	-4.184301	4.22E-05	0.0003394	1.6466286
GCLM	0.1519486	6.3658352	4.1837498	4.23E-05	0.00034	1.64453
YJEFN3	-0.359806	4.6050029	-4.18374	4.23E-05	0.00034	1.6444944
PIGM	-0.10153	6.0008573	-4.183684	4.23E-05	0.00034	1.6442809
SMC1B	-0.930696	3.1400803	-4.183375	4.24E-05	0.0003403	1.6431027
ANXA8L1	1.3642011	0.9530444	4.1833598	4.24E-05	0.0003403	1.6430448
MACROD1	0.216265	5.8794135	4.1832039	4.24E-05	0.0003404	1.6424511
C1orf140	1.325119	0.4225485	4.1832024	4.24E-05	0.0003404	1.6424453
C2CD2	0.1330005	5.9172185	4.1830467	4.25E-05	0.0003406	1.6418527
RP11-485F1	-1.337157	-0.09751	-4.182895	4.25E-05	0.0003407	1.6412768
U1	-0.934941	1.9120597	-4.181826	4.27E-05	0.0003421	1.6372043
LRRC17	0.2923866	4.7250936	4.1815946	4.27E-05	0.0003423	1.6363239
ZGPAT	-0.127018	5.6850708	-4.181294	4.28E-05	0.0003427	1.6351802
AP000445.1	1.3086048	1.0587876	4.181122	4.28E-05	0.0003428	1.6345251
MIR663AHG	-1.376635	-0.067827	-4.181098	4.28E-05	0.0003428	1.6344338
GHRLOS	-0.509522	3.201901	-4.180899	4.28E-05	0.000343	1.6336748
ZNF391	-0.716713	3.810969	-4.180627	4.29E-05	0.0003433	1.6326402
ACTN3	-1.028216	0.5771928	-4.180325	4.29E-05	0.0003437	1.6314909
TUBB8P7	-1.081008	0.3380594	-4.180012	4.30E-05	0.000344	1.6303013
RP11-165H4	-0.877202	0.1325165	-4.179618	4.31E-05	0.0003445	1.6288011
RP11-215P8	-0.824322	-0.396833	-4.178925	4.32E-05	0.0003454	1.6261651
AGAP7P	-1.035359	1.4395041	-4.178863	4.32E-05	0.0003454	1.6259282
ARHGAP1	0.0666665	6.5492939	4.178758	4.32E-05	0.0003455	1.625529
SLC8B1	0.1203383	6.1346927	4.1785181	4.32E-05	0.0003457	1.624616
AC079776.2	0.9213009	-0.365634	4.1784953	4.32E-05	0.0003457	1.6245293
RP11-347H1	-1.250046	-0.577387	-4.177786	4.34E-05	0.0003465	1.6218318
RP11-679B1	0.7101197	2.4701088	4.17778	4.34E-05	0.0003465	1.6218085
LRRC1	-0.366958	5.6234881	-4.177604	4.34E-05	0.0003467	1.6211394
TEX19	-1.110648	1.8021665	-4.177424	4.34E-05	0.0003469	1.6204526
RP11-476H1	-0.817991	1.7100757	-4.177316	4.35E-05	0.000347	1.6200442
RP11-458J1	-0.126713	5.0517145	-4.177163	4.35E-05	0.0003471	1.6194615
OSBPL11	0.1159303	5.9800978	4.1756946	4.37E-05	0.000349	1.6138777
RXFP4	-0.984343	-0.396695	-4.175687	4.37E-05	0.000349	1.6138491
LIMK2	0.1486477	6.2239634	4.1756661	4.37E-05	0.000349	1.6137692
SH3BP5	0.2990097	5.1239692	4.1752797	4.38E-05	0.0003494	1.6123001
RP4-785G19	-0.939734	1.6029018	-4.174018	4.40E-05	0.0003512	1.6075055
ARMC3	-1.144383	0.2118946	-4.173088	4.42E-05	0.0003524	1.6039678
PWAR1	-0.985036	-0.126176	-4.172826	4.43E-05	0.0003527	1.6029737
RP11-763B2	-0.563622	-1.098545	-4.172764	4.43E-05	0.0003527	1.6027387
ZKSCAN4	-0.164373	5.2101542	-4.172518	4.43E-05	0.000353	1.6018026
TSPO2	-0.807617	3.5415007	-4.172393	4.43E-05	0.0003531	1.6013277
HSPA1L	-0.188527	4.6640079	-4.172229	4.44E-05	0.0003533	1.6007066
RP11-468E2	-0.340306	3.2831019	-4.172116	4.44E-05	0.0003533	1.6002776
AC004540.4	1.2272455	1.7688699	4.1719976	4.44E-05	0.0003534	1.599826
HCP5	0.2933394	5.9528693	4.1713677	4.45E-05	0.0003543	1.5974327
RP11-401F2	-0.640366	3.1613913	-4.171206	4.45E-05	0.0003544	1.5968183
AC073321.4	1.0710789	-0.189495	4.1710869	4.46E-05	0.0003545	1.5963663

WDR87	-0.852002	-0.771137	-4.170681	4.46E-05	0.000355	1.5948258
AKAP12	0.2890498	5.9131388	4.1703481	4.47E-05	0.0003554	1.5935599
AKR1B15	1.4836811	3.3942598	4.1701988	4.47E-05	0.0003555	1.5929929
AHNAK2	0.7947926	4.5545151	4.1697165	4.48E-05	0.0003562	1.5911612
TMBIM6	0.0913042	7.3784983	4.1695574	4.48E-05	0.0003563	1.5905571
RP11-30504	-0.895247	0.8898456	-4.169458	4.49E-05	0.0003564	1.5901789
KCNQ1OT1	-0.353453	4.7849573	-4.168536	4.50E-05	0.0003576	1.586678
KCNE3	-0.196411	5.4052348	-4.16852	4.50E-05	0.0003576	1.586616
HSD17B7P2	-0.341655	4.5580819	-4.167775	4.52E-05	0.0003586	1.5837894
AMICA1	-0.208033	5.7787388	-4.167427	4.52E-05	0.000359	1.5824671
CNTN1	1.479515	1.8398634	4.1666498	4.54E-05	0.00036	1.5795185
EBNA1BP2	0.1049551	6.3445354	4.1666348	4.54E-05	0.00036	1.5794616
GAS5	-0.145638	6.6182414	-4.16631	4.54E-05	0.0003604	1.5782293
RP1-278C19	-0.890414	1.6878481	-4.165909	4.55E-05	0.0003609	1.5767082
HIBADH	0.1263847	6.522453	4.1657261	4.55E-05	0.0003611	1.5760132
CXorf38	0.0856406	5.9474904	4.1645472	4.58E-05	0.0003626	1.5715401
LINGO1	-0.345645	5.3540781	-4.16454	4.58E-05	0.0003626	1.5715128
LINC00883	0.45312	3.9024889	4.1645366	4.58E-05	0.0003626	1.5715001
AC079807.2	-0.252732	3.9418134	-4.164498	4.58E-05	0.0003626	1.5713552
RP11-1260E	0.8106727	2.5600746	4.1642536	4.58E-05	0.0003629	1.5704265
RP11-452D2	-1.11482	0.3360673	-4.163585	4.59E-05	0.0003638	1.5678917
ANKRD29	0.4942113	5.2035912	4.1628868	4.61E-05	0.0003647	1.5652422
LINC-ROR	-1.132077	0.0789317	-4.162709	4.61E-05	0.0003649	1.5645679
MS4A14	0.5152369	3.8490395	4.162658	4.61E-05	0.0003649	1.5643747
FBXO45	-0.086702	5.8994305	-4.162543	4.61E-05	0.0003649	1.563939
GINS4	-0.28388	4.9534357	-4.162528	4.61E-05	0.0003649	1.5638832
LY86	0.2618663	5.0632406	4.1620785	4.62E-05	0.0003655	1.5621773
ANXA5	0.1191553	6.8354362	4.1608743	4.64E-05	0.0003672	1.5576117
RP11-213H1	1.0504389	0.8692278	4.1604192	4.65E-05	0.0003678	1.5558863
ZNF696	-0.141166	5.5693034	-4.160361	4.65E-05	0.0003678	1.5556677
RGMB	0.2182642	5.1932292	4.1602621	4.66E-05	0.0003679	1.5552909
PTPRU	0.2641914	5.9486732	4.1601024	4.66E-05	0.000368	1.5546857
SPEG	0.5767653	4.0732714	4.1600784	4.66E-05	0.000368	1.5545949
ARL4D	-0.243013	6.0394186	-4.159937	4.66E-05	0.0003681	1.5540571
CTD-2104P1	-0.923204	2.1480447	-4.159489	4.67E-05	0.0003687	1.5523619
ETV6	0.0877984	6.0648215	4.1590182	4.68E-05	0.0003693	1.5505767
IDH1-AS1	-0.475424	3.6609858	-4.158931	4.68E-05	0.0003694	1.5502459
ATP8B3	0.3529724	4.7752742	4.15881	4.68E-05	0.0003695	1.5497877
MBOAT1	0.4349736	5.0055118	4.1580079	4.70E-05	0.0003706	1.5467489
MLLT10	-0.094461	6.0803724	-4.157807	4.70E-05	0.0003708	1.5459861
RP5-908M14	-0.691838	2.9810658	-4.157668	4.70E-05	0.000371	1.5454625
HIAT1	0.0853343	6.1430375	4.1576152	4.71E-05	0.000371	1.545261
BDNF-AS	0.2758922	4.6628791	4.1571972	4.71E-05	0.0003715	1.5436774
CTC-480C2.	-1.30921	0.1284853	-4.157022	4.72E-05	0.0003717	1.543013
TCF7L1	-0.163096	5.9715314	-4.156763	4.72E-05	0.0003719	1.5420342
INAFM2	0.1810839	5.0937378	4.1567491	4.72E-05	0.0003719	1.54198
AC003006.1	-0.697425	-0.97527	-4.156436	4.73E-05	0.0003723	1.5407951
LINC01456	-0.70067	-1.176248	-4.155968	4.74E-05	0.0003729	1.5390204
LINC01162	-0.665024	-1.175669	-4.155783	4.74E-05	0.0003731	1.538321
VKORC1	0.1378088	6.3669892	4.1556775	4.74E-05	0.0003732	1.5379218
DDX28	0.1140584	5.7809463	4.1549075	4.76E-05	0.0003743	1.5350061

TOMM40L	-0.125159	6.226721	-4.154859	4.76E-05	0.0003743	1.5348215
IRF8	0.1970754	5.852949	4.1545142	4.77E-05	0.0003747	1.533517
CABLES2	-0.158834	5.6005186	-4.154038	4.77E-05	0.0003753	1.5317154
CTD-3014M2	-0.773142	2.4623192	-4.15403	4.77E-05	0.0003753	1.5316853
RP11-264I1	0.4216916	3.9854373	4.1539961	4.78E-05	0.0003753	1.5315554
CFTR	1.5038968	3.0659526	4.1537928	4.78E-05	0.0003755	1.5307861
MTMR6	0.1015997	6.0024678	4.153694	4.78E-05	0.0003756	1.5304121
SULT1B1	0.4993923	5.2335513	4.1535047	4.78E-05	0.0003758	1.5296953
RAP2A	-0.120307	6.2566144	-4.152929	4.80E-05	0.0003766	1.5275166
RP11-38L15	-0.854147	2.4171991	-4.152349	4.81E-05	0.0003774	1.5253209
CCDC186	0.1280506	5.601275	4.1521262	4.81E-05	0.0003776	1.5244785
ZNF567	-0.14701	5.0840316	-4.151602	4.82E-05	0.0003783	1.5224952
RP6-109B7.	-0.82979	2.2684034	-4.151381	4.83E-05	0.0003786	1.5216575
RP11-94I2.	-1.03681	1.0502189	-4.150894	4.84E-05	0.0003793	1.519815
GDF10	-1.404601	2.0464774	-4.150728	4.84E-05	0.0003794	1.5191885
RP11-89F3.	-0.822875	-0.542342	-4.150608	4.84E-05	0.0003795	1.5187354
RPL4P3	-0.84673	0.8716676	-4.150299	4.85E-05	0.0003799	1.5175635
SERPINB6	0.0980083	6.455378	4.1498541	4.86E-05	0.0003805	1.5158824
ACOX3	0.1265778	6.0495449	4.1491201	4.87E-05	0.0003816	1.5131065
SSR2	-0.092399	6.942631	-4.148655	4.88E-05	0.0003822	1.5113464
AADAT	0.4351583	5.083853	4.1478912	4.89E-05	0.0003833	1.5084594
GADD45G	0.262749	6.159902	4.1477376	4.90E-05	0.0003835	1.5078785
RP11-683L2	-1.183494	0.3659063	-4.147622	4.90E-05	0.0003836	1.5074413
MEIS3P2	0.9446217	0.0995696	4.1472195	4.91E-05	0.0003841	1.5059201
SPATA31C2	-0.871767	-0.917697	-4.146235	4.93E-05	0.0003855	1.5021996
RP11-543G1	-0.849942	-0.733525	-4.14623	4.93E-05	0.0003855	1.5021783
CERS5	-0.07691	6.037539	-4.145469	4.94E-05	0.0003866	1.4993046
ZBTB39	-0.128905	5.4399639	-4.145368	4.94E-05	0.0003866	1.4989222
C9	0.9579988	5.9023813	4.1453221	4.95E-05	0.0003866	1.4987484
RP11-129B9	-0.909965	1.3253905	-4.145314	4.95E-05	0.0003866	1.4987179
RPL14P3	-0.825354	1.2809663	-4.14488	4.95E-05	0.0003871	1.4970791
AC012358.4	-0.277573	3.873955	-4.144833	4.95E-05	0.0003871	1.4969022
RP5-901A4.	-0.839058	1.937064	-4.143793	4.98E-05	0.0003887	1.4929725
CCDC154	-0.53054	3.9766703	-4.14346	4.98E-05	0.0003891	1.4917121
RP13-977J1	0.7407317	-0.864834	4.1420249	5.01E-05	0.0003913	1.4862929
ENTPD2	-0.428604	5.012017	-4.141948	5.01E-05	0.0003913	1.4860007
LRRC2-AS1	-0.916728	1.2951027	-4.140864	5.03E-05	0.000393	1.4819092
DDN	-1.131968	1.2209666	-4.140351	5.05E-05	0.0003937	1.4799725
FAM184B	-0.588732	2.6759534	-4.140295	5.05E-05	0.0003937	1.4797625
TUSC3	0.4003071	5.3840905	4.140145	5.05E-05	0.0003939	1.4791951
ADAMTS4	0.1912392	5.8676139	4.1398909	5.05E-05	0.0003942	1.478236
HDGFRP2	-0.081813	6.4088809	-4.13936	5.07E-05	0.0003949	1.4762314
AF196972.9	-0.791486	1.9932823	-4.139161	5.07E-05	0.0003952	1.4754814
DLG2	0.4546466	4.1484985	4.1388829	5.08E-05	0.0003955	1.4744314
CHTOP	-0.060302	6.5067555	-4.138752	5.08E-05	0.0003956	1.4739378
HLX	0.1657092	5.7899179	4.1382633	5.09E-05	0.0003963	1.4720931
CTA-363E6.	1.1271582	0.0102757	4.1381568	5.09E-05	0.0003964	1.4716914
MIP	0.8863832	2.386179	4.1378107	5.10E-05	0.0003969	1.4703853
ZNF554	-0.134833	5.2816859	-4.137613	5.10E-05	0.0003971	1.4696411
RP11-141C7	-0.954693	0.3013279	-4.137047	5.11E-05	0.0003979	1.4675033
RP11-513G1	-1.056476	0.8189131	-4.136723	5.12E-05	0.0003984	1.4662837

UBXN7	-0.078655	6.0463323	-4.136651	5.12E-05	0.0003984	1.4660104
TNFRSF11B	0.5056824	5.1828203	4.1365873	5.12E-05	0.0003984	1.4657699
RP11-707G1	-0.854511	1.8404985	-4.1365	5.12E-05	0.0003985	1.4654399
ZNF295-AS1	0.9349215	1.7085907	4.1363942	5.13E-05	0.0003986	1.4650414
GP2	-1.673325	1.9040355	-4.13607	5.13E-05	0.000399	1.4638192
LRRC29	0.2060653	4.8039912	4.1360395	5.13E-05	0.000399	1.4637036
RP11-143K1	-0.767594	-0.815367	-4.135876	5.14E-05	0.0003991	1.4630873
VHL	-0.09301	6.0884037	-4.135578	5.14E-05	0.0003995	1.4619627
NDUFS1	0.0838212	6.6580816	4.1355153	5.14E-05	0.0003995	1.4617267
VASH1	0.1613207	5.7498225	4.1350829	5.15E-05	0.0004002	1.4600959
AC093716.1	-0.467285	-1.325606	-4.134957	5.16E-05	0.0004003	1.4596222
ZNF300	-0.418126	4.68795	-4.13478	5.16E-05	0.0004005	1.4589532
PRKAR2B	0.4764006	4.4386062	4.1346198	5.16E-05	0.0004006	1.4583498
RPL5P5	-0.79822	-0.289616	-4.134557	5.16E-05	0.0004007	1.4581136
CATIP-AS2	-0.907441	-0.1196	-4.134469	5.17E-05	0.0004007	1.4577826
SPATA24	-0.193526	4.88903	-4.134426	5.17E-05	0.0004007	1.4576193
RP5-864K19	0.4331708	3.7476013	4.1340383	5.17E-05	0.0004012	1.4561572
AIM1	0.2786202	5.6682369	4.1336717	5.18E-05	0.0004017	1.4547752
SOX6	0.5495299	5.1816351	4.1336428	5.18E-05	0.0004017	1.4546662
RP4-568F9.	-0.819171	-0.877852	-4.133323	5.19E-05	0.0004021	1.4534612
AC008753.3	-0.894245	0.6629472	-4.133043	5.20E-05	0.0004025	1.4524034
FAR1	0.1976597	5.4955913	4.1330033	5.20E-05	0.0004025	1.4522556
RP11-534L2	-0.897061	0.7830924	-4.132947	5.20E-05	0.0004025	1.4520419
NACA2	-0.781796	1.8330332	-4.132847	5.20E-05	0.0004026	1.4516656
C12orf56	-1.45705	0.9704489	-4.131697	5.22E-05	0.0004044	1.4473315
ASH1L-AS1	-0.235463	4.2008885	-4.131552	5.23E-05	0.0004045	1.4467857
WDR82P1	-0.973815	-0.746965	-4.131411	5.23E-05	0.0004046	1.4462537
IL12A	-0.855154	2.5144715	-4.130486	5.25E-05	0.0004061	1.4427702
RP11-18707	-0.907668	0.6094526	-4.130424	5.25E-05	0.0004061	1.4425359
HDAC2	-0.080371	6.3319763	-4.130135	5.26E-05	0.0004064	1.4414477
U73166.2	-0.616358	3.1704857	-4.130116	5.26E-05	0.0004064	1.4413766
RP11-977G1	-0.320755	3.8208569	-4.130086	5.26E-05	0.0004064	1.4412623
CTD-3035D6	-0.663657	2.8006632	-4.129961	5.26E-05	0.0004064	1.4407908
FAM114A2	0.0835706	5.9424039	4.1299507	5.26E-05	0.0004064	1.4407527
ALOX5AP	0.4411522	5.0181052	4.1298213	5.26E-05	0.0004065	1.4402653
WDR75	-0.069576	6.1140896	-4.129663	5.27E-05	0.0004067	1.4396675
LINC01189	-0.54904	-1.285515	-4.129458	5.27E-05	0.000407	1.4388966
ZXDA	0.2345546	4.7038818	4.1293782	5.27E-05	0.000407	1.4385961
CHST11	0.2484926	5.7054381	4.1292349	5.28E-05	0.0004072	1.4380564
KLHDC3	-0.089994	6.6557282	-4.128788	5.29E-05	0.0004078	1.4363725
SPTA1	1.1988959	1.5731084	4.1285895	5.29E-05	0.000408	1.4356255
SUCLG2P2	0.6980938	3.0277705	4.1284375	5.29E-05	0.0004082	1.4350532
BCAM	-0.15964	6.7855209	-4.128258	5.30E-05	0.0004084	1.4343775
RP4-583P15	-0.332852	4.0338821	-4.127985	5.30E-05	0.0004088	1.4333508
RP11-295P9	-0.146507	5.3837763	-4.127123	5.32E-05	0.0004101	1.4301033
CTC-428G20	0.4988416	2.6402182	4.1271006	5.32E-05	0.0004101	1.4300192
TRIM23	0.1122224	5.7082668	4.1259741	5.35E-05	0.0004118	1.4257784
IDH3A	0.165066	5.7611715	4.1258055	5.35E-05	0.000412	1.4251441
AC010148.1	-1.049606	2.0501368	-4.125594	5.35E-05	0.0004123	1.4243495
RPL17	-0.110684	6.1025707	-4.12502	5.37E-05	0.0004131	1.4221895
MYCBP2	0.1129695	6.0584934	4.1245814	5.38E-05	0.0004138	1.4205372

ZSWIM6	0.1794206	5.5683349	4.1244745	5.38E-05	0.0004139	1.4201349
MED15P8	-0.69973	-1.147039	-4.124095	5.39E-05	0.0004144	1.4187081
CTD-2260A1	0.5113143	2.9529069	4.1237107	5.39E-05	0.000415	1.4172612
SOD3	0.3663154	5.6770986	4.1233916	5.40E-05	0.0004154	1.4160608
GLO1	-0.092609	6.6475503	-4.122296	5.42E-05	0.0004172	1.4119408
RP11-215A2	-0.422052	3.3485734	-4.12221	5.43E-05	0.0004172	1.4116163
KIAA0825	0.4213081	3.650717	4.1221307	5.43E-05	0.0004173	1.4113177
RP11-39901	0.5425535	3.2190606	4.1220228	5.43E-05	0.0004174	1.410912
RP11-286H1	0.966032	2.0617197	4.1211366	5.45E-05	0.0004188	1.4075791
VDAC1P8	-0.20927	4.6505635	-4.120322	5.47E-05	0.00042	1.4045167
RP11-44K6.	0.7591754	-1.036398	4.1197675	5.48E-05	0.0004207	1.4024319
CTD-2369P2	-0.545245	2.5522845	-4.11972	5.48E-05	0.0004207	1.4022547
COX20	-0.133918	5.5659731	-4.119698	5.48E-05	0.0004207	1.4021714
RP11-568A7	-0.937276	-0.542247	-4.119692	5.48E-05	0.0004207	1.4021475
RP11-15001	-1.336687	2.2276806	-4.119683	5.48E-05	0.0004207	1.4021145
DNAJC27	0.160821	4.9273705	4.1195586	5.48E-05	0.0004208	1.4016466
ZKSCAN8	-0.102044	5.9353228	-4.119426	5.49E-05	0.0004209	1.4011469
GARNL3	0.305911	4.8426242	4.1192486	5.49E-05	0.0004211	1.4004815
NIPAL3	0.1040744	5.7363447	4.1191133	5.49E-05	0.0004213	1.3999728
ZNF280A	-1.000893	-0.641076	-4.118893	5.50E-05	0.0004215	1.3991438
GBP2	0.1605518	6.5217554	4.1188693	5.50E-05	0.0004215	1.3990557
AFAP1L2	0.2215268	5.0656932	4.1186233	5.51E-05	0.0004218	1.398131
STAT6	0.1031502	6.6940528	4.1185894	5.51E-05	0.0004218	1.3980037
ATP10A	0.3598699	4.8523187	4.1185138	5.51E-05	0.0004219	1.3977197
MIR7111	-0.943068	0.1504015	-4.118388	5.51E-05	0.000422	1.397247
FSIP2-AS1	-0.996442	0.3575847	-4.117977	5.52E-05	0.0004226	1.3957007
MT-ATP8	0.14443	7.0807031	4.1176382	5.53E-05	0.0004231	1.3944293
KRCC1	0.1131241	6.0447172	4.1172229	5.54E-05	0.0004236	1.3928686
ABI3	0.169017	5.5940127	4.1172011	5.54E-05	0.0004236	1.3927867
HLA-DMB	0.2181134	5.9670666	4.1170191	5.54E-05	0.0004239	1.3921031
CPT1B	-0.321509	4.4974505	-4.116927	5.54E-05	0.0004239	1.3917588
MTA1	-0.081418	6.1884974	-4.116272	5.56E-05	0.0004249	1.3892954
ARL8A	0.0961912	6.4052715	4.1159392	5.56E-05	0.0004254	1.3880462
RPL38	-0.098053	6.9651678	-4.115378	5.58E-05	0.0004263	1.3859372
RP11-108K3	-0.85939	-0.739059	-4.115117	5.58E-05	0.0004266	1.3849569
GRAMD4	0.1726698	6.3882286	4.1142185	5.60E-05	0.0004281	1.381584
CTD-2134A5	-0.832317	2.4035478	-4.11338	5.62E-05	0.0004294	1.3784355
ZNF543	-0.200716	4.979776	-4.113368	5.62E-05	0.0004294	1.3783924
RP11-234P3	-0.549736	-1.263268	-4.113124	5.63E-05	0.0004297	1.377476
RAB11FIP5	0.1524178	5.9707289	4.1130661	5.63E-05	0.0004297	1.3772573
CTD-2373N4	0.56564	3.5879056	4.1128771	5.63E-05	0.00043	1.3765477
DIXDC1	0.192048	5.5660953	4.1127747	5.64E-05	0.00043	1.3761635
AC159540.1	-0.932577	1.6527585	-4.112458	5.64E-05	0.0004305	1.3749751
C1GALT1	-0.111766	6.0475841	-4.11196	5.65E-05	0.0004313	1.3731067
ERCC2	-0.10382	6.1465074	-4.111503	5.66E-05	0.000432	1.371389
RP11-428K3	-0.537083	4.0437503	-4.111378	5.67E-05	0.0004321	1.3709222
INSIG1	0.2304864	6.898181	4.1112612	5.67E-05	0.0004322	1.3704827
RASSF8	0.2385454	5.5390351	4.1106912	5.68E-05	0.000433	1.3683441
AC006050.2	-1.034278	-0.799236	-4.110678	5.68E-05	0.000433	1.3682939
RP11-108K3	-0.767355	-0.961312	-4.110598	5.69E-05	0.0004331	1.3679962
CTA-268H5.	-0.607213	-1.126227	-4.110548	5.69E-05	0.0004331	1.3678065

AC111186.1	-0.72261	3.8993056	-4.110443	5.69E-05	0.0004332	1.3674128
RP11-602N2	-0.864801	-0.13572	-4.109351	5.71E-05	0.0004349	1.363316
GIPC3	0.1914177	5.2735439	4.1093287	5.71E-05	0.0004349	1.3632324
ZMAT1	0.3278924	5.2568286	4.1090546	5.72E-05	0.0004353	1.3622044
RP3-441A12	-0.794423	-0.461956	-4.108153	5.74E-05	0.0004368	1.3588236
NTRK3	0.8891782	2.9175909	4.1075595	5.75E-05	0.0004377	1.3565973
TMEM92	1.132509	4.571668	4.1074821	5.76E-05	0.0004378	1.356307
ANKRD11	0.0729613	6.3531422	4.1068844	5.77E-05	0.0004387	1.3540657
CTD-2536I1	0.9800668	1.4891091	4.1067111	5.77E-05	0.000439	1.3534161
EPB41L3	0.2704255	5.3367232	4.1061561	5.79E-05	0.0004398	1.3513354
HNRNPA3P3	-0.890004	0.961353	-4.105957	5.79E-05	0.0004401	1.3505879
F2RL2	0.6997502	4.1296291	4.1054478	5.80E-05	0.0004409	1.3486803
CHGA	-1.322211	1.3088842	-4.105299	5.81E-05	0.0004411	1.3481225
AC244230.1	-1.05614	1.4817387	-4.10493	5.82E-05	0.0004416	1.3467406
METRNL	0.2380376	5.7514704	4.1048663	5.82E-05	0.0004416	1.346501
RP11-424M2	-0.82993	-0.49287	-4.104831	5.82E-05	0.0004416	1.3463683
FPGT-TNNI3	0.8714577	2.0454489	4.1045085	5.83E-05	0.0004421	1.3451601
CAPZA2	0.0701706	6.5464254	4.1040195	5.84E-05	0.0004429	1.3433279
RTEL1-TNFR	-0.258323	4.4472842	-4.10374	5.84E-05	0.0004433	1.3422822
PAX5	-0.906667	3.2085838	-4.102886	5.86E-05	0.0004447	1.3390827
PPARGC1B	0.2707235	5.0354675	4.1027323	5.87E-05	0.0004449	1.3385052
RP11-314N1	-1.414626	0.283232	-4.102685	5.87E-05	0.0004449	1.3383264
ACVRL1	0.134068	5.8500217	4.1021982	5.88E-05	0.0004456	1.3365046
GNRHR2P1	-0.714956	-0.932411	-4.101927	5.89E-05	0.000446	1.3354903
HES6	-0.201909	5.4442661	-4.101702	5.89E-05	0.0004463	1.334647
SLC26A10	0.9114836	1.7882138	4.1016666	5.89E-05	0.0004463	1.3345134
RP11-710C1	0.9572421	1.249458	4.1015261	5.90E-05	0.0004465	1.3339874
CH25H	0.6068615	4.0514214	4.1013607	5.90E-05	0.0004467	1.333368
DRD4	-0.539799	3.8560519	-4.101061	5.91E-05	0.0004471	1.3322457
RP11-794M8	-0.463287	-1.275757	-4.100741	5.91E-05	0.0004476	1.3310458
PAGE2B	-1.342185	0.2142485	-4.100477	5.92E-05	0.000448	1.3300587
CTD-2574D2	-0.20282	4.5655332	-4.100393	5.92E-05	0.000448	1.3297449
C1orf162	0.2054189	5.5177588	4.1003531	5.92E-05	0.000448	1.3295949
TPD52L1	0.2845608	5.9033372	4.1003145	5.92E-05	0.000448	1.3294502
GSTM4	0.1630508	6.0791196	4.1000601	5.93E-05	0.0004483	1.3284979
NR4A2	0.2919881	5.3055171	4.1000357	5.93E-05	0.0004483	1.3284064
AC034228.3	-0.690278	-1.157492	-4.09939	5.95E-05	0.0004494	1.3259891
PRKD1	0.3502064	4.8848775	4.0988795	5.96E-05	0.0004502	1.3240781
PWAR6	-0.52382	4.8002571	-4.098611	5.96E-05	0.0004506	1.3230722
RPL23AP68	-0.437267	-1.340327	-4.09837	5.97E-05	0.0004509	1.322173
RBM12	-0.061669	6.2843251	-4.098148	5.98E-05	0.0004512	1.3213406
PDZK1P2	-0.450529	-1.268108	-4.09799	5.98E-05	0.0004514	1.3207504
RP11-173P1	-0.958869	0.5472169	-4.097884	5.98E-05	0.0004515	1.3203535
ZFP90	0.102606	5.677037	4.0972843	6.00E-05	0.0004525	1.3181082
RP11-297L1	-0.644511	4.6771045	-4.097165	6.00E-05	0.0004526	1.3176607
KPNA5	-0.190744	5.1367921	-4.096593	6.01E-05	0.0004536	1.3155205
TCTE3	-0.24712	4.475527	-4.096141	6.02E-05	0.0004543	1.3138297
FPR1	0.5018747	4.6437784	4.0960902	6.02E-05	0.0004543	1.3136409
RPL7P23	-0.50122	3.5501284	-4.096025	6.03E-05	0.0004543	1.3133957
COTL1	0.1601464	6.3256816	4.0953602	6.04E-05	0.0004554	1.3109101
RCVRN	0.7975017	-0.523368	4.0939745	6.08E-05	0.0004579	1.3057277

UROD	0.1003631	6.5384333	4.0939304	6.08E-05	0.0004579	1.305563
CTA-14H9.5	0.6319917	2.4898988	4.0936643	6.08E-05	0.0004582	1.304568
ABCC9	0.3332202	5.6656365	4.0935653	6.09E-05	0.0004583	1.3041978
PUS7L	-0.088657	5.8015683	-4.092991	6.10E-05	0.0004593	1.3020494
METTL23	-0.088818	6.0680225	-4.09218	6.12E-05	0.0004607	1.2990184
ZNF14	-0.321904	4.7962393	-4.092055	6.12E-05	0.0004608	1.2985522
AGAP4	-0.269689	4.2804199	-4.091905	6.13E-05	0.0004609	1.2979901
FAM150B	1.2308954	2.3627623	4.0918572	6.13E-05	0.0004609	1.2978123
AVP	-0.957731	-0.633847	-4.09183	6.13E-05	0.0004609	1.2977113
RP11-713C5	0.8515948	-0.722872	4.0913955	6.14E-05	0.0004616	1.296087
SOD1	0.1304506	7.0686311	4.0908676	6.15E-05	0.0004625	1.2941142
CALR3	-0.936309	0.1508834	-4.090602	6.16E-05	0.0004629	1.2931209
H3F3A	-0.085768	6.04827	-4.09029	6.17E-05	0.0004633	1.2919566
AP000936.1	-0.857887	1.4828313	-4.090277	6.17E-05	0.0004633	1.2919069
GOLGA6L9	-0.325017	4.5230701	-4.090236	6.17E-05	0.0004633	1.2917539
RP11-655G2	-0.695677	-1.056006	-4.089618	6.18E-05	0.0004644	1.2894444
HOMEZ	-0.092802	5.7535839	-4.089398	6.19E-05	0.0004647	1.2886224
ESRRG	1.1235856	2.6545925	4.0891477	6.19E-05	0.000465	1.2876884
ADAMTSL4-A	0.4500764	2.9727347	4.0891087	6.19E-05	0.000465	1.2875426
NEURL3	-0.662027	4.9900598	-4.0889	6.20E-05	0.0004653	1.2867632
ZNF773	-0.282369	4.8954118	-4.088841	6.20E-05	0.0004653	1.2865443
RP11-545I5	0.275721	4.3311149	4.088324	6.21E-05	0.0004662	1.2846115
RP11-120D5	-0.7688	2.3472835	-4.08672	6.25E-05	0.0004691	1.2786214
RP4-660H19	-1.029049	-0.634645	-4.086508	6.26E-05	0.0004694	1.2778309
HORMAD2	1.3633359	1.9635933	4.0863804	6.26E-05	0.0004695	1.2773539
RP11-351J2	-0.746989	3.595497	-4.08612	6.27E-05	0.0004699	1.2763829
MARK1	0.4578187	4.1529904	4.0849814	6.30E-05	0.0004719	1.2721317
TNNI3	-1.368088	0.7569278	-4.084971	6.30E-05	0.0004719	1.2720916
RP11-795F1	-0.358143	3.8708907	-4.084439	6.31E-05	0.0004728	1.2701091
AC006037.2	-1.088103	-0.016296	-4.083844	6.33E-05	0.0004738	1.2678873
ZNF350	-0.179569	5.3381427	-4.083703	6.33E-05	0.000474	1.26736
GTDC1	0.1102035	5.6956366	4.0834705	6.34E-05	0.0004743	1.2664933
G3BP2	0.0801918	6.4620611	4.0834442	6.34E-05	0.0004743	1.2663951
APBA1	0.3058914	5.5782839	4.0832021	6.34E-05	0.0004746	1.2654922
AC109829.1	-0.939757	1.1846111	-4.082223	6.37E-05	0.0004764	1.2618385
RP11-307C1	-0.37075	3.641505	-4.081916	6.37E-05	0.0004768	1.260696
KLF7	0.2099045	5.5744719	4.0818356	6.38E-05	0.0004768	1.2603943
MAP7	0.2452999	5.9972466	4.0818268	6.38E-05	0.0004768	1.2603617
RP3-523E19	-0.990727	0.6337189	-4.08175	6.38E-05	0.0004769	1.2600735
RP11-89B16	0.7955426	-0.572067	4.0814022	6.39E-05	0.0004775	1.258778
DOCK9	0.1517627	6.0155229	4.0807308	6.41E-05	0.0004786	1.2562744
FAM89A	0.210546	5.5740814	4.080664	6.41E-05	0.0004787	1.2560251
JMJD4	-0.115453	6.0195084	-4.080054	6.42E-05	0.0004797	1.2537511
BDNF	0.9074004	2.2711842	4.0796349	6.43E-05	0.0004804	1.2521881
AC006539.3	-0.874098	-0.38265	-4.07927	6.44E-05	0.000481	1.2508279
SRPK1	-0.090579	6.3039585	-4.078362	6.47E-05	0.0004827	1.2474433
HMGCR	-0.14712	6.4337279	-4.078173	6.47E-05	0.0004829	1.2467399
ZNF83	-0.263714	5.7225466	-4.078033	6.47E-05	0.0004831	1.2462187
RP1-124C6.	-0.86551	-0.479443	-4.077963	6.48E-05	0.0004831	1.2459557
ZSCAN25	-0.080873	5.7009708	-4.077921	6.48E-05	0.0004831	1.2457979
RP11-477I4	-0.760924	2.2412415	-4.077814	6.48E-05	0.0004832	1.2454011

GABRA3	-1.545765	0.6014733	-4.077046	6.50E-05	0.0004846	1.2425395
FDX1	0.1333812	6.296409	4.0765694	6.51E-05	0.0004854	1.240763
AC005077.1	0.646721	4.5554174	4.0762502	6.52E-05	0.000486	1.2395739
RP11-177H1	0.7714872	1.9674676	4.0761439	6.52E-05	0.000486	1.239178
ZNF597	0.2684383	5.0011763	4.0761131	6.52E-05	0.000486	1.2390632
TAPBPL	0.1737078	6.1319549	4.0758729	6.53E-05	0.0004863	1.2381681
RP11-329J1	0.4850627	-1.263109	4.0758631	6.53E-05	0.0004863	1.2381318
RP11-93209	-0.903934	1.7456054	-4.07538	6.54E-05	0.0004871	1.2363305
NMT2	0.124442	6.0757436	4.0751103	6.55E-05	0.0004876	1.2353275
ZNF492	-1.233041	0.9852609	-4.074762	6.56E-05	0.0004881	1.2340287
C10orf2	-0.14266	5.7530337	-4.074626	6.56E-05	0.0004883	1.2335257
KRTAP6-3	-0.478801	-1.319959	-4.074595	6.56E-05	0.0004883	1.2334093
RNU6-925P	-0.884749	0.4359031	-4.074279	6.57E-05	0.0004888	1.2322298
SYT5	-1.040115	2.0315569	-4.074141	6.58E-05	0.0004889	1.2317162
RP11-408E5	-1.231036	-0.464162	-4.073863	6.58E-05	0.0004894	1.2306843
AC007364.1	0.6467044	-0.958026	4.0734791	6.59E-05	0.00049	1.2292529
VIPR1	0.5854881	4.4180877	4.0727761	6.61E-05	0.0004913	1.2266356
GAS2	-0.348921	5.6603907	-4.072041	6.63E-05	0.0004926	1.223901
HEYL	0.2609609	5.7734586	4.0715771	6.64E-05	0.0004935	1.2221726
OAS2	0.2094241	6.093361	4.0714706	6.65E-05	0.0004936	1.2217762
USP12	0.1094632	6.074119	4.0713684	6.65E-05	0.0004937	1.2213958
REXO4	-0.094053	6.1489917	-4.071224	6.65E-05	0.0004938	1.2208601
NDEL1	0.0946421	6.0469442	4.0710627	6.66E-05	0.0004941	1.2202581
RP11-498D1	1.042569	0.3716771	4.0706671	6.67E-05	0.0004947	1.2187861
LPIN2	0.1456399	6.7262777	4.0705841	6.67E-05	0.0004948	1.218477
TJP1	0.0973247	6.4389995	4.0703062	6.68E-05	0.0004952	1.2174432
BAALC	0.4806802	4.7268282	4.0700409	6.68E-05	0.0004957	1.2164561
FAM86JP	-0.227589	4.7073526	-4.069461	6.70E-05	0.0004967	1.2143
POLR2A	0.0729608	6.5988495	4.0693125	6.70E-05	0.0004967	1.2137458
DARS2	-0.106559	6.2059357	-4.069301	6.70E-05	0.0004967	1.2137017
BAATP1	1.1054038	2.7099328	4.0692913	6.70E-05	0.0004967	1.2136672
SKA2	-0.108293	6.0079026	-4.068717	6.72E-05	0.0004978	1.21153
RP11-417E7	1.1839164	0.9824731	4.0683196	6.73E-05	0.0004985	1.2100527
POLR2H	-0.084154	6.276714	-4.068224	6.73E-05	0.0004985	1.2096984
FGF23	0.9780851	-0.700708	4.0674921	6.75E-05	0.0004999	1.2069752
TMCC3	0.1848833	5.6620402	4.06705	6.76E-05	0.0005007	1.2053313
RP11-701H2	-1.106142	1.1041478	-4.066854	6.77E-05	0.000501	1.2046006
SNRPA	-0.084215	6.3300767	-4.066655	6.77E-05	0.0005012	1.2038629
MYO19	-0.099298	6.1697263	-4.066642	6.77E-05	0.0005012	1.203813
RP11-307C1	0.9209495	1.4342346	4.0657863	6.80E-05	0.0005028	1.2006331
ATP6V1B2	0.0905959	6.3927031	4.0655687	6.80E-05	0.0005031	1.1998241
CIITA	0.2237451	5.6807295	4.0654988	6.80E-05	0.0005032	1.199564
CXCL14	1.030371	3.9188424	4.0650974	6.82E-05	0.0005038	1.1980721
ANKRD24	0.2658873	5.0978234	4.0650648	6.82E-05	0.0005038	1.197951
AC007461.2	-1.004791	0.6955321	-4.064879	6.82E-05	0.0005041	1.1972617
RP11-122K1	-0.851786	-0.376099	-4.064586	6.83E-05	0.0005046	1.1961716
HRH4	0.9031388	0.5665404	4.0638373	6.85E-05	0.000506	1.1933892
DYDC2	-0.988315	3.8414869	-4.06364	6.86E-05	0.0005063	1.1926573
CLDN15	-0.271656	6.0932	-4.062832	6.88E-05	0.0005078	1.1896536
RP11-21401	-0.662166	3.8963635	-4.0618	6.91E-05	0.0005098	1.1858197
PRRC2A	-0.065494	6.7941768	-4.061544	6.91E-05	0.0005102	1.1848701

GPI	0.0944119	7.0543154	4.0607315	6.93E-05	0.0005117	1.1818523
RP11-94C24	-0.590536	3.6765588	-4.060599	6.94E-05	0.0005119	1.1813615
HNRNPA1P54	-0.886892	-0.087192	-4.060227	6.95E-05	0.0005125	1.1799791
CTC-526N19	0.4736964	3.4724573	4.0601933	6.95E-05	0.0005125	1.1798538
TMEM191B	-1.045392	1.6567418	-4.059998	6.96E-05	0.0005128	1.1791298
KB-1572G7.	-0.469903	3.4925657	-4.059811	6.96E-05	0.0005131	1.1784346
RP11-4204.	-0.990125	1.0462445	-4.059658	6.96E-05	0.0005133	1.1778668
SLC38A9	-0.109297	5.9434124	-4.059488	6.97E-05	0.0005134	1.1772342
RP11-78F17	-0.974086	-0.388265	-4.059484	6.97E-05	0.0005134	1.1772199
RP11-498P1	-1.161201	2.405983	-4.059138	6.98E-05	0.000514	1.1759354
TMEM127	0.0596055	6.6361525	4.0581781	7.01E-05	0.0005159	1.1723725
RP4-669H2.	-0.973016	1.0464964	-4.056288	7.06E-05	0.0005197	1.1653595
KCNC4	0.2230928	5.1732232	4.0561537	7.06E-05	0.0005198	1.1648607
TXLNG	-0.079182	5.9362228	-4.055825	7.07E-05	0.0005204	1.1636415
FBXL21	-1.203198	2.7537672	-4.054185	7.12E-05	0.0005236	1.1575595
RP11-116B1	1.0892128	1.1431996	4.0541619	7.12E-05	0.0005236	1.1574728
RNPEP	0.0867389	6.5315524	4.0536433	7.13E-05	0.0005246	1.1555499
LINC01108	-1.198161	1.788329	-4.0536	7.13E-05	0.0005246	1.1553895
RP5-1186N2	-0.93447	-0.318527	-4.053433	7.14E-05	0.0005248	1.1547689
RP11-192H2	-0.272006	4.1083143	-4.05261	7.16E-05	0.0005263	1.151719
RP1-68D18.	0.8242658	-0.618385	4.0525701	7.16E-05	0.0005263	1.1515709
AC006126.4	0.523791	3.9456702	4.0525565	7.16E-05	0.0005263	1.1515205
FAM186B	0.3048215	3.6502678	4.0521378	7.18E-05	0.0005271	1.1499685
GTF3C2	-0.060421	6.1908354	-4.05163	7.19E-05	0.0005281	1.148085
FOXO1	0.1561375	6.0434749	4.0515317	7.19E-05	0.0005282	1.1477218
DEFB126	-0.652229	-1.186523	-4.051343	7.20E-05	0.0005284	1.1470239
SPRY1	0.1475	5.960249	4.0513189	7.20E-05	0.0005284	1.1469332
CCNE2	-0.315987	4.7499348	-4.050708	7.22E-05	0.0005296	1.1446702
IMPACT	0.1057132	6.0048191	4.0505434	7.22E-05	0.0005298	1.1440595
RP11-147L1	-0.19345	5.2597816	-4.050451	7.22E-05	0.0005298	1.1437186
SOX17	0.2752745	4.8403681	4.0504501	7.22E-05	0.0005298	1.1437138
RP11-214K3	-0.891536	0.2958809	-4.050325	7.23E-05	0.0005298	1.1432507
ZNF252P-AS	-0.547308	3.1749395	-4.050324	7.23E-05	0.0005298	1.1432477
CTD-2203K1	-0.559183	3.1199518	-4.050285	7.23E-05	0.0005298	1.1431022
AC005355.2	-0.555542	4.6933306	-4.050213	7.23E-05	0.0005299	1.142835
UTP3	0.0792051	6.0522937	4.0497908	7.24E-05	0.0005306	1.1412706
KCNQ1	0.2331478	5.4109308	4.0497465	7.24E-05	0.0005306	1.1411066
LINC01125	-0.26111	4.4205937	-4.049651	7.25E-05	0.0005307	1.1407512
P3H4	-0.197017	5.7661121	-4.049499	7.25E-05	0.0005309	1.1401898
CAMK1G	0.8546543	2.6855014	4.0486277	7.28E-05	0.0005327	1.1369621
TMEM237	-0.136905	5.5602886	-4.048382	7.28E-05	0.0005331	1.1360507
AC009413.2	-0.734639	-0.824396	-4.048124	7.29E-05	0.0005335	1.1350966
CARD18	-1.233063	0.040661	-4.047911	7.30E-05	0.0005339	1.134309
HNF1A-AS1	-0.497429	5.5635038	-4.047368	7.31E-05	0.0005349	1.1322957
TPTE	-1.129808	-0.495765	-4.047104	7.32E-05	0.0005354	1.1313186
LINC00491	-1.283377	0.0184636	-4.046385	7.34E-05	0.0005367	1.1286556
PKMYT1	-0.28008	5.3160347	-4.04638	7.34E-05	0.0005367	1.1286379
SMARCD1	-0.069758	6.2950203	-4.04546	7.37E-05	0.0005385	1.1252336
RALB	0.0784487	6.2317163	4.045418	7.37E-05	0.0005385	1.1250771
AAK1	0.0984468	6.0968627	4.0452972	7.37E-05	0.0005386	1.1246299
C2orf27AP3	-0.901945	-0.476528	-4.045261	7.37E-05	0.0005386	1.1244979

SIX5	-0.14131	5.6780083	-4.044882	7.38E-05	0.0005393	1.1230921
NOL3	0.2035813	5.9370084	4.0440379	7.41E-05	0.000541	1.1199692
PLRG1	0.0682949	6.2775592	4.0438247	7.42E-05	0.0005414	1.1191803
RP3-509I19	-0.433893	3.2729343	-4.043422	7.43E-05	0.0005421	1.1176904
NTF3	0.7423239	3.3374885	4.0430939	7.44E-05	0.0005426	1.1164766
PHKA1	0.4640684	4.8416187	4.0430881	7.44E-05	0.0005426	1.116455
PIGZ	-0.225416	5.2804597	-4.042722	7.45E-05	0.0005433	1.1151012
GOT1	0.1715697	6.8858471	4.0426603	7.45E-05	0.0005433	1.1148722
RPL7AP66	-0.574359	3.0937024	-4.042624	7.45E-05	0.0005433	1.1147373
KB-208E9.1	-0.910771	2.099871	-4.041298	7.49E-05	0.000546	1.1098318
NETO1	1.001727	-0.263844	4.0405671	7.51E-05	0.0005475	1.1071305
LINC00479	-1.023975	2.041542	-4.040502	7.51E-05	0.0005475	1.1068899
AC004490.1	-0.793746	-0.400437	-4.040371	7.52E-05	0.0005476	1.1064045
MGAT5	0.1400177	6.0642382	4.0403669	7.52E-05	0.0005476	1.1063902
MT-ND4	0.1063813	7.9004892	4.0399995	7.53E-05	0.0005483	1.1050316
CDK7	-0.094537	5.8994992	-4.03986	7.53E-05	0.0005485	1.1045149
VPS13C	0.1102369	6.1852555	4.0393731	7.55E-05	0.0005494	1.102716
LINC00494	-1.344667	1.9091128	-4.038952	7.56E-05	0.0005502	1.1011578
C1QTNF1-AS	1.2121654	2.167756	4.0389412	7.56E-05	0.0005502	1.1011193
RP11-17M15	-0.630705	-1.245467	-4.038845	7.56E-05	0.0005503	1.100762
CBX7	0.1267986	6.041852	4.0387037	7.57E-05	0.0005504	1.1002412
FDPSP8	-0.733819	-0.630563	-4.038654	7.57E-05	0.0005504	1.1000566
ZNF202	-0.097349	5.4992366	-4.037908	7.59E-05	0.000552	1.0972996
DRGX	1.2085539	-0.03252	4.0372643	7.61E-05	0.0005533	1.0949218
CREG2	-0.996853	2.425025	-4.037078	7.62E-05	0.0005536	1.0942322
ARRDC4	0.3169563	5.8171548	4.0368917	7.62E-05	0.0005538	1.093545
KIAA1211	0.8372051	3.8000068	4.0366422	7.63E-05	0.0005543	1.0926232
SSSCA1-AS1	-0.183675	4.2272483	-4.036451	7.63E-05	0.0005546	1.0919178
RP4-803J11	-0.894003	0.5060226	-4.036188	7.64E-05	0.0005551	1.0909462
ANP32E	-0.080603	6.3693034	-4.035827	7.65E-05	0.0005557	1.0896112
EML4	-0.081305	6.617907	-4.035671	7.66E-05	0.000556	1.0890345
NCR3LG1	0.8624219	3.347508	4.0353094	7.67E-05	0.0005567	1.0876993
RPL13AP23	-0.73993	-0.643603	-4.035145	7.67E-05	0.0005569	1.0870913
METTL25	-0.103063	5.0981919	-4.034994	7.68E-05	0.0005571	1.086534
GAREM	0.1972133	5.6880448	4.0337384	7.72E-05	0.0005598	1.0818977
MORC2-AS1	-0.748596	1.8861581	-4.033437	7.73E-05	0.0005603	1.0807846
DEK	-0.086893	6.5342273	-4.033418	7.73E-05	0.0005603	1.080715
ARPP19	0.0655545	6.5492247	4.0331013	7.74E-05	0.0005609	1.0795452
ASH2L	0.0962643	6.0741314	4.0317853	7.78E-05	0.0005637	1.0746874
AC007292.7	-0.902751	0.800257	-4.031653	7.78E-05	0.0005638	1.0741992
CES4A	0.5443712	4.4904856	4.0315133	7.78E-05	0.000564	1.0736837
PARVB	0.1717078	6.0867983	4.0314922	7.79E-05	0.000564	1.0736055
PPOX	-0.111616	5.9593219	-4.031174	7.79E-05	0.0005646	1.0724301
GIMAP4	0.1563965	5.981655	4.031086	7.80E-05	0.0005647	1.0721064
RP11-338N1	-1.005869	-0.31988	-4.031002	7.80E-05	0.0005647	1.071795
RP11-199F1	-0.371471	3.7245249	-4.030688	7.81E-05	0.0005653	1.0706362
VRK3	0.0890977	6.246855	4.0306247	7.81E-05	0.0005654	1.0704041
DMRTB1	-0.791285	-0.98175	-4.029694	7.84E-05	0.0005673	1.0669698
LGI1	1.0905157	-0.053846	4.0294529	7.85E-05	0.0005677	1.0660809
AHCTF1	-0.085831	6.3085587	-4.028434	7.88E-05	0.0005699	1.0623222
EGFL8	-0.327152	4.1226771	-4.02841	7.88E-05	0.0005699	1.0622338

CUL5	0.0721898	6.1273244	4.028183	7.89E-05	0.0005703	1.0613965
MAFA	-1.244134	2.0416517	-4.028101	7.89E-05	0.0005703	1.0610941
RP11-573D1	-0.48029	3.0208167	-4.027874	7.90E-05	0.0005707	1.060257
AC005076.5	-0.4535	3.4482932	-4.027581	7.91E-05	0.0005713	1.0591766
RP11-324I2	-0.31644	4.2053431	-4.027296	7.92E-05	0.0005718	1.058124
MRPL9	-0.08314	6.2983146	-4.026801	7.93E-05	0.0005728	1.0563005
CES5A	1.1512747	3.4422454	4.0266047	7.94E-05	0.0005731	1.0555767
CTD-2083E4	-0.579482	2.7467025	-4.02628	7.95E-05	0.0005738	1.0543802
RPL7P37	-0.638298	-0.957146	-4.026066	7.95E-05	0.0005741	1.0535902
RPL29P14	-0.922089	0.2984171	-4.026044	7.95E-05	0.0005741	1.053511
TRIM60P17	-0.954284	-0.280456	-4.025806	7.96E-05	0.0005745	1.0526323
PYY	-0.948818	0.9154417	-4.025241	7.98E-05	0.0005757	1.0505499
RPS10	-0.107971	6.6717112	-4.024972	7.99E-05	0.0005761	1.0495599
NAGLU	0.1085143	6.4765919	4.0241494	8.01E-05	0.0005779	1.0465266
ZFP37	-0.48153	4.1413663	-4.0236	8.03E-05	0.000579	1.0445037
ZBTB46-AS1	-0.824691	-0.990942	-4.023177	8.04E-05	0.0005799	1.0429442
KDM4A	0.0802998	6.2020196	4.022902	8.05E-05	0.0005804	1.0419306
PDE7A	-0.135638	5.7637632	-4.02254	8.06E-05	0.0005811	1.0405986
WDR5	-0.086441	6.3406198	-4.022128	8.08E-05	0.000582	1.0390786
MYOZ1	0.9505682	2.2469001	4.0216781	8.09E-05	0.0005829	1.0374223
U82695.9	-0.870458	-0.694896	-4.021098	8.11E-05	0.0005841	1.0352841
RP11-96020	-0.882177	0.5805177	-4.021026	8.11E-05	0.0005841	1.0350192
ZSCAN12	-0.170862	5.1091074	-4.020827	8.12E-05	0.0005845	1.0342893
HGS	-0.077549	6.4366498	-4.018882	8.18E-05	0.0005888	1.0271284
RP11-295D4	-0.854117	0.1028318	-4.018874	8.18E-05	0.0005888	1.0270983
RP11-305D1	0.793751	-0.403403	4.0181105	8.21E-05	0.0005904	1.0242876
LRRC75A-AS	-0.124324	6.7174405	-4.017992	8.21E-05	0.0005906	1.0238528
KCNS2	1.0415364	0.1914654	4.0178059	8.22E-05	0.0005909	1.0231668
YWHAG	0.0606042	6.7678323	4.0177039	8.22E-05	0.000591	1.0227913
RP11-268G1	-1.318278	0.8366336	-4.017649	8.22E-05	0.000591	1.0225888
ASIC1	-0.65564	4.3128094	-4.017465	8.23E-05	0.0005912	1.0219121
C3AR1	0.2393419	5.4100738	4.0174622	8.23E-05	0.0005912	1.0219018
VENTX	0.4941367	3.4853343	4.0169721	8.24E-05	0.0005922	1.0200986
CT83	-1.165065	-0.501482	-4.016604	8.26E-05	0.000593	1.0187459
GANC	0.1076213	5.7090645	4.0156362	8.29E-05	0.0005951	1.0151841
FOSL1	0.373826	4.3354066	4.0154107	8.29E-05	0.0005955	1.0143544
LIPH	0.9168417	3.8274582	4.0152911	8.30E-05	0.0005957	1.0139146
CDX2	-1.28899	0.3943126	-4.015233	8.30E-05	0.0005957	1.0137021
MRPL37	0.0906127	6.5863777	4.0151532	8.30E-05	0.0005958	1.0134076
FCN2	1.302716	2.5607853	4.0149576	8.31E-05	0.0005961	1.0126881
DERL1	0.0894017	6.6394007	4.014939	8.31E-05	0.0005961	1.0126197
LYRM2	-0.080068	6.0783606	-4.014838	8.31E-05	0.0005962	1.012247
SLC26A6	-0.169281	5.9710274	-4.014535	8.32E-05	0.0005968	1.0111356
RABGEF1	-0.134113	5.4112771	-4.014373	8.33E-05	0.000597	1.0105398
RP11-18707	0.9382392	1.3589984	4.0140016	8.34E-05	0.0005978	1.0091725
PRR16	0.4914253	3.9867144	4.0138569	8.35E-05	0.000598	1.0086403
LUC7L3	-0.060618	6.4529813	-4.013676	8.35E-05	0.0005983	1.0079757
RP11-332J1	-1.17954	-0.423989	-4.013313	8.36E-05	0.0005991	1.0066423
GOT2	0.1391468	6.8667541	4.013247	8.37E-05	0.0005991	1.0063982
UBE2N	-0.052527	6.3954095	-4.013086	8.37E-05	0.0005994	1.0058045
RASGRF2	0.1993675	5.3467765	4.0130398	8.37E-05	0.0005994	1.0056361

SIGLEC9	0.303204	4.6638122	4.0129466	8.37E-05	0.0005995	1.0052936
KCNMB1	0.3247849	4.3395334	4.0128162	8.38E-05	0.0005996	1.0048142
IFI27	0.3109027	6.4524429	4.0119471	8.41E-05	0.0006015	1.0016196
RP11-432J2	-1.151792	0.0218989	-4.011941	8.41E-05	0.0006015	1.0015955
RP11-87H9.	-0.22744	4.4763261	-4.011397	8.43E-05	0.0006026	0.9995967
CTB-50E14.	0.4226343	3.5544332	4.010637	8.45E-05	0.0006043	0.996805
RP11-448A1	-0.17295	4.7030698	-4.010566	8.45E-05	0.0006043	0.9965443
PAQR9-AS1	-0.582603	5.0925445	-4.010506	8.46E-05	0.0006043	0.9963247
YTHDC2	0.0909803	6.0810672	4.0104827	8.46E-05	0.0006043	0.9962382
STK17B	0.1720943	5.7228405	4.0103718	8.46E-05	0.0006045	0.9958307
MYO1F	0.1631588	5.836863	4.01021	8.47E-05	0.0006047	0.9952361
STARD6	-0.8743	-0.616872	-4.01011	8.47E-05	0.0006049	0.9948691
IPO13	0.0795459	6.2043968	4.0097839	8.48E-05	0.0006055	0.9936708
TSGA10IP	-0.964283	1.1602705	-4.009726	8.48E-05	0.0006055	0.9934592
CARD6	0.2402018	5.4728155	4.0095657	8.49E-05	0.0006058	0.9928692
ATP5A1P3	0.8711224	2.0364293	4.0094306	8.49E-05	0.000606	0.9923728
LGALS3	0.2028825	6.339283	4.009272	8.50E-05	0.0006063	0.9917901
LGALSL	-0.10086	5.8769915	-4.009058	8.50E-05	0.0006067	0.9910041
RP11-1143G	-0.929437	0.3919718	-4.008922	8.51E-05	0.0006068	0.9905053
SUSD5	0.8627074	3.39837	4.0089065	8.51E-05	0.0006068	0.9904477
RP4-621N11	-0.743315	-0.6397	-4.008593	8.52E-05	0.0006073	0.9892963
TLL2	-0.796966	2.8155329	-4.008577	8.52E-05	0.0006073	0.9892375
IL34	0.2760142	5.1852382	4.0081368	8.53E-05	0.0006083	0.9876209
HAS2	0.8974651	3.5484853	4.007515	8.56E-05	0.0006096	0.9853376
RGAG4	0.2600474	5.1088973	4.0073038	8.56E-05	0.00061	0.9845619
BAZ2B	0.1293316	5.8540908	4.0071938	8.57E-05	0.0006102	0.9841579
PRODH	0.7667892	4.5795966	4.0058904	8.61E-05	0.0006132	0.9793731
RP11-348J2	-1.177336	1.3800106	-4.005546	8.62E-05	0.0006139	0.9781096
SCLY	-0.16283	5.345158	-4.00538	8.63E-05	0.0006141	0.977499
RP1-46F2.3	-0.875174	-0.845682	-4.005371	8.63E-05	0.0006141	0.9774674
ATPAF1	0.0982505	6.4024547	4.0050309	8.64E-05	0.0006148	0.9762183
RNF144A-AS	-1.049579	2.0913747	-4.004566	8.66E-05	0.0006158	0.9745123
STXBP5L	-0.924507	-0.567739	-4.004474	8.66E-05	0.0006159	0.9741759
AC008271.1	-0.759719	-1.103907	-4.003421	8.69E-05	0.0006183	0.9703124
SCN8A	-0.291244	5.3672734	-4.002929	8.71E-05	0.0006194	0.9685059
ZNF343	-0.105269	5.561664	-4.002354	8.73E-05	0.0006207	0.9663979
RP11-7M8.2	1.2975855	1.4019453	4.0021604	8.74E-05	0.000621	0.9656866
CTBP2P1	-0.59593	-1.232616	-4.001898	8.75E-05	0.0006215	0.964726
PHLDB1	0.1813203	5.9078767	4.0018053	8.75E-05	0.0006216	0.9643844
TMEM97	-0.179383	6.5189406	-4.001626	8.76E-05	0.0006219	0.9637276
TMEM41B	-0.084906	6.3383445	-4.000886	8.78E-05	0.0006236	0.9610147
AP006216.5	1.198688	1.9504502	3.9998129	8.82E-05	0.0006261	0.9570787
HIST1H2BH	-1.049049	0.6335883	-3.999792	8.82E-05	0.0006261	0.9570029
ASIC2	1.0819001	0.8840355	3.9996889	8.82E-05	0.0006262	0.9566242
RRP1B	-0.086906	6.2076503	-3.999611	8.83E-05	0.0006263	0.9563402
CTD-2547L1	0.6915225	2.4607127	3.9980964	8.88E-05	0.0006299	0.9507874
SLCO4C1	-1.078538	3.9665787	-3.998012	8.88E-05	0.00063	0.9504778
CTB-186H2.	-0.854531	2.1934795	-3.997419	8.90E-05	0.0006313	0.9483051
UBE2D3	0.0661147	6.7984888	3.995703	8.96E-05	0.0006353	0.9420186
MT1P3	0.892578	-0.713371	3.9956912	8.96E-05	0.0006353	0.9419753
ZNF681	-0.520819	4.3151666	-3.995638	8.96E-05	0.0006354	0.9417794

BLMH	-0.166837	6.0631154	-3.995135	8.98E-05	0.0006364	0.9399385
MLLT3	0.34556	4.9886712	3.995134	8.98E-05	0.0006364	0.9399347
ENPP2	-0.237834	6.3096382	-3.994901	8.99E-05	0.0006368	0.9390807
RP13-12804	0.782434	2.6059382	3.994797	8.99E-05	0.000637	0.9387007
SPTBN4	0.4535433	4.2019301	3.9944154	9.01E-05	0.0006378	0.9373032
SPRY4	0.1245948	6.0557421	3.9939615	9.02E-05	0.0006388	0.9356414
ZNF714	-0.464241	4.4171179	-3.99381	9.03E-05	0.000639	0.9350848
DUSP5	0.2194973	5.9174969	3.9937961	9.03E-05	0.000639	0.9350357
RP5-1142A6	0.8000614	1.8380836	3.9932427	9.05E-05	0.0006402	0.9330098
RP6-65G23.	-0.609926	3.5333869	-3.993062	9.05E-05	0.0006406	0.9323494
TIMP2	0.164053	6.6154692	3.9927682	9.06E-05	0.0006412	0.9312726
PLAG1	-0.58104	4.4796401	-3.992376	9.08E-05	0.000642	0.9298366
HAGLR	-0.684845	4.6341445	-3.992162	9.09E-05	0.0006424	0.9290546
SEMA6A	0.2975153	5.7477702	3.991985	9.09E-05	0.0006428	0.928406
UCP3	-0.288214	3.8302165	-3.991642	9.11E-05	0.0006435	0.9271498
MMP15	0.1219927	6.6122016	3.9914921	9.11E-05	0.0006437	0.9266024
C19orf45	-0.975529	1.968822	-3.991464	9.11E-05	0.0006437	0.9265005
ADPRHL1	-0.340051	4.7623159	-3.9908	9.14E-05	0.0006453	0.9240687
AC015922.6	-0.636158	-1.189825	-3.990492	9.15E-05	0.0006459	0.9229423
PPP2R3A	0.2701155	5.0079541	3.9897068	9.17E-05	0.0006478	0.9200703
RP11-61L23	0.9482065	1.8446449	3.9895694	9.18E-05	0.0006479	0.9195676
RPS18	-0.094042	7.2981625	-3.989543	9.18E-05	0.0006479	0.919471
FAM101B	0.141079	5.6332739	3.9892774	9.19E-05	0.0006485	0.9184996
DUSP27	0.985763	0.0092466	3.989019	9.20E-05	0.000649	0.9175545
RP11-154J2	-0.773084	2.2685098	-3.988527	9.22E-05	0.0006501	0.9157547
DLX5	-1.242385	1.338451	-3.987846	9.24E-05	0.0006517	0.9132633
SFTA2	1.1217469	-0.050919	3.9878102	9.24E-05	0.0006517	0.913134
OR56B4	-0.772049	-0.589947	-3.987324	9.26E-05	0.0006528	0.9113569
RP11-701H2	-0.889348	-0.406194	-3.987238	9.26E-05	0.0006529	0.9110436
FAM87A	-1.1508	0.9982891	-3.987169	9.27E-05	0.000653	0.9107901
MKRN2	-0.07192	6.0373054	-3.986881	9.28E-05	0.0006536	0.9097378
MNDA	0.2994496	4.9942203	3.9868286	9.28E-05	0.0006536	0.9095452
IQCJ-SCHIP	0.4167212	4.2125982	3.9863236	9.30E-05	0.0006548	0.9076989
NLE1	-0.121191	5.8698675	-3.986156	9.30E-05	0.0006551	0.9070873
PIH1D1	-0.08221	6.4315402	-3.986109	9.30E-05	0.0006551	0.9069156
LRAT	0.585261	4.2621508	3.9859883	9.31E-05	0.0006552	0.9064735
KCNIP1	1.1121986	1.2254046	3.9859282	9.31E-05	0.0006553	0.9062537
TMTC2	0.4327919	4.590666	3.9856332	9.32E-05	0.0006559	0.9051757
CHIC2	0.1036035	5.5970136	3.9854942	9.33E-05	0.0006561	0.9046674
MMADHC	0.0784765	6.5057575	3.9853902	9.33E-05	0.0006563	0.9042874
RP11-234G1	-1.03958	0.4650575	-3.985206	9.34E-05	0.0006565	0.903614
FCGR2A	0.2021901	5.74964	3.9852035	9.34E-05	0.0006565	0.9036052
RP13-514E2	0.9307018	0.023224	3.9850407	9.34E-05	0.0006567	0.9030102
RP11-161I6	-1.128065	0.2172995	-3.985034	9.34E-05	0.0006567	0.9029852
AC098617.1	-1.093531	0.038843	-3.984925	9.35E-05	0.0006568	0.9025887
MAP3K7CL	0.1838732	5.3595153	3.9848231	9.35E-05	0.000657	0.9022152
TIA1	-0.087132	6.2644289	-3.984546	9.36E-05	0.0006575	0.9012007
ETFA	0.1121755	6.7916417	3.9845078	9.36E-05	0.0006575	0.9010628
RP11-867G2	0.9510215	0.4965211	3.984441	9.37E-05	0.0006576	0.9008188
FZD9	-1.124116	2.3225409	-3.984315	9.37E-05	0.0006578	0.9003602
RNF175	0.636972	3.1005432	3.9840793	9.38E-05	0.0006582	0.8994974

RP11-184E9	-1.109055	-0.300055	-3.983909	9.38E-05	0.0006586	0.8988738
ABHD14B	0.1231522	6.6300924	3.9835853	9.40E-05	0.0006593	0.8976925
NALCN	0.8835564	3.8329252	3.9834161	9.40E-05	0.0006596	0.8970742
IRF2BP1	-0.106626	6.2492175	-3.982415	9.44E-05	0.000662	0.8934178
DDIAS	-0.291314	4.7734279	-3.981949	9.46E-05	0.0006631	0.8917175
RP11-197N1	-0.511543	3.2011487	-3.981626	9.47E-05	0.0006638	0.8905379
FDPSP1	-0.795685	-0.406794	-3.981227	9.48E-05	0.0006647	0.8890789
RP11-94P11	-0.856979	-0.690182	-3.980937	9.49E-05	0.0006653	0.8880202
KIAA0141	0.0830199	6.4337329	3.9808976	9.50E-05	0.0006653	0.8878763
DENND3	0.157838	5.6644026	3.9807346	9.50E-05	0.0006656	0.8872812
GABRB1	-1.021812	-0.368493	-3.980538	9.51E-05	0.000666	0.8865625
RP11-792A8	-0.271191	4.3167782	-3.980337	9.52E-05	0.0006664	0.8858296
RP11-636O2	-0.998969	-0.522261	-3.979984	9.53E-05	0.0006672	0.8845427
C1orf64	-1.302141	0.6807173	-3.979872	9.53E-05	0.0006674	0.8841326
RASGRP4	0.2900908	4.3507981	3.9798227	9.54E-05	0.0006674	0.8839525
SEMA3C	0.6189444	4.4637738	3.9784667	9.59E-05	0.0006708	0.8790034
FDXACB1	-0.165079	4.7928589	-3.977984	9.60E-05	0.0006719	0.8772424
GAREML	-0.333278	4.8578317	-3.977698	9.62E-05	0.0006725	0.8761988
RP11-552F3	-0.554266	2.8217065	-3.977646	9.62E-05	0.0006725	0.8760074
CNGB3	-1.084798	0.5244739	-3.977365	9.63E-05	0.0006731	0.8749849
ARL15	0.1073247	5.9257563	3.9772326	9.63E-05	0.0006734	0.8745004
RP11-400G3	1.0966536	1.5192853	3.977091	9.64E-05	0.0006736	0.8739839
HOXA11	-1.289311	0.2820186	-3.977014	9.64E-05	0.0006737	0.8737019
EIF2S2P3	-0.772223	1.4681003	-3.976959	9.64E-05	0.0006737	0.8735012
RPL18AP16	-0.811098	-0.225198	-3.976031	9.68E-05	0.000676	0.8701186
CKS1BP7	-0.653562	-0.781226	-3.975139	9.71E-05	0.0006782	0.8668629
PHTF1	0.1308053	5.6128046	3.9750764	9.71E-05	0.0006783	0.8666358
EDNRA	0.260731	5.1997936	3.9744184	9.74E-05	0.0006799	0.8642366
FKBPL	-0.12376	5.4628925	-3.974303	9.74E-05	0.0006801	0.8638157
NUDT18	0.1629681	5.293677	3.9742061	9.75E-05	0.0006802	0.8634626
SMARCA1	-0.134065	6.5778368	-3.973927	9.76E-05	0.0006808	0.8624443
RP11-124N3	-0.790973	-1.001761	-3.972343	9.82E-05	0.0006848	0.8566701
DIS3L2P1	0.8017988	-0.674978	3.9723409	9.82E-05	0.0006848	0.8566636
TAF1D	-0.099812	6.2766465	-3.972206	9.82E-05	0.000685	0.8561704
CXCR2	0.9031547	2.4755148	3.9718029	9.84E-05	0.0006859	0.8547031
ZNF436	0.1059731	5.5664844	3.9708229	9.88E-05	0.0006884	0.8511324
ENKUR	0.7649147	2.8017229	3.9702316	9.90E-05	0.0006899	0.8489784
RP11-83M16	-0.46496	-1.251872	-3.969996	9.91E-05	0.0006904	0.848121
CTPS2	-0.090588	5.9952172	-3.969782	9.92E-05	0.0006908	0.8473396
IL17RB	-0.215011	6.3104068	-3.969694	9.92E-05	0.000691	0.8470196
EXOC1	0.0798347	5.8931287	3.969641	9.92E-05	0.000691	0.8468272
IGHVII-44-	-0.4948	-1.268303	-3.969395	9.93E-05	0.0006915	0.8459327
MIR3646	-0.866413	1.7621898	-3.9692	9.94E-05	0.0006919	0.8452204
LRRC9	-0.912425	0.7060482	-3.968907	9.95E-05	0.0006925	0.8441543
RP11-498P1	-0.574952	-1.184141	-3.966888	0.0001003	0.0006979	0.8368045
HSPB1P2	-0.799093	1.9560953	-3.966567	0.0001004	0.0006986	0.8356341
LINC-PINT	0.1946323	5.1252862	3.9662228	0.0001006	0.0006994	0.8343816
PRKAR2A-AS	-0.304819	4.2700016	-3.966119	0.0001006	0.0006995	0.8340041
TMEM150A	-0.113576	6.2161142	-3.966094	0.0001006	0.0006995	0.8339122
PELP1	-0.087862	6.382117	-3.965895	0.0001007	0.0006999	0.8331882
CTD-2655K5	-0.883352	1.8011395	-3.965653	0.0001008	0.0007004	0.8323082

AC009245.3	-0.603878	2.8432314	-3.965614	0.0001008	0.0007004	0.8321645
RP11-365D9	-0.561697	-1.191688	-3.965495	0.0001008	0.0007006	0.8317333
CLINT1	0.080057	6.563078	3.9653786	0.0001009	0.0007008	0.8313095
CTSK	0.2276951	5.6871803	3.9653131	0.0001009	0.0007008	0.831071
TUBE1	0.1640001	5.6033692	3.9650059	0.000101	0.0007015	0.8299531
SLC12A9	-0.085479	6.2839753	-3.964558	0.0001012	0.0007026	0.8283236
TRAPPC3L	0.913694	0.5276945	3.9644674	0.0001012	0.0007028	0.8279939
CRP	0.6879898	6.877536	3.963893	0.0001015	0.0007042	0.8259042
C1QB	0.1872247	6.602151	3.9637371	0.0001015	0.0007045	0.825337
EIF4A2P2	-0.838702	0.2371021	-3.963511	0.0001016	0.000705	0.8245155
SZRD1	0.0697373	6.5481758	3.9633298	0.0001017	0.0007053	0.8238557
ZCWPW1	0.2082904	5.1867707	3.9625846	0.000102	0.0007073	0.8211453
RP11-306B9	-0.520815	-1.202577	-3.962473	0.000102	0.0007074	0.8207393
RP11-643C9	0.9189633	0.1229363	3.9623817	0.0001021	0.0007075	0.8204074
SYT11	0.2074463	5.3799099	3.9621633	0.0001022	0.000708	0.819613
RP11-909N1	-1.257542	0.6601587	-3.961864	0.0001023	0.0007086	0.8185259
CADPS2	0.189553	5.9837113	3.9618449	0.0001023	0.0007086	0.8184552
FRA10AC1	-0.090983	5.9329953	-3.961758	0.0001023	0.0007087	0.8181409
RP11-539I5	0.60684	4.3682032	3.9607233	0.0001027	0.0007114	0.8143775
CTSB	0.099211	7.3080562	3.9602313	0.0001029	0.0007127	0.8125891
HOMER2	0.2841701	6.1353261	3.9601296	0.000103	0.0007128	0.8122194
BTN1A1	-0.937835	-0.272592	-3.959423	0.0001033	0.0007146	0.8096528
H3F3AP6	-0.729677	1.3401134	-3.959262	0.0001033	0.000715	0.8090652
NPM1P46	-0.854455	0.0964288	-3.959068	0.0001034	0.0007154	0.8083598
AC006116.1	-0.846783	1.110576	-3.958707	0.0001035	0.0007162	0.8070507
REM1	0.451555	3.8639146	3.9579631	0.0001038	0.0007182	0.8043465
CLIC1P1	-0.810319	-0.168469	-3.957734	0.0001039	0.0007187	0.8035136
RP11-324H9	-0.808951	-0.925267	-3.957664	0.000104	0.0007187	0.803259
SPIRE2	-0.161228	5.8974876	-3.957247	0.0001041	0.0007198	0.8017436
NCKAP5	0.4630115	4.645417	3.9571113	0.0001042	0.00072	0.801252
TTTY14	1.515335	1.8560793	3.9568168	0.0001043	0.0007207	0.8001823
ZNF414	-0.094957	5.7100032	-3.956742	0.0001043	0.0007208	0.7999103
XAGE1B	-0.773771	-0.903681	-3.956632	0.0001044	0.0007208	0.7995129
NRK	1.0875491	1.9739448	3.9566	0.0001044	0.0007208	0.7993949
TSPAN11	0.7238373	3.7674519	3.9565846	0.0001044	0.0007208	0.7993389
CLIP3	0.2212219	5.3120986	3.9562567	0.0001045	0.0007216	0.798148
MTMR9LP	0.1845915	5.0809213	3.9557573	0.0001047	0.0007229	0.7963345
HRSP12	0.2093811	6.7659191	3.9555004	0.0001048	0.0007234	0.7954017
ZNF548	-0.129667	5.4978632	-3.954779	0.0001051	0.0007253	0.7927817
COA6	-0.117149	6.1929942	-3.954386	0.0001053	0.0007262	0.7913559
RP11-113I2	0.7885448	-0.951168	3.954365	0.0001053	0.0007262	0.7912792
PPM1D	-0.08746	5.7498349	-3.953626	0.0001056	0.0007282	0.7885955
BHMG1	-0.84955	-0.521345	-3.953369	0.0001057	0.0007288	0.7876653
FAM9A	-1.048866	-0.484482	-3.953155	0.0001058	0.0007292	0.7868885
STIP1	-0.078163	6.6990089	-3.952315	0.0001061	0.0007315	0.7838401
RP11-309H2	-0.568069	-1.212841	-3.952268	0.0001062	0.0007315	0.7836693
FIGNL2	-0.845777	3.5605195	-3.951932	0.0001063	0.0007323	0.7824484
RP1-52J10.	-0.698825	-1.160252	-3.951637	0.0001064	0.000733	0.7813795
CTA-992D9.	0.8855129	-0.261062	3.9514742	0.0001065	0.0007333	0.780788
F2RL3	0.4450779	4.9380985	3.9513332	0.0001065	0.0007336	0.7802768
RP11-585P4	0.8146245	2.0797044	3.9508831	0.0001067	0.0007347	0.7786438

HOXC-AS1	-1.163766	-0.164437	-3.950772	0.0001068	0.0007349	0.7782411
ZNF431	-0.227348	5.2294963	-3.950735	0.0001068	0.0007349	0.7781061
KB-1732A1.	-0.323826	4.4264013	-3.950303	0.000107	0.000736	0.7765404
PTN	0.3769936	4.6758091	3.9492308	0.0001074	0.0007389	0.772651
MSX2	1.1739753	0.8241088	3.9471231	0.0001083	0.0007448	0.7650099
INTS10	0.0922806	6.2242583	3.947099	0.0001083	0.0007448	0.7649226
AC080125.1	-0.690375	-0.931461	-3.947053	0.0001083	0.0007448	0.7647571
LINC00571	0.9348787	0.8887349	3.9467259	0.0001085	0.0007455	0.7635703
HTR1D	-1.29721	2.55334	-3.946689	0.0001085	0.0007455	0.7634365
CDH10	-1.187363	-0.316715	-3.946134	0.0001087	0.000747	0.7614261
BPIFB4	-1.220522	1.0838528	-3.945902	0.0001088	0.0007475	0.7605847
WAC-AS1	-0.089174	5.938218	-3.945795	0.0001089	0.0007477	0.7601981
RPL4P1	-0.900867	0.6953673	-3.945661	0.0001089	0.000748	0.7597125
RP5-89003.	-0.202186	4.3102595	-3.945598	0.0001089	0.000748	0.7594826
GBP1	0.1971881	6.2561782	3.9453128	0.0001091	0.0007487	0.7584496
XAF1	0.3779865	5.2424275	3.9452092	0.0001091	0.0007487	0.7580743
SLC19A1	-0.141452	6.1425076	-3.945208	0.0001091	0.0007487	0.7580713
NFXL1	-0.129849	5.5535217	-3.944839	0.0001093	0.0007496	0.7567333
AFF4	0.0979375	6.5430918	3.9441744	0.0001096	0.0007514	0.7543257
RP11-372E1	-1.022363	2.0735651	-3.944132	0.0001096	0.0007514	0.7541724
IQCB1	-0.095135	5.6840982	-3.943894	0.0001097	0.000752	0.7533107
IGFBPL1	-1.303304	1.9209152	-3.943552	0.0001098	0.0007528	0.7520721
RECQL5	-0.07962	6.1181294	-3.943363	0.0001099	0.0007532	0.7513867
SUGT1P3	-0.535578	2.9632021	-3.942711	0.0001102	0.000755	0.7490264
RAB30-AS1	-0.138245	5.373104	-3.942595	0.0001102	0.0007552	0.7486066
LDLRAD2	0.3412895	3.9640092	3.9422395	0.0001104	0.0007561	0.7473182
CTD-208602	-0.72176	-0.670114	-3.941987	0.0001105	0.0007567	0.7464053
RP11-1267H	0.5913852	-1.136128	3.9417078	0.0001106	0.0007572	0.7453934
AC007365.4	-0.860235	1.7891176	-3.941702	0.0001106	0.0007572	0.7453738
DRICH1	-0.766093	2.2145862	-3.94152	0.0001107	0.0007576	0.7447142
BMP7	-1.407783	1.335718	-3.940802	0.000111	0.0007596	0.7421137
SORBS2	0.2078792	6.4441452	3.9407513	0.000111	0.0007596	0.741931
CAD	-0.095777	6.2377908	-3.94061	0.0001111	0.0007599	0.7414178
DNAJA3	0.0933966	6.5057266	3.9399423	0.0001114	0.0007617	0.7390028
EPAS1	0.0991318	6.8618688	3.9390746	0.0001117	0.0007641	0.7358632
COMP	1.2068842	3.7002411	3.9389112	0.0001118	0.0007645	0.7352718
PHLDB2	0.1305358	6.1350927	3.9388618	0.0001118	0.0007645	0.7350932
SSTR2	-0.385108	5.0753595	-3.938593	0.0001119	0.0007651	0.7341191
MMRN1	0.3770924	4.8354144	3.938515	0.000112	0.0007652	0.7338383
PCDHB12	0.6635688	3.4832119	3.9381946	0.0001121	0.000766	0.7326793
KCTD7	-0.127015	5.5582795	-3.937743	0.0001123	0.0007672	0.731046
EGF	1.4424238	1.6694314	3.9376619	0.0001124	0.0007673	0.7307523
FBXW11	0.077538	6.3078293	3.9374273	0.0001125	0.0007679	0.7299038
C2orf15	-0.32572	4.618001	-3.936981	0.0001127	0.0007691	0.7282911
RP11-109A6	-0.936732	-0.033711	-3.936553	0.0001128	0.0007702	0.7267428
NOV	0.3748009	4.8582339	3.9360355	0.0001131	0.0007716	0.7248704
NOTCH2	0.1110929	6.490304	3.9358857	0.0001131	0.0007719	0.7243289
MDH1	0.0784082	6.7969341	3.935589	0.0001133	0.0007726	0.7232562
NR1H2	0.0743418	6.5130207	3.9354972	0.0001133	0.0007728	0.7229243
DPEP2	0.2577142	4.704204	3.9351035	0.0001135	0.0007738	0.721501
GAPDHP22	-0.56815	-1.145614	-3.93505	0.0001135	0.0007738	0.7213091

SMARCA5	0.0710553	6.387967	3.9343586	0.0001138	0.0007758	0.7188084
SNORA40	-0.842058	1.2418066	-3.933734	0.0001141	0.0007775	0.7165511
RP11-1267H	1.0932507	0.6904861	3.933193	0.0001143	0.000779	0.7145958
NKPD1	-1.03289	1.0537212	-3.932517	0.0001146	0.0007809	0.7121519
ZNF32-AS2	-0.612556	2.8056584	-3.932475	0.0001146	0.0007809	0.712001
PRUNE	-0.095035	6.1494021	-3.932399	0.0001147	0.0007809	0.7117281
LINC01242	-0.897171	-0.736267	-3.931998	0.0001148	0.000782	0.7102795
RP11-254B1	-0.725998	2.341547	-3.931556	0.000115	0.0007832	0.708683
LAPTM5	0.1351518	6.5688822	3.9313654	0.0001151	0.0007836	0.707993
PTPLAD2	0.2355161	4.8109675	3.9311627	0.0001152	0.0007841	0.7072608
TPM1	0.1100276	6.6895564	3.9311239	0.0001152	0.0007841	0.7071206
CHST3	0.2484934	5.3958023	3.9310631	0.0001153	0.0007841	0.7069009
MED9	0.1049302	5.9648421	3.9308622	0.0001154	0.0007846	0.7061755
CTD-2251F1	-0.718489	-1.108013	-3.930749	0.0001154	0.0007848	0.7057651
TIMM8AP1	-0.886439	1.7666367	-3.930592	0.0001155	0.000785	0.7052005
RP11-98D18	-0.910926	0.6367948	-3.930585	0.0001155	0.000785	0.7051733
HSPA12B	0.2169916	5.3284393	3.9299236	0.0001158	0.0007868	0.7027856
FAM160B1	0.0913839	6.1334044	3.9295291	0.0001159	0.0007879	0.7013612
ANKHD1	-0.102117	5.2678865	-3.927938	0.0001167	0.0007926	0.6956157
RPL7AP15	-0.87258	0.2797674	-3.927861	0.0001167	0.0007927	0.69534
RP11-478C6	-0.820361	1.3706294	-3.927679	0.0001168	0.0007931	0.6946827
CTD-3006G1	-1.134371	-0.133936	-3.927523	0.0001169	0.0007934	0.6941189
LINC00628	-0.950834	1.4302474	-3.926851	0.0001172	0.0007952	0.691695
DIAPH3	-0.345795	4.9205068	-3.926806	0.0001172	0.0007952	0.6915321
ANKRD52	-0.099535	6.2429571	-3.926791	0.0001172	0.0007952	0.6914762
GRHPR	0.1341416	6.8474113	3.9263628	0.0001174	0.0007964	0.6899326
RP11-191N8	0.5531271	-1.254741	3.9255224	0.0001178	0.0007988	0.6869006
RPL24P2	-0.302795	3.9829728	-3.925505	0.0001178	0.0007988	0.6868388
ROPN1B	1.0543271	2.107723	3.9243519	0.0001183	0.0008022	0.6826785
LLNLR-470E	1.0627081	1.7381913	3.9239687	0.0001185	0.0008031	0.6812966
THEM4	-0.113223	5.9697314	-3.923949	0.0001185	0.0008031	0.6812269
ADARB1	0.1315613	5.7304482	3.9234725	0.0001187	0.0008045	0.6795073
JAM3	0.1483622	5.6740521	3.9231404	0.0001188	0.0008053	0.6783097
CLEC1A	0.2899586	4.4429471	3.9228364	0.000119	0.0008061	0.6772136
RP11-71602	-0.787142	-0.781958	-3.92268	0.0001191	0.0008065	0.6766498
AC010547.9	0.7660521	-0.390823	3.9223548	0.0001192	0.0008072	0.6754775
RP11-32D4.	-0.671211	-1.225604	-3.922342	0.0001192	0.0008072	0.6754312
RP11-203H1	-0.874641	1.1682779	-3.921812	0.0001195	0.0008087	0.6735207
RAD51-AS1	-0.190365	4.665999	-3.92115	0.0001198	0.0008107	0.671135
OR8G3P	-0.803023	-0.913615	-3.92059	0.00012	0.0008123	0.6691151
RP11-203J2	0.6963849	3.449821	3.9194615	0.0001205	0.0008157	0.6650503
HLA-DRB1	0.1763757	6.8166738	3.919283	0.0001206	0.0008161	0.6644072
ZNF77	-0.194575	4.9245528	-3.919046	0.0001207	0.0008167	0.6635532
RPL7AP28	-0.974735	-0.381633	-3.918962	0.0001208	0.0008168	0.663249
AC092171.2	-0.796003	-0.062273	-3.918397	0.000121	0.0008184	0.661214
ADAMTS9-AS	0.7059766	2.8461725	3.9183655	0.0001211	0.0008184	0.6611022
ANKRD30BP2	-0.64694	-1.072917	-3.917562	0.0001214	0.0008208	0.6582086
ATP6V0B	0.09243	6.7046834	3.9175	0.0001215	0.0008208	0.657985
RP11-643G1	-0.816826	-0.133144	-3.917254	0.0001216	0.0008214	0.6570982
BBX	0.1214504	6.0657988	3.9171874	0.0001216	0.0008215	0.6568594
AC003988.1	0.4574274	-1.303511	3.9170007	0.0001217	0.0008219	0.656187

AP001189.4	1.0119494	1.9300813	3.9156338	0.0001223	0.0008261	0.6512656
PDCD7	-0.06611	5.8410181	-3.915013	0.0001226	0.0008279	0.6490305
PPP2R5D	-0.076239	6.3446376	-3.914922	0.0001227	0.0008281	0.6487029
CPEB4	0.1364223	6.3426561	3.9147647	0.0001228	0.0008284	0.6481376
LINC01143	-0.840869	-0.755246	-3.914382	0.0001229	0.0008294	0.6467595
RPS24	-0.092445	7.1976317	-3.914363	0.0001229	0.0008294	0.6466928
ACKR3	0.2378856	5.8228867	3.9143219	0.000123	0.0008294	0.646544
CTD-3025N2	-0.37264	4.1709599	-3.914259	0.000123	0.0008294	0.6463162
PCSK1N	-1.342926	2.89611	-3.913998	0.0001231	0.00083	0.6453782
AKR1C6P	0.9835288	4.0861998	3.9139829	0.0001231	0.00083	0.6453243
PUS1	-0.107602	6.1305163	-3.913538	0.0001233	0.0008313	0.6437224
MTMR9	0.1213475	5.4663738	3.9132424	0.0001235	0.0008321	0.6426596
MLK7-AS1	0.7504278	2.6340539	3.9127333	0.0001237	0.0008336	0.6408282
FOPNL	0.074906	6.107022	3.9124796	0.0001238	0.0008342	0.6399155
AC009133.1	0.1463033	5.0762203	3.9122433	0.000124	0.0008346	0.6390655
AK4P3	-0.855184	-0.12909	-3.912228	0.000124	0.0008346	0.6390089
RP11-474P2	-0.786487	1.8569892	-3.912222	0.000124	0.0008346	0.6389897
BMS1P1	-0.221517	4.5887163	-3.911769	0.0001242	0.0008358	0.637359
TRMU	-0.097952	6.0788592	-3.911712	0.0001242	0.0008358	0.6371556
RP11-44N11	-0.907503	0.8831162	-3.911707	0.0001242	0.0008358	0.6371377
POLR3E	0.0732168	6.1517745	3.9107766	0.0001247	0.0008386	0.6337905
GPR20	1.0772991	1.6873329	3.9100078	0.000125	0.0008408	0.6310262
SPRR3	1.0174328	-0.533099	3.9099989	0.000125	0.0008408	0.6309942
AC073283.4	-0.900651	1.5958172	-3.909726	0.0001252	0.0008416	0.6300137
TNS3	0.0852192	6.707498	3.9095261	0.0001253	0.0008419	0.6292944
ENTPD1	0.0862793	6.1048265	3.9095225	0.0001253	0.0008419	0.6292815
EFNA5	0.9211866	3.8214853	3.9094115	0.0001253	0.0008421	0.6288826
FCGR3A	0.2171937	6.0683783	3.9090198	0.0001255	0.0008432	0.6274745
NAT10	-0.060516	6.2839088	-3.908922	0.0001256	0.0008434	0.6271217
FJX1	0.2208022	5.1366728	3.9083819	0.0001258	0.000845	0.6251817
KDM4A-AS1	-0.357484	3.8638158	-3.908206	0.0001259	0.0008454	0.6245505
CTD-2288F1	0.8393135	1.8651476	3.9080724	0.000126	0.0008457	0.6240693
CLIC5	0.4008285	4.7342907	3.9077783	0.0001261	0.0008465	0.6230125
WDR27	-0.156633	5.4544789	-3.907506	0.0001262	0.0008472	0.6220356
CRKL	-0.070016	6.4933394	-3.90745	0.0001263	0.0008473	0.6218331
VN1R81P	-1.028022	1.2928415	-3.907337	0.0001263	0.0008475	0.6214261
B4GAT1	-0.105516	6.19954	-3.907235	0.0001264	0.0008477	0.6210609
PLCB2	0.1782276	5.6253232	3.9068834	0.0001265	0.0008485	0.6197971
PRKCE	0.1572336	5.713737	3.9068686	0.0001265	0.0008485	0.6197439
ARHGAP20	0.6580416	3.9573729	3.9063264	0.0001268	0.0008502	0.6177961
HMGB3	0.1790587	6.3541178	3.9060771	0.0001269	0.0008508	0.6169006
SCPEP1	0.1916175	6.2677236	3.9056996	0.0001271	0.0008519	0.6155445
ZNF461	-0.154536	4.9753393	-3.905612	0.0001272	0.000852	0.6152315
LMLN	0.2104084	5.1435549	3.9050007	0.0001275	0.0008539	0.6130345
ZNF688	0.1238014	5.604593	3.9039921	0.000128	0.0008569	0.6094125
ARHGAP27	0.135721	5.8259109	3.9039891	0.000128	0.0008569	0.6094017
HIGD1A	0.1340898	6.4399484	3.9037642	0.0001281	0.0008575	0.6085942
LINC01089	-0.173717	5.2638702	-3.903714	0.0001281	0.0008575	0.6084123
KLF3-AS1	-0.353418	4.1061434	-3.903429	0.0001282	0.0008583	0.6073911
RP11-624M8	0.988117	1.567352	3.9030516	0.0001284	0.0008594	0.6060361
AC005387.3	-0.852643	1.6036481	-3.902752	0.0001286	0.0008602	0.6049617

RP11-15A1.	-0.925406	2.1281384	-3.902441	0.0001287	0.000861	0.6038446
AVIL	-0.209071	5.0721728	-3.902417	0.0001287	0.000861	0.6037572
ZNF91	-0.120354	5.7433702	-3.900785	0.0001295	0.0008663	0.5979028
MIR4292	-0.843281	1.7684637	-3.900614	0.0001296	0.0008667	0.5972889
MOGS	-0.061696	6.5198256	-3.900549	0.0001297	0.0008667	0.5970553
RP11-180M1	-0.471068	-1.258036	-3.899785	0.00013	0.000869	0.5943145
POU2AF1	-0.497508	4.5948852	-3.899691	0.0001301	0.000869	0.5939785
SCARA3	-0.301379	5.8600573	-3.89967	0.0001301	0.000869	0.5939001
VPS25	-0.077286	6.3908309	-3.89967	0.0001301	0.000869	0.5939
AF196972.3	-0.460248	-1.285247	-3.8991	0.0001304	0.0008708	0.5918582
FAM76A	0.0909999	5.4803145	3.8990034	0.0001304	0.0008709	0.5915104
TMEM9B	0.075431	6.265051	3.8987062	0.0001306	0.0008718	0.5904445
CTB-113I20	-0.3058	3.8763874	-3.89737	0.0001313	0.0008761	0.5856516
ATXN1L	0.0864248	6.0707787	3.8972808	0.0001313	0.0008762	0.5853333
PRSS27	0.3958357	3.7363184	3.8971124	0.0001314	0.0008767	0.5847296
PTER	0.1485216	5.8591541	3.8965804	0.0001317	0.0008783	0.5828224
NFE2L2	0.0820591	6.5318933	3.8963612	0.0001318	0.0008789	0.5820367
KIAA1755	0.4876421	4.6515105	3.8960177	0.0001319	0.0008799	0.5808054
AIFM1	0.1143906	6.6543122	3.8955195	0.0001322	0.0008814	0.5790198
CT55	-0.996456	-0.726404	-3.895163	0.0001324	0.0008824	0.5777422
USP3-AS1	-0.205135	4.2163997	-3.894887	0.0001325	0.0008832	0.5767546
RP11-890B1	0.1941883	5.0661948	3.8941111	0.0001329	0.0008857	0.5739734
MIR99AHG	0.4901654	4.5295074	3.893979	0.000133	0.000886	0.5735001
CTC-510F12	-0.972535	1.3824134	-3.893636	0.0001332	0.000887	0.5722725
RP11-462G1	0.7351537	1.9165431	3.893277	0.0001333	0.000888	0.5709852
ZNF282	-0.072117	6.1666026	-3.893028	0.0001335	0.0008887	0.5700928
ZNF124	-0.226266	4.943695	-3.892423	0.0001338	0.0008906	0.5679281
MKRN9P	-0.952936	-0.651643	-3.89232	0.0001338	0.0008908	0.5675587
RP11-332H1	-0.64745	-0.959066	-3.892155	0.0001339	0.0008912	0.5669662
RP11-202K2	-1.079059	-0.537333	-3.89205	0.000134	0.0008914	0.5665896
BCL6B	0.1662464	5.7529451	3.8916199	0.0001342	0.0008927	0.5650506
ZBTB32	-0.622217	3.0485554	-3.891533	0.0001342	0.0008929	0.5647393
SH2D3C	0.1384054	5.8201975	3.8912155	0.0001344	0.0008938	0.5636027
RP11-867G2	-0.245841	5.1344395	-3.891046	0.0001345	0.0008942	0.5629973
RP11-74J13	-0.417273	3.1010464	-3.890371	0.0001348	0.0008962	0.5605804
RP11-53019	-0.545577	3.1219014	-3.89037	0.0001348	0.0008962	0.5605756
RP11-579D7	-0.599976	3.3678599	-3.890299	0.0001349	0.0008963	0.5603222
CHMP7	0.0757915	6.131508	3.8900615	0.000135	0.000897	0.5594716
TRMT1	-0.087578	6.2090475	-3.889346	0.0001354	0.0008993	0.5569091
RP13-516M1	-0.877876	1.4304273	-3.888999	0.0001355	0.0009001	0.55567
LINC01194	-1.459275	0.4758777	-3.888969	0.0001356	0.0009001	0.5555596
DBH-AS1	-0.31688	5.6034493	-3.888964	0.0001356	0.0009001	0.5555417
TMEM213	-0.741244	-0.772713	-3.888892	0.0001356	0.0009002	0.5552859
SMC2-AS1	-0.897448	1.116191	-3.887911	0.0001361	0.0009034	0.5517739
EXOC6B	0.0907513	5.8420155	3.8868807	0.0001367	0.0009068	0.5480897
CACNB3	0.2141466	5.0711116	3.8862804	0.000137	0.0009087	0.5459428
TMEM165	0.0958018	6.1755685	3.8861111	0.0001371	0.0009091	0.5453372
TM4SF1	0.1715716	6.5944511	3.886086	0.0001371	0.0009091	0.5452474
RP11-247C2	1.1068699	0.8869782	3.8853618	0.0001375	0.0009115	0.5426577
CCDC73	-0.382123	3.6142547	-3.885254	0.0001375	0.0009117	0.5422711
RP11-310E2	-0.877292	-0.129359	-3.884968	0.0001377	0.0009125	0.5412479

MYADML2	-0.979015	0.0830679	-3.884852	0.0001377	0.0009127	0.5408334
LIN28A	-0.880158	-0.76593	-3.884732	0.0001378	0.000913	0.5404074
DLL3	-1.040751	0.2757968	-3.884182	0.0001381	0.0009145	0.5384399
SERPINA9	-1.008829	1.4826018	-3.884158	0.0001381	0.0009145	0.5383522
LINC00619	0.8228155	-0.371816	3.8841153	0.0001381	0.0009145	0.5382013
RP13-870H1	1.1719376	1.3243252	3.8841117	0.0001381	0.0009145	0.5381883
AIM1L	-0.478162	4.7504821	-3.883142	0.0001386	0.0009177	0.5347232
SHPK	0.1611293	5.3795887	3.8829921	0.0001387	0.0009181	0.5341866
GTF2IRD1	-0.151635	5.812159	-3.882458	0.000139	0.0009198	0.5322762
RP11-1114I	-0.768332	-0.791951	-3.882024	0.0001392	0.0009212	0.5307256
ROCK1P1	-0.657435	3.2209616	-3.881748	0.0001394	0.000922	0.5297399
CCDC67	-0.939608	0.3836757	-3.881275	0.0001396	0.0009235	0.5280513
AK1	0.1615258	5.6547743	3.8810086	0.0001398	0.0009243	0.5270994
FAAH2	0.2851721	5.457801	3.8806979	0.0001399	0.0009252	0.5259895
RP11-492D6	1.1867346	1.3841788	3.8806207	0.00014	0.0009252	0.5257137
C1orf198	0.1164274	6.4210841	3.8806124	0.00014	0.0009252	0.5256839
TADA2A	-0.078614	5.6337682	-3.880497	0.00014	0.0009253	0.5252727
DLK1	-1.71233	2.5450096	-3.880495	0.00014	0.0009253	0.5252642
ZNF107	-0.215331	5.1595812	-3.880011	0.0001403	0.0009268	0.5235352
KRT223P	0.5996515	-1.231031	3.8799648	0.0001403	0.0009268	0.5233708
RP11-457K1	-0.488252	-1.28884	-3.879887	0.0001404	0.0009269	0.5230928
ROMO1	-0.109101	6.4665219	-3.879569	0.0001405	0.0009279	0.521957
GLIDR	0.5785468	3.1988033	3.8794762	0.0001406	0.000928	0.5216262
NLK	-0.071755	6.0503159	-3.87926	0.0001407	0.0009286	0.5208531
UQCRQ	0.1200101	6.8633698	3.8787673	0.000141	0.0009302	0.5190947
ZNF319	0.0828372	5.6586794	3.8786626	0.000141	0.0009304	0.518721
RP11-982M1	-0.944603	0.0451482	-3.878587	0.0001411	0.0009305	0.5184517
AC005229.7	0.6927166	-0.757397	3.8785237	0.0001411	0.0009306	0.518225
KRTAP5-5	-1.180706	0.1169981	-3.877835	0.0001415	0.0009329	0.5157657
KCTD11	0.1242204	5.5525348	3.8776518	0.0001416	0.0009334	0.5151121
TTC7B	0.1486408	5.7847193	3.8771991	0.0001418	0.0009348	0.5134963
SEPHS1	-0.061301	6.4312766	-3.876903	0.000142	0.0009357	0.5124388
CEP104	0.098125	6.0389476	3.8762835	0.0001423	0.0009378	0.5102287
GDNF-AS1	1.2049404	1.2690464	3.8754749	0.0001428	0.0009405	0.5073433
RWDD2B	0.1034129	5.87526	3.8748129	0.0001431	0.0009427	0.5049815
DCUN1D2	-0.113158	5.5829565	-3.874636	0.0001432	0.0009432	0.5043508
RP11-398C1	-0.907715	2.3651755	-3.873656	0.0001438	0.0009466	0.5008542
IGHV7-40	-0.756683	-0.882547	-3.872671	0.0001443	0.00095	0.4973426
NPBWR1	1.3768664	1.9017504	3.8723789	0.0001445	0.0009508	0.4963011
TNFAIP1	0.068354	6.5233997	3.8710796	0.0001452	0.0009554	0.4916691
TEX10	-0.085956	5.8859921	-3.870473	0.0001455	0.0009575	0.4895058
PLEKHM2	0.0725985	6.3547926	3.8700578	0.0001457	0.0009588	0.4880275
GLMP	-0.110582	6.6024549	-3.869609	0.000146	0.0009603	0.4864278
BLOC1S3	-0.092289	5.9299039	-3.868899	0.0001464	0.0009627	0.4838972
TRIM41	0.0800854	6.1781553	3.8685579	0.0001466	0.0009638	0.4826831
LTF	0.5654258	4.4573269	3.8684186	0.0001467	0.0009641	0.4821872
SLC6A1-AS1	-0.991544	2.1082826	-3.868052	0.0001469	0.0009653	0.4808805
MARVELD1	0.2077944	5.6287385	3.8677681	0.000147	0.0009662	0.4798698
ZNF565	-0.123268	4.9176777	-3.867569	0.0001471	0.0009668	0.4791594
TRMT10B	-0.093492	5.3684712	-3.867234	0.0001473	0.0009678	0.4779673
HIST1H3J	-0.821959	-0.191262	-3.866931	0.0001475	0.0009688	0.4768894

FKBP9P1	0.659995	3.3361407	3.8664749	0.0001478	0.0009703	0.4752644
CTD-228701	-0.243365	4.1300343	-3.866175	0.0001479	0.0009712	0.4741974
MLLT4-AS1	-0.417274	4.4809562	-3.866122	0.000148	0.0009712	0.4740088
AC004076.9	-0.829146	0.0117668	-3.864759	0.0001487	0.0009761	0.4691557
RP11-20I20	-0.424183	4.7237661	-3.864558	0.0001488	0.0009767	0.468439
ETV4	-0.456488	5.6752327	-3.864395	0.0001489	0.0009771	0.4678608
AP001412.1	-0.777067	1.7218977	-3.863722	0.0001493	0.0009795	0.4654637
BRAP	-0.048162	5.9798782	-3.863183	0.0001496	0.0009813	0.4635464
HMGCLL1	1.1200742	1.0331077	3.8630595	0.0001497	0.0009816	0.4631077
RP11-264F2	-0.797988	-0.151912	-3.863023	0.0001497	0.0009816	0.4629768
GUCY2D	-0.866415	3.107994	-3.862631	0.0001499	0.0009829	0.4615822
PODNL1	0.5146462	3.5736681	3.862315	0.0001501	0.0009839	0.4604588
RPL21P131	-0.727643	-0.497471	-3.862179	0.0001502	0.0009842	0.4599753
ZNF488	-1.038821	1.1612071	-3.861631	0.0001505	0.0009861	0.4580251
XRCC1	-0.075466	6.1478325	-3.860874	0.000151	0.0009888	0.4553341
ANKS6	-0.280506	5.7216035	-3.860632	0.0001511	0.0009895	0.454474
CTC-542B22	-0.656073	2.3246462	-3.860242	0.0001513	0.0009908	0.4530869
MT-ND5	0.1291211	7.5204301	3.859141	0.000152	0.0009948	0.4491717
RP11-501C1	-0.891836	0.5576434	-3.858952	0.0001521	0.0009953	0.4485001
MT-CO3	0.1022594	7.8261507	3.8589112	0.0001521	0.0009953	0.4483546
LA16c-380A	0.7118656	-0.77085	3.8587763	0.0001522	0.0009956	0.4478751
SPANXA2-OT	-0.641749	-1.074137	-3.85871	0.0001522	0.0009957	0.4476385
TAS2R43	-0.570579	-0.996762	-3.858468	0.0001523	0.0009965	0.4467788
OSBP2	-0.305597	5.1577351	-3.858154	0.0001525	0.0009975	0.4456626
SMARCE1	-0.056808	6.3616745	-3.857916	0.0001527	0.0009982	0.444819
RN7SKP22	-0.744533	-0.599068	-3.857402	0.000153	0.001	0.4429902
MT-CO1	0.1020729	7.9604263	3.8572458	0.0001531	0.0010004	0.4424359
ZMYND19	-0.104064	5.8785819	-3.856812	0.0001533	0.0010019	0.4408955
AP001046.5	0.5145364	3.521767	3.8565699	0.0001535	0.0010026	0.4400342
SMPD1	0.1085593	6.4775708	3.8560947	0.0001537	0.0010043	0.4383462
RP11-39201	-0.595412	3.0142911	-3.855968	0.0001538	0.0010046	0.4378962
ADCY1	0.4174323	5.2086317	3.8556394	0.000154	0.0010056	0.4367287
CYBB	0.2369841	5.8627707	3.8553779	0.0001542	0.0010065	0.4357998
DSCR8	-1.438702	0.3804385	-3.854924	0.0001544	0.0010078	0.4341881
RAB18	0.0660217	6.4242781	3.8548542	0.0001545	0.0010078	0.4339396
RP11-173E2	-1.000554	0.1782501	-3.854836	0.0001545	0.0010078	0.4338758
EPM2A	0.1519263	5.2637158	3.8548137	0.0001545	0.0010078	0.4337958
CTD-2528L1	-0.270736	4.3766173	-3.854784	0.0001545	0.0010078	0.4336897
ZFAND4	-0.123466	5.4561347	-3.854661	0.0001546	0.0010081	0.433253
PCCB	0.1404485	6.6102656	3.854371	0.0001547	0.001009	0.4322238
ST6GALNAC4	-0.760181	1.6144948	-3.854304	0.0001548	0.001009	0.4319858
PPFIA4	-0.817051	3.1835951	-3.854289	0.0001548	0.001009	0.4319315
TNS1	0.0959809	6.6180164	3.8542335	0.0001548	0.0010091	0.4317354
PDGFD	0.2652554	5.5096403	3.8526736	0.0001557	0.0010149	0.4261972
PAGE1	-1.376898	0.2018467	-3.851965	0.0001562	0.0010175	0.4236833
RIOK3	0.077631	6.4479042	3.8518547	0.0001562	0.0010177	0.4232906
ASRGL1	-0.264456	5.3778926	-3.851682	0.0001563	0.0010182	0.4226764
LIG3	-0.078558	6.0153681	-3.851628	0.0001564	0.0010182	0.4224857
AUTS2	0.2720677	5.8589431	3.8514068	0.0001565	0.0010189	0.4217011
RP11-20024	-0.271115	3.9587596	-3.850905	0.0001568	0.0010205	0.4199216
MIR6129	-0.428739	-1.256889	-3.850898	0.0001568	0.0010205	0.4198938

C1orf100	-0.911773	0.9271397	-3.850817	0.0001569	0.0010205	0.4196095
TRAPPC2B	-0.177586	5.3373891	-3.850797	0.0001569	0.0010205	0.4195373
LCN6	0.9834793	0.4387221	3.8507146	0.0001569	0.0010207	0.4192446
CTB-1202.1	-0.834683	-0.354647	-3.850229	0.0001572	0.0010223	0.4175202
RP11-872D1	0.8072998	3.5324363	3.8502018	0.0001572	0.0010223	0.4174255
DENND2A	0.1749043	5.3591276	3.8500943	0.0001573	0.0010225	0.4170438
HSD17B10	0.1091437	6.6199718	3.8493918	0.0001577	0.0010251	0.414552
LYRM4	-0.10731	6.0255558	-3.84827	0.0001584	0.0010293	0.4105717
RP11-463J1	-0.983402	-0.237139	-3.848186	0.0001584	0.0010295	0.4102755
CLCC1	0.0932861	5.9540745	3.8477057	0.0001587	0.0010312	0.4085722
RP11-477D1	-0.276966	3.9143536	-3.847532	0.0001588	0.0010317	0.4079563
MTPN	0.0596043	6.5458691	3.8474537	0.0001589	0.0010318	0.4076785
RPL3P7	-0.504392	3.0015872	-3.846989	0.0001592	0.0010334	0.4060306
AC144449.1	0.5352824	2.9191282	3.8454934	0.0001601	0.0010391	0.4007298
EFCAB12	0.527425	4.7030373	3.8453202	0.0001602	0.0010396	0.4001157
IER2	0.1078876	6.4366223	3.8450602	0.0001603	0.0010402	0.3991944
IPO5P1	-0.226907	5.364369	-3.845053	0.0001603	0.0010402	0.3991691
PLCH2	0.4999464	4.9911793	3.8450442	0.0001603	0.0010402	0.3991377
WIF1	-1.215345	0.0380022	-3.844748	0.0001605	0.0010412	0.3980893
RPL36A	-0.121441	6.3195882	-3.844018	0.000161	0.0010439	0.3955022
RNF141	0.0718774	6.1093336	3.8438908	0.000161	0.0010442	0.3950508
RPL23AP2	-0.370934	3.2952863	-3.84381	0.0001611	0.0010443	0.3947664
AC011515.2	0.7878577	-0.478922	3.8437454	0.0001611	0.0010444	0.3945357
RPS7P3	-0.827731	1.1721214	-3.843193	0.0001615	0.0010464	0.3925803
ZNF610	-0.531013	3.7996291	-3.842376	0.000162	0.0010495	0.3896845
RPL21P136	-0.496605	-1.126829	-3.842233	0.0001621	0.0010499	0.389178
AC104843.3	-0.752026	-0.464069	-3.841507	0.0001625	0.0010526	0.3866093
MT-CO2	0.1029804	7.7772586	3.8406899	0.000163	0.0010557	0.3837149
CDKN2AIPNL	-0.101893	5.9993817	-3.840468	0.0001632	0.0010564	0.38293
CASC8	-1.130551	0.7598897	-3.840186	0.0001633	0.0010573	0.3819294
COL4A2-AS1	0.8069954	-0.242402	3.8399303	0.0001635	0.0010582	0.3810259
DNAJB11	-0.077821	6.6651369	-3.839622	0.0001637	0.0010591	0.3799333
AL590431.1	-0.750334	1.955098	-3.839592	0.0001637	0.0010591	0.3798292
LINC01202	-1.005546	-0.494962	-3.838491	0.0001644	0.0010634	0.3759326
MATK	0.247826	4.9620256	3.8382773	0.0001645	0.0010641	0.3751762
SSC4D	-0.292908	5.1368301	-3.837897	0.0001648	0.0010654	0.3738298
CALY	-1.069384	1.4410718	-3.83782	0.0001648	0.0010655	0.3735578
CYGB	0.1801455	5.8242599	3.83746	0.000165	0.0010668	0.3722846
RP4-595K12	-1.073755	-0.23358	-3.836361	0.0001657	0.0010711	0.3683973
CTD-2231E1	-0.636396	2.785708	-3.835977	0.000166	0.0010725	0.3670386
FAM83C-AS1	-0.897309	0.7438376	-3.835767	0.0001661	0.0010731	0.366298
TMCC1-AS1	-0.310414	4.2907957	-3.835173	0.0001665	0.0010754	0.3641955
SERPINB9	0.1648772	5.7829416	3.8349529	0.0001666	0.0010761	0.3634177
RPRD1A	-0.072501	6.2865323	-3.834795	0.0001667	0.0010765	0.3628591
LRRC37A7P	0.9881626	3.6365827	3.8345531	0.0001669	0.0010773	0.3620042
MTOR	0.096465	6.4396349	3.8339166	0.0001673	0.0010797	0.3597544
RPL7P1	-0.3001	4.3160665	-3.833664	0.0001674	0.0010806	0.3588609
LRRC37A6P	-0.905635	3.2481691	-3.832522	0.0001682	0.0010851	0.3548239
MT-CYB	0.1104585	7.7609479	3.8321792	0.0001684	0.0010863	0.3536141
RP11-603J2	-0.7373	-0.495364	-3.831656	0.0001687	0.0010883	0.3517655
KCTD17	-0.212013	5.7271694	-3.831552	0.0001688	0.0010885	0.3513963

AIRE	-1.027188	0.5770925	-3.831376	0.0001689	0.001089	0.3507763
RP11-167N5	-0.874223	0.945571	-3.831312	0.0001689	0.0010891	0.3505496
PRDM13	-0.655457	-1.176102	-3.831008	0.0001691	0.0010902	0.3494761
AC246787.1	-0.610115	-1.067745	-3.830702	0.0001693	0.0010913	0.3483939
RP11-43505	-0.949034	0.3684188	-3.830549	0.0001694	0.0010917	0.3478538
SLC39A14	0.1353388	6.9244503	3.8303286	0.0001696	0.0010922	0.3470764
LRRC8C	0.1540338	5.4835408	3.8303105	0.0001696	0.0010922	0.3470125
AL162151.3	-0.646595	2.8104354	-3.830286	0.0001696	0.0010922	0.3469254
AC009362.2	-0.701822	-0.672627	-3.830047	0.0001697	0.0010929	0.3460825
RP11-563D1	1.2407268	1.3522489	3.8300274	0.0001698	0.0010929	0.3460125
FLJ41941	-0.817927	-0.469175	-3.829866	0.0001699	0.0010934	0.3454426
DDX58	0.1120015	5.8905598	3.8297129	0.00017	0.0010936	0.344902
LGI2	0.4094155	4.5691173	3.8296415	0.00017	0.0010936	0.3446498
RPS23P1	-0.829078	0.1055896	-3.829637	0.00017	0.0010936	0.3446344
RP11-146F1	-0.82916	0.5747375	-3.829627	0.00017	0.0010936	0.3445978
CNTD1	-0.230258	4.082043	-3.829418	0.0001701	0.0010943	0.3438621
CTA-445C9.	-0.285254	4.5687928	-3.829311	0.0001702	0.0010945	0.3434836
RP11-158L1	-0.616589	-1.083031	-3.82912	0.0001703	0.0010951	0.3428096
SBNO1	-0.065072	6.1674942	-3.829042	0.0001704	0.0010952	0.3425337
PDRG1	-0.092389	6.019036	-3.828909	0.0001705	0.0010956	0.3420631
NPFFR2	-1.429607	1.1284441	-3.82881	0.0001705	0.0010958	0.3417145
CHCHD10	0.1541596	6.5809345	3.8281999	0.0001709	0.0010982	0.3395596
PRKCI	-0.082764	5.9498533	-3.827794	0.0001712	0.0010996	0.3381264
ITGB8	0.8864379	3.8736109	3.8277547	0.0001712	0.0010996	0.3379882
MEF2A	0.104574	6.1342662	3.8276789	0.0001713	0.0010998	0.3377207
PCMTD2	-0.092006	6.3270428	-3.827487	0.0001714	0.0011004	0.3370423
INPP5F	-0.111066	5.5156529	-3.82714	0.0001716	0.0011016	0.3358169
RP11-475I2	-0.539851	-1.268116	-3.827092	0.0001717	0.0011016	0.3356475
TMSB4X	0.0908617	7.1695216	3.8269108	0.0001718	0.0011021	0.3350097
RP11-123J1	-0.939204	0.2405008	-3.826881	0.0001718	0.0011021	0.3349054
RPL5P24	-0.817686	0.8626158	-3.825486	0.0001727	0.0011078	0.3299826
ATP2B4	0.1343354	6.4431293	3.8249728	0.000173	0.0011097	0.3281715
ZNF221	-0.401135	3.5684551	-3.824835	0.0001731	0.0011101	0.327684
ZDHHC8P1	-1.210107	1.8313331	-3.824476	0.0001734	0.0011114	0.3264184
RP11-69M1.	-0.320816	3.7614569	-3.824402	0.0001734	0.0011115	0.3261565
CENPP	-0.14911	5.0003051	-3.82428	0.0001735	0.0011119	0.3257262
HNRNPA1P38	-0.601791	2.0689737	-3.823521	0.000174	0.0011149	0.3230493
RPL27AP5	-0.904	0.1417527	-3.823091	0.0001743	0.0011165	0.3215332
SENP8	0.2155477	4.4695311	3.8225697	0.0001746	0.0011185	0.3196963
EDN2	1.1159186	2.136196	3.8221141	0.0001749	0.0011202	0.3180899
RANGRF	-0.171473	5.4529737	-3.822067	0.000175	0.0011202	0.3179235
SPATC1L	-0.479394	5.1842237	-3.821966	0.000175	0.0011205	0.317569
NSMF	0.1305979	6.2032497	3.8217853	0.0001751	0.001121	0.3169311
GPR89B	-0.154337	5.1344582	-3.821751	0.0001752	0.001121	0.3168094
AASS	0.390407	5.3819741	3.8213676	0.0001754	0.0011224	0.3154584
CREG1	0.1412321	6.8662422	3.8213195	0.0001754	0.0011224	0.3152889
RP11-313P1	-0.826469	0.7831395	-3.821169	0.0001755	0.0011229	0.3147585
SDC3	0.1059467	6.4098237	3.8210734	0.0001756	0.0011231	0.3144215
RP11-230B2	-0.785815	2.0882094	-3.820621	0.0001759	0.0011248	0.3128272
FAM182B	-0.851983	2.4451286	-3.820458	0.000176	0.0011253	0.3122534
RP11-522B1	0.7494301	-0.484612	3.8200876	0.0001763	0.0011267	0.3109475

SLC35A1	-0.099249	5.685985	-3.819876	0.0001764	0.0011274	0.3102014
SLC13A2	-1.007809	4.1724294	-3.819469	0.0001767	0.0011289	0.3087695
RP11-132N1	-0.77656	-0.604487	-3.819212	0.0001769	0.0011298	0.3078625
UBE2E1	-0.065599	6.2813703	-3.818831	0.0001771	0.0011313	0.3065205
SIGLEC8	0.642019	3.6799717	3.8186925	0.0001772	0.0011317	0.3060323
SH3BGRL3	0.1003922	6.6072644	3.8183516	0.0001774	0.0011329	0.3048313
MYL3	0.8010523	2.712124	3.8174583	0.000178	0.0011366	0.3016852
ZNF248	-0.098313	5.4918203	-3.817402	0.0001781	0.0011366	0.3014861
RPL7AP60	-0.827564	0.1606077	-3.817337	0.0001781	0.0011367	0.3012569
RP11-318A1	-0.854195	1.5603001	-3.817248	0.0001782	0.0011369	0.3009446
COL6A5	0.8682662	-0.384029	3.8172084	0.0001782	0.0011369	0.300805
LYPD5	0.447949	3.9364647	3.8170159	0.0001783	0.0011375	0.3001272
PLEKHA3	0.0708984	5.8631494	3.8165355	0.0001787	0.0011394	0.2984356
CTC-281F24	-0.779238	1.7073646	-3.816322	0.0001788	0.0011401	0.2976831
CTBP2	0.2255805	5.6071088	3.8161636	0.0001789	0.0011406	0.2971263
RAPGEF3	0.1763625	5.4179253	3.8159845	0.000179	0.0011411	0.2964959
NQO2	0.1422912	6.4417622	3.8155224	0.0001793	0.0011429	0.2948691
FAM129A	0.1995405	5.6473617	3.815156	0.0001796	0.0011443	0.2935792
CACNA1B	-1.006933	-0.049619	-3.814928	0.0001797	0.0011451	0.2927758
AC007773.2	-0.528338	3.4991855	-3.814785	0.0001798	0.0011454	0.2922729
CLDN10	1.4078168	2.500101	3.8147771	0.0001798	0.0011454	0.2922458
ZNF675	-0.23631	4.9716328	-3.814658	0.0001799	0.0011457	0.2918264
RP11-436H1	-0.85382	1.3649136	-3.814214	0.0001802	0.0011474	0.2902656
GPRIN3	0.2271166	5.6399635	3.8140663	0.0001803	0.0011478	0.289744
LRRC55	0.8872387	2.6831768	3.813091	0.000181	0.0011519	0.2863125
SPECC1	0.2543344	5.4578692	3.8130406	0.000181	0.0011519	0.2861351
AGAP6	-0.146659	5.2399239	-3.812982	0.0001811	0.0011519	0.2859294
MPRIP	0.0747652	6.5171114	3.81222	0.0001816	0.0011551	0.2832486
U82670.9	-0.570369	-1.006623	-3.811805	0.0001819	0.0011567	0.2817878
RP11-731J8	-0.951885	-0.000776	-3.811718	0.0001819	0.0011567	0.2814818
CCL15	-0.651017	5.6019856	-3.81171	0.0001819	0.0011567	0.2814534
P3H3	0.3124609	5.2380994	3.8113439	0.0001822	0.0011581	0.2801672
AHSG	-0.476444	7.236659	-3.810863	0.0001825	0.00116	0.2784773
SYK	0.2111165	5.5901849	3.8102266	0.000183	0.0011626	0.2762384
LINC00564	-0.5808	-1.211178	-3.81016	0.000183	0.0011627	0.2760052
CTD-2666L2	-0.874431	0.6384084	-3.810082	0.0001831	0.0011628	0.2757295
GRB7	-0.373692	5.7250465	-3.810032	0.0001831	0.0011628	0.2755539
LRRC70	0.6494276	2.8613401	3.8097449	0.0001833	0.0011638	0.2745447
DDX26B	0.2956187	4.7430691	3.8097263	0.0001833	0.0011638	0.2744793
RP1-102K2.	0.6686096	-0.790973	3.8091244	0.0001837	0.0011661	0.2723634
SC22CB-1E7	-0.492503	3.3660672	-3.809112	0.0001837	0.0011661	0.2723187
RP11-411B1	-0.855161	-0.090792	-3.808728	0.000184	0.0011676	0.2709712
RPS14P8	-1.197445	1.6043997	-3.808153	0.0001844	0.0011697	0.2689492
RPL13AP6	-0.800589	1.7557681	-3.808149	0.0001844	0.0011697	0.2689368
RP5-836N17	-0.831037	0.1847642	-3.807962	0.0001845	0.0011703	0.2682772
SRC	-0.170535	6.2013093	-3.80745	0.0001849	0.0011724	0.2664777
CTSD	0.0959045	7.4087643	3.8071141	0.0001851	0.0011737	0.2652991
SLC39A12	0.7106896	-0.80575	3.8069883	0.0001852	0.001174	0.2648569
NKD2	0.6924088	4.3677765	3.8052886	0.0001864	0.0011814	0.2588866
PIK3R5	0.2102351	5.2676499	3.8049626	0.0001866	0.0011826	0.2577416
RP11-118B1	1.267756	1.4673301	3.8048474	0.0001867	0.001183	0.2573371

SAV1	0.1219866	5.8503943	3.8045692	0.0001869	0.001184	0.2563601
GAL3ST3	-0.572776	-1.20816	-3.804388	0.000187	0.0011846	0.2557256
SOCS4	0.0855468	5.7661314	3.804317	0.0001871	0.0011846	0.2554748
NFIX	0.1221801	6.3612142	3.8042617	0.0001871	0.0011846	0.2552806
ADAP2	0.1543132	5.5714298	3.8042525	0.0001871	0.0011846	0.2552482
AC023347.1	-0.967699	-0.546127	-3.803312	0.0001878	0.0011886	0.2519455
MIR570	-0.496871	3.2670814	-3.803211	0.0001879	0.0011888	0.2515902
AC069277.2	-1.261207	0.2668918	-3.802992	0.000188	0.0011896	0.2508245
AC096574.5	-0.947042	1.1121123	-3.802923	0.0001881	0.0011897	0.2505802
POP5	-0.094818	6.0376267	-3.802868	0.0001881	0.0011898	0.2503877
ZNF787	-0.090393	6.3830315	-3.802607	0.0001883	0.0011907	0.2494727
CTA-253N17	-0.459339	3.0305492	-3.802539	0.0001884	0.0011908	0.249232
RP11-334C1	-0.487742	3.222397	-3.802364	0.0001885	0.0011914	0.2486171
PITX1	-1.223155	3.9371426	-3.802272	0.0001885	0.0011916	0.2482947
LINC01037	-0.569016	-1.188099	-3.802188	0.0001886	0.0011918	0.2480001
RP11-467L2	-0.713369	-1.056341	-3.801953	0.0001888	0.0011925	0.2471781
MAFG-AS1	-0.412293	4.5523436	-3.801941	0.0001888	0.0011925	0.2471336
ALG1L8P	-0.827467	0.118367	-3.801641	0.000189	0.0011934	0.2460827
RP11-388K1	-1.253883	0.2938373	-3.801635	0.000189	0.0011934	0.2460618
RP11-131L1	-0.652464	2.4085732	-3.801536	0.0001891	0.0011937	0.2457142
PRKAG3	-0.907278	0.7989745	-3.801289	0.0001892	0.0011946	0.2448469
RP11-797A1	-0.932448	0.9553058	-3.800996	0.0001895	0.0011957	0.2438169
RPS12	-0.096144	7.1452124	-3.800723	0.0001897	0.0011967	0.2428614
LZTS1	0.2201487	5.3885559	3.8006436	0.0001897	0.0011969	0.2425821
HNRNPL	-0.042944	6.7390845	-3.800118	0.0001901	0.001199	0.2407399
RP11-678G1	-0.961343	0.4842207	-3.800087	0.0001901	0.001199	0.2406312
PEF1	0.0788871	6.4322651	3.7999825	0.0001902	0.0011992	0.2402632
PCDH1	0.1605475	6.247442	3.7992957	0.0001907	0.0012021	0.2378541
VAC14-AS1	-0.649125	4.0259795	-3.798708	0.0001911	0.0012045	0.2357916
RILPL2	0.1188211	5.7489205	3.7986884	0.0001911	0.0012045	0.2357245
DNAH17	-0.323558	4.4677594	-3.798576	0.0001912	0.0012048	0.2353316
AC124789.1	0.5573777	2.8210018	3.7983981	0.0001913	0.0012053	0.2347063
SLC1A4	-0.134432	6.1769745	-3.798364	0.0001913	0.0012053	0.2345859
RP11-872J2	-0.20997	4.2867324	-3.798101	0.0001915	0.0012063	0.2336639
MT1CP	0.9536073	-0.468601	3.7977823	0.0001918	0.0012076	0.2325473
AC018865.5	0.7505831	-0.038446	3.7972998	0.0001921	0.0012095	0.2308559
ITGA7	0.1486996	6.0123658	3.7972595	0.0001921	0.0012095	0.2307147
RNF185	0.0885854	6.3099674	3.7969542	0.0001924	0.0012107	0.2296445
MB21D2	0.2730954	4.5848323	3.7964413	0.0001927	0.0012128	0.2278467
RP11-402G3	-0.614621	-0.974006	-3.795948	0.0001931	0.0012149	0.2261165
GDAP1L1	-0.988717	0.0075459	-3.795616	0.0001933	0.0012162	0.2249553
RP11-214L1	-0.719108	-0.939898	-3.794549	0.0001941	0.0012209	0.2212141
ILDR2	0.7545473	4.5755249	3.7943463	0.0001943	0.0012216	0.2205056
NNT-AS1	0.1054753	5.7561556	3.7937346	0.0001947	0.0012242	0.2183629
SLC2A10	0.2287464	6.176939	3.7937029	0.0001947	0.0012242	0.2182518
ZNF816-ZNF	-0.646443	3.502174	-3.793381	0.000195	0.0012254	0.217126
SYT15	-0.834958	3.445297	-3.792716	0.0001955	0.0012282	0.2147969
OGFOD2	-0.178145	4.7646326	-3.792685	0.0001955	0.0012282	0.2146863
NAP1L5	0.3006393	4.9117438	3.7919997	0.000196	0.0012312	0.2122874
MAP2K6	-0.274623	5.2111379	-3.791338	0.0001965	0.001234	0.2099716
CCDC110	0.7316024	3.0921022	3.79131	0.0001965	0.001234	0.2098725

RP11-521B2	-0.294725	4.1914062	-3.791094	0.0001967	0.0012347	0.2091172
PSAPL1	-1.208264	0.4423636	-3.790699	0.000197	0.0012364	0.2077339
RPL4P2	-0.891051	0.5211287	-3.790567	0.000197	0.0012365	0.2072705
C19orf33	-0.871949	3.3758864	-3.790539	0.0001971	0.0012365	0.2071724
AL078471.5	-0.827137	-0.841248	-3.790528	0.0001971	0.0012365	0.2071347
AC104654.2	1.0063228	1.6938713	3.7900529	0.0001974	0.0012385	0.2054723
DLEU1	-0.139907	5.1538256	-3.790021	0.0001975	0.0012385	0.2053615
TMEM64	0.1981698	6.1415691	3.7895622	0.0001978	0.0012404	0.2037552
ZNF738	-0.328624	4.4795752	-3.789307	0.000198	0.0012414	0.2028637
ARFGAP1	-0.067761	6.4916469	-3.789211	0.0001981	0.0012416	0.2025246
RPL31P52	-0.746601	-0.197091	-3.78892	0.0001983	0.0012428	0.2015097
ZNF720	0.1041274	5.4677925	3.7887735	0.0001984	0.0012432	0.2009956
APOBEC3H	0.5606052	3.5557759	3.7884407	0.0001986	0.0012446	0.1998313
GREM1	0.7395995	4.5781151	3.7881679	0.0001988	0.0012456	0.1988768
VCX3B	-0.915151	-0.62383	-3.787938	0.000199	0.0012465	0.1980709
DMKN	-0.765778	4.7840862	-3.787853	0.0001991	0.0012467	0.1977752
RP11-32K4.	-0.755497	-0.97771	-3.787078	0.0001997	0.0012501	0.1950632
TNFRSF10A	0.1705369	5.3992069	3.7870167	0.0001997	0.0012502	0.1948503
EFCAB8	-0.895859	1.109502	-3.786941	0.0001998	0.0012503	0.1945871
DHDH	-0.798829	2.9184169	-3.785508	0.0002008	0.0012569	0.1895733
PRAMEF11	0.6719818	-1.081728	3.7845765	0.0002015	0.0012611	0.1863186
RP11-57H14	-0.222712	4.617524	-3.784291	0.0002018	0.0012622	0.1853213
ZSWIM8-AS1	-0.744615	-0.306374	-3.784036	0.0002019	0.0012632	0.1844286
DNAJC5G	-0.832378	-0.254201	-3.783831	0.0002021	0.0012639	0.1837128
SPAG9	0.0688331	6.3860059	3.7837396	0.0002022	0.0012642	0.1833934
RP11-26P13	0.4843919	-1.209863	3.7835094	0.0002023	0.001265	0.1825889
RP11-132F7	-0.802222	3.468496	-3.783449	0.0002024	0.0012651	0.182377
TSC22D4	0.1043837	6.2567869	3.7832399	0.0002026	0.0012659	0.1816473
CDC42EP5	0.22395	4.7645192	3.7821086	0.0002034	0.0012711	0.1776947
LAGE3P1	-0.78642	1.0691433	-3.781967	0.0002035	0.0012713	0.1772
AC012485.2	-0.921588	1.2900143	-3.781937	0.0002035	0.0012713	0.177094
RP11-29H23	-0.823172	0.1792947	-3.78193	0.0002036	0.0012713	0.1770707
KIF12	-0.458409	5.9035202	-3.781324	0.000204	0.0012739	0.1749544
SUN2	0.10317	6.7426336	3.7808276	0.0002044	0.0012761	0.1732204
RCHY1	0.0934217	5.7241602	3.7805326	0.0002046	0.0012773	0.1721902
SRSF2	-0.050367	6.5799003	-3.780387	0.0002047	0.0012778	0.1716812
HIPK2	0.129708	6.4405418	3.7792961	0.0002056	0.0012828	0.1678729
QRFPR	1.1242709	0.7620643	3.7790886	0.0002057	0.0012836	0.1671482
LINC00624	-0.677014	3.2170679	-3.778329	0.0002063	0.001287	0.1644972
PTPDC1	-0.176031	5.1120079	-3.778222	0.0002064	0.0012873	0.1641225
ZNF578	-0.868698	1.8638168	-3.778152	0.0002065	0.0012875	0.1638785
CTD-2537I9	-0.351734	4.0943753	-3.777769	0.0002068	0.0012891	0.1625415
CASC16	-0.801445	-0.512752	-3.777642	0.0002069	0.0012893	0.1621008
DHRS7B	0.1075028	6.0621175	3.7775875	0.0002069	0.0012893	0.161909
ZNF573	-0.258419	4.1696842	-3.777586	0.0002069	0.0012893	0.1619028
C1QA	0.1718555	6.600283	3.7775054	0.000207	0.0012895	0.1616226
B3GNT2	0.0947832	5.9464058	3.777266	0.0002072	0.0012904	0.160787
AC007743.1	0.8712767	1.9336435	3.7764468	0.0002078	0.0012942	0.1579287
LIN28B	-1.582227	0.8596972	-3.776343	0.0002079	0.0012945	0.1575656
RP11-124N3	-0.676992	-1.107861	-3.774051	0.0002097	0.0013054	0.1495722
LRRK1	0.2117967	5.2026051	3.7738293	0.0002098	0.0013061	0.1487996

RP11-276H1	0.947328	0.8423595	3.7738284	0.0002098	0.0013061	0.1487964
RP11-490M8	0.631647	3.5703737	3.7735974	0.00021	0.001307	0.1479908
RP4-781L3.	-0.584045	-1.001076	-3.773325	0.0002102	0.0013081	0.1470403
RP11-138H1	0.7773175	-0.832863	3.7730744	0.0002104	0.0013091	0.1461674
ZEB1	0.114884	5.9428661	3.7725925	0.0002108	0.0013112	0.1444876
SCD	-0.198418	7.2873384	-3.771886	0.0002114	0.0013145	0.1420243
VSIG10L	0.3017341	5.3985868	3.7716085	0.0002116	0.0013156	0.1410582
RPL9P18	-0.860815	0.7363793	-3.771127	0.000212	0.0013178	0.1393802
CARS	0.076728	6.2998729	3.7706551	0.0002124	0.0013199	0.1377358
MEST	-0.142994	6.2404653	-3.770572	0.0002124	0.00132	0.1374462
AC016739.2	-0.256657	4.6177312	-3.770517	0.0002125	0.00132	0.1372565
NOSTRIN	0.1971207	5.2761629	3.7704892	0.0002125	0.00132	0.1371578
PRRT3	-0.18484	4.6535208	-3.770403	0.0002126	0.0013202	0.1368584
HSD17B7	-0.157804	5.908288	-3.770369	0.0002126	0.0013202	0.136739
AC074289.1	-0.337442	4.2297834	-3.770095	0.0002128	0.0013213	0.1357839
NFATC2IP	0.0742528	6.0690882	3.7693759	0.0002134	0.0013247	0.1332797
PIGH	-0.088989	5.8424009	-3.769009	0.0002137	0.0013262	0.1320021
RP11-196G1	-0.196798	5.3112026	-3.767864	0.0002146	0.0013317	0.1280154
BTBD3	-0.112673	6.010189	-3.767684	0.0002147	0.0013324	0.1273862
IFT43	0.1018163	5.9678203	3.7672166	0.0002151	0.0013345	0.1257605
CDK9	0.0764783	6.3822454	3.7668374	0.0002154	0.0013361	0.1244403
RNA5SP334	0.7825483	-0.866618	3.7668003	0.0002154	0.0013361	0.1243112
HAUS6	-0.109358	5.7738204	-3.766621	0.0002156	0.0013368	0.123687
CELF3	-0.88977	0.2062242	-3.766217	0.0002159	0.0013386	0.1222812
XAGE1A	-0.612497	-1.05753	-3.764647	0.0002172	0.0013459	0.116817
FAM214B	0.0984162	5.7625361	3.7646436	0.0002172	0.0013459	0.1168054
PRLR	-0.359218	6.0247079	-3.764624	0.0002172	0.0013459	0.1167373
SUPT3H	-0.186951	5.2382965	-3.764488	0.0002173	0.0013464	0.1162624
CAPN14	-0.983753	1.0460342	-3.763862	0.0002178	0.0013493	0.1140865
FARSB	-0.07357	6.2359989	-3.763522	0.0002181	0.0013508	0.1129031
CD274	0.3286282	4.5874332	3.7629914	0.0002185	0.0013533	0.1110579
APOLD1	0.1794746	6.0092463	3.7621696	0.0002192	0.0013572	0.1081997
MITD1	-0.076578	5.7736791	-3.762101	0.0002193	0.0013573	0.1079616
AL662800.1	-0.53557	2.4886873	-3.761876	0.0002195	0.0013582	0.10718
AC144831.1	0.6533796	2.9887644	3.7618297	0.0002195	0.0013582	0.1070178
RP11-190J1	-0.879051	-0.789025	-3.761702	0.0002196	0.0013586	0.1065754
TMEM216	-0.120355	5.2982681	-3.761635	0.0002196	0.0013588	0.1063393
RP1-12G14.	0.5747014	2.2748664	3.7614766	0.0002198	0.0013593	0.1057902
RNF14	0.0750739	6.3413581	3.7613911	0.0002198	0.0013595	0.1054929
PLA2G15	0.10037	5.978917	3.7611271	0.0002201	0.0013607	0.104575
ARHGAP17	0.0649017	6.2327734	3.7610416	0.0002201	0.0013609	0.104278
CACNG1	-0.981119	-0.248633	-3.760733	0.0002204	0.0013622	0.1032051
TK1	-0.180971	6.0853749	-3.760561	0.0002205	0.0013628	0.1026062
AC008132.1	-0.654435	-0.917424	-3.760447	0.0002206	0.0013632	0.1022099
RP11-290F2	-0.412959	3.4904319	-3.760254	0.0002208	0.0013639	0.1015417
SHCBP1	-0.359214	5.0149473	-3.759946	0.000221	0.0013652	0.1004699
CTC-444N24	-0.871987	0.5380568	-3.759909	0.0002211	0.0013652	0.1003418
C1orf195	-0.643502	-0.934329	-3.758827	0.000222	0.0013705	0.0965825
CDSN	0.4097091	-1.378116	3.7587365	0.000222	0.0013708	0.0962665
TPD52L2	-0.067248	6.5577196	-3.7585	0.0002222	0.0013718	0.0954465
LDB3	0.4406602	3.7985825	3.7583482	0.0002224	0.0013723	0.0949172

RHBDD2	0.0899943	6.5672089	3.7581852	0.0002225	0.0013729	0.0943509
ZNF624	-0.178359	4.7297631	-3.757758	0.0002229	0.0013749	0.0928653
MTMR7	-0.359862	4.7652116	-3.75662	0.0002238	0.0013805	0.0889128
PARP10	0.1242653	6.6080592	3.7565687	0.0002238	0.0013805	0.0887361
ST3GAL5	0.1425575	5.8056833	3.7563154	0.0002241	0.0013816	0.0878565
CCDC66	-0.096399	5.455545	-3.756203	0.0002242	0.001382	0.0874678
RP3-425C14	-0.786819	-0.07557	-3.755775	0.0002245	0.0013839	0.0859786
MTATP6P1	0.1311397	7.1330783	3.7553974	0.0002248	0.0013857	0.0846688
TRIM52-AS1	-0.146084	5.3687335	-3.75535	0.0002249	0.0013857	0.0845055
RP11-321P1	0.5910412	-1.027924	3.7549502	0.0002252	0.0013875	0.0831164
LINC01107	-0.525365	-1.196925	-3.754915	0.0002252	0.0013875	0.0829931
RP11-108F1	-0.843182	0.4159803	-3.753988	0.000226	0.001392	0.0797768
IL22RA1	-0.430924	5.6075232	-3.753727	0.0002262	0.001393	0.0788711
RP11-76C10	-0.939734	-0.71898	-3.753717	0.0002262	0.001393	0.0788364
TAX1BP1	0.0597402	6.6802859	3.7529896	0.0002269	0.0013965	0.0763118
CEP192	-0.102754	5.7940928	-3.752957	0.0002269	0.0013965	0.0761967
DIDO1	-0.061634	6.37813	-3.75269	0.0002271	0.0013976	0.075272
MND1	-0.287463	4.7695903	-3.75228	0.0002275	0.0013995	0.0738512
AC012370.3	-0.477592	-1.176542	-3.752148	0.0002276	0.0013999	0.0733917
CTD-3064M3	0.7504467	3.4104695	3.7521188	0.0002276	0.0013999	0.0732903
LDLRAD4-AS	0.9876397	-0.208937	3.7520658	0.0002276	0.0013999	0.0731063
GPANK1	-0.095076	6.0754326	-3.751939	0.0002278	0.0014004	0.072668
RP11-36501	-0.63253	2.1168729	-3.751763	0.0002279	0.0014009	0.0720571
SLC6A10P	-0.812804	-0.998298	-3.751709	0.000228	0.0014009	0.071867
EFEMP2	0.1788787	5.8443511	3.7517019	0.000228	0.0014009	0.071844
PET117	-0.118966	5.2819382	-3.751646	0.000228	0.001401	0.0716501
RP11-179A1	-0.816745	0.279062	-3.751274	0.0002283	0.0014027	0.0703588
ZACN	-0.32185	3.2881075	-3.75082	0.0002287	0.0014048	0.0687866
RP11-996F1	-0.864635	1.7809406	-3.75051	0.000229	0.0014062	0.0677092
ANGPT2	-0.217574	5.3736261	-3.750403	0.0002291	0.0014065	0.0673376
MRPS17P1	-0.81926	-0.292887	-3.750319	0.0002291	0.0014067	0.0670474
VPS33B	-0.064204	5.7988578	-3.749792	0.0002296	0.0014093	0.0652215
SEPT4	0.1944226	5.8790137	3.749524	0.0002298	0.0014104	0.0642909
NUP50-AS1	0.1637752	5.6219029	3.7494198	0.0002299	0.0014107	0.0639296
RP1-71H19.	-0.62339	-0.818241	-3.749146	0.0002301	0.001412	0.0629799
CEP295	-0.127916	5.4384259	-3.748926	0.0002303	0.0014129	0.0622183
PDE4B	0.243839	5.5015713	3.7486901	0.0002305	0.0014136	0.0613997
CILP2	0.7016113	3.7813569	3.7486452	0.0002306	0.0014136	0.0612441
NXT1	-0.101557	5.7579949	-3.748645	0.0002306	0.0014136	0.0612431
RP11-707M3	-0.48019	3.1094577	-3.748071	0.0002311	0.0014164	0.0592522
LRP4-AS1	-0.861281	0.5452682	-3.748003	0.0002311	0.0014166	0.0590178
GPR173	0.534325	3.4087914	3.747336	0.0002317	0.0014199	0.0567065
SNHG22	-0.286922	4.0494769	-3.746819	0.0002321	0.0014224	0.0549137
ZNF775	-0.122117	5.8571241	-3.746619	0.0002323	0.0014232	0.0542208
bP-2171C21	-1.11227	0.6023173	-3.74537	0.0002334	0.0014296	0.0498944
MFI2	-0.334798	5.4763163	-3.745314	0.0002335	0.0014296	0.0497008
RP1-315G1.	-0.807646	1.3956346	-3.744529	0.0002341	0.0014336	0.0469807
KIF6	-0.834361	3.2792523	-3.744462	0.0002342	0.0014337	0.046749
RP1-68D18.	0.819364	-0.389665	3.7443958	0.0002343	0.0014338	0.0465211
AC012442.6	-0.511635	2.3890422	-3.742962	0.0002355	0.0014413	0.0415556
HAUS3	-0.082742	5.6678738	-3.742773	0.0002357	0.001442	0.0409024

MIR222HG	-0.403141	3.971212	-3.742643	0.0002358	0.0014425	0.0404525
RP11-402J6	-0.572745	2.1989947	-3.742504	0.0002359	0.001443	0.0399723
RP11-297A1	-0.805578	0.1635243	-3.741908	0.0002364	0.001446	0.0379094
ZSCAN9	-0.111235	5.5248001	-3.741843	0.0002365	0.0014461	0.037682
PARD6G	-0.143696	5.4092157	-3.741503	0.0002368	0.0014477	0.0365081
PPID	0.0932311	6.2268355	3.7406788	0.0002375	0.0014519	0.0336546
RP11-268G1	-1.162109	0.5624531	-3.739591	0.0002385	0.0014575	0.0298918
LPAR6	0.1244683	5.8138816	3.739161	0.0002389	0.0014596	0.0284039
RPL3L	-0.549853	3.188865	-3.739053	0.000239	0.00146	0.0280303
PRMT1	-0.080927	6.4917392	-3.738883	0.0002391	0.0014606	0.0274416
GEMIN2	-0.109917	5.2840034	-3.738794	0.0002392	0.0014609	0.0271359
NMRK1	0.1221397	5.9731217	3.7383036	0.0002396	0.0014633	0.0254384
RP11-253E3	-0.741306	1.7788432	-3.736532	0.0002412	0.0014727	0.0193135
SCML1	-0.152917	6.0642325	-3.736437	0.0002413	0.001473	0.0189859
NTM	0.8365202	4.063424	3.736243	0.0002415	0.0014738	0.0183143
ARAF	0.0783174	6.5188772	3.7361915	0.0002415	0.0014739	0.0181363
FYTTD1	0.0668091	6.2507071	3.7358474	0.0002418	0.0014755	0.0169468
RP5-875H18	-0.686964	3.7235051	-3.735259	0.0002424	0.0014785	0.0149118
PPDPF	0.1604891	6.643303	3.7350076	0.0002426	0.0014796	0.0140446
ANKRD65	-0.434616	4.958887	-3.734932	0.0002427	0.0014798	0.013782
IMPDH2	-0.089043	6.5921068	-3.734832	0.0002428	0.0014801	0.0134392
RIOK1	-0.089044	5.8893632	-3.734716	0.0002429	0.0014805	0.0130383
PCP2	-0.603632	2.9153135	-3.734388	0.0002432	0.001482	0.011904
CD34	0.1313535	6.3135592	3.7342516	0.0002433	0.001482	0.0114324
RP11-173P1	-0.853033	1.0860056	-3.734238	0.0002433	0.001482	0.0113866
RASL11A	0.2722502	5.2432842	3.7342255	0.0002433	0.001482	0.0113425
SNRPB2	-0.069287	6.2840689	-3.734212	0.0002433	0.001482	0.0112969
TRIM9	0.7526493	3.6337965	3.7341271	0.0002434	0.0014823	0.0110025
CCDC58	-0.09748	5.9181935	-3.733653	0.0002438	0.0014846	0.0093635
GABBR2	-1.252877	2.5717925	-3.733166	0.0002443	0.0014871	0.0076838
PTPN23	-0.063506	6.3952552	-3.732634	0.0002447	0.0014898	0.0058461
DHX9	-0.049707	6.6481726	-3.732018	0.0002453	0.0014929	0.0037195
CTB-1I21.1	-0.856819	-0.69762	-3.731474	0.0002458	0.0014957	0.001839
IPO4	-0.134598	5.3475889	-3.731381	0.0002459	0.001496	0.0015171
PHOSPHO2	-0.269252	4.6753615	-3.731291	0.000246	0.0014962	0.0012088
CTD-2008P7	-0.77061	-0.571493	-3.731012	0.0002462	0.0014975	0.0002435
ITGB1BP2	-0.326704	3.9000629	-3.730957	0.0002463	0.0014976	5.53E-05
TMEM27	0.5195767	4.535957	3.7306556	0.0002466	0.001499	-0.000986
FAM221A	0.3480422	5.0523328	3.7302549	0.0002469	0.001501	-0.002369
CA9	-1.156897	3.7173441	-3.729942	0.0002472	0.0015025	-0.003449
KCNK15	1.2601156	2.1463547	3.729501	0.0002476	0.0015047	-0.004971
NR1H3	-0.101078	6.4305943	-3.729407	0.0002477	0.001505	-0.005296
CCDC160	0.9110874	-0.402044	3.7288066	0.0002483	0.0015081	-0.007367
BMP2K	0.1519296	5.2014184	3.7287324	0.0002483	0.0015083	-0.007623
CTD-2017F1	-0.642654	-0.734912	-3.728248	0.0002488	0.0015107	-0.009296
CACNA2D2	-0.351379	4.2770071	-3.727999	0.000249	0.0015118	-0.010155
RP11-1038A	-0.929264	-0.468754	-3.727975	0.000249	0.0015118	-0.010236
ZNF366	0.5786621	3.8085778	3.7272807	0.0002497	0.0015154	-0.012631
RP11-745A2	-0.836584	0.559606	-3.727228	0.0002497	0.0015155	-0.012812
ADD3	-0.137503	6.1288549	-3.726899	0.00025	0.0015171	-0.013948
GPR75-ASB3	-0.12862	4.797797	-3.726572	0.0002503	0.0015187	-0.015075

RP11-80I15	0.8161202	0.6294239	3.7263547	0.0002505	0.0015196	-0.015825
AP000240.9	-0.652879	2.7446276	-3.726082	0.0002508	0.0015209	-0.016766
SPSB4	1.0200627	0.9217258	3.7256899	0.0002511	0.0015229	-0.018117
HCFC1-AS1	-0.45281	-1.2186	-3.724866	0.0002519	0.0015273	-0.020959
TMEM88B	-1.068167	0.3217265	-3.72478	0.000252	0.0015275	-0.021256
CTC-351M12	-0.25601	4.315692	-3.724148	0.0002526	0.0015309	-0.023433
SNHG14	-0.288704	5.8785244	-3.723973	0.0002528	0.0015316	-0.024035
RP13-516M1	-0.875348	1.4838066	-3.723541	0.0002532	0.0015338	-0.025523
NUCB1	0.0818096	7.0999795	3.7233694	0.0002533	0.0015345	-0.026116
RP11-10017	-0.613895	2.208283	-3.722291	0.0002543	0.0015404	-0.029834
RP11-514F3	-0.541164	-1.289377	-3.721614	0.000255	0.0015441	-0.032166
TSC1	-0.102702	6.0335899	-3.721112	0.0002554	0.0015467	-0.033892
CHORDC1	-0.110314	5.8940883	-3.720054	0.0002565	0.0015525	-0.037538
GNA15	0.2363417	4.9900034	3.7199329	0.0002566	0.0015529	-0.037954
PPP1R2P10	-0.736407	-1.018134	-3.719822	0.0002567	0.0015533	-0.038336
SBNO2	0.077878	6.3494768	3.7196796	0.0002568	0.0015539	-0.038826
COG7	0.0791148	5.9400801	3.7196066	0.0002569	0.0015541	-0.039078
CCKBR	-0.849439	-0.733001	-3.718995	0.0002575	0.0015571	-0.041185
GPC4	0.4245635	5.2039163	3.7189863	0.0002575	0.0015571	-0.041213
CHD7	-0.13051	5.9807606	-3.71892	0.0002575	0.0015572	-0.04144
RFPL1S	0.9058931	2.1430727	3.7186042	0.0002578	0.0015588	-0.042529
FAM71E1	-0.280213	4.7360718	-3.718321	0.0002581	0.0015602	-0.043504
AC008154.5	0.604811	-1.10518	3.7177676	0.0002586	0.0015631	-0.045408
NUDCD1	-0.121726	5.8625513	-3.717607	0.0002588	0.0015638	-0.045961
SOHLH1	-0.908135	-0.51045	-3.717446	0.0002589	0.0015645	-0.046514
RP11-345K9	-0.69491	-1.087801	-3.717022	0.0002594	0.0015667	-0.047975
CTD-2562J1	-0.76021	-0.746253	-3.716115	0.0002602	0.0015717	-0.051094
RRP15	-0.087447	6.0282302	-3.715853	0.0002605	0.001573	-0.051997
ANXA6	0.1362239	6.7866372	3.7157721	0.0002606	0.0015731	-0.052274
CREBRF	0.0918589	5.9540064	3.7157481	0.0002606	0.0015731	-0.052356
CTD-2302E2	-0.777239	1.0296851	-3.715701	0.0002606	0.0015731	-0.052517
GNPAT	-0.081759	6.5168792	-3.715428	0.0002609	0.0015744	-0.053457
IL33	0.2986467	5.5058083	3.7151846	0.0002611	0.0015754	-0.054295
SLITRK3	1.35612	0.8844199	3.715172	0.0002611	0.0015754	-0.054338
RP11-284B1	0.8660153	0.8611806	3.7150679	0.0002612	0.0015757	-0.054696
CCDC108	-0.894088	1.9968158	-3.714917	0.0002614	0.0015764	-0.055217
POTEH	-0.542976	-1.253579	-3.714814	0.0002615	0.0015767	-0.05557
WNK4	-0.610584	4.8163676	-3.714716	0.0002616	0.001577	-0.055907
GLDN	-0.707289	4.3213334	-3.714491	0.0002618	0.0015778	-0.056682
RYR3	-0.297319	3.8370524	-3.714455	0.0002618	0.0015778	-0.056804
RHOXF2	-0.956254	-0.789588	-3.714442	0.0002618	0.0015778	-0.056848
CTB-75G16.	-0.709973	1.7130509	-3.714105	0.0002622	0.0015795	-0.058009
DLEU7-AS1	-0.931835	1.2091164	-3.713278	0.000263	0.0015841	-0.060852
PPP2R4	0.0752539	6.6811879	3.7131902	0.0002631	0.0015844	-0.061153
TUFT1	-0.140222	5.8695392	-3.712904	0.0002633	0.0015858	-0.062137
LGALS1	0.1195265	6.6290541	3.7128038	0.0002634	0.0015861	-0.062481
ARF4	0.0668792	6.7927405	3.7126372	0.0002636	0.0015868	-0.063054
BAI3	1.0235117	2.0430418	3.7122831	0.0002639	0.0015887	-0.064271
BTBD19	0.1786826	5.1061227	3.7115549	0.0002647	0.0015927	-0.066774
AC074183.3	-0.93382	0.1291503	-3.711418	0.0002648	0.0015932	-0.067244
NACAP1	-0.453009	2.869062	-3.711116	0.0002651	0.0015948	-0.068281

RCCD1	-0.099359	5.7828319	-3.710939	0.0002653	0.0015955	-0.068892
SMYD5	-0.079595	6.1081964	-3.710677	0.0002655	0.0015968	-0.069792
RP11-445P1	-0.620329	-1.120451	-3.710533	0.0002657	0.0015974	-0.070284
ADI1	0.1398738	6.9205038	3.7097813	0.0002664	0.001601	-0.072868
PSMB2	0.077814	6.7003778	3.7097712	0.0002664	0.001601	-0.072902
POTEF	-0.931704	0.0056624	-3.709768	0.0002664	0.001601	-0.072912
TMEM169	-0.32468	4.7907286	-3.709748	0.0002664	0.001601	-0.072984
HDAC4	-0.101927	5.6996162	-3.709432	0.0002668	0.0016025	-0.074066
A2MP1	0.6967328	2.7973399	3.7094107	0.0002668	0.0016025	-0.07414
RP11-230C9	-0.635382	2.4530194	-3.70918	0.000267	0.0016036	-0.074934
RBPMS-AS1	0.3692358	3.9274603	3.7091006	0.0002671	0.0016038	-0.075206
ZBTB40	-0.080139	5.9234654	-3.708655	0.0002675	0.0016062	-0.076734
RP11-728K2	0.8743546	0.7279018	3.7078381	0.0002683	0.0016108	-0.079541
CTD-2521M2	0.3095663	5.2313413	3.7076325	0.0002685	0.0016117	-0.080247
ZNF100	-0.190776	5.0030893	-3.707205	0.000269	0.001614	-0.081716
BLOC1S4	-0.086798	5.7869209	-3.706141	0.00027	0.0016201	-0.085366
AC090286.2	-0.681309	-0.490799	-3.705675	0.0002705	0.0016226	-0.086967
ST7-OT4	-0.827059	0.8525782	-3.705612	0.0002705	0.0016228	-0.087184
AMN	-0.62505	5.5904518	-3.705371	0.0002708	0.0016239	-0.08801
AC002076.1	-0.830647	-0.631069	-3.705242	0.0002709	0.0016243	-0.088451
RPL5P9	-0.82014	0.6891397	-3.705222	0.0002709	0.0016243	-0.088522
METTL12	-0.192304	4.7683687	-3.704953	0.0002712	0.0016257	-0.089444
AC005822.1	-0.828222	0.8846209	-3.704702	0.0002715	0.0016269	-0.090304
MEGF10	1.0657038	1.0937075	3.7045555	0.0002716	0.0016275	-0.090808
TRIM60P18	-0.485934	3.6828736	-3.704357	0.0002718	0.0016284	-0.09149
RAB12	0.0711827	6.1259589	3.7042221	0.0002719	0.001629	-0.091952
WI2-89031E	-0.848255	-0.080076	-3.703947	0.0002722	0.0016304	-0.092894
EGFL7	0.1453116	6.3042839	3.7036784	0.0002725	0.0016317	-0.093817
RND2	-0.649384	4.5964183	-3.703185	0.000273	0.0016342	-0.09551
ACACB	0.1607235	6.4236436	3.7031818	0.000273	0.0016342	-0.09552
TMEM40	1.087311	1.7867834	3.7028922	0.0002733	0.0016357	-0.096513
TMEM183A	-0.070348	6.1776812	-3.702712	0.0002735	0.0016363	-0.09713
CARD16	0.1951818	5.2036993	3.7027066	0.0002735	0.0016363	-0.09715
RP11-787P2	-0.584671	-1.16089	-3.702627	0.0002736	0.0016365	-0.097424
ACKR1	1.0264899	3.8642169	3.702149	0.000274	0.0016391	-0.099062
RPL37P23	-0.749095	1.7789165	-3.702104	0.0002741	0.0016391	-0.099218
TMEM219	0.0886976	6.5750787	3.7019889	0.0002742	0.0016395	-0.099611
ZNF37A	-0.091915	5.8471138	-3.701836	0.0002744	0.0016402	-0.100137
SPIN3	-0.182419	4.8266754	-3.701344	0.0002749	0.0016429	-0.101821
MSTO1	-0.119401	5.7951188	-3.701167	0.000275	0.0016437	-0.102429
RP5-1085F1	-0.163535	5.2216054	-3.70112	0.0002751	0.0016437	-0.102588
RP11-344P1	-0.565768	3.733058	-3.701049	0.0002752	0.0016439	-0.102833
SFRP4	0.5658735	4.8811773	3.7007187	0.0002755	0.0016456	-0.103966
RP11-143N1	1.0153984	0.6855336	3.7003195	0.0002759	0.0016478	-0.105334
PKD1	0.081767	6.2467855	3.7002697	0.0002759	0.0016478	-0.105505
RNU1-138P	0.6665962	-0.971914	3.7001588	0.0002761	0.0016482	-0.105884
RP11-6F2.5	1.0254345	1.7332922	3.7000279	0.0002762	0.0016488	-0.106333
DDX49	-0.0802	6.3652808	-3.699979	0.0002762	0.0016488	-0.1065
C19orf47	-0.085164	5.7786734	-3.699937	0.0002763	0.0016488	-0.106644
AC092933.3	-0.656705	-0.721969	-3.699584	0.0002766	0.0016506	-0.107855
SLBP	-0.071193	6.2431034	-3.699521	0.0002767	0.0016508	-0.108071

PA2G4	-0.063618	6.6556402	-3.699313	0.0002769	0.0016518	-0.108783
LAMB2	0.1057551	6.769055	3.6991357	0.0002771	0.0016525	-0.109391
RP4-584D14	-0.846237	0.4847578	-3.699101	0.0002771	0.0016525	-0.109509
PHLDA3	0.3205542	5.7534755	3.6987664	0.0002775	0.0016543	-0.110656
TPBG	0.5026042	4.5789979	3.698726	0.0002775	0.0016543	-0.110794
LYVE1	0.3533215	4.9341973	3.6985572	0.0002777	0.001655	-0.111373
CLIC2	0.196011	5.464388	3.6985174	0.0002777	0.001655	-0.111509
IGF2	-0.502664	6.7160224	-3.698215	0.000278	0.0016566	-0.112546
IGHV5-78	-0.966861	0.2915252	-3.697894	0.0002784	0.0016583	-0.113643
HEATR1	-0.103916	6.1365	-3.697293	0.000279	0.0016617	-0.115705
NUP62	-0.072527	6.3606127	-3.697049	0.0002792	0.0016629	-0.11654
TNFRSF10B	0.0940398	6.3715156	3.6969318	0.0002794	0.0016634	-0.11694
GPR65	0.2840228	4.7537531	3.6963269	0.00028	0.0016668	-0.119012
DOC2GP	-0.623552	2.8836346	-3.696208	0.0002801	0.0016673	-0.11942
ZNF595	-0.162174	5.2930159	-3.695953	0.0002804	0.0016686	-0.120294
ARHGAP5	0.0793407	6.2736002	3.6951183	0.0002812	0.0016734	-0.12315
SRGN	0.1666387	6.1936657	3.6947534	0.0002816	0.0016752	-0.124399
CISH	0.1713993	6.0856815	3.6947444	0.0002816	0.0016752	-0.12443
ANKRD26P1	-1.099534	-0.439935	-3.694621	0.0002818	0.0016757	-0.124851
AC007292.3	-0.306499	3.273798	-3.694342	0.0002821	0.0016771	-0.125807
CCDC114	-0.736467	2.7114402	-3.694185	0.0002822	0.0016778	-0.126344
RP11-248E9	0.8746867	-0.508967	3.6938827	0.0002825	0.0016794	-0.127379
GS1-279B7.	-0.618102	2.1608446	-3.693124	0.0002833	0.0016837	-0.129974
TBCC	-0.081608	5.9529854	-3.693106	0.0002833	0.0016837	-0.130037
FZD10-AS1	0.8909472	2.1226477	3.6929656	0.0002835	0.0016843	-0.130517
WWC2	0.1304317	6.0047716	3.6926597	0.0002838	0.0016859	-0.131564
NAA16	-0.100013	5.5449411	-3.692511	0.000284	0.0016866	-0.132071
SHC4	0.7183522	2.4391172	3.6914992	0.000285	0.0016926	-0.135534
FRMD1	-1.121553	2.0376215	-3.691174	0.0002854	0.0016944	-0.136645
RHBG	-0.907998	4.68975	-3.69099	0.0002856	0.0016953	-0.137274
RP11-234K1	0.3730085	-1.351291	3.6909267	0.0002856	0.0016954	-0.137491
AC005355.3	-0.798454	-0.047741	-3.690256	0.0002863	0.0016992	-0.139784
CTD-2270N2	-0.470428	3.1709926	-3.690228	0.0002864	0.0016992	-0.139882
PAXBP1	-0.079958	5.9262437	-3.689508	0.0002871	0.0017034	-0.142343
JAK2	0.1509713	5.4439587	3.6893192	0.0002873	0.0017043	-0.142988
TBRG1	0.0749249	5.9303569	3.6890546	0.0002876	0.0017057	-0.143893
LRCH1	0.110849	5.8601098	3.6888509	0.0002878	0.0017065	-0.144589
INPP5A	-0.086002	6.0018807	-3.688832	0.0002878	0.0017065	-0.144655
RP11-80F22	-0.551781	-1.205451	-3.688726	0.000288	0.0017065	-0.145017
RP11-136F1	0.3313918	-1.440792	3.688723	0.000288	0.0017065	-0.145026
RP11-388C1	-0.683541	2.4973695	-3.688711	0.000288	0.0017065	-0.145066
USP44	-0.829107	2.1745741	-3.688441	0.0002883	0.0017079	-0.14599
DLG5-AS1	-0.440903	3.409735	-3.687707	0.000289	0.0017123	-0.148497
RP11-464D2	-0.505953	3.0391397	-3.68721	0.0002896	0.0017149	-0.150196
PNRC1	0.0956368	6.6003582	3.6871975	0.0002896	0.0017149	-0.150239
PTGFRN	0.1214399	6.3127906	3.6870947	0.0002897	0.0017151	-0.150591
RP11-366H4	-0.72031	-1.030179	-3.687088	0.0002897	0.0017151	-0.150615
C12orf49	-0.116989	6.1197982	-3.686877	0.0002899	0.0017161	-0.151333
CHN1	0.2374456	4.9991852	3.6865451	0.0002903	0.0017177	-0.152468
UBOX5-AS1	-0.620942	2.1599737	-3.686544	0.0002903	0.0017177	-0.152471
RASSF2	0.1708949	5.643086	3.686037	0.0002908	0.0017205	-0.154204

ANO3	1.0091122	2.7537836	3.6860126	0.0002908	0.0017205	-0.154287
KRT4	1.0234457	-0.410525	3.6857457	0.0002911	0.0017219	-0.155199
CCHCR1	-0.108315	6.1237378	-3.685433	0.0002915	0.0017236	-0.156266
TATDN2	-0.079292	6.0036279	-3.685313	0.0002916	0.0017241	-0.156675
LTC4S	0.7113353	-0.493173	3.685038	0.0002919	0.0017256	-0.157616
TLR7	0.4092786	4.3180039	3.6844729	0.0002925	0.0017289	-0.159546
CPM	0.145635	6.340604	3.6839307	0.0002931	0.001732	-0.161397
AGTPBP1	0.1114266	5.5368548	3.6835989	0.0002934	0.0017339	-0.16253
TTLL7	0.4135963	4.7722728	3.6834753	0.0002936	0.0017344	-0.162952
IL1RL2	0.8091654	4.0711752	3.6833621	0.0002937	0.0017348	-0.163338
TRIM36	0.54334	3.5736114	3.6831665	0.0002939	0.0017358	-0.164006
RORA	0.1548488	6.0649074	3.6827519	0.0002944	0.0017382	-0.165421
SCARNA12	-0.732583	1.2682621	-3.682372	0.0002948	0.0017403	-0.166716
TM9SF4	-0.057152	6.5955169	-3.682211	0.0002949	0.0017411	-0.167267
GOLGA8B	-0.173918	5.9089387	-3.681888	0.0002953	0.0017429	-0.168371
ANKK1	-0.906201	1.3440675	-3.681575	0.0002956	0.0017446	-0.169438
STARD4-AS1	-0.443868	4.2647508	-3.681295	0.0002959	0.0017461	-0.170392
CMPK2	0.2240861	5.350802	3.6812039	0.000296	0.0017463	-0.170704
TMEM229B	0.2359993	5.0553494	3.6811562	0.0002961	0.0017463	-0.170866
PCDHB15	0.4369977	4.2353287	3.6811271	0.0002961	0.0017463	-0.170966
RP11-218C1	-0.603012	2.4978047	-3.681026	0.0002962	0.0017467	-0.171311
MAGEF1	-0.102705	6.0751378	-3.680941	0.0002963	0.001747	-0.171602
AC007750.5	0.9223189	0.8206173	3.6808841	0.0002964	0.0017471	-0.171795
C1QC	0.16612	6.5525257	3.6804671	0.0002968	0.0017495	-0.173217
TMEM207	-0.675792	-1.122817	-3.680388	0.0002969	0.0017497	-0.173487
CTD-2545H1	-0.71604	4.5661032	-3.679969	0.0002974	0.0017521	-0.174916
CTD-2571L2	-0.651819	1.7400348	-3.679749	0.0002976	0.001753	-0.175668
DCHS1	0.168626	5.6153287	3.679735	0.0002976	0.001753	-0.175715
RP11-426C2	0.5128502	3.1284463	3.6795438	0.0002979	0.001754	-0.176367
SFRP2	1.3397741	1.6951682	3.679294	0.0002981	0.0017553	-0.177219
EXOC6	-0.081632	6.0340794	-3.678949	0.0002985	0.0017573	-0.178395
IPO5	-0.083019	6.613085	-3.678883	0.0002986	0.0017574	-0.178619
VWA1	0.121591	6.6420833	3.6785183	0.000299	0.0017595	-0.179864
UGT8	-1.189206	1.5743725	-3.678169	0.0002994	0.0017615	-0.181053
LAIR1	0.2068828	5.7002276	3.6779841	0.0002996	0.0017624	-0.181686
RP11-464F9	-0.706289	2.1571624	-3.677903	0.0002997	0.0017626	-0.181961
ARHGAP39	-0.192949	5.2586854	-3.677478	0.0003001	0.0017651	-0.183412
LNPEP	0.0841104	6.0916776	3.6770653	0.0003006	0.0017675	-0.184818
LZTS3	-0.145132	5.9448165	-3.676717	0.000301	0.0017695	-0.186003
MSNP1	0.8239014	-0.010904	3.6761686	0.0003016	0.0017728	-0.187874
MT-ND4L	0.1381894	7.1532789	3.6752155	0.0003026	0.0017784	-0.191122
AKT1	0.069172	6.5903667	3.675173	0.0003027	0.0017784	-0.191266
ST6GAL2-IT	0.3910285	-1.342108	3.6751672	0.0003027	0.0017784	-0.191286
GPT2	0.221203	6.5898224	3.67506	0.0003028	0.0017787	-0.191651
HIPK1	0.0865754	6.2711577	3.6750418	0.0003028	0.0017787	-0.191713
TNFSF8	0.4076779	4.1504482	3.6744526	0.0003035	0.0017823	-0.193721
PPAT	-0.114259	5.6770482	-3.674262	0.0003037	0.0017832	-0.194371
NAA40	-0.10124	5.8194402	-3.673383	0.0003047	0.0017883	-0.197363
RP11-218F4	-0.813715	-0.90181	-3.67337	0.0003047	0.0017883	-0.197409
AC002310.1	-0.762971	-0.49762	-3.673358	0.0003047	0.0017883	-0.197449
RP11-135A1	-0.789997	2.570972	-3.673223	0.0003049	0.0017887	-0.197908

ZNF709	-0.562992	2.4059788	-3.673209	0.0003049	0.0017887	-0.197957
SRRM2	0.0585287	6.9539411	3.6731672	0.0003049	0.0017887	-0.198098
TRIM44	0.066211	6.4412841	3.6728754	0.0003053	0.0017902	-0.199092
AJ239322.1	-0.569953	-1.17964	-3.672854	0.0003053	0.0017902	-0.199164
OSBPL10	0.2710456	5.1149529	3.6720688	0.0003062	0.001795	-0.201839
RP11-296A1	-0.772926	1.0736932	-3.671753	0.0003065	0.0017968	-0.202912
RP11-25K21	-0.414356	-1.329657	-3.671367	0.0003069	0.001799	-0.204227
CTD-2017D1	-0.399694	4.2408391	-3.671333	0.000307	0.001799	-0.204344
NECAP2	0.06614	6.328052	3.6711125	0.0003072	0.0018002	-0.205094
RAD1P1	-0.541969	-1.208315	-3.670648	0.0003078	0.001803	-0.206675
EFCAB1	1.0129036	1.508339	3.6705894	0.0003078	0.0018031	-0.206874
RBM12B-AS1	-0.664568	2.2894162	-3.670401	0.000308	0.001804	-0.207516
PAPD5	0.094632	5.8513465	3.6701467	0.0003083	0.001805	-0.208381
RPS7P14	-0.705971	1.4413233	-3.670142	0.0003083	0.001805	-0.208397
AC009014.3	-1.344109	3.0499685	-3.670119	0.0003084	0.001805	-0.208476
XXbac-BPGE	-0.318859	3.7931885	-3.669996	0.0003085	0.0018056	-0.208893
USP36	-0.064836	6.2750924	-3.669935	0.0003086	0.0018057	-0.209101
NCBP2	-0.056706	6.3709888	-3.669667	0.0003089	0.0018072	-0.210013
AC007277.3	-1.025758	0.7439549	-3.669607	0.0003089	0.0018073	-0.210217
TP53TG3D	-0.774412	-0.714171	-3.669455	0.0003091	0.001808	-0.210734
ACTR3B	-0.11625	5.4632038	-3.668889	0.0003098	0.0018114	-0.212661
AC024937.6	-0.869283	1.6630516	-3.668856	0.0003098	0.0018114	-0.212774
RP11-12K22	-0.756441	-0.88249	-3.668454	0.0003102	0.0018138	-0.21414
ZNHIT3	-0.073337	6.052354	-3.668071	0.0003107	0.001816	-0.215444
RP11-104J2	-0.833492	1.9385823	-3.667613	0.0003112	0.0018188	-0.217
AC006116.1	-0.816975	0.0534978	-3.667556	0.0003113	0.0018189	-0.217195
RNA5SP383	-0.796669	0.6132751	-3.667373	0.0003115	0.0018198	-0.217817
PSG5	0.7821492	-0.785247	3.6672241	0.0003117	0.0018205	-0.218323
LINC00528	-0.324987	4.307354	-3.667122	0.0003118	0.0018209	-0.218669
RP11-666A8	-0.824906	1.1551438	-3.667032	0.0003119	0.001821	-0.218976
AC005307.1	-0.737157	-0.999597	-3.667022	0.0003119	0.001821	-0.219009
SRBD1	0.0815862	5.7623517	3.6664274	0.0003126	0.0018247	-0.221032
SEL1L3	0.2270734	6.1628918	3.6658912	0.0003132	0.0018279	-0.222855
RP13-644M1	-0.972174	-0.214475	-3.665856	0.0003132	0.0018279	-0.222975
EHBP1	0.0968069	6.3402132	3.665803	0.0003133	0.001828	-0.223155
KIAA1045	0.8046539	2.2965945	3.6656594	0.0003134	0.0018287	-0.223643
AMOT	0.3521972	5.1098918	3.6648854	0.0003143	0.0018336	-0.226274
RPS27P29	-0.801398	0.1238802	-3.66474	0.0003145	0.001834	-0.226769
B4GALNT1	0.4810533	4.9916206	3.6647373	0.0003145	0.001834	-0.226777
ACO1	0.1150471	6.7064072	3.6646175	0.0003146	0.0018345	-0.227184
OTUD3	-0.150021	5.1613463	-3.664484	0.0003148	0.0018351	-0.227637
SP110	0.1237253	5.3537715	3.6641235	0.0003152	0.0018372	-0.228863
snoZ196	-0.760547	0.0422049	-3.663975	0.0003154	0.0018379	-0.229368
RP11-396F2	0.5277216	3.6985336	3.6638952	0.0003155	0.0018382	-0.229639
CTC-429P9.	-0.160442	5.1340246	-3.663584	0.0003158	0.00184	-0.230695
HNRNPH1	-0.059935	6.7780664	-3.663386	0.0003161	0.001841	-0.231367
TAF1L	-0.547054	-1.09409	-3.663201	0.0003163	0.001842	-0.231998
CTD-2085J2	-0.492223	-1.155462	-3.663025	0.0003165	0.0018429	-0.232597
HMGN2P15	-0.697048	2.7209855	-3.662613	0.000317	0.0018454	-0.233995
HOXC10	-1.328275	0.4991503	-3.662526	0.0003171	0.0018457	-0.234291
NAAA	0.163811	5.9036442	3.6624758	0.0003171	0.0018457	-0.234461

ATP5B	0.0738756	7.1952564	3.6622682	0.0003174	0.0018468	-0.235166
TPRG1L	0.1133116	6.4827793	3.6621098	0.0003176	0.0018476	-0.235704
MOCOS	0.1654696	6.1382181	3.6620203	0.0003177	0.0018479	-0.236008
RP11-78L16	-1.197741	0.162483	-3.661552	0.0003182	0.0018508	-0.237598
CACFD1	-0.094532	6.5193576	-3.661283	0.0003185	0.0018523	-0.238511
ZNF680P1	-0.528143	-1.151753	-3.661122	0.0003187	0.0018531	-0.239057
RP11-653B1	-0.436485	-1.318805	-3.66096	0.0003189	0.0018539	-0.239609
RP11-91K8.	-0.800055	-0.160667	-3.660673	0.0003192	0.0018556	-0.240584
TRPA1	1.0822277	1.4184547	3.6605047	0.0003194	0.0018564	-0.241155
RP11-285F1	-0.886017	1.0233755	-3.660422	0.0003195	0.0018567	-0.241435
SPRED1	0.1358295	5.8382442	3.6602052	0.0003198	0.0018579	-0.242172
RP11-367N1	-0.652148	2.2019923	-3.660138	0.0003199	0.001858	-0.242401
CARD10	-0.111698	6.3063325	-3.659878	0.0003202	0.0018595	-0.243284
DCX	1.1287401	1.4697543	3.6597343	0.0003203	0.0018601	-0.24377
CMKLR1	0.2010128	5.5223264	3.6597008	0.0003204	0.0018601	-0.243884
CTD-2012K1	0.363602	4.777399	3.6596165	0.0003205	0.0018604	-0.24417
MTF2	-0.088161	5.7707514	-3.658829	0.0003214	0.0018655	-0.246844
RP11-80H5.	-0.79145	1.1761761	-3.658531	0.0003217	0.0018672	-0.247855
CTC-471J1.	-0.62609	2.6419878	-3.658443	0.0003218	0.0018673	-0.248154
FBXW8	-0.083203	5.7376345	-3.65843	0.0003219	0.0018673	-0.248197
CTD-2532D1	-0.625346	-0.992945	-3.657881	0.0003225	0.0018705	-0.250059
AQP4-AS1	0.9403552	1.5419551	3.657877	0.0003225	0.0018705	-0.250073
TTC12	0.1112837	5.4412523	3.6575287	0.0003229	0.0018726	-0.251255
CLEC7A	0.3460473	4.963469	3.6571401	0.0003234	0.001875	-0.252574
GNGT1	-1.250157	0.1749097	-3.656891	0.0003237	0.0018764	-0.253417
HIVEP2	0.1146811	5.8184953	3.6567723	0.0003238	0.0018769	-0.253821
RP11-159J3	-0.811819	3.7250729	-3.656271	0.0003244	0.0018801	-0.25552
AC079776.1	-0.617018	-0.969344	-3.65609	0.0003246	0.001881	-0.256136
SSH1	0.06376	6.1917862	3.6555281	0.0003253	0.0018841	-0.258041
AC004447.2	-0.121551	5.6435907	-3.655506	0.0003253	0.0018841	-0.258115
LMO1	0.8067524	-0.758948	3.6555046	0.0003253	0.0018841	-0.258121
ZNF438	0.1055416	5.5363496	3.6554232	0.0003254	0.0018844	-0.258397
NUB1	0.0627857	6.4651355	3.6552059	0.0003257	0.0018856	-0.259134
EXOSC1	-0.082655	5.9794604	-3.654902	0.000326	0.0018874	-0.260165
FNBP4	-0.065509	6.2005133	-3.654293	0.0003268	0.0018913	-0.262228
AC069513.4	-0.81996	1.6352443	-3.654247	0.0003268	0.0018913	-0.262386
ELOVL3	-0.998497	1.5561622	-3.654106	0.000327	0.0018918	-0.262864
ZBED1	0.0810104	6.4840334	3.6541004	0.000327	0.0018918	-0.262882
TRIM14	0.1019888	6.2354377	3.6538468	0.0003273	0.0018932	-0.263742
RP11-20J15	0.9596383	1.3909371	3.653248	0.000328	0.0018971	-0.265771
EIF4BP7	-0.153878	4.9166111	-3.653028	0.0003283	0.0018983	-0.266517
TGFB2	0.4629488	4.8897254	3.6524355	0.000329	0.0019021	-0.268525
TRAF7	0.0717464	6.4847509	3.6521432	0.0003294	0.0019038	-0.269515
SSRP1	-0.05691	6.6340988	-3.651605	0.00033	0.0019073	-0.271338
RPL27A	-0.083521	7.1456896	-3.65108	0.0003306	0.0019107	-0.273117
LAMB3	0.2568803	5.8273971	3.650974	0.0003308	0.0019111	-0.273476
MIER2	-0.117429	5.7949556	-3.650838	0.0003309	0.0019117	-0.273935
APOM	-0.289469	6.7218129	-3.650716	0.0003311	0.0019123	-0.274349
KIAA0196-A	-0.765899	1.1275049	-3.648329	0.000334	0.0019287	-0.282432
BEGAIN	0.8583638	3.3218641	3.6480433	0.0003343	0.0019305	-0.2834
SNX24	0.0874571	5.7108465	3.6478255	0.0003346	0.0019317	-0.284137

HTRA2	-0.068787	5.9924305	-3.647643	0.0003348	0.0019322	-0.284754
C1DP1	-1.303402	1.3305668	-3.647634	0.0003348	0.0019322	-0.284786
RPL7P15	-0.771593	-0.25818	-3.647626	0.0003348	0.0019322	-0.284813
PDHA1	0.0809055	6.5904264	3.6472956	0.0003352	0.0019342	-0.285931
C9orf91	0.1167081	5.6694533	3.6470215	0.0003356	0.0019358	-0.286859
SCGN	-0.629173	4.9488074	-3.646928	0.0003357	0.001936	-0.287176
AC005498.4	-0.579881	-1.038503	-3.646915	0.0003357	0.001936	-0.287217
CDC123	-0.063779	6.3633214	-3.646644	0.000336	0.0019376	-0.288135
RP11-592N2	-0.410909	3.2619642	-3.64598	0.0003369	0.001942	-0.290382
TOMM20	-0.075872	6.8703022	-3.645728	0.0003372	0.0019435	-0.291237
SNORA73B	-0.818163	2.0768553	-3.645652	0.0003373	0.0019437	-0.291493
EPSTI1	0.2560862	5.5662651	3.6454073	0.0003376	0.0019451	-0.29232
DPY19L2P2	-0.652723	3.0960771	-3.645114	0.0003379	0.0019469	-0.293313
UHRF2	-0.077572	5.9777862	-3.644578	0.0003386	0.0019504	-0.295126
VWA8-AS1	-0.821462	0.474712	-3.644126	0.0003391	0.0019533	-0.296654
LRRC28	-0.136526	6.1405345	-3.644068	0.0003392	0.0019534	-0.29685
LINC00238	1.1035851	0.7197819	3.643438	0.00034	0.0019576	-0.298981
EEF1A2	0.7779344	5.3967105	3.6433839	0.0003401	0.0019577	-0.299163
RP5-983L19	-0.533832	-1.155915	-3.64277	0.0003408	0.0019618	-0.301241
BRWD1-AS2	-0.400969	3.4290773	-3.642527	0.0003411	0.0019628	-0.302061
RP11-729I1	0.8666179	0.0549789	3.6425199	0.0003411	0.0019628	-0.302085
HIST3H2BB	-1.169315	1.1683493	-3.642502	0.0003412	0.0019628	-0.302146
AC092198.1	-0.954652	0.1063849	-3.642191	0.0003416	0.0019647	-0.303196
RNF216	-0.062033	6.2863577	-3.64184	0.000342	0.0019669	-0.304383
SEPT7	0.0474839	6.5326372	3.6413645	0.0003426	0.00197	-0.30599
CDC42	0.0581126	6.7389934	3.6412965	0.0003427	0.0019702	-0.30622
CD9	0.1455921	6.2990308	3.6412132	0.0003428	0.0019705	-0.306501
ZNF674-AS1	-0.149225	4.8465147	-3.641085	0.0003429	0.0019711	-0.306935
SMCO3	0.4640068	3.6491504	3.6408199	0.0003433	0.0019727	-0.30783
LINC00601	0.8395355	-0.837476	3.6401733	0.0003441	0.001977	-0.310015
TMEM223	-0.08537	5.9904939	-3.639786	0.0003446	0.0019795	-0.311324
CTD-3214H1	-0.746859	2.0281619	-3.639702	0.0003447	0.0019798	-0.311607
ANGPTL7	0.8407124	-0.742598	3.6392849	0.0003452	0.0019825	-0.313017
RP11-466P2	-1.112427	0.8560347	-3.63868	0.000346	0.0019865	-0.315059
GPR64	0.5166326	4.5496545	3.6386461	0.000346	0.0019865	-0.315174
AC098973.2	-1.516996	1.0685424	-3.638092	0.0003467	0.0019902	-0.317044
RP11-527J8	-0.352406	3.1713983	-3.638015	0.0003468	0.0019903	-0.317304
AC007182.6	1.0401098	1.5583512	3.6379941	0.0003468	0.0019903	-0.317376
TMEM55B	-0.070107	6.1027705	-3.637709	0.0003472	0.0019921	-0.318339
RP11-62F24	0.8017329	-0.490862	3.6375312	0.0003474	0.0019931	-0.318939
RP11-466H1	-0.189614	5.2914609	-3.63689	0.0003482	0.0019974	-0.321103
ZSWIM3	-0.101774	5.1642758	-3.636852	0.0003483	0.0019974	-0.32123
SCRG1	0.8731061	1.0222052	3.6367101	0.0003485	0.0019978	-0.321711
CEPT1	0.100735	5.9368214	3.6366617	0.0003485	0.0019978	-0.321874
COG4	0.0695535	6.3462576	3.6366249	0.0003486	0.0019978	-0.321999
PEX5L	-0.861885	1.3999946	-3.63662	0.0003486	0.0019978	-0.322015
RAMP2	0.1742561	5.7780022	3.6364127	0.0003488	0.001999	-0.322715
MSL1	-0.049363	6.4247358	-3.636239	0.0003491	0.0019998	-0.323302
UBQLN4	-0.089016	6.3262368	-3.636224	0.0003491	0.0019998	-0.323352
LSMEM1	0.263259	4.3809849	3.6355303	0.00035	0.0020044	-0.325693
CTD-2541J1	1.0278302	1.5562936	3.6354962	0.00035	0.0020044	-0.325808

ZGRF1	-0.152127	4.8695902	-3.635078	0.0003505	0.0020072	-0.32722
PJA2	0.0834594	6.5584075	3.6339436	0.000352	0.0020152	-0.331047
RP11-521B2	-0.255874	3.9400806	-3.633679	0.0003523	0.0020168	-0.33194
RP11-465I4	-0.480813	-1.284062	-3.63356	0.0003525	0.0020173	-0.332339
BTNL10	-0.821101	0.8557603	-3.633528	0.0003525	0.0020173	-0.332449
CTD-252902	-1.092792	1.1218407	-3.633234	0.0003529	0.0020191	-0.333441
AC005306.3	-0.710992	1.7164234	-3.632913	0.0003533	0.0020212	-0.334524
TBX10	-0.816892	3.3081974	-3.63204	0.0003544	0.0020273	-0.337468
C3orf35	-0.354486	3.508632	-3.631792	0.0003548	0.0020287	-0.338304
ZNF251	-0.110808	5.8421471	-3.631768	0.0003548	0.0020287	-0.338386
CHML	-0.174437	5.7952249	-3.631574	0.000355	0.0020298	-0.339039
HCN3	-0.209837	6.0812962	-3.630854	0.000356	0.0020348	-0.341466
UCKL1-AS1	-0.538749	2.9907772	-3.630091	0.000357	0.0020401	-0.344039
C2-AS1	-0.672174	3.2274906	-3.629812	0.0003573	0.0020419	-0.344979
ATP5A1	0.0760139	7.0962093	3.6296855	0.0003575	0.0020425	-0.345405
PCSK9	-0.285977	6.2683534	-3.629445	0.0003578	0.002044	-0.346216
NAT14	-0.193974	5.4117025	-3.629224	0.0003581	0.0020453	-0.34696
SNORD7	-0.80052	0.3647157	-3.629029	0.0003583	0.0020464	-0.347617
RP11-464F9	-0.591113	2.7101357	-3.628983	0.0003584	0.0020465	-0.347772
NPM1P27	-0.230382	4.5570589	-3.62888	0.0003585	0.0020468	-0.34812
NGFRAP1	-0.175275	6.4438291	-3.628854	0.0003586	0.0020468	-0.348208
HOXC13	-1.091745	0.0428203	-3.628461	0.0003591	0.0020492	-0.349531
IFT57	0.176878	5.5890089	3.6284411	0.0003591	0.0020492	-0.349598
ANKRD30BP3	-0.703626	-1.090352	-3.628235	0.0003594	0.0020504	-0.350293
SCARNA9	-0.573369	2.5520654	-3.627956	0.0003597	0.0020522	-0.351233
GMNC	1.1185433	3.2000993	3.6272706	0.0003606	0.002057	-0.353541
RP11-50C13	-0.608352	-0.925648	-3.626978	0.000361	0.0020589	-0.354526
GTF2IRD2	0.1996056	4.6680429	3.6265705	0.0003616	0.0020616	-0.355898
VENTXP5	-0.753753	-0.843539	-3.626098	0.0003622	0.0020645	-0.357491
CTDSPL2	-0.075011	5.9644705	-3.626096	0.0003622	0.0020645	-0.357495
KMT2E-AS1	-0.178988	5.0190955	-3.62581	0.0003626	0.0020663	-0.358459
PABPC3	-0.491426	3.1840922	-3.625704	0.0003627	0.0020668	-0.358816
LYRM9	0.1524779	5.3165149	3.6250594	0.0003636	0.0020712	-0.360986
CANT1	-0.061537	6.3627666	-3.62504	0.0003636	0.0020712	-0.36105
RP11-113C1	-0.641867	-0.657367	-3.624876	0.0003638	0.0020721	-0.361604
LARP1BP1	-0.529571	-1.295062	-3.623723	0.0003653	0.0020805	-0.365485
EGFR	0.1505066	6.5964041	3.623138	0.0003661	0.0020846	-0.367453
RP11-504P2	-0.211909	4.5838413	-3.62306	0.0003662	0.0020848	-0.367713
STON1	0.2531822	4.963973	3.6226831	0.0003667	0.0020874	-0.368983
MDH2	0.0870526	6.9571324	3.6226259	0.0003668	0.0020875	-0.369176
CTD-2036P1	-0.682775	1.338325	-3.622543	0.0003669	0.0020878	-0.369454
RP11-30P6.	-0.688385	4.7825401	-3.621872	0.0003678	0.0020925	-0.371713
WIPF1	0.1347318	6.035988	3.6218338	0.0003678	0.0020925	-0.37184
AC253576.2	-0.870785	1.0691017	-3.621218	0.0003687	0.0020969	-0.373913
ECSCR	0.2072228	4.8241505	3.6211646	0.0003687	0.002097	-0.374091
FBXO28	0.0742777	6.1484045	3.6209863	0.000369	0.002098	-0.374691
MIR7-3HG	-0.734725	-0.832724	-3.620873	0.0003691	0.0020985	-0.375071
SMURF1	0.0796977	6.2504081	3.6207618	0.0003693	0.0020991	-0.375445
RP11-57B24	0.3756347	-1.367958	3.6206793	0.0003694	0.0020994	-0.375723
FUT2	-0.51647	4.3752244	-3.620398	0.0003698	0.0021012	-0.376668
MAP2	0.2804985	5.7459831	3.6202754	0.0003699	0.0021018	-0.377081

CTA-481E9.	-0.497496	-1.310791	-3.619945	0.0003704	0.002104	-0.378192
RP11-326C3	0.6632907	3.1016387	3.6196402	0.0003708	0.002106	-0.379217
CH17-13I23	-0.980095	1.0487954	-3.619324	0.0003712	0.0021078	-0.380279
SMLR1	-0.44085	6.0388515	-3.619317	0.0003712	0.0021078	-0.380303
RP11-336K2	-0.520649	-1.128944	-3.618987	0.0003717	0.00211	-0.381413
RBM12B	-0.104825	5.6066971	-3.618464	0.0003724	0.0021137	-0.38317
TP53AIP1	-0.806799	-0.063214	-3.617991	0.000373	0.002117	-0.38476
RP11-87C12	-0.735724	1.4620579	-3.617563	0.0003736	0.00212	-0.386199
ITPKA	-0.325541	5.3827574	-3.616863	0.0003745	0.002125	-0.38855
SLFN12	0.3448752	4.4807539	3.6168004	0.0003746	0.0021252	-0.38876
SCG3	-1.038993	1.1942397	-3.616501	0.000375	0.0021271	-0.389765
CH507-396I	0.5833249	-1.049554	3.6164099	0.0003752	0.0021273	-0.390072
MPZL2	-0.1372	6.1003576	-3.616401	0.0003752	0.0021273	-0.390103
AC011899.9	0.3509259	4.1399324	3.6163214	0.0003753	0.0021275	-0.39037
EML6	-0.38507	4.5784678	-3.616223	0.0003754	0.002128	-0.390702
GNG2	0.1516059	5.5010084	3.6160167	0.0003757	0.0021292	-0.391393
BRAF	-0.075762	5.9639038	-3.61502	0.0003771	0.0021366	-0.39474
RBM47	0.0994443	6.4464433	3.6145194	0.0003777	0.0021402	-0.396421
SAMD10	-0.12329	5.5553859	-3.614292	0.0003781	0.0021416	-0.397186
VSTM4	0.2313181	5.6607804	3.6141373	0.0003783	0.0021425	-0.397704
SLC30A2	1.0314678	2.644971	3.6132077	0.0003795	0.0021494	-0.400825
PAXBP1-AS1	-0.196341	4.1061271	-3.61317	0.0003796	0.0021494	-0.400953
RP11-366M4	0.8035052	1.5275136	3.6131122	0.0003797	0.0021495	-0.401146
DANCR	-0.184402	5.9987949	-3.613009	0.0003798	0.0021496	-0.401492
CTD-255501	0.2254262	3.9493781	3.6129728	0.0003799	0.0021496	-0.401614
CTA-342B11	-0.770396	0.1976194	-3.612967	0.0003799	0.0021496	-0.401632
1-Dec	0.9180476	0.4183377	3.6124557	0.0003806	0.0021533	-0.403349
TIAL1	-0.05434	6.3964637	-3.612396	0.0003807	0.0021534	-0.403551
SENP2	-0.071733	6.1630549	-3.61227	0.0003808	0.002154	-0.403972
RNMT	-0.061744	6.1018617	-3.612189	0.000381	0.0021543	-0.404242
MMP25-AS1	0.1782448	5.199265	3.6115892	0.0003818	0.0021587	-0.406257
RP11-40801	-0.774253	0.2085689	-3.611542	0.0003818	0.0021587	-0.406415
ZNF350-AS1	-0.850934	1.1033259	-3.611258	0.0003822	0.0021606	-0.407369
GRK5	0.1096369	5.87835	3.6111927	0.0003823	0.0021608	-0.407587
RP11-267M2	-0.28688	4.2383137	-3.610352	0.0003835	0.0021671	-0.410408
RP11-30H9.	-0.868416	-0.606662	-3.610163	0.0003838	0.0021682	-0.41104
AC000089.3	-0.313922	3.8967164	-3.609757	0.0003843	0.0021711	-0.412402
HIST1H3I	-0.590073	-0.970422	-3.609658	0.0003845	0.0021715	-0.412735
BUB3	-0.057308	6.4481277	-3.608691	0.0003858	0.0021788	-0.415978
HCG4	0.9844928	2.4582891	3.6083806	0.0003862	0.0021809	-0.417018
RNASE1	0.1557612	6.3300693	3.6082633	0.0003864	0.0021815	-0.417411
BBOX1	0.6860663	4.8271307	3.6080553	0.0003867	0.0021825	-0.418109
LINC01159	0.3851726	-1.39062	3.6080489	0.0003867	0.0021825	-0.41813
WBP11	-0.059766	6.435394	-3.607905	0.0003869	0.0021833	-0.418614
RP11-382F2	-1.061759	-0.149938	-3.607738	0.0003871	0.0021843	-0.419172
RP11-517I3	0.2229538	4.3934269	3.6076828	0.0003872	0.0021844	-0.419358
RP11-134K1	-0.816672	0.2621985	-3.607386	0.0003876	0.0021864	-0.420353
GPR132	0.3160167	4.6052623	3.6067846	0.0003885	0.0021909	-0.422368
OASL	0.2736308	5.7615466	3.6064858	0.0003889	0.0021926	-0.42337
TRPV6	1.2293126	2.4343045	3.6064813	0.0003889	0.0021926	-0.423385
MICU3	0.3838258	4.9025249	3.6063391	0.0003891	0.0021934	-0.423861

TOX3	-0.575141	5.3905392	-3.606179	0.0003893	0.0021943	-0.424397
LLNLR-285E	-0.583287	-1.044044	-3.605897	0.0003897	0.0021962	-0.425343
RP1-40E16.	-0.448371	3.1943641	-3.605789	0.0003899	0.0021967	-0.425705
UQCRC1	0.0866171	6.9199189	3.6055441	0.0003902	0.0021981	-0.426525
NIF3L1	-0.059421	5.9597022	-3.605535	0.0003903	0.0021981	-0.426557
APLN	-0.293404	5.2586398	-3.604564	0.0003916	0.0022055	-0.42981
CIDECP	-0.108487	5.502526	-3.604503	0.0003917	0.0022056	-0.430013
RP11-567G1	-1.152788	1.2767512	-3.604313	0.000392	0.0022068	-0.430648
KIAA1919	-0.112199	5.3038785	-3.603551	0.0003931	0.0022126	-0.4332
RP11-307N1	0.3870316	-1.298617	3.6031608	0.0003936	0.0022153	-0.434507
FBXO2	0.2824931	5.9032985	3.6028372	0.0003941	0.0022176	-0.435591
MEOX1	0.7396033	3.3734835	3.6027613	0.0003942	0.0022179	-0.435845
RP11-379F4	-0.817654	1.1881256	-3.601779	0.0003956	0.0022254	-0.439132
SMIM17	-0.901085	0.4600714	-3.601531	0.000396	0.0022271	-0.439965
TRABD2B	0.4392928	5.068574	3.6012197	0.0003964	0.0022292	-0.441005
CLIC6	1.0204119	3.3880873	3.6008405	0.0003969	0.002232	-0.442275
HSP90B2P	-0.377459	4.2072516	-3.600766	0.000397	0.0022322	-0.442525
ZNF611	-0.279411	4.9977465	-3.600292	0.0003977	0.0022357	-0.44411
RP11-76E17	0.8610745	0.2637471	3.6001362	0.000398	0.0022366	-0.444631
DOCK11	0.2273584	5.3259288	3.5997167	0.0003986	0.0022397	-0.446035
CEP112	0.1437961	5.2803679	3.5989476	0.0003997	0.0022453	-0.448608
RP11-1060G	-0.769988	0.2427871	-3.598943	0.0003997	0.0022453	-0.448625
SETD8	-0.069027	5.9219807	-3.59876	0.0003999	0.0022464	-0.449234
RP5-827C21	-0.528146	3.0227007	-3.598685	0.0004	0.0022467	-0.449485
INA	0.8942486	0.138055	3.5986092	0.0004002	0.0022469	-0.44974
RP11-84C10	-0.689379	1.8803048	-3.598565	0.0004002	0.002247	-0.449887
CYP2A7P1	0.7042925	-0.91708	3.5984664	0.0004004	0.0022474	-0.450217
RPL7AP11	-0.673529	2.2663641	-3.598284	0.0004006	0.0022485	-0.450826
TSPAN18	0.1818998	5.7442812	3.5981175	0.0004009	0.0022496	-0.451384
UBE2W	0.0732426	6.1034208	3.597972	0.0004011	0.0022504	-0.451871
C20orf194	0.1315058	5.6920764	3.5978774	0.0004012	0.0022508	-0.452187
C11orf84	-0.151671	5.6424823	-3.597027	0.0004025	0.0022574	-0.455032
NEK2P4	-0.550153	-1.123634	-3.596845	0.0004027	0.0022585	-0.455639
GGCT	-0.085429	6.1437851	-3.596469	0.0004033	0.0022613	-0.456896
PNPO	0.1302293	6.3288661	3.5961498	0.0004037	0.0022635	-0.457963
SLC44A2	0.1293193	6.2982684	3.5958856	0.0004041	0.0022653	-0.458846
RP11-4N23.	0.3617997	-1.425295	3.595727	0.0004043	0.0022663	-0.459376
RP11-138I1	-0.715012	1.9540165	-3.595363	0.0004049	0.0022689	-0.460591
NDST4	-0.729915	-0.987445	-3.595155	0.0004052	0.0022703	-0.461287
RBM39	-0.048379	6.7411778	-3.594795	0.0004057	0.0022729	-0.46249
SORCS3	-0.986739	-0.516618	-3.594638	0.0004059	0.0022738	-0.463017
CCR10	-0.469765	3.3239655	-3.594558	0.0004061	0.0022741	-0.463282
IKBKG	0.1871245	5.5927189	3.5942213	0.0004066	0.0022766	-0.464408
CTC-260E6.	-0.682646	3.1479817	-3.593891	0.000407	0.0022789	-0.465511
INADL	0.1211573	6.1951313	3.5936061	0.0004075	0.0022809	-0.466463
PHF12	-0.052455	6.1611662	-3.593514	0.0004076	0.0022813	-0.46677
SCYL1	0.0672932	6.605265	3.5933102	0.0004079	0.0022826	-0.467452
RP11-32D17	-0.829462	-0.047503	-3.593273	0.000408	0.0022826	-0.467576
ZNF16	-0.101081	5.6503472	-3.59288	0.0004085	0.0022855	-0.468888
POLR3C	-0.083579	6.0133388	-3.592696	0.0004088	0.0022866	-0.469503
KIAA1161	-0.15118	6.5096777	-3.592659	0.0004089	0.0022866	-0.469627

PDLIM2	0.1259134	5.8755952	3.5924561	0.0004092	0.002288	-0.470304
KCNK3	0.856314	3.1697405	3.5923339	0.0004093	0.0022886	-0.470712
AC067959.1	0.8851947	0.0523271	3.5916999	0.0004103	0.0022934	-0.47283
RP11-178C3	0.5318804	3.1675797	3.5916709	0.0004103	0.0022934	-0.472926
RP11-689C9	-0.808406	-0.599067	-3.59159	0.0004104	0.0022937	-0.473197
TLE1	-0.101871	6.5549313	-3.591525	0.0004105	0.0022939	-0.473413
RP11-44F21	-1.104773	0.0450694	-3.591412	0.0004107	0.0022943	-0.473791
SLC48A1	0.0964869	6.1282117	3.5913735	0.0004108	0.0022943	-0.473919
GP6	0.8693274	0.858979	3.59135	0.0004108	0.0022943	-0.473998
RPP25	0.2089803	5.4762074	3.5911741	0.0004111	0.0022954	-0.474585
RP11-444E1	-0.786979	1.5903704	-3.591124	0.0004111	0.0022955	-0.474754
SUPT4H1	-0.062754	6.4336885	-3.590819	0.0004116	0.0022977	-0.475769
BCCIP	-0.069711	6.3467725	-3.590748	0.0004117	0.0022979	-0.476007
AC006486.1	-0.453208	2.9511496	-3.59059	0.0004119	0.0022987	-0.476535
STK32A	0.8957019	2.0694367	3.5905638	0.000412	0.0022987	-0.476622
RP11-2N1.2	0.6678194	-0.979638	3.5904412	0.0004121	0.0022991	-0.477032
GBP6	0.929424	0.8775412	3.5904318	0.0004122	0.0022991	-0.477063
LYSMD2	0.1038899	5.7832603	3.5903794	0.0004122	0.0022992	-0.477238
ZNF224	-0.112109	5.4659747	-3.590257	0.0004124	0.0022999	-0.477648
SAR1B	0.1165313	6.6654483	3.5898798	0.000413	0.0023026	-0.478905
C19orf80	-0.566311	6.1325095	-3.589391	0.0004137	0.0023064	-0.480537
OVCH1-AS1	0.8876544	0.0840748	3.5892075	0.000414	0.0023075	-0.481149
MED30	-0.11554	5.6381542	-3.588645	0.0004148	0.0023119	-0.483027
AC015922.5	-0.598738	-1.156365	-3.588361	0.0004153	0.0023139	-0.483975
SNORA45B	-0.833476	0.1889567	-3.588316	0.0004153	0.0023139	-0.484124
RP11-680F2	-0.706796	-0.726351	-3.588048	0.0004157	0.0023158	-0.485019
HOXA6	-1.032995	0.1894178	-3.587759	0.0004162	0.0023179	-0.485983
RP11-206L1	-0.424929	3.2033258	-3.587493	0.0004166	0.0023197	-0.486868
RP11-372E1	-0.727527	2.0784414	-3.587014	0.0004173	0.0023234	-0.488466
LINC00664	-1.031615	1.2592379	-3.586586	0.0004179	0.0023266	-0.489893
FAM228A	0.912738	1.0632279	3.585975	0.0004188	0.0023314	-0.491932
NUDT7	0.1993741	5.5517957	3.5857488	0.0004192	0.002333	-0.492686
ZNF615	-0.167385	5.1875054	-3.585506	0.0004195	0.0023346	-0.493494
ARHGAP22	0.264418	4.7008974	3.5853749	0.0004197	0.0023354	-0.493932
RNF40	0.0888691	6.2917281	3.5852785	0.0004199	0.0023358	-0.494254
LA16c-429E	0.8049016	1.7086735	3.5850308	0.0004203	0.0023376	-0.49508
PROSC	0.1047041	6.3084118	3.5846945	0.0004208	0.0023401	-0.4962
SUCLG2-AS1	0.4176152	3.8838314	3.5845003	0.0004211	0.0023413	-0.496848
AC009961.2	-0.637693	-0.563172	-3.584004	0.0004218	0.0023452	-0.498502
RP3-500L14	-0.890152	0.8841114	-3.583719	0.0004223	0.0023472	-0.49945
UPP1	0.1218757	5.9166953	3.5829659	0.0004234	0.0023533	-0.501961
MIR568	0.5690614	3.6822418	3.5827543	0.0004237	0.0023547	-0.502666
SMOC2	0.2647966	5.6174536	3.5823894	0.0004243	0.0023574	-0.503882
AC010524.2	-1.086009	3.1363598	-3.581988	0.0004249	0.0023605	-0.505219
MAGI1	0.0967529	6.1542677	3.581925	0.000425	0.0023607	-0.505429
LRCH3	-0.057649	6.0577112	-3.581792	0.0004252	0.0023614	-0.505871
OMG	0.6996453	2.3465615	3.5813678	0.0004259	0.0023647	-0.507285
RP11-283I3	-0.823859	-0.049212	-3.58112	0.0004262	0.0023664	-0.508109
MECOM	0.2146184	5.4318373	3.5810035	0.0004264	0.0023671	-0.508498
RP11-363E6	-0.833473	0.1206538	-3.580861	0.0004266	0.0023679	-0.508972
RP11-104J2	-0.751086	3.2291571	-3.580608	0.000427	0.0023697	-0.509816

CTA-481E9.	-0.499796	-1.309663	-3.580562	0.0004271	0.0023697	-0.509969
RP3-324017	-0.75478	0.3278527	-3.580528	0.0004271	0.0023697	-0.510082
ANO5	0.7747892	4.7636773	3.5802778	0.0004275	0.0023714	-0.510915
RP11-443C1	0.9167913	0.717222	3.5802105	0.0004276	0.0023714	-0.511139
PRKAR1B	0.1720522	5.8945412	3.580198	0.0004277	0.0023714	-0.51118
NUP43	-0.071394	6.0582774	-3.579693	0.0004284	0.0023754	-0.512861
FRY	0.1500384	6.0693155	3.5793565	0.000429	0.0023776	-0.513982
RP11-426C2	0.775656	1.7465694	3.5793538	0.000429	0.0023776	-0.513991
SEMA4B	0.1074589	6.2787	3.5792452	0.0004291	0.0023781	-0.514352
RP13-192B1	-0.70825	-0.525054	-3.578745	0.0004299	0.0023821	-0.516017
ABHD17AP4	-0.665338	-0.87542	-3.578618	0.0004301	0.0023825	-0.516441
PDK3	0.1887575	5.1577529	3.5786084	0.0004301	0.0023825	-0.516472
CHSY1	0.1393108	5.913439	3.5780871	0.0004309	0.0023866	-0.518207
RP11-766H1	-0.653218	-0.628729	-3.577926	0.0004312	0.0023877	-0.518744
ALOX5	0.2842781	5.4141326	3.5778202	0.0004313	0.0023882	-0.519095
PECAM1	0.1031926	6.5866812	3.5776642	0.0004316	0.0023892	-0.519614
SIGLEC14	1.1270925	3.3184654	3.5775498	0.0004318	0.0023898	-0.519995
GPATCH2	-0.088492	5.7167285	-3.576823	0.0004329	0.0023957	-0.522413
AP3S2	0.0906407	6.0248142	3.576558	0.0004333	0.0023976	-0.523295
PLA2G2A	0.6900183	5.958188	3.576227	0.0004338	0.0024001	-0.524396
VSNL1	-0.662471	5.2767596	-3.575799	0.0004345	0.0024033	-0.525818
AC022182.2	-0.749808	-0.166946	-3.575772	0.0004345	0.0024033	-0.525909
LINC00355	-1.343156	1.0182573	-3.575579	0.0004348	0.0024044	-0.526551
FLJ20021	0.1667917	4.9115755	3.5755667	0.0004348	0.0024044	-0.526592
TACSTD2	0.7106917	4.4011376	3.5753844	0.0004351	0.0024055	-0.527198
KMO	0.355289	5.6754414	3.5753551	0.0004352	0.0024055	-0.527296
ZNF579	-0.132244	5.6795014	-3.57513	0.0004355	0.0024071	-0.528044
AKIRIN1	0.0799708	6.2902809	3.5747369	0.0004361	0.0024101	-0.529351
PSAP	0.0626931	7.4093065	3.5746745	0.0004362	0.0024103	-0.529559
SEC14L2	0.2617147	6.3402705	3.5744333	0.0004366	0.002412	-0.530361
ARL2BP	0.2552704	5.0575238	3.5743189	0.0004368	0.0024124	-0.530741
HOXD9	-0.929441	3.3774334	-3.574299	0.0004368	0.0024124	-0.530808
RP11-855A2	-0.809987	-0.292432	-3.574258	0.0004369	0.0024124	-0.530945
LINC00523	-0.582871	-1.141815	-3.573934	0.0004374	0.0024149	-0.532022
C19orf66	0.1165371	6.4369923	3.5738285	0.0004376	0.0024154	-0.532371
NKAPL	0.766467	2.2198059	3.5735607	0.000438	0.0024174	-0.533262
LINC00298	-0.860019	0.1842347	-3.573348	0.0004383	0.0024189	-0.53397
ACTBP11	-0.709457	2.2880676	-3.572709	0.0004393	0.0024241	-0.536092
RPS3	-0.079812	7.1714588	-3.572483	0.0004397	0.0024257	-0.536843
RAB14	0.0673479	6.6112625	3.5719452	0.0004405	0.00243	-0.538631
WFDC21P	0.6566532	3.5951843	3.5718456	0.0004407	0.0024305	-0.538962
RP11-83J16	-0.79694	1.1066324	-3.571327	0.0004415	0.0024347	-0.540686
PSMB9	0.1667772	6.2221327	3.5712585	0.0004416	0.0024349	-0.540912
AC096664.1	-0.586377	-0.876244	-3.570933	0.0004422	0.0024374	-0.541994
C12orf5	0.1345921	5.4070053	3.5707258	0.0004425	0.0024388	-0.542682
RNF139-AS1	-0.267151	4.3260318	-3.570635	0.0004426	0.0024393	-0.542983
RPS15AP38	-0.783376	0.3606897	-3.570582	0.0004427	0.0024393	-0.54316
RP11-38902	-0.567324	-0.886746	-3.570507	0.0004428	0.0024393	-0.543409
TEPP	0.7384262	-0.536888	3.5705045	0.0004428	0.0024393	-0.543417
RP4-669P10	-0.511463	2.8992731	-3.570407	0.000443	0.0024397	-0.54374
RP11-520H1	-0.764119	2.4672784	-3.570381	0.000443	0.0024397	-0.543829

HOXD-AS2	-1.090729	0.9869503	-3.57027	0.0004432	0.0024403	-0.544195
RP13-415G1	-0.833889	0.0628423	-3.570081	0.0004435	0.0024413	-0.544824
LMBRD2	0.1138498	5.9747589	3.570019	0.0004436	0.0024413	-0.54503
ZNF829	-0.351945	4.5717838	-3.570014	0.0004436	0.0024413	-0.545045
RP11-440D1	0.5830599	2.8935941	3.5699784	0.0004437	0.0024413	-0.545165
RP11-243A1	0.8730201	3.0872752	3.569595	0.0004443	0.0024443	-0.546438
ZNF70	-0.199687	4.8954934	-3.568859	0.0004455	0.0024505	-0.548882
HOXB13	-1.338891	0.7568885	-3.568653	0.0004458	0.0024519	-0.549566
RP11-112J3	-0.651387	2.4895994	-3.567885	0.000447	0.0024583	-0.552116
SOWAHA	-0.469114	5.4681175	-3.567792	0.0004472	0.0024585	-0.552424
ACAD8	0.0897203	6.1013307	3.5677838	0.0004472	0.0024585	-0.552451
COQ7	0.0777772	5.8295124	3.5677328	0.0004473	0.0024585	-0.552621
SLC51B	-0.486611	4.9755855	-3.567627	0.0004474	0.0024586	-0.552972
RPL13AP5	-0.166038	5.456226	-3.567626	0.0004474	0.0024586	-0.552976
RPSAP47	-0.712084	1.9553654	-3.567601	0.0004475	0.0024586	-0.553059
AC139100.4	-0.496938	3.6291573	-3.567297	0.000448	0.0024604	-0.554067
ANKRD50	0.1298452	5.7960994	3.567278	0.000448	0.0024604	-0.55413
ZNF235	-0.124383	4.8474292	-3.567271	0.000448	0.0024604	-0.554153
GJA10	-1.188594	0.1906714	-3.567018	0.0004484	0.0024623	-0.554994
BTN2A2	0.1382778	5.7163226	3.5668884	0.0004486	0.0024629	-0.555423
ZFAND2A	-0.12455	6.2271532	-3.566865	0.0004487	0.0024629	-0.555503
RP11-72304	-0.537007	3.2785046	-3.566823	0.0004487	0.0024629	-0.555639
ARHGDIB	0.0997036	6.4783606	3.5667349	0.0004489	0.0024633	-0.555933
F13A1	0.3406043	5.4236734	3.5665141	0.0004492	0.0024647	-0.556665
IGFBP7-AS1	0.8254994	0.2874323	3.56649	0.0004493	0.0024647	-0.556745
RPL4P5	-0.351825	3.7090125	-3.566027	0.00045	0.0024684	-0.55828
RP11-175D1	-0.766115	0.7743088	-3.565983	0.0004501	0.0024685	-0.558427
LINC00399	0.8704551	0.0464882	3.5659064	0.0004502	0.0024688	-0.558682
KIAA0195	-0.063588	6.4514033	-3.565795	0.0004504	0.0024694	-0.55905
RP11-63A2.	-0.581505	-1.091101	-3.565645	0.0004506	0.0024701	-0.559549
AC005082.1	0.8251542	1.235075	3.5656274	0.0004507	0.0024701	-0.559608
RP11-244M2	-1.094317	2.3706589	-3.564919	0.0004518	0.002476	-0.561957
DOCK10	0.2032611	5.4705054	3.5648796	0.0004519	0.002476	-0.562088
AC098828.3	0.7494614	-0.605872	3.5646731	0.0004522	0.0024775	-0.562773
ERHP1	-0.517091	-1.047648	-3.564119	0.0004531	0.0024821	-0.564612
RP11-364L4	-0.789665	1.6862979	-3.564068	0.0004532	0.0024821	-0.56478
RP11-693J1	-0.927517	-0.603126	-3.563591	0.000454	0.002486	-0.566363
UXS1	-0.076378	6.2522784	-3.563392	0.0004543	0.0024871	-0.567022
LINC00683	1.0056883	0.0521964	3.563382	0.0004543	0.0024871	-0.567055
CAPNS1	0.0675766	6.9047571	3.563221	0.0004546	0.0024882	-0.567589
SAGE1	-0.967256	-0.610583	-3.562938	0.000455	0.0024904	-0.568526
PFAS	-0.086463	5.986002	-3.562412	0.0004559	0.0024947	-0.570272
RP3-416H24	1.1314049	1.8004317	3.5620869	0.0004564	0.0024972	-0.571349
LINC01545	-0.514895	2.5270388	-3.561187	0.0004579	0.0025049	-0.574333
RP11-448G1	-0.330383	3.9861142	-3.560953	0.0004583	0.0025067	-0.575108
RP1-69M21.	-0.60084	-1.094198	-3.560658	0.0004588	0.0025089	-0.576084
AC004386.4	-0.574218	-0.96166	-3.560548	0.000459	0.0025092	-0.576449
RP11-1246C	-0.295625	4.5289299	-3.560541	0.000459	0.0025092	-0.576472
LACTB	0.0868837	6.1928839	3.5603562	0.0004593	0.0025102	-0.577084
AC144530.1	-0.290597	4.5857025	-3.560351	0.0004593	0.0025102	-0.5771
SNHG6	-0.1238	6.3297318	-3.560219	0.0004595	0.002511	-0.57754

RP11-291L2	-0.469229	3.8046126	-3.560164	0.0004596	0.0025111	-0.577721
HRH1	0.2472428	4.6661627	3.5600547	0.0004598	0.0025117	-0.578083
RPL29P12	-0.73353	1.2022787	-3.559733	0.0004603	0.0025143	-0.579149
RP11-388C1	-0.513771	3.033076	-3.559588	0.0004605	0.002515	-0.57963
FLII	0.0656108	6.6149562	3.559573	0.0004606	0.002515	-0.579679
RP11-401P9	-0.771184	-0.758003	-3.55915	0.0004613	0.0025184	-0.581079
KDM3A	-0.073003	6.1785972	-3.559047	0.0004614	0.0025189	-0.581422
ZNF560	-1.021225	-0.424962	-3.558835	0.0004618	0.0025205	-0.582123
THBS1	0.1836629	6.5807449	3.5584442	0.0004624	0.0025236	-0.583418
ZNF566	-0.100182	5.3456605	-3.55828	0.0004627	0.0025247	-0.58396
CXCL8	0.558391	5.3216373	3.5581056	0.000463	0.002526	-0.584539
CCDC144NL	-0.95324	-0.557288	-3.557663	0.0004637	0.0025296	-0.586005
ARHGEF11	-0.07973	6.4257128	-3.55703	0.0004648	0.0025349	-0.588099
BUD13	-0.060015	5.8435214	-3.556227	0.0004661	0.0025419	-0.590758
CNOT6	-0.081074	5.9967333	-3.555963	0.0004666	0.0025439	-0.591632
SCN7A	1.0593764	2.7803665	3.5555042	0.0004673	0.0025471	-0.593151
PHF2P2	-0.908005	-0.838266	-3.555494	0.0004673	0.0025471	-0.593183
PLVAP	0.1245547	6.5950655	3.5554934	0.0004674	0.0025471	-0.593187
CHP1	0.0998601	6.8259572	3.5553174	0.0004676	0.0025483	-0.593769
FBF1	-0.173375	4.9606997	-3.554862	0.0004684	0.0025521	-0.595276
MYO16	0.5200072	4.2984305	3.5542308	0.0004695	0.0025575	-0.597364
DRAXINP1	-0.736815	-1.020351	-3.553989	0.0004699	0.0025593	-0.598162
CHAD	-0.373764	5.5499403	-3.55375	0.0004703	0.0025611	-0.598953
RP11-103J1	-1.2023	0.1859438	-3.55325	0.0004711	0.0025653	-0.600609
RP5-1057J7	-0.480163	3.8812668	-3.552167	0.000473	0.0025749	-0.604188
WDR47	0.1148595	5.3275687	3.5520143	0.0004732	0.0025758	-0.604695
RP11-358D1	0.7508943	-0.198168	3.5519532	0.0004733	0.0025758	-0.604897
GRID1	0.6460856	4.3435472	3.5519394	0.0004733	0.0025758	-0.604942
NDUFAB1	0.0977012	6.3773665	3.5518703	0.0004735	0.0025761	-0.605171
RP11-305L7	-0.584436	3.3136242	-3.551656	0.0004738	0.0025777	-0.605879
CTA-215D11	-0.593442	-0.842986	-3.551545	0.000474	0.0025782	-0.606247
RP11-33H15	-0.868466	-0.551945	-3.55152	0.000474	0.0025782	-0.60633
RPL37P6	-0.466227	3.1832218	-3.550948	0.000475	0.0025831	-0.608221
TAT-AS1	0.6847177	2.9745892	3.5507072	0.0004754	0.0025849	-0.609016
CTD-2540B1	-0.541314	-1.132059	-3.55057	0.0004757	0.0025858	-0.609469
RP11-122M1	-0.815929	0.0678799	-3.550372	0.000476	0.002587	-0.610125
FAM35BP	-0.741679	3.0388863	-3.550359	0.000476	0.002587	-0.610169
RP11-295M3	0.429312	-1.208778	3.5500907	0.0004765	0.0025891	-0.611054
RPS4XP13	-0.784585	0.6121186	-3.549823	0.0004769	0.0025912	-0.611938
RP11-138B4	-0.980196	-0.078076	-3.549685	0.0004772	0.0025921	-0.612395
EED	-0.079097	5.8094399	-3.549398	0.0004777	0.0025943	-0.613341
NMRAL1	0.1838098	6.2190211	3.5492172	0.000478	0.0025956	-0.61394
ALKBH2	-0.131798	5.972309	-3.549167	0.0004781	0.0025957	-0.614106
NLGN2	0.1885109	5.488008	3.5488742	0.0004786	0.0025981	-0.615074
RP11-888D1	0.7688376	-0.287406	3.5487976	0.0004787	0.0025984	-0.615326
XX-CR54.1	-1.056621	-0.406392	-3.548583	0.0004791	0.0026	-0.616036
ZNF367	-0.154514	5.5112112	-3.548447	0.0004793	0.0026009	-0.616484
ZNF501	-0.329868	4.3015571	-3.548405	0.0004794	0.0026009	-0.616625
NET1	-0.085135	6.3626235	-3.548296	0.0004796	0.0026015	-0.616985
RCSD1	0.1706979	5.5464448	3.5479358	0.0004802	0.0026045	-0.618174
MICALCL	0.7093055	2.7702217	3.5475327	0.0004809	0.0026078	-0.619505

PRKCG	-0.829255	-0.393532	-3.547324	0.0004812	0.0026094	-0.620193
LINC00649	-0.35583	5.0123992	-3.546765	0.0004822	0.0026142	-0.622042
RP11-5106.	-0.177112	4.969654	-3.545841	0.0004838	0.0026225	-0.625091
RP11-276H7	0.7138329	2.1171382	3.5453844	0.0004846	0.0026264	-0.626598
RP11-468E2	0.8101065	0.5351211	3.5450624	0.0004851	0.0026289	-0.627661
RP11-31H15	-0.515976	-1.279824	-3.545039	0.0004852	0.0026289	-0.62774
KHSRPP1	-0.684701	-0.690915	-3.544967	0.0004853	0.0026291	-0.627977
GUSBP4	-0.572602	2.0429295	-3.544686	0.0004858	0.0026314	-0.628903
CRB2	-0.889768	1.7776888	-3.544197	0.0004866	0.0026356	-0.630519
NSUN4	0.0699273	5.9380024	3.5437023	0.0004875	0.0026399	-0.63215
ALDOA	0.1032517	7.0575138	3.5434478	0.0004879	0.0026419	-0.63299
SPATA20	0.104665	6.4076811	3.5430934	0.0004886	0.0026449	-0.634159
FNDC3A	0.112975	6.4486964	3.5429496	0.0004888	0.0026458	-0.634633
PPP2R5B	0.0752069	5.8487728	3.5428085	0.0004891	0.0026468	-0.635099
SIGLEC1	0.2089292	5.6491096	3.5416755	0.000491	0.0026571	-0.638836
APOL3	0.1870774	6.2668984	3.5413944	0.0004915	0.0026594	-0.639763
LCP2	0.1573635	5.8208294	3.5413059	0.0004917	0.0026595	-0.640055
ZFP36L1	0.0803576	6.8736744	3.5413015	0.0004917	0.0026595	-0.640069
RP11-31K23	-0.659904	-1.041012	-3.541255	0.0004918	0.0026595	-0.640222
PIGV	0.1101886	5.9187996	3.5411063	0.000492	0.0026605	-0.640713
RP5-1042K1	-0.748138	0.2777231	-3.54086	0.0004925	0.0026625	-0.641524
RP1-39G22.	-0.143072	5.0307679	-3.54069	0.0004928	0.0026632	-0.642085
SNORD15B	-0.774486	-0.042447	-3.540671	0.0004928	0.0026632	-0.642149
RP11-334C1	-0.190041	5.0847211	-3.540663	0.0004928	0.0026632	-0.642173
TMEM163	0.8445312	3.7092073	3.5406023	0.0004929	0.0026634	-0.642375
RP11-597D1	0.4759126	3.2111173	3.5404809	0.0004931	0.0026641	-0.642775
CSNK1G3	0.0720302	5.9887636	3.5401097	0.0004938	0.0026673	-0.643999
HPD	0.4759438	6.6760575	3.539944	0.0004941	0.0026684	-0.644545
FUS	-0.056999	6.7966022	-3.539904	0.0004942	0.0026684	-0.644675
IFITM3	0.0986209	7.3056892	3.5395214	0.0004948	0.0026715	-0.645938
CTD-2384A1	-1.135705	-0.101872	-3.539503	0.0004949	0.0026715	-0.645997
RP11-395G2	0.5761077	3.2634138	3.5391712	0.0004954	0.0026742	-0.647092
RP11-403.1	0.3685939	-1.421833	3.5389533	0.0004958	0.0026759	-0.64781
SEMA6D	0.3413707	4.7251808	3.5388417	0.000496	0.0026766	-0.648178
RP4-580N22	1.0104497	1.2097516	3.5386229	0.0004964	0.0026783	-0.648899
SZT2	0.0700028	6.0735512	3.5385174	0.0004966	0.0026789	-0.649247
RP11-230C9	-0.793816	0.4488433	-3.538009	0.0004975	0.002683	-0.650922
VGLL4	0.136961	5.8352673	3.5380031	0.0004975	0.002683	-0.650942
COPS8	0.0555726	6.1796643	3.5379408	0.0004976	0.0026831	-0.651147
RPS15AP40	-0.65236	-0.616492	-3.53791	0.0004977	0.0026831	-0.65125
HERC2P8	-0.631832	-0.892396	-3.53775	0.000498	0.0026843	-0.651776
EIF2B1	-0.055786	6.3206701	-3.53762	0.0004982	0.0026851	-0.652205
DHX37	-0.08169	6.0595684	-3.537375	0.0004986	0.0026871	-0.653011
HAUS1	-0.109938	5.6448098	-3.537053	0.0004992	0.0026898	-0.65407
DPH6-AS1	0.8008995	2.0929816	3.5369922	0.0004993	0.0026899	-0.654272
YARS	0.0823879	6.5262443	3.5367901	0.0004997	0.0026915	-0.654938
ADAMTS3	0.7111381	3.5203435	3.5365705	0.0005001	0.0026932	-0.655661
PCDHB1	-0.754155	-0.688022	-3.53647	0.0005002	0.0026938	-0.655992
MAGEA8-AS1	-0.539851	-1.179448	-3.53634	0.0005005	0.0026946	-0.65642
TOM1L2	0.0981177	6.0907133	3.5352119	0.0005025	0.0027051	-0.660135
RP11-318E3	-0.690591	1.8646268	-3.534948	0.000503	0.0027072	-0.661004

AC090602.2	-0.689242	-0.388278	-3.53469	0.0005034	0.0027093	-0.661855
PTBP1P	-0.667161	-0.682836	-3.534447	0.0005039	0.0027113	-0.662654
MAGEA6	-1.59082	1.1265316	-3.534206	0.0005043	0.0027132	-0.663448
RP11-43P8.	-0.602777	-0.831769	-3.533955	0.0005048	0.0027152	-0.664272
LINC00618	-0.625302	-0.844947	-3.533329	0.0005059	0.0027209	-0.666334
ANKRD20A19	-0.878608	-0.69603	-3.532289	0.0005078	0.0027306	-0.669756
IVD	0.1195413	6.693407	3.532042	0.0005082	0.0027326	-0.670569
AL163953.3	-0.775677	2.2710129	-3.531896	0.0005085	0.0027335	-0.671049
CTB-47B8.1	-0.743308	-0.020308	-3.531874	0.0005085	0.0027335	-0.671122
TTC37	0.0788556	6.3745106	3.5315956	0.000509	0.0027357	-0.672037
FAM167A	0.7646063	3.5390384	3.5315608	0.0005091	0.0027357	-0.672152
IGLL1	-0.810576	-0.386262	-3.530887	0.0005103	0.0027419	-0.674368
RHOXF2B	-0.767721	-1.078714	-3.530577	0.0005109	0.0027445	-0.675386
LYPD2	1.0325864	-0.057889	3.5293292	0.0005132	0.0027563	-0.679491
RPL7AP31	-0.695638	-0.481048	-3.528598	0.0005145	0.0027631	-0.681894
GALNT7	0.291877	5.0224065	3.5285431	0.0005146	0.0027633	-0.682075
RP11-203B9	-0.408859	3.2042193	-3.52829	0.0005151	0.0027653	-0.682906
PABPN1	-0.064468	6.4940719	-3.528165	0.0005153	0.0027662	-0.683318
LINC01341	-0.410729	4.4699216	-3.527985	0.0005156	0.0027675	-0.683911
TF	-0.273588	7.6128007	-3.527812	0.0005159	0.0027688	-0.684476
GPR141	0.8482779	1.7982323	3.5277459	0.0005161	0.0027691	-0.684695
LPP	0.0714911	6.4934812	3.5275127	0.0005165	0.002771	-0.685462
ZNF432	-0.149001	5.4140894	-3.527362	0.0005168	0.0027721	-0.685958
WWOX	0.1211982	5.6909354	3.5270486	0.0005173	0.0027747	-0.686987
SLAIN2	0.0675632	6.2349047	3.5268151	0.0005178	0.0027766	-0.687754
RP1-179N16	-0.52302	3.2192292	-3.526301	0.0005187	0.0027813	-0.689444
QKI	0.0756357	6.4711157	3.5259516	0.0005194	0.0027844	-0.690591
GUCA2A	-1.242494	1.2533152	-3.525875	0.0005195	0.0027847	-0.690842
EXOC3L4	-0.319158	5.8984446	-3.525451	0.0005203	0.0027878	-0.692234
CST8	-0.636088	-1.144576	-3.525446	0.0005203	0.0027878	-0.692253
RP11-39404	0.3248169	4.6910257	3.5254377	0.0005203	0.0027878	-0.692279
SENCR	0.3255324	3.5213562	3.5250135	0.0005211	0.0027916	-0.693672
AL022326.1	-0.72368	-0.272264	-3.524737	0.0005216	0.002794	-0.694579
SPATA31D1	-0.816478	-0.930311	-3.52458	0.0005219	0.0027948	-0.695096
LSM7	-0.090862	6.1614565	-3.524575	0.0005219	0.0027948	-0.695111
KIAA2012	0.834548	2.7578429	3.5238876	0.0005232	0.0028012	-0.697369
RP11-336A1	0.9096814	-0.082113	3.5238179	0.0005233	0.0028015	-0.697598
HIVEP1	0.1020819	5.9041771	3.5237067	0.0005235	0.0028022	-0.697963
BCL2L13	0.0671583	6.386576	3.5235866	0.0005238	0.002803	-0.698358
RTKN	-0.088574	6.5333842	-3.523438	0.000524	0.002804	-0.698844
FDPS	-0.116288	6.8016001	-3.523275	0.0005243	0.0028049	-0.69938
PHOSPHO1	0.5863336	2.7579736	3.5232659	0.0005244	0.0028049	-0.69941
TRIM45	-0.224262	5.0959538	-3.523198	0.0005245	0.0028052	-0.699635
EEF2K	0.0678286	6.1187179	3.5228385	0.0005251	0.0028079	-0.700813
XYLB	-0.207264	6.1213872	-3.522793	0.0005252	0.0028079	-0.700964
RFXAP	-0.167116	4.6854045	-3.522789	0.0005252	0.0028079	-0.700975
RP1-96H9.5	-0.757341	-0.230858	-3.522765	0.0005253	0.0028079	-0.701055
KDELR3	0.2052923	5.9951769	3.5222858	0.0005262	0.0028123	-0.702627
CTB-30L5.1	0.835106	0.073678	3.5221929	0.0005264	0.0028124	-0.702932
FAIM2	0.8366116	2.5780658	3.5221905	0.0005264	0.0028124	-0.70294
RP11-577H5	-0.563325	2.7794478	-3.520635	0.0005293	0.0028274	-0.708045

CCNB3	-0.346966	3.9150164	-3.520609	0.0005293	0.0028274	-0.708131
ARMCX1	0.2210184	5.068851	3.5201947	0.0005301	0.0028312	-0.709488
AP001429.1	-0.683091	-0.33198	-3.519982	0.0005305	0.0028329	-0.710187
RP11-722M1	-0.858503	-0.768363	-3.519559	0.0005313	0.0028368	-0.711574
RP11-184A2	-0.61663	-0.994256	-3.519328	0.0005317	0.0028387	-0.71233
CTB-51J22.	0.9624427	1.8854523	3.5192827	0.0005318	0.0028387	-0.71248
CD300C	0.4132855	4.0089562	3.5192277	0.0005319	0.0028389	-0.71266
RP11-434D9	0.9487784	3.4404111	3.518915	0.0005325	0.0028413	-0.713685
LINC00200	-0.885836	-0.75444	-3.518892	0.0005326	0.0028413	-0.713761
RP3-412A9.	-0.7443	0.7910956	-3.51886	0.0005326	0.0028413	-0.713867
RFXANK	-0.090115	6.1493889	-3.518722	0.0005329	0.0028423	-0.714318
METTL18	-0.108619	5.4819768	-3.518433	0.0005334	0.0028448	-0.715267
RP11-136B1	-0.462578	-1.276104	-3.517422	0.0005354	0.0028546	-0.71858
RP11-95H3.	0.3878904	-1.384476	3.5170819	0.000536	0.0028577	-0.719695
AC126365.1	-0.660175	-1.022426	-3.516676	0.0005368	0.0028614	-0.721026
RP11-972P1	-0.85207	1.3018504	-3.516634	0.0005369	0.0028614	-0.721163
MIR4263	0.7291854	1.4550343	3.5165499	0.000537	0.0028618	-0.721439
THOP1	-0.102653	6.3449944	-3.516453	0.0005372	0.0028624	-0.721757
NCEH1	0.2028142	5.6161413	3.5158948	0.0005383	0.0028677	-0.723585
RP4-761J14	-0.567097	-0.975051	-3.51569	0.0005387	0.0028693	-0.724255
TMEM184C	0.0894192	6.0420557	3.5152258	0.0005396	0.0028736	-0.725778
RP11-618M2	0.5959183	-0.969457	3.5147435	0.0005405	0.0028781	-0.727357
SLC25A25	0.1729593	6.3937659	3.5142464	0.0005414	0.0028828	-0.728986
CTD-2532N2	-0.769688	0.1762645	-3.513966	0.000542	0.0028853	-0.729905
RP11-3P17.	-0.519817	3.0707617	-3.513788	0.0005423	0.0028867	-0.730486
LRFN1	-0.305063	4.7335277	-3.513429	0.000543	0.0028899	-0.731663
SLC2A11	0.1227942	5.3562234	3.5133085	0.0005432	0.0028907	-0.732058
BRAFP1	-0.627101	-1.024434	-3.513241	0.0005434	0.002891	-0.732278
LRIG3	-0.143725	5.8340176	-3.512639	0.0005445	0.0028968	-0.73425
PLAC4	0.9712748	-0.124634	3.5125424	0.0005447	0.0028973	-0.734566
RP11-20F18	-0.694561	-0.972317	-3.512288	0.0005452	0.0028995	-0.735398
AMOTL1	0.211809	6.0424974	3.5118577	0.000546	0.0029036	-0.736807
AC068533.7	0.9005085	1.7140418	3.5118147	0.0005461	0.0029036	-0.736948
RP13-585F2	-0.73586	1.3278944	-3.511538	0.0005467	0.002906	-0.737855
KIRREL2	-1.022595	0.4480242	-3.510582	0.0005485	0.0029155	-0.740983
RP11-120K2	-0.631245	-1.001836	-3.510218	0.0005492	0.0029188	-0.742175
PPAP2A	0.1299876	6.3913117	3.5093677	0.0005509	0.0029272	-0.744955
RP11-727F1	-0.417306	2.9995053	-3.509283	0.0005511	0.0029276	-0.745231
PI16	0.9861436	1.7525938	3.5086296	0.0005523	0.002934	-0.74737
CEBPZOS	-0.058625	6.2580633	-3.508372	0.0005528	0.0029363	-0.748211
ZNF451	-0.06347	6.0721984	-3.507823	0.0005539	0.0029416	-0.750007
GOLGA2P7	-0.550344	3.0335368	-3.507756	0.0005541	0.0029418	-0.750228
ZNF571-AS1	-0.667856	2.5302331	-3.50748	0.0005546	0.0029439	-0.751129
HNRNPH2	0.063716	6.511508	3.5074777	0.0005546	0.0029439	-0.751137
RP1-35C21.	0.7302141	-0.13897	3.5073085	0.0005549	0.0029452	-0.75169
MOGAT1	0.9196229	2.0047581	3.5072587	0.000555	0.0029453	-0.751853
GATA6	0.1833242	5.8052211	3.5068353	0.0005559	0.0029493	-0.753237
DEFA10P	-0.617515	-1.075815	-3.506597	0.0005563	0.0029514	-0.754015
RP11-15F12	0.3544829	-1.383496	3.5064638	0.0005566	0.0029523	-0.754451
SLC6A16	0.3210887	4.9129278	3.5060006	0.0005575	0.0029568	-0.755965
GTPBP2	-0.078919	6.2655486	-3.505834	0.0005578	0.0029581	-0.756511

RP11-299J3	-0.236182	3.8154545	-3.505764	0.000558	0.0029584	-0.756738
RP4-706A16	-0.11925	5.8084755	-3.505305	0.0005589	0.0029628	-0.758239
CH17-472G2	0.2636971	4.7461473	3.5052381	0.000559	0.0029628	-0.758458
RP11-18H7.	-0.429502	3.4321241	-3.505218	0.0005591	0.0029628	-0.758523
EID1	0.0749416	6.5693958	3.5051768	0.0005591	0.0029628	-0.758658
RBFA	0.096438	6.0199904	3.5050931	0.0005593	0.0029633	-0.758932
FGR	0.1728328	5.3610954	3.5047312	0.00056	0.0029666	-0.760114
ARHGEF17	0.1461971	5.9595943	3.5045849	0.0005603	0.0029678	-0.760592
RP11-712L6	-0.140938	4.6285572	-3.504474	0.0005605	0.0029685	-0.760956
FADS1	-0.214698	6.4670904	-3.503845	0.0005618	0.0029747	-0.763011
KALRN	0.1227181	6.0474347	3.5037746	0.0005619	0.002975	-0.763239
C19orf35	0.5448485	2.8020051	3.5037307	0.000562	0.002975	-0.763383
CA7	-0.724176	-0.744867	-3.50346	0.0005626	0.0029774	-0.764268
PLEKHN1	0.6334439	3.8707486	3.5033595	0.0005628	0.0029774	-0.764595
CTNNBIP1	-0.0872	6.0311833	-3.503344	0.0005628	0.0029774	-0.764646
RN7SKP80	-0.802628	0.6894359	-3.503303	0.0005629	0.0029774	-0.764781
MSL3P1	-0.676111	3.3556161	-3.503298	0.0005629	0.0029774	-0.764796
RASL10B	-0.604275	4.4724217	-3.503035	0.0005634	0.0029798	-0.765656
AP001469.7	-0.805616	0.5396947	-3.501953	0.0005656	0.0029908	-0.769188
SMPD4P1	-0.829246	-0.533517	-3.501864	0.0005657	0.0029911	-0.769479
RP11-1041F	-1.113135	-0.250095	-3.501839	0.0005658	0.0029911	-0.769562
LINC01535	-1.040036	2.0802915	-3.501323	0.0005668	0.0029958	-0.771244
MPP1	0.1379604	6.192034	3.5013114	0.0005669	0.0029958	-0.771283
RP4-593M8.	-0.509966	-1.022284	-3.50056	0.0005684	0.003003	-0.773738
RP11-45506	-0.833489	1.5547782	-3.500558	0.0005684	0.003003	-0.773743
RP11-212P7	-0.569559	2.8146352	-3.500329	0.0005688	0.003005	-0.774491
MUC20	0.4273312	5.1280794	3.5002318	0.000569	0.0030056	-0.774807
RSL24D1P6	-0.684102	1.4613892	-3.500112	0.0005693	0.0030064	-0.7752
NT5C1B	0.7558987	2.1336381	3.4999148	0.0005697	0.0030081	-0.775842
VAMP7	0.0878806	6.3365053	3.4998107	0.0005699	0.0030088	-0.776182
WDR92	-0.142057	4.4069403	-3.49937	0.0005708	0.003013	-0.77762
ZNF225	-0.122389	5.0716585	-3.499116	0.0005713	0.0030153	-0.778449
BMPR1B	0.95916	2.4445435	3.499042	0.0005714	0.0030156	-0.77869
LHX3	1.2586585	0.7867001	3.4989777	0.0005716	0.0030159	-0.7789
RNA5SP498	0.5436993	-1.064854	3.4986173	0.0005723	0.0030193	-0.780076
LINC01444	0.4194453	-1.395918	3.4984859	0.0005725	0.0030203	-0.780504
CD7	-0.277908	5.4148763	-3.498443	0.0005726	0.0030203	-0.780645
ALDH2	0.1380388	7.1053411	3.4974729	0.0005746	0.0030303	-0.783809
CNTNAP3	0.8258463	3.32137	3.4971099	0.0005753	0.0030337	-0.784993
MED24	-0.056088	6.4683694	-3.496826	0.0005759	0.0030363	-0.785918
GNS	0.0663962	6.8146045	3.4967186	0.0005761	0.003037	-0.786269
TLX1	-0.844002	4.1279115	-3.496675	0.0005762	0.0030371	-0.786411
MARCKS	-0.087428	6.620762	-3.496608	0.0005764	0.0030373	-0.78663
RUSC1	-0.109011	6.0778027	-3.496426	0.0005767	0.0030389	-0.787224
RP13-638C3	-0.753196	1.264164	-3.49626	0.0005771	0.0030402	-0.787762
RP11-430H1	-0.519409	-1.273561	-3.495856	0.0005779	0.0030441	-0.78908
ACADVL	0.0982279	7.0889388	3.495754	0.0005781	0.0030448	-0.789414
FER1L6-AS2	-0.784846	-0.792374	-3.495291	0.0005791	0.0030493	-0.790924
KCNH6	-1.150512	0.751845	-3.494747	0.0005802	0.0030548	-0.792697
CPXM1	0.3296029	4.9676649	3.4945897	0.0005805	0.003056	-0.793209
MIR497HG	0.4148138	3.7854992	3.4944175	0.0005808	0.0030574	-0.79377

RP11-815M8	0.7664972	-0.587435	3.4941899	0.0005813	0.0030595	-0.794512
TMEM134	0.0876165	6.1421083	3.4941252	0.0005814	0.0030597	-0.794722
TBCE	-0.087099	6.2385233	-3.493996	0.0005817	0.0030607	-0.795142
SESN3	0.2105674	5.7657945	3.4938693	0.000582	0.0030616	-0.795556
ANAPC11	-0.087348	6.5696798	-3.493775	0.0005822	0.0030618	-0.795863
GPC6-AS1	-0.728154	-0.664458	-3.493767	0.0005822	0.0030618	-0.795891
TRAPPC11	0.0816705	6.0216145	3.4935192	0.0005827	0.0030641	-0.796697
NAMPTP1	0.4657665	4.9124252	3.4930977	0.0005836	0.003068	-0.79807
IP6K2	-0.068833	6.3070237	-3.493076	0.0005836	0.003068	-0.798143
THOC3	-0.101708	5.5803825	-3.492809	0.0005842	0.0030704	-0.799012
HBP1	0.0971311	6.1942283	3.4922457	0.0005853	0.0030761	-0.800846
RP11-1398F	0.4096505	3.4016291	3.4921801	0.0005855	0.0030764	-0.80106
LA16c-360H	-0.428662	2.7169819	-3.491633	0.0005866	0.0030819	-0.802843
RP11-397G5	-0.510493	-1.104019	-3.491559	0.0005867	0.0030823	-0.803083
SNORD116-4	-0.806436	-0.302519	-3.491198	0.0005875	0.0030858	-0.804257
RP11-4M23.	-0.513414	-1.065617	-3.49038	0.0005892	0.0030942	-0.806923
RP11-243M5	-0.794859	-0.472548	-3.490048	0.0005899	0.0030974	-0.808001
HIST1H2BF	-0.872422	0.7273047	-3.489587	0.0005909	0.003102	-0.809504
HNRNPA3P5	-0.577318	2.2691882	-3.489045	0.000592	0.0031075	-0.811268
PCMTD1P3	-0.461015	-1.306776	-3.488864	0.0005924	0.0031091	-0.811856
TFPI2	0.4362268	4.3829169	3.4886588	0.0005928	0.0031109	-0.812524
CRTC3-AS1	-0.596059	2.2049635	-3.488543	0.000593	0.0031117	-0.812899
RAB11FIP3	0.0957769	6.2478759	3.4884079	0.0005933	0.0031127	-0.81334
RP11-172E9	0.9778061	-0.072906	3.4882418	0.0005937	0.0031141	-0.81388
EDA	0.3412159	5.0969292	3.4880296	0.0005941	0.0031157	-0.814571
HMOX1	0.1557558	6.3934246	3.4880183	0.0005941	0.0031157	-0.814608
BZRAP1-AS1	-0.52678	3.0129691	-3.487768	0.0005947	0.003118	-0.815422
SMPD3	0.2800782	4.577788	3.4871863	0.0005959	0.0031239	-0.817314
RP5-1103B4	0.8055284	2.4824365	3.4871266	0.000596	0.0031242	-0.817509
PTPRD	0.7024271	4.7984949	3.4865579	0.0005972	0.00313	-0.819359
RGS18	0.3951113	4.2213911	3.4864016	0.0005975	0.0031313	-0.819867
RP11-148E1	-0.992381	-0.344597	-3.485889	0.0005986	0.0031364	-0.821535
AC005150.1	-1.074624	-0.384091	-3.48586	0.0005987	0.0031364	-0.821629
CETN1	-0.572285	-1.247631	-3.485621	0.0005992	0.0031385	-0.822406
RP11-285G1	-0.767873	1.443413	-3.485436	0.0005996	0.0031402	-0.823008
CEP152	-0.169413	4.9400182	-3.485156	0.0006002	0.0031426	-0.823919
FAM175A	-0.136919	5.1149213	-3.485132	0.0006002	0.0031426	-0.823995
TRIM56	0.0622338	6.3680563	3.4850602	0.0006004	0.003143	-0.824229
RP11-279F6	0.7247041	4.8937334	3.4846027	0.0006013	0.0031476	-0.825716
RP1-35C21.	-0.853513	-0.508786	-3.484177	0.0006022	0.0031519	-0.827099
BOLA3	-0.096746	5.890872	-3.483616	0.0006034	0.0031577	-0.828925
PIK3IP1	0.1452242	6.0282544	3.4833809	0.0006039	0.0031598	-0.829688
RP11-527L4	-0.660526	-0.991794	-3.483293	0.0006041	0.0031604	-0.829973
AC002480.2	-0.627154	-1.198063	-3.481789	0.0006073	0.0031767	-0.83486
HFE	0.1352893	6.0132147	3.4816564	0.0006076	0.0031777	-0.835291
RP11-863P1	0.8660972	0.0744308	3.4807989	0.0006095	0.0031867	-0.838076
YWHAB	0.0430806	6.8228274	3.4807777	0.0006095	0.0031867	-0.838145
CYP2A7	1.1437475	4.0141921	3.4802858	0.0006106	0.0031918	-0.839742
PCDH9	0.5241971	3.9328634	3.4798217	0.0006116	0.0031962	-0.841249
RP3-333H23	-0.839855	0.0257712	-3.479812	0.0006116	0.0031962	-0.841281
HMGN1	-0.071275	6.5359113	-3.479562	0.0006121	0.0031983	-0.842093

RBM7	0.0747371	5.8894147	3.479545	0.0006122	0.0031983	-0.842148
FNIP1	0.082729	6.0884337	3.4792181	0.0006129	0.0032015	-0.843209
IGSF21	0.3573614	4.4839728	3.4785433	0.0006143	0.0032087	-0.845399
FLJ42102	-0.511088	-1.111302	-3.478264	0.0006149	0.0032114	-0.846306
GJA5	0.2582577	5.490779	3.4781392	0.0006152	0.0032123	-0.846711
MT-ND6	0.1403956	7.2556152	3.4780615	0.0006154	0.0032127	-0.846963
ALOX12P2	0.6995813	4.222452	3.4779594	0.0006156	0.0032134	-0.847294
PGM3	-0.092189	6.3086655	-3.477562	0.0006165	0.0032175	-0.848585
STARD3	-0.059205	6.3550418	-3.477453	0.0006167	0.0032182	-0.848936
FGL2	0.1876985	5.8863588	3.4766951	0.0006183	0.0032264	-0.851397
IFT140	0.1308564	5.5588365	3.4759036	0.0006201	0.0032349	-0.853964
SGK223	0.1553688	5.6861816	3.4757615	0.0006204	0.0032358	-0.854425
CYP39A1	0.5717971	5.0760437	3.4757404	0.0006204	0.0032358	-0.854493
NRBF2	0.0856477	6.0612774	3.4757059	0.0006205	0.0032358	-0.854606
FBXL19-AS1	-0.347462	4.3422144	-3.475426	0.0006211	0.0032385	-0.855513
RSAD2	0.258585	5.1314572	3.4752378	0.0006215	0.0032402	-0.856124
RASL10A	0.4183927	3.1818516	3.4747891	0.0006225	0.0032449	-0.857579
CERS3	-0.783358	-0.2727	-3.474687	0.0006227	0.0032455	-0.857908
ZBTB1	0.0849619	5.8752323	3.4746561	0.0006228	0.0032455	-0.85801
RP11-554E2	-0.673187	1.3369663	-3.474001	0.0006242	0.0032525	-0.860133
TMEM200A	0.4169092	4.3216801	3.4737396	0.0006248	0.003255	-0.860982
PDIA2	-1.061937	2.5179058	-3.473416	0.0006255	0.0032583	-0.86203
RPS23P8	-0.315229	4.0520123	-3.472427	0.0006277	0.003269	-0.865237
WNT11	0.5429494	4.5389503	3.4724015	0.0006278	0.003269	-0.865319
CAPZA3	0.9135286	0.3866791	3.4719568	0.0006287	0.0032736	-0.86676
CTD-3051D2	-0.727665	1.4981841	-3.471877	0.0006289	0.0032741	-0.867018
MME	0.91314	4.3477503	3.4717828	0.0006291	0.0032743	-0.867324
C2orf73	0.5174957	-1.107704	3.4717804	0.0006291	0.0032743	-0.867331
HEXA	0.0842897	6.5169096	3.4710686	0.0006307	0.003282	-0.869638
ANKRD23	-0.222462	3.8955512	-3.470824	0.0006312	0.003284	-0.870431
CTD-3229J4	-0.458099	-1.330111	-3.470818	0.0006313	0.003284	-0.870451
AF277315.1	-0.986751	-0.540533	-3.470694	0.0006315	0.0032849	-0.870852
MED12	-0.066348	6.1883062	-3.47061	0.0006317	0.0032854	-0.871122
ACTL6B	-0.912751	1.340788	-3.470433	0.0006321	0.003287	-0.871696
TBX20	0.8014568	-0.363015	3.470062	0.0006329	0.0032906	-0.872898
PDE4DIP	0.1314609	6.3843442	3.4700407	0.000633	0.0032906	-0.872967
CTD-2620I2	-0.548653	-1.062242	-3.46996	0.0006332	0.0032911	-0.873229
AC097468.4	-0.949592	1.1803	-3.469653	0.0006339	0.0032942	-0.874223
LRRC16B	-0.330507	4.2532055	-3.469242	0.0006348	0.0032985	-0.875554
TMEM208	0.0965171	6.3899353	3.4689437	0.0006354	0.0033015	-0.876519
RP5-1056L3	-0.314926	3.8089307	-3.467611	0.0006384	0.0033165	-0.880834
CTC-338M12	-0.560088	-1.001404	-3.467554	0.0006385	0.0033167	-0.881017
RP11-750H9	0.5975	3.2692229	3.4675144	0.0006386	0.0033167	-0.881147
SNORA70	-0.661783	-0.590153	-3.466944	0.0006399	0.0033228	-0.882992
RP11-546D6	-0.13831	5.0618661	-3.466737	0.0006404	0.0033248	-0.883662
C2orf40	0.8696597	2.3312735	3.4666238	0.0006406	0.0033252	-0.884029
RP11-422P2	-0.193168	4.3250722	-3.466618	0.0006407	0.0033252	-0.884049
MPG	0.0906774	6.0279612	3.4665793	0.0006407	0.0033252	-0.884173
ADCY5	0.2719904	5.3470514	3.4663967	0.0006412	0.0033269	-0.884764
ZNF568	-0.299237	5.0905297	-3.466007	0.000642	0.003331	-0.886026
MAU2	-0.0594	6.2526314	-3.465921	0.0006422	0.0033315	-0.886302

CTD-2151A2	-0.408584	-1.354392	-3.465391	0.0006434	0.0033372	-0.888017
PITPNC1	0.1128458	5.918115	3.4653399	0.0006435	0.0033374	-0.888183
AC006946.1	-1.001126	0.0266294	-3.464976	0.0006444	0.0033412	-0.889361
RP11-736K2	0.4718649	3.7301809	3.4648969	0.0006445	0.0033416	-0.889616
DGKZP1	-0.280569	3.3836132	-3.464271	0.000646	0.0033485	-0.891639
PGRMC1	0.1186619	6.9525665	3.4638145	0.000647	0.0033534	-0.893116
RP11-140K1	-0.174938	4.9370964	-3.463383	0.000648	0.003358	-0.894511
C12orf42	-0.886558	0.9763247	-3.463283	0.0006482	0.0033587	-0.894836
CTC-251I16	0.4320952	3.9097492	3.4631382	0.0006485	0.0033599	-0.895303
TMEM37	0.1530666	6.4605615	3.4627803	0.0006493	0.0033637	-0.89646
RP11-541F9	-0.89909	-0.750196	-3.462466	0.0006501	0.0033669	-0.897475
GATA3	0.3807268	4.3985381	3.4623631	0.0006503	0.0033677	-0.897809
RP11-479J7	0.6929005	-0.594995	3.4622801	0.0006505	0.0033682	-0.898077
SLC25A11	0.0849791	6.4548176	3.4618223	0.0006515	0.0033726	-0.899557
NECAB2	0.6035071	5.0029264	3.4618217	0.0006515	0.0033726	-0.899559
ILF2	-0.063379	6.7226508	-3.461491	0.0006523	0.0033761	-0.900628
RP11-613D1	0.879947	1.2764188	3.461229	0.0006529	0.0033787	-0.901474
MAX	0.0629478	6.2214804	3.4607005	0.0006541	0.0033845	-0.903182
MSL3	0.0571825	5.8812576	3.4597182	0.0006564	0.0033957	-0.906355
SNAP47-AS1	-0.62688	-0.758122	-3.458904	0.0006582	0.0034045	-0.908984
TEX11	-1.053985	2.2033757	-3.458897	0.0006583	0.0034045	-0.909006
TXK	0.3782246	3.4714733	3.4586722	0.0006588	0.0034067	-0.909734
STXBP1	0.192721	5.5834834	3.4585431	0.0006591	0.0034075	-0.91015
CNP	-0.061671	6.6073103	-3.458528	0.0006591	0.0034075	-0.910199
RP11-120B7	-0.744982	1.248432	-3.458468	0.0006593	0.0034077	-0.910394
MORC2	-0.061947	6.2918517	-3.457927	0.0006605	0.0034137	-0.91214
CHD1L	-0.09117	6.4520842	-3.45699	0.0006627	0.0034245	-0.915164
RP11-468N1	-0.978218	0.518533	-3.456935	0.0006628	0.0034246	-0.915342
MYH7B	-0.286594	4.5739564	-3.456848	0.000663	0.0034252	-0.915623
RPS4XP8	-0.598065	-0.78521	-3.456355	0.0006642	0.0034306	-0.917214
EIF2S2P2	-0.644552	-0.680563	-3.456301	0.0006643	0.0034308	-0.917389
VAT1	-0.08754	6.7616569	-3.456261	0.0006644	0.0034308	-0.917517
RMI1	-0.107841	5.5837272	-3.456158	0.0006646	0.0034311	-0.91785
RP11-307O1	-0.647091	-0.539109	-3.456119	0.0006647	0.0034311	-0.917976
TRPV3	0.6977154	3.3268866	3.4561153	0.0006647	0.0034311	-0.917988
RP11-313M3	-0.967211	0.0981094	-3.455877	0.0006653	0.0034335	-0.918757
RP11-33N14	-0.650346	-0.443455	-3.455677	0.0006657	0.0034354	-0.919403
DBT	0.1063945	6.1594215	3.4553909	0.0006664	0.0034384	-0.920325
RP5-1074L1	-0.212544	4.8794965	-3.455296	0.0006666	0.003439	-0.920631
PVALB	0.975786	0.8666009	3.4549785	0.0006674	0.0034422	-0.921656
RP11-632K2	0.4536439	-1.198009	3.4549148	0.0006675	0.0034422	-0.921862
RP11-620J1	-0.660529	-0.771992	-3.454885	0.0006676	0.0034422	-0.921959
RP11-435F1	-0.753026	0.7921565	-3.454835	0.0006677	0.0034422	-0.922119
RP11-488I2	-0.534608	-1.203925	-3.454829	0.0006677	0.0034422	-0.922137
MDGA1	0.3353611	4.8801009	3.4546868	0.0006681	0.0034435	-0.922597
LINC00160	0.7530538	-0.789511	3.4541696	0.0006693	0.0034489	-0.924266
DNMBP-AS1	0.6357939	3.7008391	3.4541538	0.0006693	0.0034489	-0.924317
RP11-505E2	-0.788615	-0.419794	-3.45398	0.0006697	0.0034505	-0.924876
HSPD1P7	-0.526429	-0.964774	-3.453831	0.0006701	0.0034518	-0.925358
RP11-549K2	-0.99705	-0.36647	-3.453462	0.0006709	0.0034558	-0.926549
GIP	-0.777813	-0.789973	-3.453103	0.0006718	0.0034597	-0.927704

FABP5	-0.19412	5.4438241	-3.452527	0.0006731	0.0034662	-0.929564
BNC1	0.9197791	0.9512202	3.4524439	0.0006733	0.0034667	-0.929831
SLC22A12	1.3710264	2.2103559	3.4522838	0.0006737	0.0034682	-0.930347
NID2	0.2401016	5.3268928	3.4519897	0.0006744	0.0034712	-0.931295
HNRNPA1P7	-0.723127	-0.004545	-3.451902	0.0006746	0.0034718	-0.931579
RANBP3L	0.7088984	3.2539186	3.451822	0.0006748	0.0034723	-0.931836
RP11-640L9	0.6428143	2.6934641	3.4514543	0.0006757	0.0034763	-0.933021
BMP5	1.0230397	2.8166392	3.4513757	0.0006758	0.0034768	-0.933274
CHST8	1.0462183	1.5226832	3.4507966	0.0006772	0.0034833	-0.935141
RP11-219E7	0.7680871	1.6150068	3.45063	0.0006776	0.0034849	-0.935678
NPM1P50	-0.568126	-0.939747	-3.450307	0.0006784	0.0034879	-0.936718
RP13-638C3	-0.725411	-0.144518	-3.450302	0.0006784	0.0034879	-0.936736
CTD-3007L5	-0.521748	-1.233183	-3.450226	0.0006786	0.0034883	-0.93698
PIN1P1	-0.489421	-1.222552	-3.450039	0.000679	0.0034901	-0.937582
C1QTNF6	-0.129675	5.9676305	-3.449895	0.0006794	0.0034914	-0.938046
RSPH14	-0.756577	2.8095837	-3.44976	0.0006797	0.0034925	-0.938482
CTD-3162L1	-0.887257	1.4963066	-3.449393	0.0006806	0.0034965	-0.939662
GPR87	0.6833163	-0.886763	3.4492045	0.000681	0.0034984	-0.940271
LRRC8A	0.073821	6.5220674	3.4489865	0.0006815	0.0035005	-0.940973
RP11-389C8	0.2042066	4.8643708	3.4489472	0.0006816	0.0035005	-0.9411
RP11-391H1	-0.818679	1.7215491	-3.448843	0.0006819	0.0035013	-0.941436
NGDN	-0.064983	6.0691619	-3.448507	0.0006827	0.0035049	-0.942517
ESCO1	-0.06969	5.9287052	-3.448115	0.0006836	0.0035093	-0.943779
RPS10P3	-0.687937	2.0153454	-3.448046	0.0006838	0.0035096	-0.944003
CLDN19	-1.103868	2.1939708	-3.447837	0.0006843	0.0035117	-0.944676
SNORD72	-0.613788	-0.694673	-3.447705	0.0006846	0.0035128	-0.9451
RNU2-22P	-0.622804	-0.574138	-3.447428	0.0006853	0.0035157	-0.945991
RP11-686G8	-0.442905	2.7567514	-3.446818	0.0006867	0.0035226	-0.947957
RP11-396B1	0.7983567	2.1554797	3.4467865	0.0006868	0.0035226	-0.948058
CTBP1-AS2	-0.08496	5.8435644	-3.446469	0.0006876	0.0035256	-0.949081
PSPC1	-0.071479	6.0085789	-3.446467	0.0006876	0.0035256	-0.949086
CRACR2A	-0.278846	4.6723994	-3.446201	0.0006882	0.0035284	-0.949943
UPP2	0.8029345	4.0893266	3.4461551	0.0006883	0.0035284	-0.950091
KLHDC8A	-0.930873	2.8135187	-3.446114	0.0006884	0.0035285	-0.950222
RP11-220D1	-0.7041	-0.313015	-3.44553	0.0006898	0.0035352	-0.952101
PPM1N	0.4012562	3.9857149	3.4448911	0.0006914	0.0035426	-0.954159
CTD-2297D1	-1.277824	1.1269376	-3.444739	0.0006917	0.003544	-0.954648
RP11-468N1	-0.674956	-0.730586	-3.444426	0.0006925	0.003547	-0.955655
RHOQP2	0.7294019	0.5822904	3.4444165	0.0006925	0.003547	-0.955686
NDUFV2	0.1034668	6.002919	3.4440692	0.0006933	0.0035508	-0.956803
DEFA6	-0.621884	-1.079411	-3.443982	0.0006936	0.0035514	-0.957084
TWF2	0.0969778	6.3121901	3.4439195	0.0006937	0.0035517	-0.957285
EIF4G1	0.0594239	7.0745926	3.443774	0.0006941	0.003553	-0.957753
OSCP1	0.2587638	4.731191	3.4436725	0.0006943	0.0035537	-0.958079
QDPR	0.1369435	6.5314979	3.4430413	0.0006958	0.0035611	-0.96011
FEZ2	0.0778344	6.1172307	3.4428855	0.0006962	0.0035625	-0.960611
CTD-2001C1	-0.746888	0.0444807	-3.442552	0.000697	0.0035662	-0.961685
MYT1	-0.866766	2.5226227	-3.442233	0.0006978	0.0035697	-0.962711
AACSP1	-1.056567	-0.089358	-3.441916	0.0006986	0.0035731	-0.963728
RPL37P15	-0.563564	-1.166661	-3.44148	0.0006997	0.0035781	-0.96513
HSPB9	-0.324932	4.4291237	-3.441378	0.0006999	0.0035788	-0.965458

SLX1A	-0.728512	-0.3473	-3.441125	0.0007005	0.0035815	-0.966272
CHRNA6	0.8473107	0.0815108	3.4408732	0.0007011	0.0035841	-0.967081
RP11-480N2	-0.777764	0.0458972	-3.44057	0.0007019	0.0035874	-0.968056
ORC3	-0.089627	5.9840642	-3.440012	0.0007033	0.0035939	-0.96985
VWA8	0.121969	6.1545649	3.4399646	0.0007034	0.003594	-0.970001
CASC5	-0.159804	5.771578	-3.439849	0.0007037	0.003595	-0.970373
RERGL	0.7978561	3.4379018	3.4397824	0.0007038	0.0035953	-0.970587
NFKBIB	0.0845837	6.2180348	3.4396747	0.0007041	0.0035962	-0.970933
CTD-3220F1	-0.877534	0.7520851	-3.439593	0.0007043	0.0035967	-0.971197
TECRP1	-0.245683	4.2576642	-3.439345	0.0007049	0.0035993	-0.971994
RP11-669N7	-1.047345	-0.281279	-3.438326	0.0007074	0.0036116	-0.975268
SMG5	-0.078708	6.7540555	-3.438237	0.0007076	0.0036122	-0.975553
ANXA10	0.6140419	5.0841906	3.4381266	0.0007079	0.0036131	-0.975907
BCOR	-0.086156	6.1204154	-3.438055	0.0007081	0.0036132	-0.976139
INHA	-1.048847	2.5372023	-3.438043	0.0007081	0.0036132	-0.976176
MTUS2	1.0397881	1.9022072	3.4363943	0.0007122	0.0036335	-0.981471
ZBTB8A	0.1723193	5.1025191	3.436085	0.000713	0.0036369	-0.982464
ZNF222	-0.188026	4.7100077	-3.434707	0.0007164	0.003654	-0.986886
GLG1	0.0548023	6.6751725	3.4346161	0.0007166	0.0036546	-0.987179
RPL6P27	-0.155233	5.3609589	-3.434275	0.0007175	0.0036585	-0.988274
KPTN	-0.100105	5.7091724	-3.434148	0.0007178	0.0036596	-0.988679
SOS1-IT1	-0.18608	4.0383374	-3.43405	0.000718	0.0036603	-0.988995
ATF4P4	-0.597881	2.0790833	-3.434012	0.0007181	0.0036603	-0.989117
RP11-459A1	-0.57347	-0.827768	-3.433054	0.0007205	0.003672	-0.992192
TLE6	0.3397366	4.7833401	3.4327607	0.0007213	0.0036753	-0.993132
ZNF101	-0.081098	5.5265547	-3.432629	0.0007216	0.0036764	-0.993555
XXYLT1	-0.116587	5.5129087	-3.432353	0.0007223	0.0036795	-0.99444
RP11-109M1	-1.195361	0.526781	-3.432094	0.000723	0.0036823	-0.995272
IGHVIII-38	-0.378047	-1.312978	-3.431982	0.0007232	0.0036832	-0.99563
RPL6P30	-0.477762	-1.08768	-3.431922	0.0007234	0.0036835	-0.995823
KCNJ12	0.4622301	3.5260075	3.4316074	0.0007242	0.003687	-0.996831
INPP4A	0.0717087	5.8585419	3.4312803	0.000725	0.0036905	-0.99788
KIAA1614	-0.264578	4.7546915	-3.431216	0.0007252	0.0036905	-0.998085
RP11-97C16	-0.188599	4.4821818	-3.431214	0.0007252	0.0036905	-0.998091
SCAMP3	-0.078821	6.6385987	-3.43112	0.0007254	0.0036912	-0.998394
VCX2	-0.610977	-1.141037	-3.430688	0.0007265	0.0036963	-0.99978
CHST9	0.8819258	4.913935	3.4304607	0.0007271	0.0036987	-1.000508
FAM214A	0.1007371	6.1018846	3.4301043	0.000728	0.0037026	-1.00165
TLR1	0.2471751	5.1345388	3.4300789	0.0007281	0.0037026	-1.001732
PCNA	-0.091084	6.465077	-3.430034	0.0007282	0.0037026	-1.001875
XIAP	0.0731291	6.3531158	3.4292799	0.0007301	0.0037113	-1.004293
FLOT2	0.0736422	6.6675837	3.4292547	0.0007302	0.0037113	-1.004373
LY6G5B	-0.320239	4.344614	-3.429244	0.0007302	0.0037113	-1.004407
DOK4	0.107339	6.2818175	3.4291179	0.0007305	0.0037124	-1.004812
PPP6R1	-0.060565	6.5519873	-3.429046	0.0007307	0.0037126	-1.005042
HNRNPAB	0.0632572	6.6631511	3.4290163	0.0007308	0.0037126	-1.005137
AP001065.1	-0.881389	4.0531369	-3.428979	0.0007309	0.0037126	-1.005256
LHFPL4	-1.297672	0.9748677	-3.428382	0.0007324	0.0037199	-1.00717
SSX2	-0.51544	-1.18684	-3.428261	0.0007327	0.0037209	-1.007559
RP11-407B7	0.8945591	0.9126084	3.427982	0.0007334	0.003724	-1.008452
RPL3P2	-0.353772	3.5068032	-3.427762	0.000734	0.0037264	-1.009157

RRM2B	0.0829441	6.1950347	3.427664	0.0007342	0.0037271	-1.00947
UBE2F-SCLY	-0.689333	-0.142585	-3.427485	0.0007347	0.0037289	-1.010045
ANKRD26	-0.11931	5.3040327	-3.42724	0.0007353	0.0037316	-1.010828
RP11-432I5	0.4769959	-1.106667	3.426312	0.0007377	0.0037431	-1.013801
RP11-667F1	0.6609138	1.2118677	3.4261139	0.0007382	0.0037452	-1.014435
ABCG4	0.7004649	1.6639123	3.4258421	0.0007389	0.0037482	-1.015306
CTC-359D24	-0.397433	4.0594182	-3.425668	0.0007393	0.00375	-1.015862
CTC-255N20	0.8698107	-0.458827	3.4256086	0.0007395	0.0037502	-1.016053
RPL14	-0.081289	7.0674411	-3.425487	0.0007398	0.0037513	-1.016444
SOWAHC	0.1116331	6.2042585	3.4253798	0.0007401	0.0037522	-1.016786
LRRK2	0.2397038	5.2846927	3.4252413	0.0007404	0.0037535	-1.017229
ALG1	0.0813558	6.0441762	3.4251725	0.0007406	0.0037538	-1.017449
RP11-800A1	-1.124064	1.0523585	-3.425114	0.0007408	0.0037541	-1.017635
MAGEA2	-0.532749	-1.128489	-3.424974	0.0007411	0.0037554	-1.018084
ANAPC10	0.0727699	5.4387628	3.4247067	0.0007418	0.0037584	-1.018941
LA16c-360A	0.6580133	-0.574759	3.4244418	0.0007425	0.0037613	-1.019789
RP11-365H8	0.6751874	-0.801218	3.4243984	0.0007426	0.0037614	-1.019928
RP11-94B19	-0.823786	-0.551172	-3.424264	0.000743	0.0037626	-1.020358
CCDC91	0.0679499	5.896199	3.4237485	0.0007443	0.0037688	-1.022008
SNORD114-3	-0.446847	-1.291819	-3.423702	0.0007444	0.0037689	-1.022156
RP11-57H14	-0.671277	0.9454579	-3.423557	0.0007448	0.0037698	-1.022621
RP11-363E6	-0.814766	0.1771911	-3.423556	0.0007448	0.0037698	-1.022624
POLE	-0.077782	6.2432503	-3.423269	0.0007455	0.003773	-1.023541
RP11-399J1	-0.737085	3.2905363	-3.423233	0.0007456	0.003773	-1.023658
RP11-111H3	0.3919226	-1.32382	3.4229986	0.0007462	0.0037755	-1.024407
AHI1	0.1619177	5.3666783	3.4229364	0.0007464	0.0037758	-1.024606
CASP16	0.8607313	3.5465098	3.422633	0.0007472	0.0037793	-1.025577
NADK	0.0865201	6.4882403	3.4224457	0.0007477	0.0037812	-1.026176
SKIV2L	-0.067578	6.4530497	-3.422215	0.0007483	0.0037838	-1.026915
PLD2	0.0859222	5.9399611	3.4221532	0.0007484	0.003784	-1.027112
CCDC89	0.796422	0.6795264	3.4211739	0.000751	0.0037964	-1.030244
WNT4	0.6003948	4.7580048	3.4210945	0.0007512	0.003797	-1.030498
TSPAN17	0.07723	6.2805246	3.4209767	0.0007515	0.003798	-1.030875
RPS27P23	-0.654302	-0.50252	-3.420147	0.0007537	0.0038084	-1.033527
ZNF195	-0.082573	5.7818634	-3.419899	0.0007543	0.0038112	-1.034321
COG2	-0.075522	6.113442	-3.419778	0.0007546	0.0038123	-1.034708
RPL13AP7	-0.32631	3.5324948	-3.419703	0.0007548	0.0038123	-1.034949
RAB27A	0.1224077	6.1333544	3.4196958	0.0007549	0.0038123	-1.034971
GTF3C3	-0.05587	5.9938568	-3.419571	0.0007552	0.0038134	-1.035369
ACSM5P1	0.9469861	2.653696	3.4191959	0.0007562	0.0038179	-1.036569
CCDC77	-0.109017	5.3506071	-3.418938	0.0007569	0.0038208	-1.037393
RP11-129K1	-0.639726	-0.538992	-3.418486	0.000758	0.0038263	-1.038837
MASTL	-0.093638	5.6307637	-3.418297	0.0007585	0.003828	-1.039443
ZNF440	-0.123002	5.2710337	-3.418274	0.0007586	0.003828	-1.039516
YIPF5	0.0577036	6.2456494	3.41814	0.000759	0.0038293	-1.039943
AC083899.3	-0.227794	4.368654	-3.416522	0.0007632	0.0038503	-1.045113
HOXB3	0.2560646	5.0009319	3.4160168	0.0007646	0.003856	-1.046726
AC011294.3	-0.91404	3.21031	-3.415981	0.0007647	0.003856	-1.046841
TRAF3IP2-A	-0.147535	4.4496136	-3.415922	0.0007648	0.003856	-1.047031
NLRP1	0.1493123	5.7455701	3.4159088	0.0007649	0.003856	-1.047071
LURAP1L-AS	0.7553167	-0.044287	3.4158998	0.0007649	0.003856	-1.0471

MIR3125	-0.763609	-0.719364	-3.415843	0.000765	0.0038562	-1.04728
RPS4XP23	-0.729752	-0.417926	-3.415771	0.0007652	0.0038567	-1.047511
NAALADL2	0.5085641	4.613481	3.4154391	0.0007661	0.0038604	-1.048571
HNRNPU	-0.04152	6.9772762	-3.415413	0.0007662	0.0038604	-1.048654
GFY	-0.798428	-0.614352	-3.414732	0.000768	0.003869	-1.05083
M1AP	0.679001	2.3358387	3.4139923	0.00077	0.0038784	-1.053191
RAB8A	0.0731281	6.2866646	3.4139334	0.0007701	0.0038786	-1.053379
MIR219A1	-0.570058	-0.784541	-3.413904	0.0007702	0.0038786	-1.053474
MAGED1	-0.103079	6.806219	-3.413772	0.0007706	0.0038798	-1.053893
RP4-585I14	-0.753405	0.0784963	-3.413632	0.0007709	0.0038811	-1.05434
SAMD14	0.202836	4.544675	3.4135565	0.0007711	0.0038816	-1.054582
ZNF623	-0.084445	6.0551432	-3.413271	0.0007719	0.0038849	-1.055492
TMEM81	-0.136109	5.103411	-3.413224	0.000772	0.003885	-1.055644
POM121C	-0.063767	6.1421567	-3.412788	0.0007732	0.0038904	-1.057035
RP3-331H24	0.7262943	-0.443379	3.4125443	0.0007739	0.0038931	-1.057812
MGST3	0.0885873	6.5459661	3.4123516	0.0007744	0.0038952	-1.058427
RP1-267D11	-0.189561	4.3297082	-3.412166	0.0007749	0.0038972	-1.059021
AL022344.2	-0.546525	-1.178548	-3.41194	0.0007755	0.0038997	-1.059742
HNRNPA0	-0.052926	6.6763017	-3.411662	0.0007762	0.0039029	-1.060628
RPL15P3	-0.175974	4.9350335	-3.411478	0.0007767	0.0039049	-1.061215
ZNF92	-0.120877	5.3080858	-3.411261	0.0007773	0.0039073	-1.061905
LINC00401	-0.446979	-1.247944	-3.411031	0.0007779	0.0039097	-1.062641
PDCD1LG2	0.3748378	4.2371394	3.4109818	0.0007781	0.0039097	-1.062797
RP11-93B14	0.7691973	2.4899467	3.4109654	0.0007781	0.0039097	-1.062849
SCRN1	0.2358236	5.5583961	3.4107466	0.0007787	0.0039121	-1.063547
AC068282.3	-0.247047	4.7220631	-3.410633	0.000779	0.0039131	-1.063911
SOCS7	-0.11714	5.6913588	-3.410598	0.0007791	0.0039131	-1.06402
RP11-364B6	-0.511749	-1.108771	-3.409496	0.0007821	0.0039275	-1.067536
MPPED2	0.5124646	4.0770191	3.4094022	0.0007823	0.0039283	-1.067834
TMEM87A	0.0665018	6.1682438	3.4092005	0.0007829	0.0039305	-1.068477
CTD-2315E1	0.8371305	-0.388662	3.4091267	0.0007831	0.0039309	-1.068713
RP1-278E11	-0.437615	3.2404436	-3.408935	0.0007836	0.003933	-1.069325
NIT2	0.0894313	6.5012005	3.4086969	0.0007843	0.0039357	-1.070083
C4orf3	0.0790353	6.5237159	3.4082394	0.0007855	0.003941	-1.071541
GDPD4	-0.829706	2.6424457	-3.408235	0.0007855	0.003941	-1.071557
NKAPP1	-0.179035	4.1944883	-3.408191	0.0007856	0.003941	-1.071695
C16orf58	0.1045619	6.2455347	3.408025	0.0007861	0.0039421	-1.072225
MLPH	0.3091731	5.9336745	3.4080245	0.0007861	0.0039421	-1.072226
MIR647	-0.51748	3.1255735	-3.407994	0.0007862	0.0039421	-1.072323
LIMD2	-0.156266	5.9008569	-3.407881	0.0007865	0.0039431	-1.072682
RP1-90G24.	-0.927857	0.1538696	-3.407587	0.0007873	0.0039466	-1.073621
RP11-361D1	0.7687218	0.8022877	3.4074425	0.0007877	0.003948	-1.074081
LYSMD3	0.0950646	6.0329262	3.4073612	0.0007879	0.0039486	-1.07434
MAP1A	0.3140783	5.0028966	3.4070209	0.0007888	0.0039527	-1.075424
SERINC1	0.0748151	6.7751628	3.4068641	0.0007893	0.0039543	-1.075924
MAP3K7	-0.062609	6.1292378	-3.406573	0.0007901	0.0039577	-1.076853
ZNF628	-0.084236	5.506893	-3.406486	0.0007903	0.0039584	-1.077127
MAP1LC3C	0.7478542	1.5681853	3.4062755	0.0007909	0.0039607	-1.077799
RNU6-909P	-0.406428	-1.285155	-3.405927	0.0007918	0.003965	-1.078909
BRWD3	-0.100031	5.7737683	-3.405643	0.0007926	0.0039684	-1.079816
RPL9P7	-0.427152	2.8038592	-3.405234	0.0007937	0.0039734	-1.081117

PAXIP1	-0.08038	5.8018464	-3.405012	0.0007943	0.0039759	-1.081824
RP4-616B8.	-0.741754	-0.091527	-3.404899	0.0007946	0.003977	-1.082183
NDUFB10	0.0882147	6.5178889	3.4047786	0.000795	0.0039781	-1.082567
TFEC	0.294822	4.7636325	3.404556	0.0007956	0.0039806	-1.083276
CTD-2005H7	0.6334502	-0.730377	3.4044934	0.0007958	0.0039809	-1.083475
ROR1-AS1	0.7473569	-0.555111	3.4042544	0.0007964	0.0039837	-1.084236
CLYBL	0.1700895	6.0953683	3.403636	0.0007981	0.0039916	-1.086205
C2CD4B	0.5193586	3.563007	3.4036011	0.0007982	0.0039916	-1.086316
RP11-332H1	-0.22972	4.2310054	-3.403265	0.0007992	0.0039957	-1.087385
TLL1	0.7329105	2.5564281	3.4028114	0.0008004	0.0040014	-1.08883
RP11-180I4	-0.755719	-0.383096	-3.402663	0.0008008	0.004003	-1.089304
POU6F2-AS2	-0.79532	-0.717121	-3.402333	0.0008017	0.004007	-1.090352
RP11-286E1	0.70422	-0.274418	3.4021621	0.0008022	0.0040088	-1.090897
CALR4P	0.7855835	-0.135648	3.4018536	0.0008031	0.0040124	-1.091878
LINC01449	-0.621104	-0.790748	-3.401814	0.0008032	0.0040124	-1.092005
AC013463.2	-0.518099	3.48396	-3.401782	0.0008033	0.0040124	-1.092106
PCGF3	-0.068703	6.2022181	-3.401701	0.0008035	0.004013	-1.092364
BASP1	0.256845	5.4822894	3.4016063	0.0008038	0.0040138	-1.092665
RP11-384M2	-0.734395	1.165229	-3.401368	0.0008044	0.0040166	-1.093424
LINC00997	0.1491783	5.0325608	3.4012395	0.0008048	0.0040178	-1.093832
VEGFB	-0.144734	6.2832886	-3.400896	0.0008057	0.004022	-1.094926
NRBF2P2	-0.627858	-0.888247	-3.400859	0.0008059	0.004022	-1.095044
RPL27	-0.074538	7.0775733	-3.399662	0.0008092	0.0040379	-1.09885
ZNF7	-0.085429	5.947507	-3.399643	0.0008093	0.0040379	-1.09891
TXNDC11	0.0790477	6.5279778	3.3994879	0.0008097	0.0040395	-1.099404
RP11-305K5	-0.230505	4.5543327	-3.399442	0.0008098	0.0040396	-1.099549
GMNN	-0.134454	6.2592819	-3.399298	0.0008102	0.004041	-1.100008
FGF13	0.3485807	5.0425502	3.3992161	0.0008104	0.0040416	-1.100268
ETF1	0.0546063	6.5539269	3.399022	0.000811	0.0040438	-1.100885
LLGL2	-0.107229	6.5321584	-3.398459	0.0008126	0.0040511	-1.102676
HSPA4L	0.2197002	5.7881741	3.398402	0.0008127	0.0040514	-1.102856
FTSJ3	-0.055639	6.3944762	-3.398355	0.0008129	0.0040515	-1.103006
TMEM114	0.4646595	-1.249423	3.3982724	0.0008131	0.0040521	-1.103268
MMP11	-0.255505	5.8349303	-3.398223	0.0008132	0.0040522	-1.103424
EBF1	0.2225416	5.0986482	3.3981044	0.0008136	0.0040529	-1.103802
HIST1H2BO	-0.827828	1.7950532	-3.398065	0.0008137	0.0040529	-1.103927
HNRNPA1P12	-0.682101	1.2775412	-3.398057	0.0008137	0.0040529	-1.103954
OPTN	0.0756983	6.7016753	3.3977582	0.0008145	0.0040565	-1.104903
RNU6-403P	0.6059183	-0.979747	3.3976683	0.0008148	0.0040572	-1.105189
RP11-396C2	-0.704795	1.3954897	-3.397545	0.0008151	0.0040581	-1.10558
RP11-10J21	-0.65765	-0.678147	-3.397524	0.0008152	0.0040581	-1.105648
RP4-607I7.	0.890217	-0.110103	3.3973003	0.0008158	0.0040607	-1.106358
CTSZ	0.0838668	6.9767991	3.3970434	0.0008166	0.0040634	-1.107175
RP4-639F20	0.2634454	5.3256969	3.3970347	0.0008166	0.0040634	-1.107202
AC011747.6	-0.892444	0.179624	-3.396857	0.0008171	0.0040653	-1.107768
RPS6KB1	-0.059897	6.0651961	-3.396423	0.0008183	0.0040709	-1.109146
IAH1	0.0692126	6.2062677	3.3962161	0.0008189	0.0040732	-1.109804
KRT19P4	0.2174124	-1.498877	3.3959301	0.0008197	0.0040767	-1.110713
RASSF5	0.1485882	5.8646601	3.3956087	0.0008206	0.0040807	-1.111734
AC022182.1	-0.756495	0.0015375	-3.395553	0.0008208	0.0040809	-1.111912
TIMM17B	-0.078767	6.3056989	-3.39533	0.0008214	0.0040835	-1.11262

PSTPIP2	0.2002482	5.7249492	3.3950521	0.0008222	0.0040869	-1.113501
RP11-355N1	-0.827942	0.8141984	-3.394923	0.0008226	0.0040881	-1.11391
IPP	0.0913592	5.602669	3.3945394	0.0008237	0.0040928	-1.11513
PSG8	0.6515813	-1.009876	3.3945116	0.0008237	0.0040928	-1.115218
CTC-204F22	-0.580902	2.1240653	-3.394438	0.000824	0.0040933	-1.115451
RP11-618I1	-1.254107	1.3236828	-3.393916	0.0008254	0.0040995	-1.11711
LAMA1	0.858309	3.2165977	3.3938885	0.0008255	0.0040995	-1.117197
LIMD1-AS1	-0.701152	1.1823512	-3.393884	0.0008255	0.0040995	-1.117211
IGF2BP3	-0.984287	3.8579304	-3.393629	0.0008263	0.0041025	-1.11802
RP11-263G2	-1.117875	1.9004732	-3.393171	0.0008276	0.0041085	-1.119475
SRD5A2	0.6740503	4.796175	3.393076	0.0008278	0.0041093	-1.119777
CADM3-AS1	0.8541059	0.0648051	3.3926034	0.0008292	0.0041154	-1.121277
CTD-2575K1	-0.960601	1.3400249	-3.392535	0.0008294	0.0041159	-1.121495
RP11-483A2	0.3804258	-1.370275	3.3923368	0.00083	0.0041181	-1.122123
RP11-216L1	-0.818652	0.5288159	-3.39204	0.0008308	0.0041216	-1.123065
RP11-527L4	-0.633378	-0.806032	-3.392014	0.0008309	0.0041216	-1.123148
SPATA45	-0.772681	1.0850059	-3.391897	0.0008312	0.0041227	-1.123519
ANKRD16	-0.09448	5.5392595	-3.391814	0.0008315	0.0041233	-1.123783
RP11-845M1	0.8448434	-0.341227	3.3914383	0.0008325	0.0041281	-1.124975
C1orf132	0.3092294	4.3301864	3.3910538	0.0008337	0.004133	-1.126195
MFAP5	0.8495281	2.4330262	3.3910152	0.0008338	0.004133	-1.126318
C4A	0.1841415	6.7430262	3.3905773	0.000835	0.0041386	-1.127707
RP11-427M2	-0.738762	-0.65054	-3.390548	0.0008351	0.0041386	-1.127801
NCMAP	-0.637656	4.7866246	-3.390229	0.000836	0.0041426	-1.128812
SRP68	-0.051848	6.5417912	-3.390008	0.0008367	0.0041452	-1.129512
ZBTB46	-0.099746	5.7247537	-3.389579	0.0008379	0.0041508	-1.130873
CBLN3	0.2671909	4.6737645	3.3893652	0.0008385	0.0041533	-1.131552
MYOZ2	0.7782784	0.0339712	3.3891335	0.0008392	0.004156	-1.132287
RP11-196G1	0.7819104	0.6322104	3.3889681	0.0008397	0.0041576	-1.132811
RNF144A	-0.177168	5.4531103	-3.388949	0.0008397	0.0041576	-1.13287
PDE4D	0.2522361	5.2273953	3.3881388	0.0008421	0.0041686	-1.135441
PPP2R3B	-0.102392	5.6446299	-3.387774	0.0008432	0.0041733	-1.136598
CTD-3105H1	-0.643947	-0.648172	-3.387668	0.0008435	0.0041743	-1.136934
MINOS1	0.0986523	5.9445423	3.3875347	0.0008438	0.0041757	-1.137356
RP11-114H2	-0.605758	-0.825414	-3.387482	0.000844	0.0041758	-1.137523
DBP	-0.136855	5.917666	-3.387147	0.000845	0.0041801	-1.138584
MRO	0.4060104	4.3662501	3.387021	0.0008453	0.0041814	-1.138984
FILIP1	0.2382835	5.1456028	3.3867868	0.000846	0.0041842	-1.139727
EFTUD2	-0.05367	6.550838	-3.386419	0.0008471	0.0041889	-1.140892
RP4-765C7.	-1.331765	2.1484842	-3.386063	0.0008481	0.0041935	-1.142021
KIF3A	0.098092	5.3939633	3.3859226	0.0008486	0.0041939	-1.142465
RTN1	0.3037321	4.8728109	3.3859205	0.0008486	0.0041939	-1.142472
LARP6	0.3487282	5.0265124	3.3859191	0.0008486	0.0041939	-1.142476
SDF4	0.068913	6.77871	3.3858549	0.0008488	0.0041942	-1.14268
OSGEPL1-AS	-0.407426	2.5415027	-3.385601	0.0008495	0.0041974	-1.143486
FAM107A	0.2481741	5.2834429	3.3853421	0.0008503	0.0042005	-1.144305
RPL9	-0.076542	6.9942248	-3.385198	0.0008507	0.0042021	-1.14476
C3orf33	-0.111386	5.1823926	-3.385059	0.0008511	0.0042035	-1.1452
AC025165.8	0.8895338	1.2423805	3.3847835	0.0008519	0.0042069	-1.146075
GBP1P1	0.4093205	4.1124676	3.3847427	0.000852	0.0042069	-1.146204
RP11-403A3	1.1952556	1.4416342	3.384709	0.0008521	0.0042069	-1.14631

RPL6	-0.068833	7.1273574	-3.384387	0.0008531	0.004211	-1.14733
AGAP3	0.0673938	6.4503893	3.3843471	0.0008532	0.004211	-1.147457
RP11-148L2	0.804642	-0.142445	3.384052	0.0008541	0.0042144	-1.148391
PROS1	0.1457547	6.7110779	3.3840354	0.0008541	0.0042144	-1.148444
C12orf65	-0.058013	5.9259172	-3.383972	0.0008543	0.0042147	-1.148644
RP11-97012	-0.654913	-0.29678	-3.383783	0.0008548	0.0042169	-1.149242
ACBD4	0.1502408	6.2247472	3.3833903	0.000856	0.0042221	-1.150487
ALDH3A1	0.8326204	4.6774277	3.3829121	0.0008574	0.0042285	-1.152001
SFXN1	0.0962311	6.5098977	3.3827673	0.0008578	0.00423	-1.15246
RPL18AP3	-0.164131	5.3137581	-3.382672	0.0008581	0.0042308	-1.152762
HNRNPA1P28	-0.495258	-0.90934	-3.382523	0.0008586	0.0042324	-1.153232
RPL32P31	-0.435671	-1.20052	-3.381799	0.0008607	0.0042419	-1.155524
TMPPE	-0.223125	4.2154701	-3.381799	0.0008607	0.0042419	-1.155524
AP000648.5	-0.21891	4.456862	-3.381476	0.0008617	0.004246	-1.156547
PTGES2-AS1	-0.831482	1.9230149	-3.380956	0.0008632	0.0042531	-1.158192
GAPDHP1	-0.405377	5.0828879	-3.380303	0.0008652	0.0042615	-1.160259
RP11-543P1	-0.189398	5.3649486	-3.380302	0.0008652	0.0042615	-1.160264
MMP23B	0.8459719	1.3205584	3.3800515	0.0008659	0.0042646	-1.161055
CTC-459F4.	-0.605677	2.5681185	-3.379777	0.0008667	0.0042681	-1.161923
IKBKB	0.0746743	6.0986872	3.3796484	0.0008671	0.0042694	-1.16233
RP4-671G15	-0.680689	1.8335035	-3.379323	0.0008681	0.0042732	-1.163361
RP11-587P2	-1.220835	0.1862063	-3.379298	0.0008682	0.0042732	-1.163437
LINC01511	-0.744873	-0.842711	-3.379271	0.0008682	0.0042732	-1.163524
CD300A	0.1922502	5.3595537	3.3791584	0.0008686	0.0042743	-1.16388
CTD-2192J1	-0.738469	0.5855243	-3.379035	0.000869	0.0042755	-1.16427
MCMBP	-0.05611	6.2959396	-3.378867	0.0008695	0.0042774	-1.1648
RP11-177H2	0.7545505	1.1904967	3.3786454	0.0008701	0.0042801	-1.165503
RP4-616B8.	-0.726501	2.0755301	-3.378609	0.0008702	0.0042801	-1.165619
RPL7P38	-0.509695	-1.0855	-3.378288	0.0008712	0.0042841	-1.166632
DUS2	0.0967984	5.6751491	3.3782586	0.0008713	0.0042841	-1.166726
SNHG24	-1.003434	-0.09357	-3.378014	0.000872	0.0042871	-1.167501
KLHL24	0.0775992	6.2644611	3.3779239	0.0008723	0.0042879	-1.167785
PAGE2	-1.196872	-0.072622	-3.377593	0.0008733	0.0042921	-1.16883
USHBP1	0.1763579	5.0693212	3.3775587	0.0008734	0.0042921	-1.168939
NR2E3	0.808629	1.2879831	3.3775046	0.0008735	0.0042924	-1.16911
RP11-783K1	-0.745378	0.3993523	-3.377168	0.0008746	0.0042968	-1.170176
RP11-338N1	-1.006897	0.3975518	-3.376904	0.0008754	0.0043001	-1.17101
RP11-328K4	1.070599	3.6620298	3.3767939	0.0008757	0.0043011	-1.171357
FCGR1A	0.3645674	4.441335	3.3767586	0.0008758	0.0043011	-1.171469
NANP	-0.082764	5.5311424	-3.376419	0.0008768	0.0043055	-1.172541
HSPA8	0.0761077	7.2338347	3.3755923	0.0008793	0.0043172	-1.175155
FRAS1	-0.609665	4.9200464	-3.375422	0.0008798	0.0043192	-1.175694
LINC00589	0.7166596	-0.587318	3.375283	0.0008803	0.0043207	-1.176132
CTD-3064H1	-0.548199	-0.965372	-3.375209	0.0008805	0.0043212	-1.176367
HLA-DPB1	0.1377732	6.6841781	3.3745947	0.0008823	0.0043298	-1.178307
RASA3	0.1662203	5.7128382	3.3741053	0.0008838	0.0043365	-1.179853
IGF1R	0.2708626	5.3141635	3.3739413	0.0008843	0.0043383	-1.180371
CTD-2340E1	-0.601188	-0.974974	-3.373829	0.0008847	0.0043394	-1.180726
CYB5R3	0.0666745	6.9579774	3.3736583	0.0008852	0.0043414	-1.181265
ANGPTL2	0.1601155	5.8837778	3.3733738	0.0008861	0.0043451	-1.182163
FAM47E-STB	0.1786524	5.7416585	3.372902	0.0008875	0.0043515	-1.183654

MRPL42	-0.052038	6.2746826	-3.372682	0.0008882	0.0043542	-1.184347
TMTC1	0.2561848	5.374153	3.3726177	0.0008884	0.0043546	-1.184551
AC019118.4	-0.552165	-0.921234	-3.37225	0.0008895	0.0043596	-1.185711
BMX	0.5928824	3.5024179	3.3719459	0.0008904	0.0043635	-1.186672
RP11-165F2	-0.646245	-0.6959	-3.371753	0.000891	0.0043658	-1.18728
RPL37P2	-0.635075	2.6569881	-3.371624	0.0008914	0.004367	-1.187689
FAM86EP	-0.127631	4.9400231	-3.371595	0.0008915	0.004367	-1.18778
C11orf24	0.0909311	6.3898243	3.3713974	0.0008921	0.0043694	-1.188404
SNORD100	-0.687224	1.8117145	-3.371092	0.000893	0.0043734	-1.189368
MIR143HG	0.4650521	3.4233261	3.3708605	0.0008938	0.0043763	-1.190098
RP11-440I1	0.97284	0.7261352	3.3704861	0.0008949	0.0043814	-1.19128
AC009236.2	-0.671937	-0.65524	-3.370343	0.0008953	0.004383	-1.191732
LDHAL6A	-0.787884	0.4332187	-3.37024	0.0008957	0.0043839	-1.192057
ARL6IP1	0.0707577	6.827745	3.3696522	0.0008975	0.0043922	-1.193911
PRR34	0.441345	2.924146	3.369557	0.0008978	0.004393	-1.194212
APEX1	-0.056524	6.6581392	-3.367825	0.0009031	0.0044187	-1.199674
MFGE8	0.1278753	6.145511	3.3671381	0.0009053	0.0044285	-1.201841
RP11-297C4	-0.804145	0.402917	-3.366785	0.0009064	0.0044333	-1.202953
MOB4	-0.054457	5.935303	-3.366541	0.0009071	0.0044365	-1.203724
RP11-521C2	-0.759186	0.0871646	-3.366174	0.0009083	0.0044415	-1.204882
GPR35	-0.426228	4.7895965	-3.364974	0.000912	0.0044592	-1.208661
C17orf82	-0.42979	3.4594154	-3.364881	0.0009123	0.00446	-1.208954
RP11-74J13	-0.375675	3.0540635	-3.364735	0.0009128	0.0044617	-1.209414
TM2D1	0.0731646	5.8780441	3.3639289	0.0009153	0.0044734	-1.211956
CTD-2619J1	-0.725521	-0.569134	-3.363884	0.0009154	0.0044735	-1.212097
CSDE1	0.0562961	7.002555	3.3637596	0.0009158	0.0044746	-1.212489
RUNDC3A	-0.639799	3.1842858	-3.363734	0.0009159	0.0044746	-1.212571
RP11-334C1	-0.673497	2.1581439	-3.36362	0.0009163	0.0044758	-1.21293
MAGED4	-0.337261	-1.367463	-3.363489	0.0009167	0.0044772	-1.213343
RP11-532F6	0.5876014	2.8478826	3.3632187	0.0009175	0.0044808	-1.214193
CLEC4E	0.5762234	3.8302658	3.363065	0.000918	0.0044825	-1.214677
DNAJC25	0.113389	5.8755026	3.3628358	0.0009187	0.0044855	-1.215398
EIF2B3	0.0877486	6.0059805	3.3626873	0.0009192	0.0044871	-1.215866
ZNF493	-0.191117	5.0943482	-3.362609	0.0009195	0.0044877	-1.216112
RP11-586D1	-0.749448	-0.506164	-3.36202	0.0009213	0.0044962	-1.217968
C22orf39	-0.075666	5.8998858	-3.361975	0.0009215	0.0044963	-1.218109
YBX3	0.1536494	6.166849	3.3616497	0.0009225	0.0045007	-1.219133
SCN1A	-1.094545	0.172909	-3.361523	0.0009229	0.0045021	-1.219533
KRT27	0.9043638	0.2876266	3.3614574	0.0009231	0.0045024	-1.219739
QSER1	-0.07211	5.9965311	-3.361424	0.0009232	0.0045024	-1.219843
HRH2	0.7733036	2.2261681	3.361201	0.0009239	0.0045053	-1.220546
RP11-278C7	-0.27561	3.2806225	-3.360404	0.0009264	0.004517	-1.223054
MOSPD1	0.1284912	5.6836348	3.3599899	0.0009278	0.0045228	-1.224358
UCHL1	0.5322745	4.5809524	3.359833	0.0009283	0.0045247	-1.224852
ITPRIPL2	0.1343857	5.9661665	3.3591399	0.0009305	0.0045348	-1.227033
CHMP5	0.0626737	6.3452017	3.3589919	0.0009309	0.0045365	-1.227498
ADAM19	0.1264198	5.7774258	3.3587422	0.0009317	0.0045398	-1.228284
LINC01503	0.2911443	4.5926818	3.3586939	0.0009319	0.0045399	-1.228436
LPHN2	0.1710751	6.2260817	3.3585661	0.0009323	0.0045413	-1.228838
BMS1P4	-0.315049	3.7004304	-3.358499	0.0009325	0.0045418	-1.229049
SLC25A24	0.2372627	5.3692473	3.3580229	0.000934	0.004548	-1.230547

SNN	0.0937774	5.9932544	3.3580226	0.000934	0.004548	-1.230547
FAM174A	0.0739045	5.8448604	3.3578279	0.0009347	0.0045504	-1.23116
KRTAP1-1	0.6975571	-0.80838	3.3577766	0.0009348	0.0045506	-1.231321
LINC01185	-0.521567	-0.976058	-3.357641	0.0009353	0.0045521	-1.231749
ZNF214	0.5837599	3.7651601	3.3573754	0.0009361	0.0045557	-1.232583
ILKAP	-0.064597	6.0167297	-3.357057	0.0009371	0.00456	-1.233584
CTB-60B18.	0.3108271	-1.451272	3.3570171	0.0009373	0.00456	-1.23371
ZNF418	-0.383466	4.5058051	-3.356713	0.0009382	0.0045642	-1.234667
DRAM2	0.0612165	6.2240934	3.3563576	0.0009394	0.0045691	-1.235783
ADAL	0.1333807	5.4460437	3.356099	0.0009402	0.0045726	-1.236597
RP11-94C24	-0.797825	0.2004569	-3.355681	0.0009416	0.0045785	-1.23791
TCF20	-0.068669	6.2302369	-3.355425	0.0009424	0.0045819	-1.238715
FAM173A	0.1139511	5.7608302	3.3550468	0.0009436	0.0045873	-1.239904
POLDIP3	-0.065354	6.4283455	-3.354827	0.0009443	0.0045901	-1.240596
CTD-3252C9	0.2326872	5.039375	3.3542602	0.0009462	0.0045984	-1.242376
CSNK1G1	-0.077344	5.6890947	-3.353773	0.0009477	0.0046055	-1.243908
CCSER2	0.0758666	6.102622	3.353583	0.0009484	0.0046079	-1.244504
RPS15P5	-0.725984	0.9272425	-3.352148	0.000953	0.00463	-1.24901
LINC00963	0.0875181	6.4562226	3.3519987	0.0009535	0.0046317	-1.24948
RP11-775G2	-0.701581	-1.019447	-3.351822	0.0009541	0.0046339	-1.250036
LMCD1-AS1	-0.448547	3.0046917	-3.351378	0.0009556	0.0046403	-1.251428
AC005152.3	-0.928776	0.0732533	-3.350918	0.0009571	0.004647	-1.252872
JPX	-0.089106	5.8274065	-3.35073	0.0009577	0.0046494	-1.253463
ASB9P1	-0.910753	1.215437	-3.349827	0.0009606	0.0046632	-1.256298
SLC6A6	0.2225942	5.7111881	3.3497772	0.0009608	0.0046634	-1.256454
RPSAP14	-0.664982	1.2739979	-3.349405	0.000962	0.0046682	-1.257621
NUP205	-0.072741	6.1657057	-3.349394	0.0009621	0.0046682	-1.257655
SPINK4	-0.988962	-0.178705	-3.349259	0.0009625	0.0046698	-1.258081
CASS4	0.2664331	4.4852267	3.3489674	0.0009635	0.0046738	-1.258995
RSU1	0.0602424	6.3637725	3.3487356	0.0009642	0.0046769	-1.259723
FAH	0.1373118	6.7107877	3.3485905	0.0009647	0.0046786	-1.260178
APOA2	-0.330319	7.6319011	-3.34812	0.0009663	0.0046855	-1.261653
RP6-218J18	-0.655086	-0.257465	-3.347895	0.000967	0.0046881	-1.26236
NDRG4	0.320131	4.5997041	3.3478768	0.0009671	0.0046881	-1.262417
CTD-2270P1	-0.468225	3.3804503	-3.347808	0.0009673	0.0046881	-1.262632
RP11-483L5	0.9048037	1.6097692	3.3478013	0.0009673	0.0046881	-1.262654
SLC25A43	0.1041334	5.923144	3.3475183	0.0009683	0.0046921	-1.263541
OR7C1	-0.675604	-1.013725	-3.347208	0.0009693	0.0046964	-1.264516
ARL6	0.1818197	4.5301091	3.3467809	0.0009707	0.0047027	-1.265854
PARP1	-0.068736	6.7699954	-3.346658	0.0009711	0.004704	-1.26624
LINC01225	-0.984498	1.1639578	-3.34657	0.0009714	0.0047048	-1.266514
LRRC25	0.1803222	5.3310892	3.3459553	0.0009734	0.0047141	-1.268443
TNNT2	-0.910317	2.8144683	-3.345823	0.0009739	0.0047156	-1.268858
RP11-333I1	-0.729699	1.7347729	-3.345739	0.0009742	0.0047163	-1.269121
NT5DC1	0.1057103	6.1489439	3.3453364	0.0009755	0.0047222	-1.270383
RP11-863P1	0.3231453	-1.376702	3.3451131	0.0009763	0.0047252	-1.271083
RP1-163G9.	-0.740034	-0.704868	-3.344991	0.0009767	0.0047263	-1.271466
RP11-49014	-0.807906	2.1308305	-3.344966	0.0009767	0.0047263	-1.271545
CSAG4	-0.947541	-0.564118	-3.344721	0.0009776	0.0047296	-1.272312
AGGF1P2	-0.71466	-0.535433	-3.34466	0.0009778	0.00473	-1.272502
ZDHHC6	-0.068728	6.1749262	-3.344335	0.0009789	0.0047346	-1.273522

RP11-49907	0.5928305	3.3205368	3.3442022	0.0009793	0.0047361	-1.273938
USP26	-0.461154	-1.240993	-3.344169	0.0009794	0.0047361	-1.274043
RP11-184E9	-0.922798	-0.477918	-3.343854	0.0009805	0.0047405	-1.27503
FAM69B	-0.191806	5.6946081	-3.343553	0.0009815	0.0047446	-1.275973
GTSE1-AS1	-0.534164	2.3209754	-3.343527	0.0009816	0.0047446	-1.276053
AF131215.2	0.7226613	2.700047	3.3431088	0.000983	0.0047507	-1.277364
TGM5	0.6973943	-0.818102	3.3430567	0.0009831	0.0047509	-1.277527
SNORD17	-0.681301	2.1183622	-3.342802	0.000984	0.0047544	-1.278324
WARS2	0.0893335	5.7847201	3.3425432	0.0009849	0.0047575	-1.279136
ABHD12B	-0.553032	3.5378648	-3.342537	0.0009849	0.0047575	-1.279156
C1GALT1C1	0.0839502	6.1229483	3.3421258	0.0009863	0.0047636	-1.280443
ADORA2A-AS	-0.420802	5.7358612	-3.34075	0.0009909	0.0047854	-1.284751
RP11-100E5	-0.768446	0.0425139	-3.340505	0.0009918	0.0047882	-1.285519
LINC01307	0.3619667	-1.374923	3.3404945	0.0009918	0.0047882	-1.285551
RP11-609D2	0.8281324	1.5848917	3.3404607	0.0009919	0.0047882	-1.285657
AC021224.1	-0.737034	0.7852999	-3.340337	0.0009923	0.0047896	-1.286045
IZUMO1	-0.697559	2.1196215	-3.34018	0.0009929	0.0047915	-1.286534
GRM8	1.1053056	2.174674	3.3395964	0.0009948	0.0048004	-1.288362
SEPN1	0.0650587	6.491668	3.3395442	0.000995	0.0048007	-1.288526
AP003900.6	-0.795549	-0.676795	-3.339142	0.0009964	0.0048066	-1.289786
AC016995.3	-0.821767	1.2862678	-3.338152	0.0009998	0.0048223	-1.292882
RPL12L3	-0.573549	1.6417061	-3.337456	0.0010021	0.0048331	-1.295061
GUK1	0.0835758	6.840522	3.3368267	0.0010043	0.0048426	-1.297028
RP6-105D16	-0.729156	1.543243	-3.336806	0.0010044	0.0048426	-1.297093
RP11-4B16.	0.8135745	0.230107	3.3367298	0.0010046	0.0048432	-1.297331
SNORA33	-0.370417	3.6198227	-3.336436	0.0010056	0.0048474	-1.298251
GAPDHP69	-0.775038	0.1435642	-3.336293	0.0010061	0.0048491	-1.298698
RP1-90G24.	-1.025598	0.7556758	-3.335408	0.0010092	0.0048631	-1.301465
CTC-297N7.	-0.608163	-0.588648	-3.335311	0.0010095	0.0048641	-1.301768
LAMC2	0.6937439	4.4241239	3.3347792	0.0010113	0.0048723	-1.30343
ZNF417	-0.125472	5.0269603	-3.334742	0.0010115	0.0048723	-1.303548
IGDCC4	-0.6614	3.9185116	-3.334428	0.0010125	0.0048768	-1.304527
CGA	-1.082349	0.5997314	-3.334294	0.001013	0.0048784	-1.304948
RN7SL8P	-0.941891	-0.217767	-3.334149	0.0010135	0.0048796	-1.3054
RP11-1151B	-0.575664	5.4229815	-3.334145	0.0010135	0.0048796	-1.305411
RP5-1125A1	-0.556629	2.3496054	-3.334042	0.0010139	0.0048802	-1.305734
WDR86	0.357382	3.7441944	3.3340353	0.0010139	0.0048802	-1.305755
ANKRD62	-0.659395	-0.710278	-3.333693	0.0010151	0.0048852	-1.306827
RP5-1086L2	0.6509304	-0.822413	3.3334517	0.0010159	0.0048886	-1.307579
BTAF1	-0.096193	6.0716859	-3.333307	0.0010164	0.0048901	-1.308031
PSMA3-AS1	-0.076959	6.0955245	-3.333287	0.0010165	0.0048901	-1.308094
GAPDHP62	-0.732259	0.3745631	-3.332471	0.0010193	0.004903	-1.310642
SLC7A9	-0.420774	5.6566201	-3.332021	0.0010209	0.0049099	-1.312049
FCF1P2	-0.096735	5.0908715	-3.331857	0.0010215	0.004912	-1.312561
FXYD2	-0.739812	4.8287478	-3.331769	0.0010218	0.0049128	-1.312835
MIB1	-0.083378	6.2682377	-3.331487	0.0010227	0.0049169	-1.313717
RP13-39P12	-0.750971	1.6106987	-3.331338	0.0010233	0.0049188	-1.314181
TMEM121	0.4976302	3.6491085	3.3309575	0.0010246	0.0049245	-1.31537
SARS	0.0647715	6.6894246	3.3308659	0.0010249	0.0049254	-1.315656
ZBTB45	-0.070684	5.7387722	-3.330736	0.0010254	0.0049264	-1.316063
HIST1H2AA	-0.594336	-0.844642	-3.330731	0.0010254	0.0049264	-1.316077

CTD-2587H1	-0.866501	-0.273664	-3.330534	0.0010261	0.004929	-1.316692
SSX9	-0.556234	-1.202855	-3.330365	0.0010267	0.0049312	-1.317222
GABRG2	-1.097474	-0.034734	-3.330259	0.001027	0.0049323	-1.31755
RP4-800G7.	-0.335757	4.399938	-3.330178	0.0010273	0.0049331	-1.317804
FOXA3	-0.372696	6.2445954	-3.329927	0.0010282	0.0049366	-1.318589
RP11-517P1	0.3170136	3.9401898	3.3294742	0.0010298	0.004943	-1.320001
PPP1R3B	0.1621861	6.4025396	3.3294695	0.0010298	0.004943	-1.320016
NID1	-0.101533	6.7733352	-3.329165	0.0010309	0.0049472	-1.320967
GNAI2	0.0582298	6.8772263	3.3291358	0.001031	0.0049472	-1.321057
PZP	0.4858697	4.2798867	3.3290866	0.0010311	0.0049472	-1.321211
TKTL2	-0.6085	-1.057675	-3.329065	0.0010312	0.0049472	-1.32128
RAB38	0.6140473	3.5900123	3.3290115	0.0010314	0.0049475	-1.321445
RP11-289K1	-0.41647	-1.214515	-3.328945	0.0010316	0.0049479	-1.321653
PDCL3P4	-0.411502	3.6144695	-3.328908	0.0010318	0.0049479	-1.321768
TRIP11	0.0755718	6.1226392	3.3282701	0.001034	0.004958	-1.323759
CTB-167G5.	-0.497276	-1.119659	-3.327929	0.0010352	0.0049627	-1.324823
CTD-2521M2	-0.559608	2.3868944	-3.327919	0.0010353	0.0049627	-1.324855
CTD-2337I7	0.612085	-1.009364	3.3277888	0.0010357	0.0049642	-1.325261
PBX2P1	-0.736218	0.6120464	-3.327498	0.0010367	0.0049685	-1.326168
AC093323.3	-0.111303	5.7455536	-3.326936	0.0010387	0.0049774	-1.327922
GALR3	-0.925477	1.3369848	-3.326871	0.0010389	0.0049778	-1.328123
PLAC9	0.2457495	4.9743362	3.3260186	0.001042	0.0049912	-1.330783
ITGA9-AS1	-0.164257	4.7594173	-3.326003	0.001042	0.0049912	-1.330831
FAM47C	0.3983223	-1.302112	3.3259398	0.0010422	0.0049913	-1.331028
APOL2	0.0991057	6.5002596	3.3259039	0.0010424	0.0049913	-1.33114
RP11-675F6	-0.734676	-0.711345	-3.325886	0.0010424	0.0049913	-1.331197
AC008069.2	-0.856454	0.1472168	-3.325681	0.0010432	0.0049941	-1.331835
SSB	-0.057278	6.4626562	-3.325089	0.0010453	0.0050035	-1.333682
AP000797.3	0.5770303	-0.915839	3.3249733	0.0010457	0.0050048	-1.334042
ESYT1	0.0637024	6.7263245	3.3248759	0.001046	0.0050058	-1.334346
UBQLN1	0.0571549	6.6392799	3.3244029	0.0010477	0.0050132	-1.33582
CTH	0.3063683	5.9708077	3.3242713	0.0010482	0.0050148	-1.33623
CTSC	0.1397807	6.5016035	3.3240394	0.001049	0.0050181	-1.336953
RP11-1070N	-0.946303	-0.237453	-3.323993	0.0010492	0.0050182	-1.337099
HBB	0.2772122	5.5035339	3.3238701	0.0010496	0.0050197	-1.337481
SCFD2	0.0728166	5.7578704	3.3234065	0.0010513	0.0050269	-1.338926
RP11-64B16	0.6634722	2.511096	3.3232046	0.001052	0.0050297	-1.339555
RP11-66N24	-0.953632	2.3532146	-3.323042	0.0010526	0.0050319	-1.340062
KB-68A7.2	-0.983388	0.9365061	-3.322794	0.0010535	0.0050354	-1.340834
MSH3	0.0914201	5.8572542	3.3227194	0.0010537	0.0050361	-1.341067
RP1-137D17	0.8293505	0.2554211	3.3219677	0.0010564	0.0050478	-1.343408
RP11-57407	0.3783875	-1.340236	3.3219585	0.0010565	0.0050478	-1.343437
RP11-32B5.	0.2772086	-1.468404	3.3219082	0.0010566	0.005048	-1.343594
RTP4	0.2204845	5.451393	3.3216265	0.0010577	0.0050522	-1.344471
NOX3	-0.486153	-1.233982	-3.320878	0.0010604	0.0050644	-1.346801
RP11-100N2	-0.636934	1.9877789	-3.320805	0.0010606	0.005065	-1.347028
HTR7	0.6887668	2.4945131	3.3205957	0.0010614	0.0050679	-1.347682
TKTL1	-1.006966	1.5668144	-3.320443	0.0010619	0.0050699	-1.348158
PRRT3-AS1	-0.522522	3.786195	-3.320256	0.0010626	0.0050725	-1.34874
IQGAP3	-0.208658	5.8819144	-3.320139	0.001063	0.0050738	-1.349104
LAMTOR3	0.0642627	6.066074	3.3195165	0.0010653	0.0050839	-1.351042

RP11-701H2	-0.943851	0.6827857	-3.319365	0.0010658	0.0050858	-1.351512
AC004221.2	-0.678303	-0.626712	-3.319104	0.0010668	0.0050897	-1.352327
CTD-2555K7	-0.640983	1.8168129	-3.31857	0.0010687	0.0050983	-1.353988
SLC39A8	0.1510984	6.0442153	3.3177408	0.0010717	0.005112	-1.356568
LINC01580	-0.648007	-1.059942	-3.317472	0.0010727	0.005116	-1.357404
SELT	0.0535458	6.4559162	3.3171898	0.0010737	0.0051202	-1.358282
PI4KAP1	-0.3025	4.817749	-3.316865	0.0010749	0.0051252	-1.359292
RNU4ATAC	-0.738883	-0.264952	-3.316366	0.0010767	0.0051332	-1.360847
LPL	0.2673237	4.9989246	3.3162256	0.0010773	0.005135	-1.361282
CITF22-49E	0.8728642	1.6348717	3.3158472	0.0010786	0.0051403	-1.362459
VNN1	0.3460927	6.4027628	3.3158434	0.0010787	0.0051403	-1.362471
WRAP53	-0.095826	5.5741531	-3.31566	0.0010793	0.0051429	-1.36304
RP5-940J5.	-0.332827	3.2896305	-3.315491	0.0010799	0.0051452	-1.363567
RP11-104L2	0.7009801	2.6090945	3.31507	0.0010815	0.0051518	-1.364875
RP11-351D1	-0.513409	2.7690321	-3.314937	0.001082	0.0051533	-1.36529
B3GALT5-AS	0.8833558	-0.17614	3.3149097	0.0010821	0.0051533	-1.365374
RP11-3L10.	-0.687009	-0.179252	-3.314415	0.0010839	0.0051613	-1.366913
NPTXR	0.3913051	4.9200176	3.3140559	0.0010852	0.0051669	-1.368028
BOLA2B	-0.396201	3.4709796	-3.313242	0.0010882	0.0051805	-1.370558
RP1-273G13	-0.724134	-0.435289	-3.312865	0.0010896	0.0051862	-1.37173
IL10RA	0.1672259	5.8374788	3.3128432	0.0010897	0.0051862	-1.371797
RP11-626P1	-0.830503	-0.451359	-3.312697	0.0010902	0.0051881	-1.372252
RP11-10K16	0.7186415	1.8347479	3.3126028	0.0010906	0.0051891	-1.372544
ZCCHC24	0.1170755	6.2112191	3.312462	0.0010911	0.0051909	-1.372982
INF2	0.0740154	6.646625	3.3123617	0.0010915	0.0051914	-1.373293
GAPDHP59	-0.556411	-0.80801	-3.312355	0.0010915	0.0051914	-1.373313
MIR6071	0.4170302	-1.241683	3.3122508	0.0010919	0.0051926	-1.373638
AC093388.3	-0.566804	2.2580723	-3.312015	0.0010928	0.0051961	-1.374372
MSANTD4	0.2117414	5.379939	3.3118939	0.0010932	0.0051975	-1.374747
CYP2A6	0.7998123	5.8857233	3.3113232	0.0010953	0.0052069	-1.376519
NTHL1	0.1356057	6.0658647	3.3111499	0.001096	0.0052093	-1.377058
CTB-129P6.	-0.661355	1.6079162	-3.311	0.0010965	0.0052113	-1.377523
CCL22	0.5333391	3.7168521	3.3108132	0.0010972	0.0052139	-1.378103
MIR324	-0.729778	-0.143621	-3.310712	0.0010976	0.0052149	-1.378417
DNAJB9	0.1168304	6.4897653	3.3106791	0.0010977	0.0052149	-1.37852
ABCC3	0.1554939	6.7159224	3.3105925	0.001098	0.0052151	-1.378789
KCNA5	0.7463201	3.0148917	3.3105617	0.0010982	0.0052151	-1.378885
SPHK1	-0.309435	5.6620217	-3.310551	0.0010982	0.0052151	-1.378917
RP11-439C1	0.336626	-1.361519	3.3104063	0.0010987	0.005217	-1.379367
RP11-715I4	-0.519479	-0.993508	-3.310334	0.001099	0.0052176	-1.379593
RP2	0.1023648	5.7596908	3.3100027	0.0011002	0.0052228	-1.380621
GPNMB	0.1658627	6.3276716	3.3098283	0.0011009	0.0052252	-1.381162
RUNX2	0.3790572	4.438465	3.3097203	0.0011013	0.0052264	-1.381497
AP000351.3	0.9192249	3.0404164	3.3093857	0.0011025	0.0052317	-1.382536
RP11-480G7	-0.713065	-0.439022	-3.309141	0.0011035	0.0052354	-1.383297
ATXN7L2	-0.120202	5.221292	-3.309009	0.001104	0.005237	-1.383706
SPHK2	-0.103435	5.9772403	-3.308964	0.0011041	0.0052371	-1.383844
RP11-849F2	-0.528051	-0.889838	-3.308802	0.0011047	0.0052391	-1.384349
HCFC2	0.0965423	5.8092196	3.3087796	0.0011048	0.0052391	-1.384418
SPRR1A	0.7216349	-0.904552	3.3087083	0.0011051	0.0052396	-1.384639
MRPS14	-0.073796	6.1542737	-3.308313	0.0011066	0.0052454	-1.385865

ALG13	0.0758304	6.0350812	3.3083072	0.0011066	0.0052454	-1.385884
RP11-554A1	-0.861406	0.3670791	-3.308071	0.0011075	0.0052489	-1.386616
FAM154A	-0.733982	0.5804232	-3.308024	0.0011076	0.0052491	-1.386761
ARMCX4	0.1298682	5.188045	3.3079549	0.0011079	0.0052496	-1.386977
LPIN1	0.1321259	6.1596935	3.3078756	0.0011082	0.0052504	-1.387223
RP11-430H1	-0.522562	-1.077718	-3.307424	0.0011099	0.0052577	-1.388625
GLYAT	0.8997815	5.0608916	3.3072851	0.0011104	0.0052595	-1.389055
OR2C1	-0.647129	-0.408064	-3.307216	0.0011107	0.0052601	-1.38927
ZNF814	-0.123768	5.5479027	-3.30715	0.0011109	0.0052606	-1.389476
AL109763.2	-1.104287	-0.095359	-3.306914	0.0011118	0.0052641	-1.390205
XRCC6P2	-0.552582	2.2321168	-3.306833	0.0011121	0.0052648	-1.390458
SYT10	0.5905529	-1.012691	3.3064794	0.0011135	0.0052705	-1.391555
Vax2os1_3	-0.545242	-1.100227	-3.306427	0.0011137	0.0052707	-1.391719
PTPN4	0.0770264	5.7220654	3.3059718	0.0011154	0.0052782	-1.39313
PDE9A	-0.282584	5.5211133	-3.305695	0.0011164	0.0052825	-1.393989
AC018890.6	-1.106612	1.2705718	-3.305188	0.0011183	0.0052908	-1.39556
RP11-101E1	-0.092566	5.6922324	-3.305105	0.0011187	0.0052917	-1.395819
ANGEL2	-0.071236	6.0248701	-3.304331	0.0011216	0.0053049	-1.398218
ACSL1	0.1875232	6.9471178	3.3041994	0.0011221	0.0053065	-1.398626
PPHLN1	-0.043523	6.1862807	-3.304056	0.0011226	0.0053084	-1.399069
LINC01443	0.5009402	-1.21551	3.3039684	0.001123	0.0053093	-1.399342
CMAHP	0.2429653	5.1971411	3.303834	0.0011235	0.005311	-1.399758
MAGEB2	-1.289209	0.3431294	-3.303405	0.0011251	0.0053181	-1.401087
AL050310.1	0.4737928	-1.062945	3.3033504	0.0011253	0.0053184	-1.401257
AC004941.3	-0.524483	-1.113158	-3.30329	0.0011256	0.0053188	-1.401446
CSNK1A1	0.0714238	6.2565919	3.3032338	0.0011258	0.0053191	-1.401619
RCN1	0.1317449	6.5239227	3.3030285	0.0011266	0.0053221	-1.402255
WWC1	0.142107	6.2868447	3.3029198	0.001127	0.0053234	-1.402592
NLRC3	0.1758197	4.9904539	3.3025887	0.0011282	0.0053287	-1.403618
KIFC3	0.0863938	6.4280461	3.3022705	0.0011295	0.0053337	-1.404604
C1QL3	0.7531391	3.2487013	3.3017395	0.0011315	0.0053421	-1.406249
GOSR2	-0.057384	5.9812364	-3.301728	0.0011315	0.0053421	-1.406286
RP11-488L1	-0.16816	5.5376459	-3.301454	0.0011326	0.0053464	-1.407133
CLGN	-0.55342	5.3418272	-3.301295	0.0011332	0.0053486	-1.407625
STRN4	-0.053758	6.447611	-3.300233	0.0011373	0.0053671	-1.410914
RP11-35G9.	-0.175887	4.5990822	-3.300129	0.0011377	0.0053675	-1.411239
RP11-517C1	0.3213328	-1.372867	3.3001173	0.0011377	0.0053675	-1.411274
UBE3C	0.0644435	6.4773639	3.3001012	0.0011378	0.0053675	-1.411324
RP11-350G1	-0.753965	0.1387131	-3.299916	0.0011385	0.0053701	-1.411897
C1orf194	-0.694602	-0.247139	-3.299592	0.0011397	0.0053753	-1.412899
HIST1H2BM	-0.50575	-1.00572	-3.299556	0.0011399	0.0053753	-1.413011
AC064875.2	0.7912039	0.9633606	3.2989305	0.0011423	0.005386	-1.414948
CTD-2331H1	-0.830773	0.1315368	-3.298549	0.0011438	0.0053922	-1.416128
SARNP	-0.12338	4.6740978	-3.29833	0.0011446	0.0053955	-1.416806
RPL31P63	-0.763806	0.8464111	-3.298237	0.001145	0.0053965	-1.417094
RP11-336K2	-0.708012	2.5490802	-3.2976	0.0011474	0.0054075	-1.419065
MRPL35	0.0610653	6.2380222	3.2974447	0.001148	0.0054096	-1.419547
RPL29P11	-0.556002	2.3454316	-3.296704	0.0011509	0.0054225	-1.421839
AKR1C2	0.3301508	6.6116775	3.2965577	0.0011515	0.0054245	-1.422291
RP1-97D16.	-0.767491	-0.377817	-3.296428	0.001152	0.0054261	-1.422693
CTC-260E6.	-0.700093	2.1944432	-3.295312	0.0011564	0.0054454	-1.426144

SERPINB11	-0.703856	-0.912267	-3.295305	0.0011564	0.0054454	-1.426165
DAND5	-0.846248	1.6223442	-3.295214	0.0011567	0.0054464	-1.426449
RP6-7406.2	-0.535744	2.6888611	-3.294819	0.0011583	0.0054529	-1.427668
RP11-173C1	0.7369177	1.1694155	3.2946987	0.0011588	0.0054538	-1.428041
KRTCAP2	-0.095399	6.1676882	-3.294695	0.0011588	0.0054538	-1.428053
ARL17A	-0.349569	3.3714668	-3.294502	0.0011595	0.005456	-1.428648
NPM1P9	-0.749019	0.5166907	-3.294501	0.0011595	0.005456	-1.428653
IRF1	0.1090661	6.3991816	3.2944063	0.0011599	0.005457	-1.428945
NPHP3	-0.085975	5.3411459	-3.29419	0.0011607	0.0054601	-1.429613
GTPBP8	0.08417	5.3495037	3.2941632	0.0011609	0.0054601	-1.429697
CTD-2194A8	0.8643703	2.1248472	3.2941072	0.0011611	0.0054604	-1.42987
AC093627.8	0.4926942	-1.088583	3.2940663	0.0011612	0.0054605	-1.429996
SLC30A9	0.0702655	6.2964352	3.2939815	0.0011616	0.0054613	-1.430259
SEC61A2	-0.10225	5.5305601	-3.293699	0.0011627	0.0054658	-1.431131
RP11-326I1	-0.601663	2.3097978	-3.293451	0.0011636	0.0054697	-1.431898
HBE1	-0.954223	-0.266991	-3.293134	0.0011649	0.0054749	-1.432879
CTNNA3	0.6979761	3.2982656	3.2929204	0.0011657	0.0054781	-1.433538
RP11-252A2	0.1720072	4.2582555	3.2928392	0.0011661	0.0054789	-1.433789
UGT2B29P	0.4643402	-1.206567	3.2926498	0.0011668	0.0054817	-1.434375
RP11-366M4	-0.692102	-0.153486	-3.292566	0.0011671	0.005482	-1.434634
GS1-393G12	-0.639582	1.7399713	-3.29256	0.0011672	0.005482	-1.434653
DDX51	-0.069834	5.9865451	-3.292384	0.0011678	0.0054844	-1.435195
KCNRG	-0.613382	1.7896503	-3.292351	0.001168	0.0054844	-1.435298
RP11-115I9	-0.440187	-1.338894	-3.292175	0.0011687	0.005487	-1.435842
KIAA1143	-0.081942	5.9450191	-3.291915	0.0011697	0.0054911	-1.436644
ATP5HP2	-0.664521	-0.29579	-3.291857	0.0011699	0.0054915	-1.436824
LINC01094	0.5134102	3.4329461	3.2914837	0.0011714	0.0054977	-1.437978
AOAH	0.2177421	5.3326838	3.291342	0.001172	0.0054996	-1.438416
CCER2	-0.572247	2.9880403	-3.291174	0.0011726	0.005502	-1.438935
BAIAP2-AS1	-0.118784	6.0085763	-3.290878	0.0011738	0.0055068	-1.43985
CFAP20	0.0705282	6.0373655	3.290631	0.0011748	0.00551	-1.440612
RPL32P3	-0.11426	5.2010044	-3.29063	0.0011748	0.00551	-1.440616
SPACA7	0.8720583	-0.334135	3.2904234	0.0011756	0.0055125	-1.441253
AC005884.1	-0.73541	1.0457746	-3.290419	0.0011756	0.0055125	-1.441265
CTA-544A11	-0.584252	-0.705172	-3.290321	0.001176	0.0055136	-1.441569
USP30	-0.084958	6.0429512	-3.29016	0.0011767	0.0055159	-1.442068
NOP2	-0.076699	6.2192319	-3.289945	0.0011775	0.0055192	-1.442729
RPL7P32	-0.587549	2.1923558	-3.289871	0.0011778	0.0055197	-1.442959
RP11-80202	-0.404462	2.6028977	-3.289841	0.0011779	0.0055197	-1.44305
SUPT16H	-0.052372	6.472079	-3.289096	0.0011809	0.0055329	-1.445352
ZNF526	-0.067257	5.6947488	-3.289046	0.0011811	0.0055329	-1.445505
HINFP	-0.05915	5.7721413	-3.289021	0.0011812	0.0055329	-1.445584
ANP32B	-0.070702	6.6178372	-3.288465	0.0011834	0.0055426	-1.447301
TEX26	0.6789147	-0.779135	3.2876989	0.0011865	0.0055562	-1.449665
TMEM187	0.126438	5.6972832	3.2873464	0.0011879	0.0055621	-1.450752
MOV10	-0.06702	6.5423592	-3.287228	0.0011884	0.0055636	-1.451118
RP1-273G13	-0.585418	-0.681139	-3.286945	0.0011895	0.0055682	-1.45199
TAS2R10	-0.676362	-0.188665	-3.286861	0.0011898	0.0055691	-1.452251
FAM230C	-1.024086	-0.333522	-3.286695	0.0011905	0.0055715	-1.452763
ASLP1	-0.64826	2.2624308	-3.286627	0.0011908	0.0055721	-1.452973
FAM217A	-0.791942	0.3543613	-3.286126	0.0011928	0.0055808	-1.454519

SP140L	0.1247597	5.8222221	3.285355	0.0011959	0.0055937	-1.456896
CCDC102B	0.1646362	5.1398514	3.2853294	0.001196	0.0055937	-1.456975
RP11-392P7	-0.72998	1.3092118	-3.285326	0.001196	0.0055937	-1.456985
RP11-496H1	-0.71285	0.6278244	-3.285255	0.0011963	0.0055943	-1.457206
MIR3131	-0.773484	-0.170517	-3.284892	0.0011978	0.0056005	-1.458323
CYP4B1	0.9036509	0.9772351	3.2846599	0.0011987	0.0056041	-1.45904
CTD-2291D1	-0.944307	0.2011022	-3.284584	0.001199	0.0056048	-1.459273
POLD4	0.1011797	6.240969	3.2844633	0.0011995	0.0056064	-1.459646
TULP3	-0.079956	5.8221995	-3.284351	0.0011999	0.005607	-1.459994
RP11-127B2	-0.403746	2.8716098	-3.284341	0.0012	0.005607	-1.460023
SYNJ2	-0.106648	6.0795616	-3.284317	0.0012001	0.005607	-1.460097
DDX3X	0.0507185	6.7973248	3.2835928	0.001203	0.00562	-1.46233
DNAI1	-0.940924	1.5796582	-3.283364	0.0012039	0.0056236	-1.463037
RP11-85B7.	-0.581757	-0.692163	-3.2833	0.0012042	0.0056241	-1.463232
THSD7A	0.2775458	5.2263169	3.2831581	0.0012048	0.0056261	-1.46367
PARVG	0.1681664	5.5098894	3.2824556	0.0012076	0.005638	-1.465835
MIR135A1	-0.507945	4.1171972	-3.282453	0.0012076	0.005638	-1.465842
RP11-36I17	-0.741206	-0.037777	-3.282241	0.0012085	0.0056413	-1.466497
RP11-613F7	-0.806349	1.2985443	-3.281966	0.0012096	0.0056458	-1.467344
CAPN10	-0.07577	5.8263589	-3.281827	0.0012102	0.0056478	-1.467772
CH507-513H	0.5122407	3.201183	3.2817401	0.0012105	0.0056487	-1.46804
PRSS38	-0.450635	-1.311866	-3.281452	0.0012117	0.0056535	-1.468927
ZNF827	0.3558661	4.5549365	3.28116	0.0012129	0.005658	-1.469827
CTD-2256P1	-0.681194	-0.380672	-3.281137	0.001213	0.005658	-1.469897
CCDC28A	0.0972123	6.0363606	3.2810841	0.0012132	0.0056583	-1.470061
RP11-559M2	-0.509541	-0.976217	-3.280863	0.0012141	0.0056618	-1.470741
GET4	-0.114653	5.2537274	-3.280195	0.0012169	0.0056738	-1.472799
NDUFB2-AS1	-0.177718	3.9475695	-3.280059	0.0012174	0.0056757	-1.473218
LINC01588	0.2223979	4.6850298	3.2798821	0.0012181	0.0056784	-1.473763
RP11-592N2	0.312441	-1.400162	3.2796536	0.0012191	0.005682	-1.474467
ETV2	-0.174201	4.6958846	-3.279615	0.0012192	0.005682	-1.474587
LINC00158	-0.779234	-0.328715	-3.279435	0.00122	0.0056847	-1.475141
RGS16	0.2190987	5.3480699	3.2793688	0.0012203	0.0056853	-1.475344
FCRLA	-0.743424	3.2428046	-3.279325	0.0012204	0.0056854	-1.475479
RP11-350J2	0.8016624	0.2734911	3.2790311	0.0012216	0.0056903	-1.476384
TYROBP	0.1331509	6.1931307	3.2774655	0.0012281	0.0057196	-1.481203
RPS17P5	-0.538818	-0.849174	-3.277272	0.0012289	0.0057226	-1.481798
PKIG	0.0770929	6.145637	3.2770178	0.0012299	0.0057268	-1.482581
C10orf54	0.1090276	6.1041726	3.2768698	0.0012306	0.0057287	-1.483036
ZNF732	-0.819252	0.7783168	-3.276839	0.0012307	0.0057287	-1.483132
RPL5P12	-0.777502	0.6692553	-3.276806	0.0012308	0.0057287	-1.483231
ERAL1	-0.059538	6.3929494	-3.276709	0.0012312	0.0057298	-1.483532
CNTNAP5	-0.79402	-0.611457	-3.276223	0.0012332	0.0057385	-1.485027
LETM1P2	-0.730717	-0.6129	-3.276178	0.0012334	0.0057386	-1.485165
MARK4	-0.075879	6.3606282	-3.276018	0.0012341	0.0057407	-1.485658
INPP5J	-0.466983	4.0981183	-3.275994	0.0012342	0.0057407	-1.48573
FBXO39	0.7133464	2.7842776	3.2757611	0.0012352	0.0057445	-1.486447
CLCF1	0.2513433	5.148837	3.2755936	0.0012359	0.005747	-1.486963
MBNL1-AS1	0.2233354	4.0655869	3.275127	0.0012378	0.0057553	-1.488398
TMSB4Y	1.1556133	1.9283931	3.2747333	0.0012394	0.0057618	-1.489608
TCEA2	0.1258006	6.2371891	3.2747141	0.0012395	0.0057618	-1.489667

SLC25A36P1	-0.691725	-0.310047	-3.273352	0.0012452	0.0057875	-1.493855
RP11-700A2	-0.723864	0.314612	-3.27283	0.0012474	0.0057968	-1.495459
LPAR4	0.8039561	0.6534171	3.272797	0.0012475	0.0057968	-1.495562
ASNS	-0.266525	5.5254037	-3.272466	0.0012489	0.0058026	-1.496578
AC144831.3	0.8074357	1.2425081	3.2724009	0.0012492	0.0058031	-1.496779
PLA2G12B	-0.636756	5.912615	-3.27236	0.0012494	0.0058032	-1.496905
RP11-641D5	-0.291791	3.7866533	-3.272159	0.0012502	0.0058063	-1.497524
RP11-445P1	-0.996298	2.7872521	-3.271778	0.0012518	0.005813	-1.498692
ZNF341	-0.095318	5.4169537	-3.271456	0.0012532	0.0058183	-1.499684
CCAR1	-0.049518	6.3225564	-3.271431	0.0012533	0.0058183	-1.499759
DMBT1	-1.066389	2.2282666	-3.271389	0.0012535	0.0058184	-1.499887
RARS2	-0.077662	6.1007809	-3.271179	0.0012543	0.0058218	-1.500533
CTD-2334D1	0.7976345	0.408345	3.2709282	0.0012554	0.005826	-1.501304
DYM	-0.061366	6.1933098	-3.270713	0.0012563	0.0058287	-1.501966
RP4-583K8.	-0.528725	-1.106499	-3.270712	0.0012563	0.0058287	-1.501967
LINC01501	-0.49501	-1.168001	-3.270443	0.0012574	0.0058333	-1.502796
RP11-223I1	-0.254308	5.1931861	-3.269557	0.0012612	0.0058499	-1.505517
DPF2	-0.057716	6.1350718	-3.269442	0.0012617	0.0058514	-1.505868
RP11-629G1	0.4827738	-1.208787	3.2690132	0.0012635	0.0058591	-1.507185
RP11-136K7	1.0782704	2.6896848	3.2687855	0.0012645	0.0058624	-1.507885
KRTDAP	-0.843632	-0.225254	-3.268764	0.0012646	0.0058624	-1.50795
MYL7	0.4807857	-1.196624	3.268732	0.0012647	0.0058624	-1.508049
HS3ST4	-0.965562	-0.563589	-3.268182	0.001267	0.0058725	-1.509736
CHCHD1	-0.076847	6.1906851	-3.268016	0.0012677	0.005875	-1.510247
HNRNPA1P4	-0.659395	1.6486538	-3.267691	0.0012691	0.0058807	-1.511245
ZBTB16	0.4707547	5.0063086	3.2675667	0.0012697	0.0058824	-1.511626
PADI1	1.0978914	1.5601012	3.2674201	0.0012703	0.0058846	-1.512076
RP4-539M6.	0.5979774	2.6793636	3.2671149	0.0012716	0.0058887	-1.513013
RP11-503N1	0.3976455	-1.229848	3.2671129	0.0012716	0.0058887	-1.513019
CKB	-0.283893	6.0056088	-3.267098	0.0012717	0.0058887	-1.513066
YIPF6	0.0656531	6.3985235	3.2667573	0.0012731	0.0058947	-1.51411
SCARNA13	-0.5535	2.4707221	-3.266672	0.0012735	0.0058956	-1.514371
RP11-552F3	-0.687094	0.1239248	-3.266492	0.0012742	0.0058979	-1.514923
CCDC115	0.0751642	6.0153943	3.2664805	0.0012743	0.0058979	-1.514959
EHD3	0.1563676	5.4648727	3.2662952	0.0012751	0.0059002	-1.515528
RP11-298I3	-0.475571	2.8621854	-3.266287	0.0012751	0.0059002	-1.515553
TSNAXIP1	0.2580757	3.9074668	3.2661901	0.0012755	0.0059014	-1.51585
ZNF316	-0.063913	6.1428469	-3.266045	0.0012762	0.0059035	-1.516294
EEF1B2P6	-0.263717	4.058688	-3.265986	0.0012764	0.005904	-1.516475
KNOP1P5	-0.926183	-0.482569	-3.265836	0.0012771	0.0059062	-1.516937
ZNF507	-0.068174	5.9287572	-3.265449	0.0012787	0.0059131	-1.518124
LA16c-380A	0.5537961	-0.945762	3.2652767	0.0012795	0.0059158	-1.518652
LRRC38	-0.718361	-0.870857	-3.265188	0.0012798	0.0059168	-1.518924
B3GNT9	0.2153056	5.0415895	3.2651061	0.0012802	0.0059177	-1.519176
CTB-36H16.	-0.238431	4.4144004	-3.264919	0.001281	0.0059204	-1.519749
CSNK2B-LY6	-0.699778	1.23432	-3.264867	0.0012812	0.0059204	-1.51991
GNAI3	0.0492601	6.4713166	3.2648543	0.0012813	0.0059204	-1.519948
CREB3L4	-0.120504	5.8584985	-3.26481	0.0012815	0.0059206	-1.520085
AC245100.1	-0.230863	4.7291833	-3.26468	0.001282	0.0059224	-1.520482
RP11-138A9	-0.719946	1.7109148	-3.264319	0.0012836	0.0059288	-1.521589
GRAMD1C	0.2387239	5.4479645	3.2638795	0.0012855	0.0059368	-1.522937

KLHL29	0.2850909	5.5821619	3.2627336	0.0012904	0.0059589	-1.52645
POTEE	-0.658816	-0.691997	-3.262317	0.0012922	0.0059665	-1.527727
RP5-991G2C	-0.769924	0.8569694	-3.262129	0.001293	0.0059695	-1.528304
RP11-347E1	1.0718719	1.5354535	3.2615902	0.0012954	0.0059796	-1.529954
CHRNA10	-0.269344	3.5156289	-3.261447	0.001296	0.0059817	-1.530393
RPL17P26	-0.546059	-0.879004	-3.261337	0.0012965	0.0059831	-1.53073
DENND5B-AS	-0.700474	0.1981129	-3.261165	0.0012972	0.0059858	-1.531257
SNF8	-0.06505	6.4578002	-3.261115	0.0012974	0.0059861	-1.53141
NRG2	0.6532428	3.6533733	3.260849	0.0012986	0.0059906	-1.532225
RPL3P1	-0.689082	0.3848708	-3.260635	0.0012995	0.0059942	-1.532881
ZC3H7A	0.0531226	6.175913	3.2602277	0.0013013	0.0060016	-1.534128
DKK3	0.1746702	5.9667824	3.2596642	0.0013038	0.0060115	-1.535854
RP11-314D7	-0.540046	-1.084	-3.25966	0.0013038	0.0060115	-1.535867
TIAM2	-0.175988	5.2868342	-3.259384	0.001305	0.0060163	-1.536712
RP11-14K3.	-0.649941	-1.040761	-3.258863	0.0013073	0.0060259	-1.538307
FADS3	0.1014846	6.0482162	3.2588343	0.0013074	0.0060259	-1.538395
AEBP2	-0.066958	5.871956	-3.258628	0.0013083	0.0060293	-1.539026
LINC01029	-0.573447	-1.186826	-3.258527	0.0013087	0.0060306	-1.539335
STAT5A	0.0938141	5.9585023	3.258131	0.0013105	0.0060371	-1.540548
ERVMER34-1	-1.013048	2.3712097	-3.258129	0.0013105	0.0060371	-1.540555
CBR3	0.3405045	4.1806333	3.2580514	0.0013108	0.0060379	-1.540792
SLC31A2	0.3319895	4.2072165	3.2577051	0.0013124	0.0060442	-1.541852
ANK3	0.2871014	5.4113385	3.2576153	0.0013127	0.0060452	-1.542127
BMPR1A	-0.071677	5.8689128	-3.257354	0.0013139	0.0060497	-1.542925
DCAF8L1	-1.02592	-0.183136	-3.257103	0.001315	0.0060541	-1.543696
TMEM180	-0.141963	5.6414693	-3.256885	0.001316	0.0060572	-1.544362
CAHM	-0.424475	3.1513167	-3.256873	0.001316	0.0060572	-1.544399
NOVA1-AS1	-1.142862	0.6063491	-3.256825	0.0013162	0.0060574	-1.544545
RP11-394A1	-0.674551	-0.793338	-3.256526	0.0013175	0.0060627	-1.545461
DKFZP434IC	-0.164425	4.584837	-3.256467	0.0013178	0.0060632	-1.545642
RP11-666A8	-0.608642	2.0119162	-3.256353	0.0013183	0.0060647	-1.54599
RP11-551L1	-0.854266	0.8482648	-3.256036	0.0013197	0.0060704	-1.546959
AHSA2	-0.105735	6.0267642	-3.255965	0.00132	0.0060711	-1.547177
CFAP97	0.0918018	6.0846443	3.2556557	0.0013214	0.0060766	-1.548123
ATP6V0C	0.079251	6.1659766	3.2553562	0.0013227	0.0060819	-1.54904
CCR1	0.220987	5.2423562	3.255139	0.0013237	0.0060848	-1.549704
RP11-73301	0.645415	2.4211426	3.2551383	0.0013237	0.0060848	-1.549706
TOB2P1	-0.51118	3.3414101	-3.253823	0.0013295	0.0061109	-1.553729
VTCN1	1.0948515	2.8741243	3.2533206	0.0013318	0.0061204	-1.555265
TUBA3C	-1.23801	0.2713222	-3.253262	0.001332	0.0061204	-1.555444
RP11-12301	0.2987099	-1.434683	3.2532478	0.0013321	0.0061204	-1.555487
AP000593.5	-0.59063	-1.177987	-3.253137	0.0013326	0.0061219	-1.555827
NTAN1P3	0.5178933	-1.121835	3.2529045	0.0013336	0.0061259	-1.556537
BRI3P1	-0.577106	1.9975952	-3.252786	0.0013341	0.0061275	-1.5569
GRIP1	0.8284409	2.1769926	3.2526436	0.0013348	0.0061296	-1.557334
RP1-80N2.4	-0.595313	2.4764943	-3.25261	0.0013349	0.0061296	-1.557436
ST3GAL5-AS	0.7047672	0.8204222	3.2524506	0.0013356	0.0061321	-1.557924
SUMO2P1	-0.676	1.1840564	-3.251934	0.001338	0.0061419	-1.559502
HSF5	-0.716182	0.1174345	-3.251572	0.0013396	0.0061486	-1.560608
RP11-863P1	0.600331	-0.861803	3.2514898	0.0013399	0.0061495	-1.56086
IRF2	0.0779916	6.1316065	3.2511203	0.0013416	0.0061564	-1.561989

RP11-305E6	-0.188518	4.5020795	-3.251	0.0013421	0.0061581	-1.562358
EVI2A	0.2104616	5.0335998	3.2507995	0.001343	0.0061614	-1.562969
PXMP4	-0.09925	6.1046717	-3.250573	0.0013441	0.0061653	-1.56366
PSG1	0.2469478	-1.461062	3.2502139	0.0013457	0.0061719	-1.564758
RP11-385H1	-0.546892	-0.917826	-3.250177	0.0013458	0.0061719	-1.564871
OLFML2A	0.171614	5.7490633	3.2497952	0.0013476	0.006179	-1.566037
RABEP2	0.0875341	5.9365848	3.2493819	0.0013494	0.0061868	-1.567299
MARCO	0.7846941	3.9632381	3.2491373	0.0013505	0.0061911	-1.568046
RP11-66N11	-0.277637	4.0495299	-3.249074	0.0013508	0.0061916	-1.56824
ACTBL2	0.7488831	-0.583892	3.2489711	0.0013513	0.006193	-1.568554
TUG1	-0.052052	6.5527947	-3.248515	0.0013533	0.0062016	-1.569945
PDE8A	0.0789976	6.2016242	3.2484661	0.0013536	0.0062019	-1.570095
RP4-60503.	0.2545013	3.7929241	3.2483592	0.001354	0.0062033	-1.570422
LINC00638	-0.308612	4.089836	-3.24803	0.0013555	0.0062094	-1.571428
RP11-393K1	-0.531263	-1.237418	-3.247917	0.001356	0.0062106	-1.571771
RP11-686D2	0.5656605	3.0385348	3.2478947	0.0013562	0.0062106	-1.57184
RP11-466A1	-0.487963	-0.973153	-3.247539	0.0013578	0.0062172	-1.572926
CSE1L-AS1	-0.676764	-0.333715	-3.247136	0.0013596	0.0062248	-1.574156
AC007386.4	-0.824587	0.274073	-3.246985	0.0013603	0.0062272	-1.574615
CTD-2308L2	0.7541863	-0.277851	3.2467184	0.0013615	0.006232	-1.57543
RP11-120K2	-0.828446	-0.505911	-3.246622	0.0013619	0.0062332	-1.575725
RP11-421L2	0.1578986	5.016552	3.2463248	0.0013633	0.0062386	-1.576631
CSNK1D	-0.044951	6.6780677	-3.246188	0.0013639	0.0062407	-1.577049
RAD51AP2	-0.851126	1.7132474	-3.245848	0.0013655	0.0062464	-1.578087
PFDN6	-0.088028	6.3633239	-3.24584	0.0013655	0.0062464	-1.578109
AP003068.2	-0.240298	5.1847105	-3.245672	0.0013663	0.0062491	-1.578621
KCNH2	-0.731421	3.4631511	-3.245607	0.0013666	0.0062497	-1.57882
ASPSCR1	-0.102817	6.4074651	-3.244775	0.0013704	0.0062663	-1.581359
AC015933.2	-0.73119	0.2564528	-3.244632	0.001371	0.0062685	-1.581795
PPP1R3A	-0.614161	-1.210905	-3.244501	0.0013716	0.0062704	-1.582195
FBP1	0.2468561	6.6408006	3.2444635	0.0013718	0.0062704	-1.582309
CA13	0.2743611	4.8643008	3.2443113	0.0013725	0.0062728	-1.582773
TENM4	0.7650454	3.1340902	3.2440742	0.0013736	0.0062767	-1.583496
NRBP1	-0.051322	6.6329237	-3.244054	0.0013737	0.0062767	-1.583557
DACT2	-0.945933	4.3498012	-3.24369	0.0013753	0.0062835	-1.584669
CYSLTR1	0.2685024	4.3672706	3.2434235	0.0013766	0.0062883	-1.58548
FOXL1	0.5456661	3.8229059	3.2431819	0.0013777	0.0062926	-1.586216
PSTK	-0.129206	5.2380277	-3.243122	0.0013779	0.0062926	-1.586401
RP13-977J1	0.5086407	3.1679955	3.2431046	0.001378	0.0062926	-1.586452
SRL	0.4408726	3.4148704	3.2428475	0.0013792	0.0062973	-1.587236
PIK3CD-AS2	-0.729429	2.9870426	-3.242567	0.0013805	0.0063024	-1.588091
JAG2	0.1672557	5.7819386	3.2423208	0.0013816	0.0063068	-1.588841
RP11-390P2	-0.143422	4.9676326	-3.241888	0.0013836	0.0063151	-1.590159
MGST1	0.2141756	6.9926672	3.2416801	0.0013846	0.0063187	-1.590794
C9orf78	0.0642731	6.3999794	3.2416164	0.0013849	0.0063192	-1.590988
ST20-AS1	0.1563614	5.1385481	3.2413648	0.0013861	0.0063238	-1.591755
SDF2	-0.056863	6.2500481	-3.241184	0.0013869	0.0063268	-1.592305
GMPSP1	-0.568309	2.2361753	-3.241086	0.0013873	0.0063281	-1.592605
VMAC	-0.098675	5.1698522	-3.240495	0.0013901	0.0063398	-1.594406
GCH1	0.1801892	6.1489451	3.2400112	0.0013923	0.0063492	-1.595878
SEPT11	0.0764797	6.4381444	3.2399082	0.0013928	0.0063506	-1.596192

SERTAD4-AS	0.6574644	2.6182594	3.2397316	0.0013936	0.0063536	-1.59673
RP11-57A19	-0.894335	0.4805046	-3.239507	0.0013947	0.0063575	-1.597415
EEF1B2P2	-0.670082	1.3460496	-3.239425	0.0013951	0.0063585	-1.597663
ZFP69B	-0.236998	4.2391406	-3.239208	0.0013961	0.0063623	-1.598324
RP11-179G5	-0.482328	2.8745573	-3.238764	0.0013981	0.0063709	-1.599677
VMO1	0.184845	4.9623586	3.23842	0.0013997	0.0063774	-1.600724
AP000593.7	-0.861304	0.5853232	-3.238275	0.0014004	0.0063797	-1.601164
PCDH17	0.1664131	5.5109659	3.2381418	0.001401	0.0063818	-1.601571
RP11-246K1	0.9468354	-0.10483	3.2378315	0.0014025	0.0063876	-1.602516
PPWD1	-0.054422	5.8316331	-3.237554	0.0014038	0.0063927	-1.60336
RP4-545K15	0.8060679	0.4870735	3.2373404	0.0014048	0.0063964	-1.60401
LRRC36	-0.889177	1.4609095	-3.23688	0.0014069	0.0064055	-1.605412
IGHV3-47	-0.875056	0.137717	-3.236669	0.0014079	0.0064092	-1.606055
RP4-710M16	-0.468436	4.7712903	-3.236589	0.0014083	0.0064097	-1.606296
CPSF3	-0.049289	6.181705	-3.236568	0.0014084	0.0064097	-1.606361
FBXL8	0.1457799	5.1048394	3.2364561	0.0014089	0.0064113	-1.606701
RP11-323I1	-0.611868	-0.405545	-3.235411	0.0014138	0.0064326	-1.60988
PWRN1	0.7451492	-0.556451	3.2353893	0.0014139	0.0064326	-1.609947
PRKAB2	-0.118998	6.478832	-3.235211	0.0014148	0.0064356	-1.61049
RP11-983P1	-0.137739	5.5446088	-3.23422	0.0014195	0.0064561	-1.613505
APOC2	-0.386088	6.1703198	-3.23402	0.0014204	0.0064596	-1.614112
RP5-856G1.	-0.765413	-0.503332	-3.233515	0.0014228	0.0064696	-1.615646
RPL19P21	-0.645643	1.6572364	-3.233443	0.0014231	0.0064704	-1.615867
PCDHA1	-1.04716	0.8651278	-3.232926	0.0014256	0.0064807	-1.617437
RP11-643G1	-0.576136	-0.666904	-3.232759	0.0014264	0.0064835	-1.617946
CTA-989H11	-0.425838	3.3368044	-3.232502	0.0014276	0.0064882	-1.618725
FALEC	-0.714918	0.9893618	-3.232465	0.0014278	0.0064883	-1.61884
PPP1R13L	0.1392783	5.8309247	3.2321628	0.0014292	0.0064939	-1.619758
KCNJ4	0.9027576	3.7228055	3.2321285	0.0014294	0.0064939	-1.619862
ALDH1L1	0.3435565	6.6347523	3.2320191	0.0014299	0.0064955	-1.620194
CHCHD3	0.0680231	6.2562992	3.2316851	0.0014315	0.0065019	-1.621209
CTD-2643K1	-0.374508	-1.230514	-3.23136	0.0014331	0.0065077	-1.622197
MIR25	-0.585982	2.5305007	-3.231341	0.0014331	0.0065077	-1.622254
RP11-1055E	-0.669013	-0.225017	-3.231288	0.0014334	0.0065081	-1.622416
NPR1	0.1596411	5.6463354	3.2310513	0.0014345	0.0065124	-1.623135
SPR	0.078495	6.5049831	3.2309595	0.001435	0.0065136	-1.623414
RAD52	-0.098084	5.3444966	-3.230856	0.0014355	0.006515	-1.62373
DRD5	-0.567465	-1.033776	-3.230729	0.0014361	0.0065168	-1.624114
RP11-136I1	-0.582292	-0.886987	-3.230699	0.0014362	0.0065168	-1.624204
AC009487.6	-0.518687	2.8936701	-3.230537	0.001437	0.0065195	-1.624697
GKAP1	-0.103103	5.4920785	-3.230371	0.0014378	0.0065222	-1.625202
RP11-211G3	-0.469381	3.1640165	-3.230336	0.0014379	0.0065222	-1.625307
AC016700.6	-0.710029	0.9449553	-3.229605	0.0014415	0.0065373	-1.627529
DCLK1	0.4425914	4.2178709	3.2294005	0.0014424	0.0065409	-1.62815
HADHA	0.0659659	6.9719486	3.2293419	0.0014427	0.0065414	-1.628327
RP11-394G3	0.6221009	-0.920786	3.2291648	0.0014436	0.0065444	-1.628865
RP13-1016M	0.8314696	0.2935624	3.2285597	0.0014465	0.0065566	-1.630703
LYPLAL1-AS	0.7428826	1.1571484	3.2285327	0.0014466	0.0065566	-1.630785
ACSL4	-0.211744	6.8297085	-3.22841	0.0014472	0.0065576	-1.631156
DLST	0.0795278	6.6270918	3.2283959	0.0014473	0.0065576	-1.6312
ZCCHC18	0.5722647	2.4247709	3.2283749	0.0014474	0.0065576	-1.631264

LINC01564	0.6087169	4.0489639	3.2283236	0.0014476	0.0065579	-1.631419
TSC2	0.0718168	6.4388039	3.2280761	0.0014488	0.0065625	-1.632171
SWT1	-0.093828	5.3598211	-3.227921	0.0014495	0.0065651	-1.632642
SERPINE1	0.1959196	6.5427935	3.2278041	0.0014501	0.0065668	-1.632997
AL356585.2	-0.593833	-0.620212	-3.227692	0.0014507	0.0065684	-1.633337
EXTL2	-0.091192	5.6709893	-3.227582	0.0014512	0.00657	-1.633672
AC068831.1	-0.691518	1.4657663	-3.227316	0.0014525	0.006575	-1.634477
DDX47	-0.178537	4.2053343	-3.227276	0.0014527	0.0065751	-1.6346
RP11-172H2	-0.899264	1.665436	-3.227211	0.001453	0.0065757	-1.634797
RP11-676J1	-0.600605	1.5837909	-3.227155	0.0014532	0.0065761	-1.634965
KIF5A	-0.8743	2.0886298	-3.226941	0.0014543	0.0065797	-1.635615
RP11-412P1	-0.905539	-0.519532	-3.22691	0.0014544	0.0065797	-1.635711
HNRNPA1P52	-0.556499	-0.727068	-3.226879	0.0014546	0.0065797	-1.635805
FCAMR	0.9634817	3.8822371	3.2268183	0.0014549	0.0065802	-1.635989
AC006116.1	-0.521498	-0.894266	-3.226458	0.0014566	0.0065873	-1.637082
ZNF121	-0.079836	5.9697532	-3.226265	0.0014576	0.0065907	-1.637668
RP11-122C5	0.5414132	-0.924356	3.2262017	0.0014579	0.0065913	-1.63786
C17orf99	-0.852992	0.1860448	-3.226046	0.0014586	0.0065938	-1.638331
FAM155A	0.7005294	3.0319717	3.2258208	0.0014597	0.006598	-1.639015
WASL	0.0610519	6.5067527	3.2253759	0.0014619	0.0066069	-1.640365
KLK11	1.0647398	0.4248647	3.2253304	0.0014621	0.0066071	-1.640503
RP11-524F1	-0.618574	-0.569916	-3.225222	0.0014626	0.0066087	-1.640833
AC092670.1	-0.657997	1.1265789	-3.224567	0.0014658	0.0066223	-1.642819
TANGO6	0.1101815	5.8756229	3.2245008	0.0014661	0.0066229	-1.643019
TBC1D23	0.0665094	6.0852152	3.2243745	0.0014667	0.0066249	-1.643402
ALAS1	0.1246766	6.7741624	3.2241493	0.0014678	0.006629	-1.644085
DMBX1	-1.079166	0.9173679	-3.224023	0.0014685	0.006631	-1.644468
GALT	-0.111386	6.4714979	-3.223807	0.0014695	0.0066349	-1.645124
LINC01133	0.882353	0.9459043	3.2234604	0.0014712	0.0066417	-1.646174
POGK	-0.075922	6.3792542	-3.223206	0.0014724	0.0066465	-1.646944
RP11-493L1	-0.784038	-0.338373	-3.222581	0.0014755	0.0066588	-1.648839
CLPX	0.0799944	6.4406585	3.2225551	0.0014756	0.0066588	-1.648918
PARP11	0.1197043	5.3245986	3.2225405	0.0014757	0.0066588	-1.648963
TMOD3	0.0678284	6.3797939	3.2224954	0.0014759	0.0066589	-1.649099
PTPRD-AS1	0.9104358	1.4327475	3.2223699	0.0014765	0.0066609	-1.64948
GLP2R	0.9370749	2.3393218	3.2223306	0.0014767	0.0066609	-1.649599
RP13-580B1	-0.685906	-0.165265	-3.222249	0.0014771	0.0066613	-1.649846
SOX9	-0.255951	6.1388088	-3.222241	0.0014772	0.0066613	-1.649869
MYCBPAP	-0.614037	2.4950741	-3.222138	0.0014777	0.0066627	-1.650184
ZNF324B	-0.09548	5.0295041	-3.222018	0.0014783	0.0066646	-1.650545
KCNT1	0.6705905	1.7447602	3.2217215	0.0014797	0.0066703	-1.651445
BCKDHA	0.1823923	5.3273742	3.2216493	0.0014801	0.0066711	-1.651664
RABL3	0.0621078	5.985821	3.2211694	0.0014824	0.0066809	-1.653118
RP11-130C6	0.7497459	0.7779422	3.2209922	0.0014833	0.006684	-1.653655
HAS1	0.9887338	2.0456951	3.2202337	0.0014871	0.0067	-1.655952
CDK5RAP1	-0.054564	6.0910219	-3.219886	0.0014888	0.006707	-1.657006
KIF3C	0.197104	5.1909245	3.2197885	0.0014893	0.0067083	-1.657301
RP11-473M2	0.6976699	2.6558262	3.2192712	0.0014918	0.0067182	-1.658867
HNRNPA1P39	-0.649815	-0.1688	-3.219269	0.0014918	0.0067182	-1.658873
CH507-216K	-0.423537	-1.228375	-3.219187	0.0014922	0.0067192	-1.659123
KAT7	-0.050974	6.1204794	-3.218703	0.0014946	0.0067292	-1.660587

FZD8	0.2684331	5.1673156	3.2184912	0.0014957	0.0067331	-1.661229
RP11-525G1	-0.762765	1.4047547	-3.218385	0.0014962	0.0067346	-1.661551
DLC1	0.1229945	6.1692767	3.2180914	0.0014977	0.0067404	-1.662439
RP11-530A1	-0.680808	0.8838924	-3.21784	0.0014989	0.0067452	-1.663199
WBP2	0.0643848	6.7726664	3.217712	0.0014996	0.0067472	-1.663588
MIR3942	-0.600282	-0.458184	-3.217387	0.0015012	0.0067536	-1.664571
RP11-1100L	0.7918992	1.4388433	3.2166635	0.0015048	0.006769	-1.666761
PGAM1	0.0905255	6.1141567	3.216057	0.0015078	0.0067812	-1.668596
STK16	0.0681087	6.1810931	3.2160464	0.0015079	0.0067812	-1.668629
RPF1	0.0696007	6.0182635	3.2158989	0.0015086	0.0067837	-1.669075
KALP	0.8800481	0.0877594	3.2153075	0.0015116	0.0067958	-1.670864
C18orf54	-0.213653	4.6177206	-3.215286	0.0015117	0.0067958	-1.670928
SHISA4	0.1927252	5.6183376	3.2152396	0.0015119	0.0067959	-1.671069
RP3-476K8.	-0.680538	0.2944689	-3.21521	0.0015121	0.0067959	-1.671159
BAG3	0.0806968	6.2657741	3.2150337	0.0015129	0.006799	-1.671692
TCTN2	-0.191997	5.2033695	-3.214955	0.0015133	0.0068	-1.67193
CADM3	0.9359073	2.3196748	3.2146308	0.001515	0.0068064	-1.672911
ALKBH6	-0.140345	4.9411722	-3.214141	0.0015174	0.0068167	-1.674393
STARD4	-0.140407	5.9195411	-3.213735	0.0015195	0.006825	-1.675619
TMX3	0.0707779	6.0125243	3.2131153	0.0015226	0.0068377	-1.677493
POLG	0.070141	6.2248876	3.2131021	0.0015227	0.0068377	-1.677533
RNU4-62P	0.732784	-0.034778	3.213026	0.001523	0.0068385	-1.677763
ZNF788	-0.298706	4.4217748	-3.212713	0.0015246	0.0068448	-1.678709
ST6GALNAC6	0.0928399	6.3193167	3.2125741	0.0015253	0.0068471	-1.679129
RP11-162O1	-0.73457	0.7009501	-3.212407	0.0015262	0.0068501	-1.679635
RPS20P10	-0.696744	-0.094581	-3.212199	0.0015272	0.0068527	-1.680262
LOXL2	0.1145817	5.918277	3.2121953	0.0015272	0.0068527	-1.680274
ZNF45	-0.107344	5.50843	-3.21218	0.0015273	0.0068527	-1.68032
RPL7AP10	-0.714454	0.1339414	-3.211597	0.0015303	0.0068651	-1.682081
FAM171A1	0.2699742	6.0482611	3.2114943	0.0015308	0.0068666	-1.682392
MRPL40P1	-0.793926	0.5518699	-3.211453	0.001531	0.0068667	-1.682517
PCK1	0.4517981	6.6331088	3.2102739	0.001537	0.0068927	-1.686079
MKRN1	0.047943	6.4940927	3.2101801	0.0015375	0.006894	-1.686363
ATP6V0E1	0.0702295	6.7430643	3.2100936	0.0015379	0.0068951	-1.686624
RP11-694I1	-0.319155	3.5419113	-3.210023	0.0015383	0.0068959	-1.686837
SUMO2P3	-0.494946	-0.914498	-3.209957	0.0015386	0.0068966	-1.687037
RP11-60L3.	0.3272599	-1.415374	3.2098842	0.001539	0.0068974	-1.687256
RP11-311H1	-0.588679	-0.672343	-3.209322	0.0015419	0.0069094	-1.688954
CIDEA	0.8846043	-0.50188	3.2090971	0.001543	0.0069137	-1.689634
CTB-176F20	-0.412376	3.4701725	-3.208887	0.0015441	0.0069177	-1.690268
SLC9A3R2	0.1157361	6.7538745	3.2083172	0.001547	0.0069292	-1.691988
TMEM262	-0.168678	4.1004155	-3.208311	0.001547	0.0069292	-1.692006
RP1-257C22	0.8996118	1.5194584	3.2082409	0.0015474	0.0069299	-1.692219
KCTD9	0.0979749	5.7165739	3.2080184	0.0015485	0.0069342	-1.69289
BNIP3P39	-0.548645	-0.94799	-3.207587	0.0015507	0.0069426	-1.694191
DYNLL2	0.0604407	6.6484012	3.2075779	0.0015508	0.0069426	-1.69422
RP11-552M1	-0.567579	1.6899574	-3.207485	0.0015513	0.0069439	-1.694499
CCL20	-0.431635	5.913641	-3.207353	0.0015519	0.0069461	-1.694898
SUDS3	-0.05552	6.1669954	-3.207279	0.0015523	0.0069469	-1.695123
EIF4EBP2	-0.077089	6.8472668	-3.207137	0.0015531	0.0069493	-1.695551
SIPA1L1	0.0677095	6.2607523	3.2069522	0.001554	0.0069527	-1.696108

VN1R20P	-0.679491	-0.15131	-3.206765	0.001555	0.0069562	-1.696672
BPIFA1	-0.921886	-0.443959	-3.205664	0.0015606	0.0069803	-1.699995
RP1-209A6.	-0.906189	-0.041796	-3.205646	0.0015607	0.0069803	-1.700049
RP11-43505	-0.643666	1.4940092	-3.205606	0.0015609	0.0069803	-1.700169
ADPRHL2	0.0717528	6.1061811	3.2050057	0.001564	0.0069934	-1.701981
RP11-415J8	-0.691554	0.0110267	-3.204536	0.0015665	0.0070034	-1.703396
HNRNPH3	-0.043814	6.4566782	-3.204196	0.0015682	0.0070104	-1.704423
RAD21	-0.058958	6.7641052	-3.203956	0.0015695	0.0070151	-1.705148
EXOSC3P1	-0.822705	0.8218466	-3.203896	0.0015698	0.0070157	-1.705327
SSBP2	0.2105309	5.2769251	3.2038425	0.0015701	0.007016	-1.705489
RP13-554M1	-0.704715	0.648344	-3.203584	0.0015714	0.0070212	-1.706268
LINC01231	-0.497575	-1.266942	-3.203108	0.0015739	0.007031	-1.707704
OLFML1	0.2283944	5.4514705	3.2030886	0.001574	0.007031	-1.707761
PKN2	0.0663491	6.1032228	3.2030147	0.0015744	0.0070318	-1.707984
RP11-717F1	-0.811002	1.0545253	-3.202766	0.0015757	0.0070367	-1.708733
NUP188	-0.070028	6.1896462	-3.202717	0.0015759	0.007037	-1.708881
KCNE5	-0.716429	3.4853216	-3.2026	0.0015765	0.0070389	-1.709234
MKRN2OS	-0.7412	2.811696	-3.202338	0.0015779	0.0070441	-1.710024
STX17-AS1	-0.320099	3.6585416	-3.20223	0.0015785	0.0070454	-1.710349
PDSS1	-0.108208	5.5182164	-3.202211	0.0015786	0.0070454	-1.710407
MT1DP	0.6920161	3.0679451	3.2019555	0.0015799	0.0070505	-1.711177
MIR646HG	-1.007716	1.9794012	-3.20134	0.0015831	0.0070637	-1.713032
LINC01272	0.3974457	4.279458	3.201313	0.0015833	0.0070637	-1.713113
C4orf27	0.0689477	5.8075196	3.2003416	0.0015884	0.0070856	-1.716039
CTD-2256P1	0.8083447	-0.120336	3.1998679	0.0015908	0.0070958	-1.717466
PLAA	0.0723878	6.1687517	3.1996706	0.0015919	0.0070996	-1.71806
USP35	0.1296326	5.7146297	3.1995616	0.0015925	0.0071012	-1.718388
AC005480.1	-0.595552	-0.599885	-3.199297	0.0015938	0.0071066	-1.719184
DDX39B	-0.083373	6.342574	-3.199238	0.0015942	0.007107	-1.719363
PDLIM4	0.4205014	4.1364565	3.1992044	0.0015943	0.007107	-1.719464
HOPX	0.2566403	4.9202284	3.199156	0.0015946	0.0071073	-1.71961
RP11-401L1	-0.563323	1.9776334	-3.198819	0.0015964	0.0071143	-1.720625
SLCO4A1-AS	-1.037376	0.527222	-3.19868	0.0015971	0.007116	-1.721042
OCLM	-0.769861	1.4116872	-3.198673	0.0015971	0.007116	-1.721063
RP5-857K21	0.2154388	4.9272019	3.1985354	0.0015979	0.0071176	-1.721479
HNRNPA1P61	-0.443329	-1.08472	-3.198532	0.0015979	0.0071176	-1.721487
RP11-603J2	-0.570005	-0.629068	-3.198453	0.0015983	0.0071186	-1.721726
DTWD2	0.1229098	5.399466	3.1983133	0.001599	0.007121	-1.722147
CTD-2081C1	-0.537767	-1.176679	-3.19787	0.0016014	0.0071306	-1.72348
HIBCH	0.1049305	6.3044097	3.1976726	0.0016024	0.0071344	-1.724076
RP11-849H4	-0.422316	2.9255392	-3.197605	0.0016028	0.0071351	-1.724279
RP1-310013	0.6615322	1.1953815	3.1973245	0.0016043	0.0071405	-1.725123
PALM	-0.186766	5.7812744	-3.197285	0.0016045	0.0071405	-1.725244
RNASEH1P1	-0.599379	-1.066288	-3.197257	0.0016046	0.0071405	-1.725326
C9orf163	-0.464676	3.0519354	-3.197226	0.0016048	0.0071405	-1.72542
SMG1	0.0650281	6.3799423	3.1964892	0.0016087	0.0071571	-1.727637
RP11-440I1	0.8802426	0.6659017	3.1964416	0.0016089	0.0071573	-1.72778
RP11-894J1	-0.38329	2.6240099	-3.196192	0.0016103	0.007162	-1.728531
RP11-685B1	0.7964045	-0.211308	3.1961432	0.0016105	0.007162	-1.728678
RP11-429J1	-0.473416	2.8602282	-3.196133	0.0016106	0.007162	-1.728708
CRTC2	-0.05843	6.3853341	-3.196036	0.0016111	0.0071634	-1.729002

ARL6IP4	-0.140683	4.8900444	-3.195372	0.0016146	0.0071783	-1.730999
DMRTA1	0.4744788	5.2323206	3.1952741	0.0016152	0.0071797	-1.731293
RP11-367G1	-0.624388	2.0213378	-3.195232	0.0016154	0.0071798	-1.731419
CTB-129P6.	-0.323292	4.8807748	-3.195099	0.0016161	0.0071821	-1.731819
ASAH2	-0.356434	4.61887	-3.194816	0.0016176	0.0071879	-1.732671
RP11-385F7	-0.325909	3.7038746	-3.194379	0.0016199	0.0071974	-1.733985
SPATA31C1	-0.389738	-1.363634	-3.193769	0.0016232	0.0072111	-1.735818
C20orf27	-0.101169	6.1391314	-3.193262	0.0016259	0.0072223	-1.737342
CTD-3216D2	-0.618372	2.0037095	-3.192971	0.0016275	0.0072283	-1.738217
RP11-424N2	-0.731958	0.7670991	-3.192813	0.0016283	0.0072312	-1.738693
RP11-480I1	-0.309135	4.9853888	-3.192584	0.0016296	0.0072358	-1.739382
AKAP8	-0.048014	6.0915635	-3.192405	0.0016305	0.0072392	-1.739917
MPZL3	-0.134384	5.8167356	-3.192246	0.0016314	0.0072421	-1.740396
RP1-309F2C	-0.761869	-0.021986	-3.192156	0.0016319	0.0072434	-1.740668
WDPCP	0.1403709	4.8477399	3.1916589	0.0016345	0.0072544	-1.742161
SLC7A7	0.1582635	5.7060155	3.1913409	0.0016363	0.0072605	-1.743116
RBM5	-0.04735	6.3522848	-3.191329	0.0016363	0.0072605	-1.743152
GJB3	0.8395502	2.7896591	3.1912859	0.0016366	0.0072607	-1.743282
TAS2R15P	-0.62931	1.5762387	-3.191169	0.0016372	0.0072626	-1.743632
RPL39P3	-0.20234	4.8044743	-3.190959	0.0016383	0.0072667	-1.744264
ZNF320	-0.275199	5.3904454	-3.190885	0.0016387	0.0072674	-1.744486
CRYBB2P1	-0.108871	5.5025765	-3.190855	0.0016389	0.0072674	-1.744575
HNRNPA3P12	-0.5283	1.9703884	-3.190598	0.0016403	0.0072727	-1.745349
CTD-2012B7	-0.290086	-1.390597	-3.190136	0.0016428	0.0072829	-1.746735
NBN	0.0685929	6.2892463	3.1897712	0.0016447	0.0072908	-1.747832
RPL12P8	-0.718161	0.5914405	-3.189434	0.0016466	0.007298	-1.748846
SLC35A4	0.0497123	6.5373095	3.1890724	0.0016485	0.0073058	-1.74993
B4GALT1	0.0742589	6.7923265	3.1884816	0.0016517	0.0073192	-1.751704
EDA2R	0.6112407	3.9299264	3.1882391	0.0016531	0.0073241	-1.752432
RP11-214K3	-0.685969	-0.121847	-3.188149	0.0016536	0.0073254	-1.752702
FBXL14	0.1225752	5.3973998	3.1879221	0.0016548	0.00733	-1.753383
GIMAP7	0.1614358	5.6200217	3.1876184	0.0016565	0.0073365	-1.754295
TLCD2	0.1571604	5.6136717	3.1874433	0.0016574	0.0073398	-1.75482
UBL3	0.0930659	6.3084662	3.1872039	0.0016587	0.0073447	-1.755539
RP11-439K3	0.495997	-1.030323	3.1868806	0.0016605	0.0073517	-1.756509
ZNF227	-0.090481	5.4954668	-3.18603	0.0016651	0.0073714	-1.75906
RPS6KA2-IT	0.535477	-0.977226	3.1855575	0.0016677	0.007382	-1.760479
RP1-40E16.	-0.884569	-0.481814	-3.185	0.0016708	0.0073946	-1.762152
PSD3	0.1707472	5.9917764	3.1849324	0.0016712	0.0073954	-1.762353
ANAPC4	-0.062005	5.9548015	-3.184723	0.0016723	0.0073996	-1.762981
CTD-2308N2	0.3562918	-1.291518	3.1845148	0.0016735	0.0074038	-1.763606
AF124730.4	0.9095208	1.7432269	3.1842821	0.0016748	0.0074085	-1.764304
IKZF1	0.2065343	5.3613314	3.1841212	0.0016756	0.0074116	-1.764786
ATG9B	0.2496666	4.1398246	3.1837418	0.0016777	0.0074199	-1.765924
NEK5	-0.209943	3.8621793	-3.183471	0.0016792	0.0074256	-1.766736
SNRPEP6	-0.588739	-0.591499	-3.183279	0.0016803	0.0074294	-1.76731
DGKD	-0.07629	6.0018974	-3.182978	0.001682	0.0074359	-1.768213
NEU1	-0.081829	6.6732195	-3.182539	0.0016844	0.0074457	-1.769528
RP11-232D9	-0.601511	-1.157431	-3.181857	0.0016882	0.0074609	-1.771573
IGFL1	0.6628795	-0.945863	3.1818459	0.0016882	0.0074609	-1.771606
RP11-259P1	-0.43369	-1.171162	-3.181588	0.0016897	0.0074664	-1.772379

CIB2	-0.265099	4.8481491	-3.181452	0.0016904	0.0074688	-1.772787
CTAGE16P	-0.480475	-1.05144	-3.181182	0.0016919	0.0074745	-1.773595
PPEF1	-0.828886	1.6627432	-3.181107	0.0016923	0.0074755	-1.773821
MTX1	-0.073594	5.9970656	-3.181023	0.0016928	0.0074766	-1.774071
LINC00222	-0.839107	1.7874902	-3.180888	0.0016936	0.007479	-1.774474
PPP3CC	0.0837279	5.6703792	3.180832	0.0016939	0.0074795	-1.774643
OR5G5P	0.3061939	-1.430869	3.1807249	0.0016945	0.0074813	-1.774964
ZNF276	0.073896	5.7306168	3.1806129	0.0016951	0.0074831	-1.7753
DAG1	0.0636228	6.7477558	3.1805087	0.0016957	0.0074848	-1.775612
LIF	0.3947098	5.0940435	3.1804069	0.0016962	0.0074861	-1.775917
ADAMTS20	-0.861897	-0.44	-3.180365	0.0016965	0.0074861	-1.776043
AC002075.4	-0.653231	1.5044258	-3.180344	0.0016966	0.0074861	-1.776104
RP11-64P14	-0.495674	-1.04065	-3.180296	0.0016969	0.0074864	-1.776248
IGLJ1	-0.425399	-1.271273	-3.179693	0.0017002	0.0075003	-1.778056
RAB32	0.1212735	6.0384188	3.1795391	0.0017011	0.0075032	-1.778516
RP11-523H2	-0.556691	2.1811898	-3.179006	0.0017041	0.0075155	-1.780112
GRASP	0.1648932	5.422767	3.1788136	0.0017052	0.0075184	-1.780688
RP5-1065J2	-0.56366	-0.603519	-3.178759	0.0017055	0.0075184	-1.780851
ARL16	-0.083856	5.9920533	-3.178727	0.0017056	0.0075184	-1.780946
TP53I11	0.103265	6.2201381	3.1787228	0.0017057	0.0075184	-1.78096
AC018804.6	-0.832647	1.4032893	-3.178703	0.0017058	0.0075184	-1.781018
NSUN5	-0.072232	6.1042898	-3.17851	0.0017069	0.0075223	-1.781596
TEAD3	-0.070671	6.0809722	-3.178368	0.0017077	0.0075246	-1.782022
RP11-333E1	-0.718006	0.6952361	-3.178342	0.0017078	0.0075246	-1.782099
RP11-513M1	-0.17003	4.1561626	-3.178084	0.0017092	0.0075298	-1.782871
TEP1	0.0801128	5.9678075	3.1780612	0.0017094	0.0075298	-1.78294
RP11-600F2	-0.699426	0.3513399	-3.177884	0.0017104	0.0075332	-1.78347
ERGIC1	0.0757694	6.9062639	3.1778391	0.0017106	0.0075334	-1.783605
KRT85	-1.086997	1.1433015	-3.177553	0.0017122	0.0075395	-1.784461
YAF2	0.0677166	5.7900515	3.17748	0.0017126	0.0075395	-1.78468
TUBAP2	-0.549091	2.5097519	-3.177463	0.0017127	0.0075395	-1.784731
HIST1H3F	-0.435355	-1.205255	-3.177446	0.0017128	0.0075395	-1.78478
RP11-387M2	-0.675202	0.824909	-3.177402	0.0017131	0.0075397	-1.784913
ADD3-AS1	-0.670976	1.6256019	-3.17719	0.0017143	0.0075441	-1.785549
CTD-2006H1	-0.194821	4.3996007	-3.176898	0.0017159	0.0075504	-1.786423
FOXJ3	0.0540844	6.1168339	3.176729	0.0017169	0.0075531	-1.786927
C10orf91	-1.049549	0.745455	-3.176717	0.0017169	0.0075531	-1.786962
MFSD4	-0.248034	4.5570639	-3.176291	0.0017194	0.0075627	-1.788238
RP11-91P24	-0.687669	0.4717742	-3.175868	0.0017217	0.0075723	-1.789502
NDUFA5	0.0769897	6.368097	3.175332	0.0017248	0.0075848	-1.791106
EPB41L4A-A	-0.11559	5.7119765	-3.175133	0.0017259	0.0075888	-1.791701
SDC1	0.1085324	7.1816005	3.1750567	0.0017263	0.0075898	-1.79193
CYP8B1	0.5954494	5.9268042	3.1750116	0.0017266	0.0075898	-1.792064
AC061992.2	0.8412386	0.588368	3.1749859	0.0017267	0.0075898	-1.792141
POM121L4P	-0.70997	-0.401277	-3.174932	0.001727	0.0075902	-1.792303
TPRG1-AS2	0.4214618	-1.172705	3.174767	0.001728	0.0075934	-1.792796
SLFNL1-AS1	0.3421511	3.9487729	3.1746899	0.0017284	0.0075944	-1.793027
CTD-2270F1	0.2681768	-1.450243	3.1742693	0.0017308	0.007604	-1.794284
RP5-1021I2	0.5729004	-0.935151	3.173884	0.001733	0.0076127	-1.795436
RP11-141B1	-0.693338	0.2773821	-3.173771	0.0017336	0.0076146	-1.795774
NPSR1	-0.506584	-1.069441	-3.173668	0.0017342	0.0076163	-1.796082

LINC01546	0.5225362	-0.925489	3.1736295	0.0017344	0.0076163	-1.796197
ITGA4	0.2384511	5.0928358	3.1733727	0.0017359	0.0076218	-1.796965
A4GNT	-1.08891	0.8020709	-3.173233	0.0017367	0.0076244	-1.797383
ARID2	-0.06996	5.8600897	-3.173026	0.0017379	0.0076287	-1.798001
ZCCHC14	0.0789555	6.2950557	3.1726532	0.00174	0.0076369	-1.799115
ARMCX3	0.2155582	5.9983163	3.1726221	0.0017402	0.0076369	-1.799208
QPCTL	-0.099278	5.9161053	-3.172574	0.0017405	0.0076372	-1.799353
OR2H1	-0.746924	-0.740512	-3.172252	0.0017423	0.0076444	-1.800314
SMARCE1P6	-0.758283	0.1936272	-3.172005	0.0017437	0.0076497	-1.801052
RP11-632C1	-0.234731	5.0163479	-3.171784	0.001745	0.0076543	-1.801712
PRAMEF2	0.7393753	-0.747715	3.171312	0.0017477	0.0076645	-1.803123
RP11-4K16.	-0.738381	0.545749	-3.171303	0.0017477	0.0076645	-1.803149
NR2C1	-0.062846	5.9099823	-3.17121	0.0017483	0.007666	-1.803428
FABP5P1	-0.496587	-1.017591	-3.170583	0.0017519	0.0076803	-1.805301
HIST1H2BG	-0.623673	3.5144586	-3.170567	0.001752	0.0076803	-1.805349
RP11-344E1	-0.686227	-0.9205	-3.170431	0.0017528	0.0076828	-1.805754
LINC00907	0.8500698	1.4777411	3.1699663	0.0017554	0.0076936	-1.807142
APRT	0.0836998	6.5593718	3.1697474	0.0017567	0.0076982	-1.807796
AP001462.6	-0.552077	1.8854234	-3.169476	0.0017583	0.0077037	-1.808606
NR5A1	-0.836492	0.246709	-3.169458	0.0017584	0.0077037	-1.80866
BCORP1	0.8852557	0.4412652	3.1694145	0.0017586	0.0077038	-1.80879
AC009404.2	-0.237993	4.4042468	-3.169381	0.0017588	0.0077038	-1.808889
RP11-503C2	-0.828308	3.5524072	-3.169021	0.0017609	0.007712	-1.809965
SYAP1	0.0664762	6.5389843	3.1686077	0.0017633	0.0077214	-1.811198
SPRY2	0.1176854	5.7751622	3.1685596	0.0017635	0.0077214	-1.811342
Y_RNA	-0.249496	6.6784158	-3.168538	0.0017637	0.0077214	-1.811407
RFPL4B	-1.250999	0.6144945	-3.168486	0.001764	0.0077218	-1.811561
PHKG2	0.0777021	6.0775917	3.1682497	0.0017653	0.0077268	-1.812267
TMEM171	0.5047779	4.6708911	3.1680935	0.0017662	0.0077299	-1.812733
CEACAM22P	-0.798905	2.0391505	-3.16795	0.0017671	0.0077323	-1.813163
RP11-360D2	-0.652939	0.1202274	-3.167926	0.0017672	0.0077323	-1.813234
CSNK2B	-0.073739	6.4395262	-3.167792	0.001768	0.0077347	-1.813634
RP4-631H13	0.7158967	2.5435628	3.1674982	0.0017697	0.0077413	-1.81451
LTBP1	0.1927579	5.954601	3.1673689	0.0017704	0.0077436	-1.814896
RASSF8-AS1	0.2405613	5.0560293	3.1668793	0.0017733	0.0077551	-1.816357
APOO	0.0990161	5.7850735	3.1667158	0.0017742	0.0077584	-1.816845
SLC12A2	0.204451	5.9806131	3.1665974	0.0017749	0.0077604	-1.817198
FAM96B	0.0797758	6.4283486	3.1665474	0.0017752	0.0077608	-1.817347
AC104809.2	1.1744611	1.5809219	3.1664232	0.0017759	0.007763	-1.817718
HCG4P7	0.2944326	4.6035664	3.166331	0.0017765	0.0077644	-1.817993
YIPF1	0.0640961	6.2606763	3.1660855	0.0017779	0.0077698	-1.818725
CTC-265F19	0.8087573	0.403216	3.1656573	0.0017804	0.0077797	-1.820002
SLA	0.1927367	5.5992426	3.1655118	0.0017813	0.0077825	-1.820436
ZSWIM4	0.1352012	5.4880061	3.165419	0.0017818	0.0077839	-1.820713
SLC43A1	-0.11836	6.74588	-3.164969	0.0017844	0.0077943	-1.822055
CAPN11	0.3265921	3.8143987	3.1649389	0.0017846	0.0077943	-1.822145
FRYL	0.0699259	6.0181875	3.16487	0.001785	0.0077952	-1.82235
RP1-97J1.2	-0.533271	-1.141238	-3.16466	0.0017862	0.0077996	-1.822976
CX3CR1	0.2946748	4.4089992	3.1644486	0.0017875	0.0078036	-1.823606
RP11-323F2	-0.605268	-0.375582	-3.164432	0.0017876	0.0078036	-1.823657
RP11-2H3.6	-0.72563	1.1279121	-3.164307	0.0017883	0.0078054	-1.82403

RP11-291I6	0.4924857	-1.032833	3.1642886	0.0017884	0.0078054	-1.824084
RP11-216N1	-0.722691	1.0762876	-3.164206	0.0017889	0.0078065	-1.824329
RP11-229P1	-0.30695	-1.338517	-3.163859	0.0017909	0.0078138	-1.825366
RP11-338N1	-0.897283	0.1101971	-3.163851	0.001791	0.0078138	-1.825388
RP11-524H1	0.4965788	-1.194134	3.1637391	0.0017916	0.0078157	-1.825722
CTC-340D7.	0.5114445	-0.978102	3.1633648	0.0017938	0.0078243	-1.826837
GFPT1	0.0640753	6.5482873	3.1632934	0.0017942	0.0078249	-1.82705
DACH2	-0.929476	0.3208221	-3.163268	0.0017944	0.0078249	-1.827125
AC098614.1	-0.418392	-1.143279	-3.162982	0.0017961	0.0078313	-1.827977
RP11-465N4	-0.668366	-0.001647	-3.162736	0.0017975	0.0078367	-1.828712
TENM2	0.9566975	3.2887329	3.1624972	0.0017989	0.0078419	-1.829423
RP1-247F3.	-0.489908	-0.91465	-3.16226	0.0018003	0.0078471	-1.830131
SLC11A1	0.2584101	4.8428087	3.1620211	0.0018017	0.0078523	-1.830842
MTRNR2L10	0.745153	1.2590414	3.1616754	0.0018038	0.0078602	-1.831872
ACVR2B-AS1	-0.193702	4.1323369	-3.161608	0.0018042	0.007861	-1.832073
RP4-669B10	-0.656558	-0.813946	-3.161505	0.0018048	0.0078627	-1.832378
GOT2P2	0.9760884	2.0045821	3.1614544	0.0018051	0.0078631	-1.83253
ZNF780A	-0.079413	5.6373274	-3.160919	0.0018082	0.0078759	-1.834124
RP11-5809.	-0.520058	-0.880019	-3.160786	0.001809	0.0078784	-1.834522
CTD-3035K2	0.7169176	1.0774411	3.1604337	0.0018111	0.0078866	-1.83557
MAN1A2	0.0674645	6.3222103	3.1603782	0.0018115	0.0078871	-1.835736
CMSS1	-0.092721	5.8562529	-3.159805	0.0018149	0.007901	-1.837443
TAF1B	-0.077761	5.5004278	-3.159571	0.0018162	0.0079053	-1.838139
COX7B	0.0830388	6.6931509	3.159564	0.0018163	0.0079053	-1.83816
RP11-300M2	-0.491782	-1.228346	-3.15929	0.0018179	0.0079114	-1.838975
RP11-1D12.	0.7408362	0.4818323	3.159199	0.0018185	0.0079129	-1.839247
CYP2G1P	0.8040528	0.3054626	3.1589186	0.0018201	0.0079192	-1.840082
CASC9	-1.374039	1.8812231	-3.158746	0.0018212	0.0079227	-1.840596
SHISA8	-0.832436	0.1427788	-3.158165	0.0018246	0.0079368	-1.842323
RP11-218M2	-0.138779	5.6394534	-3.157165	0.0018306	0.0079619	-1.845301
RP11-397A1	-0.32496	-1.35159	-3.157074	0.0018311	0.0079633	-1.845571
ZNF546	-0.103969	5.1473471	-3.156977	0.0018317	0.0079647	-1.845859
PSG4	0.7764426	-0.543113	3.1569497	0.0018319	0.0079647	-1.845941
SLC10A1	0.6388868	5.7083117	3.1566983	0.0018334	0.0079703	-1.846689
TCN2	0.1224513	6.0247229	3.1559752	0.0018377	0.0079881	-1.84884
TRAPPC3	0.0578163	6.3011978	3.1559425	0.0018379	0.0079881	-1.848937
MICU2	0.0772904	6.1135397	3.1557296	0.0018392	0.007992	-1.84957
ATP10D	0.1390229	5.5701328	3.1557177	0.0018393	0.007992	-1.849606
RNFT1	-0.096464	5.8039741	-3.155384	0.0018413	0.0079998	-1.850597
WDYHV1	-0.108043	5.6699698	-3.155158	0.0018426	0.0080048	-1.851269
CTD-3162L1	-0.776448	0.2685262	-3.154857	0.0018445	0.0080117	-1.852166
DPH6	-0.094845	5.4340043	-3.154719	0.0018453	0.0080143	-1.852574
CMC2	0.0769298	6.0871692	3.1544725	0.0018468	0.0080186	-1.853309
CTC-457E21	-0.410438	-1.230953	-3.154472	0.0018468	0.0080186	-1.853311
RP11-699A5	-0.829017	0.8880225	-3.154435	0.001847	0.0080186	-1.853421
WDR45	0.070091	6.3163981	3.1544123	0.0018471	0.0080186	-1.853488
KATNAL2	0.215183	4.9350682	3.1542739	0.001848	0.0080213	-1.853899
FYB	0.2018966	5.5575534	3.1541811	0.0018485	0.0080227	-1.854175
RP11-346D1	-0.5588	-1.052372	-3.153967	0.0018498	0.0080274	-1.85481
GRINA	0.0852922	7.0474276	3.153636	0.0018518	0.0080351	-1.855795
TCAIM	0.0840693	6.2588588	3.1534929	0.0018527	0.0080379	-1.85622

LINC01510	-0.826229	-0.231415	-3.153362	0.0018535	0.0080404	-1.856611
RP11-180P8	0.3101031	-1.415362	3.1531219	0.001855	0.0080448	-1.857323
AC127904.2	-0.601887	1.822973	-3.153122	0.001855	0.0080448	-1.857324
ERVMER61-1	-0.912574	-0.409517	-3.15297	0.0018559	0.0080479	-1.857776
CHM	0.0886871	5.7927756	3.1526823	0.0018576	0.0080545	-1.85863
SERPIND1	-0.32676	7.0375665	-3.15241	0.0018593	0.0080607	-1.859439
LRFN4	0.2494155	4.8033235	3.1522054	0.0018605	0.0080651	-1.860047
ULK4	0.1847275	5.2318761	3.1513692	0.0018656	0.0080862	-1.862531
KCNK7	-0.472038	2.7962825	-3.151081	0.0018674	0.0080928	-1.863386
ACADS	0.1285583	6.5139668	3.1505639	0.0018705	0.0081056	-1.864923
AC068831.3	0.6124656	-0.512808	3.1500397	0.0018737	0.0081185	-1.866479
LINC00471	-0.538485	2.4307796	-3.149556	0.0018767	0.0081304	-1.867917
SGK494	-0.246913	4.2944598	-3.148892	0.0018807	0.008147	-1.869886
RPL21P40	-0.509961	-0.963691	-3.148538	0.0018829	0.0081555	-1.870936
RBM4	-0.084719	5.5461009	-3.148221	0.0018849	0.0081629	-1.871877
KCTD21-AS1	-0.202129	4.5750463	-3.147969	0.0018864	0.0081687	-1.872625
MOGAT3	-0.61225	5.5456336	-3.147925	0.0018867	0.0081689	-1.872756
TLE4	0.17164	5.3824462	3.1474972	0.0018893	0.0081793	-1.874026
NDST3	0.8731834	0.4312491	3.1472039	0.0018911	0.0081855	-1.874896
RWDD2A	-0.087133	5.5097585	-3.147192	0.0018912	0.0081855	-1.87493
RP11-44D19	-0.568119	-1.154054	-3.147151	0.0018915	0.0081857	-1.875054
ZBTB49	-0.082674	5.1112231	-3.146527	0.0018953	0.0082014	-1.876904
SMIM11	-0.455759	-0.993525	-3.146072	0.0018981	0.0082126	-1.878254
AC097523.1	-0.522788	1.9147507	-3.145982	0.0018987	0.008214	-1.878522
GPX1P2	0.6130252	2.1266494	3.1459416	0.0018989	0.0082141	-1.87864
RP11-31H5.	-0.475163	2.744967	-3.145667	0.0019006	0.0082198	-1.879454
BST1	0.2297046	4.8889339	3.1456587	0.0019007	0.0082198	-1.879479
RP1-249H1.	-0.382372	-1.265888	-3.145577	0.0019012	0.0082203	-1.879721
RP11-372K1	0.6124992	2.2188955	3.1455647	0.0019013	0.0082203	-1.879758
RP11-474B1	0.6828061	-0.772224	3.1455148	0.0019016	0.0082207	-1.879906
RP11-174G6	-0.257684	4.1561974	-3.145157	0.0019038	0.0082284	-1.880968
RP11-403P1	0.3523669	-1.285205	3.1451543	0.0019038	0.0082284	-1.880975
LINC00485	-0.760902	-0.038067	-3.144965	0.001905	0.0082325	-1.881535
AC079780.3	-0.459832	-1.15127	-3.144826	0.0019059	0.0082353	-1.881949
MTFR1L	0.0695117	6.3191444	3.1445017	0.0019079	0.008243	-1.88291
RN7SL832P	-0.406403	2.8915462	-3.144455	0.0019082	0.0082433	-1.883048
KRT8P38	-0.383642	-1.344718	-3.14427	0.0019093	0.0082473	-1.883596
RP11-718G2	-0.599804	-0.796733	-3.143668	0.0019131	0.0082625	-1.885382
PRDX1	0.0863656	6.9844747	3.1435797	0.0019136	0.0082638	-1.885643
ZNF730	-0.908222	0.7774568	-3.143549	0.0019138	0.0082638	-1.885735
FOXJ2	0.1018697	5.8188234	3.1434416	0.0019145	0.0082657	-1.886052
RPL30P14	-0.489399	-1.024425	-3.143348	0.0019151	0.0082672	-1.886331
LPPR5	-0.585184	-1.137628	-3.143019	0.0019171	0.0082745	-1.887304
AC114730.5	-0.916086	0.2874197	-3.143006	0.0019172	0.0082745	-1.887342
ONECUT3	-0.783314	-0.216233	-3.142891	0.0019179	0.0082766	-1.887684
RING1	-0.066925	6.3754169	-3.1428	0.0019185	0.0082781	-1.887953
RP11-404P2	0.3240148	-1.376259	3.1425581	0.00192	0.0082829	-1.88867
CTD-2333K2	-0.300761	-1.407267	-3.142551	0.0019201	0.0082829	-1.888692
FUCA1	0.0961346	6.4871578	3.1421451	0.0019226	0.0082928	-1.889894
GHc-857G6.	-0.653111	1.8054794	-3.141732	0.0019252	0.008303	-1.891118
MRAP2	-0.734156	4.2483162	-3.141522	0.0019265	0.0083077	-1.891739

SLC30A10	-0.390472	6.0947409	-3.140554	0.0019326	0.008333	-1.894606
GDI1	0.0568842	6.5969624	3.1404236	0.0019334	0.0083355	-1.894993
C2orf54	-0.608523	5.3565794	-3.1397	0.001938	0.0083542	-1.897135
HMGB3P7	0.5426597	-0.858984	3.1395932	0.0019386	0.0083561	-1.897451
2-Mar	0.0907451	6.2797912	3.1394903	0.0019393	0.0083579	-1.897756
RP11-570P1	-0.63806	-0.263671	-3.138986	0.0019425	0.0083707	-1.899248
chr22-38_2	-0.12205	5.2190043	-3.138889	0.0019431	0.0083723	-1.899535
FMO3	0.2882596	6.639661	3.1388041	0.0019436	0.0083737	-1.899787
UBE2K	-0.056299	6.5483023	-3.138507	0.0019455	0.0083808	-1.900665
TMEM242	0.0919471	5.8246816	3.1381716	0.0019476	0.008389	-1.901659
LINC00173	-0.805794	1.7592369	-3.137717	0.0019505	0.0084004	-1.903004
AKR7A2	0.0899954	6.3244015	3.1376783	0.0019508	0.0084005	-1.903118
CTC-559E9.	-0.282199	4.0895246	-3.137311	0.0019531	0.0084095	-1.904204
RP11-815I9	-0.391971	2.9184421	-3.137059	0.0019547	0.0084154	-1.904951
CPN1	-0.501504	6.0268197	-3.136607	0.0019576	0.0084268	-1.906287
CALM1	0.0592608	6.8414393	3.1362112	0.0019601	0.0084367	-1.907458
BAGE2	-1.131565	0.2428315	-3.135975	0.0019616	0.0084422	-1.908157
RP11-573G6	-0.705339	0.3795377	-3.135415	0.0019652	0.0084556	-1.909813
PARD3	-0.069234	6.3505805	-3.135405	0.0019652	0.0084556	-1.909843
RP11-736N1	-0.800289	2.3227245	-3.135379	0.0019654	0.0084556	-1.90992
MRPL30	-0.054747	6.1473033	-3.135205	0.0019665	0.0084594	-1.910434
CTD-2319I1	-0.551029	2.0938046	-3.135113	0.0019671	0.0084609	-1.910706
NT5E	-0.169879	6.2633949	-3.135019	0.0019677	0.0084625	-1.910984
TPPP3	0.2023579	5.3674927	3.1349361	0.0019682	0.0084638	-1.911228
BRIX1	-0.071276	6.0399525	-3.134868	0.0019687	0.0084646	-1.911431
RP11-244J1	-0.703991	1.3522508	-3.134399	0.0019717	0.0084749	-1.912816
RP11-467D6	-0.217062	3.706641	-3.134392	0.0019717	0.0084749	-1.912837
RP11-839D1	-0.805994	0.8769969	-3.134377	0.0019718	0.0084749	-1.912881
ZNF713	-0.175207	4.4775387	-3.134351	0.001972	0.0084749	-1.912959
TCEB3	0.0684984	6.3412102	3.1342405	0.0019727	0.0084769	-1.913284
GFM1	0.0639791	6.4013147	3.1341755	0.0019731	0.0084777	-1.913477
RNF39	-0.374285	3.7853875	-3.133545	0.0019772	0.0084941	-1.91534
WASH2P	-0.120133	4.943813	-3.133439	0.0019779	0.0084961	-1.915653
RP11-34D15	-0.464655	-1.098428	-3.133333	0.0019785	0.008498	-1.915966
RP11-348A7	-0.783567	-0.043539	-3.133298	0.0019788	0.008498	-1.91607
RP11-941H1	-0.700238	0.9825722	-3.13308	0.0019802	0.0085027	-1.916713
ALG10	-0.143663	4.7605557	-3.133054	0.0019803	0.0085027	-1.91679
SLC52A3	0.5462113	4.1729942	3.1328558	0.0019816	0.0085069	-1.917377
PTRHD1	-0.081895	5.8358111	-3.13282	0.0019818	0.0085069	-1.917481
SST	-0.67252	-1.076373	-3.132772	0.0019821	0.0085069	-1.917624
RP4-781K5.	-0.584206	-1.145541	-3.132757	0.0019822	0.0085069	-1.917668
TBC1D31	-0.108288	5.4674198	-3.132703	0.0019826	0.0085074	-1.917829
HIST1H2BA	-0.492771	-1.010025	-3.132201	0.0019858	0.0085203	-1.919312
MBD6	-0.055213	6.4263429	-3.131931	0.0019876	0.0085268	-1.92011
RP11-445N1	-0.587455	-0.645767	-3.131868	0.001988	0.0085275	-1.920293
KSR2	-0.855328	2.0297493	-3.131656	0.0019893	0.0085323	-1.92092
RP11-249C2	0.6301732	-0.94401	3.1316218	0.0019896	0.0085323	-1.921022
TIFAB	-0.663789	2.9845908	-3.13139	0.0019911	0.0085378	-1.921707
SLC6A20	0.9739791	0.980543	3.1312087	0.0019922	0.0085418	-1.922242
RP11-214N9	-0.455514	2.960426	-3.131018	0.0019935	0.0085458	-1.922805
IL2RB	0.212376	5.4698439	3.1309656	0.0019938	0.0085458	-1.92296

RP11-462G1	0.7056084	0.4572642	3.1309557	0.0019939	0.0085458	-1.922989
HIST1H3PS1	-0.600387	1.4831827	-3.130525	0.0019967	0.0085568	-1.92426
LINC00501	-0.845915	-0.065748	-3.130049	0.0019998	0.008569	-1.925665
PNMA5	-1.02417	0.7942684	-3.129965	0.0020003	0.0085703	-1.925915
CCDC171	0.2565557	4.2276531	3.1297923	0.0020014	0.0085741	-1.926424
RP11-227F1	0.4841612	-1.032547	3.1297506	0.0020017	0.0085743	-1.926547
DOLPP1	-0.082691	6.083632	-3.129641	0.0020024	0.0085763	-1.926869
RP11-176N1	-0.454957	-1.229079	-3.129442	0.0020037	0.0085809	-1.927457
RP11-50B3.	-0.424452	-1.211076	-3.129151	0.0020056	0.0085871	-1.928317
PCYT1A	0.0479382	6.3445222	3.1291487	0.0020056	0.0085871	-1.928324
EVL	0.1158236	6.2954005	3.129071	0.0020061	0.0085881	-1.928553
SLC5A9	-0.348792	5.754163	-3.129038	0.0020063	0.0085881	-1.92865
RP6-7406.6	0.6765025	-0.001618	3.1289339	0.002007	0.00859	-1.928957
RP11-146E1	-0.860309	0.4278168	-3.12877	0.0020081	0.0085936	-1.929442
RP11-242D8	-0.299248	3.9075145	-3.128495	0.0020099	0.0086003	-1.930252
ROGDI	0.0971434	6.0424949	3.1283575	0.0020108	0.0086031	-1.930658
RPS6KB2	-0.063751	6.3548022	-3.128305	0.0020111	0.0086035	-1.930812
LARP1B	0.0952955	6.068418	3.1279587	0.0020134	0.0086122	-1.931835
RP11-500G1	-0.678858	1.3667865	-3.127859	0.002014	0.008614	-1.932128
C10orf90	1.0544862	0.7963761	3.1278238	0.0020143	0.008614	-1.932233
CXXC1	-0.055009	6.2338961	-3.12763	0.0020155	0.0086162	-1.932804
ACTR3	0.0424318	6.6472411	3.1276041	0.0020157	0.0086162	-1.932881
NEO1	0.1030411	6.1600222	3.1276037	0.0020157	0.0086162	-1.932882
AC096669.2	-0.508988	-1.160805	-3.127601	0.0020157	0.0086162	-1.932889
RP4-564F22	-0.645087	-0.287269	-3.12733	0.0020175	0.0086227	-1.933689
NMD3	0.065464	6.3778161	3.1270695	0.0020192	0.008629	-1.934458
AP000354.2	-0.708562	0.8770443	-3.126823	0.0020208	0.0086349	-1.935186
ARPC3P2	-0.496365	-0.926289	-3.126776	0.0020211	0.0086352	-1.935324
RPS27	-0.07644	7.1047266	-3.126698	0.0020216	0.0086355	-1.935555
TNFAIP6	0.7315028	2.9926483	3.1266936	0.0020217	0.0086355	-1.935567
RPL24P4	-0.206447	4.7085767	-3.126579	0.0020224	0.0086377	-1.935905
LINC00685	-0.487813	2.940115	-3.125768	0.0020278	0.0086595	-1.938297
GAGE1	-0.843513	-0.680423	-3.125619	0.0020287	0.0086627	-1.938735
RP11-727F1	-0.611827	1.2770081	-3.125565	0.0020291	0.0086631	-1.938894
UGT1A8	0.9544944	0.8271025	3.1254212	0.00203	0.0086662	-1.939319
CEP290	-0.08233	5.6256741	-3.124907	0.0020334	0.0086796	-1.940835
PSMA5	0.0696793	6.6317172	3.124533	0.0020359	0.0086892	-1.941937
RP11-790I1	-0.572726	-1.012293	-3.124253	0.0020378	0.0086961	-1.942763
TMEM102	0.1389906	5.2465655	3.1239679	0.0020396	0.0087031	-1.943603
PINLYP	0.2959514	3.7397652	3.1238626	0.0020403	0.008705	-1.943913
SLC22A8	-1.010136	0.1910375	-3.123666	0.0020416	0.0087096	-1.944492
TOX	0.4277814	4.5469017	3.1233935	0.0020434	0.0087162	-1.945295
RP11-186N1	-0.328595	3.510908	-3.123271	0.0020443	0.0087181	-1.945657
RP11-438F1	-0.445428	-1.30642	-3.123257	0.0020443	0.0087181	-1.945699
RP11-428J1	-0.216205	4.3331256	-3.123216	0.0020446	0.0087182	-1.945817
RP11-1099M	-0.805346	0.9734399	-3.122658	0.0020483	0.008733	-1.947462
KIAA1715	0.0692785	6.1205144	3.1222638	0.0020509	0.0087425	-1.948623
PPIL2	-0.056663	6.322178	-3.122249	0.002051	0.0087425	-1.948668
MRPL28	0.0804495	6.4329244	3.1219458	0.0020531	0.0087501	-1.94956
TMEM79	-0.090293	5.4662026	-3.12148	0.0020562	0.0087623	-1.950931
ZFX	-0.080849	6.1000894	-3.121317	0.0020572	0.0087659	-1.951411

DGKK	-1.098086	0.5640324	-3.120997	0.0020594	0.008774	-1.952353
TMEM71	0.2592535	4.0089202	3.1207578	0.002061	0.0087798	-1.953058
EIF4BP2	-0.496792	-0.974044	-3.120548	0.0020624	0.0087847	-1.953674
RANBP1	-0.071908	6.3794089	-3.120167	0.0020649	0.0087945	-1.954797
SULT1C2	-0.52885	5.4652208	-3.120094	0.0020654	0.0087956	-1.955013
RPRD2	-0.067396	6.3495514	-3.119953	0.0020664	0.0087986	-1.955426
RP11-444D3	0.8479889	1.0798229	3.1197827	0.0020675	0.0088022	-1.955929
HSD17B12	0.0843836	6.503453	3.1197542	0.0020677	0.0088022	-1.956013
PPIAP30	-0.576021	-0.672167	-3.119437	0.0020698	0.0088102	-1.956947
C12orf4	-0.060305	5.6719021	-3.119271	0.0020709	0.0088136	-1.957435
DNAJC8P1	-0.514733	-0.925282	-3.119246	0.0020711	0.0088136	-1.957508
ABCB8	0.0788097	6.3372075	3.1188146	0.002074	0.0088249	-1.958778
LINC01091	-0.552357	3.1928445	-3.118732	0.0020745	0.0088263	-1.95902
JKAMP	0.0609402	6.1113223	3.1185999	0.0020754	0.008829	-1.959409
SPDYA	-0.384312	3.3169877	-3.118401	0.0020768	0.0088337	-1.959995
RP11-156E6	-0.070907	5.5731373	-3.118103	0.0020788	0.0088412	-1.960872
RPL7P26	-0.6771	0.2075086	-3.117903	0.0020801	0.0088459	-1.961458
PDZD2	0.2098763	4.8630979	3.117122	0.0020854	0.0088672	-1.963757
AC104653.1	0.738302	0.2192852	3.1167628	0.0020878	0.0088765	-1.964814
C11orf91	0.7884146	0.6434453	3.116562	0.0020892	0.0088809	-1.965404
TUBB7P	-0.475597	-1.058523	-3.116539	0.0020893	0.0088809	-1.965471
PKDREJ	-0.549688	2.694845	-3.116369	0.0020905	0.0088846	-1.965973
TREX1	-0.417918	2.6109376	-3.116338	0.0020907	0.0088846	-1.966063
PCDH15	-0.745156	-0.508498	-3.116085	0.0020924	0.0088909	-1.966807
PHKA2	-0.074227	6.485259	-3.115654	0.0020953	0.0089021	-1.968074
THY1	0.1218547	6.3894135	3.1156231	0.0020955	0.0089021	-1.968164
GBA3	0.6283169	5.2680842	3.115446	0.0020967	0.0089057	-1.968685
RP5-907D15	0.7892381	-0.236729	3.1154115	0.002097	0.0089057	-1.968787
ZXDC	-0.061839	6.1070559	-3.115389	0.0020971	0.0089057	-1.968852
SAMD3	0.4365456	3.6321015	3.1153153	0.0020976	0.0089068	-1.969069
FOXG1	-0.665596	-1.015245	-3.115257	0.002098	0.0089075	-1.96924
DZANK1	0.1779671	4.3540757	3.115204	0.0020984	0.008908	-1.969397
ZFYVE1	0.0755336	6.0101007	3.1149443	0.0021002	0.0089144	-1.97016
MAEL	0.9130955	2.7021299	3.1141701	0.0021054	0.008935	-1.972435
SPPL2A	0.0677587	6.5567008	3.1141244	0.0021057	0.008935	-1.972569
PRRX1	0.3693188	4.8162973	3.1141222	0.0021057	0.008935	-1.972576
DLX2-AS1	-0.589954	-1.01977	-3.113352	0.002111	0.0089563	-1.974838
RP11-14C10	-0.556687	3.6440557	-3.112987	0.0021135	0.0089658	-1.975911
RPL36AP45	-0.555784	-0.656671	-3.112634	0.0021159	0.008975	-1.976948
RP11-283I3	-0.097115	5.1839616	-3.112008	0.0021202	0.0089922	-1.978787
USP4	0.0605708	6.1809285	3.1115683	0.0021232	0.0090039	-1.980078
MDGA2	-1.106361	0.4342515	-3.111352	0.0021247	0.0090092	-1.980712
YRDC	0.0710848	5.843412	3.1111583	0.002126	0.0090134	-1.981282
ARL4A	-0.126305	5.7759507	-3.111137	0.0021262	0.0090134	-1.981344
OR2A20P	0.8723384	0.2859143	3.1109359	0.0021276	0.0090182	-1.981935
DOCK2	0.1906737	5.5626094	3.1108982	0.0021278	0.0090182	-1.982045
PICALM	0.0513001	6.6959219	3.1107349	0.0021289	0.0090219	-1.982524
AC008991.1	-0.753248	-0.621655	-3.110511	0.0021305	0.0090274	-1.983182
ZNF230	-0.114049	4.9988539	-3.109793	0.0021354	0.0090474	-1.985289
MEP1B	0.9904187	0.9400156	3.1096194	0.0021366	0.0090514	-1.985799
NDOR1	-0.081001	5.6648738	-3.109556	0.0021371	0.0090522	-1.985986

RPL7P59	-0.496168	-0.927378	-3.109393	0.0021382	0.0090559	-1.986462
P2RY1	0.1898616	4.7872162	3.1093354	0.0021386	0.0090566	-1.986632
GRPEL1	0.0807937	6.3458537	3.1092626	0.0021391	0.0090576	-1.986846
RARRES2P6	-0.748142	-0.859093	-3.109224	0.0021394	0.0090577	-1.986959
PQBP1	-0.069431	6.4103028	-3.109115	0.0021401	0.0090599	-1.98728
CTD-2547G2	-0.22512	4.3097818	-3.108964	0.0021412	0.0090632	-1.987722
PPP1R3G	-0.344128	5.4201062	-3.108848	0.002142	0.0090656	-1.988064
FLT1	0.0999482	6.2377523	3.1083095	0.0021457	0.0090791	-1.989642
KRT83	0.7139981	-0.670433	3.1082892	0.0021458	0.0090791	-1.989702
SUMF2	0.0749394	6.7319828	3.1082773	0.0021459	0.0090791	-1.989736
CCT3	-0.064014	7.026851	-3.108143	0.0021469	0.0090811	-1.99013
LINC00094	0.120507	5.4257511	3.1081086	0.0021471	0.0090811	-1.990231
OSTF1	0.069863	6.1248465	3.1080755	0.0021473	0.0090811	-1.990329
LINC00968	0.6096089	-0.689797	3.1080444	0.0021475	0.0090811	-1.99042
RP11-26L2C	0.2571483	-1.478627	3.1080324	0.0021476	0.0090811	-1.990455
TMEM212	-0.467465	-1.116108	-3.107811	0.0021492	0.0090858	-1.991103
ANK1	0.5854305	3.243052	3.1078003	0.0021492	0.0090858	-1.991136
BNIP3P11	-0.622945	2.6282747	-3.107645	0.0021503	0.0090893	-1.991591
RBM10	-0.045809	6.4290571	-3.107569	0.0021508	0.0090905	-1.991813
RP11-146I2	-0.695724	-0.395051	-3.107488	0.0021514	0.0090918	-1.992051
TPM4	0.0739473	6.7751972	3.1074011	0.002152	0.0090933	-1.992307
RPS27A	-0.064861	7.0350913	-3.107365	0.0021523	0.0090933	-1.992413
RP11-131L1	-0.459056	2.9491426	-3.106752	0.0021565	0.0091103	-1.994209
RP11-191L9	-1.015136	0.320337	-3.106673	0.0021571	0.0091115	-1.994442
POLM	-0.057966	5.9403338	-3.106528	0.0021581	0.0091147	-1.994866
RPL32	-0.074653	7.1607279	-3.106281	0.0021598	0.009121	-1.995591
RP11-573D1	-0.330159	5.0314845	-3.106054	0.0021614	0.0091266	-1.996258
RP11-5C23.	0.1441927	4.9245884	3.1058454	0.0021629	0.0091317	-1.996868
AP001437.1	-0.676753	1.1498063	-3.10564	0.0021643	0.0091367	-1.99747
AP000347.4	-0.514673	3.2857053	-3.105231	0.0021671	0.0091477	-1.998668
RPL3P3	-0.666831	-0.151285	-3.105021	0.0021686	0.0091524	-1.999285
RP11-404P2	0.3331498	-1.340526	3.105002	0.0021687	0.0091524	-1.99934
PLXNB1	-0.083954	6.6947939	-3.10477	0.0021704	0.0091582	-2.00002
MAST3	-0.090451	6.1002727	-3.104565	0.0021718	0.0091632	-2.000619
ITM2C	-0.100336	6.4421094	-3.104042	0.0021755	0.0091776	-2.002152
HS3ST6	-0.834305	-0.091189	-3.103779	0.0021773	0.0091843	-2.002924
MTR	-0.082342	6.2292632	-3.103382	0.0021801	0.0091951	-2.004088
ZNF701	-0.28461	4.7466211	-3.103042	0.0021825	0.0092041	-2.005083
RSPH9	-0.149086	4.0567178	-3.103002	0.0021828	0.0092042	-2.0052
RP11-241K1	-0.523731	-0.859734	-3.102444	0.0021867	0.0092197	-2.006834
RP11-101E1	1.093965	1.0541872	3.1024106	0.002187	0.0092197	-2.006932
LINC00310	0.5684904	2.1463492	3.1022624	0.002188	0.009223	-2.007366
ZDHHC1	0.203951	5.1956856	3.1020442	0.0021896	0.009228	-2.008005
RP1-232L24	-0.538373	-1.006479	-3.101989	0.0021899	0.009228	-2.008167
RN7SL825P	-0.445072	-1.147996	-3.101969	0.0021901	0.009228	-2.008226
NCKAP1L	0.1625433	5.7965184	3.10195	0.0021902	0.009228	-2.00828
WASF3	0.3847754	5.1681106	3.1015258	0.0021932	0.0092396	-2.009522
TXNRD1	0.1195062	6.7056887	3.1013635	0.0021944	0.0092413	-2.009998
RP11-148B1	0.7960633	0.6891831	3.1013525	0.0021944	0.0092413	-2.01003
PAQR8	-0.179351	5.3482227	-3.101326	0.0021946	0.0092413	-2.010106
NME1	-0.092448	6.3751852	-3.101325	0.0021946	0.0092413	-2.010109

LINC00648	-1.163234	0.5016198	-3.101087	0.0021963	0.0092474	-2.010806
KCNJ15	0.4586342	4.0126922	3.1006584	0.0021994	0.0092591	-2.012062
GDF11	-0.157572	5.2709781	-3.100562	0.0022	0.0092609	-2.012343
BLOC1S5-TX	0.7185222	1.8725451	3.1004262	0.002201	0.0092628	-2.012741
MCEE	0.1107486	5.7675087	3.1004256	0.002201	0.0092628	-2.012743
POLR3GL	-0.101836	6.185798	-3.100209	0.0022026	0.0092682	-2.013377
SNORD114-1	-0.365279	-1.345723	-3.099621	0.0022067	0.0092848	-2.015097
LRRN2	0.3512465	4.8502084	3.0995273	0.0022074	0.0092865	-2.015372
RRAGC	0.075198	5.9488535	3.0994315	0.0022081	0.0092883	-2.015652
CTA-280A3.	-0.708995	-0.535361	-3.098814	0.0022125	0.0093053	-2.017457
HLA-L	0.2551672	5.2098778	3.0987942	0.0022126	0.0093053	-2.017517
FRZB	0.2020925	5.5380121	3.098565	0.0022143	0.0093111	-2.018187
OR12D2	-0.647207	-0.873269	-3.098475	0.0022149	0.0093127	-2.01845
APC	0.0772445	5.9670257	3.0980164	0.0022182	0.0093254	-2.019792
MIR30C2	0.5442437	-0.857759	3.0978679	0.0022192	0.0093288	-2.020226
RP1-40G4P.	-0.616629	1.2251642	-3.097814	0.0022196	0.0093293	-2.020383
GCLC	0.101874	6.528685	3.0976294	0.0022209	0.0093338	-2.020924
RP11-319E1	-0.425289	-1.200718	-3.097459	0.0022222	0.0093379	-2.021423
SCG5	-0.254478	5.1189543	-3.097395	0.0022226	0.0093387	-2.021607
SNCAIP	0.3403451	4.6557858	3.0972879	0.0022234	0.0093405	-2.021922
PHF5A	-0.069383	6.0030174	-3.097263	0.0022236	0.0093405	-2.021995
CYP26A1	0.9162726	3.0795703	3.0961478	0.0022316	0.0093731	-2.025255
ATXN2	-0.043118	6.2068431	-3.096081	0.0022321	0.009374	-2.02545
HCCS	0.0638471	6.0282735	3.0954273	0.0022368	0.0093927	-2.027361
C16orf87	0.1076336	5.7158472	3.0950457	0.0022395	0.0094032	-2.028476
SLAIN1	0.532249	4.3172408	3.0949459	0.0022402	0.0094051	-2.028768
LHFPL3	-0.970721	0.2901706	-3.094754	0.0022416	0.0094098	-2.029329
C1orf50	0.0857899	5.4975731	3.0946061	0.0022427	0.0094132	-2.029761
ARHGEF37	0.2680569	5.3346675	3.0942667	0.0022451	0.0094224	-2.030752
OSTN-AS1	1.0340588	1.3031308	3.0940434	0.0022468	0.0094281	-2.031405
LYZL2	-0.58782	-0.945517	-3.093825	0.0022483	0.0094328	-2.032043
USP10	0.0523427	6.3362952	3.0938166	0.0022484	0.0094328	-2.032067
RP11-203M5	-0.717056	1.3635045	-3.093782	0.0022486	0.0094328	-2.032169
XXbac-B135	-0.385319	3.3146854	-3.093675	0.0022494	0.009435	-2.032479
RPS2P1	-0.362909	-1.236202	-3.092819	0.0022556	0.0094599	-2.034979
RP11-366M4	-0.517655	-0.926481	-3.092462	0.0022582	0.0094697	-2.036024
RP11-426A6	-0.542019	2.1161927	-3.092368	0.0022589	0.0094702	-2.036299
PFKFB4	-0.228941	5.0570258	-3.092288	0.0022595	0.0094702	-2.03653
RP11-269C2	-0.620272	-0.627344	-3.092288	0.0022595	0.0094702	-2.03653
C16orf86	0.1877459	4.7463837	3.092269	0.0022596	0.0094702	-2.036587
HIST1H3B	-0.810748	1.841028	-3.092248	0.0022598	0.0094702	-2.036648
RP11-430C7	-0.471308	3.6610455	-3.092233	0.0022599	0.0094702	-2.036692
RP11-22N19	0.5753848	2.1403317	3.0914917	0.0022653	0.0094917	-2.038856
LINC01539	-0.545753	-1.031156	-3.091352	0.0022663	0.0094949	-2.039263
CLDN23	0.1282794	5.6247112	3.090765	0.0022706	0.0095118	-2.040977
FKBP11	-0.121478	6.3842457	-3.090239	0.0022744	0.0095268	-2.042511
GPR183	0.2617271	5.1345226	3.090146	0.0022751	0.0095285	-2.042783
RP11-649A1	-1.010088	0.2053837	-3.090042	0.0022759	0.0095306	-2.043086
RP11-504P2	-0.143785	5.2267333	-3.089536	0.0022796	0.0095441	-2.044564
ST7-AS1	-0.251756	3.9760156	-3.089533	0.0022796	0.0095441	-2.044573
AC092171.4	-0.159319	5.6220516	-3.089495	0.0022799	0.0095441	-2.044683

AC116366.6	0.3258384	3.9885162	3.0893039	0.0022813	0.0095489	-2.04524
ETV1	0.2473648	5.3116294	3.0891532	0.0022824	0.0095524	-2.04568
GPR150	0.7453545	2.0378361	3.089087	0.0022829	0.0095534	-2.045873
PARK7	0.0742821	6.7866382	3.0890168	0.0022834	0.0095544	-2.046078
AC005336.4	0.6499637	4.5744221	3.0889418	0.0022839	0.0095547	-2.046297
NIFK-AS1	-0.115925	4.9831737	-3.088937	0.002284	0.0095547	-2.04631
ANGPTL4	0.2240062	6.4380746	3.0886566	0.002286	0.0095622	-2.047128
UPP2-IT1	0.9007948	-0.107995	3.088232	0.0022892	0.0095742	-2.048367
SNORD60	-0.706104	1.3416237	-3.087893	0.0022917	0.0095835	-2.049355
FKBP4P6	-0.498588	-1.163873	-3.087712	0.002293	0.0095872	-2.049882
HNRNPA1P41	-0.511116	-0.827226	-3.08767	0.0022933	0.0095872	-2.050006
DUOX1	-0.296456	4.870479	-3.087667	0.0022933	0.0095872	-2.050013
RP11-527N2	0.6327355	2.9044091	3.0874239	0.0022951	0.0095936	-2.050723
RPS29	-0.086014	6.8422837	-3.087315	0.0022959	0.0095958	-2.051039
AP000593.6	-0.453982	-1.097135	-3.087129	0.0022973	0.0095995	-2.051582
CC2D2B	0.6347173	0.6118149	3.0871262	0.0022973	0.0095995	-2.051591
CTC-444N24	-0.094078	5.2420399	-3.087051	0.0022979	0.0096003	-2.051811
MYBPHL	-1.04308	1.1916748	-3.087028	0.002298	0.0096003	-2.051878
PRPF6	-0.060277	6.7144563	-3.086638	0.0023009	0.0096113	-2.053015
RPS2P35	-0.65427	0.1786184	-3.085712	0.0023078	0.009638	-2.055712
KRT5	1.0382569	2.0099708	3.0857018	0.0023079	0.009638	-2.055743
GAPT	0.5215998	3.4321847	3.0855136	0.0023092	0.0096427	-2.056291
UEVLD	0.0615863	5.8914368	3.0852926	0.0023109	0.0096485	-2.056935
RPL13AP3	-0.500659	2.368535	-3.085228	0.0023114	0.009649	-2.057123
HRH3	-0.617972	-0.669203	-3.085204	0.0023115	0.009649	-2.057192
SNAPIN	-0.059484	6.1792748	-3.085026	0.0023129	0.0096534	-2.057712
RPLP2	-0.07048	7.1289494	-3.084856	0.0023141	0.0096576	-2.058208
RP11-526I2	-0.44535	3.8370507	-3.084789	0.0023146	0.0096586	-2.058401
C5orf24	0.0672793	6.3147951	3.0843855	0.0023176	0.00967	-2.059578
RARRES1	0.2583168	5.350697	3.0842525	0.0023186	0.0096722	-2.059965
RP13-16H11	-0.662533	0.2596121	-3.084243	0.0023187	0.0096722	-2.059993
USP37	-0.170235	5.1312816	-3.084138	0.0023195	0.0096744	-2.060297
GAPDHP28	-0.380978	-1.210015	-3.084095	0.0023198	0.0096746	-2.060424
PCDHB4	0.477405	3.7851548	3.0838926	0.0023213	0.0096798	-2.061014
VN1R51P	-0.818629	0.0289908	-3.0837	0.0023227	0.0096847	-2.061574
AJAP1	0.8374445	2.0597976	3.08316	0.0023268	0.0097004	-2.063147
UGT2B15	0.4552942	6.3963387	3.0831193	0.0023271	0.0097005	-2.063265
CYB5D2	0.1191487	5.9455065	3.0829302	0.0023285	0.0097053	-2.063816
LRRN4	0.882275	1.6132206	3.0828965	0.0023287	0.0097053	-2.063914
LANCL3	0.6850213	1.512469	3.0827963	0.0023295	0.0097065	-2.064206
ACOX1	0.1031333	6.7956703	3.082786	0.0023296	0.0097065	-2.064236
SLC30A3	-0.812074	2.6582404	-3.082699	0.0023302	0.0097081	-2.064489
AL645937.1	-0.400632	-1.244187	-3.082081	0.0023348	0.0097263	-2.066288
NRARP	0.1899672	5.1216473	3.0818549	0.0023365	0.0097322	-2.066947
RP11-449D8	0.6605283	-0.236293	3.0815317	0.002339	0.0097412	-2.067887
EDARADD	-0.531543	3.7922944	-3.081476	0.0023394	0.0097418	-2.068048
AC142119.1	-0.318643	-1.376593	-3.081062	0.0023425	0.0097534	-2.069253
BLNK	0.1338786	5.9956597	3.0810369	0.0023427	0.0097534	-2.069327
OPCML	0.8192468	1.537556	3.0802147	0.0023489	0.0097769	-2.07172
EXOC5	0.0584529	6.179639	3.0802095	0.0023489	0.0097769	-2.071735
RP11-217E1	0.2240502	-1.495495	3.0801786	0.0023491	0.0097769	-2.071825

TMEM129	0.0828127	6.4171517	3.0799547	0.0023508	0.0097821	-2.072476
CRISPLD1	0.4111576	4.1367292	3.0799425	0.0023509	0.0097821	-2.072512
COL9A3	-0.399418	4.7604345	-3.079899	0.0023513	0.0097823	-2.072637
RAD50	0.0616044	6.3013596	3.0798629	0.0023515	0.0097824	-2.072743
RP11-296P7	-0.609058	-0.495288	-3.079816	0.0023519	0.0097827	-2.07288
ITSN1	0.0875823	6.2323189	3.0797431	0.0023524	0.0097839	-2.073092
ANKLE2	-0.049275	6.3344194	-3.079686	0.0023529	0.0097846	-2.073257
AC114730.1	-0.545342	2.178381	-3.079586	0.0023536	0.0097866	-2.073548
ZSCAN18	-0.217949	5.6574629	-3.079178	0.0023567	0.0097983	-2.074735
KCNIP2	-0.332533	3.884778	-3.07909	0.0023574	0.0097999	-2.07499
ITIH5	0.4371354	5.1977859	3.07887	0.002359	0.0098049	-2.075631
RP11-337N6	-0.256852	3.579537	-3.078863	0.0023591	0.0098049	-2.075652
CLIP1	0.0774597	6.433148	3.0784856	0.0023619	0.0098156	-2.076749
REM2	-0.569945	2.1372734	-3.078194	0.0023642	0.0098237	-2.077596
AP000473.8	0.8056258	1.078996	3.0780754	0.0023651	0.0098263	-2.077942
INPP5K	0.0668848	6.0729218	3.0779967	0.0023657	0.0098277	-2.078171
RP11-91P24	-0.631829	-0.263527	-3.077921	0.0023662	0.0098289	-2.07839
CYSLTR2	0.2859917	4.2112272	3.0777947	0.0023672	0.0098318	-2.078758
RP3-393E18	0.6713566	2.7616019	3.077363	0.0023705	0.0098443	-2.080013
RP11-89K21	-1.128245	1.284484	-3.076897	0.002374	0.0098578	-2.081369
CDH18	-0.8243	-0.624425	-3.076866	0.0023742	0.0098578	-2.081459
RP11-771F2	-0.655869	0.1662759	-3.076675	0.0023757	0.0098627	-2.082013
NARFL	0.0674982	6.123213	3.0765176	0.0023769	0.0098665	-2.08247
RPS6KA6	1.0138846	1.5940837	3.076208	0.0023793	0.0098748	-2.08337
RP11-415J8	-0.670464	-0.438099	-3.076186	0.0023794	0.0098748	-2.083434
RP11-768F2	0.3646872	3.1558786	3.0758045	0.0023823	0.0098857	-2.084542
TAOK2	0.0477181	6.3832955	3.0757565	0.0023827	0.0098857	-2.084682
PHLDB3	-0.105441	6.0027036	-3.075734	0.0023829	0.0098857	-2.084749
SSX3	-0.785473	-0.754866	-3.075576	0.0023841	0.009889	-2.085208
RP11-112J1	-0.509008	2.6724648	-3.075561	0.0023842	0.009889	-2.085251
CPSF1	-0.067009	6.5904079	-3.075438	0.0023851	0.0098917	-2.085608
RPL22P19	-0.491699	-0.950427	-3.075268	0.0023864	0.0098959	-2.086102
SOS2	0.0772895	5.9861648	3.0752366	0.0023867	0.0098959	-2.086192
RAB41	-0.295896	3.4248525	-3.075023	0.0023883	0.0099015	-2.086814
RP11-687F6	-0.697075	0.793252	-3.074815	0.0023899	0.009907	-2.087416
ROBO1	-0.198692	6.3265437	-3.074744	0.0023904	0.0099071	-2.087622
PCBP4	-0.09013	6.1097416	-3.074742	0.0023905	0.0099071	-2.087629
ANXA7	0.0557316	6.7168676	3.074686	0.0023909	0.0099077	-2.087792
MPC1	0.1219079	6.4809904	3.07372	0.0023983	0.0099373	-2.090597
EHMT1	-0.050081	6.2771903	-3.073314	0.0024014	0.0099491	-2.091776
IL7	0.2132083	5.0781335	3.0731193	0.0024029	0.0099542	-2.092341
STOX1	-0.565846	3.7476473	-3.072896	0.0024046	0.0099602	-2.092988
MAML3	0.13997	5.5050103	3.0728375	0.0024051	0.0099609	-2.09316
RP5-874C20	-0.779748	0.2159533	-3.072573	0.0024071	0.0099682	-2.093928
RCL1	0.1383632	6.020611	3.0722834	0.0024094	0.0099763	-2.094768
DDX46	-0.050175	6.2892754	-3.07219	0.0024101	0.0099782	-2.095039
MIR765	-0.560141	-0.642717	-3.072023	0.0024114	0.0099824	-2.095524
AC107081.5	-0.631014	1.7053067	-3.07189	0.0024124	0.0099855	-2.095909
MGARP	0.6521231	1.3222864	3.0718109	0.002413	0.0099869	-2.096139
RP1-146I3.	-0.469661	-0.946429	-3.071528	0.0024152	0.0099948	-2.096959
BACH1-IT2	-0.600461	1.8134186	-3.071432	0.0024159	0.0099968	-2.097239

SLC50A1	-0.0934	6.5244791	-3.071346	0.0024166	0.0099984	-2.097489
RP11-567F1	-0.555337	-0.787452	-3.070814	0.0024207	0.0100143	-2.099032
CTD-2528L1	-0.657827	-0.370102	-3.070561	0.0024227	0.0100211	-2.099765
PMF1	-0.084609	6.2288056	-3.070499	0.0024232	0.0100211	-2.099946
AHCYL2	0.0868338	5.8120197	3.0704954	0.0024232	0.0100211	-2.099956
CTB-191K22	-0.646925	0.0329819	-3.070198	0.0024255	0.0100295	-2.10082
GXYLT1	-0.070981	6.0448651	-3.06962	0.00243	0.010047	-2.102495
ZNF880	-0.288958	4.7279316	-3.069471	0.0024312	0.0100498	-2.102927
NCBP2-AS2	-0.089464	5.7520754	-3.069438	0.0024314	0.0100498	-2.103022
MICALL2	0.1142577	6.0262212	3.0694259	0.0024315	0.0100498	-2.103058
RP11-205M3	0.935732	0.3011032	3.0692102	0.0024332	0.0100556	-2.103684
ZNF883	-0.598782	3.4833662	-3.069027	0.0024346	0.0100598	-2.104217
RP11-946L2	-1.190007	0.54624	-3.069009	0.0024348	0.0100598	-2.104268
GMIP	0.1067571	5.7285049	3.0688566	0.0024359	0.0100636	-2.104709
EGLN1	0.0760344	6.6058998	3.0688142	0.0024363	0.0100638	-2.104832
GRID2	-0.662046	-0.797442	-3.068605	0.0024379	0.0100691	-2.105439
UGT1A3	0.8103761	4.1055246	3.0685779	0.0024381	0.0100691	-2.105517
STX2	0.0867118	5.760385	3.0683397	0.00244	0.0100757	-2.106208
RP11-798M1	-0.673484	0.6489716	-3.068228	0.0024408	0.0100781	-2.10653
CRCT1	0.6685401	-0.862478	3.0678858	0.0024435	0.010088	-2.107524
CALCA	0.9750977	2.7347844	3.0678521	0.0024438	0.010088	-2.107622
MSL2	-0.048859	6.0394717	-3.067799	0.0024442	0.0100885	-2.107775
IGFN1	0.9843421	1.7843837	3.067711	0.0024449	0.0100902	-2.10803
RP11-4M23.	-0.460591	-1.009806	-3.067624	0.0024456	0.0100919	-2.108282
CTD-2619J1	-0.58659	4.0419202	-3.067407	0.0024473	0.0100978	-2.108911
RP11-309L2	-0.369807	3.7477386	-3.067063	0.00245	0.0101078	-2.109909
GNAZ	-0.278296	5.5647563	-3.06686	0.0024516	0.0101132	-2.110497
PLCZ1	0.7503508	0.7175322	3.0664394	0.0024549	0.0101256	-2.111716
ITGA8	0.2075313	4.7927792	3.0658686	0.0024593	0.010143	-2.113369
UPF2	-0.059814	6.2014273	-3.065695	0.0024607	0.0101475	-2.113872
AC010677.5	-0.607533	-0.423306	-3.065563	0.0024617	0.0101506	-2.114256
MATR3	-0.168197	5.4465731	-3.065523	0.0024621	0.0101508	-2.11437
RP5-1063M2	0.4723	-1.120776	3.0652869	0.0024639	0.0101573	-2.115054
NOMO1	0.0694596	6.256431	3.0652433	0.0024643	0.0101575	-2.115181
CCDC109B	0.204541	5.1823999	3.0650131	0.0024661	0.0101637	-2.115847
SULT1A1	0.1634958	6.4155243	3.0649636	0.0024665	0.0101637	-2.115991
RP5-1092A3	-0.266351	4.4109586	-3.064948	0.0024666	0.0101637	-2.116036
COQ2	0.093174	5.628075	3.0648506	0.0024674	0.0101657	-2.116318
HMGB3P32	-0.694736	0.1383073	-3.064693	0.0024686	0.0101695	-2.116775
AC092625.1	-0.759724	-0.548534	-3.064663	0.0024688	0.0101695	-2.116863
ANP32C	-0.475182	-0.956468	-3.064412	0.0024708	0.0101765	-2.117588
KIAA1429	-0.064881	6.3993512	-3.063662	0.0024767	0.0101998	-2.119759
LINC00475	0.7224234	0.9282716	3.0636247	0.002477	0.0101999	-2.119868
SIGLEC11	0.470125	3.5996939	3.0632545	0.00248	0.0102108	-2.12094
LINC00221	-1.337272	0.9539846	-3.063204	0.0024804	0.0102112	-2.121085
PKN1	-0.081368	6.6591176	-3.063173	0.0024806	0.0102112	-2.121176
CASP8AP2	-0.099951	5.594534	-3.063097	0.0024812	0.0102125	-2.121395
CHRAC1	-0.07077	6.155844	-3.06273	0.0024841	0.0102232	-2.122457
COL9A2	-0.306687	4.9647404	-3.062699	0.0024844	0.0102232	-2.122547
RP11-900F1	-0.786169	0.1155721	-3.062488	0.0024861	0.0102289	-2.123158
SERTAD3	0.0771318	5.9261142	3.0624339	0.0024865	0.0102295	-2.123315

RP11-141M1	-0.493688	3.2746459	-3.062008	0.0024899	0.0102423	-2.124547
AC010872.1	-0.561541	-0.629931	-3.06189	0.0024908	0.010245	-2.12489
ZNF223	-0.252866	3.8968517	-3.061843	0.0024912	0.0102454	-2.125026
RPF2P1	-0.462729	-0.998385	-3.061543	0.0024936	0.0102541	-2.125894
SF3B4	-0.068824	6.5528512	-3.061223	0.0024961	0.0102634	-2.126817
GPR137B	0.1349257	5.8600308	3.061071	0.0024973	0.0102672	-2.127258
SDC4P	0.6482343	-0.665345	3.0608364	0.0024992	0.0102737	-2.127937
CTC-534A2.	-0.110039	4.8069016	-3.060625	0.0025009	0.0102779	-2.128548
RP11-243M5	-0.817147	0.1071761	-3.060614	0.002501	0.0102779	-2.128579
DIRAS1	-0.818753	3.2044324	-3.060603	0.0025011	0.0102779	-2.128611
GNB2	0.0539959	6.7777899	3.0603976	0.0025027	0.0102835	-2.129206
PHC2	0.0567569	6.6656702	3.0603612	0.002503	0.0102836	-2.129311
LY6E	0.1788655	6.5581854	3.0603094	0.0025034	0.0102838	-2.129461
ATP6V1D	0.0585933	6.3071657	3.0602598	0.0025038	0.0102838	-2.129605
CD97	0.1186141	6.2677082	3.0602478	0.0025039	0.0102838	-2.129639
CH507-24F1	-0.723466	-0.177901	-3.060205	0.0025042	0.010284	-2.129762
BBS12	0.2084095	4.4102528	3.0600787	0.0025053	0.010287	-2.130128
SLC25A24P2	-0.479331	-1.15566	-3.06002	0.0025057	0.0102878	-2.130298
HIST1H2AI	-0.880657	1.399184	-3.059954	0.0025063	0.0102888	-2.130489
MIR4767	0.394641	-1.29264	3.0599054	0.0025066	0.0102893	-2.13063
EEF1A1P2	-0.549477	-0.616805	-3.05953	0.0025096	0.0103004	-2.131714
RPL17P25	-0.36604	-1.235825	-3.059393	0.0025107	0.0103038	-2.13211
MTNR1B	-0.951601	-0.402645	-3.058676	0.0025165	0.0103262	-2.134184
SMC4	-0.134962	5.9406824	-3.058526	0.0025177	0.01033	-2.134617
CLCN7	0.073899	6.4676694	3.0582164	0.0025202	0.0103391	-2.135513
U91328.19	-0.153399	5.2050741	-3.058138	0.0025208	0.0103405	-2.135739
DDX27	-0.054036	6.3558332	-3.058068	0.0025214	0.0103415	-2.135941
RP11-400D2	-0.903128	-0.047058	-3.058039	0.0025216	0.0103415	-2.136026
RP11-238F2	1.1510762	0.9021262	3.0579492	0.0025223	0.0103429	-2.136285
AC098820.2	0.4343847	-1.151147	3.0579242	0.0025225	0.0103429	-2.136357
RP1-182D15	-0.661628	2.8244929	-3.057865	0.002523	0.0103437	-2.136529
RP11-14101	0.5356825	-0.930626	3.0577668	0.0025238	0.010345	-2.136812
CTSF	0.1374733	6.5531643	3.0577556	0.0025239	0.010345	-2.136845
PPP4R1L	0.2052268	4.698655	3.0576851	0.0025245	0.0103451	-2.137048
RP11-439E1	-0.295988	4.4293411	-3.057635	0.0025249	0.0103451	-2.137193
CTD-2006C1	-0.522975	2.4580018	-3.05762	0.002525	0.0103451	-2.137236
SPATA7	0.1039006	5.2604281	3.0576106	0.0025251	0.0103451	-2.137264
RP11-81H14	0.9559455	0.7554784	3.0575047	0.0025259	0.0103475	-2.13757
PXDC1	0.10006	6.5496779	3.0573223	0.0025274	0.0103523	-2.138097
RAMP2-AS1	-0.388049	3.8677759	-3.05726	0.0025279	0.0103532	-2.138276
CTGLF12P	-0.786402	2.1713568	-3.057176	0.0025286	0.0103547	-2.13852
C3orf80	0.6220418	3.49335	3.0571457	0.0025288	0.0103547	-2.138607
KLHL38	-0.822317	1.5612805	-3.057023	0.0025298	0.0103553	-2.138962
AC010761.1	-0.613408	-0.268675	-3.056989	0.0025301	0.0103553	-2.13906
ALDH1B1	0.1206297	6.5887267	3.056932	0.0025305	0.0103553	-2.139225
RPL3P10	-0.486112	-0.975362	-3.056929	0.0025306	0.0103553	-2.139233
TBC1D12	-0.081762	5.60174	-3.056916	0.0025307	0.0103553	-2.139271
PTOV1	-0.060889	6.6779663	-3.056915	0.0025307	0.0103553	-2.139272
RPL15P20	-0.597018	-0.400667	-3.056194	0.0025365	0.010378	-2.141358
RP11-2E11.	-0.494052	2.5644426	-3.056069	0.0025375	0.01038	-2.141719
RP11-832N8	-0.506298	2.541603	-3.056064	0.0025376	0.01038	-2.141732

SLC35F6	0.0646179	6.5117234	3.0558725	0.0025391	0.0103852	-2.142285
PEAK1	0.0813534	5.9925314	3.0558198	0.0025395	0.0103858	-2.142438
RP11-701H2	-0.822149	0.1296699	-3.055771	0.0025399	0.0103862	-2.142579
GPAM	-0.2229	6.608799	-3.05539	0.002543	0.0103977	-2.143679
GNA12	0.0737794	6.3002327	3.0553542	0.0025433	0.0103977	-2.143782
RP11-102M1	-0.508397	-0.892838	-3.055302	0.0025437	0.0103982	-2.143933
INTS12	0.0594228	5.8473203	3.0552495	0.0025442	0.0103982	-2.144085
TFE3	0.0503654	6.376759	3.0552305	0.0025443	0.0103982	-2.144139
RP11-96B2.	0.9359347	0.6524964	3.0551979	0.0025446	0.0103982	-2.144234
MARCKSL1	-0.098123	6.4969222	-3.054946	0.0025466	0.0104054	-2.14496
SEC22C	0.0509068	6.0504182	3.0547235	0.0025484	0.0104114	-2.145604
AP001628.7	-0.342349	-1.364968	-3.054695	0.0025487	0.0104114	-2.145686
ZNF843	0.3412574	3.6351952	3.0535809	0.0025577	0.0104473	-2.148902
CWC22	-0.049024	5.9721941	-3.053462	0.0025587	0.0104494	-2.149245
HIST1H2AM	-0.715059	1.8977151	-3.053447	0.0025588	0.0104494	-2.14929
RP11-1094M	-0.517663	-0.919999	-3.053332	0.0025598	0.0104521	-2.149622
HSPA9	0.0661039	7.0715764	3.0531643	0.0025611	0.0104565	-2.150104
LINC01139	-1.207622	1.2023888	-3.05309	0.0025617	0.0104578	-2.150319
XKR3	0.6485992	-0.792127	3.0530511	0.0025621	0.0104579	-2.150431
NDFIP2	0.0856744	6.1265782	3.0527997	0.0025641	0.0104651	-2.151157
C8orf58	0.1205825	4.9413865	3.0524187	0.0025672	0.0104767	-2.152256
RAB3B	-0.49235	5.3965465	-3.051262	0.0025767	0.0105142	-2.155593
RP11-296L2	-0.440451	-1.159112	-3.050585	0.0025823	0.0105357	-2.157545
EVPLL	0.9723141	1.0812624	3.0504609	0.0025833	0.0105387	-2.157904
IBA57-AS1	-0.626721	1.3343313	-3.050422	0.0025836	0.0105388	-2.158017
LMTK3	0.6348555	3.9468355	3.0503335	0.0025843	0.0105406	-2.158271
FUK	0.0776286	6.0477529	3.0497278	0.0025893	0.0105597	-2.160017
CTD-2213F2	-0.411603	-1.117862	-3.049694	0.0025896	0.0105597	-2.160115
RP11-11N7.	-0.410121	3.3964524	-3.049627	0.0025902	0.0105608	-2.160308
ESF1	-0.07459	5.9575879	-3.049454	0.0025916	0.010565	-2.160807
ZFAS1	-0.102865	6.2719987	-3.049413	0.0025919	0.010565	-2.160926
INPP5B	0.0784673	5.716454	3.0493988	0.002592	0.010565	-2.160966
ZNF32	-0.073433	5.9880731	-3.049324	0.0025927	0.010565	-2.161181
HOTAIRM1	0.2434425	4.233301	3.0493028	0.0025928	0.010565	-2.161243
RP11-158M2	0.4207366	2.8249748	3.0492916	0.0025929	0.010565	-2.161275
ACTR1A	0.0483914	6.5667535	3.0488618	0.0025965	0.0105783	-2.162514
UBN2	-0.07378	5.8936602	-3.04875	0.0025974	0.0105809	-2.162835
IL23A	0.2173544	4.0528137	3.0486731	0.002598	0.010582	-2.163058
TSTA3	0.0837584	6.5975025	3.0486472	0.0025982	0.010582	-2.163132
RP11-650K2	-0.720621	1.1089948	-3.047991	0.0026037	0.0106029	-2.165024
KLF11	0.1058198	6.1310691	3.0478746	0.0026046	0.0106057	-2.165359
LDOC1L	0.1883819	5.5409638	3.0478177	0.0026051	0.0106064	-2.165523
ELMOD1	0.8814593	0.2720683	3.0476599	0.0026064	0.0106106	-2.165978
RP11-129H1	-0.637296	-0.429975	-3.047584	0.0026071	0.0106114	-2.166195
AP000350.8	0.8329215	1.1103718	3.0475653	0.0026072	0.0106114	-2.16625
RP11-312B8	-0.577716	2.1672393	-3.047321	0.0026092	0.0106185	-2.166953
RPL37	-0.069383	7.1491452	-3.0471	0.0026111	0.0106248	-2.167591
AC079922.2	-0.23296	4.3539167	-3.047007	0.0026118	0.0106267	-2.167859
MAGEB1	-0.949816	-0.442488	-3.046771	0.0026138	0.0106335	-2.16854
RP11-218M2	-0.383506	-1.249165	-3.046396	0.0026169	0.010645	-2.169619
RP11-16D22	0.2395762	-1.487582	3.0459808	0.0026204	0.0106579	-2.170814

RP11-313L6	-0.624842	-0.756793	-3.045899	0.0026211	0.0106595	-2.171049
CTD-220702	-0.361602	-1.238002	-3.045814	0.0026218	0.0106612	-2.171294
AC006077.3	-0.413524	2.9827948	-3.045614	0.0026235	0.0106668	-2.171872
RP1-228P16	-0.611213	2.3122071	-3.045522	0.0026242	0.0106687	-2.172137
IGHVII-40-	-0.373576	-1.305845	-3.045485	0.0026245	0.0106688	-2.172243
RP11-9502.	-0.705323	1.2019104	-3.045429	0.002625	0.0106695	-2.172403
AURKC	-0.220812	4.1808033	-3.04481	0.0026302	0.0106883	-2.174185
TGFBR1	0.0783141	6.1873589	3.0448055	0.0026302	0.0106883	-2.174199
RP11-166N6	0.4339787	-1.19834	3.0446242	0.0026317	0.0106933	-2.17472
PTOV1-AS1	-0.094526	5.3518554	-3.044268	0.0026347	0.0107036	-2.175747
RPS4XP1	-0.682572	1.0371481	-3.04425	0.0026349	0.0107036	-2.175799
CTD-237701	-0.80399	1.6701745	-3.044131	0.0026359	0.0107055	-2.176141
NUTM2B-AS1	-0.093723	5.4605852	-3.0441	0.0026361	0.0107055	-2.176228
ZFP62	-0.083637	5.8010696	-3.044091	0.0026362	0.0107055	-2.176254
PCGF6	-0.084422	5.577801	-3.043815	0.0026385	0.0107137	-2.17705
DOLK	-0.067075	6.0400085	-3.043522	0.002641	0.0107224	-2.177894
RP11-318C2	-0.30187	-1.362913	-3.04349	0.0026412	0.0107224	-2.177984
NARF	-0.077415	6.2755076	-3.043345	0.0026424	0.0107261	-2.178401
RP11-51301	-1.055258	0.4596159	-3.042844	0.0026467	0.0107409	-2.179845
SLC4A1APP1	-0.618213	-0.172696	-3.042843	0.0026467	0.0107409	-2.179846
RP11-419J1	-0.686893	-0.633156	-3.042559	0.0026491	0.0107494	-2.180663
ST20-MTHFS	0.6991997	1.0762907	3.042114	0.0026528	0.0107634	-2.181944
CDC37L1-AS	0.2713698	3.7716038	3.042041	0.0026534	0.0107647	-2.182154
SAP30BP	-0.048534	6.3440014	-3.041965	0.0026541	0.0107661	-2.182372
LLNLR-268E	-0.611057	1.957908	-3.041723	0.0026561	0.0107731	-2.183068
SLC39A4	-0.356737	5.4871494	-3.041691	0.0026564	0.0107731	-2.183162
PRPSAP1	-0.081232	6.4779204	-3.041442	0.0026585	0.0107804	-2.183878
C14orf39	-0.487831	-1.176101	-3.041264	0.00266	0.0107853	-2.184389
RP11-2I17.	0.2960302	-1.413284	3.0411173	0.0026612	0.0107891	-2.18481
C21orf59	-0.072884	5.8272015	-3.04102	0.002662	0.0107913	-2.185089
AC006026.1	-0.480203	-0.937894	-3.040731	0.0026645	0.0108	-2.185921
OR51B4	-0.536592	-1.157191	-3.040588	0.0026657	0.0108037	-2.186332
SMO	-0.145863	6.3760792	-3.040278	0.0026683	0.0108132	-2.187225
C10orf111	-0.476685	2.1588723	-3.040055	0.0026702	0.0108196	-2.187866
RP11-64K12	-0.670507	0.2859034	-3.039739	0.0026729	0.0108285	-2.188773
RP11-861E2	0.7751262	0.7830778	3.0397255	0.002673	0.0108285	-2.188812
HLA-DOA	0.2135076	5.6568571	3.0395413	0.0026746	0.0108337	-2.189341
HIST1H4B	-0.642277	-0.403464	-3.039506	0.0026749	0.0108337	-2.189442
SUGT1P2	-0.54687	-0.639988	-3.039098	0.0026784	0.0108455	-2.190615
ZNF598	0.0637672	6.2187265	3.039092	0.0026784	0.0108455	-2.190633
AFAP1	0.1494569	5.6967325	3.0387037	0.0026817	0.0108577	-2.191749
RP11-572O6	-0.670697	0.8985981	-3.038636	0.0026823	0.0108588	-2.191943
AC002511.3	-0.729323	-0.408134	-3.03847	0.0026837	0.0108634	-2.19242
ZNF683	0.5510134	3.5637575	3.0382991	0.0026852	0.0108681	-2.192911
RP11-353H3	-0.533983	-0.772487	-3.037635	0.0026908	0.0108898	-2.19482
RNF138	-0.074734	5.861833	-3.037375	0.002693	0.0108976	-2.195565
CDH3	0.7105318	2.9320671	3.0371485	0.002695	0.0109042	-2.196217
ZNF169	-0.193496	4.3160031	-3.03708	0.0026956	0.0109054	-2.196413
SMC6	-0.069141	6.1117285	-3.036877	0.0026973	0.0109112	-2.196997
DNAJC30	0.0844078	5.8900089	3.0368211	0.0026978	0.0109119	-2.197157
CCDC169	0.8076654	-0.33372	3.0364757	0.0027007	0.0109227	-2.198149

SNORD114-1	-0.386342	-1.306537	-3.036295	0.0027023	0.0109271	-2.198669
STK33	-0.966683	2.1691992	-3.036262	0.0027026	0.0109271	-2.198762
RPL10AP6	-0.193927	4.5049006	-3.036242	0.0027027	0.0109271	-2.19882
RP11-244B2	-0.747983	-0.762297	-3.035839	0.0027062	0.0109392	-2.199977
RP5-1039K5	-0.196898	4.1542055	-3.035824	0.0027063	0.0109392	-2.200021
POLR2C	0.0779476	6.4106473	3.0357706	0.0027068	0.0109399	-2.200174
CLCA2	0.798686	-0.365739	3.0356954	0.0027074	0.0109413	-2.200389
MAZ	-0.074146	6.3093218	-3.035659	0.0027078	0.0109413	-2.200492
MXRA5Y	0.8529117	0.2606169	3.0354962	0.0027092	0.0109458	-2.200961
CREBBP	0.05395	6.434374	3.0354567	0.0027095	0.0109459	-2.201075
PROC	-0.243527	6.7831214	-3.035087	0.0027127	0.0109565	-2.202135
AC097374.2	-0.61759	-0.962408	-3.035084	0.0027127	0.0109565	-2.202146
RPS24P17	-0.511087	-0.871839	-3.034994	0.0027135	0.0109584	-2.202403
AC027601.1	-0.244546	4.0748178	-3.03473	0.0027158	0.0109663	-2.203162
RN7SKP30	-0.53727	-0.742984	-3.03457	0.0027171	0.0109707	-2.20362
RP11-793H1	-0.195524	4.4580444	-3.034263	0.0027198	0.0109802	-2.2045
ECI1	0.0892423	6.6147099	3.0341911	0.0027204	0.0109815	-2.204707
TMEM30A	0.0519834	6.6542469	3.0340156	0.0027219	0.0109864	-2.205211
OLAH	0.7429949	0.2341001	3.0339054	0.0027229	0.010989	-2.205527
ELOVL1	0.0586866	6.4356364	3.0338187	0.0027236	0.0109908	-2.205776
COQ6	0.0809024	5.8029826	3.0335741	0.0027257	0.0109981	-2.206478
RAB24	-0.087602	5.6573056	-3.033398	0.0027272	0.011003	-2.206983
NFIL3	0.1080074	6.2809312	3.0332567	0.0027285	0.0110067	-2.207388
AP001062.7	-0.212288	4.4482308	-3.033217	0.0027288	0.0110069	-2.207501
QRICH1	-0.03854	6.3252545	-3.033165	0.0027293	0.0110075	-2.207651
RP11-326C3	-0.365663	-1.265915	-3.032996	0.0027307	0.0110122	-2.208137
OSR1	0.5472757	3.7694281	3.0329379	0.0027312	0.011013	-2.208303
RP11-242C2	-0.337087	-1.289254	-3.032753	0.0027328	0.0110183	-2.208833
CTC-329H14	-0.445069	-1.065382	-3.03259	0.0027342	0.0110227	-2.209299
ZDHHC8	0.1387718	5.9357369	3.0324957	0.0027351	0.0110237	-2.209571
IVL	0.5971723	-0.911857	3.0324914	0.0027351	0.0110237	-2.209584
WNT2B	0.2195987	4.4921124	3.0321916	0.0027377	0.0110328	-2.210444
RP11-986G1	0.4333515	-1.034472	3.0321629	0.0027379	0.0110328	-2.210526
PERM1	0.625285	3.4311556	3.032113	0.0027384	0.0110333	-2.210669
TMCO6	-0.111639	5.9809015	-3.031466	0.002744	0.0110548	-2.212526
HERC1	0.0698625	6.2357374	3.0314158	0.0027444	0.0110553	-2.212668
SLC23A2	0.1180658	6.4154486	3.0309662	0.0027484	0.0110698	-2.213957
MOB3B	0.2225497	5.4777436	3.0308118	0.0027497	0.011074	-2.2144
EIF5	0.0664844	6.8095944	3.0306806	0.0027508	0.0110774	-2.214776
URB2	-0.089067	5.6787169	-3.029917	0.0027575	0.011103	-2.216964
RP3-486I3.	-0.695853	0.4706067	-3.029431	0.0027618	0.0111189	-2.218358
PFDN4	-0.087657	5.7987945	-3.029242	0.0027634	0.0111243	-2.218898
RP11-344N1	-0.265865	3.6710043	-3.029006	0.0027655	0.0111314	-2.219574
ADH1B	0.3136678	6.9395083	3.0289339	0.0027661	0.0111328	-2.219782
ISM2	-0.873664	-0.283085	-3.028656	0.0027685	0.0111413	-2.220577
PWP1	-0.047882	6.2872058	-3.028589	0.0027691	0.0111425	-2.220771
ANKHD1-EIF	0.1061292	5.1452234	3.0281634	0.0027729	0.0111563	-2.221989
HNRNPA1P8	-0.657986	1.3402998	-3.027967	0.0027746	0.011162	-2.222553
ZNF849P	-0.497968	-1.061668	-3.027791	0.0027761	0.011167	-2.223055
SCG2	0.8088237	2.5210341	3.0275853	0.002778	0.011173	-2.223645
TMEM61	-0.891077	2.2393797	-3.027479	0.0027789	0.0111751	-2.223949

NPW	-0.562139	4.8497551	-3.027456	0.0027791	0.0111751	-2.224016
ZDHHC17	-0.060407	5.89976	-3.027331	0.0027802	0.0111783	-2.224374
RP11-226L1	-0.105374	5.4326152	-3.027223	0.0027811	0.0111809	-2.224682
AC093838.4	-0.083338	5.7143153	-3.027105	0.0027822	0.0111839	-2.22502
INSL6	-0.793016	-0.692652	-3.026944	0.0027836	0.0111883	-2.22548
MTND1P14	0.4655373	-1.009583	3.0268246	0.0027847	0.0111913	-2.225823
RPL35P5	-0.436404	3.186593	-3.026432	0.0027881	0.011204	-2.226947
UCN3	-0.498488	-1.145291	-3.026329	0.002789	0.0112064	-2.227242
RPS19P1	-0.70397	1.0034117	-3.026162	0.0027905	0.0112111	-2.227722
RP11-290F2	-0.387161	-1.211563	-3.026111	0.002791	0.0112117	-2.227867
AUP1	-0.053799	6.7677942	-3.025945	0.0027924	0.0112164	-2.228343
AC021016.7	-0.636423	0.8390771	-3.025712	0.0027945	0.0112234	-2.229008
TXNDC15	0.0561678	6.2926222	3.0255864	0.0027956	0.0112266	-2.229368
CEP83-AS1	-0.520182	2.2695311	-3.025491	0.0027964	0.0112288	-2.22964
RP11-849F2	-0.296617	3.4442503	-3.025375	0.0027975	0.0112317	-2.229973
RP1-197B17	-0.493417	2.4570663	-3.025104	0.0027999	0.0112401	-2.230749
TYRO3	-0.232766	5.5739002	-3.025034	0.0028005	0.0112413	-2.230949
GRAPL	0.5986114	-0.545531	3.0249943	0.0028008	0.0112415	-2.231063
C17orf78	-0.581453	-0.494134	-3.024697	0.0028035	0.0112509	-2.231913
NUPR1L	-0.409653	-1.235184	-3.023164	0.0028171	0.0113043	-2.236298
KRT18P36	-0.492378	-1.028703	-3.02293	0.0028192	0.0113114	-2.236968
TSC22D2	0.0718834	6.0803846	3.0227685	0.0028206	0.011316	-2.23743
ACSBG2	-0.719406	0.6705597	-3.022522	0.0028228	0.0113236	-2.238136
IRS2	0.1368041	6.4320555	3.0224333	0.0028236	0.0113255	-2.238389
PACS1	0.0687145	6.3690985	3.0222446	0.0028253	0.011331	-2.238928
CTB-147C13	0.352922	-1.368753	3.0220174	0.0028273	0.0113379	-2.239578
CTB-83J4.1	-0.37977	-1.21756	-3.021319	0.0028336	0.0113617	-2.241574
KB-1517D11	0.6261785	-0.727017	3.0212268	0.0028344	0.0113638	-2.241838
TMEM151B	-0.632621	0.3898158	-3.020863	0.0028377	0.0113756	-2.242877
CHKB	-0.124573	5.1255822	-3.020799	0.0028382	0.0113767	-2.243062
ATP6V1H	0.0732968	6.2552171	3.0207286	0.0028389	0.0113779	-2.243262
FAM182A	-0.785212	0.1024697	-3.02051	0.0028408	0.0113845	-2.243886
NFKBIL1	-0.075588	6.0939327	-3.020182	0.0028438	0.0113951	-2.244825
RP3-467K16	-0.657785	1.1897691	-3.01999	0.0028455	0.0114008	-2.245372
CTD-2542C2	-0.466291	-1.118317	-3.019913	0.0028462	0.0114023	-2.245593
C20orf202	-0.40614	2.7992267	-3.019744	0.0028477	0.0114071	-2.246075
CTC-537E7.	0.7116991	-0.465425	3.0196527	0.0028485	0.0114092	-2.246337
LINC00898	-0.641086	-0.866665	-3.019547	0.0028495	0.0114118	-2.24664
RP11-1105G	-0.571971	-0.695835	-3.01942	0.0028506	0.0114151	-2.247002
YBX2	-0.652731	4.2705156	-3.019197	0.0028527	0.0114219	-2.247639
MCAM	0.0931094	6.4262474	3.0191617	0.002853	0.0114219	-2.247739
SCEL	0.7319347	-0.467357	3.0185597	0.0028584	0.0114424	-2.249459
CTD-228808	0.6468707	-0.123646	3.0182771	0.002861	0.0114514	-2.250266
SNAPC3	0.0651313	5.9157907	3.0176762	0.0028664	0.0114719	-2.251982
RP3-325F22	0.7759941	2.5176431	3.0175762	0.0028673	0.0114742	-2.252268
ISY1	-0.066695	5.8308623	-3.016721	0.0028751	0.011504	-2.254709
AC004947.2	0.5979922	-0.669868	3.0165379	0.0028767	0.0115094	-2.255232
AC105399.2	-0.422265	-1.143414	-3.016303	0.0028788	0.0115167	-2.255902
LINC00397	-0.391795	-1.2291	-3.016142	0.0028803	0.0115213	-2.256362
RP11-331F9	-0.432333	-1.047516	-3.016094	0.0028808	0.0115217	-2.256499
RP11-480A1	-0.356766	3.6693063	-3.015958	0.002882	0.0115254	-2.256886

ZNRF3	-0.641367	2.4326093	-3.015846	0.002883	0.0115282	-2.257207
MIR4697HG	0.6494446	2.6513633	3.0152625	0.0028883	0.0115482	-2.258872
SMIM13	-0.071906	6.0207102	-3.015066	0.0028901	0.0115541	-2.259434
INTS2	-0.089314	5.5724641	-3.014704	0.0028934	0.0115649	-2.260465
CLDN5	0.1747963	5.8720303	3.0146717	0.0028937	0.0115649	-2.260557
SNHG4	-0.502223	3.8360481	-3.014667	0.0028938	0.0115649	-2.260569
AC008074.3	-0.614794	1.6812642	-3.014139	0.0028986	0.0115829	-2.262078
RP5-845024	0.5330874	-1.153484	3.0140634	0.0028993	0.0115844	-2.262293
COL6A6	0.7159906	1.9469195	3.0140113	0.0028998	0.011585	-2.262441
RP11-131L1	-0.679233	0.9600224	-3.013693	0.0029027	0.0115954	-2.26335
RP4-714D9.	-0.191084	4.4481637	-3.013535	0.0029041	0.0115999	-2.263801
RP1-228H13	-0.173533	4.6902672	-3.013313	0.0029061	0.0116068	-2.264432
CTD-2290C2	-0.611765	-0.356495	-3.013051	0.0029086	0.0116151	-2.265181
SLC25A4	0.1019358	6.3035725	3.0129724	0.0029093	0.0116167	-2.265404
NAPA-AS1	-0.203353	4.2679499	-3.012856	0.0029103	0.0116197	-2.265737
SNX18	0.0741915	6.142468	3.0127702	0.0029111	0.0116212	-2.26598
AC005796.2	-0.358509	-1.300228	-3.012746	0.0029114	0.0116212	-2.26605
GTF2B	0.0642973	6.0372887	3.012363	0.0029149	0.0116328	-2.267141
KBTBD6	-0.083164	5.5461225	-3.012361	0.0029149	0.0116328	-2.267146
DUSP4	0.2250953	5.2496754	3.0122957	0.0029155	0.0116339	-2.267333
EIF4A3	-0.053096	6.4300246	-3.012042	0.0029178	0.011642	-2.268057
RPL36AP13	-0.406972	-1.137459	-3.012003	0.0029182	0.0116421	-2.268169
RP11-359D2	0.2401617	-1.487284	3.0118214	0.0029199	0.0116475	-2.268685
NDUFV2P1	0.2978543	4.5616912	3.0117172	0.0029208	0.0116501	-2.268982
TPRG1-AS1	0.5616374	4.2050855	3.0114252	0.0029235	0.011659	-2.269814
GABARAP	0.0624192	6.3552059	3.0114043	0.0029237	0.011659	-2.269874
MED28P7	-0.658624	-0.27635	-3.01128	0.0029248	0.0116623	-2.270228
RP11-114H2	-0.691618	-0.14303	-3.01118	0.0029258	0.0116647	-2.270515
WI2-2118C2	-0.748583	-0.509037	-3.011138	0.0029262	0.011665	-2.270632
RP11-855A2	-0.569821	-0.393313	-3.010899	0.0029284	0.0116721	-2.271314
RP11-16N11	-0.353165	3.2989713	-3.010877	0.0029286	0.0116721	-2.271377
AC107072.2	0.4401474	-1.070516	3.0108361	0.0029289	0.0116723	-2.271493
CTD-3064H1	-0.759515	-0.168615	-3.010791	0.0029294	0.0116727	-2.27162
AP000350.7	0.6250509	-0.373704	3.0103533	0.0029334	0.0116875	-2.272869
LAG3	-0.252358	5.0217809	-3.010297	0.0029339	0.0116883	-2.273028
SELPLG	0.1561212	5.6904957	3.0101805	0.002935	0.0116913	-2.273361
XPNPEP2	-0.686342	5.2581013	-3.009934	0.0029373	0.0116989	-2.274063
SRCAP	0.0487424	6.5207383	3.0098949	0.0029376	0.0116989	-2.274175
SSTR5	-1.195504	1.3439992	-3.009873	0.0029379	0.0116989	-2.274238
FBXO24	-0.193898	4.0871497	-3.00973	0.0029392	0.0117024	-2.274645
VAV1	0.1852333	5.3056357	3.0097067	0.0029394	0.0117024	-2.274711
CSNK1A1P1	0.6750073	1.4300523	3.0095593	0.0029408	0.0117066	-2.275131
HIST2H2AB	-0.593768	-0.565136	-3.009332	0.0029429	0.0117137	-2.27578
RP11-686D2	0.4856815	3.1039774	3.0092549	0.0029436	0.0117153	-2.275998
MZT2A	-0.091792	6.1744895	-3.009203	0.0029441	0.0117159	-2.276145
ZNF304	-0.136333	5.3956729	-3.009102	0.002945	0.0117184	-2.276433
RP11-1070N	-0.840423	-0.313547	-3.008933	0.0029466	0.0117233	-2.276915
ETHE1	0.0967476	6.0220536	3.0084852	0.0029507	0.0117386	-2.27819
AC005197.2	-0.552562	-0.811845	-3.008318	0.0029523	0.0117435	-2.278665
GUSBP6	-0.531932	-1.068045	-3.007937	0.0029558	0.0117564	-2.279751
DGKG	0.3033182	4.7305351	3.0078114	0.002957	0.0117591	-2.280108

RP11-564A8	-0.662432	1.8103533	-3.007794	0.0029572	0.0117591	-2.280157
TTTY10	0.6319531	-0.442882	3.007524	0.0029597	0.0117678	-2.280926
RP11-351I2	0.5332037	-0.869323	3.0073341	0.0029615	0.0117736	-2.281467
RP11-848G1	0.5991579	2.3898567	3.007289	0.0029619	0.011774	-2.281595
GDPD1	-0.224401	4.8614951	-3.007096	0.0029637	0.0117798	-2.282143
TMED7-TICA	0.4754955	2.6842834	3.0070493	0.0029641	0.0117803	-2.282278
SLC25A12	0.1836325	5.2233482	3.006836	0.0029661	0.0117869	-2.282884
RN7SL75P	-0.496111	-0.850585	-3.006659	0.0029678	0.0117922	-2.283387
SERPINA5	-0.275015	6.9404315	-3.006257	0.0029715	0.0118059	-2.284532
WBP1L	0.0626995	6.4007846	3.0062119	0.0029719	0.0118063	-2.284661
PRH1-PRR4	-0.639545	1.1260922	-3.00608	0.0029732	0.0118099	-2.285036
CUL1	0.0563592	6.4914609	3.005999	0.0029739	0.0118116	-2.285266
LINC00622	-0.484831	3.1296795	-3.005708	0.0029767	0.0118211	-2.286094
ZNF286A	-0.156872	4.6545053	-3.005674	0.002977	0.0118211	-2.286192
PRR35	-0.430935	-1.264712	-3.005555	0.0029781	0.0118243	-2.28653
RP11-25H12	-0.996726	1.2466318	-3.004902	0.0029842	0.0118473	-2.288387
RP11-38002	-0.54637	2.3189423	-3.00478	0.0029854	0.0118506	-2.288735
LINC00533	-0.567213	-0.933574	-3.004659	0.0029865	0.0118538	-2.289079
FBRSL1	-0.0642	6.3660653	-3.004548	0.0029876	0.0118567	-2.289395
CREB5	0.2223862	4.940574	3.0044769	0.0029882	0.011858	-2.289596
PMS2P10	0.6164501	1.6801292	3.0043926	0.002989	0.0118599	-2.289836
PDCD4	-0.070672	6.3458375	-3.003848	0.0029941	0.0118789	-2.291384
RP11-490G8	-0.59341	1.9926474	-3.003812	0.0029945	0.011879	-2.291488
ODAM	-1.07243	0.8803418	-3.00349	0.0029975	0.0118897	-2.292401
RP1-239B22	0.5067329	3.9558498	3.0034502	0.0029979	0.01189	-2.292516
MYL12B	0.0595201	6.9882034	3.0032673	0.0029996	0.0118955	-2.293036
RP11-555K2	-0.640972	-0.953197	-3.002915	0.0030029	0.0119072	-2.294036
NHSL1	-0.122976	6.2094169	-3.002885	0.0030032	0.0119072	-2.294121
ZC3H8	-0.08371	5.488974	-3.00281	0.0030039	0.0119087	-2.294336
HIATL2	-0.142005	4.5130005	-3.002771	0.0030043	0.0119087	-2.294446
LTBP3	0.0930549	6.6122042	3.0027428	0.0030046	0.0119087	-2.294527
AL133493.2	-0.895963	1.697165	-3.002084	0.0030108	0.0119321	-2.2964
FAM208A	-0.047441	6.2991997	-3.001928	0.0030123	0.0119362	-2.296843
RP5-965F6.	0.6159588	-0.614552	3.0019063	0.0030125	0.0119362	-2.296904
FLJ36000	-0.73983	-0.721208	-3.001334	0.0030179	0.0119564	-2.298532
CD226	0.2777746	4.5676784	3.0010062	0.003021	0.0119674	-2.299462
PF4	0.8494175	0.4949248	3.0009629	0.0030214	0.0119678	-2.299585
TRIM4	0.0644629	6.1464515	3.0008361	0.0030227	0.0119712	-2.299945
ZNF525	-0.297757	4.9405585	-3.000801	0.003023	0.0119713	-2.300044
RP11-701P1	0.7354451	0.2776522	3.0006082	0.0030248	0.0119772	-2.300592
MST1R	0.4682942	4.292296	3.0002739	0.003028	0.0119885	-2.301542
RP11-203I2	0.8464317	2.3578928	2.9999714	0.0030309	0.0119986	-2.302401
CSF2RA	0.2274596	5.0725097	2.9997523	0.003033	0.0120056	-2.303023
CNEP1R1	0.0664181	5.5665863	2.9995917	0.0030345	0.0120104	-2.303479
CCBL2	0.081332	5.8793084	2.9994159	0.0030362	0.0120156	-2.303979
RP11-14101	0.6689108	2.6958496	2.9993843	0.0030365	0.0120156	-2.304068
ABCD1	-0.100137	6.2183893	-2.999226	0.003038	0.0120203	-2.304518
RP11-153K1	0.8644146	1.280413	2.9990657	0.0030395	0.012025	-2.304973
NPM1P47	0.4189682	-1.131596	2.998758	0.0030425	0.0120354	-2.305847
LDHA	0.0661623	7.1418995	2.9985749	0.0030442	0.012041	-2.306366
NKX6-2	-0.503189	-1.037306	-2.998206	0.0030478	0.0120519	-2.307413

CTD-3037G2	-0.868438	0.061966	-2.998193	0.0030479	0.0120519	-2.307451
RP11-713C1	-0.817463	1.4821534	-2.998183	0.003048	0.0120519	-2.307478
RP5-994D16	-0.667233	0.1363529	-2.997776	0.0030519	0.012066	-2.308635
RP11-299L1	-0.433915	-1.070266	-2.997544	0.0030541	0.0120735	-2.309292
PDCD6IPP2	-0.586038	2.5715461	-2.99689	0.0030604	0.012097	-2.311148
UBA6-AS1	0.0885445	5.3779528	2.9967789	0.0030615	0.0121	-2.311463
RP11-366L2	-0.718717	-0.129074	-2.996645	0.0030627	0.0121037	-2.311844
AC022431.3	0.403458	-1.210905	2.9965601	0.0030636	0.0121057	-2.312084
RP11-807H2	0.6807683	0.2456885	2.996095	0.003068	0.0121221	-2.313404
EEF1A1P30	-0.652898	1.3314395	-2.995789	0.003071	0.0121324	-2.314272
RP11-256K7	-0.677678	-0.011425	-2.995641	0.0030724	0.0121367	-2.314693
ADAMTS8	0.4492104	2.9723221	2.9952836	0.0030759	0.0121486	-2.315705
ZNF793-AS1	-0.70641	2.9873042	-2.995249	0.0030762	0.0121486	-2.315803
MICU1	0.0697873	6.4962636	2.9952264	0.0030764	0.0121486	-2.315867
EIF2B4	-0.052717	6.2187124	-2.995106	0.0030776	0.0121519	-2.316208
ACLY	-0.057634	6.6440432	-2.99507	0.0030779	0.0121519	-2.316311
CAPZB	0.0502775	6.7444168	2.9948366	0.0030802	0.0121595	-2.316973
ZNF383	-0.094295	5.4042208	-2.994791	0.0030806	0.0121599	-2.317101
PIM2	-0.114249	5.8913978	-2.994591	0.0030826	0.0121663	-2.317669
RNU6-796P	0.5122815	-0.907733	2.9943106	0.0030853	0.0121751	-2.318464
AKAP1	-0.072267	6.5877369	-2.994245	0.0030859	0.0121751	-2.31865
CTD-2240J1	-0.732445	0.9142636	-2.994232	0.003086	0.0121751	-2.318688
CDK5R1	-0.192409	4.8127589	-2.994222	0.0030861	0.0121751	-2.318715
XPO6	0.0497679	6.4432157	2.9940524	0.0030878	0.0121803	-2.319196
KCNC3	-0.177192	5.3694957	-2.994001	0.0030883	0.0121809	-2.319341
ASTE1	-0.062911	5.3887123	-2.992726	0.0031007	0.0122285	-2.322955
RABAC1	0.0809668	6.4164352	2.9926564	0.0031013	0.0122298	-2.323153
RP11-458K1	-0.405712	-1.216371	-2.992523	0.0031026	0.0122336	-2.32353
CDHR3	-0.159321	5.1452504	-2.991987	0.0031079	0.0122522	-2.325049
CTSLP2	-0.829489	1.4833223	-2.99197	0.003108	0.0122522	-2.325097
EIF4E2	-0.05249	6.4796615	-2.991855	0.0031092	0.0122541	-2.325423
RP11-219E7	0.3258907	-1.352539	2.9918528	0.0031092	0.0122541	-2.32543
NCLP1	-0.662194	0.2775608	-2.991789	0.0031098	0.0122552	-2.325611
NPM1P29	-0.451637	-0.933363	-2.991671	0.003111	0.0122585	-2.325946
PYGB	0.080151	6.5446265	2.991614	0.0031115	0.0122593	-2.326106
RP11-640N1	-0.851726	-0.086716	-2.991473	0.0031129	0.0122634	-2.326506
CTD-3138B1	-0.659208	1.792148	-2.991356	0.003114	0.0122666	-2.326836
RRP1	-0.071745	6.1945505	-2.991183	0.0031157	0.0122719	-2.327326
NPRL2	-0.069119	5.9912482	-2.990885	0.0031186	0.012282	-2.328172
LILRA1	0.3847111	3.8333043	2.9908517	0.003119	0.012282	-2.328266
LIMA1	0.0866015	6.1668923	2.9906117	0.0031213	0.0122891	-2.328945
XXyac-YM21	-0.929093	2.1961816	-2.990599	0.0031214	0.0122891	-2.328982
HIST3H2A	-0.568671	4.0561698	-2.99046	0.0031228	0.0122932	-2.329375
NBL1	0.2145536	5.2767321	2.9899274	0.003128	0.0123124	-2.330883
JAG1	0.1244577	6.2442036	2.9898177	0.0031291	0.0123153	-2.331194
HSD3B1	1.0154386	1.281813	2.9893918	0.0031333	0.0123304	-2.332399
CTD-2587H1	-0.513082	-0.773657	-2.989183	0.0031353	0.0123364	-2.332991
NDUFA3P1	-0.381725	-1.192065	-2.989168	0.0031355	0.0123364	-2.333034
ZFP64	-0.065417	5.8418908	-2.989029	0.0031368	0.0123405	-2.333426
TTL	0.0553618	6.1388556	2.98889	0.0031382	0.0123445	-2.33382
CRABP2	0.3855848	4.0582381	2.9885939	0.0031411	0.0123547	-2.334658

PDZD7	-0.358897	3.4417981	-2.988477	0.0031423	0.0123579	-2.334989
SERTAD4	0.648064	3.0569475	2.9884198	0.0031428	0.0123587	-2.335151
BRI3BP	-0.079556	6.0659091	-2.988221	0.0031448	0.0123651	-2.335712
NLRP4	-0.76377	-0.469429	-2.988055	0.0031464	0.0123702	-2.336182
SKAP1	0.2283617	5.2952701	2.987934	0.0031476	0.0123736	-2.336526
MACC1	0.6557762	3.3993579	2.9878642	0.0031483	0.012375	-2.336723
TMEM91	-0.155432	5.1937665	-2.987792	0.003149	0.0123764	-2.336927
RP11-582E3	0.451104	-1.189682	2.9876086	0.0031508	0.0123822	-2.337446
CALB2	0.8596431	1.0776733	2.9874137	0.0031528	0.0123884	-2.337998
CTD-2152M2	-0.427033	2.9402885	-2.98668	0.00316	0.0124147	-2.340073
GPN3	-0.068452	6.0131028	-2.98667	0.0031601	0.0124147	-2.340101
CGNL1	0.1490808	6.4420458	2.986537	0.0031614	0.0124174	-2.340478
RP11-727F1	-0.60791	1.5312328	-2.98653	0.0031615	0.0124174	-2.340496
TMEM62	0.0815071	5.9834664	2.9864599	0.0031622	0.0124188	-2.340696
LCA5	0.2575886	4.2734638	2.9863412	0.0031634	0.0124221	-2.341032
ARL14EPL	0.2711836	-1.425947	2.9861566	0.0031652	0.012428	-2.341554
NPIPP1	-0.138368	4.7683104	-2.986067	0.0031661	0.0124301	-2.341808
PDE5A	0.172402	5.2397208	2.9857784	0.003169	0.01244	-2.342623
ALX3	-0.935015	-0.152049	-2.985723	0.0031695	0.0124408	-2.342779
DUSP8P3	-0.653301	0.7207361	-2.98558	0.0031709	0.0124451	-2.343184
AC005363.1	-0.597657	-0.49085	-2.98522	0.0031745	0.0124567	-2.344203
RP11-159N1	0.2013009	-1.507088	2.9852145	0.0031746	0.0124567	-2.344217
AC110781.3	-0.593913	-0.843272	-2.985079	0.0031759	0.0124606	-2.3446
FAM120AOS	-0.053002	6.1111113	-2.984904	0.0031777	0.0124657	-2.345096
PPP6R2	-0.081089	6.707401	-2.98488	0.0031779	0.0124657	-2.345162
SNORA65	-0.425653	2.2881114	-2.984778	0.0031789	0.0124684	-2.345452
F3	0.2513837	5.0001321	2.9842485	0.0031842	0.0124873	-2.346948
TNFSF12	0.1112772	5.8212836	2.9842244	0.0031844	0.0124873	-2.347016
TRMT11	0.0891564	5.7147421	2.9838817	0.0031878	0.0124994	-2.347985
NATD1	0.1032525	5.7508758	2.9835215	0.0031914	0.0125111	-2.349003
ANO10	0.0928515	6.1087922	2.9835141	0.0031915	0.0125111	-2.349024
PAQR6	-0.247217	4.6938876	-2.98341	0.0031926	0.0125138	-2.349317
RDH5	0.265813	5.1449406	2.9832607	0.0031941	0.0125183	-2.34974
F8A1	0.1308937	5.5966724	2.983098	0.0031957	0.0125223	-2.350199
RP11-325N1	-0.595548	-0.46755	-2.98309	0.0031958	0.0125223	-2.350221
PDE4C	-0.274912	4.4889881	-2.982837	0.0031983	0.0125309	-2.350936
SLC31A1	0.0897991	6.5932806	2.9825879	0.0032008	0.0125387	-2.351641
RBM8B	-0.60434	1.795719	-2.982571	0.003201	0.0125387	-2.351688
RP11-63K6.	-0.963992	-0.063787	-2.982329	0.0032034	0.0125468	-2.352372
RNU6-863P	0.2486648	-1.460187	2.9821026	0.0032057	0.0125544	-2.353012
RP11-476H2	-0.376457	-1.274527	-2.981834	0.0032083	0.0125636	-2.353769
PPM1G	-0.044752	6.614707	-2.981347	0.0032132	0.0125814	-2.355145
WAC	-0.041946	6.5954762	-2.981057	0.0032162	0.0125915	-2.355964
FITM2	0.0815558	5.861278	2.9804876	0.0032219	0.0126126	-2.357572
GOLGB1	0.0480272	6.5742038	2.9794469	0.0032324	0.0126523	-2.36051
LRRC74A	-0.721751	-0.153227	-2.979328	0.0032336	0.0126557	-2.360846
RP11-267M2	-0.49702	2.5125179	-2.979287	0.003234	0.0126559	-2.36096
TMEM183AP1	0.337599	-1.383787	2.9791581	0.0032353	0.0126597	-2.361325
LAMA5	0.1097489	6.4628698	2.9789405	0.0032375	0.012667	-2.361939
SULT6B1	-0.41543	-1.212819	-2.978809	0.0032389	0.0126697	-2.362311
LRRC14	-0.077128	6.1360336	-2.978803	0.0032389	0.0126697	-2.362327

UGT3A1	0.8453237	5.3684752	2.9786798	0.0032402	0.0126732	-2.362675
SUCLA2	0.0779464	5.942391	2.9786203	0.0032408	0.0126742	-2.362842
ZNF845	-0.221576	5.0584279	-2.97844	0.0032426	0.01268	-2.36335
SLC16A6P1	-0.678383	0.1830898	-2.9784	0.003243	0.0126802	-2.363464
HIST1H2BPS	-0.609965	-0.748136	-2.978193	0.0032451	0.0126871	-2.364047
GALNT9	0.8068773	0.7138565	2.9778999	0.0032481	0.0126974	-2.364875
AC002056.3	-0.623252	0.1453536	-2.977521	0.0032519	0.0127111	-2.365944
GBP7	0.6029603	5.262761	2.9771663	0.0032555	0.0127234	-2.366944
NSUN5P1	-0.11382	5.5251597	-2.977135	0.0032559	0.0127234	-2.367032
CTD-2024P1	0.5921463	2.0166244	2.9771075	0.0032561	0.0127234	-2.36711
RP11-380M2	0.5863712	2.2086514	2.977015	0.0032571	0.0127257	-2.367371
SMAGP	-0.131577	5.6727828	-2.976543	0.0032619	0.0127432	-2.368703
CSF2RB	0.1933635	5.2260637	2.976505	0.0032623	0.0127433	-2.368809
CSDC2	0.7626058	2.4595094	2.9761739	0.0032657	0.0127551	-2.369742
HHEX	-0.08603	6.399009	-2.975981	0.0032676	0.0127615	-2.370286
RP11-259P1	-0.476024	-1.020297	-2.975833	0.0032691	0.0127659	-2.370704
MYL6	0.0547516	7.1504285	2.9758011	0.0032695	0.0127659	-2.370794
CCDC74A	0.3814015	4.033671	2.9747853	0.0032799	0.0128051	-2.373657
RP11-356I2	-0.400167	-1.239836	-2.974734	0.0032804	0.0128051	-2.3738
ARFIP2	-0.057822	6.3187524	-2.974718	0.0032805	0.0128051	-2.373847
DNAJC2	-0.058931	6.1278293	-2.97413	0.0032866	0.0128272	-2.375503
C18orf32	0.0761758	5.5626117	2.9738976	0.003289	0.0128345	-2.376158
RPL41	-0.07046	7.2807217	-2.973862	0.0032893	0.0128345	-2.376258
GGT2	-0.75052	0.0991128	-2.973845	0.0032895	0.0128345	-2.376307
TAS2R19	-0.639803	0.4507041	-2.973813	0.0032898	0.0128345	-2.376397
RP11-356C4	-0.831707	2.5418489	-2.973708	0.0032909	0.012837	-2.376693
RP11-1275H	-0.476503	2.846278	-2.97368	0.0032912	0.012837	-2.37677
RP11-540A2	-0.479232	3.1135972	-2.973632	0.0032917	0.0128376	-2.376905
RP11-1084J	-0.668172	-0.540775	-2.973407	0.003294	0.0128453	-2.377541
RPS12P26	-0.680951	0.735242	-2.973206	0.0032961	0.012852	-2.378107
ZNF22	-0.078756	6.0608718	-2.973085	0.0032973	0.0128549	-2.378448
RP11-703I1	-0.231096	3.9754388	-2.973064	0.0032975	0.0128549	-2.378507
AC005537.2	-0.912661	0.0576618	-2.972988	0.0032983	0.0128566	-2.378722
RFC1	-0.063324	6.4302645	-2.972676	0.0033015	0.0128677	-2.379598
LINC00113	0.5900571	-1.123897	2.9724885	0.0033035	0.0128739	-2.380127
MT-ND1	0.0827694	7.6706677	2.9723824	0.0033046	0.0128768	-2.380426
RP11-49401	-0.570112	-0.353688	-2.972116	0.0033073	0.0128862	-2.381177
RPS4XP17	-0.620029	0.1395998	-2.971827	0.0033103	0.0128964	-2.381989
RP11-1094H	0.7807259	-0.076545	2.9716568	0.0033121	0.0129015	-2.382469
TUBB8P8	-0.43253	-1.121393	-2.971631	0.0033123	0.0129015	-2.382542
RP5-101101	-1.047966	1.7554761	-2.971376	0.003315	0.0129104	-2.383261
SPI1	0.1339412	5.8151313	2.9709349	0.0033195	0.0129268	-2.384502
RP1-8B1.4	-0.730438	2.1445761	-2.970862	0.0033203	0.0129284	-2.384707
UPF3AP1	-0.510555	-0.612827	-2.970806	0.0033209	0.0129293	-2.384863
RP11-168L7	-0.877866	2.7196131	-2.970689	0.0033221	0.0129326	-2.385193
MGC27382	0.6322638	3.1601712	2.9706237	0.0033228	0.0129339	-2.385378
FKBP9	0.062826	6.3622144	2.9704339	0.0033247	0.0129402	-2.385912
CBX3P2	-0.344706	3.0826988	-2.970201	0.0033271	0.0129466	-2.386566
ZNF665	-0.450226	3.6608946	-2.970183	0.0033273	0.0129466	-2.386619
TXLNA	0.0570914	6.5180969	2.9701728	0.0033274	0.0129466	-2.386646
IL7R	0.2826086	5.4702865	2.9701116	0.0033281	0.0129477	-2.386819

ARHGAP30	0.146656	5.7432791	2.9700431	0.0033288	0.0129491	-2.387011
CFAP53	0.3203491	3.6878015	2.9699862	0.0033294	0.01295	-2.387172
TUBBP1	-0.223004	3.8170034	-2.96994	0.0033299	0.0129505	-2.387302
bP-21264C1	-0.331515	-1.299986	-2.969745	0.0033319	0.0129569	-2.38785
OR5AK4P	0.3626025	-1.312002	2.9695983	0.0033334	0.0129615	-2.388263
NEURL1B	0.1072612	6.0204622	2.969416	0.0033353	0.0129675	-2.388776
WDR24	0.0834735	5.843357	2.9693479	0.003336	0.0129687	-2.388968
RP11-795F1	-0.557758	2.9278529	-2.969319	0.0033363	0.0129687	-2.389049
ZCCHC7	-0.060195	5.8245092	-2.96894	0.0033403	0.0129826	-2.390114
RP11-84C13	0.5628371	2.7767948	2.9683655	0.0033463	0.0130035	-2.391731
RPL7A	-0.067757	7.2145383	-2.968356	0.0033463	0.0130035	-2.391757
NHSL2	0.5673414	3.0549262	2.968289	0.0033471	0.0130049	-2.391946
LCE3E	0.2803522	-1.466802	2.9671002	0.0033595	0.0130518	-2.395289
TPMT	0.0846336	6.3883909	2.9666753	0.0033639	0.0130677	-2.396484
MED6	-0.056421	5.8236163	-2.966495	0.0033658	0.0130736	-2.39699
ZNF865	-0.06934	5.8757031	-2.966296	0.0033679	0.0130804	-2.397551
BNIP3P17	-0.844304	0.3714604	-2.965957	0.0033715	0.0130928	-2.398503
CTC-33909.	-0.667408	-0.675665	-2.965811	0.003373	0.0130974	-2.398914
AC004951.5	-0.214765	3.7937818	-2.965749	0.0033737	0.0130985	-2.399086
SPRTN	-0.065576	5.6090854	-2.965659	0.0033746	0.0131008	-2.399341
HNRNPCL2	-0.332972	-1.331005	-2.965462	0.0033767	0.0131074	-2.399893
GTF2H3	-0.062348	5.9853603	-2.96541	0.0033772	0.0131082	-2.400041
TMIGD2	0.4614703	2.9234012	2.9653256	0.0033781	0.0131102	-2.400277
ITGB6	0.800272	3.2652195	2.9651867	0.0033796	0.0131145	-2.400668
GPR161	0.1875703	4.9914059	2.964942	0.0033822	0.0131231	-2.401355
RP11-254F7	-0.689246	0.6883447	-2.964904	0.0033826	0.0131233	-2.401462
RP11-708J1	-0.634946	-0.266273	-2.964769	0.003384	0.0131274	-2.401842
ORAI2	0.1261359	5.6771769	2.9644078	0.0033878	0.0131408	-2.402856
ALG1L13P	0.6155083	2.5055026	2.9643449	0.0033885	0.013142	-2.403033
MBOAT2	0.2623311	4.7910534	2.9642129	0.0033899	0.013146	-2.403404
ZBED3	-0.101763	6.1151804	-2.964079	0.0033913	0.0131501	-2.403779
RGS2	0.1890647	5.8567567	2.9638876	0.0033933	0.0131558	-2.404317
RNA5SP46	0.53142	-0.938401	2.9638458	0.0033937	0.0131558	-2.404435
LCN15	-0.809044	-0.267682	-2.963835	0.0033939	0.0131558	-2.404466
RP11-298I3	-0.190377	4.2152307	-2.963803	0.0033942	0.0131558	-2.404555
RPL35P1	-0.302212	3.5952662	-2.963751	0.0033947	0.0131559	-2.404701
UNC5B	0.1483205	5.8750907	2.9637329	0.0033949	0.0131559	-2.404752
TRMT112P6	-0.640468	1.2117631	-2.963523	0.0033971	0.0131631	-2.40534
CTD-2636A2	-0.336807	4.1454646	-2.963279	0.0033997	0.0131717	-2.406026
EPCAM	-0.679302	5.2459853	-2.963043	0.0034022	0.0131791	-2.406689
HIST1H2BE	-0.69691	1.2827691	-2.963007	0.0034026	0.0131791	-2.406791
OR1H1P	-0.414445	-1.172599	-2.962997	0.0034027	0.0131791	-2.406819
CCDC30	0.2230435	4.0897365	2.962852	0.0034043	0.0131836	-2.407226
ESYT3	-0.602854	3.9652556	-2.962812	0.0034047	0.0131839	-2.407338
GPS2	-0.118377	4.9427471	-2.962704	0.0034058	0.0131869	-2.407641
DROSHA	-0.051411	6.2207013	-2.962652	0.0034064	0.0131871	-2.407788
CD4	0.1196287	6.3284237	2.9626302	0.0034066	0.0131871	-2.407848
RBMS2P1	0.6924074	0.5181885	2.9625574	0.0034074	0.0131887	-2.408053
CDC14A	-0.174184	4.9819111	-2.962453	0.0034085	0.0131916	-2.408345
RAN	-0.053738	6.7671866	-2.962409	0.003409	0.013192	-2.408468
CTC-529G1.	0.547969	-1.062624	2.9617315	0.0034162	0.0132181	-2.410371

AC008753.6	-0.610203	-0.378261	-2.961708	0.0034164	0.0132181	-2.410439
HADHB	0.0718808	6.8476384	2.9614003	0.0034197	0.0132293	-2.411301
ASIC4	-0.719128	-0.021359	-2.961345	0.0034203	0.0132302	-2.411457
UTP20	-0.063619	5.9502403	-2.961149	0.0034224	0.0132369	-2.412005
ANKRD34B	-0.827412	-0.157185	-2.960992	0.003424	0.0132419	-2.412447
CYP2B7P	0.5584131	5.3949179	2.9607683	0.0034264	0.0132498	-2.413075
RP1-34B20.	-0.768395	2.145495	-2.96048	0.0034295	0.0132603	-2.413884
RNF217-AS1	0.6024207	2.2202244	2.9604457	0.0034299	0.0132603	-2.41398
TBK1	0.0481001	6.0816557	2.9603076	0.0034313	0.0132646	-2.414367
PPP2R5E	0.0505198	6.1763273	2.9601965	0.0034325	0.0132678	-2.414679
KBTBD8	0.2803082	4.2419354	2.9598528	0.0034362	0.0132805	-2.415643
ANXA2P2	0.2155843	4.8549912	2.9594996	0.00344	0.0132937	-2.416634
TCEAL7	0.3606716	3.5163999	2.9594109	0.0034409	0.013296	-2.416883
APOC4-APOC	-0.496356	5.6093847	-2.95915	0.0034437	0.0133054	-2.417614
PDE1C	0.4935435	3.6180263	2.9588099	0.0034474	0.0133181	-2.418568
IDH2	0.0910081	6.919849	2.9585949	0.0034497	0.0133256	-2.419171
CCDC58P1	-0.306195	-1.334308	-2.95809	0.0034551	0.0133451	-2.420587
XXbac-BPG3	-0.630801	0.1033145	-2.958007	0.003456	0.0133471	-2.420818
RP11-923I1	-0.698915	1.8878576	-2.957761	0.0034586	0.0133549	-2.421508
FZD1	0.1563523	5.5827722	2.9577515	0.0034587	0.0133549	-2.421536
RP11-381N2	-0.429569	-1.269228	-2.957625	0.0034601	0.0133578	-2.421891
TTC19	0.0834467	6.1009196	2.9576147	0.0034602	0.0133578	-2.421919
MPEG1	0.1411307	6.0649269	2.9574943	0.0034615	0.0133614	-2.422257
KCNJ1	0.6227205	-0.234136	2.9573198	0.0034634	0.0133672	-2.422746
NXNL2	0.7796738	0.7866001	2.9572002	0.0034647	0.0133707	-2.423081
CHURC1	0.0769369	6.1260427	2.9571693	0.003465	0.0133707	-2.423168
CTAG2	-1.270848	0.9489935	-2.957123	0.0034655	0.0133712	-2.423299
RP11-219G1	-0.589618	-0.555027	-2.957025	0.0034666	0.0133738	-2.423571
CCRL2	0.1908926	4.8042231	2.956851	0.0034684	0.0133797	-2.42406
LINC01096	-0.720474	-0.649445	-2.956661	0.0034705	0.0133862	-2.424592
AL138726.1	-0.447751	-1.085072	-2.95654	0.0034718	0.0133898	-2.424932
CTD-2021H9	-0.882914	0.6682714	-2.956423	0.0034731	0.0133932	-2.42526
ZNF287	-0.342674	4.0583401	-2.956251	0.0034749	0.0133983	-2.425741
AKR1C1	0.2221074	6.9058605	2.9562127	0.0034753	0.0133983	-2.425849
RP11-262A1	0.4252386	2.7790235	2.9561993	0.0034755	0.0133983	-2.425886
TIFA	0.1117402	5.6512439	2.9556669	0.0034812	0.0134191	-2.427378
TNR	-0.914103	1.7482363	-2.954894	0.0034896	0.01345	-2.429544
RP11-157J2	-0.586769	-0.791053	-2.954652	0.0034922	0.0134586	-2.430221
RP11-3D4.4	-0.601573	-0.34758	-2.954326	0.0034958	0.0134709	-2.431135
PLEKHA5	-0.086023	6.0569864	-2.953984	0.0034995	0.0134838	-2.432092
CTB-193M12	-0.1727	4.9183712	-2.953876	0.0035007	0.0134863	-2.432394
RP11-57G10	-0.522951	1.8020133	-2.953856	0.0035009	0.0134863	-2.432451
AUNIP	-0.286274	4.0330163	-2.953548	0.0035042	0.0134978	-2.433313
RP11-417E7	0.7805681	1.1555674	2.9533602	0.0035063	0.0135042	-2.433838
WASH4P	-0.197241	3.7969628	-2.95332	0.0035067	0.0135045	-2.433949
ARHGEF26	0.2262397	5.8696876	2.952974	0.0035105	0.0135176	-2.434919
NT5DC4	-0.791151	1.3836373	-2.952912	0.0035112	0.0135188	-2.435093
SCIMP	0.2280666	4.8493787	2.9527708	0.0035127	0.0135233	-2.435488
DOCK1	0.1002114	6.2011549	2.9526923	0.0035136	0.0135251	-2.435708
RPL5P4	-0.478746	2.4555297	-2.952288	0.003518	0.0135388	-2.436841
WDR53	-0.065328	5.4449306	-2.952277	0.0035181	0.0135388	-2.436871

ZNF528-AS1	-0.294101	4.3607201	-2.952266	0.0035182	0.0135388	-2.436901
LINC00441	-0.618299	1.5917022	-2.95175	0.0035239	0.0135591	-2.438345
CDH13	0.1269926	5.6257979	2.9515907	0.0035256	0.0135644	-2.438791
TXNIP	0.1181442	6.8440664	2.9512174	0.0035297	0.0135775	-2.439835
ANKRD55	0.6041573	3.2291087	2.9512098	0.0035298	0.0135775	-2.439856
TSEN34	-0.057999	6.2967818	-2.951089	0.0035311	0.0135788	-2.440195
AC010084.1	0.6273034	-0.538126	2.9510809	0.0035312	0.0135788	-2.440217
RNASEH2C	-0.078269	6.0781595	-2.951078	0.0035312	0.0135788	-2.440225
RP11-962G1	-1.016261	0.2312343	-2.950876	0.0035334	0.0135834	-2.440791
AMZ1	0.6904327	2.7743924	2.9508744	0.0035335	0.0135834	-2.440795
RP11-326C3	0.7026461	2.030253	2.9508666	0.0035335	0.0135834	-2.440817
RNF148	0.7200608	1.0656076	2.9507289	0.003535	0.0135878	-2.441202
VAC14	0.0638544	6.3305418	2.9505062	0.0035375	0.0135958	-2.441825
DKC1	-0.063289	6.3216644	-2.950004	0.003543	0.0136155	-2.443229
TMEM14D	-0.642593	0.2828537	-2.949662	0.0035468	0.0136273	-2.444186
HDAC1	-0.05616	6.5613618	-2.949641	0.003547	0.0136273	-2.444244
TCEAL3	0.1528392	5.4058377	2.949625	0.0035472	0.0136273	-2.444289
ARIH2OS	-0.223683	4.3807731	-2.949458	0.003549	0.0136329	-2.444755
FUT8	0.1723851	5.2776922	2.9491991	0.0035519	0.0136424	-2.44548
CHMP1A	0.0701036	6.4648034	2.9491338	0.0035526	0.0136438	-2.445663
AC018647.3	0.6225913	2.6614869	2.949027	0.0035538	0.0136454	-2.445961
CDK14	0.1138881	6.0205594	2.9490017	0.0035541	0.0136454	-2.446032
NMNAT2	0.5475015	3.2609492	2.9489775	0.0035543	0.0136454	-2.4461
FREM2	1.1004887	2.290256	2.9489595	0.0035545	0.0136454	-2.44615
COL17A1	0.6201973	2.8578582	2.9487833	0.0035565	0.0136514	-2.446643
C11orf74	-0.087965	5.5757174	-2.94872	0.0035572	0.0136527	-2.44682
RP11-413M3	-0.604739	-0.27707	-2.948541	0.0035591	0.0136588	-2.447319
KB-1836B5.	-0.689285	0.3639165	-2.948309	0.0035617	0.0136672	-2.447967
STRN3	0.0668343	6.040256	2.948068	0.0035644	0.013676	-2.448642
RP11-414H1	0.5737826	-0.8433	2.9478041	0.0035673	0.0136858	-2.44938
C9orf41-AS	-0.518944	-0.674618	-2.947663	0.0035689	0.0136903	-2.449774
MLLT10P1	-0.637261	0.218206	-2.947622	0.0035693	0.0136906	-2.449888
AC079250.1	-0.421467	3.1498189	-2.947321	0.0035726	0.013702	-2.450731
PSPC1P1	-0.654852	1.1622356	-2.94701	0.0035761	0.0137138	-2.4516
KLF10	0.0976289	6.3411	2.9469719	0.0035765	0.013714	-2.451705
RLIM	0.0637936	6.111874	2.9465292	0.0035814	0.0137314	-2.452942
LCA5L	0.2385764	4.0168641	2.9463808	0.0035831	0.0137357	-2.453357
PHYHIP	0.4408526	3.4484081	2.946359	0.0035833	0.0137357	-2.453417
ARHGEF16	0.3794682	5.6378097	2.9461194	0.003586	0.0137445	-2.454087
RPL29	-0.069718	7.0504367	-2.945752	0.0035901	0.0137587	-2.455112
OLFM2	0.1850099	6.3464581	2.9456088	0.0035917	0.0137622	-2.455513
NHLRC3	0.0738732	6.0198472	2.9456024	0.0035917	0.0137622	-2.455531
AC108868.6	-0.473413	-1.20629	-2.945454	0.0035934	0.0137671	-2.455944
PA2G4P2	-0.510336	-0.678058	-2.944963	0.0035989	0.0137866	-2.457315
RP11-366F6	-0.563166	-1.15501	-2.944882	0.0035998	0.0137886	-2.457541
RP11-459K2	-0.268379	-1.423147	-2.944824	0.0036004	0.0137897	-2.457704
PIRT	-0.526549	-0.943711	-2.944665	0.0036022	0.013795	-2.458147
MYLIP	0.1097807	5.6076446	2.9445903	0.003603	0.0137968	-2.458357
ERVV-2	-0.626223	-0.894494	-2.944514	0.0036039	0.0137986	-2.458569
RPL36A-HNR	-0.586123	-0.393672	-2.944165	0.0036078	0.0138108	-2.459545
RPL7P12	-0.419899	-1.09505	-2.944162	0.0036078	0.0138108	-2.459553

UBOX5	-0.068528	5.7240755	-2.944126	0.0036082	0.0138109	-2.459654
RP11-589M2	0.2545925	-1.452407	2.9438155	0.0036117	0.0138227	-2.46052
RP11-60E8.	-0.582532	-0.618048	-2.943692	0.0036131	0.0138265	-2.460864
RP11-506B6	-0.580591	-0.282095	-2.943557	0.0036146	0.0138309	-2.46124
RPL26P19	-0.287054	3.7167424	-2.943435	0.0036159	0.0138346	-2.461581
SEPT7P2	-0.097157	5.4448643	-2.943147	0.0036192	0.0138455	-2.462385
TMEM232	0.5376826	2.7313282	2.9427917	0.0036232	0.0138584	-2.463377
RPL37A	-0.071412	7.1437253	-2.94278	0.0036233	0.0138584	-2.463411
RP11-55K13	-0.638981	1.7067877	-2.942672	0.0036245	0.0138616	-2.463712
RPL18	-0.068392	7.1266765	-2.942613	0.0036252	0.0138627	-2.463875
ZNF680	0.1217976	5.5241905	2.941862	0.0036336	0.0138935	-2.465971
HS1BP3	0.0711934	6.324419	2.9418187	0.0036341	0.0138939	-2.466092
HLA-W	0.6391271	2.0449969	2.941693	0.0036355	0.0138979	-2.466443
CSRP2	0.1471225	5.9062032	2.9416219	0.0036363	0.0138995	-2.466641
CTD-2014B1	0.4275243	-1.133083	2.9412863	0.0036401	0.0139125	-2.467577
HMGA1P3	-0.574379	-0.363588	-2.94113	0.0036419	0.0139178	-2.468013
RP11-244B2	-0.650114	-0.955414	-2.940955	0.0036438	0.0139238	-2.4685
RP11-469N6	-0.953488	0.3708903	-2.940766	0.003646	0.0139306	-2.469029
SMG6	0.1107861	5.6308614	2.9406432	0.0036474	0.0139344	-2.46937
USP8	0.05583	6.239807	2.9403037	0.0036512	0.0139476	-2.470317
RP11-492I2	-0.461244	2.0945398	-2.939936	0.0036554	0.013962	-2.471343
NCKAP1	0.0493492	6.5824318	2.9396949	0.0036581	0.013969	-2.472014
ZC2HC1C	0.1473815	5.1211738	2.9396753	0.0036583	0.013969	-2.472069
AC004775.5	-0.670609	0.0186699	-2.939672	0.0036583	0.013969	-2.472078
TCAM1P	-0.978106	0.8157257	-2.939455	0.0036608	0.013977	-2.472683
JADE3	-0.077453	5.9433681	-2.939305	0.0036625	0.013982	-2.4731
ZNF708	-0.148381	4.969832	-2.939142	0.0036643	0.0139876	-2.473554
RP11-611E1	-0.152526	4.5533	-2.938702	0.0036693	0.0140052	-2.474783
TAGAP	0.2524359	4.8804688	2.9384863	0.0036718	0.0140118	-2.475383
CRHR2	-0.56473	2.864434	-2.938457	0.0036721	0.0140118	-2.475466
AC009974.1	-0.62306	-0.335514	-2.938447	0.0036722	0.0140118	-2.475492
PTMAP8	-0.620714	0.1030574	-2.938369	0.0036731	0.0140138	-2.475709
AACS	-0.102629	5.9306419	-2.938231	0.0036747	0.0140183	-2.476093
RP11-16402	0.7416629	2.2367017	2.9381835	0.0036752	0.0140183	-2.476227
PLEKHG2	-0.089571	5.9510786	-2.938164	0.0036755	0.0140183	-2.476281
LOXL3	0.1455879	4.8069956	2.938126	0.0036759	0.0140185	-2.476387
SMCO2	0.731651	1.9265708	2.9376986	0.0036808	0.0140355	-2.477578
TGS1	-0.067063	5.8467999	-2.937597	0.0036819	0.0140385	-2.47786
LINC00278	0.8703569	0.6208734	2.9375511	0.0036824	0.014039	-2.477989
MAGEL2	0.7827115	1.192783	2.9371919	0.0036865	0.0140532	-2.478989
IGFL3	0.3633731	-1.333437	2.9371393	0.0036871	0.014054	-2.479136
RP11-894J1	-0.547437	2.4963717	-2.936846	0.0036905	0.0140653	-2.479954
ANKRD1	0.7182388	3.9818032	2.9366279	0.003693	0.0140733	-2.48056
RP11-14N7.	-0.767851	1.9532427	-2.936585	0.0036935	0.0140737	-2.480679
DZIP1	0.2501461	4.9415131	2.9365042	0.0036944	0.0140757	-2.480905
AP006222.2	-0.683036	2.3000516	-2.936352	0.0036961	0.0140809	-2.481329
RAB27B	0.4510733	4.6002903	2.9362481	0.0036973	0.014084	-2.481618
RASGEF1C	-0.775754	0.5208907	-2.936166	0.0036983	0.0140861	-2.481848
CCDC15	-0.147605	4.8078536	-2.936074	0.0036993	0.0140886	-2.482102
C12orf80	0.3555668	-1.36018	2.9351191	0.0037102	0.0141288	-2.484761
MMD	-0.107675	5.9837047	-2.934729	0.0037147	0.0141444	-2.485847

PPIP5K1	0.0802644	5.6151328	2.9345742	0.0037165	0.0141495	-2.486278
PHLDA1	0.1219689	6.3774326	2.9345174	0.0037172	0.0141495	-2.486436
RP11-452L6	0.1410709	5.0806889	2.9345102	0.0037172	0.0141495	-2.486456
AL122127.2	-0.499124	-1.073359	-2.934434	0.0037181	0.0141514	-2.486669
PSD4	0.0921084	6.4518301	2.9342972	0.0037197	0.0141559	-2.487049
KIF28P	-0.601841	3.087692	-2.934172	0.0037211	0.0141599	-2.487398
MMP19	0.2568136	5.3270376	2.9341148	0.0037218	0.0141609	-2.487557
KDM5C	-0.047289	6.5122755	-2.934006	0.003723	0.0141642	-2.487859
MYO7B	0.3252604	5.0142904	2.9338735	0.0037246	0.0141685	-2.488228
RPL7P11	-0.415891	-1.104282	-2.933806	0.0037253	0.01417	-2.488415
RALY-AS1	-0.153211	4.6867329	-2.933757	0.0037259	0.0141707	-2.488551
RP5-827C21	-0.194028	4.4851842	-2.933619	0.0037275	0.0141752	-2.488935
ATP7B	-0.154009	6.0005739	-2.93351	0.0037287	0.0141785	-2.489238
OFD1	-0.06251	5.9606409	-2.933411	0.0037299	0.0141814	-2.489516
NLRP12	0.6337318	1.8497531	2.9331723	0.0037326	0.0141904	-2.490179
AC099552.3	-0.375724	-1.252982	-2.933105	0.0037334	0.0141919	-2.490367
PIH1D2	0.3512568	3.3758544	2.9325692	0.0037396	0.0142126	-2.491857
RP11-533E1	-0.320379	3.2031629	-2.932566	0.0037396	0.0142126	-2.491865
IHH	-0.526779	4.8275872	-2.932482	0.0037406	0.0142134	-2.492098
SLC16A10	-0.348584	5.0940996	-2.93248	0.0037406	0.0142134	-2.492106
TNFRSF13C	-0.466568	3.6789256	-2.932317	0.0037425	0.0142191	-2.492559
MOCS2	0.076811	6.3646861	2.9321473	0.0037445	0.0142251	-2.49303
RP11-353N1	-0.560855	-0.874418	-2.931962	0.0037466	0.0142317	-2.493544
MPPE1	0.0952486	5.8834223	2.9317255	0.0037494	0.0142406	-2.494203
C11orf65	0.2896983	3.5223	2.9313183	0.0037541	0.0142571	-2.495335
RP11-680F8	0.3122048	3.5477325	2.9311953	0.0037555	0.014261	-2.495677
RP11-91I8.	-0.830942	-0.236045	-2.931019	0.0037576	0.0142673	-2.496168
RP4-800015	-0.834406	0.5478714	-2.930801	0.0037601	0.0142753	-2.496774
FAM83F	0.8700674	2.922561	2.9307714	0.0037604	0.0142753	-2.496856
SNX13	0.063066	6.0359821	2.9306748	0.0037616	0.014278	-2.497125
CROCCP3	-0.17561	4.4288695	-2.930336	0.0037655	0.0142915	-2.498065
CTC-465D4.	0.5988489	-0.936502	2.930242	0.0037666	0.0142942	-2.498328
COG1	-0.046909	6.1429996	-2.930084	0.0037684	0.0142996	-2.498767
SLC4A5	-0.454158	3.0575818	-2.929607	0.003774	0.0143192	-2.500092
RPL41P2	-0.255159	3.5052311	-2.929536	0.0037748	0.0143209	-2.50029
TUBB3	0.7073196	2.5478459	2.929398	0.0037764	0.014324	-2.500673
IFI44L	0.2357626	5.4133002	2.9293978	0.0037764	0.014324	-2.500674
USO1	0.0683435	6.5051875	2.9290418	0.0037806	0.0143383	-2.501663
ERBB4	0.8529391	0.1556774	2.928571	0.0037861	0.0143577	-2.502971
FAM103A2P	-0.656309	-0.102197	-2.928272	0.0037896	0.0143694	-2.503802
HERC2P4	-0.872994	1.2193156	-2.928098	0.0037916	0.0143756	-2.504285
RP11-407H1	-0.349401	-1.383415	-2.928038	0.0037923	0.0143768	-2.504453
ACER3	0.078028	5.7878488	2.9278306	0.0037947	0.0143832	-2.505027
PTGER2	0.3327465	4.1607537	2.9278263	0.0037948	0.0143832	-2.505039
LRRC37B	-0.105446	5.0685668	-2.927701	0.0037962	0.0143873	-2.505386
RSPO4	-0.857342	1.2484784	-2.927664	0.0037967	0.0143875	-2.505491
RP13-379L1	0.6471894	-0.703939	2.927504	0.0037986	0.0143931	-2.505934
AC073133.2	-0.564759	-0.739693	-2.927396	0.0037998	0.0143964	-2.506234
RP11-154D6	0.4164067	-1.157074	2.9269672	0.0038049	0.0144135	-2.507425
SLC35A5	0.0620304	5.9752981	2.9269432	0.0038051	0.0144135	-2.507492
RP11-307O1	0.2121634	-1.501552	2.9266045	0.0038091	0.0144271	-2.508432

ABCB4	0.310411	6.2551808	2.9265711	0.0038095	0.0144271	-2.508525
NYNRIN	-0.180103	5.7580027	-2.926341	0.0038122	0.0144358	-2.509164
RASSF6	0.4439621	4.5264526	2.926101	0.003815	0.014445	-2.50983
USP24P1	-0.475238	-1.247991	-2.926043	0.0038157	0.0144461	-2.50999
PTOV1-AS2	-0.16854	4.6302484	-2.925949	0.0038168	0.0144488	-2.510251
RP11-192H2	-0.398675	3.0045799	-2.925878	0.0038177	0.0144504	-2.510448
TFAP2C	0.9710493	1.0190317	2.9257774	0.0038189	0.0144534	-2.510728
C5orf66	-0.34654	3.6039302	-2.925701	0.0038197	0.0144553	-2.510939
DHRS7C	0.2848641	-1.428224	2.92498	0.0038283	0.014485	-2.512941
RP11-709A2	0.3257166	-1.325105	2.9249693	0.0038284	0.014485	-2.512971
CXADR	-0.138163	6.3544794	-2.924752	0.0038309	0.0144932	-2.513572
TNFRSF4	-0.187776	5.1049667	-2.924714	0.0038314	0.0144934	-2.513679
ZNF112	-0.201196	4.6740001	-2.9244	0.0038351	0.014506	-2.514551
TLDC1	0.2008575	5.2702511	2.92424	0.003837	0.0145116	-2.514994
TAS2R1	-0.409912	-1.288026	-2.924147	0.0038381	0.0145143	-2.515254
SCX	-0.342763	4.0020163	-2.923925	0.0038407	0.0145227	-2.515868
AC115522.3	0.7180448	0.7208607	2.9238204	0.003842	0.0145245	-2.516158
CNTFR-AS1	-0.767212	-0.157656	-2.923818	0.003842	0.0145245	-2.516164
HN1	-0.089699	6.3297259	-2.923693	0.0038435	0.0145286	-2.516512
SNORA47	-0.581669	-0.467382	-2.923593	0.0038447	0.0145305	-2.516789
GTPBP4	-0.054448	6.2991613	-2.923583	0.0038448	0.0145305	-2.516817
SEPT2	0.0486376	6.7883106	2.9232296	0.003849	0.0145448	-2.517797
AC009955.8	0.3189197	-1.360582	2.9226931	0.0038553	0.0145674	-2.519285
RP11-881M1	0.4695574	-1.1473	2.9225776	0.0038567	0.0145704	-2.519605
RP11-307C1	-0.546408	-0.800934	-2.922558	0.0038569	0.0145704	-2.519659
RP11-388C1	-0.636873	0.4357516	-2.922309	0.0038599	0.0145801	-2.520349
NAT8	-0.398493	5.9782154	-2.922228	0.0038609	0.0145822	-2.520574
RP11-369K1	0.7954262	0.4021465	2.9220905	0.0038625	0.0145869	-2.520955
RP11-358N2	-0.632397	1.8572643	-2.922054	0.0038629	0.014587	-2.521056
SNORD94	-0.543217	1.8459075	-2.922003	0.0038636	0.0145878	-2.521198
LAMB4	-0.567681	2.4466026	-2.921868	0.0038652	0.0145924	-2.521573
LINC01269	0.8314686	0.2306109	2.9217637	0.0038664	0.0145956	-2.521861
IMMT	0.0489817	6.5652533	2.9215978	0.0038684	0.0145998	-2.522321
PAPD7	0.0641837	6.0631524	2.9215714	0.0038687	0.0145998	-2.522394
RPAP2	0.0586591	5.7464619	2.9215695	0.0038687	0.0145998	-2.5224
RPL21P28	-0.237873	4.0596421	-2.921307	0.0038718	0.0146101	-2.523126
RP11-514D2	0.4981222	-0.966715	2.9212322	0.0038727	0.0146104	-2.523335
SUMO2P17	-0.39461	2.7951135	-2.921232	0.0038727	0.0146104	-2.523335
RNF126	-0.065674	6.2629276	-2.921142	0.0038738	0.014613	-2.523584
RP11-363N2	-0.669223	-0.088017	-2.920927	0.0038764	0.0146212	-2.524181
DMXL1	0.0759808	6.0107081	2.9206697	0.0038795	0.0146312	-2.524894
FRG1	0.0697025	5.8971047	2.9202252	0.0038848	0.0146498	-2.526125
SIGLEC7	0.2898737	4.3706527	2.920128	0.0038859	0.0146521	-2.526395
ATG2A	0.0677003	6.3612944	2.920107	0.0038862	0.0146521	-2.526453
PACRG-AS1	0.7138609	0.3579691	2.9199405	0.0038882	0.0146581	-2.526914
RP1-56K13.	0.4231657	-1.089777	2.9194436	0.0038941	0.014679	-2.528291
RP11-573D1	-0.667603	1.6160784	-2.919173	0.0038974	0.0146897	-2.529039
EMP3	0.1372707	5.7762532	2.91898	0.0038997	0.014697	-2.529575
SATL1	-0.422339	-1.143439	-2.918776	0.0039022	0.0147044	-2.530139
MAP6	0.2804277	4.6570252	2.9187496	0.0039025	0.0147044	-2.530213
PANX2	0.3585662	5.5512295	2.9185045	0.0039054	0.0147139	-2.530892

ZNF212	-0.059683	5.7981405	-2.918388	0.0039068	0.0147177	-2.531214
APOBR	0.1201598	5.4787225	2.9181221	0.00391	0.014727	-2.53195
GSTA8P	-0.78642	0.3848487	-2.918116	0.0039101	0.014727	-2.531967
RUNX3	0.1866322	5.3372958	2.9179297	0.0039123	0.0147339	-2.532483
RP11-348H3	-0.495246	-0.726153	-2.917748	0.0039145	0.0147406	-2.532986
RP11-739N2	-0.551729	2.9698655	-2.917611	0.0039162	0.0147454	-2.533367
MIR3685	-0.47004	2.5548285	-2.916935	0.0039243	0.0147745	-2.535237
RPL13AP25	-0.158301	5.1460261	-2.916902	0.0039247	0.0147745	-2.535329
SNHG23	-0.87846	0.076504	-2.916702	0.0039271	0.0147821	-2.535882
PYCARD	0.1683901	5.7443634	2.9163843	0.003931	0.014795	-2.536761
RPL23AP10	0.4074583	-1.056677	2.9162987	0.003932	0.0147974	-2.536998
ZNF784	-0.087833	5.6635727	-2.916153	0.0039338	0.0148025	-2.537401
SRSF3	-0.040225	6.767306	-2.915995	0.0039357	0.0148081	-2.537837
RP11-612B6	-0.333666	4.3076203	-2.915677	0.0039396	0.0148211	-2.538718
CT45A5	-0.337428	-1.389286	-2.915552	0.0039411	0.0148253	-2.539064
CCND2	0.1424363	5.6610832	2.915499	0.0039417	0.0148262	-2.53921
FAF2P1	-0.317303	-1.33344	-2.915209	0.0039452	0.0148379	-2.540013
FTHL17	-0.635691	-1.021193	-2.915079	0.0039468	0.0148407	-2.540373
C17orf97	0.2647412	4.5019466	2.9150498	0.0039472	0.0148407	-2.540453
LA16c-306E	-0.473774	-0.885391	-2.915048	0.0039472	0.0148407	-2.540458
AC006538.1	-0.523162	2.8037026	-2.914761	0.0039507	0.0148523	-2.541253
ZNF260	-0.073414	5.8197301	-2.914662	0.0039519	0.0148552	-2.541527
CSPG5	-0.366757	4.15072	-2.914417	0.0039549	0.0148649	-2.542204
FBXW5	0.0635927	6.7502692	2.9140896	0.0039588	0.0148783	-2.543109
MRPL34	0.0787981	6.4041768	2.9140141	0.0039598	0.0148803	-2.543318
HEBP1	0.081824	6.4272637	2.9137887	0.0039625	0.0148891	-2.543941
MCMDC2	-0.20839	4.5125965	-2.913676	0.0039639	0.0148927	-2.544253
CBR1	0.1144212	6.7414875	2.9135585	0.0039653	0.0148965	-2.544577
TMEM9	-0.069432	6.6763728	-2.91342	0.003967	0.0149013	-2.54496
LINC01258	-0.431558	-1.286737	-2.913363	0.0039677	0.0149024	-2.545119
RABIF	-0.068381	5.9274286	-2.913299	0.0039685	0.0149038	-2.545295
RP11-77H9.	0.7638189	1.1298198	2.9130602	0.0039714	0.0149132	-2.545955
RNF126P1	-0.824231	1.2268361	-2.913014	0.003972	0.0149138	-2.546084
FBXO3	0.0580155	6.2080289	2.9129297	0.003973	0.0149161	-2.546316
RP11-466L1	0.4211464	-1.053322	2.9126732	0.0039761	0.0149264	-2.547025
ARHGAP21	0.0685632	6.2332588	2.9124399	0.003979	0.0149355	-2.54767
PLOD2	-0.102383	6.5373678	-2.912372	0.0039798	0.0149372	-2.547859
RPL35A	-0.064681	6.9770309	-2.912324	0.0039804	0.0149378	-2.54799
ZG16	-0.791256	4.7276442	-2.911833	0.0039864	0.0149579	-2.549347
RP11-303E1	0.6654004	0.5842963	2.9118202	0.0039866	0.0149579	-2.549382
RP11-672A2	0.4196406	-1.077296	2.9117704	0.0039872	0.0149586	-2.54952
FLJ44511	0.5998407	3.5408549	2.9116459	0.0039887	0.0149628	-2.549864
WDR48	-0.046723	6.1539627	-2.911586	0.0039894	0.0149641	-2.550031
LRP1B	1.0154121	2.0946094	2.9113064	0.0039929	0.0149754	-2.550802
TRAPPC13	0.0572085	5.8626925	2.9112538	0.0039935	0.0149762	-2.550947
NFRKB	-0.058424	6.0894261	-2.911119	0.0039952	0.0149809	-2.55132
RP11-681N2	-0.392661	-1.161802	-2.910694	0.0040004	0.0149989	-2.552494
WDR36	0.0579876	6.1073939	2.9105979	0.0040016	0.0150018	-2.552759
NRL	-0.123433	4.8059003	-2.91045	0.0040034	0.0150071	-2.553167
RP5-966M1.	-0.662478	1.1471354	-2.910404	0.0040039	0.0150077	-2.553296
AC012322.1	-0.31545	-1.400064	-2.91036	0.0040045	0.0150082	-2.553417

KB-1471A8.	-0.64418	1.3558664	-2.910125	0.0040074	0.0150175	-2.554065
LILRB5	0.2475723	5.2392557	2.9096571	0.0040131	0.0150375	-2.555358
RP11-329E2	-0.30008	-1.407601	-2.909461	0.0040156	0.015045	-2.555899
ARMCX6	0.2333852	5.0725082	2.909011	0.0040211	0.0150643	-2.557142
RP3-333H23	-0.473402	-0.867906	-2.908974	0.0040216	0.0150645	-2.557244
BTG3	-0.084418	5.8423109	-2.908524	0.0040271	0.0150838	-2.558486
RP11-826N1	0.4229803	-1.197727	2.908279	0.0040302	0.0150936	-2.559163
GHET1	-0.520324	2.591299	-2.90816	0.0040316	0.0150975	-2.559491
RRM1	-0.065451	6.3063235	-2.907954	0.0040342	0.0151044	-2.56006
LINC00694	-0.525988	3.6600004	-2.907922	0.0040346	0.0151044	-2.560147
RP3-428L16	-0.339083	3.400721	-2.907912	0.0040347	0.0151044	-2.560176
RP11-302F1	0.3015639	-1.360177	2.9076664	0.0040377	0.0151128	-2.560853
TOMM40	-0.069021	6.4813938	-2.907664	0.0040378	0.0151128	-2.56086
RP11-552C1	-0.757117	-0.426766	-2.906485	0.0040524	0.015166	-2.564114
PQLC1	0.0982951	6.679402	2.906406	0.0040534	0.0151682	-2.564331
S100A14	-0.370769	5.6391424	-2.906229	0.0040556	0.0151748	-2.564819
RP11-15H20	-0.309093	-1.381276	-2.906161	0.0040565	0.0151765	-2.565008
DYNLT3	0.0936744	6.1740883	2.9059255	0.0040594	0.0151859	-2.565657
AC005534.8	-0.624293	1.5811137	-2.905648	0.0040628	0.0151972	-2.566422
HLA-J	0.2803078	4.8265231	2.9055341	0.0040643	0.015201	-2.566736
VASN	0.0924002	6.3146752	2.905127	0.0040693	0.0152184	-2.567859
NLGN4Y	1.2391124	2.5188392	2.9050405	0.0040704	0.0152209	-2.568097
RP11-895M1	-0.555834	-0.637854	-2.904974	0.0040712	0.0152225	-2.56828
RPL9P32	-0.661241	0.9815785	-2.90482	0.0040732	0.0152281	-2.568704
RP11-216L1	-0.674456	0.8480981	-2.904456	0.0040777	0.0152435	-2.569708
TUSC8	-0.82007	3.9520928	-2.903679	0.0040875	0.0152784	-2.571851
FBXO44	0.0980052	6.0326936	2.9035447	0.0040891	0.0152831	-2.572221
RNU6-1136P	-0.618959	-0.203783	-2.903228	0.0040931	0.0152964	-2.573093
RP11-760D2	0.1516974	-1.532367	2.9031953	0.0040935	0.0152964	-2.573184
PTPRN2	0.1843941	5.0162552	2.9029187	0.004097	0.0153078	-2.573946
RP11-260A9	-0.67435	-0.970102	-2.902854	0.0040978	0.0153092	-2.574125
CES1P2	0.8681541	0.862037	2.9023594	0.004104	0.0153309	-2.575487
RP11-83J16	-0.514663	-0.626191	-2.902067	0.0041077	0.0153431	-2.576293
COL21A1	-0.328817	4.7792915	-2.901809	0.004111	0.0153537	-2.577005
RP11-12M9.	-0.232325	4.0136039	-2.901704	0.0041123	0.015357	-2.577292
RNA5SP118	0.4118812	-1.153097	2.9015769	0.0041139	0.0153614	-2.577643
ZNF382	-0.312109	4.4843492	-2.901478	0.0041151	0.0153639	-2.577916
HDAC5	-0.067971	6.4265207	-2.901459	0.0041154	0.0153639	-2.577969
LINC01415	0.668527	1.296993	2.9013409	0.0041169	0.0153678	-2.578293
RP3-522J7.	-0.62282	-0.242743	-2.901028	0.0041208	0.015381	-2.579155
EEPD1	0.0909061	6.1109411	2.9009198	0.0041222	0.0153845	-2.579453
NUMA1	0.0458085	6.6102924	2.9008279	0.0041233	0.0153873	-2.579706
DOCK4	0.0841322	6.030458	2.900182	0.0041315	0.0154162	-2.581484
SNX5P1	-0.63233	-0.170121	-2.899886	0.0041353	0.0154271	-2.5823
RP11-26J3.	-0.647992	-0.117954	-2.899872	0.0041354	0.0154271	-2.582338
GLS2	0.6573464	4.3592868	2.8998508	0.0041357	0.0154271	-2.582396
ZNF736	-0.151781	5.217183	-2.899683	0.0041378	0.0154335	-2.582857
MAGEA12	-1.187221	0.7283951	-2.899319	0.0041425	0.0154491	-2.583861
AC093850.2	0.6951279	1.5498525	2.8990246	0.0041462	0.0154615	-2.58467
ANXA11	0.0529614	6.7509408	2.8987156	0.0041501	0.0154745	-2.585521
CTC-498J12	0.6125971	2.8275223	2.8984731	0.0041532	0.0154839	-2.586188

RP11-106M7	0.3543748	-1.224203	2.8984513	0.0041535	0.0154839	-2.586248
FAM114A1	0.0671804	6.455224	2.8983142	0.0041552	0.0154888	-2.586625
MIR5591	-0.272254	-1.399341	-2.898234	0.0041562	0.0154911	-2.586847
ZNF506	-0.162515	5.3481342	-2.898095	0.004158	0.0154961	-2.587228
RP11-574K1	-0.147905	4.9743738	-2.897459	0.0041661	0.0155247	-2.588977
ATP6AP2	0.0459618	6.6514125	2.8973776	0.0041672	0.0155256	-2.589202
CTA-85E5.7	0.2026483	-1.483637	2.8973728	0.0041672	0.0155256	-2.589215
RP11-430L1	-0.682085	0.2654621	-2.897048	0.0041714	0.0155395	-2.590108
MOB1B	0.0891606	5.7878939	2.896964	0.0041724	0.0155419	-2.59034
TSPAN15	0.1560307	5.869459	2.8969006	0.0041732	0.0155421	-2.590514
SMIM2-AS1	0.351595	4.4533251	2.8968925	0.0041733	0.0155421	-2.590536
CDON	-0.14529	5.2577813	-2.896751	0.0041752	0.0155473	-2.590927
CD2BP2	0.0581863	6.3707227	2.894963	0.0041981	0.015631	-2.595842
CTD-2256P1	-0.641991	-0.09865	-2.894698	0.0042015	0.0156421	-2.596569
C9orf116	0.2631512	4.3274598	2.8946017	0.0042027	0.0156451	-2.596835
AP3M1	-0.047489	6.2834622	-2.894402	0.0042053	0.015653	-2.597383
ZNF311	0.5099352	3.5147842	2.8939859	0.0042106	0.0156714	-2.598527
AD000684.2	-0.454078	2.8951919	-2.893823	0.0042127	0.0156776	-2.598975
RPL21P18	-0.448022	-0.992739	-2.89333	0.0042191	0.0156997	-2.60033
RP11-164H1	-0.487793	-1.093221	-2.892865	0.0042251	0.0157204	-2.601607
RPL4P4	-0.17721	4.7945672	-2.892813	0.0042258	0.0157213	-2.60175
RP11-809C1	0.4565242	-1.098007	2.8927636	0.0042264	0.0157221	-2.601885
RP11-253E3	0.4061838	3.838705	2.8927303	0.0042268	0.0157221	-2.601976
NAP1L1	-0.059649	6.8390732	-2.892645	0.0042279	0.0157246	-2.602211
SLC26A4	0.4454586	2.6879406	2.8922054	0.0042336	0.0157441	-2.603418
RP5-857K21	0.1188493	6.6109802	2.8921038	0.0042349	0.0157474	-2.603697
CTC-459F4.	-0.575711	-0.392313	-2.892069	0.0042354	0.0157475	-2.603791
RP11-697N1	0.4977607	3.2754235	2.8919355	0.0042371	0.0157523	-2.604159
RP11-48901	0.6526211	2.0449326	2.8916433	0.0042409	0.0157648	-2.604962
TLE2	0.1723612	5.7154183	2.8915612	0.004242	0.0157659	-2.605187
SNHG16	-0.085418	5.8778534	-2.891554	0.0042421	0.0157659	-2.605206
AC007163.1	0.5010399	-0.82781	2.8912943	0.0042454	0.0157768	-2.60592
FAM160B2	0.0658955	6.1364248	2.8910383	0.0042488	0.0157875	-2.606623
RP4-621B1C	0.5437054	1.7820289	2.8908154	0.0042517	0.0157967	-2.607235
RNU6-762P	-0.625377	0.1768721	-2.890467	0.0042562	0.0158119	-2.608192
TAF9	-0.061948	7.1171399	-2.890184	0.0042599	0.0158227	-2.608967
RP11-1055E	-0.287311	4.4490542	-2.890177	0.00426	0.0158227	-2.608986
CTD-319301	0.7321059	1.8900038	2.8898969	0.0042636	0.0158336	-2.609755
GGA1	-0.056601	6.3597521	-2.889885	0.0042638	0.0158336	-2.609787
RP11-289I1	0.213782	4.3138137	2.8898353	0.0042644	0.0158344	-2.609924
RP11-519G1	-0.628396	0.0872258	-2.889733	0.0042657	0.0158377	-2.610204
C7orf31	-0.204025	5.0187777	-2.889655	0.0042668	0.0158399	-2.610418
PWAR5	-0.827468	2.1214134	-2.889307	0.0042713	0.0158552	-2.611375
AC004076.7	-0.577015	-0.417551	-2.889261	0.0042719	0.0158558	-2.6115
ALG12	-0.084625	5.9557512	-2.888966	0.0042758	0.0158685	-2.61231
CLASP1	0.048589	6.2378878	2.8887915	0.004278	0.0158753	-2.612788
LGR5	-0.921364	3.7704999	-2.888643	0.00428	0.0158809	-2.613197
AAED1	0.0951434	5.5906734	2.8885728	0.0042809	0.0158827	-2.613388
UQCC2	-0.07822	6.261198	-2.888516	0.0042816	0.0158838	-2.613545
RP11-180M1	-0.531043	2.3576523	-2.888452	0.0042825	0.0158853	-2.613719
CYP3A7	-0.43466	6.2120645	-2.888255	0.0042851	0.0158933	-2.614261

RP11-365H2	-0.580956	2.0511061	-2.88814	0.0042866	0.0158973	-2.614577
MEF2B	-0.378176	3.0728392	-2.887422	0.004296	0.0159305	-2.616544
RP11-7908.	-0.623492	-0.074432	-2.886961	0.004302	0.0159513	-2.617809
PTPRO	0.3558857	4.1508347	2.8868095	0.004304	0.0159571	-2.618224
ZNF718	-0.178997	4.9426688	-2.886357	0.00431	0.0159776	-2.619465
RP11-379K1	-0.485151	-0.872015	-2.886221	0.0043117	0.0159825	-2.619837
RP11-98D18	-0.510507	-0.717057	-2.88577	0.0043177	0.016003	-2.621074
KLHL18	0.0561174	5.6239052	2.885052	0.0043272	0.0160364	-2.623041
SLC13A1	0.6182498	-0.874264	2.8850074	0.0043278	0.016037	-2.623163
AC008427.2	-0.500223	-0.876267	-2.884661	0.0043323	0.0160523	-2.624112
RP11-206L1	-0.658415	0.0739531	-2.884564	0.0043336	0.0160554	-2.624377
CD86	0.1711021	5.3252994	2.8844732	0.0043348	0.0160583	-2.624626
SNX2	0.0550947	6.3844667	2.8841306	0.0043393	0.0160727	-2.625565
ZNF669	-0.18185	4.943916	-2.884112	0.0043396	0.0160727	-2.625616
PPBP	0.7778544	0.1517056	2.884062	0.0043403	0.0160735	-2.625753
CPA6	-0.94712	1.3317231	-2.883817	0.0043435	0.0160839	-2.626424
RP3-467N11	-0.218061	4.391187	-2.88369	0.0043452	0.0160885	-2.626772
MRPS26	-0.069401	6.3968673	-2.883261	0.0043509	0.0161079	-2.627946
RP5-942I16	-0.638982	-0.302269	-2.883226	0.0043513	0.0161081	-2.628044
RP11-156E8	-0.460347	2.5743165	-2.882894	0.0043557	0.0161227	-2.628952
STYXL1	0.093197	6.0669275	2.8823975	0.0043623	0.0161455	-2.630311
ZNF593	0.1332859	5.1867923	2.8819972	0.0043677	0.0161636	-2.631407
TBL3	0.0598176	6.3071953	2.8816009	0.004373	0.0161815	-2.632492
RP4-568B10	-0.689488	1.8650058	-2.881502	0.0043743	0.0161848	-2.632763
CYB5D1	0.2763038	4.0318329	2.8814308	0.0043752	0.0161866	-2.632957
EIF3LP2	-0.659598	0.5299268	-2.881324	0.0043766	0.0161902	-2.633248
PSMD2	0.0464907	6.7763599	2.8811796	0.0043786	0.0161953	-2.633645
RP11-314D7	-0.686206	-0.597584	-2.881156	0.0043789	0.0161953	-2.63371
LINC00934	0.1596974	-1.52829	2.880707	0.0043849	0.016214	-2.634938
H2AFZP3	-0.639611	0.3059187	-2.880682	0.0043852	0.016214	-2.635006
RP4-613B23	0.65429	0.9276993	2.8806777	0.0043853	0.016214	-2.635018
RP11-622O1	-0.317755	-1.372449	-2.879469	0.0044015	0.0162722	-2.638325
RP11-525A1	0.7900434	2.8799108	2.8794112	0.0044022	0.0162734	-2.638483
FAM171B	0.2628652	4.6323497	2.8792384	0.0044046	0.0162803	-2.638955
FBXW10	0.9147894	2.1736211	2.8790754	0.0044068	0.0162867	-2.639401
TACR3	-0.490773	-1.180297	-2.878872	0.0044095	0.0162952	-2.639957
WWC3	0.0873346	5.9775679	2.8788341	0.00441	0.0162954	-2.640061
RP5-1039K5	-0.130177	4.7652278	-2.878465	0.004415	0.0163121	-2.64107
AC000111.4	0.2989063	-1.417274	2.8782431	0.0044179	0.0163215	-2.641677
PSG3	0.3699808	-1.348075	2.8782057	0.0044184	0.0163217	-2.641779
PRDM4	-0.051409	5.9357079	-2.878068	0.0044203	0.016326	-2.642155
ZUFSP	-0.072882	5.374011	-2.878053	0.0044205	0.016326	-2.642197
SIAH1P1	-0.392811	-1.362127	-2.877665	0.0044257	0.0163436	-2.643256
KCNJ16	1.0930119	1.9667963	2.877354	0.0044299	0.0163575	-2.644107
BMP8B	-0.455693	4.5362251	-2.87723	0.0044316	0.016362	-2.644447
PAFAH1B2	0.0417861	6.4664737	2.8771964	0.004432	0.016362	-2.644538
RP11-59C5.	0.4022395	3.3302494	2.8766371	0.0044396	0.0163882	-2.646067
APOL4	0.2167203	5.0851797	2.8764892	0.0044416	0.016394	-2.646471
CTD-2335A1	0.1206334	-1.548197	2.8758605	0.0044501	0.0164237	-2.648188
RALGAPA2	0.084082	6.4594677	2.8756856	0.0044525	0.0164308	-2.648666
STK4	-0.047026	6.1490414	-2.875547	0.0044544	0.0164361	-2.649045

BTF3P2	-0.428215	-1.024357	-2.875466	0.0044555	0.0164385	-2.649266
SNRPN	-0.164664	6.2667087	-2.875003	0.0044618	0.01646	-2.650532
HLTF	-0.076349	6.2246805	-2.874911	0.004463	0.016463	-2.650783
RP11-53B2.	-0.490167	-0.84024	-2.874715	0.0044657	0.0164711	-2.651319
RP11-119F7	-0.448022	-0.964782	-2.874638	0.0044667	0.0164733	-2.651529
AC096772.6	0.0940267	5.0821935	2.8744714	0.004469	0.01648	-2.651983
SEC62	0.0584327	6.6964764	2.8741509	0.0044733	0.016494	-2.652858
DEPDC1-AS1	-0.607567	-0.52511	-2.874115	0.0044738	0.016494	-2.652955
FNBP1	-0.060309	6.2758288	-2.874093	0.0044741	0.016494	-2.653017
AC015987.1	0.7171396	-0.114708	2.8738705	0.0044772	0.0165035	-2.653623
CTB-171A8.	-0.216194	4.6383897	-2.873833	0.0044777	0.0165037	-2.653727
ZNF649	-0.181085	5.0178128	-2.873786	0.0044783	0.0165044	-2.653854
ZSWIM5	-0.443931	5.0532396	-2.873647	0.0044802	0.0165097	-2.654232
RP11-432I1	-0.465872	-1.262957	-2.873576	0.0044812	0.0165116	-2.654427
RP11-350G8	-0.631379	0.8397619	-2.873473	0.0044826	0.0165151	-2.654708
EIF4BP3	-0.244825	3.9506388	-2.873259	0.0044855	0.0165242	-2.655292
ATP6V1G3	-0.4017	-1.284053	-2.872875	0.0044907	0.0165419	-2.656342
KCND1	0.2012501	4.4692929	2.8724847	0.0044961	0.0165598	-2.657406
GAL3ST2	-0.854644	1.4760348	-2.872258	0.0044992	0.0165696	-2.658025
AC005618.6	-0.703928	1.923219	-2.872081	0.0045016	0.0165768	-2.658507
EXD1	-0.558753	-0.715021	-2.871875	0.0045044	0.0165855	-2.659069
KLRF1	0.5071061	2.8688049	2.8717616	0.004506	0.0165896	-2.659379
VPS41	0.0577439	6.2563853	2.8716078	0.0045081	0.0165957	-2.659799
MS4A13	-0.463739	-1.160042	-2.871506	0.0045095	0.0165991	-2.660075
CTD-2311B1	-0.390917	-1.298241	-2.871371	0.0045113	0.0166043	-2.660446
PARG	-0.055778	5.8536954	-2.871192	0.0045138	0.0166116	-2.660932
CLDN2	0.3699928	5.7518756	2.870653	0.0045212	0.0166361	-2.662403
U95743.1	-0.604139	-0.84453	-2.870641	0.0045213	0.0166361	-2.662435
EXOSC6	0.0617608	6.0170562	2.8703828	0.0045249	0.0166475	-2.66314
MTRNR2L6	0.6792784	0.6688212	2.8703251	0.0045257	0.0166479	-2.663298
C18orf25	0.0546758	5.8734466	2.8703089	0.0045259	0.0166479	-2.663342
C11orf72	-0.607203	0.2357152	-2.869765	0.0045334	0.0166738	-2.664825
FTCDNL1	0.1625957	5.0627974	2.869594	0.0045358	0.0166808	-2.665291
KCNK9	-1.035361	1.8176878	-2.869229	0.0045408	0.0166976	-2.666287
COL11A1	0.8838092	3.3615318	2.8691826	0.0045414	0.0166983	-2.666413
UBA6	0.0639844	6.0801223	2.8690964	0.0045426	0.0166995	-2.666648
CTC-444N24	-0.302953	3.0092553	-2.869091	0.0045427	0.0166995	-2.666661
PLGRKT	0.0778833	5.794725	2.8690257	0.0045436	0.0167012	-2.66684
RHBDD1	0.0589097	6.1186392	2.8689652	0.0045444	0.0167026	-2.667005
ANGPT4	0.7634518	0.9111773	2.868814	0.0045465	0.0167086	-2.667418
HDAC7	0.0741147	6.2422017	2.8686186	0.0045492	0.0167168	-2.66795
CTD-2161E1	-0.594768	1.4907608	-2.868498	0.0045509	0.0167213	-2.66828
ASNSP1	-1.106179	0.70226	-2.868392	0.0045524	0.016725	-2.668568
CHFR	-0.084822	5.9613172	-2.868228	0.0045546	0.0167316	-2.669016
C15orf56	-0.769562	0.1529811	-2.868039	0.0045572	0.0167395	-2.669528
RP11-310E2	-0.675677	-0.534536	-2.867724	0.0045616	0.0167539	-2.670389
RP11-318C2	-0.61184	1.678737	-2.867678	0.0045622	0.0167546	-2.670514
RPS27L	0.0790812	6.5072725	2.8675773	0.0045636	0.016758	-2.670788
SLC45A4	-0.205186	5.5804241	-2.867446	0.0045655	0.0167615	-2.671144
ITM2B	0.0627792	7.0754849	2.8674413	0.0045655	0.0167615	-2.671158
AMH	-0.764416	2.5762327	-2.866834	0.004574	0.0167908	-2.672812

IFNAR1	0.0601387	6.5611659	2.8667679	0.0045749	0.0167925	-2.672993
EIF3J-AS1	-0.107934	5.075127	-2.866576	0.0045775	0.0168006	-2.673514
H3F3BP1	-0.6228	0.8022603	-2.866328	0.004581	0.0168116	-2.674192
ZG16B	0.6484245	3.4702377	2.8662928	0.0045815	0.0168117	-2.674287
SFXN3	0.105568	5.9202993	2.8658109	0.0045882	0.0168346	-2.675599
SHB	0.1054194	5.9872691	2.8655342	0.004592	0.016847	-2.676353
MAGOHB	-0.073782	5.783386	-2.865085	0.0045983	0.0168683	-2.677576
ADM	0.1461951	5.8176236	2.8650299	0.0045991	0.0168695	-2.677726
EVI2B	0.183153	5.3891961	2.8647003	0.0046037	0.0168847	-2.678623
CTSL	0.0723986	6.7547404	2.864635	0.0046046	0.0168863	-2.678801
PARD3-AS1	-0.58898	1.795529	-2.864543	0.0046059	0.0168893	-2.679052
RP11-770E5	0.7026882	0.2657014	2.8643317	0.0046088	0.0168985	-2.679626
LINC00305	0.393955	-1.19463	2.8641635	0.0046112	0.0169038	-2.680084
RP11-308D1	-0.669839	-0.477869	-2.864162	0.0046112	0.0169038	-2.68009
ZFP91	0.0493794	6.3548932	2.8639905	0.0046136	0.0169109	-2.680555
AL365181.2	-0.416302	2.4124076	-2.863869	0.0046153	0.0169154	-2.680887
RP11-165F2	-0.701104	1.2613018	-2.863789	0.0046164	0.0169178	-2.681104
CXorf67	-0.8767	-0.315424	-2.863675	0.004618	0.0169212	-2.681414
MTRNR2L12	0.2656835	4.2769337	2.8636564	0.0046183	0.0169212	-2.681464
RP11-162D1	-0.782833	0.5061863	-2.863579	0.0046194	0.0169235	-2.681675
AP1G2	-0.13139	5.8201301	-2.863355	0.0046225	0.0169333	-2.682286
RP11-37B2.	-0.14438	5.0035591	-2.86328	0.0046236	0.0169352	-2.682488
FGD2	0.1684899	5.2853593	2.863226	0.0046243	0.0169352	-2.682635
RP11-273B2	-0.253145	4.7769063	-2.86322	0.0046244	0.0169352	-2.682652
HIST1H2BPS	-0.361552	-1.254926	-2.863128	0.0046257	0.0169372	-2.682903
TUBA1B	-0.067229	6.6288144	-2.863114	0.0046259	0.0169372	-2.682941
BAALC-AS2	0.616978	-0.621367	2.8630712	0.0046265	0.0169377	-2.683057
RNF149	-0.055502	6.3521989	-2.862684	0.0046319	0.0169559	-2.68411
TRPV5	0.6991555	-0.438002	2.8626415	0.0046325	0.0169564	-2.684226
NUDT6	0.1961318	4.9889482	2.8625566	0.0046337	0.0169591	-2.684457
RP11-950C1	-0.580765	0.1133161	-2.861855	0.0046436	0.0169935	-2.686365
CTB-23I7.1	-0.319352	-1.39815	-2.861665	0.0046463	0.0170016	-2.686883
RPL8P2	-0.546965	-0.634012	-2.861592	0.0046473	0.0170037	-2.687081
RP11-712B9	0.47431	-1.004603	2.8614769	0.0046489	0.0170079	-2.687393
RP11-138I1	-0.44035	-1.106026	-2.861222	0.0046525	0.0170194	-2.688088
SNX5	-0.047219	6.5113755	-2.861176	0.0046532	0.01702	-2.688213
RPL23AP12	-0.321119	-1.279759	-2.860645	0.0046607	0.0170457	-2.689656
GRIN3A	-0.195528	4.0367425	-2.860533	0.0046623	0.0170498	-2.689959
CNTNAP3B	0.6701876	3.4146729	2.8604288	0.0046637	0.0170533	-2.690243
OSM	0.4138827	3.7856721	2.8603989	0.0046642	0.0170533	-2.690324
NOD1	0.0794513	5.3570968	2.8602653	0.004666	0.0170585	-2.690687
RP11-379F1	0.2149016	-1.477393	2.8600611	0.0046689	0.0170669	-2.691242
FAM3C2	0.3189563	4.1587108	2.8600375	0.0046693	0.0170669	-2.691306
TAS2R4	-0.557537	2.2820795	-2.859763	0.0046732	0.0170794	-2.692054
TRIM61	0.7269413	0.3638589	2.8597163	0.0046738	0.0170801	-2.69218
ZNF98	-0.852775	-0.036018	-2.859661	0.0046746	0.0170813	-2.69233
CTA-29F11.	-0.151772	4.8004775	-2.859268	0.0046802	0.0170999	-2.693398
DEDD	-0.053829	6.2749253	-2.858899	0.0046854	0.0171173	-2.6944
HCRTR1	-0.571941	-0.227288	-2.858611	0.0046895	0.0171297	-2.695183
RP11-65L19	-0.338441	-1.388789	-2.858595	0.0046897	0.0171297	-2.695227
PIK3CA	0.0612047	5.8533914	2.8585569	0.0046903	0.0171299	-2.69533

CTD-2269E2	-0.425332	-1.164153	-2.858186	0.0046955	0.0171475	-2.696337
C12orf43	-0.055079	5.8056737	-2.858125	0.0046964	0.0171489	-2.696504
RP11-459I1	-0.548302	3.329793	-2.858003	0.0046981	0.0171535	-2.696834
C17orf100	-0.167955	4.76956	-2.857539	0.0047048	0.017176	-2.698096
LRRC75A	-0.226474	4.15848	-2.857432	0.0047063	0.0171798	-2.698385
ENTPD6	-0.062053	6.543152	-2.857238	0.0047091	0.0171882	-2.698912
TRIM65	-0.074585	5.8851243	-2.85719	0.0047097	0.0171887	-2.699043
GNRHR2	-0.195246	3.8967043	-2.857163	0.0047101	0.0171887	-2.699115
SNORD113-3	-0.491848	-1.125483	-2.857125	0.0047107	0.0171889	-2.69922
LYPD6	-0.85585	2.9528951	-2.857092	0.0047111	0.0171889	-2.699309
FAM35A	-0.07324	6.1568369	-2.856625	0.0047178	0.0172116	-2.700577
RP11-317N8	0.494834	1.9661982	2.8558836	0.0047284	0.0172481	-2.70259
WIPF2	-0.04615	6.2449841	-2.855858	0.0047288	0.0172481	-2.702659
LINC00640	0.6243251	-0.237376	2.8553453	0.0047361	0.0172732	-2.704051
NPM1P6	-0.34016	2.9168537	-2.855158	0.0047388	0.0172813	-2.704559
TRIM68	0.0895179	5.641966	2.8550535	0.0047403	0.017285	-2.704843
RPS19	-0.07155	7.200716	-2.854237	0.004752	0.0173261	-2.707059
EIF2AK4	0.0626157	6.3328371	2.854149	0.0047533	0.0173289	-2.707297
RP11-567J2	0.4481203	-0.976332	2.8540451	0.0047548	0.0173315	-2.707579
ADRB2	0.3278156	4.9167695	2.8540337	0.004755	0.0173315	-2.70761
PDCL	-0.054736	5.7277024	-2.85395	0.0047562	0.017333	-2.707837
SPATS2L	-0.054821	6.7492964	-2.853908	0.0047568	0.017333	-2.70795
AC008391.1	0.6110547	-0.472196	2.8539076	0.0047568	0.017333	-2.707952
SNORA14B	-0.620978	0.6405118	-2.853382	0.0047644	0.0173567	-2.709377
PSMA7	0.057604	6.7776811	2.8533751	0.0047645	0.0173567	-2.709396
RP11-707A1	0.5055414	-1.041949	2.8533565	0.0047647	0.0173567	-2.709447
WDR1	0.0469181	6.8377545	2.853201	0.004767	0.0173631	-2.709868
MAPRE1	-0.056736	6.4021221	-2.853034	0.0047694	0.0173702	-2.710321
CCDC88C	-0.135629	5.6586351	-2.852792	0.0047729	0.0173812	-2.710977
RPL41P5	-0.147323	5.0463067	-2.852687	0.0047744	0.017385	-2.711263
EPB41	0.0866687	6.3084152	2.8524474	0.0047778	0.0173958	-2.711912
RP3-508I15	-0.639017	0.2949556	-2.852317	0.0047797	0.0174009	-2.712264
RP3-470B24	-0.533168	-0.999203	-2.851998	0.0047844	0.017416	-2.713131
RALGDS	0.062322	6.1567857	2.8518511	0.0047865	0.017422	-2.713529
RP11-229D1	-0.571618	1.5432067	-2.851758	0.0047878	0.0174252	-2.71378
PTPN9	0.0584732	6.1122399	2.851181	0.0047962	0.0174539	-2.715345
FCGR3B	0.6784446	3.1858177	2.8509446	0.0047996	0.0174646	-2.715986
LINC01015	0.2844178	-1.437207	2.8508808	0.0048005	0.0174663	-2.716158
WEE1	-0.09772	6.1832696	-2.850376	0.0048079	0.0174912	-2.717527
FABP5P7	-0.689107	0.3418026	-2.850222	0.0048101	0.0174976	-2.717943
LINC00426	0.4125647	3.5126325	2.8500087	0.0048132	0.0175072	-2.718522
RP11-131K5	0.6230882	-0.748161	2.8499378	0.0048143	0.0175092	-2.718714
RP11-7I15.	-0.668624	0.0380428	-2.849765	0.0048168	0.0175166	-2.719183
FAM160A1	0.7104551	2.8419754	2.8495106	0.0048205	0.0175283	-2.719871
CRYGS	-0.166851	4.6607749	-2.849474	0.004821	0.0175285	-2.719971
DDX19A	0.0518602	6.1849785	2.8493648	0.0048226	0.0175309	-2.720266
RAB22A	0.0476535	6.2534189	2.8493631	0.0048226	0.0175309	-2.72027
AC007292.6	-0.240168	3.8326483	-2.848966	0.0048284	0.0175502	-2.721346
RP11-649A1	-0.650725	0.5304707	-2.848826	0.0048305	0.0175559	-2.721727
SH3GL1P2	-0.631015	1.2896043	-2.848781	0.0048311	0.0175565	-2.721848
C1orf106	-0.547105	4.8864953	-2.848598	0.0048338	0.0175645	-2.722343

CNPY4	-0.083042	5.5809289	-2.848155	0.0048403	0.0175854	-2.723542
GJA1	0.1312704	5.8697231	2.8481381	0.0048405	0.0175854	-2.723588
RP11-163G1	0.2656066	-1.378502	2.8480801	0.0048414	0.0175867	-2.723745
AC002310.1	-0.583396	-0.328365	-2.847998	0.0048426	0.0175893	-2.723967
TRAF5	-0.12351	5.5799487	-2.847861	0.0048446	0.0175949	-2.724338
RPL7P10	-0.385499	-1.176734	-2.847787	0.0048457	0.0175971	-2.724539
XXbac-BPG2	0.1633086	-1.526449	2.8477158	0.0048467	0.0175989	-2.724731
RP11-465L1	-0.640695	2.3960054	-2.847677	0.0048473	0.0175989	-2.724835
AC018766.6	-0.624321	0.5722826	-2.847654	0.0048476	0.0175989	-2.7249
FAM78B	0.2721807	4.7916349	2.8475154	0.0048496	0.0176045	-2.725274
RP11-178L8	0.5596818	-0.430818	2.847291	0.0048529	0.0176147	-2.725881
ZCCHC5	0.4855788	-0.973255	2.8471555	0.0048549	0.0176202	-2.726248
ANP32A	-0.045956	6.644737	-2.847104	0.0048557	0.0176211	-2.726386
bP-218909.	-0.715528	-0.055633	-2.847067	0.0048562	0.0176214	-2.726487
IGHA1	0.3077852	6.2223312	2.8470278	0.0048568	0.0176217	-2.726594
RP1-140A9.	-0.605895	2.7482209	-2.846927	0.0048583	0.0176253	-2.726867
TTC21A	0.1418975	4.6665706	2.8467964	0.0048602	0.0176305	-2.72722
EFCC1	0.227822	4.4131009	2.8467486	0.0048609	0.0176313	-2.727349
RP11-390K5	-0.338359	3.4972228	-2.846553	0.0048637	0.01764	-2.727878
CTA-268H5.	-0.701079	0.7059387	-2.846027	0.0048715	0.0176663	-2.729302
THRB-AS1	-0.345397	3.5934754	-2.845554	0.0048784	0.0176898	-2.730582
U47924.32	-0.646558	0.989814	-2.845349	0.0048815	0.017699	-2.731137
PRSS45	-0.826403	2.040454	-2.84523	0.0048832	0.0177034	-2.731458
CHMP3	0.0523135	6.2214666	2.8452005	0.0048837	0.0177034	-2.731538
IL13RA2	0.7314168	2.8666907	2.8449271	0.0048877	0.0177163	-2.732278
BAZ1A	0.0773392	6.0270491	2.844581	0.0048928	0.0177315	-2.733214
RP5-965G21	-0.590799	2.4222374	-2.844576	0.0048929	0.0177315	-2.733226
GLIPR1L1	0.6916606	1.2372575	2.8441277	0.0048995	0.0177538	-2.73444
CD58	0.0976597	5.5695102	2.8436997	0.0049058	0.017775	-2.735597
RP11-755B1	-0.488094	2.461491	-2.843595	0.0049074	0.0177788	-2.735881
CDKN2C	-0.136006	5.7906483	-2.843515	0.0049086	0.0177813	-2.736095
ABCA9	0.3106882	5.0666535	2.8434358	0.0049097	0.0177838	-2.73631
TRI-TAT2-2	0.6649197	0.7183133	2.8429985	0.0049162	0.0178042	-2.737493
STAG3	0.1617615	5.1008915	2.8429909	0.0049163	0.0178042	-2.737513
MIR4737	-0.569601	-0.158663	-2.842944	0.004917	0.017805	-2.737641
RP11-764K9	-0.543854	-0.842439	-2.842886	0.0049179	0.0178058	-2.737798
SMAD6	0.1453581	5.4072778	2.8428621	0.0049183	0.0178058	-2.737861
CPB1	0.8318384	1.3573685	2.8428302	0.0049187	0.0178058	-2.737948
N4BP2	-0.122833	5.7670808	-2.842463	0.0049242	0.0178237	-2.738941
SUB1P1	-0.605135	-0.158101	-2.84236	0.0049257	0.0178275	-2.739218
UNC13D	0.1533629	5.5923047	2.8422479	0.0049274	0.0178318	-2.739521
LINC00704	0.8354958	1.0589527	2.8420486	0.0049303	0.0178407	-2.74006
KB-1615E4.	0.808663	0.8868695	2.8416935	0.0049356	0.0178581	-2.741019
C1orf210	-0.443134	5.1590537	-2.841391	0.0049401	0.0178726	-2.741836
RP11-159H2	-0.590161	0.4884907	-2.841209	0.0049429	0.0178799	-2.74233
RP11-439K3	0.5900505	-0.353028	2.8411572	0.0049436	0.0178799	-2.742468
RDH16	0.3696645	6.1644597	2.8411097	0.0049443	0.0178799	-2.742597
QSOX2	-0.079083	5.9412131	-2.841101	0.0049445	0.0178799	-2.742621
HINT2	0.0980946	6.2938659	2.8410812	0.0049448	0.0178799	-2.742674
RNU6V	0.3367225	-1.220126	2.8410288	0.0049455	0.0178799	-2.742815
LEPR	0.2458463	6.3731357	2.8410256	0.0049456	0.0178799	-2.742824

LRRN3	0.5642396	2.6560407	2.8408416	0.0049483	0.0178881	-2.743321
SERTAD2	-0.064286	6.117754	-2.840533	0.0049529	0.017903	-2.744154
CECR7	-0.996438	1.3814529	-2.840474	0.0049538	0.0179044	-2.744314
RP11-411B1	-0.465638	-0.904269	-2.840431	0.0049545	0.0179049	-2.744431
EDIL3	0.2260221	5.2917788	2.8402717	0.0049568	0.0179118	-2.74486
C9orf89	0.0768326	5.7495659	2.8400004	0.0049609	0.0179246	-2.745593
CTA-984G1.	-0.417651	2.7198841	-2.839746	0.0049647	0.0179366	-2.746281
CTD-2023N9	0.4030423	-1.148026	2.8392427	0.0049722	0.0179621	-2.747639
HNRNPLP1	-0.443975	-0.950655	-2.838998	0.0049759	0.0179736	-2.7483
ZYX	0.061106	6.760024	2.838706	0.0049803	0.0179876	-2.749088
RP5-1057I2	0.6150571	-0.21857	2.8386687	0.0049809	0.0179879	-2.749189
TAB2	0.0627518	6.3377892	2.8385507	0.0049826	0.0179925	-2.749507
MRPL32	0.0577896	6.2642326	2.8384749	0.0049838	0.0179948	-2.749712
NT5C3B	-0.088539	6.219423	-2.838111	0.0049892	0.0180128	-2.750694
KMT2C	0.0696092	6.2707885	2.837924	0.004992	0.018021	-2.751199
RP5-1096J1	-0.75463	-0.520635	-2.837894	0.0049925	0.018021	-2.751279
GIMAP2	0.1537874	5.4424161	2.8378406	0.0049933	0.0180221	-2.751423
UNC80	-0.687334	1.3670018	-2.837724	0.0049951	0.0180266	-2.751737
CYP4F12	-0.34463	5.825221	-2.837664	0.004996	0.0180281	-2.751901
RP11-93B14	0.2830472	4.5691763	2.8375462	0.0049977	0.0180328	-2.752218
ATP6V1E2	0.1627004	5.0094877	2.8373662	0.0050004	0.0180408	-2.752704
TCEAL5	-0.549846	-1.008583	-2.837284	0.0050017	0.0180424	-2.752925
ZSCAN5D	-0.593911	-0.25911	-2.837271	0.0050019	0.0180424	-2.75296
NACAD	0.416748	3.3963399	2.8372202	0.0050026	0.0180433	-2.753098
CTD-2619J1	-0.496665	2.7041207	-2.837098	0.0050045	0.0180482	-2.753428
RP11-715J2	-0.627232	0.3595356	-2.836622	0.0050117	0.0180723	-2.754712
RP11-467D1	-0.587802	-0.044771	-2.836589	0.0050122	0.0180724	-2.754801
AC023137.2	0.5560657	-0.66574	2.836543	0.0050129	0.0180731	-2.754925
AC011288.2	-0.60546	-0.876169	-2.836305	0.0050165	0.0180842	-2.755565
ANKRD7	-0.729855	-0.205562	-2.836063	0.0050201	0.0180948	-2.756218
RP4-563E14	-0.310071	3.3633674	-2.836045	0.0050204	0.0180948	-2.756267
KYNU	0.2061299	6.1531472	2.8359583	0.0050217	0.018096	-2.756501
TSPAN10	-0.224467	4.8537173	-2.835957	0.0050217	0.018096	-2.756504
AC093419.1	-0.263758	-1.425413	-2.835899	0.0050226	0.0180974	-2.75666
SNX1	0.0498169	6.5540229	2.8357411	0.005025	0.0181042	-2.757087
AC010907.2	-0.794654	0.3455083	-2.835442	0.0050295	0.0181172	-2.757893
AC108142.1	0.5308691	-1.056172	2.8354379	0.0050296	0.0181172	-2.757905
RP11-22903	0.336336	-1.311379	2.8348443	0.0050386	0.0181451	-2.759505
MYCBP2-AS1	0.5814926	0.7039109	2.834838	0.0050387	0.0181451	-2.759522
AC104395.1	-0.345063	-1.299057	-2.834829	0.0050388	0.0181451	-2.759548
RP11-264L1	-0.663265	-0.296553	-2.834778	0.0050396	0.0181456	-2.759683
UBE2QL1	0.6024762	4.2400102	2.8347545	0.0050399	0.0181456	-2.759747
DHX38	0.0495833	6.2976552	2.8344622	0.0050444	0.0181587	-2.760535
GPR62	0.759781	1.527797	2.8344285	0.0050449	0.0181587	-2.760626
BAX	-0.06664	6.4131546	-2.834416	0.0050451	0.0181587	-2.76066
ZNF594	-0.251348	4.5148352	-2.834173	0.0050488	0.0181702	-2.761315
AIF1	0.1319879	5.888959	2.833965	0.0050519	0.0181798	-2.761876
P2RX7	-0.174163	5.5318924	-2.833853	0.0050536	0.0181841	-2.762177
ASL	0.1321281	6.6880377	2.8337388	0.0050554	0.0181886	-2.762485
AC012512.1	-0.703428	-0.549385	-2.833657	0.0050566	0.0181913	-2.762705
ZNF442	-0.141115	4.7688571	-2.833519	0.0050587	0.018197	-2.763077

KRT13	0.9590594	-0.052517	2.8334712	0.0050595	0.0181979	-2.763206
AC003104.1	-0.621797	0.0400952	-2.833355	0.0050612	0.0182025	-2.76352
KLHL30	0.5253849	3.2007804	2.8329663	0.0050671	0.018222	-2.764567
RNF181	-0.070089	6.5297207	-2.832865	0.0050687	0.0182246	-2.764839
RP11-156L1	0.5232101	-0.96071	2.8328138	0.0050695	0.0182246	-2.764978
DGUOK-AS1	-0.361863	3.2062529	-2.8328	0.0050697	0.0182246	-2.765016
AC004987.9	0.2398514	3.6550197	2.8327614	0.0050703	0.0182246	-2.765119
MAPK6	-0.069425	6.4381323	-2.832754	0.0050704	0.0182246	-2.765138
FAM131A	-0.072436	5.6693084	-2.832708	0.0050711	0.0182254	-2.765262
CH17-408M7	0.554382	-0.85015	2.8323231	0.005077	0.0182447	-2.7663
SNORA42	-0.604686	0.3061476	-2.832219	0.0050786	0.0182486	-2.76658
RP11-348P1	-0.303525	3.5405103	-2.832169	0.0050793	0.0182496	-2.766716
MMP25	0.2357782	4.5139765	2.8318496	0.0050842	0.0182639	-2.767575
RP11-552M1	-0.635055	-0.849827	-2.831843	0.0050843	0.0182639	-2.767592
AC097721.2	-0.416557	3.076636	-2.831745	0.0050858	0.0182675	-2.767858
CTD-3099C6	-0.639941	2.9278147	-2.831526	0.0050892	0.0182777	-2.768446
RNA5SP78	-0.495587	-0.675657	-2.831486	0.0050898	0.0182781	-2.768553
CD3EAP	-0.119569	5.2084675	-2.831174	0.0050946	0.0182935	-2.769395
RP11-3J1.1	-0.831032	0.1891181	-2.830604	0.0051033	0.0183231	-2.770929
RP11-449P1	-0.296465	4.6527974	-2.830486	0.0051051	0.0183277	-2.771246
NMI	0.089964	5.9686141	2.8304256	0.005106	0.0183293	-2.771409
TMEM14B	-0.061683	6.3725621	-2.830369	0.0051069	0.0183306	-2.771561
PLEKHJ1	-0.070754	6.353734	-2.830239	0.0051089	0.018336	-2.771911
MYO1D	0.1066796	6.3892948	2.8300768	0.0051114	0.0183431	-2.772348
ZIC2	-0.75784	4.4231538	-2.830033	0.0051121	0.0183437	-2.772465
YEATS2	-0.062029	6.0042736	-2.8297	0.0051172	0.0183603	-2.773363
C17orf67	0.2945316	4.4816191	2.8296394	0.0051181	0.0183618	-2.773526
FAM19A1	0.6550641	0.2673042	2.8295472	0.0051195	0.0183651	-2.773774
FAM83C	-0.622452	-0.140713	-2.829486	0.0051205	0.0183667	-2.77394
C7orf69	0.3240647	-1.2709	2.8293027	0.0051233	0.0183738	-2.774432
RP11-10H3.	0.3149746	-1.348584	2.829292	0.0051235	0.0183738	-2.774461
CTB-3204.3	-0.559411	-0.568104	-2.829162	0.0051255	0.0183789	-2.774811
RP11-269F1	-0.508872	1.886704	-2.829134	0.0051259	0.0183789	-2.774887
RP11-92C4.	0.713346	1.1813549	2.8289216	0.0051292	0.0183889	-2.775457
GTF3C4	-0.058755	5.9775463	-2.828853	0.0051302	0.0183909	-2.775642
KANTR	-0.411563	3.1084729	-2.828487	0.0051359	0.0184093	-2.776626
FANCM	-0.089596	5.060953	-2.82814	0.0051412	0.0184267	-2.777559
STT3A	-0.05175	6.7874606	-2.827842	0.0051459	0.0184414	-2.778363
GNGT2	0.2498267	4.1914318	2.8275844	0.0051498	0.0184539	-2.779055
RP11-355B1	-0.134022	4.6139509	-2.827524	0.0051508	0.0184554	-2.779217
XXyac-YX15	-0.471725	-0.864916	-2.827457	0.0051518	0.0184573	-2.779396
CTD-2206G1	-0.56305	-0.347045	-2.826797	0.005162	0.0184921	-2.781171
RP11-318C2	-0.593139	0.9923055	-2.826571	0.0051655	0.0185029	-2.78178
NPY4R	0.4383361	-1.200812	2.8264425	0.0051675	0.0185082	-2.782125
CCDC33	0.4747969	-1.02675	2.8262904	0.0051699	0.0185149	-2.782534
L3MBTL4-AS	0.5716978	2.1366825	2.8262084	0.0051712	0.0185176	-2.782755
RP11-439M1	-0.713623	-0.405201	-2.826163	0.0051719	0.0185183	-2.782877
ACSM6	-0.663408	0.0274659	-2.824986	0.0051902	0.0185821	-2.78604
NCF4	0.1519745	5.3873304	2.824922	0.0051912	0.0185838	-2.786212
RP11-777J2	-0.589434	-0.266066	-2.824543	0.0051971	0.0186031	-2.78723
WDR46	-0.059508	6.3563718	-2.823999	0.0052056	0.0186316	-2.788694

RP11-507K2	-0.518653	2.0114959	-2.823969	0.0052061	0.0186316	-2.788772
MB21D1	0.2320062	4.7526919	2.8236787	0.0052106	0.018646	-2.789553
TMEM72	-0.896803	2.2378689	-2.823647	0.0052111	0.018646	-2.789638
RP1-122P22	0.6144853	2.1791775	2.8235897	0.005212	0.018647	-2.789792
SORBS1	0.1078723	6.3198134	2.8235481	0.0052127	0.018647	-2.789904
NUDT11	0.7496791	1.4422014	2.8235299	0.0052129	0.018647	-2.789952
PLAUR	0.1732682	5.4634574	2.8232874	0.0052167	0.0186588	-2.790604
RP11-104D3	-0.61584	-0.070463	-2.823122	0.0052193	0.0186662	-2.791048
ZNF408	-0.062365	5.9368283	-2.823068	0.0052202	0.0186675	-2.791194
MTFMT	0.0577971	5.657566	2.8222249	0.0052334	0.0187119	-2.793457
TRAF6	0.0608769	5.5311531	2.8222095	0.0052336	0.0187119	-2.793498
RP5-933B4.	0.1731276	-1.521445	2.8219609	0.0052375	0.0187217	-2.794166
KLF17	0.5792308	-0.791532	2.8219563	0.0052376	0.0187217	-2.794178
CSPG4P8	-0.299144	4.2091235	-2.821938	0.0052379	0.0187217	-2.794227
RP11-77901	0.6150922	-0.294273	2.8218034	0.00524	0.0187274	-2.794589
RP11-1010E	-0.603052	1.1073359	-2.821762	0.0052407	0.0187279	-2.794699
MIR5195	-0.453617	-1.0039	-2.821497	0.0052448	0.018741	-2.795411
ZSCAN5C	-0.565835	-0.157942	-2.821439	0.0052458	0.0187424	-2.795566
RP11-95I16	-0.508303	-1.052627	-2.821157	0.0052502	0.0187549	-2.796323
S100A8	0.3507537	4.5804876	2.8211511	0.0052503	0.0187549	-2.796339
RP11-394B2	-0.383942	3.7086944	-2.820923	0.0052539	0.0187659	-2.796952
MAP4K4	-0.061718	6.6809947	-2.820652	0.0052581	0.0187793	-2.797678
FAM159A	-0.527934	3.3372512	-2.820246	0.0052646	0.0188004	-2.798769
CRCP	0.059978	6.2644531	2.8199141	0.0052698	0.0188173	-2.799659
RP11-379F4	-0.606298	0.6752373	-2.819825	0.0052712	0.0188205	-2.799899
RP11-1105C	0.5956162	-0.933477	2.8197328	0.0052727	0.0188238	-2.800145
THAP8	-0.090516	5.5976546	-2.819414	0.0052777	0.01884	-2.801
RP11-12N13	-0.326566	-1.350803	-2.819224	0.0052807	0.0188489	-2.80151
CNPY3	-0.075128	6.4802932	-2.818828	0.005287	0.0188694	-2.802572
RP11-299H2	-0.240665	3.6433343	-2.818638	0.00529	0.0188784	-2.803082
PRR4	-0.25686	3.5510096	-2.818393	0.0052939	0.0188888	-2.803739
AC005077.8	0.3955264	-1.212481	2.8183883	0.005294	0.0188888	-2.803751
GPR68	0.2555542	4.5266943	2.8179162	0.0053015	0.0189122	-2.805017
RN7SL444P	-0.391077	-1.066847	-2.81791	0.0053016	0.0189122	-2.805034
DHX16	-0.049315	6.3117507	-2.817635	0.0053059	0.0189259	-2.805771
RP3-486D24	-0.351103	-1.237411	-2.817547	0.0053073	0.0189291	-2.806006
B9D1	-0.123415	5.6134428	-2.817403	0.0053096	0.0189354	-2.806393
NUMBL	0.1120304	5.5491518	2.8173202	0.0053109	0.0189382	-2.806615
AC005154.6	-0.080406	5.7577484	-2.81691	0.0053175	0.0189597	-2.807715
KLRC1	0.72622	1.8726363	2.8164797	0.0053243	0.0189823	-2.808868
MTHFR	0.0818283	6.0279773	2.8162245	0.0053284	0.018995	-2.809551
MIR4297	-0.44411	-1.055645	-2.815764	0.0053358	0.0190194	-2.810786
RP11-462G2	-0.448578	-1.015272	-2.815679	0.0053371	0.0190223	-2.811013
ZNF888	-0.781788	1.5757658	-2.81545	0.0053408	0.0190335	-2.811625
CNOT6L	0.0743546	6.1114268	2.8151317	0.0053459	0.0190498	-2.812479
KRT18P66	-0.260415	-1.405147	-2.815024	0.0053476	0.0190527	-2.812769
CLEC18A	-0.675131	1.0912232	-2.815015	0.0053477	0.0190527	-2.81279
RP11-701P1	0.6695191	0.746222	2.8146452	0.0053537	0.019072	-2.813782
RPS11P7	-0.392451	-1.125669	-2.814568	0.0053549	0.0190746	-2.81399
CTD-2523D1	-0.688629	1.2459812	-2.813658	0.0053695	0.0191248	-2.816427
RP11-348J1	0.4859085	-0.906548	2.8133539	0.0053744	0.0191403	-2.81724

MYCN	-0.627493	3.989406	-2.813317	0.005375	0.0191405	-2.817338
AC005251.3	-0.510034	2.1062609	-2.81305	0.0053793	0.019154	-2.818053
TDRD5	-0.948585	0.9483993	-2.813	0.0053801	0.0191549	-2.818186
LINC00639	0.5200934	2.8944346	2.8127605	0.005384	0.0191668	-2.818828
RP4-545L17	-0.635336	-0.309449	-2.812642	0.0053859	0.0191718	-2.819146
MX1	0.1405756	6.323835	2.8125895	0.0053867	0.0191729	-2.819286
AL122127.2	-0.723964	-0.288461	-2.81252	0.0053879	0.019175	-2.819472
RP5-1068E1	-0.378523	3.261364	-2.812244	0.0053923	0.019189	-2.82021
AC013460.1	-0.586803	-0.660239	-2.811775	0.0053999	0.0192141	-2.821467
UNC45A	0.0564121	6.4466057	2.8117308	0.0054006	0.0192148	-2.821584
MAL2	-0.22371	6.486347	-2.811601	0.0054027	0.0192203	-2.82193
RP11-438D1	0.2365195	-1.438853	2.8115348	0.0054038	0.0192223	-2.822108
ANKRD6	0.0998976	5.5261009	2.8114174	0.0054057	0.0192272	-2.822422
Metazoa_SR	-0.25047	5.8996866	-2.811346	0.0054068	0.0192294	-2.822612
CTB-33G10.	-0.297089	3.0996796	-2.811187	0.0054094	0.0192367	-2.823038
FAM220CP	-0.6305	1.3455564	-2.8111	0.0054108	0.0192398	-2.823273
CYP4X1	0.3148936	4.7420807	2.8109663	0.005413	0.0192456	-2.823629
ASZ1	-0.497311	-1.12715	-2.810931	0.0054135	0.0192458	-2.823724
SNORA79	-0.509294	-0.713414	-2.810842	0.005415	0.0192491	-2.823963
RP11-50D16	-0.634925	0.5728418	-2.810779	0.005416	0.0192508	-2.824129
RP11-23P13	0.5911133	3.0033367	2.8105543	0.0054196	0.0192619	-2.824731
EFTUD1P2	-0.440144	-1.064802	-2.810372	0.0054226	0.0192705	-2.82522
RP11-296I1	-0.430036	-1.185958	-2.810249	0.0054246	0.0192757	-2.825549
AC073130.3	0.6814904	0.3912423	2.8101799	0.0054257	0.0192778	-2.825732
TUBB	-0.05421	7.1357787	-2.809749	0.0054327	0.0193008	-2.826884
AMY2B	0.1973909	5.2347396	2.8095515	0.0054359	0.0193101	-2.827413
AC137932.6	0.6183633	1.466894	2.8095232	0.0054364	0.0193101	-2.827488
RP11-1E22.	-0.68139	-0.520377	-2.809362	0.005439	0.0193175	-2.82792
MC2R	0.1864819	-1.491876	2.8091593	0.0054423	0.0193274	-2.828461
RB1	0.1004113	6.0133996	2.8087449	0.0054491	0.0193494	-2.829569
RP11-407P2	-0.580685	-0.331946	-2.808522	0.0054527	0.0193604	-2.830164
MSANTD1	-0.413496	2.7819074	-2.808486	0.0054533	0.0193606	-2.83026
CCDC57	0.0880327	5.9448903	2.8082662	0.0054569	0.0193709	-2.830849
UBLCP1	0.0472211	5.956265	2.8082441	0.0054572	0.0193709	-2.830908
SYTL3	0.2315288	4.5285679	2.808115	0.0054593	0.0193751	-2.831253
SNORD113-4	-0.351491	-1.326	-2.808107	0.0054595	0.0193751	-2.831273
RP11-659E9	0.7808059	1.5768023	2.8080391	0.0054606	0.0193771	-2.831455
AKR1B10P1	1.0379339	2.573699	2.8078587	0.0054635	0.0193857	-2.831938
SECISBP2L	0.0684598	6.138556	2.8076718	0.0054666	0.0193946	-2.832437
ADAP1	-0.26978	4.972988	-2.807526	0.0054689	0.0194012	-2.832825
AC007228.1	-0.583387	2.3104762	-2.80717	0.0054748	0.01942	-2.833778
TAP2	0.1031473	6.1155859	2.8070736	0.0054763	0.0194237	-2.834035
RP11-797H7	0.3912702	-1.227896	2.8064401	0.0054867	0.0194586	-2.835727
WFIKKN1	-0.420139	3.588144	-2.806405	0.0054873	0.0194587	-2.835821
RP3-42906.	0.1555433	-1.530407	2.8062769	0.0054894	0.0194629	-2.836163
ATP5G2P3	-0.235828	-1.439109	-2.806269	0.0054895	0.0194629	-2.836185
CADM2	-0.982258	1.5003402	-2.806135	0.0054917	0.0194688	-2.836543
RP11-378E1	-0.536815	-0.519924	-2.805735	0.0054983	0.0194902	-2.837612
NR1I2	0.6524461	5.316861	2.8056123	0.0055003	0.0194954	-2.837938
RP11-402D2	-0.649721	0.66238	-2.805356	0.0055045	0.0195085	-2.838623
FAM86LP	0.23194	-1.441187	2.8051332	0.0055082	0.0195187	-2.839217

PRKRIP1	-0.057183	6.0554716	-2.805105	0.0055087	0.0195187	-2.839293
OR8A2P	-0.366964	-1.278338	-2.805083	0.005509	0.0195187	-2.839351
KDM5B	-0.089853	6.2299503	-2.804707	0.0055152	0.0195387	-2.840354
MIR4539	-0.381892	-1.244218	-2.80447	0.0055191	0.0195507	-2.840989
AC002064.7	0.3568353	-1.240954	2.804393	0.0055204	0.0195533	-2.841193
DAPK2	0.1693737	5.579733	2.804282	0.0055222	0.0195579	-2.84149
RP11-180P8	0.2585579	-1.409617	2.8040746	0.0055256	0.0195681	-2.842043
PAPL	-0.713309	-0.1038	-2.804	0.0055269	0.0195705	-2.842242
TBPL2	-0.34496	-1.293393	-2.803911	0.0055283	0.0195715	-2.842479
SNX25	0.1030566	5.6660228	2.8038685	0.005529	0.0195715	-2.842593
TAP1	0.1038518	6.4717296	2.8038549	0.0055293	0.0195715	-2.84263
IQCE	0.0810159	5.9102228	2.8038529	0.0055293	0.0195715	-2.842635
RP11-297N6	0.6456193	1.1767975	2.8038101	0.00553	0.0195721	-2.842749
SRRM1	0.0448753	6.3170397	2.8037655	0.0055307	0.0195729	-2.842868
SDR42E1	0.5090812	4.8293954	2.8036492	0.0055327	0.0195778	-2.843179
RP4-763G1.	0.8029666	3.770589	2.8032743	0.0055389	0.0195978	-2.844179
ELOVL5	0.0723518	6.6103485	2.8030175	0.0055431	0.0196109	-2.844864
RP1-102E24	-0.585823	1.873123	-2.802771	0.0055472	0.0196235	-2.845523
SPRR2G	0.2990378	-1.45728	2.8025393	0.005551	0.0196351	-2.84614
PREPL	0.0566825	6.1204319	2.8024805	0.005552	0.0196367	-2.846297
RP5-1099D1	-0.610662	0.7433859	-2.802251	0.0055558	0.0196482	-2.846908
JMJD6	-0.063898	6.0778434	-2.802039	0.0055593	0.0196587	-2.847474
C17orf102	0.396496	-1.094545	2.8014475	0.0055691	0.0196912	-2.849052
POTEC	-0.419861	-1.238782	-2.801421	0.0055696	0.0196912	-2.849123
HNRNPA1P44	-0.412453	-0.988277	-2.80139	0.0055701	0.0196912	-2.849206
RP5-892K4.	-0.515783	-0.574454	-2.801335	0.005571	0.0196925	-2.849352
RP11-231I1	-0.672266	0.4274809	-2.801245	0.0055725	0.0196958	-2.849591
ARHGAP15	0.1452325	5.3475457	2.8011511	0.0055741	0.0196995	-2.849842
SNORD12B	-0.640911	0.2584505	-2.80103	0.0055761	0.019703	-2.850164
PNMA6B	0.872899	-0.105796	2.8010258	0.0055762	0.019703	-2.850176
RP11-331F4	0.5649246	-0.447255	2.8005664	0.0055838	0.0197281	-2.851401
RP1-22N22.	-0.48713	-0.745631	-2.800329	0.0055877	0.0197402	-2.852033
RP11-417L1	-0.5818	2.2428597	-2.799932	0.0055944	0.0197617	-2.853091
CD84	0.1891616	5.358692	2.7997661	0.0055971	0.0197695	-2.853534
OPRD1	-0.534755	2.5591952	-2.799431	0.0056027	0.0197873	-2.854426
RP4-529N6.	0.3261231	-1.324898	2.7993797	0.0056036	0.0197873	-2.854564
AC007879.2	-0.643761	0.9026044	-2.799369	0.0056038	0.0197873	-2.854593
RBM17	-0.04077	6.403386	-2.799154	0.0056074	0.0197976	-2.855166
RP11-112N1	-0.317748	-1.293732	-2.799128	0.0056078	0.0197976	-2.855233
BPGM	0.0653893	5.8766014	2.7987963	0.0056134	0.0198141	-2.856118
AC090587.2	-0.462136	3.1581297	-2.798784	0.0056136	0.0198141	-2.85615
AVL9	-0.05516	6.2443379	-2.798573	0.0056171	0.0198247	-2.856714
SH2D7	-0.657219	0.0032264	-2.798441	0.0056193	0.0198306	-2.857066
RP11-96K19	-0.317562	3.6148822	-2.798382	0.0056203	0.0198321	-2.857221
RP5-1142J1	-0.617163	-0.447098	-2.798203	0.0056233	0.0198408	-2.857698
DRG1	-0.054758	6.3557677	-2.797911	0.0056282	0.0198562	-2.858477
GLRX3P2	-0.596364	-0.13019	-2.797815	0.0056298	0.0198599	-2.858731
ZNF20	-0.304383	3.1601287	-2.797576	0.0056338	0.0198722	-2.859369
AAR2	-0.043759	6.3099948	-2.797491	0.0056352	0.0198753	-2.859594
FAM46A	0.1252829	6.0254652	2.7973603	0.0056374	0.0198811	-2.859943
IGBP1P4	-0.333775	-1.276973	-2.797216	0.0056399	0.0198877	-2.860326

NCF2	0.1652311	5.6827761	2.7970585	0.0056425	0.0198951	-2.860747
TC2N	0.2615046	5.5350478	2.7958468	0.0056629	0.0199652	-2.863972
PKP1	0.7590463	2.4721069	2.795751	0.0056645	0.0199674	-2.864228
EEF1B2P3	-0.170751	4.4578279	-2.795745	0.0056646	0.0199674	-2.864244
RP11-278J6	-0.531365	2.2379633	-2.795532	0.0056683	0.0199782	-2.864812
RPL5P34	-0.265963	3.5707458	-2.795369	0.005671	0.0199843	-2.865245
CTC-205M6.	-0.400066	3.0330891	-2.795364	0.0056711	0.0199843	-2.865257
RP11-121A1	-0.557376	1.6488338	-2.795097	0.0056756	0.0199983	-2.865968
XIRP2	-0.596534	-0.887895	-2.794794	0.0056807	0.0200144	-2.866774
RP11-421P2	0.2563486	-1.451512	2.7943143	0.0056888	0.0200411	-2.86805
MMP24-AS1	0.1305776	6.111504	2.7941788	0.0056911	0.0200472	-2.868411
RNU4-23P	0.2932276	-1.352557	2.7940661	0.0056931	0.020052	-2.868711
RP11-1072C	-0.60648	0.9506528	-2.793965	0.0056948	0.0200553	-2.86898
BCL10	0.064818	5.7624704	2.7939469	0.0056951	0.0200553	-2.869028
LPXN	0.0833733	5.6872092	2.7938422	0.0056968	0.0200596	-2.869306
CECR5	0.0728701	6.3070135	2.7935652	0.0057015	0.0200742	-2.870043
MIR3609	-0.453969	-0.967974	-2.793414	0.0057041	0.0200809	-2.870445
NKAIN1	-0.821242	0.8992253	-2.793342	0.0057053	0.0200809	-2.870636
ZFAND2B	-0.066939	6.2778102	-2.793335	0.0057055	0.0200809	-2.870656
EGOT	0.7840016	1.3046431	2.7933236	0.0057057	0.0200809	-2.870686
AL445183.1	0.2941635	-1.360394	2.7932509	0.0057069	0.0200816	-2.870879
SNORA26	-0.652392	1.4258432	-2.793247	0.0057069	0.0200816	-2.870888
DNAJB6	0.0463361	6.4055699	2.7931955	0.0057078	0.0200828	-2.871026
BSCL2	0.1035394	5.2329745	2.7930239	0.0057107	0.0200911	-2.871483
ZNF574	-0.056332	5.8592595	-2.792656	0.005717	0.0201111	-2.87246
CTB-140J7.	0.5417798	-0.630452	2.7921483	0.0057257	0.0201396	-2.873811
LL0YNC03-2	-0.428962	-1.076157	-2.791998	0.0057282	0.0201451	-2.874211
RP11-345K9	-0.282979	-1.415987	-2.791993	0.0057283	0.0201451	-2.874223
RP11-95G17	-0.353628	-1.248864	-2.791788	0.0057318	0.0201554	-2.874768
PCNX	0.0646067	6.2295795	2.7913338	0.0057395	0.0201807	-2.875976
DCAF12L1	-0.66776	-0.826299	-2.791033	0.0057447	0.0201969	-2.876774
ZAN	-0.606493	-0.271015	-2.79091	0.0057468	0.0202023	-2.877101
MIR499A	-0.459059	-0.865389	-2.790704	0.0057503	0.0202128	-2.877649
C4B	0.1581953	6.79665	2.7902764	0.0057576	0.0202365	-2.878785
SMCHD1	-0.05981	6.1243092	-2.790192	0.0057591	0.0202397	-2.87901
HTATIP2	0.106661	6.5546014	2.7901121	0.0057605	0.0202425	-2.879222
FAM117A	-0.104827	5.7637652	-2.789905	0.005764	0.020253	-2.879771
PDCL2	-0.383232	-1.323014	-2.789357	0.0057734	0.0202842	-2.881229
CTC-484M2.	-0.634735	1.2003323	-2.789283	0.0057747	0.0202867	-2.881425
CASP7	0.072837	6.0028456	2.7891115	0.0057776	0.0202951	-2.88188
AC005550.5	-0.381288	-1.227664	-2.78892	0.0057809	0.0203047	-2.882388
DYNC1I2	0.0589318	6.2604028	2.7888863	0.0057815	0.0203048	-2.882478
TBC1D22B	-0.068677	5.6968118	-2.78846	0.0057888	0.0203253	-2.883609
SEC14L3	0.9112887	2.231132	2.7884582	0.0057889	0.0203253	-2.883614
PNISR	-0.061306	6.264741	-2.78845	0.005789	0.0203253	-2.883637
CEP128	-0.146819	4.8055991	-2.788095	0.0057951	0.0203447	-2.884577
RP11-138J2	-1.034324	1.3017935	-2.787609	0.0058035	0.0203722	-2.885868
RP11-996F1	-0.361327	3.2922585	-2.787409	0.0058069	0.0203824	-2.886399
RP11-348H3	-0.549289	-0.090736	-2.787258	0.0058096	0.020388	-2.886801
RPL7P9	-0.150216	5.4423225	-2.787251	0.0058097	0.020388	-2.886818
LL22NC03-8	-0.365649	-1.248763	-2.787214	0.0058103	0.0203883	-2.886918

RPL7P7	-0.501282	-0.743633	-2.78638	0.0058247	0.0204369	-2.88913
TRAPPC2L	0.0707181	6.1757971	2.7859899	0.0058315	0.0204587	-2.890165
CYCS	0.0694989	6.6624132	2.7859429	0.0058323	0.0204596	-2.89029
TECPR2	0.0640874	5.8351694	2.7857812	0.0058351	0.0204675	-2.890719
Z69890.1	0.5249758	-0.760566	2.7855193	0.0058397	0.0204814	-2.891414
C15orf57	0.0720127	5.5120564	2.7854675	0.0058405	0.0204826	-2.891551
AC005071.4	0.438168	-0.954323	2.7853893	0.0058419	0.0204851	-2.891759
RP11-71602	-0.435317	-1.139849	-2.785362	0.0058424	0.0204851	-2.891831
AC093702.1	-0.616146	-0.724913	-2.784627	0.0058551	0.0205279	-2.89378
FSIP1	0.5529716	2.5734097	2.7844018	0.0058591	0.0205397	-2.894377
CTB-96E2.1	-0.530188	-0.567354	-2.784326	0.0058604	0.0205423	-2.894579
RECK	0.1656382	5.4133869	2.7842117	0.0058624	0.0205473	-2.894881
TRUB2	0.0658722	6.3470029	2.7838274	0.0058691	0.0205688	-2.8959
SRRD	-0.061141	5.8139994	-2.783636	0.0058724	0.0205784	-2.896408
GPR82	0.4892736	2.7974432	2.7836061	0.0058729	0.0205784	-2.896487
KARS	0.0538558	6.6107051	2.7831263	0.0058813	0.0206048	-2.897759
RP11-402P6	-0.811044	-0.500651	-2.78311	0.0058816	0.0206048	-2.897802
HSD17B1	-0.129546	4.6702443	-2.783065	0.0058824	0.020605	-2.897921
WDR66	0.2748612	4.1161068	2.7830419	0.0058828	0.020605	-2.897982
RP4-625H18	0.7421122	-0.050457	2.7829823	0.0058838	0.0206051	-2.89814
SKAP2	0.1104255	5.9880762	2.7829753	0.0058839	0.0206051	-2.898159
RNU6-1010F	0.3538046	-1.270957	2.7828691	0.0058858	0.0206096	-2.89844
GSKIP	0.0841494	6.0278056	2.782739	0.0058881	0.0206156	-2.898785
RN7SL205P	-0.29652	-1.387442	-2.782485	0.0058925	0.0206292	-2.899457
WBP1	-0.070621	5.6612787	-2.782315	0.0058955	0.0206364	-2.899908
KRT18	0.0727251	7.1883843	2.7823031	0.0058957	0.0206364	-2.89994
TBC1D7	-0.068127	5.9182275	-2.782141	0.0058985	0.0206443	-2.900371
RHPN1-AS1	-0.354924	3.8245118	-2.782005	0.0059009	0.0206507	-2.900731
HIST1H2AB	-0.55305	-0.481074	-2.781719	0.0059059	0.0206663	-2.901489
RP11-167N2	0.5758405	-0.801847	2.7816513	0.0059071	0.0206684	-2.901667
PCP4L1	-0.550072	4.136601	-2.781382	0.0059118	0.0206829	-2.90238
HLA-V	0.7191859	3.0073511	2.7812269	0.0059146	0.02069	-2.902791
NAP1L2	0.6740431	3.6645642	2.7812022	0.005915	0.02069	-2.902857
CTD-2576F9	-0.481457	2.3261387	-2.781152	0.0059159	0.0206911	-2.902989
AC103563.2	-0.662132	-0.598178	-2.781003	0.0059185	0.0206979	-2.903385
TTC30B	-0.118016	5.1475378	-2.780978	0.0059189	0.0206979	-2.903452
MRPL19	0.0549241	6.3105452	2.7809089	0.0059201	0.0207001	-2.903633
PAM16	0.0826438	5.6466639	2.7808235	0.0059216	0.0207031	-2.90386
LSM10	0.067507	6.0495882	2.7807954	0.0059221	0.0207031	-2.903934
KRTAP5-11	-0.331048	-1.348605	-2.780699	0.0059238	0.0207071	-2.90419
RP4-753P9.	-0.603747	0.9012777	-2.780525	0.0059269	0.0207157	-2.904649
AC123886.2	0.316665	-1.412018	2.7804763	0.0059278	0.0207168	-2.904779
RP11-291L2	-0.317893	3.3015237	-2.780278	0.0059312	0.0207264	-2.905305
HLA-DPB2	-0.670352	3.4806924	-2.780256	0.0059316	0.0207264	-2.905363
KCNJ10	-0.572709	3.553097	-2.779823	0.0059393	0.020751	-2.906509
HMGN2P5	-0.274932	3.2809939	-2.779678	0.0059418	0.0207579	-2.906892
TSEN15	-0.064856	6.0422166	-2.779644	0.0059424	0.020758	-2.906982
TLE1P1	-0.276287	4.3333653	-2.779238	0.0059496	0.0207811	-2.908058
RP11-137J7	-0.492619	-0.57202	-2.77912	0.0059517	0.0207864	-2.90837
CD109	0.2257994	5.5679398	2.7786978	0.0059591	0.0208104	-2.909487
RP11-7F17.	0.6821054	0.8745181	2.7785277	0.0059621	0.0208189	-2.909938

RP11-460N2	-0.58452	1.9382704	-2.778467	0.0059632	0.0208207	-2.910097
HCN4	0.883569	1.035929	2.7782512	0.005967	0.0208311	-2.910669
RP11-701I2	-0.267386	-1.357919	-2.778233	0.0059673	0.0208311	-2.910717
HMX2	-0.617695	-0.869166	-2.778155	0.0059687	0.020834	-2.910925
RP11-656D1	-0.447112	3.067432	-2.778023	0.005971	0.0208383	-2.911273
FAM161A	-0.175141	4.7810931	-2.778021	0.0059711	0.0208383	-2.91128
PTPLA	0.4883994	3.643942	2.7778848	0.0059735	0.020841	-2.911639
GUSBP1	-0.104822	5.06641	-2.777882	0.0059735	0.020841	-2.911647
RP11-369E1	0.4420789	-1.158332	2.777865	0.0059738	0.020841	-2.911691
RP11-4C20.	0.5339187	-0.771221	2.7778474	0.0059742	0.020841	-2.911738
RP11-208K4	0.3612535	-1.301341	2.7777544	0.0059758	0.0208448	-2.911984
RP11-236L1	0.6006655	1.7493499	2.7774879	0.0059805	0.020859	-2.912689
MAP3K12	0.1079949	5.1977321	2.7774596	0.005981	0.020859	-2.912764
LINC01489	0.316679	-1.361724	2.7773328	0.0059833	0.0208649	-2.913099
LRRC8B	0.1427139	5.6022627	2.7772615	0.0059845	0.0208673	-2.913288
RP11-242P2	-0.358205	-1.278898	-2.776921	0.0059906	0.0208864	-2.914187
CTD-2085J2	-0.317467	-1.311455	-2.776886	0.0059912	0.0208865	-2.914281
RP1-197B17	0.5251185	2.2757281	2.7767986	0.0059928	0.02089	-2.914512
SIX4	-0.584167	3.9184578	-2.776707	0.0059944	0.0208937	-2.914755
RP11-191L1	-0.56179	1.8427427	-2.77658	0.0059966	0.0208995	-2.915089
SRI	0.1036888	6.1382416	2.7759781	0.0060074	0.0209348	-2.916682
RP11-404P2	0.5365253	-0.891772	2.7759213	0.0060084	0.0209364	-2.916832
EXO5	-0.082341	5.3409668	-2.775745	0.0060115	0.0209439	-2.917297
RP11-15G16	0.0987285	-1.55936	2.7757346	0.0060117	0.0209439	-2.917326
PHF11	0.0657489	5.9369753	2.7755301	0.0060153	0.0209517	-2.917866
RASIP1	0.1086926	5.7184878	2.7755244	0.0060154	0.0209517	-2.917881
RP11-728F1	-0.942964	0.9049209	-2.775513	0.0060156	0.0209517	-2.917912
RP11-167N2	0.2069278	-1.50422	2.7754709	0.0060164	0.0209523	-2.918023
AZGP1P2	0.8253706	1.2064284	2.7753496	0.0060185	0.0209578	-2.918344
RP11-16801	-0.66585	-0.163666	-2.77488	0.0060269	0.0209851	-2.919586
BSN	-0.238578	4.9080681	-2.774685	0.0060304	0.0209952	-2.920101
C2orf48	-0.639285	2.2584265	-2.77452	0.0060334	0.0210029	-2.920537
USH1G	-0.787487	-0.136418	-2.774496	0.0060338	0.0210029	-2.9206
RP11-539G1	-0.298397	-1.311647	-2.77443	0.006035	0.0210044	-2.920775
CTA-223H9.	-0.658893	1.224686	-2.774408	0.0060354	0.0210044	-2.920833
RP11-162K6	-0.433115	-0.995636	-2.774102	0.0060408	0.0210214	-2.921642
RGPD8	-0.287156	3.9778186	-2.774052	0.0060417	0.0210225	-2.921772
UBA1	0.042302	6.9248425	2.7738654	0.0060451	0.0210321	-2.922266
PNPLA2	0.0673302	6.5859896	2.7738013	0.0060462	0.0210341	-2.922435
CTD-2589H1	-0.406174	3.8312694	-2.773548	0.0060508	0.0210472	-2.923106
CTD-2369P2	0.5698678	2.8351235	2.7735272	0.0060511	0.0210472	-2.92316
SNORD3B-2	-0.565963	-0.672174	-2.7734	0.0060534	0.0210531	-2.923496
EIF5AL1	0.3045465	4.3753858	2.7733276	0.0060547	0.0210556	-2.923687
C8orf44-SG	-0.610016	0.5250902	-2.773279	0.0060556	0.0210567	-2.923815
NDUFS2	0.065449	6.8455996	2.7725878	0.006068	0.0210978	-2.925641
CTD-2027I1	-0.623008	1.0011223	-2.772545	0.0060688	0.0210985	-2.925755
MIR6832	-0.372065	-1.112714	-2.772409	0.0060712	0.0211041	-2.926113
KHDC1L	-0.528939	-1.048766	-2.77239	0.0060715	0.0211041	-2.926164
HMGN2P6	-0.526081	-0.312504	-2.771834	0.0060815	0.0211368	-2.92763
RP11-22A3.	-0.520416	-0.70135	-2.770964	0.0060972	0.0211884	-2.929928
MCM9	-0.072676	5.5843591	-2.770946	0.0060975	0.0211884	-2.929975

TG	0.4721961	3.3010582	2.7709079	0.0060982	0.0211888	-2.930077
CTD-2130F2	0.2708533	-1.440565	2.7707985	0.0061002	0.0211937	-2.930365
GJB4	0.7131228	-0.303965	2.7699457	0.0061156	0.0212441	-2.932616
KCTD10	0.0462817	6.1879734	2.7699312	0.0061159	0.0212441	-2.932654
AC090154.1	-0.603974	1.289295	-2.769718	0.0061197	0.0212555	-2.933218
DUSP8P5	-0.308218	3.5191533	-2.769685	0.0061203	0.0212556	-2.933305
FHL5	0.6809838	2.5365425	2.7691542	0.00613	0.0212859	-2.934704
SNORA62	0.5152683	-0.968431	2.7691379	0.0061303	0.0212859	-2.934747
RP11-344B5	0.4873285	3.0880273	2.7690531	0.0061318	0.0212892	-2.934971
MRPL11	-0.06175	6.2539638	-2.768765	0.006137	0.0213047	-2.935731
HIST1H4D	-0.68981	0.6252854	-2.768742	0.0061374	0.0213047	-2.935791
LDLR	0.0876189	6.5308207	2.7686184	0.0061397	0.0213105	-2.936118
AC004870.4	0.8210317	-0.39972	2.7684494	0.0061428	0.0213192	-2.936564
RP11-177B4	-0.713224	0.8460835	-2.768204	0.0061472	0.0213318	-2.93721
RP5-1116H2	-0.57079	-0.098453	-2.768151	0.0061482	0.0213318	-2.93735
TGOLN2	0.0574239	6.9506181	2.7681244	0.0061487	0.0213318	-2.937421
RAB11FIP1	0.1896836	5.7458298	2.76812	0.0061487	0.0213318	-2.937432
RP11-190A1	0.2378299	-1.457395	2.7679739	0.0061514	0.021339	-2.937818
RP13-147D1	-0.74733	-0.623071	-2.767932	0.0061522	0.0213397	-2.937929
RP11-65J3.	-0.557196	-0.723235	-2.767856	0.0061535	0.02134	-2.93813
RP11-445P1	-0.685395	1.7730095	-2.767848	0.0061537	0.02134	-2.938149
CSPP1	-0.103912	5.8342643	-2.76783	0.006154	0.02134	-2.938198
MIR5690	0.2945888	-1.360177	2.7675188	0.0061597	0.0213576	-2.939018
ABAT	0.1740035	6.7351093	2.7671974	0.0061655	0.0213759	-2.939865
GPN1	-0.042848	6.1023698	-2.766991	0.0061693	0.0213869	-2.940409
ZNF559	-0.133082	5.3770085	-2.766764	0.0061734	0.0213992	-2.941009
RP11-787D1	-0.27381	-1.398578	-2.76638	0.0061804	0.0214215	-2.942019
ZNF106	0.05687	6.2140781	2.766314	0.0061817	0.0214236	-2.942194
SETP14	-0.518952	1.9495023	-2.766112	0.0061854	0.0214344	-2.942727
RP11-88E10	0.5933315	2.4566782	2.76603	0.0061868	0.0214376	-2.942942
FCER1A	0.5461477	3.7753643	2.7658947	0.0061893	0.0214441	-2.943299
AP000345.1	-0.477192	-1.06717	-2.765747	0.006192	0.0214514	-2.943688
SSX2IP	0.1440924	5.863533	2.7654042	0.0061983	0.0214711	-2.944591
XAGE5	-0.977108	0.0282954	-2.765297	0.0062003	0.0214759	-2.944873
DDX50	-0.051859	6.0832981	-2.765	0.0062057	0.0214927	-2.945657
PANK4	0.0617294	5.9104561	2.7647113	0.006211	0.021509	-2.946416
FAM127C	0.1893975	5.0395796	2.7641664	0.006221	0.0215416	-2.947852
MYH4	-1.268957	3.025191	-2.764044	0.0062233	0.0215473	-2.948173
IGLL4P	-0.740602	-0.480547	-2.76392	0.0062255	0.0215532	-2.9485
RNU4-1	-0.695199	0.3035225	-2.763761	0.0062285	0.0215613	-2.948918
SLC46A1	-0.101312	6.1364623	-2.763673	0.0062301	0.0215648	-2.949151
MYRFL	0.728465	2.1630475	2.7631739	0.0062393	0.0215946	-2.950465
RNU6-1061F	0.4257672	-1.072387	2.763041	0.0062417	0.021601	-2.950815
SETD5	-0.044869	6.4019086	-2.762931	0.0062437	0.021606	-2.951105
DDX59	-0.074167	5.9524246	-2.762691	0.0062482	0.0216193	-2.951736
LINC00645	-0.546521	-0.883931	-2.762545	0.0062509	0.0216265	-2.95212
OR1J4	-0.255861	-1.40738	-2.762401	0.0062535	0.0216332	-2.952499
ZNF85	-0.236551	4.4530668	-2.762376	0.006254	0.0216332	-2.952564
RP11-52402	-0.530348	-0.456697	-2.762139	0.0062584	0.0216463	-2.953188
AC006369.3	0.1206334	-1.548197	2.7618687	0.0062634	0.0216616	-2.953901
AC010987.6	0.3819877	-1.296428	2.7617433	0.0062657	0.0216675	-2.954231

GS1-309P15	-1.11306	0.3737644	-2.761707	0.0062663	0.0216678	-2.954326
GJB7	-0.629627	-0.57225	-2.761263	0.0062746	0.0216942	-2.955496
SLC25A5	0.0638345	6.9694173	2.7611881	0.006276	0.0216969	-2.955692
RPL18P13	-0.898046	0.651432	-2.761133	0.006277	0.0216979	-2.955838
MYO5C	-0.11637	5.8530497	-2.761108	0.0062774	0.0216979	-2.955903
CIDEC	0.6925249	3.9850276	2.7609267	0.0062808	0.0217075	-2.956379
RP11-147I3	-0.302859	3.8831874	-2.760682	0.0062853	0.0217205	-2.957022
RP13-463N1	0.5963303	-0.549811	2.7606301	0.0062863	0.0217205	-2.95716
MIR100HG	0.737556	2.3392517	2.7606268	0.0062864	0.0217205	-2.957168
ZNF808	-0.182607	5.0827992	-2.760449	0.0062897	0.0217299	-2.957636
TTC23L	-0.513934	2.3299036	-2.760267	0.006293	0.0217395	-2.958115
PSMA8	-0.653924	-0.55882	-2.759862	0.0063006	0.021762	-2.95918
RNU12	-0.613869	-0.061313	-2.759852	0.0063007	0.021762	-2.959205
CTD-233602	0.1184462	5.4151123	2.759708	0.0063034	0.0217692	-2.959585
RNF213	0.0640277	6.8174863	2.7595165	0.006307	0.0217775	-2.960089
PSMD10P2	-0.691528	2.0206293	-2.759514	0.006307	0.0217775	-2.960094
DYNLT3P2	-0.516493	-0.545974	-2.759193	0.006313	0.0217961	-2.960939
RP11-218E2	0.7820619	0.1935744	2.7591549	0.0063137	0.0217965	-2.961039
PTPRZ1	0.7857204	1.8193042	2.7589447	0.0063176	0.0218079	-2.961592
RPS13P2	-0.268508	3.687336	-2.758791	0.0063205	0.0218158	-2.961996
RP11-285G1	-0.62378	-0.244719	-2.758555	0.0063249	0.0218289	-2.962618
DPCR1	0.8106549	0.0949136	2.7585001	0.0063259	0.0218304	-2.962761
RP3-423B22	-0.560765	1.7082476	-2.75846	0.0063267	0.0218309	-2.962865
ZNF354C	-0.272226	4.487757	-2.758415	0.0063275	0.0218317	-2.962983
MTMR3	-0.093867	5.2763296	-2.758184	0.0063318	0.0218446	-2.963592
ECD	-0.044838	6.1134929	-2.758102	0.0063334	0.0218477	-2.963807
RP11-44503	-0.511454	-1.062235	-2.757988	0.0063355	0.0218529	-2.964106
TOB1	0.1003644	6.6088393	2.7579494	0.0063362	0.0218529	-2.964208
LTBR	0.0590336	6.7268387	2.7579254	0.0063367	0.0218529	-2.964271
CTD-3247F1	0.5936961	-0.315261	2.7578467	0.0063381	0.0218559	-2.964478
FAM195A	0.0930982	6.3982091	2.7576698	0.0063414	0.0218653	-2.964943
HLA-S	0.6169606	0.1682612	2.757609	0.0063426	0.0218671	-2.965103
CACTIN	-0.046979	5.9292293	-2.757544	0.0063438	0.0218693	-2.965273
RP11-60802	-0.896983	0.5141531	-2.757351	0.0063474	0.0218786	-2.965782
FBXO31	0.1044202	6.3162895	2.7573348	0.0063477	0.0218786	-2.965823
E2F5	-0.146843	5.3834494	-2.75668	0.00636	0.0219173	-2.967544
RPL7P20	-0.41025	-1.047072	-2.756672	0.0063601	0.0219173	-2.967564
ITGB2	0.1196701	6.3108703	2.7565096	0.0063632	0.0219257	-2.967991
C15orf38-A	0.5413654	1.7889474	2.7563616	0.0063659	0.0219332	-2.96838
MCF2	0.6664198	0.9248543	2.7561918	0.0063691	0.0219421	-2.968826
PRRC2C	-0.05139	6.6731819	-2.756154	0.0063698	0.0219425	-2.968925
ITLN1	0.8417125	1.9455769	2.7558977	0.0063747	0.021957	-2.969599
KIAA0319L	0.0555271	6.5740548	2.7554908	0.0063823	0.0219813	-2.970667
GPR114	-0.287891	5.0152963	-2.75534	0.0063851	0.0219876	-2.971063
FBXO36	0.1850898	4.703075	2.7553291	0.0063853	0.0219876	-2.971092
RP11-283G6	0.7469788	0.3079545	2.7548175	0.006395	0.0220187	-2.972435
CTC-510F12	-0.387639	2.7707415	-2.754779	0.0063957	0.0220191	-2.972536
CTD-3099C6	-0.474253	-1.041326	-2.754496	0.006401	0.0220354	-2.973279
EP400	-0.047794	6.2212105	-2.754376	0.0064033	0.0220411	-2.973595
CTD-2263F2	0.6895276	-0.384168	2.7543331	0.0064041	0.0220418	-2.973707
CTD-3116E2	0.667097	0.9710968	2.754061	0.0064092	0.0220574	-2.974421

RP5-902P8.	-0.281848	3.279186	-2.753996	0.0064105	0.0220585	-2.974592
LYPLA2P2	-0.510933	-0.604217	-2.753977	0.0064108	0.0220585	-2.974641
STARD13-AS	-0.534171	1.8044839	-2.753949	0.0064114	0.0220585	-2.974716
RP11-231N3	0.376267	-1.323287	2.7538735	0.0064128	0.0220613	-2.974913
NISCH	-0.04413	6.4379505	-2.75379	0.0064144	0.0220646	-2.975131
CALML3-AS1	-0.591881	2.4717693	-2.753734	0.0064154	0.0220662	-2.97528
RP11-314N2	0.5073752	-0.978916	2.7533797	0.0064221	0.0220871	-2.976209
CTD-2616J1	-0.593174	-0.090083	-2.753307	0.0064235	0.0220898	-2.976401
OVOL1	-0.898761	2.2949916	-2.752943	0.0064304	0.0221114	-2.977355
NDUFC1	0.0665503	6.244201	2.7528763	0.0064316	0.0221137	-2.97753
SUSD3	0.2533317	5.4170761	2.7528348	0.0064324	0.0221143	-2.977639
LILRB1	0.1964891	5.0916026	2.7527572	0.0064339	0.0221173	-2.977842
RP11-702B1	0.3102189	-1.310239	2.752717	0.0064347	0.0221178	-2.977948
CELSR3	-0.24383	5.1531902	-2.752611	0.0064367	0.0221226	-2.978226
HPS4	-0.054938	6.0950203	-2.752504	0.0064387	0.0221275	-2.978507
ZNF783	-0.110964	5.3600641	-2.752306	0.0064425	0.0221383	-2.979026
SLX4IP	-0.076664	5.5798886	-2.752265	0.0064432	0.0221389	-2.979133
OGG1	-0.072078	6.040307	-2.752026	0.0064478	0.0221524	-2.979762
UTP11L	0.050837	6.0147131	2.751747	0.0064531	0.0221685	-2.980492
RP11-536G4	-0.296842	-1.365379	-2.751689	0.0064542	0.0221691	-2.980644
GK	0.1354385	6.1235307	2.7516746	0.0064544	0.0221691	-2.980682
RP11-109P1	0.2858443	-1.394834	2.7513608	0.0064604	0.0221875	-2.981505
CHI3L1	0.3645601	6.4422506	2.7512612	0.0064623	0.0221919	-2.981766
CTD-3010D2	-0.763849	-0.295095	-2.751029	0.0064667	0.022205	-2.982374
LAMP1	0.0659425	7.058664	2.7508357	0.0064704	0.022214	-2.982882
CLDN18	-0.849719	1.6920021	-2.750818	0.0064707	0.022214	-2.982928
PANK2	-0.046742	6.013015	-2.750796	0.0064712	0.022214	-2.982987
CTB-35F21.	0.2850707	-1.35551	2.7506638	0.0064737	0.0222205	-2.983333
XKR9	0.6627405	3.7286776	2.7505641	0.0064756	0.0222249	-2.983594
NBEAL1	0.111613	5.4852334	2.750514	0.0064765	0.0222261	-2.983725
XXbac-BPG2	0.1995005	-1.508005	2.7503298	0.00648	0.0222361	-2.984208
KLHL6	0.1772576	5.0914468	2.7502695	0.0064812	0.0222379	-2.984366
CXCR1	0.6803803	2.2467339	2.7502331	0.0064819	0.0222382	-2.984462
MUC17	-0.594105	-0.770474	-2.750186	0.0064828	0.0222392	-2.984586
FGF14-IT1	0.5139595	-0.932599	2.749939	0.0064875	0.0222518	-2.985233
RPS27AP1	-0.57061	-0.294652	-2.74993	0.0064877	0.0222518	-2.985256
UTY	1.2429855	2.9050458	2.7498663	0.0064889	0.0222533	-2.985423
SPTSSB	-0.724327	2.9883337	-2.749843	0.0064893	0.0222533	-2.985484
CLSPN	-0.337834	4.4924267	-2.749753	0.006491	0.0222571	-2.985719
OR5H2	-0.340454	-1.343992	-2.749367	0.0064984	0.0222803	-2.986731
PUS7	-0.08001	5.7948264	-2.748746	0.0065103	0.0223189	-2.988358
RP11-440G5	-0.689842	1.510653	-2.74856	0.0065139	0.0223291	-2.988847
MIR3682	-0.402346	3.5295612	-2.747949	0.0065256	0.0223671	-2.990447
CD53	0.1469261	5.9009657	2.7478374	0.0065277	0.0223724	-2.990739
UBL7-AS1	-0.148488	4.5167712	-2.747696	0.0065304	0.0223795	-2.991108
CTD-2014B1	0.4201562	-1.107996	2.7476196	0.0065319	0.0223825	-2.99131
RP11-365F1	-0.577375	-0.277878	-2.747574	0.0065328	0.0223828	-2.99143
NPIPA1	-0.140402	4.8837914	-2.747549	0.0065333	0.0223828	-2.991495
CARM1	-0.059421	6.1974578	-2.747508	0.0065341	0.0223828	-2.991603
RP11-22B23	-0.643236	-0.00326	-2.747487	0.0065345	0.0223828	-2.991657
HOXA9	-0.755591	0.9074629	-2.747364	0.0065368	0.022388	-2.991978

RP11-767C1	-0.624527	0.7973918	-2.747345	0.0065372	0.022388	-2.992029
SH3D21	-0.183078	4.728441	-2.74698	0.0065442	0.0224099	-2.992984
AC018463.5	-0.542769	-0.292735	-2.746554	0.0065524	0.0224359	-2.994099
C5orf30	0.2900587	4.9029219	2.746281	0.0065577	0.0224518	-2.994815
PACRG	0.4573356	3.9437896	2.7461137	0.0065609	0.0224607	-2.995253
ZNRD1-AS1	-0.138743	4.8220476	-2.745611	0.0065706	0.0224918	-2.996568
JOSD1	0.0521287	6.3575054	2.7455301	0.0065722	0.0224951	-2.99678
RAB2A	0.0604265	6.7274286	2.7452037	0.0065785	0.0225145	-2.997634
LINC00941	-0.769386	2.174067	-2.745055	0.0065813	0.0225223	-2.998024
TM4SF5	-0.366661	6.5609289	-2.744994	0.0065825	0.0225242	-2.998184
QARS	-0.051466	6.7501296	-2.744761	0.006587	0.0225375	-2.998792
RNU6-678P	-0.430177	-0.880286	-2.743882	0.0066041	0.0225937	-3.001093
CERS3-AS1	0.6402778	-0.550314	2.7438495	0.0066047	0.0225937	-3.001177
RP11-290D2	-0.394788	-1.066975	-2.743669	0.0066082	0.0226036	-3.001651
SAFB	-0.037796	6.5268038	-2.743019	0.0066208	0.0226416	-3.003349
DUTP6	-0.33621	3.1117411	-2.742996	0.0066213	0.0226416	-3.003409
EIF2S2	-0.0466	6.5433695	-2.742987	0.0066214	0.0226416	-3.003432
CLIP2	0.1524116	5.887942	2.7429675	0.0066218	0.0226416	-3.003484
SSUH2	-0.448486	4.6983144	-2.742937	0.0066224	0.0226416	-3.003563
ZNF277	0.0673945	6.0433154	2.7427224	0.0066266	0.0226538	-3.004125
RP11-472G2	0.6522998	-0.085871	2.7426042	0.0066289	0.0226577	-3.004434
RPL7P47	-0.576716	2.0094759	-2.7426	0.006629	0.0226577	-3.004446
ZZZ3	0.0510281	6.114401	2.742164	0.0066375	0.0226846	-3.005585
ACY3	0.3009959	5.5945798	2.7419991	0.0066407	0.0226934	-3.006016
RNU6-118P	-0.590555	0.1072001	-2.741968	0.0066413	0.0226934	-3.006098
CACNB4	-0.464491	3.3056578	-2.741903	0.0066425	0.0226956	-3.006268
RPS20P33	-0.560692	1.2720988	-2.741781	0.0066449	0.0227016	-3.006587
RP11-424D1	0.0987285	-1.55936	2.7413751	0.0066528	0.0227245	-3.007647
TPT1P13	-0.412984	-1.060573	-2.741374	0.0066529	0.0227245	-3.007651
RP11-742B1	-0.903865	0.8255297	-2.741227	0.0066557	0.0227321	-3.008033
CTB-78F1.2	0.9066041	0.7000594	2.7407588	0.0066649	0.0227613	-3.009257
ZNF585A	-0.138963	5.0560042	-2.740651	0.006667	0.0227664	-3.00954
EEF1A1P25	-0.511482	1.897428	-2.74034	0.0066731	0.022785	-3.010352
MZT2B	-0.070488	6.4311421	-2.740285	0.0066742	0.0227866	-3.010496
RP11-93O01	0.4777617	-0.876928	2.7399891	0.00668	0.0228042	-3.011268
MAPK8IP1	0.1597304	5.3582487	2.7397163	0.0066853	0.0228187	-3.011981
RP11-102K1	-0.632067	1.3834517	-2.739709	0.0066855	0.0228187	-3.012001
ZNF584	0.0787607	5.4648208	2.7394921	0.0066897	0.0228311	-3.012567
CHIC1	0.255277	4.837619	2.739426	0.006691	0.0228334	-3.012739
GPR137	0.063159	6.1818393	2.7389991	0.0066994	0.0228599	-3.013854
CSAG1	-1.101536	1.4064211	-2.73879	0.0067035	0.0228717	-3.014399
PLA2G2E	-0.380133	-1.34186	-2.738591	0.0067074	0.022883	-3.014921
RP11-6I2.3	0.6355476	0.4093902	2.7383353	0.0067125	0.022898	-3.015587
RP11-136C2	0.7195614	0.4856009	2.7382587	0.006714	0.022901	-3.015787
H1FNT	-0.577409	-0.698782	-2.737913	0.0067208	0.0229217	-3.016689
CTB-79E8.3	-0.454796	3.2149045	-2.737886	0.0067213	0.0229217	-3.01676
CTD-2014D2	-0.647156	0.0254353	-2.737618	0.0067266	0.0229377	-3.017461
FRMPD4	0.5437405	-0.886398	2.7374562	0.0067298	0.0229464	-3.017882
ERICH3-AS1	-0.290128	-1.390576	-2.73715	0.0067358	0.0229648	-3.018681
RNU2-11P	-0.580192	-0.238357	-2.736949	0.0067398	0.0229763	-3.019207
RP11-574H6	0.1750752	-1.520453	2.7368289	0.0067422	0.0229822	-3.019519

RP13-122B2	0.4157313	3.0909809	2.736779	0.0067432	0.0229834	-3.01965
RPL23	-0.061561	7.1170023	-2.736441	0.0067498	0.023004	-3.020531
RNU1-70P	-0.480942	5.5690159	-2.736384	0.006751	0.0230058	-3.020681
USP32P1	0.8486866	0.3401251	2.736072	0.0067571	0.0230246	-3.021494
GLUD1P7	-0.32132	3.3250107	-2.735937	0.0067598	0.0230316	-3.021846
AC024995.1	-0.613614	-0.159065	-2.735844	0.0067617	0.0230347	-3.02209
RP11-264B1	0.5077389	-0.728565	2.735827	0.006762	0.0230347	-3.022133
POLE4	0.0833006	6.0203747	2.7356883	0.0067647	0.0230419	-3.022495
FTSJ2	-0.047226	6.1176128	-2.735359	0.0067713	0.02306	-3.023354
KRT79	0.524355	-0.881981	2.7353569	0.0067713	0.02306	-3.023359
RPL31P49	-0.582205	0.1600653	-2.735173	0.006775	0.0230703	-3.023839
AC018799.1	0.6328811	-0.748971	2.7350303	0.0067778	0.0230778	-3.024211
LIPM	0.8120774	0.6209156	2.7347915	0.0067825	0.0230918	-3.024834
RP11-643M1	-0.629695	0.9805685	-2.734629	0.0067858	0.0231006	-3.025257
OTUD5	0.0457626	6.4218327	2.7345231	0.0067879	0.0231056	-3.025534
KRT17P7	0.4448825	-1.052817	2.7343889	0.0067906	0.0231126	-3.025884
TACR1	0.771884	1.9730776	2.7342955	0.0067924	0.0231167	-3.026127
C20orf62	0.4404318	-1.102384	2.734133	0.0067956	0.0231256	-3.026551
ATP5F1	0.0539352	6.682973	2.7339176	0.0067999	0.023138	-3.027113
SCOC	0.0546537	6.286672	2.7338331	0.0068016	0.0231416	-3.027333
CTD-2165H1	-0.363843	3.0812155	-2.733706	0.0068041	0.0231481	-3.027664
ST6GALNAC4	0.1235688	5.6137632	2.7331879	0.0068145	0.0231811	-3.029015
PXT1	-0.582975	0.3545524	-2.733086	0.0068165	0.0231858	-3.02928
LINC00551	0.4933226	-1.011781	2.7328918	0.0068204	0.0231969	-3.029786
ALX1	-0.743007	-0.556498	-2.732806	0.0068221	0.0232005	-3.030009
CTD-3080P1	-0.565709	4.1082525	-2.732427	0.0068297	0.023223	-3.030998
PLEKHG6	-0.241263	5.4562852	-2.732411	0.00683	0.023223	-3.031039
AC093724.2	-0.466753	3.074713	-2.732252	0.0068332	0.0232317	-3.031453
RP11-467D1	0.2104042	-1.479685	2.7321688	0.0068349	0.0232352	-3.03167
TAS2R64P	-0.599727	-0.306578	-2.732104	0.0068362	0.0232374	-3.03184
RP11-793A3	0.5597211	1.3035672	2.7317707	0.0068428	0.0232579	-3.032707
RBM19	-0.049316	6.1577912	-2.73165	0.0068452	0.023264	-3.033023
MCOLN2	0.4058879	3.9282011	2.7316131	0.006846	0.0232644	-3.033118
SNRPD3	-0.055908	6.5773153	-2.731551	0.0068472	0.0232664	-3.03328
RP11-505K9	0.2633502	4.0901414	2.7313772	0.0068507	0.0232761	-3.033732
RP11-80H5.	-0.258097	-1.384379	-2.731209	0.0068541	0.0232854	-3.034172
TYW5	-0.059278	5.4671739	-2.731069	0.0068569	0.0232927	-3.034534
FTH1P14	-0.375528	-1.283883	-2.731035	0.0068576	0.0232929	-3.034624
AC079779.6	0.3044226	-1.296123	2.7309501	0.0068593	0.0232965	-3.034845
KIR3DX1	-0.630319	0.6694094	-2.730818	0.0068619	0.0233033	-3.035187
RP11-452K1	-0.580206	2.3751077	-2.730648	0.0068653	0.0233128	-3.035631
RP11-153F5	-0.456655	2.7573641	-2.730541	0.0068675	0.023318	-3.035911
AP000473.5	0.666322	2.2357978	2.7303308	0.0068717	0.0233301	-3.036457
LINC01249	-0.61577	-0.956468	-2.730066	0.006877	0.0233444	-3.037147
RP11-295D4	0.4210909	3.6634081	2.7300588	0.0068772	0.0233444	-3.037166
PLA2G4F	-0.895993	1.0034325	-2.729917	0.00688	0.0233517	-3.037534
TRMT2A	-0.058176	6.285842	-2.729878	0.0068808	0.0233517	-3.037636
IGHJ3P	-0.66398	-0.406672	-2.729856	0.0068813	0.0233517	-3.037693
PAGR1	-0.076581	5.4624487	-2.729731	0.0068838	0.0233581	-3.038019
RP11-46H11	-0.621725	1.4081195	-2.729656	0.0068853	0.0233589	-3.038214
IL36RN	0.4182197	-1.205003	2.7296553	0.0068853	0.0233589	-3.038216

EIF4A1P5	-0.434875	-0.884704	-2.729577	0.0068869	0.0233621	-3.038419
RP11-3L21.	0.6949374	-0.473603	2.729517	0.0068881	0.023364	-3.038576
TTC31	-0.065333	6.1946371	-2.729131	0.0068959	0.0233882	-3.03958
PDYN	-0.33742	-1.279765	-2.728974	0.0068991	0.0233968	-3.03999
RP11-650L1	0.7416251	1.104071	2.7287759	0.0069031	0.0234066	-3.040505
RP11-356J5	0.2787764	-1.353785	2.7287675	0.0069032	0.0234066	-3.040527
RP4-684024	-0.377012	-1.144039	-2.728692	0.0069048	0.0234096	-3.040724
PITHD1	0.0643477	6.1934596	2.7283579	0.0069115	0.0234303	-3.041593
RP11-181G1	-0.687087	0.67993	-2.728063	0.0069175	0.0234484	-3.042361
EIF1B	-0.052289	6.2231501	-2.727817	0.0069224	0.023463	-3.043
RPL24P8	-0.423487	2.9478807	-2.727474	0.0069294	0.0234844	-3.043892
RP11-439E1	-0.672603	0.1632384	-2.727048	0.006938	0.0235115	-3.044999
RP11-191G2	0.5867598	2.2070657	2.7269783	0.0069394	0.0235141	-3.045181
CH507-154E	-0.900227	1.5156512	-2.726916	0.0069407	0.0235162	-3.045344
RP11-173E2	0.1906132	-1.512535	2.7268106	0.0069428	0.0235213	-3.045618
LINC01254	-0.372078	-1.332219	-2.726734	0.0069444	0.0235243	-3.045815
JAKMIP3	0.6509281	2.9740544	2.7266641	0.0069458	0.0235257	-3.045999
AC064850.4	-0.552446	-0.406177	-2.72665	0.0069461	0.0235257	-3.046035
RP11-542B1	0.5690215	-0.713041	2.7265645	0.0069478	0.0235277	-3.046258
RP11-148K1	-0.276631	3.9347501	-2.726558	0.006948	0.0235277	-3.046274
CTD-2240J1	-0.62403	0.1549523	-2.726488	0.0069494	0.0235304	-3.046457
TSPAN13	0.2073518	6.0117416	2.7263653	0.0069519	0.0235366	-3.046776
ROPN1L	0.5451519	1.6488673	2.7262801	0.0069536	0.0235403	-3.046997
GSR	0.0740328	6.5386674	2.7261088	0.0069571	0.0235499	-3.047442
AC005329.7	-0.610319	2.9152223	-2.725958	0.0069602	0.0235581	-3.047835
RP5-877J2.	0.4734912	-0.798649	2.7258459	0.0069625	0.0235636	-3.048126
RP4-543J13	-0.322752	-1.326188	-2.725565	0.0069682	0.0235808	-3.048856
AC144652.1	0.2099497	4.4866866	2.7253858	0.0069718	0.023591	-3.049322
AP001601.2	-0.392515	-1.302462	-2.725171	0.0069762	0.0236036	-3.04988
CTD-2509G1	-0.315478	-1.26062	-2.725004	0.0069796	0.0236129	-3.050315
NPC1	-0.060642	6.2705292	-2.724889	0.0069819	0.0236186	-3.050612
AC011516.2	-0.361649	-1.310646	-2.724821	0.0069833	0.0236191	-3.050791
PTX3	0.5226883	3.4059835	2.724819	0.0069834	0.0236191	-3.050795
TMEM248	0.0365241	6.6691874	2.7246583	0.0069867	0.023628	-3.051213
C8orf59	-0.081803	6.1506565	-2.72451	0.0069897	0.0236361	-3.051598
FBXO25	0.092237	6.2159019	2.7243725	0.0069925	0.0236421	-3.051955
PCDH9-AS2	0.6374576	-0.816251	2.7243313	0.0069933	0.0236421	-3.052063
AOC3	0.1119644	5.9414392	2.7243279	0.0069934	0.0236421	-3.052071
AC009403.2	-0.111158	5.3706846	-2.724073	0.0069986	0.0236575	-3.052734
RP11-1038A	-0.939317	0.3011562	-2.723918	0.0070018	0.0236652	-3.053136
BVES	0.3313366	3.7743046	2.7238986	0.0070022	0.0236652	-3.053187
TMED3	-0.171085	6.1781691	-2.723783	0.0070046	0.023671	-3.053488
ACIN1	-0.035827	6.5865065	-2.723567	0.007009	0.0236837	-3.054047
RP11-598F7	-0.350206	3.5352633	-2.723444	0.0070115	0.02369	-3.054367
XKR8	-0.078237	5.8679678	-2.723237	0.0070157	0.0237022	-3.054906
C20orf166-	0.6959314	0.2378729	2.7231656	0.0070172	0.0237049	-3.055091
AC246787.3	-0.511561	-0.925504	-2.722702	0.0070267	0.0237348	-3.056295
ABCA7	0.1289809	5.7293247	2.7224476	0.0070319	0.0237503	-3.056956
GFAP	0.6974563	2.6664592	2.7223312	0.0070343	0.0237558	-3.057258
CCDC130	-0.062536	6.0022658	-2.722305	0.0070348	0.0237558	-3.057326
ARHGEF40	-0.067792	6.52521	-2.722116	0.0070387	0.0237667	-3.057817

AC007229.3	-0.401693	2.3893571	-2.722043	0.0070402	0.0237696	-3.058007
RPL10P15	-0.579979	1.8802229	-2.721969	0.0070418	0.0237725	-3.058199
SIMC1	0.1438697	5.6823534	2.7217924	0.0070454	0.0237826	-3.058657
TLR6	0.2108001	4.6167622	2.7216946	0.0070474	0.0237871	-3.05891
RP11-10J18	-0.338144	-1.235341	-2.721193	0.0070577	0.0238198	-3.060214
RP11-531A2	-0.337987	3.2072654	-2.721053	0.0070606	0.0238273	-3.060575
ZSCAN26	-0.079948	5.5527928	-2.720985	0.007062	0.0238299	-3.060752
ZNF317P1	-0.309495	-1.381079	-2.720942	0.0070629	0.0238307	-3.060865
RP5-855D21	-0.555167	1.7628701	-2.720703	0.0070678	0.0238451	-3.061484
LRRC41	0.0425271	6.4365076	2.7206664	0.0070686	0.0238454	-3.061579
RP5-854E16	-0.56753	0.0204155	-2.720599	0.00707	0.0238479	-3.061755
AC006373.1	0.4179425	-1.181504	2.7203077	0.007076	0.023866	-3.06251
RP11-158I9	0.3483605	-1.17962	2.7202629	0.0070769	0.0238669	-3.062626
DOK1	0.1114137	5.3153286	2.7197253	0.007088	0.0239018	-3.064021
C4orf29	0.0818389	5.6114278	2.7196983	0.0070886	0.0239018	-3.064091
PES1P1	-0.832652	0.0332313	-2.719593	0.0070907	0.0239051	-3.064364
SLC2A3	0.1511384	5.7437036	2.7195878	0.0070909	0.0239051	-3.064378
NPRL3	0.0651248	6.1716328	2.7195273	0.0070921	0.0239071	-3.064535
FMO4	0.1681318	5.8160418	2.7194352	0.007094	0.0239105	-3.064774
RP11-2E11.	-0.567107	0.0235899	-2.719416	0.0070944	0.0239105	-3.064825
RP11-430L3	0.1652877	-1.525441	2.7188694	0.0071057	0.0239464	-3.066241
CNOT8	0.0380118	6.1891812	2.7187916	0.0071074	0.0239497	-3.066443
RP11-443B7	0.6517043	0.684252	2.7183081	0.0071174	0.0239813	-3.067697
SPG11	0.0560862	6.3549628	2.7179918	0.0071239	0.0240012	-3.068517
RPL39P38	-0.619729	0.5606037	-2.717914	0.0071256	0.0240044	-3.068718
RP1-140J1.	-0.386096	-1.137881	-2.717838	0.0071271	0.0240075	-3.068915
CCDC86	-0.070824	5.9931653	-2.717784	0.0071283	0.0240091	-3.069055
SEH1L	-0.046624	6.2003888	-2.716881	0.0071471	0.0240701	-3.071397
RP11-109A6	-0.567276	-0.211098	-2.716844	0.0071478	0.0240705	-3.071493
KRT16P2	0.6225013	-0.784554	2.716587	0.0071532	0.0240863	-3.072159
RP11-452H2	-0.629388	0.7738912	-2.71651	0.0071548	0.0240895	-3.072359
LINC00271	0.6627447	1.5041891	2.716373	0.0071577	0.0240969	-3.072713
RP5-1028L1	0.1993967	-1.508058	2.7159842	0.0071658	0.024122	-3.073721
EIF3B	-0.05151	6.8412383	-2.715664	0.0071725	0.0241423	-3.07455
GAGE12J	-0.450862	-1.201182	-2.715515	0.0071756	0.0241505	-3.074935
RP11-512M8	-0.432234	-0.91656	-2.715453	0.0071769	0.0241527	-3.075096
PYGM	0.2498111	3.8947644	2.7152105	0.0071819	0.0241676	-3.075725
RP11-515E2	-0.425917	-0.95699	-2.715164	0.0071829	0.0241686	-3.075845
RP11-74M13	0.7668073	1.1715806	2.7149236	0.007188	0.0241833	-3.076468
MIR1915	-0.412583	-1.081312	-2.714824	0.00719	0.0241867	-3.076727
CTD-2161F6	-0.290564	-1.412267	-2.714812	0.0071903	0.0241867	-3.076757
AC016683.5	-0.38142	-1.075115	-2.714583	0.0071951	0.0242007	-3.077351
MAOA	0.1100931	6.6535122	2.7143328	0.0072003	0.0242161	-3.077998
PTK2B	0.091998	5.9115897	2.7139678	0.007208	0.0242396	-3.078943
RP11-268P4	-0.437357	-0.997777	-2.71385	0.0072105	0.0242444	-3.079248
HCK	0.1503366	5.6214158	2.7138358	0.0072108	0.0242444	-3.079285
FZD6	0.1795351	5.6441961	2.7131618	0.0072249	0.0242897	-3.08103
RPL30	-0.069751	7.1871578	-2.713133	0.0072255	0.0242897	-3.081105
EEF1A1P5	-0.154024	5.6704379	-2.71303	0.0072277	0.0242927	-3.081372
MGAT4C	0.7457527	2.780293	2.713027	0.0072278	0.0242927	-3.081379
SSX2B	-0.506967	-1.099255	-2.712705	0.0072346	0.0243133	-3.082214

HNRNPA1P35	-0.556565	1.4516578	-2.712419	0.0072406	0.0243313	-3.082953
FIS1	0.069626	6.6574571	2.7123829	0.0072413	0.0243316	-3.083046
ZMYM6	0.0480067	5.5856183	2.7122804	0.0072435	0.0243346	-3.083311
RPL3P12	-0.461483	-0.82497	-2.712277	0.0072436	0.0243346	-3.083321
HSPBAP1	0.0800047	5.4260892	2.7120223	0.007249	0.0243504	-3.083979
RP11-67H24	-0.264896	-1.381045	-2.711671	0.0072564	0.0243713	-3.084888
UPK3B	0.4242738	3.5553101	2.7116641	0.0072565	0.0243713	-3.084906
PKD1L2	0.5956385	3.7236639	2.7111188	0.007268	0.0244065	-3.086317
TMEM147-AS	-0.126285	5.0386516	-2.711105	0.0072683	0.0244065	-3.086352
LKAAEAR1	-0.714	-0.135677	-2.71106	0.0072693	0.0244074	-3.086467
STX6	-0.05713	6.0451596	-2.71092	0.0072723	0.0244152	-3.086832
CTD-2140G1	-0.502297	-0.939448	-2.710601	0.007279	0.0244356	-3.087656
RP4-798A10	-0.687728	0.7607368	-2.710432	0.0072826	0.0244452	-3.088093
RP11-4K3__	-0.56678	0.2436398	-2.710397	0.0072833	0.0244452	-3.088183
STARD13	0.0901269	5.7627349	2.710372	0.0072839	0.0244452	-3.088248
RP11-615J4	-0.448465	-1.234636	-2.710287	0.0072857	0.024449	-3.088469
OLIG3	-0.578497	-1.052661	-2.709896	0.0072939	0.0244745	-3.089478
DYNLL1P4	-0.450665	-0.9656	-2.709865	0.0072946	0.0244745	-3.08956
RNA5SP507	-0.399899	-1.288357	-2.709639	0.0072994	0.0244884	-3.090144
PRR7-AS1	-0.623436	1.950564	-2.709523	0.0073019	0.0244944	-3.090443
FAM85B	0.6349547	-0.172255	2.709361	0.0073053	0.0245037	-3.090862
CCDC13	0.4191522	3.4426757	2.7092027	0.0073087	0.0245127	-3.091271
KRTAP19-3	-0.289202	-1.386451	-2.709103	0.0073108	0.0245176	-3.091529
NR2F2-AS1	0.2236941	3.921666	2.7090031	0.0073129	0.0245224	-3.091787
RP4-800G7.	-0.495265	2.0829847	-2.708964	0.0073138	0.0245229	-3.091888
RP11-61011	-0.447422	-1.247727	-2.708737	0.0073186	0.0245369	-3.092475
TTC6	-0.376901	4.716857	-2.708685	0.0073197	0.0245373	-3.092608
RP11-212I2	0.7585774	1.4825485	2.7086678	0.0073201	0.0245373	-3.092654
CTD-2061E1	-0.570974	-0.257248	-2.708369	0.0073264	0.0245536	-3.093425
AC106786.1	0.7321879	0.6942154	2.7083661	0.0073265	0.0245536	-3.093434
SAMSN1	0.2054209	5.0615695	2.7082999	0.0073279	0.0245536	-3.093605
AFAP1L1	0.0922612	5.7302481	2.7082871	0.0073282	0.0245536	-3.093638
HIST3H3	0.2215671	-1.446473	2.7082816	0.0073283	0.0245536	-3.093652
RP11-128N1	-0.597891	-0.117949	-2.707388	0.0073474	0.0246153	-3.09596
TMEM88	0.171359	4.8657677	2.7071939	0.0073515	0.0246269	-3.096462
SNORA34	-0.535957	-0.287789	-2.706849	0.0073589	0.0246494	-3.097354
RHBDF1	0.0997161	5.8129262	2.7067529	0.0073609	0.0246525	-3.097601
FBXO22	-0.054931	6.1872343	-2.706742	0.0073612	0.0246525	-3.097629
LRP4	-0.282774	4.6000817	-2.706621	0.0073638	0.0246589	-3.097941
SEC14L1P1	0.4987275	2.4378184	2.7060097	0.0073769	0.0246991	-3.099521
RP11-255H2	-0.841411	2.2254989	-2.705998	0.0073771	0.0246991	-3.099551
MYO3B	0.8011292	1.7851651	2.7059021	0.0073792	0.0247037	-3.099798
RP11-354M2	-0.400323	-1.032408	-2.705196	0.0073943	0.0247505	-3.101622
LDHAP7	0.6908215	3.0122411	2.7051878	0.0073945	0.0247505	-3.101643
ANKRD20A17	-0.574428	-0.801602	-2.705135	0.0073956	0.024752	-3.101779
SLC35D1	0.1004376	6.4243675	2.7050422	0.0073976	0.0247548	-3.102018
DCP1B	-0.070318	5.7003603	-2.705008	0.0073984	0.0247548	-3.102107
TRIM31	-0.444324	5.2021895	-2.705002	0.0073985	0.0247548	-3.102122
SSTR5-AS1	-1.148519	2.1232964	-2.70497	0.0073992	0.0247548	-3.102205
AC109826.1	0.5388852	2.3315193	2.7048605	0.0074015	0.0247604	-3.102487
GRM7-AS2	-0.227249	-1.421411	-2.704803	0.0074028	0.0247623	-3.102635

GRK6	-0.060719	6.1570413	-2.704731	0.0074043	0.0247652	-3.102823
EXTL1	0.6319577	2.0965775	2.7042558	0.0074146	0.0247971	-3.104048
CISD2	0.0574265	6.3010966	2.7041535	0.0074168	0.0248022	-3.104312
RP11-244B2	-0.577619	-1.017658	-2.704122	0.0074174	0.0248022	-3.104392
RP1-29C18.	0.7341446	1.611551	2.7039532	0.0074211	0.0248121	-3.104829
ACTG1P12	-0.403102	-1.071435	-2.70352	0.0074304	0.0248402	-3.105946
AFG3L2	0.0565233	6.4020015	2.7035012	0.0074308	0.0248402	-3.105995
RP11-274H2	-0.413994	-1.094892	-2.703465	0.0074316	0.0248405	-3.106088
RP11-6E9.4	0.1252129	-1.545864	2.7033492	0.0074341	0.0248459	-3.106387
RP11-570G2	-0.53101	-0.256916	-2.703327	0.0074346	0.0248459	-3.106445
DKFZP434L1	-0.410619	-1.197515	-2.703101	0.0074395	0.02486	-3.107028
DNM3	0.1685208	4.6702214	2.7030396	0.0074408	0.0248621	-3.107186
SUCLG2P4	-0.332888	-1.255503	-2.703007	0.0074415	0.0248622	-3.107269
RP11-16E23	-0.551009	-0.146253	-2.70276	0.0074468	0.0248778	-3.107907
BOK	0.1399585	6.4302717	2.7026985	0.0074482	0.0248799	-3.108066
ATP9A	-0.10055	6.361274	-2.702652	0.0074492	0.024881	-3.108185
RP11-458F8	-0.483642	-0.661669	-2.702617	0.0074499	0.0248813	-3.108275
MED13	-0.057209	6.3909234	-2.702483	0.0074528	0.0248887	-3.108621
FAM151B	0.1469089	4.0511422	2.7015663	0.0074727	0.0249527	-3.110985
GVQW1	0.2098706	3.6031928	2.7013505	0.0074774	0.0249661	-3.111542
H1FX	-0.073099	6.5213921	-2.701152	0.0074817	0.0249782	-3.112053
KIAA1033	0.0529138	6.299474	2.7008402	0.0074885	0.0249985	-3.112857
UFM1	0.0563816	6.3860936	2.70059	0.0074939	0.0250144	-3.113502
CHORDC2P	-0.266642	-1.380188	-2.700504	0.0074958	0.0250183	-3.113724
RP11-478C6	-0.40792	-1.140658	-2.70028	0.0075006	0.0250323	-3.1143
RPS15AP1	-0.191047	4.0741058	-2.699978	0.0075072	0.025052	-3.11508
LINC01144	-0.222831	4.1381821	-2.699861	0.0075098	0.0250582	-3.11538
AMPD2	0.0681107	6.3435353	2.6996294	0.0075148	0.0250727	-3.115977
RP4-545L17	-0.419436	2.5787261	-2.699322	0.0075215	0.0250928	-3.11677
SNORD19	-0.621588	1.502338	-2.699255	0.007523	0.0250954	-3.116943
RP11-203F1	0.5041605	-0.699194	2.699121	0.0075259	0.0251018	-3.117287
TRIAP1	-0.058191	6.2079478	-2.699104	0.0075263	0.0251018	-3.117331
OBFC1	0.0683256	5.7557377	2.6988238	0.0075324	0.0251182	-3.118053
FAM109A	-0.074461	6.0406128	-2.698816	0.0075326	0.0251182	-3.118074
HSP90AA4P	-0.549058	-0.312122	-2.698737	0.0075343	0.0251199	-3.118278
RP11-445N1	-0.571668	-1.020526	-2.698704	0.007535	0.0251199	-3.118363
OSMR-AS1	0.6811352	1.6900569	2.6986987	0.0075351	0.0251199	-3.118375
AGPAT1	-0.055737	6.5044051	-2.698398	0.0075417	0.0251387	-3.11915
RP11-12J10	-0.583916	1.2231703	-2.698357	0.0075426	0.0251387	-3.119255
FOLR1	-0.853339	2.7253964	-2.698345	0.0075429	0.0251387	-3.119285
RNF4	0.0468558	6.2953289	2.6981096	0.007548	0.0251536	-3.119892
PTGR1	0.1727131	6.7161508	2.6979463	0.0075516	0.0251633	-3.120313
AC111155.1	-0.368431	-1.186263	-2.697569	0.0075599	0.0251883	-3.121284
HDAC8	0.058586	5.8973069	2.6975396	0.0075605	0.0251883	-3.12136
TDRD3	0.0688946	5.7756403	2.6974493	0.0075625	0.0251926	-3.121592
AC012487.2	-0.527357	2.1620417	-2.697397	0.0075636	0.0251941	-3.121726
RP4-694A7.	-0.493428	-0.847155	-2.697348	0.0075647	0.0251955	-3.121854
ZNF354B	-0.088306	5.2015302	-2.697137	0.0075693	0.0252086	-3.122396
USP2-AS1	0.4889392	3.7173996	2.6970591	0.007571	0.025212	-3.122597
RP1-181J22	-0.603054	-0.106661	-2.696841	0.0075758	0.0252256	-3.123159
SKA2P1	-0.581404	-0.040032	-2.696635	0.0075804	0.0252364	-3.123688

RP3-402G11	-0.25914	3.3540263	-2.696631	0.0075804	0.0252364	-3.123699
RIPK3	0.2137293	4.5938245	2.6964753	0.0075839	0.0252455	-3.124099
CAB39	0.0407704	6.4236651	2.6954172	0.0076072	0.0253207	-3.126822
RP5-875H18	-0.644147	2.585729	-2.695378	0.007608	0.0253213	-3.126924
RP11-44N11	-0.547692	-0.278126	-2.695102	0.0076141	0.0253392	-3.127633
RP5-1061H2	-0.275737	4.0031687	-2.69483	0.0076201	0.0253569	-3.128333
UGT1A12P	-0.442414	-1.042028	-2.694728	0.0076224	0.0253621	-3.128595
PDE1B	0.1355994	4.8931066	2.6946142	0.0076249	0.0253681	-3.128888
TIMM17A	0.0655189	6.4857621	2.6943471	0.0076308	0.0253854	-3.129575
CYP4A22-AS	-0.642005	1.2769662	-2.694058	0.0076372	0.0254044	-3.130318
MMP21	-0.589979	0.8964473	-2.693922	0.0076402	0.0254121	-3.130667
VN1R42P	-0.52627	-0.430237	-2.693796	0.007643	0.025419	-3.130991
AC018766.4	-0.268704	3.6723361	-2.693686	0.0076454	0.0254248	-3.131275
RP11-381E2	-0.691747	0.6952159	-2.693519	0.0076491	0.0254348	-3.131705
MTRNR2L1	0.8668431	2.9912846	2.6932826	0.0076544	0.0254499	-3.132312
CTD-2005H7	0.5026306	-0.737794	2.693243	0.0076552	0.0254505	-3.132414
CTD-2358C2	-0.535979	-0.547724	-2.692982	0.007661	0.0254674	-3.133084
NDC1	-0.065901	6.0197502	-2.692523	0.0076712	0.025499	-3.134263
C1orf226	-0.164774	5.8673924	-2.691922	0.0076846	0.02554	-3.135809
GATA4	-0.098035	6.1917433	-2.691905	0.007685	0.02554	-3.135853
RP11-111M2	-0.214807	3.8668219	-2.691733	0.0076888	0.0255504	-3.136295
RP11-282K2	-0.562968	1.4788826	-2.691677	0.00769	0.0255522	-3.136437
BEX1	-0.902823	2.6560552	-2.691396	0.0076963	0.0255707	-3.13716
CTD-2525P1	0.6849924	0.2348158	2.6912783	0.0076989	0.0255771	-3.137463
CHRDL2	0.4219018	5.2048496	2.6907933	0.0077097	0.0256095	-3.138709
RP11-305B6	-0.564906	-0.930223	-2.690777	0.0077101	0.0256095	-3.13875
RP11-718B1	0.5519225	-0.441963	2.690733	0.0077111	0.0256105	-3.138864
RP3-406A7.	0.4230518	4.3473437	2.6903409	0.0077198	0.0256362	-3.139871
ZNF141	-0.224916	4.9646134	-2.690324	0.0077202	0.0256362	-3.139915
RP11-160E2	-0.455777	-1.008949	-2.690001	0.0077274	0.0256578	-3.140743
IGHV3-32	-0.259742	-1.405477	-2.689917	0.0077293	0.0256617	-3.140959
RP11-715J2	-0.439356	2.7852261	-2.689851	0.0077308	0.0256643	-3.14113
ZNF781	-0.369532	3.5940394	-2.689574	0.007737	0.0256825	-3.141839
RP11-63P12	0.7886752	1.2713059	2.6894956	0.0077387	0.0256852	-3.142041
XIST	-1.237664	2.4771453	-2.689475	0.0077392	0.0256852	-3.142095
SLC39A3	-0.067108	6.0322592	-2.689403	0.0077408	0.0256882	-3.142279
CAMSAP3	-0.178799	6.2539761	-2.689316	0.0077427	0.0256923	-3.142502
NMRK2	0.6633102	-0.399929	2.6888903	0.0077523	0.0257216	-3.143595
CIAPIN1	0.0599227	6.2145378	2.6888538	0.0077531	0.025722	-3.143689
URM1	-0.056363	6.3984542	-2.688814	0.007754	0.0257226	-3.143791
AC090043.1	-0.29049	-1.315525	-2.688322	0.007765	0.0257569	-3.145053
RP11-91I2C	-0.49317	-0.695526	-2.688279	0.007766	0.0257578	-3.145165
XXbac-B444	-0.415622	2.4908348	-2.688209	0.0077676	0.0257607	-3.145343
RP11-456H1	0.5754716	3.0181096	2.6880563	0.007771	0.0257697	-3.145736
DHX29	0.0496628	6.2044651	2.687761	0.0077777	0.0257851	-3.146494
AOX1	0.2398727	6.8857979	2.6877495	0.0077779	0.0257851	-3.146523
REEP6	0.175282	6.7639049	2.6877267	0.0077784	0.0257851	-3.146582
POLE3	-0.045824	6.3152067	-2.687724	0.0077785	0.0257851	-3.146588
CDHR2	-0.510985	5.4632705	-2.687621	0.0077808	0.0257902	-3.146853
EIF1AD	-0.04119	6.0145314	-2.687593	0.0077814	0.0257902	-3.146926
RP5-1017F8	0.3281882	-1.31033	2.6874884	0.0077838	0.0257957	-3.147193

INSL5	0.1857482	-1.49225	2.6874279	0.0077851	0.0257967	-3.147348
LRR1	-0.083219	5.3586844	-2.687411	0.0077855	0.0257967	-3.147391
PIFO	-0.707541	2.4568388	-2.687358	0.0077867	0.0257984	-3.147527
PYCR2	-0.058688	6.4238288	-2.687234	0.0077895	0.0258053	-3.147845
NF1P6	-0.276443	-1.419192	-2.686928	0.0077964	0.0258258	-3.14863
RP1-63G5.7	-0.493131	-0.922982	-2.686465	0.0078068	0.025858	-3.149818
PADI2	0.3350269	4.3788781	2.6864234	0.0078078	0.0258588	-3.149925
RP11-30506	-0.53077	1.8464091	-2.686269	0.0078113	0.025868	-3.150321
C9orf43	-0.18437	4.0897726	-2.68617	0.0078135	0.0258719	-3.150574
RP11-1C8.6	-0.601268	1.57005	-2.686154	0.0078139	0.0258719	-3.150616
RP4-583P15	-0.724426	1.3770158	-2.686107	0.0078149	0.0258731	-3.150737
OSCAR	0.2281647	4.5623123	2.6860329	0.0078166	0.0258762	-3.150927
HPGDS	0.3777009	3.6626252	2.6858209	0.0078214	0.0258898	-3.15147
TRIM39	-0.05635	5.7758958	-2.685736	0.0078233	0.0258937	-3.151687
ARSA	0.086016	6.427722	2.6856117	0.0078261	0.0259007	-3.152007
PRAMEF17	0.4077899	-1.173054	2.6851333	0.007837	0.0259342	-3.153233
PPP1R3D	0.171767	4.797726	2.6850404	0.0078391	0.0259388	-3.153471
NKX1-2	-0.776242	-0.420874	-2.684917	0.0078419	0.0259457	-3.153787
CALCOCO2	0.0469026	6.6025633	2.6847742	0.0078451	0.0259541	-3.154154
HUS1	0.0571192	5.8472889	2.6841892	0.0078584	0.0259956	-3.155653
PKN3	-0.106775	5.7088151	-2.683939	0.007864	0.026012	-3.156293
RSPH3	0.085027	5.5268141	2.6838632	0.0078658	0.0260154	-3.156488
SIGMAR1	-0.080749	6.7921981	-2.683683	0.0078699	0.0260266	-3.156949
SEC24A	0.0785726	6.349992	2.6830786	0.0078836	0.0260658	-3.158498
AC116035.1	0.6094367	-0.010487	2.6830623	0.007884	0.0260658	-3.15854
GPRIN1	-0.214782	5.1151101	-2.683057	0.0078841	0.0260658	-3.158554
LRRC61	-0.10114	6.1007611	-2.683036	0.0078846	0.0260658	-3.158608
PKMP3	0.4478537	2.3626588	2.682822	0.0078895	0.0260768	-3.159156
RP11-349N1	-0.363936	2.3474281	-2.682802	0.0078899	0.0260768	-3.159207
LINC01399	-0.460286	-1.092026	-2.682796	0.00789	0.0260768	-3.159221
RP11-47I22	0.6043934	-0.319565	2.6827232	0.0078917	0.026079	-3.159409
ACTN2	-0.609193	3.730221	-2.682704	0.0078921	0.026079	-3.159458
CTA-293F17	0.810674	1.2610127	2.6826577	0.0078932	0.0260798	-3.159577
ZNF354A	-0.145486	5.2916619	-2.682631	0.0078938	0.0260798	-3.159645
TUBB4BP2	-0.34031	-1.239396	-2.681797	0.0079128	0.0261403	-3.161781
DHFR	-0.101397	5.8682038	-2.681643	0.0079163	0.0261495	-3.162174
RP11-436I9	-0.693748	2.7744221	-2.681602	0.0079173	0.0261503	-3.162281
SCRIB	-0.071621	6.6297516	-2.681346	0.0079231	0.0261673	-3.162936
OR2H5P	0.2580627	-1.455397	2.6812176	0.0079261	0.0261746	-3.163265
RP11-815J2	0.5399024	-0.545752	2.6810646	0.0079296	0.0261837	-3.163656
ZNF142	-0.057135	5.8290504	-2.681034	0.0079303	0.0261837	-3.163735
RP11-253M7	0.430917	-0.978175	2.6809928	0.0079312	0.0261845	-3.16384
SYNPR-AS1	-0.495965	-1.0969	-2.68094	0.0079324	0.026186	-3.163974
RP11-66N24	-0.195882	4.6446948	-2.680715	0.0079376	0.0262006	-3.164552
MRGPRE	0.3796715	-1.269886	2.6806857	0.0079383	0.0262006	-3.164626
TRAF2	-0.067671	6.1413216	-2.68032	0.0079466	0.0262259	-3.165563
CTA-363E19	-0.443619	2.6422293	-2.680148	0.0079506	0.0262347	-3.166002
RP11-254F7	0.6206887	0.2213464	2.6801399	0.0079508	0.0262347	-3.166023
ZNF41	0.0882647	5.3550718	2.679867	0.007957	0.026253	-3.166721
TDPX2	-0.611272	0.9266799	-2.67978	0.007959	0.0262572	-3.166943
RNA5SP40	0.4351215	-1.093548	2.6796747	0.0079615	0.0262629	-3.167213

RP11-355F1	0.6119661	1.7400163	2.6795732	0.0079638	0.0262682	-3.167473
RPL9P29	-0.399954	2.7652634	-2.67954	0.0079646	0.0262684	-3.167559
GALK1	-0.109425	6.5688856	-2.679373	0.0079684	0.0262786	-3.167985
TRIM52	-0.076428	5.4930442	-2.679204	0.0079723	0.0262891	-3.168419
LPO	-0.685658	-0.201613	-2.679032	0.0079762	0.0262997	-3.168858
TAS1R3	-0.375646	3.7643303	-2.678994	0.0079771	0.0263002	-3.168955
ZNF10	-0.106811	5.3069747	-2.678927	0.0079786	0.0263029	-3.169125
SNAP23P	-0.437271	-0.882056	-2.678727	0.0079832	0.0263157	-3.169637
SH2D6	-0.445953	3.2670593	-2.678194	0.0079955	0.0263539	-3.171002
TTLL11-IT1	-0.496001	-0.662183	-2.678102	0.0079977	0.0263585	-3.171238
SPAG7	0.071791	6.2450687	2.6780522	0.0079988	0.0263599	-3.171364
FAM185A	0.0799372	5.2855834	2.6775836	0.0080096	0.0263931	-3.172562
GDF5	0.5977035	-0.45266	2.6774201	0.0080134	0.0264032	-3.17298
RPL31P12	-0.269934	-1.373994	-2.677335	0.0080154	0.0264065	-3.173197
RP11-424G1	0.9005798	1.3734705	2.6773144	0.0080158	0.0264065	-3.17325
RP11-543C4	0.5705976	0.184363	2.6772082	0.0080183	0.0264122	-3.173522
SERPINF2	-0.20736	7.1782807	-2.67709	0.008021	0.026417	-3.173825
AC107983.4	-0.282342	3.6895886	-2.677082	0.0080212	0.026417	-3.173844
RBMXP4	0.6039321	0.5763599	2.6769396	0.0080245	0.0264255	-3.174209
PFKM	0.12258	5.8383242	2.6768184	0.0080273	0.0264323	-3.174518
LINC01268	-0.290156	4.4577205	-2.676771	0.0080284	0.0264335	-3.174639
HLA-DQB1	0.1539308	6.3595961	2.6767393	0.0080291	0.0264336	-3.174721
RP11-455J2	-0.611192	1.050836	-2.676214	0.0080413	0.0264693	-3.176063
LINC00703	0.1906132	-1.512535	2.6762077	0.0080414	0.0264693	-3.176079
INSRR	-0.784642	0.9878543	-2.676149	0.0080428	0.0264714	-3.176228
SLC37A2	0.1445764	5.4014395	2.6759984	0.0080463	0.0264805	-3.176614
PRPF4B	-0.044232	6.323933	-2.675752	0.008052	0.0264969	-3.177243
CELSR3-AS1	-0.581619	1.7966384	-2.675685	0.0080536	0.0264997	-3.177415
CTD-2532K1	0.4028575	-1.258596	2.6755923	0.0080557	0.0265044	-3.177652
AP000705.7	-0.514681	-0.873526	-2.675407	0.00806	0.0265161	-3.178125
SSBP4	-0.06512	6.1556359	-2.675343	0.0080615	0.0265187	-3.178289
FGF4	-0.56798	-1.054605	-2.675311	0.0080623	0.0265187	-3.17837
CRYBB3	0.4460645	2.9229653	2.6750887	0.0080674	0.0265333	-3.178939
LAMTOR5	0.0565701	6.3968479	2.6748541	0.0080729	0.0265469	-3.179538
RP11-64702	-0.516413	1.7341261	-2.674848	0.008073	0.0265469	-3.179553
RP11-177F1	-0.635848	-0.642415	-2.674639	0.0080779	0.0265606	-3.180088
NDUFA10	0.0538534	6.6178129	2.6744773	0.0080816	0.0265705	-3.1805
HMGN2P18	-0.280251	-1.330605	-2.674363	0.0080843	0.0265767	-3.180792
BZW1	0.0510796	6.6102892	2.6743343	0.008085	0.0265767	-3.180865
NCDN	0.0763825	5.9331569	2.6738199	0.008097	0.0266137	-3.182179
ITFG3	0.0810151	6.3779531	2.6735188	0.008104	0.0266344	-3.182948
TMEM123	0.0659393	6.9850776	2.6732715	0.0081098	0.026651	-3.183579
SDHAP1	-0.073669	5.3858958	-2.673166	0.0081122	0.0266554	-3.183848
CECR1	0.128281	6.1363079	2.6731086	0.0081136	0.0266554	-3.183995
AC073415.2	-0.576906	-0.224441	-2.673099	0.0081138	0.0266554	-3.184021
RNVU1-7	-0.41515	-1.106308	-2.673089	0.008114	0.0266554	-3.184045
HOXB-AS1	0.3299753	3.4342171	2.6727281	0.0081225	0.0266807	-3.184966
RP11-1069G	0.7386284	0.6803828	2.6723889	0.0081304	0.0267044	-3.185832
AC092566.1	0.189594	-1.49029	2.6719302	0.0081411	0.0267372	-3.187003
CHD8	-0.043667	6.3049787	-2.671655	0.0081476	0.026756	-3.187705
EIF3LP3	-0.559607	0.0699741	-2.671591	0.0081491	0.0267577	-3.187869

LINC01057	-0.493287	3.7868377	-2.67157	0.0081496	0.0267577	-3.187921
GFRA1	0.6276951	5.1872712	2.6714047	0.0081535	0.0267667	-3.188344
RP11-61L19	-0.544285	-0.284923	-2.67139	0.0081538	0.0267667	-3.18838
AGBL5-AS1	-0.558679	-0.079244	-2.67136	0.0081545	0.0267667	-3.188459
RP13-16H11	-0.581278	-1.021182	-2.671182	0.0081587	0.0267781	-3.188913
PVRL3	0.2066205	5.7161434	2.6711392	0.0081597	0.0267789	-3.189021
TOR1A	0.0585118	6.2501793	2.670806	0.0081675	0.0268022	-3.189871
STK36	-0.066228	5.7782688	-2.670685	0.0081704	0.0268091	-3.190181
MED23	-0.066398	5.8944197	-2.670246	0.0081807	0.0268402	-3.191299
ZMYM6NB	0.1172019	5.2405767	2.67022	0.0081813	0.0268402	-3.191366
PARD6A	0.1681264	4.9900143	2.6700115	0.0081862	0.0268539	-3.191897
MIR608	-0.268361	-1.374766	-2.669669	0.0081943	0.0268779	-3.19277
RP11-157P1	0.1933531	4.192099	2.6696224	0.0081954	0.0268791	-3.19289
RP11-531F1	-0.353472	3.0650633	-2.669476	0.0081989	0.0268881	-3.193263
EEF1A1P22	-0.432817	2.5905934	-2.669202	0.0082053	0.0269068	-3.193962
GABRA2	-1.022654	0.5640508	-2.669065	0.0082085	0.026915	-3.19431
DENND6B	0.1038613	5.3515232	2.6689555	0.0082111	0.0269211	-3.19459
CASK	0.0851251	6.2067721	2.6686293	0.0082188	0.0269439	-3.195421
NARS	0.0423895	6.6774666	2.6684754	0.0082225	0.0269534	-3.195814
CYCSP24	-0.492278	-0.59048	-2.66821	0.0082288	0.0269716	-3.19649
CTD-2521M2	0.5294197	2.4580794	2.6676637	0.0082417	0.0270116	-3.197882
RP1-59D14.	-0.411196	3.1342892	-2.667559	0.0082442	0.0270173	-3.19815
RP11-504P2	-0.632188	0.6785632	-2.667443	0.0082469	0.0270238	-3.198443
MSANTD3-TM	0.5336088	-0.2414	2.6669934	0.0082576	0.0270564	-3.19959
PKD1L3	0.4521592	2.5249041	2.66684	0.0082612	0.0270659	-3.199981
OR4K1	-0.280954	-1.41698	-2.666683	0.008265	0.0270757	-3.200382
MT-RNR1	0.0879828	7.3479841	2.6664034	0.0082716	0.027095	-3.201093
RP3-355L5.	-0.523722	1.8667957	-2.66585	0.0082848	0.0271357	-3.202503
WDR18	-0.062319	6.2483128	-2.665748	0.0082872	0.0271412	-3.202762
CTD-2619J1	-0.3219	5.2704924	-2.66533	0.0082972	0.0271714	-3.203826
CITED2	0.1021742	6.1870883	2.6650577	0.0083037	0.0271902	-3.204519
ZNF625-ZNF	-0.499485	-0.417313	-2.664852	0.0083086	0.0272039	-3.205044
RPA1	-0.044653	6.3626147	-2.664641	0.0083136	0.0272179	-3.205579
XK	0.6505276	4.2155492	2.664523	0.0083164	0.0272247	-3.20588
RP11-65J21	0.647077	-0.549857	2.6642559	0.0083228	0.0272432	-3.20656
TAF3	-0.055571	5.7063202	-2.664161	0.0083251	0.0272479	-3.206801
CHPF2	0.052883	6.3709767	2.664133	0.0083257	0.0272479	-3.206873
DPPA2P4	0.2975565	-1.36222	2.6639865	0.0083293	0.0272569	-3.207246
WDR33	-0.034861	6.4032069	-2.663906	0.0083312	0.0272608	-3.207451
CTD-210809	0.2775079	-1.445488	2.6638151	0.0083334	0.0272655	-3.207682
ZNF621	-0.064251	5.8137792	-2.663603	0.0083384	0.0272768	-3.208221
RIPPLY2	-0.757108	-0.375943	-2.663595	0.0083386	0.0272768	-3.208243
RPL7P50	-0.553089	-0.268507	-2.663576	0.0083391	0.0272768	-3.20829
KISS1	-0.406963	4.2510486	-2.663516	0.0083405	0.0272791	-3.208443
TMEM53	0.091648	6.0721504	2.6632373	0.0083472	0.0272985	-3.209152
KDM1A	-0.053586	6.2837397	-2.663111	0.0083502	0.0273059	-3.209472
IL37	-0.487771	-0.789685	-2.663029	0.0083522	0.0273099	-3.209682
HOXD11	-0.609321	-0.863538	-2.662331	0.008369	0.0273623	-3.211459
RP11-552F3	-0.576387	0.3082074	-2.661953	0.008378	0.0273895	-3.212418
YBX1P4	-0.371248	-1.031547	-2.661907	0.0083791	0.0273907	-3.212537
RP11-145E1	-0.41984	-1.230188	-2.661872	0.00838	0.027391	-3.212626

GGTA1P	0.1367462	5.4757175	2.6617681	0.0083825	0.0273967	-3.212889
RP11-267J2	-0.460997	-0.680543	-2.661717	0.0083837	0.0273983	-3.213019
H2BFWT	-0.457717	-1.22894	-2.661646	0.0083854	0.0274014	-3.213199
RP3-44501C	-0.262653	-1.35566	-2.661474	0.0083896	0.0274125	-3.213638
CHRM5	-0.441831	2.7320994	-2.661395	0.0083915	0.0274163	-3.213838
BRD9	-0.055955	6.0842308	-2.66125	0.0083949	0.0274252	-3.214206
ZNF302	-0.069052	5.979785	-2.661123	0.008398	0.0274328	-3.21453
ZNF75D	0.0796871	5.6783213	2.6609354	0.0084025	0.0274451	-3.215006
RPS7P15	-0.34606	-1.197233	-2.660743	0.0084072	0.0274577	-3.215494
CTSLP7	-0.689482	1.4095143	-2.660614	0.0084103	0.0274655	-3.215824
EEF1B2P1	-0.500381	-0.566887	-2.659923	0.008427	0.0275176	-3.21758
S1PR1	0.1168007	5.9951022	2.6598473	0.0084288	0.0275211	-3.217772
RP5-1180C1	0.2261318	-1.47167	2.6594653	0.0084381	0.0275485	-3.218742
MED26	-0.051448	5.493453	-2.659437	0.0084387	0.0275485	-3.218813
LDHAL6B	0.5958974	0.1017622	2.6588289	0.0084535	0.0275942	-3.220359
CXorf58	0.5751087	0.6272755	2.6587349	0.0084558	0.0275991	-3.220598
LRRC37A	-0.424531	3.1125928	-2.658626	0.0084584	0.0276053	-3.220875
RBMXP1	-0.676956	-0.274496	-2.65857	0.0084597	0.0276072	-3.221016
ERLIN2	0.0779753	6.373052	2.6585236	0.0084609	0.0276077	-3.221134
PDPK2P	-0.55734	2.4241397	-2.658502	0.0084614	0.0276077	-3.221189
GTSCR1	0.3819013	-1.189608	2.6584417	0.0084629	0.02761	-3.221342
TRO	0.2211812	5.2911413	2.6580549	0.0084723	0.0276367	-3.222325
RPL5P29	-0.58927	0.5262904	-2.658018	0.0084732	0.0276367	-3.22242
HLA-G	0.2549432	5.0982238	2.6580118	0.0084733	0.0276367	-3.222434
SLC44A3	-0.231039	5.8166178	-2.657881	0.0084765	0.0276421	-3.222767
WNT9B	0.6706449	0.8690804	2.6578807	0.0084765	0.0276421	-3.222767
BTK	0.1746126	5.035274	2.6577981	0.0084785	0.0276462	-3.222977
APOA1-AS	-0.647577	0.9381836	-2.657726	0.0084802	0.0276494	-3.223159
SFPQ	-0.035554	6.7645597	-2.657667	0.0084817	0.0276516	-3.223309
ETV3L	-0.432692	-0.949181	-2.657398	0.0084882	0.0276705	-3.223992
CU459211.1	-0.309557	-1.302329	-2.657295	0.0084907	0.0276762	-3.224254
RP11-474D1	-0.628054	0.3702053	-2.657262	0.0084915	0.0276764	-3.224339
PARP4	0.0675141	6.3728417	2.6570698	0.0084962	0.0276891	-3.224826
UBE2A	0.049152	6.3449028	2.6569319	0.0084996	0.0276953	-3.225176
CTD-2349P2	-0.558621	1.4547821	-2.656929	0.0084996	0.0276953	-3.225183
bP-218909.	-0.814722	0.0612589	-2.656806	0.0085027	0.0277027	-3.225497
RPL4P6	-0.313275	2.8018556	-2.656772	0.0085035	0.0277029	-3.225582
CTD-3126B1	-0.559951	0.1062311	-2.656633	0.0085069	0.0277114	-3.225934
PKD1L1	0.2944662	3.5575662	2.6565144	0.0085098	0.0277184	-3.226236
RP4-669P1C	-0.59594	2.2710567	-2.656324	0.0085144	0.027731	-3.226719
RP11-6860€	-0.497016	-0.741405	-2.655946	0.0085236	0.0277586	-3.227678
HNRNPA1P33	0.5066685	-0.852036	2.6557404	0.0085287	0.0277725	-3.2282
SPDYE6	-0.405391	2.5478757	-2.655647	0.0085309	0.0277775	-3.228436
RP11-177A2	-0.372558	-1.109366	-2.655531	0.0085338	0.0277843	-3.228733
ADAM9	0.1060877	6.2061034	2.6552691	0.0085402	0.0278026	-3.229396
RP11-783L4	0.0768235	-1.570524	2.6550529	0.0085455	0.0278174	-3.229944
GAPDHP52	-0.386601	-1.019605	-2.654942	0.0085482	0.0278237	-3.230225
MAN2A1	0.0626977	6.5610091	2.6546031	0.0085565	0.0278483	-3.231085
ASB16-AS1	-0.073243	5.4531799	-2.654331	0.0085632	0.0278675	-3.231775
ZNF430	-0.176552	4.8668308	-2.654282	0.0085644	0.0278689	-3.2319
RP11-690J1	-0.340246	-1.285073	-2.654231	0.0085656	0.0278705	-3.232029

OR2C3	-0.38636	-1.229756	-2.654165	0.0085672	0.0278733	-3.232196
RPS15	-0.06652	6.8544516	-2.654037	0.0085704	0.0278811	-3.232522
RP11-175I6	-0.448155	-1.29013	-2.653979	0.0085718	0.0278832	-3.232668
RBPMS2	0.1712469	5.6702415	2.6538795	0.0085742	0.0278886	-3.23292
RNU6-415P	-0.478696	1.1052391	-2.653617	0.0085807	0.0279071	-3.233587
AC097461.4	-0.587992	0.719002	-2.653135	0.0085926	0.0279432	-3.234809
FAM209B	-0.572593	1.216294	-2.653076	0.008594	0.0279435	-3.234958
EEF1G	-0.274155	3.4477947	-2.653069	0.0085942	0.0279435	-3.234976
RP11-502I4	-0.213709	3.9847573	-2.652935	0.0085975	0.0279518	-3.235316
BCRP3	-0.43746	3.6149227	-2.652827	0.0086001	0.0279579	-3.235589
OTOS	0.4391642	-1.159607	2.6527586	0.0086018	0.0279604	-3.235762
SNORD67	-0.490296	-0.643298	-2.652734	0.0086024	0.0279604	-3.235825
SEMA5B	-0.162693	5.2667191	-2.652386	0.008611	0.0279857	-3.236706
FMNL2	0.1556042	5.7432164	2.6520503	0.0086193	0.0280101	-3.237557
OAF	0.09363	6.7816877	2.6518247	0.0086248	0.0280257	-3.238129
RP1-28H20.	0.4041166	2.8792882	2.6517279	0.0086272	0.028031	-3.238374
RPL7AP8	-0.469341	-0.76523	-2.651691	0.0086281	0.0280315	-3.238468
PABPC1P5	0.1763134	-1.519822	2.6516363	0.0086295	0.0280334	-3.238607
ALPPL2	-0.482819	-1.025125	-2.651278	0.0086384	0.0280597	-3.239515
ASNA1	0.0530552	6.3688108	2.6512302	0.0086395	0.028061	-3.239635
BTN2A3P	0.140893	4.5867024	2.6511424	0.0086417	0.0280655	-3.239858
OTUD1	0.0695257	5.6800779	2.6509615	0.0086462	0.0280776	-3.240316
CLVS2	-0.823942	-0.377936	-2.650687	0.008653	0.0280972	-3.241012
HOXB6	0.2785076	4.0909201	2.6504156	0.0086597	0.0281165	-3.241699
CTB-88F18.	-0.477875	-0.744933	-2.650307	0.0086624	0.0281227	-3.241973
SNAP47	-0.063582	6.2470091	-2.649851	0.0086737	0.0281559	-3.243129
AC009950.2	0.3471647	3.4194044	2.6498322	0.0086742	0.0281559	-3.243176
RP11-426C2	0.6261847	1.5253336	2.649358	0.0086859	0.0281916	-3.244377
ZNF649-AS1	-0.535736	-0.453413	-2.649278	0.0086879	0.0281955	-3.244578
RORA-AS1	-0.518495	1.6713914	-2.649098	0.0086924	0.0282065	-3.245034
ERBB3	-0.153076	6.8113385	-2.649071	0.0086931	0.0282065	-3.245102
ZNF580	-0.089225	5.6919729	-2.649035	0.008694	0.0282065	-3.245195
AC073316.2	0.2343261	-1.44473	2.6490186	0.0086944	0.0282065	-3.245236
C2orf72	-0.111476	6.741678	-2.648959	0.0086958	0.0282088	-3.245386
SLC7A4	0.8303531	1.3009925	2.648925	0.0086967	0.028209	-3.245473
CEP164	-0.056384	5.8922021	-2.64882	0.0086993	0.028215	-3.245738
CAPS	-0.114098	5.7246598	-2.64824	0.0087137	0.0282593	-3.247206
KLK1	-0.712278	0.5181343	-2.64811	0.008717	0.0282673	-3.247537
LILRB2	0.1562621	5.3434253	2.6480212	0.0087192	0.028272	-3.247761
POLD3	-0.071446	5.7644895	-2.647987	0.0087201	0.0282722	-3.247847
ZNF746	0.0671833	5.9526885	2.6479051	0.0087221	0.0282763	-3.248054
SLC3A1	0.5082484	4.5502472	2.6477667	0.0087255	0.0282843	-3.248404
AL589822.1	-0.33898	-1.180126	-2.647723	0.0087266	0.0282843	-3.248515
ESD	0.0690959	6.5684887	2.647713	0.0087269	0.0282843	-3.24854
CTD-2192J1	-0.587361	1.1337275	-2.647645	0.0087286	0.0282873	-3.248711
CBX3P6	-0.303619	-1.354056	-2.64759	0.00873	0.0282893	-3.248852
RP11-1250I	-0.292342	-1.292711	-2.647401	0.0087347	0.028298	-3.24933
DUSP28	-0.069287	5.038693	-2.647389	0.008735	0.028298	-3.24936
1-Mar	0.129867	5.2193067	2.6473887	0.008735	0.028298	-3.249361
FAM104A	-0.049749	5.7578013	-2.647137	0.0087413	0.0283159	-3.249998
B3GALT5	0.866581	2.1058844	2.6470792	0.0087427	0.028318	-3.250144

OR5K2	0.3618167	-1.170297	2.6465264	0.0087565	0.0283603	-3.251542
RP11-1006G	0.1690229	-1.523537	2.6463189	0.0087617	0.0283746	-3.252067
GSTP1	0.1365191	6.1649752	2.6462341	0.0087639	0.0283789	-3.252281
JRKL	-0.089393	5.4764694	-2.646116	0.0087668	0.028386	-3.25258
AMMECR1	0.0800554	5.6239254	2.6459091	0.008772	0.0284003	-3.253103
CDH23	0.1788759	5.507532	2.6458026	0.0087747	0.0284064	-3.253372
BANF1P2	-0.48424	3.8074781	-2.645739	0.0087763	0.0284086	-3.253534
CPD	-0.07398	6.8305488	-2.645713	0.0087769	0.0284086	-3.253599
RP1-159M24	-0.580963	0.4033268	-2.645635	0.0087789	0.0284124	-3.253795
BPIFA4P	-0.494369	-1.001267	-2.645421	0.0087843	0.0284273	-3.254338
RP11-298H2	0.391687	-1.223482	2.6453016	0.0087872	0.0284345	-3.254639
SULT1C2P1	-0.747301	1.2127359	-2.645196	0.0087899	0.0284406	-3.254907
TUSC2	-0.058736	6.1111562	-2.645143	0.0087912	0.0284423	-3.255039
TAB1	-0.052137	6.1879304	-2.644942	0.0087963	0.0284562	-3.255549
SOCS5P5	-0.333331	-1.313001	-2.644861	0.0087983	0.0284602	-3.255753
CTC-436P18	0.3338365	-1.299138	2.6443873	0.0088102	0.0284962	-3.25695
RNU5B-4P	0.4716478	-0.681009	2.6441022	0.0088174	0.0285169	-3.257671
RP11-265E1	-0.629497	0.6548533	-2.643671	0.0088283	0.0285495	-3.25876
CFH	0.1375165	7.2779321	2.6435145	0.0088322	0.0285598	-3.259156
GUSBP5	0.6750456	1.7422473	2.6430933	0.0088428	0.0285901	-3.26022
HSPE1P2	-0.45268	2.4897946	-2.64308	0.0088432	0.0285901	-3.260253
HYAL3	-0.153814	5.2394067	-2.64304	0.0088442	0.0285909	-3.260355
RP5-1063M2	0.2917109	-1.378444	2.6426494	0.0088541	0.0286177	-3.261341
RP11-469J4	-0.587004	-0.084998	-2.642649	0.0088541	0.0286177	-3.261342
CTB-41I6.2	0.603189	1.23992	2.6425739	0.008856	0.0286213	-3.261532
ELFN1	0.3050119	5.4253708	2.6423931	0.0088605	0.0286335	-3.261988
SVOP	-0.644902	2.6396561	-2.642364	0.0088613	0.0286335	-3.262063
RP1-167022	0.7590993	-0.097905	2.6422931	0.0088631	0.0286367	-3.262241
RP11-340I6	-0.5631	-0.355289	-2.64206	0.008869	0.0286532	-3.26283
TRIP4	-0.042971	5.9655403	-2.642016	0.0088701	0.0286543	-3.26294
CD276	-0.056735	6.4735319	-2.641975	0.0088711	0.0286551	-3.263045
AC008268.2	-0.660151	-0.356289	-2.641805	0.0088754	0.0286665	-3.263474
MT-TL1	-0.503965	2.6309906	-2.641634	0.0088798	0.0286779	-3.263906
DISP1	0.1143723	5.7054191	2.6414087	0.0088855	0.0286938	-3.264474
PLCL1	0.1834022	4.6511743	2.6412913	0.0088884	0.0286982	-3.26477
POLR2J3	-0.144039	4.6413906	-2.641269	0.008889	0.0286982	-3.264827
GS1-24F4.1	-0.377644	-1.092966	-2.641255	0.0088894	0.0286982	-3.264861
TMC5	0.5727891	4.5811392	2.6412317	0.00889	0.0286982	-3.264921
RP11-775C2	0.2204321	3.7092173	2.6409119	0.0088981	0.0287212	-3.265728
PBX1	0.2030127	5.6070977	2.6408885	0.0088987	0.0287212	-3.265787
BHLHE40-AS	0.2819482	3.6085017	2.6408577	0.0088994	0.0287212	-3.265865
RP11-244H3	0.1350149	4.5413902	2.6408125	0.0089006	0.0287221	-3.265979
AB019438.6	-0.3239	-1.320501	-2.640785	0.0089013	0.0287221	-3.26605
RP11-154P1	-0.731488	0.8892142	-2.640681	0.0089039	0.028728	-3.26631
PPIAP21	-0.603294	1.7013191	-2.640628	0.0089053	0.0287299	-3.266446
RP11-146D1	-0.613779	0.1651847	-2.640592	0.0089062	0.0287303	-3.266535
C10orf126	0.7950795	0.7345113	2.6404129	0.0089108	0.0287424	-3.266988
CTD-2503I6	0.0768235	-1.570524	2.6403443	0.0089125	0.0287455	-3.267161
RP11-673E1	0.6051037	0.2685676	2.6402321	0.0089153	0.0287522	-3.267444
RP11-155D1	0.5276023	2.487597	2.6397969	0.0089264	0.0287854	-3.268542
AL133245.2	-0.459017	-0.610236	-2.639687	0.0089292	0.0287919	-3.268821

SGIP1	0.2948477	4.3296084	2.639606	0.0089313	0.0287959	-3.269024
NLGN1	0.8034939	0.6991222	2.6394989	0.008934	0.0288022	-3.269294
PANK3	-0.068186	6.5260365	-2.638975	0.0089474	0.0288427	-3.270616
RP11-217B1	-0.426222	2.4683236	-2.638853	0.0089505	0.0288502	-3.270923
DDX26B-AS1	0.2420405	-1.404962	2.6387867	0.0089522	0.0288531	-3.271091
RP11-307L1	-0.562806	0.4193763	-2.638747	0.0089532	0.0288538	-3.271191
LRIG2	-0.061222	5.6659907	-2.638653	0.0089556	0.0288591	-3.271429
ACTR8	-0.046907	5.8315564	-2.638619	0.0089565	0.0288593	-3.271513
LINC01435	0.43531	-1.127642	2.6382371	0.0089662	0.0288882	-3.272477
AC010095.7	-0.361172	-1.145754	-2.638186	0.0089675	0.0288898	-3.272605
LINC00691	-0.369726	-1.071099	-2.63786	0.0089759	0.0289142	-3.273428
NOTCH2NL	-0.461117	2.457498	-2.637708	0.0089798	0.0289241	-3.273811
PDCD6IP	0.0393579	6.6150452	2.6375762	0.0089831	0.0289324	-3.274143
RP9	-0.075324	5.3916948	-2.63737	0.0089884	0.0289469	-3.274663
APOOL	0.0722369	6.1637455	2.636716	0.0090052	0.0289975	-3.276312
PASD1	-0.477429	-1.229233	-2.636695	0.0090057	0.0289975	-3.276365
DDI2	0.0734615	6.3595003	2.6364908	0.009011	0.0290119	-3.276879
NPM1P12	-0.421844	-0.923861	-2.636261	0.0090169	0.0290283	-3.277459
RP11-145P1	-0.644126	-0.23351	-2.636102	0.009021	0.0290368	-3.277859
RP13-516M1	-0.13036	4.742926	-2.636097	0.0090211	0.0290368	-3.277873
AC005785.2	-0.280584	3.6241246	-2.636049	0.0090223	0.0290381	-3.277992
RP11-439E1	-0.364631	2.9312596	-2.63585	0.0090274	0.0290521	-3.278494
NTAN1P2	0.2925749	3.636079	2.6354621	0.0090374	0.0290816	-3.279471
RP11-564P9	0.1873959	-1.514174	2.6352121	0.0090439	0.0290998	-3.280101
H1F0	-0.073484	6.9974362	-2.635059	0.0090478	0.0291099	-3.280487
CMIP	0.0500249	6.2521891	2.6342309	0.0090692	0.0291761	-3.282572
LINC00605	0.8444662	1.3116489	2.6341968	0.0090701	0.0291763	-3.282658
RP11-776A1	-0.318443	-1.354786	-2.634165	0.0090709	0.0291764	-3.282738
RP11-282I1	-0.58362	-0.43269	-2.63406	0.0090736	0.0291826	-3.283002
LINC00894	-0.22299	4.204388	-2.633865	0.0090786	0.0291962	-3.283495
RP11-94D20	-0.348089	-1.278854	-2.633816	0.0090799	0.0291977	-3.283618
SLC22A3	-0.295107	5.9230972	-2.633748	0.0090817	0.0292008	-3.283789
TNNI2	-0.303226	4.0192919	-2.633689	0.0090832	0.0292032	-3.283938
PIP5K1C	0.0573693	6.1395185	2.6333588	0.0090917	0.029228	-3.284768
EEF1A1P27	-0.253378	-1.364788	-2.632916	0.0091032	0.0292623	-3.285882
LETM2	0.2376792	3.9328422	2.6327835	0.0091066	0.0292708	-3.286216
LMBRD1	0.0652469	6.4246039	2.6326506	0.0091101	0.0292793	-3.286551
RP11-579O2	0.0987285	-1.55936	2.6323855	0.0091169	0.0292988	-3.287218
NOSIP	-0.059721	6.309828	-2.632335	0.0091182	0.0293004	-3.287344
LHFPL3-AS2	-0.947039	1.9352869	-2.632261	0.0091202	0.029304	-3.287531
RP11-480C2	0.4457661	-1.038483	2.631935	0.0091286	0.0293287	-3.288352
UTP23	-0.065618	5.8443312	-2.631473	0.0091407	0.0293647	-3.289515
RP11-15001	-0.438834	-1.114187	-2.631423	0.009142	0.0293663	-3.289641
PDCD5P1	-0.487351	-0.589136	-2.63135	0.0091439	0.0293698	-3.289823
RP3-340N1.	0.5574116	3.9140878	2.6307769	0.0091588	0.0294151	-3.291265
SCGB3A1	0.4592813	2.7661047	2.6307096	0.0091605	0.0294182	-3.291434
RP5-1031J8	-0.651592	-0.517637	-2.630489	0.0091663	0.0294331	-3.291989
IMPDH1P4	-0.561216	-1.065334	-2.630445	0.0091674	0.0294331	-3.2921
RP11-510I6	-0.34626	-1.161326	-2.630439	0.0091676	0.0294331	-3.292116
RLF	0.067584	5.7583326	2.6304032	0.0091685	0.0294335	-3.292205
RP11-71L14	0.5823225	-0.372831	2.6303685	0.0091694	0.0294338	-3.292292

KANK2	0.0695792	6.5206262	2.630337	0.0091703	0.0294338	-3.292371
LINC01393	0.6670855	0.8524492	2.6301585	0.0091749	0.0294462	-3.29282
TNFRSF14	0.0726091	6.4204366	2.629629	0.0091887	0.029488	-3.294152
CIRH1A	0.0543094	6.109564	2.6295658	0.0091904	0.0294907	-3.294311
RP11-649E7	-0.581624	0.8276838	-2.629308	0.0091971	0.0295097	-3.294959
ACTN1	0.0540232	6.7545404	2.6292428	0.0091988	0.0295126	-3.295123
RP11-130C1	-0.425358	-0.921713	-2.629193	0.0092001	0.0295142	-3.295249
CRTC1	-0.064665	5.8237692	-2.62911	0.0092023	0.0295176	-3.295455
ACE2	0.6291382	3.8963015	2.6290906	0.0092028	0.0295176	-3.295505
RP11-1029J	0.5673569	-0.935868	2.6289535	0.0092064	0.0295265	-3.29585
PGM2L1	0.1431195	5.1475742	2.6287982	0.0092105	0.029537	-3.29624
RNASEH2B-A	-0.452615	2.0790707	-2.628747	0.0092118	0.0295386	-3.296368
HOXC5	-0.493787	-0.987586	-2.628673	0.0092138	0.0295423	-3.296555
RP11-115H1	0.4765991	-0.735022	2.6282777	0.0092241	0.0295729	-3.297548
AC007403.3	-0.362799	-1.21093	-2.62799	0.0092317	0.0295945	-3.298272
ARL14	0.8311987	2.8451898	2.6273612	0.0092482	0.0296449	-3.299851
ZNF18	0.073168	5.4753108	2.6269643	0.0092586	0.0296757	-3.300848
RP11-815J4	-0.591271	1.0837574	-2.62688	0.0092608	0.0296802	-3.301059
RP11-6B4.1	0.9745621	2.1679026	2.6264559	0.009272	0.0297134	-3.302125
TAS2R20	-0.508807	2.1264174	-2.626241	0.0092777	0.0297289	-3.302664
RSPO2	0.8114061	0.6543601	2.6260476	0.0092828	0.0297413	-3.30315
RP11-315I2	-0.208629	3.661841	-2.626033	0.0092832	0.0297413	-3.303188
RPL29P19	0.6055907	1.2489961	2.6256311	0.0092938	0.0297727	-3.304196
RP5-832C2.	0.2834825	3.4519232	2.6255481	0.009296	0.029776	-3.304404
RP11-711C1	-0.318292	-1.35486	-2.62553	0.0092964	0.029776	-3.304451
LCN8	-0.376472	-1.178215	-2.62533	0.0093017	0.0297903	-3.304952
RP11-675F6	-0.588718	-0.713867	-2.625282	0.009303	0.0297917	-3.305071
RP5-1051H1	-0.486274	-1.103204	-2.625168	0.009306	0.0297988	-3.305359
IDI1	-0.092863	6.6807907	-2.625013	0.0093101	0.0298093	-3.305747
HMGB1P11	-0.436368	-0.955571	-2.624703	0.0093183	0.029833	-3.306526
RNF121	-0.05462	6.0023271	-2.624647	0.0093198	0.0298351	-3.306667
UBTFL11	-0.240438	-1.414943	-2.624598	0.0093211	0.0298366	-3.30679
RP5-106502	0.4625607	-1.032896	2.6245649	0.009322	0.0298368	-3.306872
RALGAPB	-0.041019	6.274388	-2.624517	0.0093232	0.0298382	-3.306992
CTC-459F4.	-0.311556	3.4814509	-2.623633	0.0093467	0.0299106	-3.309211
RP11-417L1	-0.693813	1.9321166	-2.623554	0.0093488	0.0299147	-3.309409
AC104809.3	-0.706024	1.9161363	-2.623433	0.009352	0.0299223	-3.309711
CKMT1B	-0.943005	1.0905756	-2.623236	0.0093572	0.0299365	-3.310206
RP11-680H2	-0.655245	-0.104114	-2.62308	0.0093613	0.0299471	-3.310597
CDC42EP2	-0.110247	5.4011221	-2.621901	0.0093927	0.0300449	-3.313555
GCFC2	-0.060563	5.959858	-2.621856	0.0093939	0.0300461	-3.313667
MXI1	0.0748814	6.2094517	2.6217658	0.0093963	0.0300511	-3.313893
BET1	0.0506816	5.9943582	2.6216792	0.0093986	0.0300559	-3.31411
GMFG	0.0995739	5.7933858	2.621491	0.0094037	0.0300693	-3.314582
IRF2BP2	-0.054875	6.8144694	-2.621282	0.0094092	0.0300845	-3.315107
CBX3	-0.040931	6.6469813	-2.62111	0.0094138	0.0300965	-3.315536
MAGEB10	-0.430457	-1.208557	-2.621001	0.0094167	0.0301032	-3.315811
SMIM24	-0.669107	4.4909847	-2.620762	0.0094231	0.030121	-3.316409
ZFHX2	-0.242416	4.2758213	-2.620637	0.0094265	0.030129	-3.316722
CTD-3098H1	0.880009	2.9575848	2.6206031	0.0094274	0.0301293	-3.316807
RP11-415F2	0.3272794	2.6669205	2.6205321	0.0094293	0.0301324	-3.316986

ARHGAP33	-0.111531	5.3948766	-2.620477	0.0094307	0.0301324	-3.317123
TRAP1	0.0721728	6.5475291	2.6204742	0.0094308	0.0301324	-3.317131
IGHD3-3	-0.402038	-1.205796	-2.620275	0.0094362	0.0301468	-3.317629
DIABLO	-0.066487	5.483818	-2.620199	0.0094382	0.0301507	-3.31782
RPL28	-0.062146	7.1561141	-2.619666	0.0094525	0.0301936	-3.319155
SERINC3	0.043586	6.7374523	2.6194724	0.0094577	0.0302076	-3.319641
GNL3	-0.053303	6.4520025	-2.618166	0.0094928	0.0303171	-3.322913
RPL13AP2	-0.548548	0.0038716	-2.618133	0.0094937	0.0303172	-3.322996
KIAA1147	0.0847848	6.2752357	2.6175734	0.0095087	0.0303605	-3.324396
CXADRP3	-0.460202	-0.933397	-2.617567	0.0095089	0.0303605	-3.324411
BRMS1L	0.0653164	5.358922	2.6170272	0.0095234	0.0304044	-3.325763
TMTC4	-0.08653	5.9363851	-2.616996	0.0095243	0.0304045	-3.325842
SIGLEC22P	0.5754846	0.0098959	2.6169624	0.0095252	0.0304047	-3.325925
NCF1B	0.3772834	3.5827601	2.6169049	0.0095268	0.030407	-3.326069
RP11-229M1	-0.309125	-1.289061	-2.616708	0.0095321	0.0304213	-3.326561
ADAM24P	0.0987285	-1.55936	2.616573	0.0095357	0.030429	-3.3269
RP11-3K24.	-0.601266	-0.162744	-2.616557	0.0095362	0.030429	-3.32694
KREMEN2	-0.614201	2.6560317	-2.616106	0.0095483	0.0304652	-3.328067
KCNK13	0.4107163	3.8384298	2.6159228	0.0095533	0.0304772	-3.328527
TMLHE	0.0770248	5.6197313	2.6158959	0.009554	0.0304772	-3.328594
AC012065.1	-0.2731	3.0100407	-2.615821	0.0095561	0.0304772	-3.328782
NDUFS5	0.0637692	6.6186699	2.6157858	0.009557	0.0304772	-3.32887
RP11-415I1	-0.556711	0.6870513	-2.615775	0.0095573	0.0304772	-3.328897
RP11-477G1	-0.261109	-1.356417	-2.615756	0.0095578	0.0304772	-3.328945
ECE2	-0.095283	5.8605466	-2.615751	0.0095579	0.0304772	-3.328956
CDRT1	0.7545966	1.6115532	2.6153153	0.0095698	0.0305122	-3.330047
TCEB2	0.0636595	6.69164	2.6147228	0.0095858	0.0305597	-3.331528
RP11-503E2	-0.577905	1.4165381	-2.614705	0.0095863	0.0305597	-3.331574
RP11-175P1	-0.544163	2.8558543	-2.614582	0.0095897	0.0305677	-3.331882
IP6K1	-0.049686	6.3269008	-2.614456	0.0095931	0.0305759	-3.332195
HELQ	0.0542536	5.5045145	2.6144024	0.0095945	0.0305778	-3.33233
RAP1A	0.0459376	6.4126951	2.6143665	0.0095955	0.0305783	-3.332419
KB-1572G7.	-0.348265	-1.215684	-2.613971	0.0096063	0.0306082	-3.333408
RP13-942N8	-0.226827	3.9651965	-2.61396	0.0096066	0.0306082	-3.333436
ERICH6	-0.584252	0.8528011	-2.6139	0.0096082	0.0306107	-3.333587
RPL29P30	-0.390609	-1.017214	-2.613763	0.0096119	0.0306173	-3.333927
LINC01423	0.3140235	-1.331064	2.6137622	0.0096119	0.0306173	-3.33393
RP11-84N19	-0.797132	0.0167138	-2.613471	0.0096199	0.0306399	-3.334659
PLD4	0.3013504	4.465549	2.6131729	0.009628	0.0306629	-3.335403
BIN2	0.1518134	5.3032509	2.6131443	0.0096288	0.0306629	-3.335475
ENO1	0.0677857	7.2650112	2.6131052	0.0096298	0.0306636	-3.335573
LCTL	-0.495077	2.3459132	-2.613047	0.0096314	0.030666	-3.335719
BACH1-IT1	-0.481297	2.0067032	-2.612872	0.0096362	0.0306784	-3.336154
ARTN	-0.422245	3.2614249	-2.612596	0.0096437	0.0306998	-3.336844
KIAA1279	0.0516551	5.8802476	2.6120421	0.0096589	0.0307453	-3.338229
CTD-3064C1	-0.385092	-1.264475	-2.611846	0.0096642	0.0307574	-3.33872
PSMD4	-0.061408	6.8863164	-2.611841	0.0096644	0.0307574	-3.338732
FGF12	0.2825533	4.5897693	2.611767	0.0096664	0.0307612	-3.338916
PTBP3	-0.047818	6.4154108	-2.611551	0.0096723	0.0307773	-3.339455
ZNF443	-0.094516	4.8884653	-2.611132	0.0096838	0.0308111	-3.340503
RP11-522M2	-0.423213	-0.950229	-2.610975	0.0096881	0.0308221	-3.340895

AC007041.2	-0.56509	0.6557756	-2.610927	0.0096894	0.0308236	-3.341014
CROT	-0.10698	6.2117338	-2.610885	0.0096905	0.0308246	-3.34112
HILS1	-0.542391	-0.863861	-2.610848	0.0096916	0.0308252	-3.341213
PCDHB7	0.4029856	3.7584384	2.610548	0.0096998	0.0308478	-3.341961
CFHR3	0.4122636	5.9497026	2.6105264	0.0097004	0.0308478	-3.342015
CDC42BPB	0.051143	6.5399801	2.6102453	0.0097081	0.0308697	-3.342717
RP3-461P17	-0.454293	2.3122624	-2.609752	0.0097216	0.0309101	-3.343949
ATG101	0.0553651	6.0147009	2.6096616	0.0097241	0.030913	-3.344174
XXbac-B135	-0.346354	3.4944336	-2.609657	0.0097243	0.030913	-3.344186
CACNA2D3	0.4535018	3.2431978	2.6095269	0.0097278	0.0309217	-3.34451
NPHP3-AS1	-0.543591	-0.327002	-2.609141	0.0097385	0.0309528	-3.345474
APCDD1L	0.7591619	0.5705083	2.6090937	0.0097397	0.0309542	-3.345591
RP11-580P2	-0.384977	-1.115855	-2.608673	0.0097513	0.0309883	-3.346642
CTC-542B22	-0.592987	-0.253494	-2.608007	0.0097697	0.031044	-3.348303
CTC-265N9.	0.3292639	-1.371485	2.6078645	0.0097736	0.0310538	-3.348659
RP4-564F22	-0.560136	2.0511718	-2.607654	0.0097795	0.0310696	-3.349183
AKAP11	0.0692056	6.0426309	2.607555	0.0097822	0.0310756	-3.349431
RP11-49K24	-0.552367	-0.366437	-2.607516	0.0097833	0.0310763	-3.349528
PRH1	-0.443942	2.3321072	-2.607175	0.0097927	0.0311036	-3.350379
ANKFN1	-0.775259	-0.013951	-2.606931	0.0097995	0.0311222	-3.350986
RP13-20L14	0.1809109	4.1523645	2.6069011	0.0098003	0.0311222	-3.351062
PSORS1C1	0.5136928	3.3321691	2.6068507	0.0098017	0.031124	-3.351187
YDJC	-0.080166	5.8707375	-2.606607	0.0098084	0.0311427	-3.351794
RP11-172F4	-0.567418	0.6733214	-2.606483	0.0098119	0.0311509	-3.352103
SURF1	0.0687293	6.37757	2.6064033	0.0098141	0.0311539	-3.352303
CYTL1	0.542914	2.5111914	2.6063872	0.0098145	0.0311539	-3.352343
RP5-965G21	-0.352637	3.4342447	-2.606321	0.0098164	0.031157	-3.352508
KRT3	0.3023296	-1.360281	2.6062525	0.0098183	0.0311603	-3.352679
TSGA10	-0.133879	4.6840904	-2.60622	0.0098192	0.0311605	-3.352761
SNORD3B-1	-0.56812	-0.429567	-2.60588	0.0098286	0.0311877	-3.353607
OAZ1	0.0499888	7.0335908	2.6058315	0.00983	0.0311893	-3.353728
HNRNPLP2	-0.307302	3.1196561	-2.605473	0.0098399	0.0312182	-3.354622
RP11-10C24	-0.158737	4.1520158	-2.604984	0.0098535	0.0312586	-3.35584
IGHA2	0.4004916	5.1557554	2.6048942	0.009856	0.0312638	-3.356064
CH507-154E	-0.575119	-0.098069	-2.604752	0.00986	0.0312736	-3.356418
RP11-686D2	0.6572479	1.2714102	2.6046991	0.0098614	0.0312752	-3.356551
ALAS2	0.6790429	1.1043623	2.6046529	0.0098627	0.0312752	-3.356666
SLC46A2	-0.747474	1.0926175	-2.60462	0.0098637	0.0312752	-3.356749
RP3-395M20	-0.467927	2.434442	-2.604612	0.0098639	0.0312752	-3.356769
RP11-368M1	-0.32266	-1.327369	-2.604512	0.0098666	0.0312812	-3.357016
RP11-421M1	-0.528295	1.9256631	-2.604091	0.0098784	0.0313157	-3.358065
CTD-2353F2	0.5796057	-0.340819	2.6032548	0.0099017	0.031387	-3.360149
HSD3B2	0.7463275	0.81481	2.6031585	0.0099044	0.0313928	-3.360388
RP11-800A3	0.6294427	1.7586133	2.6031033	0.009906	0.031395	-3.360526
RPL7	-0.067716	7.1179595	-2.602976	0.0099095	0.0314036	-3.360844
RP11-407G2	0.5899024	0.2927061	2.6028852	0.0099121	0.0314089	-3.361069
FAM169A	-0.319043	4.6251553	-2.602694	0.0099174	0.0314231	-3.361546
RP11-522B1	0.5265389	-0.940191	2.6025373	0.0099218	0.0314343	-3.361935
LUZP4	-0.647638	-0.761956	-2.602392	0.0099259	0.0314445	-3.362298
POFUT2	-0.055809	6.0319374	-2.601695	0.0099454	0.0315036	-3.364033
PREX1	0.0826392	6.2070022	2.6015682	0.009949	0.03151	-3.364348

GAS7	0.1585597	5.4923305	2.6015616	0.0099492	0.03151	-3.364364
CTD-2561B2	-0.481949	-0.827259	-2.601385	0.0099541	0.0315211	-3.364803
RP11-484L8	0.4925108	-0.633148	2.6013757	0.0099544	0.0315211	-3.364827
CRAT	0.0764936	6.698723	2.601086	0.0099625	0.0315441	-3.365548
TBC1D19	0.1246726	4.8882518	2.601004	0.0099648	0.0315487	-3.365752
FBXO42	0.0517602	5.9458184	2.6008565	0.009969	0.0315591	-3.366119
VANGL1	0.0727772	5.7721528	2.6003418	0.0099835	0.0316022	-3.3674
C19orf38	0.1754345	4.5257606	2.6002836	0.0099851	0.0316046	-3.367545
PHBP9	-0.527489	2.0490341	-2.600166	0.0099884	0.0316098	-3.367836
CREB3L2	-0.057743	6.4171025	-2.600136	0.0099893	0.0316098	-3.367912
RMDN2-AS1	-0.573467	-0.16744	-2.600133	0.0099893	0.0316098	-3.367919
RP11-13P5.	-0.30153	-1.295158	-2.600059	0.0099914	0.0316136	-3.368104
NEK3	-0.113822	5.4990577	-2.600028	0.0099923	0.0316136	-3.368181
ELOVL4	-0.470715	3.0090916	-2.599998	0.0099931	0.0316136	-3.368254
PDE3A	0.1889703	5.3289729	2.5998109	0.0099984	0.0316276	-3.368721
RP11-410N8	-0.318602	-1.341703	-2.59964	0.0100032	0.0316401	-3.369146
LILRB4	0.1888967	5.3934625	2.5995882	0.0100047	0.031642	-3.369275
BBS4	0.062324	5.8031666	2.5992776	0.0100135	0.0316669	-3.370047
FAM173B	0.0640668	5.7064816	2.5982045	0.0100438	0.0317598	-3.372715
LMBR1L	-0.047493	5.9763938	-2.598178	0.0100446	0.0317598	-3.372782
DDX18P1	-0.482187	-0.560026	-2.598087	0.0100471	0.0317651	-3.373007
LINC00240	0.2140055	4.3204145	2.5977222	0.0100575	0.0317927	-3.373914
ENHO	-0.307941	5.3637236	-2.597718	0.0100576	0.0317927	-3.373925
B4GALT5	0.0617308	6.3902103	2.5975512	0.0100623	0.0318049	-3.374339
AC087499.1	-0.387231	-1.252474	-2.59746	0.0100649	0.0318103	-3.374567
GPR61	-0.587024	1.3277054	-2.597126	0.0100744	0.0318375	-3.375397
HSPD1P1	-0.346867	3.3570901	-2.596961	0.0100791	0.0318496	-3.375807
CA3	0.6407486	2.7426364	2.5967906	0.0100839	0.0318621	-3.376229
MYBPC1	-0.69526	2.1794482	-2.596502	0.0100921	0.0318853	-3.376947
TGFBR2	0.0742221	6.5575479	2.5962786	0.0100985	0.0319026	-3.377502
NCOA1	0.0527461	6.2195645	2.5961495	0.0101021	0.0319091	-3.377822
TDRG1	-0.457172	-0.97135	-2.596145	0.0101023	0.0319091	-3.377834
AP000275.6	0.5724551	0.2824716	2.5959368	0.0101082	0.031925	-3.378351
RP3-406P24	-0.394664	3.1482714	-2.595779	0.0101127	0.0319343	-3.378743
SNORD1B	-0.367044	-1.089906	-2.595743	0.0101137	0.0319343	-3.378831
GPR78	0.5883405	-0.408334	2.5957409	0.0101138	0.0319343	-3.378837
CRLF3	-0.057419	5.602394	-2.595393	0.0101237	0.0319629	-3.379702
PDGFRL	-0.224222	4.596383	-2.59533	0.0101255	0.0319658	-3.379857
RP11-244H3	0.4861032	1.6580613	2.5950464	0.0101336	0.0319864	-3.380562
AC006547.1	0.1261325	4.5445167	2.5950398	0.0101338	0.0319864	-3.380578
PTPRF	0.066586	7.0094917	2.5949649	0.0101359	0.0319889	-3.380764
DHRS3	0.082773	6.8548364	2.5949504	0.0101363	0.0319889	-3.3808
RP11-889L3	-0.271329	3.3565103	-2.59489	0.010138	0.0319916	-3.380951
HSPA8P15	-0.592175	0.5883048	-2.594589	0.0101466	0.0320128	-3.381697
PRSS8	0.7927357	4.485997	2.5945821	0.0101468	0.0320128	-3.381715
RP11-100M1	0.0768235	-1.570524	2.5945629	0.0101474	0.0320128	-3.381762
CACNA1H	0.2961688	5.735224	2.5945209	0.0101486	0.0320138	-3.381867
OR6C2	-0.262854	-1.425856	-2.59436	0.0101532	0.0320255	-3.382265
TBX2	0.1074314	5.8035268	2.5941008	0.0101606	0.0320461	-3.38291
USP51	-0.231216	4.3655394	-2.594007	0.0101633	0.0320516	-3.383143
OSBPL9	0.065111	6.5900544	2.5939784	0.0101641	0.0320516	-3.383214

ARHGEF5	0.148307	5.732922	2.5938463	0.0101679	0.0320608	-3.383541
ST6GALNAC5	-0.799512	2.1426973	-2.593702	0.010172	0.0320711	-3.383901
AC007279.2	-0.585553	0.3125789	-2.593515	0.0101774	0.0320852	-3.384365
CTC-338M12	-0.163482	4.3339703	-2.593445	0.0101794	0.0320887	-3.384538
TM4SF19-AS	-0.438075	2.7795935	-2.593367	0.0101816	0.0320925	-3.384731
SDPR	0.1650156	5.7606822	2.593342	0.0101823	0.0320925	-3.384793
GCDH	0.1095111	6.3048285	2.5933063	0.0101833	0.0320929	-3.384882
CH17-353B1	-0.656152	0.0982175	-2.593014	0.0101917	0.0321165	-3.385607
C14orf180	0.8667672	0.8862724	2.5928427	0.0101966	0.0321293	-3.386032
RP11-354K4	0.3706922	-1.289365	2.5928034	0.0101978	0.03213	-3.386129
HTR4	-0.775734	1.9409279	-2.592639	0.0102025	0.0321421	-3.386537
XAB2	-0.049888	6.3963367	-2.592593	0.0102038	0.0321436	-3.386653
POT1	0.0683894	5.8278078	2.5922081	0.0102148	0.0321756	-3.387606
ZNF549	-0.197651	4.7992189	-2.592054	0.0102193	0.0321824	-3.387988
RP11-543H2	-0.327145	-1.307609	-2.592049	0.0102194	0.0321824	-3.388001
KANSL1	-0.04613	6.1764023	-2.592039	0.0102197	0.0321824	-3.388025
ZNF563	-0.169198	5.4730372	-2.59201	0.0102206	0.0321824	-3.388099
P2RX5-TAX1	-0.25062	3.3741469	-2.591944	0.0102224	0.0321856	-3.388261
RP4-797C5.	0.2861086	-1.372544	2.5918743	0.0102244	0.0321892	-3.388434
NUP214	-0.040263	6.398115	-2.591703	0.0102294	0.0322019	-3.388859
TXLNGY	1.1852759	3.1636721	2.5914291	0.0102373	0.0322239	-3.389539
RP11-25I15	-0.584593	0.6896296	-2.591209	0.0102436	0.03224	-3.390085
RP5-1059L7	0.5603038	2.8167921	2.5911638	0.0102449	0.03224	-3.390196
CHP2	-0.837029	-0.174831	-2.591141	0.0102455	0.03224	-3.390252
ASB1	-0.067825	5.9196324	-2.59113	0.0102459	0.03224	-3.390281
PAXIP1-AS1	-0.096949	5.5249039	-2.590888	0.0102528	0.0322591	-3.390879
CTD-2561B2	0.2563492	-1.387978	2.5907506	0.0102568	0.0322661	-3.391221
RP4-612B15	-0.589111	0.7928114	-2.59075	0.0102568	0.0322661	-3.391223
AGT	-0.105052	7.5365237	-2.59065	0.0102597	0.0322717	-3.391471
RP3-483K16	-0.435712	3.1603642	-2.590626	0.0102604	0.0322717	-3.391529
RHCE	0.5106396	3.4262768	2.5903928	0.0102671	0.0322902	-3.392108
CLEC9A	0.5907274	2.5929169	2.5899058	0.0102812	0.0323316	-3.393315
KB-1615E4.	0.7294401	2.4051104	2.589566	0.010291	0.0323581	-3.394157
CH17-262A2	-0.608049	1.4645105	-2.589553	0.0102914	0.0323581	-3.394189
RP11-16C1.	-0.491586	-0.618178	-2.589431	0.0102949	0.0323664	-3.394491
FLJ12825	-0.682563	1.1803325	-2.589377	0.0102965	0.0323686	-3.394626
ZHX1-C8orf	-0.164359	4.2961276	-2.589277	0.0102994	0.0323749	-3.394873
ZXDB	0.1295868	5.9202378	2.5891661	0.0103026	0.0323803	-3.395148
ASAP3	0.1409608	5.8829807	2.5891559	0.0103029	0.0323803	-3.395174
TMEM261	0.1159501	5.8853377	2.5889971	0.0103075	0.032392	-3.395567
AF131215.8	0.5330212	-0.643877	2.5889668	0.0103084	0.032392	-3.395642
ERAS	-0.617314	0.5094335	-2.588892	0.0103106	0.0323957	-3.395828
AC012462.3	0.0768235	-1.570524	2.588865	0.0103113	0.0323957	-3.395894
RP11-320H1	-0.724011	-0.574874	-2.58851	0.0103216	0.0324209	-3.396774
GAPDHP33	-0.541275	-0.440993	-2.588502	0.0103219	0.0324209	-3.396795
PIK3CG	0.3345127	4.3494054	2.5884962	0.010322	0.0324209	-3.396808
DHRS4-AS1	0.0946955	5.9222331	2.5882088	0.0103304	0.0324443	-3.39752
DISC1	0.1510399	4.9146434	2.5879587	0.0103376	0.0324643	-3.39814
HTR2C	-0.683052	-0.546781	-2.587629	0.0103472	0.0324916	-3.398956
RP11-1072C	-0.520277	0.8983478	-2.587145	0.0103613	0.032533	-3.400154
NR0B1	-0.970951	0.6008988	-2.587107	0.0103624	0.0325337	-3.400249

SLC10A6	0.672061	1.6096953	2.5870745	0.0103634	0.0325339	-3.400329
TRHDE-AS1	1.0092127	1.2521734	2.5869257	0.0103677	0.0325447	-3.400698
PLA2G4D	-0.667759	-0.236426	-2.586836	0.0103703	0.03255	-3.400919
SNHG19	-0.159674	5.371618	-2.586776	0.0103721	0.0325528	-3.401069
SPN	0.1722415	5.2525593	2.5866468	0.0103758	0.0325618	-3.401388
ATP1A1-AS1	-0.123729	4.6534003	-2.586377	0.0103837	0.0325837	-3.402056
AC010641.1	0.3325873	-1.336054	2.5860847	0.0103922	0.0326076	-3.402779
RP11-38902	-0.567504	2.1755167	-2.58582	0.0103999	0.0326287	-3.403434
AP000350.5	0.8272191	2.0978612	2.5857938	0.0104007	0.0326287	-3.4035
SLITRK1	-0.655783	-0.669205	-2.58567	0.0104043	0.0326372	-3.403805
RP11-26001	-0.481539	-1.044647	-2.585587	0.0104068	0.0326421	-3.404013
KLHDC8B	-0.093166	5.9844291	-2.584995	0.010424	0.0326935	-3.405475
AC023115.4	0.3485795	-1.241338	2.5847603	0.0104309	0.0327101	-3.406057
MIA	-0.625298	-0.095915	-2.584754	0.0104311	0.0327101	-3.406073
CTD-2515A1	0.6146909	0.6294037	2.5843786	0.0104421	0.0327417	-3.407001
PRSS46	-0.670725	-0.176387	-2.584325	0.0104437	0.0327437	-3.407134
DENND5B	-0.081807	6.0632928	-2.584281	0.010445	0.0327437	-3.407243
SHMT2	0.0674197	6.8207139	2.5842582	0.0104456	0.0327437	-3.407299
SYNGAP1	0.1118777	5.353737	2.5842348	0.0104463	0.0327437	-3.407357
TMEM181	-0.071051	6.2389024	-2.584117	0.0104498	0.0327517	-3.407648
CKM	-0.619659	1.2867946	-2.583949	0.0104547	0.0327643	-3.408064
SPDYE1	-0.51571	1.5403853	-2.583783	0.0104596	0.0327768	-3.408474
CADPS	-0.831164	2.1897252	-2.583606	0.0104648	0.0327903	-3.408912
ABCE1	0.0507513	6.2857105	2.5835525	0.0104664	0.0327924	-3.409044
GON4L	-0.051168	6.1092903	-2.583411	0.0104705	0.0328026	-3.409394
CLEC4GP1	0.6278226	-0.432821	2.5833086	0.0104735	0.0328092	-3.409648
DHX9P1	-0.414878	-0.875209	-2.582604	0.0104942	0.0328713	-3.411389
PIK3CB	0.0511533	6.1408549	2.5825159	0.0104968	0.0328766	-3.411607
SLFN11	0.1476815	5.5047645	2.5824802	0.0104979	0.0328771	-3.411695
RP11-107I1	-0.414735	-1.157385	-2.582302	0.0105032	0.0328907	-3.412137
PIP5K1A	-0.052928	6.3027537	-2.582219	0.0105056	0.0328955	-3.41234
CD200	0.173382	5.115713	2.5821279	0.0105083	0.0329004	-3.412566
LINC00899	0.2120866	4.3505121	2.5821054	0.0105089	0.0329004	-3.412622
MIR659	-0.512036	-0.360095	-2.581869	0.0105159	0.0329194	-3.413207
RCC2P3	-0.32979	-1.178728	-2.581478	0.0105274	0.0329527	-3.414172
MYPOP	-0.070503	5.5682426	-2.581129	0.0105378	0.0329822	-3.415035
CTD-2017C7	-0.520663	1.802967	-2.580977	0.0105423	0.0329934	-3.41541
RP11-512F2	-0.499776	1.8101878	-2.580922	0.0105439	0.0329957	-3.415547
RHOD	0.1269271	6.2252321	2.5806841	0.0105509	0.0330148	-3.416134
NFKB2	0.0602228	6.4793982	2.5804843	0.0105568	0.0330305	-3.416627
STRCP1	-0.642075	1.872108	-2.580438	0.0105582	0.0330319	-3.416741
RP11-505K9	0.4479034	1.7095471	2.5799058	0.010574	0.0330784	-3.418056
SETBP1	0.1291212	5.6031082	2.5798754	0.0105749	0.0330784	-3.418131
RP11-127I2	-0.576484	0.922645	-2.57936	0.0105902	0.0331234	-3.419404
EMR4P	0.5324691	2.7473217	2.5793007	0.0105919	0.0331261	-3.41955
CATSPERG	0.1895994	4.3016375	2.5792666	0.0105929	0.0331264	-3.419634
RIF1	-0.052659	6.1845974	-2.579105	0.0105977	0.0331386	-3.420034
HIST1H2APS	-0.69329	0.395991	-2.578953	0.0106023	0.0331499	-3.420409
TPM3P6	-0.55346	2.666003	-2.578573	0.0106136	0.0331824	-3.421347
AC018738.2	-0.141101	5.2262116	-2.578483	0.0106162	0.0331878	-3.421568
TMIE	-0.445504	4.0289136	-2.578385	0.0106192	0.0331929	-3.421812

CFAP69	0.1931648	4.6350269	2.5783683	0.0106196	0.0331929	-3.421852
RNU6ATAC35	-0.579148	2.3651833	-2.578272	0.0106225	0.033199	-3.42209
RPN2	-0.043387	7.0713182	-2.577908	0.0106333	0.03323	-3.422988
LCE3D	0.2830252	-1.442676	2.5778081	0.0106363	0.0332349	-3.423235
LL0XNC01-2	0.2988265	-1.319715	2.577794	0.0106367	0.0332349	-3.42327
LHX2	0.4096798	3.7858969	2.5775801	0.0106431	0.033252	-3.423797
AC007969.5	-0.139681	5.0557803	-2.577402	0.0106484	0.0332658	-3.424238
LINC00852	-0.193014	3.5331867	-2.577144	0.0106561	0.033287	-3.424873
GPSM3	0.1038308	5.7894553	2.5769327	0.0106624	0.0333027	-3.425395
GRTP1	-0.126277	6.1143411	-2.576914	0.010663	0.0333027	-3.42544
AC087499.5	-0.459015	-1.11706	-2.576849	0.0106649	0.0333059	-3.4256
RN7SL208P	0.4956929	-0.636416	2.5766575	0.0106707	0.033319	-3.426074
RP11-310I2	0.2429724	-1.435564	2.5766447	0.010671	0.033319	-3.426105
TMEM50B	0.0545359	6.1113612	2.5766172	0.0106719	0.033319	-3.426173
RPL19P20	-0.489551	-0.715886	-2.576561	0.0106735	0.0333214	-3.42631
RP11-239E1	0.4716454	-0.84646	2.5763748	0.0106791	0.0333346	-3.426771
RPS2P46	-0.158836	4.8649395	-2.576354	0.0106797	0.0333346	-3.426821
AC022007.5	-0.192517	4.3751244	-2.576328	0.0106805	0.0333346	-3.426886
RP11-20024	-0.566469	0.2652406	-2.576015	0.0106899	0.033361	-3.427659
PKN2-AS1	0.630464	0.2896676	2.575878	0.010694	0.0333709	-3.427996
CLK4	0.0644935	5.7714516	2.575789	0.0106966	0.0333764	-3.428216
HNRNPKP2	-0.386106	2.1096852	-2.575722	0.0106986	0.0333798	-3.42838
RP5-906C1.	-0.497003	2.008626	-2.575623	0.0107016	0.0333862	-3.428624
IRX6	0.7619047	0.0098345	2.575513	0.0107049	0.0333936	-3.428896
LINC00698	-0.399712	-1.161765	-2.575468	0.0107063	0.033395	-3.429008
LINC00948	-0.545578	-0.879891	-2.575328	0.0107105	0.0334052	-3.429351
HNF1A	-0.284363	5.8374227	-2.575073	0.0107181	0.0334262	-3.42998
CEBPD	0.1030278	6.5581358	2.574839	0.0107251	0.0334453	-3.430558
RP3-434P1.	0.8727413	2.6398529	2.5745482	0.0107339	0.0334697	-3.431274
KPNB1	-0.036333	6.7684458	-2.574233	0.0107433	0.0334963	-3.43205
AC091320.1	-0.410917	-1.082758	-2.573902	0.0107533	0.0335245	-3.432867
RHO	-0.723305	1.3097903	-2.573727	0.0107586	0.0335381	-3.433299
RP9P	-0.136273	4.8842191	-2.573649	0.0107609	0.0335401	-3.433491
AP001042.1	0.4757921	-1.0379	2.5736448	0.010761	0.0335401	-3.433501
CTD-2047H1	-0.375075	3.1177469	-2.573578	0.0107631	0.0335435	-3.433666
C5orf63	-0.287903	4.852809	-2.573354	0.0107698	0.0335617	-3.434218
DLX1	-0.745682	0.588879	-2.573313	0.0107711	0.0335627	-3.434319
RPS27AP2	-0.488858	-0.474879	-2.573201	0.0107744	0.0335703	-3.434593
SRP54	0.0559747	6.3889535	2.5731232	0.0107768	0.033574	-3.434786
HDAC10	-0.084343	5.6482696	-2.5731	0.0107775	0.033574	-3.434843
DND1P1	0.783639	1.4082802	2.5730327	0.0107795	0.0335764	-3.435009
RP11-481J2	-0.551209	0.316677	-2.573014	0.0107801	0.0335764	-3.435056
RP11-400N9	-0.44659	-0.766217	-2.571996	0.0108108	0.0336693	-3.437562
RHOBTB3	-0.104118	6.3247306	-2.571909	0.0108134	0.0336746	-3.437777
RNU6-813P	0.3768585	-1.233216	2.5716667	0.0108208	0.0336946	-3.438373
AC006159.4	-0.29173	-1.341401	-2.571431	0.0108279	0.0337133	-3.438953
BRICD5	-0.150814	4.7086389	-2.571402	0.0108288	0.0337133	-3.439025
ZDHHC12	-0.06542	6.2484824	-2.571377	0.0108295	0.0337133	-3.439087
RP11-248N2	0.3875848	-1.149005	2.5712964	0.010832	0.033718	-3.439285
CTD-2515C1	-0.554178	-0.833822	-2.570901	0.010844	0.0337187	-3.440259
NECAB3	-0.078826	6.2644422	-2.570842	0.0108458	0.0337187	-3.440404

CTD-2326C4	0.0549185	-1.581687	2.5708007	0.010847	0.0337187	-3.440505
ENOX1-AS2	0.0549185	-1.581687	2.5708007	0.010847	0.0337187	-3.440505
HNRNPFP1	0.0549185	-1.581687	2.5708007	0.010847	0.0337187	-3.440505
RP1-293L6.	0.0549185	-1.581687	2.5708007	0.010847	0.0337187	-3.440505
RP11-208N2	0.0549185	-1.581687	2.5708007	0.010847	0.0337187	-3.440505
RP11-45F15	0.0549185	-1.581687	2.5708007	0.010847	0.0337187	-3.440505
RP11-562L8	0.0549185	-1.581687	2.5708007	0.010847	0.0337187	-3.440505
RP11-76E12	0.0549185	-1.581687	2.5708007	0.010847	0.0337187	-3.440505
RP11-775A1	0.0549185	-1.581687	2.5708007	0.010847	0.0337187	-3.440505
RP6-43L17.	0.0549185	-1.581687	2.5708007	0.010847	0.0337187	-3.440505
SSU72P3	0.0549185	-1.581687	2.5708007	0.010847	0.0337187	-3.440505
VN1R25P	0.0549185	-1.581687	2.5708007	0.010847	0.0337187	-3.440505
Z95114.5	0.0549185	-1.581687	2.5708007	0.010847	0.0337187	-3.440505
ZNF385D-AS	0.0549185	-1.581687	2.5708007	0.010847	0.0337187	-3.440505
C2orf69	0.0668219	5.7839791	2.5706538	0.0108515	0.033728	-3.440867
RP11-435F1	-0.56984	1.3663727	-2.570641	0.0108519	0.033728	-3.440898
FMO1	-0.455691	4.439567	-2.570611	0.0108528	0.033728	-3.440972
HEY1	-0.125992	5.3934789	-2.570498	0.0108562	0.0337358	-3.441251
RP11-202G1	-0.207623	-1.45294	-2.570443	0.0108579	0.0337381	-3.441385
RCN2	-0.056722	6.1360053	-2.5704	0.0108592	0.0337393	-3.441492
LINC01450	-0.407661	-0.983952	-2.570352	0.0108606	0.0337409	-3.441609
TUBGCP6	-0.054709	6.213141	-2.570182	0.0108658	0.0337538	-3.442029
IGLC7	0.8407087	1.897594	2.5701545	0.0108666	0.0337538	-3.442096
GABARAPL3	0.28698	-1.283779	2.5699004	0.0108743	0.0337747	-3.442721
RP11-829H1	-0.382269	-1.043468	-2.569872	0.0108752	0.0337747	-3.442791
DOK7	0.4294502	4.3431026	2.5697541	0.0108788	0.033783	-3.443081
TIMM13	-0.067122	6.4844577	-2.569324	0.0108919	0.0338208	-3.444139
GTF2H2C	-0.131454	5.0129995	-2.569219	0.0108951	0.0338278	-3.444397
SLX1A-SULT	-0.553793	0.4725382	-2.56913	0.0108978	0.0338334	-3.444616
MIR4258	-0.501041	-0.401283	-2.56909	0.010899	0.0338342	-3.444713
MT1HL1	0.3637763	-1.28388	2.5690317	0.0109008	0.0338369	-3.444858
MIR196A1	0.2613047	-1.414354	2.5688378	0.0109067	0.0338519	-3.445335
C2CD2L	0.062085	5.7751552	2.5688124	0.0109075	0.0338519	-3.445397
KIAA1551	0.0751286	6.1633505	2.5687636	0.0109089	0.0338536	-3.445518
RP11-392P7	-0.117688	5.0627174	-2.568542	0.0109157	0.0338717	-3.446062
RPL24P7	-0.449038	-0.80775	-2.568275	0.0109239	0.0338941	-3.446719
PPP1R9A	-0.395905	4.9749897	-2.568061	0.0109304	0.0339115	-3.447245
RPSAP64	0.4003503	-1.191255	2.5679768	0.010933	0.0339166	-3.447452
RP11-20B24	0.5738387	0.1719047	2.5675277	0.0109467	0.0339563	-3.448557
CTD-3187F8	0.347262	-1.128631	2.5674344	0.0109495	0.0339623	-3.448786
SLC30A6	-0.044861	6.0980886	-2.567325	0.0109529	0.0339698	-3.449055
RP11-23J9.	-0.633639	1.5555871	-2.567259	0.0109549	0.0339731	-3.449217
RP11-436K8	-0.732876	0.3951828	-2.567114	0.0109593	0.033984	-3.449574
DSTNP5	0.103308	-1.557027	2.5668505	0.0109674	0.0340055	-3.450221
EIF1AY	1.1914012	3.2304002	2.5668268	0.0109681	0.0340055	-3.45028
C19orf18	-0.45131	3.2630919	-2.566762	0.0109701	0.0340087	-3.450438
KPRP	-0.472062	-1.051185	-2.56672	0.0109714	0.0340098	-3.450542
FAIM3	0.2088131	4.9787882	2.566646	0.0109737	0.034014	-3.450724
LIPJ	0.5751362	-0.051767	2.5661931	0.0109876	0.0340542	-3.451837
AJUBA	-0.097985	6.3323925	-2.565833	0.0109986	0.0340856	-3.452723
KCNMA1-AS1	0.5649194	-0.267989	2.5657759	0.0110004	0.0340881	-3.452862

MOAP1	0.0580343	6.0735629	2.5655686	0.0110067	0.0341049	-3.453371
NOTO	-0.341798	-1.294944	-2.565498	0.0110089	0.0341087	-3.453544
RP11-37C7.	-0.504287	2.5608273	-2.565156	0.0110194	0.0341385	-3.454386
OR2B6	-0.617498	1.8578353	-2.565108	0.0110209	0.0341401	-3.454504
CTD-2378E1	-0.560691	0.485692	-2.565036	0.0110231	0.0341418	-3.45468
CTC-55802.	-0.688142	1.2995992	-2.565029	0.0110233	0.0341418	-3.454697
RP11-87501	-0.655095	2.7919384	-2.564729	0.0110326	0.0341675	-3.455433
AC024937.4	-0.587714	-0.27556	-2.564526	0.0110388	0.0341815	-3.455934
RNF224	-0.667117	0.9896981	-2.564522	0.0110389	0.0341815	-3.455942
SLC25A15P3	0.8432942	0.4909741	2.5641132	0.0110515	0.0342176	-3.456946
ADRA2C	-0.395494	4.6820231	-2.563968	0.011056	0.0342286	-3.457303
IRF7	0.1072473	6.168616	2.5636814	0.0110649	0.034253	-3.458006
HIST1H3G	-0.797072	0.6849742	-2.563628	0.0110665	0.0342552	-3.458137
ACTR5	-0.073124	5.5756347	-2.563449	0.011072	0.0342673	-3.458576
RN7SL578P	0.1067284	-1.555284	2.5634413	0.0110723	0.0342673	-3.458596
THPO	-0.310829	5.7163722	-2.563115	0.0110824	0.0342956	-3.459396
DAAM2	0.1454755	5.4233289	2.5630682	0.0110838	0.0342972	-3.459512
PRPF31	-0.049782	6.3903156	-2.562927	0.0110882	0.0343077	-3.459858
SLC9A6	0.0607631	5.8556398	2.5627822	0.0110927	0.0343187	-3.460213
RP11-30K9.	-0.557398	0.4699464	-2.562731	0.0110942	0.0343207	-3.460339
MRGPRX2	-0.400728	-1.229989	-2.562627	0.0110975	0.0343278	-3.460595
RP11-467I1	0.3918172	-1.09717	2.5622693	0.0111085	0.0343591	-3.461472
RPS4XP11	-0.549779	1.688718	-2.561811	0.0111227	0.0344001	-3.462596
RNA5SP122	-0.527964	-0.260108	-2.561484	0.0111329	0.0344286	-3.463399
CTD-3037G2	-0.745547	0.0379043	-2.561447	0.0111341	0.0344293	-3.46349
CSTF3-AS1	-0.592822	0.3763425	-2.561308	0.0111384	0.0344396	-3.46383
CASP2	-0.066156	5.984575	-2.560998	0.011148	0.0344666	-3.464592
POC1B	0.0624961	5.7025769	2.5608393	0.0111529	0.0344789	-3.46498
ZNF76	-0.049057	6.1819707	-2.56062	0.0111598	0.0344971	-3.465519
CTC-546K23	-0.494932	-1.114445	-2.560539	0.0111623	0.0345019	-3.465717
UBQLN2	0.0557749	6.2406462	2.5597987	0.0111853	0.0345702	-3.467532
RP11-333E1	-0.53588	-0.067539	-2.55974	0.0111872	0.034573	-3.467676
GP1BA	0.2657243	3.9644261	2.5595966	0.0111916	0.0345839	-3.468027
EPG5	0.0607322	5.9938478	2.5594313	0.0111968	0.0345964	-3.468432
MIR503HG	0.239352	3.9835631	2.559406	0.0111976	0.0345964	-3.468494
RP11-532F6	0.3429741	-1.201401	2.5591258	0.0112063	0.0346181	-3.469181
RP11-226L1	-0.308211	-1.252651	-2.559111	0.0112068	0.0346181	-3.469217
RP11-672A2	0.4194708	-0.865128	2.5590719	0.011208	0.0346181	-3.469313
C10orf55	0.5849788	1.3807536	2.5590591	0.0112084	0.0346181	-3.469345
IL17RA	0.0537726	6.0925198	2.5588678	0.0112144	0.0346337	-3.469813
KIFAP3	0.0695543	6.127642	2.5586468	0.0112213	0.0346521	-3.470355
RP11-359H1	-0.395671	-1.265105	-2.558593	0.011223	0.0346544	-3.470488
VIPR1-AS1	0.6324582	0.2652556	2.5585007	0.0112259	0.0346603	-3.470713
LRCOL1	0.717181	3.8530638	2.5584049	0.0112289	0.0346663	-3.470948
RP11-138E2	-0.545461	0.135056	-2.558378	0.0112297	0.0346663	-3.471015
SNTB1	-0.108972	6.7050172	-2.558143	0.0112371	0.0346861	-3.471591
LINC00882	0.5590853	1.7251419	2.5580137	0.0112411	0.0346956	-3.471906
ZNF613	-0.113406	5.0372696	-2.557838	0.0112466	0.0347096	-3.472336
RP11-452D1	0.4434745	-0.874129	2.5577303	0.01125	0.0347167	-3.472601
SNRPD2	-0.064389	6.6374411	-2.557704	0.0112508	0.0347167	-3.472665
RP11-874J1	-0.760683	-0.128239	-2.557482	0.0112578	0.0347353	-3.473209

NDUFA4L2	-0.140296	5.9811899	-2.556985	0.0112734	0.0347805	-3.474427
AC018495.3	0.0768235	-1.570524	2.5568361	0.011278	0.034792	-3.474791
ZFY	1.1328144	2.72927	2.5567917	0.0112794	0.0347933	-3.4749
KIF4B	-0.424705	-0.877273	-2.556672	0.0112832	0.034802	-3.475194
RP11-43402	-0.465473	-0.647885	-2.556434	0.0112907	0.0348221	-3.475774
RP11-594N1	-0.60255	-0.60713	-2.556336	0.0112937	0.0348286	-3.476015
AC007952.5	-0.653633	-0.106865	-2.556181	0.0112986	0.0348408	-3.476395
NXNL1	0.2608218	-1.449232	2.5560274	0.0113035	0.0348527	-3.476771
RP11-848P1	0.1952284	3.9742178	2.5559455	0.011306	0.0348576	-3.476971
JOSD2	0.0736243	5.9962279	2.5559159	0.011307	0.0348576	-3.477044
KATNB1	0.0590987	5.9343164	2.5553923	0.0113235	0.0349055	-3.478326
RP11-106J2	0.1067284	-1.555284	2.5553023	0.0113263	0.034911	-3.478546
TMX1	0.0530595	6.1780954	2.5552751	0.0113272	0.034911	-3.478613
NSRP1	0.039919	6.0014304	2.5551738	0.0113303	0.0349179	-3.47886
AC006116.2	-0.502357	-0.60188	-2.555071	0.0113336	0.034925	-3.479112
ZNF570	-0.131192	5.1267087	-2.554774	0.0113429	0.0349509	-3.479838
KNCN	0.103308	-1.557027	2.5545618	0.0113497	0.0349686	-3.480358
RP11-324D1	0.2622405	-1.353898	2.5543195	0.0113573	0.0349892	-3.480951
PKLR	-0.440762	6.2627369	-2.554232	0.0113601	0.0349948	-3.481164
AP001476.2	-0.682796	-0.108223	-2.554072	0.0113651	0.0350074	-3.481556
HSBP1	0.0467463	6.4389398	2.5537977	0.0113738	0.0350265	-3.482227
RP11-380P1	0.7095213	0.2466202	2.5537818	0.0113743	0.0350265	-3.482266
SLC27A2	0.2379408	6.4798319	2.5537761	0.0113745	0.0350265	-3.48228
RP11-157K1	-0.328719	-1.175832	-2.553755	0.0113752	0.0350265	-3.482331
ADAD1	-0.345653	-1.385252	-2.553642	0.0113788	0.0350346	-3.482609
RNF44	-0.055404	6.2420057	-2.553607	0.0113799	0.035035	-3.482694
PRDX4	0.0704512	6.687009	2.5533262	0.0113887	0.0350594	-3.483381
SCAMP5	-0.138353	5.8793959	-2.553034	0.011398	0.035085	-3.484095
RP11-400K9	-0.380089	-1.027378	-2.552893	0.0114025	0.035095	-3.484441
SLITRK4	0.6266053	2.8460802	2.5528459	0.011404	0.035095	-3.484555
ARF6	0.0451172	6.599444	2.5528409	0.0114041	0.035095	-3.484568
SRSF7	-0.035444	6.4772677	-2.552737	0.0114074	0.0351021	-3.48482
RP11-573D1	-0.617752	0.3083297	-2.55259	0.0114121	0.0351135	-3.485181
RP5-851M4.	-0.377511	-1.047182	-2.552361	0.0114194	0.0351318	-3.485741
SIGLEC10	0.1999447	4.9102149	2.5523423	0.01142	0.0351318	-3.485787
CTD-2123J1	-0.389068	-0.987165	-2.552219	0.0114239	0.0351409	-3.486087
KLHL32	0.5317055	3.0906203	2.5518049	0.011437	0.0351784	-3.4871
GORASP2	-0.040091	6.593878	-2.551511	0.0114464	0.0352042	-3.487819
AC105053.3	0.4729962	-0.804923	2.5510866	0.0114599	0.0352403	-3.488856
RP11-94H18	-0.456761	-0.93429	-2.551082	0.01146	0.0352403	-3.488866
CRHR1	0.5366384	-1.008898	2.5510337	0.0114616	0.0352421	-3.488985
CKMT2-AS1	-0.100874	5.5110775	-2.550849	0.0114675	0.0352572	-3.489437
RP1-278022	0.5438983	0.1783419	2.5508133	0.0114686	0.0352577	-3.489524
NXPH2	-0.494302	-0.983377	-2.550741	0.0114709	0.0352618	-3.4897
RNU6-713P	0.0768235	-1.570524	2.5507003	0.0114722	0.0352628	-3.4898
RP11-504I1	-0.557801	0.0045273	-2.550587	0.0114758	0.035271	-3.490078
INTS3	-0.062348	6.3559802	-2.55026	0.0114863	0.0353001	-3.490875
ANAPC1P1	-0.547205	-0.35987	-2.549933	0.0114967	0.0353292	-3.491674
CTC-543D15	-0.349331	-1.105489	-2.549689	0.0115045	0.0353502	-3.49227
RP11-357G3	-0.436736	-0.900447	-2.549462	0.0115118	0.0353695	-3.492825
ACTBP12	-0.549632	-0.877549	-2.54925	0.0115185	0.0353874	-3.493343

EEF1A1P16	-0.437325	2.3687481	-2.549197	0.0115202	0.0353896	-3.493472
3-Mar	-0.161585	5.0254261	-2.549118	0.0115228	0.0353944	-3.493665
CAPN7	-0.048006	6.0651517	-2.548608	0.0115391	0.0354398	-3.49491
CYTH1	-0.050159	6.4105584	-2.548596	0.0115395	0.0354398	-3.494939
DUSP23	0.0842567	6.5051042	2.5484732	0.0115434	0.0354461	-3.495239
RHBDL2	0.3284278	3.5415421	2.5484712	0.0115435	0.0354461	-3.495244
HEPACAM	1.0128864	1.6791193	2.5483353	0.0115479	0.0354565	-3.495576
MIR6859-3	-0.46698	-0.662265	-2.548276	0.0115498	0.0354572	-3.495721
CPSF7	-0.041015	6.4448665	-2.548268	0.01155	0.0354572	-3.49574
EEF1A1P17	-0.612572	0.8675027	-2.548121	0.0115547	0.0354687	-3.4961
CEACAM4	0.4601511	2.6104419	2.5479524	0.0115601	0.0354823	-3.496511
GS1-114I9.	-0.505738	-0.172592	-2.547841	0.0115637	0.0354904	-3.496783
AC000068.5	-0.386419	2.9731396	-2.547768	0.0115661	0.0354928	-3.496962
UBL5P2	-0.523925	-0.026938	-2.547756	0.0115665	0.0354928	-3.496991
TFAP4	-0.077839	5.6507417	-2.547678	0.011569	0.0354975	-3.497181
SLC36A3	-0.289623	-1.297577	-2.547286	0.0115816	0.0355331	-3.498138
FAM192A	0.0431109	6.3100706	2.5472153	0.0115838	0.0355371	-3.49831
RP11-789C1	0.6079412	1.7450315	2.5468217	0.0115965	0.035573	-3.49927
RP11-558O1	-0.315223	-1.206617	-2.54672	0.0115998	0.03558	-3.499518
NOL12	-0.083241	5.215947	-2.546654	0.0116019	0.0355835	-3.499679
CCDC96	0.1284829	4.4252191	2.5462681	0.0116143	0.0356187	-3.500621
RP11-482D2	0.3716329	-1.097002	2.5461602	0.0116178	0.0356264	-3.500884
FAM21EP	-0.290288	2.8951143	-2.545955	0.0116244	0.0356437	-3.501384
ANXA2P1	0.5293585	1.1727539	2.5457946	0.0116296	0.0356566	-3.501776
RP11-80H5.	-0.5752	-0.080287	-2.545703	0.0116326	0.0356626	-3.501998
CCT8P1	-0.177424	5.7287562	-2.545651	0.0116343	0.0356648	-3.502127
HSP90AB2P	-0.356352	3.6846235	-2.545422	0.0116417	0.0356845	-3.502686
RP11-61J19	-0.341722	-1.157924	-2.545229	0.0116479	0.0357006	-3.503154
RP11-772C9	0.7476384	0.5631248	2.5451308	0.0116511	0.0357073	-3.503395
VWF	0.1364458	6.390552	2.5450493	0.0116537	0.0357124	-3.503593
ECHS1	0.0885543	7.065316	2.5449023	0.0116584	0.0357222	-3.503952
DRD1	0.7178349	1.8661798	2.5448906	0.0116588	0.0357222	-3.50398
RP11-611L7	0.5852213	1.096556	2.5447917	0.011662	0.035729	-3.504221
EMILIN3	-0.583648	2.1057011	-2.544712	0.0116646	0.0357339	-3.504416
FCGR1B	0.4401994	3.0156316	2.5445326	0.0116704	0.0357487	-3.504853
TMC7	-0.183275	4.9039615	-2.544463	0.0116727	0.035751	-3.505022
HOXA2	0.4937448	2.5180203	2.5444485	0.0116731	0.035751	-3.505058
BOD1	-0.049836	6.181734	-2.544399	0.0116747	0.0357529	-3.505178
WNT9A	0.599833	3.0834714	2.5439776	0.0116884	0.0357917	-3.506206
SERGEF	-0.081121	5.8119627	-2.54389	0.0116913	0.0357975	-3.506421
UBXN2A	-0.049692	5.8869587	-2.543851	0.0116925	0.0357983	-3.506513
HNRNPA1P50	-0.489809	-0.477944	-2.543792	0.0116944	0.0358011	-3.506658
BCYRN1	-0.461332	3.1546501	-2.543612	0.0117003	0.0358161	-3.507098
SLC38A1	0.1743961	6.1484394	2.5433719	0.0117081	0.0358369	-3.507682
PDX1	-0.965394	3.3551283	-2.543127	0.011716	0.0358582	-3.508279
ALG1L15P	-0.509743	-0.172513	-2.542952	0.0117217	0.0358726	-3.508705
ATP13A3	0.0629018	6.7211781	2.5423164	0.0117424	0.0359324	-3.510254
NDUFB2	0.0563369	6.5531288	2.542291	0.0117432	0.0359324	-3.510315
ZZEF1	0.0567668	6.1601704	2.5418334	0.0117581	0.0359729	-3.51143
GOLGA2P10	-0.290667	4.4668102	-2.541824	0.0117584	0.0359729	-3.511453
SIL1	0.0726446	6.674045	2.5415151	0.0117685	0.0360008	-3.512205

DCAF6	0.0589043	6.6507215	2.5412446	0.0117773	0.0360247	-3.512864
RP11-643A5	0.523745	-0.890282	2.5411843	0.0117793	0.0360277	-3.513011
RP11-7F17.	0.4778835	3.1096916	2.5411373	0.0117808	0.0360294	-3.513125
AC006042.8	-0.5144	-0.514833	-2.541003	0.0117852	0.0360398	-3.513452
AHR	0.0892865	6.4526445	2.5406999	0.0117951	0.0360671	-3.51419
RP11-423E7	-0.596084	0.8712258	-2.540624	0.0117976	0.0360717	-3.514376
PLIN1	-0.28633	5.2035149	-2.540475	0.0118025	0.0360832	-3.514738
RP11-97012	-0.5279	1.7044829	-2.540418	0.0118043	0.0360832	-3.514876
RP11-2C24.	0.5191261	-0.355178	2.540418	0.0118043	0.0360832	-3.514876
RANGAP1	-0.060256	6.7538738	-2.540247	0.0118099	0.0360973	-3.515292
NSUN5P2	-0.165499	4.3004395	-2.54004	0.0118167	0.036115	-3.515797
AC010878.3	0.2938872	-1.255202	2.5397989	0.0118246	0.0361361	-3.516383
TCHP	-0.055679	5.837246	-2.539755	0.011826	0.0361374	-3.51649
RP11-21401	-0.665142	0.8748669	-2.539413	0.0118372	0.0361686	-3.517321
AF131215.9	0.3700492	3.3658181	2.5392123	0.0118438	0.0361857	-3.517811
RPSAP52	-0.54693	-0.565728	-2.539108	0.0118472	0.0361931	-3.518064
ARHGEF35	0.4872758	4.6762317	2.5390021	0.0118507	0.0362008	-3.518322
PPP1R16B	0.1567859	5.3299893	2.5389552	0.0118523	0.0362024	-3.518436
CTB-134H23	-0.508395	-0.241772	-2.53879	0.0118577	0.036216	-3.518838
GS1-600G8.	0.7295834	-0.176315	2.5387553	0.0118588	0.0362164	-3.518922
AC007349.4	0.4152307	-0.982755	2.5386531	0.0118622	0.0362236	-3.519171
GPRC6A	-0.513312	-0.95191	-2.538279	0.0118745	0.0362581	-3.52008
AC002398.1	-0.292848	3.2719876	-2.538013	0.0118833	0.0362819	-3.520729
HMGB1P44	-0.234528	-1.417842	-2.537934	0.0118858	0.0362828	-3.52092
TNP1	-0.571109	-0.868848	-2.537927	0.0118861	0.0362828	-3.520936
CTC-459F4.	-0.311602	-1.350141	-2.537913	0.0118865	0.0362828	-3.520971
ADAM12	0.3082199	4.690953	2.5378288	0.0118893	0.0362882	-3.521176
RP5-1139I1	-0.44178	-0.966863	-2.537661	0.0118948	0.0363021	-3.521584
SFXN4	-0.065918	6.1778304	-2.537311	0.0119063	0.0363342	-3.522434
NELFB	-0.045265	6.4351917	-2.537222	0.0119093	0.0363401	-3.522651
RP11-766N7	-0.578293	-0.529691	-2.537066	0.0119145	0.0363505	-3.523031
RP11-298D2	0.2235693	-1.450212	2.5370583	0.0119147	0.0363505	-3.523049
DAP	0.0558402	6.8242832	2.5367563	0.0119247	0.0363779	-3.523783
AC092667.2	-0.650465	2.2485381	-2.53667	0.0119275	0.0363835	-3.523993
AP001471.1	0.322781	-1.245236	2.5365246	0.0119323	0.0363951	-3.524347
SLC25A24P1	-0.6859	-0.465615	-2.536494	0.0119333	0.0363952	-3.524421
PCDHGB2	0.491309	3.7874512	2.5360986	0.0119464	0.0364312	-3.525382
RGL2	-0.060011	6.2606399	-2.536076	0.0119471	0.0364312	-3.525437
SH3BP4	0.0793001	6.4396311	2.536034	0.0119485	0.0364324	-3.525539
ALG1L10P	-0.588432	0.1442809	-2.535936	0.0119518	0.0364393	-3.525778
IFT22	0.0802617	5.6999317	2.5358536	0.0119545	0.0364445	-3.525977
ANXA13	-0.420412	5.6435362	-2.535641	0.0119615	0.036463	-3.526495
RP11-343H5	-0.796777	1.8743097	-2.535468	0.0119673	0.0364773	-3.526914
HIST1H4A	-0.379271	-1.045648	-2.535242	0.0119747	0.0364971	-3.527463
RP11-488I2	-0.480812	-1.137058	-2.535035	0.0119816	0.0365123	-3.527965
RPSAP56	-0.569432	-0.154806	-2.535031	0.0119817	0.0365123	-3.527975
FAM83B	0.8383061	0.2369821	2.5347501	0.0119911	0.0365377	-3.528658
RP11-1166F	0.0768235	-1.570524	2.5346737	0.0119936	0.0365423	-3.528844
KCTD19	-0.635983	-0.090454	-2.534205	0.0120091	0.0365867	-3.529982
SLCO6A1	-0.850021	0.2380933	-2.533475	0.0120334	0.0366576	-3.531755
RP11-468E2	-0.141026	4.5371962	-2.533424	0.0120351	0.0366597	-3.531878

IGHD3-9	-0.316058	-1.307566	-2.533353	0.0120375	0.0366636	-3.53205
AC090283.3	0.3821614	-1.203823	2.5333251	0.0120384	0.0366636	-3.532119
RP11-313D6	-0.533503	0.7651346	-2.533163	0.0120438	0.036677	-3.532513
RSBN1	0.0623145	5.6824113	2.5329682	0.0120503	0.0366894	-3.532985
OR11A1	-0.442297	-1.092895	-2.532954	0.0120508	0.0366894	-3.533021
GALNT5	0.7981255	1.7121506	2.53295	0.0120509	0.0366894	-3.53303
RFPL1	0.3640637	-1.196233	2.53227	0.0120736	0.0367524	-3.53468
HSPB1P1	-0.270788	4.2143979	-2.532228	0.012075	0.0367524	-3.534783
RP11-568K1	-0.104892	6.1816294	-2.532223	0.0120751	0.0367524	-3.534794
AC051649.1	0.1481654	-1.534167	2.5322092	0.0120756	0.0367524	-3.534827
KB-1440D3.	-0.326824	-1.158277	-2.532137	0.012078	0.0367566	-3.535002
AC083867.4	-0.220466	-1.424737	-2.531786	0.0120897	0.0367893	-3.535854
RP11-438L1	-0.35932	3.144677	-2.531578	0.0120967	0.0368073	-3.536358
ARL11	0.2259887	4.2177833	2.5312166	0.0121088	0.0368411	-3.537236
AGRN	0.0703348	6.8099405	2.5311851	0.0121098	0.0368412	-3.537312
PWWP2AP1	-0.222951	-1.445424	-2.531021	0.0121153	0.0368548	-3.53771
RP11-314B1	-0.662164	-0.577737	-2.530985	0.0121165	0.0368555	-3.537798
FAR2	0.1511195	5.0314796	2.5307421	0.0121247	0.0368771	-3.538387
ATP8A2P1	-0.493501	-1.114363	-2.530615	0.0121289	0.036887	-3.538694
RP13-726E6	0.3799779	-1.130044	2.5304413	0.0121348	0.0369016	-3.539116
RP11-20J15	0.645636	0.3350604	2.5303609	0.0121375	0.0369049	-3.539311
RP11-171I2	-0.560193	-0.02547	-2.530349	0.0121378	0.0369049	-3.53934
HAVCR1	-0.802389	2.3409351	-2.530292	0.0121398	0.0369068	-3.53948
RP11-73M18	-0.10155	5.2644978	-2.53027	0.0121405	0.0369068	-3.539531
CTD-2621I1	-0.513262	2.6334848	-2.530058	0.0121476	0.0369253	-3.540045
PSORS1C2	0.5275273	-0.623459	2.5298234	0.0121555	0.0369462	-3.540615
CTD-2036J7	0.2609967	-1.349331	2.5297693	0.0121573	0.0369487	-3.540746
GRIP2	0.5828203	3.2884692	2.52968	0.0121603	0.0369547	-3.540962
PDSS2	0.0752693	6.0749306	2.5292254	0.0121756	0.0369981	-3.542064
ARL17B	-0.409757	3.0429975	-2.529097	0.0121799	0.0370081	-3.542377
RP11-568A7	-0.299249	-1.333135	-2.528998	0.0121832	0.0370149	-3.542615
RP4-758J18	-0.149291	4.5679217	-2.52895	0.0121848	0.0370149	-3.542731
USP31	0.0783629	5.842757	2.5289402	0.0121852	0.0370149	-3.542756
VEZF1	-0.049488	6.1133598	-2.528436	0.0122022	0.0370634	-3.543979
SENP3-EIF4	-0.550246	0.1244853	-2.528381	0.012204	0.037066	-3.544112
PPP1R3C	-0.161423	6.1148472	-2.528103	0.0122134	0.0370913	-3.544784
CTC-428G20	0.5000506	0.9186557	2.5278604	0.0122216	0.0371131	-3.545372
RPS4XP2	-0.581198	0.4657128	-2.527595	0.0122305	0.0371372	-3.546015
RP5-1092A3	-0.516276	-0.216757	-2.527487	0.0122342	0.0371452	-3.546277
CYP2S1	0.2723362	4.7286381	2.5273031	0.0122404	0.0371609	-3.546723
TMEM206	-0.088383	5.3393522	-2.526723	0.01226	0.0372174	-3.548127
RARRES2P8	-0.827717	-0.114094	-2.526647	0.0122626	0.0372221	-3.548312
AC007064.2	0.4601852	-0.987806	2.5265412	0.0122662	0.0372299	-3.548568
FDXR	0.0978144	6.036563	2.5264321	0.0122699	0.037238	-3.548832
RP11-378A1	-0.556311	1.3709743	-2.526368	0.012272	0.0372415	-3.548988
CDC42EP4	-0.054179	6.428153	-2.526145	0.0122796	0.0372613	-3.549527
RP11-347I1	-0.314292	3.7658835	-2.526091	0.0122814	0.0372637	-3.549657
LSM14A	-0.035891	6.5924699	-2.525729	0.0122937	0.0372979	-3.550534
FAM63B	0.0825609	5.7658994	2.525616	0.0122975	0.0373064	-3.550808
RP5-882C2.	-0.21229	4.1010057	-2.525544	0.0123	0.0373108	-3.550983
PEX12	0.080822	5.5774339	2.5254719	0.0123024	0.0373151	-3.551157

CENPIP1	-0.281387	-1.416768	-2.525268	0.0123093	0.037333	-3.551652
KRTAP5-7	-0.687966	-0.04281	-2.524985	0.0123189	0.0373565	-3.552335
RP11-255E6	-0.576277	0.6203465	-2.52495	0.0123201	0.0373565	-3.552419
HNRNPUP1	-0.524602	1.1590197	-2.524949	0.0123202	0.0373565	-3.552422
PRAMEF6	0.3088445	-1.342344	2.5248138	0.0123248	0.0373674	-3.55275
KRT8P15	-0.341794	-1.105967	-2.524477	0.0123362	0.037397	-3.553565
NARS2	-0.06875	5.9932678	-2.524466	0.0123366	0.037397	-3.553592
ARHGAP40	-0.892681	1.154001	-2.524268	0.0123433	0.0374138	-3.554072
CCDC24	0.1301077	5.3893324	2.5242433	0.0123442	0.0374138	-3.55413
RP11-773H2	-0.571659	1.543199	-2.524176	0.0123465	0.0374176	-3.554292
GCSHP5	-0.555403	1.5383625	-2.524041	0.0123511	0.0374285	-3.55462
SLC28A3	0.8426099	1.9944812	2.5239524	0.0123541	0.0374345	-3.554834
CTC-573N18	0.8388927	0.8075707	2.5238823	0.0123565	0.0374386	-3.555004
NPM1P32	-0.474196	-0.579825	-2.523509	0.0123692	0.0374741	-3.555908
CTD-2616J1	-0.574023	0.1218036	-2.523203	0.0123796	0.0375026	-3.556648
CTC-265F19	0.3386058	-1.17622	2.5230466	0.012385	0.0375157	-3.557025
PRICKLE2	0.1956847	5.193318	2.5229985	0.0123866	0.0375175	-3.557142
AC010423.1	0.3211919	-1.250805	2.5226357	0.012399	0.037549	-3.558019
NONO	-0.043177	6.9556023	-2.522634	0.0123991	0.037549	-3.558023
CNNM2	-0.075866	5.864735	-2.522518	0.012403	0.0375579	-3.558304
AC073133.1	-0.618793	0.1659261	-2.522382	0.0124077	0.0375689	-3.558633
IRS4	0.2319063	-1.463968	2.5222831	0.0124111	0.037576	-3.558872
LINC00316	-0.589945	-0.45055	-2.522223	0.0124131	0.0375791	-3.559016
FABP5P14	0.0768235	-1.570524	2.5220228	0.01242	0.0375967	-3.559501
CTD-2587H2	-0.142857	4.311069	-2.521966	0.0124219	0.0375985	-3.559639
ADAMTS16	-0.752342	3.3872861	-2.521946	0.0124226	0.0375985	-3.559687
EI24P1	0.0768235	-1.570524	2.5218088	0.0124273	0.0376056	-3.560018
INSL4	0.4356121	-1.145073	2.5217922	0.0124279	0.0376056	-3.560058
VPS37C	-0.04772	5.9405703	-2.521787	0.0124281	0.0376056	-3.560071
SCARNA15	-0.462357	2.5662232	-2.521694	0.0124312	0.0376106	-3.560296
AC083884.8	-0.575373	0.1676326	-2.521679	0.0124318	0.0376106	-3.560333
RP11-76E17	-0.446261	-1.046554	-2.52163	0.0124334	0.0376125	-3.560451
RNA5SP490	-0.440125	-0.847534	-2.521513	0.0124374	0.0376215	-3.560733
ESRRA	0.0608242	6.5117542	2.5214422	0.0124399	0.0376257	-3.560904
RHBDF2	0.086735	6.0686222	2.52139	0.0124417	0.037628	-3.561031
AC006539.1	-0.410784	-0.931317	-2.521126	0.0124507	0.0376523	-3.561669
RP11-66602	-0.340952	3.0461402	-2.520623	0.012468	0.0377014	-3.562884
ATIC	-0.052379	6.5187499	-2.520554	0.0124703	0.0377054	-3.56305
RP11-767N6	0.0928005	4.6214787	2.5202768	0.0124799	0.0377311	-3.563721
SAE1	-0.050483	6.5191051	-2.520088	0.0124864	0.0377476	-3.564177
SLC12A7	0.0564675	6.7263841	2.5199758	0.0124902	0.0377562	-3.564448
ATP6V0E2	0.1103639	6.4181483	2.5198514	0.0124945	0.037766	-3.564748
TXNL4A	-0.054271	6.3532436	-2.519696	0.0124999	0.037779	-3.565125
HFM1	-0.647491	1.394442	-2.519627	0.0125022	0.037783	-3.56529
HNRNPA3P10	-0.511968	1.1762322	-2.519551	0.0125049	0.0377878	-3.565474
RP11-618N2	-0.28387	-1.296866	-2.519433	0.0125089	0.037794	-3.56576
AC134882.2	0.1117333	-1.552733	2.5194313	0.012509	0.037794	-3.565763
CHL1-AS2	-0.583408	-0.799877	-2.519304	0.0125134	0.0378042	-3.56607
RP11-434D2	0.7129419	-0.110235	2.5191845	0.0125175	0.0378132	-3.566359
LINC01314	-0.304577	5.5249137	-2.519157	0.0125184	0.0378132	-3.566425
SLC26A3	-0.749677	2.9671066	-2.518875	0.0125281	0.0378394	-3.567106

RP11-661P1	0.6648298	2.0724991	2.5187547	0.0125323	0.0378489	-3.567397
NPIPA3	-0.665358	1.2601312	-2.518464	0.0125424	0.0378733	-3.5681
MYB	-0.406696	3.5890774	-2.51846	0.0125425	0.0378733	-3.568108
PARM1	0.1745664	5.3619825	2.5179845	0.0125589	0.0379198	-3.569257
BCAS2	0.0533704	6.1336624	2.5171616	0.0125874	0.0380026	-3.571243
CTA-363E6.	0.1971687	-1.48643	2.5170864	0.01259	0.0380074	-3.571424
FLJ37035	-0.322788	3.1204211	-2.516879	0.0125972	0.0380259	-3.571924
RP11-656D1	-0.38922	2.3957027	-2.516748	0.0126017	0.0380356	-3.57224
NRTN	-0.163989	5.3149481	-2.516726	0.0126025	0.0380356	-3.572294
SACS	0.1369551	5.3885668	2.5165905	0.0126072	0.0380455	-3.572621
RP4-816N1.	-0.392369	2.2313665	-2.516572	0.0126078	0.0380455	-3.572665
RHOA	0.0346444	6.993396	2.5164645	0.0126116	0.0380514	-3.572925
HAND1	-0.318115	-1.354947	-2.516423	0.012613	0.0380514	-3.573025
TAF1C	0.0498923	6.0604917	2.5164178	0.0126132	0.0380514	-3.573037
KANK4	-0.729056	2.8724742	-2.516395	0.012614	0.0380514	-3.573092
RP4-756H11	0.2580455	3.6902801	2.516135	0.012623	0.0380755	-3.57372
S100A12	0.6507605	1.1520507	2.5160871	0.0126247	0.0380774	-3.573835
RP11-734B5	-0.446351	-0.681618	-2.515863	0.0126324	0.0380952	-3.574375
LINC01331	-0.371584	-1.20857	-2.515857	0.0126327	0.0380952	-3.574391
TULP3P1	-0.275369	-1.393234	-2.515738	0.0126368	0.0381003	-3.574677
IER5	0.0712573	6.0632792	2.5157355	0.0126369	0.0381003	-3.574683
RP11-278A2	-0.433366	2.7456491	-2.515702	0.012638	0.0381003	-3.574763
RP11-69M1.	-0.35421	-1.1358	-2.515688	0.0126385	0.0381003	-3.574797
TMUB2	-0.040811	6.2413446	-2.515647	0.01264	0.0381015	-3.574898
RAB15	-0.093702	6.0593587	-2.515486	0.0126456	0.0381152	-3.575285
U6	-0.507795	1.5970837	-2.515373	0.0126495	0.0381239	-3.575557
MRPL38	-0.058678	5.5628786	-2.515025	0.0126616	0.0381573	-3.576398
RNU4-21P	-0.263659	-1.325262	-2.514787	0.0126699	0.0381791	-3.576969
SLC38A3	-0.220463	6.9396594	-2.514745	0.0126713	0.0381803	-3.577072
APEH	0.0529992	6.6504298	2.514655	0.0126745	0.0381842	-3.577289
PRDM1	0.1353951	5.3369628	2.5146227	0.0126756	0.0381842	-3.577367
RP11-556I1	0.4112738	-1.200392	2.5146187	0.0126758	0.0381842	-3.577376
HM13	-0.04848	6.8841718	-2.514424	0.0126825	0.0382015	-3.577846
RP11-84H6.	-0.385844	-0.956468	-2.514366	0.0126846	0.0382044	-3.577986
CTA-796E4.	-0.682838	-0.26656	-2.514244	0.0126888	0.038214	-3.578279
RP11-358B2	-0.340817	-1.131883	-2.514193	0.0126906	0.0382163	-3.578404
FIP1L1	-0.04197	6.0756655	-2.514121	0.0126931	0.0382207	-3.578575
SLC7A5	0.1576546	5.9434822	2.5140213	0.0126966	0.038228	-3.578817
HMGB1P17	-0.24904	-1.410725	-2.513916	0.0127003	0.0382359	-3.57907
SAMD1	-0.062289	6.2147333	-2.513856	0.0127024	0.0382391	-3.579214
XCL1	-0.464475	3.5351667	-2.513797	0.0127044	0.0382422	-3.579358
PRRG2	0.2663835	4.9453196	2.5136598	0.0127092	0.0382534	-3.579688
ITIH2	-0.196794	7.4144004	-2.513604	0.0127112	0.0382562	-3.579823
PTPRN	-0.694274	0.5925983	-2.512707	0.0127425	0.0383474	-3.581983
ATF1	-0.051165	5.8393219	-2.512586	0.0127468	0.038357	-3.582276
RP11-671P2	0.2903871	-1.365873	2.512329	0.0127558	0.0383809	-3.582894
RP11-187E1	-0.757827	1.12978	-2.512286	0.0127573	0.0383823	-3.582997
RP11-290F5	0.4006864	4.7136337	2.512112	0.0127634	0.038396	-3.583417
AC005624.2	-0.549156	-0.120575	-2.512065	0.0127651	0.038396	-3.583531
CLPSL2	-0.616196	-0.567415	-2.512047	0.0127657	0.038396	-3.583573
EEF1A1P28	-0.568919	0.0083952	-2.512036	0.0127661	0.038396	-3.5836

C8orf88	0.5516407	2.5951718	2.5112555	0.0127935	0.0384753	-3.58548
RP11-258F2	-0.546062	1.8777138	-2.510949	0.0128043	0.0385045	-3.586218
RP11-36C2C	-0.225545	4.0335666	-2.510915	0.0128054	0.0385049	-3.586299
OR7A5	-0.486235	-0.962501	-2.510675	0.0128139	0.0385272	-3.586878
GNG7	0.1630302	5.4840595	2.5105212	0.0128193	0.0385403	-3.587248
RP11-426C2	0.6270638	-0.075046	2.5104235	0.0128228	0.0385447	-3.587483
PTPRS	0.2648934	5.4747116	2.5104196	0.0128229	0.0385447	-3.587492
CADM4	0.1969676	5.5169857	2.5103881	0.012824	0.0385449	-3.587568
PTDSS2	-0.067581	6.0964434	-2.510356	0.0128251	0.038545	-3.587644
AC005519.4	-0.385995	2.9518098	-2.510291	0.0128274	0.0385488	-3.587801
RP11-296E2	0.250206	-1.482165	2.5099789	0.0128384	0.0385783	-3.588553
RP11-59D5_	0.5674536	3.7525158	2.509952	0.0128394	0.0385783	-3.588618
GLCCI1	-0.099114	5.4760615	-2.509871	0.0128422	0.0385838	-3.588813
RP11-64C12	0.3186665	-1.292861	2.5096819	0.0128489	0.0386006	-3.589268
CTD-2540B1	-0.570297	-0.353769	-2.509558	0.0128533	0.0386106	-3.589567
RP11-431M7	-0.455063	-0.872324	-2.509507	0.0128551	0.0386118	-3.589688
FOXC2	0.3901277	3.5927241	2.5094867	0.0128558	0.0386118	-3.589737
RP11-394B2	-0.601411	0.7722869	-2.50939	0.0128592	0.0386188	-3.58997
KTN1-AS1	-0.137422	4.4966695	-2.509343	0.0128608	0.0386193	-3.590082
RP13-890H1	-0.453041	2.9902074	-2.509326	0.0128615	0.0386193	-3.590124
MIR579	0.2781543	-1.289538	2.5092784	0.0128631	0.0386212	-3.590239
ADCY7	0.205652	4.8519853	2.5091718	0.0128669	0.0386293	-3.590495
RP11-341D1	-0.301929	-1.311074	-2.508921	0.0128758	0.0386503	-3.591097
RP4-641G12	-0.442121	2.5048809	-2.508914	0.012876	0.0386503	-3.591115
RP11-23D5.	-0.251623	-1.431364	-2.508721	0.0128828	0.0386649	-3.591579
RP11-363E7	0.1241019	4.8672915	2.5087167	0.012883	0.0386649	-3.59159
RP11-496B1	-0.279128	-1.303771	-2.508611	0.0128867	0.0386729	-3.591845
RP11-254F7	-0.610719	-0.265782	-2.508476	0.0128915	0.0386841	-3.592169
TMEM160	-0.109386	5.3952793	-2.508312	0.0128973	0.0386983	-3.592563
DUSP3	0.0470975	6.7336589	2.5082357	0.0129	0.0387032	-3.592747
AC006548.2	-0.563925	1.1591737	-2.508132	0.0129037	0.038711	-3.592996
RP11-58A18	0.1867957	-1.491716	2.5080568	0.0129064	0.0387159	-3.593177
NLRC4	0.1148264	4.612121	2.5079141	0.0129114	0.0387279	-3.59352
RNASEH2B	-0.062962	5.8144121	-2.507868	0.012913	0.0387296	-3.593632
RP11-163E9	-0.299974	3.1980906	-2.507677	0.0129198	0.0387467	-3.594091
FADS2P1	0.3075147	-1.366836	2.5076445	0.012921	0.038747	-3.594169
MIR573	-0.559042	-0.006065	-2.507378	0.0129304	0.0387698	-3.59481
CTD-2090I1	-0.242924	-1.369914	-2.50737	0.0129307	0.0387698	-3.594829
MIR23B	0.2605714	-1.349548	2.5072829	0.0129338	0.0387759	-3.595038
AC079305.1	-0.406552	-1.069207	-2.507039	0.0129425	0.0387987	-3.595625
OR2B2	0.1891687	-1.490507	2.5067611	0.0129523	0.0388251	-3.596293
TRPS1	0.1926102	5.1722895	2.5065504	0.0129598	0.0388443	-3.596799
C8orf76	-0.084222	5.6740339	-2.506142	0.0129743	0.0388833	-3.597781
RP1-232L22	0.4266758	3.5236961	2.5061243	0.012975	0.0388833	-3.597823
SH3RF2	-0.277143	5.5020298	-2.506095	0.012976	0.0388833	-3.597893
KRT33B	0.1976229	-1.508962	2.5059957	0.0129795	0.0388902	-3.598132
CH17-125A1	0.1447449	-1.53591	2.5059705	0.0129804	0.0388902	-3.598193
SUCNR1	0.2460283	4.8342658	2.5057968	0.0129866	0.0389055	-3.59861
FAM167A-AS	0.2205465	-1.474516	2.5057418	0.0129886	0.0389082	-3.598742
TMUB1	-0.06322	6.4154364	-2.505709	0.0129898	0.0389086	-3.598822
TMED7	0.0552745	6.6143581	2.5056208	0.0129929	0.0389135	-3.599033

STAB2	0.4009455	3.8454909	2.5055954	0.0129938	0.0389135	-3.599094
EIF4A1P7	-0.504151	-0.27662	-2.505573	0.0129946	0.0389135	-3.599149
CSGALNACT2	0.0662057	5.8967619	2.505078	0.0130123	0.0389631	-3.600337
RP11-1334A	-0.52024	2.1262953	-2.504862	0.01302	0.038983	-3.600855
ZNF268	-0.099295	5.8579424	-2.504644	0.0130277	0.0390016	-3.601379
RP11-72M17	0.5476649	1.7758299	2.5046284	0.0130283	0.0390016	-3.601417
TAF9P3	-0.527208	0.2322413	-2.504584	0.0130299	0.0390031	-3.601523
DUX4L16	0.1163128	-1.550399	2.5045226	0.0130321	0.039005	-3.601672
RPSAP58	-0.203576	5.3343553	-2.50446	0.0130343	0.039005	-3.601823
CD300LB	0.3717152	3.71682	2.5044581	0.0130344	0.039005	-3.601826
PDCD2	-0.053333	6.1804421	-2.504447	0.0130348	0.039005	-3.601854
RP11-49002	-0.303236	-1.331179	-2.504383	0.0130371	0.0390059	-3.602007
SLC34A2	0.8966542	3.3851326	2.5043793	0.0130372	0.0390059	-3.602016
UBE2Q1	-0.046671	6.6151338	-2.50425	0.0130418	0.0390165	-3.602326
RPL39	-0.068309	6.5828357	-2.504194	0.0130438	0.0390172	-3.602461
NCRNA0025C	0.5625588	-0.006052	2.5041832	0.0130442	0.0390172	-3.602487
ADCY6	-0.057767	6.2311493	-2.504098	0.0130473	0.0390231	-3.602692
RNF6	0.0624381	5.9747115	2.5040691	0.0130483	0.0390231	-3.602761
MEX3B	0.2343114	4.5690179	2.5037596	0.0130594	0.039053	-3.603504
RP13-452N2	0.6557223	1.2571837	2.50368	0.0130622	0.0390583	-3.603695
RP11-467C1	-0.448213	-0.851799	-2.503533	0.0130675	0.0390709	-3.604048
LYL1	0.1127269	5.0947586	2.5034044	0.0130721	0.0390785	-3.604357
FBXW4P1	-0.29521	3.4529506	-2.503402	0.0130722	0.0390785	-3.604362
RP11-420K1	-0.527683	0.363427	-2.503245	0.0130778	0.0390921	-3.604739
AC005262.4	-0.413576	-0.901431	-2.503032	0.0130855	0.0391117	-3.605251
CTD-2116N1	-0.454585	-0.541542	-2.502976	0.0130875	0.0391143	-3.605385
CASQ2	0.3882121	4.0058755	2.5029263	0.0130892	0.0391143	-3.605504
RPSAP63	-0.391156	-1.028792	-2.502894	0.0130904	0.0391143	-3.605582
RN7SL452P	0.2130068	-1.43283	2.5028883	0.0130906	0.0391143	-3.605596
KDM7A	0.0758927	5.9199833	2.5028355	0.0130925	0.0391168	-3.605722
RP11-439I1	-0.256151	-1.407238	-2.502784	0.0130943	0.039119	-3.605845
CTD-2105E1	0.2739618	-1.424531	2.5023837	0.0131087	0.0391588	-3.606807
VOPP1	0.0601928	6.1577543	2.5022688	0.0131128	0.0391674	-3.607082
STARD7-AS1	-0.075905	4.8579344	-2.502244	0.0131137	0.0391674	-3.607141
TAS2R2P	-0.452587	-0.898171	-2.502099	0.013119	0.0391798	-3.60749
RP5-1115A1	0.4665251	1.7340626	2.5019004	0.0131261	0.0391979	-3.607966
AGBL3	-0.129112	5.1414215	-2.501722	0.0131325	0.0392138	-3.608394
RBM15B	-0.0388	6.3522813	-2.501642	0.0131354	0.0392192	-3.608586
TOP1MT	-0.078551	6.3075643	-2.501527	0.0131395	0.0392284	-3.608862
CDH8	-0.705021	1.8659754	-2.501129	0.0131539	0.039268	-3.609818
ATP6V1C2	-0.126028	4.6146609	-2.501006	0.0131583	0.0392779	-3.610111
EIF3I	0.0545547	6.7580027	2.5008863	0.0131626	0.0392876	-3.610399
CSAD	0.1174121	6.0458593	2.5006625	0.0131707	0.0393085	-3.610936
FABP1	-0.328999	6.9670837	-2.500522	0.0131757	0.0393204	-3.611272
ZNF239	-0.408474	4.296754	-2.500355	0.0131818	0.0393352	-3.611675
FOXK1	-0.070261	6.0783497	-2.499926	0.0131973	0.0393782	-3.612703
ALDH4A1	0.1085646	6.8506726	2.4996347	0.0132078	0.0394064	-3.613401
FRAT1	-0.104794	5.7844988	-2.499599	0.0132091	0.039407	-3.613486
HERC2P2	-0.116705	6.0896906	-2.499545	0.013211	0.0394096	-3.613616
GPC5-IT1	-0.319732	-1.27928	-2.49942	0.0132156	0.0394199	-3.613916
KATNA1	-0.053375	5.6886993	-2.49937	0.0132174	0.039422	-3.614034

FAM187B2P	0.5463731	0.2744311	2.4992528	0.0132216	0.0394315	-3.614316
AP001059.5	-0.555645	-0.43608	-2.49917	0.0132246	0.0394372	-3.614515
MORC3	0.0651755	5.9330739	2.4989607	0.0132322	0.0394566	-3.615016
CTD-2526M8	-0.728673	1.0876288	-2.498811	0.0132376	0.0394696	-3.615376
ZNF333	-0.068955	5.4160335	-2.498727	0.0132406	0.0394754	-3.615576
EEF1B2	-0.057652	6.880169	-2.498616	0.0132447	0.0394841	-3.615841
GPR149	0.0768235	-1.570524	2.4981105	0.013263	0.0395356	-3.617054
KCNQ3	0.4203787	3.3829348	2.4979004	0.0132706	0.0395551	-3.617557
RN7SL724P	-0.404999	-0.923633	-2.497825	0.0132734	0.0395601	-3.617738
RP11-42901	0.2019	-1.461255	2.4975733	0.0132825	0.0395809	-3.618341
RPL18A	-0.06386	6.8912671	-2.497551	0.0132833	0.0395809	-3.618395
CTD-2015B2	0.4590841	-0.5458	2.4975425	0.0132836	0.0395809	-3.618414
SQSTM1	0.0847078	7.1628139	2.4975127	0.0132847	0.0395809	-3.618486
LINC01572	-0.621429	1.8446592	-2.497191	0.0132964	0.0396126	-3.619256
AC004895.4	-0.547671	0.6560721	-2.497078	0.0133005	0.0396215	-3.619526
TMEM141	-0.080847	6.4105229	-2.497014	0.0133029	0.0396241	-3.619681
PIGR	0.440103	6.0218054	2.496995	0.0133036	0.0396241	-3.619726
RNASE4	0.1738416	5.7018312	2.4969304	0.0133059	0.0396279	-3.619881
USF2	0.0485581	6.6606942	2.4968958	0.0133072	0.0396284	-3.619964
PNPLA3	-0.19019	5.7398733	-2.49677	0.0133118	0.039637	-3.620265
TCTEX1D4	0.5967128	1.2281244	2.4967569	0.0133122	0.039637	-3.620296
LINC00393	-0.292107	-1.411511	-2.496684	0.0133149	0.0396416	-3.62047
FAM155B	-0.672544	3.9054534	-2.496574	0.0133189	0.0396503	-3.620733
CEP83	-0.062766	5.4504692	-2.496333	0.0133277	0.0396732	-3.621311
RP11-791G1	-0.352301	3.0141406	-2.496242	0.013331	0.0396799	-3.621529
RP11-620J1	-0.169226	4.7448803	-2.496115	0.0133356	0.0396847	-3.621834
RPL7P58	-0.373704	-1.107644	-2.496114	0.0133357	0.0396847	-3.621836
BEX4	-0.136702	5.5732273	-2.496109	0.0133359	0.0396847	-3.621849
RP11-67C2.	0.6720809	0.87475	2.4959406	0.013342	0.0396997	-3.622251
DENND2D	0.1168465	5.8050103	2.4958197	0.0133464	0.0397096	-3.62254
RP11-25K21	-0.213481	-1.428163	-2.495471	0.0133591	0.0397416	-3.623376
RP11-432J2	0.2338732	-1.394674	2.4954651	0.0133593	0.0397416	-3.623389
ZNF528	-0.190543	5.0557464	-2.49526	0.0133668	0.0397607	-3.62388
RP11-208G2	-0.82848	1.652629	-2.494706	0.0133871	0.0398177	-3.625206
CTNNA1	0.0394197	6.9431761	2.4944622	0.013396	0.0398405	-3.625789
KAT8	0.0512185	6.0842017	2.4944366	0.013397	0.0398405	-3.625851
DEF8	0.0549328	6.1261795	2.4943848	0.0133989	0.0398429	-3.625975
RP11-212I2	0.1858673	-1.492189	2.4932031	0.0134422	0.0399686	-3.628801
RPS3AP49	-0.544351	0.7503098	-2.492951	0.0134515	0.0399915	-3.629404
SEC23A	0.0724081	6.4534221	2.4929339	0.0134521	0.0399915	-3.629445
NPC1L1	-0.565498	5.4106846	-2.492523	0.0134672	0.0400331	-3.630428
RP5-1057J7	-0.451261	-0.699194	-2.492337	0.0134741	0.0400501	-3.630873
AC109631.1	0.2287492	-1.397285	2.4922566	0.013477	0.0400501	-3.631064
PRSS55	-0.40713	-0.998398	-2.49222	0.0134784	0.0400501	-3.631152
RP11-25K19	0.583207	3.270731	2.4922084	0.0134788	0.0400501	-3.63118
HERC2P10	-0.551219	-0.054603	-2.492205	0.0134789	0.0400501	-3.631188
FAM21C	-0.047946	6.1079659	-2.492189	0.0134795	0.0400501	-3.631227
RP11-755B1	-0.429605	-0.736243	-2.492068	0.013484	0.0400601	-3.631515
ADAMTS7P3	0.6241433	1.1925106	2.4919356	0.0134888	0.04007	-3.631832
CLEC12A	0.297053	3.9678742	2.4919175	0.0134895	0.04007	-3.631875
SPRR1B	0.5993859	-0.773476	2.4918843	0.0134907	0.0400704	-3.631954

ZNF395	-0.076414	6.0714784	-2.491797	0.0134939	0.040074	-3.632163
DNASE1	-0.08982	5.4899974	-2.491792	0.0134941	0.040074	-3.632176
SURF6	-0.048381	6.2053163	-2.491393	0.0135088	0.0401144	-3.633128
RP11-560F1	0.3374197	-1.296377	2.4912506	0.0135141	0.0401267	-3.633469
GTSF1L	-0.51498	-0.750547	-2.49118	0.0135167	0.0401312	-3.633638
CBX3P4	-0.484943	-0.372522	-2.491025	0.0135224	0.0401449	-3.634007
AC009299.3	0.5624639	4.1823901	2.4905154	0.0135412	0.0401976	-3.635226
AL022344.7	0.4329367	-0.941661	2.4900938	0.0135568	0.0402406	-3.636233
RP11-567E2	0.2696771	-1.399191	2.4900526	0.0135584	0.0402419	-3.636331
RPTOR	-0.044524	6.2452594	-2.490018	0.0135596	0.0402424	-3.636414
WEE2-AS1	0.201174	4.1026434	2.4898896	0.0135644	0.0402523	-3.636721
METTL20	0.101359	5.2749079	2.489868	0.0135652	0.0402523	-3.636773
CTD-2554C2	-0.558822	1.1787132	-2.489337	0.0135849	0.0403075	-3.638041
RPRM	-0.614859	-0.47669	-2.489003	0.0135973	0.0403409	-3.638838
ZNF154	0.1701809	4.4812055	2.4889637	0.0135987	0.040341	-3.638932
IL21-AS1	-0.695456	-0.230236	-2.488922	0.0136003	0.040341	-3.639033
RP11-377G1	0.2018316	-1.47574	2.4889132	0.0136006	0.040341	-3.639053
FOXR2	-0.342127	-1.365077	-2.488724	0.0136076	0.0403585	-3.639505
RP11-513I1	0.3236774	2.8203901	2.488676	0.0136094	0.0403605	-3.639619
FURIN	0.0848761	7.1036101	2.4886437	0.0136106	0.0403608	-3.639696
C2orf50	-0.618383	0.3785281	-2.488434	0.0136184	0.0403807	-3.640198
KCNQ4	-0.336324	3.8039632	-2.488183	0.0136277	0.0404051	-3.640797
CTD-2342J1	-0.263412	3.1587044	-2.488074	0.0136318	0.0404138	-3.641057
FAM21A	-0.052618	6.1749479	-2.487946	0.0136365	0.0404246	-3.641362
RP11-661C8	0.1927509	-1.483922	2.4876897	0.0136461	0.0404496	-3.641973
COA3	-0.061558	6.5384724	-2.487346	0.0136589	0.0404842	-3.642793
AC093627.7	0.2002753	-1.484847	2.4871363	0.0136667	0.0405041	-3.643294
RP11-407A1	0.4354249	-1.163996	2.487055	0.0136697	0.0405098	-3.643488
CRLF2	0.7806144	1.4421896	2.4870062	0.0136715	0.0405119	-3.643605
RP11-249C2	0.2028237	-1.456025	2.4869671	0.013673	0.0405129	-3.643698
PPP1R1C	-0.488938	4.2855584	-2.486907	0.0136752	0.0405163	-3.643841
LINC00989	0.5847931	0.4354311	2.4868736	0.0136765	0.0405167	-3.643921
MUSTN1	-0.632571	1.237712	-2.486752	0.013681	0.0405269	-3.644212
RP4-760C5.	-0.605251	0.4459067	-2.486661	0.0136844	0.0405337	-3.644429
RP1-159G19	0.7707285	0.563473	2.4865407	0.0136889	0.0405436	-3.644715
TCTEX1D1	0.4836696	3.410355	2.4865002	0.0136904	0.0405448	-3.644812
RP11-423C1	-0.385761	-1.03587	-2.486399	0.0136942	0.0405527	-3.645054
PANX1	0.0731408	5.8562806	2.4863133	0.0136974	0.0405589	-3.645258
ATP6V1A	0.0426134	6.4932053	2.4861161	0.0137048	0.0405774	-3.645728
HIST1H2BL	-0.615219	0.1678496	-2.485857	0.0137145	0.0406029	-3.646347
SPDYE5	-0.44808	2.2131739	-2.485818	0.0137159	0.0406039	-3.64644
SPINK14	-0.533839	-0.499669	-2.485543	0.0137262	0.040631	-3.647095
LL22NC03-6	-0.508535	-0.921893	-2.485487	0.0137283	0.0406339	-3.647229
ZBTB20	0.1679518	4.2945292	2.4853989	0.0137316	0.0406404	-3.647438
AC011933.2	-0.458115	-0.543878	-2.485087	0.0137433	0.0406701	-3.648183
TMEM68	-0.067383	5.8790224	-2.485071	0.0137439	0.0406701	-3.64822
RP11-664H1	0.6160647	-0.551756	2.4847094	0.0137574	0.0407069	-3.649082
KRT8P12	-0.095954	5.2939045	-2.484246	0.0137748	0.0407551	-3.650188
GJC2	0.3070819	3.8789418	2.4841525	0.0137783	0.0407621	-3.65041
FMN2	-0.735334	-0.179703	-2.484063	0.0137817	0.0407688	-3.650623
HNRNPA1P36	-0.457125	-0.563533	-2.484019	0.0137833	0.0407703	-3.650728

RP11-128A1	-0.661164	1.0000877	-2.48371	0.0137949	0.0408014	-3.651464
PSMA2	0.0608064	6.0048997	2.4833357	0.013809	0.0408397	-3.652356
TAC4	-0.524434	1.3960228	-2.482919	0.0138247	0.0408828	-3.653349
OPLAH	0.0934611	6.4517175	2.4827622	0.0138306	0.040897	-3.653723
RP11-342M1	0.3635159	3.3838947	2.4825433	0.0138389	0.040918	-3.654244
RP4-796I8.	0.2581254	-1.455365	2.4824853	0.0138411	0.0409212	-3.654382
RP11-610P1	0.543495	-0.295767	2.4822949	0.0138482	0.0409391	-3.654836
RP11-161D1	0.2727835	-1.366088	2.4822126	0.0138513	0.0409449	-3.655032
CRB1	0.68348	0.7115289	2.4821111	0.0138552	0.040953	-3.655274
AP005901.1	-0.601187	-0.788335	-2.48208	0.0138564	0.0409531	-3.655348
CTD-2302E2	-0.522315	0.1088211	-2.481986	0.0138599	0.0409602	-3.655571
RP11-73801	-0.494504	-1.085037	-2.481778	0.0138678	0.0409802	-3.656068
RPRD1B	0.0655385	6.3405806	2.4816087	0.0138741	0.0409957	-3.65647
LGALS17A	-0.752615	0.9175527	-2.481454	0.01388	0.0410085	-3.656838
RP11-626H1	0.7514805	1.3111973	2.4814352	0.0138807	0.0410085	-3.656883
ALOX12-AS1	-0.096157	4.8736658	-2.481383	0.0138827	0.041011	-3.657007
BCAN	0.5286772	4.2428585	2.4812052	0.0138894	0.0410275	-3.657431
AC006539.2	-0.363496	-1.151567	-2.481054	0.0138951	0.0410411	-3.657791
DOCK8	0.1300959	5.7533701	2.4810105	0.0138968	0.0410426	-3.657894
CTD-2147F2	-0.773007	-0.327612	-2.48086	0.0139024	0.0410561	-3.658253
TRAV33	0.1540793	-1.50363	2.4807059	0.0139083	0.0410688	-3.658619
RP11-343C2	0.1965554	-1.459219	2.4806872	0.013909	0.0410688	-3.658664
AP003419.1	0.2183764	-1.448099	2.4806285	0.0139112	0.0410721	-3.658804
RPS3AP6	-0.233434	3.7795607	-2.480535	0.0139148	0.0410792	-3.659026
SNRPCP2	-0.355608	-1.064542	-2.480397	0.01392	0.0410911	-3.659354
CLUHP4	0.1631179	-1.526547	2.4803694	0.013921	0.0410911	-3.65942
RP11-640L9	0.6169316	0.7516789	2.480264	0.013925	0.0410995	-3.659671
UBXN10-AS1	0.6511861	1.4409742	2.4801723	0.0139285	0.0411065	-3.659889
ZNF365	0.617184	1.5948653	2.4799039	0.0139387	0.0411331	-3.660528
HARS2	0.0487128	6.1473306	2.4798746	0.0139398	0.0411331	-3.660598
RP4-539M6.	0.5574491	1.2812971	2.4796993	0.0139464	0.0411494	-3.661015
EDN3	-0.701536	-0.049955	-2.479593	0.0139505	0.041158	-3.661267
C9orf114	-0.052806	6.147894	-2.479437	0.0139564	0.0411721	-3.661638
HOXC6	-0.843151	1.4334284	-2.479371	0.0139589	0.0411763	-3.661796
LINC01529	-0.684192	1.6240367	-2.479229	0.0139643	0.0411889	-3.662135
EIF3LP1	-0.364064	-1.047391	-2.479149	0.0139673	0.0411944	-3.662323
RCC2	-0.054205	6.4125266	-2.479022	0.0139722	0.0412054	-3.662627
OR2A1-AS1	0.2694554	4.485262	2.4788155	0.01398	0.0412252	-3.663117
RP11-423H2	-0.151674	5.4230131	-2.478779	0.0139814	0.0412259	-3.663204
GOLGA6L2	-0.386786	-1.253462	-2.478734	0.0139831	0.0412276	-3.66331
ERVH48-1	-0.619672	0.7387186	-2.477939	0.0140134	0.0413108	-3.665201
PMCH	-0.486063	-0.580036	-2.477934	0.0140136	0.0413108	-3.665213
GATAD2A	0.0428519	6.4501521	2.477829	0.0140176	0.0413184	-3.665464
EEF1GP5	-0.527457	1.2562551	-2.477807	0.0140184	0.0413184	-3.665516
SYCE2	-0.171686	3.9944764	-2.477714	0.014022	0.041325	-3.665736
RP11-408E5	-0.273867	-1.39855	-2.477689	0.0140229	0.041325	-3.665797
RP11-221N1	0.3848831	-1.050685	2.4775752	0.0140273	0.0413345	-3.666067
ZNF90	-0.396717	3.3504032	-2.477525	0.0140292	0.0413367	-3.666186
RP11-195B1	-0.43533	1.7685189	-2.477454	0.0140319	0.0413414	-3.666355
RP5-890E16	-0.244287	3.1417416	-2.477303	0.0140377	0.0413551	-3.666715
RP11-162A1	0.3125967	3.4061668	2.4772186	0.0140409	0.0413612	-3.666915

AC058791.1	0.2819905	3.5025795	2.4770871	0.0140459	0.0413727	-3.667227
STOML1	0.0719824	5.9746168	2.477054	0.0140472	0.0413731	-3.667306
RPS26P13	-0.515262	-0.032325	-2.476716	0.0140601	0.0414078	-3.66811
RP11-390N6	-0.529085	-0.66456	-2.476274	0.014077	0.0414525	-3.669161
HELT	-0.334019	-1.247658	-2.476259	0.0140775	0.0414525	-3.669195
ALG3	-0.055641	6.5402408	-2.475581	0.0141035	0.0415256	-3.670805
SNRNP27	0.0385947	5.9650159	2.4753	0.0141143	0.041554	-3.671474
RP11-390P2	0.1618128	4.6492205	2.4752541	0.0141161	0.0415559	-3.671583
ANXA4	0.0840953	6.8298141	2.4751841	0.0141188	0.0415604	-3.671749
RP11-235G2	-0.277217	-1.31624	-2.474954	0.0141276	0.0415831	-3.672297
ACA64	-0.391081	-0.937788	-2.474829	0.0141324	0.0415939	-3.672594
RCAN3	-0.114725	5.5727376	-2.474544	0.0141433	0.0416213	-3.673269
FER1L4	-0.406729	4.4055814	-2.474527	0.014144	0.0416213	-3.67331
MRRFP1	-0.43567	-0.5405	-2.47442	0.0141481	0.04163	-3.673563
CEP76	-0.074236	5.2748101	-2.474181	0.0141573	0.0416537	-3.674131
PRPS1L1	-0.384838	-1.234348	-2.473968	0.0141655	0.0416745	-3.674638
LRRC31	-0.547756	4.4766606	-2.473924	0.0141672	0.0416761	-3.674743
FGF2	0.2950415	4.6963499	2.4737221	0.014175	0.0416956	-3.675221
CTD-2535L2	-0.202044	-1.455676	-2.473449	0.0141855	0.0417232	-3.675869
MT3	-0.848804	0.9890005	-2.473384	0.014188	0.0417272	-3.676024
RP11-441F2	0.4307706	-0.864876	2.4732981	0.0141913	0.0417336	-3.676227
CTXN2	-0.344734	-1.271599	-2.473189	0.0141955	0.0417426	-3.676487
OR2B7P	0.1673049	-1.478885	2.4730207	0.014202	0.0417583	-3.676886
RP11-404F1	-0.548031	0.1523634	-2.472663	0.0142158	0.0417955	-3.677734
RP13-766D2	-0.457675	-0.936825	-2.472488	0.0142226	0.0418106	-3.678151
PTPN20CP	-0.328005	-1.25039	-2.472471	0.0142232	0.0418106	-3.678191
MRPL43	-0.049507	6.4121986	-2.472364	0.0142273	0.0418194	-3.678444
FTH1P12	-0.355388	2.8367682	-2.472154	0.0142355	0.0418399	-3.678943
RP11-16604	-0.476514	-0.502579	-2.472116	0.0142369	0.0418408	-3.679033
RP11-206P5	-0.687554	-0.371608	-2.471844	0.0142474	0.0418683	-3.679678
AC104131.1	-0.490813	-1.1043	-2.471725	0.014252	0.0418785	-3.679961
FAM193A	-0.044039	6.0683566	-2.471552	0.0142587	0.0418948	-3.680371
AP1S1	0.0579448	6.365057	2.4714025	0.0142645	0.0419069	-3.680726
SUMF1	0.0567683	6.2499754	2.4713866	0.0142651	0.0419069	-3.680763
ATP6V0A4	-0.596188	-0.304954	-2.471293	0.0142688	0.0419141	-3.680985
RP11-482E1	-0.371618	-1.096655	-2.47081	0.0142875	0.0419657	-3.68213
AC113192.1	0.3700155	-1.03023	2.4702861	0.0143078	0.0420221	-3.683373
TMEM217	0.2702277	3.9085145	2.4693467	0.0143443	0.0421235	-3.6856
UNKL	0.0890347	5.5855286	2.4693386	0.0143446	0.0421235	-3.685619
RP11-498C9	-0.330374	-1.233986	-2.469214	0.0143495	0.0421343	-3.685913
RP11-54D18	-0.343564	-1.095627	-2.469067	0.0143552	0.0421478	-3.686264
RP5-856G1.	-0.580306	-0.418233	-2.468944	0.01436	0.0421584	-3.686555
RP11-216M2	-0.792159	1.19586	-2.468703	0.0143694	0.0421826	-3.687125
MTMR10	0.0685208	6.1797519	2.468673	0.0143706	0.0421826	-3.687196
ZNF470	-0.192076	5.0570489	-2.468643	0.0143717	0.0421826	-3.687267
P2RY4	-0.565588	-0.587959	-2.46861	0.014373	0.0421831	-3.687346
RP11-550F7	0.3656729	3.0295172	2.4681604	0.0143906	0.0422311	-3.688411
RPS4X	-0.054423	7.2393617	-2.468079	0.0143937	0.0422371	-3.688604
AC008746.5	-0.557938	1.3514156	-2.467869	0.0144019	0.0422577	-3.689101
KIAA2013	0.0619225	6.4927575	2.4676361	0.014411	0.042281	-3.689653
RP11-1029M	-0.538217	0.8448671	-2.467573	0.0144135	0.0422848	-3.689802

PABPC5	0.5372868	2.4509789	2.4673995	0.0144203	0.0423014	-3.690213
C18orf42	0.5663579	-0.666629	2.467326	0.0144232	0.0423026	-3.690387
RBM22P3	0.0768235	-1.570524	2.4672835	0.0144248	0.0423026	-3.690488
SSU72P5	0.0768235	-1.570524	2.4672835	0.0144248	0.0423026	-3.690488
LSM8	-0.056292	5.9628319	-2.46727	0.0144254	0.0423026	-3.69052
RP11-428C1	-0.315607	-1.212167	-2.467211	0.0144277	0.0423058	-3.690659
TRPC3	0.7028986	0.8893712	2.467183	0.0144288	0.0423058	-3.690726
ADHFE1	0.1916013	5.9252544	2.4670802	0.0144328	0.0423142	-3.690969
FAUP1	-0.348546	2.6972856	-2.466997	0.014436	0.0423204	-3.691167
RP11-72304	-0.445441	2.0183209	-2.466953	0.0144377	0.042322	-3.69127
RP11-187E1	0.2862399	-1.36406	2.4669012	0.0144398	0.0423245	-3.691393
CTD-2162K1	-0.341657	-1.177488	-2.466864	0.0144412	0.0423254	-3.691482
SEMA3F-AS1	-0.156528	4.0079604	-2.46674	0.0144461	0.0423362	-3.691775
SEPT10	0.0583204	6.2168526	2.4665236	0.0144546	0.0423566	-3.692287
FASN	-0.09461	7.1131947	-2.466503	0.0144554	0.0423566	-3.692335
CTD-2530H1	0.557098	0.6808847	2.466436	0.014458	0.0423609	-3.692495
BTC	0.4462368	4.1489519	2.4663454	0.0144616	0.0423679	-3.692709
MAG	-0.699754	0.2805694	-2.466074	0.0144722	0.0423957	-3.693352
ELAVL3	-0.589686	0.1115918	-2.465878	0.0144799	0.0424148	-3.693815
RP3-510D11	0.2194067	3.903223	2.4656123	0.0144903	0.042442	-3.694444
CTD-2396E7	-0.358737	5.6195036	-2.465478	0.0144956	0.042454	-3.694762
TBX19	-0.113487	4.7867069	-2.465438	0.0144972	0.0424553	-3.694857
EEF1A1P3	-0.503096	1.4815991	-2.465402	0.0144986	0.0424559	-3.694941
Six3os1_1	-0.56617	-0.668108	-2.465176	0.0145074	0.0424785	-3.695476
HAR1A	0.6244472	1.7816471	2.4650697	0.0145116	0.0424874	-3.695728
CCDC178	0.5463164	-0.65152	2.4649348	0.0145169	0.0424995	-3.696047
RP5-858B6.	-0.546948	1.5039641	-2.464903	0.0145182	0.0424998	-3.696123
FTLP12	-0.60703	1.4481388	-2.464621	0.0145293	0.0425288	-3.696789
SNORD9	-0.327312	-1.156879	-2.464383	0.0145387	0.0425529	-3.697354
FZD7	0.2006806	5.0795221	2.4642548	0.0145437	0.0425642	-3.697656
ALKBH3-AS1	0.4432915	1.9703817	2.4640367	0.0145523	0.042586	-3.698172
FAM60A	-0.116341	5.7909841	-2.463636	0.0145681	0.0426288	-3.69912
ZCCHC4	-0.058299	5.4592972	-2.463349	0.0145794	0.0426585	-3.699798
PP2672	-0.418845	-0.972304	-2.46319	0.0145857	0.0426734	-3.700173
THOC2	-0.047481	6.2418695	-2.462834	0.0145998	0.0427112	-3.701017
RP11-973D8	-0.518065	-0.276192	-2.462483	0.0146137	0.0427484	-3.701847
SMIM19	0.096521	6.1167712	2.4624386	0.0146154	0.0427501	-3.701951
GOLT1B	-0.045595	6.2583767	-2.461808	0.0146404	0.0428197	-3.703441
BIVM-ERCC5	0.2161286	-1.42648	2.4616974	0.0146448	0.0428269	-3.703703
MYEF2	-0.392353	4.3242559	-2.461676	0.0146456	0.0428269	-3.703753
BOLA3-AS1	-0.318424	3.4026193	-2.461657	0.0146464	0.0428269	-3.703799
RPS17	-0.058052	7.1425523	-2.461406	0.0146563	0.0428497	-3.704391
RP11-522B1	0.4713836	-0.979181	2.4614016	0.0146565	0.0428497	-3.704402
SRA1	0.0602053	6.3043806	2.4611006	0.0146684	0.0428811	-3.705113
CTD-2649C1	-0.32847	3.5233837	-2.460907	0.0146761	0.0429002	-3.705571
MRGPRF	0.3071674	4.9501071	2.4608637	0.0146779	0.0429018	-3.705673
RP4-680D5.	-0.429538	3.6884555	-2.460805	0.0146802	0.0429052	-3.705812
C20orf24	0.0714624	6.1830667	2.4606741	0.0146854	0.0429154	-3.706121
LINC01405	-0.296434	-1.328721	-2.460658	0.014686	0.0429154	-3.706159
RP5-928E24	-0.268535	-1.349355	-2.460539	0.0146907	0.0429257	-3.706438
HDAC6	0.0686392	6.4702887	2.460407	0.014696	0.0429376	-3.706751

CCND1	0.1090784	6.6345931	2.4600904	0.0147086	0.042971	-3.707499
HNRNPA1P6	-0.382398	-0.975183	-2.459652	0.014726	0.0430155	-3.708534
AC009948.5	0.133342	4.7636407	2.4596488	0.0147262	0.0430155	-3.708542
UGT2B7	0.3022407	6.4476038	2.4595844	0.0147287	0.0430195	-3.708694
TSSC1	-0.049463	5.9869807	-2.459492	0.0147324	0.0430245	-3.708912
AC099344.2	-0.352759	-1.246894	-2.459483	0.0147328	0.0430245	-3.708934
NRN1	0.1872066	4.9676917	2.4593797	0.0147369	0.043033	-3.709177
DDX3Y	1.111947	3.6469153	2.4591752	0.0147451	0.0430534	-3.70966
TMEM59L	0.6038697	2.1917143	2.4590787	0.0147489	0.0430612	-3.709888
RP11-788M5	0.4905722	-0.743173	2.4590297	0.0147509	0.0430634	-3.710004
RP11-347C1	0.5864078	-0.10702	2.4588366	0.0147586	0.0430825	-3.71046
C8orf22	-0.51729	-1.074269	-2.458659	0.0147657	0.0430997	-3.710879
RP11-479O1	-0.467226	-1.100591	-2.458472	0.0147731	0.0431124	-3.711321
HDC	0.5281707	3.3677172	2.4584696	0.0147732	0.0431124	-3.711326
B4GALT4	-0.050025	6.0088593	-2.458462	0.0147735	0.0431124	-3.711345
TRADD	0.0760723	5.9561029	2.4583604	0.0147776	0.0431208	-3.711583
RP11-175P1	-0.356789	3.1316552	-2.458304	0.0147798	0.0431239	-3.711718
RP11-15A1.	-0.269952	3.3537321	-2.458194	0.0147842	0.0431333	-3.711977
PI4KB	-0.043193	6.4701429	-2.45811	0.0147876	0.0431396	-3.712174
WDR45BP1	-0.543941	-0.517064	-2.458009	0.0147916	0.0431479	-3.712413
RP11-60L3.	0.2504078	-1.404252	2.4579304	0.0147948	0.0431537	-3.712598
CTD-2376I4	0.2624574	3.0442996	2.4577781	0.0148009	0.043168	-3.712958
RP11-124G5	0.0768235	-1.570524	2.4576062	0.0148077	0.0431846	-3.713363
N4BP2L1	0.1031242	5.9601363	2.4574526	0.0148139	0.0431991	-3.713726
MIR210HG	-0.27753	4.612434	-2.457368	0.0148173	0.0432043	-3.713925
OSGIN2	0.0679178	6.1249161	2.4573492	0.014818	0.0432043	-3.71397
NCAPD3	-0.061442	5.8668832	-2.456945	0.0148342	0.0432447	-3.714923
MTHFSD	0.0532501	5.7118274	2.4568854	0.0148366	0.0432447	-3.715064
LRPPRC	-0.039679	6.7243733	-2.456866	0.0148374	0.0432447	-3.715108
GJB5	0.6319475	1.4746512	2.4568578	0.0148377	0.0432447	-3.715129
RP11-319G6	-0.126796	4.5329618	-2.456833	0.0148387	0.0432447	-3.715187
OXER1	-0.157142	5.8250502	-2.456826	0.014839	0.0432447	-3.715205
XXbac-BPG2	0.2499313	-1.409254	2.4566145	0.0148475	0.043266	-3.715703
AC073046.2	-0.546231	1.8999925	-2.45653	0.0148509	0.0432724	-3.715903
C1orf159	-0.065419	5.5686024	-2.456482	0.0148528	0.0432745	-3.716015
TWIST1	0.5070177	2.9336524	2.4564415	0.0148544	0.0432758	-3.716111
RP11-66A2.	0.6936633	0.6015167	2.4563562	0.0148579	0.0432824	-3.716312
LST1	0.1243242	5.5178432	2.4559556	0.0148739	0.0433258	-3.717257
PIWIL4	0.2721413	4.461356	2.4558699	0.0148774	0.0433323	-3.717459
SERPINB4	0.5710189	-0.899596	2.4552593	0.014902	0.0434004	-3.718898
TALDO1	0.0694653	6.8194556	2.4552185	0.0149036	0.0434017	-3.718994
RP11-12J10	-0.564612	1.0217291	-2.455025	0.0149114	0.0434187	-3.71945
LINC01102	-0.335095	-1.22251	-2.455014	0.0149118	0.0434187	-3.719475
RP11-350N1	0.4404186	2.0697013	2.4547418	0.0149228	0.0434473	-3.720118
MT-ND2	0.0693871	7.6809726	2.4547118	0.014924	0.0434473	-3.720189
AC079741.2	-0.343134	-1.111215	-2.454597	0.0149286	0.0434573	-3.720458
NDUFS7	0.0676336	6.4648817	2.4545278	0.0149314	0.043462	-3.720622
CD200R1	0.2565469	4.0412986	2.4544742	0.0149336	0.0434647	-3.720749
RP11-404P2	-0.568478	3.9860075	-2.454446	0.0149347	0.0434647	-3.720816
ZC3H12B	-0.275836	4.3529895	-2.454383	0.0149373	0.0434686	-3.720963
HOXB-AS4	0.4013599	-1.2304	2.454317	0.0149399	0.0434729	-3.721119

CTB-3M24.2	-0.3334	-1.215643	-2.454195	0.0149449	0.043483	-3.721408
CORO2A	-0.169357	5.5882358	-2.454128	0.0149476	0.043483	-3.721565
NRGN	0.1366206	5.063981	2.4541234	0.0149477	0.043483	-3.721575
SS18L2	-0.067673	5.8633245	-2.454113	0.0149482	0.043483	-3.7216
MTND6P5	-0.285592	-1.370896	-2.454063	0.0149502	0.0434853	-3.721716
SERPINI2	-0.511968	-0.708159	-2.45394	0.0149551	0.043492	-3.722007
D4S234E	0.427528	2.8568969	2.4539355	0.0149553	0.043492	-3.722018
RP11-81B1C	0.1607082	-1.527775	2.45391	0.0149564	0.043492	-3.722078
RP11-6N13.	-0.725702	0.2383231	-2.453889	0.0149572	0.043492	-3.722129
C17orf58	-0.073683	5.8523599	-2.453844	0.014959	0.0434938	-3.722234
RP11-149B9	0.3939463	-1.233757	2.453714	0.0149643	0.0435056	-3.72254
TBC1D8B	0.1003453	5.6712128	2.4535245	0.0149719	0.0435244	-3.722986
FTX	0.1748542	4.037079	2.453438	0.0149754	0.0435311	-3.72319
C15orf32	0.1671944	-1.524469	2.4532023	0.014985	0.0435553	-3.723745
RP11-326N1	-0.46555	-0.837702	-2.453146	0.0149872	0.0435584	-3.723877
AC092159.2	-0.554593	0.173414	-2.45291	0.0149968	0.0435828	-3.724435
JAGN1	-0.055963	6.335898	-2.452592	0.0150097	0.0436167	-3.725183
RP11-388M2	-0.545179	0.617034	-2.452556	0.0150112	0.0436175	-3.725269
RP1-71H24.	-0.282607	-1.352827	-2.452338	0.01502	0.0436397	-3.725782
USP27X	-0.18607	5.0639268	-2.452178	0.0150264	0.043655	-3.726157
RP11-797D2	-0.517593	0.3986885	-2.45214	0.015028	0.0436561	-3.726247
BECN1P1	-0.568273	-0.480543	-2.452101	0.0150296	0.0436572	-3.726339
PRR26	0.3805456	5.0339434	2.4519015	0.0150377	0.0436772	-3.726809
AC005027.3	0.2286507	-1.402094	2.4516361	0.0150484	0.043705	-3.727433
TTC29	-0.409144	-1.098698	-2.451552	0.0150519	0.0437087	-3.727632
AC009264.1	-0.586401	-0.71346	-2.451546	0.0150521	0.0437087	-3.727645
CLDN11	0.2778796	4.7263496	2.4512943	0.0150623	0.0437349	-3.728238
RP11-400F1	-0.059373	5.2459843	-2.451252	0.015064	0.0437364	-3.728337
CTD-2143L2	-0.299775	-1.307551	-2.451163	0.0150677	0.0437435	-3.728548
FCER2	-0.539558	2.5607242	-2.45105	0.0150723	0.0437525	-3.728813
CYP1A1	0.7510571	3.9586343	2.4510277	0.0150732	0.0437525	-3.728866
NRADDP	-0.532112	0.4499053	-2.450832	0.0150811	0.0437721	-3.729326
RP11-895M1	-0.529322	-0.399029	-2.450453	0.0150965	0.0438133	-3.730217
RPS13	-0.051737	6.9399732	-2.450137	0.0151094	0.0438473	-3.730961
MEA1	-0.05357	6.4566562	-2.449967	0.0151163	0.0438638	-3.73136
SYT1	-0.531791	4.1446471	-2.449883	0.0151198	0.0438704	-3.73156
AC073621.2	0.4603608	-0.836149	2.4498427	0.0151214	0.0438716	-3.731654
LL09NC01-2	-0.517197	1.408748	-2.449685	0.0151279	0.0438829	-3.732025
RP11-432I1	-0.425878	-1.211554	-2.449668	0.0151286	0.0438829	-3.732065
AC079325.6	-0.591436	0.6556872	-2.44964	0.0151297	0.0438829	-3.73213
FAM9B	0.8081381	2.5336876	2.4496291	0.0151301	0.0438829	-3.732156
HAT1	-0.048921	6.123805	-2.449499	0.0151355	0.0438949	-3.732463
ART4	0.2879805	5.6970883	2.4494567	0.0151372	0.0438964	-3.732562
NAPSB	0.1940803	5.1385641	2.4490239	0.0151549	0.0439441	-3.73358
LINC00970	0.4207121	-1.129776	2.4489867	0.0151564	0.0439451	-3.733667
C22orf34	0.2269288	4.1282207	2.4488755	0.0151609	0.0439543	-3.733928
C14orf79	0.0877915	5.3149377	2.4488464	0.0151621	0.0439543	-3.733997
RP11-482E1	-0.240344	-1.388505	-2.44882	0.0151632	0.0439543	-3.734058
RP11-70J12	0.3563505	-1.125016	2.4484601	0.0151779	0.0439935	-3.734905
VSTM1	0.4883503	-0.650502	2.4483566	0.0151821	0.0440023	-3.735149
RP11-87H9.	-0.092755	4.946044	-2.44817	0.0151898	0.044021	-3.735588

ENO1-AS1	-0.609802	0.9126431	-2.448068	0.015194	0.0440296	-3.735827
NHP2L1	-0.048179	6.585292	-2.44803	0.0151955	0.0440306	-3.735917
GSDMB	-0.11232	5.9116659	-2.447867	0.0152022	0.0440464	-3.736299
TUBA3GP	-0.282691	-1.349366	-2.447774	0.015206	0.044054	-3.736518
TOP2B	-0.036866	6.5393322	-2.447737	0.0152075	0.0440549	-3.736605
AC108456.1	-0.428632	-0.79142	-2.447662	0.0152106	0.0440602	-3.736781
CTD-2639E6	-0.211709	3.3117566	-2.447462	0.0152188	0.0440805	-3.737251
CYP51A1-AS	-0.453771	2.0818236	-2.447087	0.0152342	0.0441216	-3.738133
UXT-AS1	-0.20304	3.3988601	-2.447027	0.0152367	0.0441253	-3.738275
RP11-1060J	0.4620057	1.9680262	2.4463109	0.0152661	0.0442069	-3.739956
HEATR4	-0.217847	3.8662851	-2.446215	0.01527	0.0442148	-3.740181
ISM1	0.3198567	4.8550321	2.4458683	0.0152843	0.0442527	-3.740996
RP5-875H18	0.5239812	-0.611972	2.4458347	0.0152857	0.0442532	-3.741075
MGAT4EP	-0.464369	-0.781494	-2.445656	0.015293	0.044271	-3.741495
CDC42P3	-0.310295	-1.205188	-2.44559	0.0152958	0.0442753	-3.74165
SLC6A7	-0.670157	0.7025959	-2.445167	0.0153132	0.0443222	-3.742642
TMEM144	0.2120531	5.0209346	2.4449671	0.0153214	0.0443426	-3.743113
ANKRD30A	-0.619504	-0.473494	-2.444874	0.0153253	0.0443502	-3.743331
RP11-175B9	-0.170238	4.8408711	-2.444839	0.0153267	0.0443503	-3.743413
GREM2	0.7532311	4.1326622	2.4448142	0.0153277	0.0443503	-3.743471
TM7SF2	-0.144752	6.5943337	-2.444764	0.0153298	0.0443528	-3.743589
TMED5	0.0676783	6.6572556	2.4446901	0.0153329	0.0443581	-3.743763
LINC00641	-0.142086	4.8987106	-2.444602	0.0153365	0.0443651	-3.743969
RP11-946P6	-0.515126	1.2442872	-2.444541	0.015339	0.044369	-3.744114
RPS4XP22	0.8451591	1.4893168	2.4439879	0.0153619	0.0444315	-3.745411
STX16	-0.042787	6.3393368	-2.443949	0.0153635	0.0444327	-3.745503
SLC25A15P2	0.1117333	-1.552733	2.4439024	0.0153654	0.0444347	-3.745612
PCDHB18P	0.515451	2.0210765	2.4431791	0.0153954	0.0445162	-3.747309
AP000997.3	0.6133222	0.1403828	2.4431517	0.0153965	0.0445162	-3.747374
CH17-302M2	0.5682464	1.5022761	2.4431048	0.0153984	0.0445162	-3.747484
GCA	0.1123617	5.6757039	2.443104	0.0153985	0.0445162	-3.747485
AP000472.3	0.4367652	-0.938788	2.4430225	0.0154019	0.0445224	-3.747677
FAM73A	0.066856	5.9322199	2.4429171	0.0154062	0.0445315	-3.747924
AC141928.1	-0.722267	2.4046562	-2.442719	0.0154144	0.0445511	-3.748388
EEF1A1P19	-0.14958	4.510364	-2.442694	0.0154155	0.0445511	-3.748448
HAO2	0.5766967	5.2554406	2.4426659	0.0154166	0.0445511	-3.748513
RP11-131M1	0.431066	1.7715817	2.442627	0.0154183	0.0445522	-3.748604
FAM228B	-0.127229	4.9862973	-2.442401	0.0154276	0.0445758	-3.749135
ID2	0.0780173	6.7136778	2.4423355	0.0154304	0.0445801	-3.749288
LCAT	0.1792917	6.0996141	2.4422939	0.0154321	0.0445816	-3.749386
RP11-378J1	-0.49817	-0.277142	-2.441942	0.0154467	0.0446202	-3.75021
HCFC1R1	0.0705992	6.106241	2.4417777	0.0154535	0.0446365	-3.750597
PRAMEF12	-0.295013	-1.353271	-2.44148	0.0154659	0.0446687	-3.751294
GOLGA6L10	-0.381312	2.8481501	-2.44136	0.0154709	0.0446795	-3.751575
BRAT1	-0.044821	6.4076556	-2.441031	0.0154846	0.0447139	-3.752347
KB-1125A3.	0.6232651	1.0169735	2.4410075	0.0154856	0.0447139	-3.752403
TMEM55A	0.1477247	5.4160825	2.4409658	0.0154873	0.0447139	-3.7525
CCDC120	-0.079564	5.7463682	-2.440957	0.0154877	0.0447139	-3.752521
RP11-443C1	0.2475328	-1.364948	2.4407471	0.0154964	0.0447356	-3.753013
TPSG1	0.7537074	2.0792646	2.4404994	0.0155068	0.0447619	-3.753594
ANKRD45	-0.572311	2.4399647	-2.440359	0.0155126	0.0447718	-3.753923

RPL3P4	-0.115728	5.6538688	-2.440358	0.0155127	0.0447718	-3.753926
AL133247.2	0.2591828	-1.395784	2.4403291	0.0155139	0.0447718	-3.753993
ALMS1	-0.056514	5.8507332	-2.440268	0.0155164	0.0447755	-3.754135
MTCH1	-0.037902	6.7483156	-2.440146	0.0155215	0.0447867	-3.754422
LINC00670	0.4183158	-0.928132	2.439803	0.0155358	0.0448245	-3.755226
AC016768.1	0.6285114	3.6245916	2.439729	0.0155389	0.0448299	-3.755399
CEP350	-0.054757	6.2606811	-2.439692	0.0155405	0.0448308	-3.755486
RP11-830F9	0.5002295	-0.319335	2.4393924	0.015553	0.0448629	-3.756188
AC079586.1	-0.55704	-0.839419	-2.439367	0.0155541	0.0448629	-3.756248
CASC17	-0.27189	-1.384567	-2.439217	0.0155603	0.0448774	-3.756598
SSBP3-AS1	0.4099216	2.7741661	2.4391661	0.0155624	0.04488	-3.756718
SKP1	0.0435856	6.8228453	2.4391298	0.015564	0.0448809	-3.756803
RP11-337C1	-0.262935	4.3982902	-2.438519	0.0155895	0.0449497	-3.758234
EIF4H	0.0343187	6.7681797	2.4385007	0.0155903	0.0449497	-3.758277
HNRNPCP3	-0.463614	-0.336244	-2.43841	0.0155941	0.0449572	-3.75849
RP11-161H2	-0.559483	0.0823163	-2.438379	0.0155954	0.0449573	-3.758561
NOP58	-0.047266	6.4379843	-2.438058	0.0156089	0.0449926	-3.759313
SPECC1L	0.0556914	6.2040117	2.4379382	0.0156139	0.0450035	-3.759594
GRAMD2	0.6168507	1.1688986	2.4378661	0.0156169	0.045004	-3.759763
RP11-775L1	-0.322318	-1.358134	-2.437847	0.0156177	0.045004	-3.759809
RP11-829H1	-0.680501	0.9808997	-2.437846	0.0156177	0.045004	-3.759809
ARR3	-0.442122	2.0100453	-2.437209	0.0156445	0.0450776	-3.761302
LRRC16A	0.1490574	5.5817858	2.4370483	0.0156513	0.0450901	-3.761677
ZNF767P	-0.075765	5.3259981	-2.437046	0.0156514	0.0450901	-3.761682
DMTF1	-0.050775	6.0343996	-2.436822	0.0156608	0.0451138	-3.762208
TNPO3	0.0419543	6.360556	2.4366862	0.0156665	0.0451266	-3.762525
RP11-732A2	-0.521468	1.4337241	-2.436562	0.0156717	0.0451382	-3.762817
ADAM30	0.3366796	-1.207329	2.4363096	0.0156824	0.0451617	-3.763406
CBX2	-0.187186	5.1889928	-2.436309	0.0156824	0.0451617	-3.763408
CTD-2210P2	0.357215	-1.105765	2.4361497	0.0156891	0.0451773	-3.763781
PGD	0.0757005	6.6462077	2.4361217	0.0156903	0.0451773	-3.763846
C1orf56	-0.08852	5.7628601	-2.436085	0.0156918	0.0451782	-3.763933
ACOT1	0.2255902	5.4450797	2.4357693	0.0157051	0.0452129	-3.764671
CYP3A52P	0.2644339	-1.348022	2.4356053	0.015712	0.0452293	-3.765054
THBS4	-0.32125	5.2019459	-2.435531	0.0157152	0.0452348	-3.765229
APH1A	-0.04597	6.8942358	-2.435333	0.0157235	0.0452552	-3.76569
EIF4ENIF1	-0.045269	5.9812858	-2.435248	0.0157271	0.045262	-3.76589
TNFSF10	0.1038176	6.5207063	2.4351848	0.0157298	0.0452661	-3.766038
GINS3	-0.1118	5.1570154	-2.435113	0.0157328	0.0452712	-3.766205
MUT	0.0871087	6.5812118	2.4348432	0.0157442	0.0453005	-3.766837
SLC25A18	0.2275473	6.1204164	2.4344722	0.0157599	0.045342	-3.767705
ANKRD18CP	-0.685606	0.3970546	-2.43439	0.0157634	0.0453485	-3.767897
KCNJ11	-0.343286	4.7454324	-2.434345	0.0157653	0.0453504	-3.768003
RP1-283K11	0.2401755	-1.418985	2.4341086	0.0157753	0.0453744	-3.768555
AC074011.2	0.4708805	-0.572139	2.4340888	0.0157761	0.0453744	-3.768601
AC007250.4	-0.352307	-1.28179	-2.433786	0.015789	0.0454078	-3.769309
CFL2	0.0804111	6.3918885	2.433731	0.0157913	0.0454109	-3.769438
PCP4	-0.750117	-0.036953	-2.433345	0.0158077	0.0454544	-3.770341
RPL21P11	-0.52613	1.0919647	-2.433188	0.0158143	0.04547	-3.770708
DCXR	0.1253085	6.854058	2.4328711	0.0158278	0.045505	-3.771448
TTC25	0.2714064	3.5548271	2.4326384	0.0158377	0.0455299	-3.771992

MUC1	0.3616652	4.5662728	2.4325116	0.0158431	0.0455418	-3.772288
RP1-293L8.	0.2161286	-1.42648	2.4324414	0.015846	0.0455467	-3.772452
RP11-395A1	-0.126358	4.2459363	-2.432377	0.0158488	0.045551	-3.772602
EMC7	0.0519385	6.4597798	2.4321813	0.0158571	0.0455714	-3.77306
RP11-244F1	0.4967168	2.3208874	2.4319947	0.015865	0.0455906	-3.773496
RP11-490K7	-0.315726	-1.190204	-2.431561	0.0158835	0.0456401	-3.774509
DUSP22	-0.054846	6.2044398	-2.431203	0.0158987	0.0456803	-3.775344
RP11-404J2	0.212689	-1.501284	2.4310966	0.0159033	0.045686	-3.775593
RNU6-1231F	-0.377788	-1.045407	-2.431066	0.0159046	0.045686	-3.775664
TRAFD1	-0.047316	6.1212802	-2.431028	0.0159062	0.045686	-3.775753
PRR12	-0.057363	6.0796286	-2.431023	0.0159064	0.045686	-3.775765
FZD10	0.6618067	2.2145227	2.4310094	0.015907	0.045686	-3.775797
AL121748.1	0.2638685	-1.355535	2.4307518	0.015918	0.045714	-3.776399
RP11-723D2	0.5788904	0.1896997	2.430658	0.015922	0.0457219	-3.776618
RPL23AP67	-0.478808	-0.29194	-2.430419	0.0159322	0.0457477	-3.777176
EXOSC7	-0.053434	6.0527433	-2.4303	0.0159373	0.0457553	-3.777454
AC005150.2	-0.256188	-1.429125	-2.430298	0.0159374	0.0457553	-3.777458
ZBTB20-AS1	-0.546997	0.1933563	-2.43005	0.015948	0.0457821	-3.778037
NUP153	-0.056613	6.2757005	-2.429979	0.015951	0.0457872	-3.778202
FAM50B	0.1896096	5.6605303	2.4296343	0.0159658	0.0458259	-3.779008
RP11-361H1	-0.530465	0.5070503	-2.42945	0.0159737	0.0458432	-3.779439
RP11-91P24	-0.483884	-0.380908	-2.429427	0.0159746	0.0458432	-3.779491
FAM8A2P	-0.405766	-1.154006	-2.429406	0.0159755	0.0458432	-3.779541
RP11-36501	-0.530012	0.9952656	-2.429354	0.0159778	0.0458459	-3.779662
TRABD2A	-0.316953	4.8205771	-2.429319	0.0159793	0.0458466	-3.779743
AC006946.1	-0.434375	-1.017616	-2.42908	0.0159895	0.0458724	-3.780301
AC013275.2	0.5681945	3.7328296	2.4289348	0.0159957	0.0458867	-3.78064
ASB17	0.1954506	-1.441777	2.4289046	0.015997	0.0458868	-3.780711
AVPR2	0.5584757	2.3516599	2.428754	0.0160035	0.0459004	-3.781062
RP1-92014.	-0.387616	2.7748887	-2.428735	0.0160043	0.0459004	-3.781106
ENTPD4	0.0649412	5.9830582	2.4286285	0.0160089	0.0459099	-3.781355
RP11-129M1	0.182605	4.4406435	2.4283212	0.016022	0.0459441	-3.782072
CTD-2623N2	-0.296789	2.9261202	-2.428187	0.0160278	0.045957	-3.782384
PIP4K2A	0.053475	6.1086707	2.4281192	0.0160307	0.0459618	-3.782543
EGFLAM	0.1298046	5.1508139	2.4279843	0.0160365	0.0459748	-3.782858
PTPN11	0.0465555	6.5831121	2.4279071	0.0160398	0.0459774	-3.783038
RP11-428G5	-0.401216	-0.753165	-2.427893	0.0160405	0.0459774	-3.783071
RP11-587P2	-0.383258	-1.200053	-2.427875	0.0160412	0.0459774	-3.783113
TMEM186	0.0722339	5.6626527	2.4276861	0.0160493	0.0459971	-3.783553
RP11-361C1	0.2824136	-1.342907	2.4275075	0.016057	0.0460113	-3.78397
RP11-20I23	0.6827096	-0.032682	2.4274974	0.0160574	0.0460113	-3.783993
SLMO1	0.3220584	3.5992622	2.4274746	0.0160584	0.0460113	-3.784047
RP11-492M2	-0.525202	0.5823212	-2.427453	0.0160594	0.0460113	-3.784097
DUTP2	-0.443061	-0.843534	-2.427269	0.0160673	0.0460305	-3.784527
AMT	-0.116444	6.2156679	-2.427196	0.0160704	0.0460359	-3.784697
RP11-709A2	0.35734	-1.080956	2.4266432	0.0160942	0.0460981	-3.785985
G6PC3	0.0654571	6.0789608	2.4266326	0.0160947	0.0460981	-3.78601
RP11-331N1	-0.549208	1.0996342	-2.42646	0.0161021	0.0461157	-3.786411
RP11-405A1	0.686226	-0.002455	2.4259751	0.016123	0.0461721	-3.787543
GLDC	-0.18558	6.4045348	-2.425874	0.0161274	0.0461809	-3.787777
LINC01574	0.1892614	-1.490459	2.4257752	0.0161317	0.0461895	-3.788008

MPST	-0.07987	6.9188119	-2.425691	0.0161353	0.0461943	-3.788205
FGF17	-0.640583	1.7827358	-2.425678	0.0161359	0.0461943	-3.788236
SIPA1L2	0.1456683	6.0812316	2.4254249	0.0161468	0.046222	-3.788825
AP000351.1	0.346012	-1.278925	2.4252439	0.0161546	0.0462408	-3.789247
CTGLF10P	-0.479472	1.6608681	-2.425183	0.0161573	0.0462447	-3.789389
ZNF197-AS1	-0.572432	0.2196147	-2.424857	0.0161714	0.0462814	-3.790148
SNRPFP1	-0.367638	-1.015502	-2.424813	0.0161733	0.0462832	-3.79025
RP11-121L1	-0.524127	1.6311852	-2.424653	0.0161802	0.0462994	-3.790623
RBM6	-0.04276	6.338398	-2.424623	0.0161815	0.0462995	-3.790693
RP11-44201	0.6165333	1.7219823	2.4244584	0.0161886	0.046314	-3.791077
AC006380.3	0.1803679	-1.494992	2.4244477	0.0161891	0.046314	-3.791101
RP11-417L1	-0.287426	3.103769	-2.424145	0.0162022	0.0463479	-3.791808
CH17-360D5	0.3201858	-1.313622	2.4240059	0.0162082	0.0463615	-3.792131
CHGB	-0.750795	1.2918882	-2.423808	0.0162168	0.0463825	-3.792592
NFKBIZ	0.104208	5.9347329	2.4236564	0.0162234	0.0463976	-3.792945
GTSF1	-0.722729	2.987418	-2.422401	0.016278	0.0465501	-3.795868
MPV17L	-0.232985	6.0753761	-2.4222	0.0162867	0.0465714	-3.796334
UBXN8	0.089708	5.8410657	2.4221582	0.0162885	0.046573	-3.796432
CCT6B	0.2134535	4.3526993	2.4220774	0.0162921	0.0465794	-3.79662
RP11-49I11	-0.168182	4.5977124	-2.421922	0.0162988	0.0465951	-3.796982
MAGEA3	-1.085131	1.4970822	-2.421517	0.0163165	0.0466359	-3.797924
HSPE1P7	-0.423595	-0.782414	-2.421507	0.0163169	0.0466359	-3.797948
TTC34	-0.505204	2.509024	-2.421507	0.0163169	0.0466359	-3.797948
U52111.14	-0.526859	1.6272244	-2.421281	0.0163268	0.0466604	-3.798473
P3H1	-0.059235	6.3065162	-2.420839	0.0163461	0.0467119	-3.799501
RNF5P1	-0.29585	4.3476969	-2.420694	0.0163524	0.0467264	-3.799839
CCDC170	0.267887	4.8371144	2.4204209	0.0163644	0.0467568	-3.800474
ACSL6	-0.271798	5.1354073	-2.419905	0.016387	0.0468177	-3.801675
HAUS7	-0.118447	4.7375103	-2.419743	0.016394	0.0468343	-3.80205
ACVR1C	0.3162335	4.0861789	2.4196054	0.0164001	0.0468478	-3.802371
RP11-208N1	0.1463171	-1.535109	2.4194968	0.0164048	0.0468578	-3.802623
EZR	0.0730718	6.5773299	2.4192508	0.0164156	0.0468849	-3.803195
RP5-1132H1	0.6458632	2.0337609	2.4191098	0.0164218	0.0468989	-3.803523
FAM53A	-0.202342	4.6095192	-2.419063	0.0164239	0.0469012	-3.803633
GPS1	-0.0457	6.6763881	-2.418794	0.0164357	0.0469311	-3.804256
ACSS3	0.2199297	6.1863565	2.4185511	0.0164463	0.0469579	-3.804821
RNF207	0.085409	5.632525	2.4184892	0.0164491	0.0469588	-3.804965
B3GNT8	-0.230218	4.2928465	-2.418486	0.0164492	0.0469588	-3.804973
PRKY	1.007206	2.7018505	2.4181917	0.0164621	0.046992	-3.805657
HNRNPM	-0.033229	6.7786961	-2.417942	0.0164731	0.0470197	-3.806237
RP11-44D5.	-0.323924	-1.210037	-2.417843	0.0164775	0.0470285	-3.806467
RP11-195M1	-0.525535	-0.598987	-2.417671	0.016485	0.0470463	-3.806866
ACER1	0.5486138	-0.470239	2.4175678	0.0164896	0.0470557	-3.807106
CTA-384D8.	-0.107014	5.0608913	-2.417451	0.0164947	0.0470667	-3.807378
RP4-635E18	-0.504999	1.5346314	-2.417198	0.0165059	0.047092	-3.807964
VPREB3	0.4248209	3.4434591	2.4171529	0.0165079	0.047092	-3.80807
NDUFB5	0.048267	6.5040506	2.4171453	0.0165082	0.047092	-3.808088
LOH12CR1	0.0733997	5.5112864	2.4171327	0.0165088	0.047092	-3.808117
RP11-537E1	0.5096266	-0.067201	2.4167557	0.0165254	0.0471357	-3.808993
RPL37AP1	-0.503828	2.2161926	-2.416614	0.0165316	0.0471499	-3.809322
INPP1	0.1121456	5.9314824	2.4165169	0.0165359	0.0471584	-3.809547

APOA1	-0.238701	7.5186294	-2.416439	0.0165394	0.0471645	-3.809728
PRR14L	-0.046517	6.1123756	-2.416328	0.0165443	0.0471749	-3.809987
ZNF35	-0.11872	4.9906183	-2.416283	0.0165463	0.0471768	-3.810091
RP11-93K22	-0.428113	-0.796469	-2.416224	0.0165489	0.0471806	-3.810228
FCRL4	-0.488523	-0.866822	-2.416176	0.016551	0.0471829	-3.810339
FCRL6	0.3427192	3.7886842	2.4160536	0.0165564	0.0471946	-3.810623
RP11-94B19	-0.322377	-1.234487	-2.415884	0.0165639	0.0472123	-3.811016
RP11-10N23	-0.300358	3.2275116	-2.415579	0.0165774	0.0472471	-3.811725
TRPT1	-0.064821	6.0539242	-2.415485	0.0165815	0.0472552	-3.811942
ZNF836	-0.095695	5.0008626	-2.415306	0.0165895	0.0472742	-3.812359
DDX6	0.0354694	6.5263418	2.4150814	0.0165994	0.0472988	-3.81288
SERPINA2	-0.548641	0.001853	-2.414791	0.0166123	0.0473317	-3.813553
RAP1AP	-0.34797	-1.055449	-2.41469	0.0166167	0.0473408	-3.813787
HIST2H2BC	-0.525613	1.6111413	-2.414644	0.0166188	0.0473429	-3.813894
bP-21264C1	-0.622931	0.2506914	-2.414613	0.0166202	0.0473432	-3.813967
PAK1IP1	-0.054254	5.9031679	-2.414443	0.0166277	0.047361	-3.814362
KIAA0408	0.4026339	-0.991372	2.4141998	0.0166385	0.047388	-3.814926
AC074212.5	-0.159372	4.4636948	-2.414128	0.0166417	0.0473934	-3.815093
RP1-168L15	-0.539298	1.1311716	-2.413853	0.0166539	0.0474245	-3.815731
AP000487.5	-0.272924	3.1610751	-2.413772	0.0166575	0.047431	-3.815918
ARPP21-AS1	0.2076113	-1.465569	2.4137148	0.01666	0.0474325	-3.816051
RP11-479G2	0.12881	5.2639118	2.4136861	0.0166613	0.0474325	-3.816117
MSRB1	0.0822008	6.6226497	2.4136724	0.0166619	0.0474325	-3.816149
RNY1P13	0.3159781	-1.134883	2.4136416	0.0166633	0.0474327	-3.81622
HNRNPA1P51	-0.269437	-1.308523	-2.413603	0.016665	0.0474339	-3.816311
RP11-295P2	0.1571379	-1.529594	2.4134023	0.0166739	0.0474556	-3.816776
VGF	-0.633532	2.8119381	-2.412806	0.0167004	0.0475273	-3.818157
ULK2	0.16156	5.4064677	2.41274	0.0167034	0.047532	-3.818312
CCDC176	0.095475	4.9969032	2.4126495	0.0167074	0.0475398	-3.818521
RP11-38902	-0.348433	-1.028997	-2.412523	0.0167131	0.0475522	-3.818815
NDUFV1	0.0558789	6.8195654	2.4120539	0.016734	0.0476079	-3.819902
CTD-2545G1	-0.481162	1.6526634	-2.411971	0.0167377	0.0476148	-3.820095
RP11-153K1	-0.391165	-1.227484	-2.411915	0.0167402	0.0476181	-3.820224
CTD-2240J1	-0.551904	2.0674674	-2.411873	0.0167421	0.0476198	-3.820322
CLCN2	-0.087357	5.5223396	-2.411767	0.0167468	0.0476296	-3.820568
HYAL2	0.0534878	6.3730457	2.4117025	0.0167497	0.047634	-3.820717
SPPL2B	-0.050494	6.24384	-2.411589	0.0167547	0.0476392	-3.82098
TUBA4A	0.1054765	6.3711886	2.411587	0.0167548	0.0476392	-3.820985
RP11-818F2	0.559512	-0.385065	2.4115734	0.0167554	0.0476392	-3.821016
RP11-72304	-0.451534	-0.516449	-2.411497	0.0167589	0.0476453	-3.821194
PDZD8	0.0704968	6.2784164	2.4112981	0.0167677	0.0476668	-3.821654
HSD17B14	-0.207129	5.8528439	-2.410626	0.0167978	0.0477485	-3.823212
RP11-162A2	-0.485494	-0.385963	-2.410556	0.0168009	0.0477537	-3.823374
MIR770	-0.389698	-1.120735	-2.410489	0.0168039	0.0477585	-3.823529
RBP1	-0.181066	6.0741857	-2.410433	0.0168064	0.0477619	-3.823658
FAM132A	-0.298661	3.7535298	-2.410331	0.016811	0.0477712	-3.823895
RP11-17403	-0.460091	-0.55639	-2.41023	0.0168155	0.0477804	-3.824129
CTB-47B11.	0.5402856	0.2655357	2.4101214	0.0168204	0.0477904	-3.82438
RP5-1172A2	0.6497526	0.7087774	2.4099876	0.0168264	0.0478029	-3.82469
ZFP41	-0.109523	5.7244923	-2.409965	0.0168274	0.0478029	-3.824742
RP11-471M2	-0.706131	-0.088358	-2.409791	0.0168352	0.0478214	-3.825146

NOX4	0.1998495	4.4731309	2.4096446	0.0168418	0.0478332	-3.825485
CTB-152G17	-0.260584	3.072917	-2.409639	0.016842	0.0478332	-3.825497
GPR88	0.6480478	4.5293991	2.4095577	0.0168457	0.0478399	-3.825686
TPST2	0.0682996	6.5271942	2.4094706	0.0168496	0.0478473	-3.825888
RP11-484D2	-0.456973	-0.548279	-2.409378	0.0168537	0.0478554	-3.826102
AC004603.4	0.1958843	-1.46432	2.4093315	0.0168558	0.0478576	-3.82621
SCARB1	-0.084226	6.9835457	-2.408911	0.0168747	0.0479075	-3.827184
RP11-165A2	-0.616886	1.4872433	-2.408875	0.0168763	0.0479084	-3.827267
IGKV7-3	-0.48567	-0.86705	-2.408817	0.0168789	0.047912	-3.827401
ZNF700	-0.069716	5.584798	-2.408762	0.0168814	0.0479153	-3.827528
RPL31P61	-0.471111	-0.414431	-2.408629	0.0168874	0.0479285	-3.827835
KB-1125A3.	-0.535215	1.2240785	-2.408601	0.0168887	0.0479285	-3.827902
LRRFIP1P1	-0.445675	3.3012582	-2.408524	0.0168921	0.0479309	-3.828079
RP11-154H1	0.2253556	-1.426537	2.4085231	0.0168922	0.0479309	-3.828082
AGTRAP	0.0752625	6.0357259	2.4084912	0.0168936	0.0479313	-3.828156
FP325317.1	0.6634098	0.1999654	2.4082527	0.0169043	0.047958	-3.828708
RP1-78014.	0.6354134	1.2021059	2.40814	0.0169094	0.0479687	-3.828968
RP11-1020A	-0.081723	4.7290765	-2.408089	0.0169117	0.0479715	-3.829087
SPATA32	0.5734263	1.0531651	2.4079298	0.0169189	0.047988	-3.829455
C2orf27AP1	-0.5123	-0.746665	-2.407901	0.0169202	0.047988	-3.829521
COX6A2	0.9290041	2.2578897	2.4078297	0.0169234	0.0479934	-3.829687
KLF7-IT1	0.45998	-0.540199	2.4076402	0.0169319	0.0480139	-3.830125
EEF2KMT	0.0696325	5.5995983	2.4071754	0.0169529	0.0480696	-3.831201
RP11-84C10	-0.473876	-0.437704	-2.407139	0.0169545	0.0480706	-3.831286
RPE	0.0388407	6.0553451	2.4067169	0.0169736	0.0481208	-3.832261
SPANXB2	-0.513904	-0.838508	-2.406383	0.0169887	0.0481599	-3.833034
CHRM3-AS1	-0.47031	-0.933747	-2.406216	0.0169962	0.0481746	-3.833421
DNASE1L2	-0.389021	3.2910521	-2.40621	0.0169965	0.0481746	-3.833434
RP11-234N1	-0.54305	0.0953834	-2.406111	0.017001	0.0481835	-3.833662
USP9Y	1.0540845	2.8556783	2.4059325	0.017009	0.0482026	-3.834076
RGS1	0.2060753	5.8456201	2.4058903	0.017011	0.0482043	-3.834173
GPR180	0.1549349	5.5920969	2.405823	0.017014	0.0482092	-3.834329
NIP7	0.0529195	5.9402797	2.40569	0.01702	0.0482225	-3.834636
RP11-1055B	-0.304164	3.8889941	-2.405504	0.0170284	0.0482426	-3.835066
FAM45A	-0.078401	5.9293022	-2.405444	0.0170312	0.0482442	-3.835205
RP11-13N12	0.2819326	-1.347418	2.4054332	0.0170317	0.0482442	-3.83523
TP63	0.4838406	3.0879167	2.4053705	0.0170345	0.0482486	-3.835375
NXPH1	-0.47475	-0.820317	-2.405302	0.0170376	0.0482536	-3.835534
CTNNBL1	-0.052729	6.3784466	-2.405161	0.017044	0.0482679	-3.835859
LAMB1	0.0839014	6.5619523	2.4050469	0.0170492	0.0482789	-3.836123
CKMT2	0.362732	4.1805116	2.404772	0.0170616	0.0483104	-3.836759
RP11-346C4	0.4029264	-1.023427	2.4046956	0.0170651	0.0483165	-3.836935
AC073635.5	0.5246352	-0.071739	2.4044545	0.0170761	0.0483438	-3.837493
BX470102.3	0.6273604	0.2791535	2.4044248	0.0170774	0.0483438	-3.837561
RPS4XP3	-0.531891	-0.143739	-2.404389	0.017079	0.0483446	-3.837644
SPINK7	0.2862396	-1.30063	2.4042175	0.0170868	0.0483622	-3.83804
RP11-1110F	0.2465956	-1.410954	2.4041672	0.0170891	0.0483622	-3.838157
HMGCS1	-0.103273	6.7109728	-2.404146	0.0170901	0.0483622	-3.838206
RP11-681L8	-0.237416	-1.389941	-2.404136	0.0170905	0.0483622	-3.838228
CCDC85C	-0.082198	6.0605106	-2.404043	0.0170947	0.0483704	-3.838443
PTGDR	0.4301267	2.9482022	2.4039707	0.017098	0.048376	-3.838611

LINC01252	0.4952549	3.145856	2.4037255	0.0171092	0.0484038	-3.839177
ABCB7	0.0547688	5.9674909	2.4036049	0.0171147	0.0484156	-3.839456
SEPT12	-0.435295	-0.92653	-2.403516	0.0171187	0.0484233	-3.839662
HYOU1	0.0582982	6.8793818	2.4034782	0.0171204	0.0484244	-3.839749
RP11-141J1	0.241764	-1.384805	2.4034391	0.0171222	0.0484256	-3.839839
SLC51A	0.248681	6.0773467	2.4033033	0.0171284	0.0484394	-3.840153
TEX22	-0.329251	3.2259481	-2.403214	0.0171325	0.0484471	-3.840358
RPL21P7	-0.426926	-0.693161	-2.402739	0.0171541	0.0485046	-3.841457
HNRNPA1P27	-0.507365	-0.248287	-2.402685	0.0171566	0.0485048	-3.841582
UBE2D4	0.0592875	5.9801404	2.4026373	0.0171588	0.0485048	-3.841691
RP11-209A2	-0.350422	-1.072565	-2.402622	0.0171594	0.0485048	-3.841726
ACTR6	-0.05494	5.9219379	-2.402621	0.0171595	0.0485048	-3.841729
ANKRD63	0.1882726	-1.486204	2.4021486	0.0171811	0.048562	-3.84282
RP11-701H2	-0.70527	1.4425287	-2.402103	0.0171831	0.048564	-3.842924
RP1-207H1.	-0.365246	-1.140761	-2.402031	0.0171864	0.0485696	-3.843091
DEPTOR	-0.092889	6.0943595	-2.401653	0.0172037	0.0486147	-3.843965
RP11-544M2	0.2993845	-1.336231	2.4015403	0.0172089	0.0486255	-3.844224
RP11-509J2	0.2539039	-1.352183	2.4015063	0.0172104	0.0486261	-3.844303
STK39	0.1653953	5.6630208	2.4013638	0.0172169	0.0486381	-3.844632
CEP170B	0.0612913	6.270471	2.4013552	0.0172173	0.0486381	-3.844652
NUDT4	-0.057476	6.0943888	-2.401173	0.0172257	0.0486579	-3.845072
FOXP2	0.7840881	3.4465841	2.4009547	0.0172357	0.04868	-3.845576
FOXA2	-0.325485	6.1552799	-2.400943	0.0172362	0.04868	-3.845602
LRRC75B	-0.112086	5.9540297	-2.400702	0.0172473	0.0487076	-3.84616
RP11-272L1	0.4604714	-0.604168	2.4005997	0.0172519	0.048717	-3.846395
CTD-2006M2	0.5813457	-0.206058	2.4005498	0.0172542	0.0487197	-3.846511
NOLC1	-0.04539	6.4660903	-2.400272	0.0172669	0.0487519	-3.847152
SLC36A2	-0.542343	-0.611423	-2.400145	0.0172728	0.0487646	-3.847446
RP11-91G21	-0.351342	3.0661181	-2.399742	0.0172913	0.0488097	-3.848374
AC079807.4	-0.559744	-0.096465	-2.399733	0.0172917	0.0488097	-3.848395
SUPT20H	-0.049197	5.9412566	-2.399709	0.0172928	0.0488097	-3.84845
ADK	0.0827567	6.2799872	2.3996756	0.0172943	0.0488103	-3.848528
RP11-654E1	0.1970884	-1.481712	2.3993535	0.0173091	0.0488483	-3.849271
SCTR	0.490873	4.2084247	2.3992621	0.0173133	0.0488548	-3.849482
MTCP1	0.2111448	4.1081298	2.3992448	0.0173141	0.0488548	-3.849522
PHKG1	0.1404476	3.9935051	2.3990678	0.0173223	0.048874	-3.84993
RP11-135F9	-0.071161	5.6323467	-2.398993	0.0173257	0.04888	-3.850103
SLC27A5	0.2024458	6.6280845	2.3988594	0.0173318	0.0488935	-3.85041
ZNF267	0.069679	5.4472498	2.3987472	0.017337	0.0489043	-3.850669
RP11-451G4	-0.660689	-0.488563	-2.398523	0.0173473	0.0489296	-3.851185
ENOX2	0.0729842	5.69998	2.3983744	0.0173542	0.0489451	-3.851529
TIAM1	0.1866822	5.1429948	2.3982433	0.0173602	0.0489584	-3.851831
UBL4B	-0.543232	-0.583645	-2.398085	0.0173675	0.0489752	-3.852196
VIM-AS1	0.3389993	3.4852968	2.3979148	0.0173754	0.0489935	-3.852588
CTC-325H20	-0.525832	0.544038	-2.397728	0.017384	0.0490109	-3.853018
MRAP	-0.809823	1.43352	-2.397723	0.0173842	0.0490109	-3.85303
SHANK1	0.5688492	1.9497388	2.3976542	0.0173874	0.0490161	-3.853189
ARL13B	0.0852796	5.336448	2.3975777	0.0173909	0.0490222	-3.853365
RP11-71N10	-0.316705	-1.333733	-2.397484	0.0173952	0.0490306	-3.85358
ANO4	0.5801455	2.5641067	2.3972924	0.0174041	0.0490479	-3.854023
RP4-566L20	0.1078875	-1.554693	2.397274	0.0174049	0.0490479	-3.854065

RP11-756H6	-0.627415	2.3290323	-2.397264	0.0174054	0.0490479	-3.854088
SUMO2P19	-0.449473	-0.55222	-2.396905	0.017422	0.0490883	-3.854916
RP11-24M17	-0.529612	1.2232952	-2.396896	0.0174224	0.0490883	-3.854937
EHMT2-AS1	-0.393394	-0.916863	-2.396744	0.0174294	0.0491043	-3.855287
ATP5C1P1	-0.532233	0.0312665	-2.396705	0.0174312	0.0491055	-3.855376
RP11-804H8	-0.346239	3.0169761	-2.396674	0.0174327	0.0491058	-3.855448
GPBP1L1	0.0417138	6.5705408	2.3964518	0.017443	0.049131	-3.85596
BECN1	0.0548213	6.2360388	2.3961531	0.0174568	0.0491659	-3.856648
CD300LG	0.7170577	2.2709007	2.3961258	0.017458	0.0491659	-3.856711
RP11-847H1	0.2802332	-1.403061	2.3960548	0.0174613	0.0491714	-3.856875
ACTG1P1	-0.451592	2.1264032	-2.395129	0.0175043	0.0492884	-3.859007
PTCD3	-0.041331	6.3811338	-2.394689	0.0175247	0.0493422	-3.860019
IGLL3P	-0.572829	-0.222015	-2.394472	0.0175348	0.0493636	-3.860519
RP5-1009N1	-0.376883	-0.928159	-2.394467	0.017535	0.0493636	-3.86053
MYH1	-0.809054	0.5629011	-2.394298	0.0175429	0.0493819	-3.86092
RP11-77H9.	0.5749271	0.5725566	2.3941082	0.0175517	0.049403	-3.861357
LINC00323	0.6397111	0.1297456	2.3939458	0.0175593	0.0494205	-3.861731
RP5-828H9.	-0.586971	-0.394244	-2.393864	0.0175631	0.0494274	-3.86192
PTPN22	0.2098939	4.5273831	2.3937598	0.0175679	0.0494348	-3.862159
LINC00483	-0.60255	-0.073086	-2.393721	0.0175697	0.0494348	-3.862248
RPS16	-0.055844	7.1748836	-2.39372	0.0175698	0.0494348	-3.86225
PACSIN2	0.0534204	6.5786037	2.3932271	0.0175927	0.0494956	-3.863385
FAM78A	0.1132311	5.3512182	2.3931865	0.0175946	0.0494971	-3.863478
MGC32805	0.6159094	3.8892905	2.3930214	0.0176023	0.049515	-3.863858
RP11-254B1	-0.490084	-0.201915	-2.392862	0.0176098	0.0495321	-3.864226
TEX43	-0.359039	-1.04228	-2.392677	0.0176184	0.0495503	-3.864651
ARHGAP26	-0.071981	5.8367277	-2.392665	0.017619	0.0495503	-3.864678
ELDR	-0.536094	-0.479701	-2.392628	0.0176207	0.0495513	-3.864763
C17orf70	-0.045993	6.3014872	-2.392487	0.0176273	0.049566	-3.865087
LINC01119	0.3995816	3.213554	2.392447	0.0176292	0.0495675	-3.86518
TPT1-AS1	-0.088185	5.1614437	-2.392391	0.0176318	0.049571	-3.865308
AC022210.2	-0.504366	1.8515153	-2.39217	0.0176421	0.0495963	-3.865818
CCDC14	-0.067248	5.9417088	-2.392137	0.0176436	0.0495968	-3.865893
NPM1P25	0.2279798	4.3768432	2.3920377	0.0176483	0.049606	-3.866121
RP11-630C1	0.2572273	-1.333689	2.3918191	0.0176585	0.0496309	-3.866624
PLK5	-0.663432	0.9437141	-2.391734	0.0176625	0.0496383	-3.86682
COX5BP6	-0.525116	1.130264	-2.391597	0.0176689	0.0496525	-3.867136
ANKMY1	0.060401	5.6214046	2.39155	0.0176711	0.0496548	-3.867243
TEAD4	0.1461802	5.3547396	2.3914577	0.0176754	0.0496632	-3.867455
DHRS1	0.1073917	6.3093779	2.3914185	0.0176773	0.0496645	-3.867545
SERAC1	-0.073525	5.4849937	-2.391188	0.017688	0.049691	-3.868075
AC012442.5	-0.395288	1.9838911	-2.391112	0.0176916	0.0496972	-3.86825
CHKB-AS1	-0.178751	3.9043891	-2.390856	0.0177036	0.049727	-3.868838
RP11-429J1	0.5535844	-0.36747	2.3908252	0.0177051	0.0497273	-3.868909
WDR35	0.0976803	5.4378433	2.390588	0.0177162	0.0497512	-3.869455
CHN2	-0.129016	6.0464048	-2.390585	0.0177163	0.0497512	-3.869461
WDR11-AS1	-0.518626	0.4320857	-2.390439	0.0177232	0.0497667	-3.869797
LINGO3	0.5129727	1.3788048	2.3899869	0.0177444	0.0498225	-3.870836
LSAMP-AS1	-0.45306	-1.002549	-2.389749	0.0177556	0.04985	-3.871382
CEP164P1	-0.483971	1.40446	-2.389673	0.0177592	0.0498562	-3.871558
XXbac-BPG1	0.6295964	1.4763383	2.3894866	0.0177679	0.0498757	-3.871986

RP11-789C2	-0.7085	0.0810061	-2.389438	0.0177702	0.0498757	-3.872098
LGI4	-0.191847	5.1283847	-2.389434	0.0177704	0.0498757	-3.872107
RN7SL138P	0.3674218	2.4775656	2.3894092	0.0177716	0.0498757	-3.872164
RAC2	0.1266057	5.9230397	2.3888066	0.0177999	0.0499515	-3.873548
RNU2-23P	0.0848235	-1.566447	2.3886927	0.0178053	0.0499627	-3.87381
DNASE1L3	0.2511974	5.5301125	2.388228	0.0178272	0.0500203	-3.874877
LRRC37A11F	0.5813484	0.0973082	2.3879873	0.0178386	0.0500484	-3.87543
TOMM5	-0.064472	5.9382404	-2.387906	0.0178424	0.0500546	-3.875616
SSX5	-0.630373	-0.61065	-2.387849	0.0178451	0.0500546	-3.875748
FAM149B1	-0.050384	6.0645612	-2.387829	0.017846	0.0500546	-3.875792
SYNDIG1L	0.5621455	0.2599218	2.3878244	0.0178463	0.0500546	-3.875804
NUDT2	-0.071674	5.9164191	-2.387787	0.017848	0.0500557	-3.87589
LDB1	-0.051489	6.0656266	-2.387316	0.0178703	0.0501142	-3.876971
RP11-132A1	0.2541642	-1.367263	2.3869577	0.0178872	0.0501579	-3.877794
RP5-1166F1	-0.405036	-1.226506	-2.38662	0.0179032	0.0501988	-3.878568
SSX4B	-0.37847	-1.205822	-2.386588	0.0179048	0.0501993	-3.878643
RP1-78B3.1	-0.466903	1.3774332	-2.386008	0.0179322	0.0502697	-3.879972
RP11-798K2	0.2845548	3.3010058	2.3859994	0.0179326	0.0502697	-3.879993
SMG8	-0.062525	5.7803126	-2.385901	0.0179373	0.0502757	-3.880219
RP5-899B16	-0.48675	-0.803585	-2.385886	0.017938	0.0502757	-3.880254
OR2A7	0.5438564	2.7057337	2.3858676	0.0179389	0.0502757	-3.880295
RP11-197K6	-0.540693	-1.007432	-2.385825	0.0179409	0.0502775	-3.880392
TRAPPC8	0.0567494	6.2165957	2.3857839	0.0179429	0.0502791	-3.880487
AK7	0.228712	4.3127699	2.385707	0.0179465	0.0502855	-3.880664
LARGE	0.0923257	5.9989006	2.3855991	0.0179516	0.050296	-3.880911
OXLD1	-0.066547	5.9313706	-2.385518	0.0179555	0.0503029	-3.881098
AMER3	-0.335241	-1.294738	-2.38516	0.0179725	0.0503466	-3.881918
TSC22D1	0.0621238	6.6358506	2.3851083	0.0179749	0.0503497	-3.882037
UFC1	-0.049663	6.6043363	-2.384863	0.0179866	0.0503784	-3.882599
CH17-125A1	-0.574733	-0.724568	-2.384626	0.0179979	0.0504062	-3.883143
RP5-963E22	-0.414393	2.454765	-2.384397	0.0180088	0.0504322	-3.883669
GOLGA6B	0.6175978	-0.418667	2.384373	0.0180099	0.0504322	-3.883723
HBZ	0.1840965	-1.493092	2.3842591	0.0180153	0.0504435	-3.883985
AC144568.4	-0.437286	-0.679674	-2.38407	0.0180243	0.0504648	-3.884418
RFWD3	-0.054248	5.9170424	-2.383637	0.018045	0.0505187	-3.885411
CIRBP-AS1	-0.160393	4.1296104	-2.383329	0.0180597	0.0505559	-3.886116
SLC25A22	0.0837442	6.203961	2.3831013	0.0180705	0.0505822	-3.886639
RP5-1116H2	-0.443094	-0.589681	-2.383075	0.0180718	0.0505822	-3.886699
MPP4	-0.543302	0.9892093	-2.382996	0.0180755	0.0505888	-3.88688
UBE2FP3	-0.427509	1.8641484	-2.382775	0.0180861	0.0506145	-3.887386
TANK	0.0493824	6.1628215	2.3826487	0.0180921	0.0506275	-3.887676
RP11-982M1	0.6048008	-0.011883	2.3825343	0.0180976	0.0506359	-3.887938
RP11-888D1	-0.212815	3.9216554	-2.382528	0.0180979	0.0506359	-3.887953
RNU6-137P	-0.520889	0.0026065	-2.382166	0.0181152	0.0506796	-3.888781
RP11-478P1	0.5529296	-0.280616	2.382117	0.0181176	0.0506796	-3.888894
AP000662.4	0.5652638	1.6649692	2.382114	0.0181177	0.0506796	-3.888901
RNU6-725P	-0.435449	-1.068789	-2.382019	0.0181223	0.0506885	-3.889119
RP11-177G2	-0.449603	-0.778127	-2.381969	0.0181246	0.0506912	-3.889232
KRT18P58	-0.256424	-1.319484	-2.381907	0.0181276	0.0506956	-3.889374
CTD-2298J1	-0.450554	-0.984127	-2.381708	0.0181372	0.0507165	-3.889832
FKBP15	0.0380229	6.1977943	2.3816935	0.0181379	0.0507165	-3.889864

KRTAP19-4	-0.191431	-1.460881	-2.381641	0.0181404	0.0507197	-3.889985
RP3-404F18	-0.501654	-0.344307	-2.38129	0.0181572	0.0507628	-3.890789
AC141586.5	-0.135681	4.322826	-2.381214	0.0181608	0.0507691	-3.890962
TRPC4AP	-0.034916	6.5945976	-2.381081	0.0181672	0.0507831	-3.891268
ENOX1	0.2935066	3.6200278	2.3807239	0.0181844	0.0508261	-3.892085
TLE3	0.07603	6.1731232	2.3807022	0.0181854	0.0508261	-3.892135
RP11-461A8	-0.333838	2.7685687	-2.38058	0.0181913	0.0508387	-3.892415
ZSCAN29	-0.051356	5.8559539	-2.38047	0.0181965	0.0508494	-3.892665
SP5	-0.395349	4.8500861	-2.380357	0.018202	0.0508608	-3.892925
ZNF793	-0.278127	4.3876827	-2.380313	0.0182041	0.0508628	-3.893027
HTR3E	-0.265858	-1.380573	-2.380202	0.0182094	0.0508738	-3.893279
PLAC8L1	-0.440421	2.5958889	-2.380111	0.0182138	0.0508816	-3.893488
EML2	0.0751362	6.0390382	2.3800859	0.018215	0.0508816	-3.893545
C6orf99	-0.745421	0.9244212	-2.379913	0.0182233	0.0509009	-3.893941
PRMT5-AS1	-0.383554	2.1246106	-2.379874	0.0182252	0.0509023	-3.894031
LAPTM4B	-0.111956	6.6285731	-2.379746	0.0182313	0.0509155	-3.894323
RP11-347H1	-0.227844	-1.421119	-2.379505	0.0182429	0.0509401	-3.894874
RP11-67K19	-0.548662	1.7794256	-2.379505	0.0182429	0.0509401	-3.894874
CTD-2286N8	-0.353185	2.7844411	-2.379472	0.0182445	0.0509407	-3.89495
CTD-2022H1	-0.494216	-0.014116	-2.379324	0.0182517	0.0509532	-3.89529
DEFA4	-0.525712	-0.653726	-2.379321	0.0182518	0.0509532	-3.895296
AC025627.4	0.3427943	-1.093425	2.3792843	0.0182536	0.0509543	-3.89538
AC002064.4	0.3901886	-0.893565	2.3792361	0.0182559	0.0509568	-3.89549
ALG6	-0.054041	5.8297751	-2.37904	0.0182653	0.0509793	-3.895938
AP000866.1	-0.501397	1.4054665	-2.378723	0.0182806	0.0510181	-3.896664
MARC2	0.1094004	6.518076	2.3781044	0.0183105	0.0510967	-3.89808
PIGG	-0.047946	6.1429062	-2.378081	0.0183116	0.0510967	-3.898133
ALG1L6P	-0.260299	3.3794215	-2.377956	0.0183177	0.0511097	-3.898418
TRMT112P4	0.6139821	2.3420133	2.3779214	0.0183193	0.0511104	-3.898498
RP11-141A1	-0.229972	-1.412076	-2.377887	0.018321	0.0511112	-3.898577
RP11-1102F	0.5153994	0.1400697	2.3777797	0.0183262	0.0511205	-3.898822
AC019181.2	-0.573856	2.2743841	-2.37776	0.0183271	0.0511205	-3.898867
ATP5J2-PTC	-0.459511	1.7521143	-2.377567	0.0183365	0.0511426	-3.899308
RP11-45M22	0.3855193	-0.936148	2.3773387	0.0183475	0.0511695	-3.899831
RP11-144G6	-0.507934	1.3339445	-2.377192	0.0183546	0.0511854	-3.900166
CCL15-CCL1	-0.465736	4.3604796	-2.376965	0.0183656	0.0512122	-3.900686
RP11-804A2	-0.371375	-0.983995	-2.376877	0.0183699	0.0512202	-3.900887
RP11-76C10	-0.245006	-1.368893	-2.376771	0.018375	0.0512306	-3.90113
TBC1D5	0.0417189	6.2670603	2.3766853	0.0183792	0.0512382	-3.901324
CNPPD1	0.0411056	6.4593899	2.3763095	0.0183974	0.0512851	-3.902183
RP11-269M2	-0.5518	-0.000112	-2.37604	0.0184104	0.0513123	-3.902799
PLS3-AS1	0.2957366	3.2575129	2.3760289	0.018411	0.0513123	-3.902825
RNU6-705P	-0.209686	-1.430024	-2.376021	0.0184114	0.0513123	-3.902842
RP5-1030M6	0.081403	-1.56819	2.3759551	0.0184146	0.0513134	-3.902994
NUPL2	-0.041381	5.9848588	-2.375955	0.0184146	0.0513134	-3.902994
GCG	0.5773854	-0.612378	2.3758631	0.018419	0.0513219	-3.903204
ZNF707	-0.067105	5.6592137	-2.375668	0.0184285	0.0513445	-3.903651
CACYBPP3	-0.210934	-1.451317	-2.375576	0.018433	0.051353	-3.90386
F2	-0.246681	7.1961413	-2.375395	0.0184418	0.0513726	-3.904274
C14orf132	0.3372275	5.0997541	2.3753402	0.0184444	0.0513726	-3.904399
AC015849.1	-0.506333	3.0118567	-2.375314	0.0184457	0.0513726	-3.904458

RP11-135A1	0.6358556	0.6942075	2.3753063	0.0184461	0.0513726	-3.904476
RP13-212L9	-0.331305	-1.336338	-2.375286	0.0184471	0.0513726	-3.904522
RFT1	-0.044452	6.0035611	-2.375256	0.0184485	0.0513728	-3.904591
LMX1B	-0.867074	1.0314665	-2.37481	0.0184702	0.0514293	-3.90561
SLCO2B1	0.1211254	6.7209838	2.3744404	0.0184882	0.0514754	-3.906454
ENDOV	0.0766929	5.712895	2.3743386	0.0184932	0.0514853	-3.906687
RP11-646I6	-0.508553	0.8107802	-2.374031	0.0185082	0.0515231	-3.907389
UBAC2	-0.047256	6.501324	-2.373945	0.0185124	0.0515309	-3.907585
KCNJ6	0.8023785	1.3645194	2.3737575	0.0185215	0.0515524	-3.908014
AC005702.1	-0.376475	1.932686	-2.373715	0.0185236	0.0515543	-3.908111
AC018685.1	0.1596696	-1.500781	2.3736019	0.0185291	0.0515657	-3.908369
LCMT2	0.0609413	5.5723543	2.3734071	0.0185386	0.0515882	-3.908814
MESP1	-0.168582	4.8038712	-2.373233	0.0185471	0.0516079	-3.90921
RBKS	0.0964008	5.9379982	2.3728299	0.0185668	0.0516588	-3.910131
RP11-752G1	-0.451359	-0.307229	-2.372677	0.0185743	0.0516757	-3.910481
RP5-1142A6	0.4082437	2.2891029	2.3724324	0.0185863	0.0517051	-3.911039
CTD-2017D1	-0.445854	2.5100502	-2.372379	0.0185889	0.0517084	-3.91116
RPL27AP	-0.442482	-0.688754	-2.372167	0.0185993	0.0517334	-3.911645
MAGEA11	-0.77273	-0.174158	-2.372109	0.0186021	0.0517373	-3.911776
CTD-3195I5	-0.420524	-0.893624	-2.372065	0.0186043	0.0517394	-3.911878
DPRXP4	0.5182523	-0.149073	2.371905	0.0186121	0.0517572	-3.912242
PCDHB16	0.3335089	4.2976703	2.3718093	0.0186168	0.0517663	-3.91246
POLR1C	-0.052965	5.9913345	-2.371566	0.0186287	0.0517955	-3.913015
AARS	0.0510365	6.7886626	2.3714776	0.0186331	0.0518036	-3.913217
VAMP8	0.0653393	6.5689162	2.3712817	0.0186427	0.0518264	-3.913664
CAPN15	-0.049795	6.2445583	-2.371236	0.0186449	0.0518287	-3.913768
PIP4K2C	0.0464282	6.2019068	2.3709814	0.0186574	0.0518595	-3.914349
BCRP8	0.3368709	-1.125171	2.3709104	0.0186609	0.0518652	-3.914511
RP11-575H3	0.6253697	-0.097619	2.3707869	0.018667	0.0518781	-3.914793
MSH6	-0.047588	6.348853	-2.370584	0.018677	0.0519019	-3.915256
AC092687.5	-0.482117	-0.499775	-2.370365	0.0186877	0.0519278	-3.915754
FEV	-0.400631	-1.034223	-2.370243	0.0186937	0.0519405	-3.916032
RP4-640H8.	-0.776243	0.6371785	-2.370144	0.0186986	0.0519502	-3.916259
ADCY2	0.6548597	0.4183337	2.369844	0.0187134	0.0519872	-3.916943
NOL4L	-0.063655	5.9158589	-2.369809	0.0187151	0.0519881	-3.917023
CIZ1	-0.046361	6.344659	-2.369712	0.0187199	0.0519974	-3.917244
SLC3A2	0.0576967	6.6199425	2.3696052	0.0187251	0.052008	-3.917487
RP11-753H1	-0.51007	-0.385973	-2.369366	0.0187369	0.0520368	-3.918032
TTTY15	0.9431848	2.1908111	2.3691778	0.0187462	0.0520586	-3.918461
HSD17B4	0.0776682	6.9033221	2.3691446	0.0187478	0.0520592	-3.918537
RP11-272P1	-0.442072	-0.585275	-2.368895	0.0187601	0.0520895	-3.919106
ZNF502	-0.244599	4.5487052	-2.368786	0.0187655	0.0521004	-3.919355
NTSR2	0.47158	-1.018751	2.368711	0.0187692	0.0521065	-3.919525
SUGP1	-0.041002	6.1290749	-2.368683	0.0187706	0.0521065	-3.919588
LMAN2L	-0.048625	6.0765895	-2.368473	0.018781	0.0521315	-3.920068
GTPBP10	0.0503335	5.9608554	2.3684257	0.0187833	0.052134	-3.920175
KCNJ14	-0.208389	4.0891045	-2.368199	0.0187945	0.052161	-3.920691
RP13-58209	-0.261479	3.420617	-2.368144	0.0187972	0.0521647	-3.920818
RP11-48B14	-0.269818	2.9625746	-2.367893	0.0188096	0.0521951	-3.921388
NPM1P8	-0.414286	-0.816869	-2.367816	0.0188134	0.0521978	-3.921563
DCUN1D1	0.0540339	6.1792881	2.3678158	0.0188135	0.0521978	-3.921565

TRIM66	0.0897259	5.5448147	2.3675717	0.0188255	0.0522273	-3.922121
DNAH7	0.5134487	2.6451002	2.3673519	0.0188364	0.0522536	-3.922621
NSUN3	0.0625936	5.4103716	2.3670892	0.0188494	0.0522847	-3.92322
CCL26	0.631411	2.2366886	2.3670678	0.0188505	0.0522847	-3.923268
RP5-1091N2	0.3939773	2.7601809	2.3668607	0.0188608	0.0523075	-3.92374
PCDHGA4	0.4977556	3.5942859	2.3668437	0.0188616	0.0523075	-3.923779
ADORA2B	-0.330425	4.0304996	-2.366696	0.0188689	0.0523238	-3.924115
UCHL5	-0.057431	6.2715989	-2.366585	0.0188744	0.0523352	-3.924368
RANP1	-0.502715	1.6880824	-2.366422	0.0188825	0.0523535	-3.924738
RN7SKP287	-0.396749	-0.818384	-2.366193	0.0188939	0.0523812	-3.925261
WASF5P	0.1620147	-1.504345	2.3660718	0.0188999	0.0523938	-3.925536
CD19	-0.499254	3.3333038	-2.365907	0.0189081	0.0524126	-3.925911
GRID1-AS1	0.3905691	-1.052569	2.3654674	0.0189299	0.0524692	-3.926912
PCDHGA1	-0.544568	3.1410679	-2.365241	0.0189412	0.0524964	-3.927428
RP11-528I4	-0.481038	-0.373307	-2.365001	0.0189532	0.0525255	-3.927973
KLK15	-0.409714	-1.120002	-2.364845	0.0189609	0.052543	-3.928327
RP11-406A9	-0.372307	-1.176967	-2.364783	0.018964	0.0525444	-3.928468
AC005083.1	0.5771892	2.2144075	2.3647778	0.0189643	0.0525444	-3.928481
TMEM57	0.0591856	6.3633855	2.3646575	0.0189703	0.052557	-3.928755
KIAA0391	-0.077969	5.2364983	-2.364553	0.0189755	0.0525674	-3.928992
IRAK1	0.0610014	6.6777952	2.364173	0.0189944	0.0526159	-3.929857
LLNLR-304A	-0.491678	-0.378161	-2.364021	0.019002	0.0526329	-3.930203
GS1-594A7.	-0.312411	-1.29635	-2.363783	0.0190139	0.0526619	-3.930745
CRIP1	0.2211682	4.0850967	2.3636933	0.0190184	0.0526703	-3.930948
YY1	-0.033056	6.5810763	-2.363621	0.019022	0.0526762	-3.931111
CYP1B1-AS1	0.2411635	3.4451724	2.3632794	0.0190391	0.0527174	-3.931889
PSMD8	0.0430841	6.6992046	2.3632526	0.0190404	0.0527174	-3.93195
LINC00526	0.2323687	5.2083895	2.3632368	0.0190412	0.0527174	-3.931986
ALDH18A1	-0.061014	6.470453	-2.363146	0.0190457	0.052726	-3.932193
RP11-15B24	0.7195176	1.0054884	2.3630762	0.0190492	0.0527315	-3.932351
CATSPERB	0.6256539	2.824955	2.3630488	0.0190506	0.0527315	-3.932413
APOBEC3G	0.1408231	5.3688325	2.3627721	0.0190644	0.0527658	-3.933042
RP11-66N24	-0.246197	3.6601179	-2.362631	0.0190715	0.0527814	-3.933364
UHRF1BP1	-0.057602	5.9987646	-2.36257	0.0190746	0.0527858	-3.933502
RP3-324017	-0.381692	-0.846822	-2.362285	0.0190888	0.0528213	-3.934151
CXorf36	0.0916184	5.6807424	2.3622473	0.0190907	0.0528225	-3.934235
TGFBR3L	0.4835293	3.9106272	2.3620943	0.0190984	0.0528397	-3.934583
GPHA2	-0.587638	-0.345504	-2.361949	0.0191057	0.0528559	-3.934914
RP11-395E1	0.2062684	-1.454269	2.3619089	0.0191077	0.0528574	-3.935004
RP11-393N4	-0.523474	1.6745153	-2.361726	0.0191169	0.0528741	-3.935421
MEMO1	-0.066879	4.93367	-2.361722	0.019117	0.0528741	-3.935429
CTB-55B8.1	-0.463089	-0.355463	-2.361702	0.0191181	0.0528741	-3.935475
CD68	0.2301989	4.5150907	2.3614977	0.0191283	0.0528985	-3.935939
BCL2A1	0.2614622	4.4523812	2.3614524	0.0191306	0.0529007	-3.936042
RP11-77901	0.560606	0.1050782	2.3613962	0.0191334	0.0529031	-3.93617
CTCF	0.0321409	6.2668133	2.3613775	0.0191343	0.0529031	-3.936212
PPP4R2	-0.053426	6.4351039	-2.361275	0.0191395	0.0529133	-3.936444
CCT6P2	-0.394296	-0.83796	-2.361116	0.0191475	0.0529314	-3.936805
FAM225A	-0.520682	2.4518816	-2.360916	0.0191575	0.0529551	-3.937259
RP11-1017G	-0.420094	2.4472532	-2.360645	0.0191712	0.0529889	-3.937877
CTD-2139B1	0.2516291	-1.408389	2.3604672	0.0191801	0.0530096	-3.93828

FH	0.0775574	6.8315915	2.3602573	0.0191907	0.0530347	-3.938757
STAT4	0.1720222	5.1548957	2.3599374	0.0192068	0.0530753	-3.939483
CTC-756D1.	0.5430571	0.5454339	2.3597335	0.0192171	0.0530996	-3.939946
TAX1BP3	0.0992093	5.8205896	2.3595922	0.0192242	0.0531153	-3.940267
GLUD1	-0.088933	7.036689	-2.359455	0.0192311	0.0531304	-3.940578
CYSTM1	0.0686762	6.4815556	2.3591895	0.0192445	0.0531634	-3.941181
LINC01115	-0.397069	-1.154981	-2.359103	0.0192489	0.0531715	-3.941378
RP11-384J4	-0.265905	-1.34564	-2.359054	0.0192513	0.0531742	-3.941488
AC008703.1	0.2521482	-1.408124	2.3589623	0.019256	0.053183	-3.941697
AC139100.3	-0.36002	3.4644129	-2.358932	0.0192575	0.0531832	-3.941765
RP11-472B1	0.3332936	2.6417195	2.3588544	0.0192614	0.05319	-3.941942
RP11-563J2	0.6196525	1.2182168	2.3586256	0.019273	0.0532179	-3.942461
RPSAP1	-0.281168	-1.284398	-2.358196	0.0192947	0.0532738	-3.943436
RP4-592A1.	0.4502313	2.1094854	2.358039	0.0193026	0.0532889	-3.943792
DSTNP2	-0.108769	5.0659688	-2.35803	0.0193031	0.0532889	-3.943812
DAB2IP	0.0757027	6.3046784	2.3579501	0.0193071	0.0532961	-3.943994
RPS15AP17	-0.523877	0.5503031	-2.357702	0.0193197	0.0533264	-3.944558
OR6C70	-0.258505	-1.427989	-2.357675	0.019321	0.0533264	-3.944617
KLRD1	0.2492103	4.4589494	2.3575167	0.0193291	0.0533446	-3.944977
RPS3AP5	-0.372353	3.0652432	-2.357473	0.0193313	0.0533467	-3.945077
DLGAP1-AS3	0.6251916	-0.29814	2.3573025	0.0193399	0.0533664	-3.945463
KANSL3	-0.041777	6.3621944	-2.357064	0.019352	0.0533958	-3.946004
RP11-7011.	-0.369132	2.4334062	-2.357022	0.0193541	0.0533976	-3.946099
RP11-398M1	-0.38785	-1.084098	-2.35697	0.0193568	0.0534009	-3.946217
RP11-240G2	0.1831745	-1.516325	2.3568184	0.0193645	0.0534181	-3.946561
PTPRC	0.1485512	5.8730137	2.3567666	0.0193671	0.0534185	-3.946679
BHLHA15	-0.40986	4.1609581	-2.356758	0.0193676	0.0534185	-3.946699
RP11-229P1	-0.479405	1.4176062	-2.356693	0.0193708	0.0534235	-3.946846
RP11-1267H	0.4160916	-0.938342	2.3566003	0.0193756	0.0534324	-3.947056
RP11-341D1	-0.444358	-1.041662	-2.356556	0.0193778	0.0534346	-3.947156
PTMAP2	-0.235818	3.5348105	-2.356506	0.0193804	0.0534376	-3.94727
GS1-39E22.	0.081403	-1.56819	2.3563593	0.0193878	0.0534541	-3.947602
EIF2S3L	-0.449539	2.4883673	-2.356174	0.0193972	0.053476	-3.948022
SNX12	0.0449653	6.271443	2.3557507	0.0194187	0.0535313	-3.948982
HSPA12A	0.1768158	5.0275376	2.3556215	0.0194253	0.0535454	-3.949275
MACROD2	-0.166407	5.08698	-2.355559	0.0194285	0.05355	-3.949416
AC010890.1	0.5463875	-0.058104	2.3552375	0.0194449	0.0535881	-3.950146
RP11-347C1	-0.411913	2.1229105	-2.355231	0.0194452	0.0535881	-3.950161
RP11-118M9	-0.502346	-0.195584	-2.355021	0.0194559	0.0536135	-3.950637
CYP51A1P1	-0.516489	-0.22668	-2.354654	0.0194746	0.0536592	-3.951467
SLC45A3	-0.118827	5.7965525	-2.354638	0.0194754	0.0536592	-3.951504
SDC2	-0.078914	7.0247875	-2.354397	0.0194877	0.053689	-3.952051
RP11-53B5.	-0.28754	-1.330451	-2.354368	0.0194892	0.0536891	-3.952117
CLU	0.086171	7.6161498	2.3543072	0.0194923	0.0536935	-3.952254
EMC8	0.0485304	5.9632948	2.3539055	0.0195128	0.0537459	-3.953164
BRPF1	-0.038483	5.8654316	-2.353792	0.0195186	0.0537543	-3.953421
RP11-80I3.	0.1117333	-1.552733	2.3537884	0.0195188	0.0537543	-3.953429
AC009336.1	-0.482862	-0.595946	-2.353702	0.0195232	0.0537624	-3.953624
RP11-566K1	-0.682314	0.88231	-2.353644	0.0195261	0.0537665	-3.953755
MT-ND3	0.0751905	7.4250459	2.3534295	0.0195371	0.0537927	-3.954242
ATF7IP2	-0.123828	5.8974163	-2.353116	0.0195532	0.0538328	-3.954953

RP11-95G6.	0.4946734	1.2948768	2.3530042	0.0195589	0.0538445	-3.955205
PYURF	0.0992325	4.7365375	2.3525988	0.0195796	0.0538975	-3.956123
LYZ	-0.17072	6.6761074	-2.35249	0.0195852	0.0539052	-3.956369
BPIFB9P	-0.449418	-0.480929	-2.352487	0.0195854	0.0539052	-3.956377
RP11-450I1	0.0768235	-1.570524	2.3522878	0.0195956	0.0539292	-3.956827
EFNA3	-0.205355	4.8207552	-2.352214	0.0195994	0.0539356	-3.956995
RP11-54C4.	-0.139863	4.9628395	-2.352151	0.0196026	0.0539404	-3.957137
RP1-86C11.	-0.573937	2.336159	-2.352094	0.0196055	0.0539415	-3.957267
SAMD4A	0.0871118	5.9183531	2.3520859	0.0196059	0.0539415	-3.957284
RP11-167P1	0.505293	0.2823997	2.3520282	0.0196089	0.0539444	-3.957415
CTD-2530H1	-0.27029	-1.251715	-2.352007	0.01961	0.0539444	-3.957462
ZNF446	-0.054959	5.4737399	-2.351824	0.0196194	0.0539662	-3.957876
RAC1	0.0356973	6.8648063	2.3516286	0.0196294	0.0539898	-3.958319
RP11-98G7.	-0.569974	2.9207016	-2.351442	0.019639	0.0540121	-3.958742
RP11-70D24	0.278	3.7640878	2.3510074	0.0196613	0.0540694	-3.959725
TRAPPC2	-0.050732	5.7490439	-2.350927	0.0196655	0.0540767	-3.959907
AC017104.3	-0.498209	-0.055916	-2.350841	0.0196699	0.0540848	-3.960102
HSPE1P18	-0.368492	-0.996411	-2.350519	0.0196865	0.0541263	-3.96083
VPS53	0.0482083	6.092655	2.3503866	0.0196933	0.054141	-3.961129
ANKRD22	0.5213943	3.7625632	2.3502341	0.0197011	0.0541585	-3.961474
RPL8	-0.06012	7.3792224	-2.350057	0.0197102	0.0541794	-3.961874
AC098614.2	0.3706912	3.3100944	2.3499965	0.0197134	0.054184	-3.962012
AC010880.1	-0.32947	-1.366704	-2.349511	0.0197385	0.0542488	-3.963111
NPM1P39	-0.410708	2.3886968	-2.349076	0.0197609	0.0543064	-3.964094
DNTTIP1	-0.048441	6.03196	-2.348904	0.0197698	0.0543266	-3.964481
HMGB1P5	-0.077145	5.469304	-2.348787	0.0197758	0.0543392	-3.964745
CNN2P6	-0.240782	-1.436679	-2.348487	0.0197913	0.0543777	-3.965424
RP11-452H2	-0.448281	-0.521686	-2.34804	0.0198145	0.0544373	-3.966436
TUBA4B	0.607928	2.7437556	2.3480018	0.0198164	0.0544386	-3.966521
SNORA71A	-0.535167	0.6805864	-2.347525	0.0198412	0.0545023	-3.967599
HK3	0.2027695	4.979661	2.3474221	0.0198465	0.0545129	-3.967831
AURKAIP1	0.0640588	6.5740121	2.3471702	0.0198596	0.0545447	-3.9684
AC020983.5	-0.322773	-1.172584	-2.347058	0.0198654	0.0545565	-3.968653
RP11-325E5	-0.465622	-0.156979	-2.346726	0.0198826	0.0545998	-3.969404
FASTKD2	0.0416203	6.104945	2.3465495	0.0198918	0.0546208	-3.969802
CTD-2595P9	0.4717818	-0.610035	2.346322	0.0199036	0.0546492	-3.970316
TBL1X	-0.066092	6.2694247	-2.34624	0.0199079	0.0546569	-3.970502
OS9	0.0485402	7.0164124	2.3461847	0.0199107	0.0546606	-3.970626
Z83844.1	-0.574775	0.805859	-2.346083	0.019916	0.0546669	-3.970855
TOR1AIP1	0.0549912	6.3399625	2.3460829	0.019916	0.0546669	-3.970856
LMO3	-0.525307	2.9487749	-2.346004	0.0199201	0.0546704	-3.971035
SLTM	0.0321681	6.4114783	2.3459819	0.0199213	0.0546704	-3.971084
KIAA0556	0.0655836	5.8152702	2.3459718	0.0199218	0.0546704	-3.971107
Z69720.3	0.4897077	-0.721229	2.345855	0.0199279	0.054683	-3.971371
CENPC	0.0604139	5.6272852	2.3456478	0.0199387	0.0547085	-3.971839
XXbac-BPG2	-0.517418	1.1789707	-2.345373	0.019953	0.0547436	-3.972459
RP11-717A5	-0.340542	-1.12464	-2.345322	0.0199557	0.0547468	-3.972574
ZNF804A	0.6235171	1.9716367	2.3452381	0.01996	0.0547547	-3.972764
RP1-178F15	-0.322746	3.0084157	-2.34513	0.0199656	0.054766	-3.973007
AC083864.4	0.1675095	-1.496785	2.3449506	0.019975	0.0547876	-3.973413
RP11-27N21	-0.523158	2.7905191	-2.344824	0.0199816	0.0548016	-3.973699

CTD-2287O1	-0.111	5.301011	-2.344742	0.0199859	0.0548092	-3.973884
KCNU1	-0.895606	0.5828374	-2.34471	0.0199876	0.0548096	-3.973955
SVILP1	0.4826313	2.3871085	2.3445143	0.0199978	0.0548335	-3.974397
ZNF833P	-0.251643	3.212089	-2.344397	0.0200039	0.0548429	-3.974663
SNX18P7	-0.492452	-0.702728	-2.344391	0.0200042	0.0548429	-3.974675
PIK3CD	0.1225357	5.5442875	2.3443352	0.0200071	0.0548468	-3.974801
CTC-250I14	-0.509978	0.1608223	-2.343851	0.0200324	0.0549063	-3.975894
PSMA3	0.0492082	6.5356281	2.3438397	0.020033	0.0549063	-3.975919
WDR60	0.068846	5.5564823	2.3438333	0.0200333	0.0549063	-3.975934
RP11-715J2	-0.579592	0.5079684	-2.343729	0.0200388	0.0549171	-3.976168
GABRR1	-0.5573	-0.274805	-2.343603	0.0200454	0.0549311	-3.976453
AC091633.2	-0.420985	-0.724561	-2.343545	0.0200484	0.0549335	-3.976584
EZH1	0.0437015	6.0351401	2.3435117	0.0200502	0.0549335	-3.976659
DAK	0.1081682	6.5617625	2.3434998	0.0200508	0.0549335	-3.976686
MED29	0.0421236	6.4430677	2.3434019	0.0200559	0.0549434	-3.976907
FOXO6	-0.627202	3.3224726	-2.343246	0.0200641	0.0549616	-3.977258
RP13-20L14	-0.580256	0.799526	-2.343096	0.020072	0.0549791	-3.977598
RP11-686D2	0.2866636	3.3761541	2.3427666	0.0200892	0.0550222	-3.97834
REXO1	-0.054311	6.1218783	-2.342494	0.0201035	0.0550572	-3.978954
PCDHAC2	0.6980122	2.1205308	2.3422313	0.0201173	0.0550908	-3.979547
RP11-625I7	0.3145979	-1.318088	2.342166	0.0201207	0.0550961	-3.979694
CTD-2021A8	-0.417023	-0.637237	-2.342115	0.0201234	0.0550993	-3.97981
MDS2	-0.484104	1.4053318	-2.341966	0.0201312	0.0551166	-3.980146
CPEB1	0.6591453	1.7709469	2.3417543	0.0201423	0.0551428	-3.980622
TEX2	-0.052843	6.4271843	-2.341704	0.0201449	0.0551459	-3.980735
C15orf59-A	-0.337463	-1.220723	-2.341512	0.020155	0.0551695	-3.981169
RP11-771K4	-0.406035	2.0102702	-2.341447	0.0201584	0.0551746	-3.981314
RP11-146F1	-0.214044	3.8329127	-2.341321	0.0201651	0.0551887	-3.9816
MID1IP1-AS	-0.401813	2.9435007	-2.341222	0.0201703	0.0551987	-3.981821
AC005616.2	0.276512	-1.384928	2.3410331	0.0201802	0.0552162	-3.982248
RP11-356C4	-0.455223	-0.641994	-2.341027	0.0201806	0.0552162	-3.982262
AC005517.3	-0.499858	1.4257386	-2.341015	0.0201812	0.0552162	-3.982289
PGK2	-0.372989	-1.141788	-2.340946	0.0201848	0.0552219	-3.982444
RP11-122F2	-0.379153	-1.143344	-2.34064	0.0202009	0.0552619	-3.983135
AC007387.2	-0.454807	0.9947217	-2.340496	0.0202085	0.0552745	-3.983459
MYRF	-0.113858	6.4134737	-2.340495	0.0202086	0.0552745	-3.983461
CTD-2587M2	-0.334865	3.1549487	-2.340433	0.0202118	0.0552793	-3.983601
Z97634.3	-0.26469	3.4156204	-2.339872	0.0202414	0.055356	-3.984863
IL17RD	0.2429116	4.2056835	2.3398115	0.0202446	0.0553606	-3.985
TH	0.6583507	0.1557279	2.3396606	0.0202526	0.0553782	-3.98534
AP000469.2	-0.26517	-1.394815	-2.339575	0.0202571	0.0553864	-3.985532
ZBTB38	0.0713358	6.2849163	2.3394119	0.0202657	0.0554057	-3.9859
RP11-19N8.	-0.330393	-1.191554	-2.339384	0.0202672	0.0554057	-3.985963
TRIM37	-0.052658	5.9264109	-2.339266	0.0202734	0.0554186	-3.986229
P2RX6	0.4805807	2.8172807	2.3391831	0.0202778	0.0554264	-3.986415
AC026700.1	0.0898283	-1.563896	2.3391528	0.0202794	0.0554267	-3.986483
RPL31P7	-0.318314	-1.197102	-2.339036	0.0202855	0.0554393	-3.986746
AC026904.1	0.4730925	-0.597486	2.3386823	0.0203043	0.0554863	-3.987543
XPOT	-0.050623	6.5055199	-2.338639	0.0203065	0.0554878	-3.98764
FBXL6	-0.079496	6.1543227	-2.338603	0.0203084	0.0554878	-3.98772
CUEDC2	0.0528053	6.2603857	2.3385863	0.0203093	0.0554878	-3.987759

SULT1A3	-0.500612	0.2006708	-2.338433	0.0203174	0.0555058	-3.988104
COX6CP1	-0.906342	1.063722	-2.338215	0.020329	0.0555305	-3.988596
SNRNP70	-0.040899	6.7534842	-2.3382	0.0203298	0.0555305	-3.988628
PLLP	0.1380618	5.6463588	2.3381759	0.0203311	0.0555305	-3.988683
PNPLA1	-0.525294	-0.248548	-2.337941	0.0203435	0.0555603	-3.989211
SNORD101	-0.537666	0.5410691	-2.33768	0.0203573	0.0555939	-3.989799
BOLA1	-0.068852	5.9428498	-2.337641	0.0203594	0.0555955	-3.989887
RPL29P2	-0.335398	-1.053873	-2.337367	0.0203739	0.055631	-3.990504
CPEB2-AS1	-0.529248	0.4031741	-2.337327	0.0203761	0.0556327	-3.990594
DKKL1	-0.601367	2.1627701	-2.337204	0.0203826	0.0556463	-3.99087
NR5A2	-0.130543	6.3176595	-2.337036	0.0203915	0.0556665	-3.991249
RPL7P44	-0.497996	-0.136293	-2.337002	0.0203933	0.0556672	-3.991325
RP11-303E1	0.1088221	5.2454204	2.3369016	0.0203987	0.0556761	-3.99155
C16orf90	-0.305719	-1.172947	-2.336883	0.0203996	0.0556761	-3.991591
KCNJ8	0.1622504	5.9531412	2.3368361	0.0204021	0.0556788	-3.991698
RP11-26501	-0.327278	-1.140729	-2.336671	0.0204109	0.0556986	-3.99207
CNTFR	-0.622316	4.3859242	-2.336633	0.0204129	0.0556999	-3.992154
MBD2	0.0397029	6.3288542	2.3365881	0.0204153	0.0557023	-3.992256
RP11-332K1	0.4570889	-1.084353	2.3365554	0.020417	0.0557028	-3.992329
LMO7-AS1	0.6258898	1.4942464	2.3363529	0.0204278	0.0557251	-3.992785
RP11-89N17	0.3986514	-0.842367	2.3363447	0.0204283	0.0557251	-3.992803
AC011747.3	0.5020188	-0.538668	2.336216	0.0204351	0.0557394	-3.993093
IKZF5	-0.06216	5.875241	-2.336189	0.0204366	0.0557394	-3.993154
RP11-494I9	0.1793319	-1.518284	2.3361026	0.0204411	0.0557477	-3.993348
SYNE1-AS1	0.2415088	-1.345254	2.3357971	0.0204574	0.0557879	-3.994035
RP11-219D1	0.467864	1.4893442	2.3353058	0.0204836	0.0558551	-3.99514
GLYATL3	-0.613971	-0.237422	-2.334978	0.0205011	0.0558987	-3.995877
PCDHGA8	-0.613661	2.0039806	-2.334915	0.0205044	0.0559036	-3.996017
ZNHIT1	0.0655895	6.7017227	2.3344408	0.0205297	0.0559685	-3.997084
C9orf47	0.597998	1.5452555	2.3340811	0.020549	0.0560167	-3.997892
BTBD6	0.0866972	6.1660298	2.3338445	0.0205616	0.0560439	-3.998424
RP1-230L10	-0.242654	-1.405857	-2.333837	0.020562	0.0560439	-3.998441
AGER	-0.10701	4.9591081	-2.333768	0.0205657	0.0560497	-3.998595
RNF146	0.0460293	5.9866885	2.3336573	0.0205716	0.0560617	-3.998845
FCAR	0.6099197	0.8266718	2.3335982	0.0205748	0.0560662	-3.998977
EBI3	0.1898919	4.8509138	2.3335334	0.0205783	0.0560714	-3.999123
RP11-170K4	0.502993	-0.158059	2.3334424	0.0205831	0.0560763	-3.999327
GRPEL2	-0.045518	5.8583531	-2.333442	0.0205831	0.0560763	-3.999328
TMEM199	-0.044598	5.885271	-2.333151	0.0205988	0.0561147	-3.999983
MFRP	0.5173856	0.1615485	2.3330348	0.020605	0.0561274	-4.000243
FAM200B	-0.055547	5.8741782	-2.332961	0.0206089	0.056134	-4.000409
RPL4	-0.049535	7.3011286	-2.332931	0.0206106	0.0561343	-4.000477
NEK7	0.0564381	6.3096884	2.332726	0.0206215	0.0561599	-4.000937
RPL35	-0.06239	6.9973619	-2.332282	0.0206453	0.0562207	-4.001934
PADI4	0.6199293	0.4593389	2.3322165	0.0206489	0.056226	-4.002081
ZFAND6	0.0427072	6.4564737	2.3320643	0.020657	0.0562399	-4.002423
RP11-260A9	-0.499146	-1.055506	-2.332064	0.020657	0.0562399	-4.002423
RP5-1077I2	-0.425711	-0.803148	-2.331894	0.0206662	0.0562606	-4.002806
RP11-15B17	-0.463742	-0.474302	-2.331854	0.0206684	0.0562623	-4.002896
RP11-972P1	-0.344136	3.2260284	-2.331743	0.0206743	0.056273	-4.003144
RP4-61404.	-0.170275	4.1838801	-2.331723	0.0206753	0.056273	-4.003188

CTC-232H1C	-0.510517	0.0046541	-2.331576	0.0206833	0.0562903	-4.003518
RP11-1078H	0.33002	-1.140531	2.3315117	0.0206867	0.0562955	-4.003663
RP11-55204	-0.243099	-1.318019	-2.331078	0.0207101	0.0563549	-4.004637
CTD-2517M2	-0.452669	2.3765465	-2.330769	0.0207267	0.056396	-4.005331
RP3-466P17	-0.454243	1.7446914	-2.330709	0.0207299	0.0564005	-4.005465
RP11-460N2	-0.482379	-0.239096	-2.330659	0.0207327	0.0564037	-4.005578
RASAL3	0.1129024	5.5042032	2.3305888	0.0207364	0.0564097	-4.005735
BBS7	0.1079164	5.1796427	2.3300657	0.0207646	0.0564803	-4.006908
CRISP2	-0.68162	-0.406693	-2.33005	0.0207655	0.0564803	-4.006944
RP11-351I2	-0.399491	-0.842609	-2.329959	0.0207704	0.0564895	-4.007147
RP11-305A4	0.1153845	-1.550872	2.3299222	0.0207724	0.0564907	-4.00723
LINC00226	-0.229938	-1.420092	-2.329727	0.0207829	0.0565151	-4.007668
TRNP1	-0.207431	5.7556221	-2.329427	0.0207991	0.0565522	-4.008342
RP11-791G1	-0.522445	0.5666144	-2.329417	0.0207996	0.0565522	-4.008363
CLPS	-0.427411	-1.187849	-2.329341	0.0208037	0.0565592	-4.008533
LRP2BP	0.1325476	4.2294924	2.3290787	0.0208179	0.0565926	-4.009122
PSMD1	0.0343728	6.6183644	2.3290562	0.0208192	0.0565926	-4.009173
CLEC18B	-0.529544	1.6157491	-2.328638	0.0208418	0.0566499	-4.010111
CLUH	0.0618997	6.8675914	2.3284526	0.0208518	0.0566721	-4.010526
EPHX4	-0.475117	2.7826284	-2.32843	0.020853	0.0566721	-4.010576
LINC01044	0.3982497	-1.109124	2.3282628	0.0208621	0.0566925	-4.010952
RP11-134N1	-0.599788	-0.032575	-2.328029	0.0208748	0.0567227	-4.011476
PDGFB	0.07841	5.9275465	2.3278604	0.0208839	0.0567433	-4.011854
RRP9	-0.067233	5.9833358	-2.327827	0.0208857	0.0567439	-4.011928
TMEM136	-0.138178	5.0639083	-2.327786	0.020888	0.0567459	-4.012021
CAPZA1	0.0366022	6.5718412	2.3272072	0.0209194	0.0568263	-4.013318
PA2G4P1	-0.393741	-0.876674	-2.327183	0.0209207	0.0568263	-4.013372
VAMP5	0.081314	6.3386516	2.327123	0.0209239	0.0568309	-4.013506
CRYBB1	0.3125951	3.20797	2.3270009	0.0209306	0.0568447	-4.01378
RP11-806L2	-0.338357	-1.099652	-2.326812	0.0209408	0.0568683	-4.014203
PCGF7P	-0.517311	0.3416236	-2.326714	0.0209462	0.0568757	-4.014424
PLCD1	-0.066729	5.7428757	-2.326705	0.0209467	0.0568757	-4.014444
CD300LD	0.1813572	-1.471724	2.3265956	0.0209526	0.0568876	-4.014688
RP11-51501	-0.392174	3.6015119	-2.326506	0.0209575	0.0568951	-4.014889
SF3B6	-0.038217	6.3686104	-2.326488	0.0209585	0.0568951	-4.01493
SLURP1	0.213365	-1.432648	2.3264063	0.0209629	0.0569029	-4.015112
CTD-2313J1	-0.487609	-0.249564	-2.326277	0.0209699	0.0569177	-4.015402
AP004550.1	-0.413283	-1.09072	-2.32618	0.0209752	0.0569278	-4.015619
RP11-133K1	-0.284192	-1.249478	-2.325793	0.0209963	0.0569808	-4.016486
HSPE1P3	-0.4778	1.5419231	-2.325373	0.0210192	0.0570387	-4.017426
KRT16P6	0.3751532	-1.178713	2.3250693	0.0210358	0.0570794	-4.018107
RTCA	0.0452787	6.1176977	2.3247318	0.0210542	0.0571251	-4.018862
CTC-459M5.	0.3101163	-1.15943	2.3246427	0.021059	0.0571311	-4.019062
CTD-2047H1	-0.442693	-0.697333	-2.324634	0.0210595	0.0571311	-4.019081
SLC6A4	-0.539367	1.8116898	-2.323992	0.0210946	0.0572221	-4.020518
EIF1P6	-0.552398	0.6498503	-2.323787	0.0211058	0.0572483	-4.020977
PCDHB6	0.4897704	2.9388228	2.3236541	0.0211131	0.0572631	-4.021274
PDE6A	-0.572245	0.9558022	-2.32363	0.0211144	0.0572631	-4.021328
ACAD10	0.0641102	6.3335035	2.3234675	0.0211233	0.057283	-4.021692
AC092839.4	-0.351585	-1.005546	-2.323427	0.0211256	0.0572847	-4.021783
CYP4F26P	-0.609897	-0.227224	-2.323347	0.0211299	0.0572924	-4.021962

RNU5B-2P	-0.345177	2.4277148	-2.3231	0.0211435	0.0573249	-4.022515
CTC-260F2C	-0.11947	4.16486	-2.322871	0.021156	0.0573546	-4.023026
NEK4	-0.081491	5.8929462	-2.322772	0.0211614	0.057365	-4.023248
AC093732.1	-0.305836	-1.170517	-2.322718	0.0211644	0.0573688	-4.023368
MRPL30P1	-0.344551	-1.019031	-2.322542	0.0211741	0.0573875	-4.023762
PHBP11	-0.566213	2.6353399	-2.322535	0.0211745	0.0573875	-4.023778
CUTC	-0.06463	5.888186	-2.322441	0.0211796	0.0573972	-4.023988
RP11-676J1	0.2006156	-1.439145	2.3221082	0.0211979	0.0574425	-4.024732
ZNF716	-0.775603	-0.052868	-2.321964	0.0212058	0.0574597	-4.025054
RP1-197B17	0.3542556	2.8496261	2.3215708	0.0212274	0.057514	-4.025934
CTD-2562J1	-0.492067	0.5999473	-2.32153	0.0212297	0.0575159	-4.026026
RP11-235C2	-0.435082	-0.541716	-2.321304	0.0212421	0.0575453	-4.026531
SP4	-0.084455	5.2869057	-2.321173	0.0212493	0.0575605	-4.026823
RASSF9	0.4486175	4.204473	2.3210752	0.0212547	0.0575709	-4.027042
RP11-724N1	-0.595842	1.3544493	-2.320992	0.0212593	0.0575789	-4.027227
ZC3HAV1	0.0418333	6.2228479	2.3209429	0.021262	0.057582	-4.027338
RNA5SP477	-0.266834	-1.314379	-2.320733	0.0212736	0.0576085	-4.027806
TTC8	0.0871919	5.6354754	2.3207085	0.0212749	0.0576085	-4.027862
IGSF22	0.2388205	3.756423	2.3206772	0.0212766	0.0576089	-4.027932
EMR2	0.2027833	4.6465417	2.3205781	0.0212821	0.0576194	-4.028153
POMP	0.0555221	6.5407075	2.3205314	0.0212847	0.0576221	-4.028257
RP11-603J2	-0.252867	2.9383674	-2.320404	0.0212917	0.0576368	-4.028542
SNAP25	-0.454432	4.6419045	-2.320176	0.0213043	0.0576666	-4.029051
LINC01250	-0.413579	-0.996903	-2.320065	0.0213104	0.0576789	-4.029299
RPL13AP20	-0.149001	4.5480049	-2.320022	0.0213128	0.057681	-4.029395
RP11-556N2	0.3921361	-1.028475	2.319955	0.0213165	0.0576868	-4.029545
RP1-228H13	-0.512255	0.1710528	-2.319924	0.0213182	0.0576871	-4.029614
RP11-284N8	0.3570304	3.8444019	2.3198723	0.0213211	0.0576906	-4.02973
OFD1P2Y	0.1890466	-1.445737	2.3195248	0.0213403	0.0577383	-4.030507
LINC01504	0.4212946	2.8236958	2.3193364	0.0213507	0.0577622	-4.030927
LINC00390	0.5423594	-0.031281	2.3192541	0.0213552	0.0577702	-4.031111
NUBPL	0.0817777	5.6253141	2.3191491	0.021361	0.0577775	-4.031346
PHF21A	-0.052294	6.007157	-2.319148	0.0213611	0.0577775	-4.031349
RP11-708L7	0.2730297	-1.265831	2.3190378	0.0213672	0.057787	-4.031594
COL18A1	0.0758158	7.2362098	2.3190038	0.0213691	0.057787	-4.03167
PTGES	0.4263503	4.5195049	2.3189985	0.0213694	0.057787	-4.031682
GAPDHP23	0.6152021	0.052644	2.3186905	0.0213864	0.0578288	-4.03237
FAM86B3P	0.1721737	4.441917	2.3186518	0.0213886	0.0578304	-4.032456
AC073218.3	0.4903093	-0.705243	2.3184904	0.0213975	0.0578502	-4.032817
FTLP14	0.2728945	4.1473565	2.3183389	0.0214059	0.0578687	-4.033155
POLRMT	-0.048475	6.4773863	-2.318289	0.0214086	0.0578718	-4.033266
RP11-138I1	-0.407883	-1.191195	-2.317964	0.0214267	0.0579163	-4.033993
HP1BP3	0.0376088	6.5749361	2.317879	0.0214314	0.0579247	-4.034182
RPS4XP14	-0.524074	0.8854371	-2.317839	0.0214336	0.0579265	-4.034272
SMARCAD1	0.0732357	5.8188107	2.3176205	0.0214457	0.0579549	-4.034759
RP3-43804.	0.520722	-0.506922	2.3173	0.0214635	0.0579987	-4.035474
SPG20-AS1	0.4957019	-0.606272	2.3172135	0.0214683	0.0580074	-4.035667
CTD-2524L6	0.5043324	-0.038168	2.3171462	0.0214721	0.0580132	-4.035818
RP11-563P1	0.3238072	-1.239512	2.3171098	0.0214741	0.0580144	-4.035899
RP11-571F1	-0.488121	0.0845399	-2.31708	0.0214758	0.0580146	-4.035966
SLC35A3	0.075714	6.1363862	2.316943	0.0214834	0.0580308	-4.036271

RP11-505P4	-0.491848	1.305798	-2.316905	0.0214855	0.0580322	-4.036355
LEO1	-0.04986	6.1502751	-2.316698	0.021497	0.0580591	-4.036819
GBA2	-0.045809	6.3858462	-2.316649	0.0214997	0.0580621	-4.036928
PTPN21	0.0940154	5.7290824	2.3163314	0.0215174	0.0581055	-4.037636
RP3-453C12	-0.360592	3.0778256	-2.316132	0.0215285	0.0581312	-4.03808
NINL	-0.120748	5.851395	-2.315951	0.0215386	0.058152	-4.038484
CTB-1048E9	-0.275738	-1.270524	-2.315936	0.0215394	0.058152	-4.038517
PSIP1	-0.056462	6.1738318	-2.315297	0.021575	0.0582439	-4.039942
ZNF878	-0.584323	1.5428575	-2.315062	0.0215882	0.0582751	-4.040467
HNRNPCP6	-0.409048	-0.572068	-2.314885	0.021598	0.0582974	-4.040861
TRAV1-1	-0.548602	-0.064939	-2.314839	0.0216006	0.0583	-4.040964
RP13-15201	-0.521958	1.6812148	-2.3147	0.0216084	0.0583131	-4.041275
RPL23AP3	-0.495168	-0.053073	-2.314683	0.0216093	0.0583131	-4.041311
PAPLN	0.1947884	5.651126	2.314667	0.0216102	0.0583131	-4.041348
DESI2	-0.048955	6.230082	-2.314558	0.0216163	0.0583251	-4.04159
Z83826.1	0.4908459	-0.074289	2.314057	0.0216443	0.0583965	-4.042707
RP11-218D6	0.0898283	-1.563896	2.3138366	0.0216567	0.0584254	-4.043199
CTC-492K19	-0.466275	-0.452872	-2.313565	0.0216718	0.0584621	-4.043803
COLEC11	0.1671283	6.1073427	2.3134225	0.0216799	0.0584794	-4.044122
UBE2Q1-AS1	-0.503872	1.2508331	-2.313385	0.021682	0.0584806	-4.044206
AES	0.0488544	6.8889359	2.313357	0.0216835	0.0584806	-4.044268
PPP1R35	-0.064785	5.9127215	-2.31331	0.0216862	0.058481	-4.044372
RP11-31I22	0.1266858	-1.545113	2.3132976	0.0216869	0.058481	-4.0444
VIP	0.4387094	2.8495594	2.3132231	0.021691	0.0584879	-4.044566
AGO3	0.0526102	5.725399	2.3131602	0.0216946	0.0584931	-4.044706
OPN3	0.1410401	5.6775936	2.3128968	0.0217093	0.058526	-4.045293
ZBTB2	-0.044832	5.7591364	-2.312886	0.02171	0.058526	-4.045318
RP11-304F1	0.1897825	-1.46743	2.3128316	0.021713	0.0585298	-4.045438
RPL34P34	-0.519188	0.2467043	-2.3127	0.0217204	0.0585454	-4.045731
CTB-70G10.	0.1312653	-1.542779	2.3122027	0.0217483	0.0586164	-4.046839
RPS12P31	-0.412487	-0.762522	-2.311904	0.0217651	0.0586574	-4.047505
LINC01460	-0.426943	-0.830016	-2.311838	0.0217688	0.058663	-4.047651
MKNK1	0.0405899	6.0113908	2.311611	0.0217816	0.058693	-4.048157
PHACTR1	0.1382471	4.8414186	2.3115209	0.0217866	0.0587018	-4.048357
RP11-300E4	-0.298196	-1.292715	-2.311496	0.021788	0.0587018	-4.048413
IRAK4	0.0402756	5.8029083	2.3112829	0.0218	0.0587298	-4.048887
IQCG	0.1028	5.1880058	2.3112183	0.0218037	0.0587353	-4.049031
GOT2P3	0.6815614	1.0276453	2.3111758	0.0218061	0.0587374	-4.049126
RP11-734K2	0.2238833	-1.377001	2.3111321	0.0218085	0.0587397	-4.049223
SPIB	-0.445258	3.7858834	-2.311087	0.0218111	0.0587423	-4.049324
CCDC83	-0.260438	-1.309516	-2.311013	0.0218153	0.0587491	-4.049489
PCBP2	-0.035354	6.9258968	-2.310939	0.0218194	0.0587557	-4.049652
ISCA2	-0.055869	5.8621936	-2.310912	0.0218209	0.0587557	-4.049712
RP11-795J1	0.2221121	-1.397113	2.3108736	0.0218231	0.0587572	-4.049798
TREH	0.5053216	4.0169748	2.3108434	0.0218248	0.0587575	-4.049866
RP11-40H20	-0.346704	-1.061808	-2.310739	0.0218307	0.058769	-4.050098
SRGAP3	0.1401771	4.9975455	2.3107031	0.0218327	0.0587701	-4.050178
RP11-421P2	0.469452	-0.966279	2.3106424	0.0218361	0.058775	-4.050313
KCNH8	-0.748356	1.2192095	-2.310308	0.021855	0.0588214	-4.051058
IDH1	0.0614417	6.9417401	2.3101997	0.0218611	0.0588335	-4.051298
BRD7	0.0544302	6.2365747	2.3097561	0.0218862	0.0588966	-4.052285

ZNF197	-0.056132	5.7452042	-2.309633	0.0218931	0.058911	-4.052559
GPATCH8	-0.046256	6.1236908	-2.309552	0.0218977	0.058919	-4.05274
CTD-2195M1	0.2130068	-1.43283	2.3095207	0.0218995	0.0589194	-4.052809
NAT8L	-0.507757	3.2336563	-2.30937	0.021908	0.058938	-4.053145
RAB10	0.0391076	6.6531323	2.3093006	0.0219119	0.0589442	-4.053299
LRP1	0.066741	7.1117139	2.3092373	0.0219155	0.0589495	-4.053439
KB-1991G8.	0.2991498	-1.327864	2.3091216	0.021922	0.0589627	-4.053697
RPSAP43	-0.268956	-1.296179	-2.308914	0.0219338	0.0589879	-4.054159
SDK1	0.4228659	4.4577139	2.3088993	0.0219346	0.0589879	-4.054191
NDRG2	0.0801302	6.7192504	2.308746	0.0219433	0.0590069	-4.054532
HBQ1	-0.494148	-0.910168	-2.308416	0.021962	0.0590527	-4.055265
GPHN	0.0939154	5.9807008	2.3083712	0.0219645	0.0590553	-4.055366
CLRN1-AS1	0.6221521	-0.147791	2.3082654	0.0219705	0.0590654	-4.055601
CTD-2616J1	-0.346056	-1.087452	-2.308248	0.0219715	0.0590654	-4.055641
NKTR	-0.059351	6.1944491	-2.307637	0.0220062	0.0591541	-4.056997
LINC01483	-0.415543	-0.932367	-2.307333	0.0220235	0.0591963	-4.057675
ST8SIA3	-0.75179	1.6806527	-2.307033	0.0220405	0.0592377	-4.05834
RP11-214N1	-0.506923	0.6884291	-2.306971	0.0220441	0.0592429	-4.05848
ASAP2	0.1944494	5.079814	2.3068651	0.0220501	0.0592546	-4.058714
IGHV1-17	-0.425899	-0.985493	-2.30679	0.0220543	0.0592589	-4.058882
RP5-984P4.	-0.556303	-0.489171	-2.30678	0.0220549	0.0592589	-4.058903
ERICH4	-0.61404	-0.186859	-2.306732	0.0220576	0.0592618	-4.059009
RP11-416N2	-0.380592	-1.08322	-2.306269	0.022084	0.0593284	-4.060039
ASS1	0.1132601	7.1031361	2.3058679	0.0221068	0.0593854	-4.06093
RP4-633O19	-0.52239	0.1106178	-2.30552	0.0221267	0.0594308	-4.061702
RP4-773N10	-0.104827	5.02407	-2.305514	0.022127	0.0594308	-4.061715
OR8D1	-0.259795	-1.342109	-2.305303	0.022139	0.0594588	-4.062184
RP11-864G5	-0.425373	-0.69224	-2.304851	0.0221649	0.0595237	-4.063188
NACC2	0.0632352	6.164826	2.3047809	0.0221689	0.0595301	-4.063344
LINC00582	-0.521275	-0.524846	-2.304645	0.0221766	0.0595466	-4.063645
CD69	0.243888	4.7815858	2.3045448	0.0221824	0.0595576	-4.063868
IL23R	-0.583688	0.6538833	-2.303332	0.0222518	0.0597355	-4.06656
LINC00308	-0.423059	-0.936839	-2.30333	0.0222519	0.0597355	-4.066563
RP11-523H2	-0.304861	3.3127752	-2.303134	0.0222632	0.0597613	-4.067
CAB39L	0.0878006	5.3842764	2.303025	0.0222694	0.0597714	-4.067241
LINC01251	-0.487276	-0.26596	-2.303011	0.0222702	0.0597714	-4.067272
RP11-674N2	0.485923	-0.719604	2.3027327	0.0222862	0.0598061	-4.06789
RP1-313I6.	-0.133917	4.5114196	-2.302694	0.0222884	0.0598061	-4.067976
METTL17	-0.043652	6.1635387	-2.302683	0.0222891	0.0598061	-4.068001
ADRA1D	0.6705621	2.2520288	2.3026596	0.0222904	0.0598061	-4.068052
RP1-79C4.4	0.4695319	-0.669912	2.3026438	0.0222913	0.0598061	-4.068087
RP11-574K1	-0.432674	1.8853709	-2.302495	0.0222998	0.0598235	-4.068416
LEKR1	-0.195407	3.6691576	-2.302474	0.0223011	0.0598235	-4.068465
RP5-1031D4	0.3500006	-1.082969	2.3023752	0.0223067	0.0598342	-4.068683
STON1-GTF2	0.4811405	-0.713088	2.3021431	0.02232	0.0598656	-4.069198
ACOXL	-0.547604	1.5899914	-2.301964	0.0223304	0.0598889	-4.069595
PTP4A3	-0.128142	6.056656	-2.301917	0.0223331	0.0598895	-4.069699
CTAGE7P	-0.463206	2.3194254	-2.301902	0.0223339	0.0598895	-4.069732
DGCR8	-0.037726	6.0677602	-2.301762	0.022342	0.0599068	-4.070044
RP11-188D8	-0.453485	1.491714	-2.301549	0.0223542	0.0599319	-4.070516
CTSLP4	-0.216515	-1.426675	-2.301518	0.022356	0.0599319	-4.070585

ERN2	-0.677983	-0.015047	-2.301514	0.0223562	0.0599319	-4.070593
KIAA0232	0.0500346	6.3433098	2.3014512	0.0223599	0.0599372	-4.070732
RPS6	-0.05306	7.3024284	-2.301372	0.0223644	0.0599449	-4.070907
FOXCUT	0.4168353	-0.952285	2.3012965	0.0223688	0.0599522	-4.071075
TDGF1	-0.561892	4.3727414	-2.30125	0.0223714	0.059955	-4.071178
CTD-2031P1	-0.186477	3.5972119	-2.301147	0.0223774	0.0599665	-4.071407
RP11-370A5	-0.449838	-0.550838	-2.300958	0.0223883	0.0599913	-4.071826
RPL7P18	-0.439684	-0.723309	-2.300769	0.0223991	0.060016	-4.072244
RP11-180N1	0.6594188	0.7586906	2.3007084	0.0224027	0.060021	-4.072379
BRINP1	0.7057458	1.6554368	2.3003908	0.022421	0.0600645	-4.073083
UBE2R2-AS1	-0.467652	-0.181623	-2.300343	0.0224238	0.0600645	-4.07319
RP11-1336C	0.4511214	-0.857902	2.3003416	0.0224238	0.0600645	-4.073192
RP11-267A1	0.1692838	-1.50064	2.3000916	0.0224383	0.0600988	-4.073746
GPX7	-0.177332	5.3687085	-2.300063	0.0224399	0.0600988	-4.07381
SPRR2B	0.1929554	-1.511341	2.2999981	0.0224437	0.0601044	-4.073953
RP11-173B1	-0.515214	0.7757968	-2.299764	0.0224572	0.0601362	-4.074471
CTD-2325M2	-0.254875	3.3296936	-2.299698	0.022461	0.0601412	-4.074617
CCDC42B	-0.408721	2.0058788	-2.299675	0.0224623	0.0601412	-4.07467
SNAPC1	0.0758224	5.2535898	2.2995314	0.0224706	0.060159	-4.074988
AP002954.4	0.5826019	0.8867146	2.2994969	0.0224726	0.0601599	-4.075064
LINC00412	-0.420029	-0.746539	-2.299215	0.0224889	0.0601991	-4.075688
GS1-259H13	0.5158867	-0.350487	2.2990673	0.0224975	0.0602176	-4.076016
AC012354.8	-0.436243	-0.396949	-2.298968	0.0225033	0.0602283	-4.076237
RP11-311F1	-0.517679	-0.000702	-2.298938	0.0225049	0.0602283	-4.076301
MAGOH2P	-0.404215	2.8103436	-2.298913	0.0225064	0.0602283	-4.076358
CD33	0.1669799	4.8262939	2.2982484	0.0225449	0.0603269	-4.077829
TCTE1	-0.535552	0.8558714	-2.298102	0.0225534	0.060343	-4.078153
RP11-124G5	0.223564	-1.408568	2.298088	0.0225542	0.060343	-4.078185
ADAMTS7	0.1077275	5.2601586	2.2979838	0.0225603	0.0603547	-4.078415
GOLGA8A	-0.17503	5.2437426	-2.297952	0.0225621	0.0603553	-4.078486
PAX8-AS1	0.3925986	4.866239	2.2979002	0.0225651	0.0603589	-4.0786
RP11-359K1	-0.274566	-1.255099	-2.297834	0.022569	0.0603647	-4.078747
GTF2IRD1P1	0.5384691	1.3015789	2.2976881	0.0225774	0.0603829	-4.07907
UTRN	-0.061148	6.3939495	-2.297602	0.0225825	0.060392	-4.079261
GOLGA6A	0.5602384	-0.360329	2.2975216	0.0225871	0.0603999	-4.079439
RP11-946P6	-0.425906	-0.814946	-2.297442	0.0225917	0.0604078	-4.079614
CTD-2576D5	0.1374169	-1.539644	2.2973062	0.0225996	0.0604246	-4.079915
AC011290.4	-0.427476	-0.837931	-2.297223	0.0226045	0.0604331	-4.0801
ERH	-0.042561	6.4883645	-2.297081	0.0226127	0.0604507	-4.080414
CCDC65	-0.138505	4.3151881	-2.297011	0.0226168	0.0604572	-4.080569
EXD3	0.0690509	5.6454256	2.2969639	0.0226195	0.0604573	-4.080673
MPC2	-0.065982	6.9228953	-2.29695	0.0226203	0.0604573	-4.080704
DFFBP1	-0.486445	0.9451159	-2.296925	0.0226218	0.0604573	-4.08076
CTC-490E21	0.3282505	2.9547374	2.296896	0.0226235	0.0604573	-4.080823
IKBIP	-0.068094	5.8689648	-2.296583	0.0226417	0.0605015	-4.081515
CYP2D6	-0.273301	6.4440917	-2.296343	0.0226556	0.0605344	-4.082046
VWC2	0.3349579	-1.201432	2.2962926	0.0226586	0.0605371	-4.082159
ANKRD18EP	-0.223979	4.6042808	-2.296269	0.02266	0.0605371	-4.082211
PARD6B	-0.09786	5.6154545	-2.296219	0.0226629	0.0605405	-4.082322
CTD-2124B8	-0.131273	4.3356578	-2.296173	0.0226655	0.0605431	-4.082422
AC144833.1	0.6386894	-0.626871	2.2959592	0.022678	0.0605721	-4.082896

PTPRD-AS2	0.184064	-1.493108	2.2955492	0.0227019	0.0606315	-4.083803
ZFHX3	0.0531584	6.0056816	2.2951287	0.0227265	0.0606926	-4.084733
MAK	-0.313434	2.9445519	-2.295093	0.0227286	0.0606937	-4.084812
LINC00706	0.2405914	-1.418773	2.2948892	0.0227405	0.0607211	-4.085263
SLC35B4	0.0650285	6.0624576	2.2948568	0.0227424	0.0607217	-4.085335
HEXB	0.054996	6.7531163	2.2947145	0.0227507	0.0607373	-4.085649
RP11-118F1	-0.357938	-0.997851	-2.2947	0.0227515	0.0607373	-4.085682
RNU6-875P	-0.353161	-1.000668	-2.294275	0.0227764	0.0607991	-4.08662
MRFAP1	0.0399461	6.9138218	2.294205	0.0227805	0.0608018	-4.086776
SASH3	0.1329302	5.5311311	2.2942014	0.0227807	0.0608018	-4.086784
RP11-404K5	-0.199906	-1.43024	-2.293921	0.0227971	0.0608412	-4.087404
RP11-439L8	-0.548801	0.2662176	-2.29381	0.0228036	0.0608541	-4.08765
OR11H7	-0.370108	-1.026368	-2.293744	0.0228075	0.0608599	-4.087795
C15orf61	-0.072459	5.4661126	-2.293412	0.0228269	0.0609074	-4.088529
RUNDC3A-AS	-0.585959	0.9434273	-2.293355	0.0228303	0.0609119	-4.088655
OR1E1	0.1194986	-1.548776	2.2929166	0.022856	0.060976	-4.089623
RP11-139H1	-0.399923	2.0394383	-2.292722	0.0228674	0.061002	-4.090053
FPGT	0.0577791	5.7410309	2.2923727	0.0228879	0.0610512	-4.090825
FLJ27354	0.3628695	2.5880584	2.2923516	0.0228892	0.0610512	-4.090872
RP11-254A1	-0.29208	-1.231705	-2.292297	0.0228924	0.0610552	-4.090991
SPSB2	-0.090507	5.6278213	-2.292183	0.0228991	0.0610687	-4.091245
DCAF13P2	0.1344511	-1.541156	2.2918502	0.0229186	0.0611164	-4.091979
ZNF397	-0.040972	5.9026081	-2.291692	0.0229279	0.0611367	-4.092328
TEX264	0.0505651	6.4958719	2.2916628	0.0229297	0.0611369	-4.092393
VPS45	-0.042216	6.1842932	-2.291588	0.0229341	0.0611441	-4.092558
RASAL1	-0.515242	3.8097143	-2.291424	0.0229437	0.0611654	-4.09292
NPPB	-0.668267	-0.065083	-2.29113	0.022961	0.061207	-4.093569
RP11-53B2.	-0.522451	0.4758124	-2.291033	0.0229668	0.0612179	-4.093784
PIK3C2G	0.5124165	4.9179272	2.2908721	0.0229763	0.0612387	-4.094139
RPL7AP14	-0.401951	-0.769419	-2.290737	0.0229842	0.061253	-4.094437
CTD-2267D1	-0.484814	-0.031461	-2.290718	0.0229854	0.061253	-4.09448
LIX1L	-0.069516	6.2337246	-2.290696	0.0229867	0.061253	-4.094529
RNF133	0.5401455	0.1617005	2.2905699	0.0229941	0.0612683	-4.094806
CEACAMP7	0.2966316	-1.320985	2.2903819	0.0230052	0.0612934	-4.095221
KRT28	0.3276084	-1.330631	2.2903516	0.023007	0.0612937	-4.095288
HOXC8	-0.732017	0.0301429	-2.290157	0.0230184	0.0613198	-4.095717
ORAOV1	-0.090497	5.6913839	-2.289462	0.0230595	0.0614247	-4.09725
RP5-839B4.	-0.652449	0.1175327	-2.289194	0.0230754	0.0614625	-4.097842
USP47	0.0386202	6.4660449	2.2891321	0.023079	0.0614678	-4.097979
LINC01169	-0.66022	2.0266282	-2.28882	0.0230975	0.0615126	-4.098668
NSMCE1	0.0637031	6.3236791	2.2884738	0.023118	0.0615627	-4.099431
PANO1	0.2804369	3.0455428	2.2883747	0.0231239	0.0615736	-4.09965
LRCH2	0.3455118	3.6401228	2.2883337	0.0231263	0.0615736	-4.09974
RP11-344H1	-0.430941	-0.638864	-2.288319	0.0231272	0.0615736	-4.099772
RPSAP9	-0.195447	3.8681557	-2.288249	0.0231314	0.0615802	-4.099928
PCOLCE	0.139946	6.2589978	2.2881617	0.0231365	0.0615895	-4.100119
CWC27	-0.046349	5.8578783	-2.287878	0.0231534	0.0616261	-4.100745
TCEA1P3	-0.454901	-0.713171	-2.287873	0.0231537	0.0616261	-4.100755
SFTPA1	0.3569071	-1.283043	2.2870622	0.0232018	0.0617498	-4.102543
DGCR6	0.1740467	5.2143362	2.2870073	0.0232051	0.061754	-4.102665
ARPC5L	-0.049296	6.2557367	-2.286961	0.0232079	0.0617569	-4.102767

AC008982.2	-0.496183	1.5363593	-2.286887	0.0232123	0.061764	-4.10293
FCGR1C	0.5906273	1.3058892	2.2868404	0.023215	0.0617669	-4.103032
MYC	0.14535	6.2630308	2.2867533	0.0232202	0.0617762	-4.103224
CXorf22	-0.730767	1.1754191	-2.286652	0.0232262	0.0617876	-4.103446
CTD-2349P2	-0.504358	1.0435214	-2.286512	0.0232346	0.0618054	-4.103756
CTD-2540M1	0.1995505	-1.457693	2.2862152	0.0232523	0.0618479	-4.10441
RP11-98D18	-0.314584	3.2864849	-2.286149	0.0232562	0.0618539	-4.104555
RP11-410N8	-0.226995	-1.421535	-2.28594	0.0232687	0.0618826	-4.105017
RP4-764022	-0.370089	-1.006957	-2.285633	0.023287	0.0619267	-4.105693
LENG1	-0.059259	5.6649633	-2.284946	0.023328	0.0620313	-4.107206
GPR115	0.7157104	0.1403095	2.2845479	0.0233518	0.0620866	-4.108083
RP11-1277A	0.3830329	2.6802647	2.284529	0.0233529	0.0620866	-4.108124
VSTM2L	0.5288142	4.014292	2.2845009	0.0233546	0.0620866	-4.108186
RNU7-140P	-0.421789	-0.704013	-2.284484	0.0233556	0.0620866	-4.108223
CLDND1	0.0321787	6.1785499	2.2843817	0.0233617	0.0620984	-4.108449
RP11-96B5.	0.1944032	-1.442311	2.2841636	0.0233748	0.0621285	-4.108929
DBR1	-0.048452	5.7033365	-2.284018	0.0233835	0.0621472	-4.10925
OSBPL8	0.0557051	6.1337779	2.2836138	0.0234077	0.0622039	-4.110139
IGSF10	0.5159537	2.7169744	2.2836044	0.0234083	0.0622039	-4.11016
BCL9P1	-0.358738	-1.053276	-2.283409	0.02342	0.0622305	-4.11059
KRR1	-0.03665	6.0608632	-2.28311	0.0234379	0.0622736	-4.111247
SLC30A5	0.0417342	6.2533322	2.2829672	0.0234465	0.0622919	-4.111562
NCS1	0.1715823	5.5437221	2.282768	0.0234584	0.0623176	-4.112
ANKDD1B	-0.451855	2.9145906	-2.282749	0.0234596	0.0623176	-4.112042
DLAT	0.0689067	6.2042311	2.282684	0.0234635	0.0623235	-4.112185
RP11-53M11	0.3926388	-0.860288	2.2825719	0.0234702	0.0623368	-4.112432
VCAN-AS1	0.3695108	-1.157889	2.2824033	0.0234804	0.0623592	-4.112803
RP11-20F24	-0.364982	-1.191375	-2.282372	0.0234822	0.0623597	-4.112872
RP11-209K1	-0.431569	4.5556301	-2.281882	0.0235117	0.0624334	-4.11395
AMDHD2	0.0716715	5.981049	2.2817602	0.023519	0.0624482	-4.114217
RP11-369E1	0.3415683	-1.181469	2.2815933	0.0235291	0.0624704	-4.114585
ENO4	0.4880326	1.1403423	2.2815317	0.0235328	0.0624757	-4.11472
FAM209A	-0.51535	0.5473357	-2.281332	0.0235448	0.062503	-4.115159
RP4-671014	0.3794797	2.710467	2.2812615	0.0235491	0.0625078	-4.115314
RP11-313J2	-0.630496	2.9460329	-2.281246	0.02355	0.0625078	-4.115349
ATE1-AS1	0.5259742	2.4050543	2.281064	0.0235609	0.0625322	-4.115748
CLEC18C	-0.376006	-0.994351	-2.281036	0.0235626	0.0625322	-4.115809
KRT8P39	-0.491763	1.1276418	-2.280961	0.0235672	0.0625397	-4.115975
BOK-AS1	0.6711092	1.2481414	2.2807492	0.0235799	0.062569	-4.116441
EGLN2	-0.057864	6.0621389	-2.280559	0.0235914	0.0625833	-4.116858
AC016700.5	-0.295713	4.2820904	-2.280559	0.0235914	0.0625833	-4.116859
RP11-109E2	-0.560142	-0.501707	-2.280552	0.0235918	0.0625833	-4.116874
TPRX2P	0.2230673	-1.391867	2.2805465	0.0235922	0.0625833	-4.116886
VN1R7P	-0.191993	-1.460605	-2.280192	0.0236136	0.0626355	-4.117665
SYN1	0.2922621	4.2697695	2.2799949	0.0236255	0.0626625	-4.118098
NBPF1	0.0865611	5.5514529	2.2799069	0.0236308	0.0626721	-4.118292
PITPNB	0.0478512	6.457091	2.2798433	0.0236346	0.0626777	-4.118432
SRSF4	0.0336666	6.4462937	2.2796515	0.0236462	0.0627039	-4.118853
XRN1	0.0563954	6.1601117	2.2794523	0.0236583	0.0627313	-4.119291
CASC4P1	-0.452542	-0.724576	-2.279188	0.0236743	0.0627679	-4.119872
CHCHD7	-0.073162	6.0641866	-2.279167	0.0236755	0.0627679	-4.119917

RP11-407N1	0.1238261	3.9128707	2.2791237	0.0236781	0.0627703	-4.120013
ZNF839	-0.073547	5.3442203	-2.279008	0.0236852	0.0627844	-4.120267
NCOR1	0.0580901	6.4179514	2.2789343	0.0236896	0.0627899	-4.120429
HDGFL1	-0.615752	-0.67603	-2.278917	0.0236907	0.0627899	-4.120468
RP1-130H16	0.5085449	0.4049507	2.2787549	0.0237005	0.0628076	-4.120823
USP14	-0.036702	6.4435395	-2.278737	0.0237016	0.0628076	-4.120863
RP11-247A1	0.4646003	1.6657061	2.2787213	0.0237025	0.0628076	-4.120897
RP11-86A5.	-0.360794	-1.240152	-2.278559	0.0237123	0.0628269	-4.121253
SART1	-0.042093	6.4924554	-2.278544	0.0237133	0.0628269	-4.121286
AC008063.2	-0.525502	-0.149707	-2.278514	0.0237151	0.0628271	-4.121351
CTGF	0.1006166	6.5021594	2.2784371	0.0237197	0.0628349	-4.121521
PUS3	0.0678596	5.9560892	2.2783021	0.0237279	0.0628521	-4.121818
DDX39B-AS1	-0.519902	0.893507	-2.278185	0.023735	0.0628663	-4.122074
RAB3GAP2	-0.046917	6.2380464	-2.277879	0.0237536	0.0629109	-4.122746
RP4-612B15	0.2996998	2.6255551	2.2775663	0.0237726	0.0629567	-4.123433
AARD	-0.559551	-0.104019	-2.27749	0.0237772	0.0629568	-4.1236
KRT77	0.2528541	-1.430529	2.277489	0.0237773	0.0629568	-4.123603
RP11-297L1	-0.530583	0.3537853	-2.27747	0.0237784	0.0629568	-4.123644
IST1	0.0335146	6.5032159	2.277425	0.0237812	0.0629568	-4.123744
C19orf53	-0.058482	6.5160852	-2.277424	0.0237812	0.0629568	-4.123746
ACCSL	0.2496668	-1.423397	2.2773943	0.023783	0.0629569	-4.123811
TMEM178A	0.2386715	4.5136837	2.2771873	0.0237956	0.0629857	-4.124265
SOX21	0.6091133	-0.103456	2.2771244	0.0237994	0.0629912	-4.124403
APLP1	0.3216404	4.4540176	2.277025	0.0238055	0.0630027	-4.124622
KRTAP5-1	-0.633847	1.3161463	-2.276803	0.023819	0.0630338	-4.125108
CCDC134	-0.094615	4.9327925	-2.276629	0.0238296	0.0630573	-4.125492
RP11-560A1	0.1369334	-1.517127	2.2765929	0.0238318	0.0630583	-4.12557
ACYP1	-0.09394	5.00744	-2.276566	0.0238334	0.0630583	-4.125629
RNU6-831P	0.1859367	-1.46939	2.2763065	0.0238492	0.0630955	-4.126198
LA16c-380F	0.3996391	-0.825121	2.2761929	0.0238561	0.0631093	-4.126448
PRKCB	0.1834005	5.1253325	2.2760023	0.0238678	0.0631354	-4.126866
RNF112	0.2768165	3.7628561	2.2759225	0.0238726	0.0631437	-4.127041
IL3RA	0.0940884	5.470455	2.2758037	0.0238799	0.0631583	-4.127302
RP11-257A2	0.091776	-1.562904	2.2756419	0.0238897	0.0631776	-4.127657
TMEM252	0.716234	0.7201404	2.2756272	0.0238906	0.0631776	-4.127689
RP11-66B24	-0.334239	-1.205906	-2.275042	0.0239264	0.0632655	-4.128973
MEG9	-0.735992	1.4221784	-2.275026	0.0239273	0.0632655	-4.129008
AC007405.4	-0.349744	2.8484121	-2.274978	0.0239303	0.0632687	-4.129114
BAHCC1	0.1068138	5.7711029	2.2749277	0.0239333	0.0632722	-4.129223
RP11-95P13	-0.477298	-0.942295	-2.274522	0.0239582	0.0633332	-4.130113
UBL4A	0.0548413	6.3630608	2.2744683	0.0239614	0.0633373	-4.130231
NRSN1	-0.625111	-0.099813	-2.274073	0.0239856	0.0633966	-4.131097
ANTXRL	0.3218326	-1.227445	2.2739257	0.0239946	0.0634159	-4.13142
KCNMB4	0.1745731	4.6781787	2.2734882	0.0240214	0.0634821	-4.132379
PRKACB	0.0802451	6.024385	2.2732983	0.0240331	0.0635083	-4.132795
NSFP1	-0.608972	0.2567785	-2.273121	0.024044	0.063525	-4.133185
RP11-613M1	-0.38515	-0.714547	-2.273111	0.0240446	0.063525	-4.133207
RP5-837J1.	-0.480764	0.7194001	-2.27311	0.0240446	0.063525	-4.133208
RP5-907C10	0.6440967	-0.133845	2.2729719	0.0240531	0.0635428	-4.133511
RP11-4K3__	-0.494647	1.3178217	-2.272853	0.0240604	0.0635576	-4.133772
CTD-2373J6	-0.488479	0.3261764	-2.272596	0.0240762	0.0635945	-4.134333

ST13P19	-0.508038	0.6775694	-2.272531	0.0240802	0.0636005	-4.134477
AC005256.1	0.4526525	-0.9401	2.2719978	0.024113	0.0636825	-4.135645
TMEM25	0.2053715	5.1879606	2.2719207	0.0241177	0.0636904	-4.135814
ZNF337	-0.171703	4.4738265	-2.271763	0.0241274	0.0637113	-4.136158
RP11-497H1	-0.54026	0.8706323	-2.271632	0.0241355	0.0637277	-4.136446
RP1-140K8.	0.5247148	-0.463325	2.2715955	0.0241377	0.0637277	-4.136526
LINC01387	0.1366334	-1.540044	2.2715648	0.0241396	0.0637277	-4.136593
TTLL11	-0.083645	4.9925638	-2.271549	0.0241406	0.0637277	-4.136628
TFAP2A	-0.460186	3.7715686	-2.271064	0.0241704	0.0637969	-4.137689
ANKRD54	-0.052232	6.0219901	-2.271051	0.0241712	0.0637969	-4.137718
S100P	-0.500781	5.1749114	-2.271039	0.024172	0.0637969	-4.137746
PABPC1P3	-0.393833	2.5388921	-2.270851	0.0241836	0.0638229	-4.138157
CCL4	0.1806314	5.1678509	2.2708097	0.0241861	0.0638238	-4.138247
LINC00972	-0.324834	-1.255603	-2.270788	0.0241874	0.0638238	-4.138293
RRP7A	-0.053518	6.3221107	-2.270738	0.0241905	0.0638273	-4.138403
SLCO1A2	-0.328929	4.805908	-2.270498	0.0242054	0.0638618	-4.138929
SETDB2	-0.064778	5.7017639	-2.270456	0.0242079	0.063864	-4.13902
ZNF596	0.141933	4.5652075	2.2701748	0.0242253	0.0639052	-4.139636
KAAG1	-0.656666	2.8698383	-2.269926	0.0242406	0.0639376	-4.14018
GPR160	-0.166707	5.2114346	-2.269919	0.0242411	0.0639376	-4.140196
CTD-2139B1	0.3412467	-1.294599	2.2698345	0.0242463	0.0639468	-4.140381
RWDD4P2	0.1221517	3.9058484	2.269763	0.0242507	0.0639538	-4.140538
MIR4322	0.1918929	-1.453282	2.2693729	0.0242749	0.0640128	-4.141391
AC253572.1	0.4090641	3.288692	2.2690957	0.024292	0.0640534	-4.141998
CDH1	0.1074327	6.7828003	2.2688158	0.0243093	0.0640945	-4.14261
FAM170A	0.4668716	-0.492519	2.2681861	0.0243484	0.0641921	-4.143987
PPRC1	-0.05102	6.1672209	-2.268119	0.0243526	0.0641921	-4.144135
PLSCR5	-0.306484	-1.40446	-2.26808	0.024355	0.0641921	-4.14422
PMS2P5	0.2630143	3.7916006	2.2680789	0.024355	0.0641921	-4.144222
MFF	-0.036251	6.3909144	-2.268077	0.0243552	0.0641921	-4.144226
SET	-0.036647	6.7923733	-2.268033	0.0243579	0.0641946	-4.144322
ASGR1	-0.166643	6.9733618	-2.267534	0.0243889	0.0642712	-4.145414
RP11-32B5.	0.2166039	-1.375951	2.2675084	0.0243904	0.0642712	-4.145469
NEBL-AS1	0.5632278	-0.496937	2.2674737	0.0243926	0.0642722	-4.145545
RP11-94B19	-0.428732	-1.060372	-2.26738	0.0243985	0.0642809	-4.145751
AC092765.1	0.1277744	-1.521794	2.2673642	0.0243994	0.0642809	-4.145784
CTC-453G23	-0.502878	0.1508585	-2.267114	0.024415	0.0643172	-4.146331
CCDC38	-0.447424	3.7849843	-2.266963	0.0244243	0.0643373	-4.146662
RP11-642P1	-0.171543	3.453293	-2.266727	0.024439	0.0643713	-4.147176
RP11-274B2	0.3561045	-1.032358	2.2666076	0.0244465	0.0643863	-4.147438
RPL30P4	-0.515252	0.1667427	-2.266317	0.0244645	0.0644292	-4.148072
RP5-1099C1	-0.316564	-1.085748	-2.266068	0.0244801	0.0644654	-4.148618
RP11-352M1	-0.065984	5.4633398	-2.26604	0.0244818	0.0644654	-4.148678
FHP1	-0.51659	0.744559	-2.265918	0.0244894	0.0644773	-4.148946
AC007566.1	0.2868578	3.2427449	2.2658931	0.024491	0.0644773	-4.149
CCNB1IP1	-0.112689	6.0560721	-2.265882	0.0244916	0.0644773	-4.149023
OR12D1	-0.403043	-1.101217	-2.26565	0.0245062	0.0645109	-4.149532
PGM5P4-AS1	0.3840526	-1.023839	2.2653636	0.024524	0.0645532	-4.150156
PGM5P3-AS1	0.4975656	-0.604113	2.2652255	0.0245326	0.0645713	-4.150458
LINC01134	-0.263357	4.2190928	-2.265008	0.0245462	0.0646024	-4.150934
RPL34P33	-0.456616	-0.610241	-2.264951	0.0245498	0.0646071	-4.151058

RP11-45101	-0.360367	-1.194964	-2.26476	0.0245617	0.0646338	-4.151475
LINC00909	-0.062509	5.3746426	-2.26472	0.0245642	0.0646357	-4.151562
RP11-582E3	0.0736415	5.2778006	2.2645416	0.0245754	0.0646604	-4.151952
ITPK1	0.0662906	6.6074701	2.2644921	0.0245784	0.0646639	-4.15206
LL22NC03-2	-0.590013	0.7171803	-2.264359	0.0245868	0.0646812	-4.152351
EIF2D	-0.047061	6.4480605	-2.264291	0.024591	0.0646845	-4.152499
RP11-268G1	-0.558197	-0.420372	-2.264282	0.0245916	0.0646845	-4.152519
RP11-379B1	-0.200604	4.008869	-2.264016	0.0246082	0.0647236	-4.153099
ANP32BP1	-0.485246	-0.083008	-2.263734	0.0246259	0.0647625	-4.153716
RP5-1098D1	-0.583727	0.6520154	-2.263723	0.0246266	0.0647625	-4.153738
RP11-541P9	-0.525512	1.197122	-2.263484	0.0246416	0.0647973	-4.154261
RP11-227D2	0.2002215	-1.507638	2.2634255	0.0246452	0.0648022	-4.154388
IGLV3-7	0.1283893	-1.544245	2.2633611	0.0246493	0.0648082	-4.154529
SPAG16	0.151131	5.4720609	2.2633214	0.0246518	0.0648101	-4.154615
RNY4P19	0.295402	-1.249228	2.2632905	0.0246537	0.0648105	-4.154683
PIAS1	-0.042137	5.9981857	-2.263248	0.0246564	0.0648128	-4.154776
ST3GAL6-AS	0.3660123	3.071971	2.2631945	0.0246597	0.0648148	-4.154892
THSD7B	0.5129045	2.9074884	2.2631407	0.0246631	0.0648148	-4.15501
ZNF346-IT1	-0.490296	1.13109	-2.263129	0.0246638	0.0648148	-4.155035
ALDH6A1	0.1240577	6.6435053	2.2630951	0.0246659	0.0648148	-4.155109
RP11-458I7	-0.495849	0.208226	-2.263094	0.024666	0.0648148	-4.155111
EEF1A1P9	-0.127356	4.7344113	-2.262967	0.024674	0.0648311	-4.155388
SLC25A32	0.0614306	6.0367778	2.2627518	0.0246875	0.064862	-4.155858
RP1-137K24	0.2706832	-1.404373	2.2626965	0.024691	0.0648664	-4.155979
RP11-443B7	-0.665751	1.4930286	-2.262638	0.0246946	0.0648714	-4.156106
LINC01019	-0.779616	-0.169373	-2.262516	0.0247023	0.0648869	-4.156373
ITFG2	-0.050733	5.8285483	-2.262445	0.0247068	0.0648897	-4.156527
HOXA3	-0.305574	4.3202672	-2.262442	0.0247069	0.0648897	-4.156534
LL22NC03-N	-0.511046	-0.422558	-2.2624	0.0247096	0.064892	-4.156626
BRWD1-AS1	-0.397019	-0.74906	-2.26195	0.0247378	0.0649581	-4.157607
C9orf147	-0.310978	2.4234896	-2.261943	0.0247383	0.0649581	-4.157622
RP1-199J3.	0.5109262	0.2667895	2.2618666	0.0247431	0.064966	-4.157789
TNNT3	0.555505	1.5092771	2.2617738	0.024749	0.0649742	-4.157992
RP5-1180D1	-0.385723	3.5288383	-2.261761	0.0247498	0.0649742	-4.15802
C17orf89	-0.071326	6.0228746	-2.261563	0.0247622	0.0650022	-4.158452
SRSF1	-0.027016	6.6523895	-2.26142	0.0247712	0.0650212	-4.158764
RP11-104N1	-0.152705	4.5634162	-2.261342	0.0247761	0.0650266	-4.158933
RPL21P39	-0.472816	0.233365	-2.261313	0.024778	0.0650266	-4.158996
CTC-523E23	0.1262604	-1.54533	2.2613018	0.0247787	0.0650266	-4.159021
YPEL4	0.1888265	3.68517	2.2611108	0.0247907	0.0650535	-4.159438
ATP2A3	0.1197436	5.8186056	2.2608215	0.0248089	0.0650967	-4.160068
LINC00632	-0.69817	0.0847954	-2.260717	0.0248155	0.0651093	-4.160295
KLC3	-0.68291	2.6061808	-2.260476	0.0248307	0.0651445	-4.160821
HHATL-AS1	0.4944881	-0.681623	2.2603152	0.0248409	0.0651665	-4.161172
XKR6	0.4543123	2.3603974	2.2601293	0.0248526	0.0651926	-4.161577
RP1-151B14	-0.481828	0.07202	-2.26008	0.0248557	0.0651961	-4.161685
RP11-542G1	-0.284271	-1.282617	-2.260031	0.0248588	0.0651995	-4.161791
AC012613.2	0.4898357	-0.42055	2.2591667	0.0249135	0.0653381	-4.163675
RP11-475J5	0.356919	3.0056602	2.2591279	0.0249159	0.0653398	-4.163759
DGAT2	-0.169092	6.5822685	-2.258858	0.024933	0.0653799	-4.164347
C15orf39	-0.068316	6.1057147	-2.258675	0.0249446	0.0654057	-4.164747

RP11-10C24	-0.309872	2.8770063	-2.258452	0.0249587	0.0654332	-4.165232
UBE2MP1	-0.230232	3.2224745	-2.258444	0.0249593	0.0654332	-4.16525
RP11-29H23	-0.286613	2.8729401	-2.258424	0.0249605	0.0654332	-4.165292
RP11-468E2	-0.341584	2.6870708	-2.25839	0.0249627	0.0654343	-4.165367
RP11-354I1	0.2796656	-1.260919	2.2578812	0.0249949	0.0655141	-4.166475
SRGAP2C	0.0926128	5.1884419	2.257784	0.0250011	0.0655256	-4.166686
PMPCA	0.0600117	6.5228747	2.2576784	0.0250078	0.0655384	-4.166916
RP11-502M1	0.5403931	-0.638218	2.2574456	0.0250226	0.0655725	-4.167423
USP7	0.035817	6.545995	2.256996	0.0250512	0.0656387	-4.168402
ADD2	0.3598047	3.5414173	2.2569891	0.0250516	0.0656387	-4.168417
RP11-422P2	-0.299701	2.7617568	-2.256963	0.0250533	0.0656387	-4.168474
AK3P2	0.224155	-1.376862	2.2561625	0.0251042	0.0657642	-4.170216
RNU6-1035F	-0.434879	-0.496918	-2.256154	0.0251048	0.0657642	-4.170235
TNFAIP8L2	0.1323877	5.0256012	2.2558518	0.025124	0.0658078	-4.170892
RP11-60A8.	0.2643821	-1.374367	2.255836	0.025125	0.0658078	-4.170926
SNORD124	-0.403941	-0.696417	-2.255807	0.0251268	0.0658079	-4.170988
RN7SKP276	-0.230412	-1.441765	-2.255755	0.0251302	0.0658119	-4.171103
ZNF660	-0.236452	3.9606019	-2.255679	0.025135	0.0658198	-4.171267
RP11-27901	-0.624892	0.1344213	-2.255614	0.0251392	0.065826	-4.171409
RNU6-343P	0.2518086	-1.319901	2.2551137	0.0251711	0.0659049	-4.172498
CTD-2015H3	-0.652534	0.7496249	-2.255038	0.0251759	0.0659127	-4.172661
NXT2	-0.077748	5.7235411	-2.254771	0.025193	0.0659527	-4.173243
AATK-AS1	-0.559868	-0.553639	-2.254724	0.025196	0.0659554	-4.173346
MRPS34	0.0565529	6.4427752	2.2546983	0.0251976	0.0659554	-4.173401
CR1	0.4287912	3.7639419	2.2544317	0.0252147	0.0659953	-4.173981
METTL22	0.0540568	5.6018661	2.2540895	0.0252366	0.0660479	-4.174725
RP11-141M3	0.5567962	3.5022647	2.2537985	0.0252552	0.0660912	-4.175357
RP11-104C4	-0.304767	-1.266542	-2.253764	0.0252574	0.0660912	-4.175432
SNORD99	-0.492371	1.897975	-2.253746	0.0252585	0.0660912	-4.175471
RNU5E-1	-0.359624	-1.008242	-2.253525	0.0252727	0.0661193	-4.175952
CCDC62	0.4698396	1.1255794	2.2535221	0.0252729	0.0661193	-4.175958
ENOPH1	-0.039486	6.0985614	-2.253409	0.0252802	0.0661303	-4.176205
MTERF4	-0.043137	5.9618125	-2.2534	0.0252807	0.0661303	-4.176224
EBP	-0.086915	6.767027	-2.253259	0.0252898	0.0661492	-4.176531
CCDC58P3	-0.355492	-0.918236	-2.253131	0.0252979	0.0661658	-4.176807
HMGA1P2	-0.509948	0.3300844	-2.25289	0.0253134	0.0662015	-4.177331
ZSCAN21	-0.060772	5.4297226	-2.252853	0.0253158	0.0662031	-4.177413
DZIP3	-0.069542	5.6972668	-2.25281	0.0253186	0.0662056	-4.177506
GLRXP3	0.5156234	-0.448779	2.2526284	0.0253302	0.0662274	-4.1779
POPDC3	-0.851375	0.9058813	-2.252623	0.0253306	0.0662274	-4.177912
KIAA0895L	0.0894032	5.4077738	2.2523146	0.0253504	0.0662745	-4.178582
ZSCAN31	-0.142628	5.5869913	-2.252243	0.025355	0.0662818	-4.178738
OR7E96P	0.185042	-1.515374	2.2520762	0.0253657	0.066305	-4.1791
AXIN1	0.0669286	6.1153021	2.2518521	0.0253801	0.0663379	-4.179586
MIDN	0.0580584	6.4720267	2.251273	0.0254174	0.0664306	-4.180844
TUBA3E	-0.690926	0.3517146	-2.251094	0.0254289	0.0664559	-4.181232
RIMKLA	0.4218436	3.2910848	2.2510345	0.0254327	0.0664612	-4.181362
WDR6	-0.048346	6.5034254	-2.250964	0.0254373	0.0664683	-4.181515
SPINK5	-0.519538	3.6505969	-2.25091	0.0254407	0.0664726	-4.181631
AL022393.9	-0.546815	1.3436846	-2.250879	0.0254428	0.0664731	-4.181699
MYPN	-0.537965	0.0905617	-2.250817	0.0254468	0.0664788	-4.181835

TCL1A	-0.676142	2.092476	-2.250737	0.0254519	0.0664833	-4.182008
RP11-93H12	0.5017898	-0.72117	2.2507339	0.0254521	0.0664833	-4.182014
CTD-2553C6	0.5627379	0.0469698	2.2504767	0.0254687	0.0665219	-4.182573
AC018866.1	0.1456059	-1.535471	2.2504056	0.0254733	0.0665291	-4.182727
TSPYL4	0.0845771	5.5962873	2.250376	0.0254752	0.0665293	-4.182791
SGSM3	-0.040902	6.376583	-2.250197	0.0254867	0.0665547	-4.18318
AC007228.5	-0.261744	-1.307717	-2.250077	0.0254945	0.0665661	-4.18344
HIST2H3D	-0.500297	-0.170615	-2.250062	0.0254955	0.0665661	-4.183474
EAF2	-0.12843	4.6907002	-2.250045	0.0254966	0.0665661	-4.18351
ZCWPW2	-0.177375	3.6241377	-2.249718	0.0255177	0.0666164	-4.184219
RP11-483F1	-0.447245	2.382262	-2.249378	0.0255397	0.0666689	-4.184957
RPL5P18	-0.436761	-0.473831	-2.249351	0.0255414	0.0666689	-4.185016
RP11-640N2	-0.326353	2.7410781	-2.249259	0.0255473	0.0666795	-4.185214
RPA3	-0.069788	5.9901354	-2.249102	0.0255575	0.0667013	-4.185556
CTD-3193K9	-0.548744	0.98018	-2.248722	0.0255821	0.0667548	-4.18638
TRERF1	0.1293955	5.0359536	2.2487116	0.0255828	0.0667548	-4.186403
NECAB1	0.3215808	3.7417219	2.2487009	0.0255835	0.0667548	-4.186426
RASAL2-AS1	-0.205598	3.7050069	-2.248567	0.0255921	0.0667726	-4.186716
C21orf91	0.0869539	5.4669655	2.248314	0.0256085	0.0668106	-4.187265
AC108868.5	-0.386399	-1.177767	-2.248145	0.0256195	0.0668344	-4.187631
CYP2R1	-0.05397	5.722199	-2.248055	0.0256254	0.0668437	-4.187827
MPP2	-0.332202	3.6840037	-2.248013	0.0256281	0.0668437	-4.187918
RP11-574F2	0.4946767	1.941045	2.2480055	0.0256286	0.0668437	-4.187934
GPX3	0.1196177	7.0985843	2.2479029	0.0256352	0.0668563	-4.188157
RP11-466P2	0.4629602	2.1546994	2.2478395	0.0256393	0.0668622	-4.188294
SLC36A4	0.0942272	5.531456	2.2477249	0.0256468	0.066875	-4.188543
CTA-221G9.	0.2679439	4.4865142	2.2477077	0.0256479	0.066875	-4.18858
AC079584.2	0.0848235	-1.566447	2.2476389	0.0256523	0.0668819	-4.188729
MLXIPL	-0.148631	6.7987691	-2.247492	0.0256619	0.0669019	-4.189047
RP11-68D16	0.1182604	-1.549407	2.2473812	0.0256691	0.066912	-4.189288
RP11-548P2	0.4663133	1.2282039	2.2473762	0.0256694	0.066912	-4.189299
SAG	-0.490677	-0.165338	-2.247139	0.0256848	0.0669473	-4.189812
C1orf27	-0.060526	6.2724581	-2.246992	0.0256944	0.0669676	-4.190132
FGF3	-0.423081	-0.98955	-2.246739	0.0257108	0.0670057	-4.19068
PRPF38A	-0.04141	6.1359027	-2.246624	0.0257183	0.0670203	-4.190929
AC004754.3	0.4598046	1.7397841	2.2463061	0.025739	0.0670695	-4.191618
ELANE	0.5751502	0.3722504	2.2461868	0.0257468	0.0670849	-4.191876
DHCR7	-0.08771	6.7802184	-2.246081	0.0257536	0.0670981	-4.192106
AC072062.1	-0.588698	1.0827115	-2.246049	0.0257557	0.0670987	-4.192175
ZNF513	-0.044992	6.0317559	-2.245973	0.0257607	0.0671069	-4.19234
RPL7P6	-0.435917	2.5423247	-2.245829	0.02577	0.0671236	-4.192651
KRT39	0.6605174	0.5603007	2.2458177	0.0257708	0.0671236	-4.192676
DDX25	-0.596129	1.4878784	-2.24572	0.0257771	0.0671353	-4.192887
OR3A2	-0.220601	-1.416671	-2.245687	0.0257793	0.0671363	-4.19296
ANXA9	-0.114058	6.2717068	-2.245608	0.0257845	0.0671448	-4.19313
HCFC1	-0.032992	6.475438	-2.245543	0.0257887	0.0671511	-4.193271
UHMK1	0.0514531	6.6723897	2.2454123	0.0257972	0.0671685	-4.193554
RBPJL	-0.504742	-0.589965	-2.2452	0.0258111	0.0671997	-4.194013
RP11-241F1	-0.575168	-0.35013	-2.245048	0.025821	0.0672192	-4.194344
RN7SKP230	-0.325951	-1.09299	-2.245029	0.0258222	0.0672192	-4.194384
ATP4B	-0.286251	-1.269214	-2.244946	0.0258277	0.0672285	-4.194564

CTSH	0.0710253	6.7414717	2.2447886	0.0258379	0.0672505	-4.194905
RP11-1057E	-0.410158	-0.639067	-2.244723	0.0258423	0.0672569	-4.195048
ZNF766	-0.049048	5.7774637	-2.244651	0.0258469	0.0672643	-4.195203
CCL5	0.1454488	5.8893375	2.2445963	0.0258505	0.0672663	-4.195321
GNPTAB	0.0521462	6.0844627	2.2445827	0.0258514	0.0672663	-4.195351
RPUSD3	-0.059188	6.118762	-2.244541	0.0258541	0.0672687	-4.195442
HELZ2	0.0745042	6.2484325	2.244398	0.0258635	0.0672882	-4.195751
FOXF2	0.4411739	3.2116433	2.244218	0.0258752	0.067314	-4.196141
RP11-820K3	-0.186653	-1.441319	-2.244186	0.0258773	0.0673146	-4.196209
CFAP54	0.3918987	2.5858029	2.2441536	0.0258795	0.0673153	-4.19628
SCNN1D	-0.133857	5.2270885	-2.24379	0.0259033	0.0673725	-4.197068
KRT222	-0.508054	3.7184844	-2.243737	0.0259067	0.0673766	-4.197181
AP000265.1	-0.372792	-0.855379	-2.243467	0.0259244	0.0674178	-4.197766
PPP2R2B	-0.309204	3.982147	-2.243228	0.0259401	0.0674538	-4.198284
POLR3D	0.0653463	5.6699038	2.2428881	0.0259624	0.0675035	-4.199019
DHTKD1	0.0967667	6.6347187	2.24288	0.0259629	0.0675035	-4.199036
RP11-855A2	-0.507423	0.6750297	-2.242744	0.0259718	0.0675219	-4.199331
ZNF585B	-0.121385	5.3642375	-2.242555	0.0259842	0.0675453	-4.19974
SOX9-AS1	-0.319092	5.1240488	-2.24255	0.0259845	0.0675453	-4.199749
RP11-452N1	-0.353944	2.7210522	-2.242513	0.025987	0.0675469	-4.199831
PLCG1	-0.051827	6.2691305	-2.242377	0.0259959	0.0675653	-4.200125
SPCS2P4	-0.157528	4.0255031	-2.242274	0.0260026	0.0675752	-4.200346
RIN1	0.1330922	5.0949246	2.2422626	0.0260034	0.0675752	-4.200372
STAC	0.5027079	3.0518954	2.242173	0.0260093	0.0675857	-4.200566
DCAF8L2	-0.821793	0.2312605	-2.242144	0.0260112	0.0675858	-4.200629
ZNF19	0.0799808	4.8077248	2.2419948	0.026021	0.0676065	-4.200951
ZFAT	-0.057713	5.6293316	-2.241928	0.0260254	0.067613	-4.201095
SETP8	-0.298368	-1.127263	-2.241877	0.0260288	0.067617	-4.201207
RP11-281O1	-0.320532	-1.141489	-2.241704	0.0260402	0.0676418	-4.201581
SUMO2P6	-0.300407	-1.127422	-2.241597	0.0260472	0.0676552	-4.201812
RPL9P28	-0.456744	-0.416842	-2.241535	0.0260512	0.0676609	-4.201945
RP11-430G1	-0.498267	-0.107887	-2.241308	0.0260662	0.067695	-4.202438
CTC-398G3.	-0.544077	0.9348457	-2.241068	0.026082	0.0677312	-4.202956
PCSK6-AS1	-0.362203	-0.998071	-2.240818	0.0260984	0.067769	-4.203495
RNU6-481P	-0.485999	1.1632649	-2.240412	0.0261252	0.0678338	-4.204374
SMARCAL1	-0.038882	5.9617747	-2.240326	0.0261309	0.0678437	-4.20456
SPRY4-IT1	0.5404537	0.0959979	2.240213	0.0261383	0.0678582	-4.204804
GLMN	-0.070442	5.2471215	-2.240175	0.0261409	0.0678599	-4.204886
OR4C6	0.1968815	-1.467694	2.2399673	0.0261546	0.0678907	-4.205335
RP11-647P1	0.1393646	-1.538652	2.2398051	0.0261653	0.0679129	-4.205685
RP11-341N2	-0.488117	1.987266	-2.239776	0.0261672	0.0679129	-4.205747
MDN1	-0.063798	6.215732	-2.239753	0.0261687	0.0679129	-4.205798
CTC-261N6.	-0.695562	0.4911335	-2.239486	0.0261864	0.0679539	-4.206376
TGFB1	0.0968302	6.2403707	2.2394314	0.02619	0.0679584	-4.206493
OTX1	-0.580844	3.632627	-2.239325	0.026197	0.0679719	-4.206723
MAGEC3	-0.521155	-0.796668	-2.239204	0.026205	0.0679877	-4.206984
TPTEP1	0.4905214	2.4408683	2.2391635	0.0262077	0.0679898	-4.207072
ST3GAL2	0.0435748	6.1486968	2.2390656	0.0262142	0.0680018	-4.207283
UBE2V1P2	-0.481887	-0.015232	-2.238966	0.0262207	0.0680138	-4.207497
RP11-193H2	0.5117852	-0.443086	2.2389318	0.026223	0.0680138	-4.207572
AC096669.3	-0.394559	-1.14243	-2.238909	0.0262245	0.0680138	-4.20762

GS1-166A23	0.4306931	-0.652543	2.2388833	0.0262262	0.0680138	-4.207677
MTA2	0.0318656	6.4264321	2.2385331	0.0262494	0.0680688	-4.208433
SLC28A2	-0.504014	2.8244346	-2.238507	0.0262511	0.0680688	-4.20849
NSUN2	0.0387304	6.4442902	2.2384207	0.0262568	0.0680787	-4.208676
DEFB133	-0.28574	-1.307481	-2.238147	0.026275	0.0681188	-4.209268
RPL15	-0.042959	7.1244883	-2.238125	0.0262765	0.0681188	-4.209315
GLIPR1L2	0.3585655	3.2934808	2.238103	0.0262779	0.0681188	-4.209362
DECR2	0.0856584	6.5098484	2.2380386	0.0262822	0.068125	-4.209501
MIR4520-1	-0.364995	-0.894191	-2.237805	0.0262976	0.0681603	-4.210005
SLC25A6P5	-0.187742	-1.440785	-2.23739	0.0263252	0.0682268	-4.210901
VTI1B	0.0441327	6.3989608	2.2373107	0.0263305	0.0682357	-4.211073
SAMD8	0.048504	5.8449732	2.2366827	0.0263722	0.0683352	-4.212428
ARHGAP9	0.1393172	5.3689885	2.2366762	0.0263726	0.0683352	-4.212442
RP11-376P6	0.5089429	0.7985769	2.2364733	0.0263861	0.0683653	-4.21288
ADAM1B	-0.353003	2.6737742	-2.236273	0.0263994	0.068395	-4.213312
RPL12P4	-0.350335	2.8953107	-2.236219	0.0264031	0.0683995	-4.213429
AC104699.1	-0.525255	-0.458418	-2.235848	0.0264278	0.0684538	-4.21423
CYTIP	0.1804105	5.2053044	2.2358472	0.0264278	0.0684538	-4.214231
ABHD17AP3	-0.348095	-0.964606	-2.235815	0.02643	0.0684538	-4.214301
RP5-1147A1	-0.463042	-0.432501	-2.235791	0.0264315	0.0684538	-4.214351
Six3os1_2	-0.424055	-0.958337	-2.235657	0.0264405	0.0684721	-4.214641
CDC20B	-0.702269	1.572187	-2.235564	0.0264467	0.0684833	-4.214841
RPL21P3	-0.462213	0.4045046	-2.235437	0.0264552	0.0685005	-4.215116
RP1-288H2.	-0.527363	-0.458409	-2.234969	0.0264863	0.0685763	-4.216124
NAV2-AS4	0.5420385	-0.23436	2.2349355	0.0264886	0.0685773	-4.216197
HNRNPLL	-0.034324	5.9893675	-2.234872	0.0264928	0.0685834	-4.216333
RPS25	-0.048326	6.9995595	-2.234654	0.0265074	0.0686098	-4.216803
STPG1	0.0844604	5.2112482	2.2346424	0.0265082	0.0686098	-4.216829
SDAD1P2	-0.264457	-1.267155	-2.234635	0.0265087	0.0686098	-4.216845
BBS9	0.0735072	5.4228342	2.2343695	0.0265264	0.0686508	-4.217417
RP11-510C1	0.2155729	-1.477051	2.2342037	0.0265375	0.0686747	-4.217775
LINC00534	-0.379552	-1.12002	-2.234063	0.0265469	0.0686942	-4.218079
RP11-365N1	-0.358714	2.4598158	-2.233744	0.0265682	0.0687445	-4.218765
WDR63	0.5528009	0.4638781	2.2337155	0.0265702	0.0687446	-4.218827
CST11	-0.187364	-1.44097	-2.233683	0.0265723	0.0687453	-4.218897
RPL15P18	-0.468555	0.23212	-2.233535	0.0265822	0.0687661	-4.219216
PI4K2B	0.0763586	6.1140243	2.233464	0.026587	0.0687735	-4.219369
PPP2R5A	-0.060606	6.63794	-2.233396	0.0265915	0.0687801	-4.219515
LINC01374	-0.484953	-0.350113	-2.23337	0.0265933	0.0687801	-4.219572
SLCO1C1	0.4510879	2.6837084	2.2332359	0.0266023	0.0687954	-4.219861
TMEM106C	-0.070362	6.4518056	-2.233219	0.0266034	0.0687954	-4.219896
PLGLA	0.7362561	3.1242444	2.233197	0.0266049	0.0687954	-4.219945
IQSEC3	0.4754503	3.2974487	2.2330782	0.0266128	0.0688092	-4.220201
RP1-65P5.5	-0.496093	-0.700926	-2.233061	0.026614	0.0688092	-4.220237
ACER2	0.3552333	3.6698192	2.2327413	0.0266354	0.068851	-4.220926
RP11-1148L	0.1094456	4.8799877	2.2327006	0.0266382	0.068851	-4.221014
AC005682.7	-0.488244	-0.09575	-2.232693	0.0266386	0.068851	-4.22103
NAF1	0.06095	5.3297987	2.2326832	0.0266393	0.068851	-4.221052
TPT1P8	-0.396524	-1.074558	-2.232679	0.0266396	0.068851	-4.22106
TMEM74B	-0.157987	5.0615943	-2.232496	0.0266519	0.0688779	-4.221455
PSKH1	0.0565222	6.1316276	2.2322976	0.0266652	0.0689075	-4.221882

PPIL3	-0.05777	5.8066969	-2.231844	0.0266957	0.0689733	-4.22286
VGLL1	0.6006383	-0.424635	2.2318398	0.026696	0.0689733	-4.222868
SLC1A7	-0.332894	4.9198129	-2.231834	0.0266964	0.0689733	-4.222881
BAD	0.0528839	6.2140445	2.2316634	0.0267078	0.068998	-4.223248
CTC-429P9.	-0.299531	2.8433481	-2.231257	0.0267352	0.0690638	-4.224123
CTD-2033A1	0.6561035	0.354622	2.2310566	0.0267486	0.0690937	-4.224555
KB-318B8.7	0.5512381	0.3226179	2.2309196	0.0267579	0.0691126	-4.224849
CHRDL1	0.6930135	2.7663331	2.2308566	0.0267621	0.0691187	-4.224985
LL22NC03-3	0.5462026	0.779989	2.2305058	0.0267857	0.0691749	-4.22574
RAB34	-0.153035	5.8734024	-2.229951	0.0268232	0.0692666	-4.226935
LAMC1	-0.063715	6.7570666	-2.229907	0.0268261	0.0692694	-4.227029
CTD-2015A6	-0.265766	-1.298903	-2.229484	0.0268547	0.0693382	-4.227939
RP11-180C1	-0.744956	-0.143108	-2.229123	0.0268791	0.0693963	-4.228716
OLFML3	0.135474	5.6373227	2.2290788	0.0268821	0.0693991	-4.228811
CRYM	0.2306455	5.4317716	2.2287554	0.026904	0.0694507	-4.229506
MALL	0.4019877	3.3561244	2.2283885	0.0269288	0.0695099	-4.230295
ZNF23	0.2505954	3.8125476	2.2281864	0.0269425	0.0695403	-4.23073
KIAA1324L	0.257342	4.8113626	2.2280234	0.0269535	0.0695639	-4.231081
BZW2	-0.056804	6.190247	-2.227966	0.0269574	0.0695689	-4.231203
RP11-19G24	-0.385665	-0.781482	-2.227901	0.0269618	0.0695755	-4.231345
RP5-1057I2	-0.473591	-0.290841	-2.227841	0.0269659	0.0695811	-4.231474
HES2	0.5046869	3.0494133	2.2277958	0.026969	0.0695829	-4.23157
NKIRAS1	0.0780568	5.6211057	2.2277739	0.0269704	0.0695829	-4.231617
IKBKE	-0.126369	5.5514027	-2.227735	0.0269731	0.069583	-4.2317
RP11-537H1	-0.390852	-0.897748	-2.227717	0.0269743	0.069583	-4.231739
MGAM	0.678597	3.0288933	2.227653	0.0269786	0.0695893	-4.231877
RP3-333A15	0.2282184	-1.470606	2.2275324	0.0269868	0.0696055	-4.232136
RP11-113K2	-0.521561	1.4030014	-2.227308	0.027002	0.0696398	-4.232617
ARCN1	0.0338674	6.6865301	2.2272172	0.0270082	0.0696508	-4.232814
P2RX6P	0.3367929	-1.203347	2.2271451	0.0270131	0.0696585	-4.232969
LINC01077	-0.262413	-1.347352	-2.227101	0.0270161	0.0696613	-4.233063
RPS21P1	-0.471111	-0.20304	-2.226298	0.0270707	0.0697972	-4.23479
TMEM130	0.5986354	2.669984	2.2262304	0.0270753	0.0698041	-4.234934
RN7SK	-0.194104	4.8175233	-2.226159	0.0270801	0.0698116	-4.235086
CRYAA	0.5795825	-0.389042	2.22601	0.0270903	0.0698329	-4.235408
EXTL3-AS1	-0.347817	2.9223343	-2.225717	0.0271102	0.0698793	-4.236036
RP11-311P8	-0.402246	2.2396564	-2.225407	0.0271313	0.0699288	-4.236702
RHOQP3	0.351067	-0.938617	2.2251631	0.027148	0.0699627	-4.237226
DNAJC3	0.0554931	6.6319913	2.2251583	0.0271483	0.0699627	-4.237237
Six3os1_5	-0.346544	-1.12698	-2.225088	0.0271531	0.0699701	-4.237388
C9orf9	0.1308686	5.0662148	2.2249105	0.0271652	0.0699935	-4.237769
RNASEH1-AS	-0.098381	5.0438068	-2.224899	0.027166	0.0699935	-4.237794
AC006534.3	-0.247096	-1.316059	-2.224665	0.0271819	0.0700296	-4.238296
TTTY16	0.1754944	-1.497475	2.2244919	0.0271938	0.0700552	-4.238668
RP4-597N16	-0.268658	-1.274736	-2.224073	0.0272224	0.070124	-4.239567
RP11-25902	0.3343559	-1.274085	2.2237306	0.0272458	0.0701782	-4.240302
RP11-666F1	-0.511079	1.8439131	-2.223709	0.0272473	0.0701782	-4.240348
AC104532.4	-0.427988	2.1294916	-2.223241	0.0272793	0.0702557	-4.241353
SLMO2P11	-0.410721	-0.681316	-2.222943	0.0272997	0.0703033	-4.241992
RNF165	0.3313404	3.9408667	2.2228958	0.027303	0.0703056	-4.242093
MICAL1	0.0924471	5.9182728	2.222874	0.0273045	0.0703056	-4.24214

CTA-384D8.	0.4343005	3.1611085	2.22273	0.0273143	0.0703261	-4.242449
RP11-105N1	0.611416	1.1383246	2.2226073	0.0273228	0.0703427	-4.242712
CTB-102L5.	-0.433587	-0.488283	-2.22255	0.0273267	0.070348	-4.242835
AP001172.2	0.2254482	-1.371444	2.2224928	0.0273306	0.0703531	-4.242958
LRRC20	-0.092356	6.2605583	-2.222365	0.0273394	0.0703707	-4.243232
RP11-401L1	-0.374387	-0.827291	-2.222173	0.0273526	0.0703997	-4.243644
RP11-50102	-0.170524	-1.471133	-2.222084	0.0273587	0.0704105	-4.243835
RP11-365H2	-0.330389	-1.060065	-2.221951	0.0273678	0.0704252	-4.244119
PTMAP5	-0.136664	4.5067397	-2.221944	0.0273683	0.0704252	-4.244135
RP4-718P11	-0.340541	-1.020365	-2.221911	0.0273705	0.070426	-4.244205
RP11-58E21	0.4239285	2.2084336	2.2216701	0.0273871	0.0704637	-4.244722
RP11-583F2	-0.378658	-0.872588	-2.221393	0.0274061	0.0705077	-4.245316
RP11-3N13.	0.149025	-1.506205	2.2211467	0.0274231	0.0705463	-4.245844
RPS20P14	-0.195716	4.0509609	-2.221076	0.027428	0.0705539	-4.245996
RP11-240M1	-0.289516	-1.204383	-2.220922	0.0274385	0.0705761	-4.246326
CD1E	0.4489194	3.5295043	2.2207063	0.0274534	0.0706094	-4.246788
HSBP1P2	0.5356567	-0.388041	2.2205436	0.0274646	0.0706332	-4.247137
CTD-2026D2	-0.470251	0.0547837	-2.220407	0.027474	0.0706524	-4.247429
RAB7A	0.0307785	6.8637386	2.2203281	0.0274794	0.0706614	-4.247599
CPSF1P1	-0.687437	2.4202338	-2.220295	0.0274818	0.0706624	-4.24767
MIR3197	0.3269127	-1.202925	2.2201459	0.027492	0.0706838	-4.247989
ABCA17P	-0.469607	2.3386878	-2.219969	0.0275042	0.0707101	-4.248367
UBE2V1P1	0.3208501	-1.104435	2.2199253	0.0275072	0.0707129	-4.248462
LINC01230	-0.386059	-1.201833	-2.219759	0.0275187	0.0707375	-4.248819
POLR1D	-0.04396	6.4052889	-2.219728	0.0275209	0.070738	-4.248885
RP1-265C24	-0.479835	1.2913353	-2.219639	0.027527	0.0707488	-4.249076
GABRG3	-0.726301	0.8515096	-2.219384	0.0275445	0.0707889	-4.24962
GAPDHP25	-0.353453	-0.963994	-2.218863	0.0275806	0.0708765	-4.250737
RPL36AP26	-0.450271	-0.477943	-2.218785	0.027586	0.0708817	-4.250904
TSR3	0.0532784	6.2938059	2.2187777	0.0275865	0.0708817	-4.25092
ELMOD2	0.0420587	6.0511061	2.2187191	0.0275905	0.070886	-4.251045
LINC00936	-0.148183	4.004366	-2.218697	0.027592	0.070886	-4.251092
CTD-2616J1	0.424034	2.1931953	2.2185438	0.0276027	0.0709083	-4.251421
MTND4P9	0.3554728	-1.039339	2.2184919	0.0276063	0.0709126	-4.251532
RP11-13P5.	0.5415585	0.028139	2.2183097	0.0276189	0.07094	-4.251922
PARK2	0.1655724	4.9261277	2.2182195	0.0276251	0.070951	-4.252115
CH17-53B9.	-0.284479	-1.358862	-2.218131	0.0276313	0.0709618	-4.252306
SLC38A7	0.0497094	6.1786659	2.2178721	0.0276492	0.0710028	-4.252859
RP11-33301	0.3804638	-0.88671	2.2177787	0.0276556	0.0710116	-4.253059
RP3-395M2C	0.5217425	-0.392548	2.2177668	0.0276565	0.0710116	-4.253084
PEX11A	-0.088015	5.9585505	-2.217736	0.0276586	0.0710121	-4.253151
RUNX1	0.0884564	6.0068694	2.2176371	0.0276655	0.0710192	-4.253362
RP11-420L9	0.0986645	5.3783031	2.2176277	0.0276661	0.0710192	-4.253382
SLC4A11	-0.326951	4.2143528	-2.217612	0.0276672	0.0710192	-4.253417
IL18RAP	0.2474109	4.0726039	2.2175171	0.0276738	0.071028	-4.253619
RP11-245J9	-0.301454	2.5571185	-2.217506	0.0276745	0.071028	-4.253642
STX19	0.5357873	0.5560203	2.2174664	0.0276773	0.0710301	-4.253727
STAC2	0.5892391	0.1145612	2.2174041	0.0276816	0.0710362	-4.253861
RP11-42801	0.484046	2.0745357	2.2172849	0.0276899	0.0710524	-4.254116
AC011742.3	-0.333464	-1.050051	-2.216835	0.0277211	0.0711275	-4.255079
LLOXNC01-7	-0.174548	4.1583966	-2.216425	0.0277496	0.0711955	-4.255955

TTLL5	-0.050452	5.7335463	-2.216232	0.027763	0.0712251	-4.256369
SLC35B2	-0.048246	6.4974693	-2.21595	0.0277826	0.0712703	-4.256972
FAM107B	0.0558649	6.596519	2.2158882	0.0277869	0.0712748	-4.257104
AC092675.3	0.2001373	-1.480158	2.215869	0.0277883	0.0712748	-4.257145
CHD6	-0.047973	6.1040891	-2.215735	0.0277976	0.0712937	-4.257433
RP11-61K9.	-0.119579	4.698223	-2.215503	0.0278137	0.07133	-4.257927
SLITRK6	0.6633995	0.6222021	2.2154281	0.027819	0.0713385	-4.258088
RP11-434H6	-0.16606	3.9508873	-2.215386	0.0278219	0.071341	-4.258179
RP11-763B2	-0.269113	-1.352492	-2.21487	0.0278579	0.0714282	-4.259281
DIS3	0.0509969	6.1064424	2.2147815	0.027864	0.071439	-4.259471
AC090044.2	0.3060541	-1.205766	2.2147275	0.0278678	0.0714437	-4.259586
NSUN7	-0.670053	2.8003698	-2.214646	0.0278735	0.0714532	-4.259761
NLRP7	-0.585319	0.1704501	-2.214523	0.0278821	0.0714702	-4.260024
RP11-241I2	-0.263437	-1.325371	-2.214429	0.0278886	0.0714819	-4.260224
EMC9	-0.068054	5.7769928	-2.214259	0.0279005	0.0715074	-4.260588
MRPL4	0.0498331	6.3863416	2.2141129	0.0279107	0.0715285	-4.2609
G2E3	-0.056339	5.5676252	-2.213683	0.0279407	0.0716004	-4.261818
TAMM41	-0.058308	5.3344521	-2.213649	0.0279431	0.0716016	-4.261892
GRPR	0.6429655	1.6359123	2.2134936	0.027954	0.0716244	-4.262223
LINC01361	-0.501104	-0.558878	-2.213435	0.0279581	0.0716299	-4.262349
COG3	0.0679314	6.2398584	2.2133698	0.0279627	0.071633	-4.262488
MPC1L	0.1603524	-1.482428	2.2133526	0.0279639	0.071633	-4.262525
CTD-2050E2	0.2007507	-1.434317	2.2133332	0.0279652	0.071633	-4.262566
MT1H	0.6223538	3.8472421	2.2131635	0.0279771	0.0716549	-4.262929
RP11-111F5	-0.218187	-1.425855	-2.213155	0.0279777	0.0716549	-4.262946
SORCS2	0.3208414	5.030476	2.2129633	0.0279911	0.0716806	-4.263356
TTC3-AS1	-0.459093	0.1073944	-2.212939	0.0279928	0.0716806	-4.263409
PMPCAP1	-0.607302	-0.528589	-2.212928	0.0279936	0.0716806	-4.263432
CLVS1	0.4743436	2.7409982	2.2128727	0.0279975	0.0716854	-4.26355
UBE2J1	-0.040459	6.3807518	-2.212699	0.0280096	0.0717077	-4.263921
AC098973.1	-0.280087	-1.277131	-2.212689	0.0280103	0.0717077	-4.263942
CBY3	-0.471359	0.7073811	-2.212645	0.0280134	0.0717077	-4.264037
SLC36A1	-0.071235	5.699926	-2.212631	0.0280144	0.0717077	-4.264065
KCNN3	0.1383808	5.1043004	2.2125837	0.0280177	0.0717077	-4.264167
FAM186A	-0.324006	2.9672958	-2.21258	0.0280179	0.0717077	-4.264174
HULC	-0.389853	6.1914494	-2.212149	0.0280482	0.0717801	-4.265096
RPL31	-0.050406	7.1086137	-2.212012	0.0280578	0.0717997	-4.265389
ALDH1A2	0.2181274	5.169114	2.2119796	0.0280601	0.0718004	-4.265457
RPL5P22	-0.469141	-0.3425	-2.211942	0.0280627	0.0718022	-4.265538
IFIT5	0.0668797	5.9379515	2.2118266	0.0280708	0.0718179	-4.265784
RN7SKP173	-0.40744	-0.540634	-2.211697	0.0280799	0.0718361	-4.266061
NEB	-0.253024	5.4781599	-2.211624	0.0280851	0.0718403	-4.266217
LINC00675	0.6149636	1.224077	2.2116173	0.0280855	0.0718403	-4.266231
TICAM1	0.0688878	6.152557	2.2115238	0.0280921	0.0718521	-4.266431
ILDR1	-0.594741	3.7404482	-2.211483	0.0280949	0.0718531	-4.266518
AC016723.4	-0.599175	-0.256757	-2.211462	0.0280964	0.0718531	-4.266562
AC092301.3	-0.467651	1.2402718	-2.211232	0.0281126	0.0718818	-4.267053
CTD-2374C2	-0.71588	0.3902658	-2.211221	0.0281134	0.0718818	-4.267077
RP11-215D1	0.1884273	-1.423528	2.2112185	0.0281135	0.0718818	-4.267082
ERGIC3	-0.040714	6.8559359	-2.211181	0.0281162	0.0718835	-4.267163
IQCC	-0.115961	4.7663977	-2.21099	0.0281296	0.0719086	-4.267571

RP11-114N1	0.4491172	1.8370106	2.2109852	0.0281299	0.0719086	-4.26758
CTD-2568A1	-0.208669	-1.386712	-2.210933	0.0281336	0.0719112	-4.267691
RP11-427P5	-0.451469	0.0779773	-2.210915	0.0281349	0.0719112	-4.267731
RP11-386I2	-0.435253	-0.470969	-2.210683	0.0281512	0.0719478	-4.268224
ZNF385D	0.4897324	3.2665296	2.2106205	0.0281556	0.0719541	-4.268359
GPATCH2L	-0.049443	5.8368954	-2.210463	0.0281667	0.0719731	-4.268694
CTD-2369P2	-0.302839	-1.115911	-2.210452	0.0281675	0.0719731	-4.268719
TBC1D2	0.0796487	5.8062649	2.210431	0.0281689	0.0719731	-4.268763
RP11-53601	0.5445024	0.2451368	2.2103141	0.0281772	0.0719891	-4.269012
AC091729.7	-0.393168	-0.759905	-2.210062	0.028195	0.0720295	-4.269551
PABPC1	-0.055718	7.2867694	-2.209827	0.0282115	0.0720667	-4.270051
RP11-713H1	-0.422636	-0.433003	-2.209643	0.0282245	0.0720948	-4.270444
MAML1	-0.040719	6.102269	-2.209049	0.0282664	0.0721968	-4.271711
PPIEL	0.4981958	1.6456083	2.2089828	0.0282711	0.0722037	-4.271852
MAB21L3	0.538202	3.2123733	2.2088159	0.0282829	0.0722288	-4.272208
RP11-97012	-0.279284	3.5301923	-2.208429	0.0283103	0.0722937	-4.273034
RNU1-88P	-0.175284	-1.468799	-2.207929	0.0283456	0.0723757	-4.274099
RP11-384J4	-0.506068	-0.72265	-2.207919	0.0283463	0.0723757	-4.274121
RP11-752G1	-0.513709	0.4948942	-2.20783	0.0283526	0.0723832	-4.274309
MNX1-AS2	-0.633485	-0.287795	-2.207821	0.0283532	0.0723832	-4.274329
STX4	0.0422692	6.3184385	2.2076456	0.0283657	0.0724099	-4.274703
ORAI3	0.0748372	6.032289	2.2074324	0.0283808	0.0724416	-4.275157
WDR93	-0.463258	1.8544983	-2.207414	0.0283821	0.0724416	-4.275197
RP11-725G5	-0.553581	1.8368676	-2.207239	0.0283945	0.0724682	-4.27557
RP11-276H7	0.534849	0.4493598	2.2071718	0.0283993	0.0724753	-4.275713
LGALS2	-0.228443	4.7941742	-2.207018	0.0284102	0.0724982	-4.276041
TPSP2	0.5128958	3.7798702	2.2069721	0.0284134	0.0725013	-4.276138
RHBDL3	-0.530163	2.716009	-2.206832	0.0284233	0.0725166	-4.276436
PSMD10P1	-0.44869	-0.214013	-2.206832	0.0284234	0.0725166	-4.276438
SCARNA21	-0.530357	-0.034104	-2.206771	0.0284277	0.0725226	-4.276568
AC073072.5	0.4846957	-0.504223	2.2067379	0.02843	0.0725234	-4.276637
AC010649.1	0.2252186	-1.417089	2.2066823	0.028434	0.0725284	-4.276756
NPM1P5	-0.383866	-0.80769	-2.20638	0.0284555	0.0725782	-4.2774
LINC00244	-0.502438	0.0007161	-2.206261	0.0284639	0.0725945	-4.277652
SLC35E1	0.0401418	6.3601306	2.2062144	0.0284672	0.072598	-4.277753
ZNF663P	-0.463838	-0.688843	-2.205834	0.0284942	0.0726618	-4.278562
BMPR1APS1	-0.529126	0.2525229	-2.205779	0.0284982	0.0726633	-4.278681
OSGIN1	0.1460203	6.5889001	2.2057702	0.0284988	0.0726633	-4.278699
EEF1A1P34	-0.259286	-1.269691	-2.20565	0.0285073	0.07268	-4.278954
RPL29P33	-0.475763	0.2123384	-2.205503	0.0285178	0.0727016	-4.279267
SLA2	0.1908265	4.5603108	2.2052764	0.028534	0.0727377	-4.27975
ZNF729	-0.479701	-0.86999	-2.205227	0.0285375	0.0727415	-4.279855
RP11-66B24	0.2831821	-1.268971	2.2047867	0.0285688	0.0728165	-4.280793
RP4-798A10	-0.219454	3.2640619	-2.204678	0.0285766	0.072831	-4.281023
RP11-701I2	0.091776	-1.562904	2.2046436	0.028579	0.0728323	-4.281097
NCLN	-0.047475	6.6106182	-2.204503	0.0285891	0.0728527	-4.281396
ZNF821	-0.098229	4.7551779	-2.204467	0.0285916	0.0728542	-4.281473
ZBTB42	-0.075308	5.7210474	-2.203994	0.0286254	0.0729351	-4.28248
PRPH2	0.4127474	2.7694862	2.2039462	0.0286288	0.0729387	-4.282581
CSMD1	-0.902581	1.9286499	-2.203723	0.0286448	0.0729743	-4.283057
ITM2A	0.1849516	5.4207962	2.2036596	0.0286493	0.0729807	-4.283191

VN2R17P	-0.372176	-0.861637	-2.203366	0.0286703	0.073029	-4.283815
SCART1	-0.231694	4.3945822	-2.202738	0.0287152	0.0731385	-4.285152
GOLGA6L7P	-0.410366	-0.983874	-2.202639	0.0287223	0.0731514	-4.285363
AGTR2	-0.443658	-0.967525	-2.202576	0.0287268	0.0731579	-4.285497
LINC00115	-0.165853	3.6471177	-2.202424	0.0287377	0.0731804	-4.285819
ELF4	0.1208391	5.4570018	2.2021726	0.0287557	0.0732212	-4.286354
VTA1	-0.042458	6.1831125	-2.201838	0.0287797	0.0732764	-4.287064
FAM120B	0.0497505	6.0192737	2.201814	0.0287814	0.0732764	-4.287116
STX17	0.0656164	5.6778962	2.2017636	0.0287851	0.0732768	-4.287224
CTC-499J9.	-0.374476	-1.12654	-2.201756	0.0287856	0.0732768	-4.28724
PDF	0.1120005	4.9811243	2.2017131	0.0287887	0.0732795	-4.287331
RP11-383C5	-0.49115	0.2719718	-2.201581	0.0287982	0.0732986	-4.287613
USP9X	0.0409253	6.6911445	2.2015492	0.0288004	0.0732993	-4.28768
TNNT1	-0.665923	2.5444227	-2.201502	0.0288039	0.0733028	-4.287781
CEMIP	0.2660854	5.0190152	2.2011942	0.0288259	0.0733539	-4.288434
CCDC43	-0.047634	5.7473305	-2.201059	0.0288357	0.0733735	-4.288721
CTB-61M7.2	0.5950561	0.6141334	2.2010181	0.0288386	0.0733759	-4.288808
RP11-15E18	0.2743248	-1.302197	2.2009189	0.0288457	0.0733889	-4.289019
RP11-30J20	-0.890058	1.2507551	-2.200804	0.028854	0.0734049	-4.289264
DUXA	-0.283468	-1.340873	-2.200377	0.0288847	0.0734779	-4.290171
SP3P	-0.654966	-0.208695	-2.199909	0.0289184	0.0735584	-4.291164
BPTF	-0.045449	6.2500478	-2.199754	0.0289296	0.0735818	-4.291495
ACSM3	0.1782175	5.7696227	2.1996192	0.0289393	0.0736013	-4.29178
C11orf53	-0.725083	-0.166789	-2.199589	0.0289414	0.0736017	-4.291844
SEPP1	0.1010078	7.0569999	2.1994722	0.0289499	0.0736147	-4.292093
UCN	0.233471	4.0781641	2.1994623	0.0289506	0.0736147	-4.292114
RNU6ATAC2F	0.1312653	-1.542779	2.1993007	0.0289622	0.0736392	-4.292457
NUP210L	-0.499506	0.62497	-2.199226	0.0289676	0.0736477	-4.292615
PLXDC1	0.1330481	5.4851967	2.1991853	0.0289706	0.0736501	-4.292702
RPS7P8	-0.320678	-1.081671	-2.199043	0.0289808	0.0736702	-4.293003
RP4-612B18	-0.46493	-0.212141	-2.198986	0.0289849	0.0736702	-4.293124
AOC4P	-0.403519	5.0736863	-2.198986	0.028985	0.0736702	-4.293125
AL928761.1	-0.454426	-0.801876	-2.198964	0.0289866	0.0736702	-4.293172
SNTG1	-0.760063	0.9354992	-2.198587	0.0290138	0.0737321	-4.293973
PIR	0.1149445	6.0181917	2.1985513	0.0290163	0.0737321	-4.294048
LINC01138	-0.146265	5.1267422	-2.198522	0.0290185	0.0737321	-4.294111
ARRDC1-AS1	-0.058162	5.5749384	-2.198507	0.0290196	0.0737321	-4.294142
RP11-59E19	0.2202155	-1.497449	2.1984867	0.029021	0.0737321	-4.294185
CASQ1	-0.449818	2.0513865	-2.198433	0.0290249	0.0737369	-4.294299
RNF2	-0.06908	5.7085884	-2.198233	0.0290394	0.0737685	-4.294724
PMFBP1	-0.253892	4.0406223	-2.198123	0.0290473	0.0737836	-4.294958
DPM1	-0.043272	6.1515543	-2.197975	0.029058	0.0738056	-4.295272
KLHL10	-0.475428	0.5560337	-2.197382	0.029101	0.0739095	-4.29653
RP11-288C1	-0.195615	-1.415019	-2.197347	0.0291035	0.0739108	-4.296604
RP1-199J3.	-0.410906	1.804031	-2.197121	0.0291199	0.0739472	-4.297084
RP11-369G6	-0.298148	-1.183613	-2.196992	0.0291292	0.0739657	-4.297356
AC010127.3	-0.707463	0.3444293	-2.196956	0.0291318	0.0739673	-4.297434
GOLGA2P8	-0.537137	-0.089567	-2.196768	0.0291455	0.0739968	-4.297833
RP1-121G13	-0.184859	-1.464104	-2.196644	0.0291545	0.0740145	-4.298096
AMD1P1	0.2181147	-1.382406	2.1965451	0.0291616	0.0740275	-4.298305
PTGER4	0.1515489	5.1260023	2.1960931	0.0291944	0.0741057	-4.299264

IL12RB1	0.1632595	4.9121003	2.1958973	0.0292087	0.0741366	-4.299679
MRPS18A	-0.052084	6.0667941	-2.195745	0.0292197	0.0741595	-4.300001
TAAR9	-0.197375	-1.436061	-2.19533	0.0292499	0.074231	-4.300881
ITGAX	0.1017911	5.8086842	2.1950855	0.0292677	0.074271	-4.3014
ERVV-1	-0.332263	-1.20832	-2.195054	0.02927	0.0742717	-4.301468
F10	-0.138993	6.7073108	-2.194988	0.0292748	0.0742786	-4.301606
USP6	-0.323568	3.1359137	-2.194869	0.0292835	0.0742956	-4.30186
CCDC26	0.3898164	-0.835648	2.1945995	0.0293031	0.0743384	-4.30243
RP1-154K9.	-0.412561	-1.059345	-2.194581	0.0293044	0.0743384	-4.302469
TCF12	0.049029	6.2364629	2.1940356	0.0293442	0.0744341	-4.303625
OPHN1	0.1750041	4.6580482	2.1939548	0.0293501	0.0744439	-4.303796
RPS2P55	-0.309129	3.0448885	-2.193456	0.0293865	0.0745311	-4.304853
MBD3	-0.051193	6.5060086	-2.193407	0.0293901	0.074535	-4.304957
APPL2	0.0422406	6.1004095	2.1930547	0.0294159	0.0745948	-4.305703
LENEP	-0.411168	-0.612632	-2.193028	0.0294178	0.0745948	-4.305759
LINC00635	-0.42758	-0.928902	-2.192927	0.0294252	0.0746084	-4.305973
STARD8	0.091467	5.8123047	2.1927802	0.0294359	0.0746284	-4.306284
AC073465.1	0.1822162	-1.49405	2.1927632	0.0294372	0.0746284	-4.30632
CDKN2A-AS1	-0.548418	-0.075762	-2.192614	0.0294481	0.074649	-4.306637
ERLEC1	-0.03725	6.4426685	-2.192578	0.0294508	0.074649	-4.306713
GULP1	0.5197458	3.9190227	2.1925685	0.0294514	0.074649	-4.306733
RP13-254B1	-0.505888	0.0071259	-2.192526	0.0294545	0.0746516	-4.306822
SEL1L	0.057308	6.7346466	2.1924408	0.0294608	0.0746575	-4.307003
KCNQ5	0.5362743	1.7835195	2.1924088	0.0294631	0.0746575	-4.307071
IGHV3-33-2	-0.185022	-1.442119	-2.192399	0.0294639	0.0746575	-4.307092
TOMM20L	-0.398672	1.7374359	-2.192357	0.0294669	0.0746575	-4.307181
METTL15	0.0496241	5.6884081	2.192355	0.0294671	0.0746575	-4.307185
RP3-523K23	0.7555205	0.6038651	2.1922483	0.0294749	0.0746721	-4.307411
RP11-211G2	0.5725395	-0.559563	2.192145	0.0294824	0.0746826	-4.307629
RP1-81D8.3	-0.535958	-0.219744	-2.192136	0.0294831	0.0746826	-4.307648
URGCP-MRPS	0.4204933	-0.495143	2.1920334	0.0294906	0.0746965	-4.307866
PSMG3	-0.060142	6.0715621	-2.191932	0.029498	0.0747098	-4.30808
RP11-145M4	0.1707915	-1.46759	2.1919055	0.0295	0.0747098	-4.308136
AC005562.1	-0.117166	4.2438626	-2.191551	0.029526	0.0747705	-4.308887
HMGN2P39	-0.177662	-1.467633	-2.191469	0.029532	0.0747805	-4.309061
PLCB4	0.2897335	4.2772813	2.1912478	0.0295482	0.0748164	-4.309528
RARA-AS1	0.1053523	4.9725929	2.1910867	0.0295601	0.0748412	-4.309869
RP11-147L1	-0.422292	1.6578445	-2.190994	0.0295669	0.0748532	-4.310066
PRKCA	-0.059115	6.2340927	-2.190926	0.0295719	0.074856	-4.31021
FAM131B	0.2502357	3.33425	2.1909232	0.0295721	0.074856	-4.310215
RP11-465K1	0.31355	-1.139675	2.1908604	0.0295767	0.0748625	-4.310348
RP11-536C1	-0.214476	-1.419675	-2.190743	0.0295853	0.0748791	-4.310596
KCNG2	0.4854136	1.7613265	2.1906297	0.0295936	0.074895	-4.310836
PRDM10	-0.053845	5.5244004	-2.190563	0.0295985	0.0749022	-4.310977
MOS	0.2446628	-1.48499	2.1902694	0.0296201	0.0749516	-4.311598
RP11-353K1	-0.246843	3.2850439	-2.189962	0.0296427	0.0750036	-4.312248
AC008753.4	-0.22448	-1.444674	-2.189758	0.0296577	0.0750354	-4.312679
RP11-686D2	0.4479239	-0.469801	2.1897357	0.0296594	0.0750354	-4.312727
LL22NC03-2	-0.353823	-1.223085	-2.189662	0.0296648	0.075044	-4.312883
RP11-550H2	0.1662251	-1.474676	2.1895313	0.0296744	0.0750631	-4.313159
CYP21A2	0.2836	5.2106567	2.1892056	0.0296984	0.0751186	-4.313848

RP11-85G21	-0.362813	-1.14495	-2.189012	0.0297127	0.0751495	-4.314257
TNFRSF10C	0.2004395	4.6251233	2.1889613	0.0297165	0.0751496	-4.314365
KLK10	0.6697137	1.1360161	2.188938	0.0297182	0.0751496	-4.314414
C12orf79	-0.408966	3.1428975	-2.188925	0.0297191	0.0751496	-4.314441
HSPE1P5	-0.351193	-0.958018	-2.188897	0.0297212	0.0751496	-4.314501
AC114730.3	-0.571022	3.2564361	-2.188872	0.0297231	0.0751496	-4.314554
LINC01176	-0.148892	4.1525659	-2.188844	0.0297251	0.0751497	-4.314614
OR2AF1P	-0.222056	-1.372148	-2.188804	0.0297281	0.075152	-4.314698
SPATA6	0.1643574	4.8134618	2.1887308	0.0297335	0.0751603	-4.314852
CTD-219904	-0.197127	-1.392372	-2.188121	0.0297785	0.0752684	-4.316141
LCMT1-AS1	0.4541442	1.3567258	2.1880959	0.0297804	0.0752684	-4.316194
RNF114	-0.03461	6.4816936	-2.18806	0.029783	0.07527	-4.316271
CDC26	-0.062827	5.3962409	-2.187974	0.0297894	0.0752807	-4.316452
OPA1	0.0415685	6.3958364	2.1879421	0.0297917	0.0752807	-4.316519
ZNF274	-0.053419	6.0077701	-2.187918	0.0297935	0.0752807	-4.316569
ZADH2	0.0599031	6.0656081	2.1877057	0.0298092	0.0753153	-4.317018
RP1-228P16	-0.150226	-1.481087	-2.187566	0.0298195	0.0753362	-4.317314
MAP3K15	-0.581421	2.153939	-2.187528	0.0298224	0.0753381	-4.317395
NAP1L6	-0.733543	0.2180049	-2.187451	0.0298281	0.0753473	-4.317557
PCLO	-0.789015	2.4903237	-2.187388	0.0298327	0.0753538	-4.31769
ITGB3BP	-0.079491	5.5516815	-2.187354	0.0298352	0.0753549	-4.317761
FZD2	0.2281579	4.3007321	2.1870626	0.0298568	0.0753957	-4.318377
KIF21A	-0.058641	6.1881882	-2.187052	0.0298576	0.0753957	-4.318399
PIGF	-0.047769	5.6290413	-2.187052	0.0298576	0.0753957	-4.318399
DDX24	0.0385829	6.5963798	2.186873	0.0298708	0.075424	-4.318778
KCNV2	-0.436034	-0.571519	-2.186791	0.0298769	0.0754342	-4.318951
AMPD3	0.1269771	5.163644	2.1864353	0.0299033	0.0754955	-4.319702
RP11-420A2	-0.181969	4.28008	-2.186297	0.0299136	0.0755162	-4.319994
PLEKHA2	0.0789614	5.8113576	2.1858632	0.0299457	0.0755922	-4.32091
RASSF3	-0.073661	6.1419723	-2.185423	0.0299784	0.0756695	-4.321839
THRAP3P1	-0.217179	-1.404444	-2.185392	0.0299807	0.0756701	-4.321904
RNU6-564P	0.4225283	-0.685103	2.1852839	0.0299888	0.0756841	-4.322133
KLRC4-KLRK	0.5411589	-0.068324	2.1852619	0.0299904	0.0756841	-4.322179
SHCBP1L	-0.210029	-1.414903	-2.185151	0.0299987	0.0756997	-4.322414
C20orf195	0.55189	0.5781791	2.1850293	0.0300077	0.0757173	-4.32267
RP11-747H7	0.2502842	-1.360245	2.1849358	0.0300147	0.0757296	-4.322868
TSPYL1	0.0590089	6.4009583	2.1847651	0.0300274	0.0757564	-4.323228
AHNAK	0.050921	7.0533764	2.1845911	0.0300403	0.0757838	-4.323595
ADH5P2	-0.179199	-1.423069	-2.184516	0.0300459	0.0757928	-4.323754
AQPEP	-0.58623	1.2996692	-2.184457	0.0300503	0.0757986	-4.323878
RP5-1070G2	0.2215645	-1.360177	2.1843333	0.0300595	0.0758166	-4.324139
RNY3P16	0.4998642	1.0123515	2.1842969	0.0300622	0.0758182	-4.324216
CTC-332L22	-0.299582	2.8283555	-2.184154	0.0300729	0.0758398	-4.324517
DIAPH1	0.0544786	6.8148926	2.1837874	0.0301002	0.0759035	-4.325291
KRTAP5-6	-0.588216	2.7695859	-2.183615	0.0301131	0.0759307	-4.325655
RP11-227D1	-0.422058	-0.615263	-2.183516	0.0301205	0.075944	-4.325863
KLHL5	0.0658219	6.3677599	2.1833175	0.0301353	0.0759761	-4.326282
SLC43A2	0.0853711	5.9990042	2.1832219	0.0301424	0.0759883	-4.326483
SGSM2	-0.062835	6.1071051	-2.183197	0.0301443	0.0759883	-4.326536
SMPD2	-0.060856	5.5859589	-2.183055	0.0301549	0.0760069	-4.326835
ABCB10	-0.064963	6.1496561	-2.183043	0.0301558	0.0760069	-4.326861

FOXK2	-0.045068	6.2500211	-2.182692	0.030182	0.0760676	-4.3276
C19orf81	-0.730288	0.7942539	-2.18201	0.0302331	0.0761905	-4.329039
AP001425.1	-0.246402	-1.362156	-2.181985	0.0302349	0.0761905	-4.329091
PARD3B	-0.107861	5.7857367	-2.181789	0.0302496	0.0762222	-4.329504
LINC00052	0.1987112	-1.470105	2.1817157	0.0302551	0.0762308	-4.329659
PRKRIRP7	0.4962771	1.059849	2.1816231	0.030262	0.076243	-4.329854
MTPAP	-0.035935	5.9799069	-2.181552	0.0302673	0.0762511	-4.330003
TEDDM1	-0.407784	-0.854848	-2.181522	0.0302696	0.0762516	-4.330067
LINC01362	-0.270737	-1.299461	-2.181137	0.0302985	0.076319	-4.330878
AP1AR	0.0575089	5.9015479	2.1808236	0.030322	0.0763707	-4.331538
RP11-587P2	-0.207362	-1.404679	-2.180808	0.0303232	0.0763707	-4.331571
AC112721.1	0.3531891	-1.076996	2.1806869	0.0303322	0.0763883	-4.331826
RP11-181K3	-0.537787	0.9108965	-2.18048	0.0303478	0.0764221	-4.332261
LINC01208	-0.531223	-0.420382	-2.180415	0.0303526	0.0764291	-4.332398
PMM1	-0.066998	6.3470937	-2.180357	0.030357	0.0764348	-4.33252
CTD-3131K8	-0.488579	1.0454292	-2.180247	0.0303653	0.0764503	-4.332752
LINC00545	0.2584002	-1.317553	2.1800234	0.0303821	0.076483	-4.333224
RPS4XP20	-0.266595	-1.283432	-2.180019	0.0303824	0.076483	-4.333233
RP11-170N1	0.4426202	2.3926272	2.1799193	0.0303899	0.0764966	-4.333443
AC004980.1	-0.485799	-0.085507	-2.179887	0.0303923	0.0764974	-4.33351
IGLV1-40	0.5899228	4.3426285	2.1797161	0.0304052	0.0765206	-4.333871
MIR548D2	0.2814413	-1.230467	2.1797088	0.0304058	0.0765206	-4.333886
MKX-AS1	0.1427269	-1.514174	2.1796607	0.0304094	0.0765245	-4.333987
MAPK8IP2	-0.250813	5.2992674	-2.179569	0.0304163	0.0765341	-4.334181
RP11-296O1	-0.295641	-1.161492	-2.179554	0.0304174	0.0765341	-4.334212
CPNE6	-0.640308	0.869909	-2.179407	0.0304285	0.0765568	-4.334522
CHST2	0.1059835	5.2673884	2.1793461	0.0304331	0.0765619	-4.33465
RTP2	0.2647381	-1.305893	2.179324	0.0304347	0.0765619	-4.334696
DUSP6	0.0746868	6.4311106	2.1792925	0.0304371	0.0765625	-4.334763
ASMTL	0.0725881	6.2441477	2.1791376	0.0304488	0.0765866	-4.335089
NRROS	0.1122257	5.2161672	2.1790476	0.0304555	0.0765984	-4.335278
RP11-251G2	0.1034574	4.3901916	2.1788927	0.0304672	0.0766225	-4.335604
AC093495.4	-0.159644	4.0234619	-2.178782	0.0304755	0.0766381	-4.335837
DDC	-0.389749	5.8712358	-2.178669	0.0304841	0.0766478	-4.336074
RNU6-339P	0.4217987	-0.65638	2.1786588	0.0304849	0.0766478	-4.336097
AL161645.1	0.4224083	-0.85917	2.1786476	0.0304857	0.0766478	-4.33612
RP11-545A1	-0.610142	0.1849237	-2.178601	0.0304892	0.0766514	-4.336218
DLGAP2	0.6362256	0.6363531	2.1784239	0.0305026	0.0766797	-4.336591
AC026188.1	-0.233565	-1.339594	-2.178316	0.0305107	0.0766948	-4.336817
DLG5	-0.100972	5.8276279	-2.178172	0.0305216	0.0767169	-4.337121
RP11-526I2	-0.398716	-0.836813	-2.178123	0.0305253	0.0767182	-4.337225
RGS8	0.4271135	-0.696571	2.1781094	0.0305263	0.0767182	-4.337253
DIRAS2	0.6757469	2.5462196	2.1779071	0.0305416	0.0767513	-4.337678
RP11-467H1	-0.277016	2.8544772	-2.177858	0.0305453	0.076755	-4.337782
MLST8	0.0483809	6.2636269	2.1778321	0.0305472	0.076755	-4.337836
RP11-22L13	-0.447415	-0.701205	-2.177683	0.0305585	0.0767681	-4.338149
SLC25A23	0.0665797	6.3813841	2.177669	0.0305596	0.0767681	-4.338179
CERKL	0.1160815	5.219957	2.1776668	0.0305597	0.0767681	-4.338184
CTD-2616J1	-0.448436	1.793117	-2.177637	0.030562	0.0767681	-4.338247
GGPS1	-0.043439	6.1035424	-2.177605	0.0305644	0.0767681	-4.338314
LINC01411	0.3538437	-1.13029	2.177596	0.0305651	0.0767681	-4.338333

BAHD1	-0.046955	6.1268166	-2.177421	0.0305783	0.0767961	-4.338701
RP11-360L9	-0.189043	-1.418242	-2.177338	0.0305846	0.0768056	-4.338876
PODXL2	-0.370881	4.7327815	-2.177315	0.0305863	0.0768056	-4.338924
POP7	-0.047743	6.1700124	-2.176471	0.0306502	0.0769607	-4.340698
IGLJ3	-0.296126	-1.262898	-2.176347	0.0306596	0.0769742	-4.340959
RP11-301L8	0.5539851	2.6809918	2.1763447	0.0306598	0.0769742	-4.340964
RP11-696F1	0.313998	-1.219351	2.1763019	0.030663	0.076977	-4.341054
CLIC1	-0.056125	6.8261524	-2.176262	0.0306661	0.0769794	-4.341139
RNF215	0.0533107	5.8463717	2.1762246	0.0306689	0.0769812	-4.341217
SYNJ2BP	0.0513457	6.2246056	2.1760702	0.0306806	0.0770041	-4.341541
TAL2	-0.580295	1.5181213	-2.176048	0.0306822	0.0770041	-4.341587
CHMP1B	0.041694	6.3093638	2.1758383	0.0306982	0.0770373	-4.342029
RBBP8P1	-0.255262	-1.307475	-2.175805	0.0307007	0.0770373	-4.342098
TTC17	-0.042215	6.4785056	-2.17579	0.0307018	0.0770373	-4.34213
OR51B5	-0.256203	-1.319592	-2.175744	0.0307053	0.0770409	-4.342227
RP11-565A3	-0.629773	-0.39927	-2.175692	0.0307093	0.0770455	-4.342336
FLT3	0.4037784	3.290585	2.1754283	0.0307293	0.0770852	-4.342891
SNTN	-0.273966	-1.329008	-2.175428	0.0307293	0.0770852	-4.342891
GLTSCR1L	0.0568917	5.8270944	2.1750156	0.0307606	0.0771562	-4.343758
NAP1L1P1	-0.227046	3.0469502	-2.174982	0.0307632	0.0771562	-4.343828
INMT-FAM18	0.4080186	-0.82883	2.1749718	0.030764	0.0771562	-4.34385
C5AR2	0.182663	5.3169384	2.1749181	0.0307681	0.0771611	-4.343963
UGCG	0.061761	6.3274002	2.174554	0.0307957	0.0772253	-4.344728
AGPS	-0.043557	6.3438111	-2.174498	0.0308	0.0772307	-4.344846
RP5-1154L1	-0.725601	3.0228098	-2.17444	0.0308045	0.0772365	-4.344968
AC011290.5	-0.370499	2.5801447	-2.17427	0.0308174	0.0772636	-4.345325
RFC2	-0.051511	6.1379042	-2.174231	0.0308204	0.0772657	-4.345407
AC092614.2	-0.403783	2.1376906	-2.174142	0.0308271	0.0772774	-4.345594
RP11-142C4	0.4613909	-0.215884	2.1735746	0.0308703	0.0773804	-4.346785
CDC27	-0.031094	6.3156707	-2.173546	0.0308725	0.0773805	-4.346844
AP002381.2	-0.395323	-0.587084	-2.173461	0.030879	0.0773914	-4.347023
TXNL1	0.0578884	6.4858356	2.1734208	0.0308821	0.0773938	-4.347108
C1D	0.0462995	5.8262803	2.1732029	0.0308987	0.0774302	-4.347565
FARP1-AS1	-0.425215	-0.663411	-2.173057	0.0309098	0.0774486	-4.347871
RP11-631M2	-0.35305	-0.926386	-2.173051	0.0309103	0.0774486	-4.347885
RP11-108L7	-0.476682	0.7137427	-2.172848	0.0309258	0.0774795	-4.34831
GALNTL6	0.7284451	0.4184722	2.1728254	0.0309275	0.0774795	-4.348358
EID2B	-0.148181	4.2531145	-2.172806	0.030929	0.0774795	-4.348398
RP11-410L1	-0.20319	3.8523436	-2.172595	0.0309451	0.0775146	-4.348842
SLC15A1	-0.414363	5.6324005	-2.172403	0.0309598	0.0775425	-4.349244
INO80	0.0369429	6.0909433	2.1723932	0.0309605	0.0775425	-4.349265
RP11-53I6.	-0.322083	-1.018854	-2.17228	0.0309692	0.0775589	-4.349502
GALE	-0.064094	6.4744887	-2.172132	0.0309805	0.077582	-4.349814
BEND3P1	-0.509179	3.3542414	-2.171799	0.0310059	0.0776403	-4.350512
OR5H8	-0.37681	-1.136767	-2.171673	0.0310156	0.0776592	-4.350776
RP11-643G5	0.2326209	-1.413317	2.1715365	0.031026	0.07768	-4.351063
PCDHB14	0.2159895	4.7702412	2.1714551	0.0310323	0.0776903	-4.351234
C10orf25	-0.100983	5.0589951	-2.171295	0.0310445	0.0777156	-4.351569
TRGV10	0.5141353	1.7091794	2.1712462	0.0310483	0.0777197	-4.351672
RP11-462B1	-0.289969	-1.167191	-2.171167	0.0310543	0.0777295	-4.351837
GAS2L1P2	-0.58074	-0.154328	-2.17111	0.0310587	0.0777351	-4.351957

SIDT1	0.1986422	4.4065541	2.1708665	0.0310774	0.0777765	-4.352468
FSCN2	-0.516199	1.6751578	-2.170698	0.0310903	0.0778036	-4.352822
CCDC180	-0.321289	3.4684118	-2.17064	0.0310948	0.0778094	-4.352944
RP11-662M2	-0.258302	-1.384278	-2.170454	0.031109	0.0778397	-4.353334
ASS1P1	0.2703084	3.8656839	2.1703772	0.0311149	0.0778472	-4.353494
CTD-3195I5	-0.389667	2.1277833	-2.170335	0.0311181	0.0778472	-4.353583
RP11-21A7A	-0.363443	-0.907887	-2.170331	0.0311184	0.0778472	-4.35359
RP11-16E18	-0.115602	4.445139	-2.170253	0.0311244	0.077857	-4.353755
ZNF890P	-0.468142	-0.26873	-2.170215	0.0311274	0.0778589	-4.353834
C3orf17	-0.030485	6.0930843	-2.170078	0.0311379	0.0778742	-4.354121
G0S2	0.1703556	6.1298996	2.1700656	0.0311388	0.0778742	-4.354148
RP11-798M1	0.1826401	3.7582849	2.170029	0.0311417	0.0778742	-4.354225
RP11-283G6	-0.205286	4.1903242	-2.170024	0.031142	0.0778742	-4.354235
FLNC	0.2757307	5.3363623	2.1699416	0.0311484	0.0778848	-4.354408
AC021451.1	0.245018	-1.350821	2.1698928	0.0311521	0.0778873	-4.35451
RPS24P7	-0.422221	-0.57665	-2.169873	0.0311536	0.0778873	-4.354552
SYNGR4	-0.523863	1.6487013	-2.169814	0.0311581	0.0778932	-4.354674
FAM178B	-0.739588	1.2910491	-2.169746	0.0311634	0.077901	-4.354818
DUS3L	-0.049507	6.0442833	-2.169655	0.0311704	0.0779132	-4.35501
HELZ	-0.045538	6.2366485	-2.169498	0.0311824	0.0779379	-4.355337
NPM1	-0.045638	7.0071485	-2.169186	0.0312065	0.0779902	-4.355992
LINC01521	-0.230438	3.5580116	-2.169166	0.031208	0.0779902	-4.356033
RP11-707O2	0.5198569	0.1291682	2.1691431	0.0312098	0.0779902	-4.356082
RP11-64D24	0.2636274	-1.250847	2.1690825	0.0312144	0.0779945	-4.356209
TRAF6P1	-0.230935	-1.34473	-2.169065	0.0312157	0.0779945	-4.356244
VPS36	0.0562741	6.1643974	2.1687307	0.0312415	0.0780535	-4.356946
RP5-875O13	0.6341796	0.5596328	2.1683907	0.0312677	0.0781136	-4.357658
RP11-25D10	-0.244943	-1.408155	-2.168153	0.0312861	0.0781541	-4.358157
RP11-500G2	0.2048201	3.3200158	2.1681245	0.0312882	0.0781542	-4.358216
AC134882.3	0.0934795	-1.562035	2.1676282	0.0313265	0.0782445	-4.359256
CTD-2653D5	0.4389263	-0.641946	2.1672337	0.031357	0.0783153	-4.360082
RBM20	0.3894563	3.3436135	2.167187	0.0313606	0.0783189	-4.36018
GTF2IP3	-0.174079	-1.46939	-2.166945	0.0313793	0.0783603	-4.360687
RP11-770E5	0.2083847	-1.425668	2.1668529	0.0313864	0.0783727	-4.360879
AC018892.9	-0.467953	-0.424444	-2.166769	0.0313929	0.0783835	-4.361054
ZBED5	-0.046311	5.9239668	-2.166591	0.0314067	0.0784126	-4.361428
SUCLG1	0.0506727	6.6742049	2.1665131	0.0314127	0.0784223	-4.361591
GDF9	-0.224939	3.5829933	-2.166431	0.0314191	0.0784327	-4.361762
FBXL13	-0.330618	2.6091478	-2.165895	0.0314606	0.0785306	-4.362884
RP1-55C23.	-0.455465	-0.582762	-2.165848	0.0314642	0.0785306	-4.362983
RP11-445N2	-0.427809	-0.538658	-2.165842	0.0314647	0.0785306	-4.362996
KDM5D	0.9846713	3.4979436	2.1657662	0.0314706	0.0785398	-4.363154
CCDC181	-0.251652	3.0873787	-2.165594	0.0314839	0.0785678	-4.363514
BAZ2A	-0.03553	6.4386964	-2.165419	0.0314975	0.0785963	-4.363881
CTA-392E5.	0.2252617	-1.425708	2.1653777	0.0315007	0.0785989	-4.363967
GAPDHP65	-0.32241	3.3353117	-2.165316	0.0315055	0.0786055	-4.364096
RTN3P1	-0.479292	0.0576218	-2.165145	0.0315187	0.0786331	-4.364453
CERS6-AS1	-0.47663	0.0500322	-2.164742	0.03155	0.0787058	-4.365296
AC130469.2	0.5315301	0.4851516	2.1645618	0.0315641	0.0787354	-4.365674
NCK1-AS1	-0.074689	4.8007088	-2.164167	0.0315947	0.0788065	-4.366498
RPL23P10	-0.230175	-1.356634	-2.163893	0.031616	0.0788543	-4.367072

WDR4	-0.071725	5.6235182	-2.163857	0.0316189	0.0788561	-4.367148
C1orf54	0.0771077	5.3465568	2.1636105	0.0316381	0.0788984	-4.367663
ACSL3	-0.057361	6.5795987	-2.163401	0.0316544	0.0789338	-4.368102
ACAT2	-0.090286	6.4041193	-2.163138	0.0316749	0.0789795	-4.368651
RPS20	-0.053748	7.2227108	-2.16287	0.0316958	0.0790263	-4.369212
CLSTN3	-0.052474	6.5296889	-2.162761	0.0317043	0.079042	-4.369439
HOXB-AS2	0.441364	-0.543312	2.1626625	0.0317119	0.0790557	-4.369645
SIRT2	0.0483663	6.4021568	2.1623379	0.0317373	0.0791135	-4.370323
RP11-449G1	-0.460751	0.8622349	-2.162284	0.0317415	0.0791158	-4.370436
VWFP1	0.5948097	0.5620937	2.1622705	0.0317425	0.0791158	-4.370464
B3GNTL1	-0.086448	5.3739654	-2.162197	0.0317483	0.0791247	-4.370618
AF186192.6	-0.520278	-0.241929	-2.16208	0.0317574	0.0791421	-4.370862
KIF3B	0.0433942	6.450456	2.1619942	0.0317641	0.0791534	-4.371041
CTD-2589M5	0.2394806	-1.353513	2.1619297	0.0317692	0.0791605	-4.371176
IQSEC1	-0.054463	6.5969192	-2.161901	0.0317714	0.0791607	-4.371236
FAM136BP	-0.460552	-0.550786	-2.161787	0.0317803	0.0791775	-4.371474
MME-AS1	-0.211038	-1.429361	-2.161629	0.0317926	0.0792028	-4.371803
TMEM191A	-0.165858	4.8100223	-2.161481	0.0318042	0.0792263	-4.372113
C15orf43	0.6485413	0.5605863	2.1613598	0.0318137	0.0792445	-4.372366
RP11-839G9	0.2298395	-1.360892	2.1613042	0.0318181	0.0792473	-4.372482
RP5-1106E3	-0.269414	-1.260145	-2.16129	0.0318192	0.0792473	-4.372512
RP11-35N6.	0.4335339	4.2046714	2.1611409	0.0318309	0.079271	-4.372823
SLC25A6	0.0484592	6.9292498	2.1609996	0.0318419	0.0792931	-4.373118
TMEM60	-0.04941	5.8217779	-2.160798	0.0318577	0.0793271	-4.37354
MTM1	0.0814415	5.8383862	2.1607446	0.0318619	0.079332	-4.373651
B3GAT2	-0.326553	3.2093265	-2.159904	0.0319278	0.0794908	-4.375407
RP11-304L1	-0.43049	-0.701579	-2.159855	0.0319316	0.0794942	-4.375508
AF254982.3	-0.197296	-1.458005	-2.159831	0.0319336	0.0794942	-4.375559
N4BP1	0.0357244	6.1192642	2.1597763	0.0319378	0.0794994	-4.375673
C9orf69	-0.050274	6.2646082	-2.159671	0.0319461	0.0795145	-4.375892
AUH	0.069258	5.8912075	2.1591599	0.0319863	0.0796091	-4.376959
AC015922.7	0.1649319	-1.480094	2.1590198	0.0319973	0.0796311	-4.377251
CDK19	-0.065379	5.8955941	-2.158951	0.0320027	0.0796392	-4.377396
VN1R5	-0.306566	-1.176258	-2.158868	0.0320092	0.0796499	-4.377568
RP11-253M7	-0.465783	-0.024252	-2.158807	0.032014	0.0796564	-4.377695
FAM159B	-0.426425	-0.791687	-2.158555	0.0320339	0.0797004	-4.378222
MYLK-AS2	-0.25559	-1.262345	-2.15851	0.0320374	0.0797037	-4.378314
RP11-667K1	0.5147427	-0.061693	2.1583075	0.0320533	0.0797321	-4.378737
CTD-2517O1	-0.210883	3.8932491	-2.158284	0.0320552	0.0797321	-4.378787
RP11-510D2	-0.199312	-1.457016	-2.158282	0.0320553	0.0797321	-4.378791
GCNT2	0.1332211	5.7215098	2.1579	0.0320854	0.0798015	-4.379587
ZNF778	0.067591	5.2444446	2.1575698	0.0321115	0.0798573	-4.380276
RP11-403A3	0.5488903	-0.563719	2.1575599	0.0321122	0.0798573	-4.380296
SLC35F2	0.2160102	4.5365383	2.1575059	0.0321165	0.0798625	-4.380409
MTATP6P2	0.5467466	1.0678319	2.157276	0.0321346	0.0799021	-4.380888
C9orf139	0.2378599	3.2773489	2.1571919	0.0321413	0.0799132	-4.381064
VDAC1P2	-0.427107	1.4627138	-2.156594	0.0321885	0.0800252	-4.38231
RP4-814D15	-0.466315	0.6151023	-2.156486	0.0321971	0.080041	-4.382535
XXbac-B444	-0.479584	1.536385	-2.155978	0.0322372	0.0801353	-4.383593
CTC-448F2.	-0.524777	0.5060277	-2.155693	0.0322598	0.0801861	-4.384188
RP1-91J24.	-0.224552	-1.339434	-2.155546	0.0322714	0.0802095	-4.384494

SNORA59B	0.5904483	1.4750882	2.1554519	0.0322789	0.0802225	-4.38469
RP11-879F1	0.3915225	-0.711287	2.1554238	0.0322811	0.0802226	-4.384748
ZNF253	-0.095689	5.3545099	-2.155262	0.032294	0.0802491	-4.385086
KIF25-AS1	-0.628631	-0.0475	-2.155101	0.0323067	0.0802747	-4.38542
RP11-3D4.3	-0.301768	3.0644139	-2.155076	0.0323087	0.0802747	-4.385473
FUT4	0.1510126	5.3816897	2.1545635	0.0323493	0.0803703	-4.38654
KTN1	0.0411768	6.7503627	2.1542583	0.0323736	0.080425	-4.387176
RP11-391L3	0.2459491	-1.293909	2.1542035	0.0323779	0.0804303	-4.38729
AC027612.6	-0.288889	4.0909012	-2.154153	0.0323819	0.0804314	-4.387394
TMEM56-RWD	0.19005	4.2540675	2.1541428	0.0323828	0.0804314	-4.387416
FBXW2	-0.04651	6.4292288	-2.153985	0.0323953	0.080457	-4.387744
RP11-631N1	-0.231702	3.5910809	-2.153947	0.0323983	0.0804591	-4.387824
DENND4C	0.0742369	5.9340457	2.1538201	0.0324084	0.0804786	-4.388088
IZUMO2	-0.271996	-1.333753	-2.153754	0.0324136	0.0804862	-4.388225
AC016582.2	-0.499687	-0.51533	-2.153679	0.0324196	0.0804955	-4.388381
YWHAEP1	-0.571145	-0.069654	-2.153647	0.0324221	0.0804963	-4.388448
PRSS53	0.1941275	4.2467553	2.153616	0.0324246	0.080497	-4.388513
PAPPA2	0.6331639	3.8372686	2.1535721	0.0324281	0.0805002	-4.388604
HMGB1P14	-0.443302	-0.186261	-2.153539	0.0324308	0.0805006	-4.388674
CCNO	0.6583206	3.387361	2.1535151	0.0324327	0.0805006	-4.388723
NEAT1	0.0703462	6.9001548	2.1532868	0.0324508	0.0805402	-4.389198
RP11-417N1	0.3229812	-1.03074	2.1530561	0.0324692	0.0805803	-4.389678
NRD1	0.036323	6.693318	2.1529311	0.0324791	0.0805995	-4.389938
KB-1507C5.	0.5257302	0.8540975	2.1527907	0.0324903	0.0806218	-4.39023
ZNF480	-0.059166	5.7730141	-2.152652	0.0325014	0.0806437	-4.390518
RP11-366L5	-0.302072	2.9299628	-2.152593	0.0325061	0.0806499	-4.390641
SLC8A2	-0.561079	1.2573557	-2.152434	0.0325188	0.0806741	-4.390973
WDR38	-0.440035	-0.558768	-2.152415	0.0325202	0.0806741	-4.391011
RP11-431K2	0.6105284	0.9195796	2.1523605	0.0325246	0.0806753	-4.391125
RP11-115N1	-0.344823	-0.984011	-2.152354	0.0325252	0.0806753	-4.39114
RP11-1060J	-0.471656	-0.583637	-2.152036	0.0325505	0.0807328	-4.391801
TSTD2	0.1413145	5.2434899	2.1519881	0.0325543	0.0807367	-4.3919
GS1-358P8.	0.090591	5.6561982	2.1517722	0.0325716	0.080772	-4.392349
GH2	-0.363133	-1.173225	-2.151754	0.032573	0.080772	-4.392386
SERPINA4	-0.326306	6.4927519	-2.151017	0.0326319	0.0809126	-4.39392
VSIG8	0.5127068	-0.269542	2.1508693	0.0326437	0.0809364	-4.394226
TMEM132B	-0.47077	2.3830987	-2.150802	0.0326491	0.0809443	-4.394367
AC099344.3	0.1440613	-1.485971	2.1502592	0.0326926	0.0810465	-4.395495
RP11-297D2	-0.210746	3.4493938	-2.150163	0.0327003	0.0810601	-4.395695
ARMC4	0.667445	0.7596774	2.1501104	0.0327045	0.081065	-4.395804
CLEC2D	-0.124182	5.185258	-2.150072	0.0327076	0.081066	-4.395885
AC002480.3	0.5498031	1.0319571	2.1500502	0.0327093	0.081066	-4.395929
PHACTR2	0.0765059	5.8649432	2.1498406	0.0327261	0.0810992	-4.396365
AP000647.3	-0.288261	-1.293666	-2.149801	0.0327293	0.0810992	-4.396447
RP1-278D1.	-0.508126	-0.100353	-2.1498	0.0327294	0.0810992	-4.396449
AC017079.4	-0.257083	-1.248866	-2.149769	0.0327318	0.0810997	-4.396513
HNRNPA1P26	-0.343895	-0.882633	-2.149727	0.0327352	0.0811026	-4.3966
AC006369.2	0.5380418	1.1519251	2.1495831	0.0327468	0.0811257	-4.3969
ABI1	0.0339816	6.3139752	2.149361	0.0327646	0.0811644	-4.397361
TXNL4B	0.0569111	5.8542515	2.1490582	0.0327889	0.0812191	-4.39799
HOXB5	0.2052115	4.0157271	2.1488168	0.0328083	0.0812616	-4.398492

ZNF385D-AS	-0.556216	-0.701964	-2.148731	0.0328152	0.0812732	-4.39867
RP3-393E18	-0.460833	0.1064877	-2.14865	0.0328217	0.0812839	-4.398839
RP11-627G1	0.5130914	0.9836873	2.148336	0.0328469	0.0813408	-4.39949
DPPA2	-0.321515	-1.314718	-2.148028	0.0328717	0.0813966	-4.400129
RP11-49501	0.3471348	-1.011699	2.1479341	0.0328793	0.0814098	-4.400325
ANKRD32	-0.074061	5.1888299	-2.147471	0.0329166	0.0814966	-4.401286
PARPG1	-0.276211	3.9165415	-2.147345	0.0329267	0.081514	-4.401548
RP11-1081L	0.465028	1.8165738	2.1473285	0.0329281	0.081514	-4.401582
RP11-1084A	-0.474582	0.3533946	-2.147099	0.0329466	0.0815544	-4.402059
AC005702.3	-0.284189	-1.172232	-2.146784	0.032972	0.0816117	-4.402713
RP11-677M1	0.5274729	0.0518342	2.1462227	0.0330173	0.0817183	-4.403877
ALDH1L1-AS	0.554418	3.5750296	2.1461317	0.0330246	0.0817309	-4.404065
FERMT3	0.0874667	5.9040034	2.1459686	0.0330378	0.0817564	-4.404404
FXR2	0.0443877	6.1949036	2.1459492	0.0330394	0.0817564	-4.404444
ANKRD17	0.0503569	6.4013876	2.1458182	0.03305	0.0817763	-4.404716
RINL	-0.098362	5.6644243	-2.14578	0.0330531	0.0817763	-4.404796
CRADD	0.070312	5.895299	2.1457667	0.0330541	0.0817763	-4.404823
RP11-840I1	0.4434154	-0.154382	2.1453022	0.0330917	0.08186	-4.405786
CUL2	-0.035872	6.1867703	-2.145293	0.0330925	0.08186	-4.405805
RP11-19G24	-0.149022	-1.481677	-2.145092	0.0331087	0.0818947	-4.406222
IGLV3-6	-0.247875	-1.354482	-2.144649	0.0331447	0.081978	-4.407141
FAM168B	-0.038873	6.5776962	-2.144615	0.0331474	0.0819791	-4.40721
RP11-426L1	-0.43225	1.7534783	-2.144404	0.0331645	0.0820159	-4.407648
RP11-481J2	0.4142273	-0.867291	2.1443727	0.033167	0.0820166	-4.407713
U40455.1	-0.452195	0.1248614	-2.144237	0.033178	0.0820338	-4.407994
RP4-575N6.	-0.491184	0.2041211	-2.144213	0.03318	0.0820338	-4.408044
XKR4	-0.581004	-0.036968	-2.144204	0.0331807	0.0820338	-4.408063
ALDOAP1	-0.330754	-0.998177	-2.144146	0.0331854	0.0820399	-4.408183
DDX31	-0.046965	5.7242257	-2.144114	0.033188	0.0820408	-4.40825
IGLV2-5	-0.43757	-0.930437	-2.144068	0.0331917	0.0820413	-4.408344
KIAA1731NL	-0.485344	-0.030139	-2.144056	0.0331928	0.0820413	-4.40837
RBMY1F	0.1508966	-1.532775	2.1439738	0.0331994	0.0820522	-4.40854
EPHX2	0.1216563	6.4779014	2.1435648	0.0332326	0.0821287	-4.409388
TMEM222	0.049693	6.2515943	2.143305	0.0332537	0.0821754	-4.409926
AC003973.1	0.2025311	-1.410646	2.1432517	0.0332581	0.0821805	-4.410036
AC246787.4	-0.452273	-0.789164	-2.143009	0.0332778	0.0822214	-4.41054
RP11-690P1	0.4542202	-0.123119	2.1429926	0.0332791	0.0822214	-4.410573
IGHV30R16-	-0.397249	-0.946295	-2.142925	0.0332847	0.0822245	-4.410714
ZNF346	-0.046515	5.60318	-2.142922	0.0332849	0.0822245	-4.41072
RP11-204K1	-0.25379	-1.276966	-2.142235	0.0333408	0.0823572	-4.412143
RP11-237N1	0.1543171	-1.531032	2.1421549	0.0333473	0.0823676	-4.412309
SEPT3	-0.359245	4.0047286	-2.141835	0.0333734	0.0824264	-4.412971
TMEM161A	-0.05334	6.3774009	-2.141779	0.033378	0.0824322	-4.413087
MAPT-IT1	-0.561053	0.0333417	-2.141733	0.0333817	0.0824359	-4.413182
FER1L6-AS1	-0.210849	-1.402969	-2.141566	0.0333953	0.0824639	-4.413528
HYI	0.0676518	5.8029507	2.1410477	0.0334377	0.0825593	-4.414601
WNT10B	0.4447267	2.7459459	2.1410374	0.0334385	0.0825593	-4.414622
RUVBL2	-0.046119	6.5214378	-2.140976	0.0334435	0.082566	-4.414748
WDR19	-0.051481	5.8489381	-2.140769	0.0334604	0.0826023	-4.415178
WI2-87327B	0.6639501	0.3614655	2.1407154	0.0334648	0.0826075	-4.415289
ARL9	0.5269091	1.3267858	2.1405348	0.0334796	0.0826384	-4.415662

SERPINE2	0.1413097	5.9925675	2.1404368	0.0334876	0.0826526	-4.415865
RP4-647C14	0.3656787	2.7869049	2.1403347	0.0334959	0.0826676	-4.416076
SLC6A18	-0.381729	-1.103516	-2.140263	0.0335018	0.0826765	-4.416226
A1CF	-0.235771	6.6620958	-2.140205	0.0335065	0.0826825	-4.416344
GIN1	0.0499198	5.2265575	2.140154	0.0335107	0.0826873	-4.41645
LINC00488	0.6930726	0.2406126	2.140036	0.0335204	0.0827055	-4.416694
KIAA0226L	0.194985	4.5791865	2.1399166	0.0335302	0.0827214	-4.416941
NR1H5P	0.091776	-1.562904	2.1399021	0.0335313	0.0827214	-4.416971
INO80D	-0.048802	5.8117536	-2.139849	0.0335357	0.0827265	-4.417081
AF196970.3	-0.34479	-0.886885	-2.139764	0.0335427	0.0827381	-4.417257
SNX31	0.5553731	1.19321	2.1393366	0.0335777	0.0828184	-4.418141
RP1-286D6.	-0.217096	3.6351216	-2.139293	0.0335812	0.0828184	-4.418231
MIR3972	0.1425819	-1.537012	2.1392567	0.0335842	0.0828184	-4.418306
RP11-147L1	-0.047292	6.0732115	-2.139256	0.0335843	0.0828184	-4.418309
IRF2BPL	0.0590628	6.0844369	2.1392068	0.0335883	0.0828227	-4.41841
XKR7	-0.322917	-1.228484	-2.139179	0.0335906	0.0828229	-4.418468
FAM183DP	0.322282	-1.128359	2.1389824	0.0336067	0.0828526	-4.418874
GAPDHP21	-0.444568	-0.336661	-2.138976	0.0336072	0.0828526	-4.418887
ING4	-0.050079	6.0283369	-2.138882	0.0336149	0.082866	-4.419081
GAPDHP61	-0.498338	0.3819958	-2.138601	0.033638	0.0829173	-4.419663
FTH1P23	-0.158353	4.1734057	-2.138563	0.0336411	0.0829194	-4.419741
RP11-672A2	0.1867681	-1.441443	2.1384585	0.0336497	0.0829349	-4.419957
RP11-1H8.5	-0.326772	-1.089412	-2.138305	0.0336623	0.0829571	-4.420275
MTND1P15	0.1289334	-1.521204	2.1382939	0.0336632	0.0829571	-4.420298
RPS2P4	-0.48752	0.8154945	-2.137951	0.0336914	0.0830209	-4.421006
TMEM42	-0.06403	5.7803168	-2.137889	0.0336965	0.0830228	-4.421134
RP11-173A8	0.3377683	-0.964252	2.1378862	0.0336967	0.0830228	-4.42114
MARS	-0.039056	6.532257	-2.137689	0.033713	0.0830572	-4.421549
GPC5-AS1	-0.192014	-1.460595	-2.137641	0.0337169	0.0830612	-4.421646
RP11-216B9	-0.140311	3.452136	-2.137597	0.0337205	0.0830646	-4.421738
ISG15	0.106542	6.3114231	2.1375066	0.033728	0.0830773	-4.421925
TUBBP2	-0.415086	-0.626406	-2.137433	0.0337341	0.0830867	-4.422078
RP11-864J1	0.2947503	-1.096349	2.1371802	0.0337548	0.0831323	-4.422599
DSG4	-0.588927	-0.018757	-2.137105	0.033761	0.083141	-4.422754
UBE2L4	-0.411082	-0.530511	-2.137042	0.0337662	0.083141	-4.422885
C11orf31	-0.045136	6.4478653	-2.137032	0.0337671	0.083141	-4.422906
TIPRL	-0.040288	6.3458062	-2.137027	0.0337675	0.083141	-4.422917
AC019129.2	-0.536235	0.3024243	-2.136827	0.0337839	0.0831759	-4.423329
TUBGCP2	-0.038442	6.4240039	-2.136632	0.0338	0.0832099	-4.423732
CDK12	-0.041608	6.2868064	-2.136569	0.0338052	0.0832169	-4.423861
COX6B1P4	0.521162	-0.051801	2.1364161	0.0338178	0.0832424	-4.424178
RP11-1109M	-0.468761	-0.886177	-2.13636	0.0338224	0.0832482	-4.424294
FGF10	0.5201238	-0.463615	2.1360773	0.0338458	0.0832962	-4.424878
C9orf170	0.4015078	-0.799891	2.1360681	0.0338465	0.0832962	-4.424897
AP000442.4	-0.463091	0.1488831	-2.136028	0.0338498	0.0832987	-4.424979
WDR12	-0.042447	6.0763129	-2.135855	0.0338641	0.0833282	-4.425336
CTD-2516F1	0.1589334	4.2685949	2.1355961	0.0338855	0.0833753	-4.425871
RP11-124G5	0.1811015	-1.494618	2.1354807	0.033895	0.0833931	-4.42611
UBAP1	0.0395242	6.4127989	2.1353477	0.033906	0.0834146	-4.426384
LCT	-0.445582	-0.52752	-2.135142	0.033923	0.0834507	-4.426808
RP13-93L13	-0.452015	-0.025399	-2.135086	0.0339277	0.0834566	-4.426925

RP11-278A2	-0.409459	-0.667108	-2.134992	0.0339354	0.0834701	-4.427119
SLC26A7	0.4911061	2.4589508	2.1347831	0.0339527	0.0835069	-4.42755
RP11-477N3	-0.168937	4.0115876	-2.134747	0.0339557	0.0835087	-4.427625
HES1	0.072209	6.2633347	2.1344697	0.0339787	0.0835595	-4.428197
MCHR2	-0.221744	-1.397626	-2.134428	0.0339822	0.0835624	-4.428284
NOXRED1	-0.239636	3.1753235	-2.134232	0.0339984	0.0835967	-4.428688
TTC16	0.4329988	2.346497	2.1339563	0.0340212	0.0836472	-4.429256
LINC00839	0.3554342	3.2213729	2.1339284	0.0340235	0.0836472	-4.429314
TMEM263	0.042792	6.3300085	2.1338476	0.0340302	0.0836581	-4.42948
FITM1	0.2496758	4.1039264	2.1337476	0.0340385	0.0836728	-4.429687
NBPF4	-0.453117	-0.869383	-2.133498	0.0340592	0.0837181	-4.430202
ULK4P3	0.2590401	-1.314492	2.1334613	0.0340623	0.0837199	-4.430277
RP11-1017G	-0.151502	4.3193368	-2.133231	0.0340814	0.0837613	-4.430753
HNRNPA1P3C	-0.337552	-0.941252	-2.133176	0.034086	0.0837669	-4.430866
CTC-428H11	-0.243947	2.7594206	-2.133003	0.0341003	0.0837952	-4.431223
APCS	0.232309	6.9416719	2.1329819	0.0341021	0.0837952	-4.431266
FAM166A	-0.474842	2.0297486	-2.132952	0.0341045	0.0837957	-4.431328
BCRP4	-0.331312	-1.129808	-2.132899	0.0341089	0.0838005	-4.431437
AKR1E2	0.3285408	3.1959172	2.1328678	0.0341115	0.0838005	-4.431501
PNPLA5	0.5187421	-0.496079	2.132819	0.0341156	0.0838005	-4.431602
ZNF425	0.1458841	4.5594779	2.1328177	0.0341157	0.0838005	-4.431605
PRKDC	-0.050872	6.6525855	-2.132587	0.0341349	0.0838407	-4.43208
SNAP25-AS1	-0.451399	3.8795025	-2.132566	0.0341366	0.0838407	-4.432124
PLXNC1	-0.110566	5.8870778	-2.132475	0.0341442	0.0838535	-4.432311
LINC01049	-0.216205	-1.426827	-2.13236	0.0341537	0.0838681	-4.432548
CTA-732E4.	-0.34711	-0.923394	-2.132349	0.0341547	0.0838681	-4.432572
LONRF1	0.0708131	5.5908509	2.1323144	0.0341575	0.0838695	-4.432643
AQP7	-0.231544	5.4834181	-2.132156	0.0341708	0.0838962	-4.43297
XCR1	0.4540297	3.2836999	2.1319353	0.0341891	0.0839356	-4.433424
PGLYRP3	0.3117884	-1.206541	2.1312681	0.0342447	0.0840664	-4.434799
MIR137HG	-0.500054	-0.794075	-2.131206	0.0342499	0.0840735	-4.434928
PLTP	0.1166632	6.1683283	2.130621	0.0342987	0.0841876	-4.436133
SLC37A3	0.0513405	5.9063106	2.1305824	0.0343019	0.0841878	-4.436212
WASH1	-0.083299	5.2783815	-2.130565	0.0343034	0.0841878	-4.436248
ZNF286B	-0.261407	3.4572833	-2.130493	0.0343093	0.0841955	-4.436396
LLNLR-304A	-0.514618	0.3058818	-2.130447	0.0343132	0.0841955	-4.436491
FMR1-AS1	-0.497325	0.5004999	-2.130444	0.0343134	0.0841955	-4.436497
RP11-596C2	-0.42853	1.1379163	-2.130197	0.0343341	0.0842407	-4.437007
RP11-85I17	-0.507683	0.2956159	-2.130147	0.0343382	0.0842415	-4.437109
CTC-457E21	-0.42292	-0.887235	-2.130137	0.0343391	0.0842415	-4.437129
HRASLS	0.5651874	0.299577	2.1300076	0.0343499	0.084262	-4.437396
RP11-114H2	-0.459494	-0.079268	-2.129955	0.0343543	0.084262	-4.437505
CHPF	0.0674246	6.6068087	2.1299544	0.0343544	0.084262	-4.437506
KB-1980E6.	-0.324105	-1.044347	-2.129685	0.0343769	0.0843098	-4.438062
LINC00322	0.4890591	-0.377919	2.1296602	0.034379	0.0843098	-4.438112
CCDC185	-0.662873	0.3517588	-2.129639	0.0343808	0.0843098	-4.438156
RP13-578N3	0.1610073	-1.436566	2.1293944	0.0344012	0.0843542	-4.438659
TLK2P1	-0.358779	2.1323605	-2.129313	0.0344081	0.0843654	-4.438828
RP3-454B23	0.3756909	-0.770971	2.1292801	0.0344108	0.0843664	-4.438894
TRGV7	0.5135122	0.5041296	2.1291968	0.0344177	0.0843778	-4.439066
ZNF180	-0.058759	5.4470723	-2.129125	0.0344237	0.0843868	-4.439213

RP11-748L1	0.2459657	-1.340124	2.1288397	0.0344477	0.0844398	-4.439801
RP11-117L6	-0.302579	-1.237203	-2.128327	0.0344907	0.0845395	-4.440857
ANP32AP1	-0.433055	0.4678433	-2.128183	0.0345027	0.0845634	-4.441153
RP11-220I1	-0.352278	-1.001527	-2.128141	0.0345063	0.0845664	-4.44124
RP11-295D4	-0.307091	3.5321074	-2.127951	0.0345222	0.0845998	-4.44163
AP000705.6	-0.256287	-1.283908	-2.127759	0.0345383	0.0846335	-4.442025
SNAP23	0.0393347	6.1879688	2.1276982	0.0345434	0.0846404	-4.442151
RAB26	0.175853	5.5313776	2.1274321	0.0345658	0.0846895	-4.442698
C2orf70	0.6464268	0.2152662	2.1271435	0.03459	0.0847418	-4.443292
RP11-383G1	-0.284612	-1.147913	-2.127123	0.0345918	0.0847418	-4.443334
DKFZp434P2	0.3836514	-0.752819	2.1270458	0.0345983	0.0847513	-4.443493
EFCAB13	-0.15946	4.3915097	-2.127018	0.0346006	0.0847513	-4.443549
RP11-734E1	-0.318103	-1.01354	-2.126994	0.0346026	0.0847513	-4.443599
C1RL-AS1	-0.10406	5.369154	-2.126809	0.0346182	0.0847807	-4.443979
SEC14L4	0.3122517	5.3850082	2.1267779	0.0346208	0.0847807	-4.444044
NPM1P18	-0.3156	-1.037097	-2.126769	0.0346216	0.0847807	-4.444063
AS3MT	-0.287754	4.6119269	-2.126231	0.0346668	0.0848858	-4.445168
RP11-252I1	-0.483741	-0.686115	-2.12614	0.0346745	0.084899	-4.445355
MLF2	0.0433748	6.8788031	2.125971	0.0346888	0.0849261	-4.445703
LINC01448	-0.479037	-0.776572	-2.125945	0.0346909	0.0849261	-4.445756
METTL1	-0.062439	5.8527514	-2.125919	0.0346931	0.0849261	-4.445809
UBE2SP2	-0.469167	-0.135359	-2.125898	0.0346949	0.0849261	-4.445853
AC102953.6	-0.31574	-1.091901	-2.125851	0.0346989	0.0849302	-4.44595
DCLRE1A	-0.055618	5.7733071	-2.125796	0.0347035	0.0849357	-4.446062
RP11-274B2	-0.343965	3.282498	-2.125748	0.0347076	0.08494	-4.446161
RP11-378J1	-0.129123	4.2270222	-2.125626	0.0347179	0.0849549	-4.446412
PPIAP31	-0.347524	2.431142	-2.125595	0.0347205	0.0849549	-4.446477
UNCX	0.1492979	-1.533589	2.1255931	0.0347206	0.0849549	-4.44648
RPLP1	-0.046167	7.3058045	-2.125449	0.0347328	0.0849789	-4.446776
FLJ42969	0.4603556	-0.698426	2.1252609	0.0347487	0.0850121	-4.447163
ZNF770	0.1276962	5.7455333	2.1249701	0.0347732	0.0850665	-4.44776
AC114808.3	0.1332129	-1.541787	2.1248098	0.0347868	0.0850939	-4.44809
FAM210B	0.065217	6.5564171	2.1247122	0.034795	0.0851084	-4.44829
LINC01226	-0.605843	1.5878754	-2.124366	0.0348243	0.0851744	-4.449003
NDUFA6-AS1	-0.192379	5.1107927	-2.124286	0.0348311	0.0851851	-4.449166
LINC00993	-0.473547	-0.950499	-2.1242	0.0348383	0.0851928	-4.449343
LYPD8	-0.747426	2.1023777	-2.124194	0.0348389	0.0851928	-4.449355
RP11-271K1	0.4767488	0.6618149	2.1241141	0.0348456	0.0852036	-4.449519
C5orf38	0.4980245	-0.675922	2.1239627	0.0348584	0.0852292	-4.44983
FAM212A	0.123663	4.5875431	2.1238534	0.0348677	0.0852434	-4.450055
C12orf75	0.2042152	5.4534221	2.1238388	0.0348689	0.0852434	-4.450085
JARID2-AS1	-0.464309	0.6583646	-2.123774	0.0348744	0.0852511	-4.450218
FPR2	0.5777058	2.0458706	2.1236875	0.0348817	0.0852601	-4.450395
KRT8P40	-0.237261	-1.30935	-2.123662	0.0348839	0.0852601	-4.450449
SLC9A5	0.2257044	3.5082264	2.1236478	0.0348851	0.0852601	-4.450477
MS4A6E	0.4142967	-0.76389	2.1236	0.0348891	0.0852643	-4.450575
SPTY2D1-AS	-0.193266	3.446735	-2.123529	0.0348951	0.0852733	-4.450721
CCT8	-0.034878	6.6897649	-2.123445	0.0349023	0.085285	-4.450893
RP11-530C5	-0.394275	-0.591716	-2.123413	0.034905	0.0852859	-4.450959
RP11-814H1	-0.422157	-0.085147	-2.123385	0.0349073	0.0852859	-4.451016
AK4P2	-0.336683	-1.23411	-2.123286	0.0349157	0.0852976	-4.451219

RP11-103B5	-0.570723	1.3345277	-2.123274	0.0349168	0.0852976	-4.451245
PGBD4P8	0.1408233	-1.537908	2.1230371	0.0349369	0.0853409	-4.451731
SCN4B	0.1990815	4.7766129	2.1228532	0.0349525	0.0853693	-4.452109
NEDD1	-0.051093	5.646836	-2.122845	0.0349531	0.0853693	-4.452125
ZNF177	-0.308759	-1.117273	-2.122761	0.0349603	0.085381	-4.452298
TEX13B	-0.19254	-1.416527	-2.122671	0.0349679	0.0853889	-4.452482
TMEM254	0.0606681	6.1922901	2.1226677	0.0349682	0.0853889	-4.452489
GRAMD3	0.0555898	5.8787622	2.1224507	0.0349866	0.0854253	-4.452935
HSD17B3	-0.283184	4.4037968	-2.122414	0.0349897	0.0854253	-4.45301
ARSF	0.7630195	2.1927529	2.1224097	0.0349901	0.0854253	-4.453019
LINC00539	-0.289598	2.4919239	-2.122253	0.0350034	0.085452	-4.45334
FOXP3	0.1879983	4.9023479	2.1221979	0.0350081	0.0854578	-4.453454
RP11-85A1.	-0.473373	1.1775809	-2.122061	0.0350198	0.0854805	-4.453735
CC2D1A	0.0540104	6.29902	2.1218579	0.035037	0.0855169	-4.454152
RP11-76E17	-0.244575	-1.321615	-2.121655	0.0350542	0.0855477	-4.454567
RP11-1082L	0.3830839	-0.81358	2.1216545	0.0350543	0.0855477	-4.454569
HMGN2P21	-0.27934	-1.189562	-2.121575	0.035061	0.0855584	-4.454731
SUMO2P20	-0.237964	-1.275568	-2.121499	0.0350675	0.0855662	-4.454888
NRSN2	-0.146582	5.7370636	-2.121464	0.0350705	0.0855662	-4.454959
MAP3K14-AS	0.281677	3.5426405	2.1214551	0.0350713	0.0855662	-4.454978
CHST10	-0.174419	5.0091636	-2.121179	0.0350948	0.0856179	-4.455545
SCAMP4	-0.042923	6.3726526	-2.120963	0.0351132	0.085657	-4.455988
DACH1	0.4062557	3.2998947	2.1209167	0.0351171	0.0856608	-4.456083
AKAP5	-0.170333	4.4428316	-2.120873	0.0351208	0.0856642	-4.456172
OTX2-AS1	-0.268956	-1.303132	-2.120834	0.0351242	0.0856666	-4.456253
KANSL1L	0.0700633	5.6561135	2.1207006	0.0351355	0.0856886	-4.456526
BIK	-0.398781	3.8922503	-2.12067	0.0351381	0.0856893	-4.456589
CTD-2382E5	0.5308601	1.0835479	2.1201885	0.0351792	0.0857836	-4.457576
SPCS2	-0.042906	6.331362	-2.120121	0.035185	0.085792	-4.457715
CTD-2015G9	0.6726935	1.3397613	2.1200401	0.0351919	0.0858031	-4.45788
HMGN1P35	-0.353757	-0.851556	-2.119943	0.0352001	0.0858175	-4.45808
SNRNP48	-0.052945	5.8390663	-2.119623	0.0352274	0.0858733	-4.458735
AC068138.1	-0.355174	-1.272384	-2.11962	0.0352277	0.0858733	-4.458742
CTC-512J14	-0.322112	-1.037158	-2.119166	0.0352666	0.0859622	-4.459673
RP11-387H1	-0.485029	2.1568503	-2.11898	0.0352824	0.0859952	-4.460054
SCDP1	-0.568467	0.3680902	-2.118946	0.0352853	0.0859965	-4.460123
KIAA1217	0.061264	6.2331488	2.118768	0.0353006	0.0860279	-4.460488
SRP54-AS1	-0.132247	4.0783047	-2.118601	0.0353148	0.0860525	-4.46083
RP13-20L14	-0.466583	-0.016462	-2.118595	0.0353154	0.0860525	-4.460843
XXbac-BPG2	-0.225586	3.1358111	-2.118352	0.0353362	0.0860962	-4.461341
RP11-152C1	-0.316525	-1.035485	-2.11833	0.035338	0.0860962	-4.461385
DCAF17	-0.052716	5.6448425	-2.118257	0.0353444	0.0861059	-4.461536
LBHD1	-0.085988	5.0036652	-2.118208	0.0353485	0.0861103	-4.461635
RP11-575G1	-0.345043	-0.88644	-2.118136	0.0353547	0.0861196	-4.461783
CFL1	0.0338051	7.1518243	2.117912	0.0353739	0.0861562	-4.462242
SAP30L-AS1	-0.320475	2.3268241	-2.117905	0.0353745	0.0861562	-4.462256
FANCA	-0.088136	5.5709965	-2.117878	0.0353768	0.0861562	-4.462312
ATXN10	-0.039285	6.6126179	-2.11782	0.0353818	0.0861626	-4.462431
GNA11	0.0364165	6.4049123	2.1176077	0.0354	0.0862011	-4.462865
RP5-1119A7	-0.367127	-0.832883	-2.117302	0.0354262	0.0862592	-4.463491
RNU6-418P	-0.487469	0.0536766	-2.117271	0.0354289	0.0862599	-4.463555

LRRC56	-0.157514	4.5000422	-2.117234	0.0354321	0.0862619	-4.463631
XPA	-0.048794	5.7540203	-2.117153	0.035439	0.0862732	-4.463797
ABCC4	0.1545518	5.6818858	2.1171236	0.0354415	0.0862735	-4.463857
MRPL48P1	0.1515185	-1.532458	2.1169186	0.0354591	0.0863052	-4.464276
LECT2	-0.532259	5.6165405	-2.116917	0.0354593	0.0863052	-4.46428
RP11-296A1	0.3288228	-0.953181	2.1166056	0.035486	0.0863646	-4.464917
RP11-478K1	0.5842092	0.4626238	2.1164661	0.035498	0.086388	-4.465203
FAM160A2	-0.046271	6.2246557	-2.116329	0.0355099	0.086411	-4.465484
PMS2P4	-0.110724	4.2941345	-2.116245	0.0355171	0.0864228	-4.465656
PLA2G7	-0.148311	5.4080174	-2.116145	0.0355257	0.086438	-4.465861
SHQ1	-0.043967	5.7503889	-2.115987	0.0355393	0.0864653	-4.466184
METTL4	-0.054264	5.4400802	-2.115798	0.0355555	0.086499	-4.46657
FBXL16	0.3601438	4.1253059	2.1157168	0.0355625	0.0865092	-4.466737
RP11-777B9	-0.197376	-1.43606	-2.115695	0.0355644	0.0865092	-4.466782
DIP2B	-0.039832	6.2916739	-2.115199	0.0356071	0.0866073	-4.467796
RP11-43303	-0.376288	-0.918069	-2.114999	0.0356244	0.0866435	-4.468205
CLDN1	-0.115983	6.9301851	-2.114901	0.0356329	0.0866583	-4.468406
CTD-2561B2	-0.449765	1.3401375	-2.114839	0.0356381	0.0866654	-4.468532
RP11-145A3	0.5926015	1.8541812	2.1143004	0.0356847	0.0867728	-4.469634
RP11-12601	-0.354627	2.5613751	-2.114173	0.0356957	0.0867937	-4.469894
RP11-316K1	0.4142408	-0.496075	2.1140691	0.0357047	0.0868098	-4.470107
IGKV1OR2-1	-0.280918	-1.250399	-2.113911	0.0357183	0.0868373	-4.470431
ALOX15B	0.4541258	3.5056045	2.1137503	0.0357322	0.0868653	-4.470759
CCNA1	0.4784532	-0.47293	2.1136694	0.0357392	0.0868765	-4.470925
PSMD9	0.043892	5.958042	2.1134105	0.0357616	0.0869252	-4.471454
AF127936.5	0.5159172	0.9107155	2.1133646	0.0357656	0.086929	-4.471548
CTC-235G5.	0.2240874	-1.421336	2.1129919	0.0357979	0.0870017	-4.47231
CCAT1	-0.471749	4.3427676	-2.112938	0.0358025	0.0870072	-4.47242
RP11-316M1	-0.305324	2.7090374	-2.112649	0.0358276	0.0870574	-4.473011
AC011893.3	-0.375835	-0.883457	-2.112645	0.0358279	0.0870574	-4.473019
AC004166.6	0.1501391	3.9768549	2.1123877	0.0358502	0.0871057	-4.473544
CELP	-0.331097	-1.042294	-2.112331	0.0358551	0.0871073	-4.47366
RP5-1142A6	0.2648925	3.8949869	2.1123252	0.0358556	0.0871073	-4.473672
RNF214	-0.043331	5.6664286	-2.111925	0.0358904	0.0871858	-4.47449
SNRPGP15	-0.494042	1.288155	-2.111836	0.035898	0.0871987	-4.474671
HID1	-0.121842	5.8306743	-2.111676	0.035912	0.0872213	-4.474999
FBXL4	-0.052402	6.0298506	-2.111652	0.0359141	0.0872213	-4.475047
EPHA8	-0.383018	-0.846737	-2.111647	0.0359145	0.0872213	-4.475058
RPS4XP16	-0.314982	2.7292894	-2.111609	0.0359178	0.0872236	-4.475136
CEBPE	0.4916146	0.2008061	2.1114111	0.035935	0.0872591	-4.47554
WDR3	0.0565911	6.1187711	2.1113852	0.0359372	0.0872591	-4.475592
CRY2	0.0687686	6.4341197	2.1112064	0.0359528	0.0872876	-4.475958
BANP	0.0493365	5.7699562	2.1111952	0.0359538	0.0872876	-4.475981
RP11-231C1	0.2594218	-1.256785	2.1111623	0.0359566	0.0872887	-4.476048
AC019117.2	0.4994261	2.9717161	2.111115	0.0359607	0.0872902	-4.476144
SLC17A2	-0.416419	5.689019	-2.1111	0.035962	0.0872902	-4.476175
MRTO4	-0.049809	6.1875058	-2.110853	0.0359835	0.0873313	-4.476678
ULBP3	0.5642518	1.3954801	2.1108504	0.0359837	0.0873313	-4.476684
CTD-2062A1	0.2900234	-1.125346	2.110701	0.0359967	0.0873571	-4.47699
RP11-304F1	-0.226904	3.7172342	-2.110591	0.0360064	0.0873746	-4.477215
THAP5	0.0494109	5.9858973	2.1104003	0.0360229	0.087409	-4.477604

RP11-267D1	0.4645011	-0.351347	2.110368	0.0360257	0.08741	-4.47767
ABL2	-0.049177	6.0632012	-2.110335	0.0360286	0.0874111	-4.477736
MVD	-0.075885	6.3678488	-2.1101	0.0360491	0.0874551	-4.478217
FAM20C	0.0732159	6.5355014	2.1100428	0.0360541	0.0874613	-4.478333
DTNA	0.2010025	5.7166001	2.1099355	0.0360634	0.0874782	-4.478552
RP11-494H4	-0.434348	-0.515297	-2.109843	0.0360715	0.087492	-4.478742
MYH16	-0.4824	1.4688788	-2.109706	0.0360835	0.0875152	-4.479022
RP11-37016	0.2868975	-1.241963	2.1094591	0.036105	0.0875615	-4.479525
MAGEA2B	-0.241749	-1.335581	-2.109301	0.0361188	0.0875878	-4.479848
RN7SL25P	-0.298983	-1.101635	-2.10928	0.0361206	0.0875878	-4.479891
SRPK2	0.0449812	6.0576229	2.1092009	0.0361275	0.0875987	-4.480051
BOLA2P2	-0.429228	-0.212127	-2.10892	0.036152	0.0876471	-4.480625
FAM117B	-0.093501	5.4927802	-2.108917	0.0361523	0.0876471	-4.48063
FIGF	-0.304729	3.9477312	-2.108845	0.0361586	0.0876566	-4.480778
ZNF705E	-0.491712	0.693303	-2.108814	0.0361613	0.0876573	-4.480841
RNA5SP333	0.2612746	-1.316907	2.1087127	0.0361701	0.087673	-4.481048
ANO6	0.0476268	6.6376679	2.1086582	0.0361749	0.0876787	-4.481159
RPS26P2	-0.284093	-1.152581	-2.108561	0.0361834	0.0876935	-4.481357
RP11-359I1	-0.377864	-0.823528	-2.108506	0.0361882	0.0876939	-4.481468
CDKL1	0.2928695	3.5894501	2.1085037	0.0361884	0.0876939	-4.481474
URAD	-0.333097	-1.147516	-2.108318	0.0362046	0.0877239	-4.481852
CTB-12904.	-0.331659	3.0771663	-2.108307	0.0362056	0.0877239	-4.481874
RP11-705C1	-0.177617	4.1216257	-2.1082	0.0362149	0.0877407	-4.482093
RNU1-36P	0.2154174	-1.390564	2.107851	0.0362455	0.087809	-4.482805
DGAT2L6	-0.362669	-1.181065	-2.10766	0.0362623	0.0878409	-4.483195
LRRCC1	0.1292362	5.3249129	2.1076457	0.0362635	0.0878409	-4.483224
COX8C	-0.410257	-1.018142	-2.107532	0.0362734	0.0878591	-4.483455
PPIAP29	-0.235527	4.3131125	-2.107267	0.0362967	0.0879096	-4.483996
RP11-510J1	-0.516829	1.9168889	-2.107071	0.0363138	0.0879411	-4.484395
BLACAT1	-0.566506	-0.164182	-2.107064	0.0363145	0.0879411	-4.48441
PSMB6	0.0503533	6.5620964	2.1069913	0.0363208	0.0879506	-4.484558
NUGGC	0.3375282	5.1686117	2.1069024	0.0363286	0.0879637	-4.484739
ST13P15	-0.404663	1.9965972	-2.106871	0.0363314	0.0879644	-4.484802
AC104297.1	-0.362666	-0.769383	-2.106511	0.036363	0.0880351	-4.485536
TUBBP5	-0.751585	2.5844543	-2.106415	0.0363714	0.0880496	-4.485732
CNGB1	0.5817375	1.3253025	2.1063527	0.0363769	0.0880571	-4.485859
RP4-657E11	0.3340679	3.1055469	2.1062748	0.0363837	0.0880678	-4.486018
AC013268.3	0.3878292	-0.970866	2.1061816	0.0363919	0.0880818	-4.486208
OLA1	-0.040672	6.4390294	-2.106062	0.0364024	0.0881015	-4.486453
RPS19P3	-0.369311	1.841383	-2.105914	0.0364154	0.088127	-4.486753
C16orf91	0.0615028	5.6680567	2.1055519	0.0364472	0.0881946	-4.487491
RP11-43F13	0.6148751	1.1863108	2.1055153	0.0364505	0.0881946	-4.487565
AC007106.1	-0.249643	-1.384746	-2.105514	0.0364506	0.0881946	-4.487568
RP11-13E5.	-0.313627	-1.211557	-2.105461	0.0364552	0.0881999	-4.487675
RP1-309F20	-0.543776	1.9845493	-2.105409	0.0364598	0.0882052	-4.487782
WIBG	-0.040035	6.198382	-2.105313	0.0364683	0.0882087	-4.487978
RP3-400B16	-0.267353	-1.37526	-2.105311	0.0364685	0.0882087	-4.487982
RP11-616M2	0.2376946	-1.318798	2.1053104	0.0364685	0.0882087	-4.487983
TUBG1P	-0.375564	-0.622469	-2.105039	0.0364924	0.0882596	-4.488536
LY6H	-0.541665	2.8127506	-2.104997	0.0364961	0.0882596	-4.488622
RP11-530N7	0.5369833	0.3714946	2.1049889	0.0364968	0.0882596	-4.488638

RP11-281P2	-0.354275	-1.031484	-2.104815	0.0365121	0.0882909	-4.488993
RP11-304F1	0.1544473	-1.485437	2.1047081	0.0365215	0.0883078	-4.48921
RP11-236P2	-0.268939	-1.218941	-2.104655	0.0365262	0.0883133	-4.489318
GRP	0.5678205	-0.33813	2.1045414	0.0365362	0.0883202	-4.489549
SDCBP2	0.2014199	5.5612406	2.1045411	0.0365363	0.0883202	-4.48955
RP11-685B1	0.5523136	1.3556414	2.1045398	0.0365364	0.0883202	-4.489552
HSPA8P14	-0.371646	-0.779757	-2.104332	0.0365546	0.0883537	-4.489974
AC005330.2	-0.534536	2.2860886	-2.104302	0.0365573	0.0883537	-4.490036
RP11-654G1	-0.222811	-1.423587	-2.1043	0.0365575	0.0883537	-4.49004
TCEA1	-0.049434	6.4214286	-2.103981	0.0365857	0.0884159	-4.49069
AC016751.3	-0.193207	-1.40362	-2.103872	0.0365953	0.0884334	-4.490912
LEMD1-AS1	0.2927109	-1.167038	2.1032108	0.0366537	0.0885603	-4.492257
SLC4A7	0.1150273	5.5006162	2.1032106	0.0366537	0.0885603	-4.492258
EIF2S2P4	-0.323422	2.4571615	-2.103195	0.0366551	0.0885603	-4.49229
AC010969.1	0.676593	1.0453509	2.1030871	0.0366646	0.0885774	-4.492509
LINC01484	-0.332801	4.0880703	-2.102913	0.03668	0.0886088	-4.492864
CCDC60	-0.43208	-0.76082	-2.102622	0.0367057	0.088665	-4.493455
SH2B2	-0.111665	4.95775	-2.102368	0.0367282	0.0887135	-4.493972
RP11-638I2	-0.429574	-0.165274	-2.102275	0.0367365	0.0887227	-4.494161
LINC01296	-0.622085	2.0976398	-2.10227	0.0367369	0.0887227	-4.494171
RP11-410L1	-0.42523	-0.286283	-2.102147	0.0367478	0.0887431	-4.494421
SNTA1	-0.081563	6.0794288	-2.101971	0.0367634	0.0887749	-4.494779
RPS11	-0.045231	7.2524294	-2.101861	0.0367732	0.0887869	-4.495004
RP11-164P1	0.4885472	-0.174024	2.1018603	0.0367732	0.0887869	-4.495005
NPAP1P3	-0.257513	-1.222171	-2.101767	0.0367815	0.088801	-4.495195
SC22CB-109	-0.225143	-1.366054	-2.101728	0.0367849	0.0888034	-4.495273
RP11-517B1	-0.091746	4.4933398	-2.101603	0.036796	0.0888244	-4.495528
VN2R19P	-0.381183	-1.035217	-2.101352	0.0368183	0.0888663	-4.496038
PLAC8	0.2453348	4.4609655	2.1013521	0.0368183	0.0888663	-4.496038
RP11-290H9	0.3155943	2.5419407	2.100985	0.0368509	0.0889391	-4.496784
RP13-379O2	-0.299778	-1.265455	-2.100908	0.0368577	0.0889497	-4.496941
RP11-848P1	-0.544588	1.1620616	-2.100745	0.0368722	0.0889687	-4.497272
CTD-2385L2	0.1543171	-1.531032	2.1007282	0.0368737	0.0889687	-4.497306
RP11-1006G	0.5039997	0.5956685	2.1007164	0.0368747	0.0889687	-4.49733
LRP8	0.2287714	4.3899571	2.1007095	0.0368753	0.0889687	-4.497344
RP11-385D1	0.5712562	0.3995443	2.1004861	0.0368952	0.0890068	-4.497798
RP11-6D1.3	-0.295647	-1.181307	-2.100477	0.036896	0.0890068	-4.497817
RP1-118J21	-0.573302	0.5985629	-2.100431	0.0369001	0.0890097	-4.497911
COX14	0.0540599	6.2534519	2.1003749	0.0369051	0.0890097	-4.498024
GIGYF1	-0.036946	6.3785728	-2.100371	0.0369054	0.0890097	-4.498033
RP11-350N1	0.5673346	0.2693895	2.1003539	0.0369069	0.0890097	-4.498067
RP11-421L2	-0.354087	1.9609173	-2.100247	0.0369165	0.0890268	-4.498285
RP11-527F1	-0.451869	1.5982985	-2.100201	0.0369206	0.0890308	-4.498379
RP11-415J8	0.1651516	4.2027948	2.1000654	0.0369326	0.0890539	-4.498653
ABHD17A	-0.045476	6.0719441	-2.099885	0.0369486	0.0890813	-4.499019
ARPC3	-0.03857	6.7763904	-2.099883	0.0369488	0.0890813	-4.499025
RP11-186B7	-0.469823	0.408922	-2.099575	0.0369762	0.0891362	-4.499649
CCR7	0.2399706	4.451212	2.0995722	0.0369765	0.0891362	-4.499655
CTD-2530H1	0.4707264	0.7827103	2.0994522	0.0369872	0.0891514	-4.499899
SEC13P1	-0.316489	-0.913232	-2.099446	0.0369877	0.0891514	-4.499911
RP11-1055B	-0.529283	0.9791611	-2.099419	0.0369901	0.0891514	-4.499966

RP11-420A6	0.3490974	-0.834926	2.0993641	0.036995	0.0891574	-4.500078
ART5	-0.623446	1.5001296	-2.099208	0.0370089	0.0891849	-4.500395
H3F3AP5	-0.386072	-0.632316	-2.098811	0.0370443	0.0892644	-4.501202
ZNF192P1	-0.375729	3.0347115	-2.098693	0.0370549	0.089284	-4.501442
NDUFA5P10	0.2094405	-1.480176	2.0984777	0.0370741	0.0893242	-4.501878
AF129075.5	-0.302734	2.6401342	-2.098442	0.0370772	0.0893244	-4.501951
RP1-90G24.	-0.533257	-0.18734	-2.098422	0.037079	0.0893244	-4.501991
MCTS1	0.0510203	6.2218365	2.0983141	0.0370887	0.0893418	-4.50221
RPS15AP24	-0.438687	-0.0833	-2.098225	0.0370966	0.089355	-4.502391
C3P1	0.4096155	5.7978652	2.098008	0.037116	0.0893951	-4.502832
DSC2	-0.128743	5.7502559	-2.097984	0.0371182	0.0893951	-4.502881
FAM157C	-0.541062	1.8289085	-2.097944	0.0371217	0.0893978	-4.502962
RNA5SP141	0.091776	-1.562904	2.0978041	0.0371342	0.089422	-4.503246
SNORD36C	-0.395721	-0.590396	-2.097413	0.0371692	0.0895003	-4.504039
RP4-712E4.	0.1174014	-1.52708	2.0971365	0.0371939	0.089554	-4.504601
KRT8P36	-0.451261	2.1483278	-2.096994	0.0372067	0.0895788	-4.504891
RP5-837M10	-0.188614	-1.432357	-2.096902	0.0372149	0.0895869	-4.505076
INPP5D	0.0817981	5.8319707	2.0969015	0.037215	0.0895869	-4.505078
RP11-354E2	-0.445922	-0.262782	-2.096772	0.0372266	0.0896089	-4.50534
PVRL3-AS1	-0.495105	1.4003641	-2.096659	0.0372367	0.0896274	-4.50557
TMEM158	0.3131158	3.9607285	2.0965657	0.0372451	0.0896416	-4.505759
RP11-22C11	-0.698945	0.0165073	-2.096335	0.0372657	0.0896835	-4.506226
NIFK	-0.046053	6.1448647	-2.096316	0.0372674	0.0896835	-4.506265
TRPM6	-0.585493	1.7188264	-2.09622	0.037276	0.0896983	-4.506459
UBE2Q2P2	-0.210592	3.6985577	-2.096098	0.0372869	0.0897167	-4.506707
CARD8-AS1	0.1164191	4.7573819	2.0960688	0.0372896	0.0897167	-4.506767
GAPDHP2	-0.463096	1.0132268	-2.096053	0.037291	0.0897167	-4.506799
RP11-33O01	0.2163758	3.2289552	2.095771	0.0373163	0.0897717	-4.507371
RANBP6	0.0551217	5.9041089	2.0956418	0.0373279	0.0897921	-4.507633
RAB23	0.1582155	4.8735666	2.0956216	0.0373297	0.0897921	-4.507674
MYL1	0.274971	-1.372641	2.0951113	0.0373756	0.0898964	-4.508709
SAR1P1	0.0987285	-1.55936	2.0949983	0.0373857	0.089915	-4.508938
ZNF544	-0.092385	5.8930607	-2.094687	0.0374137	0.0899763	-4.509568
AC020915.2	-0.228658	-1.340053	-2.094572	0.0374241	0.0899953	-4.509802
ADNP-AS1	-0.127493	3.8144772	-2.094451	0.0374349	0.0900155	-4.510046
HNRNPDL	-0.030801	6.6563361	-2.094376	0.0374417	0.0900259	-4.5102
FNDC3CP	0.1411694	-1.501453	2.094268	0.0374514	0.0900406	-4.510418
CTD-2195M1	0.1678775	-1.478593	2.0942532	0.0374528	0.0900406	-4.510448
FGD1	-0.099663	5.5476479	-2.09419	0.0374585	0.0900484	-4.510577
CIC	0.0362473	6.477299	2.094143	0.0374627	0.0900526	-4.510671
CTD-2525I3	-0.339737	-0.861183	-2.093966	0.0374787	0.0900851	-4.511031
RP11-11N9.	0.4069731	3.3763816	2.0937935	0.0374942	0.0901164	-4.511379
RP11-152K4	0.1835842	-1.470589	2.0936545	0.0375067	0.0901406	-4.511661
AC003682.1	0.3148148	-1.229857	2.0936131	0.0375104	0.0901436	-4.511745
RP11-183G2	-0.421105	-0.322205	-2.09339	0.0375305	0.090186	-4.512196
RP11-45M22	-0.452643	0.1802433	-2.093269	0.0375415	0.0902063	-4.512442
CTB-41I6.1	0.4612092	0.1697617	2.0932324	0.0375448	0.0902083	-4.512516
CTA-27603.	-0.436932	0.3336436	-2.09318	0.0375495	0.0902138	-4.512623
RP11-486G1	-0.307133	3.715893	-2.093119	0.037555	0.0902172	-4.512746
RP11-305L7	-0.460327	1.7853155	-2.093109	0.0375559	0.0902172	-4.512765
RP11-471B2	0.2341771	2.8991124	2.092791	0.0375846	0.0902803	-4.51341

SYT16	0.4898232	-0.568384	2.0927262	0.0375905	0.0902884	-4.513541
HOMER2P1	-0.649275	-0.272459	-2.092274	0.0376313	0.0903749	-4.514457
GRB2	-0.027936	6.7188467	-2.092273	0.0376314	0.0903749	-4.514459
KCNH1	0.5310922	0.3675499	2.092146	0.0376429	0.0903965	-4.514716
ASCC1	0.0470289	5.906078	2.0920071	0.0376555	0.0904207	-4.514997
PTP4A2P1	-0.298641	3.1963237	-2.091609	0.0376915	0.0905014	-4.515804
AC018735.1	-0.213258	-1.383414	-2.09151	0.0377004	0.0905167	-4.516003
MAP4K2	-0.060485	5.8246792	-2.091435	0.0377072	0.0905215	-4.516155
RTCB	0.0408243	6.5414135	2.0914339	0.0377074	0.0905215	-4.516158
TRAK2	0.046321	6.2245086	2.0912982	0.0377196	0.090545	-4.516432
GPR12	0.2032844	-1.382739	2.0905228	0.0377899	0.0907019	-4.518001
RP5-1024N4	-0.461271	1.7936901	-2.090522	0.03779	0.0907019	-4.518003
RP11-715F3	-0.388858	1.7266281	-2.090369	0.0378039	0.0907293	-4.518312
TTYH1	-0.440583	3.9047783	-2.09027	0.0378128	0.0907443	-4.518512
RP11-578F2	-0.453906	-0.022315	-2.090231	0.0378164	0.0907443	-4.518592
CTB-26E19.	0.3830836	-1.013553	2.0902183	0.0378176	0.0907443	-4.518617
SNRPGP9	-0.49382	0.4485066	-2.090103	0.037828	0.0907634	-4.518851
FGF22	-0.536852	1.1377653	-2.089826	0.0378532	0.0908128	-4.519412
RBAK-RBAKD	0.4547422	0.3028738	2.0898218	0.0378536	0.0908128	-4.519419
RP3-395M20	-0.473567	1.2877574	-2.08979	0.0378565	0.0908138	-4.519484
TRIM60	-0.33939	-1.260919	-2.089567	0.0378767	0.0908564	-4.519935
PHPT1	-0.062189	6.4764659	-2.089368	0.0378948	0.0908938	-4.520337
RP11-70702	0.4880871	-0.752488	2.0892315	0.0379073	0.0909176	-4.520613
C1orf94	0.3351268	-1.233743	2.0890089	0.0379275	0.0909602	-4.521063
UMPS	-0.043199	6.264625	-2.088924	0.0379352	0.0909727	-4.521234
SNX21	0.0564164	5.6741484	2.0887674	0.0379495	0.091001	-4.521551
SNX8	-0.055697	6.1963135	-2.088666	0.0379587	0.0910137	-4.521757
R3HDM2	0.0400916	6.2576433	2.0886545	0.0379598	0.0910137	-4.52178
TMEM110-MU	-0.168424	3.9800523	-2.088561	0.0379683	0.0910282	-4.521969
CP	0.1615618	7.0468793	2.0882419	0.0379974	0.0910919	-4.522614
TOLLIP-AS1	-0.145124	4.1224242	-2.088027	0.038017	0.091133	-4.523049
RP5-1021I2	0.1415882	4.3293431	2.0876743	0.0380492	0.091204	-4.523761
RP11-115H1	0.8581038	0.9810859	2.0875516	0.0380603	0.0912249	-4.524008
RP11-272B1	0.1484222	-1.534036	2.0875164	0.0380636	0.0912266	-4.52408
LINC01355	-0.126986	4.7962104	-2.087177	0.0380945	0.0912948	-4.524765
HOXC11	-0.512738	-0.689375	-2.086778	0.038131	0.0913762	-4.525571
CNOT4	0.0349482	5.9306685	2.0866721	0.0381407	0.09139	-4.525785
AP2B1	0.0349898	6.6266253	2.0866606	0.0381418	0.09139	-4.525808
RP11-613M1	-0.156517	4.3375615	-2.08647	0.0381592	0.0914258	-4.526193
APOH	-0.178771	7.4476359	-2.086344	0.0381707	0.0914425	-4.526447
RP11-163E9	-0.391091	1.8355055	-2.086339	0.0381712	0.0914425	-4.526457
AC012123.1	-0.269595	-1.31893	-2.08622	0.0381821	0.0914626	-4.526698
DAGLB	0.0379059	6.0260758	2.0860784	0.038195	0.0914876	-4.526984
HCG27	-0.173467	4.1032398	-2.085972	0.0382048	0.091502	-4.527199
OR52E6	-0.299964	-1.19593	-2.085958	0.038206	0.091502	-4.527227
MUM1	0.0487663	6.2323739	2.0858164	0.038219	0.0915271	-4.527513
CH507-9B2.	0.2744304	4.6487961	2.0857768	0.0382227	0.0915298	-4.527593
RP11-5809.	0.4418555	-0.316047	2.0856382	0.0382354	0.0915542	-4.527873
C17orf49	-0.095229	4.5032805	-2.08541	0.0382562	0.0915982	-4.528332
MTATP8P1	0.4067901	2.6522749	2.0853378	0.0382629	0.0916081	-4.528479
RPL36AP29	0.3258703	-1.039965	2.0851049	0.0382843	0.0916533	-4.528949

DPP6	-0.541168	0.9080793	-2.084942	0.0382992	0.0916829	-4.529277
TBL1Y	0.737151	0.4013918	2.0847773	0.0383143	0.0917114	-4.52961
CTD-2063L2	-0.401387	-0.552572	-2.084758	0.0383161	0.0917114	-4.529649
LIPE-AS1	0.3063594	3.3388446	2.084707	0.0383208	0.0917166	-4.529752
RP11-613M1	-0.355053	-0.833826	-2.084678	0.0383235	0.091717	-4.529811
FUT9	-0.391337	-1.140401	-2.084311	0.0383572	0.0917916	-4.530551
RP11-571M6	-0.416659	-0.402635	-2.084159	0.0383712	0.0918192	-4.530858
ATP8B2	-0.081612	5.8778307	-2.083735	0.0384101	0.0919062	-4.531712
NDUFB11	-0.049644	6.6293621	-2.083699	0.0384134	0.0919082	-4.531785
OR5M11	-0.202516	-1.455445	-2.083539	0.0384282	0.0919375	-4.532109
XXbac-BPG1	0.4445295	-0.244729	2.0833431	0.0384462	0.0919746	-4.532503
RPL21P17	-0.313781	-1.045989	-2.083265	0.0384534	0.0919858	-4.532661
RPL7P52	-0.334466	-0.886998	-2.083182	0.0384611	0.0919981	-4.532829
ZNF174	-0.045318	5.5501408	-2.083051	0.0384731	0.0920208	-4.533092
KLHL35	-0.433896	2.4769465	-2.082901	0.0384869	0.0920478	-4.533394
RP11-697E2	-0.232251	-1.346378	-2.082822	0.0384942	0.0920592	-4.533553
GRIN2C	0.2454622	3.6353005	2.0827387	0.0385019	0.0920716	-4.533722
RP1-158P9.	0.3796663	-0.786216	2.0825358	0.0385206	0.0921103	-4.53413
MEPCE	0.0402186	6.4205609	2.0822615	0.0385459	0.0921648	-4.534683
NEUROD2	-0.354539	-0.851663	-2.082181	0.0385533	0.0921764	-4.534845
MTHFD2	0.1158356	5.4509592	2.0820341	0.0385669	0.0922029	-4.535142
CERS6	0.1105328	5.8025956	2.0813748	0.0386278	0.0923424	-4.53647
RP11-573G6	-0.357565	-0.891238	-2.081299	0.0386348	0.0923531	-4.536622
ATP11A-AS1	-0.179061	-1.445042	-2.0812	0.038644	0.0923671	-4.536822
CEP44	0.0563754	5.5870414	2.0811812	0.0386457	0.0923671	-4.53686
MARC1	-0.126293	6.2942422	-2.080977	0.0386646	0.0924061	-4.537271
EEF1A1P7	-0.369528	2.308417	-2.080841	0.0386772	0.0924302	-4.537545
RP11-214K3	-0.353475	-0.842859	-2.080762	0.0386845	0.0924362	-4.537705
RP11-462L8	-0.455415	-0.014086	-2.080756	0.038685	0.0924362	-4.537716
MYO3A	0.6744146	0.0065945	2.0807045	0.0386898	0.0924362	-4.53782
CFP	0.1863675	5.048229	2.0807044	0.0386898	0.0924362	-4.537821
CNIH1	0.053832	6.625077	2.0806386	0.0386959	0.0924447	-4.537953
RP11-270C1	-0.386782	2.2113531	-2.080581	0.0387013	0.0924515	-4.538069
LIG4	0.0742005	5.643051	2.0804897	0.0387097	0.0924656	-4.538253
RP5-837J1.	-0.411973	-0.427643	-2.080407	0.0387174	0.0924778	-4.538419
AP001257.1	0.2591252	-1.22263	2.0803089	0.0387264	0.0924935	-4.538617
PSMC2	0.0396641	6.6259546	2.0800465	0.0387508	0.0925455	-4.539145
AP000477.3	-0.173522	-1.447758	-2.079845	0.0387694	0.092584	-4.53955
LINC00657	0.0335444	6.848582	2.0794793	0.0388034	0.092659	-4.540287
AC090804.1	-0.472029	0.4268288	-2.079159	0.0388331	0.092724	-4.540932
UCP1	-0.341996	-1.042029	-2.078996	0.0388482	0.092754	-4.541259
RP11-322D1	0.4307109	-0.448214	2.0788859	0.0388585	0.0927724	-4.541482
AC005786.3	-0.194522	-1.362586	-2.078789	0.0388675	0.0927879	-4.541677
CDH19	0.6140783	3.1903366	2.0786983	0.0388759	0.0928019	-4.541859
STK11IP	-0.046275	5.8341687	-2.078614	0.0388838	0.0928145	-4.542029
SERBP1P3	-0.488186	-0.142246	-2.078414	0.0389023	0.0928528	-4.54243
CAMK4	0.1775515	4.4418145	2.0782802	0.0389148	0.0928765	-4.5427
FXN	0.0707593	5.7240352	2.0779912	0.0389417	0.0929319	-4.543282
IGHD3-22	-0.247296	-1.363191	-2.077973	0.0389434	0.0929319	-4.543318
CLCP2	-0.227892	-1.333476	-2.077949	0.0389457	0.0929319	-4.543367
COX6CP2	-0.389868	-0.70598	-2.077711	0.0389678	0.0929786	-4.543845

PRPSAP2	-0.049463	5.9872523	-2.077487	0.0389887	0.0930204	-4.544297
CTD-2531D1	-0.56659	0.2151521	-2.077465	0.0389907	0.0930204	-4.54434
RP11-79L9.	-0.405541	-0.283338	-2.077441	0.0389929	0.0930204	-4.544388
SLC10A7	0.0672153	5.4966644	2.0773809	0.0389986	0.0930277	-4.544509
RP11-159D1	-0.216627	3.7018431	-2.077315	0.0390047	0.0930363	-4.544641
ZNF138	-0.066321	5.3609233	-2.077277	0.0390083	0.0930388	-4.544719
RP11-417L1	0.5338923	0.2315225	2.0771318	0.0390218	0.0930649	-4.54501
LINC00498	0.1910869	-1.444001	2.0770638	0.0390281	0.0930739	-4.545147
MIR1295A	0.5502031	0.8548446	2.0769123	0.0390423	0.0930979	-4.545451
RPL24	-0.047589	7.0488096	-2.076901	0.0390433	0.0930979	-4.545473
RP11-42015	0.341006	4.4335976	2.0766023	0.0390712	0.0931584	-4.546075
FRMD4A	0.0838797	5.8271775	2.0765535	0.0390757	0.0931631	-4.546173
HES5	0.4762552	1.7310569	2.0762481	0.0391043	0.0932223	-4.546786
RP11-225H2	0.4543937	0.065826	2.076233	0.0391057	0.0932223	-4.546817
AC016717.1	-0.260595	-1.392054	-2.076199	0.0391088	0.0932238	-4.546885
CTC-246B18	-0.571961	0.6953309	-2.076066	0.0391213	0.0932474	-4.547153
UPF3AP2	-0.407148	1.5596027	-2.076011	0.0391264	0.0932535	-4.547263
QRSL1P3	0.1665575	-1.448188	2.0759704	0.0391302	0.0932565	-4.547345
LINC00202-	-0.383464	2.819959	-2.07594	0.0391331	0.0932572	-4.547406
IER3	0.115688	6.1583708	2.0758131	0.0391449	0.0932785	-4.547661
RP1-30M3.6	0.1521415	3.5517011	2.0757894	0.0391471	0.0932785	-4.547708
RP11-39402	-0.390533	-0.629333	-2.075591	0.0391657	0.0933166	-4.548106
LINC01500	0.2415857	-1.359223	2.0754729	0.0391767	0.0933211	-4.548344
SMIM1	-0.224935	4.7674386	-2.075471	0.0391769	0.0933211	-4.548348
OTOP1	-0.224716	-1.377796	-2.075469	0.0391771	0.0933211	-4.548351
AC007099.1	-0.679284	0.7174685	-2.075462	0.0391778	0.0933211	-4.548366
CTAG1A	-0.177609	-1.441174	-2.075421	0.0391816	0.0933241	-4.548449
RP11-84C10	0.2783245	-1.189966	2.0753387	0.0391893	0.0933364	-4.548614
AF067845.1	0.1884981	-1.44532	2.0752172	0.0392007	0.0933573	-4.548858
BTF3L4P1	-0.345993	-0.791464	-2.075145	0.0392074	0.0933673	-4.549003
KIAA0087	0.3240956	-1.142903	2.0750705	0.0392144	0.0933756	-4.549153
GRAP2	0.2156579	4.7151258	2.0750367	0.0392176	0.0933756	-4.549221
RASGRF2-AS	0.4643122	-0.057746	2.075026	0.0392186	0.0933756	-4.549242
AC093106.5	-0.436483	-0.16954	-2.074795	0.0392402	0.0934208	-4.549706
RP1-249I4.	-0.430912	-0.230066	-2.074691	0.03925	0.0934208	-4.549915
ACAD9	0.0368089	6.3610989	2.0746809	0.0392509	0.0934208	-4.549935
AC022431.2	0.4640679	-0.302721	2.0746803	0.039251	0.0934208	-4.549936
NINJ1	0.0479314	6.5358778	2.0746687	0.0392521	0.0934208	-4.54996
ANP32BP3	-0.175398	-1.446838	-2.074658	0.039253	0.0934208	-4.54998
MAST4-AS1	-0.406316	1.9388297	-2.074632	0.0392555	0.0934208	-4.550033
GCSAM	0.323397	3.4383649	2.0744973	0.0392681	0.0934448	-4.550304
HSH2D	0.2012951	5.0408324	2.074391	0.0392781	0.0934612	-4.550517
MIR4653	-0.507437	0.9364162	-2.074369	0.0392801	0.0934612	-4.550561
RRN3P3	0.0912433	4.6647439	2.0741854	0.0392974	0.0934959	-4.55093
RP11-170J3	-0.28269	-1.161436	-2.074159	0.0392998	0.0934959	-4.550982
SNORD88A	-0.332318	-0.935525	-2.074082	0.039307	0.093507	-4.551137
IL17B	-0.503058	0.5784104	-2.073975	0.0393172	0.0935249	-4.551353
RP11-569A2	0.1555933	-1.480094	2.0739469	0.0393198	0.093525	-4.551409
ASCL5	-0.521233	0.4815112	-2.073868	0.0393272	0.0935365	-4.551568
GOLGA6L4	-0.508468	0.9800942	-2.073683	0.0393445	0.0935645	-4.551939
REEP1	0.2967115	4.3532208	2.0736766	0.0393451	0.0935645	-4.551952

RP11-473A1	-0.358643	-1.200514	-2.073661	0.0393466	0.0935645	-4.551983
MAP1LC3A	0.1138971	5.9404539	2.0735206	0.0393598	0.0935897	-4.552265
TCAF2P1	0.478631	-0.067155	2.0733559	0.0393753	0.0936122	-4.552596
RP13-131K1	-0.403988	-0.392606	-2.073342	0.0393765	0.0936122	-4.552623
C2orf43	-0.045988	5.8952946	-2.073337	0.0393771	0.0936122	-4.552634
CDKN2AIP	0.0504708	5.8150133	2.073311	0.0393795	0.0936122	-4.552686
KCNK1	0.2318398	5.5709155	2.0732573	0.0393845	0.0936181	-4.552793
MIR3145	-0.293455	-1.130831	-2.073205	0.0393895	0.0936238	-4.552899
RP4-738P15	-0.3991	-0.333079	-2.072967	0.0394118	0.0936707	-4.553375
LAYN	0.1399357	4.6972651	2.0729208	0.0394162	0.093675	-4.553469
GUSBP8	-0.175974	-1.46846	-2.072862	0.0394217	0.0936821	-4.553587
IRX3	0.4047449	4.4136043	2.0727487	0.0394324	0.0937013	-4.553814
RP11-20B24	0.1471179	-1.5347	2.072689	0.039438	0.0937048	-4.553934
UBA3	0.0289138	6.1061431	2.0726784	0.039439	0.0937048	-4.553955
TSEN2	-0.055036	5.4585355	-2.072462	0.0394593	0.093747	-4.554389
RP11-309L2	0.2513684	-1.3132	2.0722624	0.0394782	0.0937857	-4.55479
RP11-541N1	0.1326518	4.3421024	2.0721368	0.03949	0.0938077	-4.555042
PDIK1L	0.054498	5.7147438	2.0719078	0.0395116	0.0938528	-4.555501
RP11-195E2	0.6133963	0.7792728	2.0718097	0.0395208	0.0938687	-4.555698
INSR	0.0556883	6.7250541	2.0716566	0.0395352	0.0938969	-4.556005
LINC00211	0.3332496	-0.949837	2.0716013	0.0395405	0.0939031	-4.556116
ZBED3-AS1	-0.182784	3.9722731	-2.071502	0.0395498	0.0939192	-4.556315
RP11-110J1	-0.367178	-0.679717	-2.07108	0.0395897	0.0940078	-4.557162
EYA1	-0.64141	0.619257	-2.071028	0.0395946	0.0940132	-4.557265
AC073043.1	-0.463649	-0.12416	-2.070973	0.0395998	0.0940195	-4.557376
BFSP2-AS1	-0.212919	-1.353564	-2.070835	0.0396128	0.0940444	-4.557653
PITX2	-0.688123	0.3811662	-2.070716	0.039624	0.0940649	-4.557891
LINC01191	-0.556086	0.2053574	-2.070675	0.0396279	0.094068	-4.557974
TBCK	0.0630149	5.7917144	2.0706016	0.0396348	0.0940783	-4.55812
GJD4	-0.350471	-0.943346	-2.070368	0.0396569	0.0941245	-4.558588
CYP2D8P	-0.277096	3.156499	-2.070137	0.0396787	0.0941703	-4.559051
LINC00381	-0.200374	-1.412685	-2.069703	0.0397199	0.0942618	-4.559922
RP11-384F7	-0.218047	-1.404018	-2.069389	0.0397496	0.0943262	-4.560551
LRRN4CL	0.5286508	1.4977937	2.0690961	0.0397773	0.0943859	-4.561137
RP11-92G12	0.5732089	1.3273181	2.0687888	0.0398065	0.0944489	-4.561753
ST13P3	-0.473343	0.8645403	-2.068705	0.0398145	0.0944616	-4.561921
RP5-1054A2	0.5293468	1.1180335	2.0685622	0.039828	0.0944828	-4.562207
RNF212	0.4652497	2.0678845	2.0685564	0.0398285	0.0944828	-4.562218
HMX3	-0.494483	-0.821899	-2.068216	0.0398609	0.0945534	-4.562901
LIPC	-0.181085	6.3771493	-2.067903	0.0398906	0.0946177	-4.563527
RP11-63B13	-0.136712	-1.487714	-2.06767	0.0399127	0.0946623	-4.563993
LUZP2	0.5577156	0.8270275	2.0676414	0.0399155	0.0946623	-4.564051
RP11-488L1	-0.499939	1.5049322	-2.067623	0.0399172	0.0946623	-4.564087
RAB4B-EGLN	0.3392109	-0.844316	2.0673796	0.0399404	0.0947111	-4.564575
RP11-276M1	-0.352943	-1.000127	-2.067331	0.039945	0.094716	-4.564672
RP11-803B1	-0.434994	1.8024337	-2.067285	0.0399494	0.0947201	-4.564764
CLP1	-0.04127	5.7018978	-2.067248	0.0399529	0.0947224	-4.564839
IL27RA	0.0901916	5.5405281	2.067024	0.0399742	0.0947667	-4.565286
SPRR2A	0.3488559	-1.149355	2.0669009	0.0399859	0.0947884	-4.565533
HOXA7	-0.547372	-0.095308	-2.066843	0.0399915	0.0947929	-4.565649
AP3S1	-0.043794	6.3220435	-2.066826	0.039993	0.0947929	-4.565682

DDX52	-0.031828	6.1003856	-2.066686	0.0400064	0.0948185	-4.565964
RP11-332E4	-0.387879	-0.60358	-2.066638	0.040011	0.0948222	-4.566059
RNU6-1098F	0.1857426	-1.419202	2.0666147	0.0400132	0.0948222	-4.566105
TRAV24	-0.468941	-0.46141	-2.06642	0.0400317	0.09486	-4.566495
TIMP4	0.3872748	3.0874548	2.066116	0.0400607	0.0949225	-4.567103
RNF217	0.1018586	5.7667457	2.0660291	0.040069	0.094936	-4.567277
AGTR1	-0.175382	6.0923525	-2.065981	0.0400736	0.0949407	-4.567373
KCTD21	0.0650166	5.97583	2.0658957	0.0400817	0.0949502	-4.567544
RP4-603I14	-0.198203	-1.383845	-2.065884	0.0400828	0.0949502	-4.567566
TSPY26P	-0.187841	4.3583392	-2.065765	0.0400943	0.0949686	-4.567806
HLA-DQA1	0.1196567	6.2921488	2.065741	0.0400965	0.0949686	-4.567853
GYS2	0.4855333	5.2473943	2.0657213	0.0400984	0.0949686	-4.567893
SIM2	-0.198063	4.3603722	-2.065587	0.0401112	0.0949869	-4.568161
RP11-436D2	-0.241001	-1.366278	-2.065576	0.0401123	0.0949869	-4.568184
U91324.1	-0.468624	3.666489	-2.065534	0.0401163	0.0949869	-4.568268
ITSN2	0.0418489	6.2471517	2.0655313	0.0401165	0.0949869	-4.568273
PLEKHG4B	-0.582108	2.5179785	-2.065423	0.0401269	0.0949995	-4.56849
RP11-961A1	0.503088	0.6217352	2.0654212	0.040127	0.0949995	-4.568493
RP11-528G1	-0.527602	-0.207331	-2.065369	0.040132	0.0950052	-4.568598
ZC3H3	-0.058581	6.2434152	-2.065324	0.0401364	0.0950092	-4.568688
CTD-2036A2	-0.165678	-1.47351	-2.065233	0.040145	0.0950236	-4.568869
LINC01220	0.4986985	0.9409222	2.0651264	0.0401552	0.0950415	-4.569082
UBE2CP3	0.0934795	-1.562035	2.0650392	0.0401635	0.0950551	-4.569257
SRPK2P	-0.424463	-0.828204	-2.064459	0.040219	0.0951802	-4.570417
SLC1A2	0.3429349	5.5254637	2.064407	0.040224	0.0951858	-4.570521
GS1-124K5.	-0.066497	5.2193877	-2.06433	0.0402314	0.0951971	-4.570675
C8orf87	0.3478769	-1.178914	2.0642037	0.0402435	0.0952196	-4.570927
PKNOX1	-0.035063	5.8433904	-2.063942	0.0402685	0.0952726	-4.571449
LINC00616	-0.403746	-1.003521	-2.063764	0.0402856	0.095307	-4.571807
CAT	0.0787375	6.9144912	2.0631066	0.0403486	0.0954498	-4.573119
RP11-712B9	0.2933155	3.0886977	2.0624206	0.0404145	0.0955994	-4.574489
FAM49A	0.1016573	5.5334205	2.0623325	0.040423	0.0956086	-4.574665
PLA2G6	-0.084946	5.8628949	-2.062326	0.0404236	0.0956086	-4.574678
C9orf172	-0.105149	5.3285761	-2.062222	0.0404336	0.095626	-4.574886
WDR70	-0.035766	5.9830621	-2.062003	0.0404547	0.0956637	-4.575324
AC009237.1	0.5045798	1.5375547	2.0620018	0.0404548	0.0956637	-4.575326
NDUFAF2	-0.068813	5.7800802	-2.06194	0.0404607	0.0956679	-4.575449
RP11-67L2.	0.079015	5.1169536	2.0619288	0.0404618	0.0956679	-4.575471
RPS26P3	-0.378575	1.5401498	-2.061776	0.0404765	0.0956965	-4.575777
CTD-2215E1	-0.432385	-0.276375	-2.061683	0.0404854	0.0957067	-4.575962
EEF1A1P29	-0.421092	1.8044071	-2.061676	0.0404861	0.0957067	-4.575976
RP11-295G2	0.4970977	1.6005128	2.0616043	0.040493	0.0957169	-4.576119
RP11-497E1	0.5352946	0.0657691	2.0615618	0.0404971	0.0957204	-4.576204
C19orf54	0.0550905	5.7316617	2.0608576	0.0405649	0.0958745	-4.57761
ALG1L7P	-0.47528	-0.279884	-2.060808	0.0405697	0.0958797	-4.577709
ACTA2-AS1	0.2767764	3.5122055	2.060739	0.0405764	0.0958876	-4.577846
ASS1P6	0.2138784	-1.348555	2.0607184	0.0405784	0.0958876	-4.577888
GVINP1	0.1691417	4.6311265	2.060626	0.0405873	0.0959025	-4.578072
BMS1P10	0.3401012	3.2445179	2.0605856	0.0405912	0.0959026	-4.578153
WAS	0.0919713	5.5898085	2.0605712	0.0405926	0.0959026	-4.578181
OCSTAMP	0.2786312	-1.207757	2.0605117	0.0405983	0.0959099	-4.5783

NPIPB1P	-0.463884	0.2528289	-2.060227	0.0406257	0.0959685	-4.578867
DPH3	-0.043147	6.1856428	-2.060166	0.0406317	0.0959764	-4.578991
THAP9-AS1	0.0656357	5.7251937	2.0599933	0.0406483	0.0960095	-4.579334
ATP4A	-0.604489	-0.049408	-2.059772	0.0406697	0.0960538	-4.579776
CTD-2616J1	-0.357377	-0.780024	-2.059739	0.0406729	0.096055	-4.579841
RP11-425A2	0.1690073	-1.432489	2.0595403	0.0406921	0.0960863	-4.580238
GPR123-AS1	-0.377578	-1.116704	-2.059537	0.0406924	0.0960863	-4.580244
GTF2H1	-0.032289	6.1550849	-2.05952	0.040694	0.0960863	-4.580278
WBP2NL	-0.241156	3.7226061	-2.059447	0.0407011	0.0960898	-4.580424
HMHB1	-0.300517	-1.132823	-2.059388	0.0407068	0.0960898	-4.58054
RP4-568F9.	-0.222939	-1.335905	-2.059384	0.0407072	0.0960898	-4.580549
POLR1A	-0.037225	6.2619108	-2.059382	0.0407074	0.0960898	-4.580554
AP000233.3	-0.188693	-1.440319	-2.059369	0.0407086	0.0960898	-4.580579
LINC00943	-0.577573	0.9136653	-2.058822	0.0407616	0.0962086	-4.581671
ITPKC	0.0500683	6.2510382	2.0587536	0.0407682	0.0962179	-4.581806
RP11-348N5	-0.456493	1.2155175	-2.058621	0.040781	0.096242	-4.582071
PTRH2	-0.048787	6.0879646	-2.058429	0.0407996	0.0962795	-4.582453
RAB4A	-0.05366	6.4173091	-2.058358	0.0408065	0.0962896	-4.582595
RP11-245P1	-0.332065	-1.024688	-2.058228	0.0408191	0.0963106	-4.582854
DNAJC14	-0.062047	5.8681342	-2.058212	0.0408206	0.0963106	-4.582886
NDRG3	-0.053766	6.1334667	-2.057899	0.040851	0.0963758	-4.583509
LINC01165	0.2556062	-1.25791	2.0577244	0.0408679	0.0964071	-4.583858
RP4-755D9.	-0.458287	2.4859451	-2.057708	0.0408695	0.0964071	-4.58389
snoZ6	0.3798622	-0.699138	2.0575486	0.040885	0.0964374	-4.584208
LINC01492	-0.180203	-1.439902	-2.057258	0.0409132	0.0964977	-4.584787
RP11-110I1	-0.35636	1.8544645	-2.057131	0.0409255	0.0965188	-4.58504
TAS2R14	-0.193215	3.6981565	-2.057112	0.0409274	0.0965188	-4.585079
AQP7P2	0.5484657	0.0918354	2.0568495	0.0409529	0.0965726	-4.585601
MIR3153	-0.313374	-1.019704	-2.056784	0.0409592	0.0965787	-4.585731
AC005355.1	-0.21184	-1.399063	-2.056769	0.0409607	0.0965787	-4.585762
FAM163A	-0.540087	1.7606288	-2.056573	0.0409798	0.0966099	-4.586152
NRBP2	0.0874371	6.5539083	2.0565553	0.0409815	0.0966099	-4.586187
OR2H2	0.4070931	-0.909514	2.056551	0.0409819	0.0966099	-4.586195
RP11-266N1	-0.463324	-0.854189	-2.056472	0.0409896	0.0966217	-4.586352
RP11-445O1	-0.425297	0.3942437	-2.055835	0.0410516	0.0967616	-4.587621
AC092155.4	0.4017997	-0.92112	2.0556951	0.0410652	0.0967813	-4.5879
GRAMD1B	0.2093368	5.3718345	2.0556949	0.0410652	0.0967813	-4.5879
SDF2L1	-0.072882	6.3361115	-2.055501	0.0410841	0.0968195	-4.588285
CTB-133G6.	-0.416558	0.4632487	-2.055222	0.0411113	0.0968773	-4.58884
CTD-2283N1	-0.425842	0.8880375	-2.055148	0.0411185	0.0968881	-4.588988
RHOF	-0.201279	4.421238	-2.055101	0.0411231	0.0968927	-4.589082
OR7E29P	0.5412506	0.0200288	2.054958	0.0411371	0.0969193	-4.589367
SNRK-AS1	-0.446247	-0.311695	-2.05491	0.0411417	0.096924	-4.589462
MUS81	-0.033145	6.0872697	-2.054788	0.0411537	0.0969459	-4.589705
GLTSCR2	-0.049333	6.8858639	-2.054723	0.04116	0.0969546	-4.589835
RP11-477H2	-0.598647	1.4420457	-2.054608	0.0411712	0.0969733	-4.590063
CH507-42P1	-0.564194	0.1484366	-2.054587	0.0411733	0.0969733	-4.590105
RP11-103C1	-0.372583	-0.666205	-2.054293	0.041202	0.0970347	-4.590691
CTA-929C8.	-0.296325	-1.099518	-2.054183	0.0412127	0.0970536	-4.590908
FEM1A	0.1202367	4.4685053	2.0540756	0.0412232	0.0970695	-4.591123
LRFN3	0.0774236	5.7644097	2.0540505	0.0412257	0.0970695	-4.591172

CD24	-0.207059	6.4837011	-2.054019	0.0412288	0.0970695	-4.591235
VN1R107P	-0.449387	-0.322786	-2.054005	0.0412301	0.0970695	-4.591262
LINC00269	-0.221077	-1.446343	-2.053905	0.0412399	0.0970857	-4.591461
RP11-44D5.	-0.422699	-0.40251	-2.053881	0.0412423	0.0970857	-4.591509
ZNF737	-0.231368	4.2743979	-2.053773	0.0412528	0.0971042	-4.591724
HIST1H1D	-0.45357	-0.481407	-2.053544	0.0412752	0.0971446	-4.59218
ANKRD39	-0.053032	5.8315965	-2.053543	0.0412753	0.0971446	-4.592181
DYRK2	-0.054231	5.9874783	-2.053499	0.0412797	0.0971487	-4.59227
TRA2B	-0.024886	6.5241751	-2.053147	0.0413141	0.0972234	-4.592969
HSPA8P4	0.5115467	0.3547029	2.0527039	0.0413575	0.0973193	-4.59385
AC073072.7	-0.418973	1.1300283	-2.052658	0.0413619	0.0973235	-4.593941
RP11-671J1	0.3048517	-1.012949	2.0524593	0.0413815	0.0973608	-4.594337
CTC-548K16	-0.515099	-0.094939	-2.052442	0.0413831	0.0973608	-4.59437
RNU4-2	-0.589103	1.4642349	-2.05234	0.0413932	0.0973781	-4.594574
RPL10AP2	-0.4326	1.9510748	-2.052274	0.0413997	0.0973858	-4.594705
RP11-795A2	-0.14623	-1.483047	-2.052246	0.0414024	0.0973858	-4.594761
HEATR5A	0.0543111	5.8571994	2.0522249	0.0414045	0.0973858	-4.594803
MKRN4P	-0.622902	-0.239043	-2.052082	0.0414184	0.0974068	-4.595086
ATP1A2	0.3153363	4.2789916	2.0520798	0.0414187	0.0974068	-4.595091
RP5-963E22	0.1319202	-1.496917	2.0517519	0.0414509	0.0974762	-4.595743
CTB-5409.9	0.4140726	-0.193188	2.0515983	0.041466	0.0975054	-4.596048
DARS	-0.033263	6.4176614	-2.051155	0.0415096	0.0976016	-4.596929
RP11-451B8	-0.321811	-1.168934	-2.051078	0.0415171	0.0976131	-4.597082
RP11-96D1.	0.4583091	3.4789566	2.050789	0.0415455	0.0976729	-4.597656
SPATA33	0.0793183	5.0863446	2.0507649	0.0415479	0.0976729	-4.597703
CD24P4	-0.49204	-0.112765	-2.050641	0.0415601	0.0976952	-4.597949
PRELID2	-0.195168	4.6001636	-2.050456	0.0415783	0.0977318	-4.598317
AQP5	-0.578819	1.0804671	-2.050336	0.0415901	0.0977514	-4.598555
ZNF862	0.0818595	5.5813649	2.0503168	0.041592	0.0977514	-4.598593
CTC-360P9.	-0.250348	-1.366943	-2.050126	0.0416108	0.0977893	-4.598972
ZDHHC20P1	0.4638749	0.8795337	2.0499636	0.0416268	0.0978206	-4.599295
RP11-218E2	-0.469155	0.0524556	-2.049881	0.0416349	0.0978333	-4.599458
RP11-284F2	0.5313029	3.6746531	2.0490027	0.0417216	0.0980307	-4.601202
RP3-486I3.	-0.406828	1.3405875	-2.048964	0.0417254	0.0980334	-4.601279
RP11-19P22	0.0934795	-1.562035	2.0487076	0.0417507	0.0980865	-4.601788
SMAD7	0.0698324	5.7724745	2.0485528	0.041766	0.0981131	-4.602095
GAPDHP74	0.5304724	0.1007108	2.0485258	0.0417687	0.0981131	-4.602148
TMEM132D	-0.600035	1.5177585	-2.048511	0.0417701	0.0981131	-4.602177
USP1	-0.050411	6.0720706	-2.048252	0.0417958	0.0981512	-4.602692
ASS1P7	0.3939065	-0.776432	2.0482401	0.0417969	0.0981512	-4.602715
AC011343.1	0.4286269	-0.50862	2.0482232	0.0417986	0.0981512	-4.602749
RP11-762E8	-0.164158	-1.45235	-2.048215	0.0417994	0.0981512	-4.602765
FAM122C	0.084614	4.6040058	2.0481916	0.0418017	0.0981512	-4.602812
C10orf10	0.0902887	6.7456157	2.0481842	0.0418025	0.0981512	-4.602826
RP11-478C1	-0.466443	0.9067315	-2.047965	0.0418242	0.0981959	-4.603262
AF064860.5	0.2028196	-1.419748	2.0477856	0.0418419	0.0982312	-4.603617
RP11-76908	-0.472843	0.1439988	-2.047697	0.0418506	0.0982454	-4.603792
GLTSCR1	-0.040002	5.8328586	-2.047615	0.0418588	0.0982581	-4.603955
RPL3P6	-0.447955	0.4475827	-2.047587	0.0418616	0.0982585	-4.604011
RP11-166A1	0.1501136	-1.482887	2.0475297	0.0418672	0.0982654	-4.604124
RPL10A	-0.045942	7.0455533	-2.047448	0.0418753	0.0982781	-4.604286

CDH16	-0.630144	3.3632092	-2.047352	0.0418848	0.0982938	-4.604476
FAM26D	-0.247669	-1.319513	-2.047326	0.0418874	0.0982938	-4.604528
DYRK1B	-0.080681	5.8392209	-2.047189	0.041901	0.0983193	-4.6048
TRMT13	-0.054346	5.5409054	-2.047002	0.0419195	0.0983564	-4.60517
RN7SKP51	0.1108743	-1.530407	2.0469737	0.0419223	0.0983568	-4.605227
RPS23	-0.047853	7.0648596	-2.046597	0.0419597	0.098438	-4.605973
C1QL2	0.2447532	-1.344094	2.0462401	0.0419951	0.0985149	-4.606681
NYAP1	-0.225199	3.5304617	-2.045906	0.0420284	0.0985865	-4.607344
QRSL1	-0.045068	5.9339495	-2.045845	0.0420344	0.0985944	-4.607465
SHISA9	0.6835665	0.7847469	2.0455915	0.0420596	0.098647	-4.607966
TNPO1	0.0337862	6.4571735	2.0455222	0.0420665	0.0986569	-4.608104
XXbac-BPG2	-0.445128	1.4048518	-2.045495	0.0420692	0.098657	-4.608158
RP11-30L15	-0.471041	1.6491556	-2.045447	0.0420739	0.0986617	-4.608253
FGF8	-0.400546	-1.008204	-2.045378	0.0420808	0.0986714	-4.608389
SCAP	-0.04379	6.689194	-2.045334	0.0420851	0.0986753	-4.608476
RN7SL749P	-0.343027	-0.912594	-2.045133	0.0421052	0.098716	-4.608876
IL10	0.4110901	2.6761788	2.045068	0.0421116	0.0987248	-4.609004
ATXN3	0.0441942	5.8354784	2.0450182	0.0421166	0.0987301	-4.609102
B4GALNT4	-0.592619	3.4114196	-2.044516	0.0421666	0.0988409	-4.610097
RP11-960L1	-0.494722	0.5515898	-2.044256	0.0421925	0.0988954	-4.610612
MTHFD2P1	-0.42931	-0.74329	-2.044181	0.0422	0.0989065	-4.610761
HMGN2P40	-0.352051	-0.935757	-2.044151	0.042203	0.0989073	-4.61082
TENM1	0.554654	4.0225239	2.0440474	0.0422133	0.098925	-4.611025
TIPIN	-0.07317	5.1971861	-2.04393	0.042225	0.098946	-4.611256
RPL34-AS1	0.4567524	1.0980566	2.043851	0.0422329	0.0989582	-4.611414
AC061961.2	-0.604448	1.1321667	-2.043675	0.0422505	0.098993	-4.611762
WI2-85898F	0.1867106	-1.446231	2.0436182	0.0422562	0.0989951	-4.611875
PPP1R26	0.0708528	5.927502	2.0436118	0.0422568	0.0989951	-4.611887
RIN3	0.0641985	6.1474305	2.0434864	0.0422693	0.0990181	-4.612136
RP11-440L1	-0.131313	4.3064209	-2.043291	0.0422888	0.0990523	-4.612522
RP11-373E1	-0.383803	-0.887766	-2.043286	0.0422893	0.0990523	-4.612533
LINC01428	-0.561397	1.4668161	-2.043167	0.0423012	0.0990738	-4.612768
RP11-155D1	-0.323883	-0.995019	-2.042995	0.0423184	0.0991077	-4.613109
KRT31	0.2259282	-1.410707	2.0428893	0.042329	0.099126	-4.613317
FOXL2	0.5072203	-0.744126	2.0427711	0.0423408	0.0991473	-4.613551
EEF1A1P13	-0.108323	4.8688701	-2.042714	0.0423465	0.0991545	-4.613665
LINC01281	-0.502746	-0.194461	-2.042544	0.0423635	0.0991843	-4.614
MIR589	-0.445143	0.2079162	-2.042532	0.0423647	0.0991843	-4.614025
AGAP1-IT1	-0.412051	3.2402521	-2.042397	0.0423782	0.0992096	-4.614292
PCDH19	0.4605953	2.1855525	2.0421158	0.0424063	0.099269	-4.614848
YBX1	0.0410316	6.9504851	2.0420493	0.042413	0.0992782	-4.614979
IL2RA	0.2708109	4.2532466	2.0419935	0.0424186	0.099285	-4.61509
RP11-407P1	-0.547406	1.6300656	-2.041674	0.0424506	0.0993441	-4.615721
RP11-735G4	0.2673346	-1.141667	2.0416683	0.0424512	0.0993441	-4.615733
RP11-110G2	-0.124102	4.4827453	-2.04166	0.042452	0.0993441	-4.615749
DICER1-AS1	-0.105067	4.8063739	-2.041629	0.0424551	0.0993449	-4.61581
MAGEA5	-0.245543	-1.338725	-2.041576	0.0424604	0.0993509	-4.615915
RP11-10J21	-0.495169	-0.060005	-2.041532	0.0424648	0.099355	-4.616002
IFITM5	-0.541707	-0.230091	-2.041498	0.0424682	0.0993565	-4.616069
AF011889.2	-0.530352	1.5670573	-2.041248	0.0424934	0.099409	-4.616565
GJB2	0.1583361	5.9812549	2.0410979	0.0425084	0.0994349	-4.616861

LBX2-AS1	-0.074483	6.0386641	-2.041083	0.0425099	0.0994349	-4.616891
RP5-826L7.	0.4203121	-0.476207	2.0407771	0.0425406	0.0995002	-4.617495
RP11-363G1	-0.212439	-1.406768	-2.040704	0.0425478	0.0995084	-4.617639
RP11-20B24	-0.308955	-1.024859	-2.040688	0.0425495	0.0995084	-4.617671
ZNF69	-0.160025	5.1655789	-2.040641	0.0425542	0.099513	-4.617763
CDC42SE2	0.0407115	6.3207647	2.0404783	0.0425706	0.0995449	-4.618086
RP11-567F1	-0.425388	1.260461	-2.040327	0.0425858	0.0995741	-4.618385
NBPF8	-0.113143	5.1065679	-2.040212	0.0425973	0.0995947	-4.618612
RAB5C	0.0311933	6.555013	2.0401046	0.0426081	0.0996136	-4.618824
RP11-575I8	-0.281881	-1.189642	-2.039985	0.0426201	0.0996353	-4.619061
RP11-3P22.	-0.189873	-1.461645	-2.039879	0.0426308	0.0996538	-4.61927
DEFB109P3	0.4195531	-0.487257	2.0398095	0.0426378	0.0996638	-4.619407
RP11-31F19	0.4698413	-0.202438	2.039404	0.0426786	0.0997528	-4.620209
MRPL41	0.0694307	6.4245848	2.0393047	0.0426886	0.0997656	-4.620405
AC105339.1	0.239159	-1.260328	2.0392952	0.0426896	0.0997656	-4.620424
EMC1	0.0439607	6.3148016	2.0392294	0.0426962	0.0997747	-4.620554
COPS4	0.0410273	5.998526	2.039194	0.0426997	0.0997767	-4.620624
EEF1A1P38	-0.39715	1.989188	-2.038933	0.042726	0.0998317	-4.621139
RP11-363J2	0.3621625	-0.688157	2.0387923	0.0427402	0.0998556	-4.621417
ZMAT5	-0.051888	5.8951467	-2.038777	0.0427417	0.0998556	-4.621447
CXorf56	0.0445065	5.8875355	2.0387223	0.0427473	0.0998622	-4.621555
RGS14	0.0674915	6.3668857	2.0384513	0.0427746	0.0999196	-4.62209
ANKRD20A11	-0.456652	-0.535543	-2.038407	0.0427791	0.0999212	-4.622178
COL18A1-AS	-0.476455	-0.359152	-2.03839	0.0427808	0.0999212	-4.622211
ZNF619	-0.07509	5.0420302	-2.038277	0.0427922	0.0999396	-4.622435
C9orf84	-0.531587	1.2437764	-2.038258	0.0427941	0.0999396	-4.622472
RP11-603B2	0.3810492	-1.010013	2.0380615	0.0428139	0.0999718	-4.62286
FTLP2	-0.194087	4.6353862	-2.038042	0.0428159	0.0999718	-4.622899
BNIP3P28	0.4885713	-0.426517	2.0380399	0.0428161	0.0999718	-4.622903
AP000347.2	-0.232582	3.7430752	-2.037641	0.0428563	0.1000593	-4.623689
ALOX12B	-0.531031	-0.235511	-2.037576	0.042863	0.1000651	-4.62382
SLC38A5	-0.21654	4.3737852	-2.037563	0.0428643	0.1000651	-4.623845
RP1-30M3.5	0.1061712	4.5517772	2.0373316	0.0428877	0.1001071	-4.624301
TMEM240	0.2637689	3.1568179	2.0373305	0.0428878	0.1001071	-4.624303
AC092484.1	0.2806074	-1.305727	2.0370416	0.042917	0.1001689	-4.624873
C5orf58	-0.573681	2.415432	-2.037	0.0429212	0.1001724	-4.624956
FSTL5	-0.780487	0.9605226	-2.036352	0.0429868	0.100319	-4.626234
RP11-470E1	-0.276706	-1.267842	-2.036275	0.0429946	0.1003309	-4.626387
LINC00900	0.3379259	3.0604198	2.0359669	0.0430258	0.1003937	-4.626994
CH17-373J2	-0.454142	0.3174752	-2.035955	0.043027	0.1003937	-4.627018
CMB9-55F22	-0.202085	4.5413475	-2.035899	0.0430327	0.1004005	-4.627128
CNTRL	-0.061403	5.7125616	-2.035779	0.0430449	0.1004225	-4.627365
CTD-2034I2	0.2848967	-1.268859	2.035704	0.0430525	0.1004337	-4.627512
PDPK1	0.0512375	6.0345469	2.0355595	0.0430671	0.1004615	-4.627797
SEPT4-AS1	0.2445328	2.859535	2.0353919	0.0430841	0.1004947	-4.628128
GAP43	0.5767119	1.8448559	2.0352605	0.0430975	0.1005194	-4.628387
SLC19A2	-0.07894	6.1162429	-2.035101	0.0431137	0.1005509	-4.628702
WFDC5	0.2408994	-1.305731	2.0350623	0.0431176	0.1005535	-4.628778
CCND2P1	0.6517318	3.9910894	2.0349645	0.0431275	0.1005702	-4.62897
PTCD1	-0.054922	5.5868487	-2.03491	0.043133	0.1005767	-4.629078
CTD-2306M5	-0.248564	-1.346144	-2.034829	0.0431413	0.1005896	-4.629238

DEFB124	0.3304838	-0.982184	2.0346858	0.0431558	0.100617	-4.62952
C20orf144	-0.409991	1.8414622	-2.034149	0.0432104	0.1007379	-4.630579
SYT13	0.7418551	3.3778406	2.0340168	0.0432239	0.1007627	-4.630838
AC108448.2	0.4104374	-0.538883	2.0338446	0.0432414	0.1007939	-4.631178
OR10Y1P	0.1016562	-1.557868	2.033831	0.0432428	0.1007939	-4.631205
RP11-145M4	-0.458615	-0.581628	-2.033494	0.0432771	0.1008676	-4.631869
PFN4	-0.345805	3.2756429	-2.033337	0.043293	0.1008983	-4.632177
AC002486.3	0.1617025	-1.527268	2.0332881	0.0432981	0.1009035	-4.632274
CATIP-AS1	0.3820456	2.8476471	2.0331596	0.0433112	0.1009276	-4.632527
AC013410.2	-0.262527	-1.244652	-2.032987	0.0433288	0.1009567	-4.632868
STAR	-0.594941	1.2704659	-2.032983	0.0433292	0.1009567	-4.632876
ZNF140	-0.055596	5.5951352	-2.032863	0.0433414	0.1009786	-4.633111
AC012370.2	-0.223976	-1.308912	-2.032794	0.0433484	0.1009886	-4.633247
PRSS30P	-0.490587	2.0975727	-2.032661	0.043362	0.1010139	-4.63351
CTC-444N24	-0.047672	5.9541358	-2.032596	0.0433687	0.1010229	-4.633638
SNTG2	0.4413629	-0.689627	2.0325121	0.0433772	0.1010363	-4.633803
AC002310.7	-0.408606	-0.403784	-2.032425	0.0433861	0.1010506	-4.633974
CTC-441N14	-0.303613	-1.233902	-2.032352	0.0433936	0.1010616	-4.634119
RP3-522D1.	-0.315651	-1.068925	-2.032244	0.0434046	0.1010778	-4.63433
THNSL1	0.0758893	5.8299226	2.0322291	0.0434061	0.1010778	-4.63436
PHKA1-AS1	0.21874	-1.352099	2.0320795	0.0434214	0.101107	-4.634654
RNF167	0.0404664	6.5971975	2.0319069	0.043439	0.1011416	-4.634994
RP11-478K1	0.1024489	-1.5347	2.0317236	0.0434578	0.1011788	-4.635355
MRPL46	0.0687858	5.956598	2.0315567	0.0434748	0.101212	-4.635684
TSHZ2	0.1466549	5.867439	2.0313544	0.0434955	0.1012538	-4.636082
SAPCD1-AS1	-0.441765	1.7510251	-2.03084	0.0435482	0.1013699	-4.637095
LINC01257	-0.499011	-0.708446	-2.030428	0.0435903	0.1014615	-4.637904
RP3-467K16	0.3310484	-0.968521	2.0302867	0.0436049	0.1014889	-4.638183
CTU2	0.0487471	5.784812	2.0301646	0.0436174	0.1015115	-4.638423
KLC2	0.0590185	5.9072864	2.0301378	0.0436201	0.1015115	-4.638476
RP11-465B2	-0.666815	1.6492215	-2.030097	0.0436243	0.1015146	-4.638556
CTBP2P7	0.2685346	-1.196486	2.0300379	0.0436304	0.1015146	-4.638672
AMZ2P1	-0.069671	5.3132797	-2.030011	0.0436332	0.1015146	-4.638726
AC007349.5	0.1610173	-1.454566	2.0300075	0.0436335	0.1015146	-4.638732
ZNF285B	-0.446085	0.5621768	-2.029989	0.0436354	0.1015146	-4.638768
RP11-355K2	0.4526248	0.3434601	2.0299421	0.0436402	0.1015194	-4.638861
TEX38	-0.438389	0.0085832	-2.029774	0.0436574	0.101553	-4.639191
CAPNS2	0.3365205	-0.956737	2.0295815	0.0436772	0.1015925	-4.63957
HMGN2P17	-0.201785	3.9648638	-2.029364	0.0436996	0.1016337	-4.639998
IGLON5	0.395391	3.5570886	2.0293551	0.0437005	0.1016337	-4.640015
RP11-545D2	0.3892897	-0.440403	2.0291123	0.0437254	0.1016852	-4.640492
PROKR1	-0.560926	0.2429214	-2.029076	0.0437292	0.1016875	-4.640565
SUGP2	-0.044083	6.1583378	-2.029016	0.0437353	0.1016952	-4.640682
RP11-666G4	-0.202973	-1.36827	-2.028844	0.043753	0.1017299	-4.64102
FCGR2B	0.2431873	4.8741919	2.0287995	0.0437576	0.101734	-4.641107
RP11-63E5.	0.0975936	-1.559939	2.0287486	0.0437628	0.1017397	-4.641207
UBE2B	0.0395907	6.413549	2.02869	0.0437688	0.1017473	-4.641323
RP11-697M1	-0.264568	-1.201552	-2.028501	0.0437883	0.101786	-4.641694
TSPAN31	0.0562024	6.1885197	2.0284492	0.0437936	0.1017919	-4.641796
RP11-680H2	-0.274052	2.8751796	-2.028369	0.0438019	0.1018046	-4.641954
GAPVD1	-0.033608	6.1264979	-2.028044	0.0438353	0.1018758	-4.642591

RP11-1096G	-0.355931	-0.564601	-2.027859	0.0438544	0.1019137	-4.642956
RPL23AP83	-0.230559	-1.283779	-2.027816	0.0438588	0.1019175	-4.643041
LINC00493	-0.047938	6.2073503	-2.02776	0.0438645	0.1019197	-4.643149
LARP1	-0.034051	6.8099053	-2.027753	0.0438653	0.1019197	-4.643165
RPS28	-0.053347	6.8850383	-2.0276	0.0438811	0.1019497	-4.643465
PRL	-0.337298	-1.169622	-2.027396	0.0439021	0.1019922	-4.643866
CTC-435M10	-0.374088	2.4807116	-2.027307	0.0439113	0.1020069	-4.64404
CLEC5A	0.5045055	3.0921747	2.0270127	0.0439416	0.102071	-4.644618
ZNF80	-0.541228	0.4605438	-2.026985	0.0439445	0.1020712	-4.644673
RP11-14501	0.0987285	-1.55936	2.0269124	0.043952	0.1020789	-4.644815
PKDCC	-0.105942	5.9895454	-2.026881	0.0439552	0.1020789	-4.644877
ASIP	0.4127929	2.4642722	2.0267994	0.0439636	0.1020789	-4.645037
ZBBX	-0.398359	-0.920629	-2.026799	0.0439637	0.1020789	-4.645038
FLJ43681	-0.469809	-0.283237	-2.02679	0.0439646	0.1020789	-4.645056
RP11-813B8	-0.159385	-1.476596	-2.02677	0.0439666	0.1020789	-4.645094
CTD-2186M1	0.1047863	4.7822039	2.0267628	0.0439674	0.1020789	-4.645109
LRFN5	-0.664516	0.1924572	-2.026654	0.0439787	0.1020985	-4.645323
RP4-598P13	0.2897599	-1.171943	2.0266045	0.0439838	0.1021038	-4.64542
GAPDHP44	-0.453124	0.3250137	-2.026578	0.0439865	0.1021038	-4.645472
HYLS1	-0.081573	5.2726947	-2.026546	0.0439898	0.102105	-4.645535
RP11-230F1	-0.332846	2.8000884	-2.026491	0.0439955	0.1021115	-4.645642
TIGD5	-0.07225	5.8063307	-2.026197	0.0440259	0.1021756	-4.64622
SYF2	0.0392193	6.2582217	2.0261014	0.0440358	0.1021921	-4.646408
PTGES3L-AA	-0.425396	-0.522794	-2.02597	0.0440494	0.1022107	-4.646666
USF1	-0.040877	6.4001416	-2.02597	0.0440494	0.1022107	-4.646666
NKX2-8	0.4805976	-0.746362	2.0254094	0.0441073	0.1023364	-4.647766
RP11-692P1	0.2678981	-1.135417	2.0253849	0.0441099	0.1023364	-4.647814
ARFGAP3	0.0407242	6.4058143	2.0253649	0.044112	0.1023364	-4.647853
TIMP3	0.2433694	4.8410782	2.0252464	0.0441242	0.1023583	-4.648086
CTD-2246P4	-0.442096	-0.069437	-2.025127	0.0441366	0.1023806	-4.648321
KIF16B	0.056675	5.8678316	2.0250329	0.0441463	0.1023966	-4.648505
RP11-380D2	-0.251653	-1.344398	-2.024813	0.0441692	0.1024431	-4.648937
RP11-308D1	0.3240458	2.9047483	2.0243852	0.0442135	0.1025393	-4.649776
IVNS1ABP	0.0460758	6.52394	2.0242968	0.0442227	0.1025541	-4.649949
MTND1P11	-0.159385	-1.476596	-2.02418	0.0442348	0.1025751	-4.650179
FAM65B	0.1430743	5.0480771	2.0241555	0.0442373	0.1025751	-4.650226
RP11-230F1	-0.474593	0.289399	-2.02384	0.04427	0.1026422	-4.650844
CTC-510F12	-0.418813	0.0368857	-2.023823	0.0442719	0.1026422	-4.650879
RP11-403A3	0.1603612	-1.4549	2.0235212	0.0443032	0.1026971	-4.65147
FP325331.1	-0.465799	-0.001004	-2.023519	0.0443034	0.1026971	-4.651475
OPN1SW	-0.116368	4.6078249	-2.023502	0.0443052	0.1026971	-4.651509
RP5-1092A1	0.5844078	0.3835037	2.0234862	0.0443068	0.1026971	-4.651539
USP15	0.0318482	6.1939689	2.0232641	0.0443299	0.1027423	-4.651975
RP4-564F22	0.452998	4.5837248	2.023243	0.0443321	0.1027423	-4.652016
RP4-790G17	-0.248564	-1.249624	-2.023218	0.0443348	0.1027423	-4.652066
RHOT2	0.0368902	6.4402096	2.0230915	0.0443479	0.1027615	-4.652313
TMEM14A	-0.069126	6.278442	-2.023083	0.0443487	0.1027615	-4.652329
RP11-778J1	-0.294319	-1.086339	-2.02304	0.0443532	0.1027654	-4.652413
RPL34P27	-0.435473	-0.206216	-2.022971	0.0443604	0.1027713	-4.652549
MIR200B	0.2056715	-1.377968	2.022937	0.0443639	0.1027713	-4.652616
PCDH8	-0.248196	-1.384655	-2.022935	0.0443642	0.1027713	-4.65262

RPS4XP7	-0.463979	0.0454839	-2.022794	0.0443788	0.1027928	-4.652895
TOB2	0.0485616	6.3356441	2.0227678	0.0443816	0.1027928	-4.652947
CYSRT1	-0.181392	3.9622405	-2.022764	0.0443819	0.1027928	-4.652954
FBXO7	0.049099	6.6418965	2.0225381	0.0444055	0.1028408	-4.653398
RP11-493P1	-0.287477	-1.104527	-2.02244	0.0444157	0.1028576	-4.65359
TP73-AS1	0.0549854	5.7651087	2.0224146	0.0444183	0.1028576	-4.65364
RP11-369E1	0.2753392	-1.306743	2.022372	0.0444228	0.1028613	-4.653723
PDLIM7	0.0655309	6.0811413	2.0223222	0.0444279	0.1028668	-4.653821
RP11-192H6	-0.23356	-1.344601	-2.022275	0.0444329	0.1028717	-4.653914
RP11-263F1	0.4195146	-0.959191	2.0220307	0.0444583	0.1029241	-4.654392
FSIP2	-0.547956	2.4937271	-2.021906	0.0444714	0.1029429	-4.654637
CTSA	0.0489203	6.9992365	2.0218985	0.0444721	0.1029429	-4.654651
CYP20A1	-0.036498	6.0722719	-2.021864	0.0444757	0.1029446	-4.654718
AF146191.4	-0.462976	0.8152694	-2.021702	0.0444926	0.1029712	-4.655036
ANKIB1	0.0467415	6.0847354	2.0217003	0.0444928	0.1029712	-4.655039
PHBP3	-0.352533	-0.778932	-2.021596	0.0445036	0.1029897	-4.655243
RP3-507I15	-0.340906	-0.85261	-2.021533	0.0445103	0.1029986	-4.655368
COMMD7	-0.041467	6.3191224	-2.021348	0.0445296	0.1030367	-4.65573
RP11-284F2	0.2920784	-1.166003	2.0212353	0.0445413	0.1030568	-4.65595
TXNDC16	-0.072532	5.6833237	-2.02121	0.0445439	0.1030568	-4.655999
NCK2	-0.088175	6.0646051	-2.020854	0.0445811	0.1031364	-4.656697
NAB2	0.0543918	6.0481495	2.0205001	0.0446181	0.1032154	-4.65739
RP4-713A8.	0.3617127	-0.871036	2.0204245	0.044626	0.1032272	-4.657538
RP1-198K11	-0.140097	4.2886063	-2.02023	0.0446463	0.1032624	-4.657919
TRBV20OR9-	-0.272369	-1.290429	-2.020225	0.0446469	0.1032624	-4.657929
RPS15AP12	-0.3334	2.4309005	-2.020129	0.0446569	0.1032789	-4.658116
GAST	-0.658438	-0.212188	-2.020065	0.0446636	0.1032879	-4.658241
CLRN3	0.6518079	4.2776668	2.0198196	0.0446893	0.1033376	-4.658722
HUNK	-0.375438	4.7378376	-2.019806	0.0446907	0.1033376	-4.658749
CYP4F11	0.2499663	6.2750534	2.0197116	0.0447006	0.1033539	-4.658934
CEACAM19	-0.114445	5.4392987	-2.019544	0.0447182	0.1033879	-4.659262
LINC00587	0.1663655	-1.524892	2.0191874	0.0447555	0.1034628	-4.65996
ATL1	0.2127343	4.0915785	2.0191806	0.0447562	0.1034628	-4.659973
CABLES1	0.1040176	5.8393715	2.0187415	0.0448023	0.1035627	-4.660832
ZP3	-0.196797	4.5866448	-2.018526	0.0448249	0.1036084	-4.661254
SPP2	-0.534525	5.5923396	-2.018364	0.0448419	0.1036394	-4.66157
XPO1	-0.026917	6.6464039	-2.018324	0.0448461	0.1036394	-4.661649
PFKFB2	-0.08614	5.8357226	-2.018317	0.0448468	0.1036394	-4.661662
AC002398.1	-0.412891	0.5593446	-2.017866	0.0448942	0.1037424	-4.662545
RP4-791C19	0.4489423	0.4982034	2.0177607	0.0449053	0.1037614	-4.662751
TAF6L	-0.044768	5.8654097	-2.017639	0.0449181	0.103779	-4.662988
AL162759.1	0.6567614	1.5087983	2.017606	0.0449215	0.103779	-4.663053
SSU72	0.0429236	6.6008987	2.0175905	0.0449232	0.103779	-4.663083
TRIM42	-0.24677	-1.370401	-2.01758	0.0449243	0.103779	-4.663104
CDK20	0.0854189	5.230783	2.0173647	0.0449469	0.1038185	-4.663525
RP11-21I4.	-0.245797	-1.35185	-2.017363	0.0449471	0.1038185	-4.663528
EIF4A1P2	-0.384411	1.6866359	-2.017323	0.0449514	0.1038218	-4.663607
RP4-630A11	0.4202254	-0.124859	2.01703	0.0449821	0.1038864	-4.664179
EMR1	0.3429653	3.5881339	2.0169061	0.0449952	0.1039099	-4.664421
AC003005.2	-0.322744	-0.970551	-2.016848	0.0450013	0.1039175	-4.664535
AC006129.1	0.490566	0.5013787	2.0167117	0.0450157	0.1039441	-4.664802

GHDC	-0.049352	6.2311964	-2.016638	0.0450234	0.1039554	-4.664945
DCUN1D5	-0.054217	5.7950846	-2.016533	0.0450345	0.1039743	-4.66515
RP11-118K6	0.4838012	-0.030303	2.0162765	0.0450615	0.1040302	-4.665652
TNFSF14	0.1964021	5.6864042	2.0158595	0.0451055	0.1041252	-4.666467
DTX2	-0.048504	5.8481186	-2.015778	0.0451141	0.1041385	-4.666627
RP11-20E24	-0.350522	2.0733808	-2.015696	0.0451227	0.1041518	-4.666786
CTD-2609K8	0.301108	-1.0037	2.0152515	0.0451697	0.1042536	-4.667655
RP4-735C1.	0.2657715	-1.361252	2.0150358	0.0451925	0.1042996	-4.668076
DOC2B	0.1812874	5.0069206	2.0149896	0.0451974	0.1043043	-4.668166
DXO	-0.054052	5.9781192	-2.014237	0.045277	0.1044815	-4.669636
SDAD1P1	0.2053744	3.8581707	2.013827	0.0453204	0.104575	-4.670436
RP11-400K9	0.55424	1.8924392	2.0133998	0.0453657	0.1046728	-4.671269
RP11-534L6	-0.237291	-1.280477	-2.013193	0.0453876	0.1047168	-4.671673
AC079613.1	-0.479529	-0.657826	-2.012964	0.0454119	0.1047663	-4.67212
SNRNP35	-0.04947	6.0285789	-2.012923	0.0454163	0.1047697	-4.6722
SDAD1	0.0545774	5.9061384	2.01288	0.0454208	0.1047736	-4.672284
PSAT1	-0.123172	6.4059959	-2.012844	0.0454246	0.1047757	-4.672354
CTD-2192J1	-0.214485	3.0587914	-2.012761	0.0454335	0.1047895	-4.672517
RP11-186B7	-0.121912	4.2585851	-2.012692	0.0454408	0.1047953	-4.672651
RP11-390G1	-0.448504	0.8224392	-2.012669	0.0454432	0.1047953	-4.672695
RRS1	-0.073916	6.1144767	-2.012656	0.0454446	0.1047953	-4.672721
LRRC43	0.4403824	2.1236581	2.0124404	0.0454675	0.1048415	-4.673141
BICC1	0.2374787	5.7338548	2.0124095	0.0454708	0.1048424	-4.673202
CH17-140K2	0.3812775	-0.857114	2.012356	0.0454765	0.1048489	-4.673306
TTI2	0.0604272	5.4281494	2.0122973	0.0454827	0.1048554	-4.673421
PLP1	0.5825783	-0.246735	2.0122757	0.045485	0.1048554	-4.673463
MTND5P28	-0.323579	-0.892869	-2.01221	0.045492	0.1048648	-4.67359
CTD-2507G9	0.275825	-1.23967	2.0121247	0.0455011	0.1048791	-4.673757
AGPAT9	0.1545162	5.6017216	2.0118816	0.0455269	0.1049321	-4.674231
LINC01151	0.6409446	2.7250859	2.0110497	0.0456154	0.1051295	-4.675853
ZDHHC18	-0.046844	6.1962425	-2.010545	0.0456692	0.1052468	-4.676837
MED16	-0.038578	6.4188862	-2.010213	0.0457046	0.1053218	-4.677484
EI24	0.047844	6.7423644	2.0101629	0.04571	0.1053239	-4.677581
PDE4A	0.107483	5.5817628	2.0101506	0.0457113	0.1053239	-4.677605
AP006285.2	-0.510779	2.5456281	-2.009854	0.0457429	0.1053885	-4.678182
NBPF11	-0.084703	5.4630239	-2.009834	0.0457452	0.1053885	-4.678223
GUSBP12	-0.283296	-1.297192	-2.009465	0.0457845	0.1054667	-4.67894
MCAT	0.0569378	5.9202149	2.0094619	0.0457849	0.1054667	-4.678947
C18orf8	0.0428784	6.0240845	2.0093199	0.0458	0.1054895	-4.679224
IFIT1B	0.3431622	-0.939902	2.0093153	0.0458005	0.1054895	-4.679232
RP11-563E2	-0.383507	-0.620633	-2.009164	0.0458167	0.1055145	-4.679527
OR7E156P	-0.19015	-1.439604	-2.009159	0.0458172	0.1055145	-4.679536
CMYA5	-0.204862	4.9699535	-2.009054	0.0458285	0.1055339	-4.679742
CST5	-0.642262	0.4110362	-2.008993	0.045835	0.1055423	-4.679861
RNF219	-0.058673	5.3718183	-2.008857	0.0458495	0.105569	-4.680125
ACBD7	-0.484726	1.9875433	-2.008657	0.0458709	0.1056116	-4.680514
RAB21	0.0301376	6.3814868	2.0084847	0.0458894	0.1056475	-4.68085
CTC-575D19	-0.213182	4.6820159	-2.008307	0.0459084	0.1056847	-4.681196
CT45A3	-0.176033	-1.468432	-2.008197	0.0459202	0.1057051	-4.68141
RP11-554F2	0.4245678	0.0137372	2.0081158	0.0459289	0.1057184	-4.681568
AC004485.3	0.182554	-1.452231	2.00787	0.0459553	0.1057724	-4.682047

PFN1P1	-0.324137	2.8749192	-2.007809	0.0459618	0.1057808	-4.682165
UBE2V1	-0.057751	5.4167108	-2.007721	0.0459713	0.1057959	-4.682337
TRIM63	0.6118653	0.333001	2.0075471	0.0459899	0.1058321	-4.682675
RP11-473E2	0.2073426	-1.41909	2.0074291	0.0460025	0.1058511	-4.682905
TERF2	0.034562	6.0815869	2.007416	0.0460039	0.1058511	-4.68293
CTD-2145A2	-0.355986	2.2062158	-2.007176	0.0460297	0.1059009	-4.683397
IFITM9P	0.4064826	1.6332392	2.0071602	0.0460314	0.1059009	-4.683428
TSPY6P	-0.316408	-1.31672	-2.007038	0.0460446	0.1059218	-4.683666
NOL10	-0.029287	6.14112	-2.007022	0.0460463	0.1059218	-4.683697
PRRT2	-0.201552	3.911759	-2.006973	0.0460515	0.1059272	-4.683793
MRPL23	-0.064411	6.2317882	-2.006791	0.046071	0.1059653	-4.684145
PLCG1-AS1	-0.38288	1.4621902	-2.006759	0.0460745	0.1059667	-4.684208
RP11-46309	-0.484171	1.8396236	-2.006574	0.0460943	0.1060056	-4.684568
RP11-39602	0.618603	0.1662562	2.0062302	0.0461313	0.106084	-4.685237
PHEX	0.4736626	3.1866236	2.0059815	0.0461581	0.1061389	-4.685721
MROH9	0.2329014	-1.258585	2.0057514	0.0461828	0.1061891	-4.686168
AL441988.1	-0.270635	-1.125171	-2.005499	0.04621	0.1062449	-4.686659
CHRNA5	0.4145981	2.9158906	2.0054615	0.0462141	0.1062475	-4.686731
RP11-395L1	0.2805765	3.0879366	2.0054123	0.0462194	0.106253	-4.686827
DDB2	0.0626608	6.0753983	2.0052969	0.0462318	0.1062749	-4.687052
SPATA17	-0.591266	2.2159294	-2.005174	0.046245	0.1062985	-4.68729
AC234582.1	0.3077839	-1.171514	2.0047971	0.0462857	0.1063853	-4.688023
AC106869.2	-0.602638	0.8500997	-2.004727	0.0462933	0.1063961	-4.68816
RP11-483E1	-0.464617	-0.345263	-2.004642	0.0463024	0.1064103	-4.688324
AC068831.6	-0.319373	2.2435147	-2.004253	0.0463444	0.1065002	-4.68908
RP11-753A2	0.4393424	-0.691567	2.0033914	0.0464375	0.1067074	-4.690754
OSBPL3	-0.083257	5.6953014	-2.003325	0.0464447	0.1067086	-4.690883
ADRA2B	0.1817056	4.5635824	2.0033242	0.0464448	0.1067086	-4.690884
CDK5RAP3	-0.044667	6.6261968	-2.003306	0.0464468	0.1067086	-4.69092
AC114271.2	-0.127034	3.893135	-2.003196	0.0464587	0.1067292	-4.691134
RP13-228J1	-0.396029	-0.625183	-2.003018	0.0464779	0.1067666	-4.691479
LINC00626	0.3291426	-1.128533	2.0027466	0.0465073	0.1068274	-4.692006
BTF3P10	-0.376956	-0.549322	-2.002667	0.0465158	0.1068385	-4.69216
CTD-2270L9	-0.405619	1.5881395	-2.002648	0.0465179	0.1068385	-4.692197
ISCA1	0.0441131	6.1646644	2.0024624	0.0465381	0.1068746	-4.692558
LINC01540	0.4237683	2.8976315	2.0024491	0.0465395	0.1068746	-4.692584
GTF2A1	0.0389817	6.1474741	2.0022037	0.0465661	0.1069289	-4.69306
EBLN2	-0.416294	2.7623064	-2.001977	0.0465907	0.1069787	-4.693501
C1orf228	0.1811406	4.5501097	2.0018913	0.0466	0.1069932	-4.693666
CCNJL	0.267344	4.1612812	2.0016903	0.0466218	0.1070366	-4.694056
NEK11	0.1624103	4.5050209	2.001604	0.0466311	0.1070464	-4.694224
NGEF	-0.208411	6.1313142	-2.001597	0.0466319	0.1070464	-4.694238
DKFZP434AC	0.4180559	-0.409811	2.0014366	0.0466493	0.1070796	-4.694549
AP000251.3	0.3140333	-1.017549	2.0013894	0.0466544	0.1070846	-4.69464
NPEPL1	-0.060161	5.7057372	-2.001267	0.0466677	0.1071083	-4.694877
AC005754.7	-0.38288	-0.892035	-2.001223	0.0466725	0.1071126	-4.694962
RP11-1016B	-0.322941	-1.073461	-2.00118	0.0466772	0.1071167	-4.695047
MANSC4	-0.402727	-0.402019	-2.001071	0.046689	0.1071371	-4.695258
S100A4	0.0986416	5.958351	2.0004822	0.046753	0.1072772	-4.6964
RN7SL471P	0.1174014	-1.52708	2.000428	0.0467589	0.107284	-4.696505
ABHD17B	0.0492966	5.7904216	2.000331	0.0467695	0.1072938	-4.696693

GMCL1	-0.049985	6.0341917	-2.000323	0.0467704	0.1072938	-4.696709
RP11-864I4	-0.070395	5.0059479	-2.000308	0.046772	0.1072938	-4.696739
ABO	0.4811348	3.1297341	2.0002588	0.0467773	0.1072992	-4.696833
H3F3AP4	-0.112276	4.8029399	-2.000109	0.0467936	0.1073298	-4.697123
WDR17	0.488471	1.4531858	1.9999919	0.0468064	0.1073524	-4.697351
RP11-578F2	-0.390519	-0.517364	-1.999907	0.0468157	0.1073669	-4.697516
RP11-379B1	0.2616978	-1.234276	1.9996858	0.0468397	0.1074093	-4.697944
PSPHP1	-0.912406	3.3856935	-1.999652	0.0468434	0.1074093	-4.698009
AL132772.1	-0.433726	2.1499178	-1.999648	0.0468438	0.1074093	-4.698017
SNRNP200	-0.028219	6.803358	-1.999629	0.0468459	0.1074093	-4.698054
ZNF774	-0.126652	4.5076172	-1.999566	0.0468528	0.1074183	-4.698176
LINC00969	-0.064544	5.8531978	-1.999537	0.0468559	0.1074187	-4.698232
SERINC2	0.0716956	6.8601427	1.9994886	0.0468612	0.1074204	-4.698327
LINC01012	-0.576296	0.5309199	-1.999459	0.0468645	0.1074204	-4.698384
RASGRP1	0.2061848	4.6360212	1.9994497	0.0468655	0.1074204	-4.698402
RPS3AP34	0.3314235	-0.882532	1.9990034	0.0469141	0.1075252	-4.699267
RPSAP6	-0.271976	-1.128359	-1.998901	0.0469253	0.1075382	-4.699465
RP11-766F1	0.5955291	0.1837682	1.9988974	0.0469257	0.1075382	-4.699472
FNDC1-IT1	0.2260959	-1.33128	1.998627	0.0469552	0.1075978	-4.699996
ASUN	-0.045032	5.9369139	-1.998605	0.0469576	0.1075978	-4.700039
RP11-254I2	-0.46847	1.6485136	-1.998291	0.0469919	0.1076657	-4.700648
TMEM89	-0.350946	-0.845739	-1.99828	0.0469932	0.1076657	-4.700669
IFNE	0.3223729	-1.179222	1.9982475	0.0469967	0.107667	-4.700732
RP11-680G2	0.3750695	2.4062569	1.9981713	0.047005	0.1076793	-4.700879
RP13-895J2	-0.407349	-0.98671	-1.997964	0.0470277	0.1077246	-4.701282
KB-1572G7.	-0.470794	0.365985	-1.99781	0.0470445	0.107755	-4.701579
GSTTP2	0.2898445	-1.112191	1.9977882	0.0470469	0.107755	-4.701621
RCC2P6	-0.416687	-0.564459	-1.997642	0.0470628	0.1077848	-4.701904
RP11-341G2	0.4657263	-0.474353	1.9975732	0.0470704	0.1077934	-4.702038
LINC00392	-0.250794	-1.409865	-1.997554	0.0470725	0.1077934	-4.702075
RP1-140K8.	-0.30937	-1.063105	-1.997471	0.0470816	0.1078074	-4.702236
PGAM1P7	0.4361942	-0.174175	1.9973617	0.0470935	0.107828	-4.702447
RP11-538D1	-0.292823	-1.231655	-1.997309	0.0470992	0.1078344	-4.702549
RP11-95I16	-0.45788	-0.103971	-1.997005	0.0471326	0.1079039	-4.703138
AC007308.6	-0.467647	0.9770514	-1.996949	0.0471387	0.1079111	-4.703246
EXT2	0.0312892	6.3885896	1.9968304	0.0471517	0.1079342	-4.703476
ZNF616	-0.052381	5.4745042	-1.996796	0.0471554	0.1079359	-4.703542
RP11-923I1	-0.497085	0.4099027	-1.99655	0.0471824	0.107991	-4.704019
VRTN	-0.213831	-1.362276	-1.996515	0.0471863	0.1079929	-4.704086
LHX9	0.5745942	3.0373675	1.9963922	0.0471997	0.1080106	-4.704324
RP4-550H1.	-0.414204	-0.480606	-1.996391	0.0471999	0.1080106	-4.704327
AL355480.1	-0.332741	-0.843609	-1.996259	0.0472143	0.1080368	-4.704581
RAB1B	0.0320858	6.6851336	1.9961257	0.047229	0.1080526	-4.70484
PFN2	0.1453379	5.6055723	1.9961241	0.0472291	0.1080526	-4.704843
SLC22A17	0.219488	5.1600524	1.9960798	0.047234	0.1080526	-4.704929
CTD-2170G1	0.5776256	0.1813061	1.9960664	0.0472355	0.1080526	-4.704955
EIF1	0.0364022	7.1240249	1.9960616	0.047236	0.1080526	-4.704964
KLHDC4	0.0457421	6.0235663	1.9957256	0.0472729	0.1081271	-4.705614
CCR5	0.1547934	5.0800766	1.9957107	0.0472745	0.1081271	-4.705643
C11orf54	0.0789923	6.4992515	1.9956838	0.0472775	0.1081271	-4.705695
MIR587	0.2203238	-1.324973	1.9955612	0.0472909	0.1081511	-4.705933

TTC7A	0.056276	6.168386	1.9954735	0.0473006	0.1081664	-4.706102
NPHP4	0.1559179	5.2195022	1.9954376	0.0473045	0.1081686	-4.706172
ZNF12	-0.037933	5.9230518	-1.995213	0.0473292	0.1082183	-4.706606
SERBP1P6	0.3905917	-0.273948	1.9950683	0.0473451	0.1082436	-4.706886
IGKJ5	-0.446173	-0.603647	-1.995058	0.0473462	0.1082436	-4.706905
S100A9	0.1765172	5.536846	1.9947976	0.0473749	0.1083024	-4.70741
RPS17P1	0.2464918	-1.302119	1.9947576	0.0473793	0.1083057	-4.707487
C19orf68	-0.049863	5.6157003	-1.994601	0.0473965	0.1083383	-4.707791
REV3L	0.0612067	5.7609634	1.9945724	0.0473996	0.1083387	-4.707845
RP11-849N1	0.4814816	0.600687	1.994488	0.0474089	0.1083531	-4.708008
RP11-184D1	-0.223872	-1.366677	-1.994349	0.0474242	0.1083793	-4.708276
RP11-448G4	-0.407381	-0.292382	-1.994308	0.0474288	0.1083793	-4.708357
RP11-255E6	0.2242745	-1.280986	1.9943029	0.0474293	0.1083793	-4.708367
RPL5P23	-0.31814	2.0095316	-1.994167	0.0474443	0.1084051	-4.70863
LHFPL5	0.4769077	-0.606645	1.9941464	0.0474465	0.1084051	-4.708669
C8orf31	0.4299515	2.7652001	1.9938076	0.0474839	0.1084836	-4.709324
RP11-546K2	-0.494363	0.108173	-1.993729	0.0474926	0.1084968	-4.709477
RP11-563M4	0.1466931	-1.48463	1.9937005	0.0474957	0.108497	-4.709531
RP11-502M1	0.1523613	-1.458977	1.9935963	0.0475072	0.1085165	-4.709733
NACA3P	-0.393572	4.2194123	-1.993471	0.047521	0.1085412	-4.709974
WI2-85898F	0.3398262	-0.961515	1.9934292	0.0475256	0.108545	-4.710055
PAX6	-0.118762	4.5967921	-1.993223	0.0475483	0.10859	-4.710453
RP11-184E9	-0.196777	-1.414449	-1.993061	0.0475662	0.1086242	-4.710767
RP11-219E7	0.2959784	-1.141367	1.9927387	0.0476018	0.1086986	-4.71139
PDP1	0.1309829	5.4683394	1.992482	0.0476302	0.1087546	-4.711886
AMIGO2	0.1488175	5.2815159	1.9924628	0.0476323	0.1087546	-4.711923
FADS2	-0.131358	6.6104759	-1.992357	0.047644	0.1087745	-4.712127
CLSTN1	-0.06013	6.4444372	-1.992086	0.0476739	0.1088361	-4.71265
OSTC	-0.041646	6.564568	-1.991997	0.0476838	0.1088518	-4.712823
SLAMF9	0.4616655	3.1926348	1.9918535	0.0476997	0.1088812	-4.7131
NMU	-0.507653	-0.085651	-1.99175	0.0477111	0.1089006	-4.7133
RPS10P18	-0.228804	-1.284639	-1.991575	0.0477305	0.108938	-4.713638
MATN1-AS1	-0.128326	4.2365185	-1.991539	0.0477345	0.1089402	-4.713707
CTD-3065J1	-0.253575	3.0858327	-1.99121	0.0477709	0.1090166	-4.714343
GAGE2A	-0.566738	-0.511692	-1.991027	0.0477912	0.1090562	-4.714697
SLC17A1	-0.333346	5.4054749	-1.990846	0.0478112	0.1090948	-4.715044
SNORD114-1	-0.186672	-1.463215	-1.99082	0.0478141	0.1090948	-4.715096
KLK2	-0.571004	-0.387079	-1.99064	0.0478341	0.1091335	-4.715444
C1orf74	-0.087065	5.1790956	-1.9902	0.0478829	0.109238	-4.716292
RP11-630I5	-0.437936	-0.069463	-1.990039	0.0479007	0.1092719	-4.716602
MRPL15	0.0510136	6.4091256	1.9899963	0.0479055	0.109276	-4.716685
DNALI1	0.2263435	5.4112738	1.9895936	0.0479503	0.1093712	-4.717462
RP11-30L15	-0.444683	0.325811	-1.988876	0.0480301	0.1095446	-4.718847
RP11-755E2	-0.483552	0.3320522	-1.988856	0.0480323	0.1095446	-4.718885
IL36B	0.2506507	-1.281995	1.9886749	0.0480524	0.1095837	-4.719234
PMS2P1	0.0522604	5.5384073	1.9885925	0.0480616	0.1095978	-4.719393
CXADRP2	0.12712	-1.517369	1.9884921	0.0480728	0.1096164	-4.719587
CTD-2270L9	-0.10097	4.6620115	-1.988312	0.0480928	0.1096552	-4.719933
KB-1184D12	0.1778003	-1.423249	1.9881209	0.0481142	0.109697	-4.720303
RP11-20G13	-0.331603	-1.076844	-1.987848	0.0481446	0.1097596	-4.720829
SHISA2	0.5231938	2.8426048	1.9878199	0.0481477	0.1097598	-4.720883

HYI-AS1	-0.338377	2.4834411	-1.987579	0.0481745	0.1098141	-4.721346
RP11-26H16	-0.440739	0.8118174	-1.98733	0.0482024	0.1098707	-4.721827
RP11-10C24	-0.209018	3.2102145	-1.987137	0.0482239	0.1099129	-4.722198
AC104777.2	0.1549139	-1.462435	1.9868226	0.048259	0.1099861	-4.722805
RP11-536C1	-0.473635	1.1893376	-1.986575	0.0482867	0.1100362	-4.723282
SPATA31E1	-0.283099	-1.162036	-1.986572	0.048287	0.1100362	-4.723287
THADA	-0.035989	6.1427521	-1.986417	0.0483044	0.110069	-4.723587
PPIC	0.0494312	6.2536628	1.9863058	0.0483168	0.1100867	-4.7238
RP11-174G1	0.487304	0.425712	1.9862932	0.0483182	0.1100867	-4.723825
RP11-279F6	0.5835401	0.4387026	1.9862482	0.0483233	0.1100913	-4.723911
FAM96A	0.0526706	6.5188342	1.9860214	0.0483486	0.1101422	-4.724348
MYOT	0.5341086	0.2538111	1.9856487	0.0483904	0.1102304	-4.725066
FAM19A4	-0.677354	0.3860907	-1.985523	0.0484045	0.1102515	-4.725308
TMPRSS11A	0.1964981	-1.441244	1.985512	0.0484057	0.1102515	-4.725329
TCIRG1	-0.05151	6.5947862	-1.98545	0.0484126	0.1102603	-4.725448
OXCT2	-0.442671	2.8031471	-1.985281	0.0484316	0.1102968	-4.725774
DCTN2	0.0319292	6.5481566	1.9851093	0.0484508	0.1103336	-4.726104
RP11-64B16	0.2003259	-1.384247	1.9848108	0.0484843	0.110403	-4.726679
MAFB	0.0875341	6.1072401	1.9844061	0.0485297	0.1104995	-4.727458
AC074389.5	-0.188063	-1.462532	-1.984337	0.0485374	0.1105102	-4.72759
RP11-100G1	-0.309501	-1.145327	-1.984279	0.048544	0.1105182	-4.727703
RP13-25801	-0.384293	-0.597428	-1.984066	0.0485678	0.1105657	-4.728112
RP11-330A1	-0.348426	-1.067824	-1.984022	0.0485729	0.1105703	-4.728198
THOC7	-0.040939	6.2377514	-1.983956	0.0485803	0.1105802	-4.728325
BX842568.4	0.1760391	-1.424147	1.9838817	0.0485886	0.1105922	-4.728467
SRPRB	-0.042101	6.5945821	-1.983796	0.0485982	0.1105981	-4.728631
TAS2R62P	0.2283973	-1.362832	1.9837814	0.0485999	0.1105981	-4.72866
BEND3P2	0.281497	-1.187376	1.983778	0.0486003	0.1105981	-4.728666
RP11-82L18	0.4210266	2.7059058	1.9837421	0.0486043	0.1106004	-4.728735
CTD-2535I1	-0.523653	-0.430619	-1.983597	0.0486206	0.1106306	-4.729015
CEP63	-0.031405	5.9557375	-1.983401	0.0486426	0.1106735	-4.729391
RP11-1M18.	-0.397286	-0.46724	-1.983375	0.0486455	0.1106735	-4.729441
SCAF11	0.031681	6.4842872	1.9830097	0.0486867	0.1107592	-4.730144
HPS3	-0.049921	6.0283445	-1.982987	0.0486893	0.1107592	-4.730188
TST	0.0788719	6.8989054	1.9828976	0.0486993	0.1107751	-4.73036
CAPN12	-0.191513	5.5982813	-1.982762	0.0487146	0.1107942	-4.730621
SYT17	0.1424085	5.7994119	1.9827363	0.0487175	0.1107942	-4.73067
CH17-258A2	-0.282307	-1.109813	-1.982728	0.0487184	0.1107942	-4.730686
SDR42E2	-0.459411	0.5555329	-1.982716	0.0487198	0.1107942	-4.73071
AP5Z1	-0.045379	6.2849628	-1.982625	0.0487301	0.110804	-4.730885
C16orf54	0.2155917	4.4718027	1.9826231	0.0487302	0.110804	-4.730888
HMGN2P4	-0.42998	1.0347468	-1.982591	0.0487338	0.1108045	-4.730949
SEMA3F	-0.05352	6.0286449	-1.982567	0.0487365	0.1108045	-4.730995
RP11-57C19	-0.295369	-1.016688	-1.982165	0.0487819	0.1109008	-4.731769
GYPA	0.3797775	-1.073619	1.9820269	0.0487974	0.1109283	-4.732034
SLC25A39P2	-0.151012	-1.480701	-1.982003	0.0488001	0.1109283	-4.732079
RP11-405A1	0.6231374	0.6702495	1.981879	0.0488141	0.1109533	-4.732318
TTF2	-0.068158	5.5427874	-1.981818	0.048821	0.1109621	-4.732436
RPL13A	-0.043894	7.3665421	-1.981713	0.0488328	0.1109821	-4.732637
GAPDHP64	-0.328326	-0.918887	-1.981534	0.0488531	0.1110212	-4.732982
CTD-2007H1	-0.301447	2.6147615	-1.981495	0.0488575	0.1110243	-4.733057

RP11-4802C	0.4177257	-0.572571	1.9814133	0.0488667	0.1110382	-4.733213
RNA5SP111	-0.488242	1.515729	-1.980991	0.0489144	0.1111397	-4.734025
CTD-3065J1	-0.150336	4.5382783	-1.980902	0.0489245	0.1111557	-4.734196
U2AF1	-0.160932	3.9917569	-1.980875	0.0489275	0.1111557	-4.734248
C17orf61-F	0.4356402	0.3852094	1.9808394	0.0489315	0.1111579	-4.734316
FMN1	0.2123333	4.5611881	1.9803872	0.0489827	0.1112671	-4.735184
CTD-2566J3	-0.617818	0.3719497	-1.980339	0.0489881	0.1112692	-4.735277
DIO2	0.5384629	3.2154432	1.9803256	0.0489896	0.1112692	-4.735303
LA16c-431H	0.4954903	0.7192753	1.9802849	0.0489942	0.1112727	-4.735381
RPL9P3	-0.420248	-0.060333	-1.979873	0.0490409	0.1113717	-4.736172
LLNLR-246C	-0.310887	2.4620999	-1.979512	0.0490818	0.1114577	-4.736866
HP	0.2041192	7.4525318	1.9793748	0.0490973	0.111486	-4.737128
USP48	0.0318253	6.1851172	1.9793196	0.0491036	0.1114933	-4.737234
PTEN	0.0501064	6.5039591	1.9790764	0.0491312	0.111549	-4.737701
SMARCA4	-0.041399	6.5737732	-1.978879	0.0491535	0.1115928	-4.738079
GS1-124K5.	-0.279905	2.6160705	-1.978765	0.0491665	0.1116153	-4.738299
RP11-47A8.	0.1301309	4.4649214	1.9784173	0.049206	0.1116932	-4.738966
GFOD1	0.0896451	5.6533675	1.9784089	0.0492069	0.1116932	-4.738982
RP11-519G1	-0.578187	0.418723	-1.978342	0.0492145	0.1117035	-4.73911
TTC14	0.0417976	5.8582873	1.97818	0.049233	0.1117384	-4.739421
AP000351.4	0.2815094	-1.151244	1.9779829	0.0492554	0.111781	-4.7398
LINC00562	-0.317403	2.4800776	-1.977961	0.0492579	0.111781	-4.739842
WDR72	-0.383172	5.5281953	-1.9779	0.0492648	0.1117897	-4.739958
SNORD123	0.2375898	-1.31021	1.9776528	0.0492929	0.1118466	-4.740433
AC016708.2	-0.3936	3.2827249	-1.977562	0.0493032	0.1118631	-4.740607
AC079907.1	-0.277929	-1.064471	-1.977511	0.049309	0.1118693	-4.740705
CCZ1	-0.087831	5.2703618	-1.977465	0.0493143	0.1118742	-4.740793
RP13-228J1	-0.227842	-1.307016	-1.977389	0.0493229	0.1118869	-4.740939
RPL12P33	-0.408042	-0.38301	-1.977319	0.0493308	0.1118979	-4.741072
RP11-48F14	0.2009546	-1.430659	1.9771829	0.0493464	0.1119262	-4.741334
OR8T1P	0.3716542	-0.687967	1.97695	0.0493729	0.1119794	-4.741781
LAMA4	0.0664723	6.1288905	1.9767574	0.0493949	0.1120222	-4.74215
APIP	-0.041587	6.0061359	-1.976631	0.0494092	0.1120478	-4.742392
AC016735.2	-0.448759	-0.402317	-1.976169	0.049462	0.1121605	-4.743278
SCLT1	0.0784219	5.203047	1.9758502	0.0494983	0.112236	-4.743889
TXNDC17	0.0574611	6.3015517	1.9754745	0.0495412	0.1123239	-4.744609
AC010894.5	-0.218539	-1.303152	-1.975457	0.0495432	0.1123239	-4.744643
DHRS2	-0.388134	5.2573568	-1.975388	0.0495511	0.1123346	-4.744774
CTC-261N6.	-0.156516	-1.478002	-1.974966	0.0495994	0.1124329	-4.745584
CTD-3193K9	-0.335844	-0.916034	-1.974955	0.0496006	0.1124329	-4.745603
MGC45922	-0.455791	0.5761358	-1.974623	0.0496386	0.112512	-4.74624
RP4-665N4.	-0.290566	-1.159085	-1.974596	0.0496417	0.112512	-4.746292
KCNC4-AS1	0.4447382	0.4119121	1.9745152	0.0496509	0.1125261	-4.746447
ABCF1	-0.034759	6.5991806	-1.974214	0.0496855	0.1125973	-4.747024
EIF3E	-0.050914	6.8809774	-1.974012	0.0497086	0.1126428	-4.747411
RP11-540B6	-0.067213	5.0376003	-1.973816	0.049731	0.1126865	-4.747785
PTPRG-AS1	-0.478852	2.2845837	-1.973626	0.0497528	0.112729	-4.74815
MCPH1	0.0537537	5.6414831	1.9732742	0.0497931	0.1128133	-4.748823
RP11-167B3	-0.462147	-0.771309	-1.973211	0.0498004	0.1128157	-4.748943
GDAP2	0.0441423	5.7452487	1.9732073	0.0498008	0.1128157	-4.748951
RP11-796G6	-0.436148	1.1309317	-1.973184	0.0498034	0.1128157	-4.748995

ITPR3	0.1617724	5.6014889	1.9730079	0.0498237	0.1128546	-4.749332
ROPN1	0.3141094	-1.101562	1.9729339	0.0498322	0.1128668	-4.749474
RP11-555K2	-0.417142	-0.954667	-1.972885	0.0498378	0.1128725	-4.749568
CCP110	0.0705382	5.4304956	1.9728351	0.0498435	0.1128725	-4.749663
MGAT2P1	-0.167392	-1.472669	-1.972831	0.049844	0.1128725	-4.74967
RP11-62H2C	-0.511565	0.4782834	-1.972787	0.0498491	0.112877	-4.749755
MTHFD2L	0.0798575	5.4945199	1.9727151	0.0498573	0.1128887	-4.749893
RP11-28002	0.4534437	0.5718432	1.9726329	0.0498668	0.1129006	-4.75005
SSR1	-0.033158	6.8344557	-1.972616	0.0498687	0.1129006	-4.750083
8-Mar	0.0432637	6.278478	1.9725532	0.0498759	0.1129098	-4.750202
PDE11A	0.2566174	4.7130055	1.9725106	0.0498808	0.1129139	-4.750284
PLD1	0.0772192	6.1663922	1.9722377	0.0499122	0.1129779	-4.750806
TRAPPC4	0.0418466	6.2049375	1.9721041	0.0499275	0.1130046	-4.751062
ZDHHC11B	0.2903186	4.6976799	1.9720813	0.0499302	0.1130046	-4.751105
RN7SL809P	-0.426116	-0.107274	-1.971987	0.049941	0.1130221	-4.751286
NKX2-4	0.2449088	-1.439337	1.9719409	0.0499463	0.1130271	-4.751374
TGIF2LX	-0.510593	-0.604488	-1.971889	0.0499523	0.1130314	-4.751474
RP11-53019	-0.061509	5.5505828	-1.971846	0.0499572	0.1130314	-4.751554
LINC00087	0.1963173	4.2870208	1.9718187	0.0499604	0.1130314	-4.751607
RP11-23F23	0.5530892	1.6237048	1.9718167	0.0499606	0.1130314	-4.751611
RANBP3	-0.033027	6.3261768	-1.971787	0.049964	0.113032	-4.751668
TMEM126A	-0.050289	5.9283372	-1.971754	0.0499678	0.1130338	-4.751732
PCYOX1	0.0514143	6.6977595	1.9711699	0.0500351	0.1131788	-4.752848
DHX32	0.0976286	5.8618159	1.9709239	0.0500634	0.1132359	-4.753319
RCOR2	-0.385766	3.5155801	-1.970541	0.0501076	0.1133288	-4.754051
TRAF3IP1	0.0351775	5.8334251	1.9704898	0.0501135	0.1133351	-4.754148
SNORA3	-0.209402	-1.316059	-1.970334	0.0501314	0.1133687	-4.754446
MMP7	0.6129082	4.4530389	1.9701444	0.0501533	0.1134112	-4.754809
SERPINA11	-0.346357	6.2551358	-1.970053	0.0501639	0.1134281	-4.754984
CLDN7	0.1734957	6.1188284	1.9699498	0.0501758	0.1134479	-4.755181
RP5-1042I8	0.1301387	4.4149832	1.9698978	0.0501818	0.1134545	-4.75528
AURKAPS1	-0.423264	-0.261734	-1.969807	0.0501923	0.1134706	-4.755453
AC007016.3	-0.369648	-0.727879	-1.969757	0.0501981	0.1134706	-4.75555
MRPL14	-0.052795	6.4655313	-1.969755	0.0501982	0.1134706	-4.755552
SPP1	0.2746903	6.5717542	1.969179	0.0502649	0.1136111	-4.756653
RAB43P1	0.1759285	4.1162872	1.9691638	0.0502666	0.1136111	-4.756682
PPP4R1	-0.031535	6.3271542	-1.968853	0.0503026	0.1136853	-4.757276
SMARCE1P2	-0.239274	-1.24618	-1.968793	0.0503095	0.1136939	-4.75739
RP11-363D1	-0.385944	-0.840551	-1.968667	0.0503242	0.11372	-4.757632
NABP2	-0.045702	6.1453609	-1.968576	0.0503346	0.1137366	-4.757804
ZFC3H1	-0.042774	5.9479661	-1.968543	0.0503385	0.1137382	-4.757868
LINC00216	-0.4263	0.9175859	-1.968396	0.0503555	0.113767	-4.758148
FAM87B	0.4072506	0.296687	1.9683791	0.0503574	0.113767	-4.758181
ZNF847P	-0.230047	-1.298179	-1.968304	0.0503661	0.1137796	-4.758324
ATP6V0D2	-0.499342	3.3304153	-1.968151	0.0503838	0.113811	-4.758615
RP11-204C2	-0.225422	-1.37745	-1.96813	0.0503863	0.113811	-4.758656
GATSL2	0.1598656	4.2720548	1.9679427	0.050408	0.1138531	-4.759014
DDX10P2	-0.250596	-1.222143	-1.967796	0.050425	0.1138845	-4.759294
RP11-563J2	0.4797266	1.9531697	1.9677682	0.0504282	0.1138847	-4.759347
RPS2P6	-0.281161	-1.032081	-1.967727	0.050433	0.1138883	-4.759425
RHD	-0.355577	2.9842386	-1.967537	0.0504551	0.1139313	-4.759789

SCO1	0.047232	6.1879632	1.967477	0.050462	0.1139397	-4.759903
RP3-426I6.	0.4078572	0.0777203	1.9674506	0.0504651	0.1139397	-4.759953
ZNF510	-0.073391	5.414996	-1.967381	0.0504732	0.1139509	-4.760086
SPRR2D	0.3745446	-1.094911	1.9670881	0.0505072	0.1140206	-4.760645
RNA5SP37	0.4045821	-0.312383	1.9669895	0.0505186	0.1140385	-4.760833
CTD-2547L1	0.3580584	-0.704133	1.966966	0.0505213	0.1140385	-4.760878
OR7E13P	0.2377049	-1.31967	1.9667987	0.0505408	0.1140754	-4.761197
RP11-403P1	-0.155805	-1.478351	-1.966726	0.0505492	0.1140874	-4.761336
ACTG1	0.0345691	7.4474735	1.9665886	0.0505652	0.1141163	-4.761598
RP11-513M1	-0.2936	2.9373362	-1.966515	0.0505737	0.1141285	-4.761738
RP11-123K3	0.4507548	0.1274979	1.9664586	0.0505803	0.114135	-4.761846
POLR3K	0.0617015	5.6990105	1.9664369	0.0505828	0.114135	-4.761888
RP11-21401	-0.246625	-1.201026	-1.966149	0.0506163	0.1142035	-4.762437
RP11-437J2	0.1240402	-1.515383	1.9660818	0.0506241	0.114214	-4.762565
DENND6A	0.0344776	5.8907592	1.9660374	0.0506293	0.1142186	-4.76265
C1orf115	-0.080774	6.69919	-1.96599	0.0506348	0.114224	-4.76274
RPSAP31	-0.419507	-0.072796	-1.965921	0.0506428	0.114235	-4.762871
AF064860.7	0.3601211	-1.065501	1.9657202	0.0506662	0.1142772	-4.763255
DNAJC15	0.088175	6.112683	1.9657068	0.0506678	0.1142772	-4.76328
RP11-335L2	0.4227408	1.003214	1.9655734	0.0506833	0.1143052	-4.763535
TUBB4A	-0.456611	4.3244249	-1.965346	0.0507097	0.1143577	-4.763967
DUXAP10	-0.538898	2.2218965	-1.965271	0.0507185	0.1143704	-4.76411
CISD1	0.0552241	6.1370578	1.9652136	0.0507252	0.1143785	-4.764221
RP11-545N8	-0.366736	-0.718788	-1.965065	0.0507425	0.1144104	-4.764503
POR	0.0661975	7.0807326	1.9647764	0.0507762	0.1144793	-4.765054
RP11-554D2	0.2500019	-1.235593	1.9646421	0.0507919	0.1145015	-4.76531
AC069294.1	-0.462865	-0.268977	-1.964638	0.0507923	0.1145015	-4.765317
CCDC28B	-0.119383	5.2212606	-1.964589	0.050798	0.1145073	-4.765411
CALM2	0.0270652	6.978506	1.9644686	0.0508121	0.114532	-4.765641
CXorf40A	0.0572274	5.6367326	1.9644187	0.0508179	0.1145381	-4.765736
RP11-1191J	0.3677495	-0.798172	1.9643241	0.050829	0.114555	-4.765916
LGALS9DP	0.3204884	-1.137465	1.9643004	0.0508317	0.114555	-4.765961
RP11-434P1	-0.415426	-0.032978	-1.964127	0.0508521	0.1145848	-4.766293
ARRDC5	0.414417	1.6789841	1.9641198	0.0508528	0.1145848	-4.766305
RP11-27M24	-0.302757	-1.046816	-1.964107	0.0508544	0.1145848	-4.766331
SEPW1	0.0705611	6.4881086	1.9640246	0.050864	0.1145993	-4.766487
ZFYVE9	0.0460724	5.9078482	1.9639854	0.0508685	0.1146025	-4.766561
AC073869.2	-0.159056	-1.450272	-1.963843	0.0508851	0.1146329	-4.766832
NBPF17P	-0.265683	-1.23207	-1.963754	0.0508955	0.1146492	-4.767001
ANKUB1	0.3799176	-0.654848	1.963504	0.0509248	0.1147081	-4.767479
RP11-360L9	0.4760433	0.6353355	1.9634251	0.050934	0.1147218	-4.767629
CCDC144A	0.5572706	0.1195165	1.963239	0.0509558	0.1147637	-4.767983
AC093901.1	-0.380443	2.7269394	-1.962917	0.0509935	0.1148415	-4.768597
ASB15	0.4934154	-0.238265	1.9628289	0.0510038	0.1148577	-4.768764
DNPH1	-0.055242	6.4409481	-1.96242	0.0510516	0.1149583	-4.769542
AC004980.9	0.3478264	-0.800739	1.9622278	0.0510742	0.1150021	-4.769909
GPT	0.161714	6.3431441	1.9620747	0.0510922	0.1150354	-4.7702
CTD-2196E1	-0.220536	3.1085189	-1.961948	0.051107	0.1150617	-4.770442
TNFRSF11A	0.1509328	4.9671535	1.9619116	0.0511113	0.1150642	-4.770511
EVI5	0.0509422	5.9570948	1.9618588	0.0511175	0.1150711	-4.770611
STEAP1B	-0.497536	2.0148519	-1.960951	0.0512241	0.115304	-4.772339

RPS4Y2	-0.307619	-1.161643	-1.960447	0.0512834	0.1154302	-4.773298
LRRC27	0.0603557	5.3421661	1.9604166	0.0512869	0.1154311	-4.773355
ALAD	0.0697227	6.7325099	1.9601871	0.0513139	0.1154848	-4.773792
GS1-24F4.2	-0.476011	-0.415731	-1.960145	0.0513189	0.1154888	-4.773872
XRN2	0.0328988	6.4558233	1.9600718	0.0513275	0.115501	-4.774011
RP11-307E1	0.4525657	1.1702718	1.960033	0.051332	0.1155042	-4.774085
RP11-944C7	-0.406957	2.4541712	-1.959981	0.0513381	0.1155108	-4.774183
RP11-946L2	-0.180373	-1.439819	-1.959882	0.0513499	0.1155301	-4.774373
TBATA	0.2238736	-1.336932	1.9596065	0.0513823	0.1155958	-4.774896
RP1-300I2.	-0.217758	-1.40416	-1.959566	0.051387	0.1155994	-4.774972
TOP3B	-0.259698	3.2836042	-1.959224	0.0514274	0.115683	-4.775623
RP11-90H3.	-0.324594	-0.86072	-1.959112	0.0514406	0.1157057	-4.775837
RP5-101101	0.1704834	-1.472506	1.9588962	0.051466	0.1157557	-4.776246
LINC00944	-0.558574	2.7540024	-1.958784	0.0514793	0.1157718	-4.77646
ATP5G2P4	-0.382963	-0.521551	-1.958782	0.0514795	0.1157718	-4.776464
SNORA71C	-0.464071	0.5657298	-1.958753	0.0514829	0.1157722	-4.776518
RBM23	0.055552	6.2934547	1.9581304	0.0515564	0.1159305	-4.777701
RGR	-0.2674	-1.24618	-1.957755	0.0516008	0.1160232	-4.778415
RP11-380J1	0.3451244	-1.053309	1.9574897	0.0516322	0.1160865	-4.778918
RP11-361I1	0.3126211	-1.031261	1.9573538	0.0516482	0.1161155	-4.779177
RPL31P28	-0.203633	-1.354272	-1.957105	0.0516777	0.1161746	-4.779649
RPS6KA4	0.0362311	6.128782	1.956964	0.0516944	0.1162049	-4.779917
EPO	-0.622528	3.2366218	-1.956847	0.0517083	0.116229	-4.78014
ZNF204P	-0.322528	4.0795355	-1.95671	0.0517245	0.1162583	-4.7804
RP11-8L2.1	-0.425074	-0.889695	-1.956578	0.0517401	0.1162862	-4.780649
RP11-386G1	-0.448325	0.7683605	-1.956445	0.0517559	0.1163092	-4.780902
WNT5A	0.2648177	5.0899235	1.9564382	0.0517567	0.1163092	-4.780915
DMXL2	-0.052623	5.9260233	-1.956093	0.0517977	0.1163906	-4.781571
ZNF702P	-0.284921	3.9366785	-1.956079	0.0517993	0.1163906	-4.781597
STAC3	0.085751	5.0465876	1.9560266	0.0518055	0.1163927	-4.781696
MGAT1	0.03813	6.86364	1.9560143	0.051807	0.1163927	-4.78172
NDUFA1	0.0529104	6.6692213	1.9559903	0.0518098	0.1163927	-4.781765
RP11-348M3	-0.225411	-1.317108	-1.955841	0.0518276	0.1164254	-4.782049
RP11-576N1	-0.281723	-0.993622	-1.955749	0.0518384	0.1164426	-4.782223
ACE	0.1007381	5.4941393	1.9550708	0.051919	0.1166165	-4.78351
CTD-2102P2	-0.302961	-0.971954	-1.954913	0.0519377	0.1166514	-4.783809
COX20P1	-0.524337	1.2981931	-1.95486	0.051944	0.1166574	-4.783909
EID2	-0.046582	5.7027123	-1.954837	0.0519468	0.1166574	-4.783954
CDHR5	-0.271133	6.4875262	-1.954639	0.0519703	0.1166998	-4.784329
RP1-90J4.1	0.5160357	0.0150997	1.9546244	0.0519721	0.1166998	-4.784357
LYPLAL1	0.0540324	6.1727586	1.9545045	0.0519863	0.116711	-4.784584
FAM110A	-0.074227	5.4743711	-1.954496	0.0519873	0.116711	-4.7846
ZNF727	-0.622748	1.1357431	-1.954482	0.051989	0.116711	-4.784627
NECAP1	0.0405651	6.0182176	1.954475	0.0519898	0.116711	-4.78464
RP11-205K6	-0.259861	-1.208015	-1.954385	0.0520005	0.1167229	-4.78481
RP11-5G9.5	-0.349771	-0.670291	-1.954377	0.0520015	0.1167229	-4.784827
RP1-296L11	-0.187349	-1.397168	-1.954225	0.0520196	0.1167562	-4.785114
PSEN2	0.0602149	6.1078887	1.9540974	0.0520348	0.1167832	-4.785356
DNAJC4	-0.061342	6.1615324	-1.954033	0.0520425	0.1167932	-4.785478
CTD-2008P7	0.5588943	-0.294197	1.9535685	0.0520978	0.1169102	-4.786359
A2M-AS1	-0.109575	4.590565	-1.953517	0.0521039	0.1169102	-4.786456

RNU6-1160F	-0.437534	-0.193208	-1.953515	0.0521042	0.1169102	-4.786461
MCHR1	-0.350437	4.4664511	-1.95347	0.0521095	0.1169149	-4.786545
TAS2R18	-0.226217	-1.264003	-1.95326	0.0521346	0.1169639	-4.786943
CTD-2005D2	-0.614939	2.3341379	-1.953103	0.0521533	0.1169987	-4.78724
RP5-1031D4	0.4676314	2.0546161	1.9530586	0.0521586	0.1170035	-4.787325
RP11-5A1.1	-0.186862	-1.463121	-1.95282	0.052187	0.1170533	-4.787777
PNLIPRP2	-0.436516	-0.876522	-1.952819	0.0521872	0.1170533	-4.78778
MIR339	-0.36232	-0.548574	-1.952709	0.0522003	0.1170754	-4.787987
GRM3	-0.527982	0.1154155	-1.952601	0.0522132	0.1170962	-4.788193
CYCTP	-0.172377	-1.470224	-1.952565	0.0522175	0.1170962	-4.78826
DTX4	0.1133446	6.0706696	1.9525512	0.0522192	0.1170962	-4.788287
RP11-659P1	0.2230746	-1.323302	1.9523725	0.0522405	0.117131	-4.788625
VCP	0.0331699	6.9819487	1.9523502	0.0522432	0.117131	-4.788667
IGHV3-35	-0.531912	-0.079262	-1.95234	0.0522443	0.117131	-4.788686
IQUB	0.4514511	1.5474472	1.9523115	0.0522478	0.1171316	-4.788741
RP11-697E2	-0.359948	-0.601366	-1.952267	0.0522531	0.1171363	-4.788825
HRASLS5	0.5704981	0.8905539	1.9522227	0.0522584	0.117141	-4.788909
RBBP5	-0.039302	6.1130961	-1.951829	0.0523055	0.1172368	-4.789656
CTA-407F11	-0.289248	-1.153751	-1.951811	0.0523076	0.1172368	-4.789688
ZNF723P	-0.507029	-0.661174	-1.951742	0.0523159	0.1172483	-4.78982
RP11-330M1	-0.507251	0.2400177	-1.951688	0.0523223	0.1172554	-4.789921
AC011239.1	0.2753966	-1.22955	1.9515277	0.0523415	0.1172913	-4.790225
COA4	-0.041637	6.4320106	-1.951394	0.0523576	0.11732	-4.790479
TFAM	-0.037783	6.1824374	-1.951258	0.0523738	0.1173492	-4.790736
MRPS36P4	0.2691214	-1.246263	1.9511611	0.0523854	0.117368	-4.79092
RP11-108P2	-0.208543	-1.338385	-1.951079	0.0523953	0.117383	-4.791076
ROCK1	0.0424376	6.2172567	1.9507259	0.0524376	0.1174704	-4.791743
RP11-638I8	-0.121303	4.2633511	-1.950515	0.0524629	0.1175199	-4.792143
SPARCL1	0.1100673	6.342024	1.9497869	0.0525502	0.1177083	-4.793521
RIBC1	-0.145107	3.8864234	-1.949752	0.0525543	0.1177102	-4.793586
RP11-413E6	0.4404479	2.5195762	1.949726	0.0525575	0.1177102	-4.793636
ST5	-0.056964	6.2538795	-1.949666	0.0525648	0.1177193	-4.79375
RP11-672A2	0.6014457	0.9860408	1.9495333	0.0525807	0.1177476	-4.794
PLXNA2	-0.05275	6.1402311	-1.94949	0.0525858	0.117752	-4.794082
HIST1H2AE	-0.322821	4.1670812	-1.94926	0.0526135	0.1178066	-4.794517
EEF1DP5	-0.480851	-0.759848	-1.949144	0.0526274	0.1178306	-4.794736
UCK1	-0.049585	6.2561063	-1.94895	0.0526507	0.1178673	-4.795103
WDFY4	0.1342559	5.2113682	1.9489288	0.0526533	0.1178673	-4.795144
GPAT2	-0.232057	4.4210426	-1.948927	0.0526535	0.1178673	-4.795146
CTD-2034I4	-0.382864	-0.651519	-1.948721	0.0526783	0.1179156	-4.795536
RP3-400B16	0.3653346	1.9809169	1.9486897	0.0526821	0.1179169	-4.795596
UBB	0.0458799	7.1785531	1.9486162	0.0526909	0.1179294	-4.795735
KB-1674E1.	-0.271943	-1.137701	-1.948476	0.0527078	0.11796	-4.796
TEX33	0.1975726	-1.38779	1.9483785	0.0527195	0.117979	-4.796185
TREML1	0.4527181	1.7294573	1.9483133	0.0527274	0.1179894	-4.796308
DNM2	0.0317215	6.6687369	1.9480252	0.0527621	0.1180594	-4.796852
RGPD2	0.420424	2.7053173	1.9479967	0.0527655	0.1180594	-4.796906
RP11-478H1	-0.475183	-0.653589	-1.947973	0.0527684	0.1180594	-4.796952
GACAT2	0.4395831	-0.813688	1.9478741	0.0527803	0.1180705	-4.797138
ZNF101P2	-0.337974	-0.814669	-1.947856	0.0527824	0.1180705	-4.797172
RP11-1010.	0.3920477	-0.858441	1.9478438	0.0527839	0.1180705	-4.797195

RP11-560A1	0.3663704	-1.033392	1.9478244	0.0527863	0.1180705	-4.797232
PFN1P8	0.2806253	-1.149329	1.9474722	0.0528287	0.1181528	-4.797898
FRK	0.0959562	6.1147016	1.9474656	0.0528295	0.1181528	-4.79791
RPP38	-0.035265	5.7352417	-1.947415	0.0528356	0.1181592	-4.798006
ARL5C	0.2377135	-1.246614	1.9472854	0.0528513	0.1181869	-4.798251
C19orf12	0.0538343	6.3066403	1.9471358	0.0528693	0.1182155	-4.798533
MKRN3-AS1	-0.367447	-0.791206	-1.947112	0.0528721	0.1182155	-4.798578
DCTD	0.0415268	6.3948627	1.9470988	0.0528738	0.1182155	-4.798603
DEFA8P	-0.381009	-0.973731	-1.94702	0.0528833	0.1182295	-4.798752
ARPC3P1	-0.314641	1.4437538	-1.946935	0.0528936	0.1182452	-4.798913
RSPO1	0.472931	-0.502298	1.9468309	0.0529061	0.1182661	-4.799109
RP11-25I15	0.3894348	-0.846802	1.9464582	0.0529511	0.1183595	-4.799814
GLIS2	0.1119549	5.7452705	1.9464051	0.0529576	0.1183649	-4.799914
GPA33	0.4833256	0.2607928	1.9463845	0.05296	0.1183649	-4.799953
EXT1	0.0508589	6.6801505	1.9462833	0.0529723	0.1183798	-4.800144
HAPLN1	-0.650959	0.698213	-1.946276	0.0529732	0.1183798	-4.800158
RP1-93H18.	0.4536339	0.3619595	1.9459956	0.0530071	0.1184482	-4.800687
CTD-2192J1	-0.104414	4.7746584	-1.94582	0.0530283	0.1184884	-4.801019
GLIS3-AS1	0.3387764	-0.894263	1.9454988	0.0530672	0.1185677	-4.801626
TMEM5-AS1	0.29206	-1.166174	1.9454734	0.0530703	0.1185677	-4.801673
SEPSECS	0.0772429	6.1215448	1.9454045	0.0530786	0.118579	-4.801803
IFI6	0.1021763	6.5310121	1.9451382	0.0531109	0.1186388	-4.802306
U91319.1	0.6846897	2.7818867	1.9451301	0.0531118	0.1186388	-4.802322
ATG7	0.0328724	5.9529625	1.945051	0.0531214	0.1186529	-4.802471
WSCD2	0.4896477	0.9070506	1.944841	0.0531469	0.1187025	-4.802867
RP11-28H5.	-0.440136	-0.116013	-1.944694	0.0531647	0.1187342	-4.803145
RPL41P1	-0.074365	5.6814528	-1.94467	0.0531676	0.1187342	-4.80319
RP11-324I2	0.2879516	2.6548785	1.9446075	0.0531752	0.1187439	-4.803308
SURF2	-0.065752	5.8298182	-1.944523	0.0531855	0.1187596	-4.803468
DICER1	-0.049068	6.2659402	-1.9444	0.0532004	0.1187857	-4.8037
MMP23A	0.6393831	0.4029013	1.9442809	0.0532148	0.1188106	-4.803924
ASB8	0.0395429	6.1548643	1.9442209	0.0532221	0.1188196	-4.804038
RGMA	0.2010171	4.2270442	1.9441296	0.0532332	0.118837	-4.80421
VNN3	-0.277459	5.5970447	-1.943999	0.053249	0.1188648	-4.804456
RP4-635E18	-0.102089	4.4456141	-1.943973	0.0532521	0.1188648	-4.804504
AK3P3	-0.417122	0.1439041	-1.943939	0.0532563	0.1188668	-4.804569
USP32P2	-0.437256	-0.582381	-1.943877	0.0532639	0.1188765	-4.804687
NAV3	0.2710881	4.7724499	1.9436119	0.053296	0.1189357	-4.805186
CTD-2323K1	-0.251089	2.8814606	-1.943605	0.0532969	0.1189357	-4.8052
TMEM30B	0.1668739	5.7809144	1.9435232	0.0533068	0.1189449	-4.805354
ERRFI1	0.0871638	6.8260309	1.9435173	0.0533075	0.1189449	-4.805365
RP4-784A16	-0.254787	-1.211662	-1.943461	0.0533143	0.1189528	-4.805471
ALDH1A1	0.1032458	7.1884224	1.9431444	0.0533529	0.1190314	-4.806068
CDH6	0.1620895	5.4368845	1.9429876	0.0533719	0.1190667	-4.806364
VIMP	-0.044835	6.4516234	-1.942884	0.0533845	0.1190875	-4.806559
ZNF402P	0.1236844	-1.523878	1.9428097	0.0533936	0.1191005	-4.806699
NUDC	0.048367	6.6957833	1.9426313	0.0534153	0.1191416	-4.807036
RP11-175K6	0.3391627	2.6641003	1.9425009	0.0534312	0.1191697	-4.807282
RP11-326A1	-0.410663	-0.165539	-1.942336	0.0534512	0.1192071	-4.807592
RAET1E-AS1	0.4634059	1.6398745	1.9421961	0.0534683	0.119238	-4.807856
RAB37	-0.099402	5.6675456	-1.942161	0.0534726	0.1192402	-4.807922

RP11-552D4	0.4442803	-0.598327	1.9420813	0.0534823	0.1192546	-4.808073
PDX1-AS1	-0.545756	0.0626825	-1.94194	0.0534995	0.1192857	-4.808339
RP11-44N12	-0.521499	-0.321123	-1.941735	0.0535245	0.1193342	-4.808726
GADD45GIP1	-0.054506	6.3510107	-1.941708	0.0535278	0.1193343	-4.808777
PPP5D1	0.2326747	2.5592282	1.9416618	0.0535334	0.1193395	-4.808863
ATXN2L	0.0306492	6.5374967	1.9410153	0.0536123	0.119508	-4.810081
RPL36	-0.049492	6.9542931	-1.940955	0.0536197	0.1195173	-4.810196
CTD-2651B2	-0.405806	0.0454118	-1.940838	0.053634	0.1195418	-4.810416
TUBB1	-0.35928	2.2511309	-1.940716	0.0536489	0.1195677	-4.810646
RP11-525K1	0.5123279	2.4638325	1.9405754	0.053666	0.1195986	-4.81091
FAM189A1	0.5135007	1.8730016	1.9404636	0.0536797	0.1196218	-4.811121
CST2	0.6232802	2.1953395	1.9403699	0.0536912	0.11964	-4.811297
AC004449.6	-0.495496	0.6733595	-1.940339	0.0536949	0.1196411	-4.811355
RP11-305F1	-0.507304	-0.567489	-1.939891	0.0537498	0.1197546	-4.812199
UGT2A1	0.7036551	2.4901494	1.9398689	0.0537524	0.1197546	-4.812241
ADAM20	0.3856028	-0.304346	1.9396489	0.0537794	0.1198073	-4.812655
HSP90B3P	-0.44332	0.844504	-1.93945	0.0538037	0.1198542	-4.813029
CYTH4	0.0904051	5.6523179	1.9393973	0.0538102	0.1198576	-4.813128
SNX27	-0.040956	6.3448179	-1.939384	0.0538118	0.1198576	-4.813153
RP6-22P16.	0.1070284	-1.532367	1.9392766	0.053825	0.1198796	-4.813356
CCBL1	-0.069878	5.8100807	-1.939109	0.0538455	0.1199124	-4.813671
JAK3	0.1156287	5.5651054	1.9391028	0.0538463	0.1199124	-4.813683
RP4-569D19	-0.276337	-1.211555	-1.938966	0.0538631	0.1199425	-4.813941
LDHC	-0.684778	1.9333216	-1.938869	0.0538749	0.1199615	-4.814122
HPSE	0.1412343	4.8672673	1.93879	0.0538846	0.1199738	-4.814271
PPP1R3F	0.1123337	5.2118473	1.9387706	0.053887	0.1199738	-4.814308
LINC00678	0.177953	-1.456149	1.9386607	0.0539005	0.1199965	-4.814515
KB-1043D8.	-0.429433	-0.266313	-1.938629	0.0539044	0.1199979	-4.814574
RP11-248B2	0.5251915	-0.097623	1.938559	0.0539129	0.1200096	-4.814706
IRF6	0.1667188	6.3887995	1.9384305	0.0539287	0.1200374	-4.814948
CR1L	0.5285609	0.8390168	1.9383644	0.0539368	0.1200481	-4.815072
NACA	-0.035076	7.1343867	-1.938269	0.0539486	0.1200552	-4.815252
RPS26P47	-0.44305	0.359175	-1.938256	0.0539501	0.1200552	-4.815275
RP4-813D12	-0.296055	-1.245391	-1.93824	0.0539521	0.1200552	-4.815306
RP11-23J18	0.4369283	1.7881407	1.9382315	0.0539531	0.1200552	-4.815322
RN7SKP26	0.3072395	-1.01916	1.9380657	0.0539735	0.1200932	-4.815634
OR1Q1	-0.195363	-1.354676	-1.937979	0.0539842	0.1201096	-4.815797
AP001891.1	-0.224385	-1.326037	-1.93786	0.0539987	0.1201301	-4.816021
RP11-85G20	-0.287855	-1.028114	-1.93785	0.054	0.1201301	-4.81604
TMEM150C	0.1108288	5.7254458	1.9375762	0.0540336	0.1201977	-4.816555
BEST3	0.5522314	-0.00679	1.9375355	0.0540386	0.1202015	-4.816631
SUPT7L	-0.023954	6.1883888	-1.937279	0.0540701	0.1202642	-4.817113
RP1-149C7.	-0.241128	-1.314405	-1.937193	0.0540808	0.1202769	-4.817276
RP11-744K1	-0.387021	-0.911047	-1.937179	0.0540824	0.1202769	-4.817301
PPIF	0.0508309	6.6675822	1.9369097	0.0541156	0.120341	-4.817808
RSL24D1P8	-0.257444	-1.173816	-1.936891	0.0541178	0.120341	-4.817842
GSTA12P	0.5257854	0.3284653	1.9366869	0.054143	0.1203852	-4.818227
LMAN1	0.0469918	6.7900247	1.9366763	0.0541443	0.1203852	-4.818247
GS1-122H1.	0.1606482	-1.427231	1.9366387	0.054149	0.1203882	-4.818317
HNRNPD	-0.028119	6.6702117	-1.93659	0.0541549	0.1203941	-4.818409
RNPEPL1	0.0399348	6.622774	1.9364377	0.0541737	0.1204286	-4.818695

EEF1A1P8	-0.375283	2.1976958	-1.936185	0.0542049	0.1204906	-4.819171
RP13-317D1	-0.295647	2.2773952	-1.936043	0.0542223	0.120522	-4.819437
AC013248.2	-0.340511	-0.984151	-1.935999	0.0542277	0.1205266	-4.819519
GRK5-IT1	-0.265894	-1.138294	-1.935586	0.0542787	0.1206297	-4.820296
GS1-388B5.	0.2308989	-1.327897	1.9355697	0.0542807	0.1206297	-4.820326
GRIN3B	-0.487194	1.662117	-1.935533	0.0542852	0.1206323	-4.820395
MYT1L	-0.464098	-0.618956	-1.935382	0.0543039	0.1206666	-4.820679
MESDC1	0.0537456	5.8908465	1.9353417	0.0543089	0.1206702	-4.820755
C22orf15	0.3456352	2.4131446	1.9352261	0.0543231	0.1206946	-4.820972
RP11-63E5.	0.1501548	-1.460102	1.9351088	0.0543376	0.1207194	-4.821192
CSF3	0.3991328	-0.776117	1.9349482	0.0543575	0.1207561	-4.821494
RUFY2	-0.047484	5.5731528	-1.934515	0.054411	0.1208676	-4.822307
AC106876.2	-0.272502	4.5370367	-1.934415	0.0544234	0.1208878	-4.822495
CTC-487M23	0.1754678	3.4681279	1.9343522	0.0544311	0.1208977	-4.822613
JPH1	0.4342888	3.9143309	1.9341359	0.0544579	0.1209498	-4.823019
RP11-99L13	-0.394198	-0.851435	-1.934012	0.0544732	0.1209763	-4.823251
AC007563.1	-0.278383	-1.120749	-1.933908	0.0544861	0.1209977	-4.823448
NUDT13	0.1161294	5.1854288	1.933782	0.0545017	0.121025	-4.823684
LINC00661	-0.571608	0.2036792	-1.933738	0.0545072	0.1210288	-4.823767
OTUD6A	-0.175797	-1.424737	-1.93371	0.0545106	0.1210288	-4.823818
RP11-330M2	-0.345844	-0.66544	-1.933688	0.0545133	0.1210288	-4.82386
PIK3C2A	0.0426252	6.200985	1.933566	0.0545284	0.1210549	-4.824089
RP11-290L1	0.3994204	-0.542502	1.9335297	0.0545329	0.1210575	-4.824157
KB-1836B5.	-0.470978	0.3680779	-1.933446	0.0545433	0.1210732	-4.824315
SNORD113	-0.182483	-1.443364	-1.933088	0.0545877	0.1211643	-4.824986
RP11-139H1	0.5091615	1.4041958	1.9329476	0.0546051	0.1211956	-4.82525
MED15P6	-0.204047	-1.432789	-1.932913	0.0546094	0.1211977	-4.825315
RP11-197P3	-0.437266	0.5957092	-1.93284	0.0546184	0.1212103	-4.825451
RP11-184I1	0.2347722	-1.312851	1.9327687	0.0546273	0.1212227	-4.825585
AL590762.7	-0.383257	-0.630432	-1.932622	0.0546455	0.1212558	-4.825861
PXMP2	0.1008392	6.2802639	1.9322669	0.0546895	0.1213461	-4.826527
EEF1A1P1	-0.465261	1.4454114	-1.932033	0.0547186	0.1214033	-4.826966
RP11-1166F	0.4414551	0.0848551	1.9319429	0.0547298	0.1214207	-4.827134
TADA1	-0.064334	6.0273983	-1.931745	0.0547543	0.1214678	-4.827505
HNRNPA1P55	-0.232587	-1.250506	-1.93139	0.0547985	0.1215584	-4.828171
RNASE3	0.2971935	-1.065943	1.9311317	0.0548306	0.1216179	-4.828655
MAPT-AS1	0.1484654	-1.51125	1.9311207	0.054832	0.1216179	-4.828676
RN7SKP281	-0.225304	-1.326745	-1.930769	0.0548758	0.1217019	-4.829336
CTD-2302E2	-0.390855	1.3990408	-1.930763	0.0548765	0.1217019	-4.829346
AC084082.3	-0.468046	0.528196	-1.930536	0.0549047	0.1217571	-4.829771
CRB3	-0.243629	5.7169865	-1.930348	0.0549283	0.1218018	-4.830125
CALML4	-0.093925	5.2271045	-1.930318	0.054932	0.1218027	-4.830181
COPE	-0.041726	6.7376765	-1.930178	0.0549494	0.1218267	-4.830443
RP11-538I1	0.2103764	-1.347605	1.9301775	0.0549495	0.1218267	-4.830444
PTPN3	0.0805435	6.2848158	1.9300307	0.0549678	0.1218598	-4.830719
PADI6	0.2062501	-1.341394	1.9299891	0.054973	0.1218639	-4.830797
PIK3R4	0.0417055	6.1077638	1.9298018	0.0549963	0.1219083	-4.831148
AC079305.1	-0.368544	1.7557653	-1.929554	0.0550272	0.1219693	-4.831611
NDUFA4	0.0456274	6.8616601	1.9294078	0.0550455	0.1220025	-4.831886
CTC-431G16	-0.535465	0.1199188	-1.929355	0.0550521	0.1220098	-4.831985
ZNF155	-0.152944	4.4807659	-1.929191	0.0550726	0.1220408	-4.832293

DUSP13	-0.620199	0.77739	-1.929189	0.0550728	0.1220408	-4.832295
UFL1-AS1	-0.316819	-0.940307	-1.929142	0.0550787	0.1220465	-4.832384
LINGO4	0.4459672	3.4302735	1.9290971	0.0550843	0.1220514	-4.832468
PLA1A	0.1370364	6.0406323	1.9290121	0.0550949	0.1220676	-4.832627
RP11-394B2	0.3986574	1.7741742	1.9288014	0.0551213	0.1221185	-4.833021
AC068522.4	-0.426287	0.6993373	-1.928668	0.0551379	0.122148	-4.833271
IGKV1D-12	-0.549226	-0.274974	-1.928638	0.0551417	0.122149	-4.833328
KIAA1324	-0.216911	4.4965843	-1.928568	0.0551504	0.1221608	-4.833458
CARKD	0.0581444	6.4078957	1.9284533	0.0551648	0.1221853	-4.833673
POFUT1	-0.044602	6.5942272	-1.928216	0.0551944	0.1222435	-4.834117
SOST	0.2695105	-1.261162	1.928152	0.0552025	0.1222539	-4.834237
MT-TW	-0.165017	-1.430024	-1.927446	0.0552908	0.1224422	-4.835558
FOXO4	0.0537908	5.8565901	1.9273643	0.0553011	0.1224575	-4.835711
RP11-415I1	-0.364271	-1.125121	-1.926918	0.0553571	0.1225741	-4.836547
PRKCQ	0.2493837	4.2081632	1.9267775	0.0553747	0.1226056	-4.836809
ANAPC13	0.0326907	6.3159539	1.9265357	0.0554051	0.1226609	-4.837261
ZNF664	-0.034611	6.6130106	-1.926525	0.0554064	0.1226609	-4.837281
HMGB1P27	0.4521812	-0.129813	1.926471	0.0554132	0.1226684	-4.837382
RP11-179B1	-0.372844	-0.524737	-1.92642	0.0554195	0.1226751	-4.837477
IL24	0.2569979	3.7843504	1.9263642	0.0554266	0.1226833	-4.837582
OXSM	0.0491753	5.8322387	1.9262574	0.05544	0.122699	-4.837782
RP11-124N1	0.4334503	0.1476769	1.9262541	0.0554404	0.122699	-4.837788
RP11-54D18	0.48157	-0.130275	1.9260862	0.0554615	0.1227382	-4.838102
MAP2K7	-0.034638	6.2452602	-1.926035	0.055468	0.1227451	-4.838198
MGAT3	0.2220206	4.3302467	1.9260069	0.0554715	0.1227454	-4.83825
PELI3	0.0927551	5.3951567	1.9259277	0.0554814	0.1227589	-4.838398
RP11-104O1	-0.449339	0.681509	-1.925905	0.0554843	0.1227589	-4.838441
DOPEY2	-0.070157	6.1091196	-1.925683	0.0555122	0.1228132	-4.838857
HOXD13	-0.473095	-0.765424	-1.925454	0.055541	0.1228694	-4.839284
HLA-DQB1-A	0.4002348	2.8817864	1.9253232	0.0555574	0.1228983	-4.839528
RPL35P2	-0.261095	2.8031858	-1.925078	0.0555882	0.1229477	-4.839986
RP11-402J6	0.1361887	-1.503991	1.9250544	0.0555913	0.1229477	-4.840031
GDF3	0.4184268	-0.336579	1.9250534	0.0555914	0.1229477	-4.840033
FAM122A	0.0444243	5.8461207	1.925039	0.0555932	0.1229477	-4.84006
RP11-309L2	0.3895721	1.1962494	1.9249558	0.0556037	0.1229634	-4.840215
FTLP1	-0.370766	-0.687295	-1.924751	0.0556295	0.123013	-4.840598
RP4-569M23	0.395092	2.7958534	1.924691	0.055637	0.1230222	-4.84071
APCDD1L-AS	0.4488332	-0.223527	1.9245878	0.05565	0.1230435	-4.840903
EBLN3	0.0447179	6.2708169	1.9244045	0.0556731	0.1230801	-4.841245
AC011477.2	0.3237972	-0.930445	1.924403	0.0556733	0.1230801	-4.841248
RP11-136C2	0.1521072	-1.441102	1.9242899	0.0556876	0.1231041	-4.841459
RPS6KA3	-0.062935	6.6081241	-1.924223	0.0556959	0.1231152	-4.841583
ZMYM5	-0.052167	5.5533155	-1.924184	0.0557009	0.1231188	-4.841657
CTD-2515H2	-0.395432	2.7413236	-1.92411	0.0557103	0.1231252	-4.841796
CFL1P1	-0.279996	2.994149	-1.924108	0.0557106	0.1231252	-4.8418
CTNNAP1	-0.416974	-0.232877	-1.924013	0.0557225	0.1231442	-4.841977
RGS13	-0.58901	1.2200345	-1.923815	0.0557474	0.1231873	-4.842346
RP11-480C1	0.309142	-0.985533	1.9238045	0.0557488	0.1231873	-4.842366
HDAC11-AS1	-0.391962	-0.374979	-1.923687	0.0557637	0.123212	-4.842586
RP11-7D5.2	-0.42533	0.1098878	-1.923662	0.0557667	0.123212	-4.842632
CDK15	0.5008559	0.4234198	1.9236087	0.0557735	0.1232195	-4.842732

FAM58A	0.0543054	5.8580747	1.9235043	0.0557867	0.1232411	-4.842927
FLJ42393	-0.470677	-0.346144	-1.923454	0.0557931	0.1232478	-4.843021
TRIP6	0.065392	6.4748787	1.9232323	0.055821	0.1233021	-4.843435
PTCHD3P2	-0.560441	1.0232917	-1.923098	0.0558381	0.1233281	-4.843686
WHAMML1	0.0945749	4.6586884	1.9230858	0.0558395	0.1233281	-4.843708
RNF223	0.4216766	-0.719188	1.923017	0.0558482	0.1233398	-4.843837
PUS10	0.0598868	5.6743178	1.9229829	0.0558525	0.1233419	-4.8439
RP11-609L3	-0.223213	-1.278222	-1.922775	0.0558788	0.1233923	-4.844288
MCOLN1	0.0405695	6.1738865	1.9223556	0.0559319	0.1235021	-4.845071
SNORA5A	-0.414501	0.2741242	-1.922173	0.0559549	0.1235456	-4.845411
RP11-44I10	-0.351242	-0.653533	-1.922114	0.0559625	0.1235548	-4.845523
CPNE3	0.0436107	6.5668118	1.9220097	0.0559756	0.1235764	-4.845717
TRIO	0.047326	6.2509673	1.9215981	0.0560278	0.123684	-4.846485
WDR31	0.1196285	4.9358319	1.9213563	0.0560584	0.1237441	-4.846936
RP11-485G7	-0.451753	-0.12697	-1.921299	0.0560657	0.1237527	-4.847043
GUCD1	0.0459368	6.7895481	1.9210503	0.0560972	0.1238148	-4.847507
IL1B	0.2424578	4.2552636	1.920931	0.0561124	0.1238407	-4.847729
ZNF160	-0.100813	5.5577368	-1.920741	0.0561365	0.1238864	-4.848084
C1QTNF8	-0.282824	-1.184175	-1.920714	0.0561398	0.1238864	-4.848133
TMBIM4	-0.044133	6.3687581	-1.920643	0.0561489	0.1238989	-4.848266
RP11-432M8	-0.204929	-1.405872	-1.920421	0.056177	0.1239535	-4.84868
PLEKHO1	0.0693696	6.0956447	1.9202654	0.0561969	0.1239897	-4.848971
HNRNPA1P15	0.2699796	-1.130532	1.9201051	0.0562172	0.1240261	-4.849269
RP3-402G11	-0.161529	4.4487452	-1.920082	0.0562201	0.1240261	-4.849312
FAM206A	-0.049633	5.7574059	-1.920052	0.056224	0.1240271	-4.849369
LINC01389	0.2785731	2.995397	1.9199278	0.0562398	0.1240544	-4.8496
AP003774.6	0.427728	0.0768901	1.9198237	0.056253	0.1240761	-4.849794
CBX3P9	-0.432221	0.4980682	-1.919692	0.0562697	0.1241055	-4.850039
HAUS2	-0.035349	5.8258732	-1.919615	0.0562796	0.1241117	-4.850184
RP11-16F15	-0.411831	1.34191	-1.919608	0.0562805	0.1241117	-4.850197
TCEB1P2	-0.388647	-0.315392	-1.91959	0.0562828	0.1241117	-4.85023
RP3-514A23	0.1864916	-1.424514	1.9193509	0.0563131	0.1241712	-4.850675
IL17RC	0.059712	6.3162653	1.9193081	0.0563186	0.1241757	-4.850755
TECR	-0.048476	6.6774859	-1.919116	0.056343	0.1242221	-4.851113
FAR2P3	-0.263275	-1.288837	-1.919075	0.0563483	0.1242263	-4.85119
EEF1A1P42	-0.208516	-1.342977	-1.918998	0.056358	0.1242345	-4.851332
CTCFL	0.5751106	0.7776468	1.9189916	0.0563589	0.1242345	-4.851345
EIF3C	0.0948771	5.2188578	1.9188962	0.056371	0.1242475	-4.851523
C20orf141	0.2142468	-1.415925	1.9188919	0.0563716	0.1242475	-4.851531
ARHGEF34P	0.2039878	4.6041183	1.91882	0.0563807	0.1242602	-4.851664
SYNPO2L	0.4586648	0.374948	1.9187914	0.0563844	0.1242607	-4.851718
ERICD	-0.191758	3.7727063	-1.918757	0.0563887	0.1242628	-4.851781
PTGES3	-0.031246	6.9342637	-1.91865	0.0564024	0.1242822	-4.851981
RP11-122K1	-0.427532	2.6910217	-1.918635	0.0564043	0.1242822	-4.85201
AC005614.3	-0.306801	-0.949046	-1.918597	0.0564092	0.1242826	-4.85208
P2RX5	-0.308266	3.8819106	-1.91858	0.0564113	0.1242826	-4.852111
NTSR1	0.5791185	1.570432	1.9180734	0.0564759	0.1244174	-4.853055
C1orf116	-0.391877	4.4644774	-1.91785	0.0565043	0.1244725	-4.85347
AC004471.9	-0.407504	1.2794113	-1.917577	0.0565392	0.1245419	-4.853979
GLB1L	0.0886268	5.4834738	1.9169643	0.0566175	0.1247068	-4.85512
CTD-2307P3	-0.177519	-1.423893	-1.916659	0.0566566	0.1247674	-4.855689

PNKD	0.0517767	6.8044149	1.9166571	0.0566568	0.1247674	-4.855692
RP11-798K3	-0.26916	5.4518999	-1.916647	0.0566582	0.1247674	-4.855712
RP11-350N1	0.3169564	-1.110801	1.9166423	0.0566587	0.1247674	-4.85572
NCBP2-AS1	-0.44034	0.571059	-1.91621	0.056714	0.1248816	-4.856524
RP11-366M4	-0.244875	-1.359042	-1.915825	0.0567633	0.1249828	-4.857241
LINC00028	0.226113	-1.28974	1.9157188	0.0567769	0.1250008	-4.857438
RP11-396J6	-0.232337	-1.318717	-1.915707	0.0567784	0.1250008	-4.857459
PRODH2	-0.333369	6.0472584	-1.915642	0.0567867	0.1250062	-4.857581
RP5-1056H1	-0.566607	1.1167345	-1.915635	0.0567877	0.1250062	-4.857595
ZNF17	-0.053412	5.0195227	-1.915601	0.056792	0.1250082	-4.857657
PTK6	0.2824584	4.8552894	1.9152932	0.0568315	0.1250875	-4.858229
RP11-807H2	0.2429067	-1.235848	1.914989	0.0568705	0.1251658	-4.858795
AC019100.3	-0.377077	-0.804473	-1.914958	0.0568744	0.1251669	-4.858852
RP11-731D1	0.3601927	-0.74196	1.9148127	0.0568931	0.1251999	-4.859123
MYBL1	-0.094092	5.1739976	-1.914774	0.0568981	0.1251999	-4.859195
RP11-290O1	0.3387201	-1.088071	1.9147613	0.0568997	0.1251999	-4.859219
HSF1	-0.047143	6.5992928	-1.914625	0.0569172	0.1252309	-4.859472
RP11-27909	0.4057693	1.7059949	1.914309	0.0569578	0.1253127	-4.86006
ZFHX4-AS1	-0.637013	1.6970389	-1.914184	0.0569739	0.1253348	-4.860292
RP11-405L1	0.4126324	-0.129956	1.9141774	0.0569747	0.1253348	-4.860304
ATG4C	0.0458288	5.6646717	1.9140576	0.0569901	0.1253611	-4.860527
F10-AS1	-0.426353	1.6132655	-1.913937	0.0570056	0.1253877	-4.860752
RP5-915N17	-0.395009	-0.327034	-1.913643	0.0570434	0.1254632	-4.861297
PDILT	-0.425769	-0.298601	-1.913559	0.0570542	0.1254794	-4.861453
AJ011931.1	0.2163302	-1.38452	1.9130945	0.057114	0.1256033	-4.862316
LMBR1	0.0412105	6.1403552	1.9128574	0.0571445	0.1256629	-4.862757
CTB-113P19	0.321735	-0.883858	1.9126075	0.0571767	0.1257166	-4.863221
RNF26	-0.036911	6.2886434	-1.912598	0.057178	0.1257166	-4.86324
SNORA20	-0.325858	-0.787174	-1.912562	0.0571827	0.1257166	-4.863306
TRIB3	-0.079197	6.483809	-1.912561	0.0571827	0.1257166	-4.863307
HCP5B	0.5248538	0.7473932	1.9124949	0.0571913	0.1257278	-4.86343
MNAT1	-0.050357	5.6972998	-1.912427	0.0572	0.1257395	-4.863557
MFSD10	-0.065548	6.1430918	-1.912382	0.0572059	0.1257447	-4.86364
TRPC2	0.3961811	1.5530617	1.9121257	0.0572389	0.1258097	-4.864116
CTD-2010I1	-0.240373	3.1469424	-1.911901	0.0572679	0.125866	-4.864534
HSPA8P1	-0.383118	2.9124919	-1.911697	0.0572942	0.1259161	-4.864912
CTD-2291D1	-0.13283	-1.489618	-1.911486	0.0573214	0.1259684	-4.865303
AC084149.2	-0.263729	-1.250243	-1.911451	0.057326	0.1259709	-4.865369
RNU2-63P	-0.21671	-1.358658	-1.911385	0.0573344	0.1259803	-4.86549
AC104655.2	-0.380358	1.2707471	-1.91136	0.0573378	0.1259803	-4.865538
UQCRFS1	0.0493708	6.5292906	1.9113376	0.0573406	0.1259803	-4.865579
TRABD	-0.04425	6.2977331	-1.911179	0.0573611	0.1260178	-4.865874
AC025335.1	-0.135641	4.5262167	-1.911084	0.0573734	0.1260371	-4.86605
TSPAN8	-0.265145	6.2229417	-1.910991	0.0573854	0.1260533	-4.866222
PMEL	0.1286572	5.2167971	1.9109735	0.0573877	0.1260533	-4.866255
TRIM8	0.038814	6.6134335	1.9107928	0.057411	0.126097	-4.86659
MOXD2P	0.141934	-1.482296	1.910501	0.0574488	0.1261724	-4.867131
S100A7A	0.2224061	-1.409766	1.9102566	0.0574804	0.1262342	-4.867585
FARS2	0.0463799	5.907702	1.9101011	0.0575006	0.1262666	-4.867873
IFFO1	-0.059179	5.9568327	-1.910089	0.0575021	0.1262666	-4.867895
RPL21P135	-0.391084	-0.341164	-1.909723	0.0575495	0.1263632	-4.868575

HNRNPA1P4C	-0.285905	-1.108049	-1.909446	0.0575854	0.1264344	-4.869088
DCLRE1B	-0.059965	5.4230334	-1.909067	0.0576346	0.1265348	-4.869792
RP11-843B1	-0.455572	0.1386622	-1.908868	0.0576604	0.1265771	-4.87016
RP11-422P2	-0.300105	-0.912107	-1.908865	0.0576608	0.1265771	-4.870165
COX6A1P2	-0.11572	4.34315	-1.908577	0.0576982	0.1266515	-4.870699
ERP29P1	-0.392424	0.7258889	-1.908182	0.0577495	0.1267565	-4.871431
FUZ	0.1175009	5.3800009	1.9080949	0.0577608	0.1267738	-4.871593
HMCN2	0.2812155	4.8626509	1.9080399	0.057768	0.1267819	-4.871695
CST6	0.5086662	0.3522158	1.9078581	0.0577916	0.1268261	-4.872032
RP11-109I1	-0.33467	-0.998944	-1.907767	0.0578035	0.1268394	-4.8722
TLDC2	-0.187416	4.8522271	-1.907758	0.0578046	0.1268394	-4.872217
RPS4Y1	0.7744673	4.5144289	1.9076762	0.0578153	0.1268552	-4.872369
TMCO3	-0.064892	6.3088454	-1.907609	0.057824	0.1268667	-4.872493
CTNND1	0.0368605	6.813013	1.9075168	0.057836	0.1268854	-4.872664
ATP5G1	-0.053638	6.6082881	-1.907156	0.057883	0.1269809	-4.873333
P2RY2	0.1603946	4.8472863	1.9068601	0.0579215	0.1270577	-4.87388
SIRT3	0.0458677	6.0909081	1.9067918	0.0579304	0.1270696	-4.874007
CTC-524C5.	-0.126685	4.5689568	-1.906678	0.0579453	0.1270946	-4.874218
AC114812.1	0.4712461	-0.17145	1.906591	0.0579566	0.1271071	-4.874379
RP11-236L1	0.2902646	-1.226439	1.9065806	0.057958	0.1271071	-4.874398
GSS	0.0381687	6.4183219	1.9064963	0.057969	0.1271236	-4.874554
ADCY8	0.5900863	0.3222546	1.9063818	0.0579839	0.1271487	-4.874766
WBP11P1	-0.256211	-1.097285	-1.906347	0.0579884	0.1271509	-4.87483
INSM1	-0.510297	-0.264286	-1.906245	0.0580017	0.1271724	-4.875018
DDX23	-0.026367	6.5528238	-1.905955	0.0580395	0.1272453	-4.875555
RP11-63N8.	0.318001	-0.969241	1.905925	0.0580435	0.1272453	-4.875612
RP11-255H2	-0.512917	-0.414048	-1.90591	0.0580454	0.1272453	-4.875639
NKX2-6	0.1875263	-1.477829	1.9055952	0.0580865	0.1273279	-4.876222
RP11-444D1	0.151015	-1.50995	1.905491	0.0581001	0.12735	-4.876415
CBLN2	0.491217	-0.497314	1.9053535	0.0581181	0.1273762	-4.876669
OR1L8	0.2898119	-1.019409	1.9053463	0.0581191	0.1273762	-4.876683
C14orf1	-0.062409	6.4766678	-1.905243	0.0581325	0.127398	-4.876873
LINC00284	0.4324696	-0.461848	1.9052167	0.058136	0.127398	-4.876922
SEC22A	-0.035907	5.7866579	-1.905036	0.0581596	0.1274421	-4.877256
EEF1A1P12	-0.151036	4.2082752	-1.904955	0.0581702	0.1274578	-4.877407
RP11-616M1	-0.245051	-1.230655	-1.904724	0.0582004	0.1275162	-4.877834
RHOT1	-0.030795	6.0389417	-1.904686	0.0582054	0.1275195	-4.877905
RP11-65D17	-0.29548	-1.175595	-1.904515	0.0582278	0.1275609	-4.878221
RRAGB	-0.057687	5.5396318	-1.904373	0.0582463	0.1275939	-4.878483
USP6NL	-0.047695	6.0804655	-1.904281	0.0582584	0.1276126	-4.878654
RP11-357N1	0.18186	-1.367339	1.9040468	0.058289	0.1276721	-4.879086
C2	-0.100296	7.062363	-1.903811	0.0583199	0.1277321	-4.879522
RP3-337H4.	0.4299538	0.5727684	1.9037242	0.0583313	0.1277425	-4.879683
RANBP2	0.0333749	6.4252715	1.9037214	0.0583317	0.1277425	-4.879688
MMD2	-0.142559	-1.484847	-1.903592	0.0583487	0.127772	-4.879927
CNGA4	-0.422085	1.0783203	-1.903482	0.0583631	0.127796	-4.880131
TATDN1P1	-0.428721	0.2096018	-1.903311	0.0583855	0.1278374	-4.880448
DHRS12	0.077694	5.7462847	1.9031669	0.0584044	0.1278711	-4.880713
DSC1	0.454967	0.2311495	1.9031284	0.0584095	0.1278745	-4.880785
RP11-66H6.	0.4671053	1.2037952	1.9023092	0.058517	0.1280956	-4.882298
ABCA1	-0.064528	6.4692584	-1.902306	0.0585175	0.1280956	-4.882305

CHRFAM7A	0.4909967	0.0973426	1.9022683	0.0585224	0.1280987	-4.882374
IMP4	0.0369778	6.4326071	1.9020433	0.058552	0.1281557	-4.88279
RP11-864N7	-0.126531	5.1392974	-1.901943	0.0585651	0.1281768	-4.882974
RP11-126K1	0.1389917	-1.516078	1.9018925	0.0585718	0.1281837	-4.883068
PSAT1P1	-0.159965	-1.454406	-1.901789	0.0585854	0.1282057	-4.883259
CH17-431G2	-0.285988	2.9988935	-1.901646	0.0586043	0.1282394	-4.883524
NPPA	-0.452753	1.2447889	-1.901425	0.0586333	0.1282952	-4.883931
RP11-556K1	-0.263672	3.9472591	-1.901393	0.0586375	0.1282967	-4.88399
GRM5-AS1	0.3504956	-1.073088	1.9013473	0.0586435	0.1283007	-4.884075
TTLL10	0.4495558	0.4432066	1.9013262	0.0586463	0.1283007	-4.884114
DOHH	-0.047075	5.8221828	-1.901278	0.0586526	0.1283068	-4.884202
RN7SL395P	-0.266911	-1.075926	-1.900671	0.0587326	0.1284741	-4.885324
RP11-734I1	-0.808579	1.2710698	-1.900591	0.0587432	0.1284896	-4.885472
CD22	0.1888094	4.6349827	1.9005333	0.0587508	0.1284961	-4.885578
CSNK1G2	-0.034323	6.3428185	-1.900494	0.058756	0.1284961	-4.885651
NEK10	-0.311403	4.0924973	-1.900449	0.0587619	0.1284961	-4.885734
PAIP1	0.0328234	6.31334	1.9004397	0.0587631	0.1284961	-4.885751
IRF3	-0.044957	6.4391353	-1.900435	0.0587638	0.1284961	-4.885761
KLHL9	0.0578801	6.0401025	1.9003944	0.0587691	0.1285	-4.885835
ARPC5	0.0324119	6.7819744	1.9002987	0.0587817	0.1285199	-4.886011
ALDH1L1-AS	0.4632494	0.1425457	1.8998996	0.0588344	0.1286256	-4.886748
CACNA2D3-A	0.1398372	-1.484569	1.8998789	0.0588371	0.1286256	-4.886786
RP11-47909	-0.425614	0.1351886	-1.899672	0.0588644	0.1286776	-4.887167
PLCD3	0.1142405	5.4037579	1.8996182	0.0588715	0.1286806	-4.887267
CTD-2114J1	0.4085781	3.9252247	1.8996081	0.0588729	0.1286806	-4.887286
RP11-403P1	-0.21178	3.1678434	-1.899538	0.0588822	0.1286872	-4.887416
RP11-153I2	-0.401505	0.737208	-1.899532	0.0588829	0.1286872	-4.887426
RP11-30101	-0.250204	3.0569825	-1.899249	0.0589204	0.1287613	-4.887949
STIP1P3	-0.323472	-0.857356	-1.899072	0.0589437	0.1288046	-4.888275
LINC01474	0.4292393	2.9038499	1.8989777	0.0589562	0.1288242	-4.888449
CAPG	-0.108071	6.1474378	-1.898854	0.0589726	0.1288523	-4.888678
GS1-115G20	0.1776432	-1.409814	1.8987525	0.058986	0.1288738	-4.888864
RP13-672B3	0.4566008	1.6653425	1.8986693	0.058997	0.1288902	-4.889017
EEFSEC	-0.045638	6.1249506	-1.89854	0.0590141	0.1289199	-4.889256
REPIN1	-0.039837	6.7230176	-1.898495	0.0590201	0.1289252	-4.889339
KRT18P4	-0.320675	1.9213699	-1.898365	0.0590373	0.128955	-4.889579
RP11-297P1	-0.175726	-1.442098	-1.898238	0.059054	0.1289839	-4.889812
GLB1L3	-0.597773	1.1129228	-1.898174	0.0590625	0.1289947	-4.88993
TOX4	-0.029184	6.3840124	-1.897863	0.0591038	0.1290702	-4.890505
PHACTR4	0.0348586	6.2695228	1.8978599	0.0591042	0.1290702	-4.89051
TMEM170A	0.0419326	5.8492831	1.897772	0.0591158	0.1290861	-4.890672
PEX11B	-0.04228	6.2199872	-1.897724	0.0591222	0.1290861	-4.89076
UNC13C	-0.472797	-0.441295	-1.897717	0.0591231	0.1290861	-4.890773
FAM205A	0.2503448	-1.254627	1.8976891	0.0591268	0.1290861	-4.890825
RP11-2L8.2	0.3089582	-1.166156	1.8976531	0.0591316	0.1290861	-4.890891
OR56A3	-0.458893	-0.753665	-1.897636	0.0591338	0.1290861	-4.890922
RP11-887P2	0.3980815	-0.466319	1.8976184	0.0591362	0.1290861	-4.890955
UBXN6	0.0388985	6.6464524	1.8975485	0.0591454	0.1290986	-4.891084
RP11-434D1	-0.198001	-1.326229	-1.897274	0.0591818	0.1291702	-4.891589
EPHA5-AS1	-0.44137	-0.904957	-1.897202	0.0591913	0.1291833	-4.891722
RP11-202G1	-0.361322	-0.832318	-1.897088	0.0592065	0.1292087	-4.891932

STMND1	-0.593553	2.6238165	-1.896399	0.0592979	0.1294005	-4.893201
RP3-368B9.	-0.184664	-1.412389	-1.8962	0.0593245	0.1294507	-4.893569
RP11-614F1	-0.168712	-1.445537	-1.896125	0.0593345	0.1294647	-4.893707
CAP2P1	-0.350823	-0.47786	-1.895721	0.0593882	0.1295743	-4.894452
PRKAR1A	0.0334649	6.8111967	1.8955606	0.0594095	0.129613	-4.894746
RP11-278H7	-0.444435	-0.502936	-1.895373	0.0594345	0.1296598	-4.895092
TOX4P1	-0.240601	-1.20056	-1.89518	0.0594601	0.1297079	-4.895446
SLC6A17	0.4971619	1.9032386	1.8948209	0.059508	0.1298046	-4.896108
RP11-661A1	-0.493266	1.5837287	-1.894782	0.0595132	0.1298081	-4.896179
AD000864.6	0.288204	3.1687051	1.8947207	0.0595214	0.1298182	-4.896292
GSAP	0.0581018	5.9807912	1.8946529	0.0595304	0.1298302	-4.896417
ANKRD20A4	-0.398381	-0.529773	-1.894364	0.0595689	0.1299063	-4.896948
RP11-314C1	0.4306903	1.5350259	1.8942953	0.0595781	0.1299187	-4.897075
RPL32P34	-0.175884	-1.408039	-1.894229	0.0595869	0.1299302	-4.897197
RP4-681N2C	-0.402924	1.7499063	-1.894061	0.0596094	0.1299713	-4.897507
KRT75	0.3468157	-1.085222	1.893966	0.059622	0.1299912	-4.897681
RP11-91J19	0.275547	2.9365179	1.893769	0.0596483	0.1300408	-4.898044
ERCC8	-0.0472	5.2969318	-1.893688	0.0596591	0.1300566	-4.898192
HOXA11-AS	-0.596641	0.5994076	-1.893132	0.0597334	0.1302107	-4.899215
RGMB-AS1	0.3455429	3.1255908	1.8927676	0.0597822	0.1303092	-4.899885
WDR77	-0.044143	5.9997155	-1.892476	0.0598212	0.1303865	-4.900421
PDPR	0.0554229	5.963167	1.8922888	0.0598463	0.1304317	-4.900766
AC087380.1	0.439873	0.5406591	1.8922677	0.0598491	0.1304317	-4.900804
DHFRL1	0.0616528	5.5008982	1.891977	0.059888	0.1305088	-4.901339
CMC1	-0.047128	6.0096278	-1.891741	0.0599196	0.1305699	-4.901772
NFKBID	0.106634	4.7784131	1.891662	0.0599302	0.1305852	-4.901917
LRRC45	-0.058851	6.1213855	-1.891398	0.0599657	0.1306546	-4.902403
RPL21P119	-0.279324	2.9182602	-1.891255	0.0599848	0.1306885	-4.902666
RP11-158I9	-0.132673	4.433819	-1.891082	0.060008	0.1307238	-4.902983
MVB12B	-0.058216	5.8813691	-1.891081	0.0600082	0.1307238	-4.902985
LINC01531	-0.298687	-1.06509	-1.891001	0.0600189	0.1307392	-4.903131
RXFP1	0.3583091	2.9089233	1.8908512	0.060039	0.1307754	-4.903407
CTD-2026G2	0.2363945	-1.27481	1.8905179	0.0600838	0.1308651	-4.904019
ZBTB43	0.0538327	5.7195654	1.8904201	0.0600969	0.1308859	-4.904199
RP11-51L5.	-0.216682	-1.360878	-1.890353	0.060106	0.1308978	-4.904323
RP11-103J8	-0.212871	-1.328262	-1.890076	0.0601432	0.1309709	-4.904831
DAPP1	0.225271	4.3665737	1.8899638	0.0601583	0.130996	-4.905037
RP5-119802	0.4161161	2.3494659	1.8898788	0.0601697	0.1310131	-4.905193
AC018712.2	0.4702568	-0.340436	1.8898098	0.060179	0.1310214	-4.90532
ZKSCAN2	0.0697221	5.2638921	1.8897971	0.0601807	0.1310214	-4.905343
OR1J2	-0.171567	-1.410156	-1.889438	0.060229	0.1311188	-4.906002
IGFBP2	0.1433263	6.739617	1.8893699	0.0602382	0.1311309	-4.906127
RPL23AP6	-0.24307	-1.208675	-1.889207	0.0602602	0.1311709	-4.906427
SMIM4	-0.078547	5.7454249	-1.889159	0.0602665	0.1311769	-4.906513
DNAJC13	0.033468	6.2403145	1.8889831	0.0602903	0.1312208	-4.906837
CHRNA7	0.4988292	0.1656001	1.8889438	0.0602956	0.1312245	-4.906909
H2AFV	-0.029626	6.6318885	-1.888751	0.0603215	0.1312732	-4.907263
RP4-803A2.	0.3604283	-0.679139	1.8883435	0.0603765	0.131385	-4.908011
RP11-79P5.	-0.265747	3.0085229	-1.888277	0.0603855	0.1313915	-4.908133
NOL8	-0.033795	5.997751	-1.888268	0.0603867	0.1313915	-4.908149
RP4-738P15	0.2828066	-1.18154	1.88823	0.0603918	0.1313948	-4.908219

RP4-665N4.	-0.414547	0.1504391	-1.888171	0.0603998	0.1314044	-4.908328
C8orf34	0.4583912	-0.380882	1.8879558	0.0604288	0.1314498	-4.908722
PYDC1	0.4763585	-0.591082	1.8879481	0.0604298	0.1314498	-4.908736
CTD-3214K2	-0.443246	0.8405278	-1.887936	0.0604315	0.1314498	-4.908758
AP000439.2	0.1565071	-1.4341	1.8878591	0.0604419	0.1314646	-4.908899
CUBN	0.1720657	3.9337204	1.8877197	0.0604607	0.131486	-4.909155
EEF1A1P10	-0.326235	2.3449971	-1.887709	0.0604622	0.131486	-4.909175
AARS2	-0.040163	6.0308718	-1.887706	0.0604625	0.131486	-4.90918
AMZ2	0.0369708	6.1789579	1.8873061	0.0605166	0.1315957	-4.909913
SLC18A2	0.3717423	2.5906151	1.8870687	0.0605487	0.1316576	-4.910349
ENDOU	0.4568917	0.733179	1.8869831	0.0605602	0.1316749	-4.910506
RAD9B	-0.228686	3.404776	-1.886914	0.0605696	0.1316812	-4.910633
HDHD1	-0.061754	5.9005508	-1.886909	0.0605703	0.1316812	-4.910642
RP11-181E1	0.4757881	0.6614023	1.8865947	0.0606128	0.1317656	-4.911218
RP11-1151E	-0.314114	-1.161443	-1.886527	0.0606219	0.1317777	-4.911341
RP11-554A1	0.5761343	1.910803	1.8862514	0.0606593	0.131851	-4.911847
RP11-864I4	-0.419434	0.931839	-1.886148	0.0606732	0.1318734	-4.912035
RP11-641D5	0.3086168	-1.097066	1.8860181	0.0606908	0.131904	-4.912274
DCBLD2	0.0537751	6.0468281	1.8859626	0.0606984	0.1319124	-4.912376
LINC01058	0.4309095	-0.207717	1.8859039	0.0607063	0.1319219	-4.912484
RP11-2906.	-0.151209	-1.4587	-1.885713	0.0607322	0.1319702	-4.912833
BATF3	0.1458631	4.3801816	1.8854997	0.0607611	0.1320252	-4.913224
CTD-2280E9	0.5850567	0.9105223	1.8853067	0.0607873	0.1320669	-4.913578
RP11-234K2	0.3809754	-0.565632	1.8853048	0.0607875	0.1320669	-4.913581
RP1-153P14	-0.402792	-0.349207	-1.885153	0.0608081	0.1321038	-4.913859
MRPS5	0.0356813	6.4583815	1.8850896	0.0608167	0.1321091	-4.913975
RP11-789C1	0.3799406	-0.520166	1.8850817	0.0608178	0.1321091	-4.91399
HMG20A	0.0680473	5.8699656	1.8849676	0.0608333	0.1321349	-4.914199
RP11-386B1	-0.361811	-1.03424	-1.88492	0.0608397	0.1321362	-4.914285
PSMD6-AS2	-0.358094	2.5376585	-1.884863	0.0608474	0.1321362	-4.914389
SUGCT	-0.112367	5.8580991	-1.884845	0.06085	0.1321362	-4.914424
KPNA3	0.0425508	6.2177827	1.8848384	0.0608508	0.1321362	-4.914435
SETX	0.033142	6.3678363	1.88483	0.060852	0.1321362	-4.914451
RP4-736L20	-0.247841	-1.204692	-1.884677	0.0608727	0.1321733	-4.91473
RP11-495P1	-0.365891	-0.910178	-1.88432	0.0609213	0.1322709	-4.915384
ZNF280C	-0.080098	4.9383578	-1.884259	0.0609295	0.132281	-4.915496
AC006273.4	-0.503054	1.2350051	-1.88358	0.0610219	0.1324736	-4.916739
CNTROB	-0.040739	6.0183747	-1.883538	0.0610277	0.1324782	-4.916816
NUMB	0.0386982	6.3722735	1.8834353	0.0610416	0.1325006	-4.917004
YTHDF1	-0.025371	6.4049189	-1.882867	0.061119	0.1326606	-4.918043
RP11-794G2	-0.500944	1.0354288	-1.882748	0.0611352	0.132688	-4.918261
RNU6-1048F	-0.353594	-0.649207	-1.882659	0.0611474	0.1327065	-4.918424
CBWD7	-0.111029	4.4784111	-1.882319	0.0611937	0.1327991	-4.919045
TMA16	0.0447128	5.7368814	1.8822378	0.0612048	0.1328081	-4.919194
RP11-182J1	0.4158702	0.1246213	1.8822356	0.0612051	0.1328081	-4.919198
CD177P1	0.258532	-1.331705	1.8819279	0.0612471	0.1328914	-4.919761
RP11-762H8	-0.355752	2.3321772	-1.881849	0.0612579	0.1329069	-4.919906
RP11-12K11	-0.200498	-1.34233	-1.881779	0.0612675	0.1329197	-4.920034
DDX42	-0.023803	6.5453429	-1.881693	0.0612791	0.1329371	-4.92019
POMT1	-0.039609	6.1814475	-1.881595	0.0612925	0.1329583	-4.920369
GRM7	-0.615531	0.6240454	-1.881188	0.0613482	0.1330712	-4.921115

OR1N2	0.2693423	-1.357758	1.8810333	0.0613693	0.1331091	-4.921397
RP11-473C1	-0.278778	-1.003345	-1.880958	0.0613796	0.1331235	-4.921534
RP11-352B1	-0.270667	-1.27391	-1.880788	0.0614028	0.1331576	-4.921844
PET100	-0.058435	6.0646168	-1.880786	0.0614032	0.1331576	-4.921849
TMPRSS9	-0.258805	4.9330933	-1.880742	0.0614091	0.1331576	-4.921928
TBC1D10B	0.0359983	6.1707873	1.8807363	0.0614099	0.1331576	-4.921939
AC097523.3	-0.1679	-1.406705	-1.880672	0.0614188	0.133162	-4.922058
CLTA	-0.039544	6.6706132	-1.880668	0.0614193	0.133162	-4.922064
CTD-2058B2	0.1499657	-1.456643	1.8805455	0.0614361	0.1331905	-4.922288
MINA	0.063029	6.0111904	1.8804817	0.0614448	0.1332015	-4.922405
RP11-677M2	-0.234136	-1.365176	-1.880263	0.0614747	0.1332585	-4.922804
MAB21L1	0.4989539	0.5252408	1.8801921	0.0614844	0.1332717	-4.922934
MEG8	-0.574322	0.5012298	-1.880159	0.061489	0.1332736	-4.922994
TMEM8B	-0.060991	5.7561519	-1.879951	0.0615175	0.1333244	-4.923375
RP11-571M6	-0.133228	3.8985035	-1.879934	0.0615197	0.1333244	-4.923404
EIF3EP1	-0.278317	2.8443196	-1.879862	0.0615296	0.1333379	-4.923536
RP6-159A1.	0.3168401	3.2688804	1.879805	0.0615375	0.133347	-4.923641
RP11-564A8	-0.278977	-1.128661	-1.879744	0.0615459	0.1333552	-4.923753
RP11-133K1	0.4226616	0.8347174	1.8797242	0.0615485	0.1333552	-4.923788
PRR29	0.113397	4.439754	1.8796879	0.0615535	0.133358	-4.923855
RP11-332L8	-0.346973	-0.769223	-1.879239	0.0616151	0.1334836	-4.924675
TCL1B	-0.229413	-1.38544	-1.879065	0.061639	0.1335274	-4.924993
C22orf23	-0.192068	3.6816521	-1.878775	0.0616788	0.1336057	-4.925522
RP11-21401	-0.253634	-1.158359	-1.87861	0.0617015	0.1336325	-4.925823
RP11-334G2	-0.245645	-1.206561	-1.878607	0.0617019	0.1336325	-4.925829
TRAF1	0.0589054	5.7015855	1.8786045	0.0617022	0.1336325	-4.925833
TMEM104	-0.043414	6.1910937	-1.878501	0.0617164	0.1336554	-4.926022
ANKRD13D	-0.05357	5.9319062	-1.878394	0.0617312	0.1336766	-4.926218
SNAPC4	-0.050467	5.9047595	-1.878377	0.0617335	0.1336766	-4.926249
RP5-855F14	0.312753	-0.979505	1.8782583	0.0617498	0.1337013	-4.926465
RP11-285A1	-0.414673	0.1701756	-1.87824	0.0617522	0.1337013	-4.926498
Z82214.2	0.1910285	-1.361462	1.8781113	0.06177	0.1337317	-4.926733
RP11-102L1	-0.418902	0.0269144	-1.87804	0.0617797	0.1337375	-4.926863
ENTPD3	0.5774375	1.6237076	1.8780386	0.06178	0.1337375	-4.926866
RP5-864K19	-0.408402	0.5174383	-1.877896	0.0617996	0.1337721	-4.927126
CSN1S1	-0.23247	-1.375041	-1.877347	0.0618751	0.1339275	-4.928127
NPM1P21	-0.402457	0.0532369	-1.877182	0.0618979	0.1339689	-4.928429
ATCAY	-0.366872	-0.767151	-1.877121	0.0619063	0.1339792	-4.928541
RP11-676F2	-0.483921	-0.809819	-1.876873	0.0619404	0.134045	-4.928992
RP11-149I9	-0.408144	-0.718846	-1.876597	0.0619785	0.1341196	-4.929496
SLC38A11	-0.206379	4.6794883	-1.876559	0.0619837	0.134123	-4.929566
RP11-108M9	0.5723031	1.9195054	1.8765035	0.0619913	0.1341315	-4.929666
SOX21-AS1	0.4815584	-0.29626	1.8761491	0.0620402	0.1342216	-4.930312
RP11-181G1	-0.182796	3.9009377	-1.876148	0.0620404	0.1342216	-4.930314
MAP3K11	-0.03894	6.7439885	-1.876023	0.0620577	0.1342499	-4.930543
CTD-2369P2	0.3400245	-0.841558	1.876	0.0620608	0.1342499	-4.930584
FAM151A	-0.536323	3.2946945	-1.875772	0.0620923	0.1343101	-4.931
FREM1	0.5501975	2.744421	1.8757014	0.062102	0.1343232	-4.931128
ABCA6	0.1688489	6.1191895	1.8756289	0.062112	0.1343369	-4.931261
NDUFA5P6	0.3624214	-1.089383	1.8752516	0.0621642	0.1344417	-4.931948
ARMC4P1	0.4836958	0.0255892	1.8751748	0.0621748	0.1344567	-4.932088

FLRT3	0.1705773	5.5851006	1.8751284	0.0621812	0.1344626	-4.932173
NAP1L1P3	-0.401899	1.2211242	-1.875015	0.0621968	0.1344884	-4.932379
CFAP52	-0.440743	1.0590643	-1.874959	0.0622046	0.1344973	-4.932481
TSPAN14	0.0389127	6.4437608	1.8749252	0.0622093	0.1344994	-4.932543
AC053503.4	-0.395125	2.3520127	-1.874886	0.0622148	0.1345033	-4.932615
SYNGR1	-0.162457	5.6032207	-1.874646	0.0622479	0.1345552	-4.933052
MRPL1	0.0482278	5.921928	1.8746455	0.062248	0.1345552	-4.933052
AP001205.1	-0.429235	0.3529767	-1.874632	0.0622498	0.1345552	-4.933077
OBP2A	-0.360601	-0.844128	-1.874578	0.0622573	0.1345606	-4.933175
CT62	0.4072178	-0.625698	1.8745609	0.0622597	0.1345606	-4.933207
HNRNPU-AS1	-0.109729	5.242876	-1.874295	0.0622965	0.1346322	-4.933691
AC004901.1	0.171974	-1.444223	1.8742452	0.0623034	0.1346391	-4.933782
RP11-686O6	-0.157779	3.8237605	-1.874082	0.062326	0.13468	-4.934079
DPP3P2	-0.365866	-1.042607	-1.873828	0.0623612	0.1347481	-4.934542
RP5-1186P1	-0.356315	-0.594521	-1.873663	0.062384	0.1347894	-4.934841
RBSN	-0.032986	6.0382605	-1.87354	0.0624012	0.134807	-4.935066
RP11-730A1	0.2695611	2.6259072	1.873493	0.0624076	0.134807	-4.935151
ACTRT1	-0.153423	-1.449614	-1.873487	0.0624084	0.134807	-4.935162
BCKDHB	0.08143	6.0392477	1.8734723	0.0624105	0.134807	-4.935189
RP11-363J2	-0.419361	0.0968559	-1.873471	0.0624107	0.134807	-4.935191
SGSM1	-0.254422	4.2717363	-1.873009	0.0624747	0.1349374	-4.936032
RP11-494M8	-0.210081	-1.2984	-1.872836	0.0624988	0.134974	-4.936347
AL022341.3	-0.423288	1.0913319	-1.872824	0.0625005	0.134974	-4.93637
MRPS17	-0.057336	5.8551616	-1.872807	0.0625027	0.134974	-4.936399
CPPED1	0.0557528	6.4180071	1.8727266	0.062514	0.1349902	-4.936546
AC084809.3	-0.334375	-0.801961	-1.872648	0.0625249	0.1349984	-4.936689
CTD-2561B2	-0.464754	0.9516613	-1.872646	0.0625251	0.1349984	-4.936693
SUB1P3	-0.412822	0.4743434	-1.8726	0.0625316	0.1350043	-4.936777
UBALD2	0.0564006	6.2431823	1.8724682	0.0625499	0.1350358	-4.937017
SNRPGP4	-0.447473	-0.011252	-1.872417	0.062557	0.1350431	-4.93711
PERP	0.0711465	6.7416786	1.8723042	0.0625727	0.135069	-4.937315
RP11-79P5.	-0.309739	-0.906447	-1.872257	0.0625792	0.1350752	-4.937401
BRD8	-0.033416	6.1876145	-1.872218	0.0625846	0.1350788	-4.937471
KLF13	0.0500673	6.4446324	1.8721409	0.0625953	0.135094	-4.937612
XXbac-BPGE	-0.41943	-0.510256	-1.872093	0.062602	0.1351004	-4.937699
CAMTA1	0.043247	6.0832802	1.8719771	0.0626181	0.1351272	-4.93791
RP11-403A2	0.4467197	0.434004	1.871779	0.0626457	0.1351787	-4.938271
NBPF26	0.2157032	4.0960322	1.8717178	0.0626542	0.1351891	-4.938382
KIDINS220	0.0413953	6.2791418	1.8715427	0.0626786	0.1352337	-4.9387
NTF4	0.3438932	-1.067681	1.8713499	0.0627054	0.1352751	-4.939051
AC104076.3	-0.342195	-0.805772	-1.871349	0.0627055	0.1352751	-4.939053
IGKV3D-7	-0.278092	-1.140738	-1.871325	0.0627089	0.1352751	-4.939097
WNT1	0.439208	-0.165324	1.8712748	0.0627158	0.1352821	-4.939187
RP11-34E5.	-0.399379	-0.346125	-1.871121	0.0627372	0.1353203	-4.939467
ABHD17AP1	-0.414831	0.3638117	-1.870793	0.062783	0.1354109	-4.940063
EWSAT1	-0.427879	2.735083	-1.870604	0.0628094	0.1354598	-4.940408
MDM2	0.0451871	6.5032084	1.8705634	0.062815	0.1354639	-4.940481
RP11-1365D	-0.421465	1.3094566	-1.870112	0.0628779	0.1355916	-4.941301
GAPDHP67	-0.258607	-1.168814	-1.870056	0.0628857	0.1356005	-4.941403
CAGE1	-0.497692	0.1702227	-1.869955	0.0628998	0.1356214	-4.941586
PGRMC2	0.0418771	6.5976739	1.8699334	0.0629028	0.1356214	-4.941626

RP11-732A1	0.1879831	-1.38654	1.869895	0.0629082	0.1356249	-4.941696
IGLV3-9	0.6255716	2.2111302	1.8698613	0.0629129	0.1356271	-4.941757
AC008940.1	0.1230201	-1.501453	1.869823	0.0629183	0.1356306	-4.941827
POTEM	-0.265347	-1.214909	-1.86975	0.0629284	0.1356444	-4.941959
RP11-99E15	0.3927601	-0.357399	1.8696815	0.062938	0.1356572	-4.942084
MBOAT7	-0.036657	6.4282122	-1.86957	0.0629537	0.1356828	-4.942287
AC002398.1	0.4741014	-0.031865	1.869292	0.0629924	0.1357584	-4.942791
ZC3H4	-0.02842	6.1665129	-1.869023	0.06303	0.1358313	-4.943279
KRT32	0.176844	-1.406427	1.868907	0.0630463	0.1358446	-4.943491
PPFIA2	0.4209313	2.0555795	1.868906	0.0630464	0.1358446	-4.943493
LIX1	-0.442436	-0.808104	-1.8689	0.0630473	0.1358446	-4.943504
CTD-3099C6	-0.350771	3.0351308	-1.868864	0.0630523	0.135847	-4.943569
RP11-600F2	-0.178161	3.9550428	-1.868838	0.0630559	0.135847	-4.943616
ACTN4	0.0299142	7.0104951	1.8686518	0.063082	0.1358952	-4.943954
AC006145.4	0.2010693	-1.425841	1.8685529	0.0630958	0.1359169	-4.944134
RP11-517B1	0.3204537	-0.866237	1.8684489	0.0631104	0.1359403	-4.944323
SPOCK3	0.5596184	-0.155856	1.8679268	0.0631835	0.1360864	-4.945271
RP11-442J1	-0.175957	-1.468469	-1.867911	0.0631856	0.1360864	-4.945299
SNAI3-AS1	-0.102825	4.7643506	-1.867586	0.0632313	0.1361766	-4.94589
AP002954.6	-0.471351	-0.207761	-1.867534	0.0632385	0.1361841	-4.945983
GORASP1	0.0281961	6.2367781	1.867238	0.06328	0.1362656	-4.946521
LINC01004	-0.158089	4.1672599	-1.867159	0.0632911	0.1362814	-4.946664
CTD-3032J1	-0.209125	-1.342679	-1.86673	0.0633514	0.1364031	-4.947443
RPGRIP1	-0.161962	4.0079647	-1.86633	0.0634076	0.136516	-4.948169
SLC5A12	-0.666943	1.5671882	-1.866267	0.0634164	0.1365271	-4.948283
CTA-351J1.	-0.396994	1.5963069	-1.866113	0.063438	0.1365655	-4.948562
RP11-1018N	-0.149708	-1.459436	-1.865917	0.0634656	0.1366169	-4.948918
AC092835.2	-0.453604	0.9235999	-1.865498	0.0635244	0.1367354	-4.949676
EEF1A1P6	-0.08207	5.345017	-1.865341	0.0635465	0.1367749	-4.949961
LTV1	-0.049412	5.925212	-1.865193	0.0635675	0.1368067	-4.95023
RP11-58A17	-0.272655	-1.014347	-1.865183	0.0635688	0.1368067	-4.950248
DHRS13	-0.135395	5.0472756	-1.864994	0.0635955	0.136852	-4.950591
RP5-1139B1	0.412641	-0.389966	1.8649805	0.0635974	0.136852	-4.950615
STK26	-0.126385	5.4578343	-1.864937	0.0636035	0.1368523	-4.950694
THRAP3	0.0249361	6.6021217	1.8649067	0.0636078	0.1368523	-4.950749
RP11-831A1	0.1549931	-1.457636	1.8648998	0.0636087	0.1368523	-4.950762
RP11-499E1	-0.382283	-0.687359	-1.864633	0.0636463	0.1369251	-4.951245
SCAMP1	0.0473877	6.0936387	1.8643696	0.0636835	0.1369969	-4.951722
RP11-182J1	0.224744	-1.254159	1.8635572	0.0637981	0.1372355	-4.953194
MIR181A2HG	-0.445713	1.8354128	-1.863478	0.0638093	0.1372488	-4.953338
EIF3D	-0.034485	6.770845	-1.86346	0.0638118	0.1372488	-4.95337
AC073869.1	-0.382571	-0.685498	-1.863355	0.0638267	0.1372728	-4.953561
AJ011932.1	0.4233082	-0.531939	1.8632871	0.0638363	0.1372852	-4.953683
LRRC4B	0.1243356	4.5771925	1.8632428	0.0638425	0.1372906	-4.953764
PGK1P2	-0.419463	1.1003392	-1.862868	0.0638955	0.1373963	-4.954442
RP13-395E1	0.2106043	-1.334685	1.8626344	0.0639285	0.1374594	-4.954865
UGT1A9	0.4944301	5.2647255	1.8624684	0.063952	0.1375018	-4.955166
PMPCB	0.0383252	6.4832009	1.8622648	0.0639809	0.1375557	-4.955534
RPL22L1	-0.085738	6.1890407	-1.861785	0.0640488	0.1376935	-4.956402
PRAP1	-0.233768	6.744965	-1.86168	0.0640636	0.1377174	-4.956592
SNX7	0.1417048	5.6855344	1.8616	0.064075	0.1377338	-4.956738

RERG-IT1	0.2332204	-1.290877	1.8611714	0.0641358	0.1378563	-4.957513
LLOXNC01-1	0.405687	-0.297872	1.8611091	0.0641446	0.1378672	-4.957626
AC072052.7	-0.480596	-0.060308	-1.861012	0.0641585	0.1378888	-4.957802
VLDLR-AS1	0.510684	1.0351647	1.860893	0.0641753	0.1379168	-4.958017
RP11-674P1	0.4370658	0.0614102	1.8608602	0.06418	0.1379187	-4.958076
RP13-467H1	-0.478328	1.442813	-1.860822	0.0641853	0.1379222	-4.958144
AL353997.3	0.2287288	-1.282598	1.860685	0.0642048	0.1379559	-4.958393
CDIP1	0.055147	6.312765	1.8606121	0.0642152	0.1379642	-4.958525
HERC2P3	-0.397255	3.9807388	-1.860605	0.0642163	0.1379642	-4.958538
RP11-365P1	-0.420459	1.5371947	-1.860553	0.0642235	0.1379717	-4.958631
MNT	0.0391426	5.8130513	1.8603977	0.0642456	0.1380067	-4.958912
SLC7A2	0.1119507	6.6196538	1.8603856	0.0642474	0.1380067	-4.958934
ZSCAN1	-0.486568	0.3782718	-1.860215	0.0642716	0.1380425	-4.959243
RP11-513O1	0.4552575	0.0457861	1.860215	0.0642716	0.1380425	-4.959243
RP11-433A1	-0.153159	-1.453164	-1.860153	0.0642805	0.1380499	-4.959355
ZNF670	-0.157751	4.930885	-1.860138	0.0642826	0.1380499	-4.959383
JRKL-AS1	0.1019741	-1.534942	1.8599349	0.0643114	0.1381036	-4.959749
RGS9BP	-0.470407	0.8620966	-1.859881	0.0643191	0.138112	-4.959847
KIF1C	0.0440041	6.7993661	1.8597492	0.0643378	0.1381441	-4.960085
RP11-613M5	-0.36181	1.3893817	-1.859567	0.0643638	0.1381917	-4.960415
RP11-452L6	0.3898842	1.4560087	1.8593414	0.0643958	0.1382497	-4.960822
RAB7B	0.1145181	5.1117159	1.8593238	0.0643983	0.1382497	-4.960854
RP11-3J10.	-0.287295	-0.942612	-1.859242	0.06441	0.1382666	-4.961002
LY6G6C	-0.350689	-0.80163	-1.859209	0.0644147	0.1382685	-4.961061
UBQLNL	0.3425423	2.9106668	1.8587638	0.0644781	0.1383839	-4.961866
CCDC93	-0.03933	6.0615038	-1.858762	0.0644784	0.1383839	-4.961869
CCND2-AS2	0.2375465	-1.251189	1.8587518	0.0644798	0.1383839	-4.961887
RP1-241P17	-0.407082	0.8574687	-1.858722	0.064484	0.1383847	-4.961941
RP11-69E11	0.1330203	4.4255719	1.8586927	0.0644882	0.1383856	-4.961994
RP3-335N17	0.3866484	-0.785929	1.8585772	0.0645047	0.1384079	-4.962203
OVOL2	-0.651246	1.5929328	-1.858551	0.0645085	0.1384079	-4.962251
EXOSC4	-0.064068	6.1677321	-1.85854	0.06451	0.1384079	-4.96227
RP11-363H1	-0.216501	-1.264188	-1.858449	0.064523	0.1384277	-4.962435
MECP2	0.0350047	6.1782235	1.8583754	0.0645335	0.1384388	-4.962567
ACTG1P10	-0.393983	0.0333332	-1.858359	0.0645357	0.1384388	-4.962596
RP11-561N1	-0.170359	-1.44473	-1.858191	0.0645598	0.1384823	-4.962901
RP11-483C6	0.2587221	-1.145025	1.8580585	0.0645787	0.1385146	-4.96314
RP11-250B2	0.1656354	4.0416651	1.857994	0.0645879	0.1385262	-4.963256
AC013470.6	-0.246063	-1.161812	-1.857857	0.0646075	0.1385601	-4.963504
ABCC5	-0.047169	5.961371	-1.857753	0.0646222	0.1385836	-4.963691
CABP4	0.2167638	3.8408894	1.8576511	0.0646368	0.1386067	-4.963875
GS1-184P14	-0.391777	1.5106915	-1.857568	0.0646486	0.1386238	-4.964025
SDHC	0.0417237	6.7329968	1.8573296	0.0646827	0.1386889	-4.964456
NUP35	-0.041574	5.7179438	-1.856828	0.0647544	0.1388343	-4.965361
LA16c-352F	-0.28012	-1.150303	-1.856723	0.0647695	0.1388586	-4.965552
RNU6-485P	-0.425162	-0.153254	-1.856688	0.0647745	0.1388611	-4.965615
RP11-889L3	-0.140434	4.1978188	-1.856252	0.0648368	0.1389867	-4.966401
RP11-725D2	-0.179488	-1.436832	-1.855686	0.0649179	0.1391524	-4.967423
AC090505.5	-0.296635	-1.059877	-1.855545	0.064938	0.1391872	-4.967676
NUCKS1	-0.037081	7.0013815	-1.855443	0.0649527	0.1392106	-4.967861
ASPG	0.3969225	5.4032907	1.8551767	0.0649909	0.1392813	-4.968341

CTD-2331H1	-0.516125	2.7479509	-1.855159	0.0649934	0.1392813	-4.968372
ANAPC16	0.033587	6.5625956	1.8550737	0.0650057	0.1392995	-4.968527
RP11-545P7	-0.44761	0.5133829	-1.855021	0.0650132	0.1393075	-4.968621
SUMO1P4	0.2246819	-1.217419	1.8548289	0.0650408	0.1393584	-4.968968
RP4-814D15	-0.256527	-1.100551	-1.854722	0.0650561	0.1393831	-4.969161
RP11-445K1	-0.296384	-1.165161	-1.854636	0.0650685	0.1394015	-4.969317
LINC01023	-0.11503	4.3415478	-1.854468	0.0650925	0.1394447	-4.969618
SPIN2A	0.4023817	-0.222092	1.8543978	0.0651027	0.139457	-4.969745
RP5-864K19	-0.300234	-0.848813	-1.854375	0.0651059	0.139457	-4.969785
RP11-297B1	-0.21472	-1.339935	-1.85402	0.065157	0.1395583	-4.970427
DYNLRB1	-0.04013	6.5580934	-1.853464	0.0652369	0.1397212	-4.971428
SLC12A5	-0.217009	4.027052	-1.853283	0.065263	0.1397688	-4.971754
QRFP	-0.416533	1.7236282	-1.853247	0.0652681	0.1397717	-4.971819
BTBD2	-0.041463	6.4525488	-1.853203	0.0652744	0.139777	-4.971898
MRI1	-0.049467	5.901517	-1.85271	0.0653455	0.139921	-4.972787
PRKCA-AS1	-0.290546	-0.945597	-1.852565	0.0653664	0.1399575	-4.973048
RP1-149A16	-0.416297	0.4492737	-1.8524	0.0653902	0.1400002	-4.973345
RP11-819C2	-0.097459	4.3679496	-1.852287	0.0654065	0.1400268	-4.973548
RPS14	-0.042888	7.1399053	-1.852174	0.0654228	0.1400536	-4.973752
RP11-790I1	0.4849719	-0.185304	1.8517709	0.0654809	0.1401697	-4.974477
SLC23A1	-0.204713	5.8693298	-1.851554	0.0655123	0.1402287	-4.974869
NLGN3	0.2224158	3.3522756	1.8513842	0.0655367	0.1402728	-4.975173
ITK	0.1944537	4.7715494	1.8511953	0.065564	0.140322	-4.975513
CTDNEP1	0.0377278	6.3613329	1.8511666	0.0655682	0.140322	-4.975565
RPL10AP1	-0.247787	-1.122162	-1.851145	0.0655712	0.140322	-4.975603
DNTTIP2	0.0321332	6.3142721	1.850739	0.06563	0.1404394	-4.976334
RP11-141J1	-0.249527	-1.244572	-1.850616	0.0656478	0.1404693	-4.976556
PTGER1	0.4961745	2.317773	1.8505867	0.065652	0.1404701	-4.976609
TRIL	0.1047338	4.812364	1.8504019	0.0656787	0.140519	-4.976941
RP11-351M1	-0.154714	-1.430497	-1.850201	0.0657077	0.140568	-4.977302
RP11-1167A	0.2861155	-0.989665	1.8501874	0.0657098	0.140568	-4.977327
CRYBB2	-0.440911	1.4726039	-1.850148	0.0657155	0.140568	-4.977398
PLOD1	0.0484577	6.8648728	1.8501372	0.065717	0.140568	-4.977417
TULP2	-0.446388	0.5594107	-1.850032	0.0657322	0.1405887	-4.977606
MUC15	-0.690406	0.3632357	-1.850017	0.0657344	0.1405887	-4.977633
RP11-627K1	0.1295472	-1.498127	1.8499075	0.0657503	0.1406145	-4.97783
TMEM246	-0.21391	5.3029441	-1.849825	0.0657622	0.1406317	-4.977978
ZHX2	-0.04964	6.3113753	-1.849654	0.065787	0.1406716	-4.978286
MAD2L2	0.0632309	6.1556261	1.8496434	0.0657885	0.1406716	-4.978305
NPB	-0.433163	0.437317	-1.849564	0.0658	0.1406872	-4.978447
AC005775.2	-0.437681	0.8986468	-1.84954	0.0658036	0.1406872	-4.978492
SPRYD7	0.0584965	5.8624053	1.8494358	0.0658186	0.1407111	-4.978678
RP11-336N8	0.4380105	-0.02659	1.8494073	0.0658227	0.1407117	-4.97873
DEPDC5	0.0507954	5.8912113	1.8492001	0.0658528	0.1407677	-4.979102
BMI1	-0.039688	6.2858983	-1.848987	0.0658837	0.1408255	-4.979485
RP11-431M3	-0.343637	-1.020228	-1.848691	0.0659267	0.1409092	-4.980018
RP11-379F4	0.1982474	2.906915	1.8482572	0.0659896	0.1410355	-4.980797
CRH	-0.292716	-1.265933	-1.848184	0.0660003	0.14105	-4.980929
AC019186.1	-0.284672	2.2370841	-1.848137	0.0660071	0.1410562	-4.981013
NKAIN3	0.3929924	-0.669191	1.8480963	0.066013	0.1410606	-4.981086
SNORA54	-0.18102	-1.351882	-1.848067	0.0660173	0.1410616	-4.981139

RP11-442N2	-0.290531	2.7284921	-1.847505	0.0660989	0.1412277	-4.982148
CTC-378H22	-0.381527	3.2752799	-1.847434	0.0661093	0.1412416	-4.982276
DEFA5	-0.26753	-1.378705	-1.846865	0.0661921	0.1414102	-4.983298
SCOCP1	0.2719778	-1.019633	1.8466929	0.0662172	0.1414555	-4.983607
LIN37	-0.055677	5.1611574	-1.846538	0.0662398	0.1414955	-4.983885
RNVU1-10	0.2480719	-1.194719	1.8464063	0.0662589	0.1415282	-4.984121
LINC00847	0.0556206	5.6993947	1.846341	0.0662685	0.1415402	-4.984239
RDH14	0.0306434	5.9063831	1.846302	0.0662741	0.1415441	-4.984308
PABPC1P2	-0.208882	-1.345171	-1.846208	0.0662879	0.1415591	-4.984477
SPATA2	-0.036699	5.8933524	-1.846185	0.0662912	0.1415591	-4.984519
SMAP1	-0.04922	5.611281	-1.846152	0.066296	0.1415591	-4.984578
AC091633.3	-0.410994	0.2851867	-1.846147	0.0662967	0.1415591	-4.984586
RP1-117P20	-0.469755	-0.32309	-1.846093	0.0663046	0.1415677	-4.984683
RNU6-100P	-0.246916	-1.120216	-1.84592	0.0663299	0.1416134	-4.984995
DENND2C	-0.109815	5.471449	-1.84583	0.0663429	0.1416329	-4.985155
RP11-413H2	0.1961419	-1.314532	1.8455304	0.0663867	0.1417181	-4.985693
RP3-526F5.	-0.171582	-1.448709	-1.845163	0.0664404	0.1418245	-4.986353
SLC2A2	-0.241132	6.7053119	-1.845076	0.0664531	0.1418433	-4.986509
IGSF6	0.0954227	5.6438581	1.8450231	0.0664608	0.1418514	-4.986603
FAM27E3	-0.462261	0.9136125	-1.844809	0.0664921	0.1419099	-4.986987
RP11-156K1	0.4651133	1.7856074	1.8447739	0.0664972	0.1419125	-4.98705
RN7SKP44	0.1428659	-1.4758	1.8443624	0.0665574	0.1420326	-4.987788
RP11-87G24	0.2625855	-1.249848	1.8442673	0.0665713	0.142054	-4.987959
RP11-1000E	0.4006911	2.0309388	1.8439818	0.0666131	0.1421318	-4.988471
CTA-363E6.	0.2568164	-1.193024	1.8439653	0.0666155	0.1421318	-4.9885
RP11-807E1	0.4896385	0.484325	1.8439374	0.0666196	0.1421321	-4.98855
RP11-51701	-0.380443	0.8676711	-1.843888	0.0666269	0.1421394	-4.98864
RNVU1-20	-0.355115	-0.769901	-1.843822	0.0666365	0.1421515	-4.988757
SPANXN1	-0.158664	-1.476949	-1.84379	0.0666412	0.1421534	-4.988815
XPR1	0.0499449	6.1139179	1.8436725	0.0666584	0.1421817	-4.989025
ATP6V1E1	0.032313	6.5901704	1.8433537	0.0667051	0.142273	-4.989597
ANKS1A	0.0391373	6.0117448	1.8430512	0.0667494	0.1423592	-4.990139
RP11-90H3.	-0.258191	-1.238761	-1.843024	0.0667533	0.1423593	-4.990187
XXbac-BPG2	-0.117495	4.3982268	-1.842919	0.0667688	0.1423797	-4.990375
SPATA21	-0.524828	3.3450351	-1.842906	0.0667707	0.1423797	-4.990399
RP11-286N2	-0.41448	0.660815	-1.84267	0.0668053	0.1424451	-4.990822
SNX6P1	-0.217896	-1.308473	-1.842369	0.0668496	0.1425312	-4.991362
AC074212.6	-0.11075	4.3732717	-1.842337	0.0668542	0.1425327	-4.991418
SNORA2A	-0.394359	-0.031365	-1.842227	0.0668703	0.1425588	-4.991615
CTC-499B15	-0.335033	-0.688723	-1.842172	0.0668785	0.1425679	-4.991715
ROBO3	0.146378	5.0470328	1.8420969	0.0668895	0.1425772	-4.991849
DGCR6L	0.0613568	6.3672886	1.8420888	0.0668907	0.1425772	-4.991863
DPM2	-0.045749	6.3116929	-1.842028	0.0668996	0.1425879	-4.991972
SLC30A1	0.0674196	6.5439944	1.8417766	0.0669365	0.1426583	-4.992422
ANKRD54P1	-0.134361	-1.488867	-1.841567	0.0669674	0.1427157	-4.992798
FIRRE	-0.555444	1.6581813	-1.841537	0.0669718	0.1427167	-4.992851
CTD-2325A1	-0.484877	2.0426176	-1.841487	0.0669792	0.1427242	-4.992942
CTB-167G5.	-0.306925	-0.802225	-1.841173	0.0670253	0.1428142	-4.993503
THUMPD3	-0.028574	6.159182	-1.8407	0.0670949	0.142951	-4.994349
ZNF644	0.03646	6.0885261	1.8406834	0.0670974	0.142951	-4.994379
SLC35F4	0.3082775	-0.976078	1.8404827	0.067127	0.1430057	-4.994739

RP11-611L7	-0.195004	-1.366929	-1.840406	0.0671382	0.1430214	-4.994876
MPL	0.3586159	1.7824823	1.8402262	0.0671647	0.1430695	-4.995198
AC012451.1	-0.286788	-1.058968	-1.83991	0.0672113	0.1431603	-4.995763
RP11-327I2	-0.159781	-1.476401	-1.839828	0.0672235	0.1431779	-4.99591
RP13-392I1	-0.495051	3.1234903	-1.839676	0.0672459	0.1431999	-4.996182
ZSWIM7	0.0566364	5.7466384	1.8396692	0.0672469	0.1431999	-4.996194
RP11-328N1	-0.668882	1.7446705	-1.839639	0.0672513	0.1431999	-4.996248
CXXC4	-0.221416	4.4795493	-1.839629	0.0672528	0.1431999	-4.996265
RP11-885N1	0.1234186	-1.510499	1.839625	0.0672534	0.1431999	-4.996273
RP11-9E17.	0.1215323	4.7238037	1.8394969	0.0672723	0.1432249	-4.996502
AC005702.4	-0.31883	-0.819003	-1.839492	0.067273	0.1432249	-4.996511
RP11-40109	-0.338961	-0.969755	-1.839457	0.0672782	0.1432277	-4.996574
AC006378.2	-0.229038	2.6390955	-1.839356	0.0672931	0.1432509	-4.996754
ZNF460	0.1840669	3.8883621	1.8391947	0.0673169	0.1432933	-4.997043
KLHL25	0.0607571	5.8411137	1.8391648	0.0673214	0.1432944	-4.997096
KDM6A	-0.054839	5.9951839	-1.839053	0.0673379	0.1433119	-4.997297
RP11-25G10	0.5116007	1.3647073	1.8390431	0.0673393	0.1433119	-4.997314
RAP2B	0.0433482	6.1069578	1.8390294	0.0673413	0.1433119	-4.997338
RP5-1086K1	0.2742208	3.474594	1.8389144	0.0673583	0.1433397	-4.997544
GALR2	-0.432265	1.5749569	-1.838872	0.0673647	0.1433448	-4.997621
AC128709.4	-0.453495	0.1327141	-1.838842	0.0673691	0.1433459	-4.997674
JAZF1-AS1	0.45684	1.1211801	1.8383796	0.0674374	0.1434828	-4.9985
snoU13	-0.455099	0.1394706	-1.838323	0.0674458	0.1434923	-4.998602
LINC01579	-0.492916	-0.568781	-1.838263	0.0674546	0.1435027	-4.998708
PTCH1	0.138018	5.3878726	1.8381332	0.0674738	0.1435352	-4.998941
CD164L2	0.3887995	-0.927593	1.8380782	0.067482	0.1435442	-4.999039
COMMD5	-0.047265	6.1837865	-1.837728	0.0675337	0.143646	-4.999664
SQRDL	0.0508364	6.3254077	1.8376109	0.0675512	0.1436735	-4.999874
RP11-65N13	-0.31375	-0.885995	-1.837588	0.0675546	0.1436735	-4.999915
LINC01050	0.2190265	-1.343639	1.8374911	0.0675689	0.1436891	-5.000088
CTC-548K16	-0.376019	-0.424233	-1.837485	0.0675698	0.1436891	-5.000099
RP11-266L9	0.1450021	4.4349123	1.8374023	0.067582	0.1436999	-5.000247
DPRXP2	0.2730542	-1.13384	1.8373978	0.0675827	0.1436999	-5.000255
GRHL1	-0.112567	5.4594538	-1.837068	0.0676315	0.1437953	-5.000843
RP11-70501	-0.363387	1.8440127	-1.837	0.0676416	0.1438084	-5.000965
RP11-574K1	-0.23197	-1.247333	-1.836899	0.0676567	0.1438321	-5.001147
SELL	0.1298063	5.2212964	1.8367729	0.0676754	0.1438633	-5.001371
RP11-697K2	0.3133679	-1.112787	1.8366133	0.067699	0.1439053	-5.001656
RP11-84A19	0.3650095	-0.442414	1.8364475	0.0677236	0.1439479	-5.001952
THRA	0.0455534	6.0322208	1.8364249	0.067727	0.1439479	-5.001993
TRAV8-3	-0.49114	0.9116558	-1.836329	0.0677412	0.1439697	-5.002163
DNMT3L	-0.605963	1.6023319	-1.836017	0.0677876	0.14406	-5.002722
FADS6	-0.607479	3.9305934	-1.835837	0.0678143	0.1441084	-5.003043
KRT18P59	0.4050685	1.2131429	1.8357263	0.0678307	0.1441337	-5.00324
SCARNA7	-0.448422	1.2079161	-1.835703	0.0678341	0.1441337	-5.003281
UQCR11	0.047057	6.5257686	1.8355042	0.0678638	0.1441883	-5.003636
KIAA1107	0.1088273	4.764499	1.8354479	0.0678721	0.1441976	-5.003737
AC137723.1	-0.171052	-1.383254	-1.835391	0.0678805	0.1442071	-5.003838
AC114765.1	0.1231812	-1.493057	1.8353603	0.0678852	0.1442085	-5.003893
DUX4L27	-0.453262	1.1773268	-1.834824	0.0679649	0.1443668	-5.00485
RNU2-27P	-0.352491	1.8089984	-1.834806	0.0679675	0.1443668	-5.004882

RP11-323I1	-0.302182	-1.040233	-1.83477	0.067973	0.14437	-5.004947
NKX6-3	-0.360541	-1.016887	-1.834622	0.067995	0.1444082	-5.00521
AC124312.1	-0.238386	-1.218002	-1.83449	0.0680146	0.1444416	-5.005446
ASGR2	-0.132176	7.0235391	-1.834206	0.068057	0.1445164	-5.005953
AC114730.7	-0.269482	-1.014318	-1.8342	0.0680578	0.1445164	-5.005963
HRK	-0.503616	0.046746	-1.834132	0.0680681	0.1445298	-5.006086
RP1-56K13.	0.3088522	-0.851989	1.8340456	0.0680809	0.1445452	-5.006239
GABRG1	0.5096291	0.3070163	1.8340299	0.0680832	0.1445452	-5.006267
NPM1P38	-0.172077	-1.400077	-1.834002	0.0680874	0.1445457	-5.006317
SLC39A1	-0.036711	6.7948039	-1.833782	0.0681202	0.1446068	-5.006709
GCNT7	-0.404576	0.7145882	-1.833446	0.0681703	0.1447048	-5.007308
ERGIC2	-0.033134	6.2070793	-1.833243	0.0682006	0.1447607	-5.00767
LINC00926	-0.155362	4.2333266	-1.833161	0.0682129	0.1447783	-5.007817
RP11-15H2C	-0.119757	4.9545252	-1.833039	0.0682311	0.1448058	-5.008034
PEX26	0.0338009	6.3693159	1.8329931	0.0682379	0.1448058	-5.008116
GAB1	0.0569006	5.6939706	1.8329814	0.0682397	0.1448058	-5.008136
TMEM255A	-0.294474	3.7714851	-1.832968	0.0682417	0.1448058	-5.008161
PRKX	0.1229806	5.5271495	1.8329415	0.0682456	0.1448058	-5.008208
KIF1A	-0.570811	3.2639836	-1.832856	0.0682584	0.1448245	-5.008361
RP11-587D2	-0.418546	0.607698	-1.832745	0.068275	0.1448453	-5.008558
FRY-AS1	-0.445107	0.4399874	-1.83272	0.0682788	0.1448453	-5.008603
AC019097.7	-0.396351	0.9907076	-1.8327	0.0682817	0.1448453	-5.008638
MIR559	-0.323625	-1.007816	-1.832684	0.0682841	0.1448453	-5.008666
RP11-1090M	0.2704923	-1.111004	1.8326538	0.0682886	0.1448464	-5.00872
COMTD1	0.0832331	5.8450804	1.8324847	0.0683139	0.1448916	-5.009022
AC004893.1	0.2986924	-0.86009	1.8322755	0.0683452	0.1449427	-5.009395
RP11-252A2	0.3789463	1.2539722	1.8322706	0.0683459	0.1449427	-5.009403
NPM1P19	-0.294951	-0.915109	-1.832116	0.068369	0.1449833	-5.009679
PIP	0.4508045	-0.544907	1.8320888	0.0683731	0.1449835	-5.009727
CFB	0.1033798	6.9124655	1.8316488	0.0684389	0.1451147	-5.010511
USP13	-0.050164	5.9085877	-1.831597	0.0684467	0.1451227	-5.010603
AC015971.2	-0.448614	1.5437817	-1.83146	0.0684672	0.1451577	-5.010847
LINC00266-	0.143188	-1.4984	1.8311269	0.0685171	0.1452475	-5.011441
PAPD4	0.0318312	6.1714091	1.8311244	0.0685175	0.1452475	-5.011445
PDDC1	-0.039944	6.3396167	-1.831074	0.068525	0.1452551	-5.011535
ZNF264	-0.057529	5.8757937	-1.830859	0.0685572	0.1453149	-5.011917
ONECUT1	-0.278349	5.4957451	-1.830616	0.0685937	0.1453838	-5.01235
FNTB	-0.036796	5.5996857	-1.830107	0.0686701	0.1455373	-5.013257
RNA5SP163	-0.465197	0.3580964	-1.830001	0.068686	0.1455624	-5.013445
RP11-496I9	-0.183158	4.4771401	-1.829736	0.0687258	0.1456383	-5.013917
RPL21P133	0.1641027	-1.480517	1.8296944	0.068732	0.1456431	-5.013991
MAT2A	-0.039587	6.5411412	-1.829531	0.0687566	0.1456831	-5.014282
AC112229.1	0.4958653	0.8377026	1.8295156	0.0687589	0.1456831	-5.014309
CACNA1C-AS	0.4238654	0.631949	1.829437	0.0687707	0.1456997	-5.014449
POLR3G	0.1144188	4.7955812	1.8293275	0.0687872	0.1457215	-5.014643
RP11-63L7.	-0.330157	-0.733145	-1.829315	0.068789	0.1457215	-5.014665
IER5L	0.1039309	5.6111231	1.8292481	0.0687991	0.1457345	-5.014785
RP11-227D1	-0.241282	-1.165316	-1.829195	0.068807	0.1457403	-5.014879
AC005758.1	0.15839	-1.460664	1.8291768	0.0688098	0.1457403	-5.014911
IL4I1	-0.162348	5.0336407	-1.829123	0.0688179	0.1457489	-5.015007
IL1RAPL1	-0.492939	0.4245169	-1.828921	0.0688483	0.1458048	-5.015366

DSP	-0.068595	6.8495858	-1.828826	0.0688626	0.1458268	-5.015536
RP11-147L1	-0.416247	0.4507957	-1.828724	0.0688779	0.1458507	-5.015717
RP11-583F2	-0.413593	0.3244259	-1.828558	0.068903	0.1458952	-5.016012
RP11-529G2	0.26791	-1.041735	1.8285294	0.0689073	0.1458958	-5.016063
AC018755.1	-0.400106	2.1938729	-1.82848	0.0689147	0.1459031	-5.016151
SPDEF	-0.587822	2.1079674	-1.828293	0.0689428	0.1459542	-5.016483
FAM8A6P	0.2548298	-1.252293	1.8281944	0.0689577	0.1459773	-5.016659
RP11-33A14	0.3826003	-0.692115	1.8280461	0.06898	0.1460161	-5.016922
RP11-536L3	-0.435647	0.2824075	-1.827617	0.0690447	0.1461445	-5.017685
TRAV20	-0.443861	-0.179871	-1.82757	0.0690518	0.1461509	-5.017768
PRKXP1	-0.452139	1.8318916	-1.827285	0.0690949	0.1462337	-5.018276
KCTD4	0.2452476	-1.235968	1.8272129	0.0691057	0.1462481	-5.018403
SETP4	-0.182454	-1.373084	-1.827161	0.0691135	0.1462563	-5.018496
TMEM229A	0.3019909	-1.210047	1.8269097	0.0691515	0.146328	-5.018942
TRBV18	-0.483915	1.1159411	-1.826769	0.0691728	0.1463646	-5.019193
CA5A	-0.382353	5.2260095	-1.82662	0.0691953	0.146398	-5.019457
C1QL1	-0.412423	4.3236492	-1.826611	0.0691966	0.146398	-5.019473
NKAP	-0.041508	6.0291978	-1.826482	0.069216	0.1464307	-5.019702
CREB1	-0.029172	6.0030458	-1.82641	0.0692269	0.1464452	-5.019829
RP11-367J1	-0.243346	3.3544663	-1.826267	0.0692486	0.1464797	-5.020084
ELL	-0.033969	5.9705124	-1.826249	0.0692512	0.1464797	-5.020115
PRMT7	0.0367531	6.0565861	1.8262213	0.0692555	0.1464802	-5.020165
CASC19	-0.520452	2.3884404	-1.826172	0.0692629	0.1464875	-5.020253
SLC2A3P2	0.2788642	-1.062319	1.8260448	0.0692822	0.1465197	-5.020478
AC092839.3	-0.310325	-0.91125	-1.825998	0.0692892	0.1465262	-5.020562
CHMP4B	0.029993	6.6429889	1.8257002	0.0693343	0.1466129	-5.021091
TP73	-0.231234	4.5102353	-1.825634	0.0693444	0.1466258	-5.021209
RP11-119F1	0.4556955	1.7229642	1.8255811	0.0693523	0.146634	-5.021302
CKLF	-0.065907	5.4285766	-1.825542	0.0693582	0.146638	-5.021371
RP11-27I1.	-0.389875	1.3240108	-1.82542	0.0693767	0.1466686	-5.021588
UBE3B	-0.028666	6.3025227	-1.825158	0.0694163	0.1467439	-5.022053
RP11-3P17.	-0.406762	-0.259105	-1.824971	0.0694447	0.1467954	-5.022385
RP11-535M1	0.4565605	0.862947	1.824831	0.0694659	0.1468318	-5.022634
FER	-0.066303	5.611589	-1.824738	0.06948	0.1468529	-5.022798
TIMM50	-0.039432	6.4354686	-1.824614	0.0694988	0.1468842	-5.023018
RP11-475J5	0.4232336	-0.183295	1.8245414	0.0695098	0.146899	-5.023147
CTD-2135D7	0.3776551	-0.077581	1.8242678	0.0695513	0.1469782	-5.023633
SH3KBP1	0.0632587	6.1703431	1.8242237	0.069558	0.1469838	-5.023711
RP1-34L19.	-0.34984	-0.759562	-1.824097	0.0695773	0.1470068	-5.023936
RP11-446H1	0.3796815	-0.366565	1.8240968	0.0695773	0.1470068	-5.023936
SMNDC1	-0.025786	6.0002898	-1.824067	0.0695818	0.1470068	-5.02399
SRRM2-AS1	-0.129661	4.3371288	-1.824046	0.069585	0.1470068	-5.024026
UBE3A	0.0333689	6.3703747	1.8235881	0.0696545	0.1471452	-5.024839
CEP85L	0.091946	4.9880247	1.8233993	0.0696832	0.1471972	-5.025174
LYRM1	0.0562205	6.214378	1.8233331	0.0696933	0.1472043	-5.025291
ZNF625	-0.422305	1.2382217	-1.823324	0.0696946	0.1472043	-5.025307
RP11-456H1	-0.220179	-1.280869	-1.823269	0.0697031	0.1472122	-5.025405
ARHGAP32	0.0584974	5.7345974	1.8232466	0.0697064	0.1472122	-5.025444
RFPL3S	-0.384958	1.8396166	-1.82318	0.0697166	0.1472251	-5.025563
RP11-220C2	-0.308564	-1.120071	-1.823143	0.0697222	0.1472285	-5.025629
RN7SL838P	0.2154156	-1.254423	1.8229215	0.0697558	0.1472884	-5.026021

AP000697.6	0.2200469	-1.301145	1.8229032	0.0697586	0.1472884	-5.026053
SPRED3	0.2593626	3.6835149	1.8228278	0.0697701	0.1473041	-5.026187
DDX50P1	-0.395868	1.3732599	-1.822756	0.069781	0.1473187	-5.026315
SLC29A1	-0.061865	6.6444778	-1.822709	0.0697881	0.1473247	-5.026397
DCAF13P3	-0.341937	-0.5034	-1.822684	0.069792	0.1473247	-5.026442
AC034243.1	-0.523851	1.8708416	-1.822643	0.0697982	0.1473293	-5.026514
RP11-204L2	-0.411151	0.0382083	-1.822594	0.0698057	0.1473367	-5.026602
CCDC172	-0.173565	-1.469642	-1.822544	0.0698132	0.147344	-5.02669
RPL26L1	0.0472582	5.9509777	1.8224497	0.0698276	0.1473642	-5.026857
EXOC4	0.0331616	6.3301537	1.8224283	0.0698309	0.1473642	-5.026895
RP11-516C1	0.2970394	-0.9331	1.8223795	0.0698383	0.1473714	-5.026982
TOMM20P4	-0.388085	-0.082229	-1.822279	0.0698537	0.1473953	-5.027161
C11orf94	-0.409654	0.7364562	-1.822184	0.069868	0.1474171	-5.027328
GEMIN8P4	-0.116197	4.382843	-1.82199	0.0698976	0.1474605	-5.027672
ANKRD30BL	-0.190325	-1.383128	-1.821989	0.0698978	0.1474605	-5.027674
RP11-231P2	0.2976376	-0.951032	1.8219696	0.0699007	0.1474605	-5.027708
KRR1P1	-0.41295	0.016354	-1.821918	0.0699086	0.1474685	-5.0278
RNU6-353P	-0.328399	-0.83158	-1.821888	0.0699131	0.1474695	-5.027852
AC091729.8	-0.376236	-0.639891	-1.821851	0.0699189	0.1474732	-5.027919
RP11-529P9	-0.215203	-1.383508	-1.821774	0.0699305	0.147481	-5.028055
GRHL3	0.3654924	3.1492104	1.8217734	0.0699306	0.147481	-5.028056
CTC-512J14	-0.207469	2.6271473	-1.821741	0.0699355	0.1474827	-5.028113
NCSTN	-0.033143	6.7263554	-1.821572	0.0699613	0.1475286	-5.028412
TMCO5A	0.3152329	-1.056816	1.821098	0.0700336	0.1476726	-5.029253
AC007326.9	0.3937984	-0.753847	1.8210007	0.0700485	0.1476922	-5.029425
TSPY3	-0.276588	-1.314342	-1.820984	0.070051	0.1476922	-5.029454
AP000997.1	0.4097447	-0.466908	1.8209308	0.0700591	0.1477008	-5.029549
LL22NC03-3	-0.392772	-0.364887	-1.820754	0.0700861	0.1477491	-5.029862
RP11-452I5	-0.121089	5.0260475	-1.820555	0.0701166	0.1478048	-5.030215
TVP23C-CDR	-0.410452	0.6495176	-1.820244	0.0701641	0.1478964	-5.030766
PAX9	0.516033	0.3514378	1.8201575	0.0701773	0.1479128	-5.030918
LA16c-431H	0.4317213	0.0015985	1.8201398	0.07018	0.1479128	-5.030949
GOLT1A	-0.213633	6.4208056	-1.819907	0.0702156	0.1479793	-5.031362
ACOT2	0.0737142	6.1205651	1.8198662	0.0702218	0.1479839	-5.031434
PABPC1P10	-0.238639	-1.153132	-1.81972	0.0702442	0.1480225	-5.031693
RP11-437B1	-0.237177	2.7977187	-1.819654	0.0702543	0.1480353	-5.03181
RP11-143A1	-0.148941	-1.459812	-1.819599	0.0702627	0.1480444	-5.031907
TASP1	-0.055142	5.3450895	-1.819441	0.0702868	0.1480803	-5.032186
CBR3-AS1	-0.15344	4.1576984	-1.819416	0.0702907	0.1480803	-5.032231
ZNF500	0.0375094	5.6065738	1.8194079	0.0702919	0.1480803	-5.032245
SLAMF8	0.1227182	5.4363727	1.8192057	0.0703229	0.1481314	-5.032603
ZNHIT6	0.0458792	5.6866931	1.8191747	0.0703276	0.1481314	-5.032658
JMY	0.0544322	5.9624233	1.8191702	0.0703283	0.1481314	-5.032666
C7orf25	-0.10872	3.7715187	-1.818848	0.0703777	0.1482268	-5.033236
NAV2	0.0780579	6.2570748	1.8187932	0.0703861	0.1482359	-5.033333
PRB3	-0.33999	-0.753593	-1.818545	0.070424	0.1483073	-5.033771
PARL	-0.033032	6.2679371	-1.818496	0.0704315	0.1483145	-5.033858
HMGA1P4	0.4475579	1.5430523	1.8184326	0.0704413	0.1483266	-5.033971
RP11-660M5	-0.266554	-1.038819	-1.818308	0.0704604	0.1483582	-5.034191
RP11-142A2	0.4559688	-0.316396	1.8182652	0.070467	0.1483635	-5.034267
RP5-1050D4	-0.380672	0.061925	-1.817859	0.0705293	0.1484861	-5.034985

RP11-206L1	-0.312942	1.6923562	-1.817769	0.0705431	0.1485066	-5.035144
NUTM2A-AS1	-0.072581	5.4659682	-1.817575	0.0705729	0.1485607	-5.035487
RP13-150K1	-0.233668	-1.303112	-1.816969	0.0706661	0.1487484	-5.036559
FOLR2	0.1309186	5.7325144	1.8169124	0.0706747	0.148758	-5.036659
NKAIN2	-0.613419	2.020227	-1.816827	0.0706878	0.148777	-5.036809
RNVU1-19	-0.267077	-1.060726	-1.816777	0.0706955	0.1487846	-5.036898
ASTN1	0.4251686	2.6860138	1.816727	0.0707032	0.1487923	-5.036986
RP11-470M1	0.3683987	-0.669565	1.8166793	0.0707106	0.1487991	-5.037071
RP11-379F4	0.1522785	3.6075636	1.8164895	0.0707398	0.148852	-5.037406
CTD-3222D1	-0.177338	3.5564683	-1.81644	0.0707474	0.1488564	-5.037493
FAXDC2	0.0974093	6.3785141	1.8164067	0.0707525	0.1488564	-5.037552
IRX2	0.4549502	-0.257463	1.8163965	0.0707541	0.1488564	-5.03757
RP11-873E2	-0.204058	3.4128793	-1.816369	0.0707583	0.1488567	-5.037619
AP000439.5	0.2319258	-1.300786	1.8162968	0.0707694	0.1488715	-5.037746
RP11-282A1	-0.360697	-0.793071	-1.815941	0.0708242	0.1489781	-5.038375
LONRF3	0.1222821	5.292517	1.8157651	0.0708513	0.1490265	-5.038686
RP11-734K2	-0.116069	3.9859559	-1.815565	0.0708822	0.1490829	-5.03904
RP5-940J5.	-0.424083	0.4784494	-1.815169	0.0709431	0.1492025	-5.039738
AC005487.2	0.3555503	-0.784704	1.8150737	0.0709579	0.1492249	-5.039907
RPP40	-0.069577	5.4879944	-1.814827	0.0709959	0.1492964	-5.040342
RP11-446H1	0.4049735	-0.080464	1.8147612	0.0710061	0.1493091	-5.040458
GSDMC	0.4909903	2.6169109	1.8146567	0.0710222	0.1493302	-5.040643
LINC01090	-0.33503	-1.075091	-1.814643	0.0710243	0.1493302	-5.040667
AC004129.9	-0.226762	-1.237251	-1.814499	0.0710466	0.1493666	-5.040922
OR2W6P	-0.273313	-1.10024	-1.814478	0.0710498	0.1493666	-5.040958
SFTA1P	-0.424833	2.2565481	-1.814391	0.0710633	0.1493864	-5.041113
RP11-958N2	0.6117036	1.389552	1.8143144	0.0710751	0.1494025	-5.041247
DRP2	-0.507372	1.308789	-1.814242	0.0710862	0.1494174	-5.041375
NLRP11	0.5520826	3.0985454	1.8140698	0.0711129	0.1494648	-5.041679
LMF2	-0.036	6.6311712	-1.813757	0.0711612	0.1495577	-5.04223
ADH4	0.3412721	6.3420269	1.8137037	0.0711695	0.1495665	-5.042325
RP11-134L1	0.1218047	4.5816735	1.8136614	0.071176	0.1495716	-5.042399
HMGN2P3	-0.26463	2.8511526	-1.813342	0.0712254	0.1496668	-5.042963
IGHV3-72	0.5860122	2.1312903	1.8132901	0.0712334	0.1496751	-5.043055
GOLGA1	0.035331	6.0369274	1.8130723	0.0712671	0.1497373	-5.043439
FLJ31356	0.4846068	0.5332513	1.812732	0.0713198	0.1498394	-5.044039
HNF4A	-0.186554	6.805253	-1.812633	0.0713352	0.1498629	-5.044213
RP5-849H19	-0.263005	-1.057096	-1.812539	0.0713497	0.1498715	-5.044379
SEC24B-AS1	-0.146493	3.3442048	-1.812538	0.0713499	0.1498715	-5.044381
TRAV8-2	-0.466705	1.452214	-1.812527	0.0713515	0.1498715	-5.0444
DNM1P51	-0.463945	0.9404215	-1.812464	0.0713613	0.1498801	-5.044511
MRPL10	-0.03583	6.2667392	-1.812448	0.0713639	0.1498801	-5.04454
MKL1	0.0324782	6.1712941	1.8122918	0.0713881	0.1499223	-5.044815
TUBGCP5	0.0414269	5.7642159	1.8120197	0.0714302	0.1500023	-5.045295
RP11-359B1	0.120739	4.2811896	1.8119357	0.0714433	0.1500155	-5.045443
KLK6	-0.509326	-0.254626	-1.811902	0.0714484	0.1500155	-5.045501
LINC01555	-0.154378	-1.452567	-1.8119	0.0714489	0.1500155	-5.045506
RPL12P15	-0.339929	-0.510787	-1.811869	0.0714536	0.1500168	-5.04556
LINC01180	-0.20654	-1.269072	-1.811479	0.0715142	0.1501353	-5.046248
AC096579.1	-0.363654	-0.859394	-1.811187	0.0715595	0.1502218	-5.046762
MRAS	0.0869573	5.841104	1.811154	0.0715646	0.150224	-5.04682

AC102948.2	-0.164089	-1.474289	-1.811101	0.0715729	0.1502327	-5.046914
ZC3H18	0.0283427	6.1802359	1.810974	0.0715926	0.1502621	-5.047138
DCDC1	0.5259486	3.2197103	1.8109309	0.0715993	0.1502621	-5.047214
LINC00086	0.2408647	3.4805466	1.8109291	0.0715996	0.1502621	-5.047217
CXorf57	-0.202324	4.6498222	-1.810905	0.0716034	0.1502621	-5.04726
RP11-685M7	-0.312994	2.0454003	-1.810546	0.0716591	0.1503704	-5.047891
MIR23A	0.2450584	-1.1927	1.8103937	0.0716828	0.1504032	-5.04816
SLC39A2	0.4230888	-0.123594	1.8103926	0.071683	0.1504032	-5.048162
MYH8	-0.435976	-0.508912	-1.810363	0.0716876	0.1504042	-5.048214
MFAP3	0.0898882	5.4719436	1.8098823	0.0717624	0.1505526	-5.04906
RP11-315O6	0.4060729	-0.53612	1.8097719	0.0717796	0.1505799	-5.049255
RP5-968D22	-0.527503	2.7073102	-1.809612	0.0718044	0.1506187	-5.049536
FAM66D	0.3974011	-0.324565	1.8096004	0.0718063	0.1506187	-5.049557
CSRNP2	-0.040145	5.9105436	-1.80952	0.0718188	0.1506363	-5.049698
ATPIF1	0.0485146	6.5973716	1.8094433	0.0718308	0.1506469	-5.049833
DPY30	-0.036162	6.2007926	-1.809435	0.0718321	0.1506469	-5.049849
SNORA5C	-0.358716	1.7875943	-1.809354	0.0718447	0.1506646	-5.04999
ISCA1P3	0.0885439	-1.541787	1.8093171	0.0718504	0.150668	-5.050055
BCL2L1	0.039979	6.6376111	1.8092503	0.0718608	0.1506812	-5.050173
MIR6810	-0.202937	-1.302804	-1.809146	0.0718771	0.1507065	-5.050356
RP11-306I1	-0.314485	-0.877928	-1.809012	0.071898	0.1507418	-5.050593
USP50	0.3927786	0.5904079	1.8089616	0.0719058	0.1507495	-5.050681
RP11-119D9	0.4615349	3.8633528	1.8089066	0.0719144	0.1507589	-5.050778
UGT1A4	0.5062033	5.0875592	1.8088119	0.0719292	0.1507781	-5.050945
RP11-1260E	0.3736238	-0.423104	1.8087948	0.0719319	0.1507781	-5.050975
RP1-12208.	-0.315541	-0.645376	-1.808661	0.0719528	0.1508133	-5.051211
RP11-720L8	0.4989824	-0.059931	1.8084855	0.0719801	0.150862	-5.051519
TRAF4	-0.042478	6.4042141	-1.80836	0.0719997	0.150888	-5.051739
CXCL3	0.3745053	3.4279507	1.8083529	0.0720008	0.150888	-5.051752
CEACAMP3	-0.304032	-0.964294	-1.808134	0.072035	0.1509499	-5.052137
AC007405.6	-0.132826	4.7197196	-1.808111	0.0720386	0.1509499	-5.052178
IGLVI-63	0.1272154	-1.476551	1.8080818	0.0720432	0.1509507	-5.052229
TRIM40	0.5916446	1.6913586	1.8079679	0.072061	0.1509793	-5.052429
C15orf62	-0.126388	4.5809077	-1.807657	0.0721096	0.1510725	-5.052977
UPRT	0.0424554	5.5912631	1.8074767	0.0721377	0.1511229	-5.053293
CCR2	0.1874493	4.5961308	1.8074473	0.0721423	0.1511238	-5.053345
RP3-399L15	-0.27701	2.3736961	-1.807407	0.0721486	0.1511283	-5.053416
LINC00239	0.434975	2.4173906	1.8072474	0.0721736	0.151172	-5.053697
RP11-74E24	-0.284859	-0.948589	-1.807206	0.0721801	0.1511756	-5.05377
TCEA1P4	0.3601796	1.3372709	1.8071834	0.0721836	0.1511756	-5.053809
BRD4	-0.031025	6.5602314	-1.807137	0.0721909	0.1511756	-5.053891
AC073465.3	-0.321697	-0.724972	-1.80713	0.0721919	0.1511756	-5.053902
RP11-162J8	0.3049694	-0.993317	1.8068422	0.072237	0.1512614	-5.054409
RP11-259K5	0.1848384	-1.365379	1.8066819	0.0722621	0.1513053	-5.054691
C15orf40	-0.038837	5.8612175	-1.806621	0.0722717	0.1513131	-5.054798
CDK6	0.1191933	6.044009	1.8066016	0.0722747	0.1513131	-5.054832
BYSL	-0.045057	6.0042073	-1.806579	0.0722783	0.1513131	-5.054872
ADAMTS9	0.0839206	5.7473575	1.8061416	0.0723467	0.1514477	-5.05564
LINC01470	0.1856492	-1.426474	1.8060863	0.0723554	0.1514572	-5.055738
TPO	0.322303	-0.72504	1.8060344	0.0723635	0.1514655	-5.055829
CKS1BP3	-0.278379	-0.96773	-1.805961	0.0723751	0.1514809	-5.055958

NBEA	0.1990736	4.5526763	1.8058285	0.0723958	0.1515157	-5.056191
RP11-834C1	-0.378051	-0.606186	-1.80576	0.0724066	0.1515295	-5.056311
AP000431.2	-0.197106	-1.414288	-1.805725	0.072412	0.1515323	-5.056372
RAB3C	0.4441527	3.704745	1.8056952	0.0724167	0.1515328	-5.056425
RP11-626G3	0.3479754	-0.709661	1.8056705	0.0724206	0.1515328	-5.056468
ITGAD	0.2813682	4.024296	1.8055559	0.0724386	0.1515603	-5.056669
F7	-0.194552	6.5535668	-1.805534	0.072442	0.1515603	-5.056708
C11orf73	-0.045366	5.9226376	-1.805192	0.0724958	0.151664	-5.057309
RASEF	0.3563683	5.0735998	1.8049305	0.0725368	0.1517411	-5.057768
LFNG	0.0873977	5.4726616	1.80484	0.072551	0.1517622	-5.057927
PTK2	-0.041381	6.5407883	-1.804564	0.0725943	0.1518441	-5.058411
RP11-373D2	0.4130768	1.8050396	1.8044563	0.0726113	0.1518682	-5.058601
EIF5A	0.0400725	6.9734075	1.804438	0.0726142	0.1518682	-5.058633
RPLP0P1	-0.235564	-1.185871	-1.804277	0.0726394	0.1519123	-5.058914
ZDHHC5	0.0268571	6.5774066	1.80418	0.0726547	0.1519356	-5.059086
AC060834.2	-0.190511	-1.351807	-1.804144	0.0726604	0.1519388	-5.059149
VPS26A	-0.026598	6.3092552	-1.8039	0.0726988	0.1520104	-5.059577
RP11-405K6	-0.258718	-1.283191	-1.803859	0.0727052	0.1520151	-5.059649
RGP1	-0.038229	6.1138803	-1.803394	0.0727784	0.1521593	-5.060464
VAMP1	0.0778949	5.3592885	1.8031608	0.0728152	0.1522247	-5.060874
IGKV1-37	-0.175753	-1.42018	-1.803143	0.072818	0.1522247	-5.060905
LRP12	0.1390008	5.1357173	1.8031147	0.0728225	0.1522253	-5.060955
HIST1H2APS	-0.247831	-1.19834	-1.802833	0.0728669	0.1523095	-5.06145
HMGN1P37	-0.315595	2.5032439	-1.802538	0.0729134	0.152397	-5.061966
MIR3193	0.0909169	-1.540577	1.8025143	0.0729171	0.152397	-5.062008
SELK	0.0435133	6.2949822	1.8024872	0.0729214	0.1523973	-5.062056
AC007036.6	0.450643	-0.051189	1.8024095	0.0729337	0.1524142	-5.062192
CTB-75G16.	-0.436034	1.2921236	-1.802346	0.0729437	0.1524263	-5.062303
RP3-341D10	0.2730965	-1.021341	1.8022851	0.0729533	0.1524377	-5.06241
RPLP1P6	-0.146962	3.9884581	-1.802057	0.0729894	0.1525044	-5.062811
GLIS1	0.4816303	0.3625376	1.8020013	0.0729981	0.1525139	-5.062908
RP11-40E6.	-0.304301	-0.929748	-1.801881	0.073017	0.1525447	-5.063118
TPP1	0.0369173	6.9139193	1.8018265	0.0730257	0.1525541	-5.063214
CAPRIN2	-0.048981	5.7850585	-1.801795	0.0730307	0.1525557	-5.063269
RP11-293P2	-0.34257	-0.844313	-1.801739	0.0730395	0.1525654	-5.063367
RNU6-695P	-0.177863	-1.414565	-1.801661	0.0730518	0.1525825	-5.063504
IL21R-AS1	-0.370973	-0.424744	-1.801317	0.0731062	0.1526868	-5.064107
MGC39584	-0.713972	0.7235826	-1.801292	0.0731101	0.1526868	-5.064151
C9orf72	0.0724698	5.4652233	1.8011163	0.0731379	0.1527361	-5.064459
MSANTD2	-0.05757	5.2422068	-1.801026	0.0731522	0.1527572	-5.064618
RNASET2	-0.05671	6.2539726	-1.800856	0.0731792	0.1528048	-5.064916
BOP1	-0.062761	6.4757015	-1.800786	0.0731903	0.1528156	-5.065039
NPIPA5	0.3956714	3.4079676	1.80077	0.0731927	0.1528156	-5.065066
RP1-29C18.	-0.45313	0.8545879	-1.800654	0.0732111	0.1528451	-5.065269
SOCS5P2	0.2041761	-1.328001	1.800471	0.0732401	0.1528969	-5.06559
OFD1P1Y	0.1657777	-1.421062	1.8004208	0.073248	0.1528976	-5.065678
CD2AP	-0.038632	6.3565517	-1.800416	0.0732488	0.1528976	-5.065687
RP4-665J23	0.1086913	5.4033786	1.7999888	0.0733164	0.1530301	-5.066435
RMDN2	0.0778809	5.5919519	1.7999369	0.0733247	0.1530385	-5.066526
C6orf141	0.5108973	3.2332999	1.7997886	0.0733482	0.1530788	-5.066785
RP11-687E1	-0.380732	-0.178923	-1.79955	0.073386	0.1531405	-5.067203

RP11-53I6.	-0.388112	-0.122937	-1.799549	0.0733861	0.1531405	-5.067205
RP3-394A18	0.1405368	4.9036955	1.7995211	0.0733906	0.1531411	-5.067254
FASTKD3	-0.040264	5.6562378	-1.799439	0.0734037	0.1531596	-5.067398
IL6R	-0.081893	6.5613302	-1.79927	0.0734305	0.1532068	-5.067694
C10orf71-A	0.3296051	-1.070373	1.7991642	0.0734472	0.1532329	-5.067879
NXPE3	0.1066149	5.1769764	1.7990654	0.0734629	0.1532569	-5.068052
IGHV2-26	0.6284707	1.8966834	1.7989312	0.0734842	0.1532926	-5.068286
EEF1GP8	-0.233479	-1.190147	-1.798885	0.0734915	0.153299	-5.068367
RP1-313L4.	-0.379757	1.1968016	-1.798762	0.073511	0.153331	-5.068582
CTB-181F24	-0.149811	-1.459385	-1.798716	0.0735183	0.1533374	-5.068662
SSX7	-0.182883	-1.422397	-1.798682	0.0735237	0.15334	-5.068722
RP11-111I1	-0.325747	-0.920061	-1.798563	0.0735427	0.1533709	-5.068932
NPM1P42	-0.192767	-1.336962	-1.798421	0.0735653	0.1534091	-5.06918
RP11-415F2	-0.204854	2.8485982	-1.798322	0.073581	0.1534331	-5.069353
HSD3BP4	-0.398544	-0.498019	-1.798295	0.0735853	0.1534334	-5.069401
RP11-712L6	-0.324581	-0.646667	-1.79819	0.0736019	0.1534572	-5.069584
PLCH1	-0.383929	3.2730071	-1.79817	0.0736051	0.1534572	-5.069619
SLC9A2	-0.513637	1.3054464	-1.797977	0.0736359	0.1535125	-5.069957
THBS3	0.0616567	5.8576113	1.7975266	0.0737075	0.153653	-5.070744
LINC01559	0.6181518	0.5825999	1.7974964	0.0737123	0.1536542	-5.070797
TXNP6	-0.415989	0.4866605	-1.797386	0.0737298	0.1536821	-5.07099
COL20A1	-0.413002	-0.397092	-1.797319	0.0737405	0.1536955	-5.071107
COX4I1P2	-0.158382	-1.411372	-1.79722	0.0737562	0.1537195	-5.07128
AC006960.7	0.1761354	-1.443307	1.7971191	0.0737724	0.1537444	-5.071457
PRICKLE3	0.047396	5.3554513	1.7970395	0.073785	0.153762	-5.071596
LRP2	-0.61794	2.8535818	-1.796743	0.0738323	0.1538516	-5.072114
RP11-182J2	-0.427075	0.0745153	-1.79666	0.0738455	0.1538704	-5.072259
RP11-114M1	0.1058694	-1.532957	1.796486	0.0738733	0.1539195	-5.072564
WHSC1L1	0.0461608	6.1520307	1.7963679	0.0738921	0.1539456	-5.07277
TCAP	-0.137063	4.183503	-1.796311	0.0739012	0.1539456	-5.072871
CALM2P2	-0.35631	2.2983827	-1.796305	0.0739022	0.1539456	-5.072881
NMUR2	0.1875683	-1.401202	1.7963017	0.0739026	0.1539456	-5.072886
ARL3	-0.047067	5.8620267	-1.796209	0.0739174	0.153962	-5.073048
GGNBP2	-0.025838	6.4105895	-1.796199	0.073919	0.153962	-5.073065
ACTR2	0.0255842	6.7634757	1.7960535	0.0739423	0.1539947	-5.07332
RP11-278H7	-0.310825	-0.805751	-1.796048	0.0739431	0.1539947	-5.073329
HMGXB4	-0.039946	5.8722267	-1.795954	0.0739581	0.1540171	-5.073493
APC2	0.1179628	4.9769847	1.795927	0.0739624	0.1540174	-5.073541
SHROOM2	0.1458717	5.626226	1.7957773	0.0739863	0.1540584	-5.073803
ATP9B	0.0460939	5.9009208	1.7956895	0.0740004	0.1540788	-5.073956
RP11-542A1	0.1428074	-1.491369	1.7956542	0.074006	0.1540817	-5.074018
LINC01233	-0.172434	-1.413382	-1.795563	0.0740206	0.1540976	-5.074178
MYO6	0.0503028	6.3124371	1.7955293	0.0740259	0.1540976	-5.074236
RP11-760H2	-0.099889	5.0771371	-1.795527	0.0740263	0.1540976	-5.07424
AGMO	-0.167326	6.1639972	-1.795285	0.074065	0.1541693	-5.074663
RNU6-312P	-0.398276	0.2314352	-1.795246	0.0740712	0.1541734	-5.07473
MAD2L1BP	-0.039099	6.0012285	-1.795058	0.0741013	0.1542221	-5.075059
RP11-151N1	0.3759707	0.7480684	1.7950225	0.0741069	0.1542221	-5.075121
AP000770.1	-0.447732	-0.418954	-1.795021	0.0741072	0.1542221	-5.075125
AC025016.1	-0.526966	-0.397208	-1.794981	0.0741135	0.1542256	-5.075193
PACRGL	0.0491854	5.2655884	1.7949575	0.0741173	0.1542256	-5.075235

C1QTNF3-AM	-0.401687	0.1333506	-1.794829	0.0741378	0.1542459	-5.075459
RP11-114H7	-0.318792	-0.793159	-1.794826	0.0741383	0.1542459	-5.075464
RP11-274B2	0.3663963	3.2545645	1.7947843	0.074145	0.1542459	-5.075537
ADNP2	-0.04266	5.8988793	-1.794778	0.074146	0.1542459	-5.075548
RP13-516M1	-0.214867	3.3834401	-1.794764	0.0741482	0.1542459	-5.075573
NAV2-IT1	-0.216	-1.251687	-1.794617	0.0741718	0.1542861	-5.07583
CTC-490G23	0.280366	-1.07654	1.7945574	0.0741813	0.1542971	-5.075934
RP4-613B23	0.3168271	-0.758361	1.7942665	0.0742279	0.1543852	-5.076442
AC246787.5	-0.192074	-1.372946	-1.79423	0.0742337	0.1543886	-5.076506
FOXA1	-0.15364	6.1496303	-1.794125	0.0742504	0.1544145	-5.076688
CDADC1	0.0556772	5.5238881	1.7940952	0.0742553	0.1544158	-5.076741
CD200R1L	-0.27623	-1.096683	-1.793987	0.0742726	0.154443	-5.076929
IGKV1-27	0.5929772	2.9165259	1.7939314	0.0742815	0.1544528	-5.077027
TBC1D32	0.1737761	4.100995	1.793835	0.074297	0.1544761	-5.077195
KDF1	-0.249492	5.0210162	-1.793609	0.0743332	0.1545426	-5.077589
EHHADH	0.1188928	6.591239	1.7933043	0.074382	0.1546353	-5.078121
USP32	0.043407	6.0724122	1.793248	0.0743911	0.1546453	-5.078219
PRY	0.1136664	-1.50622	1.7931248	0.0744108	0.1546776	-5.078435
HSPB1	-0.051427	7.0946303	-1.792921	0.0744436	0.1547368	-5.07879
CDK5RAP2	-0.036349	6.410577	-1.792762	0.074469	0.154781	-5.079068
CPXCR1	0.2057773	-1.320402	1.7927202	0.0744758	0.1547861	-5.07914
RP11-48B3.	0.4828743	0.0070861	1.7926029	0.0744946	0.1548164	-5.079345
RP11-321C2	-0.157302	-1.477617	-1.792573	0.0744994	0.1548175	-5.079397
DIS3L	0.043895	6.0377	1.7925209	0.0745078	0.1548262	-5.079488
PLEKHA8P1	-0.070279	4.9610224	-1.792314	0.074541	0.1548865	-5.07985
CTD-2325P2	-0.332347	-0.662276	-1.792202	0.0745589	0.1549148	-5.080044
RNASE10	-0.404044	-0.133153	-1.792135	0.0745697	0.1549285	-5.080161
AP000354.4	0.3699055	-0.494899	1.7916658	0.0746452	0.1550764	-5.080979
FOXP1	-0.040201	6.3807923	-1.791484	0.0746745	0.1551284	-5.081297
RPL37AP8	-0.308691	-0.764787	-1.791226	0.074716	0.1552058	-5.081747
TBC1D3K	0.1245424	-1.500677	1.7911485	0.0747284	0.1552125	-5.081881
STRA6	0.517074	3.1060152	1.7911133	0.0747341	0.1552125	-5.081943
PSMD8P1	-0.396678	-0.135756	-1.791087	0.0747382	0.1552125	-5.081988
CNNM3	-0.043789	6.3055861	-1.79108	0.0747394	0.1552125	-5.082
RP1-65J11.	0.4899321	1.0227845	1.7910737	0.0747405	0.1552125	-5.082012
AC139530.1	-0.330255	-0.695414	-1.790987	0.0747544	0.1552327	-5.082163
MORF4	-0.339327	-0.559165	-1.790932	0.0747633	0.1552423	-5.082259
PHGR1	-0.434388	-0.478508	-1.790581	0.0748199	0.1553509	-5.082871
RPS3AP44	-0.404768	0.4103588	-1.790543	0.0748259	0.1553546	-5.082936
RP11-334L9	-0.250007	2.9546801	-1.790393	0.0748502	0.1553961	-5.083199
ALK	0.4294627	0.5347521	1.7902634	0.074871	0.1554306	-5.083424
MTDH	0.0421444	6.7631738	1.7901766	0.074885	0.1554444	-5.083575
AC090505.1	0.1836557	-1.371183	1.7901487	0.0748895	0.1554444	-5.083624
RP11-554A1	-0.280462	-1.038106	-1.790143	0.0748905	0.1554444	-5.083634
METTL13	-0.036911	6.2598618	-1.790091	0.0748988	0.1554529	-5.083724
BAG6	-0.030118	6.9375184	-1.790057	0.0749044	0.1554556	-5.083784
MANBA	0.0403951	6.034036	1.7899692	0.0749185	0.155476	-5.083936
ZNF205-AS1	0.1407973	3.5145605	1.7899176	0.0749268	0.1554845	-5.084026
KCTD18	0.0424031	5.7532408	1.7897718	0.0749503	0.1555244	-5.08428
TRPC6	0.1407398	4.17089	1.789547	0.0749866	0.1555909	-5.084672
HSD17B6	0.202258	6.6798983	1.789497	0.0749946	0.1555988	-5.084759

RP11-697N1	-0.433812	0.9880226	-1.789268	0.0750317	0.1556668	-5.085158
OCIAD1	0.0335104	6.684814	1.7892032	0.0750421	0.1556727	-5.08527
U51561.1	0.2089946	-1.249382	1.7891971	0.0750431	0.1556727	-5.085281
RBM26-AS1	0.1564032	4.2334312	1.7887034	0.0751229	0.1558294	-5.086141
LRRC58	-0.042932	6.205193	-1.788368	0.075177	0.155924	-5.086724
RGL3	-0.098419	5.8441603	-1.788342	0.0751813	0.155924	-5.086769
PCED1B-AS1	0.1303844	4.9964035	1.7883419	0.0751813	0.155924	-5.08677
CXorf21	0.170883	4.1195619	1.7881023	0.0752201	0.1559956	-5.087187
CTD-2589M5	0.4814588	-0.396123	1.7880285	0.0752321	0.1560115	-5.087315
RP11-364B6	-0.541272	0.4602731	-1.787904	0.0752521	0.1560443	-5.087531
AC004893.1	-0.07165	4.8405135	-1.787594	0.0753024	0.1561396	-5.088071
AADACL3	-0.208704	-1.36479	-1.787487	0.0753197	0.156161	-5.088257
RP11-315O6	-0.216501	3.9616754	-1.787478	0.0753213	0.156161	-5.088274
AC087650.1	-0.374682	-0.229867	-1.787359	0.0753405	0.15619	-5.08848
CHRNA1	-0.528363	1.1494093	-1.787339	0.0753438	0.15619	-5.088516
LINC01436	0.5999518	2.4711809	1.7872902	0.0753516	0.1561974	-5.0886
STK24P1	-0.36532	1.4296188	-1.78716	0.0753728	0.1562323	-5.088827
TINAG	0.7515995	1.9208525	1.7870775	0.0753861	0.1562511	-5.08897
IL1R2	-0.099394	5.5313185	-1.786933	0.0754095	0.1562809	-5.089221
GPR3	0.1821241	3.7055505	1.7869119	0.075413	0.1562809	-5.089258
CLNK	0.4024813	2.2193849	1.7869098	0.0754133	0.1562809	-5.089262
CLDN14	-0.190686	5.691045	-1.786853	0.0754226	0.1562911	-5.089361
RP11-369C8	0.1818317	-1.466723	1.7868266	0.0754268	0.1562911	-5.089406
RP11-656G2	0.4513314	-0.064186	1.7867705	0.0754359	0.1563011	-5.089504
ZEB2-AS1	0.3650265	1.6659103	1.7867045	0.0754466	0.1563119	-5.089618
RP11-533K9	-0.33047	-0.762777	-1.786686	0.0754497	0.1563119	-5.089651
ISY1-RAB43	0.2366094	-1.155032	1.7866181	0.0754606	0.1563257	-5.089769
DAZL	0.3095774	-1.006353	1.7861513	0.0755364	0.1564738	-5.09058
SMAD2	0.0274838	6.3613807	1.786111	0.075543	0.1564785	-5.09065
PHEX-AS1	0.1824474	-1.408331	1.7859689	0.075566	0.1565165	-5.090897
FSD1	0.3374994	2.5604427	1.7859452	0.0755699	0.1565165	-5.090939
SLC19A3	0.1552055	5.631856	1.78576	0.0756	0.15657	-5.091261
PTGDS	0.2344045	5.9509181	1.7857015	0.0756095	0.1565808	-5.091362
IGHV3-53	0.5756246	2.5248695	1.7856234	0.0756222	0.1565982	-5.091498
RP11-862G1	-0.210557	-1.30054	-1.785534	0.0756367	0.1566193	-5.091653
MGAT3-AS1	0.2000368	-1.36163	1.7854468	0.0756509	0.1566399	-5.091805
RP11-34P13	-0.433218	0.5530415	-1.785392	0.0756598	0.1566493	-5.0919
CECR2	-0.260195	5.2766249	-1.785345	0.0756675	0.1566564	-5.091982
RP13-977J1	0.2918679	-1.14717	1.7853164	0.0756721	0.1566571	-5.092032
RP5-867C24	-0.320352	-0.644161	-1.785237	0.075685	0.1566748	-5.092169
SLX1B	0.3494235	-0.73256	1.7851971	0.0756915	0.1566795	-5.092239
UNC93B5	-0.224192	-1.209507	-1.785108	0.0757059	0.1567005	-5.092393
FAM26F	0.1449462	5.1000606	1.7850591	0.075714	0.1567082	-5.092478
RP11-384E2	0.1039218	-1.53395	1.7849938	0.0757246	0.1567213	-5.092592
ACSL5	0.1078629	6.4544169	1.7849084	0.0757385	0.1567412	-5.09274
RP11-1000B	-0.223411	-1.18282	-1.784808	0.0757548	0.1567661	-5.092914
RP3-495K2.	0.1552629	-1.471507	1.7847799	0.0757594	0.1567667	-5.092964
AC003991.3	0.3439433	-0.741584	1.7846035	0.0757881	0.1568153	-5.09327
RP11-362F1	0.3985323	-0.558681	1.7845829	0.0757915	0.1568153	-5.093306
IMPAD1	0.0426263	6.6353563	1.7844729	0.0758094	0.1568435	-5.093497
AC016745.3	0.407563	-0.281182	1.7844158	0.0758187	0.1568481	-5.093596

RP11-482M8	0.1208219	-1.525337	1.7844064	0.0758202	0.1568481	-5.093612
TET2	0.0615578	5.551362	1.7842012	0.0758537	0.1569084	-5.093969
RP11-774D1	-0.711795	0.8319029	-1.784036	0.0758806	0.1569535	-5.094256
BIN3	0.0488775	5.5006489	1.7840146	0.0758841	0.1569535	-5.094293
LSM3	-0.037917	6.2770667	-1.783829	0.0759144	0.1570073	-5.094615
AFAP1-AS1	0.6109059	2.84688	1.7837572	0.0759261	0.1570225	-5.09474
RNU6-882P	-0.390334	0.2109631	-1.783714	0.0759331	0.1570282	-5.094815
RP11-382D8	-0.280671	-0.963444	-1.783572	0.0759563	0.15706	-5.095061
MORN1	0.0745391	5.0298554	1.783567	0.0759571	0.15706	-5.09507
AC008746.1	-0.269131	3.0206334	-1.783432	0.0759791	0.1570966	-5.095304
RPL12P38	-0.392941	0.836495	-1.783331	0.0759956	0.1571218	-5.095479
MIR143	0.1669766	-1.42521	1.7830255	0.0760455	0.1572161	-5.09601
RP11-864N7	0.4396589	0.2866745	1.7828912	0.0760674	0.1572525	-5.096243
TECTA	0.1630929	4.029999	1.7828633	0.076072	0.157253	-5.096291
MED22	-0.043113	6.0074426	-1.782729	0.0760938	0.1572819	-5.096524
WDFY2	0.0490281	5.8238223	1.7827123	0.0760966	0.1572819	-5.096553
SNORA9	-0.346507	-0.432015	-1.782699	0.0760988	0.1572819	-5.096577
CTC-260E6.	-0.423439	-0.375464	-1.782506	0.0761303	0.1573381	-5.096911
TMC8	0.0927446	5.5818757	1.7824792	0.0761347	0.1573382	-5.096958
RP13-15M17	-0.397123	1.9950594	-1.782311	0.0761623	0.1573862	-5.09725
RP11-397E7	0.2881789	2.4923189	1.781992	0.0762144	0.157485	-5.097803
RPS2P48	-0.391838	0.5648071	-1.781714	0.07626	0.1575703	-5.098286
RP11-164P1	0.3976269	3.251753	1.7815322	0.0762897	0.1576227	-5.0986
RP4-622L5.	-0.420233	1.0409464	-1.781304	0.076327	0.1576909	-5.098996
KIZ-AS1	-0.392265	0.6592691	-1.781227	0.0763397	0.1577083	-5.09913
RP11-51L5.	-0.244127	-1.178251	-1.781111	0.0763586	0.1577384	-5.09933
CTD-2647E9	0.1810527	-1.394562	1.7807215	0.0764225	0.1578614	-5.100006
RNU7-103P	-0.142003	-1.463215	-1.780637	0.0764364	0.1578812	-5.100153
RGPD3	0.3978416	1.838859	1.7804028	0.0764748	0.1579515	-5.100558
AP000439.3	0.4179335	-0.67618	1.7802823	0.0764945	0.1579834	-5.100767
EVC2	-0.224558	4.6801	-1.780229	0.0765033	0.1579926	-5.100859
HAP1	-0.418667	2.5342944	-1.77975	0.0765819	0.1581459	-5.101689
EIF4A1P10	-0.21475	3.254507	-1.779685	0.0765926	0.158159	-5.101802
RP11-248J2	-0.230025	-1.205746	-1.779613	0.0766044	0.1581744	-5.101926
AP000295.9	0.4247839	0.1537462	1.7795849	0.076609	0.1581752	-5.101975
RP11-432J2	-0.278887	2.8397999	-1.779538	0.0766168	0.1581822	-5.102057
CELA2B	0.3862753	-0.177184	1.7794062	0.0766384	0.1582179	-5.102285
CTC-499B15	-0.343157	-0.527216	-1.779277	0.0766596	0.1582527	-5.102508
FUNDC1	-0.046023	5.6276069	-1.77911	0.0766872	0.1583007	-5.102798
SLC17A9	-0.087544	6.5024982	-1.778844	0.0767309	0.158382	-5.103259
RP11-574K1	-0.349235	-0.445794	-1.778731	0.0767495	0.1584114	-5.103454
GLCE	-0.059485	6.1924292	-1.778445	0.0767965	0.1584994	-5.103949
RP5-1085F1	-0.347314	-0.477222	-1.778358	0.0768108	0.1585142	-5.104099
ZNF79	-0.051327	5.1755974	-1.778332	0.0768152	0.1585142	-5.104145
RNF166	0.0379788	5.8190286	1.7783064	0.0768193	0.1585142	-5.104189
AC004985.1	-0.264319	2.5619131	-1.77829	0.076822	0.1585142	-5.104217
CDKN2B	0.1014323	5.4620164	1.7782698	0.0768254	0.1585142	-5.104252
MOG	-0.193689	-1.354995	-1.778115	0.0768508	0.1585578	-5.10452
FAM127A	0.0567709	6.370634	1.7780154	0.0768673	0.1585827	-5.104693
NUDCD3	0.0295572	6.5037232	1.7779489	0.0768782	0.1585964	-5.104808
RP11-617B3	0.2223455	-1.293953	1.7778864	0.0768885	0.1586087	-5.104916

PCMTD1	0.0430948	6.2359489	1.7778403	0.0768961	0.1586154	-5.104996
RP11-567J2	-0.209156	-1.301227	-1.77775	0.076911	0.158637	-5.105152
KLHDC1	0.1195395	4.3985186	1.7773494	0.0769771	0.1587644	-5.105845
MYOC	0.3676165	-0.886929	1.7771456	0.0770107	0.1588248	-5.106198
RP11-496I2	-0.474483	0.4345811	-1.776931	0.0770461	0.1588888	-5.106569
HEPACAM2	-0.497015	-0.1177	-1.776774	0.0770721	0.1589289	-5.106841
ARMC1	-0.038618	6.2479915	-1.776744	0.077077	0.1589289	-5.106892
THRB	-0.079297	6.098743	-1.776734	0.0770786	0.1589289	-5.106909
ONECUT2	-0.141801	6.3225974	-1.77669	0.0770859	0.1589351	-5.106986
FBXO4	-0.049784	5.6260978	-1.776414	0.0771316	0.1590201	-5.107464
RPL34P18	-0.388713	0.9535093	-1.776231	0.0771617	0.1590733	-5.107779
RP11-461F1	0.182976	-1.329556	1.7761799	0.0771702	0.1590818	-5.107868
TNF	0.3356788	3.069681	1.7760364	0.0771939	0.1591217	-5.108116
VAT1L	-0.409999	3.86613	-1.77582	0.0772297	0.1591865	-5.108491
AP003774.1	-0.43431	0.603087	-1.775545	0.0772752	0.1592712	-5.108965
FKBP1B	-0.22512	4.8738706	-1.7755	0.0772826	0.1592775	-5.109043
RP11-81A1.	-0.086245	4.691164	-1.775444	0.0772918	0.1592876	-5.109139
POLR3GP1	0.2561684	-1.184745	1.7752659	0.0773214	0.1593221	-5.109448
OR10AC1	0.1709397	-1.425657	1.7752438	0.077325	0.1593221	-5.109486
AB019441.2	-0.12345	4.610163	-1.77524	0.0773256	0.1593221	-5.109492
RP11-17E2.	0.4529929	0.6484525	1.7752377	0.077326	0.1593221	-5.109497
IGHV4OR15-	-0.263478	-1.174389	-1.775042	0.0773585	0.15938	-5.109835
RP4-712E4.	0.2364233	-1.278465	1.7749591	0.0773722	0.1593992	-5.109978
ATP6V0A2	-0.03328	5.8583102	-1.774932	0.0773767	0.1593994	-5.110025
CTD-2583A1	-0.41391	1.1283854	-1.774881	0.0773851	0.1594008	-5.110112
SNX20	0.1305796	5.0017275	1.7748752	0.0773861	0.1594008	-5.110123
RNF145	0.0536323	6.1046632	1.774755	0.077406	0.1594328	-5.110331
TMEM198B	0.0558296	5.6461867	1.7744475	0.0774569	0.1595288	-5.110862
RP11-96801	0.3190584	3.1264179	1.7744074	0.0774636	0.1595332	-5.110931
OR7E36P	0.1458341	-1.462304	1.774382	0.0774678	0.1595332	-5.110975
IL12A-AS1	-0.408999	-0.214626	-1.774229	0.0774931	0.1595673	-5.111239
RP11-680F8	-0.162586	5.4141414	-1.774229	0.0774931	0.1595673	-5.111239
ADCK2	-0.046117	6.1640604	-1.774162	0.0775042	0.1595743	-5.111354
C10orf71	0.3438884	-1.020083	1.774156	0.0775053	0.1595743	-5.111365
RFX7	-0.046365	5.7069915	-1.773996	0.0775319	0.1596201	-5.111642
MAATS1	0.3136925	3.9997944	1.7736946	0.0775819	0.159714	-5.112162
RP11-256I2	-0.354119	-0.54897	-1.77348	0.0776174	0.1597782	-5.112532
RTL1	-0.528772	0.0406286	-1.773277	0.0776512	0.1598388	-5.112883
BOLA2P3	-0.342628	-0.754041	-1.773106	0.0776795	0.159888	-5.113178
RGSL1	0.5967637	0.9726023	1.773065	0.0776864	0.1598932	-5.113249
TRMT44	-0.036058	5.5991546	-1.773026	0.0776929	0.1598975	-5.113316
TSR1	0.0380775	6.0655648	1.7729024	0.0777135	0.1599308	-5.11353
RP11-295P9	-0.372682	1.21547	-1.772744	0.0777397	0.1599758	-5.113802
CABYRP1	0.1958396	-1.379051	1.7726711	0.0777519	0.1599894	-5.113929
IGHV3-33	0.565251	3.0990076	1.7726519	0.0777551	0.1599894	-5.113962
TMEM253	-0.33076	2.096987	-1.772423	0.0777931	0.1600581	-5.114356
GLDCP1	-0.365059	3.0364234	-1.772399	0.0777972	0.1600581	-5.114399
ANKS1B	-0.413107	3.3925624	-1.77231	0.077812	0.1600794	-5.114552
AC006445.7	-0.276505	-0.982554	-1.772132	0.0778417	0.160112	-5.11486
TMEFF2	-0.426515	-0.406816	-1.77212	0.0778437	0.160112	-5.114881
CTD-2383M3	0.3925479	0.6479383	1.7721032	0.0778464	0.160112	-5.114909

CTD-307407	0.1466093	-1.461909	1.7720955	0.0778477	0.160112	-5.114922
RP11-527D7	-0.180763	-1.395818	-1.772083	0.0778498	0.160112	-5.114944
THAP6	0.0400982	5.6286741	1.7719952	0.0778644	0.160133	-5.115095
NPM1P24	-0.384195	0.8458285	-1.771942	0.0778733	0.160136	-5.115187
RPL39P40	-0.415589	2.9377146	-1.771934	0.0778746	0.160136	-5.115201
SEC11A	-0.032864	6.5897116	-1.771818	0.0778939	0.1601666	-5.1154
CFAP36	0.0512995	5.8027883	1.771721	0.0779101	0.1601909	-5.115568
XIRP1	0.441458	1.4347166	1.7716011	0.07793	0.1602229	-5.115775
COX7B2	-0.818069	1.3948057	-1.771493	0.0779481	0.160251	-5.115962
IFT52	-0.042058	5.8246001	-1.77142	0.0779602	0.1602669	-5.116087
MICAL3	0.0728133	5.9162552	1.7710168	0.0780274	0.1603961	-5.116782
KLRB1	0.1414193	4.988941	1.7709264	0.0780425	0.160418	-5.116938
SNCG	0.1634525	5.4794446	1.770862	0.0780533	0.1604258	-5.117049
RP5-1049G1	-0.22506	-1.177117	-1.770851	0.0780551	0.1604258	-5.117068
SNORA59A	0.383886	0.8672039	1.770809	0.0780621	0.1604312	-5.117141
MOV10L1	-0.295729	3.7275692	-1.770668	0.0780856	0.1604705	-5.117384
RP11-403B2	0.1394514	-1.456801	1.7704624	0.07812	0.160532	-5.117738
DDHD2	0.0545646	5.8523853	1.7703677	0.0781358	0.1605481	-5.117901
ELL3	-0.166087	3.402757	-1.770363	0.0781366	0.1605481	-5.11791
ZNF213	0.0645869	5.5580508	1.7699408	0.0782071	0.1606839	-5.118637
RP11-879F1	0.3372211	2.1123416	1.7698541	0.0782216	0.1607046	-5.118786
LINC01587	-0.590323	1.3354305	-1.769612	0.0782621	0.1607684	-5.119204
GPR142	0.3082502	-0.920128	1.7695853	0.0782665	0.1607684	-5.119249
WASF2	0.0339979	6.4769911	1.7695712	0.0782689	0.1607684	-5.119274
SNHG8	-0.059155	6.1643787	-1.769563	0.0782702	0.1607684	-5.119288
EEF1DP4	-0.467305	0.3621269	-1.769332	0.0783089	0.1608387	-5.119686
AL031768.1	0.1546335	-1.403536	1.7692559	0.0783216	0.1608477	-5.119817
SPANXD	-0.229624	-1.363857	-1.769253	0.0783221	0.1608477	-5.119822
RP11-84G21	-0.171598	3.6403855	-1.769169	0.0783361	0.1608618	-5.119966
UQCC1	0.0370289	6.1941717	1.7691586	0.0783379	0.1608618	-5.119985
CTNNA2	-0.676271	1.7330583	-1.769133	0.0783421	0.1608618	-5.120028
RP11-64C12	0.080544	-1.545864	1.7690417	0.0783574	0.1608842	-5.120186
AC068587.2	0.1982438	-1.300388	1.7690122	0.0783624	0.1608853	-5.120237
FBXO34	0.0394871	5.8709965	1.7689114	0.0783792	0.1609109	-5.12041
MIR1254-1	-0.40462	0.943075	-1.768855	0.0783887	0.1609212	-5.120508
RP11-823P9	-0.302449	-0.918022	-1.768618	0.0784284	0.1609937	-5.120916
MRPL45P1	-0.465422	-0.156208	-1.768488	0.0784501	0.1610292	-5.121139
RP11-85I21	-0.523276	0.5801019	-1.768452	0.0784562	0.1610327	-5.121202
RP11-52L5.	-0.382946	-0.68711	-1.767954	0.0785397	0.16118	-5.122059
CXCL5	0.6274427	3.1483531	1.7679371	0.0785425	0.16118	-5.122088
EIF2S1	0.0341214	6.357109	1.7679149	0.0785462	0.16118	-5.122126
VPS13A	-0.057075	6.0428651	-1.767889	0.0785506	0.16118	-5.122171
LRP11	-0.05379	6.1401981	-1.767871	0.0785535	0.16118	-5.122201
MUM1L1	0.5998424	2.7127432	1.7678615	0.0785552	0.16118	-5.122218
RP11-386I8	-0.272707	-0.998211	-1.767839	0.0785589	0.16118	-5.122256
SPATA20P1	0.2114559	-1.330671	1.7674233	0.0786287	0.1613141	-5.122972
RP13-455A7	-0.196185	-1.33144	-1.767015	0.0786973	0.1614458	-5.123675
RP1-151F17	-0.111519	5.0504745	-1.766937	0.0787104	0.161455	-5.123809
CAMSAP1	0.0380708	5.9602809	1.7669354	0.0787106	0.161455	-5.123811
IGJ	0.2498081	5.4730469	1.7669064	0.0787155	0.1614559	-5.123861
NRDE2	-0.049869	5.9016426	-1.766777	0.0787373	0.1614914	-5.124084

PROSER1	-0.041522	5.8806573	-1.766559	0.0787738	0.1615506	-5.124458
GNB5	0.0621246	6.2427284	1.7665528	0.0787749	0.1615506	-5.12447
NAALAD2	-0.191944	4.0544337	-1.766474	0.0787882	0.1615688	-5.124606
C7orf33	-0.274554	-1.162905	-1.766402	0.0788002	0.1615777	-5.124728
HSPB3	0.4039136	-0.825713	1.7663951	0.0788014	0.1615777	-5.124741
SLFNL1	0.3486039	2.7661175	1.7661919	0.0788356	0.1616387	-5.12509
NGRN	-0.038698	6.0873261	-1.766	0.0788678	0.1616891	-5.12542
CCT5	-0.032748	6.706584	-1.765993	0.0788691	0.1616891	-5.125432
RP11-723G8	-0.293078	-1.196738	-1.765933	0.0788792	0.1617007	-5.125535
AP4E1	-0.040113	5.6637648	-1.765837	0.0788953	0.1617247	-5.1257
KRT8P9	-0.423872	0.2667305	-1.765644	0.0789277	0.1617821	-5.126031
BX255923.3	-0.272268	-1.120309	-1.765431	0.0789637	0.1618389	-5.126399
HEXDC	-0.044866	6.0581887	-1.765427	0.0789643	0.1618389	-5.126405
MEFV	0.2013024	3.4060273	1.765318	0.0789827	0.1618676	-5.126593
RPL18AP7	-0.35001	-0.256531	-1.765119	0.0790162	0.1619123	-5.126935
TRMT12	-0.048315	5.6643568	-1.765099	0.0790196	0.1619123	-5.126969
LINC01482	-0.360566	2.2956725	-1.765091	0.079021	0.1619123	-5.126983
RP11-548H1	0.4182503	2.0283025	1.7650832	0.0790223	0.1619123	-5.126996
RP11-574M7	0.0935488	-1.539236	1.7650132	0.0790341	0.1619274	-5.127116
TNMD	0.4007721	-0.498519	1.7649067	0.079052	0.161955	-5.127299
ARIH1	0.0293163	6.3320886	1.7648696	0.0790583	0.161957	-5.127363
RP11-166B2	-0.396104	0.5956593	-1.764848	0.0790619	0.161957	-5.1274
RP11-128B1	0.1674754	-1.392674	1.764737	0.0790806	0.1619864	-5.127591
EWSR1	-0.022407	6.7984897	-1.764606	0.0791028	0.1620215	-5.127817
DLG4	0.0630326	5.3972402	1.7645828	0.0791066	0.1620215	-5.127856
RP11-29P20	-0.259364	-1.141783	-1.764439	0.0791309	0.162062	-5.128103
AC016894.1	-0.183121	-1.347431	-1.764256	0.0791617	0.1621046	-5.128417
RNU4ATAC18	-0.375957	0.0118769	-1.764252	0.0791625	0.1621046	-5.128424
PCAT4	0.1429264	-1.486549	1.7642371	0.079165	0.1621046	-5.12845
TESK2	-0.079238	5.3171423	-1.76416	0.079178	0.1621221	-5.128582
RP11-617F2	-0.315154	-0.81051	-1.764057	0.0791953	0.1621485	-5.128759
C11orf95	-0.049986	5.9379361	-1.764002	0.0792047	0.1621586	-5.128854
RAB8B	0.0521855	5.9789658	1.7639475	0.0792139	0.1621605	-5.128947
KIAA1328	-0.058585	5.1041079	-1.763944	0.0792145	0.1621605	-5.128954
CALML6	-0.332706	2.9562834	-1.763901	0.0792217	0.1621662	-5.129027
RP1-102E24	-0.283778	-0.856961	-1.763826	0.0792344	0.1621831	-5.129156
MFSD5	-0.035358	6.1339353	-1.763542	0.0792824	0.1622722	-5.129644
SLC35G2	0.1222322	5.1036656	1.7635111	0.0792876	0.1622737	-5.129697
ZSCAN30	-0.04634	5.5894356	-1.763441	0.0792995	0.162289	-5.129818
SUCLA2P1	0.1846662	-1.346699	1.7631641	0.0793462	0.1623756	-5.130292
RP11-374P2	0.4311497	1.2199334	1.7629474	0.0793829	0.1624415	-5.130664
XXbac-B562	-0.373354	2.5022207	-1.762618	0.0794387	0.1625466	-5.131231
NUBP2	0.0391021	6.3998501	1.7625571	0.0794489	0.1625584	-5.131334
AC093642.1	-0.477371	0.4800385	-1.762501	0.0794584	0.1625657	-5.13143
AC002117.1	-0.388288	0.6590671	-1.762483	0.0794614	0.1625657	-5.131461
LPCAT4	0.0843973	5.5403554	1.7623725	0.0794802	0.1625859	-5.131651
RNFT1P3	-0.445442	0.3198551	-1.762372	0.0794802	0.1625859	-5.131651
HSPA13	-0.045484	6.0273029	-1.762336	0.0794863	0.1625893	-5.131713
RP11-415F2	0.2950534	2.128468	1.7622638	0.0794986	0.1626053	-5.131838
CPT1A	0.0615963	6.752667	1.7621216	0.0795227	0.1626455	-5.132082
GRK1	-0.277712	-1.15728	-1.761824	0.0795731	0.1627395	-5.132592

TTC23	-0.060442	5.8859455	-1.761619	0.079608	0.1628016	-5.132945
RP1-137D17	-0.39385	0.9375218	-1.761575	0.0796154	0.1628076	-5.13302
ABCC6P1	-0.21406	5.7275346	-1.76142	0.0796416	0.1628521	-5.133285
RP11-80901	-0.359	3.0796253	-1.761245	0.0796714	0.1629039	-5.133586
C15orf59	-0.347446	3.5957091	-1.760878	0.0797336	0.163022	-5.134214
LINC01550	0.430161	2.0818421	1.7606648	0.0797698	0.1630869	-5.13458
KIRREL3-AS	-0.185199	-1.420127	-1.760497	0.0797984	0.1631362	-5.134868
DUSP18	0.0847856	4.6492278	1.7603046	0.079831	0.1631937	-5.135198
RP11-88I18	0.1713292	-1.395723	1.7601458	0.079858	0.163238	-5.13547
MFSD7	0.1384327	5.4032305	1.7601245	0.0798616	0.163238	-5.135506
CTD-2541M1	-0.239912	2.8453732	-1.760043	0.0798754	0.1632571	-5.135645
SNORD46	-0.388574	0.4306378	-1.759978	0.0798865	0.1632706	-5.135757
KB-1980E6.	-0.415584	-0.537584	-1.759902	0.0798995	0.1632881	-5.135888
TRGV3	-0.410682	0.3586048	-1.759775	0.079921	0.1633229	-5.136105
RP1-66N13.	-0.177995	-1.46747	-1.759597	0.0799513	0.1633704	-5.13641
C2CD3	-0.036686	5.803839	-1.759586	0.0799532	0.1633704	-5.136429
GLYATL1	0.2690101	5.9849583	1.759502	0.0799675	0.1633904	-5.136573
RP1-100J12	-0.377506	1.0877886	-1.759468	0.0799733	0.1633931	-5.136631
RNU1-120P	-0.187047	-1.344347	-1.759368	0.0799902	0.1634186	-5.136802
APLP2	0.032971	7.1639227	1.7590892	0.0800378	0.1635065	-5.13728
LNX1	-0.085177	5.4395909	-1.758594	0.0801221	0.1636697	-5.138128
RP11-540K1	-0.249316	-1.14911	-1.758514	0.0801357	0.1636884	-5.138265
RP11-661C3	-0.353348	-0.419757	-1.758334	0.0801665	0.163742	-5.138573
AC002056.5	-0.252858	-1.076599	-1.758274	0.0801766	0.1637535	-5.138675
FLRT1	-0.337353	3.017919	-1.758103	0.0802059	0.1638042	-5.138969
STK11	-0.038917	6.3953392	-1.758031	0.0802182	0.163818	-5.139092
CSPG4P12	-0.355502	2.3958347	-1.758011	0.0802216	0.163818	-5.139127
AC011524.2	0.0759645	-1.548197	1.757919	0.0802373	0.1638407	-5.139284
CTD-3088G3	0.1859782	4.3298456	1.7577741	0.080262	0.1638821	-5.139532
DDI1	0.1425478	-1.459219	1.7575512	0.0803	0.1639506	-5.139913
CLPP	0.0409775	6.3298563	1.7574661	0.0803146	0.1639711	-5.140059
CYB561	0.1067051	6.1119848	1.7574189	0.0803226	0.1639784	-5.14014
MORF4L2	0.0306785	6.8226713	1.7571703	0.0803651	0.1640559	-5.140565
AC004967.7	-0.073702	4.7753109	-1.75698	0.0803977	0.1641133	-5.140891
PINX1	0.0569624	5.382779	1.7568613	0.0804179	0.1641454	-5.141094
HAAO	0.1020151	6.4962838	1.7567426	0.0804382	0.1641721	-5.141297
AOX2P	-0.449086	0.7906266	-1.756732	0.08044	0.1641721	-5.141315
PPARA	0.0675761	6.4750463	1.7566763	0.0804495	0.1641824	-5.14141
RP4-775C13	-0.395865	0.0680903	-1.756649	0.0804542	0.1641828	-5.141457
ERN1	0.0518785	6.2426983	1.7565149	0.0804772	0.1642204	-5.141686
ARIH2P1	-0.321959	-0.612568	-1.756391	0.0804984	0.1642546	-5.141899
RP11-73B2.	-0.452905	0.7988479	-1.756174	0.0805356	0.1643212	-5.14227
WDR54	-0.091791	5.2234937	-1.756115	0.0805455	0.1643323	-5.14237
HECTD4	-0.03891	6.1525411	-1.756045	0.0805576	0.1643478	-5.14249
HAPLN3	0.141589	5.0934512	1.7559769	0.0805692	0.1643561	-5.142606
RP6-186E3.	-0.134558	-1.48877	-1.755968	0.0805707	0.1643561	-5.142621
RPL7P16	-0.347013	-0.771268	-1.75568	0.0806201	0.1644476	-5.143114
CALU	0.0371083	6.6238729	1.7554337	0.0806623	0.1645219	-5.143535
CTA-212A2.	0.3047596	2.8007304	1.755415	0.0806655	0.1645219	-5.143567
AC025811.3	-0.237847	-1.31533	-1.755136	0.0807133	0.1646103	-5.144044
RP11-1007C	-0.106951	4.0052182	-1.755028	0.0807318	0.1646329	-5.144228

CTLA4	-0.291832	4.0485117	-1.755019	0.0807334	0.1646329	-5.144244
TFDP2	-0.034432	6.1263526	-1.754903	0.0807534	0.1646643	-5.144443
KIAA1024	-0.359265	2.8774962	-1.754741	0.0807811	0.1647117	-5.144719
CTSW	0.1575597	4.9664187	1.7546506	0.0807966	0.1647277	-5.144874
NANOGP7	0.2898231	-1.110465	1.7546426	0.080798	0.1647277	-5.144887
TOR4A	0.0954063	5.5622656	1.7545899	0.080807	0.1647369	-5.144977
GOLGA8IP	-0.453414	0.4335878	-1.754559	0.0808123	0.1647385	-5.14503
PTBP2	-0.050941	5.6719217	-1.754504	0.0808217	0.1647484	-5.145123
C9orf152	-0.426463	3.5739466	-1.754433	0.0808339	0.1647554	-5.145245
RP11-222K1	0.3733091	2.5993285	1.7544319	0.0808341	0.1647554	-5.145247
TRBV12-5	-0.314646	-0.973203	-1.754269	0.080862	0.1648031	-5.145525
EIF3S5P1	-0.362612	-0.33974	-1.754227	0.0808693	0.1648087	-5.145597
RP5-1139B1	-0.38871	-0.585	-1.754186	0.0808763	0.1648138	-5.145667
ABHD11	0.0634997	5.9179375	1.7540188	0.0809051	0.1648632	-5.145953
PPP1R9B	0.038099	6.2790377	1.7539661	0.0809142	0.1648725	-5.146043
EDRF1-AS1	-0.399722	0.6526788	-1.753713	0.0809577	0.164952	-5.146476
AC007880.1	-0.216069	-1.291784	-1.753531	0.080989	0.1650016	-5.146786
NRIP2	0.0858829	5.1133155	1.7535184	0.0809911	0.1650016	-5.146808
ATP11B	0.0439716	6.1621461	1.7534101	0.0810097	0.1650304	-5.146992
RFX3	-0.071823	5.1781942	-1.753257	0.0810361	0.165075	-5.147255
NIPAL1	-0.164574	5.3226436	-1.753052	0.0810713	0.1651315	-5.147604
GPR56	0.0986881	6.0518343	1.7530314	0.0810749	0.1651315	-5.147639
RP1-92C8.3	-0.128993	-1.491499	-1.753016	0.0810775	0.1651315	-5.147665
RP11-20J1.	-0.183677	-1.416294	-1.752935	0.0810915	0.1651508	-5.147804
TXNRD3	0.0544702	5.6292075	1.7528131	0.0811125	0.1651759	-5.148012
RP1-266L20	-0.258297	2.628506	-1.752793	0.0811159	0.1651759	-5.148046
LINC00578	0.4638874	0.1851739	1.752761	0.0811214	0.1651759	-5.148101
RP4-737E23	0.3802364	0.7531605	1.7527584	0.0811219	0.1651759	-5.148105
PGAP3	-0.050605	6.3637918	-1.752509	0.0811648	0.165254	-5.14853
RP11-820I1	-0.2108	-1.249658	-1.752338	0.0811944	0.165305	-5.148823
SNHG12	-0.07492	5.4273409	-1.752191	0.0812196	0.1653471	-5.149073
HMHA1	0.0583141	6.1370636	1.7521096	0.0812337	0.1653666	-5.149212
RP11-295D4	-0.363871	-0.235517	-1.75187	0.081275	0.1654414	-5.149621
MAGEA9B	-0.244942	-1.355107	-1.7518	0.081287	0.1654567	-5.14974
IGHV3-75	-0.296727	-1.133002	-1.75176	0.0812939	0.1654614	-5.149808
AC016831.7	-0.278686	3.6515037	-1.75168	0.0813077	0.1654805	-5.149945
SNORA74B	-0.261804	-1.168257	-1.751448	0.0813477	0.1655526	-5.150341
PRRX2	-0.490015	1.9458213	-1.751276	0.0813775	0.1655941	-5.150634
RPL7P57	-0.311317	-0.562676	-1.751262	0.0813799	0.1655941	-5.150658
hsa-mir-45	-0.261533	-1.182295	-1.751251	0.0813817	0.1655941	-5.150677
PPIAP16	-0.36522	-0.056185	-1.75118	0.081394	0.1656098	-5.150797
RPS3AP47	-0.175496	3.3966599	-1.751137	0.0814014	0.1656157	-5.150871
NBPF6	-0.294397	-1.148679	-1.750945	0.0814347	0.1656742	-5.151199
RP11-482D2	0.4240042	0.4238267	1.7508599	0.0814493	0.1656947	-5.151344
AL133243.2	-0.127676	3.4615454	-1.750798	0.0814601	0.1656983	-5.15145
LINC00939	-0.447903	3.3419821	-1.750797	0.0814601	0.1656983	-5.151451
RPL5P17	-0.39233	0.0762315	-1.750288	0.0815481	0.165868	-5.152318
KB-1507C5.	0.1178927	4.7156524	1.7502417	0.0815562	0.1658751	-5.152398
RNU1-11P	-0.159631	-1.428086	-1.749892	0.0816166	0.1659889	-5.152994
RP11-327P2	0.1024218	4.0470447	1.7497989	0.0816328	0.1660125	-5.153153
PRR7	-0.136241	4.9072622	-1.749756	0.0816402	0.1660183	-5.153226

RP11-722E2	-0.159377	3.568461	-1.74972	0.0816465	0.1660217	-5.153287
PCDHGA9	0.344196	3.0709139	1.749681	0.0816532	0.1660262	-5.153353
RP11-467L1	-0.105161	4.7030428	-1.749581	0.0816704	0.166052	-5.153523
SKIL	-0.044511	6.198171	-1.749348	0.0817108	0.1661249	-5.153921
RP11-285E9	0.4177098	0.3936187	1.7491325	0.0817482	0.1661916	-5.154288
CCDC61	-0.048911	5.7346195	-1.749016	0.0817684	0.1662235	-5.154487
NTNG1	-0.412638	-0.51582	-1.748837	0.0817994	0.1662772	-5.154791
PILRA	0.0940155	5.1525748	1.7487898	0.0818076	0.1662793	-5.154872
PXDNL	-0.324529	3.2328225	-1.748778	0.0818096	0.1662793	-5.154891
RP11-91P17	-0.179557	-1.426629	-1.748479	0.0818615	0.1663666	-5.155401
HOXD8	-0.348605	3.6172384	-1.748478	0.0818616	0.1663666	-5.155402
RPL6P2	-0.190257	-1.308122	-1.74841	0.0818734	0.1663812	-5.155518
SPSB3	0.0839158	4.7142762	1.7482554	0.0819003	0.1664224	-5.155782
CTD-2631K1	0.2432347	-1.143989	1.7482412	0.0819028	0.1664224	-5.155806
AC083862.6	-0.337599	-0.659233	-1.74799	0.0819464	0.1664998	-5.156234
AC012354.6	-0.286227	-1.054675	-1.747969	0.08195	0.1664998	-5.156269
DLGAP1-AS2	-0.148758	4.2676274	-1.747911	0.0819601	0.1665111	-5.156368
ARMC7	-0.044608	5.9150733	-1.747807	0.0819782	0.1665385	-5.156545
SRRM5	-0.185856	3.5971104	-1.747606	0.082013	0.1665885	-5.156886
RP11-58A18	0.2096692	-1.320884	1.7475626	0.0820206	0.1665885	-5.156961
RP5-1139B1	0.3984629	1.7721101	1.7475602	0.082021	0.1665885	-5.156965
AC008850.3	-0.17106	-1.356766	-1.747519	0.0820282	0.1665885	-5.157036
CTD-2339F6	-0.394488	-0.402379	-1.747514	0.082029	0.1665885	-5.157043
ERC1	0.0394549	6.1398559	1.7475077	0.0820301	0.1665885	-5.157054
RP11-876N2	0.1598881	3.9418197	1.747481	0.0820348	0.1665886	-5.1571
ZBTB44	0.040604	6.1952767	1.7472607	0.082073	0.1666571	-5.157475
RP11-1136G	-0.347636	-0.843684	-1.747095	0.0821018	0.1667062	-5.157756
TMIGD1	-0.378485	-0.691985	-1.747055	0.0821088	0.1667112	-5.157825
RP11-168L2	0.1403134	-1.44849	1.746717	0.0821676	0.1668175	-5.1584
RP5-991C6.	-0.253503	-1.139244	-1.746702	0.0821703	0.1668175	-5.158426
HORMAD2-AS	0.6954242	3.2456779	1.746628	0.0821831	0.1668342	-5.158551
MRPL55	-0.054418	6.3987171	-1.746577	0.082192	0.1668429	-5.158638
10-Mar	0.4492213	-0.211869	1.7465041	0.0822047	0.1668594	-5.158762
NTNG2	0.3020943	3.4745011	1.7462411	0.0822505	0.166943	-5.159209
RP13-487K5	0.2126446	-1.225846	1.7461641	0.0822639	0.166961	-5.15934
RP11-109N2	-0.345063	1.5712156	-1.746131	0.0822697	0.1669634	-5.159397
BAIAP3	0.1094838	5.5940137	1.7460343	0.0822865	0.1669883	-5.159561
DOK3	0.0867476	5.2520138	1.746003	0.0822919	0.1669901	-5.159614
GRTP1-AS1	0.3173895	2.8426018	1.7459763	0.0822966	0.1669902	-5.15966
C3orf18	0.091766	5.3289012	1.7457747	0.0823317	0.1670522	-5.160002
RP11-368I7	0.1404342	3.9807801	1.7457157	0.082342	0.1670638	-5.160103
STOX2	0.1500492	5.145188	1.7455505	0.0823708	0.1671078	-5.160384
ARSH	0.1866325	-1.405675	1.7455388	0.0823728	0.1671078	-5.160403
DCLK3	-0.411379	2.2919713	-1.745501	0.0823794	0.1671119	-5.160468
CCDC27	-0.287817	-1.008943	-1.745417	0.0823941	0.1671324	-5.160611
CNR1	-0.502688	2.651005	-1.745246	0.0824238	0.1671834	-5.1609
AP000525.1	-0.375865	-0.51943	-1.745155	0.0824397	0.1672063	-5.161055
TRIP12	0.0238548	6.6225755	1.744963	0.0824733	0.1672584	-5.161382
ARF4P2	-0.465026	0.0752233	-1.744956	0.0824746	0.1672584	-5.161395
C1orf147	-0.414856	0.5971602	-1.744911	0.0824823	0.1672603	-5.16147
PI4K2A	0.0417729	6.1403151	1.7448957	0.082485	0.1672603	-5.161497

CXCR4	0.0767537	6.1532293	1.7448469	0.0824935	0.1672603	-5.16158
TRIB1	0.0615991	6.5444807	1.7448452	0.0824938	0.1672603	-5.161582
NUTM2HP	-0.36372	-0.224479	-1.744773	0.0825065	0.1672767	-5.161706
RP11-626H1	-0.436827	1.4621169	-1.744717	0.0825162	0.1672871	-5.1618
IGF2BP2-AS	-0.492871	0.7680742	-1.744663	0.0825257	0.1672929	-5.161893
RNF123	0.0453931	6.2126575	1.7446483	0.0825282	0.1672929	-5.161917
STT3B	-0.031179	6.7536127	-1.744576	0.0825408	0.1673091	-5.16204
LINC01465	-0.136718	3.709223	-1.744361	0.0825784	0.1673737	-5.162406
SLC25A21-A	-0.317049	3.5382606	-1.744341	0.0825818	0.1673737	-5.162439
FLJ16779	0.4166846	-0.476659	1.7442851	0.0825917	0.1673843	-5.162534
ETNK1	-0.034761	6.3468559	-1.744227	0.0826018	0.1673955	-5.162633
RPF2	-0.044245	6.1521447	-1.744039	0.0826347	0.167453	-5.162953
AL138898.1	-0.288059	-0.906076	-1.743977	0.0826456	0.1674565	-5.163058
RP11-725P1	-0.394712	-0.056629	-1.743976	0.0826456	0.1674565	-5.163059
RP11-327F2	-0.265154	-1.043037	-1.743936	0.0826526	0.1674604	-5.163127
RP11-216N1	-0.324391	-0.667766	-1.743913	0.0826567	0.1674604	-5.163167
MRPL48	-0.037806	6.0069081	-1.743792	0.0826778	0.1674845	-5.163371
RP11-443K8	-0.346828	-0.76346	-1.743792	0.0826778	0.1674845	-5.163371
ASCL4	-0.27901	-1.054385	-1.743718	0.0826909	0.1675017	-5.163498
PPIAL4C	-0.248645	-1.066399	-1.743626	0.0827069	0.1675249	-5.163654
RPL7P8	-0.336338	-0.515759	-1.743553	0.0827197	0.1675415	-5.163778
SCARNA11	-0.177286	-1.358292	-1.743455	0.0827368	0.1675649	-5.163944
RAB1A	0.0256605	6.7785852	1.7434342	0.0827404	0.1675649	-5.163979
OVOL3	-0.378987	-0.204769	-1.743345	0.0827561	0.1675804	-5.164131
RP13-644M1	-0.474003	0.5192816	-1.743338	0.0827573	0.1675804	-5.164143
LBX2	-0.146764	4.9524968	-1.743261	0.0827708	0.1675984	-5.164274
AC007950.2	-0.385968	0.7433661	-1.743118	0.0827958	0.1676398	-5.164516
DDAH1	0.0694736	6.5240431	1.7430763	0.0828031	0.1676452	-5.164587
SUCO	-0.044924	6.2754152	-1.743037	0.0828099	0.1676498	-5.164653
RPL17P11	0.5076256	0.8957041	1.7429655	0.0828225	0.1676659	-5.164775
CALCOCO1	0.0334191	6.3348898	1.7428602	0.0828409	0.167694	-5.164954
REC114	-0.323986	-0.780465	-1.742832	0.0828459	0.1676948	-5.165002
MIR4482	0.4251962	1.7230298	1.7427915	0.082853	0.1676998	-5.165071
UBA52P8	0.3968049	1.1905987	1.7427079	0.0828676	0.1677201	-5.165213
RP11-706P1	0.2911666	2.7354876	1.742633	0.0828807	0.1677373	-5.165339
RP11-705C1	-0.207533	-1.287314	-1.742276	0.0829433	0.1678479	-5.165946
NSD1	-0.032235	6.3222341	-1.742269	0.0829445	0.1678479	-5.165957
MYO1H	-0.375209	1.1344146	-1.742208	0.0829552	0.1678557	-5.16606
RP5-1025A1	-0.405755	-0.448962	-1.742195	0.0829576	0.1678557	-5.166084
AC005753.1	0.3868136	-0.233573	1.7419489	0.0830007	0.1679335	-5.1665
AC010243.1	0.1669301	-1.378502	1.741921	0.0830056	0.1679342	-5.166548
GOLGA6L17F	-0.214056	-1.266546	-1.741843	0.0830194	0.1679527	-5.166681
CPNE7	0.280006	4.452003	1.7417489	0.0830358	0.1679766	-5.16684
CTC-461F20	0.2193593	-1.301381	1.74156	0.0830689	0.1680344	-5.16716
AC083843.1	-0.10617	5.2155876	-1.74136	0.0831041	0.1680961	-5.1675
TSN	-0.026676	6.480287	-1.741115	0.0831472	0.168174	-5.167916
L29074.3	-0.287947	-0.908052	-1.741028	0.0831623	0.1681953	-5.168062
RP11-819M1	-0.387366	-0.251457	-1.740907	0.0831836	0.168229	-5.168267
C12orf71	0.2936903	-0.839549	1.7408441	0.0831947	0.1682422	-5.168374
AGAP5	-0.317912	2.2125275	-1.74081	0.0832007	0.1682449	-5.168432
SMIM6	-0.264307	4.7092625	-1.740766	0.0832084	0.1682513	-5.168507

MRPS18C	0.0410136	5.7370185	1.7405813	0.0832409	0.1683076	-5.16882
CACNA1C-AS	0.4110346	0.5464866	1.7404948	0.0832561	0.1683291	-5.168967
MIR31HG	0.2665789	-1.218579	1.740042	0.0833358	0.1684808	-5.169734
RP11-562A8	-0.373094	0.028885	-1.739943	0.0833532	0.1685066	-5.169901
OTUB2	-0.103403	4.8817685	-1.739916	0.083358	0.168507	-5.169948
EIF2AK3	0.0404105	5.8868736	1.7397269	0.0833913	0.168565	-5.170268
RP11-826F1	-0.402556	1.81797	-1.739679	0.0833996	0.1685726	-5.170349
RP11-61N20	-0.282981	3.4222709	-1.739613	0.0834113	0.1685868	-5.170461
RP3-51008.	0.4205424	0.4877	1.7394211	0.0834451	0.1686458	-5.170786
MIR6797	-0.341449	-0.434788	-1.739348	0.0834581	0.1686626	-5.170911
ZHX1	0.0540314	6.4152	1.7392862	0.0834689	0.1686752	-5.171015
RP11-454F8	0.2162966	-1.234881	1.7392583	0.0834738	0.1686758	-5.171062
LY75	0.1375647	4.7477796	1.739193	0.0834853	0.1686897	-5.171173
FKBP3	-0.03454	6.3098774	-1.739161	0.083491	0.1686918	-5.171227
BTF3P12	-0.255795	-1.04794	-1.738821	0.0835509	0.1688035	-5.171803
RP11-167N2	0.080544	-1.545864	1.7387058	0.0835713	0.1688353	-5.171998
AL031587.1	-0.324332	2.1659004	-1.73864	0.0835829	0.1688496	-5.17211
TDRD6	-0.161723	4.3803367	-1.738603	0.0835894	0.1688532	-5.172172
IGLV3-17	-0.193439	-1.423039	-1.738326	0.0836382	0.1689426	-5.172641
AC044907.1	0.485561	0.6533585	1.7382204	0.0836569	0.168971	-5.17282
GAB2	0.056564	5.9316955	1.7381918	0.083662	0.1689718	-5.172868
RP11-834C1	0.2834461	-0.907621	1.7380723	0.0836831	0.1690051	-5.173071
PGM2	0.0741029	5.9027919	1.7379135	0.0837111	0.1690524	-5.17334
FAM219B	-0.058634	5.345946	-1.737652	0.0837573	0.1691362	-5.173782
ZNF292	-0.059142	5.9060839	-1.737562	0.0837733	0.1691536	-5.173935
LRIG1	0.0523403	6.2749183	1.7375513	0.0837751	0.1691536	-5.173953
RFTN2	-0.105058	4.8906561	-1.737524	0.08378	0.1691541	-5.174
LSAMP	0.4309979	3.2025734	1.7373891	0.0838038	0.1691928	-5.174227
CTD-3131K8	-0.117432	3.8667434	-1.737242	0.0838298	0.1692359	-5.174476
RBBP6	0.0252698	6.2300096	1.736982	0.0838758	0.1693194	-5.174916
ZBED2	-0.503683	2.2676151	-1.736867	0.0838962	0.1693511	-5.175111
RFPL4AP6	0.1848567	-1.361052	1.7367286	0.0839207	0.1693912	-5.175345
RP11-8P13.	-0.415659	1.0662852	-1.73656	0.0839505	0.16944	-5.175629
BPIFA2	-0.597272	0.75151	-1.73654	0.0839541	0.16944	-5.175664
RNVU1-15	-0.243269	-1.133111	-1.736483	0.0839642	0.1694408	-5.175761
FAM171A2	-0.187989	4.3750576	-1.736473	0.083966	0.1694408	-5.175778
TBC1D3L	-0.319927	3.4325052	-1.736459	0.0839684	0.1694408	-5.175801
GBX2	-0.429405	-0.06668	-1.736411	0.0839769	0.1694484	-5.175882
RP3-332B22	0.3941635	-0.578002	1.7363149	0.0839939	0.1694688	-5.176045
RBBP8	0.053905	5.8884891	1.7363019	0.0839962	0.1694688	-5.176067
AC016712.1	0.2767966	-1.024403	1.7361746	0.0840188	0.1695049	-5.176282
GSTA9P	-0.232926	-1.291943	-1.736066	0.084038	0.1695342	-5.176465
AKAP10	0.0396473	5.7531947	1.7360281	0.0840448	0.1695386	-5.17653
RP4-717I23	-0.076172	5.2751468	-1.735965	0.0840558	0.1695474	-5.176635
CRBN	0.0336355	6.1114563	1.735951	0.0840584	0.1695474	-5.17666
APOB	-0.118241	7.5809616	-1.735843	0.0840776	0.1695662	-5.176843
RP4-761J14	-0.384223	0.7616513	-1.735827	0.0840804	0.1695662	-5.17687
SLC25A42	-0.087753	6.3834492	-1.735812	0.0840831	0.1695662	-5.176895
CICP3	-0.277329	-0.95524	-1.735794	0.0840863	0.1695662	-5.176926
PCDHA5	-0.477742	0.086052	-1.73563	0.0841153	0.1696153	-5.177203
CBX1P2	0.1510686	-1.414108	1.7354154	0.0841534	0.1696827	-5.177566

CTA-292E1C	0.1746718	4.1845415	1.734937	0.0842383	0.1698446	-5.178374
AC002128.5	-0.407684	0.8203183	-1.734895	0.0842457	0.1698502	-5.178445
G6PD	-0.092712	6.1808001	-1.734865	0.0842511	0.1698515	-5.178496
RP11-17E13	-0.195504	4.080759	-1.734788	0.0842648	0.1698698	-5.178626
RP11-20G13	-0.415993	-0.398523	-1.734627	0.0842934	0.1699061	-5.178898
RP11-354K1	-0.393886	-0.352968	-1.734622	0.0842943	0.1699061	-5.178907
FAM105A	0.1138198	5.0387119	1.7346078	0.0842968	0.1699061	-5.17893
CTC-436P18	0.3668085	0.3437801	1.7345509	0.0843069	0.1699171	-5.179027
C7orf43	0.0416678	5.874787	1.7344595	0.0843231	0.1699384	-5.179181
RPL5	-0.035987	7.1585182	-1.734439	0.0843268	0.1699384	-5.179216
RP11-432I5	0.1374088	-1.466597	1.7342646	0.0843578	0.1699915	-5.17951
GAPDHP58	-0.238719	-1.155466	-1.734096	0.0843878	0.1700427	-5.179796
RP11-185B1	-0.142426	-1.441102	-1.733892	0.0844241	0.1701064	-5.18014
RP11-390K5	-0.380951	1.9253434	-1.733846	0.0844322	0.1701132	-5.180217
RP11-569A1	-0.358591	1.3573606	-1.733731	0.0844528	0.1701453	-5.180412
C1orf146	-0.310121	-0.720946	-1.733655	0.0844663	0.1701632	-5.180541
RNF157	-0.147645	5.5956729	-1.733584	0.0844788	0.1701791	-5.18066
CCDC169-SC	0.1084661	-1.518119	1.7332916	0.0845309	0.1702676	-5.181154
QTRT1	-0.042802	6.2421599	-1.733252	0.0845379	0.1702676	-5.18122
AP006216.1	-0.232101	-1.137854	-1.733242	0.0845398	0.1702676	-5.181238
C7orf71	-0.289841	-1.067299	-1.733232	0.0845414	0.1702676	-5.181254
SPIN1	-0.038154	6.2976801	-1.733178	0.0845511	0.1702777	-5.181345
RP5-1004I9	0.3404568	-0.730976	1.7330398	0.0845758	0.170318	-5.181579
NCL	-0.024644	7.0711704	-1.732734	0.0846303	0.1704184	-5.182095
SEC14L1	0.0401654	6.293781	1.7326214	0.0846503	0.170445	-5.182285
MRPL45	-0.038971	6.251134	-1.732607	0.0846528	0.170445	-5.182309
OR7E87P	-0.165785	-1.451552	-1.732467	0.0846778	0.1704859	-5.182546
RP11-554I8	0.5173447	-0.226265	1.7323809	0.0846932	0.1705036	-5.182691
LINC01581	-0.208863	-1.333648	-1.732365	0.084696	0.1705036	-5.182717
EMC3	0.0379762	6.5198394	1.7321685	0.0847311	0.1705649	-5.183049
RP11-466F5	-0.361272	-0.219061	-1.732129	0.0847381	0.1705695	-5.183115
CD8BP	-0.383778	-0.808735	-1.732101	0.0847431	0.1705703	-5.183163
GRIPAP1	0.0277477	6.3295527	1.7320362	0.0847547	0.1705773	-5.183273
TRMT112	-0.036075	6.6361735	-1.732027	0.0847563	0.1705773	-5.183288
CCDC50	0.029691	6.5587763	1.732003	0.0847606	0.1705773	-5.183329
RP11-259K1	0.1556106	-1.468571	1.7319337	0.084773	0.1705928	-5.183446
AC008074.1	-0.377406	0.7260987	-1.731907	0.0847778	0.1705931	-5.183491
PRSS12	0.4707617	3.3650705	1.7318366	0.0847903	0.1706089	-5.183609
SEPHS1P4	-0.274809	-0.93915	-1.731803	0.0847963	0.1706115	-5.183666
RP11-517C1	0.4423479	0.1664035	1.7317195	0.0848112	0.1706287	-5.183807
RP11-92K15	-0.305786	2.9542072	-1.731703	0.0848142	0.1706287	-5.183835
DUX4L19	0.1422383	-1.483345	1.7316328	0.0848267	0.1706445	-5.183953
RP5-943J3.	0.3262436	-0.64669	1.7315758	0.0848369	0.1706556	-5.184049
RP11-326C3	0.1954875	-1.310107	1.7313904	0.08487	0.1707084	-5.184362
ATP5G1P5	-0.196631	-1.331647	-1.731376	0.0848725	0.1707084	-5.184386
AC138969.4	-0.295269	2.5809126	-1.731224	0.0848997	0.1707536	-5.184642
LINC01164	-0.395553	-0.849681	-1.730878	0.0849615	0.1708686	-5.185226
RP3-388E23	0.4080433	1.1234898	1.7307035	0.0849928	0.1709221	-5.185521
CTB-113P19	0.1887577	3.7642507	1.7306029	0.0850108	0.1709488	-5.18569
LINC01229	0.4263414	0.8236719	1.7303886	0.0850491	0.1710165	-5.186051
IRG1	-0.309038	-0.985998	-1.729835	0.0851482	0.1712063	-5.186984

NELL1	-0.426561	-0.689535	-1.72965	0.0851814	0.1712571	-5.187296
CYB5A	0.0783356	6.9512478	1.729642	0.0851828	0.1712571	-5.18731
GLOD4	0.0373253	6.3230857	1.7295913	0.0851919	0.1712659	-5.187395
DMC1	-0.350785	2.6641272	-1.729463	0.085215	0.1713027	-5.187612
MAP3K10	0.0520671	5.6584679	1.7292256	0.0852575	0.1713788	-5.188011
LINC01513	-0.12312	-1.472474	-1.729043	0.0852903	0.1714308	-5.188319
C8orf74	0.2498034	-1.244451	1.7290291	0.0852927	0.1714308	-5.188342
SPEN	0.0346889	6.3627131	1.7289661	0.085304	0.171444	-5.188448
CD247	0.1179996	5.0935933	1.7287314	0.0853461	0.1715119	-5.188844
PRLHR	-0.26977	-1.258923	-1.728726	0.0853472	0.1715119	-5.188854
RP3-326L13	-0.17804	-1.427372	-1.728681	0.0853551	0.1715184	-5.188928
RP11-345M2	0.451199	2.3359299	1.7286268	0.0853649	0.1715287	-5.18902
RP11-274M1	-0.19111	-1.344727	-1.728311	0.0854217	0.1716185	-5.189552
SLC14A2-AS	0.3035374	-0.880226	1.7283032	0.085423	0.1716185	-5.189565
ZNF347	-0.133489	4.9354223	-1.728299	0.0854237	0.1716185	-5.189571
BTG2	-0.065118	6.2692447	-1.728019	0.0854742	0.1717021	-5.190044
RP11-252A2	-0.22768	-1.213849	-1.728015	0.0854747	0.1717021	-5.19005
AF064858.8	0.3883512	1.8505106	1.7279379	0.0854887	0.1717206	-5.19018
RP11-291L1	-0.241807	-1.06816	-1.727879	0.0854993	0.1717325	-5.19028
RIPK1	0.0301444	6.2879934	1.7275495	0.0855585	0.1718327	-5.190834
LRRC63	-0.428676	1.6512534	-1.727549	0.0855586	0.1718327	-5.190835
TBX1	0.4446054	2.7150759	1.7274175	0.0855822	0.1718707	-5.191056
AQP7P1	0.475701	3.2572043	1.7273646	0.0855917	0.1718794	-5.191145
RPS26P8	-0.385058	0.2043132	-1.727318	0.0856002	0.1718794	-5.191224
RP11-342C2	0.1433472	-1.469509	1.7273075	0.085602	0.1718794	-5.191241
TENM3	0.5613146	1.5704208	1.7272885	0.0856054	0.1718794	-5.191273
CLLU1OS	0.4593137	-0.49617	1.727135	0.085633	0.1719255	-5.191531
FLJ38122	0.4280205	0.9190093	1.7270319	0.0856516	0.1719532	-5.191705
AC092798.2	-0.300552	-0.748766	-1.726937	0.0856687	0.1719782	-5.191865
RNA5SP154	-0.303511	-0.95907	-1.726835	0.0856871	0.1720055	-5.192036
RRH	0.3902422	0.9999683	1.726601	0.0857292	0.1720806	-5.19243
TIMD4	0.4285808	3.1296999	1.7263852	0.0857681	0.1721492	-5.192793
S100Z	0.3893901	0.8072599	1.7263544	0.0857736	0.1721509	-5.192844
MRPS16	-0.03195	6.5395973	-1.726024	0.0858332	0.1722528	-5.1934
RP11-457I1	0.0954964	-1.538244	1.7260203	0.0858338	0.1722528	-5.193406
HIST1H4C	-0.384797	1.2237796	-1.725984	0.0858403	0.1722563	-5.193467
FOLH1	0.1379915	5.4069358	1.725884	0.0858584	0.1722832	-5.193635
C9orf16	0.0566756	6.1756556	1.7257283	0.0858865	0.17233	-5.193897
SLC37A4	0.0629131	6.6757795	1.7256527	0.0859001	0.1723479	-5.194024
RP11-262H1	0.3383589	2.34764	1.7251025	0.0859995	0.1725377	-5.194949
RP3-476K8.	-0.360575	0.7432978	-1.724901	0.0860358	0.1726011	-5.195287
RP11-1145L	-0.131586	-1.490228	-1.724787	0.0860564	0.172633	-5.195479
RP11-267M2	-0.286714	-0.919495	-1.72439	0.0861281	0.1727581	-5.196146
RP11-310E2	-0.206771	-1.265539	-1.72439	0.0861282	0.1727581	-5.196147
CLLU1	0.322194	-1.034606	1.7242099	0.0861608	0.1728075	-5.196449
HNRNPA1P34	-0.230115	-1.138828	-1.724201	0.0861623	0.1728075	-5.196464
RP11-666A2	-0.389378	0.7776483	-1.723732	0.0862472	0.1729683	-5.197252
PTMAP4	-0.156336	3.2065969	-1.723598	0.0862716	0.1730076	-5.197477
POLR2L	0.0411006	6.5945052	1.7233623	0.0863142	0.1730835	-5.197873
P4HB	0.0385062	7.4974726	1.7232078	0.0863422	0.1731301	-5.198132
AC007050.1	-0.224981	-1.379848	-1.722935	0.0863917	0.1732199	-5.198591

RP4-601P9.	-0.261062	-1.117711	-1.722907	0.0863967	0.1732205	-5.198637
ELOVL6	0.1021907	6.1840341	1.7225513	0.0864612	0.1733349	-5.199234
RP11-4G2.1	0.1975995	-1.376272	1.7225396	0.0864633	0.1733349	-5.199254
AC063976.3	-0.531827	0.713661	-1.721919	0.0865759	0.1735512	-5.200295
GPR179	-0.414659	0.4124736	-1.721739	0.0866087	0.1736074	-5.200598
CSPG4	0.0831819	5.7629033	1.7216477	0.0866252	0.1736309	-5.20075
CYB5R4	0.0426595	5.7249275	1.7215076	0.0866506	0.1736682	-5.200985
PDE6D	0.03357	5.8405319	1.7214929	0.0866533	0.1736682	-5.20101
KMT2D	-0.036045	6.3479722	-1.721328	0.0866833	0.1737188	-5.201287
LINC00705	0.3500743	-0.746555	1.7212718	0.0866935	0.1737297	-5.201381
RP11-428G5	0.0975557	-1.52888	1.7212228	0.0867024	0.173738	-5.201463
RP11-153F1	0.1679007	-1.392457	1.7208252	0.0867747	0.1738733	-5.20213
HKR1	-0.064548	5.9096658	-1.720667	0.0868034	0.1739213	-5.202394
TMA7	-0.044558	6.4607408	-1.720509	0.0868322	0.1739695	-5.20266
RP11-622J8	0.3965379	-0.928929	1.7202728	0.0868752	0.1740441	-5.203056
PCDHB9	0.410783	2.8638051	1.720252	0.086879	0.1740441	-5.203091
RP11-15L13	-0.309983	2.1016435	-1.720205	0.0868877	0.1740519	-5.20317
FAM19A2	-0.161591	3.6597755	-1.720162	0.0868955	0.174058	-5.203242
RP11-181K1	0.1732653	-1.394041	1.7201177	0.0869035	0.1740645	-5.203316
RP11-227D1	0.3938328	0.2662413	1.7200389	0.0869178	0.174073	-5.203448
RP11-430C7	-0.429239	0.5936162	-1.720032	0.0869192	0.174073	-5.20346
ARSDP1	-0.290412	-1.030171	-1.720016	0.086922	0.174073	-5.203486
TMEM33	0.0388149	6.5415797	1.7199576	0.0869327	0.1740847	-5.203584
ARFGAP2	-0.031633	6.6009947	-1.719725	0.0869749	0.1741598	-5.203973
MROH3P	-0.548758	0.5977635	-1.719601	0.0869976	0.1741896	-5.204181
PGM5-AS1	0.4051865	-0.29608	1.7195914	0.0869994	0.1741896	-5.204198
KBTBD7	-0.055115	5.5571877	-1.719547	0.0870075	0.1741963	-5.204272
FAM101A	0.3910674	3.7522611	1.7194566	0.087024	0.1742191	-5.204423
RP11-38G5.	-0.393309	0.0640177	-1.719414	0.0870318	0.1742191	-5.204495
RNASE11	0.080544	-1.545864	1.719406	0.0870332	0.1742191	-5.204508
CTD-3107M8	0.2029659	-1.317075	1.7193556	0.0870424	0.1742279	-5.204593
GNL2	0.0327274	6.3505963	1.7190523	0.0870977	0.174329	-5.205101
SNORD93	-0.247437	-1.133321	-1.719024	0.0871029	0.174329	-5.205149
AP000281.1	-0.335861	-0.294166	-1.719	0.0871072	0.174329	-5.205188
SNORD116-6	-0.147454	-1.438636	-1.718966	0.0871134	0.1743319	-5.205245
ARHGAP18	-0.05542	5.8140444	-1.718576	0.0871847	0.174465	-5.205899
CCDC88A	-0.06806	6.015947	-1.718508	0.0871971	0.1744729	-5.206013
LCE1B	0.1860192	-1.426613	1.7185016	0.0871982	0.1744729	-5.206023
TMPRSS11E	0.3526305	-0.847253	1.7182506	0.0872441	0.1745551	-5.206443
ST13P6	-0.298937	2.3447945	-1.718169	0.0872589	0.1745753	-5.206579
CTD-2006C1	-0.228765	-1.143225	-1.717976	0.0872943	0.1746365	-5.206903
PRPF38AP1	0.1899014	-1.396035	1.717932	0.0873023	0.1746429	-5.206977
LRRIQ4	-0.460565	1.3202656	-1.717841	0.0873189	0.1746666	-5.207129
AC145124.2	0.403951	1.4767369	1.717547	0.0873727	0.1747646	-5.207621
NRN1L	-0.221216	-1.223971	-1.717512	0.0873792	0.174768	-5.20768
RP5-1021I2	-0.331002	-0.32408	-1.717266	0.0874242	0.1748484	-5.208092
SHARPIN	-0.044377	6.5332208	-1.716793	0.0875107	0.1749972	-5.208882
AC068641.1	-0.261752	-0.975625	-1.716759	0.087517	0.1749972	-5.20894
AASDH	0.045791	5.4872998	1.7167538	0.0875179	0.1749972	-5.208948
RPL21P123	-0.213197	-1.217419	-1.716727	0.0875228	0.1749972	-5.208993
TMEM74	-0.337791	4.0592173	-1.716721	0.087524	0.1749972	-5.209004

DBF4P1	-0.364696	0.0703545	-1.716702	0.0875274	0.1749972	-5.209035
LINC00266-	-0.177209	-1.336425	-1.716663	0.0875345	0.1750019	-5.2091
ANO8	0.073931	5.6690264	1.7165897	0.087548	0.1750193	-5.209223
MFSD8	0.0429878	5.7622368	1.7165026	0.0875639	0.1750416	-5.209368
LINC01573	-0.220951	3.672519	-1.716366	0.087589	0.175082	-5.209597
RP5-1024G6	0.1550424	3.0598221	1.716293	0.0876024	0.1750992	-5.209719
TPM3	-0.025535	6.9175713	-1.716244	0.0876113	0.1751074	-5.2098
TMC1	0.3884693	0.6943223	1.7161752	0.087624	0.1751232	-5.209916
GLRX3	-0.033963	6.3008486	-1.716086	0.0876404	0.1751464	-5.210066
AC125634.1	-0.128857	-1.491566	-1.71592	0.0876707	0.1751974	-5.210342
ABCC2	-0.158308	6.5242163	-1.715828	0.0876877	0.1752204	-5.210497
PVR	0.041865	6.4585162	1.7158053	0.0876918	0.1752204	-5.210535
PTPN14	0.0936176	5.7103207	1.7152929	0.0877859	0.1753902	-5.211391
INVS	-0.045319	5.6602875	-1.71529	0.0877864	0.1753902	-5.211396
SEC31B	0.0956793	5.1337952	1.7151918	0.0878044	0.1754166	-5.21156
RPAP3	-0.026875	5.9959868	-1.71504	0.0878323	0.1754628	-5.211814
RP11-405M1	0.3218103	2.2689912	1.7150009	0.0878395	0.1754675	-5.211879
ARPC2	0.0234084	6.7313345	1.7144676	0.0879375	0.1756537	-5.21277
C1orf158	-0.432863	-0.591407	-1.714277	0.0879726	0.1757141	-5.213089
TPTE2	-0.322072	-0.665601	-1.714091	0.0880068	0.1757728	-5.213399
RP11-770E5	0.1576539	-1.433516	1.7139138	0.0880394	0.1758283	-5.213695
RP1-193H18	0.2756277	-1.105332	1.7138315	0.0880546	0.175849	-5.213833
IGHV1OR16-	-0.282577	-1.165267	-1.713726	0.088074	0.1758782	-5.214009
NUPL1	0.0376913	6.0733344	1.7136923	0.0880802	0.1758809	-5.214065
RP11-618I1	-0.198504	-1.300167	-1.713644	0.0880891	0.1758892	-5.214146
SLC27A3	-0.072812	6.4372607	-1.713585	0.0880999	0.175901	-5.214244
RP11-260M2	-0.194192	3.4942264	-1.713492	0.088117	0.1759255	-5.214399
GZMA	0.1356884	5.1169021	1.7133626	0.0881409	0.1759636	-5.214616
RPL17P22	-0.379154	0.7669464	-1.713232	0.0881649	0.176002	-5.214833
RP11-7F17.	-0.252337	-1.07972	-1.713117	0.0881862	0.1760349	-5.215026
TRMT61A	-0.044464	6.0620582	-1.712895	0.0882271	0.1761069	-5.215397
RP4-669L17	-0.34202	1.7876585	-1.712833	0.0882386	0.1761101	-5.2155
EIF4A2	-0.033859	6.871174	-1.712819	0.0882411	0.1761101	-5.215523
EEF1A1P4	-0.23059	2.9673409	-1.712808	0.0882432	0.1761101	-5.215542
ELK4	0.039657	6.3809719	1.7127393	0.0882558	0.1761248	-5.215656
SPTB	0.206831	4.2733442	1.7127062	0.0882619	0.1761248	-5.215711
RP11-310P5	-0.32666	-0.694816	-1.712689	0.0882651	0.1761248	-5.21574
RP3-413H6.	0.3851161	-0.620096	1.7124893	0.0883019	0.1761887	-5.216073
STARD10	0.0634357	6.8741679	1.7124076	0.088317	0.1762091	-5.21621
LRGUK	-0.326257	2.8558381	-1.712382	0.0883218	0.1762091	-5.216253
MKS1	-0.0423	5.5773964	-1.712189	0.0883573	0.1762698	-5.216575
MRPS36	0.0454611	6.0301699	1.7121646	0.0883618	0.1762698	-5.216615
RP11-1072C	-0.182208	-1.333974	-1.712087	0.0883762	0.1762876	-5.216745
CTB-5506.8	0.1281204	5.4751666	1.7120583	0.0883815	0.1762876	-5.216793
LINC00421	-0.188123	-1.440598	-1.712038	0.0883852	0.1762876	-5.216827
ERI1	0.0527519	5.6142301	1.7119979	0.0883926	0.1762926	-5.216893
HSPE1P4	-0.396937	0.2340763	-1.711891	0.0884123	0.1763222	-5.217071
SEMA4A	-0.106768	5.1927717	-1.711643	0.0884582	0.1763965	-5.217486
DNER	0.6346871	0.9517866	1.7116375	0.0884592	0.1763965	-5.217495
RPL29P7	-0.22653	-1.16707	-1.711268	0.0885275	0.1765149	-5.218111
CRMP1	-0.136209	5.3498859	-1.71125	0.0885308	0.1765149	-5.218141

TRAPPC10	0.0559877	5.751902	1.7112377	0.0885331	0.1765149	-5.218161
LINC00398	0.4294829	0.2315194	1.7110894	0.0885605	0.17656	-5.218409
PRAF2	0.053955	5.7697549	1.711043	0.0885691	0.1765675	-5.218486
CASP3	-0.043769	6.1234033	-1.711016	0.088574	0.1765677	-5.218531
TMEM9B-AS1	0.1216116	4.2470184	1.7109179	0.0885922	0.1765943	-5.218695
ATG4D	0.056687	5.733772	1.7108907	0.0885973	0.1765947	-5.21874
GS1-304P7.	0.367453	-0.415502	1.7105819	0.0886544	0.1766989	-5.219255
SCAF1	-0.028471	6.5305407	-1.710514	0.088667	0.1767144	-5.219369
LTB4R2	0.1577955	4.1427828	1.7101021	0.0887433	0.1768567	-5.220055
CTB-193M12	-0.364565	0.0890795	-1.709899	0.088781	0.1769222	-5.220394
CENPCP1	0.2840911	-0.765253	1.7098198	0.0887956	0.1769416	-5.220525
SIAH1	0.0363574	5.7120878	1.7097556	0.0888075	0.1769557	-5.220632
THAP1	0.0410951	5.5323183	1.709469	0.0888606	0.1770519	-5.221109
RP11-553L6	-0.283963	-0.960628	-1.709115	0.0889264	0.1771732	-5.2217
RP11-168E1	-0.122261	-1.4948	-1.709015	0.0889449	0.1772005	-5.221866
HMGA1P1	-0.305858	-0.787034	-1.708959	0.0889552	0.1772114	-5.221959
RPL30P7	-0.32433	-0.721193	-1.70893	0.0889606	0.1772123	-5.222007
RP11-499F3	-0.267883	-1.119187	-1.708902	0.0889658	0.1772131	-5.222054
RP11-863P1	0.4539289	0.5412963	1.7088332	0.0889786	0.1772289	-5.222168
RPL19	-0.034449	7.2725876	-1.708807	0.0889835	0.177229	-5.222212
RP11-72304	-0.252648	3.4271073	-1.708629	0.0890165	0.1772851	-5.222508
LHFPL3-AS1	-0.374276	-0.764482	-1.708465	0.0890471	0.1773363	-5.222782
POPDC2	0.1067789	4.3681733	1.7083909	0.0890607	0.1773539	-5.222905
ARHGEF38	-0.314976	3.4957295	-1.708304	0.089077	0.1773681	-5.22305
CCR4	0.3214505	3.6069434	1.7082855	0.0890803	0.1773681	-5.22308
RP11-330L1	-0.221353	-1.147704	-1.708274	0.0890825	0.1773681	-5.2231
S100A16	0.0623584	6.6509384	1.7081971	0.0890968	0.1773869	-5.223228
TNNI3K	0.349743	-0.604807	1.7081693	0.0891019	0.1773874	-5.223274
TAF4B	0.2410773	3.9289241	1.7080326	0.0891273	0.1774242	-5.223501
LIPF	0.1356407	-1.472257	1.7080177	0.0891301	0.1774242	-5.223526
CTD-2173L2	0.3203187	2.6950458	1.7078468	0.0891619	0.1774778	-5.223811
RP11-297C4	-0.222122	-1.221942	-1.707816	0.0891677	0.1774796	-5.223862
RPL12P37	-0.254586	-1.108844	-1.707683	0.0891923	0.1775191	-5.224083
SLC20A1	0.0416951	6.3342562	1.7075028	0.0892259	0.1775761	-5.224383
MGAT4A	-0.043021	6.1526678	-1.70737	0.0892506	0.1776131	-5.224604
VWA3A	-0.384501	0.4224866	-1.707351	0.0892542	0.1776131	-5.224636
AC066614.1	0.3107838	-1.234782	1.707102	0.0893005	0.1776955	-5.22505
RP11-340I6	-0.207751	-1.233828	-1.706859	0.0893457	0.1777758	-5.225454
LSS	-0.060789	6.5851048	-1.706803	0.0893562	0.1777869	-5.225547
RP11-224P1	0.0978694	-1.537034	1.7066763	0.0893798	0.1778242	-5.225758
ASS1P5	0.3419029	-0.617492	1.7064914	0.0894142	0.1778831	-5.226066
RP11-247A1	0.41228	-0.115545	1.706445	0.0894229	0.1778906	-5.226143
CYCSP34	-0.391703	1.9447414	-1.706106	0.089486	0.1780065	-5.226706
RP1-60019.	0.3482531	-0.707169	1.7060077	0.0895044	0.1780334	-5.22687
GEMIN8	0.0401562	5.7106095	1.7058336	0.0895369	0.1780883	-5.22716
RP4-717I23	-0.367829	0.5907375	-1.705751	0.0895524	0.1781093	-5.227297
AC009227.2	-0.266403	-1.201386	-1.705704	0.0895611	0.178117	-5.227375
KRT16P1	0.3441502	-1.075133	1.7056611	0.0895691	0.1781232	-5.227446
CEP120	0.0417638	5.7695254	1.7056208	0.0895766	0.1781285	-5.227513
SYCE3	-0.305176	3.2119724	-1.705352	0.0896268	0.1782186	-5.22796
HLA-DOB	0.1975111	4.4367921	1.7052962	0.0896372	0.1782296	-5.228053

UPK2	0.4508059	0.496941	1.7052188	0.0896517	0.1782486	-5.228181
CTD-2196E1	-0.350654	-0.248086	-1.705031	0.0896868	0.1783088	-5.228494
PC	0.089975	6.7394842	1.7049501	0.0897019	0.1783212	-5.228628
SLC4A1AP	-0.02291	6.0727401	-1.704935	0.0897048	0.1783212	-5.228654
RP11-35609	0.3492734	-0.519305	1.7049188	0.0897077	0.1783212	-5.22868
SHMT1P1	0.2678056	-1.019025	1.704862	0.0897184	0.1783326	-5.228774
P3H2-AS1	0.2584138	-1.18917	1.704661	0.0897559	0.1783976	-5.229108
LINC00469	-0.142445	-1.462998	-1.704521	0.0897821	0.1784399	-5.229341
CA15P1	0.3753733	-0.327783	1.7041974	0.0898426	0.1785505	-5.229878
RP11-89C3.	0.2673792	-1.034019	1.704092	0.0898624	0.17858	-5.230054
HOXA1	0.3251484	2.50359	1.7040314	0.0898737	0.1785928	-5.230154
DOCK3	-0.236535	3.335504	-1.70383	0.0899114	0.1786579	-5.230488
EMC2	0.0400535	6.2747487	1.7031683	0.0900354	0.1788946	-5.231587
RP11-326I1	-0.415383	0.8638804	-1.703079	0.090052	0.1789179	-5.231735
RP11-302F1	0.2949282	-1.11239	1.7030277	0.0900617	0.1789275	-5.231821
ST13P18	-0.378587	0.8845117	-1.702991	0.0900687	0.1789316	-5.231882
KRT78	0.2647107	-1.103344	1.7029105	0.0900837	0.1789516	-5.232015
CYP4F25P	-0.266841	-1.023103	-1.702687	0.0901256	0.1790251	-5.232386
MIR93	-0.315538	-0.586165	-1.702449	0.0901704	0.1791043	-5.232782
CICP13	-0.175662	-1.390319	-1.702164	0.0902239	0.1792008	-5.233255
DHRS7	-0.056607	6.6412916	-1.702044	0.0902462	0.1792336	-5.233452
PAX2	0.5267873	0.2709216	1.7020233	0.0902502	0.1792336	-5.233487
ARL4C	0.0733699	6.061267	1.7019741	0.0902594	0.1792422	-5.233569
OIP5-AS1	0.0372363	6.5498946	1.7018708	0.0902789	0.179271	-5.23374
AGMAT	-0.122537	6.3354121	-1.70159	0.0903317	0.1793662	-5.234207
RNU6-130P	0.1629434	-1.394542	1.7014477	0.0903584	0.1794094	-5.234442
MRPS11	0.0371201	6.0921024	1.7012844	0.0903891	0.1794606	-5.234713
CYP2F1	-0.310094	-0.868237	-1.700917	0.0904582	0.1795798	-5.235322
AC008155.1	-0.383315	0.2290426	-1.700913	0.0904589	0.1795798	-5.235329
SIGLEC15	-0.270846	4.1802013	-1.700828	0.0904748	0.1796016	-5.235469
GPR1	0.4711166	1.2731149	1.700782	0.0904836	0.1796092	-5.235546
NRAS	-0.034198	6.3980902	-1.70063	0.0905122	0.1796561	-5.235797
ASS1P11	0.4705753	0.5205938	1.7004735	0.0905416	0.1797049	-5.236057
RP11-596D2	-0.369511	0.1972006	-1.700278	0.0905785	0.1797682	-5.236381
RP11-810P1	0.1034964	-1.534167	1.7001969	0.0905937	0.1797887	-5.236516
LCE2D	-0.240825	-1.230017	-1.699927	0.0906446	0.1798799	-5.236963
SLC25A15	0.1223583	5.9795017	1.6998511	0.0906589	0.1798985	-5.237088
RP11-134D3	-0.189031	-1.347953	-1.699665	0.0906939	0.1799582	-5.237396
SAMHD1	0.0486173	6.4465022	1.6995219	0.090721	0.1800021	-5.237634
RP11-29B2.	-0.379818	1.055206	-1.699423	0.0907396	0.1800292	-5.237797
IGKV1OR10-	-0.269478	-1.148973	-1.699188	0.0907841	0.1801077	-5.238188
RP3-461F17	-0.256139	2.3579651	-1.69904	0.090812	0.1801532	-5.238433
EEF1DP1	-0.167859	3.5375903	-1.699011	0.0908173	0.180154	-5.238479
RP11-6B6.3	-0.344767	0.4663673	-1.698815	0.0908544	0.1802116	-5.238805
GSTM5P1	0.1075729	-1.532089	1.6988053	0.0908562	0.1802116	-5.238821
CTC-336P14	-0.339568	1.8890278	-1.698743	0.090868	0.1802252	-5.238924
CXCL11	0.2334158	4.4630318	1.698588	0.0908972	0.1802647	-5.239181
CTD-2525I3	0.2584449	-0.973508	1.6985852	0.0908978	0.1802647	-5.239185
RNVU1-3	-0.403249	0.2735338	-1.698489	0.090916	0.1802911	-5.239345
RP1-37C10.	0.1420991	-1.441443	1.6983763	0.0909372	0.1803234	-5.239531
SHC3	0.2174445	3.9220825	1.698348	0.0909426	0.1803242	-5.239578

STK24	-0.047315	6.2305103	-1.698311	0.0909495	0.1803281	-5.239639
RP11-59K5.	-0.329321	-0.501191	-1.698245	0.090962	0.1803431	-5.239748
LINC00326	0.0935136	-1.525739	1.6982172	0.0909673	0.1803438	-5.239794
ESPN	-0.138482	6.3675746	-1.698042	0.0910005	0.1803998	-5.240085
RP11-157I4	-0.135431	-1.466437	-1.698007	0.091007	0.1804029	-5.240142
CTA-243E7.	-0.394471	0.8229075	-1.697867	0.0910336	0.1804458	-5.240375
LRRC66	-0.418895	2.3820787	-1.697809	0.0910445	0.1804577	-5.24047
RPL7P24	-0.30069	-0.618538	-1.697558	0.0910921	0.1805421	-5.240886
SERPINB12	0.0969693	-1.537493	1.6973768	0.0911263	0.1805973	-5.241185
RP11-707P1	-0.314291	2.6623629	-1.697358	0.0911298	0.1805973	-5.241216
RP11-342A2	0.4378682	1.348957	1.6972769	0.0911452	0.1806081	-5.24135
SAP25	-0.380676	1.4676967	-1.697257	0.0911489	0.1806081	-5.241383
SENP6	-0.029569	6.2902723	-1.697251	0.0911501	0.1806081	-5.241393
FTCD-AS1	-0.443242	1.1820171	-1.697192	0.0911612	0.1806181	-5.241491
CETP	0.1313567	5.1368022	1.697172	0.091165	0.1806181	-5.241524
RP11-155G1	-0.423309	0.45967	-1.697045	0.0911892	0.1806561	-5.241735
SULT1E1	-0.437033	4.3321113	-1.696876	0.0912211	0.1807031	-5.242014
SYCE1	0.5799049	0.2447074	1.696867	0.0912228	0.1807031	-5.242028
TCP1	-0.035239	6.733329	-1.696803	0.0912349	0.1807173	-5.242134
RP11-102M1	-0.362411	0.2830276	-1.696669	0.0912603	0.1807578	-5.242356
UCA1	0.6084456	2.2631868	1.6965044	0.0912915	0.1808098	-5.242628
RPL21P1	-0.291769	-0.811416	-1.69625	0.0913397	0.1808955	-5.243049
PGPEP1	-0.049984	6.4038368	-1.696146	0.0913595	0.1809249	-5.243221
RP11-666E1	0.1915228	-1.397162	1.6960616	0.0913755	0.1809466	-5.24336
OR13J1	0.2637457	-0.992366	1.6960335	0.0913808	0.1809474	-5.243407
XRCC6	-0.027818	6.8877449	-1.695966	0.0913935	0.1809628	-5.243518
RP11-264B1	-0.245752	-1.070191	-1.695852	0.0914153	0.180996	-5.243707
IGHV3-74	0.4594373	3.7252635	1.695647	0.0914541	0.1810463	-5.244046
AP000318.2	0.3834055	-0.188417	1.6956344	0.0914566	0.1810463	-5.244066
RP11-47909	-0.394777	0.2216357	-1.695631	0.0914572	0.1810463	-5.244073
PPP3R2	-0.297087	-1.066922	-1.695614	0.0914605	0.1810463	-5.244101
RP3-462C17	0.3619127	-0.753991	1.6955414	0.0914742	0.1810574	-5.24422
MIR6745	-0.199323	-1.233381	-1.695532	0.091476	0.1810574	-5.244236
CYP2C60P	0.0759645	-1.548197	1.6952817	0.0915235	0.1811415	-5.244649
ITGA1	0.0471038	6.6396015	1.6951512	0.0915483	0.1811808	-5.244865
RP11-680G2	0.3999897	0.7857051	1.6951055	0.091557	0.1811881	-5.24494
MYLK3	-0.281958	2.8036691	-1.694676	0.0916387	0.1813297	-5.245651
ZMYM4	0.0310805	6.1531216	1.6946673	0.0916403	0.1813297	-5.245665
SATB2-AS1	-0.396173	2.3019454	-1.694651	0.0916434	0.1813297	-5.245692
SPOPL	-0.041427	6.0230448	-1.69457	0.0916588	0.1813504	-5.245826
GS1-257G1.	-0.340829	2.0290528	-1.694467	0.0916784	0.1813792	-5.245995
MMAB	-0.059398	6.4057714	-1.694248	0.0917201	0.1814518	-5.246357
RP11-568J2	-0.238348	-1.13479	-1.69422	0.0917255	0.1814527	-5.246404
AMBP	-0.121764	7.5982397	-1.694131	0.0917424	0.1814763	-5.246551
CUBNP3	-0.201743	-1.375156	-1.694018	0.0917639	0.181509	-5.246737
ATG3	0.0280378	6.2552412	1.6939578	0.0917753	0.1815217	-5.246836
UBR3	0.0410242	6.2678274	1.6939305	0.0917805	0.1815221	-5.246881
RP11-407G2	0.331045	-0.353569	1.6936564	0.0918327	0.1816155	-5.247334
PTCD2	0.0458917	5.565119	1.6936203	0.0918396	0.1816193	-5.247394
AC091654.7	-0.361245	1.3431929	-1.693458	0.0918704	0.1816704	-5.247661
LINC00559	-0.252225	-1.161312	-1.693286	0.0919033	0.181718	-5.247946

SUV420H1	-0.035807	6.2443717	-1.693255	0.0919092	0.181718	-5.247997
KCNK5	0.1631255	5.4564428	1.6932539	0.0919094	0.181718	-5.247998
RPS3A	-0.037986	6.8326501	-1.693189	0.0919217	0.1817325	-5.248105
RAPH1	0.1199146	5.2028258	1.6926957	0.0920158	0.1819087	-5.24892
ZMIZ2	0.0307232	6.4599511	1.6925675	0.0920403	0.1819449	-5.249131
RP11-383C5	-0.364561	-0.070258	-1.692548	0.092044	0.1819449	-5.249164
DHX40	0.0370556	5.9849028	1.692378	0.0920764	0.181999	-5.249444
AC005387.2	-0.382678	0.8698346	-1.692327	0.0920861	0.1820082	-5.249527
AL158069.1	0.1979597	-1.344684	1.6921762	0.092115	0.1820555	-5.249777
AC011899.1	0.0989169	-1.5365	1.6920177	0.0921452	0.1821054	-5.250038
APELA	-0.463316	-0.482418	-1.691474	0.092249	0.1823007	-5.250934
HYPK	-0.071377	4.5518108	-1.691342	0.0922743	0.1823409	-5.251152
COL18A1-AS	-0.31656	-0.832359	-1.691029	0.0923341	0.1824474	-5.251668
NUTF2P6	-0.24473	-1.062526	-1.691008	0.0923383	0.1824474	-5.251703
CDK16	-0.036044	6.4135269	-1.69096	0.0923475	0.1824557	-5.251783
SOX5	-0.157594	5.4077647	-1.690927	0.0923537	0.1824581	-5.251836
RP1-240B8.	-0.11257	-1.499553	-1.690877	0.0923633	0.1824673	-5.251919
RP11-297J2	0.2917228	-0.951035	1.6908084	0.0923764	0.182479	-5.252032
CTD-2278I1	-0.369114	0.9907948	-1.690794	0.0923792	0.182479	-5.252056
ERCC3	-0.02255	6.1932957	-1.690726	0.0923921	0.1824946	-5.252167
RTN4RL2	-0.105356	5.9259522	-1.690636	0.0924095	0.182519	-5.252317
THRB-IT1	-0.361913	0.0557698	-1.690485	0.0924383	0.182566	-5.252565
MANBAL	-0.034009	6.3091963	-1.690287	0.0924762	0.1826221	-5.252891
RP5-884M6.	-0.413366	-0.641305	-1.690285	0.0924767	0.1826221	-5.252895
RP11-118H4	-0.287447	-0.809376	-1.690205	0.0924919	0.1826422	-5.253026
METTL8	-0.039355	5.635157	-1.689964	0.0925381	0.1827236	-5.253423
RNU6-1161F	0.3670756	-0.730161	1.6898679	0.0925566	0.1827501	-5.253582
NUDT15	-0.043302	5.8576868	-1.689074	0.0927089	0.1830344	-5.25489
HMGB1P39	0.1341637	-1.440728	1.6890651	0.0927105	0.1830344	-5.254904
IGKV2D-30	-0.340794	-1.018598	-1.688626	0.0927949	0.1831911	-5.255627
LOH12CR2	-0.117179	4.019146	-1.68858	0.0928038	0.1831986	-5.255703
CHL1	0.5842781	2.3478951	1.6885295	0.0928134	0.1832077	-5.255786
HARS	0.0320786	6.3592177	1.6884692	0.092825	0.1832207	-5.255885
C16orf70	0.0584473	6.1419603	1.6883599	0.092846	0.1832522	-5.256065
AC008592.5	0.4046131	0.1804598	1.6883087	0.0928558	0.1832617	-5.256149
RP11-151A6	-0.384107	1.7286755	-1.688261	0.092865	0.1832684	-5.256228
TBCAP3	-0.222363	-1.191185	-1.688239	0.0928692	0.1832684	-5.256264
GEN1	-0.045218	5.7023945	-1.688186	0.0928794	0.1832786	-5.256352
MCIDAS	-0.522978	0.3118333	-1.688028	0.0929097	0.1833285	-5.256611
RNU6-844P	-0.260276	-1.005353	-1.687989	0.0929173	0.1833334	-5.256675
CD37	0.0859417	5.6408747	1.687871	0.09294	0.1833683	-5.25687
ARHGEF7-AS	0.380116	-0.542175	1.6877862	0.0929563	0.1833906	-5.257009
PPP1R8	0.0254258	6.1130306	1.6875543	0.0930009	0.18346	-5.257391
4-Mar	0.3742504	2.2087052	1.6875512	0.0930015	0.18346	-5.257396
TCEA3	-0.083183	6.6075226	-1.687514	0.0930087	0.1834642	-5.257457
CCSER1	0.3984492	1.8860198	1.6872646	0.0930566	0.1835489	-5.257867
WTAPP1	-0.545806	0.6765797	-1.686813	0.0931437	0.1837107	-5.25861
LGALS3BP	0.0723986	7.0446024	1.6866575	0.0931736	0.1837597	-5.258866
CCL3	0.1404483	5.0107607	1.6866221	0.0931804	0.1837633	-5.258924
DIO3OS	0.4744475	3.6896738	1.6864605	0.0932116	0.1838088	-5.259189
WDR5B	-0.040217	5.5118821	-1.68645	0.0932136	0.1838088	-5.259207

DMRT2	-0.434153	-0.629994	-1.686424	0.0932186	0.1838088	-5.25925
RP11-640A1	0.1104211	-1.503115	1.6863647	0.09323	0.1838214	-5.259347
C7orf26	-0.030716	6.047152	-1.686318	0.0932391	0.1838292	-5.259424
RP11-81A1.	-0.419501	-0.473484	-1.686235	0.0932551	0.1838509	-5.259561
KB-68A7.1	0.4128547	3.7162667	1.6861737	0.0932669	0.1838607	-5.259661
RP5-1184F4	0.3297514	-0.317189	1.6861568	0.0932701	0.1838607	-5.259689
RP11-435P2	-0.329559	-0.529622	-1.686055	0.0932897	0.1838791	-5.259856
RP11-528A4	-0.482803	1.73506	-1.686031	0.0932944	0.1838791	-5.259895
FCHO1	0.1588942	4.8009565	1.6860302	0.0932945	0.1838791	-5.259897
RP11-35609	0.3539824	-0.430945	1.6860021	0.0933	0.1838798	-5.259943
HCRT	-0.280897	-0.966495	-1.685905	0.0933186	0.1839067	-5.260102
BMS1P16	-0.261809	-1.052055	-1.68585	0.0933293	0.1839178	-5.260193
DKFZp434JC	-0.479584	-0.022712	-1.685807	0.0933377	0.1839243	-5.260264
LINC01243	-0.139101	-1.464637	-1.685692	0.0933598	0.183958	-5.260453
EEF2	0.0323823	7.4450361	1.6855556	0.0933861	0.184	-5.260677
MICA	0.0973397	5.7818642	1.6854877	0.0933992	0.1840159	-5.260789
AC104395.2	0.1346021	-1.445264	1.6854208	0.0934122	0.1840314	-5.260898
RP11-553P9	-0.246618	-1.224827	-1.685288	0.0934379	0.1840722	-5.261117
MIF-AS1	0.3271361	3.5668892	1.6852095	0.093453	0.1840919	-5.261246
CYP27B1	-0.202869	4.0839049	-1.685044	0.0934849	0.184145	-5.261517
USP41	-0.344079	-0.690885	-1.684728	0.0935461	0.1842554	-5.262037
HOXB-AS3	0.4299505	1.348761	1.6843256	0.0936239	0.1843987	-5.262697
RP13-492C1	-0.260726	-1.141282	-1.684277	0.0936332	0.1844071	-5.262777
TINCR	-0.41389	1.5105495	-1.68409	0.0936695	0.184455	-5.263085
RNU6-516P	-0.311407	2.1904923	-1.684089	0.0936696	0.184455	-5.263086
LILRA4	0.3936504	2.5485371	1.6840502	0.0936771	0.184455	-5.26315
RP11-146E1	-0.14196	-1.463235	-1.684047	0.0936777	0.184455	-5.263154
RP11-1003J	0.0989169	-1.5365	1.6840178	0.0936834	0.1844563	-5.263203
ASPA	0.1828183	4.6318636	1.6838076	0.0937241	0.1845264	-5.263548
AC007238.1	-0.16415	3.3223521	-1.683774	0.0937307	0.1845294	-5.263604
POTEI	-0.248233	-1.043224	-1.68367	0.0937508	0.1845591	-5.263774
RP11-740C1	-0.366242	3.2970041	-1.683365	0.0938098	0.1846653	-5.264274
CT47B1	-0.312715	-1.051924	-1.683205	0.0938409	0.1847165	-5.264537
SMARCE1P5	-0.355371	0.4756247	-1.683147	0.0938521	0.1847198	-5.264632
TRIM34	0.2714772	2.639568	1.6831439	0.0938527	0.1847198	-5.264637
EPN2	-0.044164	5.8218374	-1.682949	0.0938904	0.1847841	-5.264957
RP11-85L21	0.1449751	-1.439977	1.6827474	0.0939296	0.1848415	-5.265288
B3GALTL	0.0468901	5.5291211	1.6827468	0.0939297	0.1848415	-5.265289
RNU6-505P	-0.124234	-1.493833	-1.682623	0.0939538	0.1848789	-5.265492
HS3ST3B1	-0.136716	5.9512216	-1.682576	0.0939628	0.1848868	-5.265569
PSG9	0.3798337	-0.802279	1.6824754	0.0939824	0.1849152	-5.265734
ADCYAP1R1	-0.414042	2.5255811	-1.682093	0.0940565	0.1850512	-5.266361
RNU6-2	-0.331432	-0.454505	-1.681941	0.0940862	0.1850996	-5.266611
DUSP16	0.0584349	6.4787535	1.6818433	0.0941051	0.1851268	-5.266771
LINC00477	-0.1378	-1.48718	-1.681696	0.0941337	0.1851732	-5.267013
PHF1	-0.035199	6.3001929	-1.681658	0.0941411	0.1851776	-5.267074
ZNF786	-0.046031	5.3991631	-1.681441	0.0941832	0.1852505	-5.26743
REC8	-0.093529	5.1340689	-1.681277	0.0942152	0.1853034	-5.267699
RP11-323P1	0.3509494	-0.502994	1.6811345	0.0942429	0.1853479	-5.267933
C3orf62	-0.051611	5.5352587	-1.681102	0.0942492	0.1853504	-5.267986
GRM5	0.393249	-0.672872	1.6809324	0.0942822	0.1854052	-5.268264

DUX4L18	0.2105788	-1.396944	1.6807717	0.0943135	0.1854567	-5.268527
RP11-702H2	-0.38821	0.0742264	-1.680669	0.0943334	0.1854859	-5.268695
RP11-210K2	-0.266171	-0.888525	-1.68038	0.0943897	0.1855866	-5.269169
TARS2	0.0468647	6.3454387	1.6798514	0.0944927	0.185765	-5.270035
CTD-2651B2	-0.261506	-0.957995	-1.679845	0.094494	0.185765	-5.270046
AE000658.2	-0.135184	-1.488463	-1.679836	0.0944957	0.185765	-5.27006
HTN3	-0.261081	-1.224809	-1.67974	0.0945144	0.1857919	-5.270218
TSPAN3	0.0355019	6.5546235	1.6796907	0.094524	0.1858007	-5.270298
PIK3R6	-0.109717	4.6007742	-1.679526	0.0945561	0.1858538	-5.270568
MIR205HG	0.4155295	-0.525812	1.6793411	0.0945922	0.1859147	-5.270871
AAGAB	0.0281949	6.1733673	1.6792284	0.0946142	0.1859479	-5.271055
RP11-10022	-0.256743	-1.315549	-1.679199	0.09462	0.1859492	-5.271104
MIPEPP3	-0.226047	3.0841723	-1.679142	0.094631	0.1859609	-5.271196
AC104306.1	-0.211766	-1.240025	-1.678991	0.0946606	0.1860091	-5.271445
ZBTB18	0.0535901	6.231145	1.6787897	0.0946998	0.1860761	-5.271774
LINC00652	-0.411997	0.7057969	-1.678694	0.0947185	0.1861029	-5.27193
RP11-91A18	0.1753512	-1.341928	1.6786609	0.094725	0.1861055	-5.271984
TGDS	0.0619398	5.760309	1.6785888	0.0947391	0.1861222	-5.272102
CHAT	0.1392662	-1.48486	1.6785533	0.094746	0.1861222	-5.27216
RP11-345J4	0.08793	5.0258747	1.6785288	0.0947508	0.1861222	-5.272201
NANOGP4	-0.241972	-1.127512	-1.678513	0.0947539	0.1861222	-5.272226
KIAA1549	0.2111221	4.9619159	1.6784114	0.0947737	0.1861512	-5.272393
DYNC2LI1	-0.040033	5.6792354	-1.678308	0.0947938	0.1861751	-5.272561
RP11-563N1	-0.15457	-1.457052	-1.678297	0.0947961	0.1861751	-5.27258
HSPA1B	0.0915896	6.2992827	1.6780532	0.0948437	0.1862586	-5.272979
RP6-114E22	-0.359025	-0.889294	-1.677648	0.094923	0.1864043	-5.273642
C12orf74	-0.42848	0.8845363	-1.677594	0.0949335	0.1864148	-5.27373
IGHV3-71	-0.446198	-0.236825	-1.677549	0.0949423	0.1864193	-5.273804
MESTP1	-0.283623	-0.874118	-1.677531	0.0949459	0.1864193	-5.273834
CTD-2537I9	-0.322557	2.2734136	-1.677417	0.0949683	0.186453	-5.27402
RP11-533K9	-0.169525	-1.364471	-1.67738	0.0949753	0.1864569	-5.274079
C11orf42	-0.393977	0.5000845	-1.677268	0.0949973	0.1864901	-5.274263
AC091849.1	-0.413649	0.9279825	-1.6769	0.0950693	0.1866136	-5.274865
RP11-103H7	-0.148377	-1.438184	-1.676894	0.0950705	0.1866136	-5.274874
ZNF57	-0.127005	5.0260593	-1.676625	0.0951233	0.1867073	-5.275315
GRM1	-0.435038	0.1040724	-1.676582	0.0951318	0.1867138	-5.275386
PIK3AP1	0.0671485	6.5806549	1.6764613	0.0951554	0.1867501	-5.275582
RP5-933K21	0.3214261	1.8941685	1.6763163	0.0951838	0.1867958	-5.275819
SPATA41	0.2398322	4.4642636	1.6762147	0.0952037	0.1868248	-5.275985
RP13-884E1	-0.336284	-0.736108	-1.67608	0.09523	0.1868583	-5.276205
SNORA51	-0.431009	-0.100081	-1.676076	0.095231	0.1868583	-5.276213
LL22NC03-2	-0.376174	-0.649583	-1.676018	0.0952422	0.1868703	-5.276306
RP11-159H3	-0.377163	0.2515682	-1.675899	0.0952656	0.1869062	-5.276501
RPL18AP8	-0.144048	-1.418402	-1.67581	0.095283	0.1869303	-5.276646
AC114776.1	-0.26345	-0.953899	-1.675713	0.0953022	0.1869578	-5.276806
SELO	0.0569143	6.4041328	1.6756483	0.0953148	0.1869725	-5.276911
RP1-41C23.	-0.329176	1.6945421	-1.675554	0.0953334	0.186999	-5.277066
MRPS21	-0.045172	6.4946034	-1.675376	0.0953682	0.1870572	-5.277355
INSC	0.4166478	2.6260945	1.6753023	0.0953827	0.1870756	-5.277476
RP11-1023L	-0.344856	-0.411462	-1.675262	0.0953906	0.187081	-5.277541
MIR4768	0.2278027	-1.236453	1.6749335	0.0954552	0.1871976	-5.278079

FN1	0.0444046	7.6547283	1.6748854	0.0954647	0.1872061	-5.278157
RP11-635N1	0.1318681	4.5506706	1.6747299	0.0954952	0.187256	-5.278411
BLOC1S6	0.0301249	6.2602571	1.6743097	0.0955778	0.1873991	-5.279097
ZNF75A	-0.038889	5.7651857	-1.674306	0.0955785	0.1873991	-5.279102
C9orf85	0.0392239	5.4820252	1.6742472	0.0955901	0.1874119	-5.279199
GEMIN6	-0.03871	5.8534925	-1.674165	0.0956063	0.1874335	-5.279333
M6PR	0.0427326	6.2192183	1.673987	0.0956414	0.1874922	-5.279624
C15orf53	-0.325228	-0.531552	-1.673841	0.0956701	0.1875384	-5.279862
GAFA3	-0.384443	1.6459481	-1.673679	0.095702	0.1875835	-5.280127
MAGEE1	-0.169557	4.4718299	-1.673655	0.0957068	0.1875835	-5.280166
TNK2-AS1	0.4187192	1.4673826	1.673646	0.0957085	0.1875835	-5.280181
FGFBP1	0.4263787	-0.523527	1.6735667	0.0957241	0.1875895	-5.28031
DNAJC11	0.0471845	6.0102361	1.6735448	0.0957284	0.1875895	-5.280346
LINC00676	0.2949721	-1.068271	1.673517	0.0957339	0.1875895	-5.280391
RXRA	0.0530882	6.8107174	1.6735125	0.0957348	0.1875895	-5.280398
TMEM179	-0.614768	1.0959473	-1.6735	0.0957372	0.1875895	-5.280419
RP11-65709	0.3807891	-0.818237	1.6733119	0.0957743	0.1876521	-5.280726
BFAR	0.0248635	6.3108392	1.6730879	0.0958185	0.1877285	-5.281091
FTO-IT1	-0.335335	1.7819566	-1.673035	0.0958289	0.1877333	-5.281178
RP4-654C18	-0.236897	-1.103539	-1.673023	0.0958312	0.1877333	-5.281197
SEMA4C	-0.037963	6.2153111	-1.672927	0.0958502	0.1877604	-5.281354
GNRH2	-0.510776	0.7487799	-1.672875	0.0958605	0.1877705	-5.281439
AC090051.1	0.1852504	-1.379689	1.6727614	0.0958828	0.1878042	-5.281624
LTB4R	0.0806954	5.3530017	1.6726316	0.0959084	0.1878442	-5.281836
RP11-292E2	-0.234884	-1.153242	-1.672445	0.0959452	0.1879062	-5.28214
TBC1D24	0.0550541	6.1259808	1.6723418	0.0959656	0.1879361	-5.282308
EDDM3A	-0.230309	-1.355812	-1.67213	0.0960074	0.1880079	-5.282654
IDI2-AS1	-0.385587	1.0755086	-1.671932	0.0960465	0.1880743	-5.282976
RP11-322J2	-0.139201	-1.486494	-1.671858	0.0960612	0.188093	-5.283098
HIST1H2BC	0.1596984	5.039774	1.6714883	0.0961342	0.1882235	-5.2837
CHCHD2P7	0.3091877	-0.931316	1.6714681	0.0961382	0.1882235	-5.283733
KCNH3	-0.334786	3.4391945	-1.671297	0.096172	0.1882618	-5.284011
IRAK1BP1	-0.111937	4.6770955	-1.671296	0.0961722	0.1882618	-5.284013
STAU2-AS1	-0.337115	1.9409922	-1.671291	0.0961732	0.1882618	-5.284021
PSMC6	0.0292854	6.4094448	1.6711199	0.096207	0.1883179	-5.2843
RP11-1006G	-0.305001	-0.626169	-1.670989	0.096233	0.1883586	-5.284514
PDZD9	0.3453214	0.0095865	1.6707135	0.0962874	0.1884551	-5.284962
RASSF7	-0.057504	6.3789432	-1.67047	0.0963356	0.1885394	-5.285359
RP11-729I1	0.3547021	-0.473473	1.6702192	0.0963853	0.1886264	-5.285768
C2orf71	0.2673599	-1.232046	1.6701428	0.0964004	0.1886403	-5.285892
Z95114.4	0.1015899	-1.512374	1.6701311	0.0964027	0.1886403	-5.285911
PBRM1	-0.034297	6.1816097	-1.670043	0.0964202	0.1886644	-5.286055
RP11-196G1	0.3492691	2.2502614	1.6699116	0.0964462	0.1886949	-5.286268
PCDHA4	-0.509235	1.0507257	-1.669896	0.0964494	0.1886949	-5.286294
CGREF1	-0.126662	5.8273247	-1.669886	0.0964513	0.1886949	-5.28631
RP11-535C2	-0.18589	-1.283779	-1.669856	0.0964572	0.1886963	-5.286359
RP11-663P9	-0.282568	-0.793339	-1.669784	0.0964716	0.1887073	-5.286477
RP11-669E1	-0.395624	0.9059124	-1.669773	0.0964738	0.1887073	-5.286495
LATS1	-0.039597	5.9038924	-1.66975	0.0964783	0.1887073	-5.286532
RP3-354N19	-0.348602	-0.231418	-1.669524	0.0965231	0.1887848	-5.2869
AC138035.2	-0.36497	1.8891004	-1.669457	0.0965364	0.188792	-5.287009

UBTD2	-0.029782	5.996483	-1.669453	0.0965371	0.188792	-5.287015
PCGEM1	-0.163827	-1.439507	-1.669273	0.0965727	0.1888515	-5.287308
RP11-264J4	0.1396944	-1.465432	1.6692304	0.0965813	0.1888581	-5.287377
SNORD3A	-0.335716	-0.63204	-1.669049	0.0966172	0.1889183	-5.287672
SEMA3G	0.1034341	5.6675244	1.6687051	0.0966855	0.1890417	-5.288232
DCDC2	-0.340337	5.626226	-1.668579	0.0967106	0.1890787	-5.288438
PPM1A	0.040251	6.4143446	1.6685431	0.0967177	0.1890787	-5.288496
RP11-2C24.	0.1716657	3.1030113	1.6685316	0.09672	0.1890787	-5.288515
RPS26P31	-0.363858	1.1918927	-1.668283	0.0967694	0.1891651	-5.288919
PDGFRB	0.0590763	6.5337534	1.668211	0.0967837	0.1891829	-5.289036
RP11-235D1	-0.275142	-0.881439	-1.667986	0.0968284	0.1892601	-5.289402
RBCK1	-0.037265	6.6817389	-1.667889	0.0968476	0.1892858	-5.28956
SUN1	-0.029289	6.50257	-1.667859	0.0968536	0.1892858	-5.289608
PAN2	-0.043221	6.2115219	-1.667842	0.096857	0.1892858	-5.289637
AC010492.2	-0.417738	0.4285146	-1.667753	0.0968748	0.1893103	-5.289782
RP11-418J1	0.4926276	1.9969191	1.6676076	0.0969036	0.1893528	-5.290018
SMIM18	0.2313691	-1.117145	1.6675828	0.0969086	0.1893528	-5.290058
IGHD	0.4675081	3.5507038	1.6675626	0.0969126	0.1893528	-5.290091
CELF4	-0.355931	2.3569713	-1.667539	0.0969172	0.1893528	-5.290129
RP11-676B1	-0.298844	-1.121039	-1.667425	0.09694	0.1893871	-5.290315
RP11-430H1	-0.450884	0.1027177	-1.667337	0.0969575	0.1894112	-5.290458
RGS17	-0.247561	3.3314531	-1.66728	0.0969688	0.189423	-5.29055
RP11-304L1	-0.141731	-1.441443	-1.667239	0.096977	0.1894289	-5.290617
CTD-2086L1	-0.319741	-0.55481	-1.667167	0.0969913	0.1894395	-5.290734
NANOS3	0.4001315	0.8475095	1.6671597	0.0969928	0.1894395	-5.290746
CTD-2007H1	0.1671197	-1.429019	1.6671308	0.0969985	0.1894406	-5.290793
MTMR14	-0.030476	6.3221674	-1.667104	0.0970038	0.1894407	-5.290836
CHKB-CPT1E	-0.235512	3.7471786	-1.666861	0.0970523	0.1895253	-5.291232
PPP1R37	-0.041735	6.2235122	-1.666825	0.0970595	0.1895261	-5.291291
SSPO	-0.179732	4.6180956	-1.666796	0.0970651	0.1895261	-5.291337
RASSF1-AS1	0.2667116	2.7429045	1.6667805	0.0970683	0.1895261	-5.291363
OTOGL	-0.437576	1.1237653	-1.666639	0.0970965	0.1895711	-5.291593
RP11-171I2	-0.26566	-0.983587	-1.666416	0.0971408	0.1896475	-5.291955
GPR50	-0.304444	-1.14055	-1.666347	0.0971546	0.1896643	-5.292067
RP11-416N2	-0.412439	0.9462237	-1.666029	0.097218	0.1897779	-5.292584
CTD-2095E4	0.243158	3.164652	1.6659598	0.0972319	0.1897948	-5.292696
CTD-2533K2	-0.161065	-1.449287	-1.665876	0.0972486	0.1898172	-5.292832
AC018462.2	-0.361275	0.8915868	-1.665779	0.0972679	0.1898448	-5.29299
ESPNL	-0.228392	4.2710909	-1.665721	0.0972795	0.1898574	-5.293085
TIMM10B	-0.02946	6.0885335	-1.665646	0.0972944	0.1898762	-5.293206
RP11-1090M	0.1992175	-1.284237	1.6654105	0.0973415	0.1899579	-5.293589
RP5-858B6.	0.4700226	0.3913552	1.6648024	0.0974629	0.1901848	-5.294576
AGFG1	0.0277104	6.3815529	1.6647253	0.0974783	0.1901958	-5.294701
TPT1	0.0398595	7.5082741	1.664722	0.097479	0.1901958	-5.294707
AC005042.2	0.3503276	-0.549848	1.664696	0.0974842	0.1901958	-5.294749
ARHGAP26-A	0.1437935	-1.437025	1.664579	0.0975076	0.1902312	-5.294939
RNF20	-0.029418	6.2252764	-1.664284	0.0975666	0.1903363	-5.295418
PPP1R2P9	-0.14633	-1.461093	-1.663865	0.0976504	0.1904896	-5.296098
RP11-177G2	0.3191203	-0.881259	1.6637515	0.0976731	0.1905237	-5.296282
TMPRSS11D	0.2624067	-1.08274	1.6637249	0.0976785	0.1905239	-5.296325
CTD-2144E2	-0.224343	-1.326372	-1.663688	0.0976857	0.1905279	-5.296384

LYPD1	-0.308078	4.7869678	-1.663639	0.0976956	0.1905369	-5.296464
RP11-834C1	-0.199079	4.5716896	-1.663525	0.0977185	0.1905715	-5.29665
RP11-933H2	0.2992225	2.3396707	1.6634774	0.097728	0.1905798	-5.296726
GLS	0.0587457	6.3064887	1.6633568	0.0977522	0.1906116	-5.296922
ADAM2	-0.237905	-1.292067	-1.663334	0.0977568	0.1906116	-5.29696
ALG2	-0.037734	6.2659445	-1.663318	0.09776	0.1906116	-5.296986
TM9SF3	0.0254391	6.7873931	1.6629794	0.0978278	0.1907336	-5.297534
RP11-285F7	-0.087304	5.1043647	-1.662844	0.0978548	0.1907762	-5.297753
KHDRBS1	-0.021273	6.6184249	-1.662664	0.097891	0.1908365	-5.298046
C9orf50	-0.397602	0.4519126	-1.662501	0.0979238	0.1908902	-5.298311
DPY19L2	0.4568498	2.0662021	1.6621526	0.0979936	0.1910161	-5.298875
TMEM254-AS	-0.132186	4.3250446	-1.662042	0.0980158	0.1910491	-5.299054
AC005740.6	0.2781749	1.8880795	1.6619986	0.0980245	0.191056	-5.299125
RP11-553N1	-0.18039	-1.32186	-1.661961	0.0980321	0.1910605	-5.299186
NMD3P1	-0.348345	-0.295853	-1.661636	0.0980973	0.1911774	-5.299712
GOLGA4	0.0357975	6.5372769	1.6616038	0.0981038	0.1911799	-5.299765
AC139103.1	-0.243104	-1.083133	-1.661378	0.0981492	0.1912581	-5.30013
SERPINH1P1	-0.231636	-1.151414	-1.661185	0.0981879	0.1913234	-5.300443
RPL34P31	-0.310204	-0.696124	-1.661143	0.0981964	0.1913297	-5.300511
GNB3	-0.313531	2.4723315	-1.661096	0.0982059	0.1913381	-5.300588
P2RY11	-0.071525	5.2142501	-1.660822	0.098261	0.1914351	-5.301031
IGHG4	0.3637185	5.2980948	1.6607809	0.0982693	0.191441	-5.301098
AC007278.3	0.4093899	0.1099512	1.6606662	0.0982923	0.1914757	-5.301283
RP11-488P3	-0.353222	1.7806286	-1.660612	0.0983032	0.1914866	-5.301371
CTD-2192J1	-0.167957	3.7202668	-1.660429	0.09834	0.1915482	-5.301667
MRPL54	0.0490909	6.2163723	1.6604008	0.0983457	0.1915491	-5.301713
LINC00525	0.3272118	2.7132567	1.6602553	0.098375	0.1915959	-5.301949
TMPRSS13	0.3480287	3.0435278	1.6602145	0.0983833	0.1916017	-5.302015
RP11-517A5	0.1098357	-1.503413	1.660062	0.098414	0.1916513	-5.302262
SLC37A1	0.0655396	5.7848657	1.6599098	0.0984446	0.1917008	-5.302508
LSM4	-0.040514	6.6102177	-1.659751	0.0984766	0.1917528	-5.302765
CTD-2583P5	-0.343261	-0.438267	-1.659415	0.0985443	0.1918744	-5.303309
CTD-2165H1	0.1591988	-1.405206	1.6593776	0.0985519	0.1918779	-5.30337
CST3	0.0442876	6.9713108	1.6593376	0.09856	0.1918779	-5.303435
GCM1	-0.453857	0.3375948	-1.659328	0.0985619	0.1918779	-5.30345
CMTM7	-0.063019	5.7035339	-1.659247	0.0985782	0.1918994	-5.303581
RPS26P55	-0.16663	-1.363518	-1.659084	0.0986112	0.1919534	-5.303846
LPP-AS2	0.0765276	4.8363522	1.6589953	0.098629	0.1919779	-5.303988
JAKMIP2-AS	0.4391404	-0.087093	1.658913	0.0986456	0.1919956	-5.304122
CTC-786C10	0.4265266	1.9426835	1.6588981	0.0986486	0.1919956	-5.304146
EGR4	0.4296715	0.0282977	1.6588404	0.0986602	0.192008	-5.304239
KLKB1	0.1259832	6.3010267	1.6588079	0.0986668	0.1920105	-5.304292
MCF2L2	0.3246655	3.4452195	1.6586942	0.0986898	0.1920449	-5.304476
LY6K	-0.470848	1.6828479	-1.658467	0.0987357	0.192124	-5.304843
LRRC37A15P	-0.409987	0.8677508	-1.658352	0.0987588	0.1921588	-5.305029
AKIRIN2	-0.044109	6.2267873	-1.658189	0.0987918	0.1922127	-5.305293
RP11-345P4	-0.323443	2.8476741	-1.658098	0.0988101	0.1922382	-5.30544
RP5-1153D9	-0.331034	-0.3207	-1.658033	0.0988233	0.1922536	-5.305545
LIM2	-0.240925	-1.226626	-1.657913	0.0988476	0.1922906	-5.30574
ZNRF2	0.0456858	6.0877986	1.6578615	0.098858	0.1923005	-5.305823
PARP14	0.0442056	6.5731086	1.6578003	0.0988703	0.1923143	-5.305921

IFT46	0.062133	5.5244451	1.6573635	0.0989587	0.1924759	-5.306628
DLEU1-AS1	0.1977144	-1.351588	1.6571359	0.0990047	0.1925552	-5.306995
KPNA7	-0.305011	3.9190797	-1.657036	0.0990249	0.1925842	-5.307157
RPL17P43	-0.333375	-0.387307	-1.656531	0.0991273	0.192773	-5.307973
RP11-327J1	0.2182371	-1.189183	1.6562463	0.0991849	0.1928748	-5.308433
ETV7	0.1691875	4.9428647	1.656215	0.0991912	0.1928768	-5.308484
RP11-483E2	-0.140497	-1.463953	-1.655945	0.099246	0.1929731	-5.30892
RPSAP55	-0.35627	0.2676862	-1.655806	0.0992741	0.1930174	-5.309144
NME8	0.3633664	1.7524897	1.6557416	0.0992872	0.1930326	-5.309248
CTD-2218G2	-0.29872	-0.798221	-1.655608	0.0993142	0.1930749	-5.309463
RP11-651P2	-0.187941	2.9358356	-1.655422	0.0993521	0.1931382	-5.309764
GFRA4	-0.170887	-1.370756	-1.655377	0.0993612	0.1931457	-5.309837
LA16c-325D	0.3941461	1.6723945	1.6552247	0.0993921	0.1931937	-5.310083
MAPK6PS2	-0.17923	-1.339755	-1.655193	0.0993986	0.1931937	-5.310135
RP11-440K2	-0.295773	-0.821599	-1.655177	0.0994018	0.1931937	-5.31016
RBFOX3	-0.389043	0.4595461	-1.655079	0.0994217	0.193222	-5.310318
RP11-598F7	0.4227521	0.5818879	1.6549949	0.0994388	0.193245	-5.310454
RP11-503C2	0.2421673	-1.177456	1.6545709	0.0995249	0.1934021	-5.311138
PVRIG	0.3990392	0.6178005	1.6544005	0.0995596	0.1934591	-5.311413
RP11-366O2	-0.267787	-0.91611	-1.654366	0.0995666	0.1934625	-5.311469
AC137932.5	-0.273785	2.9524207	-1.65434	0.0995719	0.1934625	-5.311511
RP11-156K2	0.1146484	4.2542481	1.6542672	0.0995867	0.1934808	-5.311628
RP11-120M1	-0.398722	1.4199285	-1.654141	0.0996124	0.1935205	-5.311832
PRDM12	-0.371682	1.7164627	-1.653998	0.0996413	0.1935664	-5.312062
RP11-1228E	-0.405307	0.3601561	-1.653837	0.0996742	0.19362	-5.312323
RP11-147L1	-0.110184	4.3690579	-1.6536	0.0997225	0.1937034	-5.312705
BDP1	0.0407339	5.9355039	1.653525	0.0997377	0.1937227	-5.312826
C16orf13	0.0461426	6.2028428	1.6534968	0.0997434	0.1937235	-5.312871
LONP1	0.0365387	6.7245587	1.6533602	0.0997712	0.1937672	-5.313092
DUPD1	0.1270771	-1.494626	1.653179	0.0998081	0.1938286	-5.313384
GOLGA2P11	0.4843693	0.0949597	1.6529945	0.0998457	0.1938906	-5.313681
PRDX6	0.0465093	7.1063794	1.6529541	0.099854	0.1938906	-5.313746
TBC1D29	-0.326599	-0.553771	-1.652944	0.099856	0.1938906	-5.313762
CCDC175	0.1320735	-1.46056	1.6529074	0.0998635	0.1938948	-5.313822
RNU1-122P	-0.353375	-0.45042	-1.652578	0.0999307	0.1940149	-5.314353
CTA-217C2.	-0.333444	2.0069853	-1.652376	0.0999717	0.1940843	-5.314678
EMP2	0.0568304	6.4696984	1.652337	0.0999798	0.1940896	-5.314741
RP11-452G1	-0.187481	-1.287578	-1.652184	0.1000111	0.1941401	-5.314989
RBM28	-0.029988	6.016804	-1.651757	0.1000981	0.1942924	-5.315675
LIN52	-0.043772	5.6278901	-1.651747	0.1001002	0.1942924	-5.315692
MANEA-AS1	-0.253826	2.7143509	-1.651595	0.1001312	0.1943322	-5.315937
TRNAU1AP	0.0452691	5.7444286	1.6515942	0.1001314	0.1943322	-5.315938
RP11-49G2.	-0.227455	-1.226594	-1.651344	0.1001825	0.1944211	-5.316341
LARP4B	-0.023781	6.3581409	-1.651237	0.1002044	0.1944533	-5.316514
SPTBN1	0.0356922	7.0958723	1.6511038	0.1002316	0.1944957	-5.316728
RP11-75A9.	-0.349572	0.45294	-1.650811	0.1002914	0.1946014	-5.317199
NQO1	0.1936204	6.0982418	1.6505973	0.1003351	0.1946738	-5.317544
AP2M1	0.028645	6.8912294	1.6505766	0.1003394	0.1946738	-5.317577
RPL7AP3	-0.188093	-1.278118	-1.65039	0.1003774	0.1947374	-5.317877
KRT8P14	-0.359723	-0.010842	-1.650247	0.1004067	0.1947839	-5.318107
WSB1	-0.038435	6.3379413	-1.649931	0.1004715	0.1948991	-5.318616

CTD-2330K9	-0.353256	-0.308785	-1.649547	0.1005501	0.1950304	-5.319234
TRAV10	-0.369062	-0.308609	-1.649536	0.1005525	0.1950304	-5.319252
TMEM17	-0.066116	4.8190467	-1.649522	0.1005552	0.1950304	-5.319274
TYW1	0.0320097	5.9280851	1.6494685	0.1005663	0.1950415	-5.319361
WDR25	0.0441948	5.5610153	1.6493993	0.1005804	0.1950586	-5.319472
RP11-316N2	0.1581684	-1.394951	1.6493085	0.100599	0.1950843	-5.319618
CMBL	0.1178435	6.4986746	1.6492704	0.1006068	0.1950891	-5.319679
RP11-51501	-0.220507	-1.236898	-1.649148	0.1006319	0.1951272	-5.319876
KB-1125A3.	-0.352521	0.7356553	-1.649088	0.1006443	0.1951411	-5.319974
HIST1H3H	-0.265534	4.4873888	-1.648903	0.1006823	0.1952043	-5.320271
SPATA1	0.1185015	3.6796207	1.6488081	0.1007017	0.1952315	-5.320423
LINC01569	0.1144254	4.8178817	1.648743	0.100715	0.1952388	-5.320528
EZR-AS1	-0.292456	-0.747423	-1.648737	0.1007162	0.1952388	-5.320537
RP11-73M18	-0.338483	1.0582177	-1.648685	0.1007269	0.1952433	-5.320621
HIST2H2BF	-0.271187	3.387114	-1.64866	0.1007321	0.1952433	-5.320661
LCORL	-0.055529	5.5263886	-1.648648	0.1007345	0.1952433	-5.32068
RP11-597M1	0.1190627	-1.50347	1.6485506	0.1007545	0.1952717	-5.320837
COPG1	0.0252628	6.8609741	1.6485101	0.1007628	0.1952774	-5.320902
CD209	0.1436738	5.1231833	1.6484084	0.1007837	0.1953075	-5.321066
RP11-320M2	-0.106139	4.241297	-1.648198	0.1008269	0.195381	-5.321404
CTD-2012J1	-0.391342	0.3492512	-1.648076	0.100852	0.1954133	-5.321601
RP11-320A1	-0.229197	-1.220532	-1.648064	0.1008543	0.1954133	-5.321619
RP1-20208.	0.2705741	-0.979484	1.6478265	0.1009032	0.1954976	-5.322001
NES	0.0589249	6.2837949	1.6477206	0.100925	0.1955294	-5.322172
RP11-10L7.	0.2726469	-1.080344	1.6476522	0.100939	0.1955463	-5.322281
CTD-3051D2	-0.384108	0.2369864	-1.647575	0.1009548	0.1955662	-5.322405
ANGPT1	0.1470685	4.9620793	1.6475501	0.10096	0.1955662	-5.322446
ABRA	0.2769283	-0.972844	1.6475191	0.1009664	0.1955682	-5.322495
RBM42	-0.035468	6.5596554	-1.647441	0.1009825	0.1955817	-5.322621
PLA2G3	-0.327325	-0.919451	-1.64741	0.1009888	0.1955817	-5.32267
AC007386.3	-0.211677	-1.195526	-1.647407	0.1009895	0.1955817	-5.322676
ALS2CR12	0.3084537	2.4486183	1.6472801	0.1010156	0.1956218	-5.32288
XXyac-YX65	-0.395194	-0.399961	-1.647137	0.101045	0.1956685	-5.32311
RP11-96H17	-0.401439	-0.740468	-1.646987	0.1010758	0.1957177	-5.32335
AC004067.5	0.3822046	1.6523481	1.6468598	0.101102	0.1957581	-5.323555
AGPAT2	0.0646577	6.6737641	1.6465111	0.1011738	0.1958868	-5.324115
GLTPD2	-0.161626	5.8705711	-1.646305	0.1012162	0.1959584	-5.324446
ADSSL1	0.1071253	5.6724467	1.6462399	0.1012297	0.1959741	-5.324551
RP11-157D2	0.2275993	-1.216948	1.6461534	0.1012475	0.1959982	-5.32469
CTB-40H15.	-0.377048	0.5657701	-1.646076	0.1012635	0.1960189	-5.324815
RP11-62H7.	0.3153917	2.20195	1.6459888	0.1012814	0.1960347	-5.324954
MLN	-0.254572	-1.247891	-1.645984	0.1012824	0.1960347	-5.324962
RP11-80P20	-0.329618	2.4753833	-1.645636	0.1013541	0.196163	-5.32552
RP4-651E10	0.1400838	-1.459909	1.6455752	0.1013667	0.196177	-5.325618
CTB-5E10.3	-0.215599	-1.191815	-1.645465	0.1013894	0.1962106	-5.325795
SCGB2B2	0.2551959	2.7413209	1.6452402	0.1014358	0.1962878	-5.326156
CECR9	0.3230638	-0.892757	1.6452195	0.1014401	0.1962878	-5.326189
MAGEB16	-0.357526	-0.950163	-1.645056	0.1014738	0.1963334	-5.326451
RP11-760D2	-0.419359	-0.613745	-1.645053	0.1014744	0.1963334	-5.326456
DOC2A	0.3433746	2.5753823	1.6449462	0.1014965	0.1963657	-5.326627
SLC25A33	-0.079671	5.9999185	-1.644883	0.1015095	0.1963804	-5.326729

CABYR	-0.262163	4.6048438	-1.644731	0.101541	0.1964309	-5.326973
NCF1	0.1585335	4.4581527	1.6444389	0.1016013	0.1965372	-5.327441
RNU5A-1	-0.237596	-1.14822	-1.644217	0.1016471	0.1966154	-5.327797
CH17-140K2	0.3373463	-0.699894	1.6441584	0.1016592	0.1966231	-5.327891
TYW1B	-0.112714	4.8438048	-1.644146	0.1016618	0.1966231	-5.327912
RP11-197N1	0.3858821	-0.589591	1.644091	0.1016732	0.1966346	-5.328
AQP2	0.1972005	-1.349561	1.6438788	0.101717	0.196709	-5.32834
AC103563.3	-0.22865	-1.357382	-1.643722	0.1017495	0.1967612	-5.328591
RP11-547D2	-0.354666	2.3806251	-1.643458	0.1018041	0.1968565	-5.329015
VPS37A	0.0468822	6.1029148	1.6434037	0.1018154	0.1968678	-5.329102
TMPOP1	0.1654469	-1.380462	1.643274	0.1018422	0.1969063	-5.32931
TMEM52	-0.201734	4.7665072	-1.643255	0.101846	0.1969063	-5.329339
TIMM22	0.0386487	5.8145227	1.6431473	0.1018684	0.1969392	-5.329513
FANK1	0.3358306	2.5128194	1.6430996	0.1018783	0.1969478	-5.329589
DIO3	0.5494688	2.371436	1.6430736	0.1018837	0.1969478	-5.329631
RP11-211A1	0.1420069	-1.429622	1.6430176	0.1018953	0.1969598	-5.329721
CCDC121	-0.069112	4.8083869	-1.642633	0.1019751	0.1970898	-5.330338
GOLPH3	0.0340596	6.7309079	1.6426169	0.1019783	0.1970898	-5.330363
AC104532.2	0.3487341	0.0107583	1.6426147	0.1019788	0.1970898	-5.330366
UBQLN4P1	-0.370703	1.0711515	-1.64238	0.1020275	0.1971735	-5.330743
RP11-420H1	0.1993094	-1.407074	1.6421764	0.1020696	0.1972446	-5.331069
ZNF34	-0.052242	5.4021624	-1.642084	0.1020889	0.1972713	-5.331217
RP11-488C1	-0.379011	1.4976755	-1.641984	0.1021096	0.197301	-5.331378
NENF	-0.050599	6.4556179	-1.641805	0.1021467	0.1973621	-5.331664
RP1-167F1.	0.3469771	-0.636047	1.6411201	0.1022889	0.1976188	-5.33276
ALDOC	-0.110163	6.2471937	-1.641113	0.1022904	0.1976188	-5.332772
FAM195CP	-0.414985	-0.182323	-1.640779	0.1023597	0.1977423	-5.333306
NUTM2A	-0.322714	2.5889214	-1.640742	0.1023676	0.197747	-5.333366
C10orf99	0.3061437	-1.082658	1.6406066	0.1023956	0.1977907	-5.333582
RP11-109N2	-0.297629	2.1105671	-1.640466	0.1024248	0.1978366	-5.333807
N6AMT2	0.0651447	5.3980026	1.6403199	0.1024552	0.197885	-5.334041
FCGR2C	0.2328698	4.0488253	1.6402922	0.102461	0.1978856	-5.334086
RP13-52K8.	0.2150761	-1.239762	1.640195	0.1024812	0.1979094	-5.334241
ACAA1	0.0629589	6.7308971	1.640181	0.1024841	0.1979094	-5.334264
FTH1P4	-0.328531	2.1656817	-1.640139	0.102493	0.1979097	-5.334332
ADARB2-AS1	-0.211809	-1.372593	-1.640126	0.1024955	0.1979097	-5.334351
AC067968.3	-0.115478	-1.498127	-1.640102	0.1025006	0.1979097	-5.33439
BHLHB9	0.0921312	5.0515472	1.6399828	0.1025254	0.1979471	-5.334581
GLRA4	0.1805425	-1.326037	1.6399303	0.1025363	0.1979578	-5.334665
CPA2	-0.515448	-0.111146	-1.639833	0.1025566	0.1979864	-5.33482
RP11-190A1	0.4321143	1.4945623	1.6397839	0.1025668	0.1979957	-5.334899
RP11-155G1	-0.198091	-1.286696	-1.639723	0.1025795	0.1980098	-5.334997
ZNF799	-0.046482	5.0793334	-1.639533	0.102619	0.1980755	-5.3353
ZNF840P	-0.190349	-1.336934	-1.639479	0.1026303	0.1980868	-5.335386
RP11-118F2	-0.148691	-1.416125	-1.639147	0.1026994	0.1982098	-5.335917
RP11-737F9	0.1002513	-1.508297	1.6390755	0.1027144	0.1982262	-5.336032
SIAH2-AS1	0.3652079	1.9432666	1.6390542	0.1027188	0.1982262	-5.336066
RAD23BP1	-0.291053	-0.7672	-1.638993	0.1027316	0.1982405	-5.336164
DNAJC17	0.0407983	5.8696404	1.638619	0.1028095	0.1983718	-5.336762
RP4-550H1.	-0.260454	-0.963682	-1.638614	0.1028105	0.1983718	-5.33677
ACVR1B	0.0414646	6.3813765	1.6385113	0.102832	0.1984028	-5.336934

LINC01385	0.163284	-1.448537	1.6381147	0.1029148	0.1985519	-5.337568
CDK13	-0.027672	6.2714053	-1.63793	0.1029533	0.1986157	-5.337863
RP11-619A1	0.4460249	1.7355972	1.6378581	0.1029684	0.1986343	-5.337978
HVCN1	0.0902846	5.0246773	1.6376702	0.1030076	0.1986995	-5.338278
SCGB2A2	0.2985463	-1.127586	1.63763	0.103016	0.1987037	-5.338343
RP11-613E4	-0.357058	-0.344907	-1.637608	0.1030207	0.1987037	-5.338378
SAA3P	0.2270164	-1.238207	1.6374701	0.1030494	0.1987487	-5.338598
RP11-83B2C	-0.13725	-1.44364	-1.637429	0.1030581	0.1987548	-5.338664
DOCK4-AS1	0.2057417	-1.195551	1.6373674	0.1030709	0.1987691	-5.338762
PRR13P5	-0.096953	4.1198117	-1.637208	0.1031041	0.1988211	-5.339016
RP11-104L2	0.3667807	1.7861359	1.6371863	0.1031088	0.1988211	-5.339052
Z83851.4	-0.100267	4.4005702	-1.637106	0.1031255	0.1988429	-5.33918
PIGFP1	-0.331849	-0.187522	-1.636995	0.1031488	0.1988772	-5.339357
IGKV1-13	-0.387254	-0.757842	-1.636936	0.1031612	0.1988904	-5.339452
RP11-445N1	-0.257077	-1.227745	-1.63691	0.1031665	0.1988904	-5.339492
GLYATL1P4	0.4900398	0.6834032	1.6368504	0.103179	0.198904	-5.339588
PAPPA-AS2	0.1052562	-1.505747	1.6366864	0.1032133	0.1989596	-5.33985
ABCA9-AS1	-0.356715	-0.580914	-1.636226	0.1033096	0.1991347	-5.340584
MIIP	-0.047832	6.0398397	-1.636092	0.1033377	0.1991784	-5.340798
FBXW9	-0.053951	5.4480052	-1.635968	0.1033637	0.1992179	-5.340996
RP11-50D9.	0.3708578	2.1205007	1.6359264	0.1033725	0.1992243	-5.341063
AHCYP2	0.4039253	1.2006428	1.6358679	0.1033847	0.1992374	-5.341157
DYNC1H1	0.0241331	6.8506116	1.6358184	0.1033951	0.1992433	-5.341236
SPOP	-0.025322	6.3461501	-1.635801	0.1033987	0.1992433	-5.341263
TRBV10-3	-0.420041	0.7306105	-1.635741	0.1034113	0.1992548	-5.341359
ENPP1	-0.078348	6.3714676	-1.635721	0.1034156	0.1992548	-5.341392
RP11-893F2	-0.237742	-1.239978	-1.635648	0.1034308	0.1992698	-5.341508
AF015720.3	0.0969341	-1.523996	1.6356314	0.1034343	0.1992698	-5.341534
AC005740.5	0.2963332	-0.676864	1.6355381	0.1034538	0.199297	-5.341683
AC008686.1	-0.251594	-1.062779	-1.635473	0.1034674	0.1993054	-5.341786
AC007919.1	0.1684399	-1.322686	1.6354652	0.1034691	0.1993054	-5.341799
LA16c-60H5	-0.451427	1.4492056	-1.635303	0.1035032	0.1993456	-5.342059
RNF5	-0.04223	6.532792	-1.635291	0.1035056	0.1993456	-5.342077
MTND6P3	0.3681012	-0.314859	1.6352877	0.1035063	0.1993456	-5.342083
AC110611.1	0.2154861	-1.190585	1.6351737	0.1035303	0.1993811	-5.342265
AF064858.1	0.3870011	0.9106405	1.6351327	0.1035388	0.1993871	-5.34233
RP11-305L7	0.3299156	-0.733405	1.635053	0.1035556	0.1994088	-5.342457
SH3PXD2A	-0.043253	6.2627458	-1.634959	0.1035753	0.1994363	-5.342607
RP11-171I2	-0.320347	2.5258055	-1.634892	0.1035893	0.1994527	-5.342714
TTC24	-0.455369	1.1336482	-1.634832	0.1036019	0.1994664	-5.342809
RP11-383J2	0.5332498	0.7968272	1.6346435	0.1036415	0.1995322	-5.34311
AC013717.3	-0.320679	-0.476189	-1.634364	0.1037003	0.1996348	-5.343557
CTC-498M16	0.2238093	-1.155708	1.6342905	0.1037156	0.1996538	-5.343673
VPS13A-AS1	0.1213103	-1.47956	1.6340694	0.1037621	0.1997328	-5.344026
AREG	0.3844149	3.620294	1.6338313	0.1038122	0.199802	-5.344405
PROSER2	-0.083008	6.0915434	-1.633814	0.1038157	0.199802	-5.344433
RP11-44N22	0.3567791	1.8470906	1.6337992	0.1038189	0.199802	-5.344456
RP11-505E2	-0.389418	0.7355979	-1.633784	0.1038221	0.199802	-5.344481
C6orf201	-0.224759	2.7368456	-1.633768	0.1038254	0.199802	-5.344506
UGT2A3P7	-0.406381	0.9850113	-1.633479	0.1038863	0.1999085	-5.344967
PSMC1	0.0503238	5.7740752	1.633274	0.1039294	0.1999809	-5.345294

LINC01348	0.3124033	5.1068778	1.6332385	0.1039368	0.1999847	-5.34535
UNC119	-0.046417	6.0627382	-1.633099	0.1039662	0.2000307	-5.345573
TAF8	-0.029715	6.0436473	-1.633059	0.1039746	0.2000363	-5.345636
RP3-333A15	0.2917801	-0.983205	1.6329119	0.1040056	0.2000853	-5.345871
RP11-259G1	-0.318906	2.3542719	-1.632801	0.1040289	0.2001197	-5.346047
PRDM5	0.2649474	3.5601402	1.6326713	0.1040562	0.2001606	-5.346254
CTC-523E23	0.3800445	1.6213801	1.6326481	0.1040611	0.2001606	-5.346291
ERVH-1	0.3111285	-0.889351	1.632596	0.1040721	0.2001711	-5.346374
CTD-2514C3	0.4056756	2.2381253	1.6325447	0.1040829	0.2001814	-5.346456
SSH3	-0.047159	6.0830828	-1.632391	0.1041154	0.2002333	-5.346701
CTD-228701	-0.294397	-0.695087	-1.632315	0.1041313	0.2002534	-5.346822
MXD1	-0.057612	5.7291567	-1.632282	0.1041383	0.2002563	-5.346875
IGLJCOR18	-0.296207	-1.036793	-1.63222	0.1041513	0.2002707	-5.346973
CTD-2373N4	0.2092622	-1.225277	1.6321356	0.1041691	0.2002844	-5.347107
GOLGA8R	0.376139	1.8473073	1.6321313	0.10417	0.2002844	-5.347114
RP11-626H1	-0.193704	-1.293852	-1.632108	0.1041749	0.2002844	-5.347151
CTD-2311M2	0.4317387	1.2220907	1.6319083	0.104217	0.2003549	-5.347469
TUFMP1	-0.418002	-0.191385	-1.631868	0.1042256	0.2003608	-5.347534
XPOTP1	-0.36598	0.2641948	-1.631132	0.1043809	0.2006435	-5.348705
CH17-437K3	0.3723655	2.948175	1.6311188	0.1043836	0.2006435	-5.348726
RP11-486B1	0.4140803	0.221986	1.6310175	0.104405	0.200674	-5.348887
RP11-525K1	-0.216136	-1.291752	-1.630953	0.1044187	0.2006898	-5.34899
DCAF12L2	-0.300551	-1.111802	-1.630671	0.1044782	0.2007936	-5.349439
RP11-275I4	-0.273633	-1.013183	-1.630469	0.1045209	0.2008651	-5.34976
RP1-223B1.	-0.306327	-0.818313	-1.630347	0.1045468	0.2009042	-5.349955
HIST1H4PS1	-0.300619	-0.841941	-1.630137	0.1045912	0.200979	-5.350289
GMDS-AS1	-0.048327	5.117961	-1.630053	0.1046089	0.2010024	-5.350422
RP11-338K1	0.2329035	-1.112482	1.6299952	0.1046211	0.201013	-5.350514
TATDN2P2	-0.326496	2.6517214	-1.629975	0.1046254	0.201013	-5.350546
GAPDHP15	0.1538801	-1.393582	1.6299452	0.1046317	0.2010144	-5.350593
LINC01310	-0.26277	-1.275453	-1.629877	0.1046461	0.2010316	-5.350702
CTD-2591A1	0.3772066	0.3612843	1.6296249	0.1046995	0.2011235	-5.351103
RP11-35501	-0.239644	-1.04359	-1.629344	0.1047589	0.2012271	-5.351549
ZFPM2-AS1	0.4598182	3.4347206	1.6291268	0.1048049	0.2013049	-5.351895
RPL12P41	-0.219593	-1.114083	-1.62901	0.1048296	0.2013417	-5.35208
NAPSA	-0.147592	3.7663571	-1.628983	0.1048353	0.201342	-5.352123
APOD	0.19777	4.4714396	1.6288709	0.1048591	0.2013772	-5.352301
RNU6-438P	-0.152477	-1.414268	-1.628796	0.1048751	0.2013973	-5.352421
RP5-1065J2	-0.123995	4.2418028	-1.62858	0.1049207	0.2014743	-5.352763
SLC13A4	-0.187256	3.7963472	-1.628532	0.1049309	0.2014832	-5.352839
GAPDHP68	-0.329292	-0.359634	-1.628263	0.104988	0.2015824	-5.353268
D2HGDH	0.0487122	6.145345	1.6282173	0.1049977	0.2015903	-5.35334
NDUFB1P2	0.1588285	-1.400635	1.6281864	0.1050043	0.2015923	-5.353389
SYCP3	-0.222665	2.4995216	-1.628109	0.1050208	0.2016022	-5.353513
RP11-1360M	0.0842102	-1.539236	1.6281072	0.1050211	0.2016022	-5.353515
CTD-2506P8	-0.204169	3.1053491	-1.628084	0.105026	0.2016022	-5.353552
CTHRC1	0.1840267	5.2547267	1.6280365	0.1050361	0.201611	-5.353627
LRRC40	0.0491104	5.7511896	1.6279219	0.1050604	0.2016471	-5.353809
NAGK	0.0297855	6.2339508	1.6278673	0.105072	0.2016587	-5.353896
ICA1	-0.111307	5.715946	-1.627634	0.1051216	0.2017433	-5.354267
RP11-68I3.	-0.341295	-0.22098	-1.627579	0.1051333	0.2017552	-5.354355

RP1-15D7.1	-0.319315	-0.613195	-1.627525	0.1051446	0.2017663	-5.354439
RP5-1165K1	-0.297811	1.9128493	-1.62746	0.1051584	0.2017821	-5.354542
IPO7	-0.029	6.5901325	-1.627418	0.1051673	0.2017886	-5.354609
PEX1	0.0443914	5.9581849	1.6273274	0.1051867	0.2018131	-5.354754
DHX30	-0.024783	6.5030069	-1.627306	0.1051912	0.2018131	-5.354787
RP11-677N1	-0.227344	-1.082638	-1.62715	0.1052243	0.2018661	-5.355035
RP11-568J2	0.3636058	0.7294992	1.6270009	0.105256	0.2019163	-5.355272
SMN2	-0.314107	4.7012791	-1.626862	0.1052855	0.2019622	-5.355492
OR10V3P	0.0907374	-1.53591	1.6267783	0.1053034	0.2019802	-5.355625
RP11-513O1	-0.263957	-1.189578	-1.626737	0.1053121	0.2019802	-5.355691
RP11-647F2	0.2653223	-0.881297	1.6267127	0.1053173	0.2019802	-5.35573
RP11-491H1	-0.27513	-1.046671	-1.626702	0.1053197	0.2019802	-5.355747
SMAP2	0.0393033	6.5361854	1.6266883	0.1053225	0.2019802	-5.355768
NLRP6	0.2779611	4.2464339	1.6265932	0.1053427	0.2020083	-5.355919
LURAP1	0.2902967	2.6364003	1.626226	0.1054208	0.2021349	-5.356502
RP11-472F1	-0.375579	-0.177526	-1.626223	0.1054214	0.2021349	-5.356506
LINC00371	-0.268513	-1.07039	-1.626205	0.1054253	0.2021349	-5.356536
AC007099.2	-0.227529	-1.17206	-1.626037	0.1054612	0.202193	-5.356803
RP11-238I1	-0.104698	-1.503413	-1.625947	0.1054803	0.202219	-5.356945
ZNF734P	0.1091395	-1.522977	1.625765	0.105519	0.2022826	-5.357233
ERVWE2	-0.213366	-1.391362	-1.625736	0.1055251	0.2022838	-5.357279
C2orf91	0.3956139	0.5035075	1.6256358	0.1055465	0.2023141	-5.357438
RP3-407E4.	0.3179834	-0.900014	1.625487	0.1055782	0.2023643	-5.357675
PRPF40B	-0.058423	5.2823008	-1.625392	0.1055985	0.2023924	-5.357825
AC005264.2	0.3573248	0.9881149	1.6251258	0.1056552	0.2024906	-5.358247
CMTR1	-0.028664	6.3576045	-1.624959	0.1056907	0.2025467	-5.358511
RP11-42A4.	0.1335244	-1.482585	1.6249365	0.1056956	0.2025467	-5.358548
DRAP1	-0.037297	6.5172128	-1.624741	0.1057373	0.202616	-5.358858
NMUR1	0.1765384	3.9288702	1.6245635	0.1057751	0.2026779	-5.359139
PPIHP1	-0.342307	-0.416221	-1.624521	0.1057841	0.2026845	-5.359206
AC073254.1	-0.135119	4.0845855	-1.624415	0.1058068	0.2027172	-5.359374
RP11-108M1	-0.321326	-0.965489	-1.624293	0.1058329	0.2027567	-5.359568
AC009495.3	-0.326991	-0.629701	-1.624208	0.1058511	0.2027809	-5.359703
ATG9A	-0.028681	6.5667706	-1.624135	0.1058666	0.2027999	-5.359818
TRIM75P	-0.202631	-1.22718	-1.623921	0.1059124	0.2028769	-5.360158
LASP1	-0.030137	6.9666251	-1.623736	0.1059519	0.202942	-5.360451
PSMB3P2	0.1295842	-1.443062	1.6232905	0.1060471	0.2031136	-5.361156
AASDHPPT	0.0302939	6.1543251	1.6232274	0.1060606	0.2031288	-5.361256
C1orf86	0.0415067	6.197136	1.6231755	0.1060717	0.2031394	-5.361339
WDR73	-0.060363	4.9580943	-1.62294	0.106122	0.2032251	-5.361711
RP11-7F17.	0.0953119	3.9836906	1.6228949	0.1061317	0.2032331	-5.361783
MFNG	0.0524802	5.6347995	1.6225891	0.1061972	0.2033477	-5.362267
MYO18A	0.046414	6.5752501	1.622547	0.1062062	0.2033543	-5.362334
RP11-454E5	-0.123902	3.8407837	-1.622486	0.1062193	0.2033688	-5.362431
RP11-321G1	-0.428232	-0.044639	-1.622456	0.1062256	0.2033702	-5.362478
ATG12	0.0284055	6.220199	1.6221961	0.1062813	0.2034662	-5.36289
RP11-669B1	-0.32112	-0.538651	-1.622081	0.1063059	0.2035026	-5.363071
GPATCH4	-0.042788	6.2536852	-1.621996	0.1063241	0.2035268	-5.363206
RP11-203B7	-0.200403	-1.317051	-1.621916	0.1063413	0.2035491	-5.363333
PRAC2	-0.42432	-0.570828	-1.621881	0.1063489	0.2035528	-5.363389
PCBP2-OT1	-0.376075	0.382869	-1.621599	0.1064093	0.2036578	-5.363835

U3	-0.20189	3.7607969	-1.62145	0.1064411	0.203708	-5.36407
CTD-213801	-0.238751	-1.215723	-1.621259	0.1064821	0.2037758	-5.364372
ATAD1	0.040108	6.2054186	1.6211507	0.1065054	0.2038097	-5.364544
CARF	0.0639436	5.118061	1.6208257	0.1065752	0.2039325	-5.365058
HIGD1B	-0.162964	4.2334335	-1.620645	0.1066141	0.2039963	-5.365345
SEPT7P9	-0.333634	1.0853624	-1.620514	0.1066421	0.2040287	-5.365551
RP4-61404.	-0.357695	0.6972432	-1.620514	0.1066422	0.2040287	-5.365552
CTD-2357A8	-0.425609	0.1363215	-1.620369	0.1066733	0.2040775	-5.365781
RP11-671C1	-0.147612	-1.482369	-1.620324	0.1066829	0.2040852	-5.365851
TDRD12	-0.366412	1.3560951	-1.620235	0.1067021	0.2041103	-5.365992
C7orf57	0.3220396	-0.644815	1.6202109	0.1067072	0.2041103	-5.366031
CCL17	0.3203621	2.6816021	1.6200896	0.1067333	0.2041495	-5.366222
UBE2L5P	-0.32656	1.218349	-1.620049	0.1067421	0.2041556	-5.366287
ZBTB12P1	-0.392372	1.0389988	-1.619987	0.1067554	0.2041703	-5.366384
EGLN3	-0.159355	5.390692	-1.61991	0.106772	0.2041913	-5.366506
ZNF226	-0.049037	5.6383204	-1.619791	0.1067975	0.2042295	-5.366695
CTA-414D7.	-0.306966	-0.565619	-1.619732	0.1068101	0.2042379	-5.366787
LINC01431	-0.23777	3.0774314	-1.619718	0.1068131	0.2042379	-5.366809
RP11-73B2.	-0.301329	-0.574295	-1.619678	0.1068217	0.2042437	-5.366872
PTPN18	0.0292621	6.3579634	1.6191753	0.10693	0.20444	-5.367668
WI2-3308P1	0.24822	-1.281982	1.6190541	0.1069561	0.2044791	-5.367859
AL109761.5	-0.294306	-0.953251	-1.618974	0.1069734	0.2045016	-5.367986
RP3-329E20	-0.292714	-0.999954	-1.618855	0.1069989	0.2045397	-5.368174
RP1-101G11	-0.181173	-1.351807	-1.618803	0.1070102	0.2045505	-5.368256
IGHV3-16	-0.186913	-1.370897	-1.618752	0.1070212	0.2045608	-5.368337
RP11-526F3	0.1911675	-1.38604	1.6186012	0.1070536	0.204612	-5.368575
AC234917.1	-0.387231	-0.4807	-1.618512	0.1070728	0.204638	-5.368715
FMR1NB	-0.39255	-0.41776	-1.61828	0.1071227	0.2047227	-5.369081
RP11-545G3	-0.204716	-1.301931	-1.618166	0.1071475	0.2047593	-5.369262
CACUL1	0.0272223	6.3051355	1.6180607	0.1071701	0.2047918	-5.369428
RFPL4A	0.4550735	0.3538328	1.6177626	0.1072344	0.204904	-5.369899
RP11-271C2	0.2416121	-0.997951	1.6173632	0.1073206	0.2050579	-5.37053
RNASE2	-0.41596	2.4149317	-1.617287	0.1073371	0.2050787	-5.37065
SNPH	0.140487	4.4436935	1.6172094	0.1073538	0.2050999	-5.370772
MAGEB6	-0.367855	-0.754103	-1.617148	0.107367	0.2051144	-5.370869
KEAP1	0.0321146	6.521562	1.6166256	0.10748	0.2053183	-5.371694
RP11-651L5	-0.239252	-1.027307	-1.616594	0.1074868	0.2053183	-5.371744
CTB-39G8.2	0.4110833	1.0341206	1.6165761	0.1074906	0.2053183	-5.371772
RAB40C	0.0375819	5.9903632	1.6165062	0.1075058	0.2053365	-5.371882
RN7SL272P	-0.14117	-1.463623	-1.616373	0.1075346	0.2053808	-5.372093
CTD-3222D1	-0.366757	0.4808951	-1.616298	0.1075508	0.2054011	-5.372211
FAM220A	-0.033967	5.8119841	-1.616251	0.107561	0.2054097	-5.372285
PRRC1	0.0295943	6.4425998	1.6162098	0.1075699	0.2054159	-5.37235
RP11-15B24	-0.248912	-1.187256	-1.616086	0.1075966	0.2054562	-5.372545
MAGED4B	-0.166204	-1.368473	-1.615895	0.1076379	0.205518	-5.372845
ZKSCAN5	-0.030379	5.7938775	-1.615885	0.1076402	0.205518	-5.372863
RP11-350E1	-0.329836	-0.643356	-1.615709	0.1076781	0.2055796	-5.373139
CTC-559E9.	-0.185818	3.5672052	-1.615501	0.1077233	0.2056551	-5.373468
GS1-25M2.1	-0.25399	-1.045105	-1.615443	0.1077358	0.2056575	-5.373559
RP3-468B3.	-0.383958	1.3432844	-1.615443	0.1077358	0.2056575	-5.373559
RP3-414A15	0.2344683	3.675497	1.6153456	0.1077569	0.205687	-5.373712

ARHGEF1	-0.029522	6.5063279	-1.615287	0.1077697	0.2057006	-5.373805
SEPHS1P6	0.4014456	1.0384738	1.6150887	0.1078125	0.2057717	-5.374117
PSMD7P1	0.3483415	-0.120567	1.6148496	0.1078644	0.2058598	-5.374494
RP11-314A2	-0.331393	-0.210356	-1.6148	0.1078751	0.2058695	-5.374572
AC114776.3	0.0792054	-1.541787	1.614732	0.1078899	0.2058869	-5.37468
RNU6-383P	0.238663	-1.174653	1.6146392	0.10791	0.2059146	-5.374826
MIR4435-1H	0.0707187	5.8666131	1.6145269	0.1079343	0.2059502	-5.375003
CEACAMP5	0.123956	-1.485437	1.6141744	0.1080108	0.2060854	-5.375558
TDGF1P5	-0.174251	-1.311391	-1.613963	0.1080567	0.2061623	-5.375892
MBOAT4	0.3727864	3.2506746	1.6135276	0.1081512	0.2063318	-5.376577
EIF4A2P3	-0.258704	-0.947048	-1.613434	0.1081715	0.2063597	-5.376724
VDAC3	0.0408152	6.479311	1.6133643	0.1081867	0.2063774	-5.376834
CPLX4	-0.307179	-0.642377	-1.61334	0.1081921	0.2063774	-5.376873
OR7E126P	-0.393194	0.6093175	-1.613272	0.1082068	0.2063946	-5.37698
RP11-345J1	0.3990847	1.4614059	1.6129823	0.1082697	0.2064941	-5.377436
RP1-84015.	-0.132056	-1.463513	-1.612961	0.1082743	0.2064941	-5.377469
UPF3B	-0.042186	5.7507458	-1.612954	0.1082759	0.2064941	-5.377481
RPL35P9	-0.138935	-1.438234	-1.612906	0.1082863	0.2065031	-5.377556
POLI	0.0486786	5.5762217	1.6126782	0.1083358	0.2065868	-5.377914
TRAV6	-0.373169	-0.230672	-1.612553	0.108363	0.2066278	-5.378111
RP11-3K16.	0.25357	-0.947265	1.6123946	0.1083975	0.2066829	-5.378361
CTA-221G9.	0.353187	-0.196803	1.6123334	0.1084109	0.2066975	-5.378457
CBWD5	-0.066812	5.1326086	-1.612232	0.1084329	0.2067183	-5.378616
RP11-271K1	-0.123979	-1.450148	-1.612231	0.1084331	0.2067183	-5.378618
PCDHB10	0.2660232	3.5677037	1.6119947	0.1084846	0.2068029	-5.37899
CTD-3105H1	-0.19514	-1.249581	-1.611975	0.1084888	0.2068029	-5.37902
AP000783.1	0.3824736	0.6917346	1.6118255	0.1085214	0.2068427	-5.379256
NDUFB8P2	-0.348548	0.8507934	-1.61182	0.1085225	0.2068427	-5.379264
AC003986.6	0.1988211	-1.385013	1.6118013	0.1085267	0.2068427	-5.379294
AC105760.2	-0.19363	3.1048175	-1.611484	0.1085958	0.2069636	-5.379793
PVRL1	-0.063523	5.9629121	-1.6113	0.108636	0.2070294	-5.380083
RP11-269F2	0.2670586	-0.904554	1.6112734	0.1086418	0.2070296	-5.380125
ERV3-1	0.092883	5.0774223	1.6110897	0.1086818	0.2070951	-5.380414
RP11-507K1	-0.326996	1.5509503	-1.610958	0.1087105	0.2071319	-5.38062
RPS15AP11	-0.371817	0.7141949	-1.610949	0.1087125	0.2071319	-5.380635
CLPTM1L	0.0327417	6.732189	1.6108612	0.1087316	0.2071519	-5.380773
DNAJA1P3	-0.360466	0.0729002	-1.610849	0.1087343	0.2071519	-5.380792
PRKAA1	0.0346882	6.4148368	1.6107803	0.1087493	0.2071641	-5.3809
RNF139	0.0352779	6.2176717	1.6107676	0.1087521	0.2071641	-5.38092
RP11-158J3	-0.166312	-1.443294	-1.610666	0.1087742	0.2071955	-5.38108
RP1-199J3.	0.2733095	2.4320986	1.6104533	0.1088206	0.2072731	-5.381414
BACH1-AS1	-0.37272	0.573901	-1.610214	0.1088728	0.2073606	-5.38179
ALDOB	0.1577438	7.3051914	1.6101883	0.1088785	0.2073606	-5.381831
NEDD8	-0.032489	6.4191381	-1.610165	0.1088836	0.2073606	-5.381868
WWC2-AS1	0.1878196	-1.370322	1.6099818	0.1089236	0.2074259	-5.382155
RP11-1149C	0.1418766	3.7918455	1.609602	0.1090066	0.2075731	-5.382752
FMO5	-0.116087	6.7445567	-1.609282	0.1090765	0.2076954	-5.383254
RP11-179B1	0.1800587	-1.352718	1.609125	0.1091109	0.2077426	-5.383501
NLRP8	0.1753087	-1.424519	1.609117	0.1091127	0.2077426	-5.383514
UPF1	-0.028868	6.6661196	-1.609047	0.109128	0.2077527	-5.383624
RP11-269G2	-0.330907	-0.556521	-1.609041	0.1091293	0.2077527	-5.383634

RP11-9E13.	-0.220301	-1.131006	-1.608984	0.1091418	0.2077656	-5.383723
GCNT4	0.1387804	5.1632855	1.6088842	0.1091636	0.2077962	-5.38388
RP11-21M24	0.24017	-1.088327	1.6087941	0.1091833	0.2078229	-5.384021
MED27	-0.040575	5.6799814	-1.608671	0.1092102	0.2078463	-5.384214
CEND1	0.315098	2.5316724	1.6086418	0.1092166	0.2078463	-5.38426
RAB43	0.0773489	5.2512729	1.6086351	0.1092181	0.2078463	-5.384271
RP11-50D9.	-0.342276	1.1360631	-1.608634	0.1092184	0.2078463	-5.384273
POP1	-0.047028	5.4174886	-1.608598	0.1092263	0.2078504	-5.384329
RP11-770G2	0.2120893	-1.179243	1.608524	0.1092424	0.2078704	-5.384445
RYR2	0.3788014	3.4227212	1.6084153	0.1092662	0.2079049	-5.384616
SNORA66	-0.323615	1.4225885	-1.608243	0.1093039	0.2079657	-5.384886
LINC01198	-0.268642	-1.230439	-1.608128	0.1093292	0.2080003	-5.385067
RP11-10A14	0.3168102	3.4134429	1.6081083	0.1093335	0.2080003	-5.385098
GTF2IRD2P1	0.3294769	1.8237688	1.6077004	0.1094229	0.2081595	-5.385738
MAGIX	0.1085532	5.6339872	1.6072346	0.109525	0.208343	-5.386469
EEF1A1P24	-0.30886	2.2711927	-1.60706	0.1095634	0.2084051	-5.386743
SLC22A6	-0.405357	-0.521872	-1.606837	0.1096123	0.2084872	-5.387092
OOEP	-0.509114	0.0903952	-1.606791	0.1096225	0.2084958	-5.387165
STX1A	-0.09079	4.8652871	-1.606638	0.109656	0.2085486	-5.387405
RP11-70601	0.5533222	1.9346756	1.6065907	0.1096664	0.208555	-5.387479
RN7SL132P	-0.132039	-1.446195	-1.606571	0.1096708	0.208555	-5.38751
LINC01118	-0.348316	-0.007317	-1.606527	0.1096805	0.2085626	-5.38758
RPS6P12	-0.209781	-1.24978	-1.606446	0.1096983	0.2085856	-5.387706
RP11-65I12	0.5012784	2.151663	1.6062632	0.1097384	0.2086509	-5.387993
STAU1	-0.03224	6.8327425	-1.606085	0.1097775	0.2087146	-5.388272
AC037459.4	0.2941073	-0.629193	1.6060474	0.1097858	0.2087193	-5.388331
PFDN1	0.0289895	6.1646286	1.6059453	0.1098082	0.2087512	-5.388491
RP11-598D1	-0.244035	-1.205275	-1.605779	0.1098448	0.2088013	-5.388752
LOXL1-AS1	0.2787819	3.5707335	1.6057733	0.109846	0.2088013	-5.388761
SPATA12	0.4280683	0.5091942	1.6057096	0.10986	0.208817	-5.38886
SLC35B1	-0.032929	6.5163651	-1.605593	0.1098857	0.208855	-5.389043
RNU6-20P	0.144841	-1.436491	1.6055592	0.1098931	0.2088582	-5.389096
TREM1	0.3537819	3.4727128	1.6053884	0.1099307	0.2089126	-5.389364
CTB-134F13	0.3024405	-0.532207	1.6053771	0.1099332	0.2089126	-5.389381
CCDC168	-0.372521	1.7603507	-1.605142	0.1099849	0.2089999	-5.389749
RP4-798A17	0.2861628	3.26765	1.605073	0.1100001	0.209018	-5.389858
IGKV1-5	0.3883904	4.7532764	1.604984	0.1100198	0.2090444	-5.389998
VMA21	0.0345225	6.1555619	1.6045414	0.1101172	0.2092187	-5.390691
SIGIRR	0.0676529	6.2769285	1.6045064	0.110125	0.2092225	-5.390746
GS1-21A4.1	-0.385847	0.2588805	-1.604301	0.1101701	0.2092975	-5.391067
TPTE2P5	-0.318393	2.3475707	-1.604174	0.1101982	0.2093399	-5.391266
PROX2	-0.365513	0.9736716	-1.604058	0.1102237	0.2093775	-5.391447
TMEM214	0.0242543	6.6393611	1.6039777	0.1102415	0.2094003	-5.391574
RPL5P3	-0.366211	0.5515545	-1.603863	0.1102668	0.2094374	-5.391753
PLEKHA7	-0.073114	5.891806	-1.603802	0.1102802	0.209452	-5.391848
RP11-299H2	0.5555798	2.1160424	1.6036689	0.1103096	0.2094971	-5.392057
LINC00672	-0.18808	3.6648962	-1.603554	0.110335	0.2095343	-5.392237
YWHAE	0.0275465	6.9384461	1.6033274	0.110385	0.2096184	-5.392592
RP11-174G6	-0.352389	-0.520245	-1.603266	0.1103985	0.209633	-5.392687
LL22NC03-N	-0.474045	1.169044	-1.603061	0.1104438	0.2097083	-5.393009
RP11-123G9	0.1417327	-1.478844	1.6028876	0.1104821	0.2097701	-5.39328

RP1-140K8.	0.4106909	3.2807829	1.6026082	0.1105439	0.2098764	-5.393717
RP11-734K2	-0.186753	-1.365851	-1.602548	0.1105572	0.2098907	-5.393811
TUBG2	-0.047782	5.6768611	-1.602451	0.1105786	0.2099206	-5.393963
NLRP9P	-0.399274	0.1387422	-1.602312	0.1106094	0.209968	-5.39418
RP1L1	0.3760285	0.8299885	1.6019937	0.1106798	0.2100907	-5.394678
SRP9	-0.033726	6.7587994	-1.60167	0.1107513	0.2102131	-5.395184
ARMC8	-0.022482	6.0713404	-1.60165	0.1107558	0.2102131	-5.395215
RP11-443B7	-0.136036	4.7606453	-1.601434	0.1108036	0.2102929	-5.395553
ITGA6	-0.044906	6.419093	-1.601342	0.1108241	0.2103193	-5.395698
C19orf60	0.0491963	6.1711759	1.6012889	0.1108358	0.2103193	-5.39578
C11orf45	0.1831734	4.1883196	1.6012522	0.1108439	0.2103193	-5.395838
RP1-151F17	-0.214264	3.5384062	-1.601233	0.1108482	0.2103193	-5.395868
MIS18BP1	-0.053539	5.5721031	-1.601231	0.1108487	0.2103193	-5.395871
SULT2B1	0.4519821	2.4109597	1.6011933	0.110857	0.2103193	-5.39593
RP11-11601	0.272507	-0.962518	1.6011896	0.1108578	0.2103193	-5.395935
LANCL1-AS1	-0.377841	1.2788775	-1.601062	0.1108861	0.2103621	-5.396135
ZNRF2P2	0.3845065	0.6469824	1.600946	0.1109118	0.2103998	-5.396316
CTD-2012K1	0.278489	-0.917215	1.6008312	0.1109372	0.2104371	-5.396496
SP3	-0.030279	6.3815577	-1.6007	0.1109663	0.2104814	-5.396701
PCYT2	-0.05439	6.7291152	-1.600524	0.1110053	0.2105444	-5.396976
OR5BA1P	0.31319	-0.876358	1.6003853	0.1110361	0.2105827	-5.397192
RP3-393E18	0.2319842	-1.112681	1.600381	0.111037	0.2105827	-5.397199
RP11-762I7	-0.363139	0.8702304	-1.600314	0.1110518	0.2105926	-5.397303
C1QTNF9B	-0.346181	-0.391522	-1.600306	0.1110538	0.2105926	-5.397317
RP11-284H1	0.1206278	-1.475149	1.6001428	0.1110899	0.2106501	-5.397571
BAMBI	-0.102874	6.1050258	-1.59985	0.1111548	0.2107589	-5.398028
KIAA0020	0.0428481	5.9410396	1.5998322	0.1111588	0.2107589	-5.398056
CABP2	0.1111039	-1.484762	1.5997599	0.1111749	0.2107784	-5.398169
LINC00261	-0.20523	6.4958186	-1.599689	0.1111906	0.2107973	-5.39828
RP11-79C23	-0.338267	-0.730777	-1.599606	0.111209	0.2108213	-5.39841
RP11-13J8.	0.2398534	-1.067633	1.5995335	0.1112251	0.2108409	-5.398523
RP1-178F10	-0.259004	2.2489142	-1.599376	0.1112601	0.2108963	-5.398769
SLC22A10	-0.339759	5.3212518	-1.599178	0.111304	0.2109667	-5.399077
LINC00462	-0.340303	-0.923978	-1.599129	0.1113151	0.2109667	-5.399155
PBLD	0.098891	6.3804226	1.5991042	0.1113205	0.2109667	-5.399193
LINC00886	0.1793501	4.7268302	1.5990806	0.1113258	0.2109667	-5.39923
FAM168A	-0.031976	6.3219647	-1.599079	0.1113262	0.2109667	-5.399233
STX16-NPEF	-0.206097	3.7145249	-1.599032	0.1113366	0.210971	-5.399306
ZNF322	-0.065311	5.0106387	-1.599017	0.11134	0.210971	-5.39933
GAS2L2	0.2544386	-1.1564	1.598891	0.1113679	0.2110071	-5.399526
GATAD2B	-0.031277	6.1740701	-1.598879	0.1113706	0.2110071	-5.399545
SHFM1P1	-0.378715	0.1695787	-1.598528	0.1114486	0.211131	-5.400092
LILRA5	0.1809253	4.2181374	1.5985171	0.1114511	0.211131	-5.40011
ANKRD2	-0.450128	1.8909847	-1.598507	0.1114534	0.211131	-5.400125
RP11-680B3	-0.178099	-1.340309	-1.598383	0.1114809	0.2111721	-5.400318
RP11-739B2	-0.356174	0.2641761	-1.598078	0.1115487	0.2112897	-5.400794
NAT16	0.4106504	0.8090175	1.5980394	0.1115574	0.2112952	-5.400855
GSDMA	0.387845	2.6176596	1.5979459	0.1115782	0.2113237	-5.401001
BTF3P7	-0.311755	-0.392392	-1.597871	0.111595	0.2113356	-5.401118
RP11-326C3	0.1177427	-1.473065	1.5978655	0.1115961	0.2113356	-5.401126
C8orf37-AS	0.4025003	1.4291803	1.597248	0.1117337	0.2115852	-5.402089

IFI27L2	0.09599	5.4384867	1.5971631	0.1117526	0.21161	-5.402222
CTC-436P18	0.2975639	2.1225344	1.5970906	0.1117688	0.2116297	-5.402335
FHAD1	-0.237172	3.6391548	-1.596742	0.1118465	0.2117658	-5.402878
AC023669.1	0.1146869	-1.492627	1.596574	0.1118841	0.2118259	-5.40314
SMG7	-0.02817	6.5510459	-1.59652	0.1118961	0.2118378	-5.403224
AP001604.3	0.2281589	-1.155515	1.5958846	0.112038	0.2120954	-5.404215
RP11-353N1	-0.426518	1.7819804	-1.59584	0.1120479	0.2121032	-5.404284
HSPA8P8	-0.440616	0.9730255	-1.595798	0.1120574	0.2121062	-5.40435
C19orf84	-0.403513	0.6952134	-1.595781	0.1120611	0.2121062	-5.404376
RP11-136C2	0.161071	-1.340719	1.595612	0.1120989	0.2121666	-5.404639
CTB-114C7.	0.3446457	-0.271732	1.5954317	0.1121392	0.2122256	-5.40492
SLC9A3P2	-0.16689	-1.414153	-1.595421	0.1121417	0.2122256	-5.404937
IGKV2-24	0.5025988	2.5902044	1.5952936	0.1121701	0.2122602	-5.405135
NFIA-AS2	-0.420309	0.2738906	-1.595287	0.1121716	0.2122602	-5.405146
PHC3	0.0406326	5.9196637	1.5952071	0.1121894	0.212283	-5.40527
LINC00636	-0.258901	-1.122394	-1.595142	0.1122041	0.2122997	-5.405372
AC005102.1	-0.312799	-0.894564	-1.595109	0.1122115	0.2123018	-5.405423
SGPL1	0.0301174	6.4685977	1.5950845	0.1122168	0.2123018	-5.405461
PEBP1P2	-0.322476	2.1466039	-1.595017	0.1122321	0.2123196	-5.405567
ZNF487	0.0578939	4.8595011	1.5948737	0.112264	0.212369	-5.405789
PRTFDC1	-0.117971	4.9096955	-1.594739	0.1122942	0.2124084	-5.405999
RP11-785G1	-0.230419	-1.166064	-1.594729	0.1122965	0.2124084	-5.406015
LCMT1	0.0405799	5.8855947	1.5946545	0.1123131	0.2124132	-5.40613
VTI1BP2	0.2682762	-0.86264	1.5946315	0.1123182	0.2124132	-5.406166
ATAD2B	-0.039393	5.6813433	-1.594613	0.1123224	0.2124132	-5.406195
LINC00612	-0.319545	2.0116407	-1.594601	0.1123251	0.2124132	-5.406214
LINC01446	-0.61872	0.9389714	-1.594573	0.1123313	0.2124132	-5.406257
KEL	0.3497738	3.3630796	1.5945556	0.1123352	0.2124132	-5.406284
CCDC140	0.1287558	-1.493771	1.5945133	0.1123447	0.2124132	-5.40635
RP11-114F3	-0.359602	0.1914685	-1.59451	0.1123455	0.2124132	-5.406356
RP5-1050E1	0.418989	-0.302904	1.5944601	0.1123566	0.2124231	-5.406433
RP11-29G8.	0.167159	3.6498978	1.5943437	0.1123827	0.2124614	-5.406614
METAP1	-0.030645	6.1312618	-1.594132	0.1124301	0.2125401	-5.406944
MUC13	0.3318867	5.7873996	1.5940874	0.1124401	0.2125479	-5.407013
ZFR	-0.023292	6.4379508	-1.594035	0.1124517	0.2125589	-5.407094
NAP1L3	0.2407674	3.7851173	1.5939007	0.1124819	0.2126049	-5.407304
AC084219.3	-0.387244	1.1033402	-1.593793	0.1125061	0.2126396	-5.407471
STRC	-0.439835	1.2060718	-1.593678	0.1125318	0.2126771	-5.40765
RP11-259G1	0.4894897	-0.180909	1.5935621	0.1125578	0.2127059	-5.407831
MT-TE	0.3115898	-0.71099	1.5935585	0.1125586	0.2127059	-5.407836
AC007204.2	-0.261703	-1.118372	-1.593513	0.1125689	0.2127143	-5.407907
RP11-983P1	-0.304271	-0.496032	-1.593474	0.1125776	0.2127197	-5.407968
OR52E8	0.2979378	-1.15238	1.5931598	0.112648	0.2128409	-5.408456
PTTG1IP	0.0272684	6.8422766	1.593136	0.1126534	0.2128409	-5.408493
FAM174B	0.0958549	5.5449601	1.5929219	0.1127014	0.2129206	-5.408826
Six3os1_4	-0.119072	-1.474459	-1.592894	0.1127077	0.2129214	-5.40887
TTLL2	-0.458547	2.9879731	-1.592741	0.112742	0.2129753	-5.409108
PSG7	0.1054006	-1.496917	1.5927031	0.1127506	0.2129804	-5.409167
RPL21	-0.04161	6.7234747	-1.592608	0.1127718	0.2130095	-5.409314
MAN2C1	0.0397856	6.3372937	1.5925061	0.1127948	0.2130419	-5.409473
RP11-192C2	-0.378961	0.8718409	-1.592087	0.112889	0.2132089	-5.410125

RP11-358B2	-0.281234	-0.789895	-1.591836	0.1129453	0.2133041	-5.410514
RORB-AS1	0.170421	-1.399487	1.5917297	0.1129693	0.2133383	-5.41068
TPGS1	0.0825618	5.1217441	1.5915954	0.1129995	0.2133843	-5.410889
LINC00967	-0.31097	-0.958232	-1.59152	0.1130165	0.2133983	-5.411006
AC096670.3	-0.35839	0.2796497	-1.591511	0.1130185	0.2133983	-5.41102
SERPINH1	-0.043504	6.6576664	-1.591111	0.1131085	0.2135571	-5.411641
RPS2P41	-0.215427	-1.143769	-1.590891	0.113158	0.2136297	-5.411983
RP11-319E1	0.1958241	-1.341845	1.5908882	0.1131586	0.2136297	-5.411987
RP11-475J5	0.3925521	2.2146876	1.5908515	0.1131669	0.2136342	-5.412044
RP11-176D1	-0.31549	-0.580057	-1.590637	0.1132152	0.2137144	-5.412378
AP4M1	-0.040443	5.704485	-1.590388	0.1132714	0.2138035	-5.412765
LDHAL6CP	-0.134894	-1.462121	-1.590375	0.1132741	0.2138035	-5.412784
RP11-66D17	0.3229353	-0.402412	1.5900949	0.1133374	0.213911	-5.413219
RP11-641C1	-0.176897	-1.305514	-1.590071	0.1133428	0.213911	-5.413257
RP11-447E2	0.1192934	-1.503352	1.5899	0.1133813	0.2139726	-5.413522
CTC-510F12	-0.186843	3.3155674	-1.589797	0.1134046	0.2140054	-5.413682
JMJD1C	0.0413965	6.2216569	1.5897205	0.1134218	0.2140269	-5.413801
ATP13A2	0.044628	6.0193177	1.589678	0.1134314	0.2140339	-5.413866
PRDX2P1	-0.173946	-1.325446	-1.589433	0.1134866	0.214127	-5.414246
RP11-513G1	0.468309	3.3592637	1.5893281	0.1135104	0.2141566	-5.41441
PCAT14	-0.48698	0.1685829	-1.589312	0.113514	0.2141566	-5.414435
CCT4	-0.026428	6.6952288	-1.589251	0.1135278	0.2141715	-5.414529
RP11-863P1	0.1861397	-1.341189	1.5891365	0.1135536	0.2142092	-5.414707
RP11-22P6.	-0.330953	0.9949795	-1.589074	0.1135677	0.2142247	-5.414804
PDHA2	-0.18185	-1.380332	-1.588998	0.1135848	0.2142459	-5.414921
SSU72P1	-0.157959	-1.41248	-1.588843	0.1136198	0.2142923	-5.415162
PIP5K1P1	0.1500981	-1.387573	1.5888373	0.1136212	0.2142923	-5.415171
MTRNR2L8	0.2123984	3.3960276	1.5887808	0.113634	0.2143053	-5.415259
FAM49B	-0.042715	6.2616756	-1.588694	0.1136536	0.2143312	-5.415393
RP1-229K20	0.1277667	-1.46998	1.588453	0.113708	0.2144229	-5.415767
ANAPC2	-0.032118	6.3117307	-1.588357	0.1137297	0.2144527	-5.415916
OR8G7P	-0.121276	-1.468799	-1.588324	0.1137372	0.2144558	-5.415967
LINC00846	-0.361658	-0.2717	-1.58826	0.1137517	0.2144599	-5.416067
LRRC3C	-0.349698	-0.531332	-1.588245	0.1137552	0.2144599	-5.41609
OR10J3	0.1880593	-1.322576	1.5882362	0.1137571	0.2144599	-5.416104
NUP62CL	-0.340026	3.3417219	-1.587875	0.1138387	0.2146028	-5.416663
MRPL44	0.0325681	6.2053393	1.5878266	0.1138497	0.2146124	-5.416739
HIST1H2BN	-0.173894	4.5083452	-1.58773	0.1138716	0.2146426	-5.416889
RP11-1007C	-0.257956	2.3404835	-1.587701	0.1138781	0.2146439	-5.416933
RP11-217B7	-0.208088	-1.233404	-1.587487	0.1139266	0.2147241	-5.417265
RP11-109G2	-0.230859	-1.033259	-1.587359	0.1139555	0.2147668	-5.417463
RP11-229A1	-0.177171	-1.319285	-1.587313	0.1139659	0.2147668	-5.417534
RP11-584P2	-0.347997	-0.262836	-1.587309	0.1139669	0.2147668	-5.417541
ENPP7P9	-0.194541	-1.296862	-1.587264	0.113977	0.2147747	-5.41761
AC091180.1	-0.312774	-0.448786	-1.587157	0.1140014	0.2148096	-5.417777
STEAP1	0.2060438	5.4817029	1.5870984	0.1140146	0.2148233	-5.417867
RP11-161H2	-0.387913	-0.205638	-1.586966	0.1140447	0.214869	-5.418073
FYTTD1P1	0.4512697	2.7149318	1.5868697	0.1140664	0.2148988	-5.418222
COQ5	-0.038831	6.2558869	-1.586752	0.1140931	0.2149381	-5.418404
RP11-12601	-0.366356	-0.458802	-1.58669	0.1141071	0.2149534	-5.4185
C10orf107	0.4130233	1.3502483	1.5866498	0.1141162	0.214958	-5.418562

RP11-255H2	-0.228777	-1.137704	-1.586627	0.1141213	0.214958	-5.418597
RP11-690G1	0.2024375	-1.296874	1.5865545	0.1141378	0.2149737	-5.41871
AC019172.2	0.3041939	-1.013458	1.5865321	0.1141429	0.2149737	-5.418745
DNAJC24	0.0513218	5.323307	1.5865124	0.1141474	0.2149737	-5.418775
RP11-111E1	0.2543691	-1.061273	1.58644	0.1141638	0.2149936	-5.418887
MCOLN3	-0.452697	3.2295801	-1.586125	0.1142351	0.2151168	-5.419375
FOXD3-AS1	0.3878453	-0.645396	1.5859038	0.1142854	0.2152004	-5.419718
MAPK14	-0.028746	6.4659782	-1.585829	0.1143023	0.2152211	-5.419833
RP3-473B4.	0.2293745	-1.163608	1.5856799	0.1143363	0.2152739	-5.420065
LDLRAD4	-0.062904	6.0462248	-1.585451	0.1143882	0.2153606	-5.420419
RP11-677I1	-0.328788	1.3253857	-1.585356	0.1144099	0.2153904	-5.420567
RP11-182J1	-0.380869	1.1458798	-1.585252	0.1144334	0.2154234	-5.420727
C1orf233	0.1443764	4.6847696	1.5851579	0.1144548	0.2154527	-5.420873
AC011524.1	-0.360018	-0.8036	-1.58496	0.1144998	0.2155263	-5.421179
bP-21264C1	-0.290873	2.6457501	-1.58479	0.1145384	0.2155783	-5.421442
KCND3-IT1	0.0878614	-1.537375	1.5847655	0.114544	0.2155783	-5.42148
ACKR2	0.1798501	5.1887388	1.5847357	0.1145508	0.2155783	-5.421526
ATP5G2P1	-0.212345	-1.139117	-1.584734	0.1145511	0.2155783	-5.421528
ELAVL1	-0.024546	6.4445884	-1.584702	0.1145585	0.2155811	-5.421578
PRSS21	-0.494186	1.3565531	-1.58464	0.1145726	0.2155965	-5.421675
AF131216.6	0.3633645	0.783642	1.5844435	0.1146172	0.2156694	-5.421978
RP11-680C2	0.2712029	-0.953626	1.5843767	0.1146324	0.2156814	-5.422082
NINJ2	0.1216022	5.3568643	1.5843638	0.1146354	0.2156814	-5.422102
WDR34	-0.052055	6.2423038	-1.584262	0.1146586	0.2157139	-5.42226
BCL2L2-PAB	-0.270115	2.6150948	-1.584236	0.1146645	0.2157139	-5.4223
FLOT1	0.0296365	6.7916025	1.5840578	0.114705	0.215779	-5.422575
IFNWP19	0.2047815	-1.360044	1.5838696	0.1147478	0.2158485	-5.422866
TOP1	0.0236485	6.4745799	1.583841	0.1147544	0.2158496	-5.42291
HEPHL1	-0.413253	1.9017619	-1.583692	0.1147884	0.2159025	-5.423142
RP11-522B1	0.1746074	-1.317193	1.5835175	0.114828	0.2159618	-5.423411
RP11-253I1	0.3199353	-0.333349	1.583501	0.1148318	0.2159618	-5.423436
AC006272.1	-0.364889	0.4674817	-1.583428	0.1148484	0.215982	-5.423549
LGALS9C	0.5002436	2.0058772	1.5833342	0.1148698	0.2160111	-5.423694
LINC01273	-0.242477	3.8750069	-1.583296	0.1148785	0.2160163	-5.423753
RP11-1055B	-0.288614	-0.999063	-1.583244	0.1148903	0.2160187	-5.423833
RPL17P35	-0.127489	-1.470332	-1.583239	0.1148916	0.2160187	-5.423842
STK32B	0.238811	3.4481929	1.5831779	0.1149054	0.216033	-5.423936
EIF4EBP3	0.0865927	5.410668	1.5831532	0.114911	0.216033	-5.423974
SIRPB1	0.216672	3.909322	1.5830578	0.1149328	0.2160628	-5.424121
SGK2	-0.124605	6.2009872	-1.582877	0.1149741	0.2161293	-5.424402
RPL7P3	0.1065283	-1.524308	1.5827334	0.1150068	0.2161764	-5.424623
AC009120.4	0.2964748	-0.732167	1.5827149	0.115011	0.2161764	-5.424651
AL121578.2	0.2708097	-0.951515	1.5826107	0.1150348	0.2162099	-5.424812
RP11-294J2	-0.304589	-0.294995	-1.582527	0.1150538	0.2162346	-5.424942
MPDU1	0.0417162	6.5064751	1.5824738	0.115066	0.2162463	-5.425024
DNAJC22	-0.093517	6.3938521	-1.582331	0.1150987	0.2162966	-5.425245
CRY1	0.0423577	5.8777964	1.5822937	0.1151071	0.2163013	-5.425302
IDE	0.0333994	6.2747537	1.5822234	0.1151231	0.2163203	-5.425411
RP4-593H12	-0.319015	-0.367346	-1.581996	0.1151751	0.2164068	-5.425763
EIF2B2	0.031487	6.0452193	1.5818457	0.1152094	0.2164601	-5.425994
FTH1P10	-0.236141	3.0489183	-1.581806	0.1152184	0.2164659	-5.426055

AC105398.3	0.1030257	-1.529647	1.5816868	0.1152457	0.216506	-5.42624
ETNPPL	0.3168763	5.6727165	1.5815621	0.1152742	0.2165392	-5.426432
RP11-692N5	-0.310426	-0.791664	-1.581557	0.1152752	0.2165392	-5.42644
TMEM8C	0.4128097	-0.349411	1.5813236	0.1153287	0.2166267	-5.426801
CTD-2301A4	-0.220719	-1.132015	-1.581302	0.1153336	0.2166267	-5.426834
FEZF1-AS1	0.5256103	1.879374	1.5812736	0.1153401	0.2166277	-5.426878
IKZF4	-0.049383	5.1534369	-1.581202	0.1153566	0.2166474	-5.426989
TBKBP1	-0.063818	5.5835351	-1.581054	0.1153902	0.2166995	-5.427216
AC131097.4	0.4278535	0.2315073	1.580856	0.1154356	0.2167736	-5.427523
MED14	0.0331276	6.1959942	1.5807554	0.1154586	0.2168057	-5.427678
AC110602.1	-0.202809	-1.183282	-1.580404	0.1155391	0.2169347	-5.428221
RP11-831H9	-0.227072	-1.067339	-1.580403	0.1155392	0.2169347	-5.428222
AC008079.1	-0.172151	3.5013327	-1.580282	0.1155669	0.2169754	-5.428408
GLT8D1	0.0371728	6.3620893	1.5802294	0.115579	0.2169871	-5.42849
RP1-111C20	0.1846933	-1.347179	1.5800766	0.115614	0.2170416	-5.428726
RP11-730B2	0.3096713	-0.413439	1.5800281	0.1156251	0.2170513	-5.4288
LINC01152	-0.459639	2.8358554	-1.579931	0.1156473	0.2170785	-5.42895
RP11-188C1	0.1100098	-1.530847	1.5799129	0.1156515	0.2170785	-5.428978
RP11-57C13	0.0712054	-1.545864	1.5798029	0.1156767	0.2171147	-5.429148
RARSP1	0.1342717	-1.436676	1.5793501	0.1157805	0.2172981	-5.429846
PATL2	0.1616111	4.232486	1.5793246	0.1157864	0.2172981	-5.429886
ANKRD26P3	-0.204036	-1.31527	-1.579192	0.1158167	0.2173438	-5.43009
FP671120.7	0.1454955	-1.467931	1.5789369	0.1158753	0.2174427	-5.430484
AP001630.5	-0.256035	-0.935547	-1.578753	0.1159175	0.2175106	-5.430767
SLC16A5	0.1119468	4.7359544	1.5785778	0.1159577	0.2175749	-5.431037
RP11-84A19	-0.382983	-0.136739	-1.578476	0.1159811	0.2176077	-5.431194
LINC01419	-0.739281	1.7304678	-1.578371	0.1160053	0.2176419	-5.431357
IGBP1-AS1	-0.342696	0.8774785	-1.57808	0.116072	0.2177557	-5.431804
GOLGA8O	0.2481435	-1.034826	1.5779814	0.1160947	0.2177859	-5.431956
RP11-163G1	-0.145995	-1.395542	-1.577959	0.1161	0.2177859	-5.431992
AKAP2	0.4172511	1.8460851	1.5779113	0.1161108	0.217795	-5.432064
SRPK3	-0.209204	3.5244833	-1.577836	0.1161282	0.2178164	-5.432181
MIR6814	0.1345181	-1.445306	1.5776486	0.1161712	0.2178859	-5.432469
RP11-274B2	0.3249526	2.4088166	1.5775063	0.1162039	0.2179361	-5.432688
RP11-76N22	-0.263634	-1.192336	-1.57737	0.1162353	0.2179836	-5.432898
DNAJA4	-0.112426	5.2550567	-1.577306	0.11625	0.2179896	-5.432997
AC024361.1	-0.25342	-0.95486	-1.577304	0.1162504	0.2179896	-5.433
LINC00377	-0.253424	-1.08067	-1.57725	0.1162629	0.2180018	-5.433083
AC111200.7	-0.268889	-0.824584	-1.577112	0.1162947	0.2180504	-5.433296
AC024937.1	-0.341055	1.2827561	-1.577042	0.1163107	0.2180691	-5.433404
UBE2D2	-0.024327	6.4473016	-1.576968	0.1163277	0.2180898	-5.433517
RP11-381E2	-0.259114	-0.921577	-1.576929	0.1163368	0.2180957	-5.433578
RP11-462B1	-0.210747	-1.2081	-1.57687	0.1163504	0.2180986	-5.433669
RP11-561N1	-0.349632	-1.005994	-1.576861	0.1163525	0.2180986	-5.433683
RP11-380G5	-0.332235	0.3417112	-1.576844	0.1163563	0.2180986	-5.433708
SLX4	0.0464425	5.479403	1.5767482	0.1163784	0.2181283	-5.433856
RRP12	-0.043103	6.1941982	-1.576723	0.1163841	0.2181283	-5.433894
NFAT5	0.0873343	5.693068	1.5766354	0.1164043	0.218155	-5.43403
YTHDF3	0.0329641	6.4797434	1.5764792	0.1164403	0.2182031	-5.43427
NFU1	-0.031865	6.029136	-1.576472	0.1164419	0.2182031	-5.434281
HNRNPDLP4	-0.180865	-1.37239	-1.576437	0.11645	0.2182071	-5.434335

RP11-382H2	-0.166051	-1.403032	-1.576327	0.1164753	0.2182432	-5.434504
RP11-317P1	0.3726843	1.5300307	1.5762971	0.1164823	0.2182451	-5.434551
DNAJB5	0.048644	5.4358201	1.5761388	0.1165187	0.2183022	-5.434794
AKAP3	0.2302402	3.2318879	1.576006	0.1165494	0.2183483	-5.434999
AC090696.2	-0.136408	-1.487863	-1.575907	0.1165722	0.21838	-5.435152
KRT8	0.0492549	7.2259643	1.5758504	0.1165852	0.2183931	-5.435238
RP11-538C2	0.1000923	-1.518592	1.5758168	0.116593	0.2183964	-5.43529
PRR5-ARHGA	0.3614532	-0.219471	1.5756104	0.1166406	0.2184744	-5.435608
RP11-498P1	-0.319176	2.3930582	-1.575559	0.1166524	0.2184853	-5.435686
AP002954.3	0.3709939	-0.012709	1.5755227	0.1166608	0.2184898	-5.435742
RPS24P13	-0.207649	-1.174068	-1.575272	0.1167186	0.2185869	-5.436128
CPA4	0.4380765	1.952602	1.5751028	0.1167577	0.2186489	-5.436388
BAI2	0.2808852	3.70989	1.5749672	0.116789	0.2186963	-5.436597
LINC00920	0.1785403	3.5477107	1.5749121	0.1168017	0.2187089	-5.436682
RP11-169K1	-0.284189	3.6820257	-1.574808	0.1168258	0.2187428	-5.436842
RP11-725M2	-0.115478	-1.498127	-1.574578	0.116879	0.2188311	-5.437196
AC005253.2	-0.121538	3.8002574	-1.574539	0.1168879	0.2188366	-5.437256
RP11-198M1	0.2001587	-1.184388	1.5743285	0.1169365	0.2189164	-5.437579
PCDHB19P	0.3767238	0.3785081	1.574251	0.1169544	0.2189387	-5.437698
WASIR1	-0.204104	-1.280955	-1.574181	0.1169707	0.2189579	-5.437807
RPS3AP26	-0.164126	3.8322202	-1.574068	0.1169968	0.2189956	-5.43798
KRT18P38	0.3356288	-0.239492	1.5740024	0.1170119	0.2190126	-5.438081
RPL5P11	-0.199695	-1.206714	-1.573804	0.1170577	0.2190871	-5.438385
CSNK2A2	0.0267507	6.3387708	1.5737574	0.1170686	0.2190962	-5.438457
IGKV1D-43	-0.428192	-0.283516	-1.573637	0.1170964	0.2191259	-5.438642
FAM218A	-0.42372	0.3178592	-1.573637	0.1170965	0.2191259	-5.438643
MFSD9	0.0460857	5.8281367	1.5733786	0.1171562	0.2192222	-5.439039
SLC33A1	-0.033384	6.3970908	-1.573363	0.1171599	0.2192222	-5.439064
RP11-332H1	-0.317402	-0.34518	-1.573282	0.1171787	0.219246	-5.439188
RP13-516M1	-0.29527	2.4374696	-1.573239	0.1171885	0.2192531	-5.439253
HCCAT5	0.2051117	-1.193406	1.5730921	0.1172225	0.2193057	-5.43948
LOC401913	-0.201916	-1.245656	-1.572772	0.1172966	0.219433	-5.439971
LINC01287	-0.675716	1.2450921	-1.572557	0.1173466	0.2195152	-5.440302
CTD-2132N1	-0.270282	2.7533403	-1.572349	0.1173948	0.2195941	-5.440621
AC004832.1	0.1998954	-1.261128	1.5721161	0.1174487	0.2196838	-5.440979
AC012531.2	-0.308224	-0.941727	-1.572036	0.1174674	0.2197075	-5.441102
IBSP	-0.558689	1.1577802	-1.571994	0.117477	0.2197141	-5.441166
CRTAM	0.2804871	3.4544252	1.5718615	0.1175078	0.2197604	-5.44137
RP11-28601	-0.144455	-1.462012	-1.571419	0.1176105	0.2199385	-5.442049
RBM25	-0.025499	6.3497074	-1.571399	0.117615	0.2199385	-5.442079
C2orf57	0.1370602	-1.478759	1.5712341	0.1176534	0.2199907	-5.442333
PRSS22	0.5236749	2.4136168	1.5712272	0.117655	0.2199907	-5.442343
RP3-337018	-0.345042	1.0016162	-1.571121	0.1176797	0.2200257	-5.442506
LAD1	-0.161855	6.3103865	-1.57109	0.1176868	0.2200277	-5.442553
TPT1P4	-0.1924	2.9595851	-1.571023	0.1177024	0.2200455	-5.442656
RP11-159D1	-0.265167	-0.974066	-1.570792	0.1177561	0.2201347	-5.443011
TREM2	0.1514461	5.1769768	1.5707255	0.1177716	0.2201523	-5.443113
SRGAP3-AS4	-0.173449	-1.343518	-1.570392	0.1178491	0.220271	-5.443624
MYL10	0.2330433	-1.072308	1.5703903	0.1178495	0.220271	-5.443627
NUDT16	0.0406322	6.4184643	1.5703745	0.1178532	0.220271	-5.443651
RP11-418I2	0.2916651	-0.911996	1.5702766	0.117876	0.2203013	-5.443801

RP11-552M1	-0.24019	2.9808033	-1.570253	0.1178815	0.2203013	-5.443838
RP11-49I11	-0.240774	3.0995957	-1.570218	0.1178896	0.2203052	-5.443892
MAGT1	0.0345251	6.6997869	1.570084	0.1179207	0.2203521	-5.444097
Z98750.1	-0.359357	0.208494	-1.569852	0.1179748	0.2204418	-5.444453
PAEP	0.6516571	1.4368826	1.5697157	0.1180065	0.2204897	-5.444662
DDX4	-0.360946	-0.46547	-1.569675	0.1180159	0.2204961	-5.444724
MSX2P1	-0.289164	-0.842544	-1.569311	0.1181008	0.2206433	-5.445282
CYP4F23P	-0.470071	1.3860025	-1.569276	0.1181088	0.2206471	-5.445335
AC073464.4	-0.144215	-1.454129	-1.569109	0.1181478	0.2207087	-5.445592
CNOT2	-0.019147	6.2973646	-1.568981	0.1181776	0.2207476	-5.445788
AVEN	0.047863	5.7803383	1.5689674	0.1181808	0.2207476	-5.445809
TMPRSS6	-0.163529	6.6035245	-1.568881	0.1182008	0.2207737	-5.44594
RP11-13A1.	-0.336617	-0.326695	-1.568671	0.1182499	0.2208542	-5.446263
ERMN	0.3205055	2.2772	1.5685432	0.1182797	0.2208875	-5.446459
INTS6	0.0394135	6.1144738	1.5684924	0.1182915	0.2208875	-5.446536
UNQ6494	0.3102746	2.8638922	1.5684854	0.1182931	0.2208875	-5.446547
RP11-586K1	-0.135168	-1.461987	-1.568481	0.1182941	0.2208875	-5.446553
CASZ1	0.1012941	5.0962091	1.5684547	0.1183003	0.2208875	-5.446594
RP11-547L9	-0.247218	-0.952736	-1.568438	0.1183041	0.2208875	-5.446619
KIF22	-0.040571	6.247352	-1.568329	0.1183297	0.2209241	-5.446787
UTS2	-0.501088	1.5529397	-1.568172	0.1183663	0.2209811	-5.447028
FAM153C	0.3821685	-0.020969	1.5679951	0.1184075	0.2210468	-5.447298
IGHD3-10	-0.180102	-1.384925	-1.567847	0.1184422	0.2211002	-5.447526
IGLVIV-64	0.1079561	-1.493591	1.5678167	0.1184492	0.2211019	-5.447571
TNFRSF13B	-0.436888	1.9061091	-1.567716	0.1184727	0.2211346	-5.447726
ZNF341-AS1	0.3576708	-0.09255	1.5676362	0.1184913	0.221158	-5.447848
SBK3	0.441117	1.5530538	1.5674817	0.1185274	0.221214	-5.448084
CHRND	-0.500934	0.5751666	-1.567349	0.1185585	0.2212607	-5.448288
RP11-112N2	-0.174277	-1.306799	-1.567272	0.1185764	0.2212737	-5.448405
BTNL9	0.1387086	5.246385	1.5672469	0.1185823	0.2212737	-5.448444
MAS1	0.234612	-1.197908	1.5672411	0.1185836	0.2212737	-5.448453
RP11-54015	0.1979902	-1.277312	1.567207	0.1185916	0.2212756	-5.448505
RP11-262H1	0.3595617	-0.316147	1.5671847	0.1185968	0.2212756	-5.448539
CD27-AS1	0.057569	5.6381001	1.5669904	0.1186422	0.2213416	-5.448836
RP4-777023	0.2631092	-0.877898	1.5669815	0.1186443	0.2213416	-5.44885
RP11-635L1	0.2585304	-0.972376	1.5667502	0.1186984	0.2214312	-5.449204
RP11-404P2	0.40035	0.2037293	1.5663918	0.1187822	0.2215763	-5.449752
CCT2	-0.027962	6.6171423	-1.566266	0.1188116	0.2216175	-5.449945
MYD88	0.0350886	6.3367613	1.5662455	0.1188165	0.2216175	-5.449976
CHMP2B	0.0316402	6.2273865	1.5662016	0.1188267	0.2216193	-5.450043
RP11-197B1	-0.175468	-1.424899	-1.566189	0.1188296	0.2216193	-5.450062
SPACA4	0.2387529	-1.189624	1.5661459	0.1188398	0.221627	-5.450129
RP11-1280N	-0.317269	2.2970803	-1.566095	0.1188517	0.221638	-5.450207
RP11-488L1	-0.398409	0.3780069	-1.566021	0.1188691	0.2216591	-5.45032
EIF2B5	-0.023982	6.3330383	-1.565909	0.1188951	0.221692	-5.45049
PRR31	-0.134709	-1.418402	-1.565883	0.1189013	0.221692	-5.45053
LINC01349	-0.190609	-1.29079	-1.565867	0.118905	0.221692	-5.450555
MEX3D	-0.053301	5.8020871	-1.565576	0.1189732	0.2218079	-5.451
SCT	-0.396265	1.2465835	-1.565524	0.1189854	0.2218194	-5.45108
C1orf111	0.4419586	0.8836895	1.5654853	0.1189945	0.2218249	-5.451139
CSTF3	-0.025762	6.0783232	-1.565457	0.1190012	0.2218261	-5.451183

COX7C	0.0372818	6.9139543	1.5652961	0.1190388	0.2218849	-5.451428
CLDN6	-0.468096	0.75933	-1.565142	0.119075	0.2219321	-5.451664
FBXO41	-0.154725	4.7079745	-1.565112	0.1190819	0.2219321	-5.451709
RP11-567M2	-0.162645	-1.379377	-1.56511	0.1190824	0.2219321	-5.451712
ZNF639	-0.026003	5.9992765	-1.564849	0.1191437	0.222035	-5.452112
LRRC74B	-0.247074	-1.071281	-1.564744	0.1191684	0.2220675	-5.452273
RP11-452D2	-0.125662	-1.471228	-1.564723	0.1191733	0.2220675	-5.452305
RP11-567J2	0.1558014	-1.375417	1.564622	0.1191969	0.2220857	-5.452459
PRR32	-0.171172	-1.375195	-1.564611	0.1191995	0.2220857	-5.452476
ANKRD13A	-0.030746	6.2641756	-1.564603	0.1192013	0.2220857	-5.452487
MAP3K9	-0.064155	5.4418384	-1.56444	0.1192397	0.2221459	-5.452737
RP11-85501	-0.302733	-0.897778	-1.564393	0.1192506	0.2221548	-5.452808
TWF1	0.0287871	6.2509424	1.5643052	0.1192713	0.222182	-5.452943
HSPE1	-0.038374	6.7734212	-1.564203	0.1192953	0.2222154	-5.453099
PRKRIRP5	-0.200467	-1.300054	-1.564048	0.1193317	0.222272	-5.453336
RP11-569G1	-0.210987	-1.22593	-1.563922	0.1193612	0.2223156	-5.453528
ADRBK2	-0.080434	5.7018176	-1.563887	0.1193695	0.2223197	-5.453582
RP3-326I13	-0.322828	-1.009571	-1.56378	0.1193946	0.222355	-5.453745
AJ003147.8	-0.251787	-1.070079	-1.563657	0.1194234	0.2223974	-5.453933
HNMT	0.0535044	6.5699303	1.5634919	0.1194623	0.2224584	-5.454185
RP11-277K2	-0.151961	-1.418256	-1.563449	0.1194724	0.2224658	-5.454251
RP3-508I15	0.2840473	2.1309468	1.563253	0.1195185	0.2225403	-5.45455
C7orf34	0.251011	-1.164975	1.5632069	0.1195293	0.2225491	-5.45462
RP11-283G6	0.3300003	-0.841355	1.5631166	0.1195505	0.2225567	-5.454758
RP11-57G10	-0.124469	-1.455157	-1.563082	0.1195587	0.2225567	-5.454811
RP11-167B3	-0.291814	-1.040527	-1.563082	0.1195588	0.2225567	-5.454812
TINAGL1	0.0657709	6.1345696	1.5630768	0.1195599	0.2225567	-5.454819
IGHV3-13	0.5082888	1.7824251	1.5630387	0.1195689	0.2225567	-5.454877
RP4-781K5.	-0.450602	0.156304	-1.563025	0.1195722	0.2225567	-5.454899
AC215219.2	-0.250867	-0.991644	-1.563008	0.119576	0.2225567	-5.454924
AP000235.2	-0.116567	-1.497593	-1.562956	0.1195883	0.2225681	-5.455003
LRG1	0.1122417	6.9808928	1.5628468	0.119614	0.2225943	-5.45517
RP11-10A14	-0.146225	-1.417334	-1.562845	0.1196145	0.2225943	-5.455174
NKX2-1-AS1	0.1258799	-1.495237	1.5627487	0.1196371	0.2226249	-5.45532
SHANK2-AS1	-0.221287	-1.105252	-1.562557	0.1196823	0.2226977	-5.455613
TBC1D1	0.043845	6.2955137	1.5624656	0.1197037	0.2227262	-5.455752
HNRNPH1P1	-0.357176	0.6695873	-1.562189	0.1197689	0.2228361	-5.456174
RNMTL1	0.0492781	5.8826144	1.5621532	0.1197773	0.2228403	-5.456229
AQP9	0.190576	6.4154269	1.5618146	0.1198571	0.2229774	-5.456745
C5orf15	0.0305725	6.4460231	1.5617658	0.1198686	0.2229874	-5.45682
MRPL18	-0.035023	6.3420432	-1.561382	0.1199589	0.2231442	-5.457405
COPG2	0.0360855	6.0282757	1.5613011	0.1199781	0.223159	-5.457529
RP11-475D1	-0.362344	0.8829093	-1.561297	0.1199792	0.223159	-5.457535
RP11-337C1	-0.24443	-1.011337	-1.561188	0.1200049	0.2231955	-5.457702
ANGPTL5	0.2229294	-1.23239	1.5610259	0.120043	0.2232551	-5.457948
IFITM10	-0.107379	5.6515919	-1.560957	0.1200594	0.2232741	-5.458054
EHD4-AS1	0.3564392	0.5903935	1.5609007	0.1200726	0.2232873	-5.458139
CD99	0.036682	6.8026551	1.5607916	0.1200983	0.2233236	-5.458306
CC2D1B	0.0286149	6.2461822	1.5607662	0.1201043	0.2233236	-5.458344
LINC01309	-0.122852	-1.494511	-1.56069	0.1201223	0.2233457	-5.458461
RP11-1E4.1	-0.263506	-0.815378	-1.56059	0.120146	0.2233783	-5.458613

AC005062.2	0.305449	-0.834094	1.559913	0.1203059	0.2236641	-5.459645
RP11-576C1	-0.361678	0.1949122	-1.559814	0.1203292	0.2236961	-5.459795
LINC01124	-0.212155	4.7078439	-1.559738	0.1203472	0.2237183	-5.459912
STIM2	0.0320367	5.9441118	1.5595987	0.1203802	0.2237681	-5.460124
CD96	0.1173974	5.0786387	1.5595297	0.1203965	0.223787	-5.460229
CTB-96E2.6	-0.308737	-0.680011	-1.55925	0.1204626	0.2238985	-5.460655
PILRB	-0.134551	5.0516278	-1.55921	0.120472	0.2239046	-5.460715
AC015691.1	0.2586185	-0.958576	1.5591821	0.1204787	0.2239057	-5.460758
AC007391.2	-0.268595	-0.789455	-1.55904	0.1205124	0.2239534	-5.460975
AC005754.8	-0.437776	0.8262173	-1.559022	0.1205167	0.2239534	-5.461002
RAF1	-0.030927	6.6709851	-1.558822	0.120564	0.22403	-5.461307
SMAD9	0.1238659	4.654504	1.5585611	0.1206257	0.2241311	-5.461704
JUND	0.0436439	6.7604296	1.55854	0.1206307	0.2241311	-5.461736
COMT	0.0517357	6.6701155	1.5583041	0.1206866	0.2242236	-5.462095
SNAI3	0.1143288	4.1616759	1.5579715	0.1207654	0.2243564	-5.462601
RP13-46H24	-0.303987	-0.741789	-1.557936	0.1207739	0.2243564	-5.462656
LINC00689	-0.516739	0.6905977	-1.557925	0.1207766	0.2243564	-5.462673
RNF152P1	0.1533045	-1.400418	1.5575853	0.120857	0.2244944	-5.463189
RP11-240L7	-0.39704	1.3279162	-1.557453	0.1208884	0.2245414	-5.463391
UBL5	-0.035539	6.6487315	-1.55736	0.1209105	0.2245709	-5.463532
AC009166.7	0.3569324	0.7805827	1.5573332	0.1209168	0.2245713	-5.463573
ADD1	0.0252418	6.7609827	1.5569831	0.1209999	0.2247141	-5.464105
PHF8	0.0467549	6.2338496	1.5567669	0.1210513	0.2247981	-5.464434
RP11-627K1	-0.34087	-0.2082	-1.556739	0.1210579	0.2247989	-5.464476
CTA-126B4.	0.374824	-0.166034	1.5565758	0.1210967	0.2248595	-5.464725
GP5	0.4224147	0.6435041	1.5564459	0.1211275	0.2249054	-5.464922
KIAA0355	0.0366013	6.015386	1.5557405	0.1212952	0.2252053	-5.465994
CTA-397H3.	-0.262904	-0.917788	-1.555543	0.1213423	0.2252813	-5.466295
RP11-321A1	-0.20346	3.6426659	-1.555464	0.121361	0.2253044	-5.466414
DHX33	-0.035252	5.9105232	-1.555432	0.1213687	0.2253074	-5.466464
HIST1H1PS1	-0.436664	1.1132153	-1.555214	0.1214207	0.2253923	-5.466795
GUSBP9	-0.36905	0.7399443	-1.555138	0.1214387	0.2254144	-5.466911
LGALS7B	-0.373357	-0.620526	-1.555051	0.1214593	0.2254411	-5.467042
HSPD1P5	-0.397141	1.2837698	-1.554974	0.1214776	0.2254635	-5.467159
RP11-368I2	-0.364166	-0.582241	-1.554949	0.1214837	0.2254635	-5.467197
AC144836.1	0.1423315	-1.451539	1.5548718	0.121502	0.2254761	-5.467314
IGLV2-8	0.4812474	3.2217067	1.5548683	0.1215029	0.2254761	-5.46732
UGT1A5	0.4946018	0.6694003	1.5548409	0.1215094	0.2254768	-5.467361
RP11-402G3	0.1994618	-1.231476	1.554785	0.1215227	0.22549	-5.467446
CTB-33018.	0.2981054	2.8189783	1.5545113	0.1215879	0.2255996	-5.467862
RP11-108P2	-0.274556	-0.820472	-1.554292	0.1216401	0.2256849	-5.468194
SLC26A2	-0.050689	5.6952788	-1.55371	0.121779	0.225931	-5.469078
NYAP2	-0.282808	-1.067234	-1.553614	0.121802	0.2259577	-5.469225
RP11-219A1	0.3496196	-0.68618	1.5535982	0.1218057	0.2259577	-5.469248
RN7SL684P	0.1823843	-1.30245	1.5535105	0.1218266	0.2259851	-5.469381
LUCAT1	0.5319978	2.4135412	1.5533227	0.1218715	0.2260568	-5.469666
GRN	0.0295911	7.026513	1.5532901	0.1218793	0.2260597	-5.469716
B3GNT4	-0.199552	3.2450197	-1.55289	0.1219747	0.2262222	-5.470322
ZFR2	-0.417942	1.0625953	-1.552871	0.1219793	0.2262222	-5.470351
RP11-637O1	0.0757849	-1.54353	1.5527169	0.1220162	0.2262792	-5.470585
ERBB2IP	0.0307651	6.4708527	1.5525204	0.1220632	0.2263548	-5.470883

PPAN	-0.05213	5.3736233	-1.552272	0.1221225	0.2264532	-5.47126
ZMAT2	0.0329436	6.4827899	1.5521584	0.1221498	0.2264923	-5.471433
RP11-778D9	-0.323322	1.1121547	-1.552028	0.122181	0.2265288	-5.47163
RP11-143E2	0.2140234	-1.342279	1.5520242	0.1221819	0.2265288	-5.471636
AGAP1	-0.046389	5.980998	-1.551896	0.1222126	0.2265744	-5.471831
CEP57	0.0437556	6.3141727	1.5517177	0.1222552	0.2266391	-5.472101
RP11-763B2	-0.269262	-0.923825	-1.551698	0.1222599	0.2266391	-5.472131
HIGD2A	0.0377172	6.4669496	1.5515448	0.1222966	0.2266955	-5.472363
AC016722.3	0.1770068	-1.368607	1.5513999	0.1223313	0.2267483	-5.472582
ZNF541	0.2567537	3.5519058	1.5512669	0.1223632	0.2267959	-5.472784
ELMOD3	-0.030861	5.8749044	-1.551063	0.122412	0.2268749	-5.473093
RP11-203I1	-0.194455	-1.231189	-1.550968	0.1224348	0.2269057	-5.473237
CYP2C18	0.2438037	5.905848	1.5508455	0.1224641	0.2269443	-5.473423
AKR1C4	-0.214836	6.4331663	-1.550829	0.1224681	0.2269443	-5.473448
RP11-40G16	-0.199997	-1.309743	-1.550762	0.1224841	0.2269625	-5.473549
RP5-902P8.	-0.315818	1.8356616	-1.550705	0.1224979	0.2269764	-5.473636
ATG10	0.0479795	5.4959656	1.5505601	0.1225325	0.2270291	-5.473855
LINC00910	-0.089663	4.8049439	-1.550403	0.1225703	0.2270875	-5.474093
CD48	0.1034996	5.4696824	1.5503099	0.1225926	0.2271128	-5.474234
FAM181B	0.3962392	-0.006335	1.550294	0.1225964	0.2271128	-5.474258
ARF3	0.0223529	6.6052926	1.5502424	0.1226088	0.2271242	-5.474336
DIRC3	0.3040256	2.0142735	1.5500446	0.1226562	0.2272006	-5.474636
EEF1GP2	-0.166609	-1.337044	-1.549956	0.1226775	0.2272285	-5.47477
MTND2P21	0.1133104	-1.483637	1.5499178	0.1226867	0.2272339	-5.474828
GDPD5	0.0642854	5.6741172	1.5495054	0.1227857	0.2273991	-5.475452
SPIN4	-0.105602	4.8592155	-1.549469	0.1227943	0.2273991	-5.475507
AC009948.7	-0.321174	1.388583	-1.549469	0.1227945	0.2273991	-5.475508
AC011738.4	-0.418901	0.2813752	-1.549343	0.1228246	0.2274432	-5.475697
RPL21P120	-0.356819	0.5491746	-1.549066	0.1228912	0.2275528	-5.476117
RN7SL130P	0.3201773	1.6985947	1.5490383	0.1228979	0.2275528	-5.476159
RP11-376M2	-0.264597	-0.825096	-1.549014	0.1229037	0.2275528	-5.476196
RP11-462G2	-0.442704	-0.29729	-1.548994	0.1229087	0.2275528	-5.476227
PRKG2	0.4533779	0.2650302	1.5489007	0.122931	0.2275825	-5.476367
RP11-42I10	-0.16721	3.3349731	-1.548867	0.1229391	0.227586	-5.476418
DEFA9P	-0.261812	-1.167569	-1.548791	0.1229574	0.2275941	-5.476534
AC005592.2	-0.408747	1.1752586	-1.548766	0.1229634	0.2275941	-5.476572
AF230666.2	-0.187916	2.8843937	-1.548763	0.1229641	0.2275941	-5.476576
RP3-327A19	-0.358994	0.7208096	-1.548745	0.1229684	0.2275941	-5.476603
CDRT15	-0.356019	0.0836288	-1.548699	0.1229794	0.2275941	-5.476672
EPS15L1	-0.033381	6.0697373	-1.548693	0.1229808	0.2275941	-5.476681
MIR4489	-0.344303	0.9140626	-1.548626	0.122997	0.2276124	-5.476783
USP32P3	0.398783	0.5387287	1.5484571	0.1230377	0.2276639	-5.477039
PCDHB11	0.2927729	3.6494393	1.5484351	0.123043	0.2276639	-5.477072
DPPA4	0.4465103	0.6109229	1.5484328	0.1230435	0.2276639	-5.477075
ZMYM1	-0.045693	5.4237346	-1.548298	0.1230759	0.2277123	-5.477279
CTD-2240E1	0.2296777	2.8853104	1.548214	0.1230962	0.2277303	-5.477406
RP11-484N1	0.3976367	3.7404254	1.5482057	0.1230982	0.2277303	-5.477419
CDC25B	-0.048332	6.3828483	-1.548082	0.1231279	0.2277738	-5.477606
MYADM	-0.042079	6.5028791	-1.548044	0.1231371	0.2277792	-5.477663
RP11-94B19	-0.154819	-1.37821	-1.547969	0.1231552	0.2278012	-5.477777
RNU5B-3P	0.1618337	-1.372785	1.5479144	0.1231683	0.2278139	-5.477859

AP001063.1	-0.397054	-0.278994	-1.547818	0.1231914	0.2278452	-5.478005
NBEAL2	-0.040846	6.0887851	-1.547745	0.1232091	0.2278559	-5.478116
CTD-324401	0.1211177	-1.47014	1.5477201	0.1232151	0.2278559	-5.478153
RNU6-1238F	-0.165592	-1.293732	-1.547716	0.123216	0.2278559	-5.478159
RP11-6J24.	0.2536291	-0.902911	1.5476007	0.1232438	0.2278959	-5.478334
NAGS	-0.168667	5.7666385	-1.547545	0.1232573	0.2279092	-5.478418
CTD-3187F8	-0.373024	0.3497507	-1.547337	0.1233074	0.2279811	-5.478733
PNOC	-0.459887	1.2857784	-1.547332	0.1233086	0.2279811	-5.478741
BCRP2	-0.381975	-0.468416	-1.547225	0.1233344	0.2280172	-5.478902
RPL7P46	0.2038759	-1.255103	1.5471855	0.1233439	0.2280231	-5.478962
CLTC	0.0221516	7.0170836	1.5471422	0.1233543	0.2280309	-5.479027
CTD-3116E2	-0.360657	-0.033009	-1.547097	0.1233652	0.2280395	-5.479096
PHBP13	0.2368889	-1.066627	1.5469185	0.1234082	0.2281075	-5.479365
RP11-267J2	-0.1741	3.0508472	-1.546869	0.1234201	0.2281179	-5.47944
AL356585.3	-0.2584	-0.790497	-1.546739	0.1234517	0.2281595	-5.479637
SOX18	0.0725644	5.6286005	1.546724	0.1234551	0.2281595	-5.479659
KCTD3	-0.039762	6.6615872	-1.546519	0.1235045	0.2282392	-5.479968
PKD1P5	0.2396608	3.425468	1.5464257	0.1235271	0.2282694	-5.48011
CCDC149	0.0664393	5.6259502	1.5460459	0.1236188	0.2284273	-5.480684
ANKRD9	0.0642773	5.8610443	1.5458128	0.1236751	0.2285197	-5.481036
CTD-2184D3	0.3189248	-0.744139	1.5457491	0.1236905	0.2285366	-5.481132
RN7SL574P	-0.339421	0.0523527	-1.545645	0.1237157	0.228565	-5.48129
RP1-138B7.	-0.121096	-1.473467	-1.545608	0.1237245	0.228565	-5.481345
TXNP1	-0.210983	-1.193493	-1.545608	0.1237246	0.228565	-5.481345
SHC2	0.0636815	6.4371249	1.5454512	0.1237625	0.2286233	-5.481582
CDK5R2	-0.358486	-0.664228	-1.545178	0.1238284	0.2287252	-5.481994
TIMM8B	-0.04072	6.1910623	-1.545171	0.1238302	0.2287252	-5.482005
OR9R1P	-0.119679	-1.496067	-1.545021	0.1238666	0.2287808	-5.482232
RNU7-3P	0.1421333	-1.405147	1.5449873	0.1238746	0.2287842	-5.482282
TMEM65	-0.057909	5.9940262	-1.544921	0.1238906	0.228802	-5.482382
LINC00598	-0.325046	1.5058829	-1.544774	0.1239263	0.2288565	-5.482605
FABP6	-0.527747	0.8170874	-1.544561	0.1239778	0.2289399	-5.482926
OVOL1-AS1	-0.492564	0.6789443	-1.544527	0.123986	0.2289435	-5.482977
H3F3C	-0.24292	2.247311	-1.544424	0.124011	0.2289697	-5.483133
PSAT1P3	-0.235146	-1.045731	-1.544417	0.1240127	0.2289697	-5.483144
TMPRSS4-AS	0.0881103	-1.519244	1.5443538	0.1240279	0.2289862	-5.483238
MYLK2	-0.346955	1.9994986	-1.544252	0.1240525	0.2290199	-5.483391
TSIX	-0.41268	0.2821721	-1.544067	0.1240975	0.2290915	-5.483672
SLC2A6	0.0947386	5.6652077	1.54389	0.1241403	0.2291588	-5.483938
CTB-186H2.	0.4344234	0.7304043	1.5437572	0.1241724	0.2292066	-5.484138
HUS1B	-0.354709	1.2804547	-1.54341	0.1242565	0.2293503	-5.484662
RN7SL2	0.0703485	5.8981764	1.5433645	0.1242676	0.229356	-5.484731
RPL21P134	-0.324151	1.7400101	-1.543346	0.1242722	0.229356	-5.484759
PDE6B	0.1740118	4.1848033	1.5430292	0.1243489	0.229486	-5.485236
GZMK	0.2043547	4.5286677	1.5427875	0.1244076	0.2295826	-5.485601
RP11-703M2	-0.252824	-1.177238	-1.542393	0.1245033	0.2297477	-5.486195
CEBPG	-0.038515	6.5781069	-1.542253	0.1245374	0.2297926	-5.486407
RP11-661C8	0.1850725	-1.331889	1.5422411	0.1245402	0.2297926	-5.486424
RP11-503N1	-0.281781	-0.566361	-1.541961	0.1246083	0.2299065	-5.486846
TREML3P	0.5532252	1.1339947	1.541749	0.1246598	0.22999	-5.487166
LRRIQ3	-0.236554	3.4220443	-1.541668	0.1246794	0.2299903	-5.487287

ACTL7B	-0.184725	-1.262445	-1.541651	0.1246837	0.2299903	-5.487314
RP11-219B1	-0.32173	1.7878149	-1.541645	0.124685	0.2299903	-5.487322
DOK6	0.2851785	3.9338187	1.5416447	0.1246852	0.2299903	-5.487323
ABCF2	0.0309263	6.2730307	1.5415609	0.1247055	0.2300162	-5.487449
RAB5B	0.021981	6.7122114	1.5413777	0.1247501	0.2300867	-5.487725
RP5-1182A1	0.4784734	1.5026324	1.5411525	0.1248049	0.2301762	-5.488064
AADACP1	-0.244033	4.4605433	-1.541074	0.124824	0.2301998	-5.488183
AATF	-0.030232	6.3975233	-1.54077	0.1248979	0.2303245	-5.48864
AP000487.6	-0.310766	1.6477362	-1.540302	0.1250118	0.2305229	-5.489344
CTD-2600H1	0.2277552	3.2252496	1.5400606	0.1250707	0.2306199	-5.489708
NPPA-AS1	-0.212647	3.3803221	-1.539965	0.1250939	0.2306468	-5.489851
TRIM31-AS1	-0.389423	0.3873165	-1.539949	0.1250979	0.2306468	-5.489876
AC006116.2	-0.286033	1.7037528	-1.539674	0.125165	0.2307588	-5.49029
RP11-598F7	-0.246219	2.7116489	-1.539446	0.1252206	0.2308496	-5.490633
PIWIL3	-0.240722	-1.186596	-1.539256	0.1252669	0.2309232	-5.490918
RP11-613D1	0.3134439	-0.46348	1.5391997	0.1252807	0.230937	-5.491003
PRSS37	0.1886255	-1.214234	1.5390994	0.1253051	0.2309704	-5.491154
XXbac-BPG2	-0.224707	2.521717	-1.539037	0.1253203	0.2309868	-5.491247
AP3D1	-0.034099	6.760943	-1.538999	0.1253296	0.2309921	-5.491304
CTD-2033A1	-0.287697	2.4131678	-1.538691	0.1254048	0.2311191	-5.491768
CHCHD5	0.0392922	5.9696548	1.5386036	0.1254262	0.2311369	-5.491899
IGLV3-21	0.3975861	4.4014665	1.5385997	0.1254271	0.2311369	-5.491905
RP11-713H1	-0.169456	-1.316115	-1.53832	0.1254954	0.2312436	-5.492325
CITF22-92A	-0.081019	4.6079047	-1.538311	0.1254977	0.2312436	-5.492339
TMEM185AP1	0.1527955	-1.431233	1.5380082	0.1255717	0.2313683	-5.492794
AC073464.7	-0.212937	-1.358936	-1.537979	0.1255789	0.2313699	-5.492839
CDC40	0.0417964	5.7780772	1.5379322	0.1255902	0.231372	-5.492909
STK25P1	0.1853358	-1.28376	1.5379178	0.1255938	0.231372	-5.49293
SLC2A14	-0.478125	2.523518	-1.537896	0.1255991	0.231372	-5.492963
AC136289.1	-0.323897	0.5471343	-1.537784	0.1256266	0.231411	-5.493132
KCNA3	0.3851101	2.406604	1.5377417	0.1256368	0.2314182	-5.493195
AP000472.2	0.4492221	2.2715724	1.5375726	0.1256782	0.2314824	-5.493449
TAF7	-0.035794	6.5039085	-1.537547	0.1256843	0.2314824	-5.493487
ARC	0.3475036	2.2786	1.5372316	0.1257616	0.231613	-5.493961
LCLAT1	-0.049398	5.8069668	-1.536935	0.1258343	0.2317264	-5.494407
RP11-108M9	-0.337376	-0.1072	-1.536928	0.1258359	0.2317264	-5.494417
NACAP3	-0.37508	0.6475138	-1.536766	0.1258757	0.231788	-5.494661
PCDHGB9P	-0.407494	1.0323115	-1.536725	0.1258856	0.2317945	-5.494721
ORAOV1P1	0.3768724	0.6927877	1.5366813	0.1258964	0.2318027	-5.494788
CYB5R1	0.041567	6.5118581	1.5366264	0.1259098	0.2318115	-5.49487
HS3ST5	-0.399765	-0.603279	-1.53661	0.1259138	0.2318115	-5.494895
NOMO2	0.0844359	5.4573829	1.53652	0.1259359	0.2318404	-5.49503
RP11-429J1	0.2907692	-0.90705	1.5364656	0.1259492	0.2318532	-5.495111
KLHL31	-0.162083	4.0967758	-1.536339	0.1259801	0.2318985	-5.495301
ASB6	-0.030804	5.9308524	-1.5362	0.1260144	0.2319499	-5.495511
RP5-827C21	-0.405674	0.1521111	-1.5361	0.1260387	0.2319829	-5.49566
CD1B	-0.420999	2.0722229	-1.536009	0.1260611	0.2320124	-5.495797
CLN6	-0.0345	6.3411942	-1.535947	0.1260764	0.232029	-5.495891
DMP1	0.3027724	-0.811661	1.5357931	0.1261141	0.2320865	-5.496121
PTMAP1	-0.274656	-0.688342	-1.535753	0.126124	0.2320931	-5.496182
AC007919.2	-0.107472	-1.502053	-1.535624	0.1261556	0.2321309	-5.496375

RP11-856M7	-0.287972	-0.671691	-1.535617	0.1261572	0.2321309	-5.496385
RBM33	-0.024264	6.2289378	-1.53556	0.1261712	0.2321448	-5.49647
SV2A	-0.152617	4.5491579	-1.535408	0.1262086	0.2321905	-5.496699
C22orf24	-0.336882	0.2582232	-1.535407	0.1262087	0.2321905	-5.4967
RP5-858L17	-0.306776	2.9890897	-1.535358	0.1262208	0.2322009	-5.496774
SNX18P13	-0.124735	-1.441777	-1.535279	0.1262402	0.2322251	-5.496893
RP11-398E1	-0.297791	-1.071614	-1.535236	0.1262507	0.2322326	-5.496957
PIEZO2	0.0841826	6.0617689	1.5350657	0.1262926	0.2322924	-5.497212
P2RY6	0.1476602	4.7505845	1.5350363	0.1262998	0.2322924	-5.497257
CTD-2540L5	0.3040626	-0.854801	1.5350262	0.1263023	0.2322924	-5.497272
HOXC-AS2	-0.467491	0.143254	-1.53496	0.1263185	0.2323105	-5.497371
QTRTD1	-0.031331	5.8081069	-1.53493	0.1263259	0.2323123	-5.497416
CTD-2515C1	-0.240477	-1.132274	-1.534777	0.1263635	0.2323698	-5.497645
SEPHS2P1	0.1455537	-1.403404	1.5345935	0.1264086	0.2324366	-5.497921
AC005255.3	-0.206663	3.0531228	-1.534577	0.1264126	0.2324366	-5.497945
ELK2BP	-0.211261	-1.321531	-1.534388	0.1264591	0.2325104	-5.498229
HP09025	0.3901052	0.6120064	1.5343613	0.1264656	0.2325108	-5.498269
TRIM47	0.0637715	6.172248	1.5342791	0.1264859	0.2325362	-5.498392
RP11-435J9	-0.291651	-0.565793	-1.534105	0.1265286	0.2326006	-5.498653
AP000867.1	-0.121502	-1.495173	-1.534085	0.1265336	0.2326006	-5.498683
RP13-210D1	0.3350837	-0.201783	1.5339123	0.1265761	0.2326669	-5.498942
MRPS15	0.0382529	6.5064954	1.5338019	0.1266032	0.2327051	-5.499107
ARRDC3	0.0507811	6.3539538	1.5336013	0.1266526	0.2327732	-5.499408
AC005795.1	-0.341181	0.6583887	-1.5336	0.126653	0.2327732	-5.499411
FAM213B	0.0504775	6.0439966	1.5333588	0.1267123	0.2328704	-5.499772
PDCD5	-0.037595	6.3580955	-1.533324	0.126721	0.2328719	-5.499824
BICD1	0.0930475	5.2046165	1.5333038	0.1267258	0.2328719	-5.499854
FOXE1	-0.471381	-0.099786	-1.532986	0.1268041	0.2330039	-5.50033
RPGR	-0.05489	5.3396752	-1.532898	0.1268258	0.2330321	-5.500462
COMMD3	-0.031098	6.0614788	-1.53285	0.1268376	0.2330421	-5.500534
CTB-178M22	-0.395357	1.016394	-1.53275	0.1268621	0.2330754	-5.500683
STK19	-0.034342	5.7184622	-1.532703	0.1268739	0.2330854	-5.500755
CA10	-0.348407	-0.505466	-1.532159	0.127008	0.23332	-5.501569
CTD-2046J7	-0.239413	-0.942687	-1.532011	0.1270445	0.2333751	-5.50179
FAM120C	0.0660712	5.5453504	1.531904	0.1270708	0.2334119	-5.50195
LGMN	0.039376	6.6163945	1.5318099	0.1270941	0.2334221	-5.502091
AC007318.5	-0.109426	4.6501204	-1.531803	0.1270958	0.2334221	-5.502102
SPTLC2	0.0285192	6.1434966	1.5317915	0.1270986	0.2334221	-5.502119
FLG2	-0.190385	-1.272102	-1.531778	0.127102	0.2334221	-5.502139
RPL39P5	-0.26469	-0.795507	-1.53166	0.1271311	0.2334638	-5.502316
AC087762.1	-0.250004	-1.173615	-1.531565	0.1271546	0.2334951	-5.502458
USE1	-0.044609	6.0948676	-1.531392	0.1271973	0.2335619	-5.502717
CTC-756D1.	0.3432527	-0.10599	1.5313354	0.1272112	0.2335757	-5.502802
RP11-582J1	0.1095433	3.7143793	1.5312179	0.1272402	0.2336172	-5.502977
IGKV2OR22-	-0.271198	-1.035713	-1.531105	0.1272681	0.2336532	-5.503146
GSTO1	0.0508009	6.8751568	1.5310868	0.1272726	0.2336532	-5.503174
COX4I2	-0.137417	4.4992298	-1.530955	0.1273053	0.2337013	-5.503371
TIPARP	0.0458363	6.0063956	1.5308745	0.1273251	0.233726	-5.503491
DHRSX-IT1	-0.324405	1.3109994	-1.530563	0.1274021	0.2338557	-5.503958
RP11-331F4	0.3088146	2.0862518	1.5302938	0.1274686	0.233966	-5.50436
SPTBN5	0.1179163	4.8355295	1.5301825	0.1274962	0.2340048	-5.504526

MRPS2	0.038037	6.3551562	1.5301397	0.1275068	0.2340124	-5.50459
CCDC51	0.0389645	5.8400867	1.5300523	0.1275284	0.2340404	-5.504721
PGBD4P3	0.3045274	-0.391852	1.5299687	0.1275491	0.2340644	-5.504846
MSI2	-0.040545	6.1908741	-1.529948	0.1275543	0.2340644	-5.504878
C7orf60	-0.048792	5.2635645	-1.529503	0.1276645	0.2342547	-5.505543
RP11-1151E	-0.263321	4.1684276	-1.529348	0.1277028	0.2343032	-5.505774
RP11-307C1	-0.339748	1.1226103	-1.529344	0.1277037	0.2343032	-5.50578
SLC9A8	0.0367474	6.0995224	1.5292872	0.1277178	0.2343173	-5.505865
RP11-348N5	-0.264202	2.5925315	-1.528993	0.1277907	0.234435	-5.506304
AP006621.9	0.3731829	1.7248905	1.5289764	0.1277948	0.234435	-5.506329
SERHL	-0.237602	3.0429889	-1.528902	0.1278132	0.234457	-5.50644
KLRF2	0.217393	-1.20072	1.5286936	0.127865	0.2345325	-5.506752
ATG16L1	0.0231523	6.0947828	1.5286844	0.1278672	0.2345325	-5.506766
RP11-360I2	-0.247879	-0.916417	-1.528613	0.127885	0.2345532	-5.506872
MANSC1	-0.053367	5.888647	-1.528584	0.1278921	0.2345546	-5.506916
RP11-44503	-0.300642	-1.017551	-1.528436	0.1279289	0.2346102	-5.507137
DPPA3	-0.124996	-1.493459	-1.5284	0.1279378	0.2346148	-5.507191
POU3F4	-0.323748	-0.997344	-1.528331	0.1279549	0.2346343	-5.507294
RP11-644A7	-0.209576	-1.151887	-1.528305	0.1279613	0.2346343	-5.507332
RP11-254F1	0.3790551	-0.067871	1.528275	0.1279688	0.2346362	-5.507377
CTB-50L17.	-0.291577	1.2318461	-1.528176	0.1279933	0.2346694	-5.507525
CTC-543D15	-0.336362	2.3743069	-1.528144	0.1280012	0.2346721	-5.507572
RP11-244B2	-0.134346	-1.466969	-1.527559	0.1281464	0.2349266	-5.508446
HLCS	0.0348615	6.0474733	1.527515	0.1281574	0.2349335	-5.508512
GM2A	-0.036457	6.5093986	-1.527492	0.1281631	0.2349335	-5.508546
ZNF503-AS2	-0.081053	5.0005794	-1.527253	0.1282225	0.2350305	-5.508903
RP11-238K6	0.1897066	-1.296899	1.5269145	0.1283067	0.2351731	-5.509408
C20orf196	-0.067905	4.8658347	-1.526854	0.1283216	0.2351768	-5.509498
RP13-39P12	-0.347731	0.3810836	-1.526836	0.1283262	0.2351768	-5.509526
RP11-370I1	-0.193722	-1.271512	-1.526829	0.128328	0.2351768	-5.509536
TSSC2	0.3497278	2.4401049	1.5267279	0.1283531	0.2352109	-5.509687
UBE2U	-0.52173	0.2616581	-1.526675	0.1283663	0.2352232	-5.509766
SNORD14A	-0.232429	3.0023055	-1.526638	0.1283755	0.2352284	-5.509821
SCGB1A1	-0.301648	-0.981072	-1.526231	0.1284767	0.235402	-5.510428
EXOG	-0.051586	5.2425409	-1.52604	0.1285243	0.2354774	-5.510713
RP11-1080G	0.3331043	0.032384	1.5259152	0.1285553	0.2355224	-5.510899
TIRAP	-0.049085	5.5489704	-1.525582	0.1286382	0.2356582	-5.511396
RP11-540D1	0.1319791	-1.419077	1.5255486	0.1286466	0.2356582	-5.511446
TPRA1	-0.027541	6.2769438	-1.52554	0.1286488	0.2356582	-5.511459
FEM1B	0.0293742	6.3310798	1.5254636	0.1286678	0.235666	-5.511572
CAMK2B	0.3823575	3.8838601	1.5254523	0.1286706	0.235666	-5.511589
CTD-2328D6	-0.300457	2.7819313	-1.525438	0.1286741	0.235666	-5.51161
KIR3DL1	0.3687884	-0.066584	1.5254091	0.1286814	0.235666	-5.511654
TUBGCP4	-0.037495	5.7443243	-1.525393	0.1286853	0.235666	-5.511677
PABPC4	-0.026874	6.7054515	-1.525135	0.1287496	0.2357718	-5.512062
TCEB1	-0.039312	6.3696619	-1.525053	0.1287701	0.2357976	-5.512185
SNORA28	0.2483048	-0.921452	1.5248854	0.1288119	0.2358623	-5.512434
RP11-65M17	-0.14026	-1.429585	-1.524852	0.1288203	0.2358659	-5.512485
RPL35P3	-0.16176	-1.334842	-1.524804	0.1288323	0.2358759	-5.512556
ZBTB37	-0.041522	5.8733419	-1.524761	0.1288429	0.2358835	-5.512619
UTP15	0.0315781	5.7907497	1.5247334	0.1288498	0.2358844	-5.512661

CTD-2330J2	0.137225	-1.42965	1.5245892	0.1288858	0.2359384	-5.512876
CTD-2231H1	-0.415089	-0.349332	-1.524545	0.1288967	0.2359466	-5.512941
IGHV3-22	-0.292996	-0.934664	-1.524346	0.1289465	0.2360218	-5.513238
SETP20	-0.291553	-0.466652	-1.524329	0.1289508	0.2360218	-5.513263
ZBTB24	-0.036203	5.5337817	-1.524295	0.1289593	0.2360256	-5.513314
MRPL3P1	-0.265519	-0.811137	-1.52411	0.1290054	0.236098	-5.513589
RP11-10A14	-0.371509	2.4938221	-1.524028	0.1290258	0.2361166	-5.513711
RP11-1281K	-0.208086	-1.209293	-1.524018	0.1290285	0.2361166	-5.513727
CTD-2325A1	-0.318979	-0.733841	-1.523899	0.1290581	0.2361591	-5.513904
BRD3	-0.044927	5.9867957	-1.523867	0.1290661	0.2361619	-5.513952
RP11-24M17	-0.207135	-1.26536	-1.523834	0.1290743	0.2361649	-5.514
ARID3C	-0.293072	4.4601162	-1.52378	0.1290879	0.236178	-5.514081
ST7	0.0413576	6.2024934	1.5236369	0.1291236	0.2362315	-5.514294
EML5	0.4517172	1.2322837	1.5236022	0.1291323	0.2362355	-5.514346
RPS20P24	-0.272465	-0.85425	-1.523404	0.1291817	0.2363141	-5.514641
RP11-464C1	0.1468336	-1.473778	1.5232104	0.1292302	0.2363785	-5.514929
AC021037.1	-0.197211	-1.178194	-1.523192	0.1292347	0.2363785	-5.514956
RP11-16E18	-0.165722	-1.373288	-1.523186	0.1292363	0.2363785	-5.514966
DYNC1I2P1	0.2772605	2.3645465	1.5231374	0.1292484	0.2363889	-5.515038
RP11-180I4	0.1995598	-1.251708	1.5229629	0.1292921	0.23645	-5.515298
C3	0.0682368	7.8162831	1.52293	0.1293003	0.23645	-5.515347
TMCC2	0.140584	4.1850709	1.5229259	0.1293013	0.23645	-5.515353
KRT18P34	0.2142436	3.8973656	1.5228267	0.1293261	0.2364836	-5.5155
MLH3	0.0368924	5.964976	1.5227741	0.1293393	0.2364863	-5.515579
RP11-576I2	0.2824912	2.3950322	1.5227689	0.1293406	0.2364863	-5.515587
ABC7-43041	-0.307182	-0.280914	-1.522731	0.1293502	0.236492	-5.515643
CTD-2184D3	-0.25731	-0.884278	-1.522692	0.12936	0.236498	-5.515702
DRC1	0.3908877	1.5806133	1.5223133	0.1294546	0.2366592	-5.516264
RP11-618L2	-0.294114	-0.713332	-1.521907	0.1295565	0.2368308	-5.516869
C17orf98	0.1469992	-1.417117	1.5218868	0.1295615	0.2368308	-5.516899
RP1-21018.	-0.251309	-1.037531	-1.52175	0.1295957	0.2368815	-5.517102
RANP6	-0.228482	-1.010258	-1.521664	0.1296172	0.2368942	-5.51723
PPP1R14BP3	-0.054677	5.6097601	-1.521646	0.1296217	0.2368942	-5.517257
SMAD3	-0.037465	6.3594832	-1.521645	0.1296221	0.2368942	-5.517259
RP13-188A5	0.3071052	1.8642148	1.5216043	0.1296322	0.2369009	-5.517319
RP11-235C2	-0.261921	-0.958822	-1.521451	0.1296706	0.2369396	-5.517547
TDRP	0.1175951	5.5984426	1.5214306	0.1296758	0.2369396	-5.517577
MRPL49	0.0311492	6.4041672	1.5214241	0.1296774	0.2369396	-5.517587
BCAT1	0.0944329	5.4044754	1.5214162	0.1296794	0.2369396	-5.517599
RP6-24A23.	0.1379227	-1.45758	1.5213891	0.1296862	0.2369402	-5.517639
FLJ45079	0.3082847	-0.789836	1.521154	0.1297451	0.237036	-5.517989
STMN4	-0.291305	-0.759409	-1.520985	0.1297875	0.2371016	-5.51824
CTD-2024I7	0.2053354	-1.179777	1.5208061	0.1298324	0.2371642	-5.518506
SNHG5	-0.071773	6.329657	-1.520797	0.1298348	0.2371642	-5.51852
RP13-58209	-0.095899	5.0257268	-1.520638	0.1298747	0.2372153	-5.518756
RP11-941H1	-0.276849	-0.532431	-1.520633	0.1298758	0.2372153	-5.518763
RP11-646I6	-0.33139	1.5056405	-1.52029	0.1299619	0.237354	-5.519273
EDEM2	-0.02823	6.3443708	-1.520279	0.1299647	0.237354	-5.519289
STRA13	-0.052139	6.2763447	-1.52022	0.1299796	0.2373693	-5.519377
USP42	-0.032348	5.7389939	-1.520188	0.1299876	0.237372	-5.519424
AC007403.2	0.1591965	-1.382443	1.5200885	0.1300126	0.2374058	-5.519572

VTN	-0.09981	7.6529234	-1.52002	0.1300298	0.2374253	-5.519674
BAK1	-0.048072	5.9450103	-1.519975	0.1300412	0.2374343	-5.519742
GS1-259H13	0.3269067	2.377713	1.5198044	0.130084	0.2375005	-5.519994
KCNMB2	-0.392714	0.0838615	-1.519596	0.1301364	0.2375844	-5.520305
RP11-597D1	0.2191376	-1.137233	1.5193444	0.1301996	0.2376878	-5.520678
RPS20P2	-0.319038	-0.275334	-1.519296	0.1302117	0.237698	-5.520749
RP11-428L2	-0.177361	-1.293441	-1.518595	0.1303881	0.2380048	-5.52179
RP11-347C1	0.0911666	4.6567861	1.5185525	0.1303989	0.2380048	-5.521853
CTC-325H20	-0.335843	-0.136972	-1.518551	0.1303993	0.2380048	-5.521856
RP11-104N1	0.184984	2.8456651	1.5184543	0.1304236	0.2380373	-5.521999
EEF1E1	-0.054752	5.5592632	-1.518047	0.1305261	0.2382125	-5.522603
RP11-96L7.	-0.163613	-1.404228	-1.517994	0.1305395	0.2382249	-5.522682
CTD-2154B1	-0.163654	-1.399628	-1.517953	0.1305499	0.2382302	-5.522743
EEF1B2P5	0.1235223	-1.462894	1.517931	0.1305554	0.2382302	-5.522776
AP000254.8	-0.075296	4.4742293	-1.517777	0.1305943	0.2382892	-5.523005
CCDC70	-0.151194	-1.371757	-1.517735	0.1306048	0.2382964	-5.523066
AC137723.5	0.1717294	-1.31604	1.5176606	0.1306236	0.2383017	-5.523177
YWHAQP7	-0.170818	-1.295749	-1.51765	0.1306262	0.2383017	-5.523192
RP3-334F4.	-0.096269	-1.507547	-1.517646	0.1306273	0.2383017	-5.523199
RP11-1072A	-0.344269	1.2638087	-1.517587	0.1306421	0.2383169	-5.523286
ATP5HP3	0.1252678	-1.453691	1.5174632	0.1306734	0.238362	-5.52347
CTC-412M14	-0.233266	-1.071923	-1.517369	0.1306972	0.2383936	-5.52361
DMD	0.0712573	6.0694698	1.5172408	0.1307295	0.2384405	-5.5238
RP3-473L9.	-0.377427	0.6711636	-1.517199	0.13074	0.2384479	-5.523862
RP11-58H15	-0.300787	-0.352174	-1.517086	0.1307685	0.2384878	-5.524029
HPN-AS1	-0.232852	4.043105	-1.517018	0.1307856	0.2385072	-5.52413
SPNS1	-0.073982	4.9214387	-1.51688	0.1308205	0.2385589	-5.524335
C7orf61	0.3247551	1.8617863	1.5168492	0.1308283	0.2385612	-5.52438
SRGAP2-AS1	-0.251233	-0.953329	-1.51682	0.1308356	0.2385625	-5.524423
ENTPD5	0.0738395	6.735599	1.5166291	0.1308839	0.2386387	-5.524707
TM2D2	0.0393015	6.1407257	1.5165387	0.1309067	0.2386684	-5.524841
ATF6B	-0.025742	6.5574473	-1.516423	0.130936	0.2387099	-5.525013
RIMKLBP2	-0.340118	1.7749456	-1.516129	0.1310103	0.2388333	-5.525448
RP11-168A1	-0.381813	0.3215202	-1.516055	0.131029	0.2388556	-5.525558
RP11-44B19	0.2955192	-0.792616	1.5159926	0.1310447	0.2388677	-5.52565
TTC1	0.0311427	6.3109375	1.5159766	0.1310487	0.2388677	-5.525674
IGLV4-69	0.4636823	3.3744959	1.5159307	0.1310603	0.2388769	-5.525742
CTA-292E10	-0.28021	1.8191507	-1.515871	0.1310753	0.2388849	-5.52583
MORN5	0.2286627	-1.118567	1.5158616	0.1310778	0.2388849	-5.525844
RP11-815J4	0.3660836	0.8404923	1.5158027	0.1310927	0.2389001	-5.525931
HOOK1	-0.106682	6.158874	-1.515731	0.1311108	0.2389212	-5.526038
PIGS	-0.038642	6.0500063	-1.515628	0.1311369	0.2389504	-5.52619
SLC5A1	0.5571473	2.351448	1.5156158	0.13114	0.2389504	-5.526208
B4GALNT2	-0.630113	2.5639571	-1.515148	0.1312583	0.2391541	-5.526901
NUP88	-0.037552	6.2154682	-1.514885	0.1313249	0.2392635	-5.527291
ASCL2	-0.253278	3.5149701	-1.514764	0.1313554	0.2393072	-5.52747
TNXA	0.1973509	4.4356912	1.5147197	0.1313667	0.2393159	-5.527536
ERCC6	-0.038582	5.4357832	-1.514453	0.1314342	0.2394268	-5.52793
RP11-150D5	-0.248398	-1.065606	-1.514271	0.1314803	0.2394988	-5.528199
RP11-108H9	-0.185978	-1.284635	-1.514229	0.1314909	0.2395063	-5.528261
RP11-81H14	-0.196643	-1.324144	-1.51414	0.1315137	0.239524	-5.528394

ABCB6	-0.073264	5.6648823	-1.514139	0.1315138	0.239524	-5.528395
RP11-1081M	-0.127889	-1.448231	-1.514075	0.1315302	0.2395419	-5.52849
ZNF229	-0.25011	4.2112136	-1.514014	0.1315454	0.2395577	-5.52858
GCGR	0.4480497	4.7986695	1.5137572	0.1316107	0.2396646	-5.52896
RP11-148B1	0.3597512	-0.153298	1.5136921	0.1316272	0.2396826	-5.529056
GSTM1	0.4330507	4.0990598	1.5136349	0.1316417	0.2396971	-5.529141
NDUFAF4P3	-0.369021	0.5885972	-1.51331	0.1317242	0.2398313	-5.529622
TPGS2	0.0424807	6.2305267	1.5132599	0.1317368	0.2398313	-5.529696
RP5-1047A1	-0.139654	-1.398651	-1.513256	0.1317379	0.2398313	-5.529702
ASS1P9	0.3833114	0.8320736	1.5132229	0.1317462	0.2398313	-5.529751
TMED4	0.0238631	6.5513206	1.5132149	0.1317482	0.2398313	-5.529762
SETD2	-0.024368	6.3925518	-1.513099	0.1317776	0.2398729	-5.529934
AC109642.1	0.2960219	1.8368883	1.5130415	0.1317923	0.2398753	-5.530019
CLCN5	-0.062946	6.1678651	-1.51302	0.1317976	0.2398753	-5.53005
CTB-49A3.4	-0.310464	-0.819839	-1.512997	0.1318035	0.2398753	-5.530084
RP5-1049N1	-0.141542	-1.418583	-1.51299	0.1318053	0.2398753	-5.530095
CTC-43909.	0.243253	-0.999131	1.5129372	0.1318188	0.2398879	-5.530173
BTBD17	-0.24115	-1.018579	-1.51244	0.1319452	0.240106	-5.530909
PHF20L1	0.031773	6.1658144	1.5123996	0.1319553	0.2401125	-5.530968
NAA30	0.031161	6.0599989	1.512288	0.1319837	0.2401522	-5.531133
AGGF1P3	-0.245632	-1.07802	-1.51199	0.1320594	0.2402779	-5.531573
AC008073.7	-0.284814	-0.543882	-1.511865	0.1320913	0.240324	-5.531759
RELB	0.0490226	6.1066982	1.5118308	0.1320999	0.2403277	-5.531809
NOC2L	0.036368	6.4753654	1.5117605	0.1321178	0.2403483	-5.531913
RP11-45A17	-0.148507	3.0114008	-1.511706	0.1321316	0.2403612	-5.531993
RP11-143M1	0.1689203	-1.424863	1.5116808	0.1321381	0.2403612	-5.532031
C11orf68	-0.029226	6.163057	-1.511613	0.1321553	0.2403806	-5.532131
OLA1P1	-0.133003	3.7547722	-1.511437	0.1322001	0.2404501	-5.532391
IFNG	-0.450529	1.5521824	-1.511178	0.132266	0.2405486	-5.532773
AGA	0.0533596	6.0175857	1.5111725	0.1322675	0.2405486	-5.532782
RPS3P2	-0.223735	-1.016432	-1.510967	0.1323198	0.2406319	-5.533086
RP3-508I15	-0.140889	3.7293925	-1.510842	0.1323517	0.2406778	-5.53327
GPR112	0.2252276	-1.208868	1.5107385	0.132378	0.2407115	-5.533423
RP11-401E5	0.4983406	0.3788702	1.5107173	0.1323834	0.2407115	-5.533454
CTD-2582M2	0.1113765	-1.491848	1.5106367	0.1324039	0.2407368	-5.533573
C1QTNF9B-A	0.3459197	1.6079817	1.5104495	0.1324516	0.2408029	-5.533849
RP11-788A1	0.1741825	-1.439101	1.5104423	0.1324535	0.2408029	-5.53386
TECPR1	-0.035002	6.1764629	-1.510366	0.132473	0.2408265	-5.533973
OR6E1P	-0.306149	-0.359115	-1.510069	0.1325487	0.2409483	-5.534412
CD79B	-0.092033	5.0534191	-1.510031	0.1325584	0.2409483	-5.534468
RP11-78C3.	-0.166423	-1.370572	-1.510025	0.1325599	0.2409483	-5.534476
MTND4P19	0.4251995	0.6943923	1.5098595	0.1326021	0.241013	-5.53472
AC002480.5	-0.325407	-0.676308	-1.509829	0.1326099	0.2410153	-5.534766
CKS1BP6	-0.179406	-1.243818	-1.509788	0.1326202	0.2410221	-5.534826
PNLIPRP3	0.26141	-1.176989	1.5097396	0.1326327	0.2410326	-5.534897
AC096559.1	0.4225478	0.4076749	1.509695	0.132644	0.2410413	-5.534963
TRBV11-2	-0.406612	0.7051598	-1.5096	0.1326682	0.2410732	-5.535103
RPS26P15	-0.345807	0.1888597	-1.509452	0.1327062	0.2411302	-5.535322
PPAP2C	-0.2749	5.3429812	-1.509416	0.1327153	0.2411348	-5.535375
AC013404.1	-0.167159	-1.324194	-1.509249	0.1327579	0.2412002	-5.535621
SNORA16B	-0.31321	-0.343353	-1.509121	0.1327905	0.2412422	-5.53581

PACERR	0.2676204	-0.887878	1.5091067	0.1327942	0.2412422	-5.535831
GOLPH3L	-0.038702	6.0917612	-1.509018	0.1328169	0.2412714	-5.535963
RP11-409C1	-0.232761	-1.103046	-1.508915	0.1328432	0.2413071	-5.536114
UBR5	-0.031215	6.4468702	-1.508823	0.1328666	0.2413376	-5.536249
NAALADL2-A	0.1762303	-1.412535	1.508703	0.1328973	0.2413815	-5.536427
RP11-12D5.	-0.184968	-1.367383	-1.508573	0.1329306	0.2414299	-5.536619
XXbac-BPG3	-0.191406	-1.389126	-1.508483	0.1329537	0.2414598	-5.536752
RP11-541M1	0.3111167	-0.338331	1.5083761	0.1329809	0.2414972	-5.536909
RRP36	-0.027951	6.2305583	-1.508311	0.1329975	0.2415059	-5.537004
KIR3DL3	0.1118557	-1.502383	1.5083057	0.1329989	0.2415059	-5.537013
LINC00535	0.4099003	3.0370342	1.5082245	0.1330197	0.2415108	-5.537132
HNRNPA1P67	-0.221479	-1.039946	-1.50822	0.1330207	0.2415108	-5.537138
TUBB2A	-0.068797	6.2539874	-1.508217	0.1330215	0.2415108	-5.537143
AP000797.4	0.2109997	-1.210876	1.5081675	0.1330343	0.2415114	-5.537217
RP11-944L7	-0.351671	-0.025789	-1.508164	0.1330351	0.2415114	-5.537221
RP11-548H3	-0.139072	3.8165299	-1.508092	0.1330536	0.241533	-5.537328
RP4-811H24	-0.258538	-0.801702	-1.507981	0.133082	0.2415727	-5.537492
RP3-408N23	-0.157668	-1.311523	-1.507923	0.1330968	0.2415874	-5.537577
ALPK3	0.1289832	5.7195206	1.5078293	0.1331208	0.2416099	-5.537715
KCNIP4	0.1222599	3.6375937	1.507823	0.1331224	0.2416099	-5.537724
RPS15AP6	-0.162025	-1.358676	-1.507726	0.1331472	0.241643	-5.537867
HIST1H2AC	0.0736402	6.4078376	1.5075774	0.1331852	0.2416999	-5.538086
RNU6-665P	0.1555228	-1.38076	1.5071992	0.1332821	0.2418452	-5.538644
ALG1L9P	-0.181914	3.4728304	-1.507196	0.133283	0.2418452	-5.538649
WFDC10B	0.2222902	-1.181251	1.5071872	0.1332851	0.2418452	-5.538661
ATP5G3	0.0361523	6.8818271	1.5071574	0.1332928	0.241847	-5.538705
RP6-191P20	0.1682034	-1.400051	1.5070597	0.1333178	0.2418702	-5.538849
AC016712.2	-0.32225	1.4486446	-1.507056	0.1333188	0.2418702	-5.538855
RP11-121A8	0.2964045	2.393963	1.5070239	0.133327	0.241873	-5.538902
LY6D	0.433662	-0.369786	1.5068852	0.1333625	0.2419254	-5.539106
GANAB	0.0229802	7.0650683	1.5068428	0.1333734	0.2419331	-5.539169
MUC12	-0.340333	2.8430947	-1.506661	0.1334199	0.2420032	-5.539436
RP11-523L2	0.1483567	-1.407151	1.5066388	0.1334257	0.2420032	-5.539469
ANKS4B	-0.23423	5.7127415	-1.506614	0.1334319	0.2420032	-5.539505
LINC01402	-0.293958	-0.60118	-1.50648	0.1334664	0.2420506	-5.539703
OR7E128P	-0.36099	0.5736495	-1.506461	0.1334713	0.2420506	-5.539731
RP11-248E9	0.1293733	-1.482676	1.5064274	0.1334799	0.2420541	-5.539781
RP11-13N13	-0.312256	-0.406755	-1.506255	0.1335241	0.2421223	-5.540035
MAP3K3	0.0259034	6.0380527	1.5060491	0.1335769	0.242206	-5.540338
RNU6-944P	0.1511317	-1.368548	1.5057342	0.1336577	0.2423405	-5.540801
TET2-AS1	0.2379038	-1.035517	1.5052708	0.1337767	0.2425395	-5.541483
RN7SL445P	0.1107447	-1.475427	1.5052549	0.1337808	0.2425395	-5.541507
RP11-449J1	0.1668475	-1.397753	1.5050904	0.133823	0.2426041	-5.541749
RP11-1000E	-0.332606	0.4771013	-1.504916	0.133868	0.2426735	-5.542006
RP11-2L8.1	0.4166635	1.8421664	1.5048776	0.1338777	0.2426791	-5.542062
IFT81	-0.054369	5.5136174	-1.504541	0.1339644	0.2428242	-5.542558
AC005593.2	-0.274757	-0.615088	-1.504495	0.1339762	0.2428335	-5.542625
AC024937.2	-0.316291	-0.136549	-1.504353	0.1340126	0.2428572	-5.542833
RPS20P4	-0.25833	-0.788122	-1.504328	0.1340191	0.2428572	-5.542871
SPRYD4	0.0569091	6.3299705	1.5042754	0.1340326	0.2428572	-5.542948
RP11-747H7	0.3038695	3.7314475	1.5042671	0.1340347	0.2428572	-5.54296

TRAPPC6B	0.031589	6.0124874	1.504265	0.1340352	0.2428572	-5.542963
NPAS2	0.064952	6.0016436	1.5042639	0.1340355	0.2428572	-5.542965
XX-DJ76P1C	0.1128082	-1.498343	1.5042627	0.1340359	0.2428572	-5.542966
AC114273.3	0.1284382	-1.448405	1.5040284	0.1340961	0.2429544	-5.543311
RP11-47502	0.3231718	-0.590253	1.5034348	0.134249	0.2432192	-5.544184
IDNK	0.0684049	5.6809409	1.5033157	0.1342796	0.2432627	-5.544359
RP11-1086F	-0.17168	-1.28512	-1.503289	0.1342864	0.243263	-5.544397
ZNF384	-0.041447	5.7252377	-1.503219	0.1343045	0.2432836	-5.5445
SPZ1	-0.116939	-1.49741	-1.50315	0.1343223	0.2432917	-5.544602
ZBED6	0.3145827	4.2802495	1.5031501	0.1343223	0.2432917	-5.544602
SAP30L	-0.031994	6.1986739	-1.503085	0.1343391	0.2433101	-5.544698
HSPD1P10	-0.296949	-0.405921	-1.502912	0.1343836	0.2433786	-5.544952
ALDH8A1	0.1676831	6.2315355	1.5028358	0.1344033	0.2434022	-5.545064
AC005682.5	-0.173028	3.8116956	-1.502471	0.1344973	0.2435603	-5.545599
RP6-91H8.3	0.2792625	-0.800106	1.5022616	0.1345514	0.2436462	-5.545907
RP11-213G2	-0.274499	-0.728365	-1.502209	0.1345649	0.2436555	-5.545984
RP11-151A6	-0.370392	0.5447078	-1.50219	0.1345698	0.2436555	-5.546012
FGF10-AS1	0.1309287	-1.461586	1.5021535	0.1345793	0.2436605	-5.546066
C17orf96	0.1537803	5.1823006	1.5020129	0.1346156	0.2437141	-5.546273
RP11-1007I	-0.208683	-1.172402	-1.501837	0.134661	0.2437842	-5.546531
DEFB1	0.2942046	6.0514968	1.5015407	0.1347375	0.2439086	-5.546966
RP11-174B4	-0.097204	-1.507088	-1.501519	0.134743	0.2439086	-5.546997
RP11-1081M	-0.131775	-1.42442	-1.501482	0.1347528	0.2439141	-5.547053
PCDHA10	0.4238919	1.9286238	1.5010809	0.1348563	0.2440894	-5.547641
PHF21B	-0.3442	-0.604947	-1.500986	0.1348809	0.2441219	-5.547781
CPNE5	0.117971	4.8367361	1.5005872	0.134984	0.2442962	-5.548365
GAPDHP72	-0.339199	1.4030173	-1.500514	0.1350028	0.2443182	-5.548472
RABL2A	-0.051551	5.0722157	-1.500361	0.1350426	0.2443781	-5.548698
RP11-7F17.	0.0620595	-1.555284	1.5002083	0.135082	0.2444373	-5.548921
NDUFA5P11	-0.282573	-0.6195	-1.500086	0.1351137	0.2444826	-5.549101
LINC01429	0.1651249	-1.422227	1.5000577	0.135121	0.2444836	-5.549142
LINC00264	0.3226676	-0.328129	1.5000231	0.13513	0.2444877	-5.549193
GOLGA5	0.0322646	6.3940509	1.4997309	0.1352056	0.2446125	-5.549621
LINC00862	-0.309776	3.0995955	-1.499543	0.1352542	0.2446883	-5.549896
RP11-18C24	-0.231622	2.2260285	-1.499254	0.1353291	0.2448117	-5.55032
RP11-54606	0.2184335	-1.185971	1.4991617	0.1353531	0.2448296	-5.550456
FTH1P8	-0.103226	4.4388213	-1.499158	0.1353541	0.2448296	-5.550462
AIMP1P2	0.1169415	-1.463513	1.499117	0.1353647	0.2448296	-5.550521
TAS2R46	-0.170941	-1.269204	-1.499113	0.1353658	0.2448296	-5.550528
RP11-640I1	-0.357317	0.4849255	-1.499068	0.1353773	0.2448334	-5.550593
RP4-751H13	0.3422998	0.884727	1.4990526	0.1353814	0.2448334	-5.550616
AVPI1	0.064941	6.0088857	1.4989982	0.1353955	0.2448468	-5.550695
RPL10P3	-0.215982	3.355071	-1.498952	0.1354076	0.2448565	-5.550764
ATPAF2	0.0404919	5.8738363	1.4988687	0.1354291	0.2448832	-5.550885
LL0XNC01-1	0.1906716	-1.223971	1.4988302	0.135439	0.2448887	-5.550942
ARGLU1	-0.032411	6.2484808	-1.498805	0.1354455	0.2448887	-5.550978
RP3-525N1C	0.363692	0.1645052	1.4986425	0.1354877	0.2449529	-5.551217
AL132988.1	0.3144103	3.45224	1.4984867	0.1355282	0.2450139	-5.551445
CTC-248019	0.3276855	-0.431795	1.498321	0.1355712	0.2450795	-5.551688
PTK7	-0.126502	5.6793988	-1.498105	0.1356273	0.2451689	-5.552005
CBFB	0.0320467	6.0456981	1.4980785	0.1356341	0.245169	-5.552043

RP11-142L4	-0.234203	-0.94869	-1.497738	0.1357225	0.2453166	-5.552541
RP1-37N7.1	0.3914729	-0.002024	1.4976539	0.1357444	0.245344	-5.552665
RN7SL184P	0.058639	-1.557027	1.4974719	0.1357917	0.2453942	-5.552931
RP11-31401	0.381891	1.0383818	1.4974717	0.1357917	0.2453942	-5.552932
C4orf32	0.065618	5.6339661	1.4974695	0.1357923	0.2453942	-5.552935
KCNQ1DN	0.1251562	-1.4776	1.4974217	0.1358047	0.2454045	-5.553005
SEPT7P6	0.1816847	-1.259556	1.49736	0.1358208	0.2454213	-5.553095
TTC28-AS1	-0.052309	5.6430903	-1.497316	0.1358322	0.2454298	-5.55316
CTD-3148I1	-0.302529	-0.485514	-1.497277	0.1358423	0.245436	-5.553216
HYKK	-0.055076	5.216131	-1.49711	0.1358857	0.2454975	-5.553461
RP11-154H1	0.2262579	-1.187218	1.4970943	0.1358898	0.2454975	-5.553484
STOML3	-0.290903	-1.069871	-1.496869	0.1359484	0.2455833	-5.553813
MIR331	-0.350836	0.6140757	-1.49686	0.1359508	0.2455833	-5.553827
PBXIP1	0.0345396	6.682166	1.4967555	0.135978	0.2456203	-5.55398
SPAM1	0.0831054	-1.521794	1.4965906	0.1360208	0.2456856	-5.554221
RP11-677M1	0.3071223	2.2540584	1.4965479	0.136032	0.2456935	-5.554284
RP11-104G3	0.2851476	-0.666027	1.4961058	0.1361471	0.2458892	-5.554931
EFHC1	-0.057403	5.3617478	-1.495916	0.1361965	0.2459664	-5.555209
RP11-286N2	0.1940645	-1.188698	1.4958493	0.1362139	0.2459855	-5.555306
FNBP1P1	-0.322468	2.5054439	-1.495619	0.136274	0.2460819	-5.555643
IL20RA	-0.556032	2.0317637	-1.495555	0.1362906	0.2460997	-5.555736
SYNJ1	0.0428912	5.5724254	1.495507	0.136303	0.24611	-5.555806
RP11-406H2	0.2247256	-1.089496	1.495396	0.136332	0.2461381	-5.555969
RAB6C	0.3188693	-0.187314	1.4953955	0.1363321	0.2461381	-5.555969
RP11-693J1	-0.380079	1.0559836	-1.495321	0.1363515	0.2461527	-5.556078
PAIP1P1	-0.261032	2.5773401	-1.495299	0.1363572	0.2461527	-5.55611
RP11-756P1	0.4109708	1.8490759	1.4952869	0.1363604	0.2461527	-5.556128
MUC19	0.386217	-0.381509	1.4952023	0.1363825	0.2461804	-5.556252
MAST1	-0.221493	4.2068409	-1.495159	0.1363939	0.2461888	-5.556316
EFCAB14-AS	0.2614966	-0.827721	1.4950099	0.1364326	0.2462466	-5.556533
SEPT5	-0.101578	5.5294958	-1.494762	0.1364972	0.246351	-5.556895
PPIAP6	-0.300844	1.5427071	-1.494709	0.1365111	0.2463639	-5.556973
PRH2	-0.419957	1.6463814	-1.494389	0.1365947	0.2464976	-5.557441
LHCGR	0.1719401	-1.402383	1.4943736	0.1365987	0.2464976	-5.557463
ISPD-AS1	-0.295887	-0.383336	-1.494341	0.1366072	0.2465006	-5.55751
RN7SKP16	0.3651733	0.7017785	1.4941539	0.1366561	0.2465767	-5.557784
UBASH3A	0.1866034	4.1446689	1.4940996	0.1366703	0.2465901	-5.557863
RP11-524K2	-0.185182	-1.353576	-1.494026	0.1366894	0.2466124	-5.55797
HGSNAT	0.0418359	6.4560492	1.4938137	0.1367449	0.2467005	-5.558281
TSHZ1	0.0374483	6.0275737	1.4937423	0.1367636	0.2467219	-5.558385
LINC01481	-0.316071	-0.341259	-1.493577	0.1368069	0.2467879	-5.558627
RP11-78J21	-0.302513	1.8538905	-1.493513	0.1368235	0.2468009	-5.55872
RP11-671E7	-0.322091	-0.188281	-1.493497	0.1368277	0.2468009	-5.558743
AC007879.5	-0.324797	1.1162571	-1.493425	0.1368465	0.2468196	-5.558848
CCDC157	0.0724184	4.5026826	1.4934058	0.1368515	0.2468196	-5.558877
TRBV4-1	-0.418153	0.7990999	-1.493349	0.1368663	0.2468341	-5.558959
PWP2	-0.389646	3.0906371	-1.493312	0.1368762	0.2468397	-5.559014
AC096669.1	-0.201648	-1.319058	-1.493276	0.1368854	0.2468441	-5.559066
RP11-69H7.	-0.266365	-0.769122	-1.493229	0.1368978	0.2468542	-5.559135
LINC01158	0.1855916	-1.409645	1.4931164	0.1369272	0.2468943	-5.559299
TCN1	0.5097663	0.8388884	1.4930686	0.1369397	0.2468943	-5.559369

PTGES3P1	0.0688736	4.9651728	1.4930663	0.1369403	0.2468943	-5.559372
COMMD4	-0.036688	6.4964526	-1.492979	0.1369631	0.2469231	-5.559499
PLEKHA1	-0.033621	6.2943935	-1.492631	0.1370542	0.2470652	-5.560008
CACHD1	0.1152425	5.2723981	1.4926265	0.1370554	0.2470652	-5.560014
SOCS5	-0.030795	5.8811722	-1.492584	0.1370667	0.2470733	-5.560077
AC064834.1	-0.212569	-1.30917	-1.492469	0.1370967	0.247097	-5.560245
BBS1	-0.08356	4.2067482	-1.492456	0.1371002	0.247097	-5.560264
RP4-769N13	-0.059927	4.743412	-1.492406	0.1371132	0.247097	-5.560336
RP11-63N3.	-0.14924	-1.433181	-1.492404	0.1371138	0.247097	-5.56034
RP11-347C1	-0.286282	2.2834397	-1.492389	0.1371177	0.247097	-5.560362
PRG2	0.3774599	1.7938026	1.4923781	0.1371204	0.247097	-5.560377
OR2L13	0.1824995	-1.294215	1.4923264	0.137134	0.2471055	-5.560452
CTA-212A2.	-0.190548	-1.228525	-1.492287	0.1371444	0.2471055	-5.56051
RP11-261C1	-0.420568	0.5451097	-1.492244	0.1371554	0.2471055	-5.560572
RP11-314M2	-0.415761	-0.532148	-1.492233	0.1371584	0.2471055	-5.560588
RP11-489D6	0.069012	-1.55174	1.492231	0.137159	0.2471055	-5.560592
CATSPER1	0.2692777	2.4705545	1.4921219	0.1371875	0.2471335	-5.560751
SCN2A	-0.424876	1.7813214	-1.49212	0.1371881	0.2471335	-5.560754
AC011247.3	-0.317037	-0.133748	-1.492039	0.1372093	0.2471541	-5.560872
CCAR2	0.0276166	6.3114557	1.4920246	0.137213	0.2471541	-5.560893
SYNCRIP	-0.023102	6.6135168	-1.491943	0.1372345	0.2471806	-5.561012
VAX2	-0.341437	2.7103645	-1.491679	0.1373037	0.247293	-5.561398
CDS1	-0.252151	4.6563225	-1.491603	0.1373234	0.2473164	-5.561507
ODF2	0.0341798	6.0960852	1.491482	0.1373552	0.2473518	-5.561684
IGLJ2	-0.311445	-0.69703	-1.491443	0.1373654	0.2473518	-5.561741
GCNT1P4	0.2962532	-0.755604	1.4914398	0.1373663	0.2473518	-5.561746
TMCO5B	0.0722293	-1.550101	1.4914248	0.1373702	0.2473518	-5.561768
AP000230.1	0.410924	2.0380766	1.4913473	0.1373906	0.2473762	-5.561881
CAPN13	-0.543079	2.1947882	-1.491106	0.1374539	0.2474781	-5.562233
RP11-255G1	0.166309	-1.342956	1.4910501	0.1374685	0.2474837	-5.562314
LARS2	0.0375353	6.0053521	1.4910238	0.1374754	0.2474837	-5.562352
RP11-631M6	0.3040645	-0.251316	1.4910163	0.1374774	0.2474837	-5.562363
BANF1P1	0.2647962	-1.016216	1.4908821	0.1375126	0.2475349	-5.562559
RP11-12601	0.1237896	-1.492747	1.4907261	0.1375536	0.2475964	-5.562786
RP3-380B8.	-0.203096	-1.232856	-1.490595	0.1375879	0.2476461	-5.562977
GRK6P1	-0.331834	0.1342311	-1.490563	0.1375963	0.2476489	-5.563024
SDHAF2	-0.035194	5.8961793	-1.490506	0.1376113	0.2476637	-5.563107
CTD-2213F2	-0.158823	2.834077	-1.490405	0.1376378	0.2476992	-5.563254
TBPL1	0.0424117	5.6911459	1.4903234	0.1376593	0.2477256	-5.563373
CYP4Z2P	-0.273687	-0.921997	-1.490045	0.1377325	0.2478452	-5.56378
RP11-536G4	0.3045206	-0.843337	1.4899682	0.1377526	0.2478601	-5.563891
TMEM26-AS1	0.3060531	-0.306827	1.4899614	0.1377544	0.2478601	-5.563901
RN7SKP271	-0.217836	-1.05355	-1.489515	0.1378717	0.2480589	-5.564551
RP11-45A17	0.3378334	0.6487297	1.4894302	0.137894	0.248086	-5.564675
TPM3P8	-0.281743	-0.534785	-1.489406	0.1379003	0.248086	-5.56471
CRISP3	0.5444927	0.723614	1.489334	0.1379193	0.248108	-5.564815
CHCHD2P6	-0.160559	3.2612878	-1.488834	0.1380508	0.2483323	-5.565543
RP11-35J10	0.1267757	-1.442354	1.4886279	0.1381051	0.2484178	-5.565843
SNORA67	-0.26838	-0.794739	-1.488451	0.1381517	0.2484893	-5.5661
CTC-487M23	0.2228879	3.1297893	1.4883732	0.1381722	0.2485139	-5.566214
CTC-429L19	-0.329825	1.3536522	-1.488331	0.1381834	0.2485218	-5.566275

FOLR3	0.3655434	-0.366398	1.4882828	0.1381961	0.248529	-5.566345
KDELR2	0.0282834	7.0191611	1.488264	0.138201	0.248529	-5.566373
RNF6P1	-0.12223	-1.464911	-1.488165	0.1382272	0.2485638	-5.566517
PTCHD2	-0.411252	1.4078522	-1.488035	0.1382612	0.248603	-5.566705
EIF6	-0.034426	6.7621454	-1.48803	0.1382626	0.248603	-5.566713
RP11-378A1	0.0799766	4.3166147	1.4879262	0.13829	0.24864	-5.566864
BRCC3	0.0376149	5.8624923	1.48777	0.1383312	0.2487018	-5.567091
OR2I1P	0.1268759	6.2743883	1.4877341	0.1383406	0.2487066	-5.567144
CHCHD3P3	0.2738074	2.0900048	1.4875963	0.138377	0.2487596	-5.567344
PRIMA1	-0.485384	0.6604649	-1.487517	0.1383978	0.2487848	-5.567459
PEMT	0.0723308	6.2817757	1.4873355	0.1384458	0.2488588	-5.567723
RP11-74E22	-0.179734	-1.384902	-1.487229	0.1384738	0.248897	-5.567878
SNORD56	-0.331648	0.3948311	-1.487161	0.1384917	0.2489169	-5.567977
MIR7848	-0.137456	-1.408629	-1.486445	0.1386807	0.2492443	-5.569018
GGT7	-0.051641	5.8798733	-1.486374	0.1386997	0.2492661	-5.569122
SPAG17	0.4318674	0.8255375	1.4861482	0.1387593	0.2493609	-5.56945
SIM1	0.5437518	2.0427981	1.4860337	0.1387895	0.249403	-5.569616
AJ009632.3	0.158842	-1.470125	1.4858094	0.1388488	0.2494908	-5.569942
BARHL2	-0.166405	-1.405232	-1.485797	0.138852	0.2494908	-5.56996
MYLPF	0.3587241	1.4061489	1.4855465	0.1389184	0.2495977	-5.570324
CERK	-0.038218	6.4024197	-1.48546	0.1389413	0.2496265	-5.57045
AC109309.4	0.2067704	-1.292791	1.4853516	0.1389699	0.2496657	-5.570607
AC007969.4	-0.167221	-1.244544	-1.485308	0.1389814	0.249674	-5.57067
AC004019.1	0.2787282	3.437339	1.4852634	0.1389932	0.2496831	-5.570735
AC124861.1	-0.274856	-0.898826	-1.485227	0.1390029	0.2496858	-5.570788
DERA	0.0442623	6.2754152	1.485206	0.1390085	0.2496858	-5.570819
MIR210	-0.329091	-0.261245	-1.485165	0.1390192	0.2496929	-5.570878
OR10U1P	-0.12106	-1.49539	-1.485043	0.1390515	0.2497386	-5.571055
RP11-996F1	-0.100844	3.9303417	-1.484842	0.1391048	0.2498219	-5.571347
LINC01182	0.4665043	0.6882913	1.4847537	0.1391282	0.2498437	-5.571475
RP11-495K9	0.1520629	-1.44182	1.4847236	0.1391361	0.2498437	-5.571519
RP11-616M2	0.2360779	-1.147809	1.4847189	0.1391374	0.2498437	-5.571526
CH507-513H	-0.2468	2.726612	-1.484669	0.1391507	0.2498553	-5.571599
CTB-113P19	0.2858989	-0.662925	1.4845729	0.1391761	0.2498885	-5.571738
RP11-240D1	0.1279594	-1.448649	1.4845422	0.1391842	0.2498908	-5.571782
PLEK	0.0944671	5.7073103	1.4842627	0.1392583	0.2500115	-5.572188
AC005229.1	0.1385303	3.5656341	1.4839274	0.1393471	0.2501587	-5.572675
MAPKAP1	-0.023182	6.4634917	-1.483867	0.1393632	0.2501752	-5.572763
KLK4	-0.412546	-0.536525	-1.483816	0.1393766	0.250187	-5.572836
RP11-536K7	0.0896887	4.6424057	1.4837582	0.139392	0.2502024	-5.57292
NEMF	0.0395672	5.8100762	1.483691	0.1394098	0.250222	-5.573018
ALOXE3	0.3944371	-0.210705	1.4834928	0.1394624	0.2502984	-5.573305
CYP4F36P	0.1007309	-1.490048	1.4834789	0.1394661	0.2502984	-5.573326
NARF-IT1	-0.315146	1.406888	-1.483322	0.1395078	0.2503609	-5.573553
LRWD1	0.0350412	5.9894635	1.4831825	0.1395447	0.250415	-5.573756
ROCK2	-0.030646	6.3199715	-1.483113	0.1395631	0.2504301	-5.573856
XPO7	0.0334427	6.235849	1.4830991	0.1395669	0.2504301	-5.573876
CEACAM21	0.1537758	4.0723359	1.4829721	0.1396006	0.2504783	-5.574061
RP11-521D1	0.157919	-1.372314	1.4827503	0.1396595	0.2505716	-5.574382
RP11-793H1	0.1817441	-1.25851	1.4826967	0.1396737	0.2505848	-5.57446
LINC01127	-0.283548	4.816267	-1.482333	0.1397704	0.2507459	-5.574987

FLJ38576	-0.218978	2.9239647	-1.482214	0.139802	0.2507904	-5.57516
RP11-361L1	0.2140005	-1.191342	1.4820736	0.1398393	0.2508449	-5.575363
GSTA2	0.3499511	5.626817	1.4819695	0.139867	0.2508823	-5.575514
RP13-143G1	0.4870102	2.7882723	1.481894	0.1398871	0.250906	-5.575624
RP11-642A1	-0.329624	1.6150432	-1.481492	0.1399941	0.2510855	-5.576206
PNPT1	-0.027151	6.0845753	-1.481369	0.1400267	0.2511318	-5.576384
TSPAN33	-0.059303	6.3553277	-1.481303	0.1400442	0.2511508	-5.576479
AC105009.1	-0.245104	-0.906703	-1.481128	0.1400909	0.2512222	-5.576734
CSNK2A3	-0.327416	1.306402	-1.481062	0.1401086	0.2512416	-5.57683
RP11-863K1	0.3612639	1.7740408	1.4808057	0.1401767	0.2513514	-5.5772
BMP2	0.090967	5.664679	1.48055	0.1402449	0.2514612	-5.577571
RP11-1348G	0.3149084	0.0072605	1.4804348	0.1402755	0.2515038	-5.577737
RP11-157E2	0.3077364	-0.865632	1.480387	0.1402883	0.2515143	-5.577807
HHLA2	-0.469525	0.5999342	-1.480086	0.1403684	0.2516457	-5.578242
NPIPB15	-0.306786	4.4669233	-1.48005	0.1403782	0.2516508	-5.578295
SETD6	0.0300273	5.6669098	1.4798834	0.1404226	0.2517145	-5.578536
SMOC1	-0.093041	6.4125632	-1.479865	0.1404275	0.2517145	-5.578563
ZNF43	-0.113037	5.0832566	-1.479738	0.1404614	0.2517629	-5.578746
RP1-317E23	0.1261797	3.800666	1.4796901	0.1404741	0.2517733	-5.578815
EEA1	-0.035959	6.0686651	-1.479451	0.1405379	0.2518753	-5.579161
RP11-85B7.	-0.393493	0.3234703	-1.479303	0.1405775	0.2519339	-5.579376
RP11-857B2	-0.338691	-0.015338	-1.479116	0.1406275	0.252011	-5.579647
RP11-758N1	0.2306634	-1.056923	1.4789368	0.1406752	0.2520843	-5.579905
SULT1D1P	-0.133582	-1.415534	-1.478783	0.1407164	0.2521457	-5.580128
EN2	-0.521013	0.726069	-1.478749	0.1407253	0.2521492	-5.580176
PPP1R14D	-0.455934	2.3759135	-1.478705	0.1407371	0.2521579	-5.58024
SNX18P14	-0.140979	-1.441812	-1.478629	0.1407573	0.2521819	-5.58035
RP11-161D1	-0.162623	-1.387388	-1.478262	0.1408555	0.2523454	-5.580881
MYADML	-0.141123	-1.415257	-1.478061	0.1409093	0.2524294	-5.581172
MESDC2	-0.026283	6.4473288	-1.47786	0.1409631	0.2525134	-5.581463
WWTR1-IT1	0.2324032	-1.014998	1.4776892	0.1410088	0.2525827	-5.581709
KLHL17	-0.050159	5.2985438	-1.477637	0.1410227	0.2525953	-5.581784
RP11-1281K	-0.151966	-1.35355	-1.477382	0.1410909	0.2526914	-5.582152
TMEM115	-0.027508	6.3839777	-1.477363	0.1410961	0.2526914	-5.58218
RP11-28101	0.0796849	-1.523537	1.4773369	0.1411031	0.2526914	-5.582218
RP5-891H21	-0.140724	-1.398127	-1.477333	0.1411041	0.2526914	-5.582224
RPS3AP43	-0.236531	-0.878468	-1.477251	0.141126	0.2527184	-5.582342
ZBP1	0.2080287	4.0349158	1.4771293	0.1411586	0.2527644	-5.582518
SSX4	-0.117426	-1.475267	-1.477025	0.1411865	0.2528019	-5.582668
RP1-72A23.	-0.211991	-1.117811	-1.476977	0.1411995	0.2528127	-5.582738
RP11-116D2	-0.244708	5.978846	-1.476933	0.1412112	0.2528213	-5.582802
AC009473.1	-0.336726	0.1279378	-1.476836	0.1412373	0.2528522	-5.582942
RP11-677M1	0.2398805	2.6692605	1.476817	0.1412423	0.2528522	-5.582969
TDGF1P6	0.1573815	-1.34233	1.4766923	0.1412757	0.2528996	-5.583149
RP11-488L1	-0.244342	-0.905946	-1.476653	0.1412862	0.252906	-5.583206
RP11-128A6	0.0748296	-1.548776	1.4764046	0.1413528	0.2530048	-5.583565
ZFP2	-0.167283	3.8147857	-1.476396	0.1413553	0.2530048	-5.583578
RP11-15501	-0.307747	1.4204919	-1.476273	0.1413881	0.2530512	-5.583755
RP11-470B2	0.1717563	-1.321112	1.476118	0.1414297	0.2531089	-5.583979
RP11-61I13	-0.174986	3.6383977	-1.476101	0.1414343	0.2531089	-5.584003
CDR1	-0.267217	-1.090244	-1.476071	0.1414423	0.253111	-5.584047

TMEM185A	0.0463324	5.341816	1.4759324	0.1414795	0.2531533	-5.584246
RP11-113K2	-0.362628	0.9697411	-1.475931	0.1414799	0.2531533	-5.584249
CTD-2647L4	0.2162433	3.1696349	1.4757195	0.1415366	0.2532424	-5.584554
RP11-726G2	-0.123593	-1.494147	-1.475675	0.1415485	0.2532512	-5.584618
LPA	-0.332073	5.3939359	-1.475542	0.1415842	0.2533028	-5.58481
EPOR	-0.055851	5.4771994	-1.475312	0.1416459	0.2534008	-5.585142
RP11-489E7	0.3351422	0.4469109	1.4748764	0.141763	0.2535894	-5.58577
RP11-298A8	-0.116651	-1.467647	-1.474868	0.1417652	0.2535894	-5.585782
CTB-58E17.	0.2766018	-0.618549	1.4748194	0.1417783	0.2536003	-5.585853
ANAPC15	0.035466	5.5834453	1.4747116	0.1418073	0.2536397	-5.586008
RPL12P2	-0.179711	-1.221094	-1.474634	0.1418282	0.2536647	-5.586121
RP13-216E2	-0.263499	-0.74584	-1.474522	0.1418583	0.2537061	-5.586282
EIF4E2P2	-0.208572	-1.239216	-1.474452	0.1418771	0.2537273	-5.586383
RP11-615I2	0.3480313	-0.176487	1.4743972	0.1418918	0.2537411	-5.586461
CTD-319301	0.1242429	-1.457768	1.4743126	0.1419145	0.2537694	-5.586584
RP4-802A10	-0.245341	-0.920818	-1.474222	0.1419389	0.2538005	-5.586714
CRIP3	-0.229947	5.1822239	-1.473884	0.1420298	0.2539504	-5.587201
RP5-1102E8	0.1217769	-1.456559	1.4738587	0.1420366	0.2539504	-5.587238
RAET1G	0.2643501	2.1960942	1.4737473	0.1420666	0.2539916	-5.587399
CTC-28708.	-0.214357	-1.116078	-1.473633	0.1420973	0.2540339	-5.587563
RP11-73702	0.3193359	-0.093368	1.4735084	0.1421309	0.254073	-5.587743
RP11-221J2	0.3153452	-0.569134	1.4735005	0.1421331	0.254073	-5.587754
CUX2	0.2119523	5.6624793	1.4733849	0.1421642	0.2541162	-5.587921
RPL5P1	-0.317023	1.6973463	-1.473188	0.1422171	0.2541911	-5.588204
RP11-324H7	-0.13511	-1.444689	-1.473178	0.14222	0.2541911	-5.58822
SSX8	-0.200395	-1.322339	-1.473083	0.1422455	0.2542243	-5.588356
GSTT2B	0.2802017	3.9760101	1.4728962	0.1422958	0.2543017	-5.588625
ATP5LP3	-0.170043	-1.288129	-1.472717	0.1423441	0.2543756	-5.588883
PRSS58	0.1030986	-1.515487	1.4725543	0.142388	0.2544415	-5.589117
CCNG1P1	0.1805217	-1.26947	1.4722493	0.1424702	0.254576	-5.589557
BANF1P3	-0.292472	1.8987198	-1.472176	0.14249	0.2545989	-5.589662
LRRC3	0.1305371	5.8124726	1.4721469	0.1424979	0.2546005	-5.589704
HIST4H4	-0.158712	3.2857134	-1.471887	0.142568	0.2547134	-5.590079
RP3-406P24	-0.260868	-0.752143	-1.471831	0.1425831	0.2547278	-5.590159
PTGER4P2	0.128984	-1.420604	1.4717015	0.142618	0.2547773	-5.590345
RP11-964E1	0.169233	-1.395922	1.4716766	0.1426248	0.2547773	-5.590381
SCN5A	-0.389888	1.2466557	-1.471616	0.1426412	0.2547941	-5.590469
RP11-629N8	-0.278176	-0.396754	-1.471361	0.1427099	0.2549045	-5.590835
RP11-213G2	-0.051531	4.8818159	-1.471219	0.1427482	0.2549605	-5.591039
CSN2	-0.250663	-1.265434	-1.471149	0.1427674	0.2549702	-5.591141
METTL2B	-0.029076	5.9973577	-1.471147	0.1427677	0.2549702	-5.591143
LINC01438	-0.102315	-1.504582	-1.471001	0.1428074	0.2550186	-5.591354
RP5-855D21	-0.230783	2.4910758	-1.470995	0.1428087	0.2550186	-5.591362
AATBC	-0.171917	3.3743128	-1.470909	0.1428321	0.2550415	-5.591486
UQCR10	0.0373373	6.5631245	1.470883	0.1428391	0.2550415	-5.591523
RPL21P75	-0.210543	2.9926821	-1.47087	0.1428425	0.2550415	-5.591541
LINC00511	-0.30587	4.4646471	-1.470844	0.1428497	0.2550418	-5.591579
RP4-539M6.	0.396828	1.1227846	1.4706699	0.1428967	0.2551011	-5.59183
TRBV6-5	-0.372639	1.5933126	-1.470662	0.1428988	0.2551011	-5.591841
TMEM44	-0.055261	5.4733305	-1.470644	0.1429039	0.2551011	-5.591868
RPS23P3	-0.143126	-1.38779	-1.47052	0.1429372	0.255146	-5.592045

CTD-2154I1	0.3272071	0.1458785	1.4704989	0.142943	0.255146	-5.592076
IGHV1-69-2	0.4929557	3.180316	1.4702387	0.1430134	0.2552591	-5.59245
C19orf26	-0.192316	3.5218181	-1.469886	0.1431089	0.2554171	-5.592958
RP11-288A5	0.2710642	-0.801748	1.4698439	0.1431202	0.2554248	-5.593018
NR0B2	-0.226654	6.1439226	-1.469524	0.1432067	0.2555569	-5.593477
CTC-278L1.	-0.270115	-1.065912	-1.469519	0.1432082	0.2555569	-5.593485
ARHGAP28	0.2067507	4.2365419	1.4694421	0.143229	0.2555815	-5.593595
RP11-533E1	-0.220227	-1.003988	-1.469378	0.1432463	0.255592	-5.593687
RP11-396K3	0.0666188	5.3623073	1.4693627	0.1432505	0.255592	-5.59371
TCTN1	0.0537088	5.8102878	1.4693428	0.1432559	0.255592	-5.593738
RP11-18B16	0.38967	1.7172315	1.4691441	0.1433097	0.2556756	-5.594024
AMIGO3	-0.284429	-0.311521	-1.469074	0.1433287	0.2556831	-5.594124
RP11-299G2	-0.191948	4.9568455	-1.469056	0.1433335	0.2556831	-5.59415
RP11-507K2	-0.203684	-1.121884	-1.469051	0.1433349	0.2556831	-5.594157
RP11-567C2	-0.492991	1.2057757	-1.468984	0.1433531	0.255703	-5.594254
AC021087.1	0.3653185	0.1944717	1.468895	0.1433772	0.2557335	-5.594382
LINC01342	-0.306561	-0.569927	-1.468742	0.1434188	0.2557951	-5.594602
RP11-526N1	-0.256966	-1.004143	-1.468635	0.1434478	0.2558343	-5.594755
IGKV1-33	-0.36997	-0.529599	-1.468568	0.1434659	0.2558541	-5.594851
ATXN7	0.0321898	5.9050124	1.4684132	0.1435078	0.2559165	-5.595074
RP11-382J2	-0.293795	-0.261644	-1.468276	0.1435451	0.2559624	-5.595271
SMIM20	0.0391832	6.0465543	1.4682665	0.1435476	0.2559624	-5.595284
IPMK	-0.043445	5.6373726	-1.468178	0.1435715	0.2559908	-5.595411
RP11-61A14	-0.199014	-1.208207	-1.468156	0.1435776	0.2559908	-5.595443
MEGF11	-0.346409	1.237248	-1.468123	0.1435867	0.2559945	-5.595491
RP11-114H2	0.3006877	1.7139942	1.4680852	0.1435969	0.2559952	-5.595545
UGT2B11	-0.244042	5.4263801	-1.46807	0.1436011	0.2559952	-5.595567
TMEM45B	-0.170429	6.1823958	-1.467952	0.1436331	0.2560398	-5.595737
VN1R71P	-0.12403	-1.445544	-1.467551	0.1437419	0.2562213	-5.596312
SCAND1	-0.040035	6.3989399	-1.467121	0.1438587	0.2564169	-5.596929
RAB9B	0.2612387	2.9815195	1.4670678	0.1438731	0.2564301	-5.597005
LINC00865	0.2309987	3.1479819	1.46699	0.1438943	0.2564553	-5.597117
RP11-149I2	-0.224024	-1.029129	-1.466867	0.1439278	0.2565024	-5.597294
FAR2P2	0.3975805	0.6545161	1.4668147	0.1439419	0.2565152	-5.597369
CSTA	-0.141053	5.514999	-1.466665	0.1439827	0.2565744	-5.597584
UNC93B7	-0.138809	-1.389907	-1.466641	0.1439892	0.2565744	-5.597618
RP11-474I1	0.3450381	0.1938155	1.4665371	0.1440174	0.2566121	-5.597767
LSP1	0.0677672	6.0712839	1.4663739	0.1440618	0.2566687	-5.598001
WIPI1	0.0496448	5.8867165	1.4663687	0.1440632	0.2566687	-5.598008
AC092013.1	-0.113755	-1.477067	-1.466321	0.1440762	0.2566793	-5.598077
MGAT5B	0.327647	2.3690812	1.4660588	0.1441476	0.2567939	-5.598453
PCNT	0.0328623	6.0917107	1.4658307	0.1442097	0.2568919	-5.59878
HSPA8P16	-0.209371	-1.059907	-1.465791	0.1442206	0.2568955	-5.598837
TPTE2P6	0.1214557	-1.469968	1.4657718	0.1442257	0.2568955	-5.598864
JADE2	-0.037393	6.0358589	-1.465679	0.1442509	0.2569189	-5.598997
RP11-720L2	0.0540595	-1.55936	1.4656718	0.144253	0.2569189	-5.599008
RP5-1107A1	-0.286036	-0.449597	-1.46541	0.1443243	0.2570327	-5.599383
PCNXL2	0.1237785	5.1845306	1.4653856	0.1443309	0.2570327	-5.599418
INSIG2	0.0438182	6.2053598	1.4652105	0.1443786	0.2571051	-5.599669
CCDC53	0.0433327	5.8969466	1.4651756	0.1443881	0.2571095	-5.599719
CLDN12	0.0376758	6.3704611	1.4649794	0.1444416	0.2571922	-5.6

RP11-478C6	-0.344697	0.9665403	-1.46488	0.1444688	0.257228	-5.600143
RPL10P1	-0.337147	0.3659739	-1.464655	0.14453	0.2573244	-5.600465
CTD-2015H3	-0.306824	-0.574649	-1.464615	0.144541	0.2573314	-5.600522
ZNF208	0.3244235	2.4273776	1.4645124	0.144569	0.2573687	-5.600669
ACTBP1	-0.248133	-0.777256	-1.464445	0.1445873	0.2573889	-5.600766
LPPR4	-0.358795	2.8799913	-1.464285	0.144631	0.2574541	-5.600995
METTL24	-0.344195	3.0042025	-1.464022	0.1447028	0.2575582	-5.601372
LINC00707	0.3700816	-0.492005	1.4640189	0.1447036	0.2575582	-5.601376
DNAJA1	0.035711	6.8469109	1.463797	0.1447642	0.2576535	-5.601694
EAPP	0.0314826	6.141821	1.4636545	0.1448031	0.2577102	-5.601898
ZDHHC13	-0.083518	5.3479789	-1.463264	0.1449098	0.2578874	-5.602457
AC002519.8	0.298486	-0.593745	1.4631923	0.1449294	0.2578984	-5.60256
ADCY10	-0.162329	4.9196861	-1.46319	0.1449301	0.2578984	-5.602563
GPR25	-0.333385	-0.168315	-1.463143	0.144943	0.2579088	-5.602631
RPGRIP1L	0.0661431	4.9226885	1.4630616	0.1449651	0.2579356	-5.602747
PRDX2	0.0348387	6.8303329	1.4626447	0.1450791	0.2581259	-5.603343
WDR55	0.0279504	6.2019178	1.4623944	0.1451476	0.258222	-5.603702
ECE1	0.0300488	6.6845654	1.4623702	0.1451543	0.258222	-5.603736
RP11-297K8	-0.231847	-0.907508	-1.46237	0.1451544	0.258222	-5.603737
LA16c-358E	-0.340659	0.524048	-1.46206	0.1452391	0.2583602	-5.60418
HM13-IT1	-0.266137	2.099714	-1.461976	0.1452621	0.2583885	-5.6043
RWDD3	-0.039506	5.3427769	-1.46186	0.145294	0.2584276	-5.604466
CEP41	0.0451586	5.5476817	1.4618442	0.1452983	0.2584276	-5.604489
COX7A2L	-0.025574	6.5859459	-1.461562	0.1453754	0.2585522	-5.604891
RP11-112L6	0.1956347	-1.18205	1.4613421	0.1454358	0.258647	-5.605206
CRABP1	-0.353003	-0.645806	-1.461205	0.1454734	0.2587013	-5.605402
RP13-49I15	-0.339791	-0.081616	-1.460945	0.1455446	0.2588137	-5.605774
RPL3	-0.031684	7.4431169	-1.460923	0.1455508	0.2588137	-5.605806
RP11-46F15	-0.284288	2.3868413	-1.46076	0.1455954	0.2588803	-5.606038
SLAMF6	0.1610108	4.6653812	1.4606544	0.1456244	0.2589193	-5.606189
RP11-123B3	0.3129021	-0.853588	1.46055	0.145653	0.2589576	-5.606338
PPIG	-0.0232	6.3727674	-1.460397	0.145695	0.2590197	-5.606557
MIR6772	0.3621756	0.5434854	1.4603594	0.1457053	0.2590253	-5.606611
SSX6	-0.203394	-1.299851	-1.460283	0.1457263	0.25905	-5.60672
AP001596.6	0.343483	-0.057486	1.4602532	0.1457345	0.2590519	-5.606762
SH3BP2	0.0460431	6.288612	1.4601217	0.1457706	0.2591035	-5.60695
RP11-845M1	0.132501	-1.415257	1.4600758	0.1457832	0.2591091	-5.607016
HBA2	0.1693323	4.9236565	1.4600586	0.1457879	0.2591091	-5.60704
DYNC1I1	-0.196856	4.2195114	-1.459842	0.1458474	0.2591776	-5.60735
CTD-3088G3	-0.340771	-0.065249	-1.459836	0.145849	0.2591776	-5.607358
TMEM178B	-0.261818	4.1665256	-1.459818	0.145854	0.2591776	-5.607384
MTFR1	-0.048034	6.2924357	-1.459803	0.145858	0.2591776	-5.607405
RP11-632K2	0.1003359	4.3372669	1.4597888	0.145862	0.2591776	-5.607426
RP11-144L1	-0.341183	0.9547333	-1.459644	0.1459017	0.2592355	-5.607632
CDC34	-0.034299	6.5050228	-1.459536	0.1459315	0.2592672	-5.607787
RP11-478H1	-0.104611	-1.503456	-1.459528	0.1459337	0.2592672	-5.607799
HDGF	-0.027385	7.0507074	-1.459496	0.1459425	0.2592703	-5.607845
RSL1D1	0.0259166	6.6367931	1.4594151	0.1459646	0.259297	-5.607959
LINC01568	0.1242512	-1.500826	1.4591024	0.1460506	0.259437	-5.608406
CTC-360G5.	0.1764548	4.4945801	1.4590616	0.1460618	0.2594443	-5.608464
NEDD8-MDP1	0.3172694	-0.149247	1.4587888	0.1461368	0.259565	-5.608853

RP11-571L1	-0.146186	-1.355959	-1.458558	0.1462003	0.2596525	-5.609182
RPL7L1	-0.024036	6.6000817	-1.458558	0.1462003	0.2596525	-5.609183
STAMBP	-0.022169	6.1885747	-1.458501	0.1462162	0.2596603	-5.609265
COL26A1	-0.288352	4.46101	-1.45847	0.1462246	0.2596603	-5.609309
GHSR	-0.129952	-1.44264	-1.458464	0.1462261	0.2596603	-5.609316
UBA52P5	-0.308405	0.2112388	-1.458247	0.146286	0.2597541	-5.609627
LRRC47	0.03046	6.3590871	1.4581932	0.1463008	0.2597676	-5.609703
RP11-374M1	-0.14173	-1.385054	-1.458086	0.1463303	0.2598074	-5.609856
MIRLET7D	-0.355706	0.4943182	-1.457985	0.146358	0.2598366	-5.61
RP4-535B2C	-0.19875	-1.119558	-1.457975	0.1463609	0.2598366	-5.610015
RPL7P22	-0.170168	-1.297202	-1.4577	0.1464367	0.2599585	-5.610407
RP11-400D2	-0.133406	-1.400668	-1.457632	0.1464554	0.259979	-5.610504
DYNLL1P1	-0.30023	0.153691	-1.457421	0.1465135	0.2600695	-5.610805
CTD-2196E1	0.1400059	-1.471212	1.4572619	0.1465573	0.2601346	-5.611031
LCE1F	-0.219755	-1.275346	-1.457202	0.1465739	0.2601515	-5.611117
LGR6	0.2416313	4.5106599	1.457017	0.1466248	0.2602291	-5.61138
CENPT	-0.029313	6.0190532	-1.456757	0.1466965	0.2603436	-5.611751
HHATL	0.4071981	0.1897881	1.4566219	0.1467338	0.2603973	-5.611944
U82695.5	-0.141144	-1.485541	-1.456525	0.1467606	0.2604207	-5.612082
NUAK1	-0.045667	5.9579248	-1.456522	0.1467613	0.2604207	-5.612086
UQCRHL	-0.082619	4.719419	-1.456442	0.1467836	0.2604475	-5.6122
DNAJC19P9	-0.32393	0.21695	-1.456396	0.1467963	0.2604574	-5.612266
CNN2P9	-0.306998	1.0115926	-1.456341	0.1468114	0.2604593	-5.612344
PSMC1P10	-0.342686	-0.326233	-1.45634	0.1468116	0.2604593	-5.612345
RP11-230G5	0.2370206	-0.875807	1.4561427	0.1468661	0.2605254	-5.612626
RPS26P34	-0.130289	-1.454006	-1.456134	0.1468686	0.2605254	-5.612639
TMEM63A	0.0450917	6.4651318	1.4561194	0.1468726	0.2605254	-5.61266
HTR5A	-0.217787	-1.343731	-1.456102	0.1468774	0.2605254	-5.612685
FAM217B	-0.092269	5.3099549	-1.456031	0.146897	0.2605416	-5.612786
RP1-102E24	0.1397771	4.0337243	1.456017	0.1469008	0.2605416	-5.612805
EPHA5	-0.397069	-0.538931	-1.455969	0.1469141	0.2605524	-5.612874
SLU7	0.0248609	6.2257968	1.4558568	0.1469451	0.2605948	-5.613034
NUFIP1	-0.040115	5.4453126	-1.455755	0.1469731	0.2606318	-5.613178
LANCL2	0.0309406	6.0191007	1.4554808	0.147049	0.2607537	-5.613569
MYSM1	0.0499584	5.7691619	1.4554485	0.1470579	0.2607569	-5.613615
IGHV3-11	0.4586159	3.4178941	1.4553824	0.1470762	0.2607682	-5.613709
AC004383.3	0.2637443	-0.734192	1.4553738	0.1470786	0.2607682	-5.613722
RP11-455G1	-0.254625	-0.894062	-1.455134	0.1471449	0.2608731	-5.614063
PSMD14	0.0283638	6.4175545	1.4550418	0.1471704	0.2609032	-5.614194
HSPB11	-0.03816	5.9605182	-1.455021	0.1471762	0.2609032	-5.614224
CDKL5	-0.064456	5.510671	-1.454927	0.1472021	0.2609365	-5.614358
RP5-881L22	0.3664652	2.0811775	1.4548492	0.1472236	0.260962	-5.614468
RPL36AP21	-0.252538	-0.778153	-1.454729	0.1472569	0.2610083	-5.614639
RP11-193J6	-0.136661	-1.387114	-1.454652	0.1472783	0.2610336	-5.614749
LINC01398	0.1244045	-1.422937	1.454547	0.1473072	0.2610722	-5.614898
RP11-359P5	0.322306	0.5636066	1.4545103	0.1473174	0.2610775	-5.614951
RNU6-526P	0.0886967	-1.510631	1.4544835	0.1473248	0.261078	-5.614989
PTGES3P2	-0.289313	3.5571642	-1.454297	0.1473766	0.261157	-5.615255
U8	0.4744165	0.428453	1.4542598	0.1473868	0.2611624	-5.615307
AJ003147.1	-0.259027	2.1212366	-1.454129	0.1474229	0.2612137	-5.615493
ZNF219	-0.046527	6.1646471	-1.454081	0.1474363	0.2612247	-5.615561

MMP9	-0.157521	5.6212456	-1.454028	0.147451	0.2612381	-5.615637
DNAJC6	0.1365502	5.1364979	1.4538983	0.1474869	0.2612848	-5.615821
NR1H4	-0.120268	6.3958502	-1.453856	0.1474986	0.2612848	-5.615882
TMF1	0.0294932	6.0577392	1.4538553	0.1474988	0.2612848	-5.615883
HCN1	-0.455035	0.0611589	-1.453723	0.1475355	0.2613371	-5.616071
AQP7P4	0.308699	-0.707491	1.4534401	0.1476139	0.2614633	-5.616473
C16orf71	0.1079603	3.9867233	1.4532653	0.1476623	0.2615356	-5.616721
RP11-161H2	-0.28539	-0.643059	-1.453241	0.147669	0.2615356	-5.616756
CNTNAP4	-0.599923	1.2820751	-1.453179	0.1476862	0.2615533	-5.616844
ZC3H12D	0.1350719	4.2556133	1.4530991	0.1477084	0.26158	-5.616958
THAP2	-0.041256	5.2103792	-1.453038	0.1477254	0.2615973	-5.617045
SEPHS2	0.0440579	7.0661658	1.4529053	0.1477622	0.2616498	-5.617233
RP11-258F1	-0.280269	-0.405424	-1.45287	0.1477718	0.2616524	-5.617283
RP11-295P9	0.3067002	1.7133736	1.4528483	0.147778	0.2616524	-5.617314
UQCC3	-0.04062	6.0360935	-1.452641	0.1478354	0.2617312	-5.617608
RPAIN	-0.03806	5.8981922	-1.452617	0.1478423	0.2617312	-5.617644
KLHL6-AS1	-0.424133	2.2488202	-1.452601	0.1478466	0.2617312	-5.617666
CBY1	-0.034654	5.9164604	-1.452585	0.1478512	0.2617312	-5.617689
ACRC	0.1077044	4.0412813	1.4524724	0.1478823	0.2617664	-5.617849
RP11-203F1	-0.319453	0.8848312	-1.452461	0.1478854	0.2617664	-5.617864
ZNF706	-0.036111	6.4901598	-1.452396	0.1479036	0.2617859	-5.617958
AC015884.1	0.1420652	-1.446219	1.4522173	0.1479531	0.2618609	-5.618211
HSP90AB3P	-0.150872	3.8401444	-1.452166	0.1479672	0.2618732	-5.618283
MMP12	-0.459483	3.1281627	-1.452062	0.1479964	0.261912	-5.618432
CLEC4D	0.3662234	0.1705501	1.4519314	0.1480326	0.2619634	-5.618617
RNU6-247P	0.2321213	-1.128281	1.4517183	0.1480918	0.2620555	-5.61892
RP11-34P13	-0.346329	2.4797364	-1.45164	0.1481135	0.2620811	-5.619031
AC092684.1	0.069012	-1.55174	1.4515403	0.1481412	0.2621176	-5.619173
AC005722.4	-0.142003	-1.463215	-1.451433	0.1481711	0.2621577	-5.619325
CTD-3030D2	-0.318978	-0.556076	-1.451163	0.148246	0.2622664	-5.619708
AC012368.1	-0.309558	-0.188966	-1.45116	0.1482469	0.2622664	-5.619712
RPS20P35	-0.357312	0.2754554	-1.451089	0.1482666	0.2622886	-5.619813
MYO5B	0.0581319	6.18412	1.4509249	0.1483124	0.2623568	-5.620047
TCEB2P2	-0.32133	-0.086837	-1.450845	0.1483345	0.2623832	-5.620159
DLGAP4-AS1	-0.176344	3.1010832	-1.450727	0.1483673	0.2624286	-5.620327
NPFFR1	-0.391554	0.7590481	-1.45064	0.1483917	0.262459	-5.620451
RP11-504G3	-0.320076	0.5226237	-1.450511	0.1484276	0.2625097	-5.620634
CTD-2201E1	0.0751054	-1.525871	1.450469	0.1484393	0.2625177	-5.620694
AC069368.3	0.1751774	-1.211396	1.4502597	0.1484975	0.2625998	-5.620991
ARHGEF33	0.2607751	2.0039296	1.4502504	0.1485001	0.2625998	-5.621004
BTF3P9	-0.25549	-0.694356	-1.449905	0.1485965	0.2627575	-5.621495
RP11-20I23	0.2527563	-0.773235	1.4495427	0.1486973	0.2629231	-5.622008
RP11-3B12.	0.2228397	-1.02168	1.4493451	0.1487524	0.2630078	-5.622288
LINC00954	-0.310691	1.848863	-1.449125	0.1488138	0.2631036	-5.6226
CYP26B1	-0.288006	4.1588476	-1.44891	0.1488737	0.2631966	-5.622904
TRPM7	0.0383867	6.2298435	1.448855	0.1488891	0.2632112	-5.622983
LMO7	0.0461484	6.3752936	1.4487913	0.1489069	0.2632174	-5.623073
PM20D1	-0.327248	1.9674489	-1.448791	0.148907	0.2632174	-5.623074
AC114498.1	0.3253417	2.2059745	1.4487131	0.1489287	0.263243	-5.623184
AP002414.1	-0.131986	-1.424316	-1.448555	0.1489727	0.2633081	-5.623408
CAMSAP2	0.0390629	6.0534783	1.4484841	0.1489927	0.2633305	-5.623509

HOXA13	-0.511578	3.7966363	-1.448386	0.1490202	0.2633664	-5.623648
DEPDC7	0.1252428	5.7768999	1.4482864	0.1490479	0.2634026	-5.623789
GS1-124K5.	-0.081789	4.8845218	-1.448196	0.1490732	0.2634346	-5.623917
ATP7A	0.0509764	5.4175523	1.44813	0.1490916	0.2634429	-5.62401
RP11-309M7	-0.255348	-1.007734	-1.448127	0.1490923	0.2634429	-5.624014
TMEM151A	-0.286139	3.8078489	-1.447961	0.1491389	0.2634999	-5.62425
RP11-686D2	0.379053	0.8318977	1.44796	0.149139	0.2634999	-5.624251
AC067956.1	0.0722303	-1.541787	1.4478579	0.1491676	0.2635098	-5.624396
TTTY25P	0.1368736	-1.438258	1.447853	0.1491689	0.2635098	-5.624403
RP11-823E8	0.2721697	2.5288072	1.4478505	0.1491696	0.2635098	-5.624406
RP11-415K2	-0.344762	0.5313845	-1.447795	0.1491852	0.2635098	-5.624485
MEOX2	-0.270146	3.7765351	-1.447776	0.1491903	0.2635098	-5.624511
MYH7	0.3026592	-0.724514	1.4477636	0.1491939	0.2635098	-5.62453
FAM134C	-0.028098	6.5678042	-1.447759	0.1491952	0.2635098	-5.624536
RP11-307L1	-0.214882	2.3805919	-1.447636	0.1492296	0.2635578	-5.62471
OXCT2P1	-0.27873	2.4733071	-1.447599	0.1492399	0.2635634	-5.624763
GLUL	-0.057073	7.2478961	-1.447556	0.1492519	0.2635717	-5.624823
RP11-1070N	-0.176664	-1.319533	-1.447484	0.149272	0.2635945	-5.624925
CDK10	0.0331484	6.4004168	1.4473193	0.1493182	0.2636632	-5.625159
COL11A2	-0.166336	4.112347	-1.447158	0.1493633	0.2637302	-5.625387
HNRNPUL1	-0.02121	6.7969334	-1.447109	0.1493769	0.2637415	-5.625456
MSX1	0.1025571	4.6470832	1.4468033	0.1494625	0.2638798	-5.625889
LDHAP3	0.358703	3.0740849	1.4467283	0.1494835	0.2639041	-5.625996
RP11-493E3	-0.290948	-0.16724	-1.446565	0.1495293	0.2639723	-5.626227
DTX3L	0.0339764	6.5576219	1.4464718	0.1495553	0.2640052	-5.626359
RP11-269G2	0.1579386	-1.344897	1.4464218	0.1495693	0.2640052	-5.626429
C1orf174	-0.031827	5.9362854	-1.44642	0.1495697	0.2640052	-5.626431
TFPI	0.0798693	6.7781727	1.4463589	0.1495869	0.2640229	-5.626518
TP53BP2	-0.043017	6.4100114	-1.44626	0.1496147	0.2640592	-5.626659
CYP4F8	0.313982	-0.779617	1.4457272	0.1497639	0.2643038	-5.627412
RP5-837I24	-0.138187	-1.394791	-1.445713	0.1497678	0.2643038	-5.627432
HMGN2	-0.031654	6.6472708	-1.445665	0.1497813	0.2643148	-5.6275
RP11-554D1	-0.131697	-1.424458	-1.4456	0.1497997	0.2643345	-5.627593
CTB-7E3.1	-0.134505	-1.444986	-1.445556	0.149812	0.2643434	-5.627655
AC005498.3	-0.369529	0.4965578	-1.445513	0.1498239	0.2643517	-5.627715
C12orf45	-0.045477	5.6374583	-1.44539	0.1498585	0.2643999	-5.627889
RP4-756H11	-0.052762	5.09543	-1.444796	0.1500251	0.264681	-5.628729
RP11-578O2	0.3015603	1.8595135	1.444434	0.1501268	0.2648477	-5.629241
RP11-571O6	-0.148321	-1.367917	-1.444179	0.1501983	0.2649611	-5.629601
CTC-50503.	-0.183581	-1.170806	-1.444115	0.1502163	0.2649799	-5.629691
U47924.6	-0.093347	4.4970863	-1.444088	0.1502241	0.2649809	-5.62973
IGKV1-39	-0.444126	0.0432028	-1.443827	0.1502972	0.2650853	-5.630098
MAGEC1	-0.591238	0.7083585	-1.443825	0.1502978	0.2650853	-5.630101
CTD-2350C1	-0.181561	4.471936	-1.443742	0.1503213	0.265114	-5.630219
RP11-419C1	-0.31399	-0.926159	-1.443676	0.1503397	0.2651336	-5.630312
SNX19P2	0.3038424	-0.015841	1.4435619	0.1503718	0.2651775	-5.630473
TEKT2	-0.378476	1.1532639	-1.443482	0.1503943	0.2652043	-5.630586
RBM26	-0.026352	6.149645	-1.44332	0.15044	0.2652486	-5.630815
RP11-1012E	-0.454765	0.6657782	-1.443314	0.1504417	0.2652486	-5.630824
ARHGEF9	0.0415292	5.7234157	1.4433056	0.1504439	0.2652486	-5.630835
RP11-253M7	0.3013185	2.0226697	1.4432892	0.1504485	0.2652486	-5.630858

LINC01514	-0.11525	-1.498239	-1.443037	0.1505194	0.2653608	-5.631214
HARBI1	0.0423121	5.0302706	1.4428696	0.1505666	0.2654238	-5.631451
HTR7P1	-0.077846	4.8231398	-1.442859	0.1505697	0.2654238	-5.631466
LRRC37A4P	0.2687911	3.1908987	1.4423584	0.1507106	0.2656593	-5.632172
ZNF672	-0.032473	6.3233933	-1.441781	0.1508732	0.2659332	-5.632987
AC092159.1	-0.100356	-1.483637	-1.441711	0.150893	0.2659553	-5.633086
TAF15	-0.021867	6.6796471	-1.441487	0.1509563	0.2660539	-5.633402
RP11-981P6	-0.276105	1.9549938	-1.441346	0.1509959	0.2661108	-5.6336
FAM74A7	0.0722293	-1.550101	1.4412963	0.1510099	0.2661228	-5.633671
RP11-408H1	0.3414193	2.3990615	1.4412423	0.1510252	0.2661285	-5.633747
LDHAP4	0.2348169	4.2221799	1.4412331	0.1510278	0.2661285	-5.63376
MIR5695	-0.100356	-1.483637	-1.441199	0.1510375	0.2661328	-5.633809
MCUR1	0.0288205	6.2070499	1.4411218	0.1510592	0.2661581	-5.633917
MRPS31P4	-0.255049	2.958488	-1.440903	0.1511209	0.2662541	-5.634225
SMURF2	-0.037678	5.7752747	-1.440633	0.151197	0.2663753	-5.634606
XDH	0.2010904	5.992861	1.4405477	0.1512212	0.2664051	-5.634726
HIST2H2AA4	-0.206409	-1.20222	-1.440403	0.1512622	0.2664644	-5.634931
AC116609.2	-0.265776	-1.071364	-1.440287	0.1512949	0.2664997	-5.635094
CLDN3	-0.179986	5.9487571	-1.44028	0.1512968	0.2664997	-5.635104
ITFG1-AS1	0.3161139	-0.064689	1.4401226	0.1513413	0.2665652	-5.635325
C4orf22	0.1747904	-1.289109	1.4400793	0.1513536	0.2665739	-5.635386
RP11-702F3	-0.113039	-1.477418	-1.440044	0.1513635	0.2665786	-5.635436
CTC-344H19	-0.30555	-0.17998	-1.440001	0.1513757	0.2665824	-5.635497
CTA-31J9.2	0.139242	-1.415261	1.4399847	0.1513803	0.2665824	-5.63552
ATP6V0E2-A	0.1485733	4.0235762	1.4398993	0.1514044	0.266612	-5.63564
ALG1L2	0.2976411	1.0259093	1.4397341	0.1514511	0.2666814	-5.635873
TARDBPP2	-0.153914	-1.341796	-1.439647	0.1514758	0.2667119	-5.635996
FGF19	0.535571	2.6720549	1.4396015	0.1514886	0.2667217	-5.63606
CDCP1	0.1782114	4.8254639	1.4395628	0.1514996	0.2667281	-5.636114
MEF2C-AS1	0.3157255	1.6786033	1.4395095	0.1515146	0.2667376	-5.636189
HNRNPKP1	-0.125033	3.5308968	-1.439492	0.1515196	0.2667376	-5.636214
LRSAM1	0.0376523	6.0357246	1.4393708	0.1515539	0.2667851	-5.636385
RP4-665J23	-0.210699	-1.248863	-1.439329	0.1515657	0.266793	-5.636443
FUT10	0.0712979	4.9688909	1.439244	0.1515897	0.2668098	-5.636563
RP11-175B9	-0.289828	0.3090738	-1.439244	0.1515898	0.2668098	-5.636564
S100A7	0.4137463	-0.338915	1.4390791	0.1516364	0.2668789	-5.636796
RCBTB1	0.0403325	5.8768507	1.4389374	0.1516765	0.266932	-5.636995
RP11-10N16	-0.132229	-1.438102	-1.438921	0.1516812	0.266932	-5.637018
RP11-436D1	-0.222866	-0.930027	-1.438812	0.1517121	0.2669735	-5.637172
RP11-488C1	-0.315478	-0.140524	-1.438716	0.1517392	0.2670083	-5.637307
RP11-176H8	0.1552809	3.2521222	1.4384316	0.1518197	0.2671372	-5.637707
RP11-108M9	0.1207483	4.9351906	1.4383828	0.1518336	0.2671399	-5.637776
RP11-367E1	-0.297398	0.7132149	-1.438363	0.151839	0.2671399	-5.637803
FTLP3	-0.100861	5.369836	-1.438349	0.1518432	0.2671399	-5.637824
MROH8	0.1077862	4.3760049	1.4382638	0.1518673	0.2671662	-5.637943
CTD-3088G3	-0.178044	-1.248137	-1.438244	0.1518728	0.2671662	-5.637971
ALDH5A1	0.0648958	6.564491	1.4381225	0.1519073	0.2672041	-5.638142
EMBP1	-0.116254	4.6860632	-1.438093	0.1519156	0.2672041	-5.638183
NEUROD1	-0.240306	-1.251842	-1.438087	0.1519175	0.2672041	-5.638193
USP24	0.0223836	6.3712433	1.4380649	0.1519236	0.2672041	-5.638223
RP11-110I1	-0.123762	3.9289257	-1.437932	0.1519613	0.2672574	-5.63841

DSG2	0.1138097	6.1357241	1.4374294	0.1521038	0.267492	-5.639117
CHMP4C	-0.079768	5.7440812	-1.43741	0.1521093	0.267492	-5.639145
LETMD1	-0.027265	6.3334049	-1.437368	0.1521213	0.2675002	-5.639204
RP11-865I6	0.1770427	-1.399783	1.4372279	0.152161	0.2675571	-5.639401
GJC1	0.0853769	5.311655	1.4371585	0.1521807	0.2675788	-5.639498
HDHD2	0.0369537	5.9662812	1.4370889	0.1522004	0.2676007	-5.639596
RP11-814P5	0.0898783	-1.513584	1.4370179	0.1522206	0.2676232	-5.639696
DCPS	0.0615539	6.261177	1.4367698	0.152291	0.2677341	-5.640045
NEK4P2	-0.100784	-1.505333	-1.436693	0.1523128	0.267748	-5.640153
INPPL1	0.0309072	6.4674406	1.4366787	0.1523169	0.267748	-5.640173
PEX3	0.0510453	5.8036744	1.4366645	0.1523209	0.267748	-5.640193
RP11-152F1	0.1102548	-1.480435	1.4365345	0.1523579	0.2678	-5.640376
RP11-184M1	0.3766465	0.3188808	1.4361763	0.1524596	0.267966	-5.640879
RP11-307I1	-0.156057	-1.322686	-1.435848	0.1525529	0.268117	-5.64134
PMS2L2	0.297658	-0.586672	1.4358215	0.1525605	0.2681174	-5.641378
RP11-307C1	-0.121662	4.2804799	-1.435673	0.1526027	0.2681787	-5.641586
HMGB1P10	0.0960632	4.3765697	1.4355213	0.1526458	0.2682416	-5.6418
MED28P8	0.2922839	-0.897366	1.4354793	0.1526578	0.2682449	-5.641859
MYO15B	0.0611894	6.3083311	1.435463	0.1526624	0.2682449	-5.641881
AK8	0.2962956	3.0880629	1.4353056	0.1527072	0.2683107	-5.642103
HNRNPA2B1	-0.017434	7.1473077	-1.435157	0.1527495	0.2683489	-5.642311
LINC01265	0.3159667	-0.62211	1.4351531	0.1527506	0.2683489	-5.642317
BNIP3P30	-0.353126	-0.53503	-1.435116	0.1527611	0.2683489	-5.642369
GSG1L	0.3014746	-0.769523	1.4350906	0.1527684	0.2683489	-5.642405
RNY4P34	0.0620595	-1.555284	1.4350846	0.1527701	0.2683489	-5.642413
AC135048.1	0.2392967	3.1366831	1.4350743	0.152773	0.2683489	-5.642427
TRAV4	-0.380659	1.4280795	-1.434779	0.1528572	0.2684838	-5.642843
HNRNPA1P64	-0.180281	-1.21756	-1.434673	0.1528872	0.2685236	-5.642991
NAV2-AS3	0.2760193	-0.764369	1.4345473	0.1529231	0.2685737	-5.643168
RP11-138I1	-0.153094	-1.343671	-1.434446	0.152952	0.2686115	-5.64331
TIMM44	-0.030857	6.278634	-1.434374	0.1529723	0.2686343	-5.64341
LENG8	-0.03197	6.5101259	-1.434263	0.1530041	0.2686607	-5.643566
RPS17P2	-0.264101	-0.626094	-1.434256	0.1530061	0.2686607	-5.643576
RP11-254G1	-0.25204	-0.779563	-1.434231	0.1530131	0.2686607	-5.643611
LLNLR-249E	-0.176927	-1.306825	-1.434213	0.1530183	0.2686607	-5.643637
RAB3IP	-0.05042	5.9535754	-1.434193	0.1530241	0.2686607	-5.643665
ALDH16A1	-0.039712	6.2815364	-1.4341	0.1530506	0.2686942	-5.643796
RN7SL674P	-0.288899	-0.446607	-1.434055	0.1530632	0.2687035	-5.643858
PIPSL	-0.158823	3.4213342	-1.433989	0.1530821	0.2687236	-5.643951
AP000708.1	-0.222887	-0.946128	-1.433721	0.1531586	0.2688451	-5.644327
ANKRD66	-0.190062	-1.279212	-1.433637	0.1531827	0.2688743	-5.644446
CTD-2410N1	0.1331703	3.5613785	1.4335912	0.1531956	0.2688841	-5.644509
RP11-142G1	0.058639	-1.557027	1.4334981	0.1532222	0.2689178	-5.64464
RP11-100M1	0.1112892	-1.475149	1.4334248	0.1532431	0.2689416	-5.644743
RP11-357H1	0.3778292	-0.132243	1.4333602	0.1532615	0.268961	-5.644833
OR52K3P	0.407502	0.556024	1.4333067	0.1532768	0.2689749	-5.644908
MIR574	-0.212922	-1.012575	-1.433078	0.1533421	0.2690766	-5.64523
CTD-2538G9	-0.256349	-0.81173	-1.432954	0.1533775	0.2691257	-5.645403
INO80E	0.027194	6.1829869	1.4327895	0.1534244	0.2691802	-5.645634
L3HYPDH	0.0535759	5.6174343	1.4327753	0.1534284	0.2691802	-5.645654
MSH4	0.3815659	1.3416783	1.4327676	0.1534307	0.2691802	-5.645664

GPC6	0.0781704	6.2047503	1.4326949	0.1534514	0.2691982	-5.645766
CBLN1	-0.344118	3.9547586	-1.43268	0.1534557	0.2691982	-5.645787
FOXS1	0.1493339	4.783742	1.43252	0.1535014	0.2692654	-5.646012
TCAF1P1	0.3255945	3.1718267	1.4324372	0.153525	0.269294	-5.646128
RP1-27C22.	-0.141522	-1.436966	-1.432178	0.153599	0.2694079	-5.646491
IGKV6D-21	0.4300513	0.0704836	1.4321584	0.1536047	0.2694079	-5.646519
LRRC59	-0.025815	6.730929	-1.432063	0.153632	0.2694427	-5.646652
PSMD12	0.0232687	6.4192135	1.4319521	0.1536637	0.2694854	-5.646808
NXPH3	-0.138426	3.8893218	-1.43185	0.1536927	0.2695234	-5.64695
RP11-568J2	-0.208067	-1.085495	-1.431766	0.153717	0.269553	-5.647069
PCA3	0.2459272	-1.195285	1.4316997	0.1537358	0.2695731	-5.647161
AP000695.6	0.20038	3.4253056	1.4316196	0.1537588	0.2696004	-5.647274
ZFP42	-0.216943	-1.342432	-1.431566	0.1537742	0.2696144	-5.647349
RRAS2	0.0466165	6.0462117	1.431507	0.1537909	0.2696309	-5.647431
RP11-26J3.	-0.268855	1.7958432	-1.431466	0.1538027	0.2696385	-5.647489
AKNAD1	0.3082654	-0.393865	1.4314391	0.1538104	0.2696391	-5.647526
POM121	-0.045592	6.0603823	-1.431354	0.1538346	0.2696606	-5.647645
AP003025.2	-0.096269	-1.507547	-1.431323	0.1538435	0.2696606	-5.647689
RP11-284F2	0.2563162	5.1344651	1.4313188	0.1538448	0.2696606	-5.647695
AC004000.1	-0.175605	-1.244754	-1.431199	0.153879	0.2697075	-5.647862
EPHX3	0.273585	2.8878159	1.4308921	0.1539669	0.2698487	-5.648293
IMMTP1	-0.18021	-1.233448	-1.430755	0.1540061	0.2698965	-5.648484
RP11-83N9.	0.3246427	2.0901341	1.4307452	0.154009	0.2698965	-5.648498
LINC01551	-0.203199	-1.283434	-1.430656	0.1540345	0.2699283	-5.648623
RPS8	-0.031204	7.1925157	-1.430397	0.1541088	0.2700456	-5.648986
LINC00348	-0.525728	0.517834	-1.43035	0.1541222	0.2700559	-5.649051
RP11-416N4	-0.299569	-0.481923	-1.430278	0.1541429	0.2700794	-5.649153
AP000688.1	0.1705117	-1.326716	1.4302032	0.1541643	0.270096	-5.649257
SHISA6	0.4064796	0.7285714	1.430193	0.1541672	0.270096	-5.649271
ZNF358	-0.038938	6.2860373	-1.42975	0.1542942	0.2702965	-5.649891
CNBP	0.0240005	6.8987367	1.4297422	0.1542965	0.2702965	-5.649902
RBAK	0.0339703	5.7763766	1.4297163	0.1543039	0.2702965	-5.649939
PPIAP15	0.1796378	-1.200809	1.4288862	0.1545421	0.2706979	-5.6511
PSME1	0.0336243	6.8204236	1.4288638	0.1545485	0.2706979	-5.651131
BNIP3P27	-0.290224	-0.574095	-1.42884	0.1545553	0.2706979	-5.651164
RP11-628J1	-0.129769	-1.469214	-1.428784	0.1545716	0.2707134	-5.651243
HERC2	0.0283827	6.272879	1.4287101	0.1545927	0.2707374	-5.651346
TUBBP6	-0.208351	-1.136583	-1.428617	0.1546194	0.2707712	-5.651476
RP5-968P14	-0.114558	3.8737594	-1.428454	0.1546662	0.2708285	-5.651704
RP11-91A15	0.1043972	-1.48342	1.4284277	0.1546738	0.2708285	-5.651741
CTD-2626G1	0.1787699	-1.298013	1.4284258	0.1546744	0.2708285	-5.651743
RP11-809H1	-0.181335	-1.292607	-1.428176	0.1547462	0.2709413	-5.652093
TBX18	0.3585531	2.5291239	1.4280859	0.154772	0.2709585	-5.652219
RECQL	0.0575648	5.6978354	1.4280744	0.1547754	0.2709585	-5.652235
TRAV23DV6	-0.345025	-0.058348	-1.428064	0.1547783	0.2709585	-5.652249
RP11-57H12	-0.280691	1.7838808	-1.428032	0.1547877	0.2709619	-5.652295
Z83001.1	-0.139137	-1.363262	-1.427968	0.154806	0.2709811	-5.652384
DUXAP4	0.1374636	-1.398277	1.4278436	0.1548417	0.2710305	-5.652557
RP11-734K2	0.1227995	-1.461492	1.4278067	0.1548523	0.2710361	-5.652609
PLOD3	0.0299727	6.7195329	1.4277608	0.1548655	0.2710462	-5.652673
AC130469.1	0.3212927	1.6141276	1.4275845	0.1549163	0.271122	-5.652919

LGALS7	-0.240101	-1.101461	-1.427518	0.1549354	0.2711425	-5.653012
CD63	0.0270101	7.1577681	1.4272911	0.1550006	0.2712437	-5.653329
RP4-555D2C	0.2525646	-1.096132	1.4271893	0.15503	0.271282	-5.653471
HINT1P1	-0.266593	-0.650748	-1.427149	0.1550417	0.2712895	-5.653528
FTH1P2	-0.114132	3.9378026	-1.42705	0.1550702	0.2713263	-5.653666
CHD1	0.031285	6.0671194	1.4269696	0.1550932	0.2713537	-5.653778
RP11-1193F	-0.220922	-1.099804	-1.426806	0.1551403	0.2714231	-5.654007
SLC9C2	0.3959013	0.7224601	1.4266811	0.1551763	0.271473	-5.654181
DBIL5P2	-0.289857	-0.347634	-1.426507	0.1552264	0.2715393	-5.654424
RP11-87C12	-0.166572	-1.272672	-1.426498	0.1552291	0.2715393	-5.654437
RP11-87E22	0.2374428	-1.069349	1.4264601	0.15524	0.2715453	-5.65449
AC005104.3	-0.270366	2.2548123	-1.426313	0.1552824	0.2716066	-5.654695
FCER1G	0.0722804	5.9646895	1.4259354	0.1553912	0.2717739	-5.655222
RP11-415D1	0.1335287	-1.414733	1.4259294	0.1553929	0.2717739	-5.655231
RP11-977G1	0.2296556	-0.96482	1.4255901	0.1554908	0.271932	-5.655704
C14orf142	-0.038274	5.6413301	-1.425496	0.1555181	0.2719667	-5.655836
FLT3LG	0.0864359	4.5785172	1.4254157	0.1555411	0.2719939	-5.655947
RP11-27K13	0.2919721	1.7721554	1.4252562	0.1555871	0.2720614	-5.65617
MIA-RAB4B	-0.1473	-1.353465	-1.425185	0.1556078	0.2720807	-5.65627
WBP1LP2	0.1160142	3.4579487	1.4251663	0.1556131	0.2720807	-5.656295
ATRX	0.0316918	6.1954259	1.4250093	0.1556584	0.2721469	-5.656514
RP13-514E2	0.3222367	-0.302875	1.4247131	0.155744	0.2722834	-5.656927
TMEM38B	-0.043317	6.0684977	-1.424635	0.1557666	0.2723099	-5.657036
RP11-303G3	0.3081195	-0.172089	1.4244132	0.1558306	0.2724088	-5.657345
FBXL19	-0.041863	5.692533	-1.424315	0.1558591	0.2724375	-5.657483
PPIAP2	-0.309536	0.7212653	-1.424305	0.1558619	0.2724375	-5.657497
NATP	0.0886967	-1.510631	1.4239238	0.1559721	0.272617	-5.658028
KRT18P64	-0.173594	-1.211514	-1.423741	0.1560249	0.2726962	-5.658282
CTC-366B18	0.1138215	3.5424443	1.4236333	0.1560561	0.2727377	-5.658432
RP4-753D1C	0.0913512	-1.512833	1.4236067	0.1560638	0.2727381	-5.658469
CD6	0.1097597	5.22753	1.4235569	0.1560782	0.2727502	-5.658539
ABHD14A	-0.047262	5.8756738	-1.423509	0.1560921	0.2727613	-5.658606
RP11-346C2	0.1627017	3.1402784	1.4234815	0.1561	0.2727622	-5.658644
RP11-563H6	-0.310042	0.0600231	-1.42344	0.1561122	0.272768	-5.658702
IGHG2	0.2176004	5.9013839	1.4234184	0.1561183	0.272768	-5.658732
MIR6739	-0.249371	-0.741956	-1.42331	0.1561497	0.2728098	-5.658883
WRAP73	0.0338361	5.817836	1.4230418	0.1562273	0.2729323	-5.659256
LINC00908	-0.391994	1.0074892	-1.422537	0.1563736	0.2731748	-5.65996
AP000436.4	-0.188392	-1.155868	-1.42249	0.156387	0.2731786	-5.660024
LA16c-380H	-0.280384	2.2135118	-1.422478	0.1563907	0.2731786	-5.660042
NPHS1	-0.397215	0.3968712	-1.422446	0.1563998	0.2731792	-5.660086
RP5-827C21	-0.270517	-0.779579	-1.422425	0.156406	0.2731792	-5.660116
RP11-15F12	-0.373598	0.9090527	-1.422289	0.1564453	0.2732348	-5.660304
PAICSP4	-0.308937	0.3444823	-1.422185	0.1564756	0.2732745	-5.66045
ELMSAN1	0.0368698	5.9939484	1.4220258	0.1565217	0.273342	-5.660671
RP11-736G1	-0.152751	-1.400082	-1.421823	0.1565805	0.2734315	-5.660953
PFKFB1	0.2076038	5.2419762	1.4217096	0.1566134	0.2734708	-5.661111
S100A2	0.3300654	3.1563335	1.421694	0.1566179	0.2734708	-5.661133
ZFP69	-0.057923	5.0440326	-1.421558	0.1566574	0.2735256	-5.661322
CSRP2BP	-0.036797	5.9256581	-1.421534	0.1566643	0.2735256	-5.661355
AC092168.2	-0.25972	-0.636666	-1.421459	0.156686	0.2735504	-5.661459

IRAK2	-0.057677	5.9979945	-1.421314	0.156728	0.2736107	-5.661661
FAM20B	-0.031883	6.4533161	-1.421278	0.1567388	0.2736163	-5.661712
VTRNA1-1	0.1679064	-1.321621	1.4211685	0.1567704	0.2736585	-5.661864
AC093063.3	-0.101425	-1.505018	-1.421064	0.1568006	0.2736982	-5.662009
RP11-21A7A	-0.21119	-1.165543	-1.420321	0.1570166	0.2740621	-5.663043
RP3-395M20	0.0953766	5.3107828	1.4202444	0.1570389	0.2740878	-5.663149
GIPC2	-0.142863	5.4900037	-1.419828	0.1571601	0.2742862	-5.663728
RP11-1094M	0.2015219	3.587694	1.4195929	0.1572283	0.2743861	-5.664055
TDRD10	0.1709113	4.6068223	1.4195771	0.1572329	0.2743861	-5.664077
CREB3	0.0295776	6.2496939	1.4195533	0.1572398	0.2743861	-5.66411
HCG9	0.3546857	1.0186869	1.4194841	0.15726	0.2744081	-5.664206
LIMK1	0.0521357	6.0031129	1.4194534	0.1572689	0.2744106	-5.664248
SCAPER	-0.041828	5.6700444	-1.419325	0.1573062	0.2744625	-5.664426
STAMBPL1	-0.090525	5.2346526	-1.419271	0.1573219	0.2744768	-5.664501
AC074121.4	-0.140922	-1.39345	-1.419079	0.1573779	0.2745615	-5.664769
LINC01312	0.1292852	-1.4349	1.4188908	0.1574327	0.2746439	-5.66503
RP11-755B1	0.2353166	-0.841535	1.4188348	0.157449	0.2746532	-5.665108
RP11-123J1	-0.126139	-1.405279	-1.418821	0.1574531	0.2746532	-5.665127
FAM187B	0.1951254	-1.181186	1.4184108	0.1575725	0.2748484	-5.665696
RP5-88207.	-0.32126	0.4651886	-1.418124	0.1576563	0.2749759	-5.666095
NXPH4	-0.241832	4.533726	-1.418108	0.1576607	0.2749759	-5.666116
PDXP	-0.118391	4.7620685	-1.417966	0.1577023	0.2750321	-5.666314
SERPINB9P1	0.2130891	4.12414	1.4179462	0.157708	0.2750321	-5.666341
PWRN4	-0.159395	-1.393292	-1.417567	0.1578187	0.2752121	-5.666868
MGMT	-0.057146	6.4669821	-1.417436	0.1578569	0.2752655	-5.66705
RP11-20I23	0.2748236	-0.642592	1.4173767	0.1578741	0.2752747	-5.667132
SLFN13	0.1523934	5.1143642	1.417366	0.1578773	0.2752747	-5.667147
C14orf80	-0.062701	5.4408666	-1.417073	0.1579627	0.2753983	-5.667553
RP11-71801	-0.225628	-1.095317	-1.417072	0.1579632	0.2753983	-5.667555
RP11-39H3.	0.4026379	2.7982782	1.417033	0.1579745	0.2754048	-5.667609
IGLV2-23	0.3636819	4.4438946	1.4169914	0.1579866	0.2754128	-5.667666
KCNA10	0.1336826	-1.418209	1.4168771	0.15802	0.2754579	-5.667825
SNX14	-0.029311	6.382224	-1.4168	0.1580426	0.2754826	-5.667932
RAP2CP1	0.3800302	0.2538557	1.4167767	0.1580493	0.2754826	-5.667964
CTD-2330J2	0.0540595	-1.55936	1.4165613	0.1581123	0.2755792	-5.668263
RP11-2K6.1	-0.316498	-0.083363	-1.416271	0.158197	0.2757136	-5.668664
LINC01397	-0.267616	-0.923403	-1.416125	0.1582397	0.275775	-5.668867
BAG1	0.0378655	6.3506913	1.4160172	0.1582713	0.2758169	-5.669017
LBP	0.1396603	7.0322419	1.4159639	0.1582869	0.2758309	-5.669091
COCH	-0.325401	4.4333211	-1.415554	0.1584067	0.2760265	-5.669658
IL2RG	0.0881105	5.9038002	1.4154513	0.1584369	0.2760659	-5.669801
SLMAP	0.0278076	6.1291508	1.4152524	0.1584951	0.2761541	-5.670077
CTC-250I14	-0.300072	-0.297506	-1.415177	0.1585173	0.2761796	-5.670182
AC009234.1	0.3254885	-0.793242	1.4151046	0.1585384	0.2761982	-5.670282
RP11-553P9	-0.273997	2.1591631	-1.415088	0.1585431	0.2761982	-5.670304
LINC01141	-0.329306	0.2678478	-1.415019	0.1585636	0.2762207	-5.670401
GABRB2	0.3990152	0.0660613	1.414898	0.1585988	0.276269	-5.670568
CGRRF1	-0.034852	5.5194597	-1.414815	0.1586232	0.2762982	-5.670683
ALKBH4	-0.030369	5.694079	-1.414788	0.1586312	0.2762989	-5.67072
TRGVB	0.1073697	-1.478351	1.4147077	0.1586546	0.2763265	-5.670831
YWHAQ	-0.024034	6.6789916	-1.414538	0.1587042	0.2763997	-5.671066

RP11-575A1	0.200647	-1.207019	1.4145032	0.1587145	0.2764045	-5.671114
GTPBP1	0.0260218	6.2753984	1.4143471	0.1587603	0.276471	-5.671331
RP11-475O6	0.2694571	3.1846442	1.4142608	0.1587856	0.2765019	-5.67145
NFIA-AS1	0.2123128	-1.113527	1.4142195	0.1587977	0.2765098	-5.671507
RP11-310H4	0.1364941	-1.421535	1.4141179	0.1588275	0.2765484	-5.671648
PCTP	0.0521461	6.3600689	1.4137741	0.1589283	0.2767011	-5.672124
RNF212B	-0.320599	1.5422835	-1.413767	0.1589303	0.2767011	-5.672133
RP1-131F15	-0.297117	2.0765972	-1.413698	0.1589507	0.2767234	-5.67223
LINC01088	0.3802129	0.4082102	1.4135657	0.1589894	0.2767716	-5.672412
CTD-2299I2	0.3368111	-0.202368	1.4135517	0.1589936	0.2767716	-5.672432
SF3A1	0.0231688	6.5748428	1.4132523	0.1590814	0.2769114	-5.672846
TTC30A	-0.059467	5.3081205	-1.413152	0.159111	0.2769496	-5.672985
GPR101	-0.213786	-1.191804	-1.413098	0.1591268	0.276964	-5.67306
AC114783.1	0.1919271	-1.312658	1.4128881	0.1591884	0.2770579	-5.67335
RARA	-0.031689	6.4345074	-1.412762	0.1592253	0.277109	-5.673524
AC001226.7	-0.252519	-0.678488	-1.412441	0.1593198	0.2772602	-5.673968
RP11-404K5	-0.182202	-1.36199	-1.412292	0.1593636	0.2773111	-5.674174
DUOX2	0.3732976	4.2395098	1.4122896	0.1593642	0.2773111	-5.674177
RP11-96H17	-0.406397	-0.480453	-1.411957	0.159462	0.2774681	-5.674637
RP11-49K24	-0.312349	1.9225676	-1.411913	0.1594751	0.2774775	-5.674698
SLC2A1-AS1	0.3609144	1.5605591	1.4117267	0.1595298	0.2775595	-5.674955
CCT6A	-0.025244	6.7726568	-1.411689	0.159541	0.2775657	-5.675008
TRIM6-TRIM	0.0965004	-1.501453	1.4116425	0.1595545	0.2775761	-5.675072
AVPR1B	-0.234669	-1.067457	-1.411579	0.1595733	0.2775955	-5.67516
RPS26P6	-0.310397	0.9348799	-1.411503	0.1595957	0.2776136	-5.675265
POU2F3	-0.350337	2.3844897	-1.411492	0.1595989	0.2776136	-5.67528
CCDC12	-0.034364	6.1974142	-1.411365	0.1596363	0.2776562	-5.675456
WARS	0.0403699	6.479679	1.4113123	0.1596517	0.2776562	-5.675528
SPDYE2	-0.329624	0.5428584	-1.411264	0.1596659	0.2776562	-5.675594
GRM7-AS3	-0.184007	-1.2884	-1.411258	0.1596678	0.2776562	-5.675604
LINC01264	0.0735915	-1.549407	1.4112446	0.1596717	0.2776562	-5.675621
RP4-781K5.	0.0950023	-1.510972	1.4112347	0.1596746	0.2776562	-5.675635
RP11-468O2	-0.273565	-0.38644	-1.411228	0.1596766	0.2776562	-5.675645
AL365331.2	-0.134011	-1.401419	-1.410695	0.1598336	0.2779158	-5.676381
BCS1L	-0.029049	6.0560583	-1.410534	0.1598808	0.2779816	-5.676602
RP11-48B3.	-0.076365	4.932652	-1.410515	0.1598866	0.2779816	-5.676629
GSTA7P	0.3998353	3.3159436	1.41032	0.159944	0.2780682	-5.676898
CAPN3	0.1581311	3.9575637	1.4099998	0.1600384	0.278219	-5.67734
RP11-355N1	-0.328606	0.1337317	-1.409692	0.1601291	0.2783634	-5.677765
RP11-325O2	0.0670643	-1.552733	1.4095368	0.160175	0.27843	-5.677979
AC243945.1	0.0959718	-1.487714	1.4094471	0.1602015	0.2784627	-5.678103
RNU6-524P	-0.261548	-0.77496	-1.409361	0.1602269	0.2784936	-5.678222
MRPL16	0.0315311	6.3341306	1.4092634	0.1602557	0.2785304	-5.678357
AP005135.2	0.0670643	-1.552733	1.4091206	0.1602979	0.2785904	-5.678554
PLA2G2F	0.2620617	-0.886836	1.4090622	0.1603151	0.2786072	-5.678634
CACNA1E	-0.463348	1.824477	-1.408971	0.160342	0.2786406	-5.67876
ACP1	-0.022909	6.5486913	-1.408874	0.1603706	0.278677	-5.678893
NANOS2	-0.120701	-1.425271	-1.408683	0.1604271	0.2787619	-5.679157
OR13Z3P	-0.130337	-1.434025	-1.408507	0.1604791	0.2788391	-5.6794
CTD-3035K2	0.3405422	0.0288193	1.4084676	0.1604908	0.278846	-5.679454
RP11-745C1	-0.194751	-1.168602	-1.408358	0.1605232	0.278889	-5.679605

CEACAM5	-0.457264	0.1206099	-1.408173	0.1605778	0.2789707	-5.67986
LARP1P1	-0.218373	-0.982448	-1.408039	0.1606174	0.279014	-5.680045
CADM1	0.0643165	6.4410759	1.4080372	0.160618	0.279014	-5.680048
RP11-59H7.	-0.1133	-1.47729	-1.408009	0.1606262	0.279015	-5.680086
RBBP9	0.0441646	6.1203169	1.4077612	0.1606997	0.2791266	-5.680428
RP11-6L6.2	-0.327096	-0.370075	-1.40774	0.1607058	0.2791266	-5.680457
CCND2-AS1	0.3261069	-0.127492	1.4076043	0.1607461	0.2791833	-5.680644
LY6G6D	-0.160105	-1.427853	-1.407225	0.1608584	0.2793651	-5.681167
CTC-251D13	-0.240718	2.2808125	-1.407092	0.1608976	0.27942	-5.68135
PGM5P4	0.259976	-0.848277	1.4069502	0.1609397	0.2794735	-5.681545
HMBOX1	0.0373494	5.5496565	1.4069157	0.1609499	0.2794735	-5.681593
RP11-567M1	-0.187745	-1.156185	-1.406902	0.160954	0.2794735	-5.681612
TRAK1	0.0281027	6.2214987	1.4068645	0.1609651	0.2794735	-5.681663
FSD1L	-0.088559	4.8543309	-1.406859	0.1609668	0.2794735	-5.681671
RP11-763B2	-0.11945	-1.474274	-1.406759	0.1609964	0.2795117	-5.681809
CINP	-0.034273	6.0728304	-1.406708	0.1610114	0.2795244	-5.681879
FZD3	0.1441756	4.7877769	1.4066721	0.1610221	0.2795297	-5.681928
DUSP15	-0.234089	3.4336368	-1.406512	0.1610694	0.2795985	-5.682148
CMAS	-0.032908	6.3094897	-1.406422	0.1610963	0.2796319	-5.682273
RP4-613A2.	-0.236496	-0.926764	-1.40639	0.1611057	0.2796349	-5.682317
GPRIN2	0.5116855	1.1426523	1.4063124	0.1611287	0.27965	-5.682424
ADA	-0.055346	5.5602687	-1.406309	0.1611297	0.27965	-5.682428
HSP90AB1	-0.024699	7.3435974	-1.40627	0.1611413	0.2796569	-5.682482
FMNL1	0.0587889	5.8320754	1.406159	0.1611742	0.279696	-5.682635
RP11-406A2	-0.1477	-1.355642	-1.406142	0.1611792	0.279696	-5.682658
TAAR3	0.4333352	1.9758219	1.4061111	0.1611884	0.2796987	-5.682701
DCAF13	-0.04335	6.2605136	-1.406054	0.1612052	0.2797049	-5.682779
IGHV3-41	-0.360528	-0.242914	-1.406036	0.1612108	0.2797049	-5.682805
AP001062.9	-0.267535	-0.511943	-1.406021	0.161215	0.2797049	-5.682824
RP11-327J1	0.3383781	0.6258618	1.4054482	0.161385	0.2799867	-5.683613
CTB-31O20.	-0.263711	-0.610997	-1.405312	0.1614255	0.2800436	-5.683801
PURA	0.0303223	6.0474983	1.4052827	0.1614342	0.2800453	-5.683841
RP11-139K1	-0.327659	0.0268103	-1.405246	0.1614452	0.280051	-5.683892
RP11-157G2	-0.315323	-0.386644	-1.405152	0.1614731	0.2800862	-5.684021
IKBKGP1	0.316442	-0.087509	1.4050279	0.1615098	0.2801366	-5.684191
MTCL1	0.1483929	5.2066926	1.4048539	0.1615615	0.2802129	-5.684431
RP1-257I20	-0.248072	2.021905	-1.404802	0.1615769	0.2802264	-5.684502
TOMM22	-0.029	6.4884879	-1.404602	0.1616363	0.2803066	-5.684777
EFNA1	-0.054483	6.9458654	-1.404595	0.1616385	0.2803066	-5.684787
OXGR1	-0.22746	-1.133508	-1.404545	0.1616534	0.2803189	-5.684856
AC000123.2	0.0728542	4.533253	1.4044009	0.1616961	0.2803798	-5.685054
RP11-218F1	-0.21348	2.8770642	-1.404235	0.1617453	0.2804518	-5.685281
KCNG3	-0.358519	-0.357158	-1.404147	0.1617716	0.280484	-5.685403
PPIAP9	-0.34527	1.4182644	-1.404112	0.1617819	0.2804886	-5.685451
LA16c-381G	-0.242306	-1.051458	-1.404057	0.1617983	0.2804967	-5.685526
CCDC13-AS1	-0.325298	2.0503211	-1.404045	0.1618019	0.2804967	-5.685543
RNF111	0.0339964	5.902658	1.4037938	0.1618766	0.2806068	-5.685888
GPC2	-0.238913	3.2190007	-1.40378	0.1618808	0.2806068	-5.685908
RP1-241P17	-0.15462	-1.308439	-1.403626	0.1619267	0.2806729	-5.68612
RP13-977J1	-0.317489	0.130715	-1.403445	0.1619806	0.280753	-5.686368
RP11-8H2.1	-0.324601	0.2518691	-1.403388	0.1619974	0.2807689	-5.686446

EMD	0.0317393	6.3762385	1.4032074	0.1620512	0.2808487	-5.686694
SNORA14A	-0.275344	-0.535995	-1.403138	0.1620717	0.280871	-5.686789
GYPB	0.1336616	-1.477148	1.402922	0.1621361	0.2809692	-5.687086
RP4-769N13	-0.274635	-0.664979	-1.402614	0.1622278	0.2811148	-5.687509
CPNE9	-0.339751	1.1582607	-1.402559	0.1622444	0.2811301	-5.687585
BTD	0.0494914	6.309674	1.4024564	0.1622749	0.2811696	-5.687726
PTHLH	-0.301751	3.9316815	-1.402431	0.1622825	0.2811696	-5.687761
PLD3	0.0260142	6.8323656	1.4020763	0.1623882	0.2813392	-5.688247
HNRNPA1P59	-0.310484	1.3560328	-1.402039	0.1623992	0.2813449	-5.688298
FABP4	0.2288795	4.8620164	1.4019657	0.1624212	0.2813569	-5.688399
TUT1	0.0393183	5.9982186	1.4019472	0.1624267	0.2813569	-5.688425
NTPCR	-0.035481	6.2900602	-1.401939	0.1624292	0.2813569	-5.688436
KCNV1	-0.280089	-1.047903	-1.401866	0.162451	0.2813814	-5.688537
TELO2	0.028809	6.2067374	1.401809	0.1624679	0.2813915	-5.688614
RP11-693J1	0.1190932	-1.499527	1.4017943	0.1624723	0.2813915	-5.688634
CTB-129P6.	-0.255775	1.5369921	-1.401725	0.1624931	0.2814141	-5.68873
RP11-241F1	-0.129544	-1.405982	-1.401687	0.1625043	0.2814202	-5.688782
ECEL1	-0.444541	2.5724408	-1.40138	0.162596	0.2815558	-5.689203
MPRIPP1	-0.336928	1.1191044	-1.401361	0.1626015	0.2815558	-5.689228
TRAV29DV5	-0.375485	0.5315446	-1.401347	0.1626057	0.2815558	-5.689248
RP11-759L5	-0.195338	-1.138668	-1.401293	0.1626219	0.2815704	-5.689322
DCD	-0.096058	-1.50765	-1.401131	0.1626701	0.2816243	-5.689544
RP11-193H5	0.1880906	-1.409691	1.4011138	0.1626754	0.2816243	-5.689568
MIR4677	-0.204095	-1.032045	-1.401111	0.1626762	0.2816243	-5.689572
ZC3H11B	-0.293247	-0.227519	-1.401052	0.1626939	0.2816418	-5.689653
CDK11B	0.0282551	6.0270399	1.4007005	0.1627988	0.2818054	-5.690135
LINC01270	-0.120772	5.0758934	-1.400663	0.16281	0.2818054	-5.690186
IGLV1-41	-0.377611	-0.196707	-1.400648	0.1628145	0.2818054	-5.690207
AC007381.3	-0.112014	-1.477921	-1.400632	0.1628193	0.2818054	-5.690229
ATF2	-0.024595	6.2660074	-1.40053	0.1628499	0.2818449	-5.690369
HOOK2	-0.051121	5.9947414	-1.400407	0.1628864	0.2818948	-5.690537
CSN1S2AP	-0.087513	-1.51184	-1.400372	0.162897	0.2818997	-5.690586
RP11-473M2	0.2960311	1.5806923	1.4000522	0.1629926	0.2820518	-5.691024
RP11-627G2	-0.326721	4.5513226	-1.399843	0.1630553	0.2821385	-5.691311
PDK1	0.0554568	5.8331993	1.399833	0.1630582	0.2821385	-5.691324
EPGN	0.2217945	-1.050394	1.399782	0.1630734	0.2821515	-5.691394
RP11-973H7	-0.430595	0.6227499	-1.399682	0.1631034	0.2821899	-5.691531
RP11-34P1.	-0.306787	-0.187491	-1.399652	0.1631122	0.2821919	-5.691572
AC003984.1	0.1275248	-1.467465	1.3995553	0.1631412	0.2822287	-5.691705
RP11-470C1	-0.168441	-1.399574	-1.399427	0.1631797	0.2822818	-5.691881
RBM14-RBM4	-0.062243	4.1143752	-1.39929	0.1632206	0.2823392	-5.692068
RP11-443N2	-0.129076	-1.443069	-1.399202	0.1632469	0.2823713	-5.692188
CTD-3162L1	-0.268116	-0.860477	-1.399095	0.1632791	0.282409	-5.692336
SLC4A10	0.3395903	2.1957377	1.399078	0.1632841	0.282409	-5.692359
LINC00237	-0.261875	-1.160888	-1.399027	0.1632994	0.2824097	-5.692429
C4BPB	-0.120152	6.7439552	-1.399025	0.1633	0.2824097	-5.692432
SIGLEC16	0.1922761	3.3738419	1.3989068	0.1633354	0.2824524	-5.692593
LINC01193	-0.233864	-1.246626	-1.398891	0.1633402	0.2824524	-5.692615
ZNF136	-0.042934	5.3319749	-1.398837	0.1633562	0.2824667	-5.692689
CTC-508F8.	-0.173234	-1.325795	-1.398683	0.1634025	0.2825333	-5.6929
LINC01135	0.3431119	0.6534336	1.3986116	0.1634239	0.2825569	-5.692998

CD244	0.1709866	4.0805758	1.3985421	0.1634447	0.2825795	-5.693093
RP11-343L5	-0.172442	3.3865239	-1.398424	0.1634802	0.2826276	-5.693255
ENO3	-0.101739	5.7963362	-1.398199	0.1635474	0.2827304	-5.693562
RP11-380M2	0.1117839	-1.452133	1.3980159	0.1636024	0.2828121	-5.693813
KRT18P3	0.1864276	-1.19408	1.397966	0.1636174	0.2828245	-5.693882
RP11-730G2	-0.080173	4.8857685	-1.397934	0.1636269	0.2828276	-5.693925
XX-C00717C	-0.303682	-0.040261	-1.397766	0.1636773	0.2829014	-5.694155
PAF1	0.0267974	6.485267	1.3976679	0.1637068	0.2829389	-5.69429
TATDN2P3	-0.178843	-1.295087	-1.397421	0.163781	0.2830538	-5.694628
CTB-5506.1	-0.275303	-0.600239	-1.397381	0.1637929	0.2830609	-5.694682
PRPF40A	-0.020241	6.4515371	-1.397333	0.1638073	0.2830724	-5.694748
RP11-264B1	-0.125747	3.5662987	-1.397149	0.1638625	0.2831544	-5.694999
CNN2	0.047282	6.4344882	1.3970488	0.1638926	0.283185	-5.695137
DOK2	0.0827058	5.4347369	1.3970245	0.1638999	0.283185	-5.69517
DOCK6	0.0292948	6.2481012	1.3970126	0.1639035	0.283185	-5.695186
VDR	-0.109854	5.1914268	-1.396901	0.1639371	0.2832297	-5.695339
RP11-133F8	-0.280722	-0.85328	-1.396775	0.1639747	0.2832813	-5.69551
AC008065.1	-0.240885	-0.826896	-1.396679	0.1640038	0.2833181	-5.695643
KRT8P10	-0.286412	-0.276754	-1.396614	0.1640232	0.283338	-5.695731
TEX41	-0.297884	3.8172747	-1.396589	0.1640309	0.283338	-5.695766
TTLL10-AS1	0.1952055	-1.179534	1.3963455	0.1641039	0.2834508	-5.696098
RASSF4	-0.045187	6.4628462	-1.396161	0.1641595	0.2835281	-5.696351
DAPK1	0.0440139	6.360555	1.3961451	0.1641642	0.2835281	-5.696372
RP11-622C2	0.3359273	0.4755903	1.3960943	0.1641795	0.283538	-5.696442
ZCCHC10	-0.028411	5.7176131	-1.396074	0.1641855	0.283538	-5.696469
ATE1	0.0323392	6.1461657	1.3960365	0.1641968	0.2835442	-5.696521
EIF1AXP1	-0.233202	2.4001653	-1.395983	0.164213	0.2835588	-5.696594
CTB-33018.	0.1810306	-1.278799	1.3958158	0.1642632	0.283632	-5.696822
RP11-757G1	-0.314163	0.9177454	-1.395666	0.1643084	0.2836966	-5.697028
F5	-0.091209	6.9777348	-1.39559	0.1643311	0.2837225	-5.697131
KHK	-0.088758	6.6220242	-1.395458	0.1643708	0.2837738	-5.697311
CFAP43	-0.182614	3.8246449	-1.39544	0.1643764	0.2837738	-5.697337
C11orf49	-0.061526	5.6274013	-1.39541	0.1643854	0.2837758	-5.697377
CXXC5	-0.030018	6.6540524	-1.395259	0.1644307	0.2838407	-5.697583
FAM50A	0.0434805	6.5230929	1.3951277	0.1644703	0.2838956	-5.697763
SMAD5	-0.032876	6.4127648	-1.395054	0.1644926	0.2839175	-5.697864
AC131056.3	-0.29634	2.5605482	-1.395034	0.1644985	0.2839175	-5.697891
FAF2	0.0199544	6.3930714	1.3949796	0.1645149	0.2839323	-5.697965
YPEL3	0.0384997	6.2314981	1.3948981	0.1645394	0.28395	-5.698076
RP11-478C6	-0.145799	-1.347563	-1.394894	0.1645407	0.28395	-5.698082
MT1B	0.3781279	-0.643797	1.3947633	0.1645801	0.2840045	-5.69826
FMO8P	-0.296804	-0.971042	-1.394734	0.1645889	0.2840063	-5.698301
HLA-F-AS1	-0.107556	4.6349079	-1.394684	0.164604	0.284019	-5.698369
RP11-182B2	-0.170507	-1.217607	-1.394654	0.1646129	0.2840209	-5.698409
RP1-20N18.	0.1590993	-1.370624	1.3944479	0.1646751	0.2841147	-5.698691
TTC21B-AS1	-0.237889	-0.884738	-1.39438	0.1646954	0.2841364	-5.698783
CITF22-92A	0.2819115	-0.712369	1.3942452	0.1647362	0.2841933	-5.698968
CNBD1	-0.185473	-1.263878	-1.39418	0.1647559	0.2842093	-5.699057
SLC16A8	0.2137221	3.0463956	1.3941627	0.1647611	0.2842093	-5.69908
CYCSP45	0.2807412	-0.482893	1.3940928	0.1647821	0.2842322	-5.699176
PNMA6A	-0.187226	5.5151156	-1.393986	0.1648145	0.2842746	-5.699322

THAP10	0.1033918	4.5489588	1.3939013	0.1648399	0.284305	-5.699437
RP11-53B2.	-0.300371	1.3355401	-1.393352	0.1650057	0.2845775	-5.700187
CTD-3128G1	-0.331016	1.6194685	-1.393309	0.1650186	0.2845864	-5.700246
INTS4P2	-0.2966	0.1299194	-1.393134	0.1650715	0.2846608	-5.700484
RP6-24A23.	0.1703164	-1.384187	1.3931143	0.1650774	0.2846608	-5.700511
PPIAP3	-0.308162	0.1884132	-1.39295	0.1651269	0.2847328	-5.700735
CTD-2619J1	-0.054115	5.2343687	-1.392877	0.1651491	0.2847576	-5.700835
RP11-274H2	-0.345892	0.5881471	-1.392749	0.1651877	0.2848108	-5.70101
PRICKLE2-A	0.1876942	-1.170442	1.3926913	0.1652051	0.2848273	-5.701088
RP11-336N8	-0.125877	-1.400828	-1.392524	0.1652556	0.2849009	-5.701316
ERCC5	0.035267	6.036707	1.3924625	0.1652743	0.2849196	-5.7014
SLC25A20	0.0606739	6.3874956	1.3923941	0.1652949	0.2849407	-5.701493
AC004112.4	0.3336677	1.200539	1.3923704	0.1653021	0.2849407	-5.701526
PSME3	-0.020111	6.530434	-1.391967	0.165424	0.2851372	-5.702075
RP11-1182F	0.3912259	1.6560646	1.3916391	0.1655232	0.2852949	-5.702522
DR1	0.024178	6.2897518	1.3915625	0.1655464	0.2853214	-5.702627
RP11-102G1	0.3384996	1.322378	1.3912869	0.1656298	0.2854517	-5.703002
GOLGA6L5P	-0.266986	2.5916821	-1.391206	0.1656542	0.2854802	-5.703112
SGTB	0.0522284	5.3028405	1.3909203	0.1657408	0.285616	-5.703502
FAM126A	0.0530678	5.7300009	1.3908765	0.1657541	0.2856254	-5.703561
PLGLB2	0.2381884	4.5967729	1.3907267	0.1657995	0.2856901	-5.703765
ABCB11	0.350904	5.1478955	1.3904696	0.1658774	0.2858108	-5.704115
FOXI1	-0.217438	-1.216851	-1.390305	0.1659274	0.2858835	-5.70434
C3orf70	0.0946714	4.5023369	1.390135	0.1659788	0.285951	-5.704571
RP11-145B3	0.0751054	-1.525871	1.3901236	0.1659823	0.285951	-5.704587
RP11-330H6	-0.304196	-0.05886	-1.389992	0.1660222	0.2860064	-5.704766
ADIRF-AS1	0.1731527	3.6209714	1.3896918	0.1661132	0.2861496	-5.705174
MCF2L	0.0600287	5.8921997	1.3895095	0.1661685	0.2862314	-5.705422
RP11-173A6	-0.317264	0.5301672	-1.38941	0.1661988	0.2862677	-5.705558
RP4-587D13	0.0980579	-1.514174	1.3893884	0.1662053	0.2862677	-5.705587
FBXO27	0.1104355	5.5976022	1.3891028	0.166292	0.2863897	-5.705976
RP11-244B2	-0.104802	-1.476878	-1.38908	0.1662989	0.2863897	-5.706006
CTD-2231E1	0.314824	0.6073613	1.3890624	0.1663042	0.2863897	-5.70603
HCG17	-0.317394	1.9323855	-1.389052	0.1663075	0.2863897	-5.706045
RP11-426C2	0.3138224	2.3821951	1.3888353	0.1663732	0.2864815	-5.706339
RP11-120E1	0.1950446	2.6421647	1.3888245	0.1663765	0.2864815	-5.706354
NHLH1	-0.323998	1.1187811	-1.388652	0.166429	0.2865585	-5.706589
LRRC6	0.1727927	3.8005609	1.3886104	0.1664415	0.2865664	-5.706645
PROL1	-0.505542	1.8254187	-1.388439	0.1664936	0.2866363	-5.706878
MMP1	-0.344834	3.6019926	-1.388416	0.1665007	0.2866363	-5.70691
RP11-67L3.	0.2046247	3.1929325	1.3883994	0.1665057	0.2866363	-5.706932
LINC00881	0.3498802	0.7769373	1.3882897	0.166539	0.2866802	-5.707081
LINC00940	0.3835458	-0.13473	1.3882151	0.1665617	0.2867057	-5.707183
RP11-1114A	-0.119155	3.869063	-1.388163	0.1665774	0.2867192	-5.707253
FFAR2	-0.265889	3.0633487	-1.388028	0.1666185	0.2867764	-5.707437
RP11-314D7	-0.239926	-1.081597	-1.387738	0.1667068	0.2869149	-5.707831
OSTN	0.3216223	-0.461924	1.3875947	0.1667503	0.2869664	-5.708026
IZUMO1R	0.3129775	-0.550861	1.3875879	0.1667524	0.2869664	-5.708035
OXCT1	0.1260645	5.0057409	1.3875569	0.1667618	0.2869691	-5.708077
DCAF12	-0.024012	6.2831647	-1.38753	0.1667701	0.2869698	-5.708114
PPP1R3E	-0.045889	5.5419033	-1.38745	0.1667945	0.2869948	-5.708223

FRMPD2L2	-0.349511	-0.224813	-1.38743	0.1668004	0.2869948	-5.708249
CCDC85B	0.0501713	5.759171	1.3872051	0.1668689	0.2870739	-5.708555
RP1-276N6.	0.4828828	1.0952848	1.3871959	0.1668717	0.2870739	-5.708568
ANKRD12	0.0321069	6.0889497	1.3871912	0.1668732	0.2870739	-5.708574
XXbac-B33L	0.0963829	-1.522253	1.3871759	0.1668778	0.2870739	-5.708595
RP11-108B1	-0.267211	-0.542218	-1.387085	0.1669054	0.2871079	-5.708718
SLC25A1	-0.038533	6.8210463	-1.386933	0.1669516	0.2871738	-5.708924
RP11-63K6.	-0.094738	-1.508297	-1.386857	0.166975	0.2872004	-5.709028
RP5-1059H1	-0.179481	-1.244437	-1.386793	0.1669944	0.2872203	-5.709115
RP1-170019	-0.456364	1.0034886	-1.386579	0.1670596	0.287319	-5.709406
WT1	0.465881	1.6275178	1.3865096	0.1670807	0.2873417	-5.7095
WI2-1959D1	0.1073697	-1.478351	1.3864053	0.1671125	0.2873828	-5.709641
SHANK2	-0.15174	5.9959554	-1.386341	0.167132	0.2874028	-5.709728
BZW1P1	0.1779597	-1.222135	1.3862854	0.1671491	0.2874158	-5.709804
LINC00242	-0.140861	4.5651731	-1.386265	0.1671553	0.2874158	-5.709832
AC147651.1	0.158026	-1.374726	1.3861811	0.1671809	0.2874462	-5.709946
GUCA1C	0.0916003	-1.52469	1.386102	0.167205	0.2874741	-5.710053
AP000282.2	-0.101028	-1.505213	-1.385563	0.1673694	0.2877433	-5.710785
GNB2L1	-0.029661	7.2464971	-1.385527	0.1673804	0.2877486	-5.710834
RP11-81H3.	-0.576862	1.2150715	-1.385474	0.1673964	0.2877626	-5.710905
AC012065.5	0.2145045	-0.964176	1.3853985	0.1674196	0.2877889	-5.711008
AC073343.1	0.326108	1.1859272	1.3853319	0.1674399	0.2878063	-5.711098
PLIN3	0.0327104	6.2941449	1.3852941	0.1674514	0.2878063	-5.711149
AC145676.2	0.3577534	1.1164847	1.3852831	0.1674548	0.2878063	-5.711164
SHD	-0.330171	4.382182	-1.385262	0.1674613	0.2878063	-5.711193
FBRS	0.0201312	6.3738075	1.3851572	0.1674932	0.2878477	-5.711335
GS1-44D20.	0.2644699	2.3487246	1.3850895	0.1675139	0.2878696	-5.711427
EBAG9P1	-0.314767	0.4196177	-1.385015	0.1675367	0.2878953	-5.711528
FASLG	0.2351186	3.4958482	1.3848987	0.1675722	0.2879427	-5.711686
ST3GAL3	0.0457215	5.7712475	1.3848233	0.1675952	0.2879621	-5.711788
YARS2	-0.026951	5.7928474	-1.384786	0.1676066	0.2879621	-5.711839
FRMD6-AS1	0.3076796	1.619072	1.3847841	0.1676071	0.2879621	-5.711841
CTD-2650P2	-0.271843	2.4118115	-1.384643	0.1676502	0.2880226	-5.712032
VN1R110P	0.2881002	-0.812856	1.3844755	0.1677014	0.288097	-5.71226
C4BPA	0.1306622	7.0631499	1.3842499	0.1677704	0.2882019	-5.712565
CYP11B1	0.0749259	-1.521204	1.38411	0.1678132	0.2882618	-5.712755
LA16c-390E	-0.173738	-1.223178	-1.383991	0.1678494	0.288301	-5.712916
AC063976.7	0.299096	-0.292181	1.3839837	0.1678518	0.288301	-5.712926
USP3	-0.023801	6.1009856	-1.38374	0.1679264	0.2884156	-5.713257
RP11-400L8	0.2951223	0.3624909	1.3836594	0.167951	0.2884442	-5.713366
RP11-544A1	-0.302004	-0.09061	-1.383547	0.1679852	0.2884895	-5.713518
ORMDL1	-0.023806	6.1209644	-1.383466	0.1680101	0.2885186	-5.713628
RP11-294C1	-0.162178	-1.356542	-1.383435	0.1680195	0.2885212	-5.713669
RP11-351I2	0.3116239	0.7536933	1.3834048	0.1680288	0.2885237	-5.713711
RP11-72L22	0.0813003	-1.545478	1.3833523	0.1680449	0.2885377	-5.713782
AC005009.1	-0.20942	-1.118758	-1.383239	0.1680795	0.2885834	-5.713935
MIR3649	-0.257648	-0.823583	-1.383198	0.1680921	0.2885916	-5.713991
RP11-115J1	0.2889009	-0.678231	1.3831673	0.1681015	0.2885942	-5.714033
PAPSS1	-0.035128	5.9908731	-1.382938	0.1681718	0.2887013	-5.714343
RP11-346D6	-0.255541	-0.894705	-1.382722	0.1682378	0.2888009	-5.714635
PRUNE2	0.1539697	4.4954703	1.3824936	0.1683079	0.2889076	-5.714945

AC005014.5	-0.216425	-1.020261	-1.382463	0.1683172	0.28891	-5.714986
PSMB7	0.0309284	6.6872416	1.3822997	0.1683673	0.2889825	-5.715207
GSTM2P1	0.1411567	-1.40362	1.3822594	0.1683796	0.2889875	-5.715262
RP11-345K2	-0.143819	3.4714673	-1.382239	0.168386	0.2889875	-5.71529
KRT8P28	-0.127778	-1.440285	-1.382195	0.1683993	0.2889925	-5.715349
UBE2G1	0.0302311	6.2230212	1.3821773	0.1684048	0.2889925	-5.715373
AC068196.1	-0.257206	-0.708799	-1.381994	0.168461	0.2890689	-5.715621
NOVA1	0.2168822	4.3161481	1.3819753	0.1684667	0.2890689	-5.715646
SULT1C3	0.3417286	-0.693773	1.3819547	0.168473	0.2890689	-5.715674
RNU6-1297F	0.0670643	-1.552733	1.3818744	0.1684977	0.2890919	-5.715783
LEFTY1	-0.329651	3.3937072	-1.381859	0.1685023	0.2890919	-5.715803
RP13-638C3	-0.304736	-0.314292	-1.381828	0.168512	0.2890949	-5.715846
KIAA2026	0.0379965	5.7852647	1.3817649	0.1685313	0.2890969	-5.715931
KRT18P10	0.1919703	2.8474083	1.3817508	0.1685356	0.2890969	-5.71595
TMEM63C	-0.350533	2.830357	-1.381747	0.1685369	0.2890969	-5.715956
HCAR1	-0.369834	1.6928579	-1.381202	0.168704	0.2893699	-5.716693
SLMO2	-0.024762	6.3177867	-1.381093	0.1687373	0.2894135	-5.71684
C9orf129	0.1722244	-1.317203	1.3808875	0.1688005	0.2895083	-5.717118
CFAP70	0.1411359	4.3474655	1.3808235	0.1688202	0.2895284	-5.717205
AC010980.2	0.3995825	0.2826214	1.3807681	0.1688372	0.2895439	-5.71728
TEN1	-0.217798	2.0582121	-1.380734	0.1688477	0.2895484	-5.717326
ABHD11-AS1	0.3713688	1.1376418	1.3805419	0.1689067	0.2896359	-5.717585
RP11-759A2	0.1560524	-1.312198	1.3803464	0.1689667	0.2897253	-5.71785
CORO1A	0.0656668	6.0930635	1.3802907	0.1689839	0.2897411	-5.717925
RP11-1334A	0.3009066	1.6083186	1.380164	0.1690228	0.2897942	-5.718096
RP11-536C5	-0.170871	-1.242754	-1.379974	0.1690811	0.2898806	-5.718353
ZDBF2	0.1666173	4.7185263	1.3798502	0.1691193	0.2899324	-5.71852
RP11-42D20	-0.182786	-1.188522	-1.379822	0.1691279	0.2899335	-5.718558
CD79A	-0.253416	4.327483	-1.379694	0.1691673	0.2899874	-5.718731
CCDC82	0.0321334	5.8294091	1.3793538	0.169272	0.2901533	-5.719191
CTC-523E23	-0.310047	2.2538839	-1.379279	0.169295	0.2901791	-5.719292
RP11-330L1	-0.29988	1.5695642	-1.379222	0.1693126	0.2901957	-5.71937
RP13-638C3	-0.302546	0.2414581	-1.37916	0.1693317	0.2902147	-5.719453
PPP1R13B	-0.033212	6.042024	-1.379007	0.1693786	0.2902816	-5.719659
RP11-510C1	0.2240906	-1.310916	1.3789519	0.1693957	0.2902972	-5.719734
AC093906.1	-0.08073	-1.515167	-1.37883	0.1694334	0.2903424	-5.719899
AC010894.3	0.0886412	4.3929924	1.3788146	0.169438	0.2903424	-5.719919
FAM86B1	0.2749733	2.852898	1.3786921	0.1694757	0.2903934	-5.720085
RP11-31I22	0.2039676	-1.190195	1.3785321	0.169525	0.2904642	-5.720301
SPX	0.3032996	4.3546876	1.3784851	0.1695395	0.2904669	-5.720364
RP11-401E9	-0.206343	-1.077245	-1.378475	0.1695425	0.2904669	-5.720377
RP11-274B2	0.3346567	2.2943156	1.3782941	0.1695983	0.290549	-5.720622
RP11-744D1	-0.185633	2.8876557	-1.37824	0.169615	0.290564	-5.720695
SLC2A8	-0.040243	6.1473872	-1.378199	0.1696278	0.2905721	-5.720751
AC067945.3	-0.266149	-0.572371	-1.377807	0.1697485	0.2907538	-5.721279
CTA-38K21.	-0.293797	0.0071534	-1.377803	0.1697498	0.2907538	-5.721285
STK38	0.0286063	6.259326	1.3777351	0.1697707	0.2907664	-5.721377
AC084809.2	-0.30799	0.1548391	-1.37769	0.1697846	0.2907664	-5.721437
RP11-452B1	-0.093559	-1.508876	-1.377687	0.1697855	0.2907664	-5.721441
NHP2P1	-0.293456	0.2101648	-1.377654	0.1697956	0.2907664	-5.721486
BTF3P5	-0.286383	-0.132654	-1.37765	0.169797	0.2907664	-5.721492

RP11-506B6	0.3152933	0.0149082	1.37753	0.1698339	0.2908161	-5.721653
AC114813.1	-0.202419	-1.221502	-1.377485	0.1698478	0.2908262	-5.721714
RN7SKP79	-0.122217	-1.446433	-1.377238	0.169924	0.2909431	-5.722047
RP11-152F1	-0.297501	0.0355188	-1.377157	0.1699491	0.2909724	-5.722157
STAT2	0.0326243	6.5913307	1.3771052	0.169965	0.290986	-5.722226
UCN2	-0.378716	0.1456422	-1.376917	0.1700231	0.2910627	-5.72248
PLCB3	-0.028802	6.0215498	-1.376908	0.1700258	0.2910627	-5.722492
RPL3P9	-0.32024	0.7391198	-1.376804	0.1700579	0.2911041	-5.722632
RPL15P2	-0.307429	0.5126338	-1.376571	0.17013	0.2912138	-5.722947
RP11-1148L	0.0545887	5.0518333	1.3765452	0.170138	0.2912138	-5.722982
RSRC2	-0.019681	6.3187002	-1.376436	0.1701716	0.2912576	-5.723128
CTB-107G13	0.2035185	-1.179375	1.3761827	0.17025	0.2913773	-5.72347
FOXN3P1	0.0834956	-1.508081	1.3761339	0.1702651	0.2913773	-5.723536
LHPP	0.0674019	6.1812556	1.3761326	0.1702654	0.2913773	-5.723538
ALG1L3P	-0.180143	-1.231824	-1.376009	0.1703037	0.2914219	-5.723705
OLFML2B	0.0830699	5.6225794	1.3759967	0.1703075	0.2914219	-5.723721
RP11-507K2	-0.241372	2.0901083	-1.375839	0.1703563	0.2914918	-5.723934
TCTEX1D2	0.0998479	4.3641474	1.3756722	0.1704078	0.2915663	-5.724158
RPL7P36	-0.158784	-1.275166	-1.375566	0.1704407	0.291609	-5.724302
ULBP2	0.2135555	3.5223511	1.3753618	0.1705038	0.2917032	-5.724576
LEUTX	-0.090248	-1.510499	-1.375272	0.1705315	0.2917285	-5.724697
RPL35AP21	-0.125204	-1.405737	-1.375262	0.1705346	0.2917285	-5.72471
ZNF471	-0.18327	4.4628408	-1.375114	0.1705806	0.2917936	-5.72491
FICD	0.0474593	5.6345169	1.3747655	0.1706884	0.291962	-5.725379
AC093698.1	-0.138643	-1.369242	-1.374744	0.1706951	0.291962	-5.725408
RP5-1000K2	0.2212573	2.259828	1.3746353	0.1707287	0.2920059	-5.725555
RP4-740C4.	0.2071467	2.5509818	1.3745577	0.1707528	0.2920333	-5.725659
TRBV9	-0.389473	1.2904255	-1.374362	0.1708134	0.2921233	-5.725922
ARMCX5	0.0422898	5.5597294	1.3741235	0.1708873	0.292236	-5.726244
RP11-483I1	-0.294956	-0.25109	-1.373904	0.1709553	0.2923387	-5.726539
RELT	0.0685629	5.1384797	1.3736934	0.1710206	0.2924366	-5.726822
COPS2	0.0224492	6.3532139	1.3736493	0.1710343	0.2924464	-5.726882
IFNGR2	-0.033026	6.4013669	-1.373554	0.1710638	0.2924643	-5.72701
ARPC3P5	-0.248845	-0.820706	-1.373547	0.1710662	0.2924643	-5.72702
TRAT1	0.2598994	3.5170175	1.3735379	0.1710689	0.2924643	-5.727031
NME5	0.3656952	2.520453	1.3731899	0.1711769	0.2926352	-5.7275
PRR33	0.2402846	2.9107072	1.3730627	0.1712163	0.292689	-5.727671
PRR5L	0.0836532	5.0694938	1.3729974	0.1712366	0.29271	-5.727759
CTD-2014B1	-0.257889	-0.598851	-1.372576	0.1713675	0.2929167	-5.728325
RP3-418A9.	-0.239071	-0.758225	-1.372556	0.1713736	0.2929167	-5.728352
ZNF667-AS1	-0.147466	4.8325225	-1.37252	0.1713848	0.2929222	-5.7284
RP11-551L1	-0.174986	-1.230104	-1.372466	0.1714016	0.2929371	-5.728473
DCAF4L2	-0.627123	1.1037477	-1.372269	0.1714628	0.2930281	-5.728738
AC073641.2	-0.215779	-0.983563	-1.371906	0.1715756	0.293207	-5.729225
CTD-307407	-0.074769	4.7714664	-1.371821	0.171602	0.2932205	-5.729339
RFX8	-0.202182	3.2846214	-1.37182	0.1716023	0.2932205	-5.729341
EEF1A1P11	-0.104596	4.5653374	-1.371803	0.1716076	0.2932205	-5.729364
L1CAM	0.3167072	2.836805	1.3716221	0.1716639	0.293303	-5.729607
CTB-3204.2	-0.303691	1.0585462	-1.371459	0.1717147	0.2933762	-5.729827
CTC-559E9.	-0.120685	-1.407954	-1.371388	0.1717369	0.2934002	-5.729922
PHLDA2	0.117819	5.338394	1.3712214	0.1717886	0.2934748	-5.730145

RP11-486M2	0.1951716	-1.152272	1.3710907	0.1718293	0.2935306	-5.730321
RP11-91K9.	-0.182664	4.7591889	-1.37105	0.1718418	0.2935383	-5.730375
RP11-268J1	-0.14187	3.9788447	-1.370838	0.171908	0.2936376	-5.730661
MIR3134	-0.115589	-1.432357	-1.370731	0.1719411	0.2936804	-5.730803
ATP13A4	0.3680094	1.4557624	1.3706881	0.1719546	0.2936807	-5.730861
SHROOM1	0.0563666	6.4522895	1.3706792	0.1719573	0.2936807	-5.730873
RP11-21305	0.2244938	-0.935198	1.3706094	0.1719791	0.2937041	-5.730967
RP11-264J4	-0.256072	-0.73742	-1.37057	0.1719914	0.2937114	-5.73102
RP11-43F13	-0.082709	5.4317085	-1.370531	0.1720036	0.2937131	-5.731073
HMGN2P7	-0.200122	-1.065916	-1.370515	0.1720085	0.2937131	-5.731094
RP11-502F1	-0.106133	-1.502709	-1.370446	0.1720301	0.2937362	-5.731187
AC097499.1	-0.167791	-1.342369	-1.370368	0.1720543	0.2937637	-5.731291
AKR1D1	-0.294703	5.6540793	-1.370169	0.1721164	0.2938561	-5.731559
MST1	-0.093488	6.7058015	-1.370017	0.1721636	0.2939146	-5.731762
RP11-259A2	-0.106999	-1.4758	-1.370007	0.1721668	0.2939146	-5.731776
PTPN5	0.3850009	1.4678477	1.369918	0.1721945	0.2939482	-5.731895
SMIM3	0.0789495	5.4212845	1.3697717	0.1722401	0.2940087	-5.732091
UBTFL10	0.229056	-1.093637	1.3697526	0.1722461	0.2940087	-5.732117
CTC-340A15	-0.208627	-1.191006	-1.369627	0.1722853	0.2940522	-5.732286
NFKBIE	0.0473931	6.0290847	1.3696193	0.1722876	0.2940522	-5.732296
RP11-485M7	-0.282158	0.1493277	-1.369169	0.172428	0.294278	-5.732899
ATR	0.0266726	5.9500817	1.3690514	0.1724648	0.2943271	-5.733058
RP13-34902	0.3245827	0.0660629	1.3690042	0.1724796	0.2943385	-5.733121
LINC00919	-0.145171	-1.386787	-1.36897	0.1724903	0.2943431	-5.733167
PRR23B	0.092937	-1.539548	1.3688389	0.1725312	0.294396	-5.733343
AC007040.6	-0.180848	-1.133083	-1.368819	0.1725375	0.294396	-5.73337
RP11-903H1	-0.225432	3.2384805	-1.36869	0.1725775	0.2944506	-5.733542
IGHVII-60-	-0.132499	-1.419485	-1.368631	0.172596	0.2944683	-5.733621
RAD21-AS1	-0.283802	1.9501828	-1.368443	0.1726547	0.2945435	-5.733873
HFE2	-0.217736	6.3532385	-1.368438	0.1726562	0.2945435	-5.733879
TXNP4	-0.312641	1.4838825	-1.368383	0.1726735	0.2945593	-5.733954
ACMSD	0.1959857	6.0752721	1.3679215	0.1728177	0.2947915	-5.734572
SULT4A1	0.5021798	1.2882768	1.3678555	0.1728384	0.294813	-5.734661
RP11-463I2	-0.115478	-1.498127	-1.36741	0.1729778	0.295037	-5.735258
RP11-589C2	-0.239152	-0.666299	-1.367372	0.1729897	0.2950434	-5.735309
ADIG	-0.349827	-0.000568	-1.367228	0.1730347	0.2951064	-5.735501
TSSC1-IT1	0.2921571	1.4993426	1.3669855	0.1731105	0.2952219	-5.735826
OR7E43P	-0.111161	-1.478339	-1.366878	0.1731441	0.2952655	-5.73597
ISG20	0.0626054	5.9009403	1.366781	0.1731745	0.2953035	-5.7361
IFT74-AS1	-0.249407	-0.806319	-1.366745	0.1731857	0.2953088	-5.736147
AC073850.6	-0.32754	-0.094716	-1.366423	0.1732865	0.2954669	-5.736578
RP11-13K12	0.3238151	-0.216763	1.3663788	0.1733005	0.2954769	-5.736638
PRR27	-0.233815	-1.137794	-1.36628	0.1733314	0.2955065	-5.73677
PICK1	0.0338579	6.0907322	1.3662717	0.173334	0.2955065	-5.736781
SNORD116-2	-0.168252	-1.275744	-1.366154	0.1733708	0.2955553	-5.736938
RNU2-51P	-0.259345	-0.66989	-1.366004	0.1734179	0.2956219	-5.73714
ZDHHC9	-0.035737	6.6058122	-1.365808	0.1734794	0.2957129	-5.737402
VCPIP1	0.030302	6.1226263	1.3656438	0.1735308	0.2957808	-5.737622
SIRPG-AS1	-0.250666	-0.823343	-1.365629	0.1735354	0.2957808	-5.737641
PKP4	0.0296182	6.4505089	1.3655653	0.1735554	0.295801	-5.737727
APMAP	0.0427778	6.9534122	1.3653666	0.1736177	0.2958934	-5.737992

LPAL2	-0.160409	4.8400353	-1.365165	0.173681	0.2959875	-5.738262
RP1-34H18.	0.2633746	-0.831269	1.3649445	0.1737502	0.2960915	-5.738557
CTA-256D12	-0.142527	-1.366179	-1.364725	0.1738189	0.2961949	-5.73885
FBXO38	0.0262473	6.1579078	1.3642979	0.1739532	0.2963932	-5.739421
TBX6	-0.10334	4.2039599	-1.364266	0.1739632	0.2963932	-5.739464
PRSS41	-0.251071	-1.135453	-1.364264	0.1739639	0.2963932	-5.739467
LAMTOR5P1	0.0996923	-1.463054	1.3642515	0.1739678	0.2963932	-5.739483
PPFIA3	-0.058314	5.5569729	-1.364162	0.1739959	0.2964272	-5.739603
MAN2B1	0.0290281	6.5420163	1.3639982	0.1740474	0.2965011	-5.739822
AC073052.1	-0.284534	-0.184433	-1.363868	0.1740882	0.2965568	-5.739995
TNFRSF25	-0.073803	5.4445887	-1.36381	0.1741064	0.296574	-5.740073
FAM35CP	-0.248402	-0.659927	-1.363672	0.1741499	0.2966243	-5.740257
RSPH6A	-0.181558	-1.253792	-1.363665	0.1741522	0.2966243	-5.740267
CTD-2527I2	-0.369225	2.1161091	-1.363172	0.1743072	0.2968744	-5.740925
TMEM72-AS1	0.3428361	0.6493447	1.363102	0.1743292	0.296878	-5.741019
NOMO3	0.2400821	4.4906428	1.3630879	0.1743336	0.296878	-5.741037
LINC00319	-0.292739	-0.703439	-1.363088	0.1743337	0.296878	-5.741038
RP11-1137G	-0.18365	-1.294948	-1.362963	0.1743728	0.2969308	-5.741204
SEMG1	0.2000823	-1.242875	1.3626457	0.1744728	0.2970871	-5.741628
RP11-51J9.	0.2650826	1.898855	1.3624331	0.1745397	0.2971873	-5.741912
CECR3	-0.216077	-1.08327	-1.362272	0.1745905	0.2972599	-5.742127
CTD-2210P1	0.2835338	-0.186425	1.3622432	0.1745995	0.2972614	-5.742165
AC003986.7	0.1422009	-1.449854	1.3622159	0.1746081	0.2972622	-5.742201
RP11-219B4	-0.304454	-0.170591	-1.361795	0.1747407	0.297474	-5.742763
CDK5	0.0381242	5.9115169	1.3617148	0.174766	0.2975032	-5.74287
KRTAP5-4	-0.217226	-1.06362	-1.36162	0.1747959	0.2975401	-5.742996
RAET1K	-0.343087	1.2979261	-1.361477	0.174841	0.2976031	-5.743187
HIPK1-AS1	-0.229069	2.4035249	-1.361175	0.1749362	0.2977512	-5.74359
HIGD2B	0.208548	-1.050601	1.3610787	0.1749666	0.2977892	-5.743718
GABRE	-0.134029	5.6992715	-1.361024	0.1749839	0.2978047	-5.743791
FBXO40	0.3360771	-0.422159	1.3609878	0.1749953	0.2978102	-5.743839
AP002884.2	0.2282882	-0.928745	1.3608931	0.1750252	0.2978472	-5.743966
RP11-439A1	0.1490885	-1.347761	1.3608296	0.1750452	0.2978674	-5.74405
RP11-1E11.	-0.281221	-0.596795	-1.360804	0.1750534	0.2978674	-5.744085
KRT18P1	-0.291398	-0.080028	-1.360673	0.1750947	0.2979238	-5.744259
TPM2	0.0552468	6.2878575	1.3606094	0.1751148	0.297944	-5.744344
GPR55	0.2517845	2.7171831	1.3605471	0.1751344	0.2979636	-5.744427
RP11-752L2	0.2130413	2.9947687	1.3604388	0.1751686	0.2980079	-5.744571
RP1-101D8.	0.108993	-1.463769	1.3603054	0.1752107	0.2980656	-5.744749
NBAT1	-0.240424	-0.874039	-1.36027	0.1752218	0.2980706	-5.744796
GSPT2	0.1636251	5.2530431	1.3602268	0.1752356	0.2980801	-5.744854
PRSS44	-0.361813	-0.147647	-1.360066	0.1752865	0.2981528	-5.745069
GJD3	0.2923528	-0.260363	1.3600153	0.1753024	0.2981659	-5.745136
SH3GL1P1	-0.245	2.3571212	-1.359902	0.1753381	0.2982128	-5.745286
CETN2	0.0329546	6.1856055	1.3597839	0.1753755	0.2982625	-5.745444
PRKAR2A	-0.02698	6.3625441	-1.359588	0.1754375	0.298354	-5.745705
GLRB	0.3917152	3.8190586	1.3594522	0.1754804	0.2984131	-5.745886
MTMR4	-0.039215	6.3684048	-1.359316	0.1755233	0.2984723	-5.746067
ACOT11	-0.238346	3.9455125	-1.359289	0.175532	0.298473	-5.746103
UBE2E3	-0.026011	6.2271281	-1.359191	0.1755629	0.2985118	-5.746233
CTD-2007L1	-0.30511	1.099112	-1.359137	0.1755801	0.2985271	-5.746306

TRIM13	0.0377446	5.7470603	1.3589975	0.1756242	0.2985881	-5.746491
RP11-861E2	0.1210521	-1.425139	1.3589572	0.1756369	0.2985959	-5.746545
RP11-603K1	-0.121118	-1.403162	-1.358908	0.1756524	0.2986082	-5.74661
CTC-471J1.	-0.29907	0.0661728	-1.358725	0.1757105	0.2986932	-5.746854
ASS1P4	0.1684536	-1.272885	1.358497	0.1757826	0.2987927	-5.747157
TFDP1	-0.042767	6.2403829	-1.358488	0.1757854	0.2987927	-5.747169
MORC1	-0.311991	-0.612786	-1.358426	0.1758051	0.2988123	-5.747252
RP11-439A1	0.2272561	-0.90951	1.3582621	0.175857	0.2988812	-5.74747
TCEAL1	0.0413934	5.7266085	1.3582459	0.1758621	0.2988812	-5.747491
NADK2-AS1	-0.371553	2.2421642	-1.358154	0.1758912	0.2989168	-5.747614
SLFN14	0.2859387	-0.265644	1.35801	0.1759368	0.2989804	-5.747805
ULK3	0.0319456	6.1995359	1.3579394	0.1759592	0.2990045	-5.747899
AC097724.3	-0.146134	3.2176658	-1.357897	0.1759725	0.2990133	-5.747955
RP4-61404.	0.3131796	1.5287975	1.3578658	0.1759825	0.2990163	-5.747997
CTC-559E9.	-0.268496	-0.632064	-1.357798	0.176004	0.2990389	-5.748087
RP11-1348G	-0.141408	3.1876752	-1.357691	0.1760378	0.2990824	-5.748229
SBF1	-0.031863	6.4118315	-1.357607	0.1760646	0.2991141	-5.748342
CCT7	-0.021749	6.8227059	-1.3574	0.1761301	0.299195	-5.748617
RP11-250B2	0.3054161	1.6810391	1.3573929	0.1761324	0.299195	-5.748626
AC006994.1	-0.17802	-1.176728	-1.357379	0.1761369	0.299195	-5.748645
RP1-111B22	-0.304706	0.5630101	-1.357254	0.1761764	0.2992283	-5.74881
TMSB4XP8	0.1423854	4.0247259	1.3572538	0.1761765	0.2992283	-5.748811
TMED10	0.0268701	6.911054	1.3572393	0.1761811	0.2992283	-5.74883
TCEAL4	0.0319038	6.4632793	1.3572136	0.1761893	0.2992283	-5.748865
CTD-2161F6	-0.107222	-1.502176	-1.357146	0.1762107	0.2992508	-5.748954
RP11-73M18	-0.187816	-1.125086	-1.357077	0.1762327	0.2992743	-5.749047
NPM1P40	-0.185278	-1.201094	-1.356785	0.1763252	0.2994174	-5.749434
TGFB2-AS1	0.3944897	1.3266406	1.3566896	0.1763555	0.2994549	-5.749561
RP11-87H9.	-0.347975	0.6223187	-1.35658	0.1763904	0.2995003	-5.749707
SNORA80E	-0.176951	-1.216017	-1.356419	0.1764414	0.2995607	-5.749921
METTL7B	0.0677811	6.6261236	1.3564158	0.1764424	0.2995607	-5.749925
ATP5J	0.0351573	6.6632405	1.356046	0.1765599	0.2997461	-5.750416
RP11-529F4	-0.201477	2.9467423	-1.355979	0.1765813	0.2997685	-5.750506
DSCAM	0.377284	0.4413972	1.3557085	0.1766671	0.2998945	-5.750865
GFER	0.0355165	6.0063066	1.3556935	0.1766719	0.2998945	-5.750885
RP11-148B1	0.325646	-0.395974	1.3555151	0.1767286	0.2999767	-5.751121
RNF222	-0.217731	-0.918492	-1.355342	0.1767836	0.3000563	-5.751351
HIST2H2BD	-0.310707	0.9823898	-1.355048	0.176877	0.3002007	-5.751741
RP11-20B7.	0.178491	-1.284514	1.3550013	0.176892	0.3002122	-5.751804
MYBPH	0.414485	1.4559622	1.354935	0.1769131	0.300234	-5.751892
IFT88	0.0409734	5.7733569	1.3548818	0.17693	0.3002488	-5.751962
IPCEF1	0.139121	4.3708411	1.354447	0.1770683	0.3004696	-5.752539
LSM12	-0.029452	5.473987	-1.354375	0.1770913	0.3004809	-5.752635
MATN3	-0.299202	3.5406485	-1.354374	0.1770914	0.3004809	-5.752636
RP11-521M1	0.1848712	-1.313096	1.3541616	0.1771592	0.3005819	-5.752918
SHC1	0.0279672	6.7805948	1.3540939	0.1771808	0.3006045	-5.753008
RP1-40E16.	-0.252847	-0.674389	-1.353971	0.17722	0.300657	-5.753171
SLC11A2	-0.030744	6.3039876	-1.353822	0.1772675	0.3007169	-5.753369
NIPAL4	0.3433842	0.1236263	1.3537837	0.1772796	0.3007169	-5.753419
HS3ST3A1	0.3175957	2.7446543	1.3537827	0.1772799	0.3007169	-5.753421
KLK12	0.198986	-1.232782	1.3537524	0.1772896	0.3007193	-5.753461

MAPK4	-0.372159	3.6778155	-1.353723	0.1772989	0.3007211	-5.7535
MADCAM1	-0.163161	3.6802179	-1.353621	0.1773313	0.3007621	-5.753634
RP1-168P16	0.1964284	-1.10372	1.3534753	0.1773779	0.3008271	-5.753828
CECR5-AS1	-0.212731	2.4641305	-1.353047	0.1775145	0.3010447	-5.754396
RP11-298C3	-0.18759	2.797218	-1.352989	0.177533	0.3010622	-5.754473
RP11-114M1	-0.373769	0.0056291	-1.352752	0.1776084	0.3011761	-5.754787
XPO4	-0.031565	5.9071294	-1.352185	0.1777896	0.3014694	-5.755539
ZNF815P	-0.086119	4.3032398	-1.351696	0.1779458	0.3017179	-5.756186
PHKA2-AS1	-0.152748	3.4443182	-1.351674	0.1779527	0.3017179	-5.756215
ZFYVE19	0.0347626	6.1719854	1.3515757	0.1779842	0.3017572	-5.756345
RP11-363L2	0.1151692	-1.436893	1.35116	0.1781171	0.3019685	-5.756896
LSR	-0.062133	6.81305	-1.350908	0.1781975	0.3020908	-5.757229
ADAT3	-0.082077	4.6311329	-1.350845	0.1782179	0.3021114	-5.757313
HDDC3	-0.04094	5.7330689	-1.350796	0.1782335	0.3021198	-5.757378
RP11-1060J	-0.299224	2.7135182	-1.350777	0.1782395	0.3021198	-5.757402
RP11-429K1	0.3193072	0.0765615	1.3505031	0.1783272	0.3022545	-5.757765
RP5-999L4.	-0.303784	0.1120994	-1.350286	0.1783968	0.3023584	-5.758053
RP11-561B1	-0.287803	-0.105326	-1.349862	0.1785325	0.3025744	-5.758613
KRT8P32	-0.301923	0.2689554	-1.349765	0.1785637	0.3026132	-5.758742
RP13-444K1	-0.174991	-1.168344	-1.349678	0.1785915	0.3026464	-5.758857
CTD-2620I2	-0.203983	-1.022586	-1.349425	0.1786726	0.3027696	-5.759191
LAMTOR1	0.0247977	6.5115014	1.3492326	0.1787342	0.30286	-5.759445
ACOT7	0.0383458	6.1313974	1.3491772	0.1787519	0.3028741	-5.759519
UGT2B28	-0.415554	1.2037278	-1.349155	0.1787591	0.3028741	-5.759548
CYP2C9	0.1813616	6.5229862	1.3491001	0.1787766	0.3028897	-5.759621
PRAMEF25	0.2816307	-0.972301	1.3490208	0.178802	0.3029187	-5.759725
KRT18P63	0.299513	0.1252379	1.3489906	0.1788117	0.3029211	-5.759765
GUCA2B	0.3819506	3.339238	1.3489587	0.178822	0.3029244	-5.759808
RP11-803B1	-0.141915	-1.349153	-1.348832	0.1788625	0.302979	-5.759974
ARRDC3-AS1	0.2154707	2.5396371	1.3487509	0.1788886	0.3030091	-5.760082
NHLRC1	-0.090382	5.0187066	-1.348654	0.1789196	0.3030475	-5.76021
KBTBD3	0.0416795	5.1460131	1.3485297	0.1789596	0.3031012	-5.760374
RNF31	-0.038511	5.7559319	-1.348475	0.1789771	0.3031168	-5.760446
CTDSP2	-0.023802	6.7087768	-1.348364	0.1790127	0.3031519	-5.760593
SMAD5-AS1	0.2485566	2.5264722	1.3483378	0.1790212	0.3031519	-5.760628
LA16c-312E	-0.199227	-1.158328	-1.348333	0.1790227	0.3031519	-5.760634
PROK1	-0.373764	0.2438723	-1.348206	0.1790634	0.3032068	-5.760802
PQLC2	-0.038674	6.0463208	-1.348126	0.1790891	0.3032274	-5.760907
RP11-321E2	-0.112645	-1.499516	-1.348116	0.1790922	0.3032274	-5.76092
RP11-102N1	0.1913102	3.210518	1.3480471	0.1791145	0.3032511	-5.761012
RP11-467H1	0.2654	-0.390833	1.3477126	0.1792219	0.3034189	-5.761454
RP11-1C1.4	0.3807367	-0.509167	1.347655	0.1792404	0.3034361	-5.76153
RP11-264B1	-0.20362	2.57726	-1.347575	0.1792662	0.3034656	-5.761635
RP11-195C7	-0.273445	1.9121834	-1.347496	0.1792914	0.3034942	-5.761739
LINC01271	0.1756409	3.6568029	1.3473918	0.179325	0.3035371	-5.761877
RAB2B	0.026146	5.7377255	1.347206	0.1793847	0.30362	-5.762122
RP6-65G23.	-0.327833	1.658617	-1.347188	0.1793906	0.30362	-5.762147
RP11-473N1	-0.278599	1.7072647	-1.347128	0.1794098	0.3036369	-5.762226
KCNJ13	0.2951578	-0.706116	1.3471047	0.1794173	0.3036369	-5.762256
RP11-362K2	-0.245006	-0.993152	-1.347001	0.1794506	0.3036745	-5.762393
CHRD	-0.073359	6.1592279	-1.346984	0.1794561	0.3036745	-5.762415

RP11-568N6	0.1530032	3.5386412	1.3468687	0.1794932	0.3037231	-5.762568
TM9SF2	0.030839	6.8637547	1.3466322	0.1795693	0.3038378	-5.76288
SND1-IT1	-0.198963	-1.01947	-1.346508	0.1796091	0.3038911	-5.763043
SNW1	-0.021855	6.3197723	-1.346444	0.1796298	0.3039121	-5.763128
CD99P1	0.057074	4.7505394	1.3463325	0.1796657	0.3039587	-5.763275
ELF5	-0.375561	0.6197259	-1.346166	0.1797194	0.3040354	-5.763495
MGAT2	0.0959822	5.0166792	1.3461396	0.1797278	0.3040356	-5.76353
RBBP8NL	-0.431112	0.0336014	-1.346101	0.1797401	0.3040424	-5.76358
RP11-347C1	-0.194411	-1.047293	-1.345907	0.1798027	0.3041341	-5.763836
DPY19L2P1	0.3382954	-0.215664	1.3457403	0.1798564	0.3042108	-5.764056
CTC-1337H2	-0.310856	1.9818745	-1.345639	0.179889	0.3042427	-5.76419
RSL24D1	-0.024586	6.3955425	-1.345624	0.179894	0.3042427	-5.76421
RP11-140L2	-0.25266	-0.599865	-1.345604	0.1799003	0.3042427	-5.764236
DLGAP1-AS5	-0.298104	-0.974707	-1.345524	0.179926	0.3042604	-5.764341
ASAH2B	-0.053505	5.2117361	-1.345501	0.1799333	0.3042604	-5.764371
OGFOD1P1	-0.288508	-0.269648	-1.345494	0.1799357	0.3042604	-5.764381
CTD-2003C8	0.2748297	-0.685481	1.3453054	0.1799965	0.3043463	-5.764629
AL109763.1	-0.196771	-1.257752	-1.345285	0.1800032	0.3043463	-5.764657
DPF1	-0.292172	1.9759589	-1.345227	0.1800217	0.3043626	-5.764733
RAI1-AS1	0.1036586	-1.488556	1.3451925	0.1800329	0.3043626	-5.764778
RP11-195F1	-0.261167	1.7590408	-1.345175	0.1800385	0.3043626	-5.764801
AIMP2	0.0333242	6.0686831	1.3451429	0.1800489	0.3043626	-5.764844
LINC00540	-0.426908	0.5436477	-1.345125	0.1800545	0.3043626	-5.764867
EDEM3	-0.040956	6.4332347	-1.344859	0.1801403	0.3044934	-5.765217
RNF168	-0.029227	5.8547165	-1.344784	0.1801646	0.3045204	-5.765317
COMMD9	-0.027223	6.2354311	-1.344629	0.1802144	0.3045873	-5.76552
IGLV1-47	0.3628214	4.0178909	1.3446096	0.1802208	0.3045873	-5.765546
HOXA4	0.1506278	3.5667926	1.3445706	0.1802334	0.3045937	-5.765598
DFFA	0.0274004	6.2047582	1.3445364	0.1802444	0.3045937	-5.765643
RP11-96D1.	0.1555537	2.794915	1.3445203	0.1802496	0.3045937	-5.765664
FGF5	0.279605	-0.988266	1.3444163	0.1802832	0.3046363	-5.765801
SRXN1	0.1049112	5.0029694	1.3443788	0.1802953	0.3046426	-5.76585
RP11-388M2	-0.153795	-1.255707	-1.344351	0.1803044	0.3046439	-5.765888
CTD-260009	0.2528862	3.1915414	1.344287	0.1803249	0.3046645	-5.765971
S1PR5	0.0993154	4.2324377	1.3440626	0.1803973	0.3047727	-5.766267
RP11-338K1	-0.284468	0.8553892	-1.344009	0.1804147	0.304788	-5.766338
RPL5P8	-0.197434	-1.090298	-1.343869	0.1804598	0.30485	-5.766522
FUT11	0.0368069	5.6141719	1.3436961	0.1805157	0.3049172	-5.766749
SUN3	-0.345464	-0.335101	-1.343694	0.1805162	0.3049172	-5.766752
RN7SL180P	0.1913292	-1.169563	1.3436649	0.1805257	0.3049191	-5.766791
RP11-47L3.	0.2536489	2.375502	1.3436111	0.1805431	0.3049344	-5.766861
LINC00837	-0.156871	-1.306909	-1.343549	0.180563	0.3049407	-5.766943
AC012445.1	0.117983	-1.490947	1.3435478	0.1805636	0.3049407	-5.766945
YWHAH	0.0236194	6.6207471	1.3434123	0.1806074	0.3050005	-5.767123
AC083873.4	-0.195538	2.2456573	-1.343138	0.1806962	0.305129	-5.767485
RP11-188P1	-0.275178	1.5460125	-1.343125	0.1807001	0.305129	-5.767501
AC007285.6	0.2548268	-0.757518	1.3430986	0.1807087	0.3051294	-5.767536
CWC25	0.0202397	5.9059674	1.3429117	0.1807691	0.3052172	-5.767782
RP11-338I2	0.2788513	1.9170245	1.3427979	0.1808059	0.3052652	-5.767932
HAUS4	-0.043617	5.8587548	-1.342568	0.1808802	0.3053638	-5.768234
GPR26	0.1460269	-1.362811	1.3425651	0.1808812	0.3053638	-5.768238

BEST2	-0.200122	-1.185065	-1.342527	0.1808937	0.3053638	-5.768288
IFITM4P	0.3323157	1.6790681	1.342514	0.1808978	0.3053638	-5.768305
DPP4	-0.080455	6.3765099	-1.34241	0.1809315	0.3054067	-5.768442
RND1	0.0916062	6.2445633	1.3423628	0.1809467	0.3054181	-5.768504
C1orf145	-0.307119	2.3720283	-1.342231	0.1809892	0.3054757	-5.768677
NBPF8P	0.0989752	5.1060974	1.3420489	0.1810483	0.3055549	-5.768917
RP13-608F4	-0.315819	-0.050408	-1.342035	0.1810528	0.3055549	-5.768935
RP11-216B9	-0.294955	1.419874	-1.341948	0.181081	0.3055882	-5.76905
RPS28P1	-0.105392	-1.454684	-1.341841	0.1811155	0.3056324	-5.76919
POU5F1P3	-0.275013	1.3311914	-1.341741	0.1811478	0.3056729	-5.769321
ERI3	0.0294012	6.2969255	1.3415857	0.1811983	0.3057438	-5.769526
RP11-370A5	0.1993412	3.9480342	1.3414816	0.181232	0.3057865	-5.769663
PRCD	-0.11851	3.6321219	-1.34125	0.181307	0.3058991	-5.769967
HMG20B	0.0252464	6.3557283	1.3412104	0.1813198	0.3059065	-5.770019
RP11-50C13	-0.070931	4.5144339	-1.34118	0.1813297	0.305909	-5.770059
ABTB2	0.0543497	5.9245033	1.3410504	0.1813717	0.3059657	-5.770229
NDUFB7	-0.039095	6.5772093	-1.341005	0.1813864	0.3059704	-5.770289
RP5-907C10	0.3643395	3.3829717	1.34099	0.1813912	0.3059704	-5.770309
MCU	0.0302168	5.965571	1.3408941	0.1814224	0.3060088	-5.770435
PRDM8	0.2146182	3.2207952	1.3408418	0.1814393	0.3060232	-5.770504
XXbac-B476	0.0905208	4.1206747	1.3405812	0.1815238	0.3061488	-5.770846
FTLP17	-0.336235	0.7846526	-1.340561	0.1815305	0.3061488	-5.770873
BRF1	-0.024754	6.1115313	-1.340379	0.1815895	0.3062341	-5.771112
NME1P1	0.0791056	-1.533082	1.3401933	0.1816497	0.3063214	-5.771355
CDK17	0.0272265	5.9608707	1.3400679	0.1816903	0.3063758	-5.77152
PTPN20A	-0.455481	1.8659457	-1.339965	0.1817237	0.3064179	-5.771655
STX18-AS1	-0.103106	4.041737	-1.339555	0.1818568	0.3066202	-5.772193
NDUFC2	-0.036698	6.4211761	-1.339544	0.1818605	0.3066202	-5.772208
RP11-446H1	0.188718	-1.188899	1.3394983	0.1818753	0.306631	-5.772268
UBXN4	0.0245014	6.6789748	1.3393299	0.18193	0.3067091	-5.772489
RP11-248J1	0.1493378	3.461643	1.3392381	0.1819598	0.3067452	-5.772609
HAL	-0.180885	6.2948272	-1.339057	0.1820186	0.3068302	-5.772847
AC092155.1	-0.297862	1.1339282	-1.338956	0.1820514	0.3068712	-5.772979
RP11-715J2	-0.305108	1.5209239	-1.338823	0.1820947	0.3069243	-5.773154
NPTN	0.0242842	6.4894527	1.3388077	0.1820997	0.3069243	-5.773174
RPL39L	-0.151936	5.0983033	-1.338671	0.182144	0.3069848	-5.773353
C21orf62	0.3417649	0.6713551	1.338541	0.1821864	0.3070421	-5.773524
SCARNA8	-0.188238	-1.134038	-1.338386	0.1822369	0.3071021	-5.773728
ARID4B	-0.029117	6.189268	-1.33838	0.1822388	0.3071021	-5.773735
CCDC94	-0.032584	6.0049452	-1.338275	0.182273	0.3071322	-5.773873
CUTA	-0.033155	6.7661223	-1.338267	0.1822756	0.3071322	-5.773884
ZSCAN16-AS	-0.056289	5.7371962	-1.338247	0.1822819	0.3071322	-5.773909
KHDRBS3	0.0695097	5.76936	1.3381799	0.1823039	0.307155	-5.773997
ZNF326	0.0279971	5.9409018	1.3377595	0.1824407	0.3073713	-5.774549
RN7SL239P	-0.187973	-1.063874	-1.337687	0.1824641	0.3073966	-5.774643
ITGB4	0.0866778	5.8519476	1.337605	0.182491	0.3074277	-5.774751
RP11-544A1	-0.260646	-0.384543	-1.337507	0.1825229	0.3074673	-5.77488
LIPE	0.1154642	4.6464972	1.3373954	0.1825592	0.3075143	-5.775026
NUDT5	-0.028856	6.5540199	-1.337345	0.1825756	0.3075276	-5.775092
RARRES2P1	-0.308635	-0.573314	-1.337173	0.1826317	0.307608	-5.775318
RP11-69L16	-0.266103	-0.09298	-1.337114	0.1826509	0.3076262	-5.775395

NANS	-0.031784	6.2572458	-1.336962	0.1827004	0.3076952	-5.775593
C2orf16	0.102372	4.5362006	1.3368636	0.1827325	0.3077351	-5.775723
RP11-358B2	-0.216639	-0.923377	-1.336741	0.1827726	0.3077853	-5.775884
AC034220.3	0.1025353	4.6281316	1.3367163	0.1827805	0.3077853	-5.775916
AC004156.3	-0.285459	1.5430789	-1.336692	0.1827884	0.3077853	-5.775947
RP3-467N11	-0.287633	0.2270764	-1.336669	0.182796	0.3077853	-5.775978
UVRAG	0.0245471	5.933699	1.336204	0.1829476	0.3080263	-5.776586
PTGES3P3	-0.117291	3.8303021	-1.336146	0.1829666	0.3080441	-5.776663
EPPIN	0.1206619	-1.438853	1.336108	0.1829789	0.3080506	-5.776712
ATP1B1P1	-0.269115	-0.332187	-1.336081	0.1829878	0.3080514	-5.776748
POLR2J2	-0.38226	0.8675984	-1.336054	0.1829965	0.3080518	-5.776783
S100A5	0.2672047	-0.500814	1.3360037	0.1830129	0.3080653	-5.776849
CSH2	-0.104987	-1.481367	-1.335812	0.1830754	0.3081563	-5.777099
XAGE3	0.350787	0.7551771	1.335612	0.1831408	0.3082485	-5.777361
RP11-496N1	-0.285182	-0.199898	-1.335593	0.1831471	0.3082485	-5.777387
C4BPAP1	0.3368448	0.2424881	1.335547	0.183162	0.3082594	-5.777447
RP11-247L2	0.2639829	-0.604912	1.3354168	0.1832045	0.3083148	-5.777617
KIAA1875	-0.117007	4.6103311	-1.335394	0.1832119	0.3083148	-5.777646
CTD-3137H5	-0.212758	-0.921978	-1.335249	0.1832594	0.3083806	-5.777837
CTC-459F4.	-0.101873	-1.504799	-1.335221	0.1832684	0.3083816	-5.777873
GRK7	-0.256876	-0.616207	-1.335154	0.1832905	0.3084045	-5.777961
CYB561D2	0.0407445	6.2749883	1.3350098	0.1833374	0.3084693	-5.778149
snoU109	0.2430053	1.9202685	1.3348992	0.1833736	0.3085005	-5.778294
RP11-1212A	-0.278739	-0.017886	-1.33488	0.1833798	0.3085005	-5.778319
AC010642.2	-0.251993	-0.591864	-1.334875	0.1833814	0.3085005	-5.778325
B3GNT7	0.1303897	4.8210472	1.3347702	0.1834157	0.3085379	-5.778463
TRIM46	-0.153227	3.6703268	-1.334731	0.1834285	0.3085379	-5.778514
AC011752.1	0.1891288	-1.241591	1.3347299	0.1834289	0.3085379	-5.778516
PPP1R1B	-0.460173	2.031024	-1.334553	0.1834868	0.3086089	-5.778747
LINC00487	0.3508405	0.2929216	1.3345491	0.183488	0.3086089	-5.778752
AP001052.1	0.2871107	0.4237521	1.3344763	0.1835118	0.3086347	-5.778847
RP11-159D1	0.1918154	-1.038541	1.3343785	0.1835438	0.3086742	-5.778975
AC005037.3	-0.30631	1.2338846	-1.334258	0.1835832	0.3087263	-5.779133
AC079630.4	0.2341315	2.6261283	1.3341242	0.183627	0.3087705	-5.779308
TVP23C	-0.07449	4.2452906	-1.334098	0.1836355	0.3087705	-5.779342
RP11-454L9	0.3169394	0.0436834	1.334077	0.1836424	0.3087705	-5.779369
RP11-320N7	0.4717433	1.8379635	1.3340742	0.1836433	0.3087705	-5.779373
GAPDHP32	-0.300805	-0.220526	-1.333872	0.1837095	0.3088676	-5.779637
AP000304.1	0.1646385	-1.281758	1.3338428	0.1837191	0.3088694	-5.779676
PCBD2	0.039369	5.6132094	1.3337873	0.1837372	0.3088857	-5.779748
SCUBE2	0.1843643	4.3442429	1.3337101	0.1837625	0.3089044	-5.779849
BRD1	-0.026519	6.0751143	-1.333702	0.1837653	0.3089044	-5.77986
CYP2C19	0.3388841	2.7603938	1.3336108	0.183795	0.3089262	-5.779979
RP11-1136G	-0.085047	-1.51305	-1.33361	0.1837952	0.3089262	-5.77998
IGHV3-23	0.3234485	4.5547492	1.3335794	0.1838053	0.3089289	-5.78002
RP11-730B2	0.2018213	-0.984685	1.3335496	0.183815	0.3089311	-5.780059
FXYD3	-0.330517	4.0778815	-1.333478	0.1838385	0.3089563	-5.780152
RP11-214N1	-0.223548	-0.82002	-1.333437	0.1838518	0.3089645	-5.780206
MRPS24	-0.040913	5.7518727	-1.333057	0.1839762	0.3091593	-5.780702
CTD-2586B1	0.1041246	-1.452482	1.3329327	0.1840171	0.3092119	-5.780865
RP4-665J23	-0.210474	-1.08065	-1.33291	0.1840245	0.3092119	-5.780894

ALS2	-0.030768	6.1950152	-1.332789	0.184064	0.3092642	-5.781052
HMGB3P22	-0.236099	2.5793445	-1.332713	0.1840892	0.3092922	-5.781152
VEGFA	-0.033203	6.7459951	-1.332631	0.1841159	0.3093228	-5.781259
DUSP8	0.0815622	5.7203192	1.3325575	0.18414	0.3093491	-5.781355
RFX5	0.0343886	6.2467546	1.3323765	0.1841994	0.3094346	-5.781591
RSRC1	-0.026533	5.9152715	-1.332172	0.1842664	0.3095329	-5.781858
DST	0.035033	6.6860714	1.3319747	0.1843311	0.3096212	-5.782116
ZNF184	-0.045351	5.2751593	-1.33194	0.1843424	0.3096212	-5.78216
CTC-1337H2	0.2550819	-0.447534	1.3319344	0.1843444	0.3096212	-5.782168
TEX40	-0.149268	-1.297158	-1.331775	0.1843968	0.3096919	-5.782377
LINC01563	0.1890411	-1.200833	1.3317544	0.1844034	0.3096919	-5.782403
PMAIP1	0.1899121	4.0191028	1.3316614	0.1844339	0.3097289	-5.782525
MYO7A	-0.080252	6.0299821	-1.33162	0.1844475	0.3097375	-5.782579
RP4-635E18	0.2473753	2.0173173	1.3315077	0.1844844	0.3097851	-5.782725
TRPM2	0.0835349	5.2251202	1.3309138	0.1846794	0.3100984	-5.7835
RNU6-431P	-0.207327	-0.975599	-1.330842	0.1847032	0.310124	-5.783594
MYO18B	-0.423702	2.6509563	-1.330676	0.1847575	0.3102009	-5.78381
RP11-126H7	-0.160825	-1.318875	-1.33054	0.1848024	0.3102619	-5.783988
NOG	0.3346677	-0.156708	1.330468	0.1848259	0.3102647	-5.784081
CYP46A1	0.1728999	3.6672708	1.3304562	0.1848298	0.3102647	-5.784097
RP1-76B20.	-0.130865	-1.408866	-1.330413	0.184844	0.3102647	-5.784153
AJ006998.2	0.3970446	2.46026	1.330413	0.184844	0.3102647	-5.784153
AC067969.2	-0.152968	-1.410582	-1.330398	0.1848489	0.3102647	-5.784172
KCNJ2-AS1	0.1984825	2.4688551	1.3303796	0.184855	0.3102647	-5.784197
RP11-702H2	-0.269654	0.9109843	-1.33035	0.1848648	0.3102669	-5.784235
STAM2	0.0245759	6.0467489	1.3303178	0.1848753	0.3102703	-5.784277
EIF3G	-0.027154	6.6949767	-1.330244	0.1848995	0.3102966	-5.784373
RP11-327L3	0.2822705	-0.383348	1.3302051	0.1849124	0.3103039	-5.784424
RP11-642C5	-0.196859	-1.221966	-1.330162	0.1849265	0.3103134	-5.78448
CTC-270D5.	-0.138638	-1.43038	-1.329613	0.1851072	0.3106024	-5.785196
MBLAC1	-0.064102	4.8306564	-1.329579	0.1851183	0.3106066	-5.78524
MAPK8	-0.026269	6.0811295	-1.329519	0.1851382	0.3106258	-5.785319
RFFL	-0.044271	6.2333476	-1.329489	0.1851479	0.3106278	-5.785357
FAM91A1	0.0290168	6.4115735	1.329342	0.1851963	0.3106947	-5.785549
MOCS1	0.0515269	6.2571101	1.3292073	0.1852407	0.3107549	-5.785724
RP11-793H1	-0.106344	4.174192	-1.329105	0.1852745	0.3107787	-5.785858
RP11-412B1	0.1849943	-1.292456	1.329104	0.1852747	0.3107787	-5.785859
DLX3	-0.40236	0.050437	-1.329087	0.1852804	0.3107787	-5.785881
ATP1A3	0.2691302	3.0307897	1.3288198	0.1853683	0.3109098	-5.786229
FAM13C	0.1246115	4.5734664	1.3287977	0.1853756	0.3109098	-5.786258
RP11-709D2	0.2883462	-0.029289	1.3286693	0.1854179	0.3109538	-5.786425
RP11-419C1	-0.145132	-1.386918	-1.328653	0.1854233	0.3109538	-5.786446
AC007246.3	0.0416124	5.6080879	1.3286404	0.1854274	0.3109538	-5.786463
RNF183	-0.398608	1.4113373	-1.328563	0.185453	0.3109825	-5.786564
ANKRD18DP	0.3651532	0.454854	1.3284811	0.1854799	0.3110133	-5.78667
PUSL1	-0.046742	5.7259843	-1.328281	0.185546	0.3111006	-5.786931
AP2A2	0.0199191	6.5494767	1.3282714	0.185549	0.3111006	-5.786943
RP11-359N1	0.1779776	-1.292745	1.3281837	0.1855779	0.3111348	-5.787057
CSTF2	-0.040798	5.6672913	-1.327982	0.1856445	0.311232	-5.78732
KRT18P22	0.1712477	-1.229873	1.327862	0.185684	0.3112841	-5.787476
PAPOLG	-0.024912	5.619279	-1.327811	0.185701	0.3112914	-5.787543

CTB-58E17.	-0.262194	1.5460587	-1.327797	0.1857055	0.3112914	-5.78756
RP11-334J6	-0.334464	0.7636789	-1.327672	0.1857466	0.3113321	-5.787723
RN7SL583P	-0.22768	-1.172987	-1.327672	0.1857468	0.3113321	-5.787723
WNT16	0.3314165	0.590803	1.3275069	0.1858012	0.3114089	-5.787938
RP11-71H17	-0.187178	-1.078643	-1.327436	0.1858245	0.3114338	-5.78803
RP11-41L14	-0.170348	-1.230431	-1.327281	0.1858758	0.3114977	-5.788232
RP11-945A1	-0.182299	-1.286808	-1.327245	0.1858877	0.3114977	-5.788279
SNORA4	0.1191998	-1.38958	1.3272431	0.1858883	0.3114977	-5.788281
SLC25A45	-0.052368	5.8546453	-1.327205	0.1859008	0.3115044	-5.78833
PYCARD-AS1	0.306256	0.2866551	1.3271488	0.1859194	0.3115212	-5.788404
TM2D3	0.0331872	6.2294655	1.327035	0.185957	0.3115699	-5.788552
RGS10	0.068289	5.5139831	1.3267763	0.1860424	0.3116987	-5.788888
PKP4P1	0.1178691	-1.424737	1.3267431	0.1860534	0.3117028	-5.788931
RP11-244O1	-0.085979	4.5097573	-1.326702	0.186067	0.3117112	-5.788985
RP11-443P1	-0.398902	4.0997417	-1.326545	0.1861189	0.3117754	-5.789189
LINC01179	-0.195932	-1.233135	-1.326534	0.1861224	0.3117754	-5.789203
CTD-2187J2	-0.146193	-1.346008	-1.326356	0.1861812	0.3118501	-5.789434
RP3-508I15	0.1823697	-1.098843	1.3263296	0.18619	0.3118501	-5.789469
ATP6V1B1	-0.275086	3.1477887	-1.326313	0.1861954	0.3118501	-5.78949
FAM166B	0.1554203	3.2614661	1.3262961	0.1862011	0.3118501	-5.789513
ADH5P3	-0.160828	-1.236259	-1.326244	0.1862184	0.3118648	-5.789581
AL450992.2	0.1572883	4.7146754	1.326204	0.1862316	0.3118725	-5.789632
RP11-193E1	-0.261884	-0.305703	-1.326169	0.1862431	0.3118776	-5.789678
GSTA5	0.2299444	-0.924115	1.3261269	0.186257	0.3118865	-5.789732
CICP27	-0.262479	-0.635027	-1.326031	0.1862889	0.311921	-5.789858
CHD5	-0.34669	0.9470427	-1.325999	0.1862995	0.311921	-5.789899
SACM1L	0.0234974	6.1710698	1.3259872	0.1863032	0.311921	-5.789914
TPMTP1	0.2968434	1.5611685	1.3255905	0.1864344	0.3121263	-5.790429
FLJ33581	0.3707823	-0.229343	1.3254958	0.1864658	0.312156	-5.790552
RP4-794I6.	0.2926272	2.3169184	1.3254852	0.1864693	0.312156	-5.790566
AC073069.2	-0.175551	-1.129942	-1.325392	0.1865002	0.3121936	-5.790688
MIRLET7BHG	0.1426388	3.6521412	1.3253613	0.1865103	0.312196	-5.790727
RP11-115D1	0.4576729	1.0376794	1.3248082	0.1866934	0.3124882	-5.791445
PPP6R3	-0.026129	6.4820413	-1.324712	0.1867253	0.3125273	-5.791571
USP19	-0.020734	6.3965959	-1.324519	0.1867892	0.3126199	-5.791821
RP11-78J21	-0.079199	-1.515917	-1.324389	0.1868321	0.3126757	-5.791989
LINC00337	-0.220397	3.1007254	-1.324367	0.1868397	0.3126757	-5.792019
RPL21P110	-0.236048	-0.773113	-1.324211	0.1868911	0.3127475	-5.79222
ATP1B4	0.1560583	-1.315866	1.3241104	0.1869246	0.3127892	-5.792351
RP11-168K1	0.2531873	-0.526013	1.3239848	0.1869662	0.3128445	-5.792514
MTND2P28	0.0660638	6.7103945	1.3238966	0.1869955	0.3128764	-5.792629
RP11-767I2	0.4014268	1.0411134	1.3238756	0.1870024	0.3128764	-5.792656
RAB29	-0.033319	6.2913433	-1.323726	0.1870521	0.3129452	-5.79285
MIR642A	0.1262532	-1.42769	1.3236269	0.1870849	0.3129857	-5.792979
RP11-386M2	0.1403203	-1.360985	1.3234113	0.1871564	0.3130758	-5.793258
RPS6KA5	0.0683259	4.953007	1.3233981	0.1871608	0.3130758	-5.793275
DMRTA2	-0.274205	-0.840251	-1.323387	0.1871645	0.3130758	-5.79329
CLN8	0.0467444	5.8187953	1.323216	0.1872212	0.3131564	-5.793512
ADCK1	0.0387562	5.6169106	1.32317	0.1872365	0.3131675	-5.793571
ITGB7	0.1268474	4.713658	1.3231104	0.1872563	0.3131863	-5.793649
TNFSF15	-0.142147	4.729417	-1.323069	0.18727	0.3131949	-5.793702

JAKMIP2	-0.180146	4.1997088	-1.323017	0.1872873	0.3132095	-5.79377
RP11-384C4	-0.305362	0.156448	-1.322983	0.1872987	0.3132142	-5.793814
RP11-297L6	-0.085489	-1.512833	-1.322677	0.1874002	0.3133696	-5.794211
UFD1L	-0.027132	6.4527426	-1.322419	0.1874857	0.3134982	-5.794544
IL26	-0.260423	-0.633769	-1.322124	0.1875839	0.3136367	-5.794927
CYP2A13	0.4640244	2.2269351	1.3221186	0.1875857	0.3136367	-5.794934
RP11-554A1	-0.105442	-1.481144	-1.321949	0.1876421	0.3137166	-5.795154
LINC01105	0.2784599	-1.010604	1.3219045	0.1876569	0.3137269	-5.795212
RP11-99H20	-0.128937	-1.45009	-1.321847	0.187676	0.3137446	-5.795286
RP11-394I1	0.3026725	-0.291269	1.3218117	0.1876877	0.3137497	-5.795332
AC003002.6	-0.284303	-0.049886	-1.321755	0.1877065	0.3137572	-5.795405
UTS2B	0.3328767	2.3028448	1.3217465	0.1877094	0.3137572	-5.795416
SNRNP40	-0.029424	6.0009835	-1.321701	0.1877247	0.3137684	-5.795476
RP11-354K4	-0.083959	-1.513584	-1.321661	0.1877378	0.313776	-5.795527
OTOG	-0.4132	0.2387259	-1.321582	0.1877641	0.3138056	-5.795629
RP11-386I1	-0.32172	0.9303423	-1.321526	0.1877826	0.3138221	-5.795701
DAB1	-0.19933	4.6595357	-1.3215	0.1877915	0.3138227	-5.795736
HNRNPA1P66	0.3144115	-0.391579	1.3212603	0.1878712	0.3139413	-5.796046
TGM1	-0.133454	3.5458496	-1.320981	0.1879641	0.3140823	-5.796408
RNU6-181P	-0.139018	-1.347153	-1.320844	0.1880097	0.3141302	-5.796585
CDK2AP1	0.0275053	6.154785	1.3208431	0.18801	0.3141302	-5.796586
RP5-1126H1	0.1202554	3.5790335	1.3206083	0.1880882	0.3142465	-5.79689
RP11-10A14	0.3447493	1.4720364	1.3204638	0.1881363	0.3143125	-5.797077
RP11-52401	-0.277117	-0.11007	-1.320382	0.1881634	0.3143434	-5.797182
BTBD7P1	-0.35619	0.5882724	-1.320159	0.1882377	0.3144531	-5.797471
ZSCAN10	-0.256133	-0.707787	-1.32013	0.1882476	0.3144552	-5.797509
RP11-686G8	-0.266434	-0.193793	-1.320003	0.18829	0.3145117	-5.797674
NPHP1	0.1062269	4.3524955	1.3198915	0.188327	0.3145591	-5.797818
IL18BP	0.0548886	5.7991244	1.3196072	0.1884218	0.3146888	-5.798185
SLC25A30-A	0.2020739	2.8853396	1.319607	0.1884219	0.3146888	-5.798186
SGSH	-0.025303	6.1344281	-1.319466	0.188469	0.3147531	-5.798369
TCEB2P1	0.1458417	-1.28512	1.3193473	0.1885085	0.3148046	-5.798522
GAL	-0.430959	0.7927477	-1.319277	0.1885321	0.3148296	-5.798613
CTTN	0.0238921	6.7521848	1.3191496	0.1885744	0.3148859	-5.798777
HNRNPA1P14	-0.260787	-0.475549	-1.319092	0.1885936	0.3149036	-5.798852
EIF3F	-0.02538	6.6511965	-1.319025	0.188616	0.3149265	-5.798938
AC024619.2	-0.096219	-1.481087	-1.318743	0.1887103	0.3150695	-5.799303
UBE2DNL	0.2381587	-1.156788	1.3186124	0.1887537	0.3151277	-5.799472
ABI2	-0.032203	6.0573028	-1.318479	0.1887984	0.3151879	-5.799645
AC116618.1	-0.183494	-1.150381	-1.318418	0.1888185	0.315207	-5.799722
RPS10P28	-0.251603	-0.625562	-1.318171	0.1889011	0.3153175	-5.800042
RP11-26F2.	-0.373433	0.623829	-1.318144	0.1889102	0.3153175	-5.800077
TNIP2	0.0256445	6.1815416	1.3181427	0.1889106	0.3153175	-5.800079
DSG2-AS1	0.2850539	2.6106524	1.3180353	0.1889465	0.315361	-5.800217
LIPN	0.2204877	-1.068144	1.3180129	0.188954	0.315361	-5.800246
CATSPER3	-0.102679	3.7980631	-1.317975	0.1889667	0.3153679	-5.800295
GJB1	-0.107608	6.9178241	-1.317938	0.1889792	0.3153742	-5.800344
MIR8071-2	-0.360033	0.0484074	-1.317863	0.1890042	0.3154006	-5.80044
C1orf52	-0.029477	5.6631087	-1.317819	0.1890188	0.3154006	-5.800497
AC118344.1	-0.254703	-0.342889	-1.317813	0.1890209	0.3154006	-5.800505
TSSK1A	-0.28036	0.0839543	-1.317784	0.1890303	0.315402	-5.800541

ACAD11	0.1536833	4.3402794	1.3177394	0.1890454	0.3154127	-5.8006
RAB6B	0.0706707	5.2272853	1.3175053	0.1891237	0.3155163	-5.800902
DTD2	-0.030657	5.682685	-1.317497	0.1891264	0.3155163	-5.800912
RN7SKP259	-0.193025	-1.096154	-1.317476	0.1891334	0.3155163	-5.80094
TMEM189	-0.033625	6.2667918	-1.317448	0.1891427	0.3155173	-5.800975
EFR3B	-0.116613	3.9790433	-1.317112	0.1892552	0.3156905	-5.801409
RP11-230L2	-0.087284	-1.490048	-1.317032	0.1892819	0.3157206	-5.801513
RP11-7306.	-0.301884	0.1712828	-1.316865	0.189338	0.3157998	-5.801729
RP11-17P16	0.138726	3.2752523	1.3168347	0.189348	0.315802	-5.801768
RBP3	-0.147411	-1.369263	-1.316731	0.1893829	0.3158458	-5.801902
SLC14A2	-0.359312	1.1749172	-1.316651	0.1894096	0.3158736	-5.802005
SCARNA24	-0.286	0.0458296	-1.316629	0.1894169	0.3158736	-5.802033
SARDH	-0.091362	6.4191762	-1.316527	0.1894509	0.3159147	-5.802164
TAB3	-0.036691	6.3130926	-1.316504	0.1894588	0.3159147	-5.802195
SERTM1	0.1823918	-1.32207	1.3164754	0.1894683	0.315916	-5.802231
KRT8P31	0.2250977	-0.801069	1.3163627	0.189506	0.3159645	-5.802377
RP11-169D4	0.3019451	1.373811	1.3162851	0.189532	0.3159934	-5.802477
EPHA1	-0.098885	6.0391043	-1.316249	0.1895441	0.3159953	-5.802524
RP11-748H2	0.1728959	-1.185971	1.3162302	0.1895504	0.3159953	-5.802548
RP11-350D1	-0.264174	-0.915264	-1.316091	0.189597	0.3160585	-5.802727
SPANXC	-0.144658	-1.390773	-1.31593	0.189651	0.316134	-5.802935
PREX2	0.1021019	5.0417706	1.3158659	0.1896725	0.3161554	-5.803018
RP11-324L1	0.2485816	-0.94681	1.3156477	0.1897456	0.3162629	-5.803299
CH507-9B2.	-0.059697	4.5512132	-1.315445	0.1898134	0.3163614	-5.80356
NFYC-AS1	-0.144143	3.62431	-1.315315	0.1898572	0.3164201	-5.803728
RP11-434I1	-0.198348	-1.170518	-1.314504	0.1901293	0.316859	-5.804774
CTD-3138F1	0.239921	-1.197167	1.3141077	0.1902623	0.3170662	-5.805284
TOR2A	-0.031086	5.8675598	-1.313916	0.1903266	0.3171589	-5.805531
ACSM1	0.2553533	5.2802511	1.3138652	0.1903438	0.317173	-5.805596
MRPL13	-0.037582	6.3506916	-1.313815	0.1903607	0.3171868	-5.805661
RP11-520B1	-0.247956	-0.834702	-1.313699	0.1903996	0.317237	-5.80581
RP11-71B7.	-0.126426	-1.433996	-1.313561	0.190446	0.3172998	-5.805988
RP11-23J9.	-0.266177	3.1461682	-1.313479	0.1904734	0.3173311	-5.806093
KRT9	-0.196167	-1.230681	-1.313398	0.1905007	0.317362	-5.806197
RP11-333E1	-0.289068	0.5950979	-1.313323	0.190526	0.3173897	-5.806294
EREG	0.4566855	1.7475283	1.3132693	0.1905441	0.3174053	-5.806363
RP11-490B1	0.2491355	-0.696107	1.313183	0.1905731	0.3174392	-5.806475
RP11-474D1	-0.377722	-0.107694	-1.313122	0.1905937	0.3174591	-5.806554
IGLV2-14	0.3279879	4.5011455	1.3130629	0.1906135	0.3174752	-5.806629
MTHFS	0.0679212	6.2161112	1.3130043	0.1906332	0.3174752	-5.806704
RP11-212D1	0.2561635	-0.510756	1.3129943	0.1906365	0.3174752	-5.806717
RP11-195C7	-0.100011	-1.461902	-1.312989	0.1906384	0.3174752	-5.806724
RP11-16402	0.2695702	-0.523743	1.3129636	0.1906469	0.3174752	-5.806757
C14orf183	0.1882565	-1.038388	1.3127994	0.1907021	0.3175527	-5.806968
RP11-213G2	-0.195965	-0.982135	-1.312632	0.1907583	0.3176317	-5.807183
LINC01212	-0.294017	-0.625941	-1.312313	0.1908658	0.3177962	-5.807594
KB-1410C5.	-0.205778	2.4331491	-1.312248	0.1908877	0.3178173	-5.807678
HAX1	-0.030259	6.6189748	-1.312224	0.1908958	0.3178173	-5.807709
S100A3	0.2618193	3.1161438	1.3120668	0.1909487	0.3178908	-5.807911
TBC1D20	0.0220702	6.2443082	1.3119193	0.1909984	0.317959	-5.8081
AC093822.1	0.1170041	-1.423154	1.3117631	0.191051	0.3180289	-5.808301

ECH1	0.0430186	6.9391631	1.311743	0.1910578	0.3180289	-5.808327
IP6K3	0.33426	4.2625689	1.3115627	0.1911186	0.3181155	-5.808559
CTD-2033C1	-0.193598	2.378654	-1.311402	0.1911727	0.3181911	-5.808765
CROCCP2	0.0445395	5.9969126	1.3113438	0.1911923	0.3181975	-5.80884
RP11-109L1	-0.467767	1.9658894	-1.31132	0.1912005	0.3181975	-5.808872
SSR4	-0.033237	6.7891161	-1.311313	0.1912027	0.3181975	-5.80888
RP11-266L9	-0.091668	3.7340884	-1.311247	0.1912251	0.3182204	-5.808965
TTC9C	-0.023122	5.7982201	-1.311205	0.1912393	0.3182231	-5.809019
LCN2	0.1946221	6.0481596	1.3111899	0.1912442	0.3182231	-5.809038
PLXNA1	-0.048485	6.0930184	-1.311002	0.1913077	0.3183143	-5.80928
MEF2BNB	-0.031559	5.7895932	-1.310818	0.1913697	0.3184029	-5.809516
CMTM2	0.2894844	1.719747	1.3107665	0.191387	0.3184172	-5.809582
AC108479.2	-0.269641	-0.362742	-1.310617	0.1914374	0.3184865	-5.809774
CEP170P1	0.3086157	0.3292858	1.3105287	0.1914672	0.3185114	-5.809888
SEPT1	0.0649451	5.4903704	1.310521	0.1914698	0.3185114	-5.809898
BRWD1	0.0280664	6.2631584	1.3104194	0.1915041	0.3185539	-5.810028
RFPL2	-0.320828	1.3346047	-1.310379	0.1915179	0.3185623	-5.810081
CPA3	0.2948607	3.7770418	1.3103509	0.1915272	0.3185634	-5.810116
ST13P11	-0.166371	-1.182833	-1.310279	0.1915514	0.318589	-5.810208
RHEBL1	-0.102212	4.0346508	-1.310135	0.1916	0.3186553	-5.810393
SIPA1	0.0254128	6.331275	1.3100523	0.1916281	0.3186875	-5.8105
C1orf167	-0.296268	0.0892324	-1.309913	0.1916752	0.3187346	-5.810679
RP11-109D9	0.3492196	0.4743751	1.3099051	0.1916777	0.3187346	-5.810689
FAM196B	0.2944395	-0.497902	1.3098909	0.1916826	0.3187346	-5.810707
TAAR6	-0.174848	-1.286142	-1.309821	0.1917062	0.3187592	-5.810797
KRT16P3	0.2054222	-1.251696	1.3097953	0.1917148	0.3187592	-5.81083
HIST2H2BA	0.1926043	-1.185946	1.3097624	0.191726	0.3187632	-5.810872
RP11-495P1	-0.313921	1.578153	-1.309605	0.1917793	0.3188373	-5.811075
NUP54	0.0246343	5.9667545	1.309497	0.1918156	0.3188832	-5.811213
RSPH1	0.2875559	3.4930922	1.3092187	0.1919097	0.319025	-5.81157
LPCAT3	-0.066064	6.0326232	-1.309036	0.1919713	0.319113	-5.811804
ST8SIA6-AS	0.5367115	2.5109332	1.3089943	0.1919856	0.3191161	-5.811858
RP11-159F2	0.2936364	0.503653	1.308979	0.1919907	0.3191161	-5.811877
CTA-228A9.	-0.240418	2.0891189	-1.308772	0.1920609	0.3192182	-5.812144
SAMD4B	0.0208051	6.4880538	1.3086641	0.1920972	0.3192641	-5.812281
RP11-505P4	-0.279037	0.1558883	-1.308627	0.1921099	0.3192706	-5.812329
C14orf169	-0.029862	5.7172588	-1.308556	0.1921339	0.3192959	-5.81242
PI15	-0.376145	3.3874358	-1.308424	0.1921784	0.3193554	-5.812589
EIF2S3	-0.022678	6.7285794	-1.308243	0.1922398	0.3194403	-5.812822
RAB5A	0.020465	6.2938447	1.3082215	0.192247	0.3194403	-5.812849
PPIH	-0.037533	5.7913268	-1.308139	0.192275	0.3194723	-5.812955
ACSM4	0.3388156	0.5257767	1.308036	0.1923098	0.3195156	-5.813087
CYP21A1P	0.1354449	5.176685	1.3079183	0.1923496	0.3195666	-5.813238
E2F3P1	0.2662851	-0.660422	1.3078935	0.192358	0.3195666	-5.81327
CTD-307601	-0.277157	-0.582384	-1.30778	0.1923964	0.3196158	-5.813415
WDR43	-0.024275	6.1727876	-1.307712	0.1924195	0.3196396	-5.813502
ZNF396	-0.063533	4.6118651	-1.307547	0.1924753	0.3197179	-5.813714
LCP1	0.0540677	6.4721824	1.307427	0.192516	0.3197709	-5.813868
DNAH12	-0.287124	2.9673926	-1.307396	0.1925266	0.3197738	-5.813908
CD38	0.1356711	5.0085148	1.3073615	0.1925382	0.3197787	-5.813952
STEAP2-AS1	-0.308271	-0.090204	-1.307231	0.1925824	0.3198375	-5.814119

CNOT1	0.026223	6.5161498	1.3072031	0.1925919	0.3198387	-5.814155
SGMS1	-0.035863	6.1304792	-1.307143	0.1926124	0.31985	-5.814232
DGKB	-0.346028	1.7116883	-1.307131	0.1926162	0.31985	-5.814247
HSPE1P8	-0.215108	-0.884869	-1.307022	0.1926532	0.3198768	-5.814386
SLC4A9	-0.310299	0.282307	-1.307014	0.1926561	0.3198768	-5.814397
AC004837.5	-0.301943	0.05156	-1.307006	0.1926587	0.3198768	-5.814407
AC004692.5	-0.135414	-1.388151	-1.30695	0.1926778	0.3198882	-5.814479
DSCAM-AS1	0.1908177	-1.316157	1.3069343	0.192683	0.3198882	-5.814499
MLLT4	0.0342596	6.5700638	1.3067085	0.1927596	0.3199964	-5.814788
ZMYM2	-0.029271	6.1954157	-1.30669	0.1927657	0.3199964	-5.814812
RP11-679C8	0.2912357	0.4357663	1.3062833	0.1929038	0.3202111	-5.815333
ORC5	0.0254423	5.9040424	1.3062153	0.1929269	0.3202348	-5.81542
RP11-167P2	-0.100181	-1.505628	-1.306065	0.1929778	0.3203047	-5.815612
RP11-757A1	0.3245533	-0.422947	1.3060386	0.1929869	0.3203052	-5.815646
RP4-744I24	0.1152234	-1.41886	1.3059727	0.1930092	0.3203278	-5.815731
RP11-524F1	-0.18401	3.6107282	-1.305902	0.1930332	0.320353	-5.815821
CTBP2P4	0.1183487	-1.406488	1.3057549	0.1930832	0.3204214	-5.816009
PSORS1C3	0.3721708	2.297774	1.3056359	0.1931236	0.3204739	-5.816162
CGB7	0.2746212	-0.424734	1.3053023	0.1932369	0.3206284	-5.816589
FLAD1	-0.032845	6.3960723	-1.305295	0.1932394	0.3206284	-5.816598
AC093159.1	0.3093759	-0.054905	1.3052841	0.1932431	0.3206284	-5.816612
RP11-431J2	0.3760854	1.3366947	1.305176	0.1932799	0.3206699	-5.81675
TGFA	0.1392872	5.1498665	1.3051589	0.1932856	0.3206699	-5.816772
RP1-315G1.	-0.218905	-0.84472	-1.305083	0.1933115	0.3206781	-5.81687
RP11-706D8	-0.094142	-1.486685	-1.305063	0.1933184	0.3206781	-5.816895
A2M	0.0592139	7.4928733	1.3050557	0.1933207	0.3206781	-5.816904
KDM4E	0.136063	-1.34488	1.3050233	0.1933317	0.3206781	-5.816946
GABRA4	-0.245842	-0.964316	-1.305015	0.1933345	0.3206781	-5.816956
SNX18P24	-0.160045	-1.382052	-1.304952	0.1933561	0.3206994	-5.817037
PNP	0.0410163	6.2138462	1.3048667	0.193385	0.3207285	-5.817146
AC006547.1	0.1985377	2.6192755	1.3048482	0.1933912	0.3207285	-5.81717
TISP43	-0.079199	-1.515917	-1.304763	0.1934203	0.3207621	-5.817279
CGB	0.1098885	-1.462348	1.304731	0.1934311	0.3207654	-5.81732
RP11-863H1	-0.16848	-1.204437	-1.304648	0.1934594	0.3207978	-5.817426
RP11-63701	0.162687	-1.268222	1.3043249	0.1935692	0.3209653	-5.817839
ZNF92P3	-0.361664	0.4868211	-1.303862	0.1937268	0.321212	-5.818431
RP11-532L1	-0.292735	-0.462235	-1.303808	0.1937451	0.3212278	-5.8185
RP11-883G1	-0.369904	1.6801903	-1.303615	0.1938109	0.3213222	-5.818747
B4GALT6	-0.073842	5.0131848	-1.303518	0.1938439	0.3213623	-5.81887
ATXN1	-0.036299	6.0846797	-1.303465	0.1938619	0.3213776	-5.818938
ADAM10	0.0258587	6.5275916	1.3032653	0.1939299	0.3214586	-5.819193
PCGF1	-0.026945	5.8360468	-1.303248	0.1939359	0.3214586	-5.819216
POLR2J	-0.03175	6.2779481	-1.303244	0.1939372	0.3214586	-5.81922
MEG3	-0.132322	5.6824813	-1.303143	0.1939717	0.3215013	-5.81935
CLEC2A	0.136824	-1.411283	1.3029496	0.1940375	0.3215957	-5.819596
TLK1	-0.025471	6.3475947	-1.302896	0.1940559	0.3216116	-5.819666
CTD-2076M1	-0.096666	-1.507352	-1.302804	0.1940873	0.321649	-5.819783
WTIP	0.0899114	5.1305753	1.3027073	0.1941201	0.3216744	-5.819906
FAM90A2P	0.1640721	-1.217991	1.3027069	0.1941202	0.3216744	-5.819907
PTCHD3	-0.377445	-0.310262	-1.302675	0.194131	0.3216777	-5.819947
GUCA1B	0.1898094	3.3356942	1.3025136	0.1941861	0.3217544	-5.820153

MTND5P6	0.1119151	-1.47483	1.3020167	0.1943556	0.3220206	-5.820788
ABCB10P1	-0.151699	-1.37516	-1.301654	0.1944795	0.3222113	-5.821251
REG3A	-0.590564	2.3871535	-1.301603	0.1944969	0.3222255	-5.821316
PTP4A1	0.0370478	7.136105	1.3014126	0.1945618	0.3223139	-5.821559
TGIF1	0.0350606	6.2575945	1.3013947	0.194568	0.3223139	-5.821582
RP11-1113L	0.2612735	3.2531315	1.3012971	0.1946013	0.3223294	-5.821706
ASCC3	0.0328926	6.2900505	1.3012961	0.1946016	0.3223294	-5.821707
RP11-575L7	-0.28299	2.220607	-1.30129	0.1946038	0.3223294	-5.821715
RP11-1277A	0.0831494	4.433782	1.3012605	0.1946138	0.3223313	-5.821753
FBP2	-0.312016	1.1120214	-1.301215	0.1946294	0.3223425	-5.821811
PPIE	0.0283115	6.1502915	1.3009965	0.194704	0.3224514	-5.822089
RP11-629B1	0.2699672	1.7106178	1.3009153	0.1947317	0.3224827	-5.822193
RP11-626A5	-0.158019	-1.302082	-1.300816	0.1947655	0.3225183	-5.822319
MIR4312	0.1116585	-1.429433	1.3008007	0.1947709	0.3225183	-5.822339
FAXC	0.4107283	1.7195707	1.3007312	0.1947946	0.3225307	-5.822428
DNAI2	0.2646781	-0.502511	1.3007178	0.1947992	0.3225307	-5.822445
GPR31	0.2978825	-0.401648	1.3006899	0.1948088	0.3225307	-5.822481
SETD4	-0.031311	5.6548757	-1.300675	0.1948137	0.3225307	-5.822499
STX7	0.025261	6.217736	1.3004283	0.1948982	0.3226559	-5.822814
KIAA1210	0.2806263	-0.457328	1.3003783	0.1949153	0.3226696	-5.822878
RAD21L1	0.1151589	-1.423652	1.3002817	0.1949483	0.3227096	-5.823001
LYPLA2P1	-0.278778	0.4033235	-1.300134	0.1949987	0.3227683	-5.823189
RN7SL356P	0.0848089	-1.520926	1.3001264	0.1950014	0.3227683	-5.823199
RP11-19J5.	-0.160484	-1.305137	-1.300095	0.1950121	0.3227713	-5.823239
OPA3	0.0258889	6.1991576	1.2999697	0.195055	0.3228197	-5.823399
PGLYRP4	0.3222059	-0.287963	1.299958	0.195059	0.3228197	-5.823413
ZNF263	0.0234695	6.0779193	1.299746	0.1951316	0.3229251	-5.823684
RP11-517A5	-0.236323	-0.718561	-1.299611	0.1951779	0.3229871	-5.823856
RP11-304L1	-0.127057	3.6213699	-1.299514	0.1952109	0.3230239	-5.823979
RP1-187B23	0.2815725	-0.366087	1.2994941	0.1952178	0.3230239	-5.824005
MATN1	-0.279131	1.314357	-1.299409	0.1952469	0.3230574	-5.824113
RP11-267C1	-0.383518	-0.195985	-1.299308	0.1952814	0.3230998	-5.824241
RP13-494C2	0.1867297	-1.111891	1.2992638	0.1952966	0.3231104	-5.824298
C6orf58	-0.284148	-0.137141	-1.299178	0.1953261	0.3231392	-5.824408
RP11-330C7	0.0703464	-1.523537	1.2991612	0.1953318	0.3231392	-5.824429
EIF4A2P4	-0.258347	-0.46725	-1.299095	0.1953543	0.3231619	-5.824512
CA5B	0.0595958	5.0547849	1.2990287	0.1953771	0.323185	-5.824597
IL16	0.0632428	5.5715878	1.2989318	0.1954103	0.3232253	-5.824721
MIR4740	-0.290706	0.5247333	-1.298797	0.1954566	0.3232872	-5.824893
RP11-849I1	-0.146618	-1.397609	-1.298701	0.1954895	0.323327	-5.825015
BTBD8	0.2480585	2.2749048	1.2984296	0.1955824	0.3234583	-5.82536
ST8SIA1	-0.15736	3.7743126	-1.298417	0.1955867	0.3234583	-5.825376
RP5-1029F2	0.1683512	-1.297617	1.2983305	0.1956164	0.3234906	-5.825486
RN7SL738P	0.2773833	0.5236648	1.2983013	0.1956264	0.3234906	-5.825524
KB-1958F4.	-0.184498	-1.249551	-1.298283	0.1956328	0.3234906	-5.825547
AC073325.2	0.246478	-0.815372	1.2981195	0.1956888	0.3235685	-5.825755
HTN1	-0.183042	-1.318057	-1.298006	0.1957278	0.3236184	-5.8259
UBBP2	-0.233643	-0.699184	-1.297871	0.195774	0.3236689	-5.826071
SERPINB13	0.2045329	-1.181366	1.297865	0.1957761	0.3236689	-5.826079
MUC7	0.0772577	-1.542779	1.2976634	0.1958452	0.3237686	-5.826335
URB1	0.0320838	6.0976773	1.2973925	0.1959382	0.3239076	-5.82668

RBMX2	-0.028825	5.8739033	-1.297253	0.1959861	0.3239722	-5.826857
RP11-284M1	0.2866232	1.7752069	1.2971156	0.1960333	0.3240354	-5.827032
RP11-681L4	-0.194481	-1.031021	-1.29692	0.1961005	0.3241166	-5.827281
RTCA-AS1	-0.118841	3.6879733	-1.296899	0.1961075	0.3241166	-5.827307
TNIK	0.1015614	5.4305413	1.2968951	0.196109	0.3241166	-5.827312
LINC01163	-0.224527	-0.920276	-1.296811	0.196138	0.3241499	-5.82742
ANO1-AS2	0.2790597	-0.534047	1.2966757	0.1961844	0.3242118	-5.827591
ZNF257	-0.289678	3.1973729	-1.296599	0.1962109	0.3242172	-5.827689
PCMT1	0.0289051	6.3147515	1.2965972	0.1962113	0.3242172	-5.827691
COX15	0.025823	6.267781	1.2965886	0.1962143	0.3242172	-5.827702
RP1-161N1C	0.1108837	-1.452592	1.2965499	0.1962276	0.3242245	-5.827751
OR1F12	-0.12174	-1.398277	-1.296483	0.1962505	0.3242476	-5.827836
RP11-757F1	0.4278936	0.8142077	1.2963572	0.1962938	0.3243046	-5.827996
RP11-419C5	-0.197797	2.652084	-1.296309	0.1963106	0.3243176	-5.828058
RP11-509J2	0.2450151	-0.716294	1.2962725	0.1963229	0.3243233	-5.828104
RP4-758J24	-0.162636	-1.182403	-1.296223	0.19634	0.3243368	-5.828167
ATP5SL	-0.025507	6.2499148	-1.296192	0.1963506	0.3243396	-5.828206
DTNB	-0.032185	5.6920519	-1.296153	0.1963641	0.3243473	-5.828256
LY9	0.1445799	4.4303749	1.2958918	0.1964538	0.3244808	-5.828588
RP11-422J1	-0.257444	-0.787422	-1.295811	0.1964815	0.3245119	-5.82869
AC012501.3	-0.293083	-0.930411	-1.295615	0.196549	0.3246087	-5.828939
MIR641	-0.202036	-1.016566	-1.295515	0.1965834	0.3246429	-5.829066
AC007365.1	-0.204977	-1.006573	-1.295503	0.1965875	0.3246429	-5.829081
DDT	0.0536296	6.5743335	1.2954561	0.1966037	0.3246549	-5.829141
ZSCAN32	0.0720549	4.4021797	1.2954036	0.1966218	0.3246701	-5.829208
TRBV13	-0.309566	-0.337482	-1.295352	0.1966397	0.3246849	-5.829274
AC006033.2	-0.401702	0.9876417	-1.295303	0.1966563	0.3246977	-5.829335
GATA3-AS1	0.2560922	-0.730421	1.2952265	0.1966827	0.3247267	-5.829433
RP11-326E2	0.1805443	-1.205211	1.295178	0.1966994	0.3247396	-5.829494
METTL15P1	0.2223037	3.1060051	1.2949976	0.1967616	0.3248274	-5.829723
ATP5LP2	-0.166792	-1.201111	-1.294939	0.1967818	0.3248461	-5.829798
GPR146	0.1262721	4.4623457	1.2947627	0.1968424	0.3249316	-5.830022
MRPL33	-0.029652	6.0988289	-1.294723	0.1968563	0.3249397	-5.830073
AP003068.1	-0.136842	3.850066	-1.294579	0.1969058	0.3249957	-5.830255
UNC93B6	-0.224035	-0.977613	-1.294572	0.196908	0.3249957	-5.830263
CYP2D7	-0.150705	5.3475022	-1.294525	0.1969245	0.3250082	-5.830324
DMRTC2	-0.161022	-1.32941	-1.294482	0.1969393	0.325018	-5.830379
PORCN	0.0540324	5.2568144	1.2944418	0.196953	0.3250259	-5.830429
GS1-259H13	0.2517971	-0.409693	1.2943011	0.1970015	0.3250912	-5.830608
ZNF818P	-0.321826	2.470908	-1.294231	0.1970257	0.3251164	-5.830697
ELAC2	0.0275305	6.4260096	1.2941134	0.1970662	0.3251461	-5.830846
NR2F1-AS1	0.0856322	4.8486081	1.2941056	0.1970689	0.3251461	-5.830856
RNY1P16	-0.242855	-0.631457	-1.294101	0.1970704	0.3251461	-5.830861
RP11-167H9	-0.14019	-1.358899	-1.293857	0.1971546	0.3252562	-5.831171
LCE1C	0.2015022	-1.219787	1.2938561	0.1971549	0.3252562	-5.831172
BMS1P2	-0.303691	0.5281417	-1.293819	0.1971678	0.3252627	-5.831219
BEND5	-0.17351	3.5449629	-1.29377	0.1971846	0.3252756	-5.831281
SIN3A	-0.021016	6.2846571	-1.29368	0.1972156	0.3253121	-5.831395
RP5-887A1C	-0.344574	0.1677459	-1.293564	0.1972555	0.3253633	-5.831542
CTD-2366F1	0.213262	2.4455861	1.2935167	0.197272	0.3253757	-5.831603
RP11-113K2	-0.311297	0.3085698	-1.293367	0.1973235	0.325426	-5.831792

LINC01508	0.3786082	-0.470341	1.2933549	0.1973278	0.325426	-5.831808
CTD-3023L1	0.0493004	-1.557027	1.2933509	0.1973292	0.325426	-5.831813
PPCDC	-0.036202	5.6607985	-1.293246	0.1973652	0.3254708	-5.831945
MIEF1	-0.026112	6.3692449	-1.293097	0.1974169	0.3255412	-5.832135
TMEM202	-0.126097	-1.471014	-1.293033	0.1974387	0.3255626	-5.832215
XXbac-BPG3	0.0903306	-1.525337	1.2930037	0.197449	0.3255647	-5.832253
CTBP1	-0.020732	6.6717221	-1.292753	0.1975354	0.3256877	-5.83257
RP11-284G1	-0.46185	0.3324425	-1.292729	0.197544	0.3256877	-5.832602
RPL35AP32	-0.188415	-1.036859	-1.292685	0.1975589	0.3256877	-5.832657
TOPORS	0.0300524	5.9037009	1.2926754	0.1975623	0.3256877	-5.832669
RP11-284F2	0.3052904	3.9268221	1.2926586	0.1975682	0.3256877	-5.832691
RP11-80I15	0.2091987	-0.965714	1.292378	0.1976651	0.3258328	-5.833046
AKAP17A	-0.025492	6.2550742	-1.292207	0.1977242	0.3259155	-5.833263
IGKV2-26	-0.270498	-0.907179	-1.292067	0.1977726	0.3259805	-5.83344
RP11-1101H	-0.300016	-0.342711	-1.291977	0.1978035	0.3260168	-5.833554
RP11-352E6	0.3139791	1.7078846	1.2919336	0.1978187	0.326027	-5.833609
MAPK12	0.0893011	5.551223	1.2918925	0.1978329	0.3260357	-5.833661
SLC25A3	0.0236049	7.0494307	1.2917057	0.1978975	0.3261274	-5.833898
RP11-426L1	0.165051	-1.217934	1.2915363	0.197956	0.3262051	-5.834112
RP4-676L2.	0.1642143	-1.328292	1.2915178	0.1979624	0.3262051	-5.834136
TFIP11	0.0392538	6.1919031	1.2912714	0.1980477	0.3263308	-5.834448
CTC-303L1.	-0.282042	-0.301989	-1.291121	0.1980998	0.3263958	-5.834638
IFI30	-0.095384	4.369472	-1.291106	0.198105	0.3263958	-5.834658
GJC3	-0.226047	3.2302648	-1.291079	0.1981141	0.3263961	-5.834691
UBE2D3P2	0.2702497	0.6053583	1.2907463	0.1982294	0.326565	-5.835112
LINC00504	0.2919414	2.6120297	1.2907265	0.1982363	0.326565	-5.835137
CNTNAP1	-0.086899	5.1608445	-1.290682	0.1982518	0.326565	-5.835194
LCE3A	0.1119038	-1.466522	1.2906786	0.1982529	0.326565	-5.835198
CTRC	-0.297887	0.0455808	-1.290654	0.1982614	0.326565	-5.835229
RP11-763E3	0.2827234	-0.294612	1.2906217	0.1982726	0.3265687	-5.83527
RP5-915N17	0.0707848	-1.528073	1.2905609	0.1982936	0.3265884	-5.835347
RP11-169K1	0.3235716	0.3599418	1.2905355	0.1983024	0.3265884	-5.835379
DPYD-AS1	-0.291025	-0.268322	-1.290493	0.198317	0.3265976	-5.835432
AC005682.6	-0.225988	3.1013202	-1.290365	0.1983614	0.3266561	-5.835595
RP11-158H5	0.3039625	1.6214516	1.2902697	0.1983945	0.3266958	-5.835716
ABCA3	0.0845269	5.4791263	1.2902275	0.1984091	0.3267051	-5.835769
RP11-7807.	-0.179689	-1.124178	-1.289996	0.1984894	0.326808	-5.836062
ADSS	-0.027655	6.2804108	-1.289996	0.1984895	0.326808	-5.836062
RP4-800J21	-0.284367	0.9442185	-1.289949	0.1985056	0.3268197	-5.836121
FAM133B	0.0397425	5.3164048	1.2897893	0.198561	0.3268962	-5.836323
RP11-572M1	0.2662625	1.0733195	1.2897487	0.198575	0.3269046	-5.836375
PFDN2	-0.033171	6.405778	-1.289706	0.1985899	0.3269143	-5.836429
CTD-2245F1	-0.291875	2.332389	-1.289645	0.1986108	0.3269341	-5.836505
SPECC1L-AD	-0.210066	-0.890633	-1.28959	0.1986299	0.3269508	-5.836575
TMA16P2	-0.161963	-1.187312	-1.289507	0.1986588	0.3269835	-5.83668
RP11-600L4	-0.081492	-1.514793	-1.28941	0.1986924	0.3270241	-5.836803
FNTA	-0.031213	6.2521164	-1.289331	0.19872	0.3270547	-5.836903
IGHV5-51	0.3439814	4.1728678	1.2892494	0.1987482	0.3270864	-5.837006
SLC7A13	-0.314199	-0.086042	-1.289155	0.198781	0.327118	-5.837125
GUCY2EP	0.4029027	1.1261089	1.2891424	0.1987853	0.327118	-5.837141
RBMY1A1	-0.126402	-1.426211	-1.288957	0.1988496	0.3271955	-5.837375

TMEM176B	0.0569186	7.2444609	1.288955	0.1988503	0.3271955	-5.837378
SCML2P2	0.2463897	-0.544943	1.288913	0.1988649	0.3271988	-5.837431
NAP1L4	-0.019116	6.6060894	-1.288898	0.1988703	0.3271988	-5.837451
SUMO3	-0.026059	6.5073424	-1.288825	0.1988955	0.3272257	-5.837543
SLC25A38	0.0348773	6.2239893	1.2887793	0.1989113	0.3272265	-5.8376
GS1-124K5.	-0.146087	-1.313782	-1.288772	0.198914	0.3272265	-5.83761
OTOA	0.1292414	3.4894377	1.2886908	0.198942	0.3272579	-5.837712
PIK3R1	0.0462197	6.4914174	1.2886304	0.198963	0.3272776	-5.837788
RP11-257P3	-0.213311	-0.937866	-1.288213	0.1991081	0.3275015	-5.838316
PPIAP26	-0.174047	-1.174434	-1.288171	0.1991224	0.3275103	-5.838368
EIF3FP3	-0.075073	5.0395229	-1.288118	0.199141	0.3275262	-5.838436
ZSCAN4	0.2488025	-0.78767	1.2880386	0.1991685	0.3275567	-5.838536
HNRNPA1P2	-0.20657	-0.931491	-1.287824	0.1992431	0.3276646	-5.838807
ITPA	-0.031161	6.3062391	-1.287676	0.1992944	0.3277341	-5.838993
RP5-1052M9	-0.248427	-0.578563	-1.287639	0.1993075	0.3277342	-5.839041
NCOR1P1	0.0493004	-1.557027	1.2876246	0.1993124	0.3277342	-5.839059
RP11-170L3	-0.098332	-1.48463	-1.287456	0.1993712	0.3278161	-5.839272
RP11-77K12	-0.318347	0.7635988	-1.287389	0.1993942	0.3278365	-5.839356
TDGF1P4	0.1447079	-1.361977	1.2873681	0.1994016	0.3278365	-5.839382
BCL2L12P1	-0.237184	-0.94726	-1.287169	0.1994708	0.3279356	-5.839634
RP11-629N8	-0.144866	-1.272832	-1.287053	0.199511	0.3279869	-5.839779
DPY19L1P1	-0.067851	4.4580306	-1.287007	0.199527	0.3279984	-5.839838
AC112198.1	0.3581393	0.4928843	1.2869304	0.1995538	0.3280192	-5.839935
AIF1L	0.1110378	5.5025749	1.2869196	0.1995576	0.3280192	-5.839948
RP11-377D9	-0.244751	1.7620955	-1.286889	0.1995682	0.3280219	-5.839987
CTB-180A7.	0.1563254	-1.341991	1.286826	0.1995901	0.3280431	-5.840066
LHX8	0.2523493	-1.16576	1.2867088	0.1996309	0.3280942	-5.840214
CTD-2290P7	-0.207982	-0.927312	-1.286685	0.1996392	0.3280942	-5.840244
LYPD4	-0.179274	-1.225006	-1.286457	0.1997184	0.3282097	-5.840532
CYCSP49	-0.117168	-1.436832	-1.28614	0.1998288	0.3283763	-5.840931
RP11-137L1	0.1125362	-1.417117	1.2860449	0.1998621	0.3284037	-5.841052
PRR15	0.3624491	3.1380432	1.2860409	0.1998635	0.3284037	-5.841057
CTD-204901	0.3308806	0.2169455	1.2858814	0.199919	0.3284668	-5.841258
AC011499.1	0.2027968	-0.956781	1.2858789	0.1999199	0.3284668	-5.841261
RP11-187A9	-0.286521	0.9359199	-1.285817	0.1999413	0.3284872	-5.841339
RP11-638I2	-0.274608	-0.145113	-1.285711	0.1999782	0.3285331	-5.841472
WFS1	-0.038569	6.3024375	-1.285613	0.2000126	0.3285748	-5.841597
MIR378A	0.2460132	-0.742039	1.2854221	0.2000791	0.3286641	-5.841837
EMR3	-0.350707	1.0698471	-1.285402	0.200086	0.3286641	-5.841862
KRT18P20	0.2524965	-0.580865	1.2853793	0.200094	0.3286641	-5.841891
RP11-589M4	0.267627	-0.563359	1.2852449	0.2001409	0.3287263	-5.84206
RP11-217E2	-0.177797	-1.372515	-1.285046	0.2002101	0.3288026	-5.842311
FBXO16	0.195657	4.1115919	1.2850408	0.200212	0.3288026	-5.842317
RP11-490K7	0.2969213	0.687814	1.2850342	0.2002143	0.3288026	-5.842326
XXbac-BPGE	-0.218939	-0.832178	-1.284833	0.2002845	0.3289031	-5.842579
PNMA1	-0.042189	5.8985937	-1.284778	0.2003036	0.3289197	-5.842648
DNAJC7	-0.020502	6.4257749	-1.28463	0.2003554	0.3289898	-5.842835
ACAP2	0.025847	6.1138802	1.2845453	0.2003849	0.3290235	-5.842942
CA14	-0.137644	4.9503415	-1.284507	0.2003982	0.3290306	-5.84299
RP11-17M16	-0.112393	3.7544079	-1.284368	0.2004466	0.3290888	-5.843164
STOML2	-0.02902	6.5826293	-1.284353	0.2004521	0.3290888	-5.843184

RP11-400K9	-0.154649	-1.323967	-1.284328	0.2004607	0.3290888	-5.843215
RIMBP2	0.3523706	-0.235928	1.2840538	0.2005565	0.3292312	-5.843561
AC005301.8	-0.183496	-1.327715	-1.28389	0.2006136	0.3293101	-5.843767
RP11-130F1	-0.211602	2.5295755	-1.28384	0.2006313	0.3293244	-5.84383
RP11-54C4.	-0.21092	-1.057367	-1.283674	0.2006893	0.3294048	-5.844039
RP11-1101K	0.137085	-1.39847	1.2835253	0.2007411	0.329475	-5.844226
AC073464.1	-0.147268	-1.419196	-1.283383	0.2007909	0.3295419	-5.844405
TRIM54	-0.416373	2.438173	-1.283261	0.2008333	0.32959	-5.844558
LINC00935	0.1173558	-1.454053	1.2832474	0.2008382	0.32959	-5.844576
RP11-452J6	-0.187315	-1.0898	-1.283144	0.2008743	0.3296208	-5.844705
RNU6-968P	0.1645429	-1.258846	1.283142	0.2008751	0.3296208	-5.844708
ZNF324	-0.031808	5.4483995	-1.283029	0.2009146	0.3296707	-5.84485
RPL7AP34	-0.241558	2.8170735	-1.282696	0.2010312	0.3298473	-5.84527
RP3-424M6.	-0.091662	3.7073358	-1.282608	0.2010618	0.3298826	-5.84538
RP11-344N1	-0.184746	-1.287758	-1.282543	0.2010845	0.3299052	-5.845462
RP5-878I13	0.2015669	-1.040188	1.2825018	0.201099	0.3299141	-5.845514
RP11-234K2	0.3297565	0.5455422	1.2824638	0.2011123	0.329921	-5.845561
AC010900.2	-0.294233	0.3542491	-1.282386	0.2011396	0.3299478	-5.84566
RP5-1009E2	0.2971363	-0.141826	1.2823655	0.2011467	0.3299478	-5.845685
RP11-111K1	-0.270366	1.2659256	-1.282329	0.2011596	0.3299541	-5.845731
IL31	-0.181409	-1.260392	-1.282291	0.2011728	0.3299556	-5.845779
RP11-130L8	-0.185738	4.4698906	-1.282274	0.2011786	0.3299556	-5.8458
RP11-332H1	0.2291953	2.5904569	1.2822226	0.2011967	0.3299705	-5.845865
GPD1L	-0.061496	5.6177856	-1.28173	0.2013691	0.3302385	-5.846484
HAR1B	0.3286599	0.1599788	1.2814624	0.2014629	0.3303775	-5.84682
AGBL4	0.3944135	1.3232758	1.2813883	0.2014889	0.3304025	-5.846913
TDP2	-0.027883	6.3547084	-1.28135	0.2015022	0.3304025	-5.846961
RP11-648L3	-0.142475	-1.322394	-1.281341	0.2015054	0.3304025	-5.846973
REG3G	-0.305571	-0.866601	-1.281219	0.2015482	0.3304579	-5.847126
COL28A1	-0.407263	1.9892159	-1.281189	0.2015586	0.3304602	-5.847163
RP11-74501	0.1560941	-1.239126	1.2811439	0.2015745	0.3304714	-5.84722
SSTR1	-0.231927	4.8910688	-1.280934	0.201648	0.3305771	-5.847484
TSSK5P	-0.243126	2.9606954	-1.28073	0.2017198	0.3306798	-5.847741
RP11-1033H	0.1090162	-1.440298	1.2806817	0.2017366	0.3306829	-5.847801
ECHDC3	0.1323692	6.2191789	1.2806727	0.2017397	0.3306829	-5.847812
RP11-322E1	-0.232714	-0.740758	-1.280564	0.2017779	0.3307305	-5.847949
E2F4	-0.026063	6.2426372	-1.280525	0.2017916	0.3307382	-5.847998
AC006483.5	0.181498	-1.13949	1.2802891	0.2018743	0.3308589	-5.848294
RP11-501M7	-0.125659	-1.423155	-1.280048	0.2019588	0.3309705	-5.848596
LINC00521	0.2509015	-1.004763	1.2800433	0.2019606	0.3309705	-5.848603
RP11-756J1	-0.247056	-0.713912	-1.27991	0.2020072	0.3310322	-5.84877
PCK2	0.0709186	6.8415697	1.2792968	0.2022227	0.3313704	-5.849539
MAP1S	-0.025082	6.1676798	-1.279239	0.2022432	0.331389	-5.849612
OR7E4P	-0.087513	-1.51184	-1.279082	0.2022981	0.3314538	-5.849808
ARHGAP25	0.0552806	5.5894396	1.2790744	0.2023009	0.3314538	-5.849818
RP11-746B8	-0.087211	-1.511989	-1.279011	0.2023233	0.3314756	-5.849898
KLRG2	0.2526342	-0.604512	1.278915	0.2023569	0.3315158	-5.850018
AC114877.3	0.1286272	-1.359719	1.2788671	0.2023737	0.3315285	-5.850078
SLC25A35	-0.05172	5.1579615	-1.278791	0.2024004	0.3315573	-5.850173
VN1R2	0.0624496	-1.54157	1.2787186	0.2024259	0.3315843	-5.850265
RP11-457P1	-0.07444	-1.518251	-1.278496	0.202504	0.3316974	-5.850543

RP11-229P1	0.2505996	-0.635359	1.2784556	0.2025184	0.331706	-5.850594
KAT6B	0.0355967	5.8216731	1.2783452	0.2025572	0.3317547	-5.850733
TTYH3	0.0462164	6.5166861	1.2782998	0.2025732	0.331766	-5.85079
DUSP26	-0.253435	2.7205302	-1.277993	0.2026813	0.3319281	-5.851175
KLF14	0.2608783	-0.859678	1.2779223	0.202706	0.3319538	-5.851263
ZDHHC14	-0.037083	6.0722139	-1.277862	0.2027272	0.3319675	-5.851338
BICD2	0.024598	6.0181891	1.2778285	0.202739	0.3319675	-5.85138
RP11-823P9	-0.076211	-1.517383	-1.277821	0.2027417	0.3319675	-5.85139
FAM90A12P	0.1049233	-1.502362	1.2777558	0.2027647	0.3319771	-5.851471
RPS16P9	-0.155126	-1.347563	-1.277753	0.2027658	0.3319771	-5.851475
AL022476.2	-0.251193	1.6171381	-1.277692	0.2027871	0.3319971	-5.851551
AP4B1	0.0304028	5.7039525	1.2776423	0.2028046	0.3320109	-5.851614
ZC3H11A	-0.023294	6.5918703	-1.277485	0.2028601	0.3320736	-5.851811
EPC1	-0.021921	6.0524992	-1.277482	0.2028611	0.3320736	-5.851815
SNX11	-0.024848	6.0107814	-1.277387	0.2028944	0.3321133	-5.851933
KLHL15	0.0445978	5.7081389	1.2772163	0.2029546	0.3321969	-5.852147
CTC-367J11	-0.326507	1.9042194	-1.277052	0.2030123	0.3322647	-5.852352
RPS2P32	-0.200658	2.9772501	-1.277047	0.2030143	0.3322647	-5.852359
CCDC36	-0.27668	1.9273936	-1.276916	0.2030604	0.3323114	-5.852523
TP53INP1	0.0509327	6.7631084	1.2769145	0.203061	0.3323114	-5.852525
AC016912.3	-0.104074	-1.459909	-1.276887	0.2030707	0.3323123	-5.85256
PHF3	-0.029875	6.437654	-1.276768	0.2031127	0.3323539	-5.852709
MLX	0.0255895	6.394199	1.2767633	0.2031143	0.3323539	-5.852715
AC093616.4	-0.197555	3.0737288	-1.276538	0.2031937	0.3324689	-5.852997
IGKV2D-26	-0.150498	-1.387281	-1.276368	0.2032536	0.332552	-5.853209
LRIT3	0.2812628	1.8403853	1.276274	0.2032868	0.3325915	-5.853327
TRBJ2-2	-0.204674	-0.981464	-1.276204	0.2033114	0.3326168	-5.853414
RP11-542M1	-0.178058	-1.204745	-1.276063	0.2033613	0.3326835	-5.853591
LRRC23	0.0642076	5.07061	1.2759264	0.2034094	0.3327474	-5.853762
CCDC87	-0.204552	3.2152014	-1.275856	0.2034341	0.3327729	-5.85385
RP11-344P1	-0.283698	1.1804944	-1.275667	0.2035008	0.3328642	-5.854086
MIR92B	0.0796849	-1.523537	1.2756465	0.2035082	0.3328642	-5.854112
RAB5CP1	-0.158094	-1.267979	-1.275355	0.2036111	0.3330177	-5.854477
FAM25A	0.0907208	-1.511624	1.275329	0.2036203	0.3330177	-5.85451
MEOX2-AS1	-0.325446	-0.3725	-1.275057	0.2037165	0.3331601	-5.85485
RP11-440E5	-0.204468	-0.980911	-1.274913	0.2037672	0.333228	-5.855029
PTCHD1	0.3321515	0.0140373	1.2746459	0.2038616	0.3333675	-5.855364
RP11-429D1	0.2534434	-0.633666	1.2745106	0.2039094	0.3334308	-5.855533
MOBP	-0.2956	-0.028937	-1.274451	0.2039305	0.3334462	-5.855607
CTD-2201E9	0.1306484	-1.420588	1.2744323	0.2039371	0.3334462	-5.855631
CTC-425023	-0.332918	1.0294759	-1.274403	0.2039474	0.3334481	-5.855667
RP11-504P2	-0.155546	-1.267706	-1.274231	0.2040081	0.3335113	-5.855882
NIPAL2	0.0562905	6.1624991	1.2742178	0.2040129	0.3335113	-5.855899
VTI1A	-0.021661	5.8005486	-1.274216	0.2040134	0.3335113	-5.8559
RP11-113D6	-0.088602	-1.511307	-1.274075	0.2040636	0.3335783	-5.856078
IGKV1D-35	-0.122634	-1.434152	-1.274032	0.2040787	0.33358	-5.856131
RP11-866E2	-0.10864	-1.479575	-1.274011	0.204086	0.33358	-5.856157
SDCCAG8	0.0270715	5.8804052	1.2739834	0.2040958	0.33358	-5.856192
CTB-120L21	0.1185725	-1.427114	1.2739683	0.2041012	0.33358	-5.85621
RP5-944M2.	0.1651859	-1.349248	1.2738894	0.2041291	0.3335853	-5.856309
CRYZL1	0.0254161	5.7349489	1.2738891	0.2041292	0.3335853	-5.856309

TSGA13	-0.096456	-1.463645	-1.273882	0.2041318	0.3335853	-5.856319
RNA5SP201	0.1789814	-1.079067	1.2737481	0.2041791	0.3336475	-5.856485
NEDD9	-0.049368	6.2099725	-1.273706	0.2041941	0.3336572	-5.856539
RP11-874G1	0.2298393	-0.627818	1.2736797	0.2042033	0.3336572	-5.856571
RP11-64C12	0.1563061	-1.336857	1.273534	0.2042548	0.3337265	-5.856753
CNN2P8	-0.160205	-1.351716	-1.273438	0.2042887	0.333767	-5.856872
RP11-261N1	0.4789113	1.2618695	1.2732912	0.2043408	0.3338292	-5.857056
DOCK9-AS1	-0.191951	-1.064485	-1.273279	0.2043451	0.3338292	-5.857071
FGFR1OP	-0.039218	5.8687979	-1.273215	0.2043678	0.3338514	-5.857152
RP4-683M8.	0.0573004	-1.55295	1.2731408	0.204394	0.3338792	-5.857244
RP4-620E11	-0.214574	1.9414232	-1.273009	0.2044405	0.3339403	-5.857408
HSPD1	-0.02553	7.1552659	-1.272924	0.2044706	0.3339639	-5.857514
DKK4	-0.500377	1.7258272	-1.272917	0.2044733	0.3339639	-5.857523
MTND4P31	-0.105974	-1.458977	-1.272845	0.2044989	0.3339907	-5.857613
RP11-273G1	0.334033	3.026856	1.2727325	0.2045386	0.3340403	-5.857754
NLRP14	0.3521312	0.890623	1.2727075	0.2045475	0.3340403	-5.857785
GAS8-AS1	0.2946771	0.7920434	1.2724678	0.2046324	0.3341599	-5.858084
RP11-492E3	0.2838438	0.4297935	1.2724491	0.2046391	0.3341599	-5.858107
TP53INP2	0.042757	6.4293302	1.2724005	0.2046563	0.3341659	-5.858168
DNAJB7	-0.286634	0.3605599	-1.272387	0.204661	0.3341659	-5.858185
EGLN3-AS1	-0.218394	-1.120726	-1.272091	0.2047661	0.3343224	-5.858554
AC005532.5	-0.323357	0.3588099	-1.272036	0.2047854	0.3343389	-5.858622
RPL5P25	-0.146816	-1.29494	-1.271999	0.2047988	0.3343459	-5.858669
RP11-326L1	0.220582	-0.91028	1.2719534	0.2048148	0.3343501	-5.858726
AF131216.1	0.2858582	1.003381	1.2719398	0.2048196	0.3343501	-5.858743
SLC25A5P7	0.1871675	-1.069343	1.2718764	0.2048421	0.3343718	-5.858822
ZNF674	-0.03952	4.9819941	-1.27178	0.2048763	0.3344127	-5.858942
DPP3	0.0276606	6.305687	1.2717358	0.204892	0.3344233	-5.858997
LINC01006	-0.060392	5.5074068	-1.271652	0.2049217	0.3344367	-5.859102
SPCS2P1	-0.14492	-1.290865	-1.271643	0.2049249	0.3344367	-5.859113
ADAD2	0.3045183	0.4963085	1.2716351	0.2049277	0.3344367	-5.859123
RSF1	0.0245498	6.0616854	1.2714924	0.2049783	0.3345044	-5.859301
ZNF518B	-0.093578	5.1744386	-1.271271	0.2050568	0.3346174	-5.859576
PPP1CA	-0.022858	6.7506564	-1.271216	0.2050763	0.3346344	-5.859645
LINC01033	-0.222179	-0.916602	-1.270856	0.2052042	0.3348281	-5.860094
SNX17	0.0189673	6.656446	1.270812	0.2052199	0.3348387	-5.860149
WNT5A-AS1	0.3630313	1.6595967	1.270781	0.2052309	0.3348417	-5.860188
CIB4	0.1386356	-1.40276	1.2706133	0.2052904	0.3349239	-5.860397
FLJ33360	0.3281383	1.2159433	1.2705511	0.2053126	0.334945	-5.860474
ISM1-AS1	-0.274077	-0.388175	-1.270523	0.2053225	0.3349463	-5.860509
SMKR1	0.3424533	2.2020636	1.2704021	0.2053655	0.3350014	-5.86066
RERE	0.0271573	6.4038616	1.2703121	0.2053975	0.3350386	-5.860772
RP11-11N5.	0.1913731	-1.255634	1.2702863	0.2054066	0.3350386	-5.860804
RP11-313F2	-0.304329	0.1253096	-1.27024	0.2054229	0.3350502	-5.860861
RP11-106M3	-0.072689	4.2316894	-1.270178	0.2054453	0.3350717	-5.86094
PRPF4	-0.024288	6.0827824	-1.270132	0.2054613	0.335077	-5.860996
OXA1L	-0.024719	6.7048681	-1.270101	0.2054726	0.335077	-5.861035
RP11-652L8	0.2923235	1.3790222	1.270091	0.2054761	0.335077	-5.861047
RP11-88I18	-0.307959	0.9763165	-1.269932	0.2055325	0.3351463	-5.861245
RP11-352D1	-0.301192	-0.309167	-1.26992	0.205537	0.3351463	-5.861261
AC010524.4	-0.284574	-0.202279	-1.269888	0.2055481	0.3351496	-5.8613

RP11-1376F	0.1317646	-1.360413	1.2697338	0.2056031	0.3352242	-5.861492
PRKAB1	-0.02411	6.1987276	-1.269687	0.2056197	0.3352364	-5.861551
NPIPB7	-0.203326	-0.86558	-1.269452	0.2057032	0.3353576	-5.861843
ITIH1	-0.107408	7.1989277	-1.269402	0.2057209	0.3353714	-5.861905
RP11-545A1	-0.282443	-0.500921	-1.269348	0.2057401	0.3353878	-5.861972
RP11-14101	-0.235206	-0.630712	-1.269143	0.2058134	0.3354921	-5.862228
SCML4	0.1316089	4.5901083	1.2690202	0.205857	0.3355482	-5.862381
HYAL1	0.0694699	6.5339805	1.268698	0.2059716	0.3357202	-5.862782
NGF	0.1598001	3.9429999	1.2686473	0.2059897	0.3357258	-5.862845
ZYG11A	-0.302647	3.8645872	-1.268637	0.2059935	0.3357258	-5.862858
RP11-1217F	0.0876849	-1.51946	1.2685858	0.2060116	0.3357403	-5.862921
SCNN1B	0.3308524	2.9116838	1.2684577	0.2060573	0.3357997	-5.863081
CH17-472G2	0.1220889	4.823231	1.2681215	0.206177	0.3359799	-5.863499
RP11-689P1	0.2265628	3.1484666	1.2680129	0.2062157	0.336028	-5.863634
TMEM5	-0.026555	5.8166073	-1.267785	0.2062968	0.3361385	-5.863917
SLC38A6	-0.045366	5.6058561	-1.267771	0.206302	0.3361385	-5.863935
IARS	-0.026491	6.474917	-1.267737	0.206314	0.336143	-5.863977
UTP14C	0.0393588	5.9151946	1.2676704	0.2063378	0.3361669	-5.86406
RP11-553K8	-0.100722	-1.461553	-1.267495	0.2064005	0.3362539	-5.864278
AC009274.6	-0.118409	-1.40107	-1.267421	0.2064269	0.3362722	-5.86437
RP11-555H2	-0.188807	-1.038139	-1.267412	0.2064301	0.3362722	-5.864381
CXCR5	-0.291972	-0.407966	-1.267378	0.206442	0.3362737	-5.864423
LINC01372	-0.161108	2.9626205	-1.267357	0.2064494	0.3362737	-5.864449
LINC01166	0.2436628	-0.958657	1.2672971	0.2064709	0.3362937	-5.864524
APLF	0.0608328	4.8264088	1.2672468	0.2064889	0.3362945	-5.864586
TAS1R1	-0.29418	0.5686542	-1.267244	0.2064898	0.3362945	-5.86459
RP11-76H14	-0.144686	-1.2775	-1.267043	0.2065616	0.3363836	-5.86484
AC010149.4	0.2869192	-0.231286	1.267015	0.2065716	0.3363836	-5.864874
U47924.27	-0.299496	-0.859417	-1.267013	0.2065722	0.3363836	-5.864876
H1FX-AS1	-0.089754	4.4572955	-1.26692	0.2066054	0.3364028	-5.864992
FAM71D	-0.253286	1.3290377	-1.266915	0.2066072	0.3364028	-5.864998
RP11-160H1	0.1345162	-1.367497	1.2668792	0.2066201	0.3364028	-5.865043
ARHGEF10L	0.031785	6.624378	1.2668771	0.2066208	0.3364028	-5.865046
RP11-43505	-0.314709	1.4383621	-1.266716	0.2066785	0.3364725	-5.865246
XPC	0.0285199	6.1954592	1.2667055	0.2066821	0.3364725	-5.865259
DNAH10	-0.155832	3.4919152	-1.266552	0.2067369	0.336536	-5.86545
RP1-56J10.	-0.208149	-1.123043	-1.266544	0.2067396	0.336536	-5.865459
SMG1P6	0.27844	0.9538525	1.2663784	0.2067989	0.3366176	-5.865665
RP11-762I7	-0.141908	2.4516169	-1.26619	0.2068661	0.336712	-5.865899
MIR23C	-0.131981	-1.396362	-1.266132	0.2068869	0.3367308	-5.865971
RNF151	-0.238709	-0.650382	-1.266043	0.2069188	0.3367557	-5.866082
RP11-62I21	-0.22087	-1.052146	-1.266038	0.2069206	0.3367557	-5.866088
AC097635.4	0.148146	-1.292259	1.2658894	0.2069736	0.3368268	-5.866272
ZNF280B	-0.30205	2.4780083	-1.265727	0.2070316	0.3369063	-5.866474
C2CD4D	0.2289614	2.8639808	1.2655416	0.2070979	0.3369991	-5.866704
RP11-692E1	0.0761529	-1.525337	1.2654012	0.2071481	0.3370658	-5.866879
CYCSP10	-0.272936	-0.046014	-1.265268	0.2071956	0.337128	-5.867043
RP11-470L1	0.1103858	-1.475725	1.265192	0.2072229	0.3371575	-5.867138
AC139712.2	-0.179551	-1.234708	-1.265126	0.2072465	0.3371809	-5.86722
CLEC17A	-0.336132	1.0604195	-1.265045	0.2072756	0.3372132	-5.867321
NICN1	0.0328814	5.7908808	1.2649315	0.2073161	0.337262	-5.867461

LRRC71	-0.284227	0.922291	-1.264909	0.2073241	0.337262	-5.867489
RP11-28201	-0.237257	-0.571857	-1.264844	0.2073474	0.3372849	-5.86757
NANOGNB	0.1148755	-1.447003	1.2648068	0.2073607	0.3372915	-5.867616
PPIL6	0.1494601	3.5323477	1.2647374	0.2073855	0.3373168	-5.867702
RP3-337H4.	-0.145706	3.1650201	-1.264628	0.2074246	0.3373653	-5.867838
RPS2P25	-0.136681	-1.343294	-1.264436	0.2074935	0.3374624	-5.868076
TRAM2	0.0305214	6.1438928	1.26436	0.2075206	0.3374914	-5.86817
TRDV3	0.1604247	-1.271667	1.2642805	0.2075491	0.3375227	-5.868269
FCRL2	-0.377769	1.6077066	-1.264254	0.2075585	0.337523	-5.868302
RP11-805F1	0.1249822	-1.433539	1.2637852	0.2077265	0.3377812	-5.868883
RP11-331F9	-0.252184	-0.504221	-1.263732	0.2077455	0.337797	-5.868949
RP11-12D24	-0.236259	-0.64577	-1.263583	0.207799	0.3378687	-5.869134
ENPP7P4	-0.276391	-0.11809	-1.263558	0.2078081	0.3378687	-5.869165
KPNA4	0.0182593	6.4610503	1.2634264	0.2078551	0.3379301	-5.869328
RP11-287F9	-0.127363	-1.395519	-1.263134	0.20796	0.3380856	-5.86969
TCP11L2	0.0667805	4.9373003	1.2629345	0.2080315	0.3381867	-5.869937
MTMR2	-0.03629	5.8140847	-1.262894	0.208046	0.3381952	-5.869987
NT5C1A	0.1776019	-1.203928	1.262789	0.2080837	0.3382414	-5.870117
RP13-126P2	-0.151731	-1.28453	-1.262645	0.2081354	0.3383104	-5.870296
RPS18P9	-0.056894	4.2688993	-1.262536	0.2081743	0.3383586	-5.87043
FXR1	-0.020204	6.5757387	-1.262383	0.2082295	0.3384332	-5.87062
CTB-39G8.3	0.2653045	2.2813719	1.2623262	0.2082498	0.3384378	-5.87069
SIGLECL1	-0.169826	-1.310385	-1.262319	0.2082523	0.3384378	-5.870699
RP11-806L2	-0.27018	-0.270664	-1.262297	0.2082601	0.3384378	-5.870726
ENSAP2	-0.282415	0.4829112	-1.262212	0.2082909	0.3384644	-5.870832
RP3-496C20	-0.254711	-0.332584	-1.2622	0.2082951	0.3384644	-5.870847
IL21	-0.163708	-1.294971	-1.262004	0.2083654	0.3385637	-5.871089
RP11-820I1	0.2670892	-0.547771	1.2617808	0.2084456	0.3386789	-5.871366
GALNT12	0.21963	3.8179188	1.2616107	0.2085067	0.3387631	-5.871576
SARS2	-0.039172	5.3974667	-1.261481	0.2085534	0.3388239	-5.871737
KLK14	-0.31117	1.0904956	-1.261269	0.2086295	0.3389275	-5.871999
AC093818.1	-0.120932	3.4953105	-1.261252	0.2086358	0.3389275	-5.87202
CTD-2105E1	-0.241445	-0.808927	-1.261201	0.2086538	0.3389418	-5.872082
TUBA3FP	0.2059181	2.2695718	1.2610596	0.2087048	0.3390095	-5.872258
CCDC132	0.0234651	5.8747612	1.2609815	0.2087329	0.3390401	-5.872355
GYG1	0.0290499	6.0372669	1.2608294	0.2087876	0.3391012	-5.872543
GNL3L	0.0542904	5.3026569	1.2608252	0.2087891	0.3391012	-5.872548
RP11-212I2	-0.2948	0.5673517	-1.260795	0.2088001	0.339104	-5.872586
PCCA-AS1	0.2075138	-0.908254	1.2603035	0.2089768	0.3393758	-5.873193
RP11-94C24	0.1309967	-1.338976	1.2602414	0.2089992	0.339397	-5.87327
AL023806.1	0.2734159	1.5402992	1.2601932	0.2090165	0.33941	-5.873329
CALB1	0.3361704	1.5019981	1.2601345	0.2090376	0.3394293	-5.873402
HTR5A-AS1	-0.177827	-1.362017	-1.260039	0.2090719	0.3394698	-5.873519
FZR1	-0.027742	6.350903	-1.259648	0.2092128	0.3396835	-5.874003
RPL9P31	0.0820668	-1.4948	1.2589514	0.2094638	0.3400759	-5.874863
TRIM29	0.2285518	3.6463616	1.2587353	0.2095417	0.3401873	-5.87513
C6orf195	-0.321018	0.1451002	-1.258708	0.2095514	0.3401879	-5.875163
TRAV22	-0.305917	-0.175041	-1.258678	0.2095623	0.3401905	-5.875201
RP11-318G2	0.3168837	-0.689435	1.258632	0.209579	0.340194	-5.875258
ASCL1	0.4438359	0.9743128	1.258607	0.209588	0.340194	-5.875289
PHBP4	-0.116375	-1.431972	-1.258595	0.2095925	0.340194	-5.875304

CD320	-0.041527	6.0960344	-1.258337	0.2096855	0.3403223	-5.875622
CTB-91J4.1	-0.187931	-1.078302	-1.258305	0.2096969	0.3403223	-5.875661
TFAP2B	-0.230655	-1.119978	-1.258291	0.2097021	0.3403223	-5.875679
ZMAT4	-0.319724	-0.593679	-1.258272	0.2097088	0.3403223	-5.875702
VASP	0.0258409	6.4999517	1.2580748	0.20978	0.3404227	-5.875946
IGBP1P1	-0.227259	-0.577049	-1.257955	0.2098231	0.3404775	-5.876093
SCARNA5	-0.269986	-0.422942	-1.257811	0.2098753	0.3405471	-5.876271
FBXO18	0.0197073	6.3069705	1.2577223	0.2099073	0.3405838	-5.87638
CICP8	-0.150914	-1.296706	-1.257555	0.2099676	0.3406666	-5.876587
RAB17	0.1065907	6.3505257	1.2574688	0.2099988	0.340702	-5.876693
RPL13P2	-0.273097	0.0081138	-1.257409	0.2100204	0.3407219	-5.876767
UBFD1	0.0251271	6.3018546	1.2572053	0.210094	0.3408262	-5.877018
AC009120.5	0.2862955	1.3306269	1.2568747	0.2102134	0.3409994	-5.877426
RPS15A	-0.028574	6.9468352	-1.256858	0.2102194	0.3409994	-5.877446
RP1-63G5.5	-0.357601	0.3220422	-1.256779	0.210248	0.3410305	-5.877544
SNORA77	0.2572083	-0.478479	1.2566603	0.2102909	0.3410795	-5.87769
RP11-486I1	-0.161809	3.2280943	-1.256644	0.2102968	0.3410795	-5.87771
RP11-87G24	0.1289738	-1.399049	1.2566094	0.2103093	0.3410846	-5.877753
RP11-196G1	-0.139034	3.0921569	-1.256397	0.2103861	0.3411939	-5.878014
SV2B	-0.239286	3.9886913	-1.256349	0.2104035	0.3412071	-5.878074
PLET1	0.1715138	-1.262635	1.2562777	0.2104293	0.3412336	-5.878162
RASA1	0.0399428	5.8898547	1.256191	0.2104607	0.3412584	-5.878269
CTD-2035E1	0.2262754	2.8340073	1.2561838	0.2104633	0.3412584	-5.878277
RP11-274B1	0.1465796	-1.332565	1.2556966	0.2106395	0.3415281	-5.878878
PPTC7	0.0312472	6.1384302	1.2556724	0.2106483	0.3415281	-5.878907
LGR4	-0.042703	6.526853	-1.255513	0.210706	0.3415992	-5.879104
KIAA1468	0.0245707	5.9517858	1.2554996	0.2107109	0.3415992	-5.87912
C3orf52	0.3065644	3.2813249	1.2554217	0.2107391	0.3416298	-5.879216
KRT72	-0.24952	-0.796502	-1.25538	0.2107543	0.3416393	-5.879268
RP3-369A17	0.2287785	-0.748021	1.2549201	0.2109208	0.3418939	-5.879834
AC005540.3	-0.293714	0.3414871	-1.254823	0.210956	0.3419359	-5.879954
KCNH7	-0.369883	0.749173	-1.254792	0.2109671	0.3419387	-5.879991
RP4-547N15	-0.063661	-1.523537	-1.254727	0.2109908	0.3419619	-5.880072
FMO10P	-0.158893	-1.407017	-1.254595	0.2110386	0.3420243	-5.880234
IKZF3	0.1006475	5.3064215	1.2544374	0.2110957	0.3421016	-5.880428
RP11-151F5	-0.259234	1.61506	-1.254347	0.2111285	0.3421129	-5.880539
TLR8	0.2025979	4.1566	1.2543421	0.2111302	0.3421129	-5.880545
C12orf10	-0.024617	6.2157438	-1.254341	0.2111308	0.3421129	-5.880547
NAV2-AS2	0.2404981	-0.855185	1.2542778	0.2111536	0.3421346	-5.880624
RP5-908D6.	0.1818129	-1.16929	1.254234	0.2111695	0.3421451	-5.880678
VAPA	0.0205535	6.6718244	1.2539557	0.2112704	0.3422846	-5.88102
AC010468.1	-0.212945	2.3118019	-1.253937	0.2112772	0.3422846	-5.881043
H2AFB1	0.1234244	-1.401877	1.2539192	0.2112837	0.3422846	-5.881065
RP11-552F3	0.1540293	3.5591154	1.2538666	0.2113027	0.3423003	-5.88113
NAB1	0.0278869	6.2021595	1.2537342	0.2113508	0.3423536	-5.881293
TBC1D14	0.0621829	5.701468	1.2537241	0.2113544	0.3423536	-5.881305
ASB14	-0.091122	3.7259662	-1.253668	0.2113749	0.3423716	-5.881375
RP4-800G7.	-0.265417	0.058587	-1.253537	0.2114223	0.3424197	-5.881535
SLC39A7	-0.025399	6.8498712	-1.253534	0.2114233	0.3424197	-5.881539
OR7E28P	0.1454038	-1.439759	1.2534698	0.2114467	0.3424251	-5.881618
RP11-35015	0.1301911	-1.372806	1.2534636	0.211449	0.3424251	-5.881626

GNLY	0.0975328	4.9050464	1.2534475	0.2114548	0.3424251	-5.881645
HNRNPA1P45	-0.150658	-1.231076	-1.253273	0.211518	0.3425123	-5.88186
AC008697.1	0.2102317	-1.047162	1.2532395	0.2115303	0.342517	-5.881901
PRPS1	0.0328105	6.2821683	1.2531054	0.211579	0.3425806	-5.882066
SNORA71B	-0.29347	0.8475743	-1.253039	0.2116031	0.3426034	-5.882148
CES3	-0.152688	5.7824056	-1.252986	0.2116222	0.3426034	-5.882212
SCAMP1-AS1	-0.037673	5.21139	-1.252982	0.2116238	0.3426034	-5.882218
PRKRIRP8	-0.120924	-1.442487	-1.252963	0.2116306	0.3426034	-5.882241
RP11-227G1	0.184798	-1.024112	1.2528698	0.2116646	0.3426432	-5.882356
MUTYH	-0.038378	5.6682825	-1.252659	0.2117411	0.3427443	-5.882614
CLCNKB	0.3389087	0.2076142	1.25263	0.2117517	0.3427443	-5.88265
RP11-1059L	-0.093803	-1.508756	-1.25262	0.2117552	0.3427443	-5.882662
CTD-2066L2	0.0859637	-1.488055	1.2524876	0.2118035	0.3428072	-5.882825
COX7BP2	-0.086348	-1.490507	-1.252452	0.2118164	0.3428129	-5.882869
C19orf71	0.09903	4.1104186	1.2523207	0.2118641	0.342875	-5.88303
IGHV3-15	0.3682863	3.785379	1.2519807	0.2119878	0.3430598	-5.883448
RP11-700N1	-0.099191	-1.462304	-1.251847	0.2120364	0.3431234	-5.883612
PHRF1	0.0205905	6.3675024	1.2517262	0.2120803	0.3431792	-5.88376
AP000223.4	-0.273718	1.2041311	-1.251552	0.2121438	0.3432667	-5.883975
ZFPL1	-0.027911	5.5095809	-1.251454	0.2121795	0.3433093	-5.884095
MAOB	0.0701517	6.7881656	1.2514092	0.2121957	0.3433202	-5.88415
MEGF8	0.034812	6.2923854	1.2513642	0.212212	0.3433314	-5.884205
COPS7A	-0.024124	6.4717496	-1.251248	0.2122542	0.3433844	-5.884347
AC010492.5	0.1582279	-1.242971	1.2510788	0.2123159	0.3434691	-5.884555
UBA52	-0.026215	7.064377	-1.251025	0.2123355	0.3434772	-5.884621
NRCAM	-0.235836	4.7093128	-1.251013	0.2123397	0.3434772	-5.884635
CLDN8	0.2325592	-1.011138	1.2508788	0.2123887	0.3435412	-5.884801
MAPK9	-0.023904	6.2427036	-1.250833	0.2124055	0.3435531	-5.884857
IGHV3-62	-0.261816	-0.680678	-1.250534	0.2125143	0.3437071	-5.885224
ZNF763	-0.10723	4.2123612	-1.25052	0.2125195	0.3437071	-5.885241
LINC01031	0.2048084	-0.997378	1.2503891	0.2125671	0.3437688	-5.885401
RP11-82019	-0.190501	-0.975795	-1.250143	0.2126568	0.3438986	-5.885703
BTG4	0.0872161	-1.48342	1.2499686	0.2127204	0.3439862	-5.885917
BCL11A	-0.188149	4.0493929	-1.249836	0.2127688	0.3440492	-5.88608
LINC01412	0.1651531	-1.234625	1.2496948	0.2128202	0.3441171	-5.886253
PAUPAR	0.0994082	-1.473413	1.2496153	0.2128492	0.3441488	-5.88635
RP11-532L1	-0.310805	0.1944864	-1.249534	0.2128789	0.3441745	-5.88645
CTC-471C19	0.1991579	-1.159399	1.24952	0.2128839	0.3441745	-5.886467
RP11-51B23	0.2463821	-0.641464	1.2494496	0.2129096	0.3442008	-5.886553
SLC25A17	0.0253756	6.0186303	1.2492931	0.2129667	0.3442778	-5.886745
POLR1E	0.0354054	5.9123624	1.2492404	0.212986	0.3442863	-5.88681
AP000568.2	0.1629933	-1.253809	1.2492271	0.2129908	0.3442863	-5.886826
HCG4P11	0.2760036	2.1380405	1.2489261	0.2131007	0.3444486	-5.887195
VN1R28P	0.1610182	-1.344585	1.2488674	0.2131221	0.344468	-5.887267
CTD-2349P2	-0.292881	0.5329458	-1.248759	0.2131615	0.3445165	-5.887399
PJA1	0.0418245	5.8302363	1.2487273	0.2131732	0.3445201	-5.887438
RP11-113I2	-0.207186	-1.140683	-1.248647	0.2132026	0.3445523	-5.887537
CBLL1	-0.027071	6.0107132	-1.248216	0.2133602	0.3447917	-5.888065
RP11-350G8	0.1520387	3.4037892	1.2481431	0.2133867	0.3448192	-5.888154
UNC79	0.2077058	2.7046657	1.248049	0.2134211	0.3448576	-5.888269
AC023115.2	-0.300141	-0.220771	-1.248026	0.2134293	0.3448576	-5.888297

TMEM235	0.1125904	-1.399085	1.2479545	0.2134556	0.3448848	-5.888385
RP11-166N1	-0.234245	1.7267462	-1.247879	0.2134832	0.3448931	-5.888478
CTD-3105H1	-0.17999	-1.113399	-1.247877	0.213484	0.3448931	-5.88848
PRKACA	0.0318599	6.6720969	1.247863	0.213489	0.3448931	-5.888497
RP11-800A1	0.2335712	-0.655051	1.2477916	0.2135151	0.3449199	-5.888584
AL121893.2	-0.108314	-1.40944	-1.247704	0.213547	0.3449561	-5.888691
CXCR6	0.1313058	4.8685806	1.2473476	0.2136775	0.3451517	-5.889128
RP11-359J1	-0.307054	0.6973296	-1.247258	0.2137104	0.3451895	-5.889238
PRDM14	-0.161285	-1.30517	-1.247206	0.2137293	0.3452048	-5.889301
PSMC1P9	0.2573843	-0.06357	1.2471716	0.2137419	0.3452098	-5.889343
SNORD116-2	-0.247138	-0.679264	-1.247046	0.2137878	0.3452688	-5.889497
GAS5-AS1	-0.066057	4.1095507	-1.246986	0.2138099	0.3452892	-5.889571
LL22NC03-8	-0.106992	-1.502288	-1.246947	0.213824	0.3452967	-5.889618
MRPL51	-0.029084	6.5433383	-1.246892	0.2138441	0.3453138	-5.889685
DSCAML1	-0.334547	3.0583191	-1.246853	0.2138583	0.3453214	-5.889732
RP11-597A1	-0.104226	-1.48174	-1.246811	0.2138738	0.3453312	-5.889784
CTD-2350J1	-0.138299	-1.396353	-1.24676	0.2138925	0.3453462	-5.889847
LIN7B	-0.064161	4.9956079	-1.246703	0.2139134	0.3453647	-5.889916
LINC00235	-0.177195	3.2972339	-1.246636	0.2139379	0.3453889	-5.889998
RP11-430C1	0.1084405	-1.463355	1.2465039	0.2139862	0.3454517	-5.89016
NUDT19	0.0518335	5.7878549	1.2464758	0.2139965	0.345453	-5.890194
LINC01021	0.4207763	2.9761728	1.2463391	0.2140466	0.3454936	-5.890361
GPR171	0.1984405	3.9088684	1.2463309	0.2140496	0.3454936	-5.890371
RP11-993B2	-0.229422	-0.938616	-1.24633	0.21405	0.3454936	-5.890373
FAM230A	-0.10812	-1.43602	-1.246187	0.2141022	0.345556	-5.890547
AC007386.2	-0.174098	-1.179777	-1.246172	0.2141077	0.345556	-5.890565
C11orf44	0.0816077	-1.528012	1.245982	0.2141774	0.3456533	-5.890798
LINC00431	0.2778953	-0.703897	1.2459088	0.2142042	0.3456812	-5.890887
FCRL3	-0.291464	3.4050754	-1.245758	0.2142594	0.345755	-5.891071
TNIP3	0.3506954	1.5724924	1.2456237	0.2143087	0.3458193	-5.891236
SOCS6	0.0331153	6.1322919	1.2455754	0.2143264	0.3458287	-5.891295
WDR26	-0.023163	6.642794	-1.245556	0.2143335	0.3458287	-5.891318
AL713999.1	-0.248266	-0.551668	-1.245487	0.2143589	0.3458545	-5.891403
BCL2L15	0.2415848	2.802043	1.2452877	0.2144319	0.3459569	-5.891646
RGS5	0.0426622	6.8597966	1.2450968	0.2145019	0.3460421	-5.89188
COX6CP16	-0.22647	-0.99887	-1.245074	0.2145104	0.3460421	-5.891908
ERICH3	0.3561661	1.4970912	1.2450662	0.2145131	0.3460421	-5.891917
LILRA6	0.1223843	4.1466377	1.2448135	0.2146058	0.3461592	-5.892225
HDDC2	0.0336701	5.9031723	1.2448096	0.2146073	0.3461592	-5.89223
RP11-9D8.1	-0.267617	-0.227595	-1.244778	0.2146188	0.3461592	-5.892269
CLTCL1	0.0657366	5.6704813	1.244765	0.2146236	0.3461592	-5.892285
SNORA55	-0.281831	0.0958135	-1.244664	0.2146608	0.3462038	-5.892408
RP11-739N1	-0.163343	-1.308586	-1.244581	0.214691	0.3462362	-5.892509
RP11-442J2	0.1673701	-1.334107	1.2445573	0.2146998	0.3462362	-5.892538
RP11-47A17	-0.314083	1.1152969	-1.244305	0.2147924	0.3463618	-5.892846
ZNF846	0.0433167	5.2389468	1.244288	0.2147987	0.3463618	-5.892867
RN7SL336P	-0.266804	-0.130625	-1.244268	0.2148061	0.3463618	-5.892892
SFMBT1	-0.033219	5.738642	-1.244114	0.2148626	0.3464269	-5.89308
RBM45	-0.019854	5.5665536	-1.244106	0.2148655	0.3464269	-5.893089
REPS1	0.0307283	5.9947869	1.2437033	0.2150135	0.3466501	-5.893581
SLIRP	-0.035266	6.2140186	-1.243314	0.2151564	0.3468652	-5.894055

RP11-447H1	-0.207933	-0.860665	-1.243136	0.2152221	0.3469558	-5.894273
RP11-843P1	0.2742892	2.1183612	1.2430517	0.215253	0.3469852	-5.894376
RP11-492E3	-0.384625	2.5561605	-1.243021	0.2152641	0.3469852	-5.894413
ZDHHC24	0.0328562	5.9860335	1.2430084	0.2152689	0.3469852	-5.894428
MAP2K1P1	-0.192702	-1.01107	-1.242787	0.2153504	0.3471014	-5.894699
ZBTB40-IT1	-0.232247	-0.619054	-1.24267	0.2153933	0.3471552	-5.894841
AC034228.4	-0.094182	-1.486665	-1.242627	0.2154091	0.3471654	-5.894893
RPL17P33	-0.191687	-1.004504	-1.242535	0.2154432	0.3471844	-5.895006
SENP3	-0.028318	5.8360499	-1.242507	0.2154533	0.3471844	-5.895039
PLA2G4B	-0.233607	2.4490272	-1.242493	0.2154584	0.3471844	-5.895056
C3orf49	-0.248297	1.1812509	-1.242492	0.215459	0.3471844	-5.895059
INHBC	-0.175704	5.941353	-1.242271	0.2155403	0.3473001	-5.895328
UBIAD1	0.0288013	6.0945957	1.2421531	0.2155836	0.3473545	-5.895471
USP25	0.0270901	6.0759178	1.242035	0.2156271	0.3473943	-5.895615
KRT8P46	-0.297712	1.3312433	-1.242034	0.2156273	0.3473943	-5.895616
RN7SL580P	0.1078882	-1.446327	1.2419781	0.215648	0.3474014	-5.895684
MPO	0.3191573	1.4712688	1.2419706	0.2156508	0.3474014	-5.895693
FRMD7	0.3468409	-0.077143	1.2418876	0.2156813	0.3474353	-5.895795
GLYATL1P2	-0.140653	-1.319442	-1.241823	0.2157051	0.3474583	-5.895873
RP11-510I2	0.0573004	-1.55295	1.2417743	0.2157231	0.3474692	-5.895933
SOHLH2	-0.447248	0.0861766	-1.241753	0.2157309	0.3474692	-5.895959
SNORD88	-0.181658	-1.118892	-1.241604	0.2157858	0.3475373	-5.89614
AC004549.6	-0.215617	-0.774652	-1.241586	0.2157923	0.3475373	-5.896161
RP11-459D2	0.3401236	0.9257734	1.2415383	0.21581	0.3475505	-5.89622
GPRC5C	0.0318294	6.7039528	1.2414659	0.2158367	0.3475781	-5.896308
RN7SKP269	-0.275599	-0.071103	-1.241286	0.215903	0.3476696	-5.896527
AKR7A2P1	0.3007563	0.2187381	1.2410446	0.2159919	0.3477975	-5.896821
CTB-174021	-0.199669	-0.904711	-1.241001	0.216008	0.347808	-5.896874
FAM86C1	-0.039055	5.2201605	-1.240949	0.2160271	0.3478097	-5.896938
GS1-21A4.2	-0.361627	1.9655459	-1.240947	0.2160281	0.3478097	-5.896941
PAK6	-0.281013	-0.534603	-1.240876	0.2160541	0.3478362	-5.897027
SLC6A5	-0.174185	-1.22954	-1.240743	0.2161031	0.3478893	-5.897188
RIOK2	0.02385	5.9330404	1.2407348	0.2161061	0.3478893	-5.897198
RP11-4C20.	0.3009678	0.3135123	1.2406487	0.2161379	0.3479251	-5.897303
PRKCSH	-0.022396	7.0522134	-1.240009	0.2163741	0.34829	-5.898082
RP11-14802	0.31247	-0.250948	1.239812	0.2164466	0.3483914	-5.898321
AC004069.2	-0.273767	0.8133759	-1.239683	0.2164944	0.3484529	-5.898479
RP11-17G12	0.1739504	-1.176358	1.2396104	0.2165211	0.3484805	-5.898567
RP11-638F5	0.298593	-0.806566	1.2395197	0.2165546	0.3485191	-5.898677
RP4-612B18	-0.117307	-1.387705	-1.239489	0.2165659	0.3485219	-5.898714
ELF1	0.0348664	6.1733189	1.2393378	0.2166218	0.3485964	-5.898898
LRRC18	-0.234531	-0.915992	-1.239024	0.2167378	0.3487677	-5.89928
SLC25A13	-0.04152	6.6094147	-1.238673	0.2168674	0.3489504	-5.899706
RPS29P12	0.1230438	-1.410116	1.238665	0.2168704	0.3489504	-5.899716
EEF1D	-0.029188	6.713363	-1.238572	0.2169047	0.3489902	-5.899829
SEC23IP	-0.021589	6.232613	-1.238483	0.2169378	0.3490281	-5.899937
NDUFA11	-0.032631	6.4264076	-1.238421	0.2169608	0.3490482	-5.900013
RP11-83C7.	-0.185143	-1.172414	-1.238397	0.2169695	0.3490482	-5.900041
RP11-10102	0.0995244	-1.482349	1.2383682	0.2169802	0.3490501	-5.900077
CTB-134H23	0.2633038	-0.549954	1.2383234	0.2169968	0.3490614	-5.900131
TMEM238	0.0946778	5.1396015	1.238287	0.2170102	0.3490676	-5.900175

ZNF527	-0.049577	5.1279282	-1.238188	0.2170468	0.3491111	-5.900295
RP11-387H1	-0.160347	-1.177935	-1.238097	0.2170806	0.3491501	-5.900406
PARD6G-AS1	-0.10609	4.6154616	-1.237896	0.2171551	0.3492544	-5.900651
RCOR1	-0.024974	6.0504887	-1.237851	0.2171715	0.3492643	-5.900705
RP11-1191J	0.2747165	1.6812014	1.2378248	0.2171812	0.3492643	-5.900737
TREML4	0.3678205	0.0451329	1.2378014	0.2171899	0.3492643	-5.900765
RP11-3304.	-0.287933	1.6096039	-1.23767	0.2172385	0.3493213	-5.900925
OR11N1P	-0.145844	-1.311039	-1.237606	0.2172624	0.3493213	-5.901003
GLIS2-AS1	-0.270992	3.0841184	-1.237604	0.2172629	0.3493213	-5.901005
GPR133	-0.149157	5.452929	-1.237602	0.2172636	0.3493213	-5.901007
AC005785.5	-0.263435	1.3784377	-1.237522	0.2172932	0.3493535	-5.901104
TMC3	-0.270726	1.7669049	-1.237352	0.2173563	0.3494396	-5.901311
KRT18P28	0.2436452	1.7136515	1.237215	0.217407	0.3495057	-5.901477
TFDP3	-0.393166	-0.478296	-1.237068	0.2174614	0.3495777	-5.901655
GAPDHP63	-0.266475	2.2452622	-1.236947	0.2175064	0.3496346	-5.901803
PEX5	-0.03011	6.4012859	-1.23687	0.2175348	0.349665	-5.901896
DGCR12	0.2609929	-0.115768	1.2367432	0.2175818	0.349725	-5.90205
RP11-122M1	-0.127228	-1.443975	-1.236623	0.2176265	0.3497816	-5.902196
AC007163.6	0.1702584	-1.187488	1.2365891	0.2176389	0.3497861	-5.902237
RP11-761E2	0.2956679	0.1662544	1.2363664	0.2177215	0.3499033	-5.902507
FERMT2	0.0344125	6.3818331	1.2362566	0.2177622	0.3499534	-5.90264
RP11-360O1	-0.11797	-1.394332	-1.235865	0.2179076	0.3501716	-5.903115
SNCB	-0.286796	-0.369501	-1.235824	0.2179227	0.3501805	-5.903165
NSUN6	0.0477019	5.9938897	1.2357879	0.217936	0.3501865	-5.903208
HRCT1	-0.147228	4.8628458	-1.235748	0.217951	0.3501951	-5.903257
IGHD2-2	-0.11935	-1.408608	-1.235623	0.2179972	0.3502358	-5.903408
GOLGA8VP	0.284177	-0.167584	1.2356059	0.2180036	0.3502358	-5.903429
RP11-425D1	-0.265563	1.0298851	-1.235581	0.218013	0.3502358	-5.90346
RP11-421E1	-0.11955	-1.36012	-1.235576	0.2180147	0.3502358	-5.903465
RP11-491F9	-0.322911	0.5024302	-1.235395	0.2180819	0.3503283	-5.903685
IRGQ	-0.027902	6.0847305	-1.235361	0.2180946	0.3503333	-5.903726
ADGB	-0.234805	-0.921185	-1.235334	0.2181043	0.3503336	-5.903758
SGCG	0.2564767	-0.706905	1.2351639	0.2181677	0.3504199	-5.903965
BCRP1	-0.192927	-1.068956	-1.234993	0.2182311	0.3505064	-5.904172
CTD-2545G1	-0.225207	-0.682541	-1.234927	0.2182557	0.3505304	-5.904252
RP11-508N1	0.127475	-1.378427	1.2347933	0.2183053	0.3505947	-5.904414
ZNF711	-0.183624	4.3595648	-1.234625	0.2183678	0.3506796	-5.904618
SLC25A6P4	-0.153791	-1.253503	-1.234552	0.2183949	0.3506953	-5.904706
HIST1H2BB	-0.175197	-1.152256	-1.234547	0.2183968	0.3506953	-5.904712
RP1-212P9.	-0.195246	-0.992664	-1.234507	0.2184117	0.3507038	-5.904761
GEMIN5	-0.02457	5.9691042	-1.234436	0.2184381	0.3507239	-5.904847
TTF1	-0.026879	5.5431803	-1.234413	0.2184466	0.3507239	-5.904874
SLC10A4	-0.325363	0.3648991	-1.234396	0.218453	0.3507239	-5.904895
C21orf62-A	-0.135406	3.4861628	-1.234248	0.218508	0.3507968	-5.905075
DCAF8	0.0275713	6.5583497	1.2341401	0.2185481	0.3508456	-5.905205
IPO11	0.023595	5.8712452	1.2340702	0.218574	0.3508719	-5.90529
RP11-55609	0.2018191	-1.058409	1.2337057	0.2187096	0.3510741	-5.905731
KLHL33	0.2685534	-0.148443	1.2335398	0.2187713	0.3511577	-5.905932
CNTLN	-0.052584	5.7803619	-1.233463	0.2188	0.3511685	-5.906025
TRAV14DV4	-0.321365	0.5137914	-1.233458	0.2188019	0.3511685	-5.906031
RP11-115D7	-0.128051	-1.324888	-1.233444	0.2188069	0.3511685	-5.906048

DPY19L4	-0.031454	6.1114982	-1.233412	0.2188189	0.3511723	-5.906087
IGLV2-11	0.3235732	4.0345686	1.2333589	0.2188386	0.3511784	-5.906151
SPTLC1	0.0334788	5.9644617	1.2333499	0.218842	0.3511784	-5.906162
TOMM34	0.0274786	6.1672866	1.2332791	0.2188683	0.3512053	-5.906247
RIT1	-0.026766	5.9157342	-1.233202	0.2188971	0.351236	-5.906341
TMEM212-AS	-0.12103	-1.453967	-1.233135	0.2189218	0.3512603	-5.906421
RP11-43N5.	-0.296749	-0.071713	-1.232973	0.2189823	0.3513225	-5.906618
CTC-529I10	-0.188974	2.3755655	-1.232963	0.2189862	0.3513225	-5.90663
AC004623.3	0.232657	2.0732014	1.2329216	0.2190014	0.3513225	-5.90668
HIST2H3C	-0.24621	-0.869354	-1.232921	0.2190018	0.3513225	-5.906681
RP11-650P1	-0.266388	-0.71431	-1.232902	0.2190087	0.3513225	-5.906704
CTC-296K1.	-0.36127	0.2648983	-1.232866	0.2190222	0.3513287	-5.906747
CTD-2521M2	0.116579	-1.459208	1.232783	0.2190531	0.3513401	-5.906848
RAB25	0.4170583	2.5453675	1.2327802	0.2190541	0.3513401	-5.906851
RP11-114H2	0.233482	-0.694895	1.2327691	0.2190582	0.3513401	-5.906864
LINC00592	0.2212241	-0.993091	1.232721	0.2190761	0.3513534	-5.906922
MARVELD2	-0.067326	6.1859622	-1.232677	0.2190927	0.3513645	-5.906976
AP3B2	-0.391206	1.425492	-1.23262	0.2191137	0.3513829	-5.907045
FAM3A	0.0326896	6.4331214	1.2324894	0.2191624	0.3514453	-5.907203
RPL7AP26	-0.235246	-0.563792	-1.232464	0.2191719	0.3514453	-5.907233
RP11-342D1	0.1541184	-1.245535	1.2323742	0.2192053	0.3514834	-5.907342
RNU4-52P	0.1856456	-1.036926	1.2322952	0.2192347	0.3515151	-5.907437
CCDC54	0.2421277	-0.6208	1.2321398	0.2192927	0.3515926	-5.907625
FAF1	0.0237719	6.2925948	1.2320325	0.2193326	0.3516412	-5.907755
DDR1	-0.085165	6.1413315	-1.232	0.2193449	0.3516454	-5.907795
RP5-952N6.	0.188315	-1.224577	1.2317416	0.2194411	0.3517842	-5.908107
CTD-2006C1	-0.080832	4.4988502	-1.231715	0.219451	0.3517846	-5.908139
RP1-111C20	0.1712409	2.9148948	1.2315393	0.2195166	0.3518743	-5.908351
RP11-416H1	-0.147877	-1.244871	-1.231488	0.2195357	0.3518895	-5.908413
TUBB8P2	-0.147066	-1.264487	-1.23144	0.2195536	0.3519027	-5.908471
SMG1P3	0.1795352	3.3657781	1.2312155	0.2196373	0.3520215	-5.908742
TNFAIP8L1	-0.040907	6.4097363	-1.231137	0.2196666	0.3520529	-5.908837
CTC-551A13	0.1131167	-1.430148	1.2309281	0.2197446	0.3521625	-5.90909
RNU6-388P	0.1502645	-1.236896	1.2308129	0.2197876	0.3522159	-5.909229
PTCSC3	0.0783819	-1.538652	1.2306501	0.2198484	0.3522979	-5.909425
C6orf229	0.2495646	-0.577069	1.230505	0.2199026	0.3523692	-5.9096
AL844165.1	0.1122863	-1.443563	1.2304417	0.2199262	0.3523917	-5.909677
SBDSP1	0.0364684	5.6439333	1.2303298	0.219968	0.3524293	-5.909812
IL9R	0.2994722	0.8966856	1.2303272	0.219969	0.3524293	-5.909815
RP11-5407.	-0.133052	-1.353499	-1.230221	0.2200086	0.3524772	-5.909943
ANKRD27	-0.035266	5.9823433	-1.230167	0.2200287	0.352487	-5.910008
SNX18P26	-0.232658	-0.950117	-1.230153	0.220034	0.352487	-5.910025
RP11-368I2	-0.358289	0.0216925	-1.23001	0.2200876	0.3525574	-5.910198
CTD-2553L1	-0.085059	4.0791438	-1.229846	0.2201487	0.3526399	-5.910396
RP11-20I23	0.2204189	-0.71886	1.2298007	0.2201657	0.3526437	-5.910451
IGKV1OR2-1	-0.221902	-1.121526	-1.229788	0.2201705	0.3526437	-5.910466
CCL1	0.1578621	-1.265239	1.2297615	0.2201804	0.3526441	-5.910498
SEMA3B-AS1	-0.259123	2.6808534	-1.229732	0.2201915	0.3526464	-5.910534
CCDC174	-0.019605	5.8816285	-1.229572	0.2202513	0.3527267	-5.910727
CTC-325H20	-0.121409	3.2142192	-1.229444	0.2202992	0.352788	-5.910881
INSM2	-0.184043	-1.15957	-1.229323	0.2203443	0.352831	-5.911027

RP5-988G15	-0.097902	-1.449931	-1.229308	0.2203498	0.352831	-5.911044
XXbac-BPG3	-0.132189	-1.335753	-1.229287	0.2203577	0.352831	-5.91107
RP11-588G2	0.2468174	-0.531546	1.2292685	0.2203647	0.352831	-5.911093
BEST1	-0.084816	4.8106981	-1.229057	0.2204438	0.3529422	-5.911348
MINK1	0.0233556	6.3873472	1.2289686	0.2204769	0.3529797	-5.911454
RP11-981G7	0.2609396	-0.6315	1.2287973	0.220541	0.3530668	-5.911661
MIR5006	-0.150539	-1.218974	-1.228725	0.220568	0.3530945	-5.911748
MIR4450	-0.110357	-1.430344	-1.228466	0.2206649	0.3532342	-5.91206
CRYM-AS1	0.2757956	0.337001	1.2282469	0.2207471	0.3533502	-5.912324
THAP11	-0.023653	5.9418961	-1.228065	0.2208152	0.3534355	-5.912544
RP4-724E13	-0.280031	1.5532077	-1.228053	0.2208198	0.3534355	-5.912558
TRBV15	-0.311663	0.130035	-1.227904	0.2208754	0.3535092	-5.912737
COPZ1	-0.019661	6.7390337	-1.227831	0.2209029	0.3535377	-5.912826
RP11-122C2	-0.092272	-1.509507	-1.227801	0.2209142	0.3535403	-5.912862
RLN3	-0.283236	-0.789427	-1.227659	0.2209674	0.3536099	-5.913033
RHAG	0.2697018	-0.835915	1.2273828	0.2210708	0.3537471	-5.913365
EXD2	0.027077	5.8179112	1.2273717	0.221075	0.3537471	-5.913379
SVIL-AS1	-0.0523	5.7028388	-1.227352	0.2210823	0.3537471	-5.913402
ARIH2	-0.024929	6.3724637	-1.22728	0.2211094	0.3537751	-5.913489
SPATA8	-0.234123	-1.052114	-1.227136	0.2211634	0.353846	-5.913663
AC005901.1	-0.086348	-1.490507	-1.22711	0.2211733	0.3538462	-5.913694
ST7-AS2	-0.174379	-1.155068	-1.226834	0.2212767	0.3539961	-5.914026
CTD-2012K1	0.2679607	0.4308156	1.2267919	0.2212924	0.3540058	-5.914077
RN7SL39P	0.079778	-1.525956	1.2266545	0.221344	0.3540727	-5.914242
RP11-817J1	0.1912407	-1.156209	1.2263636	0.2214531	0.3542206	-5.914592
AC016753.7	0.1532453	-1.248892	1.2263565	0.2214558	0.3542206	-5.914601
TMEM150B	-0.131707	5.3506507	-1.226123	0.2215435	0.3543383	-5.914882
RP11-57C13	-0.296231	0.9916954	-1.226109	0.2215488	0.3543383	-5.914899
RP11-849F2	-0.154485	2.8363371	-1.226018	0.2215827	0.354377	-5.915008
TSPY1	-0.22337	-1.200765	-1.22593	0.2216158	0.3543859	-5.915114
ADIPOR2	0.030738	6.6684699	1.2259222	0.2216189	0.3543859	-5.915124
RP11-401P9	-0.252858	3.7111064	-1.225906	0.221625	0.3543859	-5.915143
TRIM16L	0.1063074	5.6625087	1.2258964	0.2216285	0.3543859	-5.915155
HINT1	0.0290209	6.9734759	1.2258744	0.2216368	0.3543859	-5.915181
TPBGL	-0.200611	3.5154789	-1.225552	0.221758	0.3545642	-5.915569
RNU1-75P	0.2712309	-0.46408	1.225211	0.2218861	0.3547534	-5.915979
IFT20	-0.02363	5.992956	-1.22505	0.2219465	0.3548345	-5.916172
OTUD6B	-0.04132	5.6398141	-1.224959	0.2219809	0.3548738	-5.916282
RNFT1P2	-0.179541	-1.141354	-1.224843	0.2220243	0.3549277	-5.916421
AC093620.5	-0.265345	1.0823924	-1.224713	0.2220734	0.3549907	-5.916578
RHOBTB1	-0.078645	5.7663951	-1.224499	0.2221539	0.3551038	-5.916835
RP4-601P9.	-0.381655	1.8000795	-1.224045	0.2223248	0.3553615	-5.917381
RP11-350E1	-0.354759	1.2214899	-1.223971	0.2223524	0.35539	-5.917468
RN7SL652P	-0.167824	-1.091814	-1.223563	0.2225062	0.3556202	-5.917959
MAPK1	-0.019903	6.6015478	-1.223525	0.2225204	0.3556273	-5.918004
CORIN	-0.212937	3.2546186	-1.22347	0.2225414	0.3556453	-5.918071
RP11-455F5	-0.093207	4.0719203	-1.223427	0.2225575	0.3556555	-5.918123
LINC00929	0.0652778	-1.54533	1.2233934	0.22257	0.35566	-5.918162
AKT2	0.0250127	6.6866095	1.2232647	0.2226186	0.3557219	-5.918317
EIF3J	0.022405	6.4031742	1.2231835	0.2226491	0.3557552	-5.918414
CD1C	0.1599949	4.3221794	1.2230769	0.2226893	0.3558039	-5.918542

IGHV3-60	-0.264755	-0.725361	-1.222914	0.2227507	0.3558863	-5.918738
ATP13A5	0.1631767	-1.217003	1.2228181	0.2227869	0.3559285	-5.918853
LA16c-313D	-0.181636	2.900305	-1.222755	0.2228106	0.3559509	-5.918929
RP11-206L1	-0.285252	1.5506113	-1.222569	0.2228809	0.3560353	-5.919152
ADAM7	0.1031471	-1.470543	1.2225446	0.22289	0.3560353	-5.919181
AL008723.1	0.1923636	-0.95919	1.2225375	0.2228927	0.3560353	-5.91919
AATK	-0.11967	4.8182846	-1.222502	0.2229061	0.3560411	-5.919232
RP11-556H2	0.1639734	-1.152541	1.2224235	0.2229356	0.3560621	-5.919326
RP11-474J1	-0.167851	-1.181563	-1.222415	0.2229387	0.3560621	-5.919336
SLC24A1	0.0490063	5.4842971	1.2219297	0.223122	0.3563323	-5.919919
RP11-184M1	0.1903961	-1.140976	1.2219106	0.2231292	0.3563323	-5.919942
AC092687.4	0.0662053	-1.530407	1.2218655	0.2231462	0.3563323	-5.919996
SREK1IP1	-0.024707	6.0022425	-1.221864	0.2231469	0.3563323	-5.919998
RP11-452L6	0.2670118	-0.459237	1.2218278	0.2231604	0.3563382	-5.920041
IGHV3-38	-0.312228	-0.180742	-1.221591	0.2232499	0.3564609	-5.920325
AC025048.1	0.159979	-1.173344	1.2215615	0.2232609	0.3564609	-5.92036
SLC25A47	0.2593141	5.6035966	1.2215466	0.2232665	0.3564609	-5.920378
C19orf57	-0.087571	4.6848493	-1.221513	0.2232792	0.3564655	-5.920418
PRTG	0.2716107	3.1703226	1.2214838	0.2232903	0.3564676	-5.920453
RP11-446E2	-0.218083	-0.903104	-1.221374	0.2233317	0.3565181	-5.920585
PPP4C	0.0253408	6.5773055	1.2213172	0.2233532	0.3565368	-5.920653
RNF103	0.0315402	6.4215975	1.2212505	0.2233784	0.3565615	-5.920733
JTB	-0.026542	6.8022092	-1.221201	0.2233971	0.3565758	-5.920792
NRIP1	0.0425505	6.107633	1.2210724	0.2234457	0.3566377	-5.920946
RPL12	-0.030126	7.0431559	-1.220925	0.2235014	0.356711	-5.921123
RIC3	0.3296985	2.8275192	1.2208146	0.223543	0.3567619	-5.921255
RP11-539L1	-0.146843	4.1834743	-1.220701	0.223586	0.3568148	-5.921391
RNU4-47P	-0.266339	1.2402198	-1.220592	0.223627	0.3568648	-5.921521
RP4-816N1.	-0.23541	-0.611009	-1.2205	0.2236619	0.3569048	-5.921632
RP11-27G24	-0.18222	-1.085889	-1.220428	0.2236892	0.3569327	-5.921718
TTPA	0.1653669	5.9904838	1.2203254	0.223728	0.356979	-5.921841
SEL1L2	0.1947344	-1.089807	1.2202338	0.2237626	0.3570187	-5.921951
LINC00114	-0.346259	0.3618086	-1.220106	0.223811	0.3570684	-5.922104
TERF1P4	-0.134783	-1.30884	-1.2201	0.2238133	0.3570684	-5.922111
PBX4	0.2129913	3.3464259	1.2198767	0.2238977	0.3571874	-5.922378
OR7E5P	-0.091184	-1.51004	-1.219672	0.223975	0.3572951	-5.922623
RP11-16005	0.123716	4.5138215	1.2196135	0.2239973	0.357315	-5.922693
PRKAA2	-0.178203	5.4473927	-1.219361	0.224093	0.3574521	-5.922996
CD40LG	0.1623012	3.9765698	1.2193225	0.2241075	0.3574538	-5.923042
RP11-249L2	-0.331283	0.2524201	-1.219306	0.2241136	0.3574538	-5.923061
PBDC1	0.0307971	5.9184384	1.219247	0.224136	0.3574557	-5.923132
TTC39C-AS1	0.2783162	2.4347518	1.2192399	0.2241387	0.3574557	-5.923141
RP11-490D1	-0.124158	-1.37134	-1.219202	0.224153	0.3574557	-5.923186
LINC00508	-0.143396	-1.41134	-1.2192	0.224154	0.3574557	-5.923189
RP11-662I1	0.1793989	-1.226415	1.2191406	0.2241763	0.3574671	-5.923259
GSTO3P	-0.15696	-1.213932	-1.219129	0.2241808	0.3574671	-5.923273
CCDC47	0.0238039	6.6948785	1.2188763	0.2242764	0.3575868	-5.923576
MFN1	0.0205145	6.0602433	1.2188755	0.2242767	0.3575868	-5.923576
RNA5-8SP3	-0.110891	-1.478471	-1.218853	0.2242852	0.3575868	-5.923603
RP11-668G1	0.2616202	-0.280278	1.2187317	0.2243312	0.3576445	-5.923749
RP11-158M2	0.2902421	0.97274	1.218606	0.2243788	0.3576734	-5.923899

CYP4F30P	-0.130884	-1.355232	-1.218606	0.2243788	0.3576734	-5.923899
RP11-1L9.1	-0.22691	-1.178871	-1.218589	0.2243853	0.3576734	-5.923919
RP4-539M6.	0.267021	0.357506	1.2185567	0.2243975	0.3576734	-5.923958
YKT6	0.0233268	6.3847764	1.2185546	0.2243983	0.3576734	-5.92396
LINC00923	-0.381263	0.8912432	-1.218484	0.2244252	0.3577007	-5.924045
AC108479.3	0.1749347	-1.035275	1.218428	0.2244463	0.3577149	-5.924112
AC027612.1	-0.138562	-1.294408	-1.218408	0.2244538	0.3577149	-5.924135
RP11-356I2	0.0685454	4.2379717	1.2183687	0.2244688	0.3577232	-5.924183
S100A10	-0.047777	6.6534605	-1.218134	0.2245578	0.3578495	-5.924463
SLC16A13	-0.070919	6.0354423	-1.218079	0.2245788	0.3578672	-5.924529
RP11-542A1	0.1205588	-1.406892	1.2177468	0.2247046	0.3580521	-5.924926
FP236383.6	-0.120003	-1.408288	-1.217698	0.2247232	0.3580556	-5.924985
S100PBP	-0.027649	5.7657082	-1.217689	0.2247264	0.3580556	-5.924995
AC017104.6	0.2421598	2.3953878	1.2173997	0.2248363	0.3582151	-5.925341
GJA3	-0.347769	1.2558021	-1.217334	0.2248613	0.3582393	-5.92542
AC139099.4	-0.102254	-1.434317	-1.217283	0.2248807	0.3582546	-5.925481
EFTUD1P1	-0.158123	-1.223339	-1.217167	0.2249246	0.3583089	-5.925619
WNT7A	0.3418373	0.2369792	1.2170551	0.2249671	0.3583609	-5.925752
DEAF1	-0.029883	6.0289829	-1.217005	0.2249861	0.3583755	-5.925812
ACOX2	-0.09711	6.3733921	-1.216967	0.2250007	0.3583831	-5.925858
DUS4L	-0.029267	5.4666335	-1.216837	0.22505	0.3584461	-5.926013
AP000560.3	-0.285493	0.4821366	-1.216771	0.2250751	0.3584704	-5.926092
RP11-218C1	-0.086995	-1.49019	-1.216721	0.2250941	0.3584813	-5.926152
GTF2E1	-0.028093	5.6531633	-1.216701	0.2251016	0.3584813	-5.926175
KB-173C10.	0.2107602	-1.010844	1.2165614	0.2251546	0.35855	-5.926342
GABPB1-AS1	0.0530038	5.5142912	1.2165085	0.2251746	0.3585663	-5.926405
GS1-309P15	0.1924265	-0.884103	1.2164188	0.2252087	0.358605	-5.926512
AC098828.2	-0.313767	1.3191709	-1.216367	0.2252286	0.3586209	-5.926575
TRDC	0.1658682	3.9836294	1.2162111	0.2252876	0.3586993	-5.92676
VAV2	-0.03008	6.5549052	-1.216165	0.2253051	0.3587115	-5.926815
MDFI	0.1515819	4.8188607	1.216094	0.2253322	0.3587389	-5.9269
U4	-0.268742	0.2560864	-1.216049	0.2253492	0.3587504	-5.926953
KCNK2	0.4275627	0.5246747	1.2159939	0.2253702	0.3587682	-5.927019
AC007036.4	0.1098651	-1.426792	1.2159288	0.2253949	0.3587817	-5.927097
CTB-25B13.	-0.241208	1.8802288	-1.21592	0.2253983	0.3587817	-5.927108
RP11-401P9	-0.114067	-1.428524	-1.215482	0.2255649	0.3590312	-5.92763
SCO2	0.0318907	6.0177183	1.2153133	0.225629	0.3591176	-5.927831
SHOX	-0.172832	-1.124157	-1.215222	0.2256636	0.359157	-5.92794
FBN2	0.2095522	3.0868342	1.2151425	0.225694	0.3591897	-5.928035
CMTM6	0.0297397	6.5742555	1.2150398	0.2257331	0.3592362	-5.928157
CLEC4A	0.0714492	4.5350689	1.2148425	0.2258082	0.3593338	-5.928393
VENTXP7	-0.095172	-1.473175	-1.214827	0.2258141	0.3593338	-5.928411
NAPG	0.0243942	6.0789536	1.21469	0.2258662	0.3594011	-5.928574
CNIH3	0.0886949	4.8086203	1.2145686	0.2259125	0.3594496	-5.928719
RP6-91H8.2	-0.179337	-1.194546	-1.214558	0.2259164	0.3594496	-5.928732
RP11-349K1	0.2002889	-1.258669	1.2144239	0.2259676	0.3595154	-5.928892
SOGA1	-0.043968	5.9896354	-1.214366	0.2259895	0.3595346	-5.92896
RP11-807H1	-0.101395	-1.456643	-1.214143	0.2260745	0.359641	-5.929226
LINC00950	-0.129694	3.8067446	-1.214139	0.2260761	0.359641	-5.929231
RP11-760D2	-0.180264	-1.037993	-1.213928	0.2261565	0.3597532	-5.929483
RP11-295G2	-0.071493	5.5825624	-1.213779	0.2262132	0.3598278	-5.92966

SLC25A53	0.0475273	4.8406927	1.2137215	0.2262352	0.359832	-5.929729
B9D2	0.0533721	5.0410192	1.2137204	0.2262356	0.359832	-5.92973
LLNLR-304G	0.2177286	-0.938538	1.2136479	0.2262632	0.3598603	-5.929816
SERBP1	0.0186533	6.8419373	1.213548	0.2263013	0.3599046	-5.929935
RP11-667K1	-0.124231	3.1851392	-1.213523	0.2263108	0.3599046	-5.929965
RP11-185E8	-0.221238	-0.911039	-1.213497	0.2263209	0.3599049	-5.929996
ADAMDEC1	0.2604011	3.9690126	1.2134291	0.2263467	0.3599213	-5.930077
HEBP2	0.0337201	6.4109418	1.213418	0.2263509	0.3599213	-5.93009
AC010976.2	-0.133832	3.7398911	-1.213354	0.2263753	0.3599445	-5.930166
TBC1D25	0.0238835	5.8766637	1.2131736	0.2264441	0.3600382	-5.930381
RP11-665C1	-0.119511	-1.382045	-1.213108	0.2264693	0.3600625	-5.93046
USP40	-0.025322	6.3049918	-1.21289	0.2265525	0.360179	-5.930719
RP11-419C2	0.1363898	-1.38721	1.2127123	0.2266201	0.3602585	-5.93093
RP11-432I5	-0.30246	-0.238371	-1.212707	0.2266222	0.3602585	-5.930937
GMDS	0.0451467	6.1406805	1.2126544	0.2266422	0.3602747	-5.930999
AP000479.1	0.116826	-1.480968	1.2125594	0.2266785	0.3603166	-5.931112
RP11-475J5	-0.111689	-1.412365	-1.212522	0.2266928	0.3603238	-5.931157
GAPDHP14	0.3829201	1.3808521	1.212441	0.2267237	0.360357	-5.931253
CTC-265F19	-0.146277	-1.281299	-1.212381	0.2267466	0.3603778	-5.931325
NCF1C	0.1150079	4.1903562	1.2123203	0.2267697	0.3603989	-5.931397
DCSTAMP	0.2893721	2.3618915	1.2122415	0.2267998	0.3604073	-5.93149
RSRP1	0.0349712	6.0879155	1.2122368	0.2268016	0.3604073	-5.931496
GLYATL1P3	0.2036286	-1.027969	1.2122289	0.2268046	0.3604073	-5.931505
CTD-2026K1	0.2352209	1.6722326	1.2121634	0.2268297	0.3604205	-5.931583
TECRL	0.217738	-1.134821	1.2121555	0.2268327	0.3604205	-5.931593
RP5-1068H6	0.2171632	-0.790351	1.2119126	0.2269255	0.3605522	-5.931882
HNRNPR	-0.016556	6.5320091	-1.211847	0.2269503	0.360576	-5.931959
SLC25A2	-0.210822	-0.839636	-1.211602	0.2270441	0.3607094	-5.932251
RP11-295H2	-0.278639	0.6511742	-1.211501	0.2270827	0.3607549	-5.932371
STRN	0.0230613	5.9614153	1.2114734	0.2270933	0.360756	-5.932404
NPAP1P2	0.1295081	-1.482608	1.2114249	0.2271118	0.3607698	-5.932462
NLRX1	0.0332243	5.9664041	1.211397	0.2271225	0.360771	-5.932495
ZNF860	0.3311102	1.4895118	1.2113665	0.2271341	0.3607724	-5.932531
C2orf74	0.0571934	4.9257617	1.211343	0.2271431	0.3607724	-5.932559
RNF25	-0.024012	5.9535458	-1.211208	0.2271946	0.3608386	-5.932719
SETMAR	0.0319193	5.6275818	1.211171	0.2272089	0.3608454	-5.932763
STARD9	0.0571446	5.1113242	1.2111277	0.2272254	0.3608561	-5.932815
RP1-20C7.6	-0.187582	2.6669322	-1.210996	0.2272758	0.3609203	-5.932971
SPRR2E	0.1934139	-1.244035	1.2109396	0.2272973	0.3609264	-5.933038
RP11-1902.	-0.112399	-1.447235	-1.210934	0.2272994	0.3609264	-5.933045
AFMID	-0.03711	6.5646586	-1.210873	0.2273228	0.3609479	-5.933118
OMP	-0.300984	1.3320172	-1.210813	0.2273457	0.3609632	-5.933189
RP11-252I1	0.1638584	-1.152485	1.2107747	0.2273604	0.3609632	-5.933234
XBP1P1	0.2721357	0.8738252	1.2107703	0.2273621	0.3609632	-5.93324
ADH6	-0.113902	6.5412939	-1.210607	0.2274244	0.3610464	-5.933433
KAT6A	0.0346305	6.0639903	1.2104701	0.227477	0.3611142	-5.933596
RP11-893F2	-0.176547	-1.139702	-1.210433	0.2274912	0.3611211	-5.933641
RP11-28001	-0.199717	-1.19618	-1.210338	0.2275275	0.3611629	-5.933753
C5orf47	-0.277247	0.3883277	-1.210277	0.2275507	0.3611841	-5.933825
RP11-554D1	-0.158918	-1.304273	-1.210195	0.2275823	0.3612057	-5.933923
AC011286.1	0.1540207	-1.259392	1.2101901	0.2275841	0.3612057	-5.933929

SNORD63	-0.246594	2.0534397	-1.210122	0.2276102	0.3612314	-5.93401
MIR202HG	0.2262323	-1.076681	1.2099227	0.2276865	0.3613368	-5.934247
CTD-2035E1	-0.220763	2.159506	-1.209708	0.2277686	0.3614429	-5.934501
KCTD8	0.2852101	-0.800952	1.2096964	0.2277732	0.3614429	-5.934515
RP11-362K1	-0.256561	1.9896048	-1.2096	0.22781	0.3614856	-5.934629
AC011524.3	0.0778564	-1.524469	1.2095461	0.2278308	0.3615029	-5.934694
LINC00933	0.1642803	2.969685	1.2094665	0.2278613	0.3615239	-5.934788
ATP1B3	0.0457321	6.166369	1.2094585	0.2278643	0.3615239	-5.934798
RNU7-123P	-0.198847	-0.870253	-1.209432	0.2278746	0.3615239	-5.934829
TMEM132C	0.3129449	-0.480046	1.2093888	0.227891	0.3615239	-5.93488
RP3-453C12	0.2249399	-0.849824	1.2093641	0.2279005	0.3615239	-5.93491
MINOS1-NBL	0.1482063	-1.229631	1.2093293	0.2279139	0.3615239	-5.934951
AC009410.1	-0.381881	0.2374699	-1.209312	0.2279204	0.3615239	-5.934972
SIX3	-0.335288	-0.430519	-1.209305	0.2279233	0.3615239	-5.93498
RP11-431K2	-0.259732	1.9022827	-1.209228	0.2279525	0.361539	-5.935071
KRT18P48	-0.226188	-0.566091	-1.209228	0.2279526	0.361539	-5.935071
RN7SKP68	-0.078833	-1.516097	-1.209172	0.227974	0.3615484	-5.935137
RP4-597J3.	-0.163186	-1.158755	-1.209161	0.2279783	0.3615484	-5.935151
CSF3R	0.0901353	5.3512084	1.20906	0.2280171	0.3615941	-5.935271
LINC00863	-0.105922	4.4772831	-1.208994	0.2280423	0.3616018	-5.935349
DUTP1	0.1201793	-1.376008	1.208986	0.2280454	0.3616018	-5.935358
AC007000.1	-0.255778	1.1082411	-1.20897	0.2280516	0.3616018	-5.935378
LMCD1	0.0429374	5.9486158	1.2087592	0.2281324	0.3617142	-5.935628
RP11-60A24	-0.235102	-0.644521	-1.208566	0.2282065	0.3618159	-5.935857
SGTA	-0.022206	6.5427796	-1.208469	0.2282438	0.3618594	-5.935972
RP11-365F1	-0.170164	-1.111265	-1.208259	0.2283243	0.3619712	-5.936221
FAM179A	0.1648999	3.3952392	1.2081863	0.2283521	0.3619975	-5.936307
CTB-118N6.	0.2203319	-0.783047	1.2081641	0.2283607	0.3619975	-5.936333
RP11-236B1	0.1810284	-1.307389	1.2081091	0.2283818	0.3620098	-5.936399
AC091053.2	-0.154505	-1.202131	-1.208092	0.2283883	0.3620098	-5.936419
RGS7	-0.300043	-0.52333	-1.207964	0.2284375	0.3620722	-5.936571
USMG5P1	-0.246757	-0.236256	-1.207832	0.228488	0.3621364	-5.936727
SULT1A2	0.1208238	5.6094324	1.2076868	0.2285439	0.3622093	-5.936899
HNRNPUL2	-0.021407	6.3273326	-1.207546	0.228598	0.3622793	-5.937066
GDA	0.2632035	5.7556334	1.2074939	0.2286179	0.3622952	-5.937128
IGKV2D-29	0.402165	1.74887	1.2072947	0.2286945	0.3624007	-5.937364
CD300LF	0.0952465	4.5783909	1.2072542	0.22871	0.3624012	-5.937412
RPS18P13	-0.241036	-0.46367	-1.207242	0.2287147	0.3624012	-5.937426
TEDDM2P	-0.194169	-1.234058	-1.207161	0.2287459	0.362435	-5.937523
IGHJ1	-0.259831	-0.793033	-1.206821	0.2288765	0.3626262	-5.937925
TBL1XR1	0.0199156	6.5174972	1.2067191	0.2289157	0.3626725	-5.938046
MYO15A	-0.14256	3.7813627	-1.206688	0.2289275	0.3626755	-5.938082
SNX18P25	0.0662053	-1.530407	1.2066185	0.2289543	0.3627022	-5.938165
TMX4	-0.029788	6.4970086	-1.206593	0.2289642	0.3627022	-5.938195
HIGD1AP18	-0.120696	-1.365464	-1.206441	0.2290225	0.3627788	-5.938375
CTC-428G20	-0.229405	-0.532402	-1.206387	0.2290434	0.3627962	-5.938439
AC008154.4	0.1225966	-1.414979	1.2060508	0.2291727	0.3629748	-5.938837
CFL1P2	-0.248867	-0.157189	-1.206042	0.2291761	0.3629748	-5.938848
MTCYBP4	0.0947991	-1.457234	1.2059471	0.2292126	0.3630169	-5.93896
RP4-584D14	-0.153965	3.583555	-1.205786	0.2292744	0.363099	-5.93915
GPR135	0.0795183	4.1755833	1.2055737	0.2293563	0.3632129	-5.939402

CD207	0.2847182	2.9578427	1.2054458	0.2294055	0.3632752	-5.939553
AC104650.2	0.247869	-0.537868	1.2053653	0.2294365	0.3633085	-5.939648
RP11-481K9	0.1838753	-0.997175	1.2053247	0.2294521	0.3633174	-5.939696
ATP1B2	-0.10153	5.0421302	-1.205093	0.2295413	0.3634429	-5.93997
MRPL52	-0.029627	6.2518731	-1.20495	0.2295963	0.3635141	-5.940139
SOX15	0.1173802	3.2739063	1.2048652	0.2296291	0.3635483	-5.94024
RP11-733C7	0.1765537	-1.295954	1.2048427	0.2296378	0.3635483	-5.940267
RP11-278L1	-0.198138	4.5209262	-1.204729	0.2296815	0.3636018	-5.940401
RP11-464D2	-0.237378	-0.425648	-1.204533	0.2297572	0.3637059	-5.940633
FAM225B	-0.298435	0.4334128	-1.204432	0.2297961	0.3637516	-5.940752
SPTY2D1	0.0237565	6.0100159	1.2043706	0.2298197	0.3637733	-5.940825
PCNXL4	-0.030271	6.0185629	-1.204235	0.2298719	0.3638402	-5.940985
CLN3	-0.036517	6.2170792	-1.204154	0.2299033	0.3638585	-5.941081
C14orf178	0.1660685	-1.167382	1.2041534	0.2299035	0.3638585	-5.941082
AC002310.1	-0.10409	3.9821032	-1.204076	0.2299333	0.3638899	-5.941173
MAN1B1-AS1	0.0841023	4.2379631	1.2039416	0.2299851	0.3639562	-5.941332
WNT5B	0.141636	4.9240657	1.2038716	0.2300122	0.3639832	-5.941415
RP11-545M1	-0.212539	-0.730113	-1.203793	0.2300425	0.3640155	-5.941508
RP11-753H1	-0.282482	0.1985393	-1.203659	0.2300943	0.3640816	-5.941666
GSX1	-0.096231	-1.507565	-1.203475	0.2301653	0.3641628	-5.941883
ZIC1	0.5336614	3.079451	1.203474	0.2301656	0.3641628	-5.941884
WEE2	-0.264066	0.536445	-1.203403	0.230193	0.3641877	-5.941968
GALNTL5	-0.113999	-1.423978	-1.203382	0.2302012	0.3641877	-5.941993
RP11-342K6	0.2217844	-0.620811	1.2031597	0.2302869	0.3643036	-5.942255
LINC00152	0.0627979	5.7181474	1.2031401	0.2302945	0.3643036	-5.942279
FTLP8	-0.15502	-1.211297	-1.203024	0.2303394	0.3643588	-5.942416
RPE65	-0.218339	-0.729572	-1.202998	0.2303494	0.3643589	-5.942447
RP11-404E1	0.1370439	-1.311925	1.20287	0.2303988	0.3644212	-5.942598
TRGV5P	-0.145314	-1.256613	-1.202753	0.230444	0.3644644	-5.942736
SLC16A11	-0.133178	5.4445193	-1.202747	0.2304461	0.3644644	-5.942742
RAP1GAP	0.0650404	6.3183307	1.2026629	0.2304787	0.3645003	-5.942842
GBF1	-0.021015	6.5052323	-1.2026	0.2305029	0.3645227	-5.942916
RP5-966M1.	-0.177719	-1.016833	-1.202548	0.2305232	0.364539	-5.942978
KRTAP7-1	-0.206458	-1.221839	-1.202209	0.2306542	0.3647206	-5.943378
NOC4L	-0.027225	6.0348144	-1.202199	0.230658	0.3647206	-5.94339
AC012358.7	0.2380494	-0.563422	1.2019204	0.2307657	0.3648751	-5.943718
FTH1	0.0276943	7.1683471	1.2017899	0.2308161	0.364939	-5.943872
ARV1	-0.032173	6.0311728	-1.201388	0.2309714	0.3651687	-5.944346
RP11-1094M	0.2811014	1.8737674	1.2010799	0.2310908	0.3653417	-5.94471
PLCXD2-AS1	0.1197424	-1.389476	1.2009156	0.2311544	0.3654024	-5.944903
LRRC34	0.1171849	3.561882	1.2009076	0.2311575	0.3654024	-5.944913
RP11-697E2	-0.189833	-1.029685	-1.200872	0.2311715	0.3654024	-5.944955
CTD-2147F2	0.1653101	-1.271456	1.200866	0.2311736	0.3654024	-5.944962
AC017074.2	0.2201219	2.5159606	1.2008513	0.2311793	0.3654024	-5.944979
RP11-197K3	0.1106523	-1.398868	1.2007554	0.2312164	0.3654453	-5.945092
AC005863.2	-0.106268	-1.465786	-1.200578	0.2312852	0.3655254	-5.945301
RP1-309I22	0.1939225	3.4004171	1.2005728	0.2312871	0.3655254	-5.945307
CGB8	0.1201719	-1.477098	1.200382	0.231361	0.3656263	-5.945532
CTD-3203P2	0.2180048	3.0538911	1.2002297	0.23142	0.3657037	-5.945711
NPY2R	0.1151521	-1.46295	1.2001472	0.231452	0.3657384	-5.945809
EEF1GP7	-0.134132	-1.328314	-1.200111	0.231466	0.3657447	-5.945851

SUMO1P1	-0.185952	-1.169385	-1.200021	0.2315008	0.3657719	-5.945957
GSK3A	-0.019879	6.3754957	-1.200015	0.2315032	0.3657719	-5.945964
CTD-2530N2	-0.259976	1.5834632	-1.199847	0.2315683	0.3658588	-5.946162
RP11-707G1	-0.231028	-0.628309	-1.199817	0.2315801	0.3658617	-5.946198
PID1	0.0622358	6.1916314	1.1997339	0.2316122	0.365882	-5.946295
ZIK1	-0.131605	4.3187887	-1.199732	0.231613	0.365882	-5.946298
TPT1P9	-0.173018	2.9087852	-1.199691	0.2316287	0.3658909	-5.946346
KRT17P3	0.1127926	-1.407295	1.1996356	0.2316503	0.365908	-5.946411
RP11-215P8	-0.236605	1.1122167	-1.199612	0.2316596	0.365908	-5.946439
RPL7P4	-0.176104	-1.013138	-1.199574	0.2316742	0.3659153	-5.946484
RP11-266L9	0.2770346	-0.05323	1.19954	0.2316873	0.3659198	-5.946524
COA1	-0.021457	6.0730795	-1.199515	0.2316971	0.3659198	-5.946553
SPEM1	0.0762226	-1.509763	1.1994508	0.2317219	0.3659292	-5.946629
RP11-472K2	-0.083329	-1.513892	-1.199448	0.2317231	0.3659292	-5.946632
RABGAP1L	-0.029331	6.0871304	-1.199235	0.2318057	0.3660438	-5.946883
NGB	-0.190451	-1.237485	-1.199082	0.2318648	0.3661213	-5.947062
TP53I13	-0.057204	6.0879157	-1.198955	0.2319143	0.3661702	-5.947213
SLC39A9	0.0258403	6.5701571	1.198951	0.2319158	0.3661702	-5.947217
YWHABP2	-0.259762	0.3881296	-1.198922	0.2319272	0.3661724	-5.947252
HACE1	-0.050903	5.213435	-1.198829	0.2319631	0.3662132	-5.947361
SLC23A3	0.136406	3.9936447	1.1987953	0.2319762	0.366218	-5.9474
CHMP1B2P	-0.401752	0.2170729	-1.198732	0.2320009	0.3662287	-5.947475
TRAV2	-0.319061	0.7654562	-1.198726	0.2320031	0.3662287	-5.947482
TMEM221	0.1973766	2.7071643	1.1986543	0.2320309	0.3662569	-5.947566
RP11-50B3.	-0.257797	-0.15535	-1.198519	0.2320834	0.3663238	-5.947725
C19orf43	0.0278205	6.6226683	1.1984258	0.2321197	0.3663653	-5.947835
CTD-2044J1	0.2753325	0.2964231	1.1983948	0.2321317	0.3663684	-5.947872
RP11-835E1	-0.252974	-0.292516	-1.198351	0.2321489	0.3663797	-5.947924
FAM53B	-0.033397	5.951755	-1.198245	0.2321898	0.3664285	-5.948048
LLNLF-18A1	-0.087513	-1.51184	-1.198124	0.2322368	0.3664868	-5.94819
NAP1L4P2	-0.088552	-1.484847	-1.198087	0.2322512	0.3664936	-5.948234
RP1-13D10.	-0.26755	-0.330393	-1.198051	0.2322653	0.3665001	-5.948276
RP11-462D1	-0.234528	-0.855679	-1.197896	0.2323253	0.366579	-5.948458
ZNF250	-0.03469	5.4979227	-1.197803	0.2323615	0.3666202	-5.948567
RP11-678G1	-0.131691	-1.365699	-1.197742	0.2323851	0.3666415	-5.948639
RP11-242D8	-0.258135	-0.11136	-1.197663	0.2324161	0.3666746	-5.948733
NOP14-AS1	-0.034467	5.5622358	-1.197306	0.2325547	0.366874	-5.949152
ARGFXP2	-0.208187	-0.746466	-1.197286	0.2325626	0.366874	-5.949176
SERPINA7	-0.241432	6.3539482	-1.197153	0.2326141	0.3669394	-5.949331
SCARNA6	-0.248138	-0.140095	-1.19707	0.2326464	0.3669745	-5.949429
CACNA1G	-0.347403	1.0677425	-1.197001	0.2326732	0.367001	-5.94951
RP11-661A1	-0.300141	0.9850176	-1.19694	0.232697	0.3670227	-5.949582
SETP12	-0.134679	-1.331697	-1.196559	0.2328452	0.3672406	-5.950029
EIF3H	-0.027818	6.8011041	-1.196315	0.2329402	0.367359	-5.950316
FKSG48	0.2112682	-0.720411	1.1963143	0.2329405	0.367359	-5.950317
LRRC37A5P	0.2218187	2.6686407	1.1960794	0.2330319	0.3674735	-5.950593
DNPEP	0.022393	6.483707	1.1960762	0.2330332	0.3674735	-5.950596
RP11-692C2	-0.103286	-1.411906	-1.196022	0.2330542	0.3674907	-5.95066
RP11-71L14	0.1978025	-1.08285	1.1956989	0.2331801	0.3676734	-5.951039
AC010761.9	0.2781986	0.4633583	1.195609	0.2332151	0.3677127	-5.951145
TKT	0.0433382	6.8877919	1.1955798	0.2332265	0.3677148	-5.951179

PPP1R1A	-0.141351	6.1050262	-1.195443	0.2332798	0.3677729	-5.95134
MANF	0.033523	6.5837547	1.1954244	0.2332871	0.3677729	-5.951361
RP1-159A19	-0.234141	-0.627926	-1.195389	0.233301	0.3677729	-5.951403
GIT1	-0.022988	6.2352575	-1.195382	0.2333036	0.3677729	-5.951411
RP11-299J5	0.0918308	-1.470731	1.1953289	0.2333243	0.3677895	-5.951474
HTRA4	0.3334413	1.8835694	1.1952773	0.2333444	0.3678053	-5.951534
ZNF536	-0.260941	-0.796493	-1.195079	0.2334215	0.367911	-5.951766
RP11-331D5	0.0811226	-1.50929	1.195041	0.2334365	0.3679187	-5.951811
RP11-392O1	0.2556366	-0.689752	1.1948432	0.2335136	0.3680244	-5.952043
POLR3B	-0.02657	5.9025766	-1.194767	0.2335435	0.3680555	-5.952133
PROSP	0.2176453	-0.715015	1.1947055	0.2335673	0.3680772	-5.952205
MUCL1	0.3189271	-0.532634	1.1946097	0.2336047	0.3681202	-5.952317
IGKV3-11	0.2489011	5.0320143	1.194434	0.2336732	0.3681965	-5.952523
CAMK2G	-0.023329	6.06465	-1.194434	0.2336733	0.3681965	-5.952523
HS2ST1	-0.025907	6.2109625	-1.194392	0.2336895	0.3682062	-5.952572
CTD-3105H1	-0.18971	2.5470896	-1.194265	0.2337392	0.3682527	-5.952721
AC017078.1	0.177811	-1.238633	1.1942649	0.2337392	0.3682527	-5.952721
RP11-669M2	-0.197153	-1.144014	-1.194012	0.2338378	0.3683921	-5.953018
RP11-159M1	0.1184956	-1.387762	1.1938327	0.2339079	0.3684867	-5.953228
C5orf17	-0.414807	0.5100579	-1.193668	0.2339722	0.3685721	-5.953421
CNNM4	0.0331821	5.7435357	1.1935835	0.2340052	0.3686081	-5.95352
DGKZ	-0.020388	6.2835274	-1.193527	0.2340271	0.368615	-5.953586
ZNF236	-0.041431	5.5118088	-1.193521	0.2340297	0.368615	-5.953594
PLGLB1	0.1619198	4.5504641	1.1934604	0.2340532	0.3686361	-5.953664
SNORD116-7	-0.087553	-1.489916	-1.193227	0.2341445	0.368764	-5.953938
SLC10A5	-0.084976	4.7561016	-1.193059	0.23421	0.3688512	-5.954134
RP11-536C5	-0.180831	2.2395931	-1.193032	0.2342204	0.3688517	-5.954166
RP11-61E11	0.201254	-0.864203	1.1929644	0.234247	0.3688777	-5.954245
AC123023.1	0.122752	-1.471155	1.1929315	0.2342599	0.368882	-5.954284
RP11-46021	-0.195707	-0.837518	-1.192646	0.2343713	0.3690318	-5.954618
SMCO4	-0.040975	6.2504183	-1.192636	0.2343752	0.3690318	-5.954629
RP11-455B3	0.2112118	-0.921947	1.1926014	0.2343889	0.3690374	-5.95467
AMBRA1	-0.020013	6.1632983	-1.192123	0.2345762	0.3693163	-5.955231
CTC-412M14	0.1096826	-1.44489	1.1920435	0.2346071	0.3693491	-5.955323
TIMM21	0.0279721	5.9522385	1.1919373	0.2346486	0.3693986	-5.955448
RYR1	0.1350162	4.331066	1.1918322	0.2346898	0.3694474	-5.955571
RP11-32B5.	0.1359858	-1.356462	1.191763	0.2347168	0.3694741	-5.955651
CTA-440B3.	-0.184068	-0.981497	-1.191736	0.2347274	0.3694748	-5.955683
IGSF3	-0.100279	5.6724063	-1.191623	0.2347716	0.3695284	-5.955815
EEF1A1P33	-0.240897	-0.428598	-1.191515	0.2348139	0.3695791	-5.955942
DNAJC5	-0.023769	6.4828499	-1.191447	0.2348405	0.369605	-5.956021
OR10Q1	0.1807643	-1.21352	1.1913576	0.2348756	0.3696442	-5.956126
RP11-474C8	-0.24223	-0.219006	-1.191099	0.234977	0.3697879	-5.956429
MRGPRD	-0.139173	-1.372403	-1.190965	0.2350292	0.3698541	-5.956584
AC023672.2	0.1259918	-1.408883	1.1906498	0.2351528	0.3700327	-5.956953
RP11-893F2	0.1790129	-1.091157	1.1904776	0.2352203	0.3701229	-5.957155
ZFP3	0.122426	4.5416869	1.190296	0.2352915	0.3702189	-5.957367
RP3-340B19	-0.243465	1.5937075	-1.190057	0.2353852	0.3703505	-5.957646
ARHGEF38-I	-0.22234	-0.920013	-1.189995	0.2354095	0.3703727	-5.957718
GNG13	-0.189838	-1.073441	-1.189826	0.2354759	0.3704612	-5.957916
RP3-388N13	-0.138922	-1.354397	-1.189448	0.2356241	0.3706785	-5.958357

MPHOSPH9	-0.036799	5.739615	-1.189385	0.2356488	0.3707014	-5.958431
RP11-967K2	0.0886893	4.1246767	1.189278	0.2356908	0.3707376	-5.958556
RPL5P6	-0.2126	-0.813548	-1.189274	0.2356922	0.3707376	-5.95856
RP11-108M9	0.1459998	4.1667219	1.1892477	0.2357027	0.3707382	-5.958591
ELP5	-0.031033	6.1487103	-1.189099	0.2357611	0.3708141	-5.958765
CTD-2035E1	-0.282509	0.2142114	-1.189018	0.2357927	0.3708379	-5.958859
DUX4	0.0784158	-1.52665	1.1890085	0.2357966	0.3708379	-5.95887
CTB-5506.4	-0.324428	-0.413872	-1.188863	0.2358539	0.370912	-5.959041
C1orf186	-0.274597	4.1344672	-1.188651	0.235937	0.3710267	-5.959288
RASA4	-0.164425	3.0298014	-1.188573	0.2359675	0.3710588	-5.959378
AWAT2	0.2079542	-1.040261	1.1885431	0.2359794	0.3710614	-5.959413
SERPINA10	-0.136856	6.6896312	-1.18846	0.236012	0.3710967	-5.95951
C1orf177	-0.236162	2.707747	-1.188429	0.2360243	0.3711001	-5.959547
LA16c-380H	0.2759091	0.4402563	1.1883554	0.2360531	0.3711071	-5.959633
IFT172	-0.033967	5.7419215	-1.188345	0.2360574	0.3711071	-5.959645
SPON2	-0.047513	6.7883118	-1.18834	0.2360592	0.3711071	-5.959651
RP11-467K1	0.0805714	-1.507547	1.1881985	0.2361148	0.3711784	-5.959816
RP11-429P3	-0.195117	-0.863978	-1.187894	0.2362346	0.3713508	-5.960171
C19orf70	0.0330897	6.3183267	1.1876982	0.2363115	0.3714465	-5.960399
RTEL1	-0.131782	3.8721256	-1.187687	0.2363159	0.3714465	-5.960412
UMOD	-0.124481	-1.407227	-1.187644	0.2363329	0.3714574	-5.960463
TFCP2L1	0.1873026	3.873192	1.1876153	0.2363441	0.3714589	-5.960496
CICP14	0.1315524	4.5741181	1.1874502	0.236409	0.3715449	-5.960688
KDM4C	-0.026293	5.7164473	-1.187413	0.2364236	0.371547	-5.960731
NEUROD6	-0.103866	-1.503821	-1.187395	0.2364306	0.371547	-5.960752
CTC-490G23	0.1263417	-1.423155	1.1873085	0.2364647	0.3715733	-5.960853
CTD-2588C8	0.264528	-0.15409	1.1873009	0.2364678	0.3715733	-5.960862
ALCAM	-0.040001	6.5914993	-1.187143	0.2365298	0.3716507	-5.961046
GLYATL2	-0.275152	-0.35553	-1.187124	0.2365374	0.3716507	-5.961068
GAD2	0.2145563	-1.207072	1.1869999	0.2365862	0.3717114	-5.961213
PRICKLE4	-0.0472	5.6252812	-1.186793	0.2366676	0.3718233	-5.961454
TPSB2	0.2372435	3.9629202	1.1867497	0.2366847	0.3718341	-5.961504
TPCN2	-0.041775	6.0207569	-1.186711	0.2366998	0.3718418	-5.961549
STXBP2	-0.031811	6.2019324	-1.186677	0.2367135	0.3718474	-5.96159
RP13-379O2	0.0746664	-1.529976	1.1865949	0.2367456	0.3718819	-5.961685
C9orf64	-0.03027	5.980717	-1.186488	0.2367875	0.3719317	-5.961809
RP13-926M1	0.1470055	-1.303294	1.186447	0.2368039	0.3719413	-5.961857
CHIAP2	-0.114064	-1.418153	-1.186383	0.236829	0.3719649	-5.961931
AP001043.1	0.0707848	-1.528073	1.1863006	0.2368615	0.3719999	-5.962028
CTD-2228K2	0.05778	-1.5347	1.186162	0.2369161	0.3720696	-5.962189
LINC01098	-0.181488	-1.206492	-1.186008	0.2369768	0.3721489	-5.962368
TUBB8P4	-0.083959	-1.513584	-1.185945	0.2370017	0.3721719	-5.962442
SNX19P3	0.1723713	-1.079888	1.1858048	0.2370569	0.3722426	-5.962605
LINC00315	-0.253325	1.3336858	-1.185613	0.2371324	0.3723452	-5.962828
MIR217HG	0.3743369	0.2208773	1.1854917	0.2371803	0.3724044	-5.962969
MLLT6	-0.02282	6.4240527	-1.185433	0.2372035	0.3724247	-5.963038
RANBP9	0.0223925	6.2661092	1.1851778	0.2373041	0.3725667	-5.963335
RP1-90L6.3	0.2376506	-0.549888	1.1849752	0.237384	0.3726761	-5.96357
OAS1	0.0560932	6.2067182	1.184414	0.2376055	0.3730078	-5.964223
SFTA3	-0.127515	-1.439255	-1.184376	0.2376206	0.3730155	-5.964268
AC073150.6	-0.225475	-0.661405	-1.184191	0.2376934	0.3731137	-5.964482

RP11-383C6	-0.210206	-0.53781	-1.18391	0.2378045	0.3732575	-5.964809
NOP56P1	-0.398034	0.6333918	-1.183908	0.2378054	0.3732575	-5.964812
AC092652.1	-0.148724	-1.315568	-1.183628	0.2379158	0.3734146	-5.965136
GLYCTK	-0.061397	6.6152172	-1.183077	0.2381337	0.3737407	-5.965777
RP11-573M3	-0.112786	-1.407723	-1.18302	0.2381562	0.3737599	-5.965843
NOTUM	-0.221666	5.1351869	-1.182767	0.2382562	0.3738906	-5.966136
DENND5A	0.0222644	6.2770729	1.1827578	0.23826	0.3738906	-5.966148
EIF4A1	-0.05511	5.0995348	-1.182491	0.2383655	0.3740401	-5.966457
ZCCHC12	-0.336749	1.033956	-1.18234	0.2384251	0.3740834	-5.966632
FLJ43879	0.1292514	-1.378168	1.1823402	0.2384252	0.3740834	-5.966632
RP11-474P2	-0.201264	-0.772974	-1.182322	0.2384325	0.3740834	-5.966654
HMGN2P47	-0.309942	1.7520997	-1.182318	0.2384341	0.3740834	-5.966658
RP11-632K2	0.2803504	0.8031553	1.1822419	0.2384641	0.3741144	-5.966746
TFF3	-0.145309	5.062675	-1.182156	0.2384982	0.3741517	-5.966846
APOBEC3D	0.1091616	4.7054482	1.1820922	0.2385234	0.3741752	-5.96692
HNF1B	-0.128218	5.9260966	-1.181866	0.2386129	0.3742995	-5.967183
PROKR2	-0.13631	-1.401426	-1.181683	0.2386852	0.3743952	-5.967395
GABRR2	-0.281762	1.4387666	-1.18166	0.2386944	0.3743952	-5.967421
LINC01301	0.1766861	3.3452152	1.1813854	0.2388033	0.3745499	-5.96774
C6orf15	0.1434071	-1.388959	1.1813484	0.2388179	0.3745568	-5.967783
RP11-52101	0.1724047	-1.253219	1.1812964	0.2388385	0.374573	-5.967844
SPG21	0.0228213	6.6082243	1.1812177	0.2388697	0.374596	-5.967935
LINC01239	0.2692188	2.8516809	1.1812076	0.2388737	0.374596	-5.967946
ABCD1P2	-0.078034	-1.494584	-1.181027	0.2389455	0.3746924	-5.968156
OR10AD1	0.1374568	-1.279702	1.1809187	0.2389882	0.3747434	-5.968281
MTND5P10	0.2247579	-0.748949	1.1808431	0.2390182	0.3747743	-5.968369
LINC00116	0.0546914	5.7855493	1.1806209	0.2391063	0.3748869	-5.968627
RP11-510N1	-0.280747	3.0244139	-1.18061	0.2391105	0.3748869	-5.968639
C1orf204	0.2280725	2.7150212	1.1805381	0.2391391	0.3749156	-5.968723
RP13-92302	-0.135069	3.3320535	-1.180494	0.2391568	0.3749272	-5.968774
CTD-3185P2	-0.053335	4.2692457	-1.180218	0.2392662	0.3750803	-5.969094
RP11-85021	0.1784974	-1.051709	1.1801956	0.239275	0.3750803	-5.96912
IL1RN	-0.08755	6.172433	-1.180148	0.239294	0.375094	-5.969175
AC000029.1	0.0992546	-1.454964	1.1800442	0.2393351	0.3751423	-5.969295
AC002331.1	0.3020684	0.3702502	1.1799531	0.2393712	0.3751828	-5.969401
MAPKAPK3	0.0279064	6.2158663	1.1798909	0.2393959	0.3752054	-5.969473
RP11-12602	-0.148188	-1.380414	-1.179619	0.2395038	0.3753583	-5.969787
CTC-224D3.	-0.090991	-1.48823	-1.179572	0.2395226	0.3753718	-5.969842
AMER1	-0.059766	5.4062761	-1.179542	0.2395344	0.375374	-5.969877
RIMKLBP1	0.2114433	-0.741824	1.1795077	0.239548	0.3753793	-5.969916
DIP2A	-0.027178	5.9601653	-1.179386	0.2395964	0.375439	-5.970058
NSMCE2	-0.037169	6.0252223	-1.179357	0.2396078	0.3754407	-5.970091
RP11-297H3	0.4001203	0.5337001	1.1792746	0.2396406	0.375476	-5.970186
RP11-466F5	-0.225402	-0.50931	-1.179092	0.2397132	0.3755737	-5.970398
RP11-574K1	-0.124858	3.1441079	-1.179052	0.2397289	0.3755822	-5.970444
SEMA4D	0.0572378	5.6304967	1.1790103	0.2397456	0.3755922	-5.970492
RBM41	0.0372272	5.547257	1.1788749	0.2397994	0.3756603	-5.970649
SNX15	-0.09709	3.757855	-1.178817	0.2398225	0.3756804	-5.970716
RP11-609N1	-0.291637	2.0053811	-1.178671	0.2398804	0.3757361	-5.970885
RP11-247I1	-0.231134	1.3848151	-1.178656	0.2398863	0.3757361	-5.970902
AD001527.7	0.2575924	0.2305781	1.1786495	0.239889	0.3757361	-5.97091

ODF2-AS1	-0.151889	-1.157602	-1.17856	0.2399244	0.3757755	-5.971013
XXbac-BPG1	-0.19209	-0.96581	-1.1785	0.2399485	0.3757971	-5.971083
RP11-430H1	0.3069441	0.2997639	1.1783902	0.2399921	0.3758492	-5.97121
RNU6-33P	0.1134021	-1.360252	1.1783012	0.2400274	0.3758885	-5.971313
IGLC3	0.2731276	5.0905041	1.1782623	0.2400429	0.3758966	-5.971358
AL118496.1	-0.086528	-1.485839	-1.178166	0.2400811	0.3759403	-5.971469
AL590762.1	-0.267059	0.3712726	-1.177978	0.240156	0.3760415	-5.971687
SIKE1	0.0234244	6.0551668	1.1779216	0.2401784	0.3760604	-5.971752
FAM204A	-0.018816	6.0543428	-1.177689	0.2402711	0.3761894	-5.972021
RP11-64D22	0.3435447	0.6845321	1.1775651	0.2403203	0.3762503	-5.972164
TRBV27	-0.309969	0.0299534	-1.17751	0.2403422	0.3762684	-5.972228
ABCC8	-0.352629	2.0166934	-1.177442	0.2403691	0.3762945	-5.972306
RP1-15D23.	0.1763304	-1.139047	1.1773386	0.2404104	0.3763371	-5.972426
RP11-748L1	-0.245823	-0.652886	-1.177322	0.2404169	0.3763371	-5.972445
CD163L1	0.1053972	5.2413627	1.1770693	0.2405176	0.3764785	-5.972737
CLRN2	0.1841461	-1.132229	1.1769886	0.2405498	0.3765127	-5.972831
LINC01085	0.2370846	-0.891145	1.1768404	0.2406088	0.3765889	-5.973002
TSPAN12	0.063845	5.8508239	1.1767514	0.2406442	0.3766283	-5.973105
HOMER2P2	-0.118254	-1.443797	-1.176703	0.2406635	0.3766423	-5.973161
RP11-566J3	-0.149075	-1.338311	-1.176633	0.2406914	0.3766698	-5.973242
RNA5SP194	0.0880956	-1.496487	1.1765496	0.2407247	0.3767057	-5.973338
RP3-460G2.	0.3795085	1.5439889	1.1765043	0.2407427	0.3767178	-5.97339
AC011816.1	-0.129652	-1.303357	-1.176329	0.2408125	0.3768083	-5.973592
EXOSC5	-0.035809	6.0472503	-1.176244	0.2408465	0.3768083	-5.973691
AP000253.1	0.28632	1.2146114	1.1762095	0.2408602	0.3768083	-5.973731
CCDC117	-0.108468	4.8910802	-1.176207	0.240861	0.3768083	-5.973733
HEXA-AS1	0.2278487	2.4357819	1.1762039	0.2408624	0.3768083	-5.973737
RP5-886K2.	-0.182536	-0.917692	-1.176182	0.2408712	0.3768083	-5.973763
RN7SL481P	-0.226527	-0.500376	-1.176178	0.2408728	0.3768083	-5.973767
RP11-76C10	-0.128269	-1.324237	-1.176106	0.2409015	0.376837	-5.97385
RP11-804L2	-0.106307	-1.442814	-1.175993	0.2409464	0.3768911	-5.97398
ORAI1	-0.027049	6.0546475	-1.175771	0.241035	0.3769884	-5.974237
RP11-905K4	-0.148994	-1.229057	-1.175762	0.2410387	0.3769884	-5.974248
RP3-334F4.	-0.137677	-1.313819	-1.17574	0.2410474	0.3769884	-5.974273
RP11-886D1	-0.111307	-1.404552	-1.175734	0.2410499	0.3769884	-5.97428
ADAM3A	0.1037819	-1.465729	1.1755129	0.241138	0.37711	-5.974535
AC017060.1	0.3480983	1.2912165	1.1754694	0.2411553	0.3771197	-5.974585
RPL21P33	-0.08697	-1.485622	-1.175446	0.2411649	0.3771197	-5.974613
RP11-44M6.	-0.224218	3.322101	-1.17537	0.2411949	0.3771356	-5.974699
RP11-326C3	-0.247173	2.2402051	-1.175368	0.2411957	0.3771356	-5.974702
CTD-2314G2	0.1945361	-1.144986	1.1749845	0.2413489	0.3773589	-5.975145
GATA1	-0.264287	0.0529614	-1.174941	0.2413663	0.37737	-5.975195
MIR6503	0.1457428	-1.232417	1.1748878	0.2413874	0.3773869	-5.975256
RP1-122K4.	-0.212202	-0.842286	-1.174667	0.2414755	0.3775083	-5.97551
RP11-128A1	0.1897484	-0.988981	1.1745723	0.2415135	0.3775516	-5.97562
RHPN1	-0.072591	5.3984997	-1.174523	0.241533	0.377566	-5.975677
RP11-57C13	0.1646556	-1.177578	1.1744455	0.2415641	0.3775925	-5.975766
NDUFA12	-0.024773	6.2971051	-1.174429	0.2415706	0.3775925	-5.975785
SPRYD3	0.0216268	6.3358425	1.1743951	0.2415842	0.3775975	-5.975824
RP11-350N1	0.186869	-1.088775	1.174283	0.241629	0.3776513	-5.975954
C22orf46	-0.029561	5.8685805	-1.174238	0.2416468	0.3776631	-5.976005

IL36A	-0.082794	-1.49225	-1.174194	0.2416647	0.3776748	-5.976057
VPS35	0.0207987	6.4624615	1.1738546	0.2418002	0.3778704	-5.976448
AC098817.5	-0.08073	-1.515167	-1.173792	0.241825	0.377893	-5.976519
UQCRBP1	-0.256522	1.3773614	-1.173594	0.2419044	0.3780009	-5.976748
MIR4537	-0.154273	-1.265958	-1.173545	0.2419239	0.3780151	-5.976804
C16orf74	0.1513191	3.8099118	1.1735043	0.2419402	0.3780245	-5.976851
BLOC1S5	-0.026752	5.8695645	-1.173427	0.2419713	0.3780568	-5.976941
CTC-518B2.	0.0737381	-1.53045	1.1732385	0.2420466	0.3781434	-5.977157
HMGN1P4	-0.241724	-0.689884	-1.173236	0.2420474	0.3781434	-5.97716
BNIP3P1	-0.199953	2.8809041	-1.173018	0.2421346	0.3782635	-5.977411
MTND5P30	-0.096609	-1.46357	-1.172875	0.2421919	0.3783246	-5.977576
CARTPT	-0.122366	-1.413034	-1.172869	0.2421945	0.3783246	-5.977583
TLR8-AS1	0.1101282	-1.462495	1.1727544	0.2422403	0.3783799	-5.977715
PEX10	0.035297	5.8059904	1.1727226	0.242253	0.3783836	-5.977752
AC005324.6	0.0623595	-1.532367	1.1726591	0.2422784	0.3784071	-5.977825
C14orf119	-0.020639	6.1891557	-1.172415	0.2423761	0.3785347	-5.978106
MIA3	0.0329962	6.6734115	1.1724033	0.2423808	0.3785347	-5.978119
RP11-383M4	-0.250836	0.308141	-1.172342	0.2424052	0.3785477	-5.978189
AHSA1	-0.023826	6.5334595	-1.172331	0.2424099	0.3785477	-5.978203
PRPF38B	0.0203479	6.19582	1.1721457	0.2424839	0.3786471	-5.978416
LRRC10B	0.1936874	3.3946163	1.1720797	0.2425104	0.3786722	-5.978492
CTB-147N14	-0.294613	1.6509866	-1.171975	0.2425523	0.3786979	-5.978612
CTD-2619J1	-0.174466	2.5822379	-1.171942	0.2425655	0.3786979	-5.97865
TRAV12-3	-0.310808	1.1231893	-1.171917	0.2425754	0.3786979	-5.978678
FMO7P	-0.229722	-0.879748	-1.171903	0.2425811	0.3786979	-5.978695
ALS2CR11	-0.25741	2.0647896	-1.171877	0.2425917	0.3786979	-5.978725
RP4-751H13	0.1414458	3.1483111	1.1718671	0.2425956	0.3786979	-5.978736
RP11-158D2	-0.081589	-1.49284	-1.171833	0.242609	0.3786979	-5.978775
LINC00538	-0.27393	0.2823978	-1.171831	0.2426098	0.3786979	-5.978777
LRRTM2	0.2591336	1.5673598	1.1717223	0.2426536	0.378734	-5.978903
LINC01290	0.2706219	0.3900178	1.171722	0.2426537	0.378734	-5.978903
MEMO1P1	-0.098662	4.0718636	-1.171665	0.2426766	0.3787535	-5.978969
AC145110.1	-0.286407	0.7362396	-1.171579	0.2427111	0.3787899	-5.979068
RP1-128M12	0.1443861	-1.245586	1.171555	0.2427206	0.3787899	-5.979095
AC079834.1	-0.129627	-1.303369	-1.171311	0.2428184	0.3789263	-5.979376
NTN1	0.1220609	5.5726748	1.1710721	0.2429142	0.379037	-5.979651
MARS2	-0.041681	5.6507702	-1.171061	0.2429187	0.379037	-5.979664
SNORD51	-0.221187	-0.636261	-1.171056	0.2429205	0.379037	-5.979669
RP11-250B2	-0.221962	2.4685315	-1.170998	0.2429441	0.3790576	-5.979737
RP11-700F1	-0.113017	-1.411714	-1.170925	0.2429731	0.3790856	-5.97982
GAK	0.0215187	6.5617318	1.1709011	0.2429828	0.3790856	-5.979847
ASNSD1	-0.020007	6.2589461	-1.170852	0.2430025	0.3791001	-5.979904
SLC24A2	-0.261187	-0.599814	-1.17071	0.2430596	0.379173	-5.980067
AC007278.2	0.1222445	-1.354216	1.1706737	0.243074	0.3791748	-5.980109
SOWAHB	-0.167236	5.5412436	-1.170655	0.2430816	0.3791748	-5.98013
MTRNR2L3	0.2772073	0.6947416	1.1703721	0.2431951	0.3793264	-5.980456
FCF1	0.0214867	6.0961607	1.1703411	0.2432075	0.3793264	-5.980491
FABP5P3	-0.189856	-0.905817	-1.170335	0.2432099	0.3793264	-5.980498
ANKRD28	0.024919	6.2426741	1.170227	0.2432533	0.3793682	-5.980622
ZNF804B	-0.120266	-1.430064	-1.170217	0.2432575	0.3793682	-5.980634
AC005546.2	-0.21606	-0.738207	-1.170063	0.2433189	0.3794478	-5.98081

CCZ1B	0.0425188	5.196501	1.1698186	0.2434173	0.3795849	-5.981091
RP5-921G16	0.1226049	-1.424687	1.1697539	0.2434433	0.3796093	-5.981166
RP4-800M22	-0.202716	2.0638844	-1.169671	0.2434767	0.3796452	-5.981261
TTC3	-0.022834	6.6278704	-1.169541	0.2435287	0.3796978	-5.98141
MSRB2	0.0317611	6.3150599	1.1695348	0.2435313	0.3796978	-5.981417
SOCS5P4	-0.245286	1.3396739	-1.169026	0.2437357	0.3800004	-5.982002
CH17-53B9.	-0.073505	-1.51871	-1.168321	0.2440194	0.3804264	-5.982811
PON1	0.166488	6.5196355	1.1681298	0.2440962	0.3805299	-5.98303
ARNT2	0.1796779	4.7907657	1.1680163	0.2441419	0.3805848	-5.98316
DFFB	-0.035613	5.0525542	-1.167904	0.2441872	0.3806393	-5.983289
DHRSX	0.0398998	6.1999991	1.1677478	0.2442499	0.3807136	-5.983468
RP11-351O2	0.2667764	-0.947246	1.167719	0.2442615	0.3807136	-5.983501
RP11-399K2	0.0720346	-1.531318	1.1677075	0.2442662	0.3807136	-5.983514
TICAM2	0.2535427	1.106309	1.1675638	0.244324	0.3807874	-5.983679
MYH10	0.0432594	6.3264085	1.1675032	0.2443484	0.3808092	-5.983748
CDRT4	-0.21793	2.1057792	-1.167467	0.2443631	0.3808159	-5.98379
ALOX15	0.2662079	2.068944	1.1674109	0.2443856	0.3808346	-5.983854
LINC00272	-0.179411	-1.218231	-1.167357	0.2444071	0.380852	-5.983916
AFG3L1P	0.0310499	5.4507697	1.1673283	0.2444188	0.3808539	-5.983949
ARSG	0.0545685	5.2445153	1.1672434	0.244453	0.380891	-5.984046
RP3-469D22	0.2396997	-0.424737	1.1670365	0.2445364	0.3810046	-5.984283
RP11-757F1	-0.224765	-0.818107	-1.166938	0.244576	0.3810501	-5.984396
HOTAIR	0.2434598	-1.043313	1.1667911	0.2446353	0.3811261	-5.984565
AL158801.1	-0.087951	3.2356518	-1.166735	0.2446581	0.3811454	-5.984629
IGKV2D-28	-0.198698	-1.111324	-1.16667	0.244684	0.3811695	-5.984703
SLC35C2	-0.022331	6.4432939	-1.166586	0.2447181	0.3812064	-5.9848
LAIR2	-0.297325	2.1085915	-1.166523	0.2447433	0.3812152	-5.984871
RP11-785H5	-0.026299	6.131606	-1.166493	0.2447554	0.3812152	-5.984906
RP11-616M2	0.2366456	-0.882714	1.1664865	0.2447581	0.3812152	-5.984914
ZNF576	0.025524	5.7830928	1.1664679	0.2447656	0.3812152	-5.984935
PROSER2-AS	-0.184284	3.1880968	-1.166405	0.244791	0.3812386	-5.985007
C1orf168	-0.169204	5.3009041	-1.166314	0.2448278	0.3812796	-5.985112
AC007163.8	0.1414773	-1.316283	1.1660483	0.2449348	0.38143	-5.985415
ELP6	-0.024182	5.9610476	-1.165972	0.2449654	0.3814613	-5.985502
AC006128.2	-0.099292	4.5433294	-1.165936	0.2449802	0.3814682	-5.985544
AC002463.3	-0.146571	-1.320385	-1.165881	0.2450021	0.381486	-5.985607
RP1-17K7.2	0.1155397	-1.378626	1.1657842	0.2450413	0.3815146	-5.985718
ZNF557	-0.030114	5.3844537	-1.165781	0.2450426	0.3815146	-5.985721
GGTLC1	0.3060625	0.5783762	1.1657582	0.2450518	0.3815146	-5.985747
RP11-182L2	-0.099202	3.98271	-1.165593	0.2451184	0.3816019	-5.985936
RP11-431N1	-0.166554	-1.13746	-1.165506	0.2451538	0.3816305	-5.986037
TMEM43	0.0224035	6.2894266	1.165496	0.2451576	0.3816305	-5.986048
KCNAB1-AS2	0.1276841	-1.430784	1.1654106	0.2451921	0.3816678	-5.986145
POU4F1	-0.306724	-0.316375	-1.165315	0.2452307	0.3817116	-5.986255
RP11-342K6	0.0284805	5.441292	1.165218	0.2452699	0.3817563	-5.986366
OR2S1P	0.1833062	-1.069166	1.1650107	0.2453536	0.3818704	-5.986603
CMA1	-0.337619	0.7625017	-1.164925	0.2453882	0.3819079	-5.986701
FOXN4	-0.34296	3.22869	-1.16462	0.2455115	0.3820552	-5.98705
RP11-650L1	-0.234808	-0.318695	-1.164618	0.2455121	0.3820552	-5.987052
RAB3IL1	0.0581643	5.6667228	1.1645954	0.2455213	0.3820552	-5.987078
EIF4EP1	0.2717857	0.1227275	1.1645872	0.2455247	0.3820552	-5.987087

RRN3P2	0.1262157	3.6420496	1.1645017	0.2455592	0.3820927	-5.987185
RP11-15I11	-0.24955	-0.534309	-1.164469	0.2455723	0.3820968	-5.987222
ASS1P10	0.2644665	-0.214277	1.1641761	0.2456908	0.3822648	-5.987557
SEPT6	0.0486118	6.3978776	1.1639075	0.2457994	0.3823978	-5.987864
C11orf16	-0.252575	0.5502134	-1.163902	0.2458018	0.3823978	-5.987871
HCG14	-0.280832	0.4271046	-1.163887	0.2458077	0.3823978	-5.987888
RP11-467I2	0.1319945	-1.30498	1.1637925	0.2458459	0.382441	-5.987996
CD164	0.0212346	6.8783571	1.163683	0.2458902	0.3824936	-5.988121
TJP2	0.0337845	6.4452809	1.1635127	0.2459591	0.3825844	-5.988315
CRTC3	0.0217514	6.1120435	1.1634017	0.246004	0.382638	-5.988442
RP11-401.2	-0.071737	4.8125116	-1.163215	0.2460796	0.3827392	-5.988656
LHX5	0.1953418	-1.10091	1.1631127	0.246121	0.3827741	-5.988772
CH17-26202	-0.136406	-1.296624	-1.163108	0.246123	0.3827741	-5.988778
ATP2B3	-0.278295	-0.309033	-1.16306	0.2461424	0.3827879	-5.988833
RP11-131N1	-0.177481	-1.109883	-1.163005	0.2461647	0.3828063	-5.988896
RP11-480D4	0.21431	-0.714231	1.1629456	0.2461886	0.3828272	-5.988963
AC019117.1	0.2527604	-0.557577	1.1628894	0.2462114	0.3828463	-5.989028
XXbac-B444	-0.182326	3.2825004	-1.16279	0.2462515	0.3828924	-5.989141
CTD-2501M5	0.1320291	-1.367594	1.1626912	0.2462916	0.3829297	-5.989254
TTLL7-IT1	0.1418584	-1.256848	1.1626648	0.2463023	0.3829297	-5.989284
POU5F1	0.1475595	4.4039819	1.1626533	0.246307	0.3829297	-5.989297
RP11-143E2	-0.103163	-1.452356	-1.162608	0.2463255	0.3829422	-5.989349
RP1-28C20.	-0.100585	-1.483525	-1.162485	0.2463751	0.383003	-5.989489
ARSK	0.039317	5.3296633	1.1623933	0.2464123	0.3830445	-5.989594
RP11-16E12	-0.088702	3.9981719	-1.162321	0.2464415	0.3830645	-5.989676
IGHG3	0.2055167	5.7937721	1.1623098	0.2464461	0.3830645	-5.989689
RP11-24F11	0.2638361	2.1288613	1.1620982	0.2465318	0.3831814	-5.989931
PLEKHH3	0.0322776	6.0002616	1.1619069	0.2466094	0.3832778	-5.990149
SLC6A12	-0.119273	5.967253	-1.161878	0.2466209	0.3832778	-5.990181
RP5-1021I2	-0.230115	-0.239146	-1.161867	0.2466254	0.3832778	-5.990194
COG6	0.0325183	5.7561507	1.1616035	0.2467324	0.3834157	-5.990495
RP11-530C5	0.2286059	2.4151342	1.1615967	0.2467351	0.3834157	-5.990503
RP11-840I1	0.241102	-0.328488	1.1614507	0.2467943	0.3834914	-5.990669
SIGLEC6	-0.302659	0.3353697	-1.161217	0.2468891	0.3836223	-5.990936
TUBGCP3	-0.028252	5.9793929	-1.161166	0.2469097	0.3836287	-5.990994
ASB11	0.3167811	-0.070215	1.1611552	0.2469142	0.3836287	-5.991006
PTAR1	0.0294405	6.0766681	1.1610871	0.2469418	0.3836553	-5.991084
CTD-2342N2	0.3027567	3.1974053	1.1610382	0.2469616	0.3836697	-5.99114
RP5-1142A6	0.1648132	-1.130285	1.1609104	0.2470135	0.383734	-5.991286
CHI3L2	0.2359861	3.4346206	1.1608146	0.2470524	0.383778	-5.991395
FES	-0.062833	6.0586515	-1.160742	0.2470817	0.3838073	-5.991477
AC006042.6	-0.236199	2.8171766	-1.160522	0.247171	0.3839219	-5.991728
HILPDA	-0.063835	5.377344	-1.160509	0.2471766	0.3839219	-5.991743
GMCL1P1	-0.314689	-0.314759	-1.160275	0.2472714	0.3840529	-5.99201
COPB1	0.0170551	6.6669257	1.1601886	0.2473065	0.3840801	-5.992108
HYDIN2	0.3062272	0.3745027	1.1601801	0.24731	0.3840801	-5.992118
RP11-235E1	-0.229888	-0.370442	-1.160154	0.2473205	0.3840801	-5.992147
RP11-91I20	-0.14652	-1.220211	-1.160101	0.2473422	0.3840975	-5.992208
RP11-384K6	0.1481213	4.455718	1.160071	0.2473543	0.3840999	-5.992242
RP11-753D2	0.2781289	-0.13159	1.1598977	0.2474247	0.3841929	-5.99244
EVX1	-0.377459	0.7289581	-1.159775	0.2474744	0.3842526	-5.992579

BCAT2	0.0406482	6.1775069	1.159705	0.247503	0.3842526	-5.992659
BTNL2	-0.112728	-1.385371	-1.1597	0.2475049	0.3842526	-5.992664
MEF2D	0.0252014	6.4429071	1.1596995	0.2475052	0.3842526	-5.992665
RNU7-49P	-0.240528	-0.31011	-1.159605	0.2475435	0.3842957	-5.992773
MSMO1	0.0498623	6.7720704	1.1595372	0.2475712	0.3843223	-5.99285
MIR6757	0.2009546	-0.786235	1.1594532	0.2476053	0.384359	-5.992946
PCAT7	-0.188106	4.0790986	-1.159382	0.2476341	0.3843873	-5.993026
A1BG	-0.164458	6.1759586	-1.159263	0.2476827	0.3844464	-5.993163
RP11-996F1	-0.121847	2.9892549	-1.159198	0.2477093	0.3844712	-5.993237
CYP4F29P	-0.336061	0.5290963	-1.159059	0.2477658	0.3845426	-5.993395
PDCD1	-0.172234	4.3900216	-1.158835	0.2478568	0.3846676	-5.99365
ATAD3A	0.0370263	6.0237974	1.1588074	0.2478679	0.3846684	-5.993681
RP11-467L1	-0.294226	3.5702356	-1.158609	0.2479486	0.3847773	-5.993907
RNU1-67P	-0.177922	-1.114672	-1.158572	0.2479636	0.3847843	-5.993949
VWA7	-0.107066	5.1834794	-1.158466	0.2480067	0.3848205	-5.994069
LSM6	-0.026819	5.66715	-1.158463	0.2480081	0.3848205	-5.994073
RP4-610C12	0.2513119	-0.23274	1.1583215	0.2480656	0.384868	-5.994234
PAIP2	0.0219194	6.3821727	1.1583138	0.2480688	0.384868	-5.994242
LINC00299	-0.216083	3.0354504	-1.15831	0.2480703	0.384868	-5.994247
DEF6	-0.05783	5.5038287	-1.158222	0.2481062	0.3849073	-5.994347
ERP29	0.0232259	6.8606499	1.1581846	0.2481213	0.3849144	-5.994389
AC104024.1	-0.172668	-1.059534	-1.158127	0.2481449	0.3849346	-5.994455
SP9	0.151035	-1.378404	1.1580901	0.2481598	0.3849414	-5.994497
RNF220	-0.020513	6.3438326	-1.15791	0.2482332	0.3850389	-5.994702
CTD-2184D3	-0.320166	1.7247347	-1.157727	0.2483078	0.3851382	-5.99491
LAMP5	0.2823941	3.299496	1.1576836	0.2483254	0.3851491	-5.994959
RP5-1120P1	-0.207622	-0.974299	-1.157541	0.2483834	0.3852227	-5.995121
CTA-268H5.	-0.235259	-0.238474	-1.157495	0.2484022	0.3852355	-5.995174
PLEKHD1	-0.300364	1.5843391	-1.157455	0.2484183	0.3852441	-5.995218
RP11-501J2	0.149892	-1.299357	1.1573649	0.2484552	0.3852744	-5.995321
RP11-47901	0.2171483	-0.781004	1.1573496	0.2484614	0.3852744	-5.995339
RP11-70D24	0.1914059	-0.86347	1.1573298	0.2484695	0.3852744	-5.995361
RP11-345P4	0.055699	5.0132562	1.1572947	0.2484838	0.3852803	-5.995401
RP11-142A5	-0.13327	-1.324813	-1.157261	0.2484975	0.3852852	-5.995439
GPX6	-0.078264	-1.516376	-1.157165	0.2485368	0.3853297	-5.995549
RP11-86H7.	-0.251789	2.7268759	-1.157095	0.2485654	0.3853554	-5.995629
PTENP1	0.1104018	3.9563088	1.1570721	0.2485745	0.3853554	-5.995654
DHX36	0.0206647	6.1139791	1.1569793	0.2486123	0.3853847	-5.99576
ZNF671	-0.071075	5.0408046	-1.156955	0.2486223	0.3853847	-5.995787
CXorf23	0.0373968	5.4798804	1.1569481	0.2486251	0.3853847	-5.995795
DDX53	-0.407517	0.1307707	-1.156878	0.2486535	0.3853964	-5.995874
RIC8B	0.0312453	5.8673284	1.1568778	0.2486537	0.3853964	-5.995875
FAM66C	0.1988679	2.8197013	1.1568423	0.2486682	0.3854025	-5.995915
AC112518.3	0.0667918	-1.521794	1.1567807	0.2486933	0.385425	-5.995985
PCDHA8	-0.272458	-0.707073	-1.156738	0.2487109	0.3854359	-5.996034
RP11-3M1.1	0.315812	0.8485662	1.1566211	0.2487584	0.3854893	-5.996167
CABP5	0.0700326	-1.515383	1.1565911	0.2487706	0.3854893	-5.996201
RP3-322G13	-0.197203	-0.695407	-1.156575	0.248777	0.3854893	-5.996218
DOPEY1	-0.038624	5.6483299	-1.156493	0.2488105	0.3855118	-5.996312
WDR20	-0.02321	5.812211	-1.156488	0.2488127	0.3855118	-5.996318
RP11-207C1	-0.267718	0.0923234	-1.156342	0.2488723	0.3855878	-5.996484

NUDT21	0.0206375	6.2789893	1.1562911	0.248893	0.3855966	-5.996541
RP11-676M6	0.0978623	4.6773082	1.1562762	0.2488991	0.3855966	-5.996558
WDFY1	-0.023004	6.1571939	-1.155964	0.2490264	0.3857682	-5.996913
MIATNB	0.0707358	4.3890188	1.1559528	0.249031	0.3857682	-5.996925
TRBV6-1	-0.299837	1.3410042	-1.155728	0.249123	0.3858943	-5.997181
PCDHGA11	0.198555	3.1509291	1.1554905	0.2492198	0.3860279	-5.99745
RP11-616M2	0.2066182	3.9842855	1.1554563	0.2492337	0.3860331	-5.997489
RP11-466A1	-0.291109	0.6156252	-1.155281	0.2493055	0.3861153	-5.997688
SLC18A1	-0.27254	-0.325644	-1.155275	0.249308	0.3861153	-5.997695
OR51J1	-0.084235	-1.486964	-1.155229	0.2493266	0.3861279	-5.997747
RP11-500G2	0.2811152	0.2687999	1.1551581	0.2493556	0.3861562	-5.997827
EIF4E1B	0.1469704	-1.309692	1.1550878	0.2493843	0.3861843	-5.997907
9-Mar	-0.061527	5.5441633	-1.154798	0.2495025	0.3863446	-5.998235
AE000662.9	0.1705964	-1.183842	1.1547827	0.2495089	0.3863446	-5.998253
OCM	-0.251035	0.1229967	-1.154549	0.2496045	0.3864633	-5.998518
RAPGEF4	0.0580108	5.8834254	1.1545433	0.2496068	0.3864633	-5.998525
CARD14	0.1464127	3.7187611	1.1543851	0.2496714	0.386547	-5.998704
RP11-421F1	0.0448626	5.7226513	1.1542239	0.2497374	0.3866327	-5.998887
RP11-128P1	0.0753113	-1.531221	1.1541956	0.2497489	0.3866342	-5.998919
RDX	0.029592	6.5245707	1.1541681	0.2497602	0.3866352	-5.99895
ASH1L	-0.030835	6.2945411	-1.154119	0.2497801	0.3866496	-5.999005
ATP5G1P6	0.0958604	-1.493709	1.1540703	0.2498002	0.3866644	-5.999061
RP11-64D24	0.1135054	-1.374208	1.1537434	0.2499339	0.386855	-5.999431
LDHAL6FP	0.1549353	-1.18811	1.1536988	0.2499521	0.3868601	-5.999482
RP11-432M8	0.1158417	-1.473661	1.1536835	0.2499584	0.3868601	-5.999499
SLC2A4	-0.13898	4.9968622	-1.153599	0.2499931	0.3868973	-5.999595
PSENEN	0.0294559	6.2455502	1.1535453	0.250015	0.3869148	-5.999656
FA2H	0.3747633	2.6257812	1.1534907	0.2500373	0.386933	-5.999718
CTC-451P13	-0.08904	4.2729455	-1.153457	0.2500513	0.3869382	-5.999756
RP4-736H5.	0.2306075	-1.0302	1.153371	0.2500863	0.386976	-5.999853
PARP9	0.0356207	6.5034224	1.1532903	0.2501194	0.3870107	-5.999945
AC000036.4	-0.19458	-1.021145	-1.153232	0.2501434	0.3870303	-6.000011
MRVI1-AS1	-0.26704	-0.21531	-1.153208	0.2501532	0.3870303	-6.000038
PAX8	0.1017419	4.9355243	1.1531492	0.2501771	0.3870509	-6.000104
MAP2K5	0.0292339	5.8504411	1.1529794	0.2502467	0.3871421	-6.000297
TCF7L2	-0.027088	6.1042985	-1.152938	0.2502638	0.3871521	-6.000344
ACTR1B	0.0207215	6.4323717	1.1526126	0.2503969	0.3873416	-6.000712
RP11-393N2	0.0623595	-1.532367	1.1522959	0.2505267	0.387526	-6.00107
HDHD1P3	-0.088356	-1.511427	-1.152181	0.2505738	0.3875825	-6.0012
CPEB1-AS1	0.2026156	-1.003031	1.1521268	0.250596	0.3876004	-6.001262
DNASE2B	0.3016054	1.8450074	1.1520407	0.2506313	0.3876385	-6.001359
RP11-24M17	-0.174236	-1.03138	-1.151989	0.2506526	0.3876551	-6.001418
RP11-511B2	-0.280024	1.2234367	-1.15176	0.2507464	0.3877837	-6.001677
CRYBA2	-0.322162	-0.161778	-1.151707	0.2507683	0.3878011	-6.001737
GOLGA3	-0.017962	6.4597874	-1.15158	0.2508204	0.3878652	-6.001881
ENDOG	0.0476049	5.5255944	1.1514597	0.2508695	0.3879249	-6.002016
RP11-469H8	-0.235708	-0.904069	-1.151125	0.2510068	0.3881206	-6.002395
CDCP2	0.2074833	-0.724118	1.150904	0.2510976	0.3882446	-6.002645
RP11-159D1	-0.068871	5.2022946	-1.150733	0.2511676	0.3883365	-6.002837
RP11-272L1	-0.110955	-1.403677	-1.150524	0.2512538	0.3884454	-6.003074
IRGM	0.2796371	-0.131886	1.15051	0.2512594	0.3884454	-6.00309

RP11-440G9	0.3561287	0.185858	1.1502948	0.2513478	0.3885656	-6.003333
NUDT22	0.0313272	6.0021123	1.1502045	0.2513849	0.3886065	-6.003435
RP5-844F9.	-0.220904	-0.725561	-1.150058	0.2514451	0.3886832	-6.003601
RP11-1033H	0.0679734	-1.524747	1.1499884	0.2514736	0.3887108	-6.003679
SCARNA10	-0.247175	-0.452296	-1.149941	0.2514933	0.3887247	-6.003733
RP11-817J1	0.4239099	1.1143104	1.1498826	0.2515171	0.3887451	-6.003799
THEM5	0.0942266	4.9887428	1.1498179	0.2515437	0.3887697	-6.003872
RP11-426D1	0.2172344	-0.666853	1.1497889	0.2515556	0.3887717	-6.003904
RP11-88H10	-0.078214	-1.489916	-1.14972	0.2515838	0.3887917	-6.003982
RP11-81M19	0.0923761	-1.484903	1.1496957	0.251594	0.3887917	-6.00401
CAPN9	-0.310465	2.667554	-1.14968	0.2516006	0.3887917	-6.004028
RN7SL81P	0.2457514	-0.339548	1.1496418	0.2516161	0.3887993	-6.004071
CIR1P2	-0.074074	-1.518431	-1.149541	0.2516575	0.3888469	-6.004184
CACNG6	-0.205879	-1.060454	-1.149489	0.2516789	0.3888634	-6.004243
RP11-6N17.	-0.207277	-0.658922	-1.149072	0.2518505	0.389112	-6.004714
AC093326.3	0.2273635	-0.764522	1.1489537	0.2518991	0.3891667	-6.004847
TSSK3	-0.216381	2.3484461	-1.148916	0.2519146	0.3891667	-6.00489
DSTYK	0.0349024	5.815757	1.1489081	0.2519179	0.3891667	-6.004899
PEX19	0.0314785	6.653957	1.1488448	0.2519439	0.3891905	-6.00497
RP4-761J14	-0.169915	2.7584417	-1.148687	0.2520089	0.3892693	-6.005148
SLC26A1	-0.064062	5.9323514	-1.148669	0.2520163	0.3892693	-6.005168
AC091878.1	0.2221341	-1.053035	1.1486378	0.2520291	0.3892727	-6.005204
CTB-46B19.	-0.123679	-1.318865	-1.148593	0.2520476	0.3892848	-6.005254
IFNLR1	-0.108084	5.301828	-1.148342	0.2521508	0.3894117	-6.005537
RP11-77K12	-0.157711	-1.084194	-1.148318	0.2521608	0.3894117	-6.005565
RP11-883A1	-0.277015	-0.368978	-1.148315	0.2521618	0.3894117	-6.005567
ARL2	-0.041495	5.9866009	-1.148229	0.2521975	0.3894403	-6.005665
AC073842.1	0.2972729	2.5809902	1.1482186	0.2522017	0.3894403	-6.005676
USP46-AS1	0.0647888	4.3158668	1.1481814	0.252217	0.3894475	-6.005718
RP11-21B23	-0.12691	-1.352247	-1.147858	0.2523502	0.3896235	-6.006083
AP001065.7	0.3459594	0.7829172	1.1478529	0.2523523	0.3896235	-6.006089
LOXHD1	-0.292016	1.4981128	-1.147599	0.252457	0.3897685	-6.006375
LINC01480	0.2688676	3.0310171	1.1475731	0.2524676	0.3897685	-6.006404
OR14I1	-0.126616	-1.399418	-1.147209	0.2526178	0.3899839	-6.006815
CAPN1	0.0216751	6.7387273	1.1471231	0.252653	0.3900021	-6.006911
RNY3P8	-0.305489	1.0072506	-1.147118	0.2526553	0.3900021	-6.006918
SIGLEC12	-0.245551	3.514104	-1.147102	0.2526616	0.3900021	-6.006935
UBE2Q2	-0.039784	5.9836608	-1.147012	0.2526987	0.3900279	-6.007036
U91328.2	0.1762525	-1.067073	1.1470059	0.2527014	0.3900279	-6.007043
CNR2	-0.280265	1.8861768	-1.146984	0.2527104	0.3900279	-6.007068
WBP4	0.0254151	5.7308562	1.1468964	0.2527466	0.3900672	-6.007167
IGHV7-27	-0.22791	-0.934251	-1.146719	0.2528198	0.3901638	-6.007367
PEBP1P3	-0.141784	-1.262923	-1.146661	0.2528436	0.390184	-6.007432
TMCO2	-0.209106	-0.820901	-1.146634	0.2528547	0.3901846	-6.007462
RP11-809H1	0.2368803	-0.79813	1.1465134	0.2529045	0.3902369	-6.007598
RP11-467L1	-0.271651	-0.481319	-1.1465	0.25291	0.3902369	-6.007613
RNF7	-0.020103	6.3561443	-1.146451	0.2529303	0.3902518	-6.007668
HCG4P8	0.1179671	-1.35107	1.1463484	0.2529726	0.3903006	-6.007784
RP11-544L8	0.1943092	-0.946362	1.1461261	0.2530644	0.3904256	-6.008034
LINC00347	0.0661701	-1.51691	1.1460611	0.2530912	0.39045	-6.008107
FHOD3	0.1997485	4.2576826	1.146036	0.2531016	0.39045	-6.008136

CTC-527H23	0.2613934	1.2355464	1.1457555	0.2532173	0.3906121	-6.008451
RP11-314P1	0.0939012	-1.485215	1.1457116	0.2532355	0.3906236	-6.008501
AIFM2	-0.035454	6.323856	-1.145677	0.2532498	0.3906293	-6.00854
VPS39	0.0189194	6.3391324	1.145399	0.2533646	0.3907897	-6.008852
TMSB15A	0.244513	1.8118772	1.145189	0.2534514	0.3908789	-6.009089
AC007000.1	-0.245889	0.783064	-1.14517	0.2534592	0.3908789	-6.00911
RP11-555G1	0.2471609	-1.012579	1.1451674	0.2534603	0.3908789	-6.009113
AL358815.1	0.1716499	-1.061244	1.1451555	0.2534652	0.3908789	-6.009126
TRBV5-4	0.2985305	1.0023568	1.1451285	0.2534763	0.3908795	-6.009157
RP11-368J2	0.1583907	3.1457316	1.1450939	0.2534906	0.3908851	-6.009196
PRR34-AS1	0.0958072	4.9106985	1.1447274	0.2536421	0.3911021	-6.009608
CCDC159	0.0369988	5.9043989	1.1446896	0.2536577	0.3911097	-6.00965
HSPBP1	-0.025894	6.2957151	-1.144465	0.2537507	0.3912364	-6.009903
RP11-6L6.4	-0.252941	0.0481981	-1.144353	0.2537969	0.3912864	-6.010028
RP11-402P6	-0.096949	-1.507213	-1.144328	0.2538075	0.3912864	-6.010057
AC006960.5	0.159123	-1.119177	1.1443088	0.2538152	0.3912864	-6.010078
TTLL1	-0.038198	5.1962747	-1.144158	0.2538774	0.3913658	-6.010247
HSD17B1P1	0.1414789	3.1770382	1.1440759	0.2539116	0.3914017	-6.01034
RP11-1100L	0.0952161	-1.479344	1.1440502	0.2539222	0.3914017	-6.010369
APAF1	-0.047724	5.6476123	-1.144016	0.2539364	0.391407	-6.010407
SAMD13	-0.166157	3.3678162	-1.143937	0.2539689	0.3914265	-6.010496
SNORA69	-0.227672	-0.34672	-1.143934	0.2539704	0.3914265	-6.0105
UBD	-0.091857	6.3833735	-1.143771	0.2540378	0.3915028	-6.010683
RP11-508M8	-0.10914	-1.434473	-1.143762	0.2540414	0.3915028	-6.010692
IFFO2	-0.039998	5.8375298	-1.143691	0.254071	0.3915319	-6.010773
CYBA	-0.059581	6.3967542	-1.143577	0.254118	0.3915878	-6.0109
MAGEB6P1	-0.110984	-1.408131	-1.143446	0.2541725	0.3916552	-6.011048
MVB12A	0.0350438	6.1022789	1.1433805	0.2541994	0.3916802	-6.011121
CTA-38K21.	-0.217157	-0.590477	-1.143196	0.2542757	0.3917812	-6.011328
AC004448.2	-0.120482	-1.390894	-1.143123	0.2543062	0.3918117	-6.011411
CEP70	0.035512	5.8957169	1.1427974	0.2544409	0.3920027	-6.011776
FAM83G	-0.042783	6.3098964	-1.142754	0.2544588	0.3920136	-6.011824
RP5-1061H2	0.2208151	-0.964849	1.1426572	0.254499	0.3920591	-6.011933
RP11-8L8.2	0.2396126	1.7881279	1.1425568	0.2545406	0.3921066	-6.012046
DYRK1A	0.0190962	6.2712914	1.1424986	0.2545648	0.3921273	-6.012111
COG5	0.0288432	5.9297534	1.1424088	0.254602	0.392168	-6.012212
RP11-90B9.	-0.244556	0.8786636	-1.142126	0.2547192	0.3923161	-6.012529
CTC-429P9.	-0.236659	-0.109333	-1.142125	0.2547196	0.3923161	-6.01253
HOGA1	-0.165084	5.5571774	-1.142038	0.2547557	0.3923551	-6.012628
C7orf65	0.177855	-1.131428	1.1419428	0.2547952	0.3923963	-6.012735
RPL21P5	-0.185163	-0.874967	-1.141922	0.2548039	0.3923963	-6.012758
RPL14P5	-0.093407	-1.46056	-1.14188	0.2548212	0.3924063	-6.012805
CTB-113I20	-0.113597	-1.425334	-1.141653	0.2549153	0.3925197	-6.01306
RP11-156G1	-0.244614	0.1284945	-1.141651	0.2549163	0.3925197	-6.013062
RGAG1	-0.340908	2.8102558	-1.141499	0.2549791	0.3925843	-6.013232
GS1-293C5.	-0.260669	0.7494267	-1.141498	0.2549797	0.3925843	-6.013234
LEMD3	-0.021231	5.8842357	-1.141217	0.2550965	0.3927474	-6.013549
LRRTM4	0.3859341	0.6186945	1.1410551	0.2551635	0.3928198	-6.01373
SF3A3P2	-0.151809	-1.147323	-1.141035	0.2551719	0.3928198	-6.013753
FAM104B	-0.029254	5.5931542	-1.141026	0.2551758	0.3928198	-6.013763
EPB41L2	0.0346044	6.1974376	1.140944	0.2552097	0.3928391	-6.013855

LINC01391	0.1527793	-1.282238	1.1409437	0.2552098	0.3928391	-6.013855
ALDH3A2	0.0395146	6.8999412	1.1405901	0.2553566	0.3930364	-6.014251
AC004870.5	-0.174304	-1.29494	-1.140583	0.2553596	0.3930364	-6.014259
RP11-10N16	0.2521125	-0.400704	1.1405152	0.2553878	0.3930633	-6.014335
SETP1	-0.108206	-1.370263	-1.140452	0.2554139	0.3930826	-6.014406
RP11-548L2	0.3917191	1.9814795	1.1404332	0.2554218	0.3930826	-6.014427
AC005779.2	-0.189058	-0.859411	-1.140357	0.2554536	0.393115	-6.014513
ADM5	-0.134402	3.5229193	-1.140315	0.2554709	0.3931249	-6.014559
C9orf171	0.1759521	-1.010615	1.1402321	0.2555054	0.3931614	-6.014652
RP11-54001	-0.248544	1.6836328	-1.140097	0.2555614	0.3932311	-6.014803
TFAP2A-AS1	-0.36334	1.7183349	-1.139994	0.2556045	0.3932808	-6.014919
DHRS4L1	0.1379769	3.7040378	1.1399091	0.2556396	0.3933053	-6.015014
ZNF486	0.1411788	4.2801551	1.1398808	0.2556514	0.3933053	-6.015046
CAV3	0.1553002	-1.197557	1.1398754	0.2556536	0.3933053	-6.015052
RPS3P7	-0.142702	-1.230083	-1.139836	0.2556701	0.3933053	-6.015096
RP11-281J9	-0.167173	-1.031478	-1.139826	0.2556743	0.3933053	-6.015107
RPS15AP16	-0.179818	-1.000195	-1.139466	0.2558238	0.3935187	-6.01551
RBM18	0.0214623	5.9751787	1.1390687	0.2559891	0.3937564	-6.015955
IGHV3-76	-0.210933	-0.992707	-1.139017	0.2560106	0.3937728	-6.016012
RP11-368I2	-0.166083	-1.13062	-1.13895	0.2560387	0.3937994	-6.016088
TRMT10C	0.0242747	6.0546682	1.1387723	0.2561125	0.3938964	-6.016286
RP11-795A2	-0.072833	-1.497134	-1.138683	0.2561495	0.3939367	-6.016386
RP11-286N2	0.3547846	1.3026089	1.138551	0.2562046	0.3940049	-6.016534
RP4-704D23	-0.107928	-1.433187	-1.138497	0.2562272	0.3940229	-6.016594
RP11-145P1	0.1370389	-1.261641	1.1384672	0.2562395	0.3940253	-6.016627
RP11-434I1	-0.238107	2.5832601	-1.138297	0.2563104	0.3941091	-6.016818
AC068831.1	-0.187154	-0.916901	-1.138284	0.2563156	0.3941091	-6.016832
RP11-414C2	0.291305	0.0920314	1.1382012	0.2563503	0.3941318	-6.016925
GMPPB	0.0269095	6.150696	1.1381972	0.2563519	0.3941318	-6.016929
ZNF48	-0.032203	5.6354558	-1.137957	0.2564519	0.3942689	-6.017198
SEPT7P3	0.1793605	-1.179192	1.1378853	0.2564819	0.3942818	-6.017278
CTA-339C12	-0.133052	-1.272832	-1.13787	0.2564883	0.3942818	-6.017295
AC069257.6	0.1772286	-1.009491	1.1378593	0.2564927	0.3942818	-6.017307
DNAH11	0.2330846	3.8805195	1.1377984	0.2565181	0.3943022	-6.017375
FLJ31104	-0.18098	2.8544968	-1.137776	0.2565276	0.3943022	-6.017401
WBSCR16	0.0237801	6.4821431	1.1376728	0.2565704	0.3943489	-6.017515
DDX11L16	0.0700869	-1.53231	1.1376509	0.2565796	0.3943489	-6.01754
RP11-399C1	-0.241219	1.7668904	-1.137516	0.2566356	0.3944184	-6.01769
CAMKMT	-0.037006	5.2050785	-1.137487	0.256648	0.3944208	-6.017723
HPCA	0.2977705	0.3737467	1.1372615	0.2567419	0.3945485	-6.017975
SLC25A36	0.0687711	5.3745951	1.137162	0.2567834	0.3945957	-6.018086
FTH1P5	-0.121099	3.3167582	-1.137028	0.2568394	0.3946652	-6.018236
ADPGK-AS1	0.2499023	0.7825834	1.1368218	0.2569253	0.3947529	-6.018466
RP11-843P1	0.2646624	1.4581926	1.1368036	0.2569329	0.3947529	-6.018486
MIR26B	-0.204004	-0.736783	-1.136788	0.2569395	0.3947529	-6.018504
WDR41	0.0249706	5.9704712	1.1367871	0.2569397	0.3947529	-6.018505
SH3BGRL2	-0.049553	6.3806773	-1.136758	0.2569519	0.3947549	-6.018537
RP11-85021	0.1218272	-1.39061	1.1367301	0.2569635	0.3947561	-6.018568
RP11-294C1	-0.145542	-1.342795	-1.136609	0.2570141	0.3948172	-6.018704
PDHX	0.023976	6.1582873	1.1365687	0.2570309	0.3948264	-6.018749
LA16c-425C	0.2184261	-0.390458	1.1365095	0.2570556	0.3948388	-6.018815

MCFD2	0.0274126	6.7549902	1.1364975	0.2570606	0.3948388	-6.018828
RP11-1017G	-0.242401	1.2781221	-1.136423	0.2570918	0.3948563	-6.018912
RP5-963E22	-0.157361	-1.109949	-1.136418	0.2570936	0.3948563	-6.018917
CHL1-AS1	-0.145095	-1.285869	-1.136367	0.2571149	0.3948725	-6.018974
RN7SKP106	-0.261438	-0.821645	-1.136267	0.2571566	0.3948952	-6.019085
CFHR4	0.2593801	5.3951776	1.1362571	0.2571609	0.3948952	-6.019097
HSPD1P11	-0.237646	-0.413258	-1.136222	0.2571754	0.3948952	-6.019135
AC097713.4	-0.112484	-1.395319	-1.136216	0.2571782	0.3948952	-6.019143
AC016747.3	-0.031639	5.6222102	-1.136202	0.2571838	0.3948952	-6.019158
NPM1P35	-0.192469	-0.837892	-1.136086	0.2572323	0.394953	-6.019287
RP11-349J5	0.1833726	-0.976183	1.1358988	0.2573105	0.3950564	-6.019496
IL31RA	-0.327909	0.5246962	-1.135708	0.2573902	0.3951622	-6.019709
RP4-785G19	-0.18487	2.7250078	-1.135617	0.2574283	0.3952041	-6.019811
PUF60	-0.027331	6.8393475	-1.135452	0.2574971	0.3952742	-6.019995
SLC26A8	0.2354879	2.1016971	1.1354455	0.2574999	0.3952742	-6.020002
FOXD4	-0.267357	2.158965	-1.135421	0.2575101	0.3952742	-6.020029
RP11-1277A	0.2847866	2.2091219	1.1354038	0.2575173	0.3952742	-6.020049
AL928970.1	-0.157785	-1.097477	-1.135363	0.2575341	0.3952835	-6.020094
[truncated: 1,079,713 more chars]
